# iSS-PC: Identifying Splicing Sites via Physical-Chemical Properties Using Deep Sparse Auto-Encoder

**DOI:** 10.1038/s41598-017-08523-8

**Published:** 2017-08-15

**Authors:** Zhao-Chun Xu, Peng Wang, Wang-Ren Qiu, Xuan Xiao

**Affiliations:** 1 Computer Department, Jing-De-Zhen Ceramic Institute, Jing-De-Zhen, 333403 China; 2 Department of Computer Science and Bond Life Science Center, University of Missouri, Columbia, MO USA; 3 Gordon Life Science Institute, Boston, Massachusetts 02478 United States of America

## Abstract

Gene splicing is one of the most significant biological processes in eukaryotic gene expression, such as RNA splicing, which can cause a pre-mRNA to produce one or more mature messenger RNAs containing the coded information with multiple biological functions. Thus, identifying splicing sites in DNA/RNA sequences is significant for both the bio-medical research and the discovery of new drugs. However, it is expensive and time consuming based only on experimental technique, so new computational methods are needed. To identify the splice donor sites and splice acceptor sites accurately and quickly, a deep sparse auto-encoder model with two hidden layers, called iSS-PC, was constructed based on minimum error law, in which we incorporated twelve physical-chemical properties of the dinucleotides within DNA into PseDNC to formulate given sequence samples via a battery of cross-covariance and auto-covariance transformations. In this paper, five-fold cross-validation test results based on the same benchmark data-sets indicated that the new predictor remarkably outperformed the existing prediction methods in this field. Furthermore, it is expected that many other related problems can be also studied by this approach. To implement classification accurately and quickly, an easy-to-use web-server for identifying slicing sites has been established for free access at: http://www.jci-bioinfo.cn/iSS-PC.

## Introduction

**Figure 1:**
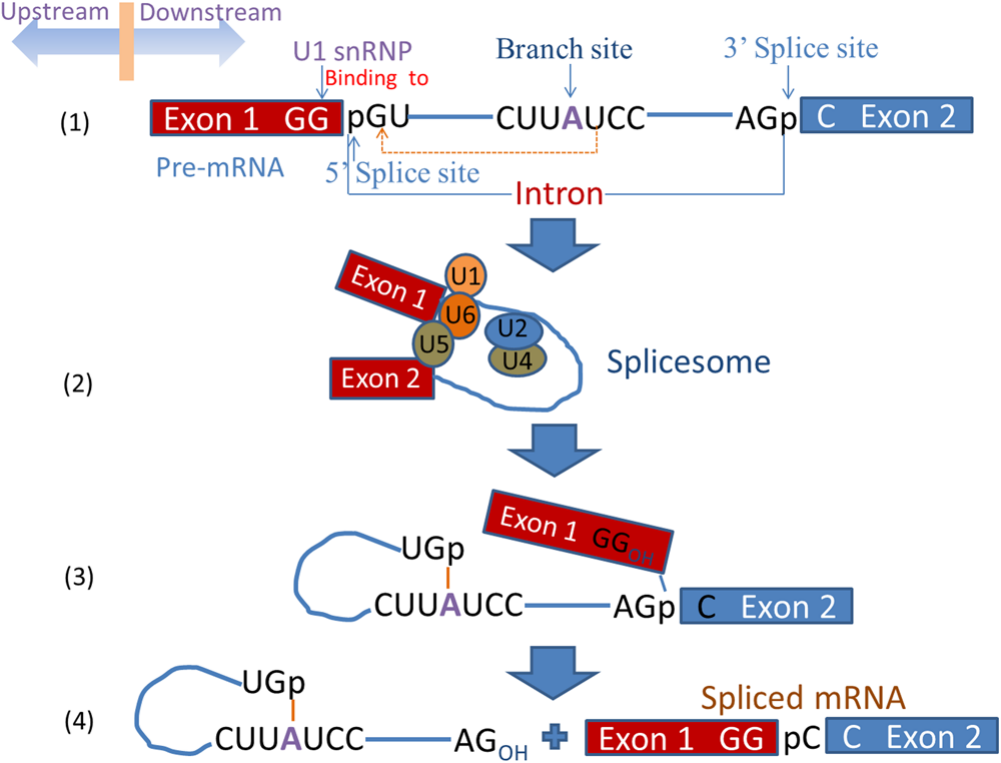
Sketch map showing the steps about the pre-mRNA how to become a mature messenger RNA.

Generally, the pre-mRNA, including exons and one or more introns, is transcribed from a eukaryotic gene’s DNA template. In the pre-mRNA, exon-intron boundaries i.e. the 5′ ends of the introns are called splice donor sites or 5′ splice sites, and intron-exon boundaries i.e. the 3′ ends of the introns are called splice acceptor sites or 3′ splice sites, as shown in Fig. 1. There are two forms of splice sites. Before the pre-mRNA becomes a mature messenger RNA (mRNA), it must go through several biological processes (Fig. 1). The final mRNA containing only remaining exons can be directly involved in the synthesis of protein. Thus, the biological process of removing introns from its 5′ splice site to its 3′ splice site in pre-mRNA and connecting exons to form mRNA plays an important role in gene regulation and expression. In this case, accurate identification of splice sites becomes increasingly important.

Although the technology of PCR has become one of the most important identification methods to accurately identify splice sites with the development of identification technology the functional sites of genes, it is very expensive and time consuming based only on experimental technique. Hence, development of an effective computational method, so as to help researchers effectively and in a timely fashion, identifying splice sites, has become the urgent need to solve a big problem. In this situation, the computational splice-site analysis tools based on the WEB took up, such as NetGene^1, 2^, SplicePredictor^3^, GeneSplicer^4^ and SplicePort^5^. Recently, Wei Chen *et al*.^6^ built a prediction model “iSS-PseDNC” which incorporated six DNA local structural properties into pseudo dinucleotide composition to identify splice donor and acceptor sites. In 2016, M Iqbal *et al*.^7^ used PseTNC and PseTetraNC methods to propose a hybrid prediction model, called iSS-Hyb-mRMR, for identifying splice sites, and Prabina Kumar Meher^8^ used a hybrid feature extraction approach, which contains positional, dependency and compositional features, to develop a predictor called HSplice for predicting the donor splice sites in eukaryotic genes. These were, on balance, successful.

Based on the above information, although the remarkable progress in identification of splice sites has been made, further study about splice-site predictors can be improved and perfected, whether it is with regard to in feature extraction, or to machine learning classification algorithms. In response to these the issue of two aspects, we have presented a solution to improve the performance of the predictive model in this paper.

On the one hand, improvement of feature extraction method is of critical importance to improve the classification performance. Since S Wold^9^ proposed the concept of auto-covariance function(ACF) and cross-covariance function(CCF) to analyze the relations between biopolymer sequences and chemical processes in 1993, this method had been applied to identify nuclear receptors and their subfamilies^10^ and N^6^-methyladenosine sites^11^ via incorporating physical-chemical properties into pseudo amino acid composition(PseAAC) or pseudo dinucleotide composition(PseDNC), respectively. Encouraged by the above successes of introducing this feature extraction approach into computational proteomics, we use twelve physical-chemical properties of the dinucleotides within DNA via a battery of cross-covariance and auto-covariance transformations to obtain a mode of PseDNC to formulate given sequence samples.

On the other hand, the improved machine learning classification algorithms that can provide a better result for classification, is one of the important factors impacting on the performance of classifiers. And in general, different classification algorithms will have different performances. Conventional classification algorithms, such as Support Vector Machine(SVM)^12–15^, random forest^16^, hidden Markov model^17^, Bayes^18^, covariance discriminant (CD)^19^, Minimax Probability Machine (MPM)^20^ and so on, have limitations in processing the original data. Recently, a novel classification algorithm, deep learning, has been proposed based on big data, and it has overcome the former limitations. Deep learning algorithm mainly includes convolutional neural network(CNN)^21^, deep belief network(DBN)^22^ and stacked auto-encoder(SAE)^23, 24^. Some remarkable progress has been made in diverse fields such as speech recognition and image recognition. In 2014, L James *et al*.^25^ firstly used SAE to predict θ and Tangles used to represent local backbone structure of proteins. In the same year, SP Nguyen *et al*.^26^ built a model “DL-Pro” that learned a SAE network as a classifier for protein structures. In 2016, J Xu *et al*.^27^ used SAE algorithm to detect on breast cancer histopathology images. W Xu *et al*.^28^ constructed a model for human promoter recognition with SAE. Inspired by these achievements, the predictor called iSS-PC is constructed by using deep sparse auto-encoder in this paper and its predication performance has been greatly improved.

**Figure 2:**
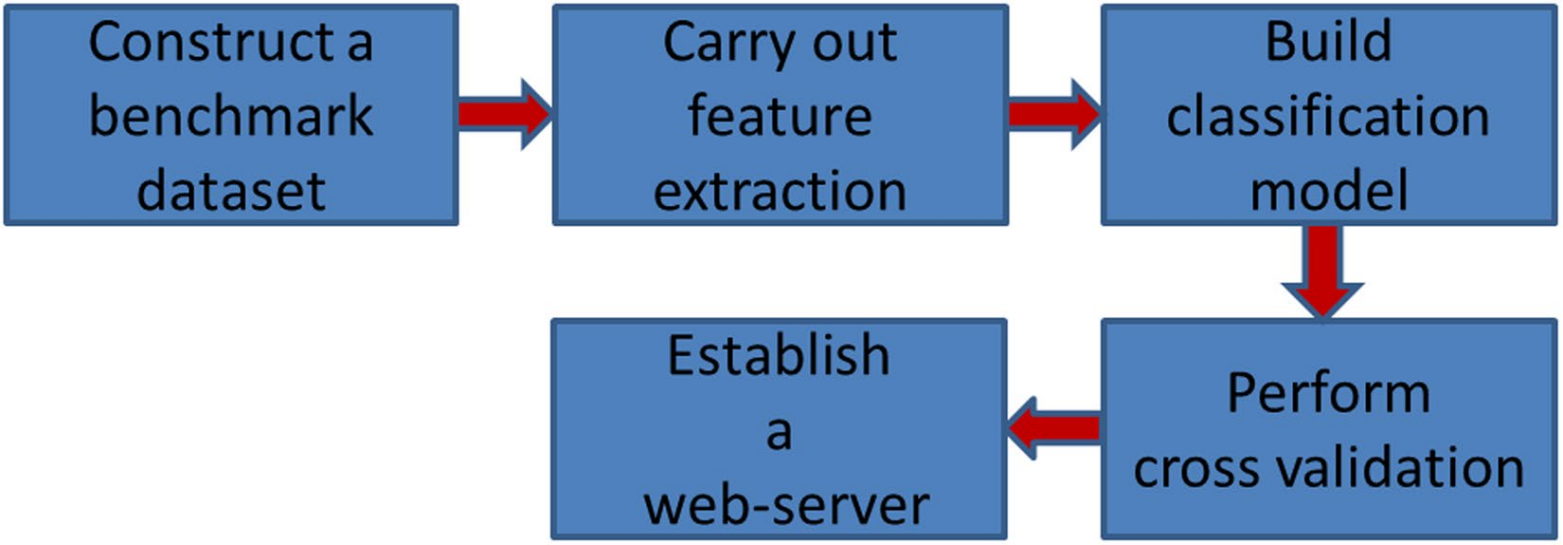
Sketch map showing the steps how to establish a predictor for biological system.

Basing on a series of recent studies^29–31^, we can draw a conclusion that we should follow the five steps^32^ shown in Fig. 2 to establish a real and effective biological predictor based on sequence. Below, we are going to discuss how to deal with these steps one by one. Of course, the order of these steps may be appropriately adjusted to be in a format that is suitable for the journal “Scientific Reports”.

## Results and Discussion

### Selection of the characteristic parameter

As described in Section Methods later in the article, we can obtain a feature vector containing 144 × *τ* components to represent the given sample sequence *D*. Here *τ* is named characteristic parameter, and its value as an integer. Obviously, the dimension *I* of the feature vector is increased with the increment of the characteristic parameter *τ*, as shown below.

1 $$I=\{\begin{array}{cc}\begin{array}{c}288\\ 432\\ \begin{array}{c}576\\ \begin{array}{c}720\\ \vdots \end{array}\end{array}\end{array} & \begin{array}{c}\tau =2\\ \tau =3\\ \begin{array}{c}\tau =4\\ \begin{array}{c}\tau =5\\ \vdots \end{array}\end{array}\end{array}\end{array}$$

However, we should notice that oversized *τ* value will lead to the problem of the curse of dimensionality. Thus, the value of *τ* is set at 2, 3, 4 and 5 to carry out experiments, respectively. And the experimental results are listed in Table 1 and Table 2. As can be seen from Table 1, *τ* = 5 gives the best results, but there is little difference between the results given by *τ* = 4 and *τ* = 5. Then, in order to reduce computation time, we fix the *τ* value into 4. As can be seen form Table 2, *τ* = 4 gives the best results. Then we can generate a feature vector containing 144 × 4 = 576 components as the input of the deep sparse auto-encoder for identifying splicing donor site and splicing acceptor site.

**Table 1:** The test results of splice donor site sequences based on different characteristic parameter *τ* values.

Predictor	ACC(%)	MCC(%)	Sn(%)	Sp(%)
*τ* = **2**	88.88	77.77	88.34	89.43
*τ* = **3**	80.58	61.15	81.01	80.14
*τ* = **4**	90.56	81.13	90.09	91.04
*τ* = **5**	90.74	81.49	90.77	90.71

**Table 2:** The test results of splice acceptor site sequences based on different characteristic parameter *τ* values.

Predictor	ACC(%)	MCC(%)	Sn(%)	Sp(%)
*τ* = **2**	89.01	78.09	87.40	96.08
*τ* = **3**	90.02	80.04	89.69	90.36
*τ* = **4**	91.11	82.24	90.14	92.11
*τ* = **5**	90.95	81.95	99.44	92.50

### Comparison with the existing methods

The four metrics i.e. accuracy (Acc), sensitivity (Sn), specificity (Sp), and Matthew correlation coefficient (Mcc) can reflect the performance of predictors clearly. Based on the benchmark dataset composed solely of splice donor site sequences, their scores obtained by the new predictor “iSS-PC” via the five-fold cross-validation test are listed in Table 3. And the results for splice acceptor site sequences, listed in Table 4. For ease of comparison between the other methods, the results obtained by the iSS-PseDNC predictor constructed by Wei Chen^6^ based on the corresponding benchmark dataset are listed in these tables, respectively.

**Table 3:** The comparison of the 5-fold cross-validation test results on benchmark data-set only containing splice donor site sequences.

Predictor	ACC(%)	MCC(%)	Sn(%)	Sp(%)
iSS-PseDNC^a^	87.71	75.46	89.56	85.86
iSS-PC^b^	90.56	81.13	90.09	91.04

^a^The prediction method developed by Wei Chen (2014).

^b^The prediction method proposed in this paper.

**Table 4:** The comparison of the 5-fold cross-validation test results on benchmark data-set only containing splice acceptor site sequences.

Predictor	ACC(%)	MCC(%)	Sn(%)	Sp(%)
iSS-PseDNC^a^	88.73	77.89	94.24	83.07
iSS-PC^b^	91.11	82.24	90.14	92.11

^a^The prediction method developed by Wei Chen (2014).

^b^The prediction method proposed in this paper.

As can be seen from Table 3, although the Sn rate of the new predictor “iSS-PC” is a little bit higher than that of the iSS-PseDNC predictor, the score of the other three metrics has been greatly improved. For example, the ACC rate of our predictor “iSS-PC” has increased by nearly three percent, the MCC rate, nearly six percent and the Sp rate, also nearly six percent. It means that better experimental effect has been acquired, and indicates that our predictor is superior to the iSS-PseDNC predictor at identifying the splice donor site sequences.

On the other hand, as can be seen from Table 4, although the Sn rate of the new iSS-PC predictor is 4% lower than that of the iSS-PseDNC predictor, the Sp rate of our predictor has increased by over 9 percent. And most importantly, the most important indicators for ranking different algorithms have different increases, ACC, nearly 2.5 percent and MCC, nearly 4.5 percent. It indicates that our predictor is also superior to the iSS-PseDNC predictor at identifying the splice acceptor site sequences.

Then through the above analyses, we can draw the conclusion that the methods of feature extraction and classification designed in this paper are very effective based on the splice site sequences. It means that the iSS-PC predictor has higher prediction precision and consumes less time than the existing predictors.

### Receiver operating characteristic (ROC) curves

Receiver operating characteristic(ROC) curve^33^ is the another important gauge of performance of a predictor. It can visually present readers’ eyes in graphical form. The area under the ROC curve(AUC) represents a popular evaluation index of the performance of a binary classifier. Studies^34, 35^ indicated that the larger the AUC meant better predictor’s performance.

**Figure 3:**
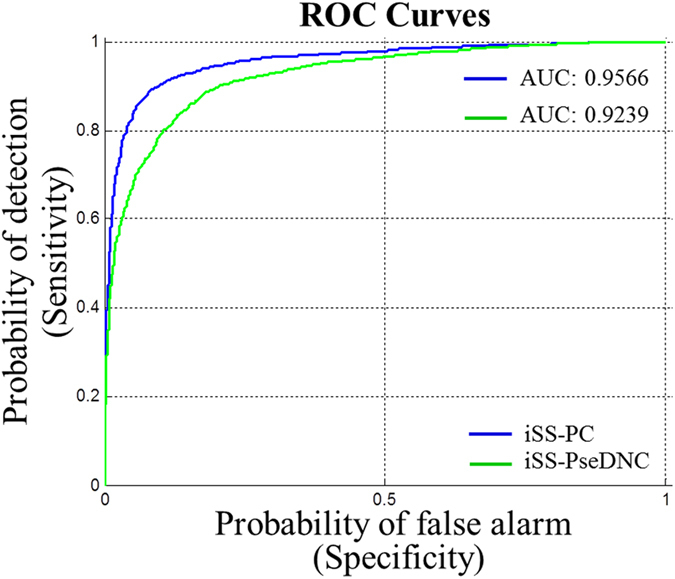
ROC curves of the two different predictors for the splice donor site sequences.

**Figure 4:**
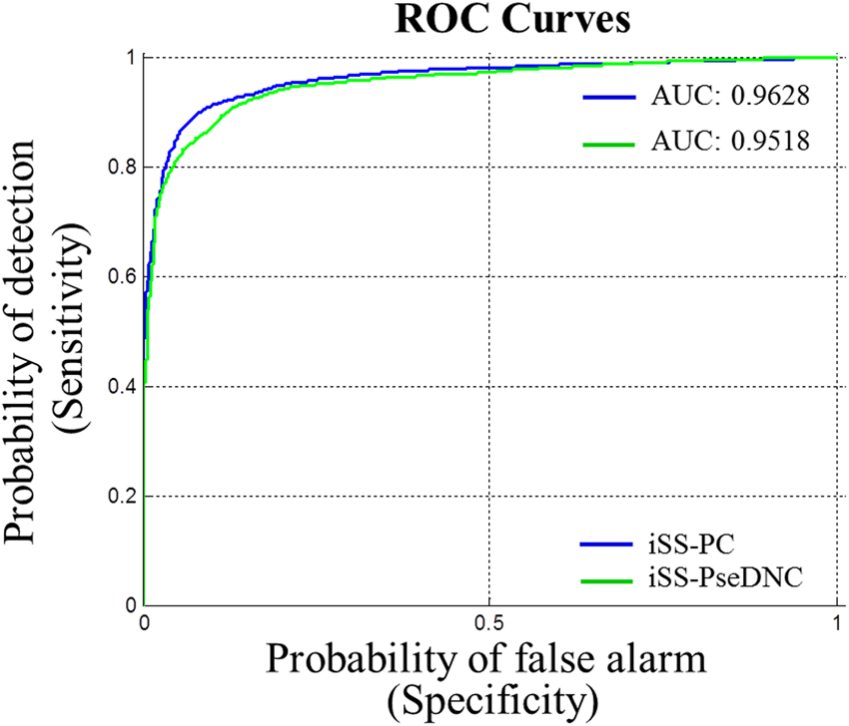
ROC curves of the two different predictors for the splice acceptor site sequences.

In the Figs 3 and 4, the blue curve is generated by new predictor “iSS-PC”, and the green curve is formed by the predictor “iSS-PseDNC” constructed by Wei Chen *et al*. The corresponding values of AUC computed over five-fold cross-validation are shown in Figs 3 and 4. From Fig. 3 it can be seen that the values of AUC are 0.9566 and 0.9239 for splice donor site sequences, respectively. On the other hand, for the splice acceptor site sequences the value of AUC generated by predictor “iSS-PC” is found to be 0.9628, whereas the value of AUC generated by predictor “iSS-PseDNC” is found to be 0.9518, as shown in Fig. 4. Obviously, it can be seen that the AUC value of the predictor “iSS-PC” is higher than that of the predictor “iSS-PseDNC” for both the splice donor and acceptor site sequences. Therefore, we can draw the conclusion that our predictor “iSS-PC” is superior to the predictor “iSS-PseDNC”, and from the experimental results, it can be proved that the predictor “iSS-PC” is accurate and stable.

### Comparison with traditional high-effectiveness machine learning algorithms

SVM and random forest (RF) are the traditional but efficient classification algorithms. In addition, Dynamic selection and Circulating Combination-based ensemble Clustering i.e. libD3C^36, 37^ is a popular tool for binary classification task, too. In order to quickly and easily perform classification prediction for users, libD3C package can be downloaded from the website: http://datamining.xmu.edu.cn/~gjs/LibD3C_1.1/index.html. Meanwhile, WEKA, a free and open source software program, should be downloaded and installed. Then, the ensemble classification model constructed by libD3C can be created in WEKA. In this paper, we compare the SAE model with these traditional machine learning algorithms to examine the performance of the new predictor. And the results are listed in Tables 5 and 6.

**Table 5:** The 5-fold cross-validation test results obtained from different classification algorithms with the same feature extraction method on benchmark data-set only containing splice donor site sequences.

Predictor	ACC(%)	MCC(%)	Sn(%)	Sp(%)
iSS-PC^a^	90.56	81.13	90.09	91.04
iSS-SVM^b^	77.59	55.25	75.68	79.50
iSS-RF^c^	83.13	66.38	80.11	86.14
iSS-libD3C^d^	83.38	67.09	78.43	88.32

^a^The predictor with SAE proposed in this paper.

^b^The predictor with SVM created in WEKA with the default parameters.

^c^The predictor with Random Forest (RF) created in WEKA.

^d^The predictor with an ensemble classifier libD3C.

**Table 6:** The 5-fold cross-validation test results obtained from different classification algorithms with the same feature extraction method on benchmark data-set only containing splice acceptor site sequences.

Predictor	ACC(%)	MCC(%)	Sn(%)	Sp(%)
iSS-PC^a^	91.11	82.24	90.14	92.11
iSS-SVM^b^	73.10	46.23	71.94	74.29
iSS-RF^c^	85.80	71.60	84.70	86.90
iSS-libD3C^d^	83.15	66.55	79.38	87.04

^a^The predictor with SAE proposed in this paper.

^b^The predictor with SVM created in WEKA with the default parameters.

^c^The predictor with Random Forest (RF) created in WEKA.

^d^The predictor with an ensemble classifier libD3C.

The results show in the Tables 5 and 6: the rates of the two most important indicators, ACC and MCC obtained from our predictor “iSS-PC” are significantly higher than those of others, respectively. It indicates the SAE classification algorithm is more effective to identify the splice sites and the new predictor “iSS-PC” would be a very useful tool in this regard.

### Web server and its user guide

**Figure 5:**
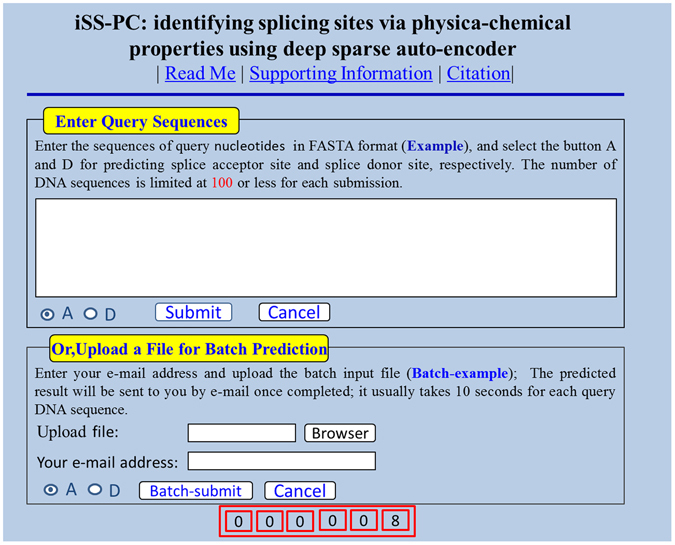
A semi-screenshot of the homepage for the web-server “iSS-PC”.

In this paper, a simple and practical network predictor shown in Fig. 5, called iSS-PC, has been developed, in order to help the researchers identify splicing sites in real-time and easily. And we provide service consumers with a Web site link http://www.jci-bioinfo.cn/iSS-PC. Below, this article provides details on how to use the network predictor “iSS-PC”.

- If you want to get the information about the network predictor, please click the Read Me button. Then you can obtain a brief introduction of our predictor and the caveats for using it.
- If you want to obtain the benchmark data-set for the iSS-PC predictor training and testing in this paper, please click the Supporting Information button. Here are a few data-sets for download, such as *S*_1_ only containing splice donor site sequences, *S*_2_ only containing splice acceptor site sequences.
- If you want to get some important references and resources in establishing the iSS-PC predictor, please click the Citation button.
- Before entering query sequences or uploading a file for batch prediction, you should choose types of splice sites: splice donor site or splice acceptor site.
- The network predictor “iSS-PC” accepts single or multiple sequence queries. But the input sequences must be in FASTA format, or the network predictor may report errors and will request you to re-input your query sequence. Click the Example button on top of the first input box to see the input format.
- If you want to obtain the prediction results, please click the Submit button. After entering query sequences in the first input box in the Example window, you will see how much you’ve been doing with the job on your screen. When the job is over, the results will be displayed in the page as “The number of DNA sequences investigated: X”, and “The DNA #xx is splice donor/acceptor site sequences” or “ The DNA #xx is non-splice donor/acceptor site sequences”.
- The lower panel of Fig. 5 offers the option for batch prediction. If you want to submit your batch of multiple sequences in FASTA format for prediction in order to avoid constantly online awaiting, please click the Browse button. The prediction results of each batch job will be sent to your e-mail address. Clicking the Batch-example button, you will see the examples of batch file in FASTA format.
- Running times of the network predictor “iSS-PC” are shown underneath the above graph in mathematical terms. And the corresponding number stands for popularity of our predictor to a certain extent.

## Conclusions

Feature extraction is the key problem in the research on bioinformatics. In this article, we incorporated twelve physical-chemical properties of the dinucleotides within DNA into PseDNC to formulate the given sequence samples via a battery of cross-covariance and auto-covariance transformations, and achieved good results. However, with the further research of feature extraction methods and the development of computer technology, more and more web servers have been emerged, such as Pse-in-One^38^, repRNA^39^, and repDNA^40^. Then, many features such as pseudo amino acid composition (PseAAC), pseudo dinucleotide composition (PseDNC), pseudo trinucleotide composition (PseTNC), dinucleotide-based auto covariance (DAC) and dinucleotide-based cross covariance (DCC) can be generated by using these web servers. Therefore, for the future, we can try to study more other similar genomic problems by using the feature extraction methods based on these web servers.

Classification algorithm design is another important step that can affect the performance of a predictor. In this paper, we used deep sparse auto-encoder to construct the iSS-PC predictor. By using the same feature extraction method on benchmark data-sets, we compared the SAE model with those traditional machine learning algorithms, and found that the SAE classification algorithm was stable and reliable. Therefore, the new approach could be used to solve many important tasks in bioinformatics, such as iRSpot-EL^41^, iDHS-EL^42^, iEnhancer-2L^43^. And these are the work which should be completed in the next phase. In fact, we had constructed a predictor called “iDHSs-PseTNC”^44^ to identify DNase I hypersensitive sites with pseudo trinucleotide component by deep sparse auto-encoder, and the results of the predictor iDHSs-PseTNC was superior to that of iDHS-EL.

In conclusion, the timely identification of the splicing sites in DNA sequence is significant for the intensive study on DNA function and the development of new drugs. The experimental results by five-fold cross-validation on the same benchmark datasets indicated that the iSS-PC predictor was superior to other predictors in this area. And the results were promising enough for our predictor to be used as an analytic solution to more genomic problems, such as DNA-binding protein prediction^45^, detection of tubule boundary^46^, methylation site prediction^47^, phosphorylation site prediction^48^, and protein-protein interaction prediction^49^.

## Methods

### Benchmark dataset

In this paper, the benchmark dataset is composed of two parts: splice donor site sequences and splice acceptor site sequences. The former can be denoted by *S*_1_, the latter can be formulated by *S*_2_, as shown below.

2 $${S}_{1}={S}_{1}^{+}\cup {S}_{1}^{-};{S}_{2}={S}_{2}^{+}\cup {S}_{2}^{-}$$

where ${S}_{1}^{+}$ represents the positive dataset containing 2796 true splice donor site sequences, while ${S}_{1}^{-}$ represents the negative dataset consisting of 2800 false splice donor site sequences. ${S}_{2}^{+}$, the positive dataset composed of 2880 true splice acceptor site sequences, while $\,{S}_{2}^{-}$, the negative dataset composed of 2800 false splice acceptor site sequences. The symbol $\cup$ denotes “union” in the Cantor set theory. Datasets *S*_1_and *S*_2_ provided by Wei Chen^6^ can be downloaded from the website: http://dx.doi.org/10.1155/2014/623149, or these datasets can be obtained from Supplementary Information.

### Feature extraction

Generally, input of nearly all the machine learning based classifiers must be numerical features but not sequences^50^, therefore, splice site sequences should be transformed into numerical feature vectors. Below, let’s describe how to formulate a sample sequence into a discrete vector model.

A sequence sample in the current benchmark dataset can be generally expressed as

3 $${\rm{D}}={N}_{1}{N}_{2}{N}_{3}{N}_{4}{N}_{5}{N}_{6}{N}_{7}\cdots {N}_{L}$$

where *N*_*i*_ (*i* = 1, 2, …, *L*) represents the *i*th nucleotide of the sequence sample. It can be any one of the four nucleotides: adenine (*A*), cytosine (*C*), guanine (*G*) and thymine (*T*), respectively. While *L* represents the length of the given sequence sample.

Some literatures have shown that among the discrete vector models for a DNA sample, nucleic acid composition (NAC) is the simplest one. According to the NAC-discrete vector model, the given sequence sample D of Eq. (3) can be defined as

4 $${\rm{D}}={[\begin{array}{cc}\begin{array}{cc}f(A) & f(C)\end{array} & \begin{array}{cc}f(G) & f(T)\end{array}\end{array}]}^{T}$$

where ${f}_{i}=f(\cdot )$, (*i* = 1, 2, 3, 4) is the normalized occurrence frequency of the corresponding descriptor in the DNA sequence. And *T* is the transpose operator. But in this way all the sequence order information of sequence D would be entirely lost.

As mentioned in the literature^51^, in order to incorporate more short-range sequence-order or local information, the *k*-tuple nucleotide composition or *k*-mers approach can be used to formulate the given sequence D into a feature vector containing 4^*k*^ components, i.e.

5 $${\rm{D}}={[\begin{array}{ccc}\begin{array}{cc}{f}_{1} & {f}_{2}\end{array} & \begin{array}{cc}{f}_{3} & \cdots \end{array} & \begin{array}{cc}{f}_{{4}^{k-1}} & {f}_{{4}^{k}}\end{array}\end{array}]}^{T}$$

where *f*_1_ is the normalized occurrence frequency of the first *k*-mer; *f*_2_, that of the second *k*-mer, and so on. It should be noted however, that *k* is usually not more than 4, otherwise it may cause over-fitting problem, “high-dimension disaster”^52^ and increase of computational run-time with the feature vector dimensions increasing.

To incorporate long-range or global sequence order information, the pseudo components were proposed to deal with not only peptide/protein sequences, but also RNA/DNA sequences. As mentioned in the recent paper^53^, the sequence D of Eq. (2) can be formulated as below by using the pseudo nucleotide composition (PseKNC).

6 $${\rm{D}}={[\begin{array}{ccc}\begin{array}{ccc}{\xi }_{1} & {\xi }_{2} & {\xi }_{3}\end{array} & \cdots  & \begin{array}{ccc}{\xi }_{\mu } & \cdots  & {\xi }_{I}\end{array}\end{array}]}^{T}$$

where subscript *I*, the vector dimension, is an integer. Its value as well as the components in Eq. (6) will depend on how to extract the desired information from the sequence D.

Below, the “physical-chemical property matrix” and “auto-covariance and covariance transformations” will be used to define the value of subscript *I* in Eq. (6).

### Physical-chemical property matrix

DNA physical-chemical(PC) property is the most intuitive feature of biochemical reactions. And it has different PC properties for each of sixteen different dinucleotides or dimers that are *AA*, *AC*, *AG*, *AT*, *CA*, …, *TT* in a DNA sequence, respectively. In this paper, the following twelve PC properties were adopted: (1) HC^1^: A-philicity^54^; (2) HC^2^: base stacking^55^; (3) HC^3^: B-DNA twist^56^; (4) HC^4^: bendability^57^; (5) HC^5^: DNA bending stiffness^58^; (6) HC^6^: DNA denaturation^59^; (7) HC^7^: duplex disrupt energy^60^; (8) HC^8^: duplex free energy^61^; (9) HC^9^: propeller twist^56^;(10) HC^10^: protein deformation^62^; (11) HC^11^: protein-DNA twist^62^; (12)HC^12^: Z-DNA^63^. The original values of the twelve descriptors for each dinucleotide are listed in Table 7. Then we can obtain a 12 × (*L* − 1) PC property matrix as shown below.

7 $${\rm{D}}=[\begin{array}{cc}\begin{array}{cc}\begin{array}{c}\begin{array}{c}P{C}^{1}({N}_{1}{N}_{2})\\ P{C}^{2}({N}_{1}{N}_{2})\end{array}\\ \begin{array}{c}\vdots \\ P{C}^{12}({N}_{1}{N}_{2})\end{array}\end{array} & \begin{array}{c}\begin{array}{c}P{C}^{1}({N}_{2}{N}_{3})\\ P{C}^{2}({N}_{2}{N}_{3})\end{array}\\ \begin{array}{c}\vdots \\ P{C}^{12}({N}_{2}{N}_{3})\end{array}\end{array}\end{array} & \begin{array}{cc}\begin{array}{c}\begin{array}{c}\cdots \\ \cdots \end{array}\\ \begin{array}{c}\vdots \\ \cdots \end{array}\end{array} & \begin{array}{c}\begin{array}{c}P{C}^{1}({N}_{L-2}{N}_{L-1})\\ P{C}^{2}({N}_{L-2}{N}_{L-1})\end{array}\\ \begin{array}{c}\vdots \\ P{C}^{12}({N}_{L-2}{N}_{L-1})\end{array}\end{array}\end{array}\end{array}]$$

where *PC* ^*i*^(*N*_*j*_ *N*_*j* + 1_) represents the *i*th (*i* = 1, 2, …, 12) PC property value for the dinucleotide *N*_*j*_ *N*_*j* + 1_ in Eq. (3). However, the data of Table 7 should be normalized by the following equation before they were substituted into Eq. (7).

8 $${y}_{k}=({x}_{k}-mean(x))/std(x)$$

where *x*_*k*_ represents the original PC property value in Table 7 of the *k*th (*k* = 1, 2, …, 16) dinucleotide. While mean (*x*) represents the average value for the sixteen dinucleotides; and std (*x*), the corresponding standard deviation; *y*_*k*_, the corresponding converted values, will remain unchanged if they go through the same conversion procedure again.

**Table 7:** The original values of the twelve PC properties for each dinucleotide.

Code	HC^1^	HC^2^	HC^3^	HC^4^	HC^5^	HC^6^	HC^7^	HC^8^	HC^9^	HC^10^	HC^11^	HC^12^
AA	0.97	−5.37	35.5	−0.27	35	66.51	1.9	−1.2	−18.66	12.1	35.1	3.9
AC	0.13	−10.5	33.1	−0.21	60	108.8	1.3	−1.5	−13.1	9.8	31.5	4.6
AG	0.33	−6.78	30.6	−0.08	60	85.12	1.6	−1.5	−14	6.3	31.9	3.4
AT	0.58	−6.57	43.2	−0.28	20	72.29	0.9	−0.9	−15.01	2.1	29.3	5.9
CA	1.04	−6.57	37.7	−0.01	60	64.92	1.9	−1.7	−9.45	6.1	37.3	1.3
CC	0.19	−8.26	35.3	−0.03	130	99.31	3.1	−2.3	−8.11	2.9	32.9	2.4
CG	0.52	−9.69	31.3	−0.03	85	88.84	3.6	−2.8	−10.03	4.5	36.1	0.7
CT	0.33	−6.78	30.6	−0.18	60	85.12	1.6	−1.5	−14	1.6	31.9	3.4
GA	0.98	−9.81	39.6	0.03	60	80.03	1.6	−1.5	−13.48	2.3	36.3	3.4
GC	0.73	−14.6	38.4	0.02	85	135.8	3.1	−2.3	−11.08	4	33.6	4
GG	0.19	−8.26	35.3	−0.06	130	99.31	3.1	−2.3	−8.11	6.1	32.9	2.4
GT	0.13	−10.51	33.1	−0.18	60	108.8	1.3	−1.5	−13.1	2.1	31.5	4.6
TA	0.73	−3.82	31.6	0.18	20	50.11	1.5	−0.9	−11.85	2.3	37.8	2.5
TC	0.98	−9.81	39.6	−0.11	60	80.03	1.6	−1.5	−13.48	4.5	36.3	3.4
TG	1.04	−6.57	37.7	0.13	60	64.92	1.9	−1.7	−9.45	9.8	37.3	1.3
TT	0.97	−5.37	35.5	−0.28	35	66.51	1.9	−1.2	−18.66	2.8	35.1	3.9

### Auto-covariance and cross covariance

The concept of auto-covariance function and cross-covariance function was proposed in 1993, when analyzing the relations between biopolymer sequences and chemical processes. Recently, according to the description to auto-covariance and cross-covariance transformations in literatures^10, 11^, these transformations could be expressed by the following mathematical expressions.

9 $${\rm{AC}}({\rm{\mu }},{\rm{\tau }})=\frac{{\sum }_{j=1}^{L-1-\tau }[P{C}^{\mu }({N}_{j}{N}_{j+1})-\overline{P{C}^{\mu }}][P{C}^{\mu }({N}_{j+\tau }{N}_{j+1+\tau })-\overline{P{C}^{\mu }}]}{L-1-\tau }\,({\rm{\mu }}=1,2,\cdots ,12)$$

where AC represents the correlation of the same PC property between two sub-sequences separated by τ dinucleotides, *τ* = 1, 2, …, *L* − 2. While $\overline{P{C}^{\mu }}=\frac{{\sum }_{j=1}^{L-1}P{C}^{\mu }({N}_{j}{N}_{j+1})}{L-1}$ is the mean of the data along the *μ*th row in the matrix of Eq. (7).

10 $${\rm{CC}}({n}_{1},{n}_{2},{\rm{\tau }})=\frac{{\sum }_{j=1}^{L-1-\tau }[P{C}^{{n}_{1}}({N}_{j}{N}_{j+1})-\overline{P{C}^{{n}_{1}}}][P{C}^{{n}_{2}}({N}_{j+\tau }{N}_{j+1+\tau })-\overline{P{C}^{{n}_{2}}}]}{L-1-\tau }\,({n}_{1}\ne {n}_{2})$$

where CC represents the correlation between two subsequences each belonging to a different PC property.

As we can see from Eq. (9), we can generate 12 × *τ* components associated with the PC properties of a sample sequence D in Eq. (3) and from Eq. (10), 12 × 11 × *τ* components. Then we can generate (12 × *τ* + 12 × 11 × *τ*) = 144 × *τ* components by ACF and CCF via 12 different PC properties. Therefore, the sample sequence *D* can be eventually formulated by

11 $${\rm{D}}={[\begin{array}{ccc}\begin{array}{ccc}{\xi }_{1} & {\xi }_{2} & {\xi }_{3}\end{array} & \cdots  & \begin{array}{ccc}{\xi }_{\mu } & \cdots  & {\xi }_{144\times \tau }\end{array}\end{array}]}^{T}$$

where *ξ*_*μ*_ represents the *μ*th of the 144 × *τ* components generated by Eqs (9) and (10) as described above.

### Deep sparse auto-encoder

In 1986, DE Rumelhart *et al*.^64^ firstly proposed the concept of an auto-encoder to process the large complex high-dimensional data. In 2006, GE Hinton *et al*.^22^ improved the prototype structure of the auto-encoder, thus making deep auto-encoder (DAE) appear. Thereafter, in 2008, Y Bengio *et al*.^65^ proposed the concept of sparse auto-encoder, therefore, the study of DAE went much deeper. And in 2010, P Vincent^24^ developed stacked de-noising auto-encoder to yield significantly lower classification error.

**Figure 6:**
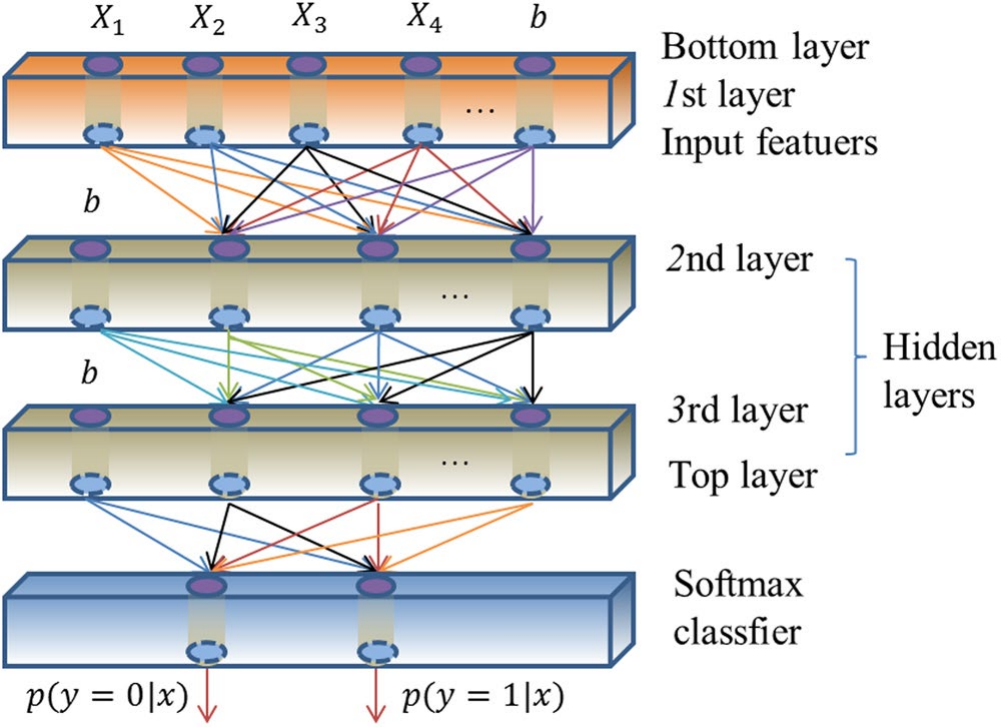
A sketch map of a deep sparse auto-encoder model with two hidden layers.

Based on the research^22^, we constructed a deep sparse auto-encoder model with two hidden layers in this paper, as shown in the Fig. 6. In order to implement classification accurately and quickly based on minimum error law, we can use deep learning software packages, including SAE and NN software, which can be obtained from the website https://github.com/rasmusbergpalm/DeepLearnToolbox. Note that, in order to optimize the effectiveness of the SAE algorithm, we should fine tune the model parameters by loop optimization. Finally, we can get the best results.

The predictor established according to the above-mentioned procedures is called ‘iSS-PC’, where ‘i’ stands for ‘identifying’, ‘SS’ for ‘splicing sites’ and ‘PC’ for ‘physical-chemical property’.

There are two issues to be dealt with: one is ‘what metrics should be used to examine the accuracy of the predictor?’ The other is ‘what validation method should be taken to calculate the metric values?’

### A set of metrics for measuring prediction quality

As mentioned in the literature, accuracy (Acc), sensitivity (Sn), specificity (Sp), and Matthew correlation coefficient (Mcc) introduced by Chou^66^ are the most frequently used metrics to evaluate the performance of the predictor in bioinformatics. To make these easier to understand for the researchers, the four metrics can be formulated as below^30, 67^.

12 $$\{\begin{array}{c}\begin{array}{c}ACC=1-\frac{{N}_{-}^{+}+{N}_{+}^{-}}{{N}^{+}+{N}^{-}}\,\\ Mcc=\frac{1-(\frac{{N}_{-}^{+}}{{N}^{+}}+\frac{{N}_{+}^{-}}{{N}^{-}})}{\sqrt{(1+\frac{{N}_{+}^{-}-{N}_{-}^{+}}{{N}^{+}})(1+\frac{{N}_{-}^{+}-{N}_{+}^{-}}{{N}^{-}})}}\,\end{array}\\ \begin{array}{c}Sn=1-\frac{{N}_{-}^{+}}{{N}^{+}}\,\\ Sp=1-\frac{{N}_{+}^{-}}{{N}^{-}}\,\end{array}\end{array}$$

where *N* ^+^ the total number of the true splice donor site sequences (true splice acceptor site sequences) detected, ${N}_{-}^{+}$ the number of the true splice donor site sequences (true splice acceptor site sequences) misidentified as the false splice donor site sequences(false splice acceptor site sequences); whereas, *N* ^−^ the total number of the false splice donor site sequences (false splice acceptor site sequences) observed, ${N}_{+}^{-}$ the number of the false splice donor site sequences (false splice acceptor site sequences) mis-predicted as the true splice donor site sequences (true splice acceptor site sequences).

However, it should be noted that the four metrics formulated in Eq. (12) are valid only for the single-label systems, but unsuitable for multi-label systems appearing frequently in system biology and system medicine. For the latter, an utterly different set of metrics is needed as elaborated in the literature^68^.

### Cross-validation

After the four well-known metrics mentioned above have been adopted to evaluate the performance of predictors, another thing we should consider at this moment is what validation method should be used to calculate the value of the four metrics. Generally speaking, there are three popular cross-validation approaches in prediction and analysis on the statistics, i.e., independent dataset test, K-fold cross-validation and jackknife test. Although the jackknife test always yielding a unique output for a given benchmark dataset seems the least arbitrary, K-fold cross-validation has more advantages in the computational time than that of the former. Therefore, in this paper, we adopt five-fold cross-validation to score the four metrics. Below, let’s introduce specific methods about five-fold cross-validation.

Firstly, for the benchmark dataset *S*_1_ of Eq. (2) consisting of splice donor site sequences, we randomly divided the data-sets ${S}_{1}^{+}$ and ${S}_{1}^{-}$ into five subsets which size was approximately equal to each other, respectively, as shown below

13 $$\{\begin{array}{c}{S}_{1}^{+}={S}_{11}^{+}\cup {S}_{12}^{+}\cup {S}_{13}^{+}\cup {S}_{14}^{+}\cup {S}_{15}^{+}\\ {S}_{1}^{-}={S}_{11}^{-}\cup {S}_{12}^{-}\cup {S}_{13}^{-}\cup {S}_{14}^{-}\cup {S}_{15}^{-}\end{array}$$

where ${S}_{1i}^{+}$, the subset of ${S}_{1}^{+}$, its label for the dividing category is set to *i*(*i* = 1, 2, …, 5). Similarly, ${S}_{1i}^{-}$, the subset of ${S}_{1}^{-}$, its label for the dividing category is set to *i*, too. Both ${S}_{1i}^{+}$ and ${S}_{1i}^{-}$ satisfied the following conditions.

14 $$\{\begin{array}{c}|{S}_{11}^{+}|\approx |{S}_{12}^{+}|\approx |{S}_{13}^{+}|\approx |{S}_{14}^{+}|\approx |{S}_{15}^{+}|\\ |{S}_{11}^{-}|\approx |{S}_{12}^{-}|\approx |{S}_{13}^{-}|\approx |{S}_{14}^{-}|\approx |{S}_{15}^{-}|\end{array}$$

where $|{S}_{11}^{+}|$ denotes the number of elements (samples) in ${S}_{11}^{+}$, and so forth.

Finally, we can obtain five subsets of the benchmark dataset *S*_1_ according to their labels for the dividing category, as shown below

15 $${S}_{1}={S}_{1}^{^{\prime} }\cup {S}_{2}^{^{\prime} }\cup {S}_{3}^{^{\prime} }\cup {S}_{4}^{^{\prime} }\cup {S}_{5}^{^{\prime} }$$

where ${S}_{1}^{^{\prime} }={S}_{11}^{+}\cup {S}_{11}^{-},{S}_{2}^{^{\prime} }={S}_{12}^{+}\cup {S}_{12}^{-}$, and so forth.

with

16 $$|{S}_{1}^{^{\prime} }|\approx |{S}_{2}^{^{\prime} }|\approx |{S}_{3}^{\text{'}}|\approx |{S}_{4}^{^{\prime} }|\approx |{S}_{5}^{^{\prime} }|$$

Therefore, we can single out each of the five subsets of Eq. (15) one by one to test the model that were trained with the remaining four subsets for identifying the splice donor site sequences. The cross validation is carried out five times, and the average scores among the output are regarded as the final outcome. It’s remarkable that the same cross-validation process can be used for the benchmark data-set *S*_2_ of Eq. (2) consisting of splice acceptor site sequences.

## Electronic supplementary material

Supplementary Information
