# Supplementary material for: iSS-PC: Identifying Splicing Sites via Physical-Chemical Properties Using Deep Sparse Auto-Encoder
Source: Sci Rep. 2017 Aug 15;7:8222. doi: 10.1038/s41598-017-08523-8 (PMC5557945; doi:10.1038/s41598-017-08523-8)
Supplement: Supplementary file 1 — Supplementary Information [file 41598_2017_8523_MOESM1_ESM.pdf]

Supplementary Information

iSS-PC: Identifying Splicing Sites via Physical-Chemical Properties Using Deep Sparse Auto-Encoder

Zhao-Chun Xu[1]*, Peng Wang[1], Wang-Ren Qiu[1,2]* and Xuan Xiao[1,3]*

[1]Computer Department, Jing-De-Zhen Ceramic Institute, Jing-De-Zhen 333403 China

[2]Department of Computer Science and Bond Life Science Center, University of Missouri, Columbia, MO, USA

[3]Gordon Life Science Institute, Boston, Massachusetts 02478, United States of America

*To whom correspondence should be addressed (jdzxuzhaochun@163.com, qiuone@163.com, or jdzxiaoxuan@163.com).

Online Supporting Information S_1^{+} represents the positive dataset containing 2796 true splice donor site sequences.

>1
CTCCTCTTTGCCTTACTCCTAGCCATGGAGCTCCCATTGGTGGCAGCCAGTGCCACCAT
GCGCGCTCAGTGTAAGTATCATTCCCTCTCACTGTCCTGGAGAGGACGAGAATTCCAC
CTGGGGTGCTGGGGGTCACTGGG
>2
AATGACTTCAACTGTCCCAACATTAGAGTATGTCCGTATCATATTAGGCGCTGTATGACA
ATCTCCATTCGTAAGTACCTCTTGGTCATTTGGACACATTGTAGATTAGTCCCCTACCTG
GGTAGTTTCTGGGGCCAGGG
>3
TGACCAGGAACTGGCGGGTGGGCGCCCTGCAGAGGCTGCTGCAGTTTGGGATCGTGG
TCTATGTGGTAGGGTAAGAGAGAAGAGCTTTTGGCCAGGCTGGAGGGGCAAGGGAAG
AGGTGGGGGGTGGGGCTTGGTCCTGC
>4
TTCCGTCACTCAGATCAAGGAGCTTGGAAACCGGCTGTGGGATGTGGCCGACTTCGTG
AAGCCACCTCAGGTGGGGGCCCTGATGTTGCTGACGGGGGCGCAAGTCCTTTCCCCAC
TGACAGCCTGAACACCCGCCATGC
>5
AGAGAACGTGTTCTTCTTGGTGACCAACTTCCTTGTGACGCCAGCCCAAGTTCAGGGC
AGATGCCCAGAGGTGAGTTTACCCAGGATCCTCCCAGCGGGTCCCTTGTTCCTCCATCA
GCCCCAGGTGGCCACCCGTGTTT
>6
TCCGTCCCACTGGCTAACTGCTGGGTCGACGAGGACTGCCCCGAAGGGGAGGGAGGC
ACACACAGCCACGGTAACTGTGGGCTCTGTCTTCCAGTGCCCCCAGCAGGGTGGGGG
CCGGGCTGGGATCCTGGGTGGCTCCT
>7
TGTTCAATGGGACCCACAGGACCTGTGAGATCTGGAGTTGGTGCCCCGTGGAGAGTG
GCGTTGTGCCCTCGTAAGTGTCCCCACAATCCCCTACCCCAACTGGCGCAGGGCCCCA
GGCCTGGCAGAGGCTGTCACCTCCC
>8
TGGCCCAGGCCCAGAACTTCACACTGTTCATCAAAAACACAGTCACCTTCAGCAAGTT
CAACTTCTCTAAGTAAGCAGAGTGGGTCTCATCTGCCCCAAGACCCTCCTTGTCCCCTA
CCTCATCTGACCTTTCCCACTCC
>9
CTGTCCCGTGTTCCGCATTGGGGACCTCGTGGCCAAGGCTGGAGGGACCTTCGAGGAC
CTGGCGTTGCTGGTGGGTCCCAAGTTGGGGGCAGGGTTCCTAGAGGGCTCTGGGAGA
GGGTCCCGGGCCCACCCACCGGTGG
>10
TGGACACCGGGGACTCTGGCTGCTGGCCTCACTACTCCTTCCAGCTGCAGGAGAAGA
GCTACAACTTCAGGTGAGGCCCCACAGCTCCCAGTGCCCAGATGCTGGGCCCATCGCC
CTCTCCCTGTGGCGGCCAGGACAGA
>11
ACCGGGTGTGGAGGCCCGCACCCTGCTCAAGCTCTATGGAATCCGCTTCGACATCCTC
GTCACCGGGCAGGTAGGCACAGGTAGGGGTCAGGCCGGGGATGGGATGGGGCAGGCA

GACAGGGCTGGAGGAGGCATGAGGC
>12
TTTCTGTGACCTGCTACTGCTGTATGTGGATAGAGAAGCCCATTTCTACTGGAGGACAA
AGTATGAGGAGGTGAGCTGAGGTCGCTCTGCTTGGACCCTGGGTTCTGCCACACTTAG
GAAGATGTTGGCTGGATCCCTGA
>13
GCCTCCTGTCGGACCCGGTTCGCCGGCGCGCTCGCCCGCCTAGTGCTGCGCCTCAA
CGCGCCGTTGTGGTGAGGACAGGGCCCGGGGAGGCGGGGACCCGAGGGCCCTGGGC
TCGCGCCGGCGGCCCCATGCGCGGTC
>14
AGCAGTTTGCGGGCGGAGACCGTGCCCGGGCTTTTGCCCGGGACTTCGCCGCCCACC
GCAAGAAGTCGGGGTGAGCGGCAGAGCAGGGCGTATGGGGCGAGCAGTGGTGGCTA
AGGCCGGGGAGCTCTGCTCAGAGGCTC
>15
AGGGCTGGAGGTCTACACGCAGAGTTTCTCCCGGAAACTGCCCTTCCCAGATGAGACC
CACGAGCGCTATGTACTGGGGGAGTGGGGTGTGCCTGGGCCCAGAAAGCACCTTGGA
GGGAGGGGTCTGGGCTGGGTATCAC
>16
CCCTGTGGCTCTGACTCTACCAACAGCCAGGCTGTGGGGCTGCTGCTGGCACTGGCTG
CCCACTTCCGGGGTGAGTCTCAGGGCTGCTGGCCGTGGAGGGGTTTGGTCAGTGCCCC
AGACCCTGCTGATCCTGCTTCTGG
>17
CTGGTAACAGAACATGACCTTCTGGGCACTGAGGCTTGGCTTGAAGCCTACCACGATG
TCAATGTCACTGGTAGGTTCTCTTGTCCTGCCTGCCCTGGTCCCCCTCTCAGGGTCACT
GTCCTCACCACGTCCTCCATTAG
>18
CCTTGACCTGCTCAATCTCTTCCAGACCTTCTGCCAGAAAGGGGGCCTGTTGTGCACG
CTTCAGGGCAAGGTGGGGCTGCCTGCCTGCCTGAGGGCTGAAGGGCACAGGGTCTGT
GGGAGGGGCTGTCTCATCCTGTCCT
>19
TGGAGGCCCTAACCCTGCGTGGCATCAATAGCTTCCGCCAGTACAAGTATGACCTGGT
GGCAGTGGGCAAGTAAGTAGTCTCTGGTCCCCCCTTTCCATGCCCAGGATGGGGTCTA
GCTGGAGCGGAGTGGGGGATGGCC
>20
TGAGGCTGGAATGGGCCTTGAGGAGCCCGGGGGTGCCCCTGGCCCCAGTGTACCCCTT
CCCCCATCACAGGTGATGGCACCCCCTTCTGTTGTTGGAATGGGCTTCTGGGGTCTGAC
TGGTGTTTAGTGTTGCAACTTAG
>21
CTGAGGCTGTGGTGCTGACACTGCTGGCGATTTATGCAGCTGGCCTGGCCCTGCCCCA
CAATACCCACCGGTAAGAGGCTGGGCTGGTTGTTGGGGGCAGGGGTAGAGGTCCCCT
GGACATGCAGACAGCTTGTGGGTTG
>22
TCTCACTGGGCTTCCTGCTGGCCACCACCATGGTGCCCACTGCTGCGCTTGCCAAGCC
TCATGGGCCCCGGTATGTATGGATCAGCCCCACTTCCCCCAATGCCTGTGCCCTCATCA
CAGACCGCACCCTCATTCAGTAG
>23

TTCGCTTTGACCCAAAGAACATCAAGGAGAGGTCACCTCTGGCTTTTATTCCCTTCTCA
GCAGGGCCCAGGTAAGGGCGGCCTGTGTTTGAGGCGGGGACGGGGAGATAGGTGCAG
GGGTCTGGGCATGACAGTGGGGAA
>24
GCCGTCATGGCGCCCCGAACCCTCGTCCTGCTACTCTCGGGGGCCCTGGCCCTGACCC
AGACCTGGGCGGGTGAGTGCGGGGTCGGGAGGGAAACGGCCTCTGTGGGGAGAAGC
AAGGGGCCCGCCCGGCGGGGGCGCAG
>25
AAGGCCCACTCACAGACTGACCGAGCGAACCTGGGGACCCTGCGCGGCTACTACAAC
CAGAGCGAGGACGGTGAGTGACCCCGGCCCGGGGCGCAGGTCACGACCCCTCATCCC
CCACGGACGGGCCAGGTCGCCCACAG
>26
CTGGAGGGCCGGTGCGTGGAGTGGCTCCGCAGATACCTGGAGAACGGGAAGGAGACG
CTGCAGCGCACGGGTACCAGGGGCCACGGGGCGCCTCCCTGATCGCCTGTAGATCTCC
CGGGCTGGCCTCCCACAAGGAGGGG
>27
GGACAGGAGCAGAGATACACCTGCCATGTGCAGCATGAGGGTCTGCCCAAGCCCCTC
ACCCTGAGATGGGGTAAGGAGGGAGACGGGGGTGTCATGTCTTTTAGGGAAAGCAGG
AGCCTCTCTGACCTTTAGCAGGGTCA
>28
CTGGTTCTCTTTGGAGCTGTGATCGCTGGAGCTGTGGTCGCTGCTGTGATGTGGAGGA
GGAAGAGCTCAGGTGGGGAAGGGATGAAGGGTGGGTCTGAGATTTCTTGTCTCACTG
AGGGTTCCAAGACCCAGGTAGAAGT
>29
CAATAAAAGGCGACGGGATGGCTACCTTTTCCAATTGCTGCGGATTGCTGATGCCCACT
TGGACAGAGTGGTGAGGAATTGCCAATGGCAGAGCTGAGTTGGGAGATTACTCACGG
GGGCAAATGTGACTCTACCCCCTA
>30
CTGTTCGGTCCTATCCAGGAAATACTGGAATGACTGTGAGCCACCTGATTCCAGACGTC
CATCTGAAATAGTAAGTAAAGAGGGCACCTTCACTCTGCTCTTTTCATTCTTATTTTCCA
TCTACTCTGTTCACTCAGCCA
>31
ATAGCTACAAGACATTCCCATGAATCTCAGGACCTCAGAGTGATTGACTTTAACTGCAC
CACAAGTTCTGGTATGGTAATCTGTTAAATTGCTAATTAATTCTTGATTAATACAAATTCA
ATCATACAGTGGTATTTGTC
>32
GAAGTACAAAGAGGAGAATGATGACTTTGCCTCTTTCAGAGTGGACCGAATCGAGAG
AGTTGCAAGAGTGGTGAGTCTCCACTAAGGTTCGGTTGGAGTCTGAAGGCCCAGGCT
CCAACCTGGCAGCAGGAACTGAATGG
>33
AGGAACTGGTTACTTCGTGGACTTCTCTGTGCGGAACTGCCCCAGACACCATTTCCCC
AGACACCCCAATGTGAGTATAAGAAATGTCTGTGATCGTTGACTAGAGTCACAGAGAA
AAAAGAAAGAGAAGAAGGAGAAGG
>34
TGATGTAGAAGCCTTGGACTTGGAAAGCCCGAAAAACCTTGTCATAAACTGTGAAGTC
TTCGACCCTCAGGTGGGTTGTCTAAGCAGACTTTGTCATGGCAGTGCCAGATTAAGTG

ACATACGTACACAAATAGTGTTGT
>35
CCCCTCTACGCCCAGCTTTCTGGCCGAACTTTTCTGCAAGGGGGGGCTGTCGAGGCTA
CACGAGCTGCAGGTAGGAAGGGACGCCTTTCCCGAGACAGAGTGCTGGGGAAACTGG
TTTTGACAGCGTCAGAAAGGACTGA
>36
AGCGCTGCAGCTTCTCGCCCTGGACGGAGCACCGCCGCTGCCGCCAGCGGGCTTGCG
GACTGCTCACTGCGTTGAGTCTTCTCTGCCCCCAGAAGCCCAGACGCCCTGCACCGGC
CCTCTCCTGCTGCGGGTCCCGAAAC
>37
GGACGTGGCACGGGGCCGCCCGCCCTGGTTCGGGACGTGGCGGGGGAATTTTACAAG
TTCGGACTGGAAGGTGAGTCCCAGGACAGAGCTGGGCAGGCGTCGGGGGCGCCCTAC
CAGAGCCTCCCGGAACCCTGACGGCG
>38
TGGCTGCGCCACCTTGTGCCTGGGCCCTGGGGCCGCCTCTGCCGAGACTGGGACCAGA
TGTTTGCATTTGGTAAGGCACAGGTCGAGGTGGAAATGGGGGAATGTAAAGCTGTCCA
GGGGTAGCGAGGTATTCACGTGCC
>39
CCGGGAAGAGTTGCCTGCCCAGTCCATCCTGGGAAATGTGACAGAGTTGCTATTGGCG
GGAGTGGACACGGTGAGGTTCTCCCTCCGTGCTGTGAGCCGGTTCCAGGGCTTAGCCT
CCGCAGACTCCGGCTCCATTTTTC
>40
GTGCCTACCCCTCAGCCACTGTTCTGTCCCAGCTGCCCCTGCTGAAGGCGGTGGTCAA
GGAAGTGCTAAGGTGAGGGGGAAGGAGAGGAGGAACAAGAGGAAATGCCAAGGAA
GGGCTGGGGAAGCAACTAGTGGATGGA
>41
TGTGGTACCTGGAAATTCTCGTGTCCCAGACAAAGACATTCATGTGGGTGACTATATTA
TCCCCAAAAATGTGAGTAAAGCCTATGCACCCTTTCTACTGAGCCATTCCTCACTATCTT
GGGCCCCAACCCTGTTCTCAA
>42
TGGCTTTGGCAAGCGCAGCTGTATGGGGAGACGCCTGGCAGAGCTTGAATTGCAAATG
GCTTTGGCCCAGGTGAGTGCTCTAGATTTTATACCTTCCCCAGACTGGAGAGACCCTAA
CCCTCTAAAGTTGTGAGCTCTTT
>43
GACTTACGGAAAATTGGAAGCTGTGCAGTATAAAACTCAAGTTGTTGCTGGAACAAAT
TACTACATTAAGGTTAGAGTTCAGCACCTACTTTAGCGCCAAAAGATGTATTTCTCATTT
TATGTAAAATATTCCCTGATTT
>44
CCAACAGCTCTGGCACCATCGAGCTCGTGAAGAAGGGCTGCTGGCTAGATGACTTCAA
CTGCTACGATAGGTACCCCAAGACTTGCCCTCCTTTCCTCTTGGACCCACCTGCGCTTAT
ACTGCCCACTGGGCCATTTGGG
>45
TTCTGCTGCTGTGAAGGCAACTTCTGCAACGAGCGCTTCACTCATTTGCCAGAGGCTG
GGGGCCCGGAAGGTAAGGGGGCAGTGTGGAAGTGGGGCCTTGAGTCAGCCGACCTG
GGCTTCTTTGGCTTGGAGCGCTTGGC
>46

CATCGTCCTGCTGGCCTTTTGGATGTACCGGCATCGCAAGCCCCCCTACGGTCATGTGG
ACATCCATGAGGTGAGACAGTGCTGGCTTGGGGCAGGGCAGGATAGAGGTGGGGAGG
ACAGGCCAGACCTTTTTAACCCTT
>47
GCGCTTTGGCTGTGTCTGGAAGGCCCAGCTCATGAATGACTTTGTAGCTGTCAAGATCT
TCCCACTCCAGGTGAGTGTTTGAGGGGCTCCATCCAGGTCCTCTGCCTCCACTGTAGCT
TGAACTGGAGGCTCTCCTGACC
>48
CATTGCTGCCGAGAAGCGAGGCTCCAACCTCGAAGTAGAGCTGTGGCTCATCACGGCC
TTCCATGACAAGGTGAGCCACACCCATCAGAATGGACTCTGAGAGGAGATGGAATGTC
CCCTGGGTGGCTCTCCATAATATG
>49
GCCTCTCATACCTGCATGAGGATGTGCCCTGGTGCCGTGGCGAGGGCCACAAGCCGTC
TATTGCCCACAGGTACCTGGGTCAGCAGGTGCCTTTCCTGCACTTGACCTGGAGCTGT
GGGAAGGGTTGGTGGGCGAGAGCA
>50
CGTGCTGGCTGACTTTGGCTTGGCTGTTCGATTTGAGCCAGGGAAACCTCCAGGGGAC
ACCCACGGACAGGTAACAGGCCTCACAGGGCAGGAAGAGAGGGACAGACCCAGGGT
AGATACTTTGCCCTTTTTGTGCTCAG
>51
CTGCGCATTGACATGTATGCCATGGGGTTGGTGCTGTGGGAGCTTGTGTCTCGCTGCAA
GGCTGCAGACGGTAAGTAGGATGGCAGCCCTGGGCATCCTAGATTGAGTGATAGGGAG
CAGTGGGACCTTAGAGTGATCCT
>52
GGAGCTGCAGGAGGTGGTGGTGCACAAGAAGATGAGGCCCACCATTAAAGATCACTG
GTTGAAACACCCGGTAAGGGGCCTGGTTCAGGCAACTTTGCAGGGGGGTGGAGAAGG
GAAAACCCTTCATGTGTAGCAGGTAA
>53
CGCTACCTTGAGATAGAGTTCTGCTTGAAACATAGGAGCTGCCCTCCTGGATTTGGAGT
GGTGCAAGCTGGTACGTGTCAATGTGCAGCAAAATTAATTAGGATCATGCAAAGTCAG
ATAGTTGTGACAGTTTAGGAGAA
>54
CAGAAAGGAAATGCAACACACGACAACATATGTTCCGGAAACAGTGAATCAACTCAA
AAATGTGGAATAGGTAATTACATTCCAAAATACGTCTTTGTACGATTTTGTAGTATCATC
TCTCTCTCTGAGTTGAACACAAG
>55
ACTTTCCAGCTGCTGAAGTTATGGAAACATCAAAACAAAGACCAAGATATAGTCAAGA
AGATCATCCAAGGTATGATAATCTAAAATAAAAAGATCAATCAGAAATCAAAGACACCT
ATTTATCATAAACCAGGAACAAG
>56
TAGGAATTTCTGATTCATTTAAAGGATTTACAAATTCATCAACCCCTGAAAACTAAAGC
AAATTGAACAGGTATGATAATAAAATAATTTTCAATTTTTTTAAGAATGTATTCCTACTGT
TAAAATTATAAAAGTATATT
>57
AAATTGAAGCAAGCATTCTATATTCCAAGAAATTTGGAACACCTATACCTACAAAATAA
TGAAATAGAAAGTATGTAGACTTTTATTGTGTTTTGAAGAGAGAAAGAATGGAATCCTG

ATCTTTTAAAGAAAAAAATATT
>58
ATCATGAAGGGCCTTGCAGCTGCCCTCCTTGTCCTCGTCTGCACCATGGCCCTCTGCTC
CTGTGCACAAGGTGAGTCTGTCATCCATGTGCTTTGATGGCTCCCTGGGCAGAAGTCA
GGCGACATCTTCCAAGTGCTGTG
>59
GGCAGATTCCACAAAAGTTCATAGTTGACTATTCTGAAACCAGCCCCCAGTGCCCCAA
GCCAGGTGTCATGTAAGTGCCAGTGCTCCTGCCCACCCCTCGGGAGGGAGGATGGGA
GGTTTGGGGTGAGGTCCCCTCAGAG
>60
TTCAGCGTGAGAAGCAGGCCAGGCCTGGGTCTGGAGCCGTCCTGGCCTTCCCAGATG
ACAAGGACCTCAGGTCAGCTTCAGCGTGAGAAGCAGGCCAGGCCTGGGTCTGGAGCC
GGGTGATGTGCACATGGGGCTCACTT
>61
ATGGTCCAGCAGAGTCTCAGAGCGCGGTCTTTGGAGATGTGTGCCCCCTACTCACTTC
TCTCTTGGATGGGTGAGTTAAAATATTTTTAAGGCCAGTCATCATAGTTACATTTGAATAT
AAAGAAGCTTCACTGTGCTGT
>62
GCCCTGTTCTGCCGCTTGACCCACAGAGTGACTTAGGAATTATCCCTAGAGTGGCTGA
GGAGCTCTTCAGGTACTGCCGATGGTGATGAGTGGGGCAGAGAAATATGGCAGGGAA
GCCAGGCGGTCAGCACCTTGGTTAT
>63
TGTCGGGGGTCAAGCGTGAGGTGGTGACAGCCAAGGATGGACGGACAGAGGTTGCGC
TGCTGGCCTCTGAGTGAGTACTGCACCTGCCCCGTGCTGCTGGCCTCTGAGTGAGAAT
GGCACCTGCCCCTCCCACCTGGTCA
>64
GCGGATTCCTCCAGGTCTCACCTGATAATTACGGTGACTCTAACCACAGCCTCCTGCTC
TGACAGCACTGGTAAGTCACCATTTGTGCTTGGTCAGTGGCAAGTAATAGAAAAACCC
ACTTAAGAAGAAAAAAATCAGGC
>65
CCCGCAGGGCATGCGGAGCAGGTGCAGGCTCGACTACAGCTCGTGGACTCGGCCGGC
AGCGAGTGCGTTGGTGAGCAGGGGCAGGCATTTCCCTGGGGGGTGGGTGCTGCAGAA
GCCTATGATCTGATGGAGCTGGATGT
>66
CCGGACCCCTTTGTGCGGCCGGAGGCGGCGGCGGGAACGGCCATGGCGGCCAACATG
TACCGGGTGGGAGGTGAGTACCGGGCGCAGGGATACGGGGCCCGGGGCGGGAGAGGT
TGGGGTAGGGACCTGAGGGGCTCGGC
>67
AAGGTTGTCTGTCTTTTCCGGCGCAGGGACATTTCTAGTAGCCTCAACAGCCTGGCTGA
TAGTAATGCCAGTGAGTATCTTCCTCCCTTTCTTTTTCTCCTACCCATGATCTGAGGCCT
GGGCATGTCCCTGCAGGTATT
>68
GCCATCAACTGAAGCACCGGGAACTTTTTCTTTCTCGGCAATTTGAATCATTACCAGCC
ACCCACATACGGTACAGGACAACTGGGGCCAGCATAGCCTGCGTGCACTTACGTAGAA
GATGGAGAGAAGGAGGTGGTATT
>69

CTCGCTGATCAGGGCGAGATTAGAGTTGGTTGCAAATACCAAGCTGAGATCCCAGATC
GCCTAGTAGAGGGTAAGCAACAGGACAAGACTGTACGGCTACCAGGTTGTCTAATTCC
AAGGGGCACTTTAACATTATAGTC
>70
AGATGAAGGTCTGGGACCCAGACAACCCTCTCACAGACCGGCAGATCGACCAGTTTC
TTGTGGTGGCCCGGTGAGGGGTGGGTGGATAGGGAAGATGGGCTGGGGGAGGTGTGG
ACATTGTTCTGAGTCCCATGGTTCTT
>71
TTGTAGCAGCTCCATTCGGCAGCCAAGCTTGCACATGAGTGCAGCTGCTGCCTCCCGA
GATATCACTCTGGTGAGCAGAAGTTGGTGGGTAGTTGGGCAGGCTGGGTTTTCTGGGG
ACTTGCAGAGCCTGGGAACAGAAG
>72
AGAGGCCATGCTATTTGAGGAGGCCCTAGAGAAGTATGGGAAGGACTTCAATGATATT
CGCCAGGATTTTGTAAGTGGATGGCACTAGGAGGAGAAGTGGGAGAGATGACAGGTG
AGGGAACCAGGACCTGGGGCCAGAT
>73
CTGGAAGTCACTTGCCAGCATAGTCCAGTTTTATTACATGTGGAAAACCACAGACCGGT
ATATTCAGCAGGTATTATCTGGGCTGGGGAGGGTGGACCATAGGCTACCTGCTGACCTC
TGCCTTTGAAGTTGATGACATT
>74
TCTGTGGGTTCAAAACCTGGCATGAATGGGGCTGGATTTCAGAAGGGCCTGACTTGTG
AGAGTTGCCACAGTGAGTTACGGGGGCACTGGGATGGTGGGGATGGGTGGAAGGCCT
GCTGTGGCTCAAGAATCTGAGGGGC
>75
GATCTACTGGAAGAAGTATGGGGGACTGAAGACCCCAACTCAGCTTGAGGGGGCCAC
TCGGGGCACCACGGTAAGGATCAGTGGGAAGAAAAGCAGGGGAGAGCAGTAAGCATA
TTGATGGTGAGCTTAGGTGAAGGCCA
>76
CTATTACAGCCAAGGAGGGCCGCCCGACGGCCTTATGCTCCTATCAATGCCAATGCCAT
CAAAGCAGAGTGTAAGGGCAGGGAGTGGTGGGACTGGTGGTGGGTTTCGGATTGCTC
TGCCCAGAGACCGTTGTTTCTCTG
>77
GCCGCCAAGACTCCATTGAAGATTCACCCTCTGGTGCGGCTGCCCCTGGCAACTATCG
TCAAAGATCTGGGTATGGGATGGGGACCAGTGAGGTTGGCATCAGGCTCCCAGTCCTT
TCCCTGTCTGACATCTCTCCCCTT
>78
GATCAACAGAAACCAGCTGTCCCAGAACCGGGGACTGGGGGGCATTATGGTGAAACG
GGCCTATGAGACTGTGAGTTGACAGAAAGGGGTGGGCCAGATTGAGTTGGGTGGGCT
TTTGTCTCTCATAGGAGTCTGAGAGG
>79
CCAAGCGTCAGAAACTAAACCCAGCTGATGCCCCCAATCCTGTGGTGTTTGTGGCCAC
AAAGGATACCAGGTAAGGCGTCTACCATGGGAGGGGCTGAGAGGGACAGTCCAGGCT
GGTGAGTGTGGGGAGTAGGAGTATC
>80
GATTGAAAATGATCAGTCGATCAGCCGAAATCATGCTGTGTTAACTGCTAACTTTTCTG
TAACCAACCTGGTATGTTACTAATTTTATTTCACTAGTTTCCAGTAAGGGGGTTGAAATC

ACAAAATATTCTCTTGGTTTG
>81
AGAATGGCTTTTCCCGAACTTTGAAGTCGGGGGATGGTATTACTTTTGGAGTGTTTGGA
AGTAAATTCAGGTAAGACATTTTAAAATTGATTTTAAAATGGACAGCTTTGTTTCAGCC
AAATCCTAAAGGAAAATGATAT
>82
ATTTACTGTAAACAATTGGACAGAAGAATGCACTCACCTTGTCATGGTATCAGTGAAAG
TTACCATTAAAGTAGGTTGAATGTCTTGTTCTTATGTTAAAAATAAGTTATTTGAATTTAA
GCTTACCCACTATACACAGA
>83
TAAAGCCAGAATATTTTACTGAATTCCTGAAAGCAGTTGAGTCCAAGAAGCAGCCTCC
ACAAATTGAAAGGTATATTATGTATTTTAAATATAACAAGATGTCACTTATATGATAGCAT
TGATTTTTATTTAAGTTAATT
>84
TCTGTCAGGACGGCAGGAAAGAAAACAAATCTTCAAAGGGAAAACATTTATATTTTTG
AATGCCAAACAGGTAATTATGTTATAAGCTAAATTTTCCTAAAGAATACATTACAAACTA
GGATACATTATTAACTCTTATC
>85
CAAACTCACAGACCTTAATTCCTGACTGTCAGAAGAAATGGATTCAGTCAATAATGGAT
ATGCTCCAAAGGTATAGAATTATTCTTTATTAGTAAAAAACTGCAAGTAGGAGATTTTAT
GTTTAACCTTTTTTGTTGCTT
>86
GAAATTGGATTGGCGGTGATTTTCATGACTACAAAGAATTACTGTGATCCTCAGGGCCA
TCCCAGTACAGGTAATTGAAGCATTATTTTATTGATTCGTTAGCAACTTTATTATGTAAAT
CGATAAGCTAAATATACGTT
>87
TAATGCCAAGCGCCCCAGTGAACACTACAACATACGTAGCTGACACAGAATCAGAGCA
AGCAGATACATGGTAAAGCTTCTTCATTACCGTACTATTGTTATTATTCTATCAATAAATT
AATAGTATATTCAGGAAGTAA
>88
AAGATAGGGCTTCTCAGCAGCAGCAGACCAACTCCATCAGAAACTACTTTCAGCCGTC
TACCAAAAAAAGGTCTGTTTTTGATAACTTCTCTCATGTCTCTGAAAAAAAAATGAAA
GTTTTTTATACTGTTGTAGAATGG
>89
GCCAAGGATGGATATAGAAACAAATGACACTTTCAGTGATGAAGCAGTACCAGAAAGT
AGCAAAATATCTGTAAGTAACATTTTAAATGAGAGGTATAGTACATAGAAGCTTAGAAT
GCTAACAGGTACTTTCGATTTTG
>90
TACTTCCAGAAATCCATCTGGCATAAATGATGATTATGGTCAACTAAAAAATTTCAAGA
AATTCAAAAAGGTAGGTATTTCAACTGTATCACAAGAGGTCAGTGTTTGAAATGTTTTT
AGAGATACCAGTGATGCTAAAC
>91
ATCAGATCTAATAGCTCATCATGCTCGAAAGAATACAGAACTAGAAGAGTGGCTAAGG
CAGGAAATGGAGGTTAGTAGGAAGTGAGGCCCAGAAACTTGCTTAAATTGCACTTTGG
TCCATTATGGTGGCCTTCAAGCAA
>92

CCGGAGGACGAGTCGACCCCGGAAAAGAGGACTGAGAAAATCTTCCGCCAAATGGAC
ACAAACAACGACGGTGAGGGGCAGGGGCGGGACGGGGTGGACGGGGCGGGCGCCTT
TCCCTCCCTCCCTCCCTGCCTCCCTTT
>93
GGCGCTGGCTCCCGGAATATGGAGCCCTGGGCGCGGGGGCGGCGTGTCAGGTGGTTG
CGGAGCTGGTAAGGTGAGGGGAAGGCAAGGTCAGGGGTCACGGCATTCCGCTCGCTG
GCAGGGAGGGGCGCGGGGCGGCTCGA
>94
TCATATTCTAAGAACTCAGCCACTCAGGTATCCACCATGGTGCTGGGTCCTGAACAGAA
GATGTCAGATGGTAATTTACCACTTTGTGTTATTGTCTAATTTTATTTGTAATTAGGTTTAT
GTTGGTAACAGAAATTTAT
>95
GTCCCCTGGGGACTCAGAGGAGTACTTCGCCACTTACTTTAATGAGAAGATCTCCATTC
CTGAGGAGGAGGTTAGTATCAGGTCCTTGGAGGAGAACACTATTGTCTGGGGAAAACC
TGTGAGTGAAAGATCTGCTGGAT
>96
GAGCATTGCCTACCTGGATCCAGGAAATATTGAATCCGATTTGCAGTCTGGAGCAGTGG
CTGGATTTAAGGTGAACATCTAGTCCTACCCCTGTCCTTTTAAGCACATAATACCCTCTC
ACATCCTTTTCTCCACCCTGC
>97
GCTTGCAGCTAGACTGGGAGTGGTTACTGGGCTGCATCTTGCTGAAGTATGTCACCGTC
AGTATCCCAAGGTGAGCAATGTATTGTCCTGTTTGTCTCTCACCCATGCCATAGGAATCT
GTGTGTCTGGCGGTATGTGCT
>98
CTATCATCGGCTCAGACATGCAAGAAGTCATTGGCTCAGCCATTGCTATCAATCTTCTGT
CTGTAGGAAGGTGAGGGGTTTTCCTCTAGAAAGTCATCAGTGTTTTGCTGGATTTCCAA
GCAGAGATAAAAACAAAAACC
>99
ATTCCTCTGTGGGGTGGCGTTCTCATCACCATTGCAGATACTTTTGTATTTCTCTTCTTG
GACAAATATGGTAAGGAGAGCAGGAATGGGAGGGTCAAAGGAGCTGCTCTCTGTCAT
GTGGCAGATGGGGAAGTCTACCT
>100
ACAGGCTGTGGGCATCGTGGGAGCTGTCATCATGCCACACAACATGTACCTGCATTCTG
CCTTAGTCAAGGTGAGCAAGGCCCTTATCTCCTTCACCTCCTAGACAGTTCATGGGATC
TTTTCCTTCAATTGCAAGGGAC
>101
CTTCATCATCAATGTCTTTGTTGTCTCAGTCTTTGCTGAAGCATTTTTTGGGAAAACCA
ACGAGCAGGTGGTGAGTAAGCCAGGGTTATGGGTGGTAGTGAATGTGAGGGTAAAGT
GAAGACTGCAATGGGATGGGGAAG
>102
TACCAGCAGTCCTCATGCTGGCCTCTTTCCTAAAGATAACTCGACACTGGCTGTGGACA
TCTACAAAGGGGTAAGCATGTTTGGATTTGTGATCTTTGTTCCTGAATCAGTATTTTTAG
CATAGTATGTGGTCTTTTTTG
>103
AGTGGGGATCCTGGCTGCAGGACAGAGCTCCACCATGACAGGAACCTATTCTGGCCAG
TTTGTCATGGAGGTACGTGCTGGTATAGGATAGAGGGAGATAACACAACCCTTTTGCTA

CTTCTTCTTCTTCTTCTTCTTTT
>104
TGCTGTCTTCCAAGATGTAGAGCATCTAACAGGGATGAATGACTTTCTGAATGTTCTAC
AGAGCTTACAGGTGAGGAGGAAGCTGAGGAAACACATCCTAGTTATTCAGGTAGCATT
ATGTACTGCCCTTGGGTACCAAG
>105
CCTTTGCTCTCATACCCATCCTCACATTTACGAGCTTGCGGCCAGTAATGAGTGACTTTG
CCAATGGACTGTGAGTGTACTCCTCTTCGTTCCCAAAGGGGATGCTGATATGGGGAAA
GGGTATCTTCATGCAGTAGGCC
>106
AGGGCATGTGGCATTATATGTGGTGGCTGCTGTGGTCAGCGTGGCTTATCTGGGCTTTG
TGTTCTACTTGGTAAGTCTAGCCTGGAAGAGCAGGGGCATAGGTATCAGGGATGGTTG
AGGTGGGGTTCACTGACATTCAG
>107
GGGCCGCGTATGGAGCCCCCGCCCCCCGGAGCTGCCAACATTGCCAACGCCACCGCCA
CGCTACACACAGGTGAGCTCTGGGCCTGGAGGGTGGAGGGCCCAGTCCGTGACCCCA
CGTATCCTTCCCGCCCCCGCGCAGA
>108
ATTCCACCCTTCCTGTACATCGTAGCGAATCAATCCGTGGCGCCGCACTCCTCCGCATC
CCTCTTTAACAGTGAGTCTACCTGAGTTTGTATTCCTGCCTCCTCCAGTCCCCCAATGCA
TCAGTCCATGGCTTTTTTCAA
>109
ACTACGCAAGAACAAGACCAACATGAATTACGACAAGCTCAGCCGGGCCTTGCGGTA
CTACTATGACAAGGTACAGTCTCTCAGACCAGGGCTGGAACCCCCCCCTGACATTTATA
TCAGAGATTTTATTTTGTTTTAAT
>110
GGGAAGTGGCTCCGGCCTCCAGGCTCCGGGGCCGGCGCTGACCCCATCCCTGCTTCCT
ACGCATACATTGGTGAGTGCTGCGGAGGGGTGGGGTGTGTGGGCGCCAGCACCAATG
GGATTGGCATGTGGCGGGTGACTGA
>111
CGCGCTGCGCGCTGGGGACCGCCTGGTCGAGGTCAACGGCGTCAACGTGGAGGGCGA
GACGCACCACCAGGTGGGGGCCAGCGCTCGCCCCCGGCCCGCCGCCCCCTCCCCGAG
CGCGTCCCCCTGGGGCGAGCAGGGGT
>112
CCACGACCCCTGGGAGCCGAAGCCAGACTGGGCACACACCGGCAGCCACAGCTCCGA
AGCTGGCAAGAAGGTATGGCGGGCTTGGCCCACAGGGACCACCCTAGCCTCTCCCAG
AGGCTCTCTCAGCTTTTGACGACGAT
>113
CATCCGCTCTGTGGACCCGGGCTCACCTGCCGCCCGCTCTGGCCTCCGCGCCCAGGAC
CGGCTCATTGAGGTACCGGCCCACCAGGGCTGCGGGGTGCCGAGTGCCCCGCACCTGT
CCACACTGGGGCCCTGGGTCCTTG
>114
GTCGTGGACCCCGAGACAGATGAACACTTCAAGCGGCTTCGGGTCACACCCACCGAG
GAGCACGTGGAAGGTGGGCCACGGCCCAGGGCACAGGGTGGGTGCGGTGTGGTGGC
TGAGCAGCCACTGACACGCTGTCCCCA
>115

CCGGGAGGCCTGGCCTCCCCCAGGGCCGCCACCTCTGCTGGTTGCTCTGTGCTTTCAC
CTTAAAGCTCTGGTAAGGAGAGTAAGGGGAGGGAAAAGAGTCACTGGAGGACAGATT
CGTTCCCCGGCCTCCGGGCTGGGGA
>116
TTTGCCATGCTGGCTGGTGGAAGAGTTTGTGGTAGCAGAAGAGTGCTCTCCATGCTCT
AATTTCCGGGCTGTGAGTATCTATTTCTCTACCCTTTTTTTTTTAAACATCTAAGCAAA
CACTTAAGCAACATCTTAACAT
>117
CTGAGTGTGGTCCCACAGGATATGTAGAGAAAATCACATGCAGCTCATCTAAGAGAAA
TGAGTTCAAAAGGTGAGTGCTGTTTCTCTTCTCTGAGATCACCTCCTTTGCTCCTATGA
CTTTAGATGGATGGTCCCACCTT
>118
GGAGCAGGAGAGCTGTCTGGATGGCTGGGAGTTCAGTCAGGACGTCTACCTGTCCAC
CATTGTGACCGAGGTGGGTGCCGGCCCCTGCTGGGGCTGAGACCAGGGCTCGGAGGA
CCTGTCGCGGTCCTTGAACCCGAGCT
>119
CACTCACAATCTCCTTGTTCTTCGTGGGTGTGCTGTTGGGCTCCTTCATTTCAGGGCAG
CTGTCAGACAGGTAAGGTGTCTGTCTTCTGGAGCACCAGGGGACCTCAGCACTGAGG
AAGAAGCGTGTGCCTGGCCCTTGA
>120
ATGTTTGTCGTGCTGTTTGTCCTTGTAGGCATGGGCCAGATCTCCAACTATGTGGCAGC
ATTTGTCCTGGGTATGGCCATCAGGTTGGAGTTGAGTACTTGATCCTGTATTTCACCATC
ATCCCATCACCTACCTTTCTG
>121
TCATCCGAGACTGGCGGATGCTGCTGGTGGCGCTGACGATGCCGGGGGTGCTGTGCGT
GGCACTCTGGTGGTGAGTGTGACCCTGTGCCCCATGTGCCCACTGGCAGGATGATTTC
TGTCTGGCCTTCACTAGAGGGCAG
>122
GGTGATCATCCGCAAGGCTGCCAAAGCCAATGGGATTGTTGTGCCTTCCACTATCTTTG
ACCCGAGTGAGGTAAGCACCATGTGGGTGTGGGTGAGAGGGACAGACTGACCGTGAT
TTGAGAGCAGCAGCACCCAGCCCT
>123
CCCACAACATTCTGGATCTGCTTCGAACCTGGAATATCCGGATGGTCACCATCATGTCC
ATAATGCTGTGGTATGTAAAAGAGACCTGCCTGAGGCTTCCAGACAAAGCTTCTTGAA
GTGGCCATTGGGCCTCTTGTTTA
>124
CGCTATTCCATGGCCACTGCCCTCTTCCTGGGTGGCAGTGTCCTTCTCTTCATGCAGCT
GGTACCCCCAGGTAGGGACCATGTGCATCTATGGTTTGGGGTCTTCACTGAGTCTCTTA
CTGTCTACCAGGCTGTCTCAAT
>125
ATGGGTGTGGGAGTCAGCTCCACAGCATCCCGCCTGGGCAGCATCCTGTCTCCCTACTT
CGTTTACCTTGGTAAGTCCCATGAGCCAAGGGCACACTAGAGCAACGGGATGGAAGTA
CTAACTGGCTTGAATGTGAGCTG
>126
CCTTGTTTCTCCCAGAGAGCTTCGGTACCCCACTCCCAGACACCATTGACCAGATGCTA
AGAGTCAAAGGGTAAGAAGACCTCCTCTGTCAGTGTTGATGCACTGGGTCTGGGTCTG

GCCAGGTCTCAGGAGCCCCTCAC
>127
CTCGGGGTCGTATGACGCACTTTTCCAGCTCGAGCCCTCACGAGGCCGTGGGTACGAC
CGGAAGCCGCAGGTGGGTGAAGCGGAAACGTTTCAAACCGGGATGTTGCGGGCCGTA
GCGCGTGGGCGGCCGTGGGGTCTTG
>128
GGGTTCAAGCGATTCTCCTGCCTCAGCCTCCCGAGTAGCTGAGATTACAGGCGCCCGC
CACCACGCCCAGGTAATTTTTGTATTTTTGGTAGAGACGGGGTTTCACCATGTTGGCCA
GGCTGGTCTCAAACTCCTGACCT
>129
ATTCTGGAACCCTAACAAGATCACATTTTTGACCAAAGTCAATATCTTGGACTATAGTTA
ATTTATCAAGGTAAATGCCACTTTGATGTGTCTTGGTTAGGAATCTTTGTTCACTTGAGG
GAATACATGGTATATGATAG
>130
GAGTGCTTTAGGAGGCCAAGGCAGGAGGACTGCTTGAGCCCAGGAGTTTGAGACCAG
CCTGGACAACAAAGTGAGATTCATCTCTACAAAAAAAAAAAAAAAAAAAAAAAATAGC
CAGGCGTGGTGGCACATGCTGGCTAA
>131
CCCGGGCTCCCAGAGGGAACTGCACTTCGGCAGAGTTGAATGAATGAAGAGAGACGC
GGAGAACTCCTAAGTGAGTAGATGCAGCCCATGCAGTTCGCCTTCTTTTATGCTTTTTC
CTTCTTTTGCACGTCTCTTCTTTC
>132
GATGGGATCCGGCTCCTCCAGCTACCGGCCCAAGGCCATCTACCTGGACATCGATGGA
CGCATTCAGAAGGTAGCCCCTCCCCCACCCAGACACCCCCTCCTCCCCCCGGGTGACA
GCGCCGGGGCCGGGCGCGGCGGGG
>133
TAATCTTCAGCAAGTACTGCAACTCCAGCGACATCATGGACCTGTTCTGCATCGCCACC
GGCCTGCCTCGGTGAGTGCGCGCTGCGGGCTCTGCCCGGTGACGCCACGCGGCCTCCT
CGCCTTTTCGGGATGGCTGGGAG
>134
CCATCTCCCTGCTGACCACCGACGACGCCATGGTCTCCATCGACCCCACCATGCCCGC
GAATTCAGAACGGTAAGAGGCTCCGGCCGCGCTCCTCGGGGTGTGCCTGGCACTTCTC
TCCATGACATGGGAGGCTTTCTGT
>135
AGTGGACAGGTAGAGCCCAGGCCCAGAGAGCCCCAGGGCTGCTACCAGGAAGGCCA
GCGCATCCCTCCAGGTAACGGGCAGCTCCTCGGCCACAGCCTCCACCCCCCAACACGT
GGGATTCGGGCTGCAATGGCCTACAC
>136
GCATTCAAAATCAATGAACTGAAAGCTGAAGTTGCAAATCACTTGGCTGTCCTAGAGA
AACGCGTGGAATGTGAGTGACGTTTCTGTTTCCTTTTTGCTTCTCTGTCCTTTAACCTCT
ATTCAAATTAACCACTTGGAAC
>137
TGGTGGAGATTGAGAAATGCAAGAGTGACATTAAGAAGATGAGGGAGGAGCTGGCGG
CCAGAAGCAGCAGGTAGGGTCTGCGCTGGGGCCACGGGCGGCCGGGCCTGGGGAGG
GCTCCCCGTACCAGTTGGGCTGGGGCT
>138

CTGTAAGTACAGTTTTTTGGATAACCACAAGAAGTTGACTCCTCGACGCGATGTTCCCA
CTTACCCCAAGGTAAGATGAGATTCCGGCCCAGAAGAAGCTGCAGCTGTGTCCCCAGC
CCCACGCCCAGCCCTGTGGCCCT
>139
GCTCTCTCCAGAGACCATCGAGGCCCTGCGGAAGCCGACCTTTGACGTCTGGCTTTGG
GAGCCCAATGAGGTAAGTGCGGGGCTTGCAGGCACCACGTCCCAGGGGGAGGCAGCT
CAGGATCTTGGACGCCAGTGGGACC
>140
GCACATGTACCACGACCTCGGGCTGGTCAGGGACTTCAGCATCAACCCTGTCACCCTC
AGGAGGTGGCTGGTGAGTGCCAAACCCGCCTTCGGTTCTTCCTGGGCACGTGGTTTCA
TCCAGTTCCACAGGAATGGAGGGA
>141
CCACAACTTCCGGCACTGCTTCTGCGTGGCCCAGATGATGTACAGCATGGTCTGGCTCT
GCAGTCTCCAGGTGGGTCCTGCCCGCTGCACACCCAGACCTCTACTCTCGGGGGTCAG
ACGGAGGCCCCCTTCCAGGGAGC
>142
AAACGGATATCCTGATCCTAATGACAGCGGCCATCTGCCACGATCTGGACCATCCCGGC
TACAACAACACGTATGTACAGGATTTTCTCTTTTTTTCCTTTTAAAAGGCACCCTGGCTA
CTGGAGGGAACCTGTCAGCCC
>143
GATCCTCGCCGAGCCTGAGTGCAACATCTTCTCCAACATCCCACCTGATGGGTTCAAG
CAGATCCGACAGGTGTGTGGGGTGAGGGCCCTCCCACCGGAGTGGGGGCACATTCAG
GGACAGAGCAGCCCCCACTCTCCAT
>144
AGAAATTATGGATTCTTTCAAAGAGAAAATGGAGAATTTTGACTACAGCAACGAGGAG
CACATGACCCTGGTGAGTGGCTTATTCTGCCTGGGTGGGCAGCCAGGCGGTGGGCTGG
CGAAGCAGGTCATCCATCCAGCTC
>145
TAACGAGGTCCGTCCAATGGAAGTCGCAGAGCCTTGGGTGGACTGTTTATTAGAGGAA
TATTTTATGCAGGTAAGAGTCTTGCAGAGCAATCAAGCCTCCAGCCACTCTTATTAGCC
CCACTTAGTAAGGGTTTGCGTTA
>146
GACCAAGGCCACAGCCCAGATTGGGTTCATCAAGTTTGTCCTGATCCCAATGTTTGAA
ACAGTGACCAAGGTGAGTAACTGTCACCACATGTCACACTTGCTTACACTCAGATACA
TGCATGCACACACAGGCACACACA
>147
GCTGCAGCCACTTTGGGAATCCCGAGATCGCTACGAGGAGCTGAAGCGGATAGATGAC
GCCATGAAAGAGGTAAAACACACTGAGAAGAGCCTGCCTTCCTTGCGGCAAGCAGGC
ACGCTGCCCTCCGCACTCCCGACTC
>148
ACTGACAGCTTGACGTCTGGGGCCACCGAGAAGTCCAGAGAGAGAAGCAGAGATGTG
AAAAACAGTGAAGGTAATGCTTGCTCTGCTGAAGTGGCATCTCAGCGCATACAATGAT
TCTGACAAAGGACAGAAGGAAAGAG
>149
ACTGGGCACCCAGTCCAGGAAAACCAACCCAAAAGGAAGCCAAGTATTAAGAAATCA
CAAGGCAGGAAGGGTAAGACAGCATTCGCAGCATATTTTATTTCAGAAATGCATTTTCT

CCTCCCAGCCTCTTAAGGATAGGG
>150
CCCGGGCTCCCAGAGGGAACTGCACTTCGGCAGAGTTGAATGAATGAAGAGAGACGC
GGAGAACTCCTAAGTGAGTAGATGCAGCCCATGCAGTTCGGCCCTTCTTTTATGCTTTT
TCCTTCTTTTGCACGTCTCTTCTT
>151
TTTGTCCTTCATTGAATTACGACACTGTCCACCTTTAATTTCCTCGAAAACGCCTGTAAC
TCGGCTGAAGGTTAGTGCAACTTCATTTCTTTCCTTTACTCTCCAGAGCTCCCCAAACA
TCAAGAAACAGGACAAGGCAA
>152
GTGTGGGGACAACGCGGCCTGCCAACACTACGGCGTGCGCACCTGTGAGGGCTGCAA
AGGCTTCTTTAAGGTGAGCAATGGCGGGAGCGGAGTAGGCAGGTAGGGAGCCCCTAG
TGCCCGGGACCTCGGAGTGTGCCCTC
>153
AAGCGTCGCCGGAATCGCTGTCAGTACTGCCGATTTCAGAAGTGCCTGGCTGTTGGGA
TGGTCAAAGAAGGTAGGCTGAGGGGAGCTGCCGACCCTCCAGTTTGCGCCTTTAGGA
AACCACTGCTCATACTCCAGCATCA
>154
TCTGATCAGTGCCCTCGTCAGGGCCCATGTCGACTCCAACCCGGCTATGACCAGCCTG
GACTATTCCAGGGTAAGAAGCTGGCGGGGGGGATATCATGTGGACAAACCGACAGATG
GGCAGGACCCCTCCCCACATCCGT
>155
CCAAAGCCGACCAAGACCTGCTTTTTGAATCAGCTTTCTTAGAACTGTTTGTCCTTCGA
TTAGCATACAGGTAATAAGGGAGGGAGGAGACAATCCAGGGAGGCTGTGAGAGAAAT
CAAGAAAGGAAAAGAAAGGGAGGA
>156
TCCAACTTGCAGAATATGAACATCGACATTTCTGCCTTCTCCTGCATTGCTGCCCTGGCT
ATGGTCACAGGTCAGTACTGCAGGCGCAGGGCGCTTCCCCTCCAGAACTGCCTAGCAG
GATTTGTCCTGAGTTTCCCTTG
>157
CAGACCGCGGACGTCTGTAATCTCAGAGGCTTGTTTGCTGAGGGTGCCTGCGCAGCTG
CGACGGCTGCTGGTGAGCGCCCCCGACCAGACCGAGGGACAGGGCGAAGGGGGACT
GGAGGAGGGAACCCCCCTTTAGGGAC
>158
AAAATTTGACCAAAATTTGGATACAAAGTGGTACCAGTGGAAGGCAACACACAGAAG
ATTATATGGCGCGGTTGGTAACATCTGAAACTGTCCAGAGGGACTCTGGAGAGAATGG
TCCTCGCTGTTGGGAGTTGATAGCC
>159
CACAATGGGGAATACAGCCAAGGGAAACATGGCTTCACAATGGCCATGAATGCTTTTG
GTGACATGGTGAGTGTGGCAAGGATTGCTGAACTCTGTGCTGCTTCTCAGTTCTTCAC
CAAAATAGTCTTGCTTTTAACATT
>160
GATCTTCCCAAATCTGTGGATTGGAGAAAGAAAGGCTACGTGACGCCAGTGAAGAATC
AGGTGAGACAGCGTCAGATTCAGACCCTGTCTCCACAGGAAAGCCAAGAAGGAATTG
GTGTCATTGCTGTGGTAGATGGTGG
>161

TAGGGCCTTCCAGTATGTCAAGGAGAACGGAGGCCTGGACTCTGAGGAATCCTATCCA
TATGTAGCAGTGGTAAATGGAGCTCCTTATCTTGTCATTCGTGCTCTGCTTTTGGAAAGT
GGAACACTTTCAGAGATAACAG
>162
GTCGCAACTGTGGGGCCCATCTCCGTTGCTATGGATGCAGGCCATTCGTCCTTCCAGTT
CTACAAATCAGGTAAGTGTCATTTTATTATAGAAATTTAGGCAGAATTGGGAAGCATCA
CTGTGATTGATTCTTGGATATG
>163
TTCTGGTGGTTGGCTACGGCTTTGAAGGAGCAAATTCGAATAACAGCAAGTATTGGCT
CGTCAAAAACAGGTATAAAACTCAAAATTGAAAAGGGAATTTTTGTTCCAAATCAGTA
TTTGGATACAAGTTCACAAAAGCT
>164
GGGGGAAAGATGGTGGGGACCAAGGCCTGGGTGTTCTCCTTCCTGGTCCTGGAAGTC
ACATCTGTGTTGGGTATAGGCTAAGTATCTGCTGGCTCCCAAATCTTCCACTTGCCACA
GTGAGGTTGTGTCTGGTCTGGGAC
>165
TCAGTAAGAAGAGTCCAGCCTGGGAAGAAGAACCCCAGCATCTTTGCCAAGCCTGCC
GACACCCTGGAGAGTGAGTTGTGGCTGAAGCAGGGAGGGGCTGGAGGGGCAGCCTTT
AACGAGCTTCCGAAGTGGATCTCTGA
>166
TGCCTCAACAGGGAGCAGCGGCCTGGCCAGAACTGCTCTAATTACACCGTACGCTTCC
TCTGCCCACCAGGTAAGCCAGAACGCCCCCACCCCCACCCGCTACTTCCCACTGGCCT
CTACTCAGGGAGAGGGAGAACCTG
>167
GCAGAGATGGTGTCGCTGTGCAGTGAGGCCAGCGAAGAGGGTCAGCACTGCATGGGC
CAGGACTGTACAGGTACCACCCTCCCTCCTGGCACTGGGCAGGGTGTGGTGGGTGAG
GCTGAAGAATGAGGAGGGAGTTAGAG
>168
CCCATTGTACTCACAATGCCCAAGACTAGCCTGAAGGCAGCCACCATCAAGGCAGAGT
TTGTGAGGGCAGGTAACTAAATTCTTTGGGATAGGGCTGGTGAGAAGGTCCAGATTTTT
AAATAAAGGGATTATAGTGCTAA
>169
GGAGAGCTGGGCAGAGCGTGTCTCTGTGCTGTAAGGCCACAGGGAAGCCCAGGCCAG
ACAAGTATTTTTGGTGCGTATTCTGCTAGCTACCCTGCCCAATTCTCCTCCTGGAAACC
AGAGCTTCTTGCATTGGGTCTGGA
>170
GAGTACTTTTGCAAGGCCCAGAGTGATGCTGGGGCTGTGAAGTCCAAGGTTGCCCAGC
TGATTGTCACAGGTAAGCCTGTCTGGGTCCCTGGGGCCCCTGGGTCTGCAACAGGGTT
GGCTGAGCACCCAGGTGGGCATAA
>171
CCGCTCCTCCTGGAGAGGGGATCTCTGCTGCTCCGACAGTTGAGCCCAGTTCCGGGGA
GGCTGAAGGCGGGTAAGAGGTCCTGCCGCCCGAGGAAGACGCGGGAGGGAGGCCCG
CCCCTCGCGAATCCCGCGGCTCCTGG
>172
GTGAGGTAGAGCTGCAATGTGGGATTTGTACAAAATGGTTCACGGCTGACACATTTGG
CATAGATACCTCGTGAGTACTTTTCATAGTTTTTGTGAGAATTGCTCGGTAAAATAAATC

TGAACATGCTCAACAGTTACTT
>173
ACCAACTACAGTTTTCATTGCAACGTCTGCCATCACAGTGGGAATACCTATTTCCTCCG
GAAGCAAGCAAGTAAGAACAAACTCTGGAGTATTTGAAGATGATTATCCACTGGAAAA
GAAAAGGGATCTGGGCGTAGCAG
>174
TTTGGCCAACCTGACATGGCAGTCCCGAACACAGGATGAACATCCGAAGACAATGTTC
TCCAAAGATAAGGTACAGGTGGAACTAATGTGATTGCAGTTATATTGAAGAGTTAGGTG
GAACTTCTAAATATACTCCCTAA
>175
CTGGGAGTGCATGACAACCAGACAGAGACCTGGGAAAATGACTTGGCCAAATAACAT
TGTTAAAACAATGGTAAGTAGATTAAAATTGATTAGACTTGACATTTAAAAAACCATTG
TTTTTTGTAAGAATTCAATAATAT
>176
AAAGGAACACCCAGATCCAGGCAGTAAAGATCCAGAAGAAGATTACCCCAAATTTGG
ACTTTTGGATCAGGTACATTAATATGTTTTACATTTTCTTTTTGATTTTAAGCCACTCGGG
GATAGGTAATCAGGTTCTGTTT
>177
AGTAACATTGGTCCTGCTTATGACAACCAAAAACAGAGCAGTGCTGTGTCTACTAGTG
GGAATTTAAATGGTAAGTGTTTACATATCTCATTGCTGAAATATTTGGAAGCTATTGAAT
CCAGTTGTGCCTAGAACTGGCC
>178
GAAAAGGACGAGGAGCCAAGCGCAAACAGCAGGATGGAGGGACCACAGGGACCACC
AAGAAGGCCCGGAGGTGGGGAGAGTGCCCACCCTGCCCCCCTCACTATTTAAGTATTA
TTTGGTGCTGGGAAAGTCCAGCAGAG
>179
AAACCTATTCCTGGAGACCTCTACAGAGCCTGCTTGTATGAACGGGTTTTGTTAGCCCT
ACATGATCGAGGTATGTAAAGTATTGGAGATCATATGGATGCAGGGTTACTTTGAACTG
CCATCATTAGCCTTGTCAGTCT
>180
TATTTTGAAATCACTGTGGATGAGATGCCACCAGATACCGCTGCCAGACTGGGTTGGTC
CCAGCCCCTAGGTAAGCTGGGGCCTTAATATGGACATCACAGCAGATAGAGAGGATAG
ACCATCTGGCCAAGGGCTAAGGC
>181
GGGATTTTATATTAATCTTCCTGAAGACACAGAGACAGCCAAGTCATTGCCAGACACAT
ACAAAGATAAGGTGAGTTTGTCCTCTCCCGGCAGATTCCTGGCTTTGAAGGCCTGTGG
CAGCCAGTGCTTATCACTGGCTT
>182
TTTGTATTTTGAGGAAAAAGACTTTGTGGATAAAGCAGAGAAGAGCCTGAAGCAGACT
CCCCATAGTGAGGTGAGTCATGGCCATAAGAACATTAGAATCATAAGGCCTTGAGCGTT
AGGACCCATTCCTTCATTATAAA
>183
AGGTGTGGCTTACAAAGATATTTTTGAGGGGGTTTACTTCCCAGCCATCTCACTGTACA
AGAGCTGCACGGTACGTACATGTTTCCATCCCATGAGCAAAACTTGAGGGAAGCAGAT
GAATGGGTTGTGATGACAAAGTT
>184

GCTGGAAGCCAAGCGGCTGCAGTGCCCCTTTGAGCAGGAGCTGCTGAGACGGCAGCA
GAGGCTGAACCAGGTGGGTGATGGGCACCCAGCAGGGACCACGCATCCTCCAGGGCT
GTCCTCCAGGGAGGAGCTCTGCTGTG
>185
GGCTAAGGTCCTCTGTTGCTTTTTGGTTCCACCTTAGAAGAGGCTCCGCTTGACTAAGA
GTAGCTTGAAGGTAAGCCAGTGGGGAGGAGGGCTCCAGGGCCAGCGGCGGGAGCGG
GAGGCCTGTTGGACATAGGGGCTGG
>186
ATGTTGACAACATTGCCGCCGATACTGCTGCTGTCTGGCTGGGCCTTTTGTAGCCAAGA
CGCCTCAGATGGTGAGTCGGGGGCACATCTCCTGCCTCAGGATGGTTCTGGAGAATCT
CAGTCTATCTGGGCACATGGCAA
>187
GGCCTGCAGTCCTACCTGCTCCAGTTCCACGGCCTCGTGCGCCTGGTGCACCAGGAGC
GGACCTTGGCCTGTGAGTAGGCGCGCAGCGGGGGCGGGGTCTGGGCGGGGCTAGTGG
GGGCGGGGCCTGGCGGGTGGGGGCG
>188
CTGCGGGAATTCCTGGAGGACACCTGTGTGCAGTATGTGCAGAAACATATTTCCGCGG
AAAACACGAAAGGTATGATGGGACGGGGCCCAGGCCTGCAAGCTGGGGAGAGGGCG
GGTTCCAGACAAATGGATGGACCTGA
>189
CAGGCTCAAGCAATCCTCCCGCCTCAGCCTTCCAAGTAACTAGGACTACAGGCATGTG
CCACCACGCCTGGTGAATTTTTTTAAATTTTGTTTTGTAGAGATAGGTTCTCACCATATG
CCCAGGCTGGTCTTGAACTCCT
>190
TCTTCACTAGTTTCCTGCTGCTGGTGTACTCCTATGCCGTGCCCCCGCTGCATGCCGGC
CTGGCCTCCACGTGAGTGGCCCAGCTGGGCAGATGATGGGTGTAGGTGGGGGAGGGA
TGGCACCCCACCCATGGGGCCCTG
>191
TCAGTGCCAGCAGCTTTCACAGAAGCTCTGCACACTGGCCTGGAGTGCACATCCACTA
GTGGGACACATGGTGAGTGGCAGCCCCTGGAGCCTAACAGGAGTCCAGGCTCTCCAA
GGCTGTGGCAGAAGACCGTGACCTT
>192
TACAAATGATGTTCCCCATATCCAGTGTGGAGATGGCTGTGACCCCCAAGGACTCAGG
GACAACAGTCAGGTACCACTGGGATGTGGCTGGGCAATGAAGGAGAGGGGACTGAGA
ACATGGCTGGGTACCATGGTAAACC
>193
TTCTCTGCTCCCTGATAGCCCTGTGGGCCAGCTTCATGCCTCCCTACTGGGCCTCAGCC
AACTCCTGCAGGTATGAAGTAGGGGCGTGGAGGATGGGGGCTTGCAGGTGTCAGAGA
CAGAGGGTTGGGGGTTAAGGGTTT
>194
GATGTGGGAGTCTTCTATGGTTCCACTGCTCCATCTCGCCTGCCCACCTGGGGCACAGG
GGCCTCAGCAGGTGAGGTGCCCAACCGCTGTGGGGAGGCATGAGCAGTCCCAGGAAA
TTCCTGCTGTGAGGGGCCGGGGAA
>195
GTCTCAGATGGGGCCCCTGCACAGCCGTGCTGCCTGCTGATTGCAGAGCGCATCCATT
CGGGTTACGAAGGTGGGCATGTCAGGGCCCTGGCCTGGTGGGGGCTGGGAGGAAGGA

CATCTCCTCATGGCCAGACATGAAT
>196
CTGGGGGCCCCAGTGCTCCTGTTCCTGCATCCTGAGGACCGACCCCTCATGCTGGCTAT
CCACAAGAAGAGTTCCTCTCGCCCTGCTCGCCCTTCCCACTGTCCTGGGCTTTTGAGT
GGCTGCCCTTGTGGTTGTGTCTC
>197
CAGCCCAGCTCCCTCCCTGGACACTGATATCCAGGAGCTGTCAGAGCAGATCCACCGG
CTGCTGCTGCAGGTGAGAGTAGCGGACAGGGAGCCTGGGAGGTGAGAAAAGGTGTG
GGAAGCCGGGTCAAGCCATCTAACCT
>198
TCTCCACAGCCCTGGGTCCTCCAGTGATAGCAACGGGGGTGATGCAGAGGGGCCTGG
GCCTCCTGCGCCAGTGAGTGACCTGCTCCTACCTCACCCTCTAATCGCCCTTTCCCCTC
TCCTTCTTGGAACCAGCACTGCCA
>199
TATACCACCTCCTCAGCCTCTGACGACGACAGGCAGAGGACAGGTCCAGTCTCTGTGG
GGACCAAGAAAGGTAAAGATCCAATGCACCCTGCTCCCACTGCCCCGTCCTGGCCTGG
TGGCCCTGTGTCTTTTCCCTTCTG
>200
AGGATCCCATTTGGCTGCTCATGGCCAATGCTGACCAGCGCGTCATGATGACCTACCAG
GTGCCCTCCAGGTGAGGCATTTCAGAGGCCTTCTTGGCCTTCCTTTCAGAGGTAGTAGT
GGGGGCAGTTTTCTTTTCTGGG
>201
GCGGCGGGAACTGGGTGCTGTGCACTCCTGGGTCCGGAAGGGCCAACTGCCTCGGGC
TCTTGATGTGATGGTGAGAGAAGCCTGGGACGGGGAGAAAAAAGAATTGAGCTCAAT
TCAAGGGGGAGAAAAAAGAATTGAGC
>202
CCTCCTCCCCGCGCTGGTGCTGCTGGCCCGGCTGGGGGCCCCGGCGGTGCCGGCCTGG
AGCGCAGCGCAGGTGAGCGGCGGGGGGGAGGCCAGGTGCCGGGTTGGGGGGGTCCT
CACGTCTGTGGGTCTGGTCTATCGGG
>203
CACGGCGGCATCCACTTCGACGACAGCGAGTACTGGGTCCTGGGCCCCACGCGCTACA
GCTGGAAGAAAGGTGACCGTCCAGGCTCCTCCTGGAGGCCTCTCCTCTGCAGCACAG
TGGGCTGCCGCGGTCGGGCTTTGGG
>204
CTGAACGCCACGCTGCGCGGCTGGAAGGCGTTGTCCCAGGACGAGCTGTGGGGGCTG
CACCGGCTCTACGGTGAGTCCCTTTGTCGGGCGGGAGGGCGGGGACCGGGCGGTCCT
GAGCCAGGCGTGCTCCCCACGCTCCC
>205
AGGGGCTTCTGCGACGCTCGCCGGCGGCTCATGAAGAGGCTCTGCCCCAGCAGCTGC
GACTTCTGCTACGGTGATGCCCACGGGCCGGGACAGGGCTGCGTGGGAGCTGGGCCT
TGGCCATGGCGCAGCTGAGGGGGCAC
>206
TGGTGCCCGAGGGCAGGAACGTGACCTTCCGCTGCGGCCAGAAGATCCTCCACAAGA
AAGGGAAAGTGTAGTGAGTGAGCGCCCCGGGCGGTCCTCGGGGTGGGCAGCCCGCGG
GCGGCCTTGGGGCAGGGGTGCGGGGC
>207

CGTCTTTCAACTGGCAAGAGTCATTTTGACCAGCAGATTAATCAACTGTAAGACGGATC
CTCACACAAAGGTCGGTACTTGGAAATATAAACTGAATATTTTTACTTTGAATATTGAAA
TTTTTCTCAAAGATAACATTT
>208
GAATGTAAGGGGAAGCGAGGTGTGAAGTATATTTATATACATATATACACACATATAGTC
AATGCCAAAAGTTTATCTTCTGTTCCCTCGGCGCCAACTTATTTCAACAACGCGAGCTT
TGCTTAGAAAATCAATTCCAC
>209
TGGAACAGCCAGGTGTACTGCACCAAGGACCAGCCACAGGGGTCCCACTTTTGGAAA
TCTTCCTTTCCAGGTAGGGGCGATGGAGAAAAGGGACCGACACGAGGGAGGGGGAGA
GAGAAGGAGAAAAGAAAGGACTAAGA
>210
GAGTGGCGACCGCGGCTGGAGTGTGGGCCACCTCAAGGCCCACCTGAGCCGCGTCTA
CCCCGAGCGTCCGGTGAGAGCGGCCCCGACTTCCCGCCCCTAGGCTGTGGCCCCCCGC
CCTCTGCTGGGGATGCCACGTCCGG
>211
GGACCAGAGGTTAATTTATTCTGGGAAGCTGTTGTTGGATCACCAATGTCTCAGGGACT
TGCTTCCAAAGGTACATCACTTACACATTAACTTCTGAATGTTTTTAAGCACTCACCAG
GTTAGGTTCAGGTCCTTAAGTA
>212
AAAACGGCATGTTTTGCATCTGGTGTGCAATGTGAAGAGTCCTTCAAAAATGCCAGAA
ATCAACGCCAAGGTGTGTCTGCCTCTTCATGATGGTAACAATTTGTATCATTCAGACTTT
CAGGGGAGTAATAAAAGAAGTT
>213
CAAGTGATGGTTTAAGGCAAAGGGAAGTTCTTCGGAACCTTTCTTCCCCTGGATGGGA
AAACATCTCAAGGTGAGTGTTATAATAAAGATCTTGGCTTATGCAACATGAATGTTCCT
CGTTTGCATCAATTTAAGAATAA
>214
ACACACCCTATGGGTGGCTTCAGCTTTCCTGGTTCCAGCAGATATATGCACGACAGTAC
TACATGCAATAGTGAGTCCTTCCCGCCATGCTGGGTGTGGCCAGGGCTCCCGGGAATT
GAAGGGAATTTTATCCATGTTAC
>215
GTATCCTCTACTTCTACTCCTCCCTGAGCAGATTCCTCATGGTCATGGGGGCCACCGTTG
TTATGTACCTGTAAGCAGATGGTTTCTCTAATATAAATTACACTACACTGTGTTCACACT
AAGCAGATTTTGCTCTTTTT
>216
GGTTCAGAACTTCCCAAATGATGGTCCTCCTCCTGACGTTGTAAATCAGGACCCCAAC
AATAACTTACAGGTATGGAGCCTCCCACGAAGCCCAGGCGAGCTTGACGTGATATGCC
AGGCTCTCCAATCCTCAACCTTTA
>217
CAGCATCACATCAGCATTAGGGATGGTCCTCTTAGGAGCCAAAGACAACACTGCACAA
CAAATTAGCAAGGTAGCTATCAGCATCATTACGTTGTCCTGTTGCAGTTTTTCTCTGGTT
CCGTCGGCTAGCACGCAGATGG
>218
ATCCACTGATGCATATGAGCTGAAGATCGCCAACAAGCTCTTCGGAGAAAAGACGTAT
CAATTTTTACAGGTAATTTCACCTGGCCTACCCACATTTCATTTGCATCCTGATGTCTGT

GTCTCTGAGTGGCCAAATGGAA
>219
ACTGATTTTGCAAATGCTCCAGAAGAAAGTCGAAAGAAGATTAACTCCTGGGTGGAAA
GTCAAACGAATGGTAGGAGAGCCACCCATTATAGAAACACCTTTGAGAAACCTATGCC
AGTGAGCCTTGTGCTTGACACTGC
>220
TTTCAAAGGGCAGTGGGAGAATAAATTTAAAAAAGAAAACACTAAAGAGGAAAAATT
TTGGCCAAACAAGGTATTGTCTATATTTTATTTATATAGTGTAATATGTTAATACATGGAAT
GTTAAACATTTCTGATGGAAT
>221
AATACCATACAAAGGCAAAGATCTAAGCATGATTGTGCTGCTGCCAAATGAAATCGATG
GTCTGCAGAAGGTAAGAACTTGCATCTACAACTCTTCCTTCTACTGCCGGACATTTTTC
CAAAGATACCAAGTTTAAACAA
>222
TTTAACGCTGACAAGTTCTGCATTTCTGCTGTTTCAGTTGTACTACTACAAGCACTATTT
ATCAACAAAGGTAATTTTATTCCTTCTTTCTTATCATTCCTCCTTTCTTATCATTGTATATT
TTCTGATCACACTTCTCT
>223
ATGCAAGTTTTTCTGTGTTCCAAGAGACTTTACTGCATTTGCACTGCAGTATCACCTATG
GAAGAATGAGGTAAGTGACTTGCTTTCAGATAATGGAATGTGTCTCTTTTTACAAAATA
GTATAGGTTTAGGCTAGTTTA
>224
AACACATTGACAGGAAATTTGTTCCCTTCCGAAAGTTACAGTTTGGTCGTTATCCAGGA
GCTTTTGACAGGTAAGTTCAGAGTCAAAACGTGAAATGTGAAATGAGTGTTGTTCAGT
CATAATTCTTGCAGTGTTTTTAC
>225
CTTTGTAGAAGAAGTACCACACTCTAGGTTTATTGAGTGTAGGTATAAAGAAGCTCGAG
CATTCTTTCAGGTTAGAGACAACCAAATGTGTACTTTTAAATTAAAGAAAATGTTGGGA
TTATTTTTAATATTTGAGGGTG
>226
CTGCAACTAGCAGCGAAAACATTAAACAAATTGGGAGTACCATTCTGGCTGAGCAGTG
GAACTTGTCTAGGTAAAATTCTTACGACTTTCCATTTGTCTTTCTGTCATTTTGTCCTCT
GGCTTTAGGGATTACTGCTTGA
>227
AGATTACAAATCTGATATTATTTTAGCATTTCAGGATGCAGGACTTCCGCTCAAACACA
AATTTGGGAAGGTCAGTAACAAAAGTCGGCTTCATTTCATAAGTAACATATCTCACTTG
TAAAGTTTACATTAAGCAACTC
>228
TTTTCTTCTATGAAGAAACTGATCACATGTGGAATGGAGGCACTCAGGCCAAAACAGG
AAAAAAATTCAAGTATGAATCAAATAAGTACTTATTTATAAAGGTACTACAGAAATAATT
TCAGGAGGTAGAAATAAAGAAG
>229
ACCGAAACCCTGCAGAGGGAACCTGGTACATCCAGTCACTTTGCCAGAGCCTGAGAG
AGCGATGTCCTCGGTAAGTTTTGCCTACTCAGCCCTCCTCACTGTTACACTACCTTCCC
CCCCTACTCCATCACACTACTATC
>230

GTGGTGGCCGTCGCTGCCAGGCACAAGTGTCCCGCCGCATCTCCTTCAGCGCGAGCCA
CCGATTGTACAGGTAGGGTGTGCACACAGGTACAGCGGCGGGCGGTGGGCGCCGGGC
CCCGGAACGTCACCGGGGCGGGGCC
>231
AGTGATGAAGAAAACTTGAAACTGTTTGGGAAATGCAACAATCCAAATGGCCATGGGC
ACAATTATAAAGGTGAGAGAAAAACTGATGACATTTCAGCCCTTCAATAAGGATGAAA
GAGTATTCAGCAAATGTAGACATA
>232
GTATTCCCGATCTGCCATGTATTCCAGAAAGGCCATGTACAAGAGGAAGTACTCAGCCG
CTAAATCCAAGGTAAGGGAAGAGGACCACGGACCATTATGTCTTAACCTTAATTGGCAT
TCTCTTACTGTTGATGCATTTG
>233
CGCAACTGTTACAAAACCAGTTGGTGGTGACAAGAACGGCGGTACCCGGGTGGTTAA
ACTTCGCAAAATGGTAAGATGTGGGGACTGTAAATTGGATTTTCTGTTTATGCTTGAAT
ACTGTGAAGATGTTTTTGAATACT
>234
GAGAAAACTGCGAGCCAGCATTACCCCCGGGACCATTCTGATCATCCTCACTGGACGC
CACAGGGGCAAGGTGAGAGTACCTGTGCTTGGGGCGCTTCACTGCAGCTGCCTGGGG
TGCCTGGTGGCAATGCGTTTGCACG
>235
TTACTTCAAGAAGAAGAAGCTGCGGAAGCCCAGACACCAGGAAGGTGAGATCTTCGA
CACAGAAAAAGAGGTAAGTTTCTACTTGTCATCTCCTGTGTTAGCACTGGCCCTTCTAC
CTGGGAGTGAAAAGAAACAGGTTG
>236
TCCCCAGCGCGCAACCACCCCCCGCCTGCCTGCCCAAAGCACCGGGACTGGAGCACG
GAGACCTACCAGGGTAAGTTGGCACTCCTTGGCCCTTCAAGCCTCAACTGCAGCCCCA
TGTTATGCGCCACTCTCAACTTGGA
>237
GATGTACCCTGCCCACATGTACCAAGTGTACAAGTCAAGACGGGGAATAAAGCGGAGC
GAGGACAGCAAGGTAAGCAAGTGCACCCCTAGGGACCCTGCGCTCAGCCCCTCGCGC
GCGCTGAGTTTCCAAGAAAAGTTTT
>238
GAGACGTGACCGGATTAACGAGTGCATCGCCCAGCTGAAGGATCTCCTACCCGAACAT
CTCAAACTTACAGTAAGTGAGAAGCTGGCCCCTTTCCAACCCAGTCCTTTGCCCAGAG
GGCGGGCGGGTCACTCGCCGCCTG
>239
AAAGCACTAACAAACCTAATTGATCAGCAGCAGCAGAAAATCATTGCCCTGCAGAGTG
GTTTACAAGCTGGTGAGTGCTGATTCTGGCTATGCTCTCTTTAAGAGTTTGAGTGCAAA
TAGAGAGCCCGTGGGCTCAACAT
>240
GGCCGGGCTGCAGGAGCTGCGATTCCCTGAGGAGAAGCCGCTGCTCCGGGGCCAGGA
CGCCACCGAGCTGGTGAGTTGGAGTCGCGCCTTTGAGGTCTTAGGGCCGAGCGCGCC
ACCTCGTGGCCAAGAGGGAGACCGCG
>241
GGCCTTTGTTTGTCCTTCCTGGTAGAGGCGGGTTCCCTCCCTCGGCAAGATGCCGGAG
TGCTGGGATGGGGTGAGTGAGGGCGCTGCGGGCATCAAGGTGGGCCGGGAGGATGGT

GCATCCTTATGACCCACCGCAGGGA
>242
GTAGTGATCCGGGGCTCCCCCAAGGGGAACCGCCCAGCCATCCTCACCTACCATGATG
TGGGCCTCAACCGTAAGTGCAGCCCAGCCTCAGTCAGCCCTCCTCTGCCTCCCATCAG
CCAGAGCGGTGAGGCCCCCCACCC
>243
CCAAGCACTTTGTGGTGTGTCACGTGGATGCCCCTGGACAACAGGTGGGGGCGTCGC
AGTTTCCTCAGGGGTAGGTACCCTGAGCCCCCTCTGCCTGTCTCCAGCTCTGCACCTG
AGGCCACACCTGGGCCCTGACCTCC
>244
GGTGGAGGGGCTGGTGCTGGTGAACATCGACCCCAATGGCAAAGGCTGGATAGACTG
GGCTGCCACCAAGGTGTGTGTGGTGACCGGGGGTGGGGTGGGTATACCTAGGGTGGG
GTGAGGGGCGGCACTCACGCTGGCGC
>245
GCTACCGGCAGCAGATTGGGAACGTGGTGAACCAGGCCAACCTGCAGCTCTTCTGGA
ACATGTACAACAGGTGCGGGTGGGATCAGCAGCCCTGGGACCCAGCACACCCCAGTG
GGGGCCCTTGCACACTGCCCCCTGAG
>246
GCTGGGACTTGAACCAGCAGTAAGATTTTCACGACACAGTGCTGTCTGCTTCTCCGTA
AGAAGTTAGAAGGTAAGAAAGCCAGAAATCCCGTGGAGAGGCAAATGTAAGGGATGT
GGCAACGACCACTCTATTCCTACAG
>247
ACCTGACGACTTTGATTCTGAAGACAAAGAGATATTAAGCTGGGATATTAATGATGTGA
AACTGCCACAGGTATGTACTTGCTATAAAAATCTGCCTTCAACATTGGGCTGTGAAGTC
TTGACTATTAACATGCGGAGAC
>248
CTCGCAGAGGAGGGGCGCAAGATCTACCTGAGACCTGCCATCTGTGACAAGGCCCGG
CAGAAGCAGCAGCGTGAGTAGAGCTTCGTGGCACCAGCAAAAAAGCTGACATCCCTC
AGATGCCCCTGCCAGATGGGTCACTG
>249
TTGCCGAGGTCATGGGGGCTTCCCGGTCACCAACTTCTGGAGGCACGACGGACGCTTT
ATATTTTTCCAGGTTGGATTTTTGAGTTAGTTACGTGAGAGTTTCATTACTGAGGAGTTT
CACCAGAGGCTAGAAATGCACC
>250
AGCCATGAAGAAAGTGAACACAGCACCTTCCTCCGTCTCATTGAGCCTGAAGGGGAG
CACAGAGACCAGGGTAATCCTTCATTATGTATGTTCAATACATGCAGTAGCTTGCCACG
CCTGTGCTGGGGGCGGTAGATTTC
>251
CTAGAGCGGTCCTTGGGGAGACGCGGGTCCCAGTCCTGCGGCTCCTACTGGGGAGTG
CGCTGGTCGGAAGGTGAGTGATCCCCTGTCGGGGACCGGGGGACTTGGGAAGGACAG
TTCCCGGACTGGACGGCCAGAACGCT
>252
GACTACGCAGGAAAGCCCCAGCCACCCATCAAATCAGAGAGAAGGAATCCACCTTCTT
ACGCTATGGCAGGTAATGATTCACTATTGTGGAGTAAGACTTTTTTTTTTTTGAGATGGG
ATTTTGTTGTATTGCCCAGGCT
>253

TGCACCGTCACAGTGTCTGATTTGTATGCCATGAACTTTGAGCCGAGGGCCACAGACA
AAGATATCACTGGTGAGTCATGGGATAAATGCTCTATTTATAAAAACTATCTTTATGTTTT
TTACTTTAAAAAATGTTGACT
>254
AAGGTCTCTGGCTAGCGACATCACTGATGAGCAGAAAAAGGTTCGGGAGGCTGACCT
AGTGATATTTCAGGTTTGTTTTTCTCTAATTAATATATTGAATCAGATTCATCTTATGTACA
AACTCTTTCTGAATATTGAGT
>255
GATGGATAGGGTGCTGTGCCAGGGCTTTGCCTTTGACATCCCAGGATTCTACGATTCCG
GTTTGCTCCAGGTATGTGCTCTTGGATAAGGATCACTATGGATAGTTGGAGGGAGGGGA
CAGAGGATGCGTCTTCTATCAA
>256
AGGCACGGCCGAGATGTACACGAAGACAGGAGTCAATGGAGATTCTCGATACTTCCTG
TGGCCACTCCAGGTAGACCAGCTGCGAGTGGCTCCCTTGCTTGTTGGCACACGCACAC
ACAGACACACACATGCACACATGC
>257
TCAGAATGACCCTTGGGCCCCCACTGTGGATTTCAGTGACTTCATCAACAATGAGACC
ATTGCTGGAAAGGTCAGCTGGCCGGGGTAGAGGGTACAGGATGAGCCTTGCTTTCTCT
TGGGAGGGTCCCTGAAAACCACCT
>258
GGCAGTGTGACCAAGAACCATGCCTGGTGGATCCAGACATGATCAAAGCCATCAACCA
GGGCAACTATGGGTGAGAGGCCCTAGAGGCACCCTCAGTGGGCACACATGCATACTCA
TGCATGTATACACGCATGCTGTGC
>259
TGAGGGCATTCGCTACCGCCTGGGCACCATCCGCCCATCTTCCTCGGTCATGAACATGC
ATGAAATTTATGTAAGTCCATCCTTCCCCACAATGCTGCCATCTCCCCATGGCTCAGAAC
CTCAGGGATGCTGGCCCTGTG
>260
TGGCCCAACCTGATTCATGAGCCTCTTGACCAAGGCAACTGTGCAGGCTCCTGGGCCT
TCTCCACAGCAGGTAAGCCAAGGGCAAGGGCTGGCGCCTGGGAGAGGAGGGCCAAG
TCCTGAGCCTCCCGACAGCCCCTCTA
>261
ACACCCACCAGCAGCAGGGCTGCCGCGGTGGGCGTCTCGATGGTGCCTGGTGGTTCCT
GCGTCGCCGAGGGTATGCAGCAACAGGGGATGTGGGCAGAGAAGAGGGCAAGGAGC
TCCGTGGGCATGGCCTGGGCCATGAT
>262
CTGCCCCAACAGCTATGTTAATAACAATGACATCTACCAGGTCACTCCTGTCTACCGCC
TCGGCTCCAACGTAAGTCAGCACTTGGGTGAGGGCGCCGAGGCAGGAGGGTTGTGAA
AGCCTGGAGTCTCACCCCTGACGT
>263
ACACGCCAGTGAGCCTTGGGAGGCCAGAGAGATACCGCCGGCATGGGACCCACTCAG
TCAAGATCACAGGGTGAGGGGCGTGTGGGCAGAGGGGGTTTGGGACAGCAGGGTTTG
TGCTAGGGGCTCTGGAGCCTGCCTTG
>264
GGTGCTGGTGTACGGCGGCAGGGGCGCTCTGGGTTCTCGATGCGTGCAGGCTTTTCGG
GCTCGCAACTGGGTAATGCTGCGGGCTGGGGCTGCTGGGTCTCCGCGGGGGTGGCCG

GGGGCTTTCGTGTGGAACCCGGCGG
>265
GGAGAATGAAGAGGCCAGCGCTAGCATCATTGTTAAAATGACAGACTCGTTCACTGAG
CAGGCTGACCAGGTAGTTTCAAAAAGGCCCCCAAACATTGTGAGGGGAACCCTGGGG
AGAGGTGACCACTGGCTGTATGTTC
>266
GGTGAAGAGAAGGTGGATGCAATTCTTTGCGTTGCTGGAGGATGGGCCGGGGGCAAT
GCCAAATCCAAGTGTGAGTCCTTTCCTGAGTCACTCATTTGTTCTGCTCTGCTCTTTTTC
CAGCTGACAAGGATTGGGTTTGC
>267
ACCAAGCATCTCAAGGAAGGAGGCCTCCTAACCTTGGCTGGCGCAAAGGCTGCCCTG
GATGGGACTCCTGGTAAGCCTTTATTTCCCTATCTGTGGAGTGGGGTTGCTTACCTTGCT
TGCTGGGGCTGCTGGGCACTTTC
>268
AGCTCTGCCAGAGCCTGGCTGGGAAGAACAGCGGCATGCCGCCCGGGGCAGCCGCCA
TCGCTGTGCTCCCGTAAGTGCTTGGGGTTCCCTGGCTGCTGCCCACCTCTGCCCCACTG
CTGCAGGTGACCGTTTGTCTGACT
>269
CCCCGATGAACAGGAAATCAATGCCTGAGGCTGACTTCAGCTCCTGGACACCCTTAGA
ATTCCTAGTTGAGTGAGTATTACAGACATGTCCATGACTGAGCAGCTTCCAGCCCCTAT
TCTCTGCCTGCTGTGGTCCAGAG
>270
CGCGCTGTTTACATCTTTGCCAAGAAGAACGACATTCCCTTCGAGCTGCGCATCGTGGA
TCTGATTAAAGGTAGGTCCAGCCTCGGGTTTGGGGAACCGAAAAGTCAGGAAGGGGA
CAGGTAGGCATACATAGCTTAGGG
>271
CCTTTGCCCAGGTGAACCCCCTCAAGAAGGTGCCAGCCTTGAAGGACGGGGACTTCA
CCTTGACGGAGAGGTAACTGGGACCCTAGGACTGCTGCCAGGCCTGCTGGAACCATCC
TGTTCTAACCCTCTATTTCATAGAC
>272
TGTGGATGAGTACCTGGCATGGCAGCACACGACTCTGCGGAGAAGCTGCCTCCGGGCC
TTGTGGCATAAGGTGAGGCTGGGAATGTGGGGGGCGGCAGCGAGAGCATTCCCCAAA
GGTGTTCAGGCACCAGTCTCTTCTT
>273
GAACAAGGCCTTCCTTACTGGTCCTCACATCTCCTTAGCTGACCTCGTAGCCATCACGG
AGCTGATGCATGTGAGTGCTGTGGGCAGGTGAACCCACTAGGCAGGGGGCCCTGGCT
AGTTGCTGAAGTCCTGCTTATGCT
>274
TTATATCCTCTGGGCCTGGGTTTCCTGAAGTCACCACACATAGAGGAGAGAGAAAATG
GCTGAGTTAAAGGTAAGAAACCATCTGACAAGTTTGCTATGGCTTCTTCAGCCAAAATT
TATGGAAGTTTCTGAGAACTTCA
>275
CATTTCTGGATTTGGGAATGAGTGTTCTTCAGAGGATCCTCGCTGCCCAGGTTCCCTGC
CAGAAGGACAGGTATGAGCAAATAAAGTGCTTAGAAGAGCTCTGATTCCTATTTCCTG
GATGACTAGCTTTCAGGCCAAAG
>276

CCTACAATCTCTATGCTGAGCAGCTCTCAGGATCGGCTTTCACTTGTCCACGGAGCACC
AATAAGAGAAGGTACAAGGATTAGATGAATTCTGACCTGCAGACTGTGGGTACTATGA
CAGGGACTTTGTGCTGCCTCCTT
>277
GCCCTTTGAATCCATTGACGAAGGCCATGTCACTCACAACTGGGATGAAGTTGATCCT
GATCCTAACCAGGTAACCTGGTCCTGTGAATTGAAGCTTATCATATACCCAAAGCCTTG
ACTAGAAATACCTAAATAGAATA
>278
CTGGAGACATAAAGTCTAACAATGGGCTTGCTATCCACATTTTCCTCTGCAATACCTCCA
TGGAGAACAGGTAAGTGGCTCAGTTTTAGGGAAGGACATGCCCATCTTCTAAGGGATT
TTGGCCAGAAGTCAAACTCCAG
>279
AGGGAACCTTCTCATTTACACCGAGTTTGGCAAGATGCTTGTACAGCCCAATGAGATCT
GCGTCATTCAGGTTGGTGATGTGTCCCCTTCTCTTCCCTGCTTCTCACTAATTTGGGAGC
AGTCAGATGTTTCAGCTGCTG
>280
GAGGAGACCAGGGGCTACATCTTGGAGGTCTATGGTGTCCACTTTGAGTTACCTGACC
TTGGACCAATTGGTAAATCTTCATTACAAGCCTCTAAGCCTCGCTTGAGATGTAGGACT
CAGATAATTGCATGAAAGTTAAA
>281
GGATCGCCAAGTACCAGGTGGTTACACGGTCATTAATAAATACCAGGGCAAGCTGTTTG
CTGCCAAACAGGTAAAGTAAAAGGGTTGGCAGGCAAGGGAGGAAATGAAGAGTGATT
GAGCACTTACTGATTGCCAAGGCG
>282
GAATTATACACCCTACAAGTACAACCTGAAGAATTTCATGGTTATCAACTCAGTGGCCT
TTGACCATGCAGTAAGTCTGAACCCTGTAGGGGCCTTGGGATGAGGCAACGCTTGGGT
GGGAGGGGTCCACAGTAGACCCC
>283
GCTGATTTTGTCATCTTCCCACCTCGATGGGGGGTTGCTGATAAGACCTTCAGGCCTCC
TTATTACCATAGTAAGTCTCTCTTTTACCAAGCCACATTTGCAAGCCAAAGAGACAACA
AAGCAGTGAGCGCTACACATTG
>284
TGATGCTGACTGCTTTGAGAAGGCCAGCAAGGTCAAGCTGGCACCTGAGAGGATTGC
CGATGGCACCATGGTAAGTATGTTAACTGCCACATTCCAGCGGCCTCTGAACTCCCCAC
CCTTACTGAGAAGGGTTGCCGTTT
>285
TCAGGACACAACTGTGGGACGTGCCGTCCTGGCTGGAGAGGAGCTGCCTGTGACCAG
AGGGTTCTCATAGGTAAGTGGAGATATGAATGAGTTCATAAGTCCTGCATGAGACTCAA
GGCTCTTAATAAAATCTTAAATCA
>286
CTCTCATGAGGGACCAGCTTTTCTCACATGGCACAGGTACCACCTCCTGCGTCTGGAG
AAAGACATGCAGGTATGTAAGAAGCATTTCAGTTTGCAGACTCTTTACAGACAAGATG
CCTTGTTTGTAAGTGATTTTAATT
>287
GTCTTTTCTCAATGGCGAGTGGTCTGTGACTCCTTGGAAGATTATGATACCCTGGGAAC
ACTTTGTAACAGTAAGTTCCAAATGATAGCTTGGAGTCAGAATTTCTTTTTAGATAAAG

AGATTAAATATGTTGCCTGAAA
>288
CCTATTTTTGTCCTCCTGCACACCTTCACAGATGCAGTCTTTGATGAATGGCTGAGGAG
ATACAATGCTGGTAAGACATTTTCATATGCTTTTTGCATGCTCAGCTGGGCGGATTGTTT
AGATGGCATAGTTATCAGTTC
>289
GTCACCAACACAGAAATGTTTGTTACTGCTCCAGACAACCTGGGATACACTTATGAAAT
TCAATGGCCAAGTGAGTGTTGAAAGTGTATTTTTACTGTGATAATTTCCAAAAGCAAAT
GTGTTATCTTTCAAGTAGAGTA
>290
CTTTTCCAGCTCCAATCTTTGATGATCAGTGTAAACTACACGATACTTTTCTTTTGTCCT
GGTTTTCCCTGTTAGGAGAGAAAGCATTGCATTTAATCTTTTTCTGCTTCTTGGAGATTG
TTTTAGTTAAAGAAATCATA
>291
ATGCTCAGCTTTGTGGATACGCGGACTTTGTTGCTGCTTGCAGTAACCTTATGCCTAGC
AACATGCCAATGTAAGTGCCTTCAGCTTGTTTGGGGGAGACTGGGTAGAGAGGTTAGA
TGGGAGGGCACCCTGCCCTGAAA
>292
AGATGGTGAAGATGGTCCCACAGGCCCTCCTGGTCCACCTGGTCCTCCTGGCCCCCCT
GGTCTCGGTGGGGTAAGGTGTCTTACGTATTGCTAACTTTTAGCTAACTTCAGTTGAAA
GAAGGTTTATTGTGGAATTTATT
>293
TCCTGCTGGTCCCGCCGGTCCCCGTGGTGAAGTGGGTCTTCCAGGCCTCTCCGGCCCC
GTTGGACCTCCTGTAAGTAGCCACTGTCTTTAAACTTTATTGAGTAAAAGAAAACAAA
GGTGGAGTATGGGGAAGAAGAAGA
>294
CCTCCCTGGACCCCGCGGTATTCCTGGCCCTGTTGGTGCTGCCGGTGCTACTGGTGCCA
GAGGACTTGTTGTAAGTGGTCATGACTGTGGTTCTCATCATCCTGAAATACCACCTCTG
CCATCATTTCATCACTATCTAG
>295
TGAAGAAGGAAAGAGAGGCCCTAATGGGGAAGCTGGATCTGCCGGCCCTCCAGGACC
TCCTGGGCTGAGAGTAGGTTTCAAATGCTCCCAACACCCTAACACACCAGAGGCAGAT
TATGATACCCCTTCATTGGGAATTG
>296
CCCTGCTGGAGTCCGAGGACCTAATGGAGATGCTGGTCGCCCTGGGGAGCCTGGTCTC
ATGGGACCCAGAGTAAGTTTCAAACTGATTCTGAGCAAATCACACCTGGCATTACTTCC
TTCTTTAAAGGGTTGGTTAATAT
>297
CCCAATTGGCCCCGTTGGAGCAAGAGGAGAGCCTGGCAACATTGGATTCCCTGGACCC
AAAGGCCCCACTGTAAGAATCACCACAACTTTCTTACCCTCAGCACTTTCTGTAGCCA
AATTTTACCAAACTCTAGTATTTA
>298
TGCTGCCGGTCCTACTGGTCCTATTGGAAGCCGAGGTCCTTCTGGACCCCCAGGGCCT
GATGGAAACAAGGTAAAATCTTATGTTTTCTATATTGCTGGTTTGGCCCAGTCTGCCTG
GAATAAGTAGACCCTTTACAATA
>299

TCCATCTGGTCCTAGTGGACTCCCAGGAGAGAGGGGTGCTGCTGGCATACCTGGAGGC
AAGGGAGAAAAGGTACGTGTTGACCCCTATTACATATTGTTGATGAACTCTAGTAAAGA
AGGCTGCACAAGGATGCCCAAGT
>300
AGGCAAAGGGCCTAAGGGTGAAAACGGTGTTGTTGGTCCCACAGGCCCCGTTGGAGC
TGCTGGCCCAGCTGTAAGTTGAATTCACTGGTGGTCCACACAGCAGCTACCCATTAGAT
CTTCCAATTAAATATATATCCGTC
>301
AGAAGTAGGTGCAGTTGGTCCCCCTGGCTTCGCTGGTGAGAAGGGTCCCTCTGGAGA
GGCTGGTACTGCTGTAAGTGATTTCCAACTCCTCTTTCTTAATACCTTATGCTGAATTAA
AATAAAGCCCCTACACAGATCTT
>302
TGCTCCTGGTATTCTGGGTCTCCCTGGCTCGAGAGGTGAACGTGGTCTACCTGGTGTTG
CTGGTGCTGTGGTGAGTGCTTGACAGTATTCTGACTCCATTAACATAAGAAAAGATTTT
AAAAGCTGCCACTTCAAATGTG
>303
GGCCCGTGGTCCTCCTGGTGCTGTGGGTAGTCCTGGAGTCAACGGTGCTCCTGGTGAA
GCTGGTCGTGATGTGAGTCCAACACTTGGTTTGTAAAATAAAACTGAGCAGGATTTCAT
TGTGTGAAACTTTATGTCCTGAG
>304
TGCTGCAGGTGCACCTGGTCCTCATGGCCCCGTGGGTCCTGCTGGCAAACATGGAAAC
CGTGGTGAAACTGTAAGTTTGTGAATACCAGTCCCTCAGTGCAGCATTCTCGTGGGCTT
CACTTCTGACTTCCCCACACTTG
>305
TGAAAAGGGGCCCAGAGGTCTTCCTGGCTTCAAGGGACACAATGGATTGCAAGGTCT
GCCTGGTATCGCTGTAAGTAAACTGTAGCCATCTCGCACATAAACTGATCCTGAAGGCC
TTCAGCTCAGAAGGATTTTCATAT
>306
TCGCACTGGACATCCTGGTACGGTTGGACCTGCTGGCATTCGAGGCCCTCAGGGTCAC
CAAGGCCCTGCTGTAAGTATGATTTGGGGAAATAATAAAGAAGATCACGGACCTAAGG
AATGTTTTCTTCAGACTAAACCAA
>307
GAAGGCTCTAGAAAGAACCCAGCTCGCACATGCCGTGACTTGAGACTCAGCCACCCA
GAGTGGAGCAGTGGTAGGTCAAGATGTCCAGACCAGACTGACCCTTCTCACAAGTTG
AGCTTTTCAAAATTAGTTTCCATTGA
>308
GAACTGGTATAGGAGCTCCAAGGACAAGAAACACGTCTGGCTAGGAGAAACTATCAAT
GCTGGCAGCCAGGTGAGGAATCCCACAAACACCTCTCCTTCTGCTAAATAATATTTTGG
TAGGACTGTTTGTTAATTATCTG
>309
TAATGATGTTGAACTTGTTGCTGAGGGCAACAGCAGGTTCACTTACACTGTTCTTGTAG
ATGGCTGCTCTGTAAGTAATAGTGAAATATGGGAATAGCTTTGGGAAGTGGGATGGAGG
GGGTTCTAACTTAGACTGCCCC
>310
CGCCGAGGGCCTGAGTGCTCCAGTAGCCACCGCATCTGGAGAACCAGCGGTTACCATG
GAGGGGATCAGTGTAAGTCCAGTTTCAACCTGCTTTGTCATAAATGTACAAACGTTTGA

ACTTAGAGCGCAGCCCCTCTCCG
>311
GAGGAGTGGAAGGAGTTCGTGTGGAACCCGAGGACGCACCAGTTTATGGGCCGCACC
GGGACCAGCTGGGGTACGCAGGGCCGGCACGCAAGGGGCGGGGGAAAGCCGCGGGG
CGACGCCTCGGGGGCGCAGGGTCCCGC
>312
ACCATGTGGGTGATGCTGCAGACTGTCTCCGACCATACCCCCAAGTACCAGGACCGAC
TGGCCACACCGGGTGAGTGTGGAGGCTCCCCCTGCCAGCTACTCTAACTGCTCTTGTG
CCCCCAAACCTCCAGAAGGAACTC
>313
GTCATTGTCAATGTCAGTGACACTGAAAGCTGGGACCAGCATGTTCAGAAGCTCAACA
AGTTCTTGGAGCGTGAGTGTGGGCCTGGTTATGTGTCAGTTCAAGACTTCGGGCAGGG
GACTGGGGACCTTGGAAGTGGAAC
>314
CGGCATTGGGGACTCCACCCACTATGGTTACAGCACTGGGCAGCCCTGTGTCTTCATCA
AGATGAACCGGGTATCTATGACCTTGGTCCCCAGGGTGAATGGAGGAAGGATCTGGGG
ACACCACCTGCAGACAATTGCAT
>315
CTTCGTCATGTTCCCCGCCAACGGCAACATCGACCTCATGTACTTCCCCTACTATGGCA
AAAAGTTCCACGTAAGTCCCAGGGGAGGCCCAGGCTGGATGGCGGGTGCGGGTGGTG
AGCTAGGGAAGGAGGCCGCGTTGC
>316
AACCAGAGCTGGGAAGATTCGAACACCGACCTCGTCCCGGCCCCTGCAGTCCGGATAC
TCACGCCAGAAGGTAAGTGAAATCTTAGAGATCCCCTCCCACCCCCCAAGCAGCCCCC
ATATCTAATCAGGGATTCCTCATC
>317
CTACGTGCTGCCCTGCGCCCGCAGCCATGTGCCGCACCCTGGCCGCCTTCCCCACCAC
CTGCCTGGAGAGGTAAGGGGAGGTTGTCCAACTGCTTGGGGGAAGTCCCACCAACTC
TTAGGGAGTGGAGCGGCAGGCCTGG
>318
AATCAGAGCTGGGCTGCGATACTGGGAGTACTGGCAAGTTCGAGTGGGGCAGTAAAC
ACAGCAAAGAGAAGTAAGTGTTGCTGACCAGCTCAGGGCTCCGAGAGGGAAGGGAG
TGGGAAGGGAGTCATGAGGAGGGAAAT
>319
AGCTACCAAGCTGGCCTCCAGGGCACACCAGATCTTTGAGGAGTTCATTTGCAGTGAG
GCCCCTAAAGAGGTTAGAGCCCCCATATGCCCAGTGAATCCTCACGACCCTGCCTGCTC
CTTCCCCTCTGAGAATCAGGGCT
>320
TTCGACGGCGAGCCGCAGCCCTACCCAACGCTGCCGCCTGGCACGGGCCGCCGCATCC
ACAGCTACCGAGGTACGGGCCCGGCGCTTAGGCCCGACCCAGCAGGGACGATAGCAC
GGTCTGAAGCCCCTCTACCGCCCCG
>321
CAAACTGAATTATTTGTGCCATCTCTCAATGTTGACGGACAGCCTATTTTTGCCAATATC
ACACTGCCAGGTACTGACGTTTTACTTTTTAAAAAGATAAGGTTGTTGTGGTAAGTACA
GGATAGACCACTTGAAAAATT
>322

TTCAGCCATGCCTCACAGCTCCGACAGCAGTGACTCCAGCTTCAGCCGCTCTCCTCCC
CCTGGCAAACAGGTAGAGTCCTCCTTTTTCTCTCTCCTACCTTCTGATTCTCCTGGGGG
ATGGAAAGAGAGCCAGGCTTCCT
>323
GGAGAAAAATCGTATTGCCGCCCAGAAGAGCCGACAGAGGCAGACACAGAAGGCCG
ACACCCTGCACCTGGTAAGTGTTCAGATCAATCAACATCCACGTGAGCTTTAGGCTTTG
CCCTCCGCCATCTGGGAACCCTTGG
>324
CTCTTAGCGGCCACCGCCCTCCTGACGCACGGCCAAGAGGAAGGCCAAGTCGAGGGC
CAAGACGAAGACAGTAAGTCCCAAACTTTTGGGAGTGCAAGGATACTCTATATCGCGC
CTTGCGCTTGGTCCCGGGGGCCGCG
>325
GAGACCAAGAACTGCCCCGGCGCCGAAGTCCCCGAGGGCGAGTGCTGTCCCGTCTGC
CCCGACGGCTCAGGTGCGGCTGCGCTCGGGGCCTGGGGCCTGGGGCTGGGGCTGGGG
GTGGTCGGCGCTCGCTGGCCCTCCGT
>326
CCCTGGACAGCCTGGACTTCCCGGACCCCCCGGACCCCCCGGACCTCCCGGACCCCCT
GGCCTCGGAGGAGTAAGTGGAGAGGCCTTGTGTGTCCACTCTCCCCTGTTTTGTTTTT
GTTTTTTGGCAGATGACATAATTT
>327
CTTTGCTCCCCAGCTGTCTTATGGCTATGATGAGAAATCAACCGGAGGAATTTCCGTGC
CTGGCCCCATGGTGAGCCAGCAGGGGGAGCATGGATGACAGAAGAGAGAATGGGTAT
CCAGAGGATGTGGGCATACGCGGC
>328
CTCTGAAGGTCCCCAGGGTGTGCGTGGTGAGCCTGGCCCCCCTGGCCCTGCTGGTGCT
GCTGGCCCTGCTGTAAGTGTCCCCGACTCAGTGTCCCCTTTGCCACTTTCTAACCTCAG
AGTCCTTGCCTGTTGCTGACACT
>329
CTTCCCTGGTGCCCGAGGCCCCTCTGGACCCCAGGGCCCCGGCGGCCCTCCTGGTCCC
AAGGGTAACAGCGTGAGTACCAAACTCTCCCTTCTGCCCACCCCATGCACTGGCTCCA
GTGCGGCTCTCATCTGGGGAGCAG
>330
AGAGGAAGGAAAGCGAGGAGCTCGAGGTGAACCCGGACCCACTGGCCTGCCCGGAC
CCCCTGGCGAGCGTGTAAGTGTCCCTGCCCGCCCCCTCCCACTCCACCCTCATTGCCTG
GCTGGTGCCTGTGTGTCGCGGAGTT
>331
CCCCGCTGGCCCCAAAGGATCTCCTGGTGAAGCTGGTCGTCCCGGTGAAGCTGGTCTG
CCTGGTGCCAAGGTGAGGCCCCAGGCTTTCAGCCTGGCTTGGCCAGGCCCTGACCATC
CCGTGTAGGGTCTGGGATGAGGCG
>332
ACCCCCAGGCCCACCTGGTGCCCGTGGTCAGGCTGGTGTGATGGGATTCCCTGGACCT
AAAGGTGCTGCTGTGAGTATTAAGTGAGGATCCATGAAGAGCCAGGGACAAACACAC
CTGAGACTTGAAGGAGTCCTGGGCT
>333
TGTGCAAGGTCCCCCTGGTCCTGCTGGTCCCCGAGGGGCCAACGGTGCTCCCGGCAA
CGATGGTGCTAAGGTGAGGGCAGCGTGGAAGGGGCTCTGGCAAGTGGCCCAGGGACC

AGGTCTCACCCCTCCTGCAGCAGGGG
>334
CGCCCCTGGCCTTCAGGGAATGCCTGGTGAACGTGGTGCAGCTGGTCTTCCAGGGCCT
AAGGGTGACAGAGTAAGTTCAACCTTCCCCCTCCCCTGAGCCCTACATGGCTCCCATC
TCTGCCTGCTTTGAATCTCTCAGC
>335
CAAAGATGGCGTCCGTGGTCTGACCGGCCCCATTGGTCCTCCTGGCCCTGCTGGTGCC
CCTGGTGACAAGGTGAGGTGGCCGCCTCCCCACCTTCTGCCCTAACACATAGCCTCCT
CAGCAGGCCTGGGCACGGTTCCGT
>336
TGATGCTGGTGCTAAAGGCGATGCTGGTCCCCCTGGCCCTGCCGGACCCGCTGGACCC
CCTGGCCCCATTGTGAGTGGCTTGGCCCTCTGTGCCCACGAGGCTGGTGGGCTGGGAC
CCAGGACGGGTCCAGGCTTGATGC
>337
TGAAGTTGGTCCCCCTGGTCCCCCTGGCCCTGCTGGCGAGAAAGGATCCCCTGGTGCT
GATGGTCCTGCTGTAAGTGCCAGCTCAGATCTCTGCAGCTCCGGAGGTGTGCAGAGCT
GGGGAGGGGTCCCTGTGCTGCTGT
>338
ACAGCGTGGTGTGGTCGGCCTGCCTGGTCAGAGAGGAGAGAGAGGCTTCCCTGGTCT
TCCTGGCCCCTCTGTAAGTGCCCCCCTCACCTTGGGGGGCCCTGAGAAAAACCATCAC
AGGACTTGGAGTGGGGCGGAGCCAA
>339
TGAACGTGGTCCCCCTGGTCCCATGGGCCCCCCTGGATTGGCTGGACCCCCTGGTGAA
TCTGGACGTGAGGTGAGCAGTCCCCAGCCCCCATGCCAGTACCCTCAGCATGGCCATT
GTGGCCTTGCCTAAGCCCTCTTCC
>340
TGCTCCTGGTGCTCCTGGTGCCCCTGGCCCCGTTGGCCCTGCTGGCAAGAGTGGTGAT
CGTGGTGAGACTGTAAGTAGCTGGGCTCCAGTTCCCTGTACCTGGTCAGGCCAGGGAC
TCTTCAGGCCTCCTTAGAGGCCTG
>341
CGAACAGGGCGACAGAGGCATAAAGGGTCACCGTGGCTTCTCTGGCCTCCAGGGTCC
CCCTGGCCCTCCTGTAAGTATGCTCAGCCCCTCCCCAGTCCCCATGCTGTGCTGTGGGA
TAGGAGGGGGAGCTTCGCCTCAGT
>342
ACTCAACGGTCTCCCTGGCCCCATTGGGCCCCCTGGTCCTCGCGGTCGCACTGGTGAT
GCTGGTCCTGTTGTATGTAGCCCCTCATCCCCTCTGCTCATGGCCCTCCAGCCCCCATAG
CACTTGGATGCCGGAATCCCCA
>343
GAGGGAAGCCGCAAGAACCCCGCCCGCACCTGCCGTGACCTCAAGATGTGCCACTCT
GACTGGAAGAGTGGTGTGGGCCTGCCCTAGCCTCTCCCTCCCTCCTACTCCTGCCATGC
CAGGGTCCCCATGCCCATATGTGC
>344
GTACATCAGCAAGAACCCCAAGGACAAGAGGCATGTCTGGTTCGGCGAGAGCATGAC
CGATGGATTCCAGGTGCGTGAGCTGGACCTCAGAGCCAGTGTTAGGAGATGGGCTAGC
CCAGTGCTCAGAAGGGACATGAAGT
>345

CAACGAGATCGAGATCCGCGCCGAGGGCAACAGCCGCTTCACCTACAGCGTCACTGTC
GATGGCTGCACGGTGAGTGCCCAGAATCCCCAGGCAGGGCCCCACCTCTCCGGCCTTG
GGCATTTTGGCCAGGCCATAGTGC
>346
CGCCGCTACGGCCTCCCCCCGCAGCCTCCTTGTTCTCCTCCAGGTGGTAGTGCTCGCTC
TGGCGCAGATTGTAAGTTTCGCAGCCCCTCCGCTGCCAGGGTCCAGGGTGCGGGGTCT
GGAGTCCGGGATGGAGGAGCTCT
>347
TCCACCGGGAGAGCGGGGCCCCCCGGGTCCCCCGGGACCGCCGGGAGTGCCTGGATC
CGACGGCATCGACGTAAGTCTCTAACCTGAGACCAGGGCGGGAGGGAGGCGGCAGAC
AAAGGAGAACTTTGTGAGCGCTGTGG
>348
TCCCCCCGGCCCTCGAGGACTGGCCGGGAACCGAGGCGTGCCAGGACAGCCCGGGAG
ACAGGGCGTGGAGGTGAGTCGGGCCCCGGGGTAGGAGGTGCTTCTTCTAGGTAGATCT
GTTCTGGGGTGCGGCTTACCCGCCA
>349
CACCCTGGCCCTCGGGGCGTTCCTGGCATCGTGGGAGCCGTGGGTCAGATCGGCAACA
CGGGGCCCAAGGGTGAGTGCTCCTCTGCGGTGGGCATGGGGGCCAGGCAGTGAGGAT
TTGTCCAGGCCGGCCCTTTCCCCAT
>350
GGAGAGAAGGGTGATCCAGGAGAAGTGGGACGGGGGCACCCCGGGATGCCTGGGCC
CCCAGGGATCCCAGGTAAGCCATTGGCCCTGCCCAGCTGCAGTGTGTTCCTCAGCTTG
GGAATTAATGACGTGTGGACACTGCG
>351
GGCTGGCACAGCTGCCTGCATCGCAGATCTCATCACCTTTCCTCTGGATACTGCTAAAG
TCCGGTTACAGGTGAGGGGATGAAGCCTGGGAGTCTTGATGGTGTCTACTCTGTTCCC
TCCCCAAAGACACAGACCCCTCA
>352
ATGAGCTTTGCCTCTGTCCGCATCGGCCTGTATGATTCTGTCAAACAGTTCTACACCAA
GGGCTCTGAGCGTGAGTATGGAGCAAGGGTGTAGGCCCCTTGGCCCTTTTTTCTCAGT
GATGATTGATCTTAGTTCATTCA
>353
AGATACCAAAGCACCGTCAATGCCTACAAGACCATTGCCCGAGAGGAAGGGTTCCGG
GGCCTCTGGAAAGGTGTGTACCAGTTGTTTTCCCTTCCCCTTTTCCTCCTCCCCGATAC
TCTGGTCTCACCCAGGATCTTCCT
>354
GTCAACTGTGCTGAGCTGGTGACCTATGACCTCATCAAGGATGCCCTCCTGAAAGCCA
ACCTCATGACAGGTGAGTCATGAGGTAGACGGTGCTGGGTCTCACCCTTCCCCCATGC
CAGGAGCAGGTGCGGGGGTCTAGC
>355
AGTACAGTAGCGCTGGCCACTGTGCCCTTACCATGCTCCAGAAGGAGGGGCCCCGAGC
CTTCTACAAAGGGTGAGCCTCTGGTTCCTCCCCACCCAGTTCAGGCCTCTTGGCTATGC
ATGTCTATTGTGGGTGGGAGAGA
>356
CCGCCCCCAATTCCTGCCCCTATTCTCTGCCTGGGAGATGGCTTCCCCGAGCCCCCCGC
CGGAGTCGAAGGTAAGTAAGCACCCCCCCGGGGATCCCGGTTGTCTCGCCGCTACCCC

AGCTAGGCCGCACCACTGCTTTC
>357
CTCCAGGTGAAGCACCATTTTTGACCTTCCCTTGTGTCTTCTGTTCCTGGCACAGTGCC
TGGACCCCAAGGTACACGCTTAGAGAACCTCAAGGAATTGAATTGAATAGAGTCACAA
GCCAGTGCAAATGGAAAATTGAA
>358
CTGGACCCAGCTCAGAGAAGCCTGTATAAAGATGTCATGATGGAGAATTATGGAAACC
TGGTCTCACTGGGTAAGAATCTGCTGTTTCTCTAATTAAAATATCTAAGAAGGTAGAGA
GAAATCAGACTAACAGAACATTT
>359
AGCCGCACTCGCCGATCCTCCAGGCATGCCCCGCTACGAGCTGGCTTTAATCCTGAAA
GCCATGCAGCGGGTAAGTGACCTTCCCTCAGAGCCGGTTTTCCCGCGCGGGCGCCCCC
TGTGCTTGTCATGACCCCTATATT
>360
TCTACTTAACCCCACCCACCAGATCTACCCTGTGACTCAGCCACTTGCCCCAGTTCATA
ACCCCATTAGTGTAAGTCCAAATTTACTGGCTTTGCTGTTTCATTCAAGATGTGTATGTG
ATGGTAGAATAAAAGAATAAA
>361
ACGGACGAGGGCGCGCTGCATGGCGCGGCTGAGGCCAATTTCCGCATCTACCCCTGGA
TGCGGTCTTCAGGTAGGCGCAGTCGCTAGGCGGGCCAGGCTGGCGGAGCGGGACCGG
GAGCGGGGAGCGCAGCGCTGGGGAG
>362
GGCTGGTACCCTGGAGGAAAGCGAGCCCTCAGCTCAGCCCAGGATCCCCAGAATGCC
CTTAGGCCCCCAGGTGGGTGTCTCCCAGCCTCATGGGGAGGAAGAAAGTGATGGCCG
GGGGCTCCCCCACCCTCCTGGAGCCT
>363
GCCCTGGGAGGGCAGGACCACGGCCCAGTGGTCCCTTCACAGGAAGCGACACCTGGC
ACGGACACTGCTGGTGAGTAGGGTGAGAGGTCCCCAGCATCAAGACCAGCCACTGGT
CATCAGAGGCCATTGTGGCTTAGGGT
>364
CAGTCTCTTAAACAAAAGCAAAAGAATAAGCTTCGGGCGCTGTAGTACCTGCCAGCTT
TCGCCACAGGAGGTAAGTGGATCTGGGAGCTGGGGGAACTGAGAAGACTAGCCAGAT
ATTACATGTATTGCCAACTCAAAAC
>365
TTGGATATCACTGATCCCCAGAGCGTCCAGCAGGCAGCCAAGTGGGTGGAGATGCACG
TTAAGGAAGCAGGTAAGTATGGTAGACCACCAGGAATATGGTGTGGGGTGTCCTGATC
CCCACAGTCACCCCAGGAGTCACC
>366
GCCTGGCAGCCAATGGTGGGGGCTACTGTGTCTCCAAATTTGGCCTGGAGGCCTTCTC
TGACAGCCTGAGGTGAGGGGTACAGGGCTCTGGGTTCCAGGACTAACAGCAGCCCAC
TCAACAAACGTGGGCCAGCAGAGGT
>367
CTGCAGGCCTGCTGGGCACGGCTGCCTCCTGCCACACAGGCCCACTATGGGGGGGCCT
TCCTCACCAAGTGTGAGTAGCCAGGCCCACACAGGGGCACATGAAGGGAAACAAGTA
CCAGAAAGGCCAGTCCTGCATAAGC
>368

GCGGTGGAAAAGACCAAGCAGGGGGTGACGGAAGCAGCTGAGAAGACCAAGGAGG
GGGTCATGTATGTGGGTAAGTGGGGCATGGCAGGGTGGGACAGTGTGGTGGCCAAAG
GGTGAGTGCCCAGTTACCTTCGCCAGAC
>369
CAACACTGTGGCCACCAAGACCGTGGAGGAGGCGGAGAACATCGCGGTCACCTCCGG
GGTGGTGCGCAAGGTGAGCCCCGGCCCTCAGACCTGCCCAGTCCTCTCCTGGGCCCA
GAAAGGCTGCTGGGCGGAGGCGGCAT
>370
GGACTTGAGGCCATCTGCCCCCCAACAGGAGGGTGTGGCATCCAAAGAGAAAGAGGA
AGTGGCAGAGGAGGTAGGAGCTGGGCTCCTGGGGTGCACCATGGGGGGTTCCTTGGT
AGGGACCCCATCCCCCACAGACGCAC
>371
TGGATGTGGAATATAATTGAATATTTTCTTGTTTAAGGGGAGCATGAAGAGGTGTTGAG
GTTATGTCAAGGTAAACAAAACCACCATTGTGGTAAAGACCACCAGGAGGGATAACTG
AATATATGGCTTACAAAATGTTT
>372
CTATGAAGGCATTGTCGTTGCAATCGACCCCAATGTGCCAGAAGATGAAACACTCATTC
AACAAATAAAGGTAAGTTCATAGTAATTATCCATACTTTTTTAAAAATAGCATAATGTTG
TGATAGAGAGAACATGAGTGC
>373
CATTTTGATTCCTGAAACATGGAAGACAAAGGCTGACTATGTGAGACCAAAACTTGAG
ACCTACAAAAATGTGAGAATATCAAGTTCTTCTTTAAAATTCCACTTTCTCCTGTAACC
AAGATTTTTTAAAATCAAAATAT
>374
AAGGGTGAAAGGATCCACCTCACTCCTGATTTCATTGCAGGAAAAAAGTTAGCTGAAT
ATGGACCACAAGGTAAGAAATATTCTACCATACTTCTCATACAATTTAACTATTTTATGTT
CAAAGTGTATCAACACTAGCA
>375
GGGGAGTATTTGACGAGTACAATAATGATGAGAAATTCTACTTATCCAATGGAAGAATA
CAAGCAGTAAGGTATGTATATTTTGATTTAACTTGTTCCAAACTATTTTAAATTGCAACC
TTTTTTTCTTGAATTGATTAC
>376
ATGTGAGTTTGTTCTCCAATCCCGCCAGACGGAGAAGGCTTCTATAATGTTTGCACAAC
ATGTTGATTCTGTAAGTACCTTGTTCTCACCCCCTCCCCCAGATTCTTATGAATTTAGTG
AGGCTTTTACTCCAAGTGCTC
>377
CACCTTCTCATTGCTGCAGATTGGACAAAGAATTGTGTGTTTAGTCCTTGACAAATCTG
GAAGCATGGCGGTATGTTCAATGAGTCTTGGTCTTCTGGATGCTGGTCACAGATATGCA
TGTTCTGGACCCTGACTCGGAA
>378
CGCCAAAAGATTACCTGCAGCAGCTTCAGGAGGGACGTCCATCTGCAGCGGGCTTCGA
TCGGCATTTACTGTGAGATGTGTTTTTCTATTGTAGTTTGGAATGTTTGCAATTTATGTTG
CTTAACAAACTTCCATTTCCA
>379
GCCATCATCCACACAGTCGCTTTGGGGCCCTCTGCAGCTCAAGAACTAGAGGAGCTGT
CCAAAATGACAGGTGAGGGATGATTTGCTGAGACCCCCGGTATTGTCTCTCCTACTGG

ACTGTGAGCTCCAGTGAACTGGGG
>380
CAATGGCCTCATTGATGCTTTTGGGGCCCTTTCATCAGGAAATGGAGCTGTCTCTCAGC
GCTCCATCCAGGTTGGAGTTCTTAATCTTTGGTTTTTCATATTTACACATAATCATAGCCA
AGATGGAGAATTTAATGGCT
>381
GAAGCAAGGTGGCTTTGTAGTGGACAAAAACACCAAAATGGCCTACCTCCAAATCCC
AGGCATTGCTAAGGTATGGAGTCAGCTTTTTTTCTTTCTCATAATTCAACAAGATAAAAT
AAAGTTTTTACAGAATAATTGTA
>382
AGTGTCACAGCCCTGATTGAATCAGTGAATGGAAAAACAGTTACCTTGGAACTACTGG
ATAATGGAGCAGGTAATCACCCAAGAAATTGGAAGATATTTATTTCTTTTTCCAAGAAG
CAACCAGGGAATGCCAAGAGGGC
>383
AACGCAGCCAGACGGAGAGTGATACCCCAGCAGAGTGGAGCACTGTACATACCTGGC
TGGATTGAGAATGGTAAGTAATTTGTAATAACATACCTGGCTTGTGCAAAAGCATTGGT
TTCAAGAGGAAACTATTAAGCCTG
>384
GAAATTCACGGGGGCAGTCTCATTAATCTGACTTGGACAGCTCCTGGGGATGATTATGA
CCATGGAACAGGTAAGCTGAACTGGTGTGGACCTGTAAAAGATTTACCAGCCAAGTAG
AAGAGCAGAAGCGGGTGAGGCAG
>385
GTCTCCGAGGCTGCCCTGTCTCTCCTTGTCCTCATCCTTATCATTACTTCGGCTTCTCGC
AGCCAGCCAAGTGAGTCCACTTGTCTCTGTAGGGCAGGAACGAGAAAGGGGGATGGA
GCAGCTGGCAGGGAGGTAGTGCC
>386
AGAAAGTGTTGCCAAGGAGACTAGTGGTGGGATACAGAAAGGCCCTCAACTGTCACC
TGCCAGCAATCATGTAAGCACGTCCTTTTAGATATTTCTGTGGAGAGGGACAGGGAGC
CCAGGGGCAGGAATGAGATACATGG
>387
AGTCAGCTTGCCGCCGGCGGCCAAGCAGCCAACCATGCTCAACTTCGGTGCCTCTCTC
CAGCAGACTGCGGTAAGTCATTTGGGGATGCCCCTGTGCTTCCTCGCCTGGTCTTGTCT
GGGGGGCCAAAGGGGGCGCGAAC
>388
TTTTTACACAATCTCTACTTTGACCAAACGAGTCTGGACAGTTTTCTTTTAATGGAAAAT
AGGAGAAATGGTGAGTAGTACCATTTTTTTTTAGCGTAGTTCAAAGAACTTTACTATAA
AACATTAGGTAGTTCTACAAA
>389
AGTATCTGATTTGTCAGAGGAAGAGAATCTGCTTCCGGGAACACCTGATTTTCATACAA
TCCCAGCATTTGTAAGTATTATTGTTTTTAAGATAGACGTAAATTATATAATTTCTTAAAA
TTTATATAATAAACTGTAGA
>390
AGCCACCCAGGATGTGGCAAGACATACTTTAAAAGTTCCCATCTGAAGGCCCACACGA
GGACGCACACAGGTACCAGCCACTTCTTATATCTTTAGTGTTTAAATGAAGAGATTTTG
GCTGTGATCACACATTTAAACCC
>391

GATCGTCAGCCTCGCAGTGGCCGACCTCCTCGTCGCCACACTGGTCATGCCCTGGGTT
GTCTACCTGGAGGTAGGTGGCCCCCTGCTTGCTCCAGCACTTTCTCCAGCAGGGCCC
TGCACTGGACACTGGGGACTCTAG
>392
TCTTCGTCACTCTGGACGTCATGATGTGCACGGCGAGCATCCTGAACTTGTGTGCCATC
AGCATCGACAGGTGAGCCCAGCCAGGGTGGAAGAGGTTGCTCTGGCCACTCACCATT
CTGGCTGCCCCAGCTTTTCTCAAA
>393
ATGATCTCCATCGTCTGGGTCCTGTCCTTCACCATCTCCTGCCCACTCCTCTTCGGACTC
AATAACGCAGGTACATTCTGCTTTTGTTTGCCTGAGGCTCAGCTGGCCCTGGGCCCCTG
CTCCCTCGAAGGGCCCTGAGG
>394
ACGCCGCAAGCGAGTCAACACCAAACGCAGCAGCCGAGCTTTCAGGGCCCACCTGAG
GGCTCCACTAAAGGTCTCAAGACACCCCCCAACCAACTCCAAGGGTCCCCACCTAACC
ATTACCAAGAGGGCTCATCTTATGC
>395
CGAGGACATGAAACTCTGCACCGTTATCATGAAGTCTAATGGGAGTTTCCCAGTGAAC
AGGCGGAGAGTGGTAAGTGCTCAGGCCAGGACCCAGAGCCAGGTCTTTCTGCCCCTA
GGGAAGCCCACTGGCCATGGTTCTG
>396
ACCATGAGCCGTAGGAAGCTCTCCCAGCAGAAGGAGAAGAAAGCCACTCAGATGCTC
GCCATTGTTCTCGGTGAGTCGGCCCTGGCTGCTGGCCACAGCCCGGTCTGTGAAAGGT
CCCATTCCCTGCCATGTCCTTCCTA
>397
CAGCATTTCATCTGCTATGGCCATGGTTTTTCTGGGGACCAGAGGTAACACGGCAGCAC
AGCTGTCCAAGGTCAGCAGAAACAAAAAAAAGGGTCTGCTTTCCTCCCATCTCCCTAC
GAATGGACAGTCTGCATGGAAAG
>398
ACGTGGAGCGTCTTATATTCTGAAACTTGCTAATAGATTATATGGAGAGAAAACTTACAA
TTTCCTTCCTGTAAGTACTTTGCCCTTCACATTCTAAAACCCCACTAGACAGTGCAGATA
TTTTCCAGCACAATCAAGTC
>399
GTGGATTTTCAGCATGCCTCTGAAGATGCAAGGAAGACCATAAACCAGTGGGTCAAAG
GACAGACAGAAGGTGAGAGCCTGGGCAGAATGGAATCATTATGCGCTGGTCATGTCAT
TCGTTTCCTCAGTCCGCCTTCCCT
>400
TTTCAAGGGAAACTGGAAGGATAAATTCATGAAAGAAGCCACGACGAATGCACCATTC
AGATTGAATAAGGTGAGGTGAGGTAACTGTACAGAGTCATGCATTGCTCTAAAAGAAT
GCAGAGAATAATTTCAGTGATTTT
>401
AGGCGAGGAGCTCAGCATGGTCATCCTGCTGCCGGATGACATTGAGGACGAGTCCACG
GGCCTGAAGAAGGTATGGCTCCTGGGCTTCATGCTTTGTGGTTCCTCTTGCATGTGTGT
GTCGCATGGTAGGTAGGTGCTTG
>402
CACCTCTCCGCCGCCTCTGGCGCCCGGGCTCTGGCGAAGCTGCTGCCGCTGCTGATGG
CGCAACTCTGGGGTAAGGTGGGGGACAGGGGGCGGGGAGAGGCGCCGGTGGGAGGC

ACGGGCGGGAGGGCAATGTGTTCCCG
>403
TCTCGTTCCACTGCGCGGGTGTCCTGGTGGACCAGAGTTGGGTGCTGACGGCCGCGCA
CTGCGGAAACAAGTAGGAGGAGATCCATCCCCGAGGACGCCACGGGGGGCTGTGGAG
GCGGCCTCCAGGGAGGAGCGGGCAG
>404
CTTCTACCCTGGCGTGGTCACCAACAACATGATATGTGCTGGACTGGACCGGGGCCAG
GACCCTTGCCAGGTAGGGTCTGAACAGGGAGAGTCTCTGACTCCTGGGAGGGAGGAC
AGGGAGGTTATGGGAAAAGAGCAGA
>405
CGGTGCACTTCCTCATCTTCCTCACCATGCACGCCAGCAGCTTCACGCTGGCCGCCGTC
TCCCTGGACAGGTGAGCCAGCGCCTTGGCCTCCCTGGGAGATGGGCATCCACGCGGG
GGATGGAGCGGGAGGCGGGACTGG
>406
GCTCCCGCCGGCACCATGGCCAAGACCGTGGCCTATTTCTACGACCCCGACGTGGGCA
ACTTCCACTACGGTGAGGAAACAGGGCTATGCATGAGGGGTTGGGACACGGAGGTTTT
GAGGCGGGGTTGCAACTCCAGGGG
>407
TATGAAGCCTCATCGCCTGGCATTGACCCATAGCCTGGTCCTGCATTACGGTCTCTATAA
GAAGATGATCGTGAGTTCCGCCGCCTCGGCCGGGGGTTGGGGGGTGGGGCGAGCTGT
GGCCGTAGGGATAGGTGGGCTGG
>408
AGAGAGTCAGCCCCACCAATATGCAAGGCTTCACCAAGAGTCTTAATGCCTTCAACGT
AGGCGATGACTGGTGAGGGAGGACTGGGAGTTTTGATGTCTAACAGTTTAATGCCAAG
CCCTTCAGTGAGGTCTTTTGGCAG
>409
CGGGCTCTTTGAGTTCTGCTCGCGTTACACAGGCGCATCTCTGCAAGGAGCAACCCAG
CTGAACAACAAGGTGACATAGTCCCGAGTCCTGTTCTTCCTTTCCTCTGGATCCCTGGA
CTCGGGATTTAACCCTGATCCTG
>410
TTCTACCTCACTGACCGGGTCATGACGGTGTCCTTCCACAAATACGGAAATTACTTCTT
CCCTGGCACAGGTATGGGAATAGGGATTACTTTCCCCTTTGCACCCTTCAGCTCTTTACT
ATGAGTGCCTCCTTCTCAGCC
>411
GAAGTCGGGGCAGAGAGTGGCCGCTACTACTGTCTGAACGTGCCCCTGCGGGATGGC
ATTGATGACCAGAGTAAGTACCGAAGTCTTCCCTCTGTTCCCTAGGCCCCTTCTTTTTCT
CCTTGAAATAACCTCTTCTGCCT
>412
CAAGCACCTTTTCCAGCCGGTTATCAACCAGGTAGTGGACTTCTACCAACCCACGTGC
ATTGTGCTCCAGGTAATACTGTCAGTTCCTCCTCAGGAGTGTTCTGCCTGGGCTTGGGG
AAAGAGAGCAAGGGAAGAGGAGG
>413
AGAGCTTCAATATCCCTCTACTCGTGCTGGGTGGTGGTGGTTATACTGTCCGAAATGTT
GCCCGCTGCTGGTGAGCACTCTCCTGATTTCCTCCCCTGTTGTCCAGTGTGAGCTAGGG
TGTCCCTCAGATATGGCCACAT
>414

GTACTTTGCCCCAGACTTCACACTTCATCCAGATGTCAGCACCCGCATCGAGAATCAGA
ACTCACGCCAGGTCAGCAGCTCAGAAAAGCTGAGCACAAACTTCTCCTGAAAGGTGG
AGCCCAGGAGAGGTCAAAGATGTG
>415
ACCTCCTGACCTATGACAGGACTGATGAGGCTGATGCAGAGGAGAGGGGTCCTGAGG
AGAACTATAGCAGGTCTGGGAGCAGGGACTTCAGCCTACTTTCAATAGGCTTGCTGGG
GAGGTTTAAAAGGGAAAAAGTAAAT
>416
GGACCATCGGAGAGCAGTGCAGCGTGTGTGGGGTCGAGCTTCCTGATAACGAGGCTG
TGGAGCAGCACAGGTAGGCCCCGCTCCAGCCCCGCACCTGATGTAGGACTTGAGGCC
CTCACACCCCTCCTTCACACCCTGGC
>417
GGGTGCGAGCTCTGCGGGAAGCGGTTCCTGGATAGTTTGCGGCTGAGAATGCACTTAC
TGGCTCATTCAGGTAGGCAAGTTCGCCTTAGTGGCCCGTTCAGATACAGGCAACCATCT
CCTGCTTGCCTTTACCCCTCCTC
>418
GTCTGTGATCAGTGCGGTGCACAGTTTTCGAAGGAGGATGCCCTGGAGACACACAGG
CAGACCCATACTGGTGAGTTGACTTGGATTCCTGTTCTCCAGGTTTTCCACAGTGTTGG
TAGCACATGGACATGAACTGTCTG
>419
ATCTGCAGTGAGTGCAACCGCACCTTCCCCAGCCACACGGCTCTCAAACGCCACCTGC
GCTCACATACAGGTAGGTCAGTCCAGCTGATGGGTGGATCTGGGTCTCTGGGAGCCAG
CGTCTATATTTACCTCCAAGGACG
>420
GAGTGCAATGGCTGTGGCAAGAAGTTCAGCCTCAAGCATCAGCTGGAGACGCACTATA
GGGTGCACACAGGTACCGAAGGCCAGGGAGGGGCCTGAGCTGGCTCTGGGACCTGGG
CGGAGGTGGGAAGCAGATAGTCTCC
>421
TGGGTGTGAGGGACACATACATCCTAAAAGCACCACAGCAGAGGAGGCCCAGGCAGT
GCCAGGAGTCAAGGTGAGTGCACGACCTGACTGTGTACCAAGGGCCGTACCCCCAGA
AACAGTGTCAGACCTGGCAGCACCGG
>422
CTGTGAGGCCCTAGAGCACCACCTTAAGAGAAGAAGAGCTGTAAGCCGGCCTTTGTC
AGAGCCATCATGGGTGAGTTTCTCAGCTGAGGCCACTGGCACTGTCCCTCTCTCCCTC
AGTCCTGTGGGATCCCATCATACCT
>423
GGAGAACAACAAACCCCGGAGCCGGAGCCAGGGGAGGCTGGACGGGACGGGATGGG
CGACAGCGGGCGGGGTGAGTGCCCCAGCAGAAGGGCAGGCGAGCCATGGACCGCTTT
ATCTGCGACCCCTCCCCCACACCCTCC
>424
TTCCATGGGGTCCCCTGGTCTGCCCCCTCCAGCTCCCCCAGGATTCTCCGGGCCTGTCA
GCAGCCCCCAGGTGAGAGGTTGTGACCAACTTACTGCCTGGCCACTTTCATTTTCTTGT
TTTCCTTGTAACCCCAGATCCT
>425
TGTCCACCCCCTCCAGGTGGCCCTGGGGCTGGCAAACGGCTATGTGCAATCTGCGGGG
ACAGAAGCTCAGGTATGTGGCTCAGAGGATGAACAGATAGGGAGAGTCTGGGCCATGT

ATCATCACCTGTGGGATTCCCAGG
>426
AAGCGCCAGCGGAACCGCTGTCAGTACTGCCGCTATCAGAAGTGCCTGGCCACTGGCA
TGAAGAGGGAGGGTAAGGACGAGTCCTGTCCACGCGTCTTAGACCACGTTCCCAGAC
TCCCTATACGCCACTCGGACTGTGT
>427
GCTTGCTGTGGAACAGAAGAGTGACCAGGGCGTTGAGGGTCCTGGGGGAACCGGGG
GTAGCGGCAGCAGCGTGAGTGTTGGGGTCAATCCACTCTCCTTCGTGATGGGGGTTGG
GGGAGGCAGTCTAGGTCTGTTAAACA
>428
GAGTGGGCGAAGAGGATCCCACACTTTTCCTCCTTGCCTCTGGATGATCAGGTCATATT
GCTGCGGGCAGGTCAGTGACCTTGGATCCCTTTGACTTCTTGACATTTGACCCCTCTTT
GACTTCCCGATCTTTAGTGACC
>429
TCCTTGCCACAGGTCTTCACGTGCACCGCAACTCAGCCCATTCAGCAGGAGTAGGAGC
CATCTTTGATCGGTCAGTGGCCCTCGGCTAGGCTGGCATGTAGATAGAGGGGGTGGGG
CTATAGGCTGGTCCGTGTCCAAGG
>430
AAAATGCGTGACATGAGGATGGACAAGACAGAGCTTGGCTGCCTGAGGGCAATCATTC
TGTTTAATCCAGGTAAGAGGAGGATTACCTTTGTCTCTCAAGGCCAAGGCAACCTTGG
AGCCTCCTCTTCTCCGAGACTCCT
>431
TCCTGCGGGAGAAAGTGTATGCATCACTGGAGACCTACTGCAAACAGAAGTACCCTGA
GCAGCAGGGACGGTGAGATGGGGCTGGGGGGCACTGAGGCTCCCTTGGGGGTGGGG
AGTGCTATGAGGATGTGTTCAGGGCC
>432
CCAGGCCCGGAGGACGAGGACTCCAGCCTGGATGAATCTGACCTCTATAGCCTGGCCC
ATTCCTACCTCGGTAAGGCCCACTCAGCCATCTCCACGGTCCTTCCTCCTCTCCCGAAA
TCAGGACCCACCCCTCTTGTTTC
>433
AAACTGGTGACCAAGCTGCAGAATTCAGAGAGGAAGAAGCGAGGGGCACGGCGCTG
AGACAGAGCTGGAGGTAAGGAGTCGGGGGGCCCAGAGAGCTCAAGGTGGTGCTTCTG
CCATGAAGGACAGGCCGGAAGGTGTGT
>434
CACCATGGGGCGCCTGCAACTGGTTGTGTTGGGCCTCACCTGCTGCTGGGCAGTGGCG
AGTGCCGCGAAGGTAAGAGCCCAGCAGAGGGGCAGGTCCTGCTGCTCTCTCGCTCAA
TCAGATCTGGAAACTTCGGGCCAGG
>435
ATCGTGGTCACCTTCAACTACCGTGTCGGCCCCCTTGGGTTCCTCAGCACTGGGGACG
CCAATCTGCCAGGTGCGTGGGTGCCTTCGGCCCTGAGGTGGGGCGACCAGCATGCTGA
GCCCAGCAGGGAGATTTTCCTCAG
>436
CAGTGTCTGAAGGTTACTGATCCCCGAGCCCTGACGCTGGCCTATAAGGTGCCGCTGG
CAGGCCTGGAGTGTGAGTAGCTGCTCGGGTTGGCCCATGGGGTCTCGAGGTGGGGGTT
GAGGGGGGTACTGCCAGGGAGTAC
>437

ACATGGACGGCCACATCTTCGCCAGCATCGACATGCCTGCCATCAACAAGGGCAACAA
GAAAGTCACGGAGTAAGCAGGGGGCACAGGACTCAGGGGCGACCCGTGCGGGAGGG
CCGCCGGGAAAGCACTGGCGAGGGGG
>438
CCCAGCCACCAATGGCTCTGAGCTGGTGACAGTACAGCTTATGGTGTCACTGGCCCAG
CTCATCAGTGTGGTGAGTAGAGGTCCCAGGCTCTCTGCCCAGCTACTGAAATCAGCCC
CGCCAAAATGTGTTAATGCTTGTG
>439
TTGACAACATGAAGAAAGTTCGGCTCCCTTCCAAACACATCTGGCTCCCAGATGTGGT
CCTGTACAACAAGTAGGTGCAATGGGAAGGTTGGGGGAGACCATTGGAGGGCTCAGG
GAGGGAAGGGGTTCTGGGGAAAATA
>440
CCTCCGGGAGGCGGTGGACGGCGTGCGCTTCATCGCAGACCACATGCGGAGCGAGGA
CGATGACCAGAGCGTGAGTGCCGCAGGCTGGGACCCCGGGCGTGAGATATGGGGTCT
GCCAGGGCCCGGGTATCTGGAAAGCA
>441
GTCACTCGGCCAGAGCACTTCGACTTTGTAAAGCCTGAGGACCTGGACGGCATAGGCA
TGGGCCGGCCTGGTGAGGGGACCCTGCCCCGAGGCCCTGGTCTCTCTGTCCACAGCCT
ATCAGTTGCCTTCCCCCACCACAA
>442
CCAGCGCAGACTGTCCGAAGCTCTGAAAAGGCTACGTTCTGGGCCTAAGTCTAAGAAC
TGGGTCTACAAGGTGTGTGTTGTAGGTGGGCAGCTTGGGCCTGGGAATGAGGTGGCTT
GAGGGGCAGGGAGGGGGGCGCAGG
>443
GGAGCTGCTGGGTTCAGGCTGCTTCGGTGTGGTGCACCGAGGGCTGTGGACGCTGCC
CAGTGGCAAGAGTGTGAGTGTCCAGGGAGCCCGCTTCATCCAGGCCAGCTGCCCCCT
CTGTTCTTCATGCCCGCAATGCCTAA
>444
CATGATGAACTTGGAGCACCCACACGTGCTGCGTCTGCACGGCCTTGTACTGGGCCAG
CCTCTGCAGATGGTGAGCAGATCCAGCCGCTGGTTCCCGGGACAGCCGTGCGGCAGG
AGCGTGGGCGGCCAGGGTCCAATGG
>445
TGGTGCGGCCTCTGGGCGGTGCCCGGGGCCGCTACGTCATGGGCGGGCCCCGCCCTAT
CCCCTACGCCTGGTGAGAGCGGGTCCGCGGGCGGTCGGGCTCTGAGCCGGGCGGATC
CGGAGGGCAGCAGCCGAGGGAGATC
>446
CTTGCGCTGCTGGGCCCCCCACCCTGCCGACCGGCCTAGCTTTTCCCACCTGGAGGGG
CTGCTGCAAGAGGTGGGAACCCCCGACCTCACCAGATACACAGTCCCTCTTCCCTCCA
TTCGCTCTCCCAGGGTTCCATGCG
>447
TGAGGGATGTCACAGAACCAGGCGCCCTGAGGATGGAGACTGGTGACCCCATCACAG
TCATCGAGGGCAGGTGACAGTCCCATACCTCCCAAGCACCCTCAGGAGGAGGATACCC
TCCCAGCCTAATTCCTGATCCCTTC
>448
TGCCAGCCACCCGTCCAGTCCACAGAGGCACCCCTGCCCGGGGAGATCAACACCCAG
GAAGCATAGATGGGTGAGGACCTGAAAGGGTGAGGGCAGGGGTTGGCTGGGAGAACT

GATGAGGTCCAGGAGCTTCTCTGGAA
>449
AAGGCAAATCTTTGGGATGCGCCCCCAGCACGGGGCCAGAGGAGGAACATGCCCCTG
GAGAGGATGAAAGGTGGGTGTGGTGGACTCCAGAGTCTCTGAGGAGATCAAGACCAG
TCCTTCAATTCCGACCCCCTGCCCTA
>450
AGCCCGTAAAGCCGCTGCCCTCTCTGGAGGCCTCTTGTCCGATCCTGAGTTGCAGAGG
AAGATTATGGAGGTGAGGTCTCACTGAAATGGCCTGGTGTCCAGAAGGGGCTACAGGC
AGGGGCAGGGGCCTGAGTGAGGCT
>451
CCACCAGGAGTGCCAGACAGCACTAGGAGCCACTGGGGGAGATGTGGTTTCTGCCAT
CCGGAACCTCAAGGTAAAGCCAGCCCCTTCTCTTGGGGTCCCTCCTCTCCTGCCCCTG
CCGCCTGGCAGTCAGCTGCAACCCA
>452
GCTCACACAGCTCCTCCTCAGGCAACGGCAAGGACTCAGCCCTGCTGGAGACCACTG
AGAGCAGCAAGAGGTGTGTATGGATGTGTATGTTGGGTCTAGGAATGGTCTTGTGAGC
AGGCAGAGCTGGGTGACAGGCCTCG
>453
GCAGTTCCATTGCCTACAGCCTCCTGAGTGCCAGCTCAGAGCAGGACAACCCGTCCAC
CAGTGGCTGCAGGTACGTCGGGTGAGGCTGGAGGAGAGGTCTGGGGGCCCAGGCCAC
ACTCTGGGACTAATGTCTTTATCTC
>454
CGGGGCAAGGGCCGCTCTGGGACCCTGGCCACGCTGCAGTACGCACTGGCCTGTGTC
AAGCAGGTGCAGGGTAAGTGGTGTGTCCCAAGAGGGAATGGCCTGGGCCTGGGCTCC
CGTGTTGAGGCAGCCTCCTTACTGAA
>455
CATGGACATGTCCACCTATACCCTGGAGGAGCTGGAGCACATCACGTCTGAGTACACA
CTTCAGAACCAGGTCAGCGGCGGCCTGGCCTCTGCATCCCGACGCACCCCTACTCCTT
CTCCCACAGCTCTTGAATCTGCTC
>456
GATGTGGGAGTCTTCTATGGTTCCACTGCTCCATCTCGCCTGCCCACCTGGGGCACAGG
GGCCTCAGCAGGTGAGGTGCCCAAGCGCTGTGGGGAGGCATGAGCAGTCCCAGGAAA
TTCCTGCTGTGAGGGGCCGGGGAA
>457
GTCTCAGATGGGGCCCCTGCACAGCCGTGCTGCCTGCTGATTGCAGAGCGCATCCATT
CGGGTTACGAAGGTGGGCAGGTCAGGGCCCTGGCCTGGTGGGGGCTGGGAGGAAGG
ACATTTCCTCATGGCCAGACATGAAT
>458
TACCCCCTGACAAGAGGATTTTCACTACGCGGCACACACCCAGCTGCCTCTTCCAGGA
TGTGGATGAAAGGTGAGGATAGGACCTAGAGGAGACGAGGGCAGCCAGGGCTGAGG
CCACGGCCACTCTGATGCCTCCTTCC
>459
CTGGGGGCCCCAGTGCTCCTGTTCCTGCATCCTGAGGACCGACCCCTCATGCTGGCTAT
CCACAAGAAGAGTGAGTTCCTCTCGCCCTGCTCGCCCTTCCCACTGTCCTGGGCTTTT
GAGTGGCTGCCCTTGTGGTTGTG
>460

GCTGGGCTGGCTTTGTGCACCCCTGGAGCCGCAAGGTAGCCTTCGTGTTGGGCCGCCA
CAAAGTACGCACGTAAGTGGGCCATGCCCCCGAGCTGGCGTTGGGGATAGGGCAGTG
CGGTGGGGGACAGGACCGGGCCAGG
>461
CAGCCCAGCTCCCTCCCTGGACACTGATATCCAGGAGCTGTCAGAGCAGATCCACCGG
CTGCTGCTGCAGGTGAGAGTAGCGGAGAGGGAGCCTGGGAGGTGAGAAAAGGTGTG
GGAAGCGGGGTCAAGCCATCTAACCT
>462
TCTCCACAGCCCTGGGTCCTCCAGTGATAGCAACGGGGGTGATGCAGAGGGGCCTGG
GCCTCCTGCGCCAGTGAGTGACCTGCTCCTACCTACCCTCTAATCGCCCTTTCCCCTCT
CCTTCTTGGAACCAGCACTGCCAG
>463
GTGAAGCACCAGGGCCAGCAGCTTTTTATTGAGTCTCGGGCCCGGCCTCAGTCCCGGC
CCCGCCTCCCTGGTGAGTTGATGATGATGTGTGGGTGAAGAGACAGACCTGGGGGGTC
CCCTGACCTTCACCCCCAACCCAT
>464
AGGAGGCCGAGAGGAAAGAAGCCTCCAGCTGCTCCTACCAGCAGATCAACTGCCTGG
ACAGCATCCTCAGGTAAGGCCTGCCAGGCATCTTCTCCACCTCGGGCTCTAACCCTGC
CCACCCCAAGCCCTGCTCTGATGCC
>465
TCCGTCACCAGTCAGTGTAGCTTCAGCTCCACCATCGTCCATGTGGGAGACAAGAAGC
CCCCGGAGTCGGGTATGGGTGTGGAATTGGGGGGCAGTGCTTGGGCTCCACCGGTCTC
CCAGCTAGAGCTCCCTCCTGCCTT
>466
TTCCGAGACCTGGGCAGGCTGCGTGGACTCGACAGCTCTTCCACAGCTCCCTCAGCCC
TTGGCGAGCGAGGTAGCCACCTGGGGCCTCCTGGAGCCTGCCCTCTGCCCAGTCTAGG
ACTGGATTGTTGGGGGGTGGGTCT
>467
AGGAGGCCCTGGGAGCAGTGCCGGGCCCCCACCTCCCAGTGCGGAGGCTGCTGAGCC
AGAGGCCAGACTGGTGAGCACTGACCCCTGCGTCTGCCTGCCAGCCCCCACCCCAGC
CCCGCCCCTCTGCCACCCTGTGCTGC
>468
GGCTCTGGCTTGGGCTCTGGGTCTGGTTCAGGCTCCCATGAAGGGGGCAGCACCTCAG
CCAGCATCACTCGTGAGTACCCCGCCTCCAGCATCTCCCAGGGTAGGGCAGTGATTGG
GGAGCCGGGAGCCCAGGCCCCGTC
>469
AGGATCCCATTTGGCTGCTCATGGCCAATGCTGACCAGCGCGTCATGATGACCTACCAG
GTGCCCTCCAGGTGAGGCATTTCAGAGGCCTTCTTGGCCTTCCTTTCAGAGGTAGAGT
GGGGGCAGTTTTCTTTTCTGGGG
>470
GCGGCGGGAACTGGGTGCTGTGCACTCCTGGGTCCGGAAGGGCCAACTGCCTCGGGC
TCTTGATGTGATGGTGAGAGAAGCCTGGGACGGGGAGAAAAAAGAATTGAGCTCAAG
TTCAAGGGGGAGAAAAAGAATTGAGC
>471
AAAGTTGCTGACCTCGCCGTCCCTAAAGCCCTCTGCAGTTAGTAGTGTGACATCGTCTA
CCTCCTTGTCAGTGAGTATCTGTCATAGCAGCCTGCCCTGCCCCACTCACAGGGGCCTT

GGGGATGAGGTTGCTTGCCTTG
>472
GCAGGGGTCTCCAAGGATGCCATCGTCACAGGCCCTGCCCCCGGGTCCTTCCACCATG
GCCTTGGCCACAGTAAGTGCTTCTTTGCTGCCTCTGGTCACTCAGGAACCTCTAGATTG
TGGCCAGGGGTCCTGCTGTTGTG
>473
TCCAGCCCGCCCCATGCAGCGCCTCTCCCACACGCTGCGGTGCCCACCCATATCCCGC
AGAGTCTGCCAGGTAATCACCCGACGGTCAGTGTGCCACGCGCACCGTGTGCCTTTGC
CCTCTCCACCCCTAGGTGCTTTGC
>474
GCTACCTGGCGGAACTGGATTTCTCTCCCGCCTGCCGGCCTGCCTGCCACAGCCGGAC
TCCGCCACTCCGGTAGGATTCCCCGCCTGTCATTCCCTAGCCCAGCTCTTGGGAAACTG
CAGAGGGGTCCAGAGGATTTGCA
>475
GCTGCCACCTTTGGGGCCGATGACTTGGTACTGACCCTGAGCAACCCCCAGATGTCAT
TGGAGGGTACAGGTGGGTCTCAGCGGGGTGGGATGGGGCACGGAGTGGGAGACAGAT
CCATCTAAGGGCCTGTTAGACAAAT
>476
GCCCTTGAGGAGCTGCGTCTGGTCTTTGGGCCTCTGGGGGACCAACTCCATGCCCAGC
TGCGAGACCTCAGTGAGTCCAGGCCCCTGGAGGCTGGGGAGCAGCTCCACATGTTGA
GCTGAGTCGAGTTCAGTGTGGCCGT
>477
AGTTGGATCATTGAGCTGCTGGAGAAGGATGGCATGGCCTTCCAGGAGGCCCTAGACC
CAGGGCCCTTTGGTGAGAACCCGTTTTCTCCTTCCTTCCCCAGCCTGTCTTGTCCCATC
CCTGCCCCTCCACAGAGTGCTAG
>478
AGCCCCTACCACCCCGGCAGCTGTGGCGCAGGAGCCCCCTCCCCTGGCAGCTCTGACG
TCTCCACCGCAGGTGAGAGCTCTCTCTGGGCCACAACCTCCCTTCCCCGAAGTGTCCC
TTGTTCCCTCTGGCTCCCAGCACC
>479
TCCCACTCCTCAGACTCCGGTGGAAGTGACGTGGACCTGGATCCCACTGATGGCAAGC
TCTTCCCCAGCGGTGAGTCGAGGGAGGTCCCCAAGAGGGCGTCCCATTTAGCAATGCA
CAGGGGGCCCGGCTCTTCCTGCAG
>480
AAACGAGGCCGGCCCCGAAAGCTGAGCAAAGAGTACTGGGACTGTCTCGAGGGCAA
GAAGAGCAAGCACGGTGAGCTCCGGGGGCACGTGGGTCCTCCCTGCGCCGGGCTGAG
CGGCTTCCTGGGGCACTGCGGGTTGTT
>481
TGGCCCAACTATGGGGCCAAAAGAAAAAGAACAGCAACATGACCTACGAGAAGCTGA
GCCGGGCCATGAGGTGAGCTGGCGGCCAGGACCCTCACGATACAGCCGGACATGGGG
ACAGGCGCTCACACTCCCACCGCCCT
>482
ACGAATTGTTCTGCTCGGGCGTCCTGGTGCATCCGCAGTGGGTGCTGTCAGCCGCACA
CTGTTTCCAGAAGTGAGTGCAGAGGTAGGGGGAGTGGGCAGGGCCTGGGTCCGGGGG
CGGGGCCTAATATCAGGCTCATCTT
>483

AGCATTGCTTCGCAGTGCCCTACCGCGGGGAACTCTTGCCTCGTTTCTGGCTGGGGTCT
GCTGGCGAACGGTGAGCTCACGGGTGTGTGTCTGCCCTCTTCAAGGAGGTCCTCTGCC
CAGTCGCGGGGGCTGACCCAGAG
>484
CTATGACCCGCTGTACCACCCCAGCATGTTCTGCGCCGGCGGAGGGCACGACCAGAAG
GACTCCTGCAACGTGAGAGAGGGGAAAGGGGAGGGCAGGCGACTCAGGGAAGGGTG
GAGAAGGGGGAGACAGAGACACACAG
>485
GGGAGGGGAGGCTTCGCGGAACGCTCTCGGCGCCAGGACTCGCGTGCAAAGCCCAG
GCCCGGGCGGCCAGGTGAGGCCAGCGTCGCTCGCGGCATCGGGGCGCCCCGCTCCTT
CCGCAGACCCCGAAGTGGGGCGCAGGG
>486
GTCATTGGCTTTGCTGTGGACCTGGCCAACGTCTGGCGGTTCCCCTACCTGTGCTACAA
AAATGGTGGCGGTAAGTCCCATCTCAGCCTCCCTGAGCATGCTGGCCGGGCGGGGGTC
GTAGGTGCAGCCGGGGCCAAGGC
>487
GAGCTGGCCCTCGGCCAGTTCAACAGGGAAGGGGCCGCTGGTGTCTGGAAGATCTGC
CCCATACTGAAAGGTAATATGCGGGTGCCTCCCTTCACCCTGGCGCAGCGCGGATCATG
GTGGTGACGCTGGAGCTTTCAAAG
>488
GTGACTCCAGTGGAGACAGCTCGGGCCTCAACGACACTTTTGGGACCACACCTGCTG
CCGAGTACTTTGAGTAAGTGGGAGTCGGGTCCTCGGGAACGGGAGAGATGGCGCAGC
CAGGTCCCCCATGGTAGCCCCTTGGT
>489
AGCCTGCCTGGTGCTGGTCATCGTGCTGCTCTACTTCAGCCTCTGGAAGGGCGTGAAG
ACCTCAGGGAAGGTGAGGCTCGGGGGTCACCAATTGGGCCTGTAGACATGGGGCCAC
CAAGTGGACATGTGGCCGTGGCTGG
>490
CCTCCCTGGAGCCATAGACGGCATCAGAGCATACCTGAGCGTTGACTTCTACCGGCTCT
GCGAGGCGTCTGTGAGTACAGTCTGCTCACTGCTTCACAGTCCACAGGGGCTCTGGCA
TTCCCAGGTTTCCATATGCATTG
>491
CCCTGGGCGTGGGGTTCGGGGTGCTGATCGCCTTCTCCAGCTACAACAAGTTCACCAA
CAACTGCTACAGGTGAGCCCCTAGCAGGGCCAGGCAGGGGCCTCATCAATACTAGGGA
GAGGTCGCTGACTTCCAGAAATAG
>492
GTCGTCTTCTCCTTCCTGGGGTACATGGCACAGAAGCACAGTGTGCCCATCGGGGACG
TGGCCAAGGACGGTGAGCCCCTCCTGCTGCACCTGGGCCTGCTCCGTGTAGCACCAGC
GCCGAGCTCTCAGCAAAGCCTTTT
>493
GCTCCCTCTGTCCTCAGCCTGGGCCGTGGTCTTCTTCATCATGCTGCTCACCCTGGGTA
TCGACAGCGCCGTGAGTAACCCGCACCGCCCGGCCCTGTCCTCGGCAGTTGGGTTCCT
TCCACCCGAGGGGGCCCTGCCCT
>494
ACACCGTGAGCTCTTCACGCTCTTCATCGTCCTGGCGACCTTCCTCCTGTCCCTGTTCT
GCGTCACCAACGTACGTACCATCGCCGGCTTCGCCTTCTCCTGGTCATGTCTCCTTAGC

ACGTGGCACGGGGCGCCCTGGC
>495
CATTTTGCAGCCGGCACGTCCATCCTCTTTGGAGTGCTCATCGAAGCCATCGGAGTGGC
CTGGTTCTATGGTGAGTCCGGGGGGAGGCAGCCAAGCCGGCACCCTCTTGTTTGGTGG
CTGTGCCACTCCCCTTCTGAAAA
>496
GATGACCGGGCAGCGGCCCAGCCTGTACTGGCGGCTGTGCTGGAAGCTGGTCAGCCC
CTGCTTTCTCCTGGTAAGGGACCTCGGGCCACCTGCCCTTCCCCCGTCTGCCCACTGCC
CCCACTCTGGCACCTGCCCCGAGG
>497
ACTGGCCTACGCCATTGCACCCGAGAAGGACCGTGAGCTGGTGGACAGAGGGGAGGT
GCGCCAGTTCACGGTGAGGTCGAGGTCCCTGCTGGGCCTCTCTCGGGGGAATTCAGAA
TGATCCCAAGCTCAGTGTTCTTAGC
>498
CGGGGAAGAGGAGAAGGCACCCGCGGAACCGACGGCTCAGGTGCCGGACGCTGGCG
GATGTGCGAGCGAGGTAGGTGCAGAGCGGGAAGCGAGCGAAGTGCGGCGTCTACGCC
GCCGGGAGCCCCCGAGGCTTGTGTCGG
>499
TGATGATGAAAACAAAATAGCAGAGAAACCTGACTGGGAGGCAGAAAAGACCACTGA
ATCTAGAAATGAGGTAAGCCTGTCAGAATATTCAAGCCTTGCCGTTTTGGTTTTAATGTA
GATTTTGTGGCATTGACAGTTTG
>500
GACAGATACTTCTTTCTCTCTGGAAGACTTATTCCAGTTGCTTTCATCACAGCCTGAAA
ATTCACTGGAGGTAATTGGAACTTTGGCTTTTATCCTCGCAGGAACATATCTGCACTGA
CCTTTTGGTGAACAAATACAAC
>501
GAACTGCTGGATTAATGGTAGCAGGGAACTGCCTCCTCTCCCCACCAGCACCATGGCT
GGCATCGCTCAGGTGGGCAGGGTAGAGTAAACAGGAGGCATAGCTGCAGCTTCTGTG
GCAGAGCTTGCCTTAACTTCTCATT
>502
GACTCATGAAGGTGTTCGTCACCCGCAGGATACCCGCCGAGGGTAGGGTCGCGCTCGC
CCGGGCGGCAGAGTAAGAGCCTCGCGCGCCGTGGAGGAGGGAGCAGGGCGGTCCCA
GGGACCGGAGAGCCGGGCGGGGCGTT
>503
GTGGCGGGGGCCCACGGCCTGCTCTGCCTCCTCTCCGACCACGTGGACAAGAGGATC
CTGGATGCTGCAGGTGCACACTGGGTGGGCAGGGGACTTGAGGGTGGCTTGGCCCTG
TGGGCCCCTCAGCCTGTGCTGCTGGC
>504
CCAATCTCAAAGTCATCAGCACCATGTCTGTGGGCATCGACCACTTGGCTTTGGATGAA
ATCAAGAAGCGGTAACTGCAGCTTGGGATCTGGAGGGGGCCTAGAGAGAGGGGTGGC
TATGAGAGAAAGAAGAGCTGTTGA
>505
CCGAACTCGCAGTCTCCCTGCTACTTACCACCTGCCGCCGGTTGCCGGAGGCCATCGA
GGAAGTGAAGAAGTAAGTGAACGCAGACCAGGTGCGGTGGCTCACGGCTGTAATCCC
AGCACTTTGGGAGGCCAAAGTGAGC
>506

AAGCCCCTCTGGCTGTGTGGCTATGGACTCACGCAGAGCACTGTCGGCATCATCGGGC
TGGGGCGCATAGGTGAGGCTCCCACCGGCCCGCTTGCCCGCCCCGGCTCTCACAGCGT
GGTTTGCATCCCTGGCACCACGTG
>507
GTCCAGAGATTTCTGTACACAGGGCGCCAGCCCAGGCCTGAGGAAGCAGCAGAATTC
CAGGCAGAGTTTGGTAATAAGCCTTGATTCCCAGAAGCCTATCTGTGCCCAGTTGAAA
GGCTGTATTTTCTCTCAATGGGGCA
>508
CCGAGGGACTCTGCAACAAGGACTTCTTCCAGAAGATGAAGGAAACAGCTGTGTTCA
TCAACATCAGCAGGTATCCTAGGGCCACCTTACCCAGCAAGCCTGGAGAGGAGCCATC
CCCACTGCTGCCATCTGGAGATTTT
>509
GCTGGACTGGATGTGACGAGCCCAGAACCACTGCCTACAAACCACCCTCTCCTGACCC
TGAAGAACTGTGGTAAGAACTGCACTTTCTGATGCAAACTCCCTGCTGCCCTGCAGGA
CCAGTGTTTTTGAGGGGGTCCCAA
>510
CCCGAGGCCGTTCCGGGTCTGAACTGTAACAGGGAGGGGCCTCGCAGGAGCAGCAGC
GGGCGAGTTAAAGGTGTGTACGTAGTTTTTCTAAATAGGACAGCGCTTTGCAAATTGGC
TGTCATGGACTTGCTGTTTTGACA
>511
CAATGCCACCTTCTGCCTCATCTCTGGCCACCGTCCCGAGGCTGCCTCGCGCTTCCTCC
AAGCCCTGCAGGTACAACCCCAATTTGAGCATCACCCATCCTCTGTCCCCCAGCACTCA
TGAGCTGGCAGGGTACCAACCT
>512
GCTGCCCTTCTCCGAGGTCATCGACTGGACCAAGGCAGCCATCGTAGCTGATGAGAGG
CTCCCACTTCAGGTAGCTCAGAGCCCTGCCCACAGGGTGGGAACTGGACCCAGGACA
GGGGAGGGACCTAGAATCCTGGATC
>513
GGGGCACTCCTGGCCCTGTCTACTTTTTCCACAAGCCCCCAGGACTTCCCCTTCTACTA
CCTGCAACAGGGTATGCCCTGGGGATGGGACAACCTGAAGTCCCCTCAGCTCTACCCA
GGGCTGCCCCAGCCTCATCTGGA
>514
GGTGGGGCCCCCAGGCCAGCCCCCTCTGAAGCTCATCCAGGCGGTGGCAGGCTCCCA
GCACTGTGCCCAGGTCTGCCCCCTTCCCAGCTGGAGTCCTGGGATCCAGGGTGGGGGT
AAGGGACAGGGCAGGGCCCTGATGA
>515
TGAGAGGCCACTCCCATCCAGGTGGCCGGAGACAGCTGTGCCCTTGACAGTCATTGAT
GGGCACAGGAAGGTAAGGGATGAGGAGAGCCATGAAAGGGGTGGGCCCATGCTGGG
AGCTGGGGGAGGGCACCCATGGGGTG
>516
CCCATATAGCACCATCAGAACAGATGCCATCCTCAGCCTCGATGCCCGCAGCAGTCTTT
CCACAAGTGAGGTGAGGGCTGGGCCCAAGAGAAGCCCAGTGTGGGTAGACACAGAG
CCAGAGGGCCCTGGCAGCCCCTCAG
>517
GCTGGGGCTACACTGCTGAGAGGACCAACGAATTCTCCATGGTTCTCACCACAGCCGC
CTTCTACCATAGGTACAGACCCTACCCTGCACAGGGATCAGAGTATCAGAGGCCAGAG

ACCACCCCCACCCCGAACGGAGCA
>518
AGCAGCAGTCACCAAGCTGCCCCCTATCAAGGTGCCCTATGGCAAGCAGCGCCAGGA
GGCTGCTCCACTGGTGAGGGCTGAGGGGGATTGGTCGGAACTGGCACGGATTGGGCG
GGGATGCCGGAGAGATCCCTCTCACT
>519
TGGGGGAATGGACCATCTTGGAGAGGCTGCTAGAAGCCGCGGTGCAGCAGCACTCCA
CTATGATCGGAAGGTAAGGGCGCCAAGCCGGCGGTCAGGGGGCCCCGTCGGGCGGCC
CCCGGGAGTCCCTTCTCACACCTGGA
>520
TTCCTCGTCAGGAAGCCCTCCGACCCCAATCGGAAGCCTAACTACAGCGAGCTGCAGG
ACTCTAATCCAGGTGCGTTGGAGGGGTTCTGGGCTCCAGGAGGTTTGGGGGAGACAG
GCGAAGGCTGCGTGGGGGGCACCTG
>521
GTCTGCGGAACCTGCGGGAAGGCCTTCTCTAGGCCCTGGCTGCTACAAGGCCATGTCC
GGACCCACACTGGTACGTGCCCCTCCAGGCGCCCCCACCGTTGCTCTCTCTGGCAGCT
TTTGTGAATCTGGGCTTGCTGTTC
>522
GCCACTTTCAGTCTTCAAAGAGAAAGATAAGAAATTCTGGATTTTCAAAATCCTTTTGA
AGCCTTTTAAGGTAAGATGAAATATCCTTTTTACTCAGAACCAACTGATTCATTTAGAA
AGAACTTTGAATTTCAAAGATG
>523
ATTATTATTCCTAAAGAAAATGAAGATAATTACATATTTTTGCATTTGGGCAGTAGCATG
GGCCATTCCAGTAAGTATGCCTTTCTTAGAAAACCTCTTCACTTTGTTATCTTTTTTAAC
CTAACATTAATACAAAATGT
>524
ACCACTGGAGAGACATGTCGAAAAATCCATGAATTTGCATCTCCTAGCAAGATCAAAT
GTGTCAGTACAGGTATAGGATGTAATATATTTCATTTTATTTCCTATTTCTGAGTTGCTAC
ATTCCATTAACTTCTCCAAGA
>525
AAGCAAACGCGTAGAAAATAGAATCACCAAAGAATCAGAGACACATGCTGTTGGGAA
GAGCCAAGATAAGGTTAGTTTGTAAAGCTGATTTCTTTCAATGGCAGTTTAAATTCTTC
CCCTCCATCTATTGATGCTAGCAC
>526
GGTGATTGTATCCAACTTCAGTCGCATCTACCACCAGAATCAACGAGCAGACAAACGA
AGGGCACAAAAGGTGCGTATTCAACTCCGTGCAACCATGGTTTAGCACTTCCCACTTT
TTATAATGAGCTTTAAAATTTCTT
>527
AGCCAAAAGCGGAAGCGCAAATGCTTACATGCAGAGCAAACGGAATGGTTTACTCAGT
AATCAGCTGCAGGTACAATCAATTACATCTCTTTTTTTAATGTTCAATTTCTTGGTTTAAT
ATTGATTTAGTTCTACTTTCC
>528
TGTTAGCAAATCCGGCTCCAGCTTTGAAACCCAGCACCACCACCTGCTTCACTGCCTG
GAAAAAACCACGGTAAGGAGACAGCATGACTGCCTTCCCTTGCTCTCTGACAGTAATT
CCATTTGCTTTTGTGCATACTTAA
>529

ATCAAGGTAGTATACAAGAACTCAGCACGATTCAGATCAGATGTGTGGAGAGAACACC
TCTGTCTAACAGGTACCTGAGATTAATCTGTGTTGTCTACACACCTGTGCTGGTTCCCA
GGGTGTGTCTTCTGCACTCATGT
>530
CTCGGTGCTCAACAGCCTGGTGGGGCCTGCCTGCATCTTCCTGCGGCCCAGCATTGCC
GCCACCCAACTCGTATGTACGGCCACCCGCCCGCCAGCCTGCCTGCCCTTGCCCCTCTC
CCACCAGCTCACCTGCCCTTGCC
>531
GGTGCCTGCATGCGGACCCTGGGCTACATGCCCACCGAGATGGAGCTCATCGAGATCT
CACAGCAAATCAGTATGTGCCTCGGACCCATCCCCATCCCCAGTCCTCCAGATGCCCCC
GCTGGGCCCATGAAGCCAAGCAG
>532
GGGCCCCAAGCTGCTGGCAGAGACGGCAGACATGATCGGTGTCCGGGAGCTACGGGA
CGCCTTCCGGGAGGTGCGGCCACTGGGGCTGAGGGCGGGCAGGGCTGCGTGTCTGCC
GTGAGTGAGGGGCTGCGCGTGTGAAT
>533
AGCCAGCGGGAGGTGGACGAGATCCTCCAGGACGTGGACCTCAATGGGGACGGTCTG
GTCGACTTCGAAGGTACCCCCTGCCGCACATAGCAACACACGCCCTGGAAGGGTCCTA
GCAGATAAGAAACCGCACAGCAAGA
>534
TATGCTGGGAGCGGTGAAGATGGAAGGGCACGAGCCGTCCGACTGGAGCAGCTACTAT
GCAGAGCCCGAGGTAAGCGCTCGTCTTTTCCAGGGGGAAGTGGAGCGGCGGCGCTCG
CTCCTTCCCCAACCTCAGGACCGCT
>535
CCAGGAAGATGTCATTGCCACAGCCAATCTGAGCCGCCGTGCTATTGCAGATATGCTTC
GGGCTTGCAAGGTAGAAATCCTTGAGGCATTTCCGTGAGGCAGCTTTGGGCCAGAACA
TAGTGGGAGAGAAACTGTAGGGA
>536
TTGACAGGACATTCAAAGCGTGTGGCTGGTTCCGTCACTGAGCTCATCCAGGCTGCTG
AAGCCATGAAGGGTGAGGGGTGACCAGGGACAGGGACGGATGGAAAACCCTTATCAT
GGTGTGATCTCTGCTTCCTGGCTGG
>537
GGGAGCTGCAGCCGCCATTGAGGCTGCAGCCAAAAAGCTAGAGCAGCTGAAGCCCCG
GGCCAAACCCAAGGTCAGTCCTGCTCCCTTCCTCTTCCACCCTCACCATGGCAGTGTC
CCCACCTTCATCTCTGACTTTACAT
>538
TGCAGCAGCCACCAGTGCACTGGTAAAGGCTGCGTCGGCTGCCCAGAGAGAACTAGT
GGCCCAAGGGAAGGTGAGTGCTTAAATACCCAGAAGAATGGGGACAGGTTGGCACTT
TGATGTCCACATCCACATCCATAGCC
>539
CACAGCCCAGCTCCTTGTGGCCTGCAAGGTCAAGGCTGACCAGGACTCGGAGGCAAT
GAAACGACTTCAGGTGAGAACCCTGACCCATCCCATTGTCCCTTGGGCTATACTGCTTC
AAACTGTCACTCCCGAGATGTATG
>540
TGCAGCCTTTGAAGAGCAGGAGAATGAGACAGTGGTGGTGAAAGAGAAGATGGTTGG
CGGCATTGCCCAGGTGAGCTTCACCCCAGATGCTGAGGGACGCTGTCAGGAGGGCAG

TCATGTTGCCTGCGCTGGTCCCAACT
>541
GGAGAAGAAGGGACCCCAGGTCAGAGACTGGAGCCATTACTTCAAGATCATCGAGGA
CCTGAGGGCTCAGGTAAGGGGTAGGAGGGACCTCAACTCCCAGCCTTGTCTGACCCTC
CAATTATACACTCCTTTGCCTCTTT
>542
CTGTGGACAATGCCCGCATCGTTCTGCAGATTGACAATGCCCGTCTTGCTGCTGATGAC
TTTAGAGTCAAGTAAGTTTGGGGGCTAGAGAGCTGGGGGTCCAGGGGTGGAGCTAAG
AAGGACTGCTCCCCAGGCTGGGTA
>543
CATCCGGGCCCAATATGACGAGCTGGCTCGGAAGAACCGAGAGGAGCTAGACAAGTA
CTGGTCTCAGCAGGTGCGTGAGGGGAGGGGATGGCTGCCAAGGTGTGGGAGGGAGG
CAGACGGAATGAGGGGCCTGATGGACT
>544
GACGACGCTCACAGAGCTGAGACGTACAGTCCAGTCCTTGGAGATCGACCTGGACTC
CATGAGAAATCTGGTGAGTGCCTTCACATCACCTGCCCAGCTCCTCCTTCACTTGGCCT
CAGACCCAACCCTGTCTCAACCCA
>545
ACATCAAGGTCAAGCTGGAGGCTGAGATCGCCACCTACCGCCGCCTGCTGGAAGATGG
CGAGGACTTTAAGTGAGTGGGGCTCTCCTACCCACACGTGCTGGGATCAGGAGATCAC
TTCTCCCCAAAGTCTGAGCTTTTG
>546
TATGTTAAATGTCTGTCTCCAGAATATGCTGGAAAGGCTTTTAAAGCATTGCATGGCTCT
TGGTTTGATGGTAAGAAATTTGAGTACTACAAACATTTTGAAAAGATAGTTATAGTTTAA
TTTTAAACTATACCAGATTT
>547
ACCGCTAACGTGCTGCGCGACATGGGCCTGCAGGAGATGGCCGGGCAGCTGCAGGCG
GCCACGCACCAGGGTGAGCCGCCCCCGTTCCCCTCCACCCCGTCTTTCCCCTCCACCC
ACACCAGCGCTTACCCCGCGGGCTC
>548
CGCCTGGAGGAAAAACAAAGACTTTCCAGAAAGAAGAGAGAAGCGGAAGCTATGAA
AGCCACAGAGGATGGTAATGAACTTCCCTTCTACTCTTCTTTCTTGATGCATGGAGAAT
GCCAGCACTAATTTTTTTCTTTCCT
>549
GGCAAGCCGGTGATGGAGCGCTGGGAGACCCCCTATATGCACGCGCTGGCCGCCGCCG
CCTCCTCCAAAGGTAGCCCCCGCCCGGCCTGCACCGCCCCCGGGGGCGCAGGGGAAA
CTGAGGCCGGGGCTGCCCCGACCCG
>550
TTGGATCATCGAGTGCAATGACGGCGTCTTCCAGCGGCTCCGGGACTGGGCCCCACGG
CAGACACACAAGGTGCCCCTCTGCCCGCAGGCCCTCCAGGACCCCCACTCCTTGGGG
TGAGGGCACTGCTTCCTGGGGGTTG
>551
GGGGCCGCCTGCTCCCCCAAAGCAGCACAGCAGTACCAGTCCCTTCCCCACCTCCACG
CCCCTCCGCAGAGTGAGTATCTCCCATGGAGTCCCTGCACATCAGTGCTCACGGATCTC
AGAGCGCTGTCAGTCTCCACTGG
>552

ATGAGAAGGAGAAGGCGGAGAAGCAGCGGCTCAAGGAGGAGCGGCGCAAGGAGAG
ACAGGAAGCCCTGGAGTGAGTGTCCTTGGAGGCCATGCTGGGCCCGCCACCCTGCTG
CTGGATTCCGCTCCTGGCCACTCTGATG
>553
TCAAAGGCCGGCAGCCCCTGAGGTCCGGACCCACGCACGTTTCCACCCGGAATGCAG
ATATTTTTAACAGGTCAGAGCCTGAGGAGGTCGGCCTTCACCCACTAGTGATGCTGGA
GGCCAGCATCCTGAACCCTGGGGTC
>554
TGCCTACTGGGGTACCTGGAATAAGAAGACGGCACTCATCCGCGCGCGAGACCCCTGG
GCCCAGGACACGGTGAGCTAGCCCCAGAGTGCCTCCGTCCCCGTACCTCCTCACTGTG
CCCCTTTCCTCCAGCCCCAAAGAC
>555
CTATGAGGTGGACAGTGATGAGGAGTGGGAAGAAGAGGAGCCTGGGGAGTCCCTGTC
CCACAGTGAGGGGGTAAGGATGTGCCCCAGCTGTCTTCACTCACAGACGGCTTGTGTT
TGAGTCTCTGTCCCACAGTGAGGGG
>556
GGATGAAGATGAGGACGATGGTTTCTTTGTGCCCCATGGGTACCTGTCTGAGGACGAA
GGTGTGACAGAGGTGAGGGAGTGAAGGGGGAGGTCACCGGCTCAGCAAAAGTTCATT
TCTGGACTGGTGCTCAGTGGCCTGA
>557
CTGGAGACCCTGCCGGCCCAGGAGGAGCAGACGCCCAAGGCCTCCAAGCGGGAGAG
GAGAGACGAGCAGAGTGAGTGTGGGCGGGGCCAGGCCACCCACCTGTTCCTGGGCCC
AGCTAATTCCAGGGCTGAGGGCAAGAG
>558
GCTGAAGAGGAAGTCAGCGGGCAGCATGTGCATCACCCAATTCATGAAGAAGCGCAG
GCACGACGGCCAGGTGAGGTGGGGTGGGCAGGTGGGGGCCTCTGCAGGGCTTTTCTT
TTTTTGTTTGTTTTTTGTTGTTTTGT
>559
CAGGCAGACACGGAGGAGGAGGAAGAGGAGGAGGGCGACTGTATGATCGTGGATGTC
CCGGATGCTGTGGGTGAGAAGGGCTGTAGATAGCAGAACGCACTGGTGAGGTGGGCG
CTGGAGGTAAACCTCAACAGAGAAGG
>560
GCATGCGTACCCTGATGGCCGTAGAGAGGAGCCACAGAGACAGAAAGTGGGAACATC
AAGCAGATACCGGGTAACTCTCACTGTTTTTTTAAAGGAGGGGAAATTCCAAGGTATC
AGTTGAGTCCTGCACATATTGGGGT
>561
CCATTCCCGATCAGCTGGATCCTGGAACTTTGATTGTGATATGTGGGCATGTTCCTAGTG
ACGCAGACAGGTAAAATCACTGTGCTAAAGGAAGGAGCATGAATAGGCTGTCTTTTTG
TGATTGTGGAATGATAACAGAG
>562
CTATGACACGCCTTTCAAAAGAGAAAAGTCTTTTGAGATCGTGATTATGGTGCTAAAGG
ACAAATTCCAGGTAGGTTTTGGAGAGGGACAGGTTGAGTCCTCATTAGTGAGCAGGAG
TGCACAGGGGGGGCCTTTCACATT
>563
AGAGAAAATAGACACTCTGGGCATTTATGGCAAAGTGAATATTCACTCAATTGGTTTTA
GCTTCAGCTCGGTGAGTGACCTTCCACAGCTTGGGGTCTTTTATGAGGATGGTTTCTGA

TGAGATGGTAGAAAAAATCTTC
>564
GGTTGAACACCCCCATGGGCCCTGGACGAACTGTCGTCGTTAAAGGAGAAGTGAATG
CAAATGCCAAAAGGTCAGTATCCTTCGGTACCAGTCACAGTGCAGATACTTCCGTGCCT
GTTACCGCCTTCTACCCGTGAACG
>565
TCAGGAGTCCTGGGGAGAAGAAGAGAGAAATATTACCTCTTTCCCATTTAGTCCTGGG
ATGTACTTTGAGGTGAGGTTCCAGTTTTTGAAAATGGGACAGCAATAAGAATCCTGGG
AGCAGGGGTGGGATAAGTGGTCCA
>566
GGGTGCACGTGTGCGGCGGGTCACTGCTCAGCCCCCAGTGGGTGCTCACAGCTGCCC
ACTGCTTCTCCGGGTGAGTGTCCGGGTGGGCAGGGGCAGTCTCAATGTCCAGGCCCCA
GTGGGGCATGCGCCAGCGCAGAGGC
>567
GAGGCCTCAGATGACTTCTGCCCTGGGATCCGGTGCTGGGTGACCGGCTGGGGCTATA
CGCGGGAGGGAGGTGAGGAGCCCCCGTGGGGATGCTGGGATGGAGGAAGGCAAGGG
CGGCCATGGAAGAGAGCTGGCTTGAG
>568
TCCCGGCCCCGGGGGCAGCATCATTCAGCCCGACATGCTGTGTGCCCGGGGCCCCGGG
GATGCCTGCCAGGTGAGCCTGCCAGGCCGTGACAGAGGATGGGGGCGGCTGAAGCCC
AGGGAGGGGCTCCCCATCTTCTCGG
>569
TGAATTTTTAGTTGGGAAGCCTCCTTTTGAGGCAAACACATACCAAGAGACCTACAAA
AGAATATCACGGGTAAGACGCAACTGAAAAGGACCCAGCACATCTGCTGGAATTCTTT
CCTTATCAGCCTGAACTGCAGGCC
>570
CTAATTAAATTTAGGGGATCCTGATGCCACTTAGCAGTGGGATTTCAGAGGACCTTTCA
GAGGAGCTAAGGTAAGATGAGATGGAGGCAGAGTGGGGCAGGAAAGATGGCTTAGGG
GCCTCTTTGGGCTTTGTGACACTC
>571
TTGCCTTGATTTTCTTCAAAAGACAACTATAATCATTCAAAAATTCCAAGTTGGGTCCA
AAATGTCAAAGGTAGGTATTGGACTCTGCCATGTGCCAGGTCATATGCCTGAGTCTGTG
TCCTTCAACAGGCTGTCCTTAT
>572
GGCGATGAGCACCACTACTGCTGCATTGTGCAGCACGCGGGGCTGGCGCAGCCCCTCA
GGGTGGAGCTGGGTGAGGTCCCGCCAGGTGGTGATGCTCCTGGTTTCCCGTTGCCTTG
TCTCACTGCTGCGCCGGTCCTTCC
>573
GTCTTGCTACTCACGGCAGCGGCTGTAGGAGGAGCTCTGTTGTGGAGAAGGATGAGG
AGTGGGCTGCCAGGTGGGGGGCAGCGGGAGGAAGAGCCTCTGAGAGAGGGACAGAG
ACCCCAAGAGAGGGGCACACAGACCTG
>574
CGGAGCAGGTTGAGGACTGTGTGCTGACTGTGGAAGGAAGTGGCCTCTTCAAGAAAC
AGCAGAAAGTCTTGTAAGTGCTGCAAGTGCTCAGCCTTCTCCTGTTTTTCCCCCCTGA
GATTAGCACTATCCTTCCTGAACAT
>575

TGGCTCTCATCCCAGACTTGGCCATGGAAACCTGGCTTCTCCTGGCTGTCAGCCTGGT
GCTCCTCTATCTGTGAGTAACTGTCCAGGCTCCTCTTCTCTGTTTCCTTGGACTTGGGG
CGCTAATCACGCCTCTCTTTCCC
>576
TTTTAAGAAGCTTGGAATTCCAGGGCCCACACCTCTGCCTTTTTTGGGAAATATTTTGT
CCTACCATAAGGTGAGTGTTTTTGAGCTCACTCTTTTGCTTCTTATGATTGCCAAGAGC
AGCTTAGTTCCATCAGTAAAAA
>577
TATCACAGATCCTGACATGATCAAAACAGTGCTAGTGAAAGAATGTTATTCTGTCTTCA
CAAACCGGAGGGTAAGCATTCATGTGTTGAAATTAAAATACTGATTGATTAAATTTATAT
TTTGAAATTCTTATATATTCA
>578
TGAGGATGAAGAATGGAAGAGATTACGATCATTGCTGTCTCCAACCTTCACCAGTGGA
AAACTCAAGGAGGTATGAAAATAACATGAGTTTTAATAAGAAACTTAAAGAATGAATC
TGGTGGGGACAGGTATAAAATAAG
>579
AGTATGGAGATGTGTTGGTGAGAAATCTGAGGCGGGAAGCAGAGACAGGCAAGCCTG
TCACCTTGAAAGAGTAAGTAGAAGCGCAGCCATGGGGTTCTGAGCTGTCATGAACCCC
TCCAGCTGCCTGCCATGGAGCTGAT
>580
GACCCCTTTGTGGAAAACACCAAGAAGCTTTTAAGATTTGATTTTTTGGATCCATTCTT
TCTCTCAATAAGTATGTGGACTACTATTTCCTTTAATTTATCTTTCTCTCTTAAAAATAAC
TGCTTTATTGAGATATAAAT
>581
AGAAGTTACAAATTTTTTAAGAAAATCTGTAAAAAGGATGAAAGAAAGTCGCCTCGAA
GATACACAAAAGGTAAAATGTGGTGGTAGTTATAGGAGGATGTTTAGTTTTTCATAATTT
TTTAGATAATATACATATGATC
>582
TGAACTGGCCACTCACCCTGATGTCCAGCAGAAACTGCAGGAGGAAATTGATGCAGTT
TTACCCAATAAGGTGAGTGGATGGTACATGGAGAAGGAGGGAGGAGGTGAAACCTTA
GCAAAAATGCCTCCTCACCACTTCC
>583
TGATTCCAAGCTATGCTCTTCACCGTGACCCAAAGTACTGGACAGAGCCTGAGAAGTT
CCTCCCTGAAAGGTACAAGGCCCCTGGGAAGGGAGCCCTCCCTGAACCAGCCTGGTT
CAAGCATATTCTGCCTCTCTTAATC
>584
CATGAACATGAAACTTGCTCTAATCAGAGTCCTTCAGAACTTCTCCTTCAAACCTTGTA
AAGAAACACAGGTTAGTCAATTTTCTATAAAAATAATGTTGTATTAATAATTCTTTTAAC
TGAGTGGTCTGTATTTTTTTAA
>585
TGTCTTGGCCATGGTCCCCTGGTGCTGCAACTCCTCTCCTTCACGCTCTTGGCTGGGCT
CCTTGTCCAAGGTCAGGGGCAGGTTCTGAGGGTGTGGGGTCCCCACGCCTGGCTTTTG
GCTGTGGACACAGCCTGGGGCTG
>586
GGTGAGCTTCCAGAGAAATCTAAGCAGCAGGAGATCTACCAGGAGCTGACCCAGCTG
AAGGCTGCAGTGGGTGAGTATCTGGAACCAGGCTTCTTGGGCCTGAGATGGTCGTTGT

GTGGTGTGAGTTTGCTTGAGTCACT
>587
GCACGACTCCATCACCGCCTGCAAAGAAGTGGGGGCCCAGCTCGTCGTAATCAAAAG
TGCTGAGGAGCAGGTACACCTGGTGGGGATCCCCGTCCTGGCCTAGGGCATGGCTTCT
GGCCAACTGGGGAGGAGGTGCTCAG
>588
GGATGGGACTTTCAGATCTAAATCAGGAAGGCACGTGGCAATGGGTGGACGGCTCACC
TCTGTTGCCCAGGTAGATCTCAGAAGGGGCACCCTATCCACTGGGGCTGGAATTGTGA
AACTCTGATTTGAACTTTCCCTGG
>589
GGCGCCCTGGTGCTGCAACTCCTCTCCTTCATGCTCTTGGCTGGGGTCCTGGTGGCCAT
CCTTGTCCAAGGTCAGGGGCAGGTTCTGAGGGTCTGGGGTCCCCAGGCCTGGCCTTTT
GGCTATGAACAGAGCCTGGAGTG
>590
GGTGAGTTGCCAGACCAGTCCAAGCAGCAGCAAATCTATCAAGAACTGACCGATTTGA
AGACTGCATTTGGTGAGTTCCTGCACATCAAGGGTCCTTGGGCCTGAGATGGTCTCTG
TGTGATGTGACTTTACTTGAGTTA
>591
GCACGACTCCGTCACCGCCTGCCAGGAAGTGAGGGCCCAGCTCGTCGTAATCAAAAC
TGCTGAGGAGCAGGTACACCTGGTGGGGGTCCTCGTCCTGGCCTGGGGCATGCTTCTG
GCCAACTGGGGAGGAAGTGCTCAGA
>592
GGATGGGACTTTCAGACCTAAATCAGGAAGGCACGTGGCAATGGGTGGACGGCTCAC
CTCTGTCACCCAGGTAGATTCTGGGAAAAAGGGACACTGTATCCAGTCCGGCTGGGTT
CCTGCAACTCTGACTTGAGCTTTCC
>593
TTCAGAGACGAATAGTTGTTTCCCTGCTAGCCTCAGCCTCCATTGTGGTATAGCAGAAC
TTCACCCACTTGTAAGCCAGCGCTTCTTCTCTCCATCCTTGGACCTTCACAAATGCCCT
GAGACGGTTCTCTGTTCGATTT
>594
GGCAGGCCCGCGCCGCTTAGGAGGGAGAGCCCACCCGCGCCAGGAGGCCGAACGCG
GACTCGCCACCCGGGTAAGCCAAACTCGGGCGAGTGGGGGCCCGGCAGGGGACGCGT
GGCCCAAGTCCTGCCGCCCAGCCCTCC
>595
TTGGATCCTTCTTTGACTCATACAATATTTAATCCAGAAGTATTTCAACCACAGATGGCA
CTGCCAACAGGTAAGAAAACTCATCCCTGTTACCCTGTTGTTCTGCTTTCAGTCTTAGT
AAAATGCAGGATTTGTTAATA
>596
AGGCCCATCCCATGGTGGACTACCTGGTGCCTCTAGTGAAAAGAACAAGAAGTCTTAC
CCTCAGGTCAAAGTAAGTTTGTGGTAGCTCTCCTTCTATTTGAATTCTGGAAATTTTGAT
TTCCTACGATTTCCAAGGAATT
>597
GCCTGGTGGGAAAACACTGTGAGGATGGGATCTGCACTGTAACTGCTGGACCCAAGG
ACATGGTGGTCGGGTAAGTAGGGGTATATGATGCTGTGGAAGGTAGGAACAGATAGAA
TATGGGATGCAGGAACTTAGAATGA
>598

CCTGGACTCTTGGTGCACCCTGACCTTGCCTATTTGCAAGCAGAAGGTGGAGGGGACC
GGCAGCTGGGAGGTAAGCATCATTTTCCTGGCCTTGATCCTCCAAGGGGTCCAGGCTT
TGGTTTTCATCTGTATGAATTATA
>599
CTTCCGGATAGCACTGGCAGCTTCACAAGGCGCCTGGAACCCGTGGTATCAGACGCCA
TCTATGACAGTAGTGAGTACTTCACTTCCAACAGGGGGCACACCAAGAATAGACTTCC
AGCCCTGCCCTGCCATTTACTTGC
>600
ATGGACAGGACAGCTGGATGTGTGACTGGAGGGGAGGAAATTTATCTTCTTTGTGACA
AAGTTCAGAAAGGTAAATACATTCTGTGATCTCTGATCTCAAGAGGTGTGATCTTGACA
CACTACAGTTCTGAGTGTGTCTG
>601
TGAAGAGGAAGAAAATGGTGGAGTCTGGGAAGGATTTGGAGATTTTTCCCCCACAGAT
GTTCATAGACAAGTAAGTGATTTATTATTATTATTAATCCTTATTATTTTTAGAGATGGGAT
CTCACTCTGACACCCAGGCT
>602
CAGCTTCGGAGGAAATCTGACTTGGAAACTAGTGAACCAAAACCTTTCCTCTACTATC
CTGAAATCAAAGGTAAGTCAGTTGTTTAAAATCTTATGCTCATATTTTATTTTATTTTATT
TTTGGTTTTTGTTTTGTTTTG
>603
GCTGGAGCTGGAGGCGGAGGCATGTTTGGTAGTGGCGGTGGAGGAGGGGGCACTGGA
AGTACAGGTCCAGGTACAAAAATACTTATTCTTCCTAAAACTTTTTCATATTGGAGAGG
GTAGAATTTTCTTTTCTTTGGTTT
>604
GGATTTCCTACTTATGGTGGGATTACTTTCCATCCTGGAACTACTAAATCTAATGCTGGG
ATGAAGCATGGTAAGTAATGCTTTGTTCTTAATACCAAGAAAGGAAAAAAATAATTAAT
GCTAAAAAGGGTTTTTAAAAT
>605
ACCGTTGGGAATGGTGAGGTCACTCTAACGTATGCAACAGGAACAAAAGAAGAGAGT
GCTGGAGTTCAGGGTAAGTGAGCACACAAATTACGTTCTGTTGGTTGGCTGGGGAGGG
GTCAGTCCTAGGTGCAGAAAGATAT
>606
CAGGAGACGTGAAGATGCTGCTGGCCGTCCAGCGCCATCTCACTGCTGTGCAGGATGA
GAATGGGGACAGGTAAGTCAGAACTTTTGCATGATAGGTTGTCCTGGGTGGGGAAGAA
GAAGAGCATCGTATAATGCATACT
>607
GGATCTACTAGAAGTCACATCTGGTTTGATTTCTGATGACATTATCAACATGAGAAATGA
TCTGTACCAGGTAAGCAGAAATCTCAAGAAAACAACTGAAGAAAAATCTGTAGTTTAC
TTTTTCTTCTGTTATGTTTGGG
>608
GATAAAGTTCTCAGTATCTTACTCAAGCACAAAAAGGCAGCACTACTTCTTGACCACC
CCAACGGGGACGGTAAGAGACAATCACACATCATTGGTGTAACTTTCTCCACCTTCTA
AATATCTACTAGGTATTTGATAAA
>609
CGGGCGCACAGCACTGCACCTGGCTGTGGAGCACGACAACATCTCATTGGCAGGCTG
CCTGCTCCTGGAGGTGAAGGGCACACTTATTTGCTTTTGCATTAAATTTCTGAGGGAGA

TTTAAGGAAATCTTTTCAAAGAAG
>610
GGAACCACACCCCTGCATATAGCAGCTGGGAGAGGGTCCACCAGGCTGGCAGCTCTTC
TCAAAGCAGCAGGTAAGATGGTGATCTGGCGGTCATTAATGAAAAATGTTACCAGGAA
TGCAAACCCAACTTCAATAGAGCA
>611
GGAAAATGCAGGAGAGGATGAAGGAGTTGTGCCTGGAACCACGCCTCTAGATATGGC
CACCAGCTGGCAGGTGAGTGCCGCTCCATCTGTCTGATGGCTGCCCCTGAGGGAGTCA
GAGGTTCAGGAATGTAAGAACAGAT
>612
TCTGGGGATACTTAATAATGCCTTCCGGCTGAGTCCTGCTCCTTCCAAAACACTTATGG
ACAACTATGAGGTAACACCTTACCTTACAGATTTAGCAATTTTCATTTGACTTTACTCTG
TTAACATCTCTGGCCAGGGCA
>613
AGCCCAGTGAAGACCACCTCTCAGGCCCACTCGCTGCCTCTCTCGCCTGCCTCCACAA
GGCAGCAAATAGGTAAAAAAAAAGACAAAAGACAGTGGAGATATTTTCCAGCTCCCC
CAGGCTGGTGTCTTCAGCTCTTTTT
>614
AGTGCAACCAATGGTCAGCCCGACCAACAGGCCGCACCAAAAGCACCCTCAAAGAAG
GAAAAAAAGAAAGGTATGGAGAAATCCACTTGAAGCCAAGGAAGGGAGCTAGAGAT
GGTTACCCCTGGAGAAATGTGAGATTC
>615
GATTGGCATTGATGATGTGCCAGATGCAAGAGGGGATAAAATGAGCCAAGACTCTATG
ATGAAACTAAAGGTAAAAGGGAAATGAATGCTTTATCTGTATATTAAGCAAAAGTCCAA
GAGAGGGAATTATATTAGAAGCT
>616
ACAACACAAACAAAGGATCTGGGTCAACATTTCCCTTTCTGGGATAAAAATAATTGATG
AGAAAACTGGGGTAAGAATTCACATTTGCTGATACCTCTCTGACCACCTGAGTCTGAC
CAACAATTTGCTACTTGAATTTC
>617
CCGGGCATTTGGTTACGTGTGTGGAGGAGAAGGCCAGCATCAGTTTTTTGCCATAAAA
ACCGGGCAACAGGTGAGCTCTAAGTCTTGACATAGACCTGGGGACTTGTCCTCTGTGG
ACATTGGAGTGTCTGTCACTCAAC
>618
ACATGACTCCATAGCCATTATCCCACCTCCACAAAGTACCAAACCAGGAAGAGGCAGA
AGGACTGCTAAGGTGAATTGTCTTCTCCACATATCCATTAGCAGAGTGCATGTTCGGTA
CCAAAGGGTGGTGTGATGCAAGC
>619
CTGCAGCCCAACCCTCTGGATCTCTTCAAAACAAGTGCTCCTGCCCCAGTGGGGCCCC
TGGTGGGTCTAGGTAGGTGCCTAGAGATAAAATTAAGTATGACTCTTTTGCTTTTGCTCA
TAAAATGATGATGCATCGGAAG
>620
GTTGGCATTCCTCAGGAGAATGCAGACCATGATGACTTTGATGCTAATCAACTATTGAA
CAAGATCAATGGTAAGCCACTCACCACTCTGCCTACTGTTCCAACAACTATCTCCACTG
CCACATGGACCTAAATGAGTTC
>621

TACCAAATCTACTGACAATGCATTTGAGAACCCTTTCTTTAAAGATTCTTTTGGTTCATC
ACAAGCCTCTGTAAGTATGAACTACTAGAAGCTCTTCTCCAAAGTACATGCCACATAGA
GCTCCAGCCTCCTGAATTAAC
>622
TGGCTTCTTCTCAACCTGTATCTTCTGAGATGTATAGGGATCCATTTGGAAATCCTTTTG
CCTAAATTCTGTAAGTAGAAGATCCTAATAAGCCTGTCTCAGGAGTTTTTGTGAGAAAG
GGTGACATGGAATAAAGAGTG
>623
GGTGTCCCCACTACCCATGGCTTCCTCGGTACCCTTGGTACCCTAGGCTCCCAGGGCAG
GTGAGGAGCAGGTGAGCATGGGCTTGGCTTGGGGGATGACAGGGAGGTTCTCCCAGT
GGGGGCCCAGCTCTCAGAATCAGA
>624
CTTGGCAACTTTCCTGGCCCTTTTGAAGAGGAAATGAAGGGTATTGCCGCTGTTACTGA
TATACCTTTAGGTAAAGTTCACTTAGAAGCTTTAAAAAAAAAAGTAATTAAATTCTTTTA
CAAGTTTCCATTGAGGTGTGA
>625
ATTATTTCATTCAATATTTTTTATGAATTATTTACCATTTGTACTTCAATAGTAGCAGAAGA
CAAAAAAGGTAATTGTGCGCTGAGGGAGGGTGGGCAGCATGAAGCACTACATAAAGA
AAAATCTTCTAAATGAAATTA
>626
AAACAACAAAACTGTCTTCAAGGCTTCAAGCTTTGCTGGCTATGTGGGCATGTTAACA
GGATTCAAACCAGTAAGGAGTCTGTTGTTACATAATTATACAGGTAGCCTTCTGTCCTG
TTACCGTTATGTAAAAGTTGAAA
>627
GGATTCTGGGAAAGAAAGATGCCATGTGGATAGGGTTCCTCACTAGAACAGTTCTGGA
AAATAGCACAAGGTAAAGTGTTTCGTTGCTTTACTATTAAATATTTATTTAAATTACTCA
TATTAAAAAATAAAAATATTT
>628
GAAACATCCCTTCTTCCTTGATGATCGCAGAACGCCTGCAAAGATGTGTTTGAACCGC
ACCAGCCAAGAGGTACCTTTGCAGGGTCATCTATAGAAAGAAACTGTTGCCATTAAAG
ATTCCCTTGGTTCTGGCATTTTTC
>629
GGAAGCCATCGCCTGGCCCCGGCTCTCTGGACCTTGTCTCGCTCGGGAGCGGAAACA
GCGGCAGCCAGAGGTGCGCGGGCGGCTTTCTCTGCCGCCTCCTGGGGACTTGGGCGC
GGGAACTGCGCGGAGCCTGCGCTGGG
>630
AATGCTTCTCACCCGGAAACAAACTTGCCAGTTGGGTATCCTCCTCAGTATCCACCGAC
AGCATTCCAAGGTAAAGCAGTTGCTGAAAGAAGAAAAAGTAAAATTAATTGAGAGAC
AGAGAGAGAGAGTGAGAGTGGGAG
>631
AGGGGTACCATGGATGCCAGCGCCACAGCCTCCATTAAACTGTCCACCTGGATTAGAAT
ATTTAAGTCAGGTAATTTCAAAGACACAAAATACTCATAAAAAACAGAACTGTGCTTC
CAGCTTGCTTAACCAGATTAGCA
>632
TCAAGAAGTCATAACTCTGGAGAGACCACTAAGATGTAGCAGCTGTTGTTGTCCCTGC
TGCCTTCAGGAGGTCTGTGATCAATTACCAGGGCACTTTGTAAAATAGTTCAGCAAGAA

TTTCTGCCCTGTCATTGAACCTT
>633
AAGAGAGGATGTACTAAAAATAAGTGGTCCATGTGTTGTGTGCAGCTGTTGTGGAGAT
GTTGATTTTGAGGTAAGAGATGTCATAATGTTTATAAGATTTGCTTTCCTAATATAAGAA
GAATGTAATTTTGCAGGACATT
>634
TGGAATCCAGTTCCCTTTAGACCTTGATGTTAAAATGAAAGCTGTAATGATTGGTGCCT
GTTTCCTCATTGTAAGTCTGTTCTTATTAATCTTGATTATAGATGCTTTCTCTAGGTTCATT
CTGAAATGATTCAGTAAAG
>635
CTGCTTACCCTGAAGCCTGGGGAGGCCATGTTTCTGGAGGCCAACGTACCCCATGCCT
ACCTGAAAGGAGGTGAGCACATTTCAGCAGTGAGCCCACTGCCATCCCTGCTGGGCCC
TGTTTCCCTATCTGGACAAAGGAA
>636
GAGTCAGGAAGACCCCTACCTCTCAATCTATGACCCCCCTGTACCAGACTTCACCATTA
TGAAGACGGAGGTGAGTGAGGGGCTATGATGGGTGTCCTTCGTGTGCCATGAAAATCT
CTGGCAAGGGTCACGATGTGGAG
>637
TCAAGATTATAAAATAACCCAACGGATGGTCTGTGCTGGCTATAAAGAAGGGGGAAAA
GATGCTTGTAAGGTAACTCATGAGATTATGAAAACACAATAGGCTGCTTGAGAAAATTC
ATTTCAAAATATATTTTCCAATA
>638
CCAACTGCTGGTGAAGGCCCACCAGGATGCCCAGCGCCACGTAGCTGCAGGCACACA
GCAGCCCTACACGGTATGTGAGCTGGTGGTGCCTCTGACAGCCTTCCTGGGGGGTCAG
GGTGTCCTCTGTGGGTGTGTGTGTG
>639
CATCCTCGCCCGGGACCCCATGAACCGCATGGAGATCTTCCGGCTCAACACCATTCCCC
TGTTTGTGCAGGTGAGTCTGGGGCAGGTGGGCAGGAGAGGTCCCCCCACCACTGCTT
AAAGGGCAAGGTTAGGAGTCCCAG
>640
GACGCCATTGATGCAGAGGGGGCCTCGGCCCCACTCATGGAGTTGCTGCACTCCCGCA
ACGAGGGCACTGGTGAGCTGGGGGAGGGCAGGAGGCTGGGTCCCACTCCCCCAGCC
ACGATGGAGGCTGGCCGACGTTTAAC
>641
CTACCGGAAGCGCGTGTCCGTGGAGCTCACCAACTCCCTCTTCAAGCATGACCCGGCT
GCCTGGGAGGCTGTGAGTATCCTAGGTTGGACCGCAGTAGTTGGTTGTGCAAGTTGGG
CACTGCCCATGGGCAACACTTTTG
>642
TCTATGGCCATGCAGGCAGCTGCACTGGGTTCCTCTATCGGAACAGCAGTTGTTGGAG
GTCTGATGACAGGTCAGCATAAAAAGTGAACACTTCCCTGTATCTTATGCTCTCAGATT
ATTTACAATAGATAATTAATGAG
>643
AATTCTAAAGTTGCCTCTCTGGGGACAGCCATCTGACCAGAACTGCTATGATGATTCTG
TTTATTGGAAGGTACTGTACAGTATTCTAAACAAGAATGTGAACGATGACTCCATTAGT
GAAAGCTTTACACACTTCAAGC
>644

AAAGGTGAAGTCTCGAGAAAGGGAGCAAATCAAGTCACTCAACAACCAATTTGCCTC
CTTCATTGACAAGGTGAGTTTCTCTCTCATTGCACTGGTAGGGCTGCCGCTGGTCCACT
TGGGATTGGTGCAGTCAAAACACA
>645
AGAGTGATCAATCTCGGTTGGATTCGGAACTGAAGAACATGCAGGACATGGTGGAGGA
TTACCGGAACAAGTAAGGGACCCTGTCTGGGCAGTTCTTAACTTTTGCTGTAAAAGAG
TTCCAGAAAGTAATAAGCTAAGAT
>646
GGTGGACCTTCAGGCCAAACTTGACAACTTGCAGCAGGAAATTGATTTCCTTACAGCA
CTCTACCAAGCAGTAAGTCTTCCAGTTTCAACCAAGTTTATCTAAATGGAGAGTTTTTA
AGCCTGAACCCACAACGATTCAG
>647
GATAGAAATTTCTGAGCTGAATCGTGTGATCCAGAGACTTAGATCTGAAATCGACAATG
TCAAGAAGCAGGTATGTGCTTTCTCCTTCTACCACTCAGCTGTATGGAATGGGGGTAAC
CCTCAGGTAAAGGGCGAGTGCT
>648
TGAACACCAAGCTGGCCCTGGATCTGGAGATTGCCACCTACAGGACCCTCCTGGAGGG
AGAAGAAAGCAGGTGAGGAAGGGACGCTGGGAGTCGAACCTCTTCTCATGGTCTTCC
TTCCTTGCAAGCTGATTGTTGTTGA
>649
GCCCACATTTATGCCACCCGCCTCATCCAAATGAGACCCTTCCTGGAGAACCGGGCCC
AGCAGCACTGGGGTAAGTGAGAGTTTGGGAAGGTGCTTCCCCCACAGCATCCCTGAA
CTTAGAAGTGTTCTGCAAGAGAATG
>650
CTGTGTGGTGGGCACTCTGTTCAAGGCCATGCCGCTGCAGCCCTCCATCCTGCGGGAG
GTCAGCGAGGAGGTGAGGCAGGGTGCTACACAGTGGGGCCGCCAGGCAGACCTGGC
CTCCCACTAGAACACCTCCCTGGAGG
>651
CTGGTCTTGGAAGATGAACTGCAGCGTATCAAACTAAAAGGCACCATTGACGTGTCAA
AGCTGGTTACGGGTAGGGAGCCCAATGAGAGGATGTGGGTGATGCAGGTGAAGAGCC
CAGCGGTGGTGTGTTAGGGATGGTG
>652
TTCTGGTGGAGGACTATTGCTTTGCTGACCTTGCTCCCCAGAAGCCCGCACCCCCACTT
GACACAGATAGGTGAGCAGCAGTTCTCGGGAGCTGGAACCAGCTCATGGTCAGTGGA
ATCTTTGAGTTGCACCTAGGAGGG
>653
CGTCTCCCGGGTTATCCTCGCTGGCAACCTCCTCAGCCACAGCACCCAGAGCAGGGAT
TCTATCAATAAGGTATGGAGCCCACCTGGCTGCATTCAGCCCCAGCCCAGGAGCCTGC
AAGCCTGTAAGACCCTCCTTCCCC
>654
CACCAAGAAAACCCAGGCAGCCAGCGTGGAGGCTGTTAAGATGCTGGATGAGATCCT
CCTGCAGCTGAGCGTGAGCGAGCTGGGGGCTGGAGGGGTGATGGGGATTGCAGTCTT
CAAAGCTGCCACTGGGCAACAGAAGG
>655
CGCTGGCCACTGCCTACTCCACGCTCCAGCTGGTCACCAACCCCTACCAGGCCACCAT
TGATGGAGTCAGGTAGCTGGCACAGCCACACTTCAGTCTGACCCAGCCTTTTGCCTCA

GGAGGCACAAAGAAGGGAGGGGAG
>656
GATCACTTGGAGATCCTGGAGTGGACCCTGCGGGTCCGTCACATCAGCCCCACAGCCC
CTGACACTCTAGGTAACAGGCTCAGCCATACAGGGTGGGAGCAGAGGGCCAGGAGGC
CTGGCAGGACCCTGAAGTGCACAGG
>657
ATCTTCCCAGAGTGCCCGCATGTCTACTTTTGTGGCAACACCCCCAGCTTTGGCTCCAA
AATCATCCGAGGTAATTTTTGTCTTCTGGGGGCCCAGGCTGATTTGCTGATTTGCTCTCA
CCTGGGGACAAGGTTCACAGA
>658
AGAGTACCCTGAATACCAGCGTTGGATGGGCCTCAACGACCGCCTCAGCTCCTGCAGA
GCTGTTCATCTGGTGAGTCTGGGGACTGTGTACTCCCTCTGTTTCCCACGCCCTCTGTC
CGCTTTCTCTTGCTTTTAGGTGA
>659
AGTTAGACTCCTTCGCTGTATCGTCTACTGATTCTTAAAATGTGACAAATCTGATTGGAC
GACTTACATGGTACGGTGCAATTTTTGATGTCCAGACTAAAGTGAGTCTTCTCTAGGCA
AACAGTAATTTTGGTTAAAAC
>660
AGTGCCGGGAGGGCAACGCAGTCGCCGTTCGCCTGTGGCTGGACAACACGGAGAACG
ACCTCAACCAGGGGTGAGCTGAAACGGTTGGTGGATGAGAGGAAGGCTAGAGATCTC
CTGGCTACGTGGAGTGGAGTGCTCAT
>661
AATGAACCGTGGGGATGACACCCCCCTGCATCTGGCAGCCAGTCATGGACACCGTGAT
ATTGTACAGAAGGTACGTACAAACTCCTTCGTCATCCACATCACATACATGCCATGAGG
GTCAGTCACAGGCACTGTAACAT
>662
TGCAGTGAATGAACACGGGAATGTGCCCCTGCACTATGCCTGTTTTTGGGGCCAAGAT
CAAGTGGCAGAGGTGAGTACTCAGCCCTTAATTCCTGAGATGGGTAGGAAGTAAAGTC
TGAGCCTTGGTGGGAGATTTTGGA
>663
AGCATCTGTAACAAGTATGGAGAGATGCCTGTGGACAAAGCCAAGGCACCCCTGAGA
GAGCTTCTCCGAGGTCCATCTCCCCATCCCCTAGCTTGTGTCCTCTCGTCCCTTCCCAC
CTGTCTTCTCCCTCTGTACCACAG
>664
ATGGGCCAGAATCTCAACCGTATTCCATACAAGGACACATTCTGGAAGGGGACCACCC
GCACTCGGCCCCGTGAGTCACCACTGTGGGAAGAAGGGTTGTAAAAGGAAATAATCC
TGGCCTCTTGGGGCTGGGTTAGGGT
>665
CAAACACTCTGGCATTGACTTCAAACAGCTTAACTTCCTGACGAAGCTCAACGAGAAT
CACTCTGGAGAGGTGACCCCTGCCCTTCTTGCCCTTCCCTCACTAAACCCCCATAAATT
ACTTGCTTTGTACCTGTTTTAAG
>666
AGGTGCTGAAGGTTCGAGACTGGAGTACAAGGAAGAGCAGGGACTTCAATGAAGAGT
GTCCCCGGCTCAGGTAGTGCAAGGCGTAACCTGGAAGCTGCTAGTTCCAAGGAACCCT
GAATAGCACTGAAAGAGATCTTTTG
>667

CCTCATCCTACTCTCATCACACACTGGATGCCGTATGGATCCCTCTACAATGTACTACAT
GAAGGCACCAGTGAGTAGGGATGTTGAATTTCCTTGGGGAGGAAATGGCAGAGAGGG
AGCCTCTCTGAACTATTTGACTT
>668
GGGCATGGCCTTCCTACACACACTAGAGCCCCTCATCCCACGACATGCACTCAATAGCC
GTAGTGTAATGGTGAGGCCACAAGCTCACTCCTGGCCCAGGCCCCAAAAGCCCTTTGC
CTATCTATGACTTACCTCCTTCT
>669
ATGGCTGATGTCAAGTTCTCTTTCCAATGTCCTGGTCGCATGTATGCACCTGCCTGGGT
AGCCCCCGAAGGTGAGTGAAGTCATCATGTCGGGAGGTAAAAAAGGACCACCTCAGA
AGTAGTGGAAGGGGGCAGAGACAG
>670
GCTTCTGTGGGAACTGGTGACACGGGAGGTACCCTTTGCTGACCTCTCCAATATGGAG
ATTGGAATGAAGGTGAGAGCACAACAGCATACATTTGTGTTGCGGGAGTGGTTGGTGA
TGGTGAAAATAACTGTAGTGGGCC
>671
TGATAATCCAAATGGAGATGGAAGATGTGTGACATGTATATATTATCTTAATAAAGACTG
GGATGCCAAGGTATGCAACTTAATTGAGCATTTATTTTTTTCTTACAGGTGTCTTTTTCT
CAAAAGCATTAAGTTTCATA
>672
TTGATAGACTGCTGTTTTTCTGGTCTGACCGTCGCAACCCTCATGAAGTACAACCAGCA
TATGCTACAAGGTAGTATTCCTTTTCAGAAAAAAATTGGTTTAGAGTATGGTAGGACAGG
AGGGAATCTGTAGAGTAAACGT
>673
CTTTCCAAAGCAATTCACAAGTCCTTCCTAGAGGTTAATGAAGAAGGCTCAGAAGCTG
CTGCTGTCTCAGGTACTGTTTGCTAGAATCTTCTCCCCTCTTCTAGATTTGTATTTTTAGA
GCAATCTTGAGTGGGGAGTGG
>674
ATGGCTGTGCTGTATCCTCAAGTTATTGTCGACCATCCATTTTTCTTTCTTATCAGAAAC
AGGAGAACTGGTAAGTTTATTATGAAAACAAAATTTTATTTAATAGGTCACAAAGAACC
TCTTTTTTAAATGGAGATTTT
>675
GACTGCCTTCGGCTTTTTTTCTGCTGACTAAGATCTCCTATAGAGAGCTACAACAATGC
CCAAAAGAAAGGTAAGTGAAATGGAAAGACAGACGGGGAATGAGCTGCTGAGTTACC
TATTTCCTAATAGGAAGAATTTAT
>676
CAAGCAAGAAGCAGTTGTTGAAGAAGACTACAATGAAAATGCTAAAAATGGAGAAGC
CAAAATTACAGAGGTATTTTCTAATGTTAACAGTTTTATCTATTTGAAAGTTTAACAATG
GGTACAACTGAAAGAAATCCTAT
>677
GGCCTTCAACCAGAGGTTCCTGTGGTTCCTGCAGAAAACAGTCCAGGAGCACTATCAG
GACTTTGACAAGGTGAGCCCGGGGTGCAGGTGGCAAGGGGCACCTTGCAGGGCCTGG
GTGCAGCCCCTCCCTCCCAGCTCCA
>678
CCTAGAGCCAGCGGCAACCTCATCCCACAGGAGAAGATTGTCAACCTTGTCAATGACA
TCTTTGGAGCAGGTAGGAACCAGAACCTTGCCCCTCCATCCAACAATGCCTGCTGTTC

ACCCACAGCCTTGCCCAGCCCCTC
>679
GCCATCTCCTGGAGCCTCATGTACCTTGTGACCAAGCCTGAGATACAGAGGAAGATCC
AGAAGGAGCTGGGTACATGGGGGCCCCCAACCCTATAGCCAGGAGAAGCCTTGAAAC
CCAGGTTGTTTGTTCAGTCTACAAA
>680
CCTACTTGGAGGCCTTCATCCTGGAGACCTTCCGACACTCCTCCTTCTTGCCCTTCACC
ATCCCCCACAGGTGAGGCCTGCCGGTTCTGCCCTCCCACCTCTAAAGTGCTTGCCATGT
TTTCTCTTCCTGGCTTCTCAGC
>681
CGCTGAATGGCTTCTACATCCCCAAGAAATGCTGTGTCTTCGTAAACCAGTGGCAGGT
CAACCATGACCCGTGAGTACATACCCCTCACGAAAAAATGTGTGCAGGTTCAGCAGTC
AGGAAGGCTGTTTGTCCCTGCTAG
>682
AAGGGGAAAGTTGCACATTTATATCCTAGAGGGAAGCGACAGCAGTGCTTCTCCCTGT
GCTGAGGTACAGGTAAGGAGGGTGGTTTGTAAAGTTCACTGGGGAGGGTGATCCCTCT
TTATTGTTCTAATATTATGCTCGC
>683
GAGGTCCGCAGCCAGGGAGGACGACAGCATCCGCCCTTTCAAGGTGGAAACGTCAGA
TGAGGAGATCCACGTAAGGCACCTTGGGCCGGGCCGGGCTGGGCAGTGGAGAGGGTG
GTGTGTCGAAGACAGGGGTTGGGTCT
>684
AATGAATTTGACTGGAAGAAGCAGGTGGAGATTCTCAACAGACACCCTCACTTCAAG
ACTAAGATTGAAGGTATGTTTGCAAAACGCCAGCCAGAGAGGGATGTATGTCATGAGA
ACAGCCTTCTTCCACAATGTGACTT
>685
GAGCACGTTTTTGAAGTCATCTGCCCTTCCATCCCTGGCTATGGCTTCTCAGAGGCATC
CTCCAAGAAGGGTACGGGGCTGCTAGAGGTTCCATAACTGCCCCGTCCTCGCCAAGGG
TGGGCCCGGTGTTCCCACCAGGC
>686
AGGAATTCTACATTCAAGGAGGGGACTGGGGGTCCCTGATCTGCACTAATATGGCCCA
GCTGGTGCCCAGGTGAGGTCACTGTTGGGGTGGTGTGTGTGTGTGTGTGTGTGTGTGT
GTGTGTGTGTGTGTCCTCTAAGAA
>687
AAGGTATTCTACAGCCTGATGAGGGAGAGCGGCTACATGCACATCCAGTGCACCAAGC
CTGACACCGTAGGTGAGTGTGCTCAGGGGTCCTCGCCCACTGCCGGCTCCACTGGGGC
AGGGAGACACCGCGGGGTAACCTC
>688
ATATTCTAGAGAAGTTTTCCACCTGGACCAATACGGAATTCCGATACCTGGAGGATGGA
GGCCTGGAAAGGTGAGGCCCTGGTTTGCCCCCTGCAGTCATGACCCTGGTCCCAGCAG
CCAACCTCCTCACCCTCTTCATC
>689
CCATCATCTCCTCCCAGCGCTTCTACAAGGAGAACCTGGGACAGGGCTGGATGACCCA
GAAGCATGAGCGGTGAGCCTGGCTGAGCCGAGAACAGGGGCCTCTGAGGCTGGAGG
CAGGGGGACGGCCAGTCTTGGGCTCC
>690

AATGATCATAGCACCTTGGATGGGTATTCCAGAAGAACCACCTTGTCTTCAAAAATGTA
TCACACCAAAGGTAAGGATGTTAAGACTCATTCTTAGCACATCAGAAGTGTCTTTTGAA
TTATTTTAGTGAAACGATGCAG
>691
GAGGGTGAGGACCGAGGAGCGCGAGCAGATCAAGACCCTCAACAATAAGTTTGCCTC
CTTCATCGACAAGGTGAGCTACGATCTTTTGTAAAAAATCACTGTGGGTCTGAAATAAA
TGCCAAAGAGAGAGAAAGAAGGAA
>692
TGGGGGAACGGGGCCGCCTGGACTCAGAGCTGAGAAACATGCAGGACCTGGTGGAA
GACTTCAAGAACAAGTGAGTTGGGGTGGAGGGTGGACACAGGGGAGGGTGGTGTCTT
CTTGGTACCAGATGGGCTTTGTTACTA
>693
GGTGGAGCTGGAGGCCAAGGTTGATGCACTGATGGATGAGATTAACTTCATGAAGATG
TTCTTTGATGCGGTAAGAAACTTATCTAAATTTTTCACATGGGTGGGTTTTTTTTCCTCA
AGTTGTCATATCACAATGAGTG
>694
GGTCAAGGCCCAGTATGAGGAGATTGCCAACCGCAGCCGGACAGAAGCCGAGTCCTG
GTATCAGACCAAGGTGGGTGCTCTGATGACTGTCTCCTGAGTGAGGGGACACACCCAT
GTCTAGGATTCTAGGCCATGACGAC
>695
GCATGAGATCACAGAGATGAACCGGATGATCCAGAGGCTGAGAGCCGAGATTGACAAT
GTCAAGAAACAGGTAGGGTGAGATTGAAAGAGGGCAAGGAAGGGGCCTGAGTTCTA
AAAGAAACACCTACTTTGTTTTATTT
>696
TGAACACCAAGCTGGCCCTGGACGTGGAGATCGCCACTTACCGCAAGCTGCTGGAGG
GCGAGGAATGCAGGTGAGTAGACAGCATGAACTCAAGATGGCCTTCAGCTGATAAAG
CGAAGCTGCTCTACTGTGGGGTGTAC
>697
GGCCCCCTGATACCCCTCTGCCTGAGGAGGTAAAGAGGTCCCAGCCTCTCCTCATCCC
AACCACCGGCAGGTACGATGGGGCGTGGGGCTTGGGGGAGGTCAGTGCTGGATAATA
CACAGAGGCTTGCAGGCCACTGCTC
>698
ACAGAGCCCAATTCTCGGGGGCCCCTCCAGTGCAAGGGGGCTGCTCCCCCGCGATGCC
AGCCGCCCCCATGTGAGTTGTCCCTCAGAAGGGAAGGGAGGGATGCACGGGTTCTGG
GTCTGTGGGAGATACACAGCCGCTT
>699
GTGCAGCGAGCTCACGCCTTGAGCGACGAGACCTGGGGGCTGGTGGAGTGCCACCCC
CACCTAGCACTGGGTAAGTCAGGTGCATGGAACTATCCGGGCTGGGAGATGATGCCTT
GCATCTTGGGCTAGGCATGTGGCTC
>700
CGTGGGCGGAGATAGCCGCTTCGTCTTCCGGAAAAACTTCGCCAAGTACGAACTGTTC
AAGAGCTCCCCAGTGAGTGCATGAGGGCTGTCTGGGCGCTGGGATGCCCTGATCCTCA
ACCTGGATGCTGGAGCCCTGATCC
>701
GTTCCCAGAAAAAATGGTCTCCAGCTGTCTCGATGCACACACTGGTATATCCCATGAAG
ACCTCATCCAGGTGGGGGGACCCCCCATTTCACTGCAGATTCACGACTCCCCAGCATT

GGCCAGTGCTTCTCCACCCTTAA
>702
GCTTTGGAAACGCTTTTTCTGTTTCTTGCGCCGATCTGGCCTCTATTACTCCACCAAGG
GCACCTCTAAGGTAAGGTCTTGAGGGTACCAGCCCCAGCCCCTCCAGTCCCTGGTCCT
TTTAGAAGTTGCCCCTTCTCTGC
>703
CAACGTGTACGTGGTGACGCAGGGCCGCAAGCTCTACGGGATGCCCACTGACTTCGGT
TTCTGTGTCAAGGTGAAGACCTGGCCAGGCCTGGCCCCTGGCCTGGGGAAGCAGGAA
CTGCTCAGGCCCCTGGATCCTGCCC
>704
GCTTCGGATCTTCTGCAGTGAAGATGAGCAGAGCCGCACCTGCTGGCTGGCTGCCTTC
CGCCTCTTCAAGGTGAGACCCTGGGAGTGGCATGGGGGGCTGGCCTGGCCAGAGGGA
TCCCCAGCTCTGCCCTCAGGAAGTC
>705
GCTGTACAAGAATTACCAGCAGGCACAGTCTCGCCATCTGCATCCATCTTGTTTGGGCT
CCCCACCCTTGGTGAGTGTGCCCAAGGGGATGGGAGGGTGGGTATGCAGGCCCTGTCT
TACGGGTACCTGGGCCCTGTTCT
>706
TGCTGGGCGTGTCATTGAGAACCCCCGGGAGGCTCTGAGTGTGGCCCTGGAGGAGGC
CCAGGCCTGGAGGGTGAGGCCTGCTGTGTGTGTGTGTGTTTGTGCTGGGGACCCACTC
TCTGGGTGGGATCCCTGAAATAGGA
>707
TCTGGTTCCACGGGCGCATTTCCCGTGAGGAGAGCCAGCGGCTTATTGGACAGCAGGG
CTTGGTAGACGGGTAAGGGGCAGGGCCGGGCAACAGACCCAGGGATAAGAGAGACTG
GGGTCCAGGTGGCAGCCATGGTCCT
>708
GCGGAACCCCCAGGGCTTTGTCCTCTCTTTGTGCCACCTGCAGAAAGTGAAGCATTAT
CTCATCCTGCCGGTGAGCTTCCCTGCGTCCCCGGAGTCCTGCAATGAGACACAGGACT
CCCAGCAACCTGTCCTCCTCACCA
>709
GATGGCAGTGGCCGGCTGCTCCAACTGGACCTCCAACTTCCTAGTCGGATTGCTCTTCC
CCTCCGCTGCTGTAAGTAAACTCACCTTATCTTAAAACCAGCCTATGTAAGCTGACATG
AAGATACTCCTTAATAATACAG
>710
TTACCCCGATGGGCGGCTAGCAGAGCCTGAGAAACCTCGGAAACAGCTCAGTTCTCA
AGGCACTGTGAGGGTAAGGACTTTAGCCATCTCTAGTTGGGGACACTTAGCAGCTGTG
GGCATTTCCAGGTACCGTTCCTGGC
>711
GATATGCTTTGTACGATGCCACATACGAAACAAAAGAGTCTAAGAAAGAAGACCTAGT
ATTTATATTCTGGTGTGTAAAAACAAAAGAAAAAAGTACTTTTAAAATGCAAGGATTAT
TATCATAGTCAGTCTTACCTTTG
>712
GAAAGTGCACCTTTAAAAAGCAAGATGATTTATGCTAGCTCTAAAGATGCCATTAAAAA
GAAATTTACAGGTACAAACTTGAATATTTGGGGCAGAGAAATTATTCTTTATTAATTATA
ATAAAGTAATAGGATAATGTT
>713

CACAAGAAGCAGCTGGACTCTCTGCGGGAGAGGCTGCAGCGGAGGCGGAAGCAGGA
CTCGGGGCACTTGGGTGAGGCACTGGGCTGTTGGGGCCGAGGCTCAACCGGGGAGGA
CGGGCGGGATCCCTAGAGAAAAAGCTC
>714
ACACCACCTCTCTGATCTGGCCTTAACATTGCCCACTGATGCTGTGTCCATCTGTCTTGC
CATCTCCACGGTAATGACCTGACAGAGTTAGGATTTACCACTTATTTTCAGTTCACGGT
CAGTGTCTTATTGCCCATACC
>715
CTCAGATGTTGATCTGGAGATAGAGAGCCTTCTGAACCAACAGTCCACTAAGGAACAA
CAGAGCAAGAAGGTAGGGGTCATGTGAAAATACTCTCACAATATGCTCTTAATTATAGC
TTTGTTTGACAGGGATTTACCCC
>716
ACTATGGAACCAAGGAGGAGTGCATGAAAGCCAGTGATGCTGATCGACCCTGTCGCAA
GCTGCACTTCAGGTCTCTTGGAAGGGAATGAGGTGGGATGGGTTTGTCAGAAGCAGC
CATTCCTTTTCAGCATATTCCTTCC
>717
AAGCCAGGAGCTTGCTCTTACACAGAGTGTCGGAGGTGATTCCAGTGCAGACCGACTC
TTCCCACCTCAGGTACCTGTCTGCTCAGAAAACTCGTCTCAACTTACGGACTTATGTTT
GATAGGGCAGCCATACACGTTAA
>718
ATGAGATGCGCAGGCTCAACATACCCGTACTACAGGATGATGGCTTTCTCTTCCTCTGG
GTCACAGGCAGGTAATGCAATTCAGTTATGTAGGTATGGACAGATACAGTACAAGAGT
GAATACTGAATGGGAGATAAAAT
>719
TCAACTGCAACGCATCATTCGGACAGGCCGTACAGGTCACTGGTTGAACCATGGGAAG
GAACACTGCTTGGTGAGCAGCAGTGGGGCCCAATTCAATAGGTGGAGCACAAAGAAG
AATCATTTGATTTCTTACTGAGAAA
>720
TTGAAAGACTATCTCCTGGCACTCGCAAGATTGAGTTATTTGGACGACCACACAATGTG
CAACCCAACTGGTAAGGTGACCAGAGAACAGGCTAGTCCTCAAGGGGCTATTATTTAT
GATTAAGGACAGAACTTAGTAGA
>721
GGCTGGGCCGAGTGGGTGTCTGGTGCCGGCGTTTGGGCTACGGTTGTTGTTGGCGACT
GTGCTTCAAGCGGTGAGTGTAGATCACCTTCGCAGCTGCCACAGGGAGAGTAGGAGT
GATTGCAGCTGAGAAAGACGCAACA
>722
CAACCTGCTTCAGCTGGATCCTGATTGCAGAGGATGCTGTCAGGAGGAAGCACAATTT
GAAACCAAAAAGGTACTGTTTTCTAGTATATTCCTTCCATTGGATGGTTTAGTTGAGAC
AAGGTAAAAATGTAAGTTGCTTA
>723
GCTGCCCCTGAACCCCAGAACAACCAGCTGGATCAGTTCTCACAGGAGCTACAGCGCT
GAGACTGGGTAAGTCAACGATCCCCAGAGCTGGGACAGAAGGGGCAGCAATGGGGCA
GCAACTGAGGGAGAAGAGAGCTGAC
>724
GCTGCCCCTGAACCCCAGAACAACCAGCTGGATCAGTTCTCACAGGAGCTACAGCGC
GGAGACTGGGTAAGTCAACGATCCCCAGAGCTGGGACAGAAGGGGCAGCAATGGGGC

AGCAACTGAGGGAGAAGAGAGCTGAC
>725
ACAGCCCCACCTCCGGCAAGTTCCGCCGCGGGAGCGGCTCGGCCTGCAGCCTTCTCTG
CTGCTGCGGAAGGTTCGAGTCCCGGGTCTGGCACATTCAGATTGGAGGTTAGGGAATG
GGGAAAGGGGAGCGAGCCAGAGAA
>726
GAAGCTGGAGTTTAACAAGGCAGAGAAGCACGTGCACAACTTCATGATGGATATCCAG
TATACCAAAGAGGTGAGATGGGCATATGGCACCCATGGGACACCCCTCTTCCAAGAAG
CAGGAGTCCAGGCGCCCCTGCTTC
>727
AACATACTCGCAGGAAGGAGTCTCATGCTGCCCGCAGGCATCAGCGCAAGCTGCTGGC
CGCCATCAACGCGTGAGGGCCGCTTTGTATGCACACGTGTCCATGTGCACCCATGTCCA
AGTCACCTCTCTGCACGGCTGTG
>728
GTTCCGCCAGGTGCGGCTGAAACACCGGAAGCTCCGGGAACAAGTGAACTCCATGGT
GGACATCTCCAAGGTACTAGGATCCCGTGGGAGGAGGTCTTGAAGAAGGGGGAGGTT
GGGGCCACAGGGAGGGAAATCTGGCC
>729
GACTGAGCTGCTTAGCACTGCCCTGGGGCCGAGGCAGCTTCCAGAACCCAGCCAGCA
GTCCAAGTAGCTGGTGAGGGGGCTGGGACTTGGGCAGGAAGGCATCCTGGAGGAAGG
GTTCCTGTGGCCAGCACCCTATGTGG
>730
CGTGGTAGCCTAGAGTCCTGGGGAGCTTCTGTCCACCTGTCCTGCAGAGGAGTCGTTT
CCAGCCCGGCTGGTGAGTGCTGGCCAGGGTGGGGCAGAGCAGGCGGGGCGGCGAAA
GTCCAGACCTGGCTGTGCTGTCTGGA
>731
CGAGAAGAAGACAACATGTGGCACCGTTTGCCTCAAGTACCTGCTGTTTACCTACAAT
TGCTGCTTCTGGGTGAGGAGGGGTCGCCTTGCCCCCACCCCCACCCCCACCCCTCCCG
GGCCACCATCAGACCTGGGCAGAT
>732
TGTCGTCATGGTGACTGGGGTCTTGGGCTGCTGCGCCACCTTCAAGGAGCGTCGGAAC
CTGCTGCGCCTGGTCAGGAGGGCGCAGGGCCACGGGGTGGGGGTGGTGCAGATGGGC
CCAAGGAGGTTGTCGCTGCAGGCTT
>733
CATCCTGCTCCTCATCATCTTTCTGCTGGAGATCATCGCTGGTATCCTCGCCTACGCCTA
CTACCAGCAGGTGAGGGGCCTGGGCAGGCCATTCAGAGACACAGACATGCACAGGCG
GGGCGGACACACACACATGCACA
>734
CATGACCAGGCGCTACCACCAGTCGGGCCATGAGGCTGTGACCAGCGCTGTGGACCA
GCTGCAGCAGGAGGTGGGTGGGTGGTGCTGGGAGGGCGCGTGCATCCCCAGGGCCTC
CCTGGACCTCTGCAGGAGCCTCTGGC
>735
CAGCTGCTGCAAGACGGTGGTGGCTCTTTGTGGACAGCGAGACCATGCCTCCAACATC
TACAAGGTGGAGGTGGGTGTGCAGCGGGATCATGCCTCCAGTGTCTACGAGGTGGTGG
GGGGGCACCCCAGTGACTGGCTGT
>736

GTTGGAGACCTTCATCCAGGAGCACCTGAGGGTCATTGGGGCTGTGGGGATCGGCATT
GCCTGTGTGCAGGTGAGGGCACATGGGGGTGGCGGTCATCTTGTTGGGGACACGGGG
CAGGGCGGTGGCTGGTGGCCCCATG
>737
GATCCTAAGGACCTTGAGCCCCGAGGAGCTAGAGCAGCTGGACTGCGAACTACAGGA
GATGGATCCTGAGGTAGGGGCTCCAGCACAGGGAACCAGAGGCAGTCCCAGGCATTT
GGGAAGGGTGTTGGGCTCTGGGTGGC
>738
TACTTGGAGCAACAGGCACTAGAAGTCAAAGAGCGTGATGACTTGGTGCCCTTCACA
GGCGAGAAGAAGGGTATGGGGACCCTGACATCCATGCCTCGTAGAACCCAAGTGGCT
GTGCTGTCTCTCACTCGTCCCCACTC
>739
ATCACCCTGGAGCCTGAGCTGGAGGAGGCACTGGCACATGCCACAGATGCTGAAATGT
GTGACATTGCAGGTGGGTAGCTGTGGGAAGTTGAGGGTGAGAGGATCAGGGGCAGGT
GGATTCTAGCCATGACATGGGACTA
>740
CTGATGAGTAACAAGCAATACTATGATGCCCTCTGCAGTGGAGAAATCTGCAACACTG
AAGGCATTAGCAGTGAGTGTGTTTGGGGAGCATGGTCCCCAGGGCTCTGGGCAGGCA
GTCCTAGTGTCTGGGAGAAGGTAGA
>741
TGAGGAGATACTAAAGAGGGTCCGAAGCAATGACAAGGAGCTGGAGGAGGTGAACTT
GAATAATATACAGGTATTTGACTTGCATCCTCCAAACCATAACACCGGATAACCACTGA
CATCCGAGAGTCCCTTAGCCCACC
>742
AATGAAGGCAAATACCTATGTGCGGAGCTTCAGTCTGGTAGCCACGAGGAGTGGTGAC
CCCATTGCCAATGTAAGGCTAGTTGACATCCCCAGCCCTCTCCCCACATCTTACATGCA
CCTGTTGCATGGCTGTCATAAAG
>743
AGGACTCATGGCTGTGCTGAAGGCAGTTCGGGAAAATGCCACACTCACTGAGCTCCGT
GTAGACAATCAGGTCAGCATTCCCTTCCCCTGATGTTAGCATTCAGTCTCATTGAGTCT
CCTCCCTGAGCCAGCCAGTGGGG
>744
GGCTACCACTTTACACAGCAGGGGCCACGAGCTCGGGCAGCCCAGGCCATGACCCGA
AACAATGAACTACGTGAGTAACTGCAGACATGATGTGTGGAGTGGTCAGGGAAGTGC
AGAAATTGGTGGATCCTCCTGAAAGC
>745
GACCAGGAGGAGACCAACACCCTGGTGGCCAATACCAGCAACTCCAACGGGCTGAAA
TTGGACCCCGCAGGTGAGCCGGGAAAGTCGCTGCCGCAGGTGGTTGGGTTTGGGGTG
CCCAGAGCCTCGCCTGGCCTTCGGCG
>746
AAAGCTATATTAGTTCACTAGCACCCCGGAAGCTCAACAGCACAAGTGATATTGAAGA
AAAGGAGAACAGGTAATCCTAAAACCAGCCAAAAGATGGTCCTCATCCTCTTAGGTCT
ACTGGACATAGTCATCTCCTGGGC
>747
ATGAAGCAAAAGGAAATGAAATCCCACAGACAATTGCTCCCTCCACCTATGTATCAAC
TTACTTGAAAAGGTACCAGGCTCAAAGGGGGTGGGAGATGTTTCTTTCACTCTAGCCA

TGGGAAATGGGTAATGATTGTATG
>748
GGGTTACAGCTACTCCTGTGCTCTCCATTACTGGAACAGATTCCTCTGTGGAAGCCAGG
GAGAAGAGGAGGTATGTTGAGTTACTGATTTCATTTGTGGGGCTCTGCTTTGAGGTAGA
CGATAAAGGAATGGGCATAAAG
>749
GGATGAGGAAGCAGAGTCTTTACGGAAAGCACGCTCCAGACAAGCTCGGCAGACACG
AAGGTCTACTCAAGTGAGTGTGGCTTTTTTTAAAATGTCATTTTGTGTATCTACCCTTCT
GTTGGAGGTACAATCAAAACACA
>750
GCCCTCAGCAGTTCCAGCAACAGAAGCTGGGGAGGGCCAGCAGCCCTGGGGCAGGA
GTCTGGATGAAGAGGTGAGCTCATTTTTGCTGGCATGTACTGTGCTAAAAGCAGGGTA
ATTAGTTTGGGGAGTATCTTCTTCAC
>751
TCAGTCCCTGATTCTGAGAGTTCAGAGACTACCACAAACACTACAACTGCAAAGGAAA
TGGACAAAAATGGTATGTAGAACTTCAAAAGACACAGGCCCCTCCACAGTGCACATCC
CCAGATTCTTAAAGTTCATACTTT
>752
GTCCATCCGAGAGAGGAGGCGGCCCAAGGAACGACGAAGAGGCACAGGCATCAATTT
CTGGACAAAGGATGTAAGTGGATTGGTCTGTGCTGAGGCATATCATATTACTCTTGGTC
ACCATTCTTTCAGTTGAGTTTAAA
>753
GGCCCGGGAGGCCCGCCTAGCCACCCTGACCAGCCGTGTAGAAGAAGACAGCAACAG
AGATTATAAAAAAGTAGGGTCTTTATTCAATTTTCCGGTTCTTTGGTGACATGTAAACCT
CTCAGCCTTCCTCCACCACACCC
>754
GAAAACAAAACTTCAGGAAGCCCAGCTAGAGCTAGCAGATATAAAGTCCAAGCTTGA
GAAGGTGGCCCAGGTAAGACGGAAGAAGAAGAAAAAAGATGAGAACCAAGGGTTGA
TGTAAGGAAGCAAACTAGACTAGGGAT
>755
GCAGGGCCCAGGCGCCCTTGGCCTTAGGACCCAACTTCTCTTACCGCCATGGAGTTCG
ACCTGGGAGCAGGTGAGCTCCTGGGGAGTGAGGACTGGAGGTGCAGGGGGCCGGAC
TCAAGCCCAGAAGCTGCCTGCACCAA
>756
GGGAGATCCCAAGCTCAGTCCCCACAAAGTTCAGGGCCGGTCGGAGGCAGGGGCAGG
TCCGGGTCCAAAGGTAAGTCGCCTCATCACCGGCTGCGGAGGGGCGGGAAGGCTGGG
GTTGCCCCTGACCCCAGGGTCCTGCC
>757
GCGCGGCCACAGCATGACCGCTCGCGGCCTGGCCCTTGGCCTCCTCCTGCTGCTACTG
TGTCCAGCGCAGGTGAGCGGTCGCCGGCCACCGGGACGCCCTGTGGGGACGTGGCGG
GGCCGAGAGCCGGGGAAGGCGACGG
>758
CAAGAGGTACAATTGCGAATATTCTGGCCCACCAAAACCATTGCCAAAGGATGGATATG
ACTTAGTGCAGGTAAGTTCATTATCTTAGGCACTGGGAACACAAAATGCTGATCACAA
GATCCTCTGTAATTATTCCTTAA
>759

TCTGTTGTGATGTTCGGCAGCTTCAGACACTAAAAGACAACCTGCAGCTGCCTCTACA
GTTTCTGTCCAGGTAGGTTCTGCTGGGGAAACAGAACAACTGGGCTGTAGATACTTGT
GAACTGGTAATGCTCAGCTTTCCA
>760
CCTGTTACAAACCAGACGAAAACAAATGTGAAAGAGTTACAATACTACGTCGGACAG
AGTTTTGCCAATGGTAAGTAAACTTTTAATTATTCCTCTTTTACAGCTGGCATCAGGACA
GCAGAGCAAATTGTTCCTTTTAG
>761
AACTGGATTGAATACATGTTCAATAAGGACAATGGACAGGCACCTTTTACCATCACTCC
TGTGTTTTCAGGTAGGTATAAAGATTCCAAGTTTGGTGTGTTTATGATATTGTTTTAAAG
CCAAGGAACTGGCTGGGCACA
>762
TCATGTGGATCACCTACATGGCGTTTTTGCTTGTGTTTTTTGGAGCATTTTTTGCAGTGT
GGTGCTACAGGTAAGCAGTTTTGTTTGTCATCCAGGGCAAAAAGAGCAAAATTGCCCA
CTGGCACTTTGAGCTTTCATTT
>763
CGGTATTTTGTCTCCGAGTACACTCCCATCGATAGCAATATAGCTTTTTCTGTTAATGCA
AGTGACAAAGGTAGGCATATTTGTCCATGAATAAAAGGGTTCAGATGATGCTGTCTGTC
ATGAGAGCATTCGTTTCCTCA
>764
TTACCAGCCATACCCTTCGGGAGCTGATGTACCCTTTGGACCTCCGCTTGACATACAGA
TACTGCACCAGGTAACCTGCTGTTTGAAGAAATAAGTCACTCTAGATGACCTTGTGGTT
TGCTGGCTTTTACATGTTGACT
>765
TGGACCACAAGAAAGGGGACGACTTCTTTGTGTATGCCGATTACCACACGCACTTTCT
GTACTGCGTACGGTAAGTGGCAAGAGACAATCATTGGTAGACACCATTCTTGGGTCTG
ACTTAGGGTACATGGGCAAAAGCT
>766
CTCCATGACCCTTGTCTGGGTACGTTTGGTGGACCAGTGTTCCCGTGGCTTGTGTTGGG
AGGCTATGATGGTAAGTAAGAGAAGCTTTGACTTTTCCTTTTAGATAAAGTCATCTCGG
TTTTGTCATTAGCATCAAGTGG
>767
TTACCTTCCCTGTCAATAATTACTATAATGATACAGAGAAGCTCCAGAGGGCCCAGGCC
TGGGAAAAAGAGTGAGTCACTCATGGGTGTGAGCAGACTGTCCTTGCATTGTGGGAA
GCTAGACACTTGCAGCAAGCACTT
>768
TAGCTATGCCATCATGTTTCTATATATTTCCCTAGCCTTGGGGCACATCAAAAGCTGTCG
CAGGCTTCTGGTAAGCCGGGGGGGGGTGGTGTATGTTCTAGAGGTTAACAGCAGTGCA
CAGTGTAACGTCATATTTTTGC
>769
TGAAGTCATCCCGTTCCTGGTGCTGGCTGTTGGAGTGGACAACATCTTCATTCTGGTGC
AGGCCTACCAGGTATACTTCCATATTCTCACAGGGCTCAGCATCTGTTCAAGTTTCAGG
GACTGTGAATGGCTCCTGGGCT
>770
AGGGTCCTAGGAGAAGTGGCTCCCAGTATGTTCCTGTCATCCTTTTCTGAGACTGTAGC
ATTTTTCTTAGGTAATTATGCTTTCAATCCTACCAGTCCTGTAGTCTGCTGAGCCTGGAG

AAAGAAGGAGCTGGCTACCTG
>771
CTTCATTGACTTTCTTCTGCAGATTACCTGTTTCGTGAGTCTCTTGGGGTTAGACATTAA
ACGTCAAGAGGTAAGTTGGTGCCAGGATTATAGTCTATTTAATTTGAGTCTAGAATCTA
GAGGAAGCAGCTAGCGGGTAT
>772
CTGTTTGTTTCGCTTCTTCAAAAACTCCTATTCTCCACTTCTGCTAAAGGACTGGATGA
GACCAATTGTGGTATGCGCTTATCTGTGGTTTTTCTCTTTCCTCTCACTGAAACAGATTA
TCACATGCCTTCTAAGAAGCC
>773
TCTGTCATTCAGCATCGCAGTCCTGAACAAAGTAGATATTGGATTGGATCAGTCTCTTT
CGATGCCAGATGTAAGATGACTTCCTTTTTTTTTTTTTTTAACTTCTTTAGCTGATGATGA
ATACATTTTTCAAAACATGC
>774
GCGGCGGCATGGGCTGCAACAATGATTCCCTGGTGCAGCAGATATTTAACGCGGCGCA
GCTGGACAACTAGTCAGTACCACCTTGTCTTGTACTTCAGAAGCAGCGGAGGAGGCTC
TCAGCCAGGCAGTCAGCTCAGGCC
>775
AGGGGGGAGACTTCATGAGATTCCTGCCCATGTTCCTTTCGGATAACCCTAACCCCAAG
TGTGGCAAAGGGTAAGTGCTGCTGCCATTGCAGATAAGCATCCACTGCAACTTTAATTT
GCAGTAGAAAACTAGGAGAGGA
>776
GACTTATAGCCAGTAATGTCACCGAAACCATGGGCATTAACGGCAGTGCCTACCGAGTA
TTTCCTTACAGGTAAAGCCTGCCCTTTTTCAATGGGGTTTACCCAGCAAAGGGCCTACA
CTGGGTGGGAGTGGGGAGGGTT
>777
CAACATGTTTGGAGTTATGTGGCTCTGGGGCATCAGTCTGAACGCTGTATCCTTGGTCA
ACCTGGTGATGGTGAGTCCTCATACAGTCTCAGTTTGTCCCTAGCTCCTGTTTCACTTG
GGGGAATTGGCTGTCTTAGATT
>778
GTTCACGGTGAGCATGAAAGGCAGCCGCGTGGAGCGCGCGGAAGAGGCACTTGCCCA
CATGGGCAGCTCCGTGAGTACCCTGAGCAGGGCCACGCCGGCAGAGTCGCACCACAA
AGTAATCCTGGTCACTGGCTGAAGAG
>779
TATTTGGCCATGGTCTTACTGGGAGCCACTCACGGATTAATATTTCTCCCTGTCTTACTC
AGTTACATAGGTAAGAGTTCTCATCTTAAAAGGGTGGCACAATGGAAGGACATTACTG
AAAGGGAATTCTATCCTGAGGC
>780
AATTGGCGGGCAACGACCTTTCTTTTATCCACCCAAAGGCCTTGTCTGGGTTGAAAGA
ACTCAAAGTTCTGTAAGTAATGCAATTTTAGAGTTTCACAGCTGGCTGTGTATTTTCTTT
CTGCGTAGTCAACATGCTTGGA
>781
CGCTCCAGAATAATCAGTTGAAAACAGTACCCAGTGAAGCCATTCGAGGGCTGAGTGC
TTTGCAGTCTTTGTAAGTAACTTCTTGGTTTCTTGACTCACCAAGAGACTACTCTGGCT
GTTAAAGATGTAAATAACTAGAA
>782

CCCTGGCTCTCAACAAGATCTCAAGCATCCCTGACTTTGCATTTACCAACCTTTCAAGC
CTGGTAGTTCTGTAAGTATCTATCCCTTTTAATTCCATATATTTGTGTTAATTGGGGTACA
AAAACAGATTTCTTCTGACA
>783
ATCTTCATAACAATAAAATTAGAGGCCTGAGTCAACACTGTTTTGATGGACTAGATAAC
CTGGAGACCTTGTAAGTATATTCATATTTTGGGCAACTGCATTGTGATATTGAAAGTATA
ATTTTATACTTCACTGCAGAA
>784
GATTTCATAGTAATTCTATTTCTGTTATCCCTGATGGAGCATTTGATGGTAATCCACTCTT
AAGAACTATGTAAGTATTTTTGAAAGTTTCTTTTAATGTATAATTATGTGAAGCTGTGTC
CGTACAATGCCAAACACCC
>785
ATTTGTATGATAATCCTCTGTCTTTTGTGGGGAACTCAGCATTTCACAATTTATCTGATCT
TCATTCCCTGTAAGTATGTGAATTTAAAAACAAACCTGGATATTTATACAGTTGTTTTAG
TGGAAAACAAGAATTTATT
>786
CTTTACAGCGTAATCAAATCTACCAAATAAAGGAAGGCACCTTTCAAGGCCTGATATCT
CTAAGGATTCTGTAAGTTTGTCTATGATTATTAAAGACAATAAATTTTTTCCAAACTATGT
TAATATGTTGTTATGATTTC
>787
ATCTGAGTAGAAACCTGATACATGAAATTCACAGTAGAGCTTTTGCCACACTTGGGCCA
ATAACTAACCTGTAAGTGGATGATTATGCAGTAATGTCATGAAAGTAGTAACTAGTGCTA
TAGAAACATCTGTTTTAACAT
>788
ATCAACTGAAACTTGTGGGCAACTTCAAGCTGAAAGAAGCCTTAGCAGCAAAAGACT
TTGTTAACCTCAGGTGGTTTTGCTTTTTCTTTTTTGTTGAAGTTATGGTAGGCTCCAAAA
TTACAGAGTGTCCTGGTTTTTTG
>789
GCAAATGTCACAAGCACTCTTGAAAATGAAGAACATAGTCAAATAATTATCCATTGTAC
ACCTTCAACAGGTATGCCTGGCCTATTTTTTCACAGTTACCCATGTTAAACAAACATTAG
GCAAATATATCTAAATGTCCT
>790
CCCTAAACCCAGTGCAAACAGTGTAACGTCACCCCACTCCAAAGAGAAAAGAATGCC
CTTCTTTAAGAAGGTAACATTAACTTCCAAGCTCCCATTGTCCACCTGCTCAACTGCAC
TGCGTCACATTTCTAGTCCTGTTG
>791
TCCTCCGTATGATGTGGTACCTTCCATGCGACCAGTGGTCCTAGTGGGCCCTTCTCTGA
AGGGCTACGAGGTGGGTAGCAGCCTTCCACAGGAAGCTTAACTTGCATGCTGAACTTC
TTTGTGATGCTGCCTCCTACTCC
>792
AAACGCTCGGTATTAAACAATCCCAGTAAGCACGCAATAATAGAAAGATCCAACACAA
GGTCAAGCTTAGGTAAGTCTGTGCAATGAGCTTAAGCTTTTTAAACTCTCCTCTCCCGA
TGTTGATGATGAGAAGACCTAAC
>793
TCATCCAGCTCAACTCAGTAAAACCTCCTTGGCCCCTATTATAGTATATGTAAAGATTTC
TTCTCCTAAGGTAAGTAGGACTGCTACTGTTTGCTCTATAATCAAACTTTCCTAAAATGT

ATTTTATGTTCTGCTTTCTA
>794
AGGGAAATCTCAAGCTAAACACCTCAACGTCCAGATGGTAGCAGCTGATAAACTGGCT
CAGTGTCCTCCAGTAAGTTATCTCTATATACAGCATAATCCAGTTACAGAGATCAGACCT
TTTTTTTTTTTTTTTTTTTTTT
>795
CCCTCTCCTTAGCCGTACATTAGCCACTTCAAGTCTGCCTCTTAGCCCCACCCTAGCCTC
TAATTCACAGGTAAGGGGAGTTTTTATATATATCTATATATACAATCTTCATAGAAAAAAG
GTTGCCTATGGTGACACCT
>796
GGGCCCCTTCCTCCTCCTCCTCCTGCTGCTGCTGCTGCTGCCACGGGGGTTCCAGCCCC
AGCCTGGCGGGGTGAGCACTGACCTTGTCGCAGTGCGCACAGAGGTTCCCAGTGGCT
ACTTGGGAGGAGGCCAGAGGGAGG
>797
GGACCCCAAAGTGGGGGGCTCCCGCCCCCGCCCAGGGCAGTTCCCTCGAGCAGTAGC
CCCCAGGCCCAAGGTGGGTCAGGTGGGCCTGGGAGGAGGTGTCGTGCTTCACCTTAG
GGCTGGGTGGGAGGAGCATGGCTGCG
>798
GGTTCCCCTTCCGCTACGGGGGCCGCATGCTGCATGCCTGCACTTCGGAGGGCAGTGC
ACACAGGAAGTGGTGGGTCCGGGCAGCCGGGGCACCCGAGCTGGGGTCACCTGCCCC
ACGCTGCCTGCTTTTCTGGGAGCCG
>799
ACTCACAACTACGACCGGGACAGGGCCTGGGGCTACTGTGTGGAGGCCACCCCGCCT
CCAGGGGGCCCAGGTGGGTGCTGGGTTGGGTAGCCTGGGGCGAGCAGGGGGCACTGG
GCCAGGCCAGAAGGGCCAAGGGGAGG
>800
AATACCCAGGACCCCCAGTCCTATCACTGCAGCTGCCCCCGGGCCTTCACCGGCAAGG
ACTGCGGCACAGGTGAGCTGGGCCCGGAGGTCCGCAGGGTCCAGGGGCCGGAGCAA
GTCCCGGAGGATGGGAGAACAGGTGG
>801
CGCCAGGGCCACGTGGAACAGTGCGAGTGCTTCGGGGGCCGGACCTGGTGCGAAGGC
ACCCGACATACAGGTGCGCCACGGGGTGTGAGCCGTGCCACTGACCCCTGACGGGTG
TCCCTGTCCACACCCGAGTGGGAGGA
>802
CTGATCGTGGCCACCGGGACCACCGTGTGTGCCTGCCCACCAGGCTTCGCTGGACGGC
TCTGCAACATCGGTGAGTGGGTCAGCCCCCCGGGGTGCCCTGGGGCAGTGCCGGGTG
GACCCACCGTGGGCCGGCCTCACTG
>803
ACCAGGAGCTGCACGTGGACTCCGTGGGCGCCGCGGCCCTGCTGGGCCTGGGCCCCC
ATGCCTACTGCCGGTCAGCACCAACGCCGCTCCAGGCCGCCGCACGCGGGGCAGGCA
GGATTTTTCCTGGTGAGAGGCCCCCC
>804
GAGAGGCCCTGGTGCTACGTGGTGAAGGACAGCGCGCTCTCCTGGGAGTACTGCCGC
CTGGAGGCCTGCGGTGCGCGGCTGGCGGGGGGTGCTGCCTTGGGCCCCACCGAGGTC
ACAGCAGTTTTCCCCGTGATCCTCCT
>805

ACAGCTTCTGCGCCGGGAGCCTGGTCCACACCTGCTGGGTGGTGTCGGCCGCCCACTG
CTTCTCCCACAGGTGCACCTCCTCTGGGCCCCAGTCACCTGCCCTGAGGCCCCACACA
CCATCCAGCGTCACTATGCGCCTG
>806
ACCTTCGGCATCGAGAAGTACATCCCGTACACCCTGTACTCGGTGTTCAACCCCAGCG
ACCACGACCTCGGTGAGCTCCGGCGTGTCGTGGCTGCACTCTGGGCAGGTGGGCCCT
GTGCTCCCCAGGCCAGGCCCAGACA
>807
CCCGAGCCCGGCAGCACCTTCCCCGCAGGACACAAGTGCCAGATTGCGGGCTGGGGC
CACTTGGATGAGAGTGAGTTGGGAGGGGGGCGCCCAGCGTCCGCCAGGTGGACAGGT
GGCCAGCCACAGTGTGGTGTCACGCT
>808
CTACGGCGCCGACATCAGCCCCAACATGCTCTGTGCCGGCTACTTCGACTGCAAGTCC
GACGCCTGCCAGGTGAGCTGGTGCCCGCCCCACCAGGACCCGACTGGTGGGGGCTCA
GCTGGTCCTGAGTCTCCGAGATGCT
>809
ACCTCAGCCATGAGACTTCTCATCCTGGCCCTCCTTGGCATCTGCTCTCTCACTGCATA
CATTGTGGAAGGTAAGTGGAGAAGCTGTCTGTGAGATAAAGAATAGGGAGGCAAGGC
AGGTGGGCACACATTTTGGGTTTG
>810
CTACCCAGCGACTGCCAGTTAGCAGAATCAAGACCTACACCATCACGGAAGGCTCCTT
GAGAGCAGTAATGTGAGTCTGCCTCCTCAGAAGTTGGGCTGGGTGGGTACCTAGAGGT
ATAGAAATACACTCTATAGAAATG
>811
ACCTCAGCCATGAGACTTCTCATCCTGGCCCTCCTTGGCATCTGCTCTCTCACTGCATA
CATTGTGGAAGGTAAGTGGAGAAGCTGTCTGTGAGATAAAGAACAGGGAGGCAAGGC
AGGTGGGCACACATTTTGGGTTTG
>812
CTACCCAGCGACTGCCGGTTAGCAGAATCAAGACCTACACCATCACGGAAGGCTCCTT
GAGAGCAGTAATGTGAGTCTGCCTCCTCAGAAGTTGTGCTGGGTGGGTATCTAGAAGT
ATAGAAATACACTCTGTAGAAATG
>813
CCCCAAGCCAAGCCCTCCAACCCCAGTAACCCTCGAGTCTTCTTTGACGTGGACATCG
GAGGGGAGCGAGGTGAGCGGAGTCTGACGCTCGCGGAAAAGCCCCGAGGAGGCGGG
GGTTCCCGGGATTTGGGGGATTCCAA
>814
ACAGGAGAAAAAGGCATTGGACACACGACTGGGAAACCTCTCCATTTCAAAGGATGC
CCTTTTCATCGAAGTATGTGTAAATTTTCTTAATCTTGTTATTCTTCATACAGACAAGTCC
TGTTCTTTAGGATTTGGGGAAG
>815
AAATCAGAATGGGACAGGTGGAGAAAGTATTTATGGTGAAAAATTTGAAGATGAAAAT
TTCCATTACAAGGTAAAGTAGAGAAAATGCATGTTATTGCTGATAAAAAGATTGTTATAT
ATCTGGAATACTTGTTGGATTA
>816
AGTAATTAAAGGAATAGGAGTGGCAAGGATATTGGAAAATGTGGAAGTGAAAGGTGA
AAAACCTGCTAAAGTAAGTAAAAAGTTACAGTGAAATACACTTATTATCTTAGTTGCTA

TCTTTTTGAGACGGAGTCTCGATC
>817
ACATTGGAAATACTTTTTTCAAATCCCAGAACTGGGAGATGGCTATTAAAAAATATGCA
GAAGTTTTAAGGTAATATTATTTCTAACAAATTATTTTTAAGAAATTATAAATTGAATTAT
AAACTAAAATTAAGAATTGC
>818
TGTACTGAATATTGGTGCTTGTAAACTGAAGATGTCAAATTGGCAGGGAGCAATTGACA
GTTGTTTAGAGGTAAGTCTGTTTGATTTTGAACTTTTTGAATAAGTTGGATTAAGACTTA
GTTTGAATAGTAGCAACTTTT
>819
ATCAAATACCAAAGCATTGTACCGCAGAGCTCAAGGATGGCAAGGATTAAAAGAATAT
GATCAAGCATTGGTAAATTTTGTTCCAAATGTTTAATTTTTTAAAATAGACAACTACCTT
TATAAATCATACACCTAACTTA
>820
CAATCAAAACCTTCCTTGGGGATACAAACACACCTTGAACCAGATTGATGAAGTAAAG
GTGTGGCCTCAGGTAAGTGGACCTGCTGTCTATGAGCTGAAATAATGTGTACATGGAGC
TCAATCAGGTGCCTCAAAAAATC
>821
GGAAACCACCTGCCATGTGCTGGACCCCACCCCTGTGGCAAGATGCAGCGTGAGGCA
GCTGAAGGAGCATGTGAGTACCCTTCTTAGGATGACTGTAGGTGGCCCTTCGGCCAGC
TCCACCGATTCACCCAGCGTCTCAG
>822
TGTGATTTCCAGCTGTTGAAACTAGATGGCAAGTTTTCCGTGGTATACGCAAAATGTGA
TTCCAGTCCAGGTACAGATGACTATTCTTATTCTCATTTTTTCCTTGTAGAGAAAGTGGG
GAAGGGATCTGAATAATTTTC
>823
GGCCGCCTTCAACGCTCAGAACAACGGCTCCAATTTTCAGCTGGAGGAAATTTCCCGG
GCTCAGCTTGTGGTAAAGACTGAGATTCTTTTGACAGGTTGGGCAGTTCGGTGGCACT
TCGGGAATGTACTGTACGTGGTGG
>824
AGTGTGTGGCACTGACTGTGTTGCTAAAGAGGCCACAGAGGCAGCCAAGTGTAACCT
GCTGGCAGAAAAGGTGAGTGGGCCGGGACCTTGGGGTGTTACCACTCGGACAGAGCT
GTTTGTGGAACAGAACATCCTTGGTT
>825
TAAGGCAACACTCAGTGAGAAGCTTGGTGGGGCAGAGGTTGCAGTGACCTGCACGGT
GTTCCAAACACAGGTAACAGCTCCGTGAATATTCTTGCCTACACCTTCAGAATACAATG
ACCCCTTCACATTTATGCAGTGCA
>826
TGTCATCAAAGGATATTCCAGATTCTTGACAGCATTCTCGTCATCTCTGAGGACATCAC
CATCATCTCAGGTGAGCACCAGGTGGAGTGCCTCTGGGTGACTGGCCGGTTTGGAGCA
GGGAGGGAGGCTTAGAGTCTCAT
>827
AGCCTTCAACATCCAGACATTTGGGGAGACCAAGATGTCCAATGCCACCCTCGTCAGC
TACATTGTGCAGGTGAGGCCAGGGCAGCCTCCCCCCAAAAGCAGAGGAGCTCTGGAG
TCTAGGGCTGGTGGGCAGGGCCAGC
>828

TCGCCCTGGTCCAGGAGGTCAGAGACAGCCACCTGACTGCCGTGGGGAAGCTGCTGG
ACAACCTCAATCAGTGGGTGACAGTGGCAGGGTCATAGGAAGGTGACATCTCGTCCAC
GGCACAGCCTCACTTCACTTGGGCC
>829
CCTATCACTACGTGGTGAGTGAGCCACTGGGACGGAACAGCTATAAGGAGCGCTACCT
GTTCGTGTACAGGTGGGTGGTCTAGAAAGCCAGGAAGCCCCTCCCTCACCTGGGAGG
GCCCCAACAGAGCAGGGAAGTAGTT
>830
TGCGAGCCCTGCGGGAACGACACCTTCAACCGAGAGCCAGCCATTGTCAGGTTCTTCT
CCCGGTTCACAGGTGGGTGCTGCCTGGGCCAGGGTGGGGCTCGGCTTGGCGCTTATGG
CCTCCACCCCCTCCTAGGGAACCT
>831
GGACGCAGTAGCCGAGATCGACGCTCTCTATGACGTCTACCTGGATGTCCAAGAGAAA
TGGGGCTTGGAGGTGAGGCCCTCCCAGGGGCAGTGGGCACCAGCGGCCTCCGCATGT
CCCAGGGCCACAGGCAGCGTTTCCT
>832
CCACCTTCCAGTGGCTGATCCCCGACAGCGCTGACACCACAGCTACACCCACGCACTG
TGCCTATGACAGGTGAGCAGGGCCTCGCGCTTAGGGCAGACTGAGGGCACCTCCAAG
GGCAGCCGTGACTCATAGGTCCGGC
>833
AGGCGCCGTTGTTCCCGACTCGGCTCTTCCCTTTAACTTCCAGGCTGCCTATGGCCTGA
GTGACCAACTGGTATGTGTCCTCCCTTGCACAGCCACATGAGGATGGGACACAGGAGC
TCAGGTAGGCTCAGCCCAGACCC
>834
CACGAAGAAGAGAAAACTGGGGAAGAACCCAGACGTTGACACAAGCTTCTTGCCTGA
TCGAGACCGTGAGGTAAAGGCTGCCTGGCCCTGACCCCGGCCTCGACTCCAGCCCTG
GTGGAGACCCTTGCTTAGGGAGCCAG
>835
AGGGCAACACCATGCAGCAGTTCCTGCAGAAGGCGCTCGAGATCCTTCGGAAAGACT
TCAGTGAGCTGAGGTGTGAGGTGTGCGTGTGTGCACGGTGGTGTGTGTGGCACCTGTG
GCTCCAGCCCGGGTGCCAGACACCC
>836
ACCACTCTTCAACTTTGATGTTCATGACGATGTGCGGTTGCTCAGTGACGCCACTGTGG
AGAAGGATGAGGTACAGCGTAGGGGGCCGTGTGAGGGGGAACGGGTGTCCCTGGGCT
ATGGAGGAGAAGCCAAGGGAAGGG
>837
GAACAAGCACATCTTTCCCGCCAGCCGCTGGGAACCCTACGACCCTGAAAAGAAGTG
GGACAAGTACACGGTGAGGAGGGGCTGGCAGGGACCCCTCCAAGTTGGGGACGGCA
GCCAGCCCCTGCTCACCCCTCGCCTTC
>838
GCATTTTGACACCCTCTCTAAGGGTGAGCTGAAGCAGCTGCTTACAAAGGAGCTTGCA
AACACCATCAAGGTAGGTGATGCCCCTCTCACTGCAAACTTGCCCATGACCCTGCACT
GAGGGGAGTGGCAAGGCCTGGCAG
>839
CTGGTCATGGCGCCCCGAACCGTCCTCCTGCTGCTCTCGGCGGCCCTGGCCCTGACCG
AGACCTGGGCCGGTGAGTGCGGGTCGGGAGGGAAATGGCCTCTGCCGGGAGGAGCG

AGGGGACCGCAGGCGGGGGCGCAGGA
>840
AAGACCAACACACAGACTGACCGAGAGAGCCTGCGGAACCTGCGCGGCTACTACAAC
CAGAGCGAGGCCGGTGAGTGACCCCGGCCCGGGGCGCAGGTCACGACTCCCCATCCC
CCACGGACGGCCCGGGTCGCCCCGAG
>841
CTGGAGGGCACGTGCGTGGAGTGGCTCCGCAGATACCTGGAGAACGGGAAGGACACG
CTGGAGCGCGCGGGTACCAGGGGCAGTGGGGAGCCTTCCCCATCTCCTATAGGTCGCC
GGGGATGGCCTCCCACGAGAAGAGG
>842
CTGGCTGTCCTAGCAGTTGTGGTCATCGGAGCTGTGGTCGCTGCTGTGATGTGTAGGA
GGAAGAGCTCAGGTAGGGAAGGGGTGAGGGGTGGGGTCTGGGTTTTCTTGTCCCACT
GGGGGTTTCAAGCCCCAGGTAGAAG
>843
CGGGTCATGGCGCCCCGAGCCCTCCTCCTGCTGCTCTCGGGAGGCCTGGCCCTGACCG
AGACCTGGGCCTGTGAGTGCGGGGTTGGGAGGGAAGCGGCCTCTGCGGAGAGGAGC
GAGGGGCCCGCCCGGCGAGGGCGCAG
>844
AAGCGCCAGGCACAGGCTGACCGAGTGAGCCTGCGGAACCTGCGCGGCTACTACAAC
CAGAGCGAGGACGGTGAGTGACCCCGGCCCGGGGCGCAGGTCACGACCCCTCCCCAT
CCCCCACGGACGGCCCGGGTCGCCCC
>845
CTGGAGGGCACGTGCGTGGAGTGGCTCCGCAGATACCTGGAGAACGGGAAGGAGACG
CTGCAGCGCGCAGGTACCAGGGGCAGTGGGGAGCCTTCCCCATCTCCTATAGATCTCC
CGGGATGGCCTCCCACGAGGAGGGG
>846
GGACAAGAGCAGAGATACACGTGCCATATGCAGCACGAGGGGCTGCAAGAGCCCCTC
ACCCTGAGCTGGGGTAAGGAGGGGAATGGGGGGTCACATCTCTTATCAGAGAAAGCA
GAAGTCCTTCTGGAGCCCTTCAGCCG
>847
GCTGTCCTGGTTGTCCTAGCTGTCCTTGGAGCTGTGGTCACCGCTATGATGTGTAGGAG
GAAGAGCTCAGGTAGGGAAGGGGTGAAGAGCGGGGTCTGGGTTTTCTTGTCCCACTG
GGAGTTTCAAGCCCCAGGTAGAAG
>848
GTGTGGCCCTGATGTGGGCCGCCCAGACCGTGGGCGCTTGGGCTTCCAGGTCTGGCTG
AAGAATGGCGTGGTGAGTGGCACCCTGGGCTAGGGCGCTGGGGGGCTGGGGTGTGAC
CCCCTGTGAGTCCTGGGCCAATCCC
>849
TTCCTGAAGGCGGCTGAGGACTATGGGGTCATCAAGACTGACATGTTCCAGACTGTTG
ACCTCTTTGAAGGTAGAGAGGAGAATGCTGGGGGAGGAGGTGGGCAGGAGGACAGG
GTGCTGGGACAGGGAGAGGGTATGAC
>850
TGGCTTTGGGCAGCTTGGCAGTGACCAAGAATGATGGGCACTACCGTGGAGATCCCAA
CTGGTTTATGAAGTATGTGGCCCCCAGGGAGCTTGAGTCTCCGCATGGGGTGGGAGGT
GGCTTGTTCTAAGGAGCTTGCGGG
>851

ATCGCTGAGGCCTACGACGTGCTCAGCGACCCGCGCAAGCGCGAGATCTTCGACCGCT
ACGGGGAGGAAGGTGTGTGCTCGCGGCCAGGGGCGCGGCCCCGCCTTTTGACAGACA
GGGAAACTGAGGCACGCTGGCCCCT
>852
CAAGCCCCACAATATCTTTAAGAGAGATGGCTCTGATGTCATTTATCCTGCCAGGATCA
GCCTCCGGGAGGTAAGGTGCCAGGTGGGGCGGTGTCTGTAGAGGGCGATGGCTGCTT
TTTGAAGCAGTTTAGTATTTGCTG
>853
ACTCCTCCCTGTGAACTCGGGCTCGAAAATGAGACGCTTTTCTGCTTGGATCAGCCCC
GTCCTTCCAAAGGTAAGTGTCGGAAGCTGTTATTTTAATTCAGGAAACTATTGAGTTGT
GGGAGAAATCAGCAAAGGCAAGG
>854
CTCATCCCCAATCTGCTCAAGGATTTCATCTTCGGGAGCGCCGTTTGCAAGACCACCAC
CTACTTCATGGGTAAGTTGCTGCTGGTGACTCTTTCTGGAGCTTGCCCCAGACCCCACT
CAGCCCCAGACCTTCCCAAACC
>855
CCAAAAATAACAACCAGACCGCGAATATGTGCCGCTTTCTACTGCCAAATGATGTTATG
CAGCAGTCCTGGTAAGGTTGCATTGTTTATTTTTAAAAATACAGCTTGATGTAATTTTGC
AATATCATGAAAAACTGAGAT
>856
TATGGATTAATCTCTTTGGAACTCTACCAGGGAATAAAATTTGAGGCTAGCCAGAAGAA
GTCTGCTAAAGGTAACTATCTTGTAGCTGTGTGTTTATGCCAAATGAGCACAAAAGCTT
GTTTTCTATCATTTAAATAGAT
>857
GCGCAGGGTCGCGATGCTGCCCGGTTTGGCACTGCTCCTGCTGGCCGCCTGGACGGCT
CGGGCGCTGGAGGTGGGTGCCGCGCCTCGGAAGGCGGGGGGAGGCTGCACGGTGGG
GACGCGATACCCCCCAAGACCTTAAC
>858
GGATTCAGATCCATCAGGGACCAAAACCTGCATTGATACCAAGGAAGGCATCCTGCAG
TATTGCCAAGAAGTAAGTCCTGTCCGGTGGCTAGCAATTCACGTTGGATCACATGCATT
TGTTTTCAAAAAATTTAACTTCT
>859
AACTGGTGCAAGCGGGGCCGCAAGCAGTGCAAGACCCATCCCCACTTTGTGATTCCCT
ACCGCTGCTTAGGTGAGCCGGCCGGCCGTGGGGCTGGTGTTGATTGGGGGCCTGGTCT
TGAGGGAAGAAAAAGAGGATGCTC
>860
CAAATTCTTACACCAGGAGAGGATGGATGTTTGCGAAACTCATCTTCACTGGCACACC
GTCGCCAAAGAGGTACCAGCCCATAAATTCTTTCTTATTGCAAAGTGAAGATTTCCTGG
GGACGTGCTTAATGGCATTTTAG
>861
CTGCTGATGCGGAGGAGGATGACTCGGATGTCTGGTGGGGCGGAGCAGACACAGACT
ATGCAGATGGGAGGTAAGGTGGCCTTTGTGTTCAGCCTCAGAGATGCTGAAACATCTT
GTATGGAGTATTTGTATCCTGTAAA
>862
ACAGAGAGAACCACCAGCATTGCCACCACCACCACCACCACCACAGAGTCTGTGGAA
GAGGTGGTTCGAGGTAATCCACCATTTGCTTGGATTCCCCCCACCCCCAAGGAAAAGA

AAGCGTAATACCAGAGTTGGAAATA
>863
GGCGGATGTGGCGGCAACCGGAACAACTTTGACACAGAAGAGTACTGCATGGCCGTG
TGTGGCAGCGCCAGTAAGTGGACCCTTCTTCGAGCCTGGCCACCTTTCGTCTCTCTCG
CCACTGACTCTGCTTTTTGTAACAG
>864
GAATGAACATGCCCATTTCCAGAAAGCCAAAGAGAGGCTTGAGGCCAAGCACCGAGA
GAGAATGTCCCAGGTAAGTCTGGCTCTTCCATCATTCAGCCCTACGATATTGGGAAACC
TGAGCTTGCCTCTGCCTCAGTCTC
>865
GAGAGAATGGGAAGAGGCAGAACGTCAAGCAAAGAACTTGCCTAAAGCTGATAAGA
AGGCAGTTATCCAGGTAAAACCTGAACCCATTTCCTACCAAACATCACATGCCAGCAG
TCTTTTTAATGAGTGTCTGCAGGTTT
>866
CATGCTCAATGACCGCCGCCGCCTGGCCCTGGAGAACTACATCACCGCTCTGCAGGCT
GTTCCTCCTCGGGTAGGTCTCGCTGCAGCCGAGTTCACACTTCAGGTCACAGCACAGA
CAGTAAGGGTGGGGCACTGGGAAC
>867
GCACACCCTAAAGCATTTCGAGCATGTGCGCATGGTGGATCCCAAGAAAGCCGCTCAG
ATCCGGTCCCAGGTAAGCGTGGGGTATAATCATCTTCTGCAGCTTTGACAATCCAGGAT
TCTTGCTTCTTGGGTTGGTGACA
>868
CGCATGAATCAGTCTCTCTCCCTGCTCTACAACGTGCCTGCAGTGGCCGAGGAGATTC
AGGATGAAGTTGGTAAGTAAGCTGTTCTTTTGATGCTGCACATGGACATGTATTTTCCC
CCAGAGGAAAATTGAGGAAGTGA
>869
AGCCTGGACGATCTCCAGCCGTGGCATTCTTTTGGGGCTGACTCTGTGCCAGCCAACA
CAGAAAACGAAGGTAAGAGTCCCCTGAGCCAGCAAGGGCGTTCTGGGAGGTATATATA
CACATACATAACGTGTGTGCAAGA
>870
GATCTCTGAAGTGAAGATGGATGCAGAATTCCGACATGACTCAGGATATGAAGTTCATC
ATCAAAAATTGGTACGTAAAATAATTTACCTCTTTCCACTACTGTTTGTCTTGCCAAATG
ACCTATTAACTCTGGTTCATC
>871
AGTGATCGTCATCACCTTGGTGATGCTGAAGAAGAAACAGTACACATCCATTCATCATG
GTGTGGTGGAGGTAGGTAAACTTGACTGCATGTTTCCAAGTGGGAATTAAGACTATGA
GAGAATTAGGCTTAGCTTTTTGC
>872
TTGACATCTGACGACGTGAAGGAGCAGATTTACAAACTGGCCAAGAAGGGCCTTACTC
CTTCACAGATCGGTGAGTGTTTGTGTCTAACATAGCCTATTTCGCCTGTCCTCGTGTGA
CTTGTAGGATCTAGTAGGTGGTA
>873
TCCTGAAGATCTCTACCATTTAATTAAGAAAGCAGTTGCTGTTCGAAAGCATCTTGAGA
GGAACAGAAAGGTAAGCTAATAAATAAAACCTGGTGCTAAGTAGGACTGCTTGTACTA
AATTTTTTCGGTAAAATGTAATG
>874

TAGAGAGCCGGATTCACCGTTTGGCTCGATATTATAAGACCAAGCGAGTCCTCCCTCCC
AATTGGAAATAGTAAGTATCAACTCTTTTGTCGTTGTTATCAAGAATAGGAGTCAGCCA
GTAGTAAAAGTCCTAGTAGTAA
>875
CGCACCTTAGTGCTGCTGGACAACCTCAACGTGCGGGAGACTCATTCGCTTTTCTTCC
GGAGCCTGAAGGGTGAGAGCGGGGTCCGAGGGGGTAGCGTGTGGGGGCCTGGGGTT
GGTACAGACATCGAGGGCATTGACCA
>876
AGCCTGTCTCTCATAAAGTATGGGGAATTCCTCTATGACAATCTCATCATTTTCTCCCCT
TCGGTAGAAGGTAAGGGTTCCGCGTCGCCCCTAGCACTGGAATTCGAGACCCGGGACT
TGATGGATTGCTTTCTTCTGGT
>877
AACGTGGAGACCATCAGTGCCTTTATTGACGGTGGAGGCAGTGTGCTGGTAGCTGCCA
GCTCCGACATTGGTGAGTCCGACCTGGGAGGTTCTGGTGCTTTCCACTTCTCCAGGGA
GTTGGAGAGCATTACAAACAGAAG
>878
GATTGAGTTTGACGAGGAGAAAACGGCTGTCATTGACCATCACAACTATGACATCTCA
GACCTTGGCCAGGTAATCAGGCCTGTCACCTTCCAGGTTTCAGGGGCATATGGCAAAG
CTCTGCTTAGCCAAAAGCCAAGAG
>879
AGAACCTGCTGAAGGCCCCAACCATCGTTGGGAAATCATCTCTAAATCCCATCCTCTTT
CGAGGTGTTGGGTGAGTGAGTGAGCAGGGAGAGGACAGCAGAAACCTGGGGGAAGA
AGGGGTCACTTAATTTTTGTGGGAA
>880
TTTGGTGCTGGACATCCTGACGGGCTCTTCCACCTCTTACTCCTTCTTCCCGGACAAGC
CTATCACCCAGGTAAGGGCTTCACAACCTTGAGGCTTCGAAATAAAAATGAAGCAGAA
CAGCACATTTGGCTGAGGCGAAG
>881
GCTCCCTCGACTTCTTCAGCGACTCCTTCTTCAACTCAGCAGTGCAGAAGGCGGCGCC
CGGCTCCCAGAGGTAGCTGCCAGGAGGCTTAGGGGCCGGGGATCCGGGTGGTGCAGT
GTGTGTATATGGGGAGGACTCAGAA
>882
GGGGCCTGTGTCCCATCATCGGGTGGGCGAGACAGCCCCACCCAATGCCTACACTGTC
ACTGACCTAGTGGTAAGAGATGTTTGGGGTGGGAGGGCCCAAAGGGTTTGCCAAGCT
GACTGAGCTCAGCTCAGAAGCACTT
>883
GATGGCGATGACATTCAGCTGGAGTTTGTCCGCATTGATCCTTTTGTGAGGACCTTCCT
GAAGAAGAAAGGTGAGTGTAGTTCCTGCACGAGGATGCTGCCAGAAAACGGCCCCAG
GCCTCACTTGCTCTCGAGGCCCAA
>884
GTATGGTGTATTCCAGTTTAAAGTGGATTACAACCGGCTAGGCTACACACACCTGTACT
CTTCCACTCAGGTAAGCACAGGTGACAGCTTCTGTTTTCAGCTAGCATGTGCCCTGCCT
GGGTTCTGCAGTGGGCAGGTCC
>885
TGTCTTTCAACTGGCAAGAGTCATTTTGACCAGCAGGTTAATCAACTCTAAGACAGATC
CTCACGCAAAGGTATGTACCTGGAAATATTAACTGAAGAAATTTTTTCTCAGACTTTTC

ATGAGCTTTTTATAGGACATTC
>886
CCACCATCCACGCCAGCCGCACCATGCCGCTCCTAAGCCTGCACAGCCGCGGCGGCAG
CAGCAGTGAGAGGTAAGTGCCCCGCCCCCGTCCGAAGCTTCCAGAAGCGCCGGCCGC
GCAGGCCTGCGGGGGGCCGTCCTCC
>887
CCTGAAGATCCCAGGTGGGCGAGGGAATAGTCAGAGGGATCACAATCTTTCAGCTAAC
TTATTCTACTCCGTGAGTATGGGGCCGGGCCTGCTGGGGCTGCTCTGGGGCCACAGGT
ACTTCTTGTGCACCTTGCTGCCCG
>888
CAGTGCTGAGTGGCGGCAGCCTCTACATCGAGATCCCGGGCGGCGCGCTGCCCGAGG
GGAGCAAGGACAGGTGAGGGGCAAGGACAGGCAGGGAGTGCTCACCCAGGGGAGCA
GGGACCAGGTGAGGGGCAAGGACAGGC
>889
GAGTTCGCTGAGGAGCAGCTGCGAGCCGACCATGTCTTCATTTGCTTCCACAAGAACC
GCGAGGACAGAGGTAGGTGACCCGTATCGCCTGCAGTAGGGTGCGTGGGGGTGCCTG
CGCCGCCCCTTCATTCTCAGCCCGC
>890
CCCTCTTGAACACGACCCTCAGCAGCAGCAGCTTTGAGATTTACCAGTGCTGCCGTGA
GGCCTTCCAGAGGTGAGGGCCTGGGCTGTGGGACCTGCAGGGCACCCACAGTGATAG
ACAGAATGCTTGAGCGCTGGGGGGG
>891
GTGGGTGATCATTTTAGCAGTTCTAGCAGGATTGTTGCTACTGGCTGTTTTGGTATTTGT
AATGTACAGGGTAAGTAACGGACTTAGAAAGAAGGAGAGAGGGAAAGCAGAAGAAA
AGGGAAAGGGAAGAAGGAAAAGAA
>892
TGATAGAAAAACAAATCAGTCATGGCAACTTCAGCACCCAGGCCCGGGCCAAGACAG
AGAACCCGGGCAGGTGAGTGAGCGTGTGTAGATAGAATGGGGCCAGGGAGGAGGGG
AGAAGGAGGGACAGAGCTCTGTAGAAC
>893
CTGGGGAATTGTCACTTATGGCAGCATTGGCAGCAGGACATCTTGTCAAAAGTCACAT
GATTCACAACAGGTAAGACTCAAAGATATATTTAACATGTTCCCCCTATACTTCAAAAA
ATATGCAGTGTAAAAACTTACTA
>894
ATCACCTTTGGTGCCTTCCTCATCGGGGCCCTGCTCACTGCTGCACTCTGGTACATCTA
CTCGCACACGCGTGAGTACCCCAGGCCCCCACAGTGAGCATGCCGGGCCCCTCCATCC
ACCCGGGGGAGCCCAGTGAAGCC
>895
GAGCAGGAGCACCAGTCCAAGCCCCTTGGAGGCCTGTCTGGAGGCTGAAGAGGCCGG
CTTGCCTCTGCAGGTAAGGACTGGAATATAGAAATTCAGTAAGAGGAGGCAGTAAAGA
GGAGGGATGTGTAATCCCCGGATGA
>896
GGGAAGTGGCTCCGGCCTCCAGGCTCCGGGGCCGGCGCTGACCCCATCCCTGCTTCCT
ACGCATACATTGGTGAGTGCCTGCGGAGGGGTGGGGTGTGTGGGCGCCAGACCAATG
GGATTGGCATGTGGCGGGTGACTGA
>897

TCCTAGCATTCACTTCTGGAGCACCCTGAGTCCCATTGCGCCCCGTAGCCCGGCCAAG
CTCTCCTTCCAGGTAGGATCCTCTTTCCCCGTTTTGGACAGGGTGAGAGACCCAAGAA
TGGCCCCCATCTGTGACCTCTCCC
>898
CCTGGGCCCTGCGTGTGATCTCACCCTCTTCGACTTCAAACAAGGCATCTTGTGCTATC
CAGCCCCCAAGGTACTTCACAACATGAGATACCTAGGTAGGGGTGGATGTGCCACCCA
ACACCCAACCACTGACCCATATT
>899
GGAAGGAGGCCCCGAGGGATGAGACCAAGGCTGACACTGACACAGCCCCAGCTTCAT
TCCCTGCTCCTGGGTGAGTGTTGGTCTAAGGTGAGCCAGATGGGTGAGGGTTGTGATT
GGGGGTGCTCTTGGAGCCCCACACC
>900
TGTGGAATCAGCTTCTCAGGGGATGGCACCCAAAGGCAGCGGCTCAAAACGTTGTGC
AAAGACTGTCGAGGTGAGTGGCCTCCAGAGGATGGGGCATCGGGAGATAGGAGCTGG
AGCAGGTCCAGTGTCATGCTACGCTG
>901
GCCCCATGATGTGGCATCGGGGCTGTTCTGCAAGTGTGAACGGAGACGCTGCCTCCGG
ATTGTGGAAAGGGTGAGTTGGGCAAGTGGAAAGAGCCCAAGGCTCACCTCGGCCCCT
GGCCCCCATGGGCTTGGCCCCATGC
>902
CATCTGCCTTCGCCCTCCCCGCCCTGGTCTCAGGCGCCAGTGGAAATGTGTCCAGCGA
CGTTGCCTACGGGTGAGTTGGGCTGGAGGGAGCAGAGCTCATTCTGCTGTTAAAATCG
TCGCTGCCTCTGCTTCCTTTCAAC
>903
CAGGCGGCGCCCCGGAGCCCAGCCACTGCCTCCACCACCCCCATCACAGTCCCCAGA
GCCCACAGAGCCGGTGAGGCCCCAGTGGGTGGAGGGAAGGCACAACCCTGACCTTAC
CCAGTACCTGCCCTGACCCCACCCCA
>904
GCCCAAATTCGGGGGCAGCAACCAGAAGCGCCAGAAGTGTCGTTGGCGCCAATGCCT
GCAGTTTGCCATGGTGGGTGGGGCAGGAAGGGTCAGGTGGACGGGATAGGTTGGGCC
AGGCGCCCCAGTGCCTGGAAGTGGTG
>905
ACGGGAAGGCGCAAGCAGTCCTGTGCAGGTGCCGGGCCCTGTTGCAGCTTCCACAGA
AGCCCTGTTGCAGGTGAGGGCCCCACCCTGTCCTGCCTTCCCAGCCTCACCCAGCTGC
ATGCCAGGGAGCTGAGACCAAGTCT
>906
CCAGGACGAGTGGACACCAGGCACAGCTGTCCTGACTTCTCCCGTATTGGTGCCTGGC
TGCCCTAGCAAGGTGGGGGCAGTGATGGATGTTCTGTTGGTGCCGTAGCGGAGTGGTC
TGTAAGCAGAGTGATGATGAGCCA
>907
GGAGAACAAGGATGATTCTGCCTCCAAATTGGCCCCAGAGGAAGAGGCAGGAGGGGC
TGGCACACCCGTGGTCAGTGCTGGGGATGCCCCACCCTGCCCTGACCCTGCTGTAGCC
CCAACAACCACATTGACCTATGGTG
>908
CCGCGGAGAGAACCCGGGCACCCCTGCTCTCTCCATAGGCTTCTCCGTGGCCCTGGGC
CACCTCCTTGGGGTAGGTCATGGCCATGGGTTCCAGCCTCCCTGGAGAAACAGACACA

CAGACCACTCCAGAGACAGACACA
>909
CGGCTGCTCTATGAATCCTGCCCGCTCCCTGGCTCCAGCTGTCGTCACTGGCAAATTTG
ATGACCACTGGGTAATGGCTGAAACCCCCTGCCCTCCCCTTCTCTAGAAACCCATTTTA
GAGGGAGAACAAGAGCTGGAAT
>910
AAATCATTCCATTCCGAGTGGCTGCCTACAACAAAGCCAAAAAAGCCATGGACTTCTA
TGATCCAGCAAGGTAGGTTTTCAGAGGAAAATTCTTTTATCAACATCTGTATAAAAGAA
ATGATGAGGCAGAATTTGGGCCC
>911
ACCTGCAAAAGTCAGGGCAAGACGTGTCCCAGGCCCAACGCCAGATCAAGCGGGGG
GCGCCGCCCCGCAGGTAAGCGGGGGTCCCCGGGGCTGGGCGGGGTCGAGCGGGGGC
GCACCACGGGTTCGCTCTGTCTAGGCCA
>912
AGCGTCCGTTTTCTGCAGCAGCGACGCCGCCAGGCGCGGCCGGGACCGCAGAGCCCC
GGGAGCCCGCTCGGTGAGTGCAGCGGAGAGGGCAGGAAGGGCAAGCCGTAGAGGCG
GAGTCAGCGGGAGAGGCGGGGCCAGAG
>913
ACCATCCCACGAACACAGATAGAAAGAAGGAAAACAAGCCTGTATTTTTCCAGCAAAT
ATAACAAAGAAGGTATCCCTTTCCCAATCAGAACAGCAAATTCTAATTCCATTTTGGGT
TTTCAATTCTGATGCACTATGTT
>914
GTTCAAGAAACACTTTTTCATGATCCATGGAAGCTTCTCATCGCTACTATATTTCTCAAT
CGGACCTCAGGTTTGGGGATTATTATCATCTTTGTCTTAGTAGAGACAGTGTGGTAGGG
AGAAAGCACTGAATTGAGGCC
>915
GATGTGTCAGAACTTCTTAAACCTCTTGGTCTCTACGATCTTCGGGCAAAAACCATTGT
CAAGTTCTCAGGTATTTTCCTATATACCCAAAGGAAAAACATAATACATTGTGCTTATTT
AAGAGAGCCACACCTTAAACT
>916
TGAGCTTCATGGGATTGGTAAATATGGCAACGACTCTTACCGAATTTTTTGTGTCAATG
AGTGGAAGCAGGTGAGGCTCACTCCCATCCATAATTCAGCACATTTGGTCTCTGAGGC
AAAATAAGTCCACCATTATGGTT
>917
AGTTGGGGAAGCTCCAGATGGACCAGCCAGTGCCTACTTGGCCGACACCACCCATACC
TCCTTGTACATGGTGAGGCCCCAAGGATGGGTGTAGACCAGCGCAGGGGGTAGAAAC
GATTTTTAGAAACGAAAGCTTTTTA
>918
TGTCTCCTCCAGGCCTGGAGGCCTTGGTGTCTGAACTCTGTGCTGTCCTGAAGCCTCG
CCTCCAGCCAGGGTGAGTGAGGTCATCACCCCAGGGTGTGAGAAAAGCCTGCTTAGA
ACATCCCTGGAGGAGCAGCAGCCAG
>919
CCCCCCAGGCTGTGGGAAGACCACAGTAGTTGCTGCTGCCTGTAGTCACCTTGGGCTC
CACTTACTGAAGGTGAGGGTCTCTGGAGAGTAGAGCCCCAGCTACTTCCCCTCCCCAG
CTCATGTTCTCCCTCTCTGCTGCT
>920

GGCTGGGTGAGGATGCCCGTGTGATGGCTGTGCTGCGTCACCTCCTCCTCAATGAGGA
CCCCCTCAACAGGTACTTATACAGAGCTGGGCCTCTCAAAGCTGGGGGGGCCGGCTGG
GCGCGGTGGCTCACACTTATAATC
>921
GCGGGCCCTCACTGCCCACCTTCCCCTGGGCCAGGAGGTGAACTTGGCACAGCTAGC
ACGGCGGTGTGCAGTGAGTATCAAGGGAGAGTACTGGCCCTCCCTAGGAGGTCCCTGG
CTTTTGTTACAAGAACTCAGTCCTG
>922
TGGTAGGGGATCTCTATGCCCTTCTGACCCACAGCAGCCGGGCAGCCTGCACCAGGAT
CAAGAACTCAGGGTAAGAGGACGTGGGCACCGGAAAGGCGGAAAGAGGAAATGGAT
GTAGATGGGTGGGTGGTGAGGAAGGG
>923
GGCTGAGGACTTTGGGCAGGCACTGGAGCAACTGCAGACAGCTCACTCCCAGGCCGT
TGGAGCCCCCAAGGTGGAGACCAAGTCCTTGGAGGTGGGACAGGAGCCCCTCCAAGA
GCATATCCCAACCACGCACTTTCCAT
>924
GGCCCCCTGGCACCGGCAAGACCCTTCTGGCCAAGGCAGTAGCCACTGAGTGCAGCC
TTACCTTCCTCAGGTGGCCAGGGGGCCTGGAAGGGATGGTGGGTGGATCAGGGGCTG
CAGCAGGCTGGGGAGCAGGAGGGGCT
>925
TCTTTGATGAACTGGACTCTTTGGCCCCAAGCCGGGGGCGAAGTGGAGATTCTGGAGG
AGTGATGGACAGGTAGGGGTCTGAGTGGGAATGAAAGCTGAGGGGTCCAGGGTGGGA
AGGATATGAGAAAGCTCACAGCAGT
>926
AGGATGTGTTTGTGATTGGAGCCACCAACAGACCAGATCTCCTGGACCCTGCCCTTCT
GCGGCCTGGCAGGTGGGCCCCTTGTACTGCTCATCTCCACCCTCATTTTGTGCCTCCTT
CACCCCTCATGGCTGGGCTCCCT
>927
AGCTGGTGTTTGTGGGGGCAAATGAGGACCGGGCCTCCCAGCTACGCGTTCTAAGTGC
CATCACACGCAAGTATGCTCTGTTAGAGGAAGACTGCGAAGGGCTTGGGCCTTGGGAT
GCTTAGGGGATGCATGCAACATGC
>928
GACCTCTACTCTCTCTGCTCTGATGCTATGACAGCTGCCCTCAAACGCAGGGTTCATGA
CCTGGAGGAAGGTGAGCCACTTGCCGGTCTGAGAGGTCAACCAAAAAGCTAGTGGGG
AGCTGGGTGCACCTGAGTCCCAGC
>929
TGGGCCGCGACACTAAGAAACATCTAGTGGTGCCGGGGGATACAATCACTACGGACAC
AGGATTCATGCGGTACGTGGGGACTTGGGGGAGTCGAGGCTTCAGAGAGCGGCTTCA
GGGCTCTCTGCACGTGGTCCAGGCC
>930
TCATTGCATCTGTTGCTGGCTCTGTGGAGAGAGTAAACAAGTTGATCTGTGTGAAAGC
TTTGAAAACCAGGTGAGAACAAAAGGTGTGTATTCCCTTTCTTGCAGCATCAGGTTGT
ACAACCAGGCTTTTCCTGCCCCCT
>931
CTTCATCTGGATTTACCCAACACCTGAGCACAAAGAAGAGGAAGCAGGGGGCTTCATT
GCAAACCTGGAGGTGAGCAAACACTGTGGCCATTTTCAGTGGGATGGAGGGGGCTCA

GTCTTTGCTGTGTTTTTGTGGCCAG
>932
GGTAACTCAGAGGATGATGCTGTATGATACCAGCATCCTGTACTGCTATGAAGCATCCC
TTCCACATCAGGTACTCTCCCCAGGGCCTCTCCCTTCTTCACTGATCTGTGAGCTGCTC
TGTTGTTTGTTCAGAGACAATA
>933
ATCTGCCTGAAGCTGGTGGGCTGCAAATCCAAGAAGGGGCTGTCCTCGTCCTCCAGCT
GTTATCTGGAAGGTAAGCCCGGGCCGCACGGGTTGGGCTGAGTAGCCGCGCGCCCTCC
CGCTGCTGCTGGGCCCTTCCTAGG
>934
GACCCCAACCTTTTCGTTGCACTGTATGATTTTGTGGCCAGTGGAGATAACACTCTAAG
CATAACTAAAGGTAAAAGGGTTGTGGGCAGCTAGTGGTGGTTGCAGGAGATAGAAATC
TGGGAATTGCGGTTTGACCTACC
>935
GAGGTCCATCTCGCTGAGATACGAAGGGAGGGTGTACCATTACAGGATCAACACTGCT
TCTGATGGCAAGGTAGGGGACCCTTGGCAGGGGGCGCTGATGGGCCCAGGGCAGGGG
AACCAGAGGTCCTGCTGTCGGATTG
>936
CCAGTACGGGGAGGTGTACGAGGGCGTGTGGAAGAAATACAGCCTGACGGTGGCCGT
GAAGACCTTGAAGGTAGGCTGGGACTGCCGGGGGTGCCCAGGGTACGTGGGGCAAGG
CGTCTGCTGGCATTAGGCGATGCATC
>937
GTGGAAGAGTTCTTGAAAGAAGCTGCAGTCATGAAAGAGATCAAACACCCTAACCTG
GTGCAGCTCCTTGGTGAGTAAGCCCGGGGCTCTGAAGAGAGGGTCTCGCGCCGCACC
CCCAGGGTGACACAGGCGCTGGGGAA
>938
TGCTGCTGTACATGGCCACTCAGATCTCGTCAGCCATGGAGTACCTGGAGAAGAAAAA
CTTCATCCACAGGTAGGGGCCTGGCCAGGCAGCCTGCGCCATGGAGTCACAGGGCGT
GGAGCCGGGCAGCCTTTTACAAAAA
>939
TTCCCCATCAAATGGACTGCACCCGAGAGCCTGGCCTACAACAAGTTCTCCATCAAGT
CCGACGTCTGGGGTAAGGGCTGCTGCTGCACTGAAGTGGTCCTTCCTGACTACAGGAG
GGTTTTTTTCTGCCTCTTTCTTGC
>940
CTAGAGAAGGACTACCGCATGGAGCGCCCAGAAGGCTGCCCAGAGAAGGTCTATGAA
CTCATGCGAGCATGTAAGCCTTCCTCAGCCTGTTCTCACGAGTATATGTGGGCATTCCA
GGAAATTCAACTGTGCAGGAGTGT
>941
GACCGGCCCTCCTTTGCTGAAATCCACCAAGCCTTTGAAACAATGTTCCAGGAATCCA
GTATCTCAGACGGTAAAGTACCCATCCCGGGGTACCTGCAGTGGGGTGAAAGGGCAGC
CATGTGGGACTGCAGCCTGGGTCA
>942
AGAGCTGCAGAGCACAGAGACACCACTGACGTGCCTGAGATGCCTCACTCCAAGGGC
CAGGGAGAGAGCGGTAAGTCCCCCGCTTCCCCCAACCCCACTGCTCTTCCCTTCCCTG
CCAGAGGCTACATTCAGGCCATCAT
>943

CAAAGCTAAAATTGAACTACTTGGGAGCTATGATCCACAAAAACAACTTATTATTGAAG
ATCCCTATTATGTAAGTACAGTTCACGTTTTAGGGCTAATATGAAGACCCAACACATTTG
TATCCTGCCATATTAAATAAC
>944
ACTATAAGCTCCAGGTTGACTTCTCCTCAGACCACCTGGGCTGTTTCACCAAGTGCCG
GAAGCCATGCAGGTTAGTGTCCCCTTCCCCGGGTACAGGTTGGGTAGAATACAGGTTA
CCGAGCCAAGCATTTGGGAGAGCT
>945
AGTGGCCAAAGTCAACATCTTCTTCAAGGAGCTGAACTACAAAACCAATTCTGAGTCT
CCCTCTGTCACGGTAGGTCGTGCCTGGATGGAGCCTGTGATGCTCTGGGGGTCTTACTT
AGCAGGGCGGCTGTCGGGGAAAA
>946
GCGCCATCTACAACAACATGAAGCAGTTCATCCGCTACCTCATTTCCTCCAACGTGGGC
GAGGTGGTCTGGTGAGCAGCTGGGTGGGCGTCCAGGAGGAAGCCGGGGTTAGGGTGG
GGTGGCTGCAGGTCTGGGAGGCAG
>947
CGCCCCCCCCGGAGCCCCAAGGAGCCCCTCATCAGTGGCTGGCTCTTCTTCCGCTACA
TGGCAATCGGGGGTGAGCTGGAGGGGTTCCTCGATCCTCCCCACCCCTTGGGACTAAC
CCCCTCTCTGGGACACCAGCTCCC
>948
CACCGTGGGAGCAGCTGCCTGGTGGTTCCTGTACGCTGAGGATGGGCCTCATGTCAAC
TACAGCCAGCTGGTAGGGGGAGGCCACAAAGGAGGGGACCAGGAGGGGGGGGGGAT
GCAGGAGGGTACCAGGAGGGTGGCAT
>949
AGGCCCCCGAGCCCATGACCATGGCCCTGTCCGTGCTGGTGACCATCGAGATGTGCAA
TGCACTGAACAGGTGGGGGCCCCCCAGCTACACCCACCACCCTCCCCTGAGGCCACT
GCCCACATCCTCCACTGTGCCGCCC
>950
CTGGCTGCTGGGCTCCATCTGCCTCTCCATGTCCCTGCACTTCCTCATCCTCTATGTTGA
CCCCCTGCCGGTGAGGTTTCTTCCGCCCAGGGCCGCCCACCCCAGCACTGGGGAGCCC
ACGGGCGCCCATGACCACTCCC
>951
CTCAAGATCTCACTGCCAGTCATTGGGCTCGACGAAATCCTCAAGTTCGTTGCTCGGA
ACTACCTAGAGGGTAAGGAGTGCCCTCTCTGTCCCAAGCCCTGGCCCCACCACAGCCC
CTTCCCCATGACGTCCGTCCCCCG
>952
ATGTGAACAGGAAGAAAAGTATGAAATTCCTGAAGGTCCACGCAGAAGCAGGCTCAA
CAGAGAACAGCTGGTATTTTTCTTTTAATACAACTTTCATTGTTCTTATTATGACATACTA
TTATTATCACCATCAGGAAGAA
>953
TGTGCACCGGCTGGTCAACCTCTTCCAGCAGGGGGGCCGGGAGCTCGACATCTACAAC
AACCTACGGCAGGTGAGGCTCGGTCTGAGGGAGGAGGGCTGGGGCCTGGACTCCTGG
GTCTGACAGAGGAGGGGCTGGGGGC
>954
ACCTGCCTGCGAGCCCTGCTGGACAGCGCAGCTCCGGGCACGTTGGACCTGGAGGCC
CGCAATTATGACGGTAAGCATTTACCGCGGGGCACCGCTGGGCTGTCCAGCGGACCTG

GAGTCCATCAGCGGCCGCAAAGCCC
>955
GGCAGTGAACACCGAGTGCCAAGAAACCGTGCAGCTCTTGCTAGAGCGCGGTGCCGA
CATCGACGCAGTGGTGAGCGTGCACTAGGAGCTGGGAGGGAGCGGGGCCTTAGCAGG
GGCGGGGTCTTGGCGGGGGCGGGGCC
>956
GAGCGGCCGCTCCCCGCTCATCCACGCCGTGGAAAACAACAGCCTTAGCATGGTGCAG
CTGCTGCTGCAGGTGCGTACAGCCCCCCTGAGCCTCGCGCCCACCCTATCCTCTGACC
CCAACCCGGCTCTGGCCTCAGCCC
>957
CAGCGGCGCTGACAGCAGCCTCAAGAACTGCCACAACGACACGCCGCTCATGGTGGC
GCGCAGCCGCAGGGTGAGCCGGGGCAGCTGTGGACATGCCCCTTGCACACGTGTGTC
CGCGGGCTGGTTGCAGGCGAGTGTGC
>958
CAGCCAGACCCCTCCCCTGACCGGAGCGCCAACACCTCCCCCGAGAGCAGCAGCCGC
CTCAGCTCCAATGGTGAGAAACCGTTCCCAACCTCCAGCCCTGGCGCCTCCTCCCTCA
GACCCAGGAATCCCAACCCACAGCC
>959
AGCTGCTGAAGAACCACTGATTGAGCCCCTGATGGAGCCAGAAGGGGAGAGTTATGA
GGACCCACCCCAGGTGAGGGGGCAGCAGGGCTGGGCGGGACTTTGGGAATCCAGGAT
GATTGCTGCCGTCCCTAGCTGGGGTG
>960
CTTCATGCCGTCTCCCCAGGTGGTCAGGTGGAACCCATGCCTGGAGCCTCACCGCTTC
AATGACACAGAGGTGATTCCTGCCTTTTTCCTCAGTAGGCTGAGGGTACACAGAGGTG
ATCTAAGTCAGGGACCAGAAGCTG
>961
CAGTATCTCTGCTGCTCTCAGACAGCCAAAAATGCTATGGGCTGCCAAATTTTGGAGA
ACAGGAATGGAAGTAAGCAATCTGATCGATATAATTTCTTTCTCCTTTGACTGTTTCCAT
CACTCTCCAAGGGTCTGTCAAG
>962
GGAAGGGTGATGAATATTTTATCTTGGAGGAAAGCAACTTACCATGGTGGAGAGCACG
AGATAAAAATGGGTGAGTCCACACCAGCCCTTCCTGAGCCTGGTGCCTGCCCACTCCC
TACACGCAAAAAGTGCTGCGGCAT
>963
GAGGTTTCATTGTCAGAGACTCCAGCAAAGCTGGCAAATATACAGTGTCTGTGTTTGCT
AAATCCACAGGGTGAGTGCTACTATTCCAAGGCCCTGAGGACAAAGAACAGGGGTAC
CCTCCTAATAGCTCCTTGATGCTG
>964
TACCTGGCTGAGAAGCACCTTTTCAGCACCATCCCTGAGCTCATTAACTACCATCAGCA
CAACTCTGCAGGTGAGTACCAGGGGCAACTGAGAAGGGAGTTACAGACACCAGGATA
AGCAATGCAGGACAAGGTGATAAG
>965
ATATCCAGGCTCAAATATCCAGTGTCTCAACAAAACAAGAATGCACCTTCCACTGCAG
GCCTGGGATACGGTAACTCCTTATTTCTCTGGGGTAGGGTGGACTGGCCAGTTGCAAA
AACTACCTTTGCTGGCCTTGCTTA
>966

TGGCCATCAAGATGATCAAAGAAGGCTCCATGTCTGAAGATGAATTCATTGAAGAAGC
CAAAGTCATGATGTGAGTTATAGCCCAAACTCAACTCTCAATCTATTTGCTGGAGTCTA
GGAATTCACACAACAACCCACTG
>967
AGAGATGTGCAAGGATGTCTGTGAAGCCATGGAATACCTGGAGTCAAAGCAGTTCCTT
CACCGAGACCTGGTGGGACCTTAGAAGGATTGGCCTAGGACAAAGGGCTGATGGGGT
GGAAGTAGCAGTGGAAGATATATCA
>968
GTCCGGTGGTCCCCACCGGAAGTCCTGATGTATAGCAAGTTCAGCAGCAAATCTGACA
TTTGGGCTTTTGGTAAGTGGATAAGATTACACAGATTATACAGCTCAAAGGATAAGAAA
TGCAATGGGGAAAATTCACACTT
>969
CCTACGTCTCTACAGGCCTCATCTGGCTTCAGAGAAGGTATATACCATCATGTACAGTTG
CTGGCATGAGGTAAGTGCTTTATTAGGATCTCTTAAATTATTTCCTTGAATCTACTTGCC
CATTTAGCTGGCCATTCAGC
>970
ATGAGATCTCTTGGGCAGAATCCCACAGAAGCAGAGTTACAGGACATGATTAATGAAG
TAGATGCTGATGGTAAGTCTTCAATTTTGTGGGGCTGGGAGGGGTTGAAGAAGTGATA
CTTAGATGTTTCCACTAAACCTTT
>971
GATGGCAAGAAAAATGAAAGACACAGACAGTGAAGAAGAAATTAGAGAAGCATTCCG
TGTGTTTGATAAGGTACGTCAAGGACTGGATATGTTAAATTTTGAGGATGAAATGAAGA
TACCAGGACTCATTATGAAGACTT
>972
ACAGATGAAGAAGTTGATGAAATGATCAGGGAAGCAGATATTGATGGTGATGGTCAAG
TAAACTATGAAGGTAAATGTTTCGGTCTCACGCCTCATTCTTCACTTTGATTTTTAAGAT
AAGAGGCTCTTTGTTAGGGAAC
>973
ATGAGGTCACTGGGTCAGAACCCAACAGAAGCTGAATTGCAGGATATGATCAATGAAG
TGGATGCTGATGGTAAGAGCTTTAAAACCATGAATGAGGGCCATTGTTGTGTAATTCAA
GTTCAGACATGTTACAGGATTGT
>974
GATGGCTAGAAAAATGAAAGATACAGATAGTGAAGAAGAAATCCGTGAGGCATTCCGA
GTCTTTGACAAGGTAATCCAGCATCTACATAGCAGATGGTACTTAAGTATGGCTTCTTCC
GCTTTCACTTCTAAAAGCTATA
>975
ACAGATGAAGAAGTAGATGAAATGATCAGAGAAGCAGATATTGATGGAGACGGACAA
GTCAACTATGAAGGTAAAACTAAATTCTCTGAGCTCAGTGTTTCATAGTCTTACCTTTA
GATCTGTAAGCAAGCCAACTGCTT
>976
TCAAAGAACAGGACATAAAAACAAAGTCACAATGACATTCCATAGTAAATTTGGAATC
AGAACTCCAAATGTGAGTATAATTTCTTCTAGGTTTAAATGCTCTGTAGGTTGGCACTGT
TACACTGCATGTATGCAAAGAA
>977
GACACGGACGCCTTCCGCAGCTGCAAGTTCCCCACCAAGAGGTCCAAGAAAGCTGGC
CGCCATTAAACAGGTGGGGGCCTAGGTAAACAGGTCCCCTATGTCCCTCTGGGTGATTC

CAGCAAGTGAGCTGGTATCTTCCA
>978
GCCCCCCGGTCCTCTGGGCCTGCAGGGCGTCCCGGGTGTTCCTGGCATCACGGGGAAG
CCGGGAGTTCCGGTACGTCGCTTTTCCGGCTTTTCCAGCTTTCACAGGGTTGAGATCGT
GTTTTTTCCGGAAGGAAGTTACT
>979
CACCCTGGCGCTCGAGGACCCCCCGGATACCGCGGTCCCACTGGGGAGCTGGGAGAC
CCCGGGCCCAGAGGTGAGTGTTTGACCCCATGACACGGTCACCCTGCTGTAAAAATCC
CTGAGACTGACTTGTTAGTAGGCGC
>980
GGTGACAGAGGAGACAAAGGCGCGGCAGGAGCAGGGCTGGACGGGCCTGAAGGAG
ACCAGGGGCCCCAAGGTACGAGTCCACGGCCAGCAAGGCTTCACTGGGTGACATCTC
TTCCGCCATCTTGTAGCTTTATGTGGGG
>981
AGGACCGTGTACCACGCAGAAGAGGTGCAGTGTGATGGCAGGAGCTTCCACCGCTGC
TGCTTTCTCTGCAGTAAGTAGCTCTCCATGCTCCACCTTTGACGGAATTTGTATGTACAG
AGCTTCAATATGTCTATTTAAGG
>982
ACGGTTATGGCCAGGGCGCTGGCACGCTTAACATGGACCGTGGCGAGAGGCTGGGCAT
CAAACCAGAGAGGTGAGTTGGGAGACCGTTGGTTACCTTCAGAAATGAGTTGCGACA
GTTAGTGCCGCTTGCTGAACAGGAA
>983
AGGTGCTGAGAAGTGTTCCAGATGTGGGGATTCTGTATATGCTGCCGAGAAGATAATTG
GAGCTGGAAAGGTAAAGTGCTATTTGGAATTAATAACAGCAAATGACTTCTCTCTGCTC
CCTTTCTCCCCTAACCCTTCCA
>984
TGTGCAAAGTGTGGGAAGAGTCTTGAATCAACAACTCTGACTGAAAAAGAAGGTGAA
ATCTATTGTAAAGGTAAAATTCATTTCAGTTACTGCTGTCCATATGAATATCCACACTGG
TTCTTGTTTAATGAAATGTCACT
>985
AGTATCTCAGCGCCCTGGAGCCACAGTACCCACTGACTTTGCCACCTTCCCTTCATCAG
CCTTCCTCAGGGTGAGGGGGAGCATGGGGTGGAGGAGGTGGGACACGTGTGGAGAG
CTCAGGCGGCCCCCCTCTCCTCCAG
>986
TGCAGAGCAGATGGTTATGGTGATCAAAGCCCCTCCTGAGACCCAGCTCCAAGCCGTG
GACTCTTCGGAGGTGAGATCTGGGAACTCCGGGCCCAACCTGGGCTGGGCTGGGCTG
GGCTGGTGGTCACCTGGGCCTCAGT
>987
CCACCACCATCATCTCCCCCCTCATCCCTCACCACAGATCCCAGCCAGTCTCTACTCAG
CCTGGAGCAAGGTGGGTGATGGGTAGGTGGGTGGGGTGGGGCAGGGCCCCTCTTCTG
GGGGGTGGGCAGGCACGACAGCCC
>988
AGGTGGACGTGGGTTTGGCTGCCGATGTAGGAACACTGGAGCGCCTGCCCAAGGTCAT
CGGGAACCAGAGGTGGCGTGCAGGGGGTGTGGGGGTGTAAACAGAGACCCCAGAGC
ATCTCCCAGCCCCGGGGTGGCAGCCT
>989

TGGTCAACGAGCTGGCCTTCACCGCCCACAAGATGATGGCTGACGAGGCCCTGGACA
GTGGGCTGGTCAGGTAGGGCCATGGGCTGGTAGCTCAGTGCTGGGGGCAGCCAGGCC
TGGAGGGAAGGAGGCCTCTCCGGGTC
>990
GGTGCAGAGCACCAAGGTCAACCTGCTGTATTCCCGCGACCATTCGGTGGCCGAGAGC
CTCAACTACGTGGTAAGGTGCACGCTCCGACCAATCACAGCCCTCCTCTAACCCCAGG
CGACCAACCAGGGTGCAGGGTGCT
>991
ACCGGGTGCGCATCAATGTACCTCCACACAGAGGGCTTCTCTGGGCCCTCTCCAGGTG
ACGGGGCCATGGGTAAGGCAGCCCCCCTTCCCCTGCCAAGCCCTCCATGTTGGGGGAG
GGAGCGCTGCCATGGGGGAGGGTC
>992
CCCAACAAATGCCCATTTCATTGCCTGGACGGGCCGGGGAATGGAGTTCAAGCTCATT
GAGCCTGAGGAGGTGGGCCTCTCGGATGTTCCCAGCCCTCCTTTCCAAAGTTTACAGC
CTGGAGGTGGGAGAACCTGGGAAA
>993
GCCAGCCATGAATTACGACAAGCTGAGCCGCTCGCTCCGATACTATTATGAGAAAGGCA
TCATGCAGAAGGTGGGGGCTGTGGGTCTAGGGACAAGGTGGTGGGGGGCAGTGGCTG
TGAGAAGCTGACTGGGGAGAGGGT
>994
ATAGCCAACGAGAGTGCCCCTCCCAACTTGTGTGGCACCTCCCACGCCATTGTGGGAC
TCATGGCCAATGGTAAGTGTTAACTTCTAGAGTCTCTCCCTCACCCCCACCTGTGCTTT
GCATATAGACCAGTGAGAAGCTG
>995
AGCATGAGATCACCATGCCCAACCAGATGGTCCGCATCTACAAGGATGGCAAGGTGTT
GTACACAATTAGGTATGTCAAGCCTCTGGAGTCTCACTTCCTGGAATTCTCTCTCCCCTT
CTGATAATTTTAGCTAAAGATC
>996
GCCGGATGCTCACTCCACATGCTCAGATTTCCAATGGATTCTCACTCTTGCCCTCTATCT
TTCTCTAGCTGTGAGTACCTTCTTAAGTTTCTGGGGCCCCAGAAACATGCTGGGCTCCT
TCTTTTTCTCATCCTTGCCAT
>997
TCCTGGAAGCTCTTCCAGTTTGATTTTACAGGAGTGAGCAACAAAACTGAAATAATCA
CAACCCCAGTTGGTAAGCGTGCCAGGGCTTGGCGGAAGTCCAGGAAGGTGGTAGGGA
TGATTGGAGATGGCCATCCATACAA
>998
GTGACCACGATGCTCTCCTGGGTTTCCTTTTGGATCAAGACAGAGTCTGCTCCAGCCC
GGACCTCTCTAGGTAAGAGGAGAAACAGGTATACGCATAGGCACATGGCTGGGAGTTG
GCTGGGCCAGGGCAGAGTTGCCTT
>999
GGAGTTTGCTGTGCTCAACTTCCTGATCTACAACCAGACAAAAGCCCATGCTTCTCCTA
AACTCCGCCATGTATGAGCTGGGTATGGGAGTGGTGGCAAGGCTTTGGAGTGTAGAGA
CATGCTAGCAAGGGTACTGGGGT
>1000
CAAGACCAGCTGCTCACACCCGAGGAGGTCGTGGACAGGATCTTCCTCCTGGTGGATG
AGAATGGAGATGGTAAGAGGGGCAGAGATGGGGAGAGTGCTGTCCACTCTGCATCATC

GCCACTTTCTGGCCGCACGTCCTT
>1001
GATGGCATTTCCCATGCACCTAATGCTGTGAAAACCGCATTTTATTTAGCAGAGTTTTTT
GTTAATGAAGGTAATATGAGGGTACATAATTTTGTTATTCTGGGGTAGTTTTGAAGAAAA
TTGCCCTTATCTGAACTTTT
>1002
ATCATGTCTTTTCCCTCTACTTCATGATACCCTTTTTTTTCACCACTTTGGGATTGCTCTA
CCACAACTGGTAAGTAGGCCTGTGGATAAGGGGACAACTACCTGAACACATGGCAAA
GATGGCCCTTATCATAACCCAC
>1003
TGTTCAACTTCCTCTACTCACTGCCTCAGCTCCTGCATATCATCCCCTGCCCTCGCCACC
GCATACCCAGGTAGCCGCTTTTTGGCTTGAAATGGACATCATAGCCTTTTCACTTGGGA
TATCTAATGCCAGCCTATACA
>1004
CAAACTGGAGATGAGCTATTCCAAGTTCAAGACCAAGAGCCTCTCTTTCTTGGGCACC
TTTATTTTAAAGGTAACAGGGTAACAAGGAGGTAAGGCCCTAGGCTGCCATCCTGACC
TTGAGGAATGGGGAACCTAGTCCT
>1005
CAACTTGCTACTTAAAGTCCTTGGGCCCATACATGAGAGAAACCTCACATTGCTCCTGC
TGCTGCTGCAGGTGAGGATGGGAATCGAGTTTATACCTCCGTGTCTCCCTTTCTGCGTG
ATTCTTACTCCAGTCCATTTCT
>1006
TCCTACTGGGGAGCCCTGCCCAGAGCCCCTGCGTACCTATGGGCTTCCTTGCCACTGTC
CCTTCAAAGAAGTAAGTACTTAGGGAGGAGAGAGCGTTACCCCTGTGGCTAAAGAGAT
GGGGTTTGGAGAGAAGGGTCTTT
>1007
AATTCACCACCTCGGACTCCTGCGACCGCATCACAGACGAATTTCAGCTACTGCAAGC
TCAGTACCACAGGTGAGCTGGGGGACTGGGGGTCCCTGCGTCCCGAGAATGTGAGTC
CCCCCATTTCTTCCTGCGCAAGAGG
>1008
GCAGCAGGTCTTGGGAGCCATTGAGAGGGCCAAGCAGGTCACCGCTCCCGAGCTGAA
CTCTATCATCCGAGTATGTATGGGGACCTGGGCGGGTGGGCGGGGGACAGGTGGGCCC
CAGGCAGCTAATGTTCCCCTCCCCC
>1009
CCCCTGCCCGACGTCAGGCTGTTCAAGTACCTCCCCCATCATTCTGCACACTCCTCCCA
ACTGGACGCTGGTAAGGACTTTGGAAGAAAAAGTTTACCTTCAGTATTTGAGCAGGTG
AGCACTTTGTGCGAGAGGTGATA
>1010
GTTCAACAAGTACCTGTCGCGGCCCAAGCGCTTCGAGGTGGCCACCTCGCTCATGCTC
ACCGAGACCCAGGTGCGCGCCCTCCCCGCCCCCGACGCATCCCGGCCTCCCAGGGGA
CACTGTTACCTTTGCACTTGTGACC
>1011
GAACTACGTATCCAGGCCGCGGAGATGTGAGCTGGCGGCCGCCCTAAACCTGCCGGAA
ACCACCATCAAGGTATGCGGGGTCCAGGCTGGGGAGGCGGGTGTGCACCTATTTAGCG
GGAAGTAAATGCCAACTGCCAACT
>1012

GAAGTGTTACTTTCGGAAGGAGAACCTCCTGGAGCATGAAGCCCGGAATTGCATGAAC
CGCTCGGAACAGGTACTTGGGAGCTGGCCCAGGTACTTGTGGGCAGGGCACACTGGC
CTCTCTGTCTTCGTGCCATCCGGGA
>1013
GGAGACATTCCGCCGAAGGATGGAGCTGCGGGTGCACATGGTGTCTCACACAGGGGA
GATGCCCTACAAGGTCAGGCTTGGCCTGTCTCCAGGGCCAGGGTTGGGTGGCCCCAGG
ATCCTTCCTGCTGAGCCCTTTACGT
>1014
GCAGTTCATGCAGAAGAAGGACTTGCAGAGCCACATGATCAAACTTCATGGAGCCCCC
AAGCCCCATGCAGTAAGTGACAGGGAGGGCTGGGCATGCTTTGATGCTGGCATGAGCA
AGAGTGAGCTGTGCCCAGAGTGGG
>1015
TGTGTGAGGAGTGTGGGCACCGGGCCTCGAGCCGGAATGGCCTGCAGATGCACATCA
AGGCCAAGCACAGGTGCGTGTCGCCCGTTCTCTCTTGGGGCCCAGTCCTGCTGCCAGC
CCAGGCTTGCACCGGCAGGGAAGAC
>1016
CACCTGCGCACACACACGGGTGAGAAGCCCTTCCAGTGCCACCTCTGTGGCAAGACC
TTCCGAACCCAAGGTGAGGTACGCCCTGCCCCTCCCCTCGTCTCCCCATCCTGAGGTC
AAGGCCACAGGCTGAGCTCTTGCCT
>1017
AGGCACGTGGCCAGCCGCCATCAGGAGGGCCGGCCCCACTTCTGCCAGATATGCGGCA
AGACCTTCAAAGGTACCTGGGCGGCCCTGGGAGAGCCATTTCCTGCTCATCCGAGTTG
GAGGGTCTCTGAGGAGGAAACGCT
>1018
GCACGTCAGACGGCACAAGGGGGTGAGGAAGTTTGAGTGCACCGAGTGTGGCTACAA
GTTTACCCGACAGGTAGGCCAGGGCCTGGCCCCCTTCCCCTACCCTAGGATCCCCCAA
AGTTCTGAGCTCACCCTCCCCGCCA
>1019
GTCAGGTTTAAGATTCGGGACATGGACTCAGGCACTGTCCTCTTTGAAATCAAGAAGC
CCCCAGTCTCAGGTGAGTGGCTGGGTGGGCCATTGATGGGGAGGCAGATAGGAGCTG
CGGAGTGGGCTGCCCAGGGAATCCT
>1020
CCAATGCTGGGCGCTTTGTCCGCTACCAGTTCACGCCTGCCTTCCTCCGCCTGAGGCA
GGTGGGAGCCACGTGAGTCGCTGGGCGATGGGTGGTGGTGGGGGTGAGGGAGAGGG
TGTGAGTGGGCCCTTCTCAGCCTCTC
>1021
TTCTGCATCCCCAGCAGCAAGAACACCTGCGAGCACATTTACGACTTCCCCCCTCTCTC
CGAGGAGCTGAGTGCGCGGGCAGGGTCTTCTGGGAGTGGGGAAGTGGGGGCGTCTAG
AGACTGAGCTGCGGGAGGGAGAGG
>1022
TGGATCGCGTTGTCCCGCCTGGCGCGCCCGCAGCGCCTGCCGGTGGCCACTCGCGCGG
TGCTCATCACCGGTGAGTGCGCGGGTCGCGGAGCGCGGGGACTCCAGGCTCGAGGGC
GGGACTGGACACTCAACAGGACTGA
>1023
ATGGACCTGACCAAACCAGGAGACATTAGCCGCTTTCTAGAGTTCACCAAGGCCCACA
CCACCAGCACCGGTCAGTGGCAAGTGTCCACCAGGCAAGGGCGTGGCAGGGGAGTG

GGAAGGACACGGGGACTGGAAGTTTG
>1024
GCTGACCAAGGGCCTCCTGCCCCTGCTGCGCAGCTCAAGGGGCCGCATCGTGACTGTG
GGGAGCCCAGCGGTGAGTGCCCCCCCCCACTGGAGCAAAAAGGAGCCCCCTGGGGTG
GGGGAGGGCTTAGGGAGCCCCTTGC
>1025
ACATTCAGCTGTGAACTCCTTCCCTGGGGGGTCAAGGTCAGCATCATCCAGCCTGGCT
GCTTCAAGACAGGTGGGAATCAGGGCTGGGGGTGGGGTGGGGGTGGGGAATGGGGCT
GGGAATGGTCTTATGGGGGCAGGTC
>1026
CCAAGAGTGTGGCCCGCCGGGTCCTCCAAGCCCTGGAGGGGCTGAAAATGGTGGAAA
AGGACCAAGATGGGTAAGCAGGGTAGGGGGGGCTGCATTGATGGAGTAGCCTTGAGG
CCCGGTCATCAATTCCCCAACGAATG
>1027
GCCTATCGTATGGCCCAGTCTCCCCGATCATAAGAAGACTCTGGAACATCTTTGTAAGA
AACCAAGAAAAGTGAGTGTTTTTGGTGCTTAAAAAGTGTTGTGTTGGCAACATCCCAG
TGGCCAAGAATGATATTCCAGGA
>1028
TGCTCCCCGCCGCTTCCGTTTCTCGAGGGAAAGGCTGCTGCCTCCTGCTCTGTCCTCAT
CCCCGGCTTAGGTAACACGTTTTCCTCCTTACGACACCCACCACAGGGTCCCCGCGCC
GCTGCCCCTCCTCCTTGGATCTG
>1029
TGCAGTTGACTTCAAGACGCTGGCTGATGTACTGGTACAGGAAGTTATAAAACAGAAT
ATGGAGAACAAGGTAAGAAAGGTCATAGCCAAGGTAACTGCAAAATAGTTGCATCTGT
GGCTTTCTCCATCATAGAATAGCA
>1030
GACCCAACTCTGGATTCCACAGAGATCAATGTTCCACAGGACATTTTGGGAATTTGGG
TGGACCCCATAGGTAAGTATAGGAAAGTATGTTTGTTCTTATTTGCAATCTTTTTTTTGG
TGGTGGGGTTGGGGATGGGTGT
>1031
TACAGACAGGGGTTCCCCTGATGGGAGTCATCAATCAACCTTTTGTGTCACGAGATCC
AAACACCCTCAGGTAAAAGGCAAATATTTTTGGTATATTATGGTTGTTAGAACTTACGA
CCTCAGCTTTGCTTGTTGTTAGG
>1032
ATCCATGCTCAGCCATCTGATAAAGAGAGGCTTACTTCTGATGCATAGTATTGATCTACT
TCTGTAATTGGTAATTGATCAATGTTTTTTAATTGCTAGCTGTGGGACCCGCTATGGTTG
TGGTGGCTAACTTTAAGAAC
>1033
CAGGGCCTGGGGATCTGGCCGAGGAGAGGACTGAGTTCCTGCACAGCCAGAACTCGC
TGTCACCACGCAGGTGCACACTCTTGGCCTTCCCTCTCCTCCAGGCCTCTGGGAAAGC
GGCTTCCCTTTCTGAGCCACCCCTG
>1034
GGACCTCCTCCTGGCCACCACAGCCAAGCCATCAGTACCCAGTGCTGACAGTGAGAC
ACCCCTGACCCAGGTGAGCAGAAACCAGGCTCTGGAGCCCAACAGCACAGTGGTGAC
AGTGGGCATCACTGAATCTGTATCCA
>1035

AACCCGGCGCCGGAGCTCAGGGCCTCCTTTCTGCCACGTACCTTGTCTCTGCGGAACT
CCATCAGCAGGAGTGAGTCTGGACCCAGGAGAGCAGGGGAGGGGTTGGGGGGTGCT
CCTGGGCTTCCCTAGCTCAGCCTGCT
>1036
GACGAGTGGCAGGCCATCTCGGAGATTGCCTCTACTTGCAACACCATTCTGGAGTCGC
TGTCCCGAGAGGGTAGGCCACCAGGGTGCTGCGGTAAGCCTGGGCAGTGCTCTTGCC
CCCTTTCTGACCTGCCCACCTGATC
>1037
TGGGAACCTCTCAGAGAAGGTCTCTCACTTGGAGTCCATGCTCAGGAAGCTGCAGGA
GGACCTGCAGAAGGTGAGGGGTGGGCTCAGCTTGGGGGGAGTCACAGGGCTGCCCC
AGGAAAGGGGCTGGCGGCTCTGACTCC
>1038
TCAGTGGTCATCATCCCCTTTGCTATCCTCCTCTTCTTTCTCCTTCATCGCTGGTGTGCC
AACAAAAAGAGTAAGTCTCACGAAGCAGAAGCCAGAGAGCTCAGGGCCATGTGGGG
AAGCAGGATGGGAGCACTCAGGTG
>1039
TCAGTGGCCATCATCCTCTTTACCATCCTTCCCTTCTTTCTCCTTCATCGCTGGTGCTCC
AAAAAAAAAAGTAAGCCTCACGAAGCAGAGGCCAGAGAACTCAGGGCCCTGTGCGG
AAGCAGGATGGGAGCACGCAGGTA
>1040
GGCAGCAGCGACCCCTTCCAGCAGGGCCTCACGCCGCCCCAAATGCCAGGTGACCAC
ATGAACCCCTATGGTAAGCCGCCCTACCCCCACCCGCCCGCCCCAGCACAGCCCCTGC
CCCCTGCCAGGCCAGGCAGGCCTGG
>1041
GTCAACCTGACAGGGGGGCCTGAGCCACGCCTCTGACGTGGTCCTTTACCATCAGCACA
AGCAGCCCGAAGGTGGGGGCAGAGGGGAGCCTGGGCTGGGGAAGGGAGGCCTGTGG
GCATTGAGTCTCCAAGCTGTGGCCCT
>1042
TGTCCCAATGGGAAGCGGCTGGACAACGGCACATGCGTGCCTGTGCCCTCTCCAACGC
CCCCCCCAGATGGTATGCTTATGCCCTCCCAGTCCCAGCCATGCCTCAGGACCATCTCC
TTCCTGTGGCTCCTGACTTCCCC
>1043
GACAAGTGTGAACTGGACCAGTGCTGGGAGCACTGTCGCAATGGGGGCACCTGTGCT
GCCTCCCCCTCTGGTATGCCCCCTCATCCCGCCACGCCTGCTCCTTGCCCCTGGGCCCC
AGCCCCGCCACCCACCACCCCACC
>1044
ACTGTCAACCAGGGCAACCAGCCCCAGTGCCGATGCCTACCCGGCTTCCTGGGCGACC
GCTGCCAGTACCGTGAGTGAGCCATCCCTGGGCCCCAGGGCATGCGGGAGGGTGACG
GGGGACCCCAGAGCATGGGGTGATG
>1045
TGAACAAGTGCAGCCGCTGTCTCGAAGGGGCCTGTGTGGTCAACAAGCAGAGTGGGG
ATGTCACCTGCAAGTGAGTGGGGCCCTCCTCCACAGTTCCACCCAGCCGGGCCCCTGC
CCTGTCCTAGCCCTGCCCTGCCCCT
>1046
TGACCTGCGTCGGCCACTGCAGCAATGGCGGCTCCTGTACCATGAACAGCAAAATGAT
GCCTGAGTGCCAGTGAGTTGGGCCCGGGCTTCACCCAGGCATAGATCATCGCTCCCTT

CCCCAGATTTGAGCAGGAGGACCG
>1047
CTCTGCTGTTGCTGCTGCTGCTGGTTCTGGTGGCCGGAGTGGTATTCTGGTATAAGCGG
CGAGTCCAAGGGTGAGTCACAGGGATTCTGGAACATTCTGGTCATTATTTTGCCATCCT
AGCCTTCCCCCCCAATAATCTC
>1048
GTACGAAGGCGGAGAGCCTGATGATGTGGGAGGCCTACTGGACGCTGACTTTGCCCTG
GACCCTGACAAGGTGGGCTGGGAGGCGGGCAGGGTCAATTGCCAAAAGGCCGTGGGT
GGCCTAACCAAAGGTGTTGGGTTAG
>1049
CACTTTTATTCTCTTGGACTCTGTTTCTCAATGTTGGACCTAAGATATTGAAGACAGGCT
GGAGTCCAGAGTGAGAGACTTCAACTATTCTTTGTGTGTGTCTGTCGTGTGAGCAAAA
ATGATGTAGTTAAGAGGCTCCC
>1050
TGAATGAGGCCTTGGAACTCAAGGATGCCCAGGCTGGGAAGGAGCCAGGGGGGAGC
AGGGCTCACTCCAGGTGAGTGACCTCAGCCCCTTCCTGGCCCTTCTCCCCTGCCTTCCT
AGGTTGGAAAGCCATAGGATTCCAT
>1051
GCTCTTTCGGGACATTGCCCAAGAGTTCTCTGATGACCTGCACCATATCGCCAGCCTCA
TTGGGAAAGTAGTGAGTAGAAGGAAAAAGGGAGTGCACCCAGGGAGGTCAGGGAGA
GAGAATGCAGTGTGCAAGATGGGGA
>1052
GACTTTGAGGGCAGCCTTGCTGAAAATCGCTTCACAGTCCTCCCCAACATAGATCCTG
AAATTGATGAGAGTGAGTGTTGGGTGTGGATGGGCCTGTGAGCCCTGCGCAGTGATGG
AGTACCATCCTTGGCAGGTGGTCA
>1053
GGTTGCCCGCAAGGAGCTGGAGAATCTGGACTCCCGTATTCCTTCATGCAGTGTCATCT
ACATCCCTCTGGTGAGGGCAGGAGAGTGGGTGTAGCCTTCAGATGTCTTTTGGGGGAG
ATATTAGGCTTATGAAAGACATA
>1054
CCCGGGACTATGGCTACTCAAGGCCGCGTTACTCCCCACAAGTCCTTGGGGTACGAAT
CCAGAATGGCAGGTAAGAATAGAGGCGGGTGGAGGAATAGACATGAGGGGCCCAAAG
GCTACATCTTCTGGGGGTTCATCTA
>1055
CAGACTTGATCCGCAGTGGAAAACCCATCAAGCCTGTCAAGGATTTGCTAAAGAAGAA
CCAAATGGAAAAGTGCGTATATGGCCCCAGTGTCTTTACCCTCTCTGCATCTTCTCCTG
CAACTCTTCTCCCCTTTTCAGGG
>1056
TCTTTCTATTCCCCGCCTGCCTTCCATGGTAGAGGCCAGTGACTTTGAGATTAATGGACT
GGACTTCATGGTAAGACCCTCAACCTCTGTAAGGTGAGTGATGAGGAAAATGAGTCAG
CAGCTGAGGAAGAGCGTTACTC
>1057
CTGCACTATCGTAGTGCCCGAACCAAGGAGCTGGATGCATTGCTGGGGGACCTGCACT
GCGAGATCCGGGGTGAGGAAAAGCCAGAGGTTATATGCATTGTAAGATGTTTAAAAAA
AGCAGCAGCCAGGGGAAGGAGGGG
>1058

CCCGGGACTATGGCTACTCAAGGCCGCGTTACTCCCCACAAGTCCTTGGGGTACGAAT
CCAGAATGGCAGGTAAGAATAGAGGCGGGTGGAGGAATACACATGAGGGGCCCAAAG
GCTACATCTTCTGGGGGTTCATCTA
>1059
TGGGGACAAAGGGAGGGTCAAAGTCATCACTGGACCCAACTCATCAGGGAAGAGCAT
ATACCTCAAACAGGTGAGGAGAAGCCCTGCAGCCTGGGCCTCTGGCGTCTCCTGCATC
TACTCCACCCCTACTTGCCAGCCAA
>1060
CTTCACACGAATTCATAGCTGCGAATCCATCTCCCTTGGCCTCTCCACCTTCATGATCGA
CCTCAACCAGGTCAAAGGGAACAAAGGGAGGTGGGATTGAGGAAGGGGATAATGGG
AAAGGAACCCCTGAAAATGCTCAT
>1061
GAAAGCAGTGAACAATGCCACTGCACAGTCGCTGGTCCTTATTGATGAATTTGGAAAG
GGAACCAACACGGTGAGGGGAGAAACTGATGAGGGGAGAAACTAAGGAGGGGAAAA
TGGAGGAGGATGAAGGAGCATGACAG
>1062
TGTGGCCACCAACTTTCTGAGCCTTGTTCAGCTACAACTGCTGCCACAAGGGCCCCTG
GTGCAGTATTTGGTGAGGAGACCAATCTAGCTCCTCGGGGACCCCCAGGCTGGGCATT
TCCCAGAGGTGGGGATTGGCTCCT
>1063
GGCCAGCCATGCCTCCCACACAGCTGCCCAGGCTGGGCTTCCTGACAAGCTTGTGGCT
CGTGGCAAGGAGGTGATGAGATCCAAATGTGCAACCACCTCCACATCAGAGCTCCCTT
TCATTCCTAGTCCTACTGGGCCTG
>1064
CAGATTTGATCCGCAGTGGAAAACCCATCAAGCCTGTCAAGGATTTGCTAAAGAAGAA
CCAAATGGAAAAGTGCGTATATGGCCCCAGTGTCTTTACCCTCTCTGCATCTTCTCCTG
CAACTCTTCTCCCCTTTTCAGGG
>1065
CTGCCTCCCACATTCTTCCCTGTCTTTTTGGGTTTTGTTTTTTGTTGTTGTGGTGGTTTTT
TAACACAAAGTCTCACTCTGTTGCCCAGGCTGGAGTGCAGTGGCATGATCTTGGCTCA
CTGCAACCTCTGCCTTCCAGG
>1066
TCCACTAGGACCAGACTGCACCCGGCACACAACACTTGGTTTGCTCAACAAAGCCAA
AGCACACATCAAGGTGAGAATTTTTACTTTCAGATTTGCACAATTCCCTCAGTTCTGGT
TTAATTATTGGCACTGATTCCTAT
>1067
GAGATGGAACGAATACGAATGGACAGCATTGGATCAACTATTTCTTCAGATCGTTCTGA
TTCAGAGCGAGGTAGGCAGCTTGCCTCTTCTCTAATGAAATACTAAACATCTCTGTATC
TGGATTTAGGGAAGGTATGTTG
>1068
CATCAAGATGAAGCAGGGGGAGACCCTGGCCCTGAACGAGCGCATCATGCTCTCGCTC
GTCAGCACTGGGGTGAGCTGGGTGGGCCGGCGTGCCGCTGAGGCTCTGCAACCCCGG
GCGCCCCTGCTAACTGCCCGCCCCC
>1069
CTATGCGGCCGAGATCATCCTGGGCCTGGAGCACATGCACAACCGCTTCGTGGTCTAC
CGGGACCTGAAGGTGAGCATGCCCCTGCTGTCCCCAGGCTGGACCTCCGTGGCTGTCC

TCTCCTTCCTCTCGACATCCCGGC
>1070
AGGGGAGGTGAACGCGGCCGACGCCTTCGACATTGGCTCCTTCGATGAGGAGGACAC
AAAAGGAATCAAGGTACTGGCCTTGCCTGGCCTCTTGTACCTAGGCTGTGATCCTGGC
CTGGGGAAGGATCCCTCTCCCTTCT
>1071
CCAGTGGCAGCGGCGGTACTTCTACCTGTTCCCCAACCGCCTCGAGTGGCGGGGCGAG
GGCGAGGCCCCGGTAAGGAGCCCATGCGGGGGTCCGGGAGCCGGGCTTCCGGGTGGC
TGGCCGGCCCGCTCTTCACCTGCAC
>1072
GCAGATCAAGGAGCGCAAGTGCCTGCTCCTCAAGATCCGCGGTGGGAAACAGTTCATT
TTGCAGTGCGATGTGAGTGGGGGCTGAGCCAGGGATGGGAGGCCGAGGGGCCAGGCC
AGGCAGGCTGGGGAGAGCCCCAGCT
>1073
CAGCCAGGAGACAGAGGCTCTAGGGCGGTCCCAGGGCCGCTTGGAGGCCAAGAAGA
GAGAGCTGCTGCTGGTGACAGCGGGGGAAGCTGCTTCCATCCACTGAATCTCTGGCTG
CCCGTGTCCGTGTCCCCATGGGTGTC
>1074
CAGCAGCAGCAAGGCCACAAGGCCCCGGCTGCCCACCCTGAGGGGCAGCTCAAGTTC
CACCCAGACACAGGTGCTGGCCCTAGTGCAGGGAGGAGGGGCTGCAGGGCTGGACCC
CTGGGTCTGAGGTTGGAGGGGCTGGG
>1075
GGAAAAGAGCCAAGAGAAGTGCTTGTCTTTGGATGCCAAGTTGCTGAAAATTAATAGC
ACAGCTGATCTGGTGAGTGTTCATGGATATTTGTTGGGGTGGAGTATTGGTTGATAGGG
AAATTAATCTGGTTTAAACTGTC
>1076
GGCTGGCAACGAGCAGCGCACGCTCATCAAGGCCTATGGCATCCGCTTCGACATCATT
GTGTTTGGGAAGGTAGCTCGCCGCCACTGGCTCCCCTCCGTCACTCCCTGCAGGGACA
AGGGGCCTCTCCCTGCCCCTGCAG
>1077
CCTCTACTGCATGAAGAAAAGACTCTACTATCGGGAGAAGAAATATAAATATGTGGAAG
ATTACGAGCAGGTAGGCCCCTCCTGGCCCCCAGCAGGCACAGGCCTCTCATCTCTTGG
GTGTGGGAGCCCTGGGCGTGGGC
>1078
CGAGCGTGAGTCGGGCCTGGACATCAGTCCCATGTGTGACAAGCATACGGCCTCAGTG
GAGAAGTCCCAGGTTGGCAGGGGACTGGGCTGAACCGGTAGACACTGGGAGGAAGT
AAAAGGAGGGGCTGAGCCAACCCCAC
>1079
GATGGTGGACCTTGCCCATCTGGCTCTTGGTCTCAGAAGAGAAGCTTTGTTTATGTCTG
GAAGACCTGGGGTGAGGGACTCCCTTCTCAGCCTATCATCCACACTTGTGTTTACTTCT
TTCTACCTGATCACCTTTCTTT
>1080
GTCTACCATCGCCGGGGATCCCGGAGCTATGTGCCTCTTGCTCATTCCAGCTCAGCCTT
CACCATTACTGGTAAGGGTTCAGGAAGGGCAAGGCCAGTTGTAGGGCAAAGAGAAGG
CAGGGAGGCTTGGATGGACTGCAA
>1081

TACTGCCTCAATGTGTCTCTGGCTGATACCAACAGCCTGGCAGTGGTCAGCACCCAGC
TTATCATGCCTGGTAGGTCCTTGGACAGAGACTAAGTGAGGAGGGAAGTGGATAGAGG
GGACAGCTGGCAAGCAGCAGACAT
>1082
CCAGTTGCAGCCTGCGATGACACAGCGAATCTATGACAAGTTTATAGCTCAGTTGCAG
ACATCTATCCGGGTGAGTGGCGGGAAGCCTGGCAGGTGCTGTTGACTTGGGTTCTGTC
TCCAGATTGTCAGTCCCCTGAGGG
>1083
ACCTAGAAGCTGTCTTGAATGCCTTGGATAAAATTGTGGAAGAAGGCAAAGTCCGCAA
AGAGCCAGCCTGGTGAGAGTGGGGTGGGGAGGTGAGAAGGTACAGGAAAGAGGCAG
CAATTAAGCTTTTTTTTTTTTTTTTT
>1084
CGTCCTGGCAGGGCGGAGGCAGGTGGAGGAGCTGCAGCTACAGGTCCAGGCCCAGCA
GCAGGCCTGGCAGGTCAGTGTCCCAGCCTGCCTCTTCCTCTTCCTTCTCTAATGGGCCC
TCTGAGATCCGCAAATTGGTGGCT
>1085
GGTGAAGGAGACCAGCATGCCTGTGTACTGCACGTGCGCGGGAGACTTCGCCCTCAC
CATCCACACCCAGGTGGGCTCTCCCTCATCTGCCTCGGCTCAGCCTGGCCTCCTTGGCC
TCCTCTCTGAGTGGACTGGGGTCT
>1086
CCTGAACCAGAAGTATGGCCCTGACTTCAAGGACCGGTACCAGATTGAGCTGCTGCTG
CGGGAGCTGGATGTGAGCGATGGCCCAGGACCCCTGCTGGCCCGGGGGGCGGGGGAC
CCCTCTACACCATGCCTGCCCTCAG
>1087
CAAGTACCGCCGGGAGACTCCGCTCAAGCCCATCCCCGTGGAGGCACTCTGTGACTTT
GAGGGGGAGCAGGTGCGTGGGGCCAGCTCCTCTGTTCACATCACTACAGAGTTTTTGG
GGTAACATGGTTGGATCCCGGGCC
>1088
AGCTGATTGCTCCGGCCGTCTGTTTTGTGATCCCCCCCACAGACCCTGAGGCCCTGGCT
CTGGCTGACAGGTACAAGGGCAAAGGCGGGAGTCTGCATGGACCAGTCCCACCCCCA
GGGCTTCTCAGGCCCCATTCATAG
>1089
CAGAAGGCAGCTGGGAGCAAACGCACGCTGCAGCAGCGGTATGAGGTGCTGAAGACC
GAGAATCCCGGAGGTGGGTGCCAGGGCCACTCGCCTGAGGCTCTGGGGGTACCGGGG
AGGAGGGGCTGGACCAGGGTCTGTGC
>1090
GATCCTGCGGCCACCACTGGAGCAAGGCCGGGCTGTGCAGGACAGTGCCGAGCGGGC
CAAGGACCTCAAGGTACCTGCGGGTCAGGAGCACAGGTGGGGCCGGGGGAGCCCTGC
ACTGGCCCCCTGGGGCCCCTATTTGA
>1091
GGACCCGGGTGGAGGACACCAACCGGAAATACGAGCACCTCCTGCAGCTGCTGGACT
TGGCCCAGGAGAAGTGGGTGGCAGACAGGCAGCATGGAGGTTGGGGTGGGGTCAGG
GGGCCAGGTGGGCATCCCCCGACGCCT
>1092
CCATCTGAATCAGGATGACACAGTGCCTGAGAGCAGCCGTGTCCTGGACAGCAAGGG
GCAGGAGCTGGCGGTGAGTCTAGGGCCCAAGTCCTGGGAGTCCTTCTCTGAGCAGGT

GAGAGCATCTGGCACATCCACCCGCC
>1093
AGCGCCAGGAGGCCGAGGTGCACAAGCTGGGCCAGCGTTTCAACAACCTGCGCCAGC
AGGTGGAACGCAGGTGAGGGTGCGGTGGGTGGTGGCCATTTTTGTTTTCTGAGAATGC
GATCTAATTCCCTCTAAGCAATGCT
>1094
CATCCCCAGTTACGAGCCCCAGGAGACAGACAGCCTCAGCCAGATGGAGACCAAGCT
GAAGAACCAGAAGGTGAGATGGCCGTTTCCGCAGCCCCAGGCACACACTGGCAAGGG
AGGGTCAGAGGTCAACAAGCCCCTTT
>1095
GATAGCAAGTAGGGAGCAGGAAGTACAGAAGATCTGTGCCAATTCCCAGCAGTACCA
GCAAGCTGTAAAGGTGAGCCGGGAAACTTCCGCCCAAAGCACAAACAGCAGGGGTCT
TATTCCACCGTGCCCTGGTCAGAGAA
>1096
GAATGGAAGGAGCAGCCACGTGAGCAAGAGAGCCAGGCTCCAATCTCCTGCCACCAA
AGTGAAGGAAGAGGTGAGTTCCATGGGCCAGGCGGCCTCGGGACTGAGGTGGAGGC
CAAGCTGCCCTGCTCCCCAGGGAGCAT
>1097
CACTGAAGTTTATGCCATCAACAGACAGAGGCTGCAGAATCTGGAGTTTGCTCTGAAT
CTCCTCAGACAGGTAACTGATCTTAGTAAGAAATTCTACTTCAGGCTGTTCTAACAGGT
GACAAATACAGACTCTGTCTTAG
>1098
ACCGGCACCTGGCCCAACAACCAGTTTGACACAGAGATGCTGCAAGCCATGATCTTGG
CGTCCGCCAGTGGTAAGTGGTGTCAGTGTGTGTATGGAAGAGTGGGAGACCTGGGGTT
CTGGGGTGCATCTCACAGCCACCA
>1099
CCACGTCCGTGAGCAGATTCTCATGAAGAGGTTCGAGATCTCTTGCAACCTGTCTCTG
GACGCCATGGAGGTGAGCCGCCAGCGCGGGGCCGGATGGATGTTGCTTCCAATGCAG
GTGGAATGCGTCGGGCTCCTGCGTT
>1100
CTGGCACTAATTGATCAGGATGCTGGGCAGGAGGATGGAGTCGCGATACCACTGCCAG
GCGTCAGGAAAGGTGAGCAGAGGTTCCTCCCGCATCTGCCCACCACTCACCGGTCCTG
GGTGACCACGCATTCATGCCCCTG
>1101
CTCATGGCCCTCCGGCTCAACGAGAGCAAACTGGTGCAGGAGGCCCTGGAGGCGGTG
CCCAGGGGCGAGAGTGAGTTGGGGCTTCGGTGTCGGGCGCCGGGGTCATCCTCTCTTC
CCCTGGTTTGAGTTGGTGGCAGAAT
>1102
GCCACCTGGAATTCTACCTCCTCTGGACTCACAAACTGCTCATGTTGCACGGACAGAA
GCTGAAGTCCAGGTAGAGGGTCTCCCCCGCAGTTCATCGGTGGCTCAGGTCACTGGAC
CAGCTTGTCCTGCTGGATAAAAGG
>1103
GGACGCTGCTGCCTGTCATTCAGTTCCTCCAGAAGAGCATCCAGCGGCACCTGGACGA
CCTGTCGAAACTGTACGTGTGGGTGCAGGGCCTGGGGGTGGGTGGGGTGCACGGTCC
CCTGCTGGTCCACACTGTGGAGTGC
>1104

GTGGCTTCTTCTCCCCCAAACAGCCCGACCTGTTCAAGGACATTGTCAATATGCTCATG
CACCATGACCGGTGAGCTGGTTGGCCCGGGAATCACTCAGGTGGGGCCGCAGGGCAG
ATCGAGGCTCAGGTGTGTGGTGGG
>1105
AGGAGGCAGTGGAGGAAGTGGGGGTTATGGTGGGAGGAGCCGATACTGAGCTTCTTC
CTATTTGCCATGGGTAAGTAGCTTTTGAGTTTTACAATTATTATTATCTTGGGGAGACATA
GCTGCAGGAGTAAAAGCTTTTT
>1106
CCAGGCCCTACAGGAGCTCATCTCAGAGCTAGATCGAAGGTGCCACAGCTCAGCACTG
GAACTGCTGCAGGTGAGACAGGGAGGGGTTCCCTTCTACAATTCAGGGAATAACTGA
AAAAGACCAGAGCTATCTGGAACTG
>1107
ATTACCTCTCCAGAACTCAGGAGTGTGTGCCATGTGCCAGGGCTGAAGAAGATGCTGA
GGACATGTGCAGGTGAGGCAAGTTCTAGTTTTGCGGGGGATAATGGGGTGCAGAGTAG
ATCCCAGGGTCAGGGAGCCTGGAT
>1108
CTACGCCATCGCCATGGAGGAGATCAGCCGTGGCTGCGCCTCCACCGGAGTCATCATG
AGTGTCAACAACGTGAGCCCCCTCCCAGGCCCCTGGGACACACGGGTGGAGGGAGGC
TCCCGTGAGCGGGCAGGCTCTGGCC
>1109
CAGAAGCAGGCGTGGGTCACGCCTTTCACCAGTGGTGACAAAATTGGCTGCTTTGCCC
TCAGCGAACCAGGTACCTGCCCTGTCCCCTCACCTGTCCTTAGGGTGACAGGCCCAGA
GGGGAGGAGAGGAAGGTGCTAGGC
>1110
GATCACCAATGCCTGGGAGGCTTCGGCTGCCGTGGTCTTTGCCAGCACGGACAGAGCC
CTGCAAAACAAGGTGGGCCCACCCAGAGAGGGGTTCAGCCGGATCCTGGGCTGCTGT
CATTTCTGTTTCTAGGGCCTTCTCT
>1111
CTTTGAGGACTGTCGCATCCCCAAGGACAGCATCCTGGGGGAGCCAGGGATGGGCTTC
AAGATAGCCATGGTGAGCCCGGCAGTGGGGGTGGCACCTTGAGGCCAGGCCCGCGCC
CCGGCTGGCGGGCCACTGACCAGGG
>1112
TTGTGCTGTGAACTACGCTGAGAATCGCATGGCCTTCGGGGCGCCCCTCACCAAGCTC
CAGGTCATCCAGGTAATGGTGGCAGCTTTAGGAGCTGGGCCTAGAGGCTGGACAGCGG
GCGGAGAGTGTGGCCTCCTGACTG
>1113
CCTGGAGAGTGCCCGGCTGCTGACCTGGCGCGCTGCCATGCTGAAGGATAACAAGAA
GCCTTTCATCAAGGTGCCCACAGGGGTCCCCGAGCCATGGCCCAGAATGTGGTGGGCC
CAGGGACGGGGAGAGGTTGGGGCGG
>1114
CACAGTGAACCACAAACAGTTCTCCCTGAACTGGACTTACCAGGAGTGCAACAACTG
CTCTGAGGAGATGGTGAGTCCTGGGCTGAAGGCAGGGGCAGGGGAGGAGGTAGGAG
CCCCATGGCATGAAGCCCCTGGTCCCA
>1115
TGCTACATCATGAACCCCCCTGACCGCCACCGTGGCCATGGCAAGATCCATCTGCAGGT
CCTCATGGAAGGTGAAGGCTGGGGTGCCAGCTGCGGGGCCCTGACCTTTCCCACCCA

CCTACTCTCTGCGGCATTTGCTCA
>1116
GTATCTCAGAAAAGAAGACATGGAATATGCCCTGCGTAAACTGGATGACACCAAATTC
CGCTCTCATGAGGTGGGTCCTTGCCTTCTGTTGAGGAGCTTGTACTCAAATCACTCCAC
TTGAACCTGACCTAGCAGAGGTA
>1117
GCACCAGGACATGCACAGCTACATCGCGGCCTTCGGGGCCGTCACGGGCCTCTGCACG
CTCTTCACCCTGGTCAGCCGCGGGAGGGCAGGCCCCGGGGGCCCTCAGCCTGGAACG
TGGGAAGAGAGCCAGAGGGAAGGGG
>1118
AGTTCATGGATGGTGCCCGCCGAGAGATCGTCTGCCGTGCAGATGGCACCATGAGGCT
TGGGGAGCCCACGTAGGTGTCTTGGGGACCCAGAGGTGAAGGTACAGGGAGGAAGG
CTGAAGTGTGCGTTTACCCCAAAGGG
>1119
CTCCTACTTCCACCTGCTCACCTGGTCACTCCCCTTTGTCCTCACTGTGGCAATCCTTG
CTGTGGCGCAGGTATAGTGACTGGTAGGAACGGGAGACCTGGATGGGGTGAGTTTGA
GGGAGGGGGCCAGTAACCCACCTT
>1120
TACCGTGCGGGCTTCGTGCTGGCCCCAATCGGCCTGGTGCTCATCGTGGGAGGCTACT
TCCTCATCCGAGGTGAGTGAAGACCAGGCCAGGACCAGTTGGGCAACAAAATGTACT
GGGCACTTGCTGCCAGTACTGGGAG
>1121
AAGAGCAACCACCCCGGGCTGCTGAGTGAGAAGGCTGCCAGCAAGATCAACGAGAC
CATGCTGCGCCTGGGTGAGTGGCCCCGGGGGACTTCGGTCTGAGGTCCTGGCCAGCCC
AACACTGCACCCTCCTGGGGCTATGC
>1122
CCTTCAGCTGCCACTTCTACGACTTCTTCAACCAGGCTGAGTGGGAGCGCAGCTTCCG
GGACTATGTGCTGTGAGTGAGGGGCATGGAGGCGGCAGTGCTGGGAGCTGGAGCCAA
GGCCATAAAGACACCCACCTCCACC
>1123
CTGGCATCGCCATGAGCACCTGGGTCTGGACCAAGGCCACGCTGCTCATCTGGAGGCG
TACCTGGTGCAGGTGGGCATGGCAGCCAGCCCCTCCTGCCCTGCCCGCCTCACCCTCA
GCCTTGGGACCCCATCTTTAGGTT
>1124
GAGCTCCTGCAGAACCCAGGCCAGGAGCTGTCCTTCAGCATGCACACTGTGTCCCACG
ACGGGCCCGTGGGTGAGCCTCACCCCTCCTCTACCGGAGCCGCCTGGCCCCGCGCTGC
CCATGTGCTAGTCTCTCCCAGCCT
>1125
ACCAAGATGGTGGCTCGGAGAGGAGCCATACTGCCCCAGGATATTTCTGTCACCCCTG
TGGCAACTCCAGGTATGAGAGTTCAAGCTTCTGGAGGAAGGTGGGGGGAGCACAGAG
GCTGGGGGCTTCTGGGACTGGAGTA
>1126
CCACCGTACCCCTACTGCATGCAGCCATCCGGGAGGTGGATATCAAGGGCGTGTTTCGA
TACTGCAACACGTGAGTATGCTGTGGGTGAAGCCGGGATGCCCAGCCTCCAGCAAGAC
CATGGCAGGCCCCACTCAGCCTC
>1127

CCTTTTGGCAGTGGTTTGATGGTGTCCTGGACCTCACCAAACGCTGTCTCCGGAGCTA
CTGGTCTGACCGGTGAGTCCCCACCCTGGGTAGTTTGAGCAGCCATACACCAGTCACC
TCCATACTCACTGCCCATGCCCCA
>1128
TTTCTCCTCCGCTTCAGCGACTCAGAGATTGGGGGCATCACCATTGCCCATGTCATCCG
GGGCCAGGATGGTGAGGCCACCCCAGCCAGTCCTCTGTCTCTGTGCCTGTGCCCTCTG
GGGTTTCTTCTGGGAATGAAATG
>1129
GATCTTGCTCAGCTCAAAAATCTCTATCCCAAGAAGCCCAAGGATGAGGCTTTCCGGA
GCCACTACAAGCGTGAGCTGGAACTGGCAGCTCTGATTCCTTCCTGTCACCCACTTCC
TGCCATGCTCCCCGCTGCCATCCT
>1130
TGGTGCCTTCTTATGACCTTGGAATGGCCCCTGATTCCTCCATGAGCATGCAGCTTGGC
CCAGATATGGTGTAAGGAGCTGGAAAGACAGGAATGGGAGTGGTCTGTGCAGATGGG
CTAATCTTAGCATGGGCAGCTGGG
>1131
CTCACTCCATCCCCCCGTATCAAGGCCTCTCCCCAGAAGAATCAGTCAACGTGTTGTCA
GCCTTCCAGGAGTAAGTGAAAAACCTCATGGGGATACCATCCCACTCTAAGGGGGTGG
GCATTTGAATTGTTAGAAGAGGC
>1132
GCTCAGATGTGACCATGGTGGAAGACAGCTGCCTGAGCCAGCCAGTGACAGCGTTTC
CTCAGGGCACTTGGTGAGTGGCAGCTTGGGAGTGGAGGCTGGGTGGCATCTAGGGGA
GTGGGCGCCATGCCTACTCCACTGCT
>1133
TTACTGGTGTCATCCCCGGAGCACCCAAAACGCTGGGGCCAAGTCAGCGAGGCCTAC
GTGAGCCTCCTAGGTATGCCTTTGTCTGTCTCCGTCAGGAAAGGTGTGGGAGTGGATC
TGGGACGGGGCCATGACCTGTCAGA
>1134
CGCCATCACCCGCAAGAGAGAAGCTGACCAGGCCGGCCGCGTCCCCTGCTGTGGGCG
AGAAGCTGAAAAAGTGAGCGGGGCGGGTCTGGAGGAAGAGGGCTGCCAATCTCGGG
TGGGGAGGGTGAACATCACTGTTTTCT
>1135
CGAGAGAAGGAGAGAGAAGAGTGGGAACGCCAGTACAGCCGGCAGAGCCGCTCACC
CTCCCCCCGATACAGTGAGTGTCCCCACCAGGCTGCAGGGCTCTGGGGCTTTAAAGAA
AAGTGTCAAGCAGGGTTACCAAAAAA
>1136
TTTTTGTGTTGTTTGGAGCAGAAATACTAAAGAAGATTCCGGGCCGAGTATCCACAGA
AGTAGACGCAAGGTAAGGATGCTTGCTCCTGCACTGGATGGGCTGGTCAGGTGTCCAC
AGTTGCACACGTGGATGCCAACAT
>1137
AAGCTGGGATCAGCAAGGACCGAATTCTTATAAAGCTGTCATCAACCTGGGAAGGAAT
TCAGGCTGGAAAGTAAGTGTCCCCCACAAGGAAGGAGCTCTCAGAGATTTTTCCTCTG
TTGGAGCAGGGTCTACATGGCATC
>1138
TTGGGCGCATCCTTGATTGGCATGTGGCAAACACCGACAAGAAATCCTATGAGCCCCT
GGAAGACCCTGGGTGAGGGTCCCTCTGTGGTAATGGGGTAAGGGGAGCAGCCTCAGC

AGCACCTGAGGCAGGAAGAGCCCGA
>1139
TCACCCAAGCTCCTGGGAGAGCTGCTGCAGGACAACGCCAAGCTGGTGCCTGTGCTC
TCAGCCAAGGCGGGTGAGGCCCCACTGCCCAGCTGTGGCTCCTGCTCGGGCAAGGCC
AGCACTGCCCTGCCACGCTGCCCTGT
>1140
TGTGGAGAAGCTCTCTGACGGGATCCGCAAGTTTGCCGCTGATGCAGTGAAGCTGGA
GCGGATGCTGACAGTGAGTGTTGTGTGTGGGTACCTACATATGCCAACCCCTATGAGAG
CCCGGGCCTGGGCGTCGGGGTTCA
>1141
GAAGAAGAAAGCAAAAAAGGCCTCAACCAAAGATTCTGAGTCACCGTCCCAGAAGA
AAAAGAAGAAAAAGGTAGAGAGTTCCTGGGGTGTCTCAGGCCAGAAAACAGACCCA
AACCCAAGCCCCCTCCTACACACCCAGA
>1142
GTACATGCGACAGTTCGTGGCTCACCTGCAGGAGACCAGCCCGCTGAGGGATGCCGTC
GTCATCGAGCAGGTCTGGGCACTGCCCTGCAGGGTTGGGCACGGACTCCCAGCAGTG
GGTCCTCCCCTGGGCAATCACTGGG
>1143
AGGCCAGCAGTGGCCTCTTCGACGTCTTCCTACGCTTCATGTGCCACCACGCCGTGCG
CATCAGGGGCAAGTGAGTCAGGTGGCCAGGTGCCATTGCCCTGCGGGTGGCTGGGCG
GGCTGGCAGGGCTTCTGCTCACCTC
>1144
CCACGCTGCTCTGCAGCCTGTGCTACGGCGACATGGAGAACAAGCTGTTTGCGGGGAT
TCGGCGGGACGGGTGAGGCCTCCTCTTCCCCAGGGGGGCTTGGGTGGGGGTTGATTTG
CTTTTGATGCATTCAGTGTTAATA
>1145
TGCTCCTGCGTTTGGTGGATGATTTCTTGTTGGTGACACCTCACCTCACCCACGCGAAA
ACCTTCCTCAGGTGAGGCCCGTGCCGTGTGTCTGTGGGGACCTCCACAGCCTGTGGGC
TTTGCAGTTGAGCCCCCCGTGTC
>1146
ACGGCCTATTCCCCTGGTGCGGCCTGCTGCTGGATACCCGGACCCTGGAGGTGCAGAG
CGACTACTCCAGGTGAGCGCACCTGGCCGGAAGTGGAGCCTGTGCCCGGCTGGGGCA
GGTGCTGCTGCAGGGCCGTTGCGTC
>1147
GAGGAACATGCGTCGCAAACTCTTTGGGGTCTTGCGGCTGAAGTGTCACAGCCTGTTT
CTGGATTTGCAGGTGAGCAGGCTGATGGTCAGCACAGAGTTCAGAGTTCAGGAGGTG
TGTGCGCAAGTATGTGTGTGTGTGT
>1148
ACATTTTTCCTGCGCGTCATCTCTGACACGGCCTCCCTCTGCTACTCCATCCTGAAAGC
CAAGAACGCAGGTATGTGCAGGTGCCTGGCCTCAGTGGCAGCAGTGCCTGCCTGCTG
GTGTTAGTGTGTCAGGAGACTGAG
>1149
GAAGCCATCCACTTCTGATGATTCTGACTCTAATTTTGAGAAAATTGTTTCGAAAGCAG
TCACAAGCAAGGTGAGTGTTGATCCTAGTCAGTCCTTTTGCTGTAGATGTTCTGAAACA
CGTAACTAAGCCATTGTTCTTA
>1150

GTGGCCCTCACTTCCAGACCTGCACCGGGTCCTAGGCCAGTACCTTAGGGACACTGCA
GCCCTGAGCCCGGTGAGTGTGCTTCCCTCCCCTGTGCCCACCACCAACCCTGCCTGGT
ACTGGATCCTTGCCCCAACAATAC
>1151
GCTGGACCTGGACTTTGACCCCCTCCCGCCCGTGACGAGCCCTGTGAAGGCACCCAC
GCCCTCTGGTCAGGTTGGTTGTGCCCACCACTGCCCATGGGCCCACCAGCTTCCAGGT
GCCCAACCCTGGGCTCATGTTGCCT
>1152
GCCCAGCGCTGCCGAGGGCACCTTTGCTGTGTCCTGGCCCAGCCAGACGGCCGAGCC
GGGGCCTGCCCAAGTAAGTGCCCACCTCCAGCCCCTGTCTGGCTTGTCCCCAGTCTCT
AGGGGTGCAGCATGGAAGGAGAGCC
>1153
GGCGGGTGGGACCCAACCTGCGGCTGGAGCCCAGGAGCCAGGGGAGACGGCGGCAA
GTGAAGCAGCCTCCGTAAGACAGCAGGGACAAAGCCCTGCCTTTTCCTCCCTGCCGCC
CGCCTGCCTGTCCGGGGCTCCCCTGT
>1154
TGTGAATGGCACCGTGGAGGGCGGCTGTGGGGCCGGGCGCTTGGACCTGCCCCCAGG
TTTCATGTTCAAGGTGAGCCCACAGCCTCTGACTGCTGCAGTCCCTCGGTGCCCTGGT
GGGCAGATGACAACCCTGAGCCTCA
>1155
GGCGGACTCTGTGAGGAAACAAGAAGAGAGGCCCAAGATGGAGACGGCGGCGGCTG
TAGCGGCGTGACAGGTGAGGGCGGGCCCGGGAGGGCTCGGTTTCTGGAGCGGCTGCC
GGGCACGGGCAGGGAGCCCGGACCGAA
>1156
CAATGGGGCCGAGCCATCCCGGGCAGTGGGCACCGTCAAAGTATACCTGCCCAACAA
GCAACGCACGGTGGTGAGTCATGGAAGCGAAATGGCAGGGGCTGTGGATGGACCCAG
TTGTAACTCTGGGATCAAAAGGGTGA
>1157
CTCTAGACAAGGCCCTGAAGGTGCGGGGTCTAAATCAGGACTGCTGTGTGGTCTACCG
ACTCATCAAGGGGTGAGTGTGGCAGCCCCACGCCCACCCACTGGGTGTCCCACCTCCC
ATCCCCTCCTCAGTCATTTGATTC
>1158
TGCTCCCCTGGATGGCGAGGAGCTCATTGTCGAGGTCCTTGAAGATGTCCCGCTGACC
ATGCACAATTTTGTGAGTGCAGGGTGGACGGTGGGGGTGGACCATGGTTGGGGGTGTC
CTTGACCAGGTCTCAAACTTCCCT
>1159
ACAAGTTCCACCAGCATTGTTCCTCCAAGGTCCCCACAGTCTGTGTTGACATGAGTAC
CAACCGCCAACAGTGAGCCCAGCCTGGGGTGGGTGGGGGGATATGGGGAGCACAGAG
GCCCAGCCACGAGGCCCTTACAGAC
>1160
GAGGCTCCAGACAGCATGAGGCTCCCTCGAACCGCCCCCTGAATGAGTTGCTAACCCC
CCAGGGTCCCAGGTAGGGATGCCTTAGCTGAAGAGCTGCTGGGGGAGAAAAAGATCT
TGGGGCTCTTGTAGACCACGAGCCA
>1161
CCGCTCCACGTCCACTCCCAACGTCCATATGGTCAGCACCACGGCCCCCATGGACTCC
AACCTCATCCAGGTTGGTGCTGTGGGGGACAATGCCGGGGACCACAGGGCAGAGGGT

AGAGCCATCCCTGTTGGCCTCCATG
>1162
GTCCCCACATTCCAAGTCACCAGCAGAGCAGCGCGAGCGGAAGTCCTTGGCCGATGA
CAAGAAGAAAGTGGTATGCTCGAGGGGGATCCTTTTGGGGCCACCAAGGCTGAGTGA
CGTGGGATGAGGGATGTGGGCAGGCC
>1163
AGGTGCTCAAGGTGTCCCAGCCCACAGCTGAGCAGGCCCAGGCTTTCAAGAATGAGA
TGCAGGTGCTCAGGTGAGGTGGCATGTGTGCGTGGATGGGGGTGGCAGGCCCGGCCT
TGGCATCTCTCTGGGAAAAGGCCATG
>1164
ATGTGGCCGACACACGCTTCGACATGGTCCAGCTCATCGACGTGGCCCGGCAGACTGC
CCAGGGCATGGAGTGAGCCTCCCAGCCTGGGGAGGGGTGGGGGAAGGGTGGGGGAA
ATGAGGTAACCCCCAGCCAATCCGCC
>1165
GGCCACAGTGAAGACTCGATGGAGCGGGGCCCAGCCCTTGGAGCAGCCCTCAGGATC
TGTGCTGTGGATGGTGAGTTGGGCCAGGCTGAATGGGGGGCACATGGGGACGTGGGC
TCCGGGATTGGGGAGTGTGGGGCCAA
>1166
CTACGGGGTTGTGCTCTACGAGCTTATGACTGGCTCACTGCCTTACAGCCACATTGGCT
GCCGTGACCAGGTGAGCCCCACACCTTACCCACAGTTCCCTGGCCTGAGGGATCCCCT
CAGGCATCCATACCCCACATCCC
>1167
CAAGGCCATGCGGCGCCTGCTGTCTGACTGCCTCAAGTTCCAGCGGGAGGAGCGGCC
CCTCTTCCCCCAGGTGGGCTGGGTAGGGGGCTGGACACCTTGGGTGGGTGACTCTGGG
TTAGAGGAAGACTGAGATGGAGGCA
>1168
TGAGCACCTGGTTACCACTGCCACCTTCTCCATCGGCTCCACTGGCCTCGTGGTGTATG
ACTACCAGCAGGTGGGTATGCCAGACCTCCTGACCTGGACCAATGACAAGGCTCTGCT
AGAGCCACCCAGCTGGGCCACTT
>1169
CATCATGAAGATAGCTCCAGAGAGCATCCCCAGTCTTGAGGCTCTCACTAGAAAAGTC
CACAACTTCCAGGTGTGTGTGTGTGGGTGAAAAGAGTGGGCTGTCTCCCTCCCAGGG
CTGCTGGGAGGAGTGTCCGAATGGT
>1170
TACGTGTGCCGGGTCCAGGAGGGCAACGAGTCATACCAGCAGTCCTGCGGCACCTACC
TCCGCGTGCGCCGTGAGTGGCCCAGCCCTGGCCCCTACTCCCACTGTCCCGCTGGGCA
CTCGGTTTATCTTTGAAGTGGGGA
>1171
AATCATCACAGCCGAGGGGATCATCCTCCTGTTCTGCGCGGTGGTGCCTGGGACGCTG
CTGCTGTTCAGGGTGAGCCCCCTCGGACCTCTGAGTCAGCCGGGCGAGGGCTGGGCG
AGGGACCCCCAATACCCAGGTAGCC
>1172
AGGAACCCTGCGGAGGGACTTCAATCACATCAATGTAGAACTCAGCCTTCTTGGAAAG
AAAAAAAAGAGGGTGAGGGTTTTTCTTCTGATAATTCAGTTGCTCGTTGGAAGGTGGT
TTATAACAGCATCACATTTGGTTG
>1173

CAGAAAGGAACTGGCTACCGTTCGGACTATTTGTAGTCATGTACAGAACATGATCAAG
GGTGTTACACTGGTAAGCAGATGTATCAGACTTCCTTGTTTTGGAAAGGGAGGTTTCTC
AAACCTGTGCTTTATACAGCGTA
>1174
AATGGGTCTCTTGTTGAAATCCGAAATTTCTTGGGTGAAAAATACATCCGCAGGGTTCG
GATGAGACCAGGTATGTGCATACCCTCAGACAGAATTAAGAAATTTTTCCTTCATATTTT
TTTCCTCCTTTTCCACTCCCA
>1175
TCAGTATCTCAAGCCCAGAAAGATGAATTAATCCTTGAAGGAAATGACATTGAGCTTGT
TTCAAATTCAGGTTTGTATGTTTACTATGTCTAACTATGCCTACAAGATTTTGTCTAAATC
TTGTTTAAAATACAGTATTT
>1176
AAATTTTTGGATGGTATCTATGTCTCTGAAAAAGGAACTGTTCAGCAGGCTGATGAATA
AGATCTAAGAGGTAAGTTCTTACAGTGTCTTAAGTTTTATTACTGCTCTAGTTGAATGAA
GCTCTTAATTTACTAAAAGAC
>1177
TCAGGGGACAGGGAGAGCAAGAGGTCAAGAGCTGTGGGACACCACAGAGCAGCACT
GAAGGAGAAGACCTGTAAGTTGGCCTTTGTTAGAACCTCCAGGGTGTGGTTCTCAGCT
GTGGCCACTTACACCCTCCCTCTCTC
>1178
TTAGAGAACAGGCCAACCTGGAGGACAGGAGTCCCAGGAGAACCCAGAGGATCACTG
GAGGAGAACAAGTGTAAGTAGGCCTTTGTTAGATTCTCCATGGTTCATATCTCATCTGA
GTCTGTTCTCACGCTCCCTCTCTC
>1179
AGCTTCTGCGTCTGACTCGCAGCTTGAGACTGGCGGAGGGAAGCCCGCCCAGGCTCTA
TAAGGAGACAAGGTGAGATGCTGAGGGAGGACTCAGGAGGACCCCCACCCCACATAG
ACGACCACAAAAAATCCAACACCAC
>1180
GAGCTGCTCTGTCTGACCAGCAGCTTGGGATTGGCGGAGGGAAGCGGGCCAGGCCCT
GTGAGGAGTCAAGGTGAGACGCTGAGGGAGGACTCAGGAGGCCCCCACCCCAGATAG
ATGACCCCAAATAATCCCGCACCACT
>1181
GAGCTGCTCTGTCTGACCAGCAGCTTGGGATTGGCGGAGGGAAGCGGGCCAGGCCCT
GTGAGGAGTCAAGGTGAGACGCTGAGGGAGGACTCAGGAGGCCCCCACCCCAGATAG
ACGACCCCAAATAATCCCGCACCACT
>1182
AAGCGAGCTGCTCTGACCAGCCGCTTGGGATTGGCGGAGGGAAGCGGGCCAGGCCCT
GTGAGGAGTCAAGGTGAGATGCTGAGGGAGGACTCAGGAGGCCCCCACCCCAGATAG
ACGACCCCAAATAATCCCGCACCACT
>1183
GCGAGCTGCTGTCTGACCAGCAGCTTGGGATTGGTGGAAGGAAGCAGGCCAGGCCCT
GTGAGGAGTCAAGGTGAGACGCTGAGGAGGACTCAGGAGGCCACCACCCCAGATAG
AAGACCCCAAATAATCCCGCACCACTC
>1184
GAGCTGCTCTGTCTGACCAGCAGCTTGGGACTGGTGGAGGGAAGCGGGCCAGGCCCT
GTGAGGAGTCAAGGTGAGACGCTGAGGGAGGACTCAGGAGGCCCCCACCGCAGATA

GACAATCCCAAATAATCCCGAACCACT
>1185
TCTGAGCAGACAGGCCAACCGGAGGACAGGATTCCCTGGAGGCCACAGAGGAGCAC
CAAGGAGAAGATCTGTAAGTAAGCCTTTGTTAGAGCCTCTAAGATTTGGTTCTCAGCTG
AGGTCTCTCACATGCTCCCTCTCTC
>1186
TGACCAGGATCACCAGGAAGCTCCAGAGGATCCCCAGGAGGCCCTAGAGGAGCACCA
AAGGAGAAGATCTGTAAGTAAGCCTTTGTTAGAGCCTCCAAGGTTCAGTTTTTAGCTG
AGGCTTCTCACATGCTCCCTCTCTC
>1187
TCAGCGGACAGGCCGGCCAGGAGGTCAGAAGCCCCAGGAGGCCCCAGAGGAGCACC
GAAGGAGAAGATCTGTAAGTAGGCCTTTGTTAGGGCCTCCAGGGCGTGGTTCACAAAT
GAGGCCCCTCACAAGCTCCTTCTCTC
>1188
CTGTGAGCGAGGACCCGGTTCCGCCCGAAGGATGAAGACTTGTCTTTAAAGATGACAA
CGTACAGGAAAGGTCCATCAGCCGATCTCCCCCTGCCTGGGCCCACAGCGCCCCCCAA
ACCCTCACCACCCTCTCTCACTGC
>1189
TCTTCCTACCCATCTGCTCCCCAGAGGGCTGCCTGCTGTGCACTTGGGTCCTGGAGCCC
TTCTCCACCCGGTGAGTGGCCAGCAGGGTGTGGGGTTATGTGAGGGTAGAAAGGACA
GCAAAGAGAAATGGGCTCCCAGCT
>1190
GGCGGCACGGCTCGGAGGCAGCGGTTGGGCTCGCGGCGAGGACGGGGTCGAGTCAG
TGCGTTCGCGCGAGGTGAGAGCGGGCAGGGCGCGTGTGCGGTACCTTGGCTTGGGCC
CGGGAGAAGATGGAACTGCGCGGGGGT
>1191
GCTCAAAGGCCGGCCATGTAAGATCGTCGAGATGTCTACTTCGAAGACTGGCAAGCAC
GGCCACGCCAAGGTTAGAATTTCACCTCCGCATCTTGCCTTCCCCATGCCTCCAGTATC
TTTGGCCCCTCCCAGCAGCAAGC
>1192
GAAATATGAAGATATCTGCCCGTCAACTCATAATATGGATGTCCCCAACATCAAAAGGA
ATGACTTCCAGGTATGTAGATGGTCTGGATGAGGATGGGTTAGCGGTTTATGGTGGAGG
GAGGGGTTGGGGGATAGGGACT
>1193
CCTTCGTCTCCCTGAGGGAGACCTTGGCAAGGAGATTGAGCAGAAGTACGACTGTGG
AGAAGAGATCCTGGTATGGTGCCTCCCTCCCTGCTTTTGTGCTCAGCTTTGTTCTGTAC
GTTTCTTCCTGAGCTCAGACATCT
>1194
CGGTGCTGTCTGCCATGACAGAGGAGGCAGCTGTTGCAATCAAGGCCATGGCAAAATA
ACTGGCTCCCAGGTGAGTGTGACAAATCCCTCACTGTCCCCTTCACATTTTGTTGTCCT
CAGCAGAGCTGCTGTAGTCTTAA
>1195
TTTACTCGCACAGCCTCCTGCCTGTGGACATTCGACAGTACTTGGCTGTCTGGATTGAA
GACCAGAACTGGTGAGGCCTTCAGGAAGTTGGGGGAATGAAAAAGGTGGCCTTCCAC
TTCTGGGCCCCCGGGATCCTGGAA
>1196

CCGTTGCAGCCAGGACCCAGAGTCCTTGTTGCTGCAGCACAATTTGCGGAAATTCTGC
CGGGACATTCAGGTACTTGGAACGGTTGGGAGTGATGGGGTAGCACTGGGAGCAGAG
CATAGAGGAGTAAGGTTTGGAGAAT
>1197
GGCTGAGATGATCTTTAACCTCCTTCTGGAAGAAAAAAGAATTTTGATCCAGGCTCAG
AGGGCCCAATTGGTGAGGACAATTCAGTGGTAATGTTGGAAACTCCTGAAGTAGAGAG
GAACCATGGAAAGGACTCAGGGAG
>1198
CGAAACACCTGTGGAGAGCCAGCAACATGAGATTGAATCCCGGATCCTGGATTTAAGG
GCTATGATGGAGGTTAGTAGATGTGGTAGGAGTTAGGGTTGACAGTGTTCAGCCTAAC
ACCTCCCTGAGAAGCAGCCTCATC
>1199
GTAAAATCCATCAGCCAACTGAAAGACCAGCAGGATGTCTTCTGCTTCCGATATAAGAT
CCAGGCCAAAGGTAGGAAGCACATTGAGGGGCTGGAGAAAGATAAGTGCCTGCTGAG
AAGCCGGAGCTGGAAGTGAACAGG
>1200
GGACCCCCATCAGACCAAAGAGCAGAAGATTCTGCAGGAAACTCTCAATGAACTGGA
CAAAAGGAGAAAGGTGGGAGGCAGCAGAACAGAACATGTGGGCAACAAGGACCTGA
AAAAATGAGGGATGTTGGGAACCCTGG
>1201
GGAAGGCCCAGCAGCAAAAAGCCTGCATCAGAGCTCCCATTGACCACGGGTTGGAAC
AGCTGGAGACATGGTGAGAGGTACCACCCCAACCCTCGTCCTCGCCATGCGCTGTGAT
TTGTAAGTTGCAGTGCCCTGCATAT
>1202
ACCCTCTGACCAAAGGGGTGGACCTACGCAACGCCCAGGTCACAGAGTTGCTACAGC
GTCTGCTCCACAGGTCTAGAGGCCAGGCAGGAACCCTGGGGGAAAGAAGGAACAAG
GGAAGCCATTCTTACACATACTGAGCT
>1203
CCTGCATGCCCCAAACTCCCCATCGACCCCTCATCCTCAAGACTGGCAGCAAGTTCAC
CGTCCGAACAAGGTTGGCATTCCAGAACTCATTCCCACTTCCTTTTTCCAACCCTGCCA
CTGTGTATTTTCTGGCTTTACAG
>1204
TTCAAACCAGAAAACTTTGACCCCCGAGAAGGGGCAGAGTCAGGGTTTGATTTGGGA
CTTTGGTTACCTGGTAAGAATAGTTTGTGACCTATGCTTTTATTACTATTTTTATTTTTTC
GAGACGGAGTCTCACTCTGTCC
>1205
GACAGAGGAACTGCACATCATCAGCTTCACGGTCAAATATACCTACCAGGGTCTGAAG
CAGGAGCTGAAAGTGAGTGAAAATGGAGGGCAAGGAGAGAGAAAGCAGCTTTGGAA
GAAGGCATAAGAAGGGGATAAACAGA
>1206
CAACATGAACCAGCTCTCAATTGCCTGGGCTTCAGTTCTCTGGTTCAATTTGCTCAGCC
CAAACCTTCAGGTAGGGGAGTGGGGCCGACAGGTCCCGGCGCGAGAGCAGGGGTGT
GGAAGCTTGGTGTGATAGGTTGCTT
>1207
CAGTTCTCCTCCTATGTTGGCCGAGGCCTCAACTCAGACCAGCTGAGCATGCTGAGAA
ACAAGCTGTTCGGTACAGATTTCCTTTTCTCTCAGCCTTTCCCCAGCCTTAGTCTTTTCT

GTCCCTCTGTCCTATCTATCCC
>1208
CATTCTGGACATGGCTGGACAAAATTCTGGAGTTGGTACATGACCACCTGAAGGATCT
CTGGAATGATGGGTAAGGCCTTGGTCACCCTTCCCTCATGGGCTTGTGCTTCCGGGCTT
GAGAGTGGAGTCTCTGCACCCTC
>1209
TTTCTACTGCGCTTCAGTGAATCGTCAGAAGGGGGCATTACCTGCTCCTGGGTGGAGC
ACCAGGATGATGGTAGCTGCTCTGCCCTGCCATTCCCACAGCCTCTCCTTTCTGCCTGG
CTCTCCTCTGGCCCCTCTGCCTG
>1210
AACCCACTGCGCTTCCTCTATCCCCGAATCCCCCGGGATGAAGCTTTTGGGTGCTACTA
CCAGGAGAAAGGTGGGAATCGTTGACATACTTCATTGCTAGATTGCAGAGATCTACCA
GACATCCATAGATCCCACTCCTT
>1211
GTATCACAGCCAGTGCCAGAGCCAGATTTGCCCTGTGATCTGAGACATTTGAACACTG
AGCCAATGGAAAGTAAGTGATGAGATGGAGTGGCACACATTCCCTTTCCTACCTCTTCT
CCCTCTCCCATTACAGAAAAAGC
>1212
GGTCACCCGTGTATGGCACCCTGCCATACCAGCTCACCCTGCCCTGTGACTGCTTGTAA
AGCTAAGGTGGGTATTTCTGGCCACAGATGCAGCATTGACTGTAGTTCTGAGGCTACTA
GTGAATCCAGAACTGTCTACAG
>1213
AGTGTGAATGTGGACGAAGAAAAGAGATGGTGATTTGCTCTGAAGCATCTAGTACTTA
TCAAAGGTTAGTGTTACTTAAATGTTAACAACTGATGGCCTAGGTGAAACAGATACATG
TGTTTTGTTTTGTTTTTAAAGCT
>1214
CAGACATGCAGCTTGGAGGTTCAGTGGAGATCAGCAAGTTAATTACCAAAAAGGAAG
TTCATCAAGCCAGGTAATTTTTAAAATGCATATATGTGCCTTCTTTCTTCAATTAGACTTC
AATTAGAGGGCAGAGATTATTC
>1215
ACCTTGCGAGCCGTCTTCCCCAGGCCTGCGTCCGAATCTCCGCCGCTGCGGGCCCGCT
CCGACGCGGAAGGTGAGGGCTGGGGGAGGGGACCGGCGCTGACGGAGCCGCAGTGC
GGGGTCGGGTCTGTGGCGGACAGAGA
>1216
TGTCCTACTGGTGGAGGCCGTGGTCGTGGAATGAGAAGCCGTGGCAGAGGTGGTTTTA
CCTCGGATAGAGGTATTTTGTCGAATAGAAAAATTTGAAGTACTTCAGTATTTGTTAGTA
TCAAGACTGGTCTGACTAGCCG
>1217
CTTCCAGATATTTGTTATCGCTGTGGTGAGTCTGGTCATCTTGCCAAGGATTGTGATCTT
CAGGAGGATGGTAAGTATTTAACACTTCCTTTTCATACCCCTCTAGAGCTTGGAGAGGT
GAGCACATGCAACTGTGTATA
>1218
AGCAGAAATGCTATTCTTGTGGAGAATTCGGACACATTCAAAAAGACTGCACCAAAGT
GAAGTGCTATAGGTAAGGTGTCAGAATGTTGTTAGAAGAAAACTCATTGCAGAGATTC
TTCCAGAGATGAATTAGCTATAAA
>1219

GACACCATGAGGAACAGCGCCTCTGGCCCCGGATGGTACGGCGCCAACCCAGACCCG
CGCTTCCCCGCCAGTAAGTGAGGCCGCCCCACTGCGGGGCCGCGGGCTGAGCTCAGG
AGGTGCGGCGAGAGGCTCCAGAAGGC
>1220
CGCCGAGCTCCAGCCCCGAGCCCTGGACATCTACTCTGCCGTGGATGATGCCTCCCAC
GAGAAGGAGCTGGTCGGTATTCCCCTCGCTCTCGACCCCCTTGACGTGTCGCCTTGTCT
CTTCTCTTGCACGCCTCCCTCCT
>1221
AGAAGTCTTGAAGAAGCTCAAGAGTAAACGTGTTCCCATCTATGAGAAGAAGTATGGC
CAAGTCCCCATGGTAAGGTTTGTGGTCACTCCCTTCCCGTGTTTTTCCAAGAGAAAGTA
CACCGCCTTGAATCGTACACACA
>1222
GAATAAGGCAAGGGGGAGTGTAGAGCAGAGCAGAAGCCTGAGCCAGACGGAGAGCC
ACCTCCTCTCCCAGGTATGTGACACTCCCCATCCCCCTTCAGAGGCCACACACCCTATG
GCATTCCCACCATGTGTTAAGGATT
>1223
GCAGGAACTGAACCCAAAGGATCACCTGGTATTCCCTGAGAGTACAGATTTCTCCGGC
GTGGCCCTCAAGGTTAGTGAGTGAGCAGGTCCACAGGGGCATGATTGGATCCTGGAAT
GAATGAATCAACCATGAGAGAGTG
>1224
TCTCAATGTCTGCATGAACGCCAAGCACCACAAGGAAAAGCCAGGCCCCGAGGACAA
GTTGCATGAGCAGGTGGGCCAGGGGGTGATCTGGGGTGGTGAGGGACTGGCTCAGGA
AGAGGAAACGAGGACATGGAAATGCC
>1225
CAAACGGCATTTCATCCAGGACACCTGCCTCTACGAGTGCTCCCCCAACTTGGGGCCC
TGGATCCAGCAGGTATGCATGGCTTCCTGCAGGTACAAGACCTAGCGGAGCAGCTGAG
CTTTCCAGGCATCTCTGCAGGCTG
>1226
TGGTGGGAAGATTGTCGCACCTCCTACACCTGCAAGAGCAACTGGCACAAGGGCTGG
AACTGGACTTCAGGTGAGGGCTGGGGTGGGCAGGAATGGAGGGATTTGGAAGTGGAG
GTGTGTGGGTGTGGAACAGGTATGTG
>1227
TCTGCCTGGCAGAGGAGTTTCTTCAGGAAGAACCAGGGCAGCTTCTGCCCCTAGAGG
GCCAATGCCCTTGGTGAGTGCAGTCCCCTGGCCCCAGCCTGGTCCACCTCTGGGAAGA
GGGTGCCCAGTTGTGCAATCCAGGC
>1228
TTGGGGAGACCTGATCTGGCTGTGCCAGATGGACACTGAGAAAGAAGTAGAAGACTC
AGAATTAGAAGAGGTGAGTGGGCTTTGGTGGCGGGCTCCCTACCCCACTCCCTGCCCT
GGGCTGCCTGTGACCACACTGCTTG
>1229
CTCCCTACACCACACCCATACTCAGCCCCTGCAGGCAAGGAGAGAACAGGTCAGGTTC
CCGAGAGCTCAGGTGAGTGACACGTTGGAATGGCCCAGGGCACCTTCACCCTGCTCA
GCTTGTGGCTCCAACATTCTAGAAG
>1230
GCACTGGGGCCCCTGCAGAGCTTCCAAGCCCGACCTGATGACCTGCTCATCAACACCT
ACCCCAAGTCTGGTAAGTGAGGAGGGCCACCCACCCTCTCCCAGGCGGCAGTCCCCA

CCTTGGTCAGCAAGGTCGTGCCCTC
>1231
AAGTGTAACCGGGCTCCCATCTACGTACGGGTGCCCTTCCTTGAGGTCAATGATCCAGG
GGAACCCTCAGGTGCATGGCTGGGTCCTGGGGGTAAGGGAAGTGGAGGAAGACAGG
GCTGGGGCTTCAGCTCACCAGACCT
>1232
CCCACGGCTCATCAAGTCACACCTGCCCCTGGCTCTGCTCCCTCAGACTCTGTTGGATC
AGAAGGTCAAGGTGAGGCCGGCCTCAATGGTTCACACCTGTCATCCCAGTTTGAGACT
GAGGAGGGAGGATCCCTTGAAGG
>1233
CGTATGGAAAAGGCGCACCCTGAGCCTGGGACCTGGGACAGCTTCCTGGAAAAGTTC
ATGGCTGGAGAAGGTGGGCTTGACTGGAGGAAGGAGGGTGTGAAGCCGAGGGGTGG
TGGCTATAACGTACAGCAACCCTGTGT
>1234
CGTGCAGGAGTGGTGGGAGCTGAGCCGCACCCACCCTGTTCTCTACCTCTTCTATGAA
GACATGAAGGAGGTGAGACCGACTGTGATGCTTCCCCCCATGTGACACCTGGGGGCA
GGCACCTCACAGGGACCCACCAAGG
>1235
CCTATGACCAACTACACCACCGTCCCCCAGGAGCTCATGGACCACAGCATCTCCCCCTT
CATGAGGAAAGGTGGGTGCTGGCCAGCACGGGGGTTTGGGGCGGGTGGGAGCAGCA
GCTGCAGCCTCCCCATAGGCACTTG
>1236
AGAGACAGCATCGTCGGGACCAGACTCGTCTCAGGCCAGTTGCAGCCTTCTCAGCCA
AACGCCGACCAAGGTACAGCTTCAGTTTGCTACTGGGTTGTGCATTCAGCTGAATTTCA
TGGGGAAGTCCAAATTCTAAGGAA
>1237
ATACCCAGATGCTGTGGCCACATGGCTAAACCCTGACCCATCTCAGAAGCAGAATCTC
CTAGCCCCACAGGTATTTTTAAACTTCTCATAATTAAACTACAGTGATGAAAGATAGCC
ACACTCAGGCCATTTGGGCTGCT
>1238
TGGCCGAGGTGATAGTGTGGTTTATGGACTGAGGTCAAAATCTAAGAAGTTTCGCAGA
CCTGACATCCAGGTAAATCCTTTAACAGACACACCTGATGGTTCTGACTAGCGCTCAAG
TCTAGGAAACCACAGTTTGCATA
>1239
ATGCACGGGCAAGGCGTGTGCATGGAGCTGGCGGAGATCGAGGCCATCCAGGAAAGC
CTGCAGCCCTCTGGTAAGGTACCCCTGCCTCCCAATTCCCTCCTGAGTAGCGCTCCCTT
CCCAGCGGCTTCTTCCCCATTCCA
>1240
TCGAGACCGGAGCACCAGTGGGGGCAAGATGAAGGTCAATGGGGCGCCCCGGGAGG
ATGCCCGGCCTGTGGTAAGGACCTCCGATGCACAAATGTGCATGTGCATAGACACACA
CACACACACATGCCCCCTGCCCCCCA
>1241
CACCCACGAGGACCTCTACTTCATCCCCATCCCCAACTGCGACCGCAACGGCAACTTC
CACCCCAAGCAGGTGGGTCTCTGTCTCCCGCTGGCTTGGCCCTGGACTCAGCTCTGGG
GCATGGTCTCTTTTCCTGCGTCGG
>1242

CACCCCATTGCCCTTCATTGGAAACTACCTGCAGCTGAACACAGAGCAGATGTACAAC
TCCCTCATGAAGGTGTCCCAAGACAGGGAGATGGGTGTCTCGGGGTGGGGGCTGCCTA
GTTGGCTGGGGCTTTGTGGCAGGG
>1243
GTGGACCAGGCTGAGGAGTTCAGCGGGCGAGGCGAGCAAGCCACCTTCGACTGGGTC
TTCAAAGGCTATGGTGCCCAAGAGGGGGAAGGTGGGCAGGTGGACACGAAGGTCTCA
GTGTTCCCAGCCTTCTCCCTGACTCT
>1244
AAGCGAGGCATCGAGGAGCGCATCCAGGAGGAGTCGGGCTTCCTCATCGAGGCCATC
CGGAGCACGCACGGTGAGCAGGGGACCCCGAGTGCGGGGGCAGGAGAAGGAAAACA
CCCAGGACGAGGAACCCGCGCGCGTTC
>1245
CAAAGAGTTCCTGTCACTGTTGCGCATGATGCTAGGAATCTTCCAGTTCACGTCAACCT
CCACGGGGCAGGTAATGGTTGCAGCCCGGCCCGTGAAGGCCCTTACCAAAACCGGCA
AATTGTTCCCCTACCGGGGGAAGG
>1246
CAACCAGCGCACGCTGGATCCCAATTCCCCACGGGACTTCATTGACTCCTTTCTCATCC
GCATGCAGGAGGTACACCCCAGCAGCCACTGCGGGGAGATGCAAAGCCAGGCAGAGG
GAAATCAGTCTGGGAGTGGGGCAG
>1247
GGCACCGAGACGGTCAGCACCACCCTGCACTATGGCTTCTTACTGCTCATGAAGCACC
CAGAGGTGGAGGGTAAGGCTGGAGGGGGACGGAAGTGGAGGGCCCCAGACCCTCAA
AATTCCCCTTCGACTGGTGCAATGTC
>1248
CGTGATCCCCATGAGTTTGGCCCGCAGAGTCAAAAAGGACACCAAGTTTCGGGATTTC
TTCCTCCCTAAGGTGCTATCCGCCCCCACCCCCCAGACTACGGGGACTCCAGCCCCTCT
CTGTGTCCCCAGCATCCCACCCA
>1249
AATCCCCAGCACTTCCTGGGTGAGAAGGGGCAGTTTAAGAAGCGTGATGCTTTTGTGC
CCTTCTCCATCAGTAAGAGACCACTGTTTGGTGCCAGGCTTACTACTCACACCAGCAG
GGGCCTCCCTTACCCAGTTCCCCT
>1250
CACCCCATTGCCCTTCATTGGAAACTACCTCCAGCTGAACACAGAGCAGATGTACAAC
TCCCTCATGAAGGTGTCCTAAGGCAGGAGATGGGTGGCACGGGGTGGGGGCTGCCCA
GTTGGCTGGGGCTTAGTGGCAGGGG
>1251
GTGGACCAGGCTGAGGAGTTCAGCGGGCGAGGCGAGCAGGCCACCTTCGACTGGCTC
TTCAAAGGCTATGGTGAGGGGGTGCCAAGAGGGGGAAGGTGGTCAGGTGGATGCAAT
GGTCTCCGTGTCCCCAGCCTTCTCCC
>1252
AAGCGCGGCATCGAGGAACGCATCCAGGAGGAGGCGGGCTTCCTCATCGACGCCCTC
CGGGGCACGCACGGTGAGTAGGGGACCCCGAGTGCGAGGGCGGGAACCCGCGCTTTC
TGCCTGGGGATGGGGACTAGGTGGGG
>1253
CAAAGAGTTCCTGTCACTGTTGCGCATGATGCTGGGAAGGTTCCAGTTCACGGGAACC
TCCACGGGGCAGGTAACTGGATGCAGCCCGCCAGTGACGCCCCTACCACAACCTGCC

AACTGCTCCCCTACCTGGAGACAGG
>1254
CAACCAGCGCACGCTGGATCCCAATTCCCCACGGGACTTCATCGACTCCTTTCTCATCC
GCATGCAGGAGGTACATCCCAGCAGCCAGTGCAGGCAGGTGCAAAGCCAGGGAGAGG
GAAATCAGGATGGGAGTGGGGTGG
>1255
GGCACTGAGACCGTGAGCACCACCCTGCGCTACGGTTTCCTGCTGCTCATGAAGCACC
CAGAGGTGGAGGGTAAGACTGGAAAGGGAGGAAAGTGAAGGGCCCCAGACCCTCAA
AACTCCCCTGAGCCTGGTGCAGTGTA
>1256
CATGCTCCCCATGGGTTTGGCCCACAGGGTCAACAAGGACACCAAGTTTCGGGATTTC
TTCCTCCCTAAGGTGCTGTCTCCCCTCCACCACCACCACTCAGACTACGGGGACTTCC
AGCCTCTCTCTGTGTCCCCAGAAT
>1257
AGTCCCCAGCACTTCCTGGATGAGAAGGGGCAGTTTAAGAAGAGTGATGCTTTTGTGC
CCTTTTCCATCGGTAAGAGACACTGTTTGCTGCCAGGCCACGGCTCACACCAGCAGGG
GCCTCTCTCACCCACCTCCCCTCT
>1258
CACGCCGAACTGCATGCCGAGCCCCGGGGTGATGACTGGAGGGTCAGCCTGGAAGAC
CACAGCAGCCAAGGTGAGCATTAAGCAGGGCAGCTTTGCCCCTGGGTGGTTGAAGCG
CCAGGCTGGAATGAGTAAGGTCTCCA
>1259
ACCCGCCACCGGTCCTTATGGAGCCCAGGAAGAAACTCCGTGTAGACAAAGCCCCAC
TGACTCCCACTGGGTAAGTGGAGTCCTCACTTGGCCCTCTCAGTGTTTTACTGCTTTTC
GATTCCTTGTATCCCTAGGCTGTG
>1260
GCTGCAGTTGGAGCCGCCCACCGTGGTGGAGACCCTGCGGCGCGGGAGTAAGTTCAT
CAAATGGGACGAGGTAAGCGCGCGGGCCCCTGCTCCGCCCAAATCCCGGGACTCTTTC
AGTCAAGCTCGACCAGACGCTGGGG
>1261
CAGTCGGAACCTGGTGACCCTGCGTGTGGACCCCAATGGCTTCTTCTTGTACTGGACG
GGCCCCAACATGGTGAGGGTGGGCGCTGGTGCAGCTCGCTCAGGCCAAGGACCCCTG
TCCCAGACCCCTGCCCTGCCCGTGG
>1262
GGTGTCTGGGCCAGACCCGGTGAACACAGTGTTCTTGAACTTCATGGCCGTGCAGGAT
GACACAGCCAAGGTGGGCTGGCACCAAGGGGACGAAGGGGGAGTCACTGTCTTATTC
TGTGAGTCGGCCGTCACTTACCGAA
>1263
AGGAGCTATTCAAGCTGGCTATGAACATCCTGGCTCAGAACGCCTCCCGGAACACCTT
CCTGCGCAAAGCGTGAGCCCCAGGCCACCCGAGGGGGAGCCGGGGGGTTCACGTGG
CCGTTTTCAGGGTGTGACCTCTTCAT
>1264
GAAGATGTTCTCAGCAGACAAGAAGCGGGTGGAGACTGCGCTGGAATCCTGTGGCCT
CAAATTCAACCGGGTGTGTGGGGTGGGGACAGGGGCGGGGTGTGGTGTGTCACGGTG
GACACCCACCCTTACGGGGGTGCCCG
>1265

TGGAAATCTTTGAGCGGTTCCTGAACAAGCTGTGTCTGCGGCCGGACATTGACAAGAT
CCTGCTGGAGATGTGAGTGGCCCGGCCTGCCTCACAACCCCTGTGCTCTGTGTTTAG
CTTCTGCTTAGCGTGCCTCTAGCT
>1266
CCCCTGCGGCCCTCCCAGGCCCGGCTGCTCATCGAAAAGTATGAGCCCAACCAGCAGT
TTCTGGAGCGAGGTGAGCTGGCTGGGGTGCAGGTGGGTGGGGGCAGGTGGGACACC
AGCTGACCCTGCTGATCCTCTCCCAC
>1267
CTGAGCACGGACATGACCCAGCCACTGAGTGCCTACTTCATCAACTCCTCGCATAACA
CCTATCTCACTGGTGAGTGGGGAGCAAGGGTGGCACCCGTGACCTCTGCCCTGTGACC
CTTGGACAGCCCCACTCCTCCTGG
>1268
CCATTGCCGAGACTGCCTTCAAGACCTCGCCCTACCCCGTCATCCTCTCCTTCGAGAAC
CATGTGGACTCGTGAGTGAGCCCCTGGCATGAAACCCCATGGACTGGGGGACAGTCTT
CCAGCTTCAGTGCTGTTGGACTG
>1269
AAAGATGGCTGAGTACTGCCGCTCCATCTTTGGAGACGCGCTACTCATCGAGCCTCTG
GACAAGTACCCGGTACGGGAGCTCGGAGCGAGGGGGGTGGCATGGGGGCCAGGGTG
CTGCGGACCCGGTCCAGGGCCCTGAC
>1270
CCCCTGGAGCAGAGCAATTCTGCCCTGAGCGAGAGCTCCGCGGCCACCGAGCCCTCC
TCCCCGCAGCTGGGTAGGCCCCAGCCCGGCCCGCCACCCTGATCGACCCTAGCCTCTG
GCCTCATGTTCCAGGCGCCGTGACA
>1271
CCGTGAGGATGAGGAGGAAGATGAGGAAGAGGAGGAACAGACAGACCCCAAAAAGC
CAACTACAGATGAGGTCAGGCCCACAGGGTGGGCAGGTCGGGGAGGTAGCATCTATTT
ACCTCCCAGGGGGCTGGGTCTAACCA
>1272
ACTGAGGAGATGTCCACGCTTGTCAACTACATCGAACCTGTCAAGTTCAAGTCCTTTG
AGGCTGCTCGAAGTGAGTGGGGGTGGGTGGCAGGCATGGGAGCTTGCCCAGCTCAGC
CCTGCCAGGTCTGACGCCCTTTCTT
>1273
TCGAGATGTCGTCCTTTGTGGAGACCAAGGCCATGGAGCAACTGACCAAGAGCCCCAT
GGAGTTTGTGGAGTATCCTTTGAAGGTGCTGTGGGCGGACAGGCCAGGGTGGGGCGC
TCGGAGGCCGGCTCCGGTGTTCTAT
>1274
TCCAACTACATGCCCCAGCTCTTCTGGAACGTAGGGTGCCAGCTTGTTGCGCTCAACTT
CCAGACCCTCGGTGAGCCCTGGCCCCCTCCATCTTGACCCCGACCCTCAGTCTTACTG
ACTTCTGACCCACGATCCTGCTG
>1275
GGACAAGTCCTTCGACCCCTTCACTGAGGTCATCGTGGATGGCATCGTGGCCAATGCC
TTGCGGGTCAAGGTGGGGCTTGCGGGCGGCTCAGGCCAGGGGTGTCCTGGGGGCAGG
ACTCTCAGCATCCGCCTCACCCTCC
>1276
CCGCACCCGGACCTCTCAGGGGAACTCGTTCAACCCCGTGTGGGACGAAGAGCCCTT
CGACTTCCCCAAGGTGAGCCTGGCCCCTGCACCCGCCCAGGCACAGGCAGATCCAGC

CCAGACCGCCCCGACTCTCAGGTGGG
>1277
ATTGCAGCCTTTGAGGAGGGGGGTAAATTCGTAGGGCACCGGATCCTGCCTGTCTCTG
CCATCCGCTCCGGTGAGGCCTTGGTGGGCTCTGGGCAGCACAGCGGGCAGTGGGTAG
GGCCCCCACCTGGCCCAGCAACCCA
>1278
CAACCGCTGTGCCTGCCGGCCCTGCTCATCTACACCGAAGCCTCGGACTACATTCCTG
ACGACCACCAGGGTGAGCTGGGGGTGGGCGGGGCCTGCCTGGCCAGGGAGTGTGAG
GGACAAGGGCCACCCCCAGGGCCTGG
>1279
CCCCATTAAGCACGTCAGCCTGATGGACCAGAGGGCCCGGCAGCTGGCCGCCCTCATT
GGGGAGAGTGAGGTGAGCCGGGGCAGGGCAGGGCTCAGGCTCACTGTGACCCAGGG
GGCCGCTGGACATGACTGCTGTGGCC
>1280
CTGGATGCCTCCCCCCGCCGGCCCCCTGGCCCCACCACCTCCCCTGCCAGCACCTCCC
TCAGCAGCCCAGGTAAGGAGTGGCCTGGGTCGGGGGTGGGTTGCAGGGAGGCACCCC
CCACCCTGTCCTCCAGCTCCTCACA
>1281
CCCGCCGCCTGCTGGATGGCCTGGCTCAGGCACAGGCTGAGGGCAGGTGCCGGCTGC
GGCCAGGTGCCCTGTGAGTGTCTGGGCCGCCTGTGTGCTATGTGTGCTGGGTGTGCTG
ATGTGAACATGGGGGTCGGTGGGCA
>1282
GAGCCTGCTGGAGCTGCGGGAGGCCCAGGTGGACGCAGAGGCCCAGCGGAGGCTGG
AACACCTGAGACAGGTAGGGGGCCTGCAGTGGCCAGGGAAAGCCTGCTGGATAGACC
CGTCGTCAGCCCGGCATCACCTGTCAG
>1283
AGCGGCTCAGGGAGGTCGTCCTTGATGCAAACACAACTCAGTTCAAGAGGCTGAAAG
AGATGAACGAGAGGTGAAAGCCGAGGATTGTCTATGGGAAGGGCTGGGGACTTCTAG
TACCAGAAGGAGGGCAGAGTCTGTGC
>1284
AGATCCTGGACAGAAAGCGCCATAACAGCATCTCGGAGGCCAAGATGAGGGACAAGC
ATAAGAAGGAGGCGTAAGGGCACCGGGACCGGGGGCCATCTGGGTACTGGGGAGGCA
GGGCAGGTGTCTGGGCCCCGAGCCAT
>1285
GCAGCGGCATGACCGTCTTGTGGCTGGGCAGCAGCAGGTCCTGCAACAGCTGGCAGA
AGAGGAGCCCAAGGTGAGGCCATGGGCGAACAGGTGGGCAGACGGTGTGCAAGGCA
GCCAGGCCTCGCCTGTGATGCCCATCC
>1286
AAGCCGCACGGCCCTGCCCCACAGGCCCCTACAGCCTCCACAGCCACTAAGCCTGCA
CCCCCAAGTGAAGGTGACTGAAGGTGGGGCAGGGGGAGGAGGGAGGCAGGCATGTC
AGGGGTGGAGGGGAGCTCTTGGCCATG
>1287
TGGGAGCCCCCAGAGAGGCTGGGGAGGCTGGGCCTCCAGGGCTATGTGCTGGAGCTC
TGCAGAGAGGGAGGTGAGTCGTCTGGGCTAAGTCTTGCATACATCCAGGGGAGGTTGT
CATGGAGGGCTGCAGGGACAAGTGC
>1288

GCAGTTCGTCCTGCAGGGGCTGGCCCGCCGGCCATGCTGGACCAGCCCATCCACATCC
GAGAGAACATTGGTAAAGCCCATCCTGGCCTTCAGCTACCCAGCCCTTCCCAAACACC
TTATCTAATGGAGAAACTGAGGCC
>1289
CCCCCGCCACCTCCGTCAGACCTACATCCGCCAGGTGGGAGAGACGGTCAACCTGCA
AATCCCCTTCCAGGTGGGTATTACCCCCACCAAGGGTAACAGCCTGGGTGGGAACCAG
GGCCCCCATTTAAGGGGAGCCTGGG
>1290
GGCCGCTACGAGCTCACTGTGCGCGTGGAAGACCTGGAGGCCAAGGCAGTCATTGAC
ATCCTGGTGATTGGTATGGAGCCAGGGGAACAGTGCTGTCTCATGGGGGACCTGGAGC
CCCCAACCTCCTATCCCAGAGCTGA
>1291
GCCACCCCAGGACACAGGCAACACAGAGCTCCTGGGCTACATGGTGCAGAAGGCAGA
CAAAAAGACAGGGGTGAGGCTGGCTGAGGCAAAGTGGGGTCGGGGTACGAAGTGGT
CAAGGTGGTGGGGAAGGCCCATCCCAC
>1292
TCAGAAAACCTGTGTGGACTCAGCACCTCGGCCACCGTCACCAAGGAGCTCGCCCAC
ATCCAGAAGGCAGGTGGGTTTGGGCCCGGAGCTGGCTGTGCTAACTCTCTCCCTGTCT
CTGCTTCTTCCTCCGCGTCTCTGCA
>1293
CCTGGCTGACCACACCTCCACCCCTGGCTACAGCACCCAGTTGTTCTGCAGTGTCCGA
GCTTCACCCAAGGTGAGGGGCCAGGCCCTGTTGTGCCCTGGGCTGGTGGGGGCAGGT
GGGGACAGCAGGGCTGAGGAATCCT
>1294
GGGGTCTACACCTGCAAGGCCATAAATGTGCTGGGGGAGGCATCTGTGGACTGCCGGC
TGGAGGTCAAAGGTGAGAGTTTGAGGACGGGAGAACAGGAGTAAGCACCTCTCTGGA
ACTGGACTAGAGGCAGCAGACAGCT
>1295
GGCCTTGGCAGGAGGATGTGGCAGATGCTGAAGAGTGTGCTGGTCGCTGTGGGCTCTT
AATGGACTGCTGGTGAGTGGCCACTGGGCCTAGATAAGACTGGGGGCAGGGGAGCCT
GGGCCGTGGCGTTACCCTGTGCCTT
>1296
ACACCATGGCCACGACCGTGGGTGGCCTGTCCTGCCAGGCTTGGAGCCACAAGTTCCC
GAATGATCACCAGTGAGACAAACACCTTCCCTCCATCCCGGCCTGGGACCTTCCCCCA
GCACACACTATAGTGATGCTCTGG
>1297
GGTCCTTGGTGCCACACAACAGACCCTGCCGTGCGCTTCCAGAGCTGCGGCATCAAAT
CCTGCCTGGTGGGTAAGCGGCGCCGGGTCAAGCTGGGAGAGTGGAGGGACAAGCCCA
CGCCCATCCACGAACCCACTGGCTC
>1298
CCGAGTCAGGGCGCGAGTGCCAGCGCTGGGATCTTCAGCACCCGCACCAGCACCCCT
TCGAGCCGGGCAAGTACGCGTAGGCGGTATCGGCGCCCTGGGGGCCGGGCTAGGGAA
GGTCCAGGACTCCAGGGGCAGGGCTC
>1299
TCCCAGCGGCCATGGTGCTACACTACGGATCCGCAGATCGAGCGAGAGTTCTGTGACC
TCCCCCGCTGCGGTAGGCGGCGGGGACCAGGCCTGGGAGGGTACCTGGGAACCTTGG

GGAGGGGCGTGGCTTGGCCGGGAGG
>1300
TACCTTGCCAGCGTTGGGACGCGCAAATCCCGCATCAGCACCGATTTACGCCAGAAAA
ATACGCGTGCAAGTGAGGTGGGGGGGCGGGCGTTGGGACGTGCTGCTGCGGGTGAGA
CGGGAGGAGGGTAGTCACAGGCTTA
>1301
CTGCGGCCCGGCACGCGCGTGGGCTTTTGCTACCAGATCCGGCGTTGTACAGACGACG
TGCGGCCCCAGGGTGAGGCCCAAGCTTGGGGGCTACAGAGCCGGGGCTGGAAGCCTG
GAACCGAAGGGCCGGGGCGAGGTCT
>1302
GCACGGTCAGCAAGACCCGCAAGGGTGTCCAGTGCCAGCGCTGGTCCGCTGAGACGC
CGCACAAGCCGCAGTGAGTCCCTGGTGCTCCCGGCCCCGCCAGGGCCCTAACCCTGG
GGCGGCATGCTTTGATGTCTGGGACC
>1303
AGCCATGGGCCCTGGTGCTACACGATGGACCCAAGGACCCCATTCGACTACTGTGCCC
TGCGACGCTGCGGTGAGCACTAGTGACGCTTGCCCCATGACCCTGCCTCAGCCCCCAC
CACCAAAGGCTGGCTCCCTTAACC
>1304
GTCGTTCCAAGCTGCGCGTGGCTGGGGGCCATCCGGGCAACTCACCCTGGACAGTCA
GCTTGGGGAATCGGTGAGGCACAACTGCCTGTCTCCCACAGAGAGGAGCTGAGGTTG
TGTCCTCTGTGGTTATGCCACTGGGG
>1305
AGCATTTCTGCGGGGGGTCTCTAGTGAAGGAGCAGTGGATACTGACTGCCCGGCAGTG
CTTCTCCTCCTGGTGAGCCTCCCTTGTGTTTGGGACCCAGTCTCATCCCACCTTCCCCT
TTCCCCAGGCAAGCTAACAAGTG
>1306
AGCGGGTCCCAGTAGCCAAGATGCTGTGTGGGCCCTCAGGCTCCCAGCTTGTCCTGCT
CAAGCTGGAGAGGTATGTGGACAACCTGGGAGGGTGTGAGGTGGGGCTGAGCCTTGT
GGCCTCAGACCCTGAGTGCCCCCAT
>1307
CTGCCGCCTGAATGATATGTGGTGCCTCCAGGGACCAAGTGTGAGATTGCAGGCTGGG
GTGAGACCAAAGGTAAGAGCATAGTGCACAGGACTGCTGGCGGCCAGGAGCCCAGCC
CTGGATCTTCCTCCAGGACCGTCTC
>1308
GCACCGAGGACATGTGCGGGAGAGCGAGATGTGCACTGAGGGACTGTTGGCCCCTGT
GGGGGCCTGTGAGGTTGGTAGCAGGGCCCTGGGCCAGCCCTGGAAGGGTATGGGGGG
CTAGAAATGAACTATTTTATCATGAA
>1309
GCCTGTCCTCCGAGCCAGTCGCTGACAGCCGCGGCGCCGCGAGCTTCTCCTCTCCTCA
CGACCGAGGCAGGTAAACGCCCGGGGTGGGAGGAACGCGGGCGGGGGCAGGGGAGC
CGCGGGGGCCGAGTGAGGACCCCGGG
>1310
ATTTCAGCCATGTCTGAGCTTTCCGTCTTCCTGGAGGCACAAATCTAGTTTAGCTGAAC
CACAACAGATTGTACATATCCTGCAGAACCTCTGTGGTCTTAGGAAGGTTGAAAGTCA
CCAAATGTCACAGAAAATGAGGG
>1311

CTGCTGCCATCCTCCAGCAAGATGCTAGGGTCTTTGGGGCTTTGGGCATTACTTCCCAC
AGCTGTGGAAGGTAAGTGTCTACAGGGAGGGGAAGGGTCTCTCCATCCATCCAGCAA
GGGAAAGGGGCGCTTGAAGCAAGA
>1312
TGCCTCTACAGCCGCTGCTGCTTTGGGATCTGGAACCTGACCCAAGACCGGGCACAGG
TGGAAATGCAAGGTGAATGGCAAAGTATATGGCAGGTGATGGCTAGGGTGGGAGACA
GACACATCCTGGGGTGTGGGTGGCA
>1313
AATTACAGCCATCTGCCTCCTCCAGGGAGCCCTGGGACTCCTGGCTCCCAGGGTCCCC
AGGCTGCCCCAGGTAGCCACCCAAGGGTACTGAAGCCTGATGGGGGCTGGGGCCCAG
GTTAGGATGAGAGGTGGAACCAGGG
>1314
ATCTGGATGGCACTGGTGCTGCTGGGGCTGTTCCTCCTCCTCCTGCTGCTGCTGGGCAG
CATCATCTTGGGTACTAATCCACCCCATCCCTCCCTTGTGACCCCAAGACATTGCCCCA
AAAGCTCTGACCCCTCTCCAGG
>1315
GCCAAGGCCAGACTCAGGCAGGGACTGGAGTGTGGAGCTGCAGGAGCTGCCTGAGCT
GTGTTTCTCCCAGGTGCCCCAGGGAGGGAGAGAAGGGCTCCTCTGGGCACTCCTGGA
GGTTGTGCTGGGGAGGAATCCTGGCC
>1316
CACTGCCAGCCGGGGGGGTCCTGGCCGCCTGCTCTCTGGGCCCCTGCTGGTACTGGAA
CTGCATCCCAAGGTGAGCACCAAGGAGTGTATATGTGTGTGTGTGTGCCTGTGTGTATG
TATAGAGGTGGGGGCTACATGGC
>1317
AGTTCCCTGCGGATGGCACTGTCCCTGGCCCAGGGCCTGGCATTTCTCCATGAGGAGC
GCTGGCAGAATGGTGGGTGAGCTGGGCATAGGAAGTCAAGGGAGCCACAGTGCTATG
TTTGTGATTCTGCCTTAGTTTGGAG
>1318
GCTCCCTGGCCTCACTCAGCCCCCTGCCTGGACCCCTACTCAACCACAAGGCCCAGCT
GCCATCATGGAAGTGAGTTCTCTGGATAACTGGTGAGGCCCAGGATGATGTTGGTGCT
GCTGATGGCAATGCAGCCATTGTG
>1319
CGAGCTGATATTTACTCTTTGGCTCTGCTCCTGTGGGAGATACTGAGCCGCTGCCCAGA
TTTGAGGCCTGGTAAGGATGGGTGGTACAGTCCCCTCTCCTGGGCTCCCCCCCGCCCAT
TCTAGGTTCACCCCAACCTGAC
>1320
GCTATGGGCCTTGGCAGTGCAGGAGAGGAGGCGTCCCTACATCCCATCCACCTGGCGC
TGCTTTGCCACAGTAAGAGGCCTAGGCTGTTGGTCTGGGAACCTGGAGAGTGGGGGC
TGGGCATGGGCTTCAAGGACGTCTC
>1321
ACCTCTAGCCTACAGGGGCCTGGAGACCGGTTCCCGGGAGGTGGTCAGCCATGTAATC
AAACAAGGGAAGGTGAGTCACCACCCCCAGGACAGACCCCAACCCCTGCCTGGCCAA
CATGGTGAAACCCCATCTCTATTAA
>1322
CAAAATCATGCGGGAGCCCTGGGTAGAGCAAGACAAGTTTGGGAAGGTGAAGTTTGC
TGTGCTGCAGACGGTGAGTTCCCTTTGGTGCCCCCACCCCTCCTGCTGTCGGCACCAC

TGAGATGTCAGGAGCCCCGCAGATG
>1323
TGAACTACATCGGCCAATTCTTGCCTGGATATGAGCCCCCAGCGTTCATGGACCCCCTA
CTTCCTAAACTGTGAGTGTCCCTCAGGGCAGAGGTGGGTGGAAACCCCTGAGGGACT
GTGAAAAGTAATGCCCCTTCCTCC
>1324
GCAGTCTGGAGATGATCGACCACATTGTGGGAAACCAGCCTGATCAGGAGATGGTGTC
CGCCTCCGAATGGTGAGACCTTGGCCCTCTCCCTCTGCGTGAAGATGACGGGTCCTCA
CGGTGGACTGGCCCACTCCCTGGT
>1325
CAACTATGAAGAGTCCATCAAGATGCCCATCAATGAGCCAGCGCCTGGCAAGAAGAAG
TCCCAGATCCAGGTGAGGCCGCCCCTGGCTGGGGGAGAGGGGAGCAGGGAAGAGGT
CTGGGGGCTCTTGTAGGGTCAGGGAT
>1326
ATATGTGGACTATAACGGGGGCGCTGGGGTCCAGCACATCGCTCTCAAGACCGAAGAC
ATCATCACAGCGGTTAGAACCCCCTTCCCTGTCCGAATTCCTCTCTCATTCAGAGCCAG
GGACGGTCTCTGCAAAATTGAGA
>1327
CTACAAACAACTGCGGGAGAAGCTGAAGACGGCCAAGATCAAGGTGAAGGAGAACA
TTGATGCCCTGGAGGTGAGGCCCAGGCAGGATCCGCAGCCTTGTGGGGGGAGTATGA
GCTCTGCATTGCATCTTTTCAGAAATA
>1328
GATCTTCACCAAACCGGTGCAGGACCGGCCCACGCTCTTCCTGGAAGTCATCCAGCGC
CACAACCACCAGGTACTGCTTGTCCCCGGCAGGCCCGAGGGGACAGGCAGCTAGCAC
ACTCGGTTTTCTGGGTCCTTGGCTC
>1329
TGGCTGGAGCCCCCTCAGTGTGCAGGCTTAGAGGGACTAGGCTGGGTGTGGAGCTGC
AGCGTATCCACAGGTAAAGCAGCTCCCTGGCTGCTCTGATGCCAGGGACGGCGGGAG
AGGCTCCCCTGGGCTGGGGGGACAGG
>1330
GCTACTCCTCTGCATTGGAGCCCTCCTCGGGCACAGCAGCTGCCAGAACCCTGCCAGC
CCCCCGGAGGAGGTCAGTAGGCAGGCGGGGAGGGCGTGGTCAGCATTCCCCGCCCCT
CCTTGGCAGGCAGCACGGGAAACAG
>1331
ATGAGCCCCACGACCAACGTGCTCCTGTCTCCTCTCAGTGTGGCCACGGCCCTCTCGG
CCCTCTCGCTGGGTGAGTGCTCAGATGCAGGAAGCCCCAGGCAGACCTGGAGAGGCC
CCCTGTGGCCTCTGCGTAAACGTGG
>1332
GAGCTCCTTGACACGGTCACTGCCCCCCAGAAGAACCTCAAGAGTGCCTCCCGGATC
GTCTTTGAGAAGAGTGAGTCGCCTTTGCAGCCCAAGTTGCCTGAGGCATGTGGGCTCC
ATGCTGCAGGCTGGGGGGGTCTTTT
>1333
CTCGCCAGGTCCACAAAGGAAATTCCCGATGAGATCAGCATTCTCCTTCTCGGTGTGG
CGCACTTCAAGGGTGAGCGCGTCTCCAATTCTTTTTCATTTATTTTACTGTATTTTAACT
AATTAATTAATTCGATGGAGTC
>1334

GAGGGTCCCCATGATGTCGGACCCTAAGGCTGTTTTACGCTATGGCTTGGATTCAGATC
TCAGCTGCAAGGTCTGTGGGGATAGGGGCAGGGTGGGGGGTGGATGGAGGGAGAGGA
TAGAGAAGCAAAACAGGGTAGTGG
>1335
GCGGTCCTCACTGTCCCCAAGCTGAAGCTGAGTTACGAAGGCGAAGTCACCAAGTCC
CTGCAGGAGATGAGTATGTCTGAAGACCCTTTCGCTCTTGGTGGGTGGATGGGGTGGG
GCAGGGTCTTTGGGCCTTCCACTGT
>1336
CCCTGTGCCTCCCTCTACAGCCCTCAGGGAGCTCATTGAGGAGCTGGTCAACATCACC
CAGAACCAGAAGGTGAGTGTCGGCTAGCCAGGGTCCTAGCTATGAGGGCTCCAGGGT
GGGTGATTCCCAAGATGAGGTCATG
>1337
AGGCTGCAGTGCCATCGAGAAGACCCAGAGGATGCTGAGCGGATTCTGCCCGCACAA
GGTCTCAGCTGGGGTAAGGCATCCCCCACCCTCTCACACCCACCCTGCACCCCCTCCT
GCCAACCCTGGGCTCGCTGAAGGGA
>1338
GTCCTGGTGGCCGTGGCTACAGCCCTGGTGTCTGCCTCCTCCCCCTGCCCCCAGGCCT
GGGGCCCCCCAGGTGAGAAGAACCCTGCTCTACAACCTCCCAACTCTGACTTGTGACT
TGCCACCCTCACTGTGGCCCCCTT
>1339
GATGAGGGCACCTACATCTGCCAGACCCTGGATGGTGCACTTGGGGGCACAGTGACCC
TGCAGCTGGGCTGTGAGTTGGGGAGGGTGGCACTGATGACACATAGGGATCCTGAGG
GAGTATGGGACCTAAGTAAGCCCTC
>1340
AGAACTTCTCTTGCACTTGGAGTCCCAGCCAGATCAGCGGTTTACCCACCCGCTACCT
CACCTCCTACAGGTGTGTGTGTGATTGGGTGTGGATGCCTACACATTTGGGTATGCTGG
TGCAATGTGGGTGTGGGAGGCAG
>1341
ATTAATGTGACTGAGGTGAACCCACTGGGTGCCAGCACACGCCTGCTGGATGTGAGCT
TGCAGAGCATCTGTGAGTACCCACCCCAGACCTCCCCCAGACCCCCATCACAGAGACC
CCAAATGGACTCAGATCTCTCCAC
>1342
GTGCCAGCCCCACTTCCTGCTCAAGTTCCGTTTGCAGTACCGTCCGGCGCAGCATCCA
GCCTGGTCCACGGTGAGGCCTGGAGTGCGTCCCAACCCACGGCTGTGGTCCTGTCTCT
GATTTCACGATCCTGGGTGTTCTG
>1343
GCCCGGGACTTTCTAGATGCTGGCACCTGGAGCACCTGGAGCCCGGAGGCCTGGGGA
ACTCCGAGCACTGGTGAGAGACAAAGCCAAAGAAAAGGGCAGAGGCCCCATCCCAA
AGCATCTATCAGCTGAACCAGTTCCAA
>1344
GAGGTGGAGCCTCAGGTGGACAGCCCTGCTCCTCCAAGGCCCTCCCTCCAACCACAC
CCTCGGCTACTTGGTGAGCTTGGGCAGATGGGCAAGACTTGTAAAGGACTGAAAACC
CCAGAGAAAAGGGGACACCGAGCTTG
>1345
CGGGGTGGGAAGGATGGATCCCCAAAGCCTGGGTTCTTGGCCTCAGTGATTCCAGTGG
ACAGGCGTCCAGGTGAGTAGGACATCCAGAAGATTTGGACTTGGAGATGTTTGCCCCT

ATTTTGAGTGTCCAGATTAAGAGC
>1346
GTCCTGGGGCCTGGGCAGCTGGGGGCTGTGGCCATTCTGCTCTATCTTGGATTACTCCG
GTCGGGGACAGGTAAGTGAGGACGTGGGGGCAGGGTGGGGACAATGCACTGGAGGG
AGCCGGCGTGATCATTTTCTGGGGT
>1347
GCGTCCATGTGTGTGGTGGCTCTCTCGTGTCTGAGCAGTGGGTGCTGTCAGCTGCTCA
CTGCTTCCCCAGGTACCAAATGGTGAAAGTCAAAAGTGGGCTGGAGGTCAAAGTTAAT
GGGTCAATCCAGGTCAGAGGTTGG
>1348
GCAGCCAACGCCTCCTTCCCCAACGGCCTCCACTGCACTGTCACTGGCTGGGGTCATG
TGGCCCCCTCAGGTGAGGTGGGACGTGGGTGCCTAGAGGTGTGGAGGGGCACCTGAC
TTGGGGAGGGGCCCAGGGTAAGCCT
>1349
GGAGCCGCACTTTGTCCAAGAGGACATGGTGTGTGCTGGCTATGTGGAGGGGGGCAA
GGACGCCTGCCAGGTAAGCACAGGCCCGGGGGCAGATGACCAGTGCAACTTCGGAAA
GGAGGCCTGGCCCGGTCCTGATGGCT
>1350
GCGCCAGCAAGTTCGCGCTCGAAGGCTTATGCGAGAGTCTGGCGGTTCTGCTGCTGCC
CTTTGGGGTCCAGTGAGTCAACACCCCCGTTCCCCGAACCCTCTTAACTCTGACCTAG
AGACGCCGAGCACCCTGTCCTGCG
>1351
CTACCAATACCTCGCCCACAGCAAGCAAGTCTTTCGCGAGGCGGCGCAGAACCCTGA
GGAGGTGGCGGAGGTGAGCGCCGGGCTGGACTCCAGGAGTGGGGGCGGTGCGTCCT
CCGGCGCGCAGCGGTGGCCACAGCTCT
>1352
CAGCCTCCAGCCAGAAGGATGGGGTGGCTCCCACTCCTGCTGCTTCTGACTCAATGCT
TAGGGGTCCCTGGTAAGTGCCCCCAACCCTGATCCCCATCTGCCTTCAGGAGGGGGTT
GGCCCCATTCTCCTATTCTAGGAT
>1353
GGCCTTGGCAGGAGGATGTGGCAGATGCTGAAGAGTGTGCTGGTCGCTGTGGGCCCTT
AATGGACTGCCGGTGAGTGGCCACTGGGCTAGATAAGACTGGGGGCAGGGAAGCCTG
GGCTGTGGCGTTACCCTGTGCCTTC
>1354
GCACCATGGCCACGACCGTGGGTGGCCTGCCCTGCCAGGCTTGGAGCCACAAGTTCC
CGAATGATCACAAGTGAGACAAACACCTTCCCTCCGTCCCGGCCTGGGGCTTCCCCCA
GCACACACTATAGTGATGCTCTGGG
>1355
GGTCCTTGGTGCTACACAACAGACCCTGCTGTGCGCTTCCAGAGCTGCGGCATCAAAT
CCTGCCGGGAGGGTAAGCGGCGCCGGGTCAAGCTGGGAGAGTGGAGACAAGCCCAC
GTCCATCCACGAACCCACTGGCTCTT
>1356
CGGAGTCAGGGCGCGAGTGCCAGCGCTGGGATCTTCAGCACCCGCACCAGCACCCCT
TCGAGCCGGGCAAGTACGCGTAGGCGGTATCGGCGTCCTGGGGGCCGGGCTAGGGAA
GGTCCAGGACTCCAGGGGCAGGGCTC
>1357

TCCGAGCGGCCATGGTGCTACACTACGGATCCGCAGATCGAGCGAGAGTTCTGTGACC
TCCCCCGCTGCGGTAGGCGGCGGGGACCAGGCCTGGGAGGGTACCTGGGAACCTTGG
GGAGGGGCGTGGCTTGGCCGGGGAG
>1358
TACCTTGCCAGCGTTGGGACGCGCAAATCCCTCATCAGCACCGATTTACGCCAGAAAA
ATACGCGTGCAAGTGAGGTGGGGGGGGGGGGCGGGCGTTGGGACGTGCTGCTGCGGG
TGAGACGGGAGGAAGGTAGTCACGG
>1359
CTGCGGCCCGGCATGCGCGCGGCCTTTTGCTACCAGATCCGGCGTTGTACAGACGACG
TGCGGCCCCAGGGTGAGGCCCAAGCTTGGGGGCTACAGAGCCGGGCTGGAAGCTGGA
ACCGGAGGCCGGGGCGAGGTCTCGG
>1360
GCACGGTCAGCAAGACCCGCAAGGGTGTCCAGTGCCAGCGCTGGTCCGCTGAGACGC
CGCACAAGCCGCAGTGAGTCCCTGGTGCTCCCGGCCCCGCCAGGGCCCTAACCCTGG
GGCGGCATGCTTTGGTGTCTGGGACC
>1361
AGCCATGGGCCCTGGTGCTACACGATGGACCCAAGGACCCCATTCGACTACTGTGCCC
TGCGACGCTGCGGTGAGCACTAGTGACGCTTCCCCCATGACCCTGCCTCAGCCCCCAC
CCAAAGGCTGGCTCCCTTAACCCC
>1362
GGCGTTCCAAGCTGCGCGTGGTTGGGGGCCATCCGGGCAACTCACCCTGGACAGTCA
GCTTGCGGAATCGGTGAGGCACAACTGCCTGTCTCCCACAGAGAGGAGCTGAGGTTG
TGTCCTCTGTGGTTATCCACTGGGGC
>1363
AGCATTTCTGCGGGGGGTCTCTAGTGAAGGAGCAGTGGATACTGACTGCCCGGCAGTG
CTTCTCCTCCTGGTGAGCCTCCCTTGTGTTTGGGGACCCAGTCTCATCCCACCTTCCCC
CTTCCCCAGGCAAGCTAACAAGT
>1364
AGCGGGTCCCAGTAGCCAAGATGGTGTGTGGGCCCTCAGGCTCCCAGCTTGTCCTGCT
CAAGCTGGAGAGGTATGTGGACAACCTGGGAGGGTGTGAGGTGGGGCTGGGCCTTGT
GGCCTCAGACCCTGAGTGCCCCCAT
>1365
CTGCCCCCTGAATGGTATGTGGTGCCTCCAGGGACCAAGTGTGAGATTGCAGGCTGGG
GTGAGACCAAAGGTAAGAGCACAGTGCACAGGACTGCTGGTGGCCAGGAGGCCAGC
CCTGGATCTTCCTGCAGGACCCTCTC
>1366
GCACCGAGGACGTGTGCGGGAGAGTGAGATGTGCACTGAGGGACTGTTGGCCCCTGT
GGGGGCCTGTGAGGTTGGTGGCAGGGCCTGGGCAGCCCTGGAAGTATGGGGGGCTAG
AAATGAACTATTTTATCATGAAGCAG
>1367
TGCCTGCGGACAGGCCCCCAGGGCTAGCGGCTTTGTGGAGGGAACACTGGGTATCCTC
TCCACAGTCCAGGTAGGTGGGACCCCCACTCCTGGCTCTCAGCCTTTAGGAACAGGAG
CTTCTCTACTTGGAGCTTCTAGAC
>1368
CTCCCTGCCAGTGAGTTTCGCTGCCTCACCCCGGAGGACGCTGTCAGCGCCTTTGAGA
TCGAGCGTGAAGGTGAGTGGCCCCGCACAGGGTCAGAGGGATGCTCCACTCTGGTCC

AGTTATCCTGTGGGGAGGAGACCCT
>1369
CTGGTTCGAGGAGGGCTGCCTTGTGGCCTTCATCATCGGCTCGCTCTGGGACAAGGAG
AGACTCATGCAGGTGAGGACAGGCCTGCGACGCCCAGCTCCAGGGAGGCCTCTGAAG
ACAGAGGTCAGCCAGATGGCGGGGA
>1370
AAATTCGTCATTCGAAACATAGTGGAGGCCGCAGCAGTCAGGGACATTTCTGAAGCGA
GCGTCTTCGATGGTAAGTGGGTCACCGGCGCGAACTGTGTGAGGATCCAGTATCTTAA
AGCCTTCGCCCAACTTCGCCCTTT
>1371
CAAAGTAGTCAGGAATCGATCTCGTGAAGCCCGCAAGGACCGAACACCCCCACCCCG
ATTTAGACCTGCGGTGAGTATTTTAAAAGGAGAATGGAAGCCAGAGGAGTGATGGTTA
AAATTTCATCCTGGAGGGTCAGGGT
>1372
AGGTTCACTGCAACCGTTCAGAGGTCCCCAGGAGCTGCTGCTGGCGAGCCCGCTACTG
CAGGGACCTATGGTGAGCAAGGCTACCTGGTGAGGGGAGACAGGCAGAGGGGGTCTA
GGAGCCTCCTTGGGGGGAAGAAGCT
>1373
TTCCAGCAAGTTTGTTCAAGATTGGCTGTCAAGAATCATGGACTGTTATTATATGCCTTG
TTTTCTGTCAGTGAGTAGACACCTCTTCCTTCCCCCTCTCCGGAATTCACTCTGCCCTC
ACCACCCCTGCTCGCCGGCTG
>1374
AGCACTGGTCTTGAGTATCCTGAGCGCCCGGCCAGCCGGGCCAACACCGTGAGGAGC
TTCCACCACGAAGGTCAGTCTCTTCCCCCAGTCTGCGTGGGGGAGGGCTGGTGGGACT
GGCTAGAGGGGCAGTGAAAGCCCTG
>1375
GGAACCCACTTAGGTGGCACCAATCTTGACTTCCAGATGGAACAGTACATCTATAAAA
GGAAAAGTGATGGTTAGTCATTGCTTTAATTTTTTGTTACTCCAGCTGTAAGTACAAATT
TTGAGCTTGCTATTCTCGTGGT
>1376
AGCTCGTGCAATTGTTGCCATTGAAAACCCTGCTGATGTCAGTGTTATATCCTCCAGGA
ATACTGGCCAGGTTTGTGGAACAGTGGTTAGTTTTTATATTATAGAAATAAAGCCTTACA
GACATTGTGAGACATATAGAA
>1377
TACCATTGCGCTGTGTAACACAGATTCTCCTCTGCGCTATGTGGACATTGCCATCCCATG
CAACAACAAGGTAATGATTTTAGGATCTAGAGTTTGTGAATGCGTGCTCTAGAAAAAA
CATTCCTGTGCACATTGTTAGA
>1378
TGGCACCATTTCCCGTGAACACCCATGGGAGGTCATGCCTGATCTGTACTTCTACAGAG
ATCCTGAAGAGGTAAGCTTCTCCAAAGGCTTGTGGTTACATAAGCAAATTGGACGACT
TGGACTGCTGTCTAGGAAGCAAA
>1379
CAGCCTGAGGTTGCAGACTGGTCTGAAGGTGTACAGGTGCCCTCTGTGCCTATTCAGC
AATTCCCTACTGGTATGTATCAGGATAGAGGTGAATCAAGCTGATATTTTGCAACTTCTC
AGTTTTATTCTAACTTTAATGA
>1380

CATGGAGCTGTTCTGCTGGTCAGGGGGCTGGGGGCTGCCGTCAGTGGACCTGGACAG
CCTGGCCGTGCTGGTGAGGGGTGGCGCCGGCGCCCTCTGCTGTGCCCTGATGACTGGG
GAGGGGGCCGAACTAGCCAAGCATC
>1381
ACCTATGCCAGATTTACTGGTGCTCCACTGAAGGTACACAAGATCAGCAACCCCTGGC
AGAGCCCTTCAGGTACCCAGTATCCCTTGGGGGTAAGGAAGGGTGTGTACAAGGGGA
GAAGCAAGAAATAGGCAGGAATGTG
>1382
TGCCCTTCGGACCAGTCATGGAGAGGTCATCTCAGTTCCACACAAGATCATCACCCAC
CTTCGAAAAGAGGTAGGTGACTTGGATAGAGGGGGCTGCCAGTGAGAGAAGTTCAGT
CAATTCTATACAATACACATTTATT
>1383
GTCAGCTCGGCAAGGGGCAGACACCCTGGCCTTCATGTCTCTCCTGGAGGAGAAGTT
GCTCCCGGTGCTGGTGAGTGTGCCCAGACCTCCCAGCATCCATGGCCAGCCGGGGAG
GGTTCGGGAACACACAGACCCACACA
>1384
GTACATGGAACGGCTACAGCTGCTGACTGGGGAGCACAGGCCTGAGGACGAGGAAGA
GCTGGAGAAGGAGGTAGCTCTGAGACCGGGGGCTATTGTATGAGATGAGCCCCAAGGA
TGCTGGCCAGGAATGGGAGTGCTTA
>1385
GAGAGGCTCGGGAGTGTCTGACCCTGCTCTCTCAGCGCCTGGGCTCTCAAAAGTTCTT
CTTTGGAGATGCGTGAGTCTGACTCCAAGAGGGTAATGGGTGGCTTGGAAGAAGATAC
AGGTTCAGATGGAGCAGCTGGAGC
>1386
CTGCGTGGGCTGCACAACCTCTGTGCCTATTGTACCCACATTCTCAGTCTCTACTTCCC
CTGGGATGGAGGTAAGGGGCAGATGGGAGGGGCAGCCCTGGGGAGAGTGGGCAGGG
ATCCAAGAACTAGTTCTCCTAACAC
>1387
GGACCATCCCAGACAGTGACATCTCTGCTTCCAGCTCCTGGTCAGATTCCACTGCCGC
CCGCCACAGCAGGTACTTGGCACACCTGGCACACTTGTAGCTGCCCCGAGAGGAGCT
CCTGGGACCTCTACTTCCCCTCCAA
>1388
TTCTACCCCCGGGCTGACCGGGTCATGAGTGTCTGTCTGCGGGTAGAGCTCTATGGCTG
CCTCTGGAGGGGTGAGTGGCTCAGCTTCCTGGGAATCTGTTTCCTGAGCAGGGGACTG
GAGGGTGGGGAGTGTGGCGAATG
>1389
CAGCTTCTCCAGTGGCTATGTGGAGATGGAGTTTGAGTTTGACCGGCTGAGGGCCTTC
CAGGCTATGCAGGTGAGTGAGTCCGGCTCTCGAGGAGGGCTCTGAAGCCATGCAGGG
TGCCGTTGGGGTGCCCCCACCACTC
>1390
CGCTTTCTGCAGTGCCGCTTCCTCTTTGCGGGGCCCTGGTTACTCTTCAGCGAAATCTC
CTTCATCTCTGGTAAGCCCTGGAGTAGCCCAGTCTCCAGTCCCTGAAATTGACAACTG
AGTTCATTCCTAACCCTGCAGTG
>1391
CTGGGAGGCACCTTCCCGCCAGCCCCCTGGTGGCCGCCTGGCCCACCTCCCACCAACT
TCAGCAGCTTGGGTGAGCAATCTTGGGTGGGCGTGTGGACCCTCTGCACCCTTCTCCC

TGGGCCTCCCCCTCGGCTAGGGTG
>1392
CATCCTGCTCCTGCTGCTCATCATTGCCCTCATGCTCTGGCGGCTGCACTGGCGCAGGC
TCCTCAGCAAGGTGGGCACAGCCGTGGCATGTGGAGTGGCGGGGGGAGGCCAGGCCC
CAGCACGAGCCAGCGTCCAGTGGG
>1393
CCCCCGTACCAGGAGCCCCGGCCTCGTGGGAATCCGCCCCACTCCGCTCCCTGTGTCC
CCAATGGCTCTGGTAAGACCTGCCTTGTTCCAGTCGCACCTCTGTCCTCTCTGCTGTTT
TCTTATTGTATCCCTTTCCCATT
>1394
ACTTACGCCCGTCCCCCTCGAGGCCCGGGCCCCCCCACACCCGCCTGGGCCAAACCCA
CCAACACCCAGGGTAAGCCCCTCTGCCCCTGGGCTCCGCCAGGCTCCCCATACCTCTA
CTGGGGCAGGGAAAAGCCCTCACA
>1395
CCCCAGAGTGGATTTCCCTCGATCTCGACTCCGCTTCAAGGAGAAGCTTGGCGAGGGC
CAGTTTGGGGAGGTAAGGAGGGTGCCTACCCAGTGTCTGGCCCTATTGTGTGCTCTGA
TGCCATGCCTGCGCATCCCCCTAG
>1396
ATGTGCGTAAGGGACACCCTTTGCTGGTAGCTGTCAAGATCTTACGGCCAGATGCCAC
CAAGAATGCCAGGTGAGGACCAGGGATGGCATCTGGAAGAAGGGAGGGGAGGCCGC
GAAGAGTGGCGAGCCATCTAGAGAGA
>1397
ACCAGCTGGAGGACAAGGCAGCCGAGGGGGCCCCTGGGGACGGGCAGGCTGCGCAG
GGGCCCACCATCAGGTACCTGTTTACCCAGGCTGGGCCATGCTCAGAATTCCCCCAGG
GGATCTCCTCCTCTCCCCTCGCTTCA
>1398
GGACTATTACCGTGTGCAGGGCCGGGCAGTGCTGCCCATCCGCTGGATGGCCTGGGAG
TGCATCCTCATGGTGAGCAGCCCGAGGACAGCCAGGTTGGAGCAGGGCAGGTGGGAG
AACACTGGCCGCCACTCACAGCCCT
>1399
TGGCATCAACCAGCGGTCCTCGGATGCTAACCTCTACCGCCTCCTGGACCTGGACCCC
AGGCCCACGATGGTGAGCTTTGGGGGACATTCTGCTCTGCTCTGGCTGGGCTTGGCAC
GTGTTGTTCCTTCTGCTCCTGCTG
>1400
AGTGAAGGAGACAGTGTGCCCCAGGACGACACAGCAGTCACCAGAGGATTGTGACTT
CAAGAAGGACGGGGTGAGGCTGGGGGCTGGGGGTGTTGGTGGGTGCCTCCCAAGGA
GCTGAACAGGGGGCACCTGGGGAATAT
>1401
GGTGAAGCGGTGTATGGGGACAGTGACCCTCAACCAGGCCAGGGGCTCCTTTGACATC
AGTTGTGATAAGGTGAGTGGGCTGTTCTGGGATGCAGGGGCTGATGGGGGCATAGAGT
GTGGACCATCCAATGGGTCAATTA
>1402
TCTCTGCATGGATACTTCGTCTTCGGGCCCACAGGATGCAATTTGGAGGGCTTCTTTGC
CACCCTGGGCGGTATGAGCCGGGTGTGGGTGGGGTGTGCAGGAGCCCGGGAGCATGG
AGGGGTCTGGGAGAGTCCCGGGCT
>1403

TCATGGGCGTTGCCTTCACCTGGGTCATGGCGCTGGCCTGCGCCGCACCCCCACTCGC
CGGCTGGTCCAGGTAATGGCACTGAGCAGAAGGGAAGAAGCTCCGGGGGCTCTTTGT
AGGGTCCTCCAGTCAGGACTCAAAC
>1404
GGTCCACTTCACCATCCCCATGATTATCATCTTTTTCTGCTATGGGCAGCTCGTCTTCAC
CGTCAAGGAGGTACGGGCCGGGGGGTGGGCGGCCTCACGGCTCTGAGGGTCCAGCCC
CCAGCATGCATCTGCGGCTCCTG
>1405
CATCCCAGCGTTCTTTGCCAAGAGCGCCGCCATCTACAACCCTGTCATCTATATCATGAT
GAACAAGCAGGTGCCTACTGCGGGTGGGAGGGCCCCAGTGCCCCAGGCCACAGGCGC
TGCCTGCCAAGGACAAGCTACTC
>1406
ACAGGTTTTATTTTTTTCTTGTTTTTTTTGACTGAATCCCTAACCCTGCCCACACAACCT
CGGGATATAGGTAAGAAATTTACTTGTATATCAGCTAAAGCATGACCAGTTAGGATAATT
TCTATTTTTAAAATTTTGTT
>1407
GAAAGACATGGTAGAATACAAAGACAGATGTATGGCTGACAAGACGCTCCCAGAGTGT
TCAAAATTACCTGTAAGTAAATTGCTTGTGTTTCTTTTCCTTTTATCTTATGTCTTTCTTT
CTCTTTCTTTCTTTCTTTCTT
>1408
CTTTCCCTACCCTGGATCCCGAAGAGAAATGCCAGGCTTATGAAAGTAACAGAGAATC
CCTTTTAAATCAGTAAGTTTAATCTTAGTAAAAAATGATCCAGTTGAAGAATTAGTCTTA
GGCTGAATCTAATATCTATCTC
>1409
TGTTCATTTTGAGGAGGTGGCCAAATCATGTTGTGAAGAACAAAACAAAGTCAACTGC
CTTCAAACAAGGGTGGGTATAGCATTTGTTCCATGAAGAGGATAAGAAATCACTCAATA
CCACAGATCCAGACCCTGACTTA
>1410
CATTTTCTTCTTATCAAAAACATGTCTGTGGGGCACTTTTGAAATTTGGAACCAAAGTT
GTACACTTTATGTGAGTTTTATACTATATGTCTTGTCTCTGCTTGCATTTCCTTGTCTGTG
TCCCATTTTTGTTAAATGGT
>1411
TGTAGAAGATGTTTCTTCCAACTATGATGGATGCTGTGAAGGGGATGTTGTGCAGTGCA
TCCGTGACACGGTGAATATTCTCTAAAACCAAGTTAAAATAGTGATTTTTGGCAATTATC
ATTTTTTTATTCTCAAGTTGC
>1412
AAGGAAAATTTACTGACAGTGAAAATGTGTGTCAAGAACGAGATGCTGACCCAGACA
CCTTCTTTGCGAAGTAATATAACTCTTTATTGAATTTATAAGGGAAACAGAAAGAAACT
TGTAAGATTTGACTTTTGTTGCTA
>1413
TCAAATATACAAAGATCTCCTGAGAAATTGCTGCAACACAGAAAACCCTCCAGGTTGT
TACCGTTACGCGGTAGGTTCCATTGTTGTAGGTTCAGAAAATCAAAAAAGAACAACTA
GAAAACACTTAAAAATTGATATCA
>1414
AAAGCCTCAAGATGGTACAACAAGAATGTAAACATTTCCAGAATTTGGGGAAGGATGG
TTTGAAATACCAGTATGTTGTTTGCACAAGTGGGCTAACACTTGCCACTAGAATTGAAT

ACATAATCCCTCGAAGCGTAAAA
>1415
CGAGAAAATGGTGACAGCTTTCACTACTTGCTGTACGCTAAGTGAAGAGTTTGCCTGT
GTTGATAATTTGGTGAGCATGGCCTGTGTACCAGACTACTCTTTTTTTTTTTTTTTAAAA
AAAAGAATATTTGCACTCATTG
>1416
AAGATTTATTTACCTTTCACGCAGACATGTGTCAATCTCAGAATGAGGAGCTTCAGAGG
AAGACAGACAGGTACAAATAATCTCTTCCATTCTTCTTTTTCTTTGGTTTGAAGACACA
CCATGTATTAGTGAGAAGGTTT
>1417
TACAAATTTCGCAAATGTAGTGGATAAGTGCTGCAAAGCAGAGAGTCCTGAAGTCTGC
TTTAATGAAGAGGTAGTTTTATTTCTTTTCTACTGAAATTCAACTGGTTTTCCTGGTCAA
ATAAAATAAAACAGAACCCTGC
>1418
ACAACCACAGTTGCAGTTACTCCACCTACGGCATAAAAACACCAAGGAAATATCAAAG
AACACAACGCAGGTAAATTAACAGTATATAAAATGAGTAATTCCGACAAGAAGCATGG
ATTTATGAATACAACCATAAATTC
>1419
GTGGATTCCCAGGGACTGTCGGAGGTGTGGACTCTGCCTGCCTACCTGGTCTGGGAAG
ATGTTCTACCATGTGAGCAGGGCTCAGGGTGGCGGCAAGGGCTGGGTGGAAAGGAAG
AGCAAGGCCAGGCGCCGGCGCGAGA
>1420
TCGGCCCCAACTTGCTCAACACGGTGAAGCAGAAGCTCTTCACCGAGGTGGAGGGGA
CCTGCACAGGGAAGTGAGTGTCGAGCTCACCGCACCGCCAGATCGTTCGATGCGCAC
ACGGGGCGCTATACCTGCAACCCCTC
>1421
GTACAAGGCCATTGTTTTCCGGCCATTTAAAGGGGAGGTCGTGGATGCTGTTGTCACTC
AGGTCAACAAGGTGAGACCATACATAGGGGAGGCAGTGGGGTAGTCTCTCGGAAGAT
CTGGGTTGGGTTCTGGCTCCACTT
>1422
TATTGTGATTCAGCAGGACGATGAGATCCGCTTAAAGATTGTGGGGACCCGTGTGGAC
AAGAATGACATTGTGATCTTTTCCCCACCTCTCTACCTATACCAGGTTGAGCTGAGAAC
TGTCGATTTCTTTTTTCTTTTTT
>1423
GCACTGGGGCCCCTGCAGAGCTTCCAGGCCCGGCCTGATGACCTGCTCATCAGCACCT
ACCCCAAGTCCGGTAAGTGAGGAGGGCCACCCACCCTCTCCCAGGTGGCAGTCCCCA
CCTTGGCCAGCGAGGTCGTGCCCTC
>1424
AAGTGTCACCGAGCTCCCATCTTCATGCGGGTGCCCTTCCTTGAGTTCAAAGCCCCAG
GGATTCCCTCAGGTGTGTGAGTGTGTCCTGGGTGCAAGGGGAGTGGAGGAAGACAGG
GCTGGGGCTTCAGCTCACCAGACCT
>1425
CCCACGACTCCTGAAGACACACCTGCCCCTGGCTCTGCTCCCCCAGACTCTGTTGGAT
CAGAAGGTCAAGGTGAGGCAGGGCACAGTGTTTCACATCCATAATCCCAGCACTTTGG
GAGGCTGAGGCAGGCAGATCACCT
>1426

CACATGGCCAAGGTGCACCCTGAGCCTGGGACCTGGGACAGCTTCCTGGAGAAGTTC
ATGGTCGGAGAAGGTGGGTTTGATGGGAGGAAGGAAAGTGTGGAGCCGAGGGGTGGT
GGCTACAACGCACAGCAACCCTGTGT
>1427
CGTGCAGGAGTGGTGGGAGCTGAGCCGCACCCACCCTGTTCTCTACCTCTTCTATGAA
GACATGAAGGAGGTGAGACCACCTGTGAAGCTTCCCTCCATGTGACACCTGGGGGCC
GGCACCTCACAGGGACCCACCAGGG
>1428
CCTATGACCAACTACACCACCGTCCCCCAGGAGTTCATGGACCACAGCATCTCCCCCTT
CATGAGGAAAGGTGGGTGCTGGCCAGTACGGGGGTTTGGGGCGGGTGGGAGCAGCAG
CTGCAGCCTCCCCATAGGCACTCG
>1429
AGCTGTAACGGATACTTCGTCTTCGGTCGCCATGTTTGTGCTTTGGAGGGCTTCCTGGG
CACTGTAGCAGGTACTGCAGGGGAAAAAGGGGTTAGGGGAAGGCAAAGGTTGCTACT
CCGCTGGAGGGGGTTCCTAAAGAG
>1430
TGACGGTGGTCCTGGCTACCTGGACCATTGGTATTGGCGTCTCCATCCCACCCTTCTTT
GGCTGGAGCCGGTGAGAGTGCAGGGCAGTGGTGCTGAGTTAACTAGGAGCTCAGGTT
GATGTGGGTGGAAAGAGAGCTTGG
>1431
CTTCTGCTTCATTGTGCCTCTCTCCCTCATCTGCTTCTCCTACACTCAGCTGCTGAGGGC
CCTGAAAGCTGTGAGTGGCATTTGATAGTCAGGGAAGAAGGGGTTCGGGGCTCCACAT
GAGAAGGAAGAGTGCTCTGAAA
>1432
CATTCCTTCATTCTTCTCCAAGAGTGCTTGCATCTACAATCCCATCATCTACTGCTTCAT
GAATAAGCAGGTAAAGCTCTTTATTCACATTCCTATGGTCCAGAAGACCCTGGTTCTTT
TCTCACCATTGACTTTTAACT
>1433
GTGGACGCTTTCCTGGGCACCTGGAAGCTAGTGGACAGCAAGAATTTCGATGACTACA
TGAAGTCACTCGGTGAGCAAGCCGCGGGGCTCAGGATGTTGGCTTGGGGACTGGCTG
GTGGCGTGCCTAGCCCCACGCAGCA
>1434
GAACACAGAGATCAGCTTTAAGTTGGGGGTGGAGTTCGATGAGACAACAGCAGATGA
CAGGAAGGTCAAGGTAAGTCAGGGAAACAGGGGTGGGGAATGGAGAGTGCTGAGAC
TCTAAAAGAGAATAGGCTGGTAGTCTT
>1435
TCACCTGCAGAAATGGGACGGGCAAGAGACCACACTTGTGCGGGAGCTAATTGATGG
AAAACTCATCCTGGTAAGATGGGCAACTTTGGAGCTATATCTGATTGGTTATTACTACTG
CTCTTTCAGCCAAGCCTGTTCTA
>1436
GGGCCGAGGGCTGCCGGTCCGGGCTCCGCTCAGCACCCTCAACGGCGAGATCAGCGC
CCTGACGGCCGAGGTGAGATCCAGATCCGACCACTAGATCATCCTTATACCGACGGGG
AAACGGAGGCCAGAGAGGGCGTGGG
>1437
ACCGACGGTACCATGAAGGACGAGGTAGCTCTACTGGCTGCTGTCACCCTCCTGGGAG
TCCTGCTGCAAGGTGGGCTGGTTCCTATCTAGGAAGAGGGTGGGCCTTAGATCCCTAC

AGCTTGCCCTCTGCCCCCTAGGCC
>1438
GCAGGGCCTTCCGCGTGTCGCCGCCGCTCACCACCGGCCCACCCGAGTTCGAGCGCG
TCTACCGAGCCCAGTGAGGCGCGGCGGGAGGGCGCGGGGCGGGGAGCGAGCCCCAG
GCGGGTCCGGGTCGCAGGACCATCCCG
>1439
GTGAACTGCAGCGAGTACTTCCCGCTGTTCCTCGCCACGCTCTGGGTCGCCGGCATCT
TCTTTCATGAAGGTCGGGGTGTGGGGCAGGGGCGCACGCGCTGGACCCCCGGGACCC
GCGCAGGGCGCTCACCAGGCCCGTG
>1440
TGTGCGGCCTGGTCTACCTGTTCGCGCGCCTCCGCTACTTCCAGGGCTACGCGCGCTCC
GCGCAGCTCAGGTGAGGGCCGGGCGGGGAGCGGGGCGGGGCCGGGGAAAGATCGCG
GGCGGGCGGGGCTCCTGGGGAGCGG
>1441
ATGGGGCTGGGCCGGGTCCTGCTGTTTCTGGCCGTCGCCTTCCCTTTTGCACCCCCGGC
AGCCGCCGCTGGTGAGTGGGGTTCCTGGCGGTCCCCGGCGGAGCGGGAGCGGCGGGG
CGTTTCCGGGGGTCCGGGTGGGTT
>1442
ACCGAGGACTTGACAGAGAATGGGCAAGACCTCAGGAGGACCCTGACTCATATCAAG
GACCAGAAAGGAGGTGAGAGTCGGCAGGGGCAAGAGTAATGGGAGGCCTTCTCCAG
GAAAGTTGGAGACAGAGAGCAGGGACC
>1443
GCTATGCAGGCAGACTGCCTGCAGAAACTACAGCGATATCTGAAATCCGGGGTGGCCA
TCAGGAGAACAGGTACCGACCCTGGCCAGGGGCTCTACTGTTCCCGCAATTCTGCTAG
AGTTGCCTCGCCTCCCAGCTCTGT
>1444
GGAGAGGAGCAGAGGTTCACCTGCTACATGGAACACAGCGGGAATCACGGCACTCAC
CCTGTGCCCTCTGGTGAGCCTGGGGTGACCCTGGAGAGGGTCAGGCCAGGGTAGGAA
CAGCAGGGACGGCTGTGGCTCTCTGC
>1445
TGTTTTGTTATTATTATTATTCTCTGTGTCCCTTGTTGCAAGAAGAAAACATCAGCGGCA
GAGGGTCCAGGTGAGAAAAGGGGACAGTTTCTGGAGATGGGAAAGCTCCTTTCTAGG
CAGTAGGGTCTCCTCATTGCTCC
>1446
CAGCCGCGGTCCAGAGCGCGCGAGGTTCGGGGAGCTCCGCCAGGCTGCTGGTACCTG
CGTCCGCCCGGCGGTGAGTCCGCGCGGCCCCGGCGGGACGCCCCCGCCACCTGCGCG
CACCGTCAGACCCGGCGGCCTCGGCT
>1447
ACGGCCATCCTCTCCAGAATGAAGATCTTCTTGCCAGTGCTGCTGGCTGCCCTTCTGGG
TGTGGAGCGAGGTGAGGTGCCCTTGGGGACCCCAGACCTTTGTCCAGCTGTGCCCTGC
TCCACTCCCTCTCCACCCCTCTC
>1448
TGCCTGAAGCCGACCATCTGCTCCGACCAGGACAACTACTGCGTGACTGTGTCTGCTA
GTGCCGGCATTGGTGAGTGCCAGGCTCAGACCGTGCCTTCCTCCCCTGGCCATCTCCCT
AGCCCGGGCCGGGGCTCAGCAGA
>1449

GCTTTGCCTCTGAGGCCCCTGACCCGGGGCTTGGCCAGCGCTGCCAAAGGAGGCCAC
GGAGGAGCAGGAGGTGAGTGGGGAACGGGCGGATCCGGGGGCTCCCCTACCCTGCCC
ACCTGTTCACAGGCCCGCCGCCCCAG
>1450
CTATCTCCACTCGGGCCACCGCCCGCGCCCCGAGTTCCGTCCCTACCAACACCTCCGCA
TCCGCACCAAGGTACGCGGGACGGGCGCGCGGGCGGCACGGGGGTGCTGCGGGGGC
GGGGGGGGGTGCTGCGGGGGCGGGG
>1451
CTGAAGGGGCTGAACCATACACTCCTTTTTCTACAACCAGCTTGCATTTTTTCTGCCCA
CAATGAGCGGGGTAAGATTTTTATTTTTGGCAAGGGGTATAATTTGGGTTCACTGTGGC
TACTTGAACACTACACTGCAGC
>1452
CAGCGACTATCTTTCTCCTCTGGGGACCACTTGGCTGAGGACCTGTGCGTGCAGGCTG
CCAAGGCCAGCGGTGAGTGCATCCCTAGTGGATCGGGCCAGAGGGAAGGATGGGGCT
GTGTGGGGCCAAGATTGGAAGCTGG
>1453
GCTGGTTCCCCCCGAGCCACATCTTCTCCGTGGAGGATGCCAGCACCCAAGTCCTGCT
GTACAGGATTCGGTCAGCCCCCGCACAAGGCGGATAGGACAGTTAGTATCAGGGTCCT
CTGCATCTCTGCTGGGACCCACAC
>1454
CTTCGGGCTACGCAAGGATTTGGCCAGTGCTATCCTTGACCTGCCAGTCCTGGAGCAC
CTCTTTGCCCAGGTGGGGTTCTGCCTGGGGTTTGACCAGGGGGTTGGGGGTCCAATGG
GCAACATGAGGACTGGCATGCAAT
>1455
CCGTGTTGGACCTGGCCCGGATGGCGCGAGAGCAGGCCCAGCGGCCGGGAGAGCTGC
TGAAGACTGTCAGGTGAGAGCCACCAGGCTGTGGGGACGGGCTCTGTTTGGGAGTGA
GCAACGTGGGCTCCATCGGGGGTTTG
>1456
GGCCACGACGGGCTGGGGCTGTTCCGCGTGGCTGGTGACGGCGGCATCGCCTGGACC
CAGGGAGAACAGGGTGAGGACGGCTTCCCCCCTGGCGGGGCCAAATGGGGGGGGGT
TTGGGGAGGGGCCGGAGATTTGTAGGG
>1457
GCCCCGCGCGTTGGCCCGGCCGGAGAGCACCGCCTGGTCACTGTTACCAGGACAGAC
AACCAGATTTTAGGTGGTGCAGGATTCCCCTCCCCCTTCAGCCTTACCCCGAAGGGCG
GGACCGGCACCCTCGGGTTTCACTG
>1458
TCCTGGCTCCTATGTTCTCCGCCGCAGCCCCCAGGACTTTGACAGCTTCCTCCTCACTG
TCTGTGTCCAGGTCGGTCTACTGCTAGGGTGGGTAGTGGAGGGCTGCCTGGACAAGAG
GTTACCTCTGAATTGAGATTTAC
>1459
TGGGATGGGGGGCTGCACGTAGATGGGGTGGCAGTGACCCTCACTTCCTGCTGTATCC
CCAGACCCAAAGGTGAGGCCCCTTGGTCCCCTGGAATGAGTGGCTGATCTGGGACCCT
GGTTTCCTATGTCTGATAACAGCT
>1460
TCAGCCCCAATCCCAATACCAGCTGAGTCAGATGACATTTCACAAGATCCCTGCTGACA
GCCTGGAGTGGGTAAGAGGCCCTGGGAAATGAGGCGATACCTCAGTCTGGGGTCCAG

AGACTCAGATGCGTGGCCTCAGGC
>1461
GGATGGGGAGGCCCGAAAGACAGAAGTGCTGCTGAAGGTCATGGATGCCAAGCACAA
GAACTGCATGGAGGTGAGAGGCAATGTGGACCAGACTTTTGGAGTCGGGGGCTGGCT
TGAGCGGGGCTCGTGGATGCAGAGAC
>1462
CGAGCTTGATGAGCCAAGTGTCGTACCGGCATCTCGTGCTGCTCCACGGCGTGTGCAT
GGCTGGAGACAGGTGAGTGCTCCTAGGGGGTGTTAAAGGGGAGAGGGAGGAAAAGA
GGGCGAGGGGGGAAAGAGAGCTAGGG
>1463
TGGCCACCTGGTGCCAGCCAGCTGGAAGCTGCAGGTGGTCAAACAGCTGGCCTACGC
CCTCAACTATCTGGTGAGTGCTCCTCCCCCCAGGGGATTTGGTTTGGGGGGAGGGGGG
GGGTTTAAAAAAACCCAACCCCCTA
>1464
GGCTGATGGGAGCCCGCCCTTCATCAAGCTGAGTGACCCTGGGGTCAGCCCCGCTGTG
TTAAGCCTGGAGGTAAGTTCCTGGAGGTGGAGGAGGGAGGGGCTGAACAGGGCAAG
GAAGTGGATCCCTGATCCCACTTTCA
>1465
CTTCGGCGCCACGGTCTGGGAAGTGTTTAGTGGCGTCACCATGCCCATCAGTGCCCTG
GATCCTGCTAAGGTCAGAGCCCCTCACCCGGCATCGGTCTCCGAACCCCCACTTTGAC
AGCCGGGCAGACTGACATCCAGTC
>1466
GCCTATGAGCCGGTCCAGAGGCCCTCCTTCCGAGCCGTCATTCGTGACCTCAATAGCCT
CATCTCTTCAGGTGCCCGCTGGGACGGGTTGGGTGGGGAGGGCTGTGATGTCATATTG
GGCCCAGTGGAAGGAGCGTGGTT
>1467
GCTCTATGCCTGCCAAGACCCCACGATCTTCGAGGAGAGACACCTCAAGTACATCTCA
CAGCTGGGCAAGGTAAGGTGGGCAGGGCCAGGGTGGGTTGGAGAGGCAGGGCAGCA
TCCAGGTACCTGGACATCAGTGCCGC
>1468
CTTCCTGCAGCGGCACCGCGCGCGCCTCGATGCCAGCCGCCTCCTTCTCTATTCCTCGC
AGATCTGCAAGGTGAGAGGGGGCGCCCCGGGACTTGTGGGGATTCAGCTGGCACGGC
CTGGGCAGGGGTCTGCTTGGAGGT
>1469
CTGTGGGCAGACAGCCATGCACCTGCCCCCAACTCTGGCAGCACCCGGCCAGCCTCTG
CGCGTTCTCAAGGTAAGAGCAGGCCAGCCCTCCTCCCTCCTGCCCGGGGCTGGAGAG
GCTGGTGTGGACAGGACCCTGTGCC
>1470
CCGCTCCTTCAGCTCCACCGCCCGGAACCGCTTTCAGAACCGAGTGCGCGAGAAACA
GAAGCTCTTCCAGGTGGGGGGTCGGGGGGGGTGGGGATCCGACGCGCCAGCCGGGA
GCGCGCCGAGCCGGGGCAGGCGGGGCG
>1471
CCGTTGTACCTGAAGGGCGGCATCGTTGACAACATCCTGTACCGAGTGACAATGACGC
TGTGTCTGGGCGGTGAGCGCAGGGCCCGTCTGGGCTGCGGGGGAGGCGGGGCTGGAC
CCAGAGTAAGAGGTGGCTGGTTTCT
>1472

TCCTGCCTTGGCCATGAGGGAAATCGTGCTCACGCAGACCGGGCAGTGCGGGAACCA
GATCGGGGCCAAGGTTGGCAGCCGGGGCTCTGAGGGCCCAGCCCGGCCTGCCGGGTG
GCCGGGGAAGATGTTGGCAGCGGCGG
>1473
GCTGGCACCTACCACGGGGACAGCCACCTGCAGCTGGAGCGCATCAACGTGCACCAC
CACGAGGCCAGCGGTGCGACCCCCGTCCTTCCCCCACCGCCCTCCTGGGAACGCGGC
CCTCCCCTCGCTCATGCCCTCCCGCC
>1474
CCGGGCACCATGGACTCTGTGCGCTCGGGGCCCTTCGCGGAGGTCTTCAGGCCAGAC
AACTTCATTTCCCGTGAGCTGCGGGCGAGGACTGGGGTGCGGCTCCTTAGCCAGGGCA
GCTCGAAATCCAGGAACGCTCCAAG
>1475
CGCTGCAAGCCCTTCTACTGCGACAGGCCATGGCAGCGGGCCACTGCCCGGGAATCCC
ACGCCTGCCTCGGTGAGGCCTTGGAGGGTGGCCTGGGGACCTTGGACACAACCAGCC
TGCCCCTGACCCATCCCTCCCTGCA
>1476
TGCCACTACTGCCGGGAGGGCTTCTATCGAGACCCTGGCCGTGCCCTGAGTGACCGTC
GGGCTTGCAGGGGTGAGCCACCACCGGCCACCTGCAGGCCCTCACCCTCTGACTTCCC
AGATCCCCAGACAGGCTTCTGACC
>1477
ACTGGCCTCACCTGCAACCGCTGCGCGCCTGGCTTCCAGCAAAGCCGCTCCCCAGTGG
CGCCCTGTGTTAGTGAGTGACCCTGCCCCGCCTCAGCCACCAAGCCAAGGCCACCCCA
GCTCCCTGCTGTTGTCCCGTCTAT
>1478
GACTCGCACTGCAAACCTGCCCGTGGCAGCTACCGCATCAGCCTAAAGAAGTTCTGCA
AGAAGGACTATGGTAGGTGCCCTCAGGCCTCCCGCGGACCTTCCCACCTTCCTCCTCT
CCCTACCTTCCCTCCTCCGCCAGC
>1479
GCGGCGGGTGGCAGCGATCCGCGGGCTGGCGACGTAGAGGAGGACGCCTCACAGCTC
ATCTTTCCTAAAGGTTGGGCTCGGGGCTGCCAAACTCCCCCGCGCCACTTCGCGTGGT
CGCCGCAGGCCTGGCGTTATGCGCG
>1480
TGAAAACATTAAACTACACAGCCCGTTTCAGTCGTTTCAAAAACAGAGAGACCATTGC
CAGTGTTCGTAGGTGAGTGCTAAAAGAAAGTTTTTATTAATCCAAACCATTGGACAACT
GCATGTAGAATGCTGTCCCCTCC
>1481
AGTTTGAGTTGGCCTGTTTGGCCAACCTTTGCCCAGAGACTGCTGAGGAGTCCAAGGC
TCTAATCCCAAGGTTAGTACCAGTTGTGATAAGTTCTCTGTATACTGGATAAATTCCTAC
AAATAGAATTGGTGTGTCTAAA
>1482
GCTCCGGGGACACTTTGCGTTCGGGCTGGGAGCGTGCTTTCCACGACGGTGACACGCT
TCCCTGGATTGGGTAAGCTCCTGACTGAACTTGATGAGTCCTCTCTGAGTCACGGGCTC
TCGGCTCCGTGTATTTTCAGCTC
>1483
AGGAGCCGCAGTCAGATCCTAGCGTCGAGCCCCCTCTGAGTCAGGAAACATTTTCAGA
CCTATGGAAACTGTGAGTGGATCCATTGGAAGGGCAGGCCACCACCCCGACCCCAACC

CCAGCCCCCTAGCAGAGACCTGTG
>1484
CTACCAGGGCAGCTACGGTTTCCGTCTGGGCTTCTTGCATTCTGGGACAGCCAAGTCT
GTGACTTGCACGGTCAGTTGCCCTGAGGGGCTGGCTTCCATGAGACTTCAATGCCTGG
CCGTATCCCCCTGCATTTCTTTTG
>1485
AAGCAGTCACAGCACATGACGGAGGTTGTGAGGCGCTGCCCCCACCATGAGCGCTGC
TCAGATAGCGATGGTGAGCAGCTGGGGCTGGAGAGACGACAGGGCTGGTTGCCCAGG
GTCCCCAGGCCTCTGATTCCTCACTG
>1486
GCGTGTGGAGTATTTGGATGACAGAAACACTTTTCGACATAGTGTGGTGGTGCCCTATG
AGCCGCCTGAGGTCTGGTTTGCAACTGGGGTCTCTGGGAGGAGGGGTTAAGGGTGGT
TGTCAGTGGCCCTCCGGGTGAGCA
>1487
GTAACAGTTCCTGCATGGGCGGCATGAACCGGAGGCCCATCCTCACCATCATCACACT
GGAAGACTCCAGGTCAGGAGCCACTTGCCACCCTGCACACTGGCCTGCTGTGCCCCA
GCCTCTGCTTGCCGCTGACCCCTGG
>1488
ACAGAGGAAGAGAATCTCCGCAAGAAAGGGGAGCCTCACCACGAGCTGCCCCCAGG
GAGCACTAAGCGAGGTAAGCAAGCAGGACAAGAAGCGGTGGAGGAGACCAAGGGTG
CAGTTATGCCTCAGATTCACTTTTATCA
>1489
GCCCAACAACACCAGCTCCTCTCCCCAGCCAAAGAAGAAACCACTGGATGGAGAATA
TTTCACCCTTCAGGTACTAAGTCTTGGGACCTCTTATCAAGTGGAAAGTTTCCAGTCTA
ACACTCAAAATGCCGTTTTCTTCT
>1490
TGAATGAGGCCTTGGAACTCAAGGATGCCCAGGCTGGGAAGGAGCCAGGGGGGAGC
AGGGCTCACTCCAGGTGAGTGACCTCAGCCCCTTCCTGGCCCTACTCCCCTGCCTTCCT
AGGTTGGAAAGCCATAGGATTCCAT
>1491
TGCGCCTGCTGCTGCTGCTGCGGCCCCCGCGCGGCGCCCTGCGGGCCCCCAGAGGCG
GCCGCCGGGCTGGGTGAGTGGGCGCGGAGCGGGGCGCGGGGCCCGGCGGAGCCCGG
GGCGCGGCGCAGTGGGTGCCGAGCTTG
>1492
CCAGTATCCTGACGTGAGTACGCGCGAGCGCCTGGCCGGCCGCATCCGCCTTCGCGAG
GAGCGCGTGGAGGTGAGTGCCCCGCCCAGCCTTTCCCCGGAGCGCGCGGGCCGCGGC
TACACTGGACTGGGGTCCTGGCGGG
>1493
CCCGAGGAGGTGATCGCCACCATCTTTTCCTCCGCCTGGTGGGTCCCTCCCTGCTGCG
GGACAGCAGCTGGTGAGTACCCCTCCTGGTTCTTCTGGAAGTAAAAAGGGTGTCTTTT
CGGTTGAAAGTACTTTTCAGCCTA
>1494
ATTTAAGAGAGAATGGGCCAGTGTCATGCGCTGCATAGCAGGTTTTGGTGGCATTAACC
ACGCCAGTGCTGTATCCTTAATTTTCTGTGCTACGTCCAGAGTATCTTCTTAGGTTATATA
TTGAAGCATTTTGTTTTGTT
>1495

GGCTGGGGATCACCATAGCTTTTCTAGCTACGCTGATCACGCAGTTTCTCGTGTATAATG
GTGTCTATCAGTATACAGTAAGTGTGTGTTTTCAAATATTGGCTTTGGAAAGCTTAGATA
TTTTCATATTTTATTTGTTT
>1496
TTCTTGGCTCCCTTGTATATTTTTCTCAGGAGGCGTCACGGTGGGGAACATAGGACGAC
AGTTAGCTATGGTAAGTGAAATGATCATATTATCTTCTAAAACTTGCTTCTCTTTACCTTG
ATAGAATGACTTTACATGAT
>1497
GGCCGGAAGATGAAACTGAGCTTCACCCTACTCTGAATGCAGATGACCAAGAATGCAA
ACGAAATCTCAGGTGAGCAGTATTTTAAGAAGGTCCTGTTGTCAGTTTTTCAGATTTTC
TTATTCCCAAGGCCTTTAAACTG
>1498
ACAGAGCACCTGTGGGAGGACTAGGAAAATTAAAGATGATTATAGCCAAAAATGGCCC
AGACACAGAAAGGTAGGTAATTATTAACTTGTGACTGTATACCTACCGAAAACCTTGCA
TTCCTCGTCACATACATATGAAC
>1499
AGGTCAGGAGGAGACTGTGAGAATTGTTGCTCTGGATCGAAGTTTTCACATTGGCTGT
TACAAGTGCGAGGTCAGGGGCCCCCAGCACGTGCAAGGGGCTGGCAGTGTCTAGGGT
GCTGGGTAGAGCATGAGGGGGAACA
>1500
CTGCCCTTCTTCCCCGAGAGCCCCAGGTACCTGCTGATTCAGAAGAAAGACGAAGCGG
CCGCCAAGAAAGGTAACGAGGGCTCTGGCGGGGGCGGCCTCCAGGTTCTGCTCCCCA
GCCCTGGTCCGACGGTGCCGCTTCT
>1501
CTCGCTGCGCTGGCAGCTGCTGTCCATCATCGTCCTCATGGGCGGCCAGCAGCTGTCG
GGCGTCAACGCTGTATGGGCCGGGCTGCGGCTCGGGAGCTGGGGAGGGCTCGGGGGG
CGCGGGGCGCAGGTCCTGGGGCGCC
>1502
GCCGGAGGAGCACGTGCAGTACGTGACGGCCGGCACCGGGGCCGTGAACGTGGTCAT
GACCTTCTGCGCCGTGAGTGTCCTGGGAGCTGCCCACAGCCTCCACCCAAGGCCCCTC
GGGCCCCACGCCCCAGCCTCGGCGC
>1503
GCTGCTGCTGCTGGGCTTCTCCATCTGCCTCATAGCCTGCTGCGTGCTCACTGCAGCTC
TGGCACTGCAGGTGAGGACAGGCCAGGGTCCCAGGAGCCCGGAGCTGCACCAGGAA
GACCAGTTGCCCCTCCCATCCTGCG
>1504
GTGTCCTGGATGCCATACATCAGCATCGTCTGTGTCATCTCCTACGTCATAGGACATGCC
CTCGGGCCCAGTACGTACCCAAAGCTGGCCTCAGGGTGTCACAACCCCCCCTGGTCCC
AGAAGCTCCGGGCTGGAGCTCC
>1505
CTTCATGGTGGGGGGCAGTGTGCACTGGCTCTCCAACTTCACCGTGGGCTTGATCTTC
CCGTTCATCCAGGTGAGTGGGGCCCGGGGCCCACGCTCACCACCAGATCCCCATGCAG
TACAGTGCGAGCCCCTGGGCAAAC
>1506
GGCTGTTCCTCAGGTATTGGCCTGCACTTGGCAATACATCTGGCTTTGGACCCATCCCA
GAGCTTCAAAGGTATAGATAGGTAGGGACAGGGAGGGAGAGAAGGGAAAAGCCCTTG

GAGGCCAGAAGAGAAGTCAGATCT
>1507
GATGTAAGGAACTCAAGCTCCCTGGCCGCTGCCCGGGAACGCGTGACCGAGGGCCGT
GTGGATGTGCTGGGTGAGCCTCCTGGAAGCATATGGGCTCCTAGGAGCCTTCTCCGCC
CTGCGTTGAAACCAACATGTCCCCA
>1508
TTCCTGCCAGACATGAAGCGGCCGGGTTCGGGACGCGTGTTGGTGACTGGGAGCATG
GGAGGATTGATGGGTGAGTGGCAGGGACCGGGCCCCGAGCTCCAGATTCTTTGTGTGC
AGAGCTGAGCCTTGAAGGCAGGCTC
>1509
GCGCCAGCAAGTTCGCGCTCGAAGGCTTATGCGAGAGTCTGGCGGTTCTGCTGCTGCC
CTTTGGGGTCCAGTGAGTCAACACCCCCGTCTCCTCAACCCTCTTAACTCTGACCTAG
AGATGCCGAGCACCCTGTCCTGCG
>1510
TTATGACCTCACCCGGGAGCGCAAAGCACGCGACATGGTGGGGCAGGTGGCCATCAC
AAGGATTGAGCAGGTGAGGGCAGGTGGTGCCATCAGGGTGTCCCCCCAGCGGGGTTC
AGGGCTCTGGTTCCTTCACAGAACAG
>1511
AGAACCAAGGCTACTATGACTACGTGAAGCCAAGACTTCGGACCACCATCAGCCGCGC
CAAGCCCGTCTGGTAAGGCTTCAGTCCCTGCCAGGAAGGCTGTGGGACCCTCCCCTCA
GGCCAGTAGGCAGTTAGCCAGGCA
>1512
ATTGTGGATTTGGTCAAGGAGCTTGACAGAGACACAGTTTTTGCTCTGGTGAATTACAT
CTTCTTTAAAGGTAAGGTTGCTCAACCAGCCTGAGCTGTTTCCCATAGAAACAAGCAA
AAATATTTCTCAAACCATCAGTT
>1513
AACTACAGCACCTGGTAAATGAACTCACCCACGATATCATCACCAAGTTCCTGGAAAAT
GAAGACAGAAGGTGATTCCCCAACCTGAGGGTGACCAAGAAGCTGCCCACACCTCTT
AGCCATGTTGGGACTGAGGCCCAT
>1514
CACTAAGGTCTTCAGCAATGGGGCTGACCTCTCCGGGGTCACAGAGGAGGCACCCCT
GAAGCTCTCCAAGGTGAGATCACCCTGACGACCTTGTTGCACCATGGTATCTGTAGGG
AAGAATGTGTGGGGGCTGCAGCACT
>1515
GGCCGGCTTCGCGGGCGACGATGCCCCCCGGGCCGTCTTCCCCTCCATCGTGGGGCGC
CCCAGGCACCAGGTAGGGGAGCTGGCTGGGTGGGGCAGCCCCGGGAGCGGGCGGGA
GGCAAGGGCGCTTTCTCTGCACAGGA
>1516
CGAGGAGCACCCCGTGCTGCTGACCGAGGCCCCCCTGAACCCCAAGGCCAACCGCGA
GAAGATGACCCAGGTGAGTGGCCCGCTACCTCTTCTGGTGGCCGCCTCCCTCCTTCCT
GGCCTCCCGGAGCTGCGCCCTTTCT
>1517
GGCCAGGTCATCACCATTGGCAATGAGCGGTTCCGCTGCCCTGAGGCACTCTTCCAGC
CTTCCTTCCTGGGTGAGTGGAGACTGTCTCCCGGCTCTGCCTGACATGAGGGTTACCC
CTCGGGGCTGTGCTGTGGAAGCTA
>1518

GTACCCTGGCATTGCCGACAGGATGCAGAAGGAGATCACTGCCCTGGCACCCAGCACA
ATGAAGATCAAGGTGGGTGTCTTTCCTGCCTGAGCTGACCTGGGCAGGTCAGCTGTGG
GGTCCTGTGGTGTGTGGGGAGCTG
>1519
AGCTGGTTTTGCTGGGGACGACGCTCCCCGAGCCGTGTTTCCTTCCATCGTCGGGCGC
CCCAGACACCAGGTGAGTGGATGGCGCCGCGGGGCTCCTGGGTTCTGCGTTGCGGGG
TGGGTTCGGTGTCGCCGGCGAGGCT
>1520
GGAGGAGCACCCAGTGCTGCTGACCGAGGCCCCCCTGAACCCCAAGGCCAACAGAG
AGAAGATGACTCAGGTGAGGCTCGGCCGACGCCCGTGCTCCTCCCGTCCTTTCCCCAG
TCATTTTCTCGCCCGGCTTGATTTCT
>1521
GGCCAGGTCATCACCATTGGCAATGAGCGGTTCCGGTGTCCGGAGGCGCTGTTCCAGC
CTTCCTTCCTGGGTAGGTGTTGTGAGCTAAAGGTTTCTACTCTTTCATCCTCGGTGACA
CAGCATCACTAAGGGAGGGCTCT
>1522
GTACCCGGGCATTGCCGACAGGATGCAGAAGGAGATCACCGCCCTGGCGCCCAGCAC
CATGAAGATCAAGGTGAGTCGAGGGGTTGGTGGCCCTCTGCCTGGCTCGGGAGAGCT
GACTGGGGGGCGCTCTGCGAGCTGAA
>1523
TTGGCATGGACAAGCCGCTCACCCTTCCAGACTTCCTGGCCAAGTTTGACTACTACATG
CCTGCTATCGCGTGAGTTGCCCCCAACCCACAGGTCCTAGGGCAGCATTGATCCCTATG
ACTAGGACCAGGCCTGTCCCTC
>1524
TGGAGGTGCGGTACAGTCCGCACCTGCTGGCCAACTCCAAAGTGGAGCCAATCCCCTG
GAACCAGGCTGAGTGAGTGATGGGCCTGGAAGGGGCCATGCTGAGGGTGTGGCTGGG
AGGCTCAGCTCTGAGACTGGAAGGG
>1525
CTGCAGGAGGGGGAGCGAGACTTCGGGGTCAAGGCCCGGTCCATCCTGTGCTGCATG
CGCCACCAGCCCAGTGAGTAGGATCACCGCCCTGCCCAGGGCCGCCCGTCTCACCCTG
GCCCTGACCTCCTGGCCTAGCAGTG
>1526
CATTGACCTGGCTGGAGATGAGACCATCCCAGGAAGCAGCCTCTTGCCTGGACATGTC
CAGGCCTACCAGGTGGGTCCTGTGAGAAGGAATGGAGAGGCTGGCCCTGGGTGAGCT
TGTCTCCCACCCATAGTTGGGAGAA
>1527
GGCTGTGAAGAGCGGCATTCACCGTACTGTCCACGCCGGGGAGGTGGGCTCGGCCGA
AGTAGTAAAAGAGGTGAGGGCCTGGGCTGGCCATGGGGTCCCTCCTCACTGCCTCCTC
CCATACTTGGCTCTATTCTGCTTCT
>1528
ACACGGCTACCACACCCTGGAAGACCAGGCCCTTTATAACAGGCTGCGGCAGGAAAA
CATGCACTTCGAGGTAAGCGGGCCAGGGAGTGGGGAGGAACCATCCCCGGCTGTCCC
AACTTCCTGTATAGAGAGGCAGAAAG
>1529
CACCCTGGACACTGATTACCAGATGACCAAACGGGACATGGGCTTTACTGAAGAGGAG
TTTAAAAGGCTGGTGAGTGGGTGTGAGCCATACTGGCCTTGACTCGGGTTTGGGAGTA

TGGTATCTACAGGTCCAGTCCGGG
>1530
GAAGATGAAAAGAGGGAGCTTCTCGACCTGCTCTATAAAGCCTATGGGATGCCACCTT
CAGCCTCTGCAGGTAGGTTCCTGTCTGGGCTTCTGGGCAGTTGCCTGTCCTGGCCCCA
GTGTGGCTTTCTGTGGGACTTCTA
>1531
AATTTGTGACTCACAATCTGTCTTTTGATGAAATCAACAAAGCCTTTGAACTGATGCAT
TCTGGAAAGAGGTAGGCTTTCTCTTTATATATCATAGAATGTAATAATGATGTTGAGTTT
GAGGGGATGGAGAGTCGAAAA
>1532
TCAATTTTTTTAATTTTCCTACTAAATTTTACTGAATCCAGAACACTGCATAGAAATGAA
TATGGAATAGGTGAGATATTTTGTGTTTTTCTTGTCTTTTCTCTATATCAAAATTTTTTAA
ATTATAAAATTTGCATTAA
>1533
GAAAGATGCATTGACTGCAATTGAGAAACCCACTGGAGATGAACAGTCTTCAGGGTGT
TTAGAAAACCAGGTGAGTGAATAATTTTAAAAAAGCATTGTGATATTTGACAAAAATTT
AGCATGCTGAAGAGAAGATACAA
>1534
TTTTCCAAGTTCCAGAACCTGTCACAAGCTGTGAAGCATATGAAGAAGACAGGGAGA
CATTCATGAACAAGTAAGGATCCAGTTTAAAGGTAGATGCAAACCTCAGAAACACAGC
AATGGCAAGCCTAATTTAGTATTTT
>1535
TGCTCGCTATGACAAAATAATTCCATCTTGCTGCAAAGCTGAAAATGCAGTTGAATGCT
TCCAAACAAAGGTATCATATTTGCGTGGATATCTGAACCAGTACTGTAGTCTATGACTCA
TTAAAACAAAACAAAGTTAAA
>1536
AAAGCAGCTTGTTAAATCAACATGCATGTGCAGTAATGAAAAATTTTGGGACCCGAAC
TTTCCAAGCCATGTAAGTTCAAGTTCTATCTAGGGAAGAGGGTGAGAGCTACAGAACT
ACCATTTTGCAATTTGGGTTCGTT
>1537
AGTCCTGGATGTGGCCCATGTACATGAGCACTGTTGCAGAGGAGATGTGCTGGATTGT
CTGCAGGATGGGGTGAGGAGTCTTGCTTCTTAAAATAGAAGATTTTCACTCCCTTTTCT
TTCTTTTTGTCTCATTCTAAAAG
>1538
TAAACAGGTTTTTAGGAGATAGAGATTTTAACCAATTTTCTTCAGGGGAAAAAAATATC
TTCTTGGCAAGGTAACACACTCTGTAAATGCATGTTCATGCAAGTAAAAATGATTATGT
GGCTGACAGATTTGCGTTGTTG
>1539
TAAAGGATACCAGGAGTTATTGGAGAAGTGTTTCCAGACTGAAAACCCTCTTGAATGC
CAAGATAAAGGAGTAAGTTGCTCTAGAATTTTAGGGGAGTATGAAAAACTGGATTGATA
TCATCTGTTAAAAATGCTGTTTG
>1540
AGAGCCAAGCATTGGCAAAGCGAAGCTGCGGCCTCTTCCAGAAACTAGGAGAATATTA
CTTACAAAATGCGTATGTTTTTGTAAACAGTATTTTTAGTGAATTAAAATTATTAAAGAG
AATGTAGCCTTCCCCAATTCTC
>1541

AATGGCAGCCACAGCAGCCACTTGTTGCCAACTCAGTGAGGACAAACTATTGGCCTGT
GGCGAGGGAGCGGTGAGTGTCTGCTTGGTTTGGTCCCATCTCATTTCTGCCCTGTTTGA
CTTGAAATAGCCTCATAATTCCC
>1542
ATGACAAGTTCATTTTCCATAAGGATCTGTGCCAAGCTCAGGGTGTAGCGCTGCAAAC
GATGAAGCAAGAGTAAGAAACTGTTACTTGCTAGCATGGAAAAGAATGACAACCCCA
AAGAGTAACTGAGACTTCTACCTCG
>1543
TGCAGATTTCTCAGGCCTGTTGGAGAAATGCTGCCAAGGCCAGGAACAGGAAGTCTG
CTTTGCTGAAGAGGTACATGCAGCTCATTTCATACTCAAAATACTTGCTATGGAATTTTC
TGTAGTGGATAATGAAAGGAAGA
>1544
GCTGATACACGCAGACCAGATCTGGTCAGGTCCTCGGAAGCTGAGTCCAGAGCGATGC
TGCTGAAGACAGGTACCTACACCGGCTGGGGTGGCTTGGAAGGGTAGGGGTGAGTGC
TACAAGGAGTGCTTTGGCCCTTCTT
>1545
TGGGCTGGCTGGCCTGGGAACGCTTCCGCTGCAACATTAACTGTGATGAGGACCCAAA
GAACTGCATAAGGTGAGTAAGGTCCACACTCACCTGATGAGCCTTGCCCAGGGCCCTG
CAGATTCGGGGGCAGGAAGTGGAG
>1546
CGATGCCAGTGGCCGCCTGATGCCAGATCCCAAGCGCTTCCCTCATGGCATTCCTTTCC
TGGCTGACTACGTGAGCCGCACCCAGCCCTGCCCTTGCGCTCAGGGCTAGACCACTCC
TTTCACCCTACCTCGCTTAGGGC
>1547
TTCGCCGAGTGGAAGGTAGACATGCTCAAGCTGGATGGCTGCTTCTCCACCCCCGAGG
AGCGGGCCCAGGGTGAGTTACGCAGCTGGCTGGGGCACGTAGGCAAACGGGGAGGG
TGGCCATGGAGACCCACCCAGCGCCC
>1548
GAATGCCACAGGCCGCCCCATCGCCTTCTCCTGCAGCTGGCCAGCCTATGAAGGCGGC
CTCCCCCCAAGGGTAAGCCATTCTCCCCCCTGCCTGATGGCGCTTCCCACACCCAGGC
CTCCTTACCTCCTGAGTGCCAGGC
>1549
TTGGTTCGTGGAGCACCAGGACATACTGCAGCCAGTGGCCGGCCCTGGGCACTGGAAT
GACCCTGACATGGTACCAGGATGGAGGGGGATGTCCTAGGCCCACCATCTGTGACGAC
CCTGCTCCCCACTGCCCTGTGCTT
>1550
TCTGCAGAATCCACTCATGATCAAAATCAACCAGGATCCCTTAGGCATCCAGGGACGC
AGGATTCACAAGGTACTAGGGTGTGGAGGGAAGGAAGGGGAGGGCTGAGGAACTGG
GTTCTCCTGAGAGAAAGGCTGCCAGC
>1551
GACCGATATGCCTTATCGCTACCACTCCTCCCTTGGCCAGCTGAACTTCACCGGGTCTG
TGATATATGAGGTGAGCACCCGGCTGCCTGCCAGCCCGCTGGGCTTCAGATTTCTGTGG
GAGGGTATTATGGGGGGTGGTT
>1552
GGTCAGCTCAGGCTCGGCCAGGGGCAAGAAGCTGTCGGAAATCATGGAGAAGGGGCA
GCTGGTTCCACTGGTGAGTGGGCCCTGGTGGGGTGAGAGGCAGGGGATGACAGTAGC

CCTGAGTGGGTGTCCCACCAGCAGCA
>1553
TACTTCCAAAGGCTTCCTGATTGATGGCTACCCGCGGGAGGTGCAGCAAGGAGAAGA
GTTTGAGCGACGGGTAAGGCACTGACCCAAGTGGGGATCCTGGTGGGCTGGGGCAGG
ATAAGACTCCCATCCCCATGGGCGGG
>1554
GCTGGAGACCTATTACAAGGCCACAGAACCCGTCATCGCCTTCTATGAGAAACGTGGC
ATTGTGCGCAAGGTGGGCCCCGCGGGACGGGGCAGCTCCGGGAAGAACGGGTTCCTA
TGTGGCTCCGCCTCAGCTCACTGTG
>1555
TTGTGCTGCTATCTACGGCAATGAGCCTGAGATTGGGGAGGCCCTGAAGGAGGACGTG
GGACCAGGCAAGGTAAGGACTGGGGTTGTAAATAGAGGTGGATAAGAGAACTTAGAA
GCTGAAGCTAGGCTGGGGCCCAGCT
>1556
GGAAGACTCTGGCTGACCTCCAGCTGGAGTATCTGGACCTGTACCTGATGCACTGGCC
TTATGCCTTTGAGTGAGCCTTGCCAGAGCCTCATCTGGGGAATCAGGGGGTTGAGCAG
GATGGTGTTAGTAACTTATTGTAA
>1557
GTCCAACTTCAACAGTCGGCAGATTGATGACATACTCAGTGTGGCCTCCGTGCGTCCA
GCTGTCTTGCAGGTAAGACAGCAAGCAGATGAGTGGTTTAGGGGTTGTCTGCTCAAGA
GCATGAGGGAGCAGACGATGGATC
>1558
TGCTGGAGGAACCAGTAGTCCTGGCATTGGCTGAAAAGTATGGCCGATCTCCAGCTCA
GATCTTGCTCAGGTATGGGGCAGTCTTAGGGAGAGGGCCCTGGGTTGGGAGGCAAGG
GTTAAGGGATTTCTTATTTCAGTGT
>1559
GCAGGTCCAGCGGAAAGTGATCTGCATCCCCAAAAGTATCACTCCTTCTCGAATCCTTC
AGAACATCAAGGTACTTGGTAATGGGTTCTATCTTACTTTAGCTCTTTGGGACATTTTCT
TGGCCCTGACTCTACCTGGCT
>1560
TAGCCCAGAAGAGATGAAGCAGCTAAATGCCCTGAACAAAAATTGGAGATATATTGTG
CCTATGCTTACGGTGAGGATGTATCAGCCTCCTAGACTTGGGGAATGTGAGATTTGGGG
TGGGATTCTGGCCCAGGTGTGAC
>1561
AACTGCCGGCATCTGAGGTTTCCTCCAAGGCCCTCTGAAGTGCAGCCCATAATGAAGG
TCTTGGCGGCAGGTAAATACACCCGCCCCGCGCCGGCTTCGCGTCCCCGCTGCGGGGC
GCGGCGGCAACTTGGGGCGCTTGG
>1562
CCTCATGAACCAGATCAGGAGCCAACTGGCACAGCTCAATGGCAGTGCCAATGCCCTC
TTTATTCTCTATGTAAGTTACCCCTGGGATACTGACAGGAGATGGCAGGGAGGGGGCTT
GTAAATATCATTAGGGGCTGTCC
>1563
GACATGCAGGGGCCCTGGGTGCTGCTCCTGCTGGGCCTGAGGCTACAGCTCTCCCTGG
GCATCATCCCAGGTAATGAGGCTCCCCAGCCTGCCCTACACACACACACACACACACA
CCACCCCCCAGCCCAGGCTGACCT
>1564

CTGGGTGCCGCCAAGAAGCTGCAGCCTGCACAGACAGCCGCCAAGAACCTCATCATG
TTCCTGGGTGACGGTGAGTGAGCCAGCCTTCCAGCCCCGCAGCCCTCACAGCCCCGG
CGCCCGGACCCTCAGTGGTTCCAGGA
>1565
GAAGAAGGACAAACTGGGGCCTGAGACCTTCCTGGCCATGGACCGCTTCCCGTACGT
GGCTCTGTCCAAGGTAAGTGCTGGGCTACCTTAGAGTCCTCCAAGCAGAGAAGGGGA
ATCCTGGCTATGGAGTGTGGTAGGAG
>1566
CGCTTTAACCAGTGCAACACGACACGCGGCAACGAGGTCATCTCCGTGGTGAATCGGG
CCAAGAAAGCAGGTGGAGCTGGGGCCCGGCTGTGGGGTCAGGGCCAGTGACAGACC
TCTATCGCATATCCTGACCTCTATCA
>1567
GCCTGCCTCGGCCCGCCAGGAGGGGTGCCAGGACATCGCCACGCAGCTCATCTCCAA
CATGGACATTGATGTGCGACCCCCGGGCCAAGGGTGGGGCTGGGCAGAGAGTAGCAG
GGAGGGGGCACCAGCTCAGACCAGGC
>1568
TGACTACAGCCAAGGTGGGACCAGGCTGGACGGGAAGAATCTGGTGCAGGAATGGCT
GGCGAAGCACCAGGTGATGGGGGCTGGTGGGTGTGCTGGGCACAGCAGGGGGAGGG
CAGAGGTGTGGGGCTCGGGGCTGTGGG
>1569
GCCCGGTACGTGTGGAACCGCACTGAGCTCCTGCAGGCTTCCCTGGACCCGTCTGTGA
CCCATCTCATGGGTAATGACCCCCTTCCTGCCCTGGCATCCTCAGATGGCCTCAGATGG
CACTTCTGAGCCTGTGTGCACAT
>1570
ATGGAGATGACAGAGGCTGCCCTGCTCCTGCTGAGCAGGAACCCCCGCGGCTTCTTCC
TCTTCGTGGAGGGTGCGTGGTGGCCCTGGGAGTGGGGGGTTGGGGGTTGGAGCAGGG
CAGGCTCAGCATCTCCCCCCTCTGG
>1571
CTCGTCACTGCCGACCACTCCCACGTCTTCTCCTTCGGAGGCTACCCCCTGCGAGGGA
GCTCCATCTTCGGTAGGCCTGGGGATGAGTGGCAGGTGCTGCTGCAGCAATTAAGTGG
GTGAAATCTGAGCCTCAGTCTCCT
>1572
CTATACGGAAACGGTCCAGGCTATGTGCTCAAGGACGGCGCCCGGCCGGATGTTACGG
AGAGCGAGAGCGGTGAGTGCCGTGGGGTGGCCTGAGGGGGACCAGGGTGCCAAGGA
TGGGGGGCTGGCGGGAAGGGGTCACC
>1573
GACATGCAGGGGCCCTGGGTGCTGCTGCTGCTGGGCCTGAGGCTACAGCTCTCCCTGG
GCGTCATCCCAGGTAATGAGGCTCCCCAAGCTGTTCCACACACAGGGCACCCCCTCAG
CCAGGCTGACCTGATCTCTACTCT
>1574
CTGGATGCTGCCAAGAAGCTGCAGCCCATCCAGAAGGTCGCCAAGAACCTCATCCTCT
TCCTGGGCGATGGTGAGTGAGCAAGGCCTGTCCAGCCCCGTAGTCCTCACAGCCCCGG
CACCCGGGACCTTCAGTGGTTCCA
>1575
GAAGAATGGCAAACTGGGGCCTGAGACGCCCCTGGCCATGGACCGCTTCCCATACCTG
GCTCTGTCCAAGGTAAGGGCTGGGCCACCTCAGAGTCCTCCAAGCAGAGGAGAGGGA

TCAAGGATATGGAGTGTGGCAGGAG
>1576
CGCTTTAACCAGTGCAACACGACACGCGGCAATGAGGTCATCTCCGTGATGAACCGGG
CCAAGCAAGCAGGTGAGCTGGGGCCCGCTGTGGGGTCAGGACCAGGCCCAAGATCTC
GGTCACCGATCCTGACCTCTGTCAC
>1577
GCCTGCCTCAGCCCGCCAGGAGGGGTGCCAGGACATCGCCACTCAGCTCATCTCCAAC
ATGGACATTGACGTGCGACCCCCGGGCCAAGGGCTGGGGCTGGGCAGAGGGGAAGGT
GGCACAGGCTCAGATCCAGGCAACC
>1578
TGATGCCAGCCAGAATGGAATCAGGCTGGACGGGAAGAACCTGGTGCAGGAATGGCT
GGCAAAGCACCAGGTGATGGGGGCTGGTGGGTGTGGGAGGCACGGCAGGGGGAGGC
CAAGTGTGTGGGTCTCAGGGCTGTGGG
>1579
GCCTGGTATGTGTGGAACCGCACTGAGCTCATGCAGGCGTCCCTGGACCAGTCTGTGA
CCCATCTCATGGGTAATGACCCCCTTCCTGCCCTGGCATTCCTCAGACAACCTCAGAGG
GTGCCATCCGAGCCTGTGTGCCC
>1580
ATGGAGATGACAGAGGCTGCCCTGCGCCTGCTGAGCAGGAACCCCCGCGGCTTCTACC
TCTTTGTGGAGGGTGCGTGGTGGCCCCTGGGGAGTGGAGGAAGGCGGGGCGCGGCAG
GGCAGGTTCAAGCATCACCCCCCTC
>1581
CTCGTCACCGCTGACCACTCCCATGTCTTCTCCTTTGGTGGCTACACCTTGCGAGGGAG
CTCCATCTTCGGTAGGCCTGGGGAGAGTGGCAGGTGCTGCTGCATCAATTATGAGGGT
GAAGTTTGAGCCTCAGTTTCCTC
>1582
CTGTACGGCAATGGCCCGGGCTACGTGTTCAACTCAGGCGTGCGACCAGACGTGAATG
AGAGCGAGAGCGGTGAGTGAGGCTGAATGGCCCGTGCAGGGGGACCAGGGTGCCAG
GGATGGGGGCATTCGCGGGAGGGGGA
>1583
GGGCCCTGCATGCTGCTGCTGCTGCTGCTGCTGGGCCTGAGGCTACAGCTCTCCCTGG
GCATCATCCCAGGTAATGAGGCTCCCCGAGCTGCCCCTACACAACACACACACAGGGC
ACCCCCCAGCCCAGGCTGACCTGA
>1584
CTGGGTGCCGCCAAGAAGCTGCAGCCTGCACAGACAGCCGCCAAGAACCTCATCATC
TTCCTGGGCGATGGTGAGTGAGCCAGGCCTTCCAGCCCTGCAGCCCTCACAGCCCCGG
CGCCCGGACCCTCAGTGGTTCCAGG
>1585
GAAGAAGGACAAACTGGGGCCTGAGATACCCCTGGCCATGGACCGCTTCCCATATGTG
GCTCTGTCCAAGGTAAGTGCTGGGCTACCTTAGAGTCCTCCAAGCACAGAAGGGGAAT
CCTGGCTATGGAGTGTGGTAGGAG
>1586
CGCTTTAACCAGTGCAACACGACACGCGGCAACGAGGTCATCTCCGTGATGAATCGGG
CCAAGAAAGCAGGTGAGCTGGGGCCCGCTGCTGGGTCACGGCCAGGTCACAGACGTT
GGTCACATATACTGACCTCTGACAC
>1587

GCCTGCCTCGGCCCGCCAGGAGGGGTGCCAGGACATCGCTACGCAGCTCATCTCCAAC
ATGGACATTGACGTGCGACCCCCAGGACCAAGGGCTGGGGCTGGGCAGAGAGTAGCA
GGGAGGGGGCACTAGCTCAGACCCA
>1588
TGACTACAGCCAAGGTGGGACCAGGCTGGACGGGAAGAATCTGGTGCAGGAATGGCT
GGCGAAGCGCCAGGTGATGGGGGCTGGCGGGTGCAGGGGGCACAGCAGGGGGAGGG
CAGAGGTGTGGGGCTCAGGGCTGTGGG
>1589
GCCCGGTACGTGTGGAACCGCACTGAGCTCATGCAGGCTTCCCTGGACCCGTCTGTGA
CCCATCTCATGGGTAATGACCCCCTTCCTGCCCTGGCATCCCTCAGATGGCCTCAGATG
GCACCTTCTGAGCCTGTGTGCAC
>1590
ATGGAGATGACAGAGGCTGCCCTGCGCCTGCTGAGCAGGAACCCCCGCGGCTTCTTCC
TCTTCGTGGAGGGTGCGTGGTGGCCCCTGGGGAGTGGGGGTTGGGGGTTGGAGCAGG
GCAGGCTCAGCATCTCCCCCCTCTG
>1591
CTCGTCACTGCCGACCACTCCCACGTCTTCTCCTTCGGAGGCTACCCCCTGCGAGGGA
GCTCCATCTTCGGTAGGCCTGGGGAGAGTGGCAGGTGCTGCTGCAGCAATTAAGTGGG
TGAAATCTGAGCCTCAGTCTCCTC
>1592
CTATACGGAAACGGTCCAGGCTATGTGCTCAAGGACGGCGCCCGGCCGGATGTTACCG
AGAGCGAGAGCGGTGAGTGCCGCGGGGTGGCCCCCTGAGGGGGGACCAGGGTGCCA
AGGATGGGGGGCTGGCGGGAAGGGGT
>1593
GGATTTCCTTCAGAAAGGGCTAATGATCTCACTGTCTACAGATGACCCAATGCAATTCC
ACTTTACCAAGGTATAGTATGGACTTGGGCAATACAAGCATATTTTGCTGGACTGTTGTT
TCCCGTCCAGAATTTGAAAGT
>1594
CAAGCTGAGCACCTGTGATATGTGCGAAGTGGCAAGGAACAGTGTCTTGCAGTGTGG
AATTTCTCATGAGGTAGACCTGTCTTCAAATGGCTTCCCTTCTGAGTATAGGGTCTGCTA
ACTTGAATACCGAAAAAGTAGTT
>1595
GCTCCTAGGTCAGACCAGAGCTAATCCCATGTACAATGCCGTGTCCAACGCAGACCTG
ATGGATTTCAAGGTAGGGCCAGGAAAGCGGGTGCAGTCTGGGGCCAGGGGGCTTTCT
GATGCTGTGCTCACTCCTCTTGATT
>1596
AGGTGGAGTGGCCGCAGCCATCTCCAAGACGGCGGTAGCGCCCATCGAGCGGGTCAA
GCTGCTGCTGCAGGTACGTCTGGGATCCAGAGCCCAACCAGGAAGTGGGGGAAGGGT
CGCACAGAAGGCGGGCGCCGAGGGGT
>1597
TTTAACGTGTCTGTGCAGGGTATTATCATCTACCGAGCCGCCTACTTCGGTATCTATGAC
ACTGCAAAGGGTAAGTTTGCTGTGGGCTTTAACGTTGTGTTCTTAGGAGACAGTTTAA
AAGAGCATTGTACCAACCTAAC
>1598
GCCGGGTTGACTTCCTATCCATTTGACACCGTTCGCCGCCGCATGATGATGCAGTCAGG
GCGCAAAGGAAGTAAGTTCCACTTGAGACAGAAGACAAAGTTGTAGTCGTGGGGCAA

TCTGCTGCCACAAACTGGTGATAC
>1599
GCCAGCTGAGCAACAATGCCAAGGAGGCCGTGGAACATCTCCAGAAATCTGAACTCA
CCCAGCAACTCAAGTAAGAGGGACTACAGTGTGTGGTGGTGACGGGGAATTCTTAAA
GGCCATGCAATGTACTGGCAAGGGTT
>1600
ACACACTGGAGGACAAGGCTCGGGAACTCATCAGCCGCATCAAACAGAGTGAACTTT
CTGCCAAGATGCGGTTAGAACCCTTCCCAGGGCACGGGAGAGCTGGGGTGTGTTTTTG
GGTGGAGCCCTGGCAGATGGTCCAA
>1601
AGACCCTGGAGGACTACACTCGGGAATTCATCAACCGCATCACACAGAGTGAACTTCC
TGCCAAGATGTGGTTAGAACCCTTCCCAGGGCACGGGAGGGCTGGGGTGTGTTTGTGT
GGAGCCCTGGAGGATGTCCAAGAT
>1602
GCTGGGTGCAGACACTGTCTGAGCAGGTGCAGGAGGAGCTGCTCAGCTCCCAGGTCA
CCCAGGAACTGAGGTGAGTGTCCCCATCCTGGCCCTTGACCCTCCTGGTGGGCGGCTA
TACCTCCCCAGGTCCAGGTTTCATT
>1603
CCGAGCTGCAGCTGGTTGAGCAGCGGATCCGCAGCTTCCCCGACTTCCCCACCCCAGG
CGTGGTATTCAGGTGCACGCACAGGCCGCCCTCGTGGCCCCGACCTGCGGGCCTACGG
ATGGGAGCGCGTGCCCGCGACCTC
>1604
GCTCATCCGAAAGCGGGGGAAGCTGCCAGGCCCCACTCTGTGGGCCTCCTATTCCCTG
GAGTACGGGAAGGTAAGAGGGCTGGGGTGGCCGGAGGAAGGGCAGGGTCGGGTCAC
CGTGACCACTCTCCCCCAGTTCTAAA
>1605
GAGATTCAGAAAGACGCCCTGGAGCCAGGACAGAGGGTGGTCGTCGTGGATGATCTG
CTGGCCACTGGTGGTAAGGGTCTCCCCGCAGCCAACTCTGTGCTCCAAGGGCCTGGTG
GGAGTGGGACAGGACCTCGCTGTGT
>1606
GCTTGGGATCCTCCTGGTGACCTCTCCCGCTAAGGTCCCTCAGCCACTCTGCCCCAAGA
TGGGCCGTGGGGTGAGTATCCCTAAAGAGCAGGGGTCGCAGTCTAGAGGGTGAGGGA
GGCTGCTGAGGAAGGATGAAGTGG
>1607
AGAGAATGGGGGCGGCAAGAAGAAACAGAAGGAGAAGGAACTGGATGAGCTGAAGA
AGGAGGTGGCAATGGTGAGGGAACTGCTGGGCCATGGAGGAGGGGCCCCATGCTGGG
AGAGCTGTCCCTGCAGCCCATTGCACT
>1608
TGGGGCTATCCTCTGCTTCCTGGCCTACGGCATCCAGGCTGCCATGGAGGATGAACCAT
CCAACGACAATGTGAGCCCACACGCCTGACCCGGGAACAGCCCGTGACTGTCCTCCA
ACCCTGAACCCCCAACACAGTGGG
>1609
CTGCTTCTCCTACTACCAGGAGGCCAAGAGCTCCAAGATCATGGATTCCTTCAAGAAC
ATGGTACCTCAGGTAAGATGGCAGGGCTGGGCTCTGGGCTAGGCTGTAAGGTTTTGGC
AAGAGTCCAGCTCATCTTCTGTCA
>1610

CCTGGTGGAGGTGAAGGGTGGAGACCGCGTCCCTGCTGACCTCCGGATCATCTCTTCT
CATGGCTGTAAGGTGAGGAGGTCATACCAGAGCAAGCAGTTGAGTCTAAGGAGAAGG
CTGTGTGCAGAGCTGAGAGGGGCCC
>1611
CCCGAGTTCACCCATGAGAACCCCCTGGAGACCCGCAATATCTGTTTCTTCTCCACCAA
CTGTGTTGAAGGTGAGAAGCCAGGCTGCCCCCTGTAGGAAAGAGTCTGAATCCTGAAT
CCATAGTCAGGATGAAGGGCTCT
>1612
GGAGGCAGTCATCTTCCTCATCGGCATCATAGTGGCCAACGTGCCTGAGGGGCTTCTG
GCCACTGTCACTGTGAGTGGGTCAGGCTGAGGTGCCACCAGGGGAGGGTCTCACTAC
TCTTTCCTCAGAGTGATAGAGGCAC
>1613
CGCATGACCGTCGCCCACATGTGGTTCGACAACCAAATCCATGAGGCTGACACCACCG
AAGATCAGTCTGGTGATTGGGTGCTCCAGAGGGGGTGGATAGGATTAGAGGAGGCTGA
GGGCAGTGGCGTGGTGGGGTGAGT
>1614
GTCTCGAATTGCTGGTCTCTGCAACCGCGCCGTCTTCAAGGCAGGACAGGAGAACATC
TCCGTGTCTAAGGTAGGGGGTCAGGACACACACCAGGTATGTTTTGGGGGTGTCTCCA
AAGCCTCTTGCTGGCCCCAGCTTT
>1615
AGTGAGGAAAATGAGAGACAGAAACCCCAAGGTGGCAGAGATTCCTTTCAACTCTAC
CAACAAGTACCAGGTCTGCTTGGGTTGCCAGGACAGAGGAAGAGAGAGGGATATAAA
TGGGTGAGGGTGGACAAAGCCAAGGG
>1616
GACAAGGAGATGCAAGATGCCTTTCAAAATGCCTACATGGAGCTGGGGGGACTTGGG
GAGCGTGTGCTGGGTGAGAGGCCAGAAACAGGAGGCTCAGAAGGGGATTCCCAAGC
CTCTGCGGCATCCCTGGGGTGGGGGAC
>1617
GTCTATGATTGACCCTCCCCGGGCTGCTGTGCCAGATGCTGTGGGCAAGTGCCGAAGC
GCAGGCATCAAGGTACTGGCCTCCCATCCTCCCCTCCATTCTAGCCTCCCCCATGCCAG
AGTTCAAGGAGCTGCAGTGGCTG
>1618
CAGAGGGTAACGAGACTGTGGAGGACATTGCAGCCCGGCTCAACATTCCCATGAGTCA
AGTCAACCCCAGGTGAGGCCTCTGCAGGAAGCCCCTGTGCCCTAATCAACACGTCCTC
TTGCACAGAAGGCTTGGGTGTCCC
>1619
CACAGAGATCGTCTTTGCTCGAACGTCTCCCCAGCAGAAGCTCATCATTGTGGAGGGA
TGTCAGAGGCAGGTGAGCACAGCCACGGGAGGCAGATGACAGGCAGGGACCGGGGA
GGCAGGGACAGGGCCAAGACAAGCAT
>1620
TCTAAGCAGGCAGCCGACATGATCCTGCTGGATGACAACTTTGCCTCCATCGTCACGG
GGGTGGAGGAGGGTGAGGAGGCTGCATGGGTTGGGATGGTTTGCAGGATACTGAAGC
CGGCACCTCTGTTCCCTGTCCCTTT
>1621
CATCATTGCCAACATCCCCCTACCTCTGGGCACTGTGACCATCCTTTGCATTGACCTGG
GCACAGATATGGTGAGCGCAGGAGGTGGAGGAGGGGACAGGCAAGGCAATCGTGATG

GCACAGTGGCAGGGAGGAGAGGTG
>1622
CAGCCACGAAACTCCCAGACGGACAAGCTGGTGAATGAGAGGCTCATCAGCATGGCC
TACGGACAGATCGGTGCGCCAAGCCCCGGGCCTCGGGAGGGAACCCCAACAGGGTTC
TTTTCCCAGCTTTCAGAGGAATGAGC
>1623
ACTGGGAATCCGCCTCGACTGGGATGACCGGACCATGAATGATCTGGAGGACAGCTAT
GGACAGGAGTGGGTGAGTGGTGCTGTGTAAACACAGCGCACATGTGTGAAGGTACGG
GAAGCTGAATGCCTGGCAGGAGTGG
>1624
TGGTGGTGCAGTGGGCTGACCTCATCATCTGCAAGACCCGCCGCAACTCAGTCTTCCA
GCAGGGCATGAAGTGAGTGCCCACCCCCATGGCACCTACCCACCCAGGCTCTGGGCAC
ACTCACCAACCCACACAGGCCAAG
>1625
AGACGGCGTTGGCTGCCTTTCTCTCTTACTGCCCAGGCATGGGTGTAGCCCTCCGCATG
TACCCGCTCAAGTGAGTGTCTCTTTCGGGCGGCCTGAGTAGTCATACGGGGGGCCTTC
AGCCCCCTCCAGGATCCAAGTTC
>1626
TTCCCCTACAGCCTCCTCATCTTCATCTATGATGAGGTCCGAAAGCTCATCCTGCGGCG
GTATCCTGGTGGTAAGCCCCTCCACATTCCCCCCAGCAAAGTGCAAGCCCCACCACCA
GCTCCTCCCTCCAGGACCACACG
>1627
GTGCCGCACGCCATCCTGATCTGGGTGTATGATGAGGTGCGGAAGCTCTTCATCAGGCT
CTACCCTGGAAGTGAGTAGCCTATGATTTTAGAGGCTCTGTTTACCCACTTCAACAGAC
TCTCCATTTCTGATGTACTTTT
>1628
CGCATGACTGTGTCCCATCTTTGGTTTGACAACCACATCCACACAGCTGACACCACGG
AAGACCAGTCAGGTGTGGGGGAGGGAGGGCACAGGCGCCAGGGCAGGATCTGGTGG
ATGGTGGAGACCTGAAGAGGGATAGG
>1629
CATCACCGTCAGCGTGCCCCTGCCCCTCGGGTGCATCACCATCCTCTTCATCGAACTCT
GCACTGACATTGTAAGTCCACAGCCCAGGGTACCCATCCACAGGGTGCCCAGAGACAC
TTGCTTGCAGATGGACAGGAGGG
>1630
AACTGCAAAGATGCTGTCCGTGCGCGTTGCTGCGGCCGTGGTCCGCGCCCTTCCTCGG
CGGGCCGGACTGGTGAGCACCGAAGGCCGGCATGATGCAGGCGGCCGGGTGGGGCTG
CAGGGTGGTGGTGCGCCGGCTCGGG
>1631
AATGCTTTGGGTTCATCTTTCATTGCTGCAAGGAACTTCCATGCCTCTAACACTCATCTT
CAAAAGACTGGTAAGTTATTATTTCTCAGTCTACGCCGCACTTACTAGATGAAGATATAA
ATTACATACATCGTATAACT
>1632
TATTGCCCGCGTACATGGGCTGAGGAATGTTCAAGCAGAAGAAATGGTAGAGTTTTCTT
CAGGCTTAAAGGTAAATTATAAAATAAGATTTTGGTGTTTTAGGATCTAAGTCTTCTCAG
TTATTCATTTAGCGATTCAAA
>1633

GGACGTTCCAGTTGGTGAGGAGCTGTTGGGTCGTGTAGTTGATGCCCTTGGTAATGCTA
TTGATGGAAAGGTAGGTTTAATGTTCGTTAGATATAGTTGCAGCTTCTAACAAACATCA
AAACTGATTATGCTTAGGGTTT
>1634
AGGCTGTGGATAGCTTGGTGCCAATTGGTCGTGGTCAGCGTGAACTGATTATTGGTGAC
CGACAGACTGGGTAAAGACTCAAAAGCATATTAAAAGTTCTAACTAAAATTTGCTCAAT
GGAAGGATTATATTTTAGTCTT
>1635
TGTATTTATGTTGCTATTGGTCAAAAGAGATCCACTGTTGCCCAGTTGGTGAAGAGACT
TACAGATGCAGGTATTAAAAGAAATTTAGTCCCATTGCTATTATCTTAAGTCCCATTGCT
GCTGATTGTAGAATAGCTTCT
>1636
TTCCATGGGAGAGTATTTTAGAGACAATGGCAAACATGCTTTGATCATCTATGACGACT
TATCCAAACAGGTCAAAGGAAATAAATTTTTAGAATCCATTTATTTGTACTGAAGTAAA
AGTTCACATATGCAACTTCTAT
>1637
CATAGAAACACAGGCTGGTGATGTGTCTGCTTACATTCCAACAAATGTCATTTCCATCA
CTGACGGACAGGTATTATTTTAATGATTAAAAGAAATGTTAAAAAAGATTTGGATAATTC
TAAGGCATTGGTTGAGACTAA
>1638
CCCTGCAATTAACGTTGGTCTGTCTGTATCTCGTGTCGGATCCGCTGCCCAAACCAGGG
CTATGAAGCAGGTAATTTTGCATCACTTTGGCAGTAAGGGGGCTATCATTAGATTAGCAT
CTTAAGGTATTAATATATTAA
>1639
GATGCTGCCACTCAACAACTTTTGAGTCGTGGCGTGCGTCTAACTGAGTTGCTGAAGC
AAGGACAGTATTGTAAGTTGTTCTTTTCTTCATTTGGTCAGGTTCCTATTGTGTATCAGG
GTCTTCCCAGGCCTACAGAGCT
>1640
TTACAAAGTTTGAGAATGCTTTCTTGTCTCATGTCGTCAGCCAGCACCAAGCCTTGTTG
GGCACTATCAGGTATGAATGCAATTGTTGGCATCTTTTTTTAAAGTTATGTTTAAGATAT
GAAGTTAAAATTATTTTCAAA
>1641
CTCACCCCTTCAGCGTCGCTGCCCCCAGCTCAGCTCTTACTGCGGGCCGCTCCGACGG
CGGTCCATCCTGGTAAGTGCTTTTCTCTAGGAGCTAACGTTCCATTTTGCCGCCCCATG
ACCTTGAGCCGGGGAACGATGGT
>1642
CCAATTCTAAATGCCCTGGAAGTGCAAGGCAGGGAGACCAGACTGGTTTTGGAGGTG
GCCCAGCATTTGGGTAAGTAGAGTTTGTTAGGAATAGTAAAGACCTTGTGTAGCCCAAA
TATCCCCAAAACCAAGCTGTCTGC
>1643
AGACTTTGGGCAGAATCATGAATGTCATTGGAGAACCTATTGATGAAAGAGGTCCCATC
AAAACCAAACAGTAAGTTGCTCTTATTGCATCTATGCACAAAGGAACTTCTGTTATTTG
CAGTTTTACACATTAAGGTGAC
>1644
CAGGAAATTCTGGTGACTGGTATCAAGGTTGTCGATCTGCTAGCTCCCTATGCCAAGGG
TGGCAAAATTGGTATGTTATTAAGGGGTACATATTTTCCAAACCTAATATGGGAGGAAA

AACTCATACAATGTTATGAGTG
>1645
TGAAGGCAATGATTTATACCATGAAATGATTGAATCTGGTGTTATCAACTTAAAAGATGC
CACCTCTAAGGTAAGCATTGAGCTAATTGATGTCCACGTGGTAGTTACGCCAGGAAACT
ATTGCCAACTGAAGCCATGTT
>1646
AGACCAAGAAGGTCAAGATGTACTGCTATTTATTGATAACATCTTTCGCTTCACCCAGG
CTGGTTCAGAGGTAAGAGGGAAGGCTTGAGAGGACCTGGTTCATCTGGCCTTTCTTCG
GATGAGGCTAAGGATGTAGACAC
>1647
GGCCACTGACATGGGTACTATGCAGGAAAGAATTACCACTACCAAGAAGGGATCTATC
ACCTCTGTACAGGTAAGAAAAATTACATAGATGAAGATCTGATTTGTATAAAGGCAGGG
TGCAGTGGTGCATCTCAGCTACT
>1648
TATCATGGATCCCAACATTGTTGGCAGTGAGCATTACGATGTTGCCCGTGGGGTGCAAA
AGATCCTGCAGGTGAGTATATTACTATGTGGGATCAGTGTCCGGAAAGTCAAAAAGAG
GGCCTGTGGGGACTATATGAGGA
>1649
TTCACAGGTCATATGGGGAAGCTGGTACCCCTGAAGGAGACCATCAAAGGATTCCAGC
AGATTTTGGCAGGTGAGATTTTGAGTACAAATCTTGAATGTTTACTGTGCTGTGGTCCC
ATTCCAACAACCGTCAAGCAATA
>1650
GGCACTTCTGCGGGGGTGCCCTGATCCATGCCCGCTTCGTGATGACCGCGGCCAGCTG
CTTCCAAAGCCAGTGAGGGGTCCTGGGGAGGGGGCCTAGGGGGCATTGGGGCTCAGA
GAAGGGGCTTGGGGGGCTTAGGCAT
>1651
TTCCATCAGCAGCATGAGCGAGAATGGCTACGACCCCCAGCAGAACCTGAACGACCTG
ATGCTGCTTCAGGTGAGAGGATGGTGCCACCTGTGATCCCAGCACCTCGGGAGGCCGA
CGTTAGCCAGGGAAACAAGTCCAA
>1652
CCCCGAGGACCAGTGTCGCCCCAACAACGTGTGCACCGGTGTGCTCACCCGCCGCGG
TGGCATCTGCAATGTGAGTGCTCCCTGTGGCGGGAGGAGGGGTCCTGAGAGGTACTGA
GCTCTCCGTGGCAGGAGAAAGCAAG
>1653
CAAGGGCACCTTTGCCACACTGAGTGAGCTGCACTGTGACAAGCTGCACGTGGATCCT
GAGAACTTCAGGGTGAGTCTATGGGACGCTTGATGTTTTCTTTCCCCTTCTTTTCTATGG
TTAAGTTCATGTCATAGGAAGG
>1654
GAAAGTGGAAGACAAGAAGAGCAAACCACGTGTACAACAGAAGGCTGGTCTCCAGA
GCCAAGGTGCTTCAGTAAGTCAGCTGGATATGTCACTCAATGTTTCAATACTCAAAGAA
ATTTGTATATAAAAATAGGGGTCCA
>1655
AAGGATGAAGAAGTGGTTCAATGTCTCTCTGATGGATGGTCTTCTCAACCAACCTGTA
GGAAAGAACATGGTATAAGAATCATTTCCTAAACTGAAGATAAGTGCTTATCAAGCTTA
TATTGAATATATTGAAGCTTATA
>1656

GCTGGAGGAAAGAAGACAGAGGAGGTAGAATGTCTCACATACGGATGGTCTCTCACA
CCAAAATGTACCAGTAGGTTCTCATTTGGAATAAAATCTACTCTCACTCTGAAAGCTTT
TTCTCATAGAGAAGTGTATATAAG
>1657
TATCTAAGTGGATCTGATTTAATTCAATGCTATAACTTTGGTTGGTACCCAGAATCTCCT
GTATGCGAAGGTAAATTTTTAAATTTAATACTCGCAAAATGCCATGTCTTTATCAGCTTT
AATCTCTATTAGCTCTGACA
>1658
TTTGAGATCCATGGGTCAGCAGAAATACGTTGTGAAGATGGAAAATCGACAGAACCTC
CAAAATGCATTGGTTAGTAACACCTTGAAGAAGCTTTGCTAAAATGAAATCTGCATGTG
TAGCTAAATGGTCAGTTCTCTTT
>1659
TACCTTCTCCATGGATCGAATGAGATAACTTGTAATCGTGGAAAATGGACACTTCCTCC
TGAGTGTGTTGGTATGTATGCTACATTTACCATCAGTAGCTAAACTTATGGTGTAAAATT
TTCCATTCTGTTACTTTAATC
>1660
TACTTACTGAGGGGATCAAAAATATCTCGTTGCGAACAAGGAAAATGGTCATCCCCAC
CTGTTTGCTTGGGTAAGAAAGAGAACACATGGAATGTCTACGTTTGTACTTTTATGTGA
TTTTCTTACAGTGTTTTATATCA
>1661
ACCCCATTGTCTGAATTATCTGTGCAGTGCAACAGAGGAGAAGTGAAATATCCTTTATG
TACTAGAAAAGGTAAAATAATAATGTTCTCTGCAAGTATCTTTACTTGACCAATATCTGC
TACCTCGGTTGAAAATTTATT
>1662
CATTTCCTAGAAGGATCTAGGGAGGCCTATTGTTTAGATGGAATGTGGACTACACCACC
ATTGTGTTTAGGTATGTACTACTAAATATGCCTCTAACAAAGTAAAACTATATATTTTTAA
TTTGCTGTCATTTTTGAGTT
>1663
CTGGATCTATACTTAGAATGCAATGTGACAGAGGGCAGTTAAAATATCCAAGATGTATT
CCAAGACAAAGGTAAGAAGTTTTTTTTTTTTGGTCAGATTATTTTTTTTGGTCAGATTGT
TATTCAACATTTCAGAAATGT
>1664
GAGAAGGAGCTGAAGGAGATCAGGGTCTTGGACAAGGCCTTCGGACCAGAATTGAGA
GAGGAATTTTCTAGTAAGTAAACTTGGGTCATGGGTGTGTGGTTCCATCTTAAACACTG
CATGTATGTGGGGGGAGATGGACC
>1665
CACACCGCCTGTATCATTGATGTCTTCGGTGTCACTCACAGAGAGTCTATCATGAATGT
CAATGTGAAAGGTATGGTAGGCTGGGGAGGAGATGCAGCAAGGTGGGGAATTAAGGA
TCACAAAGAAGGGCCAGGAAGGGA
>1666
GGAGTTTGTCAAGGCCAAGATCGTGTCTCGAGAGGGTGGCAAAGTCACTGCCGAGAC
TGAGTATGGCAAGGTGGGTGTCAGGCTGATGTGAGAGTCCACCCTGGCCACCTGTACA
CCTGGGTGGACAGAGAGGGGTACAC
>1667
GCTGACCTTCCTGCATGAGCCCGCGGTGCTCTACAACCTCAAGGATCGCTACGGCTCC
TGGATGATCTACGTGAGTGCTGCACCTGGCCCTACGTTGGGATCTCTGTTCTTGCTCCA

TCCATGTCCACCCCAGGAGCCAA
>1668
AAGAAGAGGAGCGAGGCCCCGCCCCACATCTTCTCCATCTCCGACAACGCCTATCAGT
ACATGCTGACAGGTGAGAGGCCCTGGAAGGTCTTCCTGAAGGGAACTGGGATAGGCC
GGGAGGGAGAGGGAGAAGGAAGGGA
>1669
GGTCATCCAGTACTTTGCTGTTATTGCAGCCATTGGGGACCGCAGCAAGAAGGACCAG
AGCCCGGGCAAGGTAGGCCTGCTGCCCTCCAAGGTCCTGTACCGCAGAAGGGAGGGA
GAAGAGCTCTCACCTGCCTCCTTCT
>1670
CCAGGCCAACCCTGCTCTGGAGGCCTTTGGCAATGCCAAGACCGTCCGGAACGACAA
CTCCTCCCGCTTCGTGAGTGGTCCCTGACCTTGGCCTTGGGACTTGGACTGGTGGAGG
AATGGTCTCAGATATGAGCCTTCCC
>1671
CAGCTGAAAGCAGAGAGAGATTATCACATTTTCTACCAAATCCTGTCTAACAAAAAGC
CTGAGCTGCTGGGTGAGTCAGAGCCACCGACTGAGACCAACTATCTCCATGGCAACCT
GGTCCCCTCTGCTTGTGGCTGGAC
>1672
TGCATTCATCTCCCAAGGAGAGACCACCGTGGCCTCCATTGATGACGCTGAGGAGCTC
ATGGCCACTGATGTGAGTGTGTGAGGACCCAGCCAGGGGGTGGGAGGATTGGCAGTG
AGGGGCAAACAGGGGTCCAAAAGCA
>1673
ATGCACTTTGGAAACATGAAGTTCAAGCTGAAGCAGCGGGAGGAGCAGGCGGAGCCA
GACGGCACTGAAGGTGGGAGGCAGGATTCTTGGGGCAGCTGTAAGTCATGAGGCAGT
CTGCTCAGCAGTATCTCTCTTCTCTT
>1674
CAAGGGGCTGTGCCACCCTCGGGTGAAAGTGGGCAATGAGTACGTCACCAAGGGGCA
GAATGTCCAGCAGGTGGGTCCATCTTCAGATGATAATGGGTGGGCAGGGTAGGGAGAC
TGGCATGGTGGGATGAGAGTTTTCA
>1675
CCTGGAGACCAAGCAGCCACGCCAGTACTTCATAGGAGTCCTGGACATCGCTGGCTTC
GAGATCTTCGATGTGAGTTGGGACCCCTGGGAGTGGGAGAACAATCACTCACTCGCTC
CCACATTCAACAGCTATTTGCTTA
>1676
GGAGGGCATCGAGTGGACATTCATTGACTTTGGCATGGACCTGCAGGCCTGCATTGAC
CTCATCGAGAAGGTGCCTCTTTGGCCTTACCACCTGAATTCTCCCTGCACACCCAACAA
GAACACCATAGACAAAATAGCAG
>1677
GGCTTGTATCAGAAGTCTTCCCTCAAGCTGCTCAGCACCCTGTTTGCCAACTATGCTGG
GGCTGATGCGCGTAAGTAGGGACTGAGGCTCCCGGTACAGAGGAGCAGGGATTCTGC
CAAGGTTCTGAGCCAGGTCAATTG
>1678
ATGACCAACTTGCGCTCCACCCATCCCCACTTTGTACGTTGTATCATCCCTAATGAGAC
AAAGTCTCCAGGTGAGGCCACAAACTAGGGCCAACCCACTGCTGGGCATACACCGCC
CTGGGAACAGGACCTCCTAGGACA
>1679

TGCTGGAGGGCATCCGCATCTGCAGGAAAGGCTTCCCCAACCGCATCCTCTACGGGGA
CTTCCGGCAGAGGTGGGTATGAGGGTGCACCAGAGCTCATAGAACAGGGGGAGCCAG
GCTGCCCTGATGGGAATGGGATCTG
>1680
GAAGGGGGCAGAGAAGCTGCTCAGCTCCCTGGACATTGATCACAACCAGTACAAGTT
TGGCCACACCAAGGTGAGGAAAGGAGACTAATTAATTAAAGGAAGACATCTCTTTTCC
ATTGACTCCTCTGATGCTTTTCCTG
>1681
TCACGCGTATCCAGGCCCAGTCCCGAGGTGTGCTCGCCAGAATGGAGTACAAAAAGCT
GCTGGAACGTAGGTGAGAGATCTCAAGAGGAGGTTTCCCGCTTCTCTGAGGCCCAGG
CTGGTTCAGGGGCAGTGTCAGGAAA
>1682
CAAGGAGCTGGAGGAGAAGATGGTGTCCCTGCTGCAGGAGAAGAATGACCTGCAGCT
CCAAGTGCAGGCGGTGAGGCTCCTGGGCTACTGTTGGCTCTTCCACCCTGCTCTGCCT
TCACTTCCCACAACCCTGCACCTCC
>1683
AAGGGACATCGATGATCTGGAGCTGACACTGGCCAAAGTGGAGAAGGAGAAACACGC
AACAGAGAACAAGGTAAGGGCAGCTCCCTTTGGCTCCAGCCCGGGTCTCATCAGGAC
TCTCAGACCATACTGACCTTGACCCA
>1684
TCAGGCCGAGGAGGACAAGGTCAACACCCTGACTAAGGCCAAAGTCAAGCTGGAGC
AGCAAGTGGATGATGTGAGTAGATTGAGAGTTGTGGGGCCTAGATATGCCATGTCTATC
TGTGCCCAGAGCTCTGTGTGTGTGT
>1685
TGAAGCTGACCCAGGAGAGCATCATGGACCTGGAGAATGACAAGCAGCAGCTGGATG
AGCGGCTGAAAAAGTACTGTGTCCCCTCCCTGCTGCCCTTCCTCCTCCCCAGCCCATAA
CAGTACAAGCAGACCCAAGACAGC
>1686
TCTCAACGCAAGGATTGAGGATGAACAGGCCCTCGGCAGCCAGCTGCAGAAGAAGCT
CAAGGAGCTTCAGGTGAGGTCTGGACACCCACGCGGGATGCTGAGTCCCTGGGCTGG
TTCTGTTTCCCCTGCCCCCTCCTCTA
>1687
AAGGCGGAGGAGACCCAGCGTTCTGTCAACGACCTCACCAGCCAGCGGGCCAAGTTG
CAAACCGAGAATGGTGAGCCTAGAGCAGGGGCTCCATGGTTCCCACCACAGTCTCCCC
AACCTCCTGAGCCACTCCAGCCTCG
>1688
GACCCGAGGCAAGCTCACCTACACCCAGCAGCTGGAGGACCTCAAGAGGCAGCTGGA
GGAGGAGGTTAAGGTAAGGGCTAGACTCGGGCCACCTGGCCCAAGCAAGGAGCACAC
TGACTAGCCTTGCATCAAATCACTTC
>1689
AGGTGGCCCAGTGGAGGACCAAGTATGAGACGGACGCCATTCAGCGGACTGAGGAGC
TCGAGGAGGCCAAGTGAGTTCTGAGCAGCCTGACTTCTGGCTGAGGCCCCTTTGCAG
GCAGGACTCAGCCCAGCCCCAGCCTC
>1690
GATGGTGGACGTAGAGCGCTCCAATGCTGCTGCTGCAGCCCTGGACAAGAAGCAGAG
GAACTTCGACAAGGTGGGCCCTGGGTGGGGGCCGCAGCCAGCATGCAGGGCAAGGG

GGCATGAGGGGTTCAGTGAGAGGCCAG
>1691
CTCAAGAACGCCTATGAGGAGTCCCTGGAACATCTGGAGACCTTCAAGCGGGAGAAC
AAAAACCTGCAGGGTGTGCTGGGGGCCCAAGAGGCTGGGGAGGGGCTGCACTGGCA
GTGTTCCCATATGGTGCCACCCAAGGG
>1692
GCTGGAGAAGGTCCGAAAGCAGCTGGAGGCCGAGAAGATGGAGCTGCAGTCAGCCC
TGGAGGAGGCCGAGGTGTGTGTGTGTGCAGGGCACGGGGTGCAGGGAGCTGAGCTCC
AGGATTTTGGCTCCCTGTTCTCATCCC
>1693
GCTCAGCCACGCCAACCGCATGGCCGCCGAGGCCCAGAAGCAAGTCAAGAGCCTCCA
GAGCTTGTTGAAGGTACTCACCCAGAGGGGACTGGCCTCCACGTGGCCTGGCGAAGC
AGTAGTGTCTTGATACAGGCACCAGA
>1694
GCGGTCCCGGAAGCTGGCGGAGCAGGAGCTGATTGAGACTAGTGAGCGGGTGCAGCT
GCTGCATTCCCAGGTGAGCAGCTCCCCTGCTCATTCCTGAAGGGAGCACAGGCTGGGG
CTCAGCAAGCAAGGCTTGAGAGCTA
>1695
GACTGAAGTGGAGGAGGCAGTGCAGGAGTGCAGGAATGCTGAGGAGAAGGCCAAGA
AGGCCATCACGGATGTAAGTCCCCCACTCCACCGACCCGATCCAGACCAGTGTCTCTC
CGTGGGCTGGGCAGCAAGTGTGTGAG
>1696
GAAGCGCAACGCAGAGTCGGTGAAGGGCATGAGGAAGAGCGAGCGGCGCATCAAGG
AGCTCACCTACCAGGTGCGACGGGCGGTGACTCCAGGCAGAGCCCTGGCACCATAGC
CACAGTGACAACCAGCTGAGGAGAATG
>1697
GCGGCTGCAGGACCTGGTAGACAAGCTGCAGCTAAAGGTCAAGGCCTACAAGCGCCA
GGCCGAGGAGGCGGTGAGTGACCCTGCTGGGGACTAGGCCCAGGGGAGGCATAGGA
GAGCTCGTCCCCCAAGCCAGGAGTCTG
>1698
GGAGCGGGCGGACATCGCCGAGTCCCAGGTCAACAAGCTGCGGGCCAAGAGCCGTG
ACATTGGCACGAAGGTGGGTCCCTCTTTTGGGCTTTGCTAGTCACCCCCACAGCAGGC
ATACCCAGACAGAGCACCCTCAAACC
>1699
CCTGGGAGGTCGTTCCCACCCGCTGGGCAGCCCCGGTTCAGCCTCGGACTTGGAAAC
GTCCGGGTTACAGGTGAGAGCGGAGGGCAGCTCAGGGGGATTGGACAGCAGCAATGA
AAGGGTCCTCACCTGCTGTCCCAAGA
>1700
GTGCAAGGGTCTGGCTGCTTTGGGAGGAAGATGGACCGGATCAGCTCCTCCAGTGGC
CTGGGCTGCAAAGGTAAGCACCCCCTGCCACCCCGGCCGCCTTCCCCCATTCCAGTGT
GTGACACTGTTAGAGTCACTTTGGG
>1701
ATCTTAAACCAGCTTGGTGCGGATAGTCTGACTAGTTTAAGGAGACTGGCCGAAGCTC
TGCCCAAACAATGTGAGTTTCCTAGTAATGGTTTTACCAGGGAATTACTCATTTAGCAG
CTGATTTCTGATCTCAGGGCTCA
>1702

GATGGTGAGAGGCATGCCTTCATTCTGCAGGACACAAAGGCTCTGCACCAGGTCTTTG
AACATATGCTGGGTGAGTGAGCTTTGCCCTCCTTGGTGTGGGGAGGATGGTGAGGAGC
CCGCCAAAGGCCCGTTTTGGGAAC
>1703
CTGCGGGGTGGGGAACATGTCAGCAAACGCCTCTGACCAGGAGAGGACACCCTGGCA
TGTCACTATTAAGGTACCAGGAAGGAGGGGCAGGGCTTGGATTCCAGAGGTAAAAGC
GGCCATGGGCCAGACATACTGCAATC
>1704
CAATGGGTCCTGACAGCAGCTCATTGCTTCCGCGATGGCAACGACCACTCCCTGTGGA
GGGTCAATGTGGGTAAGGCAGGGGATGCACCAGCCTCCTGATCCGTGAAGCCACAGAT
CCTACCACCTCACCCAGCCTCTGG
>1705
TGGAGTTCTATGGTGATGACATAGCTCTGCTGAAGCTGGCCCAGAAAGTAAAGATGTC
CACCCATGCCAGGTGCCTGGAGTCTTGGATGGGAGGGTGCCCTGCAGGGAAGAGTGC
TCTGGAGATCCCTGGAAGAGACTAC
>1706
TGCCTTCCCTGCACGATGGAGGCCAATCTGGCTCTGCGGAGACCTCAAGGCAGCACCT
GTAGGGACCATGGTGAGTGCTGGGACTTATGGTGCTTGAGAGCTGGGGCCGGGGTTTG
GGGGTGATAACAAGGACTAGGCTG
>1707
GAGTGTTCCTGCTCATTTTGTCGCCTTGAATGGGAGCAAACTGAACATTAACCTTAAGA
TGGGAGTGGAGGTGAGGGTCTCAGGTTGGGGATGCTGGGATCCCCCTGTGACAGCTC
CCAGAATGTCTCTCTTCCTTCTCC
>1708
GATGTCAGGGAGGTGGTGACAGACCAGTTCCTATGCAGTGGGACCCAGGAGGATGAG
AGTCCCTGCAAGGGTGAGTCCCTCACCATGCCTGGATTCCCAAGGGGAAGGCCACCTG
TGTCTCTGTGGCCAGCATGCATGCC
>1709
GGGTGACAAGCACACCCTGAGCAAGAAGGAGCTGAAGGAGCTGATCCAGAAGGAGC
TCACCATTGGCTCGGTGAGTGGCCTCCTCCCCAGGACCCCTTTTCCCACCCTTGTCCTT
TGGAAGCAAGGATTAGGGGAGAGAG
>1710
AGGGCAGAAAGTGAAGGGCACCGTCCGCCGTGTGGTCCTGCGAGGGGAGGTTGCCTA
TATCGATGGGCAGGTACGCAAGTAGCCCCTGCCTGATCTCAGTAGTGCCCTCTTCTGCA
CCACGTTCATTTCTTCCCTACCCA
>1711
ACGGAAGTGGCCACAGGGGGCTGTTCCTCAGCTCCCACCCTCAGCCCCTGCCACTAGT
GAGATGACCACGGTATCCACACTACTGGGCTAGGGGGCTGGGAGGTGTTAGTCTCAGG
GCAGGATGAACAGCTTGAACATTT
>1712
CCAGGGCTTCCTGATGGCCGCTTCCATCTGCCGCCCCGAATCCATCGAGCCTCCGACCC
AGGTTTGCCAGGTAAGAGTGGGGTTCCTGTGACTCAGAGACTGTGTAGGGACAGGAT
CCACTTCTTCCCAGTGCCTCGCCT
>1713
GACCTCACCCCTGCTGCACTCATTAGTGGGCCAACATATCCTGTCCGTCCAGCAGTTCA
CCAAGGATCAGGTGCCTGGGGCAGGGAGGATGGGACCACCCAGAGCTTTGAGGATTT

GGAAAGCTGGGGCTAGGACCTTTC
>1714
TCACCTGTTCAATGTGGCACACACACTGCGTATGATGGTGCAGAAGGAGCGGAGCCTC
GACATCCTGAAGGTCAGGATCAGGGCCGGGGGTAGGGTCCAGGCCATCGCCTGCCCTT
GGGCCGCATCAGCCAGGGCCGCGC
>1715
CTCCGTGCAGACCATGAGCTGCTATGCCGACGTCGTCGTGCTCCGGCACCCCCAGCCT
GGAGCAGTGGAGGTGAGGCCAGCCTGGGTACTGAGATGGGGTTAAGAAGGCTGGACC
CAGGGGCATGAGAACCCTTCTGCCC
>1716
GCACCCCACCCAGGCCCTGCTGGACATCTTCACCATCCGTGAGGAGCTGGGAACTGTC
AATGGCATGACGGTGAGGGTGGTGGCAGGGTTTGGATCCCTGCCAGGGGACGATCTAG
AGAGGGAGGCAGGAAGTGGTTACC
>1717
CGTGGCACCTCCCAGCCTGCGCATGCCACCCACTGTGCGGGCCTTCGTGGCCTCCCGC
GGCACCAAGCAGGTGAGACCCTCACAGCCCTGCCTGGAAGCCATGGAGATGTGGGTT
GGGCAGTCAGAGCCCAGCGCTGCAT
>1718
TGACACTGATGTGCTCTACATGACTCGAATCCAGAAGGAACGATTTGGCTCTACCCAG
GAGTACGAAGCTGTGAGTGCTGGGCTTGAGGAAGAAGCCAGGGCTGCTGCCGTAGGG
CATCAGATATGAGGACAGAAAGGCT
>1719
CCCACATCATGACCCGGGCCAAGAAGAAGATGGTGGTGATGCACCCGATGCCCCGTGT
CAACGAGATAAGGTGGTGCAGCATCAGAGTCAGAGACTGCCTCGGGGCTGGTGGACG
GGAGGAGACTTAGTCCTGGACAAGC
>1720
GTCAGAGCAGATGTGGAGGGTTCCTGGTGCGAGAAGACTTTGTGCTGACAGCAGCTC
ATTGCTGGGGAAGGTGAGGAGCTAAGGAACTTCCTGGCCAGCCAGGAACACAGCCCT
GCGGAGCTCTTCGGTGGAAGAGCCAT
>1721
TGCGCGCAGAGCCATCCGCCACCCTCAATATAATCAGCGGACCATCCAGAATGACATCA
TGTTATTGCAGGTACCACCTACCTGGCCCTCTGGCTCCTTCCTAGTGTGTCCGGGGACA
ATGGAGGAGGAAGTGAGGGCAA
>1722
CTTCGGTTCCTACGACCCCCGAAGGCAGATTTGTGTGGGGGACCGGCGGGAACGGAA
GGCTGCCTTCAAGGTAAGGCATGGGCATTGGCCAACACACCCCGGGAGAGAGGGGCC
CGTGCAGAGCCAGGCAGTGCGAACAG
>1723
GAAGTACAGCACTTTTGGGGAGGCGGCCTCAGCACCTGATGGCCTGGCTGTGGTTGGT
GTTTTTTTGGAGGTGAGTGGTGGTTTGCTGGGGCCTGGAGGGGCATGGGAGGCCTGCA
CTGCCCCTAGTTCTAGAACACAGT
>1724
GACTCCCCCACTCAGTGAGAGTGTCACCTGGATTGTGCTCCGGGAGCCCATCTGCATC
TCTGAAAGGCAGGTGAGTCCTCTCAGAGGACCAGATGGAGGGACATGGCACTCAGGG
CTCACCCCAGGCAGTGTGACTCTCC
>1725

CGCGACTTCCTGCGCAAGGAGTACGGGGGCCTGGACGTGCTGGTCAACAACGCGGGC
ATCGCCTTCAAGGGTATGGGGAGGGGACGTGGCCTCCCCGAAGAAGAACCGATGCAC
TGGGGCTCCTGGCGTCTGCGGGGTCC
>1726
ACGATGAAAACAAATTTCTTTGGTACCCGAGATGTGTGCACAGAATTACTCCCTCTAAT
AAAACCCCAAGGTGAGTCTGATGGGAAACAGCGCACCTTCTCTTTGGGGCTTGATTTG
GCCCCTGCTTGCCTGGGATTTCT
>1727
CTCTTCTTCCTCCTCTTCCTCACCCCCATGGAAGTCAGGCCCGAGGAACCTCTAGTGGT
GAAGGTGGAAGGTATGTCCAAAGGGCAGAAAGGGAAGGGATTGAGGCTGGAAACTT
GAGTTGTGGCTGGGTGTCCTTGGCT
>1728
CTGTGCCAGCCGGGGCCCCCCTCTGAGAAGGCCTGGCAGCCTGGCTGGACAGTCAAT
GTGGAGGGCAGCGGTGAGGGCCGGGCTGGGGCAGGGGCAGGAGGAGAGAAGGGAG
GCCACCATGGACAGAAGAGGTCCGCGGC
>1729
CCTGAGATCTGGGAGGGAGAGCCTCCGTGTGTCCCACCGAGGGACAGCCTGAACCAG
AGCCTCAGCCAGGGTATGGTGATGACTGGGGAGATGCCGGGAAGCGGGGGTCCAGAG
ACAGAGGGGAGGGGAAACTGAAGAGG
>1730
GCTGGAAAGTATTATTGTCACCGTGGCAACCTGACCATGTCATTCCACCTGGAGATCAC
TGCTCGGCCAGGTAGAGTTTCTCTCAACTGGGAGGCATCTGTGTGGGGGTACTGGGAA
GAAGTGGAAGCCAGTCAATCTTA
>1731
TCAGCTGTGACTTTGGCTTATCTGATCTTCTGCCTGTGTTCCCTTGTGGGCATTCTTCAT
CTTCAAAGAGGTGAGTCATGTCCCCAGTGGGTCTGTCCAAACCCTACTCCATCTTCCCC
AGGATAAGCCGGCTCTGGCCA
>1732
CCCCCAGGAAGCGGGCCCCAGAACCAGTACGGGAACGTGCTGTCTCTCCCCACACCC
ACCTCAGGCCTCGGTAAGAGGCACCGCCCCTCCAGCCTATAGCTCCGCCCCAGATCCG
GGGCTCCACCCCCACTCTCCTCATC
>1733
TCTTATGGAAACCCGAGCAGCGACGTCCAGGCGGATGGAGCCTTGGGGTCCCGGAGC
CCGCCGGGAGTGGGTGAATGACTGGGAGAGGGAAGGGTCGTTCCCCACATGGAGGGG
GTTGGAGCGGTCTGTGGCCCGAATAG
>1734
CCTGACAGTGAGGAGGACTCCGAGTTCTATGAGAACGACTCCAACCTTGGGCAGGAC
CAGCTCTCCCAGGGTAAGGCTGCCCTCCCCCGTGGCCCCCCACCTCTGCGGTGGCCTG
TGGACTCCCATGGACACCCCTCCTT
>1735
AGAGGAATCCTGTATGCAGCCCCCCAGCTCCGCTCCATTCGGGGCCAGCCTGGACCCA
ATCATGAGGAAGGTGGGTGCTTCTGCCGCTGTCCCCTGCTGTCCCCTGGGCTGACTTT
GCCTTCCAGCCTACTTCCAGTGCC
>1736
GCCAGACCCAGCCTGGGGAGGAGGGGGCCGCATGGGCACCTGGAGCACCAGGTGATC
CTCAGGTGGCCAGGTGAGCTGGGACTGCCCCTAGGGAAAGCGGGGAGGGAGGGAGAT

AGGCACGGATGGCAGTGGCTGCTGGC
>1737
AGTCTTGCCCCAGCCTCGGGAGGTGGTGGAGTGACCTGGCCCCAGTGCTGCGTCCTTA
TCAGCCGAGCCGGTAAGAGGGTGAGACTTGGTGGGGTAGGGGCCTCAGTGGGCCTGG
GAATGTGCCTGTGGCTTGAAAAGAC
>1738
GGTCCAGGAGTCCTCCAAGCTCTGCCTGCCACCATCTTCCTCCTCTTCCTGCTGTCTGC
TGTCTACCTGGGTATGTGGCCAAAGGGCAGGAACTGGCGGGAGGTGGGGGAAGCTGT
GGAGGCTGCAGAGAGGGCACAGGC
>1739
TACGTGTGCCGGGTCCAGGAGGGCAACGAGTCATACCAGCAGTCCTGCGGCACCTACC
TCCGCGTGCGCCGTGAGTGGCCCAGCCCTGGCCCCTACTCCCACTGTCCCGCTGGGGA
CACTCGGTTTATCTTTGAAGTGGG
>1740
AATCATCACAGCCGAGGGGATCATCCTCCTGTTCTGCGCGGTGGTGCCTGGGACGCTG
CTGCTGTTCAGGGTGAGCCCCCTCGGACCTCTGAGTCAGCCGGGCGAGGGCCTGGGC
CCGAGGGACCCCCAATACCCAGGTA
>1741
AAACGATGGCAGAACGAGAAGCTCGGGTTGGATGCCGGGGATGAATATGAAGATGAA
AACCTTTATGAAGGTGAGTGAAGGGTGGGGATGGGGTAGGGGCAGTTTTGTTAGGGGT
GGGGGTGTTCCCTCTGGGGGTGGCT
>1742
GAGACCATGGAGTCTCCCTCGGCCCCTCCCCACAGATGGTGCATCCCCTGGCAGAGGC
TCCTGCTCACAGGTGAAGGGAGGACAACCCCTGGGAGAGGGTGGGAGGAGGGAGCA
CAGAGACTGGCTGGGGTCTCCTGGGT
>1743
CACCATGGGGCGCCTGCAACTGGTTGTGTTGGGCCTCACCTGCTGCTGGGCAGTGGCG
AGTGCCGCGAAGGTAAGAGCCCAGCAGAGGGGCAGGTCCTGCTGCTCTCTCGCTCAA
TCAGATCTGGAACTTCGGGCCAGGC
>1744
TTCAAGGGCATCCCCTTCGCAGCTCCCACCAAGGCCCTGGAAAATCCTCAGCCACATC
CTGGCTGGCAAGGTGGGAGTGGGTGGTGCCGGACTGGCCCTGCGGCGGGGCGGGTGA
GGGCGGCTGCCTTCCTCATGCCAAC
>1745
CAGGACAGCACCTACGGGGATGAAGACTGCCTGTACCTCAACATTTGGGTGCCCCAGG
GCAGGAAGCAAGGTCTGCCTCCCCTCTACTCCCCAAGGGACCCTCCCATGCAGCCACT
GCCCCGGGTCTACTCCTGGCTTGA
>1746
ATCGTGGTCACCTTCAACTACCGTGTCGGCCCCCTTGGGTTCCTCAGCACTGGGGACG
CCAATCTGCCAGGTGCGTGGTGTCGGCCCTGAGGTGGGCGGACCAGCATGCTGAGCC
CAGCAGGGAGATTTTCCTCAGCACC
>1747
CTTCGGGGGGGACCCCAACAACATCACGCTCTTCGGGGAGTCTGCTGGAGGTGCCAG
CGTCTCTCTGCAGGTCTCGGGATCCCTGTGGGGAGGGCCTGCCCCACAGGTTGAGAGG
AAGCTCAAACGGGAAGGGGAGGGTG
>1748

CATCAGCCAGAGCGGCGTGGCCCTGAGTCCCTGGGTCATCCAGAAAAACCCACTCTTC
TGGGCCAAAAAGGTAAACGGAGGAGGGCAGGGCTGGGCGGGGTGGGGGCTGTCCAC
ATTTCCGTTCTTTATCCTGGACCCCA
>1749
CAGTGTCTGAAGGTTACTGATCCCCGAGCCCTGACGCTGGCCTATAAGGTGCCGCTGG
CAGGCCTGGAGTGTGAGTAGCCTGCTGGGTTGGCCCATGGGGTCTCGAGGTGGGGGTT
GAGGGGGGTACTGCCAGGGAGTAC
>1750
ACATGGACGGCCACATCTTCGCCAGCATCGACATGCCTGCCATCAACAAGGGCAACAA
GAAAGTCACGGAGTAAGCAGGGGGCACAGGACTCAGGGGCGACCCGTGCGGGAGGG
CCGCCGGGAAAGGCACTGGCGAGGGG
>1751
ACTTTGAGACCGATGTCCTCTTCCTGGTGCCCACCGAGATTGCCCTAGCCCAGCACAG
AGCCAATGCCAAGTGAGGATCTGGGCAGCGGGTGGCTCCTGGGGGCCTTCCTGGGGT
GCTGCACCTTCCAGCCGAGGCCTCG
>1752
GCTACCGGCCCCAAGACAGGACAGTCTCTAAGGCCATGATCGCCTACTGGACCAACTT
TGCCAAAACAGGGTAAGACGTGGGTTGAGTGCAGGGCGGAGGGCCACAGCCGAGAA
GGGCCTCCCACCACGAGGCCTTGTTC
>1753
GATGCGTGACCAGGACAAGAAACTGGTGGAGAAGAGCTGCAAGGATGCCCAGGGCT
GGTTCTTCAGCGTGGTGGGTGGCCGTGTGTAAGCAGGTGTGCACACGTGTGGGCCACA
TACGCCGTGTGCTGGTGCAGTTGGAA
>1754
CAGAGCGGCAGGATGGAGATGTGGAGCTCTACATCTCTGTGCAGAACCTGAGCCGTCC
CAGCTCAGCAAGGTAGGGCCCCTGTCCCCTCCCCACCCTGTACCTGTCACCCCAGGGT
GAGCGCACGCTTATAGACCCCTGC
>1755
TGGACGTGAAGACGCGGCTGGAGCAGGAGATCGCCACCTACAGCCGCTTGCTAGAGG
TTGAGGACGCCCGGTGAGTGTGCACCCAGGTACCTGCTGGGGCGGGCTGGGGGCCAC
TTCACCCAGGGAGAAGTTGGTGTCTG
>1756
AGGAAGGATTAAGGGTGGAGGGAAGGGACAGCCCCTTGTATTGGGAAAAGTTTCAAG
AATCAGCCCCTGAGTCCCTGTCACCTGGTGGCTGGTCCCTGGGAGGGCCACACATACT
TTCCAAGGACCACACCTGTTGGGGA
>1757
AGAGGCAGGCCCCGGGGCCGCCCGTGACTACAGCCAGTACTACAGGATAATCGAGGA
GCTGCAGAACAAGGTAGGGCCTGCTGGTGGGAGGGGTCTCCGGGGGGCATGACTTTT
TCCCCCCAACTCCTGCCCTGGCCAAA
>1758
CCGTGGACAATGCCAACATCCTGCTACAGAATGACAATGCCCATCTGGCTGCTGCTGA
CTTCCGCACCAAGTGAGTCCTAGCTGTGGGCTTGGGCAGCCTGGGTCAGCTGGGGGA
GGATCTCAGGTACCCCTCCTGACCC
>1759
GCGACCCGGAGATGCACATTGAGAACCTCAAGGAGGAGCTGGCCTACCTGAAGAAGA
ACCACGAGGTGAGGTGGCTGGGGCAGAAGGTCAAAGATGCTGAGGAGTGGGTGCAG

AGCCCTGGGGTTGGGCCATGGCTGAGG
>1760
GATGCGTGAGCAGTATGAGAAGATGGCAGAGAAGAACCGCAAGGATGCCGAGGATTG
GTTCTTCAGCAAGGTGGGAGCTGCTGCAGGCCAGAGGTCTCTCTTCGGGGCTGGGCTC
AGGGGCCTTAGCACTGACAGTAGGC
>1761
TGGACATGAAGATGCGGCTGGAGCTGGAGATCACCACCTACCACCGCCTGCTGGAGG
GCGAGGATGCCCAGTGAGTGGGGGAGCCTGGGGTCAGGGCTGGGGGCCTCTTGGCAG
GGGTGGGGCTCTCAGACTCACATCTA
>1762
CCTCAAAATTTTGTGGTGGTTTCCTTATAAGACGGAACTTTGTGCTGACGGCTGCTCAT
TGTGCAGGAAGGTGAGACAACAGGGTCTATTTATCTCCAAATGGGAGATGAACAACCA
GAGTAGCATCCAGGAATACACCT
>1763
CCTCAAAATTTTGTGGTGGTTTCCTTATAAGACGGAACTTTGTGCTGACGGCTGCTCAT
TGTGCAGGAAGGTGAGACAACAGGGTCTATTTATCTCCAAATGGGAGATGAACAACCA
GGTAGCATCCAGGATACACCTGC
>1764
GGTTATAAAGCAATTCCGTCATCCAAAATATAACACTTCTACTCTTCACCACGATATCAT
GTTACTAAAGGTGACAACACCTCTCTTCTCCCTTTCCACTTCCCATTCTCCTAAGCTTCT
CCTTCAGGTCCTCATTGCCC
>1765
AGCGTAGCCGCCCACGCTACGCCCTGGACTGAGGAACCGCGACACCTCACCACGGAG
GCCCAGGCTGCGGGTGAGCTTGCTCCCGCCCCTACCTCCTGGCTTTGCTGTCCTGGAG
GCCTGTGGTAGAACCTGTGGCCTGG
>1766
TCTTGGTCAGCGTCCCCATCACTCTGGCCCTGGCTGCCGCTGCCGCCACTGCCAGCAG
TCTCTTGATCTGGTAGGTTGAGTGAGGCTGGGTGGCAGAGGGAGCCTGAGGGAACCC
CCACTCCAGCCCCCTCCCTGACAGG
>1767
GCTCCCCAGTCGCTGGTGCTGCTGACGCTGCTCGTCGCCGCTGTCCTTCGGTGTCAGG
GCCAGGATGTCCGTAAGTCTTCCCCCGCCGCTGCCTGCCTGCCTGCTTTCCATGCGTCC
CTCAGCATCCTTCTCCCCGGCCC
>1768
TGCCTCAGCCCTGAGATCCCCTTCGGAGAGTGCTGCCCCATCTGCCCAACTGACCTCG
CCACTGCCAGTGGTTGTAATTTATTTATTTCCTGTTCAACATAAATAAATTACTTGCAAG
CACTGCAAACACGCTCCCATAG
>1769
ACCTGGGACCCCTGGAAATCCTGGCCCCCCTGGTCCTCCCGGCCCCCCTGGTCCCCCT
GGTCTTGGTGGAGTAAGTATCCTTACTTCCCATTCCTTCAGGCTGTCCCTCCAGAAATG
TGGCTTTTAAATTGCTGCTTGCA
>1770
TGCCCAGATGGCTGGAGGATTTGATGAAAAGGCTGGTGGCGCCCAGTTGGGAGTAATG
CAAGGACCAATGGTAAGAAAAGACACTAGTTCTTTGCAGCCAAAATGGCAGGAGGTG
GCCCTTAGCAGAGCCAGAGAGTCTG
>1771

TCCTGAAGGTGCTCAAGGTCCTCGCGGTGAACCTGGTACTCCTGGGTCCCCTGGGCCT
GCTGGTGCCTCCGTAAGTGCAGCTTCTCTTTGGCCTGGGGGGGTCTGGGGTCTGTGGC
TTTGGAACTCTTGACTCTGTACTT
>1772
CTTCCCTGGGCCACGGGGTCCTCCTGGCCCTCAAGGTGCAACTGGTCCTCTGGGCCCG
AAAGGTCAGACGGTAAGAGCCCAAAGTGACCCCCAAGTTCCACTGACATCTCTGGAG
TCAAACCCCATCACCCCTCTTTCCC
>1773
TGAAGAAGGCAAGAGAGGTGCCCGTGGAGAGCCTGGTGGCGTTGGGCCCATCGGTCC
CCCTGGAGAAAGAGTTAAGTGAATGTGGAGGCTCCATCCCATGGGGCCTGTGACCTCG
AGAGGGAAGTGGAGTCCTTGTGGTC
>1774
TCTTGCTGGCCCCAAGGGAGCCAACGGTGACCCTGGCCGTCCTGGAGAACCTGGCCT
TCCTGGAGCCCGGGTAAGTAGCAGAGCTGCTGTTGCCCTTGGCTTCAGACCCTCAGGC
CCTTCCTGGCTGGCTCCTTCCAGCC
>1775
ACCTCCAGGTCCTCAGGGGGCTCGTGGGCAGCCTGGTGTCATGGGTTTCCCTGGCCCC
AAAGGTGCCAACGTAAGTAATAATTTGCTCTTCTATTTCCTTCCATGTGGTGCTACCTAC
CTCCCTGCCCTCTTGGGGAAAG
>1776
CTCTCCCGGTGCCCAGGGCCTCCAGGGTCCCCGTGGCCTCCCCGGCACTCCTGGCACT
GATGGTCCCAAAGTAAGTGAGGCTGCATCCAGTAGGGGTCTTCGTGGTAGCCTGGAGT
CCCACTGAGCAGGAGAGAGGAGCG
>1777
CCCTCCAGGTCTTCAGGGAATGCCTGGCGAGAGGGGAGCAGCTGGTATCGCTGGGCCC
AAAGGCGACAGGGTAAGTACTGAGGTTACAGCCTCCTCACCAAAGCTGTGGCTTTGCC
AATGTCCTGCCCCTTGTGATCGCT
>1778
AGAGGCCGGCCAGAAAGGCGATGCTGGTGCCCCTGGTCCTCAGGGCCCCTCTGGAGC
ACCTGGGCCTCAGGTGGGTAACGCTGCACTCCAAGAATTGTTCCCTCAAGGAAGGGCT
CCTGGCGTGCAGATGGGAAGGCCCC
>1779
TGAACCCGGCCTCCAAGGTCCTGCTGGACCCCCTGGCGAGAAGGGAGAGCCTGGAGA
TGACGGTCCCTCTGTAAGTCCCTCACCAGGCCCATGCCAAGGTCCCTGGGAGCAGGGT
CGTGAGTGGCTTCTGAGCTCACAGA
>1780
TCAGAGAGGCATCGTCGGTCTGCCTGGGCAACGTGGTGAGAGAGGATTCCCTGGCTTG
CCTGGCCCATCGGTGAGTGTGGGGTATCCCTCCCTCCATCCAAGCTGGCCCTGCCTGCC
AAGGCTTCTACCTCCCTCAGCAC
>1781
AGACAGAGGTCCTCCTGGCCCCGTGGGTCCTCCTGGCCTGACGGGTCCTGCAGGTGA
ACCCGGACGAGAGGTGAGCAGTGAGACCCCCTGGGGTGGCCCTGATTGGGGAGAGG
GGCCCTGTGAGTCTCTGTGCTGGGTCA
>1782
AGCCCCTGGGCCCCCTGGCTCCCCTGGCCCCGCTGGTCCAACTGGCAAGCAAGGAGA
CAGAGGAGAAGCTGTAAGTATCCTGGAATTCAGTAAAAGCCGCCTTCCCCTGCGCGGT

GGGGCTGAGGCAGTCCCTGGGTTTC
>1783
AGAGCCTGGCGAGAGAGGCCTGAAGGGACACCGTGGCTTCACTGGTCTGCAGGGTCT
GCCCGGCCCTCCTGTGAGTGTCACTGCCTGCGTGGGACTTCCCGAGGCCTCCTGCCAC
ACAGAGCCCACTTGAGCTCCCTGTG
>1784
TGCTAATGGAATCCCTGGCCCCATTGGGCCTCCTGGTCCCCGTGGACGATCAGGCGAA
ACCGGTCCTGCTGTAAGTGTCCTGACTCCTTCCCTGCTGTCGAGGTGTCCCTACCATCC
GGGAGGCTTGAGCTCTTTTTTGC
>1785
GAGGGCTCCCGCAAGAACCCTGCTCGCACCTGCAGAGACCTGAAACTCTGCCACCCT
GAGTGGAAGAGTGGTAAGCTTGGAGAACAGGATCCCCTGCCCCGGGAAGCAGGGAGT
CATCCCTTAGGCCTAGCAGCAAGGGA
>1786
CTGGTGGAGCAGCAAGAGCAAGGAGAAGAAACACATCTGGTTTGGAGAAACCATCAA
TGGTGGCTTCCATGTGAGTACCTGGGTGCCCTAGATGATGAGCAGAGATGGCTCCTCA
AACTCTTTCTTTTCTTTCTCCCTGG
>1787
CAATGACGTGGAGATCCGGGCAGAGGGCAATAGCAGGTTCACGTACACTGCCCTGAA
GGATGGCTGCACGGTGAGTGGGGCTGCCAGAGAGAAGAGCTGCCTGTGCCCAAACTG
CCTGGAGCAGGGCTGAGGGTTGGCCC
>1788
CTGGTGGCCGCGCTCTGCGCCGGGATCCTGGCAGAGGCGCCCCGAGTGCGAGCCCAG
CACAGGGAGAGAGGTGGGCACCGCAAGGGAGGACCCGGCCCGGGGCACTGCACCGT
GCCACCCTCCGCTCCACTCGGCCTTCA
>1789
TGCCTTTCTCTGGAGCAGCCAGTGCACAGGGTGTGCGCTTTGCCACAGTGCAGTACAG
CGATGACCCACGGTGAGTAGGGGCCCTGGGGGCTGGGGACCACGAGATCCCCACCAA
GACTCCTTCCCCAGAGAGGGAGGAG
>1790
AGGGGCTGCAATTCTCCATGTGGCTGACCATGTCTTCCTGCCCCAGCTGGCCCGACCT
GGTGTCCCCAAGGTGATCCCTACCCCTACCATGCCTCCCAAGATGACCCCAAATGAAG
TGTCCAGGGGAACCGTGATTTGAC
>1791
AAGTCCCAGGACCTGGTGGACACAGCTGCCCAAAGGCTGAAGGGGCAGGGGGTCAA
GCTATTTGCTGTGGGTAAGGACCGAGCAGGAGTGACAGGTCAGCTGGGGGGTGGGGG
CAGTCAGAGAGCATGTGGGTGACTGAG
>1792
AGGACACTACTGCCCCTCGTTTCCCGGAGAGTGTGCACGACTGCTGGTGGCGTGCCTG
TGACCCGACCTCGTGAGTTCCTGCCCACACGGTGTACCCTGACCTAGACCCCGGACCC
CAATCCCCACTTGGCAGTGCTGAT
>1793
GACTGGCTACAAGGTCCAGTACACTCCTCTGACGGGGCTGGGACAGCCACTGCCGAG
TGAGCGGCAGGAGGTAGGATGTCAGGAGTATAGGTGGTGGCTGGGGACTTGGCTGGG
CAAGATAAAGTGACCTCTTGCCCTGG
>1794

TACCAAGTGACTGTGATTGCCCTCTACGCCAACAGCATCGGGGAGGCTGTGAGCGGGA
CAGCTCGGACCAGTGAGCAATTCTGCCAGCCTCTGACCCCATTCACCTAACCCCCGAC
CCCAGTACCCCCTCCCACTTCTGA
>1795
AGCCTCCTGGTGGCCTGGCGGAGTGTGCCAGGTGCCACTGGCTACCGTGTGACATGGC
GGGTCCTCAGTGGTGAGTGAGAGATGTGGCTGAGGGAGTCCCCGCGCCTCAGACAAG
GCTGTAGAGTCCTGAGTCTGCAAGC
>1796
TATGAGGTGACCGTGAGCACCCTATTTGGCCGCAGTGTGGGGCCCGCCACTTCCCTGA
TGGCTCGCACTGGTGAGAAGGCTGGGCACTTTCTTCAGGCTGGGACGGGCAGGCAGG
GCAAGGCCCGGAGGCCACTGACATC
>1797
TCCATCCTCCTTTCCTGGAACTTGGTGCCTGAGGCCCGTGGCTACCGGTTGGAATGGCG
GCGTGAGACTGGTCAGTGCGGGGGAAGGGATGGACAGGCAAGGGTCAGGGCATGGC
CTCTGCTGGTCTGACCCTGTTATCT
>1798
TACCGCCTCACACTCTACACTCTGCTGGAGGGCCACGAGGTGGCCACCCCTGCAACCG
TGGTTCCCACTGGTGAGGGCTCTGTGGGCTGGGCCAGTGAGTGGGGGAGGTGTCGGA
GCCCCAGGCTGCCTCTATGCTGTGC
>1799
CGGGTGCGAGTGTCCTGGAGCCCAGTCCCTGGTGCCACCCAGTACCGCATCATTGTGC
GCAGCACCCAGGGTGAGGTGGACGCAGCCAGCACCCCCACCACACACACTGAAGTTC
CAGCCTGAGGGGTCTGAGTGTACCT
>1800
TACACTGTGCGGGTGTCTGCTCGAGTGGGTCCCCGTGAGGGCAGTGCCAGTGTCCTCA
CTGTCCGCCGGGGTGAGTACTGCAGGAGGCTTGTGGAGGACAGCCTGCCTGCCTCACT
CTGGTCCTGGTTCTGACTTCTGAC
>1801
CGAGTGAGGGTGGCCTGGGGACCCGTCCCTGGAGCCAGTGGATTTCGGATTAGCTGGA
GCACAGGCAGTGGTCAGTGTGGGGTGTGTGGGGGGACTGCCAAAGGGACCAATGAG
GGTATGGGTGCCAGAGGGGACAGGCA
>1802
TACCAGGTGGCTGTGTCGGTACTGCGAGGCAGAGAGGAGGGCCCTGCTGCAGTCATC
GTGGCTCGAACGGGTCAGGCCCTGCCCCCGTCCCTTGGCTCTCTGCCTCCATTGCTCTT
TCAGACCCCCATGCCTTCCCTTGC
>1803
TCTGTCACCATTACCTGGACCAGGGTTCCTGGCGCCACAGGATACAGGGTTTCCTGGC
ACTCAGCCCACGGTGGGGACTGGGGTTTGGGAGGGGGCAGGTCAGGGTGGAATGGG
GGCTGGGGGTTTATTGGGGGTTCAGG
>1804
TATACGGTGCATGTGAGGGCCCATGTGGCTGGCGTGGATGGGCCCCCTGCCTCTGTGGT
TGTGAGGACTGGTGAGTGGACCCTGGCCAGCTACTAGCCACACCGCATTAGCTACCCT
GCCCTGCTGTGTGTTCCTGATTG
>1805
GTTCTACGGATCACCTGGGTAGGGGTCACTGGAGCCACAGCTTACAGACTGGCCTGGG
GCCGGAGTGAAGGTATGGCTCCCTGACGCCACCCCTGTCCTTCCTGGCTGGGACTGCT

CACCCCTAACCATTGCTGTATGCC
>1806
TACTCAGTGCGAGTGACTGCACTTGTCGGGGACCGCGAGGGCACACCTGTCTCCATTG
TTGTCACTACGCGTAGGCAGAGCATGCGCTGGAGAGCTTCGGATGGGTGGTGTGGATG
GTTTGGGGATCCGGGCTTGTGCTC
>1807
CACTCGCTGAGGCTGCGCTGGGAGCCGGTGCCCAGAGAGCAGGGCTTCCTTCTGCAC
TGGCAACCTGAGGGTGAGAGTGTCCCCAGAGAAGTTAGGACCATTGGGGCAGGCTGT
GGCAGACTCCGCAAGGTAGGCGAGAC
>1808
TACCGCGTGAGGCTGAGTGTCCTAGGGCCAGCTGGAGAAGGGCCCTCTGCAGAGGTG
ACTGCGCGCACTGGTAAGCCTCACCTTGGCGTGCTCCTCCCCTGCTGATGACCCACCC
TGACTTCCTGCACCCCGACTCTGAG
>1809
GCCTGGACTCCAGTGTCCAGGGCATCCAGCTACATCCTATCCTGGCGGCCACTCAGAG
GCCCTGGCCAGGGTGAGGGGGAGGCCAGGATTTGGGTGGGCTGGCAGTTGGGGCTCT
GTGGAGACAGCTTTTTAATCAAGTT
>1810
TACATCTTCTCCCTGACGCCTGTCCTGGATGGTGTGCGGGGTCCTGAGGCATCTGTCAC
ACAGACGCCAGGTATAGTGGGCATAGTGGGAAGGGCAGAAAGGTGTGTCTGGGTGGG
CTGCCCGCTTCAGTAACTTGTTCC
>1811
GGAGGCTACGAGGAGGGTCCTGGAGCGTCTGGTGTTGGCACTTGGGCCTCTTGGGCC
ACAGGCAGTTCAGGTTTGGCCCTGGGGCAGCCAGATTCTACCTCTCCCTGAGGCCCCC
CACCCAGGCTCCATACCAGTTACCA
>1812
CATGACCTTGGCATTATCTTGCAAAGGATCCGTGACATGCCCTACATGGACCCAAGTGG
GAACAACCTGGGTGAGGACTGCAGCAGGCATGGACTCCTGGGGCTATCCACTGGGAG
CTTGGTGCCCCAGGGTTCTGACAT
>1813
ATGGTTCTGCTAGTGGATGAACCCTTGAGAGGTGACATATTCAGCCCCATCCGTGAGGC
CCAGGCTTCTGGTAAGGAGTAGGCTGATGGGGAAGGGGTCTGGGAATAGGGGTGGCC
CTGAAAAGGCTATCATGCAGCCAC
>1814
TGGGCCAAGCCTGGACCAGGCAGTCAGTGGTCTGGCCACAGCCCTGTGTCAGGCATC
CTTCACTACTCAGGTTTGGAAGAGGCCTCTGGGGGACTGGGTGGTAGAATATAAGGGG
TCTGGGGGTTCTTGGTAGAAATGTT
>1815
AGCAGATGGGCGTCCAGGCAGCCCTGGCCGCGCCGGGAATCCTGGGACCCCTGGAGC
CCCTGGCCTAAAGGTGAGCAAGCCTTGTCCTGCAGGTCAGGGTGGGCGCTGCTGAGT
GGGTGGGGTGGCTCCGACTGTTCTGC
>1816
GGCTCCCGGACAAGTCATCGGAGGTGAAGGACCTGGGCTTCCTGGGCGGAAAGGGGA
CCCTGGACCATCGGTAAGTGCAGGGTATGTGGAGGCAAGTGATGTGTAGTGGGGGGAC
CAACACCAGGGGGGCGAGAGTGAGG
>1817

TGGACCTCGTGGACCACTGGGGGACCCAGGACCCCGTGGCCCCCCAGGGCTTCCTGG
AACAGCCATGAAGGTGACAGCCTCATGAGTGCCATGTGATGCGGAGACCTGGTGACCC
CATTTGAACCCACATAACCCCTGCC
>1818
GCCTCCTGGAGAGAAGGGAGACCCTGGGCGGCCTGGCCCCCCAGGACCTGTTGGCCC
CCGAGGACGAGATGTAAGAGGCTGGAGTCGGGGGAGTCATGGCGGGTAAGGGAGTAG
GGCTGTTGCCAGCATCATGGGGGTTC
>1819
GGCACCTGGAGTTCGGGGGCCTGTGGGTGAAAAGGGAGACCAGGGAGATCCTGGAG
AGGATGGACGAAATGTGAGTCCCAGCCTATGACTCCTCACCCCAACCTTAACCCTCCA
ACCAGCCAATTCCCAATACCTGATCC
>1820
GGACCGCGGACAAGAGGGTCCTCGAGGGCCCAAGGGTGATCCTGGCCTCCCTGGAGC
CCCTGGGGAAAGGGTGAGTGTGATTGGTCCTCGGGGGTGCAGGCATGGGAGGGCCTG
CTCTGATTTCTTCCTCCCCTATCCTC
>1821
TCCCCCTGGGCTGGATGGCCGGAGCGGACTGGATGGGAAACCAGGAGCCGCTGGGCC
CTCTGGGCCGAATGTGAGTCTTGGTAGTCCTGCCTGGTTGTCCCCTTCCCCTACCCCTT
CACATGAGGACCCTAGACCCCAGC
>1822
TGGAGCTCCTGGTATCCTTGGACCCCAGGGGCCTCCAGGCCTCCCAGGGCCAGTGGGC
CCTCCTGGCCAGGTGAGTGTCCTGGGTCATTCTGGGACTTCAGAGCATTAGAAGCCAT
GATGTTTCAATGGGCAACCCTCCT
>1823
CCTGCCTGTGCCCGAACGGCGTCGAGGCCCCAAGGGGGACTCAGGCGAACAGGGCCC
CCCAGGCAAGGAGGTGAGCAGAAGTGGCTCAGTGGGTTGTGCCCCGTGGAGTGGGGT
GTAGCTGTACAGCCACCAGCATTCTC
>1824
AAAGCCTGGTATTCCCGGGCTCCCAGGCAGGGCTGGGGGTGTGGGAGAGGCAGGAAG
GCCAGGAGAGAGGGTGAGGCTGGGGGCTGGCCAGGAGAGTGAGGGAAGAGGGGTTG
GGAGGGGTGGGACCCCCCATGGGCTTG
>1825
GAAGCCGGGAGCCCCAGGTCGAGATGGTGCCAGTGGAAAAGATGGAGACAGAGGGA
GCCCTGGTGTGCCAGTATGTGTTCTGGGGGCAGCTCGCTAGGGTGTGGTGCCCAGCTG
TGGGCCTGAAATATGAGGAGTGGGGC
>1826
TGTTGTTGGGTTCCCGGGTCAGACAGGCCCTCGAGGAGAGATGGGTCAGCCAGGCCCT
AGTGGAGAGCGGGTGAGGGGCTGGGAACAGCATGTAGGGGCATGGTGAATCCATGGT
GGGTGAGGAGGGGGCCCTGCCTTGG
>1827
TCTGGCAGGCCCCCCAGGGAGAGAAGGAATCCCAGGACCCCTGGGGCCACCTGGACC
ACCGGGGTCAGTGGTGAGTAGAGGTCCCTAAACCCCACGTATTTGATTTCCTGTCCTCG
TGAGGACTTAGGATGGGGCGGGCC
>1828
TGAACGAGGGCCTCGGGGCTTGGATGGTGACAAAGGACCTCGGGGAGACAATGGGGA
CCCTGGTGACAAGGTACAGGGAAGAGGGTGGTGTACTCTCTGTGGGGTCCTGTGGCT

GTGGTGGTTCCACAGCTACTGTGAGG
>1829
AAAGGATGGAGTGCCTGGTATCCGAGGAGAAAAAGGAGATGTTGGCTTCATGGGTCCC
CGGGGCCTCAAGGTAGGAAAGAAACAAGATTGGTTTTTTTCTGGTCAGGAAGGCCTAA
TGTTGAAGGGCGAGGGACCTGGGG
>1830
CTCTGCTGGTCCCCCAGGGCCACCTGGCAGTGTTGGTCCCAGAGGCCCCGAAGGACTT
CAGGGCCAGAAGGTAAGGGGCCCCAGCTCTGACTCCTGATCCCTGAACCTTCCCTACC
CTTCCAACCTCATAACCATCTTCC
>1831
AGGTCCCCCCGGAGAGAGAGTGGTGGGGGCTCCTGGGGTCCCTGGAGCTCCTGGCGA
GAGAGGGGAGCAGGTGAGTAGGGATTCCAAGGCTTGGGTCAGAGATCGGGGTGACTT
CTGTTGTCCCTGAGGTCAGAGGTCAC
>1832
GCTGCAGACACTGCCGGCTCCCAGCTCCATGCTGTGCCTGTGCTCCGCGTCTCTCATGC
AGAGGAGGAAGGTGAGGACAGCTGAACCCGTGGGGCAGCTATGGGTGGGGCCGAGA
CACGCACATGGGTGTCCATGAATGC
>1833
GAGTACTCTGAATACTCCGAGTATTCTGTGGAGGAGTACCAGGACCCTGAAGCTCCTT
GGGATAGTGATGGTGAGAATGGGGGGCTGCGCAGCGGGGTCTGGGGAGGGGCAGGCA
GGGCTGAGCCCTGCTGACCTCCCCC
>1834
AACCGTTTTGGGACCCGTGAGGCCTGCGAGCGCCGCTGCCCACCCCGGGTGGTCCAG
AGCCAGGGGACAGGTATGGGCTGAGCCCCCACCGTGGGGAACTGGGCACTGAGCCTG
CCTGGATCGGGTTCTGGGGGAGGAGT
>1835
GATCCTCCTGCTTGTCGCCCTCTCTGTGGCCTATGCAGCTCCTGGCCCCCGGGGGATCA
TTATCAACCTGGTAAGAGGGTCTTCAGGGGAGGCGCCTCCTGAGTCCCCAGATGAAGA
CTTCCTAAGCTTGGACCTTGACC
>1836
GCATTCAAGTGCGCTGGGCCTGGCCCGCTGCACATCCATGGCCAGCGAGAACAGCGA
GTGCTCTGTCAAGGTGGGTGCCTGAGGACTTGGCGTGGGTGGGGGAGAATGGGGAGT
AGGGCCCTGAACACTTGTGAGTGGGA
>1837
CTGGCCGCGCAGGCCCTGAGGGCTCGCGGCCCCAGTGGCGCGGCCGCGATGCGCTCC
ATGGCATCTGGAGGTACTCGGGTCTCCGGGCGTGCCAGGGACCAGAGTGTTGCCCTCC
CAGGGTGGTCCCAGGGCGGCAAAGC
>1838
TGTTCCCACTGATGAAGAGCAGGCGACTGGGTTGGAGAGGGAGATCATGCTGGCTGC
AAAGAAGGGACTGGTAAGAGAAACTCCCTTCTGTCTTCTGTGTAACTTATGGCCTTGG
ATGTGTTCATAGTGGTCTCCTCTCT
>1839
GCTTCAGGCACCAGGGAAGACCCTAATTTAGTCCCCTCCATCTCCAACAAGAGAATAG
TAGGCTGCATCTGTAAGTACCTCACCTCTATTTTTTATCCACTTGCTTAATATATCCTACA
ATAGTGTGTAAGCTGCCTCAA
>1840

CTCCCAATCTATCTGCTTGGCCTGACTCAGAAATTCGGGCCCATCTACAGGCTCCACCT
TGGGCTGCAAGGTGAGAGGCTGATCTCGCTCTGGCCCTCACCATAGGAGGGGGCGGA
GGTGACGGAGAGGGTCCTCTCTCC
>1841
AAGAGGACCATTGAGGAAGCCATGGTCAAAAAGTGGGCAGACTTTGCTGGCAGACCT
GAGCCACTTACCTGTAAGGGCTGGGGGCATTTTTTCTTTCTTAAAAAAATTTTTTTTAA
GAGATGGGTTCTTGCTATGCTGC
>1842
AGCCCTGCTGCTGGGCATCCGTGACTCCATGGAGCCAGTGGTGGAGCAGCTGACCCA
GGAGTTCTGTGAGGTAAGGCTGGGCTCCTGAGGCCACCTCGGGTCAGCCTTGCCTCTC
ACAGTAGCCCCCGCCCTGCCCGCTG
>1843
TCTCCGGAGGCTGAAGCAGGCCATAGAGAAGAGGGATCACATCGTGGAGATGCAGCT
GAGGCAGCACAAGGTGGGGACTGTACGTGGACGGCCTCCCCTCGGCCCACAGCCAGT
GATGCTACCGGCCTCAGCATTGCTAT
>1844
AAGAGGACCATTGAGGAAGCCATGGTCAAAAAGTGGGCAGACTTTGCTGGCAGACCT
GAGCCACTTACCTGTAAGGGCCGGGGGCATTTTTTCTTTCTTAAACAAATTTTTTTTTG
TTAGAGATGGGGTCTTGCTATGT
>1845
AGCCCTGCTGCTGGGCATCCGTGACTCCATGGAGCCAGTGGTGGAGCAGCTGACCCA
GGAGTTCTGTGAGGTAAGGCTGGGCTCCTGAGGCCACCTCGGGTCAGCCTCACCTCTC
ACAGTAGCCCCCGCCCTGCCCGCTG
>1846
CCATTGAGGAGGAATTCTCTCTCCTCACCTGCAGCATCAATGTTACCTCACCTTCGGAG
ACAAGATCAAGGTGCCTCACAGCCCCTCAGGCCCACCCCCAGCCCCTCCCTGAGCCTC
TCCTTGTCCTGAACTGAAAGTAC
>1847
GTATCCAGGAGGTGTTAAAAACCTGGAGCCACTGGTCCATCCAAATTGTGGACAGATT
CCCTTTCTCAGGGTGAGGACCTGGAGCCTAGACACCCCTGGGTTGTGGGGGAGAGGC
TGGGGTGGAGGGAGAGGCTCCTTCC
>1848
AGGTCTCCGGAGGCTGAAGCAGGCCATAGAGAAGAGGGACCACAAGAGAGAACAGC
TGAGGCAGCACAAGGTGGGGACTGTGTGTGGACGGCCTCCCCTCGGCCCACAGCCAG
TGATGCTACCGGCCTCAGCATTGCTAT
>1849
GATCGGTGGCACTGAGACCACAGCAAACACCCTCTCCTGGGCCGTGGTTTTTTTGCTT
CACCACCCTGAGGTGCGTCCTGCGGACAAGCAAAAGGCTCCTTCCCAGCAACCTGGC
CAGGGCGGTGGCCACCCTCACTCAG
>1850
CACCACAGAGATTAACTTCAAGGTTGGGGAGGAGTTTGAGGAGCAGACTGTGGATGG
GAGGCCCTGTAAGGTGAGTGCCAGAAGGGGCTCCAGGGTCATGGCGTCATTGCCCTGC
CTCTCAACCTGCCATTTTCCAGGCT
>1851
GCTCCTGAAGGGAGAGGGCCCCAAGACCTCGTGGACCAGAGAACTGACCAACGATGG
GGAACTGATCCTGGTAAGTCCTGCCTCCTCCCCACTAATAGCAAACCCAGTGCTACCTT

CCAAGATTCTCTGGGAGACCCCAG
>1852
GACGTGACCATGGAGAAGCTGTTGTGTTTCTTGGTCTTGACCAGCCTCTCTCATGCTTT
TGGCCAGACAGGTAAGGGCCACCCCAGGCTATGGGAGAGTTTTGATCTGAGGTATGGG
GGTGGGGTCTAAGACTGCATGAA
>1853
GCTTTTTGACCACGCTATGCTCCAAGCCCATCGCGCGCACCAGCTGGCCATTGACACCT
ACCAGGAGTTTGTAAGTTCTTGGGGAATGGGTGCGGGTCAGGGGTGGCAAGAAGGGG
TGACTTTCCCCCACTGGGGAAGTA
>1854
CCAGACCTCCTTCTGCTTCTCAGACTCTATTCCGACACCCTCCAACATGGAGGAAACG
CAACAGAAATCCGTGAGTGGATGCCGTCTCCCCTAGGCGGGGATGGGGAGACCTGTG
GTCAGGGCTCCCGGGCAGCACAGCC
>1855
TGACACCTCGGACAGCGATGACTATCACCTCCTAAAGGACCTAGAGGAAGGCATCCAA
ACGCTGATGGGGGTGAGGGTGGCGCCAGGGGTCACCAATCTTGGAACCCCACTGGCT
TCGAGGGCTGGGGGAGAGAAATACT
>1856
GGAGCATGTGAATGCCATCCAGGAGGCCCGGCGTCTCCTGAACCTGAGTAGAGACACT
GCTGCTGAGATGGTAAGTGAGAGAATGTGGGCCTGTGCCTAGGCCACCCAGCTGGCCC
CTGACTGGCCACGCCTGTCAGCTT
>1857
CCTCACCAAGCTCAAGGGCCCCTTGACCATGATGGCCAGCCACTACAAGCAGCACTGC
CCTCCAACCCCGGTGAGTGCCTACGGCAGGGCCTCCAGCAGGAATGTCTTAATCTAGG
GGGTGGGGTCGACATGGGGAGAGA
>1858
AGCCTTCCTGAGAAGATGCAACCAATCCTGCTTCTGCTGGCCTTCCTCCTGCTGCCCAG
GGCAGATGCAGGTGAGTGACCGTCTCCACCCTCGGGGGCCCAACCCGATCCCACAGG
TCTCCTGCCCTTTCTCCACATTCC
>1859
ACGCCATTATTACGACAGTACCATTGAGTTGTGCGTGGGGGACCCAGAGATTAAAAAG
ACTTCCTTTAAGGTAAGACTATGCACCTGCCTGGATTGGCTCTTGGGAGAAAGATCTTT
GGGGAATATCTGAGACCTGGAGA
>1860
AGCCTTCCTGAGAAGATGCAACCAATCCTGCTTCTGCTGGCCTTCCTCCTGCTGCCCAG
GGCAGATGCAGGTGAGTGACCGTCTCCACCCTCGGGGGCCCAACCCCATCCCACAGG
TCTCCTGCCCTTTCTCCACATTCC
>1861
TGTGAAAAGACCCATCCCCCATCCAGCCTATAATCCTAAGAACTTCTCCAACGACATCA
TGCTACTGCAGGTGAGGCACACTCCTGCCACTCTTGCTCTTCTTGGTCCAGTTGGTTCC
ACTCCCCCTGGAATGCCGGCCC
>1862
CTCTGAAGAGGTGCGGTGGCTTCCTGATACAAGACGACTTCGTGCTGACAGCTGCTCA
CTGTTGGGGAAGGTGAGGAGCAGAAAACAGCCCACACCCTCCTGGAAACACTCCACA
GAGACCCCTGCCTTCTTCCCAAGGA
>1863

TGTGAAAAGAGCCATCCCCCATCCAGCCTATAATCCTAAGAACTTCTCCAATGACATCA
TGCTACTGCAGGTGAGGCACACTCCTGCCACTCTTGCTCTTCTTGGTCCAGTTGGTTCC
ACTCCCCCTGGAATGCCGGCCC
>1864
ACGCCATTATTACGACAGTACCATTGAGTTGTGCGTGGGGGACCCAGAGATTAAAAAG
ACTTCCTTTAAGGTAAGACTATGCACCTGCCTGGATTGGCTCTTGGGAGAAAGATGTTT
GGGGAATATCTGAGACCTGGAGA
>1865
CATGGTCTCTTTGGGCGGAAGACAGGTCAGGCCCCTGGATACTCTTACACAGCCGCCA
ATAAGAACAAAGGTAAGAGTCACTTGTTAAATAAAACAACACAAAATGCAGGAATATA
ACATGTGGCAAACTATCAGGAGTG
>1866
CATCGTCCCCCTGGGTGTGACCCATATGACATCCCGTGACATCGAAGTACAGGGCTTCC
GCATCCCTAAGGTAGGCCTGGCGCCCTCCTCACCCCAGCTCAGCACCAGCACCTGGTG
ATAGCCCCAGCATGGCTACTGCC
>1867
CCTGCCACTGCCCGGGCTGGGCAACCTGCTGCATGTGGACTTCCAGAACACACCATAC
TGCTTCGACCAGGTGAGGGAGGAGGTCCTGGAGGGCGGCAGAGGTGCTGAGGCTCCC
CTACCAGAAGCAAACATGGATGGTG
>1868
CGGCCGACCGCCCGCCTGTGCCCATCACCCAGATCCTGGGTTTCGGGCCGCGTTCCCA
AGGCAAGCAGCGGTGGGGACAGAGACAGATTTCCGTGGGACCCGGGTGGGTGATGAC
CGTAGTCCGAGCTGGGCAGAGAGGG
>1869
AAGAAGTCGCTGGAGCAGTGGGTGACCGAGGAGGCCGCCTGCCTTTGTGCCGCCTTC
GCCAACCACTCCGGTGGGTGATGGGCAGAAGGGCACAAAGCGGGAACTGGGAAGGC
GGGGGACGGGGAAGGCGACCCCTTACC
>1870
CCCTCGCTTCCTCAGGCTGCTGGACCTAGCTCAGGAGGGACTGAAGGAGGAGTCGGG
CTTTCTGCGCGAGGTGCGGAGCGAGAGACCGAGGAGTCTCTGCAGGGCGAGCTCCCG
AGAGGTGCCGGGGCTGGACTGGGGCC
>1871
CAGGATGACCTGGGACCCAGCCCAGCCCCCCCGAGACCTGACTGAGGCCTTCCTGGC
AGAGATGGAGAAGGTGAGAGTGGCTGCCACGGTGGGGGGCAAGGGTGGTGGGTTGA
GCGTCCCAGGAGGAATGAGGGGAGGCT
>1872
GGGATGGTGACCACCTCGACCACGCTGGCCTGGGGCCTCCTGCTCATGATCCTACATCC
GGATGTGCAGCGTGAGCCCATCTGGGAAACAGTGCAGGGGCCGAGGGAGGAAGGGTA
CAGGCGGGGGCCCATGAACTTTGC
>1873
CATCGTCCCCCTGGGTGTGACCCATATGACATCCCGTGACATCGAAGTACAGGGCTTCC
GCATCCCTAAGGTAGGCCTGGCGCCCTCCTCACCCCAGCTCAGCACCAGCCCCTGGTG
ATAGCCCCAGCATGGCTACTGCC
>1874
CACCCCGAACACTTCCTGGATGCCCAGGGCCACTTTGTGAAGCCGGAGGCCTTCCTGC
CTTTCTCAGCAGGTGCCTGTGGGGAGCCCGGCTCCCTGTCCCCTTCCGTGGAGTCTTG

CAGGGGTATCACCCAGGAGCCAGG
>1875
GGGAGCAGGGTTATGAGCACCTGCACCTGGAGATGCACCAGACCTTCCAGGAGCTGG
GGCCCATTTTCAGGTAAAGCCCTCCCTGGCCCTCGCTGGGAACACCCAGATCCCTGCC
CCTGCTGCCCAGGACCCTGCCAGGC
>1876
GCAGGATGATCCTGGAGCCCTGGGTGGCCTACAGACAACATCGTGGGCACAAATGTGG
CGTGTTCTTGTTGTAAGCGGCGAGTTGGGAGCTGAGAGCTGGGAGCAGGGTGGGCAG
CCTGGGTGTAGGGGGGGAGGCGAGAG
>1877
GTGCTGCAGAACGCCCGGGGGAGCCTGACCCTGGACGTCCAGCCCAGCATCTTCCACT
ACACCATAGAAGGTGTGGGCCATGCGGGAAGGTCCAGCCCCAGAGACCCTGGAGTGG
CCAGGGATGGGGATGGAGGACTGAA
>1878
CTGTCTCGCTGGATCAGCCCCAAGGTGTGGAAGGAGCACTTTGAGGCCTGGGACTGCA
TCTTCCAGTACGGTGAGGCCAGGGACCCGGGCAGTGCTATGGGGAAGGGACACCATG
GGGGCCCAATTTCTCCTTCTCCACC
>1879
GAAGGCGGAACTGTCACTAGAAGCCATCAAGGCCAACTCTATGGAACTCACTGCAGG
GAGCGTGGACACGGTCAGGCCAGCAACCAGCCCCACCCAGAGAGGGTGATGCCAAG
CCTGCCTCCCAGGCACTGCCTGCCAAT
>1880
TCAGTGAACATCCCCAGAAGGCAACCACCGAGCTGCCCTTGCTGCGGGCGGCCCTCA
AGGAGACCTTGAGGTGGGTGCTGGCTGAGGCCTCCCTGTGGCCCTGGCCCCCTGCTG
GAGAGCAGCCCCCACTGGGTGGTGGC
>1881
TGTGGGTCTGTTTTTGGAGCGAGTGGTGAGCTCAGACTTGGTGCTTCAGAACTACCAC
ATCCCAGCTGGGGTGAGTGAGCCCCACACCCCTCGAGCTGAGAACCTCCCTCCCCAGT
CATTCCCTGATCCCTGCTCTGCAC
>1882
TGGCTTTGGCATGCGCCAGTGCCTCGGGCGGCGCCTGGCAGAGGCAGAGATGCTGCT
GCTGCTGCACCACGTAAGCAGGCCTGGGGGCGGGGGCGGGACCTGGGCAGCAGAGG
CGGGACCTGCACACTGGGGGCGGGGCT
>1883
TTTCCCGCTTCCCATCATCGGGAACCTCTTCCAGTTGGAATTGAAGAATATTCCCAAGT
CCTTCACCCGGGTAAGAGAAATAGTGTTGATTTTAGGGAGAATAACTCAGCAATTGGAT
CTGGTATGTGTGTATTCAACTC
>1884
CTGCTGGACTACAAGGACGAGTTCTCGGGCAGAGGCGACCTCCCCGCGTTCCATGCGC
ACAGGGACAGGGGTGAGTCCGCGTCCCTGGCACGGAGCGGGGGGTGCATAACACGCC
CCGGGACAGTTACGGGCGCTAGCCA
>1885
AAACAGGGCAATGAGAGCCGGATCCAGAGGGAGGCCCACTTCCTGCTGGAAGCACTC
AGGAAGACCCAAGGTGCGTATCTGCTGCCTAGCAGGGCCCAGTCCTCTTGCAGACCAG
CGGTGTGGGGAGCCCTGGCTGGGAC
>1886

TGAGAAGTTTCTAAGGCTGATGTATTTGTTTAATGAGAACTTCCACCTACTCAGCACTC
CCTGGCTCCAGGTGAAGCCACTTTCCTCTTTCATCAGTCATCAACTGTAGAGTTTACGT
TAGAAAAAGAAGGAAAATTTGG
>1887
GCACCATCAATCTCTGGACCCCAACTGTCCCCGGGACCTCACCGACTGCCTGCTCGTG
GAAATGGAGAAGGTAGGCTCGGCCTCCCATGATGTGGGCTCTCCGGGGTGGGCAGAG
AATGCACAATTTCAGATTTACAGAG
>1888
GGGACAGAGACCACCAGCACAACTCTGAGATATGGGCTCCTGATTCTCATGAAATACC
CTGAGATCGAAGGTAGGCAAGTGACTGAAGGGACACCGTGCGTGCGGCTGCATCTCC
CTGGATGGCCAGCCTTGCACATTTT
>1889
CCTCGTGCCCTCCAACCTGCCCCATGAAGCAACCCGAGACACCATTTTCAGAGGATAC
CTCATCCCCAAGGTTAAGCAATGAGCCTGCAGCACACAGCATGAACACCATCCTATCA
CTAATCGCCTTCCTGCCAGGGAGC
>1890
AAGCCAGAACACTTCCTGAATGAAAATGGAAAGTTCAAGTACAGTGACTATTTCAAGC
CATTTTCCACAGGTGAGAAAGATCAGAGGCAGTACCTTCCCTTGAGGAGCAGCCCACA
CTCCTCATCTCCCCTCCACATGTG
>1891
TCTGGGGAAGACAACCAGGACCTTGCTATCTCCTTTGCAGGAAATGGACTCTCTGCTC
TTAGAACCTCAGGTAGGAGACATCAATCTTGCACATCTGCAAAATCTAGAAAAAAAGG
ATTGGAGAAAGGATCTGGAGTCAA
>1892
CTAACCAGCGCGCGCGCGTCGACGTCGAGCGCGACAACCTGCTCGACGACCTGCAGC
GGCTCAAGGCCAAGTGAGGGCCCGGCACCCCAGACTCCTCTTTCTGCGGGCAGGGCA
CAGGAGGCTAGGCCTGGGGGCTGGGG
>1893
CATTGACCTGGAGCGCAGAATTGAATCTCTCAACGAGGAGATCGCGTTCCTTAAGAAA
GTGCATGAAGAGGTATACTTGGCCCCTCTTCCTGGGGTCACTGGGCCATTGGGGAAAG
CAGCCGGAAAGTGGGGTTTGGGGT
>1894
TATCCGGGCTCAGTATGAGACCATCGCGGCTAAGAACATTTCTGAAGCTGAGGAGTGG
TACAAGTCGAAGGTGGGTGGCCTCGCCCGGGGACTGGGATCTCCGTCCCCCTGGAATC
CCAAGGCCTTTGGATTGTTGCTTG
>1895
GCAGGAGATGATGGAATACCGACACCAGATCCAGTCCTACACCTGCGAGATTGACGCC
CTCAAGGGCACTGTGAGTCCCTGCCCACCTGGGCCAGGCCCTGCCCCTTCTCTGTCTG
CAGTTCACACCCTCACTTTGTGAC
>1896
TCAACGTGAAGATGGCCCTGGATGTGGAGATTGCCACCTACCGGAAGCTGCTGGAGGG
AGAGGAGAGCCGGTGAGGGGCCAGGCAGGAGCCGAGTGGGAGGTGCGGGGTGCTGG
GTGGTCCATTTCTGTCCCCAGGAGGC
>1897
GCAAAGGGGTTCTGAGGTCCATACCAAGAAGACGGTGATGATCAAGACCATCGAGAC
ACGGGATGGGGAGGTAAGTGGTCTGTCTGGGCTCCTTACCCTTGGTTGGGGCTATGGA

TGTGTCTGGGGGGACTGTCTTCCAC
>1898
CCAGTGTGTGAAGCTGAATGATGGTCACTTCATGCCTGTCCTGGGATTTGGCACCTATG
CGCCTGCAGAGGTAACAATTAATATTTTAGTGTTGAGAGTTCAAAGGAGCTAGAGTAA
GTGGAAGCTGACCAGGTTGTCAG
>1899
AAGGTCACTGAAAAATCTTCAATTGGACTATGCTGACCTCTATCTTATTCATTTTCCAGT
GTCTGTAAAGGTAGGCAGCTTGTGTGATCAAATTAATTTCACTTTTGTTCTCAGCATAAA
TATTGTTTTCATGGAGATTT
>1900
GGAAGTGATCCCAAAAGATGAAAATGGAAAAATACTATTTGACACAGTGGATCTCTGT
GCCACATGGGAGGTGAGTGTTTGGAGGTGAGAGAACGGATAAGAAAGATGAGGTAGG
ATACATCTGTTTCCTATCTTCTTAG
>1901
CTTCAACCACAGGCTGCTGGAGATGATCCTCAACGAGCCAGGGCTCAAGTACGAGCCT
GTCTGCAACCAGGTGAGGCGCCCTCAGCCTCCTCTGCCTTTCTGTTCTTCATGCCCCTG
CTTTCTGTCCTATTGGCCAAGTA
>1902
ATTTCTGCAAGTCAAAAGACATTGTTCTGGTTGCCTATAGTGCTCTGGGATCCCATCGA
GAAGAACCATGGTAATAAGAGATACAGGAAGTTTGCCGAAAACACTGGTATGATAAAA
AAGCTGGGAGAAAAAGGGATCAT
>1903
CCAGCTGCAGCGTGGGGTTGTGGTCCTGGCCAAGAGCTACAATGAGCAGCGCATCAG
ACAGAACGTGCAGGTGAGGAGCGGGGCTGTGGGCCTCAGGTCTCCTGCACAGTGTCC
TTCACACGTGTGCTTCTTGTAAGGCT
>1904
AGTTGACTTCAGAGGAGATGAAAGCCATAGATGGCCTAAACAGAAATGTGCGATATTT
GACCCTTGATATGTAAGTGATTTTGGAGATGGTTGTTCTAATTTATTTCAGAGGAGGAA
CGTAGGATGGGTGTTGAGAGTGT
>1905
GGCCGTGCGCACCCAGGAGAAGGAGCAGATCAAGACCCTCAACAACAAGTTTGCCTC
CTTCATAGACAAGGTGAGGGTCCCCTGCGTGGCTGACTGTGCCCCGCAGCCCCTTTCT
CCTGGTAGTCCCGGTCCCTATGCAC
>1906
GCCAGGAGAAGCTGAAGCTGGAGGCGGAGCTTGGCAACATGCAGGGGCTGGTGGAG
GACTTCAAGAACAAGTGAGCAACTCCACCCTCCACCCAACTGAAGTCACCTGCTCTCC
TCCACCCCTTGACCTTGGGACTAAGT
>1907
GGTAGAGCTGGAGTCTCGCCTGGAAGGGCTGACCGACGAGATCAACTTCCTCAGGCA
GCTATATGAAGAGGTATGTTCCTGGTCGCAGGAGAGTGAGGGTCCCCAGCCTTGTCAG
CGCCTCCACCCTGAGACTCAACCAG
>1908
GACTGAGATCTCTGAGATGAACCGGAACATCAGCCGGCTCCAGGCTGAGATTGAGGG
CCTCAAAGGCCAGGTATGGGCCGGGTTGGGGGTGGGAGGGTTCCTTGGACACAATCCT
GGTGAGAGGAGATAATGTAGGAAGA
>1909

TGAACGTCAAGCTGGCCCTGGACATCGAGATCGCCACCTACAGGAAGCTGCTGGAGG
GCGAGGAGAGCCGGTGGGTGTGGGTACCTCTGACCGGACCTGCTTCCCTATCCCTGGG
ACCTGGGGTGGGGACGGTGGGAGCC
>1910
CCACTCACTGGTCCAGAAGCAGCTGTAGGTGCCCACCAAGCCCATGACGACGCTGCTG
GCCAGGGTCCAGGTGAGCGGCGGCACCGCGGGGAACGGCCACTTTCACGTGCAGAG
GCCACCCCACCCGACCGCCGGCCGGG
>1911
AGCTGCTCTTCTGGGGTATCGCGATCCACTTAAGGATGAGGCAGACTTGGTGACAAGC
TGGTCTGAGCAGGTATGGGAGCCCCCTGGGGAGACGGAAGAAGGGAGGAAGTTGCCT
TCTGCCTGGGGAGGGTTTGAGAGGG
>1912
CGCCTTCAATGCCCAGCGGCTGACACTGGCCAAGGTGGCCAGGGAGCAGGTGATGGA
CACCTTAGTTCGGGTAAGTTCATCACTTCAGGGAAGCCGTGCCCCTAACCACCATGAG
CCAAAGCCCCCATCCCTGCCTGACC
>1913
TCATGGTGCTGCAGGAGGTGGTGGACTCTTCCGGCAGCGCCATCCCGCTCCTGCTTCG
AGAACTCAATCGGTGAGGAAGATTCTGTGGGTCTAATCAGGGGCACACCCAGAGGCTT
GAGGCCCTGACCCCGCCTCCCCTT
>1914
CCTACAGCACCCTGAGCAGCCCCCAGCTGGGGCGCAGCACCTACATGGAGACGTATGT
GTACTTCTATCGGTGAGCACGGAGCTGGCAGCGCAGCAGGGTGGGGGGCGGTCCTTG
GCCCTAATGTGAGGTAGGCTCATTA
>1915
GTGTACAACGATGAGGATGACGTCTTTGCCCGGGAGCCATTTGTGGCCCAGTTCTCTTT
GCCCAGCAATGGTAGGAAGGGAAGGGTGGGCTTGGTATGTGGGAGCAGAAGGGGAGT
TCTGGCTGGCTCCACTGGGGGCTT
>1916
GGCCGTAGAGAAGGAGCTGAACGCCCTCTACGATGTGTTTCTGGAGGTCTCCCAGCAC
TGGCAGAGCAAGGTAGGCCTGGGCAGTTTGTACAGGAGGGACAAGGATAGGGTGGCC
CAGCGGCCAGCCTGATGGCTGCTGT
>1917
CTGCCGGAGTCTGCTGCACACTGCGGCTGCCTTTGACTTCCCCACGAGCTTCCAGCTC
ACCGAGGAGGAGGTGAAGGGATGGGGCTGGCATGAGGAGGGAGGGCAGCAGCAGGG
TCACAGGGAAGCCCCTCGGCCCCGGG
>1918
GGAATGGCCAGAGTATTTTATGGCTGTGGCCTTCTTATCAGCACAGAGAAGCAAAGATC
CAAATTCCCAGGTAAATGAATTTCACGGGAGAATGCTTAGATACTCACAGGCAAAGAG
ATTACTGCACATGAGCAGAAAGG
>1919
GGGTGCAGTGATGACGTGTTGCCTTGGAGAAGGACAGCAGAGAATAAGCTGGACACC
AAATACCCGTACGGTAGGGCAAGTTTTATGTCCCATTCTGCTTAGTGACATAGCCTGGT
GAGTGCCTAATTAAGAAAGTTGGT
>1920
GTGAAAGGCTGTAGTATGTATGTTGCCTTGTTCCCTTGTAATGAATGCGCTAAGCTCATC
ATCCAGGCAGGTAGGAACCGGGTCTTTCCATTTGTCACATTTGCATTGCTGCCTGCTCG

TCAGTAGCTCATTGCTGATGT
>1921
ATAAATACCATGATAGTGACGAGGCAACTGCTGCGAGGCTCCTGTTTAATATGGCCGGG
GTGACATTCCGGTAAGTGGGGAGAATGCGGGCGGGGGGACGGGGTAGGGTGAAGGCT
GATGAAGAGATGTATTTGACCTTG
>1922
CGCCGTGATGCTGTCCCGATCCCGCTGTGTGTCTCGGGCGTTCAGCGCTCCGCTCTCCG
CCTTCCAGAAGGTACGGTCTGGCCGAGCCGGGGCCCCGACGGGTGAGGAGTCTGTTG
GCGGGCAGAGAGGCGGCCTGGAAG
>1923
GACTTGGTTACAGTCAAAACCCCAGCGTTTGCAGAATCTGTCACAGAGGGAGATGTCA
GGTGGGAGAAAGGTAAGATTTAGTTTCCTATTTTTTTTTTTTTTTTTTTGAGACAGAGT
CTTGCTCTGTTCCCAGCCTGGA
>1924
GCTCTTTTGGTACCTGATGGGGGAAAAGTCGAAGGAGGCACTCCACTTTTCACACTCA
GGAAAACTGGTGGTAAAGAAGTTCTCCTGGTGGTCAAGGTCTCCAGTGTTCCCTCTTG
GGATTGGGACTGAGCATAATGTGC
>1925
CCTGCAGCACCCATACCCACTCAGATGCCACCGGTGCCCTCGCCCTCACAGCCTCCTTC
TGGCAAACCTGGTAGGCTTCCAACTCCCACATGTCATGTGGGAGAACATCTCGTCTAAT
ATGTTCCTTCAAAGGGTAAACT
>1926
AGTAAAACCCACTGTTGCCCCACCACTAGCTGACGCAGGAGCTGGCAAAGGTCTGCG
TTCAGAACATCGGGTAAGCCTCTGAGGACCACTTTCAGGAAAGAGGCAGGGGGCAGT
GTTGAGATTAGTGGAGTATGGGTGTG
>1927
TTGCTCAGCGTCTGAAGGAGGCCCAGAATACATGTGCAATGCTGACAACTTTTAATGA
GATTGACATGAGGTAGTGTCTCTAGTCCCTCTTATCCCCTAGGCCCCTTTTTCTTAGAGA
ACACGAACTTGCCCCACTCCTT
>1928
CTAGGCTTCATGTCGGCATTTGTGAAGGCCTCAGCCTTTGCCTTGCAGGAACAGCCTGT
TGTAAATGCAGGTGAGTTGCTTGTGGCTGGAATTGGAGAGGTCCTGGGAGGTAGGTGT
ATGAGCACAGACCTGCTCCCACA
>1929
TGACGACACAACCAAAGAGGTGGTGTATACGGATTATATTGACATCAGTGTTGCAGTG
GCCACCCCACGGGTATGTTGGGGCAGGAGGTGGGGAATGTTGGTCTTAGACCCTCACC
TTATCTGTGTGAAGGAGATCACAC
>1930
AGTCATCAGGAATGTGGAAGCTATGAATTTTGCAGATATTGAACGGACCATCACTGAAC
TGGGAGAGAAGGTAAAGTAGAAAGATGTATACAAGCTGCTAAGCAGGCGAGGGAAGA
GAGCCTTCAGAAGGCTGGGCTCAC
>1931
CAACCCCCCTCAGTCTGCCATCCTGGGGATGCATGGCATCTTTGACAGGCCAGTGGCTA
TAGGAGGCAAGGTAGGAACCGTCACTTCTAAGGTCCTAGTGGCTAGGTCTCGATGAAA
GGGAAATCCAACTAAATGCTAAT
>1932

AGAATGGTGCCTGTCCTGCTGTCTCTGCTGCTGCTTCTGGGTCCTGCTGTCCCCCAGGA
GAACCAAGATGGTGAGTGGGGAAAGCAAGGGATGGGTGCTGGAGAGGACTGGAAGG
AGGTGAGGAACAGGACATGTGGCTG
>1933
CAGAAGGCCAGGGAGGACATCTTTATGGAGACCCTGAAAGACATCGTGGAGTATTACA
ACGACAGTAACGGTCAGTGAATAACAGACCACAGGGGTGGAAGGTCTAACCCAAGAG
GCAGCCCCCCCAGTGTGAGTGGCAA
>1934
CTGGAGGAGGAGTGCCCTGCGACTCTGCGGAAATACCTGAAATACAGCAAAAATATCC
TGGACCGGCAAGGTACTCACTGCTTCCTGCTCCCCAGTACTGAGCCCAGAATAAAAGA
CGATCTCAGGCTAGGAGCTCAGGC
>1935
GGCTGTTCCTCGGGCATCGGCCTGCACTTGGCCGTACGTCTGGCTTCAGATCCATCCCA
GAGCTTCAAAGGTATAGATAGGCAGGGACAGGGAGGGAGAGAAGGGAGGAGCCCTTG
GAGGCTAGAAGGGAAGTCAGATCT
>1936
GACGTAAGGGACTCAAAATCCGTGGCCGCTGCCCGGGAACGCGTGACTGAGGGCCGC
GTGGACGTGCTGGGTGAGCCTCCTGGAAGCATATGGGCTCCTAGGAGCCTTCTCCGCC
CTGCGTTGAAACCAACATGTTCCCA
>1937
TTCCTGCCAGACATGAAGAGGCGCGGTTCGGGACGCGTGTTGGTGACCGGGAGCGTG
GGAGGATTGATGGGTGAGTGGTAGGGAGTGGCCTCGGCAGCTCCAGATTCTTTGTGTG
CGGAGCTGAGCCTTGAAGGCAGGTT
>1938
GCGCCAGCAAGTTCGCGCTCGAAGGCTTATGCGAGAGTCTGGCGGTTCTGCTGCTGCC
CTTTGGGGTCCAGTGAGTCAACACCCCCGTTCCCCGAACCCTCTTAACTCTGACCTAG
AGATGCCGAGCACCCTGTCCTGCG
>1939
CTACCAATACCTCGCCCACAGCAAGCAAGTCTTTCGCGAGGCGGCGCAGAACCCTGA
GGAGGTGGCGGAGGTGAGCGCCGGGCTGGACTCCAGGAGTGGGGGCGGTGCGTCCT
CCGGCCGGCAGCGGTGGCCACAGCTCT
>1940
TTCAGAATGGATTATTTGCTCATGATTTTCTCTCTGCTGTTTGTGGCTTGCCAAGGAGCT
CCAGAAACAGGTAGGCACGCTCGTTGACTTGTAAGTCTCGGAATTACAAGTTAGTGTG
TTCTTATCCACCTTCATGCTTT
>1941
GCTCGTCCCTGATGGATAAAGAGTGTGTCTACTTCTGCCACCTGGACATCATTTGGGTC
AACACTCCCGAGTAAGTCTCTAGAGGGCATTGTAACCCTATTCATTCATTAGCGCTGGC
TCCACTGGAGCCCAGTTTTAGA
>1942
GTATTTATCAGCAGTTAGTGAGAGGAAGAAAAATCAGAAGAAGTTCAGAGGAACACCT
AAGACAAACCAGGTAAGAGGGAAGGAAGAAAAATTAGGTAAGAGGTTCACAAGAAC
AACTAGCCCCAGTCAGTGATGCCAGC
>1943
CCATCTGATCTATAAATGCGGTGGCATCGACAAAAGAACCATTGAAAAATTTGAGAAG
GAGGCTGCTGAGGTATGTTTAATACCAGAAAGGGAAAGATCAACTAAAATGAGTTTTA

CCAGCAGAATCATTAGGTGATTTC
>1944
CTATGTGACTATCATTGATGCCCCAGGACACAGAGACTTTATCAAAAACATGATTACAG
GGACATCTCAGGTTGGGATTAATAATTCTAGGTTTCTTTATCCCAAAAGGCTTGCTTTGT
ACACTGGTTTTGTCATTTGGA
>1945
CCCCGACACAGTAGCATTTGTGCCAATTTCTGGTTGGAATGGTGACAACATGCTGGAG
CCAAGTGCTAACGTAAGTGGCTTTCAAGACCATTGTTAAAAAGCTCTGGGAATGGCGA
TTTCATGCTTACACAAATTGGCAT
>1946
ATCCTACCACCAACTCGTCCAACTGACAAGCCCTTGCGCCTGCCTCTCCAGGATGTCTA
CAAAATTGGTGGTAAGTTGGCTGTAAACAAAGTTGAATTTGAGTTGATAGAGTACTGT
CTGCCTTCATAGGTATTTAGTAT
>1947
TCGTCGTGGCAACGTTGCTGGTGACAGCAAAAATGACCCACCAATGGAAGCAGCTGG
CTTCACTGCTCAGGTAACAATTTAAAGTAACATTAACTTATTGCAGAGGCTAAAGTCAT
TTGAGACTTTGGATTTGCACTGAA
>1948
GCTGCCATTGTTGATATGGTTCCTGGCAAGCCCATGTGTGTTGAGAGCTTCTCAGACTA
TCCACCTTTGGGTAAGGATGACTACTTAAATGTAAAAAAGTTGTGTTAAAGATGAAAAA
TACAACTGAACAGTACTTTGGG
>1949
AGCAGCTTCTTGATCGTGGTGGTGTTCCTCATCGCTGGGACGCTGGTTCTAGAGGCAG
CTGTCACGGGAGGTGAGTGAACAGGTGACCTGCTGGGCTGGGTTGGACTAAGGGGAG
ACCCTCTGGGACACCCTGGGCCAGG
>1950
GGGCATCCCTGGGTGCCAGACCTGCCGCTACCTCCTGGTGAGAAGTCTTCAGACGTTT
AGTCAAGCTTGGGTGAGTGGCCTATGGCTGAGGCTGAGGTGGGAGCATGGAACGGGT
GTGGGATATGCCCCCAGCATTGCTA
>1951
TCGAATCCAGTGTTCTGTCAGCGCGCTCAACCAGGGTCAAGTCTGGATTGGAGGCAGG
ATCACAGGCTCGGTAAGAGAAGTGTGAACACTAAATGGGGTGCACCTGCTGATCTCAG
CCAGCACTCAGCTTGCATCAGATT
>1952
AACTTTGCGTACTGGGCTGCTCACCAGCCCTGGTCCCGCGGTGGTCACTGCGTGGCCC
TGTGTACCCGAGGTGAGGTGGGGCTGGGGATGAACGATGGAAAGGTCTGGGAGATGG
GAAGTGCCCCAAGGAGGAGATGCTA
>1953
CCTTCGAGCTCCTTGTGCGCGCTGTTGGTGCTGCTGCTGCTGCTGACGCAGCCAGGGC
CCATCGCCAGCGGTGAGAGCGCATGGCGCGCGGGACGCACTCGCACTCGGGCACAGA
GGTGCATCCCAGCCTCTGCGGGGTC
>1954
TTCATCCCAAAATGATCAGTAATCTGCAAGTGTTCGCCATAGGCCCACAGTGCTCCAAG
GTGGAAGTGGTGTAAGTTCTGTGCTGCTGTGTCCGCTGTGACCTTGGCAAGAGAGAAA
TCCCGCAGCCTGGGTCTTCAACC
>1955

ACGGGAAGGAAATTTGTCTTGATCCAGAAGCCCCTTTTCTAAAGAAAGTCATCCAGAA
AATTTTGGACGGGTACTTGTCACTTTGATCTTTGTGGTTTCTAAATCTGATCTAGGGAGA
CCATAGACTTCACAAGGTCTTT
>1956
ACGCCTCATCTGTGACAGCCGAGTCCTGGAGAGGTACCTCTTGGAGGCCAAGGAGGC
CGAGAATATCACGGTGAGACCCCTTCCCCAGCACATTCCACAGAACTCACGCTCAGGG
CTTCAGGGAACTCCTCCCAGATCCA
>1957
CTGCAGCTTGAATGAGAATATCACTGTCCCAGACACCAAAGTTAATTTCTATGCCTGGA
AGAGGATGGAGGTGAGTTCCTTTTTTTTTTTTTTTCCTTTCTTTTGGAGAATCTCATTTG
CGAGCCTGATTTTGGATGAAA
>1958
GCTGCATGTGGATAAAGCCGTCAGTGGCCTTCGCAGCCTCACCACTCTGCTTCGGGCT
CTGGGAGCCCAGGTGAGTAGGAGCGGACACTTCTGCTTGCCCTTTCTGTAAGAAGGG
GAGAAGGGTCTTGCTAAGGAGTACA
>1959
ATAGATGCCAATGTGCTAGCCAAAAAGACAAGAAGTGCTGGAATTTTTGCCAAGCAGG
AAAAGAACTCAGGTGAGCAGAAACACCTTTGCTTTTCAATCAGTTTAACAGCCTCCTG
AACTCCTTCCTATCATGGTACTGC
>1960
AAACATTGAAGTTGTTTTTGAACTTGGTGTCACCTTTAATTACAACCTAGCAGACGGAA
CTGAACTCAGGGTAAGAATTTTTTTTTTTATGAGCAATGCATTCTTGATTTTTCTACCCA
ATATTAAAATGATTTCTGCTC
>1961
ATTCAAACGGACAGACAATGGAAACGAACTGAATACTGTCCGAGAAATTATAGGTGAT
GAACTAGTCCAGGTGAGTTGTCAAATTTATAGCTATTTTCAAAAGGCAAAAATTACTAC
AAAACAATAATTTTTGTCACTGC
>1962
CTGCTATATCCTGGATGCCATCCTGTTTCTGTATGGAATTGTCCTCACCCTCCTCTACTGT
CGACTGAAGGTAGCGCTGGGCAGGGTGGGGTAAGGGCTGGAAGGGGAAGTGGGAGG
AGGGCAGCAGCAAGGATTCGAAG
>1963
ATCATGGCAGAATCACCAGGCCTCATCACCATCTGCCTTTTAGGATATCTACTCAGTGCT
GAATGTACAGGTTTGTTTCCTTTTTTAAAATACATTGAGTATGCTTGCCTTTTAGATATAG
AAATATCTGATGCTGTCTT
>1964
GAGAGAATGTATGGAAGAAAAGTGTAGTTTTGAAGAAGCACGAGAAGTTTTTGAAAA
CACTGAAAGAACAGTGAGTATTTCCACATAATACCCTTCAGATGCAGAGCATAGAATAG
AAAATCTTTAAAAAGACACTTCTC
>1965
TGCAAGGATGACATTAATTCCTATGAATGTTGGTGTCCCTTTGGATTTGAAGGAAAGAA
CTGTGAATTAGGTAAGTAACTATTTTTTGAATACTCATGGTTCAAAGTTTCCCTCTGAAA
CAAGTTGAAACTGGAAAATGC
>1966
AACAAGGTGGTTTGCTCCTGTACTGAGGGATATCGACTTGCAGAAAACCAGAAGTCCT
GTGAACCAGCAGGTCATAATCTGAATAAGATTTTTTAAAGAAAATCTGTATCTGAAACT

TCAGCATTTTAACAAACCTACAT
>1967
CCAATCATTTAATGACTTCACTCGGGTTGTTGGTGGAGAAGATGCCAAACCAGGTCAAT
TCCCTTGGCAGGTACTTTATACTGATGGTGTGTCAAAACTGGAGCTCAGCTGGCAAGA
CACAGGCCAGGTGGGAGACTGAG
>1968
GTTAATGAAAAATGGATTGTAACTGCTGCCCACTGTGTTGAAACTGGTGTTAAAATTAC
AGTTGTCGCAGGTAAATACACAGAAAGAATAATAATCTGCAGCACCACTAGCTCTTTAA
TATGATTGGTACACCATATTTT
>1969
GGATAGCCTCTCTTACTACCACTCACCCGCAGACTCCTTCTCCAGCATGGGCTCGCCTG
TCAACGCGCAGGTAAGGCTGGCTTCCCGTCGCCGCGGGGCCGGGGGCTTGGGGTCGC
GGAGGAGGAGACACCGGGCGGGAC
>1970
GGCTGGCGTTGTGAAGACCATGACAGGAGGCCGAGCGCAGAGCATTGGCAGGAGGG
GCAAGGTGGAACAGGTGAGGAACTCTAGCGTACTCTTCCTGGGAATGTGGGGGCTGG
GTGGGAAGCAGCCCCGGAGATGCAGGA
>1971
GGAAAGGAATAAGATGGCTGCAGCCAAATGCCGCAACCGGAGGAGGGAGCTGACTGA
TACACTCCAAGCGGTAGGTACTCTGTGGGTTGCTCCTTTTTAAAACTTAAGGGAAAGTT
GGAGATTGAGCATAAGGGCCCTTG
>1972
GAGTCACTCTCCCCAGGAGACCCAGACCTAGAACTACCCAGAGCAAGACCACAGCTG
GTGAACAGTCCAGGTAAGAAACATATTCTGGGGGACTGGGTAGCTTGACATAGACACA
CTTAATACACTGGTGAACACAATAG
>1973
CATTTTTCTGCACTGGCCAGAAAATGGAGTCTTAAACCTTGAATCCCCCATAACTACCT
CAACTACAAAGGTAAAGAGGCTTAGTCAGTCTTAAGATGTAACTTTCTGTAAAGGAAT
TGGCAGATCCTTCCTTTCTTCCT
>1974
GTCTCCACTGCTGCCCTTGCCGTCCTCCTCTGCACCATGGCTCTCTGCAACCAGGTCCT
CTCTGCACCACGTGAGTCCATGTTGTTGTTGTGGGTATCACCACTCTCTGGCCATGGTT
AGACCACATCAGTCTTTTTTTG
>1975
GACAGATTCCACAGAATTTCATAGCTGACTACTTTGAGACGAGCAGCCAGTGCTCCAA
GCCCAGTGTCATGTAAGTGCCAGTCTTCCTGCTCACCTCTAGGGAGGTAGGGAGTGTC
AGGGTGGGGGCAGAAACAGGCCAG
>1976
GCGCTCCCACTCTCGGCCGACACCCCTCATGGCCAACCGTTACACCATGGATCTGACT
GCCATCTACGAGGTGAGTCCCCGCCGCACGGCATCCCCGGTACCTGCATGCCTGAGTC
CGAGTCCCCACCTCTCTAGCGCCG
>1977
GACCCATGGACAAGAGCGCAGGCAGTGGCCACAAGAGCGAGGAGAAGCGAGAAAAG
ATGAAACGGACCCTGTGAGTATGGCTTTCTTCCCTCTCCCGCCACCCCCTGCCCCACAC
TGCAAGCTGCAAACGCGGTACTTTC
>1978

TCTTACAAAATTCCTCTACTCCTGGGAAGCCCAAAACCGGCAAAAAAAGCAAACAGC
AAGCTTTCATCAAGTAAGTTGAGAATCCTGTGCTTGCAAATATCAATAGTTAGCTGCTG
AACTGAAAAGGGGAACTCTGATGT
>1979
ACCCCAAAAGCTGTCCTCAAAAGCAAGGAAAATATATACTGACTTCATAGAAAAGGAA
GCTCCAAAAGAGGTAAGGAAACAAGTTCCTAATTTCAGCACAATCTGGACATCTTTAG
CACAAAAGTGAAACAGTAATCAAG
>1980
GTCAGCGCCGGGCCGCGTCAACCTCATCGGGGAACACACGGACTACAACCAGGGCCT
GGTGCTGCCTATGGTGAGGGGCTGCACGGGGAGCCCCTAGCCCGCCGCCGCCTGTCCC
GGTCGCCGAGGAGGGCGGGCCTCGG
>1981
CAGCGCTCGCTGGAGCCTGGGACTCCTCGGTGGGCCAACTATGTCAAGGGAGTGATTC
AGTACTACCCAGGTATGGGGCCCAGGCCTGAGCCAAGTCCTCACTGATACTAGGAGTG
CCACCTCACAGCCACAGAGCCCAT
>1982
GGGGGTGGCCTGTCCAGCTCAGCATCCTTGGAAGTGGCCACGTACACCTTCCTCCAGC
AGCTCTGTCCAGGTACCAGCTAGGCCCCAGCCCTGACCCAGCCCTCCTTCCCTGAGGT
CTCCAGGTGGTCCCAGCTTCTACT
>1983
CCTGTGGCATCATGGACCAGTTCATCTCACTTATGGGACAGAAAGGCCACGCGCTGCT
CATTGACTGCAGGTTGGGCTCGCTCCCCTCGTCCCCTCCCGCCCTGCACTCAGCAGCT
CCTGGGTGGAGTGTGCCCACTGCC
>1984
CAATGTGAAGAAGTGGCCCGGGCGCTGGGCAAGGAAAGCCTCCGGGAGGTACAACTG
GAAGAGCTAGAGGGTGAGAACTGCCAGGGTGCTCTATCCTGGAGGCGGCTGTGCTCC
CTGCTGGCGCCTCAGTGTGGCCTTGA
>1985
CGGCCGCCCTGAGACGTGGCGACTACAGAGCCTTTGGCCGCCTCATGGTGGAGAGCC
ACCGCTCACTCAGGTGAGGCCCTCTGGGCGCCCCGCTCCTGCCGGGCACAGGCCGGC
CCAGGCCCACCCCTTCAATATCCTCT
>1986
TGGCTTCGGTGGCTGCACGGTGACACTGCTGGAGGCCTCCGCTGCTCCCCACGCCATG
CGGCACATCCAGGTGGGCGGGCACCAGGGCCTGGGCGGGCAGGAGCGGCAGCTTCCC
GGGGCCCTGCCACTCACCCCCAGCC
>1987
GGAACCGATCCTCAGCAACGCCAGCAGGCGTCAGAGGCGGACGCCGCAGCAGCAAC
CTTCCGGGCAAACGGTAACTGCACCGCGGCAGGGACTCGCTGGGGCGCGGAGCCGAG
CCCTCCCCTTCCTTAGGAAGCTTTCGT
>1988
TCTGAAGACAGTGCCCCGCCATGACCCTCTCAACCCTCTGTGTCCTGGGGCCATCCGA
GCCAACGGAGAGGTAAGCCTGTAGAGCCCTGCATCTGCAGGCTGGGCCACGGGGAGT
AGTTCCCTCTTAGAACTGTCCTCCA
>1989
CCCCAGTACGATAGCACCTTCCTGTTTGACAACGACTTCCCAGCTCTGCAGCCTGATGC
CCCCAGTCCAGGTAACCTGGCTCCAACTGCTGCTGGGGAGGAGGGTGGCTAGACCTCT

TGAGGGACTTCTGCTGCAGAGAT
>1990
GATCCGGGCTGTTGTTGATGCATGGGCCTCAGTCACAGAGGAGCTGGGTGCCCAGTAC
CCTTGGGTGCAGGTTTGTGAGGTCGCCCCTTCCCCTGGATGGGCAGGGAGGGGGTGAT
GAAGCTTTGGTTCTGGGGAGTAAC
>1991
GCAGGCCTATAAGAGTCAGCATGGAGAGCCCCTGCTAATGGAGTACAGCCGCCAGGAG
CTACTCAGGAAGGTGGGAGAGAGCCAAGCCCTGTGTCCCCAAGGAGTCCCTAACTTT
CTTATCCCATGAGAGAGGTGTGTAA
>1992
TACCAGACACTGCTGCTGCCCCGTCGGCATGTGCGGCGGCTACCTGAGCTGACCCCTG
CTGAGCGTGATGGTCAGTCTCCCAAGTAGGATCCTGGGGCTAGGCACTGGATGGAGGT
TGCTCCCAGTAGGGTCAGCATCTG
>1993
ATGAAGAAGCTCTTGACCAAGTATGACAACCTCTTTGAGACGTCCTTTCCCTACTCCAT
GGGCTGGCATGGTGAGGCTTTTCAAGTACCTATATTTAGCCCCAACACCATTTCTGGGC
TCCTGGGCTCAGCCTAGTGAAC
>1994
CACTGTCCGGAAATTCATGGTTGGCTACGAAATGCTTGCTCAGGCTCAGAGGGACCTC
ACCCCTGAGCAGGTCAGGACTCAGAACAGTCTGGCGTCTCCAGACTCTCACATGCAGT
ATGTGCAGGCACCTGATACTTCTG
>1995
ACAAGGCTACTATCACAAGCCTGTGGGGCAAGGTGAATGTGGAAGATGCTGGAGGAG
AAACCCTGGGAAGGTAGGCTCTGGTGACCAGGACAAGGGAGGGAAGGAAGGACCCT
GTGCCTGGCAAAAGTCCAGGTCGCTTC
>1996
ACAAGGCTACTATCACAAGCCTGTGGGGCAAGGTGAATGTGGAAGATGCTGGAGGAG
AAACCCTGGGAAGGTAGGCTCTGGTGACCAGGACAAGGGAGGGAAGGAAGGACCCT
GTGCCTGGCAAAAGTCCAGGTCGCTTC
>1997
CAAGGGCACCTTTGCCCAGCTGAGTGAACTGCACTGTGACAAGCTGCATGTGGATCCT
GAGAACTTCAAGGTGAGTCCAGGAGATGTTTCAGCACTGTTGCCTTTAGTCTCGAGGC
AACTTAGACAACTGAGTATTGATC
>1998
GGCTGCTTTTAACTCTGGTAAAGTGGATATTGTTGCCATCAATGACCCCTTCATTGACCT
CAACTACATGGTGAGTGCTACATGGTGAGCCCCAAAGCTGGTGTGGGAGGAGCCACCT
GGCTGATGGGCAGCCCCTTCAT
>1999
TCCATGGCACCGTCAAGGCTGAGAACGGGAAGCTTGTCATCAATGGAAATCCCATCAC
CATCTTCCAGGAGTGAGTGGAAGACAGAATGGAAGAAATGTGCTTTGGGGAGGCAAC
TAGGATGGTGTGGCTCCCTTGGGTA
>2000
GTGGGGCGATGCTGGCGCTGAGTACGTCGTGGAGTCCACTGGCGTCTTCACCACCATG
GAGAAGGCTGGGGTGAGTGCAGGAGGGCCCGCGGGAGGGGAAGCTGACTCAGCCCT
GCAAAGGCAGGACCCGGGTTCATAAC
>2001

CTGCTGATGCCCCCATGTTCGTCATGGGTGTGAACCATGAGAAGTATGACAACAGCCTC
AAGATCATCAGGTGAGGAAGGCAGGGCCCGTGGAGAAGCGGCCAGCCTGGCACCCTA
TGGACACGCTCCCCTGACTTGCGC
>2002
CACCACCAACTGCTTAGCACCCCTGGCCAAGGTCATCCATGACAACTTTGGTATCGTG
GAAGGACTCATGGTATGAGAGCTGGGGAATGGGACTGAGGCTCCCACCTTTCTCATCC
AAGACTGGCTCCTCCCTGCTGGGG
>2003
CCCACTCCTCCACCTTTGACGCTGGGGCTGGCATTGCCCTCAACGACCACTTTGTCAA
GCTCATTTCCTGGTATGTGGCTGGGGCCAGAGACTGGCTCTTAAAAAGTGCAGGGTCT
GGCGCCCTCTGGTGGCTGGCTCAG
>2004
CTCCTCAACAGCAGCAGTGTGGGCAACCTCAGCTGCGAGCCCCCTCGCATTCGCGGA
GCCGGGACACGAGGTGGGTGCCTCCCTCAGCCCCCCCACAAGCTATTTCTCACTGTA
CCCCAGAACACTAATAGAGTCCCCC
>2005
CTCCTGCCCAATCTCATGGGCACATTCATCTTTGGCACCGTCATCTGCAAGGCGGTTTC
CTACCTCATGGGTGGGTGAGACAACCACCCACCCCCACTTGCCACTCTCCCCGCCTAA
AGCTCTTGTGGATGTAGGGGTCT
>2006
TGCAACCAGTGGGGCCTCGTGTGCTGCAGTGCGTGCATCGCTGGCCCAGTGCGCGGGT
CCGCCAGACCTGGTGAGCTTGCCCATAAACTATCCTAGGAATTCCTTTCTCACCCCTATT
AGATGCTTACGACCATTGCCCA
>2007
GGGCTTCGCTTTGACGGCGACAGTGACAGCGACAGCCAAAGCAGGGTCCGAAACCAA
GGCGGGCTGCCAGGTGGGGCTGGACCACGTGAGCAAAATCTGGGCGAGGCGGACGTT
TGGAGGGCGACGGGGCCTGCTGGAGT
>2008
GAGCAGCAGGGCCCAGCCTCTCATCATCGAAGGCAGCTGGGACCCCAGGGTCCCCCA
CACCTCGTGGCAGGTAGGAGCTGCTGACTGCCCTGCTTGCCTCACTTGGCCATGTTTG
GCCAAGGTCTCCCCAGACTGGCTCT
>2009
CCTCAAGAACCTGAGCCCGTCGGCCAGCCAGTACGTGGAACAGATGTTTGAGACTTTT
GACTTCAACAAGGTGAGCAGGGGCCCAGTGGCAGGGAGGGGAAGTGCTGGAGGGAC
CCCTCTGGAAGCCTGACCAGCTGGGG
>2010
GTACTTCAAGCTCTATGATGTAGATGGCAACGGCTGCATTGACCGCGATGAGCTGCTCA
CCATCATCCAGGTGCAGAGGGCCCGGCACTGGGGGGCAGCGGTCTGGGGTGGGACCC
GGAACTGAGAGCCCAGGGTTAGAA
>2011
AGCGATACCACCATGACTGCAGAGGAGTTCACCGATACAGTGTTCTCCAAGATTGACG
TCAACGGGGATGGTGAGGGGGCCGAGGAGGGGCTCCCCAGCGGAGGGGTCACCATG
GATGTGGGGTCACCAGGGGTGGAAGG
>2012
TGGAACCCCTGTGGTCTTCTCTTCATCTAATGACCCTGAGGGGATGGAGTTTTCAAGTC
CTTCCAGAGAGGTAAGAGAGAGAGCTCCCAATCAGCATTGTCACAGTGCTTCTGGAAT

CCTGGCACTGGAATTTAATGAAT
>2013
AGTAGGGTAAGCATCATGGCTGGCAGCCTCACAGGATTGCTTCTACTTCAGGCAGTGT
CGTGGGCATCAGGTGAGTGAGTCAAGGCAGTGGGGAGGTAGCACAGAGCCTCCCTTC
TGCCTCATAGTCCTTTGGTAGCCTT
>2014
GAGAGTACACGCAGTGGGCGACGGATGGAGCTGAGTATGGGGCCCATCCAGGCTAATC
ACACGGGCACAGGTAACCATTACACCCCTCACCCCCTGGGCCAGGCTGGGTCCTCCTA
GAGGTAAATGGTGTCAGTGATCAC
>2015
GCTCTCAACATCCTTGCCCTGTCACCCCCTGCCCAAAATTTGCTACTTAAATCGTACTTC
TCTGAAGAAGGTGAGGAGGAAGGGGACAAGATGACATAGAGCCATTGAAACTTTTCG
TTTTTCTTTTCTTTTTTTAAAAT
>2016
ATGGGAAGGGGTCACTCAAGGGACAGCCCGGAGACATCTACCACCAGACCTGGGCCA
GATACTTTGTGAAGTAAGGGATCAGCAAGGATGTGGGATCAGGACTGGCCTCCCATTT
AGCCATGCTGATCTGTGTCCCAACC
>2017
TACTCACCACAATGTCCGCCTACTCATGCTGGATGACCAACGCTTGCTGCTGCCCCACT
GGGCAAAGGTGGTAAGGCCTGGACCTCCATGGTGCTCCAGTGACCTTCAAATCCAGCA
TCCAAATGACTGGCTCCCAAACT
>2018
GTTCTGGGAGCAGAGTGTGCGGCTAGGCTCCTGGGATCGAGGGATGCAGTACAGCCA
CAGCATCATCACGGTAAGCCACCCCAGTCTCCCTTCCTGCAAAGCAGACCTCAGACCT
CTTACTAGTTTCACCAAAGACTGAC
>2019
CCATCATTGTAGACATCACCAAGGACACGTTTTACAAACAGCCCATGTTCTACCACCTT
GGCCACTTCAGGTGAGTGGAGGGCGGGCACCCCCATTCCATACCAGGCCTATCATCTC
CTACATCGGATGGCTTACATCAC
>2020
AGAAGAACGACCTGGACGCAGTGGCACTGATGCATCCCGATGGCTCTGCTGTTGTGGT
CGTGCTAAACCGGTGAGGGCAATGGTGAGGTCTGGGAAGTGGGCTGAAGACAGCGTT
GGGGGCCTTGGCAGGATCACACTCT
>2021
TGGAACCCCTGTGCTCTTCTCCTCATCTAGTGACCCTGAGGTGATGGAGTTTTCAAGTC
CTTCCAGAGAGGTAAGAGAGAGAGCTCCCAATCAGCATTGTCACAGTGCTTCTGGAAT
CCTGGCACTGGAATTTAATGAAT
>2022
CAAGAGCAGAAGCAGTGGGCATTGGATGGAGCTGAGTACAGGACCATACAGGCTAATT
GCACCGGCACAGGTAACCATTACACCCTTCACCCCCCGGGCCAGGCTGGGTCCTCCTA
GAGGTAAACGGTGTCAGTGATCAC
>2023
CTATGCAGACACCCCTGATGATTTCCAGTTGCACAACTTCAGCCTCCCAGAGGAAGAT
ACCAAGCTCAAGGTAGGCATTCTAGCTTTTTCAGGCCCTGAGGGCCCTGATGTCTGGG
GGTTGAGAAACTGTAGGGTAGGTC
>2024

ATGGGAAGGGGCCACTCAAGGGACAGCCCAGAGACATCTACCACCAGACCTGGGCCA
GATACATTGTGAAGTAAGGGATCAACAAGGATGTGGATCAGGACTGGCCTCCCCTTT
GGCCATGCTGATCTGTGTCCCAACC
>2025
TACTCACCACAATGTCCGCCTACTCATGCTGGATGACCAACGCTTGCTGCTGCCCCACT
GGGCAAAGGTGGTAAGGCCTGGACCTCCATGGTGCTCCAGTGACCTTCAAATCCAGCA
TCCAAATGATTGGCTCCCAAACT
>2026
GTTCTGGGAGCAGAGTGTGCGGCTAGGCTCCTGGGATCGAGGGATGCAGTACAGCCA
CAGCATCATCACAGTAAGCCACCCCAGTCTCCCTTCCTGCAAAGGAGACCTCAGACCC
ATTAGTAGTCTCACCAAAGACTGAT
>2027
CCATCATTGTAGACATCACCAAGCACACGTTTTACAAACAGCCCATGTTCTACCACCTT
GGCCACTTCAGGTGAGTGGAGGGCGGGCACCCCCATTCCATACCAGGCCTATCATCTC
CTACATCGGATGGCTTACATCAC
>2028
AGAAGAACGACCCGGACGCAGTGGCACTGATGCATCCCGATGGCTCTCCTGTTGTGGT
CGTCCTAAACCGGTGAGGGCAATGGTGAGGTCTGGGAAGTGGGCTGAAGACAGCGTT
GGGGGCCTTGGCAGGATCACACTCT
>2029
AGCCTTCCTGAGAAAATGCAGCCATTCCTCCTCCTGTTGGCCTTTCTTCTGACCCCTGG
GGCTGGGACAGGTAAGTGACTATCCCTATTCCAGAGGCCTGAACCCATCTTATAAGATA
CCCTGTACCCATGAGCACTGGT
>2030
GATCCCAAGGCCCAACTCCCCGAACCACTCAGGGTCCTGTGGACAGCTCACCTAGCTG
CAATGGCTACAGGTAAGCGCCCCTAAAATCCCTTTGGCACAATGTGTCCTGAGGGGAG
AGGCAGCGACCTGTAGATGGGACG
>2031
GCTTTTTGACAACGCTATGCTCCGCGCCCATCGTCTGCACCAGCTGGCCTTTGACACCT
ACCAGGAGTTTGTAAGCTCTTGGGGAATGGGTGCGCATCAGGGGTGGCAGGAAGGGG
TGACTTTCCCCCGCTGGAAATAAG
>2032
CCAGACCTCCCTCTGTTTCTCAGAGTCTATTCCGACACCCTCCAACAGGGAGGAAACA
CAACAGAAATCCGTGAGTGGATGCCTTCTCCCCAGGCGGGGATGGGGGAGACCTGTA
GTCAGAGCCCCCGGGCAGCACAGCC
>2033
CGGCGCCTCTGACAGCAACGTCTATGACCTCCTAAAGGACCTAGAGGAAGGCATCCAA
ACGCTGATGGGGGTGAGGGTGGCGCCAGGGGTCCCCAATCCTGGAGCCCCACTGACT
TTGAGAGACTGTGTTAGAGAAACAC
>2034
GGATCCCAAGGCCCAACTCCCCGAACCACTCAGGGTCCTGTGGACAGCTCACTAGCG
GCAATGGCTGCAGGTAAGCGCCCCTAAAATCCCTTTGGCACAATGTGTCCTGAGGGGA
GAGGCGGCGTCCTGTAGATGGGACG
>2035
GCTTTTTGACAACGCTATGCTCCGCGCCCGTCGCCTGTACCAGCTGGCATATGACACCT
ATCAGGAGTTTGTAAGCTCTTGGGTAATGGGTGCGCTTCAGAGGTGGCAGGAAGGGGT

GAATTTCCCCCGCTGGGAAGTAA
>2036
CCAGACCTCCCTCTGCTTCTCAGAGTCTATTCCAACACCTTCCAACAGGGTGAAAACG
CAGCAGAAATCTGTGAGTGGATGCCTTCTCCCCAGGTGGGATGGGGTAGACCTGTGGT
CAGAGCCCCCGGGCAGCACAGCCA
>2037
TGGCGCCTCGGACAGCAACGTCTATCGCCACCTGAAGGACCTAGAGGAAGGCATCCA
AACGCTGATGTGGGTGAGGGTGGCACCAGGATCCAATCCTGGGGCCCCACTGGCTTCC
AGGGACTGGGGAGAGAAACACTGCT
>2038
GCTGCAGCGGCGCCTCGGACCCCGGGGCCTGGTGGTGCTCGGCTTCCCGTGCAACCA
GTTTGGGCATCAGGTGCGCCGGGCGGAGCGGGGCGGGGCGGGGGCGGACGTGCAGTA
GTGGCTGGGGGCGCCGGCGGTGTGCT
>2039
TTCGCCGCCCCTGCGCGTCCAGCTCTTCTAAGACGAGATGCCGTCGGGCTTCCAACAG
ATAGGCTCCGAAGTAGGATTCATCATGAGGGGGCGGGGCGGGGGGGCACGGGTCCCG
CTTTTCTTGGGCTGGGGTCGCGGTT
>2040
TGTGTTCTCTGCGGTGCTTGGCTCCCTGCAGTTTGGGTACAACATTGGGGTCATCAATG
CCCCTCAGAAGGTGAGGGCCTGCAGCTGGCAGGGTGGGGGTACCCAAACGAGGAGG
ACAGGTGTCTCGGGGGTGGTGGAAA
>2041
TGGCCATCTTTTCCGTGGGCGGCATGATTTCCTCCTTCCTCATTGGTATCATCTCTCAGT
GGCTTGGAAGGTTCGCAGCTGGAGGGCAGGGGTGGGGGAAACAGGAAGGGAGCCAC
TGCTGGGTGCCCTCACCCTCACAG
>2042
GGCCTGGCCAACGCTGCTGCCTCCTATGAAATGCTCATCCTTGGACGATTCCTCATTGG
CGCCTACTCAGGTACTCACGGGCACCACAGCCCTGCCTAGCGCCCTGTTCTCTTTCACC
ATGCCTGGGCTTTCAGATGGGA
>2043
CACTCACCTGCGGGGCGCCCTGGGGACGCTCAACCAACTGGCCATTGTTATCGGCATT
CTGATCGCCCAGGTGACCGGAGCAAGCCTCATGGGTGCCTGGGCAGTGGTTAGAGTG
GGGCTCTGGAGAATATGGTGGGCTT
>2044
CTGCCCTTCTGTCCCGAGAGCCCCCGCTACCTCTACATCATCCAGAATCTCGAGGGGCC
TGCCAGAAAGAGTAAGCTCTCCCGCTGCAGCCTGGCCCAGGCCCATGCCTCCGCCTCA
TCTTGCTAGCACCTGGCTTCCTC
>2045
TACCCACCGGCAGCCCCTGATCATTGCGGTCGTGCTGCAGCTGAGCCAGCAGCTCTCT
GGCATCAATGCTGTATGTGTGGAGCAGCCTCCAGGCAGGGCACAGCCCCGGGAGGGT
AGACGAGAGTGGGGAGCAAACCCCC
>2046
AGGGGTAGGCCAGCCTGCCTATGCCACCATAGGAGCTGGTGTGGTCAACACAGTCTTC
ACCTTGGTCTCGGTAACTGCTCACCTCTGGAATGGCCCGAGCCACTGGCTTCACCTCC
CTGGGTGTCCCGGAGGTCCTGCTC
>2047

GCTCCATCTCCTGGGCCTGGCGGGCATGTGTGGCTGTGCCATCCTGATGACTGTGGCTC
TGCTCCTGCTGGTAAGGCCTGGAGGCTAGGAGGGGCTAGCAGCCCACCCCATGGGAAT
GGTCCTGTGAGTCTCTGTGACCA
>2048
CATGGCTGTGGCTGGTTTCTCCAACTGGACGAGCAACTTCATCATTGGCATGGGTTTCC
AGTATGTTGCGGTAGGTCCCCCCGCCCCAGCCTCCCACACCGTAGGCCAGAGGTGGGC
ATCACACAGCTAGCCCACCTGCT
>2049
GCCCAGCCACCCCGGCCCCTGAGCCCAGCCGGGCCCCAGCATCCCTACTCCCACCAGC
GCCAGAACACAGGTAAGGGCCAGGCAGCTAGGAGCAGGTGGGCAACAAGGGTGGTG
TCAAGGCCTGAAGCCTGGGGCTGGGA
>2050
GTTCCCTCGTGTGAAGAACTGGGAGGTGGGGAGCATCACCTATGACACCCTCAGCGCC
CAGGCGCAGCAGGTAAGGCCGGCATGCCCTGTCCCCATCGTCTCCAGGGAAAGGGTG
GGTAAGGCCTGGCCTCAGATGGGGC
>2051
CCCCGGCCCCTGAGCAGCTGCTGAGTCAGGCCCGGGACTTCATCAACCAGTACTACAG
CTCCATTAAGAGGTGACAGCTTCCCGGACGCCACAGCCTCCCTTGTCCCACTGAGGCC
CCAGAAACCCCGTGACGACCTTCC
>2052
GTTCGGGGCTAAGCAGGCCTGGCGCAACGCTCCCCGCTGCGTGGGCCGGATCCAGTG
GGGGAAGCTGCAGGTGCGGCTGGCCAGCGACTGAGAGACCCGGGCGCTACCAAAAG
GGGAGCGGGGTGGCGGGGCAGTTCCTA
>2053
GGTCTGCACAGGAAATGTTCACCTACATCTGCAACCACATCAAGTATGCCACCAACCG
GGGCAACCTTCGGTGAGTGCCCCCCACCATGCCAGGCCCCAGCCTTCTTCCCCAAGGC
AGGGAAGGCGGGGCTCTGACCAGC
>2054
GCGCTACGCGGGCTACCGGCAGCAGGACGGCTCTGTGCGGGGGGACCCAGCCAACGT
GGAGATCACCGAGGTGGGCACCGAGGGCCACCCATGAGGGTGTCCCCAAGGTGGAGA
ATGAGGAAACCAGTGGGAGAAGGCTC
>2055
CAGATGAGCCCCCAGAACTCTTCCTTCTGCCCCCCGAGCTGGTCCTTGAGGTGCCCCT
GGAGCACCCCACGTGAGCACCAAAGGGATTGACTGGGTGGGATGGAGGGGGCCATCC
CTGAGCCTCTCAAGAAGGGCCTGCA
>2056
TGGCTGGTACATGAGCACTGAGATCGGCACGAGGAACCTGTGTGACCCTCACCGCTAC
AACATCCTGGAGGTGAGGTGCGGGATGGGGCTCGGGCACCGAATGCACCTGTCCAAG
GCAGGAGTCTGGCTCTCACTCCATC
>2057
GACCACCTCGTCCCTGTGGAAAGACAAGGCAGCAGTGGAAATCAACGTGGCCGTGCT
GCACAGTTACCAGGTGCAGAGGCCCAGACTGGCCAGGAAGGCAAAGGGTTTGCATAC
GGGGGCAGCAGGGGCGGGGGATGGAG
>2058
GGGCAGCCTCACTCCTGTTTTCCATCAGGAGATGGTCAACTATTTCCTGTCCCCGGCCT
TCCGCTACCAGGTGCCCACCCTAATGGCTCTGCCAGCCTGGGCCCAGCTCTAATTCTAA

GCAGCCCCTGGGGACCTCTAAC
>2059
ACCCCTGGAAGGGGAGTGCCGCCAAGGGCACCGGCATCACCAGGAAGAAGACCTTTA
AAGAAGTGGCCAAGTGGGTCCCCTGGGAGCCCCGCTCTCCCACACACACCCTGGGGG
CCCCACTCTCCCCCACACACCCTGGG
>2060
CGAGACCGGCCGGGCCCAGAGCTACGCACAGCAGCTGGGGAGACTCTTCCGGAAGGC
TTTTGATCCCCGGGTAGGGCTGAGCCCAGGGGAGCAGGGAGCTAGAAAGAGGGGGCT
CTATCAGCATCTTCAGGGGTGCCCTG
>2061
CGAACACGAGACGCTGGTGCTGGTGGTAACCAGCACATTTGGGAATGGGGATCCCCCG
GAGAATGGAGAGGTGAGAACTTCCAGGAAAGGGGCTGCTGGGAATGAGGAGAGACT
CAGAATTGGAGTGACTGGGCAGGAAC
>2062
TGTCCTCTTGGCGGCGGAAGAGGAAGGAGTCCAGTAACACAGACAGTGCAGGGGCCC
TGGGCACCCTCAGGTCAGGGCCTCACCAAGAGGGGTGCAACGGGTGGGCAAGCTGCC
TGGGCAAACGTGGCCTGCAAAGGGAG
>2063
GCTGGGCCAGGGCGACGAGCTGTGCGGCCAGGAGGAGGCCTTCCGAGGCTGGGCCC
AGGCTGCCTTCCAGGTGAGCCCAGCCCAGCCCCTGCTCTGACTCCTGCCCCCTGGGAT
GCCTCCTCCTGCCTCACTCTGCCCTG
>2064
CCCAAACGGAGCTGGAAGCGCCAGAGGTACCGGCTGAGCGCCCAGGCCGAGGGCCT
GCAGTTGCTGCCAGGTGGGCCCTGCCTCACCCTAACCCGGCTGGTTCTCTGAGGCCCC
CACACCCCGGGACTAAAGCACTCTGG
>2065
ACGTGCACAGGCGGAAGATGTTCCAGGCTACAATCCGCTCAGTGGAAAACCTGCAAA
GCAGCAAGTCCACGTGAGGACGACGGCTTTACCGCCCCCCAACCCCTGTCCTGAACA
CCCTGACCCTGGACCCTCCTCCTCCC
>2066
AGCCGCGTGGAGGACCCGCCGGCGCCCACTGAGCCCGTGGCAGTAGAGCAGCTGGAG
AAGGGCAGCCCTGGTGAGGGGCAGCCTGGGAAGCAACAGGGCACACCAGCCCCATG
CCCAGCCCCACCCCCGGCCCCAGGCCT
>2067
GCTCTTGCGGCTGCTCAGCACCTTGGCAGAAGAGCCCAGGGAACAGCAGGAGCTGGA
GGCCCTCAGCCAGGTTGGGGGCCACCCCAATGAGGCACAGGGGCTAGAGAGACGGGA
TGAGCTGGGGGGACCCCAGTGGCAGG
>2068
AGCTCGGCACCCAGCACCCACCCAGGAGAGATCCACCTCACTGTAGCTGTGCTGGCAT
ACAGGACTCAGGGTGAGGCAACAAGCAGGAGCAGGCCTGGCCACAGCAGGGTTGGG
ACCGGCCCCTCTCTGGCCCCTCACCG
>2069
ACTATGGAGTCTGCTCCACGTGGCTAAGCCAGCTCAAGCCCGGAGACCCTGTGCCCTG
CTTCATCCGGGGGTAAGTGAGATGGAGGACTTGGTGGGGAGCTGCCCAGGGTCAGGG
TGGCAGCTTTGGTGAGGAGTGTCAC
>2070

GGTCCAGGCACTGGCATTGCCCCCTTCCGGGGATTCTGGCAGGAGCGGCTGCATGACA
TTGAGAGCAAAGGTGAGGCTGGTGACTAAAGGACTGCCTGAAGGGAGTCACACAATC
TAGGGACAGAGGGGTGGGGCTGGAA
>2071
GAACGCCCAGCAGCGCGGGGTGTTTGGCCGAGTCCTCACCGCCTTCTCCCGGGAACC
TGACAACCCCAAGGTGTGAGACCCTGAGGGCGCAATGGTAACCTGAAGATAGGGAGA
GAGGGGAGGACTCGCGCTCTCCAGCG
>2072
AGAGTTGCCACCCAGGCCTCAGCCAGGACCTTTCAGGCCAGACAGGAGCACCTGACC
AAAGGCTTCACAGGTAAGTGGCCCTCCGTATTACCCAGCATGGCCTCCCACAGGGAGA
GAGGAGGTCAGTTCCTGTCCCTTTA
>2073
TGGGCACACTCCACCTTCCCTAGTCACCAGCTGGTTTCCCAGAGGAGAAGGCTGAGAC
CCGAGAAGGGAGGTGAGTGCACCCCGTCCCATGTCAGGCTCCGCTACATCCCCAGTGC
TTGCCGTCCCTGAGGATCGGTCCA
>2074
CCGAGGAGGAGGACAAGAAGGAGGACGTGGGCACGGTGGTCGGCATCGACTTGGGG
ACCACCTACTCCTGGTAAGTGGGGTTGCGGATGAGGGGGACGGGGCGTGGCGCTGGC
TGGCGTGAGAAGTGCGGTGCTGATGTC
>2075
CAAGCGGCTCATCGGCCGCACGTGGAATGACCCGTCTGTGCAGCAGGACATCAAGTTC
TTGCCGTTCAAGGTTCGACCGGTTTTCCTCATCCAGTTAGAGAACGGGTGGGTGGTGG
GAGTATTTAGAGTTATAAGTCTCT
>2076
TCCTGAAGAAATTTCTGCCATGGTTCTCACTAAAATGAAAGAAACCGCTGAGGCTTATT
TGGGAAAGAAGGTAAATATTTCTAGAACAATGTTAAGTATTTTTTGATCATTAGTATTCT
CGGTTGGCTGTTATGTATAGA
>2077
CCCAACGCCAAGCAACCAAAGACGCTGGAACTATTGCTGGCCTAAATGTTATGAGGAT
CATCAACGAGCCGTAAGTATGAAATTCAGGGATACGGCATATTTGCCAAATAGTGGAAA
TGTGAAGTACTGACAAAACTTTT
>2078
TGAGTCCTTCTATGAAGGAGAAGACTTTTCTGAGACCCTGACTCGGGCCAAATTTGAA
GAGCTCAACATGGTATGTTCCTTGTTTTCTGCTTTGCTAATGAGATCTCCTTAGACTCTG
AATTCAGGACATTGCATCTAGA
>2079
AACCCAGATGAAGCTGTAGCGTATGGTGCTGCTGTCCAGGCTGGTGTGCTCTCTGGTG
ATCAAGATACAGGTAGGTCATCATCGCAGCATCTTTCTTAGTGATTCAGTAGCTTGATGG
AAGAGCTCGGTACCCCTATTGC
>2080
ACCAAGAACTCTCAGATCTTTTCTACAGCTTCTGATAATCAACCAACTGTTACAATCAA
GGTCTATGAAGGTAATTACCTTAAGTTTGGTTAATATCATGGCTTTTTTTTTGAGATGAA
GTCTTGCTCTGTTGCCCAGGC
>2081
AGGCCTGTCTGCAGAATCGACACCAACCAGCATCATGTCCATGACACTGGGGTACTGG
GACATCCGCGGGGTGAGTGAGGGTCCGCTGCACTGTGGGACCGGGCGCGTGGGCGGG

AAGTGCCGAGCGGCTGGGGACCGGC
>2082
CACGCCATGCGCCTGCTCCTGGAATACACAGACTCAAGCTACGAGGAAAAGAAGTATA
CGATGGGGGACGGTAATGACACCCTTGTGTCCGGGCTCTGCCCACTCACGCTGAGTTG
GCACCAAGCAACCCATGGTGGCCA
>2083
ATTGATGGGGCTCACAAGATCACCCAGAGCAACGCCATCCTGTGCTACATTGCCCGCA
AGCACAACCTGTGTGAGTGTGGTTGGCTGCAGTGTGTGGGGGGAAGGTGGCATCCTC
CTTGGCTGGATTGGGGTGCTATGCT
>2084
GGACATTTTGGAGAACCAGGCTATGGACGTCTCCAATCAGCTGGCCAGAGTCTGCTAC
AGCCCTGACTTTGTGAGTCCCTCCCTGGTCTGGACCAGAAGCCAGGCTTGTATTCCCAT
CTACTCTGGTCCTATTCACAATT
>2085
GGAACTTCCTACAATGATGCAGCACTTCTCACAGTTCCTGGGGAAGAGGCCATGGTTT
GTTGGAGACAAGGTAATGGGGGCATGTGATGAGGACACTAGAGATTTGCCATACATCC
TATGTTACAGAGATTCCAGCCCAC
>2086
CCACCGTATATTTGAGCCCAACTGCTTGGACGCCTTCCCAAATCTGAAGGACTTCATCT
CCCGCTTTGAGGTGATGCCCCCATCCTCCTTTCTCTTTGATGCCCCTTGTTCCGTTACCT
CCTTTCAGATGCTTTCCCAGT
>2087
GGGCCAGAGCTGGAAGGAGGAGGTGGTGACCGTGGAGACGTGGCAGGAGGGCTCAC
TCAAAGCCTCCTGCGTAAGTGACCATGCCCGGGCAAGGGGAGGGGGTGCTGGGCCTT
AGGGGGCTGTGACTAGGATCGGGGGAC
>2088
AAGTTCCAGGACGGAGACCTCACCCTGTACCAGTCCAATACCATCCTGCGTCACCTGG
GCCGCACCCTTGGTGAGTCTTGAACCTCCAAGTCCAGGGCAGGCATGGGCAAGCCTCT
GCCCCCGGAGCCCTTTTGTTTAAA
>2089
GGTGGACATGGTGAATGACGGCGTGGAGGACCTCCGCTGCAAATACATCTCCCTCATC
TACACCAACTATGTGAGCATCTGCACCAGGGTTGGGCACTGGGGGCTGAACAAAGAA
AGGGGCTTCTTGTGCCCTCACCCCC
>2090
GCAACTGAAGCCTTTTGAGACCCTGCTGTCCCAGAACCAGGGAGGCAAGACCTTCATT
GTGGGAGACCAGGTGAGCATCTGGCCCCATGCTGTTCCTTCCTCGCCACCCTCTGCTTC
CAGATGGACACAGGTGTGAGCCA
>2091
GGATCTCACCGTGGGTCCGATTAGCCTTTTCTCTGCCTTGCTTGCTTGAGCTTCAGCGG
AATTCGAAATGGTAAACTTCGCAGAGACGCTCGATGACTCCGCGGGTTTTTGCCGGCG
AGAGAAAAACCCGTTACACAGAG
>2092
TAAGGCTGGAAAGGACTCCGGAAAGGCCAAGACAAAGGCGGTTTCCCGCTCGCAGAG
AGCCGGCTTGCAGGTCAGTAGTTGTTCGTGCAGTCTGGGAGTCATGATGTTTTTCTTTG
CATTTCCTTTTTTGTTATTCATCG
>2093

CAGTCATGGACGTGTGGGCGCGACTGCCGCTGTGTACAGCGCAGCCATCCTGGAGTAC
CTCACCGCAGAGGTAAATGGGTGAACGCCTATAATCCGGAGAAGGTTTGGGGGGCGG
GGAGGCGGCAAGAGGGAAGGAGGAA
>2094
CACTTGCAACTTGCTATTCGTGGAGATGAAGAATTGGATTCTCTCATCAAGGCTACAAT
TGCTGGTGGTGGTATGTTAACTTCTAACATTTTAAAAAATTTCTTCAGAGGAAGGAATT
TTTTGCTGCTTTTAATTAGTTT
>2095
AACGGCAAGCTTGAGTCAGGACCCTTAATTAAGATCCTCAATTGGCTGGAGGGCAGAT
CTCGCGAGTAGGGTACAAGGCACTATGAAATGATCTAGTTTCGTGGGTGAGGGGCTGA
AGGGCCTATGATGCACGGAGGCGG
>2096
TTCGTAACGGGAATGCCGAAGCGTGGGAAAAAGGGAGCGGTGGCGGAAGACGGGGA
TGAGCTCAGGACAGGTAAGGGAATGAAATCAGCCCTTCTTCCTAGAAGCTGCGGCGG
GGGTGTTTGTCATTCCCTTGATGTACG
>2097
TGCCACACTCAAGATCTGCTCTTGGAATGTGGATGGGCTTCGAGCCTGGATTAAGAAG
AAAGGATTAGATGTGAGTGGAATTTGAGGGAAAGAGACATTTTTTAGTATTGAATGGTC
TTAGGGTTTAGTCACCCCTTTTC
>2098
GACAAGGAAGGGTACAGTGGCGTGGGCCTGCTTTCCCGCCAGTGCCCACTCAAAGTT
TCTTACGGCATAGGTGAGACCCTATTGATGCCTAATGCCTGAACTCTTCAAAACCAATT
GCTAATTCTCTATCTCTGCCCCAC
>2099
GCCCAACGCGCTGTCCGCCCTGAGCGACCTGCACGCGCACAAGCTTCGGGTGGACCC
GGTCAACTTCAAGGTGAGCGGCGGGCCGGGAGCGATCTGGGTCGAGGGGCGAGATGG
CGCCTTCCTCTCAGGGCAGAGGATCA
>2100
AGAAGTCTGCCGTTACTGCCCTGTGGGGCAAGGTGAACGTGGATGAAGTTGGTGGTG
AGGCCCTGGGCAGGTTGGTATCAAGGTTACAAGACAGGTTTAAGGAGACCAATAGAA
ACTGGGCATGTGGAGACAGAGAAGAC
>2101
CAAGGGCACCTTTGCCACACTGAGTGAGCTGCACTGTGACAAGCTGCACGTGGATCCT
GAGAACTTCAGGGTGAGTCTATGGGACCCTTGATGTTTTCTTTCCCCTTCTTTTCTATGG
TTAAGTTCATGTCATAGGAAGG
>2102
CAAGGGCACCTTTGCCCAGCTGAGTGAACTGCACTGTGACAAGCTGCATGTGGATCCT
GAGAACTTCAAGGTGAGTCCAGGAGATGTTTCAGCCCTGTTGCCTTTAGTCTCGAGGC
AACTTAGACAACTGAGTATTGATC
>2103
ACAAGGCTACTATCACAAGCCTGTGGGGCAAGGTGAATGTGGAAGATGCTGGAGGAG
AAACCCTGGGAAGGTAGGCTCTGGTGACCAGGACAAGGGAGGGAAGGAAGGACCCT
GTGCCTGGCAAAAGTCCAGGTTGCTTC
>2104
CGCTGGTGCGCGCCCTGTGGAAGAAGCTGGGCAGCAACGTCGGCGTCTACACGACAG
AGGCCCTGGAAAGGTGCGGCAGGCTGGGCGCCCCCGCCCCCAGGGGCCCTCCCTCCC

CAAGCCCCCCGGACGCGCCTCACCCA
>2105
ACCCCACGCGCTGTCCGCGCTGAGCCACCTGCACGCGTGCCAGCTGCGAGTGGACCC
GGCCAGCTTCCAGGTGAGCGGCTGCCGTGCTGGGCCCCTGTCCCCGGGAGGGCCCCG
GCGGGGTGGGTGCGGGGGGCGTGCGG
>2106
TGAAGCTGCAAGTATTTCTCATTGTGCTCTCTGTTGCATTGAACCATCTGAAAGCTACA
CCCATTGAAAGGTTGGTAACGTTTAAAATCCTGTTTCTTTGTAACTTTTGTAAAGTGTG
AGAAAATTAGCATTAAATACTG
>2107
GAAGAAGGCGGGTGGCGGGGGTGGCAAGAGGAAGGAGAAGCTGGAGAACATGAAG
AAGGAGATGGAGATTGTGAGCTCTGACTGGGGAGGGGCGGGAGCTGGAGGGTAGGCT
GGGAACCAGGCAGTGGGCGGGGAGGGGG
>2108
TGCCGCCGCCATCTGCCTCATCGCCTTTGCCATCCAGGCTAGTGAGGGGGACCTCACC
ACCGACGACAATGTGAGCCATGTTGTGGGGACAGGCTCAGGACATGGGGACCTGGGG
ATGGAAGACACTGAGCCTGGGACTT
>2109
CTGCTTTGGCTACTACCAGGAATTCAAGAGCACCAACATCATCGCCAGCTTTAAGAAC
CTTGTGCCACAGGTGGGTTCCCCAGCACCCAGCCACTCCAGATCCCAGAGACTCCAAA
CTGCTCCTGTAGCCTCCTTCCATC
>2110
CCCGAGTGCACGCACGAGAGCCCTCTGGAGACCCGCAACATCGCCTTCTTCTCCACCA
TGTGCCTTGAGGGTCTGTGAAGCCCCCGCCTGCCTGCCCAGTAACTGCTGCCTCCACT
GGCCTCTCTCCTGCTATCTTCTAG
>2111
GCGGGCCATGGTCTTCTTCATGGCCATCGTGGTGGCCTATGTGCCTGAGGGGCTGCTGG
CCACTGTCACAGTGAGTGGGGGCAGGCGGGGATGCAGGGAGCTGAGGGGCCTGCTCA
ACCATCTGTTCTTTCCTCCACATT
>2112
CGCATGACTGTGTCCCATCTGTGGTTTGACAACCACATCCACACAGCTGACACCACGG
AAGACCAGTCAGGTGTGGGGGAGGGAGGGCACAGGCGCCAGGGCAGGATCTGGTGG
ATGGTGGAGACCTGAAGAGGGATAGG
>2113
GTGCCGGGTGCTCACCCTGTGCAACCGCGCCGCCTTCAAGTCCGGCCAGGATGCAGTG
CCTGTGCCCAAGGTGAGAGCCAGGGGGCCTCCAGGGAATCCCGGGAGGCTGGTGTAG
CCGGCTTCGCCCCTGACACCGGTCC
>2114
CGCCATGGGCTACCGGGACCGCTTCCCAAAAGTCTGCGAGATACCCTTCAACTCCACC
AACAAGTTCCAGGTGCGCAGCCGGCCCCGCCCACGGCCTGGGCCCGCCCACCTCGGG
CCCCGCCCCAGGCCACACCCACATC
>2115
GACGAGCAGTGGCGCGAGGCCTTCCAGACCGCCTACCTCAGCCTGGGAGGCCTGGGC
GAACGCGTGCTCGGTGAGACCCCTGGGCAGGGGAGGAGACTCCAGGTGCGAGGAGG
AGCTAGCACAGACCCTGCCCACCACTC
>2116

ATCCATGATTGACCCACCCCGGGCCACCGTCCCTGATGCTGTGCTCAAGTGTCGCACC
GCAGGCATCCGGGTATGCCACTGTGGGGAGGAACAAGTGGAGTGAGGACAGAGGATG
GCAGCAGGATTTGGGTTGAAGAACC
>2117
CGGAAGGCAGCGAGACAGTGGAGGACATCGCTGCCCGCCTCCGTGTGCCCGTAGACC
AGGTTAATCGCAAGTAAGCCCCCCCAGACCTCCCCGGGGTTCTCCCCACATCTGGCAC
AGACCGAGGTCCCAGCGAGGTCCTC
>2118
CCCCGAGATGGTGTTTGCGCGCACCAGCCCCCAGCAGAAGCTGGTGATCGTGGAGAG
CTGCCAGCGGCTGGTGAGCCCTGCTGATGAGGGTGGGCAGGCGGGCAGACAGGCGGG
GCTGGTCTTGGACCGGCCTCTCACTG
>2119
GCCAAAAATGCAGCTGACATGATCCTGCTGGATGACAACTTTGCCTCCATTGTGACAG
GCGTGGAGCAGGGTCCGAGCCACGCCAGGGGGAGGGCAGGCAGTGTGGGCACAGGA
GGGAGAGGGCTGTGAGGAGGCTGAGG
>2120
CATCACCGTCAGCGTGCCCCTGCCCCTCGGGTGCATCACCATCCTCTTCATCGAACTCT
GCACTGACATTGTAAGTCCACAGCCCAGGGTACCCATCCACAGGGTGCCCAGAGACAC
TTGCCTGCAGATGGACAGGAGGG
>2121
CGTCCACGCAACCCAAAGCGTGACAGATTGGTCAACGAGCCCCTGGCTGCCTACTCCT
ACTTCCAGATTGGTGGGTGCCCGTGGGCCGGGGAGCCTGGAGGTGGGGGGCTCTGCG
CTGGGCTGCTCGCCTGCCCCAGGCC
>2122
CGTGGGGCTGCGGGCGCAGTGGGAGGACCACCACCTACAAGATCTGCAGGACAGCTA
CGGCCAGGAGTGGGTGAGCCCTGCCCCGCCCCATCCCATCCCTGGGCTCCCTCCTTCC
CAGGGCAACTTGCCTTGTGTGCGGC
>2123
TGTGCCAGATCGCCGATGTCCTCATCCGCAAGACGCGCCGTCTCTCTGCCTTCCAGCA
AGGCTTCTTCAGGTGTCCCTGGCTGGCCCTCTTCCTGCCCTCCAACCCCTGGTCCCAAC
TCCCATCAGATCCTGACTGTGGT
>2124
AGGTCTGCATCGGCTGCTTCCTGTGCTACTGCCCCGGCATGCCCAACATCTTCAACTTC
ATGCCCATTCGGTGAGAGGCTGTGAGGGGCAAGGACAGGGAGGGGGCTGTAGGGCAC
AGGCCTGGGATGGCCTTCACCTTG
>2125
CTGCCCTACGGCATCCTCATCTTCGTCTATGATGAGATCCGGAAGCTTGGAGTTCGCTG
TTGCCCAGGGAGTGAGTGTGGAGACTGGGAGCCTGGGGGCAGGGGACTGAAACGTTT
GAGCAGCTGCACCCACCTTTGATC
>2126
CTCGGGTGGAGTCTTCTGACAGCTGGTGCGCCTGCCCGGGAACATCCTCCTGGACTCA
ATCATGGCTTGTGTGAGTGTGGGGACCCCCCCCCAAGGTCCAGGGGATAGGGCAGGA
ACTGATGGCCAGAGGAGAGCTGGGC
>2127
CCAGCAACCTGAATCTCAAACCTGGAGAGTGCCTTCGAGTGCGAGGCGAGGTGGCTC
CTGACGCTAAGAGGTGAGAAGTGAAGTCGGGGTGGTGGGCGGCAGGGACGGGCTTG

GTCCAGCAGGGAGGGCGTGGCCGGCCA
>2128
GGACGGCGGGGCCTGGGGGACCGAGCAGCGGGAGGCTGTCTTTCCCTTCCAGCCTGG
AAGTGTTGCAGAGGTGGGCTGCAGACCGGAACCGGGGACCAGGGACAGGGGCTGGG
TGGGCTGGGGCGGGGCTGGGTTAGTGA
>2129
GGCCGAAGTGGCTAACCAAGAAACTAAAGAAGACTTACCTGCGGAAAACGGGGAAA
CGAAGACTGAGGAGGTCAGAAGTGTGTGTGTGTGTGTGTGTGTGTGTGTGTGTGTG
TGATTGCTTTGACTCTGAACAGTAGT
>2130
CAAGAAGAAACACCCGGACTCTTCCGTCAATTTCGCGGAATTCTCCAAGAAGTGTTCG
GAGAGATGGAAGGTGAGGCAGGAGAGGGACGGAGCTCAGGGGGTGCAGGTGTGGTT
TTCGGCTAGGAGGGCCTTAGGCAGGT
>2131
AAAATTACGTTCCTCCCAAAGGTGATAAGAAGGGGAAGAAAAAGGACCCCAATGCTC
CTAAAAGGCCACCGTAAGTTTAAAATAACCCAAATTGCTCCTTGGATTTTTCCTTCAGT
TTATTAAACTCTGTTGCTTCCTTT
>2132
TGAGCAGTCAGCCAAAGATAAACAACCATATGAACAGAAAGCAGCTAAGCTAAAGGA
GAAATATGAAAAGGTACAGTGTCATCTTTTTTAAAGCCGTGGATAAGACTAGGTATAGG
TAATAACTGTAGAAAACCTGGGAA
>2133
AGCCCAGGGACAACCTGCCGCCCCAGCGCCTCAGAAGAGAGGACGCGGCCGCCCCA
GGAAGCAGCAGCAAGTCAGTACGAGGGCGCGGTGGGGGCACCAGCCCACCCCGTCCC
CACTGCCGGGGCCCAGACACGCGCGGG
>2134
CTCTCCTAAGAGACCCAGGGGAAGACCCAAAGGCAGCAAAAACAAGAGTCCCTCTAA
AGCAGCTCAAAAGGTGAGATTTCTCAAGTCAAGCTCTCCTAACTTCATCAATGACTGA
CTACAGGAGCCTGCCTGTAACTTTC
>2135
GCCGGTGCTGCGCTCCTCTAATTGGGACTCCGAGCCGGGGCTATTTCTGGCGCTGGCG
CTCCAAGAAGGCGTGAGTTCGCGGCCGCTCCGGTGGCTTCTTTTTTTTATATCTATAATT
TAATTAAATTATTTATTTATTG
>2136
TCCTCAGCGCCCAGCACCGCCGCTCCCGGCAACCCGGAGCGCGCACCGCAGGCCGGC
GGCCGAGCTCGCGGTGAGTCGTCCCCGGGGCCCGCGGCGGCGGCGGCGGAGGGGGG
CGCCCGCGCCCCGCCCGACGTCGCCCC
>2137
GGCGGGGAGAGACACGGGCTGCGGCGCGGCAGGAGAGTGGGGGGTCGGGCGCCCCC
CGCAGCTCAGGGAAGTTCTGGAAGTGAGGGAGATGGGGAGGAACCCCCAAATTCGGC
CCTACGCCCTCTCGGCCTTTCTCTCGC
>2138
GGTCTCGAACTCCTGACCTCAAGCAGTCCTCCTGCTTTGGCATCCCAAAGTGCTGGGA
TTACAGGCATGAGTCACCTCACCTGGCCCATAAATCATTTTCAAATGGTCTAGTTCTTAT
TTTGGTTAGAGTGAAGATTTCT
>2139

GAAGCGGGGCCGGGGCAGGCCGCGCAAGCAGCCTCCGGTGAGTCCCGGGACAGCGC
TGGTAGGGAGTCAGGTGGGTGTCCAAACCTTTGCTTCACTTGGTTTACCCTTTGGGGCT
AGGGAGGTGCCTGGAGTTTCATTCA
>2140
AGTGCCAACACCTAAGAGACCTCGGGGCCGACCAAAGGGAAGCAAAAACAAGGGTG
CTGCCAAGACCCGGGTGAGACTTGAGATGGGACTACCCCTGGGTGGATGACTAGCAG
AGGATCCTCTTGTTGGCACCATGGCCA
>2141
GAGCCGAGCCCGGCGCCGCCCGCCCAACCCCAGATCTACCCCTGGATGCGCAAGCTGC
ACATAAGTCATGGTAAAGCCAGCCTTTTTCTAAATCCACGCGACCGCGGGGACGCGGC
TCTCGGTCCCCCCTCTCTTCTCTG
>2142
AAGCAGATCTTCCCCTGGATGAAAGAGTCTCGACAGAACTCCAAGCAGAAGAACAGC
TGTGCCACTGCAGGTAGCTCCCTGAGGTGGCCTACTGCCAGACCAAGCCCCCTCCAGA
TTGACCCAAGGAAGCCTAGTCAGGG
>2143
AAGACATCGGGGACATTCTGCAGCAGATAATGACCATCACCGACCAGAGCCTGGACGA
GGCCCAGGCCAAGTGAGTGCCCCCACTCCGGGACCCCACACAGACCCAGCAAACCCC
GTTCACATGTTCTGAATCTTCTGGG
>2144
CACGCCCTAAACTGCCACCGAATGAAGCCTGCTCTCTTTAGCGTCCTGTGTGAAATCA
AGGAGAAAACTGGTATGTGGGCGCCCCCCGGATTGCTCAACTCTGGGAACAGAACCC
TGTTCATTATAGGGCTAGAGTGTGA
>2145
CTCGGACTATCGCAGCAAACTTGCCCAGATCCGTCACATATACCACTCGGAGCTGGAG
AAGTATGAGCAGGTAAGGAGAGGAGGCTTGGGTGGGTGGAGGGAAGGGCTCTTGCAG
GGGAATCCCATGGTCAAAGGGCTCC
>2146
TCCAGATGCAGCTGAAGCAGAGCACCTGCGAGGCTGTGATGATCCTGCGCTCCCGTTT
CCTGGATGCCAGGTGGGCCCAGGGACCCCAGGCTGGCCCCCAGCACTGGGCTCCTTC
CCATTCCTCTCCAAGACCCTGAGCT
>2147
GAGTAACCCATATCCTAGTGAGGAGGCCAAGGAGGAGCTTGCCAAGAAGTGTGGCATC
ACCGTGTCTCAGGTATTATGGAGGTTGCGGGAGGAGTTGTCAGGCAAAGTGCACGCAT
CTCAGCTAGGTGCAGTGGTGTGTT
>2148
AAGACCGCCGTGTCAGTCACCCAGGGGGGCCACAGCCGCACCAGCTCCCCGACACCC
CCTTCCTCTGCAGGTGGATCCCACTGTCACCCCGGCTGTTTTGCACACTTCCTGCTTTT
GTTCCCACTTCCTATCTAGGCAGG
>2149
TCTCTCAGGATCTGGAGACATGTTTCTGGGGATGCCTGGGCTCAACGGAGATTCCTATT
CTGCTTCCCAGGTCAGATGCCCATCTCCTCTCGAATAGGGCTTTCCCCAACTCCATTTC
CTCTACTTTAGGATACAAGACC
>2150
CTCGATGGGGCCAGGGGGCTATGGGGATAACCTCGGGGGAGGCCAGATGTACAGCCCA
CGGGAAATGAGGGTGAGTGGATCCTGAAGCTCCTCTCTGTCCAGTTCTCACAGGACAG

AGGGGCATTTTCCCTAGTAATGTT
>2151
TGGCGAGCCACTGGTGCTGAAGTGTAAGGGGGCCCCCAAGAAACCACCCCAGCGGCT
GGAATGGAAACTGGTAAGCGGGGCTCCTGTTGCAGCCTCCCAACTTCCAGGGAGACC
AGCAATGATTTGGATCCCCGTCACTC
>2152
ATTTTCCGGTGCCAGGCAATGAACAGGAATGGAAAGGAGACCAAGTCCAACTACCGA
GTCCGTGTCTACCGTAAGAATTCCAGGGTCTTCTCCAAGGCCTCCCTCTTACCTAAGAA
AAAGCCTTCAACCCCAGCCTTGGC
>2153
GAGGGAAGCTACCCTGCAGGGACTCTTAGCTGGCACTTGGATGGGAAGCCCCTGGTG
CCTAATGAGAAGGGTGAGTCCTAAGGTGCCCCCCAAGCTGCCTTCTCCCTGATCTCAC
TCCCACACCCACCCTGGGATAATTT
>2154
TGTAGCTTCAGCCCAGGCCTTCCCCGACACCGGGCCTTGCGCACAGCCCCCATCCAGC
CCCGTGTCTGGGGTGAGCATAGGTGGGGAGGGCCCCAAGCTCACGTGAGCACGTTCT
GGAAGTCTGACCCTTAGGGAAAGAG
>2155
TGGTGGAACCGTAACCCTGACCTGTGAAGTCCCTGCCCAGCCCTCTCCTCAAATCCAC
TGGATGAAGGATGTGAGTGACCTGGAGAGAGGGGCTGGGAGGTAGGGTGAACCATAA
CTAGCAACAGGGAGGGCAGAGGGCT
>2156
TACAGCTGTGTGGCCACCCATTCCAGCCACGGGCCCCAGGAAAGCCGTGCTGTCAGCA
TCAGCATCATCGGTGAGACCTCTCCCCAAGCCCTACAGACCCTGGGACTAGGGTGCAG
GACAGCACAGGCTCTAATTTCCTG
>2157
GCCTGGGGACAGCCGCCCTGCTCATTGGGGTCATCTTGTGGCAAAGGCGGCAACGCC
GAGGAGAGGAGAGGTGAGTGGAGAAAGCCAGACCCCTCAGACCTAGGGCTTCCAGG
CAGCAAGCGAAGAGGGGTCGGGGGGTG
>2158
GTCATTGCCCTCCTGCTCTGGGGACAGCTTTTTGCAGTGGACTCAGGCAATGATGTCAC
GGATATCGCAGGTCAGTCTTTGGTTGGGTAGGAGTGTGCATCCCACTCTGACCCTCTCG
GGTCTGCACTCTCTCTGAGAAC
>2159
CATGGCTATGTGGAGCACTCGGTTCGCTACCAGTGTAAGAACTACTACAAACTGCGCA
CAGAAGGAGATGGTAAGATGTGGACAACTGTCTCCATGCCCTACATACAACCCCCTTCT
CTGACATTTCCATGATGGGTGGT
>2160
TACACCTTAAATGATAAGAAGCAGTGGATAAATAAGGCTGTTGGAGATAAACTTCCTGA
ATGTGAAGCAGGTGGGTGCTGAGCACTGAGCACTTAAGAGAGCAGGCAGGCGTCCAG
CGGGGAACGTCCTAGAGGCACAGC
>2161
CATGGCTATGTGGAGCACTCGGTTCGCTACCAGTGTAAGAACTACTACAAACTGCGCA
CAGAAGGAGATGGTAAGATGTGGACAACTGTCTCCATGCCCTACATACAACCCCCTTCT
CTGACATTTCCATGATGGGTGGT
>2162

TACACCTTAAACAATGAGAAGCAGTGGATAAATAAGGCTGTTGGAGATAAACTTCCTG
AATGTGAAGCAGGTGGGTGCTGAGCACTTAAGAGAGCAGGCAGGCGTCCAGCGGGGA
ACGTCCTAGAGGCACAGCCTTCCAG
>2163
TTTGCATACCTAATCATTATGCTGAGGATTTGGAAAGGGTGTTTATTCCTCATGGACTAA
TTATGGACAGGTAAGTAAGATCTTAAAATGAGGTTTTTTACTTTTTCTTGTGTTAATTTC
AAACATCAGCAGCTGTTCTG
>2164
AGCACTGAATAGAAATAGTGATAGATCCATTCCTATGACTGTAGATTTTATCAGACTGAA
GAGCTATTGTGTGAGTATATTTAATATATGATTCTTTTTAGTGGCAACAGTAGGTTTTCTT
ATATTTTCTTTGAATCTCT
>2165
CTGGCAAAACAATGCAGACTTTGCTTTCCTTGGTCAGGCAGTATAATCCAAAGATGGTC
AAGGTCGCAAGGTATGTATGACATTTTGACACAGAATATTTTCCTCATTTGAAGGGGGA
TTAAGTGATTGCTTCTTTTTAA
>2166
ATTTGAAATTCCAGACAAGTTTGTTGTAGGATATGCCCTTGACTATAATGAATACTTCAG
GGATTTGAATGTAAGTAATTGCTTCTTTTTCTCACTCATTTTTCAAAACACGCATAAAAA
TTTAGGAAAGAGAATTGTTT
>2167
GAGAAGGAACTGAAGGAGATCAGGGCCTTGGACAAGGCCTTCAGACCAGAATTGAGA
GAGGAATTTTCTAGTAAGTAAACTTGAGTCATGGGTCTGTGGCTCCATCTTAAACTCTG
CATGGGTGTGGGGAGGTTGACCTT
>2168
CACACCGCCTGTATCATTGATGTCTTTGGTGTCACTCACAGAGAGTCCATCATGAATGT
CAATGTGAAAGGTACAGTAGCCTGGGGAGGAGATAAAACAAGTTGGTTAAATGAGGAT
CAGAAAGAAGGACAAGAAAGGGA
>2169
GCAAAAGTGGATGAGTTTCCGCTTTGTGGCCACATGGTGTCAGATGAATATGAGCAGC
TGTCCTCTGAAGGTAAGGCAGGATTCTTTGTTCGTCACCCCCCAGTCCTTCCTCCGTGC
TCCCTCAACCCCACCCACATACA
>2170
TCCGGGTGCGGCTCCACCCCTTCCACGTCATCCGCATCAACAAGATGTTGTCCTGTGCT
GGGGCTGACAGGTGAGCTTGGTCTGGGCCTTTTAAGGCAGTTGGAGTCTCTACATTATT
AGGCTTGCATTATTTTATCAGA
>2171
GCTGCAGAACAAGGAGCATGTGATTGAGGCCCTGCGCAGGGCCAAGTTCAAGTTTCC
TGGCCGCCAGAAGGTATGTAGTGCTGCAGCCCCCTTCTCCCACCTTTGCCCCAGCCTCC
TGACTCAGTTCTTTCCATTGCTCC
>2172
ATTGATCATCAATACTTTCTACTCGAACAAAGAGATCTTTCTGAGAGAGCTCATTTCAA
ATTCATCAGATGTAAGTCACTTATTAACCCAGAATCGGATTTTGGTTTCAGTGTGAACTT
CTTGGGGGTGCTGTATGCTTA
>2173
CATAACGATGATGAGCAGTACGCTTGGGAGTCCTCAGCAGGGGGATCATTCACAGTGA
GGACAGACACAGGTAGGCACCTGAACATTCACTGGTTAAGTGAGCGGTGGAAGGGTG

GGGTGCTGCAACCCTTGGATCCCTG
>2174
GGAGGAACGAAGAATAAAGGAGATTGTGAAGAAACATTCTCAGTTTATTGGATATCCC
ATTACTCTTTTTGTAAGTTTTTATGTAATTGCAGAGTGAATTTCTGTCTGTAGGTGATTG
GGGTGACTGTACTACATCCTTA
>2175
TACTAATGAGGAGTACGGAGAATTCTATAAGAGCTTGACCAATGACTGGGAAGATCAC
TTGGCAGTGAAGGTGAGTGACTGATGGGTGCTTCAAGCTTGTCCTTAGATTATCATCTT
TCTCCACCACCCCAAATATCTTC
>2176
AACATCAAATTGTATGTACGCAGAGTTTTCATCATGGATAACTGTGAGGAGCTAATCCC
TGAATATCTGAGTAAGTATAGATAGGAAAAATAATCACTGTCACTGATTAAAGAAGTAC
TTTCTGGGTGGGCATGGTGGCT
>2177
CTTTACTGAACTGGCGGAAGATAAAGAGAACTACAAGAAATTCTATGAGCAGTTCTCT
AAAAACATAAAGGTTGGTGTAAATAACCATTAGTTTTCCAATTGGCCTCTTTAGTTTTTT
TTTTTTTTTTTTAATTCAGAAA
>2178
GAGATGGTTTCTCTCAAGGACTACTGCACCAGAATGAAGGAGAACCAGAAACATATCT
ATTATATCACAGGTAAGAGAACACTATGTTACAGTCATACACGTCGTTCTTACAACCTTG
TAGGCTCTGTGGGTGTGTTTTC
>2179
AGAGAAAAAAACAAAGTTTGAGAACCTCTGCAAAATCATGAAAGACATATTGGAGAA
AAAAGTTGAAAAGGTATGTGAATACAGCATTTCCTGATCATTGATACTTCTAAGGTGCT
TTCAAGCTTAGTCATACATAGCCC
>2180
TCTGGCTTCAGTCTGGAAGATCCCCAGACACATGCTAACAGGATCTACAGGATGATCA
AACTTGGTCTGGGTAAGCCTTATACTATGTAATGTTAAAAAGAAAATAAACACACGTGA
CATTGAAGAAAATGGGTAAACTT
>2181
CCTCATCATCAATACCTTCTATTCCAACAAGGAGATTTTCCTTCGGGAGTTGATCTCTAA
TGCTTCTGATGTAGGTGCTCTGGTTTCCACATTTGGCATGGTTTTTTTTTTTGATACTCTA
GAAGGAGGGGAAAGGAGTG
>2182
AGCTGATCTCATAAATAATTTGGGAACCATTGCCAAGTCTGGTACTAAAGCATTCATGG
AGGCTCTTCAGGTATTGCAGTTCTGTAGGCATTCATACTTATCTGTGTTCTTTGGTTTTTT
GCTTCTTTAAAACTTGTGAT
>2183
CACAACGATGATGAACAGTATGCTTGGGAGTCTTCTGCTGGAGGTTCCTTCACTGTGC
GTGCTGACCATGGTAAGTTAGCTTTTCTGTTACAAGGTAGTTGGGTTAGGATTTTCTGG
GCTCACACCAGTAGCAGAAATTT
>2184
AGAAGAGAGGCGGGTCAAAGAAGTAGTGAAGAAGCATTCTCAGTTCATAGGCTATCCC
ATCACCCTTTATGTGAGTATGGACTTTTAAATCTTTTACACTTAACGTGCAGGATGTTTC
CTGTTCTGGAGAATCTCATTGT
>2185

CACCCAAGAGGAGTATGGAGAATTCTACAAGAGCCTCACTAATGACTGGGAAGACCAC
TTGGCAGTCAAGGTGTGAGAAGCCTTTGCATGTTGGCTCAACATGCACATATGGAGAG
GAATGAGTTAGGTGGAAGAGTGTT
>2186
AACATCAAACTCTATGTCCGCCGTGTGTTCATCATGGACAGCTGTGATGAGTTGATACC
AGAGTATCTCAGTGAGTATCTCCTTGGCCTAATTTAGTTGGGTGAAGTCTTGGGAGGTT
TTAGGCATTCTGCTAGGATATT
>2187
CTTCTCTGAGCTGGCAGAAGACAAGGAGAATTACAAGAAATTCTATGAGGCATTCTCT
AAAAATCTCAAGGTAAAAAGGCAAATAATGCTTATTCCCTTTACCACTTTCTTAGTAATA
ACAATAAATTATTCCATTCACA
>2188
GAGATGACATCTCTGTCAGAGTATGTTTCTCGCATGAAGGAGACACAGAAGTCCATCTA
TTACATCACTGGTGCGTTGACTCTGATTGAAGCCTTTTTGGAGGAGTGGGGAGCACAA
TTAGGGCTTCCTGGGAACTGGCA
>2189
AGAGAGCAAGGCAAAGTTTGAGAACCTCTGCAAGCTCATGAAAGAAATCTTAGATAA
GAAGGTTGAGAAGGTAAGCCATTCTGGGGCTAGGATATATTTTGTAACATCTTCGAGGT
GGGCTCCCTCACAAGCATGTTTCT
>2190
TCTGGCTTTTCCCTTGAGGATCCCCAGACCCACTCCAACCGCATCTATCGCATGATCAA
GCTAGGTCTAGGTAAGTAGCTTTGGTACTTGGTGTGGCAAGGAGTTTGTGCAACTCGT
CTCCTCTATGGATTTGACTTAAT
>2191
GGCATCGGCTTCCACCTCCAGGCGCTCCCCGACGGCCGCATCGGCGGCGCGCACGCG
GACACCCGCGACAGTGAGTGGCGCGGCCAGGCGCGAAGGGGCGGGGGCGGGGGGCA
ACGGCCGCCGGGCCAACCCGCTCAGTC
>2192
GGTGAGCATCTTCGGCGTGGCCAGCCGGTTCTTCGTGGCCATGAGCAGCAAGGGCAA
GCTCTATGGCTCGGTGAGTACCGCAGGGGTCTGGCTAGGCACCTAGTTGGGAACAGCG
GACATGGCTAGCAGGCTCGTGGCTT
>2193
ATCATCACCACAGCCCTGGTGTGCTTGCTGCTAGCTGGGATGTGGCCGGAAGATGTGG
ACAGCAAGAGCAGTGAGTGTGGCAGGCATCATTTTGCTTCTCTCTGGGGAGGGCAGA
AACGTGGTCAGCCACTCTGGGGTTG
>2194
AAGAGATTCCCCTGAGGGCAATCCTGTGTTACAGAAATACCAGCTCCATCTGCTCCAAT
GAGGGCTTAATGTAAGTGATCACCTGCTCAATCTCTCCCTAGAGAACAGAACCCCGCC
AGCCTGGAATTACAAGAGTAGAC
>2195
GGGCTCTGCGTCAACGCTAGTGCCGTCAGCCGCCTGCGCGCCTACCTGCTGCCAGCGC
CGCCAGCTCCAGGTGAGCCGCCCGCCAGGTGCGCTGCGTGCAGCACCGCCACTGGCG
CCGAAGGGCCTGGGGGTTGCTGGGT
>2196
AGTTGACTACGAGTCTCAGAGCACAGATACCCAGAACTTCTCCTCCGAGTCCAAGCGG
GAGACAGAATATGTGAGAGCTTTTCCTCTTGTTAAAGGAGGAGGGCAAGACCTGCCAA

GCCTGGGTACTCAGAGCCTCTTGA
>2197
CAATGTGCTGAGTCCCAGGGGTGTACACATTCCCAACTGTGACAAGAAGGGATTTTAT
AAGAAAAAGCAGGTGAGTGAGGTCCTCAGTGTGTTTTCTTCCTCTTCTGTTGACACAG
AGGAGAAACCCATGTCACCAGCGC
>2198
GCGCATGAGACCCTGGCTAGAGATGCAGATTAATTCCAACCAAATCCCGGGGCTCATCT
GGATTAATAAAGTGAGTGTAACTCTTTGGGTTTTCCTGCCACTGTTTTAACCCATGTACT
TCTGGAGGGACCAAAGCTTCA
>2199
CATGCTGCCAAGCATGGCTGGGACATCAACAAGGATGCCTGTTTGTTCCGGAGCTGGG
CCATTCACACAGGTGTGTGCCTGGGACTCAGGCCTAGGAAGCCCAGGGTAGAGACAA
GAGGAGGCACTCACGTTAACACAGA
>2200
AGGAACAAGGGCAGCTCAGCTGTGCGAGTGTACCGGATGCTTCCACCTCTCACCAAG
AACCAGAGAAAAGGTATCCAAGGACTCTGGGTCCTTGGGAAGCCCTCAGGGAGGGAG
GGTAGAAGGAGGTCAGCTGGGGCTGG
>2201
GATGACCACAGCAGCTACACAGTTCCAGGCTACATGCAGGACTTGGAGGTGGAGCAG
GCCCTGACTCCAGGTGAGCTGGTCCAGGTCTGGCAGGAGACCCCACAGGTCAGTGGG
ATGACTCTTTCTCTTGGAGGCATGGT
>2202
GAAGTTGTGCCGGACAGCACCAGTGATCTGTACAACTTCCAGGTGTCACCCATGCCCT
CCACCTCTGAAGGTTGGTGCTCCTGGGGCCTGGCCTGCTGCTTGACTGTCTGGGTCTG
TGAAGGGCTTCCTGAGAGAGAAAA
>2203
GGAGTCCAGCCCACCTCTGTCTATGGAGACTTTAGCTGTAAGGAGGAGCCAGAAATTG
ACAGCCCAGGGGGTAAGAAGGCCCTGGATCCTTATGGCTTCTTAGATGAGGGAGAACC
ACGTAGGGATGGAGAAAGCTTGGG
>2204
GAGTCCATGAAGAAGATGGCTCCTGCCATGGAATCCCCTACTCTACTGTGTGTAGCCTT
ACTGTTCTTCGGTAAGTAGAGATTCAATTACCCCTCCCAGGGAGGCCCAAATGAATTTG
GGGAGCAGCTGGGGTAGGAACC
>2205
AGTGGAGAATACAAATGTCAGCACCAACAAGTTAATGAGAGTGAACCTGTGTACCTGG
AAGTCTTCAGTGGTAAGTTCCAGGGATATGGAAATACAGATCTCTCATGTGAGGGATGG
CTCATCTGAAGATGGGAAAAAAC
>2206
TACTACTGTACGGGCAAAGTGTGGCAGCTGGACTATGAGTCTGAGCCCCTCAACATTA
CTGTAATAAAAGGTGAGTTGGTAAAGGAAAGGAAAAGCATCCATAGCAGGGGAAGGA
AGAGAGAACTTCTGAGCCTGAGCAG
>2207
CTGCACGCCCTCACCCGCGGCCAAGGCGCCTGCGTGCAGGAGTCTGACGCCTCCGCT
CCCCATGCTGCAGGTACCACAGTCCCGCCCCTGCTCCAGCACCTCCTGCCGCCCGCCC
CCGCCCGGCACCTCCCGCCCCCACT
>2208

CCTTTGGGACGCCATCAGTACCTATGATGGCTCGAAGGCTCTCCATGTCACCAACATCA
AAAAATGGAAGGTGAGGCCCAGCAGGTGGGTGGTGGGAACTCTCCTGTCAGAGGATG
TAGCTTGAGTCGGCTGTGACAAGC
>2209
ATCAGGAGAAGAAATTTCCAAATTTTACCTGCCAAACTGCAACAAGAATGGATTTTATC
ACAGCAGACAGGTAGGTGGCCTTGCCAGTGTGCGTCGTCAGGGTGAAAGGGACTACT
GCCCTACATTCCTGCCAAGCCACG
>2210
CTGGTTCCCTGCCCACAGACCTTCCAGGAGAATGACCTGAGCACCTTCTTTCCCTTCAT
CTTTGAAGAAGGTAGTTAGCCAAGAGCAGGCAGTAGATCTCCACTTGTGTCCTCTTGG
AAGTCATCAAGCCCCAGCCAACT
>2211
AGCTTGGTGATGTCTGGTCCATATGAACTGAAAGCTCTCCACCTCCAGGGACAGGATAT
GGAGCAACAAGGTAAATGGAAACATCCTGGTTTCCCTGCCTGGCCTCCTGGCAGCTTG
CTAATTCTCCATGTTTTAAACAA
>2212
GGGCCTCAAGGAAAAGAATCTGTACCTGTCCTGCGTGTTGAAAGATGATAAGCCCACT
CTACAGCTGGAGGTAAGTGAATGCTATGGAATGAAGCCCTTCTCAGCCTCCTGCTACCA
CTTATTCCCAGACAACCACCTTC
>2213
AAGCAAAAACTTTCACTTAAGACCCAGGGACTTAATCAGCAATATCAACGTAATAGTTC
TGGAACTAAAGGTAAGGCATTACTTTATTTGCTCTCCTGGAAATAAAAAAAAAAAAGT
CAGGGGGAAAAGTACCACATTTT
>2214
TACAAAGAAAACACAGCTACAACTGGAGCATTTACTGCTGGATTTACAGATGATTTTGA
ATGGAATTAATGTAAGTATATTTCCTTTCTTACTAAAATTATTACATTTAGTAATCTAGCTG
GAGATCATTTCTTAATAAC
>2215
AAGCAAAAACTTTCACTTAAGACCCAGGGACTTAATCAGCAATATCAACGTAATAGTTC
TGGAACTAAAGGTAAGGCATTACTTTATTTGCTCTCCTGGAAATAAAAAAAAAAAAGT
AGGGGGAAAAGTACCACATTTTA
>2216
CTGCCCCTGCTGGGAGTGGGGCTGAACACGACAATTCTGACGCCCAATGGGAATGAA
GACACCACAGCTGGTGGGAAATCTGGGACTGGAGGGGGCTGGTGAGAAGGGTGGCT
GTGGGAAGGGGCCGTACAGAGATCTGG
>2217
TCGAGTACATGAATTGCACTTGGAACAGCAGCTCTGAGCCCCAGCCTACCAACCTCAC
TCTGCATTATTGGTATGAGAAGGGACGAGGGGGAGGGGATGAAGAAGAGGTGGGTTG
GATCAGAGACCAAGAGAGAGGGTAG
>2218
GTTGTTCAGCTCCAGGACCCACGGGAACCCAGGAGACAGGCCACACAGATGCTAAAA
CTGCAGAATCTGGGTAATTTGGAAAGAAAGGGTCAAGAGACCAGGGATACTGTGGGA
CATTGGAGTCTACAGAGTAGTGTTCT
>2219
CAACAGATTCTTGAACCACTGTTTGGAGCACTTGGTGCAGTACCGGACTGACTGGGAC
CACAGCTGGACTGTGAGTGACTAGGGACGTGAATGTAGCAGCTAAGGCCAAGAAAGT

AGGGCTAAAGGATTCAACCAGACAG
>2220
CTCTGTGGAAGTGCTCAGCATTGGAGTGAATGGAGCCACCCAATCCACTGGGGGAGC
AATACTTCAAAAGGTAAAATGGGCCCACATGACCCAATCCATGAGCCCAACACCCCAG
CCTTTCTAACACCACTGTCTTTTGC
>2221
CCGTGGTTATCTCTGTTGGCTCCATGGGATTGATTATCAGCCTTCTCTGTGTGTATTTCTG
GCTGGAACGGTGAGATTTGGAGAAGCCCAGAAAAATGAGGGGAACGGTAGCTGACAA
TAGCAGAGGAGGGTTTTGCAGG
>2222
GACGATGCCCCGAATTCCCACCCTGAAGAACCTAGAGGATCTTGTTACTGAATACCAC
GGGAACTTTTCGGTGAGAACGCTGTCATAAGCATGCTGCAGTCTATCAACTGCCAACT
GCCTGCCAGCAAGACAGACAGAGT
>2223
CCACGGACACAAGTGCGATATCACCTTACAGGAGATCATCAAAACTTTGAACAGCCTC
ACAGAGCAGAAGGTGAGTACCTATCTGGCACCATCTCTCCAGATGTTCTGGTGATGCTC
TCAGTATTTCTAGGCATGAAAAC
>2224
CCACAGGCACAAGCAGCTGATCCGATTCCTGAAACGGCTCGACAGGAACCTCTGGGG
CCTGGCGGGCTTGGTAAGCTGCACTGTATTCCTGGCAAGCCGGCCGCGTGGCTCCTGG
TGGACAGCAGCCTCACTTCTAAACA
>2225
GCAAGGGGGTACTGTGGAAAGACTATTCAAAAACTTGTCCTTAATAAAGAAATACATT
GACGGCCAAAAAGTAAGTTACACACATTCAATGGAAGCTATATTTGTCCTGGCTGTGCC
TATTTCTATGGAATTGACAGTTT
>2226
CACAAGTGCATTGGTGAAAGAGACCTTGGCACTGCTTTCTACTCATCGAACTCTGCTG
ATAGCCAATGAGGTAATTTTCTTTATGATTCCTACAGTCTGTAAAGTGCATAGGTAATCA
TTTGTGATGGTTCCTTTACTAT
>2227
GCAAGGGGGTACTGTGGAAAGACTATTCAAAAACTTGTCCTTAATAAAGAAATACATT
GACGGCCAAAAAGTAAGTTACACACATTCAATGGAAGCTATATTTGTCTGGCTGTGCCT
ATTTCTATGGAATTGACAGTTTC
>2228
CGTGGCAGGCCAGGGGTGTCCAACCTTGGCGGGGATCCTGGACATCAACTTCCTCATC
AACAAGATGCAGGTAGGCTGCAGGGGGAGCCCATGGGAAAGACAGCTACTGACAAAG
TGAAATATGTATGAGGATGAAAAAA
>2229
AACAAGATACCCACTGATTTTCAGTCGGGTGAAAAAATCAGTTGAAGTACTAAAGAAC
AACAAGTGTCCAGTAAGTTTGTTTTCATATGTGATATGTTCCTGTTGGTGATTTCTATGT
GAATGGTGATGCCAACCCTGTT
>2230
GGTGACAAATCACCTCCAGGTTGGGGATGCCTCAGACTTGTGATGGGACTGGGCAGAT
GCATCTGGGAAGGTGAGTCTGTGCTTTGGGCTTCCCAACCTCTCAAGTCAGCATGAAA
TTCAGAAGGCAGAGAGGGACATGT
>2231

GCATCCCAGAGCCCCGCTCCTGGGTACCCACAACATGAACACCGTCCAGAAGCAAGG
GCAGCCTCTGCAGGTGGGGCGGGGGTTGGAAAACATTTATCAAACGGTTGAGTTGGGT
GCAGGGGATGCAACATGATCAAACA
>2232
CAGAGAACTTGCCACGGTGTTTCATGCTGTGGCTGGTGGTTCCAGGCTGCACGCTCCA
TTCTAGGAAAGGGTGAGGCTTCCTGATCATCAGTCTTAACAGGGGACTGTCCTATGGGT
ACCTGTATACGCTGCCGGGAGTG
>2233
TGGCTCCTGGCCTGCATCTGCATCTGCACCTGTGTCTGCTTGGGAGTCTCTGTCACAGG
GGAAGGACAAGGTGAGGGCTGGGCACTAATGTCTGTATGAGGTGGGTGGAGAACTAG
GGCATGTTTGGGGGACTGGGTTGT
>2234
TCAGGATCGATTGCCACTGGTCTGCCCCAGAGCTGGGACAGGGCTCCAGCCCCTGGCT
CCTCTTCACCAGGTGAGCATGGAGGGCCATGCCCACCTGGACAGGGATGAGGGTGAG
TTCCCCAGGATTGAAGCAGCTATGC
>2235
TTCCACCACTGCATGTCTGGGAGGGAGCAGGTCAGCCTGGTGGACCCGGAGTACCTGC
CCCGGAGACACGGTGAGCAGCAGCTATAGGTCTGGGGCGGGGCCGCTTGGCAAGAAC
ATCCTGGCTGCTTGGGGGTTTGGAG
>2236
TGCCTTGGAGCCAATGACCACACTTCTCAGCTATGAGCTGGCCTTCAAGAAGCAGGAA
GAGGCCTGGGAGGTAACACTTTGGCTGGCTTTCCCTGGGGGCCTCTCTCCTGGGAACA
GCAGTCCAGGGTAGACTCCCCACT
>2237
GAGGAGCGTTATACAGGCCAGTGGAGTGAGTGGAGCCAGCCTGTGTGCTTCCAGGCT
CCCCAGAGACAAGGTGGGCACTGCTGTGGCTGCTGCACTTCCAGCGGAGTCTGGGCT
GGGCGTCTTCTCCCCTGTTCACCTCA
>2238
CCCTTGTTGCTGTGTCCATCTTTCTCCTGCTGACTGGCCCGACCTACCTCCTGTTCAAG
CTGTCGCCCAGGTAGGTGGCTGATGTGTGCGTGTGTGTACATGTGTGAGCGGGCAAGA
GTGTGCATGTTAGTGTATGTGTG
>2239
CTACCAGAACGTGCCCTCTCCAGCGATGTTCTTCCAGCCCCTCTACAGTGTACACAATG
GGAACTTCCAGGTGTGTGCAGAGACCACAGAAGGACATGGGGGGCAGGGGTTGCCCA
GAGCTCTGGCCTGCCCAAGATGTT
>2240
CGTCCTACGTGCCAGACGACGGACTCACAGCACAGCAGCTCTTCAACTGCGGAGACG
GCCTCACCTACAAGTGCGGGCCTATGGGGCTCACCTGGGCGAAGAGAGGGGCGGCAC
CTGGGGAAAAGGCCAGATGGGGGCTT
>2241
TCTTAAGACCCCACTGGTTTCCTCTCCCATGGACACAGTCACAGAGGCTGGGATGGCC
ATAGCAATGGCGGTGAGCCCCATGGGGTGTGGGGGAAGGAGCAAGGACTCCATCGCC
TTTCCCCAAAGGCATTCAGTCCTGC
>2242
AGGCGGTATTGGCTTCATCCACCACAACTGTACACCTGAATTCCAGGCCAATGAAGTTC
GGAAAGTGAAGGTCAGAAGGGCAACGATCATTAGCAAGCGCTCCTGGGAATTGCACT

GAGGTGGGGTGGGGTGGGAGTAGG
>2243
GGTGGGCATCATCTCCTCCAGGGACATTGATTTTCTCAAAGAGGAGGAACATGACTGT
TTCTTGGAAGAGGTGGGTGCCACTGGCAGAGTAGATCCAAGCTCTAGCAGCCCAGGTT
GAAGACAAGGGGCTTCTGTTGTCT
>2244
GACTTGGTGGTAGCCCCTGCAGGCATCACACTGAAGGAGGCAAATGAAATTCTGCAGC
GCAGCAAGAAGGGTAAGTCCTAGAGCTGGGAAAGGGCCTGGAACTAATGCCTAGGGT
CCTGATTCATGTCCTGCCCTGACCA
>2245
GGCACTCATGAGGATGACAAGTATAGGCTGGACTTGCTCGCCCAGGCTGGTGTGGATG
TAGTGGTTTTGGGTGAGCCTGCTACACAGGTGGGTTGGGGCAATAGGGGCAGCTGCCA
CATGACAGTCTGAGACTTACTTCT
>2246
ATCTTCCAGATCAATATGATCAAGTACATCAAAGACAAATACCCTAATCTCCAAGTCATT
GGAGGCAATGGTAAGGCAAGATTGTGCCCTGAAGGATGTGTGGTGGGAGTGGGTAAG
CTGGGCTCCCACCTTAACCTTCA
>2247
CTCATTGATGCAGGTGTGGATGCCCTGCGGGTGGGCATGGGAAGTGGCTCCATCTGCA
TTACGCAGGAAGGTAAGAATATACTTTGATAGGGCCCACAGAGACCCCAGTTTCAGGC
CCCACATACCTTGGAACCAAGCAC
>2248
AGAAATATCGCGGTATGGGTTCTCTCGATGCCATGGACAAGCACCTCAGCAGCCAGAA
CAGATATTTCAGGTGGGACAGGCAAGCCAGTTACCCCACCCTAATCCTGCACTGACAG
TCTCATCTCTGCTGTGACCTTGCC
>2249
TCCCTTACCTGATTGCTGGCATCCAACACTCATGCCAGGACATTGGTGCCAAGAGCTTG
ACCCAAGTCCGGTGAGCTTGGGGAGCTGGGACATGGGTAGAGGGGCTGGTCCAGGGC
CAGCTACCCACATTTGACCTCTGC
>2250
CTGGGGAGCTTAAGTTTGAGAAGAGAACGTCCTCAGCCCAGGTGGAAGGTGGCGTCC
ATAGCCTCCATTCGTAAGTCACCCTGTCCTTGGTGGGGCCTCTGCCATACCTCATGCCTC
CTTTCTCCTGCCCTCACCTCACA
>2251
CGAGTATAACAAGGCCACCAAAGATGACTACTACAGACGTCCGCTGCGGGTACTAAGA
GCCAGGCAACAGGTAGGTGCTCCCTCCACCCCAGGGGTCCTGGGTCCCAGCCTGGTTT
GTTCCCCAACCCCCAAGAGCATTC
>2252
AACCATATGTACCAAGTCCCAGCCCAACTTGGACACCTGTGCCTTCCATGAACAGCCA
GAACTGCAGAAGGTACGTTCCTGATGCAGGTCCCGGGCCAGTCATGCACTGCAGAGG
GGTGCGTATGTGTCAGCCTCTGCCC
>2253
CCCACACCTGAAGGCAGCCCATATCCCTGAGAATGCCAAGGACCGCATTCCTGGAATT
GTGCCCATGCAGGTGAGACCCAAGAGAGACCTGGCTGAACCCAGTCCCGGGAGTGAG
TTGACCCATTGTCCGCACATGCAGG
>2254

TCTCCAGGCTGCTGGTGGAGCACATCTGGAAGAAGATCATGCACACGGATGCCATGAT
CATCGACATGAGGTCAGTGGCCAGGGGTCAGTGCTTCCTAGCCAGGACGCAGGGCTG
CCAGGGGACAGTCAAAGCTATGGGC
>2255
CTGCTGGACAAGATCTACAGCCGGCCTGATGACTCTGTCAGTGAACTCTGGACACACG
CCCAGGTTGTAGGTACGTGGAGAAGCTTTCTCCTTTCTGCTGTCATTCTAGAAGCTTCT
GGGAAACCAGGGAAAGACAGCTT
>2256
GCACTTTCCAGTGTGGGGGCATCCTGGTGCACCGCCAGTGGGTGCTCACAGCTGCTCA
TTGCATCAGCGAGTGAGTAGGGGCCTGGGGTCTGGGAGGAGGGCATCTATGCTGCCAC
AGGATTGACAGGCCAGTGGCATTC
>2257
CCCACCCAGGAACCCGAAGTGGGGAGCACCTGTTTGGCTTCCGGCTGGGGCAGCATC
GAACCAGAGAATTGTATGTGGGGGCAGACTGTGTAGCCCAAGGCGGGGATGGGGACT
CCTGCGTCCAAGGGAGAAAGGGCCAG
>2258
CCACGTCCAGAAGGTGACAGACTTCATGCTGTGTGTCGGACACCTGGAAGGTGGCAA
AGACACCTGTGTGGTGAGGCAGCCCTGCCCCCAGGGTCTGGAAGGGCTGAGGGAGGG
GACTCAGCCTCTGAACTGGCTTCTGA
>2259
GATGGGCACACTGTGGGGGTGTCCTGGTGCACCCCCAGTGGGTGCTCACAGCTGCCCA
TTGCCTAAAGAAGTAAGTAGGACCCTGGGATCTGGGGAGGGAATGGCTGTGTCCCACA
GGAATAACAGCGGGATGCTTCCCC
>2260
CCCACCCAGGAGCCAGCACTGGGGACCACCTGCTACGCCTCAGGCTGGGGCAGCATC
GAACCAGAGGAGTGTACGCCTGGGCCAGATGGTGTAGCTGGGAGCCCAGATGCCTGG
GTCTGAGGGAAGTGGGGCCAAAGAAC
>2261
TTACTCTGAGAAGGTGACAGAGTTCATGTTGTGTGCTGGGCTCTGGACAGGTGGTAAA
GACACTTGTGGGGTGAGTCATCCCTACTCCCAACATCTGGAGGGGAAAGGTGAGTGA
AGACCCTAATTCTGGGCTGCAATCT
>2262
ATTGTGGATTTGGTCAGTGAGCTCAAGAAGGACGTCTTGATGGTGCTGGTGAATTACAT
TTACTTCAAAGGTGAGAGTCAGATCATTGGTATATGCTAAATCCACACCACCGCCTCCA
ACCCACTAATTTGTTGACTGGT
>2263
AAATGAGGGAGATTGAAGAGGTTCTGACTCCAGAGATGCTAATGAGGTGGAACAACTT
GTTGCGGAAGAGGTAATCAGTGTGCTATGGGGGCTGAATCTACAGTACTATCCATCAAA
TGTAACTTTCTAATGAAAGACAG
>2264
CACGGATCTGTTCTCCAAGTGGGCTGACTTATCCGGCATCACCAAACAGCAAAAACTG
GAGGCATCCAAAGTAAGTCGTCAACAGTCAGCAATCCCTAGAGAACTCGGTAGGGCA
GTGCCCATGGGAGCTGCCAGGCGAT
>2265
GAGGCAGCGGCCTGCTGAGATCAAAGACTACAGTCCCTACTTCAAGACCATTGAGGAC
CTGAGGAACAAGGTGGGTGAATGGGCAGCAGAAGGCACCATTCCAGCTAGCTCCTTC

TGGGAACAATTCATGCCCCAGGCCG
>2266
CAGTGGACAATGCCAATGTCCTTCTGCAGATTGACAATGCCCGTCTGGCCGCGGATGA
CTTCCGCACCAAGTGAGTTTGAAATGGTGGGCCAGAACATCCAGTGTCCCCAGAGTAG
GGCATTTTTGGAGCAGTGTTTCCC
>2267
AGCTGACCTGGAGATGCAGATTGAGAGCCTGAAGGAGGAGCTGGCCTACCTGAAGAA
GAACCACGAGGAGGTGAGAACTATATGGAAAAGTCAGCTTAAAAGAAATGCAGGGAG
GCTGGGTACAGTGGTGCGTGCCCATA
>2268
GATGCGTGACCAGTATGAGAAGATGGCAGAGAAGAACCGCAAGGATGCCGAGGAATG
GTTCTTCACCAAGGTGGGTGTCATTTGAGGTGGAAGGAACCCAGACCACCTGCCTTCT
GGGGCCTTCTGGTGTGAATGGCATT
>2269
GAGCGAGATCTCGGAGCTCCGGCGCACCATGCAGAACCTGGAGATTGAGCTGCAGTC
CCAGCTCAGCATGGTAGGAATAGTGCCAGGAAGGGTGGTGCACCCAGGACTGGCAGG
GAGAGAACGGCCACACTCACTAATCG
>2270
TGGACGTGAAGACGCGGCTGGAGCAGGAGATCGCCACCTACCGCCGCCTGCTGGAGG
GCGAGGACGCCCAGTGAGTCTTGGCCCTCCCCTTAGTCCGCCCCCCCCATGGCACTCT
CACGGCCCCACCATGTATCTAATGA
>2271
AAAGGTGAAGTCTCGAGAAAGGGAGCAAATCAAGTCACTCAACAACCAATTTGCCTC
CTTCATTGACAAGGTGAGTTTCTCTCTCATTGCACTGGTAGCTGCGCCGGTCCACTCGG
GATCGGTGCAGTCAAAACACACGC
>2272
AGAGTGATCAATCTCGGTTGGATTCGGAACTGAAGAACATGCAGGACATGGTGGAGGA
TTACCGGAACAAGTAAGGGACCCTGTCTGGGCAGTTCTTAACTTTTGCTGTAAAAAAG
AGTTCCAAGAAAAGTAATAAGCTA
>2273
GGTGGACCTTCAGGCCAAACTTGACAACTTGCAGCAGGAAATTGATTTCCTTACAGCA
CTCTACCAAGCAGTAAGTCTTCCAGTTTCAACCAAGTTTATCTAAATGGAGAGTTTTTA
AGCCGGAACCCACAACGATCTAG
>2274
GGTCAAGGCCCAGAACGAGGATATAGCCCAGAAGAGCAAAGCTGAGGCCGAGTCCTT
GTACCAGAGCAAGGTGAGTGGCTGAAAGCCTAGCCAGTTTCCCTGAAATGGCTTGTCT
TGCTATCCTGTGTTATCTCATGTAT
>2275
GATAGAAATTTCTGAGCTGAATCGTGTGATCCAGAGACTTAGATCTGAAATCGACAATG
TCAAGAAGCAGGTATGTGCTTTCTCCTTCTACCACTCAGCTGTATGGAATGGGGGTAAC
CCTACAGGTAAAGGGCGAGTGC
>2276
TGAACACCAAGCTGGCCCTGGATCTGGAGATTGCCACCTACAGGACCCTCCTGGAGGG
AGAAGAAAGCAGGTGAGGAAGGGACGCTGGGAGTACTCGCCCCCTTCCCCTGGTCCT
TCCTTCCTTGCCACCGAATGATGAT
>2277

GTAATCTTCCGAGGTGCAAATTCTCAGAAAAATGCATTCCGGAAAAATCCCAGTGATCC
AAAGCTTGCACGTAAGTATTGATTTATGTTTATATTATATATATTACATGAAGGTTTTATTT
TTCTTTTTGTCTCGACATC
>2278
TGGGGCTTTGTTCGCCACCCCAATTACTTGGGTGATCTCATCATGGCCTTGGCGTGGTC
CCTCCCATGTGGTAAGCATTTCAGAAGAGTGCTAGTTTGACCCTTTTAGCCGCCAGAAA
GATGTGTGTGTGTGTCCTTTGT
>2279
TACTCGTTCCCAGAAAGGAAGTTCTGGAGACCAGGAAATGACAGCTACACTTCTCACA
GCAGGGTTCCAGGTGAGTTGTGCTCCTTCTCACTGAAACGCCATAGCTGTGCTTGTCA
GTGTTTGTGGCATCTGCCCTATAT
>2280
CTTGATCCATCACAGAAGGATCTGTGTAGAGATAACAGGCCAGAAAATTTCAGAAACA
TGTTCTCCCTGGGTAAGGAGAGGTGTGGTTATTATTGTCATTTTAGATTTTTTGTTTGTT
TGTTGTTTATGTGTATAGTAGC
>2281
CAGGTCTCCACTGCTGCCCTTGCTGTCCTCCTCTGCACCATGGCTCTCTGCAACCAGTT
CTCTGCATCACGTGAGTCTGAGTTTCGTTGTGGGTATCACCACTCTCTGGCCATGGTTA
GACCACATCAATCTTTTCTTGT
>2282
GGCAGATTCCACAGAATTTCATAGCTGACTACTTTGAGACGAGCAGCCAGTGCTCCAA
GCCCGGTGTCATGTAAGTGCCAGTCTTCCTGCTCACCTCTATGGAGGTAGGGAGGGTC
AGGGTTGGGGCAGAGACAGGCCAG
>2283
GCGTGTCCATCTGTGAGAAGGAGCCAGAGCCCAAGGGAGATGATGGAGCCTGAGGAG
TACAGAGAGAGAGGTGAGCAACGGGCATCCCTGGCCCTCTTCCCTGCTGCTACTCCTG
CAGGAACGGCTGCTGGTGGGTGGCC
>2284
GAGTGCTCCTGAGGACCCCGACAGCTGGGACAGCATCTTTGGGGACATTGAACGAATC
ATCATGCCTGGGGTGAGACACAGTGACCACAAGGGCAGTTCTGGATTTCAGGGCACA
GAAGGGTGGGTGGTGGCCTGGAGAA
>2285
CCTCACCTCTTGGCCCTCCCTGCTAGGAGACATGCTGGCTGATGCCATCAACTGCTTGG
GATTCACCTGGGTGAGTAGCAACGGCTGTAACTACTCGCAATGGGGAACGTAGCAGGG
AGTGGTATCCTAGGGCAGACATA
>2286
AAAAATGCTGGGACTTCCAGAGCACTTCTTGCACCACCACCCCAGCAGCCAGGGCGG
AGGCGTCCTGCAGGTACCTCCCTTGGCAAAGCTTCACCTAGCTTGGGGGCTAAGTAGC
GAAAGACAAGCCAAACCCTAACCTG
>2287
GAAAACGTCTGAGCCCGATGCTGATGAGTCCTGCCTAAATGCCCGACTCGTGGCCTAT
GCCTCTGACCAGGTGAGTTGCCCGACGCAGGACAGCCCTTCGGGTTGGTGCTTGGCTT
CTGGAATCTTGGTCCTTGAGCAAT
>2288
CTCACTCCGAGGGGAAGCTCTTCAGAAGGCCATCGAGGAAGACAAGCAGCGGGGCTT
GGTGCCCGTCTTTGTAAGTCAGGATATATGCCTAAGCTTAGAATGATAGAACTTGGATG

TGAGGAAGGAATTTTGGCAGAATT
>2289
AGTATGCCGACTCCTTCACCTTTAATCCTTCCAAGTGGATGATGGTGCATTTTGACTGTA
CTGGGTTCTGGTGAGTGTAGCAGCCCAGCTCCGAGCACGCAGGAACGCCTTGCCTCCT
CTGGAGAGACCTCAGCCACTAA
>2290
GCAGCAGACCTTCAGTGTGAATCCCATCTACCTCAGGCATGCCAACTCAGGCGTGGCC
ACCGACTTCATGGTGAGTGGCCAGGGACGGGCAGCCTGGTGGGCTCGGGTGGCCAGT
GGGAACTGTAGGCGTCTGACCCCCC
>2291
TCGCTCTGTTAAACTCTGGTTCGTGATTCGGTCCTTCGGGGTGAAGAATCTTCAAGCAC
ATGTCAGACATGTATGTAGCATGCTGCTGTGGGGGCTGAGCCTGTGTTCCTCTGTTTTC
TCATGGCGAGGAAGGCTGGTTT
>2292
GGTCAGAAACGACCCTTCCTTTGAAATTCCTGCCAAGAGGCACCTTGGCCTGGTGGTT
TTTCGTCTAAAGGTAATACCATCTTCCAAGCCCCTCTGTGAATGATGTCTTGTGGTGCTC
CAGAGCCTCTCGGAAACACAAA
>2293
GTAAACATGACTTCCAAGCTGGCCGTGGCTCTCTTGGCAGCCTTCCTGATTTCTGCAGC
TCTGTGTGAAGGTAAGCACATCTTTCTGACCTACAGCGTTTTCCTATGTCTAAATGTGAT
CCTTAGATAGCAAAGCTATTC
>2294
TCCACCCCAAATTTATCAAAGAACTGAGAGTGATTGAGAGTGGACCACACTGCGCCAA
CACAGAAATTATGTAAGTACTTTAAAAAAGATTAGATATTTTGTTTTAGCAAACTTAAAA
TTAAGGAAGGTGGAAATATTTA
>2295
ATGGAAGAGAGCTCTGTCTGGACCCCAAGGAAAACTGGGTGCAGAGGGTTGTGGAGA
AGTTTTTGAAGAGGTAAGTTATATATTTTTTAATTTAAATTTTTCATTTATCCTGAGACAT
ATAATCCAAAGTCAGCCTATAA
>2296
AACCACACCCTGGCCCAGACTGCCAGCCCTGACGGGACAGAGCCAGGGCACTCACCA
GGCTGCAAGAACAGTGCTGGGGTAAGAGGGGAGCGGGGGATCCCGGGCCTGGGACC
CAGCCTGCATTCCTTTGTTCATTCCTT
>2297
AACTCTCCACCCTGGGAACTGCCCCGCCCACTTTGGCCCTGCACTACCACCCTCACCC
CTTCCTCAACAGGTAGTGGGGATCTGGGGTGGGGGGCAGTGGGGATTGGGGGCCAGG
GTCCTTGCCCACAAGGACTTAGTGA
>2298
CCTTTTAGCATCTTCCCTAGCAGCCGGTTGAAGCGGAGACCAAGCCACTGTGAGCTGG
ACCTGGCTGAGGGTGAGTGTGGGTTTGTGTGCCTTGTGGGTTTGTATGCCTGTATGTGC
ACTTGTGGGTGCACAGAAGGCCT
>2299
CTTGTTGCTGGCGGTGCCTATCACTGTCCTGGCTGTGCTGGCCTTAGTGCCCCAGGATC
AGGGAGGACTGGTGAGTGGCTGCAACAGGCCCTGGTGGAGAGTTGTATCTTGCGGAT
GCTTGGCTCCCTCTGGTTGTGCCT
>2300

TTTCAGAAGCTGCCAGAGGAGGAGCCAGAAACAGATCTCAGCCCCGGGCTCCCAGCT
GCCCACCTCATAGGTAAGGACCTCCAAGACCTGAATAAGAGTGTAAATAATCCGAAGG
TTCCAGTTCTGCTCGCCCAGAGTCC
>2301
TTCAGGGGACAGGCCAACCCAGAGGACAGGATTCCCTGGAGGCCACAGAGGAGCAC
CAAGGAGAAGATCTGTAAGTAGGCCTTTGTTAGAGTCTCCAAGGTTCAGTTCTCAGCT
GAGGCCTCTCACACACTCCCTCTCTC
>2302
AGATTCTCGCCCTGAGCAACGGCCTGACGTCGGCGGAGGGAAGCAGGCGCAGGCTCC
GTGAGGAGGCAAGGTAAGATGCCGAGGGAGGACTGAGGCGGGCCTCACCCCAGACA
GAGGGCCCCCAATAATCCAGCGCTGCC
>2303
ACTTCTCAGGCTCAGTCGCCACCACCTCACCCCGCCACCCCCCGCCGCTTTAACCGCA
GGGAACTCTGGCGTAAGAGCTTTGTGTGACCAGGGCAGGGCTGGTTAGAAGTGCTCA
GGGCCCAGACTCAGCCAGGAATCAA
>2304
CAGGGGCCCCATCTGGTCGACAGATGCAGTGGTTCTAGGATCTGCCAAGCATCCAGGT
GGAGAGCCTGAGGTAGGATTGAGGGTACCCCTGGGCCAGAATGCAGCAAGGGGGCCC
CATAGAAATCTGCCCTGCCCCTGCG
>2305
GACGAAACCATGAAGAGCCTGATCCTTCTTGCCATCCTGGCCGCCTTAGCGGTAGTAA
CTTTGTGTTATGGTGAGAAACTTTTCTCCCATTTCTCTGTGTTTACTTTTCTGCCTCTGA
CTTTGGCTTACTTCTATTTTTC
>2306
TCATTAACAGGAGAAATGCAAATACCTTCATATCCCCTCAGCAGAGATGGAGAGCTAA
AGTCCAAGAGAGGTCAGTAACAAAACTTCATGAGGAGTGGTCATTTTTCCCAGTGTAG
ATCACAGATCTGAATTGGAGTGGG
>2307
GTGGTCATGGCGCCCCGAACCCTCTTCCTGCTGCTCTCGGGGGCCCTGACCCTGACCG
AGACCTGGGCGGGTGAGTGCGGGGTCAGGAGGGAAACGGCCCCTGCGCGGAGGAGG
GAGGGGCCCGCCTGGCGGGGGCGCAG
>2308
AAGGCCCACGCACAGACTGACAGAATGAACCTGCAGACCCTGCGCGGCTACTACAAC
CAGAGCGAGGCCAGTGAGTAACTCCGGCCCAGGGAGCAGATCACGACCCCCACCTCC
ATGCCCCACGGACGGCCCGGGTACTC
>2309
CTGGAGGGCACGTGCGTGGAGTGGCTCCACAGATACCTGGAGAACGGGAAGGAGATG
CTGCAGCGCGCGGGTACCAGGGGCAGTGGGGCGCCTCCCTGATCTCCTGTAGACCTCT
CAGCCTGGCCTAGCACAAGGAGAGG
>2310
GGAGAGGAGCAGAGATACACGTGCCATGTGCAGCATGAGGGGCTGCCGGAGCCCCTC
ATGCTGAGATGGAGTAAGGAGGGAGATGGAGGCATCATGTCTGTTAGGGAAAGCAGG
AGCCTCTCTGAAGACCTTTAACAGGG
>2311
CTGGTTGTCCTTGCAGCTGTAGTCACTGGAGCTGCGGTCGCTGCTGTGCTGTGGAGAA
AGAAGAGCTCAGGTAAGGAAGGGGTGACAAGTGGGGTCTGAGTTTTCTTGTCCCACT

GGGGGTTTCAAGCCCCAGGTAGAAG
>2312
GCCGTCATGGCGCCCCGAACCCTCGTCCTGCTACTCTCGGGGGCTCTGGCCCTGACCC
AGACCTGGGCGGGTGAGTGCGGGGTCGGGAGGGAAACGGCCTCTGTGGGGAGAAGC
AACGGGCCGCCTGGCGGGGGCGCAGG
>2313
AAGGCCCACTCACAGACTCACCGAGTGGACCTGGGGACCCTGCGCGGCTACTACAAC
CAGAGCGAGGCCGGTGAGTGACCCCGGCCCGGGGCGCAGGTCACGACCTCTCATCCC
CCACGGACGGGCCAGGTCGCCCACAG
>2314
CTGGAGGGCACGTGCGTGGAGTGGCTCCGCAGATACCTGGAGAACGGGAAGGAGACG
CTGCAGCGCACGGGTACCAGGGGCCACGGGGCGCCTCCCTGATCGCCTGTAGATCTCC
CGGGCTGGCCTCCCACAAGGAGGGG
>2315
GGACAGGAGCAGAGATACACCTGCCATGTGCAGCATGAGGGTTTGCCCAAGCCCCTCA
CCCTGAGATGGGGTAAGGAGGGAGACGGGGGTGTCATGTCTTTTAGGGAAAGCAGGA
GCCTCTCTGACCTTTAGCAGGGTCA
>2316
CTGGTTCTCTTTGGAGCTGTGATCACTGGAGCTGTGGTCGCTGCTGTGATGTGGAGGA
GGAAGAGCTCAGGTGGGGAAGGGGTGAAGGGTGGGTCTGAGATTTCTTGTCTCACTG
AGGGTTCCAAGACCCAGGTAGAAGT
>2317
GTGCTCATGGCGCCCCGAACCCTCCTCCTGCTGCTCTCAGGGGCCCTGGCCCTGACCC
AGACCTGGGCGCGTGAGTGCAGGGTCTGCAGGGAAATGGTCGGGAGGAGCGAGGGG
CCCGCCCGGCGGGGGCGCAGGACCCA
>2318
AAGGCCCAGGCACAGACTGAACGAGAGAACCTGCGGATCGCGCTCCGCTACTACAAC
CAGAGCGAGGGCGGTGAGTGACCCCGGCCCGGGACGCAGGTCACGACCCCTCCCCAT
CCCCCACGGAGGGCCGGGTCGCCTCG
>2319
CTGGAGGGCGAGTTCGTGGAGTGGCTCCGCAGATACCTGGAGAACGGGAAGGAGACG
CTGCAGCGCGCGGGTACCAGGGGCCACAGGGCGCCTCCCGGATGGCCTGTAGATCTCC
GGGGCTGGCCTCCCACAAGAAAGGG
>2320
GGAGAGGAGCAGAGATACACCTGCCATGTGCAGCATGAGGGTCTGCCAGAGCCCCTC
ACCCTGAGATGGGGTAAGGAGGGAGATGGGGGTGTCATGTCCCTTAGGAAAAGCCGG
AGCCTCTCTGGAGAGCTTTAGCAGGG
>2321
CTGGTTCTACTTGTAGCTGTGGTCACTGGAGCTGTGGTCGCTGCTGTAATGTGGAGGA
AGAAGAGCTCAGGTAAGGAAGGGGTGAGGAGTGTGGTCTGAGATTTCTTGTCTCACT
GAGAGTTCCAAGCCCCAGGTAGAAG
>2322
CGGGTCACGGCGCCCCGAACCCTCCTCCTGCTGCTCTGGGGGGCAGTGGCCCTGACC
GAGACCTGGGCTGGTGAGTGCGGGGTCAGGCAGGGAAATGGCCTCTGTGGGGAGGA
GCGAGGGGACGCAGGCGGGGGCGCAGG
>2323

AAGGCCAAGGCACAGACTGACCGAGAGGACCTGCGGACCCTGCTCCGCTACTACAAC
CAGAGCGAGGCCGGTGAGTGACCCCGGCCCGGGCGCAGGTCACGACTCCCCATCCCC
CACGTACGGCCCGGGTCGCCCCGAGT
>2324
GGAGAAGAGCAGAGATACACATGCCATGTACAGCATGAGGGGCTGCCGAAGCCCCTC
ACCCTGAGATGGGGTAAGGAGGGGGATGAGGGGTCATATCTCTTCTCAGGGAAAGCA
GGAGCCCTTCAGCAGGTCAGGGCCCC
>2325
CGGGTCACGGCGCCCCGAACCCTCCTCCTGCTGCTCTGGGGGGCAGTGGCCCTGACC
GAGACCTGGGCTGGTGAGTGCGGGGTCGGCAGGGAAATGGCCTCTGTGGGGAGGAGC
GAGGGGACCGCAGCGGGGCGCAGGAC
>2326
AAGGCCAAGGCACAGACTGACCGAGAGGACCTGCGGACCCTGCTCCGCTACTACAAC
CAGAGCGAGGCCGGTGAGTGACCCCGGCCCGGGGCGCAGGTCACGACTCCCCATCCC
CCACGTACGGCCCGGGTCGCCCCGAG
>2327
CTGGAGGGCGAGTGCGTGGAGTGGCTCCGCAGATACCTGGAGAACGGGAAGGAGAC
GCTGCAGCGCGCGGGTACCAGGGGCAGTGGGGAGCCTTCCCCATCTCCTATAGGTCGC
CGGGGATGGCCTCCCACGAGAAGAGG
>2328
GGAGAAGAGCAGAGATACACATGCCATGTACAGCATGAGGGGCTGCCGAAGCCCCTC
ACCCTGAGATGGGGTAAGGAGGGGGATGAGGGGTCATATCTCTTCTCAGGGAAAGCA
GGAGCCCTTCAGCAGGGTCAGGGCCC
>2329
CTGGCTGTCCTAGCAGTTGTGGTCATCGGAGCTGTGGTCGCTGCTGTGATGTGTAGGA
GGAAGAGCTCAGGTAGGGAAGGGGTGAGGGGTGGGGTCTGAGTTTTCTTGTCCCACT
GGGGGTTTCAAGCCCCAGGTAGAAG
>2330
AAGGCCTCCGCGCAGACTTACCGAGAGAACCTGCGGATCGCGCTCCGCTACTACAACC
AGAGCGAGGCCGGTGAGTGACCCCGGCCCGGGGCGCAGGTCACGACTCCCCATCCCC
CACGTACGGCCCGGGGTCGCCCCGA
>2331
GCGCCCACCATCGCGTCGCAGCCCCTGTCCCTGCGCCCAGAGGCGTGCCGGCCAGCG
GCGGGGGGCGCAGGTGAGGCGTGCGGGGGCCCGGGGCGGGGGCGGCACGCACCGCG
GGGAGAGCCCATGCCAGGGACAGCCCT
>2332
TGGGCGCCCCTGGCCGGGACTTGTGGGGTCCTTCTCCTGTCACTGGTTATCACCCTTTA
CTGCAACCACAGTAAGTCCCGAGGAATCGCGGCGGGAAGGAGTCGGCCCCGCTCCTG
CCCTTGGCTCTGCTAGCTCCAGGC
>2333
CGGGTCACGGCGCCCCGAACCGTCCTCCTGCTGCTCTGGGGGGCAGTGGCCCTGACC
GAGACCTGGGCCGGTGAGTGCGGGGTCGGGAGGGAAATGGCCTCTGTGGGGAGGAG
CGAGGGGACCGCAGGCGGGGGCGCAGG
>2334
AAGACCAACACACAGACTTACCGAGAGAGCCTGCGGAACCTGCGCGGCTACTACAAC
CAGAGCGAGGCCGGTGAGTGACCCCGGCCCGGGGCGCAGGTCACGACTCCCCATCCC

CCACGTACGGCCCGGGGTCGCCCCGA
>2335
CTGGAGGGCCTGTGCGTGGAGTGGCTCCGCAGATACCTGGAGAACGGGAAGGAGACG
CTGCAGCGCGCGGGTACCAGGGGCAGTGGGGAGCCTTCCCCATCTCCTATAGGTCGCC
GGGGATGGCCTCCCACGAGAAGAGG
>2336
GGAGAAGAGCAGAGATACACATGCCATGTACAGCATGAGGGGCTGCCGAAGCCCCTC
ACCCTGAGATGGGGTAAGGAGGGGGATGAGGGGTCATATCTCTTCTCAGGGAAAGCA
GGAGCCCTTCTGGAGCCCTTCAGCAG
>2337
CTGGCTGTCCTAGCAGTTGTGGTCATCGGAGCTGTGGTCGCTACTGTGATGTGTAGGAG
GAAGAGCTCAGGTAGGGAAGGGGTGAGGGGTGGGGTCTGGGTTTTCTTGTCCCACTG
GGGGTTTCAAGCCCCAGGTAGAAG
>2338
CTCCCCATCTATCTGCTTGGCCTGACTCAGAAATTCGGGCCCATCTACAGGCTCCACCT
TGGGCTGCAAGGTGAGAGGCTGATCTCGCTCTGGCCCTCACCATAGGAGGGGGCGGA
GGTGACGGAGAGGGTCCTCTCTCC
>2339
AAGAGGACCATTGAGGAAGCCATGGTCAAAAAGTGGGCAGACTTTGCTGGCAGACCT
GAGCCACTTACCTGTAAGGGCTGGGGGCATTTTTTCTTTCTTAAACAAATTTTTTTTTAA
GAGATGGGTTCTTGCTATGCTGC
>2340
AGCCCTGCTGCTGGGCATCCGTGACTCCATGGAGCCAGTGGTGGAGCAGCTGACCCA
GGAGTTCTGTGAGGTAAGGCTGGGCTCCTGAGGCCACCTCGGGTCAGCCTCGCCTCTC
ACAGTAGCCCCCGCCCTGCCCGCTG
>2341
CATTGAGGAGGAATTCTCTCTCCTCACCTGCAGCATCATCTGTTACCTCACCTTCGGAG
ACAAGATCAAGGTGCCTCACAGCCCCTCAGGCCCACCCCCAGCCCCTCCCTGAGCCTC
TCCTTGTCCTGAACTGAAAGTAC
>2342
TATCCAGGAGGTGTTAAAAACCTGGAGCCACTGGTCCATCCAAATTGTGGACGTGATT
CCCTTTCTCAGGGTGAGGACCTGGAGCCTAGACACCCCTGGGTTGTAGGGGAGAGGC
TGGGGTGGAGGGAGAGGCTCCTTCC
>2343
TCTCCGGAGGCTGAAGCAGGCCATAGAGAAGAGGGATCACATCGTGGAGATGCAGCT
GAGGCAGCACAAGGTGGGGACTCTACGTGGACGGCCTCCCCTCGGCCCACAGCCAGT
GATGCTACCGGCCTCAGCATTGCTAT
>2344
GATCGGTGGCACTGAGACCACAGCAAACACCCTCTCCTGGGCCGTGGTTTTTTTGCTT
CACCACCCTGAGGTGCGTCCTGGGGACAAGCAAAAGGCTCCTTCCCAGCAACCTGGC
CAGGGCGGTGGGCACCCTCACTCAG
>2345
TCGCCGAGGTGCTGCGCCTGCGGCCCGTTGTGCCCTTAGCCTTGCCCCACCGCACCAC
ACGGCCCAGCAGGTGACTCCCGAGGGTTGGGGATGAGTGAGGAAAGCCCGAGCCCA
GGGAGGTCCTGGCCAGCCTCTAACTC
>2346

ATCATTCCGAACCTCCAAGGCGCCCACCTGGATGAGACGGTCTGGGAGAGGCCACATG
AGTTCTGGCCTGGTATGTGGGGGGCCGGGGGCCTGCCGTGAAAATGTGGTGGAGGCT
GGTCCCCGCTGCCGCTGAACGCCTC
>2347
CGGGTCATGGCGCCCCGAACCCTCATCCTGCTGCTCTCGGGAGCCCTGGCCCTGACCG
AGACCTGGGCCTGTGAGTGCGGGGTTGGGAGGGAAACGGCCTCTGCGGAGAGGAAC
GAGGTGCCCGCCCGGCAGGCGCAGGA
>2348
AAGCGCCAGGCACAGACTGACCGAGTGAGCCTGCGGAACCTGCGCGGCTACTACAAC
CAGAGCGAGGCCGGTGAGTGACCCCGGCCCGGGGCGCAGGTCACGACCCCTCCTCAT
CCCCCACGGACGGCCCGGGTCGCCCC
>2349
CTGGAGGGCACGTGCGTGGAGTGGCTCCGCAGATACCTGGAGAACGGGAAGGAGAGC
CTGCAGCGCGCGGGTACCAGGGGCAGTGGGGAGCCTTCCCCATCTCCCGTAGATCTCC
CGGCATGGCCTCCCACGAGGAGGGG
>2350
GGAGAAGAGCAGAGATACACGTGCCATGTGCAGCACGAGGGGCTGCCGGAGCCCCTC
ACCCTGAGATGGGGTAAGGAGGGGGATGAGGGGTGATGTGTCTTCTCAGGGAAAGCA
GAAGTCCTGGAGCCCTTCACGCGGGT
>2351
GCTGTCCTGGCTGTCCTAGCTGTCCTAGGAGCTGTGGTGGCTGTTGTGATGTGTAGGAG
GAAGAGCTCAGGTAGGGAAGGGGTGAGGAGTGGGGTCTGGGTTTTCTTGTTCCACTG
GGAGTTTCAAGCCCCAGGTAGAAG
>2352
CGGGTCATGGCGCCCCGAACCCTCATCCTGCTGCTCTCGGGAGCCCTGGCCCTGACCG
AGACCTGGGCCTGTGAGTGCGGGGTTGGGAGGGAAACGGCCTCTGCGGAGAGGAGC
GAGGGGCCCGCCCGGCGAGGGCCAGG
>2353
AAGCGCCAGGCACAGACTGACCGAGTGAACCTGCGGAAACTGCGCGGCTACTACAAC
CAGAGCGAGGCCGGTGAGTGACCCCGGCCCGGGCGCAGGTCACGACCCCTCCCCATC
CCCCACGGACGGCCCGGGTCGCCCCG
>2354
CTGGAGGGCGAGTGCGTGGAGTGGCTCCGCAGATACCTGGAGAACGGGAAGGAGAA
GCTGCAGCGCGCGGGTACCAGGGGCAGTGGGGAGCCTTCCCCATCTCCTGTAGATCTC
CCGGGATGGCCTCCCACGAGGAGGGG
>2355
GGAGAAGAGCAGAGATACACGTGCCATGTGCAGCACGAGGGGCTGCCGGAGCCCCTC
ACCCTGAGATGGGGTAAGGAGGGGGATGAGGGGTACTGTGTCTTCTCAGGGAAAGCA
GAAGTCCTGGAGCCCTTCACGTGGGT
>2356
GCTGTCCTGGCTGTCCTAGCTGTCCTAGGAGCTGTGGTGGCTGTTGTGATGTGTAGGAG
GAAGAGCTCAGGTAGGGAAGGGGTGAGGAGTGGGGTCTGGGTTTTCTTGTCCCACTG
GGAGTTTCAAGCCCCAGGTAGAAG
>2357
CGGGTAGCAACTGTGACCTTGATGCTGGCGATGCTGAGCACCCCGGTGGCTGAGGGCA
GAGACTCTCCCGGTAAGTGCAGGGCCACTGCTCTCCAGAGCCGCCACTCTGGGAACA

GGCTCTCCTTGGGCTGGGGTAGGGG
>2358
CGGGCGGAGTTGGACACGGTGTGCAGACACAACTACCAGTTGGAGCTCCGCACGACC
TTGCAGCGGCGAGGTGAGCGTCGTCGCCCGTCTGCGAGGCCCATCCTTGGCAGGGGC
CCAGAGTCTCTGCCGCAGGAGGGGCG
>2359
CAGCGTGGAGACGTCTACACCTGCCACGTGGAGCACCCCAGCCTCCAGAACCCCATCA
TCGTGGAGTGGCGTAAGGGGATATTGAGTTTCTGTTACTATGGGCCCCACAAGACAAA
GGGCAGAGCTCCTTCTGACCCATT
>2360
ATTGGAGGCTTCGTGCTGGGGCTGATCTTCCTCGGGCTGGGCCTTATTATCCATCACAG
GAGTCAGAAAGGTGAGGAACCCCAAGGGAAAAGGGGAAGATGGGCTGTGACCCAGA
CCCTCTGTTCAGGGAGGTCCTGTCT
>2361
AGGGACACCGCACAGATTTTCCGAGTGAACCTGCGGACGCTGCGGCGCTACTACAATC
AGAGCGAGGCCGGTGAGTGACCCCGGCCAGGAGCAGGTCACGACCCCTCCCCATCCC
CCACGGACGGCGCGGGTCCCCTCGA
>2362
CTGGAAGACACATGCGTGGAGTGGCTCCACAAATACCTGGAGAAGGGGAAGGAGACG
CTGCTTCACCTGGGTAAGAGGGTCCACAGGGCTACTCTCCCATCTCCTTCTTGGGCTAG
GACTGTGCCCACAGCTGACAGACC
>2363
GGAGAGGAGCAGAGATACACGTGCCATGTGCAGCATGAGGGGCTACCCGAGCCCGTC
ACCCTGAGATGGAGTAAGGAGGGGGATGGGAGGTCATGTCTCTTCTCAGGGAAAGCG
GGAGCCCTTCTGGAGCCCTTCCGCAG
>2364
CTGGTTCTCCTTGGATCTGTGGTCTCTGGAGCTGTGGTTGCTGCTGTGATATGGAGGAA
GAAGAGCTCAGGTGGGGAAGGGAGAAGGGTGGGGTCTGAGTTTTCTTGTCCCACTGG
GTGTTTCAAGCCCTAGGTAAAAGT
>2365
TGGGATCATGGTAGTAGGAACCCTCCTTTTACTCCTCTCGGAGGCCCGGCCCTTACCCA
GACCTGGGCGGGTGATGCGGGGTCGGGATGGAAACGGCTTTACCGGGAGTTACACAG
GGGCCGGCCGGCGGGGGCGAAGGA
>2366
AGGGAAACCGCAAAGATTTTCCGAGTGAATCTGCGGACCGTGCGGGCTTACTACAATC
AGAGCGAGGCCGGTGAGTGACCCCGGCCAAGGGAGCAGGTCACGACCCCTCCCCATC
CCCCACGGACGGCGCGGGTCCCCTC
>2367
CTGGAAGACACATGCGTGGAGTGGCTCCACAAATACCTGGAGAAGGGGAAGGAGACG
CTGCTTCACCTGGGTAAGAGGGTCCACAGGGCTACTCTCCATCTCCTTCTTGGGCTAGG
ACTGTGCCCACAGCTGACACACCT
>2368
GGAGAGGAGCAGAGATACACGTGCCATGTGCAGCATGAGGGGCTACCCGAGCCCGTC
ACCCTGAGATGGAGTAAGGAGGGGGATGGGAGGTCATGTCTCTTCTCAGGGAAAGCG
GGAACCCTTCTGGAGCCCTTCCGCAG
>2369

CTGGTTCTCCTTGGATCTGTGGTCTCTGGAGCTGTGGTTGCTGCTGTGATATGGAGGAA
GAAGAGCTCAGGTGGGGAAGGGAGAAGGGTGGGGTCTGAGTTTTCTTGTCCCACTGG
TGTTTCAAGCCCTAGGTAAAAGTG
>2370
GGGGTCATGGCGCCCCGAACCCTCCTCCTGCTGCTCTCGGGGACCCTGGCCCTGGCCG
AGACCTGGGCGGGTGAGTGCGGGGTCAGGAGGGAAACGGCCTCTGCCGTGAGGAGC
GAAAGGTCCGCCTGGCTGGGGCGCAG
>2371
GGAGAGGAACAGAGATACACATGCCATGTGCAGCACAAGGGGCTGCCCAAGCCCCTC
ATCCTGAGATGGGGTAAGGAGAGAGATGGGGGCGGTCATGTCTCTTAGGGAAAGCAG
GAGCCCCTCTGGAGACCTTTAGCAGG
>2372
GGTATCATTGCTGGCCTGGTTCTCCTTGGAGCTGTGGTCACTGCTGTGATGTGGAGGAA
GAAGAGCTCAGGTGGGGAAGGGGTGAGGAGTGGGGTTTGAGTTTTCTTGTCCCACTG
GGGGTTTCAAGCTCCAGGTAGAAA
>2373
GGGGTCATGGCGCCCCGAACCCTCCTCCTGCTGCTCTCGGGGACCCTGGCCCTGGCCG
AGACCTGGGCGGGTGAGTGCGGGGTCAGGAGGGAAACGGCCTCTGCCGTGAGGAGC
GAAAGGTCCACCTGGCTGGGGCGCAG
>2374
AAGGCCCAGGCACAGACTGACCGAGTGAACCTGCGGACCCTGCTCCGCTACTACAAC
CAGAGCGAGGCGGGTGAGTGACCCCGGCCCGGGGCGCAGATCACTTACTCCCCGCTC
CATGCCTCACGGACGGCCCTGGTCCC
>2375
CTGGAGGGCACCTGCATGGAGTGGCTCCGCAGACACCTGGAGAACGGGAAGGAGAC
GCTGCAGCGCGCGGGTACCAGGGGCCATGGGGAGCCTGCTCGATCTCCTGTAGATCTC
CCGGGCTGGCCTCGCACAAGGAGGGG
>2376
GGAGAGGAACAGAGATACACATGCCATGTGCAGCACAAGGGGCTGCCCAAGCCCCTC
ATCCTGAGATGGGGTAAGGAGAGAGATGGGGGCGGCCATGTCTCTTAGGGAAAGCAG
GAGCCCCTCTGGAGACCTTTAGCAGG
>2377
CTGGTTCTCCTTGGAGCTGTGGTCACTGGAGCTGTGGTCACTGCTGTGATGTGGAGGA
AGAAGAGCTCAGGTGGGGAAGGGGTGAGGAGTCGGGTTTGAGTTTTCTTGTCCCACT
GGGGGTTTCAAGCTCCAGGTAGAAA
>2378
GCCAGGGGCTTCATGCTGGGGCTCATCATCTGTGGAGTGGACATCTTCACGCACAGAA
GGAGCAAGAAAGGTGAGAAATCCTGTGAGGTGACCGATACCCACCTTTCTCCTGACTT
GCTCACCCTTCTTCCATGATGAGG
>2379
CTCCGTGCCGGACGGGTTCCTCTCCGAGCTCACCCAGCAGCTGGCGCAGGCCACCGG
CAAGCCCCCCCAGGTTTGCCGGGAGGGGACAGGAAGAGGGGGGTGCCCACCGGACG
AGGGGTTCCGCGCTGGGAGCTGGGGAG
>2380
CGCAGAACCGCTCCTACAGCAAGCTGCTGTGCGGCCTGCTGGCCGAGCGCCTGCGCAT
CAGCCCGGACAGGTACGCGGAGTCGCGGAGGGGCGGGGGAGGGGCGGCGGCGCGCG

GCCAGGCCCGGGACTGAGCCACCCGC
>2381
CTACGGCGGCTGGGGGCCTGGCTGCGGGACCCTGGGGGGCAGCGCCTGGTGGTCCTA
CACCTGGAGGAAGGTATGTGGGGCCCAGCCCCAAGCTTGGCACCGCCGTCTTCCTTCA
GGTGGGCCGGGTCCTCCTAGGGAAG
>2382
GCTGCTGGTGCTGTACCCTGGGCCTGGCCCTGAGGTCACTGTGACGAGGGCTGGGCTG
CCGGGTGCCCAGGTACCAGGGAGTTGCATGGGGCAGTGCCCGGGCCGTGGCGGGGGG
CATGAATTTGTTGCAGGGTCTGCAG
>2383
GCGGTGGACCGCCCTGCGGGGGCCTGGCGCGGCTCCGGGCTGGCCTTGACCCTGCAG
CCCCGCGGAGAGGGTAGGTCCGCGTGGAGAGGGACGGGGAGCCGGGTCGACTGCCC
CCGGGCCCCCAGCCCCTGAGCCAGCCG
>2384
TGCCGCGGTCCGAGCCCGCGCCGCTGCCTGCGCACGGCCAGCTGGACACCGTGCCCT
TCCCGCCGCCCAGGTGCGCGCAGGCACCGGGACACGGGGCAGGAGCGGGCGGGGGC
GGCGTGGCCTCGTGGCCGCTCTCAACT
>2385
CACCGAGGCTTCCTCCTCCTCACCCTCCTCGCCCTGCTGGCGCTCACCTCCGCGGTCG
CCAAAAAGAAAGGTTATGGGGGATGATCGAAGGAGGGCTGGGGACGGGCAGGCGAG
GCCCCTCCACTTCTGGGCTGGGCCGC
>2386
GGCACCTGCGGGGCCCAGACCCAGCGCATCCGGTGCAGGGTGCCCTGCAACTGGAAG
AAGGAGTTTGGAGGTGAGGCGGGGGGCAGTCAGAGGGCAGGAGACGGGGGCACAGC
CTCGCCGAAGCCTGGGGGGACCCTTGG
>2387
AATGCTCAGTGCCAGGAGACCATCCGCGTCACCAAGCCCTGCACCCCCAAGACCAAA
GCAAAGGCCAAAGGTCAGCGAAAGGAGAAGGGGGTGGGGCTGTCGCGGGGGGCTGC
CCCCCCCCCCCCGCCTGTGAGGGGAC
>2388
CACCGAGGCTTCCTCCTCCTCACCCTCCTCGCCCTACTGGCGCTCACCTCCGCGGTCGC
CAAAAAGAAAGGTGATGGGGGATGATCGAAGGAGGGCTGGGGACGGGCAGGCAGGC
CCCTCCACTTCTGGCTGGCCGCCTG
>2389
GGCACCTGCGGGGCCCAGACCCAGCGCATCCGGTGCAGGGTGCCCTGCAACTGGAAG
AAGGAGTTTGGAGGTAGGCGGGCGCAGTCAGAGGGCAGAGACGGGGGCACAGCCTC
GCCGAAGCCTGGGCGGACCCTTGGCGG
>2390
AATGCTCAGTGCCAGGAGACCATCCGCGTCACCAAGCCCTGCACCCCCAAGACCAAA
GCAAAGGCCAAAGGTCAGCGAAAGGAGAAGGGGGTGGGGCTGTCGCGGGGGGCTGC
CCCCCCCCCCCCGCCTGTGAGGGGACA
>2391
GCACCCAGACACCCTGAACCAGGGGGAATTCAAAGAGCTGGTGCGAAAAGATCTGCA
AAATTTTCTCAAGGTAGGGCTGGACTCTGGCAGGTCTGACCCAGCCTCACCGCAGTTT
GGGTTGACAAGGGAGGATGGGAGTA
>2392

GAATTTCCATGCCGTCTACAGGGATGACCTGAAGAAATTGCTAGAGACCGAGTGTCCT
CAGTATATCAGGGTGAGGAGGGGCTGGGTGTGGCGGGGGCTCTCTGCCTGGTCCTGGG
GCTGCCCTGGGCCAGCGGTCCTCC
>2393
GAATTAAGTGAAACTGCCAGCATACTCATGCATGCAACAGCACATTCTCTGGTGCTTGT
GGATGAATTAGGTAAGACATTAAACTTCTCATTTGAAGACTATCTTAAAAACATTTGTAC
AAATAACTATTTTTATAGAAG
>2394
ATTTTCAACTCACTACCATTCATTAGTAGAAGATTATTCTCAAAATGTTGCTGTGCGCCT
AGGACATATGGTATGTGCAAATTGTTTTTTTCCACAAATTCGGTTTTTTGAGAGGGCAC
TTCTGTTGCTAGCACATGTAT
>2395
AAGTTATTCAAAAGGGACATAGAAAAGCAAGAGAATTTGAGAAGATGAATCAGTCACT
ACGATTATTTCGGTAACTAACTAACTATAATGGAATTATAACTAACTGACCTTAAGTTTC
AAAGAAACAGTAAAAGGGGAAG
>2396
TTCCCTTCTCGCTTGGGAACTCTAGTCTCGCCTCGGGTTGCAATGGACCCCAACTGCTC
CTGTGCCGCTGGTAAGGGACGCCCGGGTTCTGTGCCTTGGAATGCCAAATTCCCAGAC
ACCATAGAGAGTGTCCCTGGGTT
>2397
CCCGCTTCTCTGAAAGGCTCTCCTTGCAGCTGCTTAGATACTGAATTTTTTTCGGGAAG
TGGAAAACCAGGTAAGCATCGAAGTCCACTTGTCTTTTAATTTACTTTTTTATCAGTTTA
ATTCTGAGATGAGTCGAATGC
>2398
GCAGCGCATCCTGGCGACGGAGGGCGACATGGAGCTGGACGAGGCCGGCGACGTCAT
CGGCGTGCTGCGGGTGCGGAGGGGCGGGCCGGGCCTGAGCGTGCGGGGTTCCTGCTA
AGGTCTCCGAGTCGGGTTCTGATCCA
>2399
CTCAGCGGCTGCCCCACCATGGCTACCTGGGCCCTCCTGCTCCTTGCAGCCATGCTCCT
GGGCAACCCAGGTAAGGCCTTCCCCTCGGGATCGATCCTGATGGCCCACCCAGCCTCG
CACTCTCAGGCTGGCTGAACCTG
>2400
CGACCTGGCAAGAGCCCACCTGCGTGATGAGGAGAAATCCTGCCCGTGCCTGGCCCA
GGAGGGCCCCCAGGTACGTGTTGGCTCTCTGCTCACCTGCCACAGTCCCTCTCCTTTCC
CTCCTCCCTGGTGGCTCCTGGGGT
>2401
GGGCCGTGACTACAGGACCTGTCTGACGATAGTCCAAAAACTGAAGAAGATGGTGGAT
AAGCCCACCCAGGTGAGGCCAAGGGGCTACAGAGCCTCCTGTCTGCTGCTCAATGGA
GGGGCCAGCCTGTGACCAGGTCGGG
>2402
CAGGGCCTCGTGGCCGGAGAAACTGCCCAGCAGATCTGTGAGGACCTCAGGTTGTGTA
TACCTTCTACAGGTGAGTGCAGAGGTGACAGCAGGGATACCTCCTGAGGGTTGGAGAC
AGCTTCCCCCAGGATATATCAAAG
>2403
ATTGCAGCGGACATCCCAGAAGTGGTTGTTTCCCTTGCATGGGACGAAAGCTTGGCTC
CAAAGCATCCAGGTGAGAGAGGCAGGCATGCAGAGCTGCTAAGTCTAGAGGGAAGGA

CGGGAGAGAGGTTCCAGAGTTGGGT
>2404
CTTCTTCTGGACTCATCTGCGCCACTTGTCCGCTTCACACTCCGCCGCCATCATGGTGA
AGCTCGCGAAGGTAAACGGCCTTGAGCGCGACGCAGACGTGTAGGCCTGCTTCCGAG
GGGCGAGCGCGGCGCCGCGGGGAG
>2405
GGAGGTAGAAGAAGATAGTGAAGATGAGGAAATGTCAGAAGATGAAGAAGATGATAG
CAGTGGAGAAGAGGTAATTTTATCCAACTTAATGCAGAATTATGTTAAAACTACAAAAT
GGAGAGTTAAGACATGAAATTGGA
>2406
CCTGCCTCAGAGGATGAGGACGATGAGGATGACGAAGATGATGAGGATGACGATGAC
GATGAGGAAGATGGTAAGGAGTTGTCTTGGTAGTTACTGGGCTTCTGATTACAAGGTAT
CTTGAGATTCTGGGATCACATATT
>2407
GACGACGACGAAGATGATGAAGATGATGATGATGAAGATGATGAGGAGGAGGAAGAA
GAGGAGGAGGAAGGTACTTAAATTAGATTCTGACATACGACATGAGTTATGTTTAAAGG
AGGCACTTAAGTGTTTGTGGCTAC
>2408
CCTGGAAAACGAAAGAAGGAAATGGCCAAACAGAAAGCAGCTCCTGAAGCCAAGAA
ACAGAAAGTGGAAGGTAACTTGCAGAATTAGGGGATATGGGGGAGATAAACAGCACA
AATGATGAATAACAAAGGGACTTAATA
>2409
AAACTGGTATCAGCGATGTTTTTGCTAAAAATGATCTTGCTGTTGTGGATGTCAGAATT
GGTATGACTAGGTAGCTGCTTCACTGCACGTTACATACCGTGGGTCTGTTAATTTTTCCT
TCCCCTGTTAGCACAGTTACT
>2410
CTCACTGGTTTGAAAGTCTTTGGCAATGAAATTAAACTAGAGAAACCAAAAGGAAAA
GACAGTAAGAAAGGTATGTAAGGCTTTATGAGTTATGCAATGAACTCAGGAGCTAGAC
TGCTAGGGAAAATGCTTTGTAACCC
>2411
ATGAATTGAAAGAAGTGTTTGAAGATGCTGCGGAGATCAGATTAGTCAGCAAGGATGG
GAAAAGTAAAGGGTATGTTCTTCTATTGAAATGTAAGGGTTTTATTAACATTAATGCACT
TCCTGCTTTATAAAAGAAATAT
>2412
TCCCTGTACTATACTGGAGAGAAAGGTCAAAATCAAGACTATAGAGGTGGAAAGAATA
GCACTTGGAGTGGTAAGAAATTAGGCTTGTTCCAAGGTTTTCAGAATTGGTTGAGGGA
ACTCTTCTAGTCTTTGTATTTCAT
>2413
CTCTTCAGGAAGTATTTGAGAAAGCAACTTTTATCAAAGTACCCCAGAACCAAAATGG
CAAATCTAAAGGGTAAGATAATACCTTTGTATCATCAGTTATAGGCCTATATATGTCTTAG
AGGTCTAAGGACGTAAGGTCA
>2414
AGGGAAATTGAGGGCAGAGCAATCAGGCTGGAGTTGCAAGGACCCAGGGGATCACCT
AATGCCAGAAGCCGTAAGTTCACCTGGTTAGGGTGCTGTGGTTGGGGGTAGCACTCTC
GGTGCTTTGTTTATTTTTGCACAAA
>2415

TAAAGGAGTCATTTGACGGCTCCGTTCGGGCAAGGATAGTTACTGACCGGGAAACTGG
GTCCTCCAAAGGGTAAGGGAAGGAAGCGTGAGTGCTGCTTCCACTTGAAGGGGTTTT
TGTTCTGTGCAGACCTTGAGTCTAA
>2416
TTTGGAGGACGAGGTGGTGGTAGAGGAGGCCGAGGAGGATTTGGTGGCAGAGGCCGG
GGAGGCTTTGGAGGTAAGGCACGCAGAGATAATGACACCACATAGCATGTGCTCTTCA
GACCCTGTGCCCTGTCACGGTTCCT
>2417
CGGCTGCGACTCAGGCTCCGGCGTCTGCGCTTCCCCATGGGGCTGGCCTGCGGCGCCT
GGGCGCTCTGAGGTGAGGGACTCCCCGGCCGCGGAGGAAGGGAGGGAGCGAGGGCG
GGAGCGGGGCGGGCTGCGGGCCCCGG
>2418
GAATTCCTGGAGAGTTGCCTTTGTGAGAAGCTGGAAATATTTCTTTCAATTCCATCTCTT
AGTTTTCCATGTAAGTATTCAGTTTACATTTATGTTGCAGGTTAATCTTAAGAATTGTATT
GCTAAGGCTTCTAAGTGAA
>2419
TCCTTATCCCAGTTGATTGTGCAGAATACACTGCCTGTCGCTTGTCTTCTATTCACCATG
GCTTCTTCTGGTAGGTAATCTATTTGGAAAATCTGAAATTGTAATGGGCTTATGATTTTA
GATTGAGATGGCTCAGGTCT
>2420
TCCAAAGAAGAAGGATCTTTCCCTGGAGGAAATTCAGAAGAAATTAGAAGCTGCAGA
AGAAAGACGCAAGGTAAACGAAGCAATTCACAGAAAGCAGGATATTAATTTATGTAAT
GGGCAGATCAATTTTATTTCTATAA
>2421
CAAAATGGAAGCTAATAAAGAGAACCGAGAGGCACAAATGGCTGCCAAACTGGAACG
TTTGCGAGAGAAGGTTGGTTTCTTACTTTGTAAAAGGGTTGAGCTTGGAGTTTGATGC
ACCAATGAGTTGGCTTGAACTAAGT
>2422
GAGCACAGCATCGTCGGGACCAGACTCGTCTCAGGCCAGTTGCAGCCTTCTCAGCCA
AACGCCGACCAAGGTACAGCTTCAGTTTGCTACTGGGTTGTGCATTCAGCTGAATTTCA
TGGGGAAGTCCAAATTCTAAGGAA
>2423
CTCCGCCTGCTACATCCAGAACTGCCCCCTGGGAGGCAAGAGGGCCGCGCCGGACCT
CGACGTGCGCAAGGTGAGTCCCCAGCCCTGGTCCCGCGGCGCTCCGGGGAGGGAGGG
ACCCGCAGCCACAGGGGCGCGCCCCG
>2424
CCCTGCCAGTCCGGCCAGAAGGCGTGCGGGAGCGGGGGCCGCTGCGCCTTGGGCCTC
TGCTGCAGCCCGGGTGAGCGGGGCAAGGCGCTCCGGGGCCAGGGGGAGGCGGGCGG
GGGTGCGGCCGGGATTCCCCTGACTCC
>2425
CACACCAGTTGAAGCAGCATGTCATCGATGGAGAAAAAACCATTATCCAGAATCCCAC
AGACCAGCAGAAGTAGGTGCCAACCCTACTTATTACCTTCTACCACACAAGACTAGTC
ATCAGGTTTTTTTCTATACTCCCA
>2426
TGAAAAAGCTGAATTTGAGGTACATGAAGTATATGCTGTGGATGTTCTCGTCAGCTCAG
GAGAGGGCAAGGTGAGGAGAGTACCAGAGTTGGCAAAGAGGGGTGACTGAGAGTGT

TCACCAGACCAAATGTTACTTAAAT
>2427
AAATGAAAACTTCACGTGCCTTCTTCAGTGAGGTGGAAAGGCGTTTTGATGCCATGCC
GTTTACTTTAAGGTAACTAAGCAAATGATAGTACTTGGAACCAGTCTGGACTCACAGAC
CATACACCCAGGAACACTTTTTC
>2428
CGGATGGGTGTGGTGGAGTGCGCCAAACATGAACTGCTGCAACCATTTAATGTTCTCTA
TGAGAAGGAGGGTGAGTTCCAAAAAGAGCTTCACTTTGGATTCCCTGATTATAAGATAT
CCTTCCTGTAAGTTCAGAAGAG
>2429
GATAACCAGTGGTCCCTTCGAGCCTGACCTCTACAAGTCTGAGATGGAGGTCCAGGAT
GCAGAGCTAAAGGTTAGTATGGAATAGAAGGTGGTGAGTATGTACATGGTATCCTGCTT
GGGAGTGAGGGCTAAATGCCAGC
>2430
CGGCCACCACCAGCTGGCCAAGGAGGTGCTTATTAAGAAGGGCAAGGACTTCTCTGG
GCGGCCTCAAATGGTAAGTGGTGCCCATCTCCTCCCTGCCCCCTTCACCACCCCCTGGG
ATTGGTTCAGGTCTTCAGACCATG
>2431
CTGCATCGAAGGCTGGCGATGGCCACCTTTGCCCTGTTCAAGGATGGCGATCAGAAGC
TGGAGAAGATCAGTGAGTGCCAGGCTGGCCCCTGGGGCTGGGGCTGGATCCCACAGG
AGCTGCTGGAGGGAGAGGAGGGTTG
>2432
GAATTACAATGAAGGCATCATAGACAACCTGAGCAAAGACAGCCTGGTGGACCTAGTC
CCCTGGTTGAAGGTGAGATGCTGCCAGCCCTGCCTTCAGGTTCTAGTAGACCCTGACA
TTGTCCCCAATCTTCCTTCCTTTT
>2433
CCTGGAAAAATTAAAGAGCCATGTTAAAATACGAAATGATCTGCTGAATAAAATACTTG
AAAATTACAAGGTAGGTGATAGAGCAGAAGAGAATATGAGTTAGGCTAAAAGGAATCA
CAAGAGCAGGGTGGAGTCCATTC
>2434
TGGGGCTGGCGTGGAGACCACCACCTCTGTGGTTAAATGGACCCTGGCCTTCCTGCTG
CACAATCCTCAGGTGTGCTTCCCCCTCATTGATCCTAGACCCCAGCCAGCCCAATCTCT
GGGCTCCAGAGAAAGGGAGAGCC
>2435
TCCGAGAGGTGCTTCGCCTCAGGCCCGTGGCCCCTATGCTCATCCCCCACAAGGCCAA
CGTTGACTCCAGGTGTGCCTGCCCTCCCAGTGACATCTAGCCCCATGATGCATTCACTG
CTTGCCAGCCCACCTGGCTCCCC
>2436
GTTATCATCAATCTGTGGGCGCTGCATCACAATGAGAAGGAGTGGCACCAGCCGGATC
AGTTCATGCCTGGTGAGTCTGTCCTGTCCTGCGCCCTGGGCCACACAGCGAGCCTGGA
CTCTGCTCCAACCACCCCAGTACC
>2437
GATGGCTTCTCATCGTCTGCTCCTCCTCTGCCTTGCTGGACTGGTATTTGTGTCTGAGGC
TGGCCCTACGGTGAGTGTTTCTGTGACATCCCATTCCTACATTTAAGATTCACGCTAAAT
GAAGTAGAAGTGACTCCTTC
>2438

CTGCCATCAATGTGGCCGTGCATGTGTTCAGAAAGGCTGCTGATGACACCTGGGAGCC
ATTTGCCTCTGGGTAAGTTGCCAAAGAACCCTCCCACAGGACTTGGTTTTATCTTCCCG
TTTGCCCCTCACTTGGTAGAGAG
>2439
CAAAGTGGAAATAGACACCAAATCTTACTGGAAGGCACTTGGCATCTCCCCATTCCAT
GAGCATGCAGAGGTGAGTATACAGACCTTCGAGGGTTGTTTTGGTTTTGGTTTTTGCTT
TTGGCATTCCAGGAAATGCACAG
>2440
CCTCCCATGGCCCTGCCCAGTGTATCTTGGATGCTGCTTTCCTGCCTCATGCTGCTGTCT
CAGGTTCAAGGTGAGATTGCTTTGCCTCTAGCACTGGGTTCCCTATGAATCCTCAGAGC
TAACAAGAGGAGGAAGGCTCC
>2441
AGGCTCCAAGGCCTATGGCTCCCACTGCTATGCCTTGTTTTTGTCACCAAAATCCTGGA
CAGATGCAGATGTGAGTGGTTAGATGTGGTGTTGGAGGTGACCGGTCTCAGGGGGAG
GAGGGTCTCCATTCAGGAGAGTTC
>2442
GTCCTCCCTGGTGAAGAGCATTGGTAACAGCTACTCATACGTCTGGATTGGGCTCCATG
ACCCCACACAGGTGCCAGTATATCCTCCCTCTCTGTTACCTCTCAAGGTGCTATTGTTGC
CCAGGCCCACTCCCTGTCCCC
>2443
TTTGCATGGGAGAGAAATCCCTCCACCATCTCAAGCCCCGGCCACTGTGCGAGCCTGT
CGAGAAGCACAGGTAAGAAACAGAGGAGCTGCCTCTTCCCAGTGTTTTCCATCTCATC
CCCCATTCCTGGGTCTGACCTTCA
>2444
TGTGGGGTGGAATGAGCTGGAAGGCCGTGATGCCATCTTCAAGCAGTTTCATTTCAAA
GACTTCAACAGGGTATGGCACCAGGGTGGGACAGAGTTTTCTGAGACGGGCTGATGG
GGTCACCTTACAAAGCATGTTCTCT
>2445
CATGACAAGAGTGGCCCTGCAGGCTGAGAAACTGGACCACCATCCTGAATGGTTTAAC
GTGTACAACAAGGTGAGTGATGCTGTGTGCCTCTGTTGTGCTCTGCCCCCATCCAAATA
CTGCGAACTCTGACACATTCTGA
>2446
GAGTGGGTCCCCCGAGCTCTGTGACCTTATGCTCCACACTAACTCTGGCAGAGCCTCC
GTTTCCTCATAGGTAAGATGGAAATAATTACACCCTCTGGATGGTGTGACTGAAGATTA
AATACAGCGGGTGCTCTCACTCA
>2447
ATTGTGGACTTGCTTAAGAACCTCGATAGCAATGCGGTCGTGATCATGGTGAATTACAT
CTTCTTTAAAGGTAAGGCCCTTGGGCCCAAACCTGCACTTTCTTTGGCTTTTCTGCTGC
TTTTATCTAAAGAATACCCAAT
>2448
AGATGCAGCAGGTGGAGAATGGACTGAGTGAGAAAACGCTGAGGAAGTGGCTTAAGA
TGTTCAAAAAGAGGTACTTTCAGACTACCCCAGGGCCAGCCTAAACCCACACAGCCCC
AGGGAGACACACACGCCCTACCAGG
>2449
CAGTAACGTCTTCACCTCCCATGCTGATCTGTCCGGCATCAGCAACCACTCAAATATCC
AGGTGTCTGAGGTGGGTTCAGAAGCTCCTATGCATCTGCTTCCCAAGATCTATTCTGT

CTATTCTTTCTATTCTACTCTA
>2450
CCCTGCGGTCTGAGGGCTTCGACACCTACCGCTGCGACCGCAACCTGGCCATGGGCGT
GAACCTCACCAGGTGAGCCTCGCGCCCCGGGAAGCCGCCCCGGCCCGCCTGCACCTC
CGGCTGTGGCGAGCGCTTCGAGCCT
>2451
AATGAAGATATCATTACACTAAGGGCCGAAGATAACGCGGATACCTTGGCGCTAGTATT
TGAAGCACCAAGTAAGTCGTACCTTTTTACCGAGTCACGAAGCTACAGGAAAATCAAA
ACTCTGTGTGAGTAGAAACTCAA
>2452
AAGTGGAGAACTTGGAAATGGAAACATTAAATTGTCACAGACAAGTAATGTCGATAAA
GAGGAGGAAGCTGTAAGTAGTTTTTAAGTAAAAAGAAAATAGTTTGAAGAGAATTATA
ATACTGCTTATTAGGTTAATTGCT
>2453
AACTTCTTTACAAAAGCCACTCCACTCTCTTCAACGGTGACACTCAGTATGTCTGCAGA
TGTACCCCTTGGTAAGATAATAAATTTGAACCTTGTTTTGATGGTAGTCATATGTGATAC
ATACTCCTCAGTAATTAACCA
>2454
TGTCCATGTGCAAGTCTGTGGTGGCCCCTGCCACGGATGGTGGCCTCAACCTGACCTC
CACCTTCCTCAGGTGGGACAGCGGGCAGGTGGGTTTCTGGGACGGAGAGGTCCAGGG
ACACCCTCGGGCCTAACCCCTGACC
>2455
CAGTGTGAGACCCGAACCATGCTGCTGCAGCCCGCGGGGTCCCTCGGCTCCTACAGCT
ACCGGAGTCCCCGTGAGTGGGGCCTCCACCGGCCCTGGGAGCCTGGGGGACACTTGC
CGGGACGACTCTGGGCCAGCCCCCT
>2456
CAGTACGCGCTGCTGTACAGCCAGGGCAGCAAGGGCCCTGGCGAGGACTTCCGCATG
GCCACCCTCTACAGTACGTGCCCCGTGGACGCCGCCCACACGCAGGCCTGACCAGAG
GGGGGCTCCCCAAGACCCAGGGCAGG
>2457
GAGAAATTCACCGCCTTCTGCAAGGCCCAGGGCTTCACAGAGGATACCATTGTCTTCC
TGCCCCAAACCGGTGAGGGGTCCTAACTGATGGGGAGAGGATCAAGGTCAGATTAGA
GGCAGGCAGGCAGTCTCCTGACTGG
>2458
TTCACCACCACCATGACACCGGGCACCCAGTCTCCTTTCTTCCTGCTGCTGCTCCTCAC
AGTGCTTACAGGTGAGGGGCACGAGGTGGGGAGTGGGCTGCCCTGCTTAGGTGGTCT
TCGTGGTCTTTCTGTGGGTTTTGC
>2459
GTTTAATTCCTCTCTGGAAGATCCCAGCACCGACTACTACCAAGAGCTGCAGAGAGAC
ATTTCTGAAATGGTGAGTATCGGCCTTTCCTTCCCCATGCTCCCCTGAAGCAGCCATCA
GAACTGTCCACACCCTTTGCATC
>2460
GAGACACAGTTCAATCAGTATAAAACGGAAGCAGCCTCTCGATATAACCTGACGATCT
CAGACGTCAGCGGTGAGGCTACTTCCCTGGCTGCAGCCAGCACCATGCCGGGGCCCCT
CTCCTTCCAGTGTCTGGGTCCCCG
>2461

CTGGGGCATCGCGCTGCTGGTGCTGGTCTGTGTTCTGGTTGCGCTGGCCATTGTCTATC
TCATTGCCTTGGTGAGTGCAGTCCCTGGCCCTGATCAGAGCCCCCCGGTAGAAGGCAC
TCCATGGCCTGCCATAACCTCCT
>2462
GTACCCCACCTACCACACCCATGGGCGCTATGTGCCCCCTAGCAGTACCGATCGTAGCC
CCTATGAGAAGGTGAGATTGGCCCCACAGGCCAGGGGAAGCAGAGGGTTTGGCTGGG
CAAGGATTCTGAAGGGGGTACTTG
>2463
TGCGGCAGTGCAGCCCTTGGGACTTCCCTCGCCTTCCACCTCCTGCTCGTCTGCTTCAC
AAGCTATCGCTGTGAGTGGCTGGGGGCTCAAGCCTGGGGAAACCCAGGCTGTTTTGTG
GGGAAACTGAGGCTGGGATGGTG
>2464
CCTCGCCGGAGGAGCTGAACCGCTACTACGCCTCCCTGCGCCACTACCTCAACCTGGT
CACCCGGCAGCGGTGAGCGCAGGCGCTGAGCGGGACCCCTGGTGCTCTCACCCTGCG
GCCCCGCTCCACCTGGGGGCGTGGC
>2465
GAGACGGCCCGGACACGCTTCTTTCCAAAACGTTCTTCCCCGACGGCGAGGACCGCC
CCGTCAGGTCGCGGTAAAAGCGCCCGTTACCACACATCCTGCATCCGAGAGAGCGGCC
TGGCCCTACCCTGGCAACATCATTT
>2466
CGCGCCACCCGCCAGGAGATGCTGTTCTTGGCGTTGCTGCTCCTGCCAGTTGTGGTCG
CCTTCGCCAGAGGTGAGAGCAGAAACCAGGCTGGGAGGGCCAGCAGCGGCGAGGGG
GAGTCCGGGAAGCCCTGGGGCTGGGA
>2467
TCCGTCCCAGGCACATCACCAGCCTGGAGGTGATCAAGGCCGGACCCCACTGCCCCAC
TGCCCAACTCATGTGAGTCCTCGCACTGCATCAGTTAGTGCTCCCGCTCCGTGCCTCCT
CTGCCCATCCCTCCCCCTTCTAA
>2468
GAGGCGCTCCTTCGACATCCCGCCGCCCCCGATGGACGAGAAGCACCCCTACTACAAC
TCCATTAGCAAGGTGGGCTGCCTTTGCTGGGAAGGCCTCTGGGAAGCTGCAGAGTGG
GGAGTCGGGTGGGGGCCCACTGGCT
>2469
AAGGCCGGCAAGCGAGTGCTCATTGCAGCCCACGGGAACAGCCTGCGGGGCATTGTC
AAGCACCTGGAAGGTAGGCCACCTTCAGGAGCCTGGGCAGGGTGGGTGGGCAGCAGC
CAGCTGGCTTCTCATCTCAGCAAAGT
>2470
AGAATGGGCGGCTTCTGGGCCAGATGGAGGAAGAGGGCATCCTCAGGCGGCTGAAGA
AGTATGACAACTGGTAAGCTCGGCCCCCGCTGCCTGTCCCAGCACCCTGCAGGCAGGG
CTCCCCTGCGTCTCCTGGGAGTTGG
>2471
CTCGGTCGCTGGATGTCAGAGGAGGATTTTGAGAAAGCGTTCAATGCCAGGTTCCCAG
GGTGCATGAAAGGTGAGCGGAACATTGATTTGATTGGGTAAAACAGCAGAGAGCCTTT
TCTTATTTACATCTATCCTAATGG
>2472
CTGGAAGCACTGGGCGATGGGGAGTTTGTCAAATGCCTCCATTCTGTGGGGTGCCCTC
TGCCTTTACAAAGTAAGTGTATTATTTCAGAATCAAAAGTCAAAATAAAAAAGAAAGC

TGAACGCAAACCCCAGTGAGTGCC
>2473
GAAGTGCTTTGCTCTCAGGAATGCCAGCCGGCTGGCCAAGGAGGAAGGGTGGCTGGC
AGAGCACATGCTGGTGAGCTGCAGGAAGCCCTGATGTGCAGATGAGAGGCCTGGGGG
GTGGCAGAAACAAACAGCATTACAGT
>2474
CCCAGCCTCCCCGGGTGGAAGGTTGAGTGCGTCGGGGATGACATTGCCTGGATGAAGT
TTGACGCACAAGGTGACTCTTTTAGACCCAACTCTTGGTAACGATTGGACTCAAGCGA
ATCGTTGGCCTTCGAAACATGTCA
>2475
ATTGATGAGCCGCTAGCTTCAGGCGTCACCATCACGTCCTGGAAGAATAAGGAGTGGA
GCTCAGAGGATGGTGTGTCCCTGCCAGAGGCCTGTGTGTGCCGGGCTGCAGGGACTG
CCTGTTTTGAGCCAGGCACTCACGA
>2476
GATGCTGCCTGGGAGTCTCCGGAAGGTGTTCCCATTGAAGGCATTATCTTTGGAGGCC
GTAGACCTGCTGGTGAGGCTCTCCTTCATTTAGGCTGGGAACATGGGTGTGCTGGGTA
CCGAAGGCATCTGTGAAATCTCTC
>2477
AGCTGGCAACATGGAGTCTTTGTGGGGGCGGCCATGAGATCAGAGGCCACAGCGGCT
GCAGAACATAAAGGTAAATCAAAGTCCTGATCTGAAACCACAGAGAAGTGGGATTAG
AGCACTCTTCGTCACTCTTATGTCTC
>2478
AAAATCAACTCGCTTGCCCACCTGCGCGCCGCGCCCTGCAACGACCTGCACGCCACCA
AGCTGGCGCCCGGTGAGAGCACCCCCCGCTCCGGCCGGGGATGCGGGGCGGCGGCGG
GATCTCCTGGGTGGGGAGCTGGCGG
>2479
GGGCCCGCTACTGGGCAGCGGCGGCTTCGGCTCGGTCTACTCAGGCATCCGCGTCTCC
GACAACTTGCCGGTGAGTGGGCGCCCCGCGGTGGGGAGGGCGCGCCGGGCGGGGGG
CGCACGGGCGTGCTTTAGCCCGGACG
>2480
GGCGAGCTCAAGCTCATCGACTTCGGGTCGGGGGCGCTGCTCAAGGACACCGTCTAC
ACGGACTTCGATGGTGAGCCAGGCCCGGGAGGGAGCTGCCCAGGTGACTCGGCCCGG
CCCGGCCCAGTCCGGAGGCCTCGGCC
>2481
GATATTCCTTTCGAGCATGACGAAGAGATCATCAGGGGCCAGGTTTTCTTCAGGCAGA
GGGTCTCTTCAGGTAACTGATGGAAACCCCTGGCCATGGGGTTATTGGTCTTAATGGGG
CTATTAGTCTTCATGGGACAGTC
>2482
TCAACTGCTCGGGCCGCGGGCTGCGGACGCTCGGTCCCGCGCTGCGCATCCCCGCGG
ACGCCACAGCGCTGTGAGTAGCGGGCCCAGCGGCACCCGGGAGAGGCCGCGGGACG
GGCGGGCGTGGGCGGGTTCCCTGGCCC
>2483
ACGTCTCCCACAACCTGCTCCGGGCGCTGGACGTTGGGCTCCTGGCGAACCTCTCGGC
GCTGGCAGAGCTGTGAGTGTCCCCCAGTCGTGCCAGCATGCGGGGCTCACTCCGGGT
GGGCTGGCGGCACCGCCTCTTGCTG
>2484

ATATAAGCAACAACAAGATTTCTACGTTAGAAGAAGGAATATTTGCTAATTTATTTAATT
TAAGTGAAATGTAAGTTGTGGTTCTTTGGGTGGGGTCCTGGCTGGACCCCAGGCCCCC
AATATCCCTTCTGCCCTCCCAG
>2485
ACGTGTGCTGGGCCTGGCTCCCTGGCTGGCCAGCCTCTGCTTGGCATCCCCTTGCTGG
ACAGTGGCTGTGGTGAGTGCCGGTGGGTGGGGCCAGCTCTGTCCTTCCCAGCCAGGT
GGGACCTGGGCCCTGCAGACACTGG
>2486
CAGAACCGCGGTGGTTCAGGCCTGGAGGCCGCCTACAGCATCGTGGCCCTGGGCGAG
GAGCCGGCCCGAGGTGAGTGTCTGCTGCCCACTCCCCTTCCTCCCCAGGGCCATCCAG
ATGGGGCAGAGCCTGGTACCCCCGT
>2487
GGGCCGGGGCCGCCCTGGCAATGGTGGACAGTCCCGCCGTGCAGCGCTTCCTGGTCTC
CCGGGTCACCAGGTGCCTGCCCCCACCCCCCGAGGGGCCATAGGTTGGGAGATCTCTG
AAGCACTGGGGCAGAGACTGCGGC
>2488
CCCACCGGGTGGTGTAACACCGACCTGTGCTCAGCGCCGCACAGCTACGTCTGCGAGC
TGCAGCCCGGAGGTGTGCGGGGGGCCAGGCAGGGGCCTGAGACGCTGGCTGTGGTTA
GGGGCCTGCCGAGCGCCCGCGGTGG
>2489
GGACCTGCAGGGACCCCTGACGCCTCTGGCACAGCAGGACGGCCTCTCAGCCCCGCA
CGAGCCCGTGGAGGTAGTCGGCCCCCCACGTTCTACAACCTGCCCTCCTGCCTGCCCC
TGGAGGCCTTGCCTGCCCTGCCCAC
>2490
TTTGGGACCCAGGAGCTCCGGCGGCCCGCCCAGCTGCGGCTGCAGGTGTACCGGCTC
CTCAGCACAGCAGGTGGGACTCTGGGTGGTGGGTGGTGGGTGGTGGGCGCCGCAGGA
CTCGGGGTGGCCTCTCTGAGCTTTCA
>2491
GCCCTGGAGCCAACATCTGCTTGCCGCTGGACGCCTCCTGCCACCCCCAGGCCTGCGC
CAATGGCTGCACGTCAGGGCCAGGGCTACCCGGGGCCCCCTATGCGCTATGGAGAGAG
TTCCTCTTCTCCGTTCCCGCGGGG
>2492
GGAGGAGCCCATCTGTGGCCTCCGCGCCACGCCCAGCCCCGAGGCCCGTGTACTGCA
GGGAGTCCTAGTGGTGAGTATGGCCGAGGCTCCACCACCAGCCCCCAGGCAGGTGCC
TGCAGACAGGGTGCTCACACAGGGCG
>2493
GCAGTCCCTGACCTTCCAGAACGTGGTCTTCAATGTCATTTATCAGAGCGCGGCGGTCT
TCAAGCTCTCAGTAGGTGGGCGGGGGTGGGGAGGGGAGGGGATGGGGCGGGGCAGG
GCGGGGGCGGGCTCCACCTTCACCT
>2494
TGTCCCCCAATGCCACGCTAGCACTGACGGCGGGCGTGCTGGTGGACTCGGCCGTGGA
GGTGGCCTTCCTGTGAGTGACTCGGGGGCCGGTTTGGGGTGGGCACCAGGCTCTTGTC
CCAGCCCCAGCCTCAGCCGAGGGA
>2495
CCGGTTCCAGACCCCTCGGTGGCCCAGGTGCTGGTGGAGCACAATGTCATGCACACCT
ACGCTGCCCCAGGTGAGGGATGAGGGGGTGAGGGGGCCACTGCCTTTCAGGCTCTGA

GCACGGGTCCCCCAGCTCCCCAGT
>2496
GCAGCAGGTGCCTGTGAGCGTGCGCGCCTCCCTGCCCTCCGTGGCTGTGGGTGTGAGT
GACGGCGTCCTGGTGGCCGGCCGGCCCGTCACCTTCTACCCGCACCCGCTGCCCTCGC
CTGGGGGTGTTCTTTACACGTGGG
>2497
TCTGGGGGACGGTGGGTGGCTCGAGGGTCCGGAGGTCACCCACGCTTACAACAGCAC
AGGTGACTTCACCGTTAGGTGGCCGGCTGGAATGAGGTGAGCCGCAGCGAGGCCTGG
CTCAATGTGACGGTGAAGCGGCGCGT
>2498
GGCGCCCGGGCAGCTGGTCCATTTTCAGATCCTGCTGGCTGCCGGCTCAGCTGTCACC
TTCCGCCTGCAGGTCGGCGGGGCCAACCCCGAGGTGCTCCCCGGGCCCCGTTTCTCCC
ACAGCTTCCCCCGCGTCGGAGACC
>2499
CGACCCCAACCTGGAGGACGGCGACCAGACGCCGCTCAGTTTCCACTGGGCCTGTGT
GGCTTCGACACAGGTCAGTGCGTGGCAGGGCCGTCCTCCATGCCCCTCACCCGTCCAC
ACCCATGAGCCCAGAGAACACCCAG
>2500
TGGCGTGGAGTACACCTTCAGCCTGACCGTGTGGAAGGCCGGCCGCAAGGAGGAGGC
CACCAACCAGACGGTGGGTGCCGCCCGCCCCTCGGCCACTTGCCTTGGACAGCCCAG
CCTCCCTGGTCATCTACTGTTTTCCG
>2501
AGTGAGCCGCAGCTCCTACGTGTACTTGGAGGGCCGCTGCCTCAATTGCAGCAGCGGC
TCCAAGCGAGGGGTGAGTGTTGAGCGGGGTGTGGGCGGGCTGGGGATGGGTCCCATG
GCCGAGGGGACGGGGCCTGCAGGCA
>2502
GGCTCTTGCCGCCTCTTCCCACTGGGCGCTGTGCACGCCCTCACCACCAAGGTGCACT
TCGAATGCACGGGTGAGTGCAGGCCTGCGTGGGGGGAGCAGCGGGATCCCCCGACTC
TGTGACGTCACGGAGCCCTCCCGTG
>2503
GCTGCTGCGGCAGGCCGATCCCCAGCACGTCATCGAGTACTCGTTGGCCCTGGTCACC
GTGCTGAACGAGGTGAGTGCAGCCTGGGAGGGGACGTCACATCTGCTGCATGCGTGC
TTGGGACCAAGACCTGTACCCCTGC
>2504
TCTGGTGTCCCTGAGGGTCCACACTGTGGATGACATCCAGCAGATCGCTGCTGCGCTG
GCCCAGTGCATGGTAGGATGGCCCCACCTGCTCACCCTGCCCCGCATGCCTGCCAGGG
CACTGGGTTCAGCCCCCCAGGGCA
>2505
CTGCAGGCAGAGACCACCGCGGGCACCGTGACGCCCACCGCCATCGGAGACAGCATC
CTCAACATCACAGGTGCCGCGGCCCGTGCCCCATGCCACCCGCCCGCCCCGTGCGGCC
CTTTCCTCTGCCTCCCTCCTCCCCC
>2506
GTGGTCACCCTGGACAGCAGCAACCCTGCGGCCGGGCTGCATCTGCAGCTCAACTATA
CGCTGCTGGACGGTGCGTGCAGCGGGTGGGGCACACGCGGCCCCCTGGCCTTGTTCTT
GGGGGGAAGGCGTTTCTCGTAGGG
>2507

GCAGGAGGATCCGCCCAGAGTCACTCCAGGGTGCTGACCACCGGCCCTACACCTTCTT
CATTTCCCCGGGGTGAGCTCTGCGGGCCAGCCTGGCAGGGCAGGGCAGGGCATCATG
GGTCAGCATTGCCTGGGTTACTGGC
>2508
CACCCGCCACCTCACCGCCTTCGGCGCCAGCCTCTTCGTGCCCCCAAGCCATGTCCGC
TTTGTGTTTCCTGTGAGTGACCCTGTGCTCCTGGGAGCCTCTGCAGAGTCGAGGAGGG
CCTGGGTGGGCTCGGCTCTATCCT
>2509
CCTTTCTGTGGGCAGCGGGGCCGCTTCAAGTACGAGATCCTCGTCAAGACAGGCTGGG
GCCGGGGCTCAGGTGAGGGGCGCAGCGGGGTGGCAGGGCCTCCCCTGCTCTCACTGG
CTGTGCTGGTTGCACCCTCTGGGAG
>2510
ATCTTCCGGATCGCCACCCCGCACAGCCTGGGTAGCGTGTGGAAGATCCGAGTGTGGC
ACGACAACAAAGGTTTGTGCGGACCCTGCCAAGCTCTGCCCCTCTGCCCCCGCATTGG
GGCGCCCTGCGAGCCTGACCTCCC
>2511
GTCAATGACTGGCTTTCGGTGGAGACGGAGGCCAACGGGGGCCTGGTGGAGAAGGAG
GTGCTGGCCGCGAGTAAGGCCTCGTTCCATGGTCCCACTCCGTGGGAGGTTGGGCAGG
GTGGTCCTGCCCCGTGGCCTCCTGC
>2512
TTCTCCTCATCTGCCTCTTCCTGGGCGCCAACGCCGTGTGGTACGGGGCTGTTGGCGAC
TCTGCCTACAGGTGGGTGCCGTAGGGGTCGGGGCAGCCTCTTCCTGCCCAGCCCTTCC
TGCCCCTCAGCCTCACCTGTGTG
>2513
GGTGTCCAGCGTGGTTGTCTATCCCGTCTACCTGGCCATCCTTTTTCTCTTCCGGATGTC
CCGGAGCAAGGTGGGCTGGGGCTGGGGACCCGGGAGTACTGGGAATGGAGCCTGGGC
CTCGGCACCATGCCTAGGGCCGC
>2514
CATCGACAGCTGCCTGGACTCGTCCGTGCTGGACAGCTCCTTCCTCACGTTCTCAGGC
CTCCACGCTGAGGTGAGGACTCTACTGGGGGTCCTGGGCTGGGCTGGGGGTCCTGCC
GCCTTGGCGCAGCTTGGACTCAAGA
>2515
CTGGGCCCAGAGGAGGACGGCTTCTCCCTGGCCAGCCCCTACTCGCCTGCCAAATCCT
TCTCAGCATCAGGTGAGCTGGGGTGAGAGGAGGGGGCTCTGAAGCTCACCCTTGCAG
CTGGGCCCACCCTATGCCTCCTGTA
>2516
TTGCCGAGGGGGTCAGCAGCCCAGCCCCTACCCAAGACACCCACATGGAAACGGACC
TGCTCAGCAGCCTGTGAGTGTCCGGCTCTCGGGGGAGGGGGGATTGCCAGAGGAGGG
GCCGGGACTCAGGCCAGGCAGCCGTG
>2517
GGGGAGCTGGGGCCACCCAGCCCAGGCCTGAACTGGGAACAGCCCCAGGCAGCGAG
GCTGTCCAGGACAGGTGTGCTTGCGTAGCCCCGGGATGCCCCTAGCCCCTCCCTGTGA
GCTGCCTCTCACAGGTCTGTCTCTGC
>2518
GAGTGTTGCGTGGCTCCTGTCCAGCAGCGCCAGCTTCCTGGCCTCATTCCTCGGCTGG
GAGCCACTGAAGGTGAGGGGGCTGCCAGGGGTAGGCTACAGGCCTCCATCACGGGGG

ACCCCTCTGAAGCCACCCCCTCCCC
>2519
CCACGGCTTTGCACTCTTCCTGGCCAAGGAAGAAGCCCGCAAGGTCAAGAGGCTACA
TGGCATGCTGCGGGTGAGCCTGGGTGCGGCCTGTGCCCCTGCCACCTCCGTCTCTTGT
CTCCCACCTCCCACCCATGCACGCA
>2520
GGCACGCCTACCGTCTGCAAAGCGCCATCAAGCAGGAGCTGCACAGCCGGGCCTTCC
TGGCCATCACGCGGTACGGGCATCCGGTGCACTGGTCTGTCTTCTGGGCTTTAGTTTTG
CCTTTAGTCCAGCCAGACCCTAGG
>2521
TACGTCCACGGGAACCAGTCCAGCCCAGAGCTGGGGCCCCCACGGCTGCGGCAGGTG
CGGCTGCAGGAAGGTGAGCTGGCAGGGCGTGCCCCAAGACTTAAATCGTTCCTCTTGT
TGAGAGAGCAGCCTTTAGCGGAGCT
>2522
ACGACGTTGGCTGGGAGAGTCCTCACAATGGCTCGGGGACGTGGGCCTATTCAGCGCC
GGATCTGCTGGGGTGAGCAGAGCGAGGGCCCCGGGCGTCTACGCCAAGGACAAGGGA
GTAGTTCTCCAGGAGTGCCGCGGCC
>2523
TGGGCCTGAGCCTGGAGGAGAGCCGCGACCGGCTGCGCTTCCTGCAGCTGCACAACT
GGCTGGACAACAGGTGGGAGCTCCCTCCCCTGCCCTCTCCGGGGTGGCCGCAGTCAC
CAGCCAGGAGCCCACCCTCACTCCTC
>2524
CGCCCTCAGCGTCCGCCCCTTTGCGCTGCGCCGCCTCAGCGCGGGCCTCTCGCTGCCT
CTGCTCACCTCGGTACGCCCGTCCCCGGCCAGACCCCGCGCCTCCCACCGGCAGCGTC
CCGCCCCCTCGCGGGGCCCCGCCC
>2525
CCAGGTGGCGCAGCTGAGCTCCGCAGCCCGTGGCCTGGCGGCCTCGCTGCTCTTCCTG
CTTTTGGTCAAGGTGAGGGCTGGGCCGGTGGGCGCGGGGCTGGGCGCACACCCCAGG
GCTGCAAGCAGACAGATTTCTCGTC
>2526
GCCAGAGCTCCTGGGGGTCACCTTGGGCCTGGTGGTGCTCGGGGTAGCCTACGCCCAG
CTGGCCATCCTGGTAGGTGACTGCGCGGCCGGGGAGGGCGTCTTAGCTCAGCTCAGCT
CAGCTGTACGCCCTCACTGGTGTC
>2527
GGACTACGAGATGGTGGAGTTGTTCCTGCGCAGGCTGCGCCTCTGGATGGGCCTCAGC
AAGGTCAAGGAGGTGGGTACGGCCCAGTGGGGGGGAGAGGGACACGCCCTGGGCTC
TGCCCAGGGTGCAGCCGGACTGACTG
>2528
GATCCCAAGGCCCAACTCCCCGAACCACTCAGGGTCCTGTGGACAGCTCACCTAGTGG
CAATGGCTGCAGGTAAGCGCCCCTAAAATCCCTTTGGCACAACGTGTCCTGAGGGGAG
AGGCAGCGCCCTGTAGATGGGACG
>2529
GCTTTTTGACCACGCTATGCTCCAAGCCCATCGCGCGCACCAGCTGGCCATTGACACCT
ACCAGGAGTTTGTAAGTTCTTGGGGAATGGGTGCGGGTCAGGGGTGGCAAGAAGGGG
TGACTTTCCCCCACTGGAAGTAAT
>2530

CCAGACCTCCTTCTGCTTCTCAGACTCTATTCCGACACCCTCCAACATGGAGGAAACG
CAACAGAAATCCGTGAGTGGATTCCGTCTCCCTAGGCGGGGATGGGGGAGACCTGTGG
TCAGGGCTCCCGGGCAGCACAGCC
>2531
TGACACCTCGGACAGCGATGACTATCACCTCCTAAAGGACCTAGAGGAAGGCATCCAA
ACGCTGATGGGGGTGAGGGTGGCGCCAGGGGTCCGCAATCCTGGAACCCCACTGGCT
TAGAGGGCTGGGGGAGAGAAACACT
>2532
GGGGACCTGTGCAACCCGAACGGCGTCGGGCCGTGGAAGCTGCGCTGCTTGGCGCAG
ACCTTCGCCACCGGTGAGCGGGGGAAACTGAGGCACGAGGGACAAGAGGTCGTCGG
GGAGTGAAAGCAGGCGCAGGGAAATAA
>2533
TGCAGGAGGAGCCGGGGGCCTTCAACTGGAGCATGTACAGCCAACATGCCTGCCTCAT
TGAGGGCAAGGGGTAAGGACTGGGGGGTGAGGGTTGGGAGGAGGCTTCCCATAGAGT
GGCTGGTTGGGGCAACAGAGGCCTG
>2534
CGTGGCCCTGGTCTGTGGTGTCCCGGCCATGGACATCCCCCAGACCAAGCAGGACCTG
GAGCTCCCAAAGGTTTGAGGCTGGGGGAGCGGGCACTTTACTGTGGGAGGCCTGGGG
CGGGTGGGAGCTGCGGGCAGGCGGG
>2535
CTCTGAGGGTCCACATCACCTCACTGTTGCCCACCCCCGAGGACAACCTGGAGATCGT
TCTGCACAGATGGTGGGTTTCTCATCATTGAGACGGGCTGGGCGGGGGCTCAGTCTCC
CCCCTCAGGGGTCCAGGACTGGGT
>2536
AACAACAGCTGTGTTGAGAAGAAGGTCCTTGGAGAGAAGACTGAGAATCCAAAGAA
GTTCAAGATCAACTGTGAGTGTCCCCAGGCCCCAAGGGCTGGCTCAGTGCTGGCATGC
TAGCCACGCTCTCCCAGAGGCGGCTC
>2537
GACAATTTCCTGTTTCTCTGCCTACAGGACACCACCACCCCCATCCAGAGCATGATGTG
CCAGTACCTGGGTGGGTCTCACAGCACATGAGCTCAACGTGGGTGAGAGGCAGCAGC
TACGTCCATGGCTGGGAACCCTGG
>2538
GGATTCATCAGGGCTTTCAGGCCCCTGCCCAGGCACCTATGGTACTTGCTGGACTTGAA
ACAGATGGAAGGTGAGCTCTGCCTAGGACACGCCCAGCCTCAGCTGGAGGAGAAGCT
GCCTCTTTCTTAGCCCGAGCCCCC
>2539
CCACACCAGAGCAGATGGCCCAGTATGCAGCTGATCTCCGTAGATACATCAACATGCTG
ACCAGGCCTAGGTGTGTGCCACAGTTGGGGAGAGAGATCCCAGCCCCTGGGACCCTG
GGCCCACTCCACATTCCTGGCCAC
>2540
ATGGGAAAAGACACAAAGAGGACACGCTGGCCTTCTCGGAGTGGGGGTCCCCGCATG
CTGCTGTCCCCAGGTGAGTTTGACTCCCTGCCCTGTCTGTCCAGGCTCCCTGGGGCTG
AAATGGGGGTGGTGGGACTGAATCA
>2541
ATGTGGCAGCTCACAAGCCTCCTGCTGTTCGTGGCCACCTGGGGAATTTCCGGCACAC
CAGCTCCTCTTGGTAAGGCCACCCCACCCCTACCCCGGGACCCTTGTGGCCTCTACAA

GGCCCTGGTGGCATCTGCCCAGGC
>2542
GCGGGAGTGCATAGAGGAGATCTGTGACTTCGAGGAGGCCAAGGAAATTTTCCAAAAT
GTGGATGACACAGTAAGGCCACCATGGGTCCAGAGGATGAGGCTCAGGGGCGAGCTG
GTAACCAGCAGGGGCCTCGAGGAGC
>2543
TGCATCGACGGCATCGGCAGCTTCAGCTGCGACTGCCGCAGCGGCTGGGAGGGCCGC
TTCTGCCAGCGCGGTGAGGGGGAGAGGTGGATGCTGGCGGGCGGCGGGGCGGGGCT
GGGGCCGGGTTGGGGGCGCGGCACCAG
>2544
GGCTGGCGGCGCTGTAGCTGTGCGCCTGGCTACAAGCTGGGGGACGACCTCCTGCAG
TGTCACCCCGCAGGTGAGAAGCCCCCAATACATCGCCCAGGAATCACGCTGGGTGCGG
GGTGGGCAGGCCCCTGACGGGCGCG
>2545
CCAAGAAGACCAAGTAGATCCGCGGCTCATTGATGGGAAGATGACCAGGCGGGGAGA
CAGCCCCTGGCAGGTGGGAGGCGAGGCAGCACCGGCTCGTCACGTGCTGGGTCCGGG
ATCACTGAGTCCATCCTGGCAGCTAT
>2546
ATCCACCCCTCCTGGGTGCTGACAGCGGCCCACTGCATGGATGAGTCCAAGAAGCTCC
TTGTCAGGCTTGGTATGGGCTGGAGCCAGGCAGAAGGGGGCTGCCAGAGGCCTGGGT
AGGGGGACCAGGCAGGCTGTTCAGG
>2547
AGCAGCTTCTTGATCGTGGTGGTGTTCCTCATCGCTGGGACGCTGGTTCTAGAGGCAG
CTGTCACGGGAGGTGAGTGAACAGGTGACCTGCTGGGCTGGGTTGGACTAAGGGGAG
ACCCTCTGGACACCCTGGGCCAGGA
>2548
ACTGACTGCCCAGGAATCAAGAAGTGCTGTGAAGGCTCTTGCGGGATGGCCTGTTTCG
TTCCCCAGTGAGGTGAGCACTAGCTGGAGAACGAGGAGACCCCTGAAGACACAAAAG
AAGGCTGAGCGGTGGGGAAGCATCC
>2549
GTTTTGCCATTGCCAGTGGGACGTCTGAGACTTTCTCCTTCAAGTACTTGGCAGATCAC
TCTCTTAGCAGGTAGGTGCCGCAGACCCTGCGGGTTAAGAGGTGGGGTGGGGGGCAG
TGCTTGCCAAGGCCCTAAACTGGG
>2550
ACCTGGGTTCGCTCGCCTTCCTAGGCGCTGACACCGCTCGGTTGGATGTCGCGTCGGA
GTTTCGAAAGAAGTGAGTCCGGGCAGCGCCTTCCCCCTTGCTGGTACCTGGCAGGCAA
GGGGAACTGACCGTTGGTCCCGAA
>2551
AGGCCGGGCCTGCCCAGACCCTTATTCGGCCCCAGGACATGAAGGGTGCCTCTCGAAG
CCCCGAAGACAGGTAACTACGCCCTGTGCTGTCCAGGGACGGGAGGGAAGGAAGGTG
TGCGGGAGGAGTTCTCTGTCTCCAC
>2552
TTGCAGCCCAGAAGACCCACCAGGACCAGTACAGCTTCAGCACTGACACGGTGGAGT
GCCGCTTCTACAGGTGAGAGCTGGGGCTAGGGGTGGGGGGCTGGAAAAGGCGCGGG
AAACTCTGGGTGGTCTAAGAGCCCTGG
>2553

TGCAAGATGCTTCTGATTCTGCTGTCAGTGGCCCTGCTGGCCTTCAGCTCAGCTCAGGA
TTTAAATGAAGGTAAGATGAATTGGGGGAAGATATTGTGACTCTGATTGGGGTTTACAG
GCAAATGCTATAGAGGAGGAAA
>2554
TCCCCAAGGGGGCCGCCCACAAGGACCTCCACAGGGGCAGTCTCCTCAGTAATCTAG
GATTCAATGACAGGTACGATTCCAGTTTATTATCCATCAAAGGCTCCAACTGCTACAGT
TCTCCAACTTCATTGTGCCAATGA
>2555
TGCAAGATGCTTCTGATTCTGCTGTCAGTGGCCCTGCTGGCCTTCAGCTCAGCTCAGG
ACTTAGATGAAGGTAAGCCGAATTGGGGGAAGATATTGTGACTCTGATTGGGGTTTACG
GGCGAATGCTATAGAGGGGGAAA
>2556
TCCCCAAGGGGGCCGCCCACAAGGACCTCCACAGGGGCAGTCTCCTCAGTAATCTAG
GATTCAATGACAGGTATGATTCCAGTTTATTATCCATCAAAGGCTCCAACTGCTACAGTT
CTCCAACTTCATTGTGCCAGTGA
>2557
GCCGCGAGCGTGACCGGGTGCAGGTGGAGCGCGACGGGCTGGCGGAGGACCTGGCG
GCGCTCAAGCAGAGGTCAGGGGGCAGGGCTGGGCCGCTGCCGTCGAGGCGAGGTCG
AAGCGGCGTCGAGGCGGCTGCTCTTGCC
>2558
CCTGGAACTAGAGCGCAAGATTGAGTCTCTGATGGATGAGATTGAGTTCCTCAAGAAG
CTGCACGAGGAGGTAAGTGGGCCCGGTATCAGGGGCGGTTTCTGAGGTTGTGGGGTG
GTCTCGCTGGAGCTGGCGGGTGGAG
>2559
CATCCGCGCGCAGTACGAGAGCATCGCCGCGAAGAACCTGCAGGAGGCGGAGGAGTG
GTACAAGTCCAAGGTGCAAGAGCCGGGAGGGCCTGCGAGGCGGGACGCTGGGGTGG
TGTCGCGCGTCCCAGCCGACTAAAGCC
>2560
GCAGGAGATGAACGAGTCCCGACGCCAGATCCAGAGTCTAACGTGCGAGGTGGACGG
GCTGCGCGGCACGGTGAGTACGAAGCTGCGCGCTCGGGCCCGGGGAGCGGACGATGA
AATGTTCTGCAACTGGCCCCTTCCAC
>2561
TCAACGTCAAGATGGCCCTGGACATCGAGATCGCCACCTACCGCAAGCTGCTGGAGGG
CGAGGAGAGCCGGTGAGGGTGGAGCTGCTGGGGCGGGGCAGGGCGGGGTCGGGACT
GGGCCGGGCAGGGCGGGGCCTGGGCA
>2562
GGAGCCTCCCCAGGACAGCCACAGCCGGAAGACGGTTCTGATCAAGACCATTGAGAC
CCGGAATGGGGAGGTGAGGCAGGTCCCCTAATGCCAGGACCCCACCATTTCCCATATTA
TTTCTGAGCCCCAGCCTGGCTGTC
>2563
TATTACCGCCAGAGACAAAGAAGTCGCAGACGAAGGAGGCGGAGCTGCCAGACACG
GAGGAGAGCCATGAGTAAGTGGGCCCAGCTGAGGGTGGGCTGGGGCTGAGGCTGGG
AGCTCTCAGGGCCCAGCCTTCCTCTCAC
>2564
GCAGGGCAGTCTGCGGCGGTGTTCTGGTGCACCCCCAGTGGGTCCTCACAGCTGCCC
ACTGCATCAGGAAGTGAGTAGGGGCCTGGGGTCTGGGGAGCAGGTGTCTGTGTCCCA

GAGGAATAACAGCTGGGCATTTTCCC
>2565
CCCACCCAGGAGCCAGCACTGGGGACCACCTGCTACGCCTCAGGCTGGGGCAGCATT
GAACCAGAGGAGTGTACGCCTGGGCCAGATGGTGCAGCCGGGAGCCCAGATGCCTGG
GTCTGAGGGAGGAGGGGACAGGACTC
>2566
TCACCCTCAGAAGGTGACCAAGTTCATGCTGTGTGCTGGACGCTGGACAGGGGGCAA
AAGCACCTGCTCGGTGAGTCATCCCTACTCCCAAGATCTTGAGGGGAAAGGTGAGTGG
GGACCTTAATTCTGGGCTGGGGTCT
>2567
ACTCCTGGATCCCACGGCCTGCCAGGCAGGCACGGGAGAGATGGTCTCAAAGGAGAC
CTGGGCCCTCCAGGTACTGTGCTGCAGACCCCACCCTCAGCTGAGGACACAGACCCCT
TTTCAGGAGGCCCATCTGTCCAGGC
>2568
CTGCCTGGAGCCCCTGGTATCCCTGGAGAGTGTGGAGAGAAGGGGGAGCCTGGCGAG
AGGGGCCCTCCAGGTGAGCAGGGTGGGGCAGGTGGGCAGTGGAAACATGGGCACAG
CGACCCTGAAGTCAGTTACACGGGGAT
>2569
GCTCATCTAGATGAGGAGCTCCAAGCCACACTCCACGACTTTAGACATCAAATCCTGC
AGACAAGGGGAGGTAAGGGGACCCCCTGGGCTCACGGGGTAGGAGTTTCCCACAAAT
TCCCCTCATTCTCAGCACCAGCTTC
>2570
GCCCTGCCCGCCGCTGCGATGCTCGCCCGCGCCCTGCTGCTGTGCGCGGTCCTGGCGC
TCAGCCATACAGGTGAGTACCTGGCGCCGCGCACCGGGGACTCCGGTTCCACGCACCC
GGGCAGAGTTTCCGCTCTGACCTC
>2571
AGTGTGGGATTTGACCAGTATAAGTGCGATTGTACCCGGACAGGATTCTATGGAGAAA
ACTGCTCAACACGTAAGTTTGTCCTTTGGTTGCCTCATTAGGAGTGGGGCTGGATACAG
TTATCATTGTATAGATTTGTGTC
>2572
AAGGGATTTTGGAACGTTGTGAATAACATTCCCTTCCTTCGAAATGCAATTATGAGTTAT
GTGTTGACATGTAAGTACAAGTGTCTTTCTAAGGTTTTTAGCCTTCTCAAAGAAAAATA
TGCTTTATAATACTGTAAGCC
>2573
AACCTCTCCTATTATACTAGAGCCCTTCCTCCTGTGCCTGATGATTGCCCGACTCCCTTG
GGTGTCAAAGGTGAGTAAGAAGAATCCATTAGAGATGTATTAACTATAAGACGGGCTG
CATTGCTGCCAAAAAAAAAAAT
>2574
CTTCACGCATCAGTTTTTCAAGACAGATCATAAGCGAGGGCCAGCTTTCACCAACGGG
CTGGGCCATGGGGTAAGATAGAGTTAATATCTTAGAGTTAGTAAAATTATACCAAATCAT
AGTCAAGGGCTAACATTAAAGG
>2575
TATTTACGGTGAAACTCTGGCTAGACAGCGTAAACTGCGCCTTTTCAAGGATGGAAAA
ATGAAATATCAGGTATGCTTCCTTTGACTATTAAGACTTAGTTATTACCGCTTATACCCAT
ATTTTAAAATCCCTAAAAATG
>2576

GTGCTTAAACAGGAGCATCCTGAATGGGGTGATGAGCAGTTGTTCCAGACAAGCAGGC
TAATACTGATAGGTAAACAAGAAAATGATTTATATAAAACCCTCTTCCCCAGGGAAAAT
TAGTGTGCTATCTTTGTTATGTT
>2577
CAACTCTATATTGCTGGAACATGGAATTACCCAGTTTGTTGAATCATTCACCAGGCAAA
TTGCTGGCAGGGTAAGCATTATTATTGAAAACCAAAACAAAAGACTAGTCAGTAACTTT
AGAATTTCTGCCACGGAAATTA
>2578
TACCAGTCTTTTAATGAGTACCGCAAACGCTTTATGCTGAAGCCCTATGAATCATTTGAA
GAACTTACAGGTAAGAAACAGTTTCTAAACTTCTTCGTTTTTTGTTTGTTTGTTTGTTTT
TGTTGTTTTTGGTTTTCTTT
>2579
AGGCGATTATGCAGCCCCTTGATCATAAGAATCTGGATATGGATGTGCCATACTTTGCAG
ATGTGGTGAGGTGGGTGGCACTGTATCTTGCCTTATGTGGATTGTAAAACAAGAATTGA
TTTGAATACTTTGATTGTTGT
>2580
AGACGATCTGCACTTCGCATTTTGGCATTGACATTTAATTTTAGGGTCCTTTATATAGAA
GGGAGAGTAGGTAAACTGATTTTTTTTTTTAACAGGGAGGGTTTGACAATCTTTGGCAG
ACTTGGAGCAAAAGATTGAGG
>2581
GTTGGAGAAATTTCACAGTACATCATCCAAAAGAGGAGTCCATGATGGAGGCAGAGGT
AAACTTGGAGAGGTAAGAAACCCTGAAGACAGGGGAGTGCTTTGTGGCAGGCTCTGC
ATATAAGAATTCAGCCTGGCCAACA
>2582
GCGCATGCTCTGGGGCAGTTCGCGGCCCGGCGGGGAGCGCCGGAGTTCCTTGTGGCC
GACGTGCACCAAGGTAGGTCTCGCCTGGGACGCGCGGAGGGTCCGGGCAGAGGGCG
GTAACGAGCGGGCCACAGCGGAGCACG
>2583
TCACCCAAGCGCATAGCTAAAAGAAGGTCCCCCCCAGCAGATGCCATCCCCAAAAGCA
AGAAGGTGAAGGGTAAGTTGGCCTTGGCCTCTTTGTGGGTACAGGTGGCCCCTTGAAA
CCCTAAGAACCCGGACTGGGCTCC
>2584
CATTCCGGAGGATGTTGTGCAGGCTGAGGCTGGGGGCATGCACACCGTGTGTCTAAGC
AAAAGTGGCCAGGTAGGTGTTGGGGACTGGCACAGGGTTGGACAAGGCCTGGGGTTG
GGTGGCTTGGGGCAGGGCTTTTGAA
>2585
GTCAGCAGGAGACAGTCACACAGCAGCCCTCACCGATGATGGCCGTGTCTTCCTCTGG
GGCTCCTTCCGGGTAAGGCTGGGTCTGAAAGTCTGCATGGTCCCGTGAAAGACAGAAT
TAATTGCGGGGCCCCAAAGATAAT
>2586
GAGCCCATGAAGAAGAGCATGGTGCCTGTGCAGGTGCAGCTGGATGTGCCTGTGGTAA
AGGTGGCCTCAGGTGGGTCTGGGGGCACTTGCTCAGGGCAGGAGTTGGAGGACCTTG
TTCTGGGGCTGGCCTAGCCTTGGGC
>2587
TGCGGGGAACAGGGCCAGCTAGGCCGTGTGCCTGAGTTATTTGCCAACCGTGGTGGCC
GGCAAGGCCTCGGTAAGTGGCCTTGGTACCTCCAGCAGGGCAAATTGGCAGGCCACC

CCCACAGTGAAGGCCAAACGGAGGA
>2588
TATTTCACCTTTGCCATCTCCCATGAGGGCCACGTGTACGGCTTCGGCCTCTCCAACTA
CCATCAGCTTGGTGAGCCCCGAGCCCAGCTTCAGGCATGACCCAGTGGCCTGCGTTCC
TGTCCTGGCTCTGCACTCATTCA
>2589
AAGAATTCCACCAAGTCCTGGGTGGGCTTCTCTGGTGGCCAGCACCATACAGTCTGCA
TGGATTCGGAAGGTAGGGCCTTTACGTCCTTCTCTAGTTTGGGGGTGGAGTGTTCCCTG
GCCTAGGCCTAGCCAGATTCCTG
>2590
ATCTCCAGGCTGCCTGCTGTCTCCTCGGTGGCTTGTGGGGCCTCTGTGGGGTATGCTGT
GACCAAGGATGGTGAGTGGGGCTGCCTACACTCTGTCTAGTTGGGACCTGGGGGTCAT
GGTTCTTACCCAATTCCCCAATA
>2591
CCGCGGTGCCATGGCCCCCGCCCGTCTGTTCGCGCTGCTGCTGTTCTTCGTAGGCGGA
GTCGCCGAGTCGGTGGGTGCTTGGAGGTTCCCGGGCTGGGGGCGAAGCGGGGGCGCA
GGCCGGTGCCTCCTTTGTTCGTCGG
>2592
GACGATGAGGATGTAGTGGGGCCCGGGCAGGAATCTGATGACTTTGAGCTGTCTGGCT
CTGGAGATCTGGGTACGGAAGGTGTGCTGGGCAGGCGTAGGCACAAAGCTGGAGGGA
GTGGTGGCTTCACCAGCCAGGAGGG
>2593
TCCAACAAGGTGTCAATGTCCAGCACTGTGCAGGGCAGCAACATCTTTGAGAGAACG
GAGGTCCTGGCAGGTAAGTCCCATGCTGCTTATAAGATGCCTTGAAGGTGGAATGGGG
CTCAGCGGGGGAGAGCACCTGCAGG
>2594
AGGCACCAATGCCTATCGCTCCTACTGCTACTACTTTAATGAAGACCGCGAGACCTGGG
TTGATGCAGATGTGAGTGAGGAGAGCAGTGTGGGAAGGGAGACTCATGAAGGGAGGG
GAAGCTGCCACTCTCCAGTGTGTT
>2595
GGCCTCACTGATTAAGGAGAGTGGCACTGATGACTTCAATGTCTGGATTGCCCTCCATG
ACCCCAAAAAGGTAGGCTGCAGCCTTCTTTATCTCCTAATGATCAGGTTTGAGAAGTAA
GAAGGAGGTTCAAGTTCTGGTC
>2596
AAGTCCTGGGGCATTGGAGCCCCAAGCAGTGTTAATCCTGGCTACTGTGTGAGCCTGA
CCTCAAGCACAGGTGAGAGGCAGAGAATCCATCCACCTGTTTCTGTTCTCTCCTGCTTA
GCTCCAGGGATGGAACTGGGACT
>2597
CTAAGCATGGCTCAGACCAACTCGTTCTTCATGCTGATCTCCTCCCTGATGTTCCTGTCT
CTGAGCCAAGGTGAGATTTTCCCCCACACTTCCCACAACCCCAACTCTGAATTCTTCCC
TCCATCCTCATGTATAAGGTT
>2598
AGGCACCAATGCCTATCGCTCCTACTGCTACTACTTTAATGAAGACCCTGAGACCTGGG
TTGATGCAGATGTGAGTGAGGAGAGCAGCAGGGGAAGGGAGGCTTATGAAGGTAGAG
GCAGCTGCTAATTTGCAGTGTGTT
>2599

GGCCTCACTGATTAAGGAGAGTAGCACTGATGACAGCAATGTCTGGATTGGCCTCCAT
GACCCAAAAAAGGTCAGTCTGCAGCCACCTCTATCTCCTTATAAACATTTTTGAGAGGT
AAGAGGGACGTTTAAGGTCTGGC
>2600
AAGTCCTGGGACACTGGATCCCCGAGCAGTGCTAATGCTGGCTACTGTGCAAGCCTGA
CTTCATGCTCAGGTGAGAGGCAGACAATCTATCCACCTGTTGCCATTTCCTTCCCACTT
ATCTCTGGGGATGAACATGGGGA
>2601
CGCCGCCGCCGCCCCCTCCTGAGGAGGACCCAGAGCAGGACAGCGGCCCGGAGGAC
CTGCCTCTCGTCAGGTGAGCGAGCAGAGCCGCCGTCGCCTCACGCGGGAAGGGCGCC
CCGGGTGTGCGTAGGGCGGGCGCAAGG
>2602
GATACCAGATCATGTCAGAGAGAGAGCTTGGTTAACTTGGGAGAAAGTTTCATCTGTG
GATGGAGTATTGGTAAGGATTTTCTTAAAACGTTTTGAAATTTTTTTTTCTCATTTTAAA
AACAACTTCAAATCACTATACA
>2603
TTATTGCAGCAGTTGACCTAGATGAGATGTCGTTCACTTTTACTGAGCTACAGAAAAAC
ATAGAAATCAGGTAAAGTTTCTTGTATAAATATAAGCCTCTGCCATAAAAGGAAATGAA
TTCTGGATTTTCCTCTCAATAG
>2604
TTGATAATGCTATGTCAAGACTGTTGAAGAAGTATGATGTATTGTTTGCACTCTTCAGCA
AATTGGAAAGGTAAAGTAAACATTTTATTAGGGTTACACTCTGATTTTTTATGTCATTGT
TCACAATTAGATTCTGGGAA
>2605
CAGTTAATGCTATGTGTCCTTGACTATTTTATTAAACTCTCACCTCCCATGTTGCTCAAA
GAACCATATAGTAAGTATTTAATTTATGCCCCTTTTACTTTCTCATTCAGCAGTTGCTTAT
TGAATGTCTAGTGGGTACC
>2606
AAAACAACTAGAAAATGATACAAGAATTATTGAAGTTCTCTGTAAAGAACATGAATGTA
ATATAGATGAGGTAATTTAACTTCATGATTTCTTTAAAACAGTTAAAGTAGATTTAGATGT
AAGTTCTCCCTAACAATATT
>2607
TGTTTATTTCAAAAATTTTATACCTTTTATGAATTCTCTTGGACTTGTAACATCTAATGGA
CTTCCAGAGGTAATCTGAAAGGAAATTTAATAAAATATTAATGTTTTGAGACTGTGGAG
GGAGGATAATTGTCTAACTT
>2608
AAAATAAAGATCTAGATGCAAGATTATTTTTGGATCATGATAAAACTCTTCAGACTGATT
CTATAGACAGGTATTGCACATGGTATATTTGATTGATTTGCTTTAGATATAGGTTGATACT
GATATAGGTAGATTATATA
>2609
CACAGAGAACACCACGAAAAAGTAACCTTGATGAAGAGGTGAATGTAATTCCTCCAC
ACACTCCAGTTAGGTATGAATTTTCCTACTTTTAATTATATTATAATTTTGTTATTCATGGC
TTTATAGTGTTTCAGATTTGT
>2610
CCAACAATTAATGATGATTTTAAATTCAGCAAGTGATCAACCTTCAGAAAATCTGATTT
CCTATTTTAACGTAAGCCATATATGAAACATTATTTATTGTAATATCTTGGCAAAGAAACT

TGAAATTAAAAGTTAAAGTA
>2611
TATAGGATACATCTTTAAAGAGAAATTTGCTAAAGCTGTGGGACAGGGTTGTGTCGAAA
TTGGATCACAGGTAACTTGAATTCATTGTAATTCGTGGTACTATAGAGTAATAATATTAA
AAGCAGCATCTTTCCAGTTCG
>2612
CTGAATGACAACATTTTTCATATGTCTTTATTGGCGTGCGCTCTTGAGGTTGTAATGGCC
ACATATAGCAGTAAGTTAAATTTTCATAAATAAACACTTTTGTTCAATTTAAAGTTAAAA
TGTGGTGTGTTTCTTTGGTC
>2613
GACAAGAGAAATGATAAAACATTTAGAACGATGTGAACATCGAATCATGGAATCCCTT
GCATGGCTCTCAGTAAGTAGCTAAATAATTGAAGAAATTCATTCATGTGCATATGGCTAA
CAAATTATTGTTAGTGAGAGGT
>2614
CAACTGATCACCTTGAATCTGCTTGTCCTCTTAATCTTCCTCTCCAGAATAATCACACTG
CAGCAGATATGTAAGCAAAATATATGTTATGTTGACCATTCAAACTGCAAATAGATTTTA
AGCATAAGTGCAATGTAACA
>2615
CAAGCAACCTCAGCCTTCCAGACCCAGAAGCCATTGAAATCTACCTCTCTTTCACTGTT
TTATAAAAAAGGTTAGTAGATGATTATTTTCAAGAGCATGGACTCTGAAACTAGGCTGA
CTGGGTTCAAATCATGTTTCTT
>2616
CATCTGGACCCTTTTCCAGCACACCCTGCAGAATGAGTATGAACTCATGAGAGACAGG
CATTTGGACCAAGTAAGAAAATCAAGCACTTCACCTTCTCTCCTCCCTACTTACTTGTT
AACTGATTTCTTTCTTTCTTTCT
>2617
GAAGAATATAGACCTTAAATTCAAAATCATTGTAACAGCATACAAGGATCTTCCTCATG
CTGTTCAGGAGGTAGGTAATTTTCCATAGTAAGTTTTTTTGATAAATCCATATCCATAACA
TAACATAGGTAATTCATTTG
>2618
TATAGTATTCTATAACTCGGTCTTCATGCAGAGACTGAAAACAAATATTTTGCAGTATGC
TTCCACCAGGGTAGGTCAAAAGTATCCTTTGATTGGAAAAATCTAATGTAATGGGTCCA
CCAAAACATTAAATAAATAAT
>2619
CACCCCTGAAGAGTCCATATAAAATTTCAGAAGGTCTGCCAACACCAACAAAAATGAC
TCCAAGATCAAGGTGTGTGTTTTCTCTTTAGGGAAGTAGTAAAGAATGAGAGGGGGAT
TATTTTGATCCAAGAATAAAAAAT
>2620
GAAGCAACCCTCCTAAACCACTGAAAAAACTACGCTTTGATATTGAAGGATCAGATGA
AGCAGATGGAAGGTAGGAACCAGTTTTGAATGTTTTCCAGTAGCTGAGATGGTCATCT
GGGGAATCCAGAGTCTCAGCACTG
>2621
CGGTTAATGAAAAAATGGACACAGAAAGTAATAGGAGAGCAAATCTTGCTCTCCCACA
GGAGCCTTCCAGGTAGGTACAAGGTATTATTTTTTTCTACCCTCAGTCACTTGTGGCAG
GGGAAGTCATAGTCACGGTGCTT
>2622

GAAGTCGGCCTCATCCCCACCACTGCATACATGGCTGACAGTTTTGAAAAAAGAGCAG
GAGTTCCTGGGGGTGAGTGAGCCTCCTCCAACTTTGACTAGAGTAAGGGTTGGGTCTA
GAAAAGAATATTGAGTTGCATCAA
>2623
TATTTCACACATTGAGGGAGACATTTTTTCATCATTTAAAGCAGGTTATCCATTCTGGGG
AGCCATATTTGTGAGTATATATCTATAATTGTTTCTGAAATAACACTGAACATAGGTTTTT
CTCTTTCTCAGATCTAACC
>2624
GGCCTATATCCACATCCACAGTTGCCAGAAATTTTTTGAGACCAAGTGCTTTATGGCTT
CCTTTTCCACTGTATGTATTTTTTTTTGTGTGGGAAGACTAAGATTCTGGGTCCTAATGT
AAGTAAGAAGCCCTCTTCTCC
>2625
CATTCTGGGACTTGGTAGTGCTGTGTCACTCACAATCTGTGGAGCTGGGGAAGAACTC
AAAGGAAACAAGGTAGATAGAAGCCCGATATAAAATCTTGAATGACAGGTTAACGAAT
TGGAGCTTTATTCCTTAAAATATG
>2626
ACAAGGATTGGTTTGGGGTCCAGTGGGTCAGCAGCCGTTACCTGGATCCCGGTGACCG
GGATGTGGCTGAGTGAGTGATTTGCGTCTCCCTTCCTCCTCCTCCTCCTCCCTGGACAG
GCTCCCTCCTGCTGCCTTGAATC
>2627
GCTGCTGGAGCACTTGCAGGACTTGGCAGGCACACTGGGTAGCATGCTGGCTGTCACC
TTCCTACTGCAGGTGAGTCAGCAAAGCATCTGACACCTCCTCCCACCCGGGACTCCTC
CCTGCCTCCAACCCTGGGCCTCTT
>2628
ACACGAACAAGCGCGTGTGCGAGGAGATCGCCATTATCCCCAGCAAAAAGCTCCGCA
ACAAGATAGCAGGGTGAGTCGGGCCTTCCTCGGCCTCCGGGCGTCCGCGATCTGTTTG
TGTGCTCTGCCGCCCTCCGACTCGG
>2629
CCCAGTAAGAGGTATCTCCATCAAGCTGCAGGAGGAGGAGAGAGAAAGGAGAGACAA
TTATGTTCCTGAGGTAAACTTTCTGGATATTTGGGCTTCTGGCTAATCCTCAAATGATAA
AGCTATGGGTTCTTGGCTTCCAA
>2630
ACTTCGTACTTTCTATGAGAAGCGTATGGCCACAGAAGTTGCTGCTGACGCTCTGGGTG
AAGAATGGAAGGTAAAAGTTGACAAATTGTTGCAGGTATTTAAGTCAGAGACGGTAAA
CGCCATTGGTAACTGGTATTTGG
>2631
AGAAAATCAGTTCGTGGTTGCATTGTGGATGCAAATCTGAGCGTTCTCAACTTGGTTAT
TGTAAAAAAAGGTGAGGGTTACTTGTGTTCAATTTTCGTTGAAATTGATTTAAAGCCAG
TTGTCCAAATGCTTATTCATTT
>2632
AAACTTTTCAATCTCTCTAAAGAAGATGATGTCCGCCAGTATGTTGTAAGAAAGCCCTT
AAATAAAGAAGGTAGGAGGGATTATGCAATTAGGGCTTGCTTAATTTTGGTAATTTGTCT
ATCATTTTGTGTGCATATCAG
>2633
GAAGCAGCGTACCAAGAAAAATAAAGAAGAGGCTGCAGAATATGCTAAACTTTTGGC
CAAGAGAATGAAGGTTAGTCTAAGATGATTTGAGGGGGTGGGAGGAGTTCGACCTGG

CTTTGGATTTGCGTCTTTAACCCAGA
>2634
ACTACTTTGATACGACACCGCATGGCTTCGAGACTCACACGCTGGAGGAGCACAACCT
GGCCAATTACATGTGAGCAGACACACTGGCCAGTCAGGAGGGTGGGGGGCATGGCTG
CCAATAGCCAGCAGTGGGGTACTTA
>2635
GTTTTCTGCTCCTTGGTCCTGGGTGTCAGCAGCCGAAGCTTCTTTTCGTTCCTTGGCGA
GGCTTTTGATGGTAAGGCTTCAGAAGGTTTGCAGGATTTCTAAGAGAAACATCGCCCT
GGACCTATAAACTGGGGAAAATG
>2636
TCCATGCTCGGGGGAACTATGATGCTGCCAAAAGGGGACCTGGGGGTGCCTGGGCTGC
AGAAGTGATCAGGTAACTGAGCTCCTGGGACGTTAGGGCTGGGTGAGCAGAGCTTGC
CTGCCTTGGACAGTCAGGAGGGAGA
>2637
AGCCGGCTTCGCCGGGGATGACGCCCCTAGGGCCGTGTTCCCGTCCATCGTGGGCCGC
CCCCGACACCAGGTCAGGCTGCCCCTCCGCAGAGGGAGCCGGCTCGGGGTCCCCGCG
TAAGCCAGCCTGGTGCCACCCGGAG
>2638
TTCAACGTGCCCGCCATGTACGTGGCCATCCAGGCCGTGCTGTCCCTCTACGCCTCCGG
AAGGACCACCGGTGAGTGCCCGCTGGCCCCCAGTCCCCTCGTCCCGCCCCCGCCCCCG
CCCCCGCCCCCGGCCGCTAGCGC
>2639
GCGGGCCGCGATCTCACCGACTACCTGATGAAGATCCTCACTGAGCGTGGCTACTCCT
TCGTGACCACAGGTGCGCGGCGCCCCTGCACCCGGGCGGAGGGCCGCGGCGGCCTGA
GTGAGGGCTCCTCTCCTGCTTCTGC
>2640
GGGCAGGTCATCACCATCGGCAACGAGCGCTTCCGCTGCCCGGAGACGCTCTTCCAGC
CCTCCTTCATCGGTGAGCCCCGCTCGCCCTCGCCCCGGCCCCCAGGCCCGCGCCCCCC
GGCCCGAGCTTCTGCTCACGCTCC
>2641
GTACCCTGGGATCGCTGACCGCATGCAGAAAGAGATCACCGCGCTGGCACCCAGCACC
ATGAAGATCAAGGTGGGTGGTGGCCTGCGCGGGCTGTCGGCGGGGTGGGCTCCAGGG
TGAGGTCTCCCCACCTCACGCGCTG
>2642
AGGAATATGAACAAGCCGCTGCTTTGGATCTCTGTCCTCACCAGCCTCCTGGAAGCCTT
TGCTCACACAGGTAAGGAGGTGAAGGAATGGTCAAGAATCATAAAGTGAGAAAATAG
GTTGAAGCTGAGATATCTTTTCCC
>2643
CCCAACATCATCTTTGTACTTTCCCTGCTCCTCATCTTGGAGAAGCAAGCAGCTGTGAT
GGGACAAAAAGGTGAGTGGAGAGGGTAAGCCTTGGGGAAAGCTGCTCAGACAGCTA
ATAATCTAAGATTATTTGGGGTGCA
>2644
ACGACCGAAACCCATTATTTACATAAACCTACCATTCGGTAACCATGTGAAAGGATGGA
CCAATATCAAGGTAATTTTTTTTTAGCAAATAGGGGAGATATCTCTCTCATTGTTTAGAA
TTGTTGGGGACTCTCCAGGAC
>2645

TCCATCATCCTCTTTGTCCTTTCCCTGCTCCTTATCTTGGAGAAGCAAGCAGCTGTGATG
GGACAAAAAGGTGAGTGGAGAGGGTAAGCCTTGGGGAAAGCTACTTTAAAAAAATGG
CCTCTAAGGATATTCAGGGTGCA
>2646
AAGACAGAAATCCAATATCTACATAGCCCTGTTGCTTAGCAACCACTTGAAAAGCTGG
ACCAATAGCAAGGTAAGTTTGCTTTTCTTACCAAATAGGAGAGGTGCCTGTCCCAAAG
TTGGGGACTCTCCAGGAACATGGT
>2647
ACTCCTGGATCCCACGGCCTGCCAGGCAGGGACGGGAGAGATGGTGTCAAAGGAGAC
CCTGGCCCTCCAGGTACTGTGCTGCAGACCCCACCCTCAGCTGAGGGACACAGACCCC
TTTTCAGGAGGCCCATCTGTCCAGG
>2648
CTGCCTGGAGCCCCTGGTGTCCCTGGAGAGCGTGGAGAGAAGGGGGAGGCTGGCGA
GAGAGGCCCTCCAGGTGAGCAGGGCGGGCAGGTGGGCAGTGGAAACATGGGCACAG
CGACCTGAAGTCAGTTACACAGGGGTGA
>2649
GCTCATCTAGATGAGGAGCTCCAAGCCACACTCCACGACTTCAGACATCAAATCCTGC
AGACAAGGGGAGGTAAGGGGACCCCCTGGGCCTCACGGGGTAGGAGTTTCCCACAAA
TTCCCCTCATTCTCAGCACCACCTT
>2650
GTGTGGCCCTGATGTGGGCCGCCCAGACCGTGGGCGCTTGGGCTTCCAGGTCTGGCTG
AAGAATGGCGTGGTGAGTGGCACCCTGGGCTAGGGCGCTGGGGGGCTGGGTGTGCCA
CCCTGTGAGTCCTGGGCCAATCCCT
>2651
TTCCTGAAGGCGGCTGAGGACTATGGGGTCATCAAGACTGACATGTTCCAGACTGTTG
ACCTCTTTGAAGGTAGAGAGGAAGAGGCTGGGGGAGGAGGTGGGCAGGAGGACAGG
GTGCTGGGACAGGGAGAGGGAATGAC
>2652
TGGCTTTGGGCAGCTTGGCAGTGACCAAGAATGATGGGCACTACCGTGGAGATCCCAA
CTGGTTTATGAAGTATGTGGCCCCCAGGGAGCTTGGGTCTCCGCATGGGGTGGGAGGT
GGCTTGTTCTAAGGAGCTTGCGGG
>2653
CTTTGGGTGGGGGAACCTCACCTGCCCAATCTGCAAAGGTCTATTCACCGCCATCAAC
CTCGGGCTGAAGGTGAGCACTGAAGGGGCTGCAGTGGAGGAGGCCGAAAGGAGTGC
TGGGGCTGGGGGCTGGGGCTGATGCT
>2654
GCTGGCTCTATGAAGCGATGGCCAAGGCTTGGGAGCCCTGGCTGCCTGCCGAAGCCCT
GCGCACCCTCAGGTACTTATCGTCCGTGGAAACCCAGGAAGGGAAAAGAAAGGTGAA
TGAAAGTGAAGGGAGAAGGGAACCT
>2655
CACGGATCCCGCAGGACAGCTCCAGTGGCTGGTGGGGGAGCTTCAGGCTGCTGAGGA
TCGAGGAGACAAAGTGAGGGCCAGTAGTGGGAACACGGTGGTGCTGGGGGACAAGC
AGGCTCCTGTTGAGCTGGAGCACCTCT
>2656
TAATTGGCCACATTCCCCCAGGGCACTGTCTGAAGAGCTGGAGCTGGAATTATTACCGA
ATTGTAGCCAGGTAGGACGGAGATGAGGGTGGGAATAGGGACAGGGTGAGTGTCTGA

AGGCTGAAAATTCCCTTGAGCATC
>2657
ACTCTGAGCCGGCCGCTGGCTGTAGCCTTCCTGGCACCCAGTGCAACTACCTACATCG
GCCTTAATCCTGGTGAGTGAGGCAGAAGGGAGCCTCCCTTATCCTGGAGTTGGTGGGA
TAGGGGAAGGAGGTTGGAGCCAGA
>2658
CCACTTCCTCGATGAAGGTTTTACTGCCAAGGACATTCTGGACCAGAAAATTAATGAA
GTTTCTTCTTCTGTAAGTATATGAGGCCCATGCTGGCAGTGCAGCTGAGAGTGCCAGGC
AAGTGGAAAACTTTGGCAAGGTC
>2659
ATGTAATGATAGCAAAGCCATCGTGAAGACCCTTGCTGCTACCGGGACAGGATTTGACT
GTGCTAGCAAGGTAAGCGATAGCAGCAGGCCTCAAAAGCGTTGTATAAAATGGGCCTG
GTATTCCCCACGAGGCAGATACA
>2660
GAGTCCAGATGATGACTTTTGATAGTGAAGTTGAGTTGATGAAAGTTGCCAGAGCACA
TCCCAAAGCAAAGTGAGTTATTCCCCCATCTGAGGGCAAGATCGGGAGCATAAGATAT
GTGGATTCTTATCAAACAAACTTA
>2661
CCACGCTCAGAACCAGCAGGCTCCTTTTGGAACGGGCGAAAGAGCTAAATATCGATGT
TGTTGGTGTCAGGTGAGATTTTGGTGGGATAGCTAGAGGTCAAGACATTGAACAGTTT
GAGTTTTACAGGCTTTCTCCTAGT
>2662
AAGCGGCTGTACCGATCCTGAGACCTTCGTGCAGGCAATCTCTGATGCCCGCTGTGTTT
TTGACATGGGGGTGAGTATACGTGACCCTGTTAGGGAAGGGCGGGACACAACTGACAA
TAACTAGTCTTAATTCTAGAGTT
>2663
CAGCATGTATCTGCTTGATATTGGCGGTGGCTTTCCTGGATCTGAGGATGTGAAACTTA
AATTTGAAGAGGTAATTTAGAACAAAACTGTAATACTCAGTAGCCGTTCTAATAAATTC
CTTTTTGGAATATTTCAAAATT
>2664
TCAGCTTTCACGCTTGCAGTTAATATCATTGCCAAGAAAATTGTATTAAAGGAACAGAC
GGGCTCTGATGGTATGTATAAAGGACGAATCACTTCATGTATAACTGAAAGCTGATGCA
AAAAGTCATTAAGATTGTTGAT
>2665
TGATGGCGTCTATGGATCATTTAATTGCATACTCTATGACCACGCACATGTAAAGCCCCT
TCTGCAAAAGGTAATTTCTGAGCATACTGTATAAAACAATTAAGAGGACTGGTCACAAC
ACGTGTAATTAAGTAGTACTT
>2666
TTGCTGCTGCCTCTACGTTCAATGGCTTCCAGAGGCCGACGATCTACTATGTGATGTCA
GGGCCTGCGTGGTAAGTAAGCCATGCATGTTGATGGTGCTGCCAAGAATAGGCACCTT
CTTGGATGTGTGCTTCTTGTCTA
>2667
GCCATGGCTGAGTCACACCTGCTGCAGTGGCTGCTGCTGCTGCTGCCCACGCTCTGTG
GCCCAGGCACTGGTGAGTCTCCCCCAGCCTCCCCTCTCCTAGGCAGCTCCACCACTCA
CTGAGCACTGCTTTGTGCTAGGCA
>2668

CCTGGAGCAAGCATTGCAGTGCAGAGCCCTAGGGCATTGCCTACAGGAAGTCTGGGG
ACATGTGGGAGCCGTGAGTACCACCAAGGATGCATGGCAACTGGGGGTCTGAAATGA
AGGGTGCTGGGTGGGCTCTGGATGGG
>2669
TGACCTATGCCAAGAGTGTGAGGACATCGTCCACATCCTTAACAAGATGGCCAAGGAG
GCCATTTTCCAGGTAATGATGCCCAGATCCTGGATGAAGGTTGGGGCCCAAGAGATGA
GGGACAGAGCAGGGAAGAGCTGAG
>2670
CATGCCCCAGTGCAACCAAGTGCTTGACGACTACTTCCCCCTGGTCATCGACTACTTCC
AGAACCAGATTGTGAGGGCTGCAAGCTCACCTCCTGCCTGCCTCCCCACGCAGGCCCC
TGTGCCCACCCATGGGGGAGCCA
>2671
TCTGCTGGACAAGCTCGTCCTCCCTGTGCTGCCCGGGGCCCTCCAGGCGAGGCCTGGG
CCTCACACACAGGTGAGGGAGGCCCCCACAGCCAGTAAAGTGGAGATCCAGAGGGCT
AGAGCCACCTCCGAAGCCCATGGGC
>2672
CCCCATTCCTCTCCCCTATTGCTGGCTCTGCAGGGCTCTGATCAAGCGGATCCAAGCCA
TGATTCCCAAGGTGAGGCATCCAGGGCCTCAAGAGCCCAGGAGCACACGCATACCTGT
AGCTCCCTGCAGCTCCCACCTCT
>2673
GGCCGCATGCTGCCCCAGCTGGTCTGCCGCCTCGTCCTCCGGTGCTCCATGGATGACA
GCGCTGGCCCAAGTGAGCCCACTGCCCCCTCCTTAGCCCAATGCCCGCTCTCCTCCTCC
CCCTACCCTGCCACTGCATGACC
>2674
CAGCAGCGAGCAGGCCATACCACAGGCAATGCTCCAGGCCTGTGTTGGCTCCTGGCTG
GACAGGGAAAAGGTATGGGCTGGGCACATGGGGACTCATGGTCAGGGCCCGTTCAAG
GCAGAAGGCTGAGCCCAGGAAAGGC
>2675
TGTGGAGCAGCACACGCCCCAGCTGCTGACCCTGGTGCCCAGGGGCTGGGATGCCCA
CACCACCTGCCAGGTACACCCAACCCCTCCCAAGTTGGTCCTAGGACTTCCCTTGGCT
CCCAGAGCCCCCACCCTTTGGGCCC
>2676
TGTCGCTGCAGGTGGAGCAGCTGCTCCACCACCGGCGCTCGCGCTACCAGGACATCCT
CGTCTTCCGCAGGTACCGCCGCTGCCCGCAGGCGCCTGCCCCCTAGGCTCAGCCCGGG
CCGCCTGCTGCCCGCCTCACGGGC
>2677
GAGAGACGAGTTCTCCTACCAGGAGATGATCGCCAACCTGCCTCTCTGCAGCCACCCC
AACCCGCGAAAGGTACCCCAGTGTCCCCTGGAACAGTGCCGGACGAGGGGCGGCCCC
AGGTGTGCTCCGGGCTCTTCCCAGA
>2678
TGGAGGTGTCCTGCGGGAGGTGGTGAAGCACCCCTCCGTGGAGTCCGTGGTCCAGTG
TGAGATCGACGAGGTGAGTGCCGGCGTAGAGCCAGGTTTGAGTCCTGGTTCTCCCAGC
GGCCAGCTGTGCCCTGAAATGGCTG
>2679
GGTTTTGAGTTCATGAAACAGAATCAGGATGCCTTCGACGTGATCATCACTGACTCCTC
AGACCCCATGGGTAAGCAGTGGATGGGCCCCAGGGTTTTCTGGCAGCTGCAGGTCTGG

AGGTCAGCCTCCCCCAGGCCTTC
>2680
CTCTTCAAGGAGTCCTATTACCAGCTCATGAAGACAGCCCTCAAGGAAGATGGTGTCC
TCTGCTGCCAGGGTGAGCCACAGGCCTGGAGCACTGGGGCGGGGCGGGGTGGGGCA
GGGCAGGCCCTGCCGGATGCTGATGC
>2681
CTATGCCTACTGCACCATCCCCACCTACCCCAGCGGCCAGATCGGCTTCATGCTGTGCA
GCAAGAACCCGGTGAGATGGGGGTGTCTGGGGGTGGGGGTTGGGGGGAAGGTGGGC
ATAAATAGAGATCCCTGCCCCTGCC
>2682
GATGCAGCTGAAGTACTACAACTCCGACGTGCACCGCGCCGCCTTTGTGCTGCCCGAG
TTTGCCCGCAAGGTGGGTGGCCTGCGGGGCTGGGTGGTGGGACCCAGGGACCCAGAG
CGCCCTCCTGACTGGCCTCATGTCC
>2683
GTCGGAAGAGGTGTGGCGGCATCCTAGTGAGAAAGGACTTTGTGCTGACAGCTGCTC
ACTGCCAGGGAAGGTAAGGAGCAGCACCAGCTCACCTCCTGAGTTCCCCCTACAGGG
ACCCTTGTTTTCTCCTGGGGACTGCA
>2684
TGTGAAAAGACCCATCCCCCATCCAGCCTATAATCCTAAGAACTTCTCCAACGACATCA
TGCTACTGCAGGTGAGGCACACTCCTGCCACTCTTGCTCTTCTTGGTCCAGTTGGTTCC
ACTCCCACTGGGATGCCGGCCC
>2685
CCATGGCAATTACAGCAGAGCCACTGAGATTTGTGTGGGGGATCCAAAGAAGACACA
GACCGGTTTCAAGGTTGGGTTTCCAGCCTCTACCCAGAAAGACCACAGGGAGAGAGG
AACTTGAGGGAGTTCTGGGGTATGAC
>2686
GCAGATCTTTCGGATACCGAGCTGACCACCTTGCTGCGCCGGTACAACATCCCGCACG
GGCCTGTAGTAGGTACGCGGCGGCGGGCGGGACCCCTTCCGGGCCCCCTTCCTTCGTG
CTCCGCCTTCGCGACCTCCCCGCT
>2687
GAGTACGAGACCCAGAGGCGGCGGCTCTCGCCCCCCAGCTCGTCCGCCGCCTCCTCTT
ATAGCTTCTCTGGTGAGAGCCTCGCCTGTGGGGTCAGCCTGGGACGCGGGGAGGATGG
GGTCGCGAGGGTGTGGCAGGGGGG
>2688
TCGACTAGAGGGGATGCAGATATGTATGATCTTCCCAAGAAAGAGGACGCTTTACTCTA
CCAGAGCAAGGGTAAGGCAGGGGTTGGGTGGGCACGCTGGCACCTTCACCCGACTTC
GTCAGGGACCCCGCTCACAGGGAG
>2689
TGCCGGCCCGTCCAGGGCTGTCCGCCAGTCAGTGACTTCATTCCCAGATGCTGACGCT
TTCCATCACCAGGTGAGCTGGCTGGCAGGCGTCCTGTACTTGGGTACAACCTAGGGGA
TCGCGGCTGTGTTTGGATAAATCC
>2690
GTTGTGGGTCTAATTCAAGACCTCAAGCCAAACACCATCATGGTCTTAGTGAACTATAT
TCACTTTAAAGGTAAGACTTTCAGATGTCAATTTTCAGTCTAGGGCTCAGATACTTGGG
TGGAGTGCTGAGGGGAGCTCAT
>2691

AGATGGAGTCAGTGGAAGCTGCCATGTCATCTAAAACACTGAAGAAGTGGAACCGCTT
ACTACAGAAGGGGTAAATGCCTTAGAGGATGTGTGGGTGGAAGGTGGGCATAAAAGT
GCAGGTGAGACAGAGTTCAGCAGAA
>2692
TCAGCATGCCTATTCTGAAAATGCTGATTTTTCTGGACTCACAGAGGACAATGGTCTGA
AACTTTCCAATGTAAGTTGATAAACTAGAGTTCTTAAATGTATGAACTGGAAGAGCAAT
TAGGAGTCAATTGATTCAACTT
>2693
GCTAACGCCTCTTTTCCCCCTAATTGTTAAAAGCTATGGACTGCAGGAAGATGGCCCGC
TTCTCTTACAGGTATGAGCTAATCTTAGAATAGTGAACTTTTTTTGATTGCTAGAGATTG
CCAGCTTAGGAAGTAATGTTC
>2694
TGGCCCCAGGAGGAGCCTGCAATTCGGCCTCGGTCTTCCCAGCGTGTGCCGCCCATGG
GGATACAGCACAGTAAGAACTGCCTGACTTCGATGCTTCTGCCCTGGCCCTTCATGTGT
CTCCTGACTATCTTTCCAACACT
>2695
TGCTGGGGTCCTTTTGTGCCTGCCCTCCCTCCTTCTACGGACGGAACTGTGAGCACGAT
GTGCGCAAAGAGTAAGCAATTCAGAGGGGCGGGGAGCCGTGGAGAGGAGAGAGAAA
GGGAAGTGGAAATTTCAGACCCAAG
>2696
AAGTGTTCCCTGTGTAAATGCTGGCACGGTCAGCTCCGCTGCTTTCCTCAGGCATTTCT
ACCCGGCTGTGGTAAGCGGAGGTTCTCCTCTTTCTTTTGCCCTTTGAAGTTACGTAGTT
GCCTTGGGGGGTGCTTAGTTAG
>2697
CGCCCCGAGACCGCCGTCGCTCGGACGCTCCTGCTCGGCTGGGTCTTCGCCCAGGTGG
CCGGCGCTTCAGGTGAGTGGCACCAGCCCCTGGAAGCCCGGGGCGCGCCACACGCAG
GAGGGAGGCGACAGTCCTGGCTGGC
>2698
CAACTAATTTCAAGACAATTTTGGAGTGGGAACCCAAACCCGTCAATCAAGTCTACAC
TGTTCAAATAAGGTAAGCTGGGTACAGAAAAAGAAAATTAAGGTCTTTGATGTTTCTAC
TGTCCTATGCTGAACAAGAATGT
>2699
GTGGAGAGCACCGGTTCTGCTGGGGAGCCTCTGTATGAGAACTCCCCAGAGTTCACAC
CTTACCTGGAGAGTAAGTGGCTTGGGCTGTAATACCGTTCATTCTTGTTAGAAACGTCT
GAACATTCTCGTGATCTTGTGCC
>2700
CCTCCGGGATGTTTTTGGCAAGGACTTAATTTATACACTTTATTATTGGAAATCTTCAAG
TTCAGGAAAGGTGAGCATTTTTTAATTTGTTTTTATGACCTGTTTTAAATTGTGAATACT
TGGTTTTACAACCCATTTCT
>2701
CGAACAGTTAACCGGAAGAGTACAGACAGCCCGGTAGAGTGTATGGGCCAGGAGAAA
GGGGAATTCAGAGGTGAGTGGCTCTGCCAGCCATTTGCCTGGGGGTATGGGTGCTGTG
GGTGACTTCTGGAGGAGTAGCTCCA
>2702
GTCCGAGGCTTGCAGCTGCCTGGCTGCCTGGCCCTGGCTGCCCTGTGTAGCCTTGTGC
ACAGCCAGCATGGTAAGGGAGTGCTTGCAGGCTGGAACAGGCTGGAGGACTGGGGTG

TGGGCCCATGGGCTGGGGTCTCCTG
>2703
GCGAGAGTGCGTGGAGGAGACGTGCAGCTACGAGGAGGCCTTCGAGGCTCTGGAGTC
CTCCACGGCTACGGTGAGCCTGGGCTGCTCGGACGGTGCCGGGGCCTCAGACCGGGC
CCAACTCTAGACACTTCCACAGAGAA
>2704
GGCATGTGAACATCACCCGGTCAGGCATTGAGTGCCAGCTATGGAGGAGTCGCTACCC
ACATAAGCCTGAGTGAGTGAGGGGTCGGCCTTCCCACCATGGGCTGAGAACAGGGAG
CAAGCGTACCTCAAGTTCAACAGCC
>2705
ACCACGGGACCCTGGTGCTACACTACAGACCCCACCGTGAGGAGGCAGGAATGCAGC
ATCCCTGTCTGTGGTAGGCTGGGGGCAGTGGGGCGACCCATGACCAAGCCCGGGGGC
TTCATGGGGCCTGGCAGCCTGGGATG
>2706
GAGGAGGGCGTGTGGTGCTATGTGGCCGGGAAGCCTGGCGACTTTGGGTACTGCGAC
CTCAACTATTGTGGTGAGCTGCCTGGGTAGGGGGCCTGAGTTGCAGGGACAAATCCTA
GTGGGAATAACAACAGCCGCTTCTG
>2707
GAAGGGCGTACCGCCACCAGTGAGTACCAGACTTTCTTCAATCCGAGGACCTTTGGCT
CGGGAGAGGCAGGTGAGGTAGTGGGCATCCGAGGGGATGCGGGGCTGCGGGGCTGGT
GGCCAGGACTTGCCCCTCACTGCTT
>2708
AGCTCCTGGAATCCTACATCGACGGGCGCATTGTGGAGGGCTCGGATGCAGAGATCGG
CATGTCACCTTGGTGTGTCCTGGAGCCCTGCGCTACCATTCACTCCTGGGGGCAGGTGT
GCTGCTGGACCCCCACCCTCAGG
>2709
ACCCGCCCTGGGACAAGAACTTCACCGAGAATGACCTTCTGGTGCGCATTGGCAAGC
ACTCCCGCACAAGGTACAGAACTGGTGGCCCGTGGGTGTCTGGCAGGGGTCTGAGTC
CTCCAAAGCGATCATGAGGGGCCTTG
>2710
TGAAGAAGCCTGTTGCCTTCAGTGACTACATTCACCCTGTGTGTCTGCCCGACAGGGA
GACGGCAGCCAGGTGGGCCACCAGATGCTTGTTAGCTGAGGGGCAGAAGCCAAGTTC
TGGGCCTGGCTCTGATACCAAGTAG
>2711
TTACAAGCCTGATGAAGGGAAACGAGGGGATGCCTGTGAAGGTGACAGTGGGGGACC
CTTTGTCATGAAGGTAAGCTTCTCTAAAGCCCAGGGCCTGGTGAACACATCTTCTGGG
GGTGGGGAGAAACTCTAGTATCTAG
>2712
TCTTCCTACCAATCTGCTCCCCAGAGGGCTGCCTGCTGTGCACTTGGGTCCTGGAGCCC
TTCTCCACCCGGTGAGTGGCCAGCAGGGTGTGGGGTTATGTGAGGGTAGAAAGGACA
GCAAAGAGAAATGGCCTCCCAGCT
>2713
GGATTCAGGGGAGAGGCCCCAAACAGGGAGCCACGCCAGCCAGACACCCCGGCCAG
AATGGAGCTGACTGGTGAGAACACACCTGAGGGGCTAGGGCCATATGGAAACATGAC
AGAAGGGGAGAGAGAAAGGAGACACGC
>2714

TCCTCCTGCTTGTGACCTCCGAGTCCTCAGTAAACTGCTTCGTGACTCCCATGTCCTTC
ACAGCAGACTGGTGAGAACTCCCAACATTATCCCCTTTATCCGCGTAACTGGTAAGAC
ACCCATACTCCCAGGAAGACACC
>2715
TCACCCTTTGCCTACACCTGTCCTGCTGCCTGCTGTGGACTTTAGCTTGGGAGAATGGA
AAACCCAGATGGTAAGAAAGCCATCCCTAACCTTGGCTTCCCTAAGTCCTGTCTTCAGT
TTCCCACTGCTTCCCATGGATT
>2716
CCTCCTGGGGCAGCTTTCTGGACAGGTCCGTCTCCTCCTTGGGGCCCTGCAGAGCCTC
CTTGGAACCCAGGTAAGTCCCCAGTCAAGGGATCTGTAGAAACTGTTCTTTTCTGACT
CAGTCCCCCTAGAAGACCTGAGGG
>2717
TACACGTGTGCACTCCACCACTCTGGCCATTCCCCACCCATCTCCTCCCAGAACGTCAC
AGTGCTCAGAGGTGAGACAAGCCCCTAACAAGGTCAAGTGAGCTGGGAGAGCCAGG
CTCGGGGACAGCAGGCAGTTCCCTT
>2718
AGTACAAGTGCCTGGTGATCAAGTATGCCAAAGACACTCGCTACAGCAGCAGCTTCTG
CACACATGACCGGTCAGTCCCTGCCCCCTGCAGTCCTGTCCAGTGGAAAATCACAAGG
CACAGGACACACTGTTAGGACTCT
>2719
CTGCCTGCTCCGAGACGTGGCCCAGGAGGCCCTGGGCGTGGCTGTCATAGGCATCGAC
GAGGGGCAGTTTGTAAGTTGGCTTGTCTTGGCATCACTCTTCCTGCCTTCCGCTGTGTC
CTCCCGTTTTCCCTCGCTGACTT
>2720
CTGCGAGGCCATGGCCAACGCCGGGAAGACCGTAATTGTGGCTGCACTGGATGGGAC
CTTCCAGAGGAAGGTAAGGCGTCTGATCCAGGTCTGGAGCTGGGATTGAGGAGGGCA
AGAGGCTTCTGGATGGGCACAGAGAC
>2721
GCTGACGGCGGTGTGCATGGAGTGCTTCCGGGAAGCCGCCTATACCAAGAGGCTCGGC
ACAGAGAAGGAGGTAGCTCCACCTGCCTTCCCTGCAGGCCGGCGGGGTGGGGGTATG
GCTCTGCCTCCTTCCTGTCCTGGCC
>2722
ATGAGGATGCTGGGCCAGACACCCACCAAGGAGGAGCTGGACGCCATCATCGAGGAG
GTGGATGAGGACGGTGAGCGGGTGTCCCTCGGAGGCAGGGGATGGTGGGGAGAGGC
GGCAGCGGCTGGGCTCAGGCTCAGTCT
>2723
GCCAGATGAAAGAGGACGCGAAAGGGAAGAGCGAGGAGGAGCTGGCCGAGTGCTTC
CGCATCTTCGACAGGTGCGCTGGGGGCCCGGGAGCCGAGGGAGGGGCTTAGCAGTCA
GAACCCGGCCGGCAGCGCTGGAAGCTT
>2724
ACGGACGAGGAGATCGAATCTCTGATGAAAGACGGCGACAAGAACAACGACGGCCGC
ATTGACTTCGACGGTGAGGGCCAACGGGAGCTTGGGAAGAGCGGGTGGGAGCCCCAG
AGGACGGCGGGCACTGGTGCCTGGCT
>2725
CCTCTGCCTGGGCCTCGGTCCCTCCTGCACCTGCTGCCTGGATCCCCGGCCTGCCTGG
GCCTGGGCCTTGGTGGGTTTGGTTTTGGTTTCCTTCTCTGTCTCTGACTCTCCATCTGTC

AGTCTCATTGTCTCTGTCACAC
>2726
AAGGGTGTGTGGCACCACCCTACACCTCCTCCTTCTGGGGCTGCTGCTGGTTCTGCTG
CCTGGGGCCCAGGTGAGGCAGCAGGAGAATGGGGGCTGCTGGGGTGGCTCAGCCAA
ACCTTGAGCCCTAGAGCCCCCCTCAA
>2727
CAGACTGCCCGTCAGCACCCCAAGATGCATCTTGCCCACAGCACCCTCAAACCTGCTG
CTCACCTCATTGGTAAACATCCACCTGACCTCCCAGACATGTCCCCACCAGCTCTCCTC
CTACCCCTGCCTCAGGAACCCAA
>2728
GATCGTGGCAGGCGCCACCACGCTCTTCTGCCTGCTGCACTTTGGAGTGATCGGCCCC
CAGAGGGAAGAGGTGAGTGCCTGGCCAGCCTTCATCCACTCTCCCACCCAAGGGGAA
ATGGAGACGCAAGAGAGGGAGAGAG
>2729
GGCAAGCTGAAAAAGAAAAAAATGGCCAAGAGGATCCAGCAGGTGTACAAAACCAA
GACGCGGAGCTCAGGTACCCTTTAAGGAGGTGGGGAAGGGCCACCGAGCCCACAGAT
GATGGAGAGCAGACCTTGGGGGCAGTG
>2730
AGCTGAGATCCTACAGGAGTCCAGGGCTGGAGAGAAAACCTCTGCGAGGAAAGGGA
AGGAGCAAGCCGTGGTAGGTCGGGTTTCTGTACCTTGGGGTCTGTCTCCTCTTCTTTCT
CTTAAAAGTCTTTCCAGCAAGCTGA
>2731
GGATGCAATGAAGAGAGGGCTCTGCTGTGTGCTGCTGCTGTGTGGAGCAGTCTTCGTT
TCGCCCAGCCAGGTTGGTGTGCAGGATCCCTGTGTCCCGCCCCTCAAGGCTGTGATGC
TTCCGGAGGCTCCAGGGGTCTGTG
>2732
AGAAGCAACCGGGTGGAATATTGCTGGTGCAACAGTGGCAGGGCACAGTGCCACTCA
GTGCCTGTCAAAAGTATGTGCTGAGGCTGGAAGGTGGTGCATGCCTGTGATCCCAGCA
CTTTAGGAGGCCAAGGTGGGAGGGT
>2733
CAGGCCCTGTACTTCTCAGATTTCGTGTGCCAGTGCCCCGAAGGATTTGCTGGGAAGT
GCTGTGAAATAGGTGAGTAGGTGAGAGCACGTGAAACGAGCTGAAATCCCACCTCTC
CAGTCTCCCCCGCCCCCGGAAGGAA
>2734
CCCAGAAGCCCTACAGCGGGCGGAGGCCAGATGCCATCAGGCTGGGCCTGGGGAACC
ACAACTACTGCAGGTGAGGGCACATCCCCAAGGAGGGATTCCCCCTGTGAGAAGCTG
CTACAGCTGGGGAAAGATTCTGTCAG
>2735
CCCTGGTGCTACGTCTTTAAGGCGGGGAAGTACAGCTCAGAGTTCTGCAGCACCCCTG
CCTGCTCTGAGGGTAAGTGGCAGCCACCCCGCCTTTCCCTGAATGTCCCTGGGGGAGG
AAAGTCCAGGAGGACAGGCCGATA
>2736
TAGGCAAGGTTTACACAGCACAGAACCCCAGTGCCCAGGCACTGGGCCTGGGCAAAC
ATAATTACTGCCGGTAGGTAGCACAGGGGTGGGGGGTTCAGGTCTTGGCAGAACGTGG
GATTAGGGTGTGAGACGGGGGAAGA
>2737

GCCAAGCCCTGGTGCCACGTGCTGAAGAACCGCAGGCTGACGTGGGAGTACTGTGAT
GTGCCCTCCTGCTGTAAGGGCTGGGCCCCGGCTGCCTCCCTGCACCTGTCTCCCTTCTT
CACCATCTCCTCGCCACTTTCTAG
>2738
GGTTCCTGTGCGGGGGCATACTCATCAGCTCCTGCTGGATTCTCTCTGCCGCCCACTGC
TTCCAGGAGAGGTAGGGGCTCGGAAACCCAGTTGGTTTTTCCACCCTCTAACGCCTAG
AAACCAAGCCCTTTAGGGGAAAT
>2739
CAGAAATTTGAAGTCGAAAAATACATTGTCCATAAGGAATTCGATGATGACACTTACGA
CAATGACATTGGTAAGAGCTCGTCATTCCCGGCTCCAGTTCATACAGATCACACAACAC
ACCCACATGCACTCACCTATGT
>2740
CCCCCGGCGGACCTGCAGCTGCCGGACTGGACGGAGTGTGAGCTCTCCGGCTACGGC
AAGCATGAGGCCTGTAAGTGGAAGGAAGCCTCGGCCCCATCCTGTCTGCGGGACAGC
AGGGGAGGCTGCGGTCCACCAGAGGA
>2741
CGACAACATGCTGTGTGCTGGAGACACTCGGAGCGGCGGGCCCCAGGCAAACTTGCA
CGACGCCTGCCAGGTAACCAGGAGTGGCGGCCCATGCTGGGCACAACCCAGGGGAGA
ATGCCCCTGGGACAACAGGTTCTTCA
>2742
CGTCAGCCTTGGCCTCATTGCTGCCCTGCAGGCCCACCACCTCCTGGCCTCAGACGAG
GAGATTCAGGATGTGAGGCCCGGATGGAAGGCTGGGCTGGAGGGGGCAAGGGGCGA
GGCTGAGACTGGATGGAGACCCCATG
>2743
TGGAATCGGTGACACCCATGACCCTCACGACCCTGGAAGGGGGCAACCTGGAAGCCA
AGGTCACCATGCTGTGAGTGTCTGCCAGCCGGCCGGGCAGCCTGCAACCTGGTCTAGG
GCCTTCCCTTTCCCCACCCAGGAGA
>2744
ATAAGTGGCCGGTGCCAGGAGGTGAAGGCCGTCCTGGAGAAAACTGACGAGCCGGGA
AAATACACGGCCGGTGAGTCCCGGGGCCTGAGCCAGAGCCTGAGCTTGAACACACGC
TGGATGTGGGGGGGCAGCCAGTGCCT
>2745
AAGGACCACTACATCTTTTACTGTGAGGGCGAGCTGCACGGGAAGCCGGTCCGAGGG
GTGAAGCTCGTGGGTGGGTCCCGCACCCTCACCCTGCAACCCATGCCTCCACCTGCCC
TCCCTCCTCCCTCGCCCTCCTGCAC
>2746
GAGGACTTTGAGAAAGCCGCAGGAGCCCGCGGACTCAGCACGGAGAGCATCCTCATC
CCCAGGCAGAGCGGTAGGAGGCATGGCCCTGCAGAGCCCCCCATGTCCCCGCGTGGG
GACATCAGCAGAGCTGCATTGCACGG
>2747
TGATGGAGCAGCATTAACTAAGAAAGACCTGAAGAACCTCCTTGAAAGGGAATTTGG
AGCTGTGCTTCGGGTAAGAACTAACAAGAAAATGAGATCTATTGACTTGAGGCTATGA
GATTTATTCTCAGAGGAGACCAGAG
>2748
ATGAGGATGCTGGGCCAGAACCCCACCCCTGAGGAGCTGCAGGAGATGATCGATGAG
GTGGACGAGGACGGTGAGCCCCCCTCCTCCCCAGGCTCCAGAAGAACCCCAGCTGGC

TGGGGGCTGGAATGCTGGCTCTGTTT
>2749
GGTGCATGAAGGACGACAGCAAAGGGAAATCTGAGGAGGAGCTGTCTGACCTCTTCC
GCATGTTTGACAAGTGAGCACGTGACCCTTGACCTCTGACCCTGACCCACACTCAAGC
CGAGCTGTACAGGAGGGCAGTCTCA
>2750
ACGGAGGACGACATCGAGGAGCTCATGAAGGACGGAGACAAGAACAACGACGGCCG
CATCGACTATGATGGTAAGCGGGTGGGTGGGCTGATCTCCTGCCTCCATGCCCTGCCCA
GCCCCTACCCTCAACCCACACCTGC
>2751
GTTTAACTGGGTGTCCCGGCTGGCAAACCTCACGCAAGGCGAAGACCAGTACTATCTG
CGGGTCACCACGGTGAGCTGTGTCCCGGCCACATGCTGTCGGTCGGAGCCCGACGTGT
GATCGAGCAGGGGCATGTGTGCTT
>2752
CCAGGAAGAACCCTAAATTTATGGAGACCGTGGCGGAGAAAGCGCTGCAGGAATACC
GCAAAAAGCACCGGTAAGCAGGCGGGCCTTTCCTGCGCGTGCAGGGCCCAGTGAGTC
TCTGGGAGCCACAAAAAAACAAACAA
>2753
AGGAAGGACGACCGCACGGGCACCGGCACCCTGTCGGTATTCGGCATGCAGGCGCGC
TACAGCCTGAGAGGTGACGCCGCGGGCCCCTGCGGGACGGGTGGCGGGAAGGAGGG
AGGCGCGGCTGGGGAGAGCGCTCGGGA
>2754
ATTCCCTCTGCTGACAACCAAACGTGTGTTCTGGAAGGGTGTTTTGGAGGAGTTGCTG
TGGTTTATCAAGGTAAAGAAGTCGCTGCTATTAGAAGTCAGTAGTCTGTTCTCAACACA
GCAGCCAGTGAGATCCTTTCAAA
>2755
GGGGACTTGGGCCCAGTTTATGGCTTCCAGTGGAGGCATTTTGGGGCAGAATACAGAG
ATATGGAATCAGGTGAGGAGATAGAACAATGCCTTCCATTTCCGGGTGCCCTTCCTAGC
ACGTGTTTGCTCCGTTGTTTTAG
>2756
AGAGTGATTGACACCATCAAAACCAACCCTGACGACAGAAGAATCATCATGTGCGCTT
GGAATCCAAGAGGTTGAAAGAACCCCGTCGTCTTCATTTATACTAACCATACTCTTAGA
GGGAAGCAATCTGGTTTTGTGCA
>2757
CGGTGTGCCTTTCAACATCGCCAGCTACGCCCTGCTCACGTACATGATTGCGCACATCA
CGGGCCTGAAGGTGGGCTGTCTCGGGAAGGGTGACTTGCCAGCCTACCACATGAGCT
CTTCAGTTCTTTAATATGGGAAAA
>2758
AGGTGACTTTATACACACTTTGGGAGATGCACATATTTACCTGAATCACATCGAGCCAC
TGAAAATTCAGGTAAGAATTAGATGTTATACTTTTGGGTTTGGTACCTTCTCTTGATAAA
AGGTTGACTGTGGAACAGGTA
>2759
CTGGTCTCCGAGAAGCTGAACGTCCCAGTGCGCCAGCAGCGGCTGCTGTTCAAGGGC
AAGGCCCTGGCAGGTACCCAGGGAGAGGAGACGCCCGGGGAGCCCCGCAGGAAGCG
GGACCGGGGTGCGCGGGCCGAGGCCAG
>2760

GATCTCCAAAGTCTTGGCCCGCCACTTCAGTGCGGCAGATGCCAGCAGGGTCCTGGAA
CAGCTACAGAGGGTGAGAAGGGTAACCCTGGGCATCCTCTTCAGGCCCAGTTGCTCCC
AGCCCCTACCTCTGTTGCAGTGTA
>2761
CGCACATCACCTCCAGGCCCTCAGAACACCTGCCCCAGCCCCACCATGCTCATGGCGT
CCACCACTTCCGGTAAGGCTTGCCCCTCCATGAGTCCGGTGGGCAGAGTGGGTTTGAC
GATTCAGGGAAGCCCCTCTTTCTA
>2762
GTGCTGTGCTGGGCACCCTTCTTCCTGGTGCAGCTGTGGGCCGCGTGGGACCCGGAGG
CACCTCTGGAAGGTGGGTGTAGCCGTGGCTAGGGCTGACGGGGCCACTTGGGCTTGG
CCGCATGCCCCTGTGCCCCACCAGC
>2763
TCTGGTAGAAAAATGAAGAGGGTCCTGGTACTACTGCTTGCTGTGGCATTTGGACATG
CTTTAGAGAGAGGTAAGATTTCTTTTGTTGTGACCATTTACAGGAATTCTTACTAGTTTA
ATTTTATATTATCACTTAAAAA
>2764
GCCGGGATTATGAAAAGAATAAAGTCTGCAAGGAATTCTCCCATCTGGGAAAGGAGGA
CTTCACATCTCTGTAAGTGTGCAGCAGCCACTCACTCTGTTGGTGATTTGATCTTGAAC
AAAACTGAGTGTGAGGTAACTTT
>2765
GAAGGAAGTTGTCTCCTTGACCGAAGCCTGCTGTGCGGAAGGGGCTGACCCTGACTG
CTATGACACCAGGGTAGGTTTCTGTGGCTGGCCGTCTCTGTGGCAGCCCAGAGAAGGA
AGCCAAAATAGGCCTTTATACCACG
>2766
CCTACGTGGAACCCACAAATGATGAAATCTGTGAGGCGTTCAGGAAAGATCCAAAGG
AATATGCTAATCAGTGAGTGCCTTCATCATAAATAGAACTTTAGGACCTAAAGTATCAGA
AATGACTCTAATCTAACCCCCTA
>2767
CAAGAGTTATCTTTCTATGGTAGGGTCCTGCTGTACCTCTGCAAGCCCAACTGTATGCTT
TTTGAAAGAGGTATGTCCCATTTTACTTATTATATGTTTACGTTTTTTTCTTCTAATAGCAT
TCCTTTTAGATAATGGTA
>2768
TTCTCACCACTCTGTCAAATAGAGTCTGCTCACAATATGCTGCTTATGGGGAGAAGAAA
TCAAGGCTCAGGTAAAGATTAAGTGCTATTGTCATATTTAGATGTTTGGTTCTGCACTGT
CTATCCATAATATTAGGGTTT
>2769
AGCTGAAGATATTACTAACATCCTCTCCAAATGCTGTGAGTCTGCCTCTGAAGATTGCA
TGGCCAAAGAGGTAAGACAAGCTTTATTATCATTATCACCATGTGCCAGGTATTGAGTG
CCAAGTACTGAGTGGCAGGTAC
>2770
AGCTTCCAGATGTAGAGTTGCCCACAAACAAAGATGTGTGTGATCCAGGAAACACCAA
AGTCATGGATAAGTAAGTAGAGGTGATGTGAAAACGTGTTCCCATTTTAAGTTCATCTG
GACAAAAAGTGGGGGCCAGCCAG
>2771
TGAGCCAACCCTAAAAAGCCTTGGTGAATGCTGTGATGTTGAAGACTCAACTACCTGT
TTTAATGCTAAGGTATATTTGTTGGATTTTCTTTATCAAGCACATAGATCAAGGGTTGGA

AAATTATAGTTCATGGGCCAAA
>2772
CTTTCATTGACAAGGGACAAGAACTATGTGCAGATTATTCAGAAAATACATTTACTGAG
TACAAGAAAAAGTAAGAAACTTGTTCTGGCTGTATCCTCCAAATTTATCAATAATATTTT
CATAGTACTATGAATTGAAAG
>2773
TAACAAGCGCTCAGACTTTGCCTCCAACTGCTGTTCCATAAACTCACCTCCTCTTTACT
GTGATTCAGAGGTAGGAAAATGTAACCCTCCACTTAACATGGCAGAATCTTTTAAGAA
CGTATGCACTCCAATCTACTCAT
>2774
CTCCGCGTGCTACTTCCAGAACTGCCCGAGGGGCGGCAAGAGGGCCATGTCCGACCT
GGAGCTGAGACAGGTACTTCCCACTGTGGGCCATCTCAGGGCAGCCATAGCGGGCAGT
GCTGACACCCTGGGTCAGGGGCTAG
>2775
TGCCAGTCCGGCCAGAAGGCGTGCGGGAGCGGGGGCCGCTGCGCCGCCTTCGGCGTT
TGCTGCAACGACGGTGCGCGGCGGGGGCGGGCCTGGGGGGGGGCGCAGACGCTTGG
GTGGGGGGGACGCGGGCCTGCGGCGGG
>2776
CGTGGGCCAGAAGACCAGGACATATCTATTTCCTTTGCATGGGATAAAAGCTCTGCTCT
TCAGGTTTCAGGTGAGAGAGGCCAGCATAAAAAAGCTACCGAGTCTAGAGAGACGGA
TGGGAGATGGGCTCTGGAATCACA
>2777
TGGGTGGTGGCTCTGCTAGTGAATCTGACCCAACTGGATTCCTCCATGACTCAAGGCA
CAGACTCTCCAGGTAAGAACAGAGCAATTGTTTTTTTCCAGTGTGTATGCAAGAATTG
GCATGGGGGAGTGATGCCTTTCTT
>2778
AGACAGGCCGTGGATGGGGTCTGTAGACACAACTACAGGCTGGGCGCACCCTTCACT
GTGGGGAGAAAAGGTGAGCTGGAAGCTGAGGTCTGGCGGGGCTCAGGAATGTCCCCC
ATGTGAACCATGGCTCTTCTTTCTTA
>2779
GAACTTGGACATGTCTACACCTGCCTTGTCGATCACTCCAGCCTGCTGAGCCCTGTTTC
TGTGGAGTGGAGTGAGAATTAGTTTCTAGTACTCTCTGGGCCTGACTCAGGACTATACT
GACTCAATACAGAGCCTGTGTC
>2780
ATTGCAGCCTTCCTACTTGGGCTAATCTTCCTTCTGGTGGGAATCGTCATCCAGCTAAG
GGCTCAGAAAGGTAATGAGCCTGTGAGGAGTGCCCTGCCACCTGTCCCAGACCTTCCC
CACTCCCACCTTCCCTAACGTCA
>2781
GCCGTCATGGCGCCCCGAACCCTCGTCCTGCTACTCTCGGGGGCCCTGGCCCTGACCC
AGACCTGGGCAGGTGAGTGCGGGGTCGGGAGGGAAACGGCCTCTGCGGGGAGAAGC
AAGGGGCCCGCCTGGCGGGGGCGCAA
>2782
AAGGCCCACTCACAGACTGACCGAGAGAACCTGCGGATCGCGCTCCGCTACTACAAC
CAGAGCGAGGCCGGTGAGTGACCCCGGCCCGGGGCGCAGGTCACGACCCCTCATCCC
CCACGGACGGGCCGGGTCGCCCACAG
>2783

CTGGAGGGCACGTGCGTGGACGGGCTCCGCAGATACCTGGAGAACGGGAAGGAGAC
GCTGCAGCGCACGGGTACCAGGGGCCACGGGGCGCCTACCTGATCGCCTGTAGGTCTC
CCGGGCTGGCCTCCCACAAGGAGGGG
>2784
GGAGAGGAGCAGAGATACACCTGCCATGTGCAGCATGAGGGTCTGCCCAAGCCCCTC
ACCCTGAGATGGGGTAAGGAGGGAGATGGGGGTGTCATGTCTCTTAGGGAAAGCAGG
AGCCTCTCTGGAGACCTTTAGCAGGG
>2785
CTGGTTCTCCTTGGAGCTGTGATCACTGGAGCTGTGGTCGCTGCTGTGATGTGGAGGA
GGAACAGCTCAGGTGGAGAAGGGGTGAAGGGTGGGGTCTGAGATTTCTTGTCTCACT
GAGGGTTCCAAGCCCCAGCTAGAAA
>2786
GGATGCACTTCTGCGGGGGCTCCCTCATCCACCCCCAGTGGGTGCTGACCGCAGCGCA
CTGCGTGGGACCGTGAGTCTCCCGGGGCCTGGAGGGGTGGGGAAGGGCTGGATGTGA
GCCCTGGCTCCCGGGTGCTCCTGGG
>2787
CCTGCCTCAGAGACCTTCCCCCCGGGGATGCCGTGCTGGGTCACTGGCTGGGGCGATG
TGGACAATGATGGTGGGTCTGGGGACAGTGGAGGTGGGGCCAGGGTCTTAGCCACAG
CCCAGCCCCTGGGTCCCTCTGGGCT
>2788
GGGAGACGACGTCCGCATCGTCCGTGACGACATGCTGTGTGCCGGGAACACCCGGAG
GGACTCATGCCAGGTGGGCCCCGCCTGTCCCCCGCCCCCCGCCCCCCAACCCCCACTC
CCAGGCCTGTTCGGCGAGCGCTGAC
>2789
CTGAAGATCTCTGAGGAGGATGAGCTGGACACCAAGCTAACCCGTATCCCCAGTGCCA
AGAAGTACAAAGGTAAGCGGCCACTCCTTTAACTAGCTGCACCTCTGCCTCGTCCCGT
TGACTATCCTTGGAGTACTTGAGT
>2790
AGACCAGTGGCACTGTTGTTTCACAAGATGATGTTTGAAACTATTCCAATGTTCAGTGG
CGGAACTTGCAGTAAGTGCTTGAAATTCTCATCCTTCCATGTATTGGAACAGTTTTCTTA
ACCATATCTAGAAGTTTACAT
>2791
TCAGCCGTTACCTGTGTGTGGTGATATCAAAGTAGAGTTCTTCCACAAACAGAACAAG
ATGCTAAAAAAGGTTTGTACTTTACTTTCATTGGGAGAAATATCCAAAATAAGGACAGA
TTAAAAGCTATATTTTATTTTAT
>2792
TTTAACAAAAAATGATCTTGACAAAGCAAATAAAGACAAAGCCAACCGATACTTTTCT
CCAAATTTTAAGGTCAGTTAAATTAAACATTTTGTGGGGGTTGGTGACTTGTATGTATGT
GATGTGTGTTTAATTCTAGGAG
>2793
AGCAAGGACTAGAGTTCCTGACCTCCAGGCCAGTCCCTGATCCCTGACCTAATGTTATC
GCGGAATGATGGTAAGTAAAGTGTCTCTTGCATCTGCATAGAGAGAGTCCTGGGAGCT
TAGGAAGTGATGGGGAACAGTGA
>2794
AAGAAAAGCCTGGAGTGCACGAAGTTGTGCCTACCCCAGATTGAGAATGTTAAGGGC
ACTGAGGACTCAGGTGAGGAGAGGTGACCTGGTGCCCATGCTCACCTGCCCTCTCCCT

CTTCTTGCCCCCACCCGTCCATCCA
>2795
CTTTTATCCCTCCTCTTCATTGGTTTAATGTATCGCTACCAACGGTGGAAGTCCAAGCTC
TACTCCATTGGTGAGTGGGGGCTTTGGGAGGGAGAGGGAGCTGGTGGGGGTGAGGGA
GGACATGGGTGGGTGCGATGGAC
>2796
GCCTCCGACCCCATCCCCAACCCCCTTCAGAAGTGGGAGGACAGCGCCCACAAGCCA
CAGAGCCTAGACAGTGCGTTTCTCCCGCGGCTGGAGACGAGGAGGCTGGGGGAGGGC
CGGGGGAGCGCGGGAGGCGTCAGAGG

Online Supporting Information S_1^- represents the negative dataset consisting of 2800 false splice donor site sequences.

>1
ACTGTGTCCTCATTCATGTCATTCGTCCTCAGCTGAAGGATGAAAGAGATTGGCAAGG
AAGCAGGAATGAGTCAGCTTTGGGGGAATATTACCTGGTGGCTCCCATCATTTATTGCT
TTAGGACATACCCTTTTACCTAC
>2
TCCTCAGCTGAAGGATGAAAGAGATTGGCAAGGAAGCAGGAATGAGTCAGCTTTGGG
GGAATATTACCTGGTGGCTCCCATCATTTATTGCTTTAGGACATACCCTTTTACCTACTTG
CTATCATGCTGGACCTCAGCAT
>3
ACCTCAGCATTTCAGGGACCAGAAAAGATGGGGACACCAATGCAAGCCCAAGACTAC
CATCCAGAGGACCGTTCCTGCCCATGCTGCTTTCTCTAGTTTTGCTTTCAGAGTGGTGT
CACCCCACTTGCTAACTCAAGAGT
>4
AGATGGGGACACCAATGCAAGCCCAAGACTACCATCCAGAGGACCGTTCCTGCCCATG
CTGCTTTCTCTAGTTTTGCTTTCAGAGTGGTGTCACCCCACTTGCTAACTCAAGAGTGC
ATCACTAGGCTTCCTGAGCAGGT
>5
CAAGCCCAAGACTACCATCCAGAGGACCGTTCCTGCCCATGCTGCTTTCTCTAGTTTTG
CTTTCAGAGTGGTGTCACCCCACTTGCTAACTCAAGAGTGCATCACTAGGCTTCCTGA
GCAGGTTTGATCAGGATTTGGCC
>6
AGCCCAAGACTACCATCCAGAGGACCGTTCCTGCCCATGCTGCTTTCTCTAGTTTTGCT
TTCAGAGTGGTGTCACCCCACTTGCTAACTCAAGAGTGCATCACTAGGCTTCCTGAGC
AGGTTTGATCAGGATTTGGCCTG
>7
CCGTTCCTGCCCATGCTGCTTTCTCTAGTTTTGCTTTCAGAGTGGTGTCACCCCACTTG
CTAACTCAAGAGTGCATCACTAGGCTTCCTGAGCAGGTTTGATCAGGATTTGGCCTGG
GTCTGTTGGTCAGGGAGGCAGAG
>8
TAGTTTTGCTTTCAGAGTGGTGTCACCCCACTTGCTAACTCAAGAGTGCATCACTAGGC
TTCCTGAGCAGGTTTGATCAGGATTTGGCCTGGGTCTGTTGGTCAGGGAGGCAGAGGT
GTGGGGAGAAGGGTGAAAGGTGA

>9
TCACCCCACTTGCTAACTCAAGAGTGCATCACTAGGCTTCCTGAGCAGGTTTGATCAG
GATTTGGCCTGGGTCTGTTGGTCAGGGAGGCAGAGGTGTGGGGAGAAGGGTGAAAGG
TGACTGAGCCAGGGTTCACAGGCTC
>10
CCCACTTGCTAACTCAAGAGTGCATCACTAGGCTTCCTGAGCAGGTTTGATCAGGATTT
GGCCTGGGTCTGTTGGTCAGGGAGGCAGAGGTGTGGGGAGAAGGGTGAAAGGTGAC
TGAGCCAGGGTTCACAGGCTCCTCA
>11
GTGCATCACTAGGCTTCCTGAGCAGGTTTGATCAGGATTTGGCCTGGGTCTGTTGGTCA
GGGAGGCAGAGGTGTGGGGAGAAGGGTGAAAGGTGACTGAGCCAGGGTTCACAGGC
TCCTCACTCACGGGGCTGAAGCTGG
>12
GATTTGGCCTGGGTCTGTTGGTCAGGGAGGCAGAGGTGTGGGGAGAAGGGTGAAAGG
TGACTGAGCCAGGGTTCACAGGCTCCTCACTCACGGGGCTGAAGCTGGGCATCGTCA
GGCTCAGAGCCCCTAGACGTGCGGCA
>13
GGGAGAAGGGTGAAAGGTGACTGAGCCAGGGTTCACAGGCTCCTCACTCACGGGGCT
GAAGCTGGGCATCGTCAGGCTCAGAGCCCCTAGACGTGCGGCAGCGGTAGCCCTTCTT
CTTGAGCACGTGGCAGATCATGAAG
>14
GAGCCAGGGTTCACAGGCTCCTCACTCACGGGGCTGAAGCTGGGCATCGTCAGGCTC
AGAGCCCCTAGACGTGCGGCAGCGGTAGCCCTTCTTCTTGAGCACGTGGCAGATCATG
AAGCCTACAGGCCCATGAGGAAGAA
>15
CACAGGCTCCTCACTCACGGGGCTGAAGCTGGGCATCGTCAGGCTCAGAGCCCCTAG
ACGTGCGGCAGCGGTAGCCCTTCTTCTTGAGCACGTGGCAGATCATGAAGCCTACAGG
CCCATGAGGAAGAAGACCAGCACAA
>16
GCTGAAGCTGGGCATCGTCAGGCTCAGAGCCCCTAGACGTGCGGCAGCGGTAGCCCTT
CTTCTTGAGCACGTGGCAGATCATGAAGCCTACAGGCCCATGAGGAAGAAGACCAGC
ACAAGCAGGAAGAGCATATATAGCC
>17
GAAGCCTACAGGCCCATGAGGAAGAAGACCAGCACAAGCAGGAAGAGCATATATAGC
CCATGTTGGGGCGGTTCCAGGTCAGGCTGTGGTTCCGACATGAGGCCCTGGGGGGCG
AGCGCGGGCCAGGGCGCCTCCTGGGT
>18
ACAGGCCCATGAGGAAGAAGACCAGCACAAGCAGGAAGAGCATATATAGCCCATGTT
GGGGCGGTTCCAGGTCAGGCTGTGGTTCCGACATGAGGCCCTGGGGGGCGAGCGCGG
GCCAGGGCGCCTCCTGGGTTTAGGGG
>19
AGGAAGAAGACCAGCACAAGCAGGAAGAGCATATATAGCCCATGTTGGGGCGGTTCC
AGGTCAGGCTGTGGTTCCGACATGAGGCCCTGGGGGGCGAGCGCGGGCCAGGGCGCC
TCCTGGGTTTAGGGGGCTGCAGCTGG
>20
CGGTTCCAGGTCAGGCTGTGGTTCCGACATGAGGCCCTGGGGGGCGAGCGCGGGCCA

GGGCGCCTCCTGGGTTTAGGGGGCTGCAGCTGGGAATGGGGGAGGGGTAAGAATGCC
GTCCTCAGGATCTGAAACAAGCACGT
>21
GCCCTGGGGGGCGAGCGCGGGCCAGGGCGCCTCCTGGGTTTAGGGGGCTGCAGCTGG
GAATGGGGGAGGGGTAAGAATGCCGTCCTCAGGATCTGAAACAAGCACGTCTAAGAC
GTCCGGCTGAGGTCCGGCCCCACCCC
>22
CGAGCGCGGGCCAGGGCGCCTCCTGGGTTTAGGGGGCTGCAGCTGGGAATGGGGGAG
GGGTAAGAATGCCGTCCTCAGGATCTGAAACAAGCACGTCTAAGACGTCCGGCTGAG
GTCCGGCCCCACCCCCTACCCAACCA
>23
GGGTTTAGGGGGCTGCAGCTGGGAATGGGGGAGGGGTAAGAATGCCGTCCTCAGGAT
CTGAAACAAGCACGTCTAAGACGTCCGGCTGAGGTCCGGCCCCACCCCCTACCCAAC
CATGGGAGCTGTCCGGGCCAACAGTG
>24
GGGCTGCAGCTGGGAATGGGGGAGGGGTAAGAATGCCGTCCTCAGGATCTGAAACAA
GCACGTCTAAGACGTCCGGCTGAGGTCCGGCCCCACCCCCTACCCAACCATGGGAGCT
GTCCGGGCCAACAGTGCTGTTTCGC
>25
GATCTGAAACAAGCACGTCTAAGACGTCCGGCTGAGGTCCGGCCCCACCCCCTACCCA
ACCATGGGAGCTGTCCGGGCCAACAGTGCTGTTTCGCCTTTCGGCAAGAGGTCAGCG
ACAACGCAAGCTCTCTACCACGACC
>26
CACGTCTAAGACGTCCGGCTGAGGTCCGGCCCCACCCCCTACCCAACCATGGGAGCTG
TCCGGGCCAACAGTGCTGTTTCGCCTTTCGGCAAGAGGTCAGCGACAACGCAAGCTC
TCTACCACGACCATGGTCCGCTTCC
>27
CCGGCCCCACCCCCTACCCAACCATGGGAGCTGTCCGGGCCAACAGTGCTGTTTCGCC
TTTCGGCAAGAGGTCAGCGACAACGCAAGCTCTCTACCACGACCATGGTCCGCTTCCC
CGACGCCCCGAGCGTTCCCCTTTC
>28
CGGGCCAACAGTGCTGTTTCGCCTTTCGGCAAGAGGTCAGCGACAACGCAAGCTCTCT
ACCACGACCATGGTCCGCTTCCCCGACGCCCCGAGCGTTCCCCTTTCCAGCTTGTTCG
CGCTTCTGTCCCTCCCAAGGACAC
>29
TTCGGCAAGAGGTCAGCGACAACGCAAGCTCTCTACCACGACCATGGTCCGCTTCCCC
GACGCCCCGAGCGTTCCCCTTTCCAGCTTGTTCGCGCTTCTGTCCCTCCCAAGGACAC
TGCAGCACGAGATGGGACGGCATT
>30
GACAACGCAAGCTCTCTACCACGACCATGGTCCGCTTCCCCGACGCCCCGAGCGTTCC
CCTTTCCAGCTTGTTCGCGCTTCTGTCCCTCCCAAGGACACTGCAGCACGAGATGGGA
CGGCATTCTTAGGCGGCGGGAGCA
>31
TCTCTACCACGACCATGGTCCGCTTCCCCGACGCCCCGAGCGTTCCCCTTTCCAGCTTG
TTCGCGCTTCTGTCCCTCCCAAGGACACTGCAGCACGAGATGGGACGGCATTCTTAGG
CGGCGGGAGCAGAAATCCTGCCT

>32
CCCTCCCAAGGACACTGCAGCACGAGATGGGACGGCATTCTTAGGCGGCGGGAGCAG
AAATCCTGCCTGAGTCAGCGCCCGCACCTCCTCCACGCCGGCCTGAGCCAGGCCTCTG
CAGCCCCTTCCTCTCAGTACTCCCG
>33
CCTGCCTGAGTCAGCGCCCGCACCTCCTCCACGCCGGCCTGAGCCAGGCCTCTGCAGC
CCCTTCCTCTCAGTACTCCCGCACCCCTCTCCCTTTTTGGTTTCTCCCACCCTGACCACC
TGGCCGACCCGGTACCGGTGAT
>34
TCCACGCCGGCCTGAGCCAGGCCTCTGCAGCCCCTTCCTCTCAGTACTCCCGCACCCC
TCTCCCTTTTTGGTTTCTCCCACCCTGACCACCTGGCCGACCCGGTACCGGTGATTTGG
TTCTGGTTCCGGCTTCTGCTGGG
>35
CCTTCCTCTCAGTACTCCCGCACCCCTCTCCCTTTTTGGTTTCTCCCACCCTGACCACCT
GGCCGACCCGGTACCGGTGATTTGGTTCTGGTTCCGGCTTCTGCTGGGGTTCCGGCTCC
AGGGGCGTCCTAGTGCGGCTC
>36
TCTCAGTACTCCCGCACCCCTCTCCCTTTTTGGTTTCTCCCACCCTGACCACCTGGCCG
ACCCGGTACCGGTGATTTGGTTCTGGTTCCGGCTTCTGCTGGGGTTCCGGCTCCAGGG
GCGTCCTAGTGCGGCTCAGGGCC
>37
GTTTCTCCCACCCTGACCACCTGGCCGACCCGGTACCGGTGATTTGGTTCTGGTTCCGG
CTTCTGCTGGGGTTCCGGCTCCAGGGGCGTCCTAGTGCGGCTCAGGGCCCCCGACGCC
CTTCCGGCGCTCATCCCTTGATT
>38
CACCTGGCCGACCCGGTACCGGTGATTTGGTTCTGGTTCCGGCTTCTGCTGGGGTTCCG
GCTCCAGGGGCGTCCTAGTGCGGCTCAGGGCCCCCGACGCCCTTCCGGCGCTCATCCC
TTGATTCATCCCCTCCCCCTTCG
>39
CCCGACGCCCTTCCGGCGCTCATCCCTTGATTCATCCCCTCCCCCTTCGCCGCCTCACC
GGCCCCGGGCTGTGGCTGCGGATACCCGCGCCGTGGCAGGCGGGGGGAGGGAAGAAC
AGAGAAGGATGCGGGCTGGCTGAG
>40
ATCCCTTGATTCATCCCCTCCCCCTTCGCCGCCTCACCGGCCCCGGGCTGTGGCTGCGG
ATACCCGCGCCGTGGCAGGCGGGGGGAGGGAAGAACAGAGAAGGATGCGGGCTGGC
TGAGAGCGGTGGCAGGCTCCGGGCA
>41
CTGCGGATACCCGCGCCGTGGCAGGCGGGGGGAGGGAAGAACAGAGAAGGATGCGG
GCTGGCTGAGAGCGGTGGCAGGCTCCGGGCAACTCCGGCCCTGGGTCAGGGAGATTG
CCTCCGCAGCCGCCCTGGCTTCCCAAA
>42
GGAGGGAAGAACAGAGAAGGATGCGGGCTGGCTGAGAGCGGTGGCAGGCTCCGGGC
AACTCCGGCCCTGGGTCAGGGAGATTGCCTCCGCAGCCGCCCTGGCTTCCCAAATGCC
TTCACCCGTCGCGGGCCCAGGACCAG
>43
CGGGCAACTCCGGCCCTGGGTCAGGGAGATTGCCTCCGCAGCCGCCCTGGCTTCCCAA

ATGCCTTCACCCGTCGCGGGCCCAGGACCAGGGGGCGGAGCGCAGGTGTGCGGGGCG
GAGAGCGTGAGCTCTGCGCGCGCGT
>44
CTCCGCAGCCGCCCTGGCTTCCCAAATGCCTTCACCCGTCGCGGGCCCAGGACCAGGG
GGCGGAGCGCAGGTGTGCGGGGCGGAGAGCGTGAGCTCTGCGCGCGCGTGACTGCG
CCGGGCCCCCCGGGGCAATGTGCGG
>45
CCGCAGCCGCCCTGGCTTCCCAAATGCCTTCACCCGTCGCGGGCCCAGGACCAGGGG
GCGGAGCGCAGGTGTGCGGGGCGGAGAGCGTGAGCTCTGCGCGCGCGTGACTGCGCC
GGGCCCCCCGGGGCAATGTGCGGCG
>46
TTCCCAAATGCCTTCACCCGTCGCGGGCCCAGGACCAGGGGGCGGAGCGCAGGTGTG
CGGGGCGGAGAGCGTGAGCTCTGCGCGCGCGTGACTGCGCCGGGCCCCCCGGGGCA
ATGTGCGGCGCCGAGTTTGGGTCCGG
>47
GCGCAGGTGTGCGGGGCGGAGAGCGTGAGCTCTGCGCGCGCGTGACTGCGCCGGGCC
CCCCGGGGCAATGTGCGGCGCCGAGTTTGGGTCCGGGTAGGGGACAGGCGGTCCGG
CGTGGGTGTTGTCCACTGGTCTATCC
>48
GGGGCGGAGAGCGTGAGCTCTGCGCGCGCGTGACTGCGCCGGGCCCCCCGGGGCAA
TGTGCGGCGCCGAGTTTGGGTCCGGGTAGGGGACAGGCGGTCCGGCGTGGGTGTTGT
CCACTGGTCTATCCGGTACGCCTGGC
>49
CGCGTGACTGCGCCGGGCCCCCCGGGGCAATGTGCGGCGCCGAGTTTGGGTCCGGG
TAGGGGACAGGCGGTCCGGCGTGGGTGTTGTCCACTGGTCTATCCGGTACGCCTGGCG
CAGCTCCTGCCCTCAGATGCCCCGA
>50
GCCGGGCCCCCCGGGGCAATGTGCGGCGCCGAGTTTGGGTCCGGGTAGGGGACAGG
CGGTCCGGCGTGGGTGTTGTCCACTGGTCTATCCGGTACGCCTGGCGCAGCTCCTGCC
CTCAGATGCCCCGACCGAGCGGAGA
>51
CGGGCCCCCCGGGGCAATGTGCGGCGCCGAGTTTGGGTCCGGGTAGGGGACAGGCG
GTCCGGCGTGGGTGTTGTCCACTGGTCTATCCGGTACGCCTGGCGCAGCTCCTGCCCTC
AGATGCCCCGACCGAGCGGAGAGT
>52
GGGGCAATGTGCGGCGCCGAGTTTGGGTCCGGGTAGGGGACAGGCGGTCCGGCGTGG
GTGTTGTCCACTGGTCTATCCGGTACGCCTGGCGCAGCTCCTGCCCTCAGATGCCCCGA
CCGAGCGGAGAGTCAGGAATCTTC
>53
GTGTTGTCCACTGGTCTATCCGGTACGCCTGGCGCAGCTCCTGCCCTCAGATGCCCCGA
CCGAGCGGAGAGTCAGGAATCTTCTCCGAGGGCTGGGGGAAGCCGGCTTTCCGCACC
TTTGAGGGACGGAGTGGCGGGATC
>54
CCGAGCGGAGAGTCAGGAATCTTCTCCGAGGGCTGGGGGAAGCCGGCTTTCCGCACC
TTTGAGGGACGGAGTGGCGGGATCTTCGGAGCTTCCTAGAGAAGTGACCACGGCCTG
CGCCCGGTTTGGGGCAAGCGGCTGGA

>55
GGCTGGGGGAAGCCGGCTTTCCGCACCTTTGAGGGACGGAGTGGCGGGATCTTCGGA
GCTTCCTAGAGAAGTGACCACGGCCTGCGCCCGGTTTGGGGCAAGCGGCTGGATGGG
ATGGCTCGGCTTCCCGAGGATCTGAC
>56
CCGCACCTTTGAGGGACGGAGTGGCGGGATCTTCGGAGCTTCCTAGAGAAGTGACCA
CGGCCTGCGCCCGGTTTGGGGCAAGCGGCTGGATGGGATGGCTCGGCTTCCCGAGGAT
CTGACCCCCGGCCGGAGACAAATGG
>57
CTCGGCTTCCCGAGGATCTGACCCCCGGCCGGAGACAAATGGCCCTCGCATCCTAGGA
GCTATGCTGCCAGTTCCCTGCCCAACCCGGGACGCTCAAAGCCCCAGATACCGGGAGA
GCTGAGCCGGGCGAGGGGCGGAGC
>58
ACCCGGGACGCTCAAAGCCCCAGATACCGGGAGAGCTGAGCCGGGCGAGGGGCGGA
GCTCCCCTGGGGGCGTGGCCTGGGTGTGGATGGAGGCGGGGCCGCAACGCCCAGTTC
CGCAGAGGCCGGCTACTTGCTTGCGCA
>59
GCTCAAAGCCCCAGATACCGGGAGAGCTGAGCCGGGCGAGGGGCGGAGCTCCCCTGG
GGGCGTGGCCTGGGTGTGGATGGAGGCGGGGCCGCAACGCCCAGTTCCGCAGAGGCC
GGCTACTTGCTTGCGCAGCACGCACG
>60
GCCGGGCGAGGGGCGGAGCTCCCCTGGGGGCGTGGCCTGGGTGTGGATGGAGGCGG
GGCCGCAACGCCCAGTTCCGCAGAGGCCGGCTACTTGCTTGCGCAGCACGCACGGCC
GGGGAGGGGCTGGCGGCGGCCGCGGGC
>61
CGGCTACTTGCTTGCGCAGCACGCACGGCCGGGGAGGGGCTGGCGGCGGCCGCGGGC
GGCGGGCGGCGGAGTCGCGGGGCCTGCTCCCGCCGGCACCATGGCCAAGACCGTGGC
CTATTTCTACGACCCCGACGTGGGCA
>62
CTGGCGGCGGCCGCGGGCGGCGGGCGGCGGAGTCGCGGGGCCTGCTCCCGCCGGCAC
CATGGCCAAGACCGTGGCCTATTTCTACGACCCCGACGTGGGCAACTTCCACTACGGT
GAGGAAACAGGGCTATGCATGAGGG
>63
CGGCGGAGTCGCGGGGCCTGCTCCCGCCGGCACCATGGCCAAGACCGTGGCCTATTTC
TACGACCCCGACGTGGGCAACTTCCACTACGGTGAGGAAACAGGGCTATGCATGAGG
GGTTGGGACACGGAGGTTTTGAGGC
>64
GTGGCCTATTTCTACGACCCCGACGTGGGCAACTTCCACTACGGTGAGGAAACAGGGC
TATGCATGAGGGGTTGGGACACGGAGGTTTTGAGGCGGGGTTGCAACTCCAGGGGTG
AGAGCTGACGAACTCTCTTCTCCCC
>65
CGACCCCGACGTGGGCAACTTCCACTACGGTGAGGAAACAGGGCTATGCATGAGGGG
TTGGGACACGGAGGTTTTGAGGCGGGGTTGCAACTCCAGGGGTGAGAGCTGACGAAC
TCTCTTCTCCCCACCCCCAGGAGCTG
>66
GGCAACTTCCACTACGGTGAGGAAACAGGGCTATGCATGAGGGGTTGGGACACGGAG

GTTTTGAGGCGGGGTTGCAACTCCAGGGGTGAGAGCTGACGAACTCTCTTCTCCCCAC
CCCCAGGAGCTGGACACCCTATGAA
>67
GGTGAGGAAACAGGGCTATGCATGAGGGGTTGGGACACGGAGGTTTTGAGGCGGGGT
TGCAACTCCAGGGGTGAGAGCTGACGAACTCTCTTCTCCCCACCCCCAGGAGCTGGA
CACCCTATGAAGCCTCATCGCCTGGC
>68
TCTCTTCTCCCCACCCCCAGGAGCTGGACACCCTATGAAGCCTCATCGCCTGGCATTGA
CCCATAGCCTGGTCCTGCATTACGGTCTCTATAAGAAGATGATCGTGAGTTCCGCCGCC
TCGGCCGGGGGTTGGGGGGTGG
>69
CCCCCAGGAGCTGGACACCCTATGAAGCCTCATCGCCTGGCATTGACCCATAGCCTGG
TCCTGCATTACGGTCTCTATAAGAAGATGATCGTGAGTTCCGCCGCCTCGGCCGGGGGT
TGGGGGGTGGGGCGAGCTGTGGC
>70
CCTGGTCCTGCATTACGGTCTCTATAAGAAGATGATCGTGAGTTCCGCCGCCTCGGCCG
GGGGTTGGGGGGTGGGGCGAGCTGTGGCCGTAGGGATAGGTGGGCTGGACTGAGTGC
ATGAAGTGCGACCCGCCCCTAGAG
>71
TTACGGTCTCTATAAGAAGATGATCGTGAGTTCCGCCGCCTCGGCCGGGGGTTGGGGG
GTGGGGCGAGCTGTGGCCGTAGGGATAGGTGGGCTGGACTGAGTGCATGAAGTGCGA
CCCGCCCCTAGAGTATCGGGGAGCT
>72
AAGATGATCGTGAGTTCCGCCGCCTCGGCCGGGGGTTGGGGGGTGGGGCGAGCTGTG
GCCGTAGGGATAGGTGGGCTGGACTGAGTGCATGAAGTGCGACCCGCCCCTAGAGTAT
CGGGGAGCTGGATAGAAGTCACCAG
>73
GGTGGGGCGAGCTGTGGCCGTAGGGATAGGTGGGCTGGACTGAGTGCATGAAGTGCG
ACCCGCCCCTAGAGTATCGGGGAGCTGGATAGAAGTCACCAGGAGTACCTTGAAAGTG
CAGGGTGTTGGGGGTGGCGTCCTCT
>74
AGGGATAGGTGGGCTGGACTGAGTGCATGAAGTGCGACCCGCCCCTAGAGTATCGGGG
AGCTGGATAGAAGTCACCAGGAGTACCTTGAAAGTGCAGGGTGTTGGGGGTGGCGTC
CTCTGCTTAATGGAAAGTACTTTCT
>75
AGTGCATGAAGTGCGACCCGCCCCTAGAGTATCGGGGAGCTGGATAGAAGTCACCAGG
AGTACCTTGAAAGTGCAGGGTGTTGGGGGTGGCGTCCTCTGCTTAATGGAAAGTACTT
TCTCACCTCAGGTCGGCCACCCTC
>76
GAAGTGCGACCCGCCCCTAGAGTATCGGGGAGCTGGATAGAAGTCACCAGGAGTACCT
TGAAAGTGCAGGGTGTTGGGGGTGGCGTCCTCTGCTTAATGGAAAGTACTTTCTCACC
TCAGGTCGGCCACCCTCTGACTGT
>77
AGTGCGACCCGCCCCTAGAGTATCGGGGAGCTGGATAGAAGTCACCAGGAGTACCTTG
AAAGTGCAGGGTGTTGGGGGTGGCGTCCTCTGCTTAATGGAAAGTACTTTCTCACCTC
AGGTCGGCCACCCTCTGACTGTAG

>78
CCCGCCCCTAGAGTATCGGGGAGCTGGATAGAAGTCACCAGGAGTACCTTGAAAGTGC
AGGGTGTTGGGGGTGGCGTCCTCTGCTTAATGGAAAGTACTTTCTCACCTCAGGTCGG
CCACCCTCTGACTGTAGGCACACT
>79
TGGATAGAAGTCACCAGGAGTACCTTGAAAGTGCAGGGTGTTGGGGGTGGCGTCCTCT
GCTTAATGGAAAGTACTTTCTCACCTCAGGTCGGCCACCCTCTGACTGTAGGCACACTA
GCCCTTGGCCTAGGGTTAGCGTG
>80
GGGTGTTGGGGGTGGCGTCCTCTGCTTAATGGAAAGTACTTTCTCACCTCAGGTCGGC
CACCCTCTGACTGTAGGCACACTAGCCCTTGGCCTAGGGTTAGCGTGATGTGGGGCTG
GTTTGAAGACAGAAGGTGTGTTCC
>81
TAATGGAAAGTACTTTCTCACCTCAGGTCGGCCACCCTCTGACTGTAGGCACACTAGC
CCTTGGCCTAGGGTTAGCGTGATGTGGGGCTGGTTTGAAGACAGAAGGTGTGTTCCTC
CTCTGCATTTTAGAGTGTGGATAG
>82
CCTCTGACTGTAGGCACACTAGCCCTTGGCCTAGGGTTAGCGTGATGTGGGGCTGGTTT
GAAGACAGAAGGTGTGTTCCTCCTCTGCATTTTAGAGTGTGGATAGGGCCTTGGCCTC
AGGAATTAGGGTTAGGGGAGCGG
>83
TCTGACTGTAGGCACACTAGCCCTTGGCCTAGGGTTAGCGTGATGTGGGGCTGGTTTG
AAGACAGAAGGTGTGTTCCTCCTCTGCATTTTAGAGTGTGGATAGGGCCTTGGCCTCA
GGAATTAGGGTTAGGGGAGCGGAT
>84
TTGGCCTAGGGTTAGCGTGATGTGGGGCTGGTTTGAAGACAGAAGGTGTGTTCCTCCT
CTGCATTTTAGAGTGTGGATAGGGCCTTGGCCTCAGGAATTAGGGTTAGGGGAGCGGAT
ATCACTGTATCAGGGTGTGAGGT
>85
TTGAAGACAGAAGGTGTGTTCCTCCTCTGCATTTTAGAGTGTGGATAGGGCCTTGGCCT
CAGGAATTAGGGTTAGGGGAGCGGATATCACTGTATCAGGGTGTGAGGTTTACCCTCA
GTTCCCACTCCTCTTTTACCACC
>86
CTCCTCTGCATTTTAGAGTGTGGATAGGGCCTTGGCCTCAGGAATTAGGGTTAGGGGAG
CGGATATCACTGTATCAGGGTGTGAGGTTTACCCTCAGTTCCCACTCCTCTTTTACCACC
TTTCTTTACCTCCAAGGAGCT
>87
GAGTGTGGATAGGGCCTTGGCCTCAGGAATTAGGGTTAGGGGAGCGGATATCACTGTAT
CAGGGTGTGAGGTTTACCCTCAGTTCCCACTCCTCTTTTACCACCTTTCTTTACCTCCA
AGGAGCTAGGGCTCCAGGGTGG
>88
TGAGGTTTACCCTCAGTTCCCACTCCTCTTTTACCACCTTTCTTTACCTCCAAGGAGCTA
GGGCTCCAGGGTGGAACGATGCTGCCGTCTTTGTAAATGTGTATGTGCGTGTGGAACA
GAGGCGATATTTTAGATATTCC
>89
TTCCCACTCCTCTTTTACCACCTTTCTTTACCTCCAAGGAGCTAGGGCTCCAGGGTGGA

ACGATGCTGCCGTCTTTGTAAATGTGTATGTGCGTGTGGAACAGAGGCGATATTTTAGA
TATTCCTTTTTCAGCCTGGAAT
>90
CTCCTCTTTTACCACCTTTCTTTACCTCCAAGGAGCTAGGGCTCCAGGGTGGAACGATG
CTGCCGTCTTTGTAAATGTGTATGTGCGTGTGGAACAGAGGCGATATTTTAGATATTCCT
TTTTCAGCCTGGAATACCTCT
>91
CACCTTTCTTTACCTCCAAGGAGCTAGGGCTCCAGGGTGGAACGATGCTGCCGTCTTT
GTAAATGTGTATGTGCGTGTGGAACAGAGGCGATATTTTAGATATTCCTTTTTCAGCCTG
GAATACCTCTACATGGTTCTGC
>92
TGTGTATGTGCGTGTGGAACAGAGGCGATATTTTAGATATTCCTTTTTCAGCCTGGAATA
CCTCTACATGGTTCTGCTTAATCGCTATCCAGTTTTTCATCTACCAGACATACACATGCT
GTCTTCCCAGCTTGGAATTT
>93
GAGGCGATATTTTAGATATTCCTTTTTCAGCCTGGAATACCTCTACATGGTTCTGCTTAAT
CGCTATCCAGTTTTTCATCTACCAGACATACACATGCTGTCTTCCCAGCTTGGAATTTAT
GGCTGTGATATGGGATAGT
>94
GCCTGGAATACCTCTACATGGTTCTGCTTAATCGCTATCCAGTTTTTCATCTACCAGACA
TACACATGCTGTCTTCCCAGCTTGGAATTTATGGCTGTGATATGGGATAGTAACAGTTAA
GCCACGCCAGGCGTGGTGGC
>95
CTTAATCGCTATCCAGTTTTTCATCTACCAGACATACACATGCTGTCTTCCCAGCTTGGA
ATTTATGGCTGTGATATGGGATAGTAACAGTTAAGCCACGCCAGGCGTGGTGGCCCATG
CCTGTAATCCCAGCACTTTGG
>96
TTCATCTACCAGACATACACATGCTGTCTTCCCAGCTTGGAATTTATGGCTGTGATATGG
GATAGTAACAGTTAAGCCACGCCAGGCGTGGTGGCCCATGCCTGTAATCCCAGCACTT
TGGGAGGTTGAAATGGGGGGGA
>97
CACATGCTGTCTTCCCAGCTTGGAATTTATGGCTGTGATATGGGATAGTAACAGTTAAGC
CACGCCAGGCGTGGTGGCCCATGCCTGTAATCCCAGCACTTTGGGAGGTTGAAATGGG
GGGGATCGCTTGAGGCCAGGAG
>98
AGCTTGGAATTTATGGCTGTGATATGGGATAGTAACAGTTAAGCCACGCCAGGCGTGGT
GGCCCATGCCTGTAATCCCAGCACTTTGGGAGGTTGAAATGGGGGGGATCGCTTGAGG
CCAGGAGTTGGAGACCAGCTTGG
>99
CGTGGTGGCCCATGCCTGTAATCCCAGCACTTTGGGAGGTTGAAATGGGGGGGATCGC
TTGAGGCCAGGAGTTGGAGACCAGCTTGGGCCACAGTGGGACCCACATCTACAAAAA
ATAAAAATAAAAAAAGTAGCCATGT
>100
CCAGCACTTTGGGAGGTTGAAATGGGGGGGATCGCTTGAGGCCAGGAGTTGGAGACC
AGCTTGGGCCACAGTGGGACCCACATCTACAAAAAATAAAAATAAAAAAAGTAGCCAT
GTGCGGTGGGTGTGCCTGTAGTCCC

>101
GAGGCCAGGAGTTGGAGACCAGCTTGGGCCACAGTGGGACCCACATCTACAAAAAAT
AAAAATAAAAAAAGTAGCCATGTGCGGTGGGTGTGCCTGTAGTCCCAACTACTTGGGA
GGCCGAGGCGAGAGGATGGCTTGGG
>102
GAGTTGGAGACCAGCTTGGGCCACAGTGGGACCCACATCTACAAAAAATAAAAATAA
AAAAAGTAGCCATGTGCGGTGGGTGTGCCTGTAGTCCCAACTACTTGGGAGGCCGAGG
CGAGAGGATGGCTTGGGCCCAGGAG
>103
TGTGCGGTGGGTGTGCCTGTAGTCCCAACTACTTGGGAGGCCGAGGCGAGAGGATGG
CTTGGGCCCAGGAGTTTAAAGTTACAGTGATCTATGATCAGCAGCCTGAGTTGACAGG
GAGGACCTTGTCTCAAAAGAAAAAA
>104
TGGGTGTGCCTGTAGTCCCAACTACTTGGGAGGCCGAGGCGAGAGGATGGCTTGGGC
CCAGGAGTTTAAAGTTACAGTGATCTATGATCAGCAGCCTGAGTTGACAGGGAGGACC
TTGTCTCAAAAGAAAAAATAAGACA
>105
TGCCTGTAGTCCCAACTACTTGGGAGGCCGAGGCGAGAGGATGGCTTGGGCCCAGGA
GTTTAAAGTTACAGTGATCTATGATCAGCAGCCTGAGTTGACAGGGAGGACCTTGTCTC
AAAAGAAAAAATAAGACAAAACAG
>106
GAGGCCGAGGCGAGAGGATGGCTTGGGCCCAGGAGTTTAAAGTTACAGTGATCTATGA
TCAGCAGCCTGAGTTGACAGGGAGGACCTTGTCTCAAAAGAAAAAATAAGACAAAAC
AGCTAAACCCAGGCTTTATGTGTTG
>107
TGGCTTGGGCCCAGGAGTTTAAAGTTACAGTGATCTATGATCAGCAGCCTGAGTTGAC
AGGGAGGACCTTGTCTCAAAAGAAAAAATAAGACAAAACAGCTAAACCCAGGCTTTA
TGTGTTGCTAGGGAAACTAGTGAAC
>108
GCCTGAGTTGACAGGGAGGACCTTGTCTCAAAAGAAAAAATAAGACAAAACAGCTAA
ACCCAGGCTTTATGTGTTGCTAGGGAAACTAGTGAACAATATGAATGAATGTTCATTGC
TTGTGCCAAGGCACAGACAAATGG
>109
CTGAGTTGACAGGGAGGACCTTGTCTCAAAAGAAAAAATAAGACAAAACAGCTAAAC
CCAGGCTTTATGTGTTGCTAGGGAAACTAGTGAACAATATGAATGAATGTTCATTGCTT
GTGCCAAGGCACAGACAAATGGAC
>110
AAATAAGACAAAACAGCTAAACCCAGGCTTTATGTGTTGCTAGGGAAACTAGTGAACA
ATATGAATGAATGTTCATTGCTTGTGCCAAGGCACAGACAAATGGACTGGTCATAGAAA
AGACTGAGGTGATGTTAGTGCAG
>111
AACAGCTAAACCCAGGCTTTATGTGTTGCTAGGGAAACTAGTGAACAATATGAATGAAT
GTTCATTGCTTGTGCCAAGGCACAGACAAATGGACTGGTCATAGAAAAGACTGAGGTG
ATGTTAGTGCAGGAATCTATATC
>112
TGCTAGGGAAACTAGTGAACAATATGAATGAATGTTCATTGCTTGTGCCAAGGCACAG

ACAAATGGACTGGTCATAGAAAAGACTGAGGTGATGTTAGTGCAGGAATCTATATCCCT
TGAGATTGTTTGCCTGTATGTAC
>113
ACAATATGAATGAATGTTCATTGCTTGTGCCAAGGCACAGACAAATGGACTGGTCATAG
AAAAGACTGAGGTGATGTTAGTGCAGGAATCTATATCCCTTGAGATTGTTTGCCTGTAT
GTACCTGTGTGCATTTTCTGGG
>114
ATGAATGAATGTTCATTGCTTGTGCCAAGGCACAGACAAATGGACTGGTCATAGAAAA
GACTGAGGTGATGTTAGTGCAGGAATCTATATCCCTTGAGATTGTTTGCCTGTATGTACC
TGTGTGCATTTTCTGGGAGAAG
>115
ATGAATGTTCATTGCTTGTGCCAAGGCACAGACAAATGGACTGGTCATAGAAAAGACT
GAGGTGATGTTAGTGCAGGAATCTATATCCCTTGAGATTGTTTGCCTGTATGTACCTGTG
TGCATTTTCTGGGAGAAGAAAA
>116
ACAGACAAATGGACTGGTCATAGAAAAGACTGAGGTGATGTTAGTGCAGGAATCTATA
TCCCTTGAGATTGTTTGCCTGTATGTACCTGTGTGCATTTTCTGGGAGAAGAAAAGCTC
TTTCTTGTGCAGTTTTCAAAGAG
>117
CATAGAAAAGACTGAGGTGATGTTAGTGCAGGAATCTATATCCCTTGAGATTGTTTGCC
TGTATGTACCTGTGTGCATTTTCTGGGAGAAGAAAAGCTCTTTCTTGTGCAGTTTTCAA
AGAGGTGTGACACATCCCCCTG
>118
CTATATCCCTTGAGATTGTTTGCCTGTATGTACCTGTGTGCATTTTCTGGGAGAAGAAAA
GCTCTTTCTTGTGCAGTTTTCAAAGAGGTGTGACACATCCCCCTGACCTTCCAAAGATA
ATAAATCTTTGATTTTAATTA
>119
TCCCTTGAGATTGTTTGCCTGTATGTACCTGTGTGCATTTTCTGGGAGAAGAAAAGCTC
TTTCTTGTGCAGTTTTCAAAGAGGTGTGACACATCCCCCTGACCTTCCAAAGATAATAA
ATCTTTGATTTTAATTAGAAAG
>120
AGTTTTCAAAGAGGTGTGACACATCCCCCTGACCTTCCAAAGATAATAAATCTTTGATT
TTAATTAGAAAGTTGACATATATCCCATATATTTGATCCAGAAAGAAGATCCAGAGGAA
AGAGGACAGAATCAGAGCTTGG
>121
AGAATCAGAGCTTGGGGAGGAGATGAGCATGGAGAGAACTGGGAATAGGAGATTCCA
AGGAACAGGGCAGGTGGATCAGGGCTCAAAGTTGAGAAGGTACGATAGGCTGAGCTG
GAGGTGGAAAGGAAAAGAAGTGGTCT
>122
AGGAGATGAGCATGGAGAGAACTGGGAATAGGAGATTCCAAGGAACAGGGCAGGTGG
ATCAGGGCTCAAAGTTGAGAAGGTACGATAGGCTGAGCTGGAGGTGGAAAGGAAAAG
AAGTGGTCTTCAGGGGGCTCATTTGT
>123
GCATGGAGAGAACTGGGAATAGGAGATTCCAAGGAACAGGGCAGGTGGATCAGGGCT
CAAAGTTGAGAAGGTACGATAGGCTGAGCTGGAGGTGGAAAGGAAAAGAAGTGGTCT
TCAGGGGGCTCATTTGTAATAACTTA

>124
GGAGATTCCAAGGAACAGGGCAGGTGGATCAGGGCTCAAAGTTGAGAAGGTACGATA
GGCTGAGCTGGAGGTGGAAAGGAAAAGAAGTGGTCTTCAGGGGGCTCATTTGTAATA
ACTTACCTGGTTAATCGGAGTCTCTC
>125
AAGTTGAGAAGGTACGATAGGCTGAGCTGGAGGTGGAAAGGAAAAGAAGTGGTCTTC
AGGGGGCTCATTTGTAATAACTTACCTGGTTAATCGGAGTCTCTCTACTGACTTAAGGA
TTGTGGGATGAGGGGAATGCTATG
>126
GATAGGCTGAGCTGGAGGTGGAAAGGAAAAGAAGTGGTCTTCAGGGGGCTCATTTGT
AATAACTTACCTGGTTAATCGGAGTCTCTCTACTGACTTAAGGATTGTGGGATGAGGGG
AATGCTATGTTTGGCTTCATATAG
>127
GTGGTCTTCAGGGGGCTCATTTGTAATAACTTACCTGGTTAATCGGAGTCTCTCTACTG
ACTTAAGGATTGTGGGATGAGGGGAATGCTATGTTTGGCTTCATATAGTCTCCAGGACT
GGTTCAGTATGGGTGGTCTGAG
>128
TGTAATAACTTACCTGGTTAATCGGAGTCTCTCTACTGACTTAAGGATTGTGGGATGAG
GGGAATGCTATGTTTGGCTTCATATAGTCTCCAGGACTGGTTCAGTATGGGTGGTCTGA
GCCTGTGGAGTGGGGCTGACCC
>129
GGTTAATCGGAGTCTCTCTACTGACTTAAGGATTGTGGGATGAGGGGAATGCTATGTTT
GGCTTCATATAGTCTCCAGGACTGGTTCAGTATGGGTGGTCTGAGCCTGTGGAGTGGG
GCTGACCCTGCCTGGGTTCATCT
>130
CTTAAGGATTGTGGGATGAGGGGAATGCTATGTTTGGCTTCATATAGTCTCCAGGACTG
GTTCAGTATGGGTGGTCTGAGCCTGTGGAGTGGGGCTGACCCTGCCTGGGTTCATCTT
GTGTATCCATCCCGAGGTCTTCA
>131
AAGGATTGTGGGATGAGGGGAATGCTATGTTTGGCTTCATATAGTCTCCAGGACTGGTT
CAGTATGGGTGGTCTGAGCCTGTGGAGTGGGGCTGACCCTGCCTGGGTTCATCTTGTG
TATCCATCCCGAGGTCTTCAAGC
>132
GGATGAGGGGAATGCTATGTTTGGCTTCATATAGTCTCCAGGACTGGTTCAGTATGGGT
GGTCTGAGCCTGTGGAGTGGGGCTGACCCTGCCTGGGTTCATCTTGTGTATCCATCCCG
AGGTCTTCAAGCCATACCAGGC
>133
TTCATATAGTCTCCAGGACTGGTTCAGTATGGGTGGTCTGAGCCTGTGGAGTGGGGCTG
ACCCTGCCTGGGTTCATCTTGTGTATCCATCCCGAGGTCTTCAAGCCATACCAGGCCTC
CCAGCATGACATGTGCCGCTTC
>134
AGTATGGGTGGTCTGAGCCTGTGGAGTGGGGCTGACCCTGCCTGGGTTCATCTTGTGTA
TCCATCCCGAGGTCTTCAAGCCATACCAGGCCTCCCAGCATGACATGTGCCGCTTCCAC
TCCGAGGACTACATTGACTTCC
>135
CCCTGCCTGGGTTCATCTTGTGTATCCATCCCGAGGTCTTCAAGCCATACCAGGCCTCC

CAGCATGACATGTGCCGCTTCCACTCCGAGGACTACATTGACTTCCTGCAGAGAGTCA
GCCCCACCAATATGCAAGGCTTC
>136
GCCATACCAGGCCTCCCAGCATGACATGTGCCGCTTCCACTCCGAGGACTACATTGACT
TCCTGCAGAGAGTCAGCCCCACCAATATGCAAGGCTTCACCAAGAGTCTTAATGCCTT
CAACGTAGGCGATGACTGGTGAG
>137
TTCCACTCCGAGGACTACATTGACTTCCTGCAGAGAGTCAGCCCCACCAATATGCAAG
GCTTCACCAAGAGTCTTAATGCCTTCAACGTAGGCGATGACTGGTGAGGGAGGACTGG
GAGTTTTGATGTCTAACAGTTTAA
>138
CATTGACTTCCTGCAGAGAGTCAGCCCCACCAATATGCAAGGCTTCACCAAGAGTCTT
AATGCCTTCAACGTAGGCGATGACTGGTGAGGGAGGACTGGGAGTTTTGATGTCTAAC
AGTTTAATGCCAAGCCCTTCAGTG
>139
AATATGCAAGGCTTCACCAAGAGTCTTAATGCCTTCAACGTAGGCGATGACTGGTGAG
GGAGGACTGGGAGTTTTGATGTCTAACAGTTTAATGCCAAGCCCTTCAGTGAGGTCTT
TTGGCAGCAGTGGGAAGATCTCAA
>140
CCAAGAGTCTTAATGCCTTCAACGTAGGCGATGACTGGTGAGGGAGGACTGGGAGTTT
TGATGTCTAACAGTTTAATGCCAAGCCCTTCAGTGAGGTCTTTTGGCAGCAGTGGGAA
GATCTCAAGGGGGGCCTACTTGTC
>141
AACGTAGGCGATGACTGGTGAGGGAGGACTGGGAGTTTTGATGTCTAACAGTTTAATG
CCAAGCCCTTCAGTGAGGTCTTTTGGCAGCAGTGGGAAGATCTCAAGGGGGGCCTACT
TGTCAATGCATCATAATTCATTTT
>142
AGGCGATGACTGGTGAGGGAGGACTGGGAGTTTTGATGTCTAACAGTTTAATGCCAAG
CCCTTCAGTGAGGTCTTTTGGCAGCAGTGGGAAGATCTCAAGGGGGGCCTACTTGTCA
ATGCATCATAATTCATTTTATTTG
>143
ACAGTTTAATGCCAAGCCCTTCAGTGAGGTCTTTTGGCAGCAGTGGGAAGATCTCAAG
GGGGGCCTACTTGTCAATGCATCATAATTCATTTTATTTGTTTATTCAATAAATATTTATTG
GTCACTTGTTCTAGGCAGGC
>144
GGTCTTTTGGCAGCAGTGGGAAGATCTCAAGGGGGGCCTACTTGTCAATGCATCATAAT
TCATTTTATTTGTTTATTCAATAAATATTTATTGGTCACTTGTTCTAGGCAGGCCCTGTGC
TAAGTGCTGGGAATGCAGTG
>145
ATCTCAAGGGGGGCCTACTTGTCAATGCATCATAATTCATTTTATTTGTTTATTCAATAAA
TATTTATTGGTCACTTGTTCTAGGCAGGCCCTGTGCTAAGTGCTGGGAATGCAGTGATG
AACAAAAACAAAATTTTAAT
>146
GGGGGGCCTACTTGTCAATGCATCATAATTCATTTTATTTGTTTATTCAATAAATATTTATT
GGTCACTTGTTCTAGGCAGGCCCTGTGCTAAGTGCTGGGAATGCAGTGATGAACAAAA
ACAAAATTTTAATATCGTAT

>147
AATGCATCATAATTCATTTTATTTGTTTATTCAATAAATATTTATTGGTCACTTGTTCTAGG
CAGGCCCTGTGCTAAGTGCTGGGAATGCAGTGATGAACAAAAACAAAATTTTAATATC
GTATTTTTGCTAAGTCTTAT
>148
CATAATTCATTTTATTTGTTTATTCAATAAATATTTATTGGTCACTTGTTCTAGGCAGGCCC
TGTGCTAAGTGCTGGGAATGCAGTGATGAACAAAAACAAAATTTTAATATCGTATTTTT
GCTAAGTCTTATAAAGAGG
>149
ACTTGTTCTAGGCAGGCCCTGTGCTAAGTGCTGGGAATGCAGTGATGAACAAAAACAA
AATTTTAATATCGTATTTTTGCTAAGTCTTATAAAGAGGAGGTGTATGACAGATTATGAG
AGTGTATACGTGGAGGATTGTT
>150
AGGCCCTGTGCTAAGTGCTGGGAATGCAGTGATGAACAAAAACAAAATTTTAATATCG
TATTTTTGCTAAGTCTTATAAAGAGGAGGTGTATGACAGATTATGAGAGTGTATACGTGG
AGGATTGTTTAGCTTGGTGGAG
>151
GCTGGGAATGCAGTGATGAACAAAAACAAAATTTTAATATCGTATTTTTGCTAAGTCTTA
TAAAGAGGAGGTGTATGACAGATTATGAGAGTGTATACGTGGAGGATTGTTTAGCTTGG
TGGAGTGGCTGGTCGGTGGGG
>152
AAAACAAAATTTTAATATCGTATTTTTGCTAAGTCTTATAAAGAGGAGGTGTATGACAGA
TTATGAGAGTGTATACGTGGAGGATTGTTTAGCTTGGTGGAGTGGCTGGTCGGTGGGG
CCTTCCTAGAGGAAGTAAAGAT
>153
AAATTTTAATATCGTATTTTTGCTAAGTCTTATAAAGAGGAGGTGTATGACAGATTATGA
GAGTGTATACGTGGAGGATTGTTTAGCTTGGTGGAGTGGCTGGTCGGTGGGGCCTTCCT
AGAGGAAGTAAAGATTGAGCT
>154
ATCGTATTTTTGCTAAGTCTTATAAAGAGGAGGTGTATGACAGATTATGAGAGTGTATAC
GTGGAGGATTGTTTAGCTTGGTGGAGTGGCTGGTCGGTGGGGCCTTCCTAGAGGAAGT
AAAGATTGAGCTGAGATCTGAA
>155
AGTCTTATAAAGAGGAGGTGTATGACAGATTATGAGAGTGTATACGTGGAGGATTGTTT
AGCTTGGTGGAGTGGCTGGTCGGTGGGGCCTTCCTAGAGGAAGTAAAGATTGAGCTG
AGATCTGAAAAAAGAGTAGAAGTA
>156
ATGAGAGTGTATACGTGGAGGATTGTTTAGCTTGGTGGAGTGGCTGGTCGGTGGGGCC
TTCCTAGAGGAAGTAAAGATTGAGCTGAGATCTGAAAAAAGAGTAGAAGTAATCAGAT
GAAAGATGGTTGGAGGGTGAGGGG
>157
CTTGGTGGAGTGGCTGGTCGGTGGGGCCTTCCTAGAGGAAGTAAAGATTGAGCTGAGA
TCTGAAAAAAGAGTAGAAGTAATCAGATGAAAGATGGTTGGAGGGTGAGGGGAGAAG
TATAGCAGGCAGTAGGGACAAGTGC
>158
GGAGTGGCTGGTCGGTGGGGCCTTCCTAGAGGAAGTAAAGATTGAGCTGAGATCTGA

AAAAAGAGTAGAAGTAATCAGATGAAAGATGGTTGGAGGGTGAGGGGAGAAGTATAG
CAGGCAGTAGGGACAAGTGCTAAGGG
>159
TAGAGGAAGTAAAGATTGAGCTGAGATCTGAAAAAAGAGTAGAAGTAATCAGATGAA
AGATGGTTGGAGGGTGAGGGGAGAAGTATAGCAGGCAGTAGGGACAAGTGCTAAGGG
TCTGTGGTAGGACTGAGTATGACTCA
>160
AGATTGAGCTGAGATCTGAAAAAAGAGTAGAAGTAATCAGATGAAAGATGGTTGGAG
GGTGAGGGGAGAAGTATAGCAGGCAGTAGGGACAAGTGCTAAGGGTCTGTGGTAGGA
CTGAGTATGACTCATTTGAGGAACAG
>161
GATCTGAAAAAAGAGTAGAAGTAATCAGATGAAAGATGGTTGGAGGGTGAGGGGAGA
AGTATAGCAGGCAGTAGGGACAAGTGCTAAGGGTCTGTGGTAGGACTGAGTATGACTC
ATTTGAGGAACAGAAAAAAGGCCAA
>162
AGATGAAAGATGGTTGGAGGGTGAGGGGAGAAGTATAGCAGGCAGTAGGGACAAGTG
CTAAGGGTCTGTGGTAGGACTGAGTATGACTCATTTGAGGAACAGAAAAAAGGCCAA
GTGCAGAGTATGAAGAGGAGGTGAAC
>163
TGGTTGGAGGGTGAGGGGAGAAGTATAGCAGGCAGTAGGGACAAGTGCTAAGGGTCT
GTGGTAGGACTGAGTATGACTCATTTGAGGAACAGAAAAAAGGCCAAGTGCAGAGTA
TGAAGAGGAGGTGAACTGAATTGAGG
>164
GTAGGGACAAGTGCTAAGGGTCTGTGGTAGGACTGAGTATGACTCATTTGAGGAACAG
AAAAAAGGCCAAGTGCAGAGTATGAAGAGGAGGTGAACTGAATTGAGGCTAAAGAAA
TAGGACCTTTTAAAAGCAAACAGCT
>165
CAAGTGCTAAGGGTCTGTGGTAGGACTGAGTATGACTCATTTGAGGAACAGAAAAAA
GGCCAAGTGCAGAGTATGAAGAGGAGGTGAACTGAATTGAGGCTAAAGAAATAGGAC
CTTTTAAAAGCAAACAGCTCTATTAT
>166
AGGAGGTGAACTGAATTGAGGCTAAAGAAATAGGACCTTTTAAAAGCAAACAGCTCTA
TTATGAATTGGGGTTTTTACCCGTAGAGTATAGGGAAGCTATCAAAGTGTTTTAAAAAG
CAAGGGATCTTAAGAGGGCTATG
>167
CTGAATTGAGGCTAAAGAAATAGGACCTTTTAAAAGCAAACAGCTCTATTATGAATTGG
GGTTTTTACCCGTAGAGTATAGGGAAGCTATCAAAGTGTTTTAAAAAGCAAGGGATCTT
AAGAGGGCTATGGGCAAGTTAA
>168
ACCTTTTAAAAGCAAACAGCTCTATTATGAATTGGGGTTTTTACCCGTAGAGTATAGGG
AAGCTATCAAAGTGTTTTAAAAAGCAAGGGATCTTAAGAGGGCTATGGGCAAGTTAAA
AAAACAAAAAAAACAACGAAGGA
>169
TACCCGTAGAGTATAGGGAAGCTATCAAAGTGTTTTAAAAAGCAAGGGATCTTAAGAG
GGCTATGGGCAAGTTAAAAAAACAAAAAAAACAACGAAGGAGTGGTTTGATATCATTA
GGTTTACATTGAGCTTGTATCATC

>170
GTGTTTTAAAAAGCAAGGGATCTTAAGAGGGCTATGGGCAAGTTAAAAAAACAAAAA
AAACAACGAAGGAGTGGTTTGATATCATTAGGTTTACATTGAGCTTGTATCATCCATAG
AAGAGTCAGGGTTAGACCCTCTGG
>171
GATCTTAAGAGGGCTATGGGCAAGTTAAAAAAACAAAAAAAACAACGAAGGAGTGGT
TTGATATCATTAGGTTTACATTGAGCTTGTATCATCCATAGAAGAGTCAGGGTTAGACCC
TCTGGGGAAGAGAGTGGACAACA
>172
ATGGGCAAGTTAAAAAAACAAAAAAAACAACGAAGGAGTGGTTTGATATCATTAGGTT
TACATTGAGCTTGTATCATCCATAGAAGAGTCAGGGTTAGACCCTCTGGGGAAGAGAG
TGGACAACAAAGATAAGCCACACT
>173
ACAAAAAAAACAACGAAGGAGTGGTTTGATATCATTAGGTTTACATTGAGCTTGTATCA
TCCATAGAAGAGTCAGGGTTAGACCCTCTGGGGAAGAGAGTGGACAACAAAGATAAG
CCACACTCTACTCTATATCCATGA
>174
ATATCATTAGGTTTACATTGAGCTTGTATCATCCATAGAAGAGTCAGGGTTAGACCCTCT
GGGGAAGAGAGTGGACAACAAAGATAAGCCACACTCTACTCTATATCCATGAAAAAAT
AGTAACCATCACAGCTACCAGT
>175
TTAGACCCTCTGGGGAAGAGAGTGGACAACAAAGATAAGCCACACTCTACTCTATATC
CATGAAAAAATAGTAACCATCACAGCTACCAGTTGCTCAATTCTACTTACTTTTGTTTTT
AAACTTTTTATTAGGGAAAATT
>176
GAGTGGACAACAAAGATAAGCCACACTCTACTCTATATCCATGAAAAAATAGTAACCAT
CACAGCTACCAGTTGCTCAATTCTACTTACTTTTGTTTTTAAACTTTTTATTAGGGAAAA
TTTCAAAAATTTCTGAAAGTG
>177
CACTCTACTCTATATCCATGAAAAAATAGTAACCATCACAGCTACCAGTTGCTCAATTCT
ACTTACTTTTGTTTTTAAACTTTTTATTAGGGAAAATTTCAAAAATTTCTGAAAGTGAAG
TAGTGCAGTGACCTCCCATA
>178
CCAGTTGCTCAATTCTACTTACTTTTGTTTTTAAACTTTTTATTAGGGAAAATTTCAAAA
ATTTCTGAAAGTGAAGTAGTGCAGTGACCTCCCATACGTCCATCACCTAGCTTCAACGC
TTATGAGCAAATGGCCACTCT
>179
TGCTCAATTCTACTTACTTTTGTTTTTAAACTTTTTATTAGGGAAAATTTCAAAAATTTCT
GAAAGTGAAGTAGTGCAGTGACCTCCCATACGTCCATCACCTAGCTTCAACGCTTATGA
GCAAATGGCCACTCTTTTTTT
>180
CCATCACCTAGCTTCAACGCTTATGAGCAAATGGCCACTCTTTTTTTTTTTTTTTTTTTTT
TGAGACAGAGTCTCACTCTGTAGCCCAGGCTGGAGTGCAGTGGTGCAGTCTTGGCTCA
CTGCAACCTCTGCCTCCTGGG
>181
GCTTCAACGCTTATGAGCAAATGGCCACTCTTTTTTTTTTTTTTTTTTTTTTTGAGACAGA

GTCTCACTCTGTAGCCCAGGCTGGAGTGCAGTGGTGCAGTCTTGGCTCACTGCAACCT
CTGCCTCCTGGGTTCAAGCGAT
>182
AGCAAATGGCCACTCTTTTTTTTTTTTTTTTTTTTTTGAGACAGAGTCTCACTCTGTAGC
CCAGGCTGGAGTGCAGTGGTGCAGTCTTGGCTCACTGCAACCTCTGCCTCCTGGGTTC
AAGCGATTCTCATGCCTCAGAC
>183
TCTTTTTTTTTTTTTTTTTTTTTTGAGACAGAGTCTCACTCTGTAGCCCAGGCTGGAGTG
CAGTGGTGCAGTCTTGGCTCACTGCAACCTCTGCCTCCTGGGTTCAAGCGATTCTCATG
CCTCAGACTCCCGAGTAGCTG
>184
AGTCTCACTCTGTAGCCCAGGCTGGAGTGCAGTGGTGCAGTCTTGGCTCACTGCAACC
TCTGCCTCCTGGGTTCAAGCGATTCTCATGCCTCAGACTCCCGAGTAGCTGGGATTACA
GGCGCCTGCCACCATGCCTGGCT
>185
TGGTGCAGTCTTGGCTCACTGCAACCTCTGCCTCCTGGGTTCAAGCGATTCTCATGCCT
CAGACTCCCGAGTAGCTGGGATTACAGGCGCCTGCCACCATGCCTGGCTAATTTTTGTA
TGTTTAGTAGAGATGGGGTTTC
>186
CGATTCTCATGCCTCAGACTCCCGAGTAGCTGGGATTACAGGCGCCTGCCACCATGCCT
GGCTAATTTTTGTATGTTTAGTAGAGATGGGGTTTCACTATGTTGGTCAAGCTGGTTTTG
AACTCCCGACCTCAGGTGATT
>187
TCTCATGCCTCAGACTCCCGAGTAGCTGGGATTACAGGCGCCTGCCACCATGCCTGGCT
AATTTTTGTATGTTTAGTAGAGATGGGGTTTCACTATGTTGGTCAAGCTGGTTTTGAACT
CCCGACCTCAGGTGATTCACC
>188
ATTACAGGCGCCTGCCACCATGCCTGGCTAATTTTTGTATGTTTAGTAGAGATGGGGTTT
CACTATGTTGGTCAAGCTGGTTTTGAACTCCCGACCTCAGGTGATTCACCAGCCTCAGC
CTCCAAAAGTGCTGGGATTAC
>189
GCCTGCCACCATGCCTGGCTAATTTTTGTATGTTTAGTAGAGATGGGGTTTCACTATGTT
GGTCAAGCTGGTTTTGAACTCCCGACCTCAGGTGATTCACCAGCCTCAGCCTCCAAAA
GTGCTGGGATTACAGACGTGAG
>190
ATTTTTGTATGTTTAGTAGAGATGGGGTTTCACTATGTTGGTCAAGCTGGTTTTGAACTC
CCGACCTCAGGTGATTCACCAGCCTCAGCCTCCAAAAGTGCTGGGATTACAGACGTGA
GCCATCGCACTCAGCTGAGCAA
>191
TTTCACTATGTTGGTCAAGCTGGTTTTGAACTCCCGACCTCAGGTGATTCACCAGCCTC
AGCCTCCAAAAGTGCTGGGATTACAGACGTGAGCCATCGCACTCAGCTGAGCAAGTG
GCCACTCTTACTTCATCTATACTC
>192
AGCTGGTTTTGAACTCCCGACCTCAGGTGATTCACCAGCCTCAGCCTCCAAAAGTGCT
GGGATTACAGACGTGAGCCATCGCACTCAGCTGAGCAAGTGGCCACTCTTACTTCATCT
ATACTCCCTCATGTTAGATTGTT

>193
GTGATTCACCAGCCTCAGCCTCCAAAAGTGCTGGGATTACAGACGTGAGCCATCGCAC
TCAGCTGAGCAAGTGGCCACTCTTACTTCATCTATACTCCCTCATGTTAGATTGTTTTAA
AGCAGATCCTAGATGTTATATC
>194
GGATTACAGACGTGAGCCATCGCACTCAGCTGAGCAAGTGGCCACTCTTACTTCATCTA
TACTCCCTCATGTTAGATTGTTTTAAAGCAGATCCTAGATGTTATATCCTTTTATCCATAA
ATATTTTATTATGTATCTCT
>195
GACGTGAGCCATCGCACTCAGCTGAGCAAGTGGCCACTCTTACTTCATCTATACTCCCT
CATGTTAGATTGTTTTAAAGCAGATCCTAGATGTTATATCCTTTTATCCATAAATATTTTAT
TATGTATCTCTAAATGATA
>196
CTGAGCAAGTGGCCACTCTTACTTCATCTATACTCCCTCATGTTAGATTGTTTTAAAGCA
GATCCTAGATGTTATATCCTTTTATCCATAAATATTTTATTATGTATCTCTAAATGATACTCT
TTTAAAAAAACATAGTA
>197
TCCCTCATGTTAGATTGTTTTAAAGCAGATCCTAGATGTTATATCCTTTTATCCATAAATAT
TTTATTATGTATCTCTAAATGATACTCTTTTAAAAAAACATAGTATCATTATCAAACCTAA
AAAGTTAACAGTGATTC
>198
GATGTTATATCCTTTTATCCATAAATATTTTATTATGTATCTCTAAATGATACTCTTTTAAAA
AAACATAGTATCATTATCAAACCTAAAAAGTTAACAGTGATTCCTTAATGTCATCAAATA
TGGATCAATATTCAAAT
>199
AAATATTTTATTATGTATCTCTAAATGATACTCTTTTAAAAAAACATAGTATCATTATCAA
ACCTAAAAAGTTAACAGTGATTCCTTAATGTCATCAAATATGGATCAATATTCAAATTTC
TGCAGTTGTCTCATAATTA
>200
TTATTATGTATCTCTAAATGATACTCTTTTAAAAAAACATAGTATCATTATCAAACCTAAA
AAGTTAACAGTGATTCCTTAATGTCATCAAATATGGATCAATATTCAAATTTCTGCAGTT
GTCTCATAATTATTTTGTA
>201
CTAAATGATACTCTTTTAAAAAAACATAGTATCATTATCAAACCTAAAAAGTTAACAGTG
ATTCCTTAATGTCATCAAATATGGATCAATATTCAAATTTCTGCAGTTGTCTCATAATTAT
TTTGTAGCTAATTTAAATC
>202
TATCAAACCTAAAAAGTTAACAGTGATTCCTTAATGTCATCAAATATGGATCAATATTCA
AATTTCTGCAGTTGTCTCATAATTATTTTGTAGCTAATTTAAATCCAGATCTAAACAAGG
TCTATACAGTGCATTTGGTT
>203
CAAACCTAAAAAGTTAACAGTGATTCCTTAATGTCATCAAATATGGATCAATATTCAAAT
TTCTGCAGTTGTCTCATAATTATTTTGTAGCTAATTTAAATCCAGATCTAAACAAGGTCTA
TACAGTGCATTTGGTTGAT
>204
ACAGTGATTCCTTAATGTCATCAAATATGGATCAATATTCAAATTTCTGCAGTTGTCTCAT

AATTATTTTGTAGCTAATTTAAATCCAGATCTAAACAAGGTCTATACAGTGCATTTGGTT
GATATGTCTCTTAAGACTT
>205
ATCAATATTCAAATTTCTGCAGTTGTCTCATAATTATTTTGTAGCTAATTTAAATCCAGAT
CTAAACAAGGTCTATACAGTGCATTTGGTTGATATGTCTCTTAAGACTTTTAATTTGTAG
CTTTTCTGTCACCCCCTTG
>206
CAAATTTCTGCAGTTGTCTCATAATTATTTTGTAGCTAATTTAAATCCAGATCTAAACAA
GGTCTATACAGTGCATTTGGTTGATATGTCTCTTAAGACTTTTAATTTGTAGCTTTTCTGT
CACCCCCTTGAATTTATTT
>207
GCAGTTGTCTCATAATTATTTTGTAGCTAATTTAAATCCAGATCTAAACAAGGTCTATAC
AGTGCATTTGGTTGATATGTCTCTTAAGACTTTTAATTTGTAGCTTTTCTGTCACCCCCTT
GAATTTATTTCTTGAAGAA
>208
CTCATAATTATTTTGTAGCTAATTTAAATCCAGATCTAAACAAGGTCTATACAGTGCATTT
GGTTGATATGTCTCTTAAGACTTTTAATTTGTAGCTTTTCTGTCACCCCCTTGAATTTATT
TCTTGAAGAAACCAGGTT
>209
ATTTAAATCCAGATCTAAACAAGGTCTATACAGTGCATTTGGTTGATATGTCTCTTAAGA
CTTTTAATTTGTAGCTTTTCTGTCACCCCCTTGAATTTATTTCTTGAAGAAACCAGGTTG
TTGATCCTGTAGAATTTCTT
>210
TATGTCTCTTAAGACTTTTAATTTGTAGCTTTTCTGTCACCCCCTTGAATTTATTTCTTGA
AGAAACCAGGTTGTTGATCCTGTAGAATTTCTTATGATAGCTACCATTTACTGAGTGCTT
ACTCTGCTAGGTATTATGC
>211
GTCTCTTAAGACTTTTAATTTGTAGCTTTTCTGTCACCCCCTTGAATTTATTTCTTGAAG
AAACCAGGTTGTTGATCCTGTAGAATTTCTTATGATAGCTACCATTTACTGAGTGCTTAC
TCTGCTAGGTATTATGCCAA
>212
TGAATTTATTTCTTGAAGAAACCAGGTTGTTGATCCTGTAGAATTTCTTATGATAGCTAC
CATTTACTGAGTGCTTACTCTGCTAGGTATTATGCCAAGGGTTGCTGGTAAATCCACTTA
ATTCTCATACCAATCCTTAG
>213
AGAAACCAGGTTGTTGATCCTGTAGAATTTCTTATGATAGCTACCATTTACTGAGTGCTT
ACTCTGCTAGGTATTATGCCAAGGGTTGCTGGTAAATCCACTTAATTCTCATACCAATCC
TTAGATGTATTATAATAGAC
>214
GTAGAATTTCTTATGATAGCTACCATTTACTGAGTGCTTACTCTGCTAGGTATTATGCCAA
GGGTTGCTGGTAAATCCACTTAATTCTCATACCAATCCTTAGATGTATTATAATAGACCTT
TTTCTGATGTTATATATA
>215
GCTTACTCTGCTAGGTATTATGCCAAGGGTTGCTGGTAAATCCACTTAATTCTCATACCA
ATCCTTAGATGTATTATAATAGACCTTTTTCTGATGTTATATATAAGTAAATTGAGGTTTG
GGAATATCTAGGTAACTTA

>216
AGGGTTGCTGGTAAATCCACTTAATTCTCATACCAATCCTTAGATGTATTATAATAGACCT
TTTTCTGATGTTATATATAAGTAAATTGAGGTTTGGGAATATCTAGGTAACTTAGCCGTAG
TCACATAACTGGTCATTG
>217
TAAATCCACTTAATTCTCATACCAATCCTTAGATGTATTATAATAGACCTTTTTCTGATGTT
ATATATAAGTAAATTGAGGTTTGGGAATATCTAGGTAACTTAGCCGTAGTCACATAACTG
GTCATTGGAGGGGGCATG
>218
TAATTCTCATACCAATCCTTAGATGTATTATAATAGACCTTTTTCTGATGTTATATATAAGT
AAATTGAGGTTTGGGAATATCTAGGTAACTTAGCCGTAGTCACATAACTGGTCATTGGA
GGGGGCATGATCCAGATCC
>219
CCTTAGATGTATTATAATAGACCTTTTTCTGATGTTATATATAAGTAAATTGAGGTTTGGG
AATATCTAGGTAACTTAGCCGTAGTCACATAACTGGTCATTGGAGGGGGCATGATCCAG
ATCCCGGGTTTTTAAACCAC
>220
TTCTGATGTTATATATAAGTAAATTGAGGTTTGGGAATATCTAGGTAACTTAGCCGTAGT
CACATAACTGGTCATTGGAGGGGGCATGATCCAGATCCCGGGTTTTTAAACCACTAATC
TGTTTTCCTGAAAGATCTTAA
>221
TGGGAATATCTAGGTAACTTAGCCGTAGTCACATAACTGGTCATTGGAGGGGGCATGAT
CCAGATCCCGGGTTTTTAAACCACTAATCTGTTTTCCTGAAAGATCTTAATGACAGAAG
CAGCCAGGGCTGTAGGATAGAG
>222
TAGCCGTAGTCACATAACTGGTCATTGGAGGGGGCATGATCCAGATCCCGGGTTTTTAA
ACCACTAATCTGTTTTCCTGAAAGATCTTAATGACAGAAGCAGCCAGGGCTGTAGGATA
GAGGCCAACTCATTAGGCTGGC
>223
CCAGATCCCGGGTTTTTAAACCACTAATCTGTTTTCCTGAAAGATCTTAATGACAGAAG
CAGCCAGGGCTGTAGGATAGAGGCCAACTCATTAGGCTGGCATTGGCACCTTTCCAAC
TTTATCCCTACACCTCAGCTGCA
>224
TTCAAGGCCTTCTCAGCCTTCACACCATCTCCATGAAACCTTTCTTGACCACACCAGCC
CATAGAGCTCTGTCTTGCACACACTGCACTGCTCATTTGGTGTTCAGTCACATGCTGTT
TGGGGAGGTTTCTTGTTTTTTT
>225
CTCCATGAAACCTTTCTTGACCACACCAGCCCATAGAGCTCTGTCTTGCACACACTGCA
CTGCTCATTTGGTGTTCAGTCACATGCTGTTTGGGGAGGTTTCTTGTTTTTTTGTTAGTA
TTCCTGCTGTTATTTCTGAGC
>226
AAACCTTTCTTGACCACACCAGCCCATAGAGCTCTGTCTTGCACACACTGCACTGCTCA
TTTGGTGTTCAGTCACATGCTGTTTGGGGAGGTTTCTTGTTTTTTTGTTAGTATTCCTGC
TGTTATTTCTGAGCTCACTTT
>227
TGACCACACCAGCCCATAGAGCTCTGTCTTGCACACACTGCACTGCTCATTTGGTGTTC

AGTCACATGCTGTTTGGGGAGGTTTCTTGTTTTTTTGTTAGTATTCCTGCTGTTATTTCT
GAGCTCACTTTCTAGCTCTGT
>228
AGCCCATAGAGCTCTGTCTTGCACACACTGCACTGCTCATTTGGTGTTCAGTCACATGC
TGTTTGGGGAGGTTTCTTGTTTTTTTGTTAGTATTCCTGCTGTTATTTCTGAGCTCACTTT
CTAGCTCTGTGAATACAGGG
>229
GTCTTGCACACACTGCACTGCTCATTTGGTGTTCAGTCACATGCTGTTTGGGGAGGTTT
CTTGTTTTTTTGTTAGTATTCCTGCTGTTATTTCTGAGCTCACTTTCTAGCTCTGTGAATA
CAGGGTCAGCCCTTACGCCA
>230
CTGTTTGGGGAGGTTTCTTGTTTTTTTGTTAGTATTCCTGCTGTTATTTCTGAGCTCACTT
TCTAGCTCTGTGAATACAGGGTCAGCCCTTACGCCACACTGGGGTCCCGTAGATGCTCT
TTGTGCGATTAGGGAATGCT
>231
GGTTTCTTGTTTTTTTGTTAGTATTCCTGCTGTTATTTCTGAGCTCACTTTCTAGCTCTGT
GAATACAGGGTCAGCCCTTACGCCACACTGGGGTCCCGTAGATGCTCTTTGTGCGATTA
GGGAATGCTAATTAGAATAC
>232
TTCCTGCTGTTATTTCTGAGCTCACTTTCTAGCTCTGTGAATACAGGGTCAGCCCTTACG
CCACACTGGGGTCCCGTAGATGCTCTTTGTGCGATTAGGGAATGCTAATTAGAATACTAT
CTCTGGGCTATTTATGCTGC
>233
AGCTCACTTTCTAGCTCTGTGAATACAGGGTCAGCCCTTACGCCACACTGGGGTCCCGT
AGATGCTCTTTGTGCGATTAGGGAATGCTAATTAGAATACTATCTCTGGGCTATTTATGCT
GCATCAGTCTTCCTGTCAAA
>234
CGTAGATGCTCTTTGTGCGATTAGGGAATGCTAATTAGAATACTATCTCTGGGCTATTTAT
GCTGCATCAGTCTTCCTGTCAAACAAGTTACAGTTGTGCTTATCTGTATTTCCGATGAG
AATCAGGAATTTTTCTTCTG
>235
CTCTTTGTGCGATTAGGGAATGCTAATTAGAATACTATCTCTGGGCTATTTATGCTGCATC
AGTCTTCCTGTCAAACAAGTTACAGTTGTGCTTATCTGTATTTCCGATGAGAATCAGGA
ATTTTTCTTCTGTTTAATGA
>236
CGATTAGGGAATGCTAATTAGAATACTATCTCTGGGCTATTTATGCTGCATCAGTCTTCCT
GTCAAACAAGTTACAGTTGTGCTTATCTGTATTTCCGATGAGAATCAGGAATTTTTCTTC
TGTTTAATGAATTTCTGAT
>237
AGTCTTCCTGTCAAACAAGTTACAGTTGTGCTTATCTGTATTTCCGATGAGAATCAGGA
ATTTTTCTTCTGTTTAATGAATTTCTGATTGGTTTCAAAGTATTCTAACATAAGTAGCCAA
ATTGTACTATAGGCCTTTAA
>238
TACAGTTGTGCTTATCTGTATTTCCGATGAGAATCAGGAATTTTTCTTCTGTTTAATGAAT
TTCTGATTGGTTTCAAAGTATTCTAACATAAGTAGCCAAATTGTACTATAGGCCTTTAAA
TGAAAAAAATAAAAAAGTA

>239
TCCGATGAGAATCAGGAATTTTTCTTCTGTTTAATGAATTTCTGATTGGTTTCAAAGTAT
TCTAACATAAGTAGCCAAATTGTACTATAGGCCTTTAAATGAAAAAAATAAAAAAGTAG
TCCCAAGCCGGGCGCAGTGGC
>240
TCAGGAATTTTTCTTCTGTTTAATGAATTTCTGATTGGTTTCAAAGTATTCTAACATAAGT
AGCCAAATTGTACTATAGGCCTTTAAATGAAAAAAATAAAAAAGTAGTCCCAAGCCGG
GCGCAGTGGCTCACGCCTGTA
>241
TTGGTTTCAAAGTATTCTAACATAAGTAGCCAAATTGTACTATAGGCCTTTAAATGAAAA
AAATAAAAAAGTAGTCCCAAGCCGGGCGCAGTGGCTCACGCCTGTAATCCCAGCACTT
TCGGAGGCCGAGGCGGGTGGAC
>242
CATAAGTAGCCAAATTGTACTATAGGCCTTTAAATGAAAAAAATAAAAAAGTAGTCCCA
AGCCGGGCGCAGTGGCTCACGCCTGTAATCCCAGCACTTTCGGAGGCCGAGGCGGGT
GGACCACCTAAGGTCAGGAGTTCA
>243
ATTGTACTATAGGCCTTTAAATGAAAAAAATAAAAAAGTAGTCCCAAGCCGGGCGCAG
TGGCTCACGCCTGTAATCCCAGCACTTTCGGAGGCCGAGGCGGGTGGACCACCTAAGG
TCAGGAGTTCAAGACCAGCCTGGC
>244
AAAAAAGTAGTCCCAAGCCGGGCGCAGTGGCTCACGCCTGTAATCCCAGCACTTTCGG
AGGCCGAGGCGGGTGGACCACCTAAGGTCAGGAGTTCAAGACCAGCCTGGCCAACAT
GGTGAAACCCCATCTCTACTAAAAA
>245
AAGCCGGGCGCAGTGGCTCACGCCTGTAATCCCAGCACTTTCGGAGGCCGAGGCGGG
TGGACCACCTAAGGTCAGGAGTTCAAGACCAGCCTGGCCAACATGGTGAAACCCCAT
CTCTACTAAAAATACAAAAATTAGTC
>246
GCGCAGTGGCTCACGCCTGTAATCCCAGCACTTTCGGAGGCCGAGGCGGGTGGACCA
CCTAAGGTCAGGAGTTCAAGACCAGCCTGGCCAACATGGTGAAACCCCATCTCTACTA
AAAATACAAAAATTAGTCATGTGTG
>247
CAGCACTTTCGGAGGCCGAGGCGGGTGGACCACCTAAGGTCAGGAGTTCAAGACCAG
CCTGGCCAACATGGTGAAACCCCATCTCTACTAAAAATACAAAAATTAGTCATGTGTGG
TGGCACGTGCCTGTAGTCCCAGCT
>248
AAGGTCAGGAGTTCAAGACCAGCCTGGCCAACATGGTGAAACCCCATCTCTACTAAAA
ATACAAAAATTAGTCATGTGTGGTGGCACGTGCCTGTAGTCCCAGCTACTCCAGGAGG
CTGAGGCAGGAGAATTGCTTGAAC
>249
CAGGAGTTCAAGACCAGCCTGGCCAACATGGTGAAACCCCATCTCTACTAAAAATACA
AAAATTAGTCATGTGTGGTGGCACGTGCCTGTAGTCCCAGCTACTCCAGGAGGCTGAG
GCAGGAGAATTGCTTGAACCCGGG
>250
ACCAGCCTGGCCAACATGGTGAAACCCCATCTCTACTAAAAATACAAAAATTAGTCATG

TGTGGTGGCACGTGCCTGTAGTCCCAGCTACTCCAGGAGGCTGAGGCAGGAGAATTGC
TTGAACCCGGGAGTTGGAGGTTG
>251
TGTGGTGGCACGTGCCTGTAGTCCCAGCTACTCCAGGAGGCTGAGGCAGGAGAATTGC
TTGAACCCGGGAGTTGGAGGTTGCAGTGACCTGAGATTGCACCACTGCACTCCAGCCT
GGGCAACAGAGTGAGACTCTGTCT
>252
GCACGTGCCTGTAGTCCCAGCTACTCCAGGAGGCTGAGGCAGGAGAATTGCTTGAACC
CGGGAGTTGGAGGTTGCAGTGACCTGAGATTGCACCACTGCACTCCAGCCTGGGCAA
CAGAGTGAGACTCTGTCTTAAAAAA
>253
GCTTGAACCCGGGAGTTGGAGGTTGCAGTGACCTGAGATTGCACCACTGCACTCCAG
CCTGGGCAACAGAGTGAGACTCTGTCTTAAAAAAAAAAAAAAAAAAAAAATTCCAGAC
CTCTCCTTTTCACCCATAAATCCCTC
>254
GGGAGTTGGAGGTTGCAGTGACCTGAGATTGCACCACTGCACTCCAGCCTGGGCAAC
AGAGTGAGACTCTGTCTTAAAAAAAAAAAAAAAAAAAAAATTCCAGACCTCTCCTTTTC
ACCCATAAATCCCTCTGCCCAGTGA
>255
CTCTGTCTTAAAAAAAAAAAAAAAAAAAAAATTCCAGACCTCTCCTTTTCACCCATAAA
TCCCTCTGCCCAGTGACAGCCACTTTCAGCTCCTTTCCCCTATGTCTTCTGGTATTTACC
TTTGGATTTCTAAATAATAATA
>256
TCCAGACCTCTCCTTTTCACCCATAAATCCCTCTGCCCAGTGACAGCCACTTTCAGCTC
CTTTCCCCTATGTCTTCTGGTATTTACCTTTGGATTTCTAAATAATAATAATATTAATTGAC
TGTTATTACTAATCTGTAA
>257
TCTCCTTTTCACCCATAAATCCCTCTGCCCAGTGACAGCCACTTTCAGCTCCTTTCCCCT
ATGTCTTCTGGTATTTACCTTTGGATTTCTAAATAATAATAATATTAATTGACTGTTATTAC
TAATCTGTAATCCTTAGG
>258
CTATGTCTTCTGGTATTTACCTTTGGATTTCTAAATAATAATAATATTAATTGACTGTTATT
ACTAATCTGTAATCCTTAGGTAATACCTGTTATATACTAGGTACTGTTCTAAGTGCTTTAC
AGCCATTAGCTTATTTA
>259
GGTATTTACCTTTGGATTTCTAAATAATAATAATATTAATTGACTGTTATTACTAATCTGTA
ATCCTTAGGTAATACCTGTTATATACTAGGTACTGTTCTAAGTGCTTTACAGCCATTAGCT
TATTTAATCTCAACAAC
>260
AAATAATAATAATATTAATTGACTGTTATTACTAATCTGTAATCCTTAGGTAATACCTGTTA
TATACTAGGTACTGTTCTAAGTGCTTTACAGCCATTAGCTTATTTAATCTCAACAACCCCT
ATGAAGCAGGGTCTATT
>261
ATACTAGGTACTGTTCTAAGTGCTTTACAGCCATTAGCTTATTTAATCTCAACAACCCCT
ATGAAGCAGGGTCTATTATTGTGTCCTTTTACCGATGAGGACACTTAAGCACAGAAAGG
TTAAACAGTTTGTACAACCTC

>262
CTGTTCTAAGTGCTTTACAGCCATTAGCTTATTTAATCTCAACAACCCCTATGAAGCAGG
GTCTATTATTGTGTCCTTTTACCGATGAGGACACTTAAGCACAGAAAGGTTAAACAGTT
TGTACAACCTCACTATCATTA
>263
GTTCTAAGTGCTTTACAGCCATTAGCTTATTTAATCTCAACAACCCCTATGAAGCAGGGT
CTATTATTGTGTCCTTTTACCGATGAGGACACTTAAGCACAGAAAGGTTAAACAGTTTG
TACAACCTCACTATCATTAGT
>264
TCAACAACCCCTATGAAGCAGGGTCTATTATTGTGTCCTTTTACCGATGAGGACACTTA
AGCACAGAAAGGTTAAACAGTTTGTACAACCTCACTATCATTAGTAGGTGTTAAAGAA
CCTAGGTAGTCTAGCTCCAGAAT
>265
CCCTATGAAGCAGGGTCTATTATTGTGTCCTTTTACCGATGAGGACACTTAAGCACAGA
AAGGTTAAACAGTTTGTACAACCTCACTATCATTAGTAGGTGTTAAAGAACCTAGGTAG
TCTAGCTCCAGAATCGGTGCTT
>266
GTGTCCTTTTACCGATGAGGACACTTAAGCACAGAAAGGTTAAACAGTTTGTACAACC
TCACTATCATTAGTAGGTGTTAAAGAACCTAGGTAGTCTAGCTCCAGAATCGGTGCTTG
TAATCATACTCCCCTCCTGTATT
>267
CCTTTTACCGATGAGGACACTTAAGCACAGAAAGGTTAAACAGTTTGTACAACCTCAC
TATCATTAGTAGGTGTTAAAGAACCTAGGTAGTCTAGCTCCAGAATCGGTGCTTGTAAT
CATACTCCCCTCCTGTATTGTCT
>268
ACACTTAAGCACAGAAAGGTTAAACAGTTTGTACAACCTCACTATCATTAGTAGGTGTT
AAAGAACCTAGGTAGTCTAGCTCCAGAATCGGTGCTTGTAATCATACTCCCCTCCTGTA
TTGTCTCTCATTGCTATTTCTT
>269
GTTTGTACAACCTCACTATCATTAGTAGGTGTTAAAGAACCTAGGTAGTCTAGCTCCAG
AATCGGTGCTTGTAATCATACTCCCCTCCTGTATTGTCTCTCATTGCTATTTCTTCGTCTG
TCAATTTTAGGCATTATCTG
>270
CATTAGTAGGTGTTAAAGAACCTAGGTAGTCTAGCTCCAGAATCGGTGCTTGTAATCAT
ACTCCCCTCCTGTATTGTCTCTCATTGCTATTTCTTCGTCTGTCAATTTTAGGCATTATCT
GTTAAGCTTTTAGTATGGGA
>271
GTAGGTGTTAAAGAACCTAGGTAGTCTAGCTCCAGAATCGGTGCTTGTAATCATACTCC
CCTCCTGTATTGTCTCTCATTGCTATTTCTTCGTCTGTCAATTTTAGGCATTATCTGTTAA
GCTTTTAGTATGGGATATGA
>272
TAGTCTAGCTCCAGAATCGGTGCTTGTAATCATACTCCCCTCCTGTATTGTCTCTCATTG
CTATTTCTTCGTCTGTCAATTTTAGGCATTATCTGTTAAGCTTTTAGTATGGGATATGAGC
ATTTAGTCTTCTTACACCA
>273
TGTAATCATACTCCCCTCCTGTATTGTCTCTCATTGCTATTTCTTCGTCTGTCAATTTTAG

GCATTATCTGTTAAGCTTTTAGTATGGGATATGAGCATTTAGTCTTCTTACACCATCACAT
TGGCTTCCTTTCCCCTTT
>274
CCCCTCCTGTATTGTCTCTCATTGCTATTTCTTCGTCTGTCAATTTTAGGCATTATCTGTT
AAGCTTTTAGTATGGGATATGAGCATTTAGTCTTCTTACACCATCACATTGGCTTCCTTT
CCCCTTTCAGCTTCATAGT
>275
ATTGCTATTTCTTCGTCTGTCAATTTTAGGCATTATCTGTTAAGCTTTTAGTATGGGATAT
GAGCATTTAGTCTTCTTACACCATCACATTGGCTTCCTTTCCCCTTTCAGCTTCATAGTA
ATTTAAAAATAAATCAGTA
>276
TAGTATGGGATATGAGCATTTAGTCTTCTTACACCATCACATTGGCTTCCTTTCCCCTTTC
AGCTTCATAGTAATTTAAAAATAAATCAGTATTCTGTGTTTACATTATTATAACTCTGAAA
GATTCATTTACTATCAAG
>277
TTAGTCTTCTTACACCATCACATTGGCTTCCTTTCCCCTTTCAGCTTCATAGTAATTTAAA
AATAAATCAGTATTCTGTGTTTACATTATTATAACTCTGAAAGATTCATTTACTATCAAGC
CAAATAGTGTAGTGTGAT
>278
AAAAATAAATCAGTATTCTGTGTTTACATTATTATAACTCTGAAAGATTCATTTACTATCA
AGCCAAATAGTGTAGTGTGATTTAATCTATTTTCTTCTAAAGCTTTTTGTTTTTCCTGGA
GCTGACAATTGCATTATTT
>279
AAATAAATCAGTATTCTGTGTTTACATTATTATAACTCTGAAAGATTCATTTACTATCAAG
CCAAATAGTGTAGTGTGATTTAATCTATTTTCTTCTAAAGCTTTTTGTTTTTCCTGGAGCT
GACAATTGCATTATTTTT
>280
CTGAAAGATTCATTTACTATCAAGCCAAATAGTGTAGTGTGATTTAATCTATTTTCTTCTA
AAGCTTTTTGTTTTTCCTGGAGCTGACAATTGCATTATTTTTTCATTTGCTTAGTTTTCTG
GTATTAATCACTAATTCT
>281
TAATCTATTTTCTTCTAAAGCTTTTTGTTTTTCCTGGAGCTGACAATTGCATTATTTTTTC
ATTTGCTTAGTTTTCTGGTATTAATCACTAATTCTTTCCAAGCCTTTTGAGAAACTGTAA
AACCCTACAATTCTTGCTT
>282
TTTCTTCTAAAGCTTTTTGTTTTTCCTGGAGCTGACAATTGCATTATTTTTTCATTTGCTT
AGTTTTCTGGTATTAATCACTAATTCTTTCCAAGCCTTTTGAGAAACTGTAAAACCCTAC
AATTCTTGCTTGAACCCGG
>283
TGCATTATTTTTTCATTTGCTTAGTTTTCTGGTATTAATCACTAATTCTTTCCAAGCCTTTT
GAGAAACTGTAAAACCCTACAATTCTTGCTTGAACCCGGGAGGCGGAGGCTGCAGTG
AGCCGAGATCGCACCATTGCA
>284
TCTTTCCAAGCCTTTTGAGAAACTGTAAAACCCTACAATTCTTGCTTGAACCCGGGAG
GCGGAGGCTGCAGTGAGCCGAGATCGCACCATTGCACCCCAGCCTGGGCGACAGAGC
AAGACCCCATCTCAAAAAACAAACA

>285
CATTGCACCCCAGCCTGGGCGACAGAGCAAGACCCCATCTCAAAAAACAAACAAACA
AAAAACCCCTACAGTTCTGTTTTTCACATGGTCAAATGTATCAGCTGCACTATTTTTTTT
TTTTGAGACCTAAAACTAGGGAT
>286
CACCCCAGCCTGGGCGACAGAGCAAGACCCCATCTCAAAAAACAAACAAACAAAAA
ACCCCTACAGTTCTGTTTTTCACATGGTCAAATGTATCAGCTGCACTATTTTTTTTTTTG
AGACCTAAAACTAGGGATTGCCT
>287
GGCGACAGAGCAAGACCCCATCTCAAAAAACAAACAAACAAAAAACCCCTACAGTTC
TGTTTTTCACATGGTCAAATGTATCAGCTGCACTATTTTTTTTTTTTGAGACCTAAAACT
AGGGATTGCCTAAGCCTTTCCTT
>288
TATCAGCTGCACTATTTTTTTTTTTTGAGACCTAAAACTAGGGATTGCCTAAGCCTTTCC
TTTTTAAAAAGTCAGGTTTATATAGGTATAACTTGCATACAGTAAAATTAACCTTTTTA
GGTGTAAGAGTTCTGAGAGG
>289
GCTGCACTATTTTTTTTTTTTGAGACCTAAAACTAGGGATTGCCTAAGCCTTTCCTTTTT
AAAAAGTCAGGTTTATATAGGTATAACTTGCATACAGTAAAATTAACCTTTTTAGGTGT
AAGAGTTCTGAGAGGTTTGA
>290
CTAAAACTAGGGATTGCCTAAGCCTTTCCTTTTTAAAAAGTCAGGTTTATATAGGTATAA
CTTGCATACAGTAAAATTAACCTTTTTAGGTGTAAGAGTTCTGAGAGGTTTGATAGAAT
ATATCAAGGTATAGGGCCAG
>291
AGCCTTTCCTTTTTAAAAAGTCAGGTTTATATAGGTATAACTTGCATACAGTAAAATTAA
CCTTTTTAGGTGTAAGAGTTCTGAGAGGTTTGATAGAATATATCAAGGTATAGGGCCA
GCGCCGATGGCTCACACCTGT
>292
AGTCAGGTTTATATAGGTATAACTTGCATACAGTAAAATTAACCTTTTTAGGTGTAAGA
GTTCTGAGAGGTTTGATAGAATATATCAAGGTATAGGGCCAGCGCCGATGGCTCACACC
TGTAGTCCCAACAATTTGGGA
>293
AACTTGCATACAGTAAAATTAACCTTTTTAGGTGTAAGAGTTCTGAGAGGTTTGATAG
AATATATCAAGGTATAGGGCCAGCGCCGATGGCTCACACCTGTAGTCCCAACAATTTGG
GAGGCCAAGGCGGGAGGATCAC
>294
AGGTGTAAGAGTTCTGAGAGGTTTGATAGAATATATCAAGGTATAGGGCCAGCGCCGAT
GGCTCACACCTGTAGTCCCAACAATTTGGGAGGCCAAGGCGGGAGGATCACTTAGGCC
CAGGAGTTTGAGACCAGCTTGGG
>295
CGCCGATGGCTCACACCTGTAGTCCCAACAATTTGGGAGGCCAAGGCGGGAGGATCAC
TTAGGCCCAGGAGTTTGAGACCAGCTTGGGCAACATAGTGAGACCCTGTCTCTACAAA
AAAAAAAAAAAAAAAGGTATCAAG
>296
CAACAATTTGGGAGGCCAAGGCGGGAGGATCACTTAGGCCCAGGAGTTTGAGACCAG

CTTGGGCAACATAGTGAGACCCTGTCTCTACAAAAAAAAAAAAAAAAAGGTATCAA
GGTACAGGAAATTTCCACTACCCAAC
>297
GGAGGCCAAGGCGGGAGGATCACTTAGGCCCAGGAGTTTGAGACCAGCTTGGGCAAC
ATAGTGAGACCCTGTCTCTACAAAAAAAAAAAAAAAAAGGTATCAAGGTACAGGAA
ATTTCCACTACCCAACAAAGGTCACT
>298
GCCCAGGAGTTTGAGACCAGCTTGGGCAACATAGTGAGACCCTGTCTCTACAAAAAA
AAAAAAAAAAAAGGTATCAAGGTACAGGAAATTTCCACTACCCAACAAAGGTCACTC
ACCCTTTTGTAGTTAATAACTTCTCT
>299
GTTTGAGACCAGCTTGGGCAACATAGTGAGACCCTGTCTCTACAAAAAAAAAAAAAA
AAAAGGTATCAAGGTACAGGAAATTTCCACTACCCAACAAAGGTCACTCACCCTTTTG
TAGTTAATAACTTCTCTGCAACCTA
>300
GACCCTGTCTCTACAAAAAAAAAAAAAAAAAAGGTATCAAGGTACAGGAAATTTCCA
CTACCCAACAAAGGTCACTCACCCTTTTGTAGTTAATAACTTCTCTGCAACCTAGATCC
AGCCAACTACTGATAAGATGTCTG
>301
AAAAAAAAAAAAAAAAAGGTATCAAGGTACAGGAAATTTCCACTACCCAACAAAGGT
CACTCACCCTTTTGTAGTTAATAACTTCTCTGCAACCTAGATCCAGCCAACTACTGATAA
GATGTCTGTCCTTGTATTTTCCA
>302
CAAAGGTCACTCACCCTTTTGTAGTTAATAACTTCTCTGCAACCTAGATCCAGCCAACT
ACTGATAAGATGTCTGTCCTTGTATTTTCCAGAATGTACTTAAATGGAGTCATACAGTAA
GTAGGCTTTTGTGTCTGGCTT
>303
GGTCACTCACCCTTTTGTAGTTAATAACTTCTCTGCAACCTAGATCCAGCCAACTACTG
ATAAGATGTCTGTCCTTGTATTTTCCAGAATGTACTTAAATGGAGTCATACAGTAAGTAG
GCTTTTGTGTCTGGCTTCAAG
>304
TTAATAACTTCTCTGCAACCTAGATCCAGCCAACTACTGATAAGATGTCTGTCCTTGTAT
TTTCCAGAATGTACTTAAATGGAGTCATACAGTAAGTAGGCTTTTGTGTCTGGCTTCAA
GCCTTTACTTTTTATAAAGAA
>305
CCAGCCAACTACTGATAAGATGTCTGTCCTTGTATTTTCCAGAATGTACTTAAATGGAGT
CATACAGTAAGTAGGCTTTTGTGTCTGGCTTCAAGCCTTTACTTTTTATAAAGAATAACA
ATGGTAATAATTATAGCTGA
>306
ACTGATAAGATGTCTGTCCTTGTATTTTCCAGAATGTACTTAAATGGAGTCATACAGTAA
GTAGGCTTTTGTGTCTGGCTTCAAGCCTTTACTTTTTATAAAGAATAACAATGGTAATAA
TTATAGCTGACATTTATTAA
>307
ATGGAGTCATACAGTAAGTAGGCTTTTGTGTCTGGCTTCAAGCCTTTACTTTTTATAAAG
AATAACAATGGTAATAATTATAGCTGACATTTATTAAAGGCTGAGCGGATACTGTATACT
GTGCTACAGGCTTTTCGTGG

>308
CTTTACTTTTTATAAAGAATAACAATGGTAATAATTATAGCTGACATTTATTAAAGGCTGA
GCGGATACTGTATACTGTGCTACAGGCTTTTCGTGGGTTATCTCATCACTGTTTCACATA
TGTAGGTATACAGTTCTTT
>309
TTTTATAAAGAATAACAATGGTAATAATTATAGCTGACATTTATTAAAGGCTGAGCGGAT
ACTGTATACTGTGCTACAGGCTTTTCGTGGGTTATCTCATCACTGTTTCACATATGTAGG
TATACAGTTCTTTCTTTAAA
>310
AATGGTAATAATTATAGCTGACATTTATTAAAGGCTGAGCGGATACTGTATACTGTGCTA
CAGGCTTTTCGTGGGTTATCTCATCACTGTTTCACATATGTAGGTATACAGTTCTTTCTTT
AAAATATGTGACTGAAAAA
>311
GTAATAATTATAGCTGACATTTATTAAAGGCTGAGCGGATACTGTATACTGTGCTACAGG
CTTTTCGTGGGTTATCTCATCACTGTTTCACATATGTAGGTATACAGTTCTTTCTTTAAAA
TATGTGACTGAAAAATATT
>312
TGACATTTATTAAAGGCTGAGCGGATACTGTATACTGTGCTACAGGCTTTTCGTGGGTTA
TCTCATCACTGTTTCACATATGTAGGTATACAGTTCTTTCTTTAAAATATGTGACTGAAA
AATATTATTGAACATTATGC
>313
AAAGGCTGAGCGGATACTGTATACTGTGCTACAGGCTTTTCGTGGGTTATCTCATCACT
GTTTCACATATGTAGGTATACAGTTCTTTCTTTAAAATATGTGACTGAAAAATATTATTGA
ACATTATGCTAAAATTTGTT
>314
TATGTAGGTATACAGTTCTTTCTTTAAAATATGTGACTGAAAAATATTATTGAACATTATG
CTAAAATTTGTTACACTGAGAAGACTTAAGAAAAACAGAGCTCATGTTTTCTTTCAACT
GATTTCCTTTTTACATTGTA
>315
CTGAAAAATATTATTGAACATTATGCTAAAATTTGTTACACTGAGAAGACTTAAGAAAA
ACAGAGCTCATGTTTTCTTTCAACTGATTTCCTTTTTACATTGTACTTGAGTACCTGGAA
GCTGCTTGCTTCTTTCCGCAG
>316
TTTGTTACACTGAGAAGACTTAAGAAAAACAGAGCTCATGTTTTCTTTCAACTGATTTC
CTTTTTACATTGTACTTGAGTACCTGGAAGCTGCTTGCTTCTTTCCGCAGCTTAGGATAC
AAACTCCCTGACAGTTAAGTG
>317
TTTACATTGTACTTGAGTACCTGGAAGCTGCTTGCTTCTTTCCGCAGCTTAGGATACAA
ACTCCCTGACAGTTAAGTGCTTCTTTACTTCCCATCCAGTGCCTTGCACAGGGCTTGGT
ACATGGTTTGGTCCAAAATATT
>318
ATTGTACTTGAGTACCTGGAAGCTGCTTGCTTCTTTCCGCAGCTTAGGATACAAACTCC
CTGACAGTTAAGTGCTTCTTTACTTCCCATCCAGTGCCTTGCACAGGGCTTGGTACATG
GTTTGGTCCAAAATATTTGTTG
>319
CTGCTTGCTTCTTTCCGCAGCTTAGGATACAAACTCCCTGACAGTTAAGTGCTTCTTTA

CTTCCCATCCAGTGCCTTGCACAGGGCTTGGTACATGGTTTGGTCCAAAATATTTGTTG
AATAAATTTGCAGATAGCCCAC
>320
GCTTAGGATACAAACTCCCTGACAGTTAAGTGCTTCTTTACTTCCCATCCAGTGCCTTG
CACAGGGCTTGGTACATGGTTTGGTCCAAAATATTTGTTGAATAAATTTGCAGATAGCC
CACGTGTGTAGGAGAGGGAGAG
>321
TTAAGTGCTTCTTTACTTCCCATCCAGTGCCTTGCACAGGGCTTGGTACATGGTTTGGT
CCAAAATATTTGTTGAATAAATTTGCAGATAGCCCACGTGTGTAGGAGAGGGAGAGAA
GGTTCTTGAAAAATGGAGATTGG
>322
GTGCCTTGCACAGGGCTTGGTACATGGTTTGGTCCAAAATATTTGTTGAATAAATTTGC
AGATAGCCCACGTGTGTAGGAGAGGGAGAGAAGGTTCTTGAAAAATGGAGATTGGAG
GAATCTAGGATGGGGAGGGAGATA
>323
GCCTTGCACAGGGCTTGGTACATGGTTTGGTCCAAAATATTTGTTGAATAAATTTGCAG
ATAGCCCACGTGTGTAGGAGAGGGAGAGAAGGTTCTTGAAAAATGGAGATTGGAGGA
ATCTAGGATGGGGAGGGAGATATG
>324
GAAAAATGGAGATTGGAGGAATCTAGGATGGGGAGGGAGATATGGGAAGGGCCCAGA
CAGAGGCAGGAGGGTTCCAACTTGTTCCTCTTCCCTGCAGCCCAGTGTTTCCCGGGCT
CTTTGAGTTCTGCTCGCGTTACACA
>325
GATTGGAGGAATCTAGGATGGGGAGGGAGATATGGGAAGGGCCCAGACAGAGGCAGG
AGGGTTCCAACTTGTTCCTCTTCCCTGCAGCCCAGTGTTTCCCGGGCTCTTTGAGTTCT
GCTCGCGTTACACAGGCGCATCTC
>326
GGAGGGAGATATGGGAAGGGCCCAGACAGAGGCAGGAGGGTTCCAACTTGTTCCTCT
TCCCTGCAGCCCAGTGTTTCCCGGGCTCTTTGAGTTCTGCTCGCGTTACACAGGCGCAT
CTCTGCAAGGAGCAACCCAGCTGA
>327
CCCAGACAGAGGCAGGAGGGTTCCAACTTGTTCCTCTTCCCTGCAGCCCAGTGTTTCC
CGGGCTCTTTGAGTTCTGCTCGCGTTACACAGGCGCATCTCTGCAAGGAGCAACCCAG
CTGAACAACAAGGTGACATAGTCC
>328
GCAGGAGGGTTCCAACTTGTTCCTCTTCCCTGCAGCCCAGTGTTTCCCGGGCTCTTTG
AGTTCTGCTCGCGTTACACAGGCGCATCTCTGCAAGGAGCAACCCAGCTGAACAACA
AGGTGACATAGTCCCGAGTCCTGTT
>329
TTGAGTTCTGCTCGCGTTACACAGGCGCATCTCTGCAAGGAGCAACCCAGCTGAACAA
CAAGGTGACATAGTCCCGAGTCCTGTTCTTCCTTTCCTCTGGATCCCTGGACTCGGGAT
TTAACCCTGATCCTGGGCTCCCA
>330
CTGCTCGCGTTACACAGGCGCATCTCTGCAAGGAGCAACCCAGCTGAACAACAAGGT
GACATAGTCCCGAGTCCTGTTCTTCCTTTCCTCTGGATCCCTGGACTCGGGATTTAACC
CTGATCCTGGGCTCCCAGCTTGAG

>331
CGCGTTACACAGGCGCATCTCTGCAAGGAGCAACCCAGCTGAACAACAAGGTGACAT
AGTCCCGAGTCCTGTTCTTCCTTTCCTCTGGATCCCTGGACTCGGGATTTAACCCTGAT
CCTGGGCTCCCAGCTTGAGGGGTG
>332
CCTGTTCTTCCTTTCCTCTGGATCCCTGGACTCGGGATTTAACCCTGATCCTGGGCTCCC
AGCTTGAGGGGTGGGCAGGAAGGACTGTGACTTAGGTGTTTGTCTTTCAGATCTGTGA
TATTGCCATTAACTGGGCTGGT
>333
TCTGGATCCCTGGACTCGGGATTTAACCCTGATCCTGGGCTCCCAGCTTGAGGGGTGG
GCAGGAAGGACTGTGACTTAGGTGTTTGTCTTTCAGATCTGTGATATTGCCATTAACTG
GGCTGGTGGTCTGCACCATGCCA
>334
CTGATCCTGGGCTCCCAGCTTGAGGGGTGGGCAGGAAGGACTGTGACTTAGGTGTTTG
TCTTTCAGATCTGTGATATTGCCATTAACTGGGCTGGTGGTCTGCACCATGCCAAGAAG
TTTGAGGTGAGTGAGGAGGTGAT
>335
GGGTGGGCAGGAAGGACTGTGACTTAGGTGTTTGTCTTTCAGATCTGTGATATTGCCAT
TAACTGGGCTGGTGGTCTGCACCATGCCAAGAAGTTTGAGGTGAGTGAGGAGGTGAT
GGGAAAGACAGTGGCCATCCTAGG
>336
TGGGCAGGAAGGACTGTGACTTAGGTGTTTGTCTTTCAGATCTGTGATATTGCCATTAA
CTGGGCTGGTGGTCTGCACCATGCCAAGAAGTTTGAGGTGAGTGAGGAGGTGATGGG
AAAGACAGTGGCCATCCTAGGGTA
>337
GTCTTTCAGATCTGTGATATTGCCATTAACTGGGCTGGTGGTCTGCACCATGCCAAGAA
GTTTGAGGTGAGTGAGGAGGTGATGGGAAAGACAGTGGCCATCCTAGGGTAGGTGTTT
AGGATGATGGTGGGGGGCAGCTG
>338
GATCTGTGATATTGCCATTAACTGGGCTGGTGGTCTGCACCATGCCAAGAAGTTTGAGG
TGAGTGAGGAGGTGATGGGAAAGACAGTGGCCATCCTAGGGTAGGTGTTTAGGATGAT
GGTGGGGGGCAGCTGGGAGGGGA
>339
CATTAACTGGGCTGGTGGTCTGCACCATGCCAAGAAGTTTGAGGTGAGTGAGGAGGTG
ATGGGAAAGACAGTGGCCATCCTAGGGTAGGTGTTTAGGATGATGGTGGGGGGCAGCT
GGGAGGGGAATTGCTCTTCTCTTT
>340
GTGGTCTGCACCATGCCAAGAAGTTTGAGGTGAGTGAGGAGGTGATGGGAAAGACAG
TGGCCATCCTAGGGTAGGTGTTTAGGATGATGGTGGGGGGCAGCTGGGAGGGGAATTG
CTCTTCTCTTTATGAGACCACTGTC
>341
AGGGTAGGTGTTTAGGATGATGGTGGGGGGCAGCTGGGAGGGGAATTGCTCTTCTCTT
TATGAGACCACTGTCTTGCCATAGGCCTCTGGCTTCTGCTATGTCAACGACATTGTGATT
GGCATCCTGGAGCTGCTCAAGT
>342
CAGCTGGGAGGGGAATTGCTCTTCTCTTTATGAGACCACTGTCTTGCCATAGGCCTCTG

GCTTCTGCTATGTCAACGACATTGTGATTGGCATCCTGGAGCTGCTCAAGTAAGTAGCC
CAGGAGGAGATGGGGGCAGTTA
>343
GAATTGCTCTTCTCTTTATGAGACCACTGTCTTGCCATAGGCCTCTGGCTTCTGCTATGT
CAACGACATTGTGATTGGCATCCTGGAGCTGCTCAAGTAAGTAGCCCAGGAGGAGATG
GGGGCAGTTACGGGATTCTTGG
>344
CTGTCTTGCCATAGGCCTCTGGCTTCTGCTATGTCAACGACATTGTGATTGGCATCCTGG
AGCTGCTCAAGTAAGTAGCCCAGGAGGAGATGGGGGCAGTTACGGGATTCTTGGCTGA
GGAGGTCTCTCAGTCTCAACCC
>345
CTATGTCAACGACATTGTGATTGGCATCCTGGAGCTGCTCAAGTAAGTAGCCCAGGAG
GAGATGGGGGCAGTTACGGGATTCTTGGCTGAGGAGGTCTCTCAGTCTCAACCCTGAC
CTTGACCTCCCTCCTCCCTGATTA
>346
CATCCTGGAGCTGCTCAAGTAAGTAGCCCAGGAGGAGATGGGGGCAGTTACGGGATTC
TTGGCTGAGGAGGTCTCTCAGTCTCAACCCTGACCTTGACCTCCCTCCTCCCTGATTAG
CTTCTCCCAAGTTCTTGATGCTT
>347
CTTGGCTGAGGAGGTCTCTCAGTCTCAACCCTGACCTTGACCTCCCTCCTCCCTGATTA
GCTTCTCCCAAGTTCTTGATGCTTTATTCAGGCTGCCTCTTCCACTGCTGCAGATTCCCT
ACTGCCTGCTCACATTTCAGC
>348
TGCCTCTTCCACTGCTGCAGATTCCCTACTGCCTGCTCACATTTCAGCCTTCTCTGCATA
TCCCTCTCCTGTCCCTGCCTCTAGCCTCTAGGCTGGAGCCCCCAGTTGCCTTTAGGGAA
CTGGTGACATTAAGTTTCAGG
>349
GCTCACATTTCAGCCTTCTCTGCATATCCCTCTCCTGTCCCTGCCTCTAGCCTCTAGGCT
GGAGCCCCCAGTTGCCTTTAGGGAACTGGTGACATTAAGTTTCAGGTGACCAGGGCAG
AAGCAGACTCTTCACAATTCGA
>350
TCTGCATATCCCTCTCCTGTCCCTGCCTCTAGCCTCTAGGCTGGAGCCCCCAGTTGCCTT
TAGGGAACTGGTGACATTAAGTTTCAGGTGACCAGGGCAGAAGCAGACTCTTCACAAT
TCGAGACCCGGTGTTTGAGAGG
>351
CCTCTCCTGTCCCTGCCTCTAGCCTCTAGGCTGGAGCCCCCAGTTGCCTTTAGGGAACT
GGTGACATTAAGTTTCAGGTGACCAGGGCAGAAGCAGACTCTTCACAATTCGAGACCC
GGTGTTTGAGAGGAGTGGGTTGG
>352
TGTCCCTGCCTCTAGCCTCTAGGCTGGAGCCCCCAGTTGCCTTTAGGGAACTGGTGACA
TTAAGTTTCAGGTGACCAGGGCAGAAGCAGACTCTTCACAATTCGAGACCCGGTGTTT
GAGAGGAGTGGGTTGGGGGGTTC
>353
TTTAGGGAACTGGTGACATTAAGTTTCAGGTGACCAGGGCAGAAGCAGACTCTTCACA
ATTCGAGACCCGGTGTTTGAGAGGAGTGGGTTGGGGGGTTCACTTCTAGCTCAGACCC
TGGCTTTGGCTTCCCTCTAGGTAC

>354
TAGGGAACTGGTGACATTAAGTTTCAGGTGACCAGGGCAGAAGCAGACTCTTCACAAT
TCGAGACCCGGTGTTTGAGAGGAGTGGGTTGGGGGGTTCACTTCTAGCTCAGACCCTG
GCTTTGGCTTCCCTCTAGGTACCA
>355
TGACATTAAGTTTCAGGTGACCAGGGCAGAAGCAGACTCTTCACAATTCGAGACCCGG
TGTTTGAGAGGAGTGGGTTGGGGGGTTCACTTCTAGCTCAGACCCTGGCTTTGGCTTC
CCTCTAGGTACCACCCTCGGGTGC
>356
TCAGGTGACCAGGGCAGAAGCAGACTCTTCACAATTCGAGACCCGGTGTTTGAGAGG
AGTGGGTTGGGGGGTTCACTTCTAGCTCAGACCCTGGCTTTGGCTTCCCTCTAGGTACC
ACCCTCGGGTGCTCTACATTGACA
>357
CCCGGTGTTTGAGAGGAGTGGGTTGGGGGGTTCACTTCTAGCTCAGACCCTGGCTTTG
GCTTCCCTCTAGGTACCACCCTCGGGTGCTCTACATTGACATTGACATCCACCATGGTG
ACGGGGTTCAAGAAGCTTTCTAC
>358
AGGAGTGGGTTGGGGGGTTCACTTCTAGCTCAGACCCTGGCTTTGGCTTCCCTCTAGG
TACCACCCTCGGGTGCTCTACATTGACATTGACATCCACCATGGTGACGGGGTTCAAGA
AGCTTTCTACCTCACTGACCGGG
>359
AGACCCTGGCTTTGGCTTCCCTCTAGGTACCACCCTCGGGTGCTCTACATTGACATTGA
CATCCACCATGGTGACGGGGTTCAAGAAGCTTTCTACCTCACTGACCGGGTCATGACG
GTGTCCTTCCACAAATACGGAAA
>360
GGTGCTCTACATTGACATTGACATCCACCATGGTGACGGGGTTCAAGAAGCTTTCTACC
TCACTGACCGGGTCATGACGGTGTCCTTCCACAAATACGGAAATTACTTCTTCCCTGGC
ACAGGTATGGGAATAGGGATTA
>361
CATTGACATTGACATCCACCATGGTGACGGGGTTCAAGAAGCTTTCTACCTCACTGACC
GGGTCATGACGGTGTCCTTCCACAAATACGGAAATTACTTCTTCCCTGGCACAGGTATG
GGAATAGGGATTACTTTCCCCT
>362
CTTCTTCCCTGGCACAGGTATGGGAATAGGGATTACTTTCCCCTTTGCACCCTTCAGCT
CTTTACTATGAGTGCCTCCTTCTCAGCCTTCTAGTCTCATCTCAGGTGTCCCCTCCTCAG
AGAAGCCTTTCCTTACCACTC
>363
GGAATAGGGATTACTTTCCCCTTTGCACCCTTCAGCTCTTTACTATGAGTGCCTCCTTCT
CAGCCTTCTAGTCTCATCTCAGGTGTCCCCTCCTCAGAGAAGCCTTTCCTTACCACTCC
AGTTATCATAATCACCTCCTT
>364
GCCTCCTTCTCAGCCTTCTAGTCTCATCTCAGGTGTCCCCTCCTCAGAGAAGCCTTTCC
TTACCACTCCAGTTATCATAATCACCTCCTTCCCCATTCCTCTGTTACATCAGCCTCATTG
TTTCCCTCAGAACACTCACT
>365
GTGTCCCCTCCTCAGAGAAGCCTTTCCTTACCACTCCAGTTATCATAATCACCTCCTTCC

CCATTCCTCTGTTACATCAGCCTCATTGTTTCCCTCAGAACACTCACTGCTATTTGTGTT
TATTGTGGTTTTTTGTTTTG
>366
AAGCCTTTCCTTACCACTCCAGTTATCATAATCACCTCCTTCCCCATTCCTCTGTTACATC
AGCCTCATTGTTTCCCTCAGAACACTCACTGCTATTTGTGTTTATTGTGGTTTTTTGTTT
TGTTTTGTTTACTGCCTCC
>367
TAATCACCTCCTTCCCCATTCCTCTGTTACATCAGCCTCATTGTTTCCCTCAGAACACTC
ACTGCTATTTGTGTTTATTGTGGTTTTTTGTTTTGTTTTGTTTACTGCCTCCCTCCTCCTC
ATGGCTATTCCCTAAGAAT
>368
TCCTCTGTTACATCAGCCTCATTGTTTCCCTCAGAACACTCACTGCTATTTGTGTTTATT
GTGGTTTTTTGTTTTGTTTTGTTTACTGCCTCCCTCCTCCTCATGGCTATTCCCTAAGAAT
ATAAGCGTGGTGGGATATC
>369
TGTTACATCAGCCTCATTGTTTCCCTCAGAACACTCACTGCTATTTGTGTTTATTGTGGT
TTTTTGTTTTGTTTTGTTTACTGCCTCCCTCCTCCTCATGGCTATTCCCTAAGAATATAAG
CGTGGTGGGATATCGTAAA
>370
CATCAGCCTCATTGTTTCCCTCAGAACACTCACTGCTATTTGTGTTTATTGTGGTTTTTT
GTTTTGTTTTGTTTACTGCCTCCCTCCTCCTCATGGCTATTCCCTAAGAATATAAGCGTG
GTGGGATATCGTAAATTTCA
>371
GTGGTTTTTTGTTTTGTTTTGTTTACTGCCTCCCTCCTCCTCATGGCTATTCCCTAAGAAT
ATAAGCGTGGTGGGATATCGTAAATTTCATTAGTTCCATTCGTTGCTGCATCCCTACTGC
CTAGTACACAGTTGGCTCA
>372
GTTTTGTTTTGTTTACTGCCTCCCTCCTCCTCATGGCTATTCCCTAAGAATATAAGCGTG
GTGGGATATCGTAAATTTCATTAGTTCCATTCGTTGCTGCATCCCTACTGCCTAGTACAC
AGTTGGCTCAGAGTGGATCC
>373
TACTGCCTCCCTCCTCCTCATGGCTATTCCCTAAGAATATAAGCGTGGTGGGATATCGTA
AATTTCATTAGTTCCATTCGTTGCTGCATCCCTACTGCCTAGTACACAGTTGGCTCAGAG
TGGATCCCCCAAAACTATTT
>374
TAAGAATATAAGCGTGGTGGGATATCGTAAATTTCATTAGTTCCATTCGTTGCTGCATCC
CTACTGCCTAGTACACAGTTGGCTCAGAGTGGATCCCCCAAAACTATTTGCCCAATGAA
TGCAAGCTGCAAGAGATTTAG
>375
ATAAGCGTGGTGGGATATCGTAAATTTCATTAGTTCCATTCGTTGCTGCATCCCTACTGC
CTAGTACACAGTTGGCTCAGAGTGGATCCCCCAAAACTATTTGCCCAATGAATGCAAG
CTGCAAGAGATTTAGTGGCTCT
>376
GGGATATCGTAAATTTCATTAGTTCCATTCGTTGCTGCATCCCTACTGCCTAGTACACAG
TTGGCTCAGAGTGGATCCCCCAAAACTATTTGCCCAATGAATGCAAGCTGCAAGAGAT
TTAGTGGCTCTAGGGAGTTTAT

>377
AGTACACAGTTGGCTCAGAGTGGATCCCCCAAAACTATTTGCCCAATGAATGCAAGCT
GCAAGAGATTTAGTGGCTCTAGGGAGTTTATGGTGAAGCTACATAGATGGAATGAGAG
GCTCCCAGAGTGCAGATTGGGGCT
>378
CTCAGAGTGGATCCCCCAAAACTATTTGCCCAATGAATGCAAGCTGCAAGAGATTTAG
TGGCTCTAGGGAGTTTATGGTGAAGCTACATAGATGGAATGAGAGGCTCCCAGAGTGC
AGATTGGGGCTGAAACAGCTGGGT
>379
TGGATCCCCCAAAACTATTTGCCCAATGAATGCAAGCTGCAAGAGATTTAGTGGCTCTA
GGGAGTTTATGGTGAAGCTACATAGATGGAATGAGAGGCTCCCAGAGTGCAGATTGGG
GCTGAAACAGCTGGGTTGTGGGT
>380
GCTGCAAGAGATTTAGTGGCTCTAGGGAGTTTATGGTGAAGCTACATAGATGGAATGAG
AGGCTCCCAGAGTGCAGATTGGGGCTGAAACAGCTGGGTTGTGGGTGCTTCCCATGTG
TGGCTACCACCACCCACTTCCCT
>381
GAGTTTATGGTGAAGCTACATAGATGGAATGAGAGGCTCCCAGAGTGCAGATTGGGGC
TGAAACAGCTGGGTTGTGGGTGCTTCCCATGTGTGGCTACCACCACCCACTTCCCTAC
AGGTGACATGTATGAAGTCGGGGC
>382
TTTATGGTGAAGCTACATAGATGGAATGAGAGGCTCCCAGAGTGCAGATTGGGGCTGA
AACAGCTGGGTTGTGGGTGCTTCCCATGTGTGGCTACCACCACCCACTTCCCTACAGG
TGACATGTATGAAGTCGGGGCAGA
>383
AGATTGGGGCTGAAACAGCTGGGTTGTGGGTGCTTCCCATGTGTGGCTACCACCACCC
ACTTCCCTACAGGTGACATGTATGAAGTCGGGGCAGAGAGTGGCCGCTACTACTGTCT
GAACGTGCCCCTGCGGGATGGCAT
>384
GGCTGAAACAGCTGGGTTGTGGGTGCTTCCCATGTGTGGCTACCACCACCCACTTCCC
TACAGGTGACATGTATGAAGTCGGGGCAGAGAGTGGCCGCTACTACTGTCTGAACGTG
CCCCTGCGGGATGGCATTGATGAC
>385
GGGTGCTTCCCATGTGTGGCTACCACCACCCACTTCCCTACAGGTGACATGTATGAAGT
CGGGGCAGAGAGTGGCCGCTACTACTGTCTGAACGTGCCCCTGCGGGATGGCATTGAT
GACCAGAGTAAGTACCGAAGTCT
>386
CACCACCCACTTCCCTACAGGTGACATGTATGAAGTCGGGGCAGAGAGTGGCCGCTAC
TACTGTCTGAACGTGCCCCTGCGGGATGGCATTGATGACCAGAGTAAGTACCGAAGTC
TTCCCTCTGTTCCCTAGGCCCCTT
>387
GAGAGTGGCCGCTACTACTGTCTGAACGTGCCCCTGCGGGATGGCATTGATGACCAGA
GTAAGTACCGAAGTCTTCCCTCTGTTCCCTAGGCCCCTTCTTTTTCTCCTTGAAATAACC
TCTTCTGCCTGGTATTGTCTGC
>388
TAAGTACCGAAGTCTTCCCTCTGTTCCCTAGGCCCCTTCTTTTTCTCCTTGAAATAACCT

CTTCTGCCTGGTATTGTCTGCTGTTGGGCCAGTTGTCCTTAAGGATGAATGGCCTTCAG
AGGGACCATGAATCTCTGGAA
>389
ACCGAAGTCTTCCCTCTGTTCCCTAGGCCCCTTCTTTTTCTCCTTGAAATAACCTCTTCT
GCCTGGTATTGTCTGCTGTTGGGCCAGTTGTCCTTAAGGATGAATGGCCTTCAGAGGGA
CCATGAATCTCTGGAAATTGT
>390
TGTTCCCTAGGCCCCTTCTTTTTCTCCTTGAAATAACCTCTTCTGCCTGGTATTGTCTGC
TGTTGGGCCAGTTGTCCTTAAGGATGAATGGCCTTCAGAGGGACCATGAATCTCTGGA
AATTGTATGCAACATATCATGT
>391
TCCCTAGGCCCCTTCTTTTTCTCCTTGAAATAACCTCTTCTGCCTGGTATTGTCTGCTGT
TGGGCCAGTTGTCCTTAAGGATGAATGGCCTTCAGAGGGACCATGAATCTCTGGAAAT
TGTATGCAACATATCATGTGTA
>392
CAGTTGTCCTTAAGGATGAATGGCCTTCAGAGGGACCATGAATCTCTGGAAATTGTATG
CAACATATCATGTGTATGTGCATTTTTCTGGGAAAAGTCATTGGCTTTCATCAGTCACTC
ATAGCAGTCTGGGATCCCTTC
>393
GTTGTCCTTAAGGATGAATGGCCTTCAGAGGGACCATGAATCTCTGGAAATTGTATGCA
ACATATCATGTGTATGTGCATTTTTCTGGGAAAAGTCATTGGCTTTCATCAGTCACTCAT
AGCAGTCTGGGATCCCTTCCA
>394
TTCAGAGGGACCATGAATCTCTGGAAATTGTATGCAACATATCATGTGTATGTGCATTTT
TCTGGGAAAAGTCATTGGCTTTCATCAGTCACTCATAGCAGTCTGGGATCCCTTCCAGA
AGTTTCAGACCAGTGATTTGA
>395
TATGCAACATATCATGTGTATGTGCATTTTTCTGGGAAAAGTCATTGGCTTTCATCAGTC
ACTCATAGCAGTCTGGGATCCCTTCCAGAAGTTTCAGACCAGTGATTTGATTCTTTTGG
GTGGCCCCTTTCCTGGGCTAC
>396
TGTGCATTTTTCTGGGAAAAGTCATTGGCTTTCATCAGTCACTCATAGCAGTCTGGGAT
CCCTTCCAGAAGTTTCAGACCAGTGATTTGATTCTTTTGGGTGGCCCCTTTCCTGGGCT
ACATGCTCGCTCAACCCCCTTT
>397
CCAGTGATTTGATTCTTTTGGGTGGCCCCTTTCCTGGGCTACATGCTCGCTCAACCCCC
TTTTTCCTTTTGTTCCCTGTCTCCCCATCCCCTTGGGGTGCCAGGTGCCCATCTTTGGCC
CTCTCCCAGGTTACAAGCACC
>398
TTTGATTCTTTTGGGTGGCCCCTTTCCTGGGCTACATGCTCGCTCAACCCCCTTTTTCCT
TTTGTTCCCTGTCTCCCCATCCCCTTGGGGTGCCAGGTGCCCATCTTTGGCCCTCTCCC
AGGTTACAAGCACCTTTTCCA
>399
CCCTTTCCTGGGCTACATGCTCGCTCAACCCCCTTTTTCCTTTTGTTCCCTGTCTCCCCA
TCCCCTTGGGGTGCCAGGTGCCCATCTTTGGCCCTCTCCCAGGTTACAAGCACCTTTTC
CAGCCGGTTATCAACCAGGTA

>400
CTGGGCTACATGCTCGCTCAACCCCCTTTTTCCTTTTGTTCCCTGTCTCCCCATCCCCTT
GGGGTGCCAGGTGCCCATCTTTGGCCCTCTCCCAGGTTACAAGCACCTTTTCCAGCCG
GTTATCAACCAGGTAGTGGACT
>401
CTTTTTCCTTTTGTTCCCTGTCTCCCCATCCCCTTGGGGTGCCAGGTGCCCATCTTTGGC
CCTCTCCCAGGTTACAAGCACCTTTTCCAGCCGGTTATCAACCAGGTAGTGGACTTCTA
CCAACCCACGTGCATTGTGCT
>402
CCCCATCCCCTTGGGGTGCCAGGTGCCCATCTTTGGCCCTCTCCCAGGTTACAAGCACC
TTTTCCAGCCGGTTATCAACCAGGTAGTGGACTTCTACCAACCCACGTGCATTGTGCTC
CAGGTAATACTGTCAGTTCCTC
>403
GGGGTGCCAGGTGCCCATCTTTGGCCCTCTCCCAGGTTACAAGCACCTTTTCCAGCCG
GTTATCAACCAGGTAGTGGACTTCTACCAACCCACGTGCATTGTGCTCCAGGTAATACT
GTCAGTTCCTCCTCAGGAGTGTT
>404
GCCCTCTCCCAGGTTACAAGCACCTTTTCCAGCCGGTTATCAACCAGGTAGTGGACTTC
TACCAACCCACGTGCATTGTGCTCCAGGTAATACTGTCAGTTCCTCCTCAGGAGTGTTC
TGCCTGGGCTTGGGGAAAGAGA
>405
TTTTCCAGCCGGTTATCAACCAGGTAGTGGACTTCTACCAACCCACGTGCATTGTGCTC
CAGGTAATACTGTCAGTTCCTCCTCAGGAGTGTTCTGCCTGGGCTTGGGGAAAGAGAG
CAAGGGAAGAGGAGGTTCTTTAG
>406
CCAGCCGGTTATCAACCAGGTAGTGGACTTCTACCAACCCACGTGCATTGTGCTCCAG
GTAATACTGTCAGTTCCTCCTCAGGAGTGTTCTGCCTGGGCTTGGGGAAAGAGAGCAA
GGGAAGAGGAGGTTCTTTAGGTTG
>407
ACCAGGTAGTGGACTTCTACCAACCCACGTGCATTGTGCTCCAGGTAATACTGTCAGTT
CCTCCTCAGGAGTGTTCTGCCTGGGCTTGGGGAAAGAGAGCAAGGGAAGAGGAGGTT
CTTTAGGTTGGTGGTGGAAGGGGT
>408
GGTAATACTGTCAGTTCCTCCTCAGGAGTGTTCTGCCTGGGCTTGGGGAAAGAGAGCA
AGGGAAGAGGAGGTTCTTTAGGTTGGTGGTGGAAGGGGTATCTGTGATGTTCTGATCA
GTTCTGATCACTGCACTTTCCTGG
>409
GTCAGTTCCTCCTCAGGAGTGTTCTGCCTGGGCTTGGGGAAAGAGAGCAAGGGAAGA
GGAGGTTCTTTAGGTTGGTGGTGGAAGGGGTATCTGTGATGTTCTGATCAGTTCTGATC
ACTGCACTTTCCTGGGGTTTTGCT
>410
GAGTGTTCTGCCTGGGCTTGGGGAAAGAGAGCAAGGGAAGAGGAGGTTCTTTAGGTT
GGTGGTGGAAGGGGTATCTGTGATGTTCTGATCAGTTCTGATCACTGCACTTTCCTGGG
GTTTTGCTTTTTGCAGTGTGGAGC
>411
TTCTTTAGGTTGGTGGTGGAAGGGGTATCTGTGATGTTCTGATCAGTTCTGATCACTGC

ACTTTCCTGGGGTTTTGCTTTTTGCAGTGTGGAGCTGACTCTCTGGGCTGTGATCGATT
GGGCTGCTTTAACCTCAGCATC
>412
GTGGAAGGGGTATCTGTGATGTTCTGATCAGTTCTGATCACTGCACTTTCCTGGGGTTT
TGCTTTTTGCAGTGTGGAGCTGACTCTCTGGGCTGTGATCGATTGGGCTGCTTTAACCT
CAGCATCCGAGGGCATGGGTGA
>413
CTGATCAGTTCTGATCACTGCACTTTCCTGGGGTTTTGCTTTTTGCAGTGTGGAGCTGA
CTCTCTGGGCTGTGATCGATTGGGCTGCTTTAACCTCAGCATCCGAGGGCATGGGTGAG
ACTTGCTCTCCTTCTTCTCCCC
>414
TGCAGTGTGGAGCTGACTCTCTGGGCTGTGATCGATTGGGCTGCTTTAACCTCAGCATC
CGAGGGCATGGGTGAGACTTGCTCTCCTTCTTCTCCCCAAGGTTTTTAAGTGGCCTTGG
ACTTCAGTACGTGGGTGGGGGT
>415
ATCGATTGGGCTGCTTTAACCTCAGCATCCGAGGGCATGGGTGAGACTTGCTCTCCTTC
TTCTCCCCAAGGTTTTTAAGTGGCCTTGGACTTCAGTACGTGGGTGGGGGTAGTTGGG
GAATGGGCCTCACCTCTTCTTGG
>416
GGCTGCTTTAACCTCAGCATCCGAGGGCATGGGTGAGACTTGCTCTCCTTCTTCTCCCC
AAGGTTTTTAAGTGGCCTTGGACTTCAGTACGTGGGTGGGGGTAGTTGGGGAATGGGC
CTCACCTCTTCTTGGAAATCAAA
>417
GCATCCGAGGGCATGGGTGAGACTTGCTCTCCTTCTTCTCCCCAAGGTTTTTAAGTGGC
CTTGGACTTCAGTACGTGGGTGGGGGTAGTTGGGGAATGGGCCTCACCTCTTCTTGGA
AATCAAAAATAGCTACAAATTGT
>418
CAGTACGTGGGTGGGGGTAGTTGGGGAATGGGCCTCACCTCTTCTTGGAAATCAAAAA
TAGCTACAAATTGTACTTGAACTAGAAATGTCATCTTGAACAGGGTTCCCTAGATGGTC
ATTCATTCACGATATATTTTTAT
>419
TAGTTGGGGAATGGGCCTCACCTCTTCTTGGAAATCAAAAATAGCTACAAATTGTACTT
GAACTAGAAATGTCATCTTGAACAGGGTTCCCTAGATGGTCATTCATTCACGATATATTT
TTATTAAGCACCTGTTATGTT
>420
CCTCACCTCTTCTTGGAAATCAAAAATAGCTACAAATTGTACTTGAACTAGAAATGTCA
TCTTGAACAGGGTTCCCTAGATGGTCATTCATTCACGATATATTTTTATTAAGCACCTGTT
ATGTTCCAGGCATCATACCA
>421
TTGGAAATCAAAAATAGCTACAAATTGTACTTGAACTAGAAATGTCATCTTGAACAGGG
TTCCCTAGATGGTCATTCATTCACGATATATTTTTATTAAGCACCTGTTATGTTCCAGGCA
TCATACCACATCATGGGGAT
>422
CTAGAAATGTCATCTTGAACAGGGTTCCCTAGATGGTCATTCATTCACGATATATTTTTAT
TAAGCACCTGTTATGTTCCAGGCATCATACCACATCATGGGGATGGAATAAGGAGTCTC
CCATGGAGTTTACAGTTTAG

>423
AATGTCATCTTGAACAGGGTTCCCTAGATGGTCATTCATTCACGATATATTTTTATTAAGC
ACCTGTTATGTTCCAGGCATCATACCACATCATGGGGATGGAATAAGGAGTCTCCCATG
GAGTTTACAGTTTAGGAAAC
>424
CACGATATATTTTTATTAAGCACCTGTTATGTTCCAGGCATCATACCACATCATGGGGATG
GAATAAGGAGTCTCCCATGGAGTTTACAGTTTAGGAAACAGACAAAAAATAAATAATC
AAATGAAATAAATAGATTCTT
>425
GCACCTGTTATGTTCCAGGCATCATACCACATCATGGGGATGGAATAAGGAGTCTCCCA
TGGAGTTTACAGTTTAGGAAACAGACAAAAAATAAATAATCAAATGAAATAAATAGATT
CTTATAAATTCTGTGAAAGAAA
>426
GGAGTTTACAGTTTAGGAAACAGACAAAAAATAAATAATCAAATGAAATAAATAGATTC
TTATAAATTCTGTGAAAGAAAAAAAGAAAGCTGAGACACAGGATACAATGGGGGACCT
ACTTGAGATAGCATGGTTTCGGA
>427
TAAATTCTGTGAAAGAAAAAAAGAAAGCTGAGACACAGGATACAATGGGGGACCTAC
TTGAGATAGCATGGTTTCGGAAGTTTTTCTGAGGAAGTAAAATTTGAGTTGAGACCTG
GAGACTAAGAAGGAGTGAGTCATAA
>428
TGAAAGAAAAAAAGAAAGCTGAGACACAGGATACAATGGGGGACCTACTTGAGATAG
CATGGTTTCGGAAGTTTTTCTGAGGAAGTAAAATTTGAGTTGAGACCTGGAGACTAAG
AAGGAGTGAGTCATAAGAGGAGCAG
>429
AAAGCTGAGACACAGGATACAATGGGGGACCTACTTGAGATAGCATGGTTTCGGAAGT
TTTTCTGAGGAAGTAAAATTTGAGTTGAGACCTGGAGACTAAGAAGGAGTGAGTCATA
AGAGGAGCAGAGAGAAGCCTGACC
>430
GAGATAGCATGGTTTCGGAAGTTTTTCTGAGGAAGTAAAATTTGAGTTGAGACCTGGA
GACTAAGAAGGAGTGAGTCATAAGAGGAGCAGAGAGAAGCCTGACCCAAGCATGGA
CAAAGGCCCACAAAAGGCTCTGAGGG
>431
TAGCATGGTTTCGGAAGTTTTTCTGAGGAAGTAAAATTTGAGTTGAGACCTGGAGACT
AAGAAGGAGTGAGTCATAAGAGGAGCAGAGAGAAGCCTGACCCAAGCATGGACAAA
GGCCCACAAAAGGCTCTGAGGGCTGG
>432
AAGAGGAGCAGAGAGAAGCCTGACCCAAGCATGGACAAAGGCCCACAAAAGGCTCT
GAGGGCTGGGCCCAGTGGCACACACCTATAATCCCAACACTTCCAGAGGCCAAGGTG
GGAGAATCGCCTGAGGCTAGGAGTTTG
>433
GCCCACAAAAGGCTCTGAGGGCTGGGCCCAGTGGCACACACCTATAATCCCAACACTT
CCAGAGGCCAAGGTGGGAGAATCGCCTGAGGCTAGGAGTTTGAGGCCAGCCTGGGCA
ACATAGTGAGAGCCATCTCTACAGA
>434
GCCCAGTGGCACACACCTATAATCCCAACACTTCCAGAGGCCAAGGTGGGAGAATCGC

CTGAGGCTAGGAGTTTGAGGCCAGCCTGGGCAACATAGTGAGAGCCATCTCTACAGAA
AATAAAAAACAAAATCAGCTGGGC
>435
CAACACTTCCAGAGGCCAAGGTGGGAGAATCGCCTGAGGCTAGGAGTTTGAGGCCAG
CCTGGGCAACATAGTGAGAGCCATCTCTACAGAAAATAAAAAACAAAATCAGCTGGGC
ATGATGGTGCATGCCTATAGTCCTA
>436
GGCCAGCCTGGGCAACATAGTGAGAGCCATCTCTACAGAAAATAAAAAACAAAATCA
GCTGGGCATGATGGTGCATGCCTATAGTCCTAGCTACTCAGGAAGCTGAGGCAGGAGG
ATCACTTGAGCCTAGGAGTTTGAGG
>437
AACATAGTGAGAGCCATCTCTACAGAAAATAAAAAACAAAATCAGCTGGGCATGATGG
TGCATGCCTATAGTCCTAGCTACTCAGGAAGCTGAGGCAGGAGGATCACTTGAGCCTA
GGAGTTTGAGGTTACAGTGAACTA
>438
GCATGATGGTGCATGCCTATAGTCCTAGCTACTCAGGAAGCTGAGGCAGGAGGATCAC
TTGAGCCTAGGAGTTTGAGGTTACAGTGAACTATGATTGTGCCACTGGACTTCAGTCTG
TTGAGGTGAGGTCCGAGAAGCTG
>439
GGTGCATGCCTATAGTCCTAGCTACTCAGGAAGCTGAGGCAGGAGGATCACTTGAGCC
TAGGAGTTTGAGGTTACAGTGAACTATGATTGTGCCACTGGACTTCAGTCTGTTGAGGT
GAGGTCCGAGAAGCTGGCCATGG
>440
TGCCTATAGTCCTAGCTACTCAGGAAGCTGAGGCAGGAGGATCACTTGAGCCTAGGAG
TTTGAGGTTACAGTGAACTATGATTGTGCCACTGGACTTCAGTCTGTTGAGGTGAGGTC
CGAGAAGCTGGCCATGGTGAAGA
>441
AGCTACTCAGGAAGCTGAGGCAGGAGGATCACTTGAGCCTAGGAGTTTGAGGTTACA
GTGAACTATGATTGTGCCACTGGACTTCAGTCTGTTGAGGTGAGGTCCGAGAAGCTGG
CCATGGTGAAGATTGTGCATGAGCC
>442
GAGGCAGGAGGATCACTTGAGCCTAGGAGTTTGAGGTTACAGTGAACTATGATTGTGC
CACTGGACTTCAGTCTGTTGAGGTGAGGTCCGAGAAGCTGGCCATGGTGAAGATTGTG
CATGAGCCTTTAAACCCTGGTAAG
>443
GGTTACAGTGAACTATGATTGTGCCACTGGACTTCAGTCTGTTGAGGTGAGGTCCGAG
AAGCTGGCCATGGTGAAGATTGTGCATGAGCCTTTAAACCCTGGTAAGGAGTTTGAATT
ATATTCCAAAGATGATGGGAAAC
>444
GAACTATGATTGTGCCACTGGACTTCAGTCTGTTGAGGTGAGGTCCGAGAAGCTGGCC
ATGGTGAAGATTGTGCATGAGCCTTTAAACCCTGGTAAGGAGTTTGAATTATATTCCAA
AGATGATGGGAAACCATTGAAGG
>445
CTTCAGTCTGTTGAGGTGAGGTCCGAGAAGCTGGCCATGGTGAAGATTGTGCATGAGC
CTTTAAACCCTGGTAAGGAGTTTGAATTATATTCCAAAGATGATGGGAAACCATTGAAG
GATTTTAAGCAGGAAAGTGACAT

>446
AACCCTGGTAAGGAGTTTGAATTATATTCCAAAGATGATGGGAAACCATTGAAGGATTT
TAAGCAGGAAAGTGACATGATCCAATTTGTGTTTGAAAGTGACAATTCTGACTGCTGA
ATAGAGAAAGGTTTGGGTAGGGG
>447
TGAATTATATTCCAAAGATGATGGGAAACCATTGAAGGATTTTAAGCAGGAAAGTGACA
TGATCCAATTTGTGTTTGAAAGTGACAATTCTGACTGCTGAATAGAGAAAGGTTTGGGT
AGGGGGGATGGAGATGGAAGTA
>448
TCCAAAGATGATGGGAAACCATTGAAGGATTTTAAGCAGGAAAGTGACATGATCCAAT
TTGTGTTTGAAAGTGACAATTCTGACTGCTGAATAGAGAAAGGTTTGGGTAGGGGGGA
TGGAGATGGAAGTAGAAGAGACCT
>449
TTTAAGCAGGAAAGTGACATGATCCAATTTGTGTTTGAAAGTGACAATTCTGACTGCTG
AATAGAGAAAGGTTTGGGTAGGGGGGATGGAGATGGAAGTAGAAGAGACCTAGTTAG
GAAGCCATGGTGTTGGTTAGGTGC
>450
TTTGTGTTTGAAAGTGACAATTCTGACTGCTGAATAGAGAAAGGTTTGGGTAGGGGGG
ATGGAGATGGAAGTAGAAGAGACCTAGTTAGGAAGCCATGGTGTTGGTTAGGTGCCCA
GACTAAGGTGGAGGGGGTGGCAAT
>451
TGACAATTCTGACTGCTGAATAGAGAAAGGTTTGGGTAGGGGGGATGGAGATGGAAGT
AGAAGAGACCTAGTTAGGAAGCCATGGTGTTGGTTAGGTGCCCAGACTAAGGTGGAG
GGGGTGGCAATGGAGAGAAGTAGAT
>452
GCTGAATAGAGAAAGGTTTGGGTAGGGGGGATGGAGATGGAAGTAGAAGAGACCTAG
TTAGGAAGCCATGGTGTTGGTTAGGTGCCCAGACTAAGGTGGAGGGGGTGGCAATGG
AGAGAAGTAGATGATCTGAGAGGTGT
>453
GGGGGATGGAGATGGAAGTAGAAGAGACCTAGTTAGGAAGCCATGGTGTTGGTTAGG
TGCCCAGACTAAGGTGGAGGGGGTGGCAATGGAGAGAAGTAGATGATCTGAGAGGTG
TGTGTGTGTGTGTGTGTATATATATA
>454
AGATGGAAGTAGAAGAGACCTAGTTAGGAAGCCATGGTGTTGGTTAGGTGCCCAGACT
AAGGTGGAGGGGGTGGCAATGGAGAGAAGTAGATGATCTGAGAGGTGTGTGTGTGTG
TGTGTGTATATATATATATATACAC
>455
GACCTAGTTAGGAAGCCATGGTGTTGGTTAGGTGCCCAGACTAAGGTGGAGGGGGTG
GCAATGGAGAGAAGTAGATGATCTGAGAGGTGTGTGTGTGTGTGTGTGTATATATATATA
TATACACACACACACACACATAT
>456
CATGGTGTTGGTTAGGTGCCCAGACTAAGGTGGAGGGGGTGGCAATGGAGAGAAGTA
GATGATCTGAGAGGTGTGTGTGTGTGTGTGTGTATATATATATATATACACACACACACA
CACATATATATGTTTTTTTTTTT
>457
ATGATCTGAGAGGTGTGTGTGTGTGTGTGTGTATATATATATATATACACACACACACAC

ACATATATATGTTTTTTTTTTTCTTTTTTGAGATGGAGTCTTGCTCTGTCACCCAGGCTGG
AGTGCAGTGGCACGATCTT
>458
TGTGTATATATATATATATACACACACACACACACATATATATGTTTTTTTTTTTCTTTTTTG
AGATGGAGTCTTGCTCTGTCACCCAGGCTGGAGTGCAGTGGCACGATCTTGGCTCAGT
GCAAGCTCTGCCTCCTGGG
>459
TATATATATACACACACACACACACATATATATGTTTTTTTTTTTCTTTTTTGAGATGGAGT
CTTGCTCTGTCACCCAGGCTGGAGTGCAGTGGCACGATCTTGGCTCAGTGCAAGCTCT
GCCTCCTGGGTTCAAGCATT
>460
ACACACACACATATATATGTTTTTTTTTTTCTTTTTTGAGATGGAGTCTTGCTCTGTCACC
CAGGCTGGAGTGCAGTGGCACGATCTTGGCTCAGTGCAAGCTCTGCCTCCTGGGTTCA
AGCATTTCTCCTGCCTCAGCC
>461
TTTTTTCTTTTTTGAGATGGAGTCTTGCTCTGTCACCCAGGCTGGAGTGCAGTGGCACG
ATCTTGGCTCAGTGCAAGCTCTGCCTCCTGGGTTCAAGCATTTCTCCTGCCTCAGCCTC
CTGAGTAGCTGGGACTACTACA
>462
AGTCTTGCTCTGTCACCCAGGCTGGAGTGCAGTGGCACGATCTTGGCTCAGTGCAAGC
TCTGCCTCCTGGGTTCAAGCATTTCTCCTGCCTCAGCCTCCTGAGTAGCTGGGACTACT
ACAAAGGCGTGAGCACCGCGCCG
>463
TGGCACGATCTTGGCTCAGTGCAAGCTCTGCCTCCTGGGTTCAAGCATTTCTCCTGCCT
CAGCCTCCTGAGTAGCTGGGACTACTACAAAGGCGTGAGCACCGCGCCGGGCGTGAG
TTATATTTTCAAGGCTAGAATTGA
>464
AGCTCTGCCTCCTGGGTTCAAGCATTTCTCCTGCCTCAGCCTCCTGAGTAGCTGGGACT
ACTACAAAGGCGTGAGCACCGCGCCGGGCGTGAGTTATATTTTCAAGGCTAGAATTGA
CAGAACTTGGGTTGATGAGAATA
>465
CAAGCATTTCTCCTGCCTCAGCCTCCTGAGTAGCTGGGACTACTACAAAGGCGTGAGC
ACCGCGCCGGGCGTGAGTTATATTTTCAAGGCTAGAATTGACAGAACTTGGGTTGATG
AGAATAGTTGATTAGCAGGCAGGG
>466
CTACTACAAAGGCGTGAGCACCGCGCCGGGCGTGAGTTATATTTTCAAGGCTAGAATT
GACAGAACTTGGGTTGATGAGAATAGTTGATTAGCAGGCAGGGTGCCAGGGAGGGTG
TTGAGCAGAACAGAGGGGGGACTGT
>467
GTGAGCACCGCGCCGGGCGTGAGTTATATTTTCAAGGCTAGAATTGACAGAACTTGGG
TTGATGAGAATAGTTGATTAGCAGGCAGGGTGCCAGGGAGGGTGTTGAGCAGAACAG
AGGGGGGACTGTGCTTTATTTTAAA
>468
CGTGAGTTATATTTTCAAGGCTAGAATTGACAGAACTTGGGTTGATGAGAATAGTTGAT
TAGCAGGCAGGGTGCCAGGGAGGGTGTTGAGCAGAACAGAGGGGGGACTGTGCTTTA
TTTTAAAATTTTTATTGAGACACA

>469
GGGTTGATGAGAATAGTTGATTAGCAGGCAGGGTGCCAGGGAGGGTGTTGAGCAGAA
CAGAGGGGGGACTGTGCTTTATTTTAAAATTTTTATTGAGACACAGCATATAAAGTATAC
CCAAATCTTAAGGGTATAACATA
>470
AGGGTGTTGAGCAGAACAGAGGGGGGACTGTGCTTTATTTTAAAATTTTTATTGAGAC
ACAGCATATAAAGTATACCCAAATCTTAAGGGTATAACATAATGAATTTCACTATCAAGA
TAAAGATTTAGAACATTTATAG
>471
AGGGGGGACTGTGCTTTATTTTAAAATTTTTATTGAGACACAGCATATAAAGTATACCCA
AATCTTAAGGGTATAACATAATGAATTTCACTATCAAGATAAAGATTTAGAACATTTATA
GTTTTCTTGTGCTCCTTTCA
>472
AGTATACCCAAATCTTAAGGGTATAACATAATGAATTTCACTATCAAGATAAAGATTTAG
AACATTTATAGTTTTCTTGTGCTCCTTTCAGGCAATACCTAAAGGTATTTACCTTGTCTTT
TGATAACCTCCTCCACATC
>473
CAAATCTTAAGGGTATAACATAATGAATTTCACTATCAAGATAAAGATTTAGAACATTTA
TAGTTTTCTTGTGCTCCTTTCAGGCAATACCTAAAGGTATTTACCTTGTCTTTTGATAAC
CTCCTCCACATCTTCCTCTG
>474
ATTTCACTATCAAGATAAAGATTTAGAACATTTATAGTTTTCTTGTGCTCCTTTCAGGCA
ATACCTAAAGGTATTTACCTTGTCTTTTGATAACCTCCTCCACATCTTCCTCTGGTTCCTT
TTAACAGGGAATGCGTTGA
>475
GTGCTCCTTTCAGGCAATACCTAAAGGTATTTACCTTGTCTTTTGATAACCTCCTCCACA
TCTTCCTCTGGTTCCTTTTAACAGGGAATGCGTTGAATATGTCAAGAGCTTCAATATCCC
TCTACTCGTGCTGGGTGGTG
>476
TAAAGGTATTTACCTTGTCTTTTGATAACCTCCTCCACATCTTCCTCTGGTTCCTTTTAAC
AGGGAATGCGTTGAATATGTCAAGAGCTTCAATATCCCTCTACTCGTGCTGGGTGGTGG
TGGTTATACTGTCCGAAATG
>477
ACATCTTCCTCTGGTTCCTTTTAACAGGGAATGCGTTGAATATGTCAAGAGCTTCAATAT
CCCTCTACTCGTGCTGGGTGGTGGTGGTTATACTGTCCGAAATGTTGCCCGCTGCTGGT
GAGCACTCTCCTGATTTCCTC
>478
CCTCTGGTTCCTTTTAACAGGGAATGCGTTGAATATGTCAAGAGCTTCAATATCCCTCTA
CTCGTGCTGGGTGGTGGTGGTTATACTGTCCGAAATGTTGCCCGCTGCTGGTGAGCACT
CTCCTGATTTCCTCCCCTGTT
>479
CGTTGAATATGTCAAGAGCTTCAATATCCCTCTACTCGTGCTGGGTGGTGGTGGTTATAC
TGTCCGAAATGTTGCCCGCTGCTGGTGAGCACTCTCCTGATTTCCTCCCCTGTTGTCCA
GTGTGAGCTAGGGTGTCCCTC
>480
TGGGTGGTGGTGGTTATACTGTCCGAAATGTTGCCCGCTGCTGGTGAGCACTCTCCTGA

TTTCCTCCCCTGTTGTCCAGTGTGAGCTAGGGTGTCCCTCAGATATGGCCACATGGGCC
CTTCAGCCCACTTGGTCAGCCT
>481
GGTGGTTATACTGTCCGAAATGTTGCCCGCTGCTGGTGAGCACTCTCCTGATTTCCTCC
CCTGTTGTCCAGTGTGAGCTAGGGTGTCCCTCAGATATGGCCACATGGGCCCTTCAGCC
CACTTGGTCAGCCTGCTCCCCT
>482
CCTCCCCTGTTGTCCAGTGTGAGCTAGGGTGTCCCTCAGATATGGCCACATGGGCCCTT
CAGCCCACTTGGTCAGCCTGCTCCCCTCTGCTGGTTGGTTTCAGGACATATGAGACATC
GCTGCTGGTAGAAGAGGCCATT
>483
GCTAGGGTGTCCCTCAGATATGGCCACATGGGCCCTTCAGCCCACTTGGTCAGCCTGCT
CCCCTCTGCTGGTTGGTTTCAGGACATATGAGACATCGCTGCTGGTAGAAGAGGCCATT
AGTGAGGAGCTTCCCTATAGTG
>484
CCTTCAGCCCACTTGGTCAGCCTGCTCCCCTCTGCTGGTTGGTTTCAGGACATATGAGA
CATCGCTGCTGGTAGAAGAGGCCATTAGTGAGGAGCTTCCCTATAGTGGTAAGGACAC
CCACAACACCCCATAACAGGAAG
>485
TCAGCCTGCTCCCCTCTGCTGGTTGGTTTCAGGACATATGAGACATCGCTGCTGGTAGA
AGAGGCCATTAGTGAGGAGCTTCCCTATAGTGGTAAGGACACCCACAACACCCCATAA
CAGGAAGGCCATTCTCACCAGAT
>486
CTGGTTGGTTTCAGGACATATGAGACATCGCTGCTGGTAGAAGAGGCCATTAGTGAGG
AGCTTCCCTATAGTGGTAAGGACACCCACAACACCCCATAACAGGAAGGCCATTCTCA
CCAGATTCCAGAATGACCTGGTTT
>487
GTTGGTTTCAGGACATATGAGACATCGCTGCTGGTAGAAGAGGCCATTAGTGAGGAGC
TTCCCTATAGTGGTAAGGACACCCACAACACCCCATAACAGGAAGGCCATTCTCACCA
GATTCCAGAATGACCTGGTTTGAA
>488
TATAGTGGTAAGGACACCCACAACACCCCATAACAGGAAGGCCATTCTCACCAGATTC
CAGAATGACCTGGTTTGAAGGCACATGGGAAATTTTACACTCTCAGAGCTCTGCAGAC
TAAACCCCTGGGCTCAGAGCAGGG
>489
TTTACACTCTCAGAGCTCTGCAGACTAAACCCCTGGGCTCAGAGCAGGGAAGAGATTG
GGCAGCTATTATGTAGGTGCTCTGGATGGGAAGGAAAAAGATTGGGAAGTCTTGTTAG
CTGCCTTTCTCAGTCTGAGCCATA
>490
CACTCTCAGAGCTCTGCAGACTAAACCCCTGGGCTCAGAGCAGGGAAGAGATTGGGC
AGCTATTATGTAGGTGCTCTGGATGGGAAGGAAAAAGATTGGGAAGTCTTGTTAGCTG
CCTTTCTCAGTCTGAGCCATACTTT
>491
GCTCAGAGCAGGGAAGAGATTGGGCAGCTATTATGTAGGTGCTCTGGATGGGAAGGAA
AAAGATTGGGAAGTCTTGTTAGCTGCCTTTCTCAGTCTGAGCCATACTTTCTCCTCTCC
TCCCCAAGAATACTTCGAGTACT

>492
GAGCAGGGAAGAGATTGGGCAGCTATTATGTAGGTGCTCTGGATGGGAAGGAAAAAG
ATTGGGAAGTCTTGTTAGCTGCCTTTCTCAGTCTGAGCCATACTTTCTCCTCTCCTCCCC
AAGAATACTTCGAGTACTTTGCC
>493
GGCAGCTATTATGTAGGTGCTCTGGATGGGAAGGAAAAAGATTGGGAAGTCTTGTTAG
CTGCCTTTCTCAGTCTGAGCCATACTTTCTCCTCTCCTCCCCAAGAATACTTCGAGTACT
TTGCCCCAGACTTCACACTTCA
>494
GGGAAGTCTTGTTAGCTGCCTTTCTCAGTCTGAGCCATACTTTCTCCTCTCCTCCCCAA
GAATACTTCGAGTACTTTGCCCCAGACTTCACACTTCATCCAGATGTCAGCACCCGCAT
CGAGAATCAGAACTCACGCCAG
>495
CCATACTTTCTCCTCTCCTCCCCAAGAATACTTCGAGTACTTTGCCCCAGACTTCACACT
TCATCCAGATGTCAGCACCCGCATCGAGAATCAGAACTCACGCCAGGTCAGCAGCTCA
GAAAAGCTGAGCACAAACTTCT
>496
CCGCATCGAGAATCAGAACTCACGCCAGGTCAGCAGCTCAGAAAAGCTGAGCACAAA
CTTCTCCTGAAAGGTGGAGCCCAGGAGAGGTCAAAGATGTGGGAAGTAGGAAAAACC
AAAGAGAAGGAAGTTCTAAGCCATCA
>497
AACTCACGCCAGGTCAGCAGCTCAGAAAAGCTGAGCACAAACTTCTCCTGAAAGGTG
GAGCCCAGGAGAGGTCAAAGATGTGGGAAGTAGGAAAAACCAAAGAGAAGGAAGTT
CTAAGCCATCACAGTCACTGTTATTGA
>498
GCAGCTCAGAAAAGCTGAGCACAAACTTCTCCTGAAAGGTGGAGCCCAGGAGAGGTC
AAAGATGTGGGAAGTAGGAAAAACCAAAGAGAAGGAAGTTCTAAGCCATCACAGTCA
CTGTTATTGACTAGGAGGCAGTCCAG
>499
ACTTCTCCTGAAAGGTGGAGCCCAGGAGAGGTCAAAGATGTGGGAAGTAGGAAAAAC
CAAAGAGAAGGAAGTTCTAAGCCATCACAGTCACTGTTATTGACTAGGAGGCAGTCCA
GTTTAAAGTAGAGGCAGTTGGGGTT
>500
GGAGCCCAGGAGAGGTCAAAGATGTGGGAAGTAGGAAAAACCAAAGAGAAGGAAGT
TCTAAGCCATCACAGTCACTGTTATTGACTAGGAGGCAGTCCAGTTTAAAGTAGAGGC
AGTTGGGGTTGGTGGGGAGGGAGAGC
>501
TGGGAAGTAGGAAAAACCAAAGAGAAGGAAGTTCTAAGCCATCACAGTCACTGTTAT
TGACTAGGAGGCAGTCCAGTTTAAAGTAGAGGCAGTTGGGGTTGGTGGGGAGGGAGA
GCTATCAGTTGGGGGACCTGAACTAG
>502
GAGAAGGAAGTTCTAAGCCATCACAGTCACTGTTATTGACTAGGAGGCAGTCCAGTTT
AAAGTAGAGGCAGTTGGGGTTGGTGGGGAGGGAGAGCTATCAGTTGGGGGACCTGAA
CTAGAGGTACCACTGAGATGAAAAC
>503
GAAGTTCTAAGCCATCACAGTCACTGTTATTGACTAGGAGGCAGTCCAGTTTAAAGTA

GAGGCAGTTGGGGTTGGTGGGGAGGGAGAGCTATCAGTTGGGGGACCTGAACTAGAG
GTACCACTGAGATGAAAACCAGTAT
>504
TGTTATTGACTAGGAGGCAGTCCAGTTTAAAGTAGAGGCAGTTGGGGTTGGTGGGGAG
GGAGAGCTATCAGTTGGGGGACCTGAACTAGAGGTACCACTGAGATGAAAACCAGTAT
GGTAGGGGTAAAAGGCAGCTAGGA
>505
TGGGGTTGGTGGGGAGGGAGAGCTATCAGTTGGGGGACCTGAACTAGAGGTACCACT
GAGATGAAAACCAGTATGGTAGGGGTAAAAGGCAGCTAGGAGATTCCATGTCCTGGGA
ATCTCTGGAAACTGCTCACTCTGTA
>506
TTGGTGGGGAGGGAGAGCTATCAGTTGGGGGACCTGAACTAGAGGTACCACTGAGAT
GAAAACCAGTATGGTAGGGGTAAAAGGCAGCTAGGAGATTCCATGTCCTGGGAATCTC
TGGAAACTGCTCACTCTGTATTCCT
>507
ACCTGAACTAGAGGTACCACTGAGATGAAAACCAGTATGGTAGGGGTAAAAGGCAGCT
AGGAGATTCCATGTCCTGGGAATCTCTGGAAACTGCTCACTCTGTATTCCTCATTTCCA
ACCAGTATCTGGACCAGATCCGC
>508
CCAGTATGGTAGGGGTAAAAGGCAGCTAGGAGATTCCATGTCCTGGGAATCTCTGGAA
ACTGCTCACTCTGTATTCCTCATTTCCAACCAGTATCTGGACCAGATCCGCCAGACAAT
CTTTGAAAACCTGAAGATGCTGA
>509
GGCAGCTAGGAGATTCCATGTCCTGGGAATCTCTGGAAACTGCTCACTCTGTATTCCTC
ATTTCCAACCAGTATCTGGACCAGATCCGCCAGACAATCTTTGAAAACCTGAAGATGCT
GAACCATGCACCTAGTGTCCAG
>510
TCCAACCAGTATCTGGACCAGATCCGCCAGACAATCTTTGAAAACCTGAAGATGCTGA
ACCATGCACCTAGTGTCCAGATTCATGACGTGCCTGCAGACCTCCTGACCTATGACAGG
ACTGATGAGGCTGATGCAGAGGA
>511
CAACCAGTATCTGGACCAGATCCGCCAGACAATCTTTGAAAACCTGAAGATGCTGAAC
CATGCACCTAGTGTCCAGATTCATGACGTGCCTGCAGACCTCCTGACCTATGACAGGAC
TGATGAGGCTGATGCAGAGGAGA
>512
CCAGATCCGCCAGACAATCTTTGAAAACCTGAAGATGCTGAACCATGCACCTAGTGTC
CAGATTCATGACGTGCCTGCAGACCTCCTGACCTATGACAGGACTGATGAGGCTGATG
CAGAGGAGAGGGGTCCTGAGGAGA
>513
CAGATTCATGACGTGCCTGCAGACCTCCTGACCTATGACAGGACTGATGAGGCTGATG
CAGAGGAGAGGGGTCCTGAGGAGAACTATAGCAGGTCTGGGAGCAGGGACTTCAGCC
TACTTTCAATAGGCTTGCTGGGGAG
>514
GTCCTGAGGAGAACTATAGCAGGTCTGGGAGCAGGGACTTCAGCCTACTTTCAATAGG
CTTGCTGGGGAGGTTTAAAAGGGAAAAAGTAAATGGGAAGGTATTAGTTTAATTGCCT
CTGGCTTACCTCCTTTAGTCTGCT

>515
TAGCAGGTCTGGGAGCAGGGACTTCAGCCTACTTTCAATAGGCTTGCTGGGGAGGTTT
AAAAGGGAAAAAGTAAATGGGAAGGTATTAGTTTAATTGCCTCTGGCTTACCTCCTTTA
GTCTGCTACAATTTCCTCACTCT
>516
GGACTTCAGCCTACTTTCAATAGGCTTGCTGGGGAGGTTTAAAAGGGAAAAAGTAAAT
GGGAAGGTATTAGTTTAATTGCCTCTGGCTTACCTCCTTTAGTCTGCTACAATTTCCTCA
CTCTTTTCTTTGTTATTTTTAA
>517
TGGGGAGGTTTAAAAGGGAAAAAGTAAATGGGAAGGTATTAGTTTAATTGCCTCTGGC
TTACCTCCTTTAGTCTGCTACAATTTCCTCACTCTTTTCTTTGTTATTTTTAATGACAAAA
GTAACACGTATTTATTGCAAA
>518
GGAAGGTATTAGTTTAATTGCCTCTGGCTTACCTCCTTTAGTCTGCTACAATTTCCTCAC
TCTTTTCTTTGTTATTTTTAATGACAAAAGTAACACGTATTTATTGCAAAATTTTAAAAC
AGCACCAAAATATGGTGTTG
>519
GCCTCTGGCTTACCTCCTTTAGTCTGCTACAATTTCCTCACTCTTTTCTTTGTTATTTTTA
ATGACAAAAGTAACACGTATTTATTGCAAAATTTTAAAACAGCACCAAAATATGGTGTT
GGCGTTCACACAATCCCAGA
>520
GCTTACCTCCTTTAGTCTGCTACAATTTCCTCACTCTTTTCTTTGTTATTTTTAATGACAA
AAGTAACACGTATTTATTGCAAAATTTTAAAACAGCACCAAAATATGGTGTTGGCGTTC
ACACAATCCCAGACCTATTC
>521
TTCTTTGTTATTTTTAATGACAAAAGTAACACGTATTTATTGCAAAATTTTAAAACAGCA
CCAAAATATGGTGTTGGCGTTCACACAATCCCAGACCTATTCCACAGGAGTAACCACTG
TTAATAGTTTTTATTGTTTTG
>522
TTGCAAAATTTTAAAACAGCACCAAAATATGGTGTTGGCGTTCACACAATCCCAGACCT
ATTCCACAGGAGTAACCACTGTTAATAGTTTTTATTGTTTTGGATAGACCTTTTTCTATAG
ATTGCAAATATACTATTCTG
>523
TTTAAAACAGCACCAAAATATGGTGTTGGCGTTCACACAATCCCAGACCTATTCCACAG
GAGTAACCACTGTTAATAGTTTTTATTGTTTTGGATAGACCTTTTTCTATAGATTGCAAAT
ATACTATTCTGTATGTACAT
>524
AGTAACCACTGTTAATAGTTTTTATTGTTTTGGATAGACCTTTTTCTATAGATTGCAAATA
TACTATTCTGTATGTACATATAAACAAACACATATACACTCATTTACAGTTTGTGTGGTTT
TTGGTTTTTCCGTAAATA
>525
ACCACTGTTAATAGTTTTTATTGTTTTGGATAGACCTTTTTCTATAGATTGCAAATATACT
ATTCTGTATGTACATATAAACAAACACATATACACTCATTTACAGTTTGTGTGGTTTTTGG
TTTTTCCGTAAATACAAA
>526
CTTTTTCTATAGATTGCAAATATACTATTCTGTATGTACATATAAACAAACACATATACAC

TCATTTACAGTTTGTGTGGTTTTTGGTTTTTCCGTAAATACAAATAGGGTCATTCTACGT
ATGCTGCTTTACATCATAT
>527
TTCTATAGATTGCAAATATACTATTCTGTATGTACATATAAACAAACACATATACACTCAT
TTACAGTTTGTGTGGTTTTTGGTTTTTCCGTAAATACAAATAGGGTCATTCTACGTATGC
TGCTTTACATCATATAGCT
>528
CTATTCTGTATGTACATATAAACAAACACATATACACTCATTTACAGTTTGTGTGGTTTTT
GGTTTTTCCGTAAATACAAATAGGGTCATTCTACGTATGCTGCTTTACATCATATAGCTGT
TTTCAGCTAGCTAATATA
>529
ACACATATACACTCATTTACAGTTTGTGTGGTTTTTGGTTTTTCCGTAAATACAAATAGG
GTCATTCTACGTATGCTGCTTTACATCATATAGCTGTTTTCAGCTAGCTAATATAGATCTG
CTTCAGTATTTTATCAGCT
>530
GTGTGGTTTTTGGTTTTTCCGTAAATACAAATAGGGTCATTCTACGTATGCTGCTTTACA
TCATATAGCTGTTTTCAGCTAGCTAATATAGATCTGCTTCAGTATTTTATCAGCTGTATAAT
AATTTACTATGTGGATAG
>531
TAGGGTCATTCTACGTATGCTGCTTTACATCATATAGCTGTTTTCAGCTAGCTAATATAGA
TCTGCTTCAGTATTTTATCAGCTGTATAATAATTTACTATGTGGATAGATTATTTTCCATTT
TTTTGGTATTACAATAA
>532
GTATGCTGCTTTACATCATATAGCTGTTTTCAGCTAGCTAATATAGATCTGCTTCAGTATT
TTATCAGCTGTATAATAATTTACTATGTGGATAGATTATTTTCCATTTTTTTGGTATTACAA
TAATAATGTGGCAATGA
>533
ATATAGCTGTTTTCAGCTAGCTAATATAGATCTGCTTCAGTATTTTATCAGCTGTATAATAA
TTTACTATGTGGATAGATTATTTTCCATTTTTTTGGTATTACAATAATAATGTGGCAATGA
ACCCCCTTACATTATAC
>534
AGATCTGCTTCAGTATTTTATCAGCTGTATAATAATTTACTATGTGGATAGATTATTTTCCA
TTTTTTTGGTATTACAATAATAATGTGGCAATGAACCCCCTTACATTATACATCTTTGCAT
TCTTGTTTGAGTGGGTT
>535
TTTATCAGCTGTATAATAATTTACTATGTGGATAGATTATTTTCCATTTTTTTGGTATTACA
ATAATAATGTGGCAATGAACCCCCTTACATTATACATCTTTGCATTCTTGTTTGAGTGGG
TTAGATAGTAGAATGGTT
>536
TTCCATTTTTTTGGTATTACAATAATAATGTGGCAATGAACCCCCTTACATTATACATCTT
TGCATTCTTGTTTGAGTGGGTTAGATAGTAGAATGGTTGCTGCTAGGTCAAAGGCTGTG
TGCATTAATTTTTTTTTTTT
>537
TTTTTTGGTATTACAATAATAATGTGGCAATGAACCCCCTTACATTATACATCTTTGCATT
CTTGTTTGAGTGGGTTAGATAGTAGAATGGTTGCTGCTAGGTCAAAGGCTGTGTGCATT
AATTTTTTTTTTTTTTTTT

>538
TTGGTATTACAATAATAATGTGGCAATGAACCCCCTTACATTATACATCTTTGCATTCTTG
TTTGAGTGGGTTAGATAGTAGAATGGTTGCTGCTAGGTCAAAGGCTGTGTGCATTAATT
TTTTTTTTTTTTTTTTTTTT
>539
ACAATAATAATGTGGCAATGAACCCCCTTACATTATACATCTTTGCATTCTTGTTTGAGT
GGGTTAGATAGTAGAATGGTTGCTGCTAGGTCAAAGGCTGTGTGCATTAATTTTTTTTTT
TTTTTTTTTTTTGAGATGGA
>540
GAACCCCCTTACATTATACATCTTTGCATTCTTGTTTGAGTGGGTTAGATAGTAGAATGG
TTGCTGCTAGGTCAAAGGCTGTGTGCATTAATTTTTTTTTTTTTTTTTTTTTTGAGATGG
AGTCTCACTCTGTTGCCAGG
>541
GTAGAATGGTTGCTGCTAGGTCAAAGGCTGTGTGCATTAATTTTTTTTTTTTTTTTTTTT
TGAGATGGAGTCTCACTCTGTTGCCAGGCTGGAGTGCAGTGGCTAATCCCGGCTCACT
GCAACCTCCGCCTCCCAGGTT
>542
TGCTGCTAGGTCAAAGGCTGTGTGCATTAATTTTTTTTTTTTTTTTTTTTTTGAGATGGA
GTCTCACTCTGTTGCCAGGCTGGAGTGCAGTGGCTAATCCCGGCTCACTGCAACCTCC
GCCTCCCAGGTTCAAGCGATTC
>543
GGAGTCTCACTCTGTTGCCAGGCTGGAGTGCAGTGGCTAATCCCGGCTCACTGCAACC
TCCGCCTCCCAGGTTCAAGCGATTCTCCTGCCTCAGCCTCCTCAGTAGCTGAGATTACA
GGCATGTGCCACCACACCCGGCT
>544
GTGGCTAATCCCGGCTCACTGCAACCTCCGCCTCCCAGGTTCAAGCGATTCTCCTGCCT
CAGCCTCCTCAGTAGCTGAGATTACAGGCATGTGCCACCACACCCGGCTAACTTTAGTA
TTTTTAGTAGAGACGGGGTTTC
>545
GCAACCTCCGCCTCCCAGGTTCAAGCGATTCTCCTGCCTCAGCCTCCTCAGTAGCTGA
GATTACAGGCATGTGCCACCACACCCGGCTAACTTTAGTATTTTTAGTAGAGACGGGGT
TTCACCATGTTGGCCAGGATGGT
>546
CGATTCTCCTGCCTCAGCCTCCTCAGTAGCTGAGATTACAGGCATGTGCCACCACACCC
GGCTAACTTTAGTATTTTTAGTAGAGACGGGGTTTCACCATGTTGGCCAGGATGGTCTT
GATCTCTTGACCTTGTGATCCA
>547
CCTCAGTAGCTGAGATTACAGGCATGTGCCACCACACCCGGCTAACTTTAGTATTTTTA
GTAGAGACGGGGTTTCACCATGTTGGCCAGGATGGTCTTGATCTCTTGACCTTGTGATC
CACCTGCCTTGGCCTCCCAAAG
>548
TGAGATTACAGGCATGTGCCACCACACCCGGCTAACTTTAGTATTTTTAGTAGAGACGG
GGTTTCACCATGTTGGCCAGGATGGTCTTGATCTCTTGACCTTGTGATCCACCTGCCTT
GGCCTCCCAAAGTGCTGGGATT
>549
ATGTGCCACCACACCCGGCTAACTTTAGTATTTTTAGTAGAGACGGGGTTTCACCATGT

TGGCCAGGATGGTCTTGATCTCTTGACCTTGTGATCCACCTGCCTTGGCCTCCCAAAGT
GCTGGGATTACAGGTGTGAGCT
>550
GGTTTCACCATGTTGGCCAGGATGGTCTTGATCTCTTGACCTTGTGATCCACCTGCCTT
GGCCTCCCAAAGTGCTGGGATTACAGGTGTGAGCTACCACGCCCGGCTTTTGAGAATA
CTTTTTCAAAAAGAAAGAAACCA
>551
GCCAGGATGGTCTTGATCTCTTGACCTTGTGATCCACCTGCCTTGGCCTCCCAAAGTGC
TGGGATTACAGGTGTGAGCTACCACGCCCGGCTTTTGAGAATACTTTTTCAAAAAGAA
AGAAACCAATATTGAGTACCTGT
>552
GATTACAGGTGTGAGCTACCACGCCCGGCTTTTGAGAATACTTTTTCAAAAAGAAAGA
AACCAATATTGAGTACCTGTTGGGCCCTGTTTTGCTTTCACATCCATTCTTTCACTATGG
GTTTATAATACCACGTCACTCT
>553
AGGTGTGAGCTACCACGCCCGGCTTTTGAGAATACTTTTTCAAAAAGAAAGAAACCAA
TATTGAGTACCTGTTGGGCCCTGTTTTGCTTTCACATCCATTCTTTCACTATGGGTTTATA
ATACCACGTCACTCTTCTTAA
>554
AAAAGAAAGAAACCAATATTGAGTACCTGTTGGGCCCTGTTTTGCTTTCACATCCATTC
TTTCACTATGGGTTTATAATACCACGTCACTCTTCTTAAAATTAAAACTCTCCTTTACCTT
GCACTTAGAAGAAAATCCAA
>555
AATATTGAGTACCTGTTGGGCCCTGTTTTGCTTTCACATCCATTCTTTCACTATGGGTTTA
TAATACCACGTCACTCTTCTTAAAATTAAAACTCTCCTTTACCTTGCACTTAGAAGAAA
ATCCAAAATTTTGACTGTGG
>556
CCACGTCACTCTTCTTAAAATTAAAACTCTCCTTTACCTTGCACTTAGAAGAAAATCCA
AAATTTTGACTGTGGCTTAAAAAGGTCCTGCATGCTAGCCCTTACCTACATCCAGGATC
TCATCTCTAACTACTCTCCTCC
>557
CTTAAAATTAAAACTCTCCTTTACCTTGCACTTAGAAGAAAATCCAAAATTTTGACTGT
GGCTTAAAAAGGTCCTGCATGCTAGCCCTTACCTACATCCAGGATCTCATCTCTAACTA
CTCTCCTCCATGCACAGTGCTC
>558
AAAAAGGTCCTGCATGCTAGCCCTTACCTACATCCAGGATCTCATCTCTAACTACTCTCC
TCCATGCACAGTGCTCCCGGCTGTCCTGGCCTCCTTGTTCTTACTGGAATACATCAGTC
TCTTTCCATTTCATGACCTCT
>559
CATGCTAGCCCTTACCTACATCCAGGATCTCATCTCTAACTACTCTCCTCCATGCACAGT
GCTCCCGGCTGTCCTGGCCTCCTTGTTCTTACTGGAATACATCAGTCTCTTTCCATTTCA
TGACCTCTGTAGTTGGTTTT
>560
CCTACATCCAGGATCTCATCTCTAACTACTCTCCTCCATGCACAGTGCTCCCGGCTGTCC
TGGCCTCCTTGTTCTTACTGGAATACATCAGTCTCTTTCCATTTCATGACCTCTGTAGTT
GGTTTTCTTGCAGCTTGGAA

>561
TCTAACTACTCTCCTCCATGCACAGTGCTCCCGGCTGTCCTGGCCTCCTTGTTCTTACTG
GAATACATCAGTCTCTTTCCATTTCATGACCTCTGTAGTTGGTTTTCTTGCAGCTTGGAA
TGGCCTTCCTGAAGTCTTTG
>562
GTGCTCCCGGCTGTCCTGGCCTCCTTGTTCTTACTGGAATACATCAGTCTCTTTCCATTT
CATGACCTCTGTAGTTGGTTTTCTTGCAGCTTGGAATGGCCTTCCTGAAGTCTTTGCCT
GCTTAGCTCCTCCTCAGACTT
>563
CTCCCGGCTGTCCTGGCCTCCTTGTTCTTACTGGAATACATCAGTCTCTTTCCATTTCAT
GACCTCTGTAGTTGGTTTTCTTGCAGCTTGGAATGGCCTTCCTGAAGTCTTTGCCTGCT
TAGCTCCTCCTCAGACTTTAG
>564
TACATCAGTCTCTTTCCATTTCATGACCTCTGTAGTTGGTTTTCTTGCAGCTTGGAATGG
CCTTCCTGAAGTCTTTGCCTGCTTAGCTCCTCCTCAGACTTTAGGTCTTATGTCCCAATA
GTCCTGTCTGAAATTTCACC
>565
GTTGGTTTTCTTGCAGCTTGGAATGGCCTTCCTGAAGTCTTTGCCTGCTTAGCTCCTCC
TCAGACTTTAGGTCTTATGTCCCAATAGTCCTGTCTGAAATTTCACCCTTTTCCTCTCAT
CACCTTCAGTCCTATCACTTC
>566
TTCTTGCAGCTTGGAATGGCCTTCCTGAAGTCTTTGCCTGCTTAGCTCCTCCTCAGACT
TTAGGTCTTATGTCCCAATAGTCCTGTCTGAAATTTCACCCTTTTCCTCTCATCACCTTC
AGTCCTATCACTTCATTTTAT
>567
CCTCAGACTTTAGGTCTTATGTCCCAATAGTCCTGTCTGAAATTTCACCCTTTTCCTCTC
ATCACCTTCAGTCCTATCACTTCATTTTATTAATTGTACTCTCCATCATCTGAAATTATTAT
ATTTATTTACTTTGTTAG
>568
AATAGTCCTGTCTGAAATTTCACCCTTTTCCTCTCATCACCTTCAGTCCTATCACTTCAT
TTTATTAATTGTACTCTCCATCATCTGAAATTATTATATTTATTTACTTTGTTAGTTACTAGT
CTCATTTATTAGGTAAG
>569
CTTCAGTCCTATCACTTCATTTTATTAATTGTACTCTCCATCATCTGAAATTATTATATTTA
TTTACTTTGTTAGTTACTAGTCTCATTTATTAGGTAAGCTTTTTGAGGATAGACAACCTT
GTCCAGCTTGTTACTCTA
>570
AGTCCTATCACTTCATTTTATTAATTGTACTCTCCATCATCTGAAATTATTATATTTATTTA
CTTTGTTAGTTACTAGTCTCATTTATTAGGTAAGCTTTTTGAGGATAGACAACCTTGTCC
AGCTTGTTACTCTACTGT
>571
ATTATATTTATTTACTTTGTTAGTTACTAGTCTCATTTATTAGGTAAGCTTTTTGAGGATAG
ACAACCTTGTCCAGCTTGTTACTCTACTGTATTGCTAAGGTTTATGACAGTGTCTGTATA
GAGTAGTTCCTTAATGTT
>572
ATTTACTTTGTTAGTTACTAGTCTCATTTATTAGGTAAGCTTTTTGAGGATAGACAACCTT

GTCCAGCTTGTTACTCTACTGTATTGCTAAGGTTTATGACAGTGTCTGTATAGAGTAGTT
CCTTAATGTTTATTTGATA
>573
CTCATTTATTAGGTAAGCTTTTTGAGGATAGACAACCTTGTCCAGCTTGTTACTCTACTG
TATTGCTAAGGTTTATGACAGTGTCTGTATAGAGTAGTTCCTTAATGTTTATTTGATACGT
GAATGGGTGAATGAATAGC
>574
GTAAGCTTTTTGAGGATAGACAACCTTGTCCAGCTTGTTACTCTACTGTATTGCTAAGG
TTTATGACAGTGTCTGTATAGAGTAGTTCCTTAATGTTTATTTGATACGTGAATGGGTGA
ATGAATAGCAGTCTCTACCAA
>575
GCTTTTTGAGGATAGACAACCTTGTCCAGCTTGTTACTCTACTGTATTGCTAAGGTTTAT
GACAGTGTCTGTATAGAGTAGTTCCTTAATGTTTATTTGATACGTGAATGGGTGAATGAA
TAGCAGTCTCTACCAACAAG
>576
GAGGATAGACAACCTTGTCCAGCTTGTTACTCTACTGTATTGCTAAGGTTTATGACAGT
GTCTGTATAGAGTAGTTCCTTAATGTTTATTTGATACGTGAATGGGTGAATGAATAGCAG
TCTCTACCAACAAGAGGAGGA
>577
CTTGTCCAGCTTGTTACTCTACTGTATTGCTAAGGTTTATGACAGTGTCTGTATAGAGTA
GTTCCTTAATGTTTATTTGATACGTGAATGGGTGAATGAATAGCAGTCTCTACCAACAAG
AGGAGGAGTGGTTCTCAACG
>578
TTTATGACAGTGTCTGTATAGAGTAGTTCCTTAATGTTTATTTGATACGTGAATGGGTGA
ATGAATAGCAGTCTCTACCAACAAGAGGAGGAGTGGTTCTCAACGGGGCCTGAGAAA
TGGCTTTGGAAAAGGGTGCTGGG
>579
GTAGTTCCTTAATGTTTATTTGATACGTGAATGGGTGAATGAATAGCAGTCTCTACCAAC
AAGAGGAGGAGTGGTTCTCAACGGGGCCTGAGAAATGGCTTTGGAAAAGGGTGCTGG
GAGAAGATTAGAACTTAGAGTGA
>580
GTTCCTTAATGTTTATTTGATACGTGAATGGGTGAATGAATAGCAGTCTCTACCAACAAG
AGGAGGAGTGGTTCTCAACGGGGCCTGAGAAATGGCTTTGGAAAAGGGTGCTGGGAG
AAGATTAGAACTTAGAGTGAGAT
>581
GAATAGCAGTCTCTACCAACAAGAGGAGGAGTGGTTCTCAACGGGGCCTGAGAAATG
GCTTTGGAAAAGGGTGCTGGGAGAAGATTAGAACTTAGAGTGAGATCCTGACCTCTCT
CCCCTTGCTTTGCAGGCCAGAGGCA
>582
AGGAGTGGTTCTCAACGGGGCCTGAGAAATGGCTTTGGAAAAGGGTGCTGGGAGAAG
ATTAGAACTTAGAGTGAGATCCTGACCTCTCTCCCCTTGCTTTGCAGGCCAGAGGCAC
CCAATGAGTTCTATGATGGAGACCA
>583
AGAAGATTAGAACTTAGAGTGAGATCCTGACCTCTCTCCCCTTGCTTTGCAGGCCAGA
GGCACCCAATGAGTTCTATGATGGAGACCATGACAATGACAAGGAAAGCGATGTGGAG
ATTTAAGAGTGGCTTGGGATGCTG

>584
CTTGCTTTGCAGGCCAGAGGCACCCAATGAGTTCTATGATGGAGACCATGACAATGAC
AAGGAAAGCGATGTGGAGATTTAAGAGTGGCTTGGGATGCTGTGTCCCAAGGAATTTC
TTTTCACCTCTTGGTTGGGCTGGA
>585
CAGAGGCACCCAATGAGTTCTATGATGGAGACCATGACAATGACAAGGAAAGCGATGT
GGAGATTTAAGAGTGGCTTGGGATGCTGTGTCCCAAGGAATTTCTTTTCACCTCTTGGT
TGGGCTGGAGGGAAAAGGAGTGG
>586
AGTTCTATGATGGAGACCATGACAATGACAAGGAAAGCGATGTGGAGATTTAAGAGTG
GCTTGGGATGCTGTGTCCCAAGGAATTTCTTTTCACCTCTTGGTTGGGCTGGAGGGAA
AAGGAGTGGCTCCTAGAGTCCTGG
>587
AAGAGTGGCTTGGGATGCTGTGTCCCAAGGAATTTCTTTTCACCTCTTGGTTGGGCTG
GAGGGAAAAGGAGTGGCTCCTAGAGTCCTGGGGGTCACCCCAGGGCTTTTGCTGACT
CTGGGAAAGAGTCTGGAGACCACAT
>588
GGATGCTGTGTCCCAAGGAATTTCTTTTCACCTCTTGGTTGGGCTGGAGGGAAAAGGA
GTGGCTCCTAGAGTCCTGGGGGTCACCCCAGGGCTTTTGCTGACTCTGGGAAAGAGTC
TGGAGACCACATTTGGTTCTCGAA
>589
CTGGAGGGAAAAGGAGTGGCTCCTAGAGTCCTGGGGGTCACCCCAGGGCTTTTGCTG
ACTCTGGGAAAGAGTCTGGAGACCACATTTGGTTCTCGAACCATCTACCTGCTTTTCCT
CTCTCTCCCAAGGCCTGACAATGG
>590
GCTCCTAGAGTCCTGGGGGTCACCCCAGGGCTTTTGCTGACTCTGGGAAAGAGTCTGG
AGACCACATTTGGTTCTCGAACCATCTACCTGCTTTTCCTCTCTCTCCCAAGGCCTGAC
AATGGTACCTATTAGGGATGAGA
>591
AGTCTGGAGACCACATTTGGTTCTCGAACCATCTACCTGCTTTTCCTCTCTCTCCCAAG
GCCTGACAATGGTACCTATTAGGGATGAGATACAGACAAGGATAGCTATCTGGGACATT
ATTGGCAGTGGGCCCTGGAGGC
>592
CAAGGCCTGACAATGGTACCTATTAGGGATGAGATACAGACAAGGATAGCTATCTGGG
ACATTATTGGCAGTGGGCCCTGGAGGCCAGTCCCTAGCCCCCCTTGCCCCTTATTTCTT
CCCTGCTTCCCTCGAACCCAGAG
>593
ACCTATTAGGGATGAGATACAGACAAGGATAGCTATCTGGGACATTATTGGCAGTGGGC
CCTGGAGGCCAGTCCCTAGCCCCCCTTGCCCCTTATTTCTTCCCTGCTTCCCTCGAACC
CAGAGATTTTTGAGGGATGAAC
>594
CCCTAGCCCCCCTTGCCCCTTATTTCTTCCCTGCTTCCCTCGAACCCAGAGATTTTTGAG
GGATGAACGGGTAGACAAGGACTGAGATTGCCTCTGACTTCCTCCTCCCCTGGGTTCT
GACTTCTTCCTCCCCTTGCTTC
>595
ACCCAGAGATTTTTGAGGGATGAACGGGTAGACAAGGACTGAGATTGCCTCTGACTTC

CTCCTCCCCTGGGTTCTGACTTCTTCCTCCCCTTGCTTCCAGGGAAGATGAAGAGAGA
GAGATTTGGAAGGGGCTCTGGCTC
>596
GGGAAGATGAAGAGAGAGAGAGATTTGGAAGGGGCTCTGGCTCCCTAACACCTGAATCC
CAGATGATGGGAAGTATGTTTTCAAGTGTGGGGAGGATATGAAAATGTTCTGTTCTCAC
TTTTGGCTTTATGTCCATTTTACC
>597
AGATGAAGAGAGAGAGAGATTTGGAAGGGGCTCTGGCTCCCTAACACCTGAATCCCAGAT
GATGGGAAGTATGTTTTCAAGTGTGGGGAGGATATGAAAATGTTCTGTTCTCACTTTTG
GCTTTATGTCCATTTTACCACTG
>598
TCTGGCTCCCTAACACCTGAATCCCAGATGATGGGAAGTATGTTTTCAAGTGTGGGGA
GGATATGAAAATGTTCTGTTCTCACTTTTGGCTTTATGTCCATTTTACCACTGTTTTTATC
CAATAAACTAAGTCGGTATTT
>599
GGTGAAATCCCAGCTCAGGTGTTATATCCTTGCTGCTTCTGACAAATGAAACCAAGGTG
TATTAATTAAGGTAATGCTAGCTACTACAATAAGCTGAAAAATATATAATAGCTAAAAGC
TAGGGTGCCAGATTTAGGAAA
>600
AAGGTGTATTAATTAAGGTAATGCTAGCTACTACAATAAGCTGAAAAATATATAATAGCT
AAAAGCTAGGGTGCCAGATTTAGGAAAATAAAAACAAACAAAAAACAATTGAAATTT
GAATTTCAGATAAACAATTTTTT
>601
GCCAGATTTAGGAAAATAAAAACAAACAAAAAACAATTGAAATTTGAATTTCAGATAA
ACAATTTTTTTAGTATAATTATGTCCCATGCAATATTTGAGACATACTAAAAGGTTATTCA
TTGTTTATCTGATATTCAAAT
>602
GGAAAATAAAAACAAACAAAAAACAATTGAAATTTGAATTTCAGATAAACAATTTTTTT
AGTATAATTATGTCCCATGCAATATTTGAGACATACTAAAAGGTTATTCATTGTTTATCTG
ATATTCAAATTTAACATTTT
>603
ATTTGAATTTCAGATAAACAATTTTTTTAGTATAATTATGTCCCATGCAATATTTGAGACA
TACTAAAAGGTTATTCATTGTTTATCTGATATTCAAATTTAACATTTTCATTAATTTATTTT
ATTTGGCAGCCCTATGG
>604
CAGATAAACAATTTTTTTAGTATAATTATGTCCCATGCAATATTTGAGACATACTAAAAG
GTTATTCATTGTTTATCTGATATTCAAATTTAACATTTTCATTAATTTATTTTATTTGGCAG
CCCTATGGAAGACATCAA
>605
TTATCTGATATTCAAATTTAACATTTTCATTAATTTATTTTATTTGGCAGCCCTATGGAAGA
CATCAAAAGTTTATTTCTTATTTATATAAAGTCCAAAATAGATGTTTTTTAGTAGGCAGG
CAGCTCTTCTCTAAGCGT
>606
ATTTTCATTAATTTATTTTATTTGGCAGCCCTATGGAAGACATCAAAAGTTTATTTCTTAT
TTATATAAAGTCCAAAATAGATGTTTTTTAGTAGGCAGGCAGCTCTTCTCTAAGCGTAAT
TCAGGGCTCTGGGTTCTGT

>607
TATTTTATTTGGCAGCCCTATGGAAGACATCAAAAGTTTATTTCTTATTTATATAAAGTCC
AAAATAGATGTTTTTTAGTAGGCAGGCAGCTCTTCTCTAAGCGTAATTCAGGGCTCTGG
GTTCTGTCCATCCTGGCCCT
>608
TTGGCAGCCCTATGGAAGACATCAAAAGTTTATTTCTTATTTATATAAAGTCCAAAATAG
ATGTTTTTTAGTAGGCAGGCAGCTCTTCTCTAAGCGTAATTCAGGGCTCTGGGTTCTGT
CCATCCTGGCCCTGACGTCTT
>609
AAGTTTATTTCTTATTTATATAAAGTCCAAAATAGATGTTTTTTAGTAGGCAGGCAGCTC
TTCTCTAAGCGTAATTCAGGGCTCTGGGTTCTGTCCATCCTGGCCCTGACGTCTTTCATT
GCTCAGCTTCTAAGATAGCT
>610
TTTTAGTAGGCAGGCAGCTCTTCTCTAAGCGTAATTCAGGGCTCTGGGTTCTGTCCATC
CTGGCCCTGACGTCTTTCATTGCTCAGCTTCTAAGATAGCTCTATGCTCATGGGGTCAA
GTCACAGGAAAGAAAAGGGAAT
>611
CTGGGTTCTGTCCATCCTGGCCCTGACGTCTTTCATTGCTCAGCTTCTAAGATAGCTCTA
TGCTCATGGGGTCAAGTCACAGGAAAGAAAAGGGAATGTGGAGTTCCACTTGGGAGT
TTTTATGGGCCAGTCCTGGAAGC
>612
TTCTGTCCATCCTGGCCCTGACGTCTTTCATTGCTCAGCTTCTAAGATAGCTCTATGCTC
ATGGGGTCAAGTCACAGGAAAGAAAAGGGAATGTGGAGTTCCACTTGGGAGTTTTTAT
GGGCCAGTCCTGGAAGCGGCAC
>613
GTCTTTCATTGCTCAGCTTCTAAGATAGCTCTATGCTCATGGGGTCAAGTCACAGGAAA
GAAAAGGGAATGTGGAGTTCCACTTGGGAGTTTTTATGGGCCAGTCCTGGAAGCGGCA
CAGAGGATTTCTGCTCACAATCT
>614
TCTAAGATAGCTCTATGCTCATGGGGTCAAGTCACAGGAAAGAAAAGGGAATGTGGAG
TTCCACTTGGGAGTTTTTATGGGCCAGTCCTGGAAGCGGCACAGAGGATTTCTGCTCA
CAATCTATTGGCCAGAACTCAATC
>615
GCGGCACAGAGGATTTCTGCTCACAATCTATTGGCCAGAACTCAATCACATGGCCTGC
ACCTAACTGCAAGTTAAGCCAGAAAAATGTAGGTGTGCCCAGGAAGAAGTTTGGGGA
ACAGCTAGCCAGTCTCTGCCACACC
>616
CTGCTCACAATCTATTGGCCAGAACTCAATCACATGGCCTGCACCTAACTGCAAGTTAA
GCCAGAAAAATGTAGGTGTGCCCAGGAAGAAGTTTGGGGAACAGCTAGCCAGTCTCT
GCCACACCTGGTAACAGCAGAGTG
>617
AGAACTCAATCACATGGCCTGCACCTAACTGCAAGTTAAGCCAGAAAAATGTAGGTGT
GCCCAGGAAGAAGTTTGGGGAACAGCTAGCCAGTCTCTGCCACACCTGGTAACAGCA
GAGTGAATCTGTCAGTACTCGTGCC
>618
GCACCTAACTGCAAGTTAAGCCAGAAAAATGTAGGTGTGCCCAGGAAGAAGTTTGGG

GAACAGCTAGCCAGTCTCTGCCACACCTGGTAACAGCAGAGTGAATCTGTCAGTACTC
GTGCCTAATGCGTGTCTCATATGCT
>619
TAAGCCAGAAAAATGTAGGTGTGCCCAGGAAGAAGTTTGGGGAACAGCTAGCCAGTC
TCTGCCACACCTGGTAACAGCAGAGTGAATCTGTCAGTACTCGTGCCTAATGCGTGTCT
CATATGCTAGTGCTGGATTCAGTC
>620
TGTGCCCAGGAAGAAGTTTGGGGAACAGCTAGCCAGTCTCTGCCACACCTGGTAACA
GCAGAGTGAATCTGTCAGTACTCGTGCCTAATGCGTGTCTCATATGCTAGTGCTGGATT
CAGTCAGCAGTCCTACATCCGCTG
>621
GGAACAGCTAGCCAGTCTCTGCCACACCTGGTAACAGCAGAGTGAATCTGTCAGTACT
CGTGCCTAATGCGTGTCTCATATGCTAGTGCTGGATTCAGTCAGCAGTCCTACATCCGCT
GGGGTCCCTAGGAGGGGTCAGA
>622
AACAGCTAGCCAGTCTCTGCCACACCTGGTAACAGCAGAGTGAATCTGTCAGTACTCG
TGCCTAATGCGTGTCTCATATGCTAGTGCTGGATTCAGTCAGCAGTCCTACATCCGCTG
GGGTCCCTAGGAGGGGTCAGATG
>623
TCTCTGCCACACCTGGTAACAGCAGAGTGAATCTGTCAGTACTCGTGCCTAATGCGTGT
CTCATATGCTAGTGCTGGATTCAGTCAGCAGTCCTACATCCGCTGGGGTCCCTAGGAGG
GGTCAGATGCTCTCCTCTTTGC
>624
CAGTACTCGTGCCTAATGCGTGTCTCATATGCTAGTGCTGGATTCAGTCAGCAGTCCTA
CATCCGCTGGGGTCCCTAGGAGGGGTCAGATGCTCTCCTCTTTGCCCTGAACTGGTTAG
CTACTTCGGACTGGGATGACAA
>625
TAATGCGTGTCTCATATGCTAGTGCTGGATTCAGTCAGCAGTCCTACATCCGCTGGGGT
CCCTAGGAGGGGTCAGATGCTCTCCTCTTTGCCCTGAACTGGTTAGCTACTTCGGACTG
GGATGACAAGGCCATTGTGAGA
>626
TCAGTCAGCAGTCCTACATCCGCTGGGGTCCCTAGGAGGGGTCAGATGCTCTCCTCTTT
GCCCTGAACTGGTTAGCTACTTCGGACTGGGATGACAAGGCCATTGTGAGAGTGATAA
GCAATGTGATTCAAACAAATTCA
>627
GGAGGGGTCAGATGCTCTCCTCTTTGCCCTGAACTGGTTAGCTACTTCGGACTGGGAT
GACAAGGCCATTGTGAGAGTGATAAGCAATGTGATTCAAACAAATTCATTTCACTTCAA
TTCAATGCAGGTTTTTTTTTTTT
>628
GTCAGATGCTCTCCTCTTTGCCCTGAACTGGTTAGCTACTTCGGACTGGGATGACAAGG
CCATTGTGAGAGTGATAAGCAATGTGATTCAAACAAATTCATTTCACTTCAATTCAATG
CAGGTTTTTTTTTTTTTTTGAG
>629
TGACAAGGCCATTGTGAGAGTGATAAGCAATGTGATTCAAACAAATTCATTTCACTTCA
ATTCAATGCAGGTTTTTTTTTTTTTTTGAGACGGGCTGTCATGCTGTTGCCAACCCTGG
AGAGCAGTGGCGCCATTACGGC

>630
GCAATGTGATTCAAACAAATTCATTTCACTTCAATTCAATGCAGGTTTTTTTTTTTTTTT
GAGACGGGCTGTCATGCTGTTGCCAACCCTGGAGAGCAGTGGCGCCATTACGGCTCAC
TGCAGCCTCCTGGGCTCAAGCA
>631
CTTCAATTCAATGCAGGTTTTTTTTTTTTTTTGAGACGGGCTGTCATGCTGTTGCCAACC
CTGGAGAGCAGTGGCGCCATTACGGCTCACTGCAGCCTCCTGGGCTCAAGCAATTCTC
CCTCCTCAGCTTCCCAAGTAGC
>632
GAGCAGTGGCGCCATTACGGCTCACTGCAGCCTCCTGGGCTCAAGCAATTCTCCCTCC
TCAGCTTCCCAAGTAGCTGGGACTACAGGTGCATGCCACCATGTTCAGCTCATTTATTT
TTTATTTTTTTGTAGACAGGAGG
>633
ACGGCTCACTGCAGCCTCCTGGGCTCAAGCAATTCTCCCTCCTCAGCTTCCCAAGTAG
CTGGGACTACAGGTGCATGCCACCATGTTCAGCTCATTTATTTTTTATTTTTTTGTAGAC
AGGAGGTCTCACTATGTTACCC
>634
TCAGCTTCCCAAGTAGCTGGGACTACAGGTGCATGCCACCATGTTCAGCTCATTTATTT
TTTATTTTTTTGTAGACAGGAGGTCTCACTATGTTACCCAGGCTGCTGTCCAAGTCCTG
GGCTCACGTGATTCTCCCGCCT
>635
AGTAGCTGGGACTACAGGTGCATGCCACCATGTTCAGCTCATTTATTTTTTATTTTTTTG
TAGACAGGAGGTCTCACTATGTTACCCAGGCTGCTGTCCAAGTCCTGGGCTCACGTGA
TTCTCCCGCCTCAGCCTCCCAA
>636
CACCATGTTCAGCTCATTTATTTTTTATTTTTTTGTAGACAGGAGGTCTCACTATGTTACC
CAGGCTGCTGTCCAAGTCCTGGGCTCACGTGATTCTCCCGCCTCAGCCTCCCAAAATG
CTGGGATTACAGGCGTAAGCC
>637
ATTTTTTATTTTTTTGTAGACAGGAGGTCTCACTATGTTACCCAGGCTGCTGTCCAAGTC
CTGGGCTCACGTGATTCTCCCGCCTCAGCCTCCCAAAATGCTGGGATTACAGGCGTAA
GCCACCATGCCCAGACAATTTA
>638
GGCTGCTGTCCAAGTCCTGGGCTCACGTGATTCTCCCGCCTCAGCCTCCCAAAATGCT
GGGATTACAGGCGTAAGCCACCATGCCCAGACAATTTAATAAATATTTATTAAAACTTCT
AGGATATTGAGAAAAGCTTGGT
>639
TAAAACTTCTAGGATATTGAGAAAAGCTTGGTTCTAGGCCACTGGGGAAGACATCAAC
TGAAACAGCCCAGTTATACTCTCAATTAGCTGTATACAATTAAATTCACAGATATCCTCA
ACCCTTATCCATATCCCATTCA
>640
AGAAAAGCTTGGTTCTAGGCCACTGGGGAAGACATCAACTGAAACAGCCCAGTTATAC
TCTCAATTAGCTGTATACAATTAAATTCACAGATATCCTCAACCCTTATCCATATCCCATT
CATCTTTCAAAGGTTGGTGAA
>641
TAGCTGTATACAATTAAATTCACAGATATCCTCAACCCTTATCCATATCCCATTCATCTTT

CAAAGGTTGGTGAAAACCTCATAGCACAAAGAATTCTGCCCTAAATGTTTGAATACAG
AATGGTCCCCTCTCTCCCTAA
>642
CTTATCCATATCCCATTCATCTTTCAAAGGTTGGTGAAAACCTCATAGCACAAAGAATTC
TGCCCTAAATGTTTGAATACAGAATGGTCCCCTCTCTCCCTAATTCCTAAAATATTTGGT
CTCTATGTTCATTCATTCAT
>643
TCATCTTTCAAAGGTTGGTGAAAACCTCATAGCACAAAGAATTCTGCCCTAAATGTTTG
AATACAGAATGGTCCCCTCTCTCCCTAATTCCTAAAATATTTGGTCTCTATGTTCATTCAT
TCATTCATGCATGCATGCTT
>644
CACAAAGAATTCTGCCCTAAATGTTTGAATACAGAATGGTCCCCTCTCTCCCTAATTCCT
AAAATATTTGGTCTCTATGTTCATTCATTCATTCATGCATGCATGCTTTCAGTTAGTTCAT
CGACAAACATTTACATCTA
>645
ATTCTGCCCTAAATGTTTGAATACAGAATGGTCCCCTCTCTCCCTAATTCCTAAAATATTT
GGTCTCTATGTTCATTCATTCATTCATGCATGCATGCTTTCAGTTAGTTCATCGACAAAC
ATTTACATCTACTCTGTTT
>646
CCCTCTCTCCCTAATTCCTAAAATATTTGGTCTCTATGTTCATTCATTCATTCATGCATGC
ATGCTTTCAGTTAGTTCATCGACAAACATTTACATCTACTCTGTTTGACAGTGTGCTAGG
CTCTGGGGCTTGGGAGTGG
>647
CTCTCCCTAATTCCTAAAATATTTGGTCTCTATGTTCATTCATTCATTCATGCATGCATGC
TTTCAGTTAGTTCATCGACAAACATTTACATCTACTCTGTTTGACAGTGTGCTAGGCTCT
GGGGCTTGGGAGTGGAAAT
>648
CTATGTTCATTCATTCATTCATGCATGCATGCTTTCAGTTAGTTCATCGACAAACATTTAC
ATCTACTCTGTTTGACAGTGTGCTAGGCTCTGGGGCTTGGGAGTGGAAATACTAAATTG
AACAAGACTGAGGCCTTGCC
>649
TTCAGTTAGTTCATCGACAAACATTTACATCTACTCTGTTTGACAGTGTGCTAGGCTCTG
GGGCTTGGGAGTGGAAATACTAAATTGAACAAGACTGAGGCCTTGCCTTCCAGAAACT
CACAAAGGAGTGGGTGGTCAGA
>650
CTGGGGCTTGGGAGTGGAAATACTAAATTGAACAAGACTGAGGCCTTGCCTTCCAGAA
ACTCACAAAGGAGTGGGTGGTCAGAATGCACATGACTCAGTCAGATATAAGGCAGATG
GTGGTAAATACTATGATAAATGTT
>651
GGCTTGGGAGTGGAAATACTAAATTGAACAAGACTGAGGCCTTGCCTTCCAGAAACTC
ACAAAGGAGTGGGTGGTCAGAATGCACATGACTCAGTCAGATATAAGGCAGATGGTGG
TAAATACTATGATAAATGTTCAGA
>652
TTGAACAAGACTGAGGCCTTGCCTTCCAGAAACTCACAAAGGAGTGGGTGGTCAGAA
TGCACATGACTCAGTCAGATATAAGGCAGATGGTGGTAAATACTATGATAAATGTTCAG
ATAGTGCTAAGGGGCTTATGGAGA

>653
CTTCCAGAAACTCACAAAGGAGTGGGTGGTCAGAATGCACATGACTCAGTCAGATATA
AGGCAGATGGTGGTAAATACTATGATAAATGTTCAGATAGTGCTAAGGGGCTTATGGAG
AATGGGAGAAATTAAATCTGACT
>654
GGAGTGGGTGGTCAGAATGCACATGACTCAGTCAGATATAAGGCAGATGGTGGTAAAT
ACTATGATAAATGTTCAGATAGTGCTAAGGGGCTTATGGAGAATGGGAGAAATTAAATC
TGACTCAAGGGTGGGTGGAGAAG
>655
GGTCAGAATGCACATGACTCAGTCAGATATAAGGCAGATGGTGGTAAATACTATGATAA
ATGTTCAGATAGTGCTAAGGGGCTTATGGAGAATGGGAGAAATTAAATCTGACTCAAG
GGTGGGTGGAGAAGATTACATCC
>656
TACTATGATAAATGTTCAGATAGTGCTAAGGGGCTTATGGAGAATGGGAGAAATTAAAT
CTGACTCAAGGGTGGGTGGAGAAGATTACATCCAAGATCCAGCAATGACTTCCAGAAG
AGACAAATGAACCTGGCCTGGCA
>657
ATGATAAATGTTCAGATAGTGCTAAGGGGCTTATGGAGAATGGGAGAAATTAAATCTGA
CTCAAGGGTGGGTGGAGAAGATTACATCCAAGATCCAGCAATGACTTCCAGAAGAGA
CAAATGAACCTGGCCTGGCAAGGG
>658
AGATTACATCCAAGATCCAGCAATGACTTCCAGAAGAGACAAATGAACCTGGCCTGGC
AAGGGAGGCAGAGTCAAAGTGTCTGGCTCAAAGCCCACCACAGCCTTGGCTCACAGG
CATCTCCCGGTCTTCCACTCACTCA
>659
CATCCAAGATCCAGCAATGACTTCCAGAAGAGACAAATGAACCTGGCCTGGCAAGGG
AGGCAGAGTCAAAGTGTCTGGCTCAAAGCCCACCACAGCCTTGGCTCACAGGCATCT
CCCGGTCTTCCACTCACTCAGTCCTT
>660
TGGCAAGGGAGGCAGAGTCAAAGTGTCTGGCTCAAAGCCCACCACAGCCTTGGCTCA
CAGGCATCTCCCGGTCTTCCACTCACTCAGTCCTTTCAGGGAAAGGCACTGAGCTTTT
CTCAAGGGAGAACTCCAAAGACGTC
>661
GTCAAAGTGTCTGGCTCAAAGCCCACCACAGCCTTGGCTCACAGGCATCTCCCGGTCT
TCCACTCACTCAGTCCTTTCAGGGAAAGGCACTGAGCTTTTCTCAAGGGAGAACTCCA
AAGACGTCTTTCATTTCATCTTAC
>662
CCGGTCTTCCACTCACTCAGTCCTTTCAGGGAAAGGCACTGAGCTTTTCTCAAGGGAG
AACTCCAAAGACGTCTTTCATTTCATCTTACAAATCCACACAAATTTGCAATTCCACTC
CACAAGATACGAGCATTTGTCTT
>663
AAGACGTCTTTCATTTCATCTTACAAATCCACACAAATTTGCAATTCCACTCCACAAGA
TACGAGCATTTGTCTTGCTATAATTTTAATATGATTGGCATAAATTACCTGTTATCAAGTA
AAATAGTTTCTTCTTATGGC
>664
TGCAATTCCACTCCACAAGATACGAGCATTTGTCTTGCTATAATTTTAATATGATTGGCAT

AAATTACCTGTTATCAAGTAAAATAGTTTCTTCTTATGGCTTGTGTGCCCCTGACATACC
ACTGCTATTTGAAAAGAGG
>665
CACTCCACAAGATACGAGCATTTGTCTTGCTATAATTTTAATATGATTGGCATAAATTACC
TGTTATCAAGTAAAATAGTTTCTTCTTATGGCTTGTGTGCCCCTGACATACCACTGCTAT
TTGAAAAGAGGCTTCTCCA
>666
CTTGCTATAATTTTAATATGATTGGCATAAATTACCTGTTATCAAGTAAAATAGTTTCTTC
TTATGGCTTGTGTGCCCCTGACATACCACTGCTATTTGAAAAGAGGCTTCTCCAGAAAC
TCAGTGTCTGAGAGTACGTG
>667
GTTTCTTCTTATGGCTTGTGTGCCCCTGACATACCACTGCTATTTGAAAAGAGGCTTCTC
CAGAAACTCAGTGTCTGAGAGTACGTGACTGGAAGGTGAGTGTTTGCTTACCCCTTGC
GTTTTCTCCCTGTCAGGATAAA
>668
TTCTTCTTATGGCTTGTGTGCCCCTGACATACCACTGCTATTTGAAAAGAGGCTTCTCCA
GAAACTCAGTGTCTGAGAGTACGTGACTGGAAGGTGAGTGTTTGCTTACCCCTTGCGT
TTTCTCCCTGTCAGGATAAATG
>669
CTTGTGTGCCCCTGACATACCACTGCTATTTGAAAAGAGGCTTCTCCAGAAACTCAGT
GTCTGAGAGTACGTGACTGGAAGGTGAGTGTTTGCTTACCCCTTGCGTTTTCTCCCTGT
CAGGATAAATGGAATATTCTGCT
>670
CTGACATACCACTGCTATTTGAAAAGAGGCTTCTCCAGAAACTCAGTGTCTGAGAGTA
CGTGACTGGAAGGTGAGTGTTTGCTTACCCCTTGCGTTTTCTCCCTGTCAGGATAAATG
GAATATTCTGCTTTAAGGACCCT
>671
CATACCACTGCTATTTGAAAAGAGGCTTCTCCAGAAACTCAGTGTCTGAGAGTACGTG
ACTGGAAGGTGAGTGTTTGCTTACCCCTTGCGTTTTCTCCCTGTCAGGATAAATGGAAT
ATTCTGCTTTAAGGACCCTAAGT
>672
AAGAGGCTTCTCCAGAAACTCAGTGTCTGAGAGTACGTGACTGGAAGGTGAGTGTTT
GCTTACCCCTTGCGTTTTCTCCCTGTCAGGATAAATGGAATATTCTGCTTTAAGGACCCT
AAGTCTGGCAATATCATGTTTTG
>673
GAGTGTTTGCTTACCCCTTGCGTTTTCTCCCTGTCAGGATAAATGGAATATTCTGCTTTA
AGGACCCTAAGTCTGGCAATATCATGTTTTGTGAACAGCCAACACAGTAACTATGGTTG
AGGCCTGTATTCGATGAATGA
>674
CCTTGCGTTTTCTCCCTGTCAGGATAAATGGAATATTCTGCTTTAAGGACCCTAAGTCTG
GCAATATCATGTTTTGTGAACAGCCAACACAGTAACTATGGTTGAGGCCTGTATTCGAT
GAATGAGGTTTTCATATGCTT
>675
GGATAAATGGAATATTCTGCTTTAAGGACCCTAAGTCTGGCAATATCATGTTTTGTGAAC
AGCCAACACAGTAACTATGGTTGAGGCCTGTATTCGATGAATGAGGTTTTCATATGCTT
CTTTCACATCAGGAGAGAGAG

>676
GAATATTCTGCTTTAAGGACCCTAAGTCTGGCAATATCATGTTTTGTGAACAGCCAACA
CAGTAACTATGGTTGAGGCCTGTATTCGATGAATGAGGTTTTCATATGCTTCTTTCACAT
CAGGAGAGAGAGAGAGAGAAAAA
>677
CTTTAAGGACCCTAAGTCTGGCAATATCATGTTTTGTGAACAGCCAACACAGTAACTAT
GGTTGAGGCCTGTATTCGATGAATGAGGTTTTCATATGCTTCTTTCACATCAGGAGAGA
GAGAGAGAAAAAAAAAATAATAA
>678
TCTGGCAATATCATGTTTTGTGAACAGCCAACACAGTAACTATGGTTGAGGCCTGTATT
CGATGAATGAGGTTTTCATATGCTTCTTTCACATCAGGAGAGAGAGAGAGAAAAAAAA
ATAATAAACTTGGTAGAGGACGT
>679
CGATGAATGAGGTTTTCATATGCTTCTTTCACATCAGGAGAGAGAGAGAGAAAAAAAA
ATAATAAACTTGGTAGAGGACGTGGAGTTGTATTTCTGGACTATGGTGGCACCTCTAAG
GAGCATGGTTTTCCTTTTGCATG
>680
AGGTTTTCATATGCTTCTTTCACATCAGGAGAGAGAGAGAGAAAAAAAAATAATAAAC
TTGGTAGAGGACGTGGAGTTGTATTTCTGGACTATGGTGGCACCTCTAAGGAGCATGGT
TTTCCTTTTGCATGGCTTTGTCC
>681
TCAGGAGAGAGAGAGAGAAAAAAAAATAATAAACTTGGTAGAGGACGTGGAGTTGTA
TTTCTGGACTATGGTGGCACCTCTAAGGAGCATGGTTTTCCTTTTGCATGGCTTTGTCCA
TTACTATAACACTCCTGTTATAA
>682
AAAAATAATAAACTTGGTAGAGGACGTGGAGTTGTATTTCTGGACTATGGTGGCACCTC
TAAGGAGCATGGTTTTCCTTTTGCATGGCTTTGTCCATTACTATAACACTCCTGTTATAA
AATAGCTGCCGTGGATTGAAA
>683
GGACGTGGAGTTGTATTTCTGGACTATGGTGGCACCTCTAAGGAGCATGGTTTTCCTTT
TGCATGGCTTTGTCCATTACTATAACACTCCTGTTATAAAATAGCTGCCGTGGATTGAAA
TCCATAATGTTAACTCAAAAG
>684
GACTATGGTGGCACCTCTAAGGAGCATGGTTTTCCTTTTGCATGGCTTTGTCCATTACTA
TAACACTCCTGTTATAAAATAGCTGCCGTGGATTGAAATCCATAATGTTAACTCAAAAG
TTAAGCAGTCCGCTATTAAAC
>685
TAAGGAGCATGGTTTTCCTTTTGCATGGCTTTGTCCATTACTATAACACTCCTGTTATAA
AATAGCTGCCGTGGATTGAAATCCATAATGTTAACTCAAAAGTTAAGCAGTCCGCTATT
AAACAGCATAAGAAACTGTTA
>686
TTTGCATGGCTTTGTCCATTACTATAACACTCCTGTTATAAAATAGCTGCCGTGGATTGA
AATCCATAATGTTAACTCAAAAGTTAAGCAGTCCGCTATTAAACAGCATAAGAAACTGT
TACCTATCCCCTCTTCCTGGC
>687
ACTATAACACTCCTGTTATAAAATAGCTGCCGTGGATTGAAATCCATAATGTTAACTCAA

AAGTTAAGCAGTCCGCTATTAAACAGCATAAGAAACTGTTACCTATCCCCTCTTCCTGG
CCCCCCAACAAAGAAACAGCA
>688
TGCCGTGGATTGAAATCCATAATGTTAACTCAAAAGTTAAGCAGTCCGCTATTAAACAG
CATAAGAAACTGTTACCTATCCCCTCTTCCTGGCCCCCCAACAAAGAAACAGCAAACT
GTTAAAAAAGCCCATGTCTCCCC
>689
GCTATTAAACAGCATAAGAAACTGTTACCTATCCCCTCTTCCTGGCCCCCCAACAAAGA
AACAGCAAACTGTTAAAAAAGCCCATGTCTCCCCATGAGGACTTGGGGTTAATTTTCA
GCATGGTGTCTGGGGATTCATCT
>690
AAGAAACTGTTACCTATCCCCTCTTCCTGGCCCCCCAACAAAGAAACAGCAAACTGTT
AAAAAAGCCCATGTCTCCCCATGAGGACTTGGGGTTAATTTTCAGCATGGTGTCTGGG
GATTCATCTGTGGGACTCCTATTA
>691
ACAAAGAAACAGCAAACTGTTAAAAAAGCCCATGTCTCCCCATGAGGACTTGGGGTT
AATTTTCAGCATGGTGTCTGGGGATTCATCTGTGGGACTCCTATTACCCATAATAGGACT
CATGCTGCTTCATTTCATGCACA
>692
AAAGAAACAGCAAACTGTTAAAAAAGCCCATGTCTCCCCATGAGGACTTGGGGTTAAT
TTTCAGCATGGTGTCTGGGGATTCATCTGTGGGACTCCTATTACCCATAATAGGACTCAT
GCTGCTTCATTTCATGCACACG
>693
GTTAAAAAAGCCCATGTCTCCCCATGAGGACTTGGGGTTAATTTTCAGCATGGTGTCTG
GGGATTCATCTGTGGGACTCCTATTACCCATAATAGGACTCATGCTGCTTCATTTCATGC
ACACGATATGGAAAATGCACC
>694
CCTATTACCCATAATAGGACTCATGCTGCTTCATTTCATGCACACGATATGGAAAATGCA
CCCTTCCCTAGTGATGAAATTTTAGTTTTGAAATTGAGGCTAGCTTTTGCAGAACAAAG
CAGATTCCCAGAAACACCAAA
>695
TAGGACTCATGCTGCTTCATTTCATGCACACGATATGGAAAATGCACCCTTCCCTAGTGA
TGAAATTTTAGTTTTGAAATTGAGGCTAGCTTTTGCAGAACAAAGCAGATTCCCAGAA
ACACCAAAAACTATTCAATGTT
>696
TTAGTTTTGAAATTGAGGCTAGCTTTTGCAGAACAAAGCAGATTCCCAGAAACACCAA
AAACTATTCAATGTTGGCCTAGAGCCTGTGTGTCCGTGCTTAGGTGAGTATGTGTATCT
GAAAGGGAGAAAATGCCTTCCTC
>697
AGGCTAGCTTTTGCAGAACAAAGCAGATTCCCAGAAACACCAAAAACTATTCAATGTT
GGCCTAGAGCCTGTGTGTCCGTGCTTAGGTGAGTATGTGTATCTGAAAGGGAGAAAAT
GCCTTCCTCTAAGTGCGTATGCCC
>698
AACAAAGCAGATTCCCAGAAACACCAAAAACTATTCAATGTTGGCCTAGAGCCTGTGT
GTCCGTGCTTAGGTGAGTATGTGTATCTGAAAGGGAGAAAATGCCTTCCTCTAAGTGCG
TATGCCCCTCTCAAAGACAATAT

>699
GGCCTAGAGCCTGTGTGTCCGTGCTTAGGTGAGTATGTGTATCTGAAAGGGAGAAAAT
GCCTTCCTCTAAGTGCGTATGCCCCTCTCAAAGACAATATTCAAATATCATGCCCGATAG
CTGACAGCGGTCAATGGTTGCT
>700
TAGAGCCTGTGTGTCCGTGCTTAGGTGAGTATGTGTATCTGAAAGGGAGAAAATGCCTT
CCTCTAAGTGCGTATGCCCCTCTCAAAGACAATATTCAAATATCATGCCCGATAGCTGAC
AGCGGTCAATGGTTGCTATGC
>701
TGCCTTCCTCTAAGTGCGTATGCCCCTCTCAAAGACAATATTCAAATATCATGCCCGATA
GCTGACAGCGGTCAATGGTTGCTATGCTTCATCCCAAAGCTTTGTTAGGAACCCCATAA
AACCTGGCACATGGTTTGTGA
>702
CTCTAAGTGCGTATGCCCCTCTCAAAGACAATATTCAAATATCATGCCCGATAGCTGACA
GCGGTCAATGGTTGCTATGCTTCATCCCAAAGCTTTGTTAGGAACCCCATAAAACCTGG
CACATGGTTTGTGACACCCAG
>703
GACAATATTCAAATATCATGCCCGATAGCTGACAGCGGTCAATGGTTGCTATGCTTCATC
CCAAAGCTTTGTTAGGAACCCCATAAAACCTGGCACATGGTTTGTGACACCCAGTGAA
ACACCGTGCTTCTTCCATAATA
>704
TGACAGCGGTCAATGGTTGCTATGCTTCATCCCAAAGCTTTGTTAGGAACCCCATAAAA
CCTGGCACATGGTTTGTGACACCCAGTGAAACACCGTGCTTCTTCCATAATATCCTGAG
CCCTGCTGCCTGTCATTAAGGG
>705
AGCGGTCAATGGTTGCTATGCTTCATCCCAAAGCTTTGTTAGGAACCCCATAAAACCTG
GCACATGGTTTGTGACACCCAGTGAAACACCGTGCTTCTTCCATAATATCCTGAGCCCT
GCTGCCTGTCATTAAGGGACCT
>706
CCTGGCACATGGTTTGTGACACCCAGTGAAACACCGTGCTTCTTCCATAATATCCTGAG
CCCTGCTGCCTGTCATTAAGGGACCTCTCTTATTAAGCATCTCTTTTTTAAATGGCTGCC
ATCATCTGGTCCCAGTGTACA
>707
AGCCCTGCTGCCTGTCATTAAGGGACCTCTCTTATTAAGCATCTCTTTTTTAAATGGCTG
CCATCATCTGGTCCCAGTGTACATTAAATCCCATTATAATTATAAACACACTTCCCCATAA
CATTCGTTGCAGATCTCAC
>708
GCTGCCTGTCATTAAGGGACCTCTCTTATTAAGCATCTCTTTTTTAAATGGCTGCCATCA
TCTGGTCCCAGTGTACATTAAATCCCATTATAATTATAAACACACTTCCCCATAACATTCG
TTGCAGATCTCACCCTCAG
>709
GCTGCCATCATCTGGTCCCAGTGTACATTAAATCCCATTATAATTATAAACACACTTCCC
CATAACATTCGTTGCAGATCTCACCCTCAGTGCCTACCCCCTTAGGGCATCTTTGCACTA
ATAACAACTCAGATTTGCTA
>710
AGTGTACATTAAATCCCATTATAATTATAAACACACTTCCCCATAACATTCGTTGCAGATC

TCACCCTCAGTGCCTACCCCCTTAGGGCATCTTTGCACTAATAACAACTCAGATTTGCTA
TCTGAGTTGACAACATTAG
>711
AGATCTCACCCTCAGTGCCTACCCCCTTAGGGCATCTTTGCACTAATAACAACTCAGAT
TTGCTATCTGAGTTGACAACATTAGGTCCCAACACCATTTGCAATATGCTGGGGAAAGA
GACAGAGAGAGAAGAAAAAGCT
>712
GTGCCTACCCCCTTAGGGCATCTTTGCACTAATAACAACTCAGATTTGCTATCTGAGTTG
ACAACATTAGGTCCCAACACCATTTGCAATATGCTGGGGAAAGAGACAGAGAGAGAA
GAAAAAGCTTTTTCTTCCTTAAT
>713
GCAATATGCTGGGGAAAGAGACAGAGAGAGAAGAAAAAGCTTTTTCTTCCTTAATCAC
GAAGATCTTGGGGTCACAGTGACCCAAGGCCTCAGGAGCTAAAAAAAAAAAAATCCCT
CCTCCTACCCTTTTGAAAAGTCACT
>714
TGCTGGGGAAAGAGACAGAGAGAGAAGAAAAAGCTTTTTCTTCCTTAATCACGAAGA
TCTTGGGGTCACAGTGACCCAAGGCCTCAGGAGCTAAAAAAAAAAAAATCCCTCCTCCT
ACCCTTTTGAAAAGTCACTCTCTAC
>715
CTTGGGGTCACAGTGACCCAAGGCCTCAGGAGCTAAAAAAAAAAAAATCCCTCCTCCT
ACCCTTTTGAAAAGTCACTCTCTACTCTACAGATGAGGGAAGGGCATACTTGGCACCT
GGGAGAGAAACTCAAGTTTGTAAAC
>716
CTTTTGAAAAGTCACTCTCTACTCTACAGATGAGGGAAGGGCATACTTGGCACCTGGG
AGAGAAACTCAAGTTTGTAAACACCACTAACCAGGTCTCTGTTGGTTGGGGCGGTGAT
GGTGGTGGTAGTGGTGGGGTGGGG
>717
TGAAAAGTCACTCTCTACTCTACAGATGAGGGAAGGGCATACTTGGCACCTGGGAGAG
AAACTCAAGTTTGTAAACACCACTAACCAGGTCTCTGTTGGTTGGGGCGGTGATGGTG
GTGGTAGTGGTGGGGTGGGGGATG
>718
TCTACAGATGAGGGAAGGGCATACTTGGCACCTGGGAGAGAAACTCAAGTTTGTAAAC
ACCACTAACCAGGTCTCTGTTGGTTGGGGCGGTGATGGTGGTGGTAGTGGTGGGGTGG
GGGATGAGGGATGATGGCAATCAC
>719
CACCTGGGAGAGAAACTCAAGTTTGTAAACACCACTAACCAGGTCTCTGTTGGTTGGG
GCGGTGATGGTGGTGGTAGTGGTGGGGTGGGGGATGAGGGATGATGGCAATCACTAAT
CAAAGAAGCTGTCTTATTTGGTTT
>720
CTGGGAGAGAAACTCAAGTTTGTAAACACCACTAACCAGGTCTCTGTTGGTTGGGGCG
GTGATGGTGGTGGTAGTGGTGGGGTGGGGGATGAGGGATGATGGCAATCACTAATCAA
AGAAGCTGTCTTATTTGGTTTGCT
>721
GAGAAACTCAAGTTTGTAAACACCACTAACCAGGTCTCTGTTGGTTGGGGCGGTGATG
GTGGTGGTAGTGGTGGGGTGGGGGATGAGGGATGATGGCAATCACTAATCAAAGAAG
CTGTCTTATTTGGTTTGCTGAGCCT

>722
GGGCGGTGATGGTGGTGGTAGTGGTGGGGTGGGGGATGAGGGATGATGGCAATCACTA
ATCAAAGAAGCTGTCTTATTTGGTTTGCTGAGCCTCAACTAACCGGAATGCATACATCA
CCTGATTCTTTCCCCCTCTGCTT
>723
GGTGGTGGTAGTGGTGGGGTGGGGGATGAGGGATGATGGCAATCACTAATCAAAGAA
GCTGTCTTATTTGGTTTGCTGAGCCTCAACTAACCGGAATGCATACATCACCTGATTCTT
TCCCCCTCTGCTTTTTGGAGGGG
>724
TAACCGGAATGCATACATCACCTGATTCTTTCCCCCTCTGCTTTTTGGAGGGGGCATCTA
AGGACATAATGTAAAAGAATTAAAATTTAAAAACAAAAACAAAATGTCAACATATGCA
GGAGTAAATTCCACTCTGCCTA
>725
CTCTGCTTTTTGGAGGGGGCATCTAAGGACATAATGTAAAAGAATTAAAATTTAAAAAC
AAAAACAAAATGTCAACATATGCAGGAGTAAATTCCACTCTGCCTAATTTTTTTTTTTTTT
TTGAAATGAGATCTTGTTCTG
>726
GGGCATCTAAGGACATAATGTAAAAGAATTAAAATTTAAAAACAAAAACAAAATGTCA
ACATATGCAGGAGTAAATTCCACTCTGCCTAATTTTTTTTTTTTTTTTGAAATGAGATCTT
GTTCTGTCACCCAGGCTGGAGG
>727
CAAAATGTCAACATATGCAGGAGTAAATTCCACTCTGCCTAATTTTTTTTTTTTTTTTGAA
ATGAGATCTTGTTCTGTCACCCAGGCTGGAGGGCAGTGGTGCAATCTCAGCTCACTGC
AGCCACCACCTCCCAGGTTCAA
>728
TGTCAACATATGCAGGAGTAAATTCCACTCTGCCTAATTTTTTTTTTTTTTTTGAAATGAG
ATCTTGTTCTGTCACCCAGGCTGGAGGGCAGTGGTGCAATCTCAGCTCACTGCAGCCA
CCACCTCCCAGGTTCAAGCGAT
>729
AATTCCACTCTGCCTAATTTTTTTTTTTTTTTTGAAATGAGATCTTGTTCTGTCACCCAGG
CTGGAGGGCAGTGGTGCAATCTCAGCTCACTGCAGCCACCACCTCCCAGGTTCAAGC
GATTCTCCCGCCTCAGCCCCCTA
>730
GATCTTGTTCTGTCACCCAGGCTGGAGGGCAGTGGTGCAATCTCAGCTCACTGCAGCC
ACCACCTCCCAGGTTCAAGCGATTCTCCCGCCTCAGCCCCCTAAGTAGCTGGTATTACA
GGCACGTGCCACCACGCCTGGCT
>731
TGGTGCAATCTCAGCTCACTGCAGCCACCACCTCCCAGGTTCAAGCGATTCTCCCGCC
TCAGCCCCCTAAGTAGCTGGTATTACAGGCACGTGCCACCACGCCTGGCTAATTTTTGT
ATTTTTAGTAGAGATAGGGTTTC
>732
GCAGCCACCACCTCCCAGGTTCAAGCGATTCTCCCGCCTCAGCCCCCTAAGTAGCTGG
TATTACAGGCACGTGCCACCACGCCTGGCTAATTTTTGTATTTTTAGTAGAGATAGGGTT
TCACCATGTTGGCCAGGCTGGT
>733
CGATTCTCCCGCCTCAGCCCCCTAAGTAGCTGGTATTACAGGCACGTGCCACCACGCCT

GGCTAATTTTTGTATTTTTAGTAGAGATAGGGTTTCACCATGTTGGCCAGGCTGGTCTCA
AACTCCTGACCTCAAGTGATC
>734
CCTAAGTAGCTGGTATTACAGGCACGTGCCACCACGCCTGGCTAATTTTTGTATTTTTAG
TAGAGATAGGGTTTCACCATGTTGGCCAGGCTGGTCTCAAACTCCTGACCTCAAGTGA
TCCGCCTGCCTCGGCCTCCTAA
>735
TGGTATTACAGGCACGTGCCACCACGCCTGGCTAATTTTTGTATTTTTAGTAGAGATAGG
GTTTCACCATGTTGGCCAGGCTGGTCTCAAACTCCTGACCTCAAGTGATCCGCCTGCCT
CGGCCTCCTAAAGTGCTGGGA
>736
ACGTGCCACCACGCCTGGCTAATTTTTGTATTTTTAGTAGAGATAGGGTTTCACCATGTT
GGCCAGGCTGGTCTCAAACTCCTGACCTCAAGTGATCCGCCTGCCTCGGCCTCCTAAA
GTGCTGGGAGTACAGGCATGAG
>737
ATTTTTGTATTTTTAGTAGAGATAGGGTTTCACCATGTTGGCCAGGCTGGTCTCAAACTC
CTGACCTCAAGTGATCCGCCTGCCTCGGCCTCCTAAAGTGCTGGGAGTACAGGCATGA
GCCACTGCGCCCAGCCTACTCT
>738
TTTCACCATGTTGGCCAGGCTGGTCTCAAACTCCTGACCTCAAGTGATCCGCCTGCCTC
GGCCTCCTAAAGTGCTGGGAGTACAGGCATGAGCCACTGCGCCCAGCCTACTCTGCCC
AATTGTTGACATCACCTACAGCT
>739
GTTGGCCAGGCTGGTCTCAAACTCCTGACCTCAAGTGATCCGCCTGCCTCGGCCTCCT
AAAGTGCTGGGAGTACAGGCATGAGCCACTGCGCCCAGCCTACTCTGCCCAATTGTTG
ACATCACCTACAGCTCTATAGTAG
>740
CCTGCCTCGGCCTCCTAAAGTGCTGGGAGTACAGGCATGAGCCACTGCGCCCAGCCTA
CTCTGCCCAATTGTTGACATCACCTACAGCTCTATAGTAGATGTCTGGAATGATGATCTG
AAGGACTGTAAGACTGTAAGAT
>741
GGGAGTACAGGCATGAGCCACTGCGCCCAGCCTACTCTGCCCAATTGTTGACATCACC
TACAGCTCTATAGTAGATGTCTGGAATGATGATCTGAAGGACTGTAAGACTGTAAGATT
TTTTGACTCCAGAAATGGACACA
>742
ACAGGCATGAGCCACTGCGCCCAGCCTACTCTGCCCAATTGTTGACATCACCTACAGC
TCTATAGTAGATGTCTGGAATGATGATCTGAAGGACTGTAAGACTGTAAGATTTTTTGA
CTCCAGAAATGGACACAATAGGC
>743
CTACTCTGCCCAATTGTTGACATCACCTACAGCTCTATAGTAGATGTCTGGAATGATGAT
CTGAAGGACTGTAAGACTGTAAGATTTTTTGACTCCAGAAATGGACACAATAGGCTTG
CGGTGTCTTCCCCTAACAGTCC
>744
CCCAATTGTTGACATCACCTACAGCTCTATAGTAGATGTCTGGAATGATGATCTGAAGG
ACTGTAAGACTGTAAGATTTTTTGACTCCAGAAATGGACACAATAGGCTTGCGGTGTCT
TCCCCTAACAGTCCCTGGCAGG

>745
GAATGATGATCTGAAGGACTGTAAGACTGTAAGATTTTTGACTCCAGAAATGGACAC
AATAGGCTTGCGGTGTCTTCCCCTAACAGTCCCTGGCAGGGTAAAGGTCTGATGCCCT
GGTACGTTCCCCAGTGGCAATCTG
>746
ATGATGATCTGAAGGACTGTAAGACTGTAAGATTTTTGACTCCAGAAATGGACACAAT
AGGCTTGCGGTGTCTTCCCCTAACAGTCCCTGGCAGGGTAAAGGTCTGATGCCCTGGT
ACGTTCCCCAGTGGCAATCTGGA
>747
GTAAGATTTTTGACTCCAGAAATGGACACAATAGGCTTGCGGTGTCTTCCCCTAACAG
TCCCTGGCAGGGTAAAGGTCTGATGCCCTGGTACGTTCCCCAGTGGCAATCTGGAGTT
CTCAACTGCAGAGAATACTTTTC
>748
TTTTTGACTCCAGAAATGGACACAATAGGCTTGCGGTGTCTTCCCCTAACAGTCCCTG
GCAGGGTAAAGGTCTGATGCCCTGGTACGTTCCCCAGTGGCAATCTGGAGTTCTCAAC
TGCAGAGAATACTTTTCCTCTAC
>749
GAAATGGACACAATAGGCTTGCGGTGTCTTCCCCTAACAGTCCCTGGCAGGGTAAAGG
TCTGATGCCCTGGTACGTTCCCCAGTGGCAATCTGGAGTTCTCAACTGCAGAGAATACT
TTTCCTCTACTCAGGGGCGTCAC
>750
CCCTGGTACGTTCCCCAGTGGCAATCTGGAGTTCTCAACTGCAGAGAATACTTTTCCTC
TACTCAGGGGCGTCACTACCTGCATCTCTCCCTGAACATGCTTCATTTTCCACTCATCAC
AAGTTTTTGGGGAAATGCAAA
>751
TTTTCCTCTACTCAGGGGCGTCACTACCTGCATCTCTCCCTGAACATGCTTCATTTTCCA
CTCATCACAAGTTTTTGGGGAAATGCAAAGTGGCATCTTCTCCCCAAAAGGTAGGGAT
CTTTGATTTTTTTAATCACGAT
>752
GTCACTACCTGCATCTCTCCCTGAACATGCTTCATTTTCCACTCATCACAAGTTTTTGGG
GAAATGCAAAGTGGCATCTTCTCCCCAAAAGGTAGGGATCTTTGATTTTTTTAATCACG
ATAGAAAAGTTTACTTTGAAA
>753
TGAACATGCTTCATTTTCCACTCATCACAAGTTTTTGGGGAAATGCAAAGTGGCATCTT
CTCCCCAAAAGGTAGGGATCTTTGATTTTTTTAATCACGATAGAAAAGTTTACTTTGAA
ATAAAAAAATTAAATATATGTT
>754
GGGGAAATGCAAAGTGGCATCTTCTCCCCAAAAGGTAGGGATCTTTGATTTTTTTAATC
ACGATAGAAAAGTTTACTTTGAAATAAAAAAATTAAATATATGTTCCAAATTAAGAGTT
GGAACTTAAAGAGTTGCAACAT
>755
AAGGTAGGGATCTTTGATTTTTTTAATCACGATAGAAAAGTTTACTTTGAAATAAAAAA
ATTAAATATATGTTCCAAATTAAGAGTTGGAACTTAAAGAGTTGCAACATTCCATTCATA
CTCAGATACTCAAAACACCAC
>756
TGATTTTTTTAATCACGATAGAAAAGTTTACTTTGAAATAAAAAAATTAAATATATGTTC

CAAATTAAGAGTTGGAACTTAAAGAGTTGCAACATTCCATTCATACTCAGATACTCAAA
ACACCACTGTATTCTTCTTCC
>757
CGATAGAAAAGTTTACTTTGAAATAAAAAAATTAAATATATGTTCCAAATTAAGAGTTG
GAACTTAAAGAGTTGCAACATTCCATTCATACTCAGATACTCAAAACACCACTGTATTC
TTCTTCCTATCTCTTTAGTTGA
>758
TTCCAAATTAAGAGTTGGAACTTAAAGAGTTGCAACATTCCATTCATACTCAGATACTC
AAAACACCACTGTATTCTTCTTCCTATCTCTTTAGTTGAAGTTTTTTTTTGTAATTCAAA
GCCCCCTCCCCAATTTCTGCC
>759
AAAGAGTTGCAACATTCCATTCATACTCAGATACTCAAAACACCACTGTATTCTTCTTC
CTATCTCTTTAGTTGAAGTTTTTTTTTGTAATTCAAAGCCCCCTCCCCAATTTCTGCCTG
AGATTCTGAGGGAGGAGGAAA
>760
CCATTCATACTCAGATACTCAAAACACCACTGTATTCTTCTTCCTATCTCTTTAGTTGAA
GTTTTTTTTTGTAATTCAAAGCCCCCTCCCCAATTTCTGCCTGAGATTCTGAGGGAGGA
GGAAAAGTGTAAGATCCTGGG
>761
TTGAAGTTTTTTTTTGTAATTCAAAGCCCCCTCCCCAATTTCTGCCTGAGATTCTGAGG
GAGGAGGAAAAGTGTAAGATCCTGGGGGATCTAAGTCTCTTTACCTGGTGACCAAGCT
GAGAACCATTTGGTCCTCAGCTT
>762
AAGCCCCCTCCCCAATTTCTGCCTGAGATTCTGAGGGAGGAGGAAAAGTGTAAGATCC
TGGGGGATCTAAGTCTCTTTACCTGGTGACCAAGCTGAGAACCATTTGGTCCTCAGCTT
GGCTGGCTGCTGCACAGATCTTG
>763
AATTTCTGCCTGAGATTCTGAGGGAGGAGGAAAAGTGTAAGATCCTGGGGGATCTAAG
TCTCTTTACCTGGTGACCAAGCTGAGAACCATTTGGTCCTCAGCTTGGCTGGCTGCTGC
ACAGATCTTGGCCTCAGCAGAGT
>764
GAGGAGGAAAAGTGTAAGATCCTGGGGGATCTAAGTCTCTTTACCTGGTGACCAAGCT
GAGAACCATTTGGTCCTCAGCTTGGCTGGCTGCTGCACAGATCTTGGCCTCAGCAGAG
TAAGAGAAGAAGGAAAATTAAAAG
>765
TGGTGACCAAGCTGAGAACCATTTGGTCCTCAGCTTGGCTGGCTGCTGCACAGATCTT
GGCCTCAGCAGAGTAAGAGAAGAAGGAAAATTAAAAGCCCTTGCTTCCTTCATCTATT
TCACTAGAGGCTACATAATCTAGA
>766
TAAGAGAAGAAGGAAAATTAAAAGCCCTTGCTTCCTTCATCTATTTCACTAGAGGCTAC
ATAATCTAGAAGTTAAGAGCATGAGCTTTGCAGTCAGAATGGTCTGATTTAAAGTTTCA
GCTCTGATGTTTAATAGATCTA
>767
AAGCCCTTGCTTCCTTCATCTATTTCACTAGAGGCTACATAATCTAGAAGTTAAGAGCAT
GAGCTTTGCAGTCAGAATGGTCTGATTTAAAGTTTCAGCTCTGATGTTTAATAGATCTAC
AGTCTGCAGTAAGGCACTTA

>768
CTTCCTTCATCTATTTCACTAGAGGCTACATAATCTAGAAGTTAAGAGCATGAGCTTTGC
AGTCAGAATGGTCTGATTTAAAGTTTCAGCTCTGATGTTTAATAGATCTACAGTCTGCA
GTAAGGCACTTAATCTCTCTG
>769
TACATAATCTAGAAGTTAAGAGCATGAGCTTTGCAGTCAGAATGGTCTGATTTAAAGTT
TCAGCTCTGATGTTTAATAGATCTACAGTCTGCAGTAAGGCACTTAATCTCTCTGAACCT
CAGTTTTCATGTCCACAGAAA
>770
TAAGAGCATGAGCTTTGCAGTCAGAATGGTCTGATTTAAAGTTTCAGCTCTGATGTTTA
ATAGATCTACAGTCTGCAGTAAGGCACTTAATCTCTCTGAACCTCAGTTTTCATGTCCA
CAGAAATTGGGAAAATAATAGC
>771
ATGAGCTTTGCAGTCAGAATGGTCTGATTTAAAGTTTCAGCTCTGATGTTTAATAGATCT
ACAGTCTGCAGTAAGGCACTTAATCTCTCTGAACCTCAGTTTTCATGTCCACAGAAATT
GGGAAAATAATAGCACCTGCA
>772
TTAAAGTTTCAGCTCTGATGTTTAATAGATCTACAGTCTGCAGTAAGGCACTTAATCTCT
CTGAACCTCAGTTTTCATGTCCACAGAAATTGGGAAAATAATAGCACCTGCATTAATAG
GATTGTTATGTGGATTAAATA
>773
AATCTCTCTGAACCTCAGTTTTCATGTCCACAGAAATTGGGAAAATAATAGCACCTGCA
TTAATAGGATTGTTATGTGGATTAAATAAAACAACATAGGTAATGTGTTGAGTTTCGTAG
TCAGTGCTCAGAATGAGTTAT
>774
CTCTGAACCTCAGTTTTCATGTCCACAGAAATTGGGAAAATAATAGCACCTGCATTAAT
AGGATTGTTATGTGGATTAAATAAAACAACATAGGTAATGTGTTGAGTTTCGTAGTCAG
TGCTCAGAATGAGTTATTATTT
>775
CACAGAAATTGGGAAAATAATAGCACCTGCATTAATAGGATTGTTATGTGGATTAAATAA
AACAACATAGGTAATGTGTTGAGTTTCGTAGTCAGTGCTCAGAATGAGTTATTATTTTCG
CTGTCTCCTTCCTGTATGAT
>776
AAATTGGGAAAATAATAGCACCTGCATTAATAGGATTGTTATGTGGATTAAATAAAACA
ACATAGGTAATGTGTTGAGTTTCGTAGTCAGTGCTCAGAATGAGTTATTATTTTCGCTGT
CTCCTTCCTGTATGATCTCTT
>777
ATTGGGAAAATAATAGCACCTGCATTAATAGGATTGTTATGTGGATTAAATAAAACAACA
TAGGTAATGTGTTGAGTTTCGTAGTCAGTGCTCAGAATGAGTTATTATTTTCGCTGTCTC
CTTCCTGTATGATCTCTTAG
>778
TAATAGCACCTGCATTAATAGGATTGTTATGTGGATTAAATAAAACAACATAGGTAATGT
GTTGAGTTTCGTAGTCAGTGCTCAGAATGAGTTATTATTTTCGCTGTCTCCTTCCTGTAT
GATCTCTTAGAACTGCTATA
>779
GGATTGTTATGTGGATTAAATAAAACAACATAGGTAATGTGTTGAGTTTCGTAGTCAGT

GCTCAGAATGAGTTATTATTTTCGCTGTCTCCTTCCTGTATGATCTCTTAGAACTGCTATA
CCCTCCTGTTCCACTAACTG
>780
AACATAGGTAATGTGTTGAGTTTCGTAGTCAGTGCTCAGAATGAGTTATTATTTTCGCTG
TCTCCTTCCTGTATGATCTCTTAGAACTGCTATACCCTCCTGTTCCACTAACTGAGCTCT
GAAAGGCTGCCAGTGGTGGG
>781
GTGCTCAGAATGAGTTATTATTTTCGCTGTCTCCTTCCTGTATGATCTCTTAGAACTGCTA
TACCCTCCTGTTCCACTAACTGAGCTCTGAAAGGCTGCCAGTGGTGGGAGGTACATGG
GTAAAGAGGGAGATGAGGGAG
>782
TCCTTCCTGTATGATCTCTTAGAACTGCTATACCCTCCTGTTCCACTAACTGAGCTCTGA
AAGGCTGCCAGTGGTGGGAGGTACATGGGTAAAGAGGGAGATGAGGGAGTAAGATTT
AGCAATGGAATTCCATGCAAAGG
>783
TTCCTGTATGATCTCTTAGAACTGCTATACCCTCCTGTTCCACTAACTGAGCTCTGAAAG
GCTGCCAGTGGTGGGAGGTACATGGGTAAAGAGGGAGATGAGGGAGTAAGATTTAGC
AATGGAATTCCATGCAAAGGCCA
>784
ATGATCTCTTAGAACTGCTATACCCTCCTGTTCCACTAACTGAGCTCTGAAAGGCTGCC
AGTGGTGGGAGGTACATGGGTAAAGAGGGAGATGAGGGAGTAAGATTTAGCAATGGA
ATTCCATGCAAAGGCCAGCTCATC
>785
TGTTCCACTAACTGAGCTCTGAAAGGCTGCCAGTGGTGGGAGGTACATGGGTAAAGAG
GGAGATGAGGGAGTAAGATTTAGCAATGGAATTCCATGCAAAGGCCAGCTCATCTATTC
CTCTGTCTCTAGCATGATCTCCC
>786
TAAAGAGGGAGATGAGGGAGTAAGATTTAGCAATGGAATTCCATGCAAAGGCCAGCTC
ATCTATTCCTCTGTCTCTAGCATGATCTCCCCCTAAGATTGAGATCTCCTGTATTTTTAGA
ACTGATACCCAGGTTTCCCTT
>787
TTCCATGCAAAGGCCAGCTCATCTATTCCTCTGTCTCTAGCATGATCTCCCCCTAAGATT
GAGATCTCCTGTATTTTTAGAACTGATACCCAGGTTTCCCTTAAAAGACGCTTCAAGAA
AAAATGTAAAAGAAAAATCAC
>788
TATTCCTCTGTCTCTAGCATGATCTCCCCCTAAGATTGAGATCTCCTGTATTTTTAGAACT
GATACCCAGGTTTCCCTTAAAAGACGCTTCAAGAAAAAATGTAAAAGAAAAATCACTC
TAAATACACGAACATGAGAGA
>789
AAGATTGAGATCTCCTGTATTTTTAGAACTGATACCCAGGTTTCCCTTAAAAGACGCTT
CAAGAAAAAATGTAAAAGAAAAATCACTCTAAATACACGAACATGAGAGAGACAGAA
AGAGAGCGGGCATGTGCGCGCGAG
>790
CAAGAAAAAATGTAAAAGAAAAATCACTCTAAATACACGAACATGAGAGAGACAGAA
AGAGAGCGGGCATGTGCGCGCGAGAGAGATTGAGTATTCTCTTGTAATTTTCCATCCTG
CTTGTTTTTAGGTGCTGCCATATG

>791
AAATCACTCTAAATACACGAACATGAGAGAGACAGAAAGAGAGCGGGCATGTGCGCG
CGAGAGAGATTGAGTATTCTCTTGTAATTTTCCATCCTGCTTGTTTTTAGGTGCTGCCAT
ATGAAATCTCTGAACTGAGGAGT
>792
AAATACACGAACATGAGAGAGACAGAAAGAGAGCGGGCATGTGCGCGCGAGAGAGA
TTGAGTATTCTCTTGTAATTTTCCATCCTGCTTGTTTTTAGGTGCTGCCATATGAAATCTC
TGAACTGAGGAGTCTCATCAGCC
>793
AGAGAGCGGGCATGTGCGCGCGAGAGAGATTGAGTATTCTCTTGTAATTTTCCATCCTG
CTTGTTTTTAGGTGCTGCCATATGAAATCTCTGAACTGAGGAGTCTCATCAGCCTTCCC
AGGACCCAAGGACATAGCTCTG
>794
GAGTATTCTCTTGTAATTTTCCATCCTGCTTGTTTTTAGGTGCTGCCATATGAAATCTCTG
AACTGAGGAGTCTCATCAGCCTTCCCAGGACCCAAGGACATAGCTCTGGCTATAAATG
GTGCCAGTTTTGGTGGGGGAG
>795
ATGAAATCTCTGAACTGAGGAGTCTCATCAGCCTTCCCAGGACCCAAGGACATAGCTC
TGGCTATAAATGGTGCCAGTTTTGGTGGGGGAGCTGCTGCTGCTTTATCTGGGCCCAAT
AAACCTTATCAAACTGCTGTGCA
>796
TCTCTGAACTGAGGAGTCTCATCAGCCTTCCCAGGACCCAAGGACATAGCTCTGGCTAT
AAATGGTGCCAGTTTTGGTGGGGGAGCTGCTGCTGCTTTATCTGGGCCCAATAAACCTT
ATCAAACTGCTGTGCAGCCAGC
>797
AAATGGTGCCAGTTTTGGTGGGGGAGCTGCTGCTGCTTTATCTGGGCCCAATAAACCTT
ATCAAACTGCTGTGCAGCCAGCCTTCAGCGCTAAGTGGAAATGCTTGGAGCCCAATGC
TGGCATTCAGCCCACAGCCTCGC
>798
GAGCTGCTGCTGCTTTATCTGGGCCCAATAAACCTTATCAAACTGCTGTGCAGCCAGCC
TTCAGCGCTAAGTGGAAATGCTTGGAGCCCAATGCTGGCATTCAGCCCACAGCCTCGC
CTGCCTTCTTCCTTTTTGTTACC
>799
CGCTAAGTGGAAATGCTTGGAGCCCAATGCTGGCATTCAGCCCACAGCCTCGCCTGCC
TTCTTCCTTTTTGTTACCTTCTCCACCTCTTAAATTCAGGCATTAATCTCTCTTTAACACT
TACTAACAAGGGCTGGGCTTT
>800
CACCTCTTAAATTCAGGCATTAATCTCTCTTTAACACTTACTAACAAGGGCTGGGCTTTT
AATGGGCAGCGTTTATGAAGCAAGGGAGATTTTCACATAGAGTGAATATGGCACGGGG
GTGGGGAGGCCAGGGCCATAGA
>801
TAACACTTACTAACAAGGGCTGGGCTTTTAATGGGCAGCGTTTATGAAGCAAGGGAGA
TTTTCACATAGAGTGAATATGGCACGGGGGTGGGGAGGCCAGGGCCATAGAGGATTGT
GCTTTCTCTCCCATTGTTTCCAGA
>802
GGCTGGGCTTTTAATGGGCAGCGTTTATGAAGCAAGGGAGATTTTCACATAGAGTGAAT

ATGGCACGGGGGTGGGGAGGCCAGGGCCATAGAGGATTGTGCTTTCTCTCCCATTGTT
TCCAGAGATTCTCTCTTTCTCTG
>803
TGAAGCAAGGGAGATTTTCACATAGAGTGAATATGGCACGGGGGTGGGGAGGCCAGG
GCCATAGAGGATTGTGCTTTCTCTCCCATTGTTTCCAGAGATTCTCTCTTTCTCTGATTT
GAGGATTAATAGGAGATTAGGAT
>804
TCACATAGAGTGAATATGGCACGGGGGTGGGGAGGCCAGGGCCATAGAGGATTGTGCT
TTCTCTCCCATTGTTTCCAGAGATTCTCTCTTTCTCTGATTTGAGGATTAATAGGAGATT
AGGATCAGGGGTTAATTGTGTG
>805
TTCTCTCCCATTGTTTCCAGAGATTCTCTCTTTCTCTGATTTGAGGATTAATAGGAGATT
AGGATCAGGGGTTAATTGTGTGCTTCTCCTGCTACTGCACTGAGGAGAGGCTGGTAGG
TGGATGTGGACAGCAAAGCGGA
>806
CCATTGTTTCCAGAGATTCTCTCTTTCTCTGATTTGAGGATTAATAGGAGATTAGGATCA
GGGGTTAATTGTGTGCTTCTCCTGCTACTGCACTGAGGAGAGGCTGGTAGGTGGATGT
GGACAGCAAAGCGGAAACCTAC
>807
AGGATTAATAGGAGATTAGGATCAGGGGTTAATTGTGTGCTTCTCCTGCTACTGCACTG
AGGAGAGGCTGGTAGGTGGATGTGGACAGCAAAGCGGAAACCTACAGCAGGCACTAT
CTAGGCAGAAGCTCAACAAGTGTA
>808
TTAATAGGAGATTAGGATCAGGGGTTAATTGTGTGCTTCTCCTGCTACTGCACTGAGGA
GAGGCTGGTAGGTGGATGTGGACAGCAAAGCGGAAACCTACAGCAGGCACTATCTAG
GCAGAAGCTCAACAAGTGTAGTGA
>809
GGCTGGTAGGTGGATGTGGACAGCAAAGCGGAAACCTACAGCAGGCACTATCTAGGC
AGAAGCTCAACAAGTGTAGTGATTCTTTCTTCTGTTTCCCTGGTGAGGCACCAGGAGG
GTCTTTTCTCCTCTCCTTACATCCC
>810
CTGGTAGGTGGATGTGGACAGCAAAGCGGAAACCTACAGCAGGCACTATCTAGGCAG
AAGCTCAACAAGTGTAGTGATTCTTTCTTCTGTTTCCCTGGTGAGGCACCAGGAGGGT
CTTTTCTCCTCTCCTTACATCCCTC
>811
GGAAACCTACAGCAGGCACTATCTAGGCAGAAGCTCAACAAGTGTAGTGATTCTTTCT
TCTGTTTCCCTGGTGAGGCACCAGGAGGGTCTTTTCTCCTCTCCTTACATCCCTCCACT
CTTGCTCTCCTTGCAGCCCAGTT
>812
CACTATCTAGGCAGAAGCTCAACAAGTGTAGTGATTCTTTCTTCTGTTTCCCTGGTGAG
GCACCAGGAGGGTCTTTTCTCCTCTCCTTACATCCCTCCACTCTTGCTCTCCTTGCAGC
CCAGTTCTTTCCTAACTTTCTT
>813
CTGGTGAGGCACCAGGAGGGTCTTTTCTCCTCTCCTTACATCCCTCCACTCTTGCTCTC
CTTGCAGCCCAGTTCTTTCCTAACTTTCTTTAAATCCCTTTCCTCTAACAGGGTGTATAG
ACCTTAGTTAGAAAAACAGGT

>814
CCCTCCACTCTTGCTCTCCTTGCAGCCCAGTTCTTTCCTAACTTTCTTTAAATCCCTTTC
CTCTAACAGGGTGTATAGACCTTAGTTAGAAAAACAGGTAGTCTCTAAATGGGATTGCT
CTTTATTGTTAATGAAATGAA
>815
TCTCCTTGCAGCCCAGTTCTTTCCTAACTTTCTTTAAATCCCTTTCCTCTAACAGGGTGT
ATAGACCTTAGTTAGAAAAACAGGTAGTCTCTAAATGGGATTGCTCTTTATTGTTAATGA
AATGAATACCCAGGGACTGG
>816
CAGTTCTTTCCTAACTTTCTTTAAATCCCTTTCCTCTAACAGGGTGTATAGACCTTAGTT
AGAAAAACAGGTAGTCTCTAAATGGGATTGCTCTTTATTGTTAATGAAATGAATACCCA
GGGACTGGGCTTCCCCTCCGC
>817
TTTCCTCTAACAGGGTGTATAGACCTTAGTTAGAAAAACAGGTAGTCTCTAAATGGGAT
TGCTCTTTATTGTTAATGAAATGAATACCCAGGGACTGGGCTTCCCCTCCGCTTGCCCT
GGGTTTGATGTGGTTGTATCCC
>818
AAATGGGATTGCTCTTTATTGTTAATGAAATGAATACCCAGGGACTGGGCTTCCCCTCC
GCTTGCCCTGGGTTTGATGTGGTTGTATCCCGTGCTATCAGAGGAGCCCTTCCTTCACT
CAAGTGTGTTCCCCTGCCCAGC
>819
ATTGCTCTTTATTGTTAATGAAATGAATACCCAGGGACTGGGCTTCCCCTCCGCTTGCCC
TGGGTTTGATGTGGTTGTATCCCGTGCTATCAGAGGAGCCCTTCCTTCACTCAAGTGTG
TTCCCCTGCCCAGCTCTCTCC
>820
TCCCCTCCGCTTGCCCTGGGTTTGATGTGGTTGTATCCCGTGCTATCAGAGGAGCCCTT
CCTTCACTCAAGTGTGTTCCCCTGCCCAGCTCTCTCCGCAGACTCCTGCTGGGCTGAG
CTTTCCCTGCTCTTAAGAGTCAG
>821
CCCTCCGCTTGCCCTGGGTTTGATGTGGTTGTATCCCGTGCTATCAGAGGAGCCCTTCC
TTCACTCAAGTGTGTTCCCCTGCCCAGCTCTCTCCGCAGACTCCTGCTGGGCTGAGCTT
TCCCTGCTCTTAAGAGTCAGGA
>822
CTCAAGTGTGTTCCCCTGCCCAGCTCTCTCCGCAGACTCCTGCTGGGCTGAGCTTTCCC
TGCTCTTAAGAGTCAGGAGTGGCTCTTGCTGGGATGGAATGACCCGTCTTTGGGGCTG
CCTCATGAGCGGCTCTTGTGAAC
>823
GTGTTCCCCTGCCCAGCTCTCTCCGCAGACTCCTGCTGGGCTGAGCTTTCCCTGCTCTT
AAGAGTCAGGAGTGGCTCTTGCTGGGATGGAATGACCCGTCTTTGGGGCTGCCTCATG
AGCGGCTCTTGTGAACCCGGATC
>824
GACTCCTGCTGGGCTGAGCTTTCCCTGCTCTTAAGAGTCAGGAGTGGCTCTTGCTGGG
ATGGAATGACCCGTCTTTGGGGCTGCCTCATGAGCGGCTCTTGTGAACCCGGATCAGT
TCCGATGTGTAAACTCTACCGCCT
>825
TTAAGAGTCAGGAGTGGCTCTTGCTGGGATGGAATGACCCGTCTTTGGGGCTGCCTCA

TGAGCGGCTCTTGTGAACCCGGATCAGTTCCGATGTGTAAACTCTACCGCCTGGCCTTC
AGCGAACAGATACAGATTTCTGC
>826
TGGCTCTTGCTGGGATGGAATGACCCGTCTTTGGGGCTGCCTCATGAGCGGCTCTTGTG
AACCCGGATCAGTTCCGATGTGTAAACTCTACCGCCTGGCCTTCAGCGAACAGATACA
GATTTCTGCCACCTTCCATGACC
>827
TGGGATGGAATGACCCGTCTTTGGGGCTGCCTCATGAGCGGCTCTTGTGAACCCGGAT
CAGTTCCGATGTGTAAACTCTACCGCCTGGCCTTCAGCGAACAGATACAGATTTCTGCC
ACCTTCCATGACCCTACAGTTCA
>828
GATGTGTAAACTCTACCGCCTGGCCTTCAGCGAACAGATACAGATTTCTGCCACCTTCC
ATGACCCTACAGTTCATGGGACTGGGTCTGGGGCAGTGCCAGAGGCACGCATGGAGGT
GTGATTCTAGGTGAGTCCTGCGG
>829
ACCGCCTGGCCTTCAGCGAACAGATACAGATTTCTGCCACCTTCCATGACCCTACAGTT
CATGGGACTGGGTCTGGGGCAGTGCCAGAGGCACGCATGGAGGTGTGATTCTAGGTG
AGTCCTGCGGAAAACCTCTGGCCC
>830
CTTCAGCGAACAGATACAGATTTCTGCCACCTTCCATGACCCTACAGTTCATGGGACTG
GGTCTGGGGCAGTGCCAGAGGCACGCATGGAGGTGTGATTCTAGGTGAGTCCTGCGG
AAAACCTCTGGCCCACCCGTGAGT
>831
CCATGACCCTACAGTTCATGGGACTGGGTCTGGGGCAGTGCCAGAGGCACGCATGGAG
GTGTGATTCTAGGTGAGTCCTGCGGAAAACCTCTGGCCCACCCGTGAGTCACGGACAG
AACATGCAGACTCGGGCCTTGGTG
>832
GACCCTACAGTTCATGGGACTGGGTCTGGGGCAGTGCCAGAGGCACGCATGGAGGTG
TGATTCTAGGTGAGTCCTGCGGAAAACCTCTGGCCCACCCGTGAGTCACGGACAGAAC
ATGCAGACTCGGGCCTTGGTGACAT
>833
GGGGCAGTGCCAGAGGCACGCATGGAGGTGTGATTCTAGGTGAGTCCTGCGGAAAAC
CTCTGGCCCACCCGTGAGTCACGGACAGAACATGCAGACTCGGGCCTTGGTGACATAA
GCTCCGCATTGCTAAAACCGCGTGA
>834
TAGGTGAGTCCTGCGGAAAACCTCTGGCCCACCCGTGAGTCACGGACAGAACATGCA
GACTCGGGCCTTGGTGACATAAGCTCCGCATTGCTAAAACCGCGTGACCTCGAGGGCT
GACTGGCCTGAGAACCCTGGATGGC
>835
ACCCGTGAGTCACGGACAGAACATGCAGACTCGGGCCTTGGTGACATAAGCTCCGCAT
TGCTAAAACCGCGTGACCTCGAGGGCTGACTGGCCTGAGAACCCTGGATGGCGCTCTC
GGCCACCCCCACCTCCCACCCCAA
>836
TGACTGGCCTGAGAACCCTGGATGGCGCTCTCGGCCACCCCCACCTCCCACCCCAACC
TCCTGGGCTTCGGTCAGAATCCACAGCCCGTGCCCGAAGAGCGCTTCCCGCCTCTGGC
ACCCTACCTTCGCTCAGCTCCAGG

>837
CTGGATGGCGCTCTCGGCCACCCCCACCTCCCACCCCAACCTCCTGGGCTTCGGTCAG
AATCCACAGCCCGTGCCCGAAGAGCGCTTCCCGCCTCTGGCACCCTACCTTCGCTCAG
CTCCAGGGAAAAGGGGAGAGGGCA
>838
AGCGCTTCCCGCCTCTGGCACCCTACCTTCGCTCAGCTCCAGGGAAAAGGGGAGAGG
GCAGCTTTCTGCAGTCAGAGGAAGAGTACATTTTCTTTGGCTGCTCTACCCTCTGAAGT
AGGGCGGCCAGCTGAAGGAGGACA
>839
CTCTGGCACCCTACCTTCGCTCAGCTCCAGGGAAAAGGGGAGAGGGCAGCTTTCTGC
AGTCAGAGGAAGAGTACATTTTCTTTGGCTGCTCTACCCTCTGAAGTAGGGCGGCCAG
CTGAAGGAGGACACACTTTTGAGGG
>840
AAAAGGGGAGAGGGCAGCTTTCTGCAGTCAGAGGAAGAGTACATTTTCTTTGGCTGCT
CTACCCTCTGAAGTAGGGCGGCCAGCTGAAGGAGGACACACTTTTGAGGGGCCCAGA
GGTTGTCCAAGCTTCCCCCTGCCCC
>841
TCTTTGGCTGCTCTACCCTCTGAAGTAGGGCGGCCAGCTGAAGGAGGACACACTTTTG
AGGGGCCCAGAGGTTGTCCAAGCTTCCCCCTGCCCCCTGAAGACTGTGCACTGAGCT
GGGCGCAGTTCTCGGGAACTGTTTC
>842
TTGGCTGCTCTACCCTCTGAAGTAGGGCGGCCAGCTGAAGGAGGACACACTTTTGAGG
GGCCCAGAGGTTGTCCAAGCTTCCCCCTGCCCCCTGAAGACTGTGCACTGAGCTGGGC
GCAGTTCTCGGGAACTGTTTCCAC
>843
CCAGCTGAAGGAGGACACACTTTTGAGGGGCCCAGAGGTTGTCCAAGCTTCCCCCTG
CCCCCTGAAGACTGTGCACTGAGCTGGGCGCAGTTCTCGGGAACTGTTTCCACCCAGA
TTGCTGGGGGGCGGGGGGGGGTGGG
>844
CTTTTGAGGGGCCCAGAGGTTGTCCAAGCTTCCCCCTGCCCCCTGAAGACTGTGCACT
GAGCTGGGCGCAGTTCTCGGGAACTGTTTCCACCCAGATTGCTGGGGGGCGGGGGGG
GGTGGGATGAGGGCAGAGCCGAGAG
>845
CAGAGGTTGTCCAAGCTTCCCCCTGCCCCCTGAAGACTGTGCACTGAGCTGGGCGCA
GTTCTCGGGAACTGTTTCCACCCAGATTGCTGGGGGGCGGGGGGGGGTGGGATGAGG
GCAGAGCCGAGAGGCTGTCCAAGGTT
>846
AGACTGTGCACTGAGCTGGGCGCAGTTCTCGGGAACTGTTTCCACCCAGATTGCTGGG
GGGCGGGGGGGGGTGGGATGAGGGCAGAGCCGAGAGGCTGTCCAAGGTTTGGGAGA
GAGAAAAGTTTCTCCCAGGACTCGAC
>847
CTCGGGAACTGTTTCCACCCAGATTGCTGGGGGGCGGGGGGGGGTGGGATGAGGGCA
GAGCCGAGAGGCTGTCCAAGGTTTGGGAGAGAGAAAAGTTTCTCCCAGGACTCGACC
TTGGCCTCCAGCAATCGCGACAGCTA
>848
ACTGTTTCCACCCAGATTGCTGGGGGGCGGGGGGGGGTGGGATGAGGGCAGAGCCGA

GAGGCTGTCCAAGGTTTGGGAGAGAGAAAAGTTTCTCCCAGGACTCGACCTTGGCCT
CCAGCAATCGCGACAGCTAAAAACGG
>849
TGCTGGGGGGCGGGGGGGGGTGGGATGAGGGCAGAGCCGAGAGGCTGTCCAAGGTT
TGGGAGAGAGAAAAGTTTCTCCCAGGACTCGACCTTGGCCTCCAGCAATCGCGACAG
CTAAAAACGGGTGTCTCGCTTCGACAA
>850
GTTTGGGAGAGAGAAAAGTTTCTCCCAGGACTCGACCTTGGCCTCCAGCAATCGCGA
CAGCTAAAAACGGGTGTCTCGCTTCGACAATAGATCCCCGCGGACCTTCTGGCACCTG
GTTCACTAGCGCCCGCGAACTCTGC
>851
TTGGGAGAGAGAAAAGTTTCTCCCAGGACTCGACCTTGGCCTCCAGCAATCGCGACA
GCTAAAAACGGGTGTCTCGCTTCGACAATAGATCCCCGCGGACCTTCTGGCACCTGGT
TCACTAGCGCCCGCGAACTCTGCCT
>852
CAGCAATCGCGACAGCTAAAAACGGGTGTCTCGCTTCGACAATAGATCCCCGCGGACC
TTCTGGCACCTGGTTCACTAGCGCCCGCGAACTCTGCCTCGGGAGACTTATTGAAATCC
GGATGCTCAAGCCGGGAGGCGCG
>853
TCACTAGCGCCCGCGAACTCTGCCTCGGGAGACTTATTGAAATCCGGATGCTCAAGCC
GGGAGGCGCGCAGTAACCAGGAGGATGAGAGGGCCGGGTTTGGGCTAGGAAAGCGG
CCTTTTAAAACAGATGTCAGGGGGAC
>854
CGGGAGACTTATTGAAATCCGGATGCTCAAGCCGGGAGGCGCGCAGTAACCAGGAGG
ATGAGAGGGCCGGGTTTGGGCTAGGAAAGCGGCCTTTTAAAACAGATGTCAGGGGGA
CTGCAGCCCCGAGCCATGAGAAAAAA
>855
GGAGGCGCGCAGTAACCAGGAGGATGAGAGGGCCGGGTTTGGGCTAGGAAAGCGGC
CTTTTAAAACAGATGTCAGGGGGACTGCAGCCCCGAGCCATGAGAAAAAAGTTAAAG
GCGAGATGACACGCACTGAATTGGGGC
>856
GTTTGGGCTAGGAAAGCGGCCTTTTAAAACAGATGTCAGGGGGACTGCAGCCCCGAG
CCATGAGAAAAAAGTTAAAGGCGAGATGACACGCACTGAATTGGGGCAAACATTGGA
AGAGGAGACAAAACTGCGTGCTTGAG
>857
GAGAAAAAAGTTAAAGGCGAGATGACACGCACTGAATTGGGGCAAACATTGGAAGAG
GAGACAAAACTGCGTGCTTGAGCACCGGGGTGCGGGGAGGGGGGGACAAAACCCGT
ATCCAGTGCAAATTAAAATCTTGGGAG
>858
GCGAGATGACACGCACTGAATTGGGGCAAACATTGGAAGAGGAGACAAAACTGCGTG
CTTGAGCACCGGGGTGCGGGGAGGGGGGGACAAAACCCGTATCCAGTGCAAATTAAA
ATCTTGGGAGTAGGTGGGGGCTGCTG
>859
GCAAACATTGGAAGAGGAGACAAAACTGCGTGCTTGAGCACCGGGGTGCGGGGAGG
GGGGGACAAAACCCGTATCCAGTGCAAATTAAAATCTTGGGAGTAGGTGGGGGCTGCT
GCGCGCCCTTCACCCTCAGTTCCCCT

>860
CGTGCTTGAGCACCGGGGTGCGGGGAGGGGGGGACAAAACCCGTATCCAGTGCAAAT
TAAAATCTTGGGAGTAGGTGGGGGCTGCTGCGCGCCCTTCACCCTCAGTTCCCCTATTA
AGGATTCTGAGTCCCCATGCACTC
>861
CTTGAGCACCGGGGTGCGGGGAGGGGGGGACAAAACCCGTATCCAGTGCAAATTAAA
ATCTTGGGAGTAGGTGGGGGCTGCTGCGCGCCCTTCACCCTCAGTTCCCCTATTAAGGA
TTCTGAGTCCCCATGCACTCCTGT
>862
CAAAACCCGTATCCAGTGCAAATTAAAATCTTGGGAGTAGGTGGGGGCTGCTGCGCGC
CCTTCACCCTCAGTTCCCCTATTAAGGATTCTGAGTCCCCATGCACTCCTGTCCTCTGGC
TCCTTCCTTCCTCTCCGCTCGG
>863
TTAAAATCTTGGGAGTAGGTGGGGGCTGCTGCGCGCCCTTCACCCTCAGTTCCCCTATT
AAGGATTCTGAGTCCCCATGCACTCCTGTCCTCTGGCTCCTTCCTTCCTCTCCGCTCGG
CCGGTGAGAGGCGGCCGCCGGC
>864
AGGTGGGGGCTGCTGCGCGCCCTTCACCCTCAGTTCCCCTATTAAGGATTCTGAGTCCC
CATGCACTCCTGTCCTCTGGCTCCTTCCTTCCTCTCCGCTCGGCCGGTGAGAGGCGGCC
GCCGGCGCCCCAGCAGCAGCTA
>865
CCCCTATTAAGGATTCTGAGTCCCCATGCACTCCTGTCCTCTGGCTCCTTCCTTCCTCTC
CGCTCGGCCGGTGAGAGGCGGCCGCCGGCGCCCCAGCAGCAGCTAGATGTCAGGCGA
AGCCCGGAGCGCGGAGCGCGGGG
>866
CTCTGGCTCCTTCCTTCCTCTCCGCTCGGCCGGTGAGAGGCGGCCGCCGGCGCCCCAG
CAGCAGCTAGATGTCAGGCGAAGCCCGGAGCGCGGAGCGCGGGGAAGGGAGGGGAA
GGGAGGGGAGGGGAAAGGAGGGGTGG
>867
AGATGTCAGGCGAAGCCCGGAGCGCGGAGCGCGGGGAAGGGAGGGGAAGGGAGGG
GAGGGGAAAGGAGGGGTGGGGCGGGGAGCGCGCGCGGGCTCGGCCAGTGCGGGGTG
GTGGGCGGGCTGAGCCCGGGGACGGCGCG
>868
CGGGGAAGGGAGGGGAAGGGAGGGGAGGGGAAAGGAGGGGTGGGGCGGGGAGCGC
GCGCGGGCTCGGCCAGTGCGGGGTGGTGGGCGGGCTGAGCCCGGGGACGGCGCGGG
AGGGGGGAGGGAAGGGGGGCAGCTGTGGG
>869
GGGAGGGGAAGGGAGGGGAGGGGAAAGGAGGGGTGGGGCGGGGAGCGCGCGCGGG
CTCGGCCAGTGCGGGGTGGTGGGCGGGCTGAGCCCGGGGACGGCGCGGGAGGGGGG
AGGGAAGGGGGGCAGCTGTGGGCAGGGAG
>870
GGCCAGTGCGGGGTGGTGGGCGGGCTGAGCCCGGGGACGGCGCGGGAGGGGGGAGG
GAAGGGGGGCAGCTGTGGGCAGGGAGCCGGGCTCGGCCGCCAGCACTAAAGATGGA
GAGGCGCCGGGGCCTTGCAGGGGAGGGG
>871
GGGAGCCGGGCTCGGCCGCCAGCACTAAAGATGGAGAGGCGCCGGGGCCTTGCAGGG

GAGGGGGCTCGGCGTTGACGTGGGACGCGGCGGAGGCGCAGCAGCCGGTGGTGATTT
GCTAACCTCGCAGCAGAGAGGAGTTG
>872
CGGGCTCGGCCGCCAGCACTAAAGATGGAGAGGCGCCGGGGCCTTGCAGGGGAGGG
GGCTCGGCGTTGACGTGGGACGCGGCGGAGGCGCAGCAGCCGGTGGTGATTTGCTAA
CCTCGCAGCAGAGAGGAGTTGAGGGCG
>873
AGAGGCGCCGGGGCCTTGCAGGGGAGGGGGCTCGGCGTTGACGTGGGACGCGGCGG
AGGCGCAGCAGCCGGTGGTGATTTGCTAACCTCGCAGCAGAGAGGAGTTGAGGGCGA
TGAGAGCGGGTACTGCGAACTGCCGGG
>874
GGCGCCGGGGCCTTGCAGGGGAGGGGGCTCGGCGTTGACGTGGGACGCGGCGGAGG
CGCAGCAGCCGGTGGTGATTTGCTAACCTCGCAGCAGAGAGGAGTTGAGGGCGATGA
GAGCGGGTACTGCGAACTGCCGGGCGA
>875
CGGCGTTGACGTGGGACGCGGCGGAGGCGCAGCAGCCGGTGGTGATTTGCTAACCTC
GCAGCAGAGAGGAGTTGAGGGCGATGAGAGCGGGTACTGCGAACTGCCGGGCGATGC
TGTCGCTGCCGCCGTGATACGGAGAG
>876
GCGGAGGCGCAGCAGCCGGTGGTGATTTGCTAACCTCGCAGCAGAGAGGAGTTGAGG
GCGATGAGAGCGGGTACTGCGAACTGCCGGGCGATGCTGTCGCTGCCGCCGTGATACG
GAGAGCAACAGTTCCCCAGCAACAC
>877
TTTGCTAACCTCGCAGCAGAGAGGAGTTGAGGGCGATGAGAGCGGGTACTGCGAACT
GCCGGGCGATGCTGTCGCTGCCGCCGTGATACGGAGAGCAACAGTTCCCCAGCAACA
CCCCTCCCCGACACAGGCACACACCC
>878
GCAGCAGAGAGGAGTTGAGGGCGATGAGAGCGGGTACTGCGAACTGCCGGGCGATGC
TGTCGCTGCCGCCGTGATACGGAGAGCAACAGTTCCCCAGCAACACCCCTCCCCGACA
CAGGCACACACCCCCCGACAGGCAC
>879
CAGTTCCCCAGCAACACCCCTCCCCGACACAGGCACACACCCCCCGACAGGCACGCA
CACCCACCCCACAGTGCCCGGCTCGGCTGCGGTGAGTGAGGGGCCGGGAAGAGGCGC
ACCGCCCAGCGAGCGCCGCGCGCCGG
>880
CCTCCCCGACACAGGCACACACCCCCCGACAGGCACGCACACCCACCCCACAGTGCC
CGGCTCGGCTGCGGTGAGTGAGGGGCCGGGAAGAGGCGCACCGCCCAGCGAGCGCC
GCGCGCCGGGGCTTCCCGGGGCTGGAG
>881
GAGTGAGGGGCCGGGAAGAGGCGCACCGCCCAGCGAGCGCCGCGCGCCGGGGCTTC
CCGGGGCTGGAGAGGTCTGGGGGGCGCGCGCTCGCTTCGGCCACTCGGCCGCTGGGC
TTGTGCCTTTTTTATTTGGCGTGTTCC
>882
GCCGGGGCTTCCCGGGGCTGGAGAGGTCTGGGGGGCGCGCGCTCGCTTCGGCCACTC
GGCCGCTGGGCTTGTGCCTTTTTTATTTGGCGTGTTCCCTTCCTGCCGCTGCAGCCGTC
GCCGCCACCGGTTGGGGGTCGGCT

>883
TGGAGAGGTCTGGGGGGCGCGCGCTCGCTTCGGCCACTCGGCCGCTGGGCTTGTGCC
TTTTTTATTTGGCGTGTTCCCTTCCTGCCGCTGCAGCCGTCGCCGCCACCGGTTGGGGG
TCGGCTAGAAGGGGGAGCCCCGGC
>884
CGCTTCGGCCACTCGGCCGCTGGGCTTGTGCCTTTTTTATTTGGCGTGTTCCCTTCCTG
CCGCTGCAGCCGTCGCCGCCACCGGTTGGGGGTCGGCTAGAAGGGGGAGCCCCGGCT
GTCAGCCTGGGCGCAGCTGCCTCC
>885
CGGCCGCTGGGCTTGTGCCTTTTTTATTTGGCGTGTTCCCTTCCTGCCGCTGCAGCCGT
CGCCGCCACCGGTTGGGGGTCGGCTAGAAGGGGGAGCCCCGGCTGTCAGCCTGGGCG
CAGCTGCCTCCCCAGCCCTTCCTC
>886
TGGGCTTGTGCCTTTTTTATTTGGCGTGTTCCCTTCCTGCCGCTGCAGCCGTCGCCGCC
ACCGGTTGGGGGTCGGCTAGAAGGGGGAGCCCCGGCTGTCAGCCTGGGCGCAGCTGC
CTCCCCAGCCCTTCCTCTTCAGGG
>887
TGTTCCCTTCCTGCCGCTGCAGCCGTCGCCGCCACCGGTTGGGGGTCGGCTAGAAGGG
GGAGCCCCGGCTGTCAGCCTGGGCGCAGCTGCCTCCCCAGCCCTTCCTCTTCAGGGCA
CGGTCGGGGTGAGAGGTGGGGGGC
>888
GCTAGAAGGGGGAGCCCCGGCTGTCAGCCTGGGCGCAGCTGCCTCCCCAGCCCTTCCT
CTTCAGGGCACGGTCGGGGTGAGAGGTGGGGGGCGTAGCGGTGTGCGGGGGCTGAG
GGCGTGAGCAGAGGGGTCGGGGATCC
>889
AGGGGGAGCCCCGGCTGTCAGCCTGGGCGCAGCTGCCTCCCCAGCCCTTCCTCTTCAG
GGCACGGTCGGGGTGAGAGGTGGGGGGCGTAGCGGTGTGCGGGGGCTGAGGGCGTG
AGCAGAGGGGTCGGGGATCCGGAGGC
>890
GTCAGCCTGGGCGCAGCTGCCTCCCCAGCCCTTCCTCTTCAGGGCACGGTCGGGGTGA
GAGGTGGGGGGCGTAGCGGTGTGCGGGGGCTGAGGGCGTGAGCAGAGGGGTCGGGG
ATCCGGAGGCTTTGTACCGCCAGGGG
>891
CTGGGCGCAGCTGCCTCCCCAGCCCTTCCTCTTCAGGGCACGGTCGGGGTGAGAGGT
GGGGGGCGTAGCGGTGTGCGGGGGCTGAGGGCGTGAGCAGAGGGGTCGGGGATCCG
GAGGCTTTGTACCGCCAGGGGCTGGCG
>892
CAGCCCTTCCTCTTCAGGGCACGGTCGGGGTGAGAGGTGGGGGGCGTAGCGGTGTGC
GGGGGCTGAGGGCGTGAGCAGAGGGGTCGGGGATCCGGAGGCTTTGTACCGCCAGGG
GCTGGCGGAGCAACAGAGCCCGTGGG
>893
TTCAGGGCACGGTCGGGGTGAGAGGTGGGGGGCGTAGCGGTGTGCGGGGGCTGAGG
GCGTGAGCAGAGGGGTCGGGGATCCGGAGGCTTTGTACCGCCAGGGGCTGGCGGAGC
AACAGAGCCCGTGGGTGCTCTTATGTA
>894
AGGGCGTGAGCAGAGGGGTCGGGGATCCGGAGGCTTTGTACCGCCAGGGGCTGGCGG

AGCAACAGAGCCCGTGGGTGCTCTTATGTATGCGGACCGGTGCGCGGGCGCAAGATAA
GGTTGTGGTTTATTTATTTGTGTGT
>895
CGTGAGCAGAGGGGTCGGGGATCCGGAGGCTTTGTACCGCCAGGGGCTGGCGGAGCA
ACAGAGCCCGTGGGTGCTCTTATGTATGCGGACCGGTGCGCGGGCGCAAGATAAGGTT
GTGGTTTATTTATTTGTGTGTTTAT
>896
GGGGCTGGCGGAGCAACAGAGCCCGTGGGTGCTCTTATGTATGCGGACCGGTGCGCG
GGCGCAAGATAAGGTTGTGGTTTATTTATTTGTGTGTTTATTCGCCGGCCGGCTGGGAA
GCTAGAATCGGAGGAGCTGACGAG
>897
GCTGGCGGAGCAACAGAGCCCGTGGGTGCTCTTATGTATGCGGACCGGTGCGCGGGC
GCAAGATAAGGTTGTGGTTTATTTATTTGTGTGTTTATTCGCCGGCCGGCTGGGAAGCT
AGAATCGGAGGAGCTGACGAGTAG
>898
CCGTGGGTGCTCTTATGTATGCGGACCGGTGCGCGGGCGCAAGATAAGGTTGTGGTTTA
TTTATTTGTGTGTTTATTCGCCGGCCGGCTGGGAAGCTAGAATCGGAGGAGCTGACGA
GTAGATCTGGGGGCGGAGGGGAG
>899
GGTTGTGGTTTATTTATTTGTGTGTTTATTCGCCGGCCGGCTGGGAAGCTAGAATCGGA
GGAGCTGACGAGTAGATCTGGGGGCGGAGGGGAGCAGGACTGGGACTGCTTACGTTT
TGTTTCTCTTTGAGAAAACGTGGT
>900
GGGAAGCTAGAATCGGAGGAGCTGACGAGTAGATCTGGGGGCGGAGGGGAGCAGGA
CTGGGACTGCTTACGTTTTGTTTCTCTTTGAGAAAACGTGGTGGGCTTTTTCTTGATTG
GACTTGATCCCCACCCCCCTTTTGC
>901
GCTAGAATCGGAGGAGCTGACGAGTAGATCTGGGGGCGGAGGGGAGCAGGACTGGG
ACTGCTTACGTTTTGTTTCTCTTTGAGAAAACGTGGTGGGCTTTTTCTTGATTGGACTT
GATCCCCACCCCCCTTTTGCAGGGG
>902
GACGAGTAGATCTGGGGGCGGAGGGGAGCAGGACTGGGACTGCTTACGTTTTGTTTCT
CTTTGAGAAAACGTGGTGGGCTTTTTCTTGATTGGACTTGATCCCCACCCCCCTTTTGC
AGGGGAGGGAGGGAAGCTCCAGA
>903
GGTGGGCTTTTTCTTGATTGGACTTGATCCCCACCCCCCTTTTGCAGGGGAGGGAGGG
AAGCTCCAGAGGGTCTGCAGCGCTGCGGGCCCTCCTCGGCTCTCGGCGGGACCGGCG
GTGACACCGGAGCTCGCCGTGCGCT
>904
AGGGGAGGGAGGGAAGCTCCAGAGGGTCTGCAGCGCTGCGGGCCCTCCTCGGCTCTC
GGCGGGACCGGCGGTGACACCGGAGCTCGCCGTGCGCTCCCGGCCGCTCTCGGTGGG
TGCCGGTCTCTGCACCTGATGCGTTC
>905
CCAGAGGGTCTGCAGCGCTGCGGGCCCTCCTCGGCTCTCGGCGGGACCGGCGGTGAC
ACCGGAGCTCGCCGTGCGCTCCCGGCCGCTCTCGGTGGGTGCCGGTCTCTGCACCTGA
TGCGTTCGGGATGCCTTTCCCACCC

>906
GGGCCCTCCTCGGCTCTCGGCGGGACCGGCGGTGACACCGGAGCTCGCCGTGCGCTC
CCGGCCGCTCTCGGTGGGTGCCGGTCTCTGCACCTGATGCGTTCGGGATGCCTTTCCC
ACCCTGGCGCGCCCGCCGCTAGCTC
>907
CCTCCTCGGCTCTCGGCGGGACCGGCGGTGACACCGGAGCTCGCCGTGCGCTCCCGG
CCGCTCTCGGTGGGTGCCGGTCTCTGCACCTGATGCGTTCGGGATGCCTTTCCCACCCT
GGCGCGCCCGCCGCTAGCTCGCAC
>908
GGCGGTGACACCGGAGCTCGCCGTGCGCTCCCGGCCGCTCTCGGTGGGTGCCGGTCT
CTGCACCTGATGCGTTCGGGATGCCTTTCCCACCCTGGCGCGCCCGCCGCTAGCTCGC
ACAGCGCCTCGCACACTCCCGCACG
>909
CACCCTGGCGCGCCCGCCGCTAGCTCGCACAGCGCCTCGCACACTCCCGCACGCGCTT
GAAATGCGCACGGTCCCGCCGGCCCGCGGAACCACCCGGACGCACGGAGCGCTCCGC
ACCGACTCGCTCGCCGCCTCCCCGA
>910
CGCGGAACCACCCGGACGCACGGAGCGCTCCGCACCGACTCGCTCGCCGCCTCCCCG
AGACGCTCGCACCGTGCTTGGGCCGGGCGCGCTGGCCGCTGGCGCCGCTGGCCAGAG
GCCTGGGACCCAGCCGGTCGCTCCCA
>911
CGCTCGCACCGTGCTTGGGCCGGGCGCGCTGGCCGCTGGCGCCGCTGGCCAGAGGCC
TGGGACCCAGCCGGTCGCTCCCAGGGGGTCACGGCCCTGGGTCGGAGAGGGAGGGC
GGGCAGACCCCTTCTCGCCTTTCCTCC
>912
TTGGGCCGGGCGCGCTGGCCGCTGGCGCCGCTGGCCAGAGGCCTGGGACCCAGCCGG
TCGCTCCCAGGGGGTCACGGCCCTGGGTCGGAGAGGGAGGGCGGGCAGACCCCTTCT
CGCCTTTCCTCCCACAACTCGCTGCG
>913
CCTGGGTCGGAGAGGGAGGGCGGGCAGACCCCTTCTCGCCTTTCCTCCCACAACTCG
CTGCGGGGCTTTTGTGCTTCCCCTTCGCCGCGGGGCGGGTCCGCCTCCCCTGCCGCTC
TCGCCGCGGAGTCCAGCCCGCCCGG
>914
AGACCCCTTCTCGCCTTTCCTCCCACAACTCGCTGCGGGGCTTTTGTGCTTCCCCTTCG
CCGCGGGGCGGGTCCGCCTCCCCTGCCGCTCTCGCCGCGGAGTCCAGCCCGCCCGGA
CTGTCGCCGTTCCTCCCCGTCTCT
>915
CGCTGCGGGGCTTTTGTGCTTCCCCTTCGCCGCGGGGCGGGTCCGCCTCCCCTGCCGC
TCTCGCCGCGGAGTCCAGCCCGCCCGGACTGTCGCCGTTCCTCCCCGTCTCTTTCGCTT
TCCCTCGTCCCTAGCTCAGCTCT
>916
CTTCCCCTTCGCCGCGGGGCGGGTCCGCCTCCCCTGCCGCTCTCGCCGCGGAGTCCAG
CCCGCCCGGACTGTCGCCGTTCCTCCCCGTCTCTTTCGCTTTCCCTCGTCCCTAGCTCA
GCTCTCCTTCTTTCAGGAGTCTA
>917
CTTCGCCGCGGGGCGGGTCCGCCTCCCCTGCCGCTCTCGCCGCGGAGTCCAGCCCGCC

CGGACTGTCGCCGTTCCTCCCCGTCTCTTTCGCTTTCCCTCGTCCCTAGCTCAGCTCTC
CTTCTTTCAGGAGTCTAGCTCCT
>918
GGGCGGGTCCGCCTCCCCTGCCGCTCTCGCCGCGGAGTCCAGCCCGCCCGGACTGTCG
CCGTTCCTCCCCGTCTCTTTCGCTTTCCCTCGTCCCTAGCTCAGCTCTCCTTCTTTCAGG
AGTCTAGCTCCTCGGGAAAAGT
>919
GGACTGTCGCCGTTCCTCCCCGTCTCTTTCGCTTTCCCTCGTCCCTAGCTCAGCTCTCCT
TCTTTCAGGAGTCTAGCTCCTCGGGAAAAGTTGCTTCCCCAAGTTTGCTGAAGTCGTC
TCCAAGTCTCGGTGGGGGTCGC
>920
CCGTCTCTTTCGCTTTCCCTCGTCCCTAGCTCAGCTCTCCTTCTTTCAGGAGTCTAGCTC
CTCGGGAAAAGTTGCTTCCCCAAGTTTGCTGAAGTCGTCTCCAAGTCTCGGTGGGGGT
CGCTGGGAACTGGGGGGGTGTG
>921
CCCTAGCTCAGCTCTCCTTCTTTCAGGAGTCTAGCTCCTCGGGAAAAGTTGCTTCCCCA
AGTTTGCTGAAGTCGTCTCCAAGTCTCGGTGGGGGTCGCTGGGAACTGGGGGGGTGT
GAGAGCGCGGTCGATCCCCGGAGC
>922
CTCTCCTTCTTTCAGGAGTCTAGCTCCTCGGGAAAAGTTGCTTCCCCAAGTTTGCTGAA
GTCGTCTCCAAGTCTCGGTGGGGGTCGCTGGGAACTGGGGGGGTGTGAGAGCGCGGT
CGATCCCCGGAGCTCGGGCGGGTT
>923
TTCTTTCAGGAGTCTAGCTCCTCGGGAAAAGTTGCTTCCCCAAGTTTGCTGAAGTCGT
CTCCAAGTCTCGGTGGGGGTCGCTGGGAACTGGGGGGGTGTGAGAGCGCGGTCGATC
CCCGGAGCTCGGGCGGGTTATCGCC
>924
GAAAAGTTGCTTCCCCAAGTTTGCTGAAGTCGTCTCCAAGTCTCGGTGGGGGTCGCTG
GGAACTGGGGGGGTGTGAGAGCGCGGTCGATCCCCGGAGCTCGGGCGGGTTATCGCC
GGCTCTTCCCCGCGCCGCCTGGACT
>925
CAAGTCTCGGTGGGGGTCGCTGGGAACTGGGGGGGTGTGAGAGCGCGGTCGATCCCC
GGAGCTCGGGCGGGTTATCGCCGGCTCTTCCCCGCGCCGCCTGGACTGGACGCTGCAG
CCAGCGCCAGCCGGATCGCGGGCGC
>926
GCTCTTCCCCGCGCCGCCTGGACTGGACGCTGCAGCCAGCGCCAGCCGGATCGCGGG
CGCCGAGCGGCGGGTGGGGGAGACGGCTGCCCCGTGGGCGCCCAGATTTCCCTCAGG
ACCCAAAAAGCTTGTTTCAGGGCGCC
>927
GACTGGACGCTGCAGCCAGCGCCAGCCGGATCGCGGGCGCCGAGCGGCGGGTGGGG
GAGACGGCTGCCCCGTGGGCGCCCAGATTTCCCTCAGGACCCAAAAAGCTTGTTTCAG
GGCGCCAAGCTTCCATTCTTTTGGCT
>928
CGCCGAGCGGCGGGTGGGGGAGACGGCTGCCCCGTGGGCGCCCAGATTTCCCTCAGG
ACCCAAAAAGCTTGTTTCAGGGCGCCAAGCTTCCATTCTTTTGGCTGTTCCCTCCCTAG
TTCTCAGAAACCCCGATGTCTGCG

>929
GTGGGCGCCCAGATTTCCCTCAGGACCCAAAAAGCTTGTTTCAGGGCGCCAAGCTTCC
ATTCTTTTGGCTGTTCCCTCCCTAGTTCTCAGAAACCCCGATGTCTGCGGGAAAGCACG
CGGTGGCTCGGCTCGGCTGAATG
>930
ATTTCCCTCAGGACCCAAAAAGCTTGTTTCAGGGCGCCAAGCTTCCATTCTTTTGGCTG
TTCCCTCCCTAGTTCTCAGAAACCCCGATGTCTGCGGGAAAGCACGCGGTGGCTCGGC
TCGGCTGAATGGTCATCCCAACC
>931
AAAGCTTGTTTCAGGGCGCCAAGCTTCCATTCTTTTGGCTGTTCCCTCCCTAGTTCTCA
GAAACCCCGATGTCTGCGGGAAAGCACGCGGTGGCTCGGCTCGGCTGAATGGTCATCC
CAACCTTGACGGCCTCCGTCCCC
>932
GTTCCCTCCCTAGTTCTCAGAAACCCCGATGTCTGCGGGAAAGCACGCGGTGGCTCGG
CTCGGCTGAATGGTCATCCCAACCTTGACGGCCTCCGTCCCCACCCCGGGGCGGGCAG
CAAAAATGTCTTGACCAGCCGTCC
>933
CCCGATGTCTGCGGGAAAGCACGCGGTGGCTCGGCTCGGCTGAATGGTCATCCCAACC
TTGACGGCCTCCGTCCCCACCCCGGGGCGGGCAGCAAAAATGTCTTGACCAGCCGTCC
TGTGAATCCTTTCTGCGTGAGCTT
>934
CTCGGCTCGGCTGAATGGTCATCCCAACCTTGACGGCCTCCGTCCCCACCCCGGGGCG
GGCAGCAAAAATGTCTTGACCAGCCGTCCTGTGAATCCTTTCTGCGTGAGCTTGGTGG
CTCCTGAACTGATAGAGCTCCTTC
>935
AATGGTCATCCCAACCTTGACGGCCTCCGTCCCCACCCCGGGGCGGGCAGCAAAAATG
TCTTGACCAGCCGTCCTGTGAATCCTTTCTGCGTGAGCTTGGTGGCTCCTGAACTGATA
GAGCTCCTTCTGGGGGTGGAGGC
>936
CGGCCTCCGTCCCCACCCCGGGGCGGGCAGCAAAAATGTCTTGACCAGCCGTCCTGTG
AATCCTTTCTGCGTGAGCTTGGTGGCTCCTGAACTGATAGAGCTCCTTCTGGGGGTGG
AGGCAAGCAAGCAGTGTTCGAAGA
>937
GACCAGCCGTCCTGTGAATCCTTTCTGCGTGAGCTTGGTGGCTCCTGAACTGATAGAG
CTCCTTCTGGGGGTGGAGGCAAGCAAGCAGTGTTCGAAGAGTGAGGGAGAGACCTTG
CTGTTCCTTGCTCTCAGTGCATTCA
>938
ATCCTTTCTGCGTGAGCTTGGTGGCTCCTGAACTGATAGAGCTCCTTCTGGGGGTGGA
GGCAAGCAAGCAGTGTTCGAAGAGTGAGGGAGAGACCTTGCTGTTCCTTGCTCTCAG
TGCATTCACTGCAATATTGGTCGCT
>939
AACTGATAGAGCTCCTTCTGGGGGTGGAGGCAAGCAAGCAGTGTTCGAAGAGTGAGG
GAGAGACCTTGCTGTTCCTTGCTCTCAGTGCATTCACTGCAATATTGGTCGCTGTAAGA
CAGACACGGGGAGGGTCCTGGGCC
>940
CTTCTGGGGGTGGAGGCAAGCAAGCAGTGTTCGAAGAGTGAGGGAGAGACCTTGCTG

TTCCTTGCTCTCAGTGCATTCACTGCAATATTGGTCGCTGTAAGACAGACACGGGGAG
GGTCCTGGGCCTGCGCGGTCTTTCT
>941
CAAGCAGTGTTCGAAGAGTGAGGGAGAGACCTTGCTGTTCCTTGCTCTCAGTGCATTC
ACTGCAATATTGGTCGCTGTAAGACAGACACGGGGAGGGTCCTGGGCCTGCGCGGTCT
TTCTAACACTCCTTTAAGCGAGGT
>942
AGACCTTGCTGTTCCTTGCTCTCAGTGCATTCACTGCAATATTGGTCGCTGTAAGACAG
ACACGGGGAGGGTCCTGGGCCTGCGCGGTCTTTCTAACACTCCTTTAAGCGAGGTTTG
GAGTCTAGGAGCCCTCTTATTGA
>943
TGCTCTCAGTGCATTCACTGCAATATTGGTCGCTGTAAGACAGACACGGGGAGGGTCC
TGGGCCTGCGCGGTCTTTCTAACACTCCTTTAAGCGAGGTTTGGAGTCTAGGAGCCCT
CTTATTGATCCCGGACAGGCTCTG
>944
TGGTCGCTGTAAGACAGACACGGGGAGGGTCCTGGGCCTGCGCGGTCTTTCTAACACT
CCTTTAAGCGAGGTTTGGAGTCTAGGAGCCCTCTTATTGATCCCGGACAGGCTCTGGG
AGAGGCCTTTGTTTCCCAGCCCTG
>945
CTCCTTTAAGCGAGGTTTGGAGTCTAGGAGCCCTCTTATTGATCCCGGACAGGCTCTGG
GAGAGGCCTTTGTTTCCCAGCCCTGTCTTCCCTTCGTACTGGTTGTAACTCACACCACA
GGTGCTGGTGCCAATATCACTT
>946
TAGGAGCCCTCTTATTGATCCCGGACAGGCTCTGGGAGAGGCCTTTGTTTCCCAGCCCT
GTCTTCCCTTCGTACTGGTTGTAACTCACACCACAGGTGCTGGTGCCAATATCACTTCC
AGCGCCTCTGAGCCATGTCCAG
>947
CCCTCTTATTGATCCCGGACAGGCTCTGGGAGAGGCCTTTGTTTCCCAGCCCTGTCTTC
CCTTCGTACTGGTTGTAACTCACACCACAGGTGCTGGTGCCAATATCACTTCCAGCGCC
TCTGAGCCATGTCCAGCATCCC
>948
CAGGCTCTGGGAGAGGCCTTTGTTTCCCAGCCCTGTCTTCCCTTCGTACTGGTTGTAAC
TCACACCACAGGTGCTGGTGCCAATATCACTTCCAGCGCCTCTGAGCCATGTCCAGCAT
CCCTTGCTGCCCCTGGTCACAG
>949
CCCTTCGTACTGGTTGTAACTCACACCACAGGTGCTGGTGCCAATATCACTTCCAGCGC
CTCTGAGCCATGTCCAGCATCCCTTGCTGCCCCTGGTCACAGGACTTGCAGACTCCTTG
GAGCCTGTGGGACACACGTGGA
>950
ACCACAGGTGCTGGTGCCAATATCACTTCCAGCGCCTCTGAGCCATGTCCAGCATCCCT
TGCTGCCCCTGGTCACAGGACTTGCAGACTCCTTGGAGCCTGTGGGACACACGTGGA
GTTCACACACCCAGCCCAGCATGA
>951
AGCGCCTCTGAGCCATGTCCAGCATCCCTTGCTGCCCCTGGTCACAGGACTTGCAGAC
TCCTTGGAGCCTGTGGGACACACGTGGAGTTCACACACCCAGCCCAGCATGAGCACT
CCTGGCTGTAGGGGACCATATTTAA

>952
GCCATGTCCAGCATCCCTTGCTGCCCCTGGTCACAGGACTTGCAGACTCCTTGGAGCC
TGTGGGACACACGTGGAGTTCACACACCCAGCCCAGCATGAGCACTCCTGGCTGTAG
GGGACCATATTTAACTATGGTCTCT
>953
GCAGACTCCTTGGAGCCTGTGGGACACACGTGGAGTTCACACACCCAGCCCAGCATG
AGCACTCCTGGCTGTAGGGGACCATATTTAACTATGGTCTCTCTCCATACCTTAATGACC
CATCCTTCTTCGTTTTTTAGATC
>954
ACACACGTGGAGTTCACACACCCAGCCCAGCATGAGCACTCCTGGCTGTAGGGGACC
ATATTTAACTATGGTCTCTCTCCATACCTTAATGACCCATCCTTCTTCGTTTTTTAGATCC
TCCTGTCCACCGGGCAGTTGTG
>955
GCACTCCTGGCTGTAGGGGACCATATTTAACTATGGTCTCTCTCCATACCTTAATGACCC
ATCCTTCTTCGTTTTTTAGATCCTCCTGTCCACCGGGCAGTTGTGGGCAGGAGGGTAGG
TACGACACCTTACGTAAGTGT
>956
GGACCATATTTAACTATGGTCTCTCTCCATACCTTAATGACCCATCCTTCTTCGTTTTTTA
GATCCTCCTGTCCACCGGGCAGTTGTGGGCAGGAGGGTAGGTACGACACCTTACGTAA
GTGTGTGTGTGTAAACACACA
>957
ACTATGGTCTCTCTCCATACCTTAATGACCCATCCTTCTTCGTTTTTTAGATCCTCCTGTC
CACCGGGCAGTTGTGGGCAGGAGGGTAGGTACGACACCTTACGTAAGTGTGTGTGTGT
AAACACACACACACTTTTTTT
>958
CCTTAATGACCCATCCTTCTTCGTTTTTTAGATCCTCCTGTCCACCGGGCAGTTGTGGGC
AGGAGGGTAGGTACGACACCTTACGTAAGTGTGTGTGTGTAAACACACACACACTTTT
TTTTTTTTTTTCTGAGACGGAG
>959
CCTTCTTCGTTTTTTAGATCCTCCTGTCCACCGGGCAGTTGTGGGCAGGAGGGTAGGTA
CGACACCTTACGTAAGTGTGTGTGTGTAAACACACACACACTTTTTTTTTTTTTTTCTG
AGACGGAGTTTTGCTCCTGTTG
>960
CTTCGTTTTTTAGATCCTCCTGTCCACCGGGCAGTTGTGGGCAGGAGGGTAGGTACGA
CACCTTACGTAAGTGTGTGTGTGTAAACACACACACACTTTTTTTTTTTTTTTCTGAGA
CGGAGTTTTGCTCCTGTTGCCCA
>961
TCGTTTTTTAGATCCTCCTGTCCACCGGGCAGTTGTGGGCAGGAGGGTAGGTACGACA
CCTTACGTAAGTGTGTGTGTGTAAACACACACACACTTTTTTTTTTTTTTTCTGAGACG
GAGTTTTGCTCCTGTTGCCCAGG
>962
TAGATCCTCCTGTCCACCGGGCAGTTGTGGGCAGGAGGGTAGGTACGACACCTTACGT
AAGTGTGTGTGTGTAAACACACACACACTTTTTTTTTTTTTTTCTGAGACGGAGTTTTG
CTCCTGTTGCCCAGGCTGGAGTG
>963
GGTACGACACCTTACGTAAGTGTGTGTGTGTAAACACACACACACTTTTTTTTTTTTT

TCTGAGACGGAGTTTTGCTCCTGTTGCCCAGGCTGGAGTGCAGTGGCGCAATCTCGGC
TCACGGCAACCTCCGCCTCCCGG
>964
TTACGTAAGTGTGTGTGTGTAAACACACACACACTTTTTTTTTTTTTTTTCTGAGACGGA
GTTTTGCTCCTGTTGCCCAGGCTGGAGTGCAGTGGCGCAATCTCGGCTCACGGCAACC
TCCGCCTCCCGGTTTCAAGTGAT
>965
TGTGTAAACACACACACACTTTTTTTTTTTTTTTTCTGAGACGGAGTTTTGCTCCTGTTG
CCCAGGCTGGAGTGCAGTGGCGCAATCTCGGCTCACGGCAACCTCCGCCTCCCGGTTT
CAAGTGATTCTCCTGCCTCAGCC
>966
AGTTTTGCTCCTGTTGCCCAGGCTGGAGTGCAGTGGCGCAATCTCGGCTCACGGCAAC
CTCCGCCTCCCGGTTTCAAGTGATTCTCCTGCCTCAGCCTCCCGAGTAGCTGGGATTAC
AGGCGGGTGCCACCGCGCCTGGC
>967
CTCCTGTTGCCCAGGCTGGAGTGCAGTGGCGCAATCTCGGCTCACGGCAACCTCCGCC
TCCCGGTTTCAAGTGATTCTCCTGCCTCAGCCTCCCGAGTAGCTGGGATTACAGGCGG
GTGCCACCGCGCCTGGCTAATTTT
>968
TGGCGCAATCTCGGCTCACGGCAACCTCCGCCTCCCGGTTTCAAGTGATTCTCCTGCCT
CAGCCTCCCGAGTAGCTGGGATTACAGGCGGGTGCCACCGCGCCTGGCTAATTTTGTAT
TTTTTGTAGAGACGGGGCTTCT
>969
GCAACCTCCGCCTCCCGGTTTCAAGTGATTCTCCTGCCTCAGCCTCCCGAGTAGCTGG
GATTACAGGCGGGTGCCACCGCGCCTGGCTAATTTTGTATTTTTTGTAGAGACGGGGCT
TCTCCATGTTGGTCAGGCTGGTC
>970
GTGATTCTCCTGCCTCAGCCTCCCGAGTAGCTGGGATTACAGGCGGGTGCCACCGCGC
CTGGCTAATTTTGTATTTTTTGTAGAGACGGGGCTTCTCCATGTTGGTCAGGCTGGTCTC
GAACTCCCGACCTCAGGTGATC
>971
CTGCCTCAGCCTCCCGAGTAGCTGGGATTACAGGCGGGTGCCACCGCGCCTGGCTAAT
TTTGTATTTTTTGTAGAGACGGGGCTTCTCCATGTTGGTCAGGCTGGTCTCGAACTCCC
GACCTCAGGTGATCCGCCTGCCT
>972
GATTACAGGCGGGTGCCACCGCGCCTGGCTAATTTTGTATTTTTTGTAGAGACGGGGCT
TCTCCATGTTGGTCAGGCTGGTCTCGAACTCCCGACCTCAGGTGATCCGCCTGCCTGG
GCCTCCCAAAGTGCTGGGATTAG
>973
CGGGTGCCACCGCGCCTGGCTAATTTTGTATTTTTTGTAGAGACGGGGCTTCTCCATGT
TGGTCAGGCTGGTCTCGAACTCCCGACCTCAGGTGATCCGCCTGCCTGGGCCTCCCAA
AGTGCTGGGATTAGAGGCGTGAG
>974
AATTTTGTATTTTTTGTAGAGACGGGGCTTCTCCATGTTGGTCAGGCTGGTCTCGAACT
CCCGACCTCAGGTGATCCGCCTGCCTGGGCCTCCCAAAGTGCTGGGATTAGAGGCGTG
AGCCACCGCGCCCTACCCCACAC

>975
CTTCTCCATGTTGGTCAGGCTGGTCTCGAACTCCCGACCTCAGGTGATCCGCCTGCCTG
GGCCTCCCAAAGTGCTGGGATTAGAGGCGTGAGCCACCGCGCCCTACCCCACACACTC
TTTCTGTCTGTCTCTGGCTGGTG
>976
GGCTGGTCTCGAACTCCCGACCTCAGGTGATCCGCCTGCCTGGGCCTCCCAAAGTGCT
GGGATTAGAGGCGTGAGCCACCGCGCCCTACCCCACACACTCTTTCTGTCTGTCTCTG
GCTGGTGTACATACACACAGTTCA
>977
CTGCCTGGGCCTCCCAAAGTGCTGGGATTAGAGGCGTGAGCCACCGCGCCCTACCCCA
CACACTCTTTCTGTCTGTCTCTGGCTGGTGTACATACACACAGTTCAGATTTTATATTAT
ACATACAATAACTTTGAGGCTT
>978
AGTGCTGGGATTAGAGGCGTGAGCCACCGCGCCCTACCCCACACACTCTTTCTGTCTG
TCTCTGGCTGGTGTACATACACACAGTTCAGATTTTATATTATACATACAATAACTTTGA
GGCTTCCGTGGGTAGAAATGAC
>979
GAGGCGTGAGCCACCGCGCCCTACCCCACACACTCTTTCTGTCTGTCTCTGGCTGGTG
TACATACACACAGTTCAGATTTTATATTATACATACAATAACTTTGAGGCTTCCGTGGGT
AGAAATGACCCAAGTTAGTGTG
>980
CTGTCTCTGGCTGGTGTACATACACACAGTTCAGATTTTATATTATACATACAATAACTTT
GAGGCTTCCGTGGGTAGAAATGACCCAAGTTAGTGTGTGGGTTGTGTGTGTTCCTGTT
AGTACCTAGGGATGGGGACAG
>981
CTCTGGCTGGTGTACATACACACAGTTCAGATTTTATATTATACATACAATAACTTTGAG
GCTTCCGTGGGTAGAAATGACCCAAGTTAGTGTGTGGGTTGTGTGTGTTCCTGTTAGTA
CCTAGGGATGGGGACAGGCCT
>982
ATACACACAGTTCAGATTTTATATTATACATACAATAACTTTGAGGCTTCCGTGGGTAGA
AATGACCCAAGTTAGTGTGTGGGTTGTGTGTGTTCCTGTTAGTACCTAGGGATGGGGAC
AGGCCTTGGGTCTGCCAGTGC
>983
ACACAGTTCAGATTTTATATTATACATACAATAACTTTGAGGCTTCCGTGGGTAGAAATG
ACCCAAGTTAGTGTGTGGGTTGTGTGTGTTCCTGTTAGTACCTAGGGATGGGGACAGG
CCTTGGGTCTGCCAGTGCTTTG
>984
ACATACAATAACTTTGAGGCTTCCGTGGGTAGAAATGACCCAAGTTAGTGTGTGGGTT
GTGTGTGTTCCTGTTAGTACCTAGGGATGGGGACAGGCCTTGGGTCTGCCAGTGCTTTG
CAGCCCTGAGAACAGTAGAAGGC
>985
GAAATGACCCAAGTTAGTGTGTGGGTTGTGTGTGTTCCTGTTAGTACCTAGGGATGGG
GACAGGCCTTGGGTCTGCCAGTGCTTTGCAGCCCTGAGAACAGTAGAAGGCCAGAGG
CCAAGGCTGATGGTGAGAGGATAGC
>986
CCAAGTTAGTGTGTGGGTTGTGTGTGTTCCTGTTAGTACCTAGGGATGGGGACAGGCC

TTGGGTCTGCCAGTGCTTTGCAGCCCTGAGAACAGTAGAAGGCCAGAGGCCAAGGCT
GATGGTGAGAGGATAGCCTTTGCGG
>987
TGTGTTCCTGTTAGTACCTAGGGATGGGGACAGGCCTTGGGTCTGCCAGTGCTTTGCA
GCCCTGAGAACAGTAGAAGGCCAGAGGCCAAGGCTGATGGTGAGAGGATAGCCTTTG
CGGAGGAGAGCCCAGTGTCCATAGA
>988
GGACAGGCCTTGGGTCTGCCAGTGCTTTGCAGCCCTGAGAACAGTAGAAGGCCAGAG
GCCAAGGCTGATGGTGAGAGGATAGCCTTTGCGGAGGAGAGCCCAGTGTCCATAGATT
TCTGTGGCCATGGCTACAGGCTCCT
>989
CCCTGAGAACAGTAGAAGGCCAGAGGCCAAGGCTGATGGTGAGAGGATAGCCTTTGC
GGAGGAGAGCCCAGTGTCCATAGATTTCTGTGGCCATGGCTACAGGCTCCTGGCCTCC
AATGAGTTTGACTCTGCTTCTGGGG
>990
AGGCCAGAGGCCAAGGCTGATGGTGAGAGGATAGCCTTTGCGGAGGAGAGCCCAGTG
TCCATAGATTTCTGTGGCCATGGCTACAGGCTCCTGGCCTCCAATGAGTTTGACTCTGC
TTCTGGGGCTCCTGTAGTGAGGCA
>991
CCTTTGCGGAGGAGAGCCCAGTGTCCATAGATTTCTGTGGCCATGGCTACAGGCTCCT
GGCCTCCAATGAGTTTGACTCTGCTTCTGGGGCTCCTGTAGTGAGGCAGAGTTTGGAA
AAACTGTTTAAAGATAAGGCTTGA
>992
CATAGATTTCTGTGGCCATGGCTACAGGCTCCTGGCCTCCAATGAGTTTGACTCTGCTT
CTGGGGCTCCTGTAGTGAGGCAGAGTTTGGAAAAACTGTTTAAAGATAAGGCTTGACA
TCCAGGGGGCTGGCGTGAAGCTG
>993
AGATTTCTGTGGCCATGGCTACAGGCTCCTGGCCTCCAATGAGTTTGACTCTGCTTCTG
GGGCTCCTGTAGTGAGGCAGAGTTTGGAAAAACTGTTTAAAGATAAGGCTTGACATCC
AGGGGGCTGGCGTGAAGCTGACA
>994
GGCCATGGCTACAGGCTCCTGGCCTCCAATGAGTTTGACTCTGCTTCTGGGGCTCCTGT
AGTGAGGCAGAGTTTGGAAAAACTGTTTAAAGATAAGGCTTGACATCCAGGGGGCTG
GCGTGAAGCTGACAAGGAAGGGGC
>995
GGCTCCTGGCCTCCAATGAGTTTGACTCTGCTTCTGGGGCTCCTGTAGTGAGGCAGAG
TTTGGAAAAACTGTTTAAAGATAAGGCTTGACATCCAGGGGGCTGGCGTGAAGCTGAC
AAGGAAGGGGCTAAAGTCTTGCTA
>996
GGGGCTCCTGTAGTGAGGCAGAGTTTGGAAAAACTGTTTAAAGATAAGGCTTGACATC
CAGGGGGCTGGCGTGAAGCTGACAAGGAAGGGGCTAAAGTCTTGCTAATTTTAAGTA
AGTGCACTTCATCATAATGAATGTT
>997
GGAAAAACTGTTTAAAGATAAGGCTTGACATCCAGGGGGCTGGCGTGAAGCTGACAA
GGAAGGGGCTAAAGTCTTGCTAATTTTAAGTAAGTGCACTTCATCATAATGAATGTTGG
CCTTGCCTAGGGAGCCCCTACTCT

>998
GATAAGGCTTGACATCCAGGGGGCTGGCGTGAAGCTGACAAGGAAGGGGCTAAAGTC
TTGCTAATTTTAAGTAAGTGCACTTCATCATAATGAATGTTGGCCTTGCCTAGGGAGCCC
CTACTCTCAGACACAGAGAAAGC
>999
GGCGTGAAGCTGACAAGGAAGGGGCTAAAGTCTTGCTAATTTTAAGTAAGTGCACTTC
ATCATAATGAATGTTGGCCTTGCCTAGGGAGCCCCTACTCTCAGACACAGAGAAAGCT
GAGGACTTATAGGACTTCACTACA
>1000
CTCAGACACAGAGAAAGCTGAGGACTTATAGGACTTCACTACAATTTTCATTTCACTTC
ATCTAACAGATGTCATAGAGTAGTGAATTAGGCATCTGCTGTGGCAGAACCTTCTTTTT
ACTCTCAGCATCCCTGGCTTGA
>1001
CAGAGAAAGCTGAGGACTTATAGGACTTCACTACAATTTTCATTTCACTTCATCTAACA
GATGTCATAGAGTAGTGAATTAGGCATCTGCTGTGGCAGAACCTTCTTTTTACTCTCAG
CATCCCTGGCTTGAAAGGCAGA
>1002
AGGACTTCACTACAATTTTCATTTCACTTCATCTAACAGATGTCATAGAGTAGTGAATTA
GGCATCTGCTGTGGCAGAACCTTCTTTTTACTCTCAGCATCCCTGGCTTGAAAGGCAGA
TTTGTAGATTTCTAGGAAGCT
>1003
GTGAATTAGGCATCTGCTGTGGCAGAACCTTCTTTTTACTCTCAGCATCCCTGGCTTGA
AAGGCAGATTTGTAGATTTCTAGGAAGCTACTTCAATTAAAATCTCTAAGATCCTCTTGC
TAAGGGCTTGGCAACAGGGAG
>1004
AGGAAGCTACTTCAATTAAAATCTCTAAGATCCTCTTGCTAAGGGCTTGGCAACAGGG
AGGAGGGGGCATGTTGTAGTGGTTGAATTCTTACTTTTAGGGACACTGATGAATTTGTC
TTTTGTTCTCTCATCTCTTTTGC
>1005
AAGCTACTTCAATTAAAATCTCTAAGATCCTCTTGCTAAGGGCTTGGCAACAGGGAGG
AGGGGGCATGTTGTAGTGGTTGAATTCTTACTTTTAGGGACACTGATGAATTTGTCTTTT
GTTCTCTCATCTCTTTTGCTTC
>1006
GCTTGGCAACAGGGAGGAGGGGGCATGTTGTAGTGGTTGAATTCTTACTTTTAGGGAC
ACTGATGAATTTGTCTTTTGTTCTCTCATCTCTTTTGCTTCTTCCCCTCTTCTTTCTCCTA
GCCTCCTCTATTGGCCCAGGA
>1007
AACAGGGAGGAGGGGGCATGTTGTAGTGGTTGAATTCTTACTTTTAGGGACACTGATG
AATTTGTCTTTTGTTCTCTCATCTCTTTTGCTTCTTCCCCTCTTCTTTCTCCTAGCCTCCT
CTATTGGCCCAGGAAGCCCAC
>1008
GGAGCACCATGGATCTGACAAAAATGGGCATGATCCAGCTGCAGAACCCTAGCCACCC
CACGGGGCTACTGTGCAAGGCCAACCAGATGCGGCTGGCCGGGACTTTGTGCGATGTG
GTCATCATGGTGGACAGCCAGGAG
>1009
AGCTGCAGAACCCTAGCCACCCCACGGGGCTACTGTGCAAGGCCAACCAGATGCGGC

TGGCCGGGACTTTGTGCGATGTGGTCATCATGGTGGACAGCCAGGAGTTCCACGCCCA
CCGGACGGTGCTGGCCTGCACCAGC
>1010
GAACCCTAGCCACCCCACGGGGCTACTGTGCAAGGCCAACCAGATGCGGCTGGCCGG
GACTTTGTGCGATGTGGTCATCATGGTGGACAGCCAGGAGTTCCACGCCCACCGGACG
GTGCTGGCCTGCACCAGCAAGATGT
>1011
CCCCACGGGGCTACTGTGCAAGGCCAACCAGATGCGGCTGGCCGGGACTTTGTGCGAT
GTGGTCATCATGGTGGACAGCCAGGAGTTCCACGCCCACCGGACGGTGCTGGCCTGC
ACCAGCAAGATGTTTGAGATCCTCT
>1012
GCGGCTGGCCGGGACTTTGTGCGATGTGGTCATCATGGTGGACAGCCAGGAGTTCCAC
GCCCACCGGACGGTGCTGGCCTGCACCAGCAAGATGTTTGAGATCCTCTTCCACCGCA
ATAGTCAACACTATACTTTGGACT
>1013
ATGTGGTCATCATGGTGGACAGCCAGGAGTTCCACGCCCACCGGACGGTGCTGGCCTG
CACCAGCAAGATGTTTGAGATCCTCTTCCACCGCAATAGTCAACACTATACTTTGGACT
TCCTCTCGCCAAAGACCTTCCAG
>1014
GAGTTCCACGCCCACCGGACGGTGCTGGCCTGCACCAGCAAGATGTTTGAGATCCTCT
TCCACCGCAATAGTCAACACTATACTTTGGACTTCCTCTCGCCAAAGACCTTCCAGCAG
ATTCTGGAGTATGCATATACAGC
>1015
TCTTCCACCGCAATAGTCAACACTATACTTTGGACTTCCTCTCGCCAAAGACCTTCCAG
CAGATTCTGGAGTATGCATATACAGCCACGCTGCAAGCCAAGGCGGAGGACCTGGATG
ACCTGCTGTATGCGGCCGAGATC
>1016
TCCAGCAGATTCTGGAGTATGCATATACAGCCACGCTGCAAGCCAAGGCGGAGGACCT
GGATGACCTGCTGTATGCGGCCGAGATCCTGGAGATCGAGTACCTGGAGGAACAGTGC
CTGAAGATGCTGGAGACCATCCAG
>1017
CAGCCACGCTGCAAGCCAAGGCGGAGGACCTGGATGACCTGCTGTATGCGGCCGAGA
TCCTGGAGATCGAGTACCTGGAGGAACAGTGCCTGAAGATGCTGGAGACCATCCAGG
CCTCAGACGACAATGACACGGAGGCC
>1018
CCAAGGCGGAGGACCTGGATGACCTGCTGTATGCGGCCGAGATCCTGGAGATCGAGTA
CCTGGAGGAACAGTGCCTGAAGATGCTGGAGACCATCCAGGCCTCAGACGACAATGA
CACGGAGGCCACCATGGCCGATGGC
>1019
GAGGAAGAAGAGGACCGCAAGGCTCGGTACCTCAAGAACATCTTCATCTCGAAGCAT
TCCAGCGAGGAGAGTGGGTATGCCAGTGTGGCTGGACAGAGCCTCCCTGGGCCCATG
GTGGACCAGAGCCCTTCAGTCTCCAC
>1020
AAGAAGAGGACCGCAAGGCTCGGTACCTCAAGAACATCTTCATCTCGAAGCATTCCAG
CGAGGAGAGTGGGTATGCCAGTGTGGCTGGACAGAGCCTCCCTGGGCCCATGGTGGA
CCAGAGCCCTTCAGTCTCCACTTCA

>1021
CATCTCGAAGCATTCCAGCGAGGAGAGTGGGTATGCCAGTGTGGCTGGACAGAGCCTC
CCTGGGCCCATGGTGGACCAGAGCCCTTCAGTCTCCACTTCATTTGGTCTTTCAGCCAT
GAGTCCCACCAAGGCTGCAGTGG
>1022
CGAGGAGAGTGGGTATGCCAGTGTGGCTGGACAGAGCCTCCCTGGGCCCATGGTGGA
CCAGAGCCCTTCAGTCTCCACTTCATTTGGTCTTTCAGCCATGAGTCCCACCAAGGCTG
CAGTGGACAGTTTGATGACCATAG
>1023
GCCAGTGTGGCTGGACAGAGCCTCCCTGGGCCCATGGTGGACCAGAGCCCTTCAGTCT
CCACTTCATTTGGTCTTTCAGCCATGAGTCCCACCAAGGCTGCAGTGGACAGTTTGATG
ACCATAGGACAGTCTCTCCTGCA
>1024
CAGAGCCTCCCTGGGCCCATGGTGGACCAGAGCCCTTCAGTCTCCACTTCATTTGGTC
TTTCAGCCATGAGTCCCACCAAGGCTGCAGTGGACAGTTTGATGACCATAGGACAGTC
TCTCCTGCAGGGAACTCTTCAGCC
>1025
CATGGTGGACCAGAGCCCTTCAGTCTCCACTTCATTTGGTCTTTCAGCCATGAGTCCCA
CCAAGGCTGCAGTGGACAGTTTGATGACCATAGGACAGTCTCTCCTGCAGGGAACTCT
TCAGCCACCTGCAGGGCCCGAGG
>1026
ATAGGACAGTCTCTCCTGCAGGGAACTCTTCAGCCACCTGCAGGGCCCGAGGAGCCA
ACTCTGGCTGGGGGTGGGCGGCACCCTGGGGTGGCTGAGGTGAAGACGGAGATGATG
CAGGTGGATGAGGTGCCCAGCCAGGA
>1027
GCAGGGAACTCTTCAGCCACCTGCAGGGCCCGAGGAGCCAACTCTGGCTGGGGGTGG
GCGGCACCCTGGGGTGGCTGAGGTGAAGACGGAGATGATGCAGGTGGATGAGGTGCC
CAGCCAGGACAGCCCTGGGGCAGCCG
>1028
TCTTCAGCCACCTGCAGGGCCCGAGGAGCCAACTCTGGCTGGGGGTGGGCGGCACCC
TGGGGTGGCTGAGGTGAAGACGGAGATGATGCAGGTGGATGAGGTGCCCAGCCAGGA
CAGCCCTGGGGCAGCCGAGTCCAGCA
>1029
AACTCTGGCTGGGGGTGGGCGGCACCCTGGGGTGGCTGAGGTGAAGACGGAGATGAT
GCAGGTGGATGAGGTGCCCAGCCAGGACAGCCCTGGGGCAGCCGAGTCCAGCATCTC
AGGAGGGATGGGGGACAAGGTTGAGG
>1030
TGGCTGAGGTGAAGACGGAGATGATGCAGGTGGATGAGGTGCCCAGCCAGGACAGCC
CTGGGGCAGCCGAGTCCAGCATCTCAGGAGGGATGGGGGACAAGGTTGAGGAAAGA
GGCAAAGAGGGGCCTGGGACCCCGACT
>1031
GGATGAGGTGCCCAGCCAGGACAGCCCTGGGGCAGCCGAGTCCAGCATCTCAGGAGG
GATGGGGGACAAGGTTGAGGAAAGAGGCAAAGAGGGGCCTGGGACCCCGACTCGAA
GCAGCGTCATCACCAGTGCTAGGGAGC
>1032
CTCAGGAGGGATGGGGGACAAGGTTGAGGAAAGAGGCAAAGAGGGGCCTGGGACCC

CGACTCGAAGCAGCGTCATCACCAGTGCTAGGGAGCTACACTATGGGCGAGAGGAGA
GTGCCGAGCAGGTGCCACCCCCAGCTG
>1033
ATGGGGGACAAGGTTGAGGAAAGAGGCAAAGAGGGGCCTGGGACCCCGACTCGAAG
CAGCGTCATCACCAGTGCTAGGGAGCTACACTATGGGCGAGAGGAGAGTGCCGAGCA
GGTGCCACCCCCAGCTGAGGCTGGCCA
>1034
GGGCCTGGGACCCCGACTCGAAGCAGCGTCATCACCAGTGCTAGGGAGCTACACTATG
GGCGAGAGGAGAGTGCCGAGCAGGTGCCACCCCCAGCTGAGGCTGGCCAGGCCCCC
ACTGGCCGACCTGAGCACCCAGCACC
>1035
CCCGACTCGAAGCAGCGTCATCACCAGTGCTAGGGAGCTACACTATGGGCGAGAGGA
GAGTGCCGAGCAGGTGCCACCCCCAGCTGAGGCTGGCCAGGCCCCCACTGGCCGACC
TGAGCACCCAGCACCCCCGCCTGAGA
>1036
TGGCCAGGCCCCCACTGGCCGACCTGAGCACCCAGCACCCCCGCCTGAGAAGCATCT
GGGCATCTACTCCGTGTTGCCCAACCACAAGGCTGACGCTGTATTGAGCATGCCGTCTT
CCGTGACCTCTGGCCTCCACGTGC
>1037
GCCAGGCCCCCACTGGCCGACCTGAGCACCCAGCACCCCCGCCTGAGAAGCATCTGG
GCATCTACTCCGTGTTGCCCAACCACAAGGCTGACGCTGTATTGAGCATGCCGTCTTCC
GTGACCTCTGGCCTCCACGTGCAG
>1038
GCACCCAGCACCCCCGCCTGAGAAGCATCTGGGCATCTACTCCGTGTTGCCCAACCAC
AAGGCTGACGCTGTATTGAGCATGCCGTCTTCCGTGACCTCTGGCCTCCACGTGCAGC
CTGCCCTGGCTGTCTCCATGGACT
>1039
GAAGCATCTGGGCATCTACTCCGTGTTGCCCAACCACAAGGCTGACGCTGTATTGAGC
ATGCCGTCTTCCGTGACCTCTGGCCTCCACGTGCAGCCTGCCCTGGCTGTCTCCATGGA
CTTCAGCACCTATGGGGGGCTGC
>1040
CTCCGTGTTGCCCAACCACAAGGCTGACGCTGTATTGAGCATGCCGTCTTCCGTGACCT
CTGGCCTCCACGTGCAGCCTGCCCTGGCTGTCTCCATGGACTTCAGCACCTATGGGGG
GCTGCTGCCCCAGGGCTTCATCC
>1041
CAAGGCTGACGCTGTATTGAGCATGCCGTCTTCCGTGACCTCTGGCCTCCACGTGCAG
CCTGCCCTGGCTGTCTCCATGGACTTCAGCACCTATGGGGGGCTGCTGCCCCAGGGCT
TCATCCAGAGGGAGCTGTTCAGCA
>1042
CCCTGGCTGTCTCCATGGACTTCAGCACCTATGGGGGGCTGCTGCCCCAGGGCTTCATC
CAGAGGGAGCTGTTCAGCAAGCTGGGGGAGCTGGCTGTGGGCATGAAGTCAGAGAGC
CGGACCATCGGAGAGCAGTGCAGC
>1043
CACCTATGGGGGGCTGCTGCCCCAGGGCTTCATCCAGAGGGAGCTGTTCAGCAAGCTG
GGGGAGCTGGCTGTGGGCATGAAGTCAGAGAGCCGGACCATCGGAGAGCAGTGCAGC
GTGTGTGGGGTCGAGCTTCCTGATA

>1044
GGCTGCTGCCCCAGGGCTTCATCCAGAGGGAGCTGTTCAGCAAGCTGGGGGAGCTGG
CTGTGGGCATGAAGTCAGAGAGCCGGACCATCGGAGAGCAGTGCAGCGTGTGTGGGG
TCGAGCTTCCTGATAACGAGGCTGTG
>1045
GGGAGCTGTTCAGCAAGCTGGGGGAGCTGGCTGTGGGCATGAAGTCAGAGAGCCGGA
CCATCGGAGAGCAGTGCAGCGTGTGTGGGGTCGAGCTTCCTGATAACGAGGCTGTGG
AGCAGCACAGGTAGGCCCCGCTCCAG
>1046
GAAGTCAGAGAGCCGGACCATCGGAGAGCAGTGCAGCGTGTGTGGGGTCGAGCTTCC
TGATAACGAGGCTGTGGAGCAGCACAGGTAGGCCCCGCTCCAGCCCCGCACCTGATGT
AGGACTTGAGGCCCTCACACCCCTC
>1047
GGGGTCGAGCTTCCTGATAACGAGGCTGTGGAGCAGCACAGGTAGGCCCCGCTCCAG
CCCCGCACCTGATGTAGGACTTGAGGCCCTCACACCCCTCCTTCACACCCTGGCTGCT
CCCAAGTTAGGGGCCTGGCTCCCCT
>1048
GCTCCAGCCCCGCACCTGATGTAGGACTTGAGGCCCTCACACCCCTCCTTCACACCCT
GGCTGCTCCCAAGTTAGGGGCCTGGCTCCCCTGTCTTGGCAATTTGTGAAAAAACCAG
AACACTTCTTCTAAAGTTCTGGCG
>1049
GTAGGACTTGAGGCCCTCACACCCCTCCTTCACACCCTGGCTGCTCCCAAGTTAGGGG
CCTGGCTCCCCTGTCTTGGCAATTTGTGAAAAAACCAGAACACTTCTTCTAAAGTTCTG
GCGGGGAGGGGAGCAGGTTTTTT
>1050
CCCTCACACCCCTCCTTCACACCCTGGCTGCTCCCAAGTTAGGGGCCTGGCTCCCCTGT
CTTGGCAATTTGTGAAAAAACCAGAACACTTCTTCTAAAGTTCTGGCGGGGAGGGGA
GCAGGTTTTTTAAAGTGTAGTGAG
>1051
TGCTCCCAAGTTAGGGGCCTGGCTCCCCTGTCTTGGCAATTTGTGAAAAAACCAGAAC
ACTTCTTCTAAAGTTCTGGCGGGGAGGGGAGCAGGTTTTTTAAAGTGTAGTGAGGGGG
CCTAGGATCTTTCCAAAAAGCACC
>1052
CTCCCCTGTCTTGGCAATTTGTGAAAAAACCAGAACACTTCTTCTAAAGTTCTGGCGG
GGAGGGGAGCAGGTTTTTTAAAGTGTAGTGAGGGGGCCTAGGATCTTTCCAAAAAGC
ACCAGGGGACACTCATGGGCGCAGC
>1053
TTGGCAATTTGTGAAAAAACCAGAACACTTCTTCTAAAGTTCTGGCGGGGAGGGGAG
CAGGTTTTTTAAAGTGTAGTGAGGGGGCCTAGGATCTTTCCAAAAAGCACCAGGGGAC
ACTCATGGGCGCAGCTTCTTAATAG
>1054
GGCCTAGGATCTTTCCAAAAAGCACCAGGGGACACTCATGGGCGCAGCTTCTTAATAG
GCCCTTCCCTTTGTTTTTTGCTTTTGGGCCCCTGTTTGTTTTTTCTGCTGTTTGGTCTGTT
CTCCTTTGCTTGGAGTGACTG
>1055
GCACCAGGGGACACTCATGGGCGCAGCTTCTTAATAGGCCCTTCCCTTTGTTTTTTGCT

TTTGGGCCCCTGTTTGTTTTTTCTGCTGTTTGGTCTGTTCTCCTTTGCTTGGAGTGACTG
ATAAAATGGAGAGAAGGAGAA
>1056
CAGGGGACACTCATGGGCGCAGCTTCTTAATAGGCCCTTCCCTTTGTTTTTTGCTTTTG
GGCCCCTGTTTGTTTTTTCTGCTGTTTGGTCTGTTCTCCTTTGCTTGGAGTGACTGATAA
AATGGAGAGAAGGAGAAAAGG
>1057
ATGGGCGCAGCTTCTTAATAGGCCCTTCCCTTTGTTTTTTGCTTTTGGGCCCCTGTTTGT
TTTTTCTGCTGTTTGGTCTGTTCTCCTTTGCTTGGAGTGACTGATAAAATGGAGAGAAG
GAGAAAAGGAGTGGGATGAGG
>1058
TTTTTTCTGCTGTTTGGTCTGTTCTCCTTTGCTTGGAGTGACTGATAAAATGGAGAGAA
GGAGAAAAGGAGTGGGATGAGGGTCTCTTGGGCCCTGCATCTGACCATGTTACTAGGT
TTCAGGTCCTCGGGTTCCCATGC
>1059
GTTTGGTCTGTTCTCCTTTGCTTGGAGTGACTGATAAAATGGAGAGAAGGAGAAAAGG
AGTGGGATGAGGGTCTCTTGGGCCCTGCATCTGACCATGTTACTAGGTTTCAGGTCCTC
GGGTTCCCATGCAGTGGTCCCTG
>1060
GTGACTGATAAAATGGAGAGAAGGAGAAAAGGAGTGGGATGAGGGTCTCTTGGGCCC
TGCATCTGACCATGTTACTAGGTTTCAGGTCCTCGGGTTCCCATGCAGTGGTCCCTGTG
AAGACAGCCTGTCCCTTTCTGCAG
>1061
TAAAATGGAGAGAAGGAGAAAAGGAGTGGGATGAGGGTCTCTTGGGCCCTGCATCTG
ACCATGTTACTAGGTTTCAGGTCCTCGGGTTCCCATGCAGTGGTCCCTGTGAAGACAG
CCTGTCCCTTTCTGCAGCTTGGGAG
>1062
GAGAGAAGGAGAAAAGGAGTGGGATGAGGGTCTCTTGGGCCCTGCATCTGACCATGT
TACTAGGTTTCAGGTCCTCGGGTTCCCATGCAGTGGTCCCTGTGAAGACAGCCTGTCC
CTTTCTGCAGCTTGGGAGAATGGGA
>1063
TGGGATGAGGGTCTCTTGGGCCCTGCATCTGACCATGTTACTAGGTTTCAGGTCCTCGG
GTTCCCATGCAGTGGTCCCTGTGAAGACAGCCTGTCCCTTTCTGCAGCTTGGGAGAAT
GGGAGGGTCTGGGGGCTGCTTAA
>1064
CCTCGGGTTCCCATGCAGTGGTCCCTGTGAAGACAGCCTGTCCCTTTCTGCAGCTTGG
GAGAATGGGAGGGTCTGGGGGCTGCTTAAGCCACAAGAGCAGCTGCAGGTTCTGAAC
CTTATTCCTGGTGAAAGTTAATCTC
>1065
CCTGTCCCTTTCTGCAGCTTGGGAGAATGGGAGGGTCTGGGGGCTGCTTAAGCCACAA
GAGCAGCTGCAGGTTCTGAACCTTATTCCTGGTGAAAGTTAATCTCCTTGCTCTGTCTC
CCTTCTCATTCTCTGATCCTGCA
>1066
TGGGAGAATGGGAGGGTCTGGGGGCTGCTTAAGCCACAAGAGCAGCTGCAGGTTCTG
AACCTTATTCCTGGTGAAAGTTAATCTCCTTGCTCTGTCTCCCTTCTCATTCTCTGATCC
TGCACATGCATTTAGGGGGAATT

>1067
GCTGCTTAAGCCACAAGAGCAGCTGCAGGTTCTGAACCTTATTCCTGGTGAAAGTTAA
TCTCCTTGCTCTGTCTCCCTTCTCATTCTCTGATCCTGCACATGCATTTAGGGGGAATTG
ATTTAAATGCAAGGGGAAAGGA
>1068
TCCCTTCTCATTCTCTGATCCTGCACATGCATTTAGGGGGAATTGATTTAAATGCAAGGG
GAAAGGATGGGTATAAGAGTCTTGGTCTTGCAGCCTGTGAAGTCTCATTCTTATCCCAT
CTCTGCTCACACTGAACTAAA
>1069
CATTCTCTGATCCTGCACATGCATTTAGGGGGAATTGATTTAAATGCAAGGGGAAAGGA
TGGGTATAAGAGTCTTGGTCTTGCAGCCTGTGAAGTCTCATTCTTATCCCATCTCTGCTC
ACACTGAACTAAATGCCGCTT
>1070
CTGATCCTGCACATGCATTTAGGGGGAATTGATTTAAATGCAAGGGGAAAGGATGGGTA
TAAGAGTCTTGGTCTTGCAGCCTGTGAAGTCTCATTCTTATCCCATCTCTGCTCACACT
GAACTAAATGCCGCTTCAGGCT
>1071
ATGCATTTAGGGGGAATTGATTTAAATGCAAGGGGAAAGGATGGGTATAAGAGTCTTG
GTCTTGCAGCCTGTGAAGTCTCATTCTTATCCCATCTCTGCTCACACTGAACTAAATGC
CGCTTCAGGCTAGCATTGGCTTC
>1072
TCTTATCCCATCTCTGCTCACACTGAACTAAATGCCGCTTCAGGCTAGCATTGGCTTCCC
TCCGGCTTCCGTTTCCTTCTCTATGAAATAACCATTCATTGAGCTCTTATGACATGTTTG
GTTCAGTACTAAGCACTTTC
>1073
TAGCATTGGCTTCCCTCCGGCTTCCGTTTCCTTCTCTATGAAATAACCATTCATTGAGCT
CTTATGACATGTTTGGTTCAGTACTAAGCACTTTCCATGAGTTGCATTTTCATTACAACA
GCCCTTTGAGATAGGTAATG
>1074
TTGGCTTCCCTCCGGCTTCCGTTTCCTTCTCTATGAAATAACCATTCATTGAGCTCTTAT
GACATGTTTGGTTCAGTACTAAGCACTTTCCATGAGTTGCATTTTCATTACAACAGCCCT
TTGAGATAGGTAATGTCCTT
>1075
CTTCTCTATGAAATAACCATTCATTGAGCTCTTATGACATGTTTGGTTCAGTACTAAGCA
CTTTCCATGAGTTGCATTTTCATTACAACAGCCCTTTGAGATAGGTAATGTCCTTCTTTT
TTTTTTGGATGTGAAGTTTC
>1076
TGACATGTTTGGTTCAGTACTAAGCACTTTCCATGAGTTGCATTTTCATTACAACAGCCC
TTTGAGATAGGTAATGTCCTTCTTTTTTTTTTGGATGTGAAGTTTCGCTCTCCTTGCCCA
GGCTGGAGTGCAATGGTGTG
>1077
TGTTTGGTTCAGTACTAAGCACTTTCCATGAGTTGCATTTTCATTACAACAGCCCTTTGA
GATAGGTAATGTCCTTCTTTTTTTTTTGGATGTGAAGTTTCGCTCTCCTTGCCCAGGCTG
GAGTGCAATGGTGTGATCTC
>1078
CTTTCCATGAGTTGCATTTTCATTACAACAGCCCTTTGAGATAGGTAATGTCCTTCTTTT

TTTTTTGGATGTGAAGTTTCGCTCTCCTTGCCCAGGCTGGAGTGCAATGGTGTGATCTC
TGCTCACCGCAACCTCTGCAT
>1079
CATGAGTTGCATTTTCATTACAACAGCCCTTTGAGATAGGTAATGTCCTTCTTTTTTTTTT
GGATGTGAAGTTTCGCTCTCCTTGCCCAGGCTGGAGTGCAATGGTGTGATCTCTGCTCA
CCGCAACCTCTGCATCCCGG
>1080
CCCTTTGAGATAGGTAATGTCCTTCTTTTTTTTTTGGATGTGAAGTTTCGCTCTCCTTGC
CCAGGCTGGAGTGCAATGGTGTGATCTCTGCTCACCGCAACCTCTGCATCCCGGGTTA
AGGTGATTCTCCTGCCTCAGCC
>1081
GTTTCGCTCTCCTTGCCCAGGCTGGAGTGCAATGGTGTGATCTCTGCTCACCGCAACCT
CTGCATCCCGGGTTAAGGTGATTCTCCTGCCTCAGCCTCCCGAGTAGCTCGGATTACAG
GCACGCACCACCATACCTGGCT
>1082
CTCTCCTTGCCCAGGCTGGAGTGCAATGGTGTGATCTCTGCTCACCGCAACCTCTGCAT
CCCGGGTTAAGGTGATTCTCCTGCCTCAGCCTCCCGAGTAGCTCGGATTACAGGCACG
CACCACCATACCTGGCTAATTTT
>1083
TGGTGTGATCTCTGCTCACCGCAACCTCTGCATCCCGGGTTAAGGTGATTCTCCTGCCT
CAGCCTCCCGAGTAGCTCGGATTACAGGCACGCACCACCATACCTGGCTAATTTTGAAT
TTTTAGTAGAGACGGGCTTTCA
>1084
CTGCCTCAGCCTCCCGAGTAGCTCGGATTACAGGCACGCACCACCATACCTGGCTAATT
TTGAATTTTTAGTAGAGACGGGCTTTCACCGTGTTGGTCAGGATGGTCTCGAACTCCCG
ACCTCAGGTGATCGGCCTGCTT
>1085
AGCTCGGATTACAGGCACGCACCACCATACCTGGCTAATTTTGAATTTTTAGTAGAGAC
GGGCTTTCACCGTGTTGGTCAGGATGGTCTCGAACTCCCGACCTCAGGTGATCGGCCT
GCTTCGGCCTCCCAAAGTGCTGG
>1086
CTCGGATTACAGGCACGCACCACCATACCTGGCTAATTTTGAATTTTTAGTAGAGACGG
GCTTTCACCGTGTTGGTCAGGATGGTCTCGAACTCCCGACCTCAGGTGATCGGCCTGC
TTCGGCCTCCCAAAGTGCTGGAT
>1087
GATTACAGGCACGCACCACCATACCTGGCTAATTTTGAATTTTTAGTAGAGACGGGCTT
TCACCGTGTTGGTCAGGATGGTCTCGAACTCCCGACCTCAGGTGATCGGCCTGCTTCG
GCCTCCCAAAGTGCTGGATTACA
>1088
CACGCACCACCATACCTGGCTAATTTTGAATTTTTAGTAGAGACGGGCTTTCACCGTGT
TGGTCAGGATGGTCTCGAACTCCCGACCTCAGGTGATCGGCCTGCTTCGGCCTCCCAA
AGTGCTGGATTACAGGTATGAGC
>1089
AATTTTGAATTTTTAGTAGAGACGGGCTTTCACCGTGTTGGTCAGGATGGTCTCGAACT
CCCGACCTCAGGTGATCGGCCTGCTTCGGCCTCCCAAAGTGCTGGATTACAGGTATGA
GCCACCGTGTCCAGCTGGTAATG

>1090
TTTCACCGTGTTGGTCAGGATGGTCTCGAACTCCCGACCTCAGGTGATCGGCCTGCTTC
GGCCTCCCAAAGTGCTGGATTACAGGTATGAGCCACCGTGTCCAGCTGGTAATGTCCTT
CTTTTACATGGAACAATACTAA
>1091
TCAGGATGGTCTCGAACTCCCGACCTCAGGTGATCGGCCTGCTTCGGCCTCCCAAAGT
GCTGGATTACAGGTATGAGCCACCGTGTCCAGCTGGTAATGTCCTTCTTTTACATGGAA
CAATACTAAGGCTCAGATGTCAA
>1092
CGAACTCCCGACCTCAGGTGATCGGCCTGCTTCGGCCTCCCAAAGTGCTGGATTACAG
GTATGAGCCACCGTGTCCAGCTGGTAATGTCCTTCTTTTACATGGAACAATACTAAGGC
TCAGATGTCAAGTCTCTTTTCTA
>1093
TACAGGTATGAGCCACCGTGTCCAGCTGGTAATGTCCTTCTTTTACATGGAACAATACT
AAGGCTCAGATGTCAAGTCTCTTTTCTAGGGTCACATAGATAGTAAGAGGCAGAGCTG
GATTCAAATTTAGGTCCTTACCC
>1094
GTATGAGCCACCGTGTCCAGCTGGTAATGTCCTTCTTTTACATGGAACAATACTAAGGC
TCAGATGTCAAGTCTCTTTTCTAGGGTCACATAGATAGTAAGAGGCAGAGCTGGATTCA
AATTTAGGTCCTTACCCCCTGG
>1095
ATGTCCTTCTTTTACATGGAACAATACTAAGGCTCAGATGTCAAGTCTCTTTTCTAGGGT
CACATAGATAGTAAGAGGCAGAGCTGGATTCAAATTTAGGTCCTTACCCCCTGGTACTT
TGCTGCCTCTTGGAGTCACTT
>1096
AGGCTCAGATGTCAAGTCTCTTTTCTAGGGTCACATAGATAGTAAGAGGCAGAGCTGG
ATTCAAATTTAGGTCCTTACCCCCTGGTACTTTGCTGCCTCTTGGAGTCACTTAGACTTG
AAGAGGATCATGTCTATGGAAC
>1097
AGTCTCTTTTCTAGGGTCACATAGATAGTAAGAGGCAGAGCTGGATTCAAATTTAGGTC
CTTACCCCCTGGTACTTTGCTGCCTCTTGGAGTCACTTAGACTTGAAGAGGATCATGTC
TATGGAACAGAGCGTTTTGAAG
>1098
TTCAAATTTAGGTCCTTACCCCCTGGTACTTTGCTGCCTCTTGGAGTCACTTAGACTTG
AAGAGGATCATGTCTATGGAACAGAGCGTTTTGAAGATTGCTTATAGGAAGTGGAGTA
AATAACAGGGGGGATAAGTAGCG
>1099
TACCCCCTGGTACTTTGCTGCCTCTTGGAGTCACTTAGACTTGAAGAGGATCATGTCTA
TGGAACAGAGCGTTTTGAAGATTGCTTATAGGAAGTGGAGTAAATAACAGGGGGGATA
AGTAGCGAGTTTGTTTTTTTTTT
>1100
CTTGGAGTCACTTAGACTTGAAGAGGATCATGTCTATGGAACAGAGCGTTTTGAAGAT
TGCTTATAGGAAGTGGAGTAAATAACAGGGGGGATAAGTAGCGAGTTTGTTTTTTTTTT
CCTTGCTTCTGTCATAAACCACG
>1101
AGTCACTTAGACTTGAAGAGGATCATGTCTATGGAACAGAGCGTTTTGAAGATTGCTTA

TAGGAAGTGGAGTAAATAACAGGGGGGATAAGTAGCGAGTTTGTTTTTTTTTTCCTTGC
TTCTGTCATAAACCACGTGCCC
>1102
GATCATGTCTATGGAACAGAGCGTTTTGAAGATTGCTTATAGGAAGTGGAGTAAATAAC
AGGGGGGATAAGTAGCGAGTTTGTTTTTTTTTTCCTTGCTTCTGTCATAAACCACGTGC
CCATCATTTTATCCTCTAGTTT
>1103
AGTGGAGTAAATAACAGGGGGGATAAGTAGCGAGTTTGTTTTTTTTTTCCTTGCTTCTG
TCATAAACCACGTGCCCATCATTTTATCCTCTAGTTTCTGGGTCAGATGAATGGAGTCAA
GAAATCCAGCTTTCTGTTGTC
>1104
ATAAGTAGCGAGTTTGTTTTTTTTTTCCTTGCTTCTGTCATAAACCACGTGCCCATCATT
TTATCCTCTAGTTTCTGGGTCAGATGAATGGAGTCAAGAAATCCAGCTTTCTGTTGTCA
TTGACGACAGCATTTGGCTGC
>1105
TTTTCCTTGCTTCTGTCATAAACCACGTGCCCATCATTTTATCCTCTAGTTTCTGGGTCA
GATGAATGGAGTCAAGAAATCCAGCTTTCTGTTGTCATTGACGACAGCATTTGGCTGCA
TACCCTGTGGTCTGCTGTTTT
>1106
AACCACGTGCCCATCATTTTATCCTCTAGTTTCTGGGTCAGATGAATGGAGTCAAGAAA
TCCAGCTTTCTGTTGTCATTGACGACAGCATTTGGCTGCATACCCTGTGGTCTGCTGTTT
TGCTGATCTTGGGTGGATTCT
>1107
CACGTGCCCATCATTTTATCCTCTAGTTTCTGGGTCAGATGAATGGAGTCAAGAAATCC
AGCTTTCTGTTGTCATTGACGACAGCATTTGGCTGCATACCCTGTGGTCTGCTGTTTTG
CTGATCTTGGGTGGATTCTCCA
>1108
GGTCAGATGAATGGAGTCAAGAAATCCAGCTTTCTGTTGTCATTGACGACAGCATTTG
GCTGCATACCCTGTGGTCTGCTGTTTTGCTGATCTTGGGTGGATTCTCCACTTCCTCCCC
CTTCACCCCCATTCCCCTGTTG
>1109
ATGGAGTCAAGAAATCCAGCTTTCTGTTGTCATTGACGACAGCATTTGGCTGCATACCC
TGTGGTCTGCTGTTTTGCTGATCTTGGGTGGATTCTCCACTTCCTCCCCCTTCACCCCCA
TTCCCCTGTTGCTCGTTCAGG
>1110
CAGCTTTCTGTTGTCATTGACGACAGCATTTGGCTGCATACCCTGTGGTCTGCTGTTTT
GCTGATCTTGGGTGGATTCTCCACTTCCTCCCCCTTCACCCCCATTCCCCTGTTGCTCGT
TCAGGTACGGGGGATGTGAGT
>1111
CCCTGTGGTCTGCTGTTTTGCTGATCTTGGGTGGATTCTCCACTTCCTCCCCCTTCACCC
CCATTCCCCTGTTGCTCGTTCAGGTACGGGGGATGTGAGTGAGAGGGGTTGGGGTGGG
GATATCTGGAAGTTGATCTCAT
>1112
GTCTGCTGTTTTGCTGATCTTGGGTGGATTCTCCACTTCCTCCCCCTTCACCCCCATTCC
CCTGTTGCTCGTTCAGGTACGGGGGATGTGAGTGAGAGGGGTTGGGGTGGGGATATCT
GGAAGTTGATCTCATCACCCCT

>1113
TGTTTTGCTGATCTTGGGTGGATTCTCCACTTCCTCCCCCTTCACCCCCATTCCCCTGTT
GCTCGTTCAGGTACGGGGGATGTGAGTGAGAGGGGTTGGGGTGGGGATATCTGGAAG
TTGATCTCATCACCCCTAGCCGG
>1114
TCTTGGGTGGATTCTCCACTTCCTCCCCCTTCACCCCCATTCCCCTGTTGCTCGTTCAGG
TACGGGGGATGTGAGTGAGAGGGGTTGGGGTGGGGATATCTGGAAGTTGATCTCATCA
CCCCTAGCCGGAATCTGCTCTG
>1115
TTCCTCCCCCTTCACCCCCATTCCCCTGTTGCTCGTTCAGGTACGGGGGATGTGAGTGA
GAGGGGTTGGGGTGGGGATATCTGGAAGTTGATCTCATCACCCCTAGCCGGAATCTGC
TCTGACCCTGCCTCCTCGACAGC
>1116
AGTTGATCTCATCACCCCTAGCCGGAATCTGCTCTGACCCTGCCTCCTCGACAGCTCAG
AGGCTTAACTTGTGGAAAGTGTGGCAGAGCTAAACGTAGCCGACTTTTTCCAGTGGGT
GCGAAAAATAACCGCCACTAAGG
>1117
CTCATCACCCCTAGCCGGAATCTGCTCTGACCCTGCCTCCTCGACAGCTCAGAGGCTTA
ACTTGTGGAAAGTGTGGCAGAGCTAAACGTAGCCGACTTTTTCCAGTGGGTGCGAAA
AATAACCGCCACTAAGGTGTGTCT
>1118
GAATCTGCTCTGACCCTGCCTCCTCGACAGCTCAGAGGCTTAACTTGTGGAAAGTGTG
GCAGAGCTAAACGTAGCCGACTTTTTCCAGTGGGTGCGAAAAATAACCGCCACTAAGG
TGTGTCTTCTTGATGCTTGTACCG
>1119
CCTCGACAGCTCAGAGGCTTAACTTGTGGAAAGTGTGGCAGAGCTAAACGTAGCCGA
CTTTTTCCAGTGGGTGCGAAAAATAACCGCCACTAAGGTGTGTCTTCTTGATGCTTGTA
CCGCTCAGAAATAGACTGAGGTTA
>1120
TGTGGAAAGTGTGGCAGAGCTAAACGTAGCCGACTTTTTCCAGTGGGTGCGAAAAATA
ACCGCCACTAAGGTGTGTCTTCTTGATGCTTGTACCGCTCAGAAATAGACTGAGGTTAT
GTTTGTTTTTGCTCAAACTTTCC
>1121
TGGAAAGTGTGGCAGAGCTAAACGTAGCCGACTTTTTCCAGTGGGTGCGAAAAATAAC
CGCCACTAAGGTGTGTCTTCTTGATGCTTGTACCGCTCAGAAATAGACTGAGGTTATGT
TTGTTTTTGCTCAAACTTTCCTA
>1122
CTAAACGTAGCCGACTTTTTCCAGTGGGTGCGAAAAATAACCGCCACTAAGGTGTGTC
TTCTTGATGCTTGTACCGCTCAGAAATAGACTGAGGTTATGTTTGTTTTTGCTCAAACTT
TCCTAAAGAGGAAACCCGCTCC
>1123
TGCGAAAAATAACCGCCACTAAGGTGTGTCTTCTTGATGCTTGTACCGCTCAGAAATAG
ACTGAGGTTATGTTTGTTTTTGCTCAAACTTTCCTAAAGAGGAAACCCGCTCCCCAAA
GTTGACCAGATAGATGTATCATC
>1124
AAAAATAACCGCCACTAAGGTGTGTCTTCTTGATGCTTGTACCGCTCAGAAATAGACTG

AGGTTATGTTTGTTTTTGCTCAAACTTTCCTAAAGAGGAAACCCGCTCCCCAAAGTTGA
CCAGATAGATGTATCATCTTTC
>1125
GCTCAGAAATAGACTGAGGTTATGTTTGTTTTTGCTCAAACTTTCCTAAAGAGGAAACC
CGCTCCCCAAAGTTGACCAGATAGATGTATCATCTTTCCGGATGTGAATTATTTCGGCTG
TGTGCTATGAGAACTGGGTAT
>1126
GAGGTTATGTTTGTTTTTGCTCAAACTTTCCTAAAGAGGAAACCCGCTCCCCAAAGTTG
ACCAGATAGATGTATCATCTTTCCGGATGTGAATTATTTCGGCTGTGTGCTATGAGAACT
GGGTATGGTGGGAAGTAATTG
>1127
TGCTCAAACTTTCCTAAAGAGGAAACCCGCTCCCCAAAGTTGACCAGATAGATGTATC
ATCTTTCCGGATGTGAATTATTTCGGCTGTGTGCTATGAGAACTGGGTATGGTGGGAAG
TAATTGTTTTTCTGGCTCTCATC
>1128
AAGAGGAAACCCGCTCCCCAAAGTTGACCAGATAGATGTATCATCTTTCCGGATGTGA
ATTATTTCGGCTGTGTGCTATGAGAACTGGGTATGGTGGGAAGTAATTGTTTTTCTGGCT
CTCATCAGGTCCTGGTTCCTGG
>1129
GATAGATGTATCATCTTTCCGGATGTGAATTATTTCGGCTGTGTGCTATGAGAACTGGGT
ATGGTGGGAAGTAATTGTTTTTCTGGCTCTCATCAGGTCCTGGTTCCTGGTTGCTTTTCT
TTACCCGAGAGGGCCACAAA
>1130
TGTATCATCTTTCCGGATGTGAATTATTTCGGCTGTGTGCTATGAGAACTGGGTATGGTG
GGAAGTAATTGTTTTTCTGGCTCTCATCAGGTCCTGGTTCCTGGTTGCTTTTCTTTACCC
GAGAGGGCCACAAAAGATAC
>1131
GAATTATTTCGGCTGTGTGCTATGAGAACTGGGTATGGTGGGAAGTAATTGTTTTTCTG
GCTCTCATCAGGTCCTGGTTCCTGGTTGCTTTTCTTTACCCGAGAGGGCCACAAAAGAT
ACCACTCTCCTGAGACTTCCAA
>1132
GTCCTGGTTCCTGGTTGCTTTTCTTTACCCGAGAGGGCCACAAAAGATACCACTCTCCT
GAGACTTCCAAGTTCCCCTTCAGCAAGACCCTCATTCCTCTGGGGAGAGAGGGAGAG
TTGGCCATGTTATATTCGAAAGAG
>1133
GATACCACTCTCCTGAGACTTCCAAGTTCCCCTTCAGCAAGACCCTCATTCCTCTGGGG
AGAGAGGGAGAGTTGGCCATGTTATATTCGAAAGAGCCTGGGTATTGGAATTGGCCATA
TTTAGTGGTGAGAGCCTGTGAT
>1134
CTCCTGAGACTTCCAAGTTCCCCTTCAGCAAGACCCTCATTCCTCTGGGGAGAGAGGG
AGAGTTGGCCATGTTATATTCGAAAGAGCCTGGGTATTGGAATTGGCCATATTTAGTGGT
GAGAGCCTGTGATACTTATTTA
>1135
TCTGGGGAGAGAGGGAGAGTTGGCCATGTTATATTCGAAAGAGCCTGGGTATTGGAAT
TGGCCATATTTAGTGGTGAGAGCCTGTGATACTTATTTAACCTTTCTGGGCCTTGTTTCA
CTCACTACATTTTTTTTTTTTT

>1136
GGGGAGAGAGGGAGAGTTGGCCATGTTATATTCGAAAGAGCCTGGGTATTGGAATTGG
CCATATTTAGTGGTGAGAGCCTGTGATACTTATTTAACCTTTCTGGGCCTTGTTTCACTC
ACTACATTTTTTTTTTTTTTTT
>1137
GCCTGGGTATTGGAATTGGCCATATTTAGTGGTGAGAGCCTGTGATACTTATTTAACCTT
TCTGGGCCTTGTTTCACTCACTACATTTTTTTTTTTTTTTTTTTTGAGACGGAGTCTCG
CTCTGTCACCGGGCTGGAGT
>1138
ATACTTATTTAACCTTTCTGGGCCTTGTTTCACTCACTACATTTTTTTTTTTTTTTTTTTT
GAGACGGAGTCTCGCTCTGTCACCGGGCTGGAGTGCAATGGCGTGATCTCAGCTCACT
GCAGTCTCCACCTCCCGGGT
>1139
AACCTTTCTGGGCCTTGTTTCACTCACTACATTTTTTTTTTTTTTTTTTTTGAGACGGA
GTCTCGCTCTGTCACCGGGCTGGAGTGCAATGGCGTGATCTCAGCTCACTGCAGTCTC
CACCTCCCGGGTTCAAGCGATT
>1140
TTGTTTCACTCACTACATTTTTTTTTTTTTTTTTTTTGAGACGGAGTCTCGCTCTGTCAC
CGGGCTGGAGTGCAATGGCGTGATCTCAGCTCACTGCAGTCTCCACCTCCCGGGTTCA
AGCGATTCTCCTGCCTCAGCC
>1141
CACTACATTTTTTTTTTTTTTTTTTTTGAGACGGAGTCTCGCTCTGTCACCGGGCTGGA
GTGCAATGGCGTGATCTCAGCTCACTGCAGTCTCCACCTCCCGGGTTCAAGCGATTCTC
CTGCCTCAGCCTCCTGAGTAG
>1142
TTTTTTTTTGAGACGGAGTCTCGCTCTGTCACCGGGCTGGAGTGCAATGGCGTGATCTC
AGCTCACTGCAGTCTCCACCTCCCGGGTTCAAGCGATTCTCCTGCCTCAGCCTCCTGA
GTAGCTGGGACTACAGGCACCTG
>1143
TGGCGTGATCTCAGCTCACTGCAGTCTCCACCTCCCGGGTTCAAGCGATTCTCCTGCCT
CAGCCTCCTGAGTAGCTGGGACTACAGGCACCTGTCACCCCGCCCAGCTAATTTTTGT
ATTTTTAGTAGAGACAGGGTTT
>1144
AGTCTCCACCTCCCGGGTTCAAGCGATTCTCCTGCCTCAGCCTCCTGAGTAGCTGGGA
CTACAGGCACCTGTCACCCCGCCCAGCTAATTTTTGTATTTTTAGTAGAGACAGGGTT
TCACCATGTTGGCCAGAATGGTC
>1145
GATTCTCCTGCCTCAGCCTCCTGAGTAGCTGGGACTACAGGCACCTGTCACCCCGCCC
AGCTAATTTTTGTATTTTTAGTAGAGACAGGGTTTCACCATGTTGGCCAGAATGGTCTC
GATCTCTTGACCTTGCGATCCA
>1146
GCCTCAGCCTCCTGAGTAGCTGGGACTACAGGCACCTGTCACCCCGCCCAGCTAATTT
TTGTATTTTTAGTAGAGACAGGGTTTCACCATGTTGGCCAGAATGGTCTCGATCTCTTG
ACCTTGCGATCCATCCACCTCG
>1147
GGGACTACAGGCACCTGTCACCCCGCCCAGCTAATTTTTGTATTTTTAGTAGAGACAG

GGTTTCACCATGTTGGCCAGAATGGTCTCGATCTCTTGACCTTGCGATCCATCCACCTC
GGCCTCCCAAAGTGCTGGGATT
>1148
GGTTTCACCATGTTGGCCAGAATGGTCTCGATCTCTTGACCTTGCGATCCATCCACCTC
GGCCTCCCAAAGTGCTGGGATTACAGCTGTGAGCCACCCCGCCCGGCCTTCACTCACT
TCTTAAAATGGAGACGGTCAACA
>1149
CAGAATGGTCTCGATCTCTTGACCTTGCGATCCATCCACCTCGGCCTCCCAAAGTGCTG
GGATTACAGCTGTGAGCCACCCCGCCCGGCCTTCACTCACTTCTTAAAATGGAGACGG
TCAACAGGGTTGGTGCTGAATTA
>1150
TCCCAAAGTGCTGGGATTACAGCTGTGAGCCACCCCGCCCGGCCTTCACTCACTTCTT
AAAATGGAGACGGTCAACAGGGTTGGTGCTGAATTAATGAAGTAAGATCCAGAAATCA
CTAGAGCTGTGATGGGCACATAGT
>1151
GCTGGGATTACAGCTGTGAGCCACCCCGCCCGGCCTTCACTCACTTCTTAAAATGGAG
ACGGTCAACAGGGTTGGTGCTGAATTAATGAAGTAAGATCCAGAAATCACTAGAGCTG
TGATGGGCACATAGTCACCGCAGC
>1152
CCACCCCGCCCGGCCTTCACTCACTTCTTAAAATGGAGACGGTCAACAGGGTTGGTGC
TGAATTAATGAAGTAAGATCCAGAAATCACTAGAGCTGTGATGGGCACATAGTCACCG
CAGCGGTGGAGAACTGACCATCTG
>1153
TCTTAAAATGGAGACGGTCAACAGGGTTGGTGCTGAATTAATGAAGTAAGATCCAGAA
ATCACTAGAGCTGTGATGGGCACATAGTCACCGCAGCGGTGGAGAACTGACCATCTGT
CCCTTGTGTTGTTTAGGCCTCATC
>1154
CGGTCAACAGGGTTGGTGCTGAATTAATGAAGTAAGATCCAGAAATCACTAGAGCTGT
GATGGGCACATAGTCACCGCAGCGGTGGAGAACTGACCATCTGTCCCTTGTGTTGTTT
AGGCCTCATCTCTGCATGGTCCTC
>1155
TTGGTGCTGAATTAATGAAGTAAGATCCAGAAATCACTAGAGCTGTGATGGGCACATAG
TCACCGCAGCGGTGGAGAACTGACCATCTGTCCCTTGTGTTGTTTAGGCCTCATCTCTG
CATGGTCCTCATTGGCTCTGAG
>1156
AGTAAGATCCAGAAATCACTAGAGCTGTGATGGGCACATAGTCACCGCAGCGGTGGAG
AACTGACCATCTGTCCCTTGTGTTGTTTAGGCCTCATCTCTGCATGGTCCTCATTGGCTC
TGAGTTCTAGTGTGTAGTGTCC
>1157
TCCAGAAATCACTAGAGCTGTGATGGGCACATAGTCACCGCAGCGGTGGAGAACTGAC
CATCTGTCCCTTGTGTTGTTTAGGCCTCATCTCTGCATGGTCCTCATTGGCTCTGAGTTC
TAGTGTGTAGTGTCCACAGTGC
>1158
CACATAGTCACCGCAGCGGTGGAGAACTGACCATCTGTCCCTTGTGTTGTTTAGGCCTC
ATCTCTGCATGGTCCTCATTGGCTCTGAGTTCTAGTGTGTAGTGTCCACAGTGCCACCC
TCCATGAGGGTTGTGTTGACTG

>1159
GGTGGAGAACTGACCATCTGTCCCTTGTGTTGTTTAGGCCTCATCTCTGCATGGTCCTC
ATTGGCTCTGAGTTCTAGTGTGTAGTGTCCACAGTGCCACCCTCCATGAGGGTTGTGTT
GACTGTGAGGTGGTGCAGAGAA
>1160
GAACTGACCATCTGTCCCTTGTGTTGTTTAGGCCTCATCTCTGCATGGTCCTCATTGGCT
CTGAGTTCTAGTGTGTAGTGTCCACAGTGCCACCCTCCATGAGGGTTGTGTTGACTGTG
AGGTGGTGCAGAGAATGTCCC
>1161
CCTTGTGTTGTTTAGGCCTCATCTCTGCATGGTCCTCATTGGCTCTGAGTTCTAGTGTGT
AGTGTCCACAGTGCCACCCTCCATGAGGGTTGTGTTGACTGTGAGGTGGTGCAGAGAA
TGTCCCTGTCCTTGTGAGCATA
>1162
GGTCCTCATTGGCTCTGAGTTCTAGTGTGTAGTGTCCACAGTGCCACCCTCCATGAGGG
TTGTGTTGACTGTGAGGTGGTGCAGAGAATGTCCCTGTCCTTGTGAGCATATGACAGC
AAGACACTGGCCCTGGAATCCTC
>1163
TCATTGGCTCTGAGTTCTAGTGTGTAGTGTCCACAGTGCCACCCTCCATGAGGGTTGTG
TTGACTGTGAGGTGGTGCAGAGAATGTCCCTGTCCTTGTGAGCATATGACAGCAAGAC
ACTGGCCCTGGAATCCTCCGTGT
>1164
TTCTAGTGTGTAGTGTCCACAGTGCCACCCTCCATGAGGGTTGTGTTGACTGTGAGGT
GGTGCAGAGAATGTCCCTGTCCTTGTGAGCATATGACAGCAAGACACTGGCCCTGGAA
TCCTCCGTGTCACATGTGGGCTGC
>1165
TGAGGTGGTGCAGAGAATGTCCCTGTCCTTGTGAGCATATGACAGCAAGACACTGGCC
CTGGAATCCTCCGTGTCACATGTGGGCTGCTGACAGGAAGAATGTGCTTTTGTTGTTGT
TGTTGTTGTTGAGGCAGAGTCTT
>1166
AGGTGGTGCAGAGAATGTCCCTGTCCTTGTGAGCATATGACAGCAAGACACTGGCCCT
GGAATCCTCCGTGTCACATGTGGGCTGCTGACAGGAAGAATGTGCTTTTGTTGTTGTTG
TTGTTGTTGAGGCAGAGTCTTAC
>1167
TGAGCATATGACAGCAAGACACTGGCCCTGGAATCCTCCGTGTCACATGTGGGCTGCT
GACAGGAAGAATGTGCTTTTGTTGTTGTTGTTGTTGTTGAGGCAGAGTCTTACTCTGTC
GCCCAGGCTGCAGTGCAGTGGCA
>1168
GACACTGGCCCTGGAATCCTCCGTGTCACATGTGGGCTGCTGACAGGAAGAATGTGCT
TTTGTTGTTGTTGTTGTTGTTGAGGCAGAGTCTTACTCTGTCGCCCAGGCTGCAGTGCA
GTGGCATGATCTTGGCTCACTGC
>1169
ACTGGCCCTGGAATCCTCCGTGTCACATGTGGGCTGCTGACAGGAAGAATGTGCTTTT
GTTGTTGTTGTTGTTGTTGAGGCAGAGTCTTACTCTGTCGCCCAGGCTGCAGTGCAGT
GGCATGATCTTGGCTCACTGCAAC
>1170
CCTCCGTGTCACATGTGGGCTGCTGACAGGAAGAATGTGCTTTTGTTGTTGTTGTTGTT

GTTGAGGCAGAGTCTTACTCTGTCGCCCAGGCTGCAGTGCAGTGGCATGATCTTGGCT
CACTGCAACCTCTACTTCCCGGG
>1171
ACATGTGGGCTGCTGACAGGAAGAATGTGCTTTTGTTGTTGTTGTTGTTGTTGAGGCA
GAGTCTTACTCTGTCGCCCAGGCTGCAGTGCAGTGGCATGATCTTGGCTCACTGCAAC
CTCTACTTCCCGGGTTCAAGTGAT
>1172
AGTCTTACTCTGTCGCCCAGGCTGCAGTGCAGTGGCATGATCTTGGCTCACTGCAACCT
CTACTTCCCGGGTTCAAGTGATTCTCCTGCCTCAGCCTCCTGAGCAGCTGGGACTACA
GATGTGTACCACCATGCCCGACT
>1173
ACTCTGTCGCCCAGGCTGCAGTGCAGTGGCATGATCTTGGCTCACTGCAACCTCTACTT
CCCGGGTTCAAGTGATTCTCCTGCCTCAGCCTCCTGAGCAGCTGGGACTACAGATGTG
TACCACCATGCCCGACTAATTTT
>1174
CTGCAACCTCTACTTCCCGGGTTCAAGTGATTCTCCTGCCTCAGCCTCCTGAGCAGCTG
GGACTACAGATGTGTACCACCATGCCCGACTAATTTTTGTATTTTTAGTAGAGATGGGGT
TTCACCATGTTGGTCAGGCTG
>1175
GCAACCTCTACTTCCCGGGTTCAAGTGATTCTCCTGCCTCAGCCTCCTGAGCAGCTGG
GACTACAGATGTGTACCACCATGCCCGACTAATTTTTGTATTTTTAGTAGAGATGGGGTT
TCACCATGTTGGTCAGGCTGGC
>1176
TGATTCTCCTGCCTCAGCCTCCTGAGCAGCTGGGACTACAGATGTGTACCACCATGCCC
GACTAATTTTTGTATTTTTAGTAGAGATGGGGTTTCACCATGTTGGTCAGGCTGGCCTG
GAACTCCTGGCCTTAAGTGATC
>1177
CCTGAGCAGCTGGGACTACAGATGTGTACCACCATGCCCGACTAATTTTTGTATTTTTA
GTAGAGATGGGGTTTCACCATGTTGGTCAGGCTGGCCTGGAACTCCTGGCCTTAAGTG
ATCCACCTGTCTCGGCCTCCCAT
>1178
TGGGACTACAGATGTGTACCACCATGCCCGACTAATTTTTGTATTTTTAGTAGAGATGGG
GTTTCACCATGTTGGTCAGGCTGGCCTGGAACTCCTGGCCTTAAGTGATCCACCTGTCT
CGGCCTCCCATAGTGCTGGGA
>1179
ATTTTTGTATTTTTAGTAGAGATGGGGTTTCACCATGTTGGTCAGGCTGGCCTGGAACT
CCTGGCCTTAAGTGATCCACCTGTCTCGGCCTCCCATAGTGCTGGGATTATAGGTGTGT
GCCACCCTGCTGGGCCGAGAAT
>1180
TTTAGTAGAGATGGGGTTTCACCATGTTGGTCAGGCTGGCCTGGAACTCCTGGCCTTAA
GTGATCCACCTGTCTCGGCCTCCCATAGTGCTGGGATTATAGGTGTGTGCCACCCTGCT
GGGCCGAGAATGTGCTTTTGAC
>1181
TTTCACCATGTTGGTCAGGCTGGCCTGGAACTCCTGGCCTTAAGTGATCCACCTGTCTC
GGCCTCCCATAGTGCTGGGATTATAGGTGTGTGCCACCCTGCTGGGCCGAGAATGTGCT
TTTGACTGAGGGTGGAAAGTCA

>1182
CAGGCTGGCCTGGAACTCCTGGCCTTAAGTGATCCACCTGTCTCGGCCTCCCATAGTGC
TGGGATTATAGGTGTGTGCCACCCTGCTGGGCCGAGAATGTGCTTTTGACTGAGGGTG
GAAAGTCAGCAGCTGCAGCCCAA
>1183
GGCCTCCCATAGTGCTGGGATTATAGGTGTGTGCCACCCTGCTGGGCCGAGAATGTGCT
TTTGACTGAGGGTGGAAAGTCAGCAGCTGCAGCCCAAGAAGTTAGAGCCAGGGGCAG
TGCCTTTGATGGGAAGCTTGGGGG
>1184
CATAGTGCTGGGATTATAGGTGTGTGCCACCCTGCTGGGCCGAGAATGTGCTTTTGACT
GAGGGTGGAAAGTCAGCAGCTGCAGCCCAAGAAGTTAGAGCCAGGGGCAGTGCCTTT
GATGGGAAGCTTGGGGGTCTAAAG
>1185
TGTGCCACCCTGCTGGGCCGAGAATGTGCTTTTGACTGAGGGTGGAAAGTCAGCAGCT
GCAGCCCAAGAAGTTAGAGCCAGGGGCAGTGCCTTTGATGGGAAGCTTGGGGGTCTA
AAGAAATCAGAAAGGGGAGAAAATA
>1186
GCCGAGAATGTGCTTTTGACTGAGGGTGGAAAGTCAGCAGCTGCAGCCCAAGAAGTT
AGAGCCAGGGGCAGTGCCTTTGATGGGAAGCTTGGGGGTCTAAAGAAATCAGAAAGG
GGAGAAAATAATCCTGCGTGTGCTCA
>1187
GGTGGAAAGTCAGCAGCTGCAGCCCAAGAAGTTAGAGCCAGGGGCAGTGCCTTTGAT
GGGAAGCTTGGGGGTCTAAAGAAATCAGAAAGGGGAGAAAATAATCCTGCGTGTGCT
CAGCCCTCACTCAGATCAACAGCCCC
>1188
CCAGGGGCAGTGCCTTTGATGGGAAGCTTGGGGGTCTAAAGAAATCAGAAAGGGGAG
AAAATAATCCTGCGTGTGCTCAGCCCTCACTCAGATCAACAGCCCCTCGGGTCCCAGC
TAGATGTACTTTGTAGAAGGCTTTT
>1189
AGGGGCAGTGCCTTTGATGGGAAGCTTGGGGGTCTAAAGAAATCAGAAAGGGGAGAA
AATAATCCTGCGTGTGCTCAGCCCTCACTCAGATCAACAGCCCCTCGGGTCCCAGCTA
GATGTACTTTGTAGAAGGCTTTTTT
>1190
AAAGAAATCAGAAAGGGGAGAAAATAATCCTGCGTGTGCTCAGCCCTCACTCAGATCA
ACAGCCCCTCGGGTCCCAGCTAGATGTACTTTGTAGAAGGCTTTTTTTCTCCCCCTCAC
CCTCAAAACTCCAGGATTAATGT
>1191
AGGGGAGAAAATAATCCTGCGTGTGCTCAGCCCTCACTCAGATCAACAGCCCCTCGGG
TCCCAGCTAGATGTACTTTGTAGAAGGCTTTTTTTCTCCCCCTCACCCTCAAAACTCCA
GGATTAATGTGCTATTCCCTTTT
>1192
GGGTCCCAGCTAGATGTACTTTGTAGAAGGCTTTTTTTCTCCCCCTCACCCTCAAAACT
CCAGGATTAATGTGCTATTCCCTTTTTTCTACTGTTCTCTGTCTCCCCCCAACCCATCAG
CAGTCTGAGAAATGCTATGGT
>1193
GTAGAAGGCTTTTTTTCTCCCCCTCACCCTCAAAACTCCAGGATTAATGTGCTATTCCCT

TTTTTCTACTGTTCTCTGTCTCCCCCCAACCCATCAGCAGTCTGAGAAATGCTATGGTG
GAAAAAAAAAAACAACAGCCC
>1194
GCTTTTTTTCTCCCCCTCACCCTCAAAACTCCAGGATTAATGTGCTATTCCCTTTTTTCT
ACTGTTCTCTGTCTCCCCCCAACCCATCAGCAGTCTGAGAAATGCTATGGTGGAAAAA
AAAAAACAACAGCCCTATAACC
>1195
TCAAAACTCCAGGATTAATGTGCTATTCCCTTTTTTCTACTGTTCTCTGTCTCCCCCCAA
CCCATCAGCAGTCTGAGAAATGCTATGGTGGAAAAAAAAAAACAACAGCCCTATAACC
AGTACAAACAAAAAGCCAAGAC
>1196
TCTCCCCCCAACCCATCAGCAGTCTGAGAAATGCTATGGTGGAAAAAAAAAAACAAC
AGCCCTATAACCAGTACAAACAAAAAGCCAAGACAAAAAAAAAAAAAAAAAAAATC
AGAAGGGGAGATTTTGAGGCCAGGAAG
>1197
AAAAAAAAAAAAAAAATCAGAAGGGGAGATTTTGAGGCCAGGAAGCCATGGCATTCAC
TGGAAATATATCAGTGATAAAATAACAGGCTACAGGGTTTATTTTATTTTATTTTTAAGTC
AATAGGCCTGAGTGTTGGTAAA
>1198
GGAGATTTTGAGGCCAGGAAGCCATGGCATTCACTGGAAATATATCAGTGATAAAATAA
CAGGCTACAGGGTTTATTTTATTTTATTTTTAAGTCAATAGGCCTGAGTGTTGGTAAAGA
CTAGATACCCAGCAATCCTTC
>1199
CATGGCATTCACTGGAAATATATCAGTGATAAAATAACAGGCTACAGGGTTTATTTTATT
TTATTTTTAAGTCAATAGGCCTGAGTGTTGGTAAAGACTAGATACCCAGCAATCCTTCTT
GCATTTCTTCTTGGGGTCGG
>1200
GAAATATATCAGTGATAAAATAACAGGCTACAGGGTTTATTTTATTTTATTTTTAAGTCAA
TAGGCCTGAGTGTTGGTAAAGACTAGATACCCAGCAATCCTTCTTGCATTTCTTCTTGG
GGTCGGGGGGCAGAAGGGAA
>1201
AATATATCAGTGATAAAATAACAGGCTACAGGGTTTATTTTATTTTATTTTTAAGTCAATA
GGCCTGAGTGTTGGTAAAGACTAGATACCCAGCAATCCTTCTTGCATTTCTTCTTGGGG
TCGGGGGGCAGAAGGGAATG
>1202
TATCAGTGATAAAATAACAGGCTACAGGGTTTATTTTATTTTATTTTTAAGTCAATAGGCC
TGAGTGTTGGTAAAGACTAGATACCCAGCAATCCTTCTTGCATTTCTTCTTGGGGTCGG
GGGGCAGAAGGGAATGGAGA
>1203
TTTAAGTCAATAGGCCTGAGTGTTGGTAAAGACTAGATACCCAGCAATCCTTCTTGCAT
TTCTTCTTGGGGTCGGGGGGCAGAAGGGAATGGAGAGGAAATGAAGGCGGATTGCTG
GTAAGTGAGGCAGGAAGGCAGGGA
>1204
ATCCTTCTTGCATTTCTTCTTGGGGTCGGGGGGCAGAAGGGAATGGAGAGGAAATGAA
GGCGGATTGCTGGTAAGTGAGGCAGGAAGGCAGGGAGTTTATTATGGAATGTCATCGG
TAAGTTTCCCCATGATTTGATCCC

>1205
TTCTTGCATTTCTTCTTGGGGTCGGGGGGCAGAAGGGAATGGAGAGGAAATGAAGGC
GGATTGCTGGTAAGTGAGGCAGGAAGGCAGGGAGTTTATTATGGAATGTCATCGGTAA
GTTTCCCCATGATTTGATCCCAAGC
>1206
GTCGGGGGGCAGAAGGGAATGGAGAGGAAATGAAGGCGGATTGCTGGTAAGTGAGG
CAGGAAGGCAGGGAGTTTATTATGGAATGTCATCGGTAAGTTTCCCCATGATTTGATCC
CAAGCTGGTGTGGAGTGAAGGGTAA
>1207
GGGAATGGAGAGGAAATGAAGGCGGATTGCTGGTAAGTGAGGCAGGAAGGCAGGGA
GTTTATTATGGAATGTCATCGGTAAGTTTCCCCATGATTTGATCCCAAGCTGGTGTGGAG
TGAAGGGTAAGCTGTTGGCTCAAC
>1208
GAGAGGAAATGAAGGCGGATTGCTGGTAAGTGAGGCAGGAAGGCAGGGAGTTTATTA
TGGAATGTCATCGGTAAGTTTCCCCATGATTTGATCCCAAGCTGGTGTGGAGTGAAGGG
TAAGCTGTTGGCTCAACAGATAGT
>1209
GAGGCAGGAAGGCAGGGAGTTTATTATGGAATGTCATCGGTAAGTTTCCCCATGATTTG
ATCCCAAGCTGGTGTGGAGTGAAGGGTAAGCTGTTGGCTCAACAGATAGTGAGAATCA
AACTTAGTGGAGTTGTAGATCCT
>1210
GAAGGCAGGGAGTTTATTATGGAATGTCATCGGTAAGTTTCCCCATGATTTGATCCCAA
GCTGGTGTGGAGTGAAGGGTAAGCTGTTGGCTCAACAGATAGTGAGAATCAAACTTAG
TGGAGTTGTAGATCCTGTTTCCT
>1211
GGGAGTTTATTATGGAATGTCATCGGTAAGTTTCCCCATGATTTGATCCCAAGCTGGTGT
GGAGTGAAGGGTAAGCTGTTGGCTCAACAGATAGTGAGAATCAAACTTAGTGGAGTTG
TAGATCCTGTTTCCTGCAGCGG
>1212
TATTATGGAATGTCATCGGTAAGTTTCCCCATGATTTGATCCCAAGCTGGTGTGGAGTGA
AGGGTAAGCTGTTGGCTCAACAGATAGTGAGAATCAAACTTAGTGGAGTTGTAGATCC
TGTTTCCTGCAGCGGACAGCAA
>1213
CGGTAAGTTTCCCCATGATTTGATCCCAAGCTGGTGTGGAGTGAAGGGTAAGCTGTTG
GCTCAACAGATAGTGAGAATCAAACTTAGTGGAGTTGTAGATCCTGTTTCCTGCAGCG
GACAGCAAGATGTACAAGCCGTCC
>1214
GTGTGGAGTGAAGGGTAAGCTGTTGGCTCAACAGATAGTGAGAATCAAACTTAGTGGA
GTTGTAGATCCTGTTTCCTGCAGCGGACAGCAAGATGTACAAGCCGTCCACTGCAGGA
ATGACGTGCTGTAAAAGGGATGCA
>1215
GGCTCAACAGATAGTGAGAATCAAACTTAGTGGAGTTGTAGATCCTGTTTCCTGCAGC
GGACAGCAAGATGTACAAGCCGTCCACTGCAGGAATGACGTGCTGTAAAAGGGATGC
AGTGTCAAGTGTAGTCATGGATGGG
>1216
GATAGTGAGAATCAAACTTAGTGGAGTTGTAGATCCTGTTTCCTGCAGCGGACAGCAA

GATGTACAAGCCGTCCACTGCAGGAATGACGTGCTGTAAAAGGGATGCAGTGTCAAGT
GTAGTCATGGATGGGTGGGAAGAC
>1217
GAGTTGTAGATCCTGTTTCCTGCAGCGGACAGCAAGATGTACAAGCCGTCCACTGCAG
GAATGACGTGCTGTAAAAGGGATGCAGTGTCAAGTGTAGTCATGGATGGGTGGGAAG
ACCAGACAAAGGCATCCAATGGGAC
>1218
GTTTCCTGCAGCGGACAGCAAGATGTACAAGCCGTCCACTGCAGGAATGACGTGCTGT
AAAAGGGATGCAGTGTCAAGTGTAGTCATGGATGGGTGGGAAGACCAGACAAAGGCA
TCCAATGGGACTGACTTACCTAAAC
>1219
TTCCTGCAGCGGACAGCAAGATGTACAAGCCGTCCACTGCAGGAATGACGTGCTGTAA
AAGGGATGCAGTGTCAAGTGTAGTCATGGATGGGTGGGAAGACCAGACAAAGGCATC
CAATGGGACTGACTTACCTAAACAA
>1220
TGTACAAGCCGTCCACTGCAGGAATGACGTGCTGTAAAAGGGATGCAGTGTCAAGTGT
AGTCATGGATGGGTGGGAAGACCAGACAAAGGCATCCAATGGGACTGACTTACCTAA
ACAAACCAGAAGGAAGTCAGAACAG
>1221
TCATGGATGGGTGGGAAGACCAGACAAAGGCATCCAATGGGACTGACTTACCTAAACA
AACCAGAAGGAAGTCAGAACAGTGGGAAGATCTGATAGATAAGCAGTATTCTGCAGG
CAGATGAAGGAGCAAGCTTTGGGCC
>1222
GGTGGGAAGACCAGACAAAGGCATCCAATGGGACTGACTTACCTAAACAAACCAGAA
GGAAGTCAGAACAGTGGGAAGATCTGATAGATAAGCAGTATTCTGCAGGCAGATGAAG
GAGCAAGCTTTGGGCCCCCAAATCT
>1223
CCAATGGGACTGACTTACCTAAACAAACCAGAAGGAAGTCAGAACAGTGGGAAGATC
TGATAGATAAGCAGTATTCTGCAGGCAGATGAAGGAGCAAGCTTTGGGCCCCCAAATC
TTCACCAGTGCATCCACATTACAGA
>1224
AGATCTGATAGATAAGCAGTATTCTGCAGGCAGATGAAGGAGCAAGCTTTGGGCCCCC
AAATCTTCACCAGTGCATCCACATTACAGAGGATAATGCCTGAGGGAGGGGAAGATCA
TCTCAGGATTCTGGAACACTGGGC
>1225
GGGAGGGGAAGATCATCTCAGGATTCTGGAACACTGGGCCCTCCCTTTCTTATTCCAA
AAGGCTGGAGCAGTTCTGCATGTAGGCTTCTCTGGAAATGGTCTAGGAACCAGCTCAT
CCAGGCCAGGAGCACCTGATCATT
>1226
AGATCATCTCAGGATTCTGGAACACTGGGCCCTCCCTTTCTTATTCCAAAAGGCTGGAG
CAGTTCTGCATGTAGGCTTCTCTGGAAATGGTCTAGGAACCAGCTCATCCAGGCCAGG
AGCACCTGATCATTCTCAGAGTT
>1227
GAACACTGGGCCCTCCCTTTCTTATTCCAAAAGGCTGGAGCAGTTCTGCATGTAGGCTT
CTCTGGAAATGGTCTAGGAACCAGCTCATCCAGGCCAGGAGCACCTGATCATTCTCAG
AGTTACGTACATATGCGATAAGA

>1228
AGGCTTCTCTGGAAATGGTCTAGGAACCAGCTCATCCAGGCCAGGAGCACCTGATCAT
TCTCAGAGTTACGTACATATGCGATAAGATTGCAGGGAACAGTATAGTTCTATAGACTTA
AGTGTGAACATAGTCCTCATTT
>1229
GCTCATCCAGGCCAGGAGCACCTGATCATTCTCAGAGTTACGTACATATGCGATAAGAT
TGCAGGGAACAGTATAGTTCTATAGACTTAAGTGTGAACATAGTCCTCATTTATAGTCCC
TAGTCTCCCATGGCTCACGCA
>1230
CCTGATCATTCTCAGAGTTACGTACATATGCGATAAGATTGCAGGGAACAGTATAGTTCT
ATAGACTTAAGTGTGAACATAGTCCTCATTTATAGTCCCTAGTCTCCCATGGCTCACGCA
GCTTGGTCTTTATGTGTTTC
>1231
TCAGAGTTACGTACATATGCGATAAGATTGCAGGGAACAGTATAGTTCTATAGACTTAA
GTGTGAACATAGTCCTCATTTATAGTCCCTAGTCTCCCATGGCTCACGCAGCTTGGTCTT
TATGTGTTTCTGAATTTAGTG
>1232
CATATGCGATAAGATTGCAGGGAACAGTATAGTTCTATAGACTTAAGTGTGAACATAGT
CCTCATTTATAGTCCCTAGTCTCCCATGGCTCACGCAGCTTGGTCTTTATGTGTTTCTGA
ATTTAGTGCTGCAGAGGCACA
>1233
GATAAGATTGCAGGGAACAGTATAGTTCTATAGACTTAAGTGTGAACATAGTCCTCATTT
ATAGTCCCTAGTCTCCCATGGCTCACGCAGCTTGGTCTTTATGTGTTTCTGAATTTAGTG
CTGCAGAGGCACAATGGGGT
>1234
GTTCTATAGACTTAAGTGTGAACATAGTCCTCATTTATAGTCCCTAGTCTCCCATGGCTC
ACGCAGCTTGGTCTTTATGTGTTTCTGAATTTAGTGCTGCAGAGGCACAATGGGGTCTG
GTTTCTGGATCTTATACAGAC
>1235
ATAGTCCTCATTTATAGTCCCTAGTCTCCCATGGCTCACGCAGCTTGGTCTTTATGTGTTT
CTGAATTTAGTGCTGCAGAGGCACAATGGGGTCTGGTTTCTGGATCTTATACAGACAAG
CACTTAGGTCAGCTGCAGTG
>1236
TAGTCTCCCATGGCTCACGCAGCTTGGTCTTTATGTGTTTCTGAATTTAGTGCTGCAGA
GGCACAATGGGGTCTGGTTTCTGGATCTTATACAGACAAGCACTTAGGTCAGCTGCAG
TGGATGTCCTGCACAGACACCGT
>1237
GTTTCTGAATTTAGTGCTGCAGAGGCACAATGGGGTCTGGTTTCTGGATCTTATACAGA
CAAGCACTTAGGTCAGCTGCAGTGGATGTCCTGCACAGACACCGTCGCAACTCTTGCC
TGGTTCCTTTTTGTTTTTGGAGA
>1238
TTAGTGCTGCAGAGGCACAATGGGGTCTGGTTTCTGGATCTTATACAGACAAGCACTTA
GGTCAGCTGCAGTGGATGTCCTGCACAGACACCGTCGCAACTCTTGCCTGGTTCCTTT
TTGTTTTTGGAGATGGAGTCTCG
>1239
GGGTCTGGTTTCTGGATCTTATACAGACAAGCACTTAGGTCAGCTGCAGTGGATGTCCT

GCACAGACACCGTCGCAACTCTTGCCTGGTTCCTTTTTGTTTTTGGAGATGGAGTCTCG
CTCTGTCTCCCAGGCTGGAGTG
>1240
CAAGCACTTAGGTCAGCTGCAGTGGATGTCCTGCACAGACACCGTCGCAACTCTTGCC
TGGTTCCTTTTTGTTTTTGGAGATGGAGTCTCGCTCTGTCTCCCAGGCTGGAGTGCAGT
GGCGCGATCTCAGCTCACTGCAA
>1241
GCTGCAGTGGATGTCCTGCACAGACACCGTCGCAACTCTTGCCTGGTTCCTTTTTGTTT
TTGGAGATGGAGTCTCGCTCTGTCTCCCAGGCTGGAGTGCAGTGGCGCGATCTCAGCT
CACTGCAAGCTCTGCCTCCCAGG
>1242
ATGTCCTGCACAGACACCGTCGCAACTCTTGCCTGGTTCCTTTTTGTTTTTGGAGATGG
AGTCTCGCTCTGTCTCCCAGGCTGGAGTGCAGTGGCGCGATCTCAGCTCACTGCAAGC
TCTGCCTCCCAGGTTCACGCTAT
>1243
AGTCTCGCTCTGTCTCCCAGGCTGGAGTGCAGTGGCGCGATCTCAGCTCACTGCAAGC
TCTGCCTCCCAGGTTCACGCTATTCTCCTGCCTCAGCCTCCCAAGTAGTTGGGACTACA
GGCACCCGCCACCACGCCCGGCT
>1244
TGGCGCGATCTCAGCTCACTGCAAGCTCTGCCTCCCAGGTTCACGCTATTCTCCTGCCT
CAGCCTCCCAAGTAGTTGGGACTACAGGCACCCGCCACCACGCCCGGCTAATTTTTTG
TATTTTTAGTAGAGACAGGGTTT
>1245
CGCGATCTCAGCTCACTGCAAGCTCTGCCTCCCAGGTTCACGCTATTCTCCTGCCTCAG
CCTCCCAAGTAGTTGGGACTACAGGCACCCGCCACCACGCCCGGCTAATTTTTTGTATT
TTTAGTAGAGACAGGGTTTCAC
>1246
TATTCTCCTGCCTCAGCCTCCCAAGTAGTTGGGACTACAGGCACCCGCCACCACGCCC
GGCTAATTTTTTGTATTTTTAGTAGAGACAGGGTTTCACCGTGTTAGCCAGTATGGTCTC
GATCTCCTGAACTCGTGATCCA
>1247
CCAAGTAGTTGGGACTACAGGCACCCGCCACCACGCCCGGCTAATTTTTTGTATTTTTA
GTAGAGACAGGGTTTCACCGTGTTAGCCAGTATGGTCTCGATCTCCTGAACTCGTGATC
CACCTGCCTCGGCCTCCCAAAG
>1248
TTGGGACTACAGGCACCCGCCACCACGCCCGGCTAATTTTTTGTATTTTTAGTAGAGAC
AGGGTTTCACCGTGTTAGCCAGTATGGTCTCGATCTCCTGAACTCGTGATCCACCTGCC
TCGGCCTCCCAAAGTGCTGGGA
>1249
CCCGCCACCACGCCCGGCTAATTTTTTGTATTTTTAGTAGAGACAGGGTTTCACCGTGT
TAGCCAGTATGGTCTCGATCTCCTGAACTCGTGATCCACCTGCCTCGGCCTCCCAAAGT
GCTGGGATTACAGGTGTGAGCC
>1250
GGTTTCACCGTGTTAGCCAGTATGGTCTCGATCTCCTGAACTCGTGATCCACCTGCCTC
GGCCTCCCAAAGTGCTGGGATTACAGGTGTGAGCCACCACGCCCGGCCTATTGCCTGG
TTCTTGAAGGGATTCTCAGGAAC

>1251
GCCAGTATGGTCTCGATCTCCTGAACTCGTGATCCACCTGCCTCGGCCTCCCAAAGTGC
TGGGATTACAGGTGTGAGCCACCACGCCCGGCCTATTGCCTGGTTCTTGAAGGGATTCT
CAGGAACCACAGGGAATGTCTG
>1252
ATCCACCTGCCTCGGCCTCCCAAAGTGCTGGGATTACAGGTGTGAGCCACCACGCCCG
GCCTATTGCCTGGTTCTTGAAGGGATTCTCAGGAACCACAGGGAATGTCTGAGCCAGC
TTTCTGGATCCGTGTGTACAGCCC
>1253
TACAGGTGTGAGCCACCACGCCCGGCCTATTGCCTGGTTCTTGAAGGGATTCTCAGGA
ACCACAGGGAATGTCTGAGCCAGCTTTCTGGATCCGTGTGTACAGCCCTTCCCAGAGG
CTTTGTGGAGCCAGCTGTACGGTG
>1254
GGCCTATTGCCTGGTTCTTGAAGGGATTCTCAGGAACCACAGGGAATGTCTGAGCCAG
CTTTCTGGATCCGTGTGTACAGCCCTTCCCAGAGGCTTTGTGGAGCCAGCTGTACGGT
GCATGAGCTTTGTGGCTAAGATAT
>1255
CCTATTGCCTGGTTCTTGAAGGGATTCTCAGGAACCACAGGGAATGTCTGAGCCAGCT
TTCTGGATCCGTGTGTACAGCCCTTCCCAGAGGCTTTGTGGAGCCAGCTGTACGGTGC
ATGAGCTTTGTGGCTAAGATATCA
>1256
TCTCAGGAACCACAGGGAATGTCTGAGCCAGCTTTCTGGATCCGTGTGTACAGCCCTT
CCCAGAGGCTTTGTGGAGCCAGCTGTACGGTGCATGAGCTTTGTGGCTAAGATATCAG
AGTTCTGCCTGGCCCTTGGCCTTG
>1257
CAGGGAATGTCTGAGCCAGCTTTCTGGATCCGTGTGTACAGCCCTTCCCAGAGGCTTT
GTGGAGCCAGCTGTACGGTGCATGAGCTTTGTGGCTAAGATATCAGAGTTCTGCCTGG
CCCTTGGCCTTGGTCTCTCTCAGA
>1258
GTACAGCCCTTCCCAGAGGCTTTGTGGAGCCAGCTGTACGGTGCATGAGCTTTGTGGC
TAAGATATCAGAGTTCTGCCTGGCCCTTGGCCTTGGTCTCTCTCAGATGGTCAGCTGCT
CTGTTTTTAGTGGTTGATCATGT
>1259
GTGGAGCCAGCTGTACGGTGCATGAGCTTTGTGGCTAAGATATCAGAGTTCTGCCTGG
CCCTTGGCCTTGGTCTCTCTCAGATGGTCAGCTGCTCTGTTTTTAGTGGTTGATCATGTA
GAAGGCCAACAGGTGTTGACCT
>1260
CTTTGTGGCTAAGATATCAGAGTTCTGCCTGGCCCTTGGCCTTGGTCTCTCTCAGATGG
TCAGCTGCTCTGTTTTTAGTGGTTGATCATGTAGAAGGCCAACAGGTGTTGACCTTGGT
TTAGGCTACTTTTGGGATTTTT
>1261
GCTAAGATATCAGAGTTCTGCCTGGCCCTTGGCCTTGGTCTCTCTCAGATGGTCAGCTG
CTCTGTTTTTAGTGGTTGATCATGTAGAAGGCCAACAGGTGTTGACCTTGGTTTAGGCT
ACTTTTGGGATTTTTGAGGTTT
>1262
GAGTTCTGCCTGGCCCTTGGCCTTGGTCTCTCTCAGATGGTCAGCTGCTCTGTTTTTAG

TGGTTGATCATGTAGAAGGCCAACAGGTGTTGACCTTGGTTTAGGCTACTTTTGGGATT
TTTGAGGTTTCCTGAGACTCTT
>1263
CTCTCTCAGATGGTCAGCTGCTCTGTTTTTAGTGGTTGATCATGTAGAAGGCCAACAGG
TGTTGACCTTGGTTTAGGCTACTTTTGGGATTTTTGAGGTTTCCTGAGACTCTTGGTTTT
CTTTCATCATCCCGTCCAGAA
>1264
TTTAGTGGTTGATCATGTAGAAGGCCAACAGGTGTTGACCTTGGTTTAGGCTACTTTTG
GGATTTTTGAGGTTTCCTGAGACTCTTGGTTTTCTTTCATCATCCCGTCCAGAACCTCTG
ATGTCAAACTAAATTCTGTTT
>1265
TAGAAGGCCAACAGGTGTTGACCTTGGTTTAGGCTACTTTTGGGATTTTTGAGGTTTCC
TGAGACTCTTGGTTTTCTTTCATCATCCCGTCCAGAACCTCTGATGTCAAACTAAATTCT
GTTTGGAAAAGGGTATGCTGT
>1266
TACTTTTGGGATTTTTGAGGTTTCCTGAGACTCTTGGTTTTCTTTCATCATCCCGTCCAG
AACCTCTGATGTCAAACTAAATTCTGTTTGGAAAAGGGTATGCTGTAGAATAATGATAG
CCACAGCAATGATATTAATAA
>1267
TGAGGTTTCCTGAGACTCTTGGTTTTCTTTCATCATCCCGTCCAGAACCTCTGATGTCA
AACTAAATTCTGTTTGGAAAAGGGTATGCTGTAGAATAATGATAGCCACAGCAATGATA
TTAATAATACCTTTTATCGGTT
>1268
AGACTCTTGGTTTTCTTTCATCATCCCGTCCAGAACCTCTGATGTCAAACTAAATTCTGT
TTGGAAAAGGGTATGCTGTAGAATAATGATAGCCACAGCAATGATATTAATAATACCTTT
TATCGGTTGCAGCATTTTCT
>1269
TCTGTTTGGAAAAGGGTATGCTGTAGAATAATGATAGCCACAGCAATGATATTAATAATA
CCTTTTATCGGTTGCAGCATTTTCTTCTTTGCAAAGCACCTCTAATGACTTTATCTCTCTT
GCCCTCACAGCCTTCCTTG
>1270
TTGCAGCATTTTCTTCTTTGCAAAGCACCTCTAATGACTTTATCTCTCTTGCCCTCACAG
CCTTCCTTGAGTGGGTACAGGTCCATAATCCCATATCTGTGATAATGAAGTCTGAAAAG
CTCTGAAAACTAAAAAGTTTG
>1271
AGCATTTTCTTCTTTGCAAAGCACCTCTAATGACTTTATCTCTCTTGCCCTCACAGCCTT
CCTTGAGTGGGTACAGGTCCATAATCCCATATCTGTGATAATGAAGTCTGAAAAGCTCT
GAAAACTAAAAAGTTTGCAGT
>1272
CTCTAATGACTTTATCTCTCTTGCCCTCACAGCCTTCCTTGAGTGGGTACAGGTCCATAA
TCCCATATCTGTGATAATGAAGTCTGAAAAGCTCTGAAAACTAAAAAGTTTGCAGTAA
ACTCATTTAGTGGCAAAACCTG
>1273
TTATCTCTCTTGCCCTCACAGCCTTCCTTGAGTGGGTACAGGTCCATAATCCCATATCTG
TGATAATGAAGTCTGAAAAGCTCTGAAAACTAAAAAGTTTGCAGTAAACTCATTTAGT
GGCAAAACCTGATCTGAACTGA

>1274
CTTGAGTGGGTACAGGTCCATAATCCCATATCTGTGATAATGAAGTCTGAAAAGCTCTG
AAAACTAAAAAGTTTGCAGTAAACTCATTTAGTGGCAAAACCTGATCTGAACTGATGT
GAGGCTATATTTATCCTGCTTAG
>1275
TAATCCCATATCTGTGATAATGAAGTCTGAAAAGCTCTGAAAACTAAAAAGTTTGCAGT
AAACTCATTTAGTGGCAAAACCTGATCTGAACTGATGTGAGGCTATATTTATCCTGCTTA
GTTTGAATATTCATATGTTTT
>1276
TCTGAAAAGCTCTGAAAACTAAAAAGTTTGCAGTAAACTCATTTAGTGGCAAAACCTG
ATCTGAACTGATGTGAGGCTATATTTATCCTGCTTAGTTTGAATATTCATATGTTTTGCTG
CAAAAATATTAATGTGTTTGA
>1277
AGTTTGCAGTAAACTCATTTAGTGGCAAAACCTGATCTGAACTGATGTGAGGCTATATT
TATCCTGCTTAGTTTGAATATTCATATGTTTTGCTGCAAAAATATTAATGTGTTTGATTAC
AGAGTGCTGCCCTAGACTCT
>1278
ATTTAGTGGCAAAACCTGATCTGAACTGATGTGAGGCTATATTTATCCTGCTTAGTTTGA
ATATTCATATGTTTTGCTGCAAAAATATTAATGTGTTTGATTACAGAGTGCTGCCCTAGA
CTCTGCTGGGGCTGTTAAAC
>1279
GAACTGATGTGAGGCTATATTTATCCTGCTTAGTTTGAATATTCATATGTTTTGCTGCAAA
AATATTAATGTGTTTGATTACAGAGTGCTGCCCTAGACTCTGCTGGGGCTGTTAAACAC
GATAGGTGTCAGAGTACCTT
>1280
TATATTTATCCTGCTTAGTTTGAATATTCATATGTTTTGCTGCAAAAATATTAATGTGTTTG
ATTACAGAGTGCTGCCCTAGACTCTGCTGGGGCTGTTAAACACGATAGGTGTCAGAGT
ACCTTTCTTTTTTTTTTTTT
>1281
TTCATATGTTTTGCTGCAAAAATATTAATGTGTTTGATTACAGAGTGCTGCCCTAGACTC
TGCTGGGGCTGTTAAACACGATAGGTGTCAGAGTACCTTTCTTTTTTTTTTTTTTTTTTT
TTTTTGAGACGGAGTCTCGC
>1282
TGCAAAAATATTAATGTGTTTGATTACAGAGTGCTGCCCTAGACTCTGCTGGGGCTGTT
AAACACGATAGGTGTCAGAGTACCTTTCTTTTTTTTTTTTTTTTTTTTTTTTGAGACGGA
GTCTCGCTCTGTCGCCCAGGT
>1283
TGGGGCTGTTAAACACGATAGGTGTCAGAGTACCTTTCTTTTTTTTTTTTTTTTTTTTTTT
TGAGACGGAGTCTCGCTCTGTCGCCCAGGTCGGACTGCGGACTGCAGTGGCGCAATCT
CGGCTCACTGCAAGCTCCGCT
>1284
AAACACGATAGGTGTCAGAGTACCTTTCTTTTTTTTTTTTTTTTTTTTTTTTGAGACGGA
GTCTCGCTCTGTCGCCCAGGTCGGACTGCGGACTGCAGTGGCGCAATCTCGGCTCACT
GCAAGCTCCGCTTCCCGGGTTC
>1285
CTTTTTTTTTTTTTTTTTTTTTTTTGAGACGGAGTCTCGCTCTGTCGCCCAGGTCGGACT

GCGGACTGCAGTGGCGCAATCTCGGCTCACTGCAAGCTCCGCTTCCCGGGTTCATGCC
ATTCTCCTGCCTCAGCCTCCCG
>1286
CTCTGTCGCCCAGGTCGGACTGCGGACTGCAGTGGCGCAATCTCGGCTCACTGCAAGC
TCCGCTTCCCGGGTTCATGCCATTCTCCTGCCTCAGCCTCCCGAGTAGCTGGGACTACA
GGCGCCCGCCACCGCGCCCGGCT
>1287
TGGCGCAATCTCGGCTCACTGCAAGCTCCGCTTCCCGGGTTCATGCCATTCTCCTGCCT
CAGCCTCCCGAGTAGCTGGGACTACAGGCGCCCGCCACCGCGCCCGGCTAATTTTTG
TATTTTTAGTAGAGACGGGGTTT
>1288
CATTCTCCTGCCTCAGCCTCCCGAGTAGCTGGGACTACAGGCGCCCGCCACCGCGCCC
GGCTAATTTTTGTATTTTTAGTAGAGACGGGGTTTCACCTTGTTAGCCAGGATGGTCTC
GATCTCCTGACCTCATGATCCA
>1289
GCCTCAGCCTCCCGAGTAGCTGGGACTACAGGCGCCCGCCACCGCGCCCGGCTAATTT
TTGTATTTTTAGTAGAGACGGGGTTTCACCTTGTTAGCCAGGATGGTCTCGATCTCCTG
ACCTCATGATCCACCCGCCTCG
>1290
CCGAGTAGCTGGGACTACAGGCGCCCGCCACCGCGCCCGGCTAATTTTTGTATTTTTA
GTAGAGACGGGGTTTCACCTTGTTAGCCAGGATGGTCTCGATCTCCTGACCTCATGATC
CACCCGCCTCGGCCTCCCAAAG
>1291
GGGACTACAGGCGCCCGCCACCGCGCCCGGCTAATTTTTGTATTTTTAGTAGAGACGG
GGTTTCACCTTGTTAGCCAGGATGGTCTCGATCTCCTGACCTCATGATCCACCCGCCTC
GGCCTCCCAAAGTGCTGGGATT
>1292
GGTTTCACCTTGTTAGCCAGGATGGTCTCGATCTCCTGACCTCATGATCCACCCGCCTC
GGCCTCCCAAAGTGCTGGGATTACAGGCGTGAGCACCGCGCCCGGCCCAGAGTACCTT
TCTAAAGTCTGAACAGTTTTGGT
>1293
CAGGATGGTCTCGATCTCCTGACCTCATGATCCACCCGCCTCGGCCTCCCAAAGTGCTG
GGATTACAGGCGTGAGCACCGCGCCCGGCCCAGAGTACCTTTCTAAAGTCTGAACAGT
TTTGGTCGTTGAAGCACCTGAAG
>1294
CTCATGATCCACCCGCCTCGGCCTCCCAAAGTGCTGGGATTACAGGCGTGAGCACCGC
GCCCGGCCCAGAGTACCTTTCTAAAGTCTGAACAGTTTTGGTCGTTGAAGCACCTGAA
GGTTTTGGATAAGGGATTATGAAC
>1295
CGCCTCGGCCTCCCAAAGTGCTGGGATTACAGGCGTGAGCACCGCGCCCGGCCCAGA
GTACCTTTCTAAAGTCTGAACAGTTTTGGTCGTTGAAGCACCTGAAGGTTTTGGATAAG
GGATTATGAACGATTATCATTCTC
>1296
CTCCCAAAGTGCTGGGATTACAGGCGTGAGCACCGCGCCCGGCCCAGAGTACCTTTCT
AAAGTCTGAACAGTTTTGGTCGTTGAAGCACCTGAAGGTTTTGGATAAGGGATTATGA
ACGATTATCATTCTCTATTGACAA

>1297
GTGAGCACCGCGCCCGGCCCAGAGTACCTTTCTAAAGTCTGAACAGTTTTGGTCGTTG
AAGCACCTGAAGGTTTTGGATAAGGGATTATGAACGATTATCATTCTCTATTGACAAAT
GAGGAAAATGAGATTTGGAGCAA
>1298
GTTTTGGATAAGGGATTATGAACGATTATCATTCTCTATTGACAAATGAGGAAAATGAGA
TTTGGAGCAAGTAAGTGACTTCCCATGGTCACACAGCTATAAATGGGTAGAACCAGGC
ACTCAGCTTGCCTTCTAATTCA
>1299
TGGATAAGGGATTATGAACGATTATCATTCTCTATTGACAAATGAGGAAAATGAGATTTG
GAGCAAGTAAGTGACTTCCCATGGTCACACAGCTATAAATGGGTAGAACCAGGCACTC
AGCTTGCCTTCTAATTCACAGT
>1300
ATGAACGATTATCATTCTCTATTGACAAATGAGGAAAATGAGATTTGGAGCAAGTAAGT
GACTTCCCATGGTCACACAGCTATAAATGGGTAGAACCAGGCACTCAGCTTGCCTTCTA
ATTCACAGTCCTGGGCGCTCTG
>1301
TATTGACAAATGAGGAAAATGAGATTTGGAGCAAGTAAGTGACTTCCCATGGTCACAC
AGCTATAAATGGGTAGAACCAGGCACTCAGCTTGCCTTCTAATTCACAGTCCTGGGCGC
TCTGCACCAGGCCATATAAGAAC
>1302
AAGTGACTTCCCATGGTCACACAGCTATAAATGGGTAGAACCAGGCACTCAGCTTGCC
TTCTAATTCACAGTCCTGGGCGCTCTGCACCAGGCCATATAAGAACATGAGCATTGCTC
TTCTGGGTCAAATCCATCTGACC
>1303
TTGCCTTCTAATTCACAGTCCTGGGCGCTCTGCACCAGGCCATATAAGAACATGAGCAT
TGCTCTTCTGGGTCAAATCCATCTGACCTAGTCTTTGGTTGCCATCACCATGCCTAAGAT
ACCACCATTGGAGGCTACAAC
>1304
CCTGGGCGCTCTGCACCAGGCCATATAAGAACATGAGCATTGCTCTTCTGGGTCAAATC
CATCTGACCTAGTCTTTGGTTGCCATCACCATGCCTAAGATACCACCATTGGAGGCTAC
AACCATCTTCCTTGACGTCAAT
>1305
GCTCTGCACCAGGCCATATAAGAACATGAGCATTGCTCTTCTGGGTCAAATCCATCTGA
CCTAGTCTTTGGTTGCCATCACCATGCCTAAGATACCACCATTGGAGGCTACAACCATC
TTCCTTGACGTCAATCCTAAAG
>1306
GACCTAGTCTTTGGTTGCCATCACCATGCCTAAGATACCACCATTGGAGGCTACAACCA
TCTTCCTTGACGTCAATCCTAAAGGTCAGGTGTAGCTTCTCTTAGTAGCTATTTATGGCT
GCCTTGTCATTCACAGATTTG
>1307
GTTGCCATCACCATGCCTAAGATACCACCATTGGAGGCTACAACCATCTTCCTTGACGT
CAATCCTAAAGGTCAGGTGTAGCTTCTCTTAGTAGCTATTTATGGCTGCCTTGTCATTCA
CAGATTTGCCAAAATCCTTTT
>1308
AACCATCTTCCTTGACGTCAATCCTAAAGGTCAGGTGTAGCTTCTCTTAGTAGCTATTTA

TGGCTGCCTTGTCATTCACAGATTTGCCAAAATCCTTTTTGAATCCATTTCCATTTCCAG
GAAGTATCCTTTTTAGGGGT
>1309
CTATTTATGGCTGCCTTGTCATTCACAGATTTGCCAAAATCCTTTTTGAATCCATTTCCAT
TTCCAGGAAGTATCCTTTTTAGGGGTGATGAATGACAAGTTTATAATCTCCTGGGCTCCT
GGAAATCTGGTCTTTTGGA
>1310
TTGTCATTCACAGATTTGCCAAAATCCTTTTTGAATCCATTTCCATTTCCAGGAAGTATC
CTTTTTAGGGGTGATGAATGACAAGTTTATAATCTCCTGGGCTCCTGGAAATCTGGTCTT
TTGGAGAGAGGTTGTTGGTT
>1311
TTTGCCAAAATCCTTTTTGAATCCATTTCCATTTCCAGGAAGTATCCTTTTTAGGGGTGA
TGAATGACAAGTTTATAATCTCCTGGGCTCCTGGAAATCTGGTCTTTTGGAGAGAGGTT
GTTGGTTGTGAAGATAGGAAG
>1312
TTTCCAGGAAGTATCCTTTTTAGGGGTGATGAATGACAAGTTTATAATCTCCTGGGCTCC
TGGAAATCTGGTCTTTTGGAGAGAGGTTGTTGGTTGTGAAGATAGGAAGATGGCAGAA
GGCTTTGTAGTGGTGAGGCCAG
>1313
CTTTTTAGGGGTGATGAATGACAAGTTTATAATCTCCTGGGCTCCTGGAAATCTGGTCTT
TTGGAGAGAGGTTGTTGGTTGTGAAGATAGGAAGATGGCAGAAGGCTTTGTAGTGGTG
AGGCCAGGTCTAGACCTAGCTC
>1314
GGCTCCTGGAAATCTGGTCTTTTGGAGAGAGGTTGTTGGTTGTGAAGATAGGAAGATG
GCAGAAGGCTTTGTAGTGGTGAGGCCAGGTCTAGACCTAGCTCTGCGTTTTTCAGCTC
TGTCACCATTGACATCGTTTTTTT
>1315
TCCTGGAAATCTGGTCTTTTGGAGAGAGGTTGTTGGTTGTGAAGATAGGAAGATGGCA
GAAGGCTTTGTAGTGGTGAGGCCAGGTCTAGACCTAGCTCTGCGTTTTTCAGCTCTGTC
ACCATTGACATCGTTTTTTTCAT
>1316
GTCTTTTGGAGAGAGGTTGTTGGTTGTGAAGATAGGAAGATGGCAGAAGGCTTTGTAG
TGGTGAGGCCAGGTCTAGACCTAGCTCTGCGTTTTTCAGCTCTGTCACCATTGACATCG
TTTTTTTCATCGTAAGCCTTAAC
>1317
GTTGGTTGTGAAGATAGGAAGATGGCAGAAGGCTTTGTAGTGGTGAGGCCAGGTCTAG
ACCTAGCTCTGCGTTTTTCAGCTCTGTCACCATTGACATCGTTTTTTTCATCGTAAGCCT
TAACTTCTTCATTTTTAAAACT
>1318
AAGGCTTTGTAGTGGTGAGGCCAGGTCTAGACCTAGCTCTGCGTTTTTCAGCTCTGTCA
CCATTGACATCGTTTTTTTCATCGTAAGCCTTAACTTCTTCATTTTTAAAACTAGGCACT
TCAGTGAGCTCTTTCTGGATT
>1319
TGGTGAGGCCAGGTCTAGACCTAGCTCTGCGTTTTTCAGCTCTGTCACCATTGACATCG
TTTTTTTCATCGTAAGCCTTAACTTCTTCATTTTTAAAACTAGGCACTTCAGTGAGCTCT
TTCTGGATTGTTGCGCTCTTT

>1320
TCTGTCACCATTGACATCGTTTTTTTCATCGTAAGCCTTAACTTCTTCATTTTTAAAACTA
GGCACTTCAGTGAGCTCTTTCTGGATTGTTGCGCTCTTTAAAAAATTCTTTTATTCTTTG
TGTACCCACTTATATCTTC
>1321
GTTTTTTTCATCGTAAGCCTTAACTTCTTCATTTTTAAAACTAGGCACTTCAGTGAGCTC
TTTCTGGATTGTTGCGCTCTTTAAAAAATTCTTTTATTCTTTGTGTACCCACTTATATCTT
CATGAAGTCCTTTTGGTTA
>1322
TTTTAAAACTAGGCACTTCAGTGAGCTCTTTCTGGATTGTTGCGCTCTTTAAAAAATTC
TTTTATTCTTTGTGTACCCACTTATATCTTCATGAAGTCCTTTTGGTTAATTGAATTTTGC
TGTTTTGGAATCTGGATTGG
>1323
CTCTTTCTGGATTGTTGCGCTCTTTAAAAAATTCTTTTATTCTTTGTGTACCCACTTATAT
CTTCATGAAGTCCTTTTGGTTAATTGAATTTTGCTGTTTTGGAATCTGGATTGGCAGTTC
AGCCAGTTAAGTAGGGGGT
>1324
GATTGTTGCGCTCTTTAAAAAATTCTTTTATTCTTTGTGTACCCACTTATATCTTCATGAA
GTCCTTTTGGTTAATTGAATTTTGCTGTTTTGGAATCTGGATTGGCAGTTCAGCCAGTTA
AGTAGGGGGTCAGTTTGAT
>1325
AAAAATTCTTTTATTCTTTGTGTACCCACTTATATCTTCATGAAGTCCTTTTGGTTAATTG
AATTTTGCTGTTTTGGAATCTGGATTGGCAGTTCAGCCAGTTAAGTAGGGGGTCAGTTT
GATTTGATTCTGTGTGTTCT
>1326
GTACCCACTTATATCTTCATGAAGTCCTTTTGGTTAATTGAATTTTGCTGTTTTGGAATCT
GGATTGGCAGTTCAGCCAGTTAAGTAGGGGGTCAGTTTGATTTGATTCTGTGTGTTCTG
ACTTTTTTTTTTTTTTTAAA
>1327
TATATCTTCATGAAGTCCTTTTGGTTAATTGAATTTTGCTGTTTTGGAATCTGGATTGGCA
GTTCAGCCAGTTAAGTAGGGGGTCAGTTTGATTTGATTCTGTGTGTTCTGACTTTTTTTT
TTTTTTTAAATACTTTAAC
>1328
CTTCATGAAGTCCTTTTGGTTAATTGAATTTTGCTGTTTTGGAATCTGGATTGGCAGTTC
AGCCAGTTAAGTAGGGGGTCAGTTTGATTTGATTCTGTGTGTTCTGACTTTTTTTTTTTT
TTTAAATACTTTAACCTGCT
>1329
AATTTTGCTGTTTTGGAATCTGGATTGGCAGTTCAGCCAGTTAAGTAGGGGGTCAGTTT
GATTTGATTCTGTGTGTTCTGACTTTTTTTTTTTTTTTAAATACTTTAACCTGCTTAGGGT
TACTGAGCCATCTGGGATCT
>1330
GGGGTCAGTTTGATTTGATTCTGTGTGTTCTGACTTTTTTTTTTTTTTTAAATACTTTAAC
CTGCTTAGGGTTACTGAGCCATCTGGGATCTAGACTGAACTCTTGGAACTTTTGAAGAT
GATTTCCTTTATTTTATTTT
>1331
TCTGGGATCTAGACTGAACTCTTGGAACTTTTGAAGATGATTTCCTTTATTTTATTTTCA

ATGGAGGAAAGTTGCCCACTCTTTCATCTCAGTGAGAAGTCCCATTTTAATTTGCCATC
ACTATTGGATGGAGCCGCTAA
>1332
TTGGAACTTTTGAAGATGATTTCCTTTATTTTATTTTCAATGGAGGAAAGTTGCCCACTC
TTTCATCTCAGTGAGAAGTCCCATTTTAATTTGCCATCACTATTGGATGGAGCCGCTAAT
TGCTGGGAGGGAGAAAGCTA
>1333
TTTTGAAGATGATTTCCTTTATTTTATTTTCAATGGAGGAAAGTTGCCCACTCTTTCATC
TCAGTGAGAAGTCCCATTTTAATTTGCCATCACTATTGGATGGAGCCGCTAATTGCTGG
GAGGGAGAAAGCTATAGTTTA
>1334
GAGAAGTCCCATTTTAATTTGCCATCACTATTGGATGGAGCCGCTAATTGCTGGGAGGG
AGAAAGCTATAGTTTAGCAACATGTTTTCCTCCTGCTCGTTCTAGGAAAACTATTATGAA
TCTTTAAAATAGTGATTATCC
>1335
TTTAATTTGCCATCACTATTGGATGGAGCCGCTAATTGCTGGGAGGGAGAAAGCTATAG
TTTAGCAACATGTTTTCCTCCTGCTCGTTCTAGGAAAACTATTATGAATCTTTAAAATAG
TGATTATCCTAAACTGTTAAA
>1336
GAAAGCTATAGTTTAGCAACATGTTTTCCTCCTGCTCGTTCTAGGAAAACTATTATGAAT
CTTTAAAATAGTGATTATCCTAAACTGTTAAAATCAAATTCTATTCACAGCTCTTTGATTT
CTTGCTAATTCAGGAGAGA
>1337
CAACATGTTTTCCTCCTGCTCGTTCTAGGAAAACTATTATGAATCTTTAAAATAGTGATT
ATCCTAAACTGTTAAAATCAAATTCTATTCACAGCTCTTTGATTTCTTGCTAATTCAGGA
GAGAAACTTTTCCAATCACT
>1338
CAGGAGAGAAACTTTTCCAATCACTTTAATTTTTTTTATTAATTTAAAGCAAGCCCTGCT
GAAAGCCCAAGTATATATGACTTAGAGTCTTACTAAATGAATACAGGTGTGCCTCAGTT
TGTCCATTGGAGGGTTTCTGA
>1339
CCAATCACTTTAATTTTTTTTATTAATTTAAAGCAAGCCCTGCTGAAAGCCCAAGTATAT
ATGACTTAGAGTCTTACTAAATGAATACAGGTGTGCCTCAGTTTGTCCATTGGAGGGTT
TCTGAATTTTCCATAACTAAA
>1340
TATTAATTTAAAGCAAGCCCTGCTGAAAGCCCAAGTATATATGACTTAGAGTCTTACTAA
ATGAATACAGGTGTGCCTCAGTTTGTCCATTGGAGGGTTTCTGAATTTTCCATAACTAA
AGATTTTCTATTCTGTTTTGG
>1341
TTAATTTAAAGCAAGCCCTGCTGAAAGCCCAAGTATATATGACTTAGAGTCTTACTAAAT
GAATACAGGTGTGCCTCAGTTTGTCCATTGGAGGGTTTCTGAATTTTCCATAACTAAAG
ATTTTCTATTCTGTTTTGGCT
>1342
AAGCCCAAGTATATATGACTTAGAGTCTTACTAAATGAATACAGGTGTGCCTCAGTTTGT
CCATTGGAGGGTTTCTGAATTTTCCATAACTAAAGATTTTCTATTCTGTTTTGGCTGGGT
GTGTGGGGTGAAAGCTCTTA

>1343
AATACAGGTGTGCCTCAGTTTGTCCATTGGAGGGTTTCTGAATTTTCCATAACTAAAGA
TTTTCTATTCTGTTTTGGCTGGGTGTGTGGGGTGAAAGCTCTTACTCTCTGGCAGTTATT
TAAGTAGTAAGAAATTTGCTA
>1344
GCCTCAGTTTGTCCATTGGAGGGTTTCTGAATTTTCCATAACTAAAGATTTTCTATTCTG
TTTTGGCTGGGTGTGTGGGGTGAAAGCTCTTACTCTCTGGCAGTTATTTAAGTAGTAAG
AAATTTGCTACAACTCTTCCC
>1345
TTTCCATAACTAAAGATTTTCTATTCTGTTTTGGCTGGGTGTGTGGGGTGAAAGCTCTTA
CTCTCTGGCAGTTATTTAAGTAGTAAGAAATTTGCTACAACTCTTCCCCACCCCCACGT
TTTACAGATAGGAAATGAGCA
>1346
CTAAAGATTTTCTATTCTGTTTTGGCTGGGTGTGTGGGGTGAAAGCTCTTACTCTCTGG
CAGTTATTTAAGTAGTAAGAAATTTGCTACAACTCTTCCCCACCCCCACGTTTTACAGAT
AGGAAATGAGCACTTTGACTG
>1347
AAGATTTTCTATTCTGTTTTGGCTGGGTGTGTGGGGTGAAAGCTCTTACTCTCTGGCAG
TTATTTAAGTAGTAAGAAATTTGCTACAACTCTTCCCCACCCCCACGTTTTACAGATAGG
AAATGAGCACTTTGACTGTCC
>1348
GTGAAAGCTCTTACTCTCTGGCAGTTATTTAAGTAGTAAGAAATTTGCTACAACTCTTC
CCCACCCCCACGTTTTACAGATAGGAAATGAGCACTTTGACTGTCCAGGTCAGACTTC
TGATTTTGCTACTTACTAACTGT
>1349
AGTAGTAAGAAATTTGCTACAACTCTTCCCCACCCCCACGTTTTACAGATAGGAAATGA
GCACTTTGACTGTCCAGGTCAGACTTCTGATTTTGCTACTTACTAACTGTGACAGTGGT
TGTGTTACTTAACTTCTGTGTG
>1350
AAGAAATTTGCTACAACTCTTCCCCACCCCCACGTTTTACAGATAGGAAATGAGCACTT
TGACTGTCCAGGTCAGACTTCTGATTTTGCTACTTACTAACTGTGACAGTGGTTGTGTT
ACTTAACTTCTGTGTGCCTGTT
>1351
ACGTTTTACAGATAGGAAATGAGCACTTTGACTGTCCAGGTCAGACTTCTGATTTTGCT
ACTTACTAACTGTGACAGTGGTTGTGTTACTTAACTTCTGTGTGCCTGTTTGCTCATTTG
TGAAACAGAAATAATAGTAGT
>1352
AGATAGGAAATGAGCACTTTGACTGTCCAGGTCAGACTTCTGATTTTGCTACTTACTAA
CTGTGACAGTGGTTGTGTTACTTAACTTCTGTGTGCCTGTTTGCTCATTTGTGAAACAG
AAATAATAGTAGTACCTACTTC
>1353
TAGGAAATGAGCACTTTGACTGTCCAGGTCAGACTTCTGATTTTGCTACTTACTAACTG
TGACAGTGGTTGTGTTACTTAACTTCTGTGTGCCTGTTTGCTCATTTGTGAAACAGAAA
TAATAGTAGTACCTACTTCATT
>1354
TGACTGTCCAGGTCAGACTTCTGATTTTGCTACTTACTAACTGTGACAGTGGTTGTGTT

ACTTAACTTCTGTGTGCCTGTTTGCTCATTTGTGAAACAGAAATAATAGTAGTACCTACT
TCATTGGGTTGTTATAAGGAT
>1355
CTGATTTTGCTACTTACTAACTGTGACAGTGGTTGTGTTACTTAACTTCTGTGTGCCTGT
TTGCTCATTTGTGAAACAGAAATAATAGTAGTACCTACTTCATTGGGTTGTTATAAGGAT
TCATCAGAAAATATTTATCC
>1356
CTGTGACAGTGGTTGTGTTACTTAACTTCTGTGTGCCTGTTTGCTCATTTGTGAAACAG
AAATAATAGTAGTACCTACTTCATTGGGTTGTTATAAGGATTCATCAGAAAATATTTATCC
TGTGTGAGGAACGGTGGCAC
>1357
GTTACTTAACTTCTGTGTGCCTGTTTGCTCATTTGTGAAACAGAAATAATAGTAGTACCT
ACTTCATTGGGTTGTTATAAGGATTCATCAGAAAATATTTATCCTGTGTGAGGAACGGTG
GCACATAGTAAGTGCTCCAT
>1358
ACTTAACTTCTGTGTGCCTGTTTGCTCATTTGTGAAACAGAAATAATAGTAGTACCTACT
TCATTGGGTTGTTATAAGGATTCATCAGAAAATATTTATCCTGTGTGAGGAACGGTGGC
ACATAGTAAGTGCTCCATAAG
>1359
TGAAACAGAAATAATAGTAGTACCTACTTCATTGGGTTGTTATAAGGATTCATCAGAAA
ATATTTATCCTGTGTGAGGAACGGTGGCACATAGTAAGTGCTCCATAAGTGTTTGCTAG
GGTATTTTTCTTGTCATTGTGT
>1360
AATAGTAGTACCTACTTCATTGGGTTGTTATAAGGATTCATCAGAAAATATTTATCCTGTG
TGAGGAACGGTGGCACATAGTAAGTGCTCCATAAGTGTTTGCTAGGGTATTTTTCTTGT
CATTGTGTGAAGTAGGTCAT
>1361
CCTACTTCATTGGGTTGTTATAAGGATTCATCAGAAAATATTTATCCTGTGTGAGGAACG
GTGGCACATAGTAAGTGCTCCATAAGTGTTTGCTAGGGTATTTTTCTTGTCATTGTGTGA
AGTAGGTCATGCTAGCCATG
>1362
TGTTATAAGGATTCATCAGAAAATATTTATCCTGTGTGAGGAACGGTGGCACATAGTAA
GTGCTCCATAAGTGTTTGCTAGGGTATTTTTCTTGTCATTGTGTGAAGTAGGTCATGCTA
GCCATGCAGGGTGAAAAATGT
>1363
TATTTATCCTGTGTGAGGAACGGTGGCACATAGTAAGTGCTCCATAAGTGTTTGCTAGG
GTATTTTTCTTGTCATTGTGTGAAGTAGGTCATGCTAGCCATGCAGGGTGAAAAATGTG
TTCATGTTGGTCCAGATCCTGC
>1364
CTGTGTGAGGAACGGTGGCACATAGTAAGTGCTCCATAAGTGTTTGCTAGGGTATTTTT
CTTGTCATTGTGTGAAGTAGGTCATGCTAGCCATGCAGGGTGAAAAATGTGTTCATGTT
GGTCCAGATCCTGCTTTCTCCC
>1365
TGAGGAACGGTGGCACATAGTAAGTGCTCCATAAGTGTTTGCTAGGGTATTTTTCTTGT
CATTGTGTGAAGTAGGTCATGCTAGCCATGCAGGGTGAAAAATGTGTTCATGTTGGTCC
AGATCCTGCTTTCTCCCTGAGA

>1366
GAACGGTGGCACATAGTAAGTGCTCCATAAGTGTTTGCTAGGGTATTTTTCTTGTCATTG
TGTGAAGTAGGTCATGCTAGCCATGCAGGGTGAAAAATGTGTTCATGTTGGTCCAGATC
CTGCTTTCTCCCTGAGACCGT
>1367
GTGCTCCATAAGTGTTTGCTAGGGTATTTTTCTTGTCATTGTGTGAAGTAGGTCATGCTA
GCCATGCAGGGTGAAAAATGTGTTCATGTTGGTCCAGATCCTGCTTTCTCCCTGAGACC
GTTCTTCCGCATAGGCGTCAA
>1368
GGGTATTTTTCTTGTCATTGTGTGAAGTAGGTCATGCTAGCCATGCAGGGTGAAAAATG
TGTTCATGTTGGTCCAGATCCTGCTTTCTCCCTGAGACCGTTCTTCCGCATAGGCGTCA
ATCCGTACAGTGCTGTAAAACT
>1369
AGGTCATGCTAGCCATGCAGGGTGAAAAATGTGTTCATGTTGGTCCAGATCCTGCTTTC
TCCCTGAGACCGTTCTTCCGCATAGGCGTCAATCCGTACAGTGCTGTAAAACTGCTGCC
TCGGGCACCTCATGTTTGGGGC
>1370
GCAGGGTGAAAAATGTGTTCATGTTGGTCCAGATCCTGCTTTCTCCCTGAGACCGTTCT
TCCGCATAGGCGTCAATCCGTACAGTGCTGTAAAACTGCTGCCTCGGGCACCTCATGTT
TGGGGCTGAGACATTGCTTCTG
>1371
CCTGAGACCGTTCTTCCGCATAGGCGTCAATCCGTACAGTGCTGTAAAACTGCTGCCTC
GGGCACCTCATGTTTGGGGCTGAGACATTGCTTCTGCGTCTTCCCCAGAAGCTGATCTC
TTGTACTGTGGTGGCCAGGGTA
>1372
TCAATCCGTACAGTGCTGTAAAACTGCTGCCTCGGGCACCTCATGTTTGGGGCTGAGA
CATTGCTTCTGCGTCTTCCCCAGAAGCTGATCTCTTGTACTGTGGTGGCCAGGGTACTC
TTTGTCCCATTGACCTGAGGCCT
>1373
TGCTGCCTCGGGCACCTCATGTTTGGGGCTGAGACATTGCTTCTGCGTCTTCCCCAGAA
GCTGATCTCTTGTACTGTGGTGGCCAGGGTACTCTTTGTCCCATTGACCTGAGGCCTGG
TGAGTCATTCCAGTTCTCGTTT
>1374
CCTCGGGCACCTCATGTTTGGGGCTGAGACATTGCTTCTGCGTCTTCCCCAGAAGCTGA
TCTCTTGTACTGTGGTGGCCAGGGTACTCTTTGTCCCATTGACCTGAGGCCTGGTGAGT
CATTCCAGTTCTCGTTTAAATT
>1375
CATGTTTGGGGCTGAGACATTGCTTCTGCGTCTTCCCCAGAAGCTGATCTCTTGTACTG
TGGTGGCCAGGGTACTCTTTGTCCCATTGACCTGAGGCCTGGTGAGTCATTCCAGTTCT
CGTTTAAATTTAGCCGGGCAGC
>1376
TCTTCCCCAGAAGCTGATCTCTTGTACTGTGGTGGCCAGGGTACTCTTTGTCCCATTGA
CCTGAGGCCTGGTGAGTCATTCCAGTTCTCGTTTAAATTTAGCCGGGCAGCGCTCCACT
GCCATCGGGTCCAAATGCCGGG
>1377
CTGATCTCTTGTACTGTGGTGGCCAGGGTACTCTTTGTCCCATTGACCTGAGGCCTGGT

GAGTCATTCCAGTTCTCGTTTAAATTTAGCCGGGCAGCGCTCCACTGCCATCGGGTCCA
AATGCCGGGGAGGCTCACTGAT
>1378
TCTTGTACTGTGGTGGCCAGGGTACTCTTTGTCCCATTGACCTGAGGCCTGGTGAGTCA
TTCCAGTTCTCGTTTAAATTTAGCCGGGCAGCGCTCCACTGCCATCGGGTCCAAATGCC
GGGGAGGCTCACTGATGCGGTG
>1379
TGACCTGAGGCCTGGTGAGTCATTCCAGTTCTCGTTTAAATTTAGCCGGGCAGCGCTCC
ACTGCCATCGGGTCCAAATGCCGGGGAGGCTCACTGATGCGGTGTCCTGTTGCCTGTG
CACGTGGCCAGGCGTGCACATCA
>1380
CTCGTTTAAATTTAGCCGGGCAGCGCTCCACTGCCATCGGGTCCAAATGCCGGGGAGG
CTCACTGATGCGGTGTCCTGTTGCCTGTGCACGTGGCCAGGCGTGCACATCACTGTGC
TTATGAGAGAGATGAAGAGCACAG
>1381
CGTTTAAATTTAGCCGGGCAGCGCTCCACTGCCATCGGGTCCAAATGCCGGGGAGGCT
CACTGATGCGGTGTCCTGTTGCCTGTGCACGTGGCCAGGCGTGCACATCACTGTGCTTA
TGAGAGAGATGAAGAGCACAGAT
>1382
CTGCCATCGGGTCCAAATGCCGGGGAGGCTCACTGATGCGGTGTCCTGTTGCCTGTGC
ACGTGGCCAGGCGTGCACATCACTGTGCTTATGAGAGAGATGAAGAGCACAGATACCA
CCTTACTGATGGGGGTCCTGGGGC
>1383
CCAAATGCCGGGGAGGCTCACTGATGCGGTGTCCTGTTGCCTGTGCACGTGGCCAGGC
GTGCACATCACTGTGCTTATGAGAGAGATGAAGAGCACAGATACCACCTTACTGATGG
GGGTCCTGGGGCTGGGCTTGTGGG
>1384
GTGGCCAGGCGTGCACATCACTGTGCTTATGAGAGAGATGAAGAGCACAGATACCACC
TTACTGATGGGGGTCCTGGGGCTGGGCTTGTGGGTGTTGGGGGTTCCAGAGCTCCTTC
CCTTTCCTGCCTGTCAGTGCAGGA
>1385
TCACTGTGCTTATGAGAGAGATGAAGAGCACAGATACCACCTTACTGATGGGGGTCCT
GGGGCTGGGCTTGTGGGTGTTGGGGGTTCCAGAGCTCCTTCCCTTTCCTGCCTGTCAG
TGCAGGATGCTTCTAAAATGTGGA
>1386
TGTGCTTATGAGAGAGATGAAGAGCACAGATACCACCTTACTGATGGGGGTCCTGGGG
CTGGGCTTGTGGGTGTTGGGGGTTCCAGAGCTCCTTCCCTTTCCTGCCTGTCAGTGCA
GGATGCTTCTAAAATGTGGACTCA
>1387
TTACTGATGGGGGTCCTGGGGCTGGGCTTGTGGGTGTTGGGGGTTCCAGAGCTCCTTC
CCTTTCCTGCCTGTCAGTGCAGGATGCTTCTAAAATGTGGACTCAGCAGACATTGTTTT
GAGTGCTTGCTACATGTCAGATT
>1388
GGCTTGTGGGTGTTGGGGGTTCCAGAGCTCCTTCCCTTTCCTGCCTGTCAGTGCAGGA
TGCTTCTAAAATGTGGACTCAGCAGACATTGTTTTGAGTGCTTGCTACATGTCAGATTC
TGTGCTAAGTGCACTCACAGCCA

>1389
GTTCCAGAGCTCCTTCCCTTTCCTGCCTGTCAGTGCAGGATGCTTCTAAAATGTGGACT
CAGCAGACATTGTTTTGAGTGCTTGCTACATGTCAGATTCTGTGCTAAGTGCACTCACA
GCCAGCATCTCTGTAAGACCTC
>1390
TCCTGCCTGTCAGTGCAGGATGCTTCTAAAATGTGGACTCAGCAGACATTGTTTTGAGT
GCTTGCTACATGTCAGATTCTGTGCTAAGTGCACTCACAGCCAGCATCTCTGTAAGACC
TCTAGCTGTCCTGTAAGGTGGG
>1391
CAGTGCAGGATGCTTCTAAAATGTGGACTCAGCAGACATTGTTTTGAGTGCTTGCTACA
TGTCAGATTCTGTGCTAAGTGCACTCACAGCCAGCATCTCTGTAAGACCTCTAGCTGTC
CTGTAAGGTGGGTATCAGTTTC
>1392
GGATGCTTCTAAAATGTGGACTCAGCAGACATTGTTTTGAGTGCTTGCTACATGTCAGA
TTCTGTGCTAAGTGCACTCACAGCCAGCATCTCTGTAAGACCTCTAGCTGTCCTGTAAG
GTGGGTATCAGTTTCACATTCT
>1393
AGCAGACATTGTTTTGAGTGCTTGCTACATGTCAGATTCTGTGCTAAGTGCACTCACAG
CCAGCATCTCTGTAAGACCTCTAGCTGTCCTGTAAGGTGGGTATCAGTTTCACATTCTC
AGATGGGCGGAGAGAAGCCCAA
>1394
GAGTGCTTGCTACATGTCAGATTCTGTGCTAAGTGCACTCACAGCCAGCATCTCTGTAA
GACCTCTAGCTGTCCTGTAAGGTGGGTATCAGTTTCACATTCTCAGATGGGCGGAGAG
AAGCCCAAGAGGTTAGTGGACTT
>1395
TGTCAGATTCTGTGCTAAGTGCACTCACAGCCAGCATCTCTGTAAGACCTCTAGCTGTC
CTGTAAGGTGGGTATCAGTTTCACATTCTCAGATGGGCGGAGAGAAGCCCAAGAGGTT
AGTGGACTTTTCTCTAGTAAAGA
>1396
ATTCTGTGCTAAGTGCACTCACAGCCAGCATCTCTGTAAGACCTCTAGCTGTCCTGTAA
GGTGGGTATCAGTTTCACATTCTCAGATGGGCGGAGAGAAGCCCAAGAGGTTAGTGGA
CTTTTCTCTAGTAAAGAGTTGTA
>1397
AGACCTCTAGCTGTCCTGTAAGGTGGGTATCAGTTTCACATTCTCAGATGGGCGGAGA
GAAGCCCAAGAGGTTAGTGGACTTTTCTCTAGTAAAGAGTTGTACAGGCCGGGCGCAG
TGGCTCATGCCTGTAATCCCAGCA
>1398
AAGGTGGGTATCAGTTTCACATTCTCAGATGGGCGGAGAGAAGCCCAAGAGGTTAGTG
GACTTTTCTCTAGTAAAGAGTTGTACAGGCCGGGCGCAGTGGCTCATGCCTGTAATCCC
AGCACTTTGGGAGGCCGAGGCGG
>1399
GTATCAGTTTCACATTCTCAGATGGGCGGAGAGAAGCCCAAGAGGTTAGTGGACTTTT
CTCTAGTAAAGAGTTGTACAGGCCGGGCGCAGTGGCTCATGCCTGTAATCCCAGCACT
TTGGGAGGCCGAGGCGGGCGGATC
>1400
AGATGGGCGGAGAGAAGCCCAAGAGGTTAGTGGACTTTTCTCTAGTAAAGAGTTGTAC

AGGCCGGGCGCAGTGGCTCATGCCTGTAATCCCAGCACTTTGGGAGGCCGAGGCGGG
CGGATCACGAGGTCAGGAGATCGAG
>1401
ACAGGCCGGGCGCAGTGGCTCATGCCTGTAATCCCAGCACTTTGGGAGGCCGAGGCG
GGCGGATCACGAGGTCAGGAGATCGAGACCATCCTGGCTAACACAGTGAAACCCCGT
CTCTACTAAAAATACAAAAAATTAGC
>1402
CCCAGCACTTTGGGAGGCCGAGGCGGGCGGATCACGAGGTCAGGAGATCGAGACCAT
CCTGGCTAACACAGTGAAACCCCGTCTCTACTAAAAATACAAAAAATTAGCCGGGCGT
GGTGGGGGGCGCCTGTAGTCCCAGC
>1403
TGGGAGGCCGAGGCGGGCGGATCACGAGGTCAGGAGATCGAGACCATCCTGGCTAAC
ACAGTGAAACCCCGTCTCTACTAAAAATACAAAAAATTAGCCGGGCGTGGTGGGGGGC
GCCTGTAGTCCCAGCTACTCGGGAG
>1404
GAGATCGAGACCATCCTGGCTAACACAGTGAAACCCCGTCTCTACTAAAAATACAAAA
AATTAGCCGGGCGTGGTGGGGGGCGCCTGTAGTCCCAGCTACTCGGGAGGCTGAGGC
AGGAGAATGGCGTGAACCCGGGAGG
>1405
TGGCTAACACAGTGAAACCCCGTCTCTACTAAAAATACAAAAAATTAGCCGGGCGTGG
TGGGGGGCGCCTGTAGTCCCAGCTACTCGGGAGGCTGAGGCAGGAGAATGGCGTGAA
CCCGGGAGGCAGAGCTTGCAGTGAG
>1406
AAAATTAGCCGGGCGTGGTGGGGGGCGCCTGTAGTCCCAGCTACTCGGGAGGCTGAG
GCAGGAGAATGGCGTGAACCCGGGAGGCAGAGCTTGCAGTGAGCCGAGATCATGCCA
CTCACTACACTCCAGCCTGGGCAAAG
>1407
CGCCTGTAGTCCCAGCTACTCGGGAGGCTGAGGCAGGAGAATGGCGTGAACCCGGGA
GGCAGAGCTTGCAGTGAGCCGAGATCATGCCACTCACTACACTCCAGCCTGGGCAAA
GGAGTGAGACTGTCTCAAAAAAAAAA
>1408
GAACCCGGGAGGCAGAGCTTGCAGTGAGCCGAGATCATGCCACTCACTACACTCCAG
CCTGGGCAAAGGAGTGAGACTGTCTCAAAAAAAAAAAAAAAAAAAAAGAGTTGTAC
CAGGATTTATGTTCAGGTCTTCTGGCT
>1409
GAGGCAGAGCTTGCAGTGAGCCGAGATCATGCCACTCACTACACTCCAGCCTGGGCA
AAGGAGTGAGACTGTCTCAAAAAAAAAAAAAAAAAAAAAGAGTTGTACCAGGATTTA
TGTTCAGGTCTTCTGGCTCCATGCTC
>1410
ATGCCACTCACTACACTCCAGCCTGGGCAAAGGAGTGAGACTGTCTCAAAAAAAAAA
AAAAAAAAAAAGAGTTGTACCAGGATTTATGTTCAGGTCTTCTGGCTCCATGCTCTCTG
CAGCTGGGTGACCAGGGGGCTCAT
>1411
CCACTCACTACACTCCAGCCTGGGCAAAGGAGTGAGACTGTCTCAAAAAAAAAAAAA
AAAAAAAAGAGTTGTACCAGGATTTATGTTCAGGTCTTCTGGCTCCATGCTCTCTGCAG
CTGGGTGACCAGGGGGCTCATGGC

>1412
AAAAAAAAAAAAAAAAGAGTTGTACCAGGATTTATGTTCAGGTCTTCTGGCTCCATGCT
CTCTGCAGCTGGGTGACCAGGGGGCTCATGGCTTCACCAGCTCTGGCCTCCTCAGTTC
CTCTGGTGGCTTCTTGCGAGGAGG
>1413
CCATGCTCTCTGCAGCTGGGTGACCAGGGGGCTCATGGCTTCACCAGCTCTGGCCTCC
TCAGTTCCTCTGGTGGCTTCTTGCGAGGAGGAAGGTTACTGCAGAGGGTTCAGCTCTT
TAGGGGCCCAGGCCGCATGGATCC
>1414
ACCAGGGGGCTCATGGCTTCACCAGCTCTGGCCTCCTCAGTTCCTCTGGTGGCTTCTTG
CGAGGAGGAAGGTTACTGCAGAGGGTTCAGCTCTTTAGGGGCCCAGGCCGCATGGAT
CCAAGGACTAGTACGTCATCTCTT
>1415
TGGCTTCACCAGCTCTGGCCTCCTCAGTTCCTCTGGTGGCTTCTTGCGAGGAGGAAGG
TTACTGCAGAGGGTTCAGCTCTTTAGGGGCCCAGGCCGCATGGATCCAAGGACTAGTA
CGTCATCTCTTACCAACACAGAGA
>1416
TTGCGAGGAGGAAGGTTACTGCAGAGGGTTCAGCTCTTTAGGGGCCCAGGCCGCATG
GATCCAAGGACTAGTACGTCATCTCTTACCAACACAGAGAGATCAGCTTCTAGTGATAG
AGGCCTTTTATGCTGACTTGAAAA
>1417
GAGGAGGAAGGTTACTGCAGAGGGTTCAGCTCTTTAGGGGCCCAGGCCGCATGGATC
CAAGGACTAGTACGTCATCTCTTACCAACACAGAGAGATCAGCTTCTAGTGATAGAGG
CCTTTTATGCTGACTTGAAAAACAG
>1418
AGGGGCCCAGGCCGCATGGATCCAAGGACTAGTACGTCATCTCTTACCAACACAGAGA
GATCAGCTTCTAGTGATAGAGGCCTTTTATGCTGACTTGAAAAACAGAAAAAGAGAGA
AAAAAGAAACCCATTTACAAAAAT
>1419
TTATGCTGACTTGAAAAACAGAAAAAGAGAGAAAAAAGAAACCCATTTACAAAAATA
AAAATCCCATGCTGTTAATTTCATGCCAGCATAATCAGAAATTCCCCTTTCTACTGTTTC
CCCTTAAAAGCCCCGGTGTCCTT
>1420
CCATTTACAAAAATAAAAATCCCATGCTGTTAATTTCATGCCAGCATAATCAGAAATTCC
CCTTTCTACTGTTTCCCCTTAAAAGCCCCGGTGTCCTTATTATGCTGGTGCAGTCTGGAA
AAACTATGGTTGGGTTTTGC
>1421
CCCATGCTGTTAATTTCATGCCAGCATAATCAGAAATTCCCCTTTCTACTGTTTCCCCTT
AAAAGCCCCGGTGTCCTTATTATGCTGGTGCAGTCTGGAAAAACTATGGTTGGGTTTTG
CCTACGTGAGGCTTATCTCTG
>1422
ATGCCAGCATAATCAGAAATTCCCCTTTCTACTGTTTCCCCTTAAAAGCCCCGGTGTCCT
TATTATGCTGGTGCAGTCTGGAAAAACTATGGTTGGGTTTTGCCTACGTGAGGCTTATCT
CTGCAGCTGCAAGGAGGGGA
>1423
CCCCTTTCTACTGTTTCCCCTTAAAAGCCCCGGTGTCCTTATTATGCTGGTGCAGTCTGG

AAAAACTATGGTTGGGTTTTGCCTACGTGAGGCTTATCTCTGCAGCTGCAAGGAGGGG
ATCCGGTACTTTATTGTCAGCA
>1424
TTCTACTGTTTCCCCTTAAAAGCCCCGGTGTCCTTATTATGCTGGTGCAGTCTGGAAAA
ACTATGGTTGGGTTTTGCCTACGTGAGGCTTATCTCTGCAGCTGCAAGGAGGGGATCCG
GTACTTTATTGTCAGCATGGAA
>1425
CCCCTTAAAAGCCCCGGTGTCCTTATTATGCTGGTGCAGTCTGGAAAAACTATGGTTGG
GTTTTGCCTACGTGAGGCTTATCTCTGCAGCTGCAAGGAGGGGATCCGGTACTTTATTG
TCAGCATGGAAACTTCCGCTAA
>1426
AGTCTGGAAAAACTATGGTTGGGTTTTGCCTACGTGAGGCTTATCTCTGCAGCTGCAAG
GAGGGGATCCGGTACTTTATTGTCAGCATGGAAACTTCCGCTAAATACTTAGAAAAACC
TTAATTCTAAATTCCTAGCTCA
>1427
AACTATGGTTGGGTTTTGCCTACGTGAGGCTTATCTCTGCAGCTGCAAGGAGGGGATCC
GGTACTTTATTGTCAGCATGGAAACTTCCGCTAAATACTTAGAAAAACCTTAATTCTAAA
TTCCTAGCTCACTTTCAACTG
>1428
TGTCAGCATGGAAACTTCCGCTAAATACTTAGAAAAACCTTAATTCTAAATTCCTAGCT
CACTTTCAACTGTGTCCTCGGGACCTGATTTTCTTCGGTGTGAACAGATTGTGTTCAGC
ATTGCAGTAGAAAGGGTCCAAG
>1429
TCAGCATGGAAACTTCCGCTAAATACTTAGAAAAACCTTAATTCTAAATTCCTAGCTCA
CTTTCAACTGTGTCCTCGGGACCTGATTTTCTTCGGTGTGAACAGATTGTGTTCAGCAT
TGCAGTAGAAAGGGTCCAAGTT
>1430
ACTTAGAAAAACCTTAATTCTAAATTCCTAGCTCACTTTCAACTGTGTCCTCGGGACCT
GATTTTCTTCGGTGTGAACAGATTGTGTTCAGCATTGCAGTAGAAAGGGTCCAAGTTA
GGTAAGAGAAAGGACTTCCTGAG
>1431
TTAATTCTAAATTCCTAGCTCACTTTCAACTGTGTCCTCGGGACCTGATTTTCTTCGGTG
TGAACAGATTGTGTTCAGCATTGCAGTAGAAAGGGTCCAAGTTAGGTAAGAGAAAGG
ACTTCCTGAGACACTGGGGTGTT
>1432
AATTCTAAATTCCTAGCTCACTTTCAACTGTGTCCTCGGGACCTGATTTTCTTCGGTGTG
AACAGATTGTGTTCAGCATTGCAGTAGAAAGGGTCCAAGTTAGGTAAGAGAAAGGAC
TTCCTGAGACACTGGGGTGTTAC
>1433
TAGCTCACTTTCAACTGTGTCCTCGGGACCTGATTTTCTTCGGTGTGAACAGATTGTGT
TCAGCATTGCAGTAGAAAGGGTCCAAGTTAGGTAAGAGAAAGGACTTCCTGAGACAC
TGGGGTGTTACTGAGAAAGTTGTT
>1434
TTCAACTGTGTCCTCGGGACCTGATTTTCTTCGGTGTGAACAGATTGTGTTCAGCATTG
CAGTAGAAAGGGTCCAAGTTAGGTAAGAGAAAGGACTTCCTGAGACACTGGGGTGTT
ACTGAGAAAGTTGTTAGAAGACTG

>1435
AGATTGTGTTCAGCATTGCAGTAGAAAGGGTCCAAGTTAGGTAAGAGAAAGGACTTCC
TGAGACACTGGGGTGTTACTGAGAAAGTTGTTAGAAGACTGTCACAGACATTTTGATG
AAGGGGGGACATTCTGGGCCATTT
>1436
ATTGTGTTCAGCATTGCAGTAGAAAGGGTCCAAGTTAGGTAAGAGAAAGGACTTCCTG
AGACACTGGGGTGTTACTGAGAAAGTTGTTAGAAGACTGTCACAGACATTTTGATGAA
GGGGGGACATTCTGGGCCATTTGA
>1437
ATTGCAGTAGAAAGGGTCCAAGTTAGGTAAGAGAAAGGACTTCCTGAGACACTGGGG
TGTTACTGAGAAAGTTGTTAGAAGACTGTCACAGACATTTTGATGAAGGGGGGACATT
CTGGGCCATTTGAAGTGGTTTTTAG
>1438
TTACTGAGAAAGTTGTTAGAAGACTGTCACAGACATTTTGATGAAGGGGGGACATTCT
GGGCCATTTGAAGTGGTTTTTAGAGTTCTTTCCTCTTTAGGGCCATTCGGTTCCCCCAG
AAATAAGAAGAAAAGACATATAC
>1439
CTGAGAAAGTTGTTAGAAGACTGTCACAGACATTTTGATGAAGGGGGGACATTCTGGG
CCATTTGAAGTGGTTTTTAGAGTTCTTTCCTCTTTAGGGCCATTCGGTTCCCCCAGAAAT
AAGAAGAAAAGACATATACACG
>1440
TTGATGAAGGGGGGACATTCTGGGCCATTTGAAGTGGTTTTTAGAGTTCTTTCCTCTTT
AGGGCCATTCGGTTCCCCCAGAAATAAGAAGAAAAGACATATACACGCACATAAACCA
CCACCACCAGGCTTGCTGAGAAC
>1441
TCCCCCAGAAATAAGAAGAAAAGACATATACACGCACATAAACCACCACCACCAGGCT
TGCTGAGAACAAGTCAAATGTGCCACCAAACCATCGTCTTAGTGGGAGATGGGGTGGC
ACTGCAGGGGCAGAAAGACTGACG
>1442
GAAATAAGAAGAAAAGACATATACACGCACATAAACCACCACCACCAGGCTTGCTGAG
AACAAGTCAAATGTGCCACCAAACCATCGTCTTAGTGGGAGATGGGGTGGCACTGCAG
GGGCAGAAAGACTGACGTCTGATG
>1443
ACATATACACGCACATAAACCACCACCACCAGGCTTGCTGAGAACAAGTCAAATGTGC
CACCAAACCATCGTCTTAGTGGGAGATGGGGTGGCACTGCAGGGGCAGAAAGACTGA
CGTCTGATGGACTTGGGTTTGAATA
>1444
AGTCAAATGTGCCACCAAACCATCGTCTTAGTGGGAGATGGGGTGGCACTGCAGGGGC
AGAAAGACTGACGTCTGATGGACTTGGGTTTGAATACTAGTGCTCCATGGTGAATTTG
GGCAAATGACTTCACCTCTGTGAA
>1445
CAAACCATCGTCTTAGTGGGAGATGGGGTGGCACTGCAGGGGCAGAAAGACTGACGT
CTGATGGACTTGGGTTTGAATACTAGTGCTCCATGGTGAATTTGGGCAAATGACTTCAC
CTCTGTGAAGCAGCAGTATCCTCT
>1446
ATGGGGTGGCACTGCAGGGGCAGAAAGACTGACGTCTGATGGACTTGGGTTTGAATAC

TAGTGCTCCATGGTGAATTTGGGCAAATGACTTCACCTCTGTGAAGCAGCAGTATCCTC
TTCTGTGAAGTGAGGTTAGCCCC
>1447
CTGACGTCTGATGGACTTGGGTTTGAATACTAGTGCTCCATGGTGAATTTGGGCAAATG
ACTTCACCTCTGTGAAGCAGCAGTATCCTCTTCTGTGAAGTGAGGTTAGCCCCTACCTC
TCAATGTGGTTTTTAAAGTATG
>1448
TGGACTTGGGTTTGAATACTAGTGCTCCATGGTGAATTTGGGCAAATGACTTCACCTCT
GTGAAGCAGCAGTATCCTCTTCTGTGAAGTGAGGTTAGCCCCTACCTCTCAATGTGGTT
TTTAAAGTATGTTAATGAGATC
>1449
CAAATGACTTCACCTCTGTGAAGCAGCAGTATCCTCTTCTGTGAAGTGAGGTTAGCCC
CTACCTCTCAATGTGGTTTTTAAAGTATGTTAATGAGATCACTTGTAACCTAAAGCACAC
AGAGTGTGAGGCCACCTCCCTA
>1450
ATGACTTCACCTCTGTGAAGCAGCAGTATCCTCTTCTGTGAAGTGAGGTTAGCCCCTAC
CTCTCAATGTGGTTTTTAAAGTATGTTAATGAGATCACTTGTAACCTAAAGCACACAGA
GTGTGAGGCCACCTCCCTACTT
>1451
CCTCTTCTGTGAAGTGAGGTTAGCCCCTACCTCTCAATGTGGTTTTTAAAGTATGTTAAT
GAGATCACTTGTAACCTAAAGCACACAGAGTGTGAGGCCACCTCCCTACTTCCTGCTT
CTCTCATGACCCCAGCCTGACA
>1452
TTAGCCCCTACCTCTCAATGTGGTTTTTAAAGTATGTTAATGAGATCACTTGTAACCTAA
AGCACACAGAGTGTGAGGCCACCTCCCTACTTCCTGCTTCTCTCATGACCCCAGCCTG
ACAAGGGAGCCGCTGAGCCCCC
>1453
AGCCCCTACCTCTCAATGTGGTTTTTAAAGTATGTTAATGAGATCACTTGTAACCTAAAG
CACACAGAGTGTGAGGCCACCTCCCTACTTCCTGCTTCTCTCATGACCCCAGCCTGAC
AAGGGAGCCGCTGAGCCCCCTG
>1454
TACTTCCTGCTTCTCTCATGACCCCAGCCTGACAAGGGAGCCGCTGAGCCCCCTGCCA
CCCTCACCCGGCGTTGTGCTCTAAGTGGATTAGCTCAGCGTTGTGATCTTAATGCCATT
AGGCCTGTTGGCACGGCTCTCCT
>1455
TTCCTGCTTCTCTCATGACCCCAGCCTGACAAGGGAGCCGCTGAGCCCCCTGCCACCC
TCACCCGGCGTTGTGCTCTAAGTGGATTAGCTCAGCGTTGTGATCTTAATGCCATTAGG
CCTGTTGGCACGGCTCTCCTTGC
>1456
GACAAGGGAGCCGCTGAGCCCCCTGCCACCCTCACCCGGCGTTGTGCTCTAAGTGGAT
TAGCTCAGCGTTGTGATCTTAATGCCATTAGGCCTGTTGGCACGGCTCTCCTTGCCATA
GTGAGAAGCAGATGTGGCCGACC
>1457
TGCCACCCTCACCCGGCGTTGTGCTCTAAGTGGATTAGCTCAGCGTTGTGATCTTAATG
CCATTAGGCCTGTTGGCACGGCTCTCCTTGCCATAGTGAGAAGCAGATGTGGCCGACC
ATAATGGCCCTTAGCGTGATCCA

>1458
TCTAAGTGGATTAGCTCAGCGTTGTGATCTTAATGCCATTAGGCCTGTTGGCACGGCTC
TCCTTGCCATAGTGAGAAGCAGATGTGGCCGACCATAATGGCCCTTAGCGTGATCCATC
GGGCCCACAGTGCTGGGGCAAC
>1459
GCTCAGCGTTGTGATCTTAATGCCATTAGGCCTGTTGGCACGGCTCTCCTTGCCATAGT
GAGAAGCAGATGTGGCCGACCATAATGGCCCTTAGCGTGATCCATCGGGCCCACAGTG
CTGGGGCAACCATTATTAGTGTT
>1460
TTAGGCCTGTTGGCACGGCTCTCCTTGCCATAGTGAGAAGCAGATGTGGCCGACCATAA
TGGCCCTTAGCGTGATCCATCGGGCCCACAGTGCTGGGGCAACCATTATTAGTGTTAAT
TTGGGGGAGCTTTTTATGGTAG
>1461
TCTCCTTGCCATAGTGAGAAGCAGATGTGGCCGACCATAATGGCCCTTAGCGTGATCCA
TCGGGCCCACAGTGCTGGGGCAACCATTATTAGTGTTAATTTGGGGGAGCTTTTTATGG
TAGCTGGGGGAAATGTGGAGGG
>1462
CAGATGTGGCCGACCATAATGGCCCTTAGCGTGATCCATCGGGCCCACAGTGCTGGGG
CAACCATTATTAGTGTTAATTTGGGGGAGCTTTTTATGGTAGCTGGGGGAAATGTGGAG
GGATTGTTGGAAATTTATTTAAG
>1463
GATGTGGCCGACCATAATGGCCCTTAGCGTGATCCATCGGGCCCACAGTGCTGGGGCA
ACCATTATTAGTGTTAATTTGGGGGAGCTTTTTATGGTAGCTGGGGGAAATGTGGAGGG
ATTGTTGGAAATTTATTTAAGCT
>1464
TAGCGTGATCCATCGGGCCCACAGTGCTGGGGCAACCATTATTAGTGTTAATTTGGGGG
AGCTTTTTATGGTAGCTGGGGGAAATGTGGAGGGATTGTTGGAAATTTATTTAAGCTGT
TCAGCAGGTTAGGTGCTGCTGG
>1465
GTTAATTTGGGGGAGCTTTTTATGGTAGCTGGGGGAAATGTGGAGGGATTGTTGGAAAT
TTATTTAAGCTGTTCAGCAGGTTAGGTGCTGCTGGGGTCTACTATGGCAAAACCCTCAG
CTCCCCCAACCCATCTCTTTAA
>1466
GGGGAGCTTTTTATGGTAGCTGGGGGAAATGTGGAGGGATTGTTGGAAATTTATTTAAG
CTGTTCAGCAGGTTAGGTGCTGCTGGGGTCTACTATGGCAAAACCCTCAGCTCCCCCA
ACCCATCTCTTTAAGGAACGTCC
>1467
GCAGGTTAGGTGCTGCTGGGGTCTACTATGGCAAAACCCTCAGCTCCCCCAACCCATC
TCTTTAAGGAACGTCCCTCCCCAACACCGAAGCCCAGGCCTGATTACTTCCAGAGTTG
GAGAGACAACTTAGCAGGCTCTTG
>1468
GCTCCCCCAACCCATCTCTTTAAGGAACGTCCCTCCCCAACACCGAAGCCCAGGCCTG
ATTACTTCCAGAGTTGGAGAGACAACTTAGCAGGCTCTTGTTTCTTGCCGATGTACCTT
ATTAGTAGCACCTGGCATCATAA
>1469
CGTCCCTCCCCAACACCGAAGCCCAGGCCTGATTACTTCCAGAGTTGGAGAGACAACT

TAGCAGGCTCTTGTTTCTTGCCGATGTACCTTATTAGTAGCACCTGGCATCATAACCCAC
TAATGGGAACCAGTGCTCTCCT
>1470
AGGCCTGATTACTTCCAGAGTTGGAGAGACAACTTAGCAGGCTCTTGTTTCTTGCCGAT
GTACCTTATTAGTAGCACCTGGCATCATAACCCACTAATGGGAACCAGTGCTCTCCTCC
TGCGTGGCCAGTGACAAGTAGC
>1471
GCAGGCTCTTGTTTCTTGCCGATGTACCTTATTAGTAGCACCTGGCATCATAACCCACTA
ATGGGAACCAGTGCTCTCCTCCTGCGTGGCCAGTGACAAGTAGCCCTGTCAGCCGGAG
CTCGCCGTGCGAGGAAGTTCCT
>1472
TTGCCGATGTACCTTATTAGTAGCACCTGGCATCATAACCCACTAATGGGAACCAGTGC
TCTCCTCCTGCGTGGCCAGTGACAAGTAGCCCTGTCAGCCGGAGCTCGCCGTGCGAGG
AAGTTCCTATTAGCTTTATGGTC
>1473
TGTACCTTATTAGTAGCACCTGGCATCATAACCCACTAATGGGAACCAGTGCTCTCCTC
CTGCGTGGCCAGTGACAAGTAGCCCTGTCAGCCGGAGCTCGCCGTGCGAGGAAGTTC
CTATTAGCTTTATGGTCAGGAGAA
>1474
TATTAGTAGCACCTGGCATCATAACCCACTAATGGGAACCAGTGCTCTCCTCCTGCGTG
GCCAGTGACAAGTAGCCCTGTCAGCCGGAGCTCGCCGTGCGAGGAAGTTCCTATTAGC
TTTATGGTCAGGAGAACTTGTCC
>1475
GCACCTGGCATCATAACCCACTAATGGGAACCAGTGCTCTCCTCCTGCGTGGCCAGTG
ACAAGTAGCCCTGTCAGCCGGAGCTCGCCGTGCGAGGAAGTTCCTATTAGCTTTATGG
TCAGGAGAACTTGTCCTGTTCAGG
>1476
GAACCAGTGCTCTCCTCCTGCGTGGCCAGTGACAAGTAGCCCTGTCAGCCGGAGCTCG
CCGTGCGAGGAAGTTCCTATTAGCTTTATGGTCAGGAGAACTTGTCCTGTTCAGGAAAT
GAATGTTGCCGCTAAAGGTCCGC
>1477
TGCGTGGCCAGTGACAAGTAGCCCTGTCAGCCGGAGCTCGCCGTGCGAGGAAGTTCC
TATTAGCTTTATGGTCAGGAGAACTTGTCCTGTTCAGGAAATGAATGTTGCCGCTAAAG
GTCCGCGGCCCTGACCCTGGCGGC
>1478
ACAAGTAGCCCTGTCAGCCGGAGCTCGCCGTGCGAGGAAGTTCCTATTAGCTTTATGG
TCAGGAGAACTTGTCCTGTTCAGGAAATGAATGTTGCCGCTAAAGGTCCGCGGCCCTG
ACCCTGGCGGCATAACCTGTGCGG
>1479
GAGCTCGCCGTGCGAGGAAGTTCCTATTAGCTTTATGGTCAGGAGAACTTGTCCTGTTC
AGGAAATGAATGTTGCCGCTAAAGGTCCGCGGCCCTGACCCTGGCGGCATAACCTGTG
CGGGCCGGGGAGACCCTGTGCAG
>1480
GAACTTGTCCTGTTCAGGAAATGAATGTTGCCGCTAAAGGTCCGCGGCCCTGACCCTG
GCGGCATAACCTGTGCGGGCCGGGGAGACCCTGTGCAGGATGCTTGGGATTTCAGCCC
CTCTCTGGTTCTTGTCATATGTAA

>1481
ATGAATGTTGCCGCTAAAGGTCCGCGGCCCTGACCCTGGCGGCATAACCTGTGCGGGC
CGGGGAGACCCTGTGCAGGATGCTTGGGATTTCAGCCCCTCTCTGGTTCTTGTCATATG
TAAAGAGGGAATCATTGTGGTAT
>1482
CCCTGGCGGCATAACCTGTGCGGGCCGGGGAGACCCTGTGCAGGATGCTTGGGATTTC
AGCCCCTCTCTGGTTCTTGTCATATGTAAAGAGGGAATCATTGTGGTATGTTGCAGCCTT
GAACTTTTGATTTATGCAGTGA
>1483
CGGCATAACCTGTGCGGGCCGGGGAGACCCTGTGCAGGATGCTTGGGATTTCAGCCCC
TCTCTGGTTCTTGTCATATGTAAAGAGGGAATCATTGTGGTATGTTGCAGCCTTGAACTT
TTGATTTATGCAGTGAGGGCGA
>1484
AGACCCTGTGCAGGATGCTTGGGATTTCAGCCCCTCTCTGGTTCTTGTCATATGTAAAG
AGGGAATCATTGTGGTATGTTGCAGCCTTGAACTTTTGATTTATGCAGTGAGGGCGACT
TAACCGTTTCCATGCCTGGACG
>1485
TCTGGTTCTTGTCATATGTAAAGAGGGAATCATTGTGGTATGTTGCAGCCTTGAACTTTT
GATTTATGCAGTGAGGGCGACTTAACCGTTTCCATGCCTGGACGGCTGTGCAGTGACA
GAGCCGTGTTGTGAGCGTATGT
>1486
GTAAAGAGGGAATCATTGTGGTATGTTGCAGCCTTGAACTTTTGATTTATGCAGTGAGG
GCGACTTAACCGTTTCCATGCCTGGACGGCTGTGCAGTGACAGAGCCGTGTTGTGAGC
GTATGTGCCTAGGTACATGTGTG
>1487
TTGCAGCCTTGAACTTTTGATTTATGCAGTGAGGGCGACTTAACCGTTTCCATGCCTGG
ACGGCTGTGCAGTGACAGAGCCGTGTTGTGAGCGTATGTGCCTAGGTACATGTGTGTG
TGGGGTGGTCAAGGGGAAGGAGA
>1488
AACTTTTGATTTATGCAGTGAGGGCGACTTAACCGTTTCCATGCCTGGACGGCTGTGCA
GTGACAGAGCCGTGTTGTGAGCGTATGTGCCTAGGTACATGTGTGTGTGGGGTGGTCA
AGGGGAAGGAGAGACCCCCACGT
>1489
TTGATTTATGCAGTGAGGGCGACTTAACCGTTTCCATGCCTGGACGGCTGTGCAGTGAC
AGAGCCGTGTTGTGAGCGTATGTGCCTAGGTACATGTGTGTGTGGGGTGGTCAAGGGG
AAGGAGAGACCCCCACGTGCTAG
>1490
TAACCGTTTCCATGCCTGGACGGCTGTGCAGTGACAGAGCCGTGTTGTGAGCGTATGT
GCCTAGGTACATGTGTGTGTGGGGTGGTCAAGGGGAAGGAGAGACCCCCACGTGCTA
GCAGGGGCTGTTGCTGGGAGCGGGT
>1491
CCGTGTTGTGAGCGTATGTGCCTAGGTACATGTGTGTGTGGGGTGGTCAAGGGGAAGG
AGAGACCCCCACGTGCTAGCAGGGGCTGTTGCTGGGAGCGGGTGGATTTCATGGGAG
GATTCCTTTTCTTTTTTCTTTTTCT
>1492
ATGTGCCTAGGTACATGTGTGTGTGGGGTGGTCAAGGGGAAGGAGAGACCCCCACGT

GCTAGCAGGGGCTGTTGCTGGGAGCGGGTGGATTTCATGGGAGGATTCCTTTTCTTTTT
TCTTTTTCTTGCATTCTTGCAGCA
>1493
ATTTCATGGGAGGATTCCTTTTCTTTTTTCTTTTTCTTGCATTCTTGCAGCATCTAAATTT
CCTGCGATTGTTGGCTGCAAAGTGGTAAGAAGCTGGAGAGCATTGTGTCTGGAATTGA
ACCTGGGTTCGAATTAATCTT
>1494
GATTCCTTTTCTTTTTTCTTTTTCTTGCATTCTTGCAGCATCTAAATTTCCTGCGATTGTT
GGCTGCAAAGTGGTAAGAAGCTGGAGAGCATTGTGTCTGGAATTGAACCTGGGTTCG
AATTAATCTTCTGTCTCTTAGA
>1495
CTTGCATTCTTGCAGCATCTAAATTTCCTGCGATTGTTGGCTGCAAAGTGGTAAGAAGC
TGGAGAGCATTGTGTCTGGAATTGAACCTGGGTTCGAATTAATCTTCTGTCTCTTAGAG
GCTGTGTGACCGTGGCAAGATT
>1496
AAATTTCCTGCGATTGTTGGCTGCAAAGTGGTAAGAAGCTGGAGAGCATTGTGTCTGG
AATTGAACCTGGGTTCGAATTAATCTTCTGTCTCTTAGAGGCTGTGTGACCGTGGCAAG
ATTCCTCACCACTCACTTCCTCA
>1497
TGGCTGCAAAGTGGTAAGAAGCTGGAGAGCATTGTGTCTGGAATTGAACCTGGGTTCG
AATTAATCTTCTGTCTCTTAGAGGCTGTGTGACCGTGGCAAGATTCCTCACCACTCACT
TCCTCATCTGTGAGATGAAGTTA
>1498
TAAGAAGCTGGAGAGCATTGTGTCTGGAATTGAACCTGGGTTCGAATTAATCTTCTGTC
TCTTAGAGGCTGTGTGACCGTGGCAAGATTCCTCACCACTCACTTCCTCATCTGTGAGA
TGAAGTTAATAACTTCTGATTC
>1499
CGAATTAATCTTCTGTCTCTTAGAGGCTGTGTGACCGTGGCAAGATTCCTCACCACTCA
CTTCCTCATCTGTGAGATGAAGTTAATAACTTCTGATTCATACTGGTGGTAGAGCAGTG
TTGTTAAGTAGGTGATGTATAG
>1500
TTCTGTCTCTTAGAGGCTGTGTGACCGTGGCAAGATTCCTCACCACTCACTTCCTCATC
TGTGAGATGAAGTTAATAACTTCTGATTCATACTGGTGGTAGAGCAGTGTTGTTAAGTA
GGTGATGTATAGAGAATACATG
>1501
CCGTGGCAAGATTCCTCACCACTCACTTCCTCATCTGTGAGATGAAGTTAATAACTTCT
GATTCATACTGGTGGTAGAGCAGTGTTGTTAAGTAGGTGATGTATAGAGAATACATGGC
CCAGCGTCCGGCCAGTGGTGTG
>1502
CCTCACCACTCACTTCCTCATCTGTGAGATGAAGTTAATAACTTCTGATTCATACTGGTG
GTAGAGCAGTGTTGTTAAGTAGGTGATGTATAGAGAATACATGGCCCAGCGTCCGGCC
AGTGGTGTGGCCTCAGGTTGTA
>1503
CACCACTCACTTCCTCATCTGTGAGATGAAGTTAATAACTTCTGATTCATACTGGTGGTA
GAGCAGTGTTGTTAAGTAGGTGATGTATAGAGAATACATGGCCCAGCGTCCGGCCAGT
GGTGTGGCCTCAGGTTGTATAC

>1504
CTCACTTCCTCATCTGTGAGATGAAGTTAATAACTTCTGATTCATACTGGTGGTAGAGC
AGTGTTGTTAAGTAGGTGATGTATAGAGAATACATGGCCCAGCGTCCGGCCAGTGGTGT
GGCCTCAGGTTGTATACTTGAA
>1505
TCATCTGTGAGATGAAGTTAATAACTTCTGATTCATACTGGTGGTAGAGCAGTGTTGTTA
AGTAGGTGATGTATAGAGAATACATGGCCCAGCGTCCGGCCAGTGGTGTGGCCTCAGG
TTGTATACTTGAAAGGATGTGT
>1506
ACTTCTGATTCATACTGGTGGTAGAGCAGTGTTGTTAAGTAGGTGATGTATAGAGAATA
CATGGCCCAGCGTCCGGCCAGTGGTGTGGCCTCAGGTTGTATACTTGAAAGGATGTGTA
TACTTCAGTATTTTTTCCATTG
>1507
AGCAGTGTTGTTAAGTAGGTGATGTATAGAGAATACATGGCCCAGCGTCCGGCCAGTG
GTGTGGCCTCAGGTTGTATACTTGAAAGGATGTGTATACTTCAGTATTTTTTCCATTGTA
AAATTAAGGGTTTTTTTTCTTT
>1508
AGTGTTGTTAAGTAGGTGATGTATAGAGAATACATGGCCCAGCGTCCGGCCAGTGGTGT
GGCCTCAGGTTGTATACTTGAAAGGATGTGTATACTTCAGTATTTTTTCCATTGTAAAAT
TAAGGGTTTTTTTTCTTTTTT
>1509
TGATGTATAGAGAATACATGGCCCAGCGTCCGGCCAGTGGTGTGGCCTCAGGTTGTATA
CTTGAAAGGATGTGTATACTTCAGTATTTTTTCCATTGTAAAATTAAGGGTTTTTTTTCTT
TTTTTCTTGAGACAGAGTCC
>1510
CGTCCGGCCAGTGGTGTGGCCTCAGGTTGTATACTTGAAAGGATGTGTATACTTCAGTA
TTTTTTCCATTGTAAAATTAAGGGTTTTTTTTCTTTTTTTCTTGAGACAGAGTCCTACTTT
GTTGCCCAGGCTTGAGTGCA
>1511
GGTGTGGCCTCAGGTTGTATACTTGAAAGGATGTGTATACTTCAGTATTTTTTCCATTGT
AAAATTAAGGGTTTTTTTTCTTTTTTTCTTGAGACAGAGTCCTACTTTGTTGCCCAGGCT
TGAGTGCAGTGGCGCGATCT
>1512
GGATGTGTATACTTCAGTATTTTTTCCATTGTAAAATTAAGGGTTTTTTTTCTTTTTTTCT
TGAGACAGAGTCCTACTTTGTTGCCCAGGCTTGAGTGCAGTGGCGCGATCTCAGCTCA
CTGCAACTTCCGCCTCCTGGG
>1513
ACTTCAGTATTTTTTCCATTGTAAAATTAAGGGTTTTTTTTCTTTTTTTCTTGAGACAGA
GTCCTACTTTGTTGCCCAGGCTTGAGTGCAGTGGCGCGATCTCAGCTCACTGCAACTTC
CGCCTCCTGGGTCCAAGTGAT
>1514
AGTCCTACTTTGTTGCCCAGGCTTGAGTGCAGTGGCGCGATCTCAGCTCACTGCAACT
TCCGCCTCCTGGGTCCAAGTGATTCTCGTGCCTCAGCCTCCCAAGTACCTGGGACCAC
AGGGGTGCACCACCACACCTGGCT
>1515
ACTTTGTTGCCCAGGCTTGAGTGCAGTGGCGCGATCTCAGCTCACTGCAACTTCCGCC

TCCTGGGTCCAAGTGATTCTCGTGCCTCAGCCTCCCAAGTACCTGGGACCACAGGGGT
GCACCACCACACCTGGCTAATTTT
>1516
TGGCGCGATCTCAGCTCACTGCAACTTCCGCCTCCTGGGTCCAAGTGATTCTCGTGCCT
CAGCCTCCCAAGTACCTGGGACCACAGGGGTGCACCACCACACCTGGCTAATTTTTGT
ATATATATATATATATATATTTA
>1517
CTGCAACTTCCGCCTCCTGGGTCCAAGTGATTCTCGTGCCTCAGCCTCCCAAGTACCTG
GGACCACAGGGGTGCACCACCACACCTGGCTAATTTTTGTATATATATATATATATATATT
TAGTAGAGATGGGGTTTCGC
>1518
TGATTCTCGTGCCTCAGCCTCCCAAGTACCTGGGACCACAGGGGTGCACCACCACACC
TGGCTAATTTTTGTATATATATATATATATATATTTAGTAGAGATGGGGTTTCGCCATGTTG
GCCAGGCTGGTCTTGAACTC
>1519
GTACCTGGGACCACAGGGGTGCACCACCACACCTGGCTAATTTTTGTATATATATATATA
TATATATTTAGTAGAGATGGGGTTTCGCCATGTTGGCCAGGCTGGTCTTGAACTCCCGA
CCTCAGGTGATCTGCCTGTCT
>1520
CACAGGGGTGCACCACCACACCTGGCTAATTTTTGTATATATATATATATATATATTTAGTA
GAGATGGGGTTTCGCCATGTTGGCCAGGCTGGTCTTGAACTCCCGACCTCAGGTGATC
TGCCTGTCTCAGCCTCCTAA
>1521
GGCTAATTTTTGTATATATATATATATATATATTTAGTAGAGATGGGGTTTCGCCATGTTGG
CCAGGCTGGTCTTGAACTCCCGACCTCAGGTGATCTGCCTGTCTCAGCCTCCTAAAGT
GCTGGGATTATAGGTGTGAG
>1522
ATATATATATATTTAGTAGAGATGGGGTTTCGCCATGTTGGCCAGGCTGGTCTTGAACTC
CCGACCTCAGGTGATCTGCCTGTCTCAGCCTCCTAAAGTGCTGGGATTATAGGTGTGAG
CCACTGCGCCTGGCCTAAGTT
>1523
TTTAGTAGAGATGGGGTTTCGCCATGTTGGCCAGGCTGGTCTTGAACTCCCGACCTCA
GGTGATCTGCCTGTCTCAGCCTCCTAAAGTGCTGGGATTATAGGTGTGAGCCACTGCGC
CTGGCCTAAGTTTTTAAAAATTA
>1524
CAGGCTGGTCTTGAACTCCCGACCTCAGGTGATCTGCCTGTCTCAGCCTCCTAAAGTG
CTGGGATTATAGGTGTGAGCCACTGCGCCTGGCCTAAGTTTTTAAAAATTAAAAAGAAT
GGCATAGTGCTTTTTTGCTCTTT
>1525
CAGGTGATCTGCCTGTCTCAGCCTCCTAAAGTGCTGGGATTATAGGTGTGAGCCACTGC
GCCTGGCCTAAGTTTTTAAAAATTAAAAAGAATGGCATAGTGCTTTTTTGCTCTTTAAC
AGATCAAGACCAAAAGTTTTCC
>1526
AAGTGCTGGGATTATAGGTGTGAGCCACTGCGCCTGGCCTAAGTTTTTAAAAATTAAAA
AGAATGGCATAGTGCTTTTTTGCTCTTTAACAGATCAAGACCAAAAGTTTTCCCCCCAT
CATAGCCCAAAGTCCTAAAGAA

>1527
GGCCTAAGTTTTTAAAAATTAAAAAGAATGGCATAGTGCTTTTTTGCTCTTTAACAGAT
CAAGACCAAAAGTTTTCCCCCCATCATAGCCCAAAGTCCTAAAGAAAGGCTACTTAAG
TTGTTCGTTTTCTTTTGTTTCTC
>1528
AGAATGGCATAGTGCTTTTTTGCTCTTTAACAGATCAAGACCAAAAGTTTTCCCCCCAT
CATAGCCCAAAGTCCTAAAGAAAGGCTACTTAAGTTGTTCGTTTTCTTTTGTTTCTCCC
ATGAGTAATGTTTCTAAGTGTG
>1529
CTCTTTAACAGATCAAGACCAAAAGTTTTCCCCCCATCATAGCCCAAAGTCCTAAAGA
AAGGCTACTTAAGTTGTTCGTTTTCTTTTGTTTCTCCCATGAGTAATGTTTCTAAGTGTG
ATTTTTGCAGAGAACAGCAGTG
>1530
TTTAACAGATCAAGACCAAAAGTTTTCCCCCCATCATAGCCCAAAGTCCTAAAGAAAG
GCTACTTAAGTTGTTCGTTTTCTTTTGTTTCTCCCATGAGTAATGTTTCTAAGTGTGATTT
TTGCAGAGAACAGCAGTGTCT
>1531
ACCAAAAGTTTTCCCCCCATCATAGCCCAAAGTCCTAAAGAAAGGCTACTTAAGTTGTT
CGTTTTCTTTTGTTTCTCCCATGAGTAATGTTTCTAAGTGTGATTTTTGCAGAGAACAGC
AGTGTCTGACCTCATTCTTGA
>1532
AAAGTCCTAAAGAAAGGCTACTTAAGTTGTTCGTTTTCTTTTGTTTCTCCCATGAGTAA
TGTTTCTAAGTGTGATTTTTGCAGAGAACAGCAGTGTCTGACCTCATTCTTGAGGGGA
ACTTTTCTGCCTGCCTCTTCTTG
>1533
TAAGTTGTTCGTTTTCTTTTGTTTCTCCCATGAGTAATGTTTCTAAGTGTGATTTTTGCA
GAGAACAGCAGTGTCTGACCTCATTCTTGAGGGGAACTTTTCTGCCTGCCTCTTCTTG
AGAAAAAAATGTTTGTGAGTTGT
>1534
AGTTGTTCGTTTTCTTTTGTTTCTCCCATGAGTAATGTTTCTAAGTGTGATTTTTGCAGA
GAACAGCAGTGTCTGACCTCATTCTTGAGGGGAACTTTTCTGCCTGCCTCTTCTTGAG
AAAAAATGTTTGTGAGTTGTCT
>1535
GCAGAGAACAGCAGTGTCTGACCTCATTCTTGAGGGGAACTTTTCTGCCTGCCTCTTC
TTGAGAAAAAAATGTTTGTGAGTTGTCTGGCAAATGGATGCGTTACTTTTGCTGCTGTTG
CTGCCGCTGCCGTTTTAAAGGCC
>1536
AGAACAGCAGTGTCTGACCTCATTCTTGAGGGGAACTTTTCTGCCTGCCTCTTCTTGA
GAAAAAAATGTTTGTGAGTTGTCTGGCAAATGGATGCGTTACTTTTGCTGCTGTTGCTGC
CGCTGCCGTTTTAAAGGCCCCTG
>1537
CTTGAGGGGAACTTTTCTGCCTGCCTCTTCTTGAGAAAAAAATGTTTGTGAGTTGTCTGG
CAAATGGATGCGTTACTTTTGCTGCTGTTGCTGCCGCTGCCGTTTTAAAGGCCCCTGGT
GACAAAGGAGTGAACTGCACTT
>1538
TTGAGAAAAAAATGTTTGTGAGTTGTCTGGCAAATGGATGCGTTACTTTTGCTGCTGTTG

CTGCCGCTGCCGTTTTAAAGGCCCCTGGTGACAAAGGAGTGAACTGCACTTTCGGGGA
AAGCATGCCGGAATCTGGCCCAG
>1539
GTGAGTTGTCTGGCAAATGGATGCGTTACTTTTGCTGCTGTTGCTGCCGCTGCCGTTTT
AAAGGCCCCTGGTGACAAAGGAGTGAACTGCACTTTCGGGGAAAGCATGCCGGAATC
TGGCCCAGAAAAGAGGGGCCGCTG
>1540
GGAGTGAACTGCACTTTCGGGGAAAGCATGCCGGAATCTGGCCCAGAAAAGAGGGGC
CGCTGGGGGAAGGGTTTGGAGATGACGCCTCCTCAGGAACTTTAAAAGAAATCTGTCC
ATCTGTCTCTGGCATTTATGGCATC
>1541
CCCAGAAAAGAGGGGCCGCTGGGGGAAGGGTTTGGAGATGACGCCTCCTCAGGAACT
TTAAAAGAAATCTGTCCATCTGTCTCTGGCATTTATGGCATCGTTGACAGCAGTTCAAG
GAAAAATAAAAATAAGTTCTTATC
>1542
AGAGGGGCCGCTGGGGGAAGGGTTTGGAGATGACGCCTCCTCAGGAACTTTAAAAGA
AATCTGTCCATCTGTCTCTGGCATTTATGGCATCGTTGACAGCAGTTCAAGGAAAAATA
AAAATAAGTTCTTATCTGATTGCG
>1543
GACGCCTCCTCAGGAACTTTAAAAGAAATCTGTCCATCTGTCTCTGGCATTTATGGCAT
CGTTGACAGCAGTTCAAGGAAAAATAAAAATAAGTTCTTATCTGATTGCGGGGCTGATT
GCAAAGCCGAAGGACTAGAGTG
>1544
AAGAAATCTGTCCATCTGTCTCTGGCATTTATGGCATCGTTGACAGCAGTTCAAGGAAA
AATAAAAATAAGTTCTTATCTGATTGCGGGGCTGATTGCAAAGCCGAAGGACTAGAGT
GGCCAGCGAGTCGGGCGGCTGGA
>1545
GCAGTTCAAGGAAAAATAAAAATAAGTTCTTATCTGATTGCGGGGCTGATTGCAAAGC
CGAAGGACTAGAGTGGCCAGCGAGTCGGGCGGCTGGATCTGGAGTTTTGGAGAAAGG
GCCACTGGCTCATTTCTGGGCACGC
>1546
AAAAATAAAAATAAGTTCTTATCTGATTGCGGGGCTGATTGCAAAGCCGAAGGACTAG
AGTGGCCAGCGAGTCGGGCGGCTGGATCTGGAGTTTTGGAGAAAGGGCCACTGGCTC
ATTTCTGGGCACGCACGCTCACGGA
>1547
ATCTGATTGCGGGGCTGATTGCAAAGCCGAAGGACTAGAGTGGCCAGCGAGTCGGGC
GGCTGGATCTGGAGTTTTGGAGAAAGGGCCACTGGCTCATTTCTGGGCACGCACGCTC
ACGGAGGCAAGTTCTCACCTCCTCG
>1548
GCGGCTGGATCTGGAGTTTTGGAGAAAGGGCCACTGGCTCATTTCTGGGCACGCACGC
TCACGGAGGCAAGTTCTCACCTCCTCGTGGGCTTCTCTTCACTTTACGTGGAAAAACT
GTGTACGTCTCAAATTAAAAGCAG
>1549
AGTTTTGGAGAAAGGGCCACTGGCTCATTTCTGGGCACGCACGCTCACGGAGGCAAG
TTCTCACCTCCTCGTGGGCTTCTCTTCACTTTACGTGGAAAAACTGTGTACGTCTCAAA
TTAAAAGCAGCTCTTGCCCCGCTT

>1550
GGCTCATTTCTGGGCACGCACGCTCACGGAGGCAAGTTCTCACCTCCTCGTGGGCTTC
TCTTCACTTTACGTGGAAAAACTGTGTACGTCTCAAATTAAAAGCAGCTCTTGCCCCGC
TTAGAGCCGGTGAAAGCTGCTTC
>1551
GGGCACGCACGCTCACGGAGGCAAGTTCTCACCTCCTCGTGGGCTTCTCTTCACTTTA
CGTGGAAAAACTGTGTACGTCTCAAATTAAAAGCAGCTCTTGCCCCGCTTAGAGCCGG
TGAAAGCTGCTTCGAAGGCGTTTG
>1552
TCTCTTCACTTTACGTGGAAAAACTGTGTACGTCTCAAATTAAAAGCAGCTCTTGCCCC
GCTTAGAGCCGGTGAAAGCTGCTTCGAAGGCGTTTGTAGAGTAGGGATTGGAAATGTT
CAATGCAAAAGTTAAATCGTGAC
>1553
AAACTGTGTACGTCTCAAATTAAAAGCAGCTCTTGCCCCGCTTAGAGCCGGTGAAAGC
TGCTTCGAAGGCGTTTGTAGAGTAGGGATTGGAAATGTTCAATGCAAAAGTTAAATCG
TGACAGTTGTCCCCTAAATGATCT
>1554
AGCAGCTCTTGCCCCGCTTAGAGCCGGTGAAAGCTGCTTCGAAGGCGTTTGTAGAGTA
GGGATTGGAAATGTTCAATGCAAAAGTTAAATCGTGACAGTTGTCCCCTAAATGATCTG
CTAAATTAAGGATCCGCTAACAT
>1555
CCGCTTAGAGCCGGTGAAAGCTGCTTCGAAGGCGTTTGTAGAGTAGGGATTGGAAATG
TTCAATGCAAAAGTTAAATCGTGACAGTTGTCCCCTAAATGATCTGCTAAATTAAGGAT
CCGCTAACATTTGTGAGTCTACC
>1556
AGCCGGTGAAAGCTGCTTCGAAGGCGTTTGTAGAGTAGGGATTGGAAATGTTCAATGC
AAAAGTTAAATCGTGACAGTTGTCCCCTAAATGATCTGCTAAATTAAGGATCCGCTAAC
ATTTGTGAGTCTACCTCCCTGCA
>1557
TCAATGCAAAAGTTAAATCGTGACAGTTGTCCCCTAAATGATCTGCTAAATTAAGGATC
CGCTAACATTTGTGAGTCTACCTCCCTGCATTCCCCCCACCCCCACCCCCGCCCCCTTG
GCAGAAGGGATAAAGATGGTAC
>1558
TGCAAAAGTTAAATCGTGACAGTTGTCCCCTAAATGATCTGCTAAATTAAGGATCCGCT
AACATTTGTGAGTCTACCTCCCTGCATTCCCCCCACCCCCACCCCCGCCCCCTTGGCAG
AAGGGATAAAGATGGTACCTAA
>1559
ATTTGTGAGTCTACCTCCCTGCATTCCCCCCACCCCCACCCCCGCCCCCTTGGCAGAAG
GGATAAAGATGGTACCTAAAAACAATGTGAAGCGGCTGCTAAATCGAGTGACTCTAAA
AACATTTTTCTTTCCTTGAAGTT
>1560
TCCCTGCATTCCCCCCACCCCCACCCCCGCCCCCTTGGCAGAAGGGATAAAGATGGTA
CCTAAAAACAATGTGAAGCGGCTGCTAAATCGAGTGACTCTAAAAACATTTTTCTTTCC
TTGAAGTTCAGTGTGGAAAACCA
>1561
CACCCCCGCCCCCTTGGCAGAAGGGATAAAGATGGTACCTAAAAACAATGTGAAGCGG

CTGCTAAATCGAGTGACTCTAAAAACATTTTTCTTTCCTTGAAGTTCAGTGTGGAAAAC
CAGACTGGACTTGTCAGGGATGC
>1562
ATGGTACCTAAAAACAATGTGAAGCGGCTGCTAAATCGAGTGACTCTAAAAACATTTTT
CTTTCCTTGAAGTTCAGTGTGGAAAACCAGACTGGACTTGTCAGGGATGCAGACCTAT
ATAGGGCCCATGTGCCTGGCCCC
>1563
ACCTAAAAACAATGTGAAGCGGCTGCTAAATCGAGTGACTCTAAAAACATTTTTCTTTC
CTTGAAGTTCAGTGTGGAAAACCAGACTGGACTTGTCAGGGATGCAGACCTATATAGG
GCCCATGTGCCTGGCCCCTTTCC
>1564
TGCTAAATCGAGTGACTCTAAAAACATTTTTCTTTCCTTGAAGTTCAGTGTGGAAAACC
AGACTGGACTTGTCAGGGATGCAGACCTATATAGGGCCCATGTGCCTGGCCCCTTTCCT
CACTTTCTAGCACAATGGTTTT
>1565
TCTTTCCTTGAAGTTCAGTGTGGAAAACCAGACTGGACTTGTCAGGGATGCAGACCTA
TATAGGGCCCATGTGCCTGGCCCCTTTCCTCACTTTCTAGCACAATGGTTTTCATTGCCA
CCTCTCCCGCGCCTTTCCCCCC
>1566
GACTTGTCAGGGATGCAGACCTATATAGGGCCCATGTGCCTGGCCCCTTTCCTCACTTT
CTAGCACAATGGTTTTCATTGCCACCTCTCCCGCGCCTTTCCCCCCTTCATAATTATCCA
CTATTTTACTTTCTGTGTCCT
>1567
CACAATGGTTTTCATTGCCACCTCTCCCGCGCCTTTCCCCCCTTCATAATTATCCACTATT
TTACTTTCTGTGTCCTGACAGCTACAAGAGTAGTTTTTGTTTTTTTGAGCTGAGTAGCT
GCTAAATATTCCCCAGCAGC
>1568
CAATGGTTTTCATTGCCACCTCTCCCGCGCCTTTCCCCCCTTCATAATTATCCACTATTTT
ACTTTCTGTGTCCTGACAGCTACAAGAGTAGTTTTTGTTTTTTTGAGCTGAGTAGCTGC
TAAATATTCCCCAGCAGCCA
>1569
CCTCTCCCGCGCCTTTCCCCCCTTCATAATTATCCACTATTTTACTTTCTGTGTCCTGACA
GCTACAAGAGTAGTTTTTGTTTTTTTGAGCTGAGTAGCTGCTAAATATTCCCCAGCAGC
CAGATTCAACGGCTGGGGGG
>1570
CTCCCGCGCCTTTCCCCCCTTCATAATTATCCACTATTTTACTTTCTGTGTCCTGACAGCT
ACAAGAGTAGTTTTTGTTTTTTTGAGCTGAGTAGCTGCTAAATATTCCCCAGCAGCCAG
ATTCAACGGCTGGGGGGGAG
>1571
CATAATTATCCACTATTTTACTTTCTGTGTCCTGACAGCTACAAGAGTAGTTTTTGTTTTT
TTGAGCTGAGTAGCTGCTAAATATTCCCCAGCAGCCAGATTCAACGGCTGGGGGGGAG
AATAGGCCAAACCTTGGCATC
>1572
CTGCTAAATATTCCCCAGCAGCCAGATTCAACGGCTGGGGGGGAGAATAGGCCAAACC
TTGGCATCCCCTGTTGCAAAAGTGAAAAATATTTCTGTTTGCAGTTAATTTTAGTGGCA
AGCAGATCATGTTAACTGGATGT

>1573
ATTCCCCAGCAGCCAGATTCAACGGCTGGGGGGGAGAATAGGCCAAACCTTGGCATCC
CCTGTTGCAAAAGTGAAAAATATTTCTGTTTGCAGTTAATTTTAGTGGCAAGCAGATCA
TGTTAACTGGATGTAATTGCTTC
>1574
GATTCAACGGCTGGGGGGGAGAATAGGCCAAACCTTGGCATCCCCTGTTGCAAAAGTG
AAAAATATTTCTGTTTGCAGTTAATTTTAGTGGCAAGCAGATCATGTTAACTGGATGTAA
TTGCTTCCAGATATGGCCGAGG
>1575
CGGCTGGGGGGGAGAATAGGCCAAACCTTGGCATCCCCTGTTGCAAAAGTGAAAAATA
TTTCTGTTTGCAGTTAATTTTAGTGGCAAGCAGATCATGTTAACTGGATGTAATTGCTTC
CAGATATGGCCGAGGGTTTTGT
>1576
CTTGGCATCCCCTGTTGCAAAAGTGAAAAATATTTCTGTTTGCAGTTAATTTTAGTGGC
AAGCAGATCATGTTAACTGGATGTAATTGCTTCCAGATATGGCCGAGGGTTTTGTGTGT
TGAGATGAAGGGTCTGGTAAGT
>1577
CTGTTGCAAAAGTGAAAAATATTTCTGTTTGCAGTTAATTTTAGTGGCAAGCAGATCAT
GTTAACTGGATGTAATTGCTTCCAGATATGGCCGAGGGTTTTGTGTGTTGAGATGAAGG
GTCTGGTAAGTGCTTACTTCCT
>1578
GTTTGCAGTTAATTTTAGTGGCAAGCAGATCATGTTAACTGGATGTAATTGCTTCCAGAT
ATGGCCGAGGGTTTTGTGTGTTGAGATGAAGGGTCTGGTAAGTGCTTACTTCCTGTGTA
AATTAGCCTATTCCATTCAGT
>1579
CAGTTAATTTTAGTGGCAAGCAGATCATGTTAACTGGATGTAATTGCTTCCAGATATGGC
CGAGGGTTTTGTGTGTTGAGATGAAGGGTCTGGTAAGTGCTTACTTCCTGTGTAAATTA
GCCTATTCCATTCAGTCTTTC
>1580
GTAATTGCTTCCAGATATGGCCGAGGGTTTTGTGTGTTGAGATGAAGGGTCTGGTAAGT
GCTTACTTCCTGTGTAAATTAGCCTATTCCATTCAGTCTTTCATTCTGGCCAGCCCTGGA
TTCTGTCATTCTGCTCCCATT
>1581
AATTGCTTCCAGATATGGCCGAGGGTTTTGTGTGTTGAGATGAAGGGTCTGGTAAGTGC
TTACTTCCTGTGTAAATTAGCCTATTCCATTCAGTCTTTCATTCTGGCCAGCCCTGGATT
CTGTCATTCTGCTCCCATTTT
>1582
GGGTTTTGTGTGTTGAGATGAAGGGTCTGGTAAGTGCTTACTTCCTGTGTAAATTAGCC
TATTCCATTCAGTCTTTCATTCTGGCCAGCCCTGGATTCTGTCATTCTGCTCCCATTTTCC
CCCTCCCCAGCCTGCAGCCT
>1583
GTAAGTGCTTACTTCCTGTGTAAATTAGCCTATTCCATTCAGTCTTTCATTCTGGCCAGC
CCTGGATTCTGTCATTCTGCTCCCATTTTCCCCCTCCCCAGCCTGCAGCCTGGTGTGAG
AAGCCGCTTGCTATGTTCCTC
>1584
TCTTTCATTCTGGCCAGCCCTGGATTCTGTCATTCTGCTCCCATTTTCCCCCTCCCCAGC

CTGCAGCCTGGTGTGAGAAGCCGCTTGCTATGTTCCTCTAAGTGTAATTCTTTTGTCAC
TTGGAGCCACTCTCCTCTTGG
>1585
GGATTCTGTCATTCTGCTCCCATTTTCCCCCTCCCCAGCCTGCAGCCTGGTGTGAGAAG
CCGCTTGCTATGTTCCTCTAAGTGTAATTCTTTTGTCACTTGGAGCCACTCTCCTCTTGG
TTCTTGATGTGAGTTTGCCCT
>1586
ATTCTGCTCCCATTTTCCCCCTCCCCAGCCTGCAGCCTGGTGTGAGAAGCCGCTTGCTA
TGTTCCTCTAAGTGTAATTCTTTTGTCACTTGGAGCCACTCTCCTCTTGGTTCTTGATGT
GAGTTTGCCCTTAAATAATGA
>1587
TTTCCCCCTCCCCAGCCTGCAGCCTGGTGTGAGAAGCCGCTTGCTATGTTCCTCTAAGT
GTAATTCTTTTGTCACTTGGAGCCACTCTCCTCTTGGTTCTTGATGTGAGTTTGCCCTTA
AATAATGAGAAGGGTTTGAAA
>1588
AGCCGCTTGCTATGTTCCTCTAAGTGTAATTCTTTTGTCACTTGGAGCCACTCTCCTCTT
GGTTCTTGATGTGAGTTTGCCCTTAAATAATGAGAAGGGTTTGAAAAGATGCCACCTG
GACCCCAGGCCACCTCCCGCCT
>1589
GCTTGCTATGTTCCTCTAAGTGTAATTCTTTTGTCACTTGGAGCCACTCTCCTCTTGGTT
CTTGATGTGAGTTTGCCCTTAAATAATGAGAAGGGTTTGAAAAGATGCCACCTGGACC
CCAGGCCACCTCCCGCCTCTTT
>1590
ATTCTTTTGTCACTTGGAGCCACTCTCCTCTTGGTTCTTGATGTGAGTTTGCCCTTAAAT
AATGAGAAGGGTTTGAAAAGATGCCACCTGGACCCCAGGCCACCTCCCGCCTCTTTCC
CAGCAAAGGCAGATCTGAAGAG
>1591
GATGCCACCTGGACCCCAGGCCACCTCCCGCCTCTTTCCCAGCAAAGGCAGATCTGAA
GAGCATCTCCTGGTCATGGGGGTTGCATCTTGGAACCCAGAACTCCTTGTTTTCTATGC
ATTTTTTTTTCCTACTGCCCTCT
>1592
TGGACCCCAGGCCACCTCCCGCCTCTTTCCCAGCAAAGGCAGATCTGAAGAGCATCTC
CTGGTCATGGGGGTTGCATCTTGGAACCCAGAACTCCTTGTTTTCTATGCATTTTTTTTT
CCTACTGCCCTCTGACCCCCGA
>1593
TCCCAGCAAAGGCAGATCTGAAGAGCATCTCCTGGTCATGGGGGTTGCATCTTGGAAC
CCAGAACTCCTTGTTTTCTATGCATTTTTTTTTCCTACTGCCCTCTGACCCCCGACTCTC
ACCTGGGAGAACACCTTCTCTT
>1594
ATTTTTTTTTCCTACTGCCCTCTGACCCCCGACTCTCACCTGGGAGAACACCTTCTCTTT
CTCTCAATTAGTCCGACTCCTCCTCAGCCTGAACTAAGCACGTTCCAGTGGGTCCGAA
ATCTCAGAAAAAATGAAACGGC
>1595
ACTCTCACCTGGGAGAACACCTTCTCTTTCTCTCAATTAGTCCGACTCCTCCTCAGCCT
GAACTAAGCACGTTCCAGTGGGTCCGAAATCTCAGAAAAAATGAAACGGCTGTTGCG
CAGAGGAGAAGGTTGTGGCCACAG

>1596
ACCTGGGAGAACACCTTCTCTTTCTCTCAATTAGTCCGACTCCTCCTCAGCCTGAACTA
AGCACGTTCCAGTGGGTCCGAAATCTCAGAAAAAATGAAACGGCTGTTGCGCAGAGG
AGAAGGTTGTGGCCACAGAAAGGA
>1597
TCCGACTCCTCCTCAGCCTGAACTAAGCACGTTCCAGTGGGTCCGAAATCTCAGAAAA
AATGAAACGGCTGTTGCGCAGAGGAGAAGGTTGTGGCCACAGAAAGGAGTTGCCTCA
TCAGACGTGAAGCCGCCACCTTGCC
>1598
CTGAACTAAGCACGTTCCAGTGGGTCCGAAATCTCAGAAAAAATGAAACGGCTGTTGC
GCAGAGGAGAAGGTTGTGGCCACAGAAAGGAGTTGCCTCATCAGACGTGAAGCCGCC
ACCTTGCCTTGCAAAGCAGGCTGCA
>1599
AACTAAGCACGTTCCAGTGGGTCCGAAATCTCAGAAAAAATGAAACGGCTGTTGCGC
AGAGGAGAAGGTTGTGGCCACAGAAAGGAGTTGCCTCATCAGACGTGAAGCCGCCAC
CTTGCCTTGCAAAGCAGGCTGCACCT
>1600
CAGAAAAAATGAAACGGCTGTTGCGCAGAGGAGAAGGTTGTGGCCACAGAAAGGAG
TTGCCTCATCAGACGTGAAGCCGCCACCTTGCCTTGCAAAGCAGGCTGCACCTCCTGA
CACACCTGCCCCTCCCTGCAAGAAAG
>1601
ACCTCCTGACACACCTGCCCCTCCCTGCAAGAAAGGGAGGGGAAATCCCACCCAAGA
GAACCTTCTCCAGGTTCCTGTGGGTTTCCAGGGTTGCAACTGAAGAAAAGTGTTAAGA
TTCATGTTCTCCTAAATTCTGCTTT
>1602
TGACACACCTGCCCCTCCCTGCAAGAAAGGGAGGGGAAATCCCACCCAAGAGAACCT
TCTCCAGGTTCCTGTGGGTTTCCAGGGTTGCAACTGAAGAAAAGTGTTAAGATTCATG
TTCTCCTAAATTCTGCTTTTGTCCT
>1603
GAGGGGAAATCCCACCCAAGAGAACCTTCTCCAGGTTCCTGTGGGTTTCCAGGGTTGC
AACTGAAGAAAAGTGTTAAGATTCATGTTCTCCTAAATTCTGCTTTTGTCCTGTGGCCA
GTCCCACATTCTTAGTGCTATTT
>1604
GGGGAAATCCCACCCAAGAGAACCTTCTCCAGGTTCCTGTGGGTTTCCAGGGTTGCAA
CTGAAGAAAAGTGTTAAGATTCATGTTCTCCTAAATTCTGCTTTTGTCCTGTGGCCAGT
CCCACATTCTTAGTGCTATTTGC
>1605
TTCCTGTGGGTTTCCAGGGTTGCAACTGAAGAAAAGTGTTAAGATTCATGTTCTCCTAA
ATTCTGCTTTTGTCCTGTGGCCAGTCCCACATTCTTAGTGCTATTTGCATAAGAGGTTGC
TGTTTATCTTCCTATATCAGC
>1606
TGAAGAAAAGTGTTAAGATTCATGTTCTCCTAAATTCTGCTTTTGTCCTGTGGCCAGTC
CCACATTCTTAGTGCTATTTGCATAAGAGGTTGCTGTTTATCTTCCTATATCAGCCCCACT
GATAACCCCTGTGGTCTGTT
>1607
TTCATGTTCTCCTAAATTCTGCTTTTGTCCTGTGGCCAGTCCCACATTCTTAGTGCTATTT

GCATAAGAGGTTGCTGTTTATCTTCCTATATCAGCCCCACTGATAACCCCTGTGGTCTGT
TTAACAACCTATGAGGAGT
>1608
CACATTCTTAGTGCTATTTGCATAAGAGGTTGCTGTTTATCTTCCTATATCAGCCCCACTG
ATAACCCCTGTGGTCTGTTTAACAACCTATGAGGAGTAGATTACTTGATAAATGCCAGT
TTTTCCAATATGCAAAGATA
>1609
ATTCTTAGTGCTATTTGCATAAGAGGTTGCTGTTTATCTTCCTATATCAGCCCCACTGATA
ACCCCTGTGGTCTGTTTAACAACCTATGAGGAGTAGATTACTTGATAAATGCCAGTTTTT
CCAATATGCAAAGATAAAC
>1610
AGGTTGCTGTTTATCTTCCTATATCAGCCCCACTGATAACCCCTGTGGTCTGTTTAACAA
CCTATGAGGAGTAGATTACTTGATAAATGCCAGTTTTTCCAATATGCAAAGATAAACTGT
TGGGGTCAGGGTGAGGGGTG
>1611
ATCAGCCCCACTGATAACCCCTGTGGTCTGTTTAACAACCTATGAGGAGTAGATTACTT
GATAAATGCCAGTTTTTCCAATATGCAAAGATAAACTGTTGGGGTCAGGGTGAGGGGT
GGTGTGTTATGAAGGAGGGGACA
>1612
TCTGTTTAACAACCTATGAGGAGTAGATTACTTGATAAATGCCAGTTTTTCCAATATGCA
AAGATAAACTGTTGGGGTCAGGGTGAGGGGTGGTGTGTTATGAAGGAGGGGACAGGT
GGGGAGCCAGGACCCTCTTTTAG
>1613
TAACAACCTATGAGGAGTAGATTACTTGATAAATGCCAGTTTTTCCAATATGCAAAGATA
AACTGTTGGGGTCAGGGTGAGGGGTGGTGTGTTATGAAGGAGGGGACAGGTGGGGAG
CCAGGACCCTCTTTTAGTGGGCT
>1614
TTTTTCCAATATGCAAAGATAAACTGTTGGGGTCAGGGTGAGGGGTGGTGTGTTATGAA
GGAGGGGACAGGTGGGGAGCCAGGACCCTCTTTTAGTGGGCTGGAGAAGTGTGAAAG
AGCCTGATGAAAGAAAACAGGTAG
>1615
TGTTGGGGTCAGGGTGAGGGGTGGTGTGTTATGAAGGAGGGGACAGGTGGGGAGCCA
GGACCCTCTTTTAGTGGGCTGGAGAAGTGTGAAAGAGCCTGATGAAAGAAAACAGGT
AGCTTGTCACATACAACAGCTGCATT
>1616
GAGGGGTGGTGTGTTATGAAGGAGGGGACAGGTGGGGAGCCAGGACCCTCTTTTAGT
GGGCTGGAGAAGTGTGAAAGAGCCTGATGAAAGAAAACAGGTAGCTTGTCACATACA
ACAGCTGCATTTATCATACGGTTGAT
>1617
ACAGGTGGGGAGCCAGGACCCTCTTTTAGTGGGCTGGAGAAGTGTGAAAGAGCCTGA
TGAAAGAAAACAGGTAGCTTGTCACATACAACAGCTGCATTTATCATACGGTTGATCAG
CTTCCTGCCCTCACCTACTTTTCT
>1618
GGGAGCCAGGACCCTCTTTTAGTGGGCTGGAGAAGTGTGAAAGAGCCTGATGAAAGA
AAACAGGTAGCTTGTCACATACAACAGCTGCATTTATCATACGGTTGATCAGCTTCCTG
CCCTCACCTACTTTTCTCCTCCTT

>1619
AGAAGTGTGAAAGAGCCTGATGAAAGAAAACAGGTAGCTTGTCACATACAACAGCTG
CATTTATCATACGGTTGATCAGCTTCCTGCCCTCACCTACTTTTCTCCTCCTTCATGTGG
ACATGCAGAGTCTCTGAGAACAT
>1620
ACATACAACAGCTGCATTTATCATACGGTTGATCAGCTTCCTGCCCTCACCTACTTTTCT
CCTCCTTCATGTGGACATGCAGAGTCTCTGAGAACATTTCATCAAGTGGGTTGCCTATG
CCCACATTAATACAAGATGGG
>1621
GCATTTATCATACGGTTGATCAGCTTCCTGCCCTCACCTACTTTTCTCCTCCTTCATGTG
GACATGCAGAGTCTCTGAGAACATTTCATCAAGTGGGTTGCCTATGCCCACATTAATAC
AAGATGGGAAAGGGTGGAGGT
>1622
GCTTCCTGCCCTCACCTACTTTTCTCCTCCTTCATGTGGACATGCAGAGTCTCTGAGAA
CATTTCATCAAGTGGGTTGCCTATGCCCACATTAATACAAGATGGGAAAGGGTGGAGGT
TATCTTTGCTTTTTAAGTTCCC
>1623
CCTGCCCTCACCTACTTTTCTCCTCCTTCATGTGGACATGCAGAGTCTCTGAGAACATT
TCATCAAGTGGGTTGCCTATGCCCACATTAATACAAGATGGGAAAGGGTGGAGGTTATC
TTTGCTTTTTAAGTTCCCATCT
>1624
CATGCAGAGTCTCTGAGAACATTTCATCAAGTGGGTTGCCTATGCCCACATTAATACAA
GATGGGAAAGGGTGGAGGTTATCTTTGCTTTTTAAGTTCCCATCTTTTTCTCCCTTGGTC
CTCTCCCACCCCTACAAGTTG
>1625
CATCAAGTGGGTTGCCTATGCCCACATTAATACAAGATGGGAAAGGGTGGAGGTTATCT
TTGCTTTTTAAGTTCCCATCTTTTTCTCCCTTGGTCCTCTCCCACCCCTACAAGTTGTTG
GCTGGTAAGCAATTGATGCTC
>1626
CACATTAATACAAGATGGGAAAGGGTGGAGGTTATCTTTGCTTTTTAAGTTCCCATCTTT
TTCTCCCTTGGTCCTCTCCCACCCCTACAAGTTGTTGGCTGGTAAGCAATTGATGCTCA
GAAAAATGATGTTCATCTGAA
>1627
GGTGGAGGTTATCTTTGCTTTTTAAGTTCCCATCTTTTTCTCCCTTGGTCCTCTCCCACC
CCTACAAGTTGTTGGCTGGTAAGCAATTGATGCTCAGAAAAATGATGTTCATCTGAAAT
CTTAAATGAGTGGACTTTTTC
>1628
TTATCTTTGCTTTTTAAGTTCCCATCTTTTTCTCCCTTGGTCCTCTCCCACCCCTACAAGT
TGTTGGCTGGTAAGCAATTGATGCTCAGAAAAATGATGTTCATCTGAAATCTTAAATGA
GTGGACTTTTTCTACCTTCC
>1629
TTTCTCCCTTGGTCCTCTCCCACCCCTACAAGTTGTTGGCTGGTAAGCAATTGATGCTC
AGAAAAATGATGTTCATCTGAAATCTTAAATGAGTGGACTTTTTCTACCTTCCCTGACT
TTGCAGGTGCTTCTTGAAGAGC
>1630
CCCCTACAAGTTGTTGGCTGGTAAGCAATTGATGCTCAGAAAAATGATGTTCATCTGAA

ATCTTAAATGAGTGGACTTTTTCTACCTTCCCTGACTTTGCAGGTGCTTCTTGAAGAGC
CAAGGAGAAAAGAAAACTTCTG
>1631
TGCTCAGAAAAATGATGTTCATCTGAAATCTTAAATGAGTGGACTTTTTCTACCTTCCCT
GACTTTGCAGGTGCTTCTTGAAGAGCCAAGGAGAAAAGAAAACTTCTGACCTCATCA
CCTAGTCAATAAGCTGTCTGAGA
>1632
ACCTTCCCTGACTTTGCAGGTGCTTCTTGAAGAGCCAAGGAGAAAAGAAAACTTCTG
ACCTCATCACCTAGTCAATAAGCTGTCTGAGAAACAAACAAAAGAGTTTAGACTGGCT
GCTAAAGCTAGGAACCCAGCTGATA
>1633
CTTTGCAGGTGCTTCTTGAAGAGCCAAGGAGAAAAGAAAACTTCTGACCTCATCACCT
AGTCAATAAGCTGTCTGAGAAACAAACAAAAGAGTTTAGACTGGCTGCTAAAGCTAG
GAACCCAGCTGATAGCTTTGTTTTG
>1634
TAAGCTGTCTGAGAAACAAACAAAAGAGTTTAGACTGGCTGCTAAAGCTAGGAACCC
AGCTGATAGCTTTGTTTTGATGTCCTTGTAATGTTTGCGGATCGCAGAGGGTGCTATTTA
GAGCAGGGGGCATCTACTGGGAC
>1635
CTGAGAAACAAACAAAAGAGTTTAGACTGGCTGCTAAAGCTAGGAACCCAGCTGATA
GCTTTGTTTTGATGTCCTTGTAATGTTTGCGGATCGCAGAGGGTGCTATTTAGAGCAGG
GGGCATCTACTGGGACAGACTCAC
>1636
GCTGCTAAAGCTAGGAACCCAGCTGATAGCTTTGTTTTGATGTCCTTGTAATGTTTGCG
GATCGCAGAGGGTGCTATTTAGAGCAGGGGGCATCTACTGGGACAGACTCACTGGGAA
GTTGAAACAGCTAGCTCAAAGTC
>1637
GTAATGTTTGCGGATCGCAGAGGGTGCTATTTAGAGCAGGGGGCATCTACTGGGACAG
ACTCACTGGGAAGTTGAAACAGCTAGCTCAAAGTCCAAGAGATGCTAAAGATAGTGA
AATACAGCCCAGGAATTGAGGGGGA
>1638
AGGGTGCTATTTAGAGCAGGGGGCATCTACTGGGACAGACTCACTGGGAAGTTGAAAC
AGCTAGCTCAAAGTCCAAGAGATGCTAAAGATAGTGAAATACAGCCCAGGAATTGAGG
GGGAGGGGGTTCCTCACTCAAGAA
>1639
GGCATCTACTGGGACAGACTCACTGGGAAGTTGAAACAGCTAGCTCAAAGTCCAAGA
GATGCTAAAGATAGTGAAATACAGCCCAGGAATTGAGGGGGAGGGGGTTCCTCACTCA
AGAAGGAGACTTTCTAGGAGGGGGC
>1640
AAACAGCTAGCTCAAAGTCCAAGAGATGCTAAAGATAGTGAAATACAGCCCAGGAATT
GAGGGGGAGGGGGTTCCTCACTCAAGAAGGAGACTTTCTAGGAGGGGGCGGGCGTG
GTATTTTCTAGGGACAGTATGGAATG
>1641
AATACAGCCCAGGAATTGAGGGGGAGGGGGTTCCTCACTCAAGAAGGAGACTTTCTA
GGAGGGGGCGGGCGTGGTATTTTCTAGGGACAGTATGGAATGAAGGGAAGAAGGTTG
GCTTTAGAAGGAGGCACCCTGTGGGA

>1642
ACAGCCCAGGAATTGAGGGGGAGGGGGTTCCTCACTCAAGAAGGAGACTTTCTAGGA
GGGGGCGGGCGTGGTATTTTCTAGGGACAGTATGGAATGAAGGGAAGAAGGTTGGCTT
TAGAAGGAGGCACCCTGTGGGAGGC
>1643
GGGGGAGGGGGTTCCTCACTCAAGAAGGAGACTTTCTAGGAGGGGGCGGGCGTGGTA
TTTTCTAGGGACAGTATGGAATGAAGGGAAGAAGGTTGGCTTTAGAAGGAGGCACCCT
GTGGGAGGCTGAGACAGGTGGGTCA
>1644
AAGAAGGAGACTTTCTAGGAGGGGGCGGGCGTGGTATTTTCTAGGGACAGTATGGAAT
GAAGGGAAGAAGGTTGGCTTTAGAAGGAGGCACCCTGTGGGAGGCTGAGACAGGTG
GGTCACCTGAGGTCAGGAGTTTGAGA
>1645
GCGGGCGTGGTATTTTCTAGGGACAGTATGGAATGAAGGGAAGAAGGTTGGCTTTAGA
AGGAGGCACCCTGTGGGAGGCTGAGACAGGTGGGTCACCTGAGGTCAGGAGTTTGAG
ACCAGCCTGGCCAACATGGTGAAAC
>1646
TAGGGACAGTATGGAATGAAGGGAAGAAGGTTGGCTTTAGAAGGAGGCACCCTGTGG
GAGGCTGAGACAGGTGGGTCACCTGAGGTCAGGAGTTTGAGACCAGCCTGGCCAACA
TGGTGAAACCCAGTCTCTACTAAAAA
>1647
GACAGTATGGAATGAAGGGAAGAAGGTTGGCTTTAGAAGGAGGCACCCTGTGGGAGG
CTGAGACAGGTGGGTCACCTGAGGTCAGGAGTTTGAGACCAGCCTGGCCAACATGGT
GAAACCCAGTCTCTACTAAAAATACA
>1648
GCACCCTGTGGGAGGCTGAGACAGGTGGGTCACCTGAGGTCAGGAGTTTGAGACCAG
CCTGGCCAACATGGTGAAACCCAGTCTCTACTAAAAATACAAAAATTAGCTGGGTGGC
AGGTGCCTGTAATCCCAGCTAGTCG
>1649
GGAGGCTGAGACAGGTGGGTCACCTGAGGTCAGGAGTTTGAGACCAGCCTGGCCAAC
ATGGTGAAACCCAGTCTCTACTAAAAATACAAAAATTAGCTGGGTGGCAGGTGCCTGT
AATCCCAGCTAGTCGGGGGAGGCTG
>1650
CAGGAGTTTGAGACCAGCCTGGCCAACATGGTGAAACCCAGTCTCTACTAAAAATACA
AAAATTAGCTGGGTGGCAGGTGCCTGTAATCCCAGCTAGTCGGGGGAGGCTGAGGCA
GGAAAATCGCTTGGGATTACAGGTG
>1651
CATGGTGAAACCCAGTCTCTACTAAAAATACAAAAATTAGCTGGGTGGCAGGTGCCTG
TAATCCCAGCTAGTCGGGGGAGGCTGAGGCAGGAAAATCGCTTGGGATTACAGGTGCC
TGTAATCCCAGCTAGTCGGGAGAG
>1652
TGGGTGGCAGGTGCCTGTAATCCCAGCTAGTCGGGGGAGGCTGAGGCAGGAAAATCG
CTTGGGATTACAGGTGCCTGTAATCCCAGCTAGTCGGGAGAGGCTGAGGCAGGAAAAT
TGCTTGAAGGAGGCGGAGGTTGCAG
>1653
ATCCCAGCTAGTCGGGGGAGGCTGAGGCAGGAAAATCGCTTGGGATTACAGGTGCCTG

TAATCCCAGCTAGTCGGGAGAGGCTGAGGCAGGAAAATTGCTTGAAGGAGGCGGAGG
TTGCAGTGAGCCCAGATCCTGCCGT
>1654
ATTACAGGTGCCTGTAATCCCAGCTAGTCGGGAGAGGCTGAGGCAGGAAAATTGCTTG
AAGGAGGCGGAGGTTGCAGTGAGCCCAGATCCTGCCGTTGCACTCCAGCCCAAGACT
TTGTCTCATTAAAAAAAAAAAAAAGA
>1655
GGTGCCTGTAATCCCAGCTAGTCGGGAGAGGCTGAGGCAGGAAAATTGCTTGAAGGA
GGCGGAGGTTGCAGTGAGCCCAGATCCTGCCGTTGCACTCCAGCCCAAGACTTTGTCT
CATTAAAAAAAAAAAAAAGAAGAAGA
>1656
TAGTCGGGAGAGGCTGAGGCAGGAAAATTGCTTGAAGGAGGCGGAGGTTGCAGTGAG
CCCAGATCCTGCCGTTGCACTCCAGCCCAAGACTTTGTCTCATTAAAAAAAAAAAAAAG
AAGAAGAAGAAGAAGGAGGCACCCT
>1657
AAAATTGCTTGAAGGAGGCGGAGGTTGCAGTGAGCCCAGATCCTGCCGTTGCACTCC
AGCCCAAGACTTTGTCTCATTAAAAAAAAAAAAAAGAAGAAGAAGAAGAAGGAGGCA
CCCTGGATGAAGACTCAGGCTCCAGAA
>1658
AAAAAAAAAAAGAAGAAGAAGAAGAAGGAGGCACCCTGGATGAAGACTCAGGCTCC
AGAATTTACTATCTGTATGTCTTGGGCAAGTGGCTTAGCTTCTCTGAGCCCCTGTTTACT
TTTCTTTTGAAAATGACCACCTTG
>1659
AAAAAAAGAAGAAGAAGAAGAAGGAGGCACCCTGGATGAAGACTCAGGCTCCAGAA
TTTACTATCTGTATGTCTTGGGCAAGTGGCTTAGCTTCTCTGAGCCCCTGTTTACTTTTC
TTTTGAAAATGACCACCTTGTCCA
>1660
AAGAAGAAGAAGGAGGCACCCTGGATGAAGACTCAGGCTCCAGAATTTACTATCTGTA
TGTCTTGGGCAAGTGGCTTAGCTTCTCTGAGCCCCTGTTTACTTTTCTTTTGAAAATGA
CCACCTTGTCCATGGTAATGAGG
>1661
ATGAAGACTCAGGCTCCAGAATTTACTATCTGTATGTCTTGGGCAAGTGGCTTAGCTTC
TCTGAGCCCCTGTTTACTTTTCTTTTGAAAATGACCACCTTGTCCATGGTAATGAGGATA
AAAAACGTGTAAAGTGCATAG
>1662
TGTATGTCTTGGGCAAGTGGCTTAGCTTCTCTGAGCCCCTGTTTACTTTTCTTTTGAAAA
TGACCACCTTGTCCATGGTAATGAGGATAAAAAACGTGTAAAGTGCATAGCAGAGTGC
AGGTGTACTGGGTAGACATGTA
>1663
CTTGGGCAAGTGGCTTAGCTTCTCTGAGCCCCTGTTTACTTTTCTTTTGAAAATGACCA
CCTTGTCCATGGTAATGAGGATAAAAAACGTGTAAAGTGCATAGCAGAGTGCAGGTGT
ACTGGGTAGACATGTATGATTGC
>1664
CTTCTCTGAGCCCCTGTTTACTTTTCTTTTGAAAATGACCACCTTGTCCATGGTAATGAG
GATAAAAAACGTGTAAAGTGCATAGCAGAGTGCAGGTGTACTGGGTAGACATGTATGA
TTGCGCTGTAGCCCTGTTATGG

>1665
CTTTTGAAAATGACCACCTTGTCCATGGTAATGAGGATAAAAAACGTGTAAAGTGCATA
GCAGAGTGCAGGTGTACTGGGTAGACATGTATGATTGCGCTGTAGCCCTGTTATGGCCC
GGGGAGAAACTATGAAAGGAAC
>1666
TTTGAAAATGACCACCTTGTCCATGGTAATGAGGATAAAAAACGTGTAAAGTGCATAG
CAGAGTGCAGGTGTACTGGGTAGACATGTATGATTGCGCTGTAGCCCTGTTATGGCCCG
GGGAGAAACTATGAAAGGAACCA
>1667
ATGACCACCTTGTCCATGGTAATGAGGATAAAAAACGTGTAAAGTGCATAGCAGAGTG
CAGGTGTACTGGGTAGACATGTATGATTGCGCTGTAGCCCTGTTATGGCCCGGGGAGAA
ACTATGAAAGGAACCAGTGAAAT
>1668
CTTGTCCATGGTAATGAGGATAAAAAACGTGTAAAGTGCATAGCAGAGTGCAGGTGTA
CTGGGTAGACATGTATGATTGCGCTGTAGCCCTGTTATGGCCCGGGGAGAAACTATGAA
AGGAACCAGTGAAATCTTTTGTT
>1669
AAAAAACGTGTAAAGTGCATAGCAGAGTGCAGGTGTACTGGGTAGACATGTATGATTG
CGCTGTAGCCCTGTTATGGCCCGGGGAGAAACTATGAAAGGAACCAGTGAAATCTTTT
GTTTTGGGCACTGCTAAATTGATG
>1670
GTACTGGGTAGACATGTATGATTGCGCTGTAGCCCTGTTATGGCCCGGGGAGAAACTAT
GAAAGGAACCAGTGAAATCTTTTGTTTTGGGCACTGCTAAATTGATGATAGTACTAACC
CTGTGCTAGCAGCCAGCCGGTG
>1671
CATGTATGATTGCGCTGTAGCCCTGTTATGGCCCGGGGAGAAACTATGAAAGGAACCA
GTGAAATCTTTTGTTTTGGGCACTGCTAAATTGATGATAGTACTAACCCTGTGCTAGCA
GCCAGCCGGTGAAATAATAGGTG
>1672
ATGGCCCGGGGAGAAACTATGAAAGGAACCAGTGAAATCTTTTGTTTTGGGCACTGCT
AAATTGATGATAGTACTAACCCTGTGCTAGCAGCCAGCCGGTGAAATAATAGGTGAATA
ATGTTAGTACATTGTGAATATAA
>1673
AGAAACTATGAAAGGAACCAGTGAAATCTTTTGTTTTGGGCACTGCTAAATTGATGATA
GTACTAACCCTGTGCTAGCAGCCAGCCGGTGAAATAATAGGTGAATAATGTTAGTACAT
TGTGAATATAAAAGTTTCCATT
>1674
CCAGTGAAATCTTTTGTTTTGGGCACTGCTAAATTGATGATAGTACTAACCCTGTGCTAG
CAGCCAGCCGGTGAAATAATAGGTGAATAATGTTAGTACATTGTGAATATAAAAGTTTC
CATTTGCAAAGTGCTACTGCT
>1675
TTTGTTTTGGGCACTGCTAAATTGATGATAGTACTAACCCTGTGCTAGCAGCCAGCCGG
TGAAATAATAGGTGAATAATGTTAGTACATTGTGAATATAAAAGTTTCCATTTGCAAAGT
GCTACTGCTGTTCTAAAGAAG
>1676
ACTAACCCTGTGCTAGCAGCCAGCCGGTGAAATAATAGGTGAATAATGTTAGTACATTG

TGAATATAAAAGTTTCCATTTGCAAAGTGCTACTGCTGTTCTAAAGAAGCCACATTTCT
AATAAATGATTGCTTAATCCTG
>1677
GCAGCCAGCCGGTGAAATAATAGGTGAATAATGTTAGTACATTGTGAATATAAAAGTTT
CCATTTGCAAAGTGCTACTGCTGTTCTAAAGAAGCCACATTTCTAATAAATGATTGCTTA
ATCCTGTGATGCTGCCACAGC
>1678
GTGAAATAATAGGTGAATAATGTTAGTACATTGTGAATATAAAAGTTTCCATTTGCAAAG
TGCTACTGCTGTTCTAAAGAAGCCACATTTCTAATAAATGATTGCTTAATCCTGTGATGC
TGCCACAGCCCCACAGAGTT
>1679
AGTTTCCATTTGCAAAGTGCTACTGCTGTTCTAAAGAAGCCACATTTCTAATAAATGATT
GCTTAATCCTGTGATGCTGCCACAGCCCCACAGAGTTTGGGAATGTTTGGGAATTCTTT
CTGAAGTGTGTGCCGCATTCT
>1680
GCTGTTCTAAAGAAGCCACATTTCTAATAAATGATTGCTTAATCCTGTGATGCTGCCACA
GCCCCACAGAGTTTGGGAATGTTTGGGAATTCTTTCTGAAGTGTGTGCCGCATTCTTTT
GCTTTGCCTTTTGGGAGGGTA
>1681
ATGATTGCTTAATCCTGTGATGCTGCCACAGCCCCACAGAGTTTGGGAATGTTTGGGAA
TTCTTTCTGAAGTGTGTGCCGCATTCTTTTGCTTTGCCTTTTGGGAGGGTAGATGTGACT
ATTGGAAATGGTCAAAATAAT
>1682
GATTGCTTAATCCTGTGATGCTGCCACAGCCCCACAGAGTTTGGGAATGTTTGGGAATT
CTTTCTGAAGTGTGTGCCGCATTCTTTTGCTTTGCCTTTTGGGAGGGTAGATGTGACTAT
TGGAAATGGTCAAAATAATGC
>1683
AGAGTTTGGGAATGTTTGGGAATTCTTTCTGAAGTGTGTGCCGCATTCTTTTGCTTTGC
CTTTTGGGAGGGTAGATGTGACTATTGGAAATGGTCAAAATAATGCAGTAGCAAATCTG
TTGACTATAGTGAGTGATAAGC
>1684
TTCTTTCTGAAGTGTGTGCCGCATTCTTTTGCTTTGCCTTTTGGGAGGGTAGATGTGACT
ATTGGAAATGGTCAAAATAATGCAGTAGCAAATCTGTTGACTATAGTGAGTGATAAGCA
CTGTCGGCTGCTGTGTCTGAC
>1685
TGTGCCGCATTCTTTTGCTTTGCCTTTTGGGAGGGTAGATGTGACTATTGGAAATGGTC
AAAATAATGCAGTAGCAAATCTGTTGACTATAGTGAGTGATAAGCACTGTCGGCTGCTG
TGTCTGACTCGGAAGGCAGATT
>1686
CTTTTGCTTTGCCTTTTGGGAGGGTAGATGTGACTATTGGAAATGGTCAAAATAATGCA
GTAGCAAATCTGTTGACTATAGTGAGTGATAAGCACTGTCGGCTGCTGTGTCTGACTCG
GAAGGCAGATTCTGGAAGGGGT
>1687
TTGGAAATGGTCAAAATAATGCAGTAGCAAATCTGTTGACTATAGTGAGTGATAAGCAC
TGTCGGCTGCTGTGTCTGACTCGGAAGGCAGATTCTGGAAGGGGTGTGTGGGGAAGG
CTTGTTGGCTCAGCACTGGGGTGA

>1688
GGAAATGGTCAAAATAATGCAGTAGCAAATCTGTTGACTATAGTGAGTGATAAGCACTG
TCGGCTGCTGTGTCTGACTCGGAAGGCAGATTCTGGAAGGGGTGTGTGGGGAAGGCT
TGTTGGCTCAGCACTGGGGTGAGT
>1689
CTGTTGACTATAGTGAGTGATAAGCACTGTCGGCTGCTGTGTCTGACTCGGAAGGCAG
ATTCTGGAAGGGGTGTGTGGGGAAGGCTTGTTGGCTCAGCACTGGGGTGAGTGTTGG
CCTCACTTCTTAGGTGGGAAGTGGA
>1690
TGATAAGCACTGTCGGCTGCTGTGTCTGACTCGGAAGGCAGATTCTGGAAGGGGTGTG
TGGGGAAGGCTTGTTGGCTCAGCACTGGGGTGAGTGTTGGCCTCACTTCTTAGGTGGG
AAGTGGACAAGGCCAAGGGGCTTT
>1691
TGCTGTGTCTGACTCGGAAGGCAGATTCTGGAAGGGGTGTGTGGGGAAGGCTTGTTG
GCTCAGCACTGGGGTGAGTGTTGGCCTCACTTCTTAGGTGGGAAGTGGACAAGGCCA
AGGGGCTTTTGATTTCCAGCTACTAT
>1692
GTGTCTGACTCGGAAGGCAGATTCTGGAAGGGGTGTGTGGGGAAGGCTTGTTGGCTC
AGCACTGGGGTGAGTGTTGGCCTCACTTCTTAGGTGGGAAGTGGACAAGGCCAAGGG
GCTTTTGATTTCCAGCTACTATGGCT
>1693
ATTCTGGAAGGGGTGTGTGGGGAAGGCTTGTTGGCTCAGCACTGGGGTGAGTGTTGG
CCTCACTTCTTAGGTGGGAAGTGGACAAGGCCAAGGGGCTTTTGATTTCCAGCTACTAT
GGCTGGACTCCCAGTGAGGAAGGA
>1694
TCACTTCTTAGGTGGGAAGTGGACAAGGCCAAGGGGCTTTTGATTTCCAGCTACTATG
GCTGGACTCCCAGTGAGGAAGGAGTCGCTGCCATGGATTGCCTGGGACTGGGAAGAC
TCCTTTTAACTGCTGTAATGTTTCT
>1695
GTGGGAAGTGGACAAGGCCAAGGGGCTTTTGATTTCCAGCTACTATGGCTGGACTCCC
AGTGAGGAAGGAGTCGCTGCCATGGATTGCCTGGGACTGGGAAGACTCCTTTTAACTG
CTGTAATGTTTCTGCATTTGTGGT
>1696
CTGGACTCCCAGTGAGGAAGGAGTCGCTGCCATGGATTGCCTGGGACTGGGAAGACT
CCTTTTAACTGCTGTAATGTTTCTGCATTTGTGGTTTGGCCTCCTGTGAGTTTGGGCTCC
GGGGCGGCACTTCTGTTGTTAAA
>1697
CTCCCAGTGAGGAAGGAGTCGCTGCCATGGATTGCCTGGGACTGGGAAGACTCCTTTT
AACTGCTGTAATGTTTCTGCATTTGTGGTTTGGCCTCCTGTGAGTTTGGGCTCCGGGGC
GGCACTTCTGTTGTTAAAAGAAC
>1698
TGGATTGCCTGGGACTGGGAAGACTCCTTTTAACTGCTGTAATGTTTCTGCATTTGTGG
TTTGGCCTCCTGTGAGTTTGGGCTCCGGGGCGGCACTTCTGTTGTTAAAAGAACAGAA
AAGAAAACAAAACAAAACAAAAA
>1699
TTGCCTGGGACTGGGAAGACTCCTTTTAACTGCTGTAATGTTTCTGCATTTGTGGTTTG

GCCTCCTGTGAGTTTGGGCTCCGGGGCGGCACTTCTGTTGTTAAAAGAACAGAAAAG
AAAACAAAACAAAACAAAAAACCA
>1700
TTAACTGCTGTAATGTTTCTGCATTTGTGGTTTGGCCTCCTGTGAGTTTGGGCTCCGGG
GCGGCACTTCTGTTGTTAAAAGAACAGAAAAGAAAACAAAACAAAACAAAAAACCA
GGACTGTCACTGTTAGATGTTGGTT
>1701
GGCTCCGGGGCGGCACTTCTGTTGTTAAAAGAACAGAAAAGAAAACAAAACAAAAC
AAAAAACCAGGACTGTCACTGTTAGATGTTGGTTGAGGCTAGAAAGAATGGATTGAGT
TTTGACCCCCTACACCCCAGGGAGTA
>1702
GGGGCGGCACTTCTGTTGTTAAAAGAACAGAAAAGAAAACAAAACAAAACAAAAAA
CCAGGACTGTCACTGTTAGATGTTGGTTGAGGCTAGAAAGAATGGATTGAGTTTTGAC
CCCCTACACCCCAGGGAGTAGGGAGG
>1703
AAACAAAACAAAACAAAAAACCAGGACTGTCACTGTTAGATGTTGGTTGAGGCTAGA
AAGAATGGATTGAGTTTTGACCCCCTACACCCCAGGGAGTAGGGAGGCCTGGGAGGC
ACAGTGGCCTGAGTTTGAATTCTGAC
>1704
ACTGTCACTGTTAGATGTTGGTTGAGGCTAGAAAGAATGGATTGAGTTTTGACCCCCTA
CACCCCAGGGAGTAGGGAGGCCTGGGAGGCACAGTGGCCTGAGTTTGAATTCTGACT
CTGCCTCTTTGGAAGGCTCTGGAA
>1705
TGAGGCTAGAAAGAATGGATTGAGTTTTGACCCCCTACACCCCAGGGAGTAGGGAGGC
CTGGGAGGCACAGTGGCCTGAGTTTGAATTCTGACTCTGCCTCTTTGGAAGGCTCTGG
AAGGTTAAAGGAGCTTGTGTGTTC
>1706
AAAGAATGGATTGAGTTTTGACCCCCTACACCCCAGGGAGTAGGGAGGCCTGGGAGG
CACAGTGGCCTGAGTTTGAATTCTGACTCTGCCTCTTTGGAAGGCTCTGGAAGGTTAA
AGGAGCTTGTGTGTTCAAATCTTAG
>1707
TAGGGAGGCCTGGGAGGCACAGTGGCCTGAGTTTGAATTCTGACTCTGCCTCTTTGGA
AGGCTCTGGAAGGTTAAAGGAGCTTGTGTGTTCAAATCTTAGCCTGGTACCTGGCATAT
ACTCAGCATGTATTTTTTCCCCT
>1708
GAGGCACAGTGGCCTGAGTTTGAATTCTGACTCTGCCTCTTTGGAAGGCTCTGGAAGG
TTAAAGGAGCTTGTGTGTTCAAATCTTAGCCTGGTACCTGGCATATACTCAGCATGTATT
TTTTCCCCTTTCCTGACAATGT
>1709
CACAGTGGCCTGAGTTTGAATTCTGACTCTGCCTCTTTGGAAGGCTCTGGAAGGTTAA
AGGAGCTTGTGTGTTCAAATCTTAGCCTGGTACCTGGCATATACTCAGCATGTATTTTTT
CCCCTTTCCTGACAATGTTCCT
>1710
GAATTCTGACTCTGCCTCTTTGGAAGGCTCTGGAAGGTTAAAGGAGCTTGTGTGTTCA
AATCTTAGCCTGGTACCTGGCATATACTCAGCATGTATTTTTTCCCCTTTCCTGACAATGT
TCCTTGCCCCGAACACTTGAA

>1711
GAAGGCTCTGGAAGGTTAAAGGAGCTTGTGTGTTCAAATCTTAGCCTGGTACCTGGCA
TATACTCAGCATGTATTTTTTCCCCTTTCCTGACAATGTTCCTTGCCCCGAACACTTGAA
TAGTGGAGAGAGGAATGTTTGC
>1712
TTGTGTGTTCAAATCTTAGCCTGGTACCTGGCATATACTCAGCATGTATTTTTTCCCCTTT
CCTGACAATGTTCCTTGCCCCGAACACTTGAATAGTGGAGAGAGGAATGTTTGCTTTTC
CTCTCCATTGAACCAACACT
>1713
ACCTGGCATATACTCAGCATGTATTTTTTCCCCTTTCCTGACAATGTTCCTTGCCCCGAA
CACTTGAATAGTGGAGAGAGGAATGTTTGCTTTTCCTCTCCATTGAACCAACACTCGCC
CATCCACTGAGCCCAAGATTC
>1714
CAGCATGTATTTTTTCCCCTTTCCTGACAATGTTCCTTGCCCCGAACACTTGAATAGTGG
AGAGAGGAATGTTTGCTTTTCCTCTCCATTGAACCAACACTCGCCCATCCACTGAGCCC
AAGATTCACCTTAGGATTGTC
>1715
AATGTTTGCTTTTCCTCTCCATTGAACCAACACTCGCCCATCCACTGAGCCCAAGATTC
ACCTTAGGATTGTCTGTTGGACTCGGCTCTTTTGTGACCTAACAGTGCAGGGGCCTTGT
GCTGAGCTGCACATGCTCGCTC
>1716
TTTGCTTTTCCTCTCCATTGAACCAACACTCGCCCATCCACTGAGCCCAAGATTCACCT
TAGGATTGTCTGTTGGACTCGGCTCTTTTGTGACCTAACAGTGCAGGGGCCTTGTGCTG
AGCTGCACATGCTCGCTCTCGC
>1717
TGAACCAACACTCGCCCATCCACTGAGCCCAAGATTCACCTTAGGATTGTCTGTTGGA
CTCGGCTCTTTTGTGACCTAACAGTGCAGGGGCCTTGTGCTGAGCTGCACATGCTCGC
TCTCGCTGGTGCTCCCTCTCTCTC
>1718
TCGCCCATCCACTGAGCCCAAGATTCACCTTAGGATTGTCTGTTGGACTCGGCTCTTTT
GTGACCTAACAGTGCAGGGGCCTTGTGCTGAGCTGCACATGCTCGCTCTCGCTGGTGC
TCCCTCTCTCTCTCTCTCTCTGT
>1719
TGGACTCGGCTCTTTTGTGACCTAACAGTGCAGGGGCCTTGTGCTGAGCTGCACATGC
TCGCTCTCGCTGGTGCTCCCTCTCTCTCTCTCTCTGTCTGCATTATTTATAGTAAACCA
TATTAGAACAGAGTGTTAAAG
>1720
CAGTGCAGGGGCCTTGTGCTGAGCTGCACATGCTCGCTCTCGCTGGTGCTCCCTCTCT
CTCTCTCTCTCTGTCTGCATTATTTATAGTAAACCATATTAGAACAGAGTGTTAAAGAGG
AAGGATGAGTCATTCCCATTGA
>1721
TGCTGAGCTGCACATGCTCGCTCTCGCTGGTGCTCCCTCTCTCTCTCTCTCTCTGTCTG
CATTATTTATAGTAAACCATATTAGAACAGAGTGTTAAAGAGGAAGGATGAGTCATTCC
CATTGAAACCATTGATGACATA
>1722
CTCTCGCTGGTGCTCCCTCTCTCTCTCTCTCTCTGTCTGCATTATTTATAGTAAACCATAT

TAGAACAGAGTGTTAAAGAGGAAGGATGAGTCATTCCCATTGAAACCATTGATGACAT
AAATAAAAGCAAAATGCTTAC
>1723
CTCGCTGGTGCTCCCTCTCTCTCTCTCTCTCTGTCTGCATTATTTATAGTAAACCATATTA
GAACAGAGTGTTAAAGAGGAAGGATGAGTCATTCCCATTGAAACCATTGATGACATAA
ATAAAAGCAAAATGCTTACTA
>1724
CTCTCTCTCTCTCTGTCTGCATTATTTATAGTAAACCATATTAGAACAGAGTGTTAAAGA
GGAAGGATGAGTCATTCCCATTGAAACCATTGATGACATAAATAAAAGCAAAATGCTTA
CTATGTGTTTTGTGCAGTATT
>1725
TTAAAGAGGAAGGATGAGTCATTCCCATTGAAACCATTGATGACATAAATAAAAGCAA
AATGCTTACTATGTGTTTTGTGCAGTATTTAAGGCCAGGTTGTGCTAATTTGAATGCTCC
CAAACTGGATCTGTGAAAGCAT
>1726
AAAGAGGAAGGATGAGTCATTCCCATTGAAACCATTGATGACATAAATAAAAGCAAAA
TGCTTACTATGTGTTTTGTGCAGTATTTAAGGCCAGGTTGTGCTAATTTGAATGCTCCCA
AACTGGATCTGTGAAAGCATAC
>1727
ATTGAAACCATTGATGACATAAATAAAAGCAAAATGCTTACTATGTGTTTTGTGCAGTAT
TTAAGGCCAGGTTGTGCTAATTTGAATGCTCCCAAACTGGATCTGTGAAAGCATACATA
TGTGGGAAAAAATTCAGGTTT
>1728
TGCTTACTATGTGTTTTGTGCAGTATTTAAGGCCAGGTTGTGCTAATTTGAATGCTCCCA
AACTGGATCTGTGAAAGCATACATATGTGGGAAAAAATTCAGGTTTAACTAGAGGGAA
GTTATTTATTGAAAATCCATTT
>1729
TGTGCAGTATTTAAGGCCAGGTTGTGCTAATTTGAATGCTCCCAAACTGGATCTGTGAA
AGCATACATATGTGGGAAAAAATTCAGGTTTAACTAGAGGGAAGTTATTTATTGAAAAT
CCATTTCAGTATCTGCTTTCTC
>1730
TGAATGCTCCCAAACTGGATCTGTGAAAGCATACATATGTGGGAAAAAATTCAGGTTTA
ACTAGAGGGAAGTTATTTATTGAAAATCCATTTCAGTATCTGCTTTCTCCCTTCTATTTAC
TTCAGATCCATATTCATATC
>1731
GAAAGCATACATATGTGGGAAAAAATTCAGGTTTAACTAGAGGGAAGTTATTTATTGAA
AATCCATTTCAGTATCTGCTTTCTCCCTTCTATTTACTTCAGATCCATATTCATATCATGCT
TGTTGTTCTGGCTAACGAG
>1732
TATTGAAAATCCATTTCAGTATCTGCTTTCTCCCTTCTATTTACTTCAGATCCATATTCATA
TCATGCTTGTTGTTCTGGCTAACGAGAGGGACTGGCTTATTCACTAGCTAAGGTTTGTT
TATTTTCGGAAGGTTGAGG
>1733
TGAAAATCCATTTCAGTATCTGCTTTCTCCCTTCTATTTACTTCAGATCCATATTCATATCA
TGCTTGTTGTTCTGGCTAACGAGAGGGACTGGCTTATTCACTAGCTAAGGTTTGTTTATT
TTCGGAAGGTTGAGGAAT

>1734
TTCAGATCCATATTCATATCATGCTTGTTGTTCTGGCTAACGAGAGGGACTGGCTTATTC
ACTAGCTAAGGTTTGTTTATTTTCGGAAGGTTGAGGAATAGCACAGAGAGGTTGTCTAA
GTGTCCCTGTCTCCATTCCTC
>1735
GATCCATATTCATATCATGCTTGTTGTTCTGGCTAACGAGAGGGACTGGCTTATTCACTA
GCTAAGGTTTGTTTATTTTCGGAAGGTTGAGGAATAGCACAGAGAGGTTGTCTAAGTG
TCCCTGTCTCCATTCCTCCTCT
>1736
CATGCTTGTTGTTCTGGCTAACGAGAGGGACTGGCTTATTCACTAGCTAAGGTTTGTTT
ATTTTCGGAAGGTTGAGGAATAGCACAGAGAGGTTGTCTAAGTGTCCCTGTCTCCATTC
CTCCTCTTGCCACCTGTTGAGG
>1737
GAGGGACTGGCTTATTCACTAGCTAAGGTTTGTTTATTTTCGGAAGGTTGAGGAATAGC
ACAGAGAGGTTGTCTAAGTGTCCCTGTCTCCATTCCTCCTCTTGCCACCTGTTGAGGAA
GGCACTCTCCACCTGCTTTGCA
>1738
TTCACTAGCTAAGGTTTGTTTATTTTCGGAAGGTTGAGGAATAGCACAGAGAGGTTGTC
TAAGTGTCCCTGTCTCCATTCCTCCTCTTGCCACCTGTTGAGGAAGGCACTCTCCACCT
GCTTTGCAGGCGTGGGCTCTCA
>1739
TCGGAAGGTTGAGGAATAGCACAGAGAGGTTGTCTAAGTGTCCCTGTCTCCATTCCTC
CTCTTGCCACCTGTTGAGGAAGGCACTCTCCACCTGCTTTGCAGGCGTGGGCTCTCAA
TGTGTCCAAGAATCACAGTCAGAG
>1740
TAAGTGTCCCTGTCTCCATTCCTCCTCTTGCCACCTGTTGAGGAAGGCACTCTCCACCT
GCTTTGCAGGCGTGGGCTCTCAATGTGTCCAAGAATCACAGTCAGAGCTGAAGGGCCC
TTGGAATCATTTATTGCAGCATG
>1741
CTCCATTCCTCCTCTTGCCACCTGTTGAGGAAGGCACTCTCCACCTGCTTTGCAGGCGT
GGGCTCTCAATGTGTCCAAGAATCACAGTCAGAGCTGAAGGGCCCTTGGAATCATTTA
TTGCAGCATGCTCATTTGGGAGG
>1742
GCCACCTGTTGAGGAAGGCACTCTCCACCTGCTTTGCAGGCGTGGGCTCTCAATGTGT
CCAAGAATCACAGTCAGAGCTGAAGGGCCCTTGGAATCATTTATTGCAGCATGCTCATT
TGGGAGGAGTAAGTGGAGGTCCA
>1743
TGTCCAAGAATCACAGTCAGAGCTGAAGGGCCCTTGGAATCATTTATTGCAGCATGCTC
ATTTGGGAGGAGTAAGTGGAGGTCCAGAGAGGTTCTGTGATTTTCCCTAGGTCACACA
GCAAGCTAGAGGCAAAACCAGGA
>1744
CAAGAATCACAGTCAGAGCTGAAGGGCCCTTGGAATCATTTATTGCAGCATGCTCATTT
GGGAGGAGTAAGTGGAGGTCCAGAGAGGTTCTGTGATTTTCCCTAGGTCACACAGCA
AGCTAGAGGCAAAACCAGGACCCT
>1745
TCATTTATTGCAGCATGCTCATTTGGGAGGAGTAAGTGGAGGTCCAGAGAGGTTCTGT

GATTTTCCCTAGGTCACACAGCAAGCTAGAGGCAAAACCAGGACCCTCACTCCCAGCC
CACTTTAATTCTTCCTTCACAAGC
>1746
CAGCAAGCTAGAGGCAAAACCAGGACCCTCACTCCCAGCCCACTTTAATTCTTCCTTC
ACAAGCCTGCCTGTGACTGCCCAGGTCTGTTCTCTGCTTCTACCAAGGGGTAGGCAAG
ACTAGAATCTGACTTGAGAGCAGT
>1747
GGCAAAACCAGGACCCTCACTCCCAGCCCACTTTAATTCTTCCTTCACAAGCCTGCCT
GTGACTGCCCAGGTCTGTTCTCTGCTTCTACCAAGGGGTAGGCAAGACTAGAATCTGA
CTTGAGAGCAGTTTCAGATCTTTG
>1748
GCCCACTTTAATTCTTCCTTCACAAGCCTGCCTGTGACTGCCCAGGTCTGTTCTCTGCT
TCTACCAAGGGGTAGGCAAGACTAGAATCTGACTTGAGAGCAGTTTCAGATCTTTGTC
TGTGAGGGATGCTGGGGGGTTTC
>1749
CTGTGACTGCCCAGGTCTGTTCTCTGCTTCTACCAAGGGGTAGGCAAGACTAGAATCT
GACTTGAGAGCAGTTTCAGATCTTTGTCTGTGAGGGATGCTGGGGGGTTTCTGGCATG
GTGACTTGCCTCCTGGGACACTCA
>1750
GGTCTGTTCTCTGCTTCTACCAAGGGGTAGGCAAGACTAGAATCTGACTTGAGAGCAG
TTTCAGATCTTTGTCTGTGAGGGATGCTGGGGGGTTTCTGGCATGGTGACTTGCCTCCT
GGGACACTCATTAACCTCTCTGC
>1751
AAGGGGTAGGCAAGACTAGAATCTGACTTGAGAGCAGTTTCAGATCTTTGTCTGTGAG
GGATGCTGGGGGGTTTCTGGCATGGTGACTTGCCTCCTGGGACACTCATTAACCTCTCT
GCTGCTGAGCCTGAGCAGACTAA
>1752
AGACTAGAATCTGACTTGAGAGCAGTTTCAGATCTTTGTCTGTGAGGGATGCTGGGGG
GTTTCTGGCATGGTGACTTGCCTCCTGGGACACTCATTAACCTCTCTGCTGCTGAGCCT
GAGCAGACTAATGTACATCAACT
>1753
TTTCTGGCATGGTGACTTGCCTCCTGGGACACTCATTAACCTCTCTGCTGCTGAGCCTG
AGCAGACTAATGTACATCAACTCCTTAGGATTTACAGACAATGCCCTATGTTGGGGGTT
TTGAGACCCAAAGATTGCTAAG
>1754
ACCTCTCTGCTGCTGAGCCTGAGCAGACTAATGTACATCAACTCCTTAGGATTTACAGA
CAATGCCCTATGTTGGGGGTTTTGAGACCCAAAGATTGCTAAGACATGGGACTTTTCCT
TGAAGAGCTTATACTTTGAGTT
>1755
TGCTGCTGAGCCTGAGCAGACTAATGTACATCAACTCCTTAGGATTTACAGACAATGCC
CTATGTTGGGGGTTTTGAGACCCAAAGATTGCTAAGACATGGGACTTTTCCTTGAAGA
GCTTATACTTTGAGTTAAAATAT
>1756
TATGTTGGGGGTTTTGAGACCCAAAGATTGCTAAGACATGGGACTTTTCCTTGAAGAG
CTTATACTTTGAGTTAAAATATGTATGTTGATATCTATGACAGAGAATAGGAAATGGAAA
GACTATGATATATATATTCAGA

>1757
GTTTTGAGACCCAAAGATTGCTAAGACATGGGACTTTTCCTTGAAGAGCTTATACTTTG
AGTTAAAATATGTATGTTGATATCTATGACAGAGAATAGGAAATGGAAAGACTATGATAT
ATATATTCAGAGTTGAAATGA
>1758
TGAGACCCAAAGATTGCTAAGACATGGGACTTTTCCTTGAAGAGCTTATACTTTGAGTT
AAAATATGTATGTTGATATCTATGACAGAGAATAGGAAATGGAAAGACTATGATATATATA
TTCAGAGTTGAAATGACTTC
>1759
GTTAAAATATGTATGTTGATATCTATGACAGAGAATAGGAAATGGAAAGACTATGATATA
TATATTCAGAGTTGAAATGACTTCTGTCTGGAGTGATTCATTTTAAATTTTAAAACCTTG
TTGAGCACTTACTGTCTGCT
>1760
TTGATATCTATGACAGAGAATAGGAAATGGAAAGACTATGATATATATATTCAGAGTTGA
AATGACTTCTGTCTGGAGTGATTCATTTTAAATTTTAAAACCTTGTTGAGCACTTACTGT
CTGCTGGGCACCTGGGTAGG
>1761
ACTATGATATATATATTCAGAGTTGAAATGACTTCTGTCTGGAGTGATTCATTTTAAATTT
TAAAACCTTGTTGAGCACTTACTGTCTGCTGGGCACCTGGGTAGGCCCTGGGAATACT
GTGATGAGCAAGATAGACAAG
>1762
ATTCAGAGTTGAAATGACTTCTGTCTGGAGTGATTCATTTTAAATTTTAAAACCTTGTTG
AGCACTTACTGTCTGCTGGGCACCTGGGTAGGCCCTGGGAATACTGTGATGAGCAAGA
TAGACAAGACGTCTACCCTCCT
>1763
CTTCTGTCTGGAGTGATTCATTTTAAATTTTAAAACCTTGTTGAGCACTTACTGTCTGCT
GGGCACCTGGGTAGGCCCTGGGAATACTGTGATGAGCAAGATAGACAAGACGTCTACC
CTCCTGGGGCTTTCCTAATGGG
>1764
CATTTTAAATTTTAAAACCTTGTTGAGCACTTACTGTCTGCTGGGCACCTGGGTAGGCC
CTGGGAATACTGTGATGAGCAAGATAGACAAGACGTCTACCCTCCTGGGGCTTTCCTAA
TGGGGAAGAAAGATCCTTACTT
>1765
TGAGCACTTACTGTCTGCTGGGCACCTGGGTAGGCCCTGGGAATACTGTGATGAGCAA
GATAGACAAGACGTCTACCCTCCTGGGGCTTTCCTAATGGGGAAGAAAGATCCTTACTT
AGCTAGCAGTCCTGTCCGTAACT
>1766
CAAGATAGACAAGACGTCTACCCTCCTGGGGCTTTCCTAATGGGGAAGAAAGATCCTT
ACTTAGCTAGCAGTCCTGTCCGTAACTGTGTAAGGGTGATTTTGATACATGTTGGGAGC
CTGGATGAAGCAGCACATGAGTA
>1767
TAGACAAGACGTCTACCCTCCTGGGGCTTTCCTAATGGGGAAGAAAGATCCTTACTTAG
CTAGCAGTCCTGTCCGTAACTGTGTAAGGGTGATTTTGATACATGTTGGGAGCCTGGAT
GAAGCAGCACATGAGTAGGATA
>1768
GTCTACCCTCCTGGGGCTTTCCTAATGGGGAAGAAAGATCCTTACTTAGCTAGCAGTCC

TGTCCGTAACTGTGTAAGGGTGATTTTGATACATGTTGGGAGCCTGGATGAAGCAGCAC
ATGAGTAGGATATTAAAGGATA
>1769
AATGGGGAAGAAAGATCCTTACTTAGCTAGCAGTCCTGTCCGTAACTGTGTAAGGGTG
ATTTTGATACATGTTGGGAGCCTGGATGAAGCAGCACATGAGTAGGATATTAAAGGATA
AGTAGGTTTTGAAGACGGGGACA
>1770
GCAGTCCTGTCCGTAACTGTGTAAGGGTGATTTTGATACATGTTGGGAGCCTGGATGAA
GCAGCACATGAGTAGGATATTAAAGGATAAGTAGGTTTTGAAGACGGGGACAGAAAGA
AGGAAAATTGTGGTCAGGGCCAG
>1771
TGTAAGGGTGATTTTGATACATGTTGGGAGCCTGGATGAAGCAGCACATGAGTAGGATA
TTAAAGGATAAGTAGGTTTTGAAGACGGGGACAGAAAGAAGGAAAATTGTGGTCAGG
GCCAGGGGAGAAAACAAGGAGTAA
>1772
GAAGCAGCACATGAGTAGGATATTAAAGGATAAGTAGGTTTTGAAGACGGGGACAGAA
AGAAGGAAAATTGTGGTCAGGGCCAGGGGAGAAAACAAGGAGTAACTTTCTTTGGCA
GGAATGAGGGTGATTCATGCTGGAA
>1773
GCAGCACATGAGTAGGATATTAAAGGATAAGTAGGTTTTGAAGACGGGGACAGAAAGA
AGGAAAATTGTGGTCAGGGCCAGGGGAGAAAACAAGGAGTAACTTTCTTTGGCAGGA
ATGAGGGTGATTCATGCTGGAATGG
>1774
ATAAGTAGGTTTTGAAGACGGGGACAGAAAGAAGGAAAATTGTGGTCAGGGCCAGGG
GAGAAAACAAGGAGTAACTTTCTTTGGCAGGAATGAGGGTGATTCATGCTGGAATGGT
AGGCTGGGGTCAGACTGTGGTGCTA
>1775
AGAAAGAAGGAAAATTGTGGTCAGGGCCAGGGGAGAAAACAAGGAGTAACTTTCTTT
GGCAGGAATGAGGGTGATTCATGCTGGAATGGTAGGCTGGGGTCAGACTGTGGTGCTA
TAAATGCGGGGAGAGAGATTAGAGA
>1776
GGTCAGGGCCAGGGGAGAAAACAAGGAGTAACTTTCTTTGGCAGGAATGAGGGTGAT
TCATGCTGGAATGGTAGGCTGGGGTCAGACTGTGGTGCTATAAATGCGGGGAGAGAGA
TTAGAGAAGGCAGCATGAAGAATGG
>1777
AGGGGAGAAAACAAGGAGTAACTTTCTTTGGCAGGAATGAGGGTGATTCATGCTGGA
ATGGTAGGCTGGGGTCAGACTGTGGTGCTATAAATGCGGGGAGAGAGATTAGAGAAGG
CAGCATGAAGAATGGAGAGAAAATG
>1778
TCAGACTGTGGTGCTATAAATGCGGGGAGAGAGATTAGAGAAGGCAGCATGAAGAATG
GAGAGAAAATGAGTTTGAAGTTGAGCAGACCCGGGTTCAGATTTTCATGCTGCCACTT
GCAAACTGTGTGATGTTGGCCATC
>1779
GTGGTGCTATAAATGCGGGGAGAGAGATTAGAGAAGGCAGCATGAAGAATGGAGAGA
AAATGAGTTTGAAGTTGAGCAGACCCGGGTTCAGATTTTCATGCTGCCACTTGCAAAC
TGTGTGATGTTGGCCATCAAGGACT

>1780
CGGGGAGAGAGATTAGAGAAGGCAGCATGAAGAATGGAGAGAAAATGAGTTTGAAGT
TGAGCAGACCCGGGTTCAGATTTTCATGCTGCCACTTGCAAACTGTGTGATGTTGGCCA
TCAAGGACTGTGAACTGGCTGGGT
>1781
GAATGGAGAGAAAATGAGTTTGAAGTTGAGCAGACCCGGGTTCAGATTTTCATGCTGC
CACTTGCAAACTGTGTGATGTTGGCCATCAAGGACTGTGAACTGGCTGGGTGCAGTGG
CTCACACCTGTCATCCCAGTGCTT
>1782
GAGAAAATGAGTTTGAAGTTGAGCAGACCCGGGTTCAGATTTTCATGCTGCCACTTGC
AAACTGTGTGATGTTGGCCATCAAGGACTGTGAACTGGCTGGGTGCAGTGGCTCACAC
CTGTCATCCCAGTGCTTTGGGAGG
>1783
GTTGAGCAGACCCGGGTTCAGATTTTCATGCTGCCACTTGCAAACTGTGTGATGTTGG
CCATCAAGGACTGTGAACTGGCTGGGTGCAGTGGCTCACACCTGTCATCCCAGTGCTT
TGGGAGGCCAAGGCAGGAGGATTG
>1784
GGGTTCAGATTTTCATGCTGCCACTTGCAAACTGTGTGATGTTGGCCATCAAGGACTGT
GAACTGGCTGGGTGCAGTGGCTCACACCTGTCATCCCAGTGCTTTGGGAGGCCAAGG
CAGGAGGATTGCGTGAGGCCAGAA
>1785
CAGATTTTCATGCTGCCACTTGCAAACTGTGTGATGTTGGCCATCAAGGACTGTGAACT
GGCTGGGTGCAGTGGCTCACACCTGTCATCCCAGTGCTTTGGGAGGCCAAGGCAGGA
GGATTGCGTGAGGCCAGAAGTTTG
>1786
CAAACTGTGTGATGTTGGCCATCAAGGACTGTGAACTGGCTGGGTGCAGTGGCTCACA
CCTGTCATCCCAGTGCTTTGGGAGGCCAAGGCAGGAGGATTGCGTGAGGCCAGAAGT
TTGAGACTGGCTTGGGCAATATAGC
>1787
TGAACTGGCTGGGTGCAGTGGCTCACACCTGTCATCCCAGTGCTTTGGGAGGCCAAGG
CAGGAGGATTGCGTGAGGCCAGAAGTTTGAGACTGGCTTGGGCAATATAGCGAGACG
ATCTTTCCAAAAAAAAAAAAAAAAA
>1788
GTGCAGTGGCTCACACCTGTCATCCCAGTGCTTTGGGAGGCCAAGGCAGGAGGATTGC
GTGAGGCCAGAAGTTTGAGACTGGCTTGGGCAATATAGCGAGACGATCTTTCCAAAAA
AAAAAAAAAAAAAATTAAAAATGC
>1789
GAGACTGGCTTGGGCAATATAGCGAGACGATCTTTCCAAAAAAAAAAAAAAAAAAATT
AAAAATGCAGCTGTGCATGCCCATAATCCCAGCTTCTCGGGTGGCTGAAGAGGGAGGA
TGGCTTGAACTTATAAGTTCAAGG
>1790
GATCTTTCCAAAAAAAAAAAAAAAAAAAATTAAAAATGCAGCTGTGCATGCCCATAATC
CCAGCTTCTCGGGTGGCTGAAGAGGGAGGATGGCTTGAACTTATAAGTTCAAGGCTGT
GGTGAGCTATGATCACGCCACTGC
>1791
ATGCAGCTGTGCATGCCCATAATCCCAGCTTCTCGGGTGGCTGAAGAGGGAGGATGGC

TTGAACTTATAAGTTCAAGGCTGTGGTGAGCTATGATCACGCCACTGCACTCCAGCCTG
GGCAACAAAGTGAGACCCTATCT
>1792
GCATGCCCATAATCCCAGCTTCTCGGGTGGCTGAAGAGGGAGGATGGCTTGAACTTATA
AGTTCAAGGCTGTGGTGAGCTATGATCACGCCACTGCACTCCAGCCTGGGCAACAAAG
TGAGACCCTATCTCTATTTCAAA
>1793
GCTTGAACTTATAAGTTCAAGGCTGTGGTGAGCTATGATCACGCCACTGCACTCCAGCC
TGGGCAACAAAGTGAGACCCTATCTCTATTTCAAAACAACAACAACAACAACAGCAA
CACACACCATGAACTGTAGCTTCT
>1794
GGCAACAAAGTGAGACCCTATCTCTATTTCAAAACAACAACAACAACAACAGCAACA
CACACCATGAACTGTAGCTTCTTCATTCCTACAGTGCAGTTATTGTGTGGGATAGCCTG
AGGATTTGGGGTCGTGTACTTAAC
>1795
TCTCTATTTCAAAACAACAACAACAACAACAGCAACACACACCATGAACTGTAGCTTC
TTCATTCCTACAGTGCAGTTATTGTGTGGGATAGCCTGAGGATTTGGGGTCGTGTACTTA
ACACACTCTGCACAGTGCCTGT
>1796
ATTTCAAAACAACAACAACAACAACAGCAACACACACCATGAACTGTAGCTTCTTCAT
TCCTACAGTGCAGTTATTGTGTGGGATAGCCTGAGGATTTGGGGTCGTGTACTTAACAC
ACTCTGCACAGTGCCTGTAACAG
>1797
AAACAACAACAACAACAACAGCAACACACACCATGAACTGTAGCTTCTTCATTCCTAC
AGTGCAGTTATTGTGTGGGATAGCCTGAGGATTTGGGGTCGTGTACTTAACACACTCTG
CACAGTGCCTGTAACAGTGTAGT
>1798
ACACACCATGAACTGTAGCTTCTTCATTCCTACAGTGCAGTTATTGTGTGGGATAGCCT
GAGGATTTGGGGTCGTGTACTTAACACACTCTGCACAGTGCCTGTAACAGTGTAGTTG
CTCAATAAATATAACTGCTGTTT
>1799
TTCCTACAGTGCAGTTATTGTGTGGGATAGCCTGAGGATTTGGGGTCGTGTACTTAACA
CACTCTGCACAGTGCCTGTAACAGTGTAGTTGCTCAATAAATATAACTGCTGTTTGTTTT
AACTGCTGTTTCTTTTTGGAC
>1800
CAGTGCAGTTATTGTGTGGGATAGCCTGAGGATTTGGGGTCGTGTACTTAACACACTCT
GCACAGTGCCTGTAACAGTGTAGTTGCTCAATAAATATAACTGCTGTTTGTTTTAACTG
CTGTTTCTTTTTGGACTTTATC
>1801
AGTTATTGTGTGGGATAGCCTGAGGATTTGGGGTCGTGTACTTAACACACTCTGCACAG
TGCCTGTAACAGTGTAGTTGCTCAATAAATATAACTGCTGTTTGTTTTAACTGCTGTTTC
TTTTTGGACTTTATCCCGGGA
>1802
TGGGGTCGTGTACTTAACACACTCTGCACAGTGCCTGTAACAGTGTAGTTGCTCAATAA
ATATAACTGCTGTTTGTTTTAACTGCTGTTTCTTTTTGGACTTTATCCCGGGAGAGTTGG
AGCCTTGGAAGGCATTTGAAA

>1803
GTAGTTGCTCAATAAATATAACTGCTGTTTGTTTTAACTGCTGTTTCTTTTTGGACTTTAT
CCCGGGAGAGTTGGAGCCTTGGAAGGCATTTGAAAAGGAGAGTGACGTGACTGGAGT
GCTCCATACTGATGTGACATCC
>1804
TTTAACTGCTGTTTCTTTTTGGACTTTATCCCGGGAGAGTTGGAGCCTTGGAAGGCATT
TGAAAAGGAGAGTGACGTGACTGGAGTGCTCCATACTGATGTGACATCCACTAGGCAT
GTGTCTGACTGCCCCCTGCTCCT
>1805
CTTTTTGGACTTTATCCCGGGAGAGTTGGAGCCTTGGAAGGCATTTGAAAAGGAGAGT
GACGTGACTGGAGTGCTCCATACTGATGTGACATCCACTAGGCATGTGTCTGACTGCCC
CCTGCTCCTTCCCTGCCTTCACG
>1806
CCCGGGAGAGTTGGAGCCTTGGAAGGCATTTGAAAAGGAGAGTGACGTGACTGGAGT
GCTCCATACTGATGTGACATCCACTAGGCATGTGTCTGACTGCCCCCTGCTCCTTCCCT
GCCTTCACGAGCCTGTCTGAAAAG
>1807
TTGGAAGGCATTTGAAAAGGAGAGTGACGTGACTGGAGTGCTCCATACTGATGTGACA
TCCACTAGGCATGTGTCTGACTGCCCCCTGCTCCTTCCCTGCCTTCACGAGCCTGTCTG
AAAAGGAGTTTAGTGCGTGATCA
>1808
CCATACTGATGTGACATCCACTAGGCATGTGTCTGACTGCCCCCTGCTCCTTCCCTGCC
TTCACGAGCCTGTCTGAAAAGGAGTTTAGTGCGTGATCATCAGCTCCTAAGGAGACCT
CTCCCCTGTATCTTGTACCTGGC
>1809
GACATCCACTAGGCATGTGTCTGACTGCCCCCTGCTCCTTCCCTGCCTTCACGAGCCTG
TCTGAAAAGGAGTTTAGTGCGTGATCATCAGCTCCTAAGGAGACCTCTCCCCTGTATCT
TGTACCTGGCACTCTACCAAGA
>1810
CTGCCTTCACGAGCCTGTCTGAAAAGGAGTTTAGTGCGTGATCATCAGCTCCTAAGGA
GACCTCTCCCCTGTATCTTGTACCTGGCACTCTACCAAGATTTGATGAAAGGACATGAA
ATGGACCCTTGCCACCTCTCGGT
>1811
CACGAGCCTGTCTGAAAAGGAGTTTAGTGCGTGATCATCAGCTCCTAAGGAGACCTCT
CCCCTGTATCTTGTACCTGGCACTCTACCAAGATTTGATGAAAGGACATGAAATGGACC
CTTGCCACCTCTCGGTGGGCGGG
>1812
CTGTATCTTGTACCTGGCACTCTACCAAGATTTGATGAAAGGACATGAAATGGACCCTT
GCCACCTCTCGGTGGGCGGGGAAGCTGGGAGAGGGGCCTGGGGAGAGGGGGTCTTC
AGCTGTCAGGATCAAAGGAAGTGAC
>1813
AGGACATGAAATGGACCCTTGCCACCTCTCGGTGGGCGGGGAAGCTGGGAGAGGGGC
CTGGGGAGAGGGGGTCTTCAGCTGTCAGGATCAAAGGAAGTGACACTCTGAAGCTGG
TCTGCCGGACTGTTACCCGTCTCTCT
>1814
ATGGACCCTTGCCACCTCTCGGTGGGCGGGGAAGCTGGGAGAGGGGCCTGGGGAGAG

GGGGTCTTCAGCTGTCAGGATCAAAGGAAGTGACACTCTGAAGCTGGTCTGCCGGAC
TGTTACCCGTCTCTCTAACTCTCTTT
>1815
TCTCGGTGGGCGGGGAAGCTGGGAGAGGGGCCTGGGGAGAGGGGGTCTTCAGCTGTC
AGGATCAAAGGAAGTGACACTCTGAAGCTGGTCTGCCGGACTGTTACCCGTCTCTCTA
ACTCTCTTTTAATAGAAACCCAGTC
>1816
GAGAGGGGGTCTTCAGCTGTCAGGATCAAAGGAAGTGACACTCTGAAGCTGGTCTGC
CGGACTGTTACCCGTCTCTCTAACTCTCTTTTAATAGAAACCCAGTCTCCCCTCTTGCCT
CCCTGATAGTGAACAGCGACGCT
>1817
GAAGTGACACTCTGAAGCTGGTCTGCCGGACTGTTACCCGTCTCTCTAACTCTCTTTTA
ATAGAAACCCAGTCTCCCCTCTTGCCTCCCTGATAGTGAACAGCGACGCTATCTCCCGC
GGGCCAGTCTCGGCTGTGGTCG
>1818
GCCGGACTGTTACCCGTCTCTCTAACTCTCTTTTAATAGAAACCCAGTCTCCCCTCTTG
CCTCCCTGATAGTGAACAGCGACGCTATCTCCCGCGGGCCAGTCTCGGCTGTGGTCGT
AGCACTACGCAATGTCTGCAAAC
>1819
TTTTAATAGAAACCCAGTCTCCCCTCTTGCCTCCCTGATAGTGAACAGCGACGCTATCT
CCCGCGGGCCAGTCTCGGCTGTGGTCGTAGCACTACGCAATGTCTGCAAACCGACAGT
GGCATGGGGAGATAAGGGCCCCA
>1820
AAACCCAGTCTCCCCTCTTGCCTCCCTGATAGTGAACAGCGACGCTATCTCCCGCGGG
CCAGTCTCGGCTGTGGTCGTAGCACTACGCAATGTCTGCAAACCGACAGTGGCATGGG
GAGATAAGGGCCCCAACGTTGTGT
>1821
CTCCCTGATAGTGAACAGCGACGCTATCTCCCGCGGGCCAGTCTCGGCTGTGGTCGTA
GCACTACGCAATGTCTGCAAACCGACAGTGGCATGGGGAGATAAGGGCCCCAACGTT
GTGTAACGAGGGGCATGGGCTGTTT
>1822
CAGCGACGCTATCTCCCGCGGGCCAGTCTCGGCTGTGGTCGTAGCACTACGCAATGTC
TGCAAACCGACAGTGGCATGGGGAGATAAGGGCCCCAACGTTGTGTAACGAGGGGCA
TGGGCTGTTTGCCCGAGTGGCTCCT
>1823
CTCGGCTGTGGTCGTAGCACTACGCAATGTCTGCAAACCGACAGTGGCATGGGGAGAT
AAGGGCCCCAACGTTGTGTAACGAGGGGCATGGGCTGTTTGCCCGAGTGGCTCCTGGC
CCCTGGGTAACTGCTAACCTCAAC
>1824
CAATGTCTGCAAACCGACAGTGGCATGGGGAGATAAGGGCCCCAACGTTGTGTAACG
AGGGGCATGGGCTGTTTGCCCGAGTGGCTCCTGGCCCCTGGGTAACTGCTAACCTCAA
CGGAGGAGCGGGCCGGCCTTGGAAA
>1825
AAACCGACAGTGGCATGGGGAGATAAGGGCCCCAACGTTGTGTAACGAGGGGCATGG
GCTGTTTGCCCGAGTGGCTCCTGGCCCCTGGGTAACTGCTAACCTCAACGGAGGAGCG
GGCCGGCCTTGGAAACGCCGTTCCA

>1826
GGAGATAAGGGCCCCAACGTTGTGTAACGAGGGGCATGGGCTGTTTGCCCGAGTGGCT
CCTGGCCCCTGGGTAACTGCTAACCTCAACGGAGGAGCGGGCCGGCCTTGGAAACGC
CGTTCCAGGGCCCCTCGCTGGTTCC
>1827
GCCCGAGTGGCTCCTGGCCCCTGGGTAACTGCTAACCTCAACGGAGGAGCGGGCCGG
CCTTGGAAACGCCGTTCCAGGGCCCCTCGCTGGTTCCACGGATGCCTTTTTGTGGTTG
GATAAAGGAAGCTCATGTAGGCCGC
>1828
CCTGGGTAACTGCTAACCTCAACGGAGGAGCGGGCCGGCCTTGGAAACGCCGTTCCA
GGGCCCCTCGCTGGTTCCACGGATGCCTTTTTGTGGTTGGATAAAGGAAGCTCATGTAG
GCCGCCCACAGCGCAGCATGGCCT
>1829
CGGAGGAGCGGGCCGGCCTTGGAAACGCCGTTCCAGGGCCCCTCGCTGGTTCCACGG
ATGCCTTTTTGTGGTTGGATAAAGGAAGCTCATGTAGGCCGCCCACAGCGCAGCATGG
CCTTGGGCTACGGGTGTTTGGTTGT
>1830
GGAAACGCCGTTCCAGGGCCCCTCGCTGGTTCCACGGATGCCTTTTTGTGGTTGGATA
AAGGAAGCTCATGTAGGCCGCCCACAGCGCAGCATGGCCTTGGGCTACGGGTGTTTGG
TTGTTTTGTTTTTCTCTTTTCTTT
>1831
TGCCTTTTTGTGGTTGGATAAAGGAAGCTCATGTAGGCCGCCCACAGCGCAGCATGGC
CTTGGGCTACGGGTGTTTGGTTGTTTTGTTTTTCTCTTTTCTTTGTCTTTTTTAAAAAAA
GAAGTTGAAAGGGGGATAGTCT
>1832
CCTTTTTGTGGTTGGATAAAGGAAGCTCATGTAGGCCGCCCACAGCGCAGCATGGCCT
TGGGCTACGGGTGTTTGGTTGTTTTGTTTTTCTCTTTTCTTTGTCTTTTTTAAAAAAAGA
AGTTGAAAGGGGGATAGTCTTG
>1833
TTGTGGTTGGATAAAGGAAGCTCATGTAGGCCGCCCACAGCGCAGCATGGCCTTGGGC
TACGGGTGTTTGGTTGTTTTGTTTTTCTCTTTTCTTTGTCTTTTTTAAAAAAAGAAGTTG
AAAGGGGGATAGTCTTGCTTGG
>1834
GGATAAAGGAAGCTCATGTAGGCCGCCCACAGCGCAGCATGGCCTTGGGCTACGGGTG
TTTGGTTGTTTTGTTTTTCTCTTTTCTTTGTCTTTTTTAAAAAAAGAAGTTGAAAGGGG
GATAGTCTTGCTTGGCACCTACC
>1835
GTAGGCCGCCCACAGCGCAGCATGGCCTTGGGCTACGGGTGTTTGGTTGTTTTGTTTTT
CTCTTTTCTTTGTCTTTTTTAAAAAAAGAAGTTGAAAGGGGGATAGTCTTGCTTGGCAC
CTACCACCTTTAACCCAAAACC
>1836
ACGGGTGTTTGGTTGTTTTGTTTTTCTCTTTTCTTTGTCTTTTTTAAAAAAAGAAGTTGA
AAGGGGGATAGTCTTGCTTGGCACCTACCACCTTTAACCCAAAACCAAATAAAACAAA
CAGTTCCTAAATATAAATTAAA
>1837
AAGAAGTTGAAAGGGGGATAGTCTTGCTTGGCACCTACCACCTTTAACCCAAAACCAA

ATAAAACAAACAGTTCCTAAATATAAATTAAACTTCAACCTGCAGTATAGACATCCTCCT
TCCCTGCCCGAAACCCACTGGC
>1838
ACCTACCACCTTTAACCCAAAACCAAATAAAACAAACAGTTCCTAAATATAAATTAAAC
TTCAACCTGCAGTATAGACATCCTCCTTCCCTGCCCGAAACCCACTGGCCACTGGAGG
CTAGCTTTAAGAAATTGGTTTTG
>1839
CCTGCAGTATAGACATCCTCCTTCCCTGCCCGAAACCCACTGGCCACTGGAGGCTAGCT
TTAAGAAATTGGTTTTGGAGACTCTGGCTTGAAAAGGAGCCATGCCCACTTGTCTCAC
GTCCCAGTCGCTGGCTCCAGGGG
>1840
TGGCCACTGGAGGCTAGCTTTAAGAAATTGGTTTTGGAGACTCTGGCTTGAAAAGGAG
CCATGCCCACTTGTCTCACGTCCCAGTCGCTGGCTCCAGGGGGCCGTGGTAGAACATAT
ATTGTACTTCAGCTGCCTTTCCC
>1841
TGGAGGCTAGCTTTAAGAAATTGGTTTTGGAGACTCTGGCTTGAAAAGGAGCCATGCC
CACTTGTCTCACGTCCCAGTCGCTGGCTCCAGGGGGCCGTGGTAGAACATATATTGTAC
TTCAGCTGCCTTTCCCCTCCAAG
>1842
TTGGAGACTCTGGCTTGAAAAGGAGCCATGCCCACTTGTCTCACGTCCCAGTCGCTGG
CTCCAGGGGGCCGTGGTAGAACATATATTGTACTTCAGCTGCCTTTCCCCTCCAAGCTT
CCTCAGCCACCCACAGCCCTTTC
>1843
AAAAGGAGCCATGCCCACTTGTCTCACGTCCCAGTCGCTGGCTCCAGGGGGCCGTGGT
AGAACATATATTGTACTTCAGCTGCCTTTCCCCTCCAAGCTTCCTCAGCCACCCACAGC
CCTTTCAGATGAGCTGAGCCCTA
>1844
TCAGCTGCCTTTCCCCTCCAAGCTTCCTCAGCCACCCACAGCCCTTTCAGATGAGCTG
AGCCCTAGCACTGTGCCCTCTCCCCTCTGCCCTCTTAGGGTGATAGAATCCACTACCCT
GACATCTGGGGTTGCCCTGTTCT
>1845
TCAGCCACCCACAGCCCTTTCAGATGAGCTGAGCCCTAGCACTGTGCCCTCTCCCCTCT
GCCCTCTTAGGGTGATAGAATCCACTACCCTGACATCTGGGGTTGCCCTGTTCTGCTGT
GGTGGGAGATGGGCTGGAAGGG
>1846
GAGCCCTAGCACTGTGCCCTCTCCCCTCTGCCCTCTTAGGGTGATAGAATCCACTACCC
TGACATCTGGGGTTGCCCTGTTCTGCTGTGGTGGGAGATGGGCTGGAAGGGACTTCGG
AGGCTCTCCCGTGTTCTGTACCA
>1847
GCACTGTGCCCTCTCCCCTCTGCCCTCTTAGGGTGATAGAATCCACTACCCTGACATCT
GGGGTTGCCCTGTTCTGCTGTGGTGGGAGATGGGCTGGAAGGGACTTCGGAGGCTCTC
CCGTGTTCTGTACCACCCAGCTT
>1848
CCTGACATCTGGGGTTGCCCTGTTCTGCTGTGGTGGGAGATGGGCTGGAAGGGACTTC
GGAGGCTCTCCCGTGTTCTGTACCACCCAGCTTCCCCTTGGATCCCTCCACCCCCGCAG
CCAGGAGAGGGAAGTAACTGTTC

>1849
TGACATCTGGGGTTGCCCTGTTCTGCTGTGGTGGGAGATGGGCTGGAAGGGACTTCGG
AGGCTCTCCCGTGTTCTGTACCACCCAGCTTCCCCTTGGATCCCTCCACCCCCGCAGCC
AGGAGAGGGAAGTAACTGTTCTT
>1850
AGGCTCTCCCGTGTTCTGTACCACCCAGCTTCCCCTTGGATCCCTCCACCCCCGCAGCC
AGGAGAGGGAAGTAACTGTTCTTTGAGCAGGACATTTCATTTTCAAAGTATTCTTGATG
CCGCCCCCCCGCCCCACCGCCA
>1851
TCCCGTGTTCTGTACCACCCAGCTTCCCCTTGGATCCCTCCACCCCCGCAGCCAGGAG
AGGGAAGTAACTGTTCTTTGAGCAGGACATTTCATTTTCAAAGTATTCTTGATGCCGCC
CCCCCGCCCCACCGCCAAGAATT
>1852
TGGATCCCTCCACCCCCGCAGCCAGGAGAGGGAAGTAACTGTTCTTTGAGCAGGACAT
TTCATTTTCAAAGTATTCTTGATGCCGCCCCCCCGCCCCACCGCCAAGAATTCTGCTCT
GTGGGTGATACTTATAAGGTAAC
>1853
GAGCAGGACATTTCATTTTCAAAGTATTCTTGATGCCGCCCCCCCGCCCCACCGCCAAG
AATTCTGCTCTGTGGGTGATACTTATAAGGTAACTATATGGCTTCCTTATTTAGGCAGCA
GTTTCTCTTCTATGATTTTAG
>1854
AGGACATTTCATTTTCAAAGTATTCTTGATGCCGCCCCCCCGCCCCACCGCCAAGAATT
CTGCTCTGTGGGTGATACTTATAAGGTAACTATATGGCTTCCTTATTTAGGCAGCAGTTT
CTCTTCTATGATTTTAGTTCC
>1855
CACCGCCAAGAATTCTGCTCTGTGGGTGATACTTATAAGGTAACTATATGGCTTCCTTAT
TTAGGCAGCAGTTTCTCTTCTATGATTTTAGTTCCTCTCATGATTCTTATAACCCTCTCTC
AGATACTTCCAGGATGATA
>1856
TGTGGGTGATACTTATAAGGTAACTATATGGCTTCCTTATTTAGGCAGCAGTTTCTCTTCT
ATGATTTTAGTTCCTCTCATGATTCTTATAACCCTCTCTCAGATACTTCCAGGATGATAAA
TCTTCTATCACTGTTTCA
>1857
ATTTTAGTTCCTCTCATGATTCTTATAACCCTCTCTCAGATACTTCCAGGATGATAAATCT
TCTATCACTGTTTCATCTGATCACATCAGGGTGATTATATTTCGTGACATAACCAGGTGG
CTTTAATCCGTTGGTGGAA
>1858
CTTATAACCCTCTCTCAGATACTTCCAGGATGATAAATCTTCTATCACTGTTTCATCTGAT
CACATCAGGGTGATTATATTTCGTGACATAACCAGGTGGCTTTAATCCGTTGGTGGAAG
AACCAGGCTCTCACCTCCAT
>1859
CTCAGATACTTCCAGGATGATAAATCTTCTATCACTGTTTCATCTGATCACATCAGGGTG
ATTATATTTCGTGACATAACCAGGTGGCTTTAATCCGTTGGTGGAAGAACCAGGCTCTC
ACCTCCATTGTTTAAGATGTT
>1860
AGGATGATAAATCTTCTATCACTGTTTCATCTGATCACATCAGGGTGATTATATTTCGTGA

CATAACCAGGTGGCTTTAATCCGTTGGTGGAAGAACCAGGCTCTCACCTCCATTGTTTA
AGATGTTATGTTTCTACTAA
>1861
TGATTATATTTCGTGACATAACCAGGTGGCTTTAATCCGTTGGTGGAAGAACCAGGCTC
TCACCTCCATTGTTTAAGATGTTATGTTTCTACTAATCTGTCCACTGAGTGTCTCCATCCT
ATTGTTTCCATTGCATGCTC
>1862
TTCGTGACATAACCAGGTGGCTTTAATCCGTTGGTGGAAGAACCAGGCTCTCACCTCC
ATTGTTTAAGATGTTATGTTTCTACTAATCTGTCCACTGAGTGTCTCCATCCTATTGTTTC
CATTGCATGCTCTCAGGTTAT
>1863
CGTTGGTGGAAGAACCAGGCTCTCACCTCCATTGTTTAAGATGTTATGTTTCTACTAATC
TGTCCACTGAGTGTCTCCATCCTATTGTTTCCATTGCATGCTCTCAGGTTATTTCCTTTGT
TCTTGGCTGTCTGATTGCT
>1864
AGGCTCTCACCTCCATTGTTTAAGATGTTATGTTTCTACTAATCTGTCCACTGAGTGTCT
CCATCCTATTGTTTCCATTGCATGCTCTCAGGTTATTTCCTTTGTTCTTGGCTGTCTGATT
GCTCCCAGAGGCTGCAGTT
>1865
AAGATGTTATGTTTCTACTAATCTGTCCACTGAGTGTCTCCATCCTATTGTTTCCATTGCA
TGCTCTCAGGTTATTTCCTTTGTTCTTGGCTGTCTGATTGCTCCCAGAGGCTGCAGTTAT
TTTATTTTATTTATTTCAT
>1866
CTGTCCACTGAGTGTCTCCATCCTATTGTTTCCATTGCATGCTCTCAGGTTATTTCCTTTG
TTCTTGGCTGTCTGATTGCTCCCAGAGGCTGCAGTTATTTTATTTTATTTATTTCATTTTT
TGGTGGGAGTAGGGGATT
>1867
ATTGTTTCCATTGCATGCTCTCAGGTTATTTCCTTTGTTCTTGGCTGTCTGATTGCTCCCA
GAGGCTGCAGTTATTTTATTTTATTTATTTCATTTTTTGGTGGGAGTAGGGGATTGATCTC
TCTCTTGCCTTTAGCTTG
>1868
TCCTTTGTTCTTGGCTGTCTGATTGCTCCCAGAGGCTGCAGTTATTTTATTTTATTTATTT
CATTTTTTGGTGGGAGTAGGGGATTGATCTCTCTCTTGCCTTTAGCTTGAATGGGTGGT
CATTTCCAGGCTTACACATT
>1869
TTTATTTTATTTATTTCATTTTTTGGTGGGAGTAGGGGATTGATCTCTCTCTTGCCTTTAG
CTTGAATGGGTGGTCATTTCCAGGCTTACACATTGCTCCATGCCCACTGTAGGGGTCTG
GCTTAGAGATGGTCGGGGGA
>1870
ATTTTATTTATTTCATTTTTTGGTGGGAGTAGGGGATTGATCTCTCTCTTGCCTTTAGCTT
GAATGGGTGGTCATTTCCAGGCTTACACATTGCTCCATGCCCACTGTAGGGGTCTGGCT
TAGAGATGGTCGGGGGAGAG
>1871
TTGATCTCTCTCTTGCCTTTAGCTTGAATGGGTGGTCATTTCCAGGCTTACACATTGCTC
CATGCCCACTGTAGGGGTCTGGCTTAGAGATGGTCGGGGGAGAGAAGGTGGTGATTTA
AAAGATTTGGAGGCGGTGGCTT

>1872
TCTCTCTTGCCTTTAGCTTGAATGGGTGGTCATTTCCAGGCTTACACATTGCTCCATGCC
CACTGTAGGGGTCTGGCTTAGAGATGGTCGGGGGAGAGAAGGTGGTGATTTAAAAGAT
TTGGAGGCGGTGGCTTACATTG
>1873
CTTGAATGGGTGGTCATTTCCAGGCTTACACATTGCTCCATGCCCACTGTAGGGGTCTG
GCTTAGAGATGGTCGGGGGAGAGAAGGTGGTGATTTAAAAGATTTGGAGGCGGTGGC
TTACATTGATTTTAGCTTATCTAA
>1874
GCCCACTGTAGGGGTCTGGCTTAGAGATGGTCGGGGGAGAGAAGGTGGTGATTTAAA
AGATTTGGAGGCGGTGGCTTACATTGATTTTAGCTTATCTAACCCTTTAGCTAGAGGCTG
CTCTTCCCTCAGAGTGTCCAACT
>1875
TTTGGAGGCGGTGGCTTACATTGATTTTAGCTTATCTAACCCTTTAGCTAGAGGCTGCTC
TTCCCTCAGAGTGTCCAACTAGCTGTAACTTAAAGCAACAGAGAAATGTATATCATCCC
TTTTCTTGCATTAATTAGGGC
>1876
TGGAGGCGGTGGCTTACATTGATTTTAGCTTATCTAACCCTTTAGCTAGAGGCTGCTCTT
CCCTCAGAGTGTCCAACTAGCTGTAACTTAAAGCAACAGAGAAATGTATATCATCCCTT
TTCTTGCATTAATTAGGGCTG
>1877
AACCCTTTAGCTAGAGGCTGCTCTTCCCTCAGAGTGTCCAACTAGCTGTAACTTAAAGC
AACAGAGAAATGTATATCATCCCTTTTCTTGCATTAATTAGGGCTGAGAAATTCATTATTT
AGAGGGATAAGAGCTCCGGA
>1878
TCCCTTTTCTTGCATTAATTAGGGCTGAGAAATTCATTATTTAGAGGGATAAGAGCTCCG
GAGGAGGAGGGTTTTTTGAACTAATGTAGAGATCTGTGGCCAGCTGTGTGATTCTCAG
GTAGCAGGTGAGGGGGTGGGGG
>1879
TAATTAGGGCTGAGAAATTCATTATTTAGAGGGATAAGAGCTCCGGAGGAGGAGGGTT
TTTTGAACTAATGTAGAGATCTGTGGCCAGCTGTGTGATTCTCAGGTAGCAGGTGAGG
GGGTGGGGGTTGATGAGAGGGGTG
>1880
ATTATTTAGAGGGATAAGAGCTCCGGAGGAGGAGGGTTTTTTGAACTAATGTAGAGATC
TGTGGCCAGCTGTGTGATTCTCAGGTAGCAGGTGAGGGGGTGGGGGTTGATGAGAGG
GGTGAGAAGGAGCAGGTGCCAGGG
>1881
CTCCGGAGGAGGAGGGTTTTTTGAACTAATGTAGAGATCTGTGGCCAGCTGTGTGATT
CTCAGGTAGCAGGTGAGGGGGTGGGGGTTGATGAGAGGGGTGAGAAGGAGCAGGTG
CCAGGGTCAGGCAGGGGAGGGAGCCC
>1882
GAGGAGGGTTTTTTGAACTAATGTAGAGATCTGTGGCCAGCTGTGTGATTCTCAGGTAG
CAGGTGAGGGGGTGGGGGTTGATGAGAGGGGTGAGAAGGAGCAGGTGCCAGGGTCA
GGCAGGGGAGGGAGCCCCCACTCCG
>1883
AATGTAGAGATCTGTGGCCAGCTGTGTGATTCTCAGGTAGCAGGTGAGGGGGTGGGGG

TTGATGAGAGGGGTGAGAAGGAGCAGGTGCCAGGGTCAGGCAGGGGAGGGAGCCCC
CACTCCGTGCATCATCCCAGAGCCCT
>1884
GGTGGGGGTTGATGAGAGGGGTGAGAAGGAGCAGGTGCCAGGGTCAGGCAGGGGAG
GGAGCCCCCACTCCGTGCATCATCCCAGAGCCCTGTTCCTCCAAGCCCAGATTAGACA
CCCTTCTCCACGTGGGGCCTCAAGTT
>1885
GTGAGAAGGAGCAGGTGCCAGGGTCAGGCAGGGGAGGGAGCCCCCACTCCGTGCAT
CATCCCAGAGCCCTGTTCCTCCAAGCCCAGATTAGACACCCTTCTCCACGTGGGGCCT
CAAGTTAGAAATCCTGGGAGGAGGCC
>1886
GGGAGCCCCCACTCCGTGCATCATCCCAGAGCCCTGTTCCTCCAAGCCCAGATTAGAC
ACCCTTCTCCACGTGGGGCCTCAAGTTAGAAATCCTGGGAGGAGGCCTTATTGCTCAG
GATCATCTGGGAATGTTGCTTTCT
>1887
TCCGTGCATCATCCCAGAGCCCTGTTCCTCCAAGCCCAGATTAGACACCCTTCTCCACG
TGGGGCCTCAAGTTAGAAATCCTGGGAGGAGGCCTTATTGCTCAGGATCATCTGGGAA
TGTTGCTTTCTGGGTTTAAAATA
>1888
CCTTCTCCACGTGGGGCCTCAAGTTAGAAATCCTGGGAGGAGGCCTTATTGCTCAGGA
TCATCTGGGAATGTTGCTTTCTGGGTTTAAAATACAGTCAATGTTTAGATGAGCTGCTGT
TCAGGCACATGGTCTGGTTAGC
>1889
GGGGCCTCAAGTTAGAAATCCTGGGAGGAGGCCTTATTGCTCAGGATCATCTGGGAAT
GTTGCTTTCTGGGTTTAAAATACAGTCAATGTTTAGATGAGCTGCTGTTCAGGCACATG
GTCTGGTTAGCAAAGGGTTAAAT
>1890
TATTGCTCAGGATCATCTGGGAATGTTGCTTTCTGGGTTTAAAATACAGTCAATGTTTAG
ATGAGCTGCTGTTCAGGCACATGGTCTGGTTAGCAAAGGGTTAAATCCTGCATCCCGTT
TTTAGTCATCAAGCTAGCTCA
>1891
CATCTGGGAATGTTGCTTTCTGGGTTTAAAATACAGTCAATGTTTAGATGAGCTGCTGTT
CAGGCACATGGTCTGGTTAGCAAAGGGTTAAATCCTGCATCCCGTTTTTAGTCATCAAG
CTAGCTCAGATCTGCCCACCT
>1892
TTTCTGGGTTTAAAATACAGTCAATGTTTAGATGAGCTGCTGTTCAGGCACATGGTCTG
GTTAGCAAAGGGTTAAATCCTGCATCCCGTTTTTAGTCATCAAGCTAGCTCAGATCTGC
CCACCTTGGAGCCCTGTGCTTT
>1893
TGTTTAGATGAGCTGCTGTTCAGGCACATGGTCTGGTTAGCAAAGGGTTAAATCCTGCA
TCCCGTTTTTAGTCATCAAGCTAGCTCAGATCTGCCCACCTTGGAGCCCTGTGCTTTCC
TGCCTTAGTGGGCAGAGTCTAG
>1894
GCAAAGGGTTAAATCCTGCATCCCGTTTTTAGTCATCAAGCTAGCTCAGATCTGCCCAC
CTTGGAGCCCTGTGCTTTCCTGCCTTAGTGGGCAGAGTCTAGGGGACCTGTGGCATGT
TCAATGATTGAGGACTCTTGTCC

>1895
TGCATCCCGTTTTTAGTCATCAAGCTAGCTCAGATCTGCCCACCTTGGAGCCCTGTGCT
TTCCTGCCTTAGTGGGCAGAGTCTAGGGGACCTGTGGCATGTTCAATGATTGAGGACT
CTTGTCCAGATTCCAAAAACCTA
>1896
TTTTTAGTCATCAAGCTAGCTCAGATCTGCCCACCTTGGAGCCCTGTGCTTTCCTGCCTT
AGTGGGCAGAGTCTAGGGGACCTGTGGCATGTTCAATGATTGAGGACTCTTGTCCAGA
TTCCAAAAACCTACTGTGATGA
>1897
AGCTAGCTCAGATCTGCCCACCTTGGAGCCCTGTGCTTTCCTGCCTTAGTGGGCAGAG
TCTAGGGGACCTGTGGCATGTTCAATGATTGAGGACTCTTGTCCAGATTCCAAAAACCT
ACTGTGATGATGCTGTTCTGAAG
>1898
TCAGATCTGCCCACCTTGGAGCCCTGTGCTTTCCTGCCTTAGTGGGCAGAGTCTAGGG
GACCTGTGGCATGTTCAATGATTGAGGACTCTTGTCCAGATTCCAAAAACCTACTGTGA
TGATGCTGTTCTGAAGCTGTGGT
>1899
CCCTGTGCTTTCCTGCCTTAGTGGGCAGAGTCTAGGGGACCTGTGGCATGTTCAATGAT
TGAGGACTCTTGTCCAGATTCCAAAAACCTACTGTGATGATGCTGTTCTGAAGCTGTGG
TAGGCGGTGATTGAGTGGAGTG
>1900
GGGCAGAGTCTAGGGGACCTGTGGCATGTTCAATGATTGAGGACTCTTGTCCAGATTC
CAAAAACCTACTGTGATGATGCTGTTCTGAAGCTGTGGTAGGCGGTGATTGAGTGGAG
TGTGTAGTGACCCTCAGGAATTCT
>1901
AGGGGACCTGTGGCATGTTCAATGATTGAGGACTCTTGTCCAGATTCCAAAAACCTAC
TGTGATGATGCTGTTCTGAAGCTGTGGTAGGCGGTGATTGAGTGGAGTGTGTAGTGAC
CCTCAGGAATTCTTCAGAAGTTAG
>1902
GGCATGTTCAATGATTGAGGACTCTTGTCCAGATTCCAAAAACCTACTGTGATGATGCT
GTTCTGAAGCTGTGGTAGGCGGTGATTGAGTGGAGTGTGTAGTGACCCTCAGGAATTC
TTCAGAAGTTAGGATTTTGACGA
>1903
CTTGTCCAGATTCCAAAAACCTACTGTGATGATGCTGTTCTGAAGCTGTGGTAGGCGGT
GATTGAGTGGAGTGTGTAGTGACCCTCAGGAATTCTTCAGAAGTTAGGATTTTGACGAT
ACCTCTGATAAGTTGGGTTTTT
>1904
ATGCTGTTCTGAAGCTGTGGTAGGCGGTGATTGAGTGGAGTGTGTAGTGACCCTCAGG
AATTCTTCAGAAGTTAGGATTTTGACGATACCTCTGATAAGTTGGGTTTTTAATTTGCTG
GTTTATCTTAGAGTGGAGGCTG
>1905
GATTGAGTGGAGTGTGTAGTGACCCTCAGGAATTCTTCAGAAGTTAGGATTTTGACGAT
ACCTCTGATAAGTTGGGTTTTTAATTTGCTGGTTTATCTTAGAGTGGAGGCTGTGGCCC
ACAGCAAGTAGTACATTTTCCA
>1906
GACCCTCAGGAATTCTTCAGAAGTTAGGATTTTGACGATACCTCTGATAAGTTGGGTTT

TTAATTTGCTGGTTTATCTTAGAGTGGAGGCTGTGGCCCACAGCAAGTAGTACATTTTC
CATCTCAGCCCAGTGCATGGAC
>1907
TTCTTCAGAAGTTAGGATTTTGACGATACCTCTGATAAGTTGGGTTTTTAATTTGCTGGT
TTATCTTAGAGTGGAGGCTGTGGCCCACAGCAAGTAGTACATTTTCCATCTCAGCCCAG
TGCATGGACAAACGTACCTCA
>1908
CGATACCTCTGATAAGTTGGGTTTTTAATTTGCTGGTTTATCTTAGAGTGGAGGCTGTGG
CCCACAGCAAGTAGTACATTTTCCATCTCAGCCCAGTGCATGGACAAACGTACCTCAA
ACCAAAGGTTCAATGAAGCTCT
>1909
TAATTTGCTGGTTTATCTTAGAGTGGAGGCTGTGGCCCACAGCAAGTAGTACATTTTCC
ATCTCAGCCCAGTGCATGGACAAACGTACCTCAAACCAAAGGTTCAATGAAGCTCTAT
TTATCTTTACTATATGCTCTGGT
>1910
ATCTTAGAGTGGAGGCTGTGGCCCACAGCAAGTAGTACATTTTCCATCTCAGCCCAGTG
CATGGACAAACGTACCTCAAACCAAAGGTTCAATGAAGCTCTATTTATCTTTACTATATG
CTCTGGTATCTTCTCTTCCAT
>1911
TGTGGCCCACAGCAAGTAGTACATTTTCCATCTCAGCCCAGTGCATGGACAAACGTAC
CTCAAACCAAAGGTTCAATGAAGCTCTATTTATCTTTACTATATGCTCTGGTATCTTCTCT
TCCATTCTATCTTATTTCATT
>1912
CAGTGCATGGACAAACGTACCTCAAACCAAAGGTTCAATGAAGCTCTATTTATCTTTAC
TATATGCTCTGGTATCTTCTCTTCCATTCTATCTTATTTCATTTAAGAACGTTAATGAAGA
CCCACTAATGGATTGCCACC
>1913
GAAGCTCTATTTATCTTTACTATATGCTCTGGTATCTTCTCTTCCATTCTATCTTATTTCATT
TAAGAACGTTAATGAAGACCCACTAATGGATTGCCACCCATGGTTCGAGGAGGTCCCC
CAAACAGTGAGGCCTTAGC
>1914
CTTCTCTTCCATTCTATCTTATTTCATTTAAGAACGTTAATGAAGACCCACTAATGGATTG
CCACCCATGGTTCGAGGAGGTCCCCCAAACAGTGAGGCCTTAGCCATAGGTATTCAATT
CAAAAAGCATTTACTGAGCA
>1915
ATTCTATCTTATTTCATTTAAGAACGTTAATGAAGACCCACTAATGGATTGCCACCCATG
GTTCGAGGAGGTCCCCCAAACAGTGAGGCCTTAGCCATAGGTATTCAATTCAAAAAGC
ATTTACTGAGCATCACTGTGTA
>1916
TGAAGACCCACTAATGGATTGCCACCCATGGTTCGAGGAGGTCCCCCAAACAGTGAGG
CCTTAGCCATAGGTATTCAATTCAAAAAGCATTTACTGAGCATCACTGTGTACTAGGTG
GTTGCTAAATGCTATGGGGGTAA
>1917
AGGAGGTCCCCCAAACAGTGAGGCCTTAGCCATAGGTATTCAATTCAAAAAGCATTTA
CTGAGCATCACTGTGTACTAGGTGGTTGCTAAATGCTATGGGGGTAATGCAGAGAAATT
AATAAATCCCTGTAAGAGTTCAC

>1918
AAACAGTGAGGCCTTAGCCATAGGTATTCAATTCAAAAAGCATTTACTGAGCATCACTG
TGTACTAGGTGGTTGCTAAATGCTATGGGGGTAATGCAGAGAAATTAATAAATCCCTGT
AAGAGTTCACTGTAAAACAGAA
>1919
ATAGGTATTCAATTCAAAAAGCATTTACTGAGCATCACTGTGTACTAGGTGGTTGCTAA
ATGCTATGGGGGTAATGCAGAGAAATTAATAAATCCCTGTAAGAGTTCACTGTAAAACA
GAAATGACAGCCATATGTGTAT
>1920
CTGAGCATCACTGTGTACTAGGTGGTTGCTAAATGCTATGGGGGTAATGCAGAGAAATT
AATAAATCCCTGTAAGAGTTCACTGTAAAACAGAAATGACAGCCATATGTGTATGACTT
TGTGTGTGTGTGTATATATATA
>1921
ATGGGGGTAATGCAGAGAAATTAATAAATCCCTGTAAGAGTTCACTGTAAAACAGAAA
TGACAGCCATATGTGTATGACTTTGTGTGTGTGTGTATATATATATATCAGACTGTATGATT
AAATATTATATAAGTGAGGT
>1922
GGGGGTAATGCAGAGAAATTAATAAATCCCTGTAAGAGTTCACTGTAAAACAGAAATG
ACAGCCATATGTGTATGACTTTGTGTGTGTGTGTATATATATATATCAGACTGTATGATTA
AATATTATATAAGTGAGGTAT
>1923
CACTGTAAAACAGAAATGACAGCCATATGTGTATGACTTTGTGTGTGTGTGTATATATAT
ATATCAGACTGTATGATTAAATATTATATAAGTGAGGTATGGGGGAGCAGAGGAGAGGA
ATTGAATTTTGAATTTTGATT
>1924
GCCATATGTGTATGACTTTGTGTGTGTGTGTATATATATATATCAGACTGTATGATTAAATA
TTATATAAGTGAGGTATGGGGGAGCAGAGGAGAGGAATTGAATTTTGAATTTTGATTGA
AGATTCAGGAGGGCTTCAT
>1925
ATGTGTATGACTTTGTGTGTGTGTGTATATATATATATCAGACTGTATGATTAAATATTATA
TAAGTGAGGTATGGGGGAGCAGAGGAGAGGAATTGAATTTTGAATTTTGATTGAAGAT
TCAGGAGGGCTTCATGGAGG
>1926
ATGGGGGAGCAGAGGAGAGGAATTGAATTTTGAATTTTGATTGAAGATTCAGGAGGGC
TTCATGGAGGAGGTAGCATTTGGCCAGGTTTTAGAGAATGGCTAGGCTTTGGACAAGT
GGAGACAGGAACTGCCATTTGGAG
>1927
AGAGGAATTGAATTTTGAATTTTGATTGAAGATTCAGGAGGGCTTCATGGAGGAGGTA
GCATTTGGCCAGGTTTTAGAGAATGGCTAGGCTTTGGACAAGTGGAGACAGGAACTGC
CATTTGGAGGTAGGAGATGAGCTA
>1928
AGATTCAGGAGGGCTTCATGGAGGAGGTAGCATTTGGCCAGGTTTTAGAGAATGGCTA
GGCTTTGGACAAGTGGAGACAGGAACTGCCATTTGGAGGTAGGAGATGAGCTAATGT
GTGGCAGTGTATGGGTGGGGGCAGG
>1929
GTAGCATTTGGCCAGGTTTTAGAGAATGGCTAGGCTTTGGACAAGTGGAGACAGGAAC

TGCCATTTGGAGGTAGGAGATGAGCTAATGTGTGGCAGTGTATGGGTGGGGGCAGGTG
CCTGTATACAAGGTAGTGTGCTGG
>1930
TTTAGAGAATGGCTAGGCTTTGGACAAGTGGAGACAGGAACTGCCATTTGGAGGTAGG
AGATGAGCTAATGTGTGGCAGTGTATGGGTGGGGGCAGGTGCCTGTATACAAGGTAGT
GTGCTGGAATGGGGCGATGGGCAT
>1931
TAGAGAATGGCTAGGCTTTGGACAAGTGGAGACAGGAACTGCCATTTGGAGGTAGGA
GATGAGCTAATGTGTGGCAGTGTATGGGTGGGGGCAGGTGCCTGTATACAAGGTAGTG
TGCTGGAATGGGGCGATGGGCATGG
>1932
GCTTTGGACAAGTGGAGACAGGAACTGCCATTTGGAGGTAGGAGATGAGCTAATGTGT
GGCAGTGTATGGGTGGGGGCAGGTGCCTGTATACAAGGTAGTGTGCTGGAATGGGGCG
ATGGGCATGGGGTAGGATGGGAGA
>1933
TGCCATTTGGAGGTAGGAGATGAGCTAATGTGTGGCAGTGTATGGGTGGGGGCAGGTG
CCTGTATACAAGGTAGTGTGCTGGAATGGGGCGATGGGCATGGGGTAGGATGGGAGAT
CAAGCAGGGAAAGTGGATTGGGGC
>1934
TGGCAGTGTATGGGTGGGGGCAGGTGCCTGTATACAAGGTAGTGTGCTGGAATGGGGC
GATGGGCATGGGGTAGGATGGGAGATCAAGCAGGGAAAGTGGATTGGGGCCAGGTCC
TAGAATGATTTGAATACCAAGCTAG
>1935
CCTGTATACAAGGTAGTGTGCTGGAATGGGGCGATGGGCATGGGGTAGGATGGGAGAT
CAAGCAGGGAAAGTGGATTGGGGCCAGGTCCTAGAATGATTTGAATACCAAGCTAGCT
TTCTGGACATTTGTAAGGCGCACT
>1936
GTGTGCTGGAATGGGGCGATGGGCATGGGGTAGGATGGGAGATCAAGCAGGGAAAGT
GGATTGGGGCCAGGTCCTAGAATGATTTGAATACCAAGCTAGCTTTCTGGACATTTGTA
AGGCGCACTTGATAGGGGTTGGAT
>1937
CAAGCAGGGAAAGTGGATTGGGGCCAGGTCCTAGAATGATTTGAATACCAAGCTAGCT
TTCTGGACATTTGTAAGGCGCACTTGATAGGGGTTGGATCATTGCGATATTATTGGAAG
ACAAAACTGGGGAGGGTGTGAGA
>1938
GGGCCAGGTCCTAGAATGATTTGAATACCAAGCTAGCTTTCTGGACATTTGTAAGGCGC
ACTTGATAGGGGTTGGATCATTGCGATATTATTGGAAGACAAAACTGGGGAGGGTGTG
AGACCATGTGGCTGCTCAGAATG
>1939
GGACATTTGTAAGGCGCACTTGATAGGGGTTGGATCATTGCGATATTATTGGAAGACAA
AACTGGGGAGGGTGTGAGACCATGTGGCTGCTCAGAATGCAGAGCCCCTCTGATGTCC
TCAGCCTTCTACGTATTTAGTGA
>1940
GGCGCACTTGATAGGGGTTGGATCATTGCGATATTATTGGAAGACAAAACTGGGGAGG
GTGTGAGACCATGTGGCTGCTCAGAATGCAGAGCCCCTCTGATGTCCTCAGCCTTCTAC
GTATTTAGTGATGGGGGTGGTGG

>1941
TATTATTGGAAGACAAAACTGGGGAGGGTGTGAGACCATGTGGCTGCTCAGAATGCAG
AGCCCCTCTGATGTCCTCAGCCTTCTACGTATTTAGTGATGGGGGTGGTGGTGGTAAAG
AGCCTTGCAGACAGCCTGGCTTG
>1942
AACTGGGGAGGGTGTGAGACCATGTGGCTGCTCAGAATGCAGAGCCCCTCTGATGTCC
TCAGCCTTCTACGTATTTAGTGATGGGGGTGGTGGTGGTAAAGAGCCTTGCAGACAGC
CTGGCTTGGGTGCTTGCTTGAAGT
>1943
AGACCATGTGGCTGCTCAGAATGCAGAGCCCCTCTGATGTCCTCAGCCTTCTACGTATT
TAGTGATGGGGGTGGTGGTGGTAAAGAGCCTTGCAGACAGCCTGGCTTGGGTGCTTGC
TTGAAGTTCTTACGCCCACTAAC
>1944
CCATGTGGCTGCTCAGAATGCAGAGCCCCTCTGATGTCCTCAGCCTTCTACGTATTTAG
TGATGGGGGTGGTGGTGGTAAAGAGCCTTGCAGACAGCCTGGCTTGGGTGCTTGCTTG
AAGTTCTTACGCCCACTAACTCC
>1945
TGTGGCTGCTCAGAATGCAGAGCCCCTCTGATGTCCTCAGCCTTCTACGTATTTAGTGA
TGGGGGTGGTGGTGGTAAAGAGCCTTGCAGACAGCCTGGCTTGGGTGCTTGCTTGAA
GTTCTTACGCCCACTAACTCCTGC
>1946
TCCTCAGCCTTCTACGTATTTAGTGATGGGGGTGGTGGTGGTAAAGAGCCTTGCAGAC
AGCCTGGCTTGGGTGCTTGCTTGAAGTTCTTACGCCCACTAACTCCTGCCCTGCTCTGT
GGAGCCTGCTGAATGGGGCATTA
>1947
GAGCCTTGCAGACAGCCTGGCTTGGGTGCTTGCTTGAAGTTCTTACGCCCACTAACTC
CTGCCCTGCTCTGTGGAGCCTGCTGAATGGGGCATTAGGCTAGTCACACACCTGTCCTT
TTTCACATTCATGCTGTACCCTT
>1948
TGCTTGAAGTTCTTACGCCCACTAACTCCTGCCCTGCTCTGTGGAGCCTGCTGAATGGG
GCATTAGGCTAGTCACACACCTGTCCTTTTTCACATTCATGCTGTACCCTTTGCAAACAT
TACGCAGCAGTGCAAACTCAG
>1949
CTTACGCCCACTAACTCCTGCCCTGCTCTGTGGAGCCTGCTGAATGGGGCATTAGGCTA
GTCACACACCTGTCCTTTTTCACATTCATGCTGTACCCTTTGCAAACATTACGCAGCAG
TGCAAACTCAGCCCTGGGCCTC
>1950
CCTGCTCTGTGGAGCCTGCTGAATGGGGCATTAGGCTAGTCACACACCTGTCCTTTTTC
ACATTCATGCTGTACCCTTTGCAAACATTACGCAGCAGTGCAAACTCAGCCCTGGGCCT
CAGAGGAGCCAGGCGTGGCCAG
>1951
GGCATTAGGCTAGTCACACACCTGTCCTTTTTCACATTCATGCTGTACCCTTTGCAAACA
TTACGCAGCAGTGCAAACTCAGCCCTGGGCCTCAGAGGAGCCAGGCGTGGCCAGGAG
AGTGGGATTTGGAACGGAAGTGC
>1952
TTCATGCTGTACCCTTTGCAAACATTACGCAGCAGTGCAAACTCAGCCCTGGGCCTCA

GAGGAGCCAGGCGTGGCCAGGAGAGTGGGATTTGGAACGGAAGTGCTTTTGTTTTTG
GCATTCACTACTCCCCCACCCCCAC
>1953
CCTTTGCAAACATTACGCAGCAGTGCAAACTCAGCCCTGGGCCTCAGAGGAGCCAGG
CGTGGCCAGGAGAGTGGGATTTGGAACGGAAGTGCTTTTGTTTTTGGCATTCACTACT
CCCCCACCCCCACTGGCACTTGGAT
>1954
AAACTCAGCCCTGGGCCTCAGAGGAGCCAGGCGTGGCCAGGAGAGTGGGATTTGGAA
CGGAAGTGCTTTTGTTTTTGGCATTCACTACTCCCCCACCCCCACTGGCACTTGGATTG
TCCTTCTTTTTCTCCTTGGTGTCT
>1955
TGGGATTTGGAACGGAAGTGCTTTTGTTTTTGGCATTCACTACTCCCCCACCCCCACTG
GCACTTGGATTGTCCTTCTTTTTCTCCTTGGTGTCTCTCATCCTGTCCAGCCTTCAAGAC
CTCCTCAAGTCAGGCTTCTCT
>1956
GCTTTTGTTTTTGGCATTCACTACTCCCCCACCCCCACTGGCACTTGGATTGTCCTTCTT
TTTCTCCTTGGTGTCTCTCATCCTGTCCAGCCTTCAAGACCTCCTCAAGTCAGGCTTCT
CTGGGAAGCGCTGTCTGTCTG
>1957
TTTTGTTTTTGGCATTCACTACTCCCCCACCCCCACTGGCACTTGGATTGTCCTTCTTTT
TCTCCTTGGTGTCTCTCATCCTGTCCAGCCTTCAAGACCTCCTCAAGTCAGGCTTCTCT
GGGAAGCGCTGTCTGTCTGTC
>1958
TGGCACTTGGATTGTCCTTCTTTTTCTCCTTGGTGTCTCTCATCCTGTCCAGCCTTCAAG
ACCTCCTCAAGTCAGGCTTCTCTGGGAAGCGCTGTCTGTCTGTCTTGGTGCTTGCTGG
CTTCCTCTTCTGTCTCCTGTGC
>1959
TTCTCCTTGGTGTCTCTCATCCTGTCCAGCCTTCAAGACCTCCTCAAGTCAGGCTTCTC
TGGGAAGCGCTGTCTGTCTGTCTTGGTGCTTGCTGGCTTCCTCTTCTGTCTCCTGTGCC
ATCTCCAGTTTGCACCATTTGA
>1960
CCTTGGTGTCTCTCATCCTGTCCAGCCTTCAAGACCTCCTCAAGTCAGGCTTCTCTGGG
AAGCGCTGTCTGTCTGTCTTGGTGCTTGCTGGCTTCCTCTTCTGTCTCCTGTGCCATCTC
CAGTTTGCACCATTTGAGTAT
>1961
GACCTCCTCAAGTCAGGCTTCTCTGGGAAGCGCTGTCTGTCTGTCTTGGTGCTTGCTG
GCTTCCTCTTCTGTCTCCTGTGCCATCTCCAGTTTGCACCATTTGAGTATAAGCTCTGCT
GCCTCATTTGAGTTTAAGTTTT
>1962
TCTCTGGGAAGCGCTGTCTGTCTGTCTTGGTGCTTGCTGGCTTCCTCTTCTGTCTCCTG
TGCCATCTCCAGTTTGCACCATTTGAGTATAAGCTCTGCTGCCTCATTTGAGTTTAAGTT
TTATCCCCCCAGCCACAACCT
>1963
GTCTGTCTGTCTTGGTGCTTGCTGGCTTCCTCTTCTGTCTCCTGTGCCATCTCCAGTTTG
CACCATTTGAGTATAAGCTCTGCTGCCTCATTTGAGTTTAAGTTTTATCCCCCCAGCCAC
AACCTAAGTTCCATGAGGAT

>1964
CTTCCTCTTCTGTCTCCTGTGCCATCTCCAGTTTGCACCATTTGAGTATAAGCTCTGCTG
CCTCATTTGAGTTTAAGTTTTATCCCCCCAGCCACAACCTAAGTTCCATGAGGATAGTG
ATTGTGGTTTATCTTCTCTCC
>1965
TTGCACCATTTGAGTATAAGCTCTGCTGCCTCATTTGAGTTTAAGTTTTATCCCCCCAGC
CACAACCTAAGTTCCATGAGGATAGTGATTGTGGTTTATCTTCTCTCCCTCAGCTGTCTG
CTGACACCATGCTGAATACA
>1966
TATAAGCTCTGCTGCCTCATTTGAGTTTAAGTTTTATCCCCCCAGCCACAACCTAAGTTC
CATGAGGATAGTGATTGTGGTTTATCTTCTCTCCCTCAGCTGTCTGCTGACACCATGCTG
AATACATAGTAGGTACTCAG
>1967
TTTTATCCCCCCAGCCACAACCTAAGTTCCATGAGGATAGTGATTGTGGTTTATCTTCTC
TCCCTCAGCTGTCTGCTGACACCATGCTGAATACATAGTAGGTACTCAGTAAATAATAG
CTAATTGAGCATTTACTATGG
>1968
TCCATGAGGATAGTGATTGTGGTTTATCTTCTCTCCCTCAGCTGTCTGCTGACACCATGC
TGAATACATAGTAGGTACTCAGTAAATAATAGCTAATTGAGCATTTACTATGGACAAAGC
ACTTTACTTGCATTAACACA
>1969
AGTGATTGTGGTTTATCTTCTCTCCCTCAGCTGTCTGCTGACACCATGCTGAATACATAG
TAGGTACTCAGTAAATAATAGCTAATTGAGCATTTACTATGGACAAAGCACTTTACTTGC
ATTAACACATCCCATCCTCA
>1970
AGCTAATTGAGCATTTACTATGGACAAAGCACTTTACTTGCATTAACACATCCCATCCTC
ACAACCACCTGTGAGGTGAATCCTATTAAAAGGAATACTTGGGTGCTTTCAGAGTTATT
CTCTCCAGCTTTGGACTTCTT
>1971
ATTGAGCATTTACTATGGACAAAGCACTTTACTTGCATTAACACATCCCATCCTCACAAC
CACCTGTGAGGTGAATCCTATTAAAAGGAATACTTGGGTGCTTTCAGAGTTATTCTCTC
CAGCTTTGGACTTCTTGTTTC
>1972
TTTACTTGCATTAACACATCCCATCCTCACAACCACCTGTGAGGTGAATCCTATTAAAA
GGAATACTTGGGTGCTTTCAGAGTTATTCTCTCCAGCTTTGGACTTCTTGTTTCATTTGC
CCTGAGAGCTCCTCAACTCTG
>1973
TAACACATCCCATCCTCACAACCACCTGTGAGGTGAATCCTATTAAAAGGAATACTTGG
GTGCTTTCAGAGTTATTCTCTCCAGCTTTGGACTTCTTGTTTCATTTGCCCTGAGAGCTC
CTCAACTCTGTTGATGCTGTT
>1974
GTGAGGTGAATCCTATTAAAAGGAATACTTGGGTGCTTTCAGAGTTATTCTCTCCAGCT
TTGGACTTCTTGTTTCATTTGCCCTGAGAGCTCCTCAACTCTGTTGATGCTGTTCTTTCA
AAGGTTGCTTGTGACCTCTTA
>1975
GGTGCTTTCAGAGTTATTCTCTCCAGCTTTGGACTTCTTGTTTCATTTGCCCTGAGAGCT

CCTCAACTCTGTTGATGCTGTTCTTTCAAAGGTTGCTTGTGACCTCTTAGTTCTCAAGA
GCACTGTATTAGGTTGGTGCA
>1976
TCCAGCTTTGGACTTCTTGTTTCATTTGCCCTGAGAGCTCCTCAACTCTGTTGATGCTG
TTCTTTCAAAGGTTGCTTGTGACCTCTTAGTTCTCAAGAGCACTGTATTAGGTTGGTGC
AAAAGTAATTGCTATTTTAGAC
>1977
TTGGACTTCTTGTTTCATTTGCCCTGAGAGCTCCTCAACTCTGTTGATGCTGTTCTTTCA
AAGGTTGCTTGTGACCTCTTAGTTCTCAAGAGCACTGTATTAGGTTGGTGCAAAAGTAA
TTGCTATTTTAGACATTACTT
>1978
GTTTCATTTGCCCTGAGAGCTCCTCAACTCTGTTGATGCTGTTCTTTCAAAGGTTGCTT
GTGACCTCTTAGTTCTCAAGAGCACTGTATTAGGTTGGTGCAAAAGTAATTGCTATTTT
AGACATTACTTTCAATGGCAAA
>1979
CTCAACTCTGTTGATGCTGTTCTTTCAAAGGTTGCTTGTGACCTCTTAGTTCTCAAGAG
CACTGTATTAGGTTGGTGCAAAAGTAATTGCTATTTTAGACATTACTTTCAATGGCAAAA
ACTGCAATTACTTTTGCGCCA
>1980
TTTTAGACATTACTTTCAATGGCAAAAACTGCAATTACTTTTGCGCCAACTAATACTTTC
CCAGACTCCAGTATTGGCTCCATTGACCCCTCCTGTTCCTGCCAACTTCTCCCCCTCCT
CCTCTGGTATTTCCTGGAACT
>1981
AAAACTGCAATTACTTTTGCGCCAACTAATACTTTCCCAGACTCCAGTATTGGCTCCATT
GACCCCTCCTGTTCCTGCCAACTTCTCCCCCTCCTCCTCTGGTATTTCCTGGAACTCCCT
CCCTAGGGTCAGTGCCATGT
>1982
CTTTCCCAGACTCCAGTATTGGCTCCATTGACCCCTCCTGTTCCTGCCAACTTCTCCCCC
TCCTCCTCTGGTATTTCCTGGAACTCCCTCCCTAGGGTCAGTGCCATGTGGTGCCTGGA
ACTCCCTGGGATCTGTGTTTG
>1983
ATTGACCCCTCCTGTTCCTGCCAACTTCTCCCCCTCCTCCTCTGGTATTTCCTGGAACTC
CCTCCCTAGGGTCAGTGCCATGTGGTGCCTGGAACTCCCTGGGATCTGTGTTTGAATCC
TGACATTGCCACTTTCTAAGT
>1984
TTCCTGCCAACTTCTCCCCCTCCTCCTCTGGTATTTCCTGGAACTCCCTCCCTAGGGTCA
GTGCCATGTGGTGCCTGGAACTCCCTGGGATCTGTGTTTGAATCCTGACATTGCCACTT
TCTAAGTGGCAAACACTTTGG
>1985
TCCTCTGGTATTTCCTGGAACTCCCTCCCTAGGGTCAGTGCCATGTGGTGCCTGGAACT
CCCTGGGATCTGTGTTTGAATCCTGACATTGCCACTTTCTAAGTGGCAAACACTTTGGC
CACCTCCCCCTCTTCCTGAGTC
>1986
CTCTGGTATTTCCTGGAACTCCCTCCCTAGGGTCAGTGCCATGTGGTGCCTGGAACTCC
CTGGGATCTGTGTTTGAATCCTGACATTGCCACTTTCTAAGTGGCAAACACTTTGGCCA
CCTCCCCCTCTTCCTGAGTCTG

>1987
GGGTCAGTGCCATGTGGTGCCTGGAACTCCCTGGGATCTGTGTTTGAATCCTGACATTG
CCACTTTCTAAGTGGCAAACACTTTGGCCACCTCCCCCTCTTCCTGAGTCTGTTTCATC
ATCTGCAAAATGCTGGTAGTTT
>1988
TCTGTGTTTGAATCCTGACATTGCCACTTTCTAAGTGGCAAACACTTTGGCCACCTCCC
CCTCTTCCTGAGTCTGTTTCATCATCTGCAAAATGCTGGTAGTTTCTCTTCTCACAGGA
AAGATGGTGGTTGTTCATCGAT
>1989
TGTTTGAATCCTGACATTGCCACTTTCTAAGTGGCAAACACTTTGGCCACCTCCCCCTC
TTCCTGAGTCTGTTTCATCATCTGCAAAATGCTGGTAGTTTCTCTTCTCACAGGAAAGA
TGGTGGTTGTTCATCGATGTCT
>1990
TTTCTAAGTGGCAAACACTTTGGCCACCTCCCCCTCTTCCTGAGTCTGTTTCATCATCT
GCAAAATGCTGGTAGTTTCTCTTCTCACAGGAAAGATGGTGGTTGTTCATCGATGTCTG
TTGAAACCGTGACCCTCTCTGC
>1991
CTCCCCCTCTTCCTGAGTCTGTTTCATCATCTGCAAAATGCTGGTAGTTTCTCTTCTCAC
AGGAAAGATGGTGGTTGTTCATCGATGTCTGTTGAAACCGTGACCCTCTCTGCTGAAT
GTAGAAGAAGATGACCTTACCA
>1992
CCCCTCTTCCTGAGTCTGTTTCATCATCTGCAAAATGCTGGTAGTTTCTCTTCTCACAGG
AAAGATGGTGGTTGTTCATCGATGTCTGTTGAAACCGTGACCCTCTCTGCTGAATGTAG
AAGAAGATGACCTTACCAGTT
>1993
GTTTCATCATCTGCAAAATGCTGGTAGTTTCTCTTCTCACAGGAAAGATGGTGGTTGTT
CATCGATGTCTGTTGAAACCGTGACCCTCTCTGCTGAATGTAGAAGAAGATGACCTTAC
CAGTTCTCCTGTCTCTCCAGTG
>1994
TTCTCTTCTCACAGGAAAGATGGTGGTTGTTCATCGATGTCTGTTGAAACCGTGACCCT
CTCTGCTGAATGTAGAAGAAGATGACCTTACCAGTTCTCCTGTCTCTCCAGTGTCTTTC
TTGACAATCTTGTGGTTTCCAC
>1995
GTGGTTGTTCATCGATGTCTGTTGAAACCGTGACCCTCTCTGCTGAATGTAGAAGAAGA
TGACCTTACCAGTTCTCCTGTCTCTCCAGTGTCTTTCTTGACAATCTTGTGGTTTCCACA
AGCAACCCTCTGTGGGTGATT
>1996
TCATCGATGTCTGTTGAAACCGTGACCCTCTCTGCTGAATGTAGAAGAAGATGACCTTA
CCAGTTCTCCTGTCTCTCCAGTGTCTTTCTTGACAATCTTGTGGTTTCCACAAGCAACC
CTCTGTGGGTGATTTCCAGATC
>1997
CTCTGCTGAATGTAGAAGAAGATGACCTTACCAGTTCTCCTGTCTCTCCAGTGTCTTTC
TTGACAATCTTGTGGTTTCCACAAGCAACCCTCTGTGGGTGATTTCCAGATCTCTACCT
CTAGTTGTTTTTCGTTTTTTGT
>1998
TGCTGAATGTAGAAGAAGATGACCTTACCAGTTCTCCTGTCTCTCCAGTGTCTTTCTTG

ACAATCTTGTGGTTTCCACAAGCAACCCTCTGTGGGTGATTTCCAGATCTCTACCTCTA
GTTGTTTTTCGTTTTTTGTCTT
>1999
GACCTTACCAGTTCTCCTGTCTCTCCAGTGTCTTTCTTGACAATCTTGTGGTTTCCACA
AGCAACCCTCTGTGGGTGATTTCCAGATCTCTACCTCTAGTTGTTTTTCGTTTTTTGTCT
TTTCTTGTGTTTCACACATTC
>2000
TGTCTTTCTTGACAATCTTGTGGTTTCCACAAGCAACCCTCTGTGGGTGATTTCCAGAT
CTCTACCTCTAGTTGTTTTTCGTTTTTTGTCTTTTCTTGTGTTTCACACATTCTTTTCCAG
TAGCCTCACGGGCACACCAG
>2001
GACAATCTTGTGGTTTCCACAAGCAACCCTCTGTGGGTGATTTCCAGATCTCTACCTCT
AGTTGTTTTTCGTTTTTTGTCTTTTCTTGTGTTTCACACATTCTTTTCCAGTAGCCTCAC
GGGCACACCAGTCCTGCAAGG
>2002
TTGTGGTTTCCACAAGCAACCCTCTGTGGGTGATTTCCAGATCTCTACCTCTAGTTGTT
TTTCGTTTTTTGTCTTTTCTTGTGTTTCACACATTCTTTTCCAGTAGCCTCACGGGCACA
CCAGTCCTGCAAGGATCTAGA
>2003
CACAAGCAACCCTCTGTGGGTGATTTCCAGATCTCTACCTCTAGTTGTTTTTCGTTTTTT
GTCTTTTCTTGTGTTTCACACATTCTTTTCCAGTAGCCTCACGGGCACACCAGTCCTGC
AAGGATCTAGAATGTGATGGT
>2004
ATTTCCAGATCTCTACCTCTAGTTGTTTTTCGTTTTTTGTCTTTTCTTGTGTTTCACACAT
TCTTTTCCAGTAGCCTCACGGGCACACCAGTCCTGCAAGGATCTAGAATGTGATGGTTT
GAGCTAGTTCAACACCTTCC
>2005
AGTTGTTTTTCGTTTTTTGTCTTTTCTTGTGTTTCACACATTCTTTTCCAGTAGCCTCAC
GGGCACACCAGTCCTGCAAGGATCTAGAATGTGATGGTTTGAGCTAGTTCAACACCTT
CCTCTCGAATCGTCTCCTGTGT
>2006
CTTTTCTTGTGTTTCACACATTCTTTTCCAGTAGCCTCACGGGCACACCAGTCCTGCAA
GGATCTAGAATGTGATGGTTTGAGCTAGTTCAACACCTTCCTCTCGAATCGTCTCCTGT
GTCCTCATGTTTTGTGGTCTCT
>2007
TTGTGTTTCACACATTCTTTTCCAGTAGCCTCACGGGCACACCAGTCCTGCAAGGATCT
AGAATGTGATGGTTTGAGCTAGTTCAACACCTTCCTCTCGAATCGTCTCCTGTGTCCTC
ATGTTTTGTGGTCTCTGCCCTC
>2008
CGGGCACACCAGTCCTGCAAGGATCTAGAATGTGATGGTTTGAGCTAGTTCAACACCT
TCCTCTCGAATCGTCTCCTGTGTCCTCATGTTTTGTGGTCTCTGCCCTCTTCCCAGGCAC
CCAACTGCAGACTTTGGCATCA
>2009
ACCAGTCCTGCAAGGATCTAGAATGTGATGGTTTGAGCTAGTTCAACACCTTCCTCTCG
AATCGTCTCCTGTGTCCTCATGTTTTGTGGTCTCTGCCCTCTTCCCAGGCACCCAACTG
CAGACTTTGGCATCATCTGTGG

>2010
CAGTCCTGCAAGGATCTAGAATGTGATGGTTTGAGCTAGTTCAACACCTTCCTCTCGAA
TCGTCTCCTGTGTCCTCATGTTTTGTGGTCTCTGCCCTCTTCCCAGGCACCCAACTGCA
GACTTTGGCATCATCTGTGGCT
>2011
ATCTAGAATGTGATGGTTTGAGCTAGTTCAACACCTTCCTCTCGAATCGTCTCCTGTGT
CCTCATGTTTTGTGGTCTCTGCCCTCTTCCCAGGCACCCAACTGCAGACTTTGGCATCA
TCTGTGGCTCCTGTCTTCCCTT
>2012
TCCTGTGTCCTCATGTTTTGTGGTCTCTGCCCTCTTCCCAGGCACCCAACTGCAGACTT
TGGCATCATCTGTGGCTCCTGTCTTCCCTTTGCCTTCCATATCCACCATTTGCCTCTCCA
GCCTACTTAACCAGATGGCTG
>2013
CTCATGTTTTGTGGTCTCTGCCCTCTTCCCAGGCACCCAACTGCAGACTTTGGCATCAT
CTGTGGCTCCTGTCTTCCCTTTGCCTTCCATATCCACCATTTGCCTCTCCAGCCTACTTA
ACCAGATGGCTGTCCTGATTT
>2014
TGTGGCTCCTGTCTTCCCTTTGCCTTCCATATCCACCATTTGCCTCTCCAGCCTACTTAA
CCAGATGGCTGTCCTGATTTCCTCACCAGCCTTCCTGCAGTGCACCCCTGCAAGGCTCA
TCACCCCACTTCCCTGAGCAG
>2015
TATCCACCATTTGCCTCTCCAGCCTACTTAACCAGATGGCTGTCCTGATTTCCTCACCAG
CCTTCCTGCAGTGCACCCCTGCAAGGCTCATCACCCCACTTCCCTGAGCAGAGTAGGG
CTCTGATGGGGTTGCTCTCCTG
>2016
TCCTGATTTCCTCACCAGCCTTCCTGCAGTGCACCCCTGCAAGGCTCATCACCCCACTT
CCCTGAGCAGAGTAGGGCTCTGATGGGGTTGCTCTCCTGCTCTGAACCCTGCAGTGGC
TCACTATGGCCTATTCTAGAGAA
>2017
AGCCTTCCTGCAGTGCACCCCTGCAAGGCTCATCACCCCACTTCCCTGAGCAGAGTAG
GGCTCTGATGGGGTTGCTCTCCTGCTCTGAACCCTGCAGTGGCTCACTATGGCCTATTC
TAGAGAAGAAACGAGAGAATTCT
>2018
GGCTCATCACCCCACTTCCCTGAGCAGAGTAGGGCTCTGATGGGGTTGCTCTCCTGCTC
TGAACCCTGCAGTGGCTCACTATGGCCTATTCTAGAGAAGAAACGAGAGAATTCTTAG
CGTGGCATTCAAAGCCATCCGGG
>2019
CTCTCCTGCTCTGAACCCTGCAGTGGCTCACTATGGCCTATTCTAGAGAAGAAACGAG
AGAATTCTTAGCGTGGCATTCAAAGCCATCCGGGATCCTAGATTCCAGCTACATTTTCA
CTCTTCATCCCTTTCTCTGCATA
>2020
AGCCATCCGGGATCCTAGATTCCAGCTACATTTTCACTCTTCATCCCTTTCTCTGCATATT
TCTTAGAATGTACACCCCTGAAGGCAGGGACCATCTTTTTCACATCTGTGTTACAACCT
TTTAGTCCACTCTCTAGCAT
>2021
CTTCATCCCTTTCTCTGCATATTTCTTAGAATGTACACCCCTGAAGGCAGGGACCATCTT

TTTCACATCTGTGTTACAACCTTTTAGTCCACTCTCTAGCATATGGCAACTCAGCAGATG
CTGCTGTTGAGTAAACTCTT
>2022
TCATCCCTTTCTCTGCATATTTCTTAGAATGTACACCCCTGAAGGCAGGGACCATCTTTT
TCACATCTGTGTTACAACCTTTTAGTCCACTCTCTAGCATATGGCAACTCAGCAGATGCT
GCTGTTGAGTAAACTCTTAT
>2023
GCATATTTCTTAGAATGTACACCCCTGAAGGCAGGGACCATCTTTTTCACATCTGTGTTA
CAACCTTTTAGTCCACTCTCTAGCATATGGCAACTCAGCAGATGCTGCTGTTGAGTAAA
CTCTTATGCTTCCCTTCCCAT
>2024
ATCTTTTTCACATCTGTGTTACAACCTTTTAGTCCACTCTCTAGCATATGGCAACTCAGC
AGATGCTGCTGTTGAGTAAACTCTTATGCTTCCCTTCCCATGCCATATCCTTCTCTGGTC
ATCGCAACAGGGCCAAATCT
>2025
TTTCACATCTGTGTTACAACCTTTTAGTCCACTCTCTAGCATATGGCAACTCAGCAGATG
CTGCTGTTGAGTAAACTCTTATGCTTCCCTTCCCATGCCATATCCTTCTCTGGTCATCGC
AACAGGGCCAAATCTCTCAT
>2026
ATGGCAACTCAGCAGATGCTGCTGTTGAGTAAACTCTTATGCTTCCCTTCCCATGCCATA
TCCTTCTCTGGTCATCGCAACAGGGCCAAATCTCTCATGCTTCAGTGTAAATGCCCTCT
CCTTGCAGCATCCTTTCCTGG
>2027
TCTTATGCTTCCCTTCCCATGCCATATCCTTCTCTGGTCATCGCAACAGGGCCAAATCTC
TCATGCTTCAGTGTAAATGCCCTCTCCTTGCAGCATCCTTTCCTGGGGCATTCTTCCTCC
TTGCATCCAGTTGGAAATAA
>2028
TTATGCTTCCCTTCCCATGCCATATCCTTCTCTGGTCATCGCAACAGGGCCAAATCTCTC
ATGCTTCAGTGTAAATGCCCTCTCCTTGCAGCATCCTTTCCTGGGGCATTCTTCCTCCTT
GCATCCAGTTGGAAATAATA
>2029
CTCATGCTTCAGTGTAAATGCCCTCTCCTTGCAGCATCCTTTCCTGGGGCATTCTTCCTC
CTTGCATCCAGTTGGAAATAATATGTCCCTCCATTGGAGCTCCCACACCAGAAGAGGTA
TAGATAAAGCCAGATACTTCT
>2030
TAAATGCCCTCTCCTTGCAGCATCCTTTCCTGGGGCATTCTTCCTCCTTGCATCCAGTTG
GAAATAATATGTCCCTCCATTGGAGCTCCCACACCAGAAGAGGTATAGATAAAGCCAGA
TACTTCTTTTTTTTTTTTTTT
>2031
GGGCATTCTTCCTCCTTGCATCCAGTTGGAAATAATATGTCCCTCCATTGGAGCTCCCAC
ACCAGAAGAGGTATAGATAAAGCCAGATACTTCTTTTTTTTTTTTTTTTTTTGATACAGA
GTCTCACTCTGTTGCCCAGG
>2032
GAGCTCCCACACCAGAAGAGGTATAGATAAAGCCAGATACTTCTTTTTTTTTTTTTTTTT
TTGATACAGAGTCTCACTCTGTTGCCCAGGCTGGAGTGTAGTGGCATGAACTTGGCTC
ACTGCAGCCTCTGCCTCTTGGG

>2033
ACCAGAAGAGGTATAGATAAAGCCAGATACTTCTTTTTTTTTTTTTTTTTTTGATACAGA
GTCTCACTCTGTTGCCCAGGCTGGAGTGTAGTGGCATGAACTTGGCTCACTGCAGCCT
CTGCCTCTTGGGTTCAAGTGAT
>2034
GATAAAGCCAGATACTTCTTTTTTTTTTTTTTTTTTTGATACAGAGTCTCACTCTGTTGCC
CAGGCTGGAGTGTAGTGGCATGAACTTGGCTCACTGCAGCCTCTGCCTCTTGGGTTCA
AGTGATTCTTGCGTCTCAGCC
>2035
AGCCAGATACTTCTTTTTTTTTTTTTTTTTTTGATACAGAGTCTCACTCTGTTGCCCAGG
CTGGAGTGTAGTGGCATGAACTTGGCTCACTGCAGCCTCTGCCTCTTGGGTTCAAGTG
ATTCTTGCGTCTCAGCCTCCCG
>2036
AGTCTCACTCTGTTGCCCAGGCTGGAGTGTAGTGGCATGAACTTGGCTCACTGCAGCC
TCTGCCTCTTGGGTTCAAGTGATTCTTGCGTCTCAGCCTCCCGAGTAGCTGAGATTACA
GACATGCGCTACCATGCCTGGCT
>2037
ACTCTGTTGCCCAGGCTGGAGTGTAGTGGCATGAACTTGGCTCACTGCAGCCTCTGCC
TCTTGGGTTCAAGTGATTCTTGCGTCTCAGCCTCCCGAGTAGCTGAGATTACAGACATG
CGCTACCATGCCTGGCTAATTTT
>2038
CAGGCTGGAGTGTAGTGGCATGAACTTGGCTCACTGCAGCCTCTGCCTCTTGGGTTCA
AGTGATTCTTGCGTCTCAGCCTCCCGAGTAGCTGAGATTACAGACATGCGCTACCATGC
CTGGCTAATTTTTGTATTTTTAG
>2039
TGGCATGAACTTGGCTCACTGCAGCCTCTGCCTCTTGGGTTCAAGTGATTCTTGCGTCT
CAGCCTCCCGAGTAGCTGAGATTACAGACATGCGCTACCATGCCTGGCTAATTTTTGTA
TTTTTAGTAGAGATGCGGTTTC
>2040
TGATTCTTGCGTCTCAGCCTCCCGAGTAGCTGAGATTACAGACATGCGCTACCATGCCT
GGCTAATTTTTGTATTTTTAGTAGAGATGCGGTTTCACCATGCTGGCCAGGCTGGTCCCC
AACTCCTCGCCTCAAGTGATC
>2041
CGTCTCAGCCTCCCGAGTAGCTGAGATTACAGACATGCGCTACCATGCCTGGCTAATTT
TTGTATTTTTAGTAGAGATGCGGTTTCACCATGCTGGCCAGGCTGGTCCCCAACTCCTC
GCCTCAAGTGATCTTCCTGCCT
>2042
ATGCGCTACCATGCCTGGCTAATTTTTGTATTTTTAGTAGAGATGCGGTTTCACCATGCT
GGCCAGGCTGGTCCCCAACTCCTCGCCTCAAGTGATCTTCCTGCCTTAGCCTCCCAGA
GTGCTAGGATTACAGGTGTGAG
>2043
ATTTTTGTATTTTTAGTAGAGATGCGGTTTCACCATGCTGGCCAGGCTGGTCCCCAACTC
CTCGCCTCAAGTGATCTTCCTGCCTTAGCCTCCCAGAGTGCTAGGATTACAGGTGTGAG
TCACTGTGCCCGGCCTGGATC
>2044
TTTCACCATGCTGGCCAGGCTGGTCCCCAACTCCTCGCCTCAAGTGATCTTCCTGCCTT

AGCCTCCCAGAGTGCTAGGATTACAGGTGTGAGTCACTGTGCCCGGCCTGGATCTTTTA
TTGTTTGTATTTCTGTTCATTT
>2045
CAGGCTGGTCCCCAACTCCTCGCCTCAAGTGATCTTCCTGCCTTAGCCTCCCAGAGTGC
TAGGATTACAGGTGTGAGTCACTGTGCCCGGCCTGGATCTTTTATTGTTTGTATTTCTGT
TCATTTCTTCCCTAAGAAACC
>2046
GGCTGGTCCCCAACTCCTCGCCTCAAGTGATCTTCCTGCCTTAGCCTCCCAGAGTGCTA
GGATTACAGGTGTGAGTCACTGTGCCCGGCCTGGATCTTTTATTGTTTGTATTTCTGTTC
ATTTCTTCCCTAAGAAACCCC
>2047
CAACTCCTCGCCTCAAGTGATCTTCCTGCCTTAGCCTCCCAGAGTGCTAGGATTACAGG
TGTGAGTCACTGTGCCCGGCCTGGATCTTTTATTGTTTGTATTTCTGTTCATTTCTTCCCT
AAGAAACCCCACGTTTTTGT
>2048
TCCTGCCTTAGCCTCCCAGAGTGCTAGGATTACAGGTGTGAGTCACTGTGCCCGGCCT
GGATCTTTTATTGTTTGTATTTCTGTTCATTTCTTCCCTAAGAAACCCCACGTTTTTGTGG
GCAGGACCTGGAATCCTATTC
>2049
GAGTCACTGTGCCCGGCCTGGATCTTTTATTGTTTGTATTTCTGTTCATTTCTTCCCTAA
GAAACCCCACGTTTTTGTGGGCAGGACCTGGAATCCTATTCAGTTATACAGCCCCGCTT
CGTCCACTGCTCCCCTTCAGA
>2050
CTGTGCCCGGCCTGGATCTTTTATTGTTTGTATTTCTGTTCATTTCTTCCCTAAGAAACC
CCACGTTTTTGTGGGCAGGACCTGGAATCCTATTCAGTTATACAGCCCCGCTTCGTCCA
CTGCTCCCCTTCAGAGTCTAG
>2051
TTTGTATTTCTGTTCATTTCTTCCCTAAGAAACCCCACGTTTTTGTGGGCAGGACCTGG
AATCCTATTCAGTTATACAGCCCCGCTTCGTCCACTGCTCCCCTTCAGAGTCTAGTGCCC
TGGACATCATAGGTATTCAGT
>2052
TCTTCCCTAAGAAACCCCACGTTTTTGTGGGCAGGACCTGGAATCCTATTCAGTTATAC
AGCCCCGCTTCGTCCACTGCTCCCCTTCAGAGTCTAGTGCCCTGGACATCATAGGTATT
CAGTACCTCTTGCTGGCTTAGG
>2053
GTTTTTGTGGGCAGGACCTGGAATCCTATTCAGTTATACAGCCCCGCTTCGTCCACTGC
TCCCCTTCAGAGTCTAGTGCCCTGGACATCATAGGTATTCAGTACCTCTTGCTGGCTTAG
GAACAGGAGGATGCCTGATGG
>2054
TCCTATTCAGTTATACAGCCCCGCTTCGTCCACTGCTCCCCTTCAGAGTCTAGTGCCCTG
GACATCATAGGTATTCAGTACCTCTTGCTGGCTTAGGAACAGGAGGATGCCTGATGGCC
AGGGTGTTCCTCTGAAGCAGT
>2055
GTGCCCTGGACATCATAGGTATTCAGTACCTCTTGCTGGCTTAGGAACAGGAGGATGCC
TGATGGCCAGGGTGTTCCTCTGAAGCAGTGGTGGCTGAGCTTTTTGAATATCTGCATTG
TGCTAAGGGTTAACTACCCAGG

>2056
GCCCTGGACATCATAGGTATTCAGTACCTCTTGCTGGCTTAGGAACAGGAGGATGCCTG
ATGGCCAGGGTGTTCCTCTGAAGCAGTGGTGGCTGAGCTTTTTGAATATCTGCATTGTG
CTAAGGGTTAACTACCCAGGCA
>2057
AGGTATTCAGTACCTCTTGCTGGCTTAGGAACAGGAGGATGCCTGATGGCCAGGGTGT
TCCTCTGAAGCAGTGGTGGCTGAGCTTTTTGAATATCTGCATTGTGCTAAGGGTTAACT
ACCCAGGCAGTACTGGTGGGGAA
>2058
TATTCAGTACCTCTTGCTGGCTTAGGAACAGGAGGATGCCTGATGGCCAGGGTGTTCCT
CTGAAGCAGTGGTGGCTGAGCTTTTTGAATATCTGCATTGTGCTAAGGGTTAACTACCC
AGGCAGTACTGGTGGGGAAGGG
>2059
CAGGAGGATGCCTGATGGCCAGGGTGTTCCTCTGAAGCAGTGGTGGCTGAGCTTTTTG
AATATCTGCATTGTGCTAAGGGTTAACTACCCAGGCAGTACTGGTGGGGAAGGGCCCC
AGAAGCAGGCCCAGACTCAGGTAT
>2060
GCCTGATGGCCAGGGTGTTCCTCTGAAGCAGTGGTGGCTGAGCTTTTTGAATATCTGCA
TTGTGCTAAGGGTTAACTACCCAGGCAGTACTGGTGGGGAAGGGCCCCAGAAGCAGG
CCCAGACTCAGGTATTGTTAAAGC
>2061
CTGAAGCAGTGGTGGCTGAGCTTTTTGAATATCTGCATTGTGCTAAGGGTTAACTACCC
AGGCAGTACTGGTGGGGAAGGGCCCCAGAAGCAGGCCCAGACTCAGGTATTGTTAAA
GCAGAGTCATCTTTGCTCTGAAGT
>2062
CATTGTGCTAAGGGTTAACTACCCAGGCAGTACTGGTGGGGAAGGGCCCCAGAAGCA
GGCCCAGACTCAGGTATTGTTAAAGCAGAGTCATCTTTGCTCTGAAGTTTTTTCTTTTAT
CTTTTCTTTTCTTTTTTAATTGG
>2063
TGCTAAGGGTTAACTACCCAGGCAGTACTGGTGGGGAAGGGCCCCAGAAGCAGGCCC
AGACTCAGGTATTGTTAAAGCAGAGTCATCTTTGCTCTGAAGTTTTTTCTTTTATCTTTT
CTTTTCTTTTTTAATTGGGGGAA
>2064
TGGTGGGGAAGGGCCCCAGAAGCAGGCCCAGACTCAGGTATTGTTAAAGCAGAGTCA
TCTTTGCTCTGAAGTTTTTTCTTTTATCTTTTCTTTTCTTTTTTAATTGGGGGAAACCTTG
TCCTAATTGGCATCTTTATTGT
>2065
AGCAGAGTCATCTTTGCTCTGAAGTTTTTTCTTTTATCTTTTCTTTTCTTTTTTAATTGGG
GGAAACCTTGTCCTAATTGGCATCTTTATTGTGGCAGGAATAGGCCAGGCAGGGCTGG
GGGAGTGTGAAGTCTGTGAAG
>2066
AAGTTTTTTCTTTTATCTTTTCTTTTCTTTTTTAATTGGGGGAAACCTTGTCCTAATTGGC
ATCTTTATTGTGGCAGGAATAGGCCAGGCAGGGCTGGGGGAGTGTGAAGTCTGTGAAG
GGAGGTGGCCTGGGGGCCCCC
>2067
TAATTGGGGGAAACCTTGTCCTAATTGGCATCTTTATTGTGGCAGGAATAGGCCAGGCA

GGGCTGGGGGAGTGTGAAGTCTGTGAAGGGAGGTGGCCTGGGGGCCCCCCACACAA
TGCCTGATTGTGCAGGCATAGGTA
>2068
AACCTTGTCCTAATTGGCATCTTTATTGTGGCAGGAATAGGCCAGGCAGGGCTGGGGG
AGTGTGAAGTCTGTGAAGGGAGGTGGCCTGGGGGCCCCCCACACAATGCCTGATTG
TGCAGGCATAGGTATGAACACATTG
>2069
TAATTGGCATCTTTATTGTGGCAGGAATAGGCCAGGCAGGGCTGGGGGAGTGTGAAGT
CTGTGAAGGGAGGTGGCCTGGGGGCCCCCCACACAATGCCTGATTGTGCAGGCATAG
GTATGAACACATTGCAGCCTGTGT
>2070
GGCAGGGCTGGGGGAGTGTGAAGTCTGTGAAGGGAGGTGGCCTGGGGGCCCCCCA
CACAATGCCTGATTGTGCAGGCATAGGTATGAACACATTGCAGCCTGTGTGCACCCAC
AGGGTGACCACGAGCAAGGCCTGGGG
>2071
GGAGGTGGCCTGGGGGCCCCCCACACAATGCCTGATTGTGCAGGCATAGGTATGAAC
ACATTGCAGCCTGTGTGCACCCACAGGGTGACCACGAGCAAGGCCTGGGGACGAGGA
CTACGAGGCCTGCAGCTGGGTCCTC
>2072
AGGTGGCCTGGGGGCCCCCCACACAATGCCTGATTGTGCAGGCATAGGTATGAACAC
ATTGCAGCCTGTGTGCACCCACAGGGTGACCACGAGCAAGGCCTGGGGACGAGGACT
ACGAGGCCTGCAGCTGGGTCCTCTT
>2073
CAGCCTGTGTGCACCCACAGGGTGACCACGAGCAAGGCCTGGGGACGAGGACTACGA
GGCCTGCAGCTGGGTCCTCTTTTCTCCCTCCTCACCCCCACCCAGTACCCACGTGATTG
GGCCACATCCCAGTAACAAGCAGT
>2074
GCAAGGCCTGGGGACGAGGACTACGAGGCCTGCAGCTGGGTCCTCTTTTCTCCCTCCT
CACCCCCACCCAGTACCCACGTGATTGGGCCACATCCCAGTAACAAGCAGTCCTTGTG
GAAGAGCCTTCATGAAGACCTTTG
>2075
TGGGGACGAGGACTACGAGGCCTGCAGCTGGGTCCTCTTTTCTCCCTCCTCACCCCCA
CCCAGTACCCACGTGATTGGGCCACATCCCAGTAACAAGCAGTCCTTGTGGAAGAGCC
TTCATGAAGACCTTTGCTGGGAGT
>2076
GCCTGCAGCTGGGTCCTCTTTTCTCCCTCCTCACCCCCACCCAGTACCCACGTGATTGG
GCCACATCCCAGTAACAAGCAGTCCTTGTGGAAGAGCCTTCATGAAGACCTTTGCTGG
GAGTGGCGGGCAGGGATCTGCGG
>2077
GGGTCCTCTTTTCTCCCTCCTCACCCCCACCCAGTACCCACGTGATTGGGCCACATCCC
AGTAACAAGCAGTCCTTGTGGAAGAGCCTTCATGAAGACCTTTGCTGGGAGTGGCGG
GCAGGGATCTGCGGGGAGCGTTGC
>2078
ACGTGATTGGGCCACATCCCAGTAACAAGCAGTCCTTGTGGAAGAGCCTTCATGAAGA
CCTTTGCTGGGAGTGGCGGGCAGGGATCTGCGGGGAGCGTTGCTGGTTCAAGCTGGG
GCTTTCCTATGGATGATGTCCTGTG

>2079
AAGCAGTCCTTGTGGAAGAGCCTTCATGAAGACCTTTGCTGGGAGTGGCGGGCAGGG
ATCTGCGGGGAGCGTTGCTGGTTCAAGCTGGGGCTTTCCTATGGATGATGTCCTGTGAA
GGGTAGATCCTGGAGGGACAGACA
>2080
TGCTGGGAGTGGCGGGCAGGGATCTGCGGGGAGCGTTGCTGGTTCAAGCTGGGGCTT
TCCTATGGATGATGTCCTGTGAAGGGTAGATCCTGGAGGGACAGACAGGTGTCTGATC
TTTGGCAGGCAGGGATGTCTCATTT
>2081
GGAGTGGCGGGCAGGGATCTGCGGGGAGCGTTGCTGGTTCAAGCTGGGGCTTTCCTAT
GGATGATGTCCTGTGAAGGGTAGATCCTGGAGGGACAGACAGGTGTCTGATCTTTGGC
AGGCAGGGATGTCTCATTTGTACA
>2082
TTGCTGGTTCAAGCTGGGGCTTTCCTATGGATGATGTCCTGTGAAGGGTAGATCCTGGA
GGGACAGACAGGTGTCTGATCTTTGGCAGGCAGGGATGTCTCATTTGTACATTATAGGT
ATTCAGTATCTCTTGCTGGCTT
>2083
ATGGATGATGTCCTGTGAAGGGTAGATCCTGGAGGGACAGACAGGTGTCTGATCTTTG
GCAGGCAGGGATGTCTCATTTGTACATTATAGGTATTCAGTATCTCTTGCTGGCTTAGGC
TTCATAATGTCTCATTTGTACA
>2084
GTCCTGTGAAGGGTAGATCCTGGAGGGACAGACAGGTGTCTGATCTTTGGCAGGCAG
GGATGTCTCATTTGTACATTATAGGTATTCAGTATCTCTTGCTGGCTTAGGCTTCATAATG
TCTCATTTGTACATCATAGCCT
>2085
CCTGGAGGGACAGACAGGTGTCTGATCTTTGGCAGGCAGGGATGTCTCATTTGTACATT
ATAGGTATTCAGTATCTCTTGCTGGCTTAGGCTTCATAATGTCTCATTTGTACATCATAGC
CTTCAGTACCTCTTGCTGAT
>2086
TGGCAGGCAGGGATGTCTCATTTGTACATTATAGGTATTCAGTATCTCTTGCTGGCTTAG
GCTTCATAATGTCTCATTTGTACATCATAGCCTTCAGTACCTCTTGCTGATTTGGTTAACC
CAGTTGTTATACCACGATA
>2087
GGGATGTCTCATTTGTACATTATAGGTATTCAGTATCTCTTGCTGGCTTAGGCTTCATAAT
GTCTCATTTGTACATCATAGCCTTCAGTACCTCTTGCTGATTTGGTTAACCCAGTTGTTAT
ACCACGATATTGATTTTT
>2088
CATTATAGGTATTCAGTATCTCTTGCTGGCTTAGGCTTCATAATGTCTCATTTGTACATCA
TAGCCTTCAGTACCTCTTGCTGATTTGGTTAACCCAGTTGTTATACCACGATATTGATTTT
TCATGCCATTTTCTTTAG
>2089
GGCTTAGGCTTCATAATGTCTCATTTGTACATCATAGCCTTCAGTACCTCTTGCTGATTTG
GTTAACCCAGTTGTTATACCACGATATTGATTTTTCATGCCATTTTCTTTAGGAAGATTGG
AAGATCATTGAGGATAGG
>2090
TTAGGCTTCATAATGTCTCATTTGTACATCATAGCCTTCAGTACCTCTTGCTGATTTGGTT

AACCCAGTTGTTATACCACGATATTGATTTTTCATGCCATTTTCTTTAGGAAGATTGGAA
GATCATTGAGGATAGGGAC
>2091
TTATACCACGATATTGATTTTTCATGCCATTTTCTTTAGGAAGATTGGAAGATCATTGAG
GATAGGGACTGTGACCTTTTCTTTTTTTCTACCCCAGTACCCAACCTGGCAGGTAGCAC
AGCGCAATGATCCACGATGTT
>2092
CCATTTTCTTTAGGAAGATTGGAAGATCATTGAGGATAGGGACTGTGACCTTTTCTTTTT
TTCTACCCCAGTACCCAACCTGGCAGGTAGCACAGCGCAATGATCCACGATGTTTAATA
GGTTGAATTGTAGAGGGGGCC
>2093
GATTGGAAGATCATTGAGGATAGGGACTGTGACCTTTTCTTTTTTTCTACCCCAGTACC
CAACCTGGCAGGTAGCACAGCGCAATGATCCACGATGTTTAATAGGTTGAATTGTAGA
GGGGGCCTCTGATTCCAAAGATG
>2094
ACTGTGACCTTTTCTTTTTTTCTACCCCAGTACCCAACCTGGCAGGTAGCACAGCGCAA
TGATCCACGATGTTTAATAGGTTGAATTGTAGAGGGGGCCTCTGATTCCAAAGATGGGG
GCAGAATATCAGTCCTCTCCAG
>2095
TTTTCTTTTTTTCTACCCCAGTACCCAACCTGGCAGGTAGCACAGCGCAATGATCCACG
ATGTTTAATAGGTTGAATTGTAGAGGGGGCCTCTGATTCCAAAGATGGGGGCAGAATAT
CAGTCCTCTCCAGACTGCCTGG
>2096
TGATCCACGATGTTTAATAGGTTGAATTGTAGAGGGGGCCTCTGATTCCAAAGATGGGG
GCAGAATATCAGTCCTCTCCAGACTGCCTGGGAATCCTCCTCTCTCCAGCCTGGGCCTG
GCCAGTGGGAGACAGAGGCTGC
>2097
GATGGGGGCAGAATATCAGTCCTCTCCAGACTGCCTGGGAATCCTCCTCTCTCCAGCCT
GGGCCTGGCCAGTGGGAGACAGAGGCTGCTGAAGTAGCTGGCAAGGAGGCAAAGAA
GATGATGACCTAGCTACGGTCCTCA
>2098
TCTCCAGACTGCCTGGGAATCCTCCTCTCTCCAGCCTGGGCCTGGCCAGTGGGAGACA
GAGGCTGCTGAAGTAGCTGGCAAGGAGGCAAAGAAGATGATGACCTAGCTACGGTCC
TCATGTGCCCACTGGGGAATCATCT
>2099
CTGGCCAGTGGGAGACAGAGGCTGCTGAAGTAGCTGGCAAGGAGGCAAAGAAGATG
ATGACCTAGCTACGGTCCTCATGTGCCCACTGGGGAATCATCTTTTTGCATCTGAGTTA
GTCAATGTGCTGTGCATTCTCTGCA
>2100
TGGGAGACAGAGGCTGCTGAAGTAGCTGGCAAGGAGGCAAAGAAGATGATGACCTAG
CTACGGTCCTCATGTGCCCACTGGGGAATCATCTTTTTGCATCTGAGTTAGTCAATGTG
CTGTGCATTCTCTGCAGAATTTGT
>2101
GAGGCAAAGAAGATGATGACCTAGCTACGGTCCTCATGTGCCCACTGGGGAATCATCT
TTTTGCATCTGAGTTAGTCAATGTGCTGTGCATTCTCTGCAGAATTTGTGAACCTGGTAT
AGGTATGAGGTGAGAGAGGCCA

>2102
CAAAGAAGATGATGACCTAGCTACGGTCCTCATGTGCCCACTGGGGAATCATCTTTTTG
CATCTGAGTTAGTCAATGTGCTGTGCATTCTCTGCAGAATTTGTGAACCTGGTATAGGTA
TGAGGTGAGAGAGGCCAGGAG
>2103
AGATGATGACCTAGCTACGGTCCTCATGTGCCCACTGGGGAATCATCTTTTTGCATCTG
AGTTAGTCAATGTGCTGTGCATTCTCTGCAGAATTTGTGAACCTGGTATAGGTATGAGG
TGAGAGAGGCCAGGAGGTCTGG
>2104
ATGACCTAGCTACGGTCCTCATGTGCCCACTGGGGAATCATCTTTTTGCATCTGAGTTA
GTCAATGTGCTGTGCATTCTCTGCAGAATTTGTGAACCTGGTATAGGTATGAGGTGAGA
GAGGCCAGGAGGTCTGGCTGCT
>2105
ATGTGCCCACTGGGGAATCATCTTTTTGCATCTGAGTTAGTCAATGTGCTGTGCATTCTC
TGCAGAATTTGTGAACCTGGTATAGGTATGAGGTGAGAGAGGCCAGGAGGTCTGGCTG
CTCCCCCTCTAGACTTCGCAGG
>2106
TTTTTGCATCTGAGTTAGTCAATGTGCTGTGCATTCTCTGCAGAATTTGTGAACCTGGTA
TAGGTATGAGGTGAGAGAGGCCAGGAGGTCTGGCTGCTCCCCCTCTAGACTTCGCAGG
TAAAACCCAAGGTGTGTGCCAC
>2107
GTCAATGTGCTGTGCATTCTCTGCAGAATTTGTGAACCTGGTATAGGTATGAGGTGAGA
GAGGCCAGGAGGTCTGGCTGCTCCCCCTCTAGACTTCGCAGGTAAAACCCAAGGTGT
GTGCCACGTAAAGGTTCAGAGGTT
>2108
TGTGAACCTGGTATAGGTATGAGGTGAGAGAGGCCAGGAGGTCTGGCTGCTCCCCCTC
TAGACTTCGCAGGTAAAACCCAAGGTGTGTGCCACGTAAAGGTTCAGAGGTTTTATAG
GCATCCAGATAGCACATGTGGGTC
>2109
ATAGGTATGAGGTGAGAGAGGCCAGGAGGTCTGGCTGCTCCCCCTCTAGACTTCGCAG
GTAAAACCCAAGGTGTGTGCCACGTAAAGGTTCAGAGGTTTTATAGGCATCCAGATAG
CACATGTGGGTCTCTGCCCCTGCT
>2110
GTGAGAGAGGCCAGGAGGTCTGGCTGCTCCCCCTCTAGACTTCGCAGGTAAAACCCA
AGGTGTGTGCCACGTAAAGGTTCAGAGGTTTTATAGGCATCCAGATAGCACATGTGGG
TCTCTGCCCCTGCTTGCAGCCTCCC
>2111
GAGGCCAGGAGGTCTGGCTGCTCCCCCTCTAGACTTCGCAGGTAAAACCCAAGGTGT
GTGCCACGTAAAGGTTCAGAGGTTTTATAGGCATCCAGATAGCACATGTGGGTCTCTGC
CCCTGCTTGCAGCCTCCCTGGGGC
>2112
GAGGTCTGGCTGCTCCCCCTCTAGACTTCGCAGGTAAAACCCAAGGTGTGTGCCACGT
AAAGGTTCAGAGGTTTTATAGGCATCCAGATAGCACATGTGGGTCTCTGCCCCTGCTTG
CAGCCTCCCTGGGGCAGCCAGCC
>2113
TTCGCAGGTAAAACCCAAGGTGTGTGCCACGTAAAGGTTCAGAGGTTTTATAGGCATC

CAGATAGCACATGTGGGTCTCTGCCCCTGCTTGCAGCCTCCCTGGGGCAGCCAGCCAG
GCTCAGGGCAGGAAGCTGCTGATT
>2114
GTCTCTGCCCCTGCTTGCAGCCTCCCTGGGGCAGCCAGCCAGGCTCAGGGCAGGAAG
CTGCTGATTCTGGGTCACTTGGCTGCTCTTGCTTGCTGCTGCTGTGATTGTTCCTACAGT
TAAGATCTTAGCTCCATAAGGGA
>2115
GCAGCCAGCCAGGCTCAGGGCAGGAAGCTGCTGATTCTGGGTCACTTGGCTGCTCTTG
CTTGCTGCTGCTGTGATTGTTCCTACAGTTAAGATCTTAGCTCCATAAGGGACATACGG
AGGTCATCTAGGTCATCTCATGG
>2116
AGCCAGGCTCAGGGCAGGAAGCTGCTGATTCTGGGTCACTTGGCTGCTCTTGCTTGCT
GCTGCTGTGATTGTTCCTACAGTTAAGATCTTAGCTCCATAAGGGACATACGGAGGTCA
TCTAGGTCATCTCATGGCTCTCA
>2117
CTGCTCTTGCTTGCTGCTGCTGTGATTGTTCCTACAGTTAAGATCTTAGCTCCATAAGGG
ACATACGGAGGTCATCTAGGTCATCTCATGGCTCTCAGGCAAGAAAAGTAGCATTATTG
CTCTCATGCCCACTTTATGGA
>2118
CTTGCTGCTGCTGTGATTGTTCCTACAGTTAAGATCTTAGCTCCATAAGGGACATACGG
AGGTCATCTAGGTCATCTCATGGCTCTCAGGCAAGAAAAGTAGCATTATTGCTCTCATG
CCCACTTTATGGATAAGTGACC
>2119
TTAAGATCTTAGCTCCATAAGGGACATACGGAGGTCATCTAGGTCATCTCATGGCTCTC
AGGCAAGAAAAGTAGCATTATTGCTCTCATGCCCACTTTATGGATAAGTGACCGAGGCC
TGCAGAGGCAGAGTGGCTATTC
>2120
ATCTAGGTCATCTCATGGCTCTCAGGCAAGAAAAGTAGCATTATTGCTCTCATGCCCAC
TTTATGGATAAGTGACCGAGGCCTGCAGAGGCAGAGTGGCTATTCCAGCCATTAAATGT
GGCTCCCTGCTATTCTGTTCCC
>2121
GGCAAGAAAAGTAGCATTATTGCTCTCATGCCCACTTTATGGATAAGTGACCGAGGCCT
GCAGAGGCAGAGTGGCTATTCCAGCCATTAAATGTGGCTCCCTGCTATTCTGTTCCCTG
CCAGGATATTCTGTGGATGGTT
>2122
CTCTCATGCCCACTTTATGGATAAGTGACCGAGGCCTGCAGAGGCAGAGTGGCTATTCC
AGCCATTAAATGTGGCTCCCTGCTATTCTGTTCCCTGCCAGGATATTCTGTGGATGGTTA
GGGGAGGGGTCTTACCTCAGC
>2123
GGATAAGTGACCGAGGCCTGCAGAGGCAGAGTGGCTATTCCAGCCATTAAATGTGGCT
CCCTGCTATTCTGTTCCCTGCCAGGATATTCTGTGGATGGTTAGGGGAGGGGTCTTACC
TCAGCTAACGAGGTGCATTTTCT
>2124
CAGAGGCAGAGTGGCTATTCCAGCCATTAAATGTGGCTCCCTGCTATTCTGTTCCCTGC
CAGGATATTCTGTGGATGGTTAGGGGAGGGGTCTTACCTCAGCTAACGAGGTGCATTTT
CTGTGGGGTGAGGCCATTCTTG

>2125
AGAGTGGCTATTCCAGCCATTAAATGTGGCTCCCTGCTATTCTGTTCCCTGCCAGGATAT
TCTGTGGATGGTTAGGGGAGGGGTCTTACCTCAGCTAACGAGGTGCATTTTCTGTGGG
GTGAGGCCATTCTTGTCACTTA
>2126
CCAGCCATTAAATGTGGCTCCCTGCTATTCTGTTCCCTGCCAGGATATTCTGTGGATGGT
TAGGGGAGGGGTCTTACCTCAGCTAACGAGGTGCATTTTCTGTGGGGTGAGGCCATTC
TTGTCACTTAGGTGTCTGAGGC
>2127
CCTGCTATTCTGTTCCCTGCCAGGATATTCTGTGGATGGTTAGGGGAGGGGTCTTACCT
CAGCTAACGAGGTGCATTTTCTGTGGGGTGAGGCCATTCTTGTCACTTAGGTGTCTGAG
GCCTGATGGTGTTGGGGAATGT
>2128
CTGCCAGGATATTCTGTGGATGGTTAGGGGAGGGGTCTTACCTCAGCTAACGAGGTGC
ATTTTCTGTGGGGTGAGGCCATTCTTGTCACTTAGGTGTCTGAGGCCTGATGGTGTTGG
GGAATGTCCTGGTTCTATTCCCA
>2129
TGTGGATGGTTAGGGGAGGGGTCTTACCTCAGCTAACGAGGTGCATTTTCTGTGGGGT
GAGGCCATTCTTGTCACTTAGGTGTCTGAGGCCTGATGGTGTTGGGGAATGTCCTGGTT
CTATTCCCAGGGCTGTAACGTGG
>2130
TTAGGGGAGGGGTCTTACCTCAGCTAACGAGGTGCATTTTCTGTGGGGTGAGGCCATT
CTTGTCACTTAGGTGTCTGAGGCCTGATGGTGTTGGGGAATGTCCTGGTTCTATTCCCA
GGGCTGTAACGTGGGGGAAGGAT
>2131
CCTCAGCTAACGAGGTGCATTTTCTGTGGGGTGAGGCCATTCTTGTCACTTAGGTGTCT
GAGGCCTGATGGTGTTGGGGAATGTCCTGGTTCTATTCCCAGGGCTGTAACGTGGGGG
AAGGATGGAGGAAGACAGAATGG
>2132
ATTTTCTGTGGGGTGAGGCCATTCTTGTCACTTAGGTGTCTGAGGCCTGATGGTGTTGG
GGAATGTCCTGGTTCTATTCCCAGGGCTGTAACGTGGGGGAAGGATGGAGGAAGACA
GAATGGCCTCTGGAACTTTTGGAT
>2133
GCCATTCTTGTCACTTAGGTGTCTGAGGCCTGATGGTGTTGGGGAATGTCCTGGTTCTA
TTCCCAGGGCTGTAACGTGGGGGAAGGATGGAGGAAGACAGAATGGCCTCTGGAACT
TTTGGATCTTTCTGAGTTGAGGCC
>2134
TCTTGTCACTTAGGTGTCTGAGGCCTGATGGTGTTGGGGAATGTCCTGGTTCTATTCCC
AGGGCTGTAACGTGGGGGAAGGATGGAGGAAGACAGAATGGCCTCTGGAACTTTTGG
ATCTTTCTGAGTTGAGGCCTTGTC
>2135
CCCAGGGCTGTAACGTGGGGGAAGGATGGAGGAAGACAGAATGGCCTCTGGAACTTT
TGGATCTTTCTGAGTTGAGGCCTTGTCTGGCCAACTCCGGGAGGCTGCTGGCATGGCA
AGCAGGGTAGGGATGCCTGGCCTAG
>2136
AACGTGGGGGAAGGATGGAGGAAGACAGAATGGCCTCTGGAACTTTTGGATCTTTCT

GAGTTGAGGCCTTGTCTGGCCAACTCCGGGAGGCTGCTGGCATGGCAAGCAGGGTAG
GGATGCCTGGCCTAGCCAGGGGAGCT
>2137
AACTTTTGGATCTTTCTGAGTTGAGGCCTTGTCTGGCCAACTCCGGGAGGCTGCTGGC
ATGGCAAGCAGGGTAGGGATGCCTGGCCTAGCCAGGGGAGCTCCCTCTCTCCCCTCTG
CTGCCCCTCTTTCCACCCCCATCA
>2138
TGCCTGGCCTAGCCAGGGGAGCTCCCTCTCTCCCCTCTGCTGCCCCTCTTTCCACCCCC
ATCATAAACACGTGTGGACTTTCTGGTGGTGAGAAGATGGTTTTGGAGGGCTGGGCTG
AGGTCTAGGCAGGCAAGAGCTTC
>2139
CCTGGCCTAGCCAGGGGAGCTCCCTCTCTCCCCTCTGCTGCCCCTCTTTCCACCCCCAT
CATAAACACGTGTGGACTTTCTGGTGGTGAGAAGATGGTTTTGGAGGGCTGGGCTGAG
GTCTAGGCAGGCAAGAGCTTCCA
>2140
GGAGCTCCCTCTCTCCCCTCTGCTGCCCCTCTTTCCACCCCCATCATAAACACGTGTGG
ACTTTCTGGTGGTGAGAAGATGGTTTTGGAGGGCTGGGCTGAGGTCTAGGCAGGCAA
GAGCTTCCAAATGTGTGGCTTTGA
>2141
TTCCACCCCCATCATAAACACGTGTGGACTTTCTGGTGGTGAGAAGATGGTTTTGGAG
GGCTGGGCTGAGGTCTAGGCAGGCAAGAGCTTCCAAATGTGTGGCTTTGATTCTCAGC
CTTTTCATCCCTCAATCCTGGGAG
>2142
GACTTTCTGGTGGTGAGAAGATGGTTTTGGAGGGCTGGGCTGAGGTCTAGGCAGGCA
AGAGCTTCCAAATGTGTGGCTTTGATTCTCAGCCTTTTCATCCCTCAATCCTGGGAGAC
TCATTTTAAAACTTAAAAAAGGAC
>2143
CTTTTCATCCCTCAATCCTGGGAGACTCATTTTAAAACTTAAAAAAGGACTTCATTCTAT
TGGATGCTATGTGGGCAGGCCCTGGGCTGTTGTAGAGAGGCCTGAAACCCACTTCAGT
TTCCTGGACAGGCTGGGAGTGT
>2144
TGGGAGACTCATTTTAAAACTTAAAAAAGGACTTCATTCTATTGGATGCTATGTGGGCA
GGCCCTGGGCTGTTGTAGAGAGGCCTGAAACCCACTTCAGTTTCCTGGACAGGCTGGG
AGTGTGTACCCATGGGTTCCCTG
>2145
GAGACTCATTTTAAAACTTAAAAAAGGACTTCATTCTATTGGATGCTATGTGGGCAGGC
CCTGGGCTGTTGTAGAGAGGCCTGAAACCCACTTCAGTTTCCTGGACAGGCTGGGAGT
GTGTACCCATGGGTTCCCTGCTG
>2146
GGACTTCATTCTATTGGATGCTATGTGGGCAGGCCCTGGGCTGTTGTAGAGAGGCCTGA
AACCCACTTCAGTTTCCTGGACAGGCTGGGAGTGTGTACCCATGGGTTCCCTGCTGCC
CTATTCCTGGAAGACACTAATAT
>2147
CTATGTGGGCAGGCCCTGGGCTGTTGTAGAGAGGCCTGAAACCCACTTCAGTTTCCTG
GACAGGCTGGGAGTGTGTACCCATGGGTTCCCTGCTGCCCTATTCCTGGAAGACACTA
ATATCCCTCCCAAACTCTTGGTTG

>2148
ATGTGGGCAGGCCCTGGGCTGTTGTAGAGAGGCCTGAAACCCACTTCAGTTTCCTGGA
CAGGCTGGGAGTGTGTACCCATGGGTTCCCTGCTGCCCTATTCCTGGAAGACACTAATA
TCCCTCCCAAACTCTTGGTTGGT
>2149
CCTGGGCTGTTGTAGAGAGGCCTGAAACCCACTTCAGTTTCCTGGACAGGCTGGGAGT
GTGTACCCATGGGTTCCCTGCTGCCCTATTCCTGGAAGACACTAATATCCCTCCCAAAC
TCTTGGTTGGTTTCTGCTTTTTT
>2150
GGGAGTGTGTACCCATGGGTTCCCTGCTGCCCTATTCCTGGAAGACACTAATATCCCTC
CCAAACTCTTGGTTGGTTTCTGCTTTTTTTCTGTAGCATTTTGTCTTTGTGGGAAATTTT
AAGAAACATATGTATGGCTTT
>2151
GTGTGTACCCATGGGTTCCCTGCTGCCCTATTCCTGGAAGACACTAATATCCCTCCCAA
ACTCTTGGTTGGTTTCTGCTTTTTTTCTGTAGCATTTTGTCTTTGTGGGAAATTTTAAGA
AACATATGTATGGCTTTTTGG
>2152
CCCTGCTGCCCTATTCCTGGAAGACACTAATATCCCTCCCAAACTCTTGGTTGGTTTCTG
CTTTTTTTCTGTAGCATTTTGTCTTTGTGGGAAATTTTAAGAAACATATGTATGGCTTTTT
GGTTTCCACTTATTTATTT
>2153
CAAACTCTTGGTTGGTTTCTGCTTTTTTTCTGTAGCATTTTGTCTTTGTGGGAAATTTTA
AGAAACATATGTATGGCTTTTTGGTTTCCACTTATTTATTTTCCCCTCTGAGTTTCAGATT
TCATCCTTCTCCACCCCTT
>2154
GGTTTCTGCTTTTTTTCTGTAGCATTTTGTCTTTGTGGGAAATTTTAAGAAACATATGTAT
GGCTTTTTGGTTTCCACTTATTTATTTTCCCCTCTGAGTTTCAGATTTCATCCTTCTCCAC
CCCTTCCCCCTGCCCTAA
>2155
GTCTTTGTGGGAAATTTTAAGAAACATATGTATGGCTTTTTGGTTTCCACTTATTTATTTT
CCCCTCTGAGTTTCAGATTTCATCCTTCTCCACCCCTTCCCCCTGCCCTAAGGTGGGGC
TAGTGCAAATCGCAGTGTGT
>2156
TTTCCACTTATTTATTTTCCCCTCTGAGTTTCAGATTTCATCCTTCTCCACCCCTTCCCCC
TGCCCTAAGGTGGGGCTAGTGCAAATCGCAGTGTGTGTGTAGTGGGATGGTTAAAGAG
CCGTACATCCTCACACCTGGT
>2157
ATTTATTTTCCCCTCTGAGTTTCAGATTTCATCCTTCTCCACCCCTTCCCCCTGCCCTAA
GGTGGGGCTAGTGCAAATCGCAGTGTGTGTGTAGTGGGATGGTTAAAGAGCCGTACAT
CCTCACACCTGGTGACGCAGAC
>2158
GTTTCAGATTTCATCCTTCTCCACCCCTTCCCCCTGCCCTAAGGTGGGGCTAGTGCAAA
TCGCAGTGTGTGTGTAGTGGGATGGTTAAAGAGCCGTACATCCTCACACCTGGTGACG
CAGACTAAGGCAATTAACCTTCT
>2159
TTCAGATTTCATCCTTCTCCACCCCTTCCCCCTGCCCTAAGGTGGGGCTAGTGCAAATC

GCAGTGTGTGTGTAGTGGGATGGTTAAAGAGCCGTACATCCTCACACCTGGTGACGCA
GACTAAGGCAATTAACCTTCTTT
>2160
AGATTTCATCCTTCTCCACCCCTTCCCCCTGCCCTAAGGTGGGGCTAGTGCAAATCGCA
GTGTGTGTGTAGTGGGATGGTTAAAGAGCCGTACATCCTCACACCTGGTGACGCAGAC
TAAGGCAATTAACCTTCTTTAGT
>2161
TCCTTCTCCACCCCTTCCCCCTGCCCTAAGGTGGGGCTAGTGCAAATCGCAGTGTGTGT
GTAGTGGGATGGTTAAAGAGCCGTACATCCTCACACCTGGTGACGCAGACTAAGGCAA
TTAACCTTCTTTAGTAAAGACCC
>2162
CCCTTCCCCCTGCCCTAAGGTGGGGCTAGTGCAAATCGCAGTGTGTGTGTAGTGGGAT
GGTTAAAGAGCCGTACATCCTCACACCTGGTGACGCAGACTAAGGCAATTAACCTTCT
TTAGTAAAGACCCTTTCAGCTGGG
>2163
TAGTGGGATGGTTAAAGAGCCGTACATCCTCACACCTGGTGACGCAGACTAAGGCAAT
TAACCTTCTTTAGTAAAGACCCTTTCAGCTGGGTGCGGTGGGAGGCACTTCGGGAGGC
CGAGGTGGAAGGATTGCTTGAGCC
>2164
CGTACATCCTCACACCTGGTGACGCAGACTAAGGCAATTAACCTTCTTTAGTAAAGACC
CTTTCAGCTGGGTGCGGTGGGAGGCACTTCGGGAGGCCGAGGTGGAAGGATTGCTTG
AGCCCTGGAGTTGGAGACCAGCTT
>2165
AAGGCAATTAACCTTCTTTAGTAAAGACCCTTTCAGCTGGGTGCGGTGGGAGGCACTT
CGGGAGGCCGAGGTGGAAGGATTGCTTGAGCCCTGGAGTTGGAGACCAGCTTGGGCA
GCATGGTGAAACCACCTCTCTAAAA
>2166
GACCCTTTCAGCTGGGTGCGGTGGGAGGCACTTCGGGAGGCCGAGGTGGAAGGATTG
CTTGAGCCCTGGAGTTGGAGACCAGCTTGGGCAGCATGGTGAAACCACCTCTCTAAAA
AAATATAAAAATTAACGGGACGTTG
>2167
AGGCACTTCGGGAGGCCGAGGTGGAAGGATTGCTTGAGCCCTGGAGTTGGAGACCAG
CTTGGGCAGCATGGTGAAACCACCTCTCTAAAAAAATATAAAAATTAACGGGACGTTG
TGGCATGCACCTATAGTCCCAGCTA
>2168
TGGAGTTGGAGACCAGCTTGGGCAGCATGGTGAAACCACCTCTCTAAAAAAATATAAA
AATTAACGGGACGTTGTGGCATGCACCTATAGTCCCAGCTACTTGGGAGGCTGGGGCG
CGAGAAGTGCTTGAACCTGAGAGA
>2169
AGTTGGAGACCAGCTTGGGCAGCATGGTGAAACCACCTCTCTAAAAAAATATAAAAAT
TAACGGGACGTTGTGGCATGCACCTATAGTCCCAGCTACTTGGGAGGCTGGGGCGCGA
GAAGTGCTTGAACCTGAGAGATGG
>2170
TATAAAAATTAACGGGACGTTGTGGCATGCACCTATAGTCCCAGCTACTTGGGAGGCTG
GGGCGCGAGAAGTGCTTGAACCTGAGAGATGGAGACTGCGGTGAGGCGAGATTGCAC
CACTGCACTTCAGCTAGGTGACAG

>2171
CACCTATAGTCCCAGCTACTTGGGAGGCTGGGGCGCGAGAAGTGCTTGAACCTGAGAG
ATGGAGACTGCGGTGAGGCGAGATTGCACCACTGCACTTCAGCTAGGTGACAGAGTG
GGACCCTGTCTCAAAACAAAACAAA
>2172
GCGAGAAGTGCTTGAACCTGAGAGATGGAGACTGCGGTGAGGCGAGATTGCACCACT
GCACTTCAGCTAGGTGACAGAGTGGGACCCTGTCTCAAAACAAAACAAAACAACAAC
AAAAAAAGCATGTTTTCCTGTCTTTC
>2173
TGCTTGAACCTGAGAGATGGAGACTGCGGTGAGGCGAGATTGCACCACTGCACTTCA
GCTAGGTGACAGAGTGGGACCCTGTCTCAAAACAAAACAAAACAACAACAAAAAAA
GCATGTTTTCCTGTCTTTCCCTTCCTC
>2174
CTGCACTTCAGCTAGGTGACAGAGTGGGACCCTGTCTCAAAACAAAACAAAACAACA
ACAAAAAAAGCATGTTTTCCTGTCTTTCCCTTCCTCTCCTCCACCCCCTGGCATGAACT
CTCAACTCTTAGCCTGTGGATGTC
>2175
CAGCTAGGTGACAGAGTGGGACCCTGTCTCAAAACAAAACAAAACAACAACAAAAA
AAGCATGTTTTCCTGTCTTTCCCTTCCTCTCCTCCACCCCCTGGCATGAACTCTCAACTC
TTAGCCTGTGGATGTCCGTTTCTA
>2176
AAAAAGCATGTTTTCCTGTCTTTCCCTTCCTCTCCTCCACCCCCTGGCATGAACTCTCA
ACTCTTAGCCTGTGGATGTCCGTTTCTATAAATGCTGGTGCTCGGTGGTCACCGTAGAC
GTCCGAGCAGCCCCTGGGGCTT
>2177
CATGTTTTCCTGTCTTTCCCTTCCTCTCCTCCACCCCCTGGCATGAACTCTCAACTCTTA
GCCTGTGGATGTCCGTTTCTATAAATGCTGGTGCTCGGTGGTCACCGTAGACGTCCGAG
CAGCCCCTGGGGCTTTGGCTT
>2178
CCTCCACCCCCTGGCATGAACTCTCAACTCTTAGCCTGTGGATGTCCGTTTCTATAAATG
CTGGTGCTCGGTGGTCACCGTAGACGTCCGAGCAGCCCCTGGGGCTTTGGCTTGTGTT
TTCTCTGTACTTGTGAGGGTGG
>2179
CCACCCCCTGGCATGAACTCTCAACTCTTAGCCTGTGGATGTCCGTTTCTATAAATGCT
GGTGCTCGGTGGTCACCGTAGACGTCCGAGCAGCCCCTGGGGCTTTGGCTTGTGTTTT
CTCTGTACTTGTGAGGGTGGTTG
>2180
CCTGGCATGAACTCTCAACTCTTAGCCTGTGGATGTCCGTTTCTATAAATGCTGGTGCTC
GGTGGTCACCGTAGACGTCCGAGCAGCCCCTGGGGCTTTGGCTTGTGTTTTCTCTGTA
CTTGTGAGGGTGGTTGCCACTT
>2181
ATGAACTCTCAACTCTTAGCCTGTGGATGTCCGTTTCTATAAATGCTGGTGCTCGGTGG
TCACCGTAGACGTCCGAGCAGCCCCTGGGGCTTTGGCTTGTGTTTTCTCTGTACTTGTG
AGGGTGGTTGCCACTTCTGGCC
>2182
GTCCGTTTCTATAAATGCTGGTGCTCGGTGGTCACCGTAGACGTCCGAGCAGCCCCTGG

GGCTTTGGCTTGTGTTTTCTCTGTACTTGTGAGGGTGGTTGCCACTTCTGGCCTTCTTT
GTGCTTTCTTTTTCTTTCCTTT
>2183
CTCGGTGGTCACCGTAGACGTCCGAGCAGCCCCTGGGGCTTTGGCTTGTGTTTTCTCT
GTACTTGTGAGGGTGGTTGCCACTTCTGGCCTTCTTTGTGCTTTCTTTTTCTTTCCTTTC
CCCAATCTTTTGTTCCTCCCTC
>2184
GGTGGTCACCGTAGACGTCCGAGCAGCCCCTGGGGCTTTGGCTTGTGTTTTCTCTGTA
CTTGTGAGGGTGGTTGCCACTTCTGGCCTTCTTTGTGCTTTCTTTTTCTTTCCTTTCCCC
AATCTTTTGTTCCTCCCTCCCT
>2185
GCAGCCCCTGGGGCTTTGGCTTGTGTTTTCTCTGTACTTGTGAGGGTGGTTGCCACTTC
TGGCCTTCTTTGTGCTTTCTTTTTCTTTCCTTTCCCCAATCTTTTGTTCCTCCCTCCCTCC
CTCCCTCCCTTCCTCCCTTC
>2186
TACTTGTGAGGGTGGTTGCCACTTCTGGCCTTCTTTGTGCTTTCTTTTTCTTTCCTTTCC
CCAATCTTTTGTTCCTCCCTCCCTCCCTCCCTCCCTTCCTCCCTTCCTTCCTTCTGCTTTC
CTGTTTAAAGTATATGGCT
>2187
CCTTTCCCCAATCTTTTGTTCCTCCCTCCCTCCCTCCCTCCCTTCCTCCCTTCCTTCCTTC
TGCTTTCCTGTTTAAAGTATATGGCTGCACCTAGCATGACACAGTTCCTATTTATTCCTC
AGACTTGCTGGGACAGTCT
>2188
CCAATCTTTTGTTCCTCCCTCCCTCCCTCCCTCCCTTCCTCCCTTCCTTCCTTCTGCTTTC
CTGTTTAAAGTATATGGCTGCACCTAGCATGACACAGTTCCTATTTATTCCTCAGACTTG
CTGGGACAGTCTCCTCTGT
>2189
TCCCTCCCTTCCTCCCTTCCTTCCTTCTGCTTTCCTGTTTAAAGTATATGGCTGCACCTA
GCATGACACAGTTCCTATTTATTCCTCAGACTTGCTGGGACAGTCTCCTCTGTATGCGG
CGACTGAGGGTGGGTTCCAGT
>2190
TCCTGTTTAAAGTATATGGCTGCACCTAGCATGACACAGTTCCTATTTATTCCTCAGACT
TGCTGGGACAGTCTCCTCTGTATGCGGCGACTGAGGGTGGGTTCCAGTGCCCCAGCCC
ACTCCCCTTCCCCTTGTTTTTG
>2191
AAGTATATGGCTGCACCTAGCATGACACAGTTCCTATTTATTCCTCAGACTTGCTGGGAC
AGTCTCCTCTGTATGCGGCGACTGAGGGTGGGTTCCAGTGCCCCAGCCCACTCCCCTT
CCCCTTGTTTTTGTCCACTTGG
>2192
TAGCATGACACAGTTCCTATTTATTCCTCAGACTTGCTGGGACAGTCTCCTCTGTATGCG
GCGACTGAGGGTGGGTTCCAGTGCCCCAGCCCACTCCCCTTCCCCTTGTTTTTGTCCA
CTTGGCTGGACAAAGAGAACAA
>2193
TGGGACAGTCTCCTCTGTATGCGGCGACTGAGGGTGGGTTCCAGTGCCCCAGCCCACT
CCCCTTCCCCTTGTTTTTGTCCACTTGGCTGGACAAAGAGAACAAAACCAAATGGCCT
CCCCTCCCCCTCTCCTAAACCGGT

>2194
AGTCTCCTCTGTATGCGGCGACTGAGGGTGGGTTCCAGTGCCCCAGCCCACTCCCCTT
CCCCTTGTTTTTGTCCACTTGGCTGGACAAAGAGAACAAAACCAAATGGCCTCCCCTC
CCCCTCTCCTAAACCGGTCAAGCA
>2195
TTGTTTTTGTCCACTTGGCTGGACAAAGAGAACAAAACCAAATGGCCTCCCCTCCCCC
TCTCCTAAACCGGTCAAGCAGTGGTATTCTGGGATGATTTTCCCTGCAACCATTTAAAC
AAGTGCTTTGATGCTTACGAGTC
>2196
GTCCACTTGGCTGGACAAAGAGAACAAAACCAAATGGCCTCCCCTCCCCCTCTCCTAA
ACCGGTCAAGCAGTGGTATTCTGGGATGATTTTCCCTGCAACCATTTAAACAAGTGCTT
TGATGCTTACGAGTCATTGGAAT
>2197
CCCTCCCCCTCTCCTAAACCGGTCAAGCAGTGGTATTCTGGGATGATTTTCCCTGCAAC
CATTTAAACAAGTGCTTTGATGCTTACGAGTCATTGGAATGACCAGGCCTGTGCGCCCC
TTCTCACTGACACCCTTAGACC
>2198
CCGGTCAAGCAGTGGTATTCTGGGATGATTTTCCCTGCAACCATTTAAACAAGTGCTTT
GATGCTTACGAGTCATTGGAATGACCAGGCCTGTGCGCCCCTTCTCACTGACACCCTTA
GACCAGGCTGGGCCTCACCCTT
>2199
GGGATGATTTTCCCTGCAACCATTTAAACAAGTGCTTTGATGCTTACGAGTCATTGGAA
TGACCAGGCCTGTGCGCCCCTTCTCACTGACACCCTTAGACCAGGCTGGGCCTCACCC
TTTTGTCTTGCTGCCCCTCGGTG
>2200
CATTGGAATGACCAGGCCTGTGCGCCCCTTCTCACTGACACCCTTAGACCAGGCTGGG
CCTCACCCTTTTGTCTTGCTGCCCCTCGGTGCCCAGTGGGCGTGGGCAGTAGGACTGA
GCAGAGGGGCCGAGGCTGTGCTGG
>2201
CCTGTGCGCCCCTTCTCACTGACACCCTTAGACCAGGCTGGGCCTCACCCTTTTGTCTT
GCTGCCCCTCGGTGCCCAGTGGGCGTGGGCAGTAGGACTGAGCAGAGGGGCCGAGGC
TGTGCTGGCCGGCAGCTCTGTTAC
>2202
TCTCACTGACACCCTTAGACCAGGCTGGGCCTCACCCTTTTGTCTTGCTGCCCCTCGGT
GCCCAGTGGGCGTGGGCAGTAGGACTGAGCAGAGGGGCCGAGGCTGTGCTGGCCGGC
AGCTCTGTTACAGTAGAGTGGAAG
>2203
GACACCCTTAGACCAGGCTGGGCCTCACCCTTTTGTCTTGCTGCCCCTCGGTGCCCAG
TGGGCGTGGGCAGTAGGACTGAGCAGAGGGGCCGAGGCTGTGCTGGCCGGCAGCTCT
GTTACAGTAGAGTGGAAGGAGGTCC
>2204
CCCTTTTGTCTTGCTGCCCCTCGGTGCCCAGTGGGCGTGGGCAGTAGGACTGAGCAGA
GGGGCCGAGGCTGTGCTGGCCGGCAGCTCTGTTACAGTAGAGTGGAAGGAGGTCCCG
GCCAGAGGGGCAGGTCAAACAGGCC
>2205
CCTCGGTGCCCAGTGGGCGTGGGCAGTAGGACTGAGCAGAGGGGCCGAGGCTGTGCT

GGCCGGCAGCTCTGTTACAGTAGAGTGGAAGGAGGTCCCGGCCAGAGGGGCAGGTCA
AACAGGCCTCTTTTATTTACAGTGCT
>2206
AGTGGGCGTGGGCAGTAGGACTGAGCAGAGGGGCCGAGGCTGTGCTGGCCGGCAGCT
CTGTTACAGTAGAGTGGAAGGAGGTCCCGGCCAGAGGGGCAGGTCAAACAGGCCTCT
TTTATTTACAGTGCTCTCTTTCACGG
>2207
GGCAGTAGGACTGAGCAGAGGGGCCGAGGCTGTGCTGGCCGGCAGCTCTGTTACAGT
AGAGTGGAAGGAGGTCCCGGCCAGAGGGGCAGGTCAAACAGGCCTCTTTTATTTACA
GTGCTCTCTTTCACGGCCTCACTGAA
>2208
GGGGCCGAGGCTGTGCTGGCCGGCAGCTCTGTTACAGTAGAGTGGAAGGAGGTCCCG
GCCAGAGGGGCAGGTCAAACAGGCCTCTTTTATTTACAGTGCTCTCTTTCACGGCCTC
ACTGAAACAGGAGACCCGAGAGGCT
>2209
GCTCTGTTACAGTAGAGTGGAAGGAGGTCCCGGCCAGAGGGGCAGGTCAAACAGGCC
TCTTTTATTTACAGTGCTCTCTTTCACGGCCTCACTGAAACAGGAGACCCGAGAGGCTC
CGGTGAAAGGGCACTGTGTTTGTG
>2210
AAACAGGCCTCTTTTATTTACAGTGCTCTCTTTCACGGCCTCACTGAAACAGGAGACC
CGAGAGGCTCCGGTGAAAGGGCACTGTGTTTGTGCATTCAGGCCTTCCAGGGCTGGCT
ACAAAGGTGGGGGCACCCGGAGCA
>2211
ATTTACAGTGCTCTCTTTCACGGCCTCACTGAAACAGGAGACCCGAGAGGCTCCGGTG
AAAGGGCACTGTGTTTGTGCATTCAGGCCTTCCAGGGCTGGCTACAAAGGTGGGGGC
ACCCGGAGCACTGAAGGGGCTTTTT
>2212
ACAGTGCTCTCTTTCACGGCCTCACTGAAACAGGAGACCCGAGAGGCTCCGGTGAAA
GGGCACTGTGTTTGTGCATTCAGGCCTTCCAGGGCTGGCTACAAAGGTGGGGGCACCC
GGAGCACTGAAGGGGCTTTTTGTCA
>2213
GAGACCCGAGAGGCTCCGGTGAAAGGGCACTGTGTTTGTGCATTCAGGCCTTCCAGG
GCTGGCTACAAAGGTGGGGGCACCCGGAGCACTGAAGGGGCTTTTTGTCATTGGCAG
TTTGGCTTAGGGGAGAAGCGAGGGAG
>2214
GTTTGTGCATTCAGGCCTTCCAGGGCTGGCTACAAAGGTGGGGGCACCCGGAGCACTG
AAGGGGCTTTTTGTCATTGGCAGTTTGGCTTAGGGGAGAAGCGAGGGAGAGCCAGTG
TGGGAGCCGCCTCTAGGATTGTGAG
>2215
TCAGGCCTTCCAGGGCTGGCTACAAAGGTGGGGGCACCCGGAGCACTGAAGGGGCTT
TTTGTCATTGGCAGTTTGGCTTAGGGGAGAAGCGAGGGAGAGCCAGTGTGGGAGCCG
CCTCTAGGATTGTGAGCAAGTGCCTG
>2216
GGCACCCGGAGCACTGAAGGGGCTTTTTGTCATTGGCAGTTTGGCTTAGGGGAGAAGC
GAGGGAGAGCCAGTGTGGGAGCCGCCTCTAGGATTGTGAGCAAGTGCCTGTTTAGAC
AGCTCCACGCTGCTCCTTAAAACCT

>2217
TTTTTGTCATTGGCAGTTTGGCTTAGGGGAGAAGCGAGGGAGAGCCAGTGTGGGAGC
CGCCTCTAGGATTGTGAGCAAGTGCCTGTTTAGACAGCTCCACGCTGCTCCTTAAAAC
CTATTTTGTGGTCTGGGGTTGGGGC
>2218
ATTGGCAGTTTGGCTTAGGGGAGAAGCGAGGGAGAGCCAGTGTGGGAGCCGCCTCTA
GGATTGTGAGCAAGTGCCTGTTTAGACAGCTCCACGCTGCTCCTTAAAACCTATTTTGT
GGTCTGGGGTTGGGGCGGGGGCAC
>2219
GGAGCCGCCTCTAGGATTGTGAGCAAGTGCCTGTTTAGACAGCTCCACGCTGCTCCTT
AAAACCTATTTTGTGGTCTGGGGTTGGGGCGGGGGCACACTGCCTCGATCCTCTTGTT
GTGTGGAGCAAAGTGATTCCTCTC
>2220
GCCGCCTCTAGGATTGTGAGCAAGTGCCTGTTTAGACAGCTCCACGCTGCTCCTTAAA
ACCTATTTTGTGGTCTGGGGTTGGGGCGGGGGCACACTGCCTCGATCCTCTTGTTGTGT
GGAGCAAAGTGATTCCTCTCTTC
>2221
TCCACGCTGCTCCTTAAAACCTATTTTGTGGTCTGGGGTTGGGGCGGGGGCACACTGC
CTCGATCCTCTTGTTGTGTGGAGCAAAGTGATTCCTCTCTTCTTGTGTTCTCCAGATGG
TTCCGTTGTCCTCACATCCATTG
>2222
AAAACCTATTTTGTGGTCTGGGGTTGGGGCGGGGGCACACTGCCTCGATCCTCTTGTT
GTGTGGAGCAAAGTGATTCCTCTCTTCTTGTGTTCTCCAGATGGTTCCGTTGTCCTCAC
ATCCATTGTGGTTGAGGATACAG
>2223
CTGGGGTTGGGGCGGGGGCACACTGCCTCGATCCTCTTGTTGTGTGGAGCAAAGTGAT
TCCTCTCTTCTTGTGTTCTCCAGATGGTTCCGTTGTCCTCACATCCATTGTGGTTGAGGA
TACAGTGCAGAGAAGACCCCGC
>2224
GGGGCACACTGCCTCGATCCTCTTGTTGTGTGGAGCAAAGTGATTCCTCTCTTCTTGTG
TTCTCCAGATGGTTCCGTTGTCCTCACATCCATTGTGGTTGAGGATACAGTGCAGAGAA
GACCCCGCTGTCCTCTGCTCCC
>2225
TGTTGTGTGGAGCAAAGTGATTCCTCTCTTCTTGTGTTCTCCAGATGGTTCCGTTGTCC
TCACATCCATTGTGGTTGAGGATACAGTGCAGAGAAGACCCCGCTGTCCTCTGCTCCC
CCTCCTTTTCTCTCCAAAGAGAA
>2226
TGTGTGGAGCAAAGTGATTCCTCTCTTCTTGTGTTCTCCAGATGGTTCCGTTGTCCTCA
CATCCATTGTGGTTGAGGATACAGTGCAGAGAAGACCCCGCTGTCCTCTGCTCCCCCTC
CTTTTCTCTCCAAAGAGAAGTT
>2227
AGTGATTCCTCTCTTCTTGTGTTCTCCAGATGGTTCCGTTGTCCTCACATCCATTGTGGT
TGAGGATACAGTGCAGAGAAGACCCCGCTGTCCTCTGCTCCCCCTCCTTTTCTCTCCAA
AGAGAAGTTTCAGGGACAGCA
>2228
TGTTCTCCAGATGGTTCCGTTGTCCTCACATCCATTGTGGTTGAGGATACAGTGCAGAG

AAGACCCCGCTGTCCTCTGCTCCCCCTCCTTTTCTCTCCAAAGAGAAGTTTCAGGGAC
AGCAATAGACTTCTGTGGGGCCT
>2229
GTGGTTGAGGATACAGTGCAGAGAAGACCCCGCTGTCCTCTGCTCCCCCTCCTTTTCTC
TCCAAAGAGAAGTTTCAGGGACAGCAATAGACTTCTGTGGGGCCTTTGTTCCTTGTAG
CTTTCATTTTTCAATTCTTCTTC
>2230
GACCCCGCTGTCCTCTGCTCCCCCTCCTTTTCTCTCCAAAGAGAAGTTTCAGGGACAG
CAATAGACTTCTGTGGGGCCTTTGTTCCTTGTAGCTTTCATTTTTCAATTCTTCTTCTCC
CTGTATTATCTTGCTAGGCCTT
>2231
TCCCCCTCCTTTTCTCTCCAAAGAGAAGTTTCAGGGACAGCAATAGACTTCTGTGGGG
CCTTTGTTCCTTGTAGCTTTCATTTTTCAATTCTTCTTCTCCCTGTATTATCTTGCTAGGC
CTTAGTTTGTGCATCTCTAAA
>2232
AGGGACAGCAATAGACTTCTGTGGGGCCTTTGTTCCTTGTAGCTTTCATTTTTCAATTCT
TCTTCTCCCTGTATTATCTTGCTAGGCCTTAGTTTGTGCATCTCTAAAATGGGCTGGGAG
GTGCTAGCCCATACCTAATA
>2233
TGGGGCCTTTGTTCCTTGTAGCTTTCATTTTTCAATTCTTCTTCTCCCTGTATTATCTTGC
TAGGCCTTAGTTTGTGCATCTCTAAAATGGGCTGGGAGGTGCTAGCCCATACCTAATATA
TAGGGCCGTCGTAAGGCTT
>2234
TTTCAATTCTTCTTCTCCCTGTATTATCTTGCTAGGCCTTAGTTTGTGCATCTCTAAAATG
GGCTGGGAGGTGCTAGCCCATACCTAATATATAGGGCCGTCGTAAGGCTTAGGTCCCAT
AACTCATGGGGTTGGCTTGC
>2235
TGCTAGGCCTTAGTTTGTGCATCTCTAAAATGGGCTGGGAGGTGCTAGCCCATACCTAA
TATATAGGGCCGTCGTAAGGCTTAGGTCCCATAACTCATGGGGTTGGCTTGCACAGGGC
TCTCTCCTTTCAGACTGGTCAG
>2236
GGGCTGGGAGGTGCTAGCCCATACCTAATATATAGGGCCGTCGTAAGGCTTAGGTCCCA
TAACTCATGGGGTTGGCTTGCACAGGGCTCTCTCCTTTCAGACTGGTCAGGCCATGGTC
CTTTTATGAGTCTGAAGAGCCA
>2237
GGGCCGTCGTAAGGCTTAGGTCCCATAACTCATGGGGTTGGCTTGCACAGGGCTCTCT
CCTTTCAGACTGGTCAGGCCATGGTCCTTTTATGAGTCTGAAGAGCCAAAATGAAGAC
CTCACTTTCTAGTATCCCCAATCT
>2238
AGGCTTAGGTCCCATAACTCATGGGGTTGGCTTGCACAGGGCTCTCTCCTTTCAGACTG
GTCAGGCCATGGTCCTTTTATGAGTCTGAAGAGCCAAAATGAAGACCTCACTTTCTAGT
ATCCCCAATCTTCCTCCCACCT
>2239
CATAACTCATGGGGTTGGCTTGCACAGGGCTCTCTCCTTTCAGACTGGTCAGGCCATGG
TCCTTTTATGAGTCTGAAGAGCCAAAATGAAGACCTCACTTTCTAGTATCCCCAATCTT
CCTCCCACCTTTCCCTGGCTCT

>2240
TCCTTTCAGACTGGTCAGGCCATGGTCCTTTTATGAGTCTGAAGAGCCAAAATGAAGA
CCTCACTTTCTAGTATCCCCAATCTTCCTCCCACCTTTCCCTGGCTCTTGAGGGTTCTTT
GGGCATGTTCCCCTCTGGTCTC
>2241
AAGAGCCAAAATGAAGACCTCACTTTCTAGTATCCCCAATCTTCCTCCCACCTTTCCCT
GGCTCTTGAGGGTTCTTTGGGCATGTTCCCCTCTGGTCTCCATTATTATATAGGGAAGAG
AAATTTTCCAGAAGAATATTT
>2242
AAGACCTCACTTTCTAGTATCCCCAATCTTCCTCCCACCTTTCCCTGGCTCTTGAGGGTT
CTTTGGGCATGTTCCCCTCTGGTCTCCATTATTATATAGGGAAGAGAAATTTTCCAGAAG
AATATTTGAAGGATTAAAGT
>2243
ATGTTCCCCTCTGGTCTCCATTATTATATAGGGAAGAGAAATTTTCCAGAAGAATATTTG
AAGGATTAAAGTTTGAGGTTGTAGAATATGTGAAAACACACACACACCACACCACACC
CCAAAATCACCCTTTTAAAGAA
>2244
CCTCTGGTCTCCATTATTATATAGGGAAGAGAAATTTTCCAGAAGAATATTTGAAGGATT
AAAGTTTGAGGTTGTAGAATATGTGAAAACACACACACACCACACCACACCCCAAAAT
CACCCTTTTAAAGAAGATTAAA
>2245
ATTATTATATAGGGAAGAGAAATTTTCCAGAAGAATATTTGAAGGATTAAAGTTTGAGG
TTGTAGAATATGTGAAAACACACACACACCACACCACACCCCAAAATCACCCTTTTAA
AGAAGATTAAAAAACCACCAGTG
>2246
TATGTGAAAACACACACACACCACACCACACCCCAAAATCACCCTTTTAAAGAAGATT
AAAAAACCACCAGTGCCTGTCCTACATTGGTAAACTCTGGCAAGCCTCATAATGTTGA
CTTTACTGACCTGCCTGACTTCAG
>2247
AAAACACACACACACCACACCACACCCCAAAATCACCCTTTTAAAGAAGATTAAAAA
ACCACCAGTGCCTGTCCTACATTGGTAAACTCTGGCAAGCCTCATAATGTTGACTTTAC
TGACCTGCCTGACTTCAGTGGCCG
>2248
CCCTTTTAAAGAAGATTAAAAAACCACCAGTGCCTGTCCTACATTGGTAAACTCTGGC
AAGCCTCATAATGTTGACTTTACTGACCTGCCTGACTTCAGTGGCCGAGTCTACATGGG
GTGTTAAGGCCCCACGTGTTTTT
>2249
AGTGCCTGTCCTACATTGGTAAACTCTGGCAAGCCTCATAATGTTGACTTTACTGACCT
GCCTGACTTCAGTGGCCGAGTCTACATGGGGTGTTAAGGCCCCACGTGTTTTTGCCTGT
GCCCTTTAATTGTATCTTCAGG
>2250
TCCTACATTGGTAAACTCTGGCAAGCCTCATAATGTTGACTTTACTGACCTGCCTGACT
TCAGTGGCCGAGTCTACATGGGGTGTTAAGGCCCCACGTGTTTTTGCCTGTGCCCTTTA
ATTGTATCTTCAGGACCATATT
>2251
CTCATAATGTTGACTTTACTGACCTGCCTGACTTCAGTGGCCGAGTCTACATGGGGTGT

TAAGGCCCCACGTGTTTTTGCCTGTGCCCTTTAATTGTATCTTCAGGACCATATTTTCAG
AACCAGAAATCACAGCCACAG
>2252
ACTTTACTGACCTGCCTGACTTCAGTGGCCGAGTCTACATGGGGTGTTAAGGCCCCAC
GTGTTTTTGCCTGTGCCCTTTAATTGTATCTTCAGGACCATATTTTCAGAACCAGAAATC
ACAGCCACAGTTTGGCAGTGGC
>2253
GCCTGACTTCAGTGGCCGAGTCTACATGGGGTGTTAAGGCCCCACGTGTTTTTGCCTGT
GCCCTTTAATTGTATCTTCAGGACCATATTTTCAGAACCAGAAATCACAGCCACAGTTT
GGCAGTGGCACAGAATGGTTCA
>2254
CGTGTTTTTGCCTGTGCCCTTTAATTGTATCTTCAGGACCATATTTTCAGAACCAGAAAT
CACAGCCACAGTTTGGCAGTGGCACAGAATGGTTCAATTTAGGCCTATCCCAGAGAGG
CTTGGGTTGTCTGTGAAGTTCT
>2255
TGCCTGTGCCCTTTAATTGTATCTTCAGGACCATATTTTCAGAACCAGAAATCACAGCC
ACAGTTTGGCAGTGGCACAGAATGGTTCAATTTAGGCCTATCCCAGAGAGGCTTGGGT
TGTCTGTGAAGTTCTGGGTGGTC
>2256
TAATTGTATCTTCAGGACCATATTTTCAGAACCAGAAATCACAGCCACAGTTTGGCAGT
GGCACAGAATGGTTCAATTTAGGCCTATCCCAGAGAGGCTTGGGTTGTCTGTGAAGTT
CTGGGTGGTCTGTATTGTGGAGA
>2257
AAATCACAGCCACAGTTTGGCAGTGGCACAGAATGGTTCAATTTAGGCCTATCCCAGA
GAGGCTTGGGTTGTCTGTGAAGTTCTGGGTGGTCTGTATTGTGGAGAGGTGAACACCG
TATTGCTGGGGGTGTGGAGGGGGG
>2258
CACAGCCACAGTTTGGCAGTGGCACAGAATGGTTCAATTTAGGCCTATCCCAGAGAGG
CTTGGGTTGTCTGTGAAGTTCTGGGTGGTCTGTATTGTGGAGAGGTGAACACCGTATTG
CTGGGGGTGTGGAGGGGGGATGT
>2259
CCACAGTTTGGCAGTGGCACAGAATGGTTCAATTTAGGCCTATCCCAGAGAGGCTTGG
GTTGTCTGTGAAGTTCTGGGTGGTCTGTATTGTGGAGAGGTGAACACCGTATTGCTGG
GGGTGTGGAGGGGGGATGTTGCTG
>2260
TGGCACAGAATGGTTCAATTTAGGCCTATCCCAGAGAGGCTTGGGTTGTCTGTGAAGT
TCTGGGTGGTCTGTATTGTGGAGAGGTGAACACCGTATTGCTGGGGGTGTGGAGGGGG
GATGTTGCTGTTTCCCTGCTACAG
>2261
AGGCTTGGGTTGTCTGTGAAGTTCTGGGTGGTCTGTATTGTGGAGAGGTGAACACCGT
ATTGCTGGGGGTGTGGAGGGGGGATGTTGCTGTTTCCCTGCTACAGAAAGGCCAGGCT
TCCTTTTCAGCCTTTTGTTCCCCG
>2262
CTGTGAAGTTCTGGGTGGTCTGTATTGTGGAGAGGTGAACACCGTATTGCTGGGGGTG
TGGAGGGGGGATGTTGCTGTTTCCCTGCTACAGAAAGGCCAGGCTTCCTTTTCAGCCT
TTTGTTCCCCGATTCCCCTCTCTT

>2263
AGTTCTGGGTGGTCTGTATTGTGGAGAGGTGAACACCGTATTGCTGGGGGTGTGGAGG
GGGGATGTTGCTGTTTCCCTGCTACAGAAAGGCCAGGCTTCCTTTTCAGCCTTTTGTTC
CCCGATTCCCCTCTCTTCCTCCA
>2264
CTGGGGGTGTGGAGGGGGGATGTTGCTGTTTCCCTGCTACAGAAAGGCCAGGCTTCCT
TTTCAGCCTTTTGTTCCCCGATTCCCCTCTCTTCCTCCACTACACTCAGCCCTGCTGCTG
CTTCCAAGGGACAACTCCCTCT
>2265
CCCCGATTCCCCTCTCTTCCTCCACTACACTCAGCCCTGCTGCTGCTTCCAAGGGACAA
CTCCCTCTCTTGTCCTTGCCCATCTTTGAGAGAGGCGGTGAATAAACACCAAGGCAAG
AAGAAGCAACGGGACCCCTAGAA
>2266
ACACTCAGCCCTGCTGCTGCTTCCAAGGGACAACTCCCTCTCTTGTCCTTGCCCATCTT
TGAGAGAGGCGGTGAATAAACACCAAGGCAAGAAGAAGCAACGGGACCCCTAGAAG
GAGATGAGTGTGTGGCCCAAGGATG
>2267
CCATCTTTGAGAGAGGCGGTGAATAAACACCAAGGCAAGAAGAAGCAACGGGACCCC
TAGAAGGAGATGAGTGTGTGGCCCAAGGATGGTGGAGCATTGTCCTGAAAGGTAGAA
CCAAGACTATAGCCTCCGAGGCTCTG
>2268
ATCTTTGAGAGAGGCGGTGAATAAACACCAAGGCAAGAAGAAGCAACGGGACCCCTA
GAAGGAGATGAGTGTGTGGCCCAAGGATGGTGGAGCATTGTCCTGAAAGGTAGAACC
AAGACTATAGCCTCCGAGGCTCTGTT
>2269
ACCAAGGCAAGAAGAAGCAACGGGACCCCTAGAAGGAGATGAGTGTGTGGCCCAAG
GATGGTGGAGCATTGTCCTGAAAGGTAGAACCAAGACTATAGCCTCCGAGGCTCTGTT
TACCATATTCTTGGCTCTTGTGCCAC
>2270
GAAGAAGCAACGGGACCCCTAGAAGGAGATGAGTGTGTGGCCCAAGGATGGTGGAGC
ATTGTCCTGAAAGGTAGAACCAAGACTATAGCCTCCGAGGCTCTGTTTACCATATTCTT
GGCTCTTGTGCCACTTCTGTGTCC
>2271
AGTGTGTGGCCCAAGGATGGTGGAGCATTGTCCTGAAAGGTAGAACCAAGACTATAGC
CTCCGAGGCTCTGTTTACCATATTCTTGGCTCTTGTGCCACTTCTGTGTCCAGCAGCTG
GAGCTTGTAGAAGAAAGATGGGA
>2272
GAGCATTGTCCTGAAAGGTAGAACCAAGACTATAGCCTCCGAGGCTCTGTTTACCATAT
TCTTGGCTCTTGTGCCACTTCTGTGTCCAGCAGCTGGAGCTTGTAGAAGAAAGATGGG
AAAGACATAGTACAGGAAATGTT
>2273
TGAAAGGTAGAACCAAGACTATAGCCTCCGAGGCTCTGTTTACCATATTCTTGGCTCTT
GTGCCACTTCTGTGTCCAGCAGCTGGAGCTTGTAGAAGAAAGATGGGAAAGACATAGT
ACAGGAAATGTTCCCATTCAGCC
>2274
ATAGCCTCCGAGGCTCTGTTTACCATATTCTTGGCTCTTGTGCCACTTCTGTGTCCAGCA

GCTGGAGCTTGTAGAAGAAAGATGGGAAAGACATAGTACAGGAAATGTTCCCATTCAG
CCAGCACTGATTTATCTGCTGC
>2275
TATTCTTGGCTCTTGTGCCACTTCTGTGTCCAGCAGCTGGAGCTTGTAGAAGAAAGATG
GGAAAGACATAGTACAGGAAATGTTCCCATTCAGCCAGCACTGATTTATCTGCTGCCTG
GTTTCTCAGGAGGCCTAATCTG
>2276
CTTGTGCCACTTCTGTGTCCAGCAGCTGGAGCTTGTAGAAGAAAGATGGGAAAGACAT
AGTACAGGAAATGTTCCCATTCAGCCAGCACTGATTTATCTGCTGCCTGGTTTCTCAGG
AGGCCTAATCTGTAAGAACAGTC
>2277
GAAGAAAGATGGGAAAGACATAGTACAGGAAATGTTCCCATTCAGCCAGCACTGATTT
ATCTGCTGCCTGGTTTCTCAGGAGGCCTAATCTGTAAGAACAGTCGAGAACTTTATTTC
TGCCCTTTAACCTATGTCCATTT
>2278
AGTACAGGAAATGTTCCCATTCAGCCAGCACTGATTTATCTGCTGCCTGGTTTCTCAGG
AGGCCTAATCTGTAAGAACAGTCGAGAACTTTATTTCTGCCCTTTAACCTATGTCCATTT
TCTAGATAGTTTCTAAAAAAG
>2279
AATGTTCCCATTCAGCCAGCACTGATTTATCTGCTGCCTGGTTTCTCAGGAGGCCTAAT
CTGTAAGAACAGTCGAGAACTTTATTTCTGCCCTTTAACCTATGTCCATTTTCTAGATAG
TTTCTAAAAAAGGCATCACAC
>2280
GCTGCCTGGTTTCTCAGGAGGCCTAATCTGTAAGAACAGTCGAGAACTTTATTTCTGCC
CTTTAACCTATGTCCATTTTCTAGATAGTTTCTAAAAAAGGCATCACACCAGCTCTTTGA
GACAACATGTCATATGCAACT
>2281
GGAGGCCTAATCTGTAAGAACAGTCGAGAACTTTATTTCTGCCCTTTAACCTATGTCCA
TTTTCTAGATAGTTTCTAAAAAAGGCATCACACCAGCTCTTTGAGACAACATGTCATAT
GCAACTGTTGGTTTTGGGTGGA
>2282
CCCTTTAACCTATGTCCATTTTCTAGATAGTTTCTAAAAAAGGCATCACACCAGCTCTTT
GAGACAACATGTCATATGCAACTGTTGGTTTTGGGTGGAATGAAACTGAATTGTCAAC
GGACATTTTTTCTTTCTGTATT
>2283
GTCCATTTTCTAGATAGTTTCTAAAAAAGGCATCACACCAGCTCTTTGAGACAACATGT
CATATGCAACTGTTGGTTTTGGGTGGAATGAAACTGAATTGTCAACGGACATTTTTTCT
TTCTGTATTCATGTGTTTGGAA
>2284
ATTTTCTAGATAGTTTCTAAAAAAGGCATCACACCAGCTCTTTGAGACAACATGTCATA
TGCAACTGTTGGTTTTGGGTGGAATGAAACTGAATTGTCAACGGACATTTTTTCTTTCT
GTATTCATGTGTTTGGAATAGT
>2285
AGATAGTTTCTAAAAAAGGCATCACACCAGCTCTTTGAGACAACATGTCATATGCAACT
GTTGGTTTTGGGTGGAATGAAACTGAATTGTCAACGGACATTTTTTCTTTCTGTATTCAT
GTGTTTGGAATAGTGACAACG

>2286
GCATCACACCAGCTCTTTGAGACAACATGTCATATGCAACTGTTGGTTTTGGGTGGAAT
GAAACTGAATTGTCAACGGACATTTTTTCTTTCTGTATTCATGTGTTTGGAATAGTGACA
ACGTCATTGGTTCAGACTTTG
>2287
AACATGTCATATGCAACTGTTGGTTTTGGGTGGAATGAAACTGAATTGTCAACGGACAT
TTTTTCTTTCTGTATTCATGTGTTTGGAATAGTGACAACGTCATTGGTTCAGACTTTGAG
CCCCCTTCTGGTTAGGCTCTT
>2288
ATATGCAACTGTTGGTTTTGGGTGGAATGAAACTGAATTGTCAACGGACATTTTTTCTT
TCTGTATTCATGTGTTTGGAATAGTGACAACGTCATTGGTTCAGACTTTGAGCCCCCTT
CTGGTTAGGCTCTTCAGTATAA
>2289
ATGCAACTGTTGGTTTTGGGTGGAATGAAACTGAATTGTCAACGGACATTTTTTCTTTC
TGTATTCATGTGTTTGGAATAGTGACAACGTCATTGGTTCAGACTTTGAGCCCCCTTCT
GGTTAGGCTCTTCAGTATAAGC
>2290
TGGTTTTGGGTGGAATGAAACTGAATTGTCAACGGACATTTTTTCTTTCTGTATTCATGT
GTTTGGAATAGTGACAACGTCATTGGTTCAGACTTTGAGCCCCCTTCTGGTTAGGCTCT
TCAGTATAAGCCCTTAAAAAT
>2291
GGTGGAATGAAACTGAATTGTCAACGGACATTTTTTCTTTCTGTATTCATGTGTTTGGAA
TAGTGACAACGTCATTGGTTCAGACTTTGAGCCCCCTTCTGGTTAGGCTCTTCAGTATA
AGCCCTTAAAAATATTTTTGA
>2292
TTTTTCTTTCTGTATTCATGTGTTTGGAATAGTGACAACGTCATTGGTTCAGACTTTGAG
CCCCCTTCTGGTTAGGCTCTTCAGTATAAGCCCTTAAAAATATTTTTGACTTTTCAGGGC
CAGGCGCGGTGGCTCACGCC
>2293
ATTCATGTGTTTGGAATAGTGACAACGTCATTGGTTCAGACTTTGAGCCCCCTTCTGGT
TAGGCTCTTCAGTATAAGCCCTTAAAAATATTTTTGACTTTTCAGGGCCAGGCGCGGTG
GCTCACGCCTGTAATCCCAGCA
>2294
AGCCCCCTTCTGGTTAGGCTCTTCAGTATAAGCCCTTAAAAATATTTTTGACTTTTCAGG
GCCAGGCGCGGTGGCTCACGCCTGTAATCCCAGCACTTTGGGAGGCCGAGGCAGGTG
GATCACGAGGTCAGGAGATCGAG
>2295
TTAGGCTCTTCAGTATAAGCCCTTAAAAATATTTTTGACTTTTCAGGGCCAGGCGCGGT
GGCTCACGCCTGTAATCCCAGCACTTTGGGAGGCCGAGGCAGGTGGATCACGAGGTC
AGGAGATCGAGACCATCCTGGCTA
>2296
TTTTTGACTTTTCAGGGCCAGGCGCGGTGGCTCACGCCTGTAATCCCAGCACTTTGGG
AGGCCGAGGCAGGTGGATCACGAGGTCAGGAGATCGAGACCATCCTGGCTAACACGG
TGAAAGCCCGTCTCTACTAAAAATA
>2297
CAGGGCCAGGCGCGGTGGCTCACGCCTGTAATCCCAGCACTTTGGGAGGCCGAGGCA

GGTGGATCACGAGGTCAGGAGATCGAGACCATCCTGGCTAACACGGTGAAAGCCCGT
CTCTACTAAAAATACAAAAAATTAGC
>2298
CCCAGCACTTTGGGAGGCCGAGGCAGGTGGATCACGAGGTCAGGAGATCGAGACCAT
CCTGGCTAACACGGTGAAAGCCCGTCTCTACTAAAAATACAAAAAATTAGCCAGGTGT
GGTGGCGGGCGCCTGTAGTCCCAGC
>2299
TGGGAGGCCGAGGCAGGTGGATCACGAGGTCAGGAGATCGAGACCATCCTGGCTAAC
ACGGTGAAAGCCCGTCTCTACTAAAAATACAAAAAATTAGCCAGGTGTGGTGGCGGGC
GCCTGTAGTCCCAGCTACTCGAGAG
>2300
GAGATCGAGACCATCCTGGCTAACACGGTGAAAGCCCGTCTCTACTAAAAATACAAAA
AATTAGCCAGGTGTGGTGGCGGGCGCCTGTAGTCCCAGCTACTCGAGAGGCTGAGGCT
GGAGAATGGCGTGAACCCGGAGGC
>2301
ATCGAGACCATCCTGGCTAACACGGTGAAAGCCCGTCTCTACTAAAAATACAAAAAAT
TAGCCAGGTGTGGTGGCGGGCGCCTGTAGTCCCAGCTACTCGAGAGGCTGAGGCTGG
AGAATGGCGTGAACCCGGAGGCAGA
>2302
TGGCTAACACGGTGAAAGCCCGTCTCTACTAAAAATACAAAAAATTAGCCAGGTGTGG
TGGCGGGCGCCTGTAGTCCCAGCTACTCGAGAGGCTGAGGCTGGAGAATGGCGTGAA
CCCGGAGGCAGAGTTTGCAGTGAGC
>2303
AAAATTAGCCAGGTGTGGTGGCGGGCGCCTGTAGTCCCAGCTACTCGAGAGGCTGAG
GCTGGAGAATGGCGTGAACCCGGAGGCAGAGTTTGCAGTGAGCTGAGGTTGTGCCAC
TGCACTCCAGCCTGGGAGACAGAGCA
>2304
GTGGCGGGCGCCTGTAGTCCCAGCTACTCGAGAGGCTGAGGCTGGAGAATGGCGTGA
ACCCGGAGGCAGAGTTTGCAGTGAGCTGAGGTTGTGCCACTGCACTCCAGCCTGGGA
GACAGAGCAAGACTCCGTCTCAAAAA
>2305
GCGCCTGTAGTCCCAGCTACTCGAGAGGCTGAGGCTGGAGAATGGCGTGAACCCGGA
GGCAGAGTTTGCAGTGAGCTGAGGTTGTGCCACTGCACTCCAGCCTGGGAGACAGAG
CAAGACTCCGTCTCAAAAAAAATAAAA
>2306
CGGAGGCAGAGTTTGCAGTGAGCTGAGGTTGTGCCACTGCACTCCAGCCTGGGAGAC
AGAGCAAGACTCCGTCTCAAAAAAAATAAAAAATAAAAAACATTAAAAAATTGACTTTT
TGAGTAAAACAAAAACCAAACGAGT
>2307
CTGGGAGACAGAGCAAGACTCCGTCTCAAAAAAAATAAAAAATAAAAAACATTAAAAA
ATTGACTTTTTGAGTAAAACAAAAACCAAACGAGTCCGTGTAAAGTCTAAGATGTTGG
TTCATCTCAACATTGGGATATTGGA
>2308
CCGTCTCAAAAAAAATAAAAAATAAAAAACATTAAAAAATTGACTTTTTGAGTAAAACA
AAAACCAAACGAGTCCGTGTAAAGTCTAAGATGTTGGTTCATCTCAACATTGGGATATT
GGAATAGGCCACTTTGGAAGGTT

>2309
CTCAAAAAAATAAAAAATAAAAAACATTAAAAAATTGACTTTTTGAGTAAAACAAAAA
CCAAACGAGTCCGTGTAAAGTCTAAGATGTTGGTTCATCTCAACATTGGGATATTGGAA
TAGGCCACTTTGGAAGGTTTCTC
>2310
CGAGTCCGTGTAAAGTCTAAGATGTTGGTTCATCTCAACATTGGGATATTGGAATAGGC
CACTTTGGAAGGTTTCTCTAATTGATGAAGGGCATGGGCCACCCCATGGATTGCAATGC
TGTGGCTGTTTGGGTGGCATCC
>2311
TGGAATAGGCCACTTTGGAAGGTTTCTCTAATTGATGAAGGGCATGGGCCACCCCATG
GATTGCAATGCTGTGGCTGTTTGGGTGGCATCCAGCTTCTAGGGGATGGTGGCAAACTT
GGGTGTGCAGGCAGTAGGAAGGA
>2312
AGGCCACTTTGGAAGGTTTCTCTAATTGATGAAGGGCATGGGCCACCCCATGGATTGC
AATGCTGTGGCTGTTTGGGTGGCATCCAGCTTCTAGGGGATGGTGGCAAACTTGGGTG
TGCAGGCAGTAGGAAGGAGGAGGT
>2313
GAAGGGCATGGGCCACCCCATGGATTGCAATGCTGTGGCTGTTTGGGTGGCATCCAGC
TTCTAGGGGATGGTGGCAAACTTGGGTGTGCAGGCAGTAGGAAGGAGGAGGTAGCTT
CCAGAGAGAGCCATTGCTGGGGCTC
>2314
CACCCCATGGATTGCAATGCTGTGGCTGTTTGGGTGGCATCCAGCTTCTAGGGGATGGT
GGCAAACTTGGGTGTGCAGGCAGTAGGAAGGAGGAGGTAGCTTCCAGAGAGAGCCAT
TGCTGGGGCTCAAGAAGTTTCTTT
>2315
CCCCATGGATTGCAATGCTGTGGCTGTTTGGGTGGCATCCAGCTTCTAGGGGATGGTGG
CAAACTTGGGTGTGCAGGCAGTAGGAAGGAGGAGGTAGCTTCCAGAGAGAGCCATTG
CTGGGGCTCAAGAAGTTTCTTTCT
>2316
TTGCAATGCTGTGGCTGTTTGGGTGGCATCCAGCTTCTAGGGGATGGTGGCAAACTTG
GGTGTGCAGGCAGTAGGAAGGAGGAGGTAGCTTCCAGAGAGAGCCATTGCTGGGGCT
CAAGAAGTTTCTTTCTGGGCTCAAG
>2317
AAACTTGGGTGTGCAGGCAGTAGGAAGGAGGAGGTAGCTTCCAGAGAGAGCCATTGC
TGGGGCTCAAGAAGTTTCTTTCTGGGCTCAAGCATGGTTGCTAGTGGTCCACACAGTG
CTTTCGTGTGAATTAGAAAAAGCAC
>2318
GAAGGAGGAGGTAGCTTCCAGAGAGAGCCATTGCTGGGGCTCAAGAAGTTTCTTTCT
GGGCTCAAGCATGGTTGCTAGTGGTCCACACAGTGCTTTCGTGTGAATTAGAAAAAGC
ACCCTTGTTGTGGATGGATGTGGCT
>2319
AGAGAGAGCCATTGCTGGGGCTCAAGAAGTTTCTTTCTGGGCTCAAGCATGGTTGCTA
GTGGTCCACACAGTGCTTTCGTGTGAATTAGAAAAAGCACCCTTGTTGTGGATGGATG
TGGCTCTTAACTAGGGTCTATCCC
>2320
ATTGCTGGGGCTCAAGAAGTTTCTTTCTGGGCTCAAGCATGGTTGCTAGTGGTCCACA

CAGTGCTTTCGTGTGAATTAGAAAAAGCACCCTTGTTGTGGATGGATGTGGCTCTTAAC
TAGGGTCTATCCCACAGTGAGCT
>2321
CTTTCTGGGCTCAAGCATGGTTGCTAGTGGTCCACACAGTGCTTTCGTGTGAATTAGAA
AAAGCACCCTTGTTGTGGATGGATGTGGCTCTTAACTAGGGTCTATCCCACAGTGAGCT
GGTTGGGGCTGGATCTCAAACC
>2322
TCTGGGCTCAAGCATGGTTGCTAGTGGTCCACACAGTGCTTTCGTGTGAATTAGAAAA
AGCACCCTTGTTGTGGATGGATGTGGCTCTTAACTAGGGTCTATCCCACAGTGAGCTGG
TTGGGGCTGGATCTCAAACCTCT
>2323
CTTTCGTGTGAATTAGAAAAAGCACCCTTGTTGTGGATGGATGTGGCTCTTAACTAGGG
TCTATCCCACAGTGAGCTGGTTGGGGCTGGATCTCAAACCTCTTTGCCCCTCAATCAGA
GGCAGGAGGTCCCTGGCCCAAG
>2324
TGAATTAGAAAAAGCACCCTTGTTGTGGATGGATGTGGCTCTTAACTAGGGTCTATCCC
ACAGTGAGCTGGTTGGGGCTGGATCTCAAACCTCTTTGCCCCTCAATCAGAGGCAGGA
GGTCCCTGGCCCAAGGGGCTTCC
>2325
GGGTCTATCCCACAGTGAGCTGGTTGGGGCTGGATCTCAAACCTCTTTGCCCCTCAATC
AGAGGCAGGAGGTCCCTGGCCCAAGGGGCTTCCATGGTTGGAGTGGTCGTCAGCCGA
AAGCTGTTCTGGTGAGATGTTTTG
>2326
GGGGCTGGATCTCAAACCTCTTTGCCCCTCAATCAGAGGCAGGAGGTCCCTGGCCCAA
GGGGCTTCCATGGTTGGAGTGGTCGTCAGCCGAAAGCTGTTCTGGTGAGATGTTTTGA
GCTATCAGTTCCAAGTTTCCAAAG
>2327
GGATCTCAAACCTCTTTGCCCCTCAATCAGAGGCAGGAGGTCCCTGGCCCAAGGGGCT
TCCATGGTTGGAGTGGTCGTCAGCCGAAAGCTGTTCTGGTGAGATGTTTTGAGCTATCA
GTTCCAAGTTTCCAAAGGCCAAG
>2328
CAGGAGGTCCCTGGCCCAAGGGGCTTCCATGGTTGGAGTGGTCGTCAGCCGAAAGCT
GTTCTGGTGAGATGTTTTGAGCTATCAGTTCCAAGTTTCCAAAGGCCAAGGCCTCTGG
GATCAGTTTCACTGATCCTTCAACC
>2329
CCCAAGGGGCTTCCATGGTTGGAGTGGTCGTCAGCCGAAAGCTGTTCTGGTGAGATGT
TTTGAGCTATCAGTTCCAAGTTTCCAAAGGCCAAGGCCTCTGGGATCAGTTTCACTGAT
CCTTCAACCAGCTTATGTGCCCT
>2330
GGCTTCCATGGTTGGAGTGGTCGTCAGCCGAAAGCTGTTCTGGTGAGATGTTTTGAGC
TATCAGTTCCAAGTTTCCAAAGGCCAAGGCCTCTGGGATCAGTTTCACTGATCCTTCAA
CCAGCTTATGTGCCCTGTTAGCC
>2331
GAAAGCTGTTCTGGTGAGATGTTTTGAGCTATCAGTTCCAAGTTTCCAAAGGCCAAGG
CCTCTGGGATCAGTTTCACTGATCCTTCAACCAGCTTATGTGCCCTGTTAGCCCAGATA
CCCCTCCAGAGTGCTTCGCACTG

>2332
GCTATCAGTTCCAAGTTTCCAAAGGCCAAGGCCTCTGGGATCAGTTTCACTGATCCTTC
AACCAGCTTATGTGCCCTGTTAGCCCAGATACCCCTCCAGAGTGCTTCGCACTGGGCTG
CAGGTCACTGTGGCCCCACACC
>2333
GTTCCAAGTTTCCAAAGGCCAAGGCCTCTGGGATCAGTTTCACTGATCCTTCAACCAG
CTTATGTGCCCTGTTAGCCCAGATACCCCTCCAGAGTGCTTCGCACTGGGCTGCAGGTC
ACTGTGGCCCCACACCATAGAAG
>2334
GCCTCTGGGATCAGTTTCACTGATCCTTCAACCAGCTTATGTGCCCTGTTAGCCCAGAT
ACCCCTCCAGAGTGCTTCGCACTGGGCTGCAGGTCACTGTGGCCCCACACCATAGAAG
CTGGGGGCCTGTCTGCATGTTCT
>2335
GATCCTTCAACCAGCTTATGTGCCCTGTTAGCCCAGATACCCCTCCAGAGTGCTTCGCA
CTGGGCTGCAGGTCACTGTGGCCCCACACCATAGAAGCTGGGGGCCTGTCTGCATGTT
CTGGCATCCATAAAGCTGCCTGC
>2336
TCAACCAGCTTATGTGCCCTGTTAGCCCAGATACCCCTCCAGAGTGCTTCGCACTGGGC
TGCAGGTCACTGTGGCCCCACACCATAGAAGCTGGGGGCCTGTCTGCATGTTCTGGCA
TCCATAAAGCTGCCTGCAAACGT
>2337
ATACCCCTCCAGAGTGCTTCGCACTGGGCTGCAGGTCACTGTGGCCCCACACCATAGA
AGCTGGGGGCCTGTCTGCATGTTCTGGCATCCATAAAGCTGCCTGCAAACGTGAGAGG
GGGTTTATGGCACCCTTTTAGCAT
>2338
CTGTGGCCCCACACCATAGAAGCTGGGGGCCTGTCTGCATGTTCTGGCATCCATAAAG
CTGCCTGCAAACGTGAGAGGGGGTTTATGGCACCCTTTTAGCATGACTGTCTCTTGTGA
CCAAGGCCAAGAAAAATAGCACA
>2339
ACACCATAGAAGCTGGGGGCCTGTCTGCATGTTCTGGCATCCATAAAGCTGCCTGCAA
ACGTGAGAGGGGGTTTATGGCACCCTTTTAGCATGACTGTCTCTTGTGACCAAGGCCA
AGAAAAATAGCACAAAGATGTAGG
>2340
GCATGTTCTGGCATCCATAAAGCTGCCTGCAAACGTGAGAGGGGGTTTATGGCACCCT
TTTAGCATGACTGTCTCTTGTGACCAAGGCCAAGAAAAATAGCACAAAGATGTAGGAG
TGGTGAGGAATGAAGATGCACGTG
>2341
CTGGCATCCATAAAGCTGCCTGCAAACGTGAGAGGGGGTTTATGGCACCCTTTTAGCAT
GACTGTCTCTTGTGACCAAGGCCAAGAAAAATAGCACAAAGATGTAGGAGTGGTGAG
GAATGAAGATGCACGTGCACGCGC
>2342
AGGGGGTTTATGGCACCCTTTTAGCATGACTGTCTCTTGTGACCAAGGCCAAGAAAAA
TAGCACAAAGATGTAGGAGTGGTGAGGAATGAAGATGCACGTGCACGCGCCCTGCAG
TCTCAGTCAATGCGCTCTTTAGGGT
>2343
ACTGTCTCTTGTGACCAAGGCCAAGAAAAATAGCACAAAGATGTAGGAGTGGTGAGG

AATGAAGATGCACGTGCACGCGCCCTGCAGTCTCAGTCAATGCGCTCTTTAGGGTTTA
GTTTGGAAAACCACAGTGTCGTGTC
>2344
AAGGCCAAGAAAAATAGCACAAAGATGTAGGAGTGGTGAGGAATGAAGATGCACGTG
CACGCGCCCTGCAGTCTCAGTCAATGCGCTCTTTAGGGTTTAGTTTGGAAAACCACAG
TGTCGTGTCTTATGTAACCAATTTC
>2345
ATGTAGGAGTGGTGAGGAATGAAGATGCACGTGCACGCGCCCTGCAGTCTCAGTCAAT
GCGCTCTTTAGGGTTTAGTTTGGAAAACCACAGTGTCGTGTCTTATGTAACCAATTTCT
TTTCTTTTCTTTTCTTTTCTTTT
>2346
GAAGATGCACGTGCACGCGCCCTGCAGTCTCAGTCAATGCGCTCTTTAGGGTTTAGTTT
GGAAAACCACAGTGTCGTGTCTTATGTAACCAATTTCTTTTCTTTTCTTTTCTTTTCTTT
TTTTTTTTTTTTTTTTGTGAT
>2347
AGATGCACGTGCACGCGCCCTGCAGTCTCAGTCAATGCGCTCTTTAGGGTTTAGTTTGG
AAAACCACAGTGTCGTGTCTTATGTAACCAATTTCTTTTCTTTTCTTTTCTTTTCTTTTTT
TTTTTTTTTTTTTGTGATGG
>2348
ACGCGCCCTGCAGTCTCAGTCAATGCGCTCTTTAGGGTTTAGTTTGGAAAACCACAGT
GTCGTGTCTTATGTAACCAATTTCTTTTCTTTTCTTTTCTTTTCTTTTTTTTTTTTTTTTTT
TGTGATGGAGTCTCGTGTCT
>2349
CCACAGTGTCGTGTCTTATGTAACCAATTTCTTTTCTTTTCTTTTCTTTTCTTTTTTTTTT
TTTTTTTTGTGATGGAGTCTCGTGTCTTGCTCTGTCACCCAGGCTGGAGTGCAGTGGCT
CGATCTCGGCTCACTGCAA
>2350
TCGTGTCTTATGTAACCAATTTCTTTTCTTTTCTTTTCTTTTCTTTTTTTTTTTTTTTTTTT
GTGATGGAGTCTCGTGTCTTGCTCTGTCACCCAGGCTGGAGTGCAGTGGCTCGATCTC
GGCTCACTGCAACCTCCGCC
>2351
AATTTCTTTTCTTTTCTTTTCTTTTCTTTTTTTTTTTTTTTTTTTTGTGATGGAGTCTCGTGT
CTTGCTCTGTCACCCAGGCTGGAGTGCAGTGGCTCGATCTCGGCTCACTGCAACCTCC
GCCTCCCAGGTTCAAGCGAC
>2352
TGTCTTGCTCTGTCACCCAGGCTGGAGTGCAGTGGCTCGATCTCGGCTCACTGCAACC
TCCGCCTCCCAGGTTCAAGCGACTATCCTGCCTCAGCCTCCTGAGTAGCTGGGACTAC
AGGCGCCCGCCACCACGCCCAGCT
>2353
TGGCTCGATCTCGGCTCACTGCAACCTCCGCCTCCCAGGTTCAAGCGACTATCCTGCCT
CAGCCTCCTGAGTAGCTGGGACTACAGGCGCCCGCCACCACGCCCAGCTAATTTTTTG
TGTATTTAGTAGAGACGGGGTTT
>2354
GACTATCCTGCCTCAGCCTCCTGAGTAGCTGGGACTACAGGCGCCCGCCACCACGCCC
AGCTAATTTTTTGTGTATTTAGTAGAGACGGGGTTTCACCGTGTTAGCCAGGATGGTCA
CAATCTCCTGACCTCGTGGTCCG

>2355
GCCTCAGCCTCCTGAGTAGCTGGGACTACAGGCGCCCGCCACCACGCCCAGCTAATTT
TTTGTGTATTTAGTAGAGACGGGGTTTCACCGTGTTAGCCAGGATGGTCACAATCTCCT
GACCTCGTGGTCCGCCCGCCTTG
>2356
CTGAGTAGCTGGGACTACAGGCGCCCGCCACCACGCCCAGCTAATTTTTGTGTATTTA
GTAGAGACGGGGTTTCACCGTGTTAGCCAGGATGGTCACAATCTCCTGACCTCGTGGT
CCGCCCGCCTTGGCCTCCCACAG
>2357
CTGGGACTACAGGCGCCCGCCACCACGCCCAGCTAATTTTTGTGTATTTAGTAGAGAC
GGGGTTTCACCGTGTTAGCCAGGATGGTCACAATCTCCTGACCTCGTGGTCCGCCCGC
CTTGGCCTCCCACAGTGCTGGGA
>2358
AATTTTTGTGTATTTAGTAGAGACGGGGTTTCACCGTGTTAGCCAGGATGGTCACAAT
CTCCTGACCTCGTGGTCCGCCCGCCTTGGCCTCCCACAGTGCTGGGATTACAGGCGTG
AGCCACCACGCCCGGCCACCAAT
>2359
TTTTGTGTATTTAGTAGAGACGGGGTTTCACCGTGTTAGCCAGGATGGTCACAATCTC
CTGACCTCGTGGTCCGCCCGCCTTGGCCTCCCACAGTGCTGGGATTACAGGCGTGAGC
CACCACGCCCGGCCACCAATTTC
>2360
GGTTTCACCGTGTTAGCCAGGATGGTCACAATCTCCTGACCTCGTGGTCCGCCCGCCTT
GGCCTCCCACAGTGCTGGGATTACAGGCGTGAGCCACCACGCCCGGCCACCAATTTCT
TATACATAGGTTATGGAGCAGGG
>2361
CAGGATGGTCACAATCTCCTGACCTCGTGGTCCGCCCGCCTTGGCCTCCCACAGTGCT
GGGATTACAGGCGTGAGCCACCACGCCCGGCCACCAATTTCTTATACATAGGTTATGGA
GCAGGGAGAAAGAGACATGCAAA
>2362
CTTGGCCTCCCACAGTGCTGGGATTACAGGCGTGAGCCACCACGCCCGGCCACCAATT
TCTTATACATAGGTTATGGAGCAGGGAGAAAGAGACATGCAAACAAGGCAGAATTGGG
ACCTTTGCAAGAAGACCTTCTTCA
>2363
ATGCAAACAAGGCAGAATTGGGACCTTTGCAAGAAGACCTTCTTCACTCGCCCACTTT
CTCATACTTTTAGTCACCAAGGCAGGGTCTTTTTAAACAACTTCGAGGCTCAGGCCTAG
GGGGTTAGGAGAGTAAACCCTAA
>2364
GAATTGGGACCTTTGCAAGAAGACCTTCTTCACTCGCCCACTTTCTCATACTTTTAGTC
ACCAAGGCAGGGTCTTTTTAAACAACTTCGAGGCTCAGGCCTAGGGGGTTAGGAGAGT
AAACCCTAAATCCCTTTCACCCT
>2365
CCCACTTTCTCATACTTTTAGTCACCAAGGCAGGGTCTTTTTAAACAACTTCGAGGCTC
AGGCCTAGGGGGTTAGGAGAGTAAACCCTAAATCCCTTTCACCCTAGCATGCTTGGGT
GCCGTGTTGACAAGGTCTCTGAA
>2366
CAACTTCGAGGCTCAGGCCTAGGGGGTTAGGAGAGTAAACCCTAAATCCCTTTCACCC

TAGCATGCTTGGGTGCCGTGTTGACAAGGTCTCTGAAGTGCCCATGGGAAGCAGACCA
GGCTCTGAGAATAATTTCCATGGA
>2367
TCGAGGCTCAGGCCTAGGGGGTTAGGAGAGTAAACCCTAAATCCCTTTCACCCTAGCA
TGCTTGGGTGCCGTGTTGACAAGGTCTCTGAAGTGCCCATGGGAAGCAGACCAGGCT
CTGAGAATAATTTCCATGGAATTAA
>2368
GACAAGGTCTCTGAAGTGCCCATGGGAAGCAGACCAGGCTCTGAGAATAATTTCCATG
GAATTAATTATTGTTCTTACTCCAATAGATTTGAAATCTATTTAACTTTTCAGGGTCCCCT
AGCTTTTTAAATGTTTCAAGT
>2369
GAGAATAATTTCCATGGAATTAATTATTGTTCTTACTCCAATAGATTTGAAATCTATTTAA
CTTTTCAGGGTCCCCTAGCTTTTTAAATGTTTCAAGTGTATTCTCTCTGATAGGTTGCCT
TTTCTCTCCCTGTAGGCTA
>2370
TTAATTATTGTTCTTACTCCAATAGATTTGAAATCTATTTAACTTTTCAGGGTCCCCTAGC
TTTTTAAATGTTTCAAGTGTATTCTCTCTGATAGGTTGCCTTTTCTCTCCCTGTAGGCTAT
AATTAGTGTCCTTTACAC
>2371
GTTCTTACTCCAATAGATTTGAAATCTATTTAACTTTTCAGGGTCCCCTAGCTTTTTAAAT
GTTTCAAGTGTATTCTCTCTGATAGGTTGCCTTTTCTCTCCCTGTAGGCTATAATTAGTGT
CCTTTACACAAAATTGGC
>2372
ATTTGAAATCTATTTAACTTTTCAGGGTCCCCTAGCTTTTTAAATGTTTCAAGTGTATTCT
CTCTGATAGGTTGCCTTTTCTCTCCCTGTAGGCTATAATTAGTGTCCTTTACACAAAATT
GGCTTAGAATTGGATTTAG
>2373
TTTTCAGGGTCCCCTAGCTTTTTAAATGTTTCAAGTGTATTCTCTCTGATAGGTTGCCTT
TTCTCTCCCTGTAGGCTATAATTAGTGTCCTTTACACAAAATTGGCTTAGAATTGGATTT
AGAGAAGATAAGAATCAAAT
>2374
CTATAATTAGTGTCCTTTACACAAAATTGGCTTAGAATTGGATTTAGAGAAGATAAGAAT
CAAATTACCAGTATTTATCTTGTAAATTCCAAAAGATCTACTTAAGACACTGTATTAAAA
AAGGAGTATAAAAGAAAAGT
>2375
GTCCTTTACACAAAATTGGCTTAGAATTGGATTTAGAGAAGATAAGAATCAAATTACCA
GTATTTATCTTGTAAATTCCAAAAGATCTACTTAAGACACTGTATTAAAAAAGGAGTATA
AAAGAAAAGTTTATTCTATGA
>2376
ATTTAGAGAAGATAAGAATCAAATTACCAGTATTTATCTTGTAAATTCCAAAAGATCTAC
TTAAGACACTGTATTAAAAAAGGAGTATAAAAGAAAAGTTTATTCTATGAAAAAGTAAA
TTCCTCATGGTCTGAGCAACC
>2377
AGAATCAAATTACCAGTATTTATCTTGTAAATTCCAAAAGATCTACTTAAGACACTGTAT
TAAAAAAGGAGTATAAAAGAAAAGTTTATTCTATGAAAAAGTAAATTCCTCATGGTCTG
AGCAACCAGGGGTATTTTAAT

>2378
ATTCCAAAAGATCTACTTAAGACACTGTATTAAAAAAGGAGTATAAAAGAAAAGTTTAT
TCTATGAAAAAGTAAATTCCTCATGGTCTGAGCAACCAGGGGTATTTTAATATCAAATCT
CCAGACTTCCCTTGGAGGCAC
>2379
ACTTAAGACACTGTATTAAAAAAGGAGTATAAAAGAAAAGTTTATTCTATGAAAAAGTA
AATTCCTCATGGTCTGAGCAACCAGGGGTATTTTAATATCAAATCTCCAGACTTCCCTTG
GAGGCACCTAAAATTGCCTTT
>2380
TAAAAAAGGAGTATAAAAGAAAAGTTTATTCTATGAAAAAGTAAATTCCTCATGGTCTG
AGCAACCAGGGGTATTTTAATATCAAATCTCCAGACTTCCCTTGGAGGCACCTAAAATT
GCCTTTTCCCTTGTCGCTCGTT
>2381
GCAACCAGGGGTATTTTAATATCAAATCTCCAGACTTCCCTTGGAGGCACCTAAAATTG
CCTTTTCCCTTGTCGCTCGTTTTTATCTCCAGTTGAGCTTCTGAAATATGTTGCCAGGCT
TTGGATATTGGAGAGCTGGTG
>2382
GGGGTATTTTAATATCAAATCTCCAGACTTCCCTTGGAGGCACCTAAAATTGCCTTTTCC
CTTGTCGCTCGTTTTTATCTCCAGTTGAGCTTCTGAAATATGTTGCCAGGCTTTGGATAT
TGGAGAGCTGGTGTGGTTTC
>2383
ATCAAATCTCCAGACTTCCCTTGGAGGCACCTAAAATTGCCTTTTCCCTTGTCGCTCGT
TTTTATCTCCAGTTGAGCTTCTGAAATATGTTGCCAGGCTTTGGATATTGGAGAGCTGGT
GTGGTTTCAGCTTTCATTAAA
>2384
CCTTGGAGGCACCTAAAATTGCCTTTTCCCTTGTCGCTCGTTTTTATCTCCAGTTGAGCT
TCTGAAATATGTTGCCAGGCTTTGGATATTGGAGAGCTGGTGTGGTTTCAGCTTTCATTA
AAATTCGGAGGTTTTGACTT
>2385
CTTGTCGCTCGTTTTTATCTCCAGTTGAGCTTCTGAAATATGTTGCCAGGCTTTGGATAT
TGGAGAGCTGGTGTGGTTTCAGCTTTCATTAAAATTCGGAGGTTTTGACTTATCTATTAT
TTCTGATTTGTGGTAAGACA
>2386
TCTGAAATATGTTGCCAGGCTTTGGATATTGGAGAGCTGGTGTGGTTTCAGCTTTCATTA
AAATTCGGAGGTTTTGACTTATCTATTATTTCTGATTTGTGGTAAGACATAAGCCTCTAA
AATATGCTATCTTAAAGAGA
>2387
TTGGAGAGCTGGTGTGGTTTCAGCTTTCATTAAAATTCGGAGGTTTTGACTTATCTATTA
TTTCTGATTTGTGGTAAGACATAAGCCTCTAAAATATGCTATCTTAAAGAGATACACACA
AATTTTATCTTAGCATGTTA
>2388
ATTTGTGGTAAGACATAAGCCTCTAAAATATGCTATCTTAAAGAGATACACACAAATTTT
ATCTTAGCATGTTAAGTTTTTAAGCCGTTGAGAGATGGCAGAATAGACCTCTGTGGTCT
CCTGTGTGCTTCTTCGTCCTA
>2389
TGGTAAGACATAAGCCTCTAAAATATGCTATCTTAAAGAGATACACACAAATTTTATCTT

AGCATGTTAAGTTTTTAAGCCGTTGAGAGATGGCAGAATAGACCTCTGTGGTCTCCTGT
GTGCTTCTTCGTCCTAAAATG
>2390
AAGCCTCTAAAATATGCTATCTTAAAGAGATACACACAAATTTTATCTTAGCATGTTAAG
TTTTTAAGCCGTTGAGAGATGGCAGAATAGACCTCTGTGGTCTCCTGTGTGCTTCTTCG
TCCTAAAATGTCAGCATGCTG
>2391
GAGATACACACAAATTTTATCTTAGCATGTTAAGTTTTTAAGCCGTTGAGAGATGGCAG
AATAGACCTCTGTGGTCTCCTGTGTGCTTCTTCGTCCTAAAATGTCAGCATGCTGGTTTT
CATGTGCTGCCTCGTACAGTA
>2392
TAGCATGTTAAGTTTTTAAGCCGTTGAGAGATGGCAGAATAGACCTCTGTGGTCTCCTG
TGTGCTTCTTCGTCCTAAAATGTCAGCATGCTGGTTTTCATGTGCTGCCTCGTACAGTAA
GTCCCCAGCAGATTTAAATAA
>2393
AGTTTTTAAGCCGTTGAGAGATGGCAGAATAGACCTCTGTGGTCTCCTGTGTGCTTCTT
CGTCCTAAAATGTCAGCATGCTGGTTTTCATGTGCTGCCTCGTACAGTAAGTCCCCAGC
AGATTTAAATAAGGTTGTGTCA
>2394
GTTGAGAGATGGCAGAATAGACCTCTGTGGTCTCCTGTGTGCTTCTTCGTCCTAAAATG
TCAGCATGCTGGTTTTCATGTGCTGCCTCGTACAGTAAGTCCCCAGCAGATTTAAATAA
GGTTGTGTCAAAACATATGCCT
>2395
AGACCTCTGTGGTCTCCTGTGTGCTTCTTCGTCCTAAAATGTCAGCATGCTGGTTTTCAT
GTGCTGCCTCGTACAGTAAGTCCCCAGCAGATTTAAATAAGGTTGTGTCAAAACATATG
CCTATGTTTTTTTTAACATCG
>2396
TCCTAAAATGTCAGCATGCTGGTTTTCATGTGCTGCCTCGTACAGTAAGTCCCCAGCAG
ATTTAAATAAGGTTGTGTCAAAACATATGCCTATGTTTTTTTTAACATCGTTGTCTCATAA
GTGTGCAGTAATGGCATATT
>2397
TAAAATGTCAGCATGCTGGTTTTCATGTGCTGCCTCGTACAGTAAGTCCCCAGCAGATT
TAAATAAGGTTGTGTCAAAACATATGCCTATGTTTTTTTTAACATCGTTGTCTCATAAGT
GTGCAGTAATGGCATATTGAG
>2398
TTTCATGTGCTGCCTCGTACAGTAAGTCCCCAGCAGATTTAAATAAGGTTGTGTCAAAA
CATATGCCTATGTTTTTTTTAACATCGTTGTCTCATAAGTGTGCAGTAATGGCATATTGAG
ACTGTTCATAGACTGGTCAG
>2399
CCCCAGCAGATTTAAATAAGGTTGTGTCAAAACATATGCCTATGTTTTTTTTAACATCGT
TGTCTCATAAGTGTGCAGTAATGGCATATTGAGACTGTTCATAGACTGGTCAGCTGCAT
TTCCTGCAGCCGAGGAACCCT
>2400
CCAGCAGATTTAAATAAGGTTGTGTCAAAACATATGCCTATGTTTTTTTTAACATCGTTG
TCTCATAAGTGTGCAGTAATGGCATATTGAGACTGTTCATAGACTGGTCAGCTGCATTTC
CTGCAGCCGAGGAACCCTTT

>2401
AGATTTAAATAAGGTTGTGTCAAAACATATGCCTATGTTTTTTTTAACATCGTTGTCTCAT
AAGTGTGCAGTAATGGCATATTGAGACTGTTCATAGACTGGTCAGCTGCATTTCCTGCA
GCCGAGGAACCCTTTATTAA
>2402
TCAAAACATATGCCTATGTTTTTTTTAACATCGTTGTCTCATAAGTGTGCAGTAATGGCA
TATTGAGACTGTTCATAGACTGGTCAGCTGCATTTCCTGCAGCCGAGGAACCCTTTATT
AAGGTGCATTTCTGGGGTTGA
>2403
TAATGGCATATTGAGACTGTTCATAGACTGGTCAGCTGCATTTCCTGCAGCCGAGGAAC
CCTTTATTAAGGTGCATTTCTGGGGTTGAACTAAAACTGCTCACCCCCATCAAATGAGG
AAACTTGAGGGGTTTGCAGCCC
>2404
AGACTGTTCATAGACTGGTCAGCTGCATTTCCTGCAGCCGAGGAACCCTTTATTAAGGT
GCATTTCTGGGGTTGAACTAAAACTGCTCACCCCCATCAAATGAGGAAACTTGAGGGG
TTTGCAGCCCAGCATTTTACGGG
>2405
CCTTTATTAAGGTGCATTTCTGGGGTTGAACTAAAACTGCTCACCCCCATCAAATGAGG
AAACTTGAGGGGTTTGCAGCCCAGCATTTTACGGGCACTTTTGCGACTGTGTACCCTT
GCGTGGCTTCCCGCTTCAGCGAC
>2406
TGCTCACCCCCATCAAATGAGGAAACTTGAGGGGTTTGCAGCCCAGCATTTTACGGGC
ACTTTTGCGACTGTGTACCCTTGCGTGGCTTCCCGCTTCAGCGACATTGTTGACTTCAG
TTTTCTAATCTAATTCTTTTTTT
>2407
TCAAATGAGGAAACTTGAGGGGTTTGCAGCCCAGCATTTTACGGGCACTTTTGCGACT
GTGTACCCTTGCGTGGCTTCCCGCTTCAGCGACATTGTTGACTTCAGTTTTCTAATCTAA
TTCTTTTTTTCTTTTTTGGTTT
>2408
TGCAGCCCAGCATTTTACGGGCACTTTTGCGACTGTGTACCCTTGCGTGGCTTCCCGCT
TCAGCGACATTGTTGACTTCAGTTTTCTAATCTAATTCTTTTTTTCTTTTTTGGTTTTGAT
GAGAGTGTTGGATACCAAAG
>2409
CATTTTACGGGCACTTTTGCGACTGTGTACCCTTGCGTGGCTTCCCGCTTCAGCGACAT
TGTTGACTTCAGTTTTCTAATCTAATTCTTTTTTTCTTTTTTGGTTTTGATGAGAGTGTTG
GATACCAAAGCACAAACGAT
>2410
TTGCGTGGCTTCCCGCTTCAGCGACATTGTTGACTTCAGTTTTCTAATCTAATTCTTTTT
TTCTTTTTTGGTTTTGATGAGAGTGTTGGATACCAAAGCACAAACGATGGATTCCTTTT
GAAAGAACAAAAACATATTTT
>2411
CCGCTTCAGCGACATTGTTGACTTCAGTTTTCTAATCTAATTCTTTTTTTCTTTTTTGGTT
TTGATGAGAGTGTTGGATACCAAAGCACAAACGATGGATTCCTTTTGAAAGAACAAAA
ACATATTTTGTGAGCAGTTTT
>2412
GTTTTGATGAGAGTGTTGGATACCAAAGCACAAACGATGGATTCCTTTTGAAAGAACA

AAAACATATTTTGTGAGCAGTTTTACTTCTGGGAGTGTTATGTGTATACACACACACAC
ACACACCCTAAATAATTGAAAGC
>2413
TGAGAGTGTTGGATACCAAAGCACAAACGATGGATTCCTTTTGAAAGAACAAAAACAT
ATTTTGTGAGCAGTTTTACTTCTGGGAGTGTTATGTGTATACACACACACACACACACC
CTAAATAATTGAAAGCAGGAACT
>2414
TGTATACACACACACACACACACACCCTAAATAATTGAAAGCAGGAACTTGAACAGATATT
TGCACACCCATGTTCATAGCAGCATTAGCCAAAATAGCCAAAAGGTGGAAATAATTCA
AATGTTCATTGACCCATGGATAA
>2415
TGAAAGCAGGAACTTGAACAGATATTTGCACACCCATGTTCATAGCAGCATTAGCCAA
AATAGCCAAAAGGTGGAAATAATTCAAATGTTCATTGACCCATGGATAAATTGACAAAA
TGTGGTATATACATATAGTGGAA
>2416
ACAGATATTTGCACACCCATGTTCATAGCAGCATTAGCCAAAATAGCCAAAAGGTGGA
AATAATTCAAATGTTCATTGACCCATGGATAAATTGACAAAATGTGGTATATACATATAGT
GGAATATTATTCAGCCTTAAC
>2417
CATTAGCCAAAATAGCCAAAAGGTGGAAATAATTCAAATGTTCATTGACCCATGGATAA
ATTGACAAAATGTGGTATATACATATAGTGGAATATTATTCAGCCTTAACAAGGAAGGAA
ATTCTGACACATGGTACAACA
>2418
TAGCCAAAATAGCCAAAAGGTGGAAATAATTCAAATGTTCATTGACCCATGGATAAATT
GACAAAATGTGGTATATACATATAGTGGAATATTATTCAGCCTTAACAAGGAAGGAAATT
CTGACACATGGTACAACACAG
>2419
GACAAAATGTGGTATATACATATAGTGGAATATTATTCAGCCTTAACAAGGAAGGAAATT
CTGACACATGGTACAACACAGATTAACTTTGAGGGCATTATACTAGGTGAAATAAGCTA
GATAGCAAAGGACAGATAGTG
>2420
TCAGCCTTAACAAGGAAGGAAATTCTGACACATGGTACAACACAGATTAACTTTGAGG
GCATTATACTAGGTGAAATAAGCTAGATAGCAAAGGACAGATAGTGTATGGTGCCATCT
ATATGAGGCATCTGTGCTTATAT
>2421
ATGGTACAACACAGATTAACTTTGAGGGCATTATACTAGGTGAAATAAGCTAGATAGCA
AAGGACAGATAGTGTATGGTGCCATCTATATGAGGCATCTGTGCTTATATAAAGCAAATT
TGTAGAAACACGAACTAGAAT
>2422
AACACAGATTAACTTTGAGGGCATTATACTAGGTGAAATAAGCTAGATAGCAAAGGAC
AGATAGTGTATGGTGCCATCTATATGAGGCATCTGTGCTTATATAAAGCAAATTTGTAGA
AACACGAACTAGAATAGATGTT
>2423
ATTATACTAGGTGAAATAAGCTAGATAGCAAAGGACAGATAGTGTATGGTGCCATCTATA
TGAGGCATCTGTGCTTATATAAAGCAAATTTGTAGAAACACGAACTAGAATAGATGTTG
CCAGGAGCTGGGGAGAGAGGG

>2424
TAGATAGCAAAGGACAGATAGTGTATGGTGCCATCTATATGAGGCATCTGTGCTTATATA
AAGCAAATTTGTAGAAACACGAACTAGAATAGATGTTGCCAGGAGCTGGGGAGAGAG
GGGAATGGGGAGTTAAGGTTGAG
>2425
ATGGTGCCATCTATATGAGGCATCTGTGCTTATATAAAGCAAATTTGTAGAAACACGAAC
TAGAATAGATGTTGCCAGGAGCTGGGGAGAGAGGGGAATGGGGAGTTAAGGTTGAGT
GGGTACAGAGTTTCAGTTGGGGG
>2426
TAAAGCAAATTTGTAGAAACACGAACTAGAATAGATGTTGCCAGGAGCTGGGGAGAG
AGGGGAATGGGGAGTTAAGGTTGAGTGGGTACAGAGTTTCAGTTGGGGGAGATGAAA
AAGTTCTGTGGCTGGATGGTAGTAAT
>2427
AAATTTGTAGAAACACGAACTAGAATAGATGTTGCCAGGAGCTGGGGAGAGAGGGGA
ATGGGGAGTTAAGGTTGAGTGGGTACAGAGTTTCAGTTGGGGGAGATGAAAAAGTTC
TGTGGCTGGATGGTAGTAATGGTTAC
>2428
GCTGGGGAGAGAGGGGAATGGGGAGTTAAGGTTGAGTGGGTACAGAGTTTCAGTTGG
GGGAGATGAAAAAGTTCTGTGGCTGGATGGTAGTAATGGTTACACAACATCAGGAGTG
TACTAAATGACACTGATCTGTACAC
>2429
GGAGAGAGGGGAATGGGGAGTTAAGGTTGAGTGGGTACAGAGTTTCAGTTGGGGGAG
ATGAAAAAGTTCTGTGGCTGGATGGTAGTAATGGTTACACAACATCAGGAGTGTACTA
AATGACACTGATCTGTACACTTAGA
>2430
AATGGGGAGTTAAGGTTGAGTGGGTACAGAGTTTCAGTTGGGGGAGATGAAAAAGTT
CTGTGGCTGGATGGTAGTAATGGTTACACAACATCAGGAGTGTACTAAATGACACTGAT
CTGTACACTTAGAATCTTTAAAAT
>2431
CAGAGTTTCAGTTGGGGGAGATGAAAAAGTTCTGTGGCTGGATGGTAGTAATGGTTAC
ACAACATCAGGAGTGTACTAAATGACACTGATCTGTACACTTAGAATCTTTAAAATGGT
AAATTTTAGTTACATAGTTTTAC
>2432
GAAAAAGTTCTGTGGCTGGATGGTAGTAATGGTTACACAACATCAGGAGTGTACTAAA
TGACACTGATCTGTACACTTAGAATCTTTAAAATGGTAAATTTTAGTTACATAGTTTTAC
TACAATAAAAATGTATTTTATG
>2433
TAGTAATGGTTACACAACATCAGGAGTGTACTAAATGACACTGATCTGTACACTTAGAA
TCTTTAAAATGGTAAATTTTAGTTACATAGTTTTACTACAATAAAAATGTATTTTATGTTA
ATTGCAATACTTGGCATTTG
>2434
ATCAGGAGTGTACTAAATGACACTGATCTGTACACTTAGAATCTTTAAAATGGTAAATTT
TAGTTACATAGTTTTACTACAATAAAAATGTATTTTATGTTAATTGCAATACTTGGCATTT
GAGAAAAAATTCTTAGAAA
>2435
ACACTGATCTGTACACTTAGAATCTTTAAAATGGTAAATTTTAGTTACATAGTTTTACTA

CAATAAAAATGTATTTTATGTTAATTGCAATACTTGGCATTTGAGAAAAAATTCTTAGAA
AATACTGAGAATATCATACT
>2436
TGTACACTTAGAATCTTTAAAATGGTAAATTTTAGTTACATAGTTTTACTACAATAAAAA
TGTATTTTATGTTAATTGCAATACTTGGCATTTGAGAAAAAATTCTTAGAAAATACTGAG
AATATCATACTGATAAAAAC
>2437
AAAATACTGAGAATATCATACTGATAAAAACCCTCTGCTAAATTTTTTTTAAAAAAAAC
ACACATCGCCTGTACTCCTGAGTATCAGCGATCAGTCTAGCGTTGCCCTTTAACTATCAT
TATTGTTATTGTTAAAGTGAT
>2438
GAATATCATACTGATAAAAACCCTCTGCTAAATTTTTTTTAAAAAAAACACACATCGCCT
GTACTCCTGAGTATCAGCGATCAGTCTAGCGTTGCCCTTTAACTATCATTATTGTTATTGT
TAAAGTGATTTTTATTAAG
>2439
ATAAAAACCCTCTGCTAAATTTTTTTTAAAAAAAACACACATCGCCTGTACTCCTGAGT
ATCAGCGATCAGTCTAGCGTTGCCCTTTAACTATCATTATTGTTATTGTTAAAGTGATTTT
TATTAAGTGCTATCTCTCAA
>2440
AAAAACACACATCGCCTGTACTCCTGAGTATCAGCGATCAGTCTAGCGTTGCCCTTTAA
CTATCATTATTGTTATTGTTAAAGTGATTTTTATTAAGTGCTATCTCTCAAGTGGTATGATT
ACTTGTTGAGGTCTTGTGT
>2441
ACACATCGCCTGTACTCCTGAGTATCAGCGATCAGTCTAGCGTTGCCCTTTAACTATCAT
TATTGTTATTGTTAAAGTGATTTTTATTAAGTGCTATCTCTCAAGTGGTATGATTACTTGT
TGAGGTCTTGTGTTGGTAT
>2442
CGCCTGTACTCCTGAGTATCAGCGATCAGTCTAGCGTTGCCCTTTAACTATCATTATTGT
TATTGTTAAAGTGATTTTTATTAAGTGCTATCTCTCAAGTGGTATGATTACTTGTTGAGGT
CTTGTGTTGGTATTTGTCA
>2443
AGTATCAGCGATCAGTCTAGCGTTGCCCTTTAACTATCATTATTGTTATTGTTAAAGTGAT
TTTTATTAAGTGCTATCTCTCAAGTGGTATGATTACTTGTTGAGGTCTTGTGTTGGTATTT
GTCATGTGATTCTAGAAA
>2444
TTAACTATCATTATTGTTATTGTTAAAGTGATTTTTATTAAGTGCTATCTCTCAAGTGGTAT
GATTACTTGTTGAGGTCTTGTGTTGGTATTTGTCATGTGATTCTAGAAAAATCTCCCCCT
TTTTGGCTCTGAGACATT
>2445
ATCATTATTGTTATTGTTAAAGTGATTTTTATTAAGTGCTATCTCTCAAGTGGTATGATTA
CTTGTTGAGGTCTTGTGTTGGTATTTGTCATGTGATTCTAGAAAAATCTCCCCCTTTTTG
GCTCTGAGACATTCTCTAG
>2446
TATTGTTATTGTTAAAGTGATTTTTATTAAGTGCTATCTCTCAAGTGGTATGATTACTTGT
TGAGGTCTTGTGTTGGTATTTGTCATGTGATTCTAGAAAAATCTCCCCCTTTTTGGCTCT
GAGACATTCTCTAGTTGAC

>2447
TATTGTTAAAGTGATTTTTATTAAGTGCTATCTCTCAAGTGGTATGATTACTTGTTGAGGT
CTTGTGTTGGTATTTGTCATGTGATTCTAGAAAAATCTCCCCCTTTTTGGCTCTGAGACA
TTCTCTAGTTGACCTCTTC
>2448
GGTCTTGTGTTGGTATTTGTCATGTGATTCTAGAAAAATCTCCCCCTTTTTGGCTCTGAG
ACATTCTCTAGTTGACCTCTTCCCAGAGAGAAGTTAAGGAAAATCTTCCGCTCTGATTT
TTTTTTCTTTCTGCACTTAAA
>2449
TGTGATTCTAGAAAAATCTCCCCCTTTTTGGCTCTGAGACATTCTCTAGTTGACCTCTTC
CCAGAGAGAAGTTAAGGAAAATCTTCCGCTCTGATTTTTTTTTCTTTCTGCACTTAAAT
AATTAAATAATATCTGTGGCC
>2450
AGAGAAGTTAAGGAAAATCTTCCGCTCTGATTTTTTTTTCTTTCTGCACTTAAATAATTA
AATAATATCTGTGGCCTTACAACACAGGGAGGTTTCAGTGAGGAAGAAAATTCAACTG
CATTTGCAAAATGTCTGAAAAT
>2451
TGATTTTTTTTTCTTTCTGCACTTAAATAATTAAATAATATCTGTGGCCTTACAACACAGG
GAGGTTTCAGTGAGGAAGAAAATTCAACTGCATTTGCAAAATGTCTGAAAATTATGGG
AGAGATGTTTCTGCTTTTAGA
>2452
AATAATATCTGTGGCCTTACAACACAGGGAGGTTTCAGTGAGGAAGAAAATTCAACTG
CATTTGCAAAATGTCTGAAAATTATGGGAGAGATGTTTCTGCTTTTAGACTTGGTACGTA
TGTGGTCAGGTTTTCAGAGGTT
>2453
CACAGGGAGGTTTCAGTGAGGAAGAAAATTCAACTGCATTTGCAAAATGTCTGAAAAT
TATGGGAGAGATGTTTCTGCTTTTAGACTTGGTACGTATGTGGTCAGGTTTTCAGAGGT
TTTCTATTCACCATCTGTGACGG
>2454
GGAAGAAAATTCAACTGCATTTGCAAAATGTCTGAAAATTATGGGAGAGATGTTTCTG
CTTTTAGACTTGGTACGTATGTGGTCAGGTTTTCAGAGGTTTTCTATTCACCATCTGTGA
CGGGCCTTGTCTCCTCCTGCAA
>2455
GAAAATTCAACTGCATTTGCAAAATGTCTGAAAATTATGGGAGAGATGTTTCTGCTTTT
AGACTTGGTACGTATGTGGTCAGGTTTTCAGAGGTTTTCTATTCACCATCTGTGACGGG
CCTTGTCTCCTCCTGCAACTGG
>2456
AATGTCTGAAAATTATGGGAGAGATGTTTCTGCTTTTAGACTTGGTACGTATGTGGTCA
GGTTTTCAGAGGTTTTCTATTCACCATCTGTGACGGGCCTTGTCTCCTCCTGCAACTGG
TGGCAGATGTAGCCAGAGATTT
>2457
GAGAGATGTTTCTGCTTTTAGACTTGGTACGTATGTGGTCAGGTTTTCAGAGGTTTTCTA
TTCACCATCTGTGACGGGCCTTGTCTCCTCCTGCAACTGGTGGCAGATGTAGCCAGAG
ATTTTGTGAGCTTCTCAACAGC
>2458
TGCTTTTAGACTTGGTACGTATGTGGTCAGGTTTTCAGAGGTTTTCTATTCACCATCTGT

GACGGGCCTTGTCTCCTCCTGCAACTGGTGGCAGATGTAGCCAGAGATTTTGTGAGCT
TCTCAACAGCATCTTTTTCCTG
>2459
CGTATGTGGTCAGGTTTTCAGAGGTTTTCTATTCACCATCTGTGACGGGCCTTGTCTCCT
CCTGCAACTGGTGGCAGATGTAGCCAGAGATTTTGTGAGCTTCTCAACAGCATCTTTTT
CCTGCTCTTCTTCCCTTCCTC
>2460
TTTTCTATTCACCATCTGTGACGGGCCTTGTCTCCTCCTGCAACTGGTGGCAGATGTAG
CCAGAGATTTTGTGAGCTTCTCAACAGCATCTTTTTCCTGCTCTTCTTCCCTTCCTCTTT
GTTTGGTTTTTATTCACATGA
>2461
GCAGATGTAGCCAGAGATTTTGTGAGCTTCTCAACAGCATCTTTTTCCTGCTCTTCTTC
CCTTCCTCTTTGTTTGGTTTTTATTCACATGAAAATTTAAAGACCCATAAGGAAATTCAA
AGTGAAAATCTATGCCCAAAT
>2462
TGTAGCCAGAGATTTTGTGAGCTTCTCAACAGCATCTTTTTCCTGCTCTTCTTCCCTTCC
TCTTTGTTTGGTTTTTATTCACATGAAAATTTAAAGACCCATAAGGAAATTCAAAGTGA
AAATCTATGCCCAAATAAGTC
>2463
CTCTTCTTCCCTTCCTCTTTGTTTGGTTTTTATTCACATGAAAATTTAAAGACCCATAAG
GAAATTCAAAGTGAAAATCTATGCCCAAATAAGTCAGGATCTCAAGATGAAAATATAAG
TTCTGGAAATGGAAGTTTCCT
>2464
TTGGTTTTTATTCACATGAAAATTTAAAGACCCATAAGGAAATTCAAAGTGAAAATCTA
TGCCCAAATAAGTCAGGATCTCAAGATGAAAATATAAGTTCTGGAAATGGAAGTTTCCT
CTGCCCCCGTGGTGGTATCATA
>2465
AAGACCCATAAGGAAATTCAAAGTGAAAATCTATGCCCAAATAAGTCAGGATCTCAAG
ATGAAAATATAAGTTCTGGAAATGGAAGTTTCCTCTGCCCCCGTGGTGGTATCATAGCC
AGCAGCCTCGTTCTGTGCTTTTG
>2466
ATTCAAAGTGAAAATCTATGCCCAAATAAGTCAGGATCTCAAGATGAAAATATAAGTTC
TGGAAATGGAAGTTTCCTCTGCCCCCGTGGTGGTATCATAGCCAGCAGCCTCGTTCTGT
GCTTTTGAAGGTTTAATGGCCT
>2467
AGATGAAAATATAAGTTCTGGAAATGGAAGTTTCCTCTGCCCCCGTGGTGGTATCATAG
CCAGCAGCCTCGTTCTGTGCTTTTGAAGGTTTAATGGCCTGCATGTGAAGGTAGGGGC
GCACTATATATATATAGTACCAGGC
>2468
AAAATATAAGTTCTGGAAATGGAAGTTTCCTCTGCCCCCGTGGTGGTATCATAGCCAGC
AGCCTCGTTCTGTGCTTTTGAAGGTTTAATGGCCTGCATGTGAAGGTAGGGGCGCACTA
TATATATAGTACCAGGCGTGTG
>2469
CCTCTGCCCCCGTGGTGGTATCATAGCCAGCAGCCTCGTTCTGTGCTTTTGAAGGTTTA
ATGGCCTGCATGTGAAGGTAGGGGCGCACTATATATATATAGTACCAGGCGTGTGGGCGCA
CTGGCACGGATCTGAAAAAGCG

>2470
AGCAGCCTCGTTCTGTGCTTTTGAAGGTTTAATGGCCTGCATGTGAAGGTAGGGGCGC
ACTATATATATAGTACCAGGCGTGTGGGCGCACTGGCACGGATCTGAAAAAGCGTAAGT
TTGGCATTTCTAGGTTTATTTTA
>2471
GTTCTGTGCTTTTGAAGGTTTAATGGCCTGCATGTGAAGGTAGGGGCGCACTATATATAT
AGTACCAGGCGTGTGGGCGCACTGGCACGGATCTGAAAAAGCGTAAGTTTGGCATTTC
TAGGTTTATTTTAAACTTACAA
>2472
TGTGAAGGTAGGGGCGCACTATATATATAGTACCAGGCGTGTGGGCGCACTGGCACGGA
TCTGAAAAAGCGTAAGTTTGGCATTTCTAGGTTTATTTTAAACTTACAACCTTTAACGC
CAGACACAGAGCTTCTTTCCCA
>2473
AAGGTAGGGGCGCACTATATATATAGTACCAGGCGTGTGGGCGCACTGGCACGGATCTG
AAAAAGCGTAAGTTTGGCATTTCTAGGTTTATTTTAAACTTACAACCTTTAACGCCAGA
CACAGAGCTTCTTTCCCACTTA
>2474
TATATATATAGTACCAGGCGTGTGGGCGCACTGGCACGGATCTGAAAAAGCGTAAGTTT
GGCATTTCTAGGTTTATTTTAAACTTACAACCTTTAACGCCAGACACAGAGCTTCTTTCC
CACTTAATGTAACTATACATT
>2475
TTGGCATTTCTAGGTTTATTTTAAACTTACAACCTTTAACGCCAGACACAGAGCTTCTTT
CCCACTTAATGTAACTATACATTGATTGCTTTTAATTAGTGTTGGATCTTTTTTGGTGTTT
TGCAGCTTGGGATATATAA
>2476
ACAACCTTTAACGCCAGACACAGAGCTTCTTTCCCACTTAATGTAACTATACATTGATT
GCTTTTAATTAGTGTTGGATCTTTTTTGGTGTTTTGCAGCTTGGGATATATAAAAAGTCT
GACTTAAATTTAAAAAAAAAA
>2477
ACACAGAGCTTCTTTCCCACTTAATGTAACTATACATTGATTGCTTTTAATTAGTGTTGG
ATCTTTTTTGGTGTTTTGCAGCTTGGGATATATAAAAAGTCTGACTTAAATTTAAAAAAA
AAAAAAGCTAACTCCTGACA
>2478
ACAGAGCTTCTTTCCCACTTAATGTAACTATACATTGATTGCTTTTAATTAGTGTTGGATC
TTTTTTGGTGTTTTGCAGCTTGGGATATATAAAAAGTCTGACTTAAATTTAAAAAAAAA
AAAAGCTAACTCCTGACATT
>2479
ACTATACATTGATTGCTTTTAATTAGTGTTGGATCTTTTTTGGTGTTTTGCAGCTTGGGAT
ATATAAAAAGTCTGACTTAAATTTAAAAAAAAAAAAAGCTAACTCCTGACATTCTCACG
AGTCTTGCAAAAACCAGCAC
>2480
AGCTTGGGATATATAAAAAGTCTGACTTAAATTTAAAAAAAAAAAAAGCTAACTCCTG
ACATTCTCACGAGTCTTGCAAAAACCAGCACGGGAGCCTTAGAAATTCTAACCTCCTC
GTCCTCTTTATTTTTATCTTGAGT
>2481
AGCTAACTCCTGACATTCTCACGAGTCTTGCAAAAACCAGCACGGGAGCCTTAGAAAT

TCTAACCTCCTCGTCCTCTTTATTTTTATCTTGAGTCAGGTGGGCCAAGAAGCAAGGGA
AGAGTCCAATATAACATACTTAA
>2482
GAGTCTTGCAAAAACCAGCACGGGAGCCTTAGAAATTCTAACCTCCTCGTCCTCTTTAT
TTTTATCTTGAGTCAGGTGGGCCAAGAAGCAAGGGAAGAGTCCAATATAACATACTTAA
TGGATGGAGGTAATTTCCTTGG
>2483
TTAGAAATTCTAACCTCCTCGTCCTCTTTATTTTTATCTTGAGTCAGGTGGGCCAAGAAG
CAAGGGAAGAGTCCAATATAACATACTTAATGGATGGAGGTAATTTCCTTGGACTGTTT
TGGCAGAAATTAAGCATCTGT
>2484
ATTTTTATCTTGAGTCAGGTGGGCCAAGAAGCAAGGGAAGAGTCCAATATAACATACTT
AATGGATGGAGGTAATTTCCTTGGACTGTTTTGGCAGAAATTAAGCATCTGTATTTAGAT
AAAGGTAGTGGTGGGTTGGAT
>2485
AGGTGGGCCAAGAAGCAAGGGAAGAGTCCAATATAACATACTTAATGGATGGAGGTAA
TTTCCTTGGACTGTTTTGGCAGAAATTAAGCATCTGTATTTAGATAAAGGTAGTGGTGG
GTTGGATTTTCATAGCATAAGCA
>2486
GAGTCCAATATAACATACTTAATGGATGGAGGTAATTTCCTTGGACTGTTTTGGCAGAA
ATTAAGCATCTGTATTTAGATAAAGGTAGTGGTGGGTTGGATTTTCATAGCATAAGCATA
GTTGCTGAAGATCAATAATAT
>2487
ATACTTAATGGATGGAGGTAATTTCCTTGGACTGTTTTGGCAGAAATTAAGCATCTGTAT
TTAGATAAAGGTAGTGGTGGGTTGGATTTTCATAGCATAAGCATAGTTGCTGAAGATCA
ATAATATCAAATAATGGGTTC
>2488
GATGGAGGTAATTTCCTTGGACTGTTTTGGCAGAAATTAAGCATCTGTATTTAGATAAA
GGTAGTGGTGGGTTGGATTTTCATAGCATAAGCATAGTTGCTGAAGATCAATAATATCAA
ATAATGGGTTCTTGGTGAGGG
>2489
AAAGGTAGTGGTGGGTTGGATTTTCATAGCATAAGCATAGTTGCTGAAGATCAATAATA
TCAAATAATGGGTTCTTGGTGAGGGGGAAAGGAAATGGGGGAGGGGTCTTTTCACTCA
ACAGTTTGGAAATGCTAATGATT
>2490
GTGGTGGGTTGGATTTTCATAGCATAAGCATAGTTGCTGAAGATCAATAATATCAAATAA
TGGGTTCTTGGTGAGGGGGAAAGGAAATGGGGGAGGGGTCTTTTCACTCAACAGTTT
GGAAATGCTAATGATTATTTGTG
>2491
TCAATAATATCAAATAATGGGTTCTTGGTGAGGGGGAAAGGAAATGGGGGAGGGGTCT
TTTCACTCAACAGTTTGGAAATGCTAATGATTATTTGTGGTTTTCAGCCCAGAATTCTTC
ATATTTGCTTGCAAGCTTCTAT
>2492
TTGGTGAGGGGGAAAGGAAATGGGGGAGGGGTCTTTTCACTCAACAGTTTGGAAATG
CTAATGATTATTTGTGGTTTTCAGCCCAGAATTCTTCATATTTGCTTGCAAGCTTCTATGG
CACTGCCGAATTCTACAGTAAA

>2493
TATTTGTGGTTTTCAGCCCAGAATTCTTCATATTTGCTTGCAAGCTTCTATGGCACTGCC
GAATTCTACAGTAAATATTTCGGACATCCTCAGCCCTGTTCTCCTTTCCCTTCAGTAAAT
TACATATTAAAGTAGCCTGG
>2494
TCATATTTGCTTGCAAGCTTCTATGGCACTGCCGAATTCTACAGTAAATATTTCGGACAT
CCTCAGCCCTGTTCTCCTTTCCCTTCAGTAAATTACATATTAAAGTAGCCTGGTAACTGA
GCACTGGTTGCTGTGAGATA
>2495
CTTCTATGGCACTGCCGAATTCTACAGTAAATATTTCGGACATCCTCAGCCCTGTTCTCC
TTTCCCTTCAGTAAATTACATATTAAAGTAGCCTGGTAACTGAGCACTGGTTGCTGTGA
GATAAGGGCTGGGAAATCAAT
>2496
AATTCTACAGTAAATATTTCGGACATCCTCAGCCCTGTTCTCCTTTCCCTTCAGTAAATT
ACATATTAAAGTAGCCTGGTAACTGAGCACTGGTTGCTGTGAGATAAGGGCTGGGAAA
TCAATATGTTTGCCATGTGGCT
>2497
ACATCCTCAGCCCTGTTCTCCTTTCCCTTCAGTAAATTACATATTAAAGTAGCCTGGTAA
CTGAGCACTGGTTGCTGTGAGATAAGGGCTGGGAAATCAATATGTTTGCCATGTGGCTG
CTTGTTTATGACACCTGCCTC
>2498
TCAGCCCTGTTCTCCTTTCCCTTCAGTAAATTACATATTAAAGTAGCCTGGTAACTGAGC
ACTGGTTGCTGTGAGATAAGGGCTGGGAAATCAATATGTTTGCCATGTGGCTGCTTGTT
TATGACACCTGCCTCTAGAAC
>2499
AAATTACATATTAAAGTAGCCTGGTAACTGAGCACTGGTTGCTGTGAGATAAGGGCTGG
GAAATCAATATGTTTGCCATGTGGCTGCTTGTTTATGACACCTGCCTCTAGAACAGTTG
AGCAATGTTCTGTTTGTTAAAG
>2500
TGAGATAAGGGCTGGGAAATCAATATGTTTGCCATGTGGCTGCTTGTTTATGACACCTG
CCTCTAGAACAGTTGAGCAATGTTCTGTTTGTTAAAGCTGCATTTCAGAAGCTTGTATC
CTCCTAAAACAACCGTGATCTT
>2501
GCTGGGAAATCAATATGTTTGCCATGTGGCTGCTTGTTTATGACACCTGCCTCTAGAAC
AGTTGAGCAATGTTCTGTTTGTTAAAGCTGCATTTCAGAAGCTTGTATCCTCCTAAAAC
AACCGTGATCTTACTGGGGGAG
>2502
TTGTTTATGACACCTGCCTCTAGAACAGTTGAGCAATGTTCTGTTTGTTAAAGCTGCAT
TTCAGAAGCTTGTATCCTCCTAAAACAACCGTGATCTTACTGGGGGAGAGAGAGGTGC
TACAACAGATATTGATTTTAGTT
>2503
CTAGAACAGTTGAGCAATGTTCTGTTTGTTAAAGCTGCATTTCAGAAGCTTGTATCCTC
CTAAAACAACCGTGATCTTACTGGGGGAGAGAGAGGTGCTACAACAGATATTGATTTT
AGTTTTTCTAAGTATGGAATTAC
>2504
TTTGTTAAAGCTGCATTTCAGAAGCTTGTATCCTCCTAAAACAACCGTGATCTTACTGG

GGGAGAGAGAGGTGCTACAACAGATATTGATTTTAGTTTTTCTAAGTATGGAATTACAC
TTCTAAACAGTGGTCTTACAGC
>2505
CTTGTATCCTCCTAAAACAACCGTGATCTTACTGGGGGAGAGAGAGGTGCTACAACAG
ATATTGATTTTAGTTTTTCTAAGTATGGAATTACACTTCTAAACAGTGGTCTTACAGCCT
GTGATTTTTTCCCCATGAGGAG
>2506
CCTAAAACAACCGTGATCTTACTGGGGGAGAGAGAGGTGCTACAACAGATATTGATTTT
AGTTTTTCTAAGTATGGAATTACACTTCTAAACAGTGGTCTTACAGCCTGTGATTTTTTC
CCCATGAGGAGGAGATGTAGA
>2507
GGGGGAGAGAGAGGTGCTACAACAGATATTGATTTTAGTTTTTCTAAGTATGGAATTAC
ACTTCTAAACAGTGGTCTTACAGCCTGTGATTTTTTCCCCATGAGGAGGAGATGTAGAT
ACTCTGAATATATATATACACA
>2508
GCTACAACAGATATTGATTTTAGTTTTTCTAAGTATGGAATTACACTTCTAAACAGTGGT
CTTACAGCCTGTGATTTTTTCCCCATGAGGAGGAGATGTAGATACTCTGAATATATATATA
CACATACACACATATATAA
>2509
TCTAAGTATGGAATTACACTTCTAAACAGTGGTCTTACAGCCTGTGATTTTTTCCCCATG
AGGAGGAGATGTAGATACTCTGAATATATATATACACATACACACATATATAAAATAAATC
TATTTTATAATGTTTATGA
>2510
GAGGAGATGTAGATACTCTGAATATATATATACACATACACACATATATAAAATAAATCTA
TTTTATAATGTTTATGATGTGGTCAGTTAAAAAAGGAGTGTAGTTTATAATAAGGAAGTG
TAGGTATTATTTAGATTTA
>2511
TAGATACTCTGAATATATATATACACATACACACATATATAAAATAAATCTATTTTATAATG
TTTATGATGTGGTCAGTTAAAAAAGGAGTGTAGTTTATAATAAGGAAGTGTAGGTATTAT
TTAGATTTATCTCCTCTC
>2512
TACACATACACACATATATAAAATAAATCTATTTTATAATGTTTATGATGTGGTCAGTTAA
AAAAGGAGTGTAGTTTATAATAAGGAAGTGTAGGTATTATTTAGATTTATCTCCTCTCTT
GGCACCTGGCTTAAAAAAA
>2513
ACATACACACATATATAAAATAAATCTATTTTATAATGTTTATGATGTGGTCAGTTAAAAA
AGGAGTGTAGTTTATAATAAGGAAGTGTAGGTATTATTTAGATTTATCTCCTCTCTTGGC
ACCTGGCTTAAAAAAAAAA
>2514
TAAAATAAATCTATTTTATAATGTTTATGATGTGGTCAGTTAAAAAAGGAGTGTAGTTTAT
AATAAGGAAGTGTAGGTATTATTTAGATTTATCTCCTCTCTTGGCACCTGGCTTAAAAAA
AAAAAAAGTAGTAACAAGA
>2515
TATAATAAGGAAGTGTAGGTATTATTTAGATTTATCTCCTCTCTTGGCACCTGGCTTAAA
AAAAAAAAAAGTAGTAACAAGAAACATAGCAGCTGCTGGGAAACATGCATTTTAATCT
AAGTCTCCAGTGTGGTTTTAGT

>2516
AATAAGGAAGTGTAGGTATTATTTAGATTTATCTCCTCTCTTGGCACCTGGCTTAAAAAA
AAAAAAAGTAGTAACAAGAAACATAGCAGCTGCTGGGAAACATGCATTTTAATCTAAG
TCTCCAGTGTGGTTTTAGTTTA
>2517
CTGGCTTAAAAAAAAAAAAAGTAGTAACAAGAAACATAGCAGCTGCTGGGAAACATG
CATTTTAATCTAAGTCTCCAGTGTGGTTTTAGTTTAACCAGTGGATGATACTGTAGTCTC
AAGGCTTAGCCTGCTGGTGAGTC
>2518
AAAAAAAAAAAAAGTAGTAACAAGAAACATAGCAGCTGCTGGGAAACATGCATTTTA
ATCTAAGTCTCCAGTGTGGTTTTAGTTTAACCAGTGGATGATACTGTAGTCTCAAGGCTT
AGCCTGCTGGTGAGTCAATGGTT
>2519
AAGTAGTAACAAGAAACATAGCAGCTGCTGGGAAACATGCATTTTAATCTAAGTCTCC
AGTGTGGTTTTAGTTTAACCAGTGGATGATACTGTAGTCTCAAGGCTTAGCCTGCTGGT
GAGTCAATGGTTCTTAAAGCTGT
>2520
CTGCTGGGAAACATGCATTTTAATCTAAGTCTCCAGTGTGGTTTTAGTTTAACCAGTGG
ATGATACTGTAGTCTCAAGGCTTAGCCTGCTGGTGAGTCAATGGTTCTTAAAGCTGTGT
GGTGTCAGCTCAAGTGAGCGAA
>2521
AATCTAAGTCTCCAGTGTGGTTTTAGTTTAACCAGTGGATGATACTGTAGTCTCAAGGC
TTAGCCTGCTGGTGAGTCAATGGTTCTTAAAGCTGTGTGGTGTCAGCTCAAGTGAGCG
AAGCAGTGTGTGGTGTGTGTGCA
>2522
TAAGTCTCCAGTGTGGTTTTAGTTTAACCAGTGGATGATACTGTAGTCTCAAGGCTTAG
CCTGCTGGTGAGTCAATGGTTCTTAAAGCTGTGTGGTGTCAGCTCAAGTGAGCGAAGC
AGTGTGTGGTGTGTGTGCATTGG
>2523
TAGTTTAACCAGTGGATGATACTGTAGTCTCAAGGCTTAGCCTGCTGGTGAGTCAATGG
TTCTTAAAGCTGTGTGGTGTCAGCTCAAGTGAGCGAAGCAGTGTGTGGTGTGTGTGCA
TTGGAGGGTGAGGGGTATGAGGA
>2524
TCAAGGCTTAGCCTGCTGGTGAGTCAATGGTTCTTAAAGCTGTGTGGTGTCAGCTCAA
GTGAGCGAAGCAGTGTGTGGTGTGTGTGCATTGGAGGGTGAGGGGTATGAGGAGGTA
GTCAGCCTTGGAGGGAGAAGGGAGA
>2525
AAGGCTTAGCCTGCTGGTGAGTCAATGGTTCTTAAAGCTGTGTGGTGTCAGCTCAAGT
GAGCGAAGCAGTGTGTGGTGTGTGTGCATTGGAGGGTGAGGGGTATGAGGAGGTAGT
CAGCCTTGGAGGGAGAAGGGAGACA
>2526
TTAGCCTGCTGGTGAGTCAATGGTTCTTAAAGCTGTGTGGTGTCAGCTCAAGTGAGCG
AAGCAGTGTGTGGTGTGTGTGCATTGGAGGGTGAGGGGTATGAGGAGGTAGTCAGCC
TTGGAGGGAGAAGGGAGACAGGCTA
>2527
AATGGTTCTTAAAGCTGTGTGGTGTCAGCTCAAGTGAGCGAAGCAGTGTGTGGTGTGT

GTGCATTGGAGGGTGAGGGGTATGAGGAGGTAGTCAGCCTTGGAGGGAGAAGGGAGA
CAGGCTAGGTCTTCTTCACGTGGCC
>2528
GGTGTCAGCTCAAGTGAGCGAAGCAGTGTGTGGTGTGTGTGCATTGGAGGGTGAGGG
GTATGAGGAGGTAGTCAGCCTTGGAGGGAGAAGGGAGACAGGCTAGGTCTTCTTCAC
GTGGCCTTCCATTTTGGAAAACAGAA
>2529
TGTGTGTGCATTGGAGGGTGAGGGGTATGAGGAGGTAGTCAGCCTTGGAGGGAGAAG
GGAGACAGGCTAGGTCTTCTTCACGTGGCCTTCCATTTTGGAAAACAGAAGCGATGAT
ACAACTTCTCTTCTTGCCCAAGGGA
>2530
TGGAGGGTGAGGGGTATGAGGAGGTAGTCAGCCTTGGAGGGAGAAGGGAGACAGGCT
AGGTCTTCTTCACGTGGCCTTCCATTTTGGAAAACAGAAGCGATGATACAACTTCTCTT
CTTGCCCAAGGGATTCAGTAGAAA
>2531
TCTTCACGTGGCCTTCCATTTTGGAAAACAGAAGCGATGATACAACTTCTCTTCTTGCC
CAAGGGATTCAGTAGAAAAAAAATACATTTCCAAACACATTGCGCTACAGTCATTGAAC
TTCAAAGTGTAAAGCATGGATTG
>2532
TGATACAACTTCTCTTCTTGCCCAAGGGATTCAGTAGAAAAAAAATACATTTCCAAACAC
ATTGCGCTACAGTCATTGAACTTCAAAGTGTAAAGCATGGATTGTGTTGGTGCACTTGT
GGGTGCCCATGACACACACTCT
>2533
CTTGCCCAAGGGATTCAGTAGAAAAAAAATACATTTCCAAACACATTGCGCTACAGTCAT
TGAACTTCAAAGTGTAAAGCATGGATTGTGTTGGTGCACTTGTGGGTGCCCATGACAC
ACACTCTTCAATGCTCCTGTGCC
>2534
AGTAGAAAAAAAATACATTTCCAAACACATTGCGCTACAGTCATTGAACTTCAAAGTGT
AAAGCATGGATTGTGTTGGTGCACTTGTGGGTGCCCATGACACACACTCTTCAATGCTC
CTGTGCCTTCCTAAGAGGTGTCT
>2535
TCCAAACACATTGCGCTACAGTCATTGAACTTCAAAGTGTAAAGCATGGATTGTGTTGG
TGCACTTGTGGGTGCCCATGACACACACTCTTCAATGCTCCTGTGCCTTCCTAAGAGGT
GTCTGTCTTTCTCAACCCCAGT
>2536
TCAAAGTGTAAAGCATGGATTGTGTTGGTGCACTTGTGGGTGCCCATGACACACACTC
TTCAATGCTCCTGTGCCTTCCTAAGAGGTGTCTGTCTTTCTCAACCCCAGTGTGTGTCC
TCTGACGTCTAGTGGATTGCTGC
>2537
TGGATTGTGTTGGTGCACTTGTGGGTGCCCATGACACACACTCTTCAATGCTCCTGTGC
CTTCCTAAGAGGTGTCTGTCTTTCTCAACCCCAGTGTGTGTCCTCTGACGTCTAGTGGA
TTGCTGCATACACAGTCAACCC
>2538
GGGTGCCCATGACACACACTCTTCAATGCTCCTGTGCCTTCCTAAGAGGTGTCTGTCTT
TCTCAACCCCAGTGTGTGTCCTCTGACGTCTAGTGGATTGCTGCATACACAGTCAACCC
TCCTTAAAAGGTTTAAAAAGAA

>2539
GTGCCCATGACACACACTCTTCAATGCTCCTGTGCCTTCCTAAGAGGTGTCTGTCTTTC
TCAACCCCAGTGTGTGTCCTCTGACGTCTAGTGGATTGCTGCATACACAGTCAACCCTC
CTTAAAAGGTTTAAAAAGAAAC
>2540
CACTCTTCAATGCTCCTGTGCCTTCCTAAGAGGTGTCTGTCTTTCTCAACCCCAGTGTG
TGTCCTCTGACGTCTAGTGGATTGCTGCATACACAGTCAACCCTCCTTAAAAGGTTTAA
AAAGAAACATGAGCTCCCTTTT
>2541
CCTAAGAGGTGTCTGTCTTTCTCAACCCCAGTGTGTGTCCTCTGACGTCTAGTGGATTG
CTGCATACACAGTCAACCCTCCTTAAAAGGTTTAAAAAGAAACATGAGCTCCCTTTTA
GAGCACCCCAAAGCAGCTGCATT
>2542
TTCTCAACCCCAGTGTGTGTCCTCTGACGTCTAGTGGATTGCTGCATACACAGTCAACC
CTCCTTAAAAGGTTTAAAAAGAAACATGAGCTCCCTTTTAGAGCACCCCAAAGCAGCT
GCATTCTTGAGTTGGTGCCCTGC
>2543
CCCTCCTTAAAAGGTTTAAAAAGAAACATGAGCTCCCTTTTAGAGCACCCCAAAGCAG
CTGCATTCTTGAGTTGGTGCCCTGCACTGGGATTCCCATGGACTGGGAACATGATCTCT
TAATGACACTAATATATTCTGAG
>2544
CCTTAAAAGGTTTAAAAAGAAACATGAGCTCCCTTTTAGAGCACCCCAAAGCAGCTGC
ATTCTTGAGTTGGTGCCCTGCACTGGGATTCCCATGGACTGGGAACATGATCTCTTAAT
GACACTAATATATTCTGAGATCT
>2545
CACTGGGATTCCCATGGACTGGGAACATGATCTCTTAATGACACTAATATATTCTGAGAT
CTGATTTTTTGTTTGTGTGTTTTTTAACCCAGGTTGGCAGGTTCTCCTTAATCAGTAGTG
GCAAAATGTCTCTTTCCTTT
>2546
GGGATTCCCATGGACTGGGAACATGATCTCTTAATGACACTAATATATTCTGAGATCTGA
TTTTTTGTTTGTGTGTTTTTTAACCCAGGTTGGCAGGTTCTCCTTAATCAGTAGTGGCAA
AATGTCTCTTTCCTTTCTCC
>2547
TAATATATTCTGAGATCTGATTTTTTGTTTGTGTGTTTTTTAACCCAGGTTGGCAGGTTCT
CCTTAATCAGTAGTGGCAAAATGTCTCTTTCCTTTCTCCACGTAATGCTAAATGGCTTAT
CCAGTCATCTCTCTCTGCT
>2548
TATATTCTGAGATCTGATTTTTTGTTTGTGTGTTTTTTAACCCAGGTTGGCAGGTTCTCCT
TAATCAGTAGTGGCAAAATGTCTCTTTCCTTTCTCCACGTAATGCTAAATGGCTTATCCA
GTCATCTCTCTCTGCTCAT
>2549
GTGTTTTTTAACCCAGGTTGGCAGGTTCTCCTTAATCAGTAGTGGCAAAATGTCTCTTT
CCTTTCTCCACGTAATGCTAAATGGCTTATCCAGTCATCTCTCTCTGCTCATGGTCAGCC
CGAGGTGACAGTTAAAATTGG
>2550
AGGTTCTCCTTAATCAGTAGTGGCAAAATGTCTCTTTCCTTTCTCCACGTAATGCTAAAT

GGCTTATCCAGTCATCTCTCTCTGCTCATGGTCAGCCCGAGGTGACAGTTAAAATTGGA
TGGAGGTAGCTTGGGGTAGGA
>2551
TGGCAAAATGTCTCTTTCCTTTCTCCACGTAATGCTAAATGGCTTATCCAGTCATCTCTC
TCTGCTCATGGTCAGCCCGAGGTGACAGTTAAAATTGGATGGAGGTAGCTTGGGGTAG
GAAGACTTGGTGATTTTGGCGG
>2552
CTCTTTCCTTTCTCCACGTAATGCTAAATGGCTTATCCAGTCATCTCTCTCTGCTCATGGT
CAGCCCGAGGTGACAGTTAAAATTGGATGGAGGTAGCTTGGGGTAGGAAGACTTGGT
GATTTTGGCGGCACTGGGTCAA
>2553
TATCCAGTCATCTCTCTCTGCTCATGGTCAGCCCGAGGTGACAGTTAAAATTGGATGGA
GGTAGCTTGGGGTAGGAAGACTTGGTGATTTTGGCGGCACTGGGTCAAACCCAGGGA
AACTGAAGCTGTGTCTGCACAGCT
>2554
CTCTCTGCTCATGGTCAGCCCGAGGTGACAGTTAAAATTGGATGGAGGTAGCTTGGGG
TAGGAAGACTTGGTGATTTTGGCGGCACTGGGTCAAACCCAGGGAAACTGAAGCTGT
GTCTGCACAGCTGGTTGAAAGGCTG
>2555
CCGAGGTGACAGTTAAAATTGGATGGAGGTAGCTTGGGGTAGGAAGACTTGGTGATTT
TGGCGGCACTGGGTCAAACCCAGGGAAACTGAAGCTGTGTCTGCACAGCTGGTTGAA
AGGCTGTGTTGTGATCCAGGGATTT
>2556
GGAGGTAGCTTGGGGTAGGAAGACTTGGTGATTTTGGCGGCACTGGGTCAAACCCAG
GGAAACTGAAGCTGTGTCTGCACAGCTGGTTGAAAGGCTGTGTTGTGATCCAGGGATT
TAAGTAAAAATATTCTTTCCTTACC
>2557
AGGTAGCTTGGGGTAGGAAGACTTGGTGATTTTGGCGGCACTGGGTCAAACCCAGGG
AAACTGAAGCTGTGTCTGCACAGCTGGTTGAAAGGCTGTGTTGTGATCCAGGGATTTA
AGTAAAAATATTCTTTCCTTACCAC
>2558
TAGGAAGACTTGGTGATTTTGGCGGCACTGGGTCAAACCCAGGGAAACTGAAGCTGT
GTCTGCACAGCTGGTTGAAAGGCTGTGTTGTGATCCAGGGATTTAAGTAAAAATATTCT
TTCCTTACCACCCTCTATGCCCCC
>2559
TGATTTTGGCGGCACTGGGTCAAACCCAGGGAAACTGAAGCTGTGTCTGCACAGCTG
GTTGAAAGGCTGTGTTGTGATCCAGGGATTTAAGTAAAAATATTCTTTCCTTACCACCC
TCTATGCCCCCTACTCCCCAGTAA
>2560
CTGTGTTGTGATCCAGGGATTTAAGTAAAAATATTCTTTCCTTACCACCCTCTATGCCCC
CTACTCCCCAGTAATATAGTAGGCATGGGGGTTGACTTATTTTGAGGGGACTAGTATGTA
TTTAATATTTCATCTAATTT
>2561
TGATCCAGGGATTTAAGTAAAAATATTCTTTCCTTACCACCCTCTATGCCCCCTACTCCC
CAGTAATATAGTAGGCATGGGGGTTGACTTATTTTGAGGGGACTAGTATGTATTTAATAT
TTCATCTAATTTTTGTAGCC

>2562
ACCACCCTCTATGCCCCCTACTCCCCAGTAATATAGTAGGCATGGGGGTTGACTTATTTT
GAGGGGACTAGTATGTATTTAATATTTCATCTAATTTTTGTAGCCCTGGCCTGGCTGGAT
GACAAAAGTGTTTAAACTAA
>2563
CCCTCTATGCCCCCTACTCCCCAGTAATATAGTAGGCATGGGGGTTGACTTATTTTGAGG
GGACTAGTATGTATTTAATATTTCATCTAATTTTTGTAGCCCTGGCCTGGCTGGATGACA
AAAGTGTTTAAACTAAGACC
>2564
AATATAGTAGGCATGGGGGTTGACTTATTTTGAGGGGACTAGTATGTATTTAATATTTCAT
CTAATTTTTGTAGCCCTGGCCTGGCTGGATGACAAAAGTGTTTAAACTAAGACCGATTT
GAGCAAGTATTTTATCCTCC
>2565
TTTGAGGGGACTAGTATGTATTTAATATTTCATCTAATTTTTGTAGCCCTGGCCTGGCTGG
ATGACAAAAGTGTTTAAACTAAGACCGATTTGAGCAAGTATTTTATCCTCCCTCTCGGG
AGACTATGGGCATAGAACGG
>2566
TGAGGGGACTAGTATGTATTTAATATTTCATCTAATTTTTGTAGCCCTGGCCTGGCTGGAT
GACAAAAGTGTTTAAACTAAGACCGATTTGAGCAAGTATTTTATCCTCCCTCTCGGGAG
ACTATGGGCATAGAACGGTG
>2567
TTCATCTAATTTTTGTAGCCCTGGCCTGGCTGGATGACAAAAGTGTTTAAACTAAGACC
GATTTGAGCAAGTATTTTATCCTCCCTCTCGGGAGACTATGGGCATAGAACGGTGTGGG
GATTTGGAGGTGCTGGCAAGTC
>2568
AGTGTTTAAACTAAGACCGATTTGAGCAAGTATTTTATCCTCCCTCTCGGGAGACTATG
GGCATAGAACGGTGTGGGGATTTGGAGGTGCTGGCAAGTCTGGCCCCTTTCCTCTTGC
GTTTACTCAGGCACAGATGGAAA
>2569
TGTTTAAACTAAGACCGATTTGAGCAAGTATTTTATCCTCCCTCTCGGGAGACTATGGG
CATAGAACGGTGTGGGGATTTGGAGGTGCTGGCAAGTCTGGCCCCTTTCCTCTTGCGT
TTACTCAGGCACAGATGGAAAAA
>2570
CGGGAGACTATGGGCATAGAACGGTGTGGGGATTTGGAGGTGCTGGCAAGTCTGGCCC
CTTTCCTCTTGCGTTTACTCAGGCACAGATGGAAAAAGATGAAACTCAATGAGGACCC
TTTTGTGAGATGCGGCACATTTCG
>2571
TCTGGCCCCTTTCCTCTTGCGTTTACTCAGGCACAGATGGAAAAAGATGAAACTCAAT
GAGGACCCTTTTGTGAGATGCGGCACATTTCGCTTTGTGGAATTATTAACCTTTTATAAG
ACTGGGATACTTACAAACTTAC
>2572
ACTCAGGCACAGATGGAAAAAGATGAAACTCAATGAGGACCCTTTTGTGAGATGCGG
CACATTTCGCTTTGTGGAATTATTAACCTTTTATAAGACTGGGATACTTACAAACTTACC
GCAGGACCAGACAGTGACTATCA
>2573
ATTTCGCTTTGTGGAATTATTAACCTTTTATAAGACTGGGATACTTACAAACTTACCGCA

GGACCAGACAGTGACTATCAGAATCCTCTCTTCTAAAGATCAGCGATCTACAGCTTTCG
CAAAACTTAAGGCTGACTTGG
>2574
TCTTCTAAAGATCAGCGATCTACAGCTTTCGCAAAACTTAAGGCTGACTTGGGAATCTA
GACAGCCCCTAGTATGGTGAGTGACCCTAGAGTGGTTTTTAATGAGGAGATACTCACG
GGAGCCAGCATCTCGGGGGTCCC
>2575
TAAAGATCAGCGATCTACAGCTTTCGCAAAACTTAAGGCTGACTTGGGAATCTAGACA
GCCCCTAGTATGGTGAGTGACCCTAGAGTGGTTTTTAATGAGGAGATACTCACGGGAG
CCAGCATCTCGGGGGTCCCCGGGG
>2576
GATCAGCGATCTACAGCTTTCGCAAAACTTAAGGCTGACTTGGGAATCTAGACAGCCC
CTAGTATGGTGAGTGACCCTAGAGTGGTTTTTAATGAGGAGATACTCACGGGAGCCAG
CATCTCGGGGGTCCCCGGGGGGTT
>2577
TACAGCTTTCGCAAAACTTAAGGCTGACTTGGGAATCTAGACAGCCCCTAGTATGGTG
AGTGACCCTAGAGTGGTTTTTAATGAGGAGATACTCACGGGAGCCAGCATCTCGGGGG
TCCCCGGGGGGTTCATGGCCAGGA
>2578
CCCTAGTATGGTGAGTGACCCTAGAGTGGTTTTTAATGAGGAGATACTCACGGGAGCC
AGCATCTCGGGGGTCCCCGGGGGGTTCATGGCCAGGAGCGTTGCAAGCTGGTTTGTTT
GTTTGTTTGTTTTGAGACAGGGTC
>2579
TGAGTGACCCTAGAGTGGTTTTTAATGAGGAGATACTCACGGGAGCCAGCATCTCGGG
GGTCCCCGGGGGGTTCATGGCCAGGAGCGTTGCAAGCTGGTTTGTTTGTTTGTTTGTTT
TGAGACAGGGTCTTACTCTGTTG
>2580
GGTTTTTAATGAGGAGATACTCACGGGAGCCAGCATCTCGGGGGTCCCCGGGGGGTTC
ATGGCCAGGAGCGTTGCAAGCTGGTTTGTTTGTTTGTTTGTTTTGAGACAGGGTCTTAC
TCTGTTGCTCAGGCTGGAGTGCA
>2581
CTCACGGGAGCCAGCATCTCGGGGGTCCCCGGGGGGTTCATGGCCAGGAGCGTTGCA
AGCTGGTTTGTTTGTTTGTTTGTTTTGAGACAGGGTCTTACTCTGTTGCTCAGGCTGGA
GTGCAGTGGCATAATCTCGGCTCA
>2582
GGGGTCCCCGGGGGGTTCATGGCCAGGAGCGTTGCAAGCTGGTTTGTTTGTTTGTTTG
TTTTGAGACAGGGTCTTACTCTGTTGCTCAGGCTGGAGTGCAGTGGCATAATCTCGGCT
CACTGCAACCTTGCTTGCTCTCC
>2583
GGGGGTTCATGGCCAGGAGCGTTGCAAGCTGGTTTGTTTGTTTGTTTGTTTTGAGACA
GGGTCTTACTCTGTTGCTCAGGCTGGAGTGCAGTGGCATAATCTCGGCTCACTGCAAC
CTTGCTTGCTCTCCCTGGCTCAAG
>2584
GGAGCGTTGCAAGCTGGTTTGTTTGTTTGTTTGTTTTGAGACAGGGTCTTACTCTGTTG
CTCAGGCTGGAGTGCAGTGGCATAATCTCGGCTCACTGCAACCTTGCTTGCTCTCCCTG
GCTCAAGCAATTCTCCCACATT

>2585
ATAATCTCGGCTCACTGCAACCTTGCTTGCTCTCCCTGGCTCAAGCAATTCTCCCACATT
AGCCTCCCAAGTAGCTGGGATTACAGGTGTATGCCACCACAGCCTGCTAATTTTTAAGT
TTTTTGTAGAGATGAGGTTTC
>2586
GCAACCTTGCTTGCTCTCCCTGGCTCAAGCAATTCTCCCACATTAGCCTCCCAAGTAGC
TGGGATTACAGGTGTATGCCACCACAGCCTGCTAATTTTTAAGTTTTTTGTAGAGATGA
GGTTTCACTGCATTGCCTAGGC
>2587
AACCTTGCTTGCTCTCCCTGGCTCAAGCAATTCTCCCACATTAGCCTCCCAAGTAGCTG
GGATTACAGGTGTATGCCACCACAGCCTGCTAATTTTTAAGTTTTTTGTAGAGATGAGG
TTTCACTGCATTGCCTAGGCTG
>2588
ATTCTCCCACATTAGCCTCCCAAGTAGCTGGGATTACAGGTGTATGCCACCACAGCCTG
CTAATTTTTAAGTTTTTTGTAGAGATGAGGTTTCACTGCATTGCCTAGGCTGATCTTGAA
CTCCTGGGCTCAAACAATCTT
>2589
TAGAGATGAGGTTTCACTGCATTGCCTAGGCTGATCTTGAACTCCTGGGCTCAAACAAT
CTTCCCTCCTTGTCCTCTTAAAGTGCTGGGATTACAGGTGTGAGCCACAGCGCGAGGC
CTTGTTTGGTGTTTTTGTTTGTT
>2590
TTTCACTGCATTGCCTAGGCTGATCTTGAACTCCTGGGCTCAAACAATCTTCCCTCCTT
GTCCTCTTAAAGTGCTGGGATTACAGGTGTGAGCCACAGCGCGAGGCCTTGTTTGGTG
TTTTTGTTTGTTTGTTTGTTTGT
>2591
TAGGCTGATCTTGAACTCCTGGGCTCAAACAATCTTCCCTCCTTGTCCTCTTAAAGTGC
TGGGATTACAGGTGTGAGCCACAGCGCGAGGCCTTGTTTGGTGTTTTTGTTTGTTTGTT
TGTTTGTTTTATGGATCATTAC
>2592
TCAAACAATCTTCCCTCCTTGTCCTCTTAAAGTGCTGGGATTACAGGTGTGAGCCACAG
CGCGAGGCCTTGTTTGGTGTTTTTGTTTGTTTGTTTGTTTGTTTTATGGATCATTACCTAT
AAAGTAATTTAGAATACCCA
>2593
CCTCCTTGTCCTCTTAAAGTGCTGGGATTACAGGTGTGAGCCACAGCGCGAGGCCTTG
TTTGGTGTTTTTGTTTGTTTGTTTGTTTGTTTTATGGATCATTACCTATAAAGTAATTTAG
AATACCCACTCATTTATGATC
>2594
CTTGTCCTCTTAAAGTGCTGGGATTACAGGTGTGAGCCACAGCGCGAGGCCTTGTTTG
GTGTTTTTGTTTGTTTGTTTGTTTGTTTTATGGATCATTACCTATAAAGTAATTTAGAATA
CCCACTCATTTATGATCCAGC
>2595
TCCTCTTAAAGTGCTGGGATTACAGGTGTGAGCCACAGCGCGAGGCCTTGTTTGGTGT
TTTTGTTTGTTTGTTTGTTTGTTTTATGGATCATTACCTATAAAGTAATTTAGAATACCCA
CTCATTTATGATCCAGCCTTC
>2596
CCACAGCGCGAGGCCTTGTTTGGTGTTTTTGTTTGTTTGTTTGTTTGTTTTATGGATCAT

TACCTATAAAGTAATTTAGAATACCCACTCATTTATGATCCAGCCTTCCCTGCATTTTCTA
GCGTTGAGGTCCTTTGGGG
>2597
GGATCATTACCTATAAAGTAATTTAGAATACCCACTCATTTATGATCCAGCCTTCCCTGCA
TTTTCTAGCGTTGAGGTCCTTTGGGGCCATAGAAGTATGTTTTTCCAAGACTGATTGCTT
CGTGTATTTAACCAGGTTT
>2598
TTACCTATAAAGTAATTTAGAATACCCACTCATTTATGATCCAGCCTTCCCTGCATTTTCT
AGCGTTGAGGTCCTTTGGGGCCATAGAAGTATGTTTTTCCAAGACTGATTGCTTCGTGT
ATTTAACCAGGTTTTTTGTG
>2599
ACCCACTCATTTATGATCCAGCCTTCCCTGCATTTTCTAGCGTTGAGGTCCTTTGGGGCC
ATAGAAGTATGTTTTTCCAAGACTGATTGCTTCGTGTATTTAACCAGGTTTTTTGTGAAG
GGTTTGATATAAACTGAGTG
>2600
TTCCCTGCATTTTCTAGCGTTGAGGTCCTTTGGGGCCATAGAAGTATGTTTTTCCAAGA
CTGATTGCTTCGTGTATTTAACCAGGTTTTTTGTGAAGGGTTTGATATAAACTGAGTGAC
TGAAGAGAGGAACATGGTGTG
>2601
TAGCGTTGAGGTCCTTTGGGGCCATAGAAGTATGTTTTTCCAAGACTGATTGCTTCGTG
TATTTAACCAGGTTTTTTGTGAAGGGTTTGATATAAACTGAGTGACTGAAGAGAGGAA
CATGGTGTGATTTGGTAGAAAGA
>2602
TATGTTTTTCCAAGACTGATTGCTTCGTGTATTTAACCAGGTTTTTTGTGAAGGGTTTGA
TATAAACTGAGTGACTGAAGAGAGGAACATGGTGTGATTTGGTAGAAAGATCTGTAAT
TTTGGAGTCAAGACTGGCCTAG
>2603
GCTTCGTGTATTTAACCAGGTTTTTTGTGAAGGGTTTGATATAAACTGAGTGACTGAAG
AGAGGAACATGGTGTGATTTGGTAGAAAGATCTGTAATTTTGGAGTCAAGACTGGCCT
AGATGGACAGCTTGGCCACTTTT
>2604
TTTTGTGAAGGGTTTGATATAAACTGAGTGACTGAAGAGAGGAACATGGTGTGATTTG
GTAGAAAGATCTGTAATTTTGGAGTCAAGACTGGCCTAGATGGACAGCTTGGCCACTT
TTAAAATTTTGTGTCCTTAGCCCC
>2605
GTTTGATATAAACTGAGTGACTGAAGAGAGGAACATGGTGTGATTTGGTAGAAAGATC
TGTAATTTTGGAGTCAAGACTGGCCTAGATGGACAGCTTGGCCACTTTTAAAATTTTGT
GTCCTTAGCCCCATAAACATTTT
>2606
TGGTAGAAAGATCTGTAATTTTGGAGTCAAGACTGGCCTAGATGGACAGCTTGGCCAC
TTTTAAAATTTTGTGTCCTTAGCCCCATAAACATTTTGAGATTCAGTTTTCTCTCCTGTG
AATTGGGGACGATATTGCCTAA
>2607
TGGCCTAGATGGACAGCTTGGCCACTTTTAAAATTTTGTGTCCTTAGCCCCATAAACAT
TTTGAGATTCAGTTTTCTCTCCTGTGAATTGGGGACGATATTGCCTAACTTGTAAGGTTC
TTGCAAAGATCAAATGAAAAA

>2608
ACAGCTTGGCCACTTTTAAAATTTTGTGTCCTTAGCCCCATAAACATTTTGAGATTCAGT
TTTCTCTCCTGTGAATTGGGGACGATATTGCCTAACTTGTAAGGTTCTTGCAAAGATCA
AATGAAAAAATACACATAGAT
>2609
TCCTTAGCCCCATAAACATTTTGAGATTCAGTTTTCTCTCCTGTGAATTGGGGACGATAT
TGCCTAACTTGTAAGGTTCTTGCAAAGATCAAATGAAAAAATACACATAGATCTCCTGG
CTATTATTGGAGTGCCCAGTA
>2610
AGCCCCATAAACATTTTGAGATTCAGTTTTCTCTCCTGTGAATTGGGGACGATATTGCCT
AACTTGTAAGGTTCTTGCAAAGATCAAATGAAAAAATACACATAGATCTCCTGGCTATT
ATTGGAGTGCCCAGTAAATGC
>2611
TGCCTAACTTGTAAGGTTCTTGCAAAGATCAAATGAAAAAATACACATAGATCTCCTGG
CTATTATTGGAGTGCCCAGTAAATGCTAGTTGCCTTATCCTCCATTTTCTTTTTGAGAGC
TGAAGACCTGATTTTATTTCC
>2612
CTTGTAAGGTTCTTGCAAAGATCAAATGAAAAAATACACATAGATCTCCTGGCTATTATT
GGAGTGCCCAGTAAATGCTAGTTGCCTTATCCTCCATTTTCTTTTTGAGAGCTGAAGAC
CTGATTTTATTTCCTTGCATT
>2613
TCTTGCAAAGATCAAATGAAAAAATACACATAGATCTCCTGGCTATTATTGGAGTGCCC
AGTAAATGCTAGTTGCCTTATCCTCCATTTTCTTTTTGAGAGCTGAAGACCTGATTTTAT
TTCCTTGCATTTTTCATGTCA
>2614
GCTAGTTGCCTTATCCTCCATTTTCTTTTTGAGAGCTGAAGACCTGATTTTATTTCCTTG
CATTTTTCATGTCATTGCCTATAGGGAGATACATTCATATATTTGTTGCTCATTTTTGTATG
TGTTACAGTGGTGATAAA
>2615
TTTTATTTCCTTGCATTTTTCATGTCATTGCCTATAGGGAGATACATTCATATATTTGTTGC
TCATTTTTGTATGTGTTACAGTGGTGATAAAGAAACTGATGGTATCCCAGATGGGGAGG
GGCAGGTAAGGTTTTATCA
>2616
ATTTCCTTGCATTTTTCATGTCATTGCCTATAGGGAGATACATTCATATATTTGTTGCTCAT
TTTTGTATGTGTTACAGTGGTGATAAAGAAACTGATGGTATCCCAGATGGGGAGGGGCA
GGTAAGGTTTTATCAGTGG
>2617
ATAGGGAGATACATTCATATATTTGTTGCTCATTTTTGTATGTGTTACAGTGGTGATAAAG
AAACTGATGGTATCCCAGATGGGGAGGGGCAGGTAAGGTTTTATCAGTGGCTTTTAATT
TCTTTATGAAATTGGATGTG
>2618
TGTTGCTCATTTTTGTATGTGTTACAGTGGTGATAAAGAAACTGATGGTATCCCAGATGG
GGAGGGGCAGGTAAGGTTTTATCAGTGGCTTTTAATTTCTTTATGAAATTGGATGTGGC
TCTAGGGAATAGATGTCTCTG
>2619
CTCATTTTTGTATGTGTTACAGTGGTGATAAAGAAACTGATGGTATCCCAGATGGGGAG

GGGCAGGTAAGGTTTTATCAGTGGCTTTTAATTTCTTTATGAAATTGGATGTGGCTCTAG
GGAATAGATGTCTCTGGGATG
>2620
ATGGTATCCCAGATGGGGAGGGGCAGGTAAGGTTTTATCAGTGGCTTTTAATTTCTTTAT
GAAATTGGATGTGGCTCTAGGGAATAGATGTCTCTGGGATGGTCATGTTTTCCCTTGCC
TTTTGGCTCCTGCAATGAGGA
>2621
GGGGCAGGTAAGGTTTTATCAGTGGCTTTTAATTTCTTTATGAAATTGGATGTGGCTCTA
GGGAATAGATGTCTCTGGGATGGTCATGTTTTCCCTTGCCTTTTGGCTCCTGCAATGAG
GACCTCTTTCTGTTGGTGTGG
>2622
GTTTTATCAGTGGCTTTTAATTTCTTTATGAAATTGGATGTGGCTCTAGGGAATAGATGT
CTCTGGGATGGTCATGTTTTCCCTTGCCTTTTGGCTCCTGCAATGAGGACCTCTTTCTGT
TGGTGTGGGTCTTGTCTGAG
>2623
GGGAATAGATGTCTCTGGGATGGTCATGTTTTCCCTTGCCTTTTGGCTCCTGCAATGAG
GACCTCTTTCTGTTGGTGTGGGTCTTGTCTGAGCATGCCAGGGATGGCAGTGAACACA
TCGATGGTGGTTGGGACGTCCTC
>2624
ATAGATGTCTCTGGGATGGTCATGTTTTCCCTTGCCTTTTGGCTCCTGCAATGAGGACCT
CTTTCTGTTGGTGTGGGTCTTGTCTGAGCATGCCAGGGATGGCAGTGAACACATCGAT
GGTGGTTGGGACGTCCTCATTC
>2625
CCTTTTGGCTCCTGCAATGAGGACCTCTTTCTGTTGGTGTGGGTCTTGTCTGAGCATGC
CAGGGATGGCAGTGAACACATCGATGGTGGTTGGGACGTCCTCATTCCCAGACCTCTG
GGACATACTAATTCCCAGACCTC
>2626
AATGAGGACCTCTTTCTGTTGGTGTGGGTCTTGTCTGAGCATGCCAGGGATGGCAGTG
AACACATCGATGGTGGTTGGGACGTCCTCATTCCCAGACCTCTGGGACATACTAATTCC
CAGACCTCTGAAACATGGGTCTC
>2627
GAGGACCTCTTTCTGTTGGTGTGGGTCTTGTCTGAGCATGCCAGGGATGGCAGTGAAC
ACATCGATGGTGGTTGGGACGTCCTCATTCCCAGACCTCTGGGACATACTAATTCCCAG
ACCTCTGAAACATGGGTCTCAGC
>2628
CTTTCTGTTGGTGTGGGTCTTGTCTGAGCATGCCAGGGATGGCAGTGAACACATCGAT
GGTGGTTGGGACGTCCTCATTCCCAGACCTCTGGGACATACTAATTCCCAGACCTCTGA
AACATGGGTCTCAGCTGCTTTGC
>2629
CGATGGTGGTTGGGACGTCCTCATTCCCAGACCTCTGGGACATACTAATTCCCAGACCT
CTGAAACATGGGTCTCAGCTGCTTTGCCCTGCAGACTTGTTCTCAGTAGCTATTTACCT
TTAGGAGAAAAAAAAAATCCTT
>2630
CAGACCTCTGGGACATACTAATTCCCAGACCTCTGAAACATGGGTCTCAGCTGCTTTGC
CCTGCAGACTTGTTCTCAGTAGCTATTTACCTTTAGGAGAAAAAAAAAATCCTTAACCA
AATGCTTTAGGAAGGCTTTCTT

>2631
CTCAGTAGCTATTTACCTTTAGGAGAAAAAAAAAAATCCTTAACCAAATGCTTTAGGAAG
GCTTTCTTATCGTGGCTGTAAGGTGCTGGTGGGTTGTTGGCAGCCAAGTTTGGGTTTAA
ATCTCACTGGGGGTTGAGGCTG
>2632
AGCTATTTACCTTTAGGAGAAAAAAAAAAATCCTTAACCAAATGCTTTAGGAAGGCTTTC
TTATCGTGGCTGTAAGGTGCTGGTGGGTTGTTGGCAGCCAAGTTTGGGTTTAAATCTCA
CTGGGGGTTGAGGCTGCTGAAC
>2633
TTTACCTTTAGGAGAAAAAAAAAAATCCTTAACCAAATGCTTTAGGAAGGCTTTCTTATC
GTGGCTGTAAGGTGCTGGTGGGTTGTTGGCAGCCAAGTTTGGGTTTAAATCTCACTGG
GGGTTGAGGCTGCTGAACTGCTA
>2634
TTTAGGAGAAAAAAAAAAATCCTTAACCAAATGCTTTAGGAAGGCTTTCTTATCGTGGCT
GTAAGGTGCTGGTGGGTTGTTGGCAGCCAAGTTTGGGTTTAAATCTCACTGGGGGTTG
AGGCTGCTGAACTGCTATTCCTA
>2635
GGAGAAAAAAAAAAATCCTTAACCAAATGCTTTAGGAAGGCTTTCTTATCGTGGCTGTA
AGGTGCTGGTGGGTTGTTGGCAGCCAAGTTTGGGTTTAAATCTCACTGGGGGTTGAGG
CTGCTGAACTGCTATTCCTAACAT
>2636
CCTTAACCAAATGCTTTAGGAAGGCTTTCTTATCGTGGCTGTAAGGTGCTGGTGGGTTG
TTGGCAGCCAAGTTTGGGTTTAAATCTCACTGGGGGTTGAGGCTGCTGAACTGCTATTC
CTAACATGCTCTTGTGTTTTTA
>2637
CTTTCTTATCGTGGCTGTAAGGTGCTGGTGGGTTGTTGGCAGCCAAGTTTGGGTTTAAA
TCTCACTGGGGGTTGAGGCTGCTGAACTGCTATTCCTAACATGCTCTTGTGTTTTTATGC
ATAGACCGTCTTCTGGATCCT
>2638
GGCAGCCAAGTTTGGGTTTAAATCTCACTGGGGGTTGAGGCTGCTGAACTGCTATTCCT
AACATGCTCTTGTGTTTTTATGCATAGACCGTCTTCTGGATCCTTGGTGAGTTTTTTTA
CTCTGGGGTTTTCAGGAGAAA
>2639
CAGCCAAGTTTGGGTTTAAATCTCACTGGGGGTTGAGGCTGCTGAACTGCTATTCCTAA
CATGCTCTTGTGTTTTTATGCATAGACCGTCTTCTGGATCCTTGGTGAGTTTTTTTACTC
TGGGGTTTTCAGGAGAAAGG
>2640
TGAGGCTGCTGAACTGCTATTCCTAACATGCTCTTGTGTTTTTATGCATAGACCGTCTTC
TGGATCCTTGGTGAGTTTTTTTACTCTGGGGTTTTCAGGAGAAAGGTTTGTCATTCAT
CATCACTCTTCTCCTTCATCA
>2641
GCTGCTGAACTGCTATTCCTAACATGCTCTTGTGTTTTTATGCATAGACCGTCTTCTGGA
TCCTTGGTGAGTTTTTTTACTCTGGGGTTTTCAGGAGAAAGGTTTGTCATTCATCATCA
CTCTTCTCCTTCATCACAGC
>2642
CCTAACATGCTCTTGTGTTTTTATGCATAGACCGTCTTCTGGATCCTTGGTGAGTTTTTT

TTACTCTGGGGTTTTCAGGAGAAAGGTTTGTCATTCATCATCACTCTTCTCCTTCATCAC
AGCAGTGGTAACAGTGGTTA
>2643
TGTTTTTATGCATAGACCGTCTTCTGGATCCTTGGTGAGTTTTTTTACTCTGGGGTTTT
CAGGAGAAAGGTTTGTCATTCATCATCACTCTTCTCCTTCATCACAGCAGTGGTAACAG
TGGTTAGAGGGAGGAGGTAGA
>2644
TTTTTTTTACTCTGGGGTTTTCAGGAGAAAGGTTTGTCATTCATCATCACTCTTCTCCTT
CATCACAGCAGTGGTAACAGTGGTTAGAGGGAGGAGGTAGAATCAGTTGTTCAAGTTC
TTTTTTCTTCCAGGTAAGTCCC
>2645
TTTTTACTCTGGGGTTTTCAGGAGAAAGGTTTGTCATTCATCATCACTCTTCTCCTTCAT
CACAGCAGTGGTAACAGTGGTTAGAGGGAGGAGGTAGAATCAGTTGTTCAAGTTCTTT
TTTCTTCCAGGTAAGTCCCCCC
>2646
GAAAGGTTTGTCATTCATCATCACTCTTCTCCTTCATCACAGCAGTGGTAACAGTGGTT
AGAGGGAGGAGGTAGAATCAGTTGTTCAAGTTCTTTTTTCTTCCAGGTAAGTCCCCCC
CACCCCCCGGCTGCCCCTGCCCT
>2647
CATCACTCTTCTCCTTCATCACAGCAGTGGTAACAGTGGTTAGAGGGAGGAGGTAGAA
TCAGTTGTTCAAGTTCTTTTTTCTTCCAGGTAAGTCCCCCCCACCCCCCGGCTGCCCCT
GCCCTTCCGCATTTTCTGGGAAG
>2648
ATCACAGCAGTGGTAACAGTGGTTAGAGGGAGGAGGTAGAATCAGTTGTTCAAGTTCT
TTTTTCTTCCAGGTAAGTCCCCCCCACCCCCCGGCTGCCCCTGCCCTTCCGCATTTTCT
GGGAAGTGAATCTATTGGGAAAT
>2649
CAGCAGTGGTAACAGTGGTTAGAGGGAGGAGGTAGAATCAGTTGTTCAAGTTCTTTTT
TCTTCCAGGTAAGTCCCCCCCACCCCCCGGCTGCCCCTGCCCTTCCGCATTTTCTGGGA
AGTGAATCTATTGGGAAATGCAT
>2650
AGTTCTTTTTTCTTCCAGGTAAGTCCCCCCCACCCCCCGGCTGCCCCTGCCCTTCCGCA
TTTTCTGGGAAGTGAATCTATTGGGAAATGCATGGGCCTGTGTTTCTGGGATGATTTTA
GCATATGTAGTAAGAGGGGTGC
>2651
CCCACCCCCCGGCTGCCCCTGCCCTTCCGCATTTTCTGGGAAGTGAATCTATTGGGAAA
TGCATGGGCCTGTGTTTCTGGGATGATTTTAGCATATGTAGTAAGAGGGGTGCTTTTTGC
TGATAGCCCAGGCGTTGGTCC
>2652
CCGCATTTTCTGGGAAGTGAATCTATTGGGAAATGCATGGGCCTGTGTTTCTGGGATGA
TTTTAGCATATGTAGTAAGAGGGGTGCTTTTTGCTGATAGCCCAGGCGTTGGTCCTGTA
ATCCTGAGGTGTGGTTTAATAA
>2653
CATTTTCTGGGAAGTGAATCTATTGGGAAATGCATGGGCCTGTGTTTCTGGGATGATTTT
AGCATATGTAGTAAGAGGGGTGCTTTTTGCTGATAGCCCAGGCGTTGGTCCTGTAATCC
TGAGGTGTGGTTTAATAAGAG

>2654
GGAAGTGAATCTATTGGGAAATGCATGGGCCTGTGTTTCTGGGATGATTTTAGCATATGT
AGTAAGAGGGGTGCTTTTTGCTGATAGCCCAGGCGTTGGTCCTGTAATCCTGAGGTGT
GGTTTAATAAGAGCTGAACAGT
>2655
ATGGGCCTGTGTTTCTGGGATGATTTTAGCATATGTAGTAAGAGGGGTGCTTTTTGCTGA
TAGCCCAGGCGTTGGTCCTGTAATCCTGAGGTGTGGTTTAATAAGAGCTGAACAGTCAT
TTAATTATTTATTGGGTGCCT
>2656
ATTTTAGCATATGTAGTAAGAGGGGTGCTTTTTGCTGATAGCCCAGGCGTTGGTCCTGT
AATCCTGAGGTGTGGTTTAATAAGAGCTGAACAGTCATTTAATTATTTATTGGGTGCCTG
GAGAATTTTATAGGCAGGAGA
>2657
TTAGCATATGTAGTAAGAGGGGTGCTTTTTGCTGATAGCCCAGGCGTTGGTCCTGTAAT
CCTGAGGTGTGGTTTAATAAGAGCTGAACAGTCATTTAATTATTTATTGGGTGCCTGGA
GAATTTTATAGGCAGGAGAGCC
>2658
GGGTGCTTTTTGCTGATAGCCCAGGCGTTGGTCCTGTAATCCTGAGGTGTGGTTTAATA
AGAGCTGAACAGTCATTTAATTATTTATTGGGTGCCTGGAGAATTTTATAGGCAGGAGA
GCCTAGAGCTATTCCAGAAAAA
>2659
CCAGGCGTTGGTCCTGTAATCCTGAGGTGTGGTTTAATAAGAGCTGAACAGTCATTTAA
TTATTTATTGGGTGCCTGGAGAATTTTATAGGCAGGAGAGCCTAGAGCTATTCCAGAAA
AAAGGCCAATCCTAGAGCTTTT
>2660
TGCCTGGAGAATTTTATAGGCAGGAGAGCCTAGAGCTATTCCAGAAAAAAGGCCAATC
CTAGAGCTTTTTGTGTGGCTTTCTTCTTGGGCTTGAAGTTTTTGATGCTCGCCAACATTC
ATAGTAGCCCCCAATGCCACCC
>2661
CCTGGAGAATTTTATAGGCAGGAGAGCCTAGAGCTATTCCAGAAAAAAGGCCAATCCT
AGAGCTTTTTGTGTGGCTTTCTTCTTGGGCTTGAAGTTTTTGATGCTCGCCAACATTCAT
AGTAGCCCCCAATGCCACCCAC
>2662
GAGCCTAGAGCTATTCCAGAAAAAAGGCCAATCCTAGAGCTTTTTGTGTGGCTTTCTTC
TTGGGCTTGAAGTTTTTGATGCTCGCCAACATTCATAGTAGCCCCCAATGCCACCCACA
CATTGGGAAGGGACAATAGAGC
>2663
GCCAATCCTAGAGCTTTTTGTGTGGCTTTCTTCTTGGGCTTGAAGTTTTTGATGCTCGCC
AACATTCATAGTAGCCCCCAATGCCACCCACACATTGGGAAGGGACAATAGAGCCAGC
TAAAGCTCTTTCTTTGTGTGTG
>2664
ATTCATAGTAGCCCCCAATGCCACCCACACATTGGGAAGGGACAATAGAGCCAGCTAA
AGCTCTTTCTTTGTGTGTGCCCATATTAACCCATCTAGTCAAATGTGGGTACCCTTCCAA
ACCATTATGTCTTGTTCCTCTC
>2665
TCATAGTAGCCCCCAATGCCACCCACACATTGGGAAGGGACAATAGAGCCAGCTAAAG

CTCTTTCTTTGTGTGTGCCCATATTAACCCATCTAGTCAAATGTGGGTACCCTTCCAAAC
CATTATGTCTTGTTCCTCTCGG
>2666
CACACATTGGGAAGGGACAATAGAGCCAGCTAAAGCTCTTTCTTTGTGTGTGCCCATAT
TAACCCATCTAGTCAAATGTGGGTACCCTTCCAAACCATTATGTCTTGTTCCTCTCGGCT
GCTGGGAACATTAAAAGAACC
>2667
TGGGAAGGGACAATAGAGCCAGCTAAAGCTCTTTCTTTGTGTGTGCCCATATTAACCCA
TCTAGTCAAATGTGGGTACCCTTCCAAACCATTATGTCTTGTTCCTCTCGGCTGCTGGG
AACATTAAAAGAACCTTATTTG
>2668
AAAGCTCTTTCTTTGTGTGTGCCCATATTAACCCATCTAGTCAAATGTGGGTACCCTTCC
AAACCATTATGTCTTGTTCCTCTCGGCTGCTGGGAACATTAAAAGAACCTTATTTGAAG
GCAAACATTACTGTATCAGTG
>2669
AACCATTATGTCTTGTTCCTCTCGGCTGCTGGGAACATTAAAAGAACCTTATTTGAAGG
CAAACATTACTGTATCAGTGATGCTGAAACTGGAGGTCTGTCAATGGCACTTCATTGCA
TTGATAATTGTTTCTGCTTTGA
>2670
TATGTCTTGTTCCTCTCGGCTGCTGGGAACATTAAAAGAACCTTATTTGAAGGCAAACA
TTACTGTATCAGTGATGCTGAAACTGGAGGTCTGTCAATGGCACTTCATTGCATTGATAA
TTGTTTCTGCTTTGAACCACA
>2671
GCTGCTGGGAACATTAAAAGAACCTTATTTGAAGGCAAACATTACTGTATCAGTGATGC
TGAAACTGGAGGTCTGTCAATGGCACTTCATTGCATTGATAATTGTTTCTGCTTTGAAC
CACAACACTGTGATTATTGAGG
>2672
CTGGGAACATTAAAAGAACCTTATTTGAAGGCAAACATTACTGTATCAGTGATGCTGAA
ACTGGAGGTCTGTCAATGGCACTTCATTGCATTGATAATTGTTTCTGCTTTGAACCACA
ACACTGTGATTATTGAGGTTCG
>2673
GGCAAACATTACTGTATCAGTGATGCTGAAACTGGAGGTCTGTCAATGGCACTTCATTG
CATTGATAATTGTTTCTGCTTTGAACCACAACACTGTGATTATTGAGGTTCGACTGTAAG
GGCTGAGGGAGCCTTGAGGCC
>2674
GCTGAAACTGGAGGTCTGTCAATGGCACTTCATTGCATTGATAATTGTTTCTGCTTTGA
ACCACAACACTGTGATTATTGAGGTTCGACTGTAAGGGCTGAGGGAGCCTTGAGGCCA
TTGAAAGCATTCTCCTGCCTTTA
>2675
GGTCTGTCAATGGCACTTCATTGCATTGATAATTGTTTCTGCTTTGAACCACAACACTGT
GATTATTGAGGTTCGACTGTAAGGGCTGAGGGAGCCTTGAGGCCATTGAAAGCATTCT
CCTGCCTTTAGGCCAGGCCTAT
>2676
AATGGCACTTCATTGCATTGATAATTGTTTCTGCTTTGAACCACAACACTGTGATTATTG
AGGTTCGACTGTAAGGGCTGAGGGAGCCTTGAGGCCATTGAAAGCATTCTCCTGCCTT
TAGGCCAGGCCTATGTTATAAG

>2677
GTTCGACTGTAAGGGCTGAGGGAGCCTTGAGGCCATTGAAAGCATTCTCCTGCCTTTA
GGCCAGGCCTATGTTATAAGTGAGAGACAGGTGTGGAGTGTGCAGGGAGGGAATTAA
GATAGATGGTGACCTGGAATTCAGA
>2678
TGTAAGGGCTGAGGGAGCCTTGAGGCCATTGAAAGCATTCTCCTGCCTTTAGGCCAGG
CCTATGTTATAAGTGAGAGACAGGTGTGGAGTGTGCAGGGAGGGAATTAAGATAGATG
GTGACCTGGAATTCAGAAGGTGGA
>2679
CTTTAGGCCAGGCCTATGTTATAAGTGAGAGACAGGTGTGGAGTGTGCAGGGAGGGAA
TTAAGATAGATGGTGACCTGGAATTCAGAAGGTGGACCCCCACCACCCCCCAGCCTGA
GACCAAAGCACAGTTTAACTCTGT
>2680
TATAAGTGAGAGACAGGTGTGGAGTGTGCAGGGAGGGAATTAAGATAGATGGTGACCT
GGAATTCAGAAGGTGGACCCCCACCACCCCCCAGCCTGAGACCAAAGCACAGTTTAA
CTCTGTCATGGGTCATGTGCAGAGG
>2681
TTAAGATAGATGGTGACCTGGAATTCAGAAGGTGGACCCCCACCACCCCCCAGCCTGA
GACCAAAGCACAGTTTAACTCTGTCATGGGTCATGTGCAGAGGGAAGGGACTGCCCC
AGAATTAGCAGTTGGTTGAACACCC
>2682
TGGTGACCTGGAATTCAGAAGGTGGACCCCCACCACCCCCCAGCCTGAGACCAAAGC
ACAGTTTAACTCTGTCATGGGTCATGTGCAGAGGGAAGGGACTGCCCCAGAATTAGCA
GTTGGTTGAACACCCTGCTTAAAAA
>2683
TGAGACCAAAGCACAGTTTAACTCTGTCATGGGTCATGTGCAGAGGGAAGGGACTGC
CCCAGAATTAGCAGTTGGTTGAACACCCTGCTTAAAAACTTTTTCTTTTCCTTTTTTTGT
GAGTACCGGTCCGCTTGGACTAA
>2684
ACCAAAGCACAGTTTAACTCTGTCATGGGTCATGTGCAGAGGGAAGGGACTGCCCCA
GAATTAGCAGTTGGTTGAACACCCTGCTTAAAAACTTTTTCTTTTCCTTTTTTTGTGAGT
ACCGGTCCGCTTGGACTAAAGAA
>2685
GGGACTGCCCCAGAATTAGCAGTTGGTTGAACACCCTGCTTAAAAACTTTTTCTTTTCC
TTTTTTTGTGAGTACCGGTCCGCTTGGACTAAAGAAGTTTTGGTCGTTGTTTTCTTAATT
TTGGGCTACAGAGTCTCTACC
>2686
GCCCCAGAATTAGCAGTTGGTTGAACACCCTGCTTAAAAACTTTTTCTTTTCCTTTTTTT
GTGAGTACCGGTCCGCTTGGACTAAAGAAGTTTTGGTCGTTGTTTTCTTAATTTTGGGC
TACAGAGTCTCTACCTACATT
>2687
GTTGAACACCCTGCTTAAAAACTTTTTCTTTTCCTTTTTTTGTGAGTACCGGTCCGCTTG
GACTAAAGAAGTTTTGGTCGTTGTTTTCTTAATTTTGGGCTACAGAGTCTCTACCTACAT
TTGTGTTTTGTGTTTTGGTA
>2688
TTTTTGTGAGTACCGGTCCGCTTGGACTAAAGAAGTTTTGGTCGTTGTTTTCTTAATTTT

GGGCTACAGAGTCTCTACCTACATTTGTGTTTTGTGTTTTGGTAACCACATGTAACAGC
CGGTGGAATCTTTTTTCTCTT
>2689
TCCGCTTGGACTAAAGAAGTTTTGGTCGTTGTTTTCTTAATTTTGGGCTACAGAGTCTC
TACCTACATTTGTGTTTTGTGTTTTGGTAACCACATGTAACAGCCGGTGGAATCTTTTTT
CTCTTTGCTTTCTCTGAACTT
>2690
GGACTAAAGAAGTTTTGGTCGTTGTTTTCTTAATTTTGGGCTACAGAGTCTCTACCTAC
ATTTGTGTTTTGTGTTTTGGTAACCACATGTAACAGCCGGTGGAATCTTTTTTCTCTTTG
CTTTCTCTGAACTTTCCAAAG
>2691
CTTAATTTTGGGCTACAGAGTCTCTACCTACATTTGTGTTTTGTGTTTTGGTAACCACAT
GTAACAGCCGGTGGAATCTTTTTTCTCTTTGCTTTCTCTGAACTTTCCAAAGAGTAGGC
CCCTATCTCTCGGGGGATTAA
>2692
TGTTTTGGTAACCACATGTAACAGCCGGTGGAATCTTTTTTCTCTTTGCTTTCTCTGAAC
TTTCCAAAGAGTAGGCCCCTATCTCTCGGGGGATTAACTCCTCGTGGCCCCTGGGTCGT
TGGTGCTCCCCCACCCTGTGT
>2693
TCTTTTTTCTCTTTGCTTTCTCTGAACTTTCCAAAGAGTAGGCCCCTATCTCTCGGGGGA
TTAACTCCTCGTGGCCCCTGGGTCGTTGGTGCTCCCCCACCCTGTGTCAGCATCTAAAC
CTCTGCACACAGGATGTTTTC
>2694
TTTGCTTTCTCTGAACTTTCCAAAGAGTAGGCCCCTATCTCTCGGGGGATTAACTCCTC
GTGGCCCCTGGGTCGTTGGTGCTCCCCCACCCTGTGTCAGCATCTAAACCTCTGCACA
CAGGATGTTTTCTCTAGCGCTTA
>2695
AAGAGTAGGCCCCTATCTCTCGGGGGATTAACTCCTCGTGGCCCCTGGGTCGTTGGTGC
TCCCCCACCCTGTGTCAGCATCTAAACCTCTGCACACAGGATGTTTTCTCTAGCGCTTA
TCAGATGCTTTAGAAGATGCGG
>2696
CTCCTCGTGGCCCCTGGGTCGTTGGTGCTCCCCCACCCTGTGTCAGCATCTAAACCTCT
GCACACAGGATGTTTTCTCTAGCGCTTATCAGATGCTTTAGAAGATGCGGGGTGGAGAT
AGAGGTAACGTTTAACCCATCC
>2697
TGTCAGCATCTAAACCTCTGCACACAGGATGTTTTCTCTAGCGCTTATCAGATGCTTTAG
AAGATGCGGGGTGGAGATAGAGGTAACGTTTAACCCATCCTGGATTCCTTTCATGAACT
CAGCCACAGAGCAGAGGGAGG
>2698
AACCTCTGCACACAGGATGTTTTCTCTAGCGCTTATCAGATGCTTTAGAAGATGCGGGG
TGGAGATAGAGGTAACGTTTAACCCATCCTGGATTCCTTTCATGAACTCAGCCACAGAG
CAGAGGGAGGCTTGGCTGGAGC
>2699
CTGCACACAGGATGTTTTCTCTAGCGCTTATCAGATGCTTTAGAAGATGCGGGGTGGAG
ATAGAGGTAACGTTTAACCCATCCTGGATTCCTTTCATGAACTCAGCCACAGAGCAGAG
GGAGGCTTGGCTGGAGCAAACA

>2700
TTAACCCATCCTGGATTCCTTTCATGAACTCAGCCACAGAGCAGAGGGAGGCTTGGCT
GGAGCAAACAATGTTGGGCCTTCTATTTCAAACAGAGGTGTGGGGGCTTGTGGTGGCA
TCAGTGGTGCACTGAACAACCCTC
>2701
GAACTCAGCCACAGAGCAGAGGGAGGCTTGGCTGGAGCAAACAATGTTGGGCCTTCT
ATTTCAAACAGAGGTGTGGGGGCTTGTGGTGGCATCAGTGGTGCACTGAACAACCCTC
CCTGACCCTAGCGTGTGGGTTTGGA
>2702
AGAGCAGAGGGAGGCTTGGCTGGAGCAAACAATGTTGGGCCTTCTATTTCAAACAGA
GGTGTGGGGGCTTGTGGTGGCATCAGTGGTGCACTGAACAACCCTCCCTGACCCTAGC
GTGTGGGTTTGGAGCCTGGGGAATC
>2703
GCAGAGGGAGGCTTGGCTGGAGCAAACAATGTTGGGCCTTCTATTTCAAACAGAGGT
GTGGGGGCTTGTGGTGGCATCAGTGGTGCACTGAACAACCCTCCCTGACCCTAGCGTG
TGGGTTTGGAGCCTGGGGAATCAAA
>2704
GGCTTGGCTGGAGCAAACAATGTTGGGCCTTCTATTTCAAACAGAGGTGTGGGGGCTT
GTGGTGGCATCAGTGGTGCACTGAACAACCCTCCCTGACCCTAGCGTGTGGGTTTGGA
GCCTGGGGAATCAAAGAGAAATCT
>2705
ATTTCAAACAGAGGTGTGGGGGCTTGTGGTGGCATCAGTGGTGCACTGAACAACCCTC
CCTGACCCTAGCGTGTGGGTTTGGAGCCTGGGGAATCAAAGAGAAATCTAACAAACTA
TTCTTTTTCACGTGTGGGCTGAAT
>2706
CCCTGACCCTAGCGTGTGGGTTTGGAGCCTGGGGAATCAAAGAGAAATCTAACAAACT
ATTCTTTTTCACGTGTGGGCTGAATGAATGTGTGGGTTATCCACATCAGTTTCTTCTCTC
TTCTCATCCTCCCTCCTTCCTC
>2707
CTGACCCTAGCGTGTGGGTTTGGAGCCTGGGGAATCAAAGAGAAATCTAACAAACTAT
TCTTTTTCACGTGTGGGCTGAATGAATGTGTGGGTTATCCACATCAGTTTCTTCTCTCTT
CTCATCCTCCCTCCTTCCTCGC
>2708
GGGTTTGGAGCCTGGGGAATCAAAGAGAAATCTAACAAACTATTCTTTTTCACGTGTG
GGCTGAATGAATGTGTGGGTTATCCACATCAGTTTCTTCTCTCTTCTCATCCTCCCTCCT
TCCTCGCAGACCAGCATTTTGG
>2709
GTTTGGAGCCTGGGGAATCAAAGAGAAATCTAACAAACTATTCTTTTTCACGTGTGGG
CTGAATGAATGTGTGGGTTATCCACATCAGTTTCTTCTCTCTTCTCATCCTCCCTCCTTC
CTCGCAGACCAGCATTTTGGCC
>2710
GGAGCCTGGGGAATCAAAGAGAAATCTAACAAACTATTCTTTTTCACGTGTGGGCTGA
ATGAATGTGTGGGTTATCCACATCAGTTTCTTCTCTCTTCTCATCCTCCCTCCTTCCTCG
CAGACCAGCATTTTGGCCAGGG
>2711
TCAAAGAGAAATCTAACAAACTATTCTTTTTCACGTGTGGGCTGAATGAATGTGTGGGT

TATCCACATCAGTTTCTTCTCTCTTCTCATCCTCCCTCCTTCCTCGCAGACCAGCATTTT
GGCCAGGGTGCCCAGTTTGAG
>2712
GGTTATCCACATCAGTTTCTTCTCTCTTCTCATCCTCCCTCCTTCCTCGCAGACCAGCAT
TTTGGCCAGGGTGCCCAGTTTGAGCTCATTTACCAAAGGTGAATAGGGAAGGGAGTAG
AAACCAAATTAGGCACCTGTCT
>2713
CACATCAGTTTCTTCTCTCTTCTCATCCTCCCTCCTTCCTCGCAGACCAGCATTTTGGCC
AGGGTGCCCAGTTTGAGCTCATTTACCAAAGGTGAATAGGGAAGGGAGTAGAAACCA
AATTAGGCACCTGTCTGACAACA
>2714
CTCATCCTCCCTCCTTCCTCGCAGACCAGCATTTTGGCCAGGGTGCCCAGTTTGAGCTC
ATTTACCAAAGGTGAATAGGGAAGGGAGTAGAAACCAAATTAGGCACCTGTCTGACAA
CACAAGGATCTTTTCTACAATGG
>2715
CAGGGTGCCCAGTTTGAGCTCATTTACCAAAGGTGAATAGGGAAGGGAGTAGAAACC
AAATTAGGCACCTGTCTGACAACACAAGGATCTTTTCTACAATGGTGCATTTGGATTAT
GCAGAGTTTTAGATTTTCTGTTTT
>2716
GGTGAATAGGGAAGGGAGTAGAAACCAAATTAGGCACCTGTCTGACAACACAAGGAT
CTTTTCTACAATGGTGCATTTGGATTATGCAGAGTTTTAGATTTTCTGTTTTATCCCAGG
GTGAACGTGTGTAGAAAACTGGG
>2717
GAAACCAAATTAGGCACCTGTCTGACAACACAAGGATCTTTTCTACAATGGTGCATTTG
GATTATGCAGAGTTTTAGATTTTCTGTTTTATCCCAGGGTGAACGTGTGTAGAAAACTG
GGACCTAACCATGTTCTTCTCT
>2718
CACCTGTCTGACAACACAAGGATCTTTTCTACAATGGTGCATTTGGATTATGCAGAGTT
TTAGATTTTCTGTTTTATCCCAGGGTGAACGTGTGTAGAAAACTGGGACCTAACCATGT
TCTTCTCTTCAGTCTTATGGTC
>2719
GGATCTTTTCTACAATGGTGCATTTGGATTATGCAGAGTTTTAGATTTTCTGTTTTATCCC
AGGGTGAACGTGTGTAGAAAACTGGGACCTAACCATGTTCTTCTCTTCAGTCTTATGGT
CATTGCTACTTTGAAGGCAG
>2720
ATTATGCAGAGTTTTAGATTTTCTGTTTTATCCCAGGGTGAACGTGTGTAGAAAACTGG
GACCTAACCATGTTCTTCTCTTCAGTCTTATGGTCATTGCTACTTTGAAGGCAGAAAGG
ATAAAAGTAGCATAGGGAGTGG
>2721
TTAGATTTTCTGTTTTATCCCAGGGTGAACGTGTGTAGAAAACTGGGACCTAACCATGT
TCTTCTCTTCAGTCTTATGGTCATTGCTACTTTGAAGGCAGAAAGGATAAAAGTAGCAT
AGGGAGTGGTTTACCATAACTG
>2722
ACTGGGACCTAACCATGTTCTTCTCTTCAGTCTTATGGTCATTGCTACTTTGAAGGCAG
AAAGGATAAAAGTAGCATAGGGAGTGGTTTACCATAACTGCAAGTAAATACCAAGCAT
GTGCGAGCCTGTAGACATTGCCT

>2723
CCATGTTCTTCTCTTCAGTCTTATGGTCATTGCTACTTTGAAGGCAGAAAGGATAAAAG
TAGCATAGGGAGTGGTTTACCATAACTGCAAGTAAATACCAAGCATGTGCGAGCCTGTA
GACATTGCCTGCTATTGGGGAA
>2724
TTATGGTCATTGCTACTTTGAAGGCAGAAAGGATAAAAGTAGCATAGGGAGTGGTTTAC
CATAACTGCAAGTAAATACCAAGCATGTGCGAGCCTGTAGACATTGCCTGCTATTGGGG
AAAGACGGAGTTAGCTGGGGAA
>2725
AGAAAGGATAAAAGTAGCATAGGGAGTGGTTTACCATAACTGCAAGTAAATACCAAGC
ATGTGCGAGCCTGTAGACATTGCCTGCTATTGGGGAAAGACGGAGTTAGCTGGGGAAA
TACATCAGTCTTCACTCCCAGCAC
>2726
ACCATAACTGCAAGTAAATACCAAGCATGTGCGAGCCTGTAGACATTGCCTGCTATTGG
GGAAAGACGGAGTTAGCTGGGGAAATACATCAGTCTTCACTCCCAGCACCCAAGATGT
AAACATTTCTTACTCTTCCTGGA
>2727
CAAGCATGTGCGAGCCTGTAGACATTGCCTGCTATTGGGGAAAGACGGAGTTAGCTGG
GGAAATACATCAGTCTTCACTCCCAGCACCCAAGATGTAAACATTTCTTACTCTTCCTG
GATCAGGCTATCTCTGCTTTGCC
>2728
TTGCCTGCTATTGGGGAAAGACGGAGTTAGCTGGGGAAATACATCAGTCTTCACTCCC
AGCACCCAAGATGTAAACATTTCTTACTCTTCCTGGATCAGGCTATCTCTGCTTTGCCTT
TTCTTTCACCTACCTCCTTCTT
>2729
GATCAGGCTATCTCTGCTTTGCCTTTTCTTTCACCTACCTCCTTCTTTCATGGCAGGAAG
GGAAAGGAAAGTACCCCCAAGCAGCCAGCAGTCAGCACTGGGGCCTCTAGGGAAGA
GTGGATTGGTGTTGTTTTGCTGTG
>2730
GCCTTTTCTTTCACCTACCTCCTTCTTTCATGGCAGGAAGGGAAAGGAAAGTACCCCC
AAGCAGCCAGCAGTCAGCACTGGGGCCTCTAGGGAAGAGTGGATTGGTGTTGTTTTGC
TGTGGTTTGCTGTGCGTTTTCCAG
>2731
TTCATGGCAGGAAGGGAAAGGAAAGTACCCCCAAGCAGCCAGCAGTCAGCACTGGGG
CCTCTAGGGAAGAGTGGATTGGTGTTGTTTTGCTGTGGTTTGCTGTGCGTTTTCCAGAA
TCTCTCTTGGATGACTATCAGATT
>2732
AAAGTACCCCCAAGCAGCCAGCAGTCAGCACTGGGGCCTCTAGGGAAGAGTGGATTG
GTGTTGTTTTGCTGTGGTTTGCTGTGCGTTTTCCAGAATCTCTCTTGGATGACTATCAGA
TTCAGTGAAGGGATGGACCTTCA
>2733
GTACCCCCAAGCAGCCAGCAGTCAGCACTGGGGCCTCTAGGGAAGAGTGGATTGGTG
TTGTTTTGCTGTGGTTTGCTGTGCGTTTTCCAGAATCTCTCTTGGATGACTATCAGATTC
AGTGAAGGGATGGACCTTCAGGA
>2734
CAAGCAGCCAGCAGTCAGCACTGGGGCCTCTAGGGAAGAGTGGATTGGTGTTGTTTTG

CTGTGGTTTGCTGTGCGTTTTCCAGAATCTCTCTTGGATGACTATCAGATTCAGTGAAG
GGATGGACCTTCAGGAAGCTGAG
>2735
GGATTGGTGTTGTTTTGCTGTGGTTTGCTGTGCGTTTTCCAGAATCTCTCTTGGATGACT
ATCAGATTCAGTGAAGGGATGGACCTTCAGGAAGCTGAGAGATAGGAGATCCTGTGGC
CTGGGAACCCAGAGGCCTGGGC
>2736
ATCTCTCTTGGATGACTATCAGATTCAGTGAAGGGATGGACCTTCAGGAAGCTGAGAG
ATAGGAGATCCTGTGGCCTGGGAACCCAGAGGCCTGGGCCCTGGGAACAACTCACCA
CAGGACTCAGCCTGCGAAATAGGAA
>2737
GGCCTGGGAACCCAGAGGCCTGGGCCCTGGGAACAACTCACCACAGGACTCAGCCTG
CGAAATAGGAAACGTCATTGTAGCTTCTGCATCATGTTTTCCATGTTCCACTGGAAAAC
CATCTGTTAAATGGACATAGACCA
>2738
GGAACCCAGAGGCCTGGGCCCTGGGAACAACTCACCACAGGACTCAGCCTGCGAAAT
AGGAAACGTCATTGTAGCTTCTGCATCATGTTTTCCATGTTCCACTGGAAAACCATCTG
TTAAATGGACATAGACCAATTCTT
>2739
GGCCCTGGGAACAACTCACCACAGGACTCAGCCTGCGAAATAGGAAACGTCATTGTA
GCTTCTGCATCATGTTTTCCATGTTCCACTGGAAAACCATCTGTTAAATGGACATAGAC
CAATTCTTGCTTTTCTTCTTTCTG
>2740
AACAACTCACCACAGGACTCAGCCTGCGAAATAGGAAACGTCATTGTAGCTTCTGCAT
CATGTTTTCCATGTTCCACTGGAAAACCATCTGTTAAATGGACATAGACCAATTCTTGCT
TTTCTTCTTTCTGGTACCAAAG
>2741
AGCCTGCGAAATAGGAAACGTCATTGTAGCTTCTGCATCATGTTTTCCATGTTCCACTG
GAAAACCATCTGTTAAATGGACATAGACCAATTCTTGCTTTTCTTCTTTCTGGTACCAA
AGAGAACGGTGGTGTCTCTTGT
>2742
GTTTTCCATGTTCCACTGGAAAACCATCTGTTAAATGGACATAGACCAATTCTTGCTTTT
CTTCTTTCTGGTACCAAAGAGAACGGTGGTGTCTCTTGTCATCTAGATGTTCTTGTAGG
GGCACCAGAGTCATTGCAACC
>2743
CTGGAAAACCATCTGTTAAATGGACATAGACCAATTCTTGCTTTTCTTCTTTCTGGTACC
AAAGAGAACGGTGGTGTCTCTTGTCATCTAGATGTTCTTGTAGGGGCACCAGAGTCAT
TGCAACCATTTTGAGTAAAGAT
>2744
ACATAGACCAATTCTTGCTTTTCTTCTTTCTGGTACCAAAGAGAACGGTGGTGTCTCTT
GTCATCTAGATGTTCTTGTAGGGGCACCAGAGTCATTGCAACCATTTTGAGTAAAGATT
GAGTCCACTGGGGCATCAGAAG
>2745
ACCAATTCTTGCTTTTCTTCTTTCTGGTACCAAAGAGAACGGTGGTGTCTCTTGTCATCT
AGATGTTCTTGTAGGGGCACCAGAGTCATTGCAACCATTTTGAGTAAAGATTGAGTCC
ACTGGGGCATCAGAAGGGGAGT

>2746
TTCTTCTTTCTGGTACCAAAGAGAACGGTGGTGTCTCTTGTCATCTAGATGTTCTTGTA
GGGGCACCAGAGTCATTGCAACCATTTTGAGTAAAGATTGAGTCCACTGGGGCATCAG
AAGGGGAGTGTGTCATTGCTGTT
>2747
AGAGAACGGTGGTGTCTCTTGTCATCTAGATGTTCTTGTAGGGGCACCAGAGTCATTGC
AACCATTTTGAGTAAAGATTGAGTCCACTGGGGCATCAGAAGGGGAGTGTGTCATTGC
TGTTGTTACCACACAGGCCGTGG
>2748
TTGTAGGGGCACCAGAGTCATTGCAACCATTTTGAGTAAAGATTGAGTCCACTGGGGC
ATCAGAAGGGGAGTGTGTCATTGCTGTTGTTACCACACAGGCCGTGGTACTTGCCTTTT
CCTGACCCATTTTGCAGAGCAGG
>2749
GTAGGGGCACCAGAGTCATTGCAACCATTTTGAGTAAAGATTGAGTCCACTGGGGCAT
CAGAAGGGGAGTGTGTCATTGCTGTTGTTACCACACAGGCCGTGGTACTTGCCTTTTC
CTGACCCATTTTGCAGAGCAGGCC
>2750
GTCATTGCAACCATTTTGAGTAAAGATTGAGTCCACTGGGGCATCAGAAGGGGAGTGT
GTCATTGCTGTTGTTACCACACAGGCCGTGGTACTTGCCTTTTCCTGACCCATTTTGCA
GAGCAGGCCAGCTACTTAACCCC
>2751
GGTACTTGCCTTTTCCTGACCCATTTTGCAGAGCAGGCCAGCTACTTAACCCCTCGCCA
CCCACTCACCTGTGGAAGGCACATTTGCAAAACAATAGACCAGTTGGTAATTTGGCAC
TTGTTAGGTAGACATAGTGGTAC
>2752
AGCAGGCCAGCTACTTAACCCCTCGCCACCCACTCACCTGTGGAAGGCACATTTGCAA
AACAATAGACCAGTTGGTAATTTGGCACTTGTTAGGTAGACATAGTGGTACATTGAGGA
AGTTCCTCCTGTTAACTAACCTA
>2753
GGCCAGCTACTTAACCCCTCGCCACCCACTCACCTGTGGAAGGCACATTTGCAAAACA
ATAGACCAGTTGGTAATTTGGCACTTGTTAGGTAGACATAGTGGTACATTGAGGAAGTT
CCTCCTGTTAACTAACCTAGTTT
>2754
CTCACCTGTGGAAGGCACATTTGCAAAACAATAGACCAGTTGGTAATTTGGCACTTGTT
AGGTAGACATAGTGGTACATTGAGGAAGTTCCTCCTGTTAACTAACCTAGTTTTTCTTG
CTGTAATCCGAATATACTTCTC
>2755
ACATTTGCAAAACAATAGACCAGTTGGTAATTTGGCACTTGTTAGGTAGACATAGTGGT
ACATTGAGGAAGTTCCTCCTGTTAACTAACCTAGTTTTTCTTGCTGTAATCCGAATATAC
TTCTCTGATTTTTTGAAAAAG
>2756
AAACAATAGACCAGTTGGTAATTTGGCACTTGTTAGGTAGACATAGTGGTACATTGAGG
AAGTTCCTCCTGTTAACTAACCTAGTTTTTCTTGCTGTAATCCGAATATACTTCTCTGATT
TTTTGAAAAAGCCTGTGAGT
>2757
GCACTTGTTAGGTAGACATAGTGGTACATTGAGGAAGTTCCTCCTGTTAACTAACCTAG

TTTTTCTTGCTGTAATCCGAATATACTTCTCTGATTTTTTGAAAAAGCCTGTGAGTGGAG
CCTGGCTCGACAGCCCAAAAC
>2758
CCTCCTGTTAACTAACCTAGTTTTTCTTGCTGTAATCCGAATATACTTCTCTGATTTTTTG
AAAAAGCCTGTGAGTGGAGCCTGGCTCGACAGCCCAAAACGCTGAATTAATTCTGTCT
CTCTCTCTCTCTCTTTTTTTT
>2759
CTGTTAACTAACCTAGTTTTTCTTGCTGTAATCCGAATATACTTCTCTGATTTTTTGAAAA
AGCCTGTGAGTGGAGCCTGGCTCGACAGCCCAAAACGCTGAATTAATTCTGTCTCTCT
CTCTCTCTCTTTTTTTTTTTT
>2760
CTTCTCTGATTTTTTGAAAAAGCCTGTGAGTGGAGCCTGGCTCGACAGCCCAAAACGC
TGAATTAATTCTGTCTCTCTCTCTCTCTCTTTTTTTTTTTTGAGACGTGGAGTCTCGCTCT
GTCACCCAGGCTGGAATGCGC
>2761
GCCTGGCTCGACAGCCCAAAACGCTGAATTAATTCTGTCTCTCTCTCTCTCTCTTTTTTT
TTTTTGAGACGTGGAGTCTCGCTCTGTCACCCAGGCTGGAATGCGCTGGCACGATCTC
GGCTCACTGCAACTTCCACCTC
>2762
AGTCTCGCTCTGTCACCCAGGCTGGAATGCGCTGGCACGATCTCGGCTCACTGCAACT
TCCACCTCCTGGGTCCAAGCAATTCTCCTGCCTCAGCCTCCCAAGTAGCTGGGACTAC
AGGCATTGCCACTACGCCCAGCTA
>2763
TGGCACGATCTCGGCTCACTGCAACTTCCACCTCCTGGGTCCAAGCAATTCTCCTGCCT
CAGCCTCCCAAGTAGCTGGGACTACAGGCATTGCCACTACGCCCAGCTAATTTTTGTAT
TTTTAATAGAGACGGGGTTTCG
>2764
GCAATTCTCCTGCCTCAGCCTCCCAAGTAGCTGGGACTACAGGCATTGCCACTACGCCC
AGCTAATTTTTGTATTTTTAATAGAGACGGGGTTTCGCCGTGTTGGCCAGGCTGGTCTC
AAACTCCTGATCTCAGGTGATC
>2765
TCCCAAGTAGCTGGGACTACAGGCATTGCCACTACGCCCAGCTAATTTTTGTATTTTTAA
TAGAGACGGGGTTTCGCCGTGTTGGCCAGGCTGGTCTCAAACTCCTGATCTCAGGTGA
TCCACCAGCCTTGGCATCCCAA
>2766
CATTGCCACTACGCCCAGCTAATTTTTGTATTTTTAATAGAGACGGGGTTTCGCCGTGTT
GGCCAGGCTGGTCTCAAACTCCTGATCTCAGGTGATCCACCAGCCTTGGCATCCCAAA
ATGCTGGGACTGCAGGAATGAG
>2767
ATTTTTGTATTTTTAATAGAGACGGGGTTTCGCCGTGTTGGCCAGGCTGGTCTCAAACT
CCTGATCTCAGGTGATCCACCAGCCTTGGCATCCCAAAATGCTGGGACTGCAGGAATG
AGCCACCGCGCCCGACCCAATTC
>2768
CACCAGCCTTGGCATCCCAAAATGCTGGGACTGCAGGAATGAGCCACCGCGCCCGAC
CCAATTCTCTTGAGTGACATGCTGAGTGGGGCTGCTTTTTGGTGGAGGTAACAAAAAA
AGAGTTGAGGTATTGATTTTAGTTT

>2769
CATCCCAAAATGCTGGGACTGCAGGAATGAGCCACCGCGCCCGACCCAATTCTCTTGA
GTGACATGCTGAGTGGGGCTGCTTTTTGGTGGAGGTAACAAAAAAAGAGTTGAGGTAT
TGATTTTAGTTTCATTTGAGACAG
>2770
GACTGCAGGAATGAGCCACCGCGCCCGACCCAATTCTCTTGAGTGACATGCTGAGTGG
GGCTGCTTTTTGGTGGAGGTAACAAAAAAAGAGTTGAGGTATTGATTTTAGTTTCATTT
GAGACAGCGATCTGTCCCCTCCC
>2771
AGGAATGAGCCACCGCGCCCGACCCAATTCTCTTGAGTGACATGCTGAGTGGGGCTGC
TTTTTGGTGGAGGTAACAAAAAAAGAGTTGAGGTATTGATTTTAGTTTCATTTGAGACA
GCGATCTGTCCCCTCCCTGAGTT
>2772
GCGCCCGACCCAATTCTCTTGAGTGACATGCTGAGTGGGGCTGCTTTTTGGTGGAGGT
AACAAAAAAAGAGTTGAGGTATTGATTTTAGTTTCATTTGAGACAGCGATCTGTCCCCT
CCCTGAGTTCCCTCGTTTGTTTG
>2773
TTGAGTGACATGCTGAGTGGGGCTGCTTTTTGGTGGAGGTAACAAAAAAAGAGTTGA
GGTATTGATTTTAGTTTCATTTGAGACAGCGATCTGTCCCCTCCCTGAGTTCCCTCGTTT
GTTTGTAAACTGCTTAAATGAAC
>2774
CTGCTTTTTGGTGGAGGTAACAAAAAAAGAGTTGAGGTATTGATTTTAGTTTCATTTGA
GACAGCGATCTGTCCCCTCCCTGAGTTCCCTCGTTTGTTTGTAAACTGCTTAAATGAAC
TTTTTCCCCTGTTCCTCCATCA
>2775
GAGGTAACAAAAAAAGAGTTGAGGTATTGATTTTAGTTTCATTTGAGACAGCGATCTGT
CCCCTCCCTGAGTTCCCTCGTTTGTTTGTAAACTGCTTAAATGAACTTTTTCCCCTGTTC
CTCCATCACTCTGCTGTTCTC
>2776
AAAAAAAGAGTTGAGGTATTGATTTTAGTTTCATTTGAGACAGCGATCTGTCCCCTCCC
TGAGTTCCCTCGTTTGTTTGTAAACTGCTTAAATGAACTTTTTCCCCTGTTCCTCCATCA
CTCTGCTGTTCTCTTCTCCTC
>2777
AGACAGCGATCTGTCCCCTCCCTGAGTTCCCTCGTTTGTTTGTAAACTGCTTAAATGAA
CTTTTTCCCCTGTTCCTCCATCACTCTGCTGTTCTCTTCTCCTCCTCTTATCCTAACAGTC
TCATAGGTAAACTTAGAAGG
>2778
CCCTGAGTTCCCTCGTTTGTTTGTAAACTGCTTAAATGAACTTTTTCCCCTGTTCCTCCA
TCACTCTGCTGTTCTCTTCTCCTCCTCTTATCCTAACAGTCTCATAGGTAAACTTAGAAG
GAAGACAAATACTTACAAAT
>2779
TGCTTAAATGAACTTTTTCCCCTGTTCCTCCATCACTCTGCTGTTCTCTTCTCCTCCTCTT
ATCCTAACAGTCTCATAGGTAAACTTAGAAGGAAGACAAATACTTACAAATTGTCACCG
AGGGTACCATAAAAGAGACA
>2780
TTCTCTTCTCCTCCTCTTATCCTAACAGTCTCATAGGTAAACTTAGAAGGAAGACAAAT

ACTTACAAATTGTCACCGAGGGTACCATAAAAGAGACATTTGGACAGTAGTTCTGGGG
AGATCCTTGTCTTTGCTTCATGT
>2781
CTCCTCTTATCCTAACAGTCTCATAGGTAAACTTAGAAGGAAGACAAATACTTACAAAT
TGTCACCGAGGGTACCATAAAAGAGACATTTGGACAGTAGTTCTGGGGAGATCCTTGT
CTTTGCTTCATGTTAATACAGAG
>2782
GGTAAACTTAGAAGGAAGACAAATACTTACAAATTGTCACCGAGGGTACCATAAAAGA
GACATTTGGACAGTAGTTCTGGGGAGATCCTTGTCTTTGCTTCATGTTAATACAGAGGG
ATTGGGGTAGCCCAGATCACCAG
>2783
AAACTTAGAAGGAAGACAAATACTTACAAATTGTCACCGAGGGTACCATAAAAGAGAC
ATTTGGACAGTAGTTCTGGGGAGATCCTTGTCTTTGCTTCATGTTAATACAGAGGGATT
GGGGTAGCCCAGATCACCAGGGG
>2784
AAATACTTACAAATTGTCACCGAGGGTACCATAAAAGAGACATTTGGACAGTAGTTCTG
GGGAGATCCTTGTCTTTGCTTCATGTTAATACAGAGGGATTGGGGTAGCCCAGATCACC
AGGGGAAAGTGGTTTTTCTTCA
>2785
AAAGAGACATTTGGACAGTAGTTCTGGGGAGATCCTTGTCTTTGCTTCATGTTAATACA
GAGGGATTGGGGTAGCCCAGATCACCAGGGGAAAGTGGTTTTTCTTCATTTAGCTTTTA
ATTGCTTTCTTTCACTTTTATG
>2786
CTGGGGAGATCCTTGTCTTTGCTTCATGTTAATACAGAGGGATTGGGGTAGCCCAGATC
ACCAGGGGAAAGTGGTTTTTCTTCATTTAGCTTTTAATTGCTTTCTTTCACTTTTATGTAT
AAAAGATTGTCCATTTAATT
>2787
GGGAGATCCTTGTCTTTGCTTCATGTTAATACAGAGGGATTGGGGTAGCCCAGATCACC
AGGGGAAAGTGGTTTTTCTTCATTTAGCTTTTAATTGCTTTCTTTCACTTTTATGTATAAA
AGATTGTCCATTTAATTAAC
>2788
GGTAGCCCAGATCACCAGGGGAAAGTGGTTTTTCTTCATTTAGCTTTTAATTGCTTTCTT
TCACTTTTATGTATAAAAGATTGTCCATTTAATTAACTCAAGAATGCAATTTCAAAAACA
CTAATGCTTTACACATGACG
>2789
CACCAGGGGAAAGTGGTTTTTCTTCATTTAGCTTTTAATTGCTTTCTTTCACTTTTATGTA
TAAAAGATTGTCCATTTAATTAACTCAAGAATGCAATTTCAAAAACACTAATGCTTTAC
ACATGACGGCTTTTAAAATA
>2790
CATTTAATTAACTCAAGAATGCAATTTCAAAAACACTAATGCTTTACACATGACGGCTT
TTAAAATAAAAGTAGCATTCTAATATTGCATAATGCTTTTCTATCCCCTTCCTCTCCCCGC
GTTCACCCCCACCCCTCCAG
>2791
TGACGGCTTTTAAAATAAAAGTAGCATTCTAATATTGCATAATGCTTTTCTATCCCCTTCC
TCTCCCCGCGTTCACCCCCACCCCTCCAGACTGTATTAACCTTTCATAGCGGAGGGTTG
TTAGGAGGAAAACGCTGTTC

>2792
GCATTCTAATATTGCATAATGCTTTTCTATCCCCTTCCTCTCCCCGCGTTCACCCCCACCC
CTCCAGACTGTATTAACCTTTCATAGCGGAGGGTTGTTAGGAGGAAAACGCTGTTCTG
GATGGTGCCTCTCTGCCTCTC
>2793
TTTCTATCCCCTTCCTCTCCCCGCGTTCACCCCCACCCCTCCAGACTGTATTAACCTTTC
ATAGCGGAGGGTTGTTAGGAGGAAAACGCTGTTCTGGATGGTGCCTCTCTGCCTCTCC
TTCCCACCAACCAAGTACAGCA
>2794
CTATCCCCTTCCTCTCCCCGCGTTCACCCCCACCCCTCCAGACTGTATTAACCTTTCATA
GCGGAGGGTTGTTAGGAGGAAAACGCTGTTCTGGATGGTGCCTCTCTGCCTCTCCTTC
CCACCAACCAAGTACAGCAGCA
>2795
CCGCGTTCACCCCCACCCCTCCAGACTGTATTAACCTTTCATAGCGGAGGGTTGTTAGG
AGGAAAACGCTGTTCTGGATGGTGCCTCTCTGCCTCTCCTTCCCACCAACCAAGTACA
GCAGCACGACAGCCGCAGGCCTT
>2796
AGCGGAGGGTTGTTAGGAGGAAAACGCTGTTCTGGATGGTGCCTCTCTGCCTCTCCTT
CCCACCAACCAAGTACAGCAGCACGACAGCCGCAGGCCTTTCTGAAGTGTTCCGTAG
CTTCTGTAAGAAAGAGACTACTCAT
>2797
GATGGTGCCTCTCTGCCTCTCCTTCCCACCAACCAAGTACAGCAGCACGACAGCCGCA
GGCCTTTCTGAAGTGTTCCGTAGCTTCTGTAAGAAAGAGACTACTCATCCCTTCCCCCT
TGGCCGGTTTCATGCCTATATTT
>2798
CTCTCCTTCCCACCAACCAAGTACAGCAGCACGACAGCCGCAGGCCTTTCTGAAGTGT
TCCGTAGCTTCTGTAAGAAAGAGACTACTCATCCCTTCCCCCTTGGCCGGTTTCATGCC
TATATTTACCCTCTTTTGGGGGT
>2799
CCGCAGGCCTTTCTGAAGTGTTCCGTAGCTTCTGTAAGAAAGAGACTACTCATCCCTTC
CCCCTTGGCCGGTTTCATGCCTATATTTACCCTCTTTTGGGGGTCACTGTGGCTCCTACT
CCCATTGCTGCCTTTTAAGAA
>2800
CTGTAAGAAAGAGACTACTCATCCCTTCCCCCTTGGCCGGTTTCATGCCTATATTTACCC
TCTTTTGGGGGTCACTGTGGCTCCTACTCCCATTGCTGCCTTTTAAGAAGCATCATTATG
ATGGATTACATGGGAAAATT

Online Supporting Information S_2^+ represents the positive dataset composed of 2880 true splice acceptor site sequences.

>1
GGCCAGGGGCATAGAGCTGGCCAAGGAGCCATGGCTCACTAACGTGTTGTATGGGGCT
CCTTCCCTTCAGGTCCAGGCTCCTGCGTGAAGTGATGCTCCTCTTTGCCTTACTCCTAG
CCATGGAGCTCCCATTGGTGGCA
>2

GAGTGAGCTGGTAATGGGTGGAAAAGGCGTAGTGGAGCAGAAGCCTGAAGCCTGCTT
TCTCCCCTCTCAGGGACTTACAGTTTGAGATGCCATGACTGTGCGGTCATAAATGACTT
CAACTGTCCCAACATTAGAGTATG
>3
TGTATGTGCCTCAATATTTACAAGCAGAAAATGTGAAATCAATTATTTTCATTGCTGCTT
CTTTTTTTAGGCATAAATTCTCGTGAACTACTTGTTTATAAGAACTGTACAAACAACTGC
ACATTTGTATATGCAGCTGA
>4
GTAGGGCTCAGCTCCGCCCCTGTCACTACACGCTGGGGACACACCACACTGCCCGACT
TCTCCTCCCCAGGTGGGCGCTCCTCGCCAAAAAAGGCTACCAGGAGCGGGACCTGGA
ACCCCAGTTTTCCATCATCACCAAA
>5
AGGCTGCCGGCTTCCGGCCTTTCCAGTCAACACGAGCCCAGCCAGGCCAACCTTGAG
ACTTGCCTCCTAGGGAGAGAACGTGTTCTTCTTGGTGACCAACTTCCTTGTGACGCCA
GCCCAAGTTCAGGGCAGATGCCCAG
>6
CCTTTGAGACTGGGCTGTGGAGGGGAGACATCCCCTGTGCCATTGGTGACTGCTCTCT
CTCCCACCTCAGCACCCGTCCGTCCCACTGGCTAACTGCTGGGTCGACGAGGACTGCC
CCGAAGGGGAGGGAGGCACACACA
>7
ACCCCCTGGCTGAAGGCCTCCCCAGGCCTGCAGAGATTTGAAGGTCTGGAGTTCATCT
TTTGTTTTCTAGGTGTAAAAACAGGCCAGTGTGTGGTGTTCAATGGGACCCACAGGAC
CTGTGAGATCTGGAGTTGGTGCCC
>8
ACAATCCCCTACCCCAACTGGCGCAGGGCCCCAGGCCTGGCAGAGGCTGTCACCTCCC
TTCCACCTGCAGGAGGCCCCTGCTGGCCCAGGCCCAGAACTTCACACTGTTCATCAAA
AACACAGTCACCTTCAGCAAGTTC
>9
AGAGTGGGTCTCATCTGCCCCAAGACCCTCCTTGTCCCCTACCTCATCTGACCTTTCCC
ACTCCTCCCAGGTCCAATGCCTTGGAGACCTGGGACCCCACCTATTTTAAGCACTGCC
GCTATGAACCACAATTCAGCCCC
>10
GGTTCCTAGAGGGCTCTGGGAGAGGGTCCCGGGCCCACCCACCGGTGGAAAAGCTAT
GTGCTATGTGCAGGGTGGCTCTGTAGGCATCAGAGTTCACTGGGATTGTGACCTGGAC
ACCGGGGACTCTGGCTGCTGGCCTC
>11
GGTCTGGCAGGGCCAGCCCCACCTGGGGGTGGGCAAAGCAGGTCACCAGAGCCTTCT
TTCCTGCCCACAGGACAGCCACTCACTGGTGGGAGCAACCGGGTGTGGAGGCCCGCA
CCCTGCTCAAGCTCTATGGAATCCGC
>12
GTGCAGGCTGGGGAGGTGGCAGGAAAGCAGGCTCGGGGGCTGGAACATGGGTTGGC
CCTGCCTCTCCCAGGTCACCTTTTTCTGTGACCTGCTACTGCTGTATGTGGATAGAGAA
GCCCATTTCTACTGGAGGACAAAGT
>13
TTGGACCCTGGGTTCTGCCACACTTAGGAAGATGTTGGCTGGATCCCTGACCTGCTGTC
CTCATCTGCAGGCCAAGGCCCCGAAAGCAACCGCCAACTCTGTGTGGAGGGAGCTGG

CCCTTGCATCCCAAGCCCGACTGG
>14
GAGGTTGTGGCCTCGGGTCGGCGTCTGAGGACCCGCTGACGCTCCGGTGCCCCTCCGT
CCTCTCTCACAGCGTGCTGAGCTACGTGGCGGGCATCGCCTGGTTCTTGGCGCTGGTTT
TCCCGCCGCTGACCCAGCGCACT
>15
GGGCGTATGGGGCGAGCAGTGGTGGCTAAGGCCGGGGAGCTCTGCTCAGAGGCTCCC
TTCGTGTCTGCAGGGCTCTGCCAGTGGCCTGGCTTGAACGGACGATGCGGTCAGTAGG
GCTGGAGGTCTACACGCAGAGTTTC
>16
GTTGTGGGGGGCCCTTCAGCCCCCTACCACAAAGTTACACTGAGGCTCCCCCCACCGA
TGCTGCATACAGATGGTGTCGGGCACCAACGTGTACGGCATCCTGCGGGCCCCGCGTG
CTGCCAGCACCGAGTCGCTTGTGC
>17
TCAGGGCTGCTGGCCGTGGAGGGGTTTGGTCAGTGCCCCAGACCCTGCTGATCCTGCT
TCTGGCCTCCAGGGCAGATTTATTGGGCCAAAGATATCGTCTTCCTGGTAACAGAACAT
GACCTTCTGGGCACTGAGGCTTG
>18
CCTGGTCCCCCTCTCAGGGTCACTGTCCTCACCACGTCCTCCATTAGCACTCATTCACT
TGCTCCTACAGGCATGCAGTCGTCTCCCCTGCAGGGCCGAGCTGGGGCCATTCAGGCA
GCCGTGGCCCTGGAGCTGAGCAG
>19
GCCTGCCTGAGGGCTGAAGGGCACAGGGTCTGTGGGAGGGGCTGTCTCATCCTGTCCT
GCACCTCTAAAGCTGCAGCCCGAGGACTGGACATCATTGGATGGACCGCTGCAGGGCC
TGCAGACACTGCTGCTCATGGTTC
>20
TCTAGCTGGAGCGGAGTGGGGGATGGCCTGGTGGCTCTGGCCAGCCGTGAACCTGCC
CCACACCTGACAGGGCTTTGGAGGGCATGTTCCGCAAGCTCAACCACCTCCTGGAGC
GCCTGCACCAGTCCTTCTTCCTCTAC
>21
TGGGATGGTGGTGGTCTTTGGGGATCCCCTGGCCCCAGGGTCCATCTCCAAGAGCCTG
TGTGCCCTGCAGGGTGTGGGGCTGGCCTCGCTCGTGGCACCTCTGCTGATCTCACAGG
CCATGGGACTGGCCCTCTATGTCC
>22
GGGGCAGGGGTAGAGGTCCCCTGGACATGCAGACAGCTTGTGGGTTGCCTCTGAGTC
CTTTGTCTTACAGGGTGGTAAGCACACAGGCCCCAGACAGGGGCTGGATGGCACTGA
AGCTGGTAGCCCTGATCTACCTAGCA
>23
AGCCCTTTCCCCCAACCTGGTGCCCATGCCCCCACCATGGAGCCTACCTTGATGTTGCT
TCTCCACCCAGGACCCTCTATGCTGCCCTGCTGGTGCTGACCAGCCCGGCAGCCACGC
TCCTTGGCAGCCTGTTCCTGTGG
>24
AGGTTCCCGGCTTGATGGGGCCAGGATGGGGCTCCTGGGTGCAGTCAGAGTTCCCACC
TCCCTCCCCTAGGAACTGCATCGGGCAGGCGTTCGCGATGGCGGAGATGAAGGTGGTC
CTGGGGCTCACGCTGCTGGCCTTC
>25

GGGGGCGCAGGACCCGGGAAGCCGCGCCTGGAGGAGGGTCGGGCGGGTCTCAGCCA
CTCCTCGCCCCCAGGCTCCCACTCCATGAGGTATTTCTACACCTCCGTGTCCCGGCCCG
GCCGCGGGGAGCCCCGCTTCATCGC
>26
AAAATCCCCCCGGGTTGGTCGGGGCGGGGCGGGGCTCGGGGGACCGGGCTGACCTCG
GGGTCCGGGCCAGGTTCTCACACCATCCAGAGGATGTATGGCTGCGACGTGGGGCCGG
ACGGGCGCTTCCTCCGCGGGTACCA
>27
CCACATGTGTGCTGGAGGAGTGTCCCATGACAGATGCAAAATGCCTGAATGTTCTGAC
TCTTCCTGACAGACGCCCCCAAGACGCATATGACTCACCACGCTGTCTCTGACCATGA
GGCCACCCTGAGGTGCTGGGCCCT
>28
TTTAATACATAAAAAAAAAAACCCGCTAGGTTTCCATGTGCTACTCACATGTTGCTTTTG
GCATTCCTCAGGAAAATACAACTGTATATTACTTAGTCTTAGATGTGCAAGAATCGGAC
TGTTCGGTCCTATCCAGGAAAT
>29
CTTTCATATCTGACCTGAGGAAGGCAGGATGCAATGACTCAGCAAGTCCTCAAGAGCC
TCTTTCTTACAGGTGATCGGACAATGTAAGGTAATAGCTACAAGACATTCCCATGAATC
TCAGGACCTCAGAGTGATTGACT
>30
CATTCTTGCCACCAAGCCACCATTAACATTTCCAGCCCTTTACTGTGACACTGCTATCTT
CTTTCTCCAGTCTCTTCAGCACTGGCCAATACCAAAGATAGTCCGGTCCTCATAGATTT
CTTTGAGGATACTGAGCGCTA
>31
AGTTATCTACTGCTTTTTTTTCCTTTTTCTTTGTCTGTTCTTGAAACTATTTTGATCCCAT
CTGTTCTAGAGAGGAGGGGAAGGAACTGGTTACTTCGTGGACTTCTCTGTGCGGAACT
GCCCCAGACACCATTTCCCCA
>32
AAGCTAACTCTGGCTGGTCATCTTCACACCACCTGGACACACACACTAACAGCTCCTC
ATTCCTTTGTAGGTCTTTGGATTCTGCAGAGCAGATTTGTTCTATGATGTAGAAGCCTTG
GACTTGGAAAGCCCGAAAAACC
>33
ATCAAGTCAAGTATCTAACATCTTCTTTTATGTTACATGATGATAGGCACTTTTCTGTGA
CCTTTTCCAGGAACATGAGAACATCAATGGTGTACCGCCTCATTTGGGACATCCCTTCC
ACTGGGGTGGGCATGAGCGTT
>34
GGCGTGGGCACAGGTCAAGCTGAAAGTCCCCGCCCAGGTATCCAAGTGTCCGCTGTGT
CCGCTCCCCCAGGTGCAGGGCGCCGCGCACTTCGGGCCGGTGTGGCTAGCCAGCTTTG
GGACAGTGCGCACCGTGTACGTGG
>35
CCTTCACTGCAGCCAGTCCCGTCGAGCCGTCCCCACCTTCCCGATGCGCACTCTCTCCT
CAACCCTGCAGGGAAGGCGAAGAATGGCAAAGGCTCCGCAGTCTCCTGGCCCCGCTC
CTCCTCCGGCCTCAAGCGGCCGCC
>36
GAGCTGGGCAGGCGTCGGGGGCGCCCTACCAGAGCCTCCCGGAACCCTGACGGCGCC
CCCTCCCGACGAGGCATCGCCGCGGTTCTGCTCGGCTCGCGCTTGGGCTGCCTGGAGG

CTCAAGTGCCACCCGACACGGAGAC
>37
GAGGTGGAAATGGGGGAATGTAAAGCTGTCCAGGGGTAGCGAGGTATTCACGTGCCTT
CTACCCACGCAGCTCAGAGGCACGTGGAGCGGCGAGAGGCAGAGGCAGCCATGAGG
AACGGAGGACAGCCCGAGAAGGACCT
>38
TCTTCCATAATCTGCACCCTCTGCTGGGTTCTCACACCCAACACCTCTCTTGCTTTCACA
TGTTTTTCAGGTGTCCAACACGCTCTCTTGGGCTCTGTATGAGCTCTCCCGGCACCCCG
AAGTCCAGACAGCACTCCACT
>39
GAAACAATGAGAAGGGGGCTGCAGCCCCTAGCCTCATCTTGTTGTCTCCATTTTGTGCT
TTGCAACCTAGACTGTACCCTGTGGTACCTGGAAATTCTCGTGTCCCAGACAAAGACA
TTCATGTGGGTGACTATATTATC
>40
TGAATATCCTCTTCTTCATGCCTGCCCTATTCTGAGCCCAAACTGACAAGTTTGTTTTCC
TTTGCACCAGACGCTGGTCACTCTGTGTCACTATGCCACTTCAAGGGACCCTGCCCAG
TTCCCAGAGCCAAATTCTTTTC
>41
ATTGGATTACCAATGAATCCCCCCATTATAGATATCTTTCATAGTAATGCTCACCTTCTTC
CCTTTCCAGATCCTAACACATTTTGAGGTGCAGCCTGAGCCAGGTGCGGCCCCAGTTA
GACCCAAGACCCGGACTGTCC
>42
GCTCTCTCACTTGATGTAGACCCATTTGAATGAATCTCCTTTTGCTTTCTCTTTCTTTAAT
ATTTTTCAGGTACGAGCAGGTGATAATAAATATATGCACTTGAAAGTATTCAAAAGTCTT
CCCGGACAAAATGAGGACT
>43
GACTGGGATTAGGGGTTGGGGTGGTGGTCCCAGGGGCATGCTCAGTGGTTCTCTTTTC
TCTGGGGCACAGGCTCTGGGCGTGGGGAGGCTGAGACACGGGAGTGCATCTACTACA
ACGCCAACTGGGAGCTGGAGCGCAC
>44
TGGCAGGGCAGGCCTGGGGGAGTCTTGCATCCCCCAGGTGAGGGGTATATGGAAAATT
CTGTGCCTCCAGGCAGGAGTGTGTGGCCACTGAGGAGAACCCCCAGGTGTACTTCTGC
TGCTGTGAAGGCAACTTCTGCAAC
>45
CCCTGTATTCTGGCTCTGGAAGTGTGAGGGTGGGCAGACTGTTTGACACAGGGCTCTG
TGTGTCCCCCAGTCACGTACGAGCCACCCCCGACAGCCCCCACCCTGCTCACGGTGCT
GGCCTACTCACTGCTGCCCATCGG
>46
TGGCTTGGGGCAGGGCAGGATAGAGGTGGGGAGGACAGGCCAGACCTTTTTAACCCT
TGCTCTCCCCCAGGACCCTGGGCCTCCACCACCATCCCCTCTGGTGGGCCTGAAGCCA
CTGCAGCTGCTGGAGATCAAGGCTC
>47
GTAGGGCAATGTGACTGCAGCAGGGGTAAGCCAGCCACTTGTCCCCCCAACCCCTCGC
CCCCGGCCTCAGGACAAGCAGTCGTGGCAGAGTGAACGGGAGATCTTCAGCACACCT
GGCATGAAGCACGAGAACCTGCTAC
>48

TGCCAGGGCTGGGCTGGGTCCTGTCCTGTACCCAGAATCTGTGCTCAAGTTCTGTGCC
GTCTTCTCTCAGGGCTCCCTCACGGATTACCTCAAGGGGAACATCATCACATGGAACG
AACTGTGTCATGTAGCAGAGACGA
>49
GTATCTTGGGAACCAAGGTGGGAGTTGGATCATGATGTTAAGCTTTATCTCTGCCCACT
TGTTTCCACAGGGACTTTAAAAGTAAGAATGTATTGCTGAAGAGCGACCTCACAGCCG
TGCTGGCTGACTTTGGCTTGGCT
>50
AACTCCCATGTCCCAGCTGTGTGTGTATGGCCAGTCACTGTAAATCCTGCCCTCCTCTG
TCCTCACATAGGTAGGCACGAGACGGTACATGGCTCCTGAGGTGCTCGAGGGAGCCAT
CAACTTCCAGAGAGATGCCTTCC
>51
CATCCTAGATTGAGTGATAGGGAGCAGTGGGACCTTAGAGTGATCCTGCCAGGCTCTCT
CTTTCTCCTAGGACCCGTGGATGAGTACATGCTGCCCTTTGAGGAAGAGATTGGCCAG
CACCCTTCGTTGGAGGAGCTGCA
>52
TCCCCCAAAGCTTTTCCTCACTGAAGGGTCCTAACAAAGGTGTCTTTCCTGTCTGCCCT
ATGCTGCTTAGGGCCTGGCCCAGCTTTGTGTGACCATCGAGGATTGCTGGGACCATGAT
GCAGAGGCTCGCTTGTCCGCGG
>53
TGTGTTAAGAGGGCATCTGCTGGGAAACGATTTGAGGAGAAGGTACTAAATTGCTTGG
TATTTTCCGTAGGAACCCCAGAGCGAAATACAGTTTGCAAAAGATGTCCAGATGGGTT
CTTCTCAAATGAGACGTCATCTAA
>54
TTTGTATTGAATAGACTCTCAGAAATCCAATTGTTGAGTAAATCTTCTGGGTTTTCTAAC
CTTTCTTTAGATGTTACCCTGTGTGAGGAGGCATTCTTCAGGTTTGCTGTTCCTACAAA
GTTTACGCCTAACTGGCTTAG
>55
GCTTGTTTTATGATGGCATTGGATGAATATAAATGATGTGAACACTTATCTGGGCTTTTG
CTTTATGCAGATATTGACCTCTGTGAAAACAGCGTGCAGCGGCACATTGGACATGCTAA
CCTCACCTTCGAGCAGCTTCG
>56
GAGAATTTATACCAAGCGTATTCTGAGTTTACTCACTTTCTCCACAATGTTTCCTTTTTT
CCTTGAACAGGAAAAAAAAAAAGAAGATGGGTTTTTTAAGTCCAATATATGTTATTTTC
TTCTTTTTTGGAGTCAAAGTA
>57
ACATAGAATTATGGCTATAACTATGGATTATACTATAATACAAGATTAATTTTTAATTTATT
TCTTTCAGAGATGAATCTTACAGTGATGTGTCCTTCTATTGACCCACTACATTACCACCA
TTTAACATACATTCGTGT
>58
AAAGGGACCAGGAGCAGCTGGCTTGCCTTGTGAACAATGACTTGGGATCTTTCTGTCC
TGTCTCTTGCAGTTGGTACCAACAAAGAGCTCTGCTGCCTCGTCTATACCTCCTGGCAG
ATTCCACAAAAGTTCATAGTTGA
>59
AAGGACGCAGGGGCCACAGGATTCCCCTGATGAATTCGTCAGTTCTTAACTCTTCCTCC
CTTCTCCACAGCCTCCTAACCAAGAGAGGCCGGCAGATCTGTGCTGACCCCAATAAGA

AGTGGGTCCAGAAATACATCAGC
>60
GCGCTGAGTGGGGCGTGTGGATGTCCCTGAGCCCAAGTGACAGAGCCTCCCCTCCCTC
GTGCTGCTGCAGGGCACGGCCCCACCCGGAGGCTGCGAGGCTTGGGGGTCCTGGCCG
GGGTGGCTCTGCTCGCTGCCCTCTG
>61
TTCTGAGTCTGCATGCTAGAGCCGCTTTCTGCTGCTTCTATTAATACAGCATTTTCACTT
TGTCTCTCAGGGTTTATGGTCCAGCAGAGTCTCAGAGCGCGGTCTTTGGAGATGTGTG
CCCCCTACTCACTTCTCTCTTG
>62
GAAAATTTTTCCTGGAATCTTCTTGGCACCCACGTGTGTTTTGCTGGACTGAATCTGCT
CTGAATTTTAGGTACAATGTTTGTGTTATGGCGTATGGACAGACGGGCAGCGGAAAGA
GCTATACCATGCTGGGACGCCAT
>63
TATGATTGGCTTACACTGAAAATCACTCTGTGAGACTTTCTGTAAGTTTCTCTTGTGTGT
TTTCCCGCAGGCTCATTTTGGAAAATACCTCAAGAAGCCCAAAGGTTGAAGTCTCCAT
AGTGGAAGTTTACAATAATGAC
>64
AGTCTTGGAAGTCGCCTTAGGTAAGGGGGGCCGCCCTCACTGTGTTCCCCACTGCTTG
CCTTGTCTGTAGGGCTGTCGGCAGCGCCTCGAAACTGATGGAGCTCGTTCATGGAGGT
CTGCAGCTCAGGGCGAAGCACCCC
>65
CTGAGGCAAAGCACAGCCTCATGGCTTCATGCAACTGTTTTCCTCCTCGTCGCTCCTTG
GTCCCTTGCAGCAGACCAAGCCTGCAGTGCCACCCTCCCCAGGGAGCAAACAGAGGC
AGGAAGGGCAGGAAGGAGCCGCAG
>66
GGAGCCGGTTTTGCTTTTCCTGCCTCCTTTCTTGGTGCAAACGGTGATGGTGCGTGGCG
GTCCTGTCCAGGTGTGTCTGGAGTGACCGGGTTGGCCCTGAGGGAGATGGCGTGCATC
AGCCGCAGCCTTGCGGCCCTGGC
>67
GCATTTCTGTTCCCAGATGATCAATTTCCACTGCTACGTTGTTTTGTTTTTGTTTTTTTCC
TCCCCTTAGACTGCAAATGGAAATGTGGAGGCAAAGGTTGTCTGTCTTTTCCGGCGCA
GGGACATTTCTAGTAGCCTCA
>68
GTTAGAGTTTCCAGGGTGGTGTTTTGCTGGATCCATTTCTGACTTGGTTTCTTCCTCTTT
AATCTCCCAGGGGAGTTTGAAGAGGAATCAAAGCAGCCAGGGGTGTCTGAGCAGCAG
CGCCATCAACTGAAGCACCGGGA
>69
CAGATACACCTCCCTCCCTGCCGCCTATGTGGATTGCTGCCTTGCTTACCTTGTCTCTCC
CCAATTCCAGGACTGCTTTTTTTACTCACTGGTGTTTGACCCCGTGCAGAAGACACTTC
TCGCTGATCAGGGCGAGATTA
>70
TTGTGAATGAAAAGACACCAACTGTTCCCCCCTGCCCTCTCCCTGTTGATGCCATTGTT
CTTTTCCCCAGGAGAATCTGATAATCGGAACCAGCAGAAGATGGAGATGAAGGTCTGG
GACCCAGACAACCCTCTCACAGA
>71

GGCTTAATTTCCCAGCCCACTTGGGCTTTTGACTTTTCTTTCGCTTTTGACTTCTCAATT
TCTCCCTTAGAGCTGTGGGAACCTTTGCAAGAGCCCTAGATTGTAGCAGCTCCATTCGG
CAGCCAAGCTTGCACATGAGT
>72
GTTTTAAGGAGCAGGAAGGGAGTGGTGCCAAAAGTTTCAGGTCCTCAGTTGCTCTCCA
TCTTCCCCATAGTTTCACGCCATGGATACCTTGCAAAGGAACGGCTACGACCTGGCTAA
GGCCATGTCGACCCTGGTACCCC
>73
GATGACAGGTGAGGGAACCAGGACCTGGGGCCAGATGCCAGCTCATTCTTCCTCCCTC
TGCCCCCCTTAGCTACCCTGGAAGTCACTTGCCAGCATAGTCCAGTTTTATTACATGTG
GAAAACCACAGACCGGTATATTC
>74
AGAGTGGAAATTGATTCTTTTCCTTGACCAGTTTCTTGTTTTTTTCCCTTCTCCTCGCCT
TCCCCGTTAGCACTAAGCCAAACCCTAACCAGATCATTTCTGTGGGTTCAAAACCTGGC
ATGAATGGGGCTGGATTTCAG
>75
GTGGCTCAAGAATCTGAGGGGCTTAGGTAGTATGCTTCTTCCCTGTCCCCCTCCTCTCC
GTACTCTACAGCCACACAGTCTGCTCAGTGGTATGCCTGGGGCCCACCTAACATGCAG
TGCCGCCTCTGTGCTTCCTGTTG
>76
GGGAATGGAAGAAAGAAGGGAGCCTCAGTGCTGATTTCTGCTCTCTCTCTTTCCTATG
CTCTTCTTCTAGGAGCCACACTCAAGGGGTCATTTATCCAGACCTGAAGCTCAAAGTCT
CTCTCCTTACACAACCAGCGCCA
>77
AGTTGGCTTCTGGCCTTAGAGGAGACCTGAATGGTGAAGCCCTTCAGAAGCTTTCTTC
TTTGTTCTCCAGGCTCCATTCGACTTCCTAAGGCCGCCAAGACTCCATTGAAGATTCAC
CCTCTGGTGCGGCTGCCCCTGGC
>78
AGAGAGATGGAAGTAACAAAGGGGAACTGCGTGGGAAGAAAAGGTGCGAAAGTTTT
CTGTTTTTTTCCAGTGGCCCAGGCACCCCTGAAACCAAAAACACCTCGGGGTACCAAG
ACACCGATCAACAGAAACCAGCTGTC
>79
TGGGCTTTTGTCTCTCATAGGAGTCTGAGAGGGTCCCTTCCCTCATTGTGCCATCCTAC
CCCTTTTCCAGATGGCAGGGGCAGGGGTTCCTTTCTCTGCCAATGGAAGGCCTCTGGC
TTCAGGGATTCGTTCAAGCTCAC
>80
GTGGGGCTTCACCTGGAGGTCTTAATGTTCTCTAAGCCTTTTCTGTTTCCCACTTGCTCT
TTCTGGCCAGGGCCCTACGGAAGGCTCTGACCCATCTGGAAATGCGGCGAGCTGCTCG
CCGACCCAACTTGCCCCTGAAG
>81
TATGTGTGTGTTCGTGTACATGTGTATGTGTCTATCAACTTATCTTTTTTTAAAAAAAAAT
TTATCTCAGGAGAACCATACAGACTTTTGACTGGCGTTGAGTACGTTGTTGGAAGGAA
AAACTGTGCCATTCTGATTGA
>82
GAAAACTTTTTCTCTGAGAAGTGAATGTACTGAGTGGTAAAAGACTTTAAATTATTTGC
TAACTTTTCAGAGTCAAACAGATGAAATCCCTGTATTGACATTAAAAGATAATTCTAAG

TATGGTACCTTTGTTAATGAGG
>83
TAAAGTACTAAAAATTGCCATCTCTGCAACTCTGATACTATGACTTTATTTAACTTATTCT
CATTTACAGAATAGAGTATGAGCCTTTGGTTGCATGCTCTTCTTGTTTAGATGTCTCTGG
GAAAACTGCTTTAAATCAA
>84
TAACTTAAAACTTTTCTTTAAAAAATGCTAGCATGTGAATATATATATTCACATGTTTTCT
TCATTGTAGACAATATGTGCACTCATTTGTGGACGTCCAATTGTAAAGCCAGAATATTTT
ACTGAATTCCTGAAAGCAG
>85
TCACTCCGTTTACAATTTAATAGCAAACATAGTAGTATATAATTAGAATATACTTTAATTAT
TTTTACAGTTTTTACCCACCTCTTGATGAACCATCTATTGGAAGTAAAAATGTTGATCTG
TCAGGACGGCAGGAAAGA
>86
CAAATCAAATTCTTATGTGTTCAAAAAATTACTACATTTTACTTTAAAAAATGTTTCCTT
CCATTTTTAGCATAAGAAATTGAGTTCCGCAGTTGTCTTTGGAGGTGGGGAAGCTAGGT
TGATAACAGAAGAGAATGAAG
>87
CTTGACATTATCTGAATAAAAGTTGCTAGTTTTAATATATTATCATACTTAAACTATTTTAT
TCTAATAGGCAAGGTCTTAGACCTATTCCTGAAGCAGAAATTGGATTGGCGGTGATTTT
CATGACTACAAAGAATTAC
>88
TTTAAGTGACCAGATGTTCATCTTCTATCTAGCAGTTGTGATTCTTTCTTTCTACTTGTGT
GATTTACAGGATTAAAGACAACAACTCCAGGACCAAGCCTTTCACAAGGCGTGTCAGT
TGATGAAAAACTAATGCCAAG
>89
GATCTTTGTTTCTCTATTAAAGTTGCTGTAAACTTGATTATTTACCTTGCATTCTTTTCTT
TTTCTACAGGGATTTGAGTGAAAGGCCAAAAGAAATCAAAGTCTCCAAAATGGAACA
AAAATTCAGAATGCTTTCACAA
>90
GCCAAACTAAATGGTTACTTAGCTGTGTTCATTTTCTTGTTTTGTTTTCTTCTTTTTTTTT
AAATCTAAGGGAAAGGGATGAAGAAAATCAAGAAATGTCTTCATGCAAATCAGCAAG
AATAGAAACGTCTTGTTCTCTT
>91
CCCAAATGACAAGTGACTTTTCTTTGTTATTTCTTCTCTTTCTTACCTATCCATCTTAACC
CCATTTAAGAACAATGACAAACTTCAGGATGATAGTGAGATGCTTCCAAAAAAGCTGT
TATTGACTGAATTTAGATCAC
>92
AATCTTTTTAAAAAAAATGGTCATTTTTGTTTGTTAATATGATTTACTGTACTACTTTTATTA
TTCAATAGGTCACATATCCTGGAGCAGGAAAACTTCCACACATCATTGGAGGATCAGAT
CTAATAGCTCATCATGCTC
>93
AGTTCTTTACCTTTAAAAGAAATACCATCCCTTATCTAAATATCATTTAACCCCATTTCTT
TCTTTGCAGATACAATCCTTATTTAAAAAGGAGAAGATAACTGAGGATTTTAAAAAGAA
GCCATGGAAAAACTTCCTAG
>94

CCTTTCCCGCTCCGCCTCCCCTCGCTAGGCTGCCGCCTCCTCCCCCATCACCGCTCCCC
TCGCCCTGCAGGCAAGCTGTCCTTGGAGGAGTTCATCCGCGGGGCCAAAAGCGACCC
GTCCATCGTGCGTCTGCTGCAGTG
>95
ACAAACTGCTGGTTCTGAACTTGTTAGTTTGTTCTTCCTAATTCTTCTCCCTCACTTTTC
TCCTTTCCAGAATCATATTCTAAGAACTCAGCCACTCAGGTATCCACCATGGTGCTGGG
TCCTGAACAGAAGATGTCAGA
>96
ATACTAAGGATGAATTGTTAGTGTATTTTGTTCATTTTTTACTAATTTATTTTCAAATCTGT
TAATTCAGACAGTGTTTCTGGAGATCATGGGGAGTCTGCCAGTCTTGGTAACATCAACC
CTGCCTATAGTAATCCCTC
>97
GATAACAGACCAATTATATCAGTAGTTCAATGGTGATGTTCAGTTTAATCTCTTTTCCTC
TCTTGTACAGTACTCTTGTTTTAGCTTTCGTAAACTCTGGGCTTTCACCGGACCAGGTT
TTCTTATGAGCATTGCCTACC
>98
CAACTAAACAAACAAACAGACAAAAACTCAGTTGTTCAATGAGTGTTACAGGAGATTT
TGTTCTTCTTAGTTGCTCTGGATCCTTCTGTTGGCCACCCTTGTGGGGCTGCTGCTCCA
GCGGCTTGCAGCTAGACTGGGAG
>99
TTCCAAATTGTGGAGGAATTTTTTAGAAATGATTAATCAGAACCGTGTCTTTCTACTGTT
TGATGTTTAGGTCCCACGAGTCATCCTGTGGCTGATGGTGGAGTTGGCTATCATCGGCT
CAGACATGCAAGAAGTCATTG
>100
GGAAGAGAGTGTTTTTCAATGTTTCCTTTCTTTCCGTCTCTCTGACTTCCATATCTCATC
TCTGACCTAGAATTCCTCTGTGGGGTGGCGTTCTCATCACCATTGCAGATACTTTTGTAT
TTCTCTTCTTGGACAAATAT
>101
GTTCCCTGGTACTATGTTACTCCCGTTTCACGTTCCTGTAAAGGCTAATCATTGTTATGT
TGGTATGTAGTATGTTACAGTGAAACCCAGCCAGAGCCAGGTACTCAAGGGCATGTTC
GTACCATCCTGTTCAGGCTGTC
>102
AACTCCTGAAATGTCACAAGGAAACTTGTTCTAACTGGTCAAAAAGTGACTGCTGTTG
TTATCTTTCTAGTCTAGACAGGTAAACCGGAACAATAAGCAGGAAGTTCGAGAAGCCA
ATAAGTACTTTTTCATTGAATCCT
>103
ATGTAGGAGAGCTCTTATTTGGTCCTTGCTTCTGAAACAAAAGTTGCTGTTGAATTGCT
TTGGGTTCTAGGTTGAAGTCTGTACAAATACCAGCAGTCCTCATGCTGGCCTCTTTCCT
AAAGATAACTCGACACTGGCTG
>104
AACTGCTGTTTTGGATATTGTCTGAGCAGATTATTGCCACAATCATATCCTTCCTCTTGG
CCTCTCACAGGGTGTTGTGCTGGGATGTTACTTTGGGCCTGCTGCACTCTACATTTGGG
CAGTGGGGATCCTGGCTGCAG
>105
CCTGAACCTCCCCAAAACTTTTTTTTGTTTCCTGTTGCAGACAGTCATCTGCTCCTATGT
TTTCTTCCAGGGATTCCTGAACCTAAAGTGGTCACGCTTTGCCCGAGTGGTTCTGACTC

GCTCTATTGCCATCATCCCCA
>106
TTAATTGTGCTGTTCTCTAGGGTGATACAAGGACCCATAGTAATCCCATCTGATCTTCTT
TCTTTTCCAGCTTCCCTTTGCTCTCATACCCATCCTCACATTTACGAGCTTGCGGCCAGT
AATGAGTGACTTTGCCAATG
>107
TCCTGTTTCCTTTTCTGTCTGTGGCCAAGTTGGATTATTCCATCAGTGACTGTGCTGATT
TCTTTTACAGAGGCTGGCGGATTGCAGGAGGAATCTTGGTCCTTATCATCTGTTCCATC
AATATGTACTTTGTAGTGGTT
>108
GGGAGCTAGCCTGTGACAGTGTTCAGCCCCCTTAATAGTAGGTGTATCTGAGTGTTTGG
ATTTCTCCTAGCCTCAACTTTCAGGAGACCCGTCCGTGGCCTTATTTATTCCACCCTTCC
TGTACATCGTAGCGAATCAAT
>109
GAATCCTCCTCCCGCTGACCTCTGCCCCTCCTATGTCCACAGCCTCTCAACTCAGCTGT
TTGCTCTCCAGGTACCCCTGGGATGGCGTGAGCACTCCCCCAGCGATGGACCCATCTG
TGACGCTGTGGCAGTTTCTGCTG
>110
GTGGCCCACAGGAGAATCCTGTGGTCCCAACAATCCTTACACACCCTCTTTGTGCCTG
CTCCTCTACCAGGTCATCCTGACCCCGCCCGAGGCCCCAAACCTGAAATCGGAAGAGC
TTAATGTGGAGCCGGGTTTGGGCC
>111
GACCAAGTCCCTTCCAGCATCCCACATCTACTTTCTACTCACTTGAGCTCCGACCACCC
TCTCCCCACAGACCCCGGTGCTGCTGACACCCAGCTCGCTGCCTCCTAGCATTCACTTC
TGGAGCACCCTGAGTCCCATTG
>112
GTGGTGCCACTGCCCATGTGCTGCCCGGAATGTGGGCCACTGACCCGCTCCTATCGCC
CATTCGCCCCAGGTGGTGCAAAGGATCAAGGCTGTGGAGGGGCAGACTCGGCTGCTG
GTGGTGGACCAGGAGACAGATGAGG
>113
ACCACCGGGGAGTGGCCTCTGGAGGCGGGAGGCTGGAGCAAGAGACTGACCCTGTCC
GTTGGGCCTGCAGGATGTCAGTGGGCCCCTGAGGGAGCTGCGCCCTCGGCTCTGCCAC
CTGCGAAAGGGACCTCAGGGCTATG
>114
CACACCTGGCCACGCGGCGTTGGGGGCTGTCTGGGCCCATGGCGGGGCTGATACATGC
TGGTGGCGGCAGGTGAACGGGCAGAATGTGGAGGGACTGCGCCATGCTGAGGTGGTG
GCCAGCATCAAGGCACGGGAGGACG
>115
GGAGGGGCCACCGTCTGGGGTGTGGGACTAGGGCTCACTCCAGACACCCCACCCACC
GTGCTCACCCCAGGATGGCAGTGCCTGGAAGCAAGATCCCTTCCAGGAGAGCGGCCT
CCACCTGAGCCCCACGGCGGCCGAGG
>116
GTTGGATGGGCAGAGCCTGGGAGAAATGTTTTCTGAATGGCCTGGGGCTGGCATTTAT
CTTTCCTTTCAGCAAGCACCTCAAATTTGCCATGCTGGCTGGTGGAAGAGTTTGTGGTA
GCAGAAGAGTGCTCTCCATGCTC
>117

TGAATGGCAGCCTTTTTGTTTATCCTGTTGCCCCTTCCTAACATGTACTTTGCTCCTTTTC
TCCTGGCAGAAAACTACCCCTGAGTGTGGTCCCACAGGATATGTAGAGAAAATCACAT
GCAGCTCATCTAAGAGAAATG
>118
AGTAGAAGGGAAATTAGAGGGAGAAGCTGTCTGGCCTTCCTTGGGAATAACTATTCCT
TCCTGCCCTCAGCTGCCGCTCAGCTTTGATGGAACAACGCTTATTTTGGAAGTTCGAAG
GGGCTGTCGTGTGTGTGGCCCTG
>119
TCCAGGATGCCTTTGCTTTAAAACCTTTTAAAAAGAAGTGAATGATACACCCCCTTTGC
TCATCTTGCAGTGGAACCTGGTGTGTGAGGACGACTGGAAGGCCCCACTCACAATCTC
CTTGTTCTTCGTGGGTGTGCTGT
>120
CCCACTTGGTGGAGCCCATTCCTGCTGCCCTTTTCCAGCTGGTTATCTGTCACTCTCCTT
TTCTTCCCAGGTTTGGCCGGAAGAATGTGCTGTTCGTGACCATGGGCATGCAGACAGG
CTTCAGCTTCCTGCAGATCTTC
>121
ATACCATAAAAAATTAATAAGGAAGGAACCCAAATTAAACTGCTAACTCGACCTCCCTT
GTTTTGAACAGGGACAGAAATTCTTGGCAAGTCAGTTCGTATAATATTCTCTACGTTAG
GAGTGTGCATATTTTATGCATT
>122
GTGCTCTACCTGGTCTGTGGGTCTGCTGTTGGCAGGGAGGCCTCACTGAGATTGGACC
TTGTACTGCCAGGTTCATCCCTGAGTCCCCCCGATGGCTCATCTCTCAGGGACGATTTG
AAGAGGCAGAGGTGATCATCCGC
>123
GTATGTTATTGTGTGCTCTGAGTCTCTGACCACCTCTTCTTCCCATACACTTATGATGTT
GTTCCTGCAGTTACAAGACCTAAGTTCCAAGAAGCAGCAGTCCCACAACATTCTGGAT
CTGCTTCGAACCTGGAATATCC
>124
TGTAAAAGTTGTACAGGTTGGGAAAGATGTGGATACTGCTTTTCCAGCTTTCTTCTGCA
CTCTGTTTCAGGATGACCATATCAGTGGGCTATTTTGGGCTTTCGCTTGATACTCCTAAC
TTGCATGGGGACATCTTTGTG
>125
TGTTTTGCTCTCAATAGCTGCATGCCATGGGTTGGTACCTACTCCTACCCTCTTTCCTTT
GCTTCTCCAGACTTGTATTATTTGGCTACAGTCCTGGTGATGGTGGGCAAGTTTGGAGT
CACGGCTGCCTTTTCCATGGT
>126
ATAAAGGGGTAGATGAGAGACCAAGTCTAACTGCAGCCCTGGGCCTGAGGCTCCGTCT
GCTTTGCCATAGGTGCCTACGACCGCTTCCTGCCCTACATTCTCATGGGAAGTCTGACC
ATCCTGACAGCCATCCTCACCTT
>127
TTGTTTGTTTGGAGACTGGGAGGCATCTTTTTAAAATGTGTTACTGACATATTTTTGCTT
GTTTTTATAGAATGAAACACAGAAAAACTCCAAGTCACACAAGGATGTTAAAAGATGG
TCAAGAAAGGCCCACAATCCTT
>128
TGCAATGTAAGTTTTCTCACATACACAGTTACAATCCTCCCTTTATCAGCTCTACATTTTT
TTTTAATAGACTGAATCTCGCTCTGTTGCCCAGCCCATCTCGGCCCACTGAAACCTCCA

CCTCCCGGGTTCAAGCGATT
>129
ATGAAAACTTTGTTGAATTAATACCTTTGTTGTGTTTTATTCACACTGTATATACAATACC
TGGATGAAGAGAGATATTTTGAGCACTATTAAAATACATGCATTGGCTCAATACATGAAT
TGGATCAATTCTGGAACCC
>130
CTTCTCTTTCTCTCACCCTAATTCAACCTATTGCCAAGTATTGTACATTCTCCATGCTTAA
AATCTCTAGGCGGTGTCCCTGAAGTATAAATCCAAAGCCTTCATGCCTGTAATCCGAGT
GCTTTAGGAGGCCAAGGCAG
>131
ATAGTTAGAAAATAGAATTTAATGTTCCTTTTCTCGATGAATTAATTTGTTTGTCTTCCAT
TTTTAAAAGCATGCTGATCCCATTTTCAATGAAGAATTGCTTCCAGTTACTTTGTAACTG
CCAGGTCCCAGCAGCTGGC
>132
GTACCCGAGCCCGGGAGAAAGTGTTCAGTTGACCCAGGCTGAGTGTGTTATCACCCTG
TTTCATTTCCAGCCATGCCTTGTGTTCAGGCGCAGTATGGGTCCTCGCCTCAAGGAGCC
AGCCCCGCTTCTCAGAGCTACAG
>133
ACGTGGCGTCTGACGTCTCCAGGGAGACCCGCCGCCCTCGCTGCCGCCTCACCGCGCT
TCTGTTTTGCAGGTAATCTTCAGCAAGTACTGCAACTCCAGCGACATCATGGACCTGTT
CTGCATCGCCACCGGCCTGCCTC
>134
ATGTGTACATTCAAGTGTACATCCCAGAGCACACTATGGGCGAAAACACGGGCTTGGG
TGTGTTTTCCAGGAACACGACCATCTCCCTGCTGACCACCGACGACGCCATGGTCTCC
ATCGACCCCACCATGCCCGCGAAT
>135
ATAAAATAGACCTGGACACTAAGAGGAAACTTCCCATATTTGGAAACCCAGTCCTGCC
TGCTTTTTATAGCTGGTGTCGAGGACAAGAGAACCACAAGCCGTGGCCAGTCTGCTGA
GAGACCACTGAGGGACAGACGGGT
>136
TGCTTAACCGGTTTTGGCTCTGAGAATTTTAAGGGTCATATTTACTCTCTCTTTTCTTTTT
CATTCCTAGAGCATTCAAAATCAATGAACTGAAAGCTGAAGTTGCAAATCACTTGGCT
GTCCTAGAGAAACGCGTGGAA
>137
TTACCAGCCTTGAGAGATCCTCCACATTCGTCCGTGGTAACCCCCTTGCTGTCTCTCTC
TCTGTCACCAGTGGAAGGACTAAAAGTGGTGGAGATTGAGAAATGCAAGAGTGACAT
TAAGAAGATGAGGGAGGAGCTGGC
>138
TTTGCTGTGCTGAGGGCTTCACCAGACTGAAACAGCAGGACCACAGCTCATCTCTGCT
CCTTCTCTCTAGGACCAACTGCCCCTGTAAGTACAGTTTTTTGGATAACCACAAGAAGT
TGACTCCTCGACGCGATGTTCCC
>139
TATCTCAGCATGCACATCACTGTCACAGGCCCACGGCGCCTGGTTAACACGGAACTCC
TGTCCTTTCTAGTACCTGCTCTCTCCAGAGACCATCGAGGCCCTGCGGAAGCCGACCTT
TGACGTCTGGCTTTGGGAGCCCA
>140

GGAAGGAAGCCAGGGGCTGGGGAGCCGGCCACACAACTGCCCAGTGCCTATCCTTCT
CCTGTGCCCACAGATGCTGAGCTGCCTGGAGCACATGTACCACGACCTCGGGCTGGTC
AGGGACTTCAGCATCAACCCTGTCA
>141
CTGGGTGGCTTTGGGAGGAGAGGTGGGCGGGCCCAGGCACAGGGTGACTCGGACCCC
CTGCCTCCCGCAGTTCTGCGTCCACGACAACTACAGAAACAACCCCTTCCACAACTTC
CGGCACTGCTTCTGCGTGGCCCAGA
>142
CACCCCCCCTCACCCCATCCCACCCTCCGAGTGAAGAGAGCAAACACCTACGCCCTGT
TTTCCAATCCAGGAGAAGTTCTCACAAACGGATATCCTGATCCTAATGACAGCGGCCAT
CTGCCACGATCTGGACCATCCCG
>143
GTGTTGCTCCCCCGTGCAGGTACCTGGTGAGGGCGCCTGAGTCTCCCCTCACTCTCTC
CTTGCCTCCCAGGTACCAGATCAATGCCCGCACAGAGCTGGCGGTCCGCTACAATGAC
ATCTCACCGCTGGAGAACCACCAC
>144
CACAGTAGGCCTCCGCTGAATAATTGTTCAGACTATACCCGTGTTAACACGTTGTTCTC
TCGCTATTTAGGGAATGATCACATTAATCTTGGCCACTGACATGGCAAGACATGCAGAA
ATTATGGATTCTTTCAAAGAGA
>145
AGACCCGAGCAGCAGCAGCAGGCCCGTGTTCTACCTCCTGTTACCTCAATTCCAAAAT
CTCTTCTTTCAGCTGAAGATGATTTTGATAAAATGCTGTGATATCTCTAACGAGGTCCGT
CCAATGGAAGTCGCAGAGCCTT
>146
CTCTGAGAACAGCGATCTGGTTATGGGGCATTTCTGTCTCTAATGTCACTGTCTGCTGC
ATTCCCTGCAGAGCGACCGTGAGAAGTCAGAAGGCCTTCCTGTGGCACCGTTCATGGA
CCGAGACAAAGTGACCAAGGCCA
>147
TGTTTTTCTCCGACGTTTCCCATTGCCTTAAGAACGAGGGACTCTGAATAAATCCGTGT
GTCTCTCCCAGCTCTTCCCCATGGTTGAGGAGATCATGCTGCAGCCACTTTGGGAATCC
CGAGATCGCTACGAGGAGCTGA
>148
TGGGAGAGAGGCAGACACTCCCCTGCTGTCTCCTGCTCCCTGACTTGGCCTTCTCTGT
ACTCTGTTCCAGTTACAGAAGAAGACTGACAGCTTGACGTCTGGGGCCACCGAGAAG
TCCAGAGAGAGAAGCAGAGATGTGA
>149
GATTAAAGGTGTGAGCCACCGCGCCCTAATTCTAACAAAAACAAATCAGTCATCGTTTC
CCTTCTTCCAGGAGACTGTGCCTGAGGAAAGCGGGGGGCGTGGCTGCAGTTCTGGAC
GGGCTGGCCGAGCTGCGCGGGATC
>150
TCCCAGTAGCAAACCCCCAGCAGGCCACTTTCCTTATTTATCACTAAAGCCCACATTAA
CCTTGTTACAGGACAAGAGGGAGAAAGTTGTCTGAGAACTTTTGACTGTGGCCCTGG
ACTTTGCTGTGCTCGTCATTTTTG
>151
TGAGTCTACATATGCCCAGCTGAATCTCTGTCGGGAAGAAACCCTGAAGCTTCCTGTGT
CTGTATTTCAGGGAGGAGATTGGACAGGCTGGACTCCCCATTGCTTTTCTAAAAATCTT

GGAAACTTTGTCCTTCATTGAA
>152
TGTACCCGAGCCCGGGAGAAAGTGTTCAGTTGACCAGGCTGAGTGTGTTATCACCCTG
TTTCATTTCCAGCCATGCCTTGTGTTCAGGCGCAGTATGGGTCCTCGCCTCAAGGAGCC
AGCCCCGCTTCTCAGAGCTACAG
>153
AATTTTCTCCGAGTTGCCTGATTTTCAAAAGAAGAAAAAAAATGTTGGTCTATATTGTC
TCCTTTTGCAGCGCACAGTGCAAAAAAATGCAAAATACGTGTGTTTAGCAAATAAAAA
CTGCCCAGTGGACAAGCGTCGCC
>154
GGGGAGGGACCGAGGCAGCAGCTGGGCCTGGGCTCGGAAAAGCGGCGCTAACAGGG
CTCTTCCTTTGCAGTGGTTCGCACAGACAGTTTAAAAGGCCGGAGAGGTCGTTTGCCC
TCGAAACCGAAGAGCCCACAGGAGCC
>155
GGGGTTAGCAATTTCACGGGTTATATATTTTAGAGAACCTCATTAAGTGCTTTTTAAAAT
GAAATTCCAGTTCCAGGCGAACCCTGACTATCAAATGAGTGGAGATGACACCCAGCAT
ATCCAGCAATTCTATGATCTCC
>156
TCAAGTAATTAGTTGTTTAGCTTTTCTCTCTCTCTTTTTCTCCCCTCTCTCTCTTTGGTAT
TAATTGCAGGTCCAACCCAGTGGAGGGTAAACTCATCTTTTGCAATGGGGTGGTCTTG
CACAGGTTGCAATGCGTTCGT
>157
CTCCTTGGTTTTGCCAACTAGCTAACTGTCTTGTACATTCTTCTTTTGTTTCTGATTATGT
TTTCTGCAGAGAGACACGGGCTCAAGGAACCCAAGAGAGTGGAAGAACTGCAAAAC
AAGATTGTAAATTGTCTCAAAGA
>158
TTCCACGGGGGCTGTAAGATGGCTTTTTAGGATTGGTCTAATCAGATACTCATTTCTTTT
CTCTTTCTAGGTTTTGAAACATGAATCTTTCGCTCGTCCTGGCTGCCTTTTGCTTGGGAA
TAGCCTCCGCTGTTCCAAAA
>159
GCAAATCGGCTTGGTGAACATGGGTTGGGTAAGTAGACATAAAGTCCACCAGCTATATG
TTTGCCTCTAGAATGAAGAAGGATGGAGGAGAGCAGTGTGGGAAAAGAATATGAAAA
TGATTGAACTGCACAATGGGGAAT
>160
CTCTGTGCTGCTTCTCAGTTCTTCACCAAAATAGTCTTGCTTTTAACATTTTATTTACTTC
TCCTTGAAGACCAATGAAGAATTCAGGCAGATGATGGGTTGCTTTCGAAACCAGAAAT
TCAGGAAGGGGAAAGTGTTCC
>161
AATTCAATTATGGGGTACTGTATTTGTATTGGTCTTGTAAATTTACAGCTTTTTTTTTCTC
CCTTTTTAGAAACAGTGTGGTTCTTGTTGGGCTTTTAGTGCGACTGGTGCTCTTGAAGG
ACAGATGTTCCGGAAAACTG
>162
GGTTCTAGAACTAATGTTCCCCAGGAGGTAGATGGTAGTGATCAAGTCTCTCCCCCTTT
AACTTTAAAAGGATGAAATCTGTAAGTACAGACCTGAGAATTCTGTTGCTAATGACACT
GGCTTCACAGTGGTCGCACCTG
>163

GGTGGTAGGGTGGGTTACTGTCACATGTCGCTTGGAGCTTCTCACCCCAGCATTAATTT
TACTCTCCCAGGCATTTATTTTGAACCAGACTGCAGCAGCAAAAACCTGGATCATGGTG
TTCTGGTGGTTGGCTACGGCTT
>164
CTATTATTATTAATGGAGCCAATTTTCCCTTGATTCCTTGTATAAAATGGAAAACCTGCTC
CTCTTTCAGCTGGGGTCCAGAATGGGGCTCGAATGGCTATGTAAAAATAGCCAAAGAC
AAGAACAACCACTGTGGAATC
>165
AAATATTTCTGCTTGAGCCATAAGCGGTGATTCAGAAATATGAAACCTGACAAGCTTTG
TTTCTTTACAGGTCACTGGATCTGAGAAACTTCCCAGGGGACCGCATTCCAGAGTCAG
TGACTCTGTGAAGCACCCACATC
>166
CCCTGCATCTTGAGGAATGCTGTAACTTCCAGTCCTGGCCTCTCACTTAGCTTCTCCTT
CTGGCCCTCAGGGAGACAGACGATGCTCACCCAGTCAGTAAGAAGAGTCCAGCCTGG
GAAGAAGAACCCCAGCATCTTTGC
>167
GGTGAGAGGGAAAGGAGAACTGGAGCGCACCAGGATCACGGCTCCTCCGCAATTTGG
CTATGTCTCTCAGGCCCTGGTGAGTGGACAACATTGTTCAACATCGACTACCCAGGCG
GGAAGGGCGACTATGAGCGGCTGGA
>168
TCAGGGTCAGATTTCCTGACAGAGTCCCCTCAAGAGGGAAATCAGACCGTCTGCTGAT
CTCACTTTGCAGGATCCCTGCGCCGAGACACAGAGCGCATCTGGAGCCCATGGTCTCC
CTGGAGCAAGTGCTCAGCTGCCTG
>169
CTAGGCTCTGTAGAACCTGTCACACACACACACAAAAACCCAAAATGCTATCTCTCAT
CTTCTGTCCCAGAGACTCCATACATGGTGATGAACCCTGAGACAAAAGCACGGAGAGC
TGGGCAGAGCGTGTCTCTGTGCTG
>170
TCCTGGCACTTTTCTAGGAATACCTGCCCTCCCACTGCCCATTCAGGTTCTACCTCTCCA
TCCCTTGCAGGTATCATAATGACACATTGCTGGATCCTTCCCTCTACAAGCATGAGAGC
AAGCTGGTGCTGAGGAAACTG
>171
GTGAGGTTGAGGTACTCTTTGTGTAGGATGTAAACTTACATCTTAATTTCCACACCATTT
CCCTACACAGCATCTGATGAGACTCCTTGCAACCCAGTTCCTGAGAGCTATCTTATCCG
GCTGCCCCATGATTGCTTTCA
>172
CTTAAAAAAATAAAATAAAGTAAAATACATTCAAGTCAACTGATTTGAGTCTCGAACTG
CTCTGACACAGGAAGGTGCTGGGGATACATCAGAGGTGATGGATACTCAGGCGGGCTC
CGTGGATGAAGAGAATGGCCGAC
>173
CTTTAAAAAAAGAAAAAGAAGTGTGTGTGTTCACAAGTTCACTAATTCGTATAATTTGG
CTCTTCGGCAGATCCTGTCTACCTTTCATGACCAACTACAGTTTTCATTGCAACGTCTG
CCATCACAGTGGGAATACCTAT
>174
ATCGAGTATTCCCTTGGTAGTAGAGATGTAGTACTACAGATAACTGTTTCTTCCTTTTTG
TTTTTGATAGACTTGAAGGAAATGTGCCTTAGTGCTTTGGCCAACCTGACATGGCAGTC

CCGAACACAGGATGAACATCC
>175
GCTGTTTTCTTTCTTTGTCAGCATATCTTTGATTGCAGCAGTAATAGTCTTAATTTATTTT
GAAAATTAGGATATTATACCATTTATTGATAAATACTGGGAGTGCATGACAACCAGACAG
AGACCTGGGAAAATGACTT
>176
TGGTTGATTATTAATTCCATACTTTTAAGTATAAAATGTTCATTAATAGTAAAACACTACT
TCTTTTTAGAGTAAAGAAAGAGATGTATTCTTGGTAAAGGAACACCCAGATCCAGGCA
GTAAAGATCCAGAAGAAGATT
>177
TCTTGTTTTATGAGCATCCAGATTGACTGACTTACGTAGACTCCTTTTTTAATTGGATGTA
TTTTTGCAGGACCTTAGTAACATTGGTCCTGCTTATGACAACCAAAAACAGAGCAGTG
CTGTGTCTACTAGTGGGAATT
>178
TATGGAATTTCAGTATCCACATGGTGTTTCCCTTGTGGCTCATTGAAAGCAATCTGCCTT
CTCTTCAAAGGGGGAATTGCAGCAGGAAGCAGCGGAAAAGGACGAGGAGCCAAGCG
CAAACAGCAGGATGGAGGGACCAC
>179
CTTACACAGTTGAATGCATTTGAGCTAAAGGGGGTTTTAGTTTTTTGATGTTATTTTTTC
CTTTCTGCAGTGACCCTTTGTTTTCTGCTCAGCGCCTTCCCCCTCATGGCTACCCATTGG
AACACCCGTTTAACAAAGAT
>180
GTTTTTGTGGCAATTGTCCCCAAGAACCTCAAATAAGTTGCAGGCCTTCTTTTAATCTC
TGTCACCGCAGCTCCCCAGTTAAAGATCTCAGATGACCGGCTGACTGTGGTTGGAGAG
AAGGGCTACTCTATGGTGAGGGC
>181
CTTTTTCCTTCAGCAAACTAGATTTGACTTGCAATCTTCTGACTTCCTGTGTATGTTTTTA
TTGGGACAGGAAACCTTCAAGCTCCTTTAGGTTATGATAAATTTAGCTATTCTTGGCGG
AGCAAAAAGGGAACCAAGTT
>182
CGGATGCGTTTTTTTCATGATGATGGAGCTGATGCTTTATTGTGCCTTTTTTTCTTTCTTG
GTTTTCAAGGCTTTGATAAAATTCAAGAGTTATTTGTATTTTGAGGAAAAAGACTTTGT
GGATAAAGCAGAGAAGAGCC
>183
TTCCTAGAGAGTTCTTTTTTTGTTTGTTCTTTTTTACAGTTCTGAAGTAAAACCATGGTT
TTTGTTTCAGATAATATTTTATAAAAATGGTGTCAATCAAGGTGTGGCTTACAAAGATAT
TTTTGAGGGGGTTTACTTCC
>184
GGCTGCATGTGTCCTCAGCATCTCCGTGGCTGCTGTAGTCACCGTGTTCTGTTTCTCATT
CCTCCTGCAGATGAGTGACATGGGCTGGGGCGCCGTGGTAGAGCACACCCTGGCTGAC
GTCTTGTATCACGTGGAGACAG
>185
TAAGGGGTGAGTGATTTGAACCAGGCTGCACAAGGAGAATCAGTCTCATGCTCTGTGC
CCCAGCTACCAGCTGGAAAAACCACCAGAGAAGGAAGAGGATCACGCCCCCGAGTTT
ATTAAAGTCAGGGAAAACCTGCGGA
>186

GGCTTGTGGGAGGAATGAGTCCCTTTGGCAGGTTCTCGGGATCCCCTGGGTGACATGC
CTTTCTCTGCAGGAGGCACCATGCAGGAGCTGCATCTGCTCTGGTGGGCGCTTCTCCT
GGGCCTGGCTCAGGCCTGCCCTGA
>187
GGGTTATTATCTTCCGCTGCCCGCCGCCCCCTCCCACGCCGGCCCAGGCTGAAGTTGAC
TCTGCCCGCAGGCCTCCAAAGACTTCATATGCTCCAGATCTCCTACTTCCGCGACCCCT
ATCACGTGTGGTACCAGGGCGC
>188
CTTGGTGGGCCTCGCCCCACACCTGGCACCCTCTCTGCACAGTCCCCTGATCCTGACT
GTCTATCCACAGTTCCTCTGACCATCCGCTGCTTCCTGGGCTGTGAGCTGCCTCCCGAG
GGCTCTAGAGCCCATGTCTTCTT
>189
TGGGGTTTGACTCAAATCATGGACTCCTTGGGGGCCTATTCTTCGGGCTAACTCTTTGC
ATGTTCTGCAGGGAGCCAAACAAGCCGCTCCTACACTTCGCTGGTCCTGGGCGTCCTG
GTGGGCAGTTTCATCATTGCTGG
>190
CCTGGGCAGCCTTGGGGGCTCCCTGGGGCCAGCTTCCTGACACTTGCCCTACTCTGTG
CTGCCCCTGCAGGTGTCTGAGATGCTGCCACCGCAGAAGAAGCCCTGGGAGTCCATG
GCTAAGGGGCTGGTGCTGGGCGCGC
>191
CTCTAACTACCAGAGTCCTGAGGGCCCCAGCACCCCCCTGAGGCTCTCCCCACCTCTC
TGTCCCCAGCAGGACCCCGGAGGCCGCAGCGTCCTGCTCTCCACCTGCACTCGAGCC
AGAGGCAGTGATCCGGGCCAACGGC
>192
TGAGGGTCCAGGTTGGCTTCAGAAGTACTATCTTACTTCTTCATTCTTTCCACCTCTTCC
TTCATTCCAGGATCTAAGAGAAGAGGGAGATGAAGAGACTACAAATGATGTTCCCCAT
ATCCAGTGTGGAGATGGCTGTG
>193
AACTGGGTGAGTCTTCAGTGAATGGAGTAGGAAGAGGGTGTCCTCTTTCATTGCTTTC
TTTTCTCCCTAGTTCTGCTTGCAAAGGATCCACCAGGGTCTGATTTTTTATGAGAAGCT
GCTAGGATCGGATATTTTCACAG
>194
GGTGTCAGAGACAGAGGGTTGGGGGTTAAGGGTTTAGAGTCTTCTCTGACTGTGTCCT
ATGTCCTTTCAGCCTGAGGGTCACCACTGGGAGACTCAGCAGATTCCAAGCCTCAGTC
CCAGCCAGCCATGGCAGCGTCTCC
>195
GTGAAGCCAGCAGGTGCTCTGGAATTAAACCAGCTTTCTGCAAGCCCTGCTTCCTGGT
CTCCCTCTCCAGGTACTGGCTGTGATCGAACTTCTCAACCCTCAGAGACTTAGATCTTC
CACCTCACTCCCTCAGCCAAGCC
>196
TAGTGGCATGGCCTCCCTCTTACTCAGCTCTCCCTCCCTTTGACCGCTCCTCCTTTCCCA
CTTCCATCAGTTCTGCAGTTGGCGGGCCAGCCCTTTGACCACTCCCCTATCCGCTTCTG
TGCCCGCAACGGGGAGTATGT
>197
GAGCCTGGGAGGTGAGAAAAGGTGTGGGAAGCCGGGTCAAGCCATCTAACCTGCCCT
CTCCCTGCTGCAGCCCGTCCACAGCCCCAGCCCCACGGGACTCTGTGGAGTCGGCGCC

GTGACATCCCCAGGCCCTCTCCACA
>198
TATTGTGCCCTTGGCCCCTGACACTGGCGACACCCCCTGGTTCTGCTCACTGTGGTGCT
GTGTCCCACAGGTGACTTTCCAGCAGATCTGTAAGGATGTGCATCTGGTGAAGCACCA
GGGCCAGCAGCTTTTTATTGAGT
>199
AGCCTCCTGGTCTCCAGACTGTTCCCTCTGTTCCCTACCACCATCCCCTTCCCCTTTTCC
CTGGGCACAGATCCGCCGTCAGCAGCGCTGTCTGGGGAGGGGGCCACCCCACGGAAG
GAGCCAGTGGTGGGAGGCACCCT
>200
GAGGGTGTGGTATGGATGTGTTGACCCCTGAAGTGGTTCTGATGACCTTTCCCTATCTC
ACTCTCCTCAGACATCATCATGATGGAGGACCTGCCTGGCCTAGCCCCAGGCCCAGCC
CCCAGCCCAGCCCCCAGCCCCAC
>201
ATTTTCCGCGGCAGTTTTTGGTCCTGGGGACCGTCACCGATGCCTCCCACGCACGCTTT
CTTCCCTGAAGGGAGACGTCGCTGCGCTGGGCCTCTCGGCGGTCCCCCCCACCCGGGT
CCCGGGCCCACTGGCCCCCCGCA
>202
TCGGGGAGCTGGCCCACGGGCGGCGGGGCTGGGCCCGGGGCTCCCAGGCGCTGACCC
CCGGGGCCCGCAGGCGTGTGGCTCACGGACCTGGTGCACGTGGCGGCCCACGAGATC
GGCCACGCGCTGGGCCTGATGCACTC
>203
TCCCTTTGTCGGGCGGGAGGGCGGGGACCGGGCGGTCCTGAGCCAGGCGTGCTCCCC
ACGCTCCCGATAGGATGCCTCGACAGGCTGTTCGTGTGCGCGTCCTGGGCGCGGAGGG
GCTTCTGCGACGCTCGCCGGCGGCT
>204
CGTGGGAGCTGGGCCTTGGCCATGGCGCAGCTGAGGGGGCACTGACGGAGCTTTTCC
CCCACCGGAGCAGAATTCCCCTTCCCCACGGTGGCCACCACCCCACCGCCCCCCAGGA
CCAAAACCAGGCTGGTGCCCGAGGG
>205
GGGGCAGGGGTGCGGGGCAGGCAGCCGGGGGGGGGGCTGTGCCTGCAGGAGACGCC
CCGCCCCCCTGCAGCTGGTACAAGGACCAGGAGCCCCTGGAGTTCTCCTACCCCGGCT
ACCTGGCCCTGGGCGAGGCGCACCTG
>206
AGAAAGCATTCACCCTTATATTTAACTGTGAAATATCTGATTTGAAATTATTTACTTCTTT
TTTCCACAGCCTGGTGAGAGTCAAAGAGGCCCCAGGGGACCATATCCACCTGGACCGC
TGGCTCCTCCTCAACCTTTTG
>207
CACACACAATCCTCAGCTAGGTGCTCCGAATATCCCAGCCTAATTTTTCTTGTGCTTTTG
TTTGTATCAGGGGATGTGGCTCTAAATCAGCCGGACATGTGCGTCTACCGAAGAGGGA
GGAAGAAGAGAGTGCCTTACAC
>208
GTTTATTCTGTGTTTAGGAACTTGTAGGGGTGAAATGCTGACCTGTGTTACTCTTTCTTT
CCATGATCAGCGTCCAGAGGACCAGAGGTTAATTTATTCTGGGAAGCTGTTGTTGGATC
ACCAATGTCTCAGGGACTTGC
>209

AACTTCTGAATGTTTTTAAGCACTCACCAGGTTAGGTTCAGGTCCTTAAGTACCTTCGT
ATTACTTTTAGCAGGAAAAACGGCATGTTTTGCATCTGGTGTGCAATGTGAAGAGTCCT
TCAAAAATGCCAGAAATCAACG
>210
AATTCCTTTTGCTTGATTTCAGACAGTTGCATCAGCTCCTTTGTTCTGTGTTCATGTTAC
ACTTATTTAGGTGGCTGAATCCACAGAGGAGCCTGCTGGTTCTAATCGGGGACAGTATC
CTGAGGATTCCTCAAGTGATG
>211
TTTAAACTTAGTTTTCATTTGTTCATACTCAACCCACTCAAAATGAGTGTTTTTTCAAAT
CTATGTATAGGCCTGAAGCTGCCCAGCAGGCATTCCAAGGCCTGGGTCCTGGTTTCTCC
GGTTACACACCCTATGGGTGG
>212
GTGTAAATAAGCCACAGACTTCACGGTGCTTTGGTCTCAACAGTATCTGCCTCTGTCGT
TTTTATTTTAGTTTAGCAGCCACTGCTGCATCAGGGGCTTTTGTTCCACCACCAAGTGC
ACAAGAGATACCTGTGGTCTCT
>213
AGCCTTGGATTCTTGAGTAAGTCTCACTGTGACATCAGCTAAGATGGACTTTTATGTGC
TTCCTTTGAAGGCATCACGTTGGGTGGTTTCCATTTAGACCGAGGCCGGTTCAGAACTT
CCCAAATGATGGTCCTCCTCCT
>214
TATTTGGTGTTCATCATAGCCCTAATTGTTTTCTCATTGTTTCATTACCTCATTGTTTCATT
ACCTGTAGGAAGGCACTGATCCTGAAACTGAAGACCCCAACCACCTCCCTCCAGACA
GGGATGTACTAGATGGCGAGC
>215
AGAAAATTGTACTACAAAATACCATTCCATTTATTAAAGTCATTCTGACAGGAATCTGAT
GCTTTTCCAGGAGTTCCAGATCACATCGAGTTCACCATGAATTCACTCAGTGAAGCCA
ACACCAAGTTCATGTTCGATCT
>216
CAGACTTAGCATGGGTTTAGTATTTACATTGATACAGCAATTGAATGATCTCCTTTTTTG
ATGTTTGAAGGTTGATAGGTCAGGAAATGTTCATCACCAGTTTCAAAAGCTTCTGACTG
AATTCAACAAATCCACTGATG
>217
TAAGTAAGTTTGGTAATATATAGTTACATAAATTACTCCTAATTCCTACTTCTTCCTTCATA
TCTCAAAGGAATATTTAGATGCCATCAAGAAATTTTACCAGACCAGTGTGGAATCTACT
GATTTTGCAAATGCTCCAG
>218
TTTGCAAAGCATCTTCCAGACAAGCCATTTGTAGCTTGTGTAAAAGACTCTTTTATTCTT
TCCCTTGCAGAAAAAATTAAAAACCTATTTCCTGATGGGACTATTGGCAATGATACGAC
ACTGGTTCTTGTGAACGCAAT
>219
ATCTCAATAATATTATCTTTTTTGTCTTGTGTTTCACGTGTTATTTGTTGGACACATTGAT
TTATTGCAGAATACATACAAATCTGTACAGATGATGAGGCAATACAATTCCTTTAATTTT
GCCTTGCTGGAGGATGTAC
>220
TCAATTCATTGTTAAGGTGTATTAGAGATCGACAGTTAGTCATATCAGTTTCTTTTTTCCA
TTTGTATAGCTTGAAGAGAAACTCACTGCTGAGAAATTGATGGAATGGACAAGTTTGC

AGAATATGAGAGAGACATGTG
>221
ATTGTTGGTTATTACAGGTACAAAATTAAAGAGGTTTTTGTTTCTTTAAAAATATCTTTT
TTGTTCACAGAAAACAAAATTATCTTCCTTTCCAAATCCAAAAAGATGAAAACGACTG
AGATACTTTCAAAAGACAACCA
>222
TTAAAATGTTATAAAATAGACTGTTGTGTTGGCTTACTGGAATTACGAGAATTCTTTTTC
TCTCAAACAGCGTGCAGTTAAAAAATTTATTATGTTAACATCCAACCAAAATGTACCAG
TGTTTCTTATTGATCCTTTGA
>223
CTAGTATTTGGCTTTAAATATTCAATGTTTAAGTGTTGTTTCTTGATGTTTGATGCTTCTT
TGGTTCTAGGAAGGCTGGTTTCGGATAGCTGAGAATATGGGATTTCAGTGCCTAAAGAT
TGAGAGTAAAGATCCCCGGC
>224
TATTTGTCCTAATGTTCTCCCTCCCTGTTTCCCCCACCCCTCATTAATAAATCTTAACTTT
TGTTTTCAGGCCAGAGTTACAGCAAGTTACTGTTGATGGACTGGAAGTTCTCATTCCAA
AGGATCCAATGCACTTTGTA
>225
CAGATGCCACAGAAAGGTTCCTAGCAATTTAGAAATGGCTGTAATAGCTGACTATTTATT
ATATCTGTAGCAGTACCTTGATGATAACACTGTGGAAGCTGTGGCCTTTCGGAAGAGTG
CAAAGGAATTACTGCAACTAG
>226
TATAATTTGTTATAATAACTAATTTTTTCCAAACCTAAATTTTGTTAAAAAAATTTAATCT
TCTTTTTAGGATGGTATCGACAATGCAACATTATTCCTTATAGCAAAGATGTTGACCTAG
GAATTTTTATACAAGATTA
>227
TTTTAAAAAAAGGTTTTTAAAAATGTATTTCCTTTGTTTCAGTGTGTGAAGGTTTTCATC
TTCCCCATAGGTAGAAGACAGCTTGGAACTATCCTTCCAGGGAAAAGATGATGTAAAA
CTTGATGTTTTTTTCTTCTATG
>228
CACTAGCAGCTAGATTCCTTAGCTAGACCTTCTTCCACTGTTGAAGCCTAATCCCTCTGT
TTTGCTGCAGATACCTGTTTCCGAAGTTTACACTGTGCTGGACTGAGTTTGTAGACATG
AAGGTCCATGTACCCTGTGAA
>229
ACAGCAGAGGAGACAGTTCATCAGTTGCGTTCCCCCACAGACAGTCACAATGTTATGT
GATGTATTTCAGAGGCGATGATATTCTCACCATCCTGACTGAAGTGAACTATGAAGTAA
GCAACAAGGATGACAAGAAAAAC
>230
TAAGGAACAGAGAAGGGGGTTTGAATGTGATACTTGTGTCATGCTGACTTTTTTTTTTT
TTTTTGGTCAGTAAATTTCTAAGTGATGAAGAAAACTTGAAACTGTTTGGGAAATGCA
ACAATCCAAATGGCCATGGGCAC
>231
ATAAGTGGAACAATTTGGAATTTGAGTCGTAAATGGAGTCAATGATATTTTCCCTTGGTT
TTGTCTCTAGGAGGCGATTATGCAGCCCCTTGATCATAAGAATCTGGATATGGATGTGCC
ATACTTTGCAGATGTGGTGA
>232

GTTCATTATTGTCTTGTTAGAGAGATGACTGGTGAAATGCCTTTTTTTATCTACAAAAAA
ATATTTTTAGATGGCGGGTGAAAAAGTTGAGAAGCCAGATACTAAAGAGAAGAAACCC
GAAGCCAAGAAGGTTGATGCTG
>233
GGACCACGGACCATTATGTCTTAACCTTAATTGGCATTCTCTTACTGTTGATGCATTTGT
GTCCTTGTAGGTTGAAAAGAAAAAGAAGGAGAAGGTTCTCGCAACTGTTACAAAACC
AGTTGGTGGTGACAAGAACGGCG
>234
GCTGACATAGCTTGCTTCTAGTAATCTGAATGGCTTATTTAAATAAGTTGGATCTATGGA
CTCTCCACAGCCTAGATATTATCCTACTGAAGATGTGCCTCGAAAGCTGTTGAGCCACG
GCAAAAAAACCCTTCAGTCAGC
>235
AATGGTCACCTAAATTGCAGGATGATGATTCTTCCCCCTTTTCCTTGCTAAATTAATTTTT
CCCCCTTAGGACCTCTGGTCCTCAATCGAGTTCCTCTACGAAGAACACACCAGAAATT
TGTCATTGCCACTTCAACCAA
>236
ACTCCTTAGTTGACCTACAAGTAATTTGGTATGTGCCTGTTTTAAAATGTTTGATTTTCT
CTTTATTTAGAAATATGAGATTACGGAGCAGCGCAAGATTGATCAGAAAGCTGTGGACT
CACAAATTTTACCAAAAATCA
>237
TTCTTGCCCGGCAGAACGCCTGCAGCCGTGCGCAAGCAGTCACGACCCTTCCTGGTCT
CTCTTCCTGCAGGATGTACCCTGCCCACATGTACCAAGTGTACAAGTCAAGACGGGGA
ATAAAGCGGAGCGAGGACAGCAAG
>238
TGCGGGGCTGGGAGGATAGGCTTCTCATCTCCTTCCCCAAGCGCCCACCGCCTCCCCG
TGCGTCTTGCAGGAGACCTACAAATTGCCGCACCGGCTCATCGAGAAAAAGAGACGT
GACCGGATTAACGAGTGCATCGCCC
>239
ATTGCTAATAAATGTCCCAAAGGTGGGACTTCTCTGACCTCACCGCTGACTAAGGCTCT
TTTCCTTCCAGACTTTGGGTCACTTGGAAAAAGCAGTGGTTCTTGAACTTACCTTGAA
GCATGTGAAAGCACTAACAAACC
>240
TTTGGAGTATAGTTTCCAGGTGCGCCACAGGCCTTCTGAAACGTTTTCCTTCGGATTTT
TCTTTCCCCAGGTGAGCTGTCAGGGAGAAATGTCGAAACAGGTCAAGAGATGTTCTGC
TCAGGTTTCCAGACATGTGCCCG
>241
TCACCTCATTCCCCAGAGGAGCCAAGAGCGGAGGCTGCCCAGAGTGTCCAGCACGGT
CTCTCCCCTTCAGGAACATGACATCGAGACACCCTACGGCCTTCTGCATGTAGTGATCC
GGGGCTCCCCCAAGGGGAACCGCC
>242
CTGGGGGCCCGGCCTTCCTTCCAGTCCCCCGGCCCCTCTGCTCAGCCATAGTGGAAGT
GTGTCTTTGCAGACAAACTATGCTTCAACACCTTCTTCAACTTCGAGGACATGCAGGA
GATCACCAAGCACTTTGTGGTGTG
>243
CTTTACAGAGTGTTTCTGCCATCTGCACCCATCCTGGCCCCGCCCGGCCCTGTTTCCCC
TCTTACTGCAGCTCATCTTCCCCGACCTGGTGGAGGGGCTGGTGCTGGTGAACATCGA

CCCCAATGGCAAAGGCTGGATAG
>244
CCAGCCCCCTCTAAGCCTGCGTCCCTCTGTCTGCCCCTCTGCATGCCTCCATCCATCTCC
CTGGGCCTAGGAGGAGCTGGTGAACAACACAGAGTTGGTGCAGAGCTACCGGCAGCA
GATTGGGAACGTGGTGAACCAGG
>245
CCTACTTTTCCCACCAGAGGCACCTGGCCCTGCCCGCCAGTCCTCAGGCCCATCCTGTC
TCTGTCCACAGTGCCCTCAGCCAGCATGACCCGCCTGGCACGCTCCCGCACTGCATCC
CTCACCAGTGCCAGCTCGGTGGA
>246
GGCAGAGGAGCATTGGGGTTTTTTTTTAAATCTCTTTAATGGCCAATACTAATATAACTT
ATTTTTTTAGCCCTAGAAAACAATCTCCTGGTCCAAGGTGCTTGAGTGGGCCGATCCAG
CTATATCAAGAACCTTTGAGA
>247
TCACTCCACAGTGGGTGACAGCTGTGTTTTCTAGCTTAAAGTATAGTCGTATCTTTTGTG
TTTCTATCAGAACGTGAAAAAAACCGACTGGTTCCAGGAGTGGCCAGATTCCTATGCC
AAACACATCTACAGCTCGGAGG
>248
AAGACTGCACAGGAGTCAACAACCGACATGGTTCAGCCAGGTGTGGTCATTTTCCTCT
GCTCTTTTGTAGGGAAACGCTGTCCCAACTGTGACGGGCCTCTGAAGCTCATCCCTTG
CCGAGGTCATGGGGGCTTCCCGGT
>249
CTGATACACCATGAAACACAGTCTAGTTAGTGTTATATCATTTCCTTTCTACTTCCTTGTC
TCATTTAAGTCAAAGGGAGAGCATGATCATCCAAAACCAGAAACCAAGTTAGAAGCTG
AGGCAAGAAGAGCCATGAAGA
>250
CATGGGTGCCTATGGCAGTGGTTTTGATTTGCTTAACTTATTAACACGAGCATTTCACTT
TCCTTTCCAGTCTCTTCCAGGTGAAACACAAAGTCAGGGGAGTTTACCTTTAACTTGG
TCTTTCCAGGAAGGCGTCCAAT
>251
GCCTAGATGTGGTACATTCGACCTCACCTATGCCTCTCCCCACCCCCTCTGGGTTCGTTT
TGTCTTCCAGATTGCTGGACTCGCTGAAGAGAGACTACGCAGGAAAGCCCCAGCCAC
CCATCAAATCAGAGAGAAGGAAT
>252
TCTTAGTCTTCATTGAATTTGGCAGAGAGAGGTTGTTCTTCAAGAGGAACTGTTTCTTA
TCCTGATTTAGGTAAGAAAGTACTCATTGTCTATGCACACCAGGAACCCAAGTCTTTCA
ACGGATCCTTGAAGAATGTGGC
>253
TACTGGGATGGTTGGGATGGGCTGTGGATGCCCACCTAGGAGTGAGAATGTTTGGCCT
CTTCCCCGACAGGTACTCTTTCTAATCCTGAGGTTTTCAATTATGGAGTGGAAACCCAC
GAAGCCTACAAGCAAAGGTCTCT
>254
CAGATAAACTAAACTAGGAAACCTGAGGCGAGCCGCAGCCTCAGTTTCTCTTTGGTGT
TGCCGCCCACAGTTCCCGCTGTACTGGTTCAGCGTGCCAGCCATCCTGAAGGGCTGGA
TGGATAGGGTGCTGTGCCAGGGCT
>255

GCTGGAGGCTCAGCAACATTTCTGTCCTCTTCTGGAGTCCACACAAATGCATCTGCTTT
CTCCCTTGCAGGGTAAACTAGCGCTCCTTTCCGTAACCACGGGAGGCACGGCCGAGAT
GTACACGAAGACAGGAGTCAATG
>256
AATCATTTAACTGAATGGTATGTAACAGGTGTAGTTTCTAATGAGTTCTTTTCTTCCCCT
GTGGCTTTAGCATGGCACATTACACTTCTGTGGATTTAAAGTCCTTGCCCCTCAGATCA
GCTTTGCTCCTGAAATTGCAT
>257
GCTGAATCCCTACCAGGGCATGTCCTCAGCAAAGCCAGACCTCGTGATCCTCTTTCTTC
TCCCCTGCTAGGATTTGGTGGCCTGGGTGACAGCTGGTTTTCTGCATATCCCACATGCA
GAGGACATTCCTAACACAGTGA
>258
CACATGCACCCACGCCAAGGACCCTGAGCCTCGGCAGATCTGTGACTTCCTTCCGTCC
CCTCTCCTTCAGCTGGCAGGCTGGGAACCACAGCGCCTTCTGGGGCATGACCCTGGAT
GAGGGCATTCGCTACCGCCTGGGC
>259
GTGCCAATGGGCACCTGAGGGGCAGGCCAGGGCAGAGCAGGAGGCAGACAGGGCAA
CCTTTATCTTGCAGACAGTGCTGAACCCAGGGGAGGTGCTTCCCACAGCCTTCGAGGC
CTCTGAGAAGTGGCCCAACCTGATTC
>260
AGGGCTGGCGCCTGGGAGAGGAGGGCCAAGTCCTGAGCCTCCCGACAGCCCCTCTAT
CTCACCCCACCAGCTGTGGCATCCGATCGTGTCTCAATCCATTCTCTGGGACACATGAC
GCCTGTCCTGTCGCCCCAGAACCT
>261
TCCGTGGGCATGGCCTGGGCCATGATGCACTGAGTCTTTCTGCCTTTGCTCCCTCTTGC
TGCCTTTGCAGGGTGGTGTCTGACCACTGCTACCCCTTCTCGGGCCGTGAACGAGACG
AGGCTGGCCCTGCGCCCCCCTGT
>262
CTCAATAGACTCAGGCTCCAGTGCCTGTGCCAACGGGCTGAGTGGACCCTACCTTGAC
ATCTGCCCACAGCCCTCATGGAGGTGCATGAGGACTTCTTCCTATACAAGGGAGGCATC
TACAGCCACACGCCAGTGAGCCT
>263
TTTCTATGTGTGACTCTTTCATGGAATAACAACTCACTGCCTCTTAACCAAAGCTGTTTT
CTCCTTCCAGTGGGTTGCCAGCGTTGATGTGGTGGAGAATGAAGAGGCCAGCGCTAGC
ATCATTGTTAAAATGACAGACT
>264
TATCCGTAAAGATGCTAGCCTGTGTTGGCTTACCTTACAGTTTATACATTTTTTTGTTCTT
GTGTTGAAGGTGACTGCTGAGGTTGGAAAGCTCTTGGGTGAAGAGAAGGTGGATGCA
ATTCTTTGCGTTGCTGGAGGAT
>265
CTGCATGAATTGAGCTCACCTTTTGGTTTCTTTCCTGGGAATGCTGAGCCCACATGTTT
TTTTCACTCAGCTCTCTTTAAGAACTGTGACCTGATGTGGAAGCAGAGCATATGGACAT
CGACCATCTCCAGCCATCTGGC
>266
GAAGATTGCATGTCCTTGCTGTCCCCCTGGCCGCCAGTGGTCACTGAGCCATCTTCTCC
CCATTTCCCAGGTATGATCGGGTACGGCATGGCCAAGGGTGCTGTTCACCAGCTCTGCC

AGAGCCTGGCTGGGAAGAACAG
>267
CAAGGGGGACTGGGCACCTGTTGTTAGAAAGGAGCGACATGAATGACGTGCTTATCTG
TGTCCATTGCAGGGTTACCCTGGATACCCCGATGAACAGGAAATCAATGCCTGAGGCT
GACTTCAGCTCCTGGACACCCTTA
>268
CTGTGAAGGTCAGCATGTGCCCAGATTTTTGCTTTGAGAATATAAAAAAAACTTTTATT
TTCTGTTCCAGAACTTTCCATGACTGGATCACAGGGAAAAACCGACCGAGCTCAGGAA
GCCTAATCCAGGTGGTAACCACA
>269
AGGGAGGGGCACAGGCCATGGCGGGCTCAGCGTTCTCCTCCCACCACCCACCATGCT
GGACTCTTCCCAGGTCAGCACTTAAGCGATGCCTTTGCCCAGGTGAACCCCCTCAAGA
AGGTGCCAGCCTTGAAGGACGGGGA
>270
AAAAGCGACTATGTATGAAATACCCAGCACAGTGCCCTTCCCTTACCCATCATGACCCC
CACACCCACAGTGTGGCCATCCTGCTCTACCTGACGCGCAAATATAAGGTCCCTGACTA
CTGGTACCCTCAGGACCTGCAG
>271
ATTAGGGAAATGCAAATCAAAACTAACTCCCTACTCCAGTAACTCCCGACTTTGCCTGC
CCAATCCCCAGGTGATGTTCCCTGTTTTCCTGGGTGAGCCAGTATCTCCCCAGACACTG
GCAGCCACCCTGGCAGAGTTGG
>272
CCCAAATCAGATGCTGCCCATCCCTGCCCTCACAACCATCCATCCCCAGTCTGTACCCT
TTTCCCCACAGCCCGTGGGTGCTGGCTGCCAAGTCTTCGAAGGCCGACCCAAGCTGGC
CACATGGCGGCAGCGCGTGGAGG
>273
AAACTGCCAATTTTCTGAAATGCTCTTTAAATCCTGCTAATGGTGGCATTTGTGTCTTTG
TCACCTACAGTACATTTCTGGATTTGGGAATGAGTGTTCTTCAGAGGATCCTCGCTGCC
CAGGTTCCCTGCCAGAAGGAC
>274
AGTTGGTTGGTGGGAAGGTGGGATGCTTTTCGGATGGGAGTAATATTGTTTATTGCATC
CTTTGTTTCAGAATAATCCTCAGGTCTGCCCCTACAATCTCTATGCTGAGCAGCTCTCAG
GATCGGCTTTCACTTGTCCAC
>275
CTGAGCCATTCTGTGTATCACTCAGAACATTACTCTAAGGCTTGTATATCTTGTATGTTTT
TCCCTCTAGCTGGCTGTATAGGATTCTACCTTCAGTTTCTCACAAGCCCTTTGAATCCAT
TGACGAAGGCCATGTCACT
>276
GAACAGTGGAAAGGCTGCCTCTTGGGCACCGTTCACATTAACCTTTCCCTCTTCTGTG
GGTCTCTCCCAGGGCCTGCATACCTTGTGTGGAGCTGGAGACATAAAGTCTAACAATG
GGCTTGCTATCCACATTTTCCTCT
>277
CCTTTCCTGTTCATGAAATGGTTATCACACCAAAAGAATTGCTCACCACTGCCCTGTCT
TTTTTTGTCAGTTCCGCAGAAAGGGAACCTTCTCATTTACACCGAGTTTGGCAAGATGC
TTGTACAGCCCAATGAGATCTG
>278

CTCTGGGCAGCTTCTTTATAACTACAGCTGTGATTTCAGAGGGCACACCAGTGTCTGCT
TTCCATTCCAGAGAGGAATGCGGTTCAGCATAGATGTCTTTGAGGAGACCAGGGGCTA
CATCTTGGAGGTCTATGGTGTCC
>279
TCATGCAACCATGGGCATCTTTCCTATGTTTTGGAAGTTTCTAAAAGACTTTTGGGTTAC
TGTTTTCTAGGGGCCAATGGCTTGGCCAATCCTCGTGATTTCTTGATACCCATTGCCTGG
TATGAGGATCGCCAAGTACC
>280
ACATATTGTGGGATCACAGCTACAAAAGCATTAGATGGTAAAATTATCCTTTCCTGCTTT
TTGTGTTTAGGATGTCTCCCCGTTCAATGTTGTGGCCTGGCACGGGAATTATACACCCT
ACAAGTACAACCTGAAGAATT
>281
ATGAAATGTGTGTCATTGTCCAGTCACTTCCCTTGCATCATGCGTTCAGTCTCTCCTTGT
GTGTTCACAGGACCCATCCATTTTCACAGTATTGACTGCTAAGTCTGTCCGCCCTGGAG
TGGCCATTGCTGATTTTGTCA
>282
GTTTAAGGGGCTTTGTGTTTTACTGGTCTTGCCTTGGATAATAAAAATAATACTGCTTCA
ACCTTCCCAGGGAACTGCATGAGTGAGTTCATGGGACTCATCCGAGGTCACTATGAGG
CAAAGCAAGGTGGGTTCCTGCC
>283
GACATCTAATCAAATGTGTTTATTATCTATCTCACCTCTTTTTTCTCATCCCCATCGTCAC
TTCCTCCAGGCATTTATGTTTGAATCATCTTTAAGTCTGGCGGTCACAAAGTGGGGACT
CAAGGCCTCCAGGTGTTTGG
>284
GGGAAGGGGGCATACCATTTTAAGTACCAAGAAAAACTTGCATAATCTCATTTTACTTT
CTCTTTTTCAGCTGGATTTTCCTCTACGTGCTTCAGTCTTCTCTACACAAAGAGCTGCA
AACCAGGTCTTTGTTTTGCACT
>285
TGATTATGCTCTTTCTCTACCCATCCCCGCAAGGCAGATGTTTTCATGCTTGAATTTTGTA
TCCCTAAAGTCAGGAGAAATCTTCTGGACTTAAGTAAAGAAGAAAAGAACCACTTTGT
CCGGGCCCTGGATATGGCAAA
>286
GAGTAGACCAAACAGAAATGAATAATTGTAAACAGAAGCAGAGAGTATTAATGTGGTT
TCTGTGATCTAGGAAATGTTGCAAGAGCCTTCTTTCTCCCTTCCTTACTGGAATTTTGCA
ACGGGGAAAAATGTCTGTGATA
>287
AGTTTTAAAGAGCGACAATAAGAACTCCAAACATTGTGTAAATGTTTCCACATCCCATT
TTTTTCTGCAGGCACCGAGGATGGGCCAATTAGGAGAAATCCAGCTGGAAATGTGGCC
AGACCAATGGTGCAACGTCTTCC
>288
CATTGCTATTACCTGGAAAAGTGAAATATTTGCAATAGTTTTACTATTCTCCTCCTTACCA
TGTGTCTAGGTTACAGTGACCCCACGGGAAAGTATGACCCTGCTGTTCGAAGTCTTCA
CAATTTGGCTCATCTATTCCT
>289
TAGAACTCAATAATGATAGGAATATTAATTTTATTATGTTTATTAATACGTTGTCTTTGGAA
TAATTTAGATATATCCACATTTCCATTGGAAAATGCCCCTATTGGACATAATAGACAATAC

AACATGGTGCCATTCTG
>290
ATGTGTCCACTTTTTGGTGATAACTATTTTAATATTTGTCTTTTTATTTTTAATCTTCCTTT
CCAAATAGGTCGGGAGTTTAGTGTACCTGAGATAATTGCCATAGCAGTAGTTGGCGCTT
TGTTACTGGTTGCACTCAT
>291
TTACCACATATAATTCTTAGGTTTCTACAGGGCCTGTCTAACCTGACCTTACTCACTTTTT
ACATAACAGGGTCCACCAGGCCCCCCAGGCAGAGATGGTGAAGATGGTCCCACAGGC
CCTCCTGGTCCACCTGGTCCTC
>292
GGCATCTTAAAAACAGATATGCTGTTTCATTATTTGCTGGTTAATTCCTTGGTTTAATTTC
CTCTTTTAGGGTGAAATTGGAGCTATTGGTAACGCTGGTCCTGCTGGTCCCGCCGGTCC
CCGTGGTGAAGTGGGTCTTC
>293
GGCATTTAATGTGTGCTGCCTCTACAGCCCATCACCTCCCTAATGGACCACACTGCATTT
TCCTTCATAGGGCCTTCCCGGCGTTGCTGGGGCTCCCGGCCTCCCTGGACCCCGCGGTA
TTCCTGGCCCTGTTGGTGCTG
>294
TTCTCCAGCTGGACATAGTATTAAAATTATTTTTTTTACTCCCTCTTCTTTTGTTCTTTTCA
TTAAACAGGGCTCTGCTGGGCCCCAAGGTCCTCCTGGTCCCAGTGGTGAAGAAGGAA
AGAGAGGCCCTAATGGGGAAG
>295
TCTGGTGGTGGGTGTGTCATTAGCTTTAGCATCCTCCTCCTCTATCTGTTTTTTTTTTTTT
TTTGAATAGGGCCCTCCTGGTAGTCGTGGTGCAAGTGGCCCTGCTGGAGTCCGAGGAC
CTAATGGAGATGCTGGTCGCC
>296
GCAGCATCATAAGCTTGAGGTTGTGAGAATATGTTGACACTGAGTAAACTTGAAATAAC
TCTGCTTTCAGGGCCTCCCTGGCATCGACGGCAGGCCTGGCCCAATTGGCCCCGTTGG
AGCAAGAGGAGAGCCTGGCAACA
>297
CTCTCATATGTAAAACAGTATCACTGAAAGTGATGAATGGTGCAACACTTCTTCTAATC
ACTTTTTTCAGGGGGAACGCGGTCCCCCAGGTGAGAGTGGTGCTGCCGGTCCTACTGG
TCCTATTGGAAGCCGAGGTCCTT
>298
TCTTCTAGAGTTTGATTCTTCATTTTCTTCTTTCTCCACTAAAATTGATTTCACATGTGTT
TGACTCAAGGGTGAACCTGGTGTGGTTGGTGCTGTGGGCACTGCTGGTCCATCTGGTC
CTAGTGGACTCCCAGGAGAGA
>299
CTGAAAACATCCTAAGTTGGGGAGTAGAGTGGGTCGGAATACCAGAGCTGTAACTGTT
TATTTCCAACAGGGTGCTGCTGGTCAACCGGGTGCTAAAGGAGAAAGAGGAGGCAAA
GGGCCTAAGGGTGAAAACGGTGTTG
>300
ATCTTTGCTGCTCTCTTCCAGGCCCTTGGTGATTAACAGAAAGGAAATGACCTTGTACA
TTTGCTCATAGGGTATTTCTGGCCCTCCTGGTCCCCCTGGTCCTGCTGGGAAAGAAGGG
CTTCGTGGTCCTCGTGGTGACC
>301

AATCTTCAAGCCAACCTGTGTTATCACCTAGGGTCTTACCCATAATACTCAGTATTTTTT
CTCTATTTAGGGACCTCCTGGCACTCCAGGTCCTCAGGGTCTTCTTGGTGCTCCTGGTA
TTCTGGGTCTCCCTGGCTCGA
>302
TGTTTTGGAGGGGAAGGTTAGCATTCCATCGAATAAGGGGAATGTCATTTTATCTTCTCT
GCCTGTTTAGGGTGAACCTGGTCCTCTTGGCATTGCCGGCCCTCCTGGGGCCCGTGGT
CCTCCTGGTGCTGTGGGTAGTC
>303
TCTTTCTCTAATTCAAAAGTGATTAAAATGCAACCCAGATTGATGCTAAGCTTCATTTTG
CCTTTGGTAGGGAGAGCGCGGTTACCCTGGCAATATTGGTCCCGTTGGTGCTGCAGGT
GCACCTGGTCCTCATGGCCCCG
>304
GTATTTGTGGTGAAGTGAGTGCCATTTTTTTAAAACGGTAAGTCTTATCCATCCTTCTGT
TTCTTTATAGGGCCCACAAGGCATTCGTGGCGATAAGGGAGAGCCCGGTGAAAAGGGG
CCCAGAGGTCTTCCTGGCTTCA
>305
TGAGCCCCAGGCTGCTGCTCCCTTGGTGGGATTCACCAGCTCACATGTACCTGGTGTCT
GTCTTCCTTAGGGCCCTGCTGGTCCTTCTGGCCCTGCTGGAAAAGATGGTCGCACTGG
ACATCCTGGTACGGTTGGACCTG
>306
TCTGCTGCCATGGATGTCTCTCACTGTAAAAAAATATAAAGCCTCTCCTATCTAACTTTC
ACCTTTGCAGGGCCCCCCTGGTCCCCCTGGCCCTCCTGGACCTCCAGGTGTAAGCGGT
GGTGGTTATGACTTTGGTTACG
>307
TTAGTTATTATTAGAATCTGTGTTCTGCTCAATGAGAAGTTTCATGATCTGAATGTTATTT
TCTTAAAAGGTTACTACTGGATTGACCCCAACCAAGGATGCACTATGGAAGCCATCAA
AGTATACTGTGATTTCCCTAC
>308
TTTTTCATGTTTGACTCTTAGTATCTGAGTCCTTCTCCACTTAACTGGAATTTCATCCTAT
TTTCTGTAGTTTGAATATAATGTTGAAGGAGTGACTTCCAAGGAAATGGCTACCCAACT
TGCCTTCATGCGCCTGCTGG
>309
CAAAGGTTCAGATATTATCAGATTCAGAAATAGTGATGCTTTGTGTATCTATTTTCTTCTC
TTTAAACAGAAAAAGACAAATGAATGGGGAAAGACAATCATTGAATACAAAACAAATA
AGCCATCACGCCTGCCCTTCC
>310
AAATTTTTTTTAACTGGGTTAATGCTTGCTGAATTGGAAGTGAATGTCCATTCCTTTGCC
TCTTTTGCAGATATACACTTCAGATAACTACACCGAGGAAATGGGCTCAGGGGACTATG
ACTCCATGAAGGAACCCTGTT
>311
GGGTGTGTGGGGATAGTTGAGGTTATGGTGGGAACCTTGGGCCCTGCTGACCCTGTTT
CCTCCTCCCTAGCCTTTATCCTCCTCTTCTACCTCGTTTTTTATGGGTTCCTCACCGCCAT
GTTCACCCTCACCATGTGGGT
>312
CCATTTTCTTCCCTCTGTGTGCAGTCCCTCATCTTATAGATCCCCCAACTTCTGCCTTTG
TTGGCTGTAGGCTTGATGATTCGCCCCAAGACTGAGAACCTTGATGTCATTGTCAATGT

CAGTGACACTGAAAGCTGGGA
>313
TCGGGCAGGGGACTGGGGACCTTGGAAGTGGAACATCTGGCCCCTGAGTCTCTCCCTC
CCACCTCTTTAGCTTACAACGACTCTATCCAAGCCCAAAAGAATGATGTCTGCCGCCCT
GGGCGCTATTACGAACAGCCAGA
>314
AAGTCTGGTGAGCTCCTGGGTGCCTGCCATCCCTAACTGGCTAACCCCCTATCTTCCTG
CACCCCCACAGCGAGATGAAGATGCTGAGAATCTCGGCAACTTCGTCATGTTCCCCGC
CAACGGCAACATCGACCTCATGT
>315
TGCGGGTGGTGAGCTAGGGAAGGAGGCCGCGTTGCCCCAGGCCTAGACCCTGCACTG
CTCTCCGGCCCAGGTGAACTACACACAGCCCCTGGTGGCTGTGAAGTTCCTGAATGTG
ACCCCCAACGTGGAGGTGAATGTAG
>316
AATGGGCACCCTCGTTCCTGGAAAACGGTAGGCCTGTGGGTGACCAGCTTCCCTATCT
GTCTTCCCACAGTGCGGCTGGGATCCGGCGGCCACCTGCACCTGCGTATCTCTCGGGC
CGCCCTTCCCGAGGGGCTCCCCGA
>317
GACCCATAAACCTCTCCACACTGGCTGAGCAAATGAGAGAATGCTTTCATGTTCCTGAT
TTGTTTTTCAGAGCCAAAGAGTTCAAGACACGTCTGGGGATCTTTCTTCACAAATCAG
AGCTGGGCTGCGATACTGGGAGT
>318
TTTCCTTTCTTCTTGTATTCCCTGCTGTCCCTCCAGCTGACCTATTTCCTCTTCTTTCCCA
CAACCCCAGATGGAGTGGCTGCCTTCCACGCTTTCCTGAAGACAGAGTTCAGTGAGG
AGAACCTGGAGTTCTGGCTGGC
>319
CCGTTGGCCCTCCCTCAGCCATGCCCACCCTCTTCCCCAACTCACCCTGTGTGTGTCCT
CCTTCCCGCAGGTCAACATTGACCATGAGACCCGCGAGCTGACGAGGATGAACCTGCA
GACTGCCACAGCCACATGCTTTG
>320
TTTGCATTCATGGAGGGCAACTAAATACATTCTAGGACTTTATAAAAGATCACTTTTTAT
TTATGCACAGGGTGGAACAAGATGGATTATCAAGTGTCAAGTCCAATCTATGACATCAA
TTATTATACATCGGAGCCCTG
>321
GATTACAGGTGTGGGCCACCGTGCCCAGCCACCGGTGTGGCTCTTTAACAACCTTTGC
TTGTCCCGATAGGTCACCTTTGGCTCTTCAGAGATGCAGGGACACACGATGGGCTTCT
GGTTAACCAAACTGAATTATTTGT
>322
AAAGCCTCTTGTTCGTTCCTTGTACTGAGACCCTAGTCTGTCACTGAGGATTTGGTTTT
TGCCCTTCCAGTGTATACTCTGAAAGAGCGATGCCTCCAGGTTGTCCGGAGCCTAGTCA
AGCCTGAGAATTACAGGAGACT
>323
CCACCAGAGCCTGCTTTCACTCAGAAGGGAGATGCAGACCCATGTAATCCTCCCCGTC
TCTGCTTCCCAGGACTCATCTGATGATGTGAGAAGAGTTCAGAGGAGGGAGAAAAATC
GTATTGCCGCCCAGAAGAGCCGAC
>324

CCCTCCGCACCCCACCCACCTTGCCTCCTTCCTAGACACTAACCTCCGGTGCTGATCCC
CACCCCTACAGGAGAGCGAAGACCTGGAGAAACAGAACGCGGCTCTACGCAAGGAG
ATCAAGCAGCTCACAGAGGAACTGA
>325
CCCGGGGGAAAGGTGGTTAAGCTGGGGGTTGCAGTCACTACTGACAACGCCCCTCTTC
CGCCTGTCCCAGTCCCACCAATCACCTGCGTACAGAACGGCCTCAGGTACCATGACCG
AGACGTGTGGAAACCCGAGCCCTG
>326
TGTGGGGGGCTGCAGGTGGGCATGGCTCTCGGCCCCACGCTCACCCCGGCCCCGCCCT
CTCCCCCTGCAGGGACCCGCAGGCCCCCCTGGCCGAGATGGCATCCCTGGACAGCCTG
GACTTCCCGGACCCCCCGGACCCC
>327
TTCCATCCTCAGTGCCGTCTTCTGCCTTTCAATTCACTGTCCTCACTCTGACTTCTCTTG
TTTGTTCTAGAACTTTGCTCCCCAGCTGTCTTATGGCTATGATGAGAAATCAACCGGAG
GAATTTCCGTGCCTGGCCCCA
>328
TCCTGCCATCCCGAGAGGCAAGGTTGGGTTTCCCAGGGTGGCTTCTGATATGTCCTTTC
TTCTGATTCAGGGTGAAGCTGGTCCCCAAGGGCCCCGAGGCTCTGAAGGTCCCCAGG
GTGTGCGTGGTGAGCCTGGCCCCC
>329
GCCGCCTGCAGCCTGCCTGCCCCTTTCCCTCTGCTCCTAGGCTCACGCCCTGGCTGTCT
GCCTCCCACAGGGTGCTCCTGGTATTGCTGGTGCTCCTGGCTTCCCTGGTGCCCGAGG
CCCCTCTGGACCCCAGGGCCCCG
>330
CAGCCGGCTCTGAGGCTGGCACAGGATGGCCCCTCACCACAGGCCGCCTCCTCCTCTC
GGCCCTCTCCAGGGCCCTGTTGGTGTTCAAGGACCCCCTGGCCCTGCTGGAGAGGAA
GGAAAGCGAGGAGCTCGAGGTGAAC
>331
CCTCTTTTGGCCCGTGGCAAAGAGCCACAAACTTGAGACCCTAACTGTTCCTGTGACT
TCCCCCAACCAGGGTCCCGCTGGTGAACGTGGTTCTCCTGGCCCCGCTGGCCCCAAAG
GATCTCCTGGTGAAGCTGGTCGTC
>332
GAACCCCCTGCCATTGTCCTGTCTGCCTCCCTGCTGTCCTCACTGCTGCTTTCGTGCCT
CCCATCCTTAGGGTCCCGCCGGTCAAGATGGTCGCCCCGGACCCCCAGGCCCACCTGG
TGCCCGTGGTCAGGCTGGTGTGA
>333
TCCGTCAAGGTGCGTTGTACTTGGCCCTATCTCCAGAGCAGCCTTCACATGCCCTGTCC
TTCCCTTCTAGGGCGAGAGAGGTTTCCCTGGCGAGCGTGGTGTGCAAGGTCCCCCTGG
TCCTGCTGGTCCCCGAGGGGCCA
>334
GCGGGGCTTAGGGCTGTGACCCACTCTTGGGGCCCCAGGCCTCACTCCAGTCTTCTTG
GTTGTCACATAGGGTGATGCTGGTGCCCCTGGAGCTCCCGGTAGCCAGGGCGCCCCTG
GCCTTCAGGGAATGCCTGGTGAAC
>335
ACACAAGCAGAACACTATAGGTCACCTCCTTGCCCCACACTGGAAATCTCAAGCTTAT
CCATGTCTTTAGGGTGATGCTGGTCCCAAAGGTGCTGATGGCTCTCCTGGCAAAGATG

GCGTCCGTGGTCTGACCGGCCCCA
>336
CTGCTCCTCCCCCAGGTAGTGGAAACTCCTGCCTCCTTCCCTCCATTCACCGCCCTGCT
TCCTCCCCCAGGGTGCTGACGGCCAACCTGGTGCTAAAGGCGAACCTGGTGATGCTGG
TGCTAAAGGCGATGCTGGTCCCC
>337
GGGCTGACTGAGGACCCAATGATGCACCAGAGCCCCCTGGAGTCTGACAGCCCCTCCT
ATCCTCATCCAGGGAAATGCTGGACCCCCTGGCCCTCCTGGTCCTGCTGGCAAAGAAG
GCGGCAAAGGTCCCCGTGGTGAGA
>338
GAGGTGTGCAGAGCTGGGGAGGGGTCCCTGTGCTGCTGTCTGGCACCTCACCCCTGTT
TGCCTCCCAAAGGGTGCTCCTGGTACTCCCGGGCCTCAAGGTATTGCTGGACAGCGTG
GTGTGGTCGGCCTGCCTGGTCAGA
>339
GAGATGACTCCAGCGGACTCAAGGGTCCTCCCAGACCCTATCTCTGGCCTGACTCTTT
CTTCTCCCTTAGGGTGAACCTGGCAAACAAGGTCCCTCTGGAGCAAGTGGTGAACGTG
GTCCCCCTGGTCCCATGGGCCCCC
>340
CTGCTGCTTCCCTGCCCCATCCTGGCCCTTCACCCGGGGCTGACCCATATTCCCCTGCT
CTCCCCGCCAGGGTGACCGTGGTGAGACCGGCCCCGCTGGACCCCCTGGTGCTCCTG
GTGCTCCTGGTGCCCCTGGCCCCG
>341
GATGCAGACAGTGCCCCACTCGATGCCAATGGAACTTCCGCCTGACAGTTTGTCCCTT
TCTCTCTTCTAGGGACCCCAAGGCCCCCGTGGTGACAAGGGTGAGACAGGCGAACAG
GGCGACAGAGGCATAAAGGGTCACC
>342
GGCCTCTCCTCCAGAGCTGGGGTTGTTCGGGCTTCTGGCAGCCTTTCTCAAACCATTTC
CCCCACTCCAGGGTCCCCCTGGCTCTGCTGGTGCTCCTGGCAAAGATGGACTCAACGG
TCTCCCTGGCCCCATTGGGCCCC
>343
CTGCTCATGGCCCTCCAGCCCCCATAGCACTTGGATGCCGGAATCCCCACTCTCTTCCC
TCTCTGTGCAGGGTCCCCCCGGCCCTCCTGGACCTCCTGGTCCCCCTGGTCCTCCCAG
CGCTGGTTTCGACTTCAGCTTCC
>344
ATATGTGCCCCTACCATATGGTGCTGGCTGCTCCCTTTCCCTGACTCCATCTTGCCCTGC
CCTACCACAGGAGAGTACTGGATTGACCCCAACCAAGGCTGCAACCTGGATGCCATCA
AAGTCTTCTGCAACATGGAGAC
>345
CCTGGACAGGAGGCCAGCAGGACTAGAGGTTCCCGCATAGCTCACTCTTCCCTCTCTC
TCCTCCCTGCAGTTCGAGTATGGCGGCCAGGGCTCCGACCCTGCCGATGTGGCCATCC
AGCTGACCTTCCTGCGCCTGATGT
>346
GGCCATAGTGCCCTCTCTCCATCACTCCCACGTGGTAATGCCCCCTCCCGTTGTCTCCG
CCCCACCCCAGAGTCACACCGGAGCCTGGGGCAAGACAGTGATTGAATACAAAACCA
CCAAGACCTCCCGCCTGCCCATCA
>347

GCTAAGGTGGGCAGGGCGTCATATCCAGGCCCCTCATTCCATTTATTCCTTTGGTTTCTT
TTCTCCTCAGGGCCCTAAGGGGGAGCAGGGCCCCCCGGAATTCCAGGGCCCCAAGG
CTTGCCAGGCGTCAAAGGAGACA
>348
CCCACCCCCCACTCTCGCCCACCCGGGCGCCTTCTCACCCGGCTCTGCTCCCACCCCC
ATCCCCCCGCAGCAAGGAGTACGTGGAGAACCCGGCTACCCTGGCCCCAGCGGGGAT
GCGGGCGCCCCAGGGGTTCAGGGCT
>349
AACATAGCAAAAAATAGAGGAAAAGCATTTTCCAATTCTGAAAAATGAAGATTTCCTT
TTTTGTCTACAGGAAAACGTGGAGAGAAGGGTGATCCAGGAGAAGTGGGACGGGGGC
ACCCCGGGATGCCTGGGCCCCCAGG
>350
TCCCCACCTTCAAAATTCTGGGCCTGAGACAAGAGGTGCCCCTAATGTCTTGACTTTCT
CCTGTTCACAGGACTCCCTGGCCGGCCTGGCCAGGCAATCAACGGCAAGGATGGAGA
TCGAGGGTCCCCAGGGGCTCCAGG
>351
GCATCGAGATGACTGGAGGTGGGAAGGGCAACATGCTTATCCCTGTAGCTACCCTGTC
TTGGCCTTGCAGATCCAAGGAGAAAGTCAGGGGCCAGTGCGCGCTACAGCCAGCGCC
CAGTACCGCGGTGTGATGGGCACCA
>352
TTGGTGAGGAGAGATGAGGTAGAAAATGAGTGCAAGCCCGCTGGCCACTGACCCCAT
GGCTCGCCCACAGATGCCAGCATTGGGAGCCGCCTCCTAGCAGGCAGCACCACAGGT
GCCCTGGCTGTGGCTGTGGCCCAGCC
>353
GTTGTTTTCCCTTCCCCTTTTCCTCCTCCCCGATACTCTGGTCTCACCCAGGATCTTCCT
CCTCCTACAGGGACCTCTCCCAATGTTGCTCGTAATGCCATTGTCAACTGTGCTGAGCT
GGTGACCTATGACCTCATCAA
>354
AAGTAATTCTCCTGCCTTCTGGAATGATGGGTGAAGATCTTGACTGCCTTGCCTGCTCC
TCCCTTGGCAGATGACCTCCCTTGCCACTTCACTTCTGCCTTTGGGGCAGGCTTCTGCA
CCACTGTCATCGCCTCCCCTGT
>355
TGAATACCAGGCCCAGTGAGGGAAGTGGGAGGTGGAGGTGCCCCCATGACCTGTGAT
TTTTCTCCTCTAGGTTCATGCCCTCCTTTCTCCGCTTGGGTTCCTGGAACGTGGTGATGT
TCGTCACCTATGAGCAGCTGAAA
>356
CCACTAGTGCATTGGGGTGCGGTTCGCGAACAAACTGCCCACTGGCTCCCTTTTTTTTT
TCTTTTTCTAGAGAGACTTGATAAATCGGCCTGAGAAAATAAGTAATGCTCCAGACTAG
CTCTCTCATTTGTGAGTTCTCC
>357
TCTGTCACTTTTAGTGGTAGAATTGTCCTTGAGACAACAGGAACTGACTACAGAAATCA
TACTATTTCAGGGGTTGCTGACATTTGAGGATGTGGCTGTGTTTTTTACCCAGGAGGAG
TGGGATTATCTGGACCCAGCTC
>358
CCATCTTACTTTGTTTCTCCTTGCTTCAAAGACCTTGGTTGATCTTTGTATTTCCTTTTTA
TTATTCCAGATGTTTTGAACAGAGATAAGGATGAGGAGCCAACTGTAAAACAAGAGAT

TGAAGAAATTGAGGAAGAAGT
>359
AAAGTTTCCTCATCACGTGGCGAGACCTCCTCTACTTCAATCTTTGTCTTTTGTCTCTGC
TGCCTTGCAGGGTTGGTACAGTAGGCTTCACTAGACTTAGCTGCAACTCAGAATTTCTC
CTCCAGCACCTGAGTAAATGC
>360
AAATGAATTCATTATGAATGGCAATTATAGCTTAATCAAGGACTCAAAGATTCTTTTTCC
TTCTTTCCAGGATGAACACCAGGATAAAATCTACCCCTCTTTCCAGCCACAGCCTCTGA
TCTATCCATTCGTTGAACCTA
>361
AAAAGTGAAGAGAGTTGAATAGTTACGTGTTATAACATAACTAATTATATATTTGCTCTC
TATTCCACAGAATTGACTGAGACTGGAAATATGATGCCTTTTCCGTCTTTGTATCACGTT
ACCCCAAATTAAGTATGTTT
>362
ACAGCAGCCTAACGAGTGCCCTGTCTACTTGTCTGTCCTTGTGTTCATGTGTCTGCATG
TCTGTTCTCAGGACCTGACAGGAAGCGGGGCCGCCAGACCTACACGCGCTACCAGAC
GCTGGAGCTGGAGAAGGAGTTCCA
>363
TGGCCCTGGAGGAAGTAGGGGGATGTGGGGGTGAGGTAAGTCTCCTTGAATGCTGTAC
CCTGTCCATTAGAGCAGCCATGGCCAGCTCCAGGCGAGGCCTCCTGCTCCTGCTGCTG
CTGACTGCCCACCTTGGACCCTCA
>364
GCCGGGGGCTCCCCCACCCTCCTGGAGCCTGAGGTCGGGGTAGGGAGGACAGCATCA
GTTCCCTTCTAAGGAAGGGCCCTGGACACTGCAGCAGGCAGCCCAGTCCAGACTGCC
CATGGCCTCCCAAGTGATGCCCTGGC
>365
CTAGGGAGGGTATTAGGGGAAAGGGCTTGAGGGCCACAGTAAACTGGACAAGTTTTT
CTGCCCAGCCTAGGCTGCCACCTGTAGGTCACTTGGGCTCCAGCTATGTGGCTGCCTCT
TCTGCTGGGTGCCTTACTCTGGGC
>366
GCTCCCAGGAAGAAGAGGGAGCTGTGGGAGTGCCTCACCTACCCCCAGCATCCTTTTC
ATCTCCCCACAGGGCTTTTTGGTCTGGTGAATAATGCTGGTGTGGCTGGTATCATCGGA
CCCACACCATGGCTGACCCGGGA
>367
AACCCATGTCCCTCAAAGTCCCCTCCCTATAGGGCAAGAACCCAGCAACTTCGCTCTG
CCCCGACTCTAGGCGGGATGTAGCTCATTTTGGGATACGAGTCTCCATCGTGGAGCCTG
GCTTCTTCCGAACCCCTGTGACC
>368
CGAGCCTGACTCCAGCAGGCCTGCCTTGGGGCTGGGGCTGGGGTGGAGGCCAGCCAG
TGTCCTCCCATAGTGGCCGAGAAGACCAAGGAGCAGGCCAACGCGGTGAGCGAGGCT
GTGGTGAGCAGCGTCAACACTGTGGC
>369
CCTGGTTGCAGGGCAGCTAGGGGTCTCTGCATTCTCCACATGGTCTCATGCCCCCTTTT
GTCCCCTACAGGAGGACTTGAGGCCATCTGCCCCCCAACAGGAGGGTGTGGCATCCAA
AGAGAAAGAGGAAGTGGCAGAGG
>370

TCAGCCCACTCAGGGTGGCCCATTAAGGCTGGGGCCTGGAGCTGGGTGTGCAGGTCAT
TCTCTCTCCCAGGCCCAGAGTGGGGGAGACTAGAGGGCTACAGGCCAGCGTGGATGA
CCTGAAGAGCGCTCCTCTGCCTTGG
>371
CGGCCAAGCTGCTTGTCACCTGTGAGTGCTTCCAAGGAACCGGATGTGGGCTCCCCCA
CCCTTACCCCAGCATCTGGCACAGCTGAAGGCAGATGGAAATATTTACAAGTACGCAAT
TTGAGACTAAGATATTGTTATCA
>372
TGAATTTGAATGACTGATAATTTAATTTATTTTCAGTCAATTGTTACGTATGTTTTCTTTTT
CTTCCCAGGACATGGTGACCCAGGCATCTCTGTATCTGTTTGAAGCTACAGGAAAGCG
ATTTTATTTCAAAAATGTTG
>373
CTCCAGTACGAATATGGTTAATTCATTTGCTGAAACTTTGTGATGATAAAAACTTTTTCT
TAAATTTCAGGCTGATGTTCTGGTTGCTGAGTCTACTCCTCCAGGTAATGATGAACCCT
ACACTGAGCAGATGGGCAACT
>374
TTTCTTCCAGTGTGAGAAGGTAATTTTGCCATTCTTATTGTAATCAGTTTTTTAAAATACT
TTCTCACAGGTAAGGCATTTGTCCATGAGTGGGCTCATCTACGATGGGGAGTATTTGAC
GAGTACAATAATGATGAGAA
>375
AACTGAGCATAGACTCTGACCCTTTGAAATGACGACATCACCTAAACATCTAACCTTCC
TATATTTTCAGATGTTCAGCAGGTATTACTGGTACAAATGTAGTAAAGAAGTGTCAGGG
AGGCAGCTGTTACACCAAAAGA
>376
GTCTAACAAATTATTAAATATTCTACCACATAGTTTACCATTATCTATATAATTTCATTGAC
AATTTCAGATAGTTGAATTCTGTACAGAACAAAACCACAACAAAGAAGCTCCAAACAA
GCAAAATCAAAAATGCAATC
>377
CCTATGGCCTTTTCTGTTATTAGTCTTTCCATCTAAACGTAACCTGTTTTTCTTTTGCTTC
TCCATTTAGACTGGTAACCGCCTCAATCGACTGAATCAAGCAGGCCAGCTTTTCCTGCT
GCAGACAGTTGAGCTGGGGT
>378
TAAGCAGAATTTTCATAAACACTTTGGCCACCCTAAGTCTGATAACTCGTGCCCTAAAT
TCTGTCCTTAGGTGATTAGGAAGAAATATCCAACTGATGGATCTGAAATTGTGCTGCTG
ACGGATGGGGAAGACAACACTA
>379
AACAACAGCTAAAATGTTGCAAATAGTTGCTTTCTGGAAAATAATTTCTGAAAATGTAA
TTGCATTTTAGGAGGTTTACAGACATATGCTTCAGATCAAGTTCAGAACAATGGCCTCA
TTGATGCTTTTGGGGCCCTTTC
>380
GGGCAGAAGGGAAAAGACCATGTGATGGGAGCTGTATTCTCCAGAAAGTAAGAGCTG
TTTTTCTTAACAGCTTGAGAGTAAGGGATTAACCCTCCAGAACAGCCAGTGGATGAAT
GGCACAGTGATCGTGGACAGCACCG
>381
CGTGACATTGAAGTCAGGTCTGTGTCACCTGAAGTTATTCATTGGAAATGTTTACATGT
GTTTTGGTCAGGTTGGCACTTGGAAATACAGTCTGCAAGCAAGCTCACAAACCTTGAC

CCTGACTGTCACGTCCCGTGCGT
>382
ACATATGAGTTGGAAATATACAAATACCCTCCCCGTTACCTATTTATTAATTCCTTCATTC
TTTATCAAGGTGCTGATGCTACTAAGGATGACGGTGTCTACTCAAGGTATTTCACAACT
TATGACACGAATGGTAGGTA
>383
AAGAAAACAGACGGAGGTGTCACCATTTAGTGATGTTGCTTACCATATTTTGAGTATTT
TTTTAATGCAGATGAAATACAATGGAATCCACCAAGACCTGAAATTAATAAGGATGATG
TTCAACACAAGCAAGTGTGTTT
>384
AAGCTCTACTGCTTAAGAAGTTTCTTTAATATTTCAGAATTAAAGTCACATTCTTCTTTT
TTCTTTTTAGCTCACAAGTATATCATTCGAATAAGTACAAGTATTCTTGATCTCAGAGAC
AAGTTCAATGAATCTCTTCA
>385
GGAGACAGAGGTGGGCAGCTGTGGTCTCCCCTCTGCTTCCCTGGCTCAGCTTGCAATG
TGATTCCTTCAGAAGTTCCTGAGTGGGTGAACACCCCATCCACCTGCTGCCTGAAGTAT
TATGAGAAAGTGTTGCCAAGGAG
>386
AGAGGTGGGGGTGGGCACCGGACTCCTGCCCGAGTCTGCAGCCCATGACGGTCTAATT
CTGCCTCTGCAGCTTCGTCACCAAGAGGAACCGAGAAGTCTGCACCAACCCCAATGA
CGACTGGGTCCAAGAGTACATCAAG
>387
GCTCATTTTTCTGTGTGTCATTTGGATGACAAAAAATATGCTTATAAGCTTTCCTCTTTT
GTTTTTATAGGAGGAAAGAATGGAAATGATTTCTGAAAGGCCAAAAGAGAGTATGTAT
TCCTGGAACAAAACTGCAGAGA
>388
TATATAATTTCTTAAAATTTATATAATAAACTGTAGATTTCTCATGCTTATGTATTTCCCTTT
TCTTTAGTGTTTGACTCCACCTTACAGTCCTTCTGACTTTGAACCCTCTCAAGTGTCAA
ATCTGATGGCACCAGCGC
>389
TTAGACAGAATTGAACATATAATTTACGGCAATCTTATGCATAATAATGATTTGTATGTCT
TTGTTTTAGGAGAAAAGCCTTTCAGCTGTAGCTGGAAAGGTTGTGAAAGGAGGTTTGC
CCGTTCTGATGAACTGTCCAG
>390
GTACCAGGCTACAGGACCTAAGCTAATCTCCCACTCCTGCTGTCCATCCATTATGTTGCT
TTGTCCCCAGGTGGTAGGTGAGTGGAAATTCAGCAGGATTCACTGTGACATCTTCGTC
ACTCTGGACGTCATGATGTGCA
>391
GTGTGTGCATATTGTGGAGTGAGGGGTCCCTGGGCCTGCACCCCAGATTCAGGGTCCC
CCGCCCTTGCAGGTACACAGCTGTGGCCATGCCCATGCTGTACAATACGCGCTACAGCT
CCAAGCGCCGGGTCACCGTCATG
>392
ATGGGCTCTTGTGGAATTATGGCCGGTGGGCCCGCTGACTCCCTGCCTGCCCGGGCTCT
CCCTCCCCCAGACCAGAACGAGTGCATCATTGCCAACCCGGCCTTCGTGGTCTACTCC
TCCATCGTCTCCTTCTACGTGCC
>393

GCACTTTGGGTTTCTGTCCCCCCTCCATCCCCACCACTTTGTGTGACCCATGTGCTGGC
TCACTCCACAGGGCAACTGTACTCACCCCGAGGACATGAAACTCTGCACCGTTATCAT
GAAGTCTAATGGGAGTTTCCCAG
>394
CCAGTGAGATGGGTGGCTGATGCCTGGGAACTTGTCCGGCTTTACCCAGAGCCCTCTG
CCTCTGGTGCAGGAGGCTGCCCGGCGAGCCCAGGAGCTGGAGATGGAGATGCTCTCC
AGCACCAGCCCACCCGAGAGGACCC
>395
CTGGGTGTGGGTGTTCCCAGCCGTGCCTCCCCGGCTCTGGGGACCAGCCTGACCATGC
CCTCTCCCCCAGGCGTGTTCATCATCTGCTGGCTGCCCTTCTTCATCACACACATCCTG
AACATACACTGTGACTGCAACAT
>396
GGAGAAATAGTGCTGCCTAATCTGCTGGCGTGGGCAGTGGGCACCATGAGGACCCTGT
TCTGTGTTGCAGTTTTCACCATGGAGCAGCTGAGCTCAGCAAACACCCGCTTCGCCTT
GGACCTGTTCCTGGCGTTGAGTGA
>397
GGAAACACCAAGGAATCACTTTATAGTTTCAAAATAAATGGTTGTAGAAAGAAGATTTT
CTTTATTTTAGACTTTCCATTTCAACACGGTTGAAGAGGTTCATTCAAGATTCCAGAGT
CTGAATGCTGATATCAACAAAC
>398
GGATTTTAGAAAACAATTTCTATATGTAGTAGGAGTTATATATATATTTCCTTTTCCTTTTT
TTTAATAGGAGTTCTTGGTTTCGACTCAGAAAACATATGGTGCTGACCTGGCCAGTGTG
GATTTTCAGCATGCCTCTG
>399
TAATATATTGTCAGTTCAAATTTGGAATTCCGATTTGGATTTTTTTTACGCTAACTTGATT
TTTTTTTAGGAAAAATTCCGGAACTGTTGGCTTCGGGCATGGTTGATAACATGACCAAA
CTTGTGCTAGTAAATGCCAT
>400
CTGTACAGAGTCATGCATTGCTCTAAAAGAATGCAGAGAATAATTTCAGTGATTTTTTA
AAACTGTTCAGAAAGACAGAAAAACTGTGAAAATGATGTATCAGAAGAAAAAATTTG
CATATGGCTACATCGAGGACCTTA
>401
AATAATACTTTATTGCCTTACTTATGTAAAATTGATATCACTTCCTCTTTCGCCTCATTTTT
AATTGCAGATTGAGGAACAGTTGACTTTGGAAAAGTTGCATGAGTGGACTAAACCTGA
GAATCTCGATTTCATTGAAG
>402
ATGAGCGCGGCGAGGTGGGGGTCTCTAACGGAGCATCTGTTTTAACCCGCCCCTGCAC
ACACCCCAGCAGATCCTGGCCATGAGAGCTCCGCACCTCCACCTCTCCGCCGCCTCTG
GCGCCCGGGCTCTGGCGAAGCTGC
>403
AGCCCAGCTCCAACCCCAAACCCAGACCCTGCCCTCCGTGCTTTGATTCCGTCCCCTTT
CTTTCTCCCAGCCGCAGAGGCGGCGCTGCTCCCCCAAAACGACACGCGCTTGGACCC
CGAAGCCTATGGCGCCCCGTGCGC
>404
GCTTCACTGCCCACCTGCTTTCCCAGTCCCTGTGAAGGGTTTGCCTTCACATGCCTCTT
CCTTCCCCCAGGCCACTGTGGGCTCGAGTAGGGGATGACCACCTGCTGCTTCTTCAGG

GCGAGCAGCTCCGCCGGACGACT
>405
GTATCTGGACTCCTGGCTCCCCAAGGATTGGGGGCTGGACCCAGGGATTACTGGCATAT
TCTCCCTTCAGTGAAGTACAACAAGGGCCTGACCTGCTCCAGCATCACTATCCTGAGC
CCTAAAGAGTGTGAGGTCTTCTA
>406
GCCCCAGATCCCAAAGTGACAGCTCTCACGTTGGCACATTTATTTGATCTCTCCTTTCT
GCCACCCCCAGAGTGACTCTGGAGGCCCCCTGGTCTGTGACGAGACCCTCCAAGGCA
TCCTCTCGTGGGGTGTTTACCCCT
>407
GCAGGGGTCGCGGCCCTCCAGCATGAATGTGCCCGCTCAGCCGACGTCTCCCTTCCCG
GTCTGACCGCAGGTATCTGGCCATCCGCTACCCGCTGCACTCCCGCGAGCTGCGCACG
CCTCGAAACGCGCTGGCAGCCATC
>408
ACGGAGGTTTTGAGGCGGGGTTGCAACTCCAGGGGTGAGAGCTGACGAACTCTCTTC
TCCCCACCCCCAGGAGCTGGACACCCTATGAAGCCTCATCGCCTGGCATTGACCCATA
GCCTGGTCCTGCATTACGGTCTCTA
>409
AGTATGGGTGGTCTGAGCCTGTGGAGTGGGGCTGACCCTGCCTGGGTTCATCTTGTGTA
TCCATCCCGAGGTCTTCAAGCCATACCAGGCCTCCCAGCATGACATGTGCCGCTTCCAC
TCCGAGGACTACATTGACTTCC
>410
ATGGGGAGGGAGATATGGGAAGGGCCCAGACAGAGGCAGGAGGGTTCCAACTTGTTC
CTCTTCCCTGCAGCCCAGTGTTTCCCGGGCTCTTTGAGTTCTGCTCGCGTTACACAGGC
GCATCTCTGCAAGGAGCAACCCAG
>411
CCCGGTGTTTGAGAGGAGTGGGTTGGGGGGTTCACTTCTAGCTCAGACCCTGGCTTTG
GCTTCCCTCTAGGTACCACCCTCGGGTGCTCTACATTGACATTGACATCCACCATGGTG
ACGGGGTTCAAGAAGCTTTCTAC
>412
AGATTGGGGCTGAAACAGCTGGGTTGTGGGTGCTTCCCATGTGTGGCTACCACCACCC
ACTTCCCTACAGGTGACATGTATGAAGTCGGGGCAGAGAGTGGCCGCTACTACTGTCT
GAACGTGCCCCTGCGGGATGGCAT
>413
CTTTTTCCTTTTGTTCCCTGTCTCCCCATCCCCTTGGGGTGCCAGGTGCCCATCTTTGGC
CCTCTCCCAGGTTACAAGCACCTTTTCCAGCCGGTTATCAACCAGGTAGTGGACTTCTA
CCAACCCACGTGCATTGTGCT
>414
CAATACCTAAAGGTATTTACCTTGTCTTTTGATAACCTCCTCCACATCTTCCTCTGGTTC
CTTTTAACAGGGAATGCGTTGAATATGTCAAGAGCTTCAATATCCCTCTACTCGTGCTG
GGTGGTGGTGGTTATACTGTC
>415
GAAAAAGATTGGGAAGTCTTGTTAGCTGCCTTTCTCAGTCTGAGCCATACTTTCTCCTC
TCCTCCCCAAGAATACTTCGAGTACTTTGCCCCAGACTTCACACTTCATCCAGATGTCA
GCACCCGCATCGAGAATCAGAA
>416

GCAGCTAGGAGATTCCATGTCCTGGGAATCTCTGGAAACTGCTCACTCTGTATTCCTCA
TTTCCAACCAGTATCTGGACCAGATCCGCCAGACAATCTTTGAAAACCTGAAGATGCT
GAACCATGCACCTAGTGTCCAGA
>417
TTGGAAAAGGGTGCTGGGAGAAGATTAGAACTTAGAGTGAGATCCTGACCTCTCTCCC
CTTGCTTTGCAGGCCAGAGGCACCCAATGAGTTCTATGATGGAGACCATGACAATGAC
AAGGAAAGCGATGTGGAGATTTAA
>418
CAGGTAGGGGATGGCCCTGCCTCTTGTCCAGCCAGAGCAGCAGCAACCTCTCTTTTCC
TTTCCCTTACAGGAAGCTGCACAGTGGGATGAAGACGTACGGGTGCGAGCTCTGCGG
GAAGCGGTTCCTGGATAGTTTGCGG
>419
TGTGGCCCAGGACCTCCCCGCTCACCAGTGGACTATTGCTCTAGTGGTAACTGCCTCTC
CTTTCTTTCAGCGGGTGCCAAAGCCTTTGTCTGTGATCAGTGCGGTGCACAGTTTTCGA
AGGAGGATGCCCTGGAGACACA
>420
CTCCAGATGGGTAAAGAATGCACCTTGTATTCCCACCTTTCTGAGGCACCCCCTCTCCT
GTCTCCCACAGGCACTGACATGGCCGTCTTCTGTCTGCTGTGTGGGAAGCGCTTCCAG
GCGCAGAGCGCACTGCAGCAGCA
>421
TGTGCCCTACTGTCCCCTGAAGAGGGGGCAGGGAGAGGCAAAGGCCTGATCCAGCCC
CTTGTCTCCACAGGCGACCACCCCTACGAGTGTGAGTTCTGTGGCAGCTGCTTCCGGG
ATGAGAGCACACTCAAGAGCCACAA
>422
AGGGTGACATGGTGCCCTCACTGCCTTCTGTCTGTCCTCACTTTCTCCTGCCCTGTCCC
TCCGCCCTCAGGTGAGAAGCCCTTTGAGTGTAAGCTCTGCCACCAGCGCTCCCGGGAC
TACTCGGCCATGATCAAGCACCT
>423
GGGACTCTGGGAGTCTGGCCAGCCCCAGCTGCCGTCTTAGCTGGAGGGGCTCCCTCCC
TTCCTCTTGCAGGTGCTCCAGAAAGCAGGAGTTGAAGACCTGGGTGTGAGGGACACA
TACATCCTAAAAGCACCACAGCAGA
>424
GTCATTCCTGGTGCCTCATGGCTCTGCCTGCCAGCTGTGCCCCGAGGTGCTTTCTCGCG
TCCTTCTACAGGTTCCCAGAAGACAAACCCCCTAGGAAGACAGGCGACCTGTGAGGC
CCTAGAGCACCACCTTAAGAGAAG
>425
TTATTCCCCTCGTCCTCCTCCTGTTTTTCTTTCTCATCCTCATCCTCCTCTCTGCTTCTGC
GTTCTCCAGGGGACAAGGATATGCCTACTGCTGGGATGCCGAGTCTTCTCCAGAGTTC
CTCTGAGAGTCCTCAGAGTTA
>426
AGACTCATTTTCTTTTTTTGTCTCTTCTGTCTCTTGTGTGTCTGTGTGCCTGTACATACCC
CTCCCTCAGACTCCCGAAGCCCAGACAGCTCCTCCCCAAATCCCCTTCCCCAGGGAGT
CCCTCCCCCTTCTCCTCCTGG
>427
TTCCATCACTTCTGTGACATTCTGCTTCCCAATAAGTCTTCCTGTGACTTCCCTATTTCC
CCCATCCCAGATTAACTCAACAGTGTCACTCCCTGGGGGTGGGTCTGGCCCCCCTGAA

GATGTGAAGCCACCAGTCTTAG
>428
ATTGGAAACAGACTAAATGGTGAAGGTGTCTCCATGCAACCTTCTTATTGCTCCTCCCC
TTTCCCTGTAGGCAAACACTACGGGGTTTACAGCTGTGAGGGTTGCAAGGGCTTCTTC
AAACGCACCATCCGCAAAGACCT
>429
GGATATTTGTGGAACCCCTTCATGGGTGGATGCTGACTGTACATTTTTTCTGTCCCCCTT
CCCCCTGCAGCGGTACAGGAGGAGCGTCAGCGGGGAAAGGACAAGGATGGGGATGG
GGAGGGGGCTGGGGGAGCCCCCGA
>430
AAAGCCTCTTACAAGGGGGCTCCCAGCATATCTCAAAATCTTCCATAACTCTTACCCCC
GTCCCCTGCAGCCAAATGACCCTGTGACTAACATCTGTCAGGCAGCTGACAAACAGCT
ATTCACGCTTGTTGAGTGGGCGA
>431
TCTCTTCCACTGATGTGCTTTGAATCCCTTGGCCTGATTTCTGGCTCCTGACCCTTGCTG
CCCCACCCAGGCTGGAATGAACTCCTCATTGCCTCCTTTTCACACCGATCCATTGATGT
TCGAGATGGCATCCTCCTTGC
>432
GCTATAGGCTGGTCCGTGTCCAAGGCTGGCTGAGCTGTGACCTTTGAGTGACCTGCAG
GTCCCTCTCCAGGGTGCTGACAGAGCTAGTGTCCAAAATGCGTGACATGAGGATGGAC
AAGACAGAGCTTGGCTGCCTGAGG
>433
CACATCTGCATGGCCATCCTGATTTGGTTTGTCTTCATTTCTCTTCCTCCTCTCTTCCCTG
CCATCCCAGATGCCAAGGGCCTCTCCAACCCTAGTGAGGTGGAGGTCCTGCGGGAGA
AAGTGTATGCATCACTGGAGAC
>434
GTGCTATGAGGATGTGTTCAGGGCCCGTGTGCTTCCAGCTCTCAATCCCCTTCTCCCAC
CTCCACCCCAGGTTTGCCAAGCTGCTGCTACGTCTTCCTGCCCTCCGGTCCATTGGCCT
TAAGTGTCTAGAGCATCTGTTT
>435
GCCTCTCCTCTGACAGGGCCTCTCATCATGCCTATGCCTACTTCACCTCTGACTCCTGCC
TTGGTTACAGGAGGTGGAGGCCGGAAAGGTCGCACCAAGAGAGAAGCTGCTGCCAAC
ACCAACCGCCCCAGCCCTGGCGG
>436
TGAGTGGGAGAGGCAGAATTAGAACCCAGATCTCTGTCCCTTTTACCACCTGCTTTTCC
TCACCCCCCAGATGAGGCCAGACCATGGACACTACACCCAGCAATAGAGACGGGACT
GCGGAGGAAGGAGGACCCAGGACA
>437
GCCTTGCCCCCTCCGGATTCAGGCCGATGGGGCTTGAGCCCCCCTGACCCTGCCCGTG
TCTCCCTCGCAGCTGGGCGCCGTGTACACAGAAGGTGGGTTCGTGGAAGGCGTCAATA
AGAAGCTCGGCCTCCTGGGTGACT
>438
GGGACAGGGGACCGGCTGGAGACAGGGCCAGGCGGGGCGTCTGGGGTCACCAGCCG
CTCCCCCATCTCAGTCTCCCGGGACCTGCCCGTTATGATCTGGATCTATGGAGGCGCCT
TCCTCATGGGGTCCGGCCATGGGGC
>439

TGATCTCCCCTCCTGGAGGCCAGGCCTGGGCCACTGGTCTCTAGCACCCCCTCCCCTG
CCCTGCCCCCAGGTAACTATGGCCTTCGGGATCAGCACATGGCCATTGCTTGGGTGAA
GAGGAATATCGCGGCCTTCGGGGG
>440
GAGGCAAGGGGCGGCTCAGGGGAAACTGGGAGGTACAAGAACCTGCTAACCTGCTG
GCTCTCCCACCCAGACCCCATGCTGCACTATGTGGGCTTCGTCCCTGTCATTGATGGAG
ACTTCATCCCCGCTGACCCGATCAA
>441
GCAGGTGGAGAGCAGGGCTTCAGCCCCCTGGGAGTCCCCAGCCCCTGCACAGCCTCT
TCTCACTCTGCAGGGACCCCAACATGGGCGACTCGGCTGTGCCCACACACTGGGAAC
CCTACACTACGGAAAACAGCGGCTAC
>442
CCTGGGGAGGGTGGGATGCTGGGTGGGCTCTCCTTGCCTGCTTACTTTCGTCCTCCTTT
CCCCTGCCCAGGGGTGTGGGGTACGGATACAGAGGAGCGGCTGGTGGAGCATCTCCT
GGATCCTTCCCGCTACAACAAGCT
>443
AGTTCGTTTCCTTAACCTTGAGCCCCACCCAGGGCACAAGTTGGTACTGCCTCCCTCTC
TCATTTCCCAGGAGTGGGAAGATTATCGCCTCACCTGGAAGCCTGAAGAGTTTGACAA
CATGAAGAAAGTTCGGCTCCCTT
>444
AGGGGCTGGGTTGATGGGTAAGGAGGAAGGAACGCTTAGGCCAGGGCTGACTGTGCC
CATCCTTTGGCAGTGCTGACGGCATGTACGAGGTGTCCTTCTATTCCAATGCCGTGGTC
TCCTATGATGGCAGCATCTTCTGG
>445
ACCCGCTTATACTCGTTTGTCTCCCATCCTGCATCATGTGACCTGGGCCTCCTCCGTCTC
CTCCATCCAGGTGAGTGAGGACTGGAAGTACGTCGCCATGGTGATCGACCGCCTCTTC
CTCTGGATCTTTGTCTTTGTCT
>446
AGTGGCAGGGATCCTGGCTGTCTCTGCTGTGTCCCTGCCTCTGTACCTGAGTGTTTCTA
ATGACTTGCAGGTGGAGCTGGAGACCTGGTCTCTCTAGGGCCTACCCTGAGCTCACCA
TCTGAAGGAGAGTGCCATCATCC
>447
TCCCTGTAAGTCTCTCCGCACTCTTCCCCACACCCACCTCCACTCCAGCCCTGATTCTG
GCCTCCCACAGCCCAGCGCAGACTGTCCGAAGCTCTGAAAAGGCTACGTTCTGGGCCT
AAGTCTAAGAACTGGGTCTACAA
>448
ATGAGGTGGCTTGAGGGGCAGGGAGGGGGGCGCAGGGCTCTGCATACCGGATTTCCC
ATCCCTGTTTCAGATCCTTGGAGGTTTTGCCCCTGAGCACAAGGAGCCCACCCTGCCC
TCGGACAGCCCACGGCACCTCCCTG
>449
TCATCCAGGCCAGCTGCCCCCTCTGTTCTTCATGCCCGCAATGCCTAAAGGCGCTTCCC
CCCACCTCCAGGTCCCAGTGGCTGTCAAGTCCCTCCGGGTAGGTCCCGAAGGCCCGAT
GGGCACAGAACTGGGGGACTTCC
>450
GTGCGGCAGGAGCGTGGGCGGCCAGGGTCCAATGGGTCCGGCTCACGCGGCGCGGTG
TTCCCTCCTGCAGGTGATGGAGCTGGCGCCACTGGGCTCCCTGCACGCGCGCCTAACG

GCCCCGGCCCCGACACCCCCGCTGC
>451
GGTGCTCCGTGGAGGGTGGGCCTCCAAGGAGCACGTGTCCCGCCCCTTTCAGCTCCAC
TTCCTTCGGCAGGTGTGCCCCAGAGAGCCTGCGCCACGGAGCCTTCTCGTCTGCCTCG
GACGTGTGGATGTTTGGGGTGACG
>452
ATTCCTGCTCCCATATTACCTATCTGACTCAGGCCACAAGCCCAGCCACCCTTTCCTCTT
GTCTCCACAGGCCGGGCCTTCGGAAGCATGTTGTGTGAGGGATGTCACAGAACCAGG
CGCCCTGAGGATGGAGACTGGTG
>453
CTGTGGGCCAGGGCCCAGCCCTAACTCTTGCCCTTATTCCCATCCTATTTACCAGCTCCT
CTTTCCACAGCCCCGACTCCACAATCTGGAAGGGCCAGAATGGTCGCACCTTCAAAGT
GGGCAGCTTCCCAGCCTCGGCA
>454
TGGGAGGGTATATTGATCTGTGGGGTGGGGAGAGTTGGTGTGGAGGATGAACTGGATC
CCTCTCCGACAGAGACAGAAAGAAGGCAAATCTTTGGGATGCGCCCCCAGCACGGGG
CCAGAGGAGGAACATGCCCCTGGAG
>455
GATCGCTTGAGCCCGGGAGGCGGAGGCTGCAGCCAGCCGAGTTCAAGTTGTTTCCTTC
TCAACTGTGCAGGCATTTCCAGGAGTCTGGAGTCAGTTCTGTCCCTCGGTCCTCGTCC
CACAGGGGGTGGTTCAAGCCCCCC
>456
GAAATGGCCTGGTGTCCAGAAGGGGCTACAGGCAGGGGCAGGGGCCTGAGTGAGGCT
TTGTCTGTCACAGATGGAGCTGAGTGTGCATGGGGTCACCCACCAGGAGTGCCAGAC
AGCACTAGGAGCCACTGGGGGAGATG
>457
TCTTGGGGTCCCTCCTCTCCTGCCCCTGCCGCCTGGCAGTCAGCTGCAACCCACCCTC
CTGCTTCCACAGGTAGATCAGCTCTTCCACCTGAGTAGCCGGTCCAGAGCTGACTGCT
GGCGCATCCTGGAGCATTACCAGT
>458
GTGAAGCCAGCAGGTGCTCTGGAGTTAAACCAGCTTTCTGCAAGCCCTGCTTCCTGGT
CTCCCTCTCCAGGTACTGGCTGTGATCGAACTTCTCAACCCTCAGAGACTTAGATCTTC
CACCTCACTCCCTCAGCCAAGCC
>459
CAGGCAGAGCTGGGTGACAGGCCTCGCACTGCTGGCCCTACCTGTAACTGGGCCTGTT
GCTGTCTCCTAGCACAAACTCTCAGAGCCCATCCCCACCCAGCAGTTCCATTGCCTACA
GCCTCCTGAGTGCCAGCTCAGAG
>460
GAGGTCTGGGGGCCCAGGCCACACTCTGGGACTAATGTCTTTATCTCTTTCTTCCCTGT
GGGCCCCGCAGCAGTGAACAGTCAGCCCGGGCAAGGACTCAGAAGGAACTCATGAC
AGCACTTCGAGAGCTCAAGCTTCGA
>461
ATGGCCTGGGCCTGGGCTCCCGTGTTGAGGCAGCCTCCTTACTGAATGCCTCACTGCCT
CCACTCTGCAGCCAACCAGGAATACTACCAGCAGTGGAGCCTGGAGGAGGGCGAGCC
TTGCTCCATGGACATGTCCACCTA
>462

GCATCCCGACGCACCCCTACTCCTTCTCCCACAGCTCTTGAATCTGCTCTTGCTCTCCC
CCTCCTGCCAGGATACCTTCTCAGTGGCTGTCTCCTTCCTGACGGGCCGAATCGTCTAC
ATTTCGGAGCAGGCAGCCGTCC
>463
GTGACCTTATTGTTGGAGGAGTGGGTGGCTCTCTGTTGAAAGTCCCTAATGCTCCTCTC
TCCTTTGCTAGAGGAGGTCCTGACCGGGATCCAGGGCCTCGGTACCAGCCATTCCGCC
TAACCCCGTATGTGACCAAGATC
>464
AGGCAGAGTGGAGGGCTGAGGAGTTGGGCATTCTGGACGAAACCCAGTGACCACACT
CTCTGTGCCTCAGCTCCCCGGATACCCCCTGACAAGAGGATTTTCACTACGCGGCACA
CACCCAGCTGCCTCTTCCAGGATGT
>465
TAGGACCTAGAGGAGACGAGGGCAGCCAGGGCTGAGGCCACGGCCACTCTGATGCCT
CCTTCCCGTTCAGGGCTGCCCCCCTGCTGGGCTACCTGCCCCAGGACCTCCTGGGGGC
CCCAGTGCTCCTGTTCCTGCATCCT
>466
GCGTTGGGGATAGGGCAGTGCGGTGGGGGACAGGACCGGGCCAGGGCTGGATTCACT
CTTCACTCTACAGGGCCCCCCTGAATGAGGACGTGTTCACTCCCCCGGCCCCCAGCCC
AGCTCCCTCCCTGGACACTGATATC
>467
GAGCCTGGGAGGTGAGAAAAGGTGTGGGAAGCGGGGTCAAGCCATCTAACCTGCCCT
CTCCCTGCTGCAGCCCGTCCACAGCCCCAGCCCCACGGGACTCTGTGGAGTCGGCGCC
GTGACATCCCCAGGCCCTCTCCACA
>468
CCAACCCATTTGCTGTTTTTTTCCATGTGAGCTTGAGAAGGCTATTTTCCTCTTTCTCTC
TTTGCCCCAGCTACAGGCACGTTCAAGGCCAAGGCCCTTCCCTGCCAATCCCCAGACC
CAGAGCTGGAGGCGGGTTCTGC
>469
CTCCACCTCGGGCTCTAACCCTGCCCACCCCAAGCCCTGCTCTGATGCCCTGTGCTGTG
TCCATCCCCAGGTACCTGGAGAGCTGCAACCTCCCCAGCACCACTAAGCGTAAATGTG
CCTCCTCCTCCTCCTATACCACC
>470
TCCCTGGGATCCTCTCTCCTGACTTCCCAGTGGGGGTGGGGATGGTAACTGGCACCATC
TCTCTGCACAGATCCGCCGTCAGCAGCGCTGTCTGGGGAGGGGGCCACCCCACGGAA
GGAGCCAGTGGTGGGAGGCACCCT
>471
GAGGGTGTGGTATGGATGTGTTGACCCCTGAAGTGGTTCTGATGACCTTTCCCTATCTC
ACTCTCCTCAGACATCATCATGATGGAGGACCTGCCTGGTCTAGCCCCAGGCCCAGCC
CCCAGCCCAGCCCCCAGCCCCAC
>472
AGGGCAGTGTGGGTAGCAGGGGTACTAACCCCAGGTTGAGGTCCTTGCTAACCCTAGT
CTCTCCCCACAGGCTGCCACCACGGCCCCGCACCCCCAAGCCGCCGACACCACTGCC
GATCCAAAGCCAAGCGCTCACGCCA
>473
GTAAGAGCAGTTGAAGGGAGGCCTAGGTGCTGACCCCTCCATCCCTCCTTGCCCCCCT
CCCCTCCTCCAGGCGGAGGTCACTGAGTCCTCCAATCAGGACGCACTTTCCGGCTCCA

GTGACCTGCTCGAACTTCTGCTGC
>474
GGCCTTGTGAGGTCCCAGGAGTGGGCATGCAGCCGGCCTGACTCCCATTGGTCTGCCC
CCCACTTCACAGGCAGCAGCCAGAGCAGCCACACAAGCAAATACTTTGGCAGCATCG
ACTCTTCCGAGGCTGAGGCTGGGGC
>475
TGCCAGCTTCATGGGTCCTGGTCCGGGTCCTGGATCCCAGCCTCTGCCTTCTGAACCCC
TTCTTGGGCAGGGACATGACCTCTGTGCTGAAGCAGGATCGGGAGCGGCTCCGAGCCA
TGCAGAAGCAGCAGCCTCGGTTT
>476
GGGAAGCTGCGCTGGCAGGTTAGCAGTGAGAACCCTGTCTGACTCTCTCATGTCCATT
TCTCTCACCAAGGCCTGTGTGGACTGTGGGAGCAGCACCCAAGATCCTGGTCACCCTG
ATGACCCACTCTTCTCAGAGCTGG
>477
CACTTGGGAGGGGGCAGGGGAGCCCAGGCAGGCCTGGTGGGGAGCTGACCCACCGC
CGTTTCTCTGGCAGGTTCTGACGCGATGAGGAAGCACCTGAGCTGGTGGTGGCTGGCC
ACTGTCTGCATGCTGCTCTTCAGCCA
>478
TATCCTCACAACAGGCCAATGTTGGCATCTTTGGACCTTTCTAAACATCAACATTTCTGT
TTTCCTTTAGGAATCGTCTACGAAGCCTGATAAAAAGGTTTCTGTCCCATCAGGACAGA
TTGGTGGCCCCATTGCTTTGC
>479
TGCCAGGTTTGGTGTGAGTGAGCCACGAACAGGACTGGAGTTCTTACCGCTTGCTGTG
TTTCTTCCCTAGAAAGGAGCGAGTGGGACTGTGCTGCTGGCCGGCTCCTCTTTGATGG
CTTCACCCTACAAATCCAGCAGCC
>480
GGAGCTGCTTTCCTGTTCCTGTCTTCAATGTGTGTGTTTTGTTCTTTTCTCCCCTCCTGT
TTTCTCTCAGGTCTTCTGGCTGGCTTGCACTCCAGCCCGCCCCATGCAGCGCCTCTCCC
ACACGCTGCGGTGCCCACCCA
>481
GTTACTACTGCCTTAAGGAAATCAGAGAGCATGAAAAACTTGCGTTCTAATTTTTCTTA
CTCTTTTCCAGGTGCTTCTCAGCTTCATGGGAAAGGGCCTGCTGTACCACGGAAATTGT
GACCGCTTCAGAGGCAAGGCTT
>482
AGTAGCTGGGAGTGTAAGGAGAGGACCCTCGTCCCCTCACCAACCTCATCCTCTCTCC
CCCTACCCACAGGTAGCCTCATGGCTGCAACCTGTGAGATTAGCAACATTTTTAGCAAC
TACTTCAGTGCGATGTACAGCTC
>483
AAAAAAAATGGGGCTGTAAGGTCTGCTGGGTGGCCTGAGCTGAGCCTGTTTCCCTGCC
TGGCCCTTGCAGAGAAGGCCAGCTGGTTGGGGGAACAGCCCCAGTTCTGGTCGAAGA
CGCAGGTTCTGGACTGGATCAGCTA
>484
GCTGCACAGCCAGAGAGAGCCCTTGAGGGAGGGATTAGGGGAGTGTGACCCTTCCTT
CCTTCCTTGTCAGCTTCCAGCTCTTCTGATGAGCTCAGTTGGATCATTGAGCTGCTGGA
GAAGGATGGCATGGCCTTCCAGGA
>485

CCATGGGCAGCATCACCTGTCCTGGTCTGGTCCCCTGAGCCCTCTCTGAGTTCTCACCT
CCTCTTCCCAGACCAGGGCAGCCCCTTTGCCCAGGAGCTGCTGGACGACGGTCAGCA
AGCCAGCCCCTACCACCCCGGCAG
>486
CCTGAGAGCCAGCAGCGTGGTTGAACAGAAGGTGGGCCGGCAGGGGACTTACTCTGA
CCCCGCCCCCCAGGGACTGGTGCTTCTCGGAGCTCCCACTCCTCAGACTCCGGTGGAA
GTGACGTGGACCTGGATCCCACTGA
>487
TCCTGTAGAGGGGCTACTCTCCCTAACTCCCCTCTTGCCCCTCCTTGACCTTCCACCAC
CGTCCCCACAGATGGTTTTCGTGACTGCAAGAAGGGGGATCCCAAGCACGGGAAGCG
GAAACGAGGCCGGCCCCGAAAGCT
>488
GGGCAGGGCTGGCTGGCCTTGGGTGAGAGGGGACACCTGGATGGCAAACTGATGGAG
GCTGGCCTTGCAGCGCCCAGAGGCACCCACCTGTGGGAGTTCATCCGGGACATCCTCA
TCCACCCGGAGCTCAACGAGGGCCT
>489
GGTGGGCGGGCTGGCAGCCTGGGGCCCATTTCCTGCCAAAGCACCTCTGACCATCCTT
CTCTTCACCCAGGTACTACTACAAACGGGAGATCCTGGAACGGGTGGATGGCCGGCGA
CTCGTCTACAAGTTTGGCAAAAAC
>490
CGGTAAGCCCACCCCCAGGAACCCTGGGCCCGCCTCCTGGTCCCGATCCCATCCCTGA
CTCCGCCCTCAGGATCGCTCGTCTCTGGTAGCTGCAGCCAAATCATAAACGGCGAGGA
CTGCAGCCCGCACTCGCAGCCCTG
>491
GGGGTTGAAGATGAGAATGGAGGTAAGGATGTAGTTGCCACAAAACTGACCTGCCCTC
CGTGGCCCACAGCTCCTACACCATCGGGCTGGGCCTGCACAGTCTTGAGGCCGACCAA
GAGCCAGGGAGCCAGATGGTGGAG
>492
TGTGTGTCTGCCCTCTTCAAGGAGGTCCTCTGCCCAGTCGCGGGGGCTGACCCAGAGC
TCTGCGTCCCAGGCAGAATGCCTACCGTGCTGCAGTGCGTGAACGTGTCGGTGGTGTC
TGAGGAGGTCTGCAGTAAGCTCTA
>493
CCTTTCTGAGCCCACATCCATTTCCACCTCTCTGTGTTTTGAATTCTGACTCTCCCTCTC
TTCACAACAGGGTGACTCTGGGGGGCCCCTGATCTGCAACGGGTACTTGCAGGGCCTT
GTGTCTTTCGGAAAAGCCCCGT
>494
GTATGTCCCTAGCCCTGGCTGCTGAATTGTTGCGTTCCTGTTGTGTGTTTATTTTTCATAT
TGGCTGAAGACCAAGAGGGAAGAAGCACAGAATTCCTCAACTCCCAGTGTGCCCATG
AGTAAGAGCAAATGCTCCGTGG
>495
GCCCAAGCTCCCACGAGGAGAGATGGGCCCTTCCGAGGCCCCAAACTAAAGGTGCTT
TTCTGCCCCACAGGTGCCTTCCTGGTCCCCTACCTGCTCTTCATGGTCATTGCTGGGAT
GCCACTTTTCTACATGGAGCTGGC
>496
AGGGACACGTTGCTGATGGTGGCTCTGTGCTCCACCTGCGTGGGCTCCATGGCCTCCC
CCTTCCCGCTAGGTGTGGGCTTCACGGTCATCCTCATCTCACTGTATGTCGGCTTCTTCT

ACAACGTCATCATCGCCTGGGC
>497
AAGCTCAGCCGTCCAGTTCCAGGTGGGTTGACAGCCACCCACAGAGTCCCCCGCTGA
CTCCCCTCTGCAGACGTGGCGTGCTGCACCTCCACCAGAGCCATGGCATCGACGACCT
GGGGCCTCCGCGGTGGCAGCTCACA
>498
CCTTGTGCCAGTGTCTGCTCCCACCAAGGGCCCTGCCTGTGCAGCCTGGTGACACTGT
CCTCTCCTGCAGGTGGTATGGATCACAGCCACCATGCCATACGTGGTCCTCACTGCCCT
GCTCCTGCGTGGGGTCACCCTCC
>499
GGTGCTCAGGTCCTTTGCCTGTGGCCTGCGTTCTCCTGGGACAGCCTCTCACCGCCCT
CTGGCTCTGCAGGTTTGGATTGACGCGGCCACCCAGGTGTGCTTCTCCCTGGGCGTGG
GGTTCGGGGTGCTGATCGCCTTCT
>500
AATAATGACTGGAAAATTGCAGCTGCTGCAGCTCAGCTGGTTCCTGAGACGGCGTGTC
TTGTTCTTACAGGGACGCGATTGTCACCACCTCCATCAACTCCCTGACGAGCTTCTCCT
CCGGCTTCGTCGTCTTCTCCTTC
>501
CGGGGTGGGGCAGGATGGGCGGGAGAGCACAGCGTGGGCTCTGTGGCAAGCAGGGC
GGGTTCTGTTTCAGGGCCAGGGCTGATCTTCATCATCTACCCGGAAGCCATCGCCACGC
TCCCTCTGTCCTCAGCCTGGGCCGT
>502
GTAGGTCTTGGCCCAGGCTGCGGTCAGGAGCGCCGCCAGCCTGCAGCCCGACCCTGT
GCTCTGTGTGCAGATGGGTGGTATGGAGTCAGTGATCACCGGGCTCATCGATGAGTTCC
AGCTGCTGCACAGACACCGTGAGC
>503
TGAATCCCCAGGCTGGGTTTACCTCTGGGGAAAGCCCTTCTGTAGGTCCACAGGCTCC
CCTCTCTTCCAGGGTGGCATCTACGTCTTCACGCTCCTGGACCATTTTGCAGCCGGCAC
GTCCATCCTCTTTGGAGTGCTCA
>504
TGCCCGCTCCCTGCTTTGTCCTGGCACCGCCGGCTGAGAGCTGCCTGACCTCCGTATCT
GCTGGTTGCAGTTCGTGGTCGTGGTCAGCATTGTGACCTTCAGACCCCCCCACTACGG
AGCCTACATCTTCCCCGACTGGG
>505
GTGAGTCAGTGGTGGGTGCTGGCAGTGGGTACTGGTCTGCACTGACCCCTGCACTCTC
TTTCTCTTTCAGAAACTGGCCTACGCCATTGCACCCGAGAAGGACCGTGAGCTGGTGG
ACAGAGGGGAGGTGCGCCAGTTCA
>506
TAGCCACCTTCAGCTGCTCTTAAATGGGGCATCGCCCCAGGGTTTCTGACTAAACCTGT
TTTCTTTCCAGCTCCGCCACTGGCTCAAGGTGTAGAGGGAGCAGAGACGAAGACCCC
AGGAAGTCATCCTGCAATGGGAGA
>507
CCCTGAACAATAAGCACAATGCAGTTTTAAAGACTGTTTGCTTATTTGATGAAATTTCG
TACTTTTATAGGAGAATGGGGTACTAAGAGAAAAGCACGAAGCTGTGGATCATAGTTC
CCAGCATGAGGAAAATGAAGAAA
>508

AGATAAAGCCTAAAGCCCCAACCAGTTCAGTCTTTTATGTGAAAGATGCACTTTTTGTG
TTTCTCTACAGAGACATCTGAATGGGACAGATACTTCTTTCTCTCTGGAAGACTTATTCC
AGTTGCTTTCATCACAGCCTG
>509
TTGTTAGGTAATAGAACTGCTTCAGAAATAGGGCTATTCATGTTTGAAGTGTTTCTCCTT
CGTTTTTCAGGGCATCTCATTGGGAGATATTCCTCTTCCAGGCAGTATCAGTGATGGCAT
GAATTCTTCAGCACATTATC
>510
AGAGTAAACAGGAGGCATAGCTGCAGCTTCTGTGGCAGAGCTTGCCTTAACTTCTCAT
TCTCTTCTTTAGCCCCCAGCCCAATTGCCATCAAGACTCCTGAAGCCAGCTGTGCTTGA
CCAAGGATGGGCCATAAACAATG
>511
TTCCCAGCTGGGAGGGGCGGGGACAGGTGTGCGGCTCCTGCTTCTCCTGAGGGCCTC
CCTTTCCCCGCAGCTGTGAGGTGGAGCAGTGGGACTCGGATGAGCCCATCCCTGCCAA
GGAGCTAGAGCGAGGTGTGGCGGGG
>512
GATAGTCCCCAGTGCTGTGGCTTTGAGTTCCTCGAGGAAAGATACTAACAATGCACTTT
CTGCACAACAGGGGCCAATCTCAAAGTCATCAGCACCATGTCTGTGGGCATCGACCAC
TTGGCTTTGGATGAAATCAAGAA
>513
AAATGTCCCAGCAGTAGACATTGGTAAATATGGTTGAGGCTGATGTTACCACCAGATGT
TTGATTCGTAGTGGGATCCGAGTTGGCTACACCCCAGATGTCCTGACAGATACCACCGC
CGAACTCGCAGTCTCCCTGCTA
>514
TGCCGGGTTCTCAGCGGCCCCATCTTGGTCCAAGGCTGGGCCTCTCACCTGCCCCTCT
CTCTCCCCTCAGTGGTGGCTGGACCTCGTGGAAGCCCCTCTGGCTGTGTGGCTATGGA
CTCACGCAGAGCACTGTCGGCATC
>515
TCCGGAAATGCTGGGTGGTGTCCCTACCCTTTGCGGGACGGGAACGAGACATGGACTC
TCCTTGCTCTAGGCCAGGCCATTGCTCGGCGTCTGAAACCATTCGGTGTCCAGAGATTT
CTGTACACAGGGCGCCAGCCCAG
>516
GCTGGTCTCCCGCCATCTGGTTGTCCCTAGCCTGGGACTCAGTGCCTGATGGAGTCCTG
CCCTCCCTCAGTGTCTACCCCTGAGCTGGCTGCCCAATCTGATTTCATCGTCGTGGCCT
GCTCCTTAACACCTGCAACCGA
>517
GTTGTTCTGCCTCAAAGGTGGCCTGGGCGGAGGGATCTTCGGGGTACCCATGTCACCA
CTGTCATTCCAGGGGCGACGTCGTAAACCAGGACGACCTGTACCAGGCCTTGGCCAGT
GGTAAGATTGCAGCTGCTGGACTG
>518
GAAAACAGCCCAGCTGAAGGCTGCTGAACCACCCTTCTTATCTCCCTCTCTCTCTCTCT
CTCCTTTCCAGTGATTCTGCCCCACATTGGCAGTGCCACCCACAGAACCCGCAACACC
ATGTCCTTGTTGGCAGCTAACAA
>519
TTGGAACTGGGCGGGGGGTGAGTATGTGCTGGGTGGGGCTGGGCTCCCCAGTGACCT
CCCCGCCCCCCAGGCCGGCTGCATCCCAGTGCTTCTCAGCCCCCGCTGGGAGCTGCCC

TTCTCCGAGGTCATCGACTGGACCA
>520
CCCTACTCAGGTCATTCTTCATGGGGTTCTGTTCCCTCCCACTCTAATAGCCTGTGTCTC
ATTCCCCCAGGCTCCCGCCCTGAGGGCAGATTCAGCGCCCTGATCTGGGTGGGGCCCC
CAGGCCAGCCCCCTCTGAAGCT
>521
GCCTCCATTTCCCTGGATGTTCTAGGCATTTAGGAGTCCAACCACACAGCTCACTTGAG
TCCCTCCCCAGGTGGACTTTGCCTTTCTGGTGTGGCAGAGCTTCCCAGAGCGGATGGT
GGGCTTCCTGACGTCGAGCCATT
>522
AGGAGGGAGGAGAATGGGGCCTGGGGATGGATTTGGCTGCAGCCTCTCCCTGTCTTTT
CCTCTTGCCCAGGTATTACCACACTCTCTTCACCCACTCCCTGCCCAAGGCTCTGAGGA
CCCTGGCAGATGAGGCACCCACC
>523
CTTGCTGCACGGGCGTGGGGAGTCGGAAGGGCAAAGAAGCCCGTTAATGCATTGCTTC
TTTCCCTCTTAGGCGCCTGGGGGCCCGGGGCCCAGGCCAAAGCCGCCTGCCCCAGCCC
CCGACTGCATCAACCAGATAGCGG
>524
GAGGAGCAGGGGTTTCCTGTCAGTGACGCAGCCCGTGAGAACCTCTCTCCCTCCCTCT
TCCTCCGTTCAGGATCCTGTTGACTGTGGTGGTGATCTTCCGGATCCTCATTGTGGCCA
TTGTGGGGGAGACGGTGTACGAT
>525
GCGGTGGGCTCATGTTTGTTGATTGAGTGAATGATTTAATTAACGCCTGACTCTGCTTTT
TCTCCCTCAGAGTTTACCTTCCAGCAGCCCTACGACCAGGCCCACCTGCTGGCAGCCA
TCCCACCTCCGGAGATCCTCAA
>526
GGGAGGGATTCCCATCACTGCCAGCCGTTGTCCCACGGCTCACTCGGCCTTTCTGGCG
TTCTCTCCCCAGGCGAGAAGCCCTTCTCCTGTCCCCACTGCAGCCGTGCCTTCGCTGA
CCGCTCCAACCTGCGGGCCCACCT
>527
CAAAATAATATAGTACTATGATTTTCCCTTTACTTTATAAGTAATTTTGTGCTGTTCCTTTT
TTATACAGCCATTGATTATTATTATTCCTAAAGAAAATGAAGATAATTACATATTTTTGCA
TTTGGGCAGTAGCATGG
>528
GTGTGTGCACGCTCACACACATATTCACAAATAAGAACCTTTTCAATAGCCAGTATTTT
CTACTTGGCAGGTTCCTCAAAGCAAACCACTGGAGAGACATGTCGAAAAATCCATGAA
TTTGCATCTCCTAGCAAGATCAA
>529
TCCAAGATTGCAATTTGCTTTCCTTCAAGATCATTGACACTCATAATTGATTGAATTGTT
TCTTTTTCAGGATGAGTTAAATGCCAGTGGAACCATCAAAGAAAGTGGTGTCCTGGTG
CATGAAGGTGATAGAGGAAGGC
>530
TTCCTCAGATCAATGCAATTTTGCAGCAAATATTCACTAGTTAATCATTCTTTCCTCCATC
CTTCCATAGGGAATAGAAATCAAGGGTCCCAGCAGTGGCAACAGAAATATTACCAAAG
AAGTTGGGAAAGGCAACGAAG
>531

AAGGGTTCATTCACTGTTTTAAGGAAACGATTTATAAATGTTAATGGGATGTTTATTTTT
TCTCCTATAGAAAGCTAGACTGGCCAGGATCCGGGCAGCCAAAAGCGGAAGCGCAAA
TGCTTACATGCAGAGCAAACGGA
>532
AATATGTAGCCGAGGTTCGAGTTGTTTGCAAATAAAATAGAAAGCTCTTCTGTCTTCTC
TTTGTTAACAGTCCTCAGAGGATGAGCAGGCTTTTGTTAGCAAATCCGGCTCCAGCTTT
GAAACCCAGCACCACCACCTGC
>533
ATAAAGCTATGTATTTCGTTTTTAAAAATGTGCTTCTCTGTTTCTTCATTTACTTACAACT
GTGGAACAGAATCACGAGTTTGTGGACGAACAAGTCTTTGAAGAAAGCTGCATGGAA
GTTGCAACTGTTAATCGTCCTT
>534
TATTCTTCAGTTGTATGAAAAACATTTTAAGTAAACAACTTACTTTCCTAAATATTTTTTT
TTCTATCAGCCGATCCAGTTTAAATGCCAAAATGGAAGAGTGTGTTAAACTAAACTGTG
AACAACCTTATGTGACTACA
>535
GGAGTGGGGAGGGCTATCAGGCCCAGAGCTCAGCGGCCACTGCCCCAGCTCACGGCC
GGCTCTGCTGCAGGACCCCTTGCAGTGGCTCGGCTCCCCACCAAGGGGCTCCTGCCCC
AGCCCCAGCTCCAGCCCCAAGGAGC
>536
AGAAGGGTAGGCGGGTCCAGGCCAGGATCAGGGGGTTGTGGGGGAAGGACTGACCC
CACCCTGCCTGCAGAGCTGCAGGTCGCCTTCCAGGAGTTTGACCGAGACCAGGACGG
CTACATTGGCTGCCGGGAGCTGGGTGC
>537
AGAGGAGGGAGGGCCAGGGAGGGGCAGGGGTCTCTGATGCCCTGGGTCGTGTAATGG
ACGGGACCCCCAGGTGGCGGAAAGGTGGACTTTGAAGACTTCGTGGAGCTGATGGGC
CCCAAGCTGCTGGCAGAGACGGCAGA
>538
TGCCGTGAGTGAGGGGCTGCGCGTGTGAATGACTGTCAGTGACCAGACGCCACAACC
AACGCCGCCACAGTTCGACACCAATGGGGACGGCCGCATCAGCGTGGGCGAGCTCCG
GGCGGCCCTCAAGGCCCTGCTGGGGG
>539
GGGTGGGGGGTGATTGCTGGTCGTTTGTTGTGGCTGTTAAATTTTAAACTGCCATGCAC
TCGGCTTCCAGTATGCTGGGAGCGGTGAAGATGGAAGGGCACGAGCCGTCCGACTGG
AGCAGCTACTATGCAGAGCCCGAG
>540
GTGGGAGGTCTGGGCACCCTCCAGCCGCGGTGCTAACGCTGTCGCCTCCCTCCCGGGG
CTCTGTCCGCAGGGCTACTCCTCCGTGAGCAACATGAACGCCGGCCTGGGGATGAACG
GCATGAACACGTACATGAGCATGT
>541
CCAGAACATAGTGGGAGAGAAACTGTAGGGATCTGCGGGGCCTAAAGCTAACATATTA
CTCTGGGGACAGGAAGCAGCTTACCACCCAGAAGTGGCCCCTGATGTGCGGCTTCGA
GCCCTGCACTATGGCCGGGAGTGTG
>542
GCCCTATTTCATAGATCAGGGTAGACCCTAAGATGTGATGCTCTTTGTCCCCTGCCCGCT
TCTTGCCCAGACCCTGCAGAAGCCAAGCCCAGAACTGAAGCAGCAGTTGACAGGACA

TTCAAAGCGTGTGGCTGGTTCCG
>543
ACCATGCTCCCTGGTGCTTTTGACCTCCCCACTCTGCCTTAGCCTCTTTCCTCTTCCTTC
ATCCCACCAGGAACAGAATGGGTAGACCCAGAGGACCCCACAGTCATTGCTGAGAAT
GAGCTCCTGGGAGCTGCAGCCGC
>544
CTCACCATGGCAGTGTCCCCACCTTCATCTCTGACTTTACATCTCTTTCTGCAACCTTCG
CTTATTGCAGGAGGCAGATGAGTCCTTGAACTTTGAGGAGCAGATACTAGAAGCTGCC
AAGTCCATTGCAGCAGCCACCA
>545
CTTGAGAAGGCTGGAGTGGGGATGGACCAGGGCAAGGGGCATATTTTTAAGCAAACT
CTCCCTGCTTTAGGCCCGGATGGTGGCTGCGGCCACCAACAATCTGTGTGAGGCAGCC
AATGCAGCTGTACAAGGCCATGCCA
>546
AGAATTGAGGTACTGTCTCAACTGGGACCGTACCTGAGTTCTCAACCTAGGCCACTTT
CTCTCTGCCCAGGCTGCTGGCAACGCAGTGAAGCGAGCCTCAGATAATCTGGTGAAAG
CAGCACAGAAGGCTGCAGCCTTTG
>547
GCTGGTCCCAACTACCCTATGCCCCTGCTGTGCATCCTCACTGAACTAAGTCCCCGATC
TCCACCCTCAGATCATCGCAGCACAGGAAGAAATGCTTCGGAAGGAACGAGAGCTGG
AAGAGGCGCGGAAGAAACTGGCCC
>548
TTTTACACACTCACCCCACCCATCCCTGGCTTTCTATTCATGGAACAACCTCTCTCTACA
ATCCCTCCAGATCTTCGCAAATACTGTGGACAATGCCCGCATCGTTCTGCAGATTGACA
ATGCCCGTCTTGCTGCTGATG
>549
GTAGGTGGCATGAGTTGAGAAGGTTCTGGATCAGAGATAGGGGCCCCTCTGATCACCT
CCACTCCTATAGGTATGAGACAGAGCTGGCCATGCGCCAGTCTGTGGAGAACGACATC
CATGGGCTCCGCAAGGTCATTGAT
>550
ATCACAGAAGAAAGGCCTTGTTGGAGCTCTGACCCTGAACCCTCCTCACTTTTGCCCC
TGTCACCTTTAGGAAGTAAAAGGCCTACAAGCCCAGATTGCCAGCTCTGGGTTGACCG
TGGAGGTAGATGCCCCCAAATCTC
>551
GGATGGCTGCCAAGGTGTGGGAGGGAGGCAGACGGAATGAGGGGCCTGATGGACTGT
CCCCATCCTGCAGATTGAGGAGAGCACCACAGTGGTCACCACACAGTCTGCTGAGGTT
GGAGCTGCTGAGACGACGCTCACAG
>552
CCAAAAGTGAAGGGATGAGCAGTCCTGGGACTCTGGGCTCACCCTGCCCCTCCTCTCT
GTGCCCCTGCAGAAGGCCAGCTTGGAGAACAGCCTGAGGGAGGTGGAGGCCCGCTAC
GCCCTACAGATGGAGCAGCTCAACG
>553
CCTCTACCTTTCTTGTCTCCCTTCTACTCCACGGGGCTGTTTATAACTTGGGCTTGGTCT
TCTGTTACAGTCTTGGTGATGCCTTGGACAGCAGCAACTCCATGCAAACCATCCAAAA
GACCACCACCCGCCGGATAGTG
>554

ACCACAGTTAATTTTCTGCAAATATTAAACAGTTTTACTGATCTAAAATATTATTTTTATC
TTACAACAGGGAAATTGGTTACAGTAAAATATTTACGACTAGATAGATACCACCATCGC
TTTCCCCAGGCTCTCACTTC
>555
AAGAACTCAAGTTCAGCAGGGACAGGCCCCACACCCTGGCTGCTGGCTCATGTTCTCC
TCCCACCCCCAGGCCTGCACTTTATAGACCAGCACCGGGCTGCGCTTATCGCGAGGGT
CACAAACGTTGAGTGGCTGCTGGA
>556
TCTACTCTTCTTTCTTGATGCATGGAGAATGCCAGCACTAATTTTTTTCTTTCCTCTCTTT
TCCTTTTAGGCACACCATATGATCCCTATAAGGCACTGTGGTTTGAGCGGAAGAAGGAC
CCTGTTACCAAGGAGTTAAC
>557
GTGCCCCTCCTCCAGGCAGCCTCCTAAGCCCGCCCCACCTAGTGATGCCAGGCGCCCT
GTGTCTGCCCAGGGGGCCGGGTCCTGGAGGTGGGCTTTGGCATGGCCATCGCAGCGTC
AAAGGTGCAGGAGGCGCCCATTGA
>558
GAGGGGTGGGCGCCAGGGTGCAAGTGACCCCCATGGCCCTCAGAGGTGACCTGGTGC
CATCCCCGACCAGGAGACGCAGGTGCCCGCGCTGCTGGAGGCCGGCTTCCGGAGGGA
GAACATCCGTACGGAGGTGATGGCGC
>559
TAATTTTCAAGCTCATGAGTGGTTGTAACTTTAAACGTTCACTAGAGATGGCTTTCTGT
CTTTGTTTCAGCCCGTCTGCCGTTTAAGCGCCTGAATCTTGTCCCAAAGGGGAAAGCC
GATGACATGTCAGACGATCAGGG
>560
TGCTGTGAACCGAGCTTCCTCCTGGGAGTTGGAGGGAGGGCCACCTGTCACTTGCCAC
ACTGTCTTGTAGGATCAGGAGCGTCTGGGCAAGCAGCTCAAGTTACGTGCAGAAAGG
GAAGAAAAGGAGAAGCTGAAAGAGG
>561
CTGTGTGCGTCCATGGTGCCTCCTTTCTCCCATTGCTCGAAGACCCCATCGGGTCTCTT
CTTGATTTCAGACCCTGGCCGGCTCCTGTGGGAAGTTTGCCCCCTTTGAAATTAAAGA
GCACATGGTCCTGGCCCCTCGGC
>562
AGGTTCCTGGGAGGTTTGTGAGCAGGTCTGTAAGGCAGCGTGATCCTGAGCCTGGGGT
TTTCCTTCTCAGTGATGTCGTCATCGTGGAGCGTGGGAAGGGCGACGGTGTTCCCGAG
AGGAGGAAGTTTGGCAGGATGAAG
>563
TACCTCCTCACTGTGCCCCTTTCCTCCAGCCCCAAAGACAGTTGTGAGTCCCATGTGTT
TCTTTTCTCAGAAGCTCCTGGACTATGAGGTGGACAGTGATGAGGAGTGGGAAGAAG
AGGAGCCTGGGGAGTCCCTGTCCC
>564
ACTTTGTTTTTAATAAGGCACACACTCTGCTTTTCTATCTGATGAACTCTTTTTTTTTCT
CCTTTTGAGGATGATGATGACGACATGGGAGAGGATGAAGATGAGGACGATGGTTTCT
TTGTGCCCCATGGGTACCTGT
>565
TTCCTCAAAAGAGTGCCTGGGCATAGGGGAGCAAGAACCCCAAATAAACTTCTGCTTT
CTAAACCACAAGGAGTGTGCCGACCCTGAGAACCATAAGGTCCGCCAGAAACTGAAG

GCCAAGGAGTGGGACGAGTTCCTGG
>566
TTTTGTTTTTTGGCCTGTGGTGATGGGTGGCTCCCCAAGCCTCATGCCCACCCATGTTC
CCTCCTGCCAGTCCTGGCCCAGCTGCTGCCGCTCCTGCACGGCAATGTGAACGGGAGC
AAGGTCATCATCCGGGAGTTCCA
>567
CCCGCTACCGCACTGGCACCAGGGTGGCTGCTGCCTGCAGTGCTGATGGCCGTGTACC
CTGTCTGTCCAGATTGGTGCTGAAGACATGGACGGCTTCCAGGCAGACACGGAGGAG
GAGGAAGAGGAGGAGGGCGACTGTA
>568
GGGTGGGGTCTTCCCCCAGGGAGCCCAGAGGGCCCAGCCAGCTCAAGACCCTCCCTC
TCTTGCCCTGCAGAGGTCCAAGCCCCGTGTGGAGCCGCTTCCGGAGCTGGGGGTGGT
GTGGGGGTGGACACCGGCAAGGCCAC
>569
GCCTTATAAACAACAGGATAGAAGATACTGCTCGACAGTGCCATCCCTATCATGGCCTT
GTTTTTTACAGGCCCAACGAAGGAACCACTTCTGGGAACTCATTGAGGAAAGAGAGA
ACAGCAACCCCTTTGACAACGATG
>570
CCTGCTGGGCCTTTACTGGTGGCTTTCCTTTCTGAGCACTCATGAGGCCCCTGTGTCTT
CCCTCATATAGATTCCAGGTGGATCTGCAGAATGGCAGCAGTGTGAAACCTCGAGCCG
ATGTGGCCTTTCATTTCAATCCT
>571
GGATAATAAGTTAATTTATTAACTGTTTTTTTTTGGTGGCGGGGGGGCTCTGTCTTCTGT
ATCTCTCTAGGTGGCTGTAAATGGAAAACATACTCTGCTCTATGGCCACAGGATCGGCC
CAGAGAAAATAGACACTCTGG
>572
TTTGGACACGAGTTTTCCCTGGAGATCGCTTTCTGCAGGCCTCTTGGTCCTGACTGTGG
CTTCTTTTCAGAGCCTGCCATTCGCTGCAAGGTTGAACACCCCCATGGGCCCTGGACG
AACTGTCGTCGTTAAAGGAGAAG
>573
CATTGCTGAAATTGCCACTACTCTGTCCTACTTGGTTAATTAAGGTTTTTTTTTCTTTCTT
TCTCAAAAGCTTTAATGTTGACCTACTAGCAGGAAAATCAAAGGATATTGCTCTACACT
TGAACCCACGCCTGAATATT
>574
GTTAGCATGTAACAAACACAAAATTTTAAACTAAGGGGAACCACTAATGGCATGTTTCC
TTTCCTTTCAGATGATAATTTACTGTGATGTTAGAGAATTCAAGGTTGCAGTAAATGGCG
TACACAGCCTGGAGTACAAAC
>575
AGGCTTCATGGAGGAAGTGCAGTGGGTTTCTTGGGATCCCCACCAGGCACCCCTTCCT
CCTTCGACTTAGGGTGTGGCCGGCCGCAGGTTTCGGATGCAGGCGGCCGGATCGTGGG
GGGTCACGCTGCCCCGGCCGGCGC
>576
CTGGGCGGGGCCGACACTGACCTCCAGCCTCCGCAGCTGGCCCCTCTCACCCAGCCA
CCTTCCCTCCCAGGTCCCTGAACTCATCCGACTACCAGGTGCACCTGGGGGAACTGGA
GATCACTTTGTCTCCCCACTTCTCC
>577

CAAGGTCTGCCATGCCCCCAGTGCACGGCCTGGTGCCCCAACCTTCTCTGAAGCCTGT
TCCCCCAACCAGAGCCTCTGCCACCTCCGTACAGCCTGCGGGAGGTGAAAGTCTCCGT
GGTGGACACAGAGACCTGCCGCCG
>578
GGGGCGGCTGAAGCCCAGGGAGGGGCTCCCCATCTTCTCGGCTCCCAATGCTCGCCTT
CTCTGTCCTCAGGACGACTCCGGGGGGCCTCTGGTCTGCCAGGTGAACGGTGCCTGG
GTGCAGGCTGGCATTGTGAGCTGGG
>579
TCCGAACATCTATACTATTTGATCTCTCTAACCTGTGTTTTGTGATATTTTATCATTTGTTG
TACTCCAGGTTGAATTCACATTCCCTGACTTTGTAACAGAGGGAGCCAGGGACCTCATT
TCAAGACTGTTGAAGCATA
>580
GGAAATTAGTTTGCTCTGTATGGGTCAAATATAGGCATATTCTCATGATTGTCATGTTTCT
TTCTCCCAGCATTTATCAGCTGCCGCTTATTCATTGCCTTGATTTTCTTCAAAAGACAAC
TATAATCATTCAAAAATTC
>581
CTCTTGAGAAATCCTGTGTCTCAGAAACCTTCGGAGACATTTAAGCCCTCTCACCCTCT
CTTTAAACCAGGACTCTGCATGCTGCTGAAAGCAAATGAATGTGGAGGGTTTCAAACA
AGACAAAAGGCATTCCTCCCTGC
>582
CCCAGTTCTGTGTCCTGACTGGGTGGGGTGGAGGGGCTGCTCCACATCTCACAGCGTT
CTCTGGCTGCAGAATCTCCAGCCAAGTCCTCCGTGCTCGTGGTGGGAATCGTCATCGG
TGTCTTGCTACTCACGGCAGCGGC
>583
TGGGGGACGCCAGTCAGACCCAGAGCGCCTCAGAGATTCTGATGACCTCTCCCTCTCT
CTCTCTCCTCAGCCCCTTGGATCTCCCTTCGTGGAGACGACACCGGGGTCCTCCTGCCC
ACCCCAGGGGAGGCCCAGGATGC
>584
TGGGTGGAAAAGGGCTCCAGGGTCATTATCATGCTTTTGGTTTTCTGACATGCTCCATT
CTCTGTTGCAGCCTTGGAGTCCTCAAACCCCAACACCAAGCAAGCATCATTCTGGAGA
CCGTCCCCTTCAAGAGTGGACAA
>585
CAATGACTCATCAACCCCTATATCTATAAAGTCACAATCGCTGTGACTTGATTTCTGTTT
CACTTTGTAGATATGGAACCCATTCACATGGACTTTTTAAGAAGCTTGGAATTCCAGGG
CCCACACCTCTGCCTTTTTTG
>586
CTTCCTGGGTTGGGCTCCAGCTGTAGAATAAGGCTGTTGATGTTTAATCAACTCTGTTT
TTTTCACACAGCTTTTATGATGGTCAACAGCCTGTGCTGGCTATCACAGATCCTGACAT
GATCAAAACAGTGCTAGTGAAA
>587
ACACATTTTCTACAACCATGGAGACCTCCACAACTGATGTAGGACAAAATGTTTCTGCT
TTGAACTCTAGCCTTTTGGTCCAGTGGGATTTATGAAAAGTGCCATCTCTATAGCTGAG
GATGAAGAATGGAAGAGATTAC
>588
GGAAAGCCATGTCCTTCTGGGACTAGAGTCTGCACATTTAACTATGGGTGGTGTTGTGT
TTTGTGCTTAGATGGTCCCTATCATTGCCCAGTATGGAGATGTGTTGGTGAGAAATCTG

AGGCGGGAAGCAGAGACAGGCA
>589
CTGTTGCATGCATAGAGGAAGGATGGTAAAAAGGTGCTGATTTTAATTTTCCACATCTT
TCTCCACTCAGCGTCTTTGGGGCCTACAGCATGGATGTGATCACTAGCACATCATTTGG
AGTGAACATCGACTCTCTCAAC
>590
ATTGCTCCAGGTAAATTTTGCACATTCACAATGAATTTCTTTTTCTGTTTTTGTTTTGTTT
TTCCTACAGCAGTCTTTCCATTCCTCATCCCAATTCTTGAAGTATTAAATATCTGTGTGTT
TCCAAGAGAAGTTACAAA
>591
TTCTCTTCATCTAAACTGTGATGCCCTACATTGATCTGATTTACCTAAAATGTCTTTCCTC
TCCTTTCAGCTCTGTCCGATCTGGAGCTCGTGGCCCAATCAATTATCTTTATTTTTGCTG
GCTATGAAACCACGAGCAG
>592
GTACTGCATGGACTGAGTTAAAAGTTAATTCAAAATCTCAATTTATCCAAATCTGTTTCG
TTCTTTCCAGGCACCACCCACCTATGATACTGTGCTACAGATGGAGTATCTTGACATGG
TGGTGAATGAAACGCTCAGAT
>593
AAACCCTAACATGTAACTCTTAGGGGTATTATGTCATTAACTTTTTAAAAATCTACCAAC
GTGGAACCAGATTCAGCAAGAAGAACAAGGACAACATAGATCCTTACATATACACACC
CTTTGGAAGTGGACCCAGAAAC
>594
TCACTTTGATGCTATACTTTCTACTTTTGTTTATTTAATGCTTCTCAATATGCTTGTTTAAC
TGTTGCAGATCCCCCTGAAATTAAGCTTAGGAGGACTTCTTCAACCAGAAAAACCCGT
TGTTCTAAAGGTTGAGTCAA
>595
TTAGACCAGGCTCTCCCTGGGCCAGGCTCCGGTGGGACACTGGCAGGCTGACGCATGT
ATCTTCTCTCAGGGTGTCTTGGCCATGGTCCCCTGGTGCTGCAACTCCTCTCCTTCACG
CTCTTGGCTGGGCTCCTTGTCCA
>596
GGGGCTCAGTTCAAGGCTTGGCACACAGTAGGTGTCTAATAATTGCAGTTCCTTTTTTA
TTTTGGCCCAGTGTCCAAGGTCCCCAGCTCCATAAGTCAGGAACAATCCAGGCAAGAC
GCGATCTACCAGAACCTGACCCA
>597
GAAGGCTGGGCAGGTACAGGAATATGGACAACCTAATCTAAGCCCAGCCCTGACCAGC
CTCCCCCAACAGAACGCCTGTGCCACCCCTGTCCCTGGGAATGGACATTCTTCCAAGG
AAACTGTTACTTCATGTCTAACTC
>598
AAGAGATGGGGAGACAGAGCCTGGAAAATGGGAAGCAGAGCTCCCTCCTGACTCCTC
CTGTACCCTCCAGAACTTCCTACAGCTGCAGTCTTCCAGAAGTAACCGCTTCACCTGGA
TGGGACTTTCAGATCTAAATCAGG
>599
GACAGAAGGTAGAAACACAGCTCATGTAGCTGGCGGGGACAGAGGCTCACATTCCGC
ATGGGCTTTGCAGCTTCAAGCAGTATTGGAACAGAGGAGAGCCCAACAACGTTGGGG
AGGAAGACTGCGCGGAATTTAGTGGC
>600

TGGGGCTCAGTTCAGGGCTTGGCACACAGTAGGTGTTTAATAATTGCAGTTCCTTTTCT
TCTTGGCCCAGTGTCCAAGGTCCCCAGCTCCCTAAGTCAGGAACAATCCGAGCAAGAC
GCAATCTACCAGAACCTGACCCA
>601
GAAGGCTGGGCAGATATGGGAATATGGACAACCTAATCTGAGCCCAGCCCTGACCACC
CTCCCCCAACAGAACGCCTGTGCCGCCACTGTCCCAAGGACTGGACATTCTTCCAAGG
AAACTGTTACTTCATGTCTAACTC
>602
CAGAGACGGGGAGGTGGAACCTGGAAAATGGGAAGCAGAGCTCCCTCCTGACTCCTC
CTGTACCCTCCAGAACTTCCTACAGCTGCAGACTTCCAGGAGTAACCGCTTCTCCTGGA
TGGGACTTTCAGACCTAAATCAGG
>603
TTACAGAAGGTAGGAGTGTGGCTCATCTACTGGGCAGGGCAGAGGCTCACATTCTGCA
TGGGCTTTGCAGCTTCCAGCGGTACTGGAACAGTGGAGAACCCAACAATAGCGGGAA
TGAAGACTGTGCGGAATTTAGTGGC
>604
TCATGTGTAGCTGCCCTGTCCCCTGGGGCTTTATCTCATCCATGCAAACTACCATCTGCT
CAACTTCCAGCTACACCCCGTGCACCCTTTTGACTGGGGACTTGCTGGTTGAAGGAGC
TCATCTTGCAGGCTGGAAGCAC
>605
CCCAACTTCAAATTTTTCATATAATATAGGAAAAATAATGATTGAAACATTTAAATGTTC
TTCTTTACAGATGTTTCATTTGGATCCTTCTTTGACTCATACAATATTTAATCCAGAAGTA
TTTCAACCACAGATGGCAC
>606
TGTACCTTTGTTGCCGTAATTTTTAATAAGTATTTCTCAAACTTAATTGGCTTAACGTTCA
CCTTTGCAGAGAGGATTTCGTTTCCGTTATGTATGTGAAGGCCCATCCCATGGTGGACT
ACCTGGTGCCTCTAGTGAAA
>607
TATCATAAACATGTTTCCATGTTGCTGGAGAGTCAGATATGCTAACTTTTGGAATGTGCT
TCTTATATAGATCTGCAACTATGTGGGACCAGCAAAGGTTATTGTTCAGTTGGTCACAA
ATGGAAAAAAATATCCACCTGC
>608
CAGATTCCCTAGACATTAAGTGCCTAACTTTTGGTTGTTGTTGCTGCTGCTGTTACTGTT
TTTTCTCCAGCTTCGCAAACCTGGGTATACTTCATGTGACAAAGAAAAAAGTATTTGAA
ACACTGGAAGCACGAATGACA
>609
TTATAAAAGCATGGTCTTTTAAATGTTCACACTTCCCTGGGCATGAATGGACTGTGCTGT
ATGGCCCTAGATCGGGAAAAAGAGCTAATCCGCCAAGCAGCTCTGCAGCAGACCAAG
GAGATGGACCTCAGCGTGGTGCG
>610
CCTACCTAAAGGACTGGCTTGTTGAGCTGAGGATTAATCATGTTATTTGTTGTTTTTCCC
CTGTGAACAGAAGCCCCCAATGCATCCAACTTGAAAATTGTAAGAATGGACAGGACAG
CTGGATGTGTGACTGGAGGGGA
>611
ATTTTTCAGCATGTTTATAAATACTATTTACACTATGTGAAATTACACACTTCAATGTGAT
TGTTTGCAGATGACATCCAGATTCGATTTTATGAAGAGGAAGAAAATGGTGGAGTCTG

GGAAGGATTTGGAGATTTTTC
>612
TGAGTAGCATTACTGCAAAAAAAAAAAAAAATGTAGTCCTACACCAACATGTGGTTCTT
CGTATCCTGCAGTTTGCCATTGTCTTCAAAACTCCAAAGTATAAAGATATTAATATTACA
AAACCAGCCTCTGTGTTTGTCC
>613
GGTCTTTCTGTGGCTAGTGGTGGGACCAAATCAATCTTTTCTCCTCTGGTTTCTCTTCCT
TTAAATACAGATAAAGAAGAAGTGCAGAGGAAACGTCAGAAGCTCATGCCCAATTTTT
CGGATAGTTTCGGCGGTGGTAG
>614
GACACTAAGTTGTGCTGAGTCTTTGTAAAATATCTTCATGATGTTTCATTTGTTTTACTT
GCCGTTTCAGGGTATAGCTTCCCACACTATGGATTTCCTACTTATGGTGGGATTACTTTC
CATCCTGGAACTACTAAATC
>615
CATTGGAATAGCGAATTATCACTAAATACATTTTACTGAGAAAAATCTGATGTTTTTGCA
TTTATCTTAGGAACCATGGACACTGAATCTAAAAAGGACCCTGAAGGTTGTGACAAAA
GTGATGACAAAAACACTGTAAA
>616
CCTTAAGCATGCCATACCCATAAAACAAAAGTAGGAACACTCAACTATGATTGTGGTCA
TTGCCTTACAGATAACCTCTTTCTAGAGAAGGCTATGCAGCTTGCAAAGAGGCATGCCA
ATGCCCTTTTCGACTACGCGGT
>617
TCACATCTTGGGAATCTTTTCTTTTTCATTAACATTTTAGTTAATTATTGCCACTGTGTTT
TCATTCCAGTGTCTTACACTTAGCAATCATCCACCTTCATTCTCAACTTGTGAGGGATCT
ACTAGAAGTCACATCTGGT
>618
CTACCAAGCTGTGAGTTGTAAGTAAGTATTCACTGCTGACCAGTGAATCTCCTGCCCTT
TCACTTTCCAGACGCCCTTGCACTTGGCAGTGATCACTAAGCAGGAAGATGTGGTGGA
GGATTTGCTGAGGGCTGGGGCCG
>619
TCAGCTTCACAAGGGCCATCTTGTGAGTTAGCCATCCCATCCTGTGACTGTCCCTTTGC
TTGGACTCTAGGTCTGAATGCCATTCATCTAGCCATGATGAGCAATAGCCTGCCATGTTT
GCTGCTGCTGGTGGCCGCTGG
>620
CACTGGGAGGTGGGGACAAAAGGGCAAAAGAACCTTCCACTAACGCTTTCTTGTGTG
GGCTGGATTGTAGGGTGATGCCCATGTGGACAGTACTACCTACGATGGAACCACACCC
CTGCATATAGCAGCTGGGAGAGGGT
>621
TGACCTTTGCAGGCAGTTGGAAGTTGACAAGATCTGGGTTTACCTAATGCTGTGTAATC
TAACTTCGCAGGAGCAGATCCCCTGGTGGAGAACTTTGAGCCTCTCTATGACCTGGAT
GACTCTTGGGAAAATGCAGGAGA
>622
GCAATGATCAGTCCCTCCAGAGTGTCTATGGCATGTTAGAACAAGTGTGTTCCTTCTTC
ATTTCCTTCAGGAGACATGAAACAGCTGGCTGAAGATGTGAAGCTGCAGCTGTATAAG
TTACTAGAAATTCCTGATCCAGA
>623

TGTCAGTGCTTACAGCCTGCACTGGGACTCGAACACAAGAACATGCTCCTCCTTCCTT
TCTTTCTCACAGGTCTCTGGGGGTACAGTCAGAGAGCTGGTGGAGGCCCTGAGACAAA
TGGGCTACACCGAAGCAATTGAAG
>624
CCACAGAATAGTCCATGAGCTTTTTAGAGCCCGGCCATTCCCCCAGTGAATTTGTGCTT
TCTCCCCTCAGACGAGCTCCGAGACAGTGACAGTGTCTGCGACAGCGGCGTGGAGAC
ATCCTTCCGCAAACTCAGCTTTAC
>625
GAGAGTCTAGAGCATATTTGGTCATAATTAGAACTAGCATTCTTCATCCTGTATTCCTTT
CTTTTCTCAGGCCCTGAAAAGACAGATGAATATCTCTTAGCAAGGTTCAAAGGCGATG
GTGTAAAATATAAGGCCAAGCT
>626
ATTTTAATATTAAAACCATTGTAGGATGTTTTTAAAAATTCAACTTCTCTTGTTTTTCCAC
CCTCATCAGGGAATGGCGGCACGTGGTCGGTCTCAGGGACAACACAAACAAAGGATC
TGGGTCAACATTTCCCTTTCTG
>627
GCGTCGGTGTGCTAGCCTGCAGATAGATTCTAGTTTTCTTAATAGATTACTCGTTATGTT
CTTTTGTGAGGTAATAGAGCATGAACATCCAGTAAATAAGATTTCTTTCATTGCCCGTGA
TGTGACAGACAACCGGGCAT
>628
ATTGATAGCAGACAAAGGAAATAAAATACTAAAATTAAAATACTGATAACAGTGCTTTC
CCTTACTGCAGGCTGAACCATTAGTTGTTGATCTTAAAGACCTTTTTCAAGTTATCTATA
ATGTAAAGAAAAAGGAAGAAG
>629
ATGTAATTTTCTCATTTGAAGTCACAACATGGACTAACACTTTCTTTTTTTTTCTTTCCA
AATGTGATAGGAAAGCAAAGATATCCTGTTAGTGGATCTAAACTCTGAAATCGACACCA
ATCAGAATTCTTTAAGAGAAA
>630
AGACAAAATGAGGCTGTATTTCACTGTTAGTGAGTAACTAAGGCTTCTCCCTCCCTCCC
TGTCCTGTCAGTCTTCAGCCAATGACTTGCTTGCATCAGACATCTTTGCTCCTCCCGTC
TCAGAACCTTCAGGCCAGGCGT
>631
TGCTCTCTGAAATTCAAGAATCTTCTTCAAGCTTGATTCCTGATAAGTATTTTTCTTCCT
CTTACTTCAGGTGGTGTAACTGTCACACTCCCTCAGGCAGGACCATGGAACACAGCAT
CTTTGGTCTTCAATCAGTCCCC
>632
GCTGACTCGGGGACTGGCCTATATTTGGGGAAAGCTTGTCTACTGATTGCCTGGATTTT
TTACCTTCTAGAACCACCAAAGCCAGCTCCCAGACAAGTTTCCCTGCCAGTTACCAAA
TCTACTGACAATGCATTTGAGAA
>633
AAGTCTTCATTGCGATTTTTTTCTTATGACTGTAACTGTATTTATTGACTAGATGATTTTA
TCTTCTTAGGTGGCTTCTTCTCAACCTGTATCTTCTGAGATGTATAGGGATCCATTTGGA
AATCCTTTTGCCTAAATTC
>634
ATATTTAATACATTGATATTCAGTTCAAAAATTCTAACCACAAGTTTCTTTCTTCTCCATA
CCTCTCTAGGAACTTGGTCTGCAGACCATCCAGAGGAATAAAAAGGTTGGCCTTAGTA

GTCAAAAACAAAGCTGATAGC
>635
AGAAAATGGTGCCTGAGAGGCTACAGCAGGACCCGGCAGTGACCCGCTGCTCTTGGC
TGTCTCCCTGCAGGCTCCACAGACTCTCCTGGGGCCCAGAGGAGCTGGTGCTGACCTA
GCTAGGGATGCAAGAGTGAGCAAGC
>636
TTTCTTAAAACAAATTTTTCTCCCATTGTCATGGTTCTGTATATTAACACAAAATTTTCTT
TTCTCATAGGAGAGATTATTTCATTCAATATTTTTTATGAATTATTTACCATTTGTACTTCA
ATAGTAGCAGAAGACAA
>637
GCCCTCATCATCATGTTGATCATTACTATCACTGTGTTTCTGAAATGTCACTGAAACATG
TCTTTTATAGGTGGAACATAAATAATGATACCTGGGTCATAACTGAGCAACTAAAACCTT
TAACAGTGAATTTGGATTTC
>638
TCTTCCGGGTCCTGACTGGTTACATAGTGTCACCTCTTCCGGTTCCTGACATTGTGTTCA
TCTCTTCCAGGTATTCTAGAATGGATTCTGGGAAAGAAAGATGCCATGTGGATAGGGTT
CCTCACTAGAACAGTTCTGGA
>639
AAAAATAAATGAAGTTATTTACTAAAATTAAAAAAAAGTTTCATTTTTCTTTTTAAATGT
TTCTATATAGTTATGAAGAAGCCAAGAATTTATTGACCAAGACCAAGATATTGGCCCCA
GCCTACTTTATCCTGGGAGGC
>640
AAATGCTAATATATATCAGAACATCTCTGAATTGAAAACGTCCTTAAAGTAAAAATAACT
TTTCTTTCAGCTGACCGTATACACAACCTTGATAGATGTTACCAAAGGTCAATTCGAAA
CTTACCTGCGGGACTGCCCTG
>641
CAATACATTGTTTCTTTCTAATATATATTTTATTTAATCAGACATCTATTTACCTCTCCTTG
AATTGCAGACTCACAGATGAATGCTTCTCACCCGGAAACAAACTTGCCAGTTGGGTAT
CCTCCTCAGTATCCACCGAC
>642
ATAACCCTAAAAAGGTTGACAATTCCATTTTTGGTGCAGTTACAAATCATTTCACTTGTT
TGTATTTCAGGACCTCCAGGATATAGTGGCTACCCTGGGCCCCAGGTCAGCTACCCACC
CCCACCAGCCGGCCATTCAGG
>643
TTATAATATCTGGATAAATTCAATATTTTTAACATAGAGGTGATTATGATTTCGTCTTTTAT
GTTTTTAGTTTTAACAGGTTTTGAAACTAATAACAAATATGAAATTAAGAACAGCTTTG
GACAGAGGGTTTACTTTGC
>644
GTCATTGAACCTTTCATCGAACCTAGGCATTATGATTATTACATTTACATATATTATTTTGC
CTTTGTAGATAGAAATCCAAGCTCCTCCTGGTGTACCAATAGGTTATGTTATTCAGACTT
GGCACCCATGTCTACCAA
>645
ACTTCAAATTTGAAAGTACTTTTGCTATAAGTTTCCTAAAAGTATTTAATACTTTTTTTTT
TCAATTTAGATTAAATCTCTTGATGAACAGTGTGTGGTTGGCAAAATTTCCAAGCACTG
GACTGGAATTTTGAGAGAGG
>646

TTTCTGCATCTGATAATTGATCTCTGTTTTGTTGTTGAAAATTCATTTGTATACTTTTGTT
TTTATCTAGGACTTCATGTTTTTTGAAAGCACTGGCAGCCAGGAACAAAAATCAGGAG
TGTGGTAGTGGATTAGTGAAA
>647
GTTAGGAGGCCCAGAACTGGAGAGCTAAGGCATACTTCATCAGCTTAGCACATGACGA
CTGTCTCTCCAGACTGCGTGGAGTGCATGGCGTGTTCAGACAACACAGTTCGTGCTGG
CCTGACACCCAAGTTCATTGATGT
>648
TCCTGGGCCCATGAGCTGATGAGAGCCAGAGTCTGAGCTGCACTGCCTTATCATTTCTT
CCTTGCCCCAGGTCCCTGGCTCTGTCACTGAATACAAGGTCTTGGCACTGGACTCTGC
CAGCATCCTCCTGATGGTACAGG
>649
TAGAGGCAGTCACTTATTTGAATCCCATTGTCGTAACTTTCTACTATTTTATTTTTCCACT
GTGACTCAGGGAGATTCAGGTGGTCCCTTAGTTTGCAAACACAACGGAATGTGGCGTT
TGGTGGGCATCACAAGCTGGG
>650
GCTGTCATGGGGAGGAGTTGTGGCCATGTCCAAGGCACCTGTCCCATGCCCCCTGCCT
CCTCTCACACAGGATGGTGTGAGGATGGAGGAGATTGTGGAGGGCTGCACCGGAGCA
CTGCACATCCTCGCCCGGGACCCCA
>651
TGGGGTGTGTTGTCCGGCCTGCCTCACCCCTTTCCAACTCTCCCCTGCTTCCCACGTCC
CCTCTCCCCAGCTCCTGTACTCGTCGGTGGAGAACATCCAGCGCGTGGCTGCCGGGGT
GCTGTGTGAGCTGGCCCAGGACA
>652
GTCCCACTCCCCCAGCCACGATGGAGGCTGGCCGACGTTTAACCTGGCTTTTCCTTTCC
TCTCTGCTCAGCCACCTACGCTGCTGCCGTCCTGTTCCGCATCTCCGAGGACAAGAAC
CCAGACTACCGGAAGCGCGTGTC
>653
AGGCTGTGCAGACTGCTCCCTGCAGGGTAGCTTGCAGACCCTAGGACCGAGCCCCACT
TTTTGTCCCCAGACATGGATGCCACCTACCGCCCCATGTACTCCAGCGATGTGCCCCTT
GACCCGCTGGAGATGCACATGGA
>654
CCCTTTCCCCGCCCCCTCTTCCCCTCGCCCCACTCCCATTCCCCTTCTCACCACCCCTCC
TTATTCCCAGACTGCGGCCAACTCCTGGGGCCCAGCCTGGGGCGAGAGGGGCCACTTC
CGCATCGTGCGCGGCGTCAATG
>655
TAAGCTACTATGCAAGTAGATTTTCCTCTTCAGCGTCACAACATGACTCATTTTCCCTCT
ACCGTCACAGGTTTAATTCTAAAGTTGCCTCTCTGGGGACAGCCATCTGACCAGAACT
GCTATGATGATTCTGTTTATTG
>656
TTCAATACTAATTTCTGACAAGAACAGTGAGAATAAACTATTAAGTGCCCTCAAACTTG
TTTTTTTAAAGGTCCCTAAGACGAGATAACTTGACAATCAGTTCCATGGACATGGTGAC
CACAGCCAGCTGGAACCTGAAG
>657
TGAGGCACAGTTCTCCATGGCTATGAGACTCCATTTATGCATCAGGAGTAAAGGGGTCT
TGTGTTTTTAGGTGAGGTTCCTGGAGCAGCAGAACCAGGTACTGCAAACAAAATGGG

AGCTGCTGCAGCAGGTAGATACCT
>658
CCCCTCACAGGTAATGAGAGGGACCTGCCCTGGAGAGAACGTGCCTTCATGATGTCCC
TTGTTCCTCTAGGATGTGGATGGTGCTTATATGACCAAGGTGGACCTTCAGGCCAAACT
TGACAACTTGCAGCAGGAAATTG
>659
ACTGCTTTCTCTGTCTAAGTTGTTTTTTGTTTTAGTGCTGTAAGAGTCACTAACCTATTA
TGTCTTGCAGGAGTTGTCTCAGATGCAGACTCAAATCAGTGAAACTAATGTCATCCTCT
CTATGGACAACAACCGCAGTC
>660
GGAGACCAATTGGGATGAGGAGGCCTAGTGGGGAATGCATATTATGAGAGGGTGTCAT
ATTCTTTTTCAGATCTCCAACTTGCAGCAGTCCATCAGTGATGCAGAGCAGCGTGGCG
AGAATGCCCTCAAGGATGCCAAGA
>661
TGAGACACCAGGAGAGCTGCTGCTTTGGGGGATGGACAGGGGCAGCAGGAATGCCTT
TGTGTTTTCGCAGTGAACCTCCTTGGCCTGGGCGAAGCTGTGTGGACCAAGCAAGTCA
GGAGTGTGGCCATGTTTTCTGAGCA
>662
CAGGGCCTCCATGGCCTCTGTCGGGCGGGGGGGCTACTTCATCTCTGAGTCTGTACCC
CTCGTGTCCCAGGCAGTGGAGTGGGAGTGAAGAAGCTGTGTGAACTGCAGCCTGAGG
AGAAGTGCTGTGTGGTGGGCACTCT
>663
CCAGCTCACTAACACCAGAGCTTTGGTGGGCTCTGGCCCCAAGATTATACCTCCTGTCT
CTGCATTCCAGCACAACCTGCTCCCCCAGCCTCCTCGGAGTAAATACATACACCCAGAT
GACGAGCTGGTCTTGGAAGATG
>664
CGTTGGGGTGACTGGAAGGAGTGGTAGCACCTGGGGCTCCCTGCTCAGGCCTGATGCC
ACTGCTCCCCAGGGACTGTCCTGGCTGTGTTTGGCTCCGTGAGAGACGACGGGAAGTT
TCTGGTGGAGGACTATTGCTTTGC
>665
TCTTTGAGTTGCACCTAGGAGGGGCTGCCTCCCTTCTCGGCACCCTGGAGGACCCCAC
CTTCTCCCGCAGGTTTGTGCTACTGGTGTCCGGCCTGGGCCTGGGTGGCGGTGGAGGC
GAGAGCCTGCTGGGCACCCAGCTG
>666
GGTGGGGCTCTACTCTCCTCTGAGCCTGGGACTCGCCTGGGCCTGATGGCCTTCTGGG
CTTCTATTCCAGGCCAAATACCTCACCAAGAAAACCCAGGCAGCCAGCGTGGAGGCTG
TTAAGATGCTGGATGAGATCCTCC
>667
GGAGTGGCCGGAGTTGGTGTAGGGGGCTCCTTCGGGGCCCTGTGAGCTCTCCCTGCCC
TGTGCCTTCCAGGCCTCAGTGCCCGTGGACGTGATGCCAGGCGAGTTTGATCCCACCA
ATTACACGCTCCCCCAGCAGCCCC
>668
CAGATGAGCTCTGGGCTGACCACTATGGGTGGCACCCAAAGCCAAGAGTCAGCTGAG
CTTTGCCTTGCAGATTTTTGGGGACATCAGGACAGAACGTGAGTGACATTTTCCGATAC
AGCAGCATGGAGGATCACTTGGAG
>669

AGCCCCACATCCTGTGCAGAGTGCTAGTGCTGAGGCGGAACCCTCCTCAGAGCTGCCC
CTTCTCCTCCAGGTTGTTACCCCTTCTACAAAACTGACCCGTTCATCTTCCCAGAGTGC
CCGCATGTCTACTTTTGTGGCAA
>670
TTCTGGCAGCTTCTCTTTGAGGCAGGACTTACTTGGAACCTACAGTGGGTCCTATGTGA
CTTCTTTGCAGGTCCTGAGGACCAGACAGTGCTGTTGGTGACTGTCCCTGACTTCAGT
GCCACGCAGACCGCCTGCCTTGT
>671
TTCTCTAAGTTCTTGACCTGCTGGTGATTTCCATAATGGTTTCTGTTCATTTTCTCCTGTG
TCTTTTCAGCCTAGTGGAGGCCAGTATAAGATTCAGATCTTTGAGAAAGGGGATTTTAG
TGGTCAGATGTATGAAACCA
>672
CTAGGCAAACAGTAATTTTGGTTAAAACCAAAATACGTGTTCTGAATTACTGTTCTGTG
TTTATTTTCAGGCTTCTGGAGTTACAGTGAATGATGAAGTCATCAAAGTTTTTAATGATA
TGAAAGTAAGGAAATCTTCTA
>673
CACAGACCCCCACTAGCCGGGGACGCAGCTCAGGCCCCCTACCCCCAACACAAACAC
TTCTCTCCTGTAGAGGATAAAGCTTGGGGTTCATCCTCCTTCCCTGGATCACTCCACAG
TCCTCAGGCTTCCCCAATCCAGGG
>674
AGTTTTTCAGGAATCAAAACCTTTGCCCCATCCCACCTCCAGCTCAATGACCATTGCCC
CTTCCTCAAAGGGACGATCATGGCTTCTCCCCCTTGCACTGGGCCTGCCGAGAGGGCC
GCTCTGCTGTGGTTGAGATGTTG
>675
TGACACCAGTATCTCATTTGGAACTGACTGTACTTTCTGCCTCTTCTTTTTGTCTGGCCA
TGGGGTCCAGCTATTGCAGTACAAGGCAGACATCAATGCAGTGAATGAACACGGGAAT
GTGCCCCTGCACTATGCCTGTT
>676
ATTCATGGTTGGTTCAGTGACTGCCAGCGAGGTAGCAGTGGCTCTCATCATAATGGCCT
TTTCATTCCAGGACCTGGTGGCAAATGGGGCCCTTGTCAGCATCTGTAACAAGTATGGA
GAGATGCCTGTGGACAAAGCCA
>677
TGTGTCCTCTCGTCCCTTCCCACCTGTCTTCTCCCTCTGTACCACAGCTTAGGTTGTTTT
TCTTCCCTAGAGCGGGCAGAGAAGATGGGCCAGAATCTCAACCGTATTCCATACAAGG
ACACATTCTGGAAGGGGACCAC
>678
AGGAAATAATCCTGGCCTCTTGGGGCTGGGTTAGGGTGAAGCTGGGTACCTGACCTGC
CCACACTCTTAGGAAATGGAACCCTGAACAAACACTCTGGCATTGACTTCAAACAGCT
TAACTTCCTGACGAAGCTCAACGA
>679
TTTCCTCCAGTTAGTGGGCAAGGAAGTGGCAGCAACATTTCAAGCCTCCTAACCCCTA
CCTGTCCTGCAGCTATGGAAGGGCCGCTGGCAGGGCAATGACATTGTCGTGAAGGTGC
TGAAGGTTCGAGACTGGAGTACAA
>680
CCAAGGAACCCTGAATAGCACTGAAAGAGATCTTTTGTACTGGGTCTCAACCACTCCC
TCCCTCTTCTAGGATTTTCTCGCATCCAAATGTGCTCCCAGTGCTAGGTGCCTGCCAGT

CTCCACCTGCTCCTCATCCTACT
>681
TGAATTTCCTTGGGGAGGAAATGGCAGAGAGGGAGCCTCTCTGAACTATTTGACTTTT
GCCTCCTCTCAGATTTCGTCGTGGACCAGAGCCAGGCTGTGAAGTTTGCTTTGGACAT
GGCAAGGGGCATGGCCTTCCTACA
>682
CTGGCCCAGGCCCCAAAAGCCCTTTGCCTATCTATGACTTACCTCCTTCTATCTGTTTTC
TCTTCCTCAGATTGATGAGGACATGACTGCCCGAATTAGCATGGCTGATGTCAAGTTCT
CTTTCCAATGTCCTGGTCGCA
>683
CAGAAGTAGTGGAAGGGGGCAGAGACAGGACAGGCAAGGGGGCCAGAACAGACAA
GCCCTATCTCTCCAGCTCTGCAGAAGAAGCCTGAAGACACAAACAGACGCTCAGCAG
ACATGTGGAGTTTTGCAGTGCTTCTGTG
>684
CAAGGGCCAGTGGCTTCTCTCTACATGACAGACTCAAATTGTGAGGCTGCTTTTTTTCT
TGTATTCGCAGGTGGCATTGGAAGGCCTTCGGCCTACCATCCCACCAGGTATTTCCCCT
CATGTGTGTAAGCTCATGAAGA
>685
TTTTTCCCCCTAAATTTAATCTTGTAGCATTTTTGGTTACTCCTGCTTACATATTTGGCTC
TTTTCCTAGGTAAGTGGAGGTATACTTCGAATTTTTCCAGAAGGCAAAGCCCAGTTTGC
TGACATTGAACCCAAATTTG
>686
GGAATGCAGTAGCAGAAGCTCTCTGGTGCTTAGTGAATGAATGAAAATTTTTTTTTTT
TTACCTTGCAGGTGAAAAAGGTGTGAGGGTTGAACTCAATAAACCTTCAGATTCGGTC
GGTAAAGACGTCTTCTAGAGCCT
>687
GGTTTTGAGGAATTACTCATTAGTCTCTGCACATCCACAGACATATAATTACTTTTTATCT
GCACTGTAGGAATGATTGCAATTAGTAGGATGGCTGTGCTGTATCCTCAAGTTATTGTCG
ACCATCCATTTTTCTTTCT
>688
AGCAAGAAGGAAGATAAATATTTTATTATTTTGTTCACCCCCTCTTTTGTTTACTTCTGA
ACCAATACAGGTACAATTCTATTCATGGGACGAGTCATGCATCCTGAAACAATGAACAC
AAGTGGACATGATTTCGAAGA
>689
ATTGTGACCATTTAATCAACACAGCAGTTAAATTGTATATTTACTTTCATTGTTTTATATT
GCTTGGCAGAAAATGAAGACAAAAAGTGATATGATGGAAGAAAACATAGATACAAGT
GCCCAAGCAGTTGCTGAAACCA
>690
GTAAATATTTAGGGGAAATGGTTCTAAATTTACAGACAGAATCTTGTTTTTCTTTTCACT
ATACTTACAGGCACCAGCTTCTGAAAAAGAAATTGTGGAAGTAAAAGAAGAAAATATT
GAAGATGCCACAGAAAAGGGAG
>691
GACTGAGGAAATGAATGAATGAATGTCTCCATCTCAACCCTCAGCCTGGTCCCTCCTTT
TTTCCCTGCAGTTGGTACAGATGGCATTGTCCCAGTCTGTTCCCTTCTCGGCCACAGAG
CTTCTCCTGGCCTCTGCCATCT
>692

GGGAGTGGAGCAACGTTCAGCCTTTGACCTTGGAAGTGCCAGAGGTGCCCCTAAGCT
TGTGCCCCCTCAGAACAGTGTCCGGGACATCACGGGTGCCCTGTTCAAGCACAGCAA
GAAGGGGCCTAGAGCCAGCGGCAACC
>693
GATACTTGGGAAATGATGGTTTCCTTGTTTCTGTCTTCCTTCTTTCCTCACCTTACACTA
CACGGTTCAGGATTTGACACAGTCACCACAGCCATCTCCTGGAGCCTCATGTACCTTGT
GACCAAGCCTGAGATACAGAG
>694
TATAAGAGGGGATAATTCATGGGGCAGTTAGGGCAGCCCCTGAGCTCTGCTTGTCCTCT
GTGTTCTACAGACACTGTGATTGGCAGGGAGCGGCGGCCCCGGCTCTCTGACAGACCC
CAGCTGCCCTACTTGGAGGCCTT
>695
GTGGAGGTAGGAGCAACACATGCCCCAGCTTTCCAGCCCTGAGCCTCACAGTGCCCTC
TTCCCTCCTCAGCACAACAAGGGACACAACGCTGAATGGCTTCTACATCCCCAAGAAA
TGCTGTGTCTTCGTAAACCAGTGG
>696
CCCTCCCCCAGGCACCTCCTCCCAGGGCCTCTCCAGCCCTGAGGTCCCATCTCCTCTGT
TCCTCTTGCAGAGAGCTGTGGGAGGACCCCTCTGAGTTCCGGCCTGAGCGGTTCCTCA
CCGCCGATGGCACTGCCATTAAC
>697
AGTCTCTGGGCTGTCAGGGCTCGGGCTGGGCTGGGCTCTCGTTGTTAATGGTCTTCCCC
TCATCTTGCAGGAGCCATGTGGCTAGAAATCCTCCTCACTTCAGTGCTGGGCTTTGCCA
TCTACTGGTTCATCTCCCGGGA
>698
GAAACAGACTTTGCTCTTGTGCTCTGTCCTTCCCATCCCTCTCAACTTGGGGTCCTGAA
TTTTGCTCCAGGACTTACACCAGAGGATCGATAAGTTCCGTTTCACCCCACCTTTGGAG
GACAGCTGCTTCCACTATGGCT
>699
GGGGGTGCCAGAGCCTGACCGTGCAGGGTCTTCTCTCTCCCTCCACCCTGACTGTGCT
CTGTCCCCCCAGGGCTGGACATCCACTTCATCCACGTGAAGCCCCCCCAGCTGCCCGC
AGGCCGTACCCCGAAGCCCTTGCT
>700
AACACCAGAGGGCCCAAGGAGCAATCTGCCTGTGACTCCGTGACTCCATGCCTTTCCC
CATCACTGCCAGGGTTCAACTCGGTGGCCACCGCCAGGATCTTTTACAAGCTGATGCT
GCGGCTGGGCTTCCAGGAATTCTA
>701
CGCCATCTCTCCTCTCTCCCTGTCCTTGACACCAGCCCAGCCTCACCCCGGCCCCTCTC
TCTGCCTTCAGCCACGTGAAAGGCCTGCACTTGAACATGGCTTTGGTTTTAAGCAACT
TCTCTACCCTGACCCTCCTCCTG
>702
CATCACACCTGAAGCTCCAGCTCTCTGGTCCCCAGGCCTGAGTCTCTCCCTTGCTCCCC
GCTCCCGCCAGGCTCTGCTCTGAATGACTCTCCTGTGGGTCTGGCTGCCTATATTCTAG
AGAAGTTTTCCACCTGGACCAA
>703
TTTCTGGCTGCCCTTTGTCACACAACTGCATGTGGCACTGAGAGTGGGGCTTTGTGTTC
TGCGTTCCCAGGAAGTTCTCCCTGGACGACCTGCTGACCAATGTCATGCTCTACTGGA

CAACAGGCACCATCATCTCCTCC
>704
ACCCCCTGCTGTGCAGACGGAGGGGCGCAGGGCCACAGCCTCACCCCTCCGTCGGCT
CTTTCACTTCCAGGATGAAGGTCTATGTGCCCACTGGCTTCTCTGCCTTCCCTTTTGAG
CTATTGCACACGCCTGAAAAGTGG
>705
TTATTTTAGTGAAACGATGCAGGTTTAACAGTAACTATGTACTTTTTTCCTACTGTCTTC
TCCTTCGTAGGACAAGAAGGTTCTGTTTGTCTCCGGTCATCAGACTGTGCCTCAGGATT
GTGTTGTGCTAGACACTTCTG
>706
GCACCTTAGTGAGTTGATCATAGAACATGAAATCCAAGTTTCTCTATCTTCAAACCCTG
CTCCCCTCCAGGTGCGGTTCCTGGAGCAGCAGAACAAGGTTCTGGACACCAAGTGGA
CCCTGCTGCAGGAGCAGGGCACCA
>707
CTCTCTAAGCGTGGAGCTCACTTGAGTAGGGTGACGGTGTGTGCAGTGCCAATCATCC
CTGTTTCCCCAGGATGTAGATGCTGCCTACATGAACAAGGTGGAGCTGGAGGCCAAGG
TTGATGCACTGATGGATGAGATTA
>708
AGATTTGAATGGAACTGACATTAAATAACACAACACAGAACCAGATGACCGACTCCAA
ATCTCCCTGCAGGAGCTGTCCCAGATGCAGACGCATGTCTCTGACACCTCAGTGGTCC
TCTCCATGGACAACAACCGCAACC
>709
CTGTGAACTTTGGGAAAGTTCTTCCCATTCTCATATAAACAGAATCTTTCCTCTTTCCTG
GCTGCATCAGTATGAGGAGCTGCAGCAGACAGCTGGCCGGCATGGCGATGACCTCCGC
AACACCAAGCATGAGATCACAG
>710
CTGACCCAGCCCCTCAGAAGGAGACACCTCAGGTCCCAAGCAACCCCACTCTCCTCCT
TTCTATCTGTAGTGCGCCAATCTGCAGAACGCCATTGCGGATGCCGAGCAGCGTGGGG
AGCTGGCCCTCAAGGATGCCAGGA
>711
GATCAAATAAATGGGCCATGCAGGATCAGCCTGGCAGATGGTCTCACTGAGTCCTCCTC
CTTTCCTGCAGCTGTTGTCACAAGCAGTGTTTCCTCTGGATATGGCAGTGGCAGTGGCT
ATGGCGGTGGCCTCGGTGGAGG
>712
ATAATACACAGAGGCTTGCAGGCCACTGCTCCCTTCCCCACACCTCTCTCCCTTTTTCT
TCTTGCCACAGGAAACTTCGAGAGGAGGAGAGGCGTGCCACCTCCCTCCCCTCTATCC
CCAACCCCTTCCCTGAGCTCTGC
>713
TAGCAGGTGCGGAAAGGGAATGAATCACCCTTTGCCTCCCCTCAAGTCGTGTGCAATT
CCTGGGGCGCAGGTAGTAAAGGTGTACAGTGAGGATGGGGCCTGCAGGTCTGTGGAG
GTGGCAGCAGGTGCCACAGCTCGCC
>714
CTAGGCATGTGGCTCATCCAGGAGATCTGGTTGATCTCCAATAACCCCTGCTTTTTGCC
CTTGTCCCCAGAGCGGGGTTTGGAGGACCACGAGTCCGTGGTGGAAGTGCAGGCTGC
CTGGCCCGTGGGCGGAGATAGCCG
>715

GGGCGCTGGGATGCCCTGATCCTCAACCTGGATGCTGGAGCCCTGATCCCTGACACTT
GTCTACCCACAGCACTCCCTGTTCCCAGAAAAAATGGTCTCCAGCTGTCTCGATGCAC
ACACTGGTATATCCCATGAAGACC
>716
AACATCCTGGTGGTAGAGGGGAAGGGAGGAGGTCAGACCTGTCACTCTCCTCTGCTCT
CCTCTGGCTCAGAACTTCCTGAATGCTGGCAGCTTTCCTGAGATCCAGGGCTTTCTGCA
GCTGCGGGGTTCAGGACGGAAGC
>717
TGGCTTGTGGGGGGAGGTAATAGTGGGCGGGATCTACCTGTACCAAGTCCTGCTCACT
CATGCTGCTTAGGATCCGAGGCACCTGCAGTACGTGGCAGATGTGAACGAGTCCAACG
TGTACGTGGTGACGCAGGGCCGCA
>718
TATGTGGAGCAGGGGCAGACATGTGGTCCTAAAGGCAGATATGGGACCAGTCAATTTC
CTCTCTCTGCAGCCCAACAAGCTTCGAAATGGACACAAGGGGCTTCGGATCTTCTGCA
GTGAAGATGAGCAGAGCCGCACCT
>719
TGAGGGGGGCGTCACAGCCACGCCCCCAGGACCTCTCGACCTCAAGCTCTCTTTCTCT
CCCCACCCCCAGTACGGGGTGCAGCTGTACAAGAATTACCAGCAGGCACAGTCTCGCC
ATCTGCATCCATCTTGTTTGGGCT
>720
GGGTATGCAGGCCCTGTCTTACGGGTACCTGGGCCCTGTTCTGACCTCTCTCCTCTCCT
TCCATCTCCAGAGAAGTGCCTCAGATAATACCCTGGTGGCCATGGACTTCTCTGGCCAT
GCTGGGCGTGTCATTGAGAACC
>721
ATTCCCAGTGGGGAGTAGATGGTATAGGGGTCCCCTCCCAAAGTGACCGCCCATGTCCT
TCCCACCACAGCCATCCACCGCACCCAACTCTGGTTCCACGGGCGCATTTCCCGTGAG
GAGAGCCAGCGGCTTATTGGACA
>722
AGAGACTGGGGTCCAGGTGGCAGCCATGGTCCTTGGGTAGTAATGCTGCCCATCTCCT
GTCTTCTGGCAGCCTGTTCCTGGTCCGGGAGAGTCAGCGGAACCCCCAGGGCTTTGTC
CTCTCTTTGTGCCACCTGCAGAAA
>723
CTGGCCCAGGAGGGAGCTTCCCAAGCCTCGGGCCCCTCCCTGAACTTCCACCCCCTTT
ACTGTACCCCAGAGCGAGGAGGAGGGTCGCCTGTACTTCAGCATGGATGATGGCCAGA
CCCGCTTCACTGACCTGCTGCAGC
>724
GTGAAGGAAATTGACTTTGTCAATGACCAGATTTTTATATCAACCTTCCTTTTTTCTGTC
CTTTCTCTAGCACTATTTAGGAGCCTACGTTTTTATTATCTTCACCGGCTTCCTCATTACC
TTCTTGGCTTTTACCTTCT
>725
ACGATCAAGGAACGCAGCCTTCCTGTTGCTCTTAGCAGTAACCAGGCATGTCCACTGG
CTGTTTTTGCAGTTCTTTAATTCAGTGCGGCTCTGGGATTTCATCGAGAACCGAGAAAG
CCGGCATGTCCCCAACAATGAAG
>726
CTAGGCAACCAGTAATTTTGGTTAAAACCAAAATACGTGTTCTGAATTACTGTTCTGTG
TTTATTTTCAGGCTTCTGGAGTTACAGTGAATGATGAAGTCATCAAAGTTTTTAATGATA

TGAAAGTAAGGAAATCTTCTA
>727
TATTATCATAGTCAGTCTTACCTTTGTTTTTCTTATAAATGGGATTCAATAATTTTTTTTT
TTCTGTAGGGCTCCTGAAAGTGCACCTTTAAAAAGCAAGATGATTTATGCTAGCTCTAA
AGATGCCATTAAAAAGAAA
>728
ATATTTGGGGCAGAGAAATTATTCTTTATTAATTATAATAAAGTAATAGGATAATGTTTTT
TCTTTTTAGGTATTAAACATGAGTGGCAAGTAAATGGCTTGGATGATATTAAGGACCGTT
CGACACTTGGAGAGAAATT
>729
TTTGACTTCTCAGAAATATATAGACCAACAGTGGTTGCCTTTTTTCATTTTCACTTCTCT
TCATTCTTAGATCTACGGAATCCAGAGGCAGCATTGTCTCCAACCTTCCGTAGTGACAG
CCCAGTGCCTACTGCACCCAC
>730
GTTAGGATTTACCACTTATTTTCAGTTCACGGTCAGTGTCTTATTGCCCATACCTCCCCT
CGTTCTCTAGCCAGATGCTCCTGCCACTCAAGATGGGGTAGAAAGCCTCCTGCAGAAG
TTTGCAGCTCAGGAGTTGATTG
>731
TTTGCGGAGTTGGTCTAAGAGCCAGTTTGATCTATAAGACATTTTAAGTCCAAGGCTAC
TTCTGTCACAGGTCAGTCAGGAGATCCTAGAGCTATTAAATACTACAACAGCCAAGGA
ACAATCCATTGTTGAAAAATTTC
>732
TCATTTGGAATTTGAACTGTTGGATCTACATCTGGAGCACCATCTTGATTCTCCCTTTTT
ATTTTCCCAGACGAATTATCAATAAACACACTGATGAGTCTTTAGGTGACTGCTCTTTCC
TTAATACATGTTTCCACATG
>733
GAAAAGAGGGACTCGGGCCATGAATCTCAGAGTTAAAGTAGGGAAGATGACCAAATA
CCACTTTATGCAGTGGATCTGTTGTGATATCCGCTACCTGGACGTCAGTATCTTGGGCA
AGTTTGCAGTTGTGATGGCTGACC
>734
TCTATAAATGGTACTACCCCTAAACTGTAAAAGTTTGGCTAAAATTCTAGTTCTTTTTTA
TGCCCCACAGGTATGAACGGGTAGATGAAATTATTTGGGTGAAGACAAATCAACTGCA
ACGCATCATTCGGACAGGCCGT
>735
TAAACATAATAGAAAGGGCTAGAATTGCAATCTTGTGTAACTTAAAAAGCAGCTAGTTT
TTATTTCCTAGGTTCGTTCCACCAGTCATAAACCAGATGAAATCTATGGCATGATTGAAA
GACTATCTCCTGGCACTCGCA
>736
CATTTTTTAACAATATTAAACCCTCTAATTCCAGGATTTCTGGTTTGCCCCTCCTTTTACA
CTGTTTCAGGATCACCCTTGGAAACCAACTGGATGGGATCCACCTACTAGACCCAGAT
GTGGTTGCACGGTTCAAGCAA
>737
TGTTCTCTTTTTGGAAAGCTTTGTAATATAAATATGGAATTGTGTTTTATTTCCCATTTTTT
ATTTTCAGGTGTCTGCTTTTGGGGCAGAGTTTTCATCGGAGGCATGCAGAGAGTTAGG
CTTTTCTAGCAACTTGCTTT
>738

TTTGTCTTAGATGAGGCTGAGAAGGTTGTTTCTGAACAGAAAGTAATGATGACGTCCC
TCTTTTTTGTAGTATGTCCGTGGTTCAGACCCTGTATTAAAGCTTTTGGACGACAATGG
GAACATTGCTGAAGAACTGAGCA
>739
TAAGACACTACAGTGTGTGTCATAACCAAGACCTGATCAGGGAGTAGTTACTTCTCTTC
TTTTCTTACAGGAAACATGGTTCCAAAACTGTTCACTTCCCAAATTTGTCTGCTTCTTCT
GTTGGGGCTTCTGGCTGTGGA
>740
TAAGACACTACAGTGTGTGTCATAACCAAGACCCGATCAGGGAGTAGTTACTTCTCTTC
TTTTCTTACAGGAAACATGGTTCCAAAACTGTTCACTTCCCAAATTTGTCTGCTTCTTCT
GTTGGGGCTTCTGGCTGTGGA
>741
TGAGACACTATAGAGTGTGTCATAACCGAGACCGGATAGGGGAGTAGTTACTTCTCTTC
TTTTCTTACAGGAAACATGGTTCCAAAACTGTTCACTTCCCAAATTTGTCTGCTTCTTCT
GTTGGGGCTTATGGGTGTGGA
>742
TGAGACACTATAGAGTGTGTCATAACCGAGACTGGATCGGGGAGTAGTTACTTCTCTTC
TTTTCTTACAGGAAACATGGTTCCAAAACTGTTCACTTCCCAAATTTGTCTGCTTCTTCT
GTTGGGGCTTATGGGTGTGGA
>743
TGAGACACTATAGAGTGTGTCATAACCGAGACCGGATCGGGGAGTAGTTACTTCTCTTC
TTTTCTTACAGGAAACATGGTTCCAAAACTGTTCACTTCCCAAATTTGTCTGCTTCTTCT
GTTGGGGCTTATGGGTGTGGA
>744
GTTAGGGAATGGGGAAAGGGGAGCGAGCCAGAGAAAACTGACGCCCCTCTTCCCTGC
TTCCTTCCTCCAGGGACCCCTCGGAGGAGCATTCGCTGCTGGTGAATTGATTCGACCTG
ACTGCCGTTGGACCGTAGGCCCTG
>745
CCAGCCCTAGCCCTGCCTTCCCAGAACCGTAAGGGTCTATCTCCACGGACACTGTGCC
ACCCACCCCCAGATGAAGGAGTCCGCTGCCCGAGTGCTACAAGAAGCCTGGATGTTCT
ACAAACATACTCGCAGGAAGGAGT
>746
TTCCTCCCCTGAGCTACAGCTACATTCAGGGACCAGGCTTGTAAAACTGACTCCCCACT
TGTTCCCACAGGTTCCGCCAGGTGCGGCTGAAACACCGGAAGCTCCGGGAACAAGTG
AACTCCATGGTGGACATCTCCAAG
>747
TGCCTTACGCTATGGAGTAGGTGGGGGTCGACCCCAGCCCCGACACCTCTGATCCTCTC
CTCCCACCCAGATGCACATGATCCTGTATGACCTGCAGCAGAATCTGAGCAGCTCACA
CCGGGCCCTGGAGAAACAGATTG
>748
CCTCGGTGTGTCCCACTCTCACTCTCCCTCTTACTGGCCTGCCTCTCCATCAGTCTCTCT
GTCTCTCCAGGGACCCACGAGGAGGGAACCAAGGCTACTTTCCCCAAGTACTGAGGT
GGTGGACATCGTCTCTGCCACTC
>749
CCCCTCCTATCCGTCTCCCAGTCAGGGGCCTGGTGCTGTGGCCCCGCTGACCCCTCCCC
TGCCTCCTCAGCCCCAGGATGGGTGAGTTCAACGAGAAGAAGACAACATGTGGCACC

GTTTGCCTCAAGTACCTGCTGTTT
>750
CCCCACCCCCACCCCTCCCGGGCCACCATCAGACCTGGGCAGATGCGGTGACCTTTGT
GACTGCTTGTAGCTGGCTGGCCTGGCTGTCATGGCAGTGGGCATCTGGACGCTGGCCC
TCAAGAGTGACTACATCAGCCTGC
>751
CTGCTCTGAGGTGCACTAGGTCTAGGCACTAGGCCTCAGAACAAGGGTGCCCTTGTGC
TGCCCCCCCCAGTACTTCATCCTGCTCCTCATCATCTTTCTGCTGGAGATCATCGCTGGT
ATCCTCGCCTACGCCTACTACC
>752
CTGGGGCTCTGCCAGCCCCACCTTGGAAGGTCTTAGACTGAGGCTGAAGTTTCCTGCA
CCCCAACCCCAGCTGAACACGGAGCTCAAGGAGAACCTGAAGGACACCATGACCAGG
CGCTACCACCAGTCGGGCCATGAGG
>753
CAGGGCCTCCCTGGACCTCTGCAGGAGCCTCTGGCCCCAGGTCTCAACCCCAGCCTTG
TTCTTGATGCAGTTCCACTGCTGTGGCAGCAACAACTCACAGGACTGGCGAGACAGTG
AGTGGATCCGCTCACAGGAGGCCG
>754
GCTGGAGGCAGTAGGGGCCAGTGGGAGGTGCCCCCTGGGCCCGCCTTCAACACCCAT
CCGCGCCCCGCAGGGCGGCTGCATCACCAAGTTGGAGACCTTCATCCAGGAGCACCT
GAGGGTCATTGGGGCTGTGGGGATCG
>755
GGGACACGGGGCAGGGCGGTGGCTGGTGGCCCCATGACGTCTGCTTACGCCCACCCG
GCTCTGCACACAGGTCTTTGGCATGATCTTCACGTGCTGCCTGTACAGGAGTCTCAAG
CTGGAGCACTACTGACCCTGCCTTG
>756
TTTTAGGGAGCCCTAGCAGTACCTAGGTCAGAGGGGAGGTCTGACTTGCTCCCCAAAA
CTCATCCCTAAGAACATGCTCCTGCCAGCTGGACTAAGACAACGTGACCAGACAAAGA
AGAGCCCAACGGGGCCACTGGACC
>757
AAGGAAACCTGGAGGCCAGGATCTTGGGAGAAATTACCTCTACCTGAGCCATCCTTCA
TCTCCCCATCAGGGAAACCCTATATTCAGCCCAAGAGGGAAATCCCAGCAGAGGAGCA
GATCACCCTGGAGCCTGAGCTGGA
>758
AATAGGTAGGAGGGAGGCTTGGAACAGGTTCCTTCTCATTCCCTGTGACTCCTCTTCTT
ACCCTCTCCAGCAATTCTGGACATGTACACACTGATGAGTAACAAGCAATACTATGATG
CCCTCTGCAGTGGAGAAATCTG
>759
GAAAAGAAAACCAATGGGGAGGAGATTATGGGGAACATGTTGGGGTTCCCTTCCTGCC
CTCATTCACCAGGTGTGGTACAGCCTGACAAGTATAAGCCAGTGCCGGATGAACCCCC
AAATCCCACAAACATTGAGGAGAT
>760
ACCATAACACCGGATAACCACTGACATCCGAGAGTCCCTTAGCCCACCATCTCCTATTC
CCCTCCGATAGGACATCCCAATACCCATGCTAAGTGAGCTGTGTGAGGCAATGAAGGC
AAATACCTATGTGCGGAGCTTCA
>761

TCCTGTTCTTTCTTTCCTCAGTCATCTGTGAAACTGTTCCCTAATCCTAACCCAAGTCCC
TACTACCCAGGCAGTGGCTGACATGTTGCGTGAGAATCGTAGCCTCCAGAGCCTAAAC
ATCGAATCCAACTTCATTAGCA
>762
GGGTCTGTGATGTGGGATGGTAAACAGCCCAGAGAGGGTGACTGTCAGCTTTTGCCTC
TCCCTCCCCCAGCGCCAGTGGCCTGGTGATGCAGTGGAGATGGAGATGGCCACCGTGC
TAGAGCAGTGTCCCTCTATTGTCC
>763
AAGAATTAGACAAGGATGTTTATATAAACACTTTAAATGTATTTGATATAAAATTTTTATT
TCTTTGTAGACCCAGAAAACCCCAGAAGTGGTGATACAGTGGAAGTACAAGTGAATGG
AAATCTTGTCAGAGAACCTGA
>764
TCTGCATTGTATCATGGACAAAAACTGGTGGTAGGAATCAGTGTTTTGTTTTCCTTTCAT
GCTTCTACAGAGAATCAGCTGTTAATCTAGTGAGGAGTGGCTCCTATACCCGGCAGCTA
TGGAGGGATGAAGCAAAAGGA
>765
TCTGTGCCAGTGCTTTGTTGTATAGCATGCCTAACCATTTCCTTTCTGCTTGTATCTTTTT
AAATTTCAGGACTCCTCACAAATCCCAGGCCGACACAACAGCAGAGAAAACAGCAGA
CAATGTCTCTTCTAGCACCCCG
>766
AACACTTTGTCATTCCTCAAGTATATGCAGCAAAGATCTTGCTTTTGCTATTACTTTTTTT
TCTCTGCAGGTCCTATCTGACTCCTGTACGGGATGAGGAAGCAGAGTCTTTACGGAAA
GCACGCTCCAGACAAGCTCGG
>767
TATTCCTGATCAATCAAGAGTTATTCCATGGTTAGTGTGAAACAGCCCTGATCTTGTGAT
ATTTTCCCAGGGTGTCACCCTAACAGACCTTCAAGAAGCAGAAAGGACATTCAGCCGG
TCGAGGGCAGAGAGGCAAGCTC
>768
TATAAACCTGCTGTCCTTACTCTTTAGATGGACTTGATTTCTAATATCTCATATTGTGGAT
TATTTCCAGCCTATCTGTCATCGCCTGAGGTGCCCAGCTCAGCCAGACAAACCCACCAC
GCCAGCATCTCCTTCTACGT
>769
AGACACAGGCCCCTCCACAGTGCACATCCCCAGATTCTTAAAGTTCATACTTTATTTTAT
CTCTGGCCAGAGAATGAAGAAGCAGATTTGGATGAGCAGTCCTCTAAGAGGCTGTCCA
TCCGAGAGAGGAGGCGGCCCAA
>770
CTGAGCATTACCTTCTCCCCACTACTTTGTTCTCAAAACCAATTTTTATCTTTAATATTAT
CTCCCACAGGTTGGAATCGGGAGGTAGTAATCCTACAACCAGTGATTCTTACGGTGAC
CGGGCTTCAGCAAGAGCCCGT
>771
GCATTCTCATGTGATGAAATGGTGTGAGTTCTAACGCGAACGCTGATCCTCTTTTTTCC
CTCTCCTCTAGCTCTATGAGAGTGCTCTGACTGAAAACCAAAAACTGAAAACAAAACT
TCAGGAAGCCCAGCTAGAGCTAG
>772
GGCCTGGTCAGGGAGGGCCAAGGAGGATCCTGCCAGGCTCTAACTCACCTTTCCCTCT
GTCACCCTACAGGTGTTAACAGAACTGAAATCCGACAACCAGAGGCTGAAAGATGAA

AATGGTGCCCTCATCAGAGTCATCA
>773
ACTGGAGGTGCAGGGGGCCGGACTCAAGCCCAGAAGCTGCCTGCACCAACCACCCAA
CTTCTCTCCACAGCCCTGGAGCCCACCTCCCAGAAGCCCGGTGTGGGGGCGGGCCAC
GGGGGAGATCCCAAGCTCAGTCCCCA
>774
AGTGGTCCCCCTGTCTCGCTTCCAGCCCAGAACCATCTCTTCTCTCCCATCCCTGCCCT
CGGCCCCACAGTCCCACGCTGCTGGCTCCAAGCAGCACGAGAGCATCCCGGGCAAGG
CCAAGAAGCCCAAAGTGAAGAAGA
>775
AGTGGGCACTTCTTGTTAAATTTACCATTGAGACCCTGGTAACACTGAAGTTTGTGTTG
ACTTTCTGTAGGTGTTTTCACAGTCCTGTGTTTGGTATGGAGAGTGTGGAATTGCATAT
GGGGACAAGAGGTACAATTGCG
>776
GTTCACTGAGGAATGTTGACCTTACTCTAACTGTTGCCTACTCCCGTGTCTGTGCTCTTT
CACCTGAAAGGAACTCTGTCCAGGATTCTTCTTTGGCAATGTCAGTCTCTGTTGTGATG
TTCGGCAGCTTCAGACACTAA
>777
TATTTAAAATCGTTCTTGCTGGCCCTATTATGTGTGAGATCATGCAGAATATAATTTACTT
TTCCTTTAGATGTCCATCCTGTTTTTATAACCTACTGAACCTGTTTTGTGAGCTGACATG
TAGCCCTCGACAGAGTCAG
>778
TTGTAAGATCTTGCCTCGTGAATTACAGCAAGCATCTTGTCTCCTTTTTCCTTTGGGTTT
CCTCTTCTAGCAATGTACAATGCCTGCCGGGATGTGGAGGCCCCCTCAAGTAATGACAA
GGCCCTGGGACTCCTGTGTGG
>779
GTTTAAGTATAATACAAAATTTTGGATGTTTTTAATTGAGATTTGTACTCAACACAATTC
CTTTCTGTAGATTTTCCAGTCCATGGGATGGAGCCCATGAACAATGCCACCAAAGGCTG
TGACGAGTCTGTGGATGAGGT
>780
TCCCTGTTCCGACTTTCAGGAACGGCTTGGCTCTTAACCTCCACCTTCCCTTCCTCTCC
CGTTCTTCCAGGAGAGGCGTCCTGCTGTGACCCTGTCAGCGCAGCATTTGAGGGCTGC
TTGAGGCGGCTGTTCACACGCTG
>781
CTGATTAATCAAGATCTGAGAGAAATGTGACGTGTTTCTGGGTTTGCTTATTTTTAAAAT
CTTCTTTCAGGTTCTTGACTTACAAATAGCCATCGAAAACATTACTGCCTCTTATGACAA
TGAGACTGTGACACTTCAAG
>782
TGTAAATGTTTAGGTCCCTGATGTCTTGAGGCCCTTCTAACTGGGAGGTGTTTTCTAAA
CTTTTTGGCAGGGCTCCTGCCTCTCTGAATGATACAAGTTTGCTCCATGACCCTTGTCT
GGGTACGTTTGGTGGACCAGTG
>783
TCCCCTGGTATGTGTCTAATTTTCTGCATGCTTGTAATCTGCTTTTTGATGTCACATTTTT
CCTTTCTAGATCAAAACTACAATAACGCCACTGCCCTTGTGATTACCTTCCCTGTCAATA
ATTACTATAATGATACAGA
>784

TTAGGGAGAAGTTTCTTACTTAGCTGTCAGTTAGTTTAACTAAAAATATAACATTTTGCT
TACCTTTTAGGTTTATTAATTTTGTGAAAAACTACAAGAATCCCAATCTGACCATTTCCT
TCACTGCTGAACGAAGTATT
>785
GACCTTTTAGTAACAAGTGGGACAGACAACCCTGTAACTAATTGGTGATTGTGTCTGTC
GCCTCTCTCAGGTGGATTCGAAGGTCTCACTAGGCATCGCGGGCATCTTGATCGTGCTG
AGCTCGGTGGCTTGCTCCTTGG
>786
CAAGGCAGCAAGAAATGGCGTTGTTATTGAGAAATTTTAATGTCGCATAATTTTTTTTT
TTTTTTTAAGAGAGATGAACGTCTTCAAGGGGAAACCCTGGATCAGCAGCTGGGCAGG
GTCCTAGGAGAAGTGGCTCCCA
>787
TGAACATAAGACCTGCAGAGAGCCCCCGGCTGTAAACAGAAGTGACGCAGAGCCCTT
CTCTCCCCGACAGGAGCATTGTCCGTGATGCCAGCCGTGCACACCTTCTCTCTCTTTGC
GGGATTGGCAGTCTTCATTGACTT
>788
TTTAATTTGAGTCTAGAATCTAGAGGAAGCAGCTAGCGGGTATAACCTTTGTTTCCCTC
TGTCCCCTCAGAAAAATCGGCTAGACATCTTTTGCTGTGTCAGAGGTGCTGAAGATGG
AACAAGCGTCCAGGCCTCAGAGA
>789
AAGATTTTATTTCTGGTCGTAAGCTTATTTACTAAGCAGTCTCTGTCTTATGATTCATTTG
TTGTTCCAGATAGCAATATTTGTGGGTGTTCTGTCATTCAGCATCGCAGTCCTGAACAA
AGTAGATATTGGATTGGATC
>790
AAGAAAAAGTACTGAAACTAAAGACTTCCTCCCTGTGGAGCAGGTCAGTAACCCTGTC
TGTCCTCTCTAGTACCCGAATAGGCTTCGCCCCCTCGTCCTGGATCGACGATTATTTCGA
CTGGGTGAAGCCACAGTCGTCT
>791
ATTTTAAAATGTTTTACAAAAAATCTGGAGACCTATTCTTCTAACAGTCCTCCCTGCATG
TCTCCGCCAGTGGTTGACCCTGCCTGCGTTCGCTGCAGGCCTCTGACTCCGGAAGGCA
AACAGAGGCCTCAGGGGGGAGA
>792
TCAAGTGCTCAGATTCTGCTGATATTTTGCAAGACCTGGACTCTCTTGACACCCAGGAT
TCTTTCCTCAGGGGACATGCTGCCTATAGTTCTGCAGTTAACATCCTCCTTGGCCATGGC
ACCAGGGTCGGAGCCACGTAC
>793
GTGACAGGATGAACACGCAGTGTTCGGGAGTGAGAGCGAGCTTTAATGAGGCCTCCC
CTCTCCCCTCCAGTGTGTTTTATGTCTTCTACGAACAGTACCTGACCATCATTGACGAC
ACTATCTTCAACCTCGGTGTGTCC
>794
TCAGAACGGGGTGGCTGCCTAGGGTCTTCAACAGGAAGAGAGCCATCCTAAAGGAAG
TGCTTTATTTCAGAGCTGTGGCATCTCCGTGGAGTTCTGCAGCCACATAACCAGAGCGT
TCACGGTGAGCATGAAAGGCAGCC
>795
TGTAAGGAAGTGTTTTTGTTCAATTACAGGTTGGTAAAAGTGGTTTCTAACACAGTATC
TCTTCTTTTAGGTGTTCAGTGGAATCACACTTACAAAATTTGGAGGGATTGTGGTGTTG

GCTTTTGCCAAATCTCAAATTT
>796
GTGAGCCACTATGCCCAGCCAACTAGCTCCCTTTCTCCTGCAGAAATAAGAAAAAGTC
TCTCTCTCATAGGGCCATCAGTAAATAAAGCCAAAAGTTGTGCCACTGAAGAGCGATA
CAAAGGAACAGAGCGCGAACGGCT
>797
GGCTCTGAATTTTTCTAAGAAAAAATGGTAACTATCACTTGTTACTCACTAACCAAAAA
TTTTCTTTCAGAACGCTCCAGAATAATCAGTTGAAAACAGTACCCAGTGAAGCCATTC
GAGGGCTGAGTGCTTTGCAGTCT
>798
GTTGTGAGACTTGAACTGACTCATTTCACTAGAATTTGTTACTGAACATGGTTGTTTTT
GTTTCTGTTAGGCGTTTAGATGCCAACCATATTACCTCAGTCCCCGAGGACAGTTTTGA
AGGACTTGTTCAGTTACGGCAT
>799
CATATAAAACAATGATAATAGTTGCCAATGGGAGATGGAAAAGTCATTTTATGCTTTTTT
TCCCCCTTAGGCATCTTCATAACAATAAAATTAGAGGCCTGAGTCAACACTGTTTTGAT
GGACTAGATAACCTGGAGACC
>800
CAGTAGATACTTGGGTTTTATTTCGCAAGAGGATAATGCATTTCAAAATACTTTTCTCCT
ATCTTTCTAGAGGATTTCATAGTAATTCTATTTCTGTTATCCCTGATGGAGCATTTGATGG
TAATCCACTCTTAAGAACT
>801
TGAACATAGAGTTAATATTAGAGTGCTTGTGAACATATGAAAATTTTACTTTTTTTGGAT
ATTTTCACAGACATTTGTATGATAATCCTCTGTCTTTTGTGGGGAACTCAGCATTTCACA
ATTTATCTGATCTTCATTCC
>802
TATATGGGAATAAATTTTGCTTACTATCTTGAGTTGAATTATTTCTTACATGAATAATGTTT
TTCCGCAGGACTTTGACAGGTACAAAGATAAGCAGCATACCTAATAATTTGTGTCAAGA
ACAAAAGATGCTTAGGACT
>803
TCTTTCCTCCTCCTCCTGTTCTCTTCTTTGTCTTTGTCTTCGTCTTCTTTCTTCTTCTTTTT
TTATTTAGTTCTTTACAGCGTAATCAAATCTACCAAATAAAGGAAGGCACCTTTCAAGG
CCTGATATCTCTAAGGATT
>804
AGTATAGTCTAAGTAAGTGGCATTCTGAGTGTTTATTTGAAAGATTAACATGTTACTCTT
ATAATTGCAGAGATCTGAGTAGAAACCTGATACATGAAATTCACAGTAGAGCTTTTGCC
ACACTTGGGCCAATAACTAAC
>805
AATACGTGTACAGATGTTTTTATGTGATTTATATATTGGGCATTAACTTGAAGTCTCATTC
ATATTTTAGAGATGTAAGTTTCAATGAATTAACTTCCTTTCCTACGGAAGGCCTGAATGG
GCTAAATCAACTGAAACTT
>806
TACTTTTACAAAACAAAACCATAACTGCGGAGTTGCTGTTCAGTGTAGATTTTACAACT
TTTTTTTCCAGGTCTTTATCAGTACCATATGCTTATCAGTGCTGTGCATTTTGGGGTTGTG
ACTCTTATGCAAATTTAAAC
>807

TTGCAGGAAAATATGGTTCTTTTGAGTTGCATAAGGTTTTAATTAAACATGCTTTGTGTT
ATATGTTCAGGTACTGCTGATGCAGCAAATGTCACAAGCACTCTTGAAAATGAAGAAC
ATAGTCAAATAATTATCCATTG
>808
GCCATATCATTTTCTAAATTGTTTTCTAAGTTATCTTTTATTTCTCTCTCTCTGTGTGTGTT
TTTTTTAGGTGCTTTTAAGCCCTGTGAATATTTACTGGGAAGCTGGATGATTCGTCTTAC
TGTGTGGTTCATTTTCTT
>809
TACCTTATACACAAAATATTTATGTTCTACCTGCCTGTGAAACGTCTAAAAGCCTCTCCT
CTCTCTGCAGACAGAGCACACTCCTCCGTATGATGTGGTACCTTCCATGCGACCAGTGG
TCCTAGTGGGCCCTTCTCTGA
>810
GGCATACACTTCTATCCCCTTTGATTAGACCAATAACCTTAAGGTCTTCTGTGACTTTTT
CCTCCAACAGGATATCCATCACAAGGGTCACCGCTGACATCTCGCTTGCCAAACGCTC
GGTATTAAACAATCCCAGTAAG
>811
AGAAACAGTATACCATTTTACTTTATCTTAAAAATACTGCACTTTAACTGAATTGTTTCG
CCCTTTACAGCGGAAGTTCAGAGTGAAATCGAAAGGATTTTTGAACTTGCAAGAACAT
TGCAGTTGGTGGTCCTTGACGC
>812
GTCAATAATGTACCTTGTACTTTACCTGGATGTAGTTATTACTCTGTTAAAAACTCATTAT
CTTTTACAGGTTTTACAAAGGTTAATAAAATCTCGAGGGAAATCTCAAGCTAAACACCT
CAACGTCCAGATGGTAGCAG
>813
TCTTATGGAGGTGGGAAGGGGGTGAAAACTGGGCTGGCATCAATGTGGTCTGGAATGT
CTGCCTCTGCAGGAGCTGTTCGATGTGATCTTGGATGAGAACCAGCTTGAGGATGCCT
GTGAGCACCTTGCCGACTATCTGG
>814
CACTTGCTCTGGGACATGTTCTTTACACACTGACCTTGGTTAACGCCTGGTGTGCTCCT
TTCGCTGCCAGGGTTCTCAAGGTGATCAGAGGACTGATCGCTCCGCTCCTATCCGTTCT
GCTTCCCAAGCTGAAGAAGAAC
>815
CGGCCGCCGCCCCCGCCCTCTGGCGGCAGCGCGGGGTACGGCGCCGCCTCTGAGCGC
CATCGTCTCACAGGTGCAGTCGGAGATCCGCCGTGGCTGGCACCACTGCCGCCTGCGC
CGCAGCCTGGGCGAGGAGCAACGCC
>816
TGACCTCCTGCCCGGCAGGACCTGAGTGTGAGGGTCTGTCCCACACTGACACCCCTTTC
TGCTCCTCCTAGAACCGTACGGAGTCCCCAGAACCTAATGCCACAGCGACCCCTGCGA
TCCCCACTATCCTGGTGACCTCTG
>817
GCGGCTGGAGGTCCTGAGGGGCTCGGGTGCCCCTCGAGGGAGCCCTGACCCTGCCAC
CCCCTCCCCACAGCACTCACCGAGGACGGGAGGCCCTGCAGGTTCCCCTTCCGCTACG
GGGGCCGCATGCTGCATGCCTGCAC
>818
CTCCAGGGTGGTGGGGTGGGGTGGGGGGCCTCTGCCTGGGACCCCCATGCACGCAGG
TGTCGCCCCCCAGGTGTGCCACAACTCACAACTACGACCGGGACAGGGCCTGGGGCT

ACTGTGTGGAGGCCACCCCGCCTCCA
>819
GCAGAGAATGTCACAGAGGGACCCTGAGCCTCCCAGTCCGCCCCTCACATCCCCTCCC
GCATGTCCCCAGCTGCCCTGGATCCCTGTGCCTCCGGCCCCTGCCTCAATGGAGGCTCC
TGCTCCAATACCCAGGACCCCCA
>820
AGTGCAGGGCAGGGGCCCTGCACGGGGACAGCAGGTGGCAGGGTGTGAGGGCTTACT
GTGCACCCCTCAGAGAAATGCTTTGATGAGACCCGCTACGAGTACCTGGAGGGGGGC
GACCGCTGGGCCCGCGTGCGCCAGGG
>821
CCCCGGCCCCGACTCCGCTGTCGTGGGGCACTGCGCGGCCCCTGGCCCAGCTCCTCGG
CCCTGCCCCCAGCTTGTCTGAGCAGCCCTTGCCTGAACGGGGGCACCTGCCACCTGAT
CGTGGCCACCGGGACCACCGTGTG
>822
CCCCGGGGTGCCCTGGGGCAGTGCCGGGTGGACCCACCGTGGGCCGGCCTCACTGCC
CCTCTGCCCGCAGAGCCTGATGAGCGCTGCTTCTTGGGGAACGGCACTGGGTACCGTG
GCGTGGCCAGCACCTCAGCCTCGGG
>823
GATGCCCCCTCCCCATGCCCTCTTCGAGGGGCAGTGTGACTCCCTGCCAGCCCCCACTT
ATGCACCGCAGGAATCCGGACAATGACGAGAGGCCCTGGTGCTACGTGGTGAAGGAC
AGCGCGCTCTCCTGGGAGTACTGC
>824
AGGGCCTGTGTGTGGAAGGGGGCTGGCCCTGTGAACCCCAGGGGTGTGACCCGGTGA
CCTTTGCTCCCAGAATCCCTCACCAGAGTCCAACTGTCACCGGATCTCCTGGCGACCC
TGCCTGAGCCAGCCTCCCCGGGGCG
>825
GGGTGGGGAGAGGTGAGCCCGGTGGCTGGGGGAGGGCTGGTCCATGCAGCCTCCAGC
CCCCCTTGCACAGCCCCCCCAGGGACAGCGTCTCCGTGGTGCTGGGCCAGCACTTCTT
CAACCGCACGACGGACGTGACGCAG
>826
ACCACGGGCCCCGACAGCCTCCCGTTCAGCCCGCACACCACAGGCTGACCCTGGCCA
CTCTTCTGATCAGTCCTGATCCGGCTGAAGAAGAAAGGGGACCGCTGTGCCACACGCT
CGCAGTTCGTGCAGCCCATCTGCCT
>827
CCCAGCGTCCGCCAGGTGGACAGGTGGCCAGCCACAGTGTGGTGTCACGCTGAGGGC
ATTGTGTCCACAGACGTGAGCGGCTACTCCAGCTCCCTGCGGGAGGCCCTGGTCCCCC
TGGTCGCCGACCACAAGTGCAGCAG
>828
CACTTGGGGTAGAGGGGTGTCCCTGAACCAGGCCCCTGGGAGGGTGGCTCTGACCAA
CGTCTCTGCCCAGGGGGACTCAGGGGGGCCCCTGGCCTGCGAGAAGAACGGCGTGGC
TTACCTCTACGGCATCATCAGCTGGG
>829
AGACGATTGTTCCTGTGATCTGGGTAATTGCTTTATTTTTAATTGTCTGTTGTTTTTTTTT
CCTCATCAGGTGTAGGGAGTGAAGTCTCACATAGGAGGACCTGTGTGAGCCTCACTAC
CCAGCGACTGCCAGTTAGCAG
>830

TGATCTTAACTCCTGACCCTGAGGCTTTGAGAACGTGGCTAACTTCATCTGTCTTTTCC
TTGCGTTACAGTTTTATTACCAAACGTGGCCTAAAAGTCTGTGCTGATCCACAAGCCAC
GTGGGTGAGAGACGTGGTCAGG
>831
AGATGATTGTTCCTGTGATCTGGATAATTGCTTTATTTTTAATTGTCTGTTGTTTTTTTTTC
CTCACCAGGTGTAGGGAGTGAAGTCTCAGATAAGAGGACCTGTGTGAGCCTCACTACC
CAGCGACTGCCGGTTAGCAG
>832
TGATCTTAACTCCTGACCCTGAGGCTTTGAGAATGTGGCTAACTTCGTCTGTCTTTTCC
TTGCGTTACAGTTTTATTACCAAACGTGGCCTAAAAGTCTGTGCTGATCCACAAGCCAC
ATGGGTGAGAGACGTGGTCAGG
>833
TACCAACCTTCTTTTTATCATTTGTCTATTTGGTTGTTTTGCATACTGACAAGATTTTCTT
TTCTTTTAGTTGGTCGAATTGTCTTAGAATTGTTTGCAGATATCGTACCCAAAACTGCGG
AAAATTTTCGTGCACTGTG
>834
GTATCCATTAGTAAGCATTGCTAAAGGCATCCATGTCTCAGTCACTAATAGCCATTTTCC
TTTTTTGTAGTTATTAAGAAATTTATGATTCAGGGTGGAGACTTCTCAAATCAGAATGGG
ACAGGTGGAGAAAGTATTTA
>835
TTCAGACTAACACACAATTCAGAAAAGAACACATCTAACCTTGGTTTTCTTTTAATTTT
TATTTCTACAGCATGATCGGGAGGGTTTACTGAGCATGGCAAATGCAGGCCGCAACAC
AAACGGTTCTCAGTTTTTTATCA
>836
CATATGATAGAATCTAGCATTAAACCACATTACTTACTAAAATCCAACATATTTGTATTTT
TTTATACAGGTAGATAAAATTTTATTAATAACAGAAGACTTAAAAAACATTGGAAATACT
TTTTTCAAATCCCAGAACT
>837
TCTGGACATAGAGAAGAGTAATTCCTGTTTATGTGGTCTTTCATACTGCTTTTATTAATG
CATTCTGCAGATACGTGGACAGTTCAAAGGCTGTTATTGAGACAGCAGATAGAGCCAA
GCTGCAACCTATAGCTTTAAGC
>838
GCAATGAATTGAAATCTGGCATATAAGCTTAATACTTTGATATAAATACTAGCTTTATACT
TTTTTGTAGGCTCTTGAACTAGACCCATCAAATACCAAAGCATTGTACCGCAGAGCTCA
AGGATGGCAAGGATTAAAAG
>839
AATAGTTTGTTAGTCACCATAATTTTTGGATATTTTGAGAAATAACTCACATTGCATATTT
ATTTTACAGCTATCCAGGCAGAATTGCTGAAAGTCAAACAAAAGATAAAGGCACAGAA
AGATAAAGAGAAGGCAGTATA
>840
CGCATACCGTGGACCGCACCACAGGATCAGGGGAATAGGTTGCTCGCGGCTTCACTCT
TTGTCTCCACAGCAGCCCTCCGGAGAGCTGTTTGAGATTGAAATAGACACCCTGGAAA
CCACCTGCCATGTGCTGGACCCCA
>841
TGCCTGGAGGGAGCCTGCCCGGGGTGCGAAGGGGAAGGGCAGCCATCCTCACGTGGG
TTTCTTTCTCCAGGCTGTCGAAGGAGACTGTGATTTCCAGCTGTTGAAACTAGATGGCA

AGTTTTCCGTGGTATACGCAAAAT
>842
TAACGCAGCAGAGCTGGGGCCATGCCAGGGAGAATGGCTGCCCACATCCTGGTTTCCT
CTCTCCGAGCAGACTCAGCCGAGGACGTGCGCAAGGTGTGCCAAGACTGCCCCCTGC
TGGCCCCGCTGAACGACACCAGGGT
>843
CGCTCTCCTTCTCTGCCCTTTTCATTGTAAGTCATCTTTCCTCAAGAGCATTTTCATGTA
CTCTTCTCAGCCCCTCCCACCTTCTACCTATGTGGAGTTTACAGTGTGTGGCACTGACT
GTGTTGCTAAAGAGGCCACAG
>844
CGCGTCAATGAAATTGGGGGGGATCCATTTTTGAAATTAGTTAAAATAAATCCTCTTTCT
CTGTGGGCAGCAATATGGCTTTTGTAAGGCAACACTCAGTGAGAAGCTTGGTGGGGCA
GAGGTTGCAGTGACCTGCACGG
>845
CCTGGGCCTGTGGGTTGTCTTGCCTGGGAGGAGGAAGCAAACTAACTGAAGGAAATG
GTCCTTTTTCCAGCCCGTGACCTCACAGCCCCAACCAGAAGGTGCCAATGAAGCAGTC
CCCACCCCCGTGGTGGACCCAGATG
>846
GAAGGCTGGAGTGCTGTGGCAGGGATGACGTCTCACTTCTGTTATGTCTCTGTGCCCT
GTGCTCTCCCAGGATGAGGGGCATGAAGCTGCTGGGGGCGCTGCTGGCACTGGCGGC
CCTACTGCAGGGGGGCCGTGTCCCTG
>847
CCCTTTGTGGCGCTGTAGGGTCCCTGGGTGGCACCAGCCCTGCTCAGTACCACTGTGG
CCCTGCCCCCAGATCCTGAGCCGCTATGACATCGCCCTGGTCCAGGAGGTCAGAGACA
GCCACCTGACTGCCGTGGGGAAGC
>848
CCTCGCTATCGGCAGCCAGAGGGGTCCCCTATGGCCCCCGCCACTGGGACCTTTTGTTT
CTTCAATCCAGGGATGCACCAGACACCTATCACTACGTGGTGAGTGAGCCACTGGGAC
GGAACAGCTATAAGGAGCGCTAC
>849
TGCCTCCTGGGAAGCAGGAGTGGGGAGCTTCCAGCCTGGGGTCACCTCCTCCTGCCC
GGCCTTCCCGCAGGCCTGACCAGGTGTCTGCGGTGGACAGCTACTACTACGATGATGG
CTGCGAGCCCTGCGGGAACGACACC
>850
GTTCCAGCTGACATGGTGACTGAACCTGCCCCCAGGGAGTGTGCCTCACACGACGTG
GCTGTCTCCACAGAGGTCAGGGAGTTTGCCATTGTTCCCCTGCATGCGGCCCCGGGGG
ACGCAGTAGCCGAGATCGACGCTCT
>851
GCCCTCCCAGGGGCAGTGGGCACCAGCGGCCTCCGCATGTCCCAGGGCCACAGGCAG
CGTTTCCTGGTAGGACGTCATGTTGATGGGCGACTTCAATGCGGGCTGCAGCTATGTGA
GACCCTCCCAGTGGTCATCCATCC
>852
GTTCCTGCGGGTGCTGAGCCAGGCCCATGTGTGAAAGGGGAACCTACTTTCTCTTCCC
AACACCCATCAGGATCGTGGTTGCAGGGATGCTGCTCCAAGGCGCCGTTGTTCCCGAC
TCGGCTCTTCCCTTTAACTTCCAG
>853

CAGCCACATGAGGATGGGACACAGGAGCTCAGGTAGGCTCAGCCCAGACCCTGTGCC
CACTTGCCTGCAGGCCCAAGCCATCAGTGACCACTATCCAGTGGAGGTGATGCTGAAG
TGAGCAGCCCCCTCCCCACACCAGT
>854
CTCTGCCTTGTTGAGAGCCCTTCTGCAGGCTTCCGCCTTCCCTTCCCAACTCCTGGATT
CCCGGATACAGATGAGAAAGGGCAACACCATGCAGCAGTTCCTGCAGAAGGCGCTCG
AGATCCTTCGGAAAGACTTCAGTG
>855
ATAGCACCTTGCCGCCGATGTTGTCACTGGGGCAGCAGCGGGACATTGGGCTGTCACT
TCTCCCCTGCAGGACCACTCTTCAACTTTGATGTTCATGACGATGTGCGGTTGCTCAGT
GACGCCACTGTGGAGAAGGATGA
>856
CTGGGCTATGGAGGAGAAGCCAAGGGAAGGGGAGGTCAGCAGGCGGCCCTGTGCCCT
CTTCCTCCCTTAGTCCCATGCAGGCAAGGTGGTGCTGAGGAGCTGGTACGAGAAGAAC
AAGCACATCTTTCCCGCCAGCCGCT
>857
GGGACGGCAGCCAGCCCCTGCTCACCCCTCGCCTTCCTTGTCTCCTCTGCCCACCTTGT
CCTCACACTAGATCCGCTGAGCATCCAGGAGCTGCGCGGCCCCGGCTCCTCAGCTCCC
TCAGTGTGCCCCGTGGTGTCACC
>858
AAGAGAGGGGAGAGTGGAGGTTGAGGGGTGGGGGAGAAACTGCCTCTAGACTTCCCT
TGTTTCCTTGAAGGTTAACATTAGGCTGGGAAGATGACAAAACTTGAAGAGCATCTGG
AGGGAATTGTCAATATCTTCCACCA
>859
ATAAGAGGCCTAAGAAAGGGTCTTGTGTGGAGGTTCTGAACTCAAGGTCTCTGTTGTC
CTTTTTTCCTAGAATATCAAAGATAAAGCTGTCATTGATGAAATATTCCAAGGCCTGGAT
GCTAATCAAGATGAACAGGTCG
>860
GGGGGCGCAGGACCTGAGGAGCCGCGCCGGGAGGAGGGTCGGGCGGGTCTCAGCCC
CTCCTCGCCCCCAGGCTCCCACTCCATGAGGTATTTCGACACCGCCATGTCCCGGCCCG
GCCGCGGGGAGCCCCGCTTCATCTC
>861
AAATCCCCGCGGGTTGGTCGGGGCGGGGCGGGGCTCGGGGGGACGGGGCTGACCGC
GGGGCCGGGGCCAGGGTCTCACACCCTCCAGAGCATGTACGGCTGCGACGTGGGGCC
GGACGGGCGCCTCCTCCGCGGGCATAA
>862
TCACATGGGTGGTCCTAGGGTGTGCCATGAGAGATGCAAAGCGCCTGAATTTTCTGAC
TCTTCCCATCAGACCCCCCAAAGACACACGTGACCCACCACCCCATCTCTGACCATGA
GGCCACCCTGAGGTGCTGGGCCCT
>863
TATCTCTTCTCAGGGAAAGCAGGAGCCCTTCAGCAGGGTCAGGGCCCCTCATCTTCCC
CTCCTTTCCCAGAGCCGTCTTCCCAGTCCACCGTCCCCATCGTGGGCATTGTTGCTGGC
CTGGCTGTCCTAGCAGTTGTGGT
>864
GTGGGCTGTTTAGAGTGTCATCACTTACCATGACTAACCAGAATTTGTTCATGACTGTT
GTTTTCTGTAGCCTGAGACAGCTGTCTTGTGAGGGACTGAGATGCAGGATTTCTTCAC

GCCTCCCCTTTGTGACTTCAAGA
>865
GAGGGCGCAGGACCCGGGGAGCCGCGCAGGGAGGTGGGTCGGGCGGGTCTCAGCCC
CTCCTCGCCCCCAGGCTCCCACTCCATGAGGTATTTCGACACCGCCGTGTCCCGGCCCG
GCCGCGGAGAGCCCCGCTTCATCTC
>866
AAAATCCCCGCGGGTTGGTCGGGGCGGGGCGGGGCTCGGGGGACTGGGCTGACCGCG
GGGGCGGGGCCAGGGTCTCACACCCTCCAGAGGATGTATGGCTGCGACCTGGGGCCC
GACGGGCGCCTCCTCCGCGGGTATGA
>867
TCACATGGGCGCTGCTGGAGTGTCCCAAGAGAGATGCAAAGTGTCTGAATTTTCTGAC
TCTTCCCGTCAGAACCCCCAAAGACACACGTGACCCACCACCCCCTCTCTGACCATGA
GGCCACCCTGAGGTGCTGGGCCCT
>868
TGGAGCCCTTCAGCCGGGTCAGGGCTGAGGCTTGGGGGTCAGGGCCCCTCACCTTCTC
CTCCTTTCCCAGAGCCATCTTCCCAGCCCACCATCCCCATCATGGGCATCGTTGCTGGC
CTGGCTGTCCTGGTTGTCCTAGC
>869
GTGGGCCGTTCAGAGTGTCATCACTTACCATGACTGACCTGAATTTGTTCATGACTATT
GTGTTCTGTAGCCTGAGACAGCTGCCTGTGTGGGACTGAGATGCAGGATTTCTTCACA
CCTCTCCTTTGTGACTTCAAGAG
>870
TGACCCCCTGTGAGTCCTGGGCCAATCCCTGAGGACTGCTAAGCTGCGTCCTATGCCCT
ATGCCTGGTAGATTCTGAGCAAGCTGGTGAACAGCCTGTACCCTGATGGCTCCAAGCC
GGTGAAGGTGCCCGAGAACCCAC
>871
AGAGGGATCAGAAAACGGGTGGAAAACACACAGTTCTACCAAGTCTTTATCCTGCTTC
CTCCTCTTCTAGGAAAGCGCAGGAGCATAAGAGGGAATTCACAGAGAGCCAGCTGCA
GGAGGGAAAGCATGTCATTGGCCTT
>872
CCCCTGTAAGGGAAGCTCGAGAGAGAGTCTGCTGTTCAGCCATCTGACTGCTTCTAAA
TCTGCTTTTCAGGCCTAAAGGGGAGTGGCCCCAGTGGCGGTAGCGGCGGTGGTGCCAA
TGGTACCTCTTTCAGCTACACATT
>873
GACGGCCTCTGGGCAAGAGCTGAGCCTCTTGAGCCAGCTTCCATCTAATGCTGTCCTTT
TGCTTTTCAAGGCTCTGTGTGGCTGCACAGTGAACGTCCCCACTCTGGACGGCAGGAC
GATACCCGTCGTATTCAAAGATG
>874
TTGGCTTCGGTTTTAGCATTCTGCTGTCATCACCTCCATCCCTCCGTCTTGCTCCTGTTT
CTCTCTGCAGAGTGGCAGCCAGCGGTGCAGATTCTCTTGTACTCCTTGATATTCCTGCT
CAGCGTGCTGGGAAACACGCT
>875
TTCAAGCTAATGTGCAAATAAACTTTGGATTTCAGAGTCAACATAACCGGCTTCTTTGT
TCCTTTCCCAGGCACCTCTGTGAGTGTATCTACCTTTAATCTGGTAGCCATATCTCTAGA
GAGATATGGTGCGATTTGCAA
>876

ATTCATTCCTTCCCATTTGAGTTTGAAGGTCTAAAGTTGCTGGTTATTGGATTTCTTTTG
ATTTCATTAGGCACACATTCCTGTTACTCATCCTCTTTCTTATTCCTGGAATTGTGATGAT
GGTGGCATATGGATTAATC
>877
GTGGGGGCTCCATGCCTTCTACCGGGCATGACCCATCCCCAGCAATGAGTGATGTCTCT
TGCCCACCCAGAAAGGAAACCTAGCACCACCAGCAGCGGCAAATATGAGGACAGCGA
TGGGTGTTACCTGCAAAAGACCAG
>878
CCCTGTTCTTCCTCCAAGCCTCTGCCTTGGAGCTATGGATACTATAACTAACTGAAGCTT
CTTCTTTCAGGTACCCACTGATGGTAATGCTGGCCTGCTGGCTGAACCCCAGATTGCCA
TGTTCTGTGGCAGACTGAACA
>879
CTTGTTAGATGCTTGTAAATGCCAGCCCCTGCCTCAAGTAACAATTGATTCTTTTTGTGT
GCTCTCCCAGGTCTACCCTGAACTGCAGATCACCAATGTGGTAGAAGCCAACCAACCA
GTGACCATCCAGAACTGGTGCA
>880
TTGATGTCTTCTGCGGTAAGAACACTGTGATACAGATGGAATGACGGGAAGTGGTTTT
CCTTTCTTTCAGTTGGTGAGTTTGTAAGTGATGCCCTTCTCGTTCCTGACAAGTGCAAA
TTCTTACACCAGGAGAGGATGGA
>881
TTATGTGGAGTTTTCTTACAATGAAACACATCTACTCTACCACTCACTGTTTTCTCCTTA
CACTTTGTAGACATGCAGTGAGAAGAGTACCAACTTGCATGACTACGGCATGTTGCTG
CCCTGCGGAATTGACAAGTTCC
>882
ACGACTTTTCTTTTCCTTCCCTGAAATGTGTTTAATTGACTTTTTCTGTTATTTGTGTTTG
CCTTCACAGTGAAGACAAAGTAGTAGAAGTAGCAGAGGAGGAAGAAGTGGCTGAGGT
GGAAGAAGAAGAAGCCGATGAT
>883
CGTGTATACATGAACAGAGAGACAGTGCCTTTTCATGCTAAATGTGGTTCCCCACATCT
CCTCTGATTAGAGGTGTGCTCTGAACAAGCCGAGACGGGGCCGTGCCGAGCAATGATC
TCCCGCTGGTACTTTGATGTGAC
>884
ATTAAACGAGTGGATTATTCTGTTGTTGTTGGCTTTTTTTTCTCAAACCTCCTTCTCTTCT
ACTTTATAGTTCCTACAACAGCAGCCAGTACCCCTGATGCCGTTGACAAGTATCTCGAG
ACACCTGGGGATGAGAATGA
>885
CCATCCTATTAAGTCTGTATTCAAAGGATGAACTGATGATTTTAAATTCAAATGTTTCCTT
AATTTATAGGTCATGAGAGAATGGGAAGAGGCAGAACGTCAAGCAAAGAACTTGCCTA
AAGCTGATAAGAAGGCAGTTA
>886
TAGAAGACTTGAAATTGCTGCTTCATCCTACTTATTCAGTCCCCATGGACATATGTGTTT
ATGATGGCAGCATTTCCAGGAGAAAGTGGAATCTTTGGAACAGGAAGCAGCCAACGA
GAGACAGCAGCTGGTGGAGACAC
>887
ACCCATCGCCAATGGAAGAAGCAGTGTTTTGCACAAACTTGAAAAAGAGTTTTTCATT
TTCCTCCCACAGCCTCGTCACGTGTTCAATATGCTAAAGAAGTATGTCCGCGCAGAACA

GAAGGACAGACAGCACACCCTAA
>888
AAAAAGAAAGAAAAAGAACCATTCCTACCCCCAGACATTGTTGACCTGGAGTTGTCAT
CCTTTGATGCAGGTTATGACACACCTCCGTGTGATTTATGAGCGCATGAATCAGTCTCT
CTCCCTGCTCTACAACGTGCCTG
>889
TGGTGGCTGCTGCTGCTGCTGCTGTTGTGATTGTTGTTACTCACCAAAGAGATGGTTTT
GTTTGGTTTAGATGAGCTGCTTCAGAAAGAGCAAAACTATTCAGATGACGTCTTGGCC
AACATGATTAGTGAACCAAGGAT
>890
CTTACAGTGTTATTATTTATGAGTAAAACTAATTGGTTGTCCTGCATACTTTAATTATGAT
GTAATACAGGTTCTGGGTTGACAAATATCAAGACGGAGGAGATCTCTGAAGTGAAGAT
GGATGCAGAATTCCGACATGA
>891
ATTGCGTTTATAAATTGTAAATTATATTGCATTTAGAAATTAAAATTCTTTTTCTTAATTTG
TTTTCAAGGTGTTCTTTGCAGAAGATGTGGGTTCAAACAAAGGTGCAATCATTGGACT
CATGGTGGGCGGTGTTGTCA
>892
ACTGCTTCTCCATGTTCACCCTTAAAAGATTGATTTTTATTTTTTACTCAGCTCTCCTCTT
GTTTTTCAGGTTGACGCCGCTGTCACCCCAGAGGAGCGCCACCTGTCCAAGATGCAGC
AGAACGGCTACGAAAATCCAA
>893
AGGAGAGACCGCTGTTCTGCGGCGCCATTCCTGGGTTCTCATCCTAAGGCTGCTTTCTA
TTCCATAACAGTGGTTGAAGTTGACATCTGACGACGTGAAGGAGCAGATTTACAAACT
GGCCAAGAAGGGCCTTACTCCTT
>894
GAGCCTTGACATCATCACATTCTCCAGTTTGTGTTTCGTGTTGAAAGTTAAAACGTGAT
TCCCTTTTCAGGTGTAATCCTGAGAGATTCACATGGTGTTGCACAAGTACGTTTTGTGA
CAGGCAATAAAATTTTAAGAAT
>895
CTTATTAATGTTTGATAATGTTAGGTCATTTTGGGTGGTTTTCTTGAATTGCACCAAATTT
TATTTTTAGGATAAGGATGCTAAATTCCGTCTGATTCTAATAGAGAGCCGGATTCACCGT
TTGGCTCGATATTATAAGA
>896
ATTCTGTTAATGAATGTGGAGGGACCAGTATTAATCCTGATTTTCTTTCTTTCCCCTTTTG
CTTTTCCAGTGAATCATCTACAGCCTCTGCCCTGGTCGCATAAATTTGTCTGTGTACTCA
AGCAATAAAATGATTGTTT
>897
CCGCGATGTCTGCAGCAGGGGTTTCCCTTCGCGTCCCGTCTTCCTCAACTACCTGTTTT
TTCTTGTCCAGACCGGGGCTTTGAGCTCACATTCAAGACCGCTGATGACCCCAGCCTG
TCTCTCATAAAGTATGGGGAATT
>898
CCTCCCTGCCAGTGTCAGAGGAGCAGGGAGAGGAGACCTCTGGGTCACTGTGTCTGG
TGCCTGCTTTCAGATTTTGGAGGCAACATCAACGTGGAGACCATCAGTGCCTTTATTGA
CGGTGGAGGCAGTGTGCTGGTAGC
>899

GGAAGCCAAGGACACTTGGCCCAAGGCCCCCCTGTAAGGGCTCAGCTAGGTGTCTCTT
CGGTCTGTGCAGGTGACCCTCTTCGAGAGCTGGGCAGTGAGTGCGGGATTGAGTTTGA
CGAGGAGAAAACGGCTGTCATTGA
>900
GTGTGAGGAGGTTCTCCCTGGCTTGGGGAACCTTTTCCTGACCACCCACCTTTCCATCT
ACTCTTTGCAGCATACGCTCATCGTGGCTGACACTGAGAACCTGCTGAAGGCCCCAAC
CATCGTTGGGAAATCATCTCTAA
>901
CTCTCGGGGATCCCATGTCCTTGGGCTCAGTTCCTGGGGCCAGTGCTGGGCTGCTCTC
GTCCTGCTGCAGGATGGTGGCCGATCCTGATAACCCTTTGGTGCTGGACATCCTGACGG
GCTCTTCCACCTCTTACTCCTTC
>902
CTTGTTCGGGAGGCGTGCAGCTTTTCCCTTACTTGGAACTGCTTGGGTCACAGCTTCGT
TTTGTTCCCAGTATCCACATGCGGTGGGGAAGAACACCCTCCTCATTGCTGGGCTCCAG
GCCAGGAACAATGCCCGCGTCA
>903
GTCCCTGCCCATCAGGCAACACTGGAAAGGTGGCTCTGGTGGCCAAGCTGCTCCTGTT
TCCCTCCTGCAGGTATTCCCAGACAGGCAACTATGAACTAGCTGTGGCCCTCTCCCGCT
GGGTGTTCAAGGAGGAGGGTGTC
>904
AGCTCTGCCTGCAGGTGGTGGAAGGCCTCCAGTCATGGCCACCCCTGGCTAACCTGCC
CTTTTATCTCAGGAGTATAGCATCGTGATCCAGCAGCTCTCAAATGCCAAATGGGTCCC
CTTTGATGGCGATGACATTCAGC
>905
TGCTCTCGAGGCCCAACCAGGAAAAGCTCTGGGAAACCCCTCCCGGTTATTCTTGTTG
GTTCTTCCGCAGGTGGCAAATACAGTGTTCAGTTCAAGTTGCCCGACGTGTATGGTGTA
TTCCAGTTTAAAGTGGATTACAA
>906
GTTTTCAGCTAGCATGTGCCCTGCCTGGGTTCTGCAGTGGGCAGGTCCTGCCACCTCTG
TCCTCCTGCAGGTATCCGTGCGGCCACTCCAGCACACGCAGTATGAGCGCTTCATCCCC
TCGGCCTACCCCTACTACGCCA
>907
GAAACCATTCACCCTTATATTTAACTGTGAAATATCTGATTTAAAATTATTTACTTCTTTG
TTTCCACAGCCTGGTGAGAGTCAAAGAGGCCCCAGGGGACCATATCCACCTGGACCAC
TGGCTCCTCCTCCTCCACCAC
>908
GCCGCCCGGGCGCCCTGGAGGGGTCTCCGCCCGGAGCCTGGCGCTGCCTCGTGACCT
GCATCATCTTCAGTTCCAGGGTCTCCCTCCACTGCTGTAGTAACCCGGGTCCGGGGCCT
CGGTGGTGCTCCTGATGCCCCTCA
>909
AGCGCTGCCGGGTTCTTGGAGTCCTGTGGGATGAGTCAGGGTTCTGAATTGGGCTGCA
CCCCCTGGACAGGATGATCGGCTGAATGTAACAGAGGAACTAACGTCCAACGACAAG
ACGAGGATTCTCAACGTCCAGTCCA
>910
AGGGTAGCTGCTAAGGCCCAGGAGGTAGGCAGGTGGGTCGGGCCAGCCGAGACGCTG
ACACTCGCTGCAGCTTTGCAGTTCTCCTGGAGTTCGCTGAGGAGCAGCTGCGAGCCGA

CCATGTCTTCATTTGCTTCCACAAG
>911
CCCTGCGGGGGTGGGGGGTGCGGGCGGGGGGTTCTGGTCGGCAGGGGTTCTGACGGC
TTCTCCCCCGCAGCCGCCTTGCTCCGAACCTTCAGCTTTTTGGGCTTTGAGATTGTGAG
ACCGGGGCATCCCCTTGTCCCCAA
>912
TTGCACAGCCTGATGGTGGCCCGCAGAACCAGGCCTGGAGCTCCCACTGCGATCTTTG
CTTTCTTTTTAGCGTCAATCCAGAGTTCAGCCCAGAGGCCCTGGACAACTGCTGTCGG
TTTTACAACCACACGATCTGTGCA
>913
GTCACCCAAAAAATACTTTTCGTATTCTACACTGATGTGACAGTAATAATGGTTTTTCTT
CAACTGACAGATGGGCTTTTTTAAACGGGTCCGGCCACCTCAAGAAGAACAAGAAAG
GGAGCAGCTTCAACCTCGTGAAA
>914
GGCTCTGTGAAGGGTGGGCCACAACACAGCTGTTCCCAGCACAACTCAGTGTTTCCA
ACCTTCTTTTCAGTATTAGGATATCCAAGCCGCCTAGCCCGAAGCCCATGCCTGTCATCC
GAGTGGTGGAAACCTGAGCTGCC
>915
CTGTAGTTCCAAGTTAAAATTCTACACTCCTGATATTTATATATTGCTACTTTGTCATTTT
CTACCATAGGTCGAAGATCAATTTACAAGACCTCCAAGGAGCTTGCACCAAGAAGACA
GCCTGAATAGCCCGACAGTTC
>916
CACTGATTTTAAAAACTGTACAGTGATCAAACCTGGCCGTTTTATCACAAAACAATTTT
TATATTTTCAGTACGTGATGGTTCAAGACATGCTCTCTCCATCCCCCATGGAACGACCTG
AAGCTATAAACATCATTGAAA
>917
AAGCCTCTGAGGGATTGAGGGGCCCTGGCAGGACCCTGACCTCCGCCCCTGCCCCCG
CTCCCGCTCCCAGGTTCCCCCAGCAAGCGGGAGCCCGTGGTGGCGGTGGCTGCCCCG
GCCTCCTCGGAGAGCAGCAGCACCAA
>918
CCCCCCTGACACCCTGCCTCCAGCCTGCCCCTTTCCTCTAAGGCCCTCTGTTCCTCTCC
TCCCTTCCTAGAACATCATCCGCAAGGTGAGCGGCCAGAAGTTCGTCTACAAGTTTGT
GTCCTACCCTGAGGTCGCAGGGT
>919
GGTGGCCACAGGAGAATCCTGTGGTCCCAACAATCCTTACACACCCTCTTTGTGCCTG
CTCCTCTACCAGGTCATCCTGACCCCGCCCGAGGCCCCAAACCTGAAATCGGAAGAGC
TTAATGTGGAGCCGGGTTTGGGCC
>920
CCCGTTTTGGACAGGGTGAGAGACCCAAGAATGGCCCCCATCTGTGACCTCTCCCTCT
TTTTGCCCCCAGTTTCCATCCAGTGGCAGCGCCCAGGTGCACATCCCTTCTATCAGCGT
GGATGGCCTCTCGACCCCCGTGG
>921
CTAGGTAGGGGTGGATGTGCCACCCAACACCCAACCACTGACCCATATTCCTCCTTCCC
TTACTTTCCAGGCCCATCCCGTGGCGGTTGCCAGCAAGAAGCGAAAGAAGCCTTCAAG
GCCAGCCAAGACTCGGAAACGTC
>922

GTGAGCCAGATGGGTGAGGGTTGTGATTGGGGGTGCTCTTGGAGCCCCACACCTGCCT
TTCCCATCCCAGGTGCTGTGAGAACTGTGGAATCAGCTTCTCAGGGGATGGCACCCAA
AGGCAGCGGCTCAAAACGTTGTGC
>923
GGTCAGTAGACATGGGAGGACCAATGCTTCTGGAGCAGGCCCGTGATCTCGTCCCTAC
CATTCCTGGCAGCGTGTGGGCTGTGGGGAGTGTGCAGCCTGCCAGGTAACAGAAGAC
TGTGGGGCCTGCTCCACCTGCCTCC
>924
TCGGCCCCTGGCCCCCATGGGCTTGGCCCCATGCTTCATGCCTCTGCTCCCACCTGCCC
CCCGCCAACAGAGCCGAGGGTGTGGAGTATGCCGGGGCTGTCAGACCCAAGAGGATT
GTGGCCATTGCCCCATCTGCCTTC
>925
TAGGGTGTGGGGAGTTTCTGTGTCTGCCTGGTGCCACCAAGCTGAAACCACCCTTTCT
GATCTTTCCCAGGGTAAACATGCCCGCCGCAAGGGAGGCTGTGACTCCAAGATGGCTG
CCAGGCGGCGCCCCGGAGCCCAGC
>926
ACTGGAATAGGCGTTGAGGATGGCCTCTCCTAGTCCTAGTTGACAGCACCACCCATTCC
CCCCACCCCAGCAGCCCTACACGAACCGCCGGCAGAACCGCAAGTGCGGGGCCTGTG
CAGCCTGCCTACGGCGGATGGACT
>927
TGGAGCCGGGCAGGGTGGATGTGATTGGTCAGGACACCAGGTAGCCATAGCACCCTAT
CTTTCCCCATAGAAGCGGCTGCTGCCCAGTGTCTGGTCAGAGTCTGAGGATGGGGCAG
GATCGCCCGCACCTTACCGTCGTC
>928
CCTTCCCAGCCTCACCCAGCTGCATGCCAGGGAGCTGAGACCAAGTCTGCTGCAATCC
TCCCTCACGCAGGAGGCCCAGTGCTCTGGCCTGAGTTGGGTTGTGGCCTTACCCCAGG
TGAAGCAAGAGAAGGCGGATACCC
>929
TTGGCTGATAGAGACTTTCCTCAGGTGAACTCTGCCCTTCTAATGGTTGCCTGTTTCCT
ACTTCTCCCAGGCAGTAGACCCAGGCCTGCCTTCTGTGAAGCAAGAGCCACCTGACCC
AGAGGAGGACAAGGAGGAGAACA
>930
GGTTTTGTAATGCTCCATTGCGCACATAGCAAACCTCATTCAACATACAGACCTGCTCC
CTTTTCCCTAGGTCCAAAGACCTTAAAAAACCTGGAGCTAGAAAGCAGTAGACTGGAG
GCTTCTACAGACTGTAGGATTCA
>931
GGTGCGGGACAAGGACTTCCTGCCCTGTCCTCACCTCCCTTCTCTCTTTGATGCCCTCC
TCCCACTGCAGATCCATTACACCGGCTGCTCTATGAATCCTGCCCGCTCCCTGGCTCCA
GCTGTCGTCACTGGCAAATTTG
>932
GCCGGGGCCTGAGGGCTCCGCGTGCCGGTGCGGGCGCGGGTGCCAAGCCGCCCTCTC
CGCTCGCCCCCAGGTCTTCTGGATCGGACCCCTGGTGGGCGCCATCCTGGGCTCCCTC
CTCTACAACTACGTGCTGTTTCCGC
>933
CCTACTCCAGGAATCCTTAAGGCAGATATCATTTACCTACACTGAGTTCTTTGTTGCCAC
CCTGTAACAGGCACAATGAGTTTGACTTCATCTCAGGAACTCGAATGAGGAAGCTCGC

CCGGGAAGGAGAGAATCCCCCA
>934
GGTGCCCATCTGAGTCCCTGGGGGCAGGGGCGCCCTCGGGCTTTGACGACGCCCCCTC
CCGCTGGGCCAGGTCGTCCATCCGCAACGTGCACAGCATCCACCAGCGGTCGCGGAA
GCGCCTCAGCCAGGACGCCTATCGC
>935
CAGAGAGCGCTGTGGGCGGGGCCAGTGTGCGGGGCGGGGCGTCTGACTCGGCCCCGC
TCTCTGCCCGCAGAGGAGGAGCGGCAGACGCAGCGCTCTAAACCGCAGCCGGCGGTG
CCCCCGCGGCCGAGCGCCGACCTCAT
>936
TTTCCCAATCAGAACAGCAAATTCTAATTCCATTTTGGGTTTTCAATTCTGATGCACTAT
GTTTGTTTAGCTCTTAGCCCCCCACGACGTAAAGCCTTTAAGAAATGGACACCTCCTCG
GTCACCTTTTAATCTCGTTCA
>937
GAGAAACTACTTGAACGTGAAATCAGCCCACCTGGAGTCTTGTAATCATTCAGTTACTT
TTACTTCCCAGGCAAAATGGCAATACCTGTGCTTTGGAAGTTTCTGGAGAAGTATCCTT
CAGCTGAGGTAGCAAGAACCGC
>938
CCATCACCACACATTTTGGGAGGGTGTCTTTAGAAGCTGACCTGATAATGTGGGATGTT
GTATTCTTCAGATGAATACCTGACAAAGCAGTGGAAGTATCCAATTGAGCTTCATGGGA
TTGGTAAATATGGCAACGACTC
>939
AATTTTAAAAAATGTATTTCTGACTAAGTTACATTAAGGTCTCTGCCTCTGTATCTTATGT
TTCTTCCAGGTGCACCCTGAAGACCACAAATTAAATAAATATCATGACTGGCTTTGGGA
AAATCATGAAAAATTAAGTC
>940
GATGGGTGTAGACCAGCGCAGGGGGTAGAAACGATTTTTAGAAACGAAAGCTTTTTAT
TACCTTTCACAGGTGGGTTCTACCCTGAGCCCTGTTCCATGGCTCCCTTCAGAGGAATC
CACTCTCTGGAGCAGTTTGTCTC
>941
ATCTTGTGTTACTGGGGACTCTAATGTTTGGGCAAAATGAAGGAGACGAATGTCTGTTC
TCTGGTTGCAGGGGTGCCCTGCTGACAGGAACTAGCAGTGTCCTTCTACGGGGCCCCC
CAGGCTGTGGGAAGACCACAGTA
>942
CCTCCCCTGATTCTCCTGCCCTTGTCATGCTTTCCCCTTGCTCACAGACCCATTGGGATC
CCCTCCCCAGGTGCCCTGCTCCAGCCTCTGTGCAGAAAGTAGTGGGGCTGTGGAGACA
AAACTGCAGGCCATCTTCTCCC
>943
TTGGGACAAGGCAGTCCACAGGAGCTGCCAGCCATGTTCTCACCCTCAGTGCCCACTC
CTCTGTCTGCAGCTGCCCTCCCCTCATGGTTGTGGCCACCACAAGCCGGGCCCAGGAC
CTGCCTGCTGATGTGCAGACAGCA
>944
CTGGGGGACGGTCCCTGAAGGTTCCTGCCTACTGTGCCCTGCTCATGTGTCCTTTCCTG
TGGTTCCCTAGGGCTTTGTGGTAGGGGATCTCTATGCCCTTCTGACCCACAGCAGCCGG
GCAGCCTGCACCAGGATCAAGA
>945

GGGTGGTGAGGAAGGGAGAAGGGAATGGGACGCTGATGGTGAGGAGGCTTCCCTGTG
TTCCTCTTTGCAGTTTGGCAGGTGGCTTGACTGAGGAGGATGAGGGGGAGCTGTGTGC
TGCCGGCTTTCCTCTCCTGGCTGAG
>946
CCAAGTCCTTGGAGGTGGGACAGGAGCCCCTCCAAGAGCATATCCCAACCACGCACTT
TCCATCTCCTAGATCCCCTCAGTGTCCTGGCATGATGTGGGTGGGCTGCAGGAGGTGA
AGAAGGAGATCCTGGAGACCATTC
>947
CTGCTCTCACCTAACTTCAACCCCCCACAAGCCGAGCTTTGCCCTCCTGTGGGCCCCTC
TCTCTCTTCAGTGTTTGCCAGGGCCAGGGCTGCAGCTCCATGCATTATCTTCTTTGATG
AACTGGACTCTTTGGCCCCAAG
>948
AGGTACCACAGGCAGGGGCAGAGTCTAAGGGGGACACCAAGGACTCTTGCCCTTGCT
CCCTTCTCTCCAGGGTGGTGTCTCAGCTCCTTGCCGAGCTAGATGGGCTGCACAGCAC
TCAGGATGTGTTTGTGATTGGAGCC
>949
CTCATTTTGTGCCTCCTTCACCCCTCATGGCTGGGCTCCCTGAAACTTATACCTGCCTAT
CTCCCTAAAGATTTGACAAGCTGGTGTTTGTGGGGGCAAATGAGGACCGGGCCTCCCA
GCTACGCGTTCTAAGTGCCATC
>950
GAGCTGGTCAGCACTGGCTTTCGAGGGAGGAGGTGATGCCAACTCTGTTTCTTCCTGT
ATCCCACAACAGATTCAAGCTAGAGCCATCTGTGAGCCTGGTAAACGTGCTAGATTGCT
GCCCTCCCCAGCTGACGGGCGCG
>951
GGAAGCCACCTTCGCAGGGCTCTCCCCTGCCTGGGCATAGACCACAGCCTCCCCACCT
TGTCATCTCCAGGGCTGGAGCCAGGTAGCTCAGCACTGATGCTCACCATGGAGGACTT
GCTGCAGGCTGCCGCCCGGCTGCA
>952
GTTCTTGAAGGGAGAGAAGTCATTTCGAGTTACCCTCGTATCCTTTCTTTGTATCTTCGC
CTTGCATCAGGGGCCATGGAACGTATATGGGAGAAGAGAAGCTCATTGCATCTGTTGCT
GGCTCTGTGGAGAGAGTAAAC
>953
ATTCTGTAGTCTCCTTTTCATTCATCCCGCCAATCTTGCAGTGACCTAGCTTCGTGATATA
TTTCTTTAGCTAGGTCAGGGGGTTTTGGTCCAGGTTTCCCCCTCCCTGGTGAAACGGCA
GAAGACCCACTTTCATGATT
>954
AATTTCTGTGGCTGGTCACGTATTTTGGTCCTGTTTGTATTTCCCTTCCCCTCTCTGTGT
CTCCTTATAGCCTGTCTCTCTTGCTGATCGAGAGGTGATATCCCGGCTTCGGAACTGCAT
CATCTCGCTGGTAACTCAGA
>955
GTTTGGATTGGCTTGGTGGTCTGTTTATGGTTGGTTTCTTTTCTGAACAAATGCCTTTTC
CCTTTTTCAGATCAAAGACATCTTAAAGCCAGAAATAATGGAGGAGATTGTGATGGAA
ACACGCCAGAGGCTTTTGGAAC
>956
AATAAAACTAATTTTTTCTCCCAATTTTCTCTTCCTTTTTCTTTTTTCTGTTCCCCCCTTT
CTCTTCCAGAAGCCCTTCAGCGGCCAGTAGCATCTGACTTTGAGCCTCAGGGTCTGAG

TGAAGCCGCTCGTTGGAACTC
>957
AAGAATCAATGAAAAAGAACGAAGCTGGTTTCCAAAGCTGATATGTCTGATTTGGTTC
CTTTCTTCTCAGGTGAAAAGCTCCGGGTCTTAGGCTATAATCACAATGGGGAATGGTGT
GAAGCCCAAACCAAAAATGGCCA
>958
AACTGGCTCACGTGAGCTCTTTGAGCTTGCCTGTCTCTGTGGGCTGAAGGCTGTTCCC
TGTTTCCTTCAGCTCTACGTCTCCTCCGAGAGCCGCTTCAACACCCTGGCCGAGTTGGT
TCATCATCATTCAACGGTGGCCG
>959
CTAGTCAAGTACTTACCCACTGAAAAGCACTTCCTGAAATAATTTCACCTTCGTTTTTTT
CCTTCTGCAGGAGGACACCATGGAGGTGGAAGAGTTCTTGAAAGAAGCTGCAGTCAT
GAAAGAGATCAAACACCCTAACC
>960
TTCTTTAGACAGTTGTTTGTTCAGTTGGGAGCGGAGCCACGTGTTGAAGTCCTCGTTGT
CTTGTTGGCAGGGGTCTGCACCCGGGAGCCCCCGTTCTATATCATCACTGAGTTCATGA
CCTACGGGAACCTCCTGGACTA
>961
CAGCAGTGGTGGATTTGTGAAGTGGAAGGTTGGCCAGGAGCTCTCATGGGTGAACATT
TTCCTTTCTTAGAGATCTTGCTGCCCGAAACTGCCTGGTAGGGGAGAACCACTTGGTG
AAGGTAGCTGATTTTGGCCTGAGC
>962
CTGATTTTAAATGTAGTGTAGTGAAATGCTACACATCTTGAACAGCCTTTCTCTTTCGGT
TTTCTTTCAGCATTTGGAGTATTGCTTTGGGAAATTGCTACCTATGGCATGTCCCCTTAC
CCGGGAATTGACCTGTCCCA
>963
TTGAGAACTGCTAGCCCCGTATTGCTAGCCAGATCTCATGGATGATCTGACTTGGGTTT
CATCTGTCCAGGTTGGCAGTGGAATCCCTCTGACCGGCCCTCCTTTGCTGAAATCCACC
AAGCCTTTGAAACAATGTTCCA
>964
CCACCACACCAAGCCAACACCAGTACTGATGGCTGCTGGATTTTTGTTTCTGTCCCTGT
ATGATTCTTAGAAGTGGAAAAGGAGCTGGGGAAACAAGGCGTCCGTGGGGCTGTGAG
TACCTTGCTGCAGGCCCCAGAGCT
>965
GTTCAAGCGATTCTCCTCTGTCAGCCTCTAGAGTTGTCTGGAGTTGTCAGCTCTTCCCC
TTGCGTTTCAGATCCTCTGGACCATGAGCCTGCCGTGTCTCCATTGCTCCCTCGAAAAG
AGCGAGGTCCCCCGGAGGGCGG
>966
GATGAGATTGTGTTAAGGATGTTTTTGTTATGCAGGTTTTGCCATTTTCTTCTTTTTCCTG
TCCATTTAGGGGAATGACTCTGACTTTGAGACGGTGTACCAGCAGTGTGTCAGGTGCT
GCAGAGCGTTCTTGGAGAAGG
>967
GGTTGATTAGAGGAAGGTCCAGGAAATTCATCCTCTACCATTTCTTCCCACCCTCTGCC
CCACACCTAAGAAATGGAGTGGCCAAAGTCAACATCTTCTTCAAGGAGCTGAACTACA
AAACCAATTCTGAGTCTCCCTCT
>968

AAGGGGGCTCCTCTGCCAGAGTCCATCCAGAACCCTCTGTCCCATCGTCAATGTACCTC
TCCTCTCACAGATGGTCACCCTCCTGTCCAACCTGGGCAGCCAGTGGAGCCTGTGGTT
CGGCTCCTCGGTGTTGTCTGTGG
>969
ATGATTCTCATGTCCTCTGAGGCTTGAGCCCCTTAGTCCAGCCTTTGATCATTTCCACCT
TGTTCCTTAGGATGGAGTGATGGTGCTCAGCGCAACCCACCGGTACAAGAAGAAGTAC
GTCACCACCTTGTTATACAAGC
>970
CGGGGTTAGGGTGGGGTGGCTGCAGGTCTGGGAGGCAGGACAGAGGCGTGACCACCT
CCTTCCCCACCAGTATCTTCCTGACCGCTGCCCTGGGGCTGCCTGAGGCCCTGATCCCG
GTGCAGCTGCTATGGGTGAACTTG
>971
TGAGGCCCTCAACCCTCGATGCCCCCTATCTCCCCAGCCCTGACCCCCGACTCCCCTCT
CTCCACCACAGGCTATGTGGGTGCAGCCACCGTGGGAGCAGCTGCCTGGTGGTTCCTG
TACGCTGAGGATGGGCCTCATGT
>972
ATGCAGGAGGGTACCAGGAGGGTGGCATGGAGGTGGCCCTGGACCTCAGTCTCCCGT
ACCTTCCCTGCAGACTCACTTCATGCAGTGCACCGAGGACAACACCCACTTTGAGGGC
ATAGACTGTGAGGTCTTCGAGGCCC
>973
CCACATCCTCCACTGTGCCGCCCACCTCCTTCTCCTCACTGTTCCTTCTCCCTCCCCTTC
CCCTCTGCAGCCTGTCCGAGAACCAGTCCCTGCTGCGGATGCCACCCTGGGTGAACAT
CTGGCTGCTGGGCTCCATCTGC
>974
GGGCTGGGCAGTGCTGGTCTCTGGCTCCCTCCCCACCCCCTCCTGAGAGGGCGCTTGT
CCCCTGCCCCAGATGATCTTCAAGCTCCGGGCCCTGGACCTCACCCAGTGGCTCATGG
TCCTCAAGATCTCACTGCCAGTCA
>975
CATTCATATACAGTGTTTTTGAATACAGTATTTTGATCTGCCTTTAACAAATGTTTTCTCA
TTATTTCAGTTGCCAAAGCTGTTTGATGGATGCTACTTCTATTTGTGGGGAACCTTCAAA
CACCATCCAAAGGACAACC
>976
AGAAAACCTGGGGGAGGCAGGACTAGAGAGCAGGGCTGGACACAGGTCCCTCACAG
TCACTGTTCCCCAGACACCGCTCCACCTGGCTGTGATCACCACATTACCGTCTGTGGTC
CGGCTCCTGGTGACAGCTGGTGCCA
>977
AGCGGACCTGGAGTCCATCAGCGGCCGCAAAGCCCGGGCCTAGGTTTCACCGAGCCC
TCCTCTCCCTCAGGGCTCACCGCCCTGCACGTGGCAGTGAACACCGAGTGCCAAGAA
ACCGTGCAGCTCTTGCTAGAGCGCGG
>978
GGACGGGATGCGGGTCGCCGGCTGCTGGAGCAGAGCTTGGAGAAACTAAGACCTTCC
CTCCCCGCCGCAGGACATTAAGAGCGGCCGCTCCCCGCTCATCCACGCCGTGGAAAAC
AACAGCCTTAGCATGGTGCAGCTGC
>979
GCCTCCAGCCTCTGTTCCCTTACCCCGGGTGGCCCGCGCGCCCTCCTGACCCGGCCCT
CCCGTCCCGCAGCACGGCGCCAACGTGAACGCGCAAATGTACTCCGGCAGCTCCGCC

CTGCACTCAGCGTCCGGCCGCGGGC
>980
GAGCTAGGAGGGTCGGGAGAGAGGACTGTGAGGCATGGGTGGCTCTATGGTCACGCC
CATCTTCCTACAGGTCATCGACATCCTGAGGGGGAAGGCCACCCGGCCTGCTTCCACC
TCCCAGCCAGACCCCTCCCCTGACC
>981
TAGGCCCCAGCCCCTGCTCTCTGGGTCTAGCCTCTCACCTCCCACCCCTCTGTCTCTCT
TCCTTCCTCAGGTCTTCTCTCCGCATCACCATCCTCCTCACCCTCCCAGTCTCCCCCCA
GGGACCCCCCTGGATTCCCCAT
>982
GACAGGTTGGTTTGGCTCATAGGCTGTAGTCTGCCACTTCCTGTTTTATTCTACCTTCTG
TTCATTTCAGGTCACTGTGCATGGCATCATCCTGGCCCCCTCTAGAGCTCCAATCCTCC
AACCAGAGCCAGCTCTTCCCT
>983
TTGGCCACTTGGTCTCAAACTCCTCCTCCTTTTTGCTCTTCTCTACTCCACCCCCATCCC
GGTGATGCAGGAGGAATATCAGGAGTATGAGCCAGAGGCGTAGGGGCCCAGGAGAGC
CCCCACCAGCAGCACAATTCTGT
>984
AGAATGAATGAATGCAGCGGTTCTTCCTGCCATCTGATAACAGACTATTCTTTGATGTTT
TCATTTTCAGGATGTACAGATTATAGTGTTTCCAGAAGATGGCATTCATGGATTCAACTT
TACAAGAACATCCATTTATC
>985
CGCCTCAGCCTCCCACAGTGCTGGGATTACAGGCAAAAACCTCATTTATTTACACCTTT
TTTTCCTCTAGGTGCTCCAGCGCCTGAGTTGTATGGCCATCAGGGGAGATATGTTCTTG
GTGGCCAATCTTGGGACAAAGG
>986
GAGTTGGGAGAGAAGAGAAGAGTGCTTGGTATCTTGACAAACCCTCCTACCTTTTCTC
CTAACTACATAGATCTTGAAAAAGCCACTACCGCCTGAGCCAGCAGCAGCACCAGTCT
CCACAAGTGAGCTGAAAAAGGTTG
>987
GTTTGGGCAGGTGGGATGCAGGTGTGAGCACCACTTCCTCCTACAGACAGCTTCTTTT
TCGTTGTTTCAGGGGAAAGAAGGAGGTTTCATTGTCAGAGACTCCAGCAAAGCTGGC
AAATATACAGTGTCTGTGTTTGCTA
>988
ATCTCACTTCACTTCCTGGGTTCTGTTGACCTTTGTGCCCAAGTTACTGACTAAGCATC
CACTTCTTCAGGGACCCTCAAGGGGTGATACGTCATTATGTTGTGTGTTCCACACCTCA
GAGCCAGTATTACCTGGCTGAG
>989
TAAGTGAGGATGTGTGAGGCATCCCACCTCCTACACCACACCAACAGCATGACCTCTC
TCTCTGTTTCAGGACTCATATCCAGGCTCAAATATCCAGTGTCTCAACAAAACAAGAAT
GCACCTTCCACTGCAGGCCTGGG
>990
GGATTTTTCGTCTATTGTAAGAATTATACATTCATGACCCCAAAGAATCACACCAAGACT
TTATTGTTAGGATCATGGGAAATTGATCCAAAGGACCTGACCTTCTTGAAGGAGCTGGG
GACTGGACAATTTGGGGTAGT
>991

CCTCCCCAGCCCCTTTATTGCTATTATGCAGAGCCAAAAGGAGAAGACTAGTTCCTTGC
CTTTCCTGTAGGAATCTTTCCCATGAGAAGCTGGTGCAGTTGTATGGCGTCTGCACCAA
GCAGCGCCCCATCTTCATCATC
>992
CGTGCCAAGAAAACGTAAGCAAATATCAAGCCTCCAAATCCTAATGCAACAAGTCCTG
AATCCCTTGCAGGTATGTCCTGGATGATGAATACACAAGCTCAGTAGGCTCCAAATTTC
CAGTCCGGTGGTCCCCACCGGAA
>993
CTAGGAAGACTAGGACCCCTGCTATCCAAAAAGACTGCAAACCAATTTAATAATTTTTT
TCACCTTCTAGGGGTTTTGATGTGGGAAATTTACTCCCTGGGGAAGATGCCATATGAGA
GATTTACTAACAGTGAGACTGC
>994
TTACTCATTGCATTTCCCTTGAATTCTGGATTCTAGCCACTCTAACACTTTACTTTTTCTT
TGGTTTTAGAAAGCAGATGAGCGTCCCACTTTCAAAATTCTTCTGAGCAATATTCTAGA
TGTCATGGATGAAGAATCCT
>995
TCCACTAAACCTTTGCTGTTTGCTTATATGTTTCCACTAAAGCTTTGATGTTTCCACTAA
ACCATTTCAGGTAATGGCACAATTGACTTCCCTGAATTTCTGACAATGATGGCAAGAAA
AATGAAAGACACAGACAGTGA
>996
TGAGTAAAGTGTATTTTTTTCTTGGTCTTTTGCTATTGACTTACTCTTTTGTGTACTTCTT
CTTTTTCAGAGTTTGTACAAATGATGACAGCAAAGTGAAGACCTTGTACAGAATGTGTT
AAATTTCTTGTACAAAATTG
>997
CCCGGCAAGTGTGTATTGTGACTGGGTTGGATAAAGACATTGATTCTAACGGGTCAACT
TTTGTTTTCAGAATTCAAGGAAGCCTTCTCCCTATTTGATAAAGATGGCGATGGCACCA
TCACAACAAAGGAACTTGGAAC
>998
GAAATGATTACTGCAGAGCAGCGTTAATGGAGTGCTTACTTTACATGAGCTTTTTGTTT
TTTAATTCGAGGTAATGGCACCATTGACTTCCCCGAATTTTTGACTATGATGGCTAGAAA
AATGAAAGATACAGATAGTGA
>999
GCATAGCTAAGTCAAAGACCTACTTCCCCACACTACATCATTAGCAATAACAATTGCTG
AATGTTCACAGGATGGCAATGGTTATATCAGTGCAGCAGAACTACGTCACGTCATGACA
AACTTAGGAGAAAAACTAACAG
>1000
TAAAGATGTTGTGTTCATTAAAGCTGTTTTCAAAGATAACCAAAAGTTACTATTATATTT
GTCTTTTCAGAATTCGTACAGATGATGACTGCAAAATGAAGACCTACTTTCAACTCCTT
TTTCCCCCCTCTAGAAGAATC
>1001
TATTTAGAAGGAGTTAAAATGTCTTTTGTAAAGTTTCTTACACAGCTTTCTTTTTTTCCC
TTTTTTACAGGCAACTTCGGGCTCGCTGGAGAACAACTAAGGGGCACCAAAGCCCTCT
GAGGTTTTACTTTAAGGTTCGC
>1002
GACTTCCACCATTTTCTTCAAGCATCATCTGTGTTGTTGGAGACTGACATCGATCTTTGC
CTCTTTGCAGGTTCTGACCCAGCCAGTCCTGGTGACTGGCCTCTGCCTTCCACCGGAG

AGACTACTGACCTTCTCACCTC
>1003
AAAGAGCACGTCGGGGTGGCTCTGGGCTCCTGGGCTGGCCCCTGACCCACCTTCCTCT
GTTCCTCTGCAGTCTGGCAGTCGAGGGGAGCTGGGCCCCAAAGGCACCCAGGGTCCC
AACGGCACCAGCGGTGTTCAGGGTG
>1004
TATGCTTTACGTAACAATACTTCTGATGATCCTCTCTCGAGTAAACGCCTGCACCCTTGT
TTTCCCAAAGAACAAATTGCACAGTTAGCCGCGCACCTAAGGAAGCCTTTGGCACCCG
GGTCCATTGGTCGGCCCGGTCC
>1005
ATCAGACACCGCTGTGGTGTGGCTGCAACAGATACTCTAACCATATGTCTGTGTCCACA
CCTGGTGACAGGAAACCAGGGTGACAGAGGAGACAAAGGCGCGGCAGGAGCAGGGC
TGGACGGGCCTGAAGGAGACCAGGG
>1006
CAGCTGGATGTCAAGTCCCCCTGCTTTCAGTCCGGGCTGCAGCTGAACTCACCTTTCT
GCTCTGCCCAAGGACCCCAAGGCGTGCCCGGCACCAGCAAGGACGGCCAGGACGGT
GCTCCCGGCGAGCCTGGGCCTCCCGG
>1007
TGTGCCACCATTGTTCTAAGGAAAAAGGCATCTTTGGAACATGTGTAAAGTCTAACTTT
TTCCCCTGCAGTGGTTTGCAGGAAAAATTTAGATAGCACAACAGTGGCAATTCACGAT
GAAGAGATCTACTGCAAATCCTG
>1008
TTCCTGCCTTGTGTACTTAAAAGGGCCCTGTGGAAACTCTTGCCTACTTTTTTTTTCCTC
TTTTCACAAGTGTTCAGCCTCACAGGCCTACAACAAATCCAAACACTTCTAAATTTGCT
CAGAAATATGGAGGTGCTGAG
>1009
TTGCCATACATCTAAATTGAACTGTAGGAAACAGGCCTCTCAAACATGCTGACACATTC
ATCTCTTCCAGCCCTGGCACAAAAACTGTTTCCGATGTGCAAAGTGTGGGAAGAGTCT
TGAATCAACAACTCTGACTGAAA
>1010
TGGGTCTTAAGCAGTGCTAGCAAAGCTTAATGGACTTGCACTAACTATCCTTCCTCCCT
TTCCTTTGTAGGATGCTATGCAAAGAACTTTGGGCCCAAGGGATTTGGCTATGGCCAAG
GAGCAGGGGCTCTTGTTCATGC
>1011
GACCCCTTCAGTGGCCCACAGCTCAAGTGAGCCCCTGACCTGGAGTCTTTTTCTCCTC
ATCAACCACCAGGCCAAGGAGGAGCAGCAGGATGACACAGTCTACATGGGCAAAGTG
ACCTTCTCATGTGCGGCTGGTTTTG
>1012
CCTGGGCTGGGCTGGGCTGGGCTGGTGGTCACCTGGGCCTCAGTTTACCCTGCCTGCT
GCTTCCACCCAGAACTTTCAGATCTCCCTTAAGAGCAAACAAGGCCCGATCGATGTTTT
CCTGTGCCCTGAGGAGACCGTAG
>1013
CAGGCACGACAGCCCCTGCCTTCCTCCCTGCTGGGGCATCCCCGGCCTGTGATGCTCC
CCGTCTCCCCAGAACCGCTGTTGTCCCGGATGGGCAGCCTGCGGGCTCCCGTGGACGA
GGACCGCCTGTCCCCGCTGGTGGC
>1014

GTAAACAGAGACCCCAGAGCATCTCCCAGCCCCGGGGTGGCAGCCTCCTGATGCACG
CTTGCCTTTGCAGCCTGGTCAACGAGCTGGCCTTCACCGCCCACAAGATGATGGCTGA
CGAGGCCCTGGACAGTGGGCTGGTC
>1015
CTTCATCTGCGGGAGGCTGGAGGAGAGAGCCCTTCCCAGCCCACCCGGTCCCTGATCT
CTTTCACTGCAGCCGGGTGTTCCCAGACAAAGAGGTCATGCTGGATGCTGCCTTACCC
CTGGCGCCCGAGATTTCCAGCAAG
>1016
TCTCATGCTTTATCCTGATTGGCCCCTGCTAATCTCTCTCCTCGTTCTCTCTCTTCACCCT
CGGTACCAGGCGTCCTGGAACATGAGCATGCTGCAGACCCAAGACCTCGTGAAGTCG
GTCCAGCCCACGACTGAGAACA
>1017
CGGGTGGGAGGCGGTTGCTTGGGGGACCAAAAGACACAAACCCCACACCTCCTATTT
TCGTGTTCCCCAGGAGACATCAAGCAGGAAGGGGTCGGTGCATTTCGAGAGGGGCCG
CCCTACCAGCGCCGGGGTGCCCTGCA
>1018
TTTCTGACCTTACCCCCATTTTTTCCTCTGCCAAGGTAGCATCTCTACCCCAAAACTGTT
TTGTTCCCAGGTCGCCAGGCTCTGGGGCATCCAGAAGAACCGGCCAGCCATGAATTAC
GACAAGCTGAGCCGCTCGCTCC
>1019
ACTCCCCCACCTTACCCCATACAAAGGCTGGGCGGCTAGCAGGCTGACCAGACCTTCT
CTCTCCCCATAGGTGGCTGGTGAGCGTTACGTGTACAAGTTTGTGTGTGAGCCCGAGG
CCCTCTTCTCTTTGGCCTTCCCGG
>1020
TTTGCATATAGACCAGTGAGAAGCTGGAGCTAGCACCACTGGCTTAGGTTCTTCCCTTT
CCCCCTGACAGTGGGCGGCTTTCTGGCTGGGCTGCCCTTCAGCACCATTGCCAAGCAC
TACAGTTGGAGCACAGCCTTCTG
>1021
CCTAGACCAAAGCTGGGTGGATACGGAAGTGCTTAGGGCCAGCCTGATGAGGCTCCTT
TCTCCCTTCCAGGATGACCATTGATGCCGGATGCTCACTCCACATGCTCAGATTTCCAA
TGGATTCTCACTCTTGCCCTCTA
>1022
TTCATGCTATTGGAGACCTCAGGTGGGCTTCCAAGTCTCATAGGACCCTCTTTCTCACA
TTCCTTTCCAGTTTCCTATCCTGAGAATGAGATGATCTACAAGTGGGAAAATTTCAAGC
TTGAAATCAATGAGAAGAACTC
>1023
TGTTCACGTCCTGCTAAGGGGGACCGTCGTGTCAGCACTGTAATGCAGTGATGTTTTTT
GTGTCTTTCAGGTGACTTCATGGTCATGACGATTTTCTTCAATGTGAGCAGGCGGTTTG
GCTATGTTGCCTTTCAAAACTA
>1024
GTCATGGAGTCTTTTAACCAATGTCGCACATAGGTCAGGAGCTGAGCCCATCACTCTTG
TGCTCTTGCAGGGATCACCTCTGTTCTGACCATGACCACGTTGGGCACCTTTTCTCGTA
AGAATTTCCCGCGTGTCTCCTA
>1025
CCGGAGGCAGCACTTAGACTTGGGTTATCTTCCCTTGACTTTTCCCCACTTCTTTCCTT
GTCCATTTTAGCCTCGTATCAATAGCCGTGCCCATGCCCGTACCCGTGCACGTTCCCGA

GCCTGTGCCCGCCAACATCAGG
>1026
TCTCTCCTCCTGCCCCCAGACCCAGGTCTCCCCTTCTTCTACATGACCACCTCTCCTCC
CCCTTGCTCAGGCCAGCTGTCTCTGAACGAGTTTGTTGAAGGTGCCCGTCGGGACAAG
TGGGTGATGAAGATGCTGCAGAT
>1027
AATATTATAAAATAGTCTCTTGAAGATTTTGTCATCTGATCTTTTTAAAAATTAACTTTTT
TTCTTGCAGCTCGGAAAAACAACCATCATTTTAAATCTGAATCTGAAGAGGAGAAAGC
ATTGATTTATCAGTTCAGTCC
>1028
AGGGTGTGATGTGAGAGAAGGGATTGGTAATGCCTCTTGCCACTGGCTCGGATCCTCT
TCCCCCACACAGGTACCCATCACGGGTGTTTGTGGGAGATACCTTCTGTTACTTTGCTG
GCATGACCTTTGCCGTGGTGGGC
>1029
CTTCCTATTAGCCACAGGGAGGAGGGTTCAAATAGTGGCCCAGTCACATGAAGCTATCT
TCCCCCCGCAGACTCAATATCAAGACAGGCAAACTGGAGATGAGCTATTCCAAGTTCA
AGACCAAGAGCCTCTCTTTCTTG
>1030
GCATTAAATAGATCCACATTCCTAAAGCATAGGTATTAGCTGAGGTTCTCTTCACCTCTG
GTCCCTCCAGGTGGCAGAGAGCCTCCAGCTGGTGACAGTACACCAGAGTGAGACTGA
AGATGGTGAATTCACTGAATGTA
>1031
GGGAATCGAGTTTATACCTCCGTGTCTCCCTTTCTGCGTGATTCTTACTCCAGTCCATTT
CTCCTTGCAGATCCTGGGCAGTGCCATCACCTTCTCCATTCGATATCAGCTCGTTCGACT
CTTCTATGATGTCTGAGTCC
>1032
GCAGTGGCTCTAGGAGAAAGACCCCATCAGTAGGCTCCCACTGACTGGCGGTCCACTG
GCTTTCCCGCAGGGAACCTACTCACTGCCCAAGAGCGAATTCGTTGTGCCTGACCTGG
AGCTGCCCAGTTGGCTCACCACCG
>1033
GGCCGTCCCCCTCTCCTCTCCCAGGATCAGGAGGAATCACTGCCTCTCCTCTCCCCCGA
CCCCCTTCCAGCACCAGCAGCAGGTCTTGGGAGCCATTGAGAGGGCCAAGCAGGTCA
CCGCTCCCGAGCTGAACTCTATCA
>1034
GGGACCTGGGCGGGTGGGCGGGGGACAGGTGGGCCCCAGGCAGCTAATGTTCCCCTC
CCCCGCCCTGCAGCAGCAGCTCCAAGCCCACCAGCTGTCCCAGCTGCAGGCCCTGGC
CCTGCCCTTGACCCCACTACCCGTGG
>1035
CCGTGTGGCTTTGCCCGTCCCCAGGTGAGTGTCGGCGCAGCTTGGGTCTAACTTCCTG
TGTGTTTTGCAGACAGCTGGATAGAAAACGACAGCCTGTCAGTAGACAAGCACGGGG
ACGACCAGCCGGACAGCAGCAGCCT
>1036
GGGGGGACTCCGGGGACAGGAAGCCCTGGGGTGGCGGTGGCCGGTTTCTCACGCCCG
CTTGTGCCCGCAGGTGAAGATTTGGTTCCAGAACCGGCGGATGAAATGGAAACGCAG
CAAAAAGGCCAAAGAGCAGGCGGCGC
>1037

GGACCAGACTACTGGCCTCTGAGCATCGGGCCAAGTCCAGCTACTGAACCTGCTCCGC
TCCTCTCCCCAGGTGTGGTTCCAGAACCGGCGCATGAAGGACAAGCGGCAGCGCCTG
GCCATGACGTGGCCGCACCCGGCGG
>1038
CGGGCAGAGGCTGCTGTCATAGGCAGAGGCCCCCGCTGATGCCGGCCCTGCTTGCCCC
TCACACTGCCAGGTCTTCACGTGCTCTGTGTGCCAGGAGACATTCCGCCGAAGGATGG
AGCTGCGGGTGCACATGGTGTCTC
>1039
AGCCCAAGGGTGGGTGGCAGGGTTTCATGGGTGCAGGCCGCCCTGGAGGTGACTGTG
GCCTCTTTTTCAGTGTTCCTCCTGCTCCCAGCAGTTCATGCAGAAGAAGGACTTGCAG
AGCCACATGATCAAACTTCATGGAG
>1040
TCCCAGGCAGCCCTTCCCTGCTCTCACCCTGGCCCTGGTCCCTCCCTCTGCCTGCCTGG
TCTGCCTGCAGTGCCCCACCTGTGCCAAGTGCTTCCTGTCTCGGACAGAGCTGCAGCT
GCATGAAGCTTTCAAGCACCGTG
>1041
CTAGCTGTAGCAGAGCAAGGGGGTCACTTCCCTTGGTGATGGCCTCTGCCCCATGTCC
CCACCTTAACAGGAATGAGAGGCCACACGTATGTGAGTTCTGCAGCCACGCCTTCACC
CAAAAGGCCAATCTCAACATGCAC
>1042
CCTGCCCCTCCCCTCGTCTCCCCATCCTGAGGTCAAGGCCACAGGCTGAGCTCTTGCC
TTGTGCCTGCAGCCAGCCTGGACAAGCACAACCGCACCCACACCGGGGAAAGGCCCT
TCAGTTGCGAGTTCTGTGAACAGCG
>1043
CCTCCTGCTGGGTTTCCTCGAGGGTCCCGGGGCTCCTGCATGATCCCCCACGGTGTTCT
CCCTCTTGCAGCCGTGGAGCAACTGCGTGTGCACGTCAGACGGCACAAGGGGGTGAG
GAAGTTTGAGTGCACCGAGTGTGG
>1044
TAGGCCAGGGCCTGGCCCCCTTCCCCTACCCTAGGATCCCCCAAAGTTCTGAGCTCAC
CCTCCCCGCCAGGCCCACCTGCGGAGGCACATGGAGATCCACGACCGGGTAGAGAAC
TACAACCCGCGGCAGCGCAAGCTCC
>1045
AAGGGTAGAACCCTGGGTGGACAAGGGCCCAGCCTGAAGCCCACTGAGCCCCAGCTG
GGGTACATTGCAGAACGGTTGCCCATCAACCGGCGGGACCTGGACCCCAATGCTGGGC
GCTTTGTCCGCTACCAGTTCACGCC
>1046
GCCTGTCCTGAGCCTGGCTGGCTAGTTGACTTCAAGGACAGACTTAGCCCTGCCTCCT
TTTCTTGCTCAGGGTGGAGTTCACAGTGGGAGACAAGCCTGTCAACAACTTCCGCATG
ATCGAGAGGCACTACTTCCGCAAC
>1047
CCTGAAGCCCCTGCAGTGGCGGGACTCAAGCTCCTGACCTTTGCCCGGCCTGGCTGGG
CCTCTCTTGCAGTCAGCGAGATGATCCGCCACCCGTATGAGACCCAGTCTGACAGCTT
CTACTTCGTGGATGACCGGCTGGT
>1048
AGGACACGGGGACTGGAAGTTTGCTGCTGGGCTGACCTAAGGCTTCCCTCCTCTGCCT
CTGTGACCCCAGGCCTGTGGGGCCTCGTCAACAACGCAGGCCACAATGAAGTAGTTG

CTGATGCGGAGCTGTCTCCAGTGGC
>1049
CCTGGGGTGGGGGAGGGCTTAGGGAGCCCCTTGCCAAAGCTGAGCTGCCCCACTCCC
AATCCATCCGCAGGGGGACATGCCATATCCGTGCTTGGGGGCCTATGGAACCTCCAAA
GCGGCCGTGGCGCTACTCATGGACA
>1050
GGTTAAACAGTCCTAATTGGCTTTGGCTCCCCTACGTGATCCCAGTCTGACCTTGCCAC
TCCTTCCCCAGAGTCAGTGAGAAACGTGGGTCAGTGGGAAAAGCGCAAGCAATTGCT
GCTGGCCAACCTGCCTCAAGAGCT
>1051
CCACCCTGACTACTGAGGTTCTGTGCACACGTCTCTACCTTCCTGCTCAAGACACTGG
GTCTTTCCACAGCCTGAAGATGACCATGGACAAGACGGGTCTCCTGCTGCTCAGTGAC
CCAGACAAAGTGACCATCGGCCTG
>1052
CCTGAACTGAACATTTGATGGTGTGTCTCTCTGGTGCCATCTTAATACCCTTTCTCCTTT
TTCTGTGCAGAATTTAAATGTGAGTTTCAATCCTGAAAGTTTCCTGGACTGCCAGATTC
ATAGGGTGGATGACATTCAAG
>1053
AGTAGTGCCAGAGGCACCCTGACATCACTCTCTGAACTGCTTTCTCTTTCACATTCTTG
TATTTTTCCAGCTGACGGCCCAGAGGGTGGGTGCCAATTCCACCAGCAGCTGCAACTG
AAAAGCAAGGTTCAGAAATGTCA
>1054
CTCTCTCTCTCTGTTCTTTCTCCACTGAAACTCTTGAAATGTAATGGCTTATCGTGTGGC
TTTACCCTAGGGGAAAAGATTACCTTGAGGTTGTGTTCAACAGAGGAGGAAACAGCA
GAGCTTCTTAGCAAAGTCCTCAA
>1055
GAAGTCATATGGAACAGAGTCTTCTAAATGATCTTTACCTGAATCCAAACACATTTGTT
TCCTTTTTCAGATTCAACTTATCAGTATATAAAAGGTTCTGCTGACATTAAATCCAACCA
GGGAATCTTCCCCTGTGGACT
>1056
GTTTGAAAGTGACATGAGTAATGAAAAACAAATGTGATAATCATCACCAATTGAAATTT
TTCTTCTACAGGTGGAAAGGACAGTGCTATTGGGGCCTTTCTTACATGGGGACCAACAT
GCATTCACTACAGCTCACCATC
>1057
CATTCCCATTTAGTGTATTTGCTTCTTTGTGAGATTAATTTGTTTCTCTTTCTCTTTCCCTC
TTCTCTAGTGTGGATTCTTTAAACGCTCTAGGTACGATGACAGTGTTCCCCGATACCATG
CTGTAAGGATCCGGAAAG
>1058
TTGGCCTTCCCTCTCCTCCAGGCCTCTGGGAAAGCGGCTTCCCTTTCTGAGCCACCCCT
GCTCTCCCCAGCTCTCTGTCGGATGAGGCCCCAGTCCTGCCCAACACCACCCCGGACC
TCCTCCTGGCCACCACAGCCAAG
>1059
GGCTCTGGAGCCCAACAGCACAGTGGTGACAGTGGGCATCACTGAATCTGTATCCACT
TCTGTGGCACAGGACAGGCCAGGCAGTCCCAGTGGCTCTGAGGACAAGGGCAACCCG
GCGCCGGAGCTCAGGGCCTCCTTTC
>1060

AGGAGAGCAGGGGAGGGGTTGGGGGGTGCTCCTGGGCTTCCCTAGCTCAGCCTGCTC
CTTGTGCCCACAGTCATGTCGGAGGCGGGCAGTGGGACCCTGGAGGACGAGTGGCAG
GCCATCTCGGAGATTGCCTCTACTTG
>1061
GGGGCTGGGAGTCAGCTGGTGCACTGCCCCCTGCCTTGGTCCTGCTGAGTCTTGACCC
CCATGTCTTCAGGCCAGAGCCTGGGAACCTCTCAGAGAAGGTCTCTCACTTGGAGTCC
ATGCTCAGGAAGCTGCAGGAGGAC
>1062
TGGGGGGAGTCACAGGGCTGCCCCAGGAAAGGGGCTGGCGGCTCTGACTCCTGTCTC
CCCGGCACCCCAGGAGAAGGCGGACAGGGCGGCCCTGGAGGAGGAGGTGCGGAGCC
TGAGACACAACAACCGGCGGCTGCAGG
>1063
CTAGTCTTATTCCCAAAGAGTCCTGGAAAATGTGAGCACCCTCCCTCACTCAGCATTTC
CCTCCCTCCAGGACTCTGATGAACAAGACCCTCAGGAGGTGACATACGCACAGTTGAA
TCACTGCGTTTTCACACAGAGAA
>1064
ACAGTCTTATTCCGAAATAGTCCTGAAAAATGTGAACACCCTCCCTCACTCAGGATTTC
CCTCTCTCCAGGACTCTGATGAACAAGACCCTCAGGAGGTGACATACGCACAGTTGGA
TCACTGCATTTTCACACAGAGAA
>1065
ACAGGGCAAACAGGGGGCCCCCAGGAGCCCCAGCCTGTACCCCGGTCCTGACACCCC
TTCTGCCCCCCAGGGAACGACTCCATCTTCCATGACATCGACAGCGATACCTCCTTAAC
CAGCCTCAGCGACTGCTTCCTCGG
>1066
TCACCCTGGGCTCACAGGGCTCAGAGAAGGGTCCTGCTCAGCATCCTCCCCACCCCAC
CCATACCTGCAGTGACCAACCCATGTGACCGCAAGAAATGCGAGTGGCTCTGCCTGCT
GAGCCCCAGTGGGCCTGTCTGCAC
>1067
CCCTGACCTTGTTGCCTGTCCCTCTCCTCCTATTCCCCTGGCTCTGCCCCTTGACGGGC
CCTTCCTGCAGCTCCCCGGCCTGGAACCTGTAACCTGCAGTGCTTCAACGGTGGCAGC
TGTTTCCTCAATGCACGGAGGCA
>1068
CCCATTCCTCTGCTATAGGGCCAGGTGGTCTCGAGGGCCACAGTCGCGCTCACACATC
CTCTCCCACCAGGCATGCCCACGTGCCGGTGCCCCACGGGCTTCACGGGCCCCAAATG
CACCCAGCAGGTGTGTGCGGGCTA
>1069
CTCTGTTCAACCTATGGAGAGCCCTCATGAGGGTGGGGCTTGAGGCACTTCTCTCCCTC
CCCAACCACAGGGCAGTGCTCTGGCTACTGTGAGAACTTTGGCACATGCCAGATGGCT
GCTGATGGCTCCCGACAATGCCG
>1070
CCCTCCCTTCCTCAGCATCCCAGGCACGCCTCTGCCAGCCCCAGCCCCAGCCTCTGATT
CCTTCCTGCAGCTGCACGGATGGCCGGGTGGCCCCCAGCTGTCTGACCTGCGTCGGCC
ACTGCAGCAATGGCGGCTCCTGT
>1071
GAGCAGGGGGACCGTGTGCCTCCTGCTTCCCTGAGCCTTGGTGACTCAGTGTCCCACC
TCTTCCCTCCAGATATAGCCTCCATCCTAATCCCTCTGCTGTTGCTGCTGCTGCTGGTTC

TGGTGGCCGGAGTGGTATTCTG
>1072
GTCATTATTTTGCCATCCTAGCCTTCCCCCCCAATAATCTCTGTCTCCTTATACTCCTGCC
TTTCCCCAGGGCTAAGGGCTTCCAGCACCAACGGATGACCAACGGGGCCATGAACGT
GGAGATTGGAAACCCCACCTAC
>1073
GTGGGGGTGGGGTAACCTGGGCTACAGGCCCAGCTCCTGAGCCCTACCTGAACCCTCT
GTCACCCTGCAGCCCACCAACTTCACCAACCCCGTGTATGCCACACTCTACATGGGGG
GCCATGGCAGTCGCCACTCCCTGG
>1074
ACCTGTCCTTGTTCTCACTCATCGAACAGATGGGAATTTAGTCACCTAATATGTTTTGTT
TCTATTATAGGCCTTCATTCAATCTCAGATTTATGAAAATAATTACTGGATTGGATTATCAT
ATGATGAAAGGGAAAGTA
>1075
GGGGCCCAAGGAGAGCTGAGGAACAGGACAGAGGGTGCCAGGTCCTAAGAAACAGT
ACTTATCTCCTCAGGTGGACTTTGAGGGCAGCCTTGCTGAAAATCGCTTCACAGTCCTC
CCCAACATAGATCCTGAAATTGATG
>1076
GCTGGGGATCTTCATAGCAACCAGGGCAGGAGACTCACTTTTGATAACCACGTGTCTT
CCACCCTCGTAGAAAAGCGAAGACTGATGGGACTTCCCAGTTTCCTTACTGAGGTTGC
CCGCAAGGAGCTGGAGAATCTGGA
>1077
TCTAGGGTGGGGAGGTGTCCAGTAAGTCTCCAAGCAGGAGAGTAGAGTATCTCCTCTT
TACTCTCCCCAGATTGGCTTCCTTCTTTCTATTCCCCGCCTGCCTTCCATGGTAGAGGCC
AGTGACTTTGAGATTAATGGAC
>1078
AGGCCAACTGTGGTCAGTGCGTTACGGGCTTCCAATACTAACTTTCCCTTGTCCACCTT
ATACCCAGCAGGTGGCGAAAGCAGTGAACAATGCCACTGCACAGTCGCTGGTCCTTAT
TGATGAATTTGGAAAGGGAACCA
>1079
ATTTCTGACCCTTATTTCCCCTTCTCTTCCCCACTCCCCTTACTCCTCCCACCTTCTTGCT
TGTTCCTAGGTCTCAGACTTGATCCGCAGTGGAAAACCCATCAAGCCTGTCAAGGATT
TGCTAAAGAAGAACCAAATGG
>1080
GTTAGAAAGATGGGGAAGGAGAGGAGGACCAAGAGATGCAAAGTCCACAGCTTTGA
ACCCCTGTACCCAGTTTCTCTCAGAGGAGAAGCTGCACTATCGTAGTGCCCGAACCAA
GGAGCTGGATGCATTGCTGGGGGACC
>1081
TAGGGGCCTCTGCCTCTCCTTCACTTTCCCCTGGAACTGACCTCCAGCTCCCTTCCTCA
CCCACTCCCAGACCAGGAGACGCTGCTGATGTACCAGCTACAGTGCCAGGTGCTGGCA
CGAGCAGCTGTCTTAACCCGAGT
>1082
GGCTACATCTTCTGGGGGTTCATCTATCTTGATCCACAAGCCATGCGAGGTGCCTCTCC
GCCCACTGCAGACATCCTCTGATGGAACTCTGTGCCCGAACCTTTGTGCCCAACTCCA
CAGAATGTGGTGGGGACAAAGGG
>1083

ATGCCTTGAGCCTCACTTTCACCTCAGCCCACGGCACCAGGCCCCAGGCCCTGTCTCC
TTCCCTATTCAGGTAGGCTTGATCACATTCATGGCCCTGGTAGGCAGCTTTGTGCCAGC
AGAGGAGGCCGAAATTGGGGCAG
>1084
AGGCCAACTGTGGTCAGTGCGTTACGGGCTTCCAATACTAACTTCCCCTTGTCCACCTT
ATACCCAGCAGGTGGCGAAAGCAGTGAACAATGCCACTGCACAGTCGCTGGTCCTTAT
TGATGAATTTGGAAAGGGAACCA
>1085
GGCAGTGCAAGTGCAGAGGGGCATATGGGGTCCCCATGGCTCCGAATGCTAACCTCTG
CCCTCTTTGCAGGTGGATGGGCTCGCGCTTCTGGCCGCTGTGCTCCGACACTGGCTGG
CACGTGGACCCACATGCCCCCACA
>1086
AGGATTGGCCAAGGGTTTCAGGACAGGAAGGAGGTGATTGATGATACACTGTCTTTTA
TTCTCTTTTAAGACCATGGAGACCTGTGAGGATGGCAACGATCTTGTCTTCTTCTATCA
GGTTTGCGAAGGTGTTGCGAAGG
>1087
GATTTCTGACCCTTATTTCCCCTTCTCTTCCCCACTCCCCTTACTCCTCCCACCTTCTTGC
TTGTCCTAGGTCTCAGATTTGATCCGCAGTGGAAAACCCATCAAGCCTGTCAAGGATTT
GCTAAAGAAGAACCAAATGG
>1088
CCTCTCCCCTCTGCCCAGGGATTTGGTTGTCCATTCTGCCATAAATCTTGCGATTTTCTC
TCTTCTTCAGTTGCCAGACATTAGTGGATAAGTTTATGAAACTGGATTTGGAAGATCCT
AACCTGGACTTGAACGTTTTC
>1089
AGGCAAGAGAATCTCTTGAACCTGTGAGCCAGAGGTTGCAGTGAGCCGAGATTCCAC
CACTGCACTCCAGCCTGGGCAACAGAGCAAAACTCTGTCTCAAAAAAAAAAAAAAA
AAGGCAAAATTAAAGAATTTCAAGTCC
>1090
TAAAGGAGGAATCAGTTTTATTGTTTGTACTGGACTATACACAAATGTAAAAATCATTTC
ATCATTTCAGAAACTTGAAGAAGCTGAAAGAAAAAGCCAGCACCAGCTCGAGAATTT
GGAACGAGAACAGAGATTTTTAA
>1091
TGTTAGTTTTTGAAGGTGCGCTATACTCGATAAATAATGTTCTTCTTCCTTTTTTTTTTC
CTTTGGCAGAGGAGATTGAAGTGGATGTTGAAAGCACAGAGTTCTCCCATGGAGAAG
TGGACAATATAAGTACCACCAG
>1092
GCCCAGGTGCCTCTGCCCCAGGGCTGGGCAGAGGCAGCCTGTGGTGACCGCAGCTGT
CGCTGCCCCTCAGCTGACCATGAATGACTTCAGCGTGCATCGCATCATTGGGCGCGGG
GGCTTTGGCGAGGTCTATGGGTGCC
>1093
GCCGCTGAGGCTCTGCAACCCCGGGCGCCCCTGCTAACTGCCCGCCCCCTCCCCTCCT
CTCCCCTCCTAGGACTGCCCATTCATTGTCTGCATGTCATACGCGTTCCACACGCCAGA
CAAGCTCAGCTTCATCCTGGACC
>1094
CCAGCTGGCATCTTGCCTGGCTGGGCCCCATCCTGAGCTGCCCAGGCAGCTCACTGGG
CTTCCTTCACAGCCAGCCAACATCCTTCTGGACGAGCATGGCCACGTGCGGATCTCGG

ACCTGGGCCTGGCCTGTGACTTCT
>1095
CCTCTCTCCCTGAGCTGGGATGGGGTCAAGGGCTCTTCCTAAGCCCTGCAATGTCCACT
CCTCTCTAAAGGCCGTGGAGCTGCCCGACTCCTTCTCCCCTGAACTACACTCCCTGCTG
GAGGGGTTGCTGCAGAGGGATG
>1096
TGCGGCAGGGACTGGGGGTGCTCTGCAGCCCCACCCCCGAGCTAATGCCCATGACCCT
GTTCTGCTGCAGTACCCTCCCCCGCTGATCCCCCCACGAGGGGAGGTGAACGCGGCCG
ACGCCTTCGACATTGGCTCCTTCG
>1097
TGGGCAGCCTGTGGCTGATGGTATTCCGGCATCTCTGTCCACCCATGTGCCCCTGCCCC
ACCTGGTAAAGTTACTGGACAGTGATCAGGAGCTCTACCGCAACTTCCCCCTCACCAT
CTCGGAGCGGTGGCAGCAGGAGG
>1098
CCGGGAGCCGGGCTTCCGGGTGGCTGGCCGGCCCGCTCTTCACCTGCACCACCCATCC
CTGGCCCTGCAGCAGAGCCTGCTGACCATGGAGGAGATCCAGTCGGTGGAGGAGACG
CAGATCAAGGAGCGCAAGTGCCTGC
>1099
GGAGGCCGAGGGGCCAGGCCAGGCAGGCTGGGGAGAGCCCCAGCTGACAGAGCTGG
GCTTGGCTGTGCAGAGCGACCCTGAGCTGGTGCAGTGGAAGAAGGAGCTGCGCGACG
CCTACCGCGAGGCCCAGCAGCTGGTGC
>1100
GACTGAGGCCTGAGCACTTGCAATGCCAGCATCACACCCTCTTGTCTGTGTGACCCCC
CACCTCCCACAGGCTGTGCTGCACATGGAGCAGCAGAAGCAGCAGCAGCAGCAGCAG
CAAGGCCACAAGGCCCCGGCTGCCC
>1101
GGGCCTCTGGGTTCGTTTTGTAGGAGGGGTTCGGATGTCCTGTGCCACCATTCCCTCCC
TCCATTTCCAGACGATGTACCTGTCCCAGCTCCAGCCGGTGACCAGAAGGAGGTGGAC
ACTTCAGAAAAGAAACTTCTCGA
>1102
CACTAACTGACTTTCAGATTCTAAAACCACCATGTGGATTCCACAAACTCAGAAACATT
ACTCCCCACAGGACTTCATCCAGCAAGCAATTTCCTATTCCAGTTTTCCATTCTGGATG
GGGCTGTCTCGGAGGAACCCCA
>1103
AAGTTTCTGAAACAAGGTGGCAGTAAACCTAAGTTTTTTTGTTGGTTTGTTTTGTTTTT
CCTACCTGCAGATTTAGAGTCCGAGGCGCTGTCTCCCAGACATACCCTTCAGGTACCTG
TGCATATATACAACGAGGAGCT
>1104
CACGCAAAGAATAAGATGGGTTGATGGGTTGCAAGCATCCTGGCTCACTCTCACCCTAT
GCTAAACTCAGGCGACCGTGCTGTGTGACATCATAGTCCTCTACTGCATGAAGAAAAG
ACTCTACTATCGGGAGAAGAAAT
>1105
GCGCAACTCCCTCCCGCCTGCCACAAGGGGTCCCAGGGGCACCTTGATCTGCTTGTGT
CCTTCTTTGCAGGGTCTTGCTAGTGAGCTGGACCAGTGAGGCCTACCCCACACCTGGG
CTCTCCACAGCCCCATCAAAGAAC
>1106

CTGGAGGCTGGATTGAGTCTAGGGTCACTGTAACTCACCAAATGCCGACATGCCCCAC
CCCTCGCCACAGGTGGGTTTCATTGACTACATTGCTCACCCACTGTGGGAGACTTGGG
CTGACCTGGTCCACCCAGATGCAC
>1107
AAGCCTGTCCCTCAGTCCCCTTTGACCAGTAATCTCTTCTTCCTTGCTTTTCATTCCAAA
AATGCTTCAGGCCAATACTGGCAAGTTCTAGGGGGCCCAGTGTCTGGGCTGAGCATTG
GGACAGGCAGGGCAATGCTGGG
>1108
GAGGGTGTGGCTGTGAAAGGAGAAGCTGTGGCTCAGGCCTGGTTCTCACCTTTTCTGG
CTCCAATCCCAGACCAGGTGCCTTTCTCCGTGAGCGTGTCCCAGTTGCGGGCCTTGGA
TGGAGGGAACAAGCACTTCCTGAG
>1109
GAAGTGGATAGAGGGGACAGCTGGCAAGCAGCAGACATGAGTGAAGCAGTGCCTGG
GATTCTTCTCACAGGTCAAGAAGCAGGCCTTGGGCAGGTTCCGCTGATCGTGGGCATC
TTGCTGGTGTTGATGGCTGTGGTCCT
>1110
CAGCAGTTTAGCATATTTTTGCCATGAGCTCATCTCGTGTCATTACAGCTCTTTCCTCCT
TTCTCTTCAGGAGGAAATCTCTGACATCAAAGAGGAGGGGAACCTAGAAGCTGTCTTG
AATGCCTTGGATAAAATTGTGG
>1111
TGGAAGCTGAACCTTCTTCCACAAGGGCCTTCCGCCTCCTTCATTCCTTGTGCCCCGTG
TGGCCCTCCAGGCGCCCCAGCGGGATCCCAGAGAAGGATCTGCACAGTGTTATAGCAC
CCTACTTCCTGCAGCAACGGGAC
>1112
GGGACAGTGGAGGGCAAGGATTTGGGTTTTCTTAGATCCCAGTTTGTCTGTTGTCTCCC
ATTGATTTCAGGCTCTACACAGAGAACAGAGGGAGCTGGTTGCTGTGCTGAGGGAGCC
TGAGTGAGGAGACCGCCAGCCCC
>1113
GCTCAGCCTGGCCTCCTTGGCCTCCTCTCTGAGTGGACTGGGGTCTCACTGTGCCTGTT
TATTCCTGCAGGTCTTCATGGAACAGATCGGAATATTCCGGAACATTGCCCAGCACTAC
GGCATGTCGTACCTCCTGGAGA
>1114
CTGCTCTGCGTCCTCCCACCAGGGGGAAGGCTGTTGGTCTCACCTGGGCCCCTGACCC
CGTTCTCTCCAGGACCAGGAGAAGGTGCTGGACAAGTATGAGGACGTGGTGCAGGGG
CTGCAGAAGCGAGGCCAGCAGGTGG
>1115
GAGATGGCAACCGGGCTCCCAGGCCCTCAGGCCCTGCCGGGATCCTGACTGTCCAGCC
TGCCCCCGCCAGGGCCTGATCTCGCGGGGCTACAGCTACACCCTGCAGAAGAACAAC
GGGGAGAGCTGGGAGCTCATGGACA
>1116
GTCCGCAGTCCCGCCTCTGTGGGTGCAGGGGGTGGTGGGCTGCCCAGTTCCCAGCTCA
TTCGTCTCACAGCCTGGGCAGCCAGTACCGGAGCGTGCGGCAGAAGGCAGCTGGGAG
CAAACGCACGCTGCAGCAGCGGTAT
>1117
TAGGTCCATCCCTGGGCCTCACACCCCTGGCCCGTTGTCACACCTGCCTGTGTCCTCCC
TCCCTCCACAGATGCCTCTGACCTACAGGGGCGGCAGCTGCTGGCTGGCTTGGACAAG

GTGGCCAGCGACCTGGACCGGCA
>1118
CCCTGGGCACATCCTCAGGGGAAGGGGCTGCCTGGCTGTGTCCAAAACTGAGGGGCC
CTGGTTCCCACAGAACATCACCAACGAGCTACTGCGGATTGAACCTGAGAAGACGCG
GAGCACGGCTGAGGGCGAAGCCTTCA
>1119
GCAGCATGGAGGTTGGGGTGGGGTCAGGGGGCCAGGTGGGCATCCCCCGACGCCTGA
TTGGTGCTCTCAGGGTTGATGTGGCCAACCGCCTGGAGAAGAGCCTGCAGCAGAGCT
GGGAGTTGCTGGCCACACACGAGAAC
>1120
CAGGGGGTGTCATCGCCTGGCTCTGGGAGCTCTGGCCGTAGCACCGCCGTCTCCTCCT
CTGCCCCGCCAGCCCATGGCCTGTGAGTTACAGGCCCAGAAGTCCCTCCTGGGTGAGG
TGGAGCAGAACTTGCAGGCGGCCA
>1121
AAAAAAAAAATGAGGGCTCGGTGAGGCATCTGTTTGCTGTGGCTGAGGCCTGAACGC
TTTGCCTTGACAGGGCGCAGAGCCTACAGAGCGCCAAGGCAGCCTACGAGCACTTCC
ACCGCGGCCATGACCACGTGCTGCAG
>1122
TACTGCATCCATGGTTGCTTTTTTAAATTTTTTTTCTGGTTAGTTTTGATTTTTAAACTCT
GTCTTACAGAACCTGCTAGATGAGATAGCAAGTAGGGAGCAGGAAGTACAGAAGATCT
GTGCCAATTCCCAGCAGTACC
>1123
GGTACAGGAACATGTCTGTCTCTTCTCAGCTGTTTTAATCTCCCCTCTGCCATGTCTCTC
CCTCATGCAGGACTATGAGTTAGAAGCAGAAAAACTAAGGTCTCTTCTCGACTTGGAG
AATGGAAGGAGCAGCCACGTGA
>1124
GTCTAGCTCTCTGTTGCTTACCCTGTTGACACCTGTTTGTTGACCGCTCTCCTAATCCTC
TTCTCTGCAGGAAGCAGCACTTGCCGCCAAGTTCACTGAAGTTTATGCCATCAACAGA
CAGAGGCTGCAGAATCTGGAGT
>1125
CAGGTGGGGACAGGCCCTACATGTGCTGCCAGAGCCTGGCTTTAACCTTGTCTTGTCT
TCTCCATCACAGCAGCCGGAAGTAGAAGTGACCCATGAGACCCTGCAAAGGAATAGG
CCGGACTCTGGAGTGGAGGAGGCGT
>1126
GGGGATATAAGACAGTGACTGTTGAGGACCCTAAGTTTAGCTCCCACCTGATCTTCCTC
TGTCTCTGCAGAAGCTGCTGATGGGAGCTCCACCCTGGGAGGGGGTGCCGGCACCAT
GGGATTGAGCGCCCGCTACGGACC
>1127
AAGGGTAGGAGACGAGGGCCAGGCGGCCAGGCCATCAGAAGCAAGGCGTCCACTGC
TTTTGCTGTTCTAGGAATTTTTGAACCGAAGAAAAATGACAGAGTTTGGCAACCTGGC
ACTAATTGATCAGGATGCTGGGCAGG
>1128
AAAAAAAAAGAAAAAAAATGTTGGGAGGAATGAGCTTAAATTTTTAGGAAAGCACTG
CTTAATTTCCCAGGGCGCTGCTGGGCGGCCACCACCACGGAGGGACTCCTCATCTACT
CCCTGGACACCCGCGTGCTCTTTGA
>1129

CCGGGGTCATCCTCTCTTCCCCTGGTTTGAGTTGGTGGCAGAATAAGCATGATTTTTTC
TCCTTTTGCAGTTGAAGTGGTCACCTCCTCCCTTCCTGAACTGTATGTGGAGAAAGTGC
TGGAGTTTTTAGCTTCCTCCTT
>1130
CAATGCAGCATGAAGTGAGCCCACTGTACCACGGGGTGAGCCCCGCACGCTTAGGGA
GTCTCCCTTTCAGAGCCGGGACGCTGCTGCCTGTCATTCAGTTCCTCCAGAAGAGCAT
CCAGCGGCACCTGGACGACCTGTCG
>1131
AGGTGGTTGGGTGCAGGACAGGCCAGGGCCTGGGCGTTGCTGGCATTGACCATCCCT
GTGTGATTGACAGCTGTAGCTGGAACCACTATAACATGCAGTACGCACTAGCAGTTTCC
AAGCAGCGGGGCACAAAACGCTCC
>1132
CTGGTTGGGATGAGGCAGAAGGTGGAGATCCTGCCAGCAGAGTGAACCAGAGCTTCC
CTTTGACCTGCAGAACCCAAGAGAGTGGACGCGGATGGTGATCCGGAACATAGCCAC
CTCTGGCAAGTTCTCCAGTGACCGCA
>1133
TCCTTAAAATCTGGTTAGATGGATAATTTCATACCTATTTTTTTTTTACTCTTTTCTTCTGT
TGAAACAGGCTTCACACTGTATAAATAGGAGAGGATGAGAGCCCAGAGGTAACAGAA
CAGCTTCAGGTTATCGAAATA
>1134
AATGGAGGTTATAGTGTTAGGGTTTGGGATAGGAGTAGGAGACAGGAGTCTCAAACTC
TCTTTCCCCCAGGAGTGAGTCCTGGAACCTGAAGGACCTGGATATTACCTCTCCAGAA
CTCAGGAGTGTGTGCCATGTGCCA
>1135
CCCCCCACCACAGGCACAGACTTAGTGAACTCCCCCCATGCAAGGCCTGACTGTGGTC
CTCTCTCTGCAGTCCACATCACTCTGGATCCAGACACAGCCAATCCGTGGCTGATACTT
TCAGAAGATCGGAGACAAGTGAG
>1136
GGTGCAGCCCGCAGGTGGGCAGGATGCGCCTGGGCCTGGGGCCTCCGACCGCTCCCC
GCTGTCCTCCTAGTCTCTCTACCTGGGGCCCATCTTGAAGTTTGGCTCCAAGGAGCAG
AAGCAGGCGTGGGTCACGCCTTTCA
>1137
GGGAGTGAGGCTGGTGCCCTTAGGTTGTGTGGGGTGGGGCGTGCGCTGAGCCCTGGG
TCTGTGTGGGCAGGGAACGGCAGTGATGCAGGAGCTGCGTCCACCACCGCCCGGGCC
GAGGGCGACTCATGGGTTCTGAATGG
>1138
TGTTGGGTGTGCTGGTGTGAGGGTGTGGCTGAGGGGCAGCTCTGAGAAAACCACCCG
CCTCTCCTTTCAGGGCATCAGTGCCTTCCTGGTCCCCATGCCAACGCCTGGGCTCACGT
TGGGGAAGAAAGAAGACAAGCTGG
>1139
GTGGGGGTGGCACCTTGAGGCCAGGCCCGCGCCCCGGCTGGCGGGCCACTGACCAGG
GCGGTCCCCACAGCAAACCCTGGACATGGGCCGCATCGGCATCGCCTCCCAGGCCCTG
GGCATTGCCCAGACCGCCCTCGATT
>1140
CCTCCTCCCCCTCCCTTCTGTCCCTGGAGGGGCAGCTGCTGACCTGTGGTGTGGGGTG
GGGCTATTGCAGTTCAAGTTGGCAGACATGGCCCTGGCCCTGGAGAGTGCCCGGCTGC

TGACCTGGCGCGCTGCCATGCTGA
>1141
CCAGCTGCCCCTTCTCCAGCTTTCCCCACGCCGGGGTCTTCTCCCTCCTGAGCCACTGT
TCTCATCTCAGGCCATCCAGATCCTGGGCGGCATGGGCTACGTGACAGAGATGCCGGC
AGAGCGGCACTACCGCGACGCCC
>1142
GGGCATCCTCACTGTCCTTGTAGTTCCTTGCTCATCCAGCCACCCATCCTGTCCCTTGCT
CCAACCCCAGTTCCTCCAGTTCCGCATGAAGATCATTAACCTGAAGCTGGAGCGGTTT
CAAGACCGCGTGGAGTTCTCAG
>1143
ACGGGTAGTGGGGTGATGGGGGAGGCCCCTTTCCCAGCCCTGGTGCTCAGTGGCTCTG
CTTCCTTTCCAGAGCCCCCTGAGCGGGACTCCACGGTGGCCGTGATTGTGGGTGCCTC
CGTCGGGGGCTTCCTGGCTGTGGT
>1144
GTTGGTTTGCTTATTCTTCTGGCTCTGATTTTTTTCCCCTTTACTCTTTTTGTTTTTGTTT
GCATTTCAGGGTGAAACTTCCTACATCCGAGTTTATCCTGAGAGAAGCACCAGCTATGG
CTACTCACGGTCTCGGTCTG
>1145
ACTGGTGTCCACAGAGGACATGGTTTAACTGGAATTCGTCAAGCCTCTGGTTCTAATTT
CTCATTTGCAGGAAATGCTGGCATAGAGCAGCACTAAATGACACCACTAAAGAAACGA
TCAGACAGATCTGGAATGTGAAG
>1146
GCTCAGTTAAGGGTGTCTGTGTCAGCAGAATAACCCTAACCTCACAGAATGGCCCACT
TCTCTCTTCTAGGCCACATTCGTGGCTGACTGGCGGAACTCGAATCGCTACCCTGCTGT
TATTCTCTTCTACGTCAATGCGT
>1147
CAGGGTGGGGAGACCAGGTAGAGGGAGTACAGAGTGACCGCCTCAAGTGACACCTCA
CCTCTCTGCACAGCTCCAATGAGACTCTGTCCTGCGTCATCATCTTTGTCATCGTGTACT
ACGCCCTGATGGCTGGTGTGGTT
>1148
GACCTGGATGGGGTGAGTTTGAGGGAGGGGGCCAGTAACCCACCTTCTGTCCCACCCC
TTCCTGCTGCAGGTGGATGGGGACTCTGTGAGTGGCATTTGTTTTGTGGGCTACAAGA
ACTACCGATACCGTGCGGGCTTCG
>1149
CCCACTCACCCATCCTTCCCAGCAGGGCAGCCTCACCCCTGCTAATGTCTGAGGTCCCC
CTTCTGTTCAGGAGTCATGACTCTGTTCTCCATCAAGAGCAACCACCCCGGGCTGCTG
AGTGAGAAGGCTGCCAGCAAGAT
>1150
ACTCTCTCCTCCCCACTGCTGCTGCGGGACTGGGCTTGGTAACGTCCTGTCCTGCCCCT
GTCCTCCACAGGCATTTTTGGCTTCCTGGCCTTTGGCTTTGTGCTCATTACCTTCAGCTG
CCACTTCTACGACTTCTTCAA
>1151
AGATCCTATCTCAAAAAAAGAGAGAGGAAAAGAAAGGAAAGCCTCACCTGTCTACGT
TCCCTCACTGTAGATGTCAGGCCAATGTGACCATCGGGCTGCCCACCAAGCAGCCCAT
CCCTGACTGTGAGATCAAGAATCGC
>1152

TGGCCCTTCCCAAGATTTGATGGGAAGTGGCAGCTTCTTCACGCTCCTTCCCTATCCCT
TCTGCTCTCAGGTTGACTGGGCAGAGTGACGATGAGCCAAAGCGGATCAAGAAGAGC
AAGATGATTGCCAAGGCCTTCTCT
>1153
ACTATGGGAGGCACTGCCAGGGACCGGGAAGTCACTATTCCTTCTCCTTTCCTTCCTTC
CATTCCCACAGCGGGCTTGGCCTTTGACCTCAATGAGCCCTCAGCTGATGTCTCCTCTG
CCTGGGCCCAGCATGTCACCAA
>1154
CTCCCAGGGGAGCGGGGGTGGCATGGACAGAGCCAGGGCCCCAGGCTCGTGTTGTCT
CTCCTCCTGTCAGTGCCCCCAGAGGAACAAGCCAACCTGTGGCTGGTTGAGGCAGAG
ATCTCCCCAGAGCTGCAGAAGCGCCT
>1155
TGCTTGGTAGTTTGGGGCACCTGGCTCTTTCCTCTTGAAGGTTGAATATAATGCTCGTG
CTCTTTTACAGGTGGCCAGTGGCGATTTCGATGCTTGCGTCCAAGTCTGTGAATGTAAA
ACCCCTCGTCACCCATAGGTTT
>1156
TTGAGCAGCCATACACCAGTCACCTCCATACTCACTGCCCATGCCCCATCCTCTCCTTC
ATCCCGGCCAGGCTGATCATTGGCTTCATCAGCAAACAGTACGTTACTAGCCTTCTTCT
CAATGAGCCCGACGGAACCTTT
>1157
CTTATGGTAGGGGTTGGGGAAGGGGAAGAAATCCGGACAGAGGGATCTCAGGGTCTC
CTTCCTACCATAGGCTCTCCACAGATAGAGAACATCCAGCCATTCTCTGCCAAAGACCT
GTCCATTCGCTCACTGGGGGACCG
>1158
TGCTTATCTGCAGGAAGGAGGGGTGGCATCAACCCTTGGTCAGTCACATGTACCTCCTT
CCCTCCTCCAGGGACCAACCACTTCCTACCCCAGAGCTCCAGATGCCTACCATGGTGC
CTTCTTATGACCTTGGAATGGCC
>1159
TGTTCAAAAGCAGGGTGTGGTGGTGTCCAGGAGAAGGGCTGGCATCAGGGGGTCTGT
TTTCTTTCCCCAGGCCCCAGGTGTACCCACCACACTCTCACTCCATCCCCCCGTATCAA
GGCCTCTCCCCAGAAGAATCAGTC
>1160
CCTGACCCATGTGCCTCTTCTTTCCTGGGCCTTGCCCGCTCTCCTTATTTCCATTGCTGG
TTCCTGGCAGGGCCTGCTGCCGTGCCAGCCTCAGGAGCATGCTGTGTCCAGCCCTGAC
CCCCTGCTCTGCTCAGATGTGA
>1161
GTGGAGGCTGGGTGGCATCTAGGGGAGTGGGCGCCATGCCTACTCCACTGCTTCTCCC
ATCTCCTTGCAGGATTGGTGAAGACATATTCCCTCCTCTGCTGCCTCCCACTGAACAGG
ACCTCACTAAGCTTCTCCTGGAG
>1162
TTCGGCCCTTCCGAGGGCAGCTCCTGTGTGCTGAGGGCTCTGCTGCTCTGAGATGCCG
CGTCCCTTCCAGACCTCACGCCCACCATCTTGGATTGGTTCTCGATCCCGTACCCCAGC
TACGCCATCTTTGGCTCGAAGAC
>1163
GAAGCACCTGCCCTCCCTGAGTTCAGTGTGTCCAGTGTTCCCGGCCAGATGCTCATGC
CATGCTTCGCAGGGAGCGGGAACGCCGAGAGAAGGAGAGAGAAGAGTGGGAACGCC

AGTACAGCCGGCAGAGCCGCTCACCC
>1164
GCATCCACGGCCCCAGGTGTGTTGATGTGTGTATGCCCCATCCTCACGGCCCCTCCCTT
TCTCTTTCCAGGCGCTCAAGGTCCCGATCCCGAAGCCCCCATTACCGACATTAGGCAG
AAGAGTGGGGGGTGGGGAGGACA
>1165
CCTTTGCCATAGTCCCACCCCACCTGCCGCCAACACTGTGTGAGGCCTCTCCTGCCCGC
TTCCCATGCAGAAGAAAATCATGATCATCATCTGCTGTGTGATCCTGGGCATCGTCATC
GCCTCCACTGTTGGGGGCATCT
>1166
GCCTCCAGCTTGCTCTGCGTGGTGGGCAACCTGCGTGATCTGAGGGGATGGGTTCTTC
TTGCTCCTACAGGCTCTCCTTTGATAAAGATGCGATGGTGGCCAGAGCCAGGCGGCTC
ATCGAGCTCTACAAGGAAGCTGGG
>1167
CGTCACCCGCCCCCGCCCTCACCCGTCCCCGCCCTCACCTGCCCCGCCCTCACCTGTC
CCCGCCCCGCAGGGAGCTCGAGGAGCAGCACGGCATCCACTGCAACATGACGTTACT
CTTCTCCTTCGCCCAGGCTGTGGCC
>1168
GGCACTGGGAGGGCAGGTGGGGTGGTACCTCTGCCGAAGCCTTCCTGGTCACAGCTT
GGTCTCTTTCCAGGGTAAAGAGTGTCACTAAAATCTACAACTACTACAAGAAGTTTAG
CTACAAAACCATTGTCATGGGCGCC
>1169
CCCTGAGCCCAAGGGGCCAAGGTGGGCAGGGACATGGAGCAGGCATGGAAGGCTGG
TTCTTGTCCCCCAGCCCAAGCCAGTGACCTGGAAAAAATCCACCTGGATGAGAAGTCT
TTCCGTTGGTTGCACAACGAGGACCA
>1170
GTGCCTTGTGAGGCTTCAAGGTGCAGAAGGTAGGGTGGGGAGACACAGCTCGTGCTC
TGTTTGTTTCTAGGAACGAATGTTCAATGCAGAGAATGGAAAGTAGCGCATCCCTGAG
GCTGGACTCCAGATCTGCACCGCCG
>1171
TGGCCACGTGGTCCTGCCTGTCTCAGCACCCACCGGCTCACTCCCATGTGTCTCCCGTC
TGCTTTCGCAGAGCTCCTCCCTGAATGAGGCCAGCAGTGGCCTCTTCGACGTCTTCCTA
CGCTTCATGTGCCACCACGCCG
>1172
GGCTCGGTTTGAGTGTACGCATGTCCAGCACATGCCCTGCCCGTCTCTCACCTGTGTCT
TCCCGCCCCAGGTCCTACGTCCAGTGCCAGGGGATCCCGCAGGGCTCCATCCTCTCCA
CGCTGCTCTGCAGCCTGTGCTAC
>1173
AGTCCCTCGTAGACAGATACTACGTAAAAAGTGTAAAGTTAACCTTGCTGTGTATTTTC
CCTTATTTTAGGCTGCTCCTGCGTTTGGTGGATGATTTCTTGTTGGTGACACCTCACCTC
ACCCACGCGAAAACCTTCCTC
>1174
ACAAGGGTGTCAGGTTACCTCCTGGGTGACGGCCCCGCATCCTGGGGCTGACATTGCC
CCTCTGCCTTAGGACCCTGGTCCGAGGTGTCCCTGAGTATGGCTGCGTGGTGAACTTG
CGGAAGACAGTGGTGAACTTCCCT
>1175

AGCAAGCTTTGCAGGAGGGGGCTGGGTGTGGGGCAGGCACCTGTGTCTGACATTCCC
CCCTGTGTCTCAGCTATGCCCGGACCTCCATCAGAGCCAGTCTCACCTTCAACCGCGG
CTTCAAGGCTGGGAGGAACATGCGT
>1176
CCATGCCCACACCCACGAGCACCGTCTGATTAGGAGGCCTTTCCTCTGACGCTGTCCG
CCATCCTCTCAGGTTTCACGCATGTGTGCTGCAGCTCCCATTTCATCAGCAAGTTTGGA
AGAACCCCACATTTTTCCTGCGC
>1177
AGCTTTCCGGTGTCTCCTGGGAGGGGAGCTGGGCTGGGCCTGTGACTCCTCAGCCTCT
GTTTTCCCCCAGGGATGTCGCTGGGGGCCAAGGGCGCCGCCGGCCCTCTGCCCTCCGA
GGCCGTGCAGTGGCTGTGCCACCA
>1178
ACGTCCTAGGAGGGTTGGAGGATGCCACCTCTGGCCTCTTCTGGAACGGAGTCTGATT
TTGGCCCCGCAGCCCAGACGCAGCTGAGTCGGAAGCTCCCGGGGACGACGCTGACTG
CCCTGGAGGCCGCAGCCAACCCGGC
>1179
ACATTTTTTTACATTACAATTATGTTGTCAGATTTACAATGTGCAAGTTACCTGGGCTTTT
CTCTTTTAGAAATCCAAGGGGGAGAGTGATGACTTCCATATGGACTTTGACTCAGCTGT
GGCTCCTCGGGCAAAATCTG
>1180
TTCCTGTACAGTCCAGCCCCTCCTCCCACAGGATCTGCTTTAATCCAGCGCCTCTCCTC
ATCTCTCCCAGCCCAAGGCCACAGTCTCAGATACCTGTGAAGAAGTGGAACCCAGCCT
CCTTGAAATCCTCCCCAAGTCCT
>1181
TCCTGCCCATTGCCTTTCCACCCCGGCCTCCCCGCCCCCTCCCTGTTCTCTCCTCGTGG
CCTGTTACCAGCCCACAGAGAGTCCAGCCGGCAGCCTGCCTTCCGGGGAGCCCAGCG
CTGCCGAGGGCACCTTTGCTGTGT
>1182
GTGGCCTGTTTCCTGTGTCCTGGCTGTGTCCTGTCCTGTGTCTGACCCCAAGCCGGCAT
TTATGTTGCAGCCAGCAGAGGCCTCGGAGGTGGCGGGTGGGACCCAACCTGCGGCTG
GAGCCCAGGAGCCAGGGGAGACGG
>1183
TTCTCCTCGGTGGCCCTGGCTGTCCTTGGACCACCTTCCCTGCTCAGCTGACCCGTACC
TCTGCCACCAGAGCTCTCTTCCTGCTGTCGTGGTGGAGACCTTCCCAGCAACTGTGAA
TGGCACCGTGGAGGGCGGCTGTG
>1184
CAAGGCCCGTGCTCTGTGCAGGGCTGGAGGTGGGACGGAAGGTCTGACTTGCGATCC
GCATCCTCTGCAGGTACAGGCCCACCACGACTACACGGCCACTGACACAGACGAGCT
GCAGCTCAAGGCTGGTGATGTGGTGC
>1185
TTTGTCCCCTGCCACTTTGCTTCTAGACTATCATTATTGGACCTCTGAATTCTTGTCTGC
CTTCTTGTAGGAGCCCCATGGCACCTGCCCAGCCCCACCTCAGCCCATCTTGACAAAAT
CTAAGGCTCCATGGAGCCACC
>1186
CAGAAGGATGGGAACATCAGCTGCGCTTCTGTTGGGCATTGAGGACCCCTACATCTGC
ACATACACACAGGTGACTGTCCGGGATGGCATGAGTGTCTACGACTCTCTAGACAAGG

CCCTGAAGGTGCGGGGTCTAAATC
>1187
TGTGGGGGTTCCCTGCCTCCCCATGGCCCCTACCCAACCTCCCACTCATTCCTTTCCAT
GCCCCCTGCAGACGAAAGACGGTCACTGCCTGGGACACAGCCATTGCTCCCCTGGATG
GCGAGGAGCTCATTGTCGAGGTC
>1188
ACGGTGGGGGTGGACCATGGTTGGGGGTGTCCTTGACCAGGTCTCAAACTTCCCTGCT
CTGTGGCATCAGGTACGGAAGACCTTCTTCAGCCTGGCGTTCTGTGACTTCTGCCTTAA
GTTTCTGTTCCATGGCTTCCGTT
>1189
GGCCCAGCCACGAGGCCCTTACAGACAGCTGACCTGTGTCCCCTTGCTTTATACCCTTC
ATGCCCTCAAGGTTCTACCACAGTGTCCAGGATTTGTCCGGAGGCTCCAGACAGCATG
AGGCTCCCTCGAACCGCCCCCTG
>1190
GGGGGTGGGGTGGGGGGCTTTCTCGGTTCTCTGATTCCTGGCAGTGATTTCACAGCCT
TTCCCCTGGCAGCCCCCGCACCCAGCACTGTGACCCGGAGCACTTCCCCTTCCCTGCC
CCAGCCAATGCCCCCCTACAGCGC
>1191
GTCCCCTGTGCCTGCACCCTGACCCCCGCTGCCCCACTTACCTCCACTCACATCCTCTC
TGCAACTGCAGCTGCCGGTAGTAGAGGAGGTAGTGATGGAACCCCCCGGGGGAGCCC
CAGCCCAGCCAGCGTGTCCTCGGG
>1192
CACCAAGGCTGAGTGACGTGGGATGAGGGATGTGGGCAGGCCTCTTAGATGCCACCCA
TCTCGCCCACAGAAGAACCTGGGGTACCGGGACTCAGGCTATTACTGGGAGGTACCAC
CCAGTGAGGTGCAGCTGCTGAAGA
>1193
TCCTGATGCTCGGTAAGTGACAGTTGCTATTTTTCCTCCCCACCTCTGACACTGCCCTC
CCTGCCTGCAGGAAGACGCGACATGTCAACATCTTGCTGTTTATGGGCTTCATGACCCG
GCCGGGATTTGCCATCATCACA
>1194
GGGATCTGGGACTGTGGACCAGCCATTTGCTGACCTCCGTGGTCCCCCTGCCTGGTCT
GGCCTGTTGTAGGCAGCTGAGGTGATCCGTATGCAGGACCCGAACCCCTACAGCTTCC
AGTCAGACGTCTATGCCTACGGGG
>1195
ATACAAAATTCCTTGGGCCTCCCAGCCCCTGACCCCAGATCACCCCTTTCCTGCCCCCG
TCTGCCCCCAGATTATCTTTATGGTGGGCCGTGGCTATCTGTCCCCGGACCTCAGCAAA
ATCTCCAGCAACTGCCCCAAGG
>1196
CCATGAGGCTGGGGCTGTTGGGATGCCCACAGCGGTCTGGACCCCCCTCCTCCCCCCC
ACCTGCCCTCAGATCCTGGCCACAATTGAGCTGCTGCAACGGTCACTCCCCAAGATTG
AGCGGAGTGCCTCGGAACCCTCCT
>1197
GTGGCTAAGGACCTGGGTCAGGGAGAGAGCAGGGCAGGACCCCGAATGATCTCCAGC
ATTCTGTGCCTAGCTGCTGATCGCCTACAAGCCAGCCCCTGGCACCTGCTGCTACATCA
TGAAGATAGCTCCAGAGAGCATCC
>1198

TTGCCTGATATACCTGAAGTCCCACAATAAGGGCTGCACATGGGATAGAAACTCACTTC
CTACATTCCAGATGGAATGCTCTCTGCAGGCCAAGCCCGCAGTGCCTACGTCTAAGCTG
GGCCAGGCAGAGGGGCGAGATG
>1199
GAGGTGGGCGGGGCAGGAGGCTAGGGAGGGCAAGAGGGGCCAGGGCTCTGAGCATA
CTACCTCCTTGCAGAGCCGCCCCCCAGGCCCTTCCTGGACATGGGGGAGGGCACCAAG
AACCGAATCATCACAGCCGAGGGGAT
>1200
CTGGGGGTGGCTGGGGGCAGGGACCCCAGGTGTCAGGGTGCTGATGTTCGCTGCCTC
ATTTCCATCCCAGGGCCTGAACCTGGACGACTGCTCCATGTATGAGGACATCTCCCGGG
GCCTCCAGGGCACCTACCAGGATG
>1201
GTCTTTTACTTACATCTTTACTGCACGTTCCAAGCGTTGTGTGGCCTGACGAGTGTGTT
CTCTCTTCTAGTCGACATTACTCTGAAGGGACGCACAGTTATCGTGAAGGGCCCCAGA
GGAACCCTGCGGAGGGACTTCAA
>1202
TCGGTAAGTGGTCTGTTGTCTTTTAAGCCTGCATATTTCTGGAAAGAATACATTTTTTGT
TCTGTTACAGCTCCGGGTTGACAAATGGTGGGGTAACAGAAAGGAACTGGCTACCGTT
CGGACTATTTGTAGTCATGTAC
>1203
TTAGAAAATTTTAAAACTATTAAAAATATTCCTCAGCATTGCTAATAGCTTGCTTATTTAT
TTAACATAGGGCTTCCGTTACAAGATGAGGTCTGTGTATGCTCACTTCCCCATCAACGT
TGTTATCCAGGAGAATGGGT
>1204
TTAGTGCCTCTGCAATTTAAATATTTTTTACACAGATTTGATGCTGTGCAAATGCCCTCT
CCCCTTTTAGGTGTTGCTTGTTCAGTATCTCAAGCCCAGAAAGATGAATTAATCCTTGA
AGGAAATGACATTGAGCTTGT
>1205
CATAATGAAATACTTCCTTTTTAAGATGGGTTAGGCTGATGTATTAATTGCTTTATCTTCA
CTCCTATAGCGGCTTTGATTCAGCAAGCCACAACAGTTAAAAACAAGGATATCAGGAA
ATTTTTGGATGGTATCTATGT
>1206
AAAAAAGATTAGTACTTTCCTGGGCAAGTGTATTGATGGGGATTTACAGATTTTTTTCT
TTTTTTACAGTTACCTGGCTACAGAAAGAAGATGCCAGATGACACTTAAGACCTACTTG
TGATATTTAAATGATGCAATA
>1207
TTGGCCTTTGTTAGAACCTCCAGGGTGTGGTTCTCAGCTGTGGCCACTTACACCCTCCC
TCTCTCCCCAGGCCTGTGGGTCCCCATCGCCCAAGTCCTGCCCACACTCCCACCTGCTA
CCCTGATCAGAGTCATCATGCC
>1208
TAGGCCTTTGTTAGATTCTCCATGGTTCATATCTCATCTGAGTCTGTTCTCACGCTCCCT
CTCTCCCCAGGCTGTGGGGCCCCATCACCCAGATATTTCCCACAGTTCGGCCTGCTGAC
CTAACCAGAGTCATCATGCCT
>1209
CCTCCACCGACCTCACTCCTCCCACCCCCATCCACGCTGAATCGGGTTCTGCTTCCGCT
TTCAACCCAAGAAAGCCGCAGGTGCCGGAATGTGATGCCACTGACTTGCGCATTGGGG

GTTAGAGAAAAGCGAGCTGCTCT
>1210
CATCAATCCTGCAGAATCGACCTCTGCTGGCCGGCTATACCCTGAGGTGCTCTCTCACT
TCCTCCTTCAGGTTCTGAGCAGACAGGCCAACCGGAGGACAGGATTCCCTGGAGGCC
ACAGAGGAGCACCAAGGAGAAGAT
>1211
TAAGCCTTTGTTAGAGCCTCTAAGATTTGGTTCTCAGCTGAGGTCTCTCACATGCTCCC
TCTCTCCGTAGGCCTGTGGGTCCCCATTGCCCAGCTTTTGCCTGCACTCTTGCCTGCTG
CCCTGACCAGAGTCATCATGTC
>1212
TAAGCCTTTGTTAGAGCCTCTAAGATTTGGTTCTCAGCTGAGGTCTCTCACATGCTCCC
TCTCTCCGTAGGCCTGTGGGTCCCCATTGCCCAGCTTTTGCCTGCACTCTTGCCTGCTG
CCCTGAGCAGAGTCATCATGTC
>1213
CTGTCAGTCCTGCAGAATCAGCCTCTGCTTGCTTGTGTACCCTGAGGTGCCCTCTCACT
TTTTCCTTCAGGTTCTCAGGGGACAGGCTGACCAGGATCACCAGGAAGCTCCAGAGGA
TCCCCAGGAGGCCCTAGAGGAGC
>1214
TAAGCCTTTGTTAGAGCCTCCAAGGTTCAGTTTTTAGCTGAGGCTTCTCACATGCTCCC
TCTCTCTCCAGGCCAGTGGGTCTCCATTGCCCAGCTCCTGCCCACACTCCTGCCTGTTG
CGGTGACCAGAGTCGTCATGTC
>1215
CTTTGTTAGAGCCTCCAAGGTTCCATTCAGTACTCAGCTGAGGTCTCTCACATGCTCCC
TCTCTCCCCAGGCCAGTGGGTCTCCATTGCCCAGCTCCTGCCCACACTCCCGCCTGTTG
CCCTGACCAGAGTCATCATGCC
>1216
TAGGCCTTTGTTAGGGCCTCCAGGGCGTGGTTCACAAATGAGGCCCCTCACAAGCTCC
TTCTCTCCCCAGATCTGTGGGTTCCTCCCCATCGCCCAGCTGCTGCCCGCACTCCAGCC
TGCTGCCCTGACCAGAGTCATCA
>1217
TAGACCTTTGTTAGGGCATCCAGGGTGTAGTACCCAGCTGAGGCCTCTCACACGCTTCC
TCTCTCCCCAGGCCTGTGGGTCTCAATTGCCCAGCTCCGGCCCACACTCTCCTGCTGCC
CTGACCTGAGTCATCATGCTTC
>1218
TCAGCCTTTGTTAGAACCTCCAAGGTTCGGTTCTCAGCTGAAGTCTCTCACACACTCCC
TCTCTCCCCAGGCCTGTGGGTCTCCATCGCCCAGCTCCTGCCCACGCTCCTGACTGCTG
CCCTGACCAGAGTCATCATGTC
>1219
CTCACATCATTGATGAGAATGCCTTGAATCCGAGCTACTAAATCACATTTCCTTCCTTCT
AACCTTCCAGTTAGATCAAACCATTGCTGAAACTGAAGAGGACATGTCAAATATTACA
GATCCACAGATGTGGGATTTTG
>1220
GGGGCTGTGACTAGGATCGGGGGACGCCCCAAGCTCAGTGCCCCTCCCTGAGCCATGC
CTCCCCCAACAGCTATACGGGCAGCTCCCCAAGTTCCAGGACGGAGACCTCACCCTGT
ACCAGTCCAATACCATCCTGCGTC
>1221

GGCCCATCCCCAGTGACTGTGTTGATCAGGCGCCCAGTCACGCGGCCTGCTCCCCTCC
ACCCAACCCCAGGGCTCTATGGGAAGGACCAGCAGGAGGCAGCCCTGGTGGACATGG
TGAATGACGGCGTGGAGGACCTCCG
>1222
TGGGGCAGACGGGGGTGTCTCAGGGGCTGGGAGGGATGAGAGTAGGATGATACATGG
TGTGTGCTGGCAGGAGGCGGGCAAGGATGACTATGTGAAGGCACTGCCCGGGCAACT
GAAGCCTTTTGAGACCCTGCTGTCCC
>1223
AGGCCCTCTGCCCCCAATTCCTCTCCAGCCTGCTCCCGCTGGCTGAGTCCCTAGCCCCC
CTGCCCTGCAGATCTCCTTCGCTGACTACAACCTGCTGGACTTGCTGCTGATCCATGAG
GTCCTAGCCCCTGGCTGCCTGG
>1224
CTTCCCCAGCAACACAAATGTCCTGCCAGATTCCTCCTGGAAAAAAACTTCTGCTCCT
GTCCCCCTCCAGGTCCCAGGTTGCCCATGTCCAGGAAAAGATGGATCCCCCTATCCAAA
TCTTCTCCGTGGTGTGTGTGGGT
>1225
GAAATGGGATAGATTGGAGGTCCTGTTGACCTCCATGCTTATTCACTTCAGTTCTCTTCT
TGGCTCTTAGTTGGATCGAAGCCTCTTAAAATGGCAGATGACTTGGACTTCGAGACAG
GAGATGCAGGGGCCTCAGCCAC
>1226
GTCAATTTGAGCCTTTATTTCCTCCGTTTTAGAGTTTGGTTGGGTTTCTCTTTGTGATGC
ATACATACAGGTCCATCTGGTTGGTATTGACATCTTTACTGGGAAGAAATATGAAGATAT
CTGCCCGTCAACTCATAATA
>1227
CAGAGCCTTTACTGTCATGTCAGCCTCCCTGGGCCTACTACCTTCAGCCTCCTTCCCTAT
CTGCCCCCAGCTGATTGGCATCCAGGATGGGTACCTATCACTGCTCCAGGACAGCGGG
GAGGTACGAGAGGACCTTCGTC
>1228
TGCTCAGCTTTGTTCTGTACGTTTCTTCCTGAGCTCAGACATCTCTTGGCTATCCCTCTT
GCTTCTCCAGATCACGGTGCTGTCTGCCATGACAGAGGAGGCAGCTGTTGCAATCAAG
GCCATGGCAAAATAACTGGCTC
>1229
TGGCTCTGTCCTCCCTATCCTTCCTGTTGAAGGTATTATCCTGTCTTACTAATTTCTCTCT
CCTACCTAGGATGGCGGTGGTGGCAGCAGTGATCCTCTGAACCTGCAGAGGCCCCCTC
CCCCAGCCTGGCCTGGCTCTG
>1230
CTCTACGCGTGGGGAAGTTTCCTGGCAGCCTGAAGTGTACACCTGACGCCTGCACTCC
CTCCCTATGCAGGGTGCAAATCCGGCCGCGATGAACGCGAGCGCCGCCTCGCTCAACG
ACTCCCAGGTGGTGGTAGTGGCGG
>1231
GAACAATCATGAAACTATGGATGATTTAGTCCAGCAAAATATCCAATTGGGAACCCTCA
TCCTTCTGCAGAGCCCAAATGGCGCAGTGGGAAATGCTGCAGAATCTTGACAGCCCCT
TTCAGGATCAGCTGCACCAGCTT
>1232
AAGGGGTTGGAAAGCCTCAGGACTACAGTAACACTGCAGAGACACTAATACTTCTTAT
TCCTGGTCCCAGGCAGGAAGCTGCACTTGGGAGTGATGATTCCAAGGCTACCATGCTA

TTCTTCCACTTCTTGGATCAGCTG
>1233
AGGGTCTAGGGCAATAGGTCATTGAGAGTGGTTGAATTGGATCAATCCTTTCTGTTTAC
CTTTCTGTTAGCCCTTTTCCCAGGATCCTACCCAGTTGGCTGAGATGATCTTTAACCTCC
TTCTGGAAGAAAAAAGAATTT
>1234
TATAATTTCAGGCCTGAACTTAAGGCATGAAAGGCCAGAGTTAAAACGTGCTCAGAGC
CTCTTTTTTCAGGAACAAGGAGAGCCAGTTCTCGAAACACCTGTGGAGAGCCAGCAA
CATGAGATTGAATCCCGGATCCTGG
>1235
TAGGGTTGACAGTGTTCAGCCTAACACCTCCCTGAGAAGCAGCCTCATCGGGGTCCTC
TCCCCTCTGCAGAAGCTGGTAAAATCCATCAGCCAACTGAAAGACCAGCAGGATGTCT
TCTGCTTCCGATATAAGATCCAGG
>1236
CTCTGACTGAGACCCCAGTCAGGAAGGAGAGGAAAGAACCCCTGGACTGACTCCTGT
TCCCACCATCCAGGGAAGACACCCTCTCTGGACCCCCATCAGACCAAAGAGCAGAAG
ATTCTGCAGGAAACTCTCAATGAACT
>1237
CTTCATCCCCAGTTGGGTGGTGGAGGGTGAAAGGGAGAGATGCTCAACACTCACATTA
TCTCTTTCCCAGGAGGTGCTGGATGCCTCCAAAGCACTGCTAGGCCGATTAACTACCCT
AATCGAGCTACTGCTGCCAAAGT
>1238
AGATCTTGGAACCTGGATGGGGGAATCCAGGAAACTCATGGATTCCTTCTTCCTGAATT
TTATCACCCAGGTTCACAGCTGGAGCAAAGCTGTTGTTTCACCTGAGGCAGCTGCTGA
AGGAGCTGAAGGGACTGAGTTGC
>1239
TTTTTTTTTTTCTGCTGAGTTTTTACAATAATTCCATTCCTTGTCTCCATGTATCTTCCTCC
TGGAACAGCTTCCGGAAGTTCAACATTCTGACTTCAAACCAGAAAACTTTGACCCCCG
AGAAGGGGCAGAGTCAGGGT
>1240
ACAGAGGACTCGAGGCAGGGGGAGCTTGCCAAAGAGCCTTCTGATGACTATGTCTTTG
CCTGTCCCAGAGGGGCCACTAGGTGTGACAGAGGAACTGCACATCATCAGCTTCACGG
TCAAATATACCTACCAGGGTCTGA
>1241
GAGCAGGGGTGTGGAAGCTTGGTGTGATAGGTTGCTTCTGAGCCAGCCTACACTGCTC
CCACCCCTGCAGAACCAGCAGTTCTTCTCCAACCCCCCCAAGGCCCCCTGGAGCTTGC
TGGGCCCTGCTCTCAGTTGGCAGT
>1242
CTTCCCACGTGCCCTCCTTCCCTACACCAGAGGCAACTCCTCAGCTTTTGCTACCTTTC
CATTCCTCCAGCGAGAGAGCCCTCCTGGCAAGTTACCATTCTGGACATGGCTGGACAA
AATTCTGGAGTTGGTACATGACC
>1243
GGAGCAGGGAGGCAGGGGCCTGGGGGGGCAGGGTGTTGGGCGGTGTCACACGTGAC
ACACTGTGCTCCAGACGCATCATGGGCTTTGTGAGTCGGAGCCAGGAGCGCCGGCTGC
TGAAGAAGACCATGTCTGGCACCTTT
>1244

CAGAGAGTCAGAGAGCTCCAAGGCCCGGCCTCTTCCCTCAAGCCCGCCTGTTCCTGCA
TTCACTCTCCAGACAAGGTGCTCATCTACTCTGTGCAACCGTACACGAAGGAGGTGCT
GCAGTCACTCCCGCTGACTGAAAT
>1245
TCCCTTGGGCTGCTGGGTTGGGAACTCCTAACTAAGATCGGGGCCTCACTTTTCTCTCT
GGATTACCTAGTCTTCAGAAACTGTGTAAAGATTGAAGAAATCATGCCGAATGGTGAC
CCACTGTTGGCTGGCCAGAACAC
>1246
GTATCCCAAGTATAAGGAGGTTCTTGTGAAACTGCAATGCCTCTTTTTCCCTTTCTTTTT
TATATTTCAGAATAGCTGCAATCTCCATGGCCTCTAAGATAACAGACATGCAGCTTGGA
GGTTCAGTGGAGATCAGCAAG
>1247
AAACATTTAATAGAGAATAAAAGAAAAGAACTTTCAGTGGTTTAATGCTGTTACTTTTA
ATATTTCAAAGATCTGACTGCAGCCATGAGCAGCAATGAGTGCTTCAAGTGTGGACGA
TCTGGCCACTGGGCCCGGGAATG
>1248
AAGTACTTCAGTATTTGTTAGTATCAAGACTGGTCTGACTAGCCGAATTCTTTGTTTTTG
CTCAAAACAGGTTTCCAGTTTGTTTCCTCGTCTCTTCCAGATATTTGTTATCGCTGTGGT
GAGTCTGGTCATCTTGCCAA
>1249
ATAAGTTAAAACGTAACACTTTGTAAAGGTATTATAGTACATGGCCTGTTTCTTTTCCTT
ATTGTTGAAGCCTGCTATAACTGCGGTAGAGGTGGCCACATTGCCAAGGACTGCAAGG
AGCCCAAGAGAGAGCGAGAGCA
>1250
ATGGTGACTGTTGCTTTGGTAGACAGTTTTTTCTGAGGCCCATTGTGAAAACTTTTAAT
TTCTTTTTTAGGTGTGGTGAAACTGGTCATGTAGCCATCAACTGCAGCAAGACAAGTG
AAGTCAACTGTTACCGCTGTGGC
>1251
ATGGCGCGGAAAACAGGGGTGGCCTGGCTCGGCCTGGCCCCGGCCGACGCTGTGCGT
TTGTCGCTTACAGTCTCCCGCTTCATGGGCCCGGCGAGCGGCATGAACATGAGCGGCA
TGGGCGGCCTGGGCTCGCTGGGGGA
>1252
CAGAGAAGACTCTTGGGTTTCTGATAGGCACTGACTCTCTCTGCCTATTGGTCTATTTTC
CCACCCTTAGGCTGCTGGTGGTCTACCCTTGGACAGAGGTTCTTTGAGTCCTTTGGGGA
TCTGTCCACTCCTGATGCTGT
>1253
GCTGGAAGTGCGCACCTGGGCTGGGCTCGCAGCAAGGCGCAACTTCAGGCTCCGAAG
CGGTGTGTTGCAGATCGAAGCGCTGCAAGAAGTCTTGAAGAAGCTCAAGAGTAAACG
TGTTCCCATCTATGAGAAGAAGTATG
>1254
GACTGTGAGACTTGCCTGTTGGGAACCTGGGTTTGTTCATACTCGATGACCACACATTT
TGTTGTTTCAGTGTGACGCCGGTGAGCAGTGTGCAGTGAGGAAAGGGGCAAGGATCG
GGAAGCTGTGTGACTGTCCCCGAG
>1255
GCATGTGGAATATTCTGGCTGTGCTGGCTGTGCTGGCTGTGGACTGAGTCCTCTGTCTT
CCCCCATCCAGTGTCGACCCTGGAGGAAGAATGCCTGCTGTTCTACCAACACCAGCCA

GGAAGCCCATAAGGATGTTTCCT
>1256
CAGTTCTATTCGGGGCTGAGTTGCTGGGATTCTTGAACCTGAGCCCTTCTTTTGTATCA
AAATCACCCAGGTGGATCAGAGCTGGCGCAAAGAGCGGGTACTGAACGTGCCCCTGT
GCAAAGAGGACTGTGAGCAATGGT
>1257
AGTTGTAGGGCTGGCAGACCTCAAGATAGTTCCGGGCCCAGTGGCTAAAGGTCTTCCC
TCCTCTCTACAGGGTTTAACAAGTGCGCAGTGGGAGCTGCCTGCCAACCTTTCCATTTC
TACTTCCCCACACCCACTGTTCT
>1258
GCAGGGCGGATACTGGAGGGGGCTTGTGGCATCTGACTCTGTATCTCCTACCTGCCCCT
CTCCTTGGTAGCTGTGAGAAGTCACTGCTTTGGGGAGACCTGATCTGGCTGTGCCAGA
TGGACACTGAGAAAGAAGTAGAA
>1259
GAGAGAATCCCACTTTCTTGCTGTTGTGATGGTGGTAAGGGAACGGGCCTGGCTCTGG
CCCCTGATGCAGGAACATGGAGCTGATCCAGGACACCTCCCGCCCGCCACTGGAGTAC
GTGAAGGGGGTCCCGCTCATCAAG
>1260
GGCAGTCCCCACCTTGGTCAGCAAGGTCGTGCCCTCAGCCTGCTCACCTCCTATCTCCC
TCCCTCTCCAGGCACCACCTGGGTGAGCCAGATACTGGACATGATCTACCAGGGCGGC
GACCTAGAGAAGTGTAACCGGGC
>1261
GGGTAAGGGAAGTGGAGGAAGACAGGGCTGGGGCTTCAGCTCACCAGACCTTCCCTG
ACCCACTACTCAGGGCTGGAGACTCTGAAAGACACACCGCCCCCACGGCTCATCAAG
TCACACCTGCCCCTGGCTCTGCTCCC
>1262
TCAACAGATGAGCTGGCCTGGGGTGGGCTGTGTGGTGATGGTGCTGGGGCTGGGTCCT
CTGCCCCTGCAGGTGGTCTATGTTGCCCGAAACCCAAAGGACGTGGCGGTCTCCTACT
ACCATTTCCACCGTATGGAAAAGG
>1263
TGTGAAGCCGAGGGGTGGTGGCTATAACGTACAGCAACCCTGTGTCGGTGCCCCCTGC
CCGCTTCTCTAGTGTCCTACGGGTCCTGGTACCAGCACGTGCAGGAGTGGTGGGAGCT
GAGCCGCACCCACCCTGTTCTCTA
>1264
AGATCCCCTCTGAGGTTAGAGAGGGGGACCCGTTTTGTTTTGCTCCACTGAGGAGCCC
TCTGCTGCTCAGAACCCCAAAAGGGAGATTCAAAAGATCCTGGAGTTTGTGGGGCGCT
CCCTGCCAGAGGAGACCATGGACT
>1265
CCTCCCCATAGGCACTTGGGGCCTCCCCTGGGATGAGACTCCAGCTTTGCTCCCTGCCT
TCCTCCCCCAGGCATGGCTGGGGACTGGAAGACCACCTTCACCGTGGCGCAGAATGA
GCGCTTCGATGCGGACTATGCGGA
>1266
AAATGCTAATGATTCAAGGATGTCCTCAGCTCTGTATGTGTTCTAAGAGTTCTATGTTTT
TTCTCCACAGAAGGCATAAGAACTAGGAGCTGCTGACATTTCAATATGAAGGGCAACT
CCACCCTTGCCACTACTAGCAA
>1267

GGAGGCTGGGTTGGTGTGACGCTCTGACCTCTTCCGGTGCTGACCTCTCCTTATCGCTA
CCTGAATACAGACAAGGACGAGGGTGACCACCCCAACAACAGCTTCAGCCCCTGTAG
CGCCCATGACCGCAGGTGCCTGCA
>1268
CTCTGACCCCAGGCCTGGGCCTCCTGCCTCTCTTCCTTCTGCTGAGCAATTTTGTCTTC
CCCTCCTCCAGCCCCAGGGCTCCTGCCAGAGCGAGCTGCACCGGGCGCTGGAGCGGC
TGGCCGCTTCACAGAGCCGCACCC
>1269
TGGACTCAGCTCTGGGGCATGGTCTCTTTTCCTGCGTCGGAACTGACCCCTCATGTCCT
TCTCTTGGCAGTGTCACCCAGCTCTGGATGGGCAGCGTGGCAAGTGCTGGTGTGTGGA
CCGGAAGACGGGGGTGAAGCTTC
>1270
ACATCTCGGTGCTGGGCCCCATTCAGAGTGGAGGGTTCTCCCTCTAACCACTCCCACC
CACCTCCATCAGATCAGTGAGCGCTATGGCCCCGTGTTCACCATTCACTTGGGGCCCCG
GCGGGTCGTGGTGCTGTGTGGAC
>1271
GGAGCTAATGCCGTGAAGCTATGTGCATCTCTCTGTCTGGCCGTACCTGGGTAATAACC
TGATCGACTAGGCGTGGTATTCAGCAACGGGGAGCGCGCCAAGCAGCTCCTGCGCTTT
GCCATCGCCACCCTGAGGGACTT
>1272
GGAATTCTGACTCTCCTCAGACCTCTGAGTTGACTCTCTCCCCAACCCCCTTCTCCCGA
CATACCCGGAGGCGCCAATATCGATCCCACCTTCTTCCTGAGCCGCACAGTCTCCAATG
TCATCAGCTCCATTGTCTTTGG
>1273
ATCCCATTCCCATCAGCTCCTGCCCCGTGACAGCTGTCCTTCCCTTCCCATCCTCTCTCT
GCAACCCCAGCTCTATGAGATGTTCTCTTCGGTGATGAAACACCTGCCAGGACCGCAG
CAACAGGCCTTTCAGTTGCTGC
>1274
GGTGAACCCCTAGATGGTGCCCTGAGGTCAAGCAGGAGTGAGGTTGTCCTAAAGCCC
CCTCTCCCTTCAGGAGGAGAAGAACCCCAACACGGAGTTCTACTTGAAGAACCTGATG
ATGAGCACGTTGAACCTCTTCATTG
>1275
GCCTATCCGGACTATCATTTCCTGCTCTGAGACCCCTAGATACCTAAACACATTCCCCCT
CCTCCCCCAGCCAAGGTCCATGAGGAGATTGACAGAGTGATCGGCAAGAACCGGCAG
CCCAAGTTTGAGGACCGGGCCAA
>1276
TCCAGCCCCTGTGTACTCTCAACAATCCCCCAACCCGCCTCATCACATACACCTTCCTC
CTCCCTCCCAGGGCATAGAAGTGTTCCCTATGTTGGGCTCCGTGCTGAGAGACCTCAG
GTTCTTCTCCAACCCCCGGGACT
>1277
AGGTCGGTACTGGGGCGAGGCTGCACTGAGAGTGGGCTTCACCTCCACCCCTCCCGCC
TCTCCTCCTCAGGAAAGCGGAACTGTTTCGGAGAAGGCCTGGCCAGAATGGAGCTCTT
TCTCTTCTTCACCACCGTCATGCA
>1278
GATATCTCAGCGCTGGGCCCATTCAGAGTGGGGGCTGCTCCCTCTAACCACTCCCACCT
GCCTCCAACAGATCAGTGAGCGCTATGGCCCTGTGTTCACCATTCACTTGGGGCCCCG

GCGGGTCGTGGTGCTGTGCGGAC
>1279
CCTTGGAACTGGTCCGCTCCTGCTACCACCACCCCCTGACCTCTCTCCACCCCCGCGTT
CACCTCCCCAGGCGTGGCGTTCAGCAACGGGGAGCGCGCCAAGCAGCTCCGGCGCTT
CTCCATCGCCACCCTAAGGGGTTT
>1280
GAATTCTGACTCTCCTCAGACCTCTGAGTTGACTCTCTCCCCAACCCCCCTTCTCCCGC
CACACCTGTAGGCGCCAATATCGATCCCACCTTCTTCCTGAGCCGCACAGTCTCCAATG
TCATCAGCTCCATTGTCTTTGG
>1281
TCCCCATTCCCATCAGCTCCTGCCCCGTGACAGCTGTCCTTCCCTTCCCATCCTCTCTCT
GCAACCCCAGCTCTATGAGATGTTCTCTTCGGTGATGAAACACCTGCCAGGACCACAG
CAACAGGCCTTTAAGGAGCTGC
>1282
AAGGAAAAGCCCTAGAAGGGCCCCAAGAGCATGGAGAGTGAGCTTGGTCTAAACCGC
CCTCTCCCTGCAGGAGGAGAAGAACCCCAACACAGAGTTCTACTTGAAGAACCTGGT
GATGACCACCCTGAACCTCTTCTTTG
>1283
CTAGGGGGGGCGATACATGCCCTGCTCTAAGACCCCTAGAGAGGGTAAAGATATTCCC
CTCCTCCGCCAGCCAAGGTCCATGAGGAGATTGACAGAGTGATCGGCAAGAACCGGC
AGCCCAAGTTTGAGGACCGGGCCAA
>1284
GGAGCCCCTGTGTACTTTCACCAATCCCCCCAACCTGGCTCATAACACACACCTTCCTC
CTCCCTCCCAGGGCACTGAAGTGTTCCCTATGCTGGGCTCCGAGCTGAGAGACCCCAG
GTTCTTCTCCAACCCCCAGGACT
>1285
GGGGTCAGTAGGGGTTGAGGCTGCACTGAGAGTGGGCTTCACCTTCACCCCTCCTGCC
TCTCCTCCTCAGGAAAGCGGTACTGTTTTGGAGAAGGCCTGGCCAGAATGGAGCTCTT
TCTCTTCTTCACCACCATCATGCA
>1286
GCCATAGGGTGGCAAGGTCTAGGCCTTCTCAGATTTCCGGTGACCCTTGTGTCTCTCTC
ACTTCCTTCAGAAATGCACGTGGCCGTCCTCGGAAGTACCCAGTGAGCGCTCCCATGG
CTCCCCCTGCAGTTGGGGCGGGG
>1287
GGGCCCCAGGGGCCAGGCCTGCACCACAGCCCTCACTTCCTGACCCCTGCTGTCCCTG
TGCTGTCCTCAGGAGACCTCCAGTCGGAACCTGGTGACCCTGCGTGTGGACCCCAATG
GCTTCTTCTTGTACTGGACGGGCC
>1288
GCCTGGGAGTGAAGACCACAGCGAGACTGTGCGGGAGCCCCCTCTTCACCCTTCGCT
GTCACCTACTCAGGACCCCAAGATCCGGGAAGTTCTGGGCTTTGGGGGTCCCGATGCC
CGGCTGGAGGAGAAGCTGATGACGG
>1289
GACCTGGGTTGTGGCTGGGCAGCCCCTGTGTCCCCCACTCACCGCCTCCCCGTGTATA
CTGGCCCCCCAGGTCTGGTCTGAGGAGCTATTCAAGCTGGCTATGAACATCCTGGCTC
AGAACGCCTCCCGGAACACCTTCC
>1290

ACCTTCCTCCTGCCCTGACCTTGGTGACCTTTGTCCTCCACTGACCCTGAACCCCTCCT
GCCCGCATCAGCATCCTGAAGATGTTCTCAGCAGACAAGAAGCGGGTGGAGACTGCG
CTGGAATCCTGTGGCCTCAAATTC
>1291
TCACGGTGGACACCCACCCTTACGGGGGTGCCCGCCCCTGGTTTCTCACCCCACACTC
CTCGACCTCCAGAGTGAGTCCATCCGGCCTGATGAGTTTTCCTTGGAAATCTTTGAGCG
GTTCCTGAACAAGCTGTGTCTGC
>1292
GGCAAGGGCTTGGCGACGGGGTGGGAGCCCTGCCAGGCTCCATCCTGACCCAGCTTC
CTGTCCCCTCCAGAGGCGCCAAGGGCAAGCCATACCTGACGCTGGAGCAGCTCATGG
ACTTCATCAACCAGAAGCAACGCGAC
>1293
CTGGCTGGGGTGCAGGTGGGTGGGGGCAGGTGGGACACCAGCTGACCCTGCTGATCC
TCTCCCACCCCAGACCAGATGTCCATGGAGGGCTTTAGCCGCTACCTGGGAGGCGAGG
AGAATGGCATCCTGCCCCTGGAAGC
>1294
CCAGGTGGGACCGGGGTGGTGAGGAGCTGGGCTGGTGGGCGGCCTCGGTGACAGAG
CCTCGCCGCCCCAGCGGGGCAGCTGGCTGGGACCTCGTCGGTGGAGATGTACCGCCA
GGCACTACTATGGGGCTGCCGCTGCGT
>1295
TGACATGTCCCGTAGACCTCAGCTTCCTCTCTGGGGGAGGGATCTCTGACCTCTGACCT
CGGTTCCGCAGGGCAAAGCAACAGGCAAAGATGGCTGAGTACTGCCGCTCCATCTTTG
GAGACGCGCTACTCATCGAGCCT
>1296
AGGGGGGTGGCATGGGGGCCAGGGTGCTGCGGACCCGGTCCAGGGCCCTGACTCGTC
CATGCCTGCCCAGCTGGCCCCAGGCGTTCCCCTGCCCAGCCCCCAGGACCTGATGGGC
CGTATCCTGGTGAAGAACAAGAAGC
>1297
AAAACCCCTCCAGACTTCCTAAGCAGCTGTGTGGTCCTGACCCCTCACCCTGTGGCCC
TGTCCTCTGCAGGGTCTCCCAGCTCTGACAGCTGCCCAGGCCTGAGCAATGGGGAGG
AGGTAGGGCTTGAGAAGCCCAGCCT
>1298
TGGGGGGCAGGTGAGGGAATGGCCAGCTGTGGCGGGAATGGCACTGCTGACCCTGGG
TTGGGGCCCACAGGGCACAGCCAGCAGCGAGGTGAATGCCACTGAGGAGATGTCCAC
GCTTGTCAACTACATCGAACCTGTCA
>1299
GTGGCAGGCATGGGAGCTTGCCCAGCTCAGCCCTGCCAGGTCTGACGCCCTTTCTTGG
CTCACCCCTAAGAGAGGAACAAATGCTTCGAGATGTCGTCCTTTGTGGAGACCAAGGC
CATGGAGCAACTGACCAAGAGCCC
>1300
GCTGAGGCCGGAGGTGGAGGCTGATGGGGTGGGCCCTGGCACCTGTGTGGCCCCTGA
CCACCAATCTCAGATACAACAAGCAGCAGCTCAGCCGCATCTACCCCAAGGGCACCCG
CGTGGACTCCTCCAACTACATGCCC
>1301
TCTCCCATTCCCCACATGGCACCGTCCTGCCTGATCCCTGCCCCTGCTCGACGTGCCCG
TGGCACCCCAGATGTGGCGATGCAGCTCAACGCGGGCGTTTTTGAGTACAACGGGCGC

AGCGGGTACCTGCTCAAGCCGGA
>1302
GGCTCAGGCCAGGGGTGTCCTGGGGGCAGGACTCTCAGCATCCGCCTCACCCTCCTTG
GCCCACCCCCAGGTGATCTCAGGGCAGTTCCTGTCCGACAGGAAGGTGGGCATCTACG
TGGAGGTGGACATGTTTGGCCTCC
>1303
GTTAGCTGTGGCCTGGTAGCCACAGTGGCCCAGGGTCAGCCCTGCGGCTCAGCTCCAG
TCTGGCCCACAGGTGGTGCTGCCCACGCTGGCTTCACTTCGCATTGCAGCCTTTGAGG
AGGGGGGTAAATTCGTAGGGCACC
>1304
CAGCACAGCGGGCAGTGGGTAGGGCCCCCACCTGGCCCAGCAACCCAACGTTGCCTT
CCTGACCACCCAGGATACCACTACGTCTGCCTGCGGAACGAGGCCAACCAACCGCTGT
GCCTGCCGGCCCTGCTCATCTACAC
>1305
ACCAGAGGTGGCGGGTGGGGGTGGCCTGGGGGCTCTGTCTCTGAGACCTTGGCCTTC
TGCCTCCCCCCAGACTATGCGGAGGCCCTGATCAACCCCATTAAGCACGTCAGCCTGAT
GGACCAGAGGGCCCGGCAGCTGGC
>1306
GTGGTAGAGGACAGAAGGGTCTGGAGGGAACAGCATTCTCTGAAAAGAGCATTCTCC
TTTCTCCCTATAGGCTCAGGCTGGCCAAGAGACGTGCCAGGACACCCAGTCTCAGCAG
CTGGGGTCTCAGCCGTCCTCAAACC
>1307
GTACCTGGAGGCAGGGGGTTGCCTTGGAGGTTCCCCCACCCCCCGCCCACCTTTGGTC
TGCCGCTCCCAGAGGTGGCCCCCACCCCGCTGGATGAGCTCCGAGGTCACAAGGCTCT
GGTCAAGCTCCGGAGCCGGCAAGA
>1308
CCCTACACACCCTCCCCATCCCAGTCCATGTGACGGGCCCAGGCATCACCCAGAGGGG
TTCTCCTCGCAGAGGTGGGGCCGCTGATGTGGAGGACACGAAGGAGGGGGAGGACG
AGGCAAAGCGGTATCAGGAGTTCCAG
>1309
GGCCAGGGAAAGCCTGCTGGATAGACCCGTCGTCAGCCCGGCATCACCTGTCAGCTCC
CTGTGTCCACAGGCTCTGCAGCGGCTCAGGGAGGTCGTCCTTGATGCAAACACAACTC
AGTTCAAGAGGCTGAAAGAGATGA
>1310
AGTACCAGAAGGAGGGCAGAGTCTGTGCTTCTGCCGCTGACCCCTCCTCTTCCCCTGC
CGGCTTCTCCAGGGAGAAGAAGGAGCTGCAGAAGATCCTGGACAGAAAGCGCCATAA
CAGCATCTCGGAGGCCAAGATGAGG
>1311
CCAGCCAGAGCCTCGAGGCTCTAGCCCCTCCCTCAGGGCCCCTCAGCTGAGCCCTTGC
CCACGCCTGTAGCTGGAGGAGGCCCAGAAGCAGCGGCATGACCGTCTTGTGGCTGGG
CAGCAGCAGGTCCTGCAACAGCTGG
>1312
GGCGAACAGGTGGGCAGACGGTGTGCAAGGCAGCCAGGCCTCGCCTGTGATGCCCAT
CCTTCTCCCACAGCTGCTGGCCCAGCTGGCCCAGGAGTGTCAGGAGCAGCGGGCGAG
GCTCCCCCAGGAGATCCGCCGGAGCC
>1313

GGGGAGGAGGGAGGCAGGCATGTCAGGGGTGGAGGGGAGCTCTTGGCCATGAACCTC
TTCATCTCCCCAGATGTCCCCAGTGCCCCACTGCTGCTGACCCTGGATGATGTGAGCAG
CAGCTCTGTGACTGTGAGCTGGGA
>1314
AAAGAAGCCCCATGGAGAGGGGCGCCTGTGGCCACATTGCAAAGACCTCACACTCTC
TCCATCCCCTCAGGCTCGGAGTGGGTGCCTGTGAGTGCCCGGCCCATGATGGTGACCC
AGCAGACTGTGCGGAACCTGGCTCT
>1315
TGCCCCATCTCAGAAACCCCAATCAGCCACCCCACTGCACTGCTAAGCCCAGACTCTT
GCTTTGATCCAGAGGCCCCCAAGATCCGTGTCCCCCGCCACCTCCGTCAGACCTACATC
CGCCAGGTGGGAGAGACGGTCAA
>1316
TCCTGGAGTTAAGGAGGTGTGGGGACTCTGGCACCTCCCTACACTCACCCTGGGGGCT
CCTCCCTGCCAGGGGAAGCCTAAGCCTCAGGCCACATGGACCCACAACGGCCATGCCC
TGGACAGCCAGCGGGTGAGCATGC
>1317
ATTTTGGGTGTGGGGGCTCTGTGCCAGCCTGGCCCTCACACTCACTTCCTCTCACACTC
TGGGCCTACAGAGAAACCTGGACCCCCCTGCTGCATCAGGCTCCTGGACGTCTGGGGC
TGCAATGCTGCTCTTCAGTGGAC
>1318
AGGTCTCCAGGGTAAGCAGGGAGGGTGAGGACAGGGGTGAGAAATGAGGGTGACAC
CCGGGGCCCTGCAGCAATGGTTCACAGTGCTGGAGCGCTACCACCCAACCACCTGCAC
CATCTCTGACCTCATCATCGGCAACT
>1319
TAGTCAGTCCTGACAGGTCTCCCTCACTCCTGTAAGCTCAGATGCACCCGACTTTCTCC
CCTGAACCCAGATATTGCTGCCAAACCTAAAGGGTTTATTGAGCGAGACTTCTCAGAA
GCCCCCTCATTCACCCAGCCCCT
>1320
CTGGGGATAGCTGTTGAGAGCCCCGCCCCAGTCCATACCTCCCCAGCTCTCTTCTGACC
ATGTCTCCCAGCCCAAGATCATCTGGATGAAAAACAAGATGGAGATCCAGGGCAACCC
CAAATACCGCGCCCTCTCTGAGC
>1321
CTCTGCACCTCTCTGGCCTGCAGCCGAGCTCTGCTCACACCAGGGGCTCAGGAAGCAT
GTCCTCCTCCAGGTGCTGCAGGCCTGAGGGGCCAGGTCCACCAGGCCTACAGTCAAA
CTCCAGAGATGCCCAAGACCCCCGC
>1322
TGGACATGCCAATACACATTACTGACATGTATGCCCACCTGACCTGCACCCACTCATGC
CCACTCTGCAGGGCAGCGCTCGCCATTGAATGACTTCCAGGTGCTCCGGGGCACAGAG
CTACCTGCTACATGCGGTGGTGC
>1323
CTGGGCCTAGATAAGACTGGGGGCAGGGGAGCCTGGGCCGTGGCGTTACCCTGTGCCT
TCTTCTCTCCAGGGCCTTCCACTACAATGTGAGCAGCCATGGTTGCCAACTGCTGCCAT
GGACTCAACACTCGCCGCACTCG
>1324
GGGGTGGAGAGGGGCAGGGTGGGAGACAGGGGACCTCAGCCCAAGTTGATCTTCTGT
CTCTTGCTCCCAGACTACATACGGACCTGCATCATGAACAATGGGGTTGGGTACCGGG

ACACCATGGCCACGACCGTGGGTGG
>1325
AACACCTTCCCTCCATCCCGGCCTGGGACCTTCCCCCAGCACACACTATAGTGATGCTC
TGGGCCCTCAGGTACATGCCCACGCTCCGGAATGGCCTGGAAGAGAACTTCTGCCGTA
ACCCTGATGGCGACCCCGGAGGT
>1326
CGGGTCAAGCTGGGAGAGTGGAGGGACAAGCCCACGCCCATCCACGAACCCACTGGC
TCTTTGTCTCCAGCCGCGTGTGTCTGGTGCAATGGCGAGGAATACCGCGGCGCGGTAG
ACCGCACCGAGTCAGGGCGCGAGTG
>1327
TAGGGCAACTGGGCGGGGCCAGATAAGCCAGAGTCCCAGGGTCTTCTTCACGCCCCAT
TACCGCCCCCAGGTTCCTCGACCAAGGTCTGGACGACAACTATTGCCGGAGTCCTGAC
GGCTCCCAGCGGCCATGGTGCTAC
>1328
CGGGAGGAGGGTAGTCACAGGCTTAGGGCTGGAGGCTGGCGGGCTAGGGCTGAGTGC
AGCGCCTGCTTAGAGACCTTCGGGAGAACTTCTGCCGGAACCCCGACGGCTCAGAGG
CGCCCTGGTGCTTCACACTGCGGCCC
>1329
GGGGCTGGAAGCCTGGAACCGAAGGGCCGGGGCGAGGTCTCGGCCTGATGGCTGCCT
GCACCCGCCGCAGACTGCTACCACGGCGCGGGGGAGCAGTACCGCGGCACGGTCAGC
AAGACCCGCAAGGGTGTCCAGTGCCA
>1330
TATGGGTCAGGCCCTTTAGCCCACGACATCCACACAGTCTGGGTTTCATCCAGCCCACC
CCATCCTACAGACCAGGTGCAGTTTGAGAAGTGTGGCAAGAGGGTGGATCGGCTGGAT
CAGCGTCGTTCCAAGCTGCGCGT
>1331
AGCCAGAAGATGGCAGGTCTAGCATCTGTCCCAGGAGTCTGTTCCCTGTCCTAATTCCC
CACTCCTCTAGGCAGGGCCAGCATTTCTGCGGGGGGTCTCTAGTGAAGGAGCAGTGGA
TACTGACTGCCCGGCAGTGCTTC
>1332
CAAGCTAACAAGTGAGCCTTGGGGCAATGGACTGAGAGTCACAAATGACCTAGCAGA
GCTTCTCTCCCAGCCATATGCCTCTCACGGGCTATGAGGTATGGTTGGGCACCCTGTTC
CAGAACCCACAACATGGAGAGCCA
>1333
ACCTGGGAGGGTGTGAGGTGGGGCTGAGCCTTGTGGCCTCAGACCCTGAGTGCCCCC
ATTCTTGCTAAAGATCTGTGACCCTGAACCAGCGTGTGGCCCTGATCTGCCTGCCGCCT
GAATGATATGTGGTGCCTCCAGGG
>1334
TGGCGGCCAGGAGCCCAGCCCTGGATCTTCCTCCAGGACCGTCTCCTTCTCCCCATTCC
CCTCACTGCAGGTACGGGTAATGACACAGTCCTAAATGTGGCCTTGCTGAACGTCATCT
CCAACCAGGAGTGTAACATCAA
>1335
AACTATTTTATCATGAAGCAGGCTAGTCATGGCTGTGGCCCGGGGCCTCATCAGTTCTC
CTACCTGCCAGAGTGACTACGGGGGCCCACTTGCCTGCTTTACCCACAACTGCTGGGT
CCTGAAAGGAATTAGAATCCCCA
>1336

TATTGGGTTTTTTAAGAATCAGTTCTTAGATTCATTTATCAATTCTAGTTTTTTGTTGTT
GTTTTTAAGGACTCCTGAATATTTTTCAAAACTGAACAATTTCAGCCATGTCTGAGCTT
TCCGTCTTCCTGGAGGCACA
>1337
ATTATGCAGGAAACATTTAGTAATTTCAACATAAATATGGGACTCTGACGTTCTCCTCTT
CATTTTGCAGAGCAGTCATTATGGCGAACCTTGGCTGCTGGATGCTGGTTCTCTTTGTG
GCCACATGGAGTGACCTGGGC
>1338
TTTGGCCAGTTTTTTGCCTCTGCATTCACTCCCACCTTGAATCTTTTCCTTTCCCCACCC
TGGGCCTCAGCACCCCCAAACAGGCGAACCTGTGTGTTCTTTGAGGCCCCTGGAGTGC
GGGGAAGCACAAAGACACTGGG
>1339
TTCCACACCCCATTGTGCTTTCTTCCTTGCCCCCCCTTTCTCTCCTCTTCCCCTAATCCCA
TCCCATCAGGATGCCGAGACAGTGATGAGCCAGGCTGTGAGTCCCTCCACTGTGACCC
AAGTCCCCGAGCCCACCCCAG
>1340
CAGGAGGGGAAGAGCAGAGAGCAGGTTTGGGTCAGTGCTCTCCAGCCTGCATTCTTG
CCTTGATGTCCAGGTGAGTCCATCTGGATGGCACTGGTGCTGCTGGGGCTGTTCCTCCT
CCTCCTGCTGCTGCTGGGCAGCAT
>1341
CTTTCCACGAAGTCCCTTTTCCTGTCCCTATGCATTTGCACCCTGACCCTAAGGCTCTTG
TCTGTTCCAGCCCTGCTACAGCGAAAGAACTACAGAGTGCGAGGTGAGCCAGTGCCA
GAGCCAAGGCCAGACTCAGGCAG
>1342
GTTGTGCTGGGGAGGAATCCTGGCCCTGTTATAGCTCAGAGGCCCACACTCAGCACAG
TGTCCCCAGCAGGTAATCCGGGAAGGAGGTCATGCAGTGGTTTGGGCCGGGCAGCTG
CAAGGAAAACTGGTTGCCATCAAGG
>1343
CTCTGCTCCCTGGGATGGATCAGCCGTCTCCAGCTTTGTGTACCATCCTTTTCTCTCTGC
GTTTCCCAAGGGCTCCCTGTGCCACTACTTGACCCAGTACACCAGTGACTGGGGAAGT
TCCCTGCGGATGGCACTGTCCC
>1344
CGCCGACTCAAAAAAAAAAAAAGAGGGAGGAAGAAAATCCATGTTCCTTCAACCTTG
GATTCCCCCACAGGCCAATATAAACCAGGTATTGCCCACCGAGATCTGAGCAGCCAGA
ATGTGCTCATTCGGGAAGATGGATC
>1345
TGCATGGACCATTGCTGCAATGAGGATTGCCACAGAGATGATTCTTGGCCCTTCGTGCC
TTGCTCTCCAGGCTGGCACCCAGAGGTACATGGCACCAGAGCTCTTGGACAAGACTCT
GGACCTACAGGATTGGGGCATGG
>1346
TGTCTTGCCCTTTCTACATGGTAGGCACCCCTAGGACTAACTGATACCCAGCCCCTCTA
CCTTCCTCCAGACAGCAGTCCACCACCCTTCCAACTGGCCTATGAGGCAGAACTGGGC
AATACCCCTACCTCTGATGAGCT
>1347
AGATGGCTTTTAACCCTGGGGCCCACTCAAGATCCTAGGGTCAACCCTTCCTCCCTGTC
ATTCCCCCCAGGACCCTGATGGGCTGAGGGAGCTCCTAGAAGACTGTTGGGATGCAGA

CCCAGAAGCACGGCTGACAGCTG
>1348
GCTCTCTTGCGGAAGTCCCCATGCTGGCCCCTTGACCCTCATGAACCCTGCTGCAAC
CCTGTGTTTCAGGCCGCGTCATTCTACTGCAGCAAGATGGGCTTTGAACCTCTAGCCTA
CAGGGGCCTGGAGACCGGTTCCC
>1349
GGAGACTAAAAGCCAGGTGAGGGGACCTAGACTCCAGACTCCAGTGCCATCCTCTCC
CTGCCGGCCACAGAGATGGGCGATCACCTGGTGAAACACGGTGACGGAGTGAAGGAC
ATTGCGTTCGAGGTGGAAGATTGTGA
>1350
TATGAAATGTGCTCTGTCCTCGGCGGGGATGGGTGCCCAGGGGCCGAGCCAGGCTGTT
TTCCACCCGTAGTATGGGGACACCACACACACCCTGGTGGAGAAGATGAACTACATCG
GCCAATTCTTGCCTGGATATGAGC
>1351
GAGGGGTCATGGAGAAGGGAGCAATCTGGGGGGTAGCCACCTTCTCACCCCCATCTTC
TTCCCTTCCCAGGCCCAAATGCAGTCTGGAGATGATCGACCACATTGTGGGAAACCAG
CCTGATCAGGAGATGGTGTCCGCC
>1352
GTGGAAATGGGTGATGGAGGCCGTCCCTGGAGGCGCTCAGAGCATATGTGTTCCACCG
CCCACCCTACAGGTACCTGAAAAACCTGCAGTTCCACCGCTTCTGGTCCGTGGATGAC
ACGCAGGTGCACACGGAATATAGC
>1353
GGGCTCTTGTAGGGTCAGGGATGTGGGTGATGGGGCAAATGACAGTGGTCTCCTTGTT
TCCCTCCCCCAGGAATATGTGGACTATAACGGGGGCGCTGGGGTCCAGCACATCGCTCT
CAAGACCGAAGACATCATCACAG
>1354
AGCACCTCCACCTGGCTAGGGGAGTTGAACTTGGAGGCCGAAGGCTGACCTCAGCTT
TGCTCTGAAACAGATTCGCCACTTGAGAGAGAGAGGCCTGGAGTTCTTATCTGTTCCC
TCCACGTACTACAAACAACTGCGGG
>1355
GCCCCTGTGGACTTTCCAAGGATCTCACCGTGCCAGCCTCCCCTAGCAGAGCCCCCTG
TCTCCCGCCTAGGAGCTGAAAATCCTGGTGGACTACGACGAGAAAGGCTACCTCCTGC
AGATCTTCACCAAACCGGTGCAGG
>1356
CGAGGGGACAGGCAGCTAGCACACTCGGTTTTCTGGGTCCTTGGCTCATCTCCTCTCTT
TCCCGCCACAGGGTTTTGGAGCCGGCAACTTCAACTCACTGTTCAAGGCTTTCGAGGA
GGAGCAGAACCTGCGGGGTAACC
>1357
GCCAGGAAGGGGTAGCGGGGCAGTGCAGTGTCGGGGGAGAGCGGCTTGCTGCCTCGT
TCTTTTCTTGCAGGCCCCAGGATGCAGGCCCTGGTGCTACTCCTCTGCATTGGAGCCCT
CCTCGGGCACAGCAGCTGCCAGAA
>1358
TGAGGAGACAGTCCCTGTGCATCTCTGCGAGTCCCTGAACTCAAACCCAAGACTTCCT
GTCTCCTGCCAGGGCTCCCCAGACCCCGACAGCACAGGGGCGCTGGTGGAGGAGGAG
GATCCTTTCTTCAAAGTCCCCGTGA
>1359

GTTCTGGGAGGGGGCTTGATTTGGGGCCCTGGTGTGCAGTTATCAACGTCCACATCCTT
GTCTCTGGCAGGAGCGGAGCAGCGAACAGAATCCATCATTCACCGGGCTCTCTACTAT
GACTTGATCAGCAGCCCAGACAT
>1360
GGCAGGCTCTCAAAGACGGGATGCTTGTCGTCGAGTCTCATACGCTAACCTCTGCTCC
GCCTCTTCTCAGAGCTGCGCATAAAATCCAGCTTTGTGGCACCTCTGGAAAAGTCATAT
GGGACCAGGCCCAGAGTCCTGAC
>1361
GGGACACAGCATGGCGCCACTGTCTTTCTGGTCTCCTGGGGCTCAGACTATGTCATACA
CTTCTTTCCAGGGCAGTGGGTAACAAAGTTTGACTCCAGAAAGACTTCCCTCGAGGAT
TTCTACTTGGATGAAGAGAGGAC
>1362
TCCTGGCTGTGTCTGTCCCCCGGCTTTTGGGCTCTGAAGGACTAACCACATGCTTTCTC
ACTTGTCTCAGATTGCCCAGCTGCCCTTGACCGGAAGCATGAGTATCATCTTCTTCCTG
CCCCTGAAAGTGACCCAGAATT
>1363
CAAAGGGATCCCTTGGTTGGGGTGTTGGGGAAGGCAGGGTTTTAACGGAAATCTCTCT
CCATCTCTACAGAGCTGCAATCCTTGTTTGATTCACCAGACTTTAGCAAGATCACAGGC
AAACCCATCAAGCTGACTCAAGG
>1364
ATCTTCCTGCAGACTCACAAAAGGCAGCTGCCCAAGCAGGGCCTGACCCCTCGGTGTC
CCCTCCCCACAGTACTGTGCAGCCCTGGAATCCCTGATCAACGTGTCAGGCTGCAGTG
CCATCGAGAAGACCCAGAGGATGC
>1365
AGCCCCTGGTTTGTGCGAGTCGTCCCGGCCTCTGGCGTTCTACTCACGTGCTGACCTCT
TTGTCCTGCAGCAGTTTTCCAGCTTGCATGTCCGAGACACCAAAATCGAGGTGGCCCA
GTTTGTAAAGGACCTGCTCTTAC
>1366
GGAGAGGAAGAGCCAGGCTTTAGCTCCCATCTCAGGGGTGGGGGATTTTTGACTCTAC
CTCTCCCCACAGATGAGCAGCAGCTGCTCAGGGCTGAGCAGGGTCCTGGTGGCCGTG
GCTACAGCCCTGGTGTCTGCCTCCT
>1367
GAGCTGTGGCATGGTCTGACTTATGCTTGACATCTTGTCTTTGTCCATTGTCACCTCATC
TCACTTCCAGGGACCCAGTGTCCTGGTTTCGGGATGGGGAGCCAAAGCTGCTCCAGG
GACCTGACTCTGGGCTAGGGCAT
>1368
CTAAGTAAGCCCTCTGGGACCAGGAGGACCTGAGGACTGCTCAGTCTCCAGGTGTACC
CTCTGCCTCTAGACCCTCCAGCCCGCCCTGTTGTCTCCTGCCAAGCAGCCGACTATGAG
AACTTCTCTTGCACTTGGAGTCC
>1369
GCATGTGGGGGCTCCAGCTGGGTCCCACAGTAGGCATTTTCAAAGCCAAAGACCACAT
CCTCCCTTCTAGGAGGAGTCCATCCACAGGGCCCTGGCCATGCCCACAGGATCCCCTA
GGGGCTGCCCGCTGTGTTGTCCAC
>1370
ACTTTGGGAAGTGGTGGGGAAGTGATGGAGACCCATAGCCTACCCTGACTTTGTGTCT
TGATGCCCTTAGTGCGCCCTGACCCACCCCAGGGCCTGCGGGTAGAGTCAGTACCAGG

TTACCCCCGACGCCTGCGAGCCAG
>1371
CTTTGTAGATGGTGGCTGGGAAGGCCCTGCACTTACAAGCTGGGTAACAGTGAGTCAT
GTTTACCCCCAGGTGGAGCCAGCTGGACTGGAGGAGGTGATCACAGATGCTGTGGCTG
GGCTGCCCCATGCTGTACGAGTCA
>1372
CCTTGAGCACAGGACGTGACCCCCGTCCCCACCAGCGTATGGACACTTATTGGGTCTT
GCCTTCCTTTAGGGACCATACCAAAGGAGATACCAGCATGGGGCCAGCTACACACGCA
GCCAGAGGTGGAGCCTCAGGTGGA
>1373
AACCCCAGAGAAAAGGGGACACCGAGCTTGGTCAGGCTTGCTCTCAGTGACATGTGG
CCCTCCCCCTCAGATCACAGGGACTCTGTGGAGCAGGTAGCTGTGCTGGCGTCTTTGG
GAATCCTTTCTTTCCTGGGACTGGT
>1374
TAATAAACCCTTTGGCGGAGACTGAGATAACCCACATTGTTGGAGAGACAGCTGCCTT
TCTATGCCCCAGGCTGAGGCTGAGACGGGGTGGGAAGGATGGATCCCCAAAGCCTGG
GTTCTTGGCCTCAGTGATTCCAGTG
>1375
TCATTCTCAGGGTGTCTGGGCCAAGATCCGGTCTTACTGTCTCTCCTGATTTGCCTCCT
GCTTCTTCTAGGAGCTCCAAACCTGTAGAGGACCCAGGAGGGCTTCGGCAGATTCCAC
CTATAATTCTGTCTTGCTGGTGT
>1376
ATCAGAGCTCCTGGGCTGCATAGGAGTGGCCAGGCTAACAACCTGACCCCTTCCCTTC
CTCCCTGGGCAGCTCCCTGCGGTGTGGCCCCCCAAGCACGCATCACAGGTGGCAGCA
GTGCAGTCGCCGGTCAGTGGCCCTG
>1377
GGGGTTGGCAGTGGGGGTTGGGGAACAAGGAGAGGTCTCCTGGGTCTCAGCCTTTGC
CCCCTGCCTGCAGCGAGCACCACAAGGAAGCCTATGAGGTCAAGCTGGGGGCCCACC
AGCTAGACTCCTACTCCGAGGACGCC
>1378
GGTGCCTAGAGGTGTGGAGGGGCACCTGACTTGGGGAGGGGCCCAGGGTAAGCCTCT
TTTACCCCCACAGTGAGCCTCCTGACGCCCAAGCCACTGCAGCAACTCGAGGTGCCTC
TGATCAGTCGTGAGACGTGTAACTG
>1379
ATGACCAGTGCAACTTCGGAAAGGAGGCCTGGCCCGGTCCTGATGGCTGCTGTGTGGC
TTCTCTCCTCAGGGTGACTCTGGGGGCCCACTCTCCTGCCCTGTGGAGGGTCTCTGGT
ACCTGACGGGCATTGTGAGCTGGG
>1380
ACTCCAGGAGTGGGGGCGGTGCGTCCTCCGGCGCGCAGCGGTGGCCACAGCTCTCCT
CCCGCCGCCGCAGGTCTTCCTCACCGCTTTGCGCGCCCCGAAGCCGACCCTGCGCTAC
TTCACCACCGAGCGCTTCCTGCCCC
>1381
TGGACATGCCAATACACATTACTGACATGTATGCCCACCTGACCTGCACCCACTCATGC
CTACTCTGCAGGGCAGCGCTCGCCATTGAATGACTTCCAAGTGCTCCGGGGCACAGAG
CTACAGCACCTGCTACATGCGGT
>1382

ACTGGGCTAGATAAGACTGGGGGCAGGGAAGCCTGGGCTGTGGCGTTACCCTGTGCCT
TCTTCTCTCCAGGGCCTTCCACTACAACGTGAGCAGCCATGGTTGCCAACTGCTGCCAT
GGACTCAACACTCGCCCCACACG
>1383
GGGGTGGAGAGGGGCAGGGTGGGAGACAGGGGACCTCAGCCCAAGTTGATCTTCTGT
CTCTTGCTCCCAGACTACGTACGGACCTGCATCATGAACAATGGGGTTGGGTACCGGG
GCACCATGGCCACGACCGTGGGTGG
>1384
AAACACCTTCCCTCCGTCCCGGCCTGGGGCTTCCCCCAGCACACACTATAGTGATGCTC
TGGGCCCTCAGGTACACGCCCACTCTCCGGAATGGCCTGGAAGAGAACTTCTGCCGTA
ACCCTGATGGCGACCCCGGAGGT
>1385
GCCGGGTCAAGCTGGGAGAGTGGAGACAAGCCCACGTCCATCCACGAACCCACTGGC
TCTTTGTCTCCAGCCGCGTGTGTCTGGTGCAATGGCGAGGAATACCGCGGCGCGGTAG
ACCGCACGGAGTCAGGGCGCGAGTG
>1386
TAGGGCAATTGGGCGGGGCCAGATAAGCCAGAGTCCCAGGGTCTTGTTCACGCCCCAT
TACCGCCCCCAGGTTCCTCGACCAAGGTCTGGACGACAACTATTGCCGGAATCCTGAC
GGCTCCGAGCGGCCATGGTGCTAC
>1387
CGGGTTTGGTCCCAGCCCCAGAGGGATCTCAGCTCTCGCTCGGGGCCCGACCTATCTC
GGTCCATCTAAGGGTCCGAGGCACAGCCCCGCCAAGAGGCCACAACTGTCAGCTGCT
TCCGCGGGAAGGGTGAGGGCTACCG
>1388
CGGGAGGAAGGTAGTCACGGGCTCAAGGCTGGAGGCTGGCGGGCTAGGGCTGAGTG
GAGCGCCTGCTTAGAGACCTTCGGGAGAACTTCTGCCGGAACCCCGACGGCTCAGAG
GCGCCCTGGTGCTTCACACTGCGGCCC
>1389
GCCGGGCTGGAAGCTGGAACCGGAGGCCGGGGCGAGGTCTCGGCCTGATGGCTGCCC
GCACCCGCCACAGACTGCTACCACGGCGCAGGGGAGCAGTACCGCGGCACGGTCAGC
AAGACCCGCAAGGGTGTCCAGTGCCA
>1390
GGTGTCTGGGACCAGAGCCTGGAAATGGTTGAGACTACCCTGCCACGATTTTGCTCCC
GCTTCCGCCTAGGTTCACGTTTACCTCCGAACCGCATGCACAACTGGAGGAGAACTTC
TGCCGGAACCCAGATGGGGATAGC
>1391
TATGGGTCAGGCCCTTTAGCCCACGACATCCACACAGTCTGGGTTTCATCCAGCCCACC
CCATCCTACAGACCAGGTGCAGTTTGAGAAGTGTGGCAAGAGGGTGGATCGGCTGGAT
CAGCGGCGTTCCAAGCTGCGCGT
>1392
CAAGAAGATGGCAGGTCTTACGAATCTGTCCCAGGAGTCTGTTACCTGTCCTAATTCCC
CACTCCTCTAGGCAGGGCCAGCATTTCTGCGGGGGGTCTCTAGTGAAGGAGCAGTGGA
TACTGACTGCCCGGCAGTGCTTC
>1393
CAAGCTAACAAGTGAGCCTTGGGGCAATGGACTGAGAGTCACAAATGACCTAGCAGA
GCTTCTCTCCCAGCCATATGCCTCTCACGGGCTATGAGGTATGGTTGGGCACCCTGTTC

CAGAACCCACAGCATGGAGAGCCA
>1394
ACCTGGGAGGGTGTGAGGTGGGGCTGGGCCTTGTGGCCTCAGACCCTGAGTGCCCCC
ATTCTTGCTAAAGATCTGTGACCCTGAACCAGCGTGTGGCCCTGATCTGCCTGCCCCCT
GAATGGTATGTGGTGCCTCCAGGG
>1395
TGGTGGCCAGGAGGCCAGCCCTGGATCTTCCTGCAGGACCCTCTCCCTCTCCCCATTC
CCCTCACTGCAGGTACGGGTAATGACACAGTCCTAAATGTGGCCTTGCTGAATGTCATC
TCCAACCAGGAGTGTAACATCAA
>1396
AACTATTTTATCATGAAGCAGGCTAGTCATTGCTGTGGCCCGGGGCCTCATCAGTTCTC
CTACCTGCCAGGGTGACTACGGGGGCCCACTTGCCTGCTTTACCCACAACTGCTGGGT
CCTGGAAGGAATTATAATCCCCA
>1397
GATACATACTCTGGGGCCTGCCTCCCTGGAGATGAGGATGAGACCCCTGTCCCTTGCTG
TTCTCCAACAGGTGCTGGGAGGCCCTCCTTGGCTTAGGAGGACACTTCCAAAGCTGGG
GCGCCCCAAGGAGGCACCAGTGG
>1398
TCGGGGTGCAGCAGACAGTGGACGCGAGGCACAGCGACTACCAGTCACCCACCTGAG
CCTCCTGCCACAGCCTTCATCTCCGTCTTGGGCGTCTGCCCCCTGTACCTGGATGAGAT
CCGGCACTTCCTGACCCTATGTCC
>1399
GGGTTGGTGGTTGGGGGGGAGCACGTGTCAGCAGAAGTGACCTGGGATCTCATCCCTT
GCTCGCTCCCAGGAGTCACTGACGCTGCACAGGTCTGGGGGCCACATAGCCCACCTGC
ATGTGCTGGCCGTGCACCGCGCCT
>1400
TGTGGCCGGAAAGGGACTGAGGCTGGGTGAGTTGCGCCGTTTTCCTAACAGTTTTCCC
ATCCTGTCGCAGACAAAGAAAAGAAGGAACAATGGTCGTGCCAAAAAGGGCCGCGG
CCACGTGCAGCCTATTCGCTGCACTA
>1401
TCTTCATTCATGTAACAAAGAAGCAAGGTAGGTATATAATACCAGTATAACTCTATTTTC
TATTCCTTAGCCTATGTGCTTCCCAAGCTGTATGTGAAGCTACATTACTGTGTGAGTTGT
GCAATTCACAGCAAAGTAGT
>1402
AAGTTCTTTGGGGGAAGGGAGTCTTGGATCCATGGGTTTTAATTTACTCTTTTGTTTCTT
TGTCTTTCAGGGTGCTGCCCCACGTCCCCCACCAAAGCCCATGTAAGGAGCTGAGTTC
TTAAAGACTGAAGACAGGCTAT
>1403
CATAAGGCAAATCTCAAAAGTTGTTCATTATGCTTTCACATAACCTTGCACTTCTCTCTC
ATAATTTCAGGATCCAGTGCAGATATTGTGATGACCCAGACTCCACTCTCTCTGTCCGT
CACCCCTGGACAGCCGGCCTC
>1404
CATAAGGCAAATCTCAAAAGTTGTTCATTATGCTTTCACATAACCTTGCACTTCTCTCTC
ATAATTTCAGGATCCAGTGCGGATATTGTGATGACCCAGACTCCACTCTCTCTGTCCGT
CACCCCTGGACAGCCGGCCTC
>1405

TCCGAGGGGCTGGAAGAAAAACAGAGCCTGTCTGCGGTGGAGTCTCATTATATTCAAA
TATTCCTTTTAGGAGCCATTCCGTAGTGCCATCCCGAGCAACGCACTGCTGCAGCTTCC
CTGAGCCTTTCCAGCAAGTTTGT
>1406
CATCTCTATTTCTCCCTATTTATTGGTGTTTGTTTATTCTTTAACCTTCCACTCCCCTCCCC
CTCCCCAGAGACACCATGATTCCTGGTAACCGAATGCTGATGGTCGTTTTATTATGCCA
AGTCCTGCTAGGAGGCGCG
>1407
CTTGACTCTTCCATCTTTGAAAAAAGTTCATATGCTTTTTCTTTTCCCCTTCCTTCCTAA
CTGTGCCTAGAACATCTGGAGAACATCCCAGGGACCAGTGAAAACTCTGCTTTTCGTT
TCCTCTTTAACCTCAGCAGCAT
>1408
ATAGAGTAAACTTGAGAAGAATGTTTGCACAGCCAGGTTAAGTGTTACAAATCCTTCT
GCCCTCACTTAGGCATCTATATCATAAATCTCAAGAGGACCTGGGAGAAGCTTCTGCTG
GCAGCTCGTGCAATTGTTGCCAT
>1409
AAGTGCTTACTGGGTGCCAGGATTGTTGCTGTGGAATATCGAGTACCACTAACTTTTAA
ATTCTTCAAAGAGGGCTGTGCTGAAGTTTGCTGCTGCCACTGGAGCCACTCCAATTGC
TGGCCGCTTCACTCCTGGAACCT
>1410
GAGCCAGGAAGGTGCTTGCTGTTTGGGTTTGACCAAGTGTCACTTTTTAATAATCTGCC
ACTCTTGGCAGGGAGCTCACTCAGTGGGTTTAATGTGGTGGATGCTGGCTCGGGAAGT
TCTGCGCATGCGTGGCACCATTT
>1411
GGACTGCTGTCTAGGAAGCAAAACTTGTCAGTCCCTGTAAGTCTCTTCCTCTTTTTTTT
TTGTAACCCAGATTGAAAAAGAAGAGCAGGCTGCTGCTGAGAAGGCAGTGACCAAGG
AGGAATTTCAGGGTGAATGGACTG
>1412
GGAGTGGGGTAAGGAAAAATACTACATTGAGGACAGAGCTGATGGGCTTTTTTTGGTA
TTCTCTTAACAGAAGACTGGAGCGCTCAGCCTGCCACGGAAGACTGGTCTGCAGCTCC
CACTGCTCAGGCCACTGAATGGGT
>1413
TATTTCTCTTCTTGATACCACTGTGGGGGACATGGCTCTGCCTCTCTGACTTCTGCTTCC
TTCCCTGCAGACCTATGCCAGATTTACTGGTGCTCCACTGAAGGTACACAAGATCAGCA
ACCCCTGGCAGAGCCCTTCAG
>1414
GGAATGTGTTGCAACTATGTATGACTAGGCAGCTAAGGGTCTGGTTGGTATACTTCCCT
CTCAATATCAGGAACTCTGCCTGCCCTTCGGACCAGTCATGGAGAGGTCATCTCAGTTC
CACACAAGATCATCACCCACCT
>1415
GACTCCTTCCTCCCTGGTGAAGTGCCCTTGCAGCTCCCTAGTGTCCACGCCATGGATAT
TTCCTCCACAGAAGTACAATGCTGATTATGATCTGTCAGCTCGGCAAGGGGCAGACAC
CCTGGCCTTCATGTCTCTCCTGG
>1416
GAAGCCCACCTTGAATCAGACAGGTGCACTGGCTCAGACCTACCTGTTTCTTCCTGCC
CACCCAATCCAGGTACATACTTTTTGGATAGACACCAAGAACTACGTGGAAGTGACCC

GGAAGTGGTATGCAGAGGCTATGC
>1417
AATGGGAGTGCTTAGGTGGGGAGGTGGCACTGTTCCCACAGCTGCAAGCCTACCTGTG
TCGCCCCTACAGCTGTACCGAGAGGCTCGGGAGTGTCTGACCCTGCTCTCTCAGCGCC
TGGGCTCTCAAAAGTTCTTCTTTG
>1418
CTCCAGCTCCATCTTTACCCCTTCTACCCCAGTGGTTCTCCTCCATCCCACCCTTCTCTC
TCTGCTCCAGCCCTGCCTCCTTGGACGCCTTCGTCTTCAGCTACTTGGCCCTGCTGCTG
CAGGCAAAGCTGCCCAGTGGG
>1419
AGCCCTGGGGAGAGTGGGCAGGGATCCAAGAACTAGTTCTCCTAACACACCTTCCTTC
CTTGACCCTCAGCTGAGGTACCACCGCAACGCCAGACACCAGCAGGCCCAGAGACTG
AGGAGGAGCCATACCGGCGCCGGAA
>1420
TGGGGTTTAAATACTGGAGATGGAGGCAGACCTGGGGCCAGATGTTCTCTGTGCCCCT
CTTCACCCTCAGGTTGGAGAGCAGTGACGGGGATGGGGCCTGGTGCCCCGCAGGGTC
GGTGTTTCCCAAGGAGGAGGAGTAC
>1421
CCAGGCCTGATGAAGGGATGGGGGATGGAGTCTTAGTGCCTCTGACCCCCACCCTCTT
ATTTTTTCCCAGATGGACTCCTGTCTTACACCGCCCCTGTGGGGCAGACAATGTATTTAT
CTGAGGCCGTGTACCTCAACGA
>1422
ATGCAGGGTGCCGTTGGGGTGCCCCCACCACTCCTAGCCTTGACCCTGTGCCCTCTTCC
CTTCCCCCCAGGTCCACTGTAACAACATGCACACGCTGGGAGCCCGTCTGCCTGGCGG
GGTGGAATGTCGCTTCCGGCGTG
>1423
CACTGCCTCTGCCTCCCTTGGGTCTCCTCCTCATTTACCTCCCTCCTTTCTTTTTGTTCCT
TCTCCCCAGATGTGGTGAACAATTCCTCTCCGGCACTGGGAGGCACCTTCCCGCCAGC
CCCCTGGTGGCCGCCTGGCCC
>1424
CTTCTCCCTGGGCCTCCCCCTCGGCTAGGGTGGGACCCTCCTGTGGTGCTGACCCTGCT
GCCTCCACCAGAGCTGGAGCCCAGAGGCCAGCAGCCCGTGGCCAAGGCCGAGGGGA
GCCCGACCGCCATCCTCATCGGCTG
>1425
ATGGACACAGCAGAGGGCCAGGCCGTGTGTGCTGAGCAACACGGGTGATGCCTCCCA
TCCCTATGACAAGGCTGAACGGAGGGTGTTGGAAGAGGAGCTGACGGTTCACCTCTCT
GTCCCTGGGGACACTATCCTCATCA
>1426
AGGTCTCCCTGTCTGTCTAGCCTTGAGTCTCATCCCTTCCCCGTGTTTCCCCTCCTCCTT
CTCCCGACAGCGTTGCTGCTCTCCAATCCAGCCTACCGCCTCCTTCTGGCCACTTACGC
CCGTCCCCCTCGAGGCCCGGG
>1427
GTGTGGGGAACTATAGCTCTTGGGCTGTTCCTGATGCCTCGTCCTGTCTTCTTTCCCCTC
ACCCCTGCAGCCTACAGTGGGGACTATATGGAGCCTGAGAAGCCAGGCGCCCCGCTTC
TGCCCCCACCTCCCCAGAACAG
>1428

CTTAGTCATTTGTAACCGTGTTAATCCGTTTGACCCTGTGACCGCCTAGCAAACGAACT
TCTTTCTCCAGGTGCACCTGTGTGAGGTCGACAGCCCTCAAGATCTGGTTAGTCTTGAT
TTCCCCCTTAATGTGCGTAAGG
>1429
CTCTCTCTCCTCTCCTGGATGGGAATCTGCGAAGCTGCCCCCAGTGACCTTCTGTCGGT
TCCCTTCTCAGCTACCCAATGCTGCTGCATGTGGCAGCCCAGATCGCCTCCGGCATGCG
CTATCTGGCCACACTCAACTTT
>1430
AGGAAGGGAGGGGGGTCAACCGTTGCCTGATGTCCCCTGTCTGTTTTTGCTGCCTTCC
TCTGCATCCCAGGGGAAGTTCACGACTGCGAGTGACGTGTGGGCCTTTGGTGTGACCG
TGTGGGAGGTGCTGATGCTCTGTA
>1431
TTGGTGGGGAGGTCATGGACACAAATCAGAAAATACAAGAATGGGCCTCCCCATTTCC
TCCTCTGACTAGGATGGGGACCCAGACACGCCAAAGCCTGTGAGCTTCACAGTGAAG
GAGACAGTGTGCCCCAGGACGACAC
>1432
TCCCACTGGGATGTGGCTGGGAGGTCATGGCAAATGGTTTCAAGTTTGACCTTGAGCT
TCTCCTTTCCAGCTGGTGAAGCGGTGTATGGGGACAGTGACCCTCAACCAGGCCAGGG
GCTCCTTTGACATCAGTTGTGATA
>1433
GGAGGGGTCTTGGATTGACCCTGGGTACATCCCCGACAAGGAACCTGTTTCTTCCTGT
ACACAACCCCAGGATAACAAGAGATTTGCCCTGCTGGGTGATTTCTTCCGGAAATCTA
AAGAGAAGATTGGCAAAGAGTTTA
>1434
TTCCCGCCCGCCTCCGCTCTGCCTCCTGGCCAGCCCTCACCTCTCCGTCTCTCCGTGCC
TTCCCCTCCAGGCCCCTTTGGCCACTGCCGCACCCCCCTGCAGTCCCCTCCTGAAGGC
GCCCTCCCCGGCTGGCCCCTTAC
>1435
CTGTCTAGGGGGGGAGTGCACCCTCCTTAGGCAGTGGGGTCTGTGCTGACCGCCTGCTG
ACTGCCTTGCAGGTGAAATTGCCCTGTGGTCCTTGGTGGTCCTGGCCATCGAGCGGTA
CGTGGTGGTGTGTAAGCCCATGAG
>1436
GCGCTCGGCAGCCACCTTGGCTGTTCCCAAGTCCCTCACAGGCAGGGTCTCCCTACCT
GCCTGTCCTCAGGTACATCCCCGAGGGCCTGCAGTGCTCGTGTGGAATCGACTACTAC
ACGCTCAAGCCGGAGGTCAACAAC
>1437
CCCAGCATGCATCTGCGGCTCCTGCTCCCTGGAGGAGCCATGGTCTGGACCCGGGTCC
CGTGTCCTGCAGGCCGCTGCCCAGCAGCAGGAGTCAGCCACCACACAGAAGGCAGAG
AAGGAGGTCACCCGCATGGTCATCA
>1438
CTCACTAACGTGCCAGTTCCAAGCACACTGTGGGCAGCCCTGGCCCTGACTCAAGCCT
CTTGCCTTCCAGTTCCGGAACTGCATGCTCACCACCATCTGCTGCGGCAAGAACCCAC
TGGGTGACGATGAGGCCTCTGCTA
>1439
GTGACTTTTTCTTGACCATAAATGGAAACTCTGCAACTGATCTTTGTATACCCCATGTGA
ACTGTTGCAGCACCATCATTGCATTTGCTCAGTATGTTCAGGAAGCAACCTTTGAAGAA

ATGGAAAAGCTGGTGAAAGAC
>1440
TTTTCTCAGTGAGTACAGAAGCTTTACTTTACCATGACTAAAAATTGTCCTTTTCTTCTC
TGTTGTATAGAATAATGTTTTACAGGAAAAAATATGTGCTATGGAGGGGCTGCCACAAA
AGCATAATTTCTCACACTGCT
>1441
CTTGTCTACATTCATTGCTTCCTGTTTCTTCCCTCACCCGTCTTCTCTCTTTCATTTTTATT
TTTTATAGCTTTTTATATGAAGTTGCCAGAAGGAACCCATTTGTCTTCGCCCCTACACTT
CTAACTGTTGCTGTTCAT
>1442
TATGACTAAAAAATAAGAGAATAGTGAGATTTTGCTATTGTTTCAAAAGAATTTTCTCTT
TCTTCTTCAGGCAATACCTGTCACACAATATTTAAAAGCATTTTCTTCTTATCAAAAACA
TGTCTGTGGGGCACTTTTGA
>1443
TAGCAAATTACTCCCACTTGTTCTACTTGCTGTTCAAATTATTTTTGTGGTTTATCAATTC
TCTTTCCAGATATATTGCGATACTCAGTCAAAAATTCCCCAAGATTGAATTTAAGGAGCT
TATTTCTCTTGTAGAAGAT
>1444
TGCCATCATTCCATAATGGAAGAAAACCCAGCCTGAAAGTAAAATAATCTCTCATTCTT
ATTTGGTACAGAGCAAGGTTATGAACCATATTTGTTCAAAACAAGATTCTATCTCCAGC
AAAATCAAAGAGTGCTGTGAAA
>1445
TACAAAATAGTGGAATTGGGTTTTTTGCTCTTTCTCTAATTTCTAATATACAAAATTTTAC
ACATTGCAGGTTTACTTTTGAATACTCAAGGAGACATCCAGACCTGTCTATACCAGAGC
TTTTAAGAATTGTTCAAATA
>1446
CACATTGATGTAAAGCTTATGGTTAAATTTTAGATAAAATGTTTGTAACCATGATTATTCT
TATTTTCAGGAAGACAAATTCAATGAGACAACTGAGAAAAGCCTCAAGATGGTACAAC
AAGAATGTAAACATTTCCAGA
>1447
TTCAGCAGTAATTCAAGGGAAAATGGGTATCTGCTTTCATTCAGAACCAAACAGACAA
ATGTTCTTTCAGTTACCTCATCAGGCTCACGAAGATAGCTCCCCAACTCTCCACTGAAG
AACTGGTGTCTCTTGGCGAGAAA
>1448
CTAACCATATTTTTAGAAATTCCATTCAAGTTTTAAGATCGTATCTCAGTTGCAACTCTT
GTTGGTACAGGCAGATTTAGTTTTTGGAGAGTTATGTGGAGTAAATGAAAATCGAACTA
TCAACCCTGCTGTGGACCACT
>1449
AGCATTTTGGAGTAGAGACGCTTCAGAAAGAAAGCGTTAATTAATTTTATTTGACATCT
TTTGGCCACAGGTTTCTTGTCAACTTAGTGAAGCTGAAGCATGAACTCACAGATGAAG
AGCTGCAGTCTTTGTTTACAAAT
>1450
GACTTTCTATCACCCAACTTTCAACATTTCATGTTTTTTTGATGCATGAAATACTATCTTC
TCTTTCCAGGGAAGGCTTCCTATCTGTGTGGTGATGAATCGCATTTCCTGAGAACAAAA
TAAAAGGATTTTTCTGTAAC
>1451

TGCAGATTTAAGGTATTTCCACATTTGGGTCTATAATAATAATATTCTGTATAATTTATTTT
TTTTGCAGTGCCATGAGAATGATGAAAGACCATTCTATCAGAAAACAGCTCCATATGTC
CCAATGTATTATGTGCCAA
>1452
AATAAATATATATCATGAGAACTGTTAACATACTATGAATAAATTTCTAAACTATCATTATT
TTTTATAGGACTTGCTGAAACCAAATTACTACTTCACACTCTCCTTCAGCCATTTGTCTG
CCTTCAGTCAACAGAAAA
>1453
CGCGAGACTCCCCTTGTCGTCCAATAACCTGCCAGCGCCTGGCTGGTCGGCATCCCAC
TTTTCTCCGCAGATCTCCCTAGAGCACGAAATCCTGCTGCACCCGCGCTACTTCGGCCC
CAACTTGCTCAACACGGTGAAGC
>1454
ACTTTTATTGCAGATGTCTTTAGAGGCTCTTGGACACACAGTGTCTTCCTGTTTCTCATC
CTCCTTGCAGGTATGGCTTTGTAATTGCTGTCACCACCATTGACAATATTGGTGCTGGTG
TGATCCAGCCAGGCCGAGGC
>1455
AGTGAAGTGATTGTGATGTAACTTCTCATAAACTGAGGAGCATCTGTACACTGTTCCCT
CCTCTCCTCAGGATATTGTGATTCAGCAGGACGATGAGATCCGCTTAAAGATTGTGGGG
ACCCGTGTGGACAAGAATGACA
>1456
GATGAGGAGTACATGGTTGTGGCTACCCTAAATTCCGGTTCTAACTGATATGCTTTTTCT
GGTTTCGCAGGGCTTGTAAGCTGAGCCTGGTGGCCTCCTACCCTTGGTCCTACTCTAGG
AAGTGTGATTGTCACACTTAT
>1457
GGCAGTCCCCACCTTGGCCAGCGAGGTCGTGCCCTCAGCCTGCTCACCCCCCATCTCC
CTCCCTCTCCAGGCACCACCTGGGTGAGCCAGATTCTGGACATGATCTACCAGGGTGG
TGACCTGGAGAAGTGTCACCGAGC
>1458
GTGCAAGGGGAGTGGAGGAAGACAGGGCTGGGGCTTCAGCTCACCAGACCTTCCCTG
ACCCACTGCTCAGGGATGGAGACTCTGAAAGACACACCGGCCCCACGACTCCTGAAG
ACACACCTGCCCCTGGCTCTGCTCCC
>1459
CCAAGAGATGAGCTGGCCTGGGGCAGGCTGTGTGGTGATGGTGCTGGGGTTGAGTCTT
CTGCCCCTGCAGGTGGTCTATGTTGCCCGCAACGCAAAGGATGTGGCAGTTTCCTACTA
CCACTTCTACCACATGGCCAAGG
>1460
TGTGGAGCCGAGGGGTGGTGGCTACAACGCACAGCAACCCTGTGTTGGCACCCCTTG
CCTGCTTCTCCAGTGTCCTACGGATCCTGGTACCAGCACGTGCAGGAGTGGTGGGAGC
TGAGCCGCACCCACCCTGTTCTCTA
>1461
GAAGGGGACCCCTTTTACTTTTCCTGAATCAGCAATCCGAGCCTCCACTGAGGAGCCC
TCTGCTGCTCAGAACCCCAAAAGGGAGATTCAAAAGATCCTGGAGTTTGTGGGGCACT
CCCTGCCAGAGGAGACCGTGGACT
>1462
CCTCCCCATAGGCACTCGGGGCCTCCCCTGGGATGAGACTCCAGCCTTGCTCCCTGCCT
TCCCCCCCCAGGCATGGCTGGGGACTGGAAGACCACCTTCACCGTGGCGCAGAATGA

GCGCTTCGATGCGGACTATGCGGA
>1463
TCTGTTTGCCACTCTGCCAGCCAGGCTGGTGGGGCTCTGTCTAGCCCATTATTCTCACA
TTTCACCACAGGTCTGGTTACAGGATGGTCACTGGCCTTCCTGGCCTTTGAGCGCTACA
TTGTCATCTGTAAGCCCTTCGG
>1464
AATCCAGGCATCTCAGCTCCCACCGCACTCTTGTGGAGAGTCCAGTGAGCAAGTGTTT
GGTCCTTTGCAGGTTCATCCCTGAGGGCCTGCAGTGTTCCTGTGGCCCTGACTGGTAC
ACCGTGGGCACCAAATACCGCAGC
>1465
AAAAAAAAAAAAAAAAGCATCCAGAGGGACAGGAAAAAGAGAGATGTGATGCTTTCC
GTGCTCCACCCCAGGTTGCAGCTCAGCAGCAGGAGTCAGCTACGACCCAGAAGGCTG
AACGGGAGGTGAGCCGCATGGTGGTTG
>1466
GAGAATAAGGTCTTTTTTCCCATACTTCCTCATCTGCCAAGGTTATCTCCAATTGTACCT
TTCTCTCCAGTTCCAAGCTTGCATCATGAAGATGGTGTGTGGGAAGGCCATGACAGAT
GAATCCGACACATGCAGCTCCC
>1467
TTCATTGAGTTTCTTGTACACTGCTTCTCTACCCCAGCTCATATACTCATAACCTTCCCCC
TACCCTCAGGTGTGGGTTTTGCTACCAGGCAGGTGGCCAGCATGACCAAGCCTACCAC
AATCATCGAAAAGAATGGGGA
>1468
GTCCAGACCCCACCCTGCCCCTCAATTCCTGACTAAGATCACAGCTCAGGCCTCTACCC
TCTTTCCACAGTCCATTGTGACACTGGATGGAGGGAAACTTGTTCACCTGCAGAAATG
GGACGGGCAAGAGACCACACTTG
>1469
AGGGAGGGTAGAATGCTCGCAATGGTGTTCCTGGCTCCCACCCCCCATCTCACTCTGTC
TTTCCTTCCAGACACTCACCCACGGCACTGCAGTTTGCACTCGCACTTACGAGAAAGA
GGCATGACCTGACTGCACTGTTG
>1470
GATCCCTCCCACTCCCATCTCTGGGGCTTCGGGTGTCCAGACCTGACTCCCGCTCCCCC
TCCTCCCCCAGCCTACTTCTCCCTGCAGGTGATCTCGGCGCGCAGGGCCTTCCGCGTGT
CGCCGCCGCTCACCACCGGCCC
>1471
GCGAGCCCCAGGCGGGTCCGGGTCGCAGGACCATCCCGGCCGGCGCGCTCATCCCAC
CCGCCCACCGCAGGGTGAACTGCAGCGAGTACTTCCCGCTGTTCCTCGCCACGCTCTG
GGTCGCCGGCATCTTCTTTCATGAA
>1472
CAGGGGCGCACGCGCTGGACCCCCGGGACCCGCGCAGGGCGCTCACCAGGCCCGTGC
GTACCTCTCGCAGGGGCGGCGGCCCTGTGCGGCCTGGTCTACCTGTTCGCGCGCCTCC
GCTACTTCCAGGGCTACGCGCGCTC
>1473
CCTGTGTGTTAAACATCAATGTGAAGTTATTTCCAGGAAGAAGTTTCACCTGTGATTTC
CTCTTCCCCAGAGCCCCACAGTCTTCGTTACAACCTCATGGTGCTGTCCCAGGATGGAT
CTGTGCAGTCAGGGTTTCTCGC
>1474

GCTGAGTTCCTCACTTGGGTGGAAAGGTGATGGGTTCGGGAATGGAGAAGTCACTGCT
GGGTGGGGGCAGGCTTGCATTCCCTCCAGGAGATTAGGGTCTGTGAGATCCATGAAGA
CAGCAGCACCAGGGGCTCCCGGCA
>1475
AGCAGCCCTGTTCCCTGCATCTCCCTTAGAGGGGAGCAGGGCTTCACTGGCTCTGCCC
TTTCTTCTCCAGTGCCCCCCATGGTGAATGTCACCTGCAGCGAGGTCTCAGAGGGCAA
CATCACCGTGACATGCAGGGCTTC
>1476
AGGCCAGGGTAGGAACAGCAGGGACGGCTGTGGCTCTCTGCCCAGTGTATAACAAGT
CCCTTTTTTTCAGGGAAGGCGCTGGTGCTTCAGAGTCAACGGACAGACTTTCCATATGT
TTCTGCTGCTATGCCATGTTTTGT
>1477
AGAAAAGTCCTTAGGGAATAAACACAACACTGCACCCAGTGGAGCATTTACCCGTTTC
CCTCTTCTCCAGAGCTTGTGAGCCTGCAGGTCCTGGATCAACACCCAGTTGGGACAGG
AGACCACAGGGATGCAGCACAGCT
>1478
GTGGGGGTGCATGGTCTTGGGGAGGAGTGAGGCACCACCCCGGGCCCCCGTAACCAG
TGTGTCTCTCCAGAGCAGGACAGGCTGCTTTGGTTTGTGACCTCCAGGCAGGACGGCC
ATCCTCTCCAGAATGAAGATCTTCT
>1479
AATGTCCACTGGGGTCAGGCCTGGGAGGGACACTGGAGGCTTCCCTGAACAGGTGTG
TCCTCCTTCGCAGCCAGCTCGCTGATGTGCTTCTCCTGCTTGAACCAGAAGAGCAATCT
GTACTGCCTGAAGCCGACCATCTG
>1480
CTCAGCAGAGGCCATTGCTGTCTGTCTGCAGCCGTCTGTCTCTCCCCTGACAGCCTCAT
TTCCCATGCAGGGAATCTCGTGACATTTGGCCACAGCCTGAGCAAGACCTGTTCCCCG
GCCTGCCCCATCCCAGAAGGCGT
>1481
CCCTACCCTGCCCACCTGTTCACAGGCCCGCCGCCCCAGGACCGCCGCGCTCACCCCG
CTCCGTCCGCAGCTCGTACCTGGCGTCTGCTGACCTTCGTGCTGGCGCTGCCCAGCGT
GGCCCTCTGCACCTTCAACTCCTA
>1482
GGCGCGCGGGGCGGGGCGCGGACTCCGGACTCACGGCTCACGCGAGCTCCCTCCCCC
GCTCTCTCCACAGCCCTACCCCTGGGGGGACGGCAACCACACTCTGTTCCACAATAGC
CACGTGAACCCTCTGCCCACGGGCT
>1483
TATAATTTGGGTTCACTGTGGCTACTTGAACACTACACTGCAGCTAACTCTATCTTTGTT
TCCTTTCCAGGAATCAATGAATTTCAGCGATGTTTTCGACTCCAGTGAAGATTATTTTGT
GTCAGTCAATACTTCATATT
>1484
GATGCTGGCACTCCTGAAGGGCACAGATGGGGTGACTCAGGAGGGACCTGATGGGAC
CATCCCCCTGTAGGCATCCTGCCTGTGTACCACTCCCTCTTTGCTCTGGCCACGGAGGA
CCTGTCCTGCTGGTTCCCCCCGAG
>1485
CCACCATAATGTCACTCCTACTGAGGGTGGGTTGCACTTTTCATCCCAGGGTTCTCTCC
TCTCCTCACAGCTCTTTTTACTTCCCCAATTGGTTTGGGCTGGAGAAGTGCCACCGCTT

CGGGCTACGCAAGGATTTGGCC
>1486
GGTCTGCAGGTTAACAACAGGGCTTGAAGTTGGGTGGCCTCAGCTGATGCTCCCTGTG
GCGGCCCCCCAGCACCGCAGTGACCTGGTGAGTGGGCGCCTCCCCGTGGGCCTCAGT
CTCAAGGAGCAGGGTGAGTGTCTCA
>1487
TTTGTGTGTGTACCCCGCGGGGACCCCTCCCGACGCTGAGGACCGGGTCCCTCCCCTC
CAACCCCTGCAGCTACAAGGCCTGCCTACCCCCAAGCCTGCGCGACCTGATCCAGGGC
CTGAGCTTCGTGACGCGGAGGGCT
>1488
TCAGCCTTACCCCGAAGGGCGGGACCGGCACCCTCGGGTTTCACTGGGCTCTGACGCT
TGTCCCTCGCAGAGGCCGAGTTCCCAGGGCTGCCCGAGGCTCTGTCGTTCGTGGCGCT
CGTGGACGGCTACTTCCGGCTGAC
>1489
AAAAGGAAGAAGGACTGAGAAGGAGAGTGTCTGTCGCTCAGTCCCACTCAGGGGCC
ACTCTTCTTTGCAGAACCCCCTTGGTCCTGATTATAAGGGCTGCCTCATCCGGCGCAGC
CCCACAGGAACCTTCCTTCTGGTTG
>1490
TGAGTTAGCTAACCCTTGGGGACTTTTCACCTCTGATTTCTGGTTTTTCTCCCTCATCCT
CTCCCCATAGAAAAGTCCAACCTGATCGTGGTCCAGAGAGGTCACAGCCCACCCACAT
CATCCTTGGTTCAGCCCCAATC
>1491
TGTCTCGCTTGGGCTGGGATCCTGCAGGAACCCCCTCACTGGCCTCTTCTGCTGTCCCC
TCACCATTCAGCATGAGAACCTGGGCCATGGGTCCTTCACCAAGATTTACCGGGGCTG
TCGCCATGAGGTGGTGGATGGGG
>1492
GAGCACAAAATTTGGGATTATTGGAGTGGAAGAAACCCACGCATCTTCTCTCCCTTCCC
ACCTTCCCCAGTCATTCCTGGAAGCAGCGAGCTTGATGAGCCAAGTGTCGTACCGGCA
TCTCGTGCTGCTCCACGGCGTGT
>1493
GATGCCCCCAGATATATTTCGGGAGTGCGCAAAGAACTAAACTTGGAATACTGAGCAA
ATATGGGCTGAGCACCATGGTGCAGGAATTTGTACACCTGGGGGCCATAGACATGTATC
TGCGAAAACGTGGCCACCTGGTG
>1494
AGGAGTTTGCCAAACAGACTCTTCATTTTATCAAACCTCCCGGGCATTTTCGTGTGTTT
GGCCCCCTTAGGAGGACAAAGGCCTGCCCCATGGCAATGTCTCTGCCCGGAAGGTGCT
CCTGGCTCGGGAGGGGGCTGATG
>1495
TGGAGGTGGAGGAGGGAGGGGCTGAACAGGGCAAGGAAGTGGATCCCTGATCCCACT
TTCATTCCCTCAGATGCTCACCGACAGGATCCCCTGGGTGGCCCCCGAGTGTCTCCGG
GAGGCGCAGACACTTAGCTTGGAAG
>1496
ACATCCAGTCTGGGGAGATTGGGGTGGGTCTATTGGGTTGGGGATTACCGACTGCTCCT
CTCACCCTCAGAAACTCCAATTTTATGAGGACCGGCAGCAGCTGCCGGCCCCCAAGTG
GACAGAGCTGGCCCTGCTGATTC
>1497

CAGGGCCAGGGTGGGTTGGAGAGGCAGGGCAGCATCCAGGTACCTGGACATCAGTGC
CGCTATCCTCGAGGGCAACTTTGGCAGCGTGGAGCTGTGCCGCTATGACCCGCTAGGC
GACAATACAGGTGCCCTGGTGGCCG
>1498
GGTCACGCTTGGGGGTACCTGCCGGATTATCCTGGGATCCTCTCTTCAAGATCACAACG
ACCCCCCCCAGGGCATGGAGTACCTGGGCTCCCGCCGCTGCGTGCACCGCGACCTGGC
CGCCCGAAACATCCTCGTGGAGA
>1499
AGAGAGCGGGGTCCCACTTCAGGACACCCCTCCAGGTGCCTCCAATACCCGCCCACCT
CCCACCCCGCAGGTGTCCCAGGCGCTGATCCGCTCCTTCAGCTCCACCGCCCGGAACC
GCTTTCAGAACCGAGTGCGCGAGA
>1500
GCCGGGGCAGGCGGGGCGCGCTCTAAGGAGCAGCCAGCACCCCTTTCTCATCAGACA
CCCCCACATCCAGGAGGACAATGACATCCCGTTGTACCTGAAGGGCGGCATCGTTGAC
AACATCCTGTACCGAGTGACAATGA
>1501
CTGTCAAAAGCCTGGGATGTCCAGGGAGGGGATTAGGTCTCATTCTGTCTCCAACGCT
TCCCCTTTCTAGGCACTGTCTACAGCTTGTACTCCCTTGGCTGGGCCTCCTTCCCCAGG
AATTAAGACCAAGAAGCCTGGGG
>1502
GGGGTGGGAGAGCGGCTGGGGCGCCTCCGTGACTCAGCCCCGGCCTGTCTGGCCCCT
CCCGTCTCCGCAGTTCTGGGAGGTGATCTCTGATGAACATGCCATCGACTCCGCTGGC
ACCTACCACGGGGACAGCCACCTGC
>1503
CTGCATTATGGTGGTGACCACTGATGACCGTATACCTGGCCGTCGAGTGACCGGCTGTA
CTGTCTTACAGGTCAGTGTGGGGCCGGAAACAACTGGGCCAAGGGACGCTACACCGA
AGGCGCGGAGCTGATGGAGTCAGT
>1504
TGAGGCCTTGGAGGGTGGCCTGGGGACCTTGGACACAACCAGCCTGCCCCTGACCCA
TCCCTCCCTGCAGCTTGCTCCTGCAACGGCCATGCCCGCCGCTGCCGCTTCAACATGG
AGCTGTACCGACTGTCCGGCCGCCG
>1505
GCCCTCACCCTCTGACTTCCCAGATCCCCAGACAGGCTTCTGACCAGGCCCTTCCCAC
CTCTGTCCTCAGCCTGCGACTGTCACCCGGTTGGTGCTGCTGGCAAGACCTGCAACCA
GACCACAGGCCAGTGTCCCTGCAA
>1506
TGGGGGAGGGGGCTCTGGGAGGAGAGGGTGGGGAAAGGGAGTCTGTGCCAGCCTCC
CACCTTCTACCCAGACTGTGACTCGCACTGCAAACCTGCCCGTGGCAGCTACCGCATC
AGCCTAAAGAAGTTCTGCAAGAAGGA
>1507
TCCCCGTGCCTCCCCCTACCGCGGGCAGGCCGCCCCTTCCTGACCCCGCCCCCTCTCG
CTCTCCCCGCAGCGGTGCAGGTGGCGGTGGGTGCGCGCGGCGAGGCGCGCGGCGCGT
GGACACGCTTCCCGGTGGCGGTGCT
>1508
CACAGTGAACATTTAGAAGCAAACTTTTGAAGGAGTATTTTCAGCTGCCATTTATGCTT
CTTACAAATAGAGTTTGAAACAGCTGAGACACTTCTAAATTCAGAAGTTCATATGCTTC

TGGAACATCGAAAGCAGCAGAA
>1509
GATTACTCTTTGATTTTTGTTGTTTATTTGTTTTTGATTATTTTGATTTTTTTTTTTTCCTTT
TGATTAGCTTGCTACTCCAGAAAAAGCTTCATAAGTTTGAGTTGGCCTGTTTGGCCAAC
CTTTGCCCAGAGACTGCT
>1510
CACGCCTCAAAGAAGAGTGCGGGGAAATTGCCAGTCGCCAAATCACTCATTTCTTTTC
ATTTTCTTGTAGCTTGGAGGGACGGTTTGAAGATGAGGAGCTGCAGCAGATTCTTGAT
GATATCCAGACAAAGCGCAGCTTT
>1511
CTTCTCTGCAGGCCCAGGTGACCCAGGGTTGGAAGTGTCTCATGCTGGATCCCCACTT
TTCCTCTTGCAGCAGCCAGACTGCCTTCCGGGTCACTGCCATGGAGGAGCCGCAGTCA
GATCCTAGCGTCGAGCCCCCTCTG
>1512
GACCTGGAGGGCTGGGGGGCTGGGGGGCTGAGGACCTGGTCCTCTGACTGCTCTTTT
CACCCATCTACAGTCCCCCTTGCCGTCCCAAGCAATGGATGATTTGATGCTGTCCCCGG
ACGATATTGAACAATGGTTCACTG
>1513
CGTGTTCCAGTTGCTTTATCTGTTCACTTGTGCCCTGACTTTCAACTCTGTCTCCTTCCT
CTTCCTACAGTACTCCCCTGCCCTCAACAAGATGTTTTGCCAACTGGCCAAGACCTGCC
CTGTGCAGCTGTGGGTTGATT
>1514
GGGCTGGAGAGACGACAGGGCTGGTTGCCCAGGGTCCCCAGGCCTCTGATTCCTCAC
TGATTGCTCTTAGGTCTGGCCCCTCCTCAGCATCTTATCCGAGTGGAAGGAAATTTGCG
TGTGGAGTATTTGGATGACAGAAA
>1515
GGCCTCCCCTGCTTGCCACAGGTCTCCCCAAGGCGCACTGGCCTCATCTTGGGCCTGT
GTTATCTCCTAGGTTGGCTCTGACTGTACCACCATCCACTACAACTACATGTGTAACAG
TTCCTGCATGGGCGGCATGAACC
>1516
TTTTTTAAATGGGACAGGTAGGACCTGATTTCCTTACTGCCTCTTGCTTCTCTTTTCCTA
TCCTGAGTAGTGGTAATCTACTGGGACGGAACAGCTTTGAGGTGCGTGTTTGTGCCTG
TCCTGGGAGAGACCGGCGCACA
>1517
GGTGGAGGAGACCAAGGGTGCAGTTATGCCTCAGATTCACTTTTATCACCTTTCCTTGC
CTCTTTCCTAGCACTGCCCAACAACACCAGCTCCTCTCCCCAGCCAAAGAAGAAACCA
CTGGATGGAGAATATTTCACCCT
>1518
GTAACTTGAACCATCTTTTAACTCAGGTACTGTGTATATACTTACTTCTCCCCCTCCTCT
GTTGCTGCAGATCCGTGGGCGTGAGCGCTTCGAGATGTTCCGAGAGCTGAATGAGGCC
TTGGAACTCAAGGATGCCCAGG
>1519
CAGGGAAAAGGGGCACAGACCCTCTCACTCATGTGATGTCATCTCTCCTCCCTGCTTCT
GTCTCCTACAGCCACCTGAAGTCCAAAAAGGGTCAGTCTACCTCCCGCCATAAAAAAC
TCATGTTCAAGACAGAAGGGCCT
>1520

CCCGGGGCGCGGCGCAGTGGGTGCCGAGCTTGGCCCCAGCCCCGCGCCTCACCGCGC
CCTCGCTCCGCAGGCGCTCGTCTGGCGTGGCCGCTGAGGCTGGGACCGGCGGTGCCC
TTGTCTCTGGGTGCGCCAGCCGGAGG
>1521
CTGTGGGACCCTCGGGAGCCGGTGGGACGCAGGACCTGGGGCTAGGGCTGAGCATTC
CCCCCATCCCCAGGTCTGGTTCAAGAACCGCCGGGCCAAATGGCGACACCAGAAGCG
CGCGTCGGCTTCCGCGAGGCTCCTGC
>1522
ATGACCACGTTGGAAATTCTGGAACTTGGAATCTGCTGTATTCTAACATTGGCAACTTT
TTTTAAAACAGCTGTTGTTGGCCTACTGTACCCCTGTATCGACAGTCACCTCGGAGAAC
CCCACAAATTTAAGAGAGAATG
>1523
CCCTTGTGGTTTTACGTTTTTTTATCTTAATAAGAGTCACCGAACTTTTCCTTAACTTTTA
TATCACCAGAAATTGGATTTTGCCAATAATGTCCAGCTGTCCTTGACTTTAGCAGCCCTA
TCTTTGGGCCTTTGGTGGA
>1524
GAGAATCTGTGATGTAGAATTGAGCCAGGTGAAATTGACTGCTAAGGTACAGTTTCTTT
TCTCGCTCCAGGTATACGTCCCCAGATTTCCTCTATATTCGTTCTTGGCTCCCTTGTATAT
TTTTCTCAGGAGGCGTCACG
>1525
TGGTAATTAAAATATTTATACATTTAGTGCTAATTTTCTCTTTCCTTTTTTTTTTCCTTTCT
ACACATAGGGTGTTCCTGAAAAGCCCCATAGTGATTGAGTCTTCAAAACCACCGATTCT
GAGAGCAAGGAAGATTTTG
>1526
TTCAGATTTTCTTATTCCCAAGGCCTTTAAACTGTTCACCTCACTGAAACCTTTGTGTTT
TTGTCCTTAGTGATATTGACCAGAGTTTCGACAAAGTAGCTGAACGTGTCTTAATGAGA
CTACAAGAGAAACTGAAAGGA
>1527
AGCATTTAATTCTAAGTGTATTTCCCATGACTTACAGTTTTCCTGTTTTTTTCCCCTTTTC
TCTATTTAGGTTACCTACATCTCATACTTGCTTTAATGTGCTTTTACTTCCGGAATACTCA
AGCAAAGAAAAACTTAAA
>1528
GCTTCTCAAAGTGCTGGGATTACAGGTGTGAGCCATCGCGCCCGGCCAATTGTTGCTTC
TTTTTCAACAGGAGTGTGGGCTGCTGCTCTCCTCTGAGGGCGAGTGTCAGGGCTGCTA
CCCGCTGGATGGGCACATCTTGT
>1529
AGGGCTCTGGCGGGGGCGGCCTCCAGGTTCTGCTCCCCAGCCCTGGTCCGACGGTGC
CGCTTCTCCCCAGCCCTACAGACGCTGCGCGGCTGGGACTCTGTGGACAGGGAGGTG
GCCGAGATCCGGCAGGAGGATGAGGC
>1530
GCCGGGCGTGGGTCCTGGCGCCGAGGCCGCCTCCTCCGGGCAGCCGCCCAGCCCCGC
GCTTGCCTTGCAGATCTACTACTACGCGGACCAGATCTACCTGAGCGCCGGCGTGCCG
GAGGAGCACGTGCAGTACGTGACGG
>1531
CCCCATTGGAGGGGGGCTGGAGGGAGGTGAAGCTGGGCCTCTCCCGCCTGAGCCCTGC
CACCCTCCTGTAGGTGTTCGTGGTGGAGCTCCTGGGTCGGAGGCTGCTGCTGCTGCTG

GGCTTCTCCATCTGCCTCATAGCCT
>1532
GGCCGCCCCCTGGGGTCCCCGTCCTACGCTTTGTCTGCTCCAGGTGAACCAACTGCTT
TCTCTCCGACAGGACACAGTGTCCTGGATGCCATACATCAGCATCGTCTGTGTCATCTC
CTACGTCATAGGACATGCCCTCG
>1533
TCTGCATGGCCAGGCCTGGGATGCTCGGGCCCGCTCTGCCTTCAGGGAGGCTGCTCTC
CTCCCCCTACAGGTCCCATACCCGCGCTGCTCATCACTGAGATCTTCCTGCAGTCCTCT
CGGCCATCTGCCTTCATGGTGGG
>1534
CCCCATGCAGTACAGTGCGAGCCCCTGGGCAAACCACGTCCCACAGTGTCACCTGCCC
CTCTTCCCGTAGGAGGGCCTCGGCCCGTACAGCTTCATTGTCTTCGCCGTGATCTGCCT
CCTCACCACCATCTACATCTTCT
>1535
GGGAGAGAAGGGAAAAGCCCTTGGAGGCCAGAAGAGAAGTCAGATCTTCCTCCTCTC
CCAAAACCTCCAGTGTATGCCACGTTGAGGGACCTGAAAACACAGGGCCGGCTGTGG
GAGGCGGCCCGGGCCCTGGCATGCCC
>1536
TGGAGCATGAGGGGACAGGCCGTGCTGAGGGTGATGCTGAGGCGGGCTGGTTGGGCC
TCTGTCCCCGCAGTGTGTAACACAGGCCTGGGCCTGCTGGGGCCGCTGGGGGAGGAC
GCCGTGGCCTCTGTACTGGATGTGAA
>1537
GAGGGGCACTGCCTGCCGGGGGATGACCCCCTGGCCGCTGCGCCTCAGGAACCTCAT
CTTCCCACCCAAGGGCTGCCTTTCAATGACGTTTATTGCGCCAGCAAGTTCGCGCTCGA
AGGCTTATGCGAGAGTCTGGCGGT
>1538
CTGGGGCAGGAGATGGGACTTGGGCCTGGGTCGCTCCGTCCCTGCCCACTTGCGGCTC
TCGGGCCAGCAGCGTGAGCCTGATCGAGTGCGGCCCTGTGGACACCGCCTTCATGCAG
AAGGTGTTGGGCGGTCCCGACCAG
>1539
TTTCCCTTTGCTGGATCTTGCCTGCCCTCTGAGTTTCCTCTTTTTTGATCTGACCCCTGC
TGTCTCCTAGCTGTCGCCATTCCCCTTTGACCTCCTGCTGAAGGAGGTGCAGAAGTACC
CCAATGCTGAGCTGGCCTGGT
>1540
TCAATCCCTGATCACTGGGAGTCATCATGTGCCTTGACTCGGGCCTGGCCCCCCCATCT
CTGTCTTGCAGGACAATGCCGTCTTCTGTCTCGTGGGGCATCCTCCTGCTGGCAGGCCT
GTGCTGCCTGGTCCCTGTCTCC
>1541
TTTCTTATTCTGCTACACTCTTCCAAACCTTCACTCACCCCTGGTGATGCCCACCTTCCC
CTCTCTCCAGGCAAATGGGAGAGACCCTTTGAAGTCAAGGACACCGAGGAAGAGGAC
TTCCACGTGGACCAGGTGACCAC
>1542
CACTGTGGTGGGTCCCAGAAGAACAAGAGGAATGCTGTGCCATGCCTTGAATTTCTTT
TCTGCACGACAGGTCTGCCAGCTTACATTTACCCAAACTGTCCATTACTGGAACCTATG
ATCTGAAGAGCGTCCTGGGTCAA
>1543

GTGTCCACGTGAGCCTTGCTCGAGGCCTGGGATCAGCCTTACAACGTGTCTCTGCTTCT
CTCCCCTCCAGGCCGTGCATAAGGCTGTGCTGACCATCGACGAGAAAGGGACTGAAG
CTGCTGGGGCCATGTTTTTAGAGG
>1544
GCACAGGAGCCTCCCGGTTTCCGGGGTGGGCTGCGCCCGTGCTCAGGGCTTCTTGTCC
TTTCCTTCCCAGGGCGTGATGGTGGGCATGGGTCAGAAGGATTCCTATGTGGGCGACG
AGGCCCAGAGCAAGAGAGGCATCC
>1545
ATGTCCCCCGTCTGGCCTGGCTGTCCCCAGTGGCTTCCCCAGTGTGACATGGTGCATCT
CTGCCTTACAGATCATGTTTGAGACCTTCAACACCCCAGCCATGTACGTTGCTATCCAG
GCTGTGCTATCCCTGTACGCCT
>1546
TGCCTGACATGAGGGTTACCCCTCGGGGCTGTGCTGTGGAAGCTAAGTCCTGCCCTCA
TTTCCCTCTCAGGCATGGAGTCCTGTGGCATCCACGAAACTACCTTCAACTCCATCATG
AAGTGTGACGTGGACATCCGCAA
>1547
GTGGGGTCCTGTGGTGTGTGGGGAGCTGTCACATCCAGGGTCCTCACTGCCTGTCCCC
TTCCCTCCTCAGATCATTGCTCCTCCTGAGCGCAAGTACTCCGTGTGGATCGGCGGCTC
CATCCTGGCCTCGCTGTCCACCT
>1548
GGGCTCCTGGGTTCTGCGTTGCGGGGTGGGTTCGGTGTCGCCGGCGAGGCTGACGGAT
CGTCCCCTGCAGGGCGTCATGGTGGGCATGGGCCAGAAGGACTCCTACGTGGGCGAC
GAGGCCCAGAGCAAGCGTGGCATCC
>1549
TTGTGGCGTGTGAGCATGGGGTGGCCGTGGGTCTCTGTCCCTGACTAAGCCGCCCCTT
GTCCCTTCTCAGATTATGTTTGAGACCTTCAACACCCCGGCCATGTACGTGGCCATCCA
GGCCGTGCTGTCCCTCTACGCCT
>1550
GTGAGCTAAAGGTTTCTACTCTTTCATCCTCGGTGACACAGCATCACTAAGGGAGGGC
TCTGTCCCCTAGGTATGGAATCTTGCGGCATCCACGAGACCACCTTCAACTCCATCATG
AAGTGTGACGTGGACATCCGCAA
>1551
TCTGCCTGGCTCGGGAGAGCTGACTGGGGGGCGCTCTGCGAGCTGAAGCCGTGCCTG
GCTGTCTTTGCAGATCATCGCACCCCCAGAGCGCAAGTACTCAGTGTGGATCGGCGGC
TCCATCCTGGCCTCACTGTCCACCT
>1552
CCATCCTGGCCTGTCAGGCCTGCCTGGATCCTGCCCCGGGTTGGTCCACCACTCACTGT
TTTGTTTCCAGGAGGAGAGGGATCGCCCTCCCAGCTAACACAGCAGAGGGGCTGCTG
AACGTCATTGGCATGGACAAGCCG
>1553
TGTCATTTGCTCCTGGAGCTCCCAAGGGACTTGGGGAAGGTTGTTCCCAACCCCTTTC
TTCCCTTCCCAGGGGCTGCCGGGAGGCTATCAAAAGGATCGCCTATGAGTTTGTAGAG
ATGAAGGCCAAAGAGGGCGTGGTG
>1554
CCCAGTCCCAGAGCTGCCCTTTAGGCCTTCCTTTTGCCTCAGGCCCATCCCTACTCCTC
TCCTCACACAGAGGGGACCTCACCCCAGACGAGGTGGTGGCCCTAGTGGGCCAGGGC

CTGCAGGAGGGGGAGCGAGACTTC
>1555
AGCAGGCAGGTAGGCTGGGAGGTCTCTCCATGGTGCTGGTGACAGAGCCTGGGTGGG
CATCTGCCCACAGACTGGTCCCCCAAGGTGGTGGAGCTGTGTAAGAAGTACCAGCAG
CAGACCGTGGTAGCCATTGACCTGGC
>1556
CAGCATGGTCAAGGCAGGCTGCCGGGGCCCTCCCGCTAGGGCACCCATGACCTGGCTC
TCCCCCTTCCAGGAGGCTGTGAAGAGCGGCATTCACCGTACTGTCCACGCCGGGGAGG
TGGGCTCGGCCGAAGTAGTAAAAG
>1557
GCCTGGGCTGGCCATGGGGTCCCTCCTCACTGCCTCCTCCCATACTTGGCTCTATTCTG
CTTCTCTACAGGCTGTGGACATACTCAAGACAGAGCGGCTGGGACACGGCTACCACAC
CCTGGAAGACCAGGCCCTTTATA
>1558
CGTCAATGTGTTCTGACTATTTATGTAAGAGTAAAAGGAGGCTGATTCTCTCCTCCTCC
CTCTTCTGCAGGCTCAAAAATGACCAGGCTAACTACTCGCTCAACACAGATGACCCGC
TCATCTTCAAGTCCACCCTGGAC
>1559
TCACTACATTGCTAAGAGCTGCCTTCTTTGTTCTCTGGTTCCATGTTGTCTGCCATTCTG
GCCTTTCCAGAACATCAATGCGGCCAAATCTAGTTTCCTCCCAGAAGATGAAAAGAGG
GAGCTTCTCGACCTGCTCTATA
>1560
AGTCGAAAAACACAATTTTAGTTGTCTTAAAGGTATCATTTAAAACTTAGTATGTTGCTT
CCTTTTACAGCATTCGAACTGTTGTAAAGATTTAATTCAAAAGAGAAAATAATGTCCAT
CCTGTCGTGATGTGATAGGAG
>1561
AACTATACTTGAGAAAAATAAGCTTGCTGCAGGTCTGTTCCTTAAGGATTCACACGTAT
TTTTGTTTCAGGGCTACCATATTTTTTGCCCAGTTTGTTCAAGAAGCCACTTACAAGGA
AGTAAGCAAAATGGTGAAAGAT
>1562
TTTCATGTGTCTTATAGATTTTTAAAGCAAAGTTAGTTGTCTTTCTCCAAACATAAAATA
TTTCTTATAGCTACCTGCCTTTCTGGAAGAACTTTGCCATGAGAAAGAAATTTTGGAGA
AGTACGGACATTCAGACTGCT
>1563
TCCAGTTCCAAGCAGTAGTAGTCCTATTTTTAGGTGTTTATAAATCTTCTAGCTCTATTTT
ATTTCACAGATTCATTTATGAGATAGCAAGAAGGCATCCCTTCCTGTATGCACCTACAAT
TCTTCTTTGGGCTGCTCGC
>1564
CTAAATATTAGAGCTTGTAAAGAAAATGTTAGTATATGCTTTCATGACATTTTGTTTCCTC
TACATCTAGGCAGCAACAGTTACAAAAGAATTAAGAGAAAGCAGCTTGTTAAATCAAC
ATGCATGTGCAGTAATGAAAA
>1565
ACTCTATAAATTCTATTTTATTTTGACAGATAACCAAGAAATTAATTTCTAATTTCTTTTTT
TTCCCTAGAACTGTTACTAAACTGAGTCAGAAGTTTACCAAAGTTAATTTTACTGAAAT
CCAGAAACTAGTCCTGGAT
>1566

AAAGCTGGCTTTGAGATCCTTTATTAAAGAATAAATCTTTAAAACTTATACTTTATTTTCC
CTGTTGCAGGAAAAAATCATGTCCTACATATGTTCTCAACAAGACACTCTGTCAAACA
AAATAACAGAATGCTGCAAAC
>1567
CTGGAGAAGTGATGGCTCCTTTTGTCTCTTAGTTCCAATAACTTGAAATATTTTTCTCCA
CATATTTCAGTTTTGTTCATGAATATTCAAGAAGACATCCTCAGCTTGCTGTCTCAGTAA
TTCTAAGAGTTGCTAAAGGA
>1568
ATCAATTTTATATACAAAGTTATGCATCCAAGAAAAGAAAAATGTATATGTAATAATTCTT
CATTTTCAGGAAGAAGAATTACAGAAATACATCCAGGAGAGCCAAGCATTGGCAAAGC
GAAGCTGCGGCCTCTTCCAGA
>1569
GAGAAGAAGCAAGGCGGCTAAAAACTCATGAATGACTCAGCAGGACTTAGTTAAAAA
ATGCTTCTTTCAGGTTTCTCGTTGCTTACACAAAGAAAGCCCCCCAGCTGACCTCGTCG
GAGCTGATGGCCATCACCAGAAAA
>1570
CTGAAAAACTGAACCAACTTTGTGACTAATGCCCAATCTCCTTACTTTTTTTTCTCATTC
TCCTAACCAGGCTGACATTATTATCGGACACTTATGTATCAGACATGAAATGACTCCAGT
AAACCCTGGTGTTGGCCAGT
>1571
TAGGAAGGCTGTAGAAAAATAGCATTGCATAACAGACTTCTCTTGTATTTTGTTTTGTTT
TAAATCACAGGTTTCTCATTAACCTTGTGAAGCAAAAGCCACAAATAACAGAGGAACA
ACTTGAGGCTGTCATTGCAGAT
>1572
TAATAGAACCAGTTACTTACTGCACCTATTAGTTTAATTAGTATTTAATATATTTTTGCTCA
TATTGCAGGGGAAGAGAAGACAAAACGAGTCTTTCATTCGGTGTGAACTTTTCTCTTT
AATTTTAACTGATTTAACAC
>1573
GGGGTGGGATGAGGGCTGGAGCTGTGGGGGCAACTGATAGTCACCATCCCCTCCGTCC
GTTGCCCGGTAGTGCTCTTGCTGGGACATGTGGCCCAGGTGCTGATGCTGGACAATGG
GCTCCTGCAGACACCACCCATGGG
>1574
AGCTGAGGCAGGGTGTGGGTGAGGGCCGGGCTGGGTCCCTGAGCTACCCCATCCCTCT
GCCCCTCACTAGTGAACAGCTCTTCATGGAGATGGCTGACCGGATGGCACAGGATGGA
TGGCGGGACATGGGCTACACATAC
>1575
GGTCAGATATGCCTTGAGGAGGCTCCTTGAGGGTCCTGCTTGAGCCCACTGTGTCCTC
AAACCCCCACAGGTTCACTCCCTGGGCCTGAAGTTGGGTATCTACGCGGACATGGGCA
ACTTCACCTGCATGGGTTACCCAG
>1576
AAAGGTCTGCTAAGGCCCCAGACTGCCTCCTGCACTTCCTCAGGACTGAAACAGGTTG
GGCTTTCTCTAGGGTACCCCAAGATGGCTGCTGCCCTGAATGCCACAGGCCGCCCCAT
CGCCTTCTCCTGCAGCTGGCCAGC
>1577
CTCCTGTGTGGGTCCCTGGGCTGCAGCGTGTGATTCTGGTGCTGGACTCTGCCTTTCCC
TCGACATCCAGGTGAACTACAGTCTGCTGGCGGACATCTGCAACCTCTGGCGTAACTAT

GATGACATCCAGGACTCCTGGT
>1578
TTTGGGGATGGTCCCTGTGTCTACCTCCTTGAGGAGATGAGAGCTGACTACTCCTCTTC
TCTGCCCCCAGCTGCTCATTGGGAACTTTGGTCTCAGCTTAGAGCAATCCCGGGCCCA
GATGGCCCTGTGGACGGTGCTGG
>1579
GGGCCAGGGAGGCAATGGTAGGTCCTGTCTGAGCAAGGGACCCCAGCCAGTAGCCAC
CTTCTGTGCCCAGGAAAAATCTCTCATCGAAGTGTACATGCGGCCTCTGTCCAACAAG
GCTAGCGCCTTAGTCTTCTTCAGCT
>1580
ATCCTCTAGCGGGGGGGGTCTCTGTGCCCCACTCTCATGGTGGCATTGGTGATGTCCCT
CCCCACTGCAGGCCCAGGACGTCTACTCAGGTGACATCATCAGTGGCCTCCGAGATGA
AACCAACTTCACAGTGATCATCA
>1581
AGACCCTGGCAGCCCCTGTTCCTCCTGGGACCCTGTGTCTCGGGGTTTATGAATGGGT
GGGCGTTTGCAGGTGGGCCTGGCTCAGGGAAGGGCACCCAGTGTGAGAAGATCGTGC
AGAAGTATGGCTACACCCACCTCTC
>1582
GTGACGAGAGGCTGGGAGAAGGGATGGGGAGGAGAGGCTCATGGCCCCTTCCTCCTT
TGCTGTGCCCCAGGAGACAGTGTTGGACATGCTCCGGGATGCCATGGTGGCCAAAGTC
AATACTTCCAAAGGCTTCCTGATTG
>1583
TGGGAAGATGGGGAGGACATGGCCCTAGGGGGCAGGAATGGGGTCCCTGTGACATGG
TGCCCGCCTGCAGATTGGACAGCCCACACTGCTGCTGTATGTGGACGCAGGCCCTGAG
ACCATGACCCAGCGGCTCTTGAAAC
>1584
TGGGGCCTGCCATGGTGAGGCCCACCCCACTCCACCTGGTGACTCAGCCTCACGGCCC
ATCTCCCCACAGGTCAACGCTGAGGGCTCCGTGGACAGTGTCTTCTCCCAGGTCTGCA
CCCACCTGGACGCCCTAAAGTAGC
>1585
TCCCAGAGTTGAGGGTGGGTGAGACCACGTGCTCATGGCTCTTCTCACTGTGCCCTGC
CCCCTGCACTAGGCGGTGCCTCGGGAGGAGCTGTTTGTGACATCCAAGCTGTGGAACA
CCAAGCACCACCCCGAGGATGTGG
>1586
GGCTGGATGGGCAGGTAGGGTAGAAGCCTGGCATTTGTGTCCACACTTGGTGGGGCTG
TCTCTCACTCAGGCGGGGAGACAACCCCTTCCCCAAGAATGCTGATGGGACTATATGCT
ACGACTCCACCCACTACAAGGAG
>1587
ATGCATGTCTGGATGGGCCAAGCAAGTCTGGGTGTCACCACTTCATGGTGATGGGTTAT
CTTTGGCTCAGGTGGAATGCCACCCATACTTGGCTCAAAATGAGCTAATTGCCCACTGC
CAAGCACGTGGCTTGGAGGTAA
>1588
TGCATGGGGAACAAAATAGTGCTTATGAATACTGACCCCTTTTCCTCATCTGTCTAATCC
CCCAACTTAGGTGGCAGGTCCAGCGGAAAGTGATCTGCATCCCCAAAAGTATCACTCC
TTCTCGAATCCTTCAGAACATC
>1589

AGGCAGTGTTGTGGAACCCCAGCTTCTGCTCACAAAGCTGGCTTTCTTGAACCCCACT
CTCCATCCTCAGGTGTTTGACTTCACCTTTAGCCCAGAAGAGATGAAGCAGCTAAATG
CCCTGAACAAAAATTGGAGATATA
>1590
GGGAAGATGCCTGGAGTCTTTGGAATGGCAACTCCTGCTGATGGAGTAATCTATCTGTC
TCTCTTTCCAGGTGGATGGGAAGAGAGTCCCAAGGGATGCAGGGCATCCTCTGTACCC
CTTTAATGACCCGTACTGAGACC
>1591
CCGGTGTGCCATGACCCCAGGCCACCCTTTCCTGCCTTTCTACTCATGGCTTCTTCCTG
ACTGTCCCCAGGAGTTGTGCCCCTGCTGTTGGTTCTGCACTGGAAACATGGGGCGGGG
AGCCCCCTCCCCATCACCCCTGT
>1592
CCCAAAGGGTTGGCGGCAGGGCTGGGACACTGACCCCTGACTCCCACGTCACCTCCC
TTCTGCCCCTCAGTACACAGCCCAGGGGGAGCCGTTCCCCAACAACCTGGACAAGCTA
TGTGGCCCCAACGTGACGGACTTCC
>1593
TACACACACACACACACACACACCACCCCCCAGCCCAGGCTGACCTGATCTTTGCTCT
CCCCCTGGCCAGTTGAGGAGGAGAACCCGGACTTCTGGAACCGCCAGGCAGCCGAGG
CCCTGGGTGCCGCCAAGAAGCTGCA
>1594
CGGCGCCCGGACCCTCAGTGGTTCCAGGACAGCCCTGGGGAGCAAGCCTCACACACT
TCTGCTCCTTCAGGGATGGGGGTGTCTACGGTGACAGCTGCCAGGATCCTAAAAGGGC
AGAAGAAGGACAAACTGGGGCCTGA
>1595
TCCCAGAGGACAGAGATCAGGGTCTTGTTTGTCTGCCCCAGAGAAGAGCTCAGAGTG
TCTCTGTCCCCAGACATACAGTGTAGACAAGCATGTGCCAGACAGTGGAGCCACAGCC
ACGGCCTACCTGTGCGGGGTCAAGG
>1596
TGGGGCCCGGCTGTGGGGTCAGGGCCAGTGACAGACCTCTATCGCATATCCTGACCTC
TATCACCCTCAGGAAAGTCAGTGGGAGTGGTAACCACCACACGGGTGCAGCATGCCTC
GCCAGCCGGCACCTACGCCCACAC
>1597
CTCTGCATGAGGAGGGGACACGGGGCCTAGCCATGGCCCAAAGTCCACCTGCCCCATC
CTCTGTTCCCAGGTGATCCTAGGTGGAGGCCGAAAGTACATGTTTCCCATGGGGACCC
CAGACCCTGAGTACCCAGATGACT
>1598
CAGCAGGGGGAGGGCAGAGGTGTGGGGCTCGGGGCTGTGGGCTGAGGCCTGGCTCTC
TCCCTCCCCGCAGGGTGCCCGGTACGTGTGGAACCGCACTGAGCTCCTGCAGGCTTCC
CTGGACCCGTCTGTGACCCATCTCA
>1599
CTGTGTGCACATCCGCCAGCACCCTCCCACCCCCAGCCTGCCAGTCACCACAGGACCC
CTTGTCCCACAGGTCTCTTTGAGCCTGGAGACATGAAATACGAGATCCACCGAGACTC
CACACTGGACCCCTCCCTGATGGA
>1600
CCCTGGGAGTGGGGGGTTGGGGGTTGGAGCAGGGCAGGCTCAGCATCTCCCCCCTCT
GGCCTTCCTGCAGGTGGTCGCATCGACCATGGTCATCATGAAAGCAGGGCTTACCGGG

CACTGACTGAGACGATCATGTTCGA
>1601
TGGCGGGAAGGGGTCACCTCTTGTCTGCCTGGAACTGAAACTTCCTACTGAAACTGAA
CCCTCCAACCAGGGAGCCCCGAGTATCGGCAGCAGTCAGCAGTGCCCCTGGACGGAG
AGACCCACGCAGGCGAGGACGTGGC
>1602
CCCAAGCTGTTCCACACACAGGGCACCCCCTCAGCCAGGCTGACCTGATCTCTACTCT
CCCCCTGGCCAGCTGAGGAGGAGAACCCGGCCTTCTGGAACCGCCAGGCAGCTGAGG
CCCTGGATGCTGCCAAGAAGCTGCA
>1603
GGCACCCGGGACCTTCAGTGGTTCCAGGACAACCCTGGGGCCCAGGACTCACACATT
TCTGCTCCTTCAGGGTTGGGGGTGCCCACGGTGACAGCCACCAGGATCCTAAAGGGGC
AGAAGAATGGCAAACTGGGGCCTGA
>1604
CAGAACCAGGTCCTTGGTTGGGGTCTGGGTGTCCGCCCCGAAGTAGAGCTCAGGGTG
TCTCCGTTCGCAGACATACAATGTGGACAGACAGGTGCCAGACAGCGCAGCCACAGC
CACGGCCTACCTGTGCGGGGTCAAGG
>1605
TGGGGCCCGCTGTGGGGTCAGGACCAGGCCCAAGATCTCGGTCACCGATCCTGACCTC
TGTCACCCTCAGGAAAGTCAGTAGGAGTGGTGACCACCACACGGGTGCAGCACGCCT
CGCCAGCCGGCACCTACGCACACAC
>1606
GGCTATGCATGAGGAGGGGGCACGGGGCCAGCCAGGCCCCCAAACCACCTGCCCCAT
CCATTGTCCTCAGGTGATCCTTGGCGGAGGCCGCAAGTACATGTTTCCCATGGGGACCC
CAGACCCTGAGTACCCAGCTGATG
>1607
GGCACGGCAGGGGGAGGCCAAGTGTGTGGGTCTCAGGGCTGTGGGCTGAAGCCTGGC
TCTGTCCCTGCAGGGTGCCTGGTATGTGTGGAACCGCACTGAGCTCATGCAGGCGTCC
CTGGACCAGTCTGTGACCCATCTCA
>1608
CTGTGTGCCCATTTGCCAGCACCCTCCCGCTCACAGCCTGCCAATCACCACCAAGCTC
CTTGTCCCACAGGCCTCTTTGAGCCCGGAGACACGAAATATGAGATCCACCGAGACCC
CACACTGGACCCCTCCCTGATGGA
>1609
CTGGGGAGTGGAGGAAGGCGGGGCGCGGCAGGGCAGGTTCAAGCATCACCCCCCTCT
GGCCTTCCTGCAGGCGGCCGCATCGACCATGGTCATCATGAGGGTGTGGCTTACCAGG
CACTCACTGAGGCGGTCATGTTCGA
>1610
TGCCTGGCACATAGGAGGCACTCCCACAGCTGTGGTCAGCTCAACTACAGGGACCCGC
ATCTCCCTACAGGGTTGGCCCCCAGCAAGGCTCAGGACAGCAAAGCCTACACGTCCAT
CCTGTACGGCAATGGCCCGGGCTA
>1611
CAGGGATGGGGGCATTCGCGGGAGGGGGACGCCGCCTGCCTGCCCTGAAGTGCACTC
ACCCTCCTACCAGGGAGCCCCGATTACCAGCAGCAGGCGGCGGTGCCCCTGTCGTCCG
AGACCCACGGAGGCGAAGACGTGGC
>1612

CCCTACACAACACACACACAGGGCACCCCCCAGCCCAGGCTGACCTGATCTTTGCTCT
CCCCCTGGCCAGTTGAGGAGGAGAACCCGGACTTCTGGAACCGCGAGGCAGCCGAGG
CCCTGGGTGCCGCCAAGAAGCTGCA
>1613
CGGCGCCCGGACCCTCAGTGGTTCCAGGAGAGCCCTGGGGCCCAAGCCTCACACATT
TCTGTTCCTTCAGGGATGGGGGTGTCTACGGTGACAGCTGCCAGGATCCTAAAAGGGC
AGAAGAAGGACAAACTGGGGCCTGA
>1614
GAGGACAGAGATCAGGGTCTGGGTCTCCGTGTCTGCCCCAGAGAAGAGCTCAGAGTG
TCTCTGTCCCCAGACATACAATGTAGACAAACATGTGCCAGACAGTGGAGCCACAGCC
ACGGCCTACCTGTGCGGGGTCAAGG
>1615
TGGGGCCCGCTGCTGGGTCACGGCCAGGTCACAGACGTTGGTCACATATACTGACCTC
TGACACCCTTAGGGAAGTCAGTGGGAGTGGTAACCACCACACGAGTGCAGCACGCCT
CGCCAGCCGGCACCTACGCCCACAC
>1616
GCTGTGCATGAGGAGGGGGCACGGGGCCAGCCAGGGCCCCAAATCCACCTGCCCCAT
CCTCTGTTCCCAGGTGATCCTAGGTGGAGGCCGAAAGTACATGTTTCGCATGGGAACC
CCAGACCCTGAGTACCCAGATGACT
>1617
CAGCAGGGGGAGGGCAGAGGTGTGGGGCTCAGGGCTGTGGGCTGAGGCCTGGCTCTC
TCCCTCCCCACAGGGTGCCCGGTACGTGTGGAACCGCACTGAGCTCATGCAGGCTTCC
CTGGACCCGTCTGTGACCCATCTCA
>1618
CTGTGTGCACATCCGCCAGCACCCGCCCACCCCCAGCCTGCCAGTCACCACAGGACCC
CTTGTCCCACAGGTCTCTTTGAGCCTGGAGACATGAAATACGAGATCCACCGAGACTC
CACACTGGACCCCTCCCTGATGGA
>1619
CCCTGGGGAGTGGGGGTTGGGGGTTGGAGCAGGGCAGGCTCAGCATCTCCCCCCTCT
GGCCTTCCTGCAGGTGGTCGCATCGACCATGGTCATCATGAAAGCAGGGCTTACCGGG
CACTGACTGAGACGATCATGTTCGA
>1620
CGGTGCCTAGCACGTGGGAGACACTCCACAGCTGTGTTCAGCTCAACCACAGGGACC
CCTCTCTCTGCAGGGCTGGCCCCTGGCAAGGCCCGGGACAGGAAGGCCTACACGGTC
CTCCTATACGGAAACGGTCCAGGCTA
>1621
CGGGAAGGGGTCACCTCCTGTCTGCCTGGAACTGAATGAACCCTCCTACCGGAACTGA
ACCCTCCAACAGGGAGCCCCGAGTATCGGCAGCAGTCAGCAGTGCCCCTGGACGAAG
AGACCCACGCAGGCGAGGACGTGGC
>1622
ATTTTTTCAGGCAGGATGCATATTATTTATTAATTCACTCCTCTTTTTTCCTCATTTAATAT
AACTGCAGGAGCCCCTAATGGAAGAATATGCTATTGCTGCACAAGTCTTCAAGCTGAG
CACCTGTGATATGTGCGAAG
>1623
AGAATGCAATTATTGTCTATACCAGGGTGAAAAGAGACAACAATCATTCTGCCATCCTT
TTCTCTTATAGGAGAAAGTAAAGTTTCTGGGCGACAATTACCTTGAGGAAGGCCCTGC

TGGAAATGATATCCGGAGGACAA
>1624
TGCTCACTCCTCTTGATTTCCTCCAAGTCAGTGAGGTTTATCCCTTTCCCTGTATTTTCC
TTTTCTAAAGAATTTGCTGGACCATTTGGAAGAAAAGATGCCTTTAGAAGATGAGGTC
GTGCCCCCACAAGTGCTCAGTG
>1625
AGATGGCATAAGCTCTTCTCTTTCCCTTCCCCATGGTTATAACTGTCCCTGTTGGCTTCC
TTCCTGTCAGGTGCAGCATGCCAGCAAGCAGATCACTGCAGATAAGCAATACAAAGGC
ATTATAGACTGCGTGGTCCGTA
>1626
TTAGGGGATGTGGGGAAAGGAAGTCAGTAAAACTTCTGCTTTTTGGTAAAGATCTCTT
TCCTATTCCTAGGAATGCTTCCGGATCCCAAGAACACTCACATCGTCATCAGCTGGATG
ATCGCACAGACTGTCACTGCTGT
>1627
TTTAGCCTAGTGAATCTTAGGATTTTTCATCGGCCTTCAGTCACTAACTCCACGTCTTTA
TTCTTTGCAGCTGACATCATGTACACAGGCACGCTTGACTGCTGGCGGAAGATTGCTC
GTGATGAAGGAGGCAAAGCTTT
>1628
TCTGCCCCACCTCTTGATTGCTTTCCCTTTTCTTATAGTTTTTTATGCTAATTCTGTTTCAT
TTCTATAGGCGAGGAGGAGCATGATCAGGAAGGCCGGGTGATTGTGGCTGAATTTGAC
TCGTTTGTGCTGGTAACAGC
>1629
AGCTCAGAGCTGGAGCAGAGGGGCCACACAGGAGACGGGGCCTCATGAATTGCTCTC
TGTTACCACCCAGGAGCCAGGGCTGAGGTCAGTGCTGACCAGGTGGCCACAGTGATG
TGGGACTACTTCAGCCAGCTGAGCAA
>1630
ATACAAGCAGACAAAGTCTTGCCGTGTAAATGCCAAATGTAACGTGGCCTCCTTGTGC
CCTTCCCCACAGTGCCCTCTTCCAGGACAAACTTGGAGAAGTGAACACTTACGCAGGT
GACCTGCAGAAGAAGCTGGTGCCC
>1631
GAGCAAGACTCCATCTCCAAAAAAAAAAAAAAAAAAAACAAATTTTGAACCCCTGCCC
ATCTTCCTGGCAGGCCCAGCCCCAGCCCAGGGGACCCCAGACGTCTCCAGTGCCTTGG
ATAAGCTGAAGGAGTTTGGAAACAC
>1632
CCGTCTCAAAACTACAACAACAACAACAACAACAACCAAATTTTGCACCCCTGCCTCA
TCTTCCTGGCAGGCCCAGCCCCAGCCTAGGGGGCTCCAGAAGTCTCCAACCCCTTTGA
TGGCCTGGAGGAGTTAGGAAAGAC
>1633
CCCCTAGGTAGCTAGATGCCTGGACGGGGTCAGAAGGACCCTGACCCGACCTTGAACT
TGTTCCACACAGGATGCCAGGCCAAGGTGGAGCAAGCGGTGGAGACAGAGCCGGAG
CCCGAGCTGCGCCAGCAGACCGAGTG
>1634
GCCCGCCCCATCCCAGCCCTTCTCCCCCGCCTCCCCACTGTGCGACACCCTCCCGCCCT
CTCGGCCGCAGGGCGCTGATGGACGAGACCATGAAGGAGTTGAAGGCCTACAAATCG
GAACTGGAGGAACAACTGACCCCG
>1635

GAACCCTCGTCTTTCGCCCCCGGGCCCTGCTCCTTCGGCCCCGCGTCACCAGGCCTGT
CCTTGGGTCCAGGGACATCTCGCCCGTCCTGAAGGACCCCGCCTCCTTCCGCGCCGCC
ATCGGCCTCCTGGCGCGACACCTG
>1636
TTGCCACCGCCGAACCCTGCCCCTGGGGATTCCCATGCTGCCGAGTCACTCCTGTCAC
TTACCCTGACAGGCCTAGACTCCCGAGGCTTCCTCTTTGGCCCCTCCCTGGCCCAGGA
GCTTGGACTGGGCTGCGTGCTCAT
>1637
AGCTGCCAAGCTGTGCAACAAAGCTGTTTTCTGCGGGAGGCTGAGGACCACACACCA
CTTCCCACTCCAGGCTGAGCTGGAGATTCAGAAAGACGCCCTGGAGCCAGGACAGAG
GGTGGTCGTCGTGGATGATCTGCTGG
>1638
CAGGGGCTGGCCTGGAGCACCTGCTCTCTGCAGCCCAGGCCAACTGGGGACCTCACC
CTCCCATCCCCAGGAACCATGAACGCTGCCTGTGAGCTGCTGGGCCGCCTGCAGGCTG
AGGTCCTGGAGTGCGTGAGCCTGGT
>1639
CCTGGCTGAGTGGTGGGAATGGAGGCCCCAGCCCCTCTCTTCCCTGACTCTCCGGCTC
TCCCTCCCTCAGGCTGGCCGTGAGTACTCACCTGCCGCCACCACGGCAGAGAATGGGG
GCGGCAAGAAGAAACAGAAGGAGA
>1640
AGGGATGGGCATGGTGACTGGCTGGGTTGGCTCCGGATGCGTGCCCCTACGCCTCTCC
TTGCTCCCTCAGGGCCTCACCAACCAGCGGGCTCAGGACGTTCTGGCTCGAGATGGGC
CCAACGCCCTCACACCACCTCCCA
>1641
TCCCAAGTGGCAGCTGCCCCTTTAGGGTTGGGGGGAAGGTCAGGTCCCTGAAACTCTT
TCTCCTTACCAGCTATATCTGGGTGTGGTGCTGGCAGCTGTGGTCATTGTCACTGGCTG
CTTCTCCTACTACCAGGAGGCCA
>1642
GGGAGACCAGCAGGAGAAGAAGGCAGGGGCAGAGACAAGCATTTCATGAGCTGCCT
GTGGCTCCCCACAGCAAGCCCTTGTGATCCGGGAGGGAGAGAAGATGCAGATCAACG
CAGAGGAAGTGGTGGTGGGAGACCTGG
>1643
GTTCAGGAGACAGCTGTGTGCATACAAGTGGCTCTGCCAGTCTGATGACTATGCACTC
CTTCCTCCTCAGGTGGATAACTCATCCTTAACAGGAGAGTCGGAGCCCCAGACCCGCT
CCCCCGAGTTCACCCATGAGAACC
>1644
AGGAAATAGGATGGGACTGCAGTCCCTGGGAGCCACAAGGCACCCAACCTGATGCCC
CACCATGTTGCAGGCACTGCCAGGGGCATTGTGATTGCCACAGGAGACCGGACGGTGA
TGGGCCGCATAGCTACTCTCGCCTC
>1645
GTCTAGGGTAAGGTTATGGCCATCTCCGGCTTCAGCCTTAACCTTTTTTATTCTCCTCTT
TCTCTACCAGGTGTGCCTGACCCTGACAGCCAAGCGCATGGCACGGAAGAACTGCCTG
GTGAAGAACCTGGAGGCGGTGG
>1646
GTGGGGTGAGTGGTTGAGATAAAGGCTCTAAAGGGAGCCACGCTCCTGGTTCCCCCTC
ATTTCCTCCCAGGGGCCACTTTTGACAAACGATCCCCTACGTGGACGGCCCTGTCTCG

AATTGCTGGTCTCTGCAACCGCGC
>1647
TGCCGCCTTCACCTGATCCTCCACTCCCTTCCCTCCCATGCTGACACTGAATTCTTGTCT
CTTCTGGCAGCGGGACACAGCTGGTGATGCCTCTGAGTCAGCTCTGCTCAAGTGCATT
GAGCTCTCCTGTGGCTCAGTGA
>1648
CAGCTGCTGCTCTATGCCGCGCTACCAAGACAAGTATGGCCCTCTCTGTAACTACCTGT
TGTCTCTCCAGCTGTCTATCCACGAGCGAGAAGACAGCCCCCAGAGCCACGTGCTGGT
GATGAAGGGGGCCCCAGAGCGCA
>1649
GGTGGGGGACTGTGGGGGCGTCCAGGAAGCCACTCTGCGGATCTCACTGATCCCTTCT
GCCCCCCTTTAGGATTCTGTCAACTGAATCTGCCATCTGGAAAGTTTCCTCGGGGCTTC
AAATTCGACACGGATGAGCTGAA
>1650
TTGGGAGAGGAACAGGAGGGGGATAAACCCTTAATACTACTTTCCTGTTGTCTCCTCTC
CTTCCCACTAGGTGATCATGGTAACCGGGGATCACCCTATCACAGCCAAGGCCATTGCC
AAAGGCGTGGGCATCATATCAG
>1651
GAGGCAGGGGGCAGGAGGGGCTGGTACAGGTGCCAGGGGTCAGCTGTCTCTGTCCCC
ACCCACCCTCCAGAGAAGCCAAGGCATGCGTGGTGCACGGCTCTGACCTGAAGGACA
TGACATCGGAGCAGCTCGATGAGATC
>1652
ATGGAGTGAGAGGCGAGGAGCCAGGCTTGGGAAGGGGTTTCGTCCTCAAGTGTGGCC
GTCTTCCCTCCAGGGAGCCATTGTGGCCGTGACGGGTGACGGGGTGAACGACTCCCCT
GCATTGAAGAAGGCTGACATTGGCA
>1653
CATGCTTCAGGGGCTGGGGGTGGGGAAGAGTCCCTCTGACCTCCCTGATGCCCTCAGA
ATCTCCCCACAGGCCGCCTGATCTTTGACAACTTGAAGAAATCCATCGCCTACACCCTG
ACCAGCAACATCCCCGAGATCAC
>1654
GATCAATTGCTTCTGTCATCTCCTACGTCCCTTCAAATGCCCTCCCTGCCCCATTTCCTA
CCCCACACAGGTCCCTGCCATCTCCTTGGCCTATGAGGCAGCTGAGAGTGATATCATGA
AGCGGCAGCCACGAAACTCCC
>1655
GCCCTGAGGGGCTGTGCCCCTTCTGCTTCCTGCTCTGACCCTGCCCCTGCCTTTGGCCC
TCTACCCACAGGGATGATCCAGGCACTGGGTGGCTTCTTCACCTACTTTGTGATCCTGG
CAGAGAACGGTTTCCTGCCATC
>1656
AACCATGCTACCTTCTGGTCCTACCCTTTCCTCCGACACTCTCATCTGTCTCTGCCCACC
CTCCCTCCAGACCTATGAGCAGCGGAAGGTGGTGGAGTTCACGTGCCACACGGCATTC
TTTGCCAGCATCGTGGTGGTGC
>1657
CCAGGGGTATCCCAGGATCTCTGCCTCCATGATCCCCCTTCACCTGCCACCTCCTTTCTT
TGCCTTTCAGGAACAAGATCCTGATTTTTGGGCTCCTGGAGGAGACGGCGTTGGCTGC
CTTTCTCTCTTACTGCCCAGGC
>1658

AATGCTCCTTTATGTGACAGCCACCAAGCCAACCTCTGATGCTGCTGACACTCTCCTCC
ATTGCTTTCAGAGTCACCTGGTGGTTCTGCGCCTTCCCCTACAGCCTCCTCATCTTCATC
TATGATGAGGTCCGAAAGCTC
>1659
CCCTAGGAAATTGATCTCTTGCCTCCTTTTAAGCTCATGCTGCAATCTCCACTCCCAATC
TCTCTAACAGGCTGGGTGGAGAAGGAGACATACTACTGACCCCATTGGAAGAAGAAC
CAGGCATGGAAAGATGGGGAGCT
>1660
ATTTTAGAGGCTCTGTTTACCCACTTCAACAGACTCTCCATTTCTGATGTACTTTTTTCT
ACCTCTACAGGCTGGTGGGATAAGAACATGTATTATTAAGACCACCTCCCTTCCTATGTC
TCTCAGCAGCACGTTGGGGC
>1661
CCCCGTTCCCTCGTCCACAGCCCCGCCCTGTTCCCCCGTCCACAGCCTCGCCTCTCCCC
CCCGCCCACAGCTGTCCATACATACGCTGGAGGACCCGCGGGACCCGCGACACTTGCT
GGTGATGAAGGGCGCCCCCGAGC
>1662
GTCCCTCTCTTCCTGTGTCCCTGTCTCTGTGTCCATCTCTGTCTCCCTGTGTCCCCTTGT
CTCTCTCCAGGTGCCATTCAGTCCTTTGCTGGCTTCACTGACTACTTCACGGCAATGGC
CCAGGAGGGCTGGTTCCCACT
>1663
TTTGTTTAAGCTTGTAAGTTTTATAAAGGCTTTGTTAAAAATCAAATTTTTAAGAATACT
TTTGCACTAGGTCTCCAGAAATGCTTTGGGTTCATCTTTCATTGCTGCAAGGAACTTCC
ATGCCTCTAACACTCATCTTC
>1664
GGATTACAGCTGTGAGCCACTGTGCCTGGCCCAGATTATTTTTTTAATTGAATTGAATTG
TCTTCCATAGGGACTGCTGAGATGTCCTCTATTCTTGAAGAGCGTATTCTTGGAGCTGAT
ACCTCTGTTGATCTTGAAGA
>1665
ATGTAGTATTGGTGTATGGGGAGGAGTGCCCATTCAGCTTCATTGATGTTCAATTGTGTC
TTTTGTGCAGGGTATGTCCTTGAACTTGGAACCTGACAATGTTGGTGTTGTCGTGTTTG
GAAATGATAAACTAATTAAGG
>1666
ATATAGTTGCAGCTTCTAACAAACATCAAAACTGATTATGCTTAGGGTTTTTCTTTTTATT
TTTTAACAGGGTCCAATTGGTTCCAAGACGCGTAGGCGAGTTGGTCTGAAAGCCCCCG
GTATCATTCCTCGAATTTCAG
>1667
TCTCTTTCTTTCCCCACAGGGAAGTCCTTTTAAGTTTAGGAAGATTTATATTTACTTTCT
CTTTTTAAAGGAAAACCTCAATTGCTATTGACACAATCATTAACCAGAAACGTTTCAAT
GATGGATCTGATGAAAAGAAG
>1668
AGGTAATTTTTATAGTAGCCTCCAATTTAAAACCACAATATTGATAAAATTGTAAAATATA
TTTTCTCAGATGCCATGAAGTACACCATTGTGGTGTCGGCTACGGCCTCGGATGCTGCC
CCACTTCAGTACCTGGCTCC
>1669
CTGAAGTAAAAGTTCACATATGCAACTTCTATTTAATAGGTTAACTTCACAAACCTATTC
TGTACCATAGGCTGTTGCTTACCGTCAGATGTCTCTGTTGCTCCGCCGACCCCCTGGTC

GTGAGGCCTATCCTGGTGATG
>1670
TGACTTAAATTTGAAGATAGTTGATTTCTTACTTTAAGCTTGCTAGCGTGCGTATTTCCTT
ACATTATAGATCTTCTTGGAAACAGAATTGTTCTACAAAGGTATCCGCCCTGCAATTAAC
GTTGGTCTGTCTGTATCTC
>1671
TAATATATTAACTATATTTGTATAGTGCTCTGTGTCACTGAGTTACAGTACAGTAATTTCT
GTATTGCAGGTAGCAGGTACCATGAAGCTGGAATTGGCTCAGTATCGTGAGGTTGCTGC
TTTTGCCCAGTTCGGTTCTG
>1672
TCCTGACCTTGGTAACATGGCATTCATTTCTTTGAGCAACTCACTTGGCATTCTTCTTGT
ATCTTTTCAGCTCCCATGGCTATTGAAGAACAAGTGGCTGTTATCTATGCGGGTGTAAG
GGGATATCTTGATAAACTGGA
>1673
TCTGTAGTTAGGCTAGTCATTAAAACTTTTTCCAGGTCAGAACTTACGACCTGCTTTTAT
TTCCAAATAGGGCTGATGGAAAGATCTCAGAACAATCAGATGCAAAGCTGAAAGAGAT
TGTAACAAATTTCTTGGCTGGA
>1674
TTAAGTCCTGGCTTCCGATGACCTTTAACTGGCCTGACCCCAGCTCCTTCCAATATCTTT
TGTCTGTTAGTCAGGGACTATGCGGCGCAAACATCTCCTTCGCCAAAAGCAGGCGCCG
CCACCGGGCGCATCGTGGCGGT
>1675
GTCTTGGTCGATTTAGGTCTTGATACCAATTTATCCTTATGTATAACCCTGCGATGCCTGT
ATCTAACAGGTGAGAGCACAGTAAGGACTATTGCTATGGATGGTACAGAAGGCTTGGT
TAGAGGCCAGAAAGTACTGGA
>1676
AGGGATATATAAGATTATCATTTGCTTTCAACAAGGTGAATAGATACTATCCTCTCTTCTA
CCTTTACAGATTTGCTCCCATTCATGCTGAGGCTCCAGAGTTCATGGAAATGAGTGTTG
AGCAGGAAATTCTGGTGACT
>1677
ACTTTTTTTGAAGTTTGCTTATTTAGGAAAGCAGAACTCTTAAATATTCTTTCTTTTCAA
CCTCTTATAGGGCTTTTTGGTGGTGCTGGAGTTGGCAAGACTGTACTGATCATGGAGTT
AATCAACAATGTCGCCAAAGC
>1678
TTTGGGAACCTTCAGTATGTGGCTTCTCTACTCCTTTGTCAGGTTTTACGATTATGTATG
ATTACTGCAGGTAGCGCTGGTATATGGTCAAATGAATGAACCACCTGGTGCTCGTGCCC
GGGTAGCTCTGACTGGGCTGA
>1679
ACCTGGTTCATCTGGCCTTTCTTCGGATGAGGCTAAGGATGTAGACACCTAAGACCTTT
TTTTCTTTTAGGTGTCTGCATTATTGGGCCGAATCCCTTCTGCTGTGGGCTATCAGCCTA
CCCTGGCCACTGACATGGGTA
>1680
AGCGAGACCCTCTCAAAAAAAAAAAAAAAAAATTTACTTTGGTTCCTGTTCTTCATGGG
ATCTCACTTTAGGCTATCTATGTGCCTGCTGATGACTTGACTGACCCTGCCCCTGCTACT
ACGTTTGCCCATTTGGATGCTA
>1681

TTTCCCTTTGTTAGCTTGTCCCAAATTAAAGGAATGAGAATACTTAACTCAGTCTTCTTT
TTTCTCATAGGACTACAAATCCCTCCAGGATATCATTGCCATCCTGGGTATGGATGAACT
TTCTGAGGAAGACAAGTTGA
>1682
TGTTACATGATGCAGAAAGTTGATATCCCTCCGCTTCTTACTCTTTTTTTTTTTCTCCCCC
ATCATACAGGTGAATATGACCATCTCCCAGAACAGGCCTTCTATATGGTGGGACCCATT
GAAGAAGCTGTGGCAAAAGC
>1683
AAGCCGGCTTGCTCTGTGCCCAGGCCCCAGCCTGGTGTCCTCCCTCTGCCCTTTCCTCC
GCTACTCTCAGGAACCCCGGGGTTAGCACCGTGGTGCTGGGTGCCTATGACCTGAGGC
GGCGGGAGAGGCAGTCCCGCCAG
>1684
TGCTCCCAGGGGTCCCCATGAGGCTCCAGTCCCCAGGGCCACCCTCCCCTGACTCCAT
TTCCTTCCCCAGCTGGACCGTGAGGCCAACCTCACCAGCAGCGTGACGATACTGCCAC
TGCCTCTGCAGAACGCCACGGTGG
>1685
GCCTCTGCAGTTCTGGGGTGGCGTGGGAGCCAGGCCCTGGGACGCCCTGACACAGCT
GCTGCCTGCCCAGGGGGACGGGGGCACCCCCCTCGTCTGCGAGGGCCTGGCCCACGG
CGTGGCCTCCTTTTCCCTGGGGCCCT
>1686
CTGGATTATTCTGAGTCCAAGCTAGGCCCTTTTGCTAATCATGTTCATACCTCTTATCTTC
CTCCCACAGCTCCTGGGCAACGTGCTGGTCTGTGTGCTGGCCCATCACTTTGGCAAAG
AATTCACCCCACCAGTGCAGG
>1687
GTCACCTGATTACAAATTTATGTTTTTAGATTTGTTTTTCCATTTTCAGCTTGACATTTAC
TCATTTCAGAGAAACCCTGTGGTTTTCCTCATGTGGAAAATGGAAGAATTGCCCAATAT
TACTATACTTTTAAAAGCTT
>1688
CTTAATTTTACAATTTAGTAAAAGACAAGCTTAGTTTCATCATTAAGTTAAATAATTTTTT
TTCCCATAGAAAAATGCACTAAGCCTGACCTGAGTAATGGTTACATCTCTGATGTAAAG
TTATTGTATAAAATTCAAGA
>1689
CATATTTTGAGATTTTTTATAATCTAGGTTATTAAAAAGTTTATAAAATAAATAATTTTTAT
AATTTTAGAAACGTGTTTGGCTCCTGAATTATATAATGGAAATTATTCCACAACACAGAA
AACATTCAAAGTGAAGGA
>1690
AATAGTGCATTTTGTTGTAGCAGAAAAAATAAACATTTGTTTGTAATTTTATTCATTCTTT
TATTTGCAGAATTAAAGTGCTCTTCTTTAAGATTAATTGAAAATGGTTATTTTCATCCTGT
AAAGCAAACCTATGAAGA
>1691
TATTTTAATTTAATCTTTATTATTTTTTGTAATTATATGACTAAAGGTGCTGATTAATTTTTT
CCATAAGGAAGAAGAAACAGATGTCCTCCTCCACCTCTGCCCATAAACTCCAAAATTC
AAACACATTCAACAACTTA
>1692
GAAGCTTTGCTAAAATGAAATCTGCATGTGTAGCTAAATGGTCAGTTCTCTTTCAACTG
TACCTTTTCAGAAGGACAGGAGAAGGTAGCCTGTGAGGAACCACCCTTCATTGAAAAT

GGTGCAGCAAATTTACACTCTAA
>1693
ACTATAGTATGAGTTACTATAGTATATTGTTATGAGCTCATATTAATTTTAAAACAACCTC
TTTTCTTAGAAAATAATGAGAATTGTAAGCATCCTCCTGTTGTAATGAATGGGGCTGTTG
CAGACGGGATATTGGCAAG
>1694
TGTTTTAAAAAATATCACTTTTATATTTGTCAGACAAGTAAAGAATTTAAAAGAGCATTT
TGTTTTACAGAACCATGTACTGTTAATGTGGATTACATGAACAGAAATAACATAGAAAT
GAAGTGGAAATATGAAGGGAA
>1695
TGTAGCACTTATAAAGTACTGGATGTTCATTATAGCAATTCATTGTATACTTTAAAACTTA
TTTTTGCAGAATCTAAAGGAATGTGCACATCTCCTCCTCTTATTAAACATGGAGTCATTA
TTAGTTCAACAGTAGACAC
>1696
TGTTCTTACCAAGATGTAACAAGATAGTTTTCTTCTTTTTCTTTCTTTTTTTTTAAATTGT
TGTTCCCAGAGCCATGCACATTATCTTTTACTGAAATGGAAAAGAATAATTTACTTCTGA
AATGGGATTTTGACAACAG
>1697
GGCTGAGATTGTAATTAACACCTGACTGCAATTGATGCTTATTTCAAAAATCTCTCTTTT
TCCCCTCAAGCACTCTGTCTTATCAAGAACCCTTAAGAACATAGAAATGAATGGCAGA
AAGAGGAGTCATATTTCAATAC
>1698
GCAAAAAAAATGGGGTGGAGGAAAATGAGGCATCTGTGTGAGTATATAACCATTTGAC
ATCTCTTTTTAGCCCTCTCCAGGGTCACCCTAGAATCAGATCTGCTCCCCAGCATCTTCT
GTTTCCTGGTGAGTGATTCCTG
>1699
TCACCTTTATCAGAAAACTTCTCAGCCAGATACAGAAATCATTCCAATGACCTGACCTG
TGTTCACACAGAACTCCAGAACAAGACCAAGCTGACAGTGCTGGAAGGAGACATTCT
GGATGAGCCATTCCTGAAGAGAGC
>1700
AGATCTGTGTTCGTGGTTGGCACCTCTTAGGGATATATCCTGACAGTGACAATATGCTCT
TCATGGACAGGTACCCAGCTCCTGTTAGAGGCCTGTGTCCAAGCTAGTGTGCCAGTCT
TCATCTACACCAGTAGCATAGA
>1701
AAGGGGAGAGCCCAGGGAGGGAAGGGAAGAATGGGCAGATGGGAGGGCAGCCAGCT
TCTGCTCACTCCAGGCACAGCCATGGGAGATTCGGAGATGGCAGTCTTTGGGGCTGCC
GCCCCCTACCTGCGCAAGTCAGAGAA
>1702
ACTCCTAACCCTCTTGAGGAAGGAGGGAAAGCCCAGGCTGACAGGAGGGCTTGGGTG
GGGGCTCTTGCAGACAGTGACCGTGAAGGAGGACCAGGTGATGCAGCAGAACCCACC
CAAGTTCGACAAAATCGAGGACATGG
>1703
CTCTAACTCCCAAAATCACCAGCCCTCCCCCTTCGCAACTGGCAAGTCACTGCTCCTTT
TCTATCCCCAGACCTACTCGGGCCTCTTCTGTGTCACCGTCAACCCTTACAAGTGGCTG
CCGGTGTACACTCCTGAGGTGG
>1704

CTCTCCTGATTTGAGGCTTGCTGGTCTCCAGTAGTATTGTTCACTGCCCAATAAGCCCCT
GTCTTCACAGCGGAGAATCCGGAGCAGGGAAGACAGTCAACACCAAGAGGGTCATCC
AGTACTTTGCTGTTATTGCAGCC
>1705
CCCTCCAAGGTCCTGTACCGCAGAAGGGAGGGAGAAGAGCTCTCACCTGCCTCCTTCT
TGGCCTCTGCAGGGCACCCTGGAGGACCAGATCATCCAGGCCAACCCTGCTCTGGAG
GCCTTTGGCAATGCCAAGACCGTCC
>1706
TATCATTAATTTTCCTTGTGCCCAAACCCTAACTTTTCTTTCTCTCCTTCTTCTCCCCACC
TGTTCACAGATCTTCTGGAAAAATCCAGAGTTATTTTCCAGCTGAAAGCAGAGAGAGA
TTATCACATTTTCTACCAAAT
>1707
GTGCTGTGGCGAGCAGCCTCCATGAGAGCCGGGGGCTTGTGTCCCACCCTAACCATGT
TTTTTCCCCTAGACATGCTGCTGATCACCAACAACCCCTACGATTATGCATTCATCTCCC
AAGGAGAGACCACCGTGGCCTC
>1708
CTTACAAGGGATCTCACTTACCCATCATACTTCTTTTTCTGGGGTCCGCCAATATGGGGC
CTCCCTACAGAACGCTTTTGATGTGCTGGGCTTCACTTCAGAGGAGAAAAACTCCATG
TATAAGCTGACAGGCGCCATCA
>1709
AGGCATGAACCACCACACCTGGCCAGCAGTCATCTCTTTACCAACTTTGCTACTTGCCT
TTTCCTTCCAGAGGCTGACAAGTCTGCCTACCTCATGGGGCTGAACTCAGCCGACCTG
CTCAAGGGGCTGTGCCACCCTCG
>1710
GGGATGAGAGTTTTCAAGTTCACTCTTCCCAACAACCCTGCTCAATATGGGTCTCTCCT
CCACCTTGCAGGTGATATATGCCACTGGGGCACTGGCCAAGGCAGTGTATGAGAGGAT
GTTCAACTGGATGGTGACGCGCA
>1711
GGACGGGCACAGCCCCAATGGCCACTCACACCCACTTTCTGACTGCTCCCACCCCTCA
TGCCCCCTGCAGTTCAACAGCTTTGAGCAGCTCTGCATCAACTTCACCAACGAGAAGC
TGCAGCAGTTCTTCAACCACCACA
>1712
TGACCATAGAGCAGAATCCATGTCCACCTGTGTGAAGGACACTCAGTGATGCTCTCTC
CTGCTTCCTCAGCCCATGGGCATCATGTCCATCCTGGAAGAGGAGTGCATGTTCCCCAA
GGCCACCGACATGACCTTCAAGG
>1713
TCTCCTCTCTTCTTCCTGCATCTCTTTCTGGCATTTTCTTTACTTTTCTCTATTGCATTTTT
GGCCACAGGAAAATCTGAACAAGCTGATGACCAACTTGCGCTCCACCCATCCCCACTT
TGTACGTTGTATCATCCCTA
>1714
AGCCAGGATCAGAACCCAGAACTTCAGTCCAGTGTTCTCACAGACTCCTCCTACTTCC
TTCTTGCCACAGGCGTGATGGACAACCCCCTGGTCATGCACCAGCTGCGCTGCAATGG
TGTGCTGGAGGGCATCCGCATCTG
>1715
GACCCTAGAATTCTATGGGCAGAGCAGATCACTGCAGAGCATGGGTGACTCTGGACAC
TTCCCTCCTCAGGTATCGCATCCTGAACCCAGCGGCCATCCCTGAGGGACAGTTCATTG

ATAGCAGGAAGGGGGCAGAGAAG
>1716
AAGGTAATCCCCACCATCTCTTTCCCTCGTACCCCTCCCTAGTCATGGCCAACACACAC
CTTGCCTGCAGGTGTTCTTCAAGGCCGGGCTGCTGGGGCTGCTGGAGGAAATGAGGG
ACGAGAGGCTGAGCCGCATCATCA
>1717
TCCCAAGGTTTCAGGACCTCAGGTAGGAAGGAGGCAGAGGCTCAGCACTCCTTTCAAT
GGGCCCCTGCAGAGACTCCCTGCTGGTAATCCAGTGGAACATTCGGGCCTTCATGGGG
GTCAAGAATTGGCCCTGGATGAAG
>1718
TCCTTCCCTCTACCTGCAAGAATGAGGACCTTACCCCCTGAACAGCCTCCCCTCTGTTC
CTCACCTTCAGGAACAAGACAACCTGGCAGATGCTGAGGAGCGCTGTGATCAGCTGAT
CAAAAACAAGATTCAGCTGGAGG
>1719
GGAGCTCCCCACAGATGGCACCAAGCTGGTGACCTTTGACCCTAAAGGAGATGGGATT
CTTGGTCGGCAGGTGAAAAACCTGACAGAGGAGATGGCTGGGCTGGATGAGATCATT
GCCAAGCTGACCAAGGAGAAGAAAG
>1720
GTAACTGAACAACAAAATCACCAAGTCAGGATGCTTTGCCTCACTGGGCCTGGCTCCT
TCTCTCTACCAGCTGGAAGGATCCCTGGAGCAAGAGAAGAAGGTGCGCATGGACCTG
GAGCGAGCGAAGCGGAAGCTGGAGG
>1721
TTCAACCACTGGTGGACCCTCCTGAGGCCCCACGAGTCTCCCTTACCTCACCATCCCTC
CTTCCCCACAGAAAAGACTTTGAGCTGAATGCTCTCAACGCAAGGATTGAGGATGAAC
AGGCCCTCGGCAGCCAGCTGCAG
>1722
CCTTTTAGAGCCGGGGGGATTCCAGTGGAGGGGTCCAGGCGGTGGGTCTGAGCCCTTT
GTGTCTGACCAGGCACGCATCGAGGAGCTGGAGGAGGAGCTGGAGTCCGAGCGCACC
GCCAGGGCTAAGGTGGAGAAGCTGC
>1723
TTGCACCTCTTACACCCCTTCATTCCCCCCGCCCCCAACATCCATCATATAACTCTCCTT
CCCTTCTCAGGCTAACCTGGAGAAGATGTGCCGGACCTTGGAAGACCAGATGAATGAG
CACCGGAGCAAGGCGGAGGAGA
>1724
ATGGGGCCTGAGGGCTAGAGGAGGAGGTGGGGATAGAGAGGAGTGCTGATCTAGAAT
GGTGCTTCCCCAGGTGAGCTGTCCCGGCAGCTGGATGAGAAGGAGGCACTGATCTCCC
AGCTGACCCGAGGCAAGCTCACCTA
>1725
GAGAAGGGCAAGGGTGGGGTTGCTTTATGGAGAAAGCTGAACCCACCTCCTGGTGCC
CACCCCTCCCCAGGCGAAGAACGCCCTGGCCCACGCACTGCAGTCGGCCCGGCATGA
CTGCGACCTGCTGCGGGAGCAGTACG
>1726
CATGTTGTTTACCTCTTGTCCCCATCCACACCCTCCATCCTCCCCACCCTCTGCCACCTC
CCCTGGGCAGGAAGAAGCTGGCCCAGCGGCTGCAGGAAGCTGAGGAGGCCGTGGAG
GCTGTTAATGCCAAGTGCTCCTCG
>1727

GGTCCTGAGACAGACCCTGGACATGGGGCTGAGGCTGGGGGGCTGAAGAGTGAGCCT
TGTCCCCGGGCAGATCCTGGCCGAGTGGAAGCAGAAGTATGAGGAGTCGCAGTCGGA
GCTGGAGTCCTCGCAGAAGGAGGCTC
>1728
AAGAGGCGTGTCCCTCCAACTCCACTGGACCTCAGCAGCCCTCAACCGAGTTACCGTG
TTCCCCACACAGAGGAGATCTCCGACTTGACTGAGCAGTTGGGTTCCAGCGGAAAGA
CTATCCATGAGCTGGAGAAGGTCCG
>1729
GTGAGCAGGCCCCCACCCTGCCCTGTGCCCTGACTGTCTGCCTGCATCCCCTCCCCCA
ACCCCTTCCCAGGCCTCCCTGGAGCACGAGGAGGGCAAGATCCTCCGGGCCCAGCTG
GAGTTCAACCAGATCAAGGCAGAGA
>1730
TCTGACAGGTGCCTTTAGTGAAGGGAACCGAGGCTGGCTCCCTGCTCATGCCCACTCT
CCTGATCCTCAGGACACCCAGATTCAGCTGGACGATGCAGTCCGTGCCAACGACGACC
TGAAGGAGAACATCGCCATCGTGG
>1731
AGCAAGGCTTGAGAGCTATGCATAGATGCTCAATGCTTTTCCTGCTCTGCCCAACCCTC
CCCCAACCCAGAACACCAGCCTCATCAACCAGAAGAAGAAGATGGATGCTGACCTGT
CCCAGCTCCAGACTGAAGTGGAGG
>1732
TCTCCGTGGGCTGGGCAGCAAGTGTGTGAGGACTTGACCAGACCATGTGCCACCTCTC
TCCTGCACACAGGCCGCCATGATGGCAGAGGAGCTGAAGAAGGAGCAGGACACCAGC
GCCCACCTGGAGCGCATGAAGAAGA
>1733
GCAGGGCGGAGGGGATGCTACCTTCTATGACTGTGCCATCTTCACCCCCTGCCTACCCT
CTGGCCCCCAGACGGAGGAGGACAGGAAAAACCTGCTGCGGCTGCAGGACCTGGTAG
ACAAGCTGCAGCTAAAGGTCAAGG
>1734
GAGCTCGTCCCCCAAGCCAGGAGTCTGAGAACCCAGGCCCCCTCTCACCTCATGCTCC
CACCTCCCGCAGGAGGAGCAAGCCAACACCAACCTGTCCAAGTTCCGCAAGGTGCAG
CACGAGCTGGATGAGGCAGAGGAGC
>1735
GTTCCCATGCCCTGTCCCTGCCCAATACCATCTCTCCAAGGACTGATTGGACTTTGTTTC
CTTTCAAAAGGGCTTGAATGAGGAGTAGCTTTGCCACATCTTGATCTGCTCAGCCCTGG
AGGTGCCAGCAAAGCCCCATG
>1736
CTTGGGTGCCATGAAGGGCTGGTGAGCCAGGGGTGGGTCCCTGAGGCTTGGACGCCC
CCATTCATTGCAGGAGCAGCGCAACCATTTGCAGGGCAAACTGTCGGAGCTGCAGGTG
GAGCAGACATCCCTGGAGCCCCTCC
>1737
GTTTAATAAAACATTTGTTTTTAAAGAGACTGGTCAGCATTGCTAATTTAAATTTTTCTT
TTCTTAATAGGTGAATATGTTTACAAACCAAGGAACAGTGATCCACTTTAACAACCCTA
AAGTTCAGGCATCTCTGGCAG
>1738
GCCCGTTTTGGGAACCTGGACACAGTGCCCCTCACTTGCCTCCTTCCCCATCTGATCCT
CACACCCACAGATGTCTCCAAGCTCACAGACACCATCTGCGGGGTGGGGAACATGTCA

GCAAACGCCTCTGACCAGGAGAG
>1739
GATTCCAGAGGTAAAAGCGGCCATGGGCCAGACATACTGCAATCTCTGAAAATCACCT
GTTCCCCTGCAGCCCAAGAGCCAAGAGACCTGCCGGGGGGCCCTCATCTCCGACCAAT
GGGTCCTGACAGCAGCTCATTGCT
>1740
CTACCACCTCACCCAGCCTCTGGCCCCTGCAGGAGCCCTGGTCTAGCCTAATCTAGTGT
ATCATTTCCAGGAGACCCCAAATCCCAGTGGGGCAAAGAATTCCTTATTGAGAAGGCG
GTGATCTCCCCAGGGTTTGATGT
>1741
AGAGTGCTCTGGAGATCCCTGGAAGAGACTACTGGGGACAGGCTGGTGTGACCCTTG
CTCTTCTCCCCAGGCCCATCTGCCTTCCCTGCACGATGGAGGCCAATCTGGCTCTGCGG
AGACCTCAAGGCAGCACCTGTAGG
>1742
AGTCCCCAAGCCAGGAACCTGGATTCTGGGTAAAAGGACCAGCACCAACATCCCCTTC
TCTTGACTATAGAGAATGAACTGCTGAACAAACAGAGTGTTCCTGCTCATTTTGTCGCC
TTGAATGGGAGCAAACTGAACAT
>1743
GGCGTGAGCCACTGCACCCCACCCGGGTCTGCTTATTCTACCCTTCTCTCTGGTTCCAC
CCCTGCTGCAGTGGACAAGCTGTGCCGAGGTTGTCTCCCAAGAAAAAACCATGTTCCC
CAACTTGACAGATGTCAGGGAGG
>1744
GATCTAGGGAGGTTGGGGCTTACAGTTGGGGCTGTGGCAGCCTCCCAGCCAGTTCTCT
CCTTTTCTCCAGGTGGGTCTGGTGAGCTGGGGTCTTTACAACCCCTGCCTTGGCTCTGC
TGACAAAAACTCCCGCAAAAGGG
>1745
GGAGGGGGAAGGGGAAGAATCCAGGGTTGTCATCACCACTGAGTATGGATTTCACATT
CTAACACATTAGAAGCTGCAGGATGCTGAAATTGCAAGGCTGATGGAAGACTTGGACC
GGAACAAGGACCAGGAGGTGAACT
>1746
GCTGACATCTACCCCTTTAGGACCTGAGTTCTCTCTGCTCCCTCCTGAGTGCCCTGCCT
TCTGCCTGCAGGTTCTGGTACCCCCGGGCTATGGACAGGATGTACGGAAGTGGCCACA
GGGGGCTGTTCCTCAGCTCCCAC
>1747
AGCGACAGAACGAGACTCCGTCTCAAAAGAAAAAAAAAAAAACAACTCTGGGCCAA
CGTTATCCCTCCAGACACCTGAAAGACCCCGCCGTGGCATCCCAGGGCTTCCTGATGG
CCGCTTCCATCTGCCGCCCCGAATCC
>1748
GGGACCTCTGATCTGGCCTTGGTAGGAGGAACCCTCTGACCAGCCTTTTCTGCCCCATC
CCTCACTGCAGAGCTGATGGGAACCCCTGATGGCACCTGCTACCCTCCACCACCAGTA
CCGAGACAGGCATCTCCCCAGAA
>1749
CCACCCAGAGCTTTGAGGATTTGGAAAGCTGGGGCTAGGACCTTTCTAGCTAACTTGG
GCTCTTTCTTAGATGTCTCACCTGTTCAATGTGGCACACACACTGCGTATGATGGTGCA
GAAGGAGCGGAGCCTCGACATCC
>1750

GTGGTGCCTAGCTGGGGTTTCCCCAGGGTGGACACGCATACGTACACCTTCCATCTTGC
TCTTTCCCTAGGGGAAGGTCATGGCCTCCATGTTCTATGAAGTGAGCACACGGACCAG
CAGCTCCTTTGCAGCAGCCATGG
>1751
CCAGAAGAGGGGGACTGGCAGTTGGGGGGCCTCTGAGCTGCAAAAGACAGGATTTTC
CCCTTTTTTCCAGCTGGCCGCCAAGCACTGCCGGAGGCCAGTGATCAATGCTGGGGAT
GGGGTCGGAGAGCACCCCACCCAGG
>1752
TAAAAGCTTGGAAATGATGTCGGGGGGCACTCAGTCTGGGATCCCTGTGGTGACTGGA
TTCCTCTCCTAGATCACGATGGTGGGTGACCTGAAGCACGGACGCACAGTACATTCCC
TGGCCTGCCTGCTCACCCAGTATC
>1753
AGCCATGGAGATGTGGGTTGGGCAGTCAGAGCCCAGCGCTGCATCCACCATGGCTCTC
CTCACCCTCCAGGAGGAATTCGAGAGCATTGAGGAGGCGCTGCCTGACACTGATGTGC
TCTACATGACTCGAATCCAGAAGG
>1754
AGCCCAGACTTGTGTCCTCTGTAGCCACTCCTACCCCAAGGCACTAATGGGGACCCCA
TCTGCTTTGCAGTGCTTTGGTCAGTTCATCCTCACTCCCCACATCATGACCCGGGCCAA
GAAGAAGATGGTGGTGATGCACC
>1755
ACTTAGTCCTGGACAAGCCATCCATGGGCTGCACGATAACACTTCCTTTTTTTTTTTTTT
TTTTTTGCAGCGTGGAAGTGGACTCGGATCCCCGCGCAGCCTACTTCCGCCAGGCTGA
GAACGGCATGTACATCCGCATG
>1756
AGCCACCAATCTGGTACCCACATTGCAGGTTCAGCAAGCATAGAGCTAAGTGCCAAAT
GCTTCCTTCCAGGGGAGATCATCGGAGGCCGGGAGAGCAGGCCCCACTCCCGCCCCTA
CATGGCGTATCTTCAGATCCAGAG
>1757
CTGTCTGCCTACCCTGCAGAGCTGGTGGACCATAGCTCCTGCAGCCCAGACCTACCTCT
TGCTTTTGCAGCAATATAAATGTCACCCTGGGCGCCCACAATATCCAGAGACGGGAAA
ACACCCAGCAACACATCACTGCG
>1758
CTGCACCAGCGACCCCCGAGCCTTGGCTGGAGGCCCCAGCTGAGCGGGAACGCCTAC
ATTCTTCCTCCAGCTGAGCAGAAGAGTCAGACGGAATCGAAACGTGAACCCAGTGGC
TCTGCCTAGAGCCCAGGAGGGACTGA
>1759
AGAGCCCGGAGCTCAGGGAAGCAGGAGCAGGGAGGCCTGTCTCAGTCTCCCTTCTCC
TCTCTACCTACAGGGGGATTCCGGAGGCCCCCTGCTGTGTAACAATGTGGCCCACGGC
ATCGTCTCCTATGGAAAGTCGTCAG
>1760
TGGAGAAGTTGCTGGTTTGGTCCCAGTGGGAGGTCTATGCTGATTCCTGGGTTCCTCCA
CCTTGTTCCAGGGCACCAAAGCCCAGTTCAGCTGCTTCAACCCCAAGTGCCTCCTGCC
TGCCAGCCGGCACTACTGGACCT
>1761
GATGCATCTGGGGTCATAGAGGACCACAGCACCCCCAATCTGCCCTGAGCATTCCTTCT
GCTTCCTCAAGATGGGGAAGTTCCGGAGCCTGCTTTTTACCTCGGAGGACGATGAGAG

GATCCACATGGTGAACAACTTCC
>1762
TAGTATCATTGTATAGAATTTTACCCCATGGGTACAATGTATTAACTATGTTCTCTTTCTC
TCCTAAAAGTTGCTGATCCCACACCCTTTCATATTCAAGCTGAAGTGACGATGAAAACA
AATTTCTTTGGTACCCGAGA
>1763
AAATTTGGCATTCACCTCTCTACGGGATTGTTGCACACCTTTCTACATAATGCTTTGTGG
TGTATCTTAGGGAGAGTGGTGAACGTATCTAGCATCATGAGCGTCAGAGCCCTTAAAAG
CTGCAGCCCAGAGCTGCAGCA
>1764
GTGTGAGGAGTCGGGGGGCTTGGAGGTCCCCCCCACCCATGCCCACACCTCTCTCCCT
CTCTCTCCACAGAGGGAGATAACGCTGTGCTGCAGTGCCTCAAGGGGACCTCAGATGG
CCCCACTCAGCAGCTGACCTGGTC
>1765
TCTGTCTTTGAGTCTCTATCTCTCTCCCTCTCCTGGGTGTCTCTGCATTTGGTTCTGGGT
CTCTTCCCAGGGGAGCTGTTCCGGTGGAATGTTTCGGACCTAGGTGGCCTGGGCTGTG
GCCTGAAGAACAGGTCCTCAGA
>1766
GGGGAGGGGAAACTGAAGAGGTGAAACCCTGAGGATCAGGCTTTCCTTGTCTTATCTC
TCCCTGTCCCAGACCTCACCATGGCCCCTGGCTCCACACTCTGGCTGTCCTGTGGGGTA
CCCCCTGACTCTGTGTCCAGGGG
>1767
CCTTGAGATCTCAGGATTGGGACGTAAGATGCCAGCCTGGGGTCCTCGTCTCATAGCCC
CTTCCCCCTAGTACTATGGCACTGGCTGCTGAGGACTGGTGGCTGGAAGGTCTCAGCT
GTGACTTTGGCTTATCTGATCTT
>1768
AGAATCTTGAGTGGGTCCGCCATTCCATTCCAAGTCGGCCCTCACCGTGCACTTCCTCT
TCTCCCGCCAGATTCTTCAAAGTGACGCCTCCCCCAGGAAGCGGGCCCCAGAACCAGT
ACGGGAACGTGCTGTCTCTCCCC
>1769
GCCGCGCGGACCCTTAGCCTTGACCGCCCCCATCTCTTCTGACCCCGTCTTACAATGCC
CCTCTCACCAGGACGCGCCCAGCGTTGGGCCGCAGGCCTGGGGGGCACTGCCCCGTC
TTATGGAAACCCGAGCAGCGACGT
>1770
GAATAGTGGACTGGGCCCTGGAGGAGAGGGGGCATGACTCGGTTCCCCATCCCCATCC
CCAAACCCCCAGGCCCAGAAGAAGAGGAAGGGGAGGGCTATGAGGAACCTGACAGT
GAGGAGGACTCCGAGTTCTATGAGAA
>1771
GTCTTCCTCCTTACCGTAGGCTGTACTTCCCATGCCCTAGCCTCCAATTCTCCATCCCCC
GCCCAAGCAGGGTCCCAGTCCTATGAGGATATGAGAGGAATCCTGTATGCAGCCCCCC
AGCTCCGCTCCATTCGGGGCCA
>1772
CTGGGCTGACTTTGCCTTCCAGCCTACTTCCAGTGCCACCCATGTTCTCCTCCTCCCTG
GTCCTATCCAGATGCAGACTCTTATGAGAACATGGATAATCCCGATGGGCCAGACCCAG
CCTGGGGAGGAGGGGGCCGCAT
>1773

GGGTTCCTAGGGCCTGGTGGGCAGGGGGAGGACTGCTGGACCCCTCCCCATCACCGTT
TCTTCTGCATAGCCTGGATCTCCTCAAGTCCCCAAGATTCACACCTGACTCTGAAATCT
GAAGACCTCGAGCAGATGATGCC
>1774
CAGAGGGGAGGCCCGGCCAGGTCCTCCGGCAACTCCCGCGTGTTCTGCTTCTCCGGCT
GCCCACCTGCAGGTCCCAGCTCTTGCTCCTGCCTGTTTGCCTGGAAATGGCCACGCTT
CTCCTTCTCCTTGGGGTGCTGGTG
>1775
GCAGGGGCCAGGGCTGGGGAAATGTGTCACCATCCCCAGTCCCTGACCCACCCACCCT
GTCTCTCCACAGGCCCTGGGTGCCAGGCCCTGTGGATGCACAAGGTCCCAGCATCATT
GATGGTGAGCCTGGGGGAAGACGC
>1776
GGTGGGCGGGGCCAGGAGGCTAGGGAGGGCAAGAGGGGCCAGGGCTCTGAGCCATA
CTACCTCCTTGCAGAGCCGCCCCCCAGGCCCTTCCTGGACATGGGGGAGGGCACCAAG
AACCGAATCATCACAGCCGAGGGGAT
>1777
ATCCAGGAGGGTCTGAAAGATATTCACCTCCCCCTGCTCACTGAGGCACCCACCCCAC
CCACCCCTACAGAAACGATGGCAGAACGAGAAGCTCGGGTTGGATGCCGGGGATGAA
TATGAAGATGAAAACCTTTATGAAG
>1778
CTGGGGGTGGCTGGGGGCAGGGACCCCAGGTGTCAGGGTGCTGATGTTCGCTGCCTC
ATTTCCATCCCAGGCCTGAACCTGGACGACTGCTCCATGTATGAGGACATCTCCCGGGG
CCTCCAGGGCACCTACCAGGATGT
>1779
CCCAGGACCCCATTTTTCCACCCTAATGCATAGGTCCCAATATTGACCGATGCTCTCTGC
TCTCTCCTAGCCTCACTTCTAACCTTCTGGAACCCGCCCACCACTGCCAAGCTCACTAT
TGAATCCACGCCGTTCAATGT
>1780
CTTGCCTTGCCCCTCCGGATTCAGGCCGATGGGTTGAGCCCCCCTGACCCTGCCCGTGT
CTCCCTCGCAGCTGGGCGCCGTGTACACAGAAGGTGGGTTCGTGGAAGGCGTCAATA
AGAAGCTCGGCCTCCTGGGTGACT
>1781
GCCGGACTGGCCCTGCGGCGGGGCGGGTGAGGGCGGCTGCCTTCCTCATGCCAACTC
CTGCCACCTGCAGGGACCCTGAAGGCCAAGAACTTCAAGAAGAGATGCCTGCAGGCC
ACCATCACCCAGGACAGCACCTACGG
>1782
AGGGACAGGGGACCGGCTGGAGACAGGGCCAGGCGGGCGTCTGGGGTCACCAGCCG
CTCCCCCATCTCAGTCTCCCGGGACCTGCCCGTTATGATCTGGATCTATGGAGGCGCCT
TCCTCATGGGGTCCGGCCATGGGGC
>1783
GATCTCCCCTCCTGGAGGCCAGCCTGGGCCACTGGTCTCTAGCACCCCCTCCCCCTGC
CCCTGCCCCCAGGTAACTATGGCCTTCGGGATCAGCACATGGCCATTGCTTGGGTGAA
GAGGAATATCGCGGCCTTCGGGGG
>1784
TGTCCTTGTCCCAGCGTGGGGTGGGCAGAGTGGGGAGCGGCCTTGGTGACGGGATTT
CTGGGTCCCGTAGACCCTCTCCCCCTACAACAAGGGCCTCATCCGGCGAGCCATCAGC

CAGAGCGGCGTGGCCCTGAGTCCCT
>1785
CTTGCCTTCAAATGGTTCTGAGCCCTGAGCTCCGGCCTCACCTACCTGCTGGCCTTGGT
TCTGCCCCCAGGTGGCTGAGAAGGTGGGTTGCCCTGTGGGTGATGCCGCCAGGATGGC
CCAGTGTCTGAAGGTTACTGATC
>1786
GGCAAGGGGCGGCTCAGGGGAAACTGGGAGGTACAAGAACCTGCTAACCTGCCTGGC
CTCTCCCACCCAGACCCCATGCTGCACTATGTGGGCTTCGTCCCTGTCATTGATGGAGA
CTTCATCCCCGCTGACCCGATCAA
>1787
GTCGGGGTGAGTATGCACACACCTTCCTGTTGGCACAGGCTGAGTGTCAGTGCCTACT
TGATTCCCCCAGGGAGGACTTCTACAAGCTGGTCAGTGAGTTCACAATCACCAAGGGG
CTCAGAGGCGCCAAGACGACCTTT
>1788
CAAGTCCGGTCTCTGGGCTGATCGGTCCCCAGTGAGCACCCTGCCTACTTGGGTGGTC
TCTCCCCTCCAGGAGTGCCAAGACCTACGCCTACCTGTTTTCCCATCCCTCTCGGATGC
CCGTCTACCCCAAATGGGTGGGG
>1789
TGCAGGTGGAGAGCAGGCTTCAGCCCCCTGGGAGTCCCCAGCCCCTGCACAGCCTCT
TCTCACTCTGCAGGGACCCCAACATGGGCGACTCGGCTGTGCCCACACACTGGGAAC
CCTACACTACGGAAAACAGCGGCTAC
>1790
GAACAGCCCCTTTCCTGTGCCCCACTTGTGAAAGTCTCCATAAACCCCTGTGGCCCAC
TCTGCCTTCCAGAGAGAGGGGCTGAACTGCAAGGTGGCCACCAACACAGAGGCCCTG
CAGAGCGGCAGGATGGAGATGTGGA
>1791
TCTCTAGTGCTCTGCCATGGGGAAGGGTCCCACGTCCATCTCATCGCACCACCTATCTC
TTGGCCCACAGAAAGCATCGCTGGAGGGCAGCCTGGTGGAGACGGAGGTGTGTTACA
GGACCCAGCTGGCCCAGCTGCAGG
>1792
GGTGGAGAGTTTATTGAGTGTTTGGGTATTGGCTTGTCACCTGCCAATCTGTTCTGCCC
ACTCCATCCAGGCCTGGGGCTCCTAGAAGACCAGAGGGGAGGAGGAGGAAGGATTAA
GGGTGGAGGGAAGGGACAGCCCCT
>1793
AACTCTGGTGTGGGCTCAGCACCCCCATCCGATGACCTGACTACTCTCCCATCTCCGCT
GATCCTCACAGCCACCGTGGACAATGCCAACATCCTGCTACAGAATGACAATGCCCAT
CTGGCTGCTGCTGACTTCCGCAC
>1794
AGGTGAGCAGCCAGGACCTGGACCTGCAATTCCAAGCAGCCTGGGCTGAAGTCCCTG
ATTCCCATGGCAGGTTTGAGACAGAGCAGGCCCTGTGCTGAGTGTGGAGGCCGACATC
AATGGCCCGTGCAGGTGCTGGATGA
>1795
GGAAGGCTTGGGAGTGAGAGATCAGGATGGGTCCAGATGCGCACATCCACATCCCCTT
TTTCCATAGGAGATGAACGCCCCGCGAGCCAGGTGGGCGGTGAGATCAATGTGGAGAT
GCGCTGCCCAGTGTGGACCTGAGC
>1796

ATTTGGGAGGAGGCCTGACTGAGGAGAGGGATCCAGGCTCACACCACCCTGTCCTGT
GTTCTGTCTGCAGACAGAGGAGCTGAACCGCGAGTGGCTACCAACAGTGAGCTGGTC
CAGAGCGGCAAGAGCGAGATTTCGGA
>1797
GCATGGGGATGGGCCCAGAAGAGGACACTGACAACCCTCACTGACCCCTGGTCTTCCT
GCCCTCCTGCAGAAAGCATCCTGGAGGGCAACCTCGGCGGAGACAGAGAACCGCTAC
TGCATGCAGCTGTCCCAGATCCAGG
>1798
TGAGCTCACCTCAGAAGAAACCCCAAGCCATCACTGTCGCCTCCTTTTCCTTCCTTCTT
CCTCACAGCAGGTCTATAACAGTCACCCTTGGAGCCCATAACATAACAGAGGAAGAAG
ACACATGGCAGAAGCTTGAGGTT
>1799
TCCCTCCCTGATCACCTCCAACTGTCCAGAGCCCATTTCGAGGACTGACAGTCCTTCAT
TCCCTTCACAGTTGAAGGAGAAAGCCAGCCTGACCCTGGCTGTGGGGACACTCCCCTT
CCCATCACAATTCAACTTTGTCC
>1800
AACCCAATTTGTCCATTCATTGGAGAGAGTATCTATGGCCGTGTTCAAACCCTGGGGTG
CTCTGTTCCAGGGGAGATCATCGGGGGCACAGAATGCAAGCCACATTCCCGCCCCTAC
ATGGCCTACCTGGAAATTGTAAC
>1801
ATGAGCTCACCTCAGAAGAAACCCCAAGCCATCACTGTCGCTCCTTTTCCTTCCTTCTT
CCTCACAACAGGTCTATAACAGTCACCCTTGGAGCCCATAACATAACAGAGGAAGAAG
ACACATGGCAGAAGCTTGAGGTT
>1802
TGCCCTCCCTGATCACCTCCAACTGTCCAGAGCCCATTTCGAGACTGACGGTCCTTCAT
TCCCTTCACAGTTGAAGGAGAAAGCCAGCCTGACCCTGGCTGTGGGGACACTCCCCTT
CCCATCACAATTCAACTTTGTCC
>1803
CAAGCTGGCTGGCAGACAGGAGTGGAGGACTTCCCGGGCTCACCTTCTTCTCTCTCTC
CTCTCTCTACAGGGAGACTCTGGGGGCCCTCTTCTGTGTGCTGGGGTGGCCCAGGGCA
TCGTATCCTATGGACGGTCGGATG
>1804
TCTCATGAGCCCCCAGATCTGGATGGGACCCAGCCCTGGCCTTGCCCTGAGTTTCCCCC
ATGTTCCCCAGAGACCACGACCAGCACCACCAGCTCCCTGGCACCCCCACCTACCACG
AAGATCTGTGACCCTGGGGAGCT
>1805
GTGGCAGAGGGAGCCTGAGGGAACCCCCACTCCAGCCCCCTCCCTGACAGGCCCCTG
CCTGTCTCTACAGAGCCCGGCACCCCATGAGGACATGCAGAGCACCTGCAGAGGAGC
AGGAGGCCGGAGCTGAGCCTGCAGAC
>1806
TTTTTCTCTCTGGCTCCCCACCCCCTCCTTCCCTTGGCTCTCTGCTTCTCCCTCCCCACC
CTTGGTGCAGAGGAGGCTGGCAGCTGTGTGCAGGATGGGCAGAGGTATAATGATAAGG
ATGTGTGGAAGCCGGAGCCCTG
>1807
GCAGCCAAGGGACAGATGGCTCTTGGAGAAACACTGCTTCCCATTGATGCCTTTTTCT
CTTTATGCCAAGGGTGCCCCTGGACCTCGTGGCAGAGATGGAGAACCTGGGACCCCTG

GAAATCCTGGCCCCCCTGGTCCTC
>1808
AGACTTACCTAGCCAAGTAGGGAAGACCTAGTGCCTTTCAACCTCCTAACGTTGTTGG
GTTTCTTTGCAGAACTTTGCTGCCCAGATGGCTGGAGGATTTGATGAAAAGGCTGGTG
GCGCCCAGTTGGGAGTAATGCAAG
>1809
ATTGCCCACCCACTAGAGGTCTGTGTCCCCTCTCTTCTTCTTCATTTCATACCTCCCTGT
CTCTTCCCAGGGTGAAGCCGGCCCCACTGGTGCCCGTGGTCCTGAAGGTGCTCAAGGT
CCTCGCGGTGAACCTGGTACTC
>1810
GCTACCCCAGGAGAGCCCAGTCCTGCCTCTCCCCTCTCCTGACACCCCTCCCTTCTTCT
CATGCCCACAGGGTGCTCCTGGCATTGCTGGTGCTCCTGGCTTCCCTGGGCCACGGGG
TCCTCCTGGCCCTCAAGGTGCAA
>1811
ACTCTAGATCGAGGCTTGCTCCAAGGCCAGGTGAAGGCTCACTCTGTTTCTCTTTTTTG
CTGGTCCTCAGGGCCCTGCTGGCCCCCAGGGAGCCCCTGGACCCGCTGGTGAAGAAG
GCAAGAGAGGTGCCCGTGGAGAGC
>1812
AGGGGCTGGGGTCCTCCCTGCATCGCTGAGGTCACATGGTATCCCACTGACTCCCTGT
GTACCCTTGTAGGGAGCCCCTGGAGAGCGAGGGCCCAGTGGTCTTGCTGGCCCCAAG
GGAGCCAACGGTGACCCTGGCCGTC
>1813
CTTAGTGGATGTTGGGTGGATGGATGTGGTCAGAGTGTCTATGTTCTGAGAATGGTGTT
CTGTCTTTCAGGGAGCCCCTGGTGAAGATGGTCGTCCTGGACCTCCAGGTCCTCAGGG
GGCTCGTGGGCAGCCTGGTGTCA
>1814
CTCCCTGCTTCTCCCTGGACCTTCTCCCCCACTGCTGTTGGTTGATCACTTCTTGGTGTC
TCTGCCGCAGGGTGAACGAGGTTTCCCAGGTGAACGTGGCTCTCCCGGTGCCCAGGG
CCTCCAGGGTCCCCGTGGCCTCC
>1815
CAGGCTCCATGAACATGGGTTCAGGGAGAGGTGCTGTCCACTACAGACTCCCTCTTAC
CTCCCTCCCCAGGGTGCATCTGGCCCAGCAGGCCCCCCTGGCGCACAGGGCCCTCCAG
GTCTTCAGGGAATGCCTGGCGAGA
>1816
CGCTCTTCTGGAAGAGCTCCAGGGTTTTAGCCTGCCCCTCATTCACCTGCTTCCTCCTT
CCCCATTATAGGGTGCTGATGGCCAGCCTGGGGCCAAGGGTGAGCAAGGAGAGGCCG
GCCAGAAAGGCGATGCTGGTGCCC
>1817
ACAGTGACTTTGGCAGCCTGGATGAGGAAGGGTGAGATGAGTCCTCACTTCCCGCATT
TTCTCCTTCTAGGGCAACCCTGGACCCCCTGGTCCCCCTGGTCCTTCTGGAAAAGATG
GTCCCAAAGGTGCTCGAGGAGACA
>1818
CAGAGCTGTGCTGAGAGGACGAAGAGGCTGGGCCACTGTCAGTTCTCATCTCCTGCCT
CTCCTCTCTCAGGGTGCCGAAGGTCCACCAGGTCCCCAGGGTCTGGCTGGTCAGAGA
GGCATCGTCGGTCTGCCTGGGCAAC
>1819

TGAAGGGTCAGTGGGCCCTGGGCAGGGGTGCTTACCACTTGCACTCATCATCCTTGTC
TCTGTCCTCCAGGGTGAGCCCGGCAAGCAGGGTGCTCCTGGAGCATCTGGAGACAGA
GGTCCTCCTGGCCCCGTGGGTCCTC
>1820
CTAGAAGGACCTTCTGTTCCTGTCTCTTCTGGAACATTCTTCTCTGAGCCTGAGACCTC
TCTCCTGACAGGGTGATCGTGGTGAGACTGGTGCTGTGGGAGCTCCTGGAGCCCCTGG
GCCCCCTGGCTCCCCTGGCCCCG
>1821
TTGGGTGGGACAGGAGGGGGCTCCTGTCCTGGCCCTGACCTGACTCAATCGGTGTCTG
TCTTGTTCCCAGGGTCCTCAAGGCCCCAGAGGTGACAAAGGAGAGGCTGGAGAGCCT
GGCGAGAGAGGCCTGAAGGGACACC
>1822
CCCAGCCCCAGCGACTCCCCAGCCTTCCCTGTGGTGACCACTCTTTCCTCACGACCTC
TCTCTCTTGCAGGGTCCTCCTGGCCCCGTCGGTCCCTCTGGCAAAGATGGTGCTAATGG
AATCCCTGGCCCCATTGGGCCTC
>1823
AGCAAATGGGGATGGACATGGAGGGGCACTTTCACACAATCCTGGCTGATCTCTCTGT
TTCCTGCTGCAGGGTCCTCCTGGAAATCCTGGGCCCCCTGGTCCTCCAGGTCCCCCTG
GCCCTGGCATCGACATGTCCGCCT
>1824
CCTGGGTCTCTCAAGCTTTTGTGTCTGTGCCTGTCTGAGCCCCCATGGGTGCTGCCTCT
TCCCCCTGCAGGAGACTACTGGATTGACCCCAACCAAGGCTGCACCTTGGACGCCATG
AAGGTTTTCTGCAACATGGAGAC
>1825
GCCCCTACAGCCCCTGGTCCCATTGAATGCCAGCTCCCAGGCCTCACACTGCCGCTCTC
TGCCCCAACAGTTCAGCTATGGAGATGACAATCTGGCTCCCAACACTGCCAACGTCCA
GATGACCTTCCTACGCCTGCTGT
>1826
GCTTCTGGGCAGCTGGAACTGGGTAGCAAGGCATCTACTGAACAGAGCCTCCTCCTTT
TTTCTCCCCTAGAAACATACCGGTAAGTGGGGCAAGACTGTTATCGAGTACCGGTCAC
AGAAGACCTCACGCCTCCCCATCA
>1827
GAGGCCACCGTGCGGGTAGGCGGGTGGTTGGCCTCCCAGAAGAGGCCCTGATCTCTC
CTGCCCGCCCCAGTGACCTGCACGCGCCTTTACGCCGCTGACATTGTGTTCTTACTGGA
TGGCTCCTCATCCATTGGCCGCAG
>1828
AGGCCTCCTCAAGGCCAGGGCCAGAAGAGATCCTGAGTCCTAGCCTGTTGCCACCCCT
ACCCTCCAACAGGACAGAGTTCGGCCTGGATGCACTTGGCTCTGGGGGTGATGTGATC
CGCGCCATCCGTGAGCTTAGCTAC
>1829
CATGCCTCCCAAGATGACCCCAAATGAAGTGTCCAGGGGAACCGTGATTTGACCCCTG
CACCTGTCCCAGGTCTGCATCCTGATCACAGACGGGAAGTCCCAGGACCTGGTGGACA
CAGCTGCCCAAAGGCTGAAGGGGC
>1830
AGGTCAGCTGGGGGGTGGGGGCAGTCAGAGAGCATGTGGGTGACTGAGTCCTGATGG
GTCGTCACTTCAGGGATCAAGAATGCTGACCCTGAGGAGCTGAAGCGAGTTGCCTCAC

AGCCCACCAGTGACTTCTTCTTCTT
>1831
CGACCCCAACCCTGCATCATACATGCCCACCCCAATCCTCCTGCCTGATCCCCTGATCC
CGCATTCCCAGCGGATGACTCGACCTCTGCTCCACGAGACCTGGTGCTGTCTGAGCCA
AGCAGCCAATCCTTGAGAGTACA
>1832
GATGTCAGGAGTATAGGTGGTGGCTGGGGACTTGGCTGGGCAAGATAAAGTGACCTCT
TGCCCTGGGCAGGTGAACGTCCCAGCTGGTGAGACCAGTGTGCGGCTGCGGGGTCTC
CGGCCACTGACCGAGTACCAAGTGA
>1833
ACCTAACCCCCGACCCCAGTACCCCCTCCCACTTCTGACTCCATGAATCCCTGGTGGGA
CTCTCCCCCAGCTGCCCTAGAAGGGCCGGAACTGACCATCCAGAATACCACAGCCCAC
AGCCTCCTGGTGGCCTGGCGGAG
>1834
CCGCGCCTCAGACAAGGCTGTAGAGTCCTGAGTCTGCAAGCCCACTGGCCCCTTGGTG
TCCCCCATGCAGGTGGGCCCACACAGCAGCAGGAGCTGGGCCCTGGGCAGGGTTCAG
TGTTGCTGCGTGACTTGGAGCCTGG
>1835
TTTCTTCAGGCTGGGACGGGCAGGCAGGGCAAGGCCCGGAGGCCACTGACATCCCAT
GTGCCCTGGGCAGACGCTTCTGTTGAGCAGACCCTGCGCCCGGTCATCCTGGGCCCCA
CATCCATCCTCCTTTCCTGGAACTT
>1836
TGCGGGGGAAGGGATGGACAGGCAAGGGTCAGGGCATGGCCTCTGCTGGTCTGACCC
TGTTATCTTGCAGGCTTGGAGCCACCGCAGAAGGTGGTACTGCCCTCTGATGTGACCC
GCTACCAGTTGGATGGGCTGCAGCC
>1837
TGGGCTGGGCCAGTGAGTGGGGGAGGTGTCGGAGCCCCAGGCTGCCTCTATGCTGTG
CTCTCTCCAACAGGACCAGAGCTGCCTGTGAGCCCTGTAACAGACCTGCAAGCCACC
GAGCTGCCCGGGCAGCGGGTGCGAGT
>1838
CATCACTGCCATCGTCCCACATCCCTGTCTCTTTCTGACCCCTGCCCACCTACCCTGACT
TCTCTCTTAGGGGTGGAGCGGACCCTGGTGCTTCCTGGGAGTCAGACAGCATTCGACT
TGGATGACGTTCAGGCTGGGCT
>1839
TGTGGAGGACAGCCTGCCTGCCTCACTCTGGTCCTGGTTCTGACTTCTGACTTCTGTCT
GTAACTCCTAGAGCTGGAAACTCCACTTGCTGTTCCAGGGCTGCGGGTTGTGGTGTCA
GATGCAACGCGAGTGAGGGTGGC
>1840
GGGACCAATGAGGGTATGGGTGCCAGAGGGGACAGGCAGGAGCCATGCCAGCATTTC
CCTCTGACCTCAGGTCCGGAGTCCAGCCAGACACTGCCCCCAGACTCTACTGCCACAG
ACATCACAGGGCTGCAGCCTGGAAC
>1841
TTGCCCCTATCAGATAGCCCCACTCCTTCCTGCCTGTTCCAGCAGACTCCCCATTGCCT
CTCCCCACTAGACCCACTGGGCCCAGTGAGGACGGTCCATGTGACTCAGGCCAGCAG
CTCATCTGTCACCATTACCTGGAC
>1842

TGGGGGTTCAGGTGGGGCTCTGGGGCACAGAGTTTGCTAGCCCTGGAGCTGCCTCCAT
CCCTGTTCCCAGGCCCAGAGAAATCCCAGTTGGTTTCTGGGGAGGCCACGGTGGCTGA
GCTGGATGGACTGGAGCCAGATAC
>1843
CCCTGCGTAACCATTCCTGCACTGCCTCTCTGTTCTCTACCAGGACCCCTTACCTTGCCT
CTGTCCCCAGCCCCTGAGCCTGTGGGTCGTGTGTCGAGGCTGCAGATCCTCAATGCTT
CCAGCGACGTTCTACGGATCAC
>1844
CTGACGCCACCCCTGTCCTTCCTGGCTGGGACTGCTCACCCCTAACCATTGCTGTATGC
CCACCTGCCAGGCGGCCCCATGAGGCACCAGATACTCCCAGGAAACACAGACTCTGC
AGAGATCCGGGGTCTCGAAGGTGG
>1845
CCAGGGTGGGCCTGGGATTGGTGCAGGGGCATGGGGGCAGAGCTCCCTGATTCCTGA
GCTTTCTCTCCAGCGCCTGAGGCTCCGCCAGCCCTGGGGACGCTTCACGTGGTGCAGC
GCGGGGAGCACTCGCTGAGGCTGCG
>1846
CAGGCCCTTTAATGCCCCCAGGTGTCACTATCCCACATGCTCTTGGCCCCCACCCTCAC
GCCTGCCCCAGGTGGCCAGGAACAGTCCCGGGTCCTGGGGCCCGAGCTCAGCAGCTA
TCACCTGGACGGGCTGGAGCCAGC
>1847
ATCTCAGATCTCTCCCTTCTGGGTTCTCAGGACTCAGCCTCTGATCCTCGATCTTTCATT
CCTCCTTCAGAGTCACCTCGTGTTCCAAGCATTGAACTACGTGTGGTGGACACCTCGAT
CGACTCGGTGACTTTGGCCTG
>1848
CAGGATTTGGGTGGGCTGGCAGTTGGGGCTCTGTGGAGACAGCTTTTTAATCAAGTTC
TGTCTCCTGCAGAAGTGCCTGGGTCCCCGCAGACACTTCCAGGGATCTCAAGCTCCCA
GCGGGTGACAGGGCTAGAGCCTGG
>1849
ATAGTGGGAAGGGCAGAAAGGTGTGTCTGGGTGGGCTGCCCGCTTCAGTAACTTGTTC
CCCTTCCTACAGTGTGCCCCCGTGGCCTGGCGGATGTGGTGTTCCTACCACATGCCACT
CAAGACAATGCTCACCGTGCGGA
>1850
CTCCAGGCTCTGTGCACCCCCATCCCTGCTCTGTCATCCTCCTCAGGCTCTGTGTTTCC
TTCCACCCCAGGTTGGCCTGCTGTCTTACAGTCATCGGCCTTCCCCACTGTTCCCACTG
AATGGCTCCCATGACCTTGGCA
>1851
AGGCATGGACTCCTGGGGCTATCCACTGGGAGCTTGGTGCCCCAGGGTTCTGACATGT
CCTTCCTTCCAGGCACAGCCGTGGTCACAGCTCACAGATACATGTTGGCACCAGATGC
TCCTGGGCGCCGCCAGCACGTACC
>1852
ATGGGGAAGGGGTCTGGGAATAGGGGTGGCCCTGAAAAGGCTATCATGCAGCCACTG
GACCCCACCTTAGGGCTTAATGTGGTGATGTTGGGAATGGCTGGAGCGGACCCAGAGC
AGCTGCGTCGCTTGGCGCCGGGTAT
>1853
GAGGCCCCTCCAGCAAACCAGGGGCCTTGGGGGAGGGTCCCTTCCTGTCCTGACCTCT
TCACCTCCTCAGGGCTTCCCTGGAGCAGATGGGCGTCCAGGCAGCCCTGGCCGCGCCG

GGAATCCTGGGACCCCTGGAGCCC
>1854
TGTCTAGGGGCTTCTTCCAGCTCAAGGCCCCCATAGCCTGAATCCTGCCCACTGCTCTC
TGTCCTTACAGGGGGCTCCCGGACAAGTCATCGGAGGTGAAGGACCTGGGCTTCCTGG
GCGGAAAGGGGACCCTGGACCAT
>1855
GGGGACCAACACCAGGGGGGCGAGAGTGAGGTCTGTGGGGTTGCACCTTATACTTTG
TCTCCTCCATCAGGGCCCCCCTGGACCTCGTGGACCACTGGGGGACCCAGGACCCCGT
GGCCCCCCAGGGCTTCCTGGAACAG
>1856
GGGGTCCCCTCCCCTTGAGAACTGCTTGCTTCGAGCGTCCTGCATCACCTCCCTCTTGC
CTCCTCCACAGGGTGACTCAGGGCCTCCTGGAGAGAAGGGAGACCCTGGGCGGCCTG
GCCCCCCAGGACCTGTTGGCCCCC
>1857
AGTGTAGCGGGTCATAGGGGCACCTGCAAAATATTGGGAGGGTCTGTCTCAGGCCAGC
TGCTCTTCTTAGGGGGCACCTGGAGTTCGGGGGCCTGTGGGTGAAAAGGGAGACCAG
GGAGATCCTGGAGAGGATGGACGAA
>1858
CCCAGCAGGTTCTACCTTGGGCATGGCTTGGCTTCAAGGATGTTCCTCAGCAAGCTTAT
CTCTGCCACAGGGAGAGCCTGGGGACCGCGGACAAGAGGGTCCTCGAGGGCCCAAG
GGTGATCCTGGCCTCCCTGGAGCCC
>1859
TCAGGACATGAGCCTGGGTTATCAGATCCACCTCAGCCTTGGTGGCCTCTTACCACTCT
ATTTTCCACAGGGTGACCGGGGTCCCCCTGGGCTGGATGGCCGGAGCGGACTGGATGG
GAAACCAGGAGCCGCTGGGCCCT
>1860
GACCCTGGGGAATATGACTCCACTCTTTTCTGAGGTCTCAGGGTGCTGATGCTGGCTGC
ATCCTTCACAGGGTCGTGATGGCCCCAAGGGTGAGCGTGGAGCTCCTGGTATCCTTGG
ACCCCAGGGGCCTCCAGGCCTCC
>1861
CTAGTGGTGCCCACAGGCATAGGGGCTGCGGCGACGCACCCCGCTCCTCTGACCTCTT
GCTGTCCCTCAGGCATCTGCCCTGCGGGAGATCGTGGAGACCTGGGATGAGAGCTCTG
GTAGCTTCCTGCCTGTGCCCGAAC
>1862
CAGTGGGTTGTGCCCCGTGGAGTGGGGTGTAGCTGTACAGCCACCAGCATTCTCTCTT
CCACTCCTGCAGGGCCCCATCGGCTTTCCTGGAGAACGCGGGCTGAAGGGCGACCGT
GGAGACCCTGGCCCTCAGGGGCCAC
>1863
TGGGGGTCAAGGGTTGGGCTCCAGGGGTCAAGGGTCGACAGGCAGCCCTGACAGAG
CTCTTCCCTCTCAGGGGGAGACAGGGAAGCCGGGAGCCCCAGGTCGAGATGGTGCCA
GTGGAAAAGATGGAGACAGAGGGAGCC
>1864
GGAGTTCTGATGAGAGTCCTGGGAGGGGTCCTGCATTGACCATTCCCTCCCTTTGCTGT
TTTTATTTCAGGGAGATCTGGGCCTCCCTGGCCTGCCCGGTGCTCCTGGTGTTGTTGGG
TTCCCGGGTCAGACAGGCCCTC
>1865

CTTGAGGGGGCAGAGGGTAGGAGGGTTCCCAAGTCACTCTCTGCCTCTCTTGCCCCTA
TTTTTCTGGTAGGGTCTGGCAGGCCCCCCAGGGAGAGAAGGAATCCCAGGACCCCTG
GGGCCACCTGGACCACCGGGGTCAG
>1866
AGAGCTCAAACTCCCAGAGGGTGAGGGACCTTCAGCCCCTTACCGTGACTTCTTACCC
AAACCAACCCAGGGTGAACGAGGGCCTCGGGGCTTGGATGGTGACAAAGGACCTCGG
GGAGACAATGGGGACCCTGGTGACA
>1867
GGCTCGTTGTATTCTAAGCCCCCAGCCTGATCTGGTCCTGACTGAATCCCTCTCCTGGT
CACTCCCACAGGGCAGCAAGGGAGAGCCTGGTGACAAGGGCTCAGCCGGGTTGCCA
GGACTGCGTGGACTCCTGGGACCCC
>1868
CTATCCAGGGCGATTCTCTTTGGTCCCTCACTGACCTCCTCTGACCTCACATGGACCCT
CTCCCTGCTAGGGATCCCCAGGAAAGGATGGAGTGCCTGGTATCCGAGGAGAAAAAG
GAGATGTTGGCTTCATGGGTCCCC
>1869
AGCGAGGACTTTGGGAGAGGGGTCCATCTCTGGGGCCTCTGATTACTGACTCTGTCCT
ATATCTCCCCAGGGAACCCCAGGAATTGGGGGCTTCCCAGGCCCCAGTGGAAATGATG
GCTCTGCTGGTCCCCCAGGGCCAC
>1870
GGCCCCCAACCTGCCTGACTCTGATCCCCACTGCCTCTGACCCTGCTCACTTGGTCCCT
GTGTCTGACAGGGTGAGCGAGGTCCCCCCGGAGAGAGAGTGGTGGGGGCTCCTGGGG
TCCCTGGAGCTCCTGGCGAGAGAG
>1871
TCAGGGGGGTTGGCTGGGATGGCTGCCCATGGTGACTTCAGGGCCCTGAGGCCCCTGC
TCTTGGCTCCAGGACCCCTCCCTAGTTATGCTGCAGACACTGCCGGCTCCCAGCTCCAT
GCTGTGCCTGTGCTCCGCGTCTC
>1872
AAATCAGTGCTGCCCACCTTGCCCCGGGGCCAGCAGCCACTGCTCCCAGCACACCCTG
CCCTACCTGCAGAGCGGGTACCCCCTGAGGATGATGAGTACTCTGAATACTCCGAGTAT
TCTGTGGAGGAGTACCAGGACCC
>1873
TGACCTCCCCCTGACCTTTCAACCCTCTCTGATTCCCACAAACCCTGCTGACTTGACCC
CATTGGCCCAGACCCCTGTTCCCTGCCACTGGATGAGGGCTCCTGCACTGCCTACACC
CTGCGCTGGTACCATCGGGCTGT
>1874
GGTGCCTCTGCCTGAGCGTCTCCGGGGAAGGTCAGATGGCTGACGACCGTTTCCAACC
TGTCCTCACCAGGTACTGCCCAGGACTGAGGCCCAGATAATGAGCTGAGATTCAGCAT
CCCCTGGAGGAGTCGGGGTCTCAG
>1875
GTGGGTACAGGTCTGAATGGAGAAGGGACCTGAGGGAGTGGGGCCTGGGCTGACTGG
CTGCTCCTGGCAGGAGAACGGTGAGCTCTGCATGAATAGTGCCCAGTGTAAGAGCAAT
TGCTGCCAGCATTCAAGTGCGCTGG
>1876
GTCAGGCAGGGACAAGTGATGTCCAAAGTATCTCCCCATAGCCTTGACTCCATTGGCT
GTGTCTCTGCAGACGCTCTATGGGATTTACTACAAGTGTCCCTGTGAGCGTGGCCTGAC

CTGTGAGGGAGACAAGACCATCG
>1877
ATCATTTCTGTTTGTAGTTTTTCTATCAGTCATTTCAGTCAGCGTCATAATTCACGTTATC
TTCCTTTAGGTGGTGTTCCCACTGATGAAGAGCAGGCGACTGGGTTGGAGAGGGAGAT
CATGCTGGCTGCAAAGAAGGG
>1878
AGGGCTTATTTGGCCTAATGGTTCAATTCTTGTTTTTTTTGTTTTGTTTTGTTTTTTGTTT
TTTCTTCAGGACCCATACAATGTACTGGCCCCAAAGGGAGCTTCAGGCACCAGGGAAG
ACCCTAATTTAGTCCCCTCCA
>1879
AAAAGAAATTCATGTTGAAAGTCTCCCTTTCTAGGTTGGATGACCATAAACCACCTTTT
TTCCCTTTTAGGTGAAGAGGACAATACCAGCGTCGTCTGGTTTTGGCTGCACAAAGGG
CAGGCCCAGCGATGCCCCCGCTG
>1880
GGCCGAAGAAGGTCAGGCCCTCAGCTGCCTTCATCAGTTCCCACCCTCCAGCCCCCAC
CTCCTCCTGCAGACAAGCTGGTGTCTAGGAACTACCCGGACCTGTCCTTGGGAGACTA
CTCCCTGCTCTGGAAAGCCCACAA
>1881
TGCCTCTCACAGTAGCCCCCGCCCTGCCCGCTGCACAGCGGCCTGCTGAACTCACACT
GTTTCTCCACAGCGCATGAGAGCCCAGCCCGGCACCCCTGTGGCCATTGAGGAGGAAT
TCTCTCTCCTCACCTGCAGCATCA
>1882
GTTGGGGATGAGTGAGGAAAGCCCGAGCCCAGGGAGGTCCTGGCCAGCCTCTAACTC
CAGCCCCCTTCAGCATCTCCGGCTACGACATCCCTGAGGGCACAGTCATCATTCCGAAC
CTCCAAGGCGCCCACCTGGATGAG
>1883
GGCAGAAGAAGGTCAGGCCCTCGGCTTCCTTGGTCAGTTCCCACCCTCCAGCCCCCAG
CTCCTCCTGCAGACAAGCTGGTGTCTAAAACTACCCGGACCTGTCGTTGGTCTCTGCT
CTGGAAAGCCCACAAGAAGCTCAC
>1884
CACCTCTCACAGTAGCCCCCGCCCTGCCCGCTGCACAGCGGCCTGCTGAACTCACACT
GTTTCTCCACAGCGCATGAGAGCCCAGCCCGGCACCCCTGTGGCCATTGAGGAGGAAT
TCTCTCTCCTCACCTGCAGCATCA
>1885
GGCCCACCCCCAGCCCCTCCCTGAGCCTCTCCTTGTCCTGAACTGAAAGTACTCCATCC
TTTCCTGGCAGGAGGACAACTTAATGCCTGCCTATTACAAATGTATCCAGGAGGTGTTA
AAAACCTGGAGCCACTGGTCCA
>1886
TGTGGGGGAGAGGCTGGGGTGGAGGGAGAGGCTCCTTCCCACAGCTGCATTCTCATG
CTTCCTGCCGCAGTTCTTCCCCAATCCAGGTCTCCGGAGGCTGAAGCAGGCCATAGAG
AAGAGGGACCACAAGAGAGAACAGC
>1887
TGGGCCTGTTGCCACTCTGTACTCCTCTCCCCAGGCCAGCCGCTCAGCCCGCTCCTTTC
ACCCTCTGCAGGAGAGCCTGGTGGCAGGCCAGTGGAGGGACATGATGGACTACATGC
TCCAAGGGGTGGCGCAGCTAGCAT
>1888

GCTGAGGGAGCGGCTGGAGGCTGGGCAGCTGTGGGCTGCTGGGGCAGGACTCCACCC
GATCATTCCCCAGATTCAGCAGCGACTGTGGAGGAGCTAGACCACGAACTGGGCCCTG
GTGCCTCCAGCTCCCGGGTCCCCTA
>1889
TCTCCCTTATCCCTCTCACTGCATTGCCCCTGCTATGGGCCCAGCTCACTTGGCCACCTG
TCTCTTGCAGAGCCTGGTGAAATGGGAGAGTGAGAATAAAATGGTCTGTGAGCAGAA
GCTCCTGAAGGGAGAGGGCCCCA
>1890
TCAACCCTCCTCTCCAAAGCCACAGGACCCCAGGGGCCTCTCAGGCTAACAACTACTT
CTGTCCTTCCAGACCATGACGGCGGATGACGTTGTGTGCACCAGGGTCTACGTCCGAG
AGTGAGTGGCCACAGGTAGAACCG
>1891
ACGTAAGTGAGGGGATAGATCTGTGTGATCTGAGAAACCTCTCACATTTGCTTGTTTTT
CTGGCTCACAGACATGTCGAGGAAGGCTTTTGTGTTTCCCAAAGAGTCGGATACTTCC
TATGTATCCCTCAAAGCACCGTT
>1892
CTCCTGGAGCAGGGAGAGCGCTGGCCTCTTCCTCTCCGGCTCCCTCCATTGCCCTCCG
GTTTCTCCCCAGGCTCCCGGACGTCCCTGCTCCTGGCTTTTGCCCTGCTCTGCCTGCCC
TGGCTTCAAGAGGCTGGTGCCGT
>1893
AAGCAACAATGGGAGCTGGTCTCCAGCATAGAAACCAGCAGTCCTTCTTGGTGGGGG
GTCCTTCTCCTAGGAAGAAACCTATATCCCAAAGGACCAGAAGTATTCATTCCTGCATG
ACTCCCAGACCTCCTTCTGCTTCT
>1894
AGGCGGGGATGGGGAGACCTGTGGTCAGGGCTCCCGGGCAGCACAGCCACTGCCGGT
CCTTCCCCTGCAGAATCTAGAGCTGCTCCGCATCTCCCTGCTGCTCATCGAGTCGTGGC
TGGAGCCCGTGCGGTTCCTCAGGA
>1895
TGGATAAATGAGAGAGGGAGGGAACAGTGCCCAAGCGCTTGGCCTCTCCTTCTCTTCC
TTCACTTTGCAGAGGCTGGAAGACGGCAGCCGCCGGACTGGGCAGATCCTCAAGCAG
ACCTACAGCAAGTTTGACACAAACT
>1896
AGTGGTGGAGAGTTCTTGTACCACTGTGGGCACTTGGCCACTGCTCACCGACGAACGA
CATTTTCCACAGGAGCCGACCTGCCTACAGACCCGCCTGGAGCTGTACAAGCAGGGCC
TGCGGGGCAGCCTCACCAAGCTCA
>1897
CTGGTGCTTCCTTCCCCCACGTTACCCACTTGCCTGGACTCAAGTGTTTTTTATTTTTCT
TTTTTTAAAGGAAACTTCCTGTGCAACCCAGATTATCACCTTTGAAAGTTTCAAAGAGA
ACCTGAAGGACTTTCTGCTTG
>1898
GGAGCCACTAAGCAGGTGCCCACCCAGGAGATTCCATGGGTGCCCACAAGCAGACAG
ACTTTTCCTTCAGGGGAGATCATCGGGGGACATGAGGCCAAGCCCCACTCCCGCCCCT
ACATGGCTTATCTTATGATCTGGGA
>1899
CTGCTCTTCACTGTCTCTGGGCTGTATACCCTGTGACTCCACCCCCATCCTCACTCTGCT
CTCTCTGCAGCTCCATAAATGTCACCTTGGGGGCCCACAATATCAAAGAACAGGAGCC

GACCCAGCAGTTTATCCCTGTG
>1900
GCCCCATCACTGTGTCGGGGCCCAGAAGTTCGTTGGCTGACCTGGACTTTCTTGCCTC
TTCCCCCACCAGCTGGAGAGAAAGGCCAAGCGGACCAGAGCTGTGCAGCCCCTCAGG
CTACCTAGCAACAAGGCCCAGGTGA
>1901
GGGCTTGAGATGGAAGAGAGGGCAGGACCCGGAGGAGCATCAGCTCAGTCCTTCCAC
TCTCTATTCACAGGGGGACTCTGGAGGCCCTCTTGTGTGTAACAAGTGTGCCCAGGGC
ATTGTCTCCTATGGACGAAACAATG
>1902
CTGCTCTTCACTGTCTCTGGGCTGTATACCCTGTGACTCCACCCCCATCCTCACTCTGCT
CTCTGTGCAGCTCCATAAATGTCACCTTGGGGGCCCACAATATCAAAGAACAGGAGCC
GACCCAGCAGTTTATCCCTGTG
>1903
GGAGCCACTAAGCAGGTGCCCACCCAGCAGATTCCATGGGTGCCCACAAGCAGACAG
ACTTTTCCTTCAGGGGAGATCATCGGGGGACATGAGGCCAAGCCCCACTCCCGCCCCT
ACATGGCTTATCTTATGATCTGGGA
>1904
CTGCTCTTCACTGTCTCTGGGCTGTATACACTGTGACTCCACCCCCATCCTCACTCTGCT
CTCTGTGCAGCTCCATAAATGTCACCTTGGGGGCCCACAATATCAAGGAACAGGAGCC
GACCCAGCAGTTTATCCCTGTG
>1905
AGCCCCATCACTGTGTCGGGGCCCAGAAGTTCGTTGGCTGACCTGGACTTTCTTGCCT
CTTCCCCACCAGCTGGAGAGAAAGGCCAAGCGGACCAGAGCTGTGCAGCCCCTCAGG
CTACCTAGCAACAAGGCCCAGGTGA
>1906
GGGCTTGAGATGGAAGAGAGGGCAGGACCCGGAGGAGCATCAGCTCAGTCCTTCCAC
TCTCTATTCACAGGGGGACTCTGGAGGCCCTCTTGTGTGTAACAAGGTGGCCCAGGGC
ATTGTCTCCTATGGACGAAACAATG
>1907
AAATGCAGGAATATAACATGTGGCAAACTATCAGGAGTGTGAAATAACCGATGCATTCT
TTCTTGTTTAGGCATCATCTGGGGAGAGGATACACTGATGGAGTATTTGGAGAATCCCA
AGAAGTACATCCCTGGAACAAA
>1908
CAAATAGGACTAGGACCTGTAGTCTGGGGTGATCCTGGCTTGACAAGAGGCCCTGACC
CTCCCTCTGCAGTTGCGGCGCCGCTTCGGGGACGTGTTCAGCCTGCAGCTGGCCTGGA
CGCCGGTGGTCGTGCTCAATGGGC
>1909
GTTGGAGTGGGTGGTGGATGGTGGGGCTAATGCCTTCATGGCCACGCGCACGTGCCCG
TCCCACCCCCAGGGGTGTTCCTGGCGCGCTATGGGCCCGCGTGGCGCGAGCAGAGGC
GCTTCTCCGTGTCCACCTTGCGCAA
>1910
GGCACAAAGCGGGAACTGGGAAGGCGGGGGACGGGGAAGGCGACCCCTTACCCGCA
TCTCCCACCCCCAGGACGCCCCTTTCGCCCCAACGGTCTCTTGGACAAAGCCGTGAGC
AACGTGATCGCCTCCCTCACCTGCGG
>1911

CAAATAGGACTAGGACCTGTAGTCTGGGGGGATCCTGGCTTGACAAGAGGCCCTGACC
CTCCCTCTGCAGTTGCGGCGCCGCTTCGGGGACGTGTTCAGCCTGCAGCTGGCCTGGA
CGCCGGTGGTCGTGCTCAATGGGC
>1912
GTTGGAGTGGGTGGTGGATGGTGGGGCTAATGCCTTCATGGCCACGCGCACGTGCCCG
TCCCACCCCCAGGGGTGTTCCTGGCGCGCTATGGGCCCGCGTGGCGCGAGCAGAGGC
GCTTCTCCGTCTCCACCTTGCGCAA
>1913
GCACAAAGCGGGAACTGGGAAGGCGGGGGACGGGGAAGGCGACCCCTTACCCGCAT
CTCCCACCCCCAAGACGCCCCTTTCGCCCCAACGGTCTCTTGGACAAAGCCGTGAGCA
ACGTGATCGCCTCCCTCACCTGCGGG
>1914
TGGGGCCTGAGACTTGTCCAGGTGAACGCAGAGCACAGGAGGGATTGAGACCCCGTT
CTGTCTGGTGTAGGTGCTGAATGCTGTCCCCGTCCTCCTGCATATCCCAGCGCTGGCTG
GCAAGGTCCTACGCTTCCAAAAGG
>1915
CAGGTGCAGAATTGGAGGTCATTTGGGGGCTACCCCGTTCTGTCCCGAGTATGCTCTCG
GCCCTGCTCAGGCCAAGGGGAACCCTGAGAGCAGCTTCAATGATGAGAACCTGCGCAT
AGTGGTGGCTGACCTGTTCTCTG
>1916
GAGGCAAGAAGGAGTGTCAGGGCCGGACCCCCTGGGTGCTGACCCATTGTGGGGACG
CATGTCTGTCCAGGCCGTGTCCAACAGGAGATCGACGACGTGATAGGGCAGGTGCGGC
GACCAGAGATGGGTGACCAGGCTCA
>1917
TGTGTTTGGTGGCAGGGGTCCCAGCATCCTAGAGTCCAGTCCCCACTCTCACCCTGCAT
CTCCTGCCCAGGGAACGACACTCATCACCAACCTGTCATCGGTGCTGAAGGATGAGGC
CGTCTGGGAGAAGCCCTTCCGCT
>1918
CCCTTCCGTGGAGTCTTGCAGGGGTATCACCCAGGAGCCAGGCTCACTGACGCCCCTC
CCCTCCCCACAGGCCGCCGTGCATGCCTCGGGGAGCCCCTGGCCCGCATGGAGCTCTT
CCTCTTCTTCACCTCCCTGCTGCA
>1919
ATTGGGACTGCAGGGTGGGGCCACGAGGGACATGACCCCGTCCAGCAGGGCCTCCTG
CTTGGCCCCACAGGTACAACTTGGGAGGACCACGCATGGTGTGTGTGATGCTGCCGGA
GGATGTGGAGAAGCTGCAACAGGTG
>1920
AGGATGGGATACGGGTCAGGGCCTGTGTCTTGCTGGGGCGGCCTCACAAGCTCTGCCC
TGGCCTCTGTAGGAATGGGCCTGAATGGCGCTTCAACCGATTGCGGCTGAACCCAGAT
GTGCTGTCGCCCAAGGCCGTGCAG
>1921
GAAGGGAGTGTGGGGAGGCAGCCAGGAGGCCTGGGGCTGCCTTGTGCTCAGCAGTGC
ATCCTCCCCGCAGCCAGCAACTTAGCTCTTTTTGGAGAGCGGCTGGGCCTGGTTGGCC
ACAGCCCCAGTTCTGCCAGCCTGAA
>1922
GCAGGAGGAGGACACTGAAGGATGCTTCCCAGCACCAAGATCTGAGGGCTGTCCCCT
GCTCCCTGGACAGGTGACAACTGTATCCAGAAAATCTACCAGGAACTGGCCTTCAACC

GCCCTCAACACTACACAGGCATCGT
>1923
CCTGGGAGGGGTGTCCCGGGGGCTGAGTCCTCCTGTGCAAGGTCTGACCCTGCAGAC
ATGGCTTCTGTAGACAGCGTTTCCCTTGCTGATGACGCTCTTTGAGCTGGCTCGGAACC
CCGACGTGCAGCAGATCCTGCGCC
>1924
GGAAGGGTGCTGAGAGCACAGGGAGCCCCATCCAGCTGAGGACCCTTTCTGTGGATG
CCCCCACCTCCAGGCTCTACCCTGTGGGTCTGTTTTTGGAGCGAGTGGTGAGCTCAGA
CTTGGTGCTTCAGAACTACCACATC
>1925
CCCCACACCCCTCGAGCTGAGAACCTCCCTCCCCAGTCATTCCCTGATCCCTGCTCTGC
ACCGTCCGCAGACATTGGTACAGGTTTTCCTCTACTCGCTGGGTCGCAATGCCGCCTTG
TTCCCGAGGCCTGAGCGGTATA
>1926
GATGGGAGGAAGCTGCCCCAGGTCCATGGGCTACTGACCAGGCCAGATGGAAACCCA
GCCTCTGTCCTAGGTGCTGAAGCACTTCCTGGTGGAGACACTAACTCAAGAGGACATA
AAGATGGTCTACAGCTTCATATTGA
>1927
CAGGTGTGGCTTAGAGCCCCGCACCTCCTCGCCGCGCGCGGGCCTGACTTCTAGCCAC
GGGTCTCCGCAGTTGGCCCAGCGCTTCGGGCCGGTGTTCACGCTGTACGTGGGCTCGC
AGCGCATGGTGGTGATGCACGGCT
>1928
GGCCCTCTGTAGCCCATTTCTCCCCAAAATGGGTATTTAGAATAACCTTCTGCTGGCCC
CTCTGCCTTAGGAATCATTTTTAATAATGGACCTACCTGGAAGGACATCCGGCGGTTTT
CCCTGACCACCCTCCGGAACTA
>1929
TGGGTGACTGAGCAGGTGGAGGAGTCTCCTCACCCCCATCTTCTGGTTGCCCTGACTG
CCTGTTTTGTAGGCCAGCCTTTCGACCCCACCTTCCTCATCGGCTGCGCGCCCTGCAAC
GTCATAGCCGACATCCTCTTCCG
>1930
TTGTTGGTCCAACACACACAATTATGTTGCATCCAGAAAAAAGTAGTAAAATATTTTTT
TCCCTCTCTAGCTTTACAATAATTTTCCCAGCTTTCTACACTACTTGCCTGGAAGCCACA
GAAAAGTCATAAAAAATGTGG
>1931
GGAGGTGGCTGGTTCTGTGCTGAAAGGAGACAAGCAGCCCCTTCTCTCCGGTCTGTCT
CCGGTATCACAGGAAAAGCACAGTGCAGAGCGCTTGTACACAATGGACGGTATCACCG
TGACTGTGGCCGACCTGTTCTTTG
>1932
TGGATGATGGGTGGATGCCCAACTGGCCAGGAACCAATCCCTGAAATTTGTCCCATTCA
TATCTTGGCAGAGAAGCTCCATGAAGAAATTGACAGGGTGATTGGGCCAAGCCGAATC
CCTGCCATCAAGGATAGGCAAGA
>1933
CTGGGGGTTTCCAGATGAAAGCCCACATTTTGTTAACATGACTCACTGAGACAGTCTTT
GTTTCTCCTAGGGCACAGTCGTAGTGCCAACTCTGGACTCTGTTTTGTATGACAACCAA
GAATTTCCTGATCCAGAAAAGT
>1934

GTCTCACTGTTAACAGTGTCGTGTCTCTGAAACTCCCTCAGTGTCTCATCAATACCATT
GTTACTTCTAGGAAAACGAGTGTGTGCTGGAGAAGGCCTGGCTCGCATGGAGTTGTTT
CTTTTGTTGTGTGCCATTTTGCA
>1935
AGTTAGTCTGGCTGTGCTTGTCCTTTTTCCCACCTCCCTCGCTGTGCCTGACCCTCTCTT
CTTTCCACAGGTTCTCAGGCAAGAGCCACCTGCTATTGCCGAACCGGCCGTTGTGCTA
CCCGTGAGTCCCTCTCCGGGGT
>1936
TGGCCTCTCGCGTTTGCTCTGCCCCACCTGGGTCGCGGAGACCATGTCCTTCTCGCTTG
GCCTCTCCCAGGACGTGGATGCAGCTACTCTAGCTCGCATTGACCTGGAGCGCAGAAT
TGAATCTCTCAACGAGGAGATCG
>1937
GGCTGGGAATAGGGGTGTGAGGGTCGTGTGTGGGCCCTGAGAGGGACTGAAGCCCAG
TCATGCCCTACAGGAGATCCGTGAGTTGCAGGCTCAGCTTCAGGAACAGCAGGTCCAG
GTGGAGATGGACATGTCTAAGCCAG
>1938
ATGCTCCCAAGCTCATCCCTACCCGTGCCCTGCATCCTTCTCATTTTTGGGCCCCTTTCT
CTGCCCTTAGGTGTCAGACCTGACCCAGGCAGCCAACAAGAACAACGACGCCCTGCG
CCAGGCCAAGCAGGAGATGATGG
>1939
GCCCCAAAGCTTTCTTTGGGCTGCTAGTGTCCTCTTCCCTTCCTTGACCTGGGTTCCCC
CTCTCCTGCAGAACGATTCCCTGATGAGGCAGATGCGGGAATTGGAGGACCGATTTGC
CAGTGAGGCCAGTGGCTACCAGG
>1940
GGACTCCCAGCCCCTGGTATAGCCCAGCCTGGACTTGGTCAGGCTGAGTGTGCGATGG
ACCCTGTTACAGAAACCAGCCCTGAGCAAAGGGGTTCTGAGGTCCATACCAAGAAGA
CGGTGATGATCAAGACCATCGAGAC
>1941
TGCGATCCTGGGTGGGATGCCTAGGCTGACGTCATCCTGCTAGCACATGTGACTGGACT
CCTCTCCCCAGGTCGTCAGTGAGGCCACACAGCAGCAGCATGAAGTGCTCTAAAGAC
GAGAGACCCTCTGCCACCAGAGAC
>1942
TTGCTGCTTGTGCCCGAACTAACTCTCAGGCACATTAGTCAGAAAATATTACGTTTGGT
TGCTCCCCGAGGTTCCTAAAAGTAAAGCTCTAGAGGCCGTCAAATTGGCAATAGAAGC
CGGGTACCACCATATTGATTCTG
>1943
TACCTCAGAGCATGGCTATGTGTGGAGAAATTGACTTGTGACATCACTAAACTGACTGC
TTCTACTTCAGCCAGGTGAGGAAGTGATCCCAAAAGATGAAAATGGAAAAATACTATT
TGACACAGTGGATCTCTGTGCCA
>1944
AGTTCTATGTTACATTTATCTTGCTCTTCAGTCGACTACACAAATGATACCTCACAATTC
CTTTTTCCAGGCCATGGAGAAGTGTAAAGATGCAGGATTGGCCAAGTCCATCGGGGTG
TCCAACTTCAACCACAGGCTGC
>1945
TACTTTGGTGATTTTAATATTAATGTAACTTTTGGATTATCTGATGCTTTTCCATCTTGCTC
GTCTGCAGGTGGAATGTCATCCTTACTTCAACCAGAGAAAACTGCTGGATTTCTGCAA

GTCAAAAGACATTGTTCTGG
>1946
CTGTTAGGGAGCCGCCTAACAAACTGTATCCCCAGCCTCAGGGCCTCAGCCTTTCTGC
CTTTCCTTCCAGGGTGGACCCGAACTCCCCGGTGCTCTTGGAGGACCCAGTCCTTTGT
GCCTTGGCAAAAAAGCACAAGCGA
>1947
TGAACTTGGTTGTTCCCAATTGGCAATGATGTAATCTAAAAATAATTAAAGTTCCCTATT
ACTGTGATAGGTGTTTGAATTCCAGTTGACTTCAGAGGAGATGAAAGCCATAGATGGC
CTAAACAGAAATGTGCGATATT
>1948
ATCACTGCGCTACCGGCTAGTAACGGAGTCATTGCCATTCAGAGTGTGCATTTTTTTTTC
CTCTTTCCAGTTTTGCTGGCCCCCCTAATTATCCGATTTCTGATGAATATTAACATGGAG
GGCATTGCATGAGGTCTGCC
>1949
TGCTTGCTTTCCTCCAGACTTTGAGCCCTGGCCTGATGTAGACCTTTTTGCTCTCTCCTC
CTTCGTATAGGTACGGTTCCTGGAGCAGCAGAACAAGATGCTGGAGACCAAGTGGAGC
CTCCTGCAGCAGCAGAAGACGG
>1950
ACCTTGGGAGCCCCTTGGTTGGGGTTGGGTGTGGAAACCCAGCTCAGGCTCCCCTCTC
CTCATCCCCCAGGATGTGGATGAAGCTTACATGAACAAGGTAGAGCTGGAGTCTCGCC
TGGAAGGGCTGACCGACGAGATCA
>1951
AAGGCTGTTGTGGAAAAGGGGGCTCCTGTGTGCAGAGACAGGGCCTCACCACTTGCC
CTCTTCCCCACAGGAGATCCGGGAGCTGCAGTCCCAGATCTCGGACACATCTGTGGTG
CTGTCCATGGACAACAGCCGCTCCC
>1952
GAGGGTGGACGCACGGAGGAAGAGAAGGCATACCCAACCTGACCTACTTACCTGTCC
CCTACCCACAGAGAGGGCTTCCCTGGAGGCCGCCATTGCAGATGCCGAGCAGCGTGG
AGAGCTGGCCATTAAGGATGCCAACG
>1953
CAGGGAGAAGGGCATGGAGCCTGCCATGGTGGGCTTCTGTACTCATGTGGCTACCTCT
GTCCCTCACCAGGTGGTCTGAGCTCGGCCTATGGGGGCTCACAAGCCGGCCTCAGCTA
CAGCCTGGGCTCCAGCTTTGGCTC
>1954
GCCCAGCCAGGCCCTTTCTGGCCGTGTGGCTCAGGAACACAGCCTCTTCCTCTTTTTTT
TCCCCTCCTAGCCCTATTCAGGCAGGAGCTGCTCTTCTGGGGTATCGCGATCCACTTAA
GGATGAGGCAGACTTGGTGACA
>1955
ATCTCGGGAGGTCACTGGGAGGCTGGTGCTGGGCAGCAGCTAGTGCCACTTCCTCTGT
CCCATATTTCAGCGCCTCCAGAGCCAGAACTGAGCCCAGTGAGAGCGCACCCTGGGGC
AGCCTGGATTCCTGGGGTGTCCCC
>1956
CCCCTGGGCCCACTGCCTCCTCTCAGGGCAGTTGGCACTGAGTCAGGCTGGCCTTCCT
GATGTCCCACAGATACTGGCTCGCTGTGACATCATGGTGCTGCAGGAGGTGGTGGACT
CTTCCGGCAGCGCCATCCCGCTCC
>1957

TTCTGTGGGTCTAATCAGGGGCACACCCAGAGGCTTGAGGCCCTGACCCCGCCTCCCC
TTTTCTTGGCAGATTTGATGGCTCTGGGCCCTACAGCACCCTGAGCAGCCCCCAGCTG
GGGCGCAGCACCTACATGGAGACG
>1958
CGCCGTTTGGCACTTGCAAGCCAAAATCAAAGACGTGGGGCCACTGCTGCCCATCCCT
TTGTCCTCCCAGGTCACACAAAACACAGGTCCTGAGTTCCTACGTGTACAACGATGAG
GATGACGTCTTTGCCCGGGAGCCA
>1959
CCTCACCCAGACGGGCTAGGGGGAGAAGGTCCAGGAGTGGATGGCAGCCCTCGGTCC
CTGGGCCTTGCAGTCCTTCCCAGCCTGGTGTTGGTCCCGCTGCACACCACTCCTAAGG
CCGTAGAGAAGGAGCTGAACGCCCT
>1960
GGGCAGTTTGTACAGGAGGGACAAGGATAGGGTGGCCCAGCGGCCAGCCTGATGGCT
GCTGTGTCTCTAGGACGTGATCCTGCTTGGGGACTTCAATGCTGACTGCGCTTCACTGA
CCAAAAAGCGCCTGGACAAGCTGG
>1961
GGGCAGCAGCAGGGTCACAGGGAAGCCCCTCGGCCCCGGGCACTGACACTGCCCCAC
CACCCATTCCCAGGCCCTCAACATCAGTGACCACTACCCCGTGGAGGTGGAGCTGAAG
CTGAGCCAGGCGCACAGCGTCCAGC
>1962
TATTCCCTGCTTCCACAGCGGCAGACCAGCCAGTTGCAGGCACCAAGCCTTTGGGCAT
GTTTCCTTCTAGGTCGGCGCCTGCATCGTGAATTCAGAAAACAAGATTGTCGGGATTGG
GTACAATGGGATGCCAAATGGGT
>1963
GTGTTAACACTTAGTAAATTCTTCATGAGCTGCAACCAAAGTTTTTCTTTCTTTCCCTTT
CAACCCACAGTGTGCCATGCGGAGCTGAATGCCATCATGAACAAAAATTCGACCGATG
TGAAAGGCTGTAGTATGTATGT
>1964
CTCGTTCCGCCTGTCTTTTGCAGCATCTGTAGGTGCGTACACTAAGGGAGCTCCCATTG
TTACCTTACAGGTATAAAAGAAGTGATTTTCATGTCTGATAAATACCATGATAGTGACGA
GGCAACTGCTGCGAGGCTCCT
>1965
GTTAAGCCAAAGTAAACAAATTGCTTTCACTACTCACCAGATAATAATTGTATTTTGTTT
TTATTTTTAGGAAATTCATACCGAAGTGCAGCAAGATTGTCATTGACTTTGATTCAATTA
ACAGCAGACCGAGTCAAAAG
>1966
TTTAATTAAAGAAAAATATTAAAAAGGAAAAATGAACTACTTATGATTTTCTTTTTTGCA
TTTTTCCTAGAGGATGACTTGGTTACAGTCAAAACCCCAGCGTTTGCAGAATCTGTCAC
AGAGGGAGATGTCAGGTGGGA
>1967
GATTCTATACAGCTAATTTTCTAATGCTGGATAAACATTTGGACTTCCTCCATCTGTCTTC
CTCTTCCAGACATCTGTGCAGGTTCCATCACCAGCAAATGGCGTGATTGAAGCTCTTTT
GGTACCTGATGGGGGAAAAG
>1968
CCACTAAATTTTGCTTGGTTGCTTCTCATTTCAGACAGTGCCAGTGGCATATACTTTTCT
TGTTTTTCAGCTGCTCCTGCTAAGGCCAAGCCGGCTGAAGCTCCTGCTGCTGCAGCCC

CAAAAGCAGAACCTACAGCAGC
>1969
AGCACTGGCACAAACTCAGCAGATGTTTCTTTCCTTGTCTGATGCAGCTTTATCCTCTT
TTCATTTTCAGTGTCTGCAGTAAAACCCACTGTTGCCCCACCACTAGCTGACGCAGGA
GCTGGCAAAGGTCTGCGTTCAGA
>1970
TCTGACTACACGGGGAATGCTTGACCCAGAGAGATCAGATTGTCAATGCTTGTTCCACT
CTTACTTTCAGGAGAAAATGAACAGGATGCGGCAACGCATTGCTCAGCGTCTGAAGGA
GGCCCAGAATACATGTGCAATGC
>1971
GGATTGAGAAACCTGCTGTGAAAGTCAAAATGCAGGCTTTGTGGTCAGTTTGTTTTGT
AATGTTTCACAGTAACATCCAGGAGATGAGGGCTCGGCACAAAGAGGCTTTTTTGAAG
AAACATAACCTCAAACTAGGCTTC
>1972
TGCACATATTACCTCATTAGTCTTGGCCTTCCTGGGGAGTTGTATGTAACATGTATTTTCT
CTCTCATAGTGATTGACGACACAACCAAAGAGGTGGTGTATACGGATTATATTGACATC
AGTGTTGCAGTGGCCACCCC
>1973
TTCAAGGGAGTTGTTAACTATAAAAGGTACTATTAATTTGCAACTGAAAACAGCTTTTC
ACCCCCTTCAGGGTCTGGTGGTTCCAGTCATCAGGAATGTGGAAGCTATGAATTTTGCA
GATATTGAACGGACCATCACTG
>1974
GGATTATGGACTGACTTTAGGTGAAGGAAACTGGGGTGGCTTAATATTTCAATTATGAA
ATCTGTTTTAGGCCCGAAAGAATGAACTTGCCATTGAAGATATGGATGGTGGTACCTTC
ACCATTAGCAATGGAGGCGTTT
>1975
GCTAAATCTCCTTCAGCGAGGCTGGCTGTGGCTTGCTGGAGACAAACCTATTTACCTTT
CCTCTCTGTAGGTAGAGGTGCGGCCCATGATGTACGTGGCACTGACCTATGATCACCGG
CTGATTGATGGCAGAGAGGCTG
>1976
CCTAGCCCTATGCAGCCCCACAGTGGGACCCTTGCATGGACCTCAACCATCAGAATCTT
TTCTTTTGCAGGTCGTTACTCTCTGACCTATATCTACACTGGGCTGTCCAAGCATGTTGA
AGACGTCCCCGCGTTTCAGGC
>1977
GACTAAGAGTTATTTTGATAAATTTCTCACCAGGCTGACTGTCTCTCTGTGGGGTCGGG
GGAGTTTTCAGGGTCTCACGTATTGCAGGGAAGGTTTGGTTGTGAGATCGAGAATAAC
AGAAGCAGCGGAGCATTCTGGAA
>1978
GGGTAGGAAGAGACCTGGGCCGGGAGAAGTCCACCTCAAGCCTGCAGTGTCACACTC
TATCCCTCCACAGATCCTCCCTCTGTGGTGGTCACCAGCCACCAGGCCCCAGGAGAAA
AGAAGAAACTGAAGTGCCTGGCCTA
>1979
GGGAGAGAAGGGAGGAGCCCTTGGAGGCTAGAAGGGAAGTCAGATCTTCCTCCTCTC
CCAAAACCTCCAGTGTATGCCACGTTGAGGGACCTGAAAACACAGGGCCGGCTGTGG
GAGGCGGCCCGGGCCCTGGCATGCCC
>1980

GGAGCACGAGGGGACAGGCCGTGCTGAGGGTGATGCTGAGGCGGGCTGGTCGGGCCT
CTTGTCTCCGCAGTGTGTAACGCAGGCCTGGGCCTGCTGGGGCCGCTGGAGGCGCTGG
GGGAGGACGCCGTGGCCTCTGTGCT
>1981
GAGGGGCACCGTCTGCCCGGGGATGACCCCCTGGCCGCTGCGCCTCAGGAACCTCGT
CTCCCCACCTAAGGGCTGCCTTTCAATGACGTTTATTGCGCCAGCAAGTTCGCGCTCGA
AGGCTTATGCGAGAGTCTGGCGGT
>1982
TGGGGTTGGGGCTGGGACTGGGGCCTGGCTGGCGTCCGCCCCCTCCCACTCGTTGCTC
TCCGGCCAGCAGCTTGAGCCTGATCGAGTGCGGCCCAGTGCACACCGCCTTCATGGAG
AAGGTGTTGGGCAGCCCAGAGGAG
>1983
ACTCCAGGAGTGGGGGCGGTGCGTCCTCCGGCCGGCAGCGGTGGCCACAGCTCTCCT
CCCGCCGCCGCAGGTCTTCCTCACCGCTTTGCGCGCCCCGAAGCCGACCCTGCGCTAC
TTCACCACCGAGCGCTTCCTGCCCC
>1984
TACTGTGATCCAGCATGTCTCTCGGCGTTTGAGGAGACATCCCCCACTGACCTGCTCTT
TCTCTCCCCAGCAGTCTTAGGCGCTGAGCTCAGCGCGGTGGGTGAGAACGGCGGGGA
GAAACCCACTCCCAGTCCACCCTG
>1985
GTGTGTCCATGTGTCATTTTAAAGACTATTAATTACACTAATATAGTTTCTTTCTCTCTTT
GGATAATAGGCACGTTGTTCCGTATGGACTTGGAAGCCCTAGGTCCAAGAGAGCCTTG
GAGAATTTACTTCCCACAAAG
>1986
TACATGTTTCTTTTGCCAAAGGGTGATTTTTTTAAAATAACATTTGTTTTCTCTTATCTTG
CTTTATTAGGTCGGAGACCATGAGAAACAGCGTCAAATCATCTTTTCATGATCCCAAGC
TGAAAGGCAAGCCCTCCAGA
>1987
GCTGGGGACAAGGAAATTGAAGCTGGAGTTTGTGTTTTAGTAACCAAGTAACGACTCT
TAATCCTTACAGATGGGAAAGGGCTCCTTCAAGTATGCCTGGGTCTTGGATAAACTGAA
AGCTGAGCGTGAACGTGGTATCA
>1988
AAGGCTTGCTTTGTACACTGGTTTTGTCATTTGGAGAGTTGACAGGGATATGTCTTTGC
TTTCTTTAAAGGCTGACTGTGCTGTCCTGATTGTTGCTGCTGGTGTTGGTGAATTTGAA
GCTGGTATCTCCAAGAATGGGC
>1989
AAGACCATTGTTAAAAAGCTCTGGGAATGGCGATTTCATGCTTACACAAATTGGCATGC
TTGTGTTTCAGATGCCTTGGTTCAAGGGATGGAAAGTCACCCGTAAGGATGGCAATGC
CAGTGGAACCACGCTGCTTGAGG
>1990
AAAGTTGAATTTGAGTTGATAGAGTACTGTCTGCCTTCATAGGTATTTAGTATGCTGTAA
ATATTTTTAGGTATTGGTACTGTTCCTGTTGGCCGAGTGGAGACTGGTGTTCTCAAACC
CGGTATGGTGGTCACCTTTGC
>1991
TTAACTTATTGCAGAGGCTAAAGTCATTTGAGACTTTGGATTTGCACTGAATGCAAATC
TTTTTTCCAAGGTGATTATCCTGAACCATCCAGGCCAAATAAGCGCCGGCTATGCCCCT

GTATTGGATTGCCACACGGCTC
>1992
AAAGTTGTGTTAAAGATGAAAAATACAACTGAACAGTACTTTGGGTAATAATTAACTTT
TTTTTTAATAGGTCGCTTTGCTGTTCGTGATATGAGACAGACAGTTGCGGTGGGTGTCA
TCAAAGCAGTGGACAAGAAGGC
>1993
GCTGTCTGTGGTTCTGGGGAGGAACAAGGGGAGATGTGAAGGAAGGACACTTAGCTA
TCCTCCCTGCCAGGGTCTGAGACTTCCACCTTTGAGACCCCTTTGGGTGCTAAGACGC
TGCCTGAGGATGAGGAGACACCAGA
>1994
GGGACTCAAGACCCATGTGAATTCATGTAGGAAGGATGCTCCATTCTTTGTCTTTTATC
CTGCCCTGTAGTTTACTTGCCGGAGGTGCTACAGGGGCAACCTGGTTTCCATCCACAA
CTTCAATATTAATTATCGAATCC
>1995
GATACGGGGCCAGGTCTGCTTCTCTGCCTTCCCTCTGCCCCATCCCTTCTTCCCCTCTTG
CTTTCTCCAGGGTCGCTGCAGACGCTTTCAGTGGGTTGACGGCAGCCGCTGGAACTTT
GCGTACTGGGCTGCTCACCAGC
>1996
GGGGTCGCTGCGTTCCAGGGAACTCTCCCAGCAACCTGCCCTATAAAGGGTGTCTCTC
TTTCTTCCCCAGCTGGTCCTGCCGCTGCTGTGTTGAGAGAGCTGCGTTGCGTTTGTTTA
CAGACCACGCAAGGAGTTCATCC
>1997
AGAGAAATCCCGCAGCCTGGGTCTTCAACCTTGGTATCTCATGAGTGTATCTTCTTTTT
CTTTCCTTCAGAGCCTCCCTGAAGAACGGGAAGGAAATTTGTCTTGATCCAGAAGCCC
CTTTTCTAAAGAAAGTCATCCAG
>1998
AAGGAAGCTGTCCTTCCACAGCCACCCTTCTCCCTCCCCGCCTGACTCTCAGCCTGGC
TATCTGTTCTAGAATGTCCTGCCTGGCTGTGGCTTCTCCTGTCCCTGCTGTCGCTCCCTC
TGGGCCTCCCAGTCCTGGGCGC
>1999
ATCCTACGGCCTGTGGGCCAGGGCCAGAGCCTTCAGGGACCCTTGACTCCCCGGGCTG
TGTGCATTTCAGACGGGCTGTGCTGAACACTGCAGCTTGAATGAGAATATCACTGTCCC
AGACACCAAAGTTAATTTCTATG
>2000
TGGGGCTGCTGAGGGGCAGGAGGGAGAGGGTGACATGGGTCAGCTGACTCCCAGAGT
CCACTCCCTGTAGGTCGGGCAGCAGGCCGTAGAAGTCTGGCAGGGCCTGGCCCTGCT
GTCGGAAGCTGTCCTGCGGGGCCAGG
>2001
AGTACAGGAACTGTCCGTATTCCTTCCCTTTCTGTGGCACTGCAGCGACCTCCTGTTTT
CTCCTTGGCAGAAGGAAGCCATCTCCCCTCCAGATGCGGCCTCAGCTGCTCCACTCCG
AACAATCACTGCTGACACTTTCC
>2002
TTCCTTCATAAAGACCCAACCCTATGACTTTAACTTCTTACAGCTACCACAGCCCCTGG
GCCCGCAACAGTTGTTACAGGTTCTGGTCATGCAAGCTCTACCCCAGGTGGAGAAAAG
GAGACTTCGGCTACCCAGAGAAG
>2003

GAGACATTGAAAGTCAGGGTAAAGCTGAATATAACATTGCTGAAATGTTTTTCCTTGTG
TATTTTAACAGGGCTGAAGACATTATGGAGAAAGACTGGAATAATCATAAGAAAGGAA
AAGACTGTTCCAAGCTTGGGAAA
>2004
CATAACTTGGAAAAATAAACACTTCCTATGGGATTTGACTTTATTTTCTCCATTGTCTTA
CCTTTTACAGGTGTTAATATAGTGAAAAGGAAGCTTGCAGCTCATGACAATTTGAAGCT
GACAATTACACAAGAAGGAAA
>2005
CATGTGTAAGACCCTAATAAATGCCATTATTATTATTATTATTATTATTATTATTATTATT
ATGTAGGGGACCTGGAGCCTTGAGGGAAATAAACTTATTGGAAAATTCAAACGGACAG
ACAATGGAAACGAACTGA
>2006
CCCTTCTCTCCTCTGAGTACCAGATCCTCCCTGATACCCCCGACCCCATGGGCATCCTCT
ATCCCCTCAGCGGCCCTGGGAGAGCCTCAGCTCTGCTATATCCTGGATGCCATCCTGTT
TCTGTATGGAATTGTCCTCAC
>2007
TAAGAGATGTAAAATTTTCATGATGTTTTCTTTTTTGCTAAAACTAAAGAATTATTCTTTT
ACATTTCAGTTTTTCTTGATCATGAAAACGCCAACAAAATTCTGAATCGGCCAAAGAG
GTATAATTCAGGTAAATTGGA
>2008
TATCTACAGGGGAGGACCGGGCATTCTAAGCAGTTTACGTGCCAATTCAATTTCTTAAC
CTATCTCAAAGATGGAGATCAGTGTGAGTCCAATCCATGTTTAAATGGCGGCAGTTGCA
AGGATGACATTAATTCCTATGA
>2009
CAGTAGTTCCATGTACTTTTTAGAAATGCATGTTAAATGATGCTGTTACTGTCTATTTTGC
TTCTTTTAGATGTAACATGTAACATTAAGAATGGCAGATGCGAGCAGTTTTGTAAAAAT
AGTGCTGATAACAAGGTGGT
>2010
ATTTTTGTAATACATGTTCCATTTGCCAATGAGAAATATCAGGTTACTAATTTTTCTTCTA
TTTTTCTAGTGCCATTTCCATGTGGAAGAGTTTCTGTTTCACAAACTTCTAAGCTCACC
CGTGCTGAGGCTGTTTTTCC
>2011
CAGCACACATATTTATTTTTTTCTAGATCAAATGTATTATGCAGTAAGAGTCTTAATTTTG
TTTTCACAGGTTGTTTTGAATGGTAAAGTTGATGCATTCTGTGGAGGCTCTATCGTTAAT
GAAAAATGGATTGTAACTG
>2012
AGTAGTCACTTAGAAAATCTGTGTATGTGAAATACTGTTTGTGACTTAAAATGAAATTTA
TTTTTAATAGGTGAACATAATATTGAGGAGACAGAACATACAGAGCAAAAGCGAAATG
TGATTCGAATTATTCCTCACCA
>2013
TGGGAACTCGCCCCACCTGTGTCCGGAACCTGCTCGCTCACGTCGGCTTTCCCCTTCT
GTTTTGTTCTAGGACTTCTGCACGGACCTGGCCGTCTCCAGTGCCAACTTCATTCCCAC
GGTCACTGCCATCTCGACCAGTC
>2014
TGGGGTGGGTGAATGGAGGTGATGGCAGACACTTTTACTGAATGTCGGTCTTTTTTTGT
GATTATTCTAGTTATCTCCAGAAGAAGAAGAGAAAAGGAGAATCCGAAGGGAAAGGA

ATAAGATGGCTGCAGCCAAATGCC
>2015
TTGGAGATTGAGCATAAGGGCCCTTGAGTAAGACTGTGTCTTATGCTTTCCTTTATCCCT
CTGTATACAGGAGACAGACCAACTAGAAGATGAGAAGTCTGCTTTGCAGACCGAGATT
GCCAACCTGCTGAAGGAGAAGG
>2016
GAAATGAGGGGGTGGGGAAATGGTACGGAGGTTCATAACTGAGGAAATGACCACGAC
TGCACTATTTCAGGAGCAGACAAGATGGAGACAAATTCCTCTCTCCCCACGAACATCT
CTGGAGGGACACCTGCTGTATCTGC
>2017
CATAGTCAGCCTGTATTCTATACTCATTAGTGCATTACCAGTTTGTTTCATTCCCTTTTGT
TTTCTGCAGATAACAATGCTGGGAATTCAAGGAGATCTGAAGTGGTCCACAGATCCAG
ATAAAGGTCTCTTCATCTCTC
>2018
CAGGGATCAGAAGCTGCCTGGCCTTTTCTTCTGAGCTGTGACTTGGGCTTATTCTCTCC
TTTCTCCGCAGTTGCTGCTGACACGCCGACCGCCTGCTGCTTCAGCTACACCTCCCGA
CAGATTCCACAGAATTTCATAGC
>2019
AGGGCCATGGTGGGCCCAGGATTCCCCGGCTGGATTCCCCAGTGCTTAACTCTTCCTCC
CTTCTCCACAGCTTCCTAACCAAGAGAGGCCGGCAGGTCTGTGCTGACCCCAGTGAGG
AGTGGGTCCAGAAATACGTCAGT
>2020
CTCTAGTTCCCTGCAGCTGGGGTGGGGCGTGCCTGCATTTTCAGGTGCCTTAACCGAC
CCATTTCCGCAGAGCCTCCTGTCGCTGAGCCCTGACGTGCCCGTGCCATCCGACCATG
GAGGGACTGAGTCCAGCCCAGGCT
>2021
AAGCGTTCAGCTGCAGTAGCATATTCAAGTGTTGCTAGTTAGTAATTATCTTTTTAATTT
TTTGTTTTAGTTTAAAAGATTGGAAGACCCGTTTGAGCTACTTCTTACAAAATTCCTCTA
CTCCTGGGAAGCCCAAAACC
>2022
GGAGAAACATAAGGAATCATTTGGTCTCTCAGTTCTTCACATTCTGCATGCTCTTTTCTC
CCCCCTTCAGATGGTCTTGCTGCATTCAGGGCTTTTTTAAAGTCGGAATTCTGTGAAGA
AAATATTGAATTCTGGCTGGC
>2023
TGTTTTCAAATACTAAATTTTAATCTTTAACTCTGAATACCAAATAAACAACTTTTTTGTT
TTATTTCAGATAAACATAGATTTTCAAACCAAAACTCTGATTGCCCAGAATATACAAGA
AGCTACAAGTGGCTGCTTTA
>2024
GAGGCAGTGTCATTGGAGAGGCTTACTCCAGCTGGCGGAAGCCTCACGTACTGCTTGT
CTCTCCTGCCAGGCTCTGGAGCTCATGACGGTGCTGGTGGGCAGCCCCCGCAAGGATG
GGCTGGTGTCTCTCCTCACCACCT
>2025
GACACCTGCCCTTCCTGTCCAATCCTCCCAGGCAGCCTCTCTTACCATCACCTGTTCTT
TCCCCCTGCAGCTGCCCCCCTCCCTGGCTTCAGTGCAGTGGTGGTCAGCTCAGTGCCC
CTGGGGGGTGGCCTGTCCAGCTC
>2026

CCCTGGAGTGTCATTGAAGCCACTGCTGCTTCCACCAGCCCTAGCCTCCCCACCTCAC
CCTGTACTGCAGACTCGGGCACAATAGCTGCCCGCGCCCAGGTGTGTCAGCAGGCCGA
GCACAGCTTCGCAGGGATGCCCTG
>2027
TGTGCCCACTGCCTGGCGCAGCAAGCACACGCTTGGCCTCGTCATCTCCCCCATTGTA
ACTCCACCCCAGGTCCTTGGAGACCAGCCTGGTGCCACTCTCGGACCCCAAGCTGGCC
GTGCTCATCACCAACTCTAATGTC
>2028
TGCTGGTCCTCACAGGCGTCGGGGCCCCAGGCAGTGAGAAGGCGGCTGCTGACTCCT
CTTTCCTCCCCAGCTGCCAGGGACCTGGTGAGCAAAGAGGGCTTCCGGCGGGCCCGG
CACGTGGTGGGGGAGATTCGGCGCAC
>2029
CCCTCTGGGCGCCCCGCTCCTGCCGGGCACAGGCCGGCCCAGGCCCACCCCTTCAATA
TCCTCTCTGCAGAGACGACTATGAGGTGAGCTGCCCAGAGCTGGACCAGCTGGTGGA
GGCTGCGCTTGCTGTGCCTGGGGTT
>2030
CAGGGCCTGGGCGGGCAGGAGCGGCAGCTTCCCGGGGCCCTGCCACTCACCCCCAGC
CCGCCTCTTACAGGAGCACTACGGCGGGACTGCCACCTTCTACCTCTCTCAAGCAGCC
GATGGAGCCAAGGTGCTGTGCTTGT
>2031
GGGTGAGACCCAGGAGAGAGGGAGCTAGAGGGGGGAGCTCTGAGGACTGATCTTGA
CTGTCTGCCCCCAGACCATCAGCATATCCGCTACAACCCGCTGCAGGATGAGTGGGTG
CTGGTGTCAGCTCACCGCATGAAGCG
>2032
AGTCTTTGAAGCCCACCAGGTAACTGGTGGTATGGGGCAGTGAGTGCTTCTAGCCTAT
CCTTGTCGGTAGGTGAATCCCCAGTACGATAGCACCTTCCTGTTTGACAACGACTTCCC
AGCTCTGCAGCCTGATGCCCCCA
>2033
TGGGGTTCGCCCTGCCCGTAGCACAGCCAAGCCCTACCTCTCGGTTATCTTTTCTCCCG
TCACCACCCAGTAAGGTCATGTGCTTCCACCCCTGGTCGGATGTAACGCTGCCACTCAT
GTCGGTCCCTGAGATCCGGGCT
>2034
ATATGCCAATGATGTGGAGGCTTGGAGGTAAAGGACCTGCCTGTTCTTCTCTGCTTTTG
CCCCTTGACAGGTATGGGCCAGCAGTTTCCTGCCAGATATTGCCCAGCGTGAGGAGCG
ATCTCAGCAGGCCTATAAGAGTC
>2035
TGCCTATTTGCTGACCACACTCCGGCTCCTATGTCACCTTGATGACTTCCTATCCATTCT
GTCTTCCTAGGAACGTCTGGTCCTAACCAGTGAGCACTGGTTAGTACTGGTCCCCTTCT
GGGCAACATGGCCCTACCAGA
>2036
ACTGGATGGAGGTTGCTCCCAGTAGGGTCAGCATCTGGACCCCAGGCTGAGAGTCAG
GCTCTGATTCCAGATCTAGCCTCCATCATGAAGAAGCTCTTGACCAAGTATGACAACCT
CTTTGAGACGTCCTTTCCCTACTC
>2037
GGGAGTAGGTGCTAACCTGGATAACTGTAAAAGGGCTCTCTCTCCCCACTGTCTCTCTT
CTTTCTGTCAGGGGCTCCCACAGGATCAGAGGCTGGGGCCAACTGGAACCATTGGCA

GCTGCACGCTCATTACTACCCTCC
>2038
TGAAGTCCATGCCACCATTCTTGGCAGCCCAGCCCTTATCCTCCTTAATTGCTCCCTGTC
CCTTTTCCAGGCTGCAGAGAGACTAAGGGCACTTCCTGAGGTTCATTACCACCTGGGG
CAGAAGGACAGGGAGACAGCAA
>2039
AAAAGTCCAGGTCGCTTCTCAGGATTTGTGGCACCTTCTGACTGTCAAACTGTTCTTGT
CAATCTCACAGGCTCCTGGTTGTCTACCCATGGACCCAGAGGTTCTTTGACAGCTTTGG
CAACCTGTCCTCTGCCTCTGCC
>2040
AAAAGTCCAGGTCGCTTCTCAGGATTTGTGGCACCTTCTGACTGTCAAACTGTTCTTGT
CAATCTCACAGGCTCCTGGTTGTCTACCCATGGACCCAGAGGTTCTTTGACAGCTTTGG
CAACCTGTCCTCTGCCTCTGCC
>2041
ATCTGGAGGCCAGGCTGGAGCTCTCAGCTCACTATGGGTTCATCTTTATTGTCTCCTTTC
ATCTCAACAGCTCCTGGGAAATGTGCTGGTGACCGTTTTGGCAATCCATTTCGGCAAA
GAATTCACCCCTGAGGTGCAGG
>2042
ATCTGGAGGCCAGGCTGGAGCTCTCAGCTCACTATGGGTTCATCTTTATTGTCTCCTTTC
ATCTCAACAGCTCCTGGGAAATGTGCTGGTGACCGTTTTGGCAATCCATTTCGGCAAA
GAATTCACCCCTGAGGTGCAGG
>2043
TGGCCTAGGGCTGCTCACATATTCTGGAGGAGCCTCCCCTCCTCATGCCTTCTTGCCTC
TTGTCTCTTAGATTTGGTCGTATTGGGCGCCTGGTCACCAGGGCTGCTTTTAACTCTGGT
AAAGTGGATATTGTTGCCATC
>2044
CCCAAAGCTGGTGTGGGAGGAGCCACCTGGCTGATGGGCAGCCCCTTCATACCCTCAC
GTATTCCCCCAGGTTTACATGTTCCAATATGATTCCACCCATGGCAAATTCCATGGCACC
GTCAAGGCTGAGAACGGGAAGC
>2045
TCCCTTGGGTATATGGTAACCTTGTGTCCCTCAATATGGTCCTGTCCCCATCTCCCCCCC
ACCCCGGTAGGCGAGATCCCTCCAAAATCAAGTGGGGCGATGCTGGCGCTGAGTACGT
CGTGGAGTCCACTGGCGTCTTC
>2046
GGGAGGGGAAGCTGACTCAGCCCTGCAAAGGCAGGACCCGGGTTCATAACTGTCTGC
TTCTCTGCTGTAGGCTCATTTGCAGGGGGGAGCCAAAAGGGTCATCATCTCTGCCCCCT
CTGCTGATGCCCCCATGTTCGTCA
>2047
AGAAGCGGCCAGCCTGGCACCCTATGGACACGCTCCCCTGACTTGCGCCCCGCTCCCT
CTTTCTTTGCAGCAATGCCTCCTGCACCACCAACTGCTTAGCACCCCTGGCCAAGGTCA
TCCATGACAACTTTGGTATCGTG
>2048
CTTTCAAGGTGGGGAGGGAGGTAGAGGGGTGATGTGGGGAGTACGCTGCAGGGCCTC
ACTCCTTTTGCAGACCACAGTCCATGCCATCACTGCCACCCAGAAGACTGTGGATGGC
CCCTCCGGGAAACTGTGGCGTGATG
>2049

AGTGCAGGGTCTGGCGCCCTCTGGTGGCTGGCTCAGAAAAAGGGCCCTGACAACTCT
TTTCATCTTCTAGGTATGACAACGAATTTGGCTACAGCAACAGGGTGGTGGACCTCATG
GCCCACATGGCCTCCAAGGAGTAA
>2050
GACGGAAGGAGGGGGATTTGACTGAATGAAGGCTGACCCCCTACTGCCACCTCTCCCT
TTTCTTACCCAGAATTGGAGCTGGCCATTAGAATCACTCTTTACGCAGTGATCTTCCTGA
TGAGCGTTGGAGGAAATATGCT
>2051
AGGAAGTCCCACTGATGCTTGTGTAGTGCAAGTTTCCTAGGTGACTCCTTCCTTCTCTT
CCCTTGTTTAGGGGTGTCTGTGAGTGTGTCCACGCTAAGCCTCGTGGCCATCGCACTG
GAGCGGTACAGCGCCATCTGCCG
>2052
CCTTGGCTTTTTCTCCATCTGTGATTACAGCTGGACAGAAACCCGAGTGATCTGTCTGT
GTTGCCTTCAGGTCCGTACTGCTGCTTCTGCTCTTGTTCTTCATCCCGGGTGTGGTTATG
GCCGTGGCCTACGGGCTTATC
>2053
TCAGGTGGGGACTGGGCTGGAGACTGGGGGGACTCGCCTTTTTCTCTGACCGCCCACC
CTTTGTGCTCAGGGGCTGTTCACCAGAACGGGCGTTGCCGGCCTGAGACTGGCGCGG
TTGGCGAAGACAGCGATGGCTGCTA
>2054
GACTGGCTCTGACTTCAGTTCCTAGAAGGTAGGCATCCTTCCCCCATTCTCGCCTCTCT
CCCCTCCTCAGACCCGTCCAAGAAGCAGGGACCATGGCTGGAGGAAGAAGAAGAAG
CCTATGGATGGATGGACTTCGGCCG
>2055
GATGGGCGGGGCCTGAGGCTGGAGTGAGCGGGGCCCGGATGGGCTCACGGCGGCCGC
GCCCCTCGCCCAGGACGGCTACATTGATTTCATGGAGTACGTGGCAGGCCTCAGCTTG
GTCCTCAAGGGGAAGGTGGAACAGA
>2056
GTCCCCAATCCTACCCCTGAGATAGGATAAGGATGGGCCCCTCTCACTTCTGCCCCTTC
TTCCCTCCCAGGCCATTCGCGCCATTAACCCCTGCAGCGATACCACCATGACTGCAGAG
GAGTTCACCGATACAGTGTTCT
>2057
CCCGGTTCTGTGCTCTGGACTGCAGAAATGAACACCCTCCTCCCCCTGATTCCCTTTCT
CTCTACCCCAGGGGAACTCTCCCTGGAAGAGTTTATAGAGGGCGTCCAGAAGGACCAG
ATGCTCCTGGACACACTGACACG
>2058
CCCTGCCCAGGAGAGTAGTTGAGGGGTGGAGAGGGGCTTGCTTTTCAGTCATTCCTCA
TTCTGTCCTCAGGAATGTCCCAAGCCTTTGAGTAGGGTAAGCATCATGGCTGGCAGCCT
CACAGGATTGCTTCTACTTCAGG
>2059
CTCCTAGCAGATGTGTCCATTCTCCATGTCTTCATCAGACCTCACTCTGCTTGTACTCCC
TCCCTCCCAGGTGCCCGCCCCTGCATCCCTAAAAGCTTCGGCTACAGCTCGGTGGTGT
GTGTCTGCAATGCCACATACTG
>2060
ATGGTGTCAGTGATCACCATGGAGTTTCCCGCTGGGTACTGATACCCTTATTCCCTGTG
GATGTCCTCAGGCCTGCTACTGACCCTGCAGCCAGAACAGAAGTTCCAGAAAGTGAA

GGGATTTGGAGGGGCCATGACAGA
>2061
AAGTGATAAGCAGAGTCCCATACTCTCCTATTGACTCGGACTACCATATCTTGATCATCC
TTTTCTGTAGGAATCGGATATAACATCATCCGGGTACCCATGGCCAGCTGTGACTTCTCC
ATCCGCACCTACACCTATGC
>2062
GTGAGCCACCACACCCAGCTGGTCTGGTCCACTTTCTTGGCCGGATCATTCATGACCTT
TCTCTTGCCAGGTTCCTGGATGCCTATGCTGAGCACAAGTTACAGTTCTGGGCAGTGAC
AGCTGAAAATGAGCCTTCTGCT
>2063
CCTACAACAGGAACTAGAAGTTCCAGAAGCCTGTGTGCAAGGTCCAGGATCAGTTGCT
CTTCCTTTGCAGGTACTGACAGACCCAGAAGCAGCTAAATATGTTCATGGCATTGCTGT
ACATTGGTACCTGGACTTTCTGG
>2064
ACCCTTACCTACACTCTCTGGGGACCCCCAGTGTTGAGCCTTTGTCTCTTTGCCTTTGT
CCTTACCCTAGAACCTCCTGTACCATGTGGTCGGCTGGACCGACTGGAACCTTGCCCTG
AACCCCGAAGGAGGACCCAATT
>2065
GGTGACTTCTTAGATGAGGGTTTCATGGGAGGTACCCCGAGGGACTCTGACCATCTGT
TCCCACATTCAGCAAGTTCATTCCTGAGGGCTCCCAGAGAGTGGGGCTGGTTGCCAGT
CAGAAGAACGACCTGGACGCAGTG
>2066
CCCTGCCCGGGAGAGTAGTTGAGGGGTGGAGAGGGACTTGCTTTTCAGTCATTCCTCA
TTCTGTCCTCAGGAATGTCCCAAGCCTTCGGGTAGGGTAAGCATCATGGCTGGCAGCC
TCACAGGATTGCTTCTACTTCAGG
>2067
TCCAAACAGCTGTGTCCGTTCTCCACATCCTTGTCAGACCTCACCTCTGCTTGTGCTCC
CTCCCTCCCAGGTGGTGCCCCTGCATCCCTAAAAGCTTCAGTACAGCTCGGTGGTCTGT
GTCTGCAATGCCACATACTGTG
>2068
GAGGTAAACGGTGTCAGTGATCACCATGGAGTTTCTCCCTGGGCACTGATAACCCTGT
GGATGTCCTCAGGCCTGCTACTGATCCTGCAGCCAGAAGTTCCAGAAAGTGAAGGGAT
TTGGAGGGGCCGTGACAGATGCAG
>2069
AAGTGATAAGCAGAGTCCCATACTCTCCTATTGACTCGGACTACCATATCTTGATCATCC
TTTTCTGTAGGAATCGGATATAACATCATCTGGGTACCCATGGCCAGCTGTGACTTCTCC
ATCCGCACCTACACCTATGC
>2070
CGGATGCACTGGTTGGGCTAGTATGTGTTCCAACTCTGGGTGCTTCTCTCTTCACTACC
TTTGTCTCTAGATACCCCTGATTCACCGAGCCCTGCAGTTGGCCCAGCGTCCCGTTTCA
CTCCTTGCCAGCCCCTGGACAT
>2071
ATCAGGCTTCCTGTTCTCTCGGTCTGGCCCACTTTCTTGGCTGGATCATTCATGACCTTT
CTCTTGCCAGGTTCCTGGATGCCTATGCTGAGCACAAGTTACAGTTCTGGGCAGTGAC
AGGTGAAAATGAGCCTTCTGCT
>2072

CCTACAAGAGGAACTAGAAGTTCCAGAAGCCTGTGGGCAGGGTCCAGGGTGACTTGT
TCTTCCTTTGCAGGTACTGACAGACCCAGAAGCAGCTAAGTATGTTCATGGTATTGCTG
TACATTGGTACCTGGACTTTCTGG
>2073
ACCCTTACCTACACTCTCTGGGGACCCCCAGTGTTGAGCCTTTGTCTCTTTGCCTTTGT
CCTTACCCTAGAACCTCCTGTACCATGTGGTCGGCTGGACCGACTGGAACCCATCATTG
TAGACATCACCAAGCACACGTT
>2074
GGCGACTTCTTAGATGAGGGTTTCATGGGAGGTACCCCGAGGGACTCTGACCATCTGT
TCCCACATTCAGCAAGTTCATTCCTGAGGGCTCCCAGAGAGTGGGGCTGGTTGCCAGT
CAGAAGAACGACCCGGACGCAGTG
>2075
AAGTGGGCTGAAGACAGCGTTGGGGGCCTTGGCAGGATCACACTCTCAGCTTCTCCTC
CCTGCTCCCTAGCTCCTCTAAGGATGTGCCTCTTACCATCAAGGATCCTGCTGTGGGCT
TCCTGGAGACAATCTCACCTGGC
>2076
CTCCTGGAGCAGGGAGAGTGTTGGCCTCTTGCTCTCCGGCTCCCTCTGTTGCCCTCTG
GTTTCTCCCCAGGCTCCCGGACGTCCCTGCTCCTGGCTTTTGGCCTGCTCTGCCTGCCC
TGGCTTCAAGAGGGCAGTGCCTT
>2077
GCTAGGTTCCCAGAAAAGTAAAATGGGAGCAGGTCTCAGCTCAGACCTTGGTGGGCG
GTCCTTCTCCTAGGAAGAAGCCTATATCCCAAAGGAACAGAAGTATTCATTCCTGCAGA
ACCCCCAGACCTCCCTCTGTTTCT
>2078
GCGGGGATGGGGGAGACCTGTAGTCAGAGCCCCCGGGCAGCACAGCCAATGCCCGTC
CTTGCCCCTGCAGAACCTAGAGCTGCTCCGCATCTCCCTGCTGCTCATCCAGTCGTGGC
TGGAGCCCGTGCAGTTCCTCAGGA
>2079
TGAATGAATGAGAAAGGGAGGGAACAGTACCCAAGCGCTTGGCCTCTCCTTCTCTTCC
TTCACTTTGCAGAGGCTGGAAGATGGCAGCCCCCGGACTGGGCAGATCTTCAAGCAG
ACCTACAGCAAGTTCGACACAAACT
>2080
GCTCCTGGAACAGGGAGAGCGCTGGCCTCTTGCTCTCCAGCTCCCTCTGTTGCCTCCG
GTTTCTCCCCAGGCTCCCGGACGTCCCTGCTCCTGGCTTTTGGCCTGCTCTGCCTGTCC
TGGCTTCAAGAGGGCAGTGCCTT
>2081
GAGGTTCCCAGAAAAGTAACAATGGGAGCAGGTCTCCAGCATAGACCTTGGTGGGCG
GTCCTTCTCCTAGGAAGAAGCCTATATCCTGAAGGAGCAGAAGTATTCATTCCTGCAGA
ACCCCCAGACCTCCCTCTGCTTCT
>2082
AGGTGGGATGGGGTAGACCTGTGGTCAGAGCCCCCGGGCAGCACAGCCACTGCCGGT
CCTTCCCCTGCAGAACCTAGAGCTGCTCCGCATCTCCCTGCTGCTCATCCAGTCATGGC
TGGAGCCCGTGCAGCTCCTCAGGA
>2083
TGGATGAATGAGAGAGGGAGGGAACAGTGCCCAAGCGCTTGGCCTCTCCTTCTCTTCC
TTCACTTTGCAGAGGCTGGAAGATGGCAGCCCCCGGACTGGGCAGATCTTCAATCAGT

CCTACAGCAAGTTTGACACAAAAT
>2084
TCCCTTGTTTGGTTAGAACGTTTCTCTCCTCCTCTTGACCCCGGGTTCTAGCTGCCCTTC
TCTCCTGTAGGAGAACGCCAAGAACGAAGAGATTCTGAATTCCCTCAAGTACGTCCGG
CCTGGTGGTGGGTTCGAGCCCA
>2085
GGGCCCCTAGCGGAAGGAAAAAAATCATGGTTCCATGTGACATGCTGTGTCTTTGTGT
CTGCCTGTTCAGGATGGGGAACCCCCTCAGCAGCGAGTGACTGGGACCCTGGTCCTTG
CTGTGTTCTCTGCGGTGCTTGGCT
>2086
GAGGACAGGTGTCTCGGGGGTGGTGGAAAGGGGACGGTCTGCAGGAAATCTGTCCTC
TGCTGTCCCCCAGGTGATTGAACAGAGCTACAATGAGACGTGGCTGGGGAGGCAGGG
GCCTGAGGGACCCAGCTCCATCCCTC
>2087
TGGGGGAAACAGGAAGGGAGCCACTGCTGGGTGCCCTCACCCTCACAGCCTCACTCT
GTCTGCCTGCCAGGAAAAGGGCCATGCTGGTCAACAATGTCCTGGCGGTGCTGGGGG
GCAGCCTCATGGGCCTGGCCAACGCT
>2088
TGGGCTTTCAGATGGGAATGGACACCTGCCCTCAGCCCTCTCTTCTTCCCTCGCCCAGG
GCTGACATCAGGGCTGGTGCCCATGTACGTGGGGGAGATTGCTCCCACTCACCTGCGG
GGCGCCCTGGGGACGCTCAACCA
>2089
ATATGGTGGGCTTCCAAGGTAAGGCAGAAGGGCTGAGTGACCTGCCTTCTTTCCCAAC
CTTCTCCCACAGGTGCTGGGCTTGGAGTCCCTCCTGGGCACTGCCAGCCTGTGGCCAC
TGCTCCTGGGCCTCACAGTGCTAC
>2090
GCCTCCGCCTCATCTTGCTAGCACCTGGCTTCCTCTCAGGTCCCCTCAGGCCTGACCTT
CCCTTCTCCAGGTCTGAAGCGCCTGACAGGCTGGGCCGATGTTTCTGGAGTGCTGGCT
GAGCTGAAGGATGAGAAGCGGAA
>2091
CCAGCCTGTTGTGGCTGGAGTAGAGGAAGGGGCATTCCTGCCATCACTTCTTCTTCTCC
CCCACCTCTAGGTTTTCTATTATTCGACCAGCATCTTCGAGACAGCAGGGGTAGGCCAG
CCTGCCTATGCCACCATAGGAG
>2092
CCTCACCCACGCGGCCCCTCCTACTTCCCGTGCCCAAAAGGCTGGGGTCAAGCTCCGA
CTCTCCCCGCAGGTGTTGTTGGTGGAGCGGGCGGGGCGCCGGACGCTCCATCTCCTGG
GCCTGGCGGGCATGTGTGGCTGTG
>2093
CTGAGTTGAGGGCAAGGGAAGATCAGAAAGGCCTCAACTGGATTCTCCACCCTCCCT
GTCTGGCCCCTAGGAGCGAGTTCCAGCCATGAGCTACGTCTCCATTGTGGCCATCTTTG
GCTTCGTGGCATTTTTTGAGATTG
>2094
CTGGTTGCCTGAAACTACCCCTTCCCTCCCCACCTCACTCCGTCAACACCTCTTTCTCC
ACCTGTCCCAGGAGGCTATGGGGCCCTACGTCTTCCTTCTATTTGCGGTCCTCCTGCTG
GGCTTCTTCATCTTCACCTTCT
>2095

CCGGGTGACATCTGGGAAGGCTGAAAGGAAACAAACCCTTCCTGATGACCCTATCCCT
GGCTCCCAACAGCCCCCCGAGCTCCCCGCTAACCCAGCCCCCAGAGGGGCCCAAGTT
CCCTCGTGTGAAGAACTGGGAGGTG
>2096
TCCCTGCCCCCAACTCCCATCCCACCCCTGCACCCTGGCCTGTCCTGACCTTTGCACTC
CCTCGACCCAGGATGGGCCCTGCACCCCAAGACGCTGCCTGGGCTCCCTGGTATTTCC
ACGGAAACTACAGGGCCGGCCCT
>2097
AGCCTGGAGGAGGGCCTCCCTGTCCCACACAACTTCCTGCTTGTCCCCTTCCCACCCC
TCTCCTCCCCAGGAGCGGCTCCCAGGCCCACGAACAGCGGCTTCAAGAGGTGGAAGC
CGAGGTGGCAGCCACAGGCACCTAC
>2098
TCTGGAGCTGATACTCAAGACCCCCCGTCTCTCTCCTCACCCTCCTCTCCCGCTGCCTC
GGCTGGCTCAGGTGTTCGATGCCCGGGACTGCAGGTCTGCACAGGAAATGTTCACCTA
CATCTGCAACCACATCAAGTATG
>2099
CAGGCCCCAGCCTTCTTCCCCAAGGCAGGGAAGGCGGGGCTCTGACCAGCTCTTTCCC
CATGCGTGCCAGCTCGGCCATCACAGTGTTCCCGCAGCGCTGCCCTGGCCGAGGAGAC
TTCCGAATCTGGAACAGCCAGCTG
>2100
GTGGATGGAGGGGTCCCTGAGGAGGGCATGAGGCTCAGCCCCAGAACCCCCTCTGGC
CCACTCCCCACAGCTCTGCATTCAGCACGGCTGGACCCCAGGAAACGGTCGCTTCGAC
GTGCTGCCCCTGCTGCTGCAGGCCC
>2101
GGGGGCCATCCCTGAGCCTCTCAAGAAGGGCCTGCAAGGGGGTGCTGATCCCACACC
CCAACACCCCCAGGCTGGAGTGGTTTGCAGCCCTGGGCCTGCGCTGGTACGCCCTCCC
GGCAGTGTCCAACATGCTGCTGGAA
>2102
AGATAGTCTCCCCACCCCACCCCCGTGATCACCTCTGTCCCTACCGATGCCACACACCC
TTCTGCCCCAGGATGTGGCTGTCTGCATGGACCTGGATACCCGGACCACCTCGTCCCTG
TGGAAAGACAAGGCAGCAGTGG
>2103
AGTGGTGGAGGAAGAATGGGCGAGGTCTGTGGGTCTGGTTTGAGCCTCTCCCCCTCTC
TCTCCCTTCCAGCTAGCCAAAGTCACCATCGTGGACCACCACGCCGCCACGGCCTCTT
TCATGAAGCACCTGGAGAATGAGC
>2104
AGCAGCCCCTGGGGACCTCTAACCTTTCCTTTTCTTTACCTCCCCTCCCAACCCCATCA
TCTCTCTGCAGCCAGACCCCTGGAAGGGGAGTGCCGCCAAGGGCACCGGCATCACCA
GGAAGAAGACCTTTAAAGAAGTGG
>2105
GTTGGACCCTTGCCTGGGGAGGCCCTGCCTCTGTGCACCCGGGACACCCTCACACCTT
CCTCTCCCGCAGCGCCGTGAAGATCTCCGCCTCGCTCATGGGCACGGTGATGGCGAAG
CGAGTGAAGGCGACAATCCTGTAT
>2106
GAGGCCAGAGTGAGGAGGGCAGGGCCTCCGGGGGCCACAGCACCCAGGACATCTGT
CTTCCCACCCACAGGTCCTGTGTATGGATGAGTATGACGTGGTGTCCCTCGAACACGA

GACGCTGGTGCTGGTGGTAACCAGCA
>2107
GGGCTGGGTCTGGGCCTAGCCTGTATCCCCAGGGCCCTGTGACAACCTTGTCTTTGTCC
TCTCTTGCCAGGAGTTATAAGATCCGCTTCAACAGCATCTCCTGCTCAGACCCACTGGT
GTCCTCTTGGCGGCGGAAGAGG
>2108
ACGGGTGGGCAAGCTGCCTGGGCAAACGTGGCCTGCAAAGGGAGCTCCACTGACGAC
CCCTGCACCCCAGGTTCTGTGTGTTCGGGCTCGGCTCCCGGGCATACCCCCACTTCTGC
GCCTTTGCTCGTGCGGTGGACACA
>2109
CGCCCAGGCGCCTCACTAGGGCGACCCCTGGTGGCGGGAGGTCCTCAGCCCTCACCG
GCCTGTCCCGCAGGCCGCCTGTGAGACCTTCTGTGTGGGAGAGGATGCCAAGGCCGC
CGCCCGAGACATCTTCAGCCCCAAAC
>2110
ACTAAAGCACTCTGGGGCCAGGCCCTGCTCCCTAGCTCAGGCTGCCTCATTTGCCCCT
CCCCGCCCCCAGGTCTGATCCACGTGCACAGGCGGAAGATGTTCCAGGCTACAATCCG
CTCAGTGGAAAACCTGCAAAGCAG
>2111
TCCTCCTCCCACATTCTCCCGCCCCCACCCCTCTCTGACTCCCCATAAGTGCCCCTCTCC
CCACCCCCAGGAGGGCCACCATCCTGGTGCGCCTGGACACCGGAGGCCAGGAGGGGC
TGCAGTACCAGCCGGGGGACCAC
>2112
ACAAACATCAGCCCAGGTACTGCAGTCCTGCTGGGCCCTGTCCTCAGAGCTCCCTGTG
CACTATCCCCAGGTGGCCCTCCCCCCGGCTGGGTGCGGGACCCCCGGCTGCCCCCGTG
CACGCTGCGCCAGGCTCTCACCTT
>2113
GAGGGGTCAAGAAGGGAGGTTACTAGGAAGGGCTATGGGGCCTCCAACCCACTGCAT
CCTGCCCCGCCAGGATCCCCGACGCTACGAGGAGTGGAAGTGGTTCCGCTGCCCCACG
CTGCTGGAGGTGCTGGAGCAGTTCC
>2114
GGCCTGGCCACAGCAGGGTTGGGACCGGCCCCTCTCTGGCCCCTCACCGGCCTCTCCT
TCCCACCCCCAGATGGGCTGGGCCCCCTGCACTATGGAGTCTGCTCCACGTGGCTAAG
CCAGCTCAAGCCCGGAGACCCTGT
>2115
CCACCCTGCATGGTGAGAATGGTGGAGCAGGAAAGGCAAAGGGGACCTGATGGAGTG
TCTCTCCTGCCAGGGCTCCCTCCTTCCGGCTGCCACCCGATCCCAGCTTGCCCTGCATC
CTGGTGGGTCCAGGCACTGGCATT
>2116
GGCCTGTGGGGAGGCCCCACTAGCACTGTGCCCCGGAGAAGAGCCTTCCCAAGCGCG
GGGTTGCTTGCAGGGCTGCAGCCCACTCCCATGACTTTGGTGTTCGGCTGCCGATGCT
CCCAACTTGACCATCTCTACCGCGA
>2117
CAGCCAGCAGCCCCGGGCTGCCCCCGCGCCCACCCCCACCAGGGCCCGCCCTAACCC
CGCCGCCCCGCAGACCTACGTGCAGGACATCCTGAGGACGGAGCTGGCTGCGGAGGT
GCACCGCGTGCTGTGCCTCGAGCGGG
>2118

GGGGGCTGGGGCCTGGGGGGATGCTTCCAGGGGATGCAGGGGGATCCACTCAAGGCT
CCCTTGCCCACAGGTCCTCATGCCTCTCCTCCTCTTGCTGCTCCTGCTGCCAAGCCCCT
TACACCCCCACCCCATCTGTGAGG
>2119
GGGATGGGGTCTCTGCTAAGGGCCAGGAAGTCCCTTCCCCTTCCCAAAAACAAACTTA
CCCAATCCACAGCCGCCCTCACCGCCCGGCCTTCTACGGTGTCCAGAGACAGTTAGCC
AGGCCTGGGCTGGGCACACTCCAC
>2120
CCCAGTGCTTGCCGTCCCTGAGGATCGGTCCAGGCTGCCAGGCCCTCCCTCACAGCCC
CTCTCTCTGCAGCCAGCCTGTCCCATGCCTGCCTGGGGAGCCCTGTTCCTGCTCTGGGC
CACAGCAGAGGCCACCAAGGACT
>2121
GGGGGACGGGGCGTGGCGCTGGCTGGCGTGAGAAGTGCGGTGCTGATGTCCCTCTGT
CGGGTTTTTGCAGCGTCGGCGTGTTCAAGAACGGCCGCGTGGAGATCATCGCCAACGA
TCAGGGCAACCGCATCACGCCGTCC
>2122
TGGTAGTGGGTCTTTTAAACTTTGAGATGTCATTGTATCTGTGTCTGAAAACAATAATTC
TTTAAAATAGGTGGTTGAAAAGAAAACTAAACCATACATTCAAGTTGATATTGGAGGTG
GGCAAACAAAGACATTTGCTC
>2123
CTGGCCAGACTGTAATTTAAATAAGGGTTAAACTATGTGACAATACACTTAATTATCTTT
ATCCTTTTAGGTTACCCATGCAGTTGTTACTGTACCAGCCTATTTTAATGATGCCCAACG
CCAAGCAACCAAAGACGCTG
>2124
ATACGGCATATTTGCCAAATAGTGGAAATGTGAAGTACTGACAAAACTTTTCCCTTTTT
CAATCTAATAGTACGGCAGCTGCTATTGCTTATGGCCTGGATAAGAGGGAGGGGGAGA
AGAACATCCTGGTGTTTGACCTG
>2125
CTCTGAATTCAGGACATTGCATCTAGATACTTAGATAACAGACATCACAGTAACCATGT
CTTTTTTCTAGGATCTGTTCCGGTCTACTATGAAGCCCGTCCAGAAAGTGTTGGAAGAT
TCTGATTTGAAGAAGTCTGATA
>2126
TTAGAGAGATAAACTTTACATAGCTCATAGGGAGAACTTGAATTAAAAGTTAAATAACT
TATCCTTACAGGTGACCTGGTACTGCTTCATGTATGTCCCCTTACACTTGGTATTGAAAC
TGTAGGAGGTGTCATGACCAA
>2127
AATGCGGGGATCTAGATGTGCTAATGCTTGTCAGTAACAACTAACAAGTTTTTCTGTAT
GTAACTTCTAGGTGAAAGACCCCTGACAAAAGACAATCATCTTCTGGGTACATTTGATC
TGACTGGAATTCCTCCTGCTCC
>2128
CTGGTGCAGAGAAAGTCACCAAGTCAGGGACCCTCCATCTCTGACACGACCTGCGGG
CCATCTCTTCCAGCTGGCCCACGCCATGCGCCTGCTCCTGGAATACACAGACTCAAGCT
ACGAGGAAAAGAAGTATACGATGG
>2129
GCATATGGGAAGGGGATGCTGGGGAGCCTGCTGGCCCCAACTGAGCTTCCCCGGTTTC
CCATCTATCCAGCTGCCCTACTTGATTGATGGGGCTCACAAGATCACCCAGAGCAACGC

CATCCTGTGCTACATTGCCCGCA
>2130
GAAGGTGGCATCCTCCTTGGCTGGATTGGGGTGCTATGCTCAGAGTGAGTCTGTGTTTT
GTGGGTGGCAGGTGGGGAGACAGAAGAGGAGAAGATTCGTGTGGACATTTTGGAGAA
CCAGGCTATGGACGTCTCCAATCA
>2131
AGCCCTGGGGAGGCCACATCTGTGCAGGGAGCTTGTGTCTGAGGGTGGTGACAGCTG
TTTTCTGCCTCAGGAGAAACTGAAGCCAGAATACTTGGAGGAACTTCCTACAATGATG
CAGCACTTCTCACAGTTCCTGGGGA
>2132
GGACACTAGAGATTTGCCATACATCCTATGTTACAGAGATTCCAGCCCACACATTCTTG
GCCTTCTGCAGATCACCTTTGTAGATTTCCTCGCCTATGATGTCCTTGACCTCCACCGTA
TATTTGAGCCCAACTGCTTGG
>2133
TGCAGTGGGGTTGTCCCAGCCCTCATGGGCAGCTGACCTTGAGTTCTGGCCTTATTTTC
CCCCCTCTCAGGGCTTGGAGAAGATCTCTGCCTACATGAAGTCCAGCCGCTTCCTCCC
AAAACCTCTGTACACAAGGGTGG
>2134
ACCCCTCCCCGGGTTGCTGCGAGGCGGAGTCGGCCCGGTCCCCACATCTCGTACTTCT
CCCTCCCCGCAGGCCGCTGCGCGGCCCTGCGCATGCTGCTGGCAGATCAGGGCCAGA
GCTGGAAGGAGGAGGTGGTGACCGT
>2135
GGGGGCTGTGACTAGGATCGGGGGACGCCCAAGCTCAGTGCCCCTCCCTGAGCCATGC
CTCCCCCAACAGCTATACGGGCAGCTCCCCAAGTTCCAGGACGGAGACCTCACCCTGT
ACCAGTCCAATACCATCCTGCGTC
>2136
CCCATCCCCAGTGACTGTGTGTTGATCAGGCGCCCAGTCACGCGGCCTGCTCCCCTCC
ACCCAACCCCAGGGCTCTATGGGAAGGACCAGCAGGAGGCAGCCCTGGTGGACATGG
TGAATGACGGCGTGGAGGACCTCCG
>2137
TGGGGCAGACGGGGGTGTCTCAGGGGCTGGGAGGGATGAGAGTAGGATGATACATGG
TGGTGTCTGGCAGGAGGCGGGCAAGGATGACTATGTGAAGGCACTGCCCGGGCAACT
GAAGCCTTTTGAGACCCTGCTGTCCC
>2138
TTAGGCCCTCTGCCCCCAATTCCTCCAGCCTGCTCCCGCTGGCTGAGTCCCTAGCCCCC
CTGCCCTGCAGATCTCCTTCGCTGACTACAACCTGCTGGACTTGCTGCTGATCCATGAG
GTCCTAGCCCCTGGCTGCCTGG
>2139
ATGGGCGCGCCGCCTTGGTAATTCTATATTCTCCGTTCCTCCTCCGCTCGCGGGCGTGTG
CGCGCCGCAGGCTGGCGGTAAGGCTGGAAAGGACTCCGGAAAGGCCAAGACAAAGG
CGGTTTCCCGCTCGCAGAGAGCCG
>2140
GCGCATATAATTTCCCCATCTCTAGTTCTCATTTTTGTGCATTTATGTTTGTGTATGCTTAA
AATTTAAGTTCCCAGTGGGCCGTATTCATCGACACCTAAAATCTAGGACGACCAGTCAT
GGACGTGTGGGCGCGACTG
>2141

GGTTTTATTCTGTTCTCTGAATCTTGGCCAAAGGAAAGTTGGAAGGCAACTGACAGAT
TCTGCCTTTTAGGTACTTGAACTGGCAGGAAATGCATCAAAAGACTTAAAGGTAAAGC
GTATTACCCCTCGTCACTTGCAAC
>2142
CTTTCTTGGTTTCAAATACTGTGATTTTTTAAAAAATCTTAAGTAGAATTAATTCCTGTC
ACTCTCCTAGGTGTCATTCCACACATCCACAAATCTCTGATTGGGAAGAAAGGACAAC
AGAAGACTGTCTAAAGGATGCC
>2143
TGCGGGGACGCTCTTGGGAGGAGTCTTCTCCCCAGCCTTAGCTGGTTTCATGATTTCTT
TGCGTCTGTAGGCAACGCGGTAAAAATATTGCTTCGGTGGGTGACGCGGTACAGCTGC
CCAAGGGCGTTCGTAACGGGAAT
>2144
TCTATAGTTAACGCCGGATCGCAGTTGGAAACCACCAGCTTTTTGTCAGTATATATTACT
CATTTTATAGAGCCAGAGGCCAAGAAGAGTAAGACGGCCGCAAAGAAAAATGACAAA
GAGGCAGCAGGAGAGGGCCCAGC
>2145
GCATATAGATTATTCAATAAATGTTCTGCTGAATTGATAATACGTTTTCCACCTTTCTTTT
CACTTACAGTGGGTAAAGGAAGAAGCCCCAGATATACTGTGCCTTCAAGAGACCAAAT
GTTCAGAGAACAAACTACCAG
>2146
TCTGCCCCACCTCTTGATTGCTTTCCCTTTTCTTATAGTTTTTTATGCTAATTCTGTTTCAT
TTCTATAGGCGATGAGGAGCATGATCAGGAAGGCCGGGTGATTGTGGCTGAATTTGAC
TCGTTTGTGCTGGTAACAGC
>2147
CCCACAGGCCACCCTCAACCGTCCTGGCCCCGGACCCAAACCCCACCCCTCACTCTGC
TTCTCCCCGCAGGATGTTCCTGTCCTTCCCCACCACCAAGACCTACTTCCCGCACTTCG
ACCTGAGCCACGGCTCTGCCCAG
>2148
CCCACAGGCCACCCTCAACCGTCCTGGCCCCGGACCCAAACCCCACCCCTCACTCTGC
TTCTCCCCGCAGGATGTTCCTGTCCTTCCCCACCACCAAGACCTACTTCCCGCACTTCG
ACCTGAGCCACGGCTCTGCCCAG
>2149
CAGAGAAGACTCTTGGGTTTCTGATAGGCACTGACTCTCTCTGCCTATTGGTCTATTTTC
CCACCCTTAGGCTGCTGGTGGTCTACCCTTGGACCCAGAGGTTCTTTGAGTCCTTTGGG
GATCTGTCCACTCCTGATGCT
>2150
CTGGATTATTCTGAGTCCAAGCTAGGCCCTTTTGCTAATCATGTTCATACCTCTTATCTTC
CTCCCACAGCTCCTGGGCAACGTGCTGGTCTGTGTGCTGGCCCATCACTTTGGCAAAG
AATTCACCCCACCAGTGCAGC
>2151
AAAAGTCCAGGTTGCTTCTCAGGATTTGTGGCACCTTCTGACTGTCAAACTGTTCTTGT
CAATCTCACAGGCTCCTGGTTGTCTACCCATGGACCCAGAGGTTCTTTGACAGCTTTGG
CAACCTGTCCTCTGCCTCTGCC
>2152
GCGCCCCCGCCCCCAGGGGCCCTCCCTCCCCAAGCCCCCCGGACGCGCCTCACCCAC
GTTCCTCTCGCAGGACCTTCCTGGCTTTCCCCGCCACGAAGACCTACTTCTCCCACCTG

GACCTGAGCCCCGGCTCCTCACAA
>2153
CCCCGGCGGGGTGGGTGCGGGGGGCGTGCGGGGCGGGTGCAGGCGAGTGAGCCTTG
AGCGCTCGCCGCAGCTCCTGGGCCACTGCCTGCTGGTAACCCTCGCCCGGCACTACCC
CGGAGACTTCAGCCCCGCGCTGCAGG
>2154
TGATGTCACATGGCTGGATCCAGCTAAAATTCTAAGGCTCTAACTTTTCACATTTGTTCC
ATGTTACCAGTCATCAGGTGGAAAAGCGGAAATGCAACACTGCCACATGTGCAACGCA
GCGCCTGGCAAATTTTTTAGTT
>2155
TTTTTGAGAAACACTTGTACTGTCATTCTCTATTCTCTGCAATTTGCTCTCTCCTTTTGT
GTGTATGCAGGAAAAGCATCATTCACATCGAGAATTTCCATTTTATGGGGACTATGGATC
AAATTATCTATATGACAATT
>2156
TTTGTGAAGCATTTTTACTGTCATTGCCTCTATTCTCTGCAATTTGCTCTCTCCTTTTGTG
TGTATGCAGGAAAAGCATCATTCACATCGAGGCTATAGATCAAATTATCTGTATGACAAT
TGATATCTTCAGTAATCAC
>2157
CGGGGTGCAGTGGGGACAGGGGTTGCAGTGGAATCTGAGTTCTCCCTCCCTCCATCCT
GTCCATTCCCAGGAGAACTATGAGCTCTACTCGGTGGAGCTGGGTCCTGGCCCTGGCG
GGGACATGGCTGCCAAGATGAGCA
>2158
GGGGCTTTCCACGGGGTGTGCATGTGGAGGAAAGCCCCCAGGACCGCCTCGCCCTGC
CCCTCTGTCCCAGGGCCTCTCTGCGAGCCTGGCTGCTGAGCTGCTGCTGCGGGATGGG
CCCAACGCACTGCGGCCACCACGGG
>2159
AGCAGGTCGGTGGCCTCAGGCAGGCAGCTTCCGGCCCCTCTGAATAACCCCACCCCTT
CCCTGTCCCCAGCTGTACCTGGCAATCGCTCTCATTGCTGTGGTTGTCGTCACCGGCTG
CTTTGGCTACTACCAGGAATTCA
>2160
TACCTCCCCACATGCAGCAGCTCCCACCCCCACCCCCGTCCACCTCAACTTTGGTGCCC
CTGCCCCGCAGCAAGCCACTGTCATCCGCGATGGAGACAAATTCCAGATCAACGCTGA
CCAACTGGTGGTGGGCGACCTGG
>2161
TCAGCTCACACGGCAGTGCACACCCCACTGCCGCCCCCTATCCCTGAGTCGCGCCTTG
GCCCCCCTGCAGGCACCGCGCAGGGCCTGGTGGTGAACACGGGCGACCGCACCATCA
TTGGGCGCATCGCATCGCTGGCGTC
>2162
TCTTTGACGCGGGGAAGAGGACTGGAAGCCAGGGTGGGTCCCCAGCCTGCGGTGCTC
ACCCCTTCCCCAGGTCTGCCTGTCCCTGACAGCCAAGCGCCTGGCCAGTAAGAACTGC
GTGGTCAAGAACCTGGAGGCGGTGG
>2163
GGAGGAGGGGGCTCGGTTCTGGTCCGGGAGAGCCGCCGTGCGGGACCCAGCCTGGAC
GCCTGGTCTGCAGGGCAGACGTTTGACCAGTCCTCGGAGACGTGGCGGGCGCTGTGC
CGGGTGCTCACCCTGTGCAACCGCGC
>2164

GGGGGCCTCCAGGGAATCCCGGGAGGCTGGTGTAGCCGGCTTCGCCCCTGACACCGG
TCCCCGCTCCCAGCGCATCGTGATTGGAGACGCATCGGAGACGGCGCTGCTCAAGTTC
TCGGAGCTGACGCTGGGCAACGCCA
>2165
CCCCGTTCCCTCGTCCACAGCCCCGCCCCGTTCCCCCGTCCACAGCCTCGCCTCTCCCC
CCCGCCCACAGCTGTCCATCCATACGCTGGAGGACCCGCGGGACCCGCGACACTTGCT
GGTGATGAAGGGCGCCCCCGAGC
>2166
ACCCATTTCCTGGCCCTGCTAAGAAAGGGTGTGCCCAGGGCTCTTCCCTGACTTCCCC
GTTTCCGGTCAGGCTTCTGCCAGCTCTACCTGAATGAGAAGGACTACCCGCCTGGCTAT
GCCTTCGACGTAGAGGCCATGAA
>2167
TGTCTCCCAGGGAGGACAGGGAGATTGGGCTGATCTGAAGGGGTCAGTGATCCACTG
GTTCCCCCTACAGGTGATCATGGTAACGGGTGACCACCCCATCACCGCCAAGGCCATT
GCAGCCAGTGTGGGCATCATCTCGG
>2168
CCTCCCCGGGGTTCTCCCCACATCTGGCACAGACCGAGGTCCCAGCGAGGTCCTCCCA
TCTCCTCCCCAGGGATGCCCGTGCCTGTGTGATCAATGGCATGCAGCTGAAGGACATG
GACCCATCGGAACTGGTCGAGGCC
>2169
ACAGGCGGGGCTGGTCTTGGACCGGCCTCTCACTGACCACCCACCCACCACCTGCAC
CCACTGCCTGCAGGGTGCGATTGTGGCCGTCACGGGGGATGGTGTGAATGACTCCCCA
GCTCTGAAGAAGGCAGACATCGGAG
>2170
GAGGGCAGGCAGTGTGGGCACAGGAGGGAGAGGGCTGTGAGGAGGCTGAGGTGCCC
ACCCTACCCCACAGGTCGACTGATCTTCGACAACCTGAAGAAGTCTATTGCCTACACAT
TGACCAAGAACATCCCAGAGCTGAC
>2171
GGGGACCCGAAGTGGAGGGGACAAGGGCCCAGCCAAACCCTCAGTCCCAGTTCCTTC
CACTCCTGGCCAGTTCCCATCTGTGTCCCTGGCATATGAAAAGGCCGAGAGTGACATCA
TGCACCTGCGTCCACGCAACCCAA
>2172
GTCCCTCTCTTCCTGTGTCCCTGTCTCTGTGTCCATCTCTGTCTCCCTGTGTGGGGTTGT
CTCTCTCCAGGTGCCATTCAGTCCTTTGCTGGCTTCACTGACTACTTCACGGCAATGGC
CCAGGAGGGCTGGTTCCCACT
>2173
TGGGCTGCAGAGGCTCTCCGCCGGGACTCAGGCTGGACTCAGGCTGTTTCCGTTTGCC
CCTGGCCTGCAGACATTCGGGCAGCGCCTGTACCAGCAGTACACCTGCTACACCGTGT
TCTTCATCAGCATTGAGGTGTGCC
>2174
GACCCACAGTATCCTGATTTCCATCACGTCCCCAATTCCTAATGGCCTGTCCCATTCTGC
CCACCCCCAGGAATAAGATCCTGGTGATCGCCATCGTGTTCCAGGTCTGCATCGGCTGC
TTCCTGTGCTACTGCCCCGGC
>2175
GGGGCAAGGACAGGGAGGGGGCTGTAGGGCACAGGCCTGGGATGGCCTTCACCTTGC
CATGTGCCTTCAGGTTCCAGTGGTGGCTGGTCCCCCTGCCCTACGGCATCCTCATCTTC

GTCTATGATGAGATCCGGAAGCTT
>2176
ACCTCTGAGCCCCTGCCCTGCCTAAGTGGCACCCCCAGCACTGAGTCTCCTTTCTCCCT
CTCTCTCCCAGGCTGGTGGGACCAGGAACTCTACTATTAGAGGGACGACTGCCTTCAA
GCATCCCTGCAACTGCCACAGCA
>2177
CCACCCCCGGACACTTCGAGCAGTGGAGGCCTTGTCCTCTAACCGGCTGGGCCGGGG
CTTGTCTGTGCAGGGTCTGGTCGCCAGCAACCTGAATCTCAAACCTGGAGAGTGCCTT
CGAGTGCGAGGCGAGGTGGCTCCTG
>2178
GTGACAGATTAGTCGGTCAGTGGGGCTGGAGCTGGCCGAGGTGGCCTCATGCCCACCC
GTTACCCCCCAGCTTCGTGCTGAACCTGGGCAAAGACAGCAACAACCTGTGCCTGCAC
TTCAACCCTCGCTTCAACGCCCAC
>2179
CACATGAGGAACGGGTTCTGGAAGGGCCCATGGCATGTGGGCCCGGCTCACTGCTCTC
CTCTACCCCCAGGTGTGCATCACCTTCGACCAGGCCAACCTGACCGTCAAGCTGCCAG
ATGGATACGAATTCAAGTTCCCCA
>2180
GGAAAAAGTAAAACAAAATGTAAATAAGGAGTAAAGCATGTACTTAAAATATATTCTTA
TTCTATCATAGGATAAATCTTCAGACAAAAAAGTGCAAACAAAAGGGAAAAGGGGAG
CAAAGGGAAAACAGGCCGAAGTGG
>2181
GCGCCCGCCCTGGCAGCTGCGGCGTCCGCGCCGACCTCCGGCTTCCCCTCTCCCCCCT
CGGCCCCGTCAGGTGGACGCGGATCTGTCAACATGGGTAAAGGAGACCCCAACAAGC
CGCGGGGCAAAATGTCCTCGTACGC
>2182
ATCTTGTCGTCTTTGGATGTTTATAGGTAACAATTTTGCTGTATTTTTGGCTCTTATTTTAT
GTCCACAGACCATGTCTGCAAAGGAGAAGTCGAAGTTTGAAGATATGGCAAAAAGTG
ACAAAGCTCGCTATGACAGGG
>2183
AGTTTAAAATAACCCAAATTGCTCCTTGGATTTTTCCTTCAGTTTATTAAACTCTGTTGC
TTCCTTTCAGATCTGCCTTCTTCCTGTTTTGCTCTGAACATCGCCCAAAGATCAAAAGT
GAACACCCTGGCCTATCCATT
>2184
TTTACAACTAAGTGGTTTTTCACAGTTGAGTAATGATACCGGATGCTTTATTTTTTTGAC
AATATTTCAGGATATTGCTGCATATCGTGCCAAGGGCAAAAGTGAAGCAGGAAAGAAG
GGCCCTGGCAGGCCAACAGGCT
>2185
CTAAAGAGTCAGGGTCAATTTCTTTCAGACATTCTTGCCACAACAGCATTTTTTTTTCC
CTCACAATTAGGAACCAACCGGTGAGCCCTCTCCTAAGAGACCCAGGGGAAGACCCA
AAGGCAGCAAAAACAAGAGTCCCT
>2186
CCTGCGGGGGGCGGGGACCCGGGAAAGGCGCGCGGTGGGGGAGGGGGCGCGCAGCT
GCGGCGAGCGCGAGTTGTGCACCCGGCGGAGCCCGGAGGAGCCCGGCGCACCTCGC
GCGGCGGCCGCTCCCGCTGGAGCCGGAG
>2187

CAAAAATGTTTTTTTCCTCTCATTGGGGTAAAAACTCTTTTTCTTCTTTTTTTTTTTTCTT
TTAAATTAGACATGGGAGTCCCACCGTATTGTCCAGGCTGGTCTCGAACTCCTGACCTC
AAGCAGTCCTCCTGCTTTGG
>2188
TGTGGGTGATGAGGGGGTAGAAAGTGGCTGAAGCGAGATGTTTGTCTAAAAGCACTTT
TCTGTCTCCCAGCATCCCAGCCATCACTCTTCCACCTGCTCCTTAGAGAAGGGAAGATG
AGTGAGTCGAGCTCGAAGTCCAG
>2189
GAGGGCTGTGTCTTCTGAGGGGGTGGAAACAGGTGATGACTGTCCCTGACTACCCCCT
CTGTCTTTACAGAAGGAGCCCAGCGAAGTGCCAACACCTAAGAGACCTCGGGGCCGA
CCAAAGGGAAGCAAAAACAAGGGTG
>2190
GGCCAGCCTCTGGGGGTGGAAGAGGGGGCCACCGGGCCAGAGCTCACACCAACAAC
TGCCCACCTCACAGGAGAAGGAGGAAGAGGAGGGCATCTCGCAGGAGTCCTCGGAG
GAGGAGCAGTGACCCATGCGTGCCGCCT
>2191
CAAGCTTGTCCCCCTGGCGGACTTTGGAAGACAGGAGTTGGTGGCTAAACCGCTGACT
TTTCTATTGCAGACAACATAGGCGGCCCGGAAGGCAAAAGGGCCCGGACGGCCTACA
CGCGCTACCAGACCCTGGAGCTGGA
>2192
CACCCCTCTCCAGTCCTGAGGGTCCCCACCCAACTCACTCAGCGCCCTCCCTCTCTCC
CTCCCTGCCCAGGAGAGAGCTGCGAGGACAAGAGCCCGCCAGGCCCAGCATCCAAGC
GGGTACGCACGGCATACACGAGCGC
>2193
TTCTCAGGCCCCCTGCTCTGTTTCTCTAGCTCCTCAACTTTTCTCTTTCCCCACTCCCAC
TCCTCCCAAGGAAACACGCCCTAAACTGCCACCGAATGAAGCCTGCTCTCTTTAGCGT
CCTGTGTGAAATCAAGGAGAAA
>2194
AAGCACCAGGAGGACTGAGCACTGGGTATTGGGGTCTCTGGGTCCCTAAGTCCACTCG
CCTGCATGCTAGGCCTCAGCATTCGGAGCTCCCAGGAGGAGGAGCCGGTGGACCCAC
AGCTGATGCGCTTGGACAACATGCT
>2195
GAAGGGCTCTTGCAGGGGAATCCCATGGTCAAAGGGCTCCTCCTCACCAGCCCACTGG
CCCCCACTACAGGCATGTAATGAGTTCACGACCCATGTCATGAACCTGCTGAGGGAGC
AGAGCCGCACCAGGCCCGTGGCCC
>2196
GGGTCATGCCATGTGACAGCCCTGCCAGGACATCAACATCCTCCTCACCGCTCTTCTCC
CTCCTCTGTAGACGAAAGCGCCGTAACTTCAGCAAACAGGCCACTGAGGTCCTAAATG
AGTATTTCTACTCCCACCTGAGT
>2197
ACTCACATCTTATTCAGTGAAGGACTTCAGAGGCAAATGTTTCTACCTGACCCTCCTTT
CTGCCCCACAGGTCTCCAACTGGTTTGGCAACAAGAGGATTCGCTATAAGAAAAACAT
CGGAAAGTTCCAAGAGGAGGCAA
>2198
AGTTGAGAGTTGTGGTTCATCCTCTACCATCACGGGCTCTATTACACTCTTCCCTCTCTG
CCCCCACAAGGCTCTGGCGGCTCTTTCAATCTCTCAGGATCTGGAGACATGTTTCTGGG

GATGCCTGGGCTCAACGGAGA
>2199
ACTTTAGGATACAAGACCTCTTTCCTCTGAGGCTTCTCTTCACTGTCATACCTTCCTCTG
CTGCCTGCAGGTGGAATCACTCCGACACTCGATGGGGCCAGGGGGCTATGGGGATAAC
CTCGGGGGAGGCCAGATGTACA
>2200
CTAGTAATGTTGTGCCCACACAGGGTTCCCAGGGCTTTCTCTGTTTTCTGTACTCTGTCT
CTCCTTTCAGGCAAATGGCAGCTGGCAAGAGGCTGTGACCCCCTCTTCAGTGACATCC
CCAACGGAGGGACCAGGGAGTG
>2201
CACCCACCCTGTCCAAATTTTGTTAGCCCTCATTCCCTTCCTACCCCTCTACCATGGTGC
TATCTCCCAGGGGCAGTAGTAGGTGCTCAAAACATCACAGCCCGGATTGGCGAGCCAC
TGGTGCTGAAGTGTAAGGGGGC
>2202
CCCGTCACTCTGCCTCACAGTCCTTTCCCAAAGGCCTTGCACTGTTTAGGCCCTGCTTC
TCAGCTTCTAGAACACAGGCCGGACAGAAGCTTGGAAGGTCCTGTCTCCCCAGGGAG
GAGGCCCCTGGGACAGTGTGGCTC
>2203
GTGTGTGTGGGTGTGGGTGTGTGGCATCTCTCATTTTCAAAGGATTCTGAGGTCACCAC
TCTTTCCCCAGGTGGGGACATGTGTGTCAGAGGGAAGCTACCCTGCAGGGACTCTTAG
CTGGCACTTGGATGGGAAGCCCC
>2204
CAAGCTGCCTTCTCCCTGATCTCACTCCCACACCCACCCTGGGATAATTTGTCTTATCCT
CCCATCATAGGAGTATCTGTGAAGGAACAGACCAGGAGACACCCTGAGACAGGGCTC
TTCACACTGCAGTCGGAGCTAAT
>2205
AGTCAAGCCCATGGCCACTGGGATCACTCACAAGTGTAACTCTCCACCTCAAAACCCT
TCCAACTCCCAGAGCCTGTGCCTCTGGAGGAGGTCCAATTGGTGGTGGAGCCAGAAG
GTGGAGCAGTAGCTCCTGGTGGAAC
>2206
GGGTATCTGAAGATATGGAGACAAAAAGACAAGGGTTTTGAAATAGTCTCCTCTCCCC
TTCCCCCACCAGGGTGTGCCCTTGCCCCTTCCCCCCAGCCCTGTGCTGATCCTCCCTGA
GATAGGGCCTCAGGACCAGGGAA
>2207
GGGATGGTCAACAAGAAAGGAATGGTGAGTGGTGGTGGCTGTGCTCTCAATTTTCCCT
GTCTCCGTACAGGCTCTGTGGGAGGATCAGGGCTGGGAACTCTAGCCCTGGCCCTGGG
GATCCTGGGAGGCCTGGGGACAGC
>2208
GGCAGCAAGCGAAGAGGGGTCGGGGGGTGGAACGACAACGTGCCGCATTCCCCCCAA
TCTTTCTCCTCAGGAAGGCCCCAGAAAACCAGGAGGAAGAGGAGGAGCGTGCAGAA
CTGAATCAGTCGGAGGAACCTGAGGCA
>2209
GTGTGGATGCATGCATGTGCTGTGAAGCAGGGAGACTAGCTTTCCACTCCTCCTTGTCT
TCTCTCTGCAGTGCCCTGGGAGCTGTCATTGCCCTCCTGCTCTGGGGACAGCTTTTTGC
AGTGGACTCAGGCAATGATGTC
>2210

ATCTGACTTTTCACGGTTCACTGGGAACAATTTCCAAATAGCAAACTCTCTGGCTTCTC
TCTCTTTGCAGATGACGGCTGCCCGAAGCCCCCCGAGATTGCACATGGCTATGTGGAG
CACTCGGTTCGCTACCAGTGTAA
>2211
ATGCCTTCTCACTCTGCTCTGGGTGCAGACTTGACTTTTCCTTTGGCTCATTTCTTGCCT
TTTGTTTCAGGAGTATACACCTTAAATGATAAGAAGCAGTGGATAAATAAGGCTGTTGG
AGATAAACTTCCTGAATGTGA
>2212
ATCTGACTTTTCACGGTTCACTGGGAACAATTTCCAAATAGCAAACTCTCTGGCTTCTC
TCTCTTTGCAGATGACGGCTGCCCGAAGCCCCCCGAGATTGCACATGGCTATGTGGAG
CACTCGGTTCGCTACCAGTGTAA
>2213
ATGCCTTCTCACTCTGCACGGGGTCTAGACTTGACTTCTCCTTTGGCTCACTTCTTGCC
TTTTGTTTCAGGAGTGTACACCTTAAACAATGAGAAGCAGTGGATAAATAAGGCTGTT
GGAGATAAACTTCCTGAATGTGA
>2214
TTTCACCCCTTTCTCAGATGGAAAGGCTCTTGCACATTTCCACTCACGAGTGTCTTGCT
CTCCTTGACAGTATGTGGGAAGCCCAAGAATCCGGCAAACCCAGTGCAGCGGATCCTG
GGTGGACACCTGGATGCCAAAGG
>2215
AACATCTTAATCCAATCAAATGTTTGTATCCTGTAATGCTCTCATTGAAACAGCTATATTT
CTTTTTCAGATTAGTGATGATGAACCAGGTTATGACCTTGATTTATTTGCATACCTAATC
ATTATGCTGAGGATTTGG
>2216
ACTAAGAGGTGTTTGTTATAAAGTTTAATGTATGAAACTTTCTATTAAATTCCTGATTTTA
TTTCTGTAGGACTGAACGTCTTGCTCGAGATGTGATGAAGGAGATGGGAGGCCATCAC
ATTGTAGCCCTCTGTGTGCTC
>2217
ACATGGGGGTTTTGGTACTTTATATTGTGACTCTGAATTTAAAGCTATGCAATGTCTTCT
TTTTTGAAAGGATATAATTGACACTGGCAAAACAATGCAGACTTTGCTTTCCTTGGTCA
GGCAGTATAATCCAAAGATGG
>2218
CACATTTGCCAGTATTAGATTTAAAAGTGATGTTTTCTTTATCTAAATGATGAATTATGAT
TCTTTTTAGTTGTTGGATTTGAAATTCCAGACAAGTTTGTTGTAGGATATGCCCTTGACT
ATAATGAATACTTCAGGGA
>2219
TTGTAAAAAATGGGGTGGAGGAAAATAAGGCATCTGCTGAGTGTATAACCATTTTACCT
CTTGTTTTTAGCCCTCTTCTGGGTCACGCTAGAATCAGATCTGCTCTCCAGCATCTTCTG
TTTCCTGGCAAGTGTTTCCTG
>2220
TCGCCTTTATCAGAAAACTTCCCAGCCAGATCCAGAAATCTTTCCAATGACCTGACCTG
TGTTCACACAGAGCTCCAGAACAGGACCAAGCTGACTGTACTTGAAGGAGACATTCT
GGATGAGCCATTCCTGAAAAGAGC
>2221
GGATCTGTGCATGTGGTTGCAGCTCCTTTGGGATATTTCCTGACACTGTCATCATGCTCT
TCGTGGGCAGGTACCCAGCTACTGTTGGAGGCCTGTGTCCAAGCCAGTGTGCCAGTCT

TCATCTACACCAGTAGCATAGA
>2222
TGCACTGGTTGGCTCTGCGGACTCCGCGTCCGTCTGTGACACCCCCTGCACACTTACC
CAATCCTTTTAGATGCCAAGATTCGCATTTTTGACCTGGGGCGGAAAAAGGCAAAAGT
GGATGAGTTTCCGCTTTGTGGCCA
>2223
TGGAGATAGTCGTGGTGAATGTTTCTCTATTATCTTCTCTCACTTTGCTGCTTTTCTTCTC
CCTACCTAGCCCTGGAGGCTGCCCGAATTTGTGCCAATAAGTACATGGTAAAAAGTTGT
GGCAAAGATGGCTTCCATAT
>2224
CTAAGGGACCTTGGAGACAGGCCTTTCAGGTGGATGTTCATGTTTCTGACCTTGCACTA
CCCCAATGTAGGCTCCAAACAGGCATGCGAGGTGCCTTTGGAAAGCCCCAGGGCACT
GTGGCCAGGGTTCACATTGGCCAA
>2225
GTAGTGCTGCAGCCCCCTTCTCCCACCTTTGCCCCAGCCTCCTGACTCAGTTCTTTCCA
TTGCTCCTTAGATCCACATCTCAAAGAAGTGGGGCTTCACCAAGTTCAATGCTGATGAA
TTTGAAGACATGGTGGCTGAAA
>2226
ACGCGATGGAATCTCGTGTTGCCTCTGTAGACGTCCTGCAAGGTTTTAAACCGGCGCG
GTGTCGTTCCAGATGCCTGAGGAAACCCAGACCCAAGACCAACCGATGGAGGAGGAG
GAGGTTGAGACGTTCGCCTTTCAGG
>2227
ATCGGATTTTGGTTTCAGTGTGAACTTCTTGGGGGTGCTGTATGCTTAAATTAATATTTT
TTGTTAACAGGCATTGGACAAAATCCGGTATGAAAGCTTGACAGATCCCAGTAAATTAG
ACTCTGGGAAAGAGCTGCATA
>2228
GGTGTTAGGCATGGCGTTGAGCACTAAATTGAATGTATTATTAAGTCAATCTTTCTTTCT
TCTCTTGCAGGTGAACCTATGGGTCGTGGAACAAAAGTTATCCTACACCTGAAAGAAG
ACCAAACTGAGTACTTGGAGGA
>2229
AATAGTGGTGCAGCGCTGTCGCTTAGTTCTGGAGTGCAGCAGTGATGTGATTTCGTGTT
TTCTTTTGAAGGTGGAGAAGGAACGTGATAAAGAAGTAAGCGATGATGAGGCTGAAG
AAAAGGAAGACAAAGAAGAAGAAA
>2230
TTCTCCACCACCCCAAATATCTTCTATAATGCATTGTTCATTGGTACTTAGTGTATCTGTT
TTATTACAGCATTTTTCAGTTGAAGGACAGTTGGAATTCAGAGCCCTTCTATTTGTCCCA
CGACGTGCTCCTTTTGATC
>2231
TTTGGGAGTTTACATAGTACCAGTTTTGTCCTTGGAATGACTCAGTGCATTTGGTTTATA
TTTTTTTCAGACTTCATTAGAGGGGTGGTAGACTCGGAGGATCTCCCTCTAAACATATC
CCGTGAGATGTTGCAACAAAG
>2232
ATTGGCCTCTTTAGTTTTTTTTTTTTTTTTTTTAATTCAGAAAGTCTTTTAAAGAACATACT
TTGTTTCAGCTTGGAATACACGAAGACTCTCAAAATCGGAAGAAGCTTTCAGAGCTGT
TAAGGTACTACACATCTGCCT
>2233

GAAAAGTCAGAGCTAGATGAAACCATTCTTAACTGTTAAAAGGTCTAAAAGTAACTTT
GTAATACCTCAGGTGAGACCAAGGACCAGGTAGCTAACTCAGCCTTTGTGGAACGTCT
TCGGAAACATGGCTTAGAAGTGAT
>2234
GATTTCATTTCACAATGAAAGTTTAGGATTAATCTTTCTAGGTTCCTAGTCATCACTTTTT
GGATTACAGGTGGTTGTGTCAAACCGATTGGTGACATCTCCATGCTGTATTGTCACAAG
CACATATGGCTGGACAGCAA
>2235
ACCAAGAGTATTACCCTAATAGCTGGCTTTAAGAAATCTTTGTAATATGAGGATTTTATT
TTGGAAACAGGTATTGATGAAGATGACCCTACTGCTGATGATACCAGTGCTGCTGTAAC
TGAAGAAATGCCACCCCTTGA
>2236
TGTAGCTTGGGCAACAGAGCAAGAGTCTGTCTTGGAAAGAGCAAAAGTAAGTTGCTG
TTTGTATTTCCAGGCCTTGGACAAGATTCGCTATGAGAGCCTGACAGACCCTTCGAAGT
TGGACAGTGGTAAAGAGCTGAAAA
>2237
TCATACTTATCTGTGTTCTTTGGTTTTTTGCTTCTTTAAAACTTGTGATTGACTTTAAACT
TGTTGGCAGGCTGGTGCAGACATCTCCATGATTGGGCAGTTTGGTGTTGGCTTTTATTC
TGCCTACTTGGTGGCAGAGA
>2238
ACTTACTAATTGCTGGTCTCAACTGCATATACTTTTTACCCTGTTACACGCTTGTAATTG
ACTCTTCTAGGTGAGCCCATTGGCAGGGGTACCAAAGTGATCCTCCATCTTAAAGAAG
ATCAGACAGAGTACCTAGAAGA
>2239
TTGTCCCTGGCTTTTGCTTTCCCTGGTAGTGTTTTGTACTCCAAGGCTAACTTCTGTTTT
TGTTACTTAGTTGGAGAAGGAACGAGAGAAGGAAATTAGTGATGATGAGGCAGAGGA
AGAGAAAGGTGAGAAAGAAGAGG
>2240
TTAGATAATTTGGTGTTGGGGCTAAAAGGTCCTCTTTTGAAATGTACCACTTATTTTTGG
TTTCTTTCAGCACTTTTCTGTAGAAGGTCAGTTGGAATTCAGGGCATTGCTATTTATTCC
TCGTCGGGCTCCCTTTGACC
>2241
CTGTTTGAATCTGGGGACCAGGTCTGGTCTAGCTGTTTTTACTGAGCTTTCTCACCCTG
GTTGATGGCAGATTTTATCCGTGGTGTGGTTGACTCTGAGGATCTGCCCCTGAACATCT
CCCGAGAAATGCTCCAGCAGAG
>2242
ATTCCATTCACATTGAAAGTGAAGTTATTGTAGTTAAGCTGGATTGTTTTTCCTCTTCCC
ACCCTTCAAGCTTGGAATCCACGAAGACTCCACTAACCGCCGCCGCCTGTCTGAGCTG
CTGCGCTATCATACCTCCCAGT
>2243
CAATTAGGGCTTCCTGGGAACTGGCAGTATGAGGCATTTTAGTCACTGAGTTCATTTAA
TTACCCTACAGGTGAGAGCAAAGAGCAGGTGGCCAACTCAGCTTTTGTGGAGCGAGT
GCGGAAACGGGGCTTCGAGGTGGT
>2244
AGGCTTGACAATGCCTGTTTTCTCTTTCAAAGTGGTAATGTCAATCTAAGGCTTTTGTG
ATCGTCCACAGGTGACAATCTCCAATAGACTTGTGTCTTCACCTTGCTGCATTGTGACC

AGCACCTACGGCTGGACAGCCA
>2245
AACTCGTCTCCTCTATGGATTTGACTTAATGCTATTTGGTCAAGTCTCACATGGCTTAAT
TTTACTTCAGGTATTGATGAAGATGAAGTGGCAGCAGAGGAACCCAATGCTGCAGTTC
CTGATGAGATCCCCCCTCTCGA
>2246
GGCGAACAAGCCGAGGGGCGGAGTGGGCCAGGGATCACCTGCCCCGCAATGACCTGC
GCCCCGCCCCCAGGCCTGCTGGAGCTCTCGCCCGTGGAGCGGGGCGTGGTGAGCATC
TTCGGCGTGGCCAGCCGGTTCTTCGT
>2247
CCCCTGGCTTCAGGCTGGGTACCTTGCCCAAGAGGTGCCCCAGCCCTGACACTGCCCT
GCTTTGCTGCAGCCCTTCTTCACCGATGAGTGCACGTTCAAGGAGATTCTCCTTCCCAA
CAACTACAACGCCTACGAGTCCT
>2248
TTTTAAAAATTGTGCTACAGGTGAGAGGTTGAGAAATGGATGCAAACCATCGTCTGTG
TTCCTCTTCTAGTGCAGGTACCCTTCTCCAGATGTTGCTTCTCATTTGCGGAGCAAGAG
ATTCCCCTGAGGGCAATCCTGTG
>2249
GGGGCTTGTGCGTTGCCCACCCAGTGGTGGCTTGGGTGGCTCAGCCTTCTCTCTTATTC
TCTGTTCACAGATTCAAGCTGAAGAGAGGCAAAGAGGCCTGCGCCTTGGACACAGTT
GGATGGGTTCAGAGGCACAGAAAA
>2250
TTAAGATTTTTACAAGACTGTGATAGATATTGATGACTCTCATGTGTTTGTCTCTCTTGG
GCGTTTTAAGGAAATGCTAGTGAGTCGGAGGAAGACCGCAGCGCCGGCAGTGTGGAG
AGCCCGTCCGTCTCCAGCACGCA
>2251
AAGATTTTTAAAGCACACATTCTAGAGTAAAAACTGTGACTCTGTTGCTCTGGTCCTTC
CTGATCCCCAGGGTCCCTGCCGTAGAGAAATGGAAGACACACTGAATCACCTGAAGTT
CCTCAATGTGCTGAGTCCCAGGG
>2252
CGCCCTGGCCACCGACCTGACACCAGACCCAGGAGCATTCACTTCTCTGTCTTCTGTT
TCTCTCCCACAGTGTCGCCCTTCCAAAGGCAGGAAGCGGGGCTTCTGCTGGTGTGTGG
ATAAGTATGGGCAGCCTCTCCCAG
>2253
CTCAGACTCAGCCCCAAAGTCTGTGGTCTCTGACCTGACACAGCCTTATGTGTATGTGT
GTATTGTTCAGGAGGAGATGATCTTCCAGATCCCATGGAAGCATGCTGCCAAGCATGGC
TGGGACATCAACAAGGATGCCT
>2254
CTTCAGCCAGGGGCAGCTGAGGATCCCTACCCATGGCCCTGACGATCCTCTTTTTTCTC
CTGCCCTCTAGGCCGATACAAAGCAGGGGAAAAGGAGCCAGATCCCAAGACGTGGAA
GGCCAACTTTCGCTGTGCCATGAA
>2255
TGTGGGCCAGGGTGAGCAGTGGAAGAAATGCTAAGGTGGCCTGGGCCTAAGCTGCTT
TCTCCCTCGACAGTCATGTGGGGATTCCAGCCCTGATACCTTCTCTGATGGACTCAGCA
GCTCCACTCTGCCTGATGACCACA
>2256

GGAGGGCGCTCGATGTCTTGCAAACTAAGAAAGCACACAACCTTGACCTGTGGCTTCT
GCTGTTCCCCAGCACTGTCGCCATGTGCTGTCAGCAGCACTCTCCCCGACTGGCACAT
CCCAGTGGAAGTTGTGCCGGACAG
>2257
ATTGAGGCAGGGCCACAGGGTGAGTCTGCACTGGAAGGGAGTTGATAGCCTCTTGCTC
TTCTGTCCCCAGCTCTTGGAGCAGTCGGAGTGGCAGCCAACAAACGTGGATGGGAAG
GGGTACCTACTCAATGAACCTGGAG
>2258
CAGGGGTAGGGGGAAGCACACCTGATGCCACCGTCCCCTACCCTCATACAACCTTCTT
CACATCTTCTAGGGGATATTGGGCTGAGTCTACAGCGTGTCTTCACAGATCTGAAGAAC
ATGGATGCCACCTGGCTGGACAG
>2259
TTAAGCCTAGACAGTTTTCAATGACTTTTTTTCTCTCTACATGTCTTTTCATATTTTTATC
TTCTTGAAGTCCCTCAGAAACCTAAGGTCTCCTTGAACCCTCCATGGAATAGAATATTT
AAAGGAGAGAATGTGACTCT
>2260
TCTCTTTTCTCTATTCATTCTCTCTCTCTTCATTTATTGTTAAATAAATAATGTAATGAATG
TTCTTCAGACTGGCTGCTCCTTCAGGCCTCTGCTGAGGTGGTGATGGAGGGCCAGCCC
CTCTTCCTCAGGTGCCATGG
>2261
AAACTCTGAACCTCATTTTTCAGTTCCCAAGATGAATTATGTTTCTCATTGCATCTGTGT
TCCACTACAGCTCCGCGTGAGAAGTACTGGCTACAATTTTTTATCCCATTGTTGGTGGT
GATTCTGTTTGCTGTGGACAC
>2262
GATCTCCTGGGGGCCGAAAGGGGCAGATGCTTTGGGAGGGCTCATGGGGTCTGCAATG
TTTCCTTTCCAGAGGCAGGGAGCCCTGAAAGCCCAGAGAGCACGGAGATAACTGAGG
AGGAGCTCCTGGATAATTTCCATCT
>2263
ATCTCCTGGGGGCCCGAAAGGGGCAGATGCTTTGGGAGGGCTCATGGGGTCTGCAATG
TTTCCTTTCCAGAGGCAGGGAGCCCTGAAAGCCCAGAGAGCACGGAGATAACTGAGG
AGGAGCTCCTGGATAATTTCCATCT
>2264
ATCAGTCATTGTCTAGGCTGATAATTCACTGTGTCTTGTTAGATATGCTAATGTCAAGTTA
TTCTCTTAGGAGCCCTGCCGAATAGAACTCTACAGAGTCGTAGAGAGTTTAGCCAAGG
CACAGGAGACATCAGGAGAAG
>2265
GCCAGCTATGGCTCTACTTTCCCTGTCAGCTTGTCATCTGCTGCTCTTGACCTTGGTCTT
GTCTTTGCAGTGTGAGACATCCATGGATGGAGAGGCGGGACTCTGCTGGTGCGTCTAC
CCTTGGAATGGGAAGAGGATCC
>2266
CTCTCTGATGTCAAAGCATGGTTCCTGGCAGGACCACCTCACCAGGCTCCCTCCCTCG
CTCTCTCCGCAGTGCTCCTTCCAGGACCTGGACCTCTGCCCTCTGGATGGCGGCATCCA
GCTACGAATCTCCGACCACCACT
>2267
TTTGCCGCCTCGCCTCACGAGGCCTGCCCTTCTGATTTTATACCTAAACAACATGTGCT
CCACATTTCAGAACCTATCTTCTTCGACACATGGGATAACGAGGCTTATGTGCACGATG

CACCTGTACGATCACTGAACTG
>2268
ATGGGGCCTAGGGGTTGGGGTGACCCTGCACTGCTGTGTCCCTAACCACAAGACCCCC
TTCTTTCTTCAGTGGTGTTCTCCATGTCCTTTGTACAAGGAGAAGAAAGTAATGACAAA
ATACCTGTGGCCTTGGGCCTCAA
>2269
CCAAAGAACATCTTTCCCATAGCATCTTTGTCCCTTGCCCCACAAAAATTCTTCTTTCTC
TTTCGTGCAGAGTGTAGATCCCAAAAATTACCCAAAGAAGAAGATGGAAAAGCGATTT
GTCTTCAACAAGATAGAAATCA
>2270
AAATAAATTGGAAATTAATTTCTGATTCTGACCTCTATGTAAACTGAGCTGATGATAATT
ATTATTCTAGGCCACAGAACTGAAACATCTTCAGTGTCTAGAAGAAGAACTCAAACCT
CTGGAGGAAGTGCTAAATTTAG
>2271
TCAATTGGGAATAACTGTATATAAGGTAAACTACTCTGTACTTTAAAAAATTAACATTTT
TCTTTTATAGGGATCTGAAACAACATTCATGTGTGAATATGCTGATGAGACAGCAACCA
TTGTAGAATTTCTGAACAGAT
>2272
CGTGCTTGGCCTCCTCCTTCTGACCATCATTTCTCTTTCCCTCCCTGCCTTCATTTTCTCC
CCAATCTAGATTTCTTCCTGACCACTATGCCCACTGACTCCCTCAGTGTTTCCACTCTGC
CCCTCCCAGAGGTTCAGTG
>2273
TAGGCTCTGGATATCTGCAGTACCCAGATTGGCCCCACTGTTCCTCTTCCTTCCAACCTT
TCTCCTCTAGGTACAAGAACTCGGATAATGATAAAGTCCAGAAGTGCAGCCACTATCTA
TTCTCTGAAGAAATCACTTCT
>2274
GGGGCACTACCTTCAGGATCCTGACTTGTCTAGGCCAGGGGAATGACCACATATGCAC
ACATATCTCCAGTGATCCCCTGGGCTCCAGAGAACCTAACACTTCACAAACTGAGTGA
ATCCCAGCTAGAACTGAACTGGAA
>2275
TTCAGGAGTATGTTTTAATTCTCCCTTCTCTCATAGACACCCACTTTCCCTCATCCTCTTT
CTCCTCAAGGAACAATCAGTGGATTATAGACATAAGTTCTCCTTGCCTAGTGTGGATGG
GCAGAAACGCTACACGTTTC
>2276
GCAACAGTGCCTGGCATGTAGTAGGGGCACAACAAATATAAGGTCCACTTTGCTTTTCT
TTTTTCTATAGAGAATCCTTTCCTGTTTGCATTGGAAGCCGTGGTTATCTCTGTTGGCTC
CATGGGATTGATTATCAGCCT
>2277
GACAACTGGGAGAAGGGTGATAAAAAAGCTTTAACCTATGTGCTCCTGCTCCCTCTTT
CTCCCCTGTCAGGACGATGCCCCGAATTCCCACCCTGAAGAACCTAGAGGATCTTGTT
ACTGAATACCACGGGAACTTTTCG
>2278
TTTGGCACAGAGCTCGGTCTTTTACTTCCTGCCCCTAATTGACCCCTGACCTGGACATA
TCTGTCTTTAGGCCTGGAGTGGTGTGTCTAAGGGACTGGCTGAGAGTCTGCAGCCAGA
CTACAGTGAACGACTCTGCCTCG
>2279

GAGGAAAAATCTCTCTCCCATCCTCCAAATGGAGCTGGCACATTGCTATCTGTGGCATT
TGTCTTTCCAGAACACAACTGAGAAGGAAACCTTCTGCAGGGCTGCGACTGTGCTCC
GGCAGTTCTACAGCCACCATGAGA
>2280
CTTGATCAAGTAGACAGGCAGGCAAGACAAATGCTTACCAATGCAAGCTAATGAAATG
TTTCTTTTGCAGAATTCCTGTCCTGTGAAGGAAGCCAACCAGAGTACGTTGGAAAACT
TCTTGGAAAGGCTAAAGACGATCA
>2281
GAATGGACAATGGACCAAATTTTTATACCTTGTCTTGATTATTTGCATTTTAAAAATTTTC
CTCATTTAGCACCAACTGTGCACTGAAGAAATCTTTCAGGGAATAGGCACACTGGAGA
GTCAAACTGTGCAAGGGGGTA
>2282
CTGGCTGTGCCTATTTCTATGGAATTGACAGTTTCCTGTAATACCTATTGTCATTTTTCTT
TTTTCACAGAAAAAGTGTGGAGAAGAAAGACGGAGAGTAAACCAATTCCTAGACTAC
CTGCAAGAGTTTCTTGGTGTAA
>2283
TAGAAGGAAGTAGCTGGCAGAGCTGTGCCTGTTGATAAAATCAATCCTTAATCACTTTT
TCCCCCAACAGGTGCAGTTTTGCCAAGGAGTGCTAAAGAACTTAGATGTCAGTGCATA
AAGACATACTCCAAACCTTTCCA
>2284
GACTTTTCAAGAACCCTACTTTCCTTCTTATTAAACATAGCTCATCTTTATATTTTTAATT
TTATTTTAGGGCTGAGAATTCATAAAAAAATTCATTCTCTGTGGTATCCAAGAATCAGTG
AAGATGCCAGTGAAACTTC
>2285
TGCTCTTTGTGATATATTTCCAAAGCTGTCCTCAGAATATTTCTTTGAATAAATCCTTACT
ATTTACCAGGACAACTGCACCAGACCATGCTTCAGTGAGAGACTGTCTCAGATGACCA
ATACCACCATGCAAACAAGAT
>2286
TAGGCTTCTGGGGCTGCTTGGCTCTACATCATTTGAGTCACTCTGTCCTTATCAACTTTC
ATCCCCACAGTATTTTTCCTGTGAACAGCCATGCAACCAAACCACGGCAGGCAACGCG
CTGACATTTCTGAAGAGTCTTC
>2287
CAGAGCTCTCACGCTACAGCTGACAGGCAGGGCAGGCGTTTTAATTATTATTAATTTTT
TTTTGAGACAGAGTTTGAGACAGTATCAAGAGTGGGTATCCTCTCAGCGTGTCTTCAA
GTAGCATCCCAGAGCCCCGCTCC
>2288
GGGCAGCAGTTGTCCATTCTGGGGCCTGGGTCAGCTCTCAGCTGTGGCCGTTGTGCCT
GTGCTTCCCCAGGTCCTGGTGGTGACTCCAACCCTGCCCTCACATATCCCAAGAGCAG
GCTGACTGCCTTCCCCATTCCCAC
>2289
AGCAATTCATGGGGAGCACCCCTCCAGAGAGGGATGATTTGCACAGGGCCCTCAGCCC
AGTCCCTTGCAGGCTGGACCTTGGAGAGTGAGGCCCTGAGGCGAGACATGGGCACCT
GGCTCCTGGCCTGCATCTGCATCTG
>2290
CTCCCCAACCCCCGAGCTCAGCTCTGGCCTGAAGTACTTACCGTGGGCTCCTGATGGT
CACTGTCTCCAGGGCCAAGGTCTAGAACCTTCACCTGCCTCACCAACAACATTCTCAG

GATCGATTGCCACTGGTCTGCCCC
>2291
TCCCCAGGATTGAAGCAGCTATGCCAGGACAGTGTAGCAGCCCCGTGGTGCTGACAAA
TGCCCTTTCCAGCAACCAGGCTCCTGGCGGCACACATAAGTGCATCTTGCGGGGCAGT
GAGTGCACCGTCGTGCTGCCACCT
>2292
TCTTGTGTGTTCTGACTGACACACCCAGACCCATGGGGCTTCAGCCTCACATGGATTCA
CTCTGTTCCAGTTAAGCTGGACCCGCCCTCTGACTTGCAGAGCAACATCAGTTCTGGC
CACTGCATCCTGACCTGGAGCAT
>2293
GTGTGTATTCTCGAGGGCTGAGGGACCCAGCCCCACCTTCACCACCTGCTAACTGTCC
CCACCCCCACAGCAGGCCCAGCACAGGGATCACATTGTCGGGGTGACCTGGCTTATAC
TTGAAGCCTTTGAGCTGGACCCTG
>2294
GCCAGGTGGGTTTGGGGGGAGCCTCCTGGCACTGAGGCTGCTCACAGCCCTGGGCCC
TTCCTGTCCACAGGCCCTCTGATCCCACCCTGGGGGTGGCCAGGCAACACCCTTGTTG
CTGTGTCCATCTTTCTCCTGCTGAC
>2295
CTCAGCCTCTGCAGTGACCTCAGGCCACCTGGGTCGCCATAGGCCTCTGACTGGCCTC
TCTTGGCCTCAGGGTGAAGAGAATCTTCTACCAGAACGTGCCCTCTCCAGCGATGTTC
TTCCAGCCCCTCTACAGTGTACAC
>2296
GGCAGCTGCTCCCAGGATGAGTTCTGAACATGCTACCTGAGCCCTTCCCTCCTCCCGTG
CTCTGTTCCAGACTTGGATGGGGGCCCACGGGGCCGGTGTGCTGTTGAGCCAGGACTG
TGCTGGCACCCCACAGGGAGCCT
>2297
GTGGGGGACTCAGTAACACCTTGAGGTGTAAGGGATGCTTTCCCACACTGGTTGACGT
TTGTCTCCTCAGGACCTGACTTCTGCTCTGACCAAGAAAATCACTCTTAAGACCCCACT
GGTTTCCTCTCCCATGGACACAG
>2298
CATCGCCTTTCCCCAAAGGCATTCAGTCCTGCTTCTTGTCACTTAGTAGTAGTCTCTTTT
TATCCTGTAGCTTACAGGCGGTATTGGCTTCATCCACCACAACTGTACACCTGAATTCC
AGGCCAATGAAGTTCGGAAAG
>2299
ATGCTTCAGATCAAGAGCCTGATGAAAGTAGTTCAAAGATGCGATGCCCTTTCTCACCA
TCCCTTTCCAGAAATATGAACAGGGATTCATCACAGACCCTGTGGTCCTCAGCCCCAA
GGATCGCGTGCGGGATGTTTTTG
>2300
GAAGGAAAGAAAAGAGTTAACTGGTCATAGTCGATGACTGGCCCTTCTTCACATCTCA
CCTCCCACGTAGATAATGACAAAGAGGGAAGACTTGGTGGTAGCCCCTGCAGGCATCA
CACTGAAGGAGGCAAATGAAATTC
>2301
AGTCCTAGAGCTGGGAAAGGGCCTGGAACTAATGCCTAGGGTCCTGATTCATGTCCTG
CCCTGACCACAGGAAAGTTGCCCATTGTAAATGAAGATGATGAGCTTGTGGCCATCATT
GCCCGGACAGACCTGAAGAAGAA
>2302

GCTACACAGGTGGGTTGGGGCAATAGGGGCAGCTGCCACATGACAGTCTGAGACTTAC
TTCTTGTCCTAGACTCTTCCCAGGGAAATTCCATCTTCCAGATCAATATGATCAAGTACA
TCAAAGACAAATACCCTAATCT
>2303
GTGGTGGGAGTGGGTAAGCTGGGCTCCCACCTTAACCTTCACATGAGCATTTTCCTTTC
CTTCACCATAGTGGTCACTGCTGCCCAGGCCAAGAACCTCATTGATGCAGGTGTGGAT
GCCCTGCGGGTGGGCATGGGAAG
>2304
AATATGGCCACAGGGTCCACTGCCCGTCCCCATTAACCCTATCCACCCATGTGTTCCTC
CATCTCAACAGTGCTGGCCTGTGGGCGGCCCCAAGCAACAGCAGTGTACAAGGTGTC
AGAGTATGCACGGCGCTTTGGTGT
>2305
CCACCCTAATCCTGCACTGACAGTCTCATCTCTGCTGTGACCTTGCCCGTGTCTCTGCC
TCTCCCTGCAGTGAAGCTGACAAAATCAAAGTGGCCCAGGGAGTGTCTGGTGCTGTG
CAGGACAAAGGGTCAATCCACAAA
>2306
GGAGCTGGGACATGGGTAGAGGGGCTGGTCCAGGGCCAGCTACCCACATTTGACCTCT
GCCCTTCTTCAGAGCCATGATGTACTCTGGGGAGCTTAAGTTTGAGAAGAGAACGTCC
TCAGCCCAGGTGGAAGGTGGCGTC
>2307
TCAGCCACAGTGAAGTGCCTGTGAGTGCATGAACTGATGGGGGCGCTGTCCTGTTTTC
TCCTGTGTGCAGACCGTTGGGGGGGTGAATTACTTCTTCGACGTAGAGGTGGGCCGAA
CCATATGTACCAAGTCCCAGCCCA
>2308
TCTCATGCCTGGGCATCTTCCCCTTTAACTGTAACCCACACTGATTGGCCCTCTCTCTTC
CCTTTCACAGAAACAGTTGTGCTCTTTCGAGATCTACGAAGTTCCCTGGGAGAACAGA
AGGTCCCTGGTGAAATCCAGGT
>2309
ACAGTAGCATCGCCTCCCTCTGAGTTCTTCTTCCCAGCCCAACTTGGACACCTGTGCCT
TCCATGAACAGCCAGAACTGCAGAAGAAACAGTTGTGCTCTTTCGAGATCTACGAAGT
TCCCTGGGAGGACAGAATGTCCC
>2310
ACTGTGTCCTCCTGTGTCCCTCTGCCTCGCCGCTGTTCCGGAACCTGCTCTGCGCGGCA
CGTCCTGGCAGTGGGGCAGGTGGAGCTGGGCGGGGGCCCTGGTGCAGGCAGCCTGCA
GCCCTTGGCCCTGGAGGGGTCCCT
>2311
GCGCCTGCTTTCCTGGGCTCTAAAACTGGCTGCTCCTCCTGACACTGAGTAGGACCTC
CAACTCTTACAGATCCCTTCCCCTGAAGTATTTGAAGAGCTGATCAAGTTTTCCTTCCA
CACTAACGTGCTTGAGGACAACA
>2312
CTCCCCGAGGGGCACACAGGGCCTCACTGTGAGCTCAGCCCCTGAACAGGCTCTGCT
TCCCATCCTTCAGGTTCAACATCGGTGGCCCCACATCCTCCATTCCCATCTTGTGCTCCT
ACTTCTTTGATGAAGGCCCTCCA
>2313
CAGGATAGAGAAGACAGGTGCTCCAGGGTCCTGACATGACCCCCATCCTGAAGGGCCT
TATGTCTTCCAGGTGAACGCTATGGCTCCAAGAAGAGCATGGTCATTCTGACCAGCAG

TGTGACGGCCGGCACCGCGGAGGA
>2314
CCCTCTCCTAGCCTTGTCCCTGGCCTCTCCTGCCAACCCTGCCCTTCCTGACCCAGCAC
CGCCTCTGCAGGTGCTGCGCCCCCGATTCAGTCCCGGATTGTGGGAGGCTGGGAGTGT
GAGCAGCATTCCCAGCCCTGGCA
>2315
CCATGCTCCCCTGCCCCATCCCCTTTTCCCCTTCCCGTCTTCTCATCCCTCCATTCCCATC
TTTCCCCAGCAATTACCAGCTCTGGCTGGGTCGCCACAACTTGTTTGACGACGAAAAC
ACAGCCCAGTTTGTTCATGTC
>2316
CGTCCAAGGGAGAAAGGGCCAGGGAAGCAGGTGAGGTCGGGCTGCAGCCCTTTTTCT
CCCGGGTTCGTAGTCTCATTTCCAGATGATCTCCAGTGTGTGGACCTCAAAATCCTGCC
TAATGATGAGTGCAAAAAAGCCCA
>2317
TGGGGCTCCCCAGGCAGAACTTGGGCCCCGTAGACCTTTCTCACTCTCCCCTCCCACC
TTGTGCTCCCAGGGTGATTCAGGGGGCCCGCTGATGTGTGATGGTGTGCTCCAAGGTG
TCACATCATGGGGCTACGTCCCTT
>2318
CACAGCTCAGCTCCCTCCCCTATCCAATTCTTTTGGGTCTGATCCCCCTGACCCAGCAC
CCCCTCCGCAGGTGCCGTGCCCCTCATCCAGTCTCGGATTGTGGGAGGCTGGGAGTGT
GAGAAGCATTCCCAACCCTGGCA
>2319
TATCCTCCCCTGCCCCATCTACCTTTCCCCATTTTCTCTCTCCTCATGCATCCACCCCCTT
CCTCCCCAGGAATAGCCAGGTCTGGCTGGGTCGGCACAACCTGTTTGAGCCTGAAGAC
ACAGGCCAGAGGGTCCCTGTC
>2320
GGTCTGAGGGAAGTGGGGCCAAAGAACCAGGTGGGGTCCGGCCACAGCCCAGTTTTT
CTCTGACCCATAGTCTTGCGCCCCAGGAGTCTTCAGTGTGTGAGCCTCCATCTCCTGTC
CAATGACATGTGTGCTAGAGCTTA
>2321
AGAGATGGAGTTGCCTAGGCAGTTATTGGGGCCAATCTTTCTCACTGTGTCTCTCCTCC
TTTACCCTTAGGGTGATTCTGGGGGTCCACTTGTCTGTAATGGGGTGCTTCAAGGTATC
ACATCATGGGGCCCTGAGCCAT
>2322
CATTCCCTGGCTGGAGGACTAGCTCTGTGGCCTGCAGATGTCCTGTACCTTCTTTTCAT
CTTCCCTTCAGCCCTGTGGGAGAAACCATTCATTTCCTCAAGGACCACTCCCAAAGAC
TTCTATGTTGATGAGAACACAAC
>2323
GGAGGGCTCTGCCCAGCAGGGATCCTGGCTTGTTCATTAATCTAATGTTCTAACTCAAT
GCCCCTTTCAGGAATTTTTACAAGAAGCTAGAGTTGCATCTTCCCAAGTTCTCCATTTC
TGGCTCCTATGTATTAGATCAG
>2324
GGCTCTCGCAGTCTTGTCCAGGCCCCCTCTCTTGCTGGCTTGGAGATAATGCTTGTGAT
TTTCCTCCCAGAGTTTCCACAAGGCCACCTTGGACGTGGATGAGGCTGGCACCGAGGC
TGCAGCAGCCACCACGTTCGCGA
>2325

TGTAGGCTACAGTGAAGTCCAGCTTGTGAAGTCCATTTGACAAATTACCTGTGCCTTTT
CCATCCTGCAGATTCTCACAGCCACAGTGGACAATGCCAATGTCCTTCTGCAGATTGAC
AATGCCCGTCTGGCCGCGGATG
>2326
GGAGAAGAGAACAAAGCCCTCGCTGTGATCAGGATGGGTGTCTCATACCTTTTCTCTG
GGGTCATTCCAGGTATGAGACAGAGTTGAACCTGCGCATGAGTGTGGAAGCCGACATC
AATGGCCTGCGCAGGGTGCTGGAC
>2327
TTCAGGCCTAAGGAACACCAATCCGCTGCCATGGTGGAACTCCTGACTGTGGACTGTC
CCTGGCTTGCAGGAGATGAATGCCCTGAGAGGCCAGGTGGGTGGAGATGTCAATGTG
GAGATGGACGCTGCACCTGGCGTGG
>2328
GAGGTGGAAGGAACCCAGACCACCTGCCTTCTGGGGCCTTCTGGTGTGAATGGCATTC
TCTTTTTTGCAGACAGAGGAGCTGAACCGCGAGGTGGCCACCAACAGCGAGCTGGTG
CAGAGCGGCAAGAGCGAGATCTCGG
>2329
GTGCACCCAGGACTGGCAGGGAGAGAACGGCCACACTCACTAATCGTTGATTCCCTTC
CCTCCCTCACAGAAAGCATCCCTGGAGAACAGCCTGGAGGAGACCAAAGGTCGCTAC
TGCATGCAGCTGGCCCAGATCCAGG
>2330
GCCTCCTTACTCCTGATTAGTCCATTGTCTGTCCACCTCTGGTAACGTCCTCTTCCCACC
TCTTCCCCAGTGACCTCCTCCAGCCGCCAAATCCGCACCAAGGTCATGGATGTGCACG
ATGGCAAGGTGGTGTCCACCCA
>2331
TGGGGCACGGTTCTCCATGGCTATGAGACTCCATTTATGCATCAGGAGTAAAGGGGTCT
TGTGTTTTTAGGTGAGGTTCCTGGAGCAGCAGAACCAGGTACTGCAAACAAAATGGG
AGCTGCTGCAGCAGGTAGATACCT
>2332
TCCCTCACAGGTAATGAGAGGGACCTGCCCTGGAGAGAACGTGCCTTCATGATGTCCC
TTGTTCCTCTAGGATGTGGATGGTGCTTATATGACCAAGGTGGACCTTCAGGCCAAACT
TGACAACTTGCAGCAGGAAATTG
>2333
GACTGCTTCTCTGTCTAAGTTGTTTTTGTTTTAGTGCTGTAAGAGTCACTAACCTATTAT
TGTCTTGCAGGAGTTGTCTCAGATGCAGACTCAAATCAGTGAAACTAATGTCATCCTCT
CTATGGACAACAACCGCAGTC
>2334
TCTCATGTATGTGTGCCTGTGCCATGCTGAGTTCTGCCTACATTTAACAAACGCTATCTA
CCATCTTTAGTATGAAGAGCTGCAGATCACTGCTGGCAGACATGGGGATAGTGTGAGA
AATTCAAAGATAGAAATTTCTG
>2335
GGGGAGACCAATTGGGATGGAGGCCTAGTGGGGAATGCATATTATGAGAGGGTGTCAT
ATTCTTTTTCAGATCTCCAACTTGCAGCAGTCCATCAGTGATGCAGAGCAGCGTGGCG
AGAATGCCCTCAAGGATGCCAAGA
>2336
GGGGCATGTGCAGGCTTTGTTGGGCTGGAAACGGAGTTGAGGTTGAAAATAACTGAG
CTTCCTCTTGCAGCTGTGAGCACAAGCCACACCACCATCAGTGGAGGTGGCAGCCGA

GGAGGTGGCGGCGGTGGCTACGGCTC
>2337
ATATTACATGAAGGTTTTATTTTTCTTTTTGTCTCGACATCATTAAAAATGTACTTTTATTT
TTATATAGATTTAAAAACCATTCATACTTCAACGGGAAAAAATCTTCTAGTTTCTGGATG
GTGGGGCTTTGTTCGCCA
>2338
GGAAAGCAGGAAAAAAATACATTGTAGTGATAATAAAAATATCTAATACTGTGTACTATTA
TTATCCACAGGTTTTAACCACATTCTGCCTTATTTCTACATAATTTATTTCACCATGTTGC
TTGTCCACCGAGAAGCTCG
>2339
CTCTCAAGCTCTATAGGTTTGAGCTGTGGAACAGTCCCAGTCCCCAAATGGGGATCTTG
TTTTGTTTCAGACTTTGGAGAAGATTGAAGACATGGCTGTGTCCCTTATTCGAGAGGAG
TGGCTTCTTGATCCATCACAGA
>2340
TCCTTTGTCCTAGCATAAAGAATAGGTTGTGACATTGGCTTGATGTTTGTTTATTACATTT
TTATTTCAGGTGGTGAGACCAGGAGTGAGAACAGGGAATTAGCTTCAAAACAGGTAAT
ATCTACTGGAATCCAGCCACA
>2341
GGGGATCAGAAGCTGCCTGGCCTTTTCTTCTGAGCTGTGACTCGGGCTCATTCTCTTCC
TTTCTCCACAGTTGCTGCTGACACGCCGACCGCCTGCTGCTTCAGCTACACCTCCCGG
CAGATTCCACAGAATTTCATAGC
>2342
GGGGCCATGGTGGGCCCAGGATTCCCCTGCTGGATTCCCCAGTGCTTAACTCTTCCTCC
CTTCTCCACAGCTTCCTAACCAAGCGAAGCCGGCAGGTCTGTGCTGACCCCAGTGAGG
AGTGGGTCCAGAAATATGTCAGC
>2343
AGAGGGATGGGCACAAATCGGTCAGGCCCAGTCAGGGGATTTCAGCCTGATTTCTCTA
TGTCACTTCTAGGGAGAGAGATGGTGGATTACATCTGCCAGTACCTGAGCACTGTGCG
GGAGAGACGTGTGACGCCAGACGT
>2344
TGGTCTCTGGAGAAAGAAAGCCTGCTGGAAATGCCTCCTGGTGCCAACTCTGTGCATG
CTCTGCCTCCAGGTGGTACATTGGCAGAGCCCCCATATGCACGCCTACTACCCAGCCCT
CACCTCTTGGCCCTCCCTGCTAG
>2345
TTTCTGGAGCTCGTGGGGGCACCTCAGTGCCCTGGGGCTGTGCAGTTTGAAGTTCCTT
TTCCTTTCTCAGGCATCCAGCCCTGCGTGTACAGAGCTGGAGATGAACGTCATGGACT
GGTTGGCAAAAATGCTGGGACTTC
>2346
ACTAGAAAGCCTGGGGAAATTTGGCAAGAGAATACAGAAAGGCTGCCATGATAATCCT
TTTCCCCTGCAGAGCACGGTCAGTGAATCCACTTTGATTGCCCTGCTGGCAGCAAGGA
AGAACAAAATCCTGGAAATGAAAA
>2347
CTTCAGCAGTTTCTGGGGGGTGATCATTCCAGGATGACTCTTTGCTGACAGGTTTCTGT
GGCCCTTCTAGGCTCACTCCTCTGTGGAAAAGGCTGGTTTGATTTCCCTTGTGAAGATG
AAATTTCTGCCTGTGGATGACA
>2348

TTGGCCAGGTGCGAGACATCTTTCCCCACTTCATACCTCCCCTCCGGCCAGGTGATGTT
TGCCTCCTCAGGTGCCCGTGAGGGGCTGTGGCTCCACATCGATGCTGCTTATGCAGGC
ACTGCCTTCCTGTGCCCCGAGTT
>2349
CGAGCACGCAGGAACGCCTTGCCTCCTCTGGAGAGACCTCAGCCACTAATGCCCATTT
GGAAACCCACAGGGTCAAGGACAAGTACAAGCTGCAGCAGACCTTCAGTGTGAATCC
CATCTACCTCAGGCATGCCAACTCA
>2350
GCCCAGAAATGCCCGAGGGAGTGGGGCACCCTTGTCTCTAACCTTGGGCACTGTGTTC
CTTTGTTTCTAGCACTGGCAGATCCCCCTGAGCCGACGGTTTCGCTCTGTTAAACTCTG
GTTCGTGATTCGGTCCTTCGGGG
>2351
AGTCTGAAAGTTTATGCATAGTCTCTTGTCTGATGATTAATCTCAGTTCTCTTTATACTTG
CAATTCCAGGGTACTGAAATGGCTAAATATTTTGAATCTCTGGTCAGAAACGACCCTTC
CTTTGAAATTCCTGCCAAGA
>2352
CACAAACTGGGCTCTGGGGATGCTGACAGGGGGTGCGATGGAGACTTCACTTCCTTTA
TTTTTCAAACAGGGTCCTAATTGTCTCACAGAAAATGTGTTAAAGGAAATAGCTAAAG
CTGGCCGTCTCTTCCTCATCCCGG
>2353
TATGGATGCAGCTGCCTCCGTAAGTATCCTCTTGCAGCTGGTCCAGCGATGCTCTCTTCT
CCCCTTCCAGGCTCTCCTTCCTTCTCAGACAAGATGTCTTATCAGAAAAAGCAGCCCAC
CCCTCAGCCCCCAGTGGACTG
>2354
AGAAAGGAAGTAGCTGGCAGAGCTGTGCCTGTTGATAAAATCAATCCTTAATCACTTTT
TCCCCCAACAGGTGCAGTTTTGCCAAGGAGTGCTAAAGAACTTAGATGTCAGTGCATA
AAGACATACTCCAAACCTTTCCA
>2355
CTGACTTAAGGAATCATGATTTGAATGCAAAAACTAAATATTAATCTGAACCATTTCTTT
CTTATTTCAGTGTAAAGCTTTCTGATGGAAGAGAGCTCTGTCTGGACCCCAAGGAAAA
CTGGGTGCAGAGGGTTGTGGAG
>2356
TGAGCTAAGCAGGTGGGAGCGGGGCACCTAGTCCTCCTCCCCACTGGCTGCCTTCTTT
CCCACAGGTGAGTACCCCCACGTCGGGGTCCATGTGCCCGCCTCAGGCACAGGCAGA
GGTGGGCCCCACCATGACTGAGAAG
>2357
TGGGGGGCAGTGGGGATTGGGGGCCAGGGTCCTTGCCCACAAGGACTTAGTGACCCA
CGACCCCTTACAGTGTCTACATTGGGCCAGCAGGACCTTTTAGCATCTTCCCTAGCAGC
CGGTTGAAGCGGAGACCAAGCCAC
>2358
CGTGTCTAGCGGGAGGGCAGGAGGGGCCGCCCTTTGCGCATGGCACCAACCCGGCCC
TCCTTGTCCACAGGGCACCAGCCCCAGAAGGTGGCCCGGCGCGTGTTCACCAACAGC
CGGGAGCGCTGGCGGCAGCAGAACGT
>2359
CTGTTCTCTCAAGGGTAGCCTGCATGGGTGGCTGCCCTGCCCCCAATCGTGGACTCTTT
GCCCCTTCCAGGGTTTCAGAAGCTGCCAGAGGAGGAGCCAGAAACAGATCTCAGCCC

CGGGCTCCCAGCTGCCCACCTCAT
>2360
GCCACTTCCCCAGAAGTCCCAAGCCTTTAGGATCGCATTCCCAAGAGCGCGTCGGCCC
GTGTCTCCGCAGGCGCTCCGCTGAAGGGGCAGGGGCTAGGCTGGGAGACGACGAAGG
AACAGGCGTTTCTGACGAGCGGGAC
>2361
TAGGCCTTTGTTAGAGTCTCCAAGGTTCAGTTCTCAGCTGAGGCCTCTCACACACTCCC
TCTCTCCCCAGGCCTGTGGGTCTTCATTGCCCAGCTCCTGCCCACACTCCTGCCTGCTG
CCCTGACGAGAGTCATCATGTC
>2362
TGTCATTCCTTCAGCCTCAGCATGTGCTGGCCGGCTGTACCCTGAGGCGCCCTCTCACT
TGTTCCTTCAGGTTCTGAGGAGACAGGCCCCGGAGCAGCACTAGCTCCTGCCCACACT
CCTACCTGCTGCCCTGACCAGAG
>2363
CAAAGGGGTCAGCCCTGGACAACGCACGTGGGGGTAACAGGATGTGGCCCCTCCTCA
CTTGTCTTTCCAGATCTCAGGGAGTTGATGACCTTGTTTTCAGAAGGTGACTCAGGTCA
ACACAGGGGCCCCATCTGGTCGAC
>2364
CTTTGTCAGAGCCTCCAAGGTTCAGTTCAGTTCTCACCTAAGGCCTCACACACGCTCC
TTCTCTCCCCAGGCCTGTGGGTCTTCATTGCCCAGCTCCTGCCCGCACTCCTGCCTGCT
GCCCTGACCAGAGTCATCATGCC
>2365
GGGCCTCTGTTGGGGAGGGAGGTCCCTGGGCAAGTTGGCTGTGACCTCTCATGCTCCT
CTTCTTCCCCAGGCTGCTGCTCCTGCTGCCCCGTGGGCTGTAGCAAGTGTGCCCAAGG
CTGTGTTTGCAAAGGGGCGTCAGA
>2366
AAGGAATATTCACGGAAATATTTCCAGCTATGTGTCGCTAAAACTGCATTTATTTATTTT
CTGTTCTAAGATCCCTTCATTAACAGGAGAAATGCAAATACCTTCATATCCCCTCAGCA
GAGATGGAGAGCTAAAGTCCA
>2367
TTCTTCTTTTCCATTTTTGTATTTTTCCACTTTATCCTTCTCAATTTTGGCCTCTTCTTCCA
CTTTCTAGGATCCGAGAACGCTCTAAGCCTGTCCACGAGCTCAATAGGGAAGCCTGTG
ATGACTACAGACTTTGCGAA
>2368
CGGGGGCGCAGGACTCGGCAGCCGCGCCGGGAGGAGGGTCGGGCGGGTCTCAACCC
CTCCTCGCCCCCAGGCTCCCACTCCATGAGGTATTTCAGCGCCGCCGTGTCCCGGCCCG
GCCGCGGGGAGCCCCGCTTCATCGC
>2369
AAATCCCCGCGGGTGGGTCCGGGCGAGGGCGAGGCTCGGTGGGCGGGGCTGACCGA
GGGGGTGGGGCCAGGTTCTCACACCCTCCAGTGGATGATTGGCTGCGACCTGGGGTCC
GACGGACGCCTCCTCCGCGGGTATGA
>2370
ACATCCAGGTGCTGCTGGAGTGTCCCATGAGAGATGCAAAGTGCTTGAATTTTCTGAC
TCTTCCTTTCAGACCCCCCCAAGACACACGTGACCCACCACCCTGTCTTTGACTATGAG
GCCACCCTGAGGTGCTGGGCCCT
>2371

TGAAGACCTTTAACAGGGTCGGTGGTGAGGGCTGGGGGTCAGAGACCCTCACCTTCA
CCTCCTTTCCCAGAGCAGTCTTCCCTGCCCACCATCCCCATCATGGGTATCGTTGCTGG
CCTGGTTGTCCTTGCAGCTGTAGT
>2372
TGATGGGCTGTTTAAAGTGTCACCCCTCACTGTGACTGATATGAATTTGTTCATGAATAT
TTTTCTGTAGTGTGAAACAGCTGCCCTGTGTGGGACTGAGTGGCAAGTCCCTTTGTGA
CTTCAAGAACCCTGACTTCTCT
>2373
GGGGGCGCAGGACCCGGGAAGCCGCGCCGGGAGGAGGGTCGGGCGGGTCTCAGCCA
CTCCTCGTCCCCAGGCTCTCACTCCATGAGGTATTTCTTCACATCCGTGTCCCGGCCCG
GCCGCGGGGAGCCCCGCTTCATCGC
>2374
AAAATCCCCCCAGGTTGGTCGGGGCGGGGCGGGGCTCGGGGGACCGGGCTGACCGCG
GGGTCCGGGCCAGGTTCTCACACCGTCCAGAGGATGTATGGCTGCGACGTGGGGTCGG
ACTGGCGCTTCCTCCGCGGGTACCA
>2375
CCACATGTGTGCTGGAGGAGTGTCCCATGACAGATCGAAAATGCCTGAATGATCTGAC
TCTTCCTGACAGACGCCCCCAAAACGCATATGACTCACCACGCTGTCTCTGACCATGA
AGCCACCCTGAGGTGCTGGGCCCT
>2376
TTTAGGGAAAGCAGGAGCCTCTCTGACCTTTAGCAGGGTCAGGGCCCCTCACCTTCCC
CTCTTTTCCCAGAGCCGTCTTCCCAGCCCACCATCCCCATCGTGGGCATCATTGCTGGC
CTGGTTCTCTTTGGAGCTGTGAT
>2377
GGGGGCGCAGGACCCAGGGAGCCGCGCAGGGAGGAGGGTCGGGCGGGTCTCAGCTC
CTCCTCGCTCCCAGGCTCCCACTCCATGAGGTATTTCTACACCACCATGTCCCGGCCCG
GCCGCGGGGAGCCCCGCTTCATCTC
>2378
CAAAATCCCCGCGGGTTGGTCGGGGCAGGGCGGGGCTCGGGGGACCGGGCTGACCGC
GGGGGCGGGCCAGGTTCTCACACCATGCAGGTGATGTATGGCTGCGACGTGGGGCCCG
ACGGGCGCTTCCTCCGCGGGTATGA
>2379
CCACATGCGTGCTGGTGGAGTGTCCCATGACAGATGCAAAATGCCTGAATTTTCTGACT
CTTCCTGTCAGACCCCCCCAAGACACATATGACCCACCACCCCATCTCTGACCATGAGG
CCACCCTGAGGTGCTGGGCCCT
>2380
AGGAAAAGCCGGAGCCTCTCTGGAGAGCTTTAGCAGGGTCAGGGTCCCTCACCTTCC
CCCCTTTTCCCAGAGCCATCTTCCCAGCCCACCATCCCCATCGTGGGCATCGTTGCTGG
CCTGGTTCTACTTGTAGCTGTGGT
>2381
GGGGGCGCAGGACCCGGGGAGCCGCGCCGGGAGGAGGGTCGGGCGGGTCTCAGCCC
CTCCTCGCCCCCAGGCTCCCACTCCATGAGGTATTTCCACACCTCCGTGTCCCGGCCCG
GCCGCGGGGAGCCCCGCTTCATCAC
>2382
ATATCTCTTCTCAGGGAAAGCAGGAGCCCTTCAGCAGGTCAGGGCCCCTCATCTTCCCT
TCCTTTCCCAGAGCCGTCTTCCCAGTCCACCGTCCCCATCGTGGGCATTGTTGCTGGCC

TGGCTGTCCTAGCAGTTGTGGT
>2383
CGGGGCGCAGGACCCGGGGAGCCGCGCCGGGAGGAGGGTCGGGCGGGTCTCAGCCC
CTCCTCGCCCCCAGGCTCCCACTCCATGAGGTATTTCCACACCTCCGTGTCCCGGCCCG
GCCGCGGGGAGCCCCGCTTCATCAC
>2384
CAAAATCCCCGCGGGTTGGTCGGGGCGGGGCGGGGCTCGGGGGGACGGGGCTGACG
CGGGGCGGGGCCAGGGTCTCACACCCTCCAGAATATGTATGGCTGCGACGTGGGGCCG
GACGGGCGCCTCCTCCGCGGGTACCA
>2385
TCACATGGGTGGTCCTAGGGTGTCCCATGAGAGATGCAAAGCGCCTGAATTTTCTGAC
TCTTCCCATCAGACCCCCCAAAGACACACGTGACCCACCACCCCATCTCTGACCATGA
GGCCACCCTGAGGTGCTGGGCCCT
>2386
TATCTCTTCTCAGGGAAAGCAGGAGCCCTTCAGCAGGGTCAGGGCCCCTCATCTTCCC
TTCCTTTCCCAGAGCCGTCTTCCCAGTCCACCGTCCCCATCGTGGGCATTGTTGCTGGC
CTGGCTGTCCTAGCAGTTGTGGT
>2387
AAAATCCCCGCGGGTTGGTCGGGGCGGGGCGGGGCTCGGGGGACGGGGCTGACCGC
GGGGCCGGGGCCAGGGTCTCACATCATCCAGAGGATGTATGGCTGCGACCTGGGGCCC
GACGGGCGCCTCCTCCGCGGGCATGA
>2388
AGGGGATGGGGCGGGCGGGGGGCGGGGGGCTCGGGACTGCTTGCTCAGCCTCGCTGC
TTGCCTCCTGCAGACGCCGCCAGGCCGAGCCAGTTCCGGGTGTCGCCGCTGGATCGGA
CCTGGAACCTGGGCGAGACAGTGGA
>2389
GCCAGGGCTAGGGCAAGAACACCTCCATAACCCCAGCCTGGCCCCCAACCACGCTCT
GTTGCTCTTGCAGCGAAGCCCACCACGACGCCAGCGCCGCGACCACCAACACCGGCG
CCCACCATCGCGTCGCAGCCCCTGTC
>2390
TCCCGGGACGGTGGGGCGGTTGGGAGGTCCCGGGCCCTCTCCTAAATGTCGGCGTCTT
TGGTGTCGTCAGTGCACACGAGGGGGCTGGACTTCGCCTGTGATATCTACATCTGGGC
GCCCCTGGCCGGGACTTGTGGGGT
>2391
GGGGGCGCAGGACCTGAGGAGCCGCGCCGGGAGGAGGGTCGGGCGGGTCTCAGCCC
CTCCTCGCCCCCAGGCTCCCACTCCATGAGGTATTTCTACACCGCCATGTCCCGGCCCG
GCCGCGGGGAGCCCCGCTTCATCGC
>2392
AAAATCCCCGCGGGTTGGTCGGGGCGGGGCGGGGCTCGGGGGACGGGGCTGACCGC
GGGGCCGGGGCCAGGGTCTCACATCATCCAGAGGATGTATGGCTGCGACCTGGGGCCC
GACGGGCGCTTCCTCCGCGGGCATAA
>2393
TCACATGGGTGGTCCTAGGGTGTCCCATGAGAGATGCAAAGCGCCTGAATTTTCTGAC
TCTTCCCATCAGACCCCCCAAAGACACACGTGACCCACCACCCCGTCTCTGACCATGA
GGCCACCCTGAGGTGCTGGGCCCT
>2394

AGGGAAAGCAGGAGCCCTTCTGGAGCCCTTCAGCAGGGTCAGGGCCCCTCGTCTTCC
CCTCCTTTCCCAGAGCCATCTTCCCAGTCCACCATCCCCATCGTGGGCATTGTTGCTGG
CCTGGCTGTCCTAGCAGTTGTGGT
>2395
CACCATAGGAGGGGGCGGAGGTGACGGAGAGGGTCCTCTCTCCGCTGACGCTGCTTT
GGCTGTCTCCCAGATGTGGTGGTGCTGAACTCCAAGAGGACCATTGAGGAAGCCATGG
TCAAAAAGTGGGCAGACTTTGCTGG
>2396
GGCCGAAGAAGGTCAGGCCCTCAGCTGCCTTCATCAGTTCCCACCCTCCAGCCCCCAA
CTCCTCCTGCAGACAAGCTGGTGTCTAAGAACTACCCGGACTTGTCCTTGGGAGACTA
CTCCCTGCTCTGGAAAGCCCACAA
>2397
GCCTCTCACAGTAGCCCCCGCCCTGCCCGCTGCACAGCGGCCTGCTGGAACTCACACT
GTTTCTCCACAGCGCATGAGAGCCCAGCCCGGCACCCCTGTGGCCATTGAGGAGGAAT
TCTCTCTCCTCACCTGCAGCATCA
>2398
GGCCCACCCCCAGCCCCTCCCTGAGCCTCTCCTTGTCCTGAACTGAAAGTACTCCCTC
CTTTTCTGGCAGGACGACAACTTAATGCCTGCCTATTACAAATGTATCCAGGAGGTGTT
AAAAACCTGGAGCCACTGGTCCA
>2399
TGTAGGGGAGAGGCTGGGGTGGAGGGAGAGGCTCCTTCCCACAGCTGCATTCTCATGC
TTCCTGCCGCAGTTCTTCCCCAATCCAGGTCTCCGGAGGCTGAAGCAGGCCATAGAGA
AGAGGGATCACATCGTGGAGATGC
>2400
TGGGCCTGTTGCCACTCTGTACTCCTCTCCCCAGGCCAGCCGCTCAGCCCGCTCCTTTC
ACCCTCTGCAGGAGAGCCTCGTGGCAGGCCAGTGGAGGGACATGATGGACTACATGCT
CCAAGGGGTGGCGCAGCCGAGCA
>2401
GCTGAGGGAGCGGCTGGAGGCTGGGCAGCTGTGGGCTGCTGGGGCAGGACTCCACCC
GATCATTCCCCAGATTCAGCAGCGACTGCAGGAGGAGCTAGACCACGAACTGGGCCCT
GGTGCCTCCAGCTCCCGGGTCCCCT
>2402
GTTGGGGATGAGTGAGGAAAGCCCGAGCCCAGGGAGGTCCTGGCCAGCCTCTAACTC
CAGCCCCCTTCAGCATCTCTGGCTACGACATCCCTGAGGGCACAGTCATCATTCCGAAC
CTCCAAGGCGCCCACCTGGATGAG
>2403
GAAAATGTGGTGGAGGCTGGTCCCCGCTGCCGCTGAACGCCTCCCCACCCACCTGTCC
ACCCGCCCGCAGATCGCTTCCTGGAGCCAGGCAAGAACTCCAGAGCTCTGGCCTTCG
GCTGCGGTGCCCGCGTGTGCCTGGG
>2404
GCAGGCGCAGGACCCGGGGAGCCGCGCAGGGAGGAGGGTCGGGCGGGTCTCAGCCC
CTCCTCGCCCCCAGGCTCCCACTCCATGAAGTATTTCTTCACATCCGTGTCCCGGCCTG
GCCGCGGAGAGCCCCGCTTCATCTC
>2405
AAATCCCCGCCGGTTGGTCGGGACTGGGGCGGGGCTCGGGGGACGGGGCTGACCACG
GGGGCGGGGCCAGGGTCTCACACCCTCCAGTGGATGTGTGGCTGCGACCTGGGGCCC

GACGGGCGCCTCCTCCGCGGGTATGA
>2406
TCACATGGGCGCTGTTGGAGTGTCGCAAGAGAGATACAAAGTGTCTGAATTTTCTGAC
TCTTCCCGTCAGAACACCCAAAGACACACGTGACCCACCATCCCGTCTCTGACCATGA
GGCCACCCTGAGGTGCTGGGCCCT
>2407
TGGAGCCCTTCACGCGGGTCAGGGCTGAGGCTTGGAGGTCAGGGCCCCTCACCTTCC
CCTCCTTTCCCAGAGCCGTCTTCCCAGCCCACCATCCCCATCGTGGGCATCGTTGCTGG
CCTGGCTGTCCTGGCTGTCCTAGC
>2408
CGAGGGCCAGGACCCGGGAGCCCGCGCAGGGAGGAGGGTCGGGCGGGTCTCAGCCC
CTCCTCTCCCCCAGGCTCCCACTCCATGAGGTATTTCTACACCGCTGTGTCCCGGCCCA
GCCGCGGAGAGCCCCACTTCATCGC
>2409
AAAATCCCCGCGGGTTGGTCGGGGCTGGGGCGGGGCTCGGGGGACGGGCTGACCACG
GGGGCGGGGCCAGGGTCTCACACCCTCCAGAGGATGTACGGCTGCGACCTGGGGCCC
GACGGGCGCCTCCTCCGCGGGTATGA
>2410
CACATGGGCGCTGTTGGAGTGTCGCAAGAGAGATACAAAGTGTCTGAATTTTCTGACT
CTTCCCGTGCAGAACACCCAAAGACACACGTGACCCACCATCCCGTCTCTGACCATGA
GGCCACCCTGAGGTGCTGGGCCCT
>2411
TGGAGCCCTTCACGTGGGTCAGGGCTGAGGCTTGGGGGTCAGGGCCCCTCACCTTCCC
CTCCTTTCCCAGAGCCATCTTCCCAGCCCACCATCCCCATCGTGGGCATCGTTGCTGGC
CTGGCTGTCCTGGCTGTCCTAGC
>2412
CGGCGGCTGCGGGGGCGGCCGGGCTGGGGCCGGGCCGGGGCCTGACTGACCGGCCG
GTGATTCCCCGCAGAGGATTTCGTGTACCAGTTTAAGGGCATGTGCTACTTCACCAACG
GGACGGAGCGCGTGCGTCTTGTGAC
>2413
CATTCCCAATGGTAGACGTGCTGTGTGGTCTCACATCTCACTCCTGTCTTTTCCTGTTTA
CTGCCCTCAGTGGAGCCCACAGTGACCATCTCCCCATCCAGGACAGAGGCCCTCAACC
ACCACAACCTGCTGGTCTGCTC
>2414
ACTTCAGCTCCTCAGCAGGGATGCTATGGGGTGTAGGGACAAACGCTGACACTCAGGC
TCTGCTTCTTAGGGGCTCAGTCTGAATCTGCCCAGAGCAAGATGCTGAGTGGCATTGG
AGGCTTCGTGCTGGGGCTGATCTT
>2415
GGGGGCGAAGGACTCGGGGAGCCGCGCCGGGAGGAGGGTCGGGCCGATCTCAGCCC
CTCCTCGCCCCCAGGCTCCCACTCCTTGAAGTATTTCCACACTTCCGTGTCCCGGCCCG
GCCGCGGGGAGCCCCGCTTCATCTC
>2416
AAAATGCCCACAGGGTGGTGGCGACGGGGGCGGGGCTTGGTGGGCGGGACTGACTAA
GGGGCGGGGCCAGGGTCTCACACCCTGCAGTGGATGCATGGCTGCGAGCTGGGGCCC
GACAGGCGCTTCCTCCGCGGGTATGA
>2417

TCACATGGGTGCTGCTGGAGTGTCCCATGAGAGATACAAAGTGCCTGAATTTTCTGACT
CTTCCCCTCAGAGCCCCCAAAGACACACGTGACTCACCACCCCATCTCTGACCATGAG
GCCACCCTGAGGTGCTGGGCCCT
>2418
TGGAGCCCTTCCGCAGGGTCAGGGCTGAGGCCTGGGGGTCAGGGCCCCTTACGTTCCC
CTCTTTTCCCAGAGCCGGCTTCCCAGCCCACCATCCCCATCGTGGGCATCATTGCTGGC
CTGGTTCTCCTTGGATCTGTGGT
>2419
GCGGGGGCGAAGGACTCGGGGAGCCGCGCCGGGAGGAGGGTCGGGCCGATCTCAAC
CCCTCCTCCCCCAGTGCTCCCACTCCTTGAAGTATATTCCACTATCCTGTCCCGGCCCGG
CCGGGGAGCCCCGCTTCATCTCTG
>2420
GCCAAAAATGCCACAGGGTGGTGGCGACGGGGGCGGGATTGTGGGCGGGACTGACTA
AGGGCGGGGCCAGGGTCTCACACCCTCCAGTGGATGCATGGCTGCGAGCTGGGGCCC
GACGGCTTCCTCCGCGGGTATGAACA
>2421
TCACATGGGTGCTGCTGGATGTCCCATGAGAGATACAAAATGCCTTAAATTTTCTGACT
CTTCCCCTCAGAGCCCCCAAAGACACACGTGACTCACCACCCCATCTCTGACCATGAG
GCCACCCTGAGGTGCTGGGCCCT
>2422
TGGAGCCCTTCCGCAGGGTCAGGGCTGAGGCCTGGGGGTCAGGGCCCCTTACGTTCCC
CTCTTTTCCCAGGAGCCGCTTCCCAGCCCACCATCCCCATCGTGGGCATCATTGCTGGC
CTGGTTCTCCTTGGATCTGTGGT
>2423
GGGCTGTTCAGAGTGTCTATCACTTACCGTGACCTGACCTGAATTTGTTCATGACTATTT
TCTTCTGTAGCCTGAGACAGCTGCCTTGTGTGCGACTGAGATGCAGGATTTCCTCACGC
CTCCCCTATGTGTCTTAGGGG
>2424
TGGGGCGCAGGACCCGGGGAGCCGCGCCGGGAGGAGGGTCGGGCGGGTCTCAGCCC
CTCCTCGCCCCCAGGCTCCCACTCCATGAGGTATTTCAGCACCGCGGTTTCCTGGCCGG
GCCGCGGGGAGCCCAGCTTCATTGC
>2425
TCACATGGGCGCTGCTGGGGTGTCCCATGAGGAATGCAAAGTGCCTGAGTTTTCTGAC
TCTTCCTTTCAGACCCCCCCAAGACACACGTGACCCACCACCCTGTCTCTGAACATGA
GGCATAACGAGGTGCTGGGTTCTG
>2426
TGGGGCGCAGGACCCGGGGAGCCGCGCCGGGAGGAGGGTCGGGCGGGTCTCAGCCC
CTCCTCGCCCCCAGGCTCCCACTCCATGAGGTATTTCAGCACCGCCGTTTCCTGGCCGG
GCCGCGGGGAGCCCAGCTTCATTGC
>2427
AAATCTCCGCAGGTTGCTAGGGGCCGGGCCAGGGCTCGGTGGGCGGGGCTGACCGCG
GGAACTGGGCCAGGGTATCACATCCTCCAGGGAATGTTTGGCTGCGACCTGGGGCCCG
ACGGGCGTCTCCTCCGCGGGTATGA
>2428
TCACATGGGCGCTGCTGGGGTGTCCCATGAGGAATGCAAAGTGCCTGAGTTTTCCGAC
TCTTCCTTTCAGACCCCCCCCAAGACACACGTGACCCACCCCCCTCTCTGAACATGAG

GCATAACGAGGTCCTGGGTTCTGG
>2429
CTGGAGACCTTTAGCAGGGTCGGGGCTGGGTCCTGGAGGTCAGAACCCTCACATTCCC
CTCCTTTCCCAGAGCCCTCTCCCCAGCCCACCATCCCCATTGTGGGTATCATTGCTGGC
CTGGTTCTCCTTGGAGCTGTGGT
>2430
CGGGCCCGAAGTGGACGTTCGGGGCCCGACGAGGTCGCTGGGGCGGGCTGACCGCGC
CCTTTCCTCGCAGTACATCGCGGTGCACGTGGTCCCGGACCAGCTCATGGCCTTCGGC
GGCTCCAGCGAGCCGTGCGCGCTCT
>2431
GGAGGGGCGGCGGCGCGCGGCCAGGCCCGGGACTGAGCCACCCGCTGAGTCCGGCC
TCCTCCCCCCGCAGGGTCTACATCAACTATTACGACATGAACGCGGCCAATGTGGGCTG
GAACAACTCCACCTTCGCCTAAGAG
>2432
ACAGATCCCAAAGATTCCCGGGGGGTGTGGCCTTCAATGGCTCAGGCGTCCCCTGCTG
TCCCGGCTGCAGTGACCTGGGAGCCAACACCCTCGCTGAGGTTCCAGGAGCCCCCGC
CTGGAGGAGCTGGCCCCCCAGAGCT
>2433
GAGGAAGGGGACCGGTAGAGCGGGGCTGGGTAAGCCTCCATCCAGCCGGGCTGAGCC
CTGGTCTCCGCAGAGCCTCTGCCCCTCCCGAGACACCCGCTACCTGGTGTTAGCGGTG
GACCGCCCTGCGGGGGCCTGGCGCG
>2434
GGACGGGGAGCCGGGTCGACTGCCCCCGGGCCCCCAGCCCCTGAGCCAGCCGCGTGC
CCACCCACCGCAGACTCCCGGCTGAGTACCGCCCGGCTGCAGGCACTGCTGTTCGGCG
ACGACCACCGCTGCTTCACACGGAT
>2435
ACGGGGCAGGAGCGGGCGGGGGCGGCGTGGCCTCGTGGCCGCTCTCAACTCCTCCAA
TTGCGGGTTCCAGGCCATCCGCGGAACTCGAGGAGTCGCCACCCAGCGCAGACCCCT
TCCTGGAGACGCTCACGCGCCTGGTG
>2436
GAGGGCCCTGCACGCGGCCCCGGTGGGGAAGGGGACGGGCCAGGGATTCAGACTCG
GGCTCTCCCCTCAGGATGCAGCACCGAGGCTTCCTCCTCCTCACCCTCCTCGCCCTGCT
GGCGCTCACCTCCGCGGTCGCCAAA
>2437
CCCAGGAAGGCGGGTCCCGAGGGAGTCTCCCCGTGCCCCAGTCCTGAACTCTGTTCCT
CGCGCGTTGTAGATAAGGTGAAGAAGGGCGGCCCGGGGAGCGAGTGCGCTGAGTGGG
CCTGGGGGCCCTGCACCCCCAGCAG
>2438
GCCTGGGGGGACCCTTGGCGGAGGGCGGGGCCGCGGGCGCGCAGCCTGACCTGGGC
CGCTCTCTCGCCAGCCGACTGCAAGTACAAGTTTGAGAACTGGGGTGCGTGTGATGGG
GGCACAGGCACCAAAGTCCGCCAAGG
>2439
GTGGAGCCGGCGGTCTGCAATGGGTCAGCCTAACTGCTGATATGGTATTAATATTCTTCT
TGTTTTACAGCCAAGAAAGGGAAGGGAAAGGACTAGACGCCAAGCCTGGATGCCAAG
GAGCCCCTGGTGTCACATGGGGC
>2440

GAGGGCCCTGCACGCGGCCCCGGTGGGAAAGGGGACGGGCCAGGGATTCAGACTCG
GGCTCTCCCCTCAGGATGCAGCACCGAGGCTTCCTCCTCACCCTCCTCGCCCTACT
GGCGCTCACCTCCGCGGTCGCCAAA
>2441
ACCCAGGAAGGCGGCTCCCGAGGGAGTCTCCCCGTGCCCCAGTCCTGAACTCTGTTCC
TCGCGCGTGTAGATAAGGTGAAGAAGGGCGGCCCGGGGAGCGAGTGCGCTGAGTGGG
CCTGGGGGCCCTGCACCCCCAGCAG
>2442
CCTGGGCGGACCCTTGGCGGAGGGCGGGGCCGCGGGCGCGCAGCGCTGACCTGGGCC
GCTCTCTCGCCAGCCGACTGCAAGTACAAGTTTGAGAACTGGGGTGCGTGTGATGGGG
GCACAGGCACCAAAGTCCGCCAAGG
>2443
GGAGCCGGCGGTCTGCAATGGGTCAGCCTAACTGCTGATATGGTATTAATATTTCTTTCT
TGTTTTACAGCCAAGAAAGGGAAGGGAAAGGACTAGACGCCAAGCCTGGATGCCAAG
GAGCCCCTGGTGTCACATGGGGC
>2444
CATTTACTGTGCTCCCAGTCCCCACCTCTGGCCACACCCAGCTCTCACAGCCTTCTCTC
CCCACCCGCAGAAGGAGAATAAGAATGAAAAGGTCATAGAACACATCATGGAGGACC
TGGACACAAATGCAGACAAGCAGC
>2445
TCATAGATGCTATGCCTCGGCTCTCTCTGAGATCTTTAAACTCTGGCTTCTTCCTCCTCA
ATCTTGACAGAAAAAGGGTGCAGACGTCTGGTTCAAAGAGTTGGATATCAACACTGAT
GGTGCAGTTAACTTCCAGGAGT
>2446
CCTAGAAGATGAATTTATGTAATATGATTTGCAAAATGAGTATTCATTTGTGATTTTTTT
TTTTTTAAGGTGAAAGTACATTTTTTGTTGAATTAAGTGAAACTGCCAGCATACTCATG
CATGCAACAGCACATTCTCT
>2447
TTTTTGTTTTAATTCCTTTTTTGTTTTAATTCCTTTGAGTTACTTCCTTATGCATATTTTAC
TTTAACAGGAAGAGGTACTGCAACATTTGATGGGACGGCAATAGCAAATGCAGTTGTT
AAAGAACTTGCTGAGACTAT
>2448
ACAAATTCGGTTTTTTGAGAGGGCACTTCTGTTGCTAGCACATGTATCGCTAATATTTTT
CTTTCTTAAGGCATGCATGGTAGAAAATGAATGTGAAGACCCCAGCCAGGAGACTATT
ACGTTCCTCTATAAATTCATTA
>2449
AGTAAAAGGGGAAGGGATGATGCACTATGAAAAAACAAAAAAACTTTTTTTTTTTTTT
TTTAATTTTAAGGGAAGTTTGCCTGGCTAGTGAAAGGTCAACTGTAGATGCTGAAGCT
GTCCATAAATTGCTGACTTTGATT
>2450
GGCCTCTGTTGGGGCAGGGAGTTCTATGATCGAGTCTGCTCTGACCTCTCAATCTCCTT
TCCTCCCCAAGGCTGCTGCTCCTGCTGCCCTGTGGGCTGTGCCAAGTGTGCCCAAGGC
TGCATCTGCAAAGGGGCATCGGA
>2451
CTGCGCCAGGTTTCCGCACCAAGACCCCTTTAACTCAAGACTGCCTCCCGCTTTGTGT
GCCCCGCTCCAGCAGCCTCCCGCGACGATGCCCCTCAACGTTAGCTTCACCAACAGGA

ACTATGACCTCGACTACGACTCGG
>2452
GCCTGAGCGTGCGGGGTTCCTGCTAAGGTCTCCGAGTCGGGTTCTGATCCACTGTGCT
CTTTTCCGACAGGATCAGCAACGCTACCACGAAGACATTTTCGGGCTCACGCTGCGCA
CCCAGGAGGTGACAAGCCGCATAC
>2453
CCAGCCAGGAGAACGGGCTTTCCCTCTCCTTCCGCCTGCGGAGGGGAAGCTGAAGTC
TGGTCTTCCTCAGGTCTGGTCTTCTCTCGTCTGAGCCCTGAGTACTACGACCTGGCAAG
AGCCCACCTGCGTGATGAGGAGAA
>2454
CTTTCCTGGGCACTTTCACGCGCTCCCAGAGTGTCTGAGAGACCATCATAAGGGCTTTC
TTTCCTGACAGGGTGACCTGTTGACCAAAACACAGGAGCTGGGCCGTGACTACAGGA
CCTGTCTGACGATAGTCCAAAAAC
>2455
ACCTGCAGGGTTGGCAGAGCTGGGCAGGACTCACCCTATAACCATGTCCACTGTGGTG
CTGCTGCTGCAGAGAAGTGTTTCCAATGCTGCGACCCGGGTGTGTAGGACGGGGAGG
TCACGATGGCGCGACGTCTGCAGAA
>2456
ATTCTGAGTGCCTGCTTCTCCTTGAGGACCCACCACATCTGCCCACAGCTAGCTGTTCT
CTCCGCTCCAGGTCCCCTCTGAGCCCTCTCACCTTGTCCTGTGGAAGAAGCACAGGCT
CCTGTCCTCAGATCCCGGGAACG
>2457
CTTCCCCGAGATTCTTTTTCTTACCCACTGCTAACTCCATACTCAATTTCTCATGCTCTCC
CTGTCCCAGGCTCAAGGAAAAACATGGCCTGCTATTGCAGAATACCAGCGTGCATTGC
AGGAGAACGTCGCTATGGAAC
>2458
CCCAGTAGAGCTATAAATCCAGGCTGGCTCCTCCCTCCCCACACAGCTGCTCCTGCTCT
CCCTCCTCCAGGTGACCCCAGCCATGAGGACCCTCGCCATCCTTGCTGCCATTCTCCTG
GTGGCCCTGCAGGCCCAGGCTG
>2459
CTTCCCCGAGATTCTTTTTCTTACCCACTGCTAACTCCATACTCAATTTCTCATGCTCTCC
CTGTCCCAGGCTCAAGGAAAAACATGGACTGCTATTGCAGAATACCAGCGTGCATTGC
AGGAGAACGTCGCTATGGAAC
>2460
CTAATGGCAAAATATGAATGGCCTGAAATGTCGAGGTGGAGGTGTAATGATCTATTTGT
GTCCATTTTAGGCAGGTAAAAATCAAGGTGACCCCAAGAAAATGGCTCCTCCTCCAAA
GGAGGTAGAAGAAGATAGTGAAG
>2461
TCATTGGGACCATGGTTATCTTTTTGGGTGTGGGGCTGGACTTACTGGTTTGGTTTTCAT
TTATCTCAAGGTCGTCATACCTCAGAAGAAAGGCAAGAAGGCTGCTGCAACCTCAGCA
AAGAAGGTGGTCGTTTCCCCAA
>2462
TTACATTAGATTTGAAAACCGATGTTGGTTTTGGGAGATTGCTGCAATTTTTAGGTGACT
TCTCTTTCAGACTCTGAAGAAGAAGCTATGGAGACTACACCAGCCAAAGGAAAGAAA
GCTGCAAAAGTTGTTCCTGTGAA
>2463

TATGTTTAAAGGAGGCACTTAAGTGTTTGTGGCTACTGATGTGTGATACATTGTTTGACA
TCTTGTCCAGAGCCTGTCAAAGAAGCACCTGGAAAACGAAAGAAGGAAATGGCCAAA
CAGAAAGCAGCTCCTGAAGCCAA
>2464
AGAAATTTTGCTTTGGAAACTAACTTTTTACCACACTACAAGTAGACTGAGTTGAGCTT
TTTTTGTGCAGGCACAGAACCGACTACGGCTTTCAATCTCTTTGTTGGAAACCTAAACT
TTAACAAATCTGCTCCTGAATT
>2465
TCTAGATACAGATTGTGATTTTGATTTGTGTTTTCACCTGTTGTAATTTTGCCCTCCTCTC
CACCTGAAGGAAATTTGGTTATGTGGATTTTGAATCTGCTGAAGACCTGGAGAAAGCG
TTGGAACTCACTGGTTTGAAA
>2466
CTCTTTTTGTTGTTGCCCCCCACCTTTGGTTTTTCAGGCCACAGAGCTCATTTTTGTTTT
TTTAATCTAGAGCGAGATGCGAGAACACTTTTGGCTAAAAATCTCCCTTACAAAGTCAC
TCAGGATGAATTGAAAGAAGT
>2467
TGTTGCTGAGGTAAAAGCATTTGTGTTAATATGGTTTTAAAAACCATGAGTTCTTCTCCC
TCCATTGCAGGATTGCTTATATTGAATTTAAGACAGAAGCTGATGCAGAGAAAACCTTT
GAAGAAAAGCAGGGAACAGAG
>2468
AGATATTACTGTTGTGACCGAAGCTGGGACACTGTTTCAGAGTCTTAGGAATGTGGCTC
TCTATTTCGAGGTGAATCAAAAACTCTGGTTTTAAGCAACCTCTCCTACAGTGCAACAG
AAGAAACTCTTCAGGAAGTATT
>2469
ATCATGTGACCAGTGGTATAGAGAGCATGTGCCTGGTAAATTACTCTAAGCATTAACTG
TTCATCCTCAGGTATGCATTTATAGAGTTTGCTTCATTCGAAGACGCTAAAGAAGCTTTA
AATTCCTGTAATAAAAGGGAA
>2470
TAGGCATTGTGCAAATTCAGTGTACGAACTGTGAATTCACATAAATCGCAAATTTTTTTT
TCCTTCCCAGAGCCATCCAAAACTCTGTTTGTCAAAGGCCTGTCTGAGGATACCACTG
AAGAGACATTAAAGGAGTCATT
>2471
CATGTACAGTTAACTTGGGACATGTGTAGTAGGTGCTTTTTATAATGGGCAATTTCATTT
GGTGTTCTAGGTTTGGTTTTGTAGACTTCAACAGTGAGGAGGATGCCAAGGAGGCCAT
GGAAGACGGTGAAATTGATGGA
>2472
GGTTCCTAATCACTGGGGAGGAGGAGCTTTGTACCCATTCTTTTAACAGTGTCTTGCCT
TCCTCCTGTAGGGCGAGGAGGCTTCCGAGGAGGCAGAGGAGGAGGAGGTGACCACA
AGCCACAAGGAAAGAAGACGAAGTT
>2473
CAGCCAGTGCCCCATCTTCAACTCGATGTGTTCCTAAGGTAGACAGCAAATTCCCTATT
TTATTTCTCAGATTGTCACTGCTGTTCCAAGGGCACACGCAGAGGGATTTGGAATTCCT
GGAGAGTTGCCTTTGTGAGAAG
>2474
TTTTTATTTTGATTGTGGCTATAAGTGAACATCTATCATGAGACATTTGCTGCACTTTCCT
TGCTTGTAGGTTGGTTTTGCGGATTGCCACTGATGATTCCAAAGCAGTCTGTCGTCTCA

GTGTGAAATTCGGTGCCACG
>2475
GGAAGTAAATACTCTCATGCCCAGTTAGGAGTGAGTCAGGGTTTTTAATATGCCACTTT
TTCTTTCTCAGGCAACTCATGCAGCAATTCCAGAACCCTGACTTCCCACCCGAAGTAG
AGGAACAGGATGCCAGCACCCTG
>2476
GATTTTTTTGGGTTGATGACATATAGAAAGGCAAAGAAAGTGATTGCATGTTTTTGAAA
ATCATTTTCAGGACTTTCCTTATCCCAGTTGATTGTGCAGAATACACTGCCTGTCGCTTG
TCTTCTATTCACCATGGCTTC
>2477
AAAATTATAGTACAGTTTATTAGAACAGGAGAATCCTGGTTTTCTGAATTATAAATATAA
TCAATTCTAGATATCCAGGTGAAAGAACTGGAGAAGCGTGCCTCAGGCCAGGCTTTTG
AGCTGATTCTCAGCCCTCGGTC
>2478
CAGGCGTGAGCCACCACACCCAGCCTGAATACATTTTAGAGTGCCTAGCCTATTAAACT
TTTTTTTCCAGTCCCATGAAGCTGAGGTCTTGAAGCAGCTGGCTGAGAAACGAGAGCA
CGAGAAAGAAGTGCTTCAGAAGG
>2479
TCGTGACTTGACAGGGCTTTTTTTTTTTTTTTTTTTTTTTGACTGATGACACCTTACCCT
TCCTTTACAGGATAAGCACATTGAAGAAGTGCGGAAGAACAAAGAATCCAAAGACCC
TGCTGACGAGACTGAAGCTGACT
>2480
AATGGTTGTTTTTTCCATTCATCCCTACATTTCTTTTTTTAATAATGATAAACATGCAACT
TTTTTGTAGCTTTACAACAAATACCCAGATGCTGTGGCCACATGGCTAAACCCTGACCC
ATCTCAGAAGCAGAATCTCC
>2481
TAATGTGCTATAAAGGCTAAGGGAAAATAAAAACCCATATATTAATTTTCCCGGCCATCT
TAATTTTCAGACCCTTCCAAGTAAGTCCAACGAAAGCCATGACCACATGGATGATATGG
ATGATGAAGATGATGATGACC
>2482
TGGAAAAGGATTACCATATTCCCATCCCTAGCCGTTCATATAATTATTCTTCATTTGTGCC
GTGATTCAGTACCCTGATGCTACAGACGAGGACATCACCTCACACATGGAAAGCGAGG
AGTTGAATGGTGCATACAAGG
>2483
GCCTCGCAGGGTCCTCCGAGCGAGTCCCCAGCGCCGCCCCGCGTCCCGCTCACCCCGC
CCGTCCCCCGAGTGCCTCCCCTGCGGCCCCGGGGGCAAAGGCCGCTGCTTCGGGCCC
AATATCTGCTGCGCGGAAGAGCTGG
>2484
GCGCTCCGGGGCCAGGGGGAGGCGGGCGGGGGTGCGGCCGGGATTCCCCTGACTCCA
CCTCTTCCTCCAGACGGCTGCCACGCCGACCCTGCCTGCGACGCGGAAGCCACCTTCT
CCCAGCGCTGAAACTTGATGGCTCC
>2485
GAAGTGGGTGGATTTGGAAACCTTTTGGGATACTGGATAGCTTGTTCCATAGGTTGTGT
ACTATTTACAGGTATGCTGTCACACCAGTTGAAGCAGCATGTCATCGATGGAGAAAAA
ACCATTATCCAGAATCCCACAGA
>2486

CAGCCTGGGTGACAGAATGAGACTTTGTGTCAAAAAAAAAAAAAAAATTCCTTTCTCTC
TATTCCTTTTAGGAAGGACCATGAAAAAGCTGAATTTGAGGTACATGAAGTATATGCTG
TGGATGTTCTCGTCAGCTCAGGA
>2487
ACCAGAGTTGGCAAAGAGGGGTGACTGAGAGTGTTCACCAGACCAAATGTTACTTAA
ATTACTCTCTCAGGCCAAGGATGCAGGACAGAGAACCACTATTTACAAACGAGACCCC
TCTAAACAGTATGGACTGAAAATGA
>2488
AAATATGAGCAAAATGGTAAGACTTGATGGGGAGTTAAAGATACCTCTGAATATCATCT
TCCCCTGCCAGAGCATTTGAAGATGAGAAGAAGGCTCGGATGGGTGTGGTGGAGTGC
GCCAAACATGAACTGCTGCAACCA
>2489
CTGTAAGTTCAGAAGAGCTAAGTATGACAAAGGAACTTTTTATCAAAACACATCTTCAT
TTTTGCCATAGGTGAATTTGTTGCCCAGTTTAAATTTACAGTTCTGCTCATGCCCAATGG
CCCCATGCGGATAACCAGTGG
>2490
CTCTGGTCCCAGACATCCCCAAGTAGTTAGAAACTGACAGGTCTTCTCCCTCTCCTTTT
CTTGCATATAGGCCTCCAAGACTGCAGAGAATGCCACCAGTGGGGAAACATTAGAAGA
AAATGAAGCTGGGGACTGAGGTG
>2491
ACCAGAGGTGTAAGGGCAAGAGTGGGGTGGATGGGTGTGAGATTCCTACAGCCTTGC
CTGCTCTCTAAAGGCAACTCTAGACATCGCGTCCAACAACCGTAAGGGTATCGCCTTC
GCTGACTCTGGCGCACACTGGCAGC
>2492
GAGGCAACTGGTACAGAGAGGGGGTAAGGGTGCTGATTCATTTCCACCCTCATGCCCC
CTCTCCCTTCAGTTTGTCAGGAAATCAGTACATTGTGTGATATGCTGGCCACCCACAAC
GGACAGTCCATAGACATCTCCTT
>2493
AGGAGAGACTCTGGCAGCTGGAGAAGCAAAATGGAAGAAGGGTGGATTTAACATTTC
TTTTTATTTCCAGATTTTCCCCAACAAAACCCTGGAAAAATTAAAGAGCCATGTTAAAA
TACGAAATGATCTGCTGAATAAAA
>2494
GAAGTATGGCAGGAGTGTCACAGATGGGGCTCCTTCCTTATTAATGTCTCCCAAACCTC
ACCCAACCCAGGAGAAATTCCGGAGTGACTCTATCACCAACATGCTGGACACACTGAT
GCAAGCCAAGATGAACTCAGATA
>2495
TTATTCTCTAAGCCCTTGCCTCTCCTGGGCTTACACACACTAGTCACCTCCAACCTACTC
TGGTCTTCAGGTGAAGAAGAAGCTCTACGAGGAGATTGACCAGAATGTGGGTTTCAGC
CGCACACCAACTATCAGTGACC
>2496
GGGCATGAGGCTGAGCAAGGAAGGGAGTACGAAGTCCCAGACCCACTTTTCCTCTTC
CACTCTGGAGCAGCATCGGTGAGTTTGCTGTGGACAAGGGCACAGAAGTTATCATCAA
TCTGTGGGCGCTGCATCACAATGAG
>2497
AACCAGGGCAGAACCATGCCTCTCCTCCCTCCTGCCCTAACCCCTGGCTGATGCCACT
CCTTGCCAGCAGAGCGTTTCTTGAATCCAGCGGGGACCCAGCTCATCTCACCGTCAGT

AAGCTATTTGCCCTTCGGAGCAGG
>2498
TACCACAAAGAATAAATCCTTTCACTCTGATCAATTTTGTTAACTTCTCACGTGTCTTCT
CTACACCCAGGGCACCGGTGAATCCAAGTGTCCTCTGATGGTCAAAGTTCTAGATGCT
GTCCCAGGCAGTCCTGCCATCA
>2499
TACCACAAAGAATAAATCCTTTCACTCTGATCAATTTTGTTAACTTCTCACGTGTCTTCT
CTACACCCAGGGCACCGGTGAATCCAAGTGTCCTCTGATGGTCAAAGTTCTAGATGCT
GTCCGAGGCAGTCCTGCCATCA
>2500
GGGTGTATTACTTTGCCATGCCATTTGTTTCCTCCATGCGTAACTTAATCCAGACTTTCA
CACCTTATAGGAAAACCAGTGAGTCTGGAGAGCTGCATGGGCTCACAACTGAGGAGG
AATTTGTAGAAGGGATATACAAA
>2501
CAGTCATGTGTGTCATCTGTCACGTTTTTCGGGCTCTGGTGGAAATGGATCTGTCTGTC
TTCTCTCATAGGTGGTATTCACAGCCAACGACTCCGGCCCCCGCCGCTACACCATTGCC
GCCCTGCTGAGCCCCTACTCCT
>2502
ACTAGAGAAGGAAAATGACATGAGGCAGTGGGGTATCTGTGTGTTCTCCCACTGACCA
CGCTTTCTTTAGTGACTCCTGATTGCCTCCTCAAGTCGCAGACACTATGCTGCCTCCCA
TGGCCCTGCCCAGTGTATCTTGG
>2503
TTCCCCAAGGTCATCTCCCACCCCAAGCCCCACTCTTCATTTACCCTCCCCTCTCTTCT
TCCACCTCAGGTGAAGAACCCCAGAGGGAACTGCCCTCTGCACGGATCCGCTGTCCC
AAAGGCTCCAAGGCCTATGGCTC
>2504
CACCTACCTTTGCCCTGCCCTCCCTCAGCAGGTCTCAGGCTCCCTCTCATTCTCTTTGTT
GCCCTCAAAGCTGGCCTGCCAGAAGCGGCCCTCTGGAAACCTGGTGTCTGTGCTCAGT
GGGGCTGAGGGATCCTTCGTGT
>2505
AGCTAAGGGGCCATGCAAGAAGAGCTGCAATGGCCATTCCCTTCACCTGGCTACCTCC
TCTACTCTACAGGGCACCGAGCCCAATGGAGAAGGTTGGGAGTGGAGTAGCAGTGAT
GTGATGAATTACTTTGCATGGGAGA
>2506
CTGGGATGCCCCTTCATTGGATTCACAAGCTTCCTCAGCCCCATTCCATTTATGTCTTCT
GTCTCTCCAGCATTTCTGAGGTGGAAAGATTATAACTGTAATGTGAGGTTACCCTATGTC
TGCAAGTTCACTGACTAGTG
>2507
GGTTGGAGCCCCTCTGTTTTGACCTATAAAGAGAAATGCCCTTGGGCCTCTGTTTTTTT
CCCTCTTCTAGGCTGGCAAAGCACACAGGCTGAGCGCTGAGGAGAGGGACCAGCTGC
TGCCAAACCTGAGGGCTGTGGGGT
>2508
CCTTGAATTGGTGATGTCCAGAAGTCCTTTCTCTTAGAACACTAACCTGGCTCTCACTC
CACTTAAATAGGCCTTTGGGTTCATGACAAGAGTGGCCCTGCAGGCTGAGAAACTGGA
CCACCATCCTGAATGGTTTAACG
>2509

CCAGCAGCTAGTGACTCCCTCCTGTTCTTAAGTGAAGTCAGTGGAAGCAGAATTTCTG
GTTTCATTTCAGGTCCACATCACGCTGAGCACCCATGAGTGTGCCGGCCTTTCAGAAC
GGGACATAAACCTGGCCAGCTTCA
>2510
GGACATCTCTGGAAAGTCAGCACCTGGACCAGCTCCACCCCTCTCTGAGGACACCTTC
TTTCCCTTTCAGAACAAAGAACAGCCACCATGCAGCTCTTCCTCCTCTTGTGCCTGGTG
CTTCTCAGCCCTCAGGGGGCCTC
>2511
TTATTTGCAGCTGAGAATTTCTAATTCTGACACAAAATTCTTTTTCATTTTTCCCTTTTTT
CATCTTTAGCTAAGTGGGAGACAAGCTTCAACCACAAAGGCACCCAAGAGCAAGACT
TCTACGTGACCTCGGAGACTGT
>2512
AATCCAAGGGGTGAGGCTCAGTGTGCCAATGCCCCAGAACAGTCTAAGAAAGCTCCTT
TTCCCTTTCCAGGCAGCTCGAGCTTTACCTTCCCAAATTCTCCATTGAGGGCTCCTATC
AGCTGGAGAAAGTCCTCCCCAGT
>2513
GCTTTGCCATTTGCTATGATGACTTCACCTGCCCCTGGTGGCCTGGTGATGCCTGGTGT
CTCCCCTGCAGATGGTGCACAAAGCTGTGGTGGAGGTGGACGAGTCGGGAACCAGAG
CAGCGGCAGCCACGGGGACAATCT
>2514
TAACCGCGTTCGAAATACAGCCCTTCAGCAGACGGCGTTGATTTTAAAGCATGTGTCTC
CTGTCTTCTAGTATGTCCAAAATACTAAAATGCGCCGGCAATGAAGATATCATTACACTA
AGGGCCGAAGATAACGCGGAT
>2515
TTAAGAAAGTGAGGGTGCCAAATCATTAAATTTCTAACAATTAACTTTTGGAAAATTTT
GTTCTTAATAGGAACAGGAGTACAGCTGTGTAGTAAAGATGCCTTCTGGTGAATTTGCA
CGTATATGCCGAGATCTCAGCC
>2516
TAAATATATAATGAGCTAATAGATTGCAACTTTCTTGATGTTTTTCAATGATGAATCTTTT
GTTTTGTAGGTTACCATAGAGATGAATGAACCAGTTCAACTAACTTTTGCACTGAGGTA
CCTGAACTTCTTTACAAAAG
>2517
GAACCTTGTTTTGATGGTAGTCATATGTGATACATACTCCTCAGTAATTAACCATCTTCCT
GTCTTTCAGTTGTAGAGTATAAAATTGCGGATATGGGACACTTAAAATACTACTTGGCTC
CCAAGATCGAGGATGAAGA
>2518
GTTTCTGGGACGGAGAGGTCCAGGGACACCCTCGGGCCTAACCCCTGACCCTGAGGT
CACCCACCTACAGGAAAAACCAGTGTGAGACCCGAACCATGCTGCTGCAGCCCGCGG
GGTCCCTCGGCTCCTACAGCTACCGG
>2519
ACCCCTTCCCTGAAGCAGAGGTGAGGTTTGGGGGGCTGGTCCCCGACAGGGTTGTCT
CTTGGGTTCCCAGACTGGGGCAGCACCTACTCCGTGTCAGTGGTGGAGACCGACTACG
ACCAGTACGCGCTGCTGTACAGCCA
>2520
CCCAAGACCCAGGGCAGGGCACACAGCATCCCCCTCCGCTGAGGCCAGCTCCGTCTC
CACCCTGTCCCAGGCCGAACCCAGACCCCCAGGGCTGAGTTAAAGGAGAAATTCACC

GCCTTCTGCAAGGCCCAGGGCTTCAC
>2521
TTCCTTCATAAAGACCCAACCCTATGACTTTAACTTCTTACAGCTACCACAGCCCCTAA
ACCCGCAACAGTTGTTACAGGTTCTGGTCATGCAAGCTCTACCCCAGGTGGAGAAAAG
GAGACTTCGGCTACCCAGAGAAG
>2522
TAAGGTTGGGGGAAGAGTGCTGAGCCAGAGCTGGGACCCGTGGCTGAAGTGCCCATT
TCCCTGTGACCAGGCCAGGATCTGTGGTGGTACAATTGACTCTGGCCTTCCGAGAAGG
TACCATCAATGTCCACGACGTGGAG
>2523
TTCCTTAGTGCTGGCAGCGGGAGGGGCGCCTCCTCTGGGAGACTGCCCTGACCACTGC
TTTTCCTTTTAGTGAGTGATGTGCCATTTCCTTTCTCTGCCCAGTCTGGGGCTGGGGTG
CCAGGCTGGGGCATCGCGCTGCT
>2524
TCCCTGGCCCTGATCAGAGCCCCCCGGTAGAAGGCACTCCATGGCCTGCCATAACCTC
CTATCTCCCCAGGCTGTCTGTCAGTGCCGCCGAAAGAACTACGGGCAGCTGGACATCT
TTCCAGCCCGGGATACCTACCATC
>2525
GGGGTGGCATCCAGGGGCTCTAGGAGGTACCTTTTGCTCCTCACCCTGGATCTCTTTTC
CTTCCACCCAGGTTTCTGCAGGTAATGGTGGCAGCAGCCTCTCTTACACAAACCCAGC
AGTGGCAGCCACTTCTGCCAACT
>2526
GGCAGCCGCAGCCTCCCGCCGTGTAGGGTCGAGGCTTGGAATGACCCACGTCCCAATG
TCGCCTTCCCAGATGGTGTTCGTGCGCAGGCCGTGGCCCGCCTTGACCACAGTGCTTC
TGGCCCTGCTCGTCTGCCTAGGGG
>2527
CACCCTGCGGCCCCGCTCCACCTGGGGGCGTGGCCAGATCTGACCACGCTCTTCCCTT
CCCCGCGCCCAGGTATGGGAAAAGAGACGGCCCGGACACGCTTCTTTCCAAAACGTT
CTTCCCCGACGGCGAGGACCGCCCC
>2528
GCAACATCATTTAACGACGTCTCCCAGGCTCGCCTCCCAAGATCCAATTCCTTCCACTT
CGCTTCCGCAGGTCGGAGGGCCCAGACCTGTGGTGAGGACCCCTGAGGCCTCCTGGG
AGATCTGCCAACCACGCCCACGTC
>2529
TATCTCTGGCTACTCAGGAGTCACAACTCCCACCCCTCCTCCTCTCTTACTCCCTCCCTT
TCCCCCTCAGCTGAAGCTGAAGAAGATGGGGACCTGCAGTGCCTGTGTGTGAAGACC
ACCTCCCAGGTCCGTCCCAGGCA
>2530
CCCTCCCCCTTCTAATGCCATTTGCAAACCCAAGGACTGAAAGTCACGTCTCTTCTCTT
TTCCCTGCCAGAGCCACGCTGAAGAATGGGAGGAAAATTTGCTTGGATCTGCAAGCCC
TGCTGTACAAGAAAATCATTAAG
>2531
GCTGCAGAGTGGGGAGTCGGGTGGGGGCCCACTGGCTTGGGAGGGAAAGCAGCGTG
CCTGTGTCCCCCAGGAGCGTCGGTACGCAGGCCTGAAGCCCGGGGAACTCCCCACCT
GCGAGAGCCTCAAGGACACCATTGCCC
>2532

TCACATAGTGTCTGCTGTGTAAACAGGCTGGGGACAGAGGGGCTAAGGACACCTATTC
CTTCCGGCATAGGGATGTCAGACCAGGCGATCATGGAGCTGAACCTGCCCACGGGGAT
CCCCATTGTGTATGAGCTGAACAA
>2533
GCCACGGTACTGAAGGAGATGGTTCGCTGCCCGTGGTGCTTGGCTGAAAGGAAGCCT
GTGATTTTTGCAGCTGGTTGGCTCTCACTGACCCCAGGGATGTGGCCAGGATCGAAAG
CAAGACGGTTATCGTCACCCAAGAG
>2534
GAAAAAAATGCTGGTCGTGTTCGAAGTCCAAGGTCATTTCTCACCAGTGCCCACCCAT
CGCACCCTGTAGGTCGCACCATGTACGTCATCCCATTCAGCATGGGGCCGCTGGGCTCA
CCTCTGTCGAAGATCGGCATCGA
>2535
CCAGTGAGTGCCTCGGGGACCCCCAACCAGGGCCCTGGCGCACTGACTTGGGAGGGG
TCCTTGTTCACAGAGCCTTTGGTCAACAACTGGCCCTGCAACCCGGAGCTGACGCTCA
TCGCCCACCTGCCTGACCGCAGAGA
>2536
CTCTGGGCTGAAGTACCAACCTCGGGGAGAAGGAAACAAAGATCACAATAAAGAATC
TTGTCCCCAACAGATTCTGGGTATAACCAACCCTGAGGGTGAGAAGAAGTACCTGGCG
GCCGCATTTCCCAGCGCCTGCGGGA
>2537
CAATATATAAGAGTATATGTTCTGCTTTGCCTGGCACTCACTACTGCTTCTCTGGTTTAA
AACTCTCCAGGTCATTTAAGGGCCATCAACCCAGAAAATGGCTTTTTCGGTGTCGCTCC
TGGGACTTCAGTGAAGACCAA
>2538
CTGTGTGTGCCGGGCTGCAGGGACTGCCTGTTTTGAGCCAGGCACTCACGAGCCTTTC
TCTGTCTTATAGGGGAACCTTGTGCCCACCCCAACTCGAGGTTCTGCACCCCTGCCAG
CCAGTGCCCCATCATTGATGCTGC
>2539
ACACCACTGGTTGTGGAGTCTGAATTTCAAAGCCTCTGATGAACATTTCTCTTTTTTTTT
CCTGCTAAAGGTGTCCCTCTAGTCTATGAAGCTCTCAGCTGGCAACATGGAGTCTTTGT
GGGGGCGGCCATGAGATCAGA
>2540
CAACAATCAATGGCGTCAGTCTTGCCTAGGAGAGCCTCATTTACTAATGAACTCCCTCT
CTGTTTAACAGGCAAAATCATCATGCATGACCCCTTTGCCATGCGGCCCTTCTTTGGCT
ACAACTTCGGCAAATACCTGGC
>2541
GGGATCTCCTGGGTGGGGAGCTGGCGGCTCGCGGGCCGGCACTGAGTCCCCGTGCTT
CCCCCTTTCCTAGGCAAGGAGAAGGAGCCCCTGGAGTCGCAGTACCAGGTGGGCCCG
CTACTGGGCAGCGGCGGCTTCGGCTC
>2542
CCCAGGATGAGTGGGTGGGGTGAGGGGAGCCCCCGACTCCCGCCCTAACGCGGCCCC
CTCGCCCCTGCAGCCTAATGGCACTCGAGTGCCCATGGAAGTGGTCCTGCTGAAGAAG
GTGAGCTCGGGTTTCTCCGGCGTCA
>2543
ATACCACCCAGCTTCTTTGTGCTTGTTTTTGCTAAAAGTGTGTTTTCTCTTCTATTCCCTT
GGCTCACAGGGACCCGAGTGTATAGCCCTCCAGAGTGGATCCGCTACCATCGCTACCAT

GGCAGGTCGGCGGCAGTCTG
>2544
ATGGGGAAGACCTTTGCAGTGTAAAAACAAGTTGAGTCATTCATAACCTCGTCTATCCT
CCTTTCTGCAGAATGTCAGCATCTCATTAGATGGTGCTTGGCCCTGAGACCATCAGATA
GGCCAACCTTCGAAGAAATCCA
>2545
GTGGGAGGCCATAGCTTGGGGATGCTGGCAATGTGTGGGATGGGCCCAGGGAAGGCC
TCTGGCCTACTAGGGGCTCTGGCCCTGACCCACGGCCACTCACTCCTCAGAGACGTCT
CCCACAACCTGCTCCGGGCGCTGGA
>2546
GCGGCACCGCCTCTTGCTGCTCAGCTGTGGGGGCTTCCATCAGCTTTGCCGAATCCCC
CGTCTCTTCCAGGGATATAAGCAACAACAAGATTTCTACGTTAGAAGAAGGAATATTTG
CTAATTTATTTAATTTAAGTGAA
>2547
TTGCCAGTGGGGGGCTGGCATAGACCCTTCCCACCAGACCTGGTCCCCAACACCTGCC
CCTGCCCTGCAGAAACCTGAGTGGGAACCCGTTTGAGTGTGACTGTGGCCTGGCGTG
GCTGCCGCGATGGGCGGAGGAGCAG
>2548
CCCCCGCCACCTCCTGCCCCCACCTGTAGGGGCCCCACCCTCCTCCAGCACGTCTTCC
CTGCCTCCCCAGGGGCCACCCTGGTGGGGCCCCACGGACCTCTGGCCTCTGGCCAGCT
AGCAGCCTTCCACATCGCTGCCCC
>2549
GGGCAGAGCCTGGTACCCCCGTCTTGGGCCCACACTGACCGTTGACACCCTCGTTCCC
ACCGGTCTCCAGCGGTGCACCCGCTCTGCCCCTCGGACACGGAGATCTTCCCTGGCAA
CGGGCACTGCTACCGCCTGGTGGT
>2550
GCGGGCTGCTGTGAGGGTGGGAGGATGGAGGAGTGCCCTGAGCCCCCTGCCATCCCA
CACCCGCCCCCAGGAGCCTAGACGTGTGGATCGGCTTCTCGACTGTGCAGGGGGTGG
AGGTGGGCCCAGCGCCGCAGGGCGAG
>2551
GGTCCCCAGTCTGTTCGTCCTGGTGTCCTGGGCCCTGGCCCGGCGCCTCACTGTGCAC
TCGCCACCCCAGGCCCAGTGCAGGATGCCGAGAACCTCCTCGTGGGAGCGCCCAGTG
GGGACCTGCAGGGACCCCTGACGCC
>2552
GGTCTGATGGCTCGCTCTCCTTCCCTCCCCTCTTCCTGGGAAGTTCGGGTAGGGGGAGT
CTGGGCTTCAGGCTGGGATGGGGTCTGTGGAGCTGAGGCGGCCCCCTGCCCACCAGG
TCATGGTATTCCCGGGCCTGCGTC
>2553
CAGGCAGTTGGGCATCTCTGACGGTGGCCTGTGGGCAAATCAGGGCCCCAACACCCTC
CCCTCCTCACAGGGACCCCGGAGAACGGCAGCGAGCCTGAGAGCAGGTCCCCGGACA
ACAGGACCCAGCTGGCCCCCGCGTG
>2554
CCTTCGTGCCCGGCTGCCCCTGGGAGACCAACGATACCCTGTTCTCAGTGGTAGCACT
GCCGTGGCTCAGTGAGGGGGAGCACGTGGTGGACGTGGTGGTGGAAAACAGCGCCA
GCCGGGCCAACCTCAGCCTGCGGGTG
>2555

GTGTCCAGGAGGCGACAGGCTAAGGGCAGAGTCCTCCACAGAGCCCAGGCTGACACC
ATTCCCCCCGCAGAGGTACAGCCCCGTGGTGGAGGCCGGCTCGGACATGGTCTTCCGG
TGGACCATCAACGACAAGCAGTCCC
>2556
GAGGGAGGGACGCCAATCAGGGCCAGGCCTCTCACCTGCCACCTGGGCTCACTGACG
CCTGTCCCTGCAGCTGACGGCCTCCAACCACGTGAGCAACGTCACCGTGAACTACAAC
GTAACCGTGGAGCGGATGAACAGGA
>2557
CAAGCACCCCCTGCCCCGCCACCGCCCACCCCTGTCCCGGTTCACTCACTGCGTCCCA
CCGCCCCGGCAGGTGGACCTTTGGGGATGGGGAGCAGGCCCTCCACCAGTTCCAGCC
TCCGTACAACGAGTCCTTCCCGGTT
>2558
GGGCTTCTGCCGAGCGGGTGGGGAGCAGGTGGGGGTGCCGCGGCTGCCCCACTCGGG
CCTGTCCCCACAGGTGAGTACCTCCTGACCGTGCTGGCATCTAATGCCTTCGAGAACC
GGACGCAGCAGGTGCCTGTGAGCGT
>2559
CCCGCTGCCCTCGCCTGGGGGTGTTCTTTACACGTGGGACTTCGGGGACGGCTCCCCT
GTCCTGACCCAGAGCCAGCCGGCTGCCAACCACACCTATGCCTCGAGGGGCACCTACC
ACGTGCGCCTGGAGGTCAACAACA
>2560
CGGTACAGCTGCAGGCCGTGGTTAGGGATGGCACCAACGTCTCCTACAGCTGGACTGC
CTGGAGGGACAGGGGCCCGGCCCTGGCCGGCAGCGGCAAAGGCTTCTCGCTCACCGT
CTCGAGGCCGGCACCTACCATGTGC
>2561
GGCCGCCACCAGCCCCAGCCCCCGGCGTGTGGCCTACCACTGGGACTTTGGGGATGG
GTCGCCAGGGCAGGACACAGATGAGCCCAGGGCCGAGCACTCCTACCTGAGGCCTGG
GGACTACCGCGTGCAGGTGAACGCCT
>2562
CTGGGCCGGGCTCTGCTTTAAAACTGGATGGGGCTCTCAGGCCACGTCGCCCCTTGTT
CTCGGCCTGCAGAGGGAGGCTGGCGGGTGTGCGCTGAACTTTGGGCCCCGCGGGAGC
AGCACGGTCACCATTCCACGGGAGC
>2563
CCATGGGTCCCCCAGTCCTTCCAGCAGGCCAAATAGACCTGTGTTGGAGGTAACCCCA
CTCCCACGCCAGGTGCTGATCCGGAGTGGCCGGGTGCCCATTGTGTCCTTGGAGTGTG
TGTCCTGCAAGGCACAGGCCGTGT
>2564
GGGCCTGCAGGCAGAAGTGGGGCTGACAGGGCAGAGGGTTGCGCCCCCTCACCACCC
CTTCTGCCTGCAGCGGTGGGCTGCACGTACGTTCAGCAACAAGACGCTGGTGCTGGAT
GAGACCACCACATCCACGGGCAGTG
>2565
GAGCAGCGGGATCCCCCGACTCTGTGACGTCACGGAGCCCTCCCGTGATGCCGTGGG
GACCGTCCCTCAGGCTGGCATGACGCGGAGGATGCTGGCGCCCCGCTGGTGTACGCCC
TGCTGCTGCGGCGCTGTCGCCAGGG
>2566
GCTGTGCCCCCTGCCTGCCCCAGTACGAGCGGGCCCTGGACGTGGCGCAGAGCCCAA
GCACGAGCGGCAGCACCGAGCCCAGATACGCAAGAACATCACGGAGACTCTGGTGTC

CCTGAGGGTCCACACTGTGGATGACA
>2567
CGTGTAGAGAGGAGGGCGTGTGCAAGGAGTGGGGCCAGGAGCGGGGCTGGACACTG
CTGGCTCCACACAGGGGCCCAGCAGGGAGCTCGTATGCCGCTCGTGCCTGAAGCAGA
CGCTGCACAAGCTGGAGGCCATGATGC
>2568
CCCCCTTTTTGCCTGCCCCCACCCTCCCTCTACCTCCCTGTCTCTGCACTGACCTCACG
CATGTCTGCAGGAGACCTCATCCACCTGGCCAGCTCGGACGTGCGGGCACCACAGCCC
TCAGAGCTGGGAGCCGAGTCACC
>2569
GGGCCATCGTGGGTGTCCCCCGGGTGGTTGAGCTTCCCGGCAGGCGTGTGACCTGCGC
GTTCTGCCCCAGGCCACTACCTGTCTGAGGAACCTGAGCCCTACCTGGCAGTCTACCT
ACACTCGGAGCCCCGGCCCAATGA
>2570
TGGGCTCTGAGACTGCGACATCCAACCTGGCGGAGCCTGGGCTCACGTCCGCTACCCC
TTCCCTGCCCAGGAGCAGAGACCCAGCGGGGAGTTACCATCTGAACCTCTCCAGCCAC
TTCCGCTGGTCGGCGCTGCAGGTG
>2571
GGCTCGGCTCTATCCTGAGAAGGCACAGCTTGCACGTGACCTCCTGGGCCCGGCGGCT
GTGTCCTCACAGGAGCCGACAGCGGATGTAAACTACATCGTCATGCTGACATGTGCTG
TGTGCCTGGTGACCTACATGGTCA
>2572
TGACTTGCCTGGGATGCCACACGTGTTGGGCAGCAGACCGAGCCTCCCACCCCTCCCT
CTTGCCTCCCAGGTACCACGGCCCACGTGGGCATCATGCTGTATGGGGTGGACAGCCG
GAGCGGCCACCGGCACCTGGACGG
>2573
CCAAGCTCTGCCCCTCTGCCCCCGCATTGGGGCGCCCTGCGAGCCTGACCTCCCTCCT
GCGCCTCTGCAGGGCTCAGCCCTGCCTGGTTCCTGCAGCACGTCATCGTCAGGGACCT
GCAGACGGCACGCAGCGCCTTCTT
>2574
CTCCGTGGGAGGTTGGGCAGGGTGGTCCTGCCCCGTGGCCTCCTGCAGTGCGGCCCTC
CCTGCCTTCTAGGCGACGCAGCCCTTTTGCGCTTCCGGCGCCTGCTGGTGGCTGAGCT
GCAGCGTGGCTTCTTTGACAAGCA
>2575
GGCAGCCTCTTCCTGCCCAGCCCTTCCTGCCCCTCAGCCTCACCTGTGTGGCCTCCTCT
CCTCCACACAGCACGGGGCATGTGTCCAGGCTGAGCCCGCTGAGCGTCGACACAGTC
GCTGTTGGCCTGGTGTCCAGCGTG
>2576
AGGTGGCTTCTCATCCCTGTCCTGAGCAGGTCTGAGCTGCCGCCCGCTGACCACTGCC
CTCGTCCTGCAGGTGGCTGGGAGCCCGAGCCCCACACCTGCCGGGCAGCAGGTGCTG
GACATCGACAGCTGCCTGGACTCGT
>2577
GTGACTGGACGGGGGTGGGCTGTGTGTGTGACACATCCCCTGGTACCTTGCTGACCCG
CGCCACCTGCAGTCTGGTGTGCTGGCCCTCCGGCGAGGGAACGCTCAGTTGGCCGGA
CCTGCTCAGTGACCCGTCCATTGTG
>2578

GGGGTGAGAGGAGGGGGCTCTGAAGCTCACCCTTGCAGCTGGGCCCACCCTATGCCT
CCTGTACCTCTAGATGAAGACCTGATCCAGCAGGTCCTTGCCGAGGGGGTCAGCAGCC
CAGCCCCTACCCAAGACACCCACAT
>2579
GTTAACATGGGCTTGGCTGCAACTGCCTCCTGGAGGCCGGGATGAATTCACAGCCTAC
CATGTCCCTCAGGTCCAGCACTCCTGGGGAGAAGACAGAGACGCTGGCGCTGCAGAG
GCTGGGGGAGCTGGGGCCACCCAGC
>2580
GCGTAGCCCCGGGATGCCCCTAGCCCCTCCCTGTGAGCTGCCTCTCACAGGTCTGTCTC
TGCTTCCCCAGGACTGGTGGAGGGTCTGCGGAAGCGCCTGCTGCCGGCCTGGTGTGC
CTCCCTGGCCCACGGGCTCAGCCT
>2581
GAGGGGGCTGCCAGGGGTAGGCTACAGGCCTCCATCACGGGGGACCCCTCTGAAGCC
ACCCCCTCCCCAGGTCTTGCTGGAAGCCCTGTACTTCTCACTGGTGGCCAAGCGGCTG
CACCCGGATGAAGATGACACCCTGG
>2582
GCCGTTGCCAAAGCCCTGCTGTCACTGTGGGCTGGGGCCAGGCTGACCACAGGGCCC
CCCCGTCCACCAGAGCCTCCTGGTGTACATGCTTTTTCTGCTGGTGACCCTGCTGGCCA
GCTATGGGGATGCCTCATGCCATG
>2583
TCTGGTGGCCGCTCACTCGAGGCGGGCATGGGGCAGTAGGGGCTGGAGCGTGTGACT
GATGCTGTGGCAGGTCTGAGGAGCTCTGGCCATGGATGGCCCACGTGCTGCTGCCCTA
CGTCCACGGGAACCAGTCCAGCCCA
>2584
GCCAGCAGGAAACACTCCTGTTGGGTTTTGATGAGGCCCTGGCCCCGGCCTGGGGCTC
TGTGTGTTTCAGCACTCTACCCAGACCCTCCCGGCCCCAGGGTCCACACGTGCTCGGC
CGCAGGAGGCTTCAGCACCAGCGA
>2585
TCCTGACCAGCCTGGCTCCGGGGTGCCGGAAGGGCTGGGGTGCGGCACCCACGCCAC
CCCTCTCCGGCAGGGCATGGTCCTGGGGCTCCTGTGCCGTGTATGACAGCGGGGGCTA
CGTGCAGGAGCTGGGCCTGAGCCTG
>2586
GCCTGCACTGCGCGGTCCCCGCAGCTCCCGCCCTGCCACCCGCTCCTACTGACCCGCA
CCCTCTGCGCAGGAGCCGCGCTGTGTTCCTGGAGCTCACGCGCTACAGCCCGGCCGTG
GGGCTGCACGCCGCCGTCACGCTG
>2587
GCGTCCCGCCCCCTCGCAGGGCCCCGCCCCGGCAGCGTCCCTCCCGCCCTCCTGACCG
CGCCCCCCACAGGTGTGCCTGCTGCTGTTCGCCGTGCACTTCGCCGTGGCCGAGGCCC
GTACTTGGCACAGGGAAGGGCGCT
>2588
GGCTGGGCCGGTGGGCGCGGGGCTGGGCGCACACCCCAGGGCTGCAAGCAGACAGA
TTTCTCGTCCGCAGGCTGCCCAGCAGCTACGCTTCGTGCGCCAGTGGTCCGTCTTTGG
CAAGACATTATGCCGAGCTCTGCCAG
>2589
CGGCCGGGGAGGGCGTCTTAGCTCAGCTCAGCTCAGCTGTACGCCCTCACTGGTGTCG
CCTTCCCCGCAGCTCGTGTCTTCCTGTGTGGACTCCCTCTGGAGCGTGGCCCAGGCCC

TGTTGGTGCTGTGCCCTGGGACTG
>2590
GGGAGAGGGACACGCCCTGGGCTCTGCCCAGGGTGCAGCCGGACTGACTGAGCCCCT
GTGCCGCCCCCAGTTCCGCCACAAAGTCCGCTTTGAAGGGATGGAGCCGCTGCCCTCT
CGCTCCTCCAGGGGCTCCAAGGTAT
>2591
GCTCCTGGAGCAGGGAGAGCGCTGGCCTCTTCCTCTCCGGCTCCCTCCATTGCCTCCG
GTTTCTCCCCAGGCTCCCGGACGTCCCTGCTCCTGGCTTTTGCCCTGCTCTGCCTGCCC
TGGCTTCAAGAGGCTGGTGCCGT
>2592
AAGCAACAATGGGACCTGGTCTCCAGCATAGAAACCAGCAGTCCTTCTTGGTGGGGG
GTCCTTCTCCTAGGAAGAAACCTATATCCCAAAGGACCAGAAGTATTCATTCCTGCATG
ACTCCCAGACCTCCTTCTGCTTCT
>2593
GGCGGGGATGGGGGAGACCTGTGGTCAGGGCTCCCGGGCAGCACAGCCACTGCCGGT
CCTTCCCCTGCAGAATCTAGAGCTGCTCCGCATCTCCCTGCTGCTCATCGAGTCGTGGC
TGGAGCCCGTGCGGTTCCTCAGGA
>2594
TGGATGAATGAGAGAGGGAGGGAACATTGCCCAAGCGCTTGGCCTCTCCTTCTCTTCC
TTCACTTTGCAGAGGCTGGAAGATGGCAGCCGCCGGACTGGGCAGATCCTCAAGCAG
ACCTACAGCAAGTTTGACACAAACT
>2595
GGGAAGGGTAAGGAGGCAGGGGCTGCCTGGGCTGGCTGGCACCAGGACCCTCTTCCT
CTGCCCTGCCCAGGTGAAGTGTCCGGACGCACCCTCATCGACATTGGTTCAGGCCCCA
CCGTGTACCAGCTGCTCAGTGCCTG
>2596
GTGGCTGGTTGGGGCAACAGAGGCCTGAGCGTAGAACAGCCTTGAGCCCTGCCTTGT
GCCTCCTGCACAGGGAATGCTGGCAGGATAAGGAGCGCCAGCTGCGAGCCAGGGTGA
AACGGGTCCTGCCCATCGACGTGCAC
>2597
CCCCGTCTGCACCGGGTGCAACGAGAGCCATGGTGGGGTGGGACCGCCGTGCAGCCC
AAGGCCCCCTCAGTTGGCAGGGACCTGGCACTCCATGGCCATGGCGACCAACAACATC
TCCCTCATGGCGACACTGAAGGCCC
>2598
GATTTTAGGAGTGACAGTGAAGGCAACTCCAATTCAAGTGGCCACTCATCCTATTGTCA
CCACCTTTCAGGGAGAACAACAGCTGTGTTGAGAAGAAGGTCCTTGGAGAGAAGACT
GAGAATCCAAAGAAGTTCAAGATC
>2599
ACACACCTGCACAGGACTCTGCTGAGACGGAGGCTTCATCTTCCTTTTGGTTCTTCTCT
TCTTTCCCCAGATACGGTGGCGAACGAGGCCACGCTGCTCGATACTGACTACGACAAT
TTCCTGTTTCTCTGCCTACAGGA
>2600
AAGGCCTCTGGAGCTCCCCAGCTCTCATGGAAGCCCCAGGGGCCCAGGACTGACCCA
GCCTCTTCCACAGCCAGAGTCCTGGTGGAGGACGATGAGATCATGCAGGGATTCATCA
GGGCTTTCAGGCCCCTGCCCAGGCA
>2601

AGGTCAGATATGAAACAGGTGGGCTGGCACCTGGGCACAGTGCTTGCCCCTGCTGCCT
CTTCCCTCCCAGGTATGGGAAAAGACACAAAGAGGACACGCTGGCCTTCTCGGAGTG
GGGGTCCCCGCATGCTGCTGTCCCC
>2602
CAGACCCCCTCATGGCCCCAGCCCCTCTTAGGCCCCTCACCAAGGTGAGCTCCCCTCC
CTCCAAAACCAGACTCAGTGTTCTCCAGCAGCGAGCGTGCCCACCAGGTGCTGCGGA
TCCGCAAACGTGCCAACTCCTTCCT
>2603
GCTGGACTACCGGCGCCCGCGCCCCTCGGGATCTCTGGCCGCTGACCCCCTACCCCGC
CTTGTGTCGCAGACGGTGACCAGTGCTTGGTCTTGCCCTTGGAGCACCCGTGCGCCAG
CCTGTGCTGCGGGCACGGCACGTG
>2604
GGGGCGGGGCTGGGGCCGGGTTGGGGGCGCGGCACCAGCACCAGCTGCCCGCGCCCT
CCCCTGCCCGCAGAGGTGAGCTTCCTCAATTGCTCTCTGGACAACGGCGGCTGCACGC
ATTACTGCCTAGAGGAGGTGGGCTG
>2605
AGGACCAAGACAGGAGGGCAGTCTCGGGAGGAGTGCCTGGCAGGCCCCTCACCACCT
CTGCCTACCTCAGTGAAGTTCCCTTGTGGGAGGCCCTGGAAGCGGATGGAGAAGAAG
CGCAGTCACCTGAAACGAGACACAGA
>2606
AACCCAGGTGCCCTGGACTGGAGGCTGTCAGGAGGCAGCCCTGTGATGTCATCATCCC
ACCCCATTCCAGGTGGTCCTGCTGGACTCAAAGAAGAAGCTGGCCTGCGGGGCAGTG
CTCATCCACCCCTCCTGGGTGCTGA
>2607
GGGCCTCAGGAAAGTGCCACTGGGGAGAGGCTCCCCGCAGCCCACTCTGACTGTGCC
CTCTGCCCTGCAGGAGAGTATGACCTGCGGCGCTGGGAGAAGTGGGAGCTGGACCTG
GACATCAAGGAGGTCTTCGTCCACCC
>2608
GAGGAAAGACATGGCAGCTGAAGCAGAGGCTTACTGGGTATAAATGTGGGCTCGTTTC
TTCTTTTAACAGTTCCTGTTAAAGGTCAAGACACTGTCAAAGGCCGTGTTCCATTCAAT
GGACAAGATCCCGTTAAAGGACA
>2609
CTGTCTTCCTCACTGCCTCTGAGTGCTTTGATGTGCTGACTCTCACCTCTGATACTCTTC
TCTTCCACAGAGGGAGCCGGTCCTTGCTGCACCTGTGCCGTCCCCAGAGCTACAGGCC
CCATCTGGTCCTAAGTCCCTGC
>2610
CCTCCGTGCCCCGCCCGGGCGGTGCAGCTGGCCCGGGTGCTCACGCTCGACTCTCTTT
CTTCTTTTCCAGGGTCTGCGCTTCGCAGCCGGGATGAAGCTGGTTTCCGTCGCCCTGAT
GTACCTGGGTTCGCTCGCCTTCC
>2611
TGAATGGGAGCAGGGACAGGCCTGGGCGTCACCTGAACGCACGCGAATCGGGTCTGC
TTGTGTTTTCCAGGTGGAATAAGTGGGCTCTGAGTCGTGGGAAGAGGGAACTGCGGAT
GTCCAGCAGCTACCCCACCGGGCTC
>2612
CGGTGTTGGGGCCAAAGCTCTGCTTGATGGGGTCTCAAGTTGCCTTTCTTCCCCCTCCC
CCCGCCCGCAGCAGTCCGGATGCCGCCCGCATCCGAGTCAAGCGCTACCGCCAGAGCA

TGAACAACTTCCAGGGCCTCCGG
>2613
TGAAGGAGTTATTTGATTGAATGGGGGAAATACTCCCCTGGGCCCAGCTGAGGTCTCT
CTCTTCTCGCAGTTTCCATGTGGTACACACTCCCCCGCTGCACCCTGACTTCAAGAGGG
CCCTCGGGGACCTGCCCCACCAC
>2614
AGACAGAGAGGTATGAAGACAGGAGGGTTTTCCAGCATGAGCTCAGCTCTTCTTGTTT
CAACTCACACAGATGGAGGAGACTCTGAGCAGTTCATAGATGAGGAGCGTCAGGGAC
CACCTTTGGGAGGACAGCAATCTCA
>2615
TGAGGGATTAGACATTTCCTGCCATGTCAAGTGTTGTCTGTAATCTTCCTTGTTTGCTTG
TCTTTTTCAGGAAGTGAATAAGAAGATAACAGTGTTTCAAATGCCGTGAAACACGGCA
TCATGCTCTAACTTCAGTATAC
>2616
AGACAGAGAGATATGAAGACAGGAGGGTTTTCCAGCATGAGCTCAGCTCTTCTTGTTT
CAACTCACACAGATGGAGGAGACTCTGAGCAGTTCATAGATGAGGAGCGTCAGGGAC
CACCTTTGGGAGGACAGCAATCTCA
>2617
TAAACCAATGAGGTATTAGACATTTCCTGCCATGTCAAGTCTTGCCTATAATCTTCCTTG
TCTTTTTCAGGAAGTGAATAAGAAGATGACAGTGTTTCAAATGCCTTGAAACATAATGT
GATCATGCTCTAACTTCAATA
>2618
GAAGGCCTGGTCCTTCCTTGGTCTGCGCAGCCCCTAACTTATCTTGAACCTCCACTGCC
ACCCCTCGAAGGACGTGGACGATGCCACTCTGTCCCGCCTGGAACTAGAGCGCAAGAT
TGAGTCTCTGATGGATGAGATTG
>2619
CTGTGCACGAACTGCGTGGCCCGCGTGGAATTTGCGCCGCTGTCCATCCTCTGCCTGC
TCCCGGCCGTAGGAGCTGCGAGACCTGCAGGTGAGTGTGGAGAGCCAGCAGGTGCAG
CAGGTGGAGGTGGAAGCCACGGTGA
>2620
GCGAGGCGGGACGCTGGGGTGGTGTCGCGCGTCCCAGCCGACTAAAGCCTGGGTTAC
CCCCACTTCTCAGTACGCGGACCTGTCCGACGCTGCCAACCGGAACCACGAGGCCCTG
CGCCAGGCCAAGCAGGAGATGAACG
>2621
TGCGCGCTCGGGCCCGGGGAGCGGACGATGAAATGTTCTGCAACTGGCCCCTTCCACT
CTCCTACCCCAGAACGAGGCGCTGCTCAGGCAGTTGAGAGAGCTGGAGGAGCAGTTC
GCCCTGGAGGCGGGGGGCTACCAGG
>2622
TAGCAGTGGGAGCCTAAGAGGAGAGATTCCCACGCCTTCCCCCTTGAACCCTTTATCCT
GCTTTCTTCAGTGCCTGAGGTGGAGCCTCCCCAGGACAGCCACAGCCGGAAGACGGT
TCTGATCAAGACCATTGAGACCCG
>2623
CTATGATCCAAAGGAGTAGGATGGAGGGTGGCGCTGAATGGCTTGTGCCCATCATATGC
CCTGTCCCCAGCAGGTGGTGACAGAGTCCCAGAAGGAGCAGCGCAGTGAGCTGGACA
AGTCTTCTGCCCACAGTTACTGAA
>2624

CATCTCCTATCCGAGTCCCCCAGTTCCCCCTGTCAACCCTGATTCCCCTGATCTAGCACC
CCCTCTGCAGGCGCTGCGCCCCTCATCCTGTCTCGGATTGTGGGAGGCTGGGAGTGCG
AGAAGCATTCCCAACCCTGGCA
>2625
TTGCTCCTCTGTCCCTTCTCTCTTTCCTTATCATCCTCGCTCCTCATTCCTGCGTCTGCTT
CCTCCCCAGCAAAAGCGTGATCTTGCTGGGTCGGCACAGCCTGTTTCATCCTGAAGAC
ACAGGCCAGGTATTTCAGGTC
>2626
GGTCTGAGGGAGGAGGGCCAAGGAACCAGGTGGGGTCCAGCCCACAACAGTGTTTTT
GCCTGGCCCGTAGTCTTGACCCCAAAGAAACTTCAGTGTGTGGACCTCCATGTTATTTC
CAATGACGTGTGTGCGCAAGTTCA
>2627
TGAAAGATAGGATTGCCCAGGCAGAAACTGGGACTGACCTATCTCACTCTCTCCCTGC
TTTTACCCTTAGGGTGATTCTGGGGGCCCACTTGTCTGTAATGGTGTGCTTCAAGGTAT
CACGTCATGGGGCAGTGAACCAT
>2628
ATCGAGTCTCACTAGCTCCAACCAGTTGTGGGTGACAGATCCTACACATCCATGTCTCT
TTTCTCTGCAGGCCCCATGGGTCCACCTGGAGAAATGCCATGTCCTCCTGGAAATGATG
GGCTGCCTGGAGCCCCTGGTAT
>2629
AACACCTCGTGGCAGCAAGTGGGAGTCTTCACTGGCCTGCCCCTCCTTCTGTGTGGGG
CACTCTCCACAGGGCTTCGAGCTCATCTAGATGAGGAGCTCCAAGCCACACTCCACGA
CTTTAGACATCAAATCCTGCAGAC
>2630
GACCCCAGGTGAGGGAGGTGGCTTAGAGACAAAGTGGTCAGTGGCCTGACCCGGACT
CCTCTGCTCTCAGCCCTCAGTCTGCAGGGCTCCATAATGACAGTAGGAGAGAAGGTCT
TCTCCAGCAATGGGCAGTCCATCAC
>2631
CAAACAATTAAATGTTTAAATTAAGATTAAGACACTAGAGAATGATTTATTTGCTGTCCT
TTAATTGCAGCAAATCCTTGCTGTTCCCACCCATGTCAAAACCGAGGTGTATGTATGAG
TGTGGGATTTGACCAGTATAA
>2632
TCATTGTATAGATTTGTGTCTTATAATGAGTCCCATTAATTTCTCCCTCCCTTTCTTCGTCT
TCTTGCAGCGGAATTTTTGACAAGAATAAAATTATTTCTGAAACCCACTCCAAACACAG
TGCACTACATACTTACCCA
>2633
AATAAAAATAATGGTTGATTTACTTAACAAATGAGAATAAATAAAACATTTTTTTCTTTG
AAAATTTCAGCCAGATCACATTTGATTGACAGTCCACCAACTTACAATGCTGACTATGG
CTACAAAAGCTGGGAAGCCTT
>2634
AGATTGACAGTCACCATCTCCTTTCTTGAATACATAGATGGATTCTTACCTTAGCTTTCT
CATTTTTCAGGTAAAAAGCAGCTTCCTGATTCAAATGAGATTGTGGAAAAATTGCTTCT
AAGAAGAAAGTTCATCCCTGA
>2635
TATATGATAGACACTTTATATAAGAAAAACTTCAACAGCAACAAATTAAAATTTTTTCAT
CATTTTCTAGGTGGACTTAAATCATATTTACGGTGAAACTCTGGCTAGACAGCGTAAAC

TGCGCCTTTTCAAGGATGGAA
>2636
ATTTTAAAATCCCTAAAAATGTGTTCCTTAACTTTTTAACTGATGTTTATTTATTTATTTAT
TTTTTTAGATAATTGATGGAGAGATGTATCCTCCCACAGTCAAAGATACTCAGGCAGAG
ATGATCTACCCTCCTCAAG
>2637
GCATTCCAGTTGCTTGAAAGCTTGTGATCAGATGCAATAAATGTTTTTATTATTTATTTTG
TGCAAATAGGAGAGACTATTAAGATTGTGATTGAAGATTATGTGCAACACTTGAGTGGC
TATCACTTCAAACTGAAATT
>2638
AAGGCAAGCTGAATACAAACAGTAAATATGCCTAAAATTCTGGTTTTACAACTAATTTT
ACTTTCCACAGGTTGCTGGTGGTAGGAATGTTCCACCCGCAGTACAGAAAGTATCACA
GGCTTCCATTGACCAGAGCAGGC
>2639
AAGAATCAAACTGTTTTTTATTTTGTGAAGTTTGACATTGGTTGTATCTCTGTCTTCATC
GCCTTCACAGGAGAAAAGGAAATGTCTGCAGAGTTGGAAGCACTCTATGGTGACATCG
ATGCTGTGGAGCTGTATCCTGC
>2640
TAAATATTAAACATGAAATTTTATTGTTTGCATTTTGAATTTTTTTTGTTTTTGTTTTTTTT
TCTTATAGCACGACTGAAAATGTAGCTGTTTATATCTGGGACAACCTCCAGAAAGTTCT
TCCTGTAGGAGTTCTTTAT
>2641
TATCTAGTATACTAGATTTTAAGTTGAAGTAGGATCTTCAGGAGTCTAATCATTATTTCTT
TTCTTTTAGGAGAGAAGACGATCTGCACTTCGCATTTTGGCATTGACATTTAATTTTAGG
GTCCTTTATATAGAAGGGA
>2642
AGTTAACAAAATAAAGGGTCTTACAAAATAGGCAATAATAATAATAATCACTTATTATTA
TTACATGAAGCTACATGAATGTGTAAGATCTTGGAGGAAGACAGCAGAGAGAGAGAG
AGAGATCAGAGATCCCAGGGTTA
>2643
ATAAATATTGTCTGATTGGCCCAGGAAAATGTTTGCCATTTCTCATATGTAGTATTTGACT
GACTTTCAGGACAGGAAGATGTCACCCAAGCGCATAGCTAAAAGAAGGTCCCCCCCA
GCAGATGCCATCCCCAAAAGCA
>2644
CCCAGAGCCTGTGACCTCTCCTCGCTGTCGTCATCCTCTACACTCAGGGTCTATCTTCT
TCACCCTTCAGTCTCACACAGGTCCCACAGCACAGAACCCGGCTTGGTGCTGACACTA
GGCCAGGGCGACGTGGGCCAGCT
>2645
GGGGAGTGGCCTACATGAGAATGGACTGCGAGGCCAGACGTTGCATTAATGAGGGCAT
CCGTGGGCACAGGTCTATTCCTTCGGCTGCAATGATGAGGGTGCCCTGGGAAGGGACA
CATCAGTGGAGGGCTCGGAGATGG
>2646
ACTGACCCTTCTGGATTTCACTGGCACGGGGCAGGCAGGACTGGCTGATAAGTGCCTG
TCCTCCTTCTAGGACAATAACGGTGTGATTGGACTGTTGGAGCCCATGAAGAAGAGCA
TGGTGCCTGTGCAGGTGCAGCTGG
>2647

CTTGTTCTGGGGCTGGCCTAGCCTTGGGCCTTACAGTTGTGGCCTGCATCCCTTACCTT
TTCATCCTTAGGAAACGACCACTTGGTGATGCTGACAGCTGATGGTGACCTCTACACCT
TGGGCTGCGGGGAACAGGGCCA
>2648
TTAAACTCGGGGCAGAGAGAAGCCATGTGTGTCTGTCTGTCACTGACCGTGGCTTTCC
CTTTGCCTGCAGAACGACTCCTGGTCCCCAAGTGTGTGATGCTGAAATCCAGGGGAAG
CCGGGGCCACGTGAGATTCCAGGA
>2649
TGTGGGAGGAGATTGAGAAGGGCAGCTCTCAGAACACCTTCACCCCTGATGGCTCCG
GCCTTTCCCCCAGGAACTCCGGGCACAGAATCTTGCTTCATACCCCAGAACCTAACATC
CTTCAAGAATTCCACCAAGTCCTG
>2650
CTAGCCTGCCACCCATTTTGCCTGTAGCACGCCCTCTTGCTATTGCTCATCTCTCTCCCT
CCTCCCATAGGAAAAGCATACAGCCTGGGCCGGGCTGAGTATGGGCGGCTGGGCCTTG
GAGAGGGTGCTGAGGAGAAGAG
>2651
GGTTTCTCCTGCTACAGAAAGGTGGGCTGGGGATCCTGGAGACAGCTGTACCCATTTC
TCTCTCTTGCAGGTCGTGTTTTCGCCTGGGGCATGGGCACCAACTACCAGCTGGGCAC
AGGGCAGGATGAGGACGCCTGGAG
>2652
TAGCTTTTGGGCATCAGCTGTCATGGAGCACTGGGGATGTTGGCTGATGTGTCTCCTTT
CTTTATCTTAGATCCGAGAGACTGAGGTCATCGACCCCCAGGACCTCCTAGAAGGCCG
ATACTTCTCCGGAGCCCTACCAG
>2653
TGTCCCTGTCCCCTGCCCTGAGTCTCTTCCAAAATACGCTGAGTGTCTCTTCTCCTTTCC
GCCCATCCAGGTGCCTCTAGATAACCATATCCCTGAGAGGGCAGGGTCTGGGAGCCAA
GTCCCCACCGAACCCAAGAAAC
>2654
AAAGAGGGGTTCATCCTTAGGTCCTGATGAGGAGCTCTGACCCCCGCCTCTTCTCTCTC
CTCCTCTCCAGCTCTGATTGTGGGTGGCATCGTGGGCATCCTCTTTGCCGTCTTCCTGAT
CCTACTGCTCATGTACCGTAT
>2655
AAGCTCTTACCTCACCTTCAAGCCTTTTCCTTACCCTGAGAGCCTCCTTTAATTGTCTCT
TCTTTTTCAGGCCAAGAGGCCCAGACAGAGTTGCCCCAGGCCCGGATCAGCTGCCCA
GAAGGCACCAATGCCTATCGCTC
>2656
CTCTTGGAGGACTCAGTATATCCGTCACAACTTCCTCCTCCACTGAGTGCTCCATTTTCT
TCTGCAACAGCTCTATTGCCAGAACATGAATTCGGGCAACCTGGTGTCTGTGCTCACCC
AGGCCGAGGGTGCCTTTGTGG
>2657
GCACACAGGCCCAGTGATTCCATGTATTTTTGAGTGACCACTGCCTCTGTTCTGGCCCT
TCCCCATCTAGAACCGCCGCTGGCACTGGAGCAGTGGGTCCCTGGTCTCCTACAAGTC
CTGGGGCATTGGAGCCCCAAGCA
>2658
CCTATTCACCTTCCAGCCTTTTCCTTGCCCTGAGATTCTTTCTTAGTTATCTCCTTTTTTT
TTTCCCCAGGCCAGGAGTCCCAGACAGAGCTGCCTAATCCCCGAATCAGCTGCCCAGA

AGGCACCAATGCCTATCGCTC
>2659
GAGTCCTAGGTTGAGGAGGGGCTACACTCCTTCTGATCCTCTATGTCTGACACTTCTCA
TTGTACTATAGGATTCAAGAAATGGAAGGATGAATCTTGTGAGAAGAAGTTCTCCTTTG
TTTGCAAGTTCAAAAACTAGAG
>2660
AGAGGATGTTAGAAAGATAACAGGTGCTCTGCTCTCTTCTTCAAACCCTCTTCCCTGTG
TTCTCCTACAGAGATTGCTGATTTGCTCCTTAAGCAAGAGATTCACTGCCGCTAAGCAT
GGCTCAGACCAACTCGTTCTTC
>2661
CTAATTCACCTTCCAGCCTTTTCCTTGCCCTGAGATTCTTTCTTAATTATCTCCTTTTTTT
TTTCCCCAGGCCAGGAGTCCCAGACAGAGCTGCCTAATCCCCGAATCAGCTGCCCAGA
AGGCACCAATGCCTATCGCTC
>2662
CTCTGGAAGGTCTCAGTATATCCCTCACACCTTCCTTCTCCACTGAGTGCTCCATTTTCT
TCTCCAACAGCTCTATTGCCAGAACATGAATTCAGGCAACCTGGTGTCTGTGCTCACCC
AGGCGGAGGGTGCCTTCGTGG
>2663
GCACACAGGCCTGGTTATTCTATTGATTTTTGAGTGACCATGGCCCCTGTTCTGGCCCTT
CTCCATCTAGAACCGCCGCTGGCACTGGAGTAGTGGGTCCCTGGTCTCCTACAAGTCC
TGGGACACTGGATCCCCGAGCA
>2664
GAGTCCTAGGTTGAGGAGGGGCTACACTCCTTCTGATCCTCTATGTCTGACACTTCTCA
TTGTACCATAGGATTCAAGAAATGGAAGGATGAATCTTGTGAGAAGAAGTTCTCCTTTG
TTTGCAAGTTCAAAAACTAGAG
>2665
AAACAAGTATGTACTGAATCAATTTGATTTATAAGTATATGCCAATTATATGATTATTTTCA
TTTGGTAGGCTTGAGTTTGAAGAAACAGAAGAACCTGATTTTACTGCATTATGTCAGAA
ATTAAAGATACCAGATCAT
>2666
ACAGTTTTAACATAGTATCCAGTGTGTGAATTATTTAATGAAATATTTGATCTTTATTTTT
TGTTCCCAGGGAGGTTATATTCAAAAGAAAAAGGAACTGTGGGGAATCTGTATCTTTAT
TGCAGCAGTTGACCTAGATG
>2667
TGATTTGAAAACGAAATAACACAAATTTTTAAGGTTACTGATTTACTTTTTTCTATTCTT
TCCTTTGTAGTGTCCATAAATTCTTTAACTTACTAAAAGAAATTGATACCAGTACCAAAG
TTGATAATGCTATGTCAAGA
>2668
CCCTGCGATTTTCTCTCATACAAAGATCTGAATCTCTAACTTTCTTTAAAAATGTACATT
TTTTTTTCAGGGGAAGTATTACAAATGGAAGATGATCTGGTGATTTCATTTCAGTTAATG
CTATGTGTCCTTGACTATTT
>2669
TAGTTAGAATACTTCATTATTTTATATGATGGATGTACAATTGTTCTTATCTAATTTACCAC
TTTTACAGAAACAGCTGTTATACCCATTAATGGTTCACCTCGAACACCCAGGCGAGGTC
AGAACAGGAGTGCACGGAT
>2670

TGTTCAAGAGTCAAGAGATTAGATTTTGTTTTAAATTTTAATGATCATGTTGTAACTTCA
TCTTTTTCAGGTGAAAAATGTTTATTTCAAAAATTTTATACCTTTTATGAATTCTCTTGGA
CTTGTAACATCTAATGGAC
>2671
CTGTGCCTCTGTGTGCTGAGAGATGTAATGACATGTAAAGGATAATTGTCAGTGACTTT
TTTCTTTCAAGGTTGAAAATCTTTCTAAACGATACGAAGAAATTTATCTTAAAAATAAA
GATCTAGATGCAAGATTATTTT
>2672
GCTTTTTTGATGCATAAAGCACAAATTGTAAATTTTCAGTATGTGAATGACTTCACTTAT
TGTTATTTAGTTTTGAAACACAGAGAACACCACGAAAAAGTAACCTTGATGAAGAGGT
GAATGTAATTCCTCCACACACT
>2673
AAAACATTTCATTTTTTCTTTTTTTCTCCCTTCATTGCTTAACACATTTTCCTATTTTTATC
CCCTCTAGGACTGTTATGAACACTATCCAACAATTAATGATGATTTTAAATTCAGCAAGT
GATCAACCTTCAGAAAAT
>2674
ATGGAGCAGAAAATATTAATTCTGATTACACAGTATCCTCGACATTGATTTCTGTTTTTA
CCTCCTAAAGAACTGCACAGTGAATCCAAAAGAAAGTATACTGAAAAGAGTGAAGGA
TATAGGATACATCTTTAAAGAGA
>2675
TACTTTTAGTAAAAAATTTTTTTCTTTTTATAGAAGTAAGTATTTTATAATCTTTTTTTTTT
TCCTTTAGCAAACTTCTGAATGACAACATTTTTCATATGTCTTTATTGGCGTGCGCTCTT
GAGGTTGTAATGGCCACA
>2676
AACTTCCAAAAAAATACCTAGCTCAAGGGTTAATATTTCATAAATAGTTACTTTTTTTTT
TCATTTTTAGGAAGTACATCTCAGAATCTTGATTCTGGAACAGATTTGTCTTTCCCATGG
ATTCTGAATGTGCTTAATTT
>2677
ATGATTTTGATATGTACCTGGGAAAATTATGCTTACTAATGTGGTTTTAATTTCATCATGT
TTCATATAGGATTCACCTTTATTTGATCTTATTAAACAATCAAAGGACCGAGAAGGACCA
ACTGATCACCTTGAATCTG
>2678
TTGAAGTGTATGTATAATCTGTGATTCTTAGCCAACTTGAAATGAAGACTTTTCCTTTAA
ATATATCTAGGTATCTTTCTCCTGTAAGATCTCCAAAGAAAAAAGGTTCAACTACGCGT
GTAAATTCTACTGCAAATGCA
>2679
TGATTTGAAAAAAATCTACTTGTAATTCAAAATGAACAGTAAAAATGACTAATTTTTCTT
ATTCCCACAGTGTATCGGCTAGCCTATCTCCGGCTAAATACACTTTGTGAACGCCTTCTG
TCTGAGCACCCAGAATTAGA
>2680
AAATGGTATTTTTTAAGAACAAAACCATGTAATAAAATTCTGACTACTTTTACATCAATT
TATTTACTAGATTATGATGTGTTCCATGTATGGCATATGCAAAGTGAAGAATATAGACCTT
AAATTCAAAATCATTGTAA
>2681
TTTTAAAATTCATTTAACAAGTAAATTTTACTTTTTTTTTTTTTTTTTTTTTTTTTACTGTTC
TTCCTCAGACATTCAAACGTGTTTTGATCAAAGAAGAGGAGTATGATTCTATTATAGTAT

TCTATAACTCGGTCTTCA
>2682
TGGAAAAATCTAATGTAATGGGTCCACCAAAACATTAAATAAATAATCTACTTTTTTGTT
TTTGCTCTAGCCCCCTACCTTGTCACCAATACCTCACATTCCTCGAAGCCCTTACAAGTT
TCCTAGTTCACCCTTACGGA
>2683
TAACTTGAGGTTGCTAACTATGAAACACTGGCATTTAATGATTTAAAGTAAAGAATTCT
GTAATTTGTAGACTTCTGAGAAGTTCCAGAAAATAAATCAGATGGTATGTAACAGCGAC
CGTGTGCTCAAAAGAAGTGCTG
>2684
TATATGGCAGCCACTTGCCAACTTACCCAGTACCATCAATGCTGTTAACAGTTCTTCATC
CTTTTTCCAGCTTCTACTCGAACACGAATGCAAAAGCAGAAAATGAATGATAGCATGG
ATACCTCAAACAAGGAAGAGAA
>2685
TGTCTGTCGAGAATGTTGCACAGGGAGTTACAGAATGTTCTCATGACTGAATTGCTTTT
AAATTTCACAGTGTGCCTGCATTTGAAGTCTTGGAAATATCTCCCCAGGAAGTATCTTC
AGGCAGACTATTGAAGTCGGCC
>2686
ATTATTAAAAAAATGATACAATCTCGGGAAAATTTTTTTGATTTTCATGAAATTCATGTGTT
TTTCTATAGGTAACACAAATTCTGACTGCTATGATATGCCTTTGTTTTGGAACAGTTGTC
TGCTCTGTACTTGATATTT
>2687
AATGAAGATAGGTTTATTGAATGTGCCAGCAAGTGCAGGCCCAGGTCTGAGTGTTCTT
CATTATTATCAGGTGAGAGGAAGCCTGGGAGCAAACACTGCCAGCAGCATAGCTGGGG
GAACGGGAATTACCATCCTGATCA
>2688
TAAGAAAATAAACTTTTGGGGCGAATACCAATGTGACTCTTTTTGTTTGTCATTTGTTGC
TGTTCAATAGGAAATTGTAGTGATGATGCTGTTTCTCACCATTCTGGGACTTGGTAGTG
CTGTGTCACTCACAATCTGTG
>2689
TCTCTTGAGCGAGACTTCTAGGGATGGGAAATTTGTGGTGATTGATATGAAATGATTTT
TCCCTTATCAGGTTCCAGAGGATCGTGTTTATGAAGAATTAAACATATATTCAGCTACTT
ACAGTGAGTTGGAAGACCCAG
>2690
GTGCCCTTCAGTCCCTCCCCCAGGCCTCTATCTCCAGACATCCTTAACCCCTCTGTCCC
TCCCTTTGCAGCCGGATCCAGAGCAATGTAGAAGGCCTATACCTGACTGATGGGGTCCC
TTTCTCCTGTTGCAACCCCCAC
>2691
CCTGCCTCCAACCCTGGGCCTCTTGGAACCGCTGACTCTCCCTGACTCTTTCCCCTTGC
TTCCCCCACAGGCTCTGGTGCTCCTTGGCCTGCGGTACCTGCAAACAGCACTGGAGGG
GCTTGGAGGGGTCATTGATGCGG
>2692
GGGCTCGGCGCCGCACTCTTCCCAGAAGGTTGCGCTGGCTCGTGAGTGATCCTGTCCT
CTCCACCCGCAGGGCCGCGTTCGCACCAAAACCGTGAAGAAGGCGGCCCGGGTCATC
ATAGAAAAGTACTACACGCGCCTGG
>2693

CATGTGCTCAGTACCAGGTGGGAGTTGATTTGTCTTCTCACTGTTCTCTTTGGCTGTGT
GTGCTTTGTAGTTATGTCACGCATCTGATGAAGCGAATTCAGAGAGGCCCAGTAAGAG
GTATCTCCATCAAGCTGCAGGAG
>2694
GTGTTTTCTTCTGGGGTCCACTCACAGATCTCACCAGTAACTGCTCACTGAATCCATTT
TCTCGTTCCAGGACTTCGGCAGTCTGTCCAACCTTCAGGTCACTCAGCCTACAGTTGG
GATGAATTTCAAAACGCCTCGGG
>2695
CTTTGTAGATGGCTCTACAATTACCTGTATAGATAGTTTCGTAAACTATTTCCCCCCTTTT
AATCCTTAGCTGAACATCTCCTTCCCAGCCACTGGCTGCCAGAAACTCATTGAAGTGG
ACGATGAACGCAAACTTCGTA
>2696
GTCACAATCCTGTACTGTATAATTTTGGAATAGAAATAGTAAATGTGTCATTCATTGTATG
CTCTTGCAGGGTTATGTGGTCCGAATCAGTGGTGGGAACGACAAACAAGGTTTCCCCA
TGAAGCAGGGTGTCTTGACCC
>2697
ATTAAACATTCTGATTTAGGATTCTTAAGGAAGTTGTTGAATTAAAATCTCTTCAACTGT
TTCTTCTAAGGAGAGAAGGATATTCCTGGACTGACTGATACTACAGTGCCTCGCCGCCT
GGGCCCCAAAAGAGCTAGCAG
>2698
GATGTTTTTAAAGTAGATGTGGATTCTAACAAAACAAATATTGAAATGATTGAGTCCTTT
GAATTTTTAGGTAAGAAACCTAGGACCAAAGCACCCAAGATTCAGCGTCTTGTTACTC
CACGTGTCCTGCAGCACAAACG
>2699
TCGACCTGGCTTTGGATTTGCGTCTTTAACCCAGAAAGGCCTTAACTGTTTGCTGTTTT
GTTTTGTTTAGGAGGCTAAGGAGAAGCGCCAGGAACAAATTGCGAAGAGACGCAGAC
TTTCCTCTCTGCGAGCTTCTACTT
>2700
TGACCCAGAGGGATTACGGGATTCAGGGGGTCAAGTGGGCCTCCACTCTGATGTCTCT
TGCCACTCACAGACCAAGTGCTTCATCTGTGGAATCGGCAGTGACTACTTTGATACGA
CACCGCATGGCTTCGAGACTCACA
>2701
AATATATATATATATATGTCTCAAGGGTTTGAAGATGTGACCAATGAACTCTTTCTATCCC
CAATCCTAGGAGTCTTATGTCTGGAAGATGTACCAAGAGAGATGTTGGGATTTCTTCCC
AGCTGGTGATTGTTTCCGTA
>2702
TCCCCTCTAAGGTGTTGTTGGAGTCTTTATGTTCTCCTGATGTCCCTTCTGCCTTTCCTT
TCCTTTCCAGGGGCTCGGGACATGTGGAGAGCCTACTCTGACATGAGAGAAGCCAATT
ACATCGGCTCAGACAAATACTT
>2703
AAGAATGGGAGGGTGAGCTGTTGCTCACTGGCCTGATGATTAATCTCCTTCTTGCCTGC
CTGGACTACAGCAATGCCAGAGAGAATATCCAGAGACTCACAGGCCGTGGTGCGGAG
GACTCGCTGGCCGATCAGGCTGCC
>2704
GTCCCCGCGTAAGCCAGCCTGGTGCCACCCGGAGCGGCGTTAACGGGTGCGTGGTGT
CTCGGCTCTGCAGGGCGTCATGGTCGGTATGGGTCAGAAAGATTCCTACGTGGGCGAC

GAGGCTCAGAGCAAGAGAGGTATCC
>2705
CGGCCGCTAGCGCTGAGCGCCTAGCCTCGGCCTCGCCCCCAGCCACTCACTCTCTCCC
GCGCGCGCACAGGCATCGTGCTGGACTCCGGCGACGGCGTCACCCACAACGTGCCCA
TTTATGAGGGCTACGCGCTGCCGCA
>2706
GCCCCTGCACCCGGGCGGAGGGCCGCGGCGGCCTGAGTGAGGGCTCCTCTCCTGCTT
CTGCCCTCCGCAGCTGAGCGCGAGATCGTGCGCGACATCAAGGAGAAGCTGTGCTAC
GTGGCCCTGGACTTCGAGAACGAGAT
>2707
CTCGCCCCGGCCCCCAGGCCCGCGCCCCCCGGCCCGAGCTTCTGCTCACGCTCCCCGC
CGCGGTCCCCAGGTATGGAGTCGGCGGGCATTCACGAGACCACCTACAACAGCATCAT
GAAGTGTGACATCGACATCAGGAA
>2708
TGGCCTGCGCGGGCTGTCGGCGGGGTGGGCTCCAGGGTGAGGTCTCCCCACCTCACG
CGCTGTCTTGCAGATCATCGCCCCGCCGGAGCGCAAATACTCGGTGTGGATCGGCGGC
TCCATCCTGGCCTCGCTGTCCACCT
>2709
GGTTGAAGCTGAGATATCTTTTCCCTGCATTTATACTGAAGGTCATTATCTTTCTTTCTTT
ATCCCGCAGACCTCAGTGGGAAGGTGTTTGTATTTCCTAGAGAATCTGTTACTGATCAT
GTAAACTTGATCACACCGCT
>2710
AGTGGGGGGTAAGAGTTGTAAGGGAGCTTTGGAGATAATGAATGCATACCTTCTTATTA
TCAATTACCAGGTGGATCAAAAGGCCGATTACCAAGTGAATTTTCCCAATTTCCACACG
GACAAAAGGGCCAGCACTATTC
>2711
GGCCCAGCTACCATCCCCACCCTTCACTTTTTACTATACTCACATCACTGATTCTCTTCT
TTCTCTCTAGGTGTCAGTTGACCTCAGTGAATTCTGTGATGTTTCTGAGATGCAGACTC
CCGTGTAGTTTCAGATTCTTG
>2712
GGTGGGAGGTAAGAGTTGCAAGAGAGCTTTGGAGATAATGAATGCATACATTTCTATTA
TCAATTACCAGGTGGATCAAAAGGCCAATTGCCAAGCGGATCTTCCCAATTTCCACATG
GACAAAAGGGCCAGCACTATTT
>2713
TCCCAGCTCTCCCATCCTCACCCCCACTCTCCACTATCCTCACATATCTGGTTGTCTTTT
TTCTCCCTAGGTGTCACCCGACCTCAGTGAAGTCTTTGATGTTTCTGAGAGGCAGACTC
CCATGTGGTCCCAGATCCTTG
>2714
CCTGACAGAGCACAGTGGGGGAAGATGTTGGCAGAGGTGGCAGATGGGCTCACGGCC
ATCCCTCCAGCAGGAGCAGCGACTGGACCCAGAGCCATGTGGCTGTGCCCTCTGGCCC
TCAACCTCATCTTGATGGCAGCCTC
>2715
ATCGAGTCTCATTAGCTCCAACCAGTTGTGGGTGACAGATCCTACACATCCATGTCTCT
TTTCTCTGCAGGCCCCATGGGTCCGCCTGGAGAAACACCATGTCCTCCTGGGAATAATG
GGCTGCCTGGAGCCCCTGGTGT
>2716

ACGCCTGCGTGGCAGCAAGTGGGAGTCTTCACTGGCCTGCCCCTCCTTCTGTGTGGGG
CACTCTCCACAGGGCTTCCAGCTCATCTAGATGAGGAGCTCCAAGCCACACTCCACGA
CTTCAGACATCAAATCCTGCAGAC
>2717
GACCCTAGGTGAGGGAGGTGGCTTAGAGACAAAGTGGTCAGTGGCCTGACCTGGACT
CCTCTGCTCTCAGCCCTCAGTCTGCAGGGCTCCATAATGACAGTAGGAGAGAAGGTCT
TCTCCAGCAATGGGCAGTCCATCAC
>2718
CTGGCACGGGTGAAAGCAGAGCTGCTCCCTGACCCTCTGCCCCTCCCTCCTCCACCCT
GGCCTGCTTTAGCTTTCCCCAGACATGGCCAACAAGGGTCCTTCCTATGGCATGAGCCG
CGAAGTGCAGTCCAAAATCGAGA
>2719
CCACCCTGTGAGTCCTGGGCCAATCCCTGAGCTGACTGCTAAGCTGCGTCCTATGCCCT
ATGCCTGGTAGATTCTGAGCAAGCTGGTGAACAGCCTGTACCCTGATGGCTCCAAGCC
GGTGAAGGTGCCCGAGAACCCAC
>2720
GGCAGGCCCTGGCTTGGAGTCTTGTTTATACGTTCTTGATGTTCATCTCCTCTCTCCTGT
CTTCTCACAGGCAAAGACATGGCAGCAGTGCAGAGGACCCTGATGGCTTTGGGCAGC
TTGGCAGTGACCAAGAATGATGG
>2721
AGAGGGATCAGAAAACGGGTGGAAAACACACAGTTCTACCAAGTCTTTATCCTGGTTC
CTCCTCTTCTAGGAAAGCGCAGGAGCATAAGAGGGAATTCACAGAGAGCCAGCTGCA
GGAGGGAAAGCATGTCATTGGCCTT
>2722
GGTGGCCAGGGGTTGGCCTGGTTCCTCTGCTCTGCCTCTGATTTCTCACCATGCGCTCC
TCCCACTGCAGAAGGAACCCAATGTGGCTCGCGTGGGCTCCGTGGCCATCAAGCTGTG
CAATCTGCTGAAGATAGCACCAC
>2723
ACCCAAATGCCCAGCACAGGAGGACCAGGATTGGAACAAGTGTTGACCTCTCATGTTT
ACTTTGTTTCAGAATTGGGGGGTTCTATGCTCTTTCCCCATACCCCGGTCTCCGCCTCAT
CTCTCTCAATATGAATTTTTGT
>2724
CCCCTTTCTCTAGCCAGGGCTGCCTGGACCCCTGGATGCCCTGATTACCATCCTTAATTC
TCCCTACTAGGTGCATATAATTGGCCACATTCCCCCAGGGCACTGTCTGAAGAGCTGGA
GCTGGAATTATTACCGAATTG
>2725
GGCATCCTATCTCCCCAGATGTCTTCCTACCCCTCCCTAGAATCTTCTGAATGTAGTACC
TTCTGGCCAGGTATGAGAACACCCTGGCTGCTCAGTTCTTTGGCCACACTCATGTGGAT
GAATTTGAGGTCTTCTATGAT
>2726
CAGGATGTGTGGCCCCTCCCTGGAGTTACCCTTGCTCCTTGCCCCTCCAGTCAGCCCCA
CATCCTTGCAGGTTACCGTGTGTACCAAATAGATGGAAACTACTCCGGGAGCTCTCACG
TGGTCCTGGACCATGAGACCTA
>2727
AGGTCGCTGGCTATTGGTGTGGTGTGTGGTGAGGCTGCTAGTGAGTCACCTGTGACCA
CTCTTGTTTCAGGATGATAAGGATGCCTTCTATGTGGCAGACCTGGGAGACATTCTAAA

GAAACATCTGAGGTGGTTAAAAG
>2728
CCCATGCTTTTCTGTTTATTCCACGTGTTAAGCCACATATGGATAGCATGACACCACTCT
TCTTTTTCAGACTGAAATACAGTTGGTGCAGAGTCTGGGGGTGCCTCCAGAGAGGATT
ATCTATGCAAATCCTTGTAAAC
>2729
TTTTTATTTTAATTGTGGCTATAAGTGAACATCTATCATGAGACATTTGCTGCACTTTCCT
TGCTTGTAGGTTGGTTTTGCGGATTGCCACTGATGATTCCAAAGCAGTCTGTCGTCTCA
GTGTGAAATTCGGTGCCACG
>2730
TTCTCCTAGTGTTTGCTATTATTTTAAGAAATACTAAGACACAGTGTCTCGTCTCTTTATT
TTACCCCAGCTTCCATGTAGGAAGCGGCTGTACCGATCCTGAGACCTTCGTGCAGGCA
ATCTCTGATGCCCGCTGTGTT
>2731
TTCAGCCTATCTGCTGCATACATTTTTGTTATTAGCTGTGGATCTGGCTGACTTATTTTCT
TGATTCTAGGCTGAGGTTGGTTTCAGCATGTATCTGCTTGATATTGGCGGTGGCTTTCCT
GGATCTGAGGATGTGAAAC
>2732
GTGATGTTACTCCCCCATGTGAATTTCCCACAATGAAGGCTGCTCTTTCTTTTCTGTTTC
ACTCTCTTAGATCACCGGCGTAATCAACCCAGCGTTGGACAAATACTTTCCGTCAGACT
CTGGAGTGAGAATCATAGCTG
>2733
GACGAATCACTTCATGTATAACTGAAAGCTGATGCAAAAAGTCATTAAGATTGTTGATC
TGCCTTTCTAGACGAAGATGAGTCGAGTGAGCAGACCTTTATGTATTATGTGAATGATG
GCGTCTATGGATCATTTAATTG
>2734
AAACAATTAAGAGGACTGGTCACAACACGTGTAATTAAGTAGTACTTCCTCTCTCCGTC
TCTTTATATAGAGACCTAAACCAGATGAGAAGTATTATTCATCCAGCATATGGGGACCAA
CATGTGATGGCCTCGATCGGA
>2735
GGAAGTAAATACTCTCATGCCCAGTTAGGAGTGAGTCAGGGTTTTTAATATGCCACTTT
TTCTTTCTCAGGCAACTCATGCAGCAATTCCAGAACCCCGACTTCCCACCCGAAGTAG
AGGAACAGGATGCCAGCACCCTG
>2736
GTGGGTGAAATCATGATGCCAGGTGTGTAGCCTGGATCCTGATTAAGGTTGCTCTGGCC
CCAAACCACAGCTGCCTGGACCACCTCATCCTTGGCCTGTGCCCAGGGCCCTGAGTTC
TGGTGCCAAAGCCTGGAGCAAGC
>2737
AACCTCCCCATTGGAGCTGGGAGCCTCCCCCACTCATGTGTCCTCCACCTTGGGGCCC
CTCCCTCCCCAGGATGACCTATGCCAAGAGTGTGAGGACATCGTCCACATCCTTAACA
AGATGGCCAAGGAGGCCATTTTCC
>2738
CTTGTGTCCAGGGACTTGGGCGGGGTAGACCTCAGAGAGGCCCAGCTGACGGCCCCC
TCTGGCCTCCCAGGACACGATGAGGAAGTTCCTGGAGCAGGAGTGCAACGTCCTCCC
CTTGAAGCTGCTCATGCCCCAGTGCA
>2739

TTGGGCCGGCCTGAATAGGGGTGGGCTTCCCTCCCAGACCCTAACACTCCCACCCTGT
GCTGTGCCCCAGGACTCAAACGGCATCTGTATGCACCTGGGCCTGTGCAAATCCCGGC
AGCCAGAGCCAGAGCAGGAGCCAG
>2740
AGAGGGGAGGAGAGCCTGGAGGACTCTTCTCCCAGCACCCAGCCTGGCCTCCACCTG
ATTCTTTCCCCAGGATCTCTCCGAGCAGCAATTCCCCATTCCTCTCCCCTATTGCTGGCT
CTGCAGGGCTCTGATCAAGCGGA
>2741
GGTGCTGGGCAGAGAGTGGAGGCTTGCCAAGTGAAGGTCCCATGCTGGCCTCTCTCC
GCCCCCGCCCCAGGGTGCGCTACGTGTGGCAGTGGCCCAGGTGTGCCGCGTGGTACCT
CTGGTGGCGGGCGGCATCTGCCAGT
>2742
AGGAGGACAGAAACCAAACACCTCTGTAGGGTGGCCTTGCCTGCTTTCCCCCTAATGC
TCACATCTCCAGGGTCGCCGACAGGAGAATGGCTGCCGCGAGACTCTGAGTGCCACCT
CTGCATGTCCGTGACCACCCAGGC
>2743
TGGAGAGGCCCAGGACCACACGACAGGAGAGTCCTGCAAAGGGACCTCCACAGCTG
TGTGTCTCCCTCAGTGCAAGCAATTTGTGGAGCAGCACACGCCCCAGCTGCTGACCCT
GGTGCCCAGGGGCTGGGATGCCCACA
>2744
TTCCCGGAAGAGCTGGGTGTGGGGAGCTGAGGGGGCCCAGGCCTCAGCCCTGGTGCT
GTCCCTGTGACAGGCCCTCGGGGTGTGTGGGACCATGTCCAGCCCTCTCCAGTGTATC
CACAGCCCCGACCTTTGATGAGAAC
>2745
GCTATCACCTGGCTGTGGGTCCCCAGGCAGGACATACACACAGTCCCTCTCTGGCCCT
CATCCTCCTCAGCTGCAAAGGAAAAGCCAAGTGAGACGGGCTCTGGGACCATGGTGA
CCAGGCTCTTCCCCTGCTCCCTGGC
>2746
CCAGGGCGGCTTGGGGTACGTTTTCCCCACCCCAGAGAACCCAGGTCCCCGACTGTCA
CTCCGCCCGCAGTAAGACCTATGGCAACGTGCTGGTGTTGGACGGTGTCATCCAGTGC
ACGGAGAGAGACGAGTTCTCCTAC
>2747
GGTCTGCTTCAGGGGCCTGGGGGCTCCCAGTCCTGCCAGGGCCCCTGCCTTGACTGCC
CCCTCCTCCCAGGTGCTGATCATCGGGGGCGGAGATGGAGGTGTCCTGCGGGAGGTGG
TGAAGCACCCCTCCGTGGAGTCCG
>2748
CCCACGTGGCTGTGCTGGCTCCAGGTCCCCCAGCCCCTTGGCCCAGACTGGTCCCTCC
CATCCCCTCCAGGATGTCATCCAAGTCTCCAAGAAGTTCCTGCCAGGCATGGCCATTGG
CTACTCTAGCTCGAAGCTGACCC
>2749
CTGTGTGGGTGACAGTGGCCCTGGAGGTCACAGCCAGACTGTTCAGTGTGTCTCCCTC
TGTCTTCCCCAGGCCCCGCCGAAAGTCTCTTCAAGGAGTCCTATTACCAGCTCATGAA
GACAGCCCTCAAGGAAGATGGTGT
>2750
TGGAGCACTGGGGCGGGGCGGGGTGGGGCAGGGCAGGCCCTGCCGGATGCTGATGCT
TAGGGGCCCCCAGGCGAGTGCCAGTGGCTGCACCTGGACCTCATCAAGGAGATGCGG

CAGTTCTGCCAGTCCCTGTTCCCCGT
>2751
ACAGTGAGACTCCGTCTCAAAAAAAAAAAAAAAAAGTGCCCCCCCTGATGTGCCCCTG
GCCCGGTCCCCAGAGCACGAACTTCCAGGAGCCGGTGCAGCCGCTGACACAGCAGCA
GGTGGCGCAGATGCAGCTGAAGTACT
>2752
GGCCTGCGGGGCTGGGTGGTGGGACCCAGGGACCCAGAGCGCCCTCCTGACTGGCCT
CATGTCCCTCCAGGCACTGAATGATGTGAGCTGAGCCCAGGCGCCACCACTGATGCCA
CCCAGGACCTCGGACCTTGGAGCCT
>2753
TGTCGTCCTAAGCCTTCTCTCCTGACTGCAGCCATCCCCAGCTACTTTATCTTCCAGTCT
TTCCTTTCAGAGGAGATCATCGGGGGCCATGAGGCCAAGCCCCACTCCCGCCCCTACA
TGGCCTTTGTTCAGTTTCTGCA
>2754
CTGCTCTTCACTGCTCCTGGGCTCTATCCCCTGTGACTCCACCCCCATCCTCACTCTGCT
CTCTGTGCAGCTCCATAAATGTCACCTTGGGGGCCCACAATATCAAGGAACAGGAGCG
GACCCAGCAGTTTATCCCTGTG
>2755
AGCCCCATCACTGTGTCGGGGCCCAGAAGGCCATTGCCTGACCTGGACTTTCTTGCTT
CTTCCCCACCAGCTGGAGAGAAAGGCCAAGTGGACCACAGCTGTGCGGCCTCTCAGG
CTACCTAGCAGCAAGGCCCAGGTGA
>2756
CACAAAATCCATAACAGAGACCACCCATCCCAGGGAACACTAGCTCAGTCTTTCCTCT
CTCTGTTCACAGGGGGACTCCGGGGGGCCCCTCGTGTGTAAGGACGTAGCCCAAGGTA
TTCTCTCCTATGGAAACAAAAAAG
>2757
TTACTGCAACTATTCAGTGAATCTTGTGTGGGCTTTTACTCTGTCTTTCTCTTCTCTCTTT
GTCCTCCAGGAAATCCACAAGGACCATCCCCACAAGGAGGCAACAAGCCCCAAGGTC
CCCCACCTCCTCCAGGAAAGCC
>2758
ATTAGAAGTGGTATTCACCATGGCTTCTTCCCTATTATTCTCTGCTTAAATCATTCTTTTC
TTTTTCCAGGACCAGCCACTGTTGCAGCATGAGTTCCCAGCAGCAGAAGCAGCCCTGC
ATCCCACCCCCTCAGCTTCAG
>2759
AATCTGTTTGGCATTGAATTTGTATCCATCTTTCTTTAATTGAATCACTGTGTCAGCTTTC
TGTCTCTAGAAAAAAACACATTTGAAGCATGAATTCTCAGCAGCAGAAGCAGCCTTGC
ACCCCACCCCCTCAGCCTCAG
>2760
CGCGACCTCCCCGCTGCCCTCCCCGCGCGCCTTCCCCGGCCCGCGGCCTGACCCGCCC
GTGTCCGGCCAGGATCAACTCGTAGGCTTTACGAGAAGAAGATCTTCGAGTACGAGAC
CCAGAGGCGGCGGCTCTCGCCCCC
>2761
GAGAGCGGCACTGGAGAAAGGGGAGGGAAGTCTGGGGGGCAAACAGTTCTGTCTCC
TCCTTTCAATCCAGACTTGAATTCGACTAGAGGGGATGCAGATATGTATGATCTTCCCA
AGAAAGAGGACGCTTTACTCTACCA
>2762

TTCAGATTAGGCCATCAGGCCAGGCGGCACACCGATGCCCCCTCTCTGACCGCTGCC
CCCCTTCCCAAGGCTACAATGACGACTACTATGAAGAGAGCTACTTCACCACCAGGAC
TTATGGGGAGCCCGAGTCTGCCGG
>2763
GGGGGAGCCCAGGGACGGGCTGGTTCTGGGTCCAGGCTCCTGGCCCACTTGCTCCCC
TCTTTTGCCTCAGGGAACGCCCCATGTACGGCCGGGACAGTGCCTACCAGAGCATCAC
GCACTACCGCCCTGTTTCAGCCTCC
>2764
TCTGTTCACCCTGCCAGGCAGTCTCTTCCTAGAAACTTGGTTAAATGTTCACTCTTCTT
GCTACTTTCAGGATAGATTCCTCACCCTTGGCCCGCCTTTGCCCCACCCTACTCTGCCC
AGAAGTGCAAGAGCCTAAGCCG
>2765
TGTCTCTGGCTCAGGGAGTGTTTCATCCAACTAAGCTCCCTCAGTGGTTGTCTCACTTC
ATCTCTTATAGATTAACTTCCTTCCAAAATGTCACCATTCCTGTATCTGGTTCTCTTGGTA
CTTGGGCTTCATGCTACAAT
>2766
TAATGGAAAAGGTCTTGTTGGTACTGATACCGGTTTAAAATAAACACCTCTCTTTTCTTT
GTATTTTAAGCCCAGTGGGCAAATCCTTTTGATCCATCCAAGACAGAAGACAGTTCCA
GCTTCTTAATAGACAAGACCAC
>2767
AGAGAGAGAAGGAGAGAATCATAAGCTTGATATGGTGATTGCCATGTGTTCCCTTCCTC
TTTTCCCACAGATGGGTTGACTTGTTTGTTCCAAAGTTTTCCATTTCTGCCACATATGAC
CTTGGAGCCACACTTTTGAAG
>2768
TACTTCTAGACCTTCCCAAAGACTGTTCCTGTTTCTTCAGAGACCCGCAGGATTTGTTC
TCCCCACACAGGCTGCCCATAAGGCTGTGCTGCACATTGGTGAAAAGGGAACTGAAG
CTGCAGCTGTCCCTGAAGTTGAAC
>2769
GTGTGTGTGTGTGTGTGTGTGTGATGAGTACAACTGTTTTCCTCATCCTGTTCTCAACTTG
TGTCTCCGCAGATCACACAGGTGCTGGAGTCTCCCAGACCCCCAGTAACAAGGTCACA
GAGAAGGGAAAAGATGTAGAGCT
>2770
TGTTTCTTTTGATCACTCTCAATTTTATGTGGCCTAAAATACAGACTCCATGAATTGATTT
GTCGTTAAGGGCTGGGCCATCAGGAATTTGCTCGTCCATCTCGGGGATACCTGGCCTTC
AGAGATGACAGCATTTGGCC
>2771
TTCGCTTACAGGAATTGCCCTTGCACTTTTCCATCCCTACACCTCAGTCATTCTGTTCTT
ACCTTTCAAGGTAAGGAGCTAAACAGAACCTGCTGCCTGAATGGGGGAACCTGCATGC
TGGGGTCCTTTTGTGCCTGCCC
>2772
TGGAGAGGAGAGAGAAAGGGAAGTGGAAATTTCAGACCCAAGCTATCGCAGCTTACC
TGTTCATTCTCAGGAACTGTGGGTCTGTGCCCCATGACACCTGGCTGCCCAAGAAGTG
TTCCCTGTGTAAATGCTGGCACGGT
>2773
CACATTTGTGGGCAGCAGGATGAACTGCCAGAGAGGTTTGCTTTAATGACCAAGCATC
CCTACCTTCCAGATGGCCTTGTGATGGATGAGCACCTCGTGGCTTCCAGGACTCCAGA

ACTACCACCGTCTGCACGTACTAC
>2774
GTTTTATACATCAATACATTCGAGTGCTCTGAAGTGCATGTAATAATAGCTGTTTCTCTG
TTGTTTAAAGGCACTACAAATACTGTGGCAGCATATAATTTAACTTGGAAATCAACTAAT
TTCAAGACAATTTTGGAGTG
>2775
AAGCAGTCTATCTTGTTGGGTGCAAATTGTAACATTCCATTTATGTTTCTTCCTTCTTTTC
TTTCTTTAGCACTAAGTCAGGAGATTGGAAAAGCAAATGCTTTTACACAACAGACACA
GAGTGTGACCTCACCGACGAG
>2776
GATAACAAAACAGGATTTGACTTTGTACAGAATTCTTTGGTTCCAACCAAGCTCATTTC
CTTTGTTTCAGCAAACCTCGGACAGCCAACAATTCAGAGTTTTGAACAGGTGGGAACA
AAAGTGAATGTGACCGTAGAAGA
>2777
ACAGTGACTTAATAAAGGGGAAAGAATTGACTCAGAGCCCAGATGAATTAAGAACTCT
ATCTTTTTACAGAAAACAGCCAAAACAAACACTAATGAGTTTTTGATTGATGTGGATAA
AGGAGAAAACTACTGTTTCAGTG
>2778
CCGTCAATTTTGTCCACTGATAAATGGGATTTGAGCTCTCCAAGTTGACTAGATGCCCT
TTATTTTTCAGAAATATTCTACATCATTGGAGCTGTGGTATTTGTGGTCATCATCCTTGTC
ATCATCCTGGCTATATCTCT
>2779
ATGCCCCCAGATGGCCAAGACTGCCTGTTCCTGAGGTCGCTGTTCCATGACCCCCCCA
CCGCCTTTACAGTGTTCCTGGCTCCTCAGCAAGCACGGTCGCTGCTCCAGCGGGTCCG
GCGAGCCAACACCTTCTTGGAGGA
>2780
AGACAACTTTGCAGGGAGAGAGGAAATAAGTCCCCAGGCTCCAAGGCTGACCGGGGT
GGGGTCTCCGCAGGTAACTGTGCTGAGGGTCTGGGTACGAACTACCGAGGGCATGTGA
ACATCACCCGGTCAGGCATTGAGTG
>2781
ATGGGCTGAGAACAGGGAGCAAGCGTACCTCAAGTTCAACAGCCTCCTGTTGGGCAA
TTTCCTCTTCCAGAATCAACTCCACTACCCATCCTGGGGCCGACCTACAGGAGAATTTC
TGCCGCAACCCCGACAGCAGCACC
>2782
ACACCCCTGTCTGTTCCGGTCCATGTGTGGTCTCACTCACTCTGCTGCCTCCTTGCCCC
TCACCCACCAGGCCAGGATCAAGTCACTGTAGCGATGACTCCACGCTCCGAAGGCTCC
AGTGTGAATCTGTCACCTCCATT
>2783
ACTGGGGGGATCTAGGGGATGGGTGAGGAATGGCCCAGCCCAGTCCCGGCCGGTGCC
TGGGTCCCAACAGAGGAGGCCGTGGAGGAGGAGACAGGAGATGGGCTGGATGAGGA
CTCAGACAGGGCCATCGAAGGGCGTAC
>2784
ATCCGAGGGGATGCGGGGCTGCGGGGCTGGTGGCCAGGACTTGCCCCTCACTGCTTG
GCTTGCTCTGCAGACTGTGGGCTGCGACCTCTGTTCGAGAAGAAGTCGCTGGAGGAC
AAAACCGAAAGAGAGCTCCTGGAATC
>2785

TACTTCTAGGGCTGGTGTAGAGGCAGCCCCCTCATCCTCAGCTCCTAATGCTTCCTGCT
GCCCCTCCCAGGCAGGTGATGCTTTTCCGGAAGAGTCCCCAGGAGCTGCTGTGTGGGG
CCAGCCTCATCAGTGACCGCTGG
>2786
GGCAGGCAGTTTCCTGCTCCTTGCTGGGTGAACCTGCAGCTTCTCCATTTCTTTCTTGG
GGTCTCTGCAGGTACGAGCGAAACATTGAAAAGATATCCATGTTGGAAAAGATCTACA
TCCACCCCAGGTACAACTGGCGG
>2787
GAAATGGCGTTGGGGCCAGGCGGCTCCTGTGGGGGTTGGCTCTCACTAGGCCCTTCTT
CCTTCCCCAAAGCTTGCTCCAGGCTGGATACAAGGGGCGGGTGACAGGCTGGGGCAA
CCTGAAGGAGACGTGGACAGCCAAC
>2788
GTGTCTCGTGAAGGGGCGTGGCTGGGCTATGAGCTATGCTCCTGAGCACAGACGGCTG
TTCTCTTTCAAGGTTACAAGCCTGATGAAGGGAAACGAGGGGATGCCTGTGAAGGTGA
CAGTGGGGGACCCTTTGTCATGAA
>2789
TTGCCTGGCAGAGGAATACTGATGTGACCTTGAACTTGACTCTATTGGAAACCTCATCT
TTCTTCTTCAGAGCCCCTTTAACAACCGCTGGTATCAAATGGGCATCGTCTCATGGGGT
GAAGGCTGTGACCGGGATGGGA
>2790
TCTGTTCACCCTGCCAGGCAGTCTCTTCCTAGAAACTTGGTTAAATGTTCACTCTTCTT
GCTACTTTCAGGATAGATTCTTCACCCTTGGTCCGCCTTTGCCCCACCCTACTCTGCCCA
GAAGTGCAAGAGCCTAAGCCG
>2791
GGGAATTCCTGGAATACCAGCTGACAATGATTTCCTCCTCATCTTTCAACCTCACCTCT
CCTCATCTAAGAATTGCTCCTCGTGGTCATGCTTCTCCTAACTGCAAGGCTAACGCTGT
CCAGCCCGGCTCCTCCTGCTTG
>2792
CCTGCTTATTAGGCTACCATAGCTCTCTCTATTTCAGCTCCCTTCTCCCCCCACCAATCTT
TTTCAACAGAGCCAGTGCCCAGAGGTTCACCCTTTGCCTACACCTGTCCTGCTGCCTG
CTGTGGACTTTAGCTTGGGAG
>2793
CTAAGGCTCCCCACCCGCTTTTAGTGTGCCCTTTGAGGCAGTGCGCTTCTCTCTTCCAT
CTCTTTCTCAGGAGGAGACCAAGGCACAGGACATTCTGGGAGCAGTGACCCTTCTGCT
GGAGGGAGTGATGGCAGCACGGG
>2794
TCCTACTTAGACAAGGGAGGCCTGAGATCTGGCCCTGGTGTTTGGCCTCAGGACCATC
CTCTGCCCTCAGCTTCCTCCACAGGGCAGGACCACAGCTCACAAGGATCCCAATGCCA
TCTTCCTGAGCTTCCAACACCTGC
>2795
CACTTCAGGTGGGTGGGAGGCCCCTGTGCCGCAGGCCCCTCCAGTTTGAAGGAGGCA
CTGCTGGTGCCAGTCTTGCAGGTCTCCCGAGGGCAGAAGGTGACCAGCCTAACGGCC
TGCCTAGTGGACCAGAGCCTTCGTCT
>2796
GCCTGTGCCTGCCTGAGTCCTGATCCGTCTTCCTTCCTACCCTGCCCCCGGCCCCCTTC
TCTTTCTGCAGACAAACTGGTCAAGTGTGAGGGCATCAGCCTGCTGGCTCAGAACACC

TCGTGGCTGCTGCTGCTCCTGCT
>2797
TGTTGGGGAGGGGTCTCCTTCTCCCACTCCTCCCGCCGTGGACCTCACCTGACCCTCT
CTCCTCTTGCAGCACAGAGTTGATGAGACGCGTCCGTCGCTTCCAGATTGCTCAGTAC
AAGTGCCTGGTGATCAAGTATGCC
>2798
ATTCCCTGGCCCCTGCATTGTGGCTTCTGGCCTCCCTGGCCCTGCTCACGCTCTGGCTT
TCTCTTCCCAGGAACACCATGGAGGCGCTGCCCGCCTGCCTGCTCCGAGACGTGGCCC
AGGAGGCCCTGGGCGTGGCTGTC
>2799
TCGCTGGGGCTCCAGGGAGCTGCTGCTGGGGTGGAAGCTCTCACACTCTTTCTCCACG
TGCCCTTTCCAGTTCCCTGACATCATGGAGTTCTGCGAGGCCATGGCCAACGCCGGGA
AGACCGTAATTGTGGCTGCACTGG
>2800
TCAGAGGGTTTAAAAGAAAAACCCACCTGGTAAGGTGCTGAGCGCCCTCTGTCTTTCC
ATGGGAGCACAGCCATTTGGGGCCATCCTGAACCTGGTGCCGCTGGCCGAGAGCGTG
GTGAAGCTGACGGCGGTGTGCATGG
>2801
GCGGGGTGGGGGTATGGCTCTGCCTCCTTCCTGTCCTGGCCCTTCACCCATCCCCTGTC
CCTGCGGCCAGGTCGAGGTGATTGGGGGAGCAGACAAGTACCACTCCGTGTGTCGGC
TCTGCTACTTCAAGAAGGCCTCAG
>2802
GTTGGGGGGGGGTAGGTGTGAGGCTGACAGTCCAGCAGGCCTCACCTCTGCCCTCTG
CCCTCCCTCGCAGAGTTCAAGGCTGCCTTTGACATGTTTGATGCTGATGGTGGTGGGG
ACATCAGCGTCAAGGAGTTGGGCAC
>2803
GAGGCAGGGGATGGTGGGGAGAGGCGGCAGCGGCTGGGCTCAGGCTCAGTCTGCCA
CCTGCTGCCCGCAGGCAGCGGCACCATCGACTTCGAGGAGTTCTTGGTCATGATGGTG
CGCCAGATGAAAGAGGACGCGAAAGG
>2804
GGGCGTCGAGTGTGGTGGCCAGGGCAGCTCGCCCCGGCCCTGAGCCCTGCCCTGTCC
CTCGGACCCGAAGGAATGCAGACGGCTACATCGACCCGGAGGAGCTGGCTGAGATTTT
CAGGGCCTCCGGGGAGCACGTGACG
>2805
GGCTGGAGCCCTGGCCTGGCCTCTGCGTCCCCAGTGGGACCCCTGACCCTCTCCCTCT
CCTTTCCCTCAGAGTTCCTGAAGATGATGGAGGGCGTGCAGTAAGGAGTGGACAGTCG
CCTCTACCAAGATCGCGTGTCCCT
>2806
ATGGGGGCTGCTGGGGTGGCTCAGCCAAACCTTGAGCCCTAGAGCCCCCCTCAACTCT
GTTCTCCCCTAGGGGCTCCCTGGTGTTGGCCTCACACCTTCAGCTGCCCAGACTGCCC
GTCAGCACCCCAAGATGCATCTTG
>2807
TTCCTCAGGGATTGAGACCTCTGATCCAGACCCCTGATCTCCCACCCCCATCCCCTATG
GCTCTTCCTAGGAGACCCCAGCAAGCAGAACTCACTGCTCTGGAGAGCAAACACGGA
CCGTGCCTTCCTCCAGGATGGTTT
>2808

AGGGCCAGGATGTGGAGAGTGAACCGACATGGCCACACTGACTCTCCTCTCCCTCTCT
CCCTCCCTCCAGCAAACCCTCAAGCTGAGGGGCAGCTCCAGTGGCTGAACCGCCGGG
CCAATGCCCTCCTGGCCAATGGCGT
>2809
GGTCAAGGAGGTCACTGACTTCATGAAGTGCTCAGCTATTAAAGGTTCCTTTCCCACTC
TTATCCCTTAGGATGGAAATCCAACTAATGAGACCGCACTCCTTGGCTTGTTCCTGCGT
GTTTCACCCAAAGGAGAAAATG
>2810
CTTATCCTCCTCTGTTGCCATCATGGACCTCTCGCACCCCGTGATCAAGCTGTTTTTTCT
CTCCTTCCAGAATTTAAGGGACGCTGTGAAGCAATCATGGATGCAATGAAGAGAGGGC
TCTGCTGTGTGCTGCTGCTGTG
>2811
AGGTGCAAGGGTAGTGAGGCATTTCATTTTGAGTCATACTCTGATGAGGCTGGCCTGTC
TTTTCTCATAGTGATCTGCAGAGATGAAAAAACGCAGATGATATACCAGCAACATCAGT
CATGGCTGCGCCCTGTGCTCAG
>2812
CTCCTGGAACCATGGCCTGAGCACACAGAAGGAGGCTGTCATTAGGGGCTTCCTTTTT
TATACTTGACAGGTTGCAGCGAGCCAAGGTGTTTCAACGGGGGCACCTGCCAGCAGG
CCCTGTACTTCTCAGATTTCGTGTG
>2813
AGGGGAGCCTCCTGAGGGCTGAATGAAAGGGGACTCCCATGTGAGGCATCCATGCCCT
GCTCTCTGTCAGATACCAGGGCCACGTGCTACGAGGACCAGGGCATCAGCTACAGGGG
CACGTGGAGCACAGCGGAGAGTGG
>2814
AGTCCTTGGGTCCTAAAGGAGAGGCTTCACCTTTGACCTACCCTGGCCCTTCCCTTGG
CTGATTTTTCAGAAACCCAGATCGAGACTCAAAGCCCTGGTGCTACGTCTTTAAGGCG
GGGAAGTACAGCTCAGAGTTCTGC
>2815
CAAGCCCCAGCCCTGACTTGCGTGCTTCAGAATTTGTTTTTGTTTCAGTTTGAGAATTC
TTTTCTTCTAGGAAACAGTGACTGCTACTTTGGGAATGGGTCAGCCTACCGTGGCACG
CACAGCCTCACCGAGTCGGGTGC
>2816
AGACCCCATCTCTACAAAAATTAAAAAATAAGAAGATAAAAAGGAAACCTTAAACTCT
CTTATCATTTAGGAATCCTGATGGGGATGCCAAGCCCTGGTGCCACGTGCTGAAGAACC
GCAGGCTGACGTGGGAGTACTGT
>2817
CTCCTCACTCTACCTACACACGTGCAGGAGGCCTGTGGGACTGACTGCCTCCTCGTCC
TTTCCTCCCCAGCCACCTGCGGCCTGAGACAGTACAGCCAGCCTCAGTTTCGCATCAA
AGGAGGGCTCTTCGCCGACATCGC
>2818
GCCTAGAAACCAAGCCCTTTAGGGGAAATCCCACAGCAAAAAAAGCATTCTAAGGCT
GTTTCTCCACCAGGTTTCCGCCCCACCACCTGACGGTGATCTTGGGCAGAACATACCG
GGTGGTCCCTGGCGAGGAGGAGCAG
>2819
CTCCTGCGTGTTCCTCCCCTCCCGGGGACGCGGCCCTCACGCCAGCTCACTCCTCCTTT
CTCCCTCCCAGCGCTGCTGCAGCTGAAATCGGATTCGTCCCGCTGTGCCCAGGAGAGC

AGCGTGGTCCGCACTGTGTGCCT
>2820
TTGGCTTTCTCTTTCAAAAACGTTTATAAATAACTTTCCCTAAAACCATATGATGGTTCT
CCCCTTTCAGTGTCTCCTTTCTATTCGGAGCGGCTGAAGGAGGCTCATGTCAGACTGTA
CCCATCCAGCCGCTGCACATC
>2821
CTACACATACTGACTTTCTCCAGATAGTCTGCTTTTTTTGCAAACGCCAAACCTGCTATC
TCCTTTGCAGGGCGATTCGGGAGGCCCCCTGGTGTGTCTGAACGATGGCCGCATGACT
TTGGTGGGCATCATCAGCTGGG
>2822
GCTGCAGGCCAGGGAAGGGGGAGGCTCTGGAGCAGTCGGCCTGAGCCTGATAGAGAG
GGGCCTTCTCCAGGTGTCAGGGACGTGGTATCTGAAGGCCATGACGGTGGACAGGGA
GTTCCCTGAGATGAATCTGGAATCGG
>2823
CAGCATGGTTGCTCCAGGGCCGGGGGAGGCACAGCCAGGGCCGCTTTGCCAGGGGGC
TTCTGTTTTCCAGGATAAGTGGCCGGTGCCAGGAGGTGAAGGCCGTCCTGGAGAAAA
CTGACGAGCCGGGAAAATACACGGCC
>2824
CACGCTGTCCCGGGATCCTCTCTGAAGTCCCTGGTGGCTAATTCAGGAATGTGCTGCTG
TCTTTCTGCAGACGGGGGCAAGCACGTGGCATACATCATCAGGTCGCACGTGAAGGAC
CACTACATCTTTTACTGTGAGGG
>2825
TCCAGACTGGGATGGGGTGCCGTCGGCCAGTGCTGCCTCGAGCACCCACATCTCGTCC
TGGCACCCACAGGCAGAGACCCCAAGAACAACCTGGAAGCCTTGGAGGACTTTGAGA
AAGCCGCAGGAGCCCGCGGACTCAG
>2826
CCCCACATTGCATCCCCCTCCCACCCTTGCTGTCCTGGCCTCACTCACCCTCCCCCCCT
TTCCAGGGCAGGGGACACCTTGGCTCCTCAGCAGCCCAAGGACGGCACCATCCAGCA
CCTCCGTCATTCACAGGGACATGG
>2827
ATAGTGTGAATGAAGCCTTCCCAAAAGCAGAAACGGGATTTTGATTCTGGATCTTATTT
TATTGTTCTAGGTTTACTTGAACTTGAAGGAAAGAAAAAAAAATGTCTCCACTTCTGA
GAAGCATCTGTGACATCACTGAA
>2828
CATGTGTGAATATAATTTTACCACCTGTAAATAGTGCATTGTAAAATTTTTGTTTTTCACC
ATTTTATAGAGACCACATGACCCTAAGACGGTAGATCTGATCCTGGAACTTCTGGATCG
TGACAGTAATGGGCGTGTCG
>2829
CAGGGTTCTGGCCCTCCGTGGTCCTGGCTGCTGAGCCCTGACTACCGTGCCCCCCAAC
CCCTGAACACAGAGTTCAAGGCAGCCTTCGACATCTTCGTGCTGGGCGCTGAGGATGG
CTGCATCAGCACCAAGGAGCTGGG
>2830
GGTTCGTGTCGAAGGTGCTGCCTGCAGTACCTACCCTGGCCTCTGTAACGGCCATGCT
GCCCACCCCCAGGCAGCGGCACGGTGGACTTTGATGAGTTCCTGGTCATGATGGTTCG
GTGCATGAAGGACGACAGCAAAGG
>2831

GCCTCCTGGGGCATGGGTGGGGCTGCCTCATGCCCTCCCCACAGCCCTACCCTGAGCC
CCCTCCCCACAGAAATGCTGATGGCTACATCGACCTGGATGAGCTGAAGATAATGCTGC
AGGCTACAGGCGAGACCATCACG
>2832
GGGCTGATCTCCTGCCTCCATGCCCTGCCCAGCCCCTACCCTCAACCCACACCTGCCCC
TCTTTCCACAGAGTTCCTGGAGTTCATGAAGGGTGTGGAGTAGATGCTGACCTTCACC
CAGAGCTGCCTATGCCCAGCCTC
>2833
CTTCTGTTGCAGAGCGCATGCATTTTGACCACAGTGTTTCAGCTCTTCCCTTTTCTCTTG
TTCCATTTAGGTGGCTTCCCACACTTCTGACTCGGACGTTCCTTCCGGTGTCACTGAGG
TGGTCGTGAAGCTCTTTGACT
>2834
AGTTACGGGGGAATTTTATTTTTCCTTTGGAGGATGTTTTACTTTGGAGGATAACTGTTT
TTTATTTCAGGGAGGAGTGAGATGTGGATGTTGCTTTTGCACCTACGGGGGCATCTGAG
TCCAGCTCCCCCCAAGATGAG
>2835
TTCCCTGAAGAAAGTTGGATGGCATGATCTGTCTTCCCATCTTGAAACCGTATGGCAAA
TTGTTTTTCAGATGAATTCCCTCTGCTGACAACCAAACGTGTGTTCTGGAAGGGTGTTT
TGGAGGAGTTGCTGTGGTTTAT
>2836
CTCGGGGGGATCAACTGAGATGGCTTAAGATTTACCTGGATGCCTGCTCTGCTCTCCCC
ATCTCTTCCAGGGATCCACAAATGCTAAAGAGCTGTCTTCCAAGGGAGTGAAAATCTG
GGATGCCAATGGATCCCGAGACT
>2837
CCTCTAAGGCCATCTCATGACATGTGTGATTAATGTATGTACCTGTCCTCTCTTTTTGAC
AATTCTACAGATTATTCAGGACAGGGAGTTGACCAACTGCAAAGAGTGATTGACACCA
TCAAAACCAACCCTGACGACAG
>2838
TTTTACTTTGCCTTTAGCTGTGGTCTTTCAAACCACCATCCCTCCTTATCTTCCTCTGCT
GGTTCCTCAGATCTTCCTCTGATGGCGCTGCCTCCATGCCATGCCCTCTGCCAGTTCTAT
GTGGTGAACAGTGAGCTGTC
>2839
TCTAATGTTTTTAAATGATGTTTTAAAGAATTGAAACTAACATACTGTTCTGCTTTCTCC
CGGTTTATAGCCAGGTGACTTTATACACACTTTGGGAGATGCACATATTTACCTGAATCA
CATCGAGCCACTGAAAATTC
>2840
CACGGACATGAGGAGCAATTACAACAGGTCGTACAATTATGGCAAAATAATGGCCTTAT
TTTGTTTTTAGCTTCAGCGAGAACCCAGACCTTTCCCAAAGCTCAGGATTCTTCGAAA
AGTTGAGAAAATTGATGACTTCA
>2841
GCGGACGCGTGGGCTTCGCCGTCTGGGGGCCGCGGGCTCAACCCTTGCCTCCCACCCT
CCGCGCGGCCAGGTGCCAGAGGACGAGCTGGTGTCCACGCTGAAGCAGCTGGTCTCC
GAGAAGCTGAACGTCCCAGTGCGCC
>2842
GGCGGCCTCGCGCCCCGGGCCACCCCCACGGCGGTGGAGGAGGAGGAACCCATCGAT
CTGTCTTTGGCAGATGGGAAACGACTCTCGGATTATAGCATCGGGCCCAACTCCAAGC

TCAACCTAGTGGTCAAACCCCTGGA
>2843
CTACCTCTGTTGCAGTGTATGCCCCCCCCCCACTGGCAGCCCCTGAATCTTCCTGCCCT
TCTCTCCCCAGGATTACGAGAGGTCCCTGAGTCGCCTGACGCTGGACGACATCGAACG
GTTGGCCAGCCGCTTCCTGCACC
>2844
CGGGAGGCAGGCCTGAGTCCCCCTGCACAGCACCCTCTCTAACCAGGCCCTCTTCCCG
ACTCCTGCCCAGCTGTGCCTGGGCATCCCTCTCTGCCCAGCCTGCCCAGCAACAGCAG
CCAGGAGAGGCCACTGGACACCCG
>2845
TGGGCTTGGCCGCATGCCCCTGTGCCCCACCAGCCATCCTGAACCCAACCTAGATCCTC
CACCTCCACAGGGGCGCCCTTTGTGCTACTCATGTTGCTGGCCAGCCTCAACAGCTGC
ACCAACCCCTGGATCTATGCATC
>2846
ATACATGTACATGTAACATAATATTCATCAAAAGGGGACACATACAAATTTTTCTATTTCT
GCCTCCTAGGCCGGGATTATGAAAAGAATAAAGTCTGCAAGGAATTCTCCCATCTGGG
AAAGGAGGACTTCACATCTCT
>2847
GAGCGAGACTCCGTCTCAAAAAAAAAAAACCTTTCAGTTCTAATTGATATATTGTCTTAT
TTTCCCCTCAGGTCACTAGTCCTGTACAGTAGAAAATTTCCCAGTGGCACGTTTGAACA
GGTCAGCCAACTTGTGAAGGAA
>2848
TATTAAATGGAAGTCTAAAAGCCTGGAAATTTACATATATATATGAGTTTCCTTTTTCCTT
CTCCTCCAGACCTCAGCACTGTCTGCCAAGTCCTGTGAAAGTAATTCTCCATTCCCCGT
TCACCCAGGCACTGCTGAGT
>2849
TGTCTCAAATAAAAAAGTAGATTTTGTTTATTCACAAATTTTTTGACCAATTCTATTTTTT
CCTCCACAGATTTATGTGGGAATATTCCACTAATTACGGACAAGCTCCTCTGTCACTTTT
AGTCAGTTACACCAAGAGT
>2850
TATTTTTGCCATTTATACAGTATTCAATGAGTCTTGACCATATAATGAGATTCTTTCACTT
GTTTTCTAGAGACTCCAGCTTAAACATTTATCACTTCTCACCACTCTGTCAAATAGAGT
CTGCTCACAATATGCTGCTT
>2851
GTGCGTTCATCACGAAACCAAGAAAACTGAGAATGTTGATAAGCTAACATATTTATTTT
TAACTGTTTAGCAATCTCATAAAGTTAGCCCAAAAAGTGCCTACTGCTGATCTGGAGGA
TGTTTTGCCACTAGCTGAAGAT
>2852
TATGTAATTCTTATTTTTAATTAGACTATCAGGAAACTAACAATGCACAAACTAACCATT
CGTTTTGTAGCTGCCTGAACACACAGTAAAACTCTGTGACAATTTATCCACAAAGAATT
CTAAGTTTGAAGACTGTTGTC
>2853
AAATAAATAAATAAATAAATAAATAAATAGGAAATATATAACACATATCTCCTTTTCTCCC
TCATGCTAGGTATACATTTGAACTAAGCAGAAGGACTCATCTTCCGGAAGTATTCCTCA
GTAAGGTACTTGAGCCAACC
>2854

GATTTAATGGCTTGATGGGACAAGGTTTCAAGACAAATAATTTTGTTATGCAATTATTGT
TTTCTTACAGGGCCCTCTACTAAAGAAGGAACTATCTTCTTTCATTGACAAGGGACAAG
AACTATGTGCAGATTATTCAG
>2855
TTATTTTGCATTAGAAATTTGTATAAAATAAATACATGTAGTAAGACCTTACATTTAAATG
GTTTTTCAGACTGGCAGAGCGACTAAAAGCAAAATTGCCTGAGGCCACACCCACGGA
ACTGGCAAAGCTGGTTAACAAG
>2856
GAGCAATATTATGGTTGTTGCCCATTAGCTCTGTTTTTCTAACATAAAATTCATTTCATTA
TTCTCATAGACCAGAGTTGGAGCCACCCAGGGGAATGATCTCTGATGACCTAACCTAA
GCAAAACCACTGAGCTTCTGG
>2857
TCGCTGCGTTCCCCTCCAACCCCTCGACTCCCGGCTCCCCTCCTCCCGCTCACCCCGCC
CGTCCCCGCAGTGCCTCCCCTGCGGCCCCGGGGGCAAAGGCCGCTGCTTCGGGCCCA
GCATCTGCTGCGCGGACGAGCTGG
>2858
AGGGTGTGGGCCCCCCGCACCCCGAGCTGCGCCCGCCCCAGGCGCCCGTGCTCACAC
GTCCTCCCGGCAGAGAGCTGCGTGACCGAGCCCGAGTGCCGCGAGGGCTTTCACCGC
CGCGCCCGCGCCAGCGACCGGAGCAA
>2859
TCCCAGAAAGCTATAAATCCAAGCTGGCTTCTCCCTCCCCACACAGCTGCTCCTGCTCT
CCCTCCTCCAGGTCACCCCAGCCATGAGGATTATCGCCCTCCTCGCTGCTATTCTCTTG
GTAGCCCTCCAGGTCCGGGCAG
>2860
TCCAAGATTCCCTTTACCACCCACCGCTGACCTTGGTGCTTAATTTCTCAGTCTTCCTCT
GTGTTCCCAGGCTCAACAAGGGGCATGGTCTGCTCTTGCAGATTAGTATTCTGCCGGCG
AACAGAACTTCGTGTTGGGAA
>2861
ACGTAATTTACCAGGCATGGGTTTTCCCAGAGGAGGGGGCTGGTTCATGGTTTTGGTTT
TGGTTTTCCAGAAGATTTTGTGATTCAGGCAAAGGCTGACTGTTACTTCACCAACGGG
ACAGAAAAGGTGCAGTTTGTGGT
>2862
TCCCTAAAGAATCTCCTGTCCCAGCTTGGTGGTTTTCTTTCACGGTGTCTCATTTTTCCT
CCCTTCCTAGTGCAACCAGAGGTGACAGTGTACCCAGAGAGGACCCCACTCCTGCACC
AGCATAATCTGCTGCACTGCTC
>2863
TTCTGGGATTCCAAGAAAGTGCTTTAAATATTGGGATATTTTTACTAATTTAAAGACCTG
TTTCCCATAGGAGCTCAGTCTGAATATTCTTGGAGAAAGATGCTGAGTGGCATTGCAGC
CTTCCTACTTGGGCTAATCTT
>2864
CCTGAGTCTCTGGAACGGCTGTGGGGGGTGGTGAGGCTGCCTCCTGAGACCTTCATCA
CTGTGCCTCCAGGTCTCAAGAGCTGTTCTGCTCCCTCAGTCATGCTAAGGTCCTCACTG
AAGCTTCTCTCTCTGGAGCCTGA
>2865
GGGGGCGCAAGACCCGGGAAGCCGCGCCGGGAGGAGGGTCGGGCGGGTCTCAGCCA
CTCCTCGTCCCCAGGCTCCCACTCCATGAGGTATTTCTCCACATCCGTGTCCCGGCCCG

GCCGCGGGGAGCCCCGCTTCATCGC
>2866
AAAATCCCCCCGGGTTGGTCGGGGCCGGGCGGGGCTCGGGGGACTGGGCTGACCGCG
GGGTCGGGGCCAGGTTCTCACACCCTCCAGATGATGTTTGGCTGCGACGTGGGGTCGG
ACGGGCGCTTCCTCCGCGGGTACCA
>2867
CCACATGCATGCTGGTGGAGTGTCCCATGACAGATGCAAAATGCCTGAATTTTCTGACT
CTTCCCGTCAGACCCCCCCAAGACACATATGACCCACCACCCCATCTCTGACCATGAG
GCCACTCTGAGATGCTGGGCCCT
>2868
AGGGAAAGCAGGAGCCTCTCTGGAGACCTTTAGCAGGGTCAGGGCCCCTCACCTTCC
CCTCTTTTCCCAGAGCCATCTTCCCAGCCCACCGTCCCCATCGTGGGCATCATTGCTGG
CCTGGTTCTCCTTGGAGCTGTGAT
>2869
CCCCCACACAGGGAAGGGCTGGGCCCAGGCCTGGGGCTGCTTCCTGGTCCTGACCTG
GCACCTGCCCCAGCCCCAGGCCAGGCCCTGCAGCGAGTGGGCATCGTCGGGGGTCAG
GAGGCCCCCAGGAGCAAGTGGCCCTG
>2870
ATGTGAGCCCTGGCTCCCGGGTGCTCCTGGGGGCTGCCCAGGGCCCTGAGTGGGATCC
TCCGCTGCCCAGGGACGTCAAGGATCTGGCCGCCCTCAGGGTGCAACTGCGGGAGCA
GCACCTCTACTACCAGGACCAGCTG
>2871
GGTCCCTCTGGGCTCCAGGTGGGGGTTGCCCGGCCCCCTCCTGAGGCTGCACCCTCTT
CCCCACCTGCAGAGCGCCTCCCACCGCCATTTCCTCTGAAGCAGGTGAAGGTCCCCAT
AATGGAAAACCACATTTGTGACGC
>2872
CCCCCGCCCCCCGCCCCCCAACCCCCACTCCCAGGCCTGTTCGGCGAGCGCTGACCTC
TGACCTTCCCAGGGCGACTCCGGAGGGCCCCTGGTGTGCAAGGTGAATGGCACCTGG
CTGCAGGCGGGCGTGGTCAGCTGGG
>2873
CAGATGCCCAATGGGCCTGCCTGGCATCTCCCACACTGCTGTCCCTGGACACATACCTT
TTTGCCCCCAGACATTATCCGGCAGCCCTCTGAGGAAGAGATCATCAAATTGGCTCCCC
CACCGAAGAAGGCCTGAGCAAG
>2874
CGTTTTTGACAGTTTGACAGTTAAAGGCATTTCCCTGTGAAATAATACTGGTATGTATTT
AACCATGCAGATCCTCAGTTTGTGGTCTGCCAGCTAAAGGTGAAGATATATTCCTCCAA
TTCAGGACCCACACGACGGGA
>2875
TAAAAAAATAAAACATCATTAATTAAATATGTCATTTCATTTCTTTTTCTTTTCTTTTTTTT
TTTTTTAGGACAAAATGTTTCACTTTTGGGTAAATACATTCTTCATACCAGGACCAGAG
GAAACCTCAGAAAAAGTAG
>2876
AAATATATTTCACTAAATAGTTAAGATGAGTCATATTTGTGGGTTTTCATTTTAAATTTTC
TTTCTCTAGGTGAAGCTGTACTTCACAAAAACAGTAGAGGAGCCGTCAAATCCAGAGG
CTAGCAGTTCAACTTCTGTAA
>2877

AACCAACCAGGAGGCTGGGGTACGCTGGAGAAGGAATGGGCTTCCTAACCTTGAGCC
CTCTTCCCTGAAGATATATGTATCTACGGGGGCCTGGGGCTGGGCGGGCTCCTGCTTCT
GGCAGTGGTCCTTCTGTCCGCCTG
>2878
CCCACCCATCCATCTATCCCTGCGGCCCCCCTCTGCCCGCTCCTCTGACCAACACCTGC
TTTGTCTGCAGGCACCACAGTGCTGTTGCCCCTGGTCATTTTCTTTGGTCTTTGCCTTTT
ATCCCTCCTCTTCATTGGTTT
>2879
CACGTAGAATAAAGAGGGCCGGGGCTGGTTTTCGCTGCCGCACTAATCTGCGCCACCT
TCTCTCTTTCAGGGGGGAGCTTGAAGGAACTACTACTAAGCCCCTGGCCCCAAACCCAA
GCTTCAGTCCCACTCCAGGCTTCA
>2880
GCGGAGGGCGCGGGATGCGGGGCGGGGCCTGGGGTTGCCGCCCGAGGCTCACCGGCC
CGCGTCCCCGCAGCTGATGACCCCGCGACGCTGTACGCCGTGGTGGAGAACGTGCCCC
CGTTGCGCTGGAAGGAATTCGTGCG

Online Supporting Information S_2^- represents the negative dataset composed of 2800 false splice acceptor site sequences.

>1
CATTCTGGATGATGCAGCGAACAATCCTCTCTACTGTGTCCTCATTCATGTCATTCGTCC
TCAGCTGAAGGATGAAAGAGATTGGCAAGGAAGCAGGAATGAGTCAGCTTTGGGGGA
ATATTACCTGGTGGCTCCCATCA
>2
ATGATGCAGCGAACAATCCTCTCTACTGTGTCCTCATTCATGTCATTCGTCCTCAGCTGA
AGGATGAAAGAGATTGGCAAGGAAGCAGGAATGAGTCAGCTTTGGGGGAATATTACCT
GGTGGCTCCCATCATTTATTGC
>3
CTCTCTACTGTGTCCTCATTCATGTCATTCGTCCTCAGCTGAAGGATGAAAGAGATTGG
CAAGGAAGCAGGAATGAGTCAGCTTTGGGGGAATATTACCTGGTGGCTCCCATCATTTA
TTGCTTTAGGACATACCCTTTT
>4
GGCAAGGAAGCAGGAATGAGTCAGCTTTGGGGGAATATTACCTGGTGGCTCCCATCAT
TTATTGCTTTAGGACATACCCTTTTACCTACTTGCTATCATGCTGGACCTCAGCATTTCA
GGGACCAGAAAAGATGGGGACA
>5
CTGGTGGCTCCCATCATTTATTGCTTTAGGACATACCCTTTTACCTACTTGCTATCATGCT
GGACCTCAGCATTTCAGGGACCAGAAAAGATGGGGACACCAATGCAAGCCCAAGACT
ACCATCCAGAGGACCGTTCCTG
>6
TCCCATCATTTATTGCTTTAGGACATACCCTTTTACCTACTTGCTATCATGCTGGACCTCA
GCATTTCAGGGACCAGAAAAGATGGGGACACCAATGCAAGCCCAAGACTACCATCCA
GAGGACCGTTCCTGCCCATGCT
>7
TTTACCTACTTGCTATCATGCTGGACCTCAGCATTTCAGGGACCAGAAAAGATGGGGAC

ACCAATGCAAGCCCAAGACTACCATCCAGAGGACCGTTCCTGCCCATGCTGCTTTCTCT
AGTTTTGCTTTCAGAGTGGTGT
>8
TGCTGGACCTCAGCATTTCAGGGACCAGAAAAGATGGGGACACCAATGCAAGCCCAA
GACTACCATCCAGAGGACCGTTCCTGCCCATGCTGCTTTCTCTAGTTTTGCTTTCAGAG
TGGTGTCACCCCACTTGCTAACTC
>9
CTGGACCTCAGCATTTCAGGGACCAGAAAAGATGGGGACACCAATGCAAGCCCAAGA
CTACCATCCAGAGGACCGTTCCTGCCCATGCTGCTTTCTCTAGTTTTGCTTTCAGAGTG
GTGTCACCCCACTTGCTAACTCAA
>10
GATGGGGACACCAATGCAAGCCCAAGACTACCATCCAGAGGACCGTTCCTGCCCATGC
TGCTTTCTCTAGTTTTGCTTTCAGAGTGGTGTCACCCCACTTGCTAACTCAAGAGTGCA
TCACTAGGCTTCCTGAGCAGGTT
>11
ACCGTTCCTGCCCATGCTGCTTTCTCTAGTTTTGCTTTCAGAGTGGTGTCACCCCACTT
GCTAACTCAAGAGTGCATCACTAGGCTTCCTGAGCAGGTTTGATCAGGATTTGGCCTG
GGTCTGTTGGTCAGGGAGGCAGA
>12
CGTTCCTGCCCATGCTGCTTTCTCTAGTTTTGCTTTCAGAGTGGTGTCACCCCACTTGCT
AACTCAAGAGTGCATCACTAGGCTTCCTGAGCAGGTTTGATCAGGATTTGGCCTGGGT
CTGTTGGTCAGGGAGGCAGAGG
>13
TTCAGAGTGGTGTCACCCCACTTGCTAACTCAAGAGTGCATCACTAGGCTTCCTGAGC
AGGTTTGATCAGGATTTGGCCTGGGTCTGTTGGTCAGGGAGGCAGAGGTGTGGGGAG
AAGGGTGAAAGGTGACTGAGCCAGG
>14
TAACTCAAGAGTGCATCACTAGGCTTCCTGAGCAGGTTTGATCAGGATTTGGCCTGGG
TCTGTTGGTCAGGGAGGCAGAGGTGTGGGGAGAAGGGTGAAAGGTGACTGAGCCAG
GGTTCACAGGCTCCTCACTCACGGGG
>15
TCAAGAGTGCATCACTAGGCTTCCTGAGCAGGTTTGATCAGGATTTGGCCTGGGTCTGT
TGGTCAGGGAGGCAGAGGTGTGGGGAGAAGGGTGAAAGGTGACTGAGCCAGGGTTC
ACAGGCTCCTCACTCACGGGGCTGA
>16
ATCAGGATTTGGCCTGGGTCTGTTGGTCAGGGAGGCAGAGGTGTGGGGAGAAGGGTG
AAAGGTGACTGAGCCAGGGTTCACAGGCTCCTCACTCACGGGGCTGAAGCTGGGCAT
CGTCAGGCTCAGAGCCCCTAGACGTG
>17
GGATTTGGCCTGGGTCTGTTGGTCAGGGAGGCAGAGGTGTGGGGAGAAGGGTGAAAG
GTGACTGAGCCAGGGTTCACAGGCTCCTCACTCACGGGGCTGAAGCTGGGCATCGTC
AGGCTCAGAGCCCCTAGACGTGCGGC
>18
CTGGGTCTGTTGGTCAGGGAGGCAGAGGTGTGGGGAGAAGGGTGAAAGGTGACTGA
GCCAGGGTTCACAGGCTCCTCACTCACGGGGCTGAAGCTGGGCATCGTCAGGCTCAG
AGCCCCTAGACGTGCGGCAGCGGTAGC

>19
AGAGGTGTGGGGAGAAGGGTGAAAGGTGACTGAGCCAGGGTTCACAGGCTCCTCACT
CACGGGGCTGAAGCTGGGCATCGTCAGGCTCAGAGCCCCTAGACGTGCGGCAGCGGT
AGCCCTTCTTCTTGAGCACGTGGCAG
>20
GAAAGGTGACTGAGCCAGGGTTCACAGGCTCCTCACTCACGGGGCTGAAGCTGGGCA
TCGTCAGGCTCAGAGCCCCTAGACGTGCGGCAGCGGTAGCCCTTCTTCTTGAGCACGT
GGCAGATCATGAAGCCTACAGGCCC
>21
AAGGTGACTGAGCCAGGGTTCACAGGCTCCTCACTCACGGGGCTGAAGCTGGGCATC
GTCAGGCTCAGAGCCCCTAGACGTGCGGCAGCGGTAGCCCTTCTTCTTGAGCACGTGG
CAGATCATGAAGCCTACAGGCCCAT
>22
CTGAGCCAGGGTTCACAGGCTCCTCACTCACGGGGCTGAAGCTGGGCATCGTCAGGC
TCAGAGCCCCTAGACGTGCGGCAGCGGTAGCCCTTCTTCTTGAGCACGTGGCAGATCA
TGAAGCCTACAGGCCCATGAGGAAG
>23
TTCACAGGCTCCTCACTCACGGGGCTGAAGCTGGGCATCGTCAGGCTCAGAGCCCCTA
GACGTGCGGCAGCGGTAGCCCTTCTTCTTGAGCACGTGGCAGATCATGAAGCCTACAG
GCCCATGAGGAAGAAGACCAGCAC
>24
GGGGCTGAAGCTGGGCATCGTCAGGCTCAGAGCCCCTAGACGTGCGGCAGCGGTAGC
CCTTCTTCTTGAGCACGTGGCAGATCATGAAGCCTACAGGCCCATGAGGAAGAAGACC
AGCACAAGCAGGAAGAGCATATATA
>25
GTCAGGCTCAGAGCCCCTAGACGTGCGGCAGCGGTAGCCCTTCTTCTTGAGCACGTGG
CAGATCATGAAGCCTACAGGCCCATGAGGAAGAAGACCAGCACAAGCAGGAAGAGCA
TATATAGCCCATGTTGGGGCGGTTC
>26
TCAGAGCCCCTAGACGTGCGGCAGCGGTAGCCCTTCTTCTTGAGCACGTGGCAGATCA
TGAAGCCTACAGGCCCATGAGGAAGAAGACCAGCACAAGCAGGAAGAGCATATATAG
CCCATGTTGGGGCGGTTCCAGGTCA
>27
CTAGACGTGCGGCAGCGGTAGCCCTTCTTCTTGAGCACGTGGCAGATCATGAAGCCTA
CAGGCCCATGAGGAAGAAGACCAGCACAAGCAGGAAGAGCATATATAGCCCATGTTG
GGGCGGTTCCAGGTCAGGCTGTGGT
>28
CAGCGGTAGCCCTTCTTCTTGAGCACGTGGCAGATCATGAAGCCTACAGGCCCATGAG
GAAGAAGACCAGCACAAGCAGGAAGAGCATATATAGCCCATGTTGGGGCGGTTCCAG
GTCAGGCTGTGGTTCCGACATGAGG
>29
ACAGGCCCATGAGGAAGAAGACCAGCACAAGCAGGAAGAGCATATATAGCCCATGTT
GGGGCGGTTCCAGGTCAGGCTGTGGTTCCGACATGAGGCCCTGGGGGGCGAGCGCGG
GCCAGGGCGCCTCCTGGGTTTAGGGG
>30
CCCATGAGGAAGAAGACCAGCACAAGCAGGAAGAGCATATATAGCCCATGTTGGGGC

GGTTCCAGGTCAGGCTGTGGTTCCGACATGAGGCCCTGGGGGGCGAGCGCGGGCCAG
GGCGCCTCCTGGGTTTAGGGGGCTGC
>31
GCACAAGCAGGAAGAGCATATATAGCCCATGTTGGGGCGGTTCCAGGTCAGGCTGTGG
TTCCGACATGAGGCCCTGGGGGGCGAGCGCGGGCCAGGGCGCCTCCTGGGTTTAGGG
GGCTGCAGCTGGGAATGGGGGAGGG
>32
CCCATGTTGGGGCGGTTCCAGGTCAGGCTGTGGTTCCGACATGAGGCCCTGGGGGGCG
AGCGCGGGCCAGGGCGCCTCCTGGGTTTAGGGGGCTGCAGCTGGGAATGGGGGAGGG
GTAAGAATGCCGTCCTCAGGATCTG
>33
CAGGTCAGGCTGTGGTTCCGACATGAGGCCCTGGGGGGCGAGCGCGGGCCAGGGCGC
CTCCTGGGTTTAGGGGGCTGCAGCTGGGAATGGGGGAGGGGTAAGAATGCCGTCCTC
AGGATCTGAAACAAGCACGTCTAAGA
>34
TGTGGTTCCGACATGAGGCCCTGGGGGGCGAGCGCGGGCCAGGGCGCCTCCTGGGTT
TAGGGGGCTGCAGCTGGGAATGGGGGAGGGGTAAGAATGCCGTCCTCAGGATCTGAA
ACAAGCACGTCTAAGACGTCCGGCTG
>35
GGCCAGGGCGCCTCCTGGGTTTAGGGGGCTGCAGCTGGGAATGGGGGAGGGGTAAGA
ATGCCGTCCTCAGGATCTGAAACAAGCACGTCTAAGACGTCCGGCTGAGGTCCGGCCC
CACCCCCTACCCAACCATGGGAGCT
>36
CCTGGGTTTAGGGGGCTGCAGCTGGGAATGGGGGAGGGGTAAGAATGCCGTCCTCAG
GATCTGAAACAAGCACGTCTAAGACGTCCGGCTGAGGTCCGGCCCCACCCCCTACCCA
ACCATGGGAGCTGTCCGGGCCAACA
>37
GGGGGCTGCAGCTGGGAATGGGGGAGGGGTAAGAATGCCGTCCTCAGGATCTGAAAC
AAGCACGTCTAAGACGTCCGGCTGAGGTCCGGCCCCACCCCCTACCCAACCATGGGA
GCTGTCCGGGCCAACAGTGCTGTTTC
>38
AGGATCTGAAACAAGCACGTCTAAGACGTCCGGCTGAGGTCCGGCCCCACCCCCTACC
CAACCATGGGAGCTGTCCGGGCCAACAGTGCTGTTTCGCCTTTCGGCAAGAGGTCAGC
GACAACGCAAGCTCTCTACCACGA
>39
ACGTCTAAGACGTCCGGCTGAGGTCCGGCCCCACCCCCTACCCAACCATGGGAGCTGT
CCGGGCCAACAGTGCTGTTTCGCCTTTCGGCAAGAGGTCAGCGACAACGCAAGCTCT
CTACCACGACCATGGTCCGCTTCCC
>40
GTCCGGCCCCACCCCCTACCCAACCATGGGAGCTGTCCGGGCCAACAGTGCTGTTTCG
CCTTTCGGCAAGAGGTCAGCGACAACGCAAGCTCTCTACCACGACCATGGTCCGCTTC
CCCGACGCCCCGAGCGTTCCCCTT
>41
CCAACCATGGGAGCTGTCCGGGCCAACAGTGCTGTTTCGCCTTTCGGCAAGAGGTCAG
CGACAACGCAAGCTCTCTACCACGACCATGGTCCGCTTCCCCGACGCCCCGAGCGTTC
CCCTTTCCAGCTTGTTCGCGCTTC

>42
TTTCGGCAAGAGGTCAGCGACAACGCAAGCTCTCTACCACGACCATGGTCCGCTTCCC
CGACGCCCCGAGCGTTCCCCTTTCCAGCTTGTTCGCGCTTCTGTCCCTCCCAAGGACA
CTGCAGCACGAGATGGGACGGCAT
>43
AGCGACAACGCAAGCTCTCTACCACGACCATGGTCCGCTTCCCCGACGCCCCGAGCGT
TCCCCTTTCCAGCTTGTTCGCGCTTCTGTCCCTCCCAAGGACACTGCAGCACGAGATG
GGACGGCATTCTTAGGCGGCGGGA
>44
CCATGGTCCGCTTCCCCGACGCCCCGAGCGTTCCCCTTTCCAGCTTGTTCGCGCTTCTG
TCCCTCCCAAGGACACTGCAGCACGAGATGGGACGGCATTCTTAGGCGGCGGGAGCA
GAAATCCTGCCTGAGTCAGCGCCC
>45
CTTCCCCGACGCCCCGAGCGTTCCCCTTTCCAGCTTGTTCGCGCTTCTGTCCCTCCCAA
GGACACTGCAGCACGAGATGGGACGGCATTCTTAGGCGGCGGGAGCAGAAATCCTGC
CTGAGTCAGCGCCCGCACCTCCTC
>46
CCTTTCCAGCTTGTTCGCGCTTCTGTCCCTCCCAAGGACACTGCAGCACGAGATGGGA
CGGCATTCTTAGGCGGCGGGAGCAGAAATCCTGCCTGAGTCAGCGCCCGCACCTCCTC
CACGCCGGCCTGAGCCAGGCCTCT
>47
TTGTTCGCGCTTCTGTCCCTCCCAAGGACACTGCAGCACGAGATGGGACGGCATTCTT
AGGCGGCGGGAGCAGAAATCCTGCCTGAGTCAGCGCCCGCACCTCCTCCACGCCGGC
CTGAGCCAGGCCTCTGCAGCCCCTT
>48
CCTCCCAAGGACACTGCAGCACGAGATGGGACGGCATTCTTAGGCGGCGGGAGCAGA
AATCCTGCCTGAGTCAGCGCCCGCACCTCCTCCACGCCGGCCTGAGCCAGGCCTCTGC
AGCCCCTTCCTCTCAGTACTCCCGC
>49
GCATTCTTAGGCGGCGGGAGCAGAAATCCTGCCTGAGTCAGCGCCCGCACCTCCTCCA
CGCCGGCCTGAGCCAGGCCTCTGCAGCCCCTTCCTCTCAGTACTCCCGCACCCCTCTC
CCTTTTTGGTTTCTCCCACCCTGA
>50
TCTTAGGCGGCGGGAGCAGAAATCCTGCCTGAGTCAGCGCCCGCACCTCCTCCACGCC
GGCCTGAGCCAGGCCTCTGCAGCCCCTTCCTCTCAGTACTCCCGCACCCCTCTCCCTTT
TTGGTTTCTCCCACCCTGACCAC
>51
CGGGAGCAGAAATCCTGCCTGAGTCAGCGCCCGCACCTCCTCCACGCCGGCCTGAGC
CAGGCCTCTGCAGCCCCTTCCTCTCAGTACTCCCGCACCCCTCTCCCTTTTTGGTTTCT
CCCACCCTGACCACCTGGCCGACC
>52
TGACCACCTGGCCGACCCGGTACCGGTGATTTGGTTCTGGTTCCGGCTTCTGCTGGGG
TTCCGGCTCCAGGGGCGTCCTAGTGCGGCTCAGGGCCCCCGACGCCCTTCCGGCGCTC
ATCCCTTGATTCATCCCCTCCCCC
>53
CCGACCCGGTACCGGTGATTTGGTTCTGGTTCCGGCTTCTGCTGGGGTTCCGGCTCCAG

GGGCGTCCTAGTGCGGCTCAGGGCCCCGACGCCCTTCCGGCGCTCATCCCTTGATTCA
TCCCCTCCCCCTTCGCCGCCTC
>54
GATTCATCCCCTCCCCCTTCGCCGCCTCACCGGCCCCGGGCTGTGGCTGCGGATACCCG
CGCCGTGGCAGGCGGGGGGAGGGAAGAACAGAGAAGGATGCGGGCTGGCTGAGAGC
GGTGGCAGGCTCCGGGCAACTCCGG
>55
CTCCCCCTTCGCCGCCTCACCGGCCCCGGGCTGTGGCTGCGGATACCCGCGCCGTGGC
AGGCGGGGGGAGGGAAGAACAGAGAAGGATGCGGGCTGGCTGAGAGCGGTGGCAGG
CTCCGGGCAACTCCGGCCCTGGGTCA
>56
GTGGCTGCGGATACCCGCGCCGTGGCAGGCGGGGGGAGGGAAGAACAGAGAAGGAT
GCGGGCTGGCTGAGAGCGGTGGCAGGCTCCGGGCAACTCCGGCCCTGGGTCAGGGAG
ATTGCCTCCGCAGCCGCCCTGGCTTCC
>57
GGCTGCGGATACCCGCGCCGTGGCAGGCGGGGGGAGGGAAGAACAGAGAAGGATGC
GGGCTGGCTGAGAGCGGTGGCAGGCTCCGGGCAACTCCGGCCCTGGGTCAGGGAGAT
TGCCTCCGCAGCCGCCCTGGCTTCCCA
>58
GAAGAACAGAGAAGGATGCGGGCTGGCTGAGAGCGGTGGCAGGCTCCGGGCAACTC
CGGCCCTGGGTCAGGGAGATTGCCTCCGCAGCCGCCCTGGCTTCCCAAATGCCTTCAC
CCGTCGCGGGCCCAGGACCAGGGGGC
>59
GCGGGCTGGCTGAGAGCGGTGGCAGGCTCCGGGCAACTCCGGCCCTGGGTCAGGGAG
ATTGCCTCCGCAGCCGCCCTGGCTTCCCAAATGCCTTCACCCGTCGCGGGCCCAGGAC
CAGGGGGCGGAGCGCAGGTGTGCGG
>60
CCCTGGGTCAGGGAGATTGCCTCCGCAGCCGCCCTGGCTTCCCAAATGCCTTCACCCG
TCGCGGGCCCAGGACCAGGGGGCGGAGCGCAGGTGTGCGGGGCGGAGAGCGTGAGC
TCTGCGCGCGCGTGACTGCGCCGGGC
>61
ATTGCCTCCGCAGCCGCCCTGGCTTCCCAAATGCCTTCACCCGTCGCGGGCCCAGGAC
CAGGGGGCGGAGCGCAGGTGTGCGGGGCGGAGAGCGTGAGCTCTGCGCGCGCGTGA
CTGCGCCGGGCCCCCCCGGGGCAATG
>62
CTCCGCAGCCGCCCTGGCTTCCCAAATGCCTTCACCCGTCGCGGGCCCAGGACCAGGG
GGCGGAGCGCAGGTGTGCGGGGCGGAGAGCGTGAGCTCTGCGCGCGCGTGACTGCG
CCGGGCCCCCCCGGGGCAATGTGCGG
>63
GGCTTCCCAAATGCCTTCACCCGTCGCGGGCCCAGGACCAGGGGGCGGAGCGCAGGT
GTGCGGGGCGGAGAGCGTGAGCTCTGCGCGCGCGTGACTGCGCCGGGCCCCCCCGGG
GCAATGTGCGGCGCCGAGTTTGGGTC
>64
GGGCGGAGAGCGTGAGCTCTGCGCGCGCGTGACTGCGCCGGGCCCCCCCGGGGCAAT
GTGCGGCGCCGAGTTTGGGTCCGGGTAGGGGACAGGCGGTCCGGCGTGGGTGTTGTC
CACTGGTCTATCCGGTACGCCTGGCG

>65
GCTCTGCGCGCGCGTGACTGCGCCGGGCCCCCCGGGGCAATGTGCGGCGCCGAGTT
TGGGTCCGGGTAGGGGACAGGCGGTCCGGCGTGGGTGTTGTCCACTGGTCTATCCGGT
ACGCCTGGCGCAGCTCCTGCCCTCA
>66
GGGTCCGGGTAGGGGACAGGCGGTCCGGCGTGGGTGTTGTCCACTGGTCTATCCGGTA
CGCCTGGCGCAGCTCCTGCCCTCAGATGCCCCGACCGAGCGGAGAGTCAGGAATCTTC
TCCGAGGGCTGGGGGAAGCCGGCT
>67
GGACAGGCGGTCCGGCGTGGGTGTTGTCCACTGGTCTATCCGGTACGCCTGGCGCAGC
TCCTGCCCTCAGATGCCCCGACCGAGCGGAGAGTCAGGAATCTTCTCCGAGGGCTGGG
GGAAGCCGGCTTTCCGCACCTTTG
>68
GCGTGGGTGTTGTCCACTGGTCTATCCGGTACGCCTGGCGCAGCTCCTGCCCTCAGATG
CCCCGACCGAGCGGAGAGTCAGGAATCTTCTCCGAGGGCTGGGGGAAGCCGGCTTTC
CGCACCTTTGAGGGACGGAGTGGC
>69
GGTGTTGTCCACTGGTCTATCCGGTACGCCTGGCGCAGCTCCTGCCCTCAGATGCCCCG
ACCGAGCGGAGAGTCAGGAATCTTCTCCGAGGGCTGGGGGAAGCCGGCTTTCCGCAC
CTTTGAGGGACGGAGTGGCGGGAT
>70
CCGGTACGCCTGGCGCAGCTCCTGCCCTCAGATGCCCCGACCGAGCGGAGAGTCAGG
AATCTTCTCCGAGGGCTGGGGGAAGCCGGCTTTCCGCACCTTTGAGGGACGGAGTGG
CGGGATCTTCGGAGCTTCCTAGAGAA
>71
GCCCCGACCGAGCGGAGAGTCAGGAATCTTCTCCGAGGGCTGGGGGAAGCCGGCTTT
CCGCACCTTTGAGGGACGGAGTGGCGGGATCTTCGGAGCTTCCTAGAGAAGTGACCA
CGGCCTGCGCCCGGTTTGGGGCAAGC
>72
CGAGCGGAGAGTCAGGAATCTTCTCCGAGGGCTGGGGGAAGCCGGCTTTCCGCACCT
TTGAGGGACGGAGTGGCGGGATCTTCGGAGCTTCCTAGAGAAGTGACCACGGCCTGC
GCCCGGTTTGGGGCAAGCGGCTGGAT
>73
ATCTTCTCCGAGGGCTGGGGGAAGCCGGCTTTCCGCACCTTTGAGGGACGGAGTGGC
GGGATCTTCGGAGCTTCCTAGAGAAGTGACCACGGCCTGCGCCCGGTTTGGGGCAAG
CGGCTGGATGGGATGGCTCGGCTTCC
>74
GGGACGGAGTGGCGGGATCTTCGGAGCTTCCTAGAGAAGTGACCACGGCCTGCGCCC
GGTTTGGGGCAAGCGGCTGGATGGGATGGCTCGGCTTCCCGAGGATCTGACCCCCGGC
CGGAGACAAATGGCCCTCGCATCCT
>75
CTAGAGAAGTGACCACGGCCTGCGCCCGGTTTGGGGCAAGCGGCTGGATGGGATGGC
TCGGCTTCCCGAGGATCTGACCCCCGGCCGGAGACAAATGGCCCTCGCATCCTAGGAG
CTATGCTGCCAGTTCCCTGCCCAAC
>76
TGCGCCCGGTTTGGGGCAAGCGGCTGGATGGGATGGCTCGGCTTCCCGAGGATCTGAC

CCCCGGCCGGAGACAAATGGCCCTCGCATCCTAGGAGCTATGCTGCCAGTTCCCTGCC
CAACCCGGGACGCTCAAAGCCCCA
>77
GCTGGATGGGATGGCTCGGCTTCCCGAGGATCTGACCCCCGGCCGGAGACAAATGGCC
CTCGCATCCTAGGAGCTATGCTGCCAGTTCCCTGCCCAACCCGGGACGCTCAAAGCCC
CAGATACCGGGAGAGCTGAGCCGG
>78
TCGGCTTCCCGAGGATCTGACCCCCGGCCGGAGACAAATGGCCCTCGCATCCTAGGAG
CTATGCTGCCAGTTCCCTGCCCAACCCGGGACGCTCAAAGCCCCAGATACCGGGAGAG
CTGAGCCGGGCGAGGGGCGGAGCT
>79
CGGAGACAAATGGCCCTCGCATCCTAGGAGCTATGCTGCCAGTTCCCTGCCCAACCCG
GGACGCTCAAAGCCCCAGATACCGGGAGAGCTGAGCCGGGCGAGGGGCGGAGCTCCC
CTGGGGGCGTGGCCTGGGTGTGGAT
>80
CAAATGGCCCTCGCATCCTAGGAGCTATGCTGCCAGTTCCCTGCCCAACCCGGGACGC
TCAAAGCCCCAGATACCGGGAGAGCTGAGCCGGGCGAGGGGCGGAGCTCCCCTGGGG
GCGTGGCCTGGGTGTGGATGGAGGC
>81
TCGCATCCTAGGAGCTATGCTGCCAGTTCCCTGCCCAACCCGGGACGCTCAAAGCCCC
AGATACCGGGAGAGCTGAGCCGGGCGAGGGGCGGAGCTCCCCTGGGGGCGTGGCCTG
GGTGTGGATGGAGGCGGGGCCGCAA
>82
AGTTCCCTGCCCAACCCGGGACGCTCAAAGCCCCAGATACCGGGAGAGCTGAGCCGG
GCGAGGGGCGGAGCTCCCCTGGGGGCGTGGCCTGGGTGTGGATGGAGGCGGGGCCGC
AACGCCCAGTTCCGCAGAGGCCGGCT
>83
AGATACCGGGAGAGCTGAGCCGGGCGAGGGGCGGAGCTCCCCTGGGGGCGTGGCCTG
GGTGTGGATGGAGGCGGGGCCGCAACGCCCAGTTCCGCAGAGGCCGGCTACTTGCTT
GCGCAGCACGCACGGCCGGGGAGGGG
>84
CCGGGCGAGGGGCGGAGCTCCCCTGGGGGCGTGGCCTGGGTGTGGATGGAGGCGGG
GCCGCAACGCCCAGTTCCGCAGAGGCCGGCTACTTGCTTGCGCAGCACGCACGGCCG
GGGAGGGGCTGGCGGCGGCCGCGGGCG
>85
TGGCCTGGGTGTGGATGGAGGCGGGGCCGCAACGCCCAGTTCCGCAGAGGCCGGCTA
CTTGCTTGCGCAGCACGCACGGCCGGGGAGGGGCTGGCGGCGGCCGCGGGCGGCGG
GCGGCGGAGTCGCGGGGCCTGCTCCCG
>86
GAGGCGGGGCCGCAACGCCCAGTTCCGCAGAGGCCGGCTACTTGCTTGCGCAGCACG
CACGGCCGGGGAGGGGCTGGCGGCGGCCGCGGGCGGCGGGCGGCGGAGTCGCGGGG
CCTGCTCCCGCCGGCACCATGGCCAAG
>87
GGCTACTTGCTTGCGCAGCACGCACGGCCGGGGAGGGGCTGGCGGCGGCCGCGGGCG
GCGGGCGGCGGAGTCGCGGGGCCTGCTCCCGCCGGCACCATGGCCAAGACCGTGGCC
TATTTCTACGACCCCGACGTGGGCAA

>88
GGGCTGGCGGCGGCCGCGGGCGGCGGGCGGCGGAGTCGCGGGGCCTGCTCCCGCCG
GCACCATGGCCAAGACCGTGGCCTATTTCTACGACCCCGACGTGGGCAACTTCCACTA
CGGTGAGGAAACAGGGCTATGCATGA
>89
CGCCGGCACCATGGCCAAGACCGTGGCCTATTTCTACGACCCCGACGTGGGCAACTTC
CACTACGGTGAGGAAACAGGGCTATGCATGAGGGGTTGGGACACGGAGGTTTTGAGG
CGGGGTTGCAACTCCAGGGGTGAGA
>90
ACCATGGCCAAGACCGTGGCCTATTTCTACGACCCCGACGTGGGCAACTTCCACTACG
GTGAGGAAACAGGGCTATGCATGAGGGGTTGGGACACGGAGGTTTTGAGGCGGGGTT
GCAACTCCAGGGGTGAGAGCTGACG
>91
CGACCCCGACGTGGGCAACTTCCACTACGGTGAGGAAACAGGGCTATGCATGAGGGG
TTGGGACACGGAGGTTTTGAGGCGGGGTTGCAACTCCAGGGGTGAGAGCTGACGAAC
TCTCTTCTCCCCACCCCCAGGAGCTG
>92
ACGTGGGCAACTTCCACTACGGTGAGGAAACAGGGCTATGCATGAGGGGTTGGGACA
CGGAGGTTTTGAGGCGGGGTTGCAACTCCAGGGGTGAGAGCTGACGAACTCTCTTCT
CCCCACCCCCAGGAGCTGGACACCCT
>93
ACGGTGAGGAAACAGGGCTATGCATGAGGGGTTGGGACACGGAGGTTTTGAGGCGGG
GTTGCAACTCCAGGGGTGAGAGCTGACGAACTCTCTTCTCCCCACCCCCAGGAGCTG
GACACCCTATGAAGCCTCATCGCCTG
>94
GAGGTTTTGAGGCGGGGTTGCAACTCCAGGGGTGAGAGCTGACGAACTCTCTTCTCC
CCACCCCCAGGAGCTGGACACCCTATGAAGCCTCATCGCCTGGCATTGACCCATAGCC
TGGTCCTGCATTACGGTCTCTATAA
>95
TTGCAACTCCAGGGGTGAGAGCTGACGAACTCTCTTCTCCCCACCCCCAGGAGCTGG
ACACCCTATGAAGCCTCATCGCCTGGCATTGACCCATAGCCTGGTCCTGCATTACGGTC
TCTATAAGAAGATGATCGTGAGTT
>96
GAACTCTCTTCTCCCCACCCCCAGGAGCTGGACACCCTATGAAGCCTCATCGCCTGGC
ATTGACCCATAGCCTGGTCCTGCATTACGGTCTCTATAAGAAGATGATCGTGAGTTCCG
CCGCCTCGGCCGGGGGTTGGGGG
>97
TGGACACCCTATGAAGCCTCATCGCCTGGCATTGACCCATAGCCTGGTCCTGCATTACG
GTCTCTATAAGAAGATGATCGTGAGTTCCGCCGCCTCGGCCGGGGGTTGGGGGGTGGG
GCGAGCTGTGGCCGTAGGGATAG
>98
ACACCCTATGAAGCCTCATCGCCTGGCATTGACCCATAGCCTGGTCCTGCATTACGGTC
TCTATAAGAAGATGATCGTGAGTTCCGCCGCCTCGGCCGGGGGTTGGGGGGTGGGGCG
AGCTGTGGCCGTAGGGATAGGTG
>99
CATTACGGTCTCTATAAGAAGATGATCGTGAGTTCCGCCGCCTCGGCCGGGGGTTGGG

GGGTGGGGCGAGCTGTGGCCGTAGGGATAGGTGGGCTGGACTGAGTGCATGAAGTGC
GACCCGCCCCTAGAGTATCGGGGAG
>100
TATAAGAAGATGATCGTGAGTTCCGCCGCCTCGGCCGGGGGTTGGGGGGTGGGGCGA
GCTGTGGCCGTAGGGATAGGTGGGCTGGACTGAGTGCATGAAGTGCGACCCGCCCCTA
GAGTATCGGGGAGCTGGATAGAAGT
>101
TCCGCCGCCTCGGCCGGGGGTTGGGGGGTGGGGCGAGCTGTGGCCGTAGGGATAGGT
GGGCTGGACTGAGTGCATGAAGTGCGACCCGCCCCTAGAGTATCGGGGAGCTGGATAG
AAGTCACCAGGAGTACCTTGAAAGT
>102
GGGTGGGGCGAGCTGTGGCCGTAGGGATAGGTGGGCTGGACTGAGTGCATGAAGTGC
GACCCGCCCCTAGAGTATCGGGGAGCTGGATAGAAGTCACCAGGAGTACCTTGAAAGT
GCAGGGTGTTGGGGGTGGCGTCCTC
>103
GTGGGGCGAGCTGTGGCCGTAGGGATAGGTGGGCTGGACTGAGTGCATGAAGTGCGA
CCCGCCCCTAGAGTATCGGGGAGCTGGATAGAAGTCACCAGGAGTACCTTGAAAGTGC
AGGGTGTTGGGGGTGGCGTCCTCTG
>104
GTAGGGATAGGTGGGCTGGACTGAGTGCATGAAGTGCGACCCGCCCCTAGAGTATCGG
GGAGCTGGATAGAAGTCACCAGGAGTACCTTGAAAGTGCAGGGTGTTGGGGGTGGCG
TCCTCTGCTTAATGGAAAGTACTTT
>105
TGAAGTGCGACCCGCCCCTAGAGTATCGGGGAGCTGGATAGAAGTCACCAGGAGTACC
TTGAAAGTGCAGGGTGTTGGGGGTGGCGTCCTCTGCTTAATGGAAAGTACTTTCTCAC
CTCAGGTCGGCCACCCTCTGACTG
>106
GGATAGAAGTCACCAGGAGTACCTTGAAAGTGCAGGGTGTTGGGGGTGGCGTCCTCT
GCTTAATGGAAAGTACTTTCTCACCTCAGGTCGGCCACCCTCTGACTGTAGGCACACTA
GCCCTTGGCCTAGGGTTAGCGTGA
>107
GAGTACCTTGAAAGTGCAGGGTGTTGGGGGTGGCGTCCTCTGCTTAATGGAAAGTACT
TTCTCACCTCAGGTCGGCCACCCTCTGACTGTAGGCACACTAGCCCTTGGCCTAGGGTT
AGCGTGATGTGGGGCTGGTTTGA
>108
GTTGGGGGTGGCGTCCTCTGCTTAATGGAAAGTACTTTCTCACCTCAGGTCGGCCACC
CTCTGACTGTAGGCACACTAGCCCTTGGCCTAGGGTTAGCGTGATGTGGGGCTGGTTT
GAAGACAGAAGGTGTGTTCCTCCT
>109
GGCGTCCTCTGCTTAATGGAAAGTACTTTCTCACCTCAGGTCGGCCACCCTCTGACTGT
AGGCACACTAGCCCTTGGCCTAGGGTTAGCGTGATGTGGGGCTGGTTTGAAGACAGAA
GGTGTGTTCCTCCTCTGCATTTT
>110
GAAAGTACTTTCTCACCTCAGGTCGGCCACCCTCTGACTGTAGGCACACTAGCCCTTG
GCCTAGGGTTAGCGTGATGTGGGGCTGGTTTGAAGACAGAAGGTGTGTTCCTCCTCTG
CATTTTAGAGTGTGGATAGGGCCT

>111
CGGCCACCCTCTGACTGTAGGCACACTAGCCCTTGGCCTAGGGTTAGCGTGATGTGGG
GCTGGTTTGAAGACAGAAGGTGTGTTCCTCCTCTGCATTTTAGAGTGTGGATAGGGCC
TTGGCCTCAGGAATTAGGGTTAGG
>112
CACCCTCTGACTGTAGGCACACTAGCCCTTGGCCTAGGGTTAGCGTGATGTGGGGCTG
GTTTGAAGACAGAAGGTGTGTTCCTCCTCTGCATTTTAGAGTGTGGATAGGGCCTTGG
CCTCAGGAATTAGGGTTAGGGGAG
>113
CTTGGCCTAGGGTTAGCGTGATGTGGGGCTGGTTTGAAGACAGAAGGTGTGTTCCTCC
TCTGCATTTTAGAGTGTGGATAGGGCCTTGGCCTCAGGAATTAGGGTTAGGGGAGCGG
ATATCACTGTATCAGGGTGTGAGG
>114
GGGCTGGTTTGAAGACAGAAGGTGTGTTCCTCCTCTGCATTTTAGAGTGTGGATAGGG
CCTTGGCCTCAGGAATTAGGGTTAGGGGAGCGGATATCACTGTATCAGGGTGTGAGGT
TTACCCTCAGTTCCCACTCCTCTT
>115
TTTGAAGACAGAAGGTGTGTTCCTCCTCTGCATTTTAGAGTGTGGATAGGGCCTTGGCC
TCAGGAATTAGGGTTAGGGGAGCGGATATCACTGTATCAGGGTGTGAGGTTTACCCTCA
GTTCCCACTCCTCTTTTACCAC
>116
GACAGAAGGTGTGTTCCTCCTCTGCATTTTAGAGTGTGGATAGGGCCTTGGCCTCAGG
AATTAGGGTTAGGGGAGCGGATATCACTGTATCAGGGTGTGAGGTTTACCCTCAGTTCC
CACTCCTCTTTTACCACCTTTCT
>117
GCATTTTAGAGTGTGGATAGGGCCTTGGCCTCAGGAATTAGGGTTAGGGGAGCGGATAT
CACTGTATCAGGGTGTGAGGTTTACCCTCAGTTCCCACTCCTCTTTTACCACCTTTCTTT
ACCTCCAAGGAGCTAGGGCTC
>118
TCACTGTATCAGGGTGTGAGGTTTACCCTCAGTTCCCACTCCTCTTTTACCACCTTTCTT
TACCTCCAAGGAGCTAGGGCTCCAGGGTGGAACGATGCTGCCGTCTTTGTAAATGTGT
ATGTGCGTGTGGAACAGAGGCG
>119
CTGTATCAGGGTGTGAGGTTTACCCTCAGTTCCCACTCCTCTTTTACCACCTTTCTTTAC
CTCCAAGGAGCTAGGGCTCCAGGGTGGAACGATGCTGCCGTCTTTGTAAATGTGTATG
TGCGTGTGGAACAGAGGCGATA
>120
CCAAGGAGCTAGGGCTCCAGGGTGGAACGATGCTGCCGTCTTTGTAAATGTGTATGTG
CGTGTGGAACAGAGGCGATATTTTAGATATTCCTTTTTCAGCCTGGAATACCTCTACATG
GTTCTGCTTAATCGCTATCCAG
>121
AAGGAGCTAGGGCTCCAGGGTGGAACGATGCTGCCGTCTTTGTAAATGTGTATGTGCG
TGTGGAACAGAGGCGATATTTTAGATATTCCTTTTTCAGCCTGGAATACCTCTACATGGT
TCTGCTTAATCGCTATCCAGTT
>122
ATGCTGCCGTCTTTGTAAATGTGTATGTGCGTGTGGAACAGAGGCGATATTTTAGATATT

CCTTTTTCAGCCTGGAATACCTCTACATGGTTCTGCTTAATCGCTATCCAGTTTTTCATCT
ACCAGACATACACATGCTG
>123
AGGCGATATTTTAGATATTCCTTTTTCAGCCTGGAATACCTCTACATGGTTCTGCTTAATC
GCTATCCAGTTTTTCATCTACCAGACATACACATGCTGTCTTCCCAGCTTGGAATTTATG
GCTGTGATATGGGATAGTA
>124
TATTCCTTTTTCAGCCTGGAATACCTCTACATGGTTCTGCTTAATCGCTATCCAGTTTTTC
ATCTACCAGACATACACATGCTGTCTTCCCAGCTTGGAATTTATGGCTGTGATATGGGAT
AGTAACAGTTAAGCCACGC
>125
CCTCTACATGGTTCTGCTTAATCGCTATCCAGTTTTTCATCTACCAGACATACACATGCT
GTCTTCCCAGCTTGGAATTTATGGCTGTGATATGGGATAGTAACAGTTAAGCCACGCCA
GGCGTGGTGGCCCATGCCTGT
>126
AGTTTTTCATCTACCAGACATACACATGCTGTCTTCCCAGCTTGGAATTTATGGCTGTGA
TATGGGATAGTAACAGTTAAGCCACGCCAGGCGTGGTGGCCCATGCCTGTAATCCCAGC
ACTTTGGGAGGTTGAAATGGG
>127
TCATCTACCAGACATACACATGCTGTCTTCCCAGCTTGGAATTTATGGCTGTGATATGGG
ATAGTAACAGTTAAGCCACGCCAGGCGTGGTGGCCCATGCCTGTAATCCCAGCACTTT
GGGAGGTTGAAATGGGGGGGAT
>128
TACCAGACATACACATGCTGTCTTCCCAGCTTGGAATTTATGGCTGTGATATGGGATAGT
AACAGTTAAGCCACGCCAGGCGTGGTGGCCCATGCCTGTAATCCCAGCACTTTGGGAG
GTTGAAATGGGGGGGATCGCTT
>129
TACACATGCTGTCTTCCCAGCTTGGAATTTATGGCTGTGATATGGGATAGTAACAGTTAA
GCCACGCCAGGCGTGGTGGCCCATGCCTGTAATCCCAGCACTTTGGGAGGTTGAAATG
GGGGGGATCGCTTGAGGCCAGG
>130
TTATGGCTGTGATATGGGATAGTAACAGTTAAGCCACGCCAGGCGTGGTGGCCCATGCC
TGTAATCCCAGCACTTTGGGAGGTTGAAATGGGGGGGATCGCTTGAGGCCAGGAGTTG
GAGACCAGCTTGGGCCACAGTGG
>131
CGCCAGGCGTGGTGGCCCATGCCTGTAATCCCAGCACTTTGGGAGGTTGAAATGGGGG
GGATCGCTTGAGGCCAGGAGTTGGAGACCAGCTTGGGCCACAGTGGGACCCACATCT
ACAAAAAATAAAAATAAAAAAAGTA
>132
GGCGTGGTGGCCCATGCCTGTAATCCCAGCACTTTGGGAGGTTGAAATGGGGGGGATC
GCTTGAGGCCAGGAGTTGGAGACCAGCTTGGGCCACAGTGGGACCCACATCTACAAA
AAATAAAAATAAAAAAAGTAGCCAT
>133
CAGCACTTTGGGAGGTTGAAATGGGGGGGATCGCTTGAGGCCAGGAGTTGGAGACCA
GCTTGGGCCACAGTGGGACCCACATCTACAAAAAATAAAAATAAAAAAAGTAGCCATG
TGCGGTGGGTGTGCCTGTAGTCCCA

>134
AGGCCAGGAGTTGGAGACCAGCTTGGGCCACAGTGGGACCCACATCTACAAAAAATA
AAAATAAAAAAAGTAGCCATGTGCGGTGGGTGTGCCTGTAGTCCCAACTACTTGGGAG
GCCGAGGCGAGAGGATGGCTTGGGC
>135
CCAGGAGTTGGAGACCAGCTTGGGCCACAGTGGGACCCACATCTACAAAAAATAAAA
ATAAAAAAAGTAGCCATGTGCGGTGGGTGTGCCTGTAGTCCCAACTACTTGGGAGGCC
GAGGCGAGAGGATGGCTTGGGCCCA
>136
CTACAAAAAATAAAAATAAAAAAAGTAGCCATGTGCGGTGGGTGTGCCTGTAGTCCCA
ACTACTTGGGAGGCCGAGGCGAGAGGATGGCTTGGGCCCAGGAGTTTAAAGTTACAG
TGATCTATGATCAGCAGCCTGAGTT
>137
AAAATAAAAATAAAAAAAGTAGCCATGTGCGGTGGGTGTGCCTGTAGTCCCAACTACT
TGGGAGGCCGAGGCGAGAGGATGGCTTGGGCCCAGGAGTTTAAAGTTACAGTGATCTA
TGATCAGCAGCCTGAGTTGACAGG
>138
CATGTGCGGTGGGTGTGCCTGTAGTCCCAACTACTTGGGAGGCCGAGGCGAGAGGATG
GCTTGGGCCCAGGAGTTTAAAGTTACAGTGATCTATGATCAGCAGCCTGAGTTGACAG
GGAGGACCTTGTCTCAAAAGAAAA
>139
GCCTGTAGTCCCAACTACTTGGGAGGCCGAGGCGAGAGGATGGCTTGGGCCCAGGAG
TTTAAAGTTACAGTGATCTATGATCAGCAGCCTGAGTTGACAGGGAGGACCTTGTCTCA
AAAGAAAAAATAAGACAAAACAGC
>140
CTACTTGGGAGGCCGAGGCGAGAGGATGGCTTGGGCCCAGGAGTTTAAAGTTACAGT
GATCTATGATCAGCAGCCTGAGTTGACAGGGAGGACCTTGTCTCAAAAGAAAAAATAA
GACAAAACAGCTAAACCCAGGCTTT
>141
CTTGGGAGGCCGAGGCGAGAGGATGGCTTGGGCCCAGGAGTTTAAAGTTACAGTGAT
CTATGATCAGCAGCCTGAGTTGACAGGGAGGACCTTGTCTCAAAAGAAAAAATAAGA
CAAAACAGCTAAACCCAGGCTTTATG
>142
AGAGGATGGCTTGGGCCCAGGAGTTTAAAGTTACAGTGATCTATGATCAGCAGCCTGA
GTTGACAGGGAGGACCTTGTCTCAAAAGAAAAAATAAGACAAAACAGCTAAACCCAG
GCTTTATGTGTTGCTAGGGAAACTA
>143
AAAGTTACAGTGATCTATGATCAGCAGCCTGAGTTGACAGGGAGGACCTTGTCTCAAA
AGAAAAAATAAGACAAAACAGCTAAACCCAGGCTTTATGTGTTGCTAGGGAAACTAGT
GAACAATATGAATGAATGTTCATT
>144
GTGATCTATGATCAGCAGCCTGAGTTGACAGGGAGGACCTTGTCTCAAAAGAAAAAAT
AAGACAAAACAGCTAAACCCAGGCTTTATGTGTTGCTAGGGAAACTAGTGAACAATAT
GAATGAATGTTCATTGCTTGTGCC
>145
ATCAGCAGCCTGAGTTGACAGGGAGGACCTTGTCTCAAAAGAAAAAATAAGACAAAA

CAGCTAAACCCAGGCTTTATGTGTTGCTAGGGAAACTAGTGAACAATATGAATGAATGT
TCATTGCTTGTGCCAAGGCACAGA
>146
ACAGGGAGGACCTTGTCTCAAAAGAAAAAATAAGACAAAACAGCTAAACCCAGGCTT
TATGTGTTGCTAGGGAAACTAGTGAACAATATGAATGAATGTTCATTGCTTGTGCCAAG
GCACAGACAAATGGACTGGTCATA
>147
AACCCAGGCTTTATGTGTTGCTAGGGAAACTAGTGAACAATATGAATGAATGTTCATTG
CTTGTGCCAAGGCACAGACAAATGGACTGGTCATAGAAAAGACTGAGGTGATGTTAGT
GCAGGAATCTATATCCCTTGAGA
>148
GGCTTTATGTGTTGCTAGGGAAACTAGTGAACAATATGAATGAATGTTCATTGCTTGTG
CCAAGGCACAGACAAATGGACTGGTCATAGAAAAGACTGAGGTGATGTTAGTGCAGG
AATCTATATCCCTTGAGATTGTTT
>149
TAGTGAACAATATGAATGAATGTTCATTGCTTGTGCCAAGGCACAGACAAATGGACTG
GTCATAGAAAAGACTGAGGTGATGTTAGTGCAGGAATCTATATCCCTTGAGATTGTTTG
CCTGTATGTACCTGTGTGCATTT
>150
TGAATGTTCATTGCTTGTGCCAAGGCACAGACAAATGGACTGGTCATAGAAAAGACTG
AGGTGATGTTAGTGCAGGAATCTATATCCCTTGAGATTGTTTGCCTGTATGTACCTGTGT
GCATTTTCTGGGAGAAGAAAAG
>151
GTTCATTGCTTGTGCCAAGGCACAGACAAATGGACTGGTCATAGAAAAGACTGAGGTG
ATGTTAGTGCAGGAATCTATATCCCTTGAGATTGTTTGCCTGTATGTACCTGTGTGCATTT
TCTGGGAGAAGAAAAGCTCTT
>152
GGCACAGACAAATGGACTGGTCATAGAAAAGACTGAGGTGATGTTAGTGCAGGAATCT
ATATCCCTTGAGATTGTTTGCCTGTATGTACCTGTGTGCATTTTCTGGGAGAAGAAAAG
CTCTTTCTTGTGCAGTTTTCAAA
>153
GATGTTAGTGCAGGAATCTATATCCCTTGAGATTGTTTGCCTGTATGTACCTGTGTGCAT
TTTCTGGGAGAAGAAAAGCTCTTTCTTGTGCAGTTTTCAAAGAGGTGTGACACATCCC
CCTGACCTTCCAAAGATAATAA
>154
GTTAGTGCAGGAATCTATATCCCTTGAGATTGTTTGCCTGTATGTACCTGTGTGCATTTT
CTGGGAGAAGAAAAGCTCTTTCTTGTGCAGTTTTCAAAGAGGTGTGACACATCCCCCT
GACCTTCCAAAGATAATAAATC
>155
CCCTTGAGATTGTTTGCCTGTATGTACCTGTGTGCATTTTCTGGGAGAAGAAAAGCTCT
TTCTTGTGCAGTTTTCAAAGAGGTGTGACACATCCCCCTGACCTTCCAAAGATAATAAA
TCTTTGATTTTAATTAGAAAGT
>156
CTGGGAGAAGAAAAGCTCTTTCTTGTGCAGTTTTCAAAGAGGTGTGACACATCCCCCT
GACCTTCCAAAGATAATAAATCTTTGATTTTAATTAGAAAGTTGACATATATCCCATATAT
TTGATCCAGAAAGAAGATCCA

>157
TGCAGTTTTCAAAGAGGTGTGACACATCCCCCTGACCTTCCAAAGATAATAAATCTTTG
ATTTTAATTAGAAAGTTGACATATATCCCATATATTTGATCCAGAAAGAAGATCCAGAGG
AAAGAGGACAGAATCAGAGCT
>158
GACCTTCCAAAGATAATAAATCTTTGATTTTAATTAGAAAGTTGACATATATCCCATATAT
TTGATCCAGAAAGAAGATCCAGAGGAAAGAGGACAGAATCAGAGCTTGGGGAGGAG
ATGAGCATGGAGAGAACTGGGAA
>159
TTCCAAAGATAATAAATCTTTGATTTTAATTAGAAAGTTGACATATATCCCATATATTTGA
TCCAGAAAGAAGATCCAGAGGAAAGAGGACAGAATCAGAGCTTGGGGAGGAGATGA
GCATGGAGAGAACTGGGAATAGG
>160
TCTTTGATTTTAATTAGAAAGTTGACATATATCCCATATATTTGATCCAGAAAGAAGATCC
AGAGGAAAGAGGACAGAATCAGAGCTTGGGGAGGAGATGAGCATGGAGAGAACTGG
GAATAGGAGATTCCAAGGAACAG
>161
CCCATATATTTGATCCAGAAAGAAGATCCAGAGGAAAGAGGACAGAATCAGAGCTTGG
GGAGGAGATGAGCATGGAGAGAACTGGGAATAGGAGATTCCAAGGAACAGGGCAGGT
GGATCAGGGCTCAAAGTTGAGAAGG
>162
ATTTGATCCAGAAAGAAGATCCAGAGGAAAGAGGACAGAATCAGAGCTTGGGGAGGA
GATGAGCATGGAGAGAACTGGGAATAGGAGATTCCAAGGAACAGGGCAGGTGGATCA
GGGCTCAAAGTTGAGAAGGTACGATA
>163
GAAGATCCAGAGGAAAGAGGACAGAATCAGAGCTTGGGGAGGAGATGAGCATGGAG
AGAACTGGGAATAGGAGATTCCAAGGAACAGGGCAGGTGGATCAGGGCTCAAAGTTG
AGAAGGTACGATAGGCTGAGCTGGAGG
>164
AGGACAGAATCAGAGCTTGGGGAGGAGATGAGCATGGAGAGAACTGGGAATAGGAGA
TTCCAAGGAACAGGGCAGGTGGATCAGGGCTCAAAGTTGAGAAGGTACGATAGGCTG
AGCTGGAGGTGGAAAGGAAAAGAAGT
>165
GGAGATGAGCATGGAGAGAACTGGGAATAGGAGATTCCAAGGAACAGGGCAGGTGGA
TCAGGGCTCAAAGTTGAGAAGGTACGATAGGCTGAGCTGGAGGTGGAAAGGAAAAG
AAGTGGTCTTCAGGGGGCTCATTTGTA
>166
TGAGCATGGAGAGAACTGGGAATAGGAGATTCCAAGGAACAGGGCAGGTGGATCAGG
GCTCAAAGTTGAGAAGGTACGATAGGCTGAGCTGGAGGTGGAAAGGAAAAGAAGTG
GTCTTCAGGGGGCTCATTTGTAATAAC
>167
GAACTGGGAATAGGAGATTCCAAGGAACAGGGCAGGTGGATCAGGGCTCAAAGTTGA
GAAGGTACGATAGGCTGAGCTGGAGGTGGAAAGGAAAAGAAGTGGTCTTCAGGGGG
CTCATTTGTAATAACTTACCTGGTTAA
>168
CAAGGAACAGGGCAGGTGGATCAGGGCTCAAAGTTGAGAAGGTACGATAGGCTGAGC

TGGAGGTGGAAAGGAAAAGAAGTGGTCTTCAGGGGGCTCATTTGTAATAACTTACCTG
GTTAATCGGAGTCTCTCTACTGACT
>169
ATCAGGGCTCAAAGTTGAGAAGGTACGATAGGCTGAGCTGGAGGTGGAAAGGAAAAG
AAGTGGTCTTCAGGGGGCTCATTTGTAATAACTTACCTGGTTAATCGGAGTCTCTCTAC
TGACTTAAGGATTGTGGGATGAGG
>170
CTGGAGGTGGAAAGGAAAAGAAGTGGTCTTCAGGGGGCTCATTTGTAATAACTTACCT
GGTTAATCGGAGTCTCTCTACTGACTTAAGGATTGTGGGATGAGGGGAATGCTATGTTT
GGCTTCATATAGTCTCCAGGACT
>171
AGAAGTGGTCTTCAGGGGGCTCATTTGTAATAACTTACCTGGTTAATCGGAGTCTCTCT
ACTGACTTAAGGATTGTGGGATGAGGGGAATGCTATGTTTGGCTTCATATAGTCTCCAG
GACTGGTTCAGTATGGGTGGTC
>172
GGGGGCTCATTTGTAATAACTTACCTGGTTAATCGGAGTCTCTCTACTGACTTAAGGATT
GTGGGATGAGGGGAATGCTATGTTTGGCTTCATATAGTCTCCAGGACTGGTTCAGTATG
GGTGGTCTGAGCCTGTGGAGT
>173
GTTAATCGGAGTCTCTCTACTGACTTAAGGATTGTGGGATGAGGGGAATGCTATGTTTG
GCTTCATATAGTCTCCAGGACTGGTTCAGTATGGGTGGTCTGAGCCTGTGGAGTGGGGC
TGACCCTGCCTGGGTTCATCTT
>174
ACTGACTTAAGGATTGTGGGATGAGGGGAATGCTATGTTTGGCTTCATATAGTCTCCAG
GACTGGTTCAGTATGGGTGGTCTGAGCCTGTGGAGTGGGGCTGACCCTGCCTGGGTTC
ATCTTGTGTATCCATCCCGAGGT
>175
GTGGGATGAGGGGAATGCTATGTTTGGCTTCATATAGTCTCCAGGACTGGTTCAGTATG
GGTGGTCTGAGCCTGTGGAGTGGGGCTGACCCTGCCTGGGTTCATCTTGTGTATCCATC
CCGAGGTCTTCAAGCCATACCA
>176
GGGGAATGCTATGTTTGGCTTCATATAGTCTCCAGGACTGGTTCAGTATGGGTGGTCTG
AGCCTGTGGAGTGGGGCTGACCCTGCCTGGGTTCATCTTGTGTATCCATCCCGAGGTCT
TCAAGCCATACCAGGCCTCCCA
>177
GGTCTGAGCCTGTGGAGTGGGGCTGACCCTGCCTGGGTTCATCTTGTGTATCCATCCCG
AGGTCTTCAAGCCATACCAGGCCTCCCAGCATGACATGTGCCGCTTCCACTCCGAGGA
CTACATTGACTTCCTGCAGAGAG
>178
CTGTGGAGTGGGGCTGACCCTGCCTGGGTTCATCTTGTGTATCCATCCCGAGGTCTTCA
AGCCATACCAGGCCTCCCAGCATGACATGTGCCGCTTCCACTCCGAGGACTACATTGA
CTTCCTGCAGAGAGTCAGCCCCA
>179
GTGTATCCATCCCGAGGTCTTCAAGCCATACCAGGCCTCCCAGCATGACATGTGCCGCT
TCCACTCCGAGGACTACATTGACTTCCTGCAGAGAGTCAGCCCCACCAATATGCAAGG
CTTCACCAAGAGTCTTAATGCCT

>180
CAAGCCATACCAGGCCTCCCAGCATGACATGTGCCGCTTCCACTCCGAGGACTACATT
GACTTCCTGCAGAGAGTCAGCCCCACCAATATGCAAGGCTTCACCAAGAGTCTTAATG
CCTTCAACGTAGGCGATGACTGGT
>181
AGCCATACCAGGCCTCCCAGCATGACATGTGCCGCTTCCACTCCGAGGACTACATTGA
CTTCCTGCAGAGAGTCAGCCCCACCAATATGCAAGGCTTCACCAAGAGTCTTAATGCC
TTCAACGTAGGCGATGACTGGTGA
>182
GACATGTGCCGCTTCCACTCCGAGGACTACATTGACTTCCTGCAGAGAGTCAGCCCCA
CCAATATGCAAGGCTTCACCAAGAGTCTTAATGCCTTCAACGTAGGCGATGACTGGTG
AGGGAGGACTGGGAGTTTTGATGT
>183
GACTTCCTGCAGAGAGTCAGCCCCACCAATATGCAAGGCTTCACCAAGAGTCTTAATG
CCTTCAACGTAGGCGATGACTGGTGAGGGAGGACTGGGAGTTTTGATGTCTAACAGTT
TAATGCCAAGCCCTTCAGTGAGGT
>184
GTCAGCCCCACCAATATGCAAGGCTTCACCAAGAGTCTTAATGCCTTCAACGTAGGCG
ATGACTGGTGAGGGAGGACTGGGAGTTTTGATGTCTAACAGTTTAATGCCAAGCCCTT
CAGTGAGGTCTTTTGGCAGCAGTG
>185
TGCCTTCAACGTAGGCGATGACTGGTGAGGGAGGACTGGGAGTTTTGATGTCTAACAG
TTTAATGCCAAGCCCTTCAGTGAGGTCTTTTGGCAGCAGTGGGAAGATCTCAAGGGGG
GCCTACTTGTCAATGCATCATAAT
>186
ACGTAGGCGATGACTGGTGAGGGAGGACTGGGAGTTTTGATGTCTAACAGTTTAATGC
CAAGCCCTTCAGTGAGGTCTTTTGGCAGCAGTGGGAAGATCTCAAGGGGGGCCTACTT
GTCAATGCATCATAATTCATTTTA
>187
GTGAGGGAGGACTGGGAGTTTTGATGTCTAACAGTTTAATGCCAAGCCCTTCAGTGAG
GTCTTTTGGCAGCAGTGGGAAGATCTCAAGGGGGGCCTACTTGTCAATGCATCATAATT
CATTTTATTTGTTTATTCAATAA
>188
CTACTTGTCAATGCATCATAATTCATTTTATTTGTTTATTCAATAAATATTTATTGGTCACT
TGTTCTAGGCAGGCCCTGTGCTAAGTGCTGGGAATGCAGTGATGAACAAAAACAAAAT
TTTAATATCGTATTTTTGCT
>189
TTGTCAATGCATCATAATTCATTTTATTTGTTTATTCAATAAATATTTATTGGTCACTTGTT
CTAGGCAGGCCCTGTGCTAAGTGCTGGGAATGCAGTGATGAACAAAAACAAAATTTTA
ATATCGTATTTTTGCTAAGT
>190
ATAATTCATTTTATTTGTTTATTCAATAAATATTTATTGGTCACTTGTTCTAGGCAGGCCCT
GTGCTAAGTGCTGGGAATGCAGTGATGAACAAAAACAAAATTTTAATATCGTATTTTTG
CTAAGTCTTATAAAGAGGA
>191
GGCCCTGTGCTAAGTGCTGGGAATGCAGTGATGAACAAAAACAAAATTTTAATATCGTA

TTTTTGCTAAGTCTTATAAAGAGGAGGTGTATGACAGATTATGAGAGTGTATACGTGGA
GGATTGTTTAGCTTGGTGGAGT
>192
TAAGTGCTGGGAATGCAGTGATGAACAAAAACAAAATTTTAATATCGTATTTTTGCTAA
GTCTTATAAAGAGGAGGTGTATGACAGATTATGAGAGTGTATACGTGGAGGATTGTTTA
GCTTGGTGGAGTGGCTGGTCGG
>193
ACAAAAACAAAATTTTAATATCGTATTTTTGCTAAGTCTTATAAAGAGGAGGTGTATGAC
AGATTATGAGAGTGTATACGTGGAGGATTGTTTAGCTTGGTGGAGTGGCTGGTCGGTGG
GGCCTTCCTAGAGGAAGTAAA
>194
TAATATCGTATTTTTGCTAAGTCTTATAAAGAGGAGGTGTATGACAGATTATGAGAGTGT
ATACGTGGAGGATTGTTTAGCTTGGTGGAGTGGCTGGTCGGTGGGGCCTTCCTAGAGG
AAGTAAAGATTGAGCTGAGATC
>195
TTTTTGCTAAGTCTTATAAAGAGGAGGTGTATGACAGATTATGAGAGTGTATACGTGGA
GGATTGTTTAGCTTGGTGGAGTGGCTGGTCGGTGGGGCCTTCCTAGAGGAAGTAAAGA
TTGAGCTGAGATCTGAAAAAAGA
>196
AGATTATGAGAGTGTATACGTGGAGGATTGTTTAGCTTGGTGGAGTGGCTGGTCGGTGG
GGCCTTCCTAGAGGAAGTAAAGATTGAGCTGAGATCTGAAAAAAGAGTAGAAGTAATC
AGATGAAAGATGGTTGGAGGGTG
>197
TGAGAGTGTATACGTGGAGGATTGTTTAGCTTGGTGGAGTGGCTGGTCGGTGGGGCCT
TCCTAGAGGAAGTAAAGATTGAGCTGAGATCTGAAAAAAGAGTAGAAGTAATCAGATG
AAAGATGGTTGGAGGGTGAGGGGA
>198
GTGTATACGTGGAGGATTGTTTAGCTTGGTGGAGTGGCTGGTCGGTGGGGCCTTCCTAG
AGGAAGTAAAGATTGAGCTGAGATCTGAAAAAAGAGTAGAAGTAATCAGATGAAAGA
TGGTTGGAGGGTGAGGGGAGAAGT
>199
ACGTGGAGGATTGTTTAGCTTGGTGGAGTGGCTGGTCGGTGGGGCCTTCCTAGAGGAA
GTAAAGATTGAGCTGAGATCTGAAAAAAGAGTAGAAGTAATCAGATGAAAGATGGTTG
GAGGGTGAGGGGAGAAGTATAGCA
>200
GCTTGGTGGAGTGGCTGGTCGGTGGGGCCTTCCTAGAGGAAGTAAAGATTGAGCTGA
GATCTGAAAAAAGAGTAGAAGTAATCAGATGAAAGATGGTTGGAGGGTGAGGGGAGA
AGTATAGCAGGCAGTAGGGACAAGTG
>201
CTAGAGGAAGTAAAGATTGAGCTGAGATCTGAAAAAAGAGTAGAAGTAATCAGATGA
AAGATGGTTGGAGGGTGAGGGGAGAAGTATAGCAGGCAGTAGGGACAAGTGCTAAGG
GTCTGTGGTAGGACTGAGTATGACTC
>202
GAAGTAAAGATTGAGCTGAGATCTGAAAAAAGAGTAGAAGTAATCAGATGAAAGATG
GTTGGAGGGTGAGGGGAGAAGTATAGCAGGCAGTAGGGACAAGTGCTAAGGGTCTGT
GGTAGGACTGAGTATGACTCATTTGA

>203
AAAGATTGAGCTGAGATCTGAAAAAAGAGTAGAAGTAATCAGATGAAAGATGGTTGG
AGGGTGAGGGGAGAAGTATAGCAGGCAGTAGGGACAAGTGCTAAGGGTCTGTGGTAG
GACTGAGTATGACTCATTTGAGGAAC
>204
ATCTGAAAAAAGAGTAGAAGTAATCAGATGAAAGATGGTTGGAGGGTGAGGGGAGAA
GTATAGCAGGCAGTAGGGACAAGTGCTAAGGGTCTGTGGTAGGACTGAGTATGACTCA
TTTGAGGAACAGAAAAAAGGCCAAG
>205
GAAAGATGGTTGGAGGGTGAGGGGAGAAGTATAGCAGGCAGTAGGGACAAGTGCTAA
GGGTCTGTGGTAGGACTGAGTATGACTCATTTGAGGAACAGAAAAAAGGCCAAGTGC
AGAGTATGAAGAGGAGGTGAACTGAA
>206
GGTTGGAGGGTGAGGGGAGAAGTATAGCAGGCAGTAGGGACAAGTGCTAAGGGTCTG
TGGTAGGACTGAGTATGACTCATTTGAGGAACAGAAAAAAGGCCAAGTGCAGAGTAT
GAAGAGGAGGTGAACTGAATTGAGGC
>207
GGAGAAGTATAGCAGGCAGTAGGGACAAGTGCTAAGGGTCTGTGGTAGGACTGAGTAT
GACTCATTTGAGGAACAGAAAAAAGGCCAAGTGCAGAGTATGAAGAGGAGGTGAACT
GAATTGAGGCTAAAGAAATAGGACC
>208
GTATAGCAGGCAGTAGGGACAAGTGCTAAGGGTCTGTGGTAGGACTGAGTATGACTCA
TTTGAGGAACAGAAAAAAGGCCAAGTGCAGAGTATGAAGAGGAGGTGAACTGAATTG
AGGCTAAAGAAATAGGACCTTTTAA
>209
AAGGGTCTGTGGTAGGACTGAGTATGACTCATTTGAGGAACAGAAAAAAGGCCAAGT
GCAGAGTATGAAGAGGAGGTGAACTGAATTGAGGCTAAAGAAATAGGACCTTTTAAA
AGCAAACAGCTCTATTATGAATTGGG
>210
AGTATGACTCATTTGAGGAACAGAAAAAAGGCCAAGTGCAGAGTATGAAGAGGAGGT
GAACTGAATTGAGGCTAAAGAAATAGGACCTTTTAAAAGCAAACAGCTCTATTATGAAT
TGGGGTTTTTACCCGTAGAGTATA
>211
CTCATTTGAGGAACAGAAAAAAGGCCAAGTGCAGAGTATGAAGAGGAGGTGAACTGA
ATTGAGGCTAAAGAAATAGGACCTTTTAAAAGCAAACAGCTCTATTATGAATTGGGGTT
TTTACCCGTAGAGTATAGGGAAGC
>212
AAAGGCCAAGTGCAGAGTATGAAGAGGAGGTGAACTGAATTGAGGCTAAAGAAATAG
GACCTTTTAAAAGCAAACAGCTCTATTATGAATTGGGGTTTTTACCCGTAGAGTATAGG
GAAGCTATCAAAGTGTTTTAAAAA
>213
ATTGAGGCTAAAGAAATAGGACCTTTTAAAAGCAAACAGCTCTATTATGAATTGGGGTT
TTTACCCGTAGAGTATAGGGAAGCTATCAAAGTGTTTTAAAAAGCAAGGGATCTTAAGA
GGGCTATGGGCAAGTTAAAAAA
>214
TGAGGCTAAAGAAATAGGACCTTTTAAAAGCAAACAGCTCTATTATGAATTGGGGTTTT

TACCCGTAGAGTATAGGGAAGCTATCAAAGTGTTTTAAAAAGCAAGGGATCTTAAGAG
GGCTATGGGCAAGTTAAAAAAAC
>215
CCTTTTAAAAGCAAACAGCTCTATTATGAATTGGGGTTTTTACCCGTAGAGTATAGGGA
AGCTATCAAAGTGTTTTAAAAAGCAAGGGATCTTAAGAGGGCTATGGGCAAGTTAAAA
AAACAAAAAAAAACAACGAAGGAG
>216
TGAATTGGGGTTTTTACCCGTAGAGTATAGGGAAGCTATCAAAGTGTTTTAAAAAGCAA
GGGATCTTAAGAGGGCTATGGGCAAGTTAAAAAAACAAAAAAAAACAACGAAGGAGTG
GTTTGATATCATTAGGTTTACATT
>217
AATTGGGGTTTTTACCCGTAGAGTATAGGGAAGCTATCAAAGTGTTTTAAAAAGCAAG
GGATCTTAAGAGGGCTATGGGCAAGTTAAAAAAACAAAAAAAAACAACGAAGGAGTGG
TTTGATATCATTAGGTTTACATTGA
>218
ACCCGTAGAGTATAGGGAAGCTATCAAAGTGTTTTAAAAAGCAAGGGATCTTAAGAGG
GCTATGGGCAAGTTAAAAAAACAAAAAAAAACAACGAAGGAGTGGTTTGATATCATTAG
GTTTACATTGAGCTTGTATCATCC
>219
AAGTGTTTTAAAAAGCAAGGGATCTTAAGAGGGCTATGGGCAAGTTAAAAAAACAAA
AAAAACAACGAAGGAGTGGTTTGATATCATTAGGTTTACATTGAGCTTGTATCATCCAT
AGAAGAGTCAGGGTTAGACCCTCT
>220
GATCTTAAGAGGGCTATGGGCAAGTTAAAAAAACAAAAAAAAACAACGAAGGAGTGGT
TTGATATCATTAGGTTTACATTGAGCTTGTATCATCCATAGAAGAGTCAGGGTTAGACCC
TCTGGGGAAGAGAGTGGACAACA
>221
GCTATGGGCAAGTTAAAAAAACAAAAAAAAACAACGAAGGAGTGGTTTGATATCATTAG
GTTTACATTGAGCTTGTATCATCCATAGAAGAGTCAGGGTTAGACCCTCTGGGGAAGAG
AGTGGACAACAAAGATAAGCCAC
>222
AAAAACAAAAAAAAACAACGAAGGAGTGGTTTGATATCATTAGGTTTACATTGAGCTTG
TATCATCCATAGAAGAGTCAGGGTTAGACCCTCTGGGGAAGAGAGTGGACAACAAAGA
TAAGCCACACTCTACTCTATATCC
>223
TTGATATCATTAGGTTTACATTGAGCTTGTATCATCCATAGAAGAGTCAGGGTTAGACCC
TCTGGGGAAGAGAGTGGACAACAAAGATAAGCCACACTCTACTCTATATCCATGAAAA
AATAGTAACCATCACAGCTACC
>224
GATATCATTAGGTTTACATTGAGCTTGTATCATCCATAGAAGAGTCAGGGTTAGACCCTC
TGGGGAAGAGAGTGGACAACAAAGATAAGCCACACTCTACTCTATATCCATGAAAAAA
TAGTAACCATCACAGCTACCAG
>225
TACATTGAGCTTGTATCATCCATAGAAGAGTCAGGGTTAGACCCTCTGGGGAAGAGAG
TGGACAACAAAGATAAGCCACACTCTACTCTATATCCATGAAAAAATAGTAACCATCAC
AGCTACCAGTTGCTCAATTCTAC

>226
TAGACCCTCTGGGGAAGAGAGTGGACAACAAAGATAAGCCACACTCTACTCTATATCC
ATGAAAAAATAGTAACCATCACAGCTACCAGTTGCTCAATTCTACTTACTTTTGTTTTTA
AACTTTTTATTAGGGAAAATTT
>227
GGAAGAGAGTGGACAACAAAGATAAGCCACACTCTACTCTATATCCATGAAAAAATAG
TAACCATCACAGCTACCAGTTGCTCAATTCTACTTACTTTTGTTTTTAAACTTTTTATTAG
GGAAAATTTCAAAAATTTCTG
>228
AGTGGACAACAAAGATAAGCCACACTCTACTCTATATCCATGAAAAAATAGTAACCATC
ACAGCTACCAGTTGCTCAATTCTACTTACTTTTGTTTTTAAACTTTTTATTAGGGAAAAT
TTCAAAAATTTCTGAAAGTGA
>229
AAAAAATAGTAACCATCACAGCTACCAGTTGCTCAATTCTACTTACTTTTGTTTTTAAAC
TTTTTATTAGGGAAAATTTCAAAAATTTCTGAAAGTGAAGTAGTGCAGTGACCTCCCAT
ACGTCCATCACCTAGCTTCAA
>230
CAGTTGCTCAATTCTACTTACTTTTGTTTTTAAACTTTTTATTAGGGAAAATTTCAAAAA
TTTCTGAAAGTGAAGTAGTGCAGTGACCTCCCATACGTCCATCACCTAGCTTCAACGCT
TATGAGCAAATGGCCACTCTT
>231
GCTCAATTCTACTTACTTTTGTTTTTAAACTTTTTATTAGGGAAAATTTCAAAAATTTCTG
AAAGTGAAGTAGTGCAGTGACCTCCCATACGTCCATCACCTAGCTTCAACGCTTATGAG
CAAATGGCCACTCTTTTTTT
>232
AAAAATTTCTGAAAGTGAAGTAGTGCAGTGACCTCCCATACGTCCATCACCTAGCTTCA
ACGCTTATGAGCAAATGGCCACTCTTTTTTTTTTTTTTTTTTTTTTGAGACAGAGTCTCA
CTCTGTAGCCCAGGCTGGAGT
>233
TACGTCCATCACCTAGCTTCAACGCTTATGAGCAAATGGCCACTCTTTTTTTTTTTTTTT
TTTTTTTGAGACAGAGTCTCACTCTGTAGCCCAGGCTGGAGTGCAGTGGTGCAGTCTT
GGCTCACTGCAACCTCTGCCTC
>234
CAACGCTTATGAGCAAATGGCCACTCTTTTTTTTTTTTTTTTTTTTTTGAGACAGAGTCT
CACTCTGTAGCCCAGGCTGGAGTGCAGTGGTGCAGTCTTGGCTCACTGCAACCTCTGC
CTCCTGGGTTCAAGCGATTCTC
>235
CTTATGAGCAAATGGCCACTCTTTTTTTTTTTTTTTTTTTTTTGAGACAGAGTCTCACTC
TGTAGCCCAGGCTGGAGTGCAGTGGTGCAGTCTTGGCTCACTGCAACCTCTGCCTCCT
GGGTTCAAGCGATTCTCATGCC
>236
CTTTTTTTTTTTTTTTTTTTTTTGAGACAGAGTCTCACTCTGTAGCCCAGGCTGGAGTGC
AGTGGTGCAGTCTTGGCTCACTGCAACCTCTGCCTCCTGGGTTCAAGCGATTCTCATGC
CTCAGACTCCCGAGTAGCTGG
>237
CTCTGTAGCCCAGGCTGGAGTGCAGTGGTGCAGTCTTGGCTCACTGCAACCTCTGCCT

CCTGGGTTCAAGCGATTCTCATGCCTCAGACTCCCGAGTAGCTGGGATTACAGGCGCC
TGCCACCATGCCTGGCTAATTTTT
>238
GAGTGCAGTGGTGCAGTCTTGGCTCACTGCAACCTCTGCCTCCTGGGTTCAAGCGATT
CTCATGCCTCAGACTCCCGAGTAGCTGGGATTACAGGCGCCTGCCACCATGCCTGGCTA
ATTTTTGTATGTTTAGTAGAGAT
>239
CACTGCAACCTCTGCCTCCTGGGTTCAAGCGATTCTCATGCCTCAGACTCCCGAGTAGC
TGGGATTACAGGCGCCTGCCACCATGCCTGGCTAATTTTTGTATGTTTAGTAGAGATGG
GGTTTCACTATGTTGGTCAAGC
>240
GCCTCAGACTCCCGAGTAGCTGGGATTACAGGCGCCTGCCACCATGCCTGGCTAATTTT
TGTATGTTTAGTAGAGATGGGGTTTCACTATGTTGGTCAAGCTGGTTTTGAACTCCCGA
CCTCAGGTGATTCACCAGCCTC
>241
TCAGACTCCCGAGTAGCTGGGATTACAGGCGCCTGCCACCATGCCTGGCTAATTTTTGT
ATGTTTAGTAGAGATGGGGTTTCACTATGTTGGTCAAGCTGGTTTTGAACTCCCGACCT
CAGGTGATTCACCAGCCTCAGC
>242
GGCGCCTGCCACCATGCCTGGCTAATTTTTGTATGTTTAGTAGAGATGGGGTTTCACTAT
GTTGGTCAAGCTGGTTTTGAACTCCCGACCTCAGGTGATTCACCAGCCTCAGCCTCCA
AAAGTGCTGGGATTACAGACGT
>243
ATTTTTGTATGTTTAGTAGAGATGGGGTTTCACTATGTTGGTCAAGCTGGTTTTGAACTC
CCGACCTCAGGTGATTCACCAGCCTCAGCCTCCAAAAGTGCTGGGATTACAGACGTGA
GCCATCGCACTCAGCTGAGCAA
>244
TTAGTAGAGATGGGGTTTCACTATGTTGGTCAAGCTGGTTTTGAACTCCCGACCTCAGG
TGATTCACCAGCCTCAGCCTCCAAAAGTGCTGGGATTACAGACGTGAGCCATCGCACT
CAGCTGAGCAAGTGGCCACTCTT
>245
CAAGCTGGTTTTGAACTCCCGACCTCAGGTGATTCACCAGCCTCAGCCTCCAAAAGTG
CTGGGATTACAGACGTGAGCCATCGCACTCAGCTGAGCAAGTGGCCACTCTTACTTCA
TCTATACTCCCTCATGTTAGATTG
>246
GTTTTGAACTCCCGACCTCAGGTGATTCACCAGCCTCAGCCTCCAAAAGTGCTGGGAT
TACAGACGTGAGCCATCGCACTCAGCTGAGCAAGTGGCCACTCTTACTTCATCTATACT
CCCTCATGTTAGATTGTTTTAAA
>247
GACCTCAGGTGATTCACCAGCCTCAGCCTCCAAAAGTGCTGGGATTACAGACGTGAGC
CATCGCACTCAGCTGAGCAAGTGGCCACTCTTACTTCATCTATACTCCCTCATGTTAGAT
TGTTTTAAAGCAGATCCTAGAT
>248
ACAGACGTGAGCCATCGCACTCAGCTGAGCAAGTGGCCACTCTTACTTCATCTATACTC
CCTCATGTTAGATTGTTTTAAAGCAGATCCTAGATGTTATATCCTTTTATCCATAAATATTT
TATTATGTATCTCTAAATG

>249
CATCGCACTCAGCTGAGCAAGTGGCCACTCTTACTTCATCTATACTCCCTCATGTTAGAT
TGTTTTAAAGCAGATCCTAGATGTTATATCCTTTTATCCATAAATATTTTATTATGTATCTC
TAAATGATACTCTTTTAA
>250
ATGTTATATCCTTTTATCCATAAATATTTTATTATGTATCTCTAAATGATACTCTTTTAAAAA
AACATAGTATCATTATCAAACCTAAAAAGTTAACAGTGATTCCTTAATGTCATCAAATAT
GGATCAATATTCAAATT
>251
AATATTTTATTATGTATCTCTAAATGATACTCTTTTAAAAAAACATAGTATCATTATCAAAC
CTAAAAAGTTAACAGTGATTCCTTAATGTCATCAAATATGGATCAATATTCAAATTTCTG
CAGTTGTCTCATAATTAT
>252
TATTATGTATCTCTAAATGATACTCTTTTAAAAAAACATAGTATCATTATCAAACCTAAAA
AGTTAACAGTGATTCCTTAATGTCATCAAATATGGATCAATATTCAAATTTCTGCAGTTG
TCTCATAATTATTTTGTAG
>253
ATCAAACCTAAAAAGTTAACAGTGATTCCTTAATGTCATCAAATATGGATCAATATTCAA
ATTTCTGCAGTTGTCTCATAATTATTTTGTAGCTAATTTAAATCCAGATCTAAACAAGGT
CTATACAGTGCATTTGGTTG
>254
TGATTCCTTAATGTCATCAAATATGGATCAATATTCAAATTTCTGCAGTTGTCTCATAATT
ATTTTGTAGCTAATTTAAATCCAGATCTAAACAAGGTCTATACAGTGCATTTGGTTGATA
TGTCTCTTAAGACTTTTAA
>255
ATCAAATATGGATCAATATTCAAATTTCTGCAGTTGTCTCATAATTATTTTGTAGCTAATTT
AAATCCAGATCTAAACAAGGTCTATACAGTGCATTTGGTTGATATGTCTCTTAAGACTTT
TAATTTGTAGCTTTTCTG
>256
AAATTTCTGCAGTTGTCTCATAATTATTTTGTAGCTAATTTAAATCCAGATCTAAACAAG
GTCTATACAGTGCATTTGGTTGATATGTCTCTTAAGACTTTTAATTTGTAGCTTTTCTGTC
ACCCCCTTGAATTTATTTC
>257
TTTTGTAGCTAATTTAAATCCAGATCTAAACAAGGTCTATACAGTGCATTTGGTTGATAT
GTCTCTTAAGACTTTTAATTTGTAGCTTTTCTGTCACCCCCTTGAATTTATTTCTTGAAG
AAACCAGGTTGTTGATCCTG
>258
AAATCCAGATCTAAACAAGGTCTATACAGTGCATTTGGTTGATATGTCTCTTAAGACTTT
TAATTTGTAGCTTTTCTGTCACCCCCTTGAATTTATTTCTTGAAGAAACCAGGTTGTTGA
TCCTGTAGAATTTCTTATGA
>259
TGGTTGATATGTCTCTTAAGACTTTTAATTTGTAGCTTTTCTGTCACCCCCTTGAATTTAT
TTCTTGAAGAAACCAGGTTGTTGATCCTGTAGAATTTCTTATGATAGCTACCATTTACTG
AGTGCTTACTCTGCTAGGT
>260
TATGTCTCTTAAGACTTTTAATTTGTAGCTTTTCTGTCACCCCCTTGAATTTATTTCTTGA

AGAAACCAGGTTGTTGATCCTGTAGAATTTCTTATGATAGCTACCATTTACTGAGTGCTT
ACTCTGCTAGGTATTATGC
>261
TTCTGTCACCCCCTTGAATTTATTTCTTGAAGAAACCAGGTTGTTGATCCTGTAGAATTT
CTTATGATAGCTACCATTTACTGAGTGCTTACTCTGCTAGGTATTATGCCAAGGGTTGCT
GGTAAATCCACTTAATTCTC
>262
GAATTTATTTCTTGAAGAAACCAGGTTGTTGATCCTGTAGAATTTCTTATGATAGCTACC
ATTTACTGAGTGCTTACTCTGCTAGGTATTATGCCAAGGGTTGCTGGTAAATCCACTTAA
TTCTCATACCAATCCTTAGA
>263
AGAAACCAGGTTGTTGATCCTGTAGAATTTCTTATGATAGCTACCATTTACTGAGTGCTT
ACTCTGCTAGGTATTATGCCAAGGGTTGCTGGTAAATCCACTTAATTCTCATACCAATCC
TTAGATGTATTATAATAGAC
>264
GTGCTTACTCTGCTAGGTATTATGCCAAGGGTTGCTGGTAAATCCACTTAATTCTCATAC
CAATCCTTAGATGTATTATAATAGACCTTTTTCTGATGTTATATATAAGTAAATTGAGGTTT
GGGAATATCTAGGTAACT
>265
AGGTATTATGCCAAGGGTTGCTGGTAAATCCACTTAATTCTCATACCAATCCTTAGATGT
ATTATAATAGACCTTTTTCTGATGTTATATATAAGTAAATTGAGGTTTGGGAATATCTAGG
TAACTTAGCCGTAGTCACA
>266
AAATCCACTTAATTCTCATACCAATCCTTAGATGTATTATAATAGACCTTTTTCTGATGTT
ATATATAAGTAAATTGAGGTTTGGGAATATCTAGGTAACTTAGCCGTAGTCACATAACTG
GTCATTGGAGGGGGCATGA
>267
TAATTCTCATACCAATCCTTAGATGTATTATAATAGACCTTTTTCTGATGTTATATATAAGT
AAATTGAGGTTTGGGAATATCTAGGTAACTTAGCCGTAGTCACATAACTGGTCATTGGA
GGGGGCATGATCCAGATCC
>268
CCTTAGATGTATTATAATAGACCTTTTTCTGATGTTATATATAAGTAAATTGAGGTTTGGG
AATATCTAGGTAACTTAGCCGTAGTCACATAACTGGTCATTGGAGGGGGCATGATCCAG
ATCCCGGGTTTTTAAACCAC
>269
AATAGACCTTTTTCTGATGTTATATATAAGTAAATTGAGGTTTGGGAATATCTAGGTAACT
TAGCCGTAGTCACATAACTGGTCATTGGAGGGGGCATGATCCAGATCCCGGGTTTTTAA
ACCACTAATCTGTTTTCCTG
>270
ATATATAAGTAAATTGAGGTTTGGGAATATCTAGGTAACTTAGCCGTAGTCACATAACTG
GTCATTGGAGGGGGCATGATCCAGATCCCGGGTTTTTAAACCACTAATCTGTTTTCCTG
AAAGATCTTAATGACAGAAGC
>271
TGAGGTTTGGGAATATCTAGGTAACTTAGCCGTAGTCACATAACTGGTCATTGGAGGGG
GCATGATCCAGATCCCGGGTTTTTAAACCACTAATCTGTTTTCCTGAAAGATCTTAATGA
CAGAAGCAGCCAGGGCTGTAG

>272
ATAACTGGTCATTGGAGGGGGCATGATCCAGATCCCGGGTTTTTAAACCACTAATCTGT
TTTCCTGAAAGATCTTAATGACAGAAGCAGCCAGGGCTGTAGGATAGAGGCCAACTCA
TTAGGCTGGCATTGGCACCTTTC
>273
GGAGGGGGCATGATCCAGATCCCGGGTTTTTAAACCACTAATCTGTTTTCCTGAAAGAT
CTTAATGACAGAAGCAGCCAGGGCTGTAGGATAGAGGCCAACTCATTAGGCTGGCATT
GGCACCTTTCCAACTTTATCCCT
>274
GGGGGCATGATCCAGATCCCGGGTTTTTAAACCACTAATCTGTTTTCCTGAAAGATCTT
AATGACAGAAGCAGCCAGGGCTGTAGGATAGAGGCCAACTCATTAGGCTGGCATTGGC
ACCTTTCCAACTTTATCCCTACA
>275
ATCCCGGGTTTTTAAACCACTAATCTGTTTTCCTGAAAGATCTTAATGACAGAAGCAGC
CAGGGCTGTAGGATAGAGGCCAACTCATTAGGCTGGCATTGGCACCTTTCCAACTTTAT
CCCTACACCTCAGCTGCACAGG
>276
TAATCTGTTTTCCTGAAAGATCTTAATGACAGAAGCAGCCAGGGCTGTAGGATAGAGG
CCAACTCATTAGGCTGGCATTGGCACCTTTCCAACTTTATCCCTACACCTCAGCTGCAC
AGGATGCCTTCCTGGAATGCCCT
>277
GGGCTGTAGGATAGAGGCCAACTCATTAGGCTGGCATTGGCACCTTTCCAACTTTATCC
CTACACCTCAGCTGCACAGGATGCCTTCCTGGAATGCCCTTAGTTCTCTATTCAACCTC
TTCAAGGCCTTCTCAGCCTTCA
>278
GGATAGAGGCCAACTCATTAGGCTGGCATTGGCACCTTTCCAACTTTATCCCTACACCT
CAGCTGCACAGGATGCCTTCCTGGAATGCCCTTAGTTCTCTATTCAACCTCTTCAAGGC
CTTCTCAGCCTTCACACCATCT
>279
TATCCCTACACCTCAGCTGCACAGGATGCCTTCCTGGAATGCCCTTAGTTCTCTATTCAA
CCTCTTCAAGGCCTTCTCAGCCTTCACACCATCTCCATGAAACCTTTCTTGACCACACC
AGCCCATAGAGCTCTGTCTTG
>280
CCTCAGCTGCACAGGATGCCTTCCTGGAATGCCCTTAGTTCTCTATTCAACCTCTTCAA
GGCCTTCTCAGCCTTCACACCATCTCCATGAAACCTTTCTTGACCACACCAGCCCATAG
AGCTCTGTCTTGCACACACTGC
>281
TCTATTCAACCTCTTCAAGGCCTTCTCAGCCTTCACACCATCTCCATGAAACCTTTCTTG
ACCACACCAGCCCATAGAGCTCTGTCTTGCACACACTGCACTGCTCATTTGGTGTTCAG
TCACATGCTGTTTGGGGAGGT
>282
AACCTCTTCAAGGCCTTCTCAGCCTTCACACCATCTCCATGAAACCTTTCTTGACCACA
CCAGCCCATAGAGCTCTGTCTTGCACACACTGCACTGCTCATTTGGTGTTCAGTCACAT
GCTGTTTGGGGAGGTTTCTTGT
>283
AACCTTTCTTGACCACACCAGCCCATAGAGCTCTGTCTTGCACACACTGCACTGCTCAT

TTGGTGTTCAGTCACATGCTGTTTGGGGAGGTTTCTTGTTTTTTTGTTAGTATTCCTGCT
GTTATTTCTGAGCTCACTTTC
>284
AGCCCATAGAGCTCTGTCTTGCACACACTGCACTGCTCATTTGGTGTTCAGTCACATGC
TGTTTGGGGAGGTTTCTTGTTTTTTTGTTAGTATTCCTGCTGTTATTTCTGAGCTCACTTT
CTAGCTCTGTGAATACAGGG
>285
GCACACACTGCACTGCTCATTTGGTGTTCAGTCACATGCTGTTTGGGGAGGTTTCTTGT
TTTTTTGTTAGTATTCCTGCTGTTATTTCTGAGCTCACTTTCTAGCTCTGTGAATACAGG
GTCAGCCCTTACGCCACACTG
>286
GGTGTTCAGTCACATGCTGTTTGGGGAGGTTTCTTGTTTTTTTGTTAGTATTCCTGCTGT
TATTTCTGAGCTCACTTTCTAGCTCTGTGAATACAGGGTCAGCCCTTACGCCACACTGG
GGTCCCGTAGATGCTCTTTGT
>287
CATGCTGTTTGGGGAGGTTTCTTGTTTTTTTGTTAGTATTCCTGCTGTTATTTCTGAGCTC
ACTTTCTAGCTCTGTGAATACAGGGTCAGCCCTTACGCCACACTGGGGTCCCGTAGATG
CTCTTTGTGCGATTAGGGAA
>288
AGGTTTCTTGTTTTTTTGTTAGTATTCCTGCTGTTATTTCTGAGCTCACTTTCTAGCTCTG
TGAATACAGGGTCAGCCCTTACGCCACACTGGGGTCCCGTAGATGCTCTTTGTGCGATT
AGGGAATGCTAATTAGAATA
>289
TTATTTCTGAGCTCACTTTCTAGCTCTGTGAATACAGGGTCAGCCCTTACGCCACACTG
GGGTCCCGTAGATGCTCTTTGTGCGATTAGGGAATGCTAATTAGAATACTATCTCTGGGC
TATTTATGCTGCATCAGTCTT
>290
CTAGCTCTGTGAATACAGGGTCAGCCCTTACGCCACACTGGGGTCCCGTAGATGCTCTT
TGTGCGATTAGGGAATGCTAATTAGAATACTATCTCTGGGCTATTTATGCTGCATCAGTC
TTCCTGTCAAACAAGTTACAG
>291
ACAGGGTCAGCCCTTACGCCACACTGGGGTCCCGTAGATGCTCTTTGTGCGATTAGGG
AATGCTAATTAGAATACTATCTCTGGGCTATTTATGCTGCATCAGTCTTCCTGTCAAACA
AGTTACAGTTGTGCTTATCTGT
>292
GTAGATGCTCTTTGTGCGATTAGGGAATGCTAATTAGAATACTATCTCTGGGCTATTTATG
CTGCATCAGTCTTCCTGTCAAACAAGTTACAGTTGTGCTTATCTGTATTTCCGATGAGAA
TCAGGAATTTTTCTTCTGT
>293
GATTAGGGAATGCTAATTAGAATACTATCTCTGGGCTATTTATGCTGCATCAGTCTTCCTG
TCAAACAAGTTACAGTTGTGCTTATCTGTATTTCCGATGAGAATCAGGAATTTTTCTTCT
GTTTAATGAATTTCTGATT
>294
GGGCTATTTATGCTGCATCAGTCTTCCTGTCAAACAAGTTACAGTTGTGCTTATCTGTAT
TTCCGATGAGAATCAGGAATTTTTCTTCTGTTTAATGAATTTCTGATTGGTTTCAAAGTA
TTCTAACATAAGTAGCCAAA

>295
TTTATGCTGCATCAGTCTTCCTGTCAAACAAGTTACAGTTGTGCTTATCTGTATTTCCGA
TGAGAATCAGGAATTTTTCTTCTGTTTAATGAATTTCTGATTGGTTTCAAAGTATTCTAA
CATAAGTAGCCAAATTGTAC
>296
GCTTATCTGTATTTCCGATGAGAATCAGGAATTTTTCTTCTGTTTAATGAATTTCTGATTG
GTTTCAAAGTATTCTAACATAAGTAGCCAAATTGTACTATAGGCCTTTAAATGAAAAAA
ATAAAAAAGTAGTCCCAAGC
>297
CCGATGAGAATCAGGAATTTTTCTTCTGTTTAATGAATTTCTGATTGGTTTCAAAGTATT
CTAACATAAGTAGCCAAATTGTACTATAGGCCTTTAAATGAAAAAAATAAAAAAGTAGT
CCCAAGCCGGGCGCAGTGGCT
>298
ATGAGAATCAGGAATTTTTCTTCTGTTTAATGAATTTCTGATTGGTTTCAAAGTATTCTA
ACATAAGTAGCCAAATTGTACTATAGGCCTTTAAATGAAAAAAATAAAAAAGTAGTCCC
AAGCCGGGCGCAGTGGCTCAC
>299
TTTCTTCTGTTTAATGAATTTCTGATTGGTTTCAAAGTATTCTAACATAAGTAGCCAAATT
GTACTATAGGCCTTTAAATGAAAAAAATAAAAAAGTAGTCCCAAGCCGGGCGCAGTGG
CTCACGCCTGTAATCCCAGCA
>300
TGGTTTCAAAGTATTCTAACATAAGTAGCCAAATTGTACTATAGGCCTTTAAATGAAAA
AAATAAAAAAGTAGTCCCAAGCCGGGCGCAGTGGCTCACGCCTGTAATCCCAGCACTT
TCGGAGGCCGAGGCGGGTGGACC
>301
ATAAGTAGCCAAATTGTACTATAGGCCTTTAAATGAAAAAAATAAAAAAGTAGTCCCAA
GCCGGGCGCAGTGGCTCACGCCTGTAATCCCAGCACTTTCGGAGGCCGAGGCGGGTG
GACCACCTAAGGTCAGGAGTTCAA
>302
AGGCCTTTAAATGAAAAAAATAAAAAAGTAGTCCCAAGCCGGGCGCAGTGGCTCACG
CCTGTAATCCCAGCACTTTCGGAGGCCGAGGCGGGTGGACCACCTAAGGTCAGGAGTT
CAAGACCAGCCTGGCCAACATGGTG
>303
TGAAAAAAATAAAAAAGTAGTCCCAAGCCGGGCGCAGTGGCTCACGCCTGTAATCCC
AGCACTTTCGGAGGCCGAGGCGGGTGGACCACCTAAGGTCAGGAGTTCAAGACCAGC
CTGGCCAACATGGTGAAACCCCATCT
>304
GGGCGCAGTGGCTCACGCCTGTAATCCCAGCACTTTCGGAGGCCGAGGCGGGTGGAC
CACCTAAGGTCAGGAGTTCAAGACCAGCCTGGCCAACATGGTGAAACCCCATCTCTAC
TAAAAATACAAAAATTAGTCATGTG
>305
CGCAGTGGCTCACGCCTGTAATCCCAGCACTTTCGGAGGCCGAGGCGGGTGGACCAC
CTAAGGTCAGGAGTTCAAGACCAGCCTGGCCAACATGGTGAAACCCCATCTCTACTAA
AAATACAAAAATTAGTCATGTGTGG
>306
GGCTCACGCCTGTAATCCCAGCACTTTCGGAGGCCGAGGCGGGTGGACCACCTAAGGT

CAGGAGTTCAAGACCAGCCTGGCCAACATGGTGAAACCCCATCTCTACTAAAAATACA
AAAATTAGTCATGTGTGGTGGCAC
>307
AGGTCAGGAGTTCAAGACCAGCCTGGCCAACATGGTGAAACCCCATCTCTACTAAAAA
TACAAAAATTAGTCATGTGTGGTGGCACGTGCCTGTAGTCCCAGCTACTCCAGGAGGC
TGAGGCAGGAGAATTGCTTGAACC
>308
CCAACATGGTGAAACCCCATCTCTACTAAAAATACAAAAATTAGTCATGTGTGGTGGCA
CGTGCCTGTAGTCCCAGCTACTCCAGGAGGCTGAGGCAGGAGAATTGCTTGAACCCGG
GAGTTGGAGGTTGCAGTGACCTG
>309
CCCATCTCTACTAAAAATACAAAAATTAGTCATGTGTGGTGGCACGTGCCTGTAGTCCC
AGCTACTCCAGGAGGCTGAGGCAGGAGAATTGCTTGAACCCGGGAGTTGGAGGTTGC
AGTGACCTGAGATTGCACCACTGC
>310
AAAATACAAAAATTAGTCATGTGTGGTGGCACGTGCCTGTAGTCCCAGCTACTCCAGG
AGGCTGAGGCAGGAGAATTGCTTGAACCCGGGAGTTGGAGGTTGCAGTGACCTGAGA
TTGCACCACTGCACTCCAGCCTGGG
>311
ATACAAAAATTAGTCATGTGTGGTGGCACGTGCCTGTAGTCCCAGCTACTCCAGGAGG
CTGAGGCAGGAGAATTGCTTGAACCCGGGAGTTGGAGGTTGCAGTGACCTGAGATTG
CACCACTGCACTCCAGCCTGGGCAA
>312
GTGGTGGCACGTGCCTGTAGTCCCAGCTACTCCAGGAGGCTGAGGCAGGAGAATTGCT
TGAACCCGGGAGTTGGAGGTTGCAGTGACCTGAGATTGCACCACTGCACTCCAGCCT
GGGCAACAGAGTGAGACTCTGTCTT
>313
CCTGTAGTCCCAGCTACTCCAGGAGGCTGAGGCAGGAGAATTGCTTGAACCCGGGAG
TTGGAGGTTGCAGTGACCTGAGATTGCACCACTGCACTCCAGCCTGGGCAACAGAGT
GAGACTCTGTCTTAAAAAAAAAAAAAA
>314
AGGCAGGAGAATTGCTTGAACCCGGGAGTTGGAGGTTGCAGTGACCTGAGATTGCAC
CACTGCACTCCAGCCTGGGCAACAGAGTGAGACTCTGTCTTAAAAAAAAAAAAAAAAAAA
AAAAATTCCAGACCTCTCCTTTTCAC
>315
TGCTTGAACCCGGGAGTTGGAGGTTGCAGTGACCTGAGATTGCACCACTGCACTCCAG
CCTGGGCAACAGAGTGAGACTCTGTCTTAAAAAAAAAAAAAAAAAAAAAAAATTCCAGAC
CTCTCCTTTTCACCCATAAATCCCT
>316
ACCACTGCACTCCAGCCTGGGCAACAGAGTGAGACTCTGTCTTAAAAAAAAAAAAAAAAA
AAAAAAATTCCAGACCTCTCCTTTTCACCCATAAATCCCTCTGCCCAGTGACAGCCACT
TTCAGCTCCTTTCCCCTATGTCTT
>317
TCTGTCTTAAAAAAAAAAAAAAAAAAAAAAAATTCCAGACCTCTCCTTTTCACCCATAAAT
CCCTCTGCCCAGTGACAGCCACTTTCAGCTCCTTTCCCCTATGTCTTCTGGTATTTACCT
TTGGATTTCTAAATAATAATAA

>318
TTAAAAAAAAAAAAAAAAAAAAAAATTCCAGACCTCTCCTTTTCACCCATAAATCCCTCT
GCCCAGTGACAGCCACTTTCAGCTCCTTTCCCCTATGTCTTCTGGTATTTACCTTTGGAT
TTCTAAATAATAATAATATTAA
>319
GGTATTTACCTTTGGATTTCTAAATAATAATAATATTAATTGACTGTTATTACTAATCTGTA
ATCCTTAGGTAATACCTGTTATATACTAGGTACTGTTCTAAGTGCTTTACAGCCATTAGCT
TATTTAATCTCAACAAC
>320
AAATAATAATAATATTAATTGACTGTTATTACTAATCTGTAATCCTTAGGTAATACCTGTTA
TATACTAGGTACTGTTCTAAGTGCTTTACAGCCATTAGCTTATTTAATCTCAACAACCCCT
ATGAAGCAGGGTCTATT
>321
TGTTATTACTAATCTGTAATCCTTAGGTAATACCTGTTATATACTAGGTACTGTTCTAAGT
GCTTTACAGCCATTAGCTTATTTAATCTCAACAACCCCTATGAAGCAGGGTCTATTATTG
TGTCCTTTTACCGATGAGG
>322
ACTAATCTGTAATCCTTAGGTAATACCTGTTATATACTAGGTACTGTTCTAAGTGCTTTAC
AGCCATTAGCTTATTTAATCTCAACAACCCCTATGAAGCAGGGTCTATTATTGTGTCCTT
TTACCGATGAGGACACTTA
>323
TTATATACTAGGTACTGTTCTAAGTGCTTTACAGCCATTAGCTTATTTAATCTCAACAACC
CCTATGAAGCAGGGTCTATTATTGTGTCCTTTTACCGATGAGGACACTTAAGCACAGAA
AGGTTAAACAGTTTGTACAA
>324
GCCATTAGCTTATTTAATCTCAACAACCCCTATGAAGCAGGGTCTATTATTGTGTCCTTTT
ACCGATGAGGACACTTAAGCACAGAAAGGTTAAACAGTTTGTACAACCTCACTATCAT
TAGTAGGTGTTAAAGAACCTA
>325
TATTTAATCTCAACAACCCCTATGAAGCAGGGTCTATTATTGTGTCCTTTTACCGATGAG
GACACTTAAGCACAGAAAGGTTAAACAGTTTGTACAACCTCACTATCATTAGTAGGTGT
TAAAGAACCTAGGTAGTCTAG
>326
CCTATGAAGCAGGGTCTATTATTGTGTCCTTTTACCGATGAGGACACTTAAGCACAGAA
AGGTTAAACAGTTTGTACAACCTCACTATCATTAGTAGGTGTTAAAGAACCTAGGTAGT
CTAGCTCCAGAATCGGTGCTTG
>327
TGTCCTTTTACCGATGAGGACACTTAAGCACAGAAAGGTTAAACAGTTTGTACAACCT
CACTATCATTAGTAGGTGTTAAAGAACCTAGGTAGTCTAGCTCCAGAATCGGTGCTTGT
AATCATACTCCCCTCCTGTATTG
>328
CCTTTTACCGATGAGGACACTTAAGCACAGAAAGGTTAAACAGTTTGTACAACCTCAC
TATCATTAGTAGGTGTTAAAGAACCTAGGTAGTCTAGCTCCAGAATCGGTGCTTGTAAT
CATACTCCCCTCCTGTATTGTCT
>329
GATGAGGACACTTAAGCACAGAAAGGTTAAACAGTTTGTACAACCTCACTATCATTAGT

AGGTGTTAAAGAACCTAGGTAGTCTAGCTCCAGAATCGGTGCTTGTAATCATACTCCCC
TCCTGTATTGTCTCTCATTGCT
>330
AAGGTTAAACAGTTTGTACAACCTCACTATCATTAGTAGGTGTTAAAGAACCTAGGTAG
TCTAGCTCCAGAATCGGTGCTTGTAATCATACTCCCCTCCTGTATTGTCTCTCATTGCTAT
TTCTTCGTCTGTCAATTTTA
>331
ATCGGTGCTTGTAATCATACTCCCCTCCTGTATTGTCTCTCATTGCTATTTCTTCGTCTGT
CAATTTTAGGCATTATCTGTTAAGCTTTTAGTATGGGATATGAGCATTTAGTCTTCTTACA
CCATCACATTGGCTTCCT
>332
CATACTCCCCTCCTGTATTGTCTCTCATTGCTATTTCTTCGTCTGTCAATTTTAGGCATTAT
CTGTTAAGCTTTTAGTATGGGATATGAGCATTTAGTCTTCTTACACCATCACATTGGCTT
CCTTTCCCCTTTCAGCTT
>333
CCCTCCTGTATTGTCTCTCATTGCTATTTCTTCGTCTGTCAATTTTAGGCATTATCTGTTA
AGCTTTTAGTATGGGATATGAGCATTTAGTCTTCTTACACCATCACATTGGCTTCCTTTCC
CCTTTCAGCTTCATAGTA
>334
TCTCTCATTGCTATTTCTTCGTCTGTCAATTTTAGGCATTATCTGTTAAGCTTTTAGTATG
GGATATGAGCATTTAGTCTTCTTACACCATCACATTGGCTTCCTTTCCCCTTTCAGCTTC
ATAGTAATTTAAAAATAAA
>335
AAGCTTTTAGTATGGGATATGAGCATTTAGTCTTCTTACACCATCACATTGGCTTCCTTT
CCCCTTTCAGCTTCATAGTAATTTAAAAATAAATCAGTATTCTGTGTTTACATTATTATAA
CTCTGAAAGATTCATTTAC
>336
AGTATGGGATATGAGCATTTAGTCTTCTTACACCATCACATTGGCTTCCTTTCCCCTTTC
AGCTTCATAGTAATTTAAAAATAAATCAGTATTCTGTGTTTACATTATTATAACTCTGAAA
GATTCATTTACTATCAAGC
>337
TAGTCTTCTTACACCATCACATTGGCTTCCTTTCCCCTTTCAGCTTCATAGTAATTTAAA
AATAAATCAGTATTCTGTGTTTACATTATTATAACTCTGAAAGATTCATTTACTATCAAGC
CAAATAGTGTAGTGTGATT
>338
CCCCTTTCAGCTTCATAGTAATTTAAAAATAAATCAGTATTCTGTGTTTACATTATTATAA
CTCTGAAAGATTCATTTACTATCAAGCCAAATAGTGTAGTGTGATTTAATCTATTTTCTTC
TAAAGCTTTTTGTTTTTC
>339
GTAATTTAAAAATAAATCAGTATTCTGTGTTTACATTATTATAACTCTGAAAGATTCATTT
ACTATCAAGCCAAATAGTGTAGTGTGATTTAATCTATTTTCTTCTAAAGCTTTTTGTTTTT
CCTGGAGCTGACAATTGC
>340
ATAACTCTGAAAGATTCATTTACTATCAAGCCAAATAGTGTAGTGTGATTTAATCTATTTT
CTTCTAAAGCTTTTTGTTTTTCCTGGAGCTGACAATTGCATTATTTTTTCATTTGCTTAGT
TTTCTGGTATTAATCACT

>341
TTACTATCAAGCCAAATAGTGTAGTGTGATTTAATCTATTTTCTTCTAAAGCTTTTTGTTT
TTCCTGGAGCTGACAATTGCATTATTTTTTCATTTGCTTAGTTTTCTGGTATTAATCACTA
ATTCTTTCCAAGCCTTTT
>342
AATCTATTTTCTTCTAAAGCTTTTTGTTTTTCCTGGAGCTGACAATTGCATTATTTTTTCA
TTTGCTTAGTTTTCTGGTATTAATCACTAATTCTTTCCAAGCCTTTTGAGAAACTGTAAA
ACCCTACAATTCTTGCTTG
>343
CTGGAGCTGACAATTGCATTATTTTTTCATTTGCTTAGTTTTCTGGTATTAATCACTAATT
CTTTCCAAGCCTTTTGAGAAACTGTAAAACCCTACAATTCTTGCTTGAACCCGGGAGG
CGGAGGCTGCAGTGAGCCGAG
>344
ACAATTGCATTATTTTTTCATTTGCTTAGTTTTCTGGTATTAATCACTAATTCTTTCCAAG
CCTTTTGAGAAACTGTAAAACCCTACAATTCTTGCTTGAACCCGGGAGGCGGAGGCTG
CAGTGAGCCGAGATCGCACCA
>345
TTAATCACTAATTCTTTCCAAGCCTTTTGAGAAACTGTAAAACCCTACAATTCTTGCTTG
AACCCGGGAGGCGGAGGCTGCAGTGAGCCGAGATCGCACCATTGCACCCCAGCCTGG
GCGACAGAGCAAGACCCCATCTC
>346
ACTAATTCTTTCCAAGCCTTTTGAGAAACTGTAAAACCCTACAATTCTTGCTTGAACCC
GGGAGGCGGAGGCTGCAGTGAGCCGAGATCGCACCATTGCACCCCAGCCTGGGCGAC
AGAGCAAGACCCCATCTCAAAAAA
>347
CCCTACAATTCTTGCTTGAACCCGGGAGGCGGAGGCTGCAGTGAGCCGAGATCGCACC
ATTGCACCCCAGCCTGGGCGACAGAGCAAGACCCCATCTCAAAAAACAAACAAACAA
AAAACCCCTACAGTTCTGTTTTTCA
>348
TGCTTGAACCCGGGAGGCGGAGGCTGCAGTGAGCCGAGATCGCACCATTGCACCCCA
GCCTGGGCGACAGAGCAAGACCCCATCTCAAAAAACAAACAAACAAAAAACCCCTAC
AGTTCTGTTTTTCACATGGTCAAATG
>349
AACCCGGGAGGCGGAGGCTGCAGTGAGCCGAGATCGCACCATTGCACCCCAGCCTGG
GCGACAGAGCAAGACCCCATCTCAAAAAACAAACAAACAAAAAACCCCTACAGTTCT
GTTTTTCACATGGTCAAATGTATCAG
>350
ATTGCACCCCAGCCTGGGCGACAGAGCAAGACCCCATCTCAAAAAACAAACAAACAA
AAAACCCCTACAGTTCTGTTTTTCACATGGTCAAATGTATCAGCTGCACTATTTTTTTTT
TTTGAGACCTAAAACTAGGGATT
>351
ACCCCATCTCAAAAAACAAACAAACAAAAAACCCCTACAGTTCTGTTTTTCACATGGT
CAAATGTATCAGCTGCACTATTTTTTTTTTTTGAGACCTAAAACTAGGGATTGCCTAAGC
CTTTCCTTTTTAAAAAGTCAGG
>352
ACAAAAAACCCCTACAGTTCTGTTTTTCACATGGTCAAATGTATCAGCTGCACTATTTTT

TTTTTTTGAGACCTAAAACTAGGGATTGCCTAAGCCTTTCCTTTTTAAAAAGTCAGGTT
TATATAGGTATAACTTGCATA
>353
TACAGTTCTGTTTTTCACATGGTCAAATGTATCAGCTGCACTATTTTTTTTTTTTGAGAC
CTAAAACTAGGGATTGCCTAAGCCTTTCCTTTTTAAAAAGTCAGGTTTATATAGGTATAA
CTTGCATACAGTAAAATTAA
>354
TTTCACATGGTCAAATGTATCAGCTGCACTATTTTTTTTTTTTGAGACCTAAAACTAGGG
ATTGCCTAAGCCTTTCCTTTTTAAAAAGTCAGGTTTATATAGGTATAACTTGCATACAGT
AAAATTAACCTTTTTTAGGT
>355
ATCAGCTGCACTATTTTTTTTTTTTGAGACCTAAAACTAGGGATTGCCTAAGCCTTTCCT
TTTTAAAAAGTCAGGTTTATATAGGTATAACTTGCATACAGTAAAATTAACCTTTTTTAG
GTGTAAGAGTTCTGAGAGGT
>356
TAAAACTAGGGATTGCCTAAGCCTTTCCTTTTTAAAAAGTCAGGTTTATATAGGTATAAC
TTGCATACAGTAAAATTAACCTTTTTTAGGTGTAAGAGTTCTGAGAGGTTTGATAGAATA
TATCAAGGTATAGGGCCAGC
>357
AGCCTTTCCTTTTTAAAAAGTCAGGTTTATATAGGTATAACTTGCATACAGTAAAATTAA
CCTTTTTTAGGTGTAAGAGTTCTGAGAGGTTTGATAGAATATATCAAGGTATAGGGCCA
GCGCCGATGGCTCACACCTGT
>358
AAAGTCAGGTTTATATAGGTATAACTTGCATACAGTAAAATTAACCTTTTTTAGGTGTAA
GAGTTCTGAGAGGTTTGATAGAATATATCAAGGTATAGGGCCAGCGCCGATGGCTCACA
CCTGTAGTCCCAACAATTTGG
>359
AACTTGCATACAGTAAAATTAACCTTTTTTAGGTGTAAGAGTTCTGAGAGGTTTGATAG
AATATATCAAGGTATAGGGCCAGCGCCGATGGCTCACACCTGTAGTCCCAACAATTTGG
GAGGCCAAGGCGGGAGGATCAC
>360
CATACAGTAAAATTAACCTTTTTTAGGTGTAAGAGTTCTGAGAGGTTTGATAGAATATAT
CAAGGTATAGGGCCAGCGCCGATGGCTCACACCTGTAGTCCCAACAATTTGGGAGGCC
AAGGCGGGAGGATCACTTAGGC
>361
GTAAGAGTTCTGAGAGGTTTGATAGAATATATCAAGGTATAGGGCCAGCGCCGATGGCT
CACACCTGTAGTCCCAACAATTTGGGAGGCCAAGGCGGGAGGATCACTTAGGCCCAG
GAGTTTGAGACCAGCTTGGGCAAC
>362
AGAATATATCAAGGTATAGGGCCAGCGCCGATGGCTCACACCTGTAGTCCCAACAATTT
GGGAGGCCAAGGCGGGAGGATCACTTAGGCCCAGGAGTTTGAGACCAGCTTGGGCAA
CATAGTGAGACCCTGTCTCTACAA
>363
ATCAAGGTATAGGGCCAGCGCCGATGGCTCACACCTGTAGTCCCAACAATTTGGGAGG
CCAAGGCGGGAGGATCACTTAGGCCCAGGAGTTTGAGACCAGCTTGGGCAACATAGT
GAGACCCTGTCTCTACAAAAAAAAA

>364
AGGGCCAGCGCCGATGGCTCACACCTGTAGTCCCAACAATTTGGGAGGCCAAGGCGG
GAGGATCACTTAGGCCCAGGAGTTTGAGACCAGCTTGGGCAACATAGTGAGACCCTGT
CTCTACAAAAAAAAAAAAAAAAAAG
>365
GGCTCACACCTGTAGTCCCAACAATTTGGGAGGCCAAGGCGGGAGGATCACTTAGGCC
CAGGAGTTTGAGACCAGCTTGGGCAACATAGTGAGACCCTGTCTCTACAAAAAAAAA
AAAAAAAAAGGTATCAAGGTACAGG
>366
GCCCAGGAGTTTGAGACCAGCTTGGGCAACATAGTGAGACCCTGTCTCTACAAAAAA
AAAAAAAAAAAAGGTATCAAGGTACAGGAAATTTCCACTACCCAACAAAGGTCACTC
ACCCTTTTGTAGTTAATAACTTCTCT
>367
GTTTGAGACCAGCTTGGGCAACATAGTGAGACCCTGTCTCTACAAAAAAAAAAAAAA
AAAAGGTATCAAGGTACAGGAAATTTCCACTACCCAACAAAGGTCACTCACCCTTTTG
TAGTTAATAACTTCTCTGCAACCTA
>368
GACCCTGTCTCTACAAAAAAAAAAAAAAAAAAAGGTATCAAGGTACAGGAAATTTCCA
CTACCCAACAAAGGTCACTCACCCTTTTGTAGTTAATAACTTCTCTGCAACCTAGATCC
AGCCAACTACTGATAAGATGTCTG
>369
AAAAAAAAAAAAAGGTATCAAGGTACAGGAAATTTCCACTACCCAACAAAGGTCACT
CACCCTTTTGTAGTTAATAACTTCTCTGCAACCTAGATCCAGCCAACTACTGATAAGATG
TCTGTCCTTGTATTTTCCAGAAT
>370
TACAGGAAATTTCCACTACCCAACAAAGGTCACTCACCCTTTTGTAGTTAATAACTTCT
CTGCAACCTAGATCCAGCCAACTACTGATAAGATGTCTGTCCTTGTATTTTCCAGAATGT
ACTTAAATGGAGTCATACAGT
>371
AACAAAGGTCACTCACCCTTTTGTAGTTAATAACTTCTCTGCAACCTAGATCCAGCCAA
CTACTGATAAGATGTCTGTCCTTGTATTTTCCAGAATGTACTTAAATGGAGTCATACAGT
AAGTAGGCTTTTGTGTCTGGC
>372
TAGTTAATAACTTCTCTGCAACCTAGATCCAGCCAACTACTGATAAGATGTCTGTCCTTG
TATTTTCCAGAATGTACTTAAATGGAGTCATACAGTAAGTAGGCTTTTGTGTCTGGCTTC
AAGCCTTTACTTTTTATAAA
>373
GCAACCTAGATCCAGCCAACTACTGATAAGATGTCTGTCCTTGTATTTTCCAGAATGTAC
TTAAATGGAGTCATACAGTAAGTAGGCTTTTGTGTCTGGCTTCAAGCCTTTACTTTTTAT
AAAGAATAACAATGGTAATA
>374
CAGCCAACTACTGATAAGATGTCTGTCCTTGTATTTTCCAGAATGTACTTAAATGGAGTC
ATACAGTAAGTAGGCTTTTGTGTCTGGCTTCAAGCCTTTACTTTTTATAAAGAATAACAA
TGGTAATAATTATAGCTGAC
>375
CCAACTACTGATAAGATGTCTGTCCTTGTATTTTCCAGAATGTACTTAAATGGAGTCATA

CAGTAAGTAGGCTTTTGTGTCTGGCTTCAAGCCTTTACTTTTTATAAAGAATAACAATG
GTAATAATTATAGCTGACATT
>376
GTCCTTGTATTTTCCAGAATGTACTTAAATGGAGTCATACAGTAAGTAGGCTTTTGTGTC
TGGCTTCAAGCCTTTACTTTTTATAAAGAATAACAATGGTAATAATTATAGCTGACATTTA
TTAAAGGCTGAGCGGATAC
>377
ATGTACTTAAATGGAGTCATACAGTAAGTAGGCTTTTGTGTCTGGCTTCAAGCCTTTACT
TTTTATAAAGAATAACAATGGTAATAATTATAGCTGACATTTATTAAAGGCTGAGCGGAT
ACTGTATACTGTGCTACAGG
>378
GTAAGTAGGCTTTTGTGTCTGGCTTCAAGCCTTTACTTTTTATAAAGAATAACAATGGTA
ATAATTATAGCTGACATTTATTAAAGGCTGAGCGGATACTGTATACTGTGCTACAGGCTT
TTCGTGGGTTATCTCATCAC
>379
GTCTGGCTTCAAGCCTTTACTTTTTATAAAGAATAACAATGGTAATAATTATAGCTGACA
TTTATTAAAGGCTGAGCGGATACTGTATACTGTGCTACAGGCTTTTCGTGGGTTATCTCA
TCACTGTTTCACATATGTAG
>380
GAATAACAATGGTAATAATTATAGCTGACATTTATTAAAGGCTGAGCGGATACTGTATAC
TGTGCTACAGGCTTTTCGTGGGTTATCTCATCACTGTTTCACATATGTAGGTATACAGTT
CTTTCTTTAAAATATGTGAC
>381
GCTGAGCGGATACTGTATACTGTGCTACAGGCTTTTCGTGGGTTATCTCATCACTGTTTC
ACATATGTAGGTATACAGTTCTTTCTTTAAAATATGTGACTGAAAAATATTATTGAACATT
ATGCTAAAATTTGTTACAC
>382
GATACTGTATACTGTGCTACAGGCTTTTCGTGGGTTATCTCATCACTGTTTCACATATGTA
GGTATACAGTTCTTTCTTTAAAATATGTGACTGAAAAATATTATTGAACATTATGCTAAAA
TTTGTTACACTGAGAAGA
>383
ACAGTTCTTTCTTTAAAATATGTGACTGAAAAATATTATTGAACATTATGCTAAAATTTG
TTACACTGAGAAGACTTAAGAAAAACAGAGCTCATGTTTTCTTTCAACTGATTTCCTTT
TTACATTGTACTTGAGTACCT
>384
GTTCTTTCTTTAAAATATGTGACTGAAAAATATTATTGAACATTATGCTAAAATTTGTTAC
ACTGAGAAGACTTAAGAAAAACAGAGCTCATGTTTTCTTTCAACTGATTTCCTTTTTAC
ATTGTACTTGAGTACCTGGA
>385
TATGTGACTGAAAAATATTATTGAACATTATGCTAAAATTTGTTACACTGAGAAGACTTA
AGAAAAACAGAGCTCATGTTTTCTTTCAACTGATTTCCTTTTTACATTGTACTTGAGTAC
CTGGAAGCTGCTTGCTTCTT
>386
TGTGACTGAAAAATATTATTGAACATTATGCTAAAATTTGTTACACTGAGAAGACTTAA
GAAAAACAGAGCTCATGTTTTCTTTCAACTGATTTCCTTTTTACATTGTACTTGAGTACC
TGGAAGCTGCTTGCTTCTTTC

>387
CTGAGAAGACTTAAGAAAAACAGAGCTCATGTTTTCTTTCAACTGATTTCCTTTTTACA
TTGTACTTGAGTACCTGGAAGCTGCTTGCTTCTTTCCGCAGCTTAGGATACAAACTCCC
TGACAGTTAAGTGCTTCTTTAC
>388
TTAAGAAAAACAGAGCTCATGTTTTCTTTCAACTGATTTCCTTTTTACATTGTACTTGAG
TACCTGGAAGCTGCTTGCTTCTTTCCGCAGCTTAGGATACAAACTCCCTGACAGTTAAG
TGCTTCTTTACTTCCCATCCA
>389
GTTTTCTTTCAACTGATTTCCTTTTTACATTGTACTTGAGTACCTGGAAGCTGCTTGCTT
CTTTCCGCAGCTTAGGATACAAACTCCCTGACAGTTAAGTGCTTCTTTACTTCCCATCC
AGTGCCTTGCACAGGGCTTGG
>390
CTTTCAACTGATTTCCTTTTTACATTGTACTTGAGTACCTGGAAGCTGCTTGCTTCTTTC
CGCAGCTTAGGATACAAACTCCCTGACAGTTAAGTGCTTCTTTACTTCCCATCCAGTGC
CTTGCACAGGGCTTGGTACAT
>391
TGCTTGCTTCTTTCCGCAGCTTAGGATACAAACTCCCTGACAGTTAAGTGCTTCTTTAC
TTCCCATCCAGTGCCTTGCACAGGGCTTGGTACATGGTTTGGTCCAAAATATTTGTTGA
ATAAATTTGCAGATAGCCCACG
>392
TCCGCAGCTTAGGATACAAACTCCCTGACAGTTAAGTGCTTCTTTACTTCCCATCCAGT
GCCTTGCACAGGGCTTGGTACATGGTTTGGTCCAAAATATTTGTTGAATAAATTTGCAG
ATAGCCCACGTGTGTAGGAGAG
>393
TCCCATCCAGTGCCTTGCACAGGGCTTGGTACATGGTTTGGTCCAAAATATTTGTTGAA
TAAATTTGCAGATAGCCCACGTGTGTAGGAGAGGGAGAGAAGGTTCTTGAAAAATGGA
GATTGGAGGAATCTAGGATGGGG
>394
ATCCAGTGCCTTGCACAGGGCTTGGTACATGGTTTGGTCCAAAATATTTGTTGAATAAA
TTTGCAGATAGCCCACGTGTGTAGGAGAGGGAGAGAAGGTTCTTGAAAAATGGAGATT
GGAGGAATCTAGGATGGGGAGGG
>395
AGGGCTTGGTACATGGTTTGGTCCAAAATATTTGTTGAATAAATTTGCAGATAGCCCAC
GTGTGTAGGAGAGGGAGAGAAGGTTCTTGAAAAATGGAGATTGGAGGAATCTAGGAT
GGGGAGGGAGATATGGGAAGGGCC
>396
TATTTGTTGAATAAATTTGCAGATAGCCCACGTGTGTAGGAGAGGGAGAGAAGGTTCTT
GAAAAATGGAGATTGGAGGAATCTAGGATGGGGAGGGAGATATGGGAAGGGCCCAGA
CAGAGGCAGGAGGGTTCCAACTTG
>397
TGAATAAATTTGCAGATAGCCCACGTGTGTAGGAGAGGGAGAGAAGGTTCTTGAAAAA
TGGAGATTGGAGGAATCTAGGATGGGGAGGGAGATATGGGAAGGGCCCAGACAGAGG
CAGGAGGGTTCCAACTTGTTCCTCT
>398
TTTGCAGATAGCCCACGTGTGTAGGAGAGGGAGAGAAGGTTCTTGAAAAATGGAGATT

GGAGGAATCTAGGATGGGGAGGGAGATATGGGAAGGGCCCAGACAGAGGCAGGAGG
GTTCCAACTTGTTCCTCTTCCCTGCA
>399
CACGTGTGTAGGAGAGGGAGAGAAGGTTCTTGAAAAATGGAGATTGGAGGAATCTAG
GATGGGGAGGGAGATATGGGAAGGGCCCAGACAGAGGCAGGAGGGTTCCAACTTGTT
CCTCTTCCCTGCAGCCCAGTGTTTCC
>400
TCTTGAAAAATGGAGATTGGAGGAATCTAGGATGGGGAGGGAGATATGGGAAGGGCC
CAGACAGAGGCAGGAGGGTTCCAACTTGTTCCTCTTCCCTGCAGCCCAGTGTTTCCCG
GGCTCTTTGAGTTCTGCTCGCGTTA
>401
GAGGGAGATATGGGAAGGGCCCAGACAGAGGCAGGAGGGTTCCAACTTGTTCCTCTT
CCCTGCAGCCCAGTGTTTCCCGGGCTCTTTGAGTTCTGCTCGCGTTACACAGGCGCATC
TCTGCAAGGAGCAACCCAGCTGAA
>402
TTCCAACTTGTTCCTCTTCCCTGCAGCCCAGTGTTTCCCGGGCTCTTTGAGTTCTGCTC
GCGTTACACAGGCGCATCTCTGCAAGGAGCAACCCAGCTGAACAACAAGGTGACATA
GTCCCGAGTCCTGTTCTTCCTTTC
>403
CTTCCCTGCAGCCCAGTGTTTCCCGGGCTCTTTGAGTTCTGCTCGCGTTACACAGGCGC
ATCTCTGCAAGGAGCAACCCAGCTGAACAACAAGGTGACATAGTCCCGAGTCCTGTTC
TTCCTTTCCTCTGGATCCCTGGA
>404
CGGGCTCTTTGAGTTCTGCTCGCGTTACACAGGCGCATCTCTGCAAGGAGCAACCCAG
CTGAACAACAAGGTGACATAGTCCCGAGTCCTGTTCTTCCTTTCCTCTGGATCCCTGGA
CTCGGGATTTAACCCTGATCCTG
>405
TGCTCGCGTTACACAGGCGCATCTCTGCAAGGAGCAACCCAGCTGAACAACAAGGTG
ACATAGTCCCGAGTCCTGTTCTTCCTTTCCTCTGGATCCCTGGACTCGGGATTTAACCCT
GATCCTGGGCTCCCAGCTTGAGG
>406
TCCCGAGTCCTGTTCTTCCTTTCCTCTGGATCCCTGGACTCGGGATTTAACCCTGATCCT
GGGCTCCCAGCTTGAGGGGTGGGCAGGAAGGACTGTGACTTAGGTGTTTGTCTTTCAG
ATCTGTGATATTGCCATTAACT
>407
GTCCTGTTCTTCCTTTCCTCTGGATCCCTGGACTCGGGATTTAACCCTGATCCTGGGCTC
CCAGCTTGAGGGGTGGGCAGGAAGGACTGTGACTTAGGTGTTTGTCTTTCAGATCTGT
GATATTGCCATTAACTGGGCTG
>408
TCCTTTCCTCTGGATCCCTGGACTCGGGATTTAACCCTGATCCTGGGCTCCCAGCTTGA
GGGGTGGGCAGGAAGGACTGTGACTTAGGTGTTTGTCTTTCAGATCTGTGATATTGCCA
TTAACTGGGCTGGTGGTCTGCA
>409
AACCCTGATCCTGGGCTCCCAGCTTGAGGGGTGGGCAGGAAGGACTGTGACTTAGGT
GTTTGTCTTTCAGATCTGTGATATTGCCATTAACTGGGCTGGTGGTCTGCACCATGCCA
AGAAGTTTGAGGTGAGTGAGGAGG

>410
GACTTAGGTGTTTGTCTTTCAGATCTGTGATATTGCCATTAACTGGGCTGGTGGTCTGCA
CCATGCCAAGAAGTTTGAGGTGAGTGAGGAGGTGATGGGAAAGACAGTGGCCATCCT
AGGGTAGGTGTTTAGGATGATGG
>411
TTAGGTGTTTGTCTTTCAGATCTGTGATATTGCCATTAACTGGGCTGGTGGTCTGCACCA
TGCCAAGAAGTTTGAGGTGAGTGAGGAGGTGATGGGAAAGACAGTGGCCATCCTAGG
GTAGGTGTTTAGGATGATGGTGG
>412
TCAGATCTGTGATATTGCCATTAACTGGGCTGGTGGTCTGCACCATGCCAAGAAGTTTG
AGGTGAGTGAGGAGGTGATGGGAAAGACAGTGGCCATCCTAGGGTAGGTGTTTAGGA
TGATGGTGGGGGGCAGCTGGGAGG
>413
GATCTGTGATATTGCCATTAACTGGGCTGGTGGTCTGCACCATGCCAAGAAGTTTGAGG
TGAGTGAGGAGGTGATGGGAAAGACAGTGGCCATCCTAGGGTAGGTGTTTAGGATGAT
GGTGGGGGGCAGCTGGGAGGGGA
>414
TGCCATTAACTGGGCTGGTGGTCTGCACCATGCCAAGAAGTTTGAGGTGAGTGAGGAG
GTGATGGGAAAGACAGTGGCCATCCTAGGGTAGGTGTTTAGGATGATGGTGGGGGGCA
GCTGGGAGGGGAATTGCTCTTCTC
>415
GGTGGTCTGCACCATGCCAAGAAGTTTGAGGTGAGTGAGGAGGTGATGGGAAAGACA
GTGGCCATCCTAGGGTAGGTGTTTAGGATGATGGTGGGGGGCAGCTGGGAGGGGAATT
GCTCTTCTCTTTATGAGACCACTGT
>416
TGAGTGAGGAGGTGATGGGAAAGACAGTGGCCATCCTAGGGTAGGTGTTTAGGATGAT
GGTGGGGGGCAGCTGGGAGGGGAATTGCTCTTCTCTTTATGAGACCACTGTCTTGCCA
TAGGCCTCTGGCTTCTGCTATGTC
>417
GGAGGTGATGGGAAAGACAGTGGCCATCCTAGGGTAGGTGTTTAGGATGATGGTGGGG
GGCAGCTGGGAGGGGAATTGCTCTTCTCTTTATGAGACCACTGTCTTGCCATAGGCCTC
TGGCTTCTGCTATGTCAACGACA
>418
CATCCTAGGGTAGGTGTTTAGGATGATGGTGGGGGGCAGCTGGGAGGGGAATTGCTCT
TCTCTTTATGAGACCACTGTCTTGCCATAGGCCTCTGGCTTCTGCTATGTCAACGACATT
GTGATTGGCATCCTGGAGCTGC
>419
TAGGATGATGGTGGGGGGCAGCTGGGAGGGGAATTGCTCTTCTCTTTATGAGACCACT
GTCTTGCCATAGGCCTCTGGCTTCTGCTATGTCAACGACATTGTGATTGGCATCCTGGA
GCTGCTCAAGTAAGTAGCCCAGG
>420
TGAGACCACTGTCTTGCCATAGGCCTCTGGCTTCTGCTATGTCAACGACATTGTGATTG
GCATCCTGGAGCTGCTCAAGTAAGTAGCCCAGGAGGAGATGGGGGCAGTTACGGGATT
CTTGGCTGAGGAGGTCTCTCAGT
>421
TGTCTTGCCATAGGCCTCTGGCTTCTGCTATGTCAACGACATTGTGATTGGCATCCTGG

AGCTGCTCAAGTAAGTAGCCCAGGAGGAGATGGGGGCAGTTACGGGATTCTTGGCTGA
GGAGGTCTCTCAGTCTCAACCCT
>422
GGCCTCTGGCTTCTGCTATGTCAACGACATTGTGATTGGCATCCTGGAGCTGCTCAAGT
AAGTAGCCCAGGAGGAGATGGGGGCAGTTACGGGATTCTTGGCTGAGGAGGTCTCTC
AGTCTCAACCCTGACCTTGACCTC
>423
CTCTGGCTTCTGCTATGTCAACGACATTGTGATTGGCATCCTGGAGCTGCTCAAGTAAG
TAGCCCAGGAGGAGATGGGGGCAGTTACGGGATTCTTGGCTGAGGAGGTCTCTCAGTC
TCAACCCTGACCTTGACCTCCCT
>424
TGGCTTCTGCTATGTCAACGACATTGTGATTGGCATCCTGGAGCTGCTCAAGTAAGTAG
CCCAGGAGGAGATGGGGGCAGTTACGGGATTCTTGGCTGAGGAGGTCTCTCAGTCTCA
ACCCTGACCTTGACCTCCCTCCT
>425
TATGTCAACGACATTGTGATTGGCATCCTGGAGCTGCTCAAGTAAGTAGCCCAGGAGG
AGATGGGGGCAGTTACGGGATTCTTGGCTGAGGAGGTCTCTCAGTCTCAACCCTGACC
TTGACCTCCCTCCTCCCTGATTAG
>426
TGGCATCCTGGAGCTGCTCAAGTAAGTAGCCCAGGAGGAGATGGGGGCAGTTACGGG
ATTCTTGGCTGAGGAGGTCTCTCAGTCTCAACCCTGACCTTGACCTCCCTCCTCCCTGA
TTAGCTTCTCCCAAGTTCTTGATG
>427
TTACGGGATTCTTGGCTGAGGAGGTCTCTCAGTCTCAACCCTGACCTTGACCTCCCTCC
TCCCTGATTAGCTTCTCCCAAGTTCTTGATGCTTTATTCAGGCTGCCTCTTCCACTGCTG
CAGATTCCCTACTGCCTGCTC
>428
TTGGCTGAGGAGGTCTCTCAGTCTCAACCCTGACCTTGACCTCCCTCCTCCCTGATTAG
CTTCTCCCAAGTTCTTGATGCTTTATTCAGGCTGCCTCTTCCACTGCTGCAGATTCCCTA
CTGCCTGCTCACATTTCAGCC
>429
TCCCTCCTCCCTGATTAGCTTCTCCCAAGTTCTTGATGCTTTATTCAGGCTGCCTCTTCC
ACTGCTGCAGATTCCCTACTGCCTGCTCACATTTCAGCCTTCTCTGCATATCCCTCTCCT
GTCCCTGCCTCTAGCCTCTA
>430
AGTTCTTGATGCTTTATTCAGGCTGCCTCTTCCACTGCTGCAGATTCCCTACTGCCTGCT
CACATTTCAGCCTTCTCTGCATATCCCTCTCCTGTCCCTGCCTCTAGCCTCTAGGCTGGA
GCCCCCAGTTGCCTTTAGGG
>431
CTGCAGATTCCCTACTGCCTGCTCACATTTCAGCCTTCTCTGCATATCCCTCTCCTGTCC
CTGCCTCTAGCCTCTAGGCTGGAGCCCCCAGTTGCCTTTAGGGAACTGGTGACATTAA
GTTTCAGGTGACCAGGGCAGAA
>432
TTCCCTACTGCCTGCTCACATTTCAGCCTTCTCTGCATATCCCTCTCCTGTCCCTGCCTCT
AGCCTCTAGGCTGGAGCCCCCAGTTGCCTTTAGGGAACTGGTGACATTAAGTTTCAGG
TGACCAGGGCAGAAGCAGACT

>433
CTCTCCTGTCCCTGCCTCTAGCCTCTAGGCTGGAGCCCCCAGTTGCCTTTAGGGAACTG
GTGACATTAAGTTTCAGGTGACCAGGGCAGAAGCAGACTCTTCACAATTCGAGACCCG
GTGTTTGAGAGGAGTGGGTTGGG
>434
TGTCCCTGCCTCTAGCCTCTAGGCTGGAGCCCCCAGTTGCCTTTAGGGAACTGGTGACA
TTAAGTTTCAGGTGACCAGGGCAGAAGCAGACTCTTCACAATTCGAGACCCGGTGTTT
GAGAGGAGTGGGTTGGGGGGTTC
>435
CTCTAGGCTGGAGCCCCCAGTTGCCTTTAGGGAACTGGTGACATTAAGTTTCAGGTGA
CCAGGGCAGAAGCAGACTCTTCACAATTCGAGACCCGGTGTTTGAGAGGAGTGGGTT
GGGGGGTTCACTTCTAGCTCAGACC
>436
CTGGTGACATTAAGTTTCAGGTGACCAGGGCAGAAGCAGACTCTTCACAATTCGAGAC
CCGGTGTTTGAGAGGAGTGGGTTGGGGGGTTCACTTCTAGCTCAGACCCTGGCTTTGG
CTTCCCTCTAGGTACCACCCTCGG
>437
GGTGACATTAAGTTTCAGGTGACCAGGGCAGAAGCAGACTCTTCACAATTCGAGACCC
GGTGTTTGAGAGGAGTGGGTTGGGGGGTTCACTTCTAGCTCAGACCCTGGCTTTGGCT
TCCCTCTAGGTACCACCCTCGGGT
>438
GGCAGAAGCAGACTCTTCACAATTCGAGACCCGGTGTTTGAGAGGAGTGGGTTGGGG
GGTTCACTTCTAGCTCAGACCCTGGCTTTGGCTTCCCTCTAGGTACCACCCTCGGGTGC
TCTACATTGACATTGACATCCACC
>439
CTTCCCTCTAGGTACCACCCTCGGGTGCTCTACATTGACATTGACATCCACCATGGTGA
CGGGGTTCAAGAAGCTTTCTACCTCACTGACCGGGTCATGACGGTGTCCTTCCACAAA
TACGGAAATTACTTCTTCCCTGG
>440
CCCTCTAGGTACCACCCTCGGGTGCTCTACATTGACATTGACATCCACCATGGTGACGG
GGTTCAAGAAGCTTTCTACCTCACTGACCGGGTCATGACGGTGTCCTTCCACAAATAC
GGAAATTACTTCTTCCCTGGCAC
>441
TTCTACCTCACTGACCGGGTCATGACGGTGTCCTTCCACAAATACGGAAATTACTTCTT
CCCTGGCACAGGTATGGGAATAGGGATTACTTTCCCCTTTGCACCCTTCAGCTCTTTACT
ATGAGTGCCTCCTTCTCAGCC
>442
GACCGGGTCATGACGGTGTCCTTCCACAAATACGGAAATTACTTCTTCCCTGGCACAG
GTATGGGAATAGGGATTACTTTCCCCTTTGCACCCTTCAGCTCTTTACTATGAGTGCCTC
CTTCTCAGCCTTCTAGTCTCAT
>443
TTCTTCCCTGGCACAGGTATGGGAATAGGGATTACTTTCCCCTTTGCACCCTTCAGCTCT
TTACTATGAGTGCCTCCTTCTCAGCCTTCTAGTCTCATCTCAGGTGTCCCCTCCTCAGAG
AAGCCTTTCCTTACCACTCC
>444
AGGTATGGGAATAGGGATTACTTTCCCCTTTGCACCCTTCAGCTCTTTACTATGAGTGCC

TCCTTCTCAGCCTTCTAGTCTCATCTCAGGTGTCCCCTCCTCAGAGAAGCCTTTCCTTA
CCACTCCAGTTATCATAATCA
>445
ACTTTCCCCTTTGCACCCTTCAGCTCTTTACTATGAGTGCCTCCTTCTCAGCCTTCTAGT
CTCATCTCAGGTGTCCCCTCCTCAGAGAAGCCTTTCCTTACCACTCCAGTTATCATAATC
ACCTCCTTCCCCATTCCTCT
>446
CCCTTCAGCTCTTTACTATGAGTGCCTCCTTCTCAGCCTTCTAGTCTCATCTCAGGTGTC
CCCTCCTCAGAGAAGCCTTTCCTTACCACTCCAGTTATCATAATCACCTCCTTCCCCATT
CCTCTGTTACATCAGCCTCA
>447
CTTCAGCTCTTTACTATGAGTGCCTCCTTCTCAGCCTTCTAGTCTCATCTCAGGTGTCCC
CTCCTCAGAGAAGCCTTTCCTTACCACTCCAGTTATCATAATCACCTCCTTCCCCATTCC
TCTGTTACATCAGCCTCATT
>448
CAGCTCTTTACTATGAGTGCCTCCTTCTCAGCCTTCTAGTCTCATCTCAGGTGTCCCCTC
CTCAGAGAAGCCTTTCCTTACCACTCCAGTTATCATAATCACCTCCTTCCCCATTCCTCT
GTTACATCAGCCTCATTGTT
>449
CCTCCTTCTCAGCCTTCTAGTCTCATCTCAGGTGTCCCCTCCTCAGAGAAGCCTTTCCTT
ACCACTCCAGTTATCATAATCACCTCCTTCCCCATTCCTCTGTTACATCAGCCTCATTGTT
TCCCTCAGAACACTCACTG
>450
CTCAGAGAAGCCTTTCCTTACCACTCCAGTTATCATAATCACCTCCTTCCCCATTCCTCT
GTTACATCAGCCTCATTGTTTCCCTCAGAACACTCACTGCTATTTGTGTTTATTGTGGTT
TTTTGTTTTGTTTTGTTTAC
>451
TACCACTCCAGTTATCATAATCACCTCCTTCCCCATTCCTCTGTTACATCAGCCTCATTGT
TTCCCTCAGAACACTCACTGCTATTTGTGTTTATTGTGGTTTTTTGTTTTGTTTTGTTTAC
TGCCTCCCTCCTCCTCAT
>452
TTTGTGTTTATTGTGGTTTTTTGTTTTGTTTTGTTTACTGCCTCCCTCCTCCTCATGGCTA
TTCCCTAAGAATATAAGCGTGGTGGGATATCGTAAATTTCATTAGTTCCATTCGTTGCTG
CATCCCTACTGCCTAGTAC
>453
TATTGTGGTTTTTTGTTTTGTTTTGTTTACTGCCTCCCTCCTCCTCATGGCTATTCCCTAA
GAATATAAGCGTGGTGGGATATCGTAAATTTCATTAGTTCCATTCGTTGCTGCATCCCTA
CTGCCTAGTACACAGTTGG
>454
ACTGCCTCCCTCCTCCTCATGGCTATTCCCTAAGAATATAAGCGTGGTGGGATATCGTAA
ATTTCATTAGTTCCATTCGTTGCTGCATCCCTACTGCCTAGTACACAGTTGGCTCAGAGT
GGATCCCCCAAAACTATTTG
>455
AAGAATATAAGCGTGGTGGGATATCGTAAATTTCATTAGTTCCATTCGTTGCTGCATCCC
TACTGCCTAGTACACAGTTGGCTCAGAGTGGATCCCCCAAAACTATTTGCCCAATGAAT
GCAAGCTGCAAGAGATTTAGT

>456
TAAGCGTGGTGGGATATCGTAAATTTCATTAGTTCCATTCGTTGCTGCATCCCTACTGCC
TAGTACACAGTTGGCTCAGAGTGGATCCCCCAAAACTATTTGCCCAATGAATGCAAGCT
GCAAGAGATTTAGTGGCTCTA
>457
TGGGATATCGTAAATTTCATTAGTTCCATTCGTTGCTGCATCCCTACTGCCTAGTACACA
GTTGGCTCAGAGTGGATCCCCCAAAACTATTTGCCCAATGAATGCAAGCTGCAAGAGA
TTTAGTGGCTCTAGGGAGTTTA
>458
CATCCCTACTGCCTAGTACACAGTTGGCTCAGAGTGGATCCCCCAAAACTATTTGCCCA
ATGAATGCAAGCTGCAAGAGATTTAGTGGCTCTAGGGAGTTTATGGTGAAGCTACATAG
ATGGAATGAGAGGCTCCCAGAG
>459
GTACACAGTTGGCTCAGAGTGGATCCCCCAAAACTATTTGCCCAATGAATGCAAGCTG
CAAGAGATTTAGTGGCTCTAGGGAGTTTATGGTGAAGCTACATAGATGGAATGAGAGG
CTCCCAGAGTGCAGATTGGGGCTG
>460
TGGCTCAGAGTGGATCCCCCAAAACTATTTGCCCAATGAATGCAAGCTGCAAGAGATT
TAGTGGCTCTAGGGAGTTTATGGTGAAGCTACATAGATGGAATGAGAGGCTCCCAGAG
TGCAGATTGGGGCTGAAACAGCTG
>461
CCCCAAAACTATTTGCCCAATGAATGCAAGCTGCAAGAGATTTAGTGGCTCTAGGGAG
TTTATGGTGAAGCTACATAGATGGAATGAGAGGCTCCCAGAGTGCAGATTGGGGCTGA
AACAGCTGGGTTGTGGGTGCTTCC
>462
TGAATGCAAGCTGCAAGAGATTTAGTGGCTCTAGGGAGTTTATGGTGAAGCTACATAGA
TGGAATGAGAGGCTCCCAGAGTGCAGATTGGGGCTGAAACAGCTGGGTTGTGGGTGC
TTCCCATGTGTGGCTACCACCACC
>463
AGAGATTTAGTGGCTCTAGGGAGTTTATGGTGAAGCTACATAGATGGAATGAGAGGCTC
CCAGAGTGCAGATTGGGGCTGAAACAGCTGGGTTGTGGGTGCTTCCCATGTGTGGCTA
CCACCACCCACTTCCCTACAGGT
>464
TAGGGAGTTTATGGTGAAGCTACATAGATGGAATGAGAGGCTCCCAGAGTGCAGATTG
GGGCTGAAACAGCTGGGTTGTGGGTGCTTCCCATGTGTGGCTACCACCACCCACTTCC
CTACAGGTGACATGTATGAAGTCG
>465
CAGCTGGGTTGTGGGTGCTTCCCATGTGTGGCTACCACCACCCACTTCCCTACAGGTG
ACATGTATGAAGTCGGGGCAGAGAGTGGCCGCTACTACTGTCTGAACGTGCCCCTGCG
GGATGGCATTGATGACCAGAGTAA
>466
TGAAGTCGGGGCAGAGAGTGGCCGCTACTACTGTCTGAACGTGCCCCTGCGGGATGG
CATTGATGACCAGAGTAAGTACCGAAGTCTTCCCTCTGTTCCCTAGGCCCCTTCTTTTT
CTCCTTGAAATAACCTCTTCTGCC
>467
AAGTCGGGGCAGAGAGTGGCCGCTACTACTGTCTGAACGTGCCCCTGCGGGATGGCAT

TGATGACCAGAGTAAGTACCGAAGTCTTCCCTCTGTTCCCTAGGCCCCTTCTTTTTCTC
CTTGAAATAACCTCTTCTGCCTG
>468
TCTGAACGTGCCCCTGCGGGATGGCATTGATGACCAGAGTAAGTACCGAAGTCTTCCC
TCTGTTCCCTAGGCCCCTTCTTTTTCTCCTTGAAATAACCTCTTCTGCCTGGTATTGTCT
GCTGTTGGGCCAGTTGTCCTTA
>469
GTTCCCTAGGCCCCTTCTTTTTCTCCTTGAAATAACCTCTTCTGCCTGGTATTGTCTGCT
GTTGGGCCAGTTGTCCTTAAGGATGAATGGCCTTCAGAGGGACCATGAATCTCTGGAA
ATTGTATGCAACATATCATGTG
>470
CCCTTCTTTTTCTCCTTGAAATAACCTCTTCTGCCTGGTATTGTCTGCTGTTGGGCCAGT
TGTCCTTAAGGATGAATGGCCTTCAGAGGGACCATGAATCTCTGGAAATTGTATGCAAC
ATATCATGTGTATGTGCATTT
>471
TGAAATAACCTCTTCTGCCTGGTATTGTCTGCTGTTGGGCCAGTTGTCCTTAAGGATGA
ATGGCCTTCAGAGGGACCATGAATCTCTGGAAATTGTATGCAACATATCATGTGTATGTG
CATTTTTCTGGGAAAAGTCAT
>472
TCAGAGGGACCATGAATCTCTGGAAATTGTATGCAACATATCATGTGTATGTGCATTTTT
CTGGGAAAAGTCATTGGCTTTCATCAGTCACTCATAGCAGTCTGGGATCCCTTCCAGAA
GTTTCAGACCAGTGATTTGAT
>473
CTCTGGAAATTGTATGCAACATATCATGTGTATGTGCATTTTTCTGGGAAAAGTCATTGG
CTTTCATCAGTCACTCATAGCAGTCTGGGATCCCTTCCAGAAGTTTCAGACCAGTGATT
TGATTCTTTTGGGTGGCCCCT
>474
TGTATGCAACATATCATGTGTATGTGCATTTTTCTGGGAAAAGTCATTGGCTTTCATCAG
TCACTCATAGCAGTCTGGGATCCCTTCCAGAAGTTTCAGACCAGTGATTTGATTCTTTT
GGGTGGCCCCTTTCCTGGGCT
>475
ATGCAACATATCATGTGTATGTGCATTTTTCTGGGAAAAGTCATTGGCTTTCATCAGTCA
CTCATAGCAGTCTGGGATCCCTTCCAGAAGTTTCAGACCAGTGATTTGATTCTTTTGGG
TGGCCCCTTTCCTGGGCTACA
>476
TATGTGCATTTTTCTGGGAAAAGTCATTGGCTTTCATCAGTCACTCATAGCAGTCTGGG
ATCCCTTCCAGAAGTTTCAGACCAGTGATTTGATTCTTTTGGGTGGCCCCTTTCCTGGG
CTACATGCTCGCTCAACCCCCT
>477
CTGGGCTACATGCTCGCTCAACCCCCTTTTTCCTTTTGTTCCCTGTCTCCCCATCCCCTT
GGGGTGCCAGGTGCCCATCTTTGGCCCTCTCCCAGGTTACAAGCACCTTTTCCAGCCG
GTTATCAACCAGGTAGTGGACT
>478
TCTCCCCATCCCCTTGGGGTGCCAGGTGCCCATCTTTGGCCCTCTCCCAGGTTACAAGC
ACCTTTTCCAGCCGGTTATCAACCAGGTAGTGGACTTCTACCAACCCACGTGCATTGTG
CTCCAGGTAATACTGTCAGTTC

>479
GGGGTGCCAGGTGCCCATCTTTGGCCCTCTCCCAGGTTACAAGCACCTTTTCCAGCCG
GTTATCAACCAGGTAGTGGACTTCTACCAACCCACGTGCATTGTGCTCCAGGTAATACT
GTCAGTTCCTCCTCAGGAGTGTT
>480
CAAGCACCTTTTCCAGCCGGTTATCAACCAGGTAGTGGACTTCTACCAACCCACGTGC
ATTGTGCTCCAGGTAATACTGTCAGTTCCTCCTCAGGAGTGTTCTGCCTGGGCTTGGGG
AAAGAGAGCAAGGGAAGAGGAGG
>481
CAGCCGGTTATCAACCAGGTAGTGGACTTCTACCAACCCACGTGCATTGTGCTCCAGGT
AATACTGTCAGTTCCTCCTCAGGAGTGTTCTGCCTGGGCTTGGGGAAAGAGAGCAAGG
GAAGAGGAGGTTCTTTAGGTTGG
>482
CAACCAGGTAGTGGACTTCTACCAACCCACGTGCATTGTGCTCCAGGTAATACTGTCA
GTTCCTCCTCAGGAGTGTTCTGCCTGGGCTTGGGGAAAGAGAGCAAGGGAAGAGGAG
GTTCTTTAGGTTGGTGGTGGAAGGG
>483
CACGTGCATTGTGCTCCAGGTAATACTGTCAGTTCCTCCTCAGGAGTGTTCTGCCTGGG
CTTGGGGAAAGAGAGCAAGGGAAGAGGAGGTTCTTTAGGTTGGTGGTGGAAGGGGTA
TCTGTGATGTTCTGATCAGTTCTG
>484
CTCCAGGTAATACTGTCAGTTCCTCCTCAGGAGTGTTCTGCCTGGGCTTGGGGAAAGA
GAGCAAGGGAAGAGGAGGTTCTTTAGGTTGGTGGTGGAAGGGGTATCTGTGATGTTCT
GATCAGTTCTGATCACTGCACTTT
>485
CCAGGTAATACTGTCAGTTCCTCCTCAGGAGTGTTCTGCCTGGGCTTGGGGAAAGAGA
GCAAGGGAAGAGGAGGTTCTTTAGGTTGGTGGTGGAAGGGGTATCTGTGATGTTCTGA
TCAGTTCTGATCACTGCACTTTCC
>486
GTCAGTTCCTCCTCAGGAGTGTTCTGCCTGGGCTTGGGGAAAGAGAGCAAGGGAAGA
GGAGGTTCTTTAGGTTGGTGGTGGAAGGGGTATCTGTGATGTTCTGATCAGTTCTGATC
ACTGCACTTTCCTGGGGTTTTGCT
>487
AGGAGTGTTCTGCCTGGGCTTGGGGAAAGAGAGCAAGGGAAGAGGAGGTTCTTTAGG
TTGGTGGTGGAAGGGGTATCTGTGATGTTCTGATCAGTTCTGATCACTGCACTTTCCTG
GGGTTTTGCTTTTTGCAGTGTGGA
>488
TGGAAGGGGTATCTGTGATGTTCTGATCAGTTCTGATCACTGCACTTTCCTGGGGTTTT
GCTTTTTGCAGTGTGGAGCTGACTCTCTGGGCTGTGATCGATTGGGCTGCTTTAACCTC
AGCATCCGAGGGCATGGGTGAG
>489
GGTATCTGTGATGTTCTGATCAGTTCTGATCACTGCACTTTCCTGGGGTTTTGCTTTTTG
CAGTGTGGAGCTGACTCTCTGGGCTGTGATCGATTGGGCTGCTTTAACCTCAGCATCCG
AGGGCATGGGTGAGACTTGCT
>490
TGGGGTTTTGCTTTTTGCAGTGTGGAGCTGACTCTCTGGGCTGTGATCGATTGGGCTGC

TTTAACCTCAGCATCCGAGGGCATGGGTGAGACTTGCTCTCCTTCTTCTCCCCAAGGTT
TTTAAGTGGCCTTGGACTTCAG
>491
TGCTTTTTGCAGTGTGGAGCTGACTCTCTGGGCTGTGATCGATTGGGCTGCTTTAACCT
CAGCATCCGAGGGCATGGGTGAGACTTGCTCTCCTTCTTCTCCCCAAGGTTTTTAAGTG
GCCTTGGACTTCAGTACGTGGG
>492
ATCGATTGGGCTGCTTTAACCTCAGCATCCGAGGGCATGGGTGAGACTTGCTCTCCTTC
TTCTCCCCAAGGTTTTTAAGTGGCCTTGGACTTCAGTACGTGGGTGGGGGTAGTTGGG
GAATGGGCCTCACCTCTTCTTGG
>493
GCTGCTTTAACCTCAGCATCCGAGGGCATGGGTGAGACTTGCTCTCCTTCTTCTCCCCA
AGGTTTTTAAGTGGCCTTGGACTTCAGTACGTGGGTGGGGGTAGTTGGGGAATGGGCC
TCACCTCTTCTTGGAAATCAAAA
>494
CATCCGAGGGCATGGGTGAGACTTGCTCTCCTTCTTCTCCCCAAGGTTTTTAAGTGGCC
TTGGACTTCAGTACGTGGGTGGGGGTAGTTGGGGAATGGGCCTCACCTCTTCTTGGAA
ATCAAAAATAGCTACAAATTGTA
>495
CTTGGACTTCAGTACGTGGGTGGGGGTAGTTGGGGAATGGGCCTCACCTCTTCTTGGA
AATCAAAAATAGCTACAAATTGTACTTGAACTAGAAATGTCATCTTGAACAGGGTTCCC
TAGATGGTCATTCATTCACGATA
>496
GGGGTAGTTGGGGAATGGGCCTCACCTCTTCTTGGAAATCAAAAATAGCTACAAATTGT
ACTTGAACTAGAAATGTCATCTTGAACAGGGTTCCCTAGATGGTCATTCATTCACGATAT
ATTTTTATTAAGCACCTGTTA
>497
GCCTCACCTCTTCTTGGAAATCAAAAATAGCTACAAATTGTACTTGAACTAGAAATGTC
ATCTTGAACAGGGTTCCCTAGATGGTCATTCATTCACGATATATTTTTATTAAGCACCTGT
TATGTTCCAGGCATCATACC
>498
TTCTTGGAAATCAAAAATAGCTACAAATTGTACTTGAACTAGAAATGTCATCTTGAACA
GGGTTCCCTAGATGGTCATTCATTCACGATATATTTTTATTAAGCACCTGTTATGTTCCAG
GCATCATACCACATCATGGG
>499
TTGAACTAGAAATGTCATCTTGAACAGGGTTCCCTAGATGGTCATTCATTCACGATATAT
TTTTATTAAGCACCTGTTATGTTCCAGGCATCATACCACATCATGGGGATGGAATAAGGA
GTCTCCCATGGAGTTTACAG
>500
TCTTGAACAGGGTTCCCTAGATGGTCATTCATTCACGATATATTTTTATTAAGCACCTGTT
ATGTTCCAGGCATCATACCACATCATGGGGATGGAATAAGGAGTCTCCCATGGAGTTTA
CAGTTTAGGAAACAGACAAA
>501
TTCACGATATATTTTTATTAAGCACCTGTTATGTTCCAGGCATCATACCACATCATGGGGA
TGGAATAAGGAGTCTCCCATGGAGTTTACAGTTTAGGAAACAGACAAAAAAATAAATAA
TCAAATGAAATAAATAGATTC

>502
TATTAAGCACCTGTTATGTTCCAGGCATCATACCACATCATGGGGATGGAATAAGGAGT
CTCCCATGGAGTTTACAGTTTAGGAAACAGACAAAAAATAAATAATCAAATGAAATAAA
TAGATTCTTATAAATTCTGTGA
>503
CACCTGTTATGTTCCAGGCATCATACCACATCATGGGGATGGAATAAGGAGTCTCCCAT
GGAGTTTACAGTTTAGGAAACAGACAAAAAATAAATAATCAAATGAAATAAATAGATTC
TTATAAATTCTGTGAAAGAAAA
>504
GTTATGTTCCAGGCATCATACCACATCATGGGGATGGAATAAGGAGTCTCCCATGGAGT
TTACAGTTTAGGAAACAGACAAAAAATAAATAATCAAATGAAATAAATAGATTCTTATA
AATTCTGTGAAAGAAAAAAAGA
>505
TCCAGGCATCATACCACATCATGGGGATGGAATAAGGAGTCTCCCATGGAGTTTACAGT
TTAGGAAACAGACAAAAAATAAATAATCAAATGAAATAAATAGATTCTTATAAATTCTG
TGAAAGAAAAAAAGAAAGCTGA
>506
TAAGGAGTCTCCCATGGAGTTTACAGTTTAGGAAACAGACAAAAAATAAATAATCAAA
TGAAATAAATAGATTCTTATAAATTCTGTGAAAGAAAAAAAGAAAGCTGAGACACAGG
ATACAATGGGGGACCTACTTGAGA
>507
ACAGTTTAGGAAACAGACAAAAAATAAATAATCAAATGAAATAAATAGATTCTTATAAA
TTCTGTGAAAGAAAAAAAGAAAGCTGAGACACAGGATACAATGGGGGACCTACTTGA
GATAGCATGGTTTCGGAAGTTTTT
>508
GGAAACAGACAAAAAATAAATAATCAAATGAAATAAATAGATTCTTATAAATTCTGTGA
AAGAAAAAAAGAAAGCTGAGACACAGGATACAATGGGGGACCTACTTGAGATAGCAT
GGTTTCGGAAGTTTTTCTGAGGAA
>509
ATAAATAATCAAATGAAATAAATAGATTCTTATAAATTCTGTGAAAGAAAAAAAGAAAG
CTGAGACACAGGATACAATGGGGGACCTACTTGAGATAGCATGGTTTCGGAAGTTTTT
CTGAGGAAGTAAAATTTGAGTTG
>510
CTTATAAATTCTGTGAAAGAAAAAAAGAAAGCTGAGACACAGGATACAATGGGGGAC
CTACTTGAGATAGCATGGTTTCGGAAGTTTTTCTGAGGAAGTAAAATTTGAGTTGAGAC
CTGGAGACTAAGAAGGAGTGAGTC
>511
GAAAGAAAAAAAGAAAGCTGAGACACAGGATACAATGGGGGACCTACTTGAGATAGC
ATGGTTTCGGAAGTTTTTCTGAGGAAGTAAAATTTGAGTTGAGACCTGGAGACTAAGA
AGGAGTGAGTCATAAGAGGAGCAGA
>512
AAGAAAGCTGAGACACAGGATACAATGGGGGACCTACTTGAGATAGCATGGTTTCGGA
AGTTTTTCTGAGGAAGTAAAATTTGAGTTGAGACCTGGAGACTAAGAAGGAGTGAGT
CATAAGAGGAGCAGAGAGAAGCCTG
>513
GGGACCTACTTGAGATAGCATGGTTTCGGAAGTTTTTCTGAGGAAGTAAAATTTGAGTT

GAGACCTGGAGACTAAGAAGGAGTGAGTCATAAGAGGAGCAGAGAGAAGCCTGACC
CAAGCATGGACAAAGGCCCACAAAA
>514
TACTTGAGATAGCATGGTTTCGGAAGTTTTTCTGAGGAAGTAAAATTTGAGTTGAGACC
TGGAGACTAAGAAGGAGTGAGTCATAAGAGGAGCAGAGAGAAGCCTGACCCAAGCAT
GGACAAAGGCCCACAAAAGGCTCT
>515
TTGAGATAGCATGGTTTCGGAAGTTTTTCTGAGGAAGTAAAATTTGAGTTGAGACCTG
GAGACTAAGAAGGAGTGAGTCATAAGAGGAGCAGAGAGAAGCCTGACCCAAGCATG
GACAAAGGCCCACAAAAGGCTCTGAG
>516
AGATAGCATGGTTTCGGAAGTTTTTCTGAGGAAGTAAAATTTGAGTTGAGACCTGGAG
ACTAAGAAGGAGTGAGTCATAAGAGGAGCAGAGAGAAGCCTGACCCAAGCATGGAC
AAAGGCCCACAAAAGGCTCTGAGGGC
>517
TTTCGGAAGTTTTTCTGAGGAAGTAAAATTTGAGTTGAGACCTGGAGACTAAGAAGGA
GTGAGTCATAAGAGGAGCAGAGAGAAGCCTGACCCAAGCATGGACAAAGGCCCACA
AAAGGCTCTGAGGGCTGGGCCCAGTG
>518
TGAGGAAGTAAAATTTGAGTTGAGACCTGGAGACTAAGAAGGAGTGAGTCATAAGAG
GAGCAGAGAGAAGCCTGACCCAAGCATGGACAAAGGCCCACAAAAGGCTCTGAGGG
CTGGGCCCAGTGGCACACACCTATAAT
>519
CTAAGAAGGAGTGAGTCATAAGAGGAGCAGAGAGAAGCCTGACCCAAGCATGGACA
AAGGCCCACAAAAGGCTCTGAGGGCTGGGCCCAGTGGCACACACCTATAATCCCAAC
ACTTCCAGAGGCCAAGGTGGGAGAATC
>520
GAGTGAGTCATAAGAGGAGCAGAGAGAAGCCTGACCCAAGCATGGACAAAGGCCCA
CAAAAGGCTCTGAGGGCTGGGCCCAGTGGCACACACCTATAATCCCAACACTTCCAGA
GGCCAAGGTGGGAGAATCGCCTGAGG
>521
AGAGGAGCAGAGAGAAGCCTGACCCAAGCATGGACAAAGGCCCACAAAAGGCTCTG
AGGGCTGGGCCCAGTGGCACACACCTATAATCCCAACACTTCCAGAGGCCAAGGTGG
GAGAATCGCCTGAGGCTAGGAGTTTGA
>522
GGACAAAGGCCCACAAAAGGCTCTGAGGGCTGGGCCCAGTGGCACACACCTATAATC
CCAACACTTCCAGAGGCCAAGGTGGGAGAATCGCCTGAGGCTAGGAGTTTGAGGCCA
GCCTGGGCAACATAGTGAGAGCCATC
>523
GCCCACAAAAGGCTCTGAGGGCTGGGCCCAGTGGCACACACCTATAATCCCAACACTT
CCAGAGGCCAAGGTGGGAGAATCGCCTGAGGCTAGGAGTTTGAGGCCAGCCTGGGCA
ACATAGTGAGAGCCATCTCTACAGA
>524
AAAGGCTCTGAGGGCTGGGCCCAGTGGCACACACCTATAATCCCAACACTTCCAGAGG
CCAAGGTGGGAGAATCGCCTGAGGCTAGGAGTTTGAGGCCAGCCTGGGCAACATAGT
GAGAGCCATCTCTACAGAAAATAAA

>525
GGGCTGGGCCCAGTGGCACACACCTATAATCCCAACACTTCCAGAGGCCAAGGTGGG
AGAATCGCCTGAGGCTAGGAGTTTGAGGCCAGCCTGGGCAACATAGTGAGAGCCATCT
CTACAGAAAATAAAAAACAAAATCA
>526
ACACCTATAATCCCAACACTTCCAGAGGCCAAGGTGGGAGAATCGCCTGAGGCTAGGA
GTTTGAGGCCAGCCTGGGCAACATAGTGAGAGCCATCTCTACAGAAAATAAAAAACAA
AATCAGCTGGGCATGATGGTGCAT
>527
TCCAGAGGCCAAGGTGGGAGAATCGCCTGAGGCTAGGAGTTTGAGGCCAGCCTGGGC
AACATAGTGAGAGCCATCTCTACAGAAAATAAAAAACAAAATCAGCTGGGCATGATGG
TGCATGCCTATAGTCCTAGCTACTC
>528
GGTGGGAGAATCGCCTGAGGCTAGGAGTTTGAGGCCAGCCTGGGCAACATAGTGAGA
GCCATCTCTACAGAAAATAAAAAACAAAATCAGCTGGGCATGATGGTGCATGCCTATAG
TCCTAGCTACTCAGGAAGCTGAGG
>529
CTAGGAGTTTGAGGCCAGCCTGGGCAACATAGTGAGAGCCATCTCTACAGAAAATAAA
AAACAAAATCAGCTGGGCATGATGGTGCATGCCTATAGTCCTAGCTACTCAGGAAGCT
GAGGCAGGAGGATCACTTGAGCCT
>530
TGAGAGCCATCTCTACAGAAAATAAAAAACAAAATCAGCTGGGCATGATGGTGCATGC
CTATAGTCCTAGCTACTCAGGAAGCTGAGGCAGGAGGATCACTTGAGCCTAGGAGTTT
GAGGTTACAGTGAACTATGATTGT
>531
ATCTCTACAGAAAATAAAAAACAAAATCAGCTGGGCATGATGGTGCATGCCTATAGTCC
TAGCTACTCAGGAAGCTGAGGCAGGAGGATCACTTGAGCCTAGGAGTTTGAGGTTACA
GTGAACTATGATTGTGCCACTGG
>532
GAAAATAAAAAACAAAATCAGCTGGGCATGATGGTGCATGCCTATAGTCCTAGCTACTC
AGGAAGCTGAGGCAGGAGGATCACTTGAGCCTAGGAGTTTGAGGTTACAGTGAACTAT
GATTGTGCCACTGGACTTCAGTC
>533
GGGCATGATGGTGCATGCCTATAGTCCTAGCTACTCAGGAAGCTGAGGCAGGAGGATC
ACTTGAGCCTAGGAGTTTGAGGTTACAGTGAACTATGATTGTGCCACTGGACTTCAGTC
TGTTGAGGTGAGGTCCGAGAAGC
>534
GCCTATAGTCCTAGCTACTCAGGAAGCTGAGGCAGGAGGATCACTTGAGCCTAGGAGT
TTGAGGTTACAGTGAACTATGATTGTGCCACTGGACTTCAGTCTGTTGAGGTGAGGTC
CGAGAAGCTGGCCATGGTGAAGAT
>535
AGGCAGGAGGATCACTTGAGCCTAGGAGTTTGAGGTTACAGTGAACTATGATTGTGCC
ACTGGACTTCAGTCTGTTGAGGTGAGGTCCGAGAAGCTGGCCATGGTGAAGATTGTGC
ATGAGCCTTTAAACCCTGGTAAGG
>536
GATCACTTGAGCCTAGGAGTTTGAGGTTACAGTGAACTATGATTGTGCCACTGGACTTC

AGTCTGTTGAGGTGAGGTCCGAGAAGCTGGCCATGGTGAAGATTGTGCATGAGCCTTT
AAACCCTGGTAAGGAGTTTGAAT
>537
AGTGAACTATGATTGTGCCACTGGACTTCAGTCTGTTGAGGTGAGGTCCGAGAAGCTG
GCCATGGTGAAGATTGTGCATGAGCCTTTAAACCCTGGTAAGGAGTTTGAATTATATTC
CAAAGATGATGGGAAACCATTGA
>538
TTGTGCCACTGGACTTCAGTCTGTTGAGGTGAGGTCCGAGAAGCTGGCCATGGTGAAG
ATTGTGCATGAGCCTTTAAACCCTGGTAAGGAGTTTGAATTATATTCCAAAGATGATGG
GAAACCATTGAAGGATTTTAAGC
>539
GTCTGTTGAGGTGAGGTCCGAGAAGCTGGCCATGGTGAAGATTGTGCATGAGCCTTTA
AACCCTGGTAAGGAGTTTGAATTATATTCCAAAGATGATGGGAAACCATTGAAGGATTT
TAAGCAGGAAAGTGACATGATCC
>540
AAGCTGGCCATGGTGAAGATTGTGCATGAGCCTTTAAACCCTGGTAAGGAGTTTGAATT
ATATTCCAAAGATGATGGGAAACCATTGAAGGATTTTAAGCAGGAAAGTGACATGATC
CAATTTGTGTTTGAAAGTGACAA
>541
TGTGCATGAGCCTTTAAACCCTGGTAAGGAGTTTGAATTATATTCCAAAGATGATGGGA
AACCATTGAAGGATTTTAAGCAGGAAAGTGACATGATCCAATTTGTGTTTGAAAGTGA
CAATTCTGACTGCTGAATAGAGA
>542
GCCTTTAAACCCTGGTAAGGAGTTTGAATTATATTCCAAAGATGATGGGAAACCATTGA
AGGATTTTAAGCAGGAAAGTGACATGATCCAATTTGTGTTTGAAAGTGACAATTCTGA
CTGCTGAATAGAGAAAGGTTTGG
>543
CCAAAGATGATGGGAAACCATTGAAGGATTTTAAGCAGGAAAGTGACATGATCCAATT
TGTGTTTGAAAGTGACAATTCTGACTGCTGAATAGAGAAAGGTTTGGGTAGGGGGGAT
GGAGATGGAAGTAGAAGAGACCTA
>544
AAGGATTTTAAGCAGGAAAGTGACATGATCCAATTTGTGTTTGAAAGTGACAATTCTG
ACTGCTGAATAGAGAAAGGTTTGGGTAGGGGGGATGGAGATGGAAGTAGAAGAGACC
TAGTTAGGAAGCCATGGTGTTGGTT
>545
AAAGTGACATGATCCAATTTGTGTTTGAAAGTGACAATTCTGACTGCTGAATAGAGAA
AGGTTTGGGTAGGGGGGATGGAGATGGAAGTAGAAGAGACCTAGTTAGGAAGCCATG
GTGTTGGTTAGGTGCCCAGACTAAG
>546
TTGTGTTTGAAAGTGACAATTCTGACTGCTGAATAGAGAAAGGTTTGGGTAGGGGGGA
TGGAGATGGAAGTAGAAGAGACCTAGTTAGGAAGCCATGGTGTTGGTTAGGTGCCCAG
ACTAAGGTGGAGGGGGTGGCAATG
>547
TGTTTGAAAGTGACAATTCTGACTGCTGAATAGAGAAAGGTTTGGGTAGGGGGGATGG
AGATGGAAGTAGAAGAGACCTAGTTAGGAAGCCATGGTGTTGGTTAGGTGCCCAGACT
AAGGTGGAGGGGGTGGCAATGGAG

>548
GAAAGTGACAATTCTGACTGCTGAATAGAGAAAGGTTTGGGTAGGGGGGATGGAGAT
GGAAGTAGAAGAGACCTAGTTAGGAAGCCATGGTGTTGGTTAGGTGCCCAGACTAAG
GTGGAGGGGGTGGCAATGGAGAGAAG
>549
ATTCTGACTGCTGAATAGAGAAAGGTTTGGGTAGGGGGGATGGAGATGGAAGTAGAA
GAGACCTAGTTAGGAAGCCATGGTGTTGGTTAGGTGCCCAGACTAAGGTGGAGGGGG
TGGCAATGGAGAGAAGTAGATGATCT
>550
GGGTAGGGGGGATGGAGATGGAAGTAGAAGAGACCTAGTTAGGAAGCCATGGTGTTG
GTTAGGTGCCCAGACTAAGGTGGAGGGGGTGGCAATGGAGAGAAGTAGATGATCTGA
GAGGTGTGTGTGTGTGTGTGTGTATA
>551
TGGAGATGGAAGTAGAAGAGACCTAGTTAGGAAGCCATGGTGTTGGTTAGGTGCCCAG
ACTAAGGTGGAGGGGGTGGCAATGGAGAGAAGTAGATGATCTGAGAGGTGTGTGTGT
GTGTGTGTGTATATATATATATATA
>552
AAGAGACCTAGTTAGGAAGCCATGGTGTTGGTTAGGTGCCCAGACTAAGGTGGAGGG
GGTGGCAATGGAGAGAAGTAGATGATCTGAGAGGTGTGTGTGTGTGTGTGTGTATATAT
ATATATATACACACACACACACACAC
>553
GAGACCTAGTTAGGAAGCCATGGTGTTGGTTAGGTGCCCAGACTAAGGTGGAGGGGG
TGGCAATGGAGAGAAGTAGATGATCTGAGAGGTGTGTGTGTGTGTGTGTGTATATATAT
ATATATACACACACACACACACACAT
>554
CATGGTGTTGGTTAGGTGCCCAGACTAAGGTGGAGGGGGTGGCAATGGAGAGAAGTA
GATGATCTGAGAGGTGTGTGTGTGTGTGTGTGTATATATATATATATACACACACACACA
CACATATATATGTTTTTTTTTTT
>555
GTGTGTGTGTATATATATATATATACACACACACACACACATATATATGTTTTTTTTTTTCT
TTTTTGAGATGGAGTCTTGCTCTGTCACCCAGGCTGGAGTGCAGTGGCACGATCTTGG
CTCAGTGCAAGCTCTGCCTC
>556
GTGTATATATATATATATACACACACACACACACATATATATGTTTTTTTTTTTCTTTTTTG
AGATGGAGTCTTGCTCTGTCACCCAGGCTGGAGTGCAGTGGCACGATCTTGGCTCAGT
GCAAGCTCTGCCTCCTGGGT
>557
ACACACACACACACACATATATATGTTTTTTTTTTTCTTTTTTGAGATGGAGTCTTGCTC
TGTCACCCAGGCTGGAGTGCAGTGGCACGATCTTGGCTCAGTGCAAGCTCTGCCTCCT
GGGTTCAAGCATTTCTCCTGCC
>558
CACACACACATATATATGTTTTTTTTTTTCTTTTTTGAGATGGAGTCTTGCTCTGTCACCC
AGGCTGGAGTGCAGTGGCACGATCTTGGCTCAGTGCAAGCTCTGCCTCCTGGGTTCAA
GCATTTCTCCTGCCTCAGCCT
>559
TTTTTCTTTTTTGAGATGGAGTCTTGCTCTGTCACCCAGGCTGGAGTGCAGTGGCACGA

TCTTGGCTCAGTGCAAGCTCTGCCTCCTGGGTTCAAGCATTTCTCCTGCCTCAGCCTCC
TGAGTAGCTGGGACTACTACAA
>560
CTCTGTCACCCAGGCTGGAGTGCAGTGGCACGATCTTGGCTCAGTGCAAGCTCTGCCT
CCTGGGTTCAAGCATTTCTCCTGCCTCAGCCTCCTGAGTAGCTGGGACTACTACAAAG
GCGTGAGCACCGCGCCGGGCGTGA
>561
GAGTGCAGTGGCACGATCTTGGCTCAGTGCAAGCTCTGCCTCCTGGGTTCAAGCATTT
CTCCTGCCTCAGCCTCCTGAGTAGCTGGGACTACTACAAAGGCGTGAGCACCGCGCCG
GGCGTGAGTTATATTTTCAAGGCT
>562
CAAGCTCTGCCTCCTGGGTTCAAGCATTTCTCCTGCCTCAGCCTCCTGAGTAGCTGGGA
CTACTACAAAGGCGTGAGCACCGCGCCGGGCGTGAGTTATATTTTCAAGGCTAGAATT
GACAGAACTTGGGTTGATGAGAA
>563
TGCCTCCTGGGTTCAAGCATTTCTCCTGCCTCAGCCTCCTGAGTAGCTGGGACTACTAC
AAAGGCGTGAGCACCGCGCCGGGCGTGAGTTATATTTTCAAGGCTAGAATTGACAGAA
CTTGGGTTGATGAGAATAGTTGA
>564
ATTTCTCCTGCCTCAGCCTCCTGAGTAGCTGGGACTACTACAAAGGCGTGAGCACCGC
GCCGGGCGTGAGTTATATTTTCAAGGCTAGAATTGACAGAACTTGGGTTGATGAGAATA
GTTGATTAGCAGGCAGGGTGCCA
>565
TCCTGAGTAGCTGGGACTACTACAAAGGCGTGAGCACCGCGCCGGGCGTGAGTTATAT
TTTCAAGGCTAGAATTGACAGAACTTGGGTTGATGAGAATAGTTGATTAGCAGGCAGG
GTGCCAGGGAGGGTGTTGAGCAGA
>566
AGGCGTGAGCACCGCGCCGGGCGTGAGTTATATTTTCAAGGCTAGAATTGACAGAACT
TGGGTTGATGAGAATAGTTGATTAGCAGGCAGGGTGCCAGGGAGGGTGTTGAGCAGA
ACAGAGGGGGGACTGTGCTTTATTT
>567
TGAGCACCGCGCCGGGCGTGAGTTATATTTTCAAGGCTAGAATTGACAGAACTTGGGT
TGATGAGAATAGTTGATTAGCAGGCAGGGTGCCAGGGAGGGTGTTGAGCAGAACAGA
GGGGGGACTGTGCTTTATTTTAAAA
>568
GCGTGAGTTATATTTTCAAGGCTAGAATTGACAGAACTTGGGTTGATGAGAATAGTTGA
TTAGCAGGCAGGGTGCCAGGGAGGGTGTTGAGCAGAACAGAGGGGGGACTGTGCTTT
ATTTTAAAATTTTTATTGAGACAC
>569
CAGAACTTGGGTTGATGAGAATAGTTGATTAGCAGGCAGGGTGCCAGGGAGGGTGTTG
AGCAGAACAGAGGGGGGACTGTGCTTTATTTTAAAATTTTTATTGAGACACAGCATATA
AAGTATACCCAAATCTTAAGGGT
>570
GCAGGGTGCCAGGGAGGGTGTTGAGCAGAACAGAGGGGGGACTGTGCTTTATTTTAA
AATTTTTATTGAGACACAGCATATAAAGTATACCCAAATCTTAAGGGTATAACATAATGA
ATTTCACTATCAAGATAAAGATT

>571
TGCCAGGGAGGGTGTTGAGCAGAACAGAGGGGGGACTGTGCTTTATTTTAAAATTTTT
ATTGAGACACAGCATATAAAGTATACCCAAATCTTAAGGGTATAACATAATGAATTTCAC
TATCAAGATAAAGATTTAGAAC
>572
GAGGGGGGACTGTGCTTTATTTTAAAATTTTTATTGAGACACAGCATATAAAGTATACCC
AAATCTTAAGGGTATAACATAATGAATTTCACTATCAAGATAAAGATTTAGAACATTTAT
AGTTTTCTTGTGCTCCTTTC
>573
TTTATTGAGACACAGCATATAAAGTATACCCAAATCTTAAGGGTATAACATAATGAATTT
CACTATCAAGATAAAGATTTAGAACATTTATAGTTTTCTTGTGCTCCTTTCAGGCAATAC
CTAAAGGTATTTACCTTGTC
>574
CAGCATATAAAGTATACCCAAATCTTAAGGGTATAACATAATGAATTTCACTATCAAGAT
AAAGATTTAGAACATTTATAGTTTTCTTGTGCTCCTTTCAGGCAATACCTAAAGGTATTT
ACCTTGTCTTTTGATAACCT
>575
GTATACCCAAATCTTAAGGGTATAACATAATGAATTTCACTATCAAGATAAAGATTTAGA
ACATTTATAGTTTTCTTGTGCTCCTTTCAGGCAATACCTAAAGGTATTTACCTTGTCTTTT
GATAACCTCCTCCACATCT
>576
TATAACATAATGAATTTCACTATCAAGATAAAGATTTAGAACATTTATAGTTTTCTTGTGC
TCCTTTCAGGCAATACCTAAAGGTATTTACCTTGTCTTTTGATAACCTCCTCCACATCTT
CCTCTGGTTCCTTTTAACA
>577
ATTTCACTATCAAGATAAAGATTTAGAACATTTATAGTTTTCTTGTGCTCCTTTCAGGCA
ATACCTAAAGGTATTTACCTTGTCTTTTGATAACCTCCTCCACATCTTCCTCTGGTTCCTT
TTAACAGGGAATGCGTTGA
>578
TGTCTTTTGATAACCTCCTCCACATCTTCCTCTGGTTCCTTTTAACAGGGAATGCGTTGA
ATATGTCAAGAGCTTCAATATCCCTCTACTCGTGCTGGGTGGTGGTGGTTATACTGTCCG
AAATGTTGCCCGCTGCTGGT
>579
TTCAATATCCCTCTACTCGTGCTGGGTGGTGGTGGTTATACTGTCCGAAATGTTGCCCGC
TGCTGGTGAGCACTCTCCTGATTTCCTCCCCTGTTGTCCAGTGTGAGCTAGGGTGTCCC
TCAGATATGGCCACATGGGCC
>580
GTGGTTATACTGTCCGAAATGTTGCCCGCTGCTGGTGAGCACTCTCCTGATTTCCTCCC
CTGTTGTCCAGTGTGAGCTAGGGTGTCCCTCAGATATGGCCACATGGGCCCTTCAGCCC
ACTTGGTCAGCCTGCTCCCCTC
>581
TGTCCGAAATGTTGCCCGCTGCTGGTGAGCACTCTCCTGATTTCCTCCCCTGTTGTCCA
GTGTGAGCTAGGGTGTCCCTCAGATATGGCCACATGGGCCCTTCAGCCCACTTGGTCA
GCCTGCTCCCCTCTGCTGGTTGG
>582
CCTGATTTCCTCCCCTGTTGTCCAGTGTGAGCTAGGGTGTCCCTCAGATATGGCCACAT

GGGCCCTTCAGCCCACTTGGTCAGCCTGCTCCCCTCTGCTGGTTGGTTTCAGGACATAT
GAGACATCGCTGCTGGTAGAAG
>583
CCTGTTGTCCAGTGTGAGCTAGGGTGTCCCTCAGATATGGCCACATGGGCCCTTCAGCC
CACTTGGTCAGCCTGCTCCCCTCTGCTGGTTGGTTTCAGGACATATGAGACATCGCTGC
TGGTAGAAGAGGCCATTAGTGA
>584
CCTCAGATATGGCCACATGGGCCCTTCAGCCCACTTGGTCAGCCTGCTCCCCTCTGCTG
GTTGGTTTCAGGACATATGAGACATCGCTGCTGGTAGAAGAGGCCATTAGTGAGGAGC
TTCCCTATAGTGGTAAGGACACC
>585
GGCCACATGGGCCCTTCAGCCCACTTGGTCAGCCTGCTCCCCTCTGCTGGTTGGTTTCA
GGACATATGAGACATCGCTGCTGGTAGAAGAGGCCATTAGTGAGGAGCTTCCCTATAGT
GGTAAGGACACCCACAACACCC
>586
CAGCCCACTTGGTCAGCCTGCTCCCCTCTGCTGGTTGGTTTCAGGACATATGAGACATC
GCTGCTGGTAGAAGAGGCCATTAGTGAGGAGCTTCCCTATAGTGGTAAGGACACCCAC
AACACCCCATAACAGGAAGGCCA
>587
CTCCCCTCTGCTGGTTGGTTTCAGGACATATGAGACATCGCTGCTGGTAGAAGAGGCCA
TTAGTGAGGAGCTTCCCTATAGTGGTAAGGACACCCACAACACCCCATAACAGGAAGG
CCATTCTCACCAGATTCCAGAAT
>588
TGGTTGGTTTCAGGACATATGAGACATCGCTGCTGGTAGAAGAGGCCATTAGTGAGGA
GCTTCCCTATAGTGGTAAGGACACCCACAACACCCCATAACAGGAAGGCCATTCTCAC
CAGATTCCAGAATGACCTGGTTTG
>589
TTTCAGGACATATGAGACATCGCTGCTGGTAGAAGAGGCCATTAGTGAGGAGCTTCCCT
ATAGTGGTAAGGACACCCACAACACCCCATAACAGGAAGGCCATTCTCACCAGATTCC
AGAATGACCTGGTTTGAAGGCAC
>590
GCTGGTAGAAGAGGCCATTAGTGAGGAGCTTCCCTATAGTGGTAAGGACACCCACAAC
ACCCCATAACAGGAAGGCCATTCTCACCAGATTCCAGAATGACCTGGTTTGAAGGCAC
ATGGGAAATTTTACACTCTCAGAG
>591
TAGTGAGGAGCTTCCCTATAGTGGTAAGGACACCCACAACACCCCATAACAGGAAGGC
CATTCTCACCAGATTCCAGAATGACCTGGTTTGAAGGCACATGGGAAATTTTACACTCT
CAGAGCTCTGCAGACTAAACCCC
>592
TAAGGACACCCACAACACCCCATAACAGGAAGGCCATTCTCACCAGATTCCAGAATGA
CCTGGTTTGAAGGCACATGGGAAATTTTACACTCTCAGAGCTCTGCAGACTAAACCCC
TGGGCTCAGAGCAGGGAAGAGATT
>593
AGGAAGGCCATTCTCACCAGATTCCAGAATGACCTGGTTTGAAGGCACATGGGAAATT
TTACACTCTCAGAGCTCTGCAGACTAAACCCCTGGGCTCAGAGCAGGGAAGAGATTG
GGCAGCTATTATGTAGGTGCTCTGG

>594
TGACCTGGTTTGAAGGCACATGGGAAATTTTACACTCTCAGAGCTCTGCAGACTAAAC
CCCTGGGCTCAGAGCAGGGAAGAGATTGGGCAGCTATTATGTAGGTGCTCTGGATGGG
AAGGAAAAAGATTGGGAAGTCTTG
>595
ACCTGGTTTGAAGGCACATGGGAAATTTTACACTCTCAGAGCTCTGCAGACTAAACCC
CTGGGCTCAGAGCAGGGAAGAGATTGGGCAGCTATTATGTAGGTGCTCTGGATGGGAA
GGAAAAAGATTGGGAAGTCTTGTT
>596
AAGGCACATGGGAAATTTTACACTCTCAGAGCTCTGCAGACTAAACCCCTGGGCTCAG
AGCAGGGAAGAGATTGGGCAGCTATTATGTAGGTGCTCTGGATGGGAAGGAAAAAGAT
TGGGAAGTCTTGTTAGCTGCCTTT
>597
AGACTAAACCCCTGGGCTCAGAGCAGGGAAGAGATTGGGCAGCTATTATGTAGGTGCT
CTGGATGGGAAGGAAAAAGATTGGGAAGTCTTGTTAGCTGCCTTTCTCAGTCTGAGCC
ATACTTTCTCCTCTCCTCCCCAAG
>598
ACCCCTGGGCTCAGAGCAGGGAAGAGATTGGGCAGCTATTATGTAGGTGCTCTGGATG
GGAAGGAAAAAGATTGGGAAGTCTTGTTAGCTGCCTTTCTCAGTCTGAGCCATACTTT
CTCCTCTCCTCCCCAAGAATACTT
>599
CTCAGAGCAGGGAAGAGATTGGGCAGCTATTATGTAGGTGCTCTGGATGGGAAGGAAA
AAGATTGGGAAGTCTTGTTAGCTGCCTTTCTCAGTCTGAGCCATACTTTCTCCTCTCCT
CCCCAAGAATACTTCGAGTACTT
>600
GGGAAGAGATTGGGCAGCTATTATGTAGGTGCTCTGGATGGGAAGGAAAAAGATTGGG
AAGTCTTGTTAGCTGCCTTTCTCAGTCTGAGCCATACTTTCTCCTCTCCTCCCCAAGAAT
ACTTCGAGTACTTTGCCCCAGA
>601
GGAAGTCTTGTTAGCTGCCTTTCTCAGTCTGAGCCATACTTTCTCCTCTCCTCCCCAAG
AATACTTCGAGTACTTTGCCCCAGACTTCACACTTCATCCAGATGTCAGCACCCGCATC
GAGAATCAGAACTCACGCCAGG
>602
GCTGCCTTTCTCAGTCTGAGCCATACTTTCTCCTCTCCTCCCCAAGAATACTTCGAGTAC
TTTGCCCCAGACTTCACACTTCATCCAGATGTCAGCACCCGCATCGAGAATCAGAACT
CACGCCAGGTCAGCAGCTCAGA
>603
TCCCCAAGAATACTTCGAGTACTTTGCCCCAGACTTCACACTTCATCCAGATGTCAGCA
CCCGCATCGAGAATCAGAACTCACGCCAGGTCAGCAGCTCAGAAAAGCTGAGCACAA
ACTTCTCCTGAAAGGTGGAGCCCA
>604
AGAATACTTCGAGTACTTTGCCCCAGACTTCACACTTCATCCAGATGTCAGCACCCGCA
TCGAGAATCAGAACTCACGCCAGGTCAGCAGCTCAGAAAAGCTGAGCACAAACTTCT
CCTGAAAGGTGGAGCCCAGGAGAG
>605
GTACTTTGCCCCAGACTTCACACTTCATCCAGATGTCAGCACCCGCATCGAGAATCAGA

ACTCACGCCAGGTCAGCAGCTCAGAAAAGCTGAGCACAAACTTCTCCTGAAAGGTGG
AGCCCAGGAGAGGTCAAAGATGTG
>606
CCGCATCGAGAATCAGAACTCACGCCAGGTCAGCAGCTCAGAAAAGCTGAGCACAAA
CTTCTCCTGAAAGGTGGAGCCCAGGAGAGGTCAAAGATGTGGGAAGTAGGAAAAACC
AAAGAGAAGGAAGTTCTAAGCCATCA
>607
CGAGAATCAGAACTCACGCCAGGTCAGCAGCTCAGAAAAGCTGAGCACAAACTTCTC
CTGAAAGGTGGAGCCCAGGAGAGGTCAAAGATGTGGGAAGTAGGAAAAACCAAAGA
GAAGGAAGTTCTAAGCCATCACAGTCA
>608
GCCAGGTCAGCAGCTCAGAAAAGCTGAGCACAAACTTCTCCTGAAAGGTGGAGCCCA
GGAGAGGTCAAAGATGTGGGAAGTAGGAAAAACCAAAGAGAAGGAAGTTCTAAGCC
ATCACAGTCACTGTTATTGACTAGGAG
>609
CAAACTTCTCCTGAAAGGTGGAGCCCAGGAGAGGTCAAAGATGTGGGAAGTAGGAAA
AACCAAAGAGAAGGAAGTTCTAAGCCATCACAGTCACTGTTATTGACTAGGAGGCAGT
CCAGTTTAAAGTAGAGGCAGTTGGG
>610
CTTCTCCTGAAAGGTGGAGCCCAGGAGAGGTCAAAGATGTGGGAAGTAGGAAAAACC
AAAGAGAAGGAAGTTCTAAGCCATCACAGTCACTGTTATTGACTAGGAGGCAGTCCAG
TTTAAAGTAGAGGCAGTTGGGGTTG
>611
TGAAAGGTGGAGCCCAGGAGAGGTCAAAGATGTGGGAAGTAGGAAAAACCAAAGAG
AAGGAAGTTCTAAGCCATCACAGTCACTGTTATTGACTAGGAGGCAGTCCAGTTTAAA
GTAGAGGCAGTTGGGGTTGGTGGGGA
>612
AAGATGTGGGAAGTAGGAAAAACCAAAGAGAAGGAAGTTCTAAGCCATCACAGTCAC
TGTTATTGACTAGGAGGCAGTCCAGTTTAAAGTAGAGGCAGTTGGGGTTGGTGGGGAG
GGAGAGCTATCAGTTGGGGGACCTG
>613
CCAAAGAGAAGGAAGTTCTAAGCCATCACAGTCACTGTTATTGACTAGGAGGCAGTCC
AGTTTAAAGTAGAGGCAGTTGGGGTTGGTGGGGAGGGAGAGCTATCAGTTGGGGGAC
CTGAACTAGAGGTACCACTGAGATG
>614
AAAGAGAAGGAAGTTCTAAGCCATCACAGTCACTGTTATTGACTAGGAGGCAGTCCAG
TTTAAAGTAGAGGCAGTTGGGGTTGGTGGGGAGGGAGAGCTATCAGTTGGGGGACCT
GAACTAGAGGTACCACTGAGATGAA
>615
AGAAGGAAGTTCTAAGCCATCACAGTCACTGTTATTGACTAGGAGGCAGTCCAGTTTA
AAGTAGAGGCAGTTGGGGTTGGTGGGGAGGGAGAGCTATCAGTTGGGGGACCTGAAC
TAGAGGTACCACTGAGATGAAAACC
>616
CATCACAGTCACTGTTATTGACTAGGAGGCAGTCCAGTTTAAAGTAGAGGCAGTTGGG
GTTGGTGGGGAGGGAGAGCTATCAGTTGGGGGACCTGAACTAGAGGTACCACTGAGA
TGAAAACCAGTATGGTAGGGGTAAA

>617
GTCCAGTTTAAAGTAGAGGCAGTTGGGGTTGGTGGGGAGGGAGAGCTATCAGTTGGG
GGACCTGAACTAGAGGTACCACTGAGATGAAAACCAGTATGGTAGGGGTAAAAGGCA
GCTAGGAGATTCCATGTCCTGGGAAT
>618
TAGAGGCAGTTGGGGTTGGTGGGGAGGGAGAGCTATCAGTTGGGGGACCTGAACTAG
AGGTACCACTGAGATGAAAACCAGTATGGTAGGGGTAAAAGGCAGCTAGGAGATTCCA
TGTCCTGGGAATCTCTGGAAACTGC
>619
GGGGTTGGTGGGGAGGGAGAGCTATCAGTTGGGGGACCTGAACTAGAGGTACCACTG
AGATGAAAACCAGTATGGTAGGGGTAAAAGGCAGCTAGGAGATTCCATGTCCTGGGAA
TCTCTGGAAACTGCTCACTCTGTAT
>620
AGAGCTATCAGTTGGGGGACCTGAACTAGAGGTACCACTGAGATGAAAACCAGTATGG
TAGGGGTAAAAGGCAGCTAGGAGATTCCATGTCCTGGGAATCTCTGGAAACTGCTCAC
TCTGTATTCCTCATTTCCAACCAG
>621
TTCCATGTCCTGGGAATCTCTGGAAACTGCTCACTCTGTATTCCTCATTTCCAACCAGTA
TCTGGACCAGATCCGCCAGACAATCTTTGAAAACCTGAAGATGCTGAACCATGCACCT
AGTGTCCAGATTCATGACGTGC
>622
CTGGGAATCTCTGGAAACTGCTCACTCTGTATTCCTCATTTCCAACCAGTATCTGGACC
AGATCCGCCAGACAATCTTTGAAAACCTGAAGATGCTGAACCATGCACCTAGTGTCCA
GATTCATGACGTGCCTGCAGACC
>623
TCACTCTGTATTCCTCATTTCCAACCAGTATCTGGACCAGATCCGCCAGACAATCTTTG
AAAACCTGAAGATGCTGAACCATGCACCTAGTGTCCAGATTCATGACGTGCCTGCAGA
CCTCCTGACCTATGACAGGACTG
>624
CCAACCAGTATCTGGACCAGATCCGCCAGACAATCTTTGAAAACCTGAAGATGCTGAA
CCATGCACCTAGTGTCCAGATTCATGACGTGCCTGCAGACCTCCTGACCTATGACAGGA
CTGATGAGGCTGATGCAGAGGAG
>625
CAGACAATCTTTGAAAACCTGAAGATGCTGAACCATGCACCTAGTGTCCAGATTCATG
ACGTGCCTGCAGACCTCCTGACCTATGACAGGACTGATGAGGCTGATGCAGAGGAGA
GGGGTCCTGAGGAGAACTATAGCAG
>626
TGAAGATGCTGAACCATGCACCTAGTGTCCAGATTCATGACGTGCCTGCAGACCTCCT
GACCTATGACAGGACTGATGAGGCTGATGCAGAGGAGAGGGGTCCTGAGGAGAACTA
TAGCAGGTCTGGGAGCAGGGACTTC
>627
CCTAGTGTCCAGATTCATGACGTGCCTGCAGACCTCCTGACCTATGACAGGACTGATGA
GGCTGATGCAGAGGAGAGGGGTCCTGAGGAGAACTATAGCAGGTCTGGGAGCAGGGA
CTTCAGCCTACTTTCAATAGGCTT
>628
TCCAGATTCATGACGTGCCTGCAGACCTCCTGACCTATGACAGGACTGATGAGGCTGAT

GCAGAGGAGAGGGGTCCTGAGGAGAACTATAGCAGGTCTGGGAGCAGGGACTTCAGC
CTACTTTCAATAGGCTTGCTGGGG
>629
TGACGTGCCTGCAGACCTCCTGACCTATGACAGGACTGATGAGGCTGATGCAGAGGAG
AGGGGTCCTGAGGAGAACTATAGCAGGTCTGGGAGCAGGGACTTCAGCCTACTTTCAA
TAGGCTTGCTGGGGAGGTTTAAAA
>630
CGTGCCTGCAGACCTCCTGACCTATGACAGGACTGATGAGGCTGATGCAGAGGAGAG
GGGTCCTGAGGAGAACTATAGCAGGTCTGGGAGCAGGGACTTCAGCCTACTTTCAATA
GGCTTGCTGGGGAGGTTTAAAAGGG
>631
CCTATGACAGGACTGATGAGGCTGATGCAGAGGAGAGGGGTCCTGAGGAGAACTATA
GCAGGTCTGGGAGCAGGGACTTCAGCCTACTTTCAATAGGCTTGCTGGGGAGGTTTAA
AAGGGAAAAAGTAAATGGGAAGGTA
>632
GTCCTGAGGAGAACTATAGCAGGTCTGGGAGCAGGGACTTCAGCCTACTTTCAATAGG
CTTGCTGGGGAGGTTTAAAAGGGAAAAAGTAAATGGGAAGGTATTAGTTTAATTGCCT
CTGGCTTACCTCCTTTAGTCTGCT
>633
AGAACTATAGCAGGTCTGGGAGCAGGGACTTCAGCCTACTTTCAATAGGCTTGCTGGG
GAGGTTTAAAAGGGAAAAAGTAAATGGGAAGGTATTAGTTTAATTGCCTCTGGCTTAC
CTCCTTTAGTCTGCTACAATTTCC
>634
GAGCAGGGACTTCAGCCTACTTTCAATAGGCTTGCTGGGGAGGTTTAAAAGGGAAAA
AGTAAATGGGAAGGTATTAGTTTAATTGCCTCTGGCTTACCTCCTTTAGTCTGCTACAAT
TTCCTCACTCTTTTCTTTGTTAT
>635
GGGGAGGTTTAAAAGGGAAAAAGTAAATGGGAAGGTATTAGTTTAATTGCCTCTGGCT
TACCTCCTTTAGTCTGCTACAATTTCCTCACTCTTTTCTTTGTTATTTTTAATGACAAAAG
TAACACGTATTTATTGCAAAA
>636
CCTCTGGCTTACCTCCTTTAGTCTGCTACAATTTCCTCACTCTTTTCTTTGTTATTTTTAA
TGACAAAAGTAACACGTATTTATTGCAAAATTTTAAAACAGCACCAAAATATGGTGTTG
GCGTTCACACAATCCCAGAC
>637
TTCCTCACTCTTTTCTTTGTTATTTTTAATGACAAAAGTAACACGTATTTATTGCAAAATT
TTAAAACAGCACCAAAATATGGTGTTGGCGTTCACACAATCCCAGACCTATTCCACAG
GAGTAACCACTGTTAATAGTT
>638
AGTAACACGTATTTATTGCAAAATTTTAAAACAGCACCAAAATATGGTGTTGGCGTTCA
CACAATCCCAGACCTATTCCACAGGAGTAACCACTGTTAATAGTTTTTATTGTTTTGGAT
AGACCTTTTTCTATAGATTGC
>639
TATTGCAAAATTTTAAAACAGCACCAAAATATGGTGTTGGCGTTCACACAATCCCAGAC
CTATTCCACAGGAGTAACCACTGTTAATAGTTTTTATTGTTTTGGATAGACCTTTTTCTAT
AGATTGCAAATATACTATTC

>640
AGCACCAAAATATGGTGTTGGCGTTCACACAATCCCAGACCTATTCCACAGGAGTAAC
CACTGTTAATAGTTTTTATTGTTTTGGATAGACCTTTTTCTATAGATTGCAAATATACTATT
CTGTATGTACATATAAACAA
>641
GGCGTTCACACAATCCCAGACCTATTCCACAGGAGTAACCACTGTTAATAGTTTTTATT
GTTTTGGATAGACCTTTTTCTATAGATTGCAAATATACTATTCTGTATGTACATATAAACA
AACACATATACACTCATTTA
>642
CCCAGACCTATTCCACAGGAGTAACCACTGTTAATAGTTTTTATTGTTTTGGATAGACCT
TTTTCTATAGATTGCAAATATACTATTCTGTATGTACATATAAACAAACACATATACACTC
ATTTACAGTTTGTGTGGTT
>643
TTTTTCTATAGATTGCAAATATACTATTCTGTATGTACATATAAACAAACACATATACACT
CATTTACAGTTTGTGTGGTTTTTGGTTTTTCCGTAAATACAAATAGGGTCATTCTACGTA
TGCTGCTTTACATCATATA
>644
CATATAAACAAACACATATACACTCATTTACAGTTTGTGTGGTTTTTGGTTTTTCCGTAA
ATACAAATAGGGTCATTCTACGTATGCTGCTTTACATCATATAGCTGTTTTCAGCTAGCTA
ATATAGATCTGCTTCAGTA
>645
TTGTGTGGTTTTTGGTTTTTCCGTAAATACAAATAGGGTCATTCTACGTATGCTGCTTTA
CATCATATAGCTGTTTTCAGCTAGCTAATATAGATCTGCTTCAGTATTTTATCAGCTGTAT
AATAATTTACTATGTGGAT
>646
TTTGGTTTTTCCGTAAATACAAATAGGGTCATTCTACGTATGCTGCTTTACATCATATAGC
TGTTTTCAGCTAGCTAATATAGATCTGCTTCAGTATTTTATCAGCTGTATAATAATTTACTA
TGTGGATAGATTATTTT
>647
AGGGTCATTCTACGTATGCTGCTTTACATCATATAGCTGTTTTCAGCTAGCTAATATAGAT
CTGCTTCAGTATTTTATCAGCTGTATAATAATTTACTATGTGGATAGATTATTTTCCATTTT
TTTGGTATTACAATAAT
>648
ACGTATGCTGCTTTACATCATATAGCTGTTTTCAGCTAGCTAATATAGATCTGCTTCAGTA
TTTTATCAGCTGTATAATAATTTACTATGTGGATAGATTATTTTCCATTTTTTTGGTATTAC
AATAATAATGTGGCAAT
>649
GTTTTCAGCTAGCTAATATAGATCTGCTTCAGTATTTTATCAGCTGTATAATAATTTACTAT
GTGGATAGATTATTTTCCATTTTTTTGGTATTACAATAATAATGTGGCAATGAACCCCCTT
ACATTATACATCTTTGC
>650
TTTTTGGTATTACAATAATAATGTGGCAATGAACCCCCTTACATTATACATCTTTGCATTC
TTGTTTGAGTGGGTTAGATAGTAGAATGGTTGCTGCTAGGTCAAAGGCTGTGTGCATTA
ATTTTTTTTTTTTTTTTTTT
>651
ATTACAATAATAATGTGGCAATGAACCCCCTTACATTATACATCTTTGCATTCTTGTTTGA

GTGGGTTAGATAGTAGAATGGTTGCTGCTAGGTCAAAGGCTGTGTGCATTAATTTTTTTT
TTTTTTTTTTTTTTGAGAT
>652
TAATAATGTGGCAATGAACCCCCTTACATTATACATCTTTGCATTCTTGTTTGAGTGGGTT
AGATAGTAGAATGGTTGCTGCTAGGTCAAAGGCTGTGTGCATTAATTTTTTTTTTTTTTT
TTTTTTTGAGATGGAGTCT
>653
CTTACATTATACATCTTTGCATTCTTGTTTGAGTGGGTTAGATAGTAGAATGGTTGCTGCT
AGGTCAAAGGCTGTGTGCATTAATTTTTTTTTTTTTTTTTTTTTTGAGATGGAGTCTCAC
TCTGTTGCCAGGCTGGAGT
>654
AGATAGTAGAATGGTTGCTGCTAGGTCAAAGGCTGTGTGCATTAATTTTTTTTTTTTTTT
TTTTTTTGAGATGGAGTCTCACTCTGTTGCCAGGCTGGAGTGCAGTGGCTAATCCCGGC
TCACTGCAACCTCCGCCTCCC
>655
TAGAATGGTTGCTGCTAGGTCAAAGGCTGTGTGCATTAATTTTTTTTTTTTTTTTTTTTTT
GAGATGGAGTCTCACTCTGTTGCCAGGCTGGAGTGCAGTGGCTAATCCCGGCTCACTG
CAACCTCCGCCTCCCAGGTTC
>656
GGCTGTGTGCATTAATTTTTTTTTTTTTTTTTTTTTTGAGATGGAGTCTCACTCTGTTGCC
AGGCTGGAGTGCAGTGGCTAATCCCGGCTCACTGCAACCTCCGCCTCCCAGGTTCAAG
CGATTCTCCTGCCTCAGCCTC
>657
GGAGTCTCACTCTGTTGCCAGGCTGGAGTGCAGTGGCTAATCCCGGCTCACTGCAACC
TCCGCCTCCCAGGTTCAAGCGATTCTCCTGCCTCAGCCTCCTCAGTAGCTGAGATTACA
GGCATGTGCCACCACACCCGGCT
>658
CACTCTGTTGCCAGGCTGGAGTGCAGTGGCTAATCCCGGCTCACTGCAACCTCCGCCT
CCCAGGTTCAAGCGATTCTCCTGCCTCAGCCTCCTCAGTAGCTGAGATTACAGGCATGT
GCCACCACACCCGGCTAACTTTA
>659
TGGCTAATCCCGGCTCACTGCAACCTCCGCCTCCCAGGTTCAAGCGATTCTCCTGCCTC
AGCCTCCTCAGTAGCTGAGATTACAGGCATGTGCCACCACACCCGGCTAACTTTAGTAT
TTTTAGTAGAGACGGGGTTTCA
>660
CTAATCCCGGCTCACTGCAACCTCCGCCTCCCAGGTTCAAGCGATTCTCCTGCCTCAGC
CTCCTCAGTAGCTGAGATTACAGGCATGTGCCACCACACCCGGCTAACTTTAGTATTTT
TAGTAGAGACGGGGTTTCACCA
>661
CACTGCAACCTCCGCCTCCCAGGTTCAAGCGATTCTCCTGCCTCAGCCTCCTCAGTAGC
TGAGATTACAGGCATGTGCCACCACACCCGGCTAACTTTAGTATTTTTAGTAGAGACGG
GGTTTCACCATGTTGGCCAGGA
>662
GATTCTCCTGCCTCAGCCTCCTCAGTAGCTGAGATTACAGGCATGTGCCACCACACCCG
GCTAACTTTAGTATTTTTAGTAGAGACGGGGTTTCACCATGTTGGCCAGGATGGTCTTG
ATCTCTTGACCTTGTGATCCAC

>663
TCAGCCTCCTCAGTAGCTGAGATTACAGGCATGTGCCACCACACCCGGCTAACTTTAGT
ATTTTTAGTAGAGACGGGGTTTCACCATGTTGGCCAGGATGGTCTTGATCTCTTGACCT
TGTGATCCACCTGCCTTGGCCT
>664
AGCCTCCTCAGTAGCTGAGATTACAGGCATGTGCCACCACACCCGGCTAACTTTAGTAT
TTTTAGTAGAGACGGGGTTTCACCATGTTGGCCAGGATGGTCTTGATCTCTTGACCTTG
TGATCCACCTGCCTTGGCCTCC
>665
AGGCATGTGCCACCACACCCGGCTAACTTTAGTATTTTTAGTAGAGACGGGGTTTCACC
ATGTTGGCCAGGATGGTCTTGATCTCTTGACCTTGTGATCCACCTGCCTTGGCCTCCCA
AAGTGCTGGGATTACAGGTGTG
>666
GTTTCACCATGTTGGCCAGGATGGTCTTGATCTCTTGACCTTGTGATCCACCTGCCTTG
GCCTCCCAAAGTGCTGGGATTACAGGTGTGAGCTACCACGCCCGGCTTTTGAGAATAC
TTTTTCAAAAAGAAAGAAACCAA
>667
GCCAGGATGGTCTTGATCTCTTGACCTTGTGATCCACCTGCCTTGGCCTCCCAAAGTGC
TGGGATTACAGGTGTGAGCTACCACGCCCGGCTTTTGAGAATACTTTTTCAAAAAGAA
AGAAACCAATATTGAGTACCTGT
>668
GTGATCCACCTGCCTTGGCCTCCCAAAGTGCTGGGATTACAGGTGTGAGCTACCACGC
CCGGCTTTTGAGAATACTTTTTCAAAAAGAAAGAAACCAATATTGAGTACCTGTTGGG
CCCTGTTTTGCTTTCACATCCATT
>669
GCCTCCCAAAGTGCTGGGATTACAGGTGTGAGCTACCACGCCCGGCTTTTGAGAATAC
TTTTTCAAAAAGAAAGAAACCAATATTGAGTACCTGTTGGGCCCTGTTTTGCTTTCACA
TCCATTCTTTCACTATGGGTTTA
>670
CCCAAAGTGCTGGGATTACAGGTGTGAGCTACCACGCCCGGCTTTTGAGAATACTTTTT
CAAAAAGAAAGAAACCAATATTGAGTACCTGTTGGGCCCTGTTTTGCTTTCACATCCAT
TCTTTCACTATGGGTTTATAAT
>671
CTTTCACTATGGGTTTATAATACCACGTCACTCTTCTTAAAATTAAAACTCTCCTTTACCT
TGCACTTAGAAGAAAATCCAAAATTTTGACTGTGGCTTAAAAAGGTCCTGCATGCTAG
CCCTTACCTACATCCAGGATC
>672
TCACTATGGGTTTATAATACCACGTCACTCTTCTTAAAATTAAAACTCTCCTTTACCTTG
CACTTAGAAGAAAATCCAAAATTTTGACTGTGGCTTAAAAAGGTCCTGCATGCTAGCC
CTTACCTACATCCAGGATCTCA
>673
CTTAAAATTAAAACTCTCCTTTACCTTGCACTTAGAAGAAAATCCAAAATTTTGACTGT
GGCTTAAAAAGGTCCTGCATGCTAGCCCTTACCTACATCCAGGATCTCATCTCTAACTA
CTCTCCTCCATGCACAGTGCTC
>674
TCTCCTTTACCTTGCACTTAGAAGAAAATCCAAAATTTTGACTGTGGCTTAAAAAGGTC

CTGCATGCTAGCCCTTACCTACATCCAGGATCTCATCTCTAACTACTCTCCTCCATGCAC
AGTGCTCCCGGCTGTCCTGGC
>675
TTAGAAGAAAATCCAAAATTTTGACTGTGGCTTAAAAAGGTCCTGCATGCTAGCCCTTA
CCTACATCCAGGATCTCATCTCTAACTACTCTCCTCCATGCACAGTGCTCCCGGCTGTCC
TGGCCTCCTTGTTCTTACTGG
>676
AAAAGGTCCTGCATGCTAGCCCTTACCTACATCCAGGATCTCATCTCTAACTACTCTCCT
CCATGCACAGTGCTCCCGGCTGTCCTGGCCTCCTTGTTCTTACTGGAATACATCAGTCT
CTTTCCATTTCATGACCTCTG
>677
CTAACTACTCTCCTCCATGCACAGTGCTCCCGGCTGTCCTGGCCTCCTTGTTCTTACTG
GAATACATCAGTCTCTTTCCATTTCATGACCTCTGTAGTTGGTTTTCTTGCAGCTTGGAA
TGGCCTTCCTGAAGTCTTTGC
>678
TCCCGGCTGTCCTGGCCTCCTTGTTCTTACTGGAATACATCAGTCTCTTTCCATTTCATG
ACCTCTGTAGTTGGTTTTCTTGCAGCTTGGAATGGCCTTCCTGAAGTCTTTGCCTGCTT
AGCTCCTCCTCAGACTTTAGG
>679
CCTCCTTGTTCTTACTGGAATACATCAGTCTCTTTCCATTTCATGACCTCTGTAGTTGGT
TTTCTTGCAGCTTGGAATGGCCTTCCTGAAGTCTTTGCCTGCTTAGCTCCTCCTCAGAC
TTTAGGTCTTATGTCCCAATA
>680
ACATCAGTCTCTTTCCATTTCATGACCTCTGTAGTTGGTTTTCTTGCAGCTTGGAATGGC
CTTCCTGAAGTCTTTGCCTGCTTAGCTCCTCCTCAGACTTTAGGTCTTATGTCCCAATAG
TCCTGTCTGAAATTTCACCC
>681
CATTTCATGACCTCTGTAGTTGGTTTTCTTGCAGCTTGGAATGGCCTTCCTGAAGTCTTT
GCCTGCTTAGCTCCTCCTCAGACTTTAGGTCTTATGTCCCAATAGTCCTGTCTGAAATTT
CACCCTTTTCCTCTCATCAC
>682
CTCTGTAGTTGGTTTTCTTGCAGCTTGGAATGGCCTTCCTGAAGTCTTTGCCTGCTTAG
CTCCTCCTCAGACTTTAGGTCTTATGTCCCAATAGTCCTGTCTGAAATTTCACCCTTTTC
CTCTCATCACCTTCAGTCCTA
>683
GTTGGTTTTCTTGCAGCTTGGAATGGCCTTCCTGAAGTCTTTGCCTGCTTAGCTCCTCC
TCAGACTTTAGGTCTTATGTCCCAATAGTCCTGTCTGAAATTTCACCCTTTTCCTCTCAT
CACCTTCAGTCCTATCACTTC
>684
CTCAGACTTTAGGTCTTATGTCCCAATAGTCCTGTCTGAAATTTCACCCTTTTCCTCTCA
TCACCTTCAGTCCTATCACTTCATTTTATTAATTGTACTCTCCATCATCTGAAATTATTATA
TTTATTTACTTTGTTAGT
>685
GTCCTATCACTTCATTTTATTAATTGTACTCTCCATCATCTGAAATTATTATATTTATTTACT
TTGTTAGTTACTAGTCTCATTTATTAGGTAAGCTTTTTGAGGATAGACAACCTTGTCCAG
CTTGTTACTCTACTGTA

>686
CACTTCATTTTATTAATTGTACTCTCCATCATCTGAAATTATTATATTTATTTACTTTGTTA
GTTACTAGTCTCATTTATTAGGTAAGCTTTTTGAGGATAGACAACCTTGTCCAGCTTGTT
ACTCTACTGTATTGCTAA
>687
ATCATCTGAAATTATTATATTTATTTACTTTGTTAGTTACTAGTCTCATTTATTAGGTAAGC
TTTTTGAGGATAGACAACCTTGTCCAGCTTGTTACTCTACTGTATTGCTAAGGTTTATGA
CAGTGTCTGTATAGAGTA
>688
CTGAAATTATTATATTTATTTACTTTGTTAGTTACTAGTCTCATTTATTAGGTAAGCTTTTT
GAGGATAGACAACCTTGTCCAGCTTGTTACTCTACTGTATTGCTAAGGTTTATGACAGT
GTCTGTATAGAGTAGTTCC
>689
TTTATTTACTTTGTTAGTTACTAGTCTCATTTATTAGGTAAGCTTTTTGAGGATAGACAAC
CTTGTCCAGCTTGTTACTCTACTGTATTGCTAAGGTTTATGACAGTGTCTGTATAGAGTA
GTTCCTTAATGTTTATTTG
>690
CTCATTTATTAGGTAAGCTTTTTGAGGATAGACAACCTTGTCCAGCTTGTTACTCTACTG
TATTGCTAAGGTTTATGACAGTGTCTGTATAGAGTAGTTCCTTAATGTTTATTTGATACGT
GAATGGGTGAATGAATAGC
>691
TGAGGATAGACAACCTTGTCCAGCTTGTTACTCTACTGTATTGCTAAGGTTTATGACAG
TGTCTGTATAGAGTAGTTCCTTAATGTTTATTTGATACGTGAATGGGTGAATGAATAGCA
GTCTCTACCAACAAGAGGAGG
>692
AGGATAGACAACCTTGTCCAGCTTGTTACTCTACTGTATTGCTAAGGTTTATGACAGTG
TCTGTATAGAGTAGTTCCTTAATGTTTATTTGATACGTGAATGGGTGAATGAATAGCAGT
CTCTACCAACAAGAGGAGGAG
>693
GGTTTATGACAGTGTCTGTATAGAGTAGTTCCTTAATGTTTATTTGATACGTGAATGGGT
GAATGAATAGCAGTCTCTACCAACAAGAGGAGGAGTGGTTCTCAACGGGGCCTGAGA
AATGGCTTTGGAAAAGGGTGCTG
>694
GTATAGAGTAGTTCCTTAATGTTTATTTGATACGTGAATGGGTGAATGAATAGCAGTCTC
TACCAACAAGAGGAGGAGTGGTTCTCAACGGGGCCTGAGAAATGGCTTTGGAAAAGG
GTGCTGGGAGAAGATTAGAACTT
>695
ATAGAGTAGTTCCTTAATGTTTATTTGATACGTGAATGGGTGAATGAATAGCAGTCTCTA
CCAACAAGAGGAGGAGTGGTTCTCAACGGGGCCTGAGAAATGGCTTTGGAAAAGGGT
GCTGGGAGAAGATTAGAACTTAG
>696
ATACGTGAATGGGTGAATGAATAGCAGTCTCTACCAACAAGAGGAGGAGTGGTTCTCA
ACGGGGCCTGAGAAATGGCTTTGGAAAAGGGTGCTGGGAGAAGATTAGAACTTAGAG
TGAGATCCTGACCTCTCTCCCCTTG
>697
CTCTACCAACAAGAGGAGGAGTGGTTCTCAACGGGGCCTGAGAAATGGCTTTGGAAA

AGGGTGCTGGGAGAAGATTAGAACTTAGAGTGAGATCCTGACCTCTCTCCCCTTGCTT
TGCAGGCCAGAGGCACCCAATGAGT
>698
TACCAACAAGAGGAGGAGTGGTTCTCAACGGGGCCTGAGAAATGGCTTTGGAAAAGG
GTGCTGGGAGAAGATTAGAACTTAGAGTGAGATCCTGACCTCTCTCCCCTTGCTTTGC
AGGCCAGAGGCACCCAATGAGTTCT
>699
GAGGAGTGGTTCTCAACGGGGCCTGAGAAATGGCTTTGGAAAAGGGTGCTGGGAGAA
GATTAGAACTTAGAGTGAGATCCTGACCTCTCTCCCCTTGCTTTGCAGGCCAGAGGCA
CCCAATGAGTTCTATGATGGAGACC
>700
AAAGGGTGCTGGGAGAAGATTAGAACTTAGAGTGAGATCCTGACCTCTCTCCCCTTGC
TTTGCAGGCCAGAGGCACCCAATGAGTTCTATGATGGAGACCATGACAATGACAAGGA
AAGCGATGTGGAGATTTAAGAGTG
>701
AGGGTGCTGGGAGAAGATTAGAACTTAGAGTGAGATCCTGACCTCTCTCCCCTTGCTT
TGCAGGCCAGAGGCACCCAATGAGTTCTATGATGGAGACCATGACAATGACAAGGAA
AGCGATGTGGAGATTTAAGAGTGGC
>702
GAAGATTAGAACTTAGAGTGAGATCCTGACCTCTCTCCCCTTGCTTTGCAGGCCAGAG
GCACCCAATGAGTTCTATGATGGAGACCATGACAATGACAAGGAAAGCGATGTGGAGA
TTTAAGAGTGGCTTGGGATGCTGT
>703
CTCTCTCCCCTTGCTTTGCAGGCCAGAGGCACCCAATGAGTTCTATGATGGAGACCATG
ACAATGACAAGGAAAGCGATGTGGAGATTTAAGAGTGGCTTGGGATGCTGTGTCCCAA
GGAATTTCTTTTCACCTCTTGGT
>704
TTGCAGGCCAGAGGCACCCAATGAGTTCTATGATGGAGACCATGACAATGACAAGGAA
AGCGATGTGGAGATTTAAGAGTGGCTTGGGATGCTGTGTCCCAAGGAATTTCTTTTCAC
CTCTTGGTTGGGCTGGAGGGAAA
>705
CCAGAGGCACCCAATGAGTTCTATGATGGAGACCATGACAATGACAAGGAAAGCGATG
TGGAGATTTAAGAGTGGCTTGGGATGCTGTGTCCCAAGGAATTTCTTTTCACCTCTTGG
TTGGGCTGGAGGGAAAAGGAGTG
>706
TGGAGACCATGACAATGACAAGGAAAGCGATGTGGAGATTTAAGAGTGGCTTGGGAT
GCTGTGTCCCAAGGAATTTCTTTTCACCTCTTGGTTGGGCTGGAGGGAAAAGGAGTGG
CTCCTAGAGTCCTGGGGGTCACCCC
>707
TGGAGATTTAAGAGTGGCTTGGGATGCTGTGTCCCAAGGAATTTCTTTTCACCTCTTGG
TTGGGCTGGAGGGAAAAGGAGTGGCTCCTAGAGTCCTGGGGGTCACCCCAGGGCTTT
TGCTGACTCTGGGAAAGAGTCTGG
>708
TTAAGAGTGGCTTGGGATGCTGTGTCCCAAGGAATTTCTTTTCACCTCTTGGTTGGGCT
GGAGGGAAAAGGAGTGGCTCCTAGAGTCCTGGGGGTCACCCCAGGGCTTTTGCTGAC
TCTGGGAAAGAGTCTGGAGACCAC

>709
GGGATGCTGTGTCCCAAGGAATTTCTTTTCACCTCTTGGTTGGGCTGGAGGGAAAAGG
AGTGGCTCCTAGAGTCCTGGGGGTCACCCCAGGGCTTTTGCTGACTCTGGGAAAGAGT
CTGGAGACCACATTTGGTTCTCGA
>710
GCTGGAGGGAAAAGGAGTGGCTCCTAGAGTCCTGGGGGTCACCCCAGGGCTTTTGCT
GACTCTGGGAAAGAGTCTGGAGACCACATTTGGTTCTCGAACCATCTACCTGCTTTTCC
TCTCTCTCCCAAGGCCTGACAATG
>711
TGGAGGGAAAAGGAGTGGCTCCTAGAGTCCTGGGGGTCACCCCAGGGCTTTTGCTGA
CTCTGGGAAAGAGTCTGGAGACCACATTTGGTTCTCGAACCATCTACCTGCTTTTCCTC
TCTCTCCCAAGGCCTGACAATGGT
>712
CTCTGGGAAAGAGTCTGGAGACCACATTTGGTTCTCGAACCATCTACCTGCTTTTCCTC
TCTCTCCCAAGGCCTGACAATGGTACCTATTAGGGATGAGATACAGACAAGGATAGCTA
TCTGGGACATTATTGGCAGTGG
>713
CACATTTGGTTCTCGAACCATCTACCTGCTTTTCCTCTCTCTCCCAAGGCCTGACAATG
GTACCTATTAGGGATGAGATACAGACAAGGATAGCTATCTGGGACATTATTGGCAGTGG
GCCCTGGAGGCCAGTCCCTAGC
>714
CGAACCATCTACCTGCTTTTCCTCTCTCTCCCAAGGCCTGACAATGGTACCTATTAGGG
ATGAGATACAGACAAGGATAGCTATCTGGGACATTATTGGCAGTGGGCCCTGGAGGCC
AGTCCCTAGCCCCCCTTGCCCCT
>715
AAGGCCTGACAATGGTACCTATTAGGGATGAGATACAGACAAGGATAGCTATCTGGGA
CATTATTGGCAGTGGGCCCTGGAGGCCAGTCCCTAGCCCCCCTTGCCCCTTATTTCTTC
CCTGCTTCCCTCGAACCCAGAGA
>716
TGGTACCTATTAGGGATGAGATACAGACAAGGATAGCTATCTGGGACATTATTGGCAGT
GGGCCCTGGAGGCCAGTCCCTAGCCCCCCTTGCCCCTTATTTCTTCCCTGCTTCCCTCG
AACCCAGAGATTTTTGAGGGAT
>717
CAGTGGGCCCTGGAGGCCAGTCCCTAGCCCCCCTTGCCCCTTATTTCTTCCCTGCTTCC
CTCGAACCCAGAGATTTTTGAGGGATGAACGGGTAGACAAGGACTGAGATTGCCTCTG
ACTTCCTCCTCCCCTGGGTTCTG
>718
GTGGGCCCTGGAGGCCAGTCCCTAGCCCCCCTTGCCCCTTATTTCTTCCCTGCTTCCCT
CGAACCCAGAGATTTTTGAGGGATGAACGGGTAGACAAGGACTGAGATTGCCTCTGAC
TTCCTCCTCCCCTGGGTTCTGAC
>719
CCCTTGCCCCTTATTTCTTCCCTGCTTCCCTCGAACCCAGAGATTTTTGAGGGATGAAC
GGGTAGACAAGGACTGAGATTGCCTCTGACTTCCTCCTCCCCTGGGTTCTGACTTCTTC
CTCCCCTTGCTTCCAGGGAAGA
>720
CCCTTATTTCTTCCCTGCTTCCCTCGAACCCAGAGATTTTTGAGGGATGAACGGGTAGA

CAAGGACTGAGATTGCCTCTGACTTCCTCCTCCCCTGGGTTCTGACTTCTTCCTCCCCT
TGCTTCCAGGGAAGATGAAGAG
>721
GGACTGAGATTGCCTCTGACTTCCTCCTCCCCTGGGTTCTGACTTCTTCCTCCCCTTGC
TTCCAGGGAAGATGAAGAGAGAGAGATTTGGAAGGGGCTCTGGCTCCCTAACACCTG
AATCCCAGATGATGGGAAGTATGT
>722
AGATTGCCTCTGACTTCCTCCTCCCCTGGGTTCTGACTTCTTCCTCCCCTTGCTTCCAG
GGAAGATGAAGAGAGAGAGATTTGGAAGGGGCTCTGGCTCCCTAACACCTGAATCCC
AGATGATGGGAAGTATGTTTTCAA
>723
CCTCTGACTTCCTCCTCCCCTGGGTTCTGACTTCTTCCTCCCCTTGCTTCCAGGGAAGA
TGAAGAGAGAGAGATTTGGAAGGGGCTCTGGCTCCCTAACACCTGAATCCCAGATGAT
GGGAAGTATGTTTTCAAGTGTGG
>724
CTTGCTTCCAGGGAAGATGAAGAGAGAGAGATTTGGAAGGGGCTCTGGCTCCCTAAC
ACCTGAATCCCAGATGATGGGAAGTATGTTTTCAAGTGTGGGGAGGATATGAAAATGTT
CTGTTCTCACTTTTGGCTTTATGT
>725
GGAAGATGAAGAGAGAGAGATTTGGAAGGGGCTCTGGCTCCCTAACACCTGAATCCC
AGATGATGGGAAGTATGTTTTCAAGTGTGGGGAGGATATGAAAATGTTCTGTTCTCACT
TTTGGCTTTATGTCCATTTTACCA
>726
GAGAGAGATTTGGAAGGGGCTCTGGCTCCCTAACACCTGAATCCCAGATGATGGGAAG
TATGTTTTCAAGTGTGGGGAGGATATGAAAATGTTCTGTTCTCACTTTTGGCTTTATGTC
CATTTTACCACTGTTTTTATCC
>727
GGTGAAATCCCAGCTCAGGTGTTATATCCTTGCTGCTTCTGACAAATGAAACCAAGGTG
TATTAATTAAGGTAATGCTAGCTACTACAATAAGCTGAAAAATATATAATAGCTAAAAGC
TAGGGTGCCAGATTTAGGAAA
>728
CAGCTCAGGTGTTATATCCTTGCTGCTTCTGACAAATGAAACCAAGGTGTATTAATTAA
GGTAATGCTAGCTACTACAATAAGCTGAAAAATATATAATAGCTAAAAGCTAGGGTGCC
AGATTTAGGAAAATAAAAACAA
>729
ACAAATGAAACCAAGGTGTATTAATTAAGGTAATGCTAGCTACTACAATAAGCTGAAAA
ATATATAATAGCTAAAAGCTAGGGTGCCAGATTTAGGAAAATAAAAACAAACAAAAAA
CAATTGAAATTTGAATTTCAGAT
>730
AAACCAAGGTGTATTAATTAAGGTAATGCTAGCTACTACAATAAGCTGAAAAATATATAA
TAGCTAAAAGCTAGGGTGCCAGATTTAGGAAAATAAAAACAAACAAAAAACAATTGA
AATTTGAATTTCAGATAAACAAT
>731
CAAGGTGTATTAATTAAGGTAATGCTAGCTACTACAATAAGCTGAAAAATATATAATAGC
TAAAAGCTAGGGTGCCAGATTTAGGAAAATAAAAACAAACAAAAAACAATTGAAATTT
GAATTTCAGATAAACAATTTTT

>732
TAAGGTAATGCTAGCTACTACAATAAGCTGAAAAATATATAATAGCTAAAAGCTAGGGT
GCCAGATTTAGGAAAATAAAAACAAACAAAAAACAATTGAAATTTGAATTTCAGATAA
ACAATTTTTTTAGTATAATTATG
>733
AGCTAAAAGCTAGGGTGCCAGATTTAGGAAAATAAAAACAAACAAAAAACAATTGAA
ATTTGAATTTCAGATAAACAATTTTTTTAGTATAATTATGTCCCATGCAATATTTGAGACA
TACTAAAAGGTTATTCATTGTT
>734
CCAGATTTAGGAAAATAAAAACAAACAAAAAACAATTGAAATTTGAATTTCAGATAAA
CAATTTTTTTAGTATAATTATGTCCCATGCAATATTTGAGACATACTAAAAGGTTATTCAT
TGTTTATCTGATATTCAAATT
>735
AAAACAATTGAAATTTGAATTTCAGATAAACAATTTTTTTAGTATAATTATGTCCCATGC
AATATTTGAGACATACTAAAAGGTTATTCATTGTTTATCTGATATTCAAATTTAACATTTT
CATTAATTTATTTTATTTG
>736
ATTTGAATTTCAGATAAACAATTTTTTTAGTATAATTATGTCCCATGCAATATTTGAGACA
TACTAAAAGGTTATTCATTGTTTATCTGATATTCAAATTTAACATTTTCATTAATTTATTTT
ATTTGGCAGCCCTATGG
>737
ACTAAAAGGTTATTCATTGTTTATCTGATATTCAAATTTAACATTTTCATTAATTTATTTTA
TTTGGCAGCCCTATGGAAGACATCAAAAGTTTATTTCTTATTTATATAAAGTCCAAAATA
GATGTTTTTTAGTAGGCA
>738
ATTCATTGTTTATCTGATATTCAAATTTAACATTTTCATTAATTTATTTTATTTGGCAGCCC
TATGGAAGACATCAAAAGTTTATTTCTTATTTATATAAAGTCCAAAATAGATGTTTTTTAG
TAGGCAGGCAGCTCTTC
>739
TATCTGATATTCAAATTTAACATTTTCATTAATTTATTTTATTTGGCAGCCCTATGGAAGA
CATCAAAAGTTTATTTCTTATTTATATAAAGTCCAAAATAGATGTTTTTTAGTAGGCAGG
CAGCTCTTCTCTAAGCGTA
>740
TTTATTTTATTTGGCAGCCCTATGGAAGACATCAAAAGTTTATTTCTTATTTATATAAAGT
CCAAAATAGATGTTTTTTAGTAGGCAGGCAGCTCTTCTCTAAGCGTAATTCAGGGCTCT
GGGTTCTGTCCATCCTGGCC
>741
TGGCAGCCCTATGGAAGACATCAAAAGTTTATTTCTTATTTATATAAAGTCCAAAATAGA
TGTTTTTTAGTAGGCAGGCAGCTCTTCTCTAAGCGTAATTCAGGGCTCTGGGTTCTGTC
CATCCTGGCCCTGACGTCTTT
>742
CAGCCCTATGGAAGACATCAAAAGTTTATTTCTTATTTATATAAAGTCCAAAATAGATGT
TTTTTAGTAGGCAGGCAGCTCTTCTCTAAGCGTAATTCAGGGCTCTGGGTTCTGTCCAT
CCTGGCCCTGACGTCTTTCAT
>743
AAAGTTTATTTCTTATTTATATAAAGTCCAAAATAGATGTTTTTTAGTAGGCAGGCAGCT

CTTCTCTAAGCGTAATTCAGGGCTCTGGGTTCTGTCCATCCTGGCCCTGACGTCTTTCAT
TGCTCAGCTTCTAAGATAGC
>744
GCTCTTCTCTAAGCGTAATTCAGGGCTCTGGGTTCTGTCCATCCTGGCCCTGACGTCTT
TCATTGCTCAGCTTCTAAGATAGCTCTATGCTCATGGGGTCAAGTCACAGGAAAGAAA
AGGGAATGTGGAGTTCCACTTGG
>745
CTAAGCGTAATTCAGGGCTCTGGGTTCTGTCCATCCTGGCCCTGACGTCTTTCATTGCT
CAGCTTCTAAGATAGCTCTATGCTCATGGGGTCAAGTCACAGGAAAGAAAAGGGAATG
TGGAGTTCCACTTGGGAGTTTTT
>746
TCTGTCCATCCTGGCCCTGACGTCTTTCATTGCTCAGCTTCTAAGATAGCTCTATGCTCA
TGGGGTCAAGTCACAGGAAAGAAAAGGGAATGTGGAGTTCCACTTGGGAGTTTTTAT
GGGCCAGTCCTGGAAGCGGCACA
>747
CATCCTGGCCCTGACGTCTTTCATTGCTCAGCTTCTAAGATAGCTCTATGCTCATGGGGT
CAAGTCACAGGAAAGAAAAGGGAATGTGGAGTTCCACTTGGGAGTTTTTATGGGCCA
GTCCTGGAAGCGGCACAGAGGAT
>748
CTGACGTCTTTCATTGCTCAGCTTCTAAGATAGCTCTATGCTCATGGGGTCAAGTCACA
GGAAAGAAAAGGGAATGTGGAGTTCCACTTGGGAGTTTTTATGGGCCAGTCCTGGAA
GCGGCACAGAGGATTTCTGCTCAC
>749
CTAAGATAGCTCTATGCTCATGGGGTCAAGTCACAGGAAAGAAAAGGGAATGTGGAGT
TCCACTTGGGAGTTTTTATGGGCCAGTCCTGGAAGCGGCACAGAGGATTTCTGCTCAC
AATCTATTGGCCAGAACTCAATCA
>750
GGTCAAGTCACAGGAAAGAAAAGGGAATGTGGAGTTCCACTTGGGAGTTTTTATGGG
CCAGTCCTGGAAGCGGCACAGAGGATTTCTGCTCACAATCTATTGGCCAGAACTCAAT
CACATGGCCTGCACCTAACTGCAAG
>751
CACAGGAAAGAAAAGGGAATGTGGAGTTCCACTTGGGAGTTTTTATGGGCCAGTCCTG
GAAGCGGCACAGAGGATTTCTGCTCACAATCTATTGGCCAGAACTCAATCACATGGCC
TGCACCTAACTGCAAGTTAAGCCA
>752
CACTTGGGAGTTTTTATGGGCCAGTCCTGGAAGCGGCACAGAGGATTTCTGCTCACAA
TCTATTGGCCAGAACTCAATCACATGGCCTGCACCTAACTGCAAGTTAAGCCAGAAAA
ATGTAGGTGTGCCCAGGAAGAAGT
>753
CGGCACAGAGGATTTCTGCTCACAATCTATTGGCCAGAACTCAATCACATGGCCTGCAC
CTAACTGCAAGTTAAGCCAGAAAAATGTAGGTGTGCCCAGGAAGAAGTTTGGGGAAC
AGCTAGCCAGTCTCTGCCACACCT
>754
CAGAGGATTTCTGCTCACAATCTATTGGCCAGAACTCAATCACATGGCCTGCACCTAAC
TGCAAGTTAAGCCAGAAAAATGTAGGTGTGCCCAGGAAGAAGTTTGGGGAACAGCTA
GCCAGTCTCTGCCACACCTGGTAA

>755
TTGGCCAGAACTCAATCACATGGCCTGCACCTAACTGCAAGTTAAGCCAGAAAAATGT
AGGTGTGCCCAGGAAGAAGTTTGGGGAACAGCTAGCCAGTCTCTGCCACACCTGGTA
ACAGCAGAGTGAATCTGTCAGTACT
>756
ATGGCCTGCACCTAACTGCAAGTTAAGCCAGAAAAATGTAGGTGTGCCCAGGAAGAA
GTTTGGGGAACAGCTAGCCAGTCTCTGCCACACCTGGTAACAGCAGAGTGAATCTGTC
AGTACTCGTGCCTAATGCGTGTCTC
>757
CCTGCACCTAACTGCAAGTTAAGCCAGAAAAATGTAGGTGTGCCCAGGAAGAAGTTTG
GGGAACAGCTAGCCAGTCTCTGCCACACCTGGTAACAGCAGAGTGAATCTGTCAGTAC
TCGTGCCTAATGCGTGTCTCATAT
>758
GAAAAATGTAGGTGTGCCCAGGAAGAAGTTTGGGGAACAGCTAGCCAGTCTCTGCCA
CACCTGGTAACAGCAGAGTGAATCTGTCAGTACTCGTGCCTAATGCGTGTCTCATATGC
TAGTGCTGGATTCAGTCAGCAGTC
>759
CCAGGAAGAAGTTTGGGGAACAGCTAGCCAGTCTCTGCCACACCTGGTAACAGCAGA
GTGAATCTGTCAGTACTCGTGCCTAATGCGTGTCTCATATGCTAGTGCTGGATTCAGTCA
GCAGTCCTACATCCGCTGGGGTC
>760
CTCTGCCACACCTGGTAACAGCAGAGTGAATCTGTCAGTACTCGTGCCTAATGCGTGTC
TCATATGCTAGTGCTGGATTCAGTCAGCAGTCCTACATCCGCTGGGGTCCCTAGGAGGG
GTCAGATGCTCTCCTCTTTGCC
>761
TGGTAACAGCAGAGTGAATCTGTCAGTACTCGTGCCTAATGCGTGTCTCATATGCTAGT
GCTGGATTCAGTCAGCAGTCCTACATCCGCTGGGGTCCCTAGGAGGGGTCAGATGCTC
TCCTCTTTGCCCTGAACTGGTTA
>762
GTGCCTAATGCGTGTCTCATATGCTAGTGCTGGATTCAGTCAGCAGTCCTACATCCGCT
GGGGTCCCTAGGAGGGGTCAGATGCTCTCCTCTTTGCCCTGAACTGGTTAGCTACTTCG
GACTGGGATGACAAGGCCATTG
>763
CCTAATGCGTGTCTCATATGCTAGTGCTGGATTCAGTCAGCAGTCCTACATCCGCTGGG
GTCCCTAGGAGGGGTCAGATGCTCTCCTCTTTGCCCTGAACTGGTTAGCTACTTCGGAC
TGGGATGACAAGGCCATTGTGA
>764
CAGCAGTCCTACATCCGCTGGGGTCCCTAGGAGGGGTCAGATGCTCTCCTCTTTGCCCT
GAACTGGTTAGCTACTTCGGACTGGGATGACAAGGCCATTGTGAGAGTGATAAGCAAT
GTGATTCAAACAAATTCATTTCA
>765
TCCCTAGGAGGGGTCAGATGCTCTCCTCTTTGCCCTGAACTGGTTAGCTACTTCGGACT
GGGATGACAAGGCCATTGTGAGAGTGATAAGCAATGTGATTCAAACAAATTCATTTCA
CTTCAATTCAATGCAGGTTTTTT
>766
GGTCAGATGCTCTCCTCTTTGCCCTGAACTGGTTAGCTACTTCGGACTGGGATGACAAG

GCCATTGTGAGAGTGATAAGCAATGTGATTCAAACAAATTCATTTCACTTCAATTCAAT
GCAGGTTTTTTTTTTTTTTTGA
>767
TGACAAGGCCATTGTGAGAGTGATAAGCAATGTGATTCAAACAAATTCATTTCACTTCA
ATTCAATGCAGGTTTTTTTTTTTTTTTGAGACGGGCTGTCATGCTGTTGCCAACCCTGG
AGAGCAGTGGCGCCATTACGGC
>768
GTGATAAGCAATGTGATTCAAACAAATTCATTTCACTTCAATTCAATGCAGGTTTTTTTT
TTTTTTTGAGACGGGCTGTCATGCTGTTGCCAACCCTGGAGAGCAGTGGCGCCATTAC
GGCTCACTGCAGCCTCCTGGGC
>769
TTCACTTCAATTCAATGCAGGTTTTTTTTTTTTTTTGAGACGGGCTGTCATGCTGTTGCC
AACCCTGGAGAGCAGTGGCGCCATTACGGCTCACTGCAGCCTCCTGGGCTCAAGCAAT
TCTCCCTCCTCAGCTTCCCAAG
>770
CACTTCAATTCAATGCAGGTTTTTTTTTTTTTTTGAGACGGGCTGTCATGCTGTTGCCAA
CCCTGGAGAGCAGTGGCGCCATTACGGCTCACTGCAGCCTCCTGGGCTCAAGCAATTC
TCCCTCCTCAGCTTCCCAAGTA
>771
TTTTTTTGAGACGGGCTGTCATGCTGTTGCCAACCCTGGAGAGCAGTGGCGCCATTAC
GGCTCACTGCAGCCTCCTGGGCTCAAGCAATTCTCCCTCCTCAGCTTCCCAAGTAGCT
GGGACTACAGGTGCATGCCACCAT
>772
CTGTCATGCTGTTGCCAACCCTGGAGAGCAGTGGCGCCATTACGGCTCACTGCAGCCT
CCTGGGCTCAAGCAATTCTCCCTCCTCAGCTTCCCAAGTAGCTGGGACTACAGGTGCA
TGCCACCATGTTCAGCTCATTTAT
>773
ACCCTGGAGAGCAGTGGCGCCATTACGGCTCACTGCAGCCTCCTGGGCTCAAGCAATT
CTCCCTCCTCAGCTTCCCAAGTAGCTGGGACTACAGGTGCATGCCACCATGTTCAGCTC
ATTTATTTTTTATTTTTTTGTAG
>774
AGCAGTGGCGCCATTACGGCTCACTGCAGCCTCCTGGGCTCAAGCAATTCTCCCTCCT
CAGCTTCCCAAGTAGCTGGGACTACAGGTGCATGCCACCATGTTCAGCTCATTTATTTT
TTATTTTTTTGTAGACAGGAGGT
>775
ACGGCTCACTGCAGCCTCCTGGGCTCAAGCAATTCTCCCTCCTCAGCTTCCCAAGTAG
CTGGGACTACAGGTGCATGCCACCATGTTCAGCTCATTTATTTTTTATTTTTTTGTAGAC
AGGAGGTCTCACTATGTTACCC
>776
GGGCTCAAGCAATTCTCCCTCCTCAGCTTCCCAAGTAGCTGGGACTACAGGTGCATGC
CACCATGTTCAGCTCATTTATTTTTTATTTTTTTGTAGACAGGAGGTCTCACTATGTTACC
CAGGCTGCTGTCCAAGTCCTG
>777
CTTCCCAAGTAGCTGGGACTACAGGTGCATGCCACCATGTTCAGCTCATTTATTTTTTAT
TTTTTTGTAGACAGGAGGTCTCACTATGTTACCCAGGCTGCTGTCCAAGTCCTGGGCTC
ACGTGATTCTCCCGCCTCAGC

>778
CCAAGTAGCTGGGACTACAGGTGCATGCCACCATGTTCAGCTCATTTATTTTTTATTTTT
TTGTAGACAGGAGGTCTCACTATGTTACCCAGGCTGCTGTCCAAGTCCTGGGCTCACG
TGATTCTCCCGCCTCAGCCTCC
>779
GCATGCCACCATGTTCAGCTCATTTATTTTTTATTTTTTTGTAGACAGGAGGTCTCACTAT
GTTACCCAGGCTGCTGTCCAAGTCCTGGGCTCACGTGATTCTCCCGCCTCAGCCTCCCA
AAATGCTGGGATTACAGGCG
>780
GACAGGAGGTCTCACTATGTTACCCAGGCTGCTGTCCAAGTCCTGGGCTCACGTGATT
CTCCCGCCTCAGCCTCCCAAAATGCTGGGATTACAGGCGTAAGCCACCATGCCCAGAC
AATTTAATAAATATTTATTAAAAC
>781
CAGGCTGCTGTCCAAGTCCTGGGCTCACGTGATTCTCCCGCCTCAGCCTCCCAAAATG
CTGGGATTACAGGCGTAAGCCACCATGCCCAGACAATTTAATAAATATTTATTAAAACTT
CTAGGATATTGAGAAAAGCTTG
>782
GGGCTCACGTGATTCTCCCGCCTCAGCCTCCCAAAATGCTGGGATTACAGGCGTAAGC
CACCATGCCCAGACAATTTAATAAATATTTATTAAAACTTCTAGGATATTGAGAAAAGCT
TGGTTCTAGGCCACTGGGGAAG
>783
AAAATGCTGGGATTACAGGCGTAAGCCACCATGCCCAGACAATTTAATAAATATTTATTA
AAACTTCTAGGATATTGAGAAAAGCTTGGTTCTAGGCCACTGGGGAAGACATCAACTG
AAACAGCCCAGTTATACTCTCA
>784
GGATTACAGGCGTAAGCCACCATGCCCAGACAATTTAATAAATATTTATTAAAACTTCTA
GGATATTGAGAAAAGCTTGGTTCTAGGCCACTGGGGAAGACATCAACTGAAACAGCCC
AGTTATACTCTCAATTAGCTGT
>785
CCACCATGCCCAGACAATTTAATAAATATTTATTAAAACTTCTAGGATATTGAGAAAAGC
TTGGTTCTAGGCCACTGGGGAAGACATCAACTGAAACAGCCCAGTTATACTCTCAATTA
GCTGTATACAATTAAATTCAC
>786
ACAATTTAATAAATATTTATTAAAACTTCTAGGATATTGAGAAAAGCTTGGTTCTAGGCC
ACTGGGGAAGACATCAACTGAAACAGCCCAGTTATACTCTCAATTAGCTGTATACAATT
AAATTCACAGATATCCTCAAC
>787
TTATTAAAACTTCTAGGATATTGAGAAAAGCTTGGTTCTAGGCCACTGGGGAAGACATC
AACTGAAACAGCCCAGTTATACTCTCAATTAGCTGTATACAATTAAATTCACAGATATCC
TCAACCCTTATCCATATCCCA
>788
CACTGGGGAAGACATCAACTGAAACAGCCCAGTTATACTCTCAATTAGCTGTATACAAT
TAAATTCACAGATATCCTCAACCCTTATCCATATCCCATTCATCTTTCAAAGGTTGGTGA
AAACCTCATAGCACAAAGAAT
>789
CAATTAGCTGTATACAATTAAATTCACAGATATCCTCAACCCTTATCCATATCCCATTCAT

CTTTCAAAGGTTGGTGAAAACCTCATAGCACAAAGAATTCTGCCCTAAATGTTTGAATA
CAGAATGGTCCCCTCTCTCC
>790
AAATTCACAGATATCCTCAACCCTTATCCATATCCCATTCATCTTTCAAAGGTTGGTGAA
AACCTCATAGCACAAAGAATTCTGCCCTAAATGTTTGAATACAGAATGGTCCCCTCTCT
CCCTAATTCCTAAAATATTTG
>791
CAGATATCCTCAACCCTTATCCATATCCCATTCATCTTTCAAAGGTTGGTGAAAACCTCA
TAGCACAAAGAATTCTGCCCTAAATGTTTGAATACAGAATGGTCCCCTCTCTCCCTAAT
TCCTAAAATATTTGGTCTCTA
>792
CCATTCATCTTTCAAAGGTTGGTGAAAACCTCATAGCACAAAGAATTCTGCCCTAAATG
TTTGAATACAGAATGGTCCCCTCTCTCCCTAATTCCTAAAATATTTGGTCTCTATGTTCAT
TCATTCATTCATGCATGCAT
>793
CCTCTCTCCCTAATTCCTAAAATATTTGGTCTCTATGTTCATTCATTCATTCATGCATGCAT
GCTTTCAGTTAGTTCATCGACAAACATTTACATCTACTCTGTTTGACAGTGTGCTAGGCT
CTGGGGCTTGGGAGTGGA
>794
TCTCCCTAATTCCTAAAATATTTGGTCTCTATGTTCATTCATTCATTCATGCATGCATGCTT
TCAGTTAGTTCATCGACAAACATTTACATCTACTCTGTTTGACAGTGTGCTAGGCTCTG
GGGCTTGGGAGTGGAAATA
>795
TTCATTCATTCATGCATGCATGCTTTCAGTTAGTTCATCGACAAACATTTACATCTACTCT
GTTTGACAGTGTGCTAGGCTCTGGGGCTTGGGAGTGGAAATACTAAATTGAACAAGAC
TGAGGCCTTGCCTTCCAGAAA
>796
TCAGTTAGTTCATCGACAAACATTTACATCTACTCTGTTTGACAGTGTGCTAGGCTCTG
GGGCTTGGGAGTGGAAATACTAAATTGAACAAGACTGAGGCCTTGCCTTCCAGAAACT
CACAAAGGAGTGGGTGGTCAGAA
>797
TTTACATCTACTCTGTTTGACAGTGTGCTAGGCTCTGGGGCTTGGGAGTGGAAATACTA
AATTGAACAAGACTGAGGCCTTGCCTTCCAGAAACTCACAAAGGAGTGGGTGGTCAG
AATGCACATGACTCAGTCAGATAT
>798
CAGTGTGCTAGGCTCTGGGGCTTGGGAGTGGAAATACTAAATTGAACAAGACTGAGGC
CTTGCCTTCCAGAAACTCACAAAGGAGTGGGTGGTCAGAATGCACATGACTCAGTCA
GATATAAGGCAGATGGTGGTAAATA
>799
CTCTGGGGCTTGGGAGTGGAAATACTAAATTGAACAAGACTGAGGCCTTGCCTTCCAG
AAACTCACAAAGGAGTGGGTGGTCAGAATGCACATGACTCAGTCAGATATAAGGCAGA
TGGTGGTAAATACTATGATAAATG
>800
TGGGGCTTGGGAGTGGAAATACTAAATTGAACAAGACTGAGGCCTTGCCTTCCAGAAA
CTCACAAAGGAGTGGGTGGTCAGAATGCACATGACTCAGTCAGATATAAGGCAGATGG
TGGTAAATACTATGATAAATGTTC

>801
AGTGGAAATACTAAATTGAACAAGACTGAGGCCTTGCCTTCCAGAAACTCACAAAGG
AGTGGGTGGTCAGAATGCACATGACTCAGTCAGATATAAGGCAGATGGTGGTAAATACT
ATGATAAATGTTCAGATAGTGCTA
>802
GAGGCCTTGCCTTCCAGAAACTCACAAAGGAGTGGGTGGTCAGAATGCACATGACTC
AGTCAGATATAAGGCAGATGGTGGTAAATACTATGATAAATGTTCAGATAGTGCTAAGG
GGCTTATGGAGAATGGGAGAAATT
>803
GGTGGTCAGAATGCACATGACTCAGTCAGATATAAGGCAGATGGTGGTAAATACTATGA
TAAATGTTCAGATAGTGCTAAGGGGCTTATGGAGAATGGGAGAAATTAAATCTGACTCA
AGGGTGGGTGGAGAAGATTACA
>804
GTCAGAATGCACATGACTCAGTCAGATATAAGGCAGATGGTGGTAAATACTATGATAAA
TGTTCAGATAGTGCTAAGGGGCTTATGGAGAATGGGAGAAATTAAATCTGACTCAAGG
GTGGGTGGAGAAGATTACATCCA
>805
ATAAGGCAGATGGTGGTAAATACTATGATAAATGTTCAGATAGTGCTAAGGGGCTTATG
GAGAATGGGAGAAATTAAATCTGACTCAAGGGTGGGTGGAGAAGATTACATCCAAGAT
CCAGCAATGACTTCCAGAAGAGA
>806
ATACTATGATAAATGTTCAGATAGTGCTAAGGGGCTTATGGAGAATGGGAGAAATTAAA
TCTGACTCAAGGGTGGGTGGAGAAGATTACATCCAAGATCCAGCAATGACTTCCAGAA
GAGACAAATGAACCTGGCCTGGC
>807
AATGTTCAGATAGTGCTAAGGGGCTTATGGAGAATGGGAGAAATTAAATCTGACTCAA
GGGTGGGTGGAGAAGATTACATCCAAGATCCAGCAATGACTTCCAGAAGAGACAAAT
GAACCTGGCCTGGCAAGGGAGGCAG
>808
CTAAGGGGCTTATGGAGAATGGGAGAAATTAAATCTGACTCAAGGGTGGGTGGAGAA
GATTACATCCAAGATCCAGCAATGACTTCCAGAAGAGACAAATGAACCTGGCCTGGCA
AGGGAGGCAGAGTCAAAGTGTCTGG
>809
GAGAAATTAAATCTGACTCAAGGGTGGGTGGAGAAGATTACATCCAAGATCCAGCAAT
GACTTCCAGAAGAGACAAATGAACCTGGCCTGGCAAGGGAGGCAGAGTCAAAGTGTC
TGGCTCAAAGCCCACCACAGCCTTG
>810
GAAATTAAATCTGACTCAAGGGTGGGTGGAGAAGATTACATCCAAGATCCAGCAATGA
CTTCCAGAAGAGACAAATGAACCTGGCCTGGCAAGGGAGGCAGAGTCAAAGTGTCTG
GCTCAAAGCCCACCACAGCCTTGGC
>811
GGGTGGAGAAGATTACATCCAAGATCCAGCAATGACTTCCAGAAGAGACAAATGAAC
CTGGCCTGGCAAGGGAGGCAGAGTCAAAGTGTCTGGCTCAAAGCCCACCACAGCCTT
GGCTCACAGGCATCTCCCGGTCTTCC
>812
AAGATTACATCCAAGATCCAGCAATGACTTCCAGAAGAGACAAATGAACCTGGCCTGG

CAAGGGAGGCAGAGTCAAAGTGTCTGGCTCAAAGCCCACCACAGCCTTGGCTCACAG
GCATCTCCCGGTCTTCCACTCACTC
>813
AGAAGAGACAAATGAACCTGGCCTGGCAAGGGAGGCAGAGTCAAAGTGTCTGGCTC
AAAGCCCACCACAGCCTTGGCTCACAGGCATCTCCCGGTCTTCCACTCACTCAGTCCT
TTCAGGGAAAGGCACTGAGCTTTTCT
>814
GAACCTGGCCTGGCAAGGGAGGCAGAGTCAAAGTGTCTGGCTCAAAGCCCACCACAG
CCTTGGCTCACAGGCATCTCCCGGTCTTCCACTCACTCAGTCCTTTCAGGGAAAGGCA
CTGAGCTTTTCTCAAGGGAGAACTC
>815
TCAAAGTGTCTGGCTCAAAGCCCACCACAGCCTTGGCTCACAGGCATCTCCCGGTCTT
CCACTCACTCAGTCCTTTCAGGGAAAGGCACTGAGCTTTTCTCAAGGGAGAACTCCAA
AGACGTCTTTCATTTCATCTTACA
>816
CAAAGCCCACCACAGCCTTGGCTCACAGGCATCTCCCGGTCTTCCACTCACTCAGTCC
TTTCAGGGAAAGGCACTGAGCTTTTCTCAAGGGAGAACTCCAAAGACGTCTTTCATTT
CATCTTACAAATCCACACAAATTT
>817
ACCACAGCCTTGGCTCACAGGCATCTCCCGGTCTTCCACTCACTCAGTCCTTTCAGGG
AAAGGCACTGAGCTTTTCTCAAGGGAGAACTCCAAAGACGTCTTTCATTTCATCTTAC
AAATCCACACAAATTTGCAATTCC
>818
GGCTCACAGGCATCTCCCGGTCTTCCACTCACTCAGTCCTTTCAGGGAAAGGCACTGA
GCTTTTCTCAAGGGAGAACTCCAAAGACGTCTTTCATTTCATCTTACAAATCCACACAA
ATTTGCAATTCCACTCCACAAGA
>819
TCCCGGTCTTCCACTCACTCAGTCCTTTCAGGGAAAGGCACTGAGCTTTTCTCAAGGG
AGAACTCCAAAGACGTCTTTCATTTCATCTTACAAATCCACACAAATTTGCAATTCCAC
TCCACAAGATACGAGCATTTGTC
>820
GGGAGAACTCCAAAGACGTCTTTCATTTCATCTTACAAATCCACACAAATTTGCAATTC
CACTCCACAAGATACGAGCATTTGTCTTGCTATAATTTTAATATGATTGGCATAAATTACC
TGTTATCAAGTAAAATAGTT
>821
CTCCAAAGACGTCTTTCATTTCATCTTACAAATCCACACAAATTTGCAATTCCACTCCA
CAAGATACGAGCATTTGTCTTGCTATAATTTTAATATGATTGGCATAAATTACCTGTTATC
AAGTAAAATAGTTTCTTCTT
>822
ACTCCACAAGATACGAGCATTTGTCTTGCTATAATTTTAATATGATTGGCATAAATTACCT
GTTATCAAGTAAAATAGTTTCTTCTTATGGCTTGTGTGCCCCTGACATACCACTGCTATT
TGAAAAGAGGCTTCTCCAG
>823
AGATACGAGCATTTGTCTTGCTATAATTTTAATATGATTGGCATAAATTACCTGTTATCAA
GTAAAATAGTTTCTTCTTATGGCTTGTGTGCCCCTGACATACCACTGCTATTTGAAAAGA
GGCTTCTCCAGAAACTCAG

>824
CCTGTTATCAAGTAAAATAGTTTCTTCTTATGGCTTGTGTGCCCCTGACATACCACTGCT
ATTTGAAAAGAGGCTTCTCCAGAAACTCAGTGTCTGAGAGTACGTGACTGGAAGGTG
AGTGTTTGCTTACCCCTTGCGTT
>825
TGTTATCAAGTAAAATAGTTTCTTCTTATGGCTTGTGTGCCCCTGACATACCACTGCTAT
TTGAAAAGAGGCTTCTCCAGAAACTCAGTGTCTGAGAGTACGTGACTGGAAGGTGAG
TGTTTGCTTACCCCTTGCGTTTT
>826
CTGACATACCACTGCTATTTGAAAAGAGGCTTCTCCAGAAACTCAGTGTCTGAGAGTA
CGTGACTGGAAGGTGAGTGTTTGCTTACCCCTTGCGTTTTCTCCCTGTCAGGATAAATG
GAATATTCTGCTTTAAGGACCCT
>827
ATACCACTGCTATTTGAAAAGAGGCTTCTCCAGAAACTCAGTGTCTGAGAGTACGTGA
CTGGAAGGTGAGTGTTTGCTTACCCCTTGCGTTTTCTCCCTGTCAGGATAAATGGAATA
TTCTGCTTTAAGGACCCTAAGTC
>828
AACTCAGTGTCTGAGAGTACGTGACTGGAAGGTGAGTGTTTGCTTACCCCTTGCGTTT
TCTCCCTGTCAGGATAAATGGAATATTCTGCTTTAAGGACCCTAAGTCTGGCAATATCAT
GTTTTGTGAACAGCCAACACAG
>829
TGGAAGGTGAGTGTTTGCTTACCCCTTGCGTTTTCTCCCTGTCAGGATAAATGGAATAT
TCTGCTTTAAGGACCCTAAGTCTGGCAATATCATGTTTTGTGAACAGCCAACACAGTAA
CTATGGTTGAGGCCTGTATTCG
>830
AGTGTTTGCTTACCCCTTGCGTTTTCTCCCTGTCAGGATAAATGGAATATTCTGCTTTAA
GGACCCTAAGTCTGGCAATATCATGTTTTGTGAACAGCCAACACAGTAACTATGGTTGA
GGCCTGTATTCGATGAATGAG
>831
CCCTGTCAGGATAAATGGAATATTCTGCTTTAAGGACCCTAAGTCTGGCAATATCATGTT
TTGTGAACAGCCAACACAGTAACTATGGTTGAGGCCTGTATTCGATGAATGAGGTTTTC
ATATGCTTCTTTCACATCAGG
>832
GATAAATGGAATATTCTGCTTTAAGGACCCTAAGTCTGGCAATATCATGTTTTGTGAACA
GCCAACACAGTAACTATGGTTGAGGCCTGTATTCGATGAATGAGGTTTTCATATGCTTCT
TTCACATCAGGAGAGAGAGA
>833
TCTGCTTTAAGGACCCTAAGTCTGGCAATATCATGTTTTGTGAACAGCCAACACAGTAA
CTATGGTTGAGGCCTGTATTCGATGAATGAGGTTTTCATATGCTTCTTTCACATCAGGAG
AGAGAGAGAGAAAAAAAAAATA
>834
TCTGGCAATATCATGTTTTGTGAACAGCCAACACAGTAACTATGGTTGAGGCCTGTATT
CGATGAATGAGGTTTTCATATGCTTCTTTCACATCAGGAGAGAGAGAGAGAAAAAAAA
ATAATAAACTTGGTAGAGGACGT
>835
GCCAACACAGTAACTATGGTTGAGGCCTGTATTCGATGAATGAGGTTTTCATATGCTTCT

TTCACATCAGGAGAGAGAGAGAGAAAAAAAAATAATAAACTTGGTAGAGGACGTGGA
GTTGTATTTCTGGACTATGGTGG
>836
AACACAGTAACTATGGTTGAGGCCTGTATTCGATGAATGAGGTTTTCATATGCTTCTTTC
ACATCAGGAGAGAGAGAGAGAGAAAAAAAAATAATAAACTTGGTAGAGGACGTGGAGTT
GTATTTCTGGACTATGGTGGCAC
>837
CACAGTAACTATGGTTGAGGCCTGTATTCGATGAATGAGGTTTTCATATGCTTCTTTCAC
ATCAGGAGAGAGAGAGAGAGAAAAAAAAATAATAAACTTGGTAGAGGACGTGGAGTTGT
ATTTCTGGACTATGGTGGCACCT
>838
AACTATGGTTGAGGCCTGTATTCGATGAATGAGGTTTTCATATGCTTCTTTCACATCAGG
AGAGAGAGAGAGAAAAAAAAATAATAAACTTGGTAGAGGACGTGGAGTTGTATTTCT
GGACTATGGTGGCACCTCTAAGG
>839
GAATGAGGTTTTCATATGCTTCTTTCACATCAGGAGAGAGAGAGAGAAAAAAAAATAA
TAAACTTGGTAGAGGACGTGGAGTTGTATTTCTGGACTATGGTGGCACCTCTAAGGAG
CATGGTTTTCCTTTTGCATGGCTT
>840
GAGAAAAAAAAATAATAAACTTGGTAGAGGACGTGGAGTTGTATTTCTGGACTATGGT
GGCACCTCTAAGGAGCATGGTTTTCCTTTTGCATGGCTTTGTCCATTACTATAACACTCC
TGTTATAAAATAGCTGCCGTGG
>841
AAAAAAAAATAATAAACTTGGTAGAGGACGTGGAGTTGTATTTCTGGACTATGGTGGC
ACCTCTAAGGAGCATGGTTTTCCTTTTGCATGGCTTTGTCCATTACTATAACACTCCTGT
TATAAAATAGCTGCCGTGGATT
>842
ACCTCTAAGGAGCATGGTTTTCCTTTTGCATGGCTTTGTCCATTACTATAACACTCCTGT
TATAAAATAGCTGCCGTGGATTGAAATCCATAATGTTAACTCAAAAGTTAAGCAGTCCG
CTATTAAACAGCATAAGAAAC
>843
GTCCATTACTATAACACTCCTGTTATAAAATAGCTGCCGTGGATTGAAATCCATAATGTT
AACTCAAAAGTTAAGCAGTCCGCTATTAAACAGCATAAGAAACTGTTACCTATCCCCTC
TTCCTGGCCCCCCAACAAAGA
>844
TTACTATAACACTCCTGTTATAAAATAGCTGCCGTGGATTGAAATCCATAATGTTAACTC
AAAAGTTAAGCAGTCCGCTATTAAACAGCATAAGAAACTGTTACCTATCCCCTCTTCCT
GGCCCCCCAACAAAGAAACAG
>845
CTATAACACTCCTGTTATAAAATAGCTGCCGTGGATTGAAATCCATAATGTTAACTCAAA
AGTTAAGCAGTCCGCTATTAAACAGCATAAGAAACTGTTACCTATCCCCTCTTCCTGGC
CCCCCAACAAAGAAACAGCAA
>846
TATAAAATAGCTGCCGTGGATTGAAATCCATAATGTTAACTCAAAAGTTAAGCAGTCCG
CTATTAAACAGCATAAGAAACTGTTACCTATCCCCTCTTCCTGGCCCCCCAACAAAGAA
ACAGCAAACTGTTAAAAAAGCC

>847
GTTAAGCAGTCCGCTATTAAACAGCATAAGAAACTGTTACCTATCCCCTCTTCCTGGCC
CCCCAACAAAGAAACAGCAAACTGTTAAAAAAGCCCATGTCTCCCCATGAGGACTTG
GGGTTAATTTTCAGCATGGTGTCT
>848
CAGTCCGCTATTAAACAGCATAAGAAACTGTTACCTATCCCCTCTTCCTGGCCCCCCAA
CAAAGAAACAGCAAACTGTTAAAAAAGCCCATGTCTCCCCATGAGGACTTGGGGTTAA
TTTTCAGCATGGTGTCTGGGGAT
>849
AGCATAAGAAACTGTTACCTATCCCCTCTTCCTGGCCCCCCAACAAAGAAACAGCAAA
CTGTTAAAAAAGCCCATGTCTCCCCATGAGGACTTGGGGTTAATTTTCAGCATGGTGTC
TGGGGATTCATCTGTGGGACTCC
>850
CCCAACAAAGAAACAGCAAACTGTTAAAAAAGCCCATGTCTCCCCATGAGGACTTGG
GGTTAATTTTCAGCATGGTGTCTGGGGATTCATCTGTGGGACTCCTATTACCCATAATAG
GACTCATGCTGCTTCATTTCATG
>851
GAGGACTTGGGGTTAATTTTCAGCATGGTGTCTGGGGATTCATCTGTGGGACTCCTATT
ACCCATAATAGGACTCATGCTGCTTCATTTCATGCACACGATATGGAAAATGCACCCTTC
CCTAGTGATGAAATTTTAGTT
>852
CTATTACCCATAATAGGACTCATGCTGCTTCATTTCATGCACACGATATGGAAAATGCAC
CCTTCCCTAGTGATGAAATTTTAGTTTTGAAATTGAGGCTAGCTTTTGCAGAACAAAGC
AGATTCCCAGAAACACCAAAA
>853
AGGACTCATGCTGCTTCATTTCATGCACACGATATGGAAAATGCACCCTTCCCTAGTGA
TGAAATTTTAGTTTTGAAATTGAGGCTAGCTTTTGCAGAACAAAGCAGATTCCCAGAA
ACACCAAAAACTATTCAATGTTG
>854
CTTCATTTCATGCACACGATATGGAAAATGCACCCTTCCCTAGTGATGAAATTTTAGTTT
TGAAATTGAGGCTAGCTTTTGCAGAACAAAGCAGATTCCCAGAAACACCAAAAACTAT
TCAATGTTGGCCTAGAGCCTGT
>855
TTTCATGCACACGATATGGAAAATGCACCCTTCCCTAGTGATGAAATTTTAGTTTTGAAA
TTGAGGCTAGCTTTTGCAGAACAAAGCAGATTCCCAGAAACACCAAAAACTATTCAAT
GTTGGCCTAGAGCCTGTGTGTC
>856
CCCTTCCCTAGTGATGAAATTTTAGTTTTGAAATTGAGGCTAGCTTTTGCAGAACAAAG
CAGATTCCCAGAAACACCAAAAACTATTCAATGTTGGCCTAGAGCCTGTGTGTCCGTG
CTTAGGTGAGTATGTGTATCTGA
>857
AATTGAGGCTAGCTTTTGCAGAACAAAGCAGATTCCCAGAAACACCAAAAACTATTCA
ATGTTGGCCTAGAGCCTGTGTGTCCGTGCTTAGGTGAGTATGTGTATCTGAAAGGGAGA
AAATGCCTTCCTCTAAGTGCGTA
>858
TTGAGGCTAGCTTTTGCAGAACAAAGCAGATTCCCAGAAACACCAAAAACTATTCAAT

GTTGGCCTAGAGCCTGTGTGTCCGTGCTTAGGTGAGTATGTGTATCTGAAAGGGAGAA
AATGCCTTCCTCTAAGTGCGTATG
>859
AGCAGATTCCCAGAAACACCAAAAACTATTCAATGTTGGCCTAGAGCCTGTGTGTCCG
TGCTTAGGTGAGTATGTGTATCTGAAAGGGAGAAAATGCCTTCCTCTAAGTGCGTATGC
CCCTCTCAAAGACAATATTCAAA
>860
CACCAAAAACTATTCAATGTTGGCCTAGAGCCTGTGTGTCCGTGCTTAGGTGAGTATGT
GTATCTGAAAGGGAGAAAATGCCTTCCTCTAAGTGCGTATGCCCCTCTCAAAGACAATA
TTCAAATATCATGCCCGATAGC
>861
AAAAACTATTCAATGTTGGCCTAGAGCCTGTGTGTCCGTGCTTAGGTGAGTATGTGTAT
CTGAAAGGGAGAAAATGCCTTCCTCTAAGTGCGTATGCCCCTCTCAAAGACAATATTCA
AATATCATGCCCGATAGCTGAC
>862
GCCTAGAGCCTGTGTGTCCGTGCTTAGGTGAGTATGTGTATCTGAAAGGGAGAAAATG
CCTTCCTCTAAGTGCGTATGCCCCTCTCAAAGACAATATTCAAATATCATGCCCGATAGC
TGACAGCGGTCAATGGTTGCTA
>863
TGCTTAGGTGAGTATGTGTATCTGAAAGGGAGAAAATGCCTTCCTCTAAGTGCGTATGC
CCCTCTCAAAGACAATATTCAAATATCATGCCCGATAGCTGACAGCGGTCAATGGTTGC
TATGCTTCATCCCAAAGCTTTG
>864
GGGAGAAAATGCCTTCCTCTAAGTGCGTATGCCCCTCTCAAAGACAATATTCAAATATC
ATGCCCGATAGCTGACAGCGGTCAATGGTTGCTATGCTTCATCCCAAAGCTTTGTTAGG
AACCCCATAAAACCTGGCACAT
>865
AATGCCTTCCTCTAAGTGCGTATGCCCCTCTCAAAGACAATATTCAAATATCATGCCCGA
TAGCTGACAGCGGTCAATGGTTGCTATGCTTCATCCCAAAGCTTTGTTAGGAACCCCAT
AAAACCTGGCACATGGTTTGT
>866
CAAAGACAATATTCAAATATCATGCCCGATAGCTGACAGCGGTCAATGGTTGCTATGCT
TCATCCCAAAGCTTTGTTAGGAACCCCATAAAACCTGGCACATGGTTTGTGACACCCA
GTGAAACACCGTGCTTCTTCCAT
>867
GTTGCTATGCTTCATCCCAAAGCTTTGTTAGGAACCCCATAAAACCTGGCACATGGTTT
GTGACACCCAGTGAAACACCGTGCTTCTTCCATAATATCCTGAGCCCTGCTGCCTGTCA
TTAAGGGACCTCTCTTATTAAG
>868
ACCCCATAAAACCTGGCACATGGTTTGTGACACCCAGTGAAACACCGTGCTTCTTCCAT
AATATCCTGAGCCCTGCTGCCTGTCATTAAGGGACCTCTCTTATTAAGCATCTCTTTTTT
AAATGGCTGCCATCATCTGGT
>869
TGGTTTGTGACACCCAGTGAAACACCGTGCTTCTTCCATAATATCCTGAGCCCTGCTGC
CTGTCATTAAGGGACCTCTCTTATTAAGCATCTCTTTTTTAAATGGCTGCCATCATCTGG
TCCCAGTGTACATTAAATCCC

>870
TGAAACACCGTGCTTCTTCCATAATATCCTGAGCCCTGCTGCCTGTCATTAAGGGACCT
CTCTTATTAAGCATCTCTTTTTTAAATGGCTGCCATCATCTGGTCCCAGTGTACATTAAAT
CCCATTATAATTATAAACAC
>871
CTGCCTGTCATTAAGGGACCTCTCTTATTAAGCATCTCTTTTTTAAATGGCTGCCATCAT
CTGGTCCCAGTGTACATTAAATCCCATTATAATTATAAACACACTTCCCCATAACATTCGT
TGCAGATCTCACCCTCAGT
>872
TCATCTGGTCCCAGTGTACATTAAATCCCATTATAATTATAAACACACTTCCCCATAACAT
TCGTTGCAGATCTCACCCTCAGTGCCTACCCCCTTAGGGCATCTTTGCACTAATAACAA
CTCAGATTTGCTATCTGAGT
>873
GTGTACATTAAATCCCATTATAATTATAAACACACTTCCCCATAACATTCGTTGCAGATCT
CACCCTCAGTGCCTACCCCCTTAGGGCATCTTTGCACTAATAACAACTCAGATTTGCTAT
CTGAGTTGACAACATTAGG
>874
TAACATTCGTTGCAGATCTCACCCTCAGTGCCTACCCCCTTAGGGCATCTTTGCACTAAT
AACAACTCAGATTTGCTATCTGAGTTGACAACATTAGGTCCCAACACCATTTGCAATAT
GCTGGGGAAAGAGACAGAGAG
>875
GATCTCACCCTCAGTGCCTACCCCCTTAGGGCATCTTTGCACTAATAACAACTCAGATT
TGCTATCTGAGTTGACAACATTAGGTCCCAACACCATTTGCAATATGCTGGGGAAAGAG
ACAGAGAGAGAAGAAAAAGCTT
>876
AACAACTCAGATTTGCTATCTGAGTTGACAACATTAGGTCCCAACACCATTTGCAATAT
GCTGGGGAAAGAGACAGAGAGAGAAGAAAAAGCTTTTTCTTCCTTAATCACGAAGAT
CTTGGGGTCACAGTGACCCAAGGC
>877
CAACTCAGATTTGCTATCTGAGTTGACAACATTAGGTCCCAACACCATTTGCAATATGC
TGGGGAAAGAGACAGAGAGAGAAGAAAAAGCTTTTTCTTCCTTAATCACGAAGATCT
TGGGGTCACAGTGACCCAAGGCCT
>878
GAGTTGACAACATTAGGTCCCAACACCATTTGCAATATGCTGGGGAAAGAGACAGAGA
GAGAAGAAAAAGCTTTTTCTTCCTTAATCACGAAGATCTTGGGGTCACAGTGACCCAA
GGCCTCAGGAGCTAAAAAAAAAAA
>879
GCTGGGGAAAGAGACAGAGAGAGAAGAAAAAGCTTTTTCTTCCTTAATCACGAAGAT
CTTGGGGTCACAGTGACCCAAGGCCTCAGGAGCTAAAAAAAAAAAAATCCCTCCTCCT
ACCCTTTTGAAAAGTCACTCTCTACT
>880
AGAGACAGAGAGAGAAGAAAAAGCTTTTTCTTCCTTAATCACGAAGATCTTGGGGTCA
CAGTGACCCAAGGCCTCAGGAGCTAAAAAAAAAAAAATCCCTCCTCCTACCCTTTTGAA
AAGTCACTCTCTACTCTACAGATG
>881
TTGGGGTCACAGTGACCCAAGGCCTCAGGAGCTAAAAAAAAAAAAATCCCTCCTCCTA

CCCTTTTGAAAAGTCACTCTCTACTCTACAGATGAGGGAAGGGCATACTTGGCACCTG
GGAGAGAAACTCAAGTTTGTAAACA
>882
AAGGCCTCAGGAGCTAAAAAAAAAAAATCCCTCCTCCTACCCTTTTGAAAAGTCACTC
TCTACTCTACAGATGAGGGAAGGGCATACTTGGCACCTGGGAGAGAAACTCAAGTTTG
TAAACACCACTAACCAGGTCTCTG
>883
TCCTCCTACCCTTTTGAAAAGTCACTCTCTACTCTACAGATGAGGGAAGGGCATACTTG
GCACCTGGGAGAGAAACTCAAGTTTGTAAACACCACTAACCAGGTCTCTGTTGGTTGG
GGCGGTGATGGTGGTGGTAGTGG
>884
CTCCTACCCTTTTGAAAAGTCACTCTCTACTCTACAGATGAGGGAAGGGCATACTTGGC
ACCTGGGAGAGAAACTCAAGTTTGTAAACACCACTAACCAGGTCTCTGTTGGTTGGGG
CGGTGATGGTGGTGGTAGTGGTG
>885
TTTTGAAAAGTCACTCTCTACTCTACAGATGAGGGAAGGGCATACTTGGCACCTGGGA
GAGAAACTCAAGTTTGTAAACACCACTAACCAGGTCTCTGTTGGTTGGGGCGGTGATG
GTGGTGGTAGTGGTGGGGTGGGGG
>886
TCTACAGATGAGGGAAGGGCATACTTGGCACCTGGGAGAGAAACTCAAGTTTGTAAAC
ACCACTAACCAGGTCTCTGTTGGTTGGGGCGGTGATGGTGGTGGTAGTGGTGGGGTGG
GGGATGAGGGATGATGGCAATCAC
>887
GAGAGAAACTCAAGTTTGTAAACACCACTAACCAGGTCTCTGTTGGTTGGGGCGGTGA
TGGTGGTGGTAGTGGTGGGGTGGGGGATGAGGGATGATGGCAATCACTAATCAAAGAA
GCTGTCTTATTTGGTTTGCTGAGC
>888
AAACACCACTAACCAGGTCTCTGTTGGTTGGGGCGGTGATGGTGGTGGTAGTGGTGGG
GTGGGGGATGAGGGATGATGGCAATCACTAATCAAAGAAGCTGTCTTATTTGGTTTGCT
GAGCCTCAACTAACCGGAATGCA
>889
GGTTGGGGCGGTGATGGTGGTGGTAGTGGTGGGGTGGGGGATGAGGGATGATGGCAA
TCACTAATCAAAGAAGCTGTCTTATTTGGTTTGCTGAGCCTCAACTAACCGGAATGCAT
ACATCACCTGATTCTTTCCCCCTC
>890
GTGGTGGGGTGGGGGATGAGGGATGATGGCAATCACTAATCAAAGAAGCTGTCTTATT
TGGTTTGCTGAGCCTCAACTAACCGGAATGCATACATCACCTGATTCTTTCCCCCTCTG
CTTTTTGGAGGGGGCATCTAAGG
>891
TTGGTTTGCTGAGCCTCAACTAACCGGAATGCATACATCACCTGATTCTTTCCCCCTCT
GCTTTTTGGAGGGGGCATCTAAGGACATAATGTAAAAGAATTAAAATTTAAAAACAAA
AACAAAATGTCAACATATGCAGG
>892
GCCTCAACTAACCGGAATGCATACATCACCTGATTCTTTCCCCCTCTGCTTTTTGGAGG
GGGCATCTAAGGACATAATGTAAAAGAATTAAAATTTAAAAACAAAAACAAAATGTCA
ACATATGCAGGAGTAAATTCCAC

>893
AATGCATACATCACCTGATTCTTTCCCCCTCTGCTTTTTGGAGGGGGCATCTAAGGACAT
AATGTAAAAGAATTAAAATTTAAAAACAAAAACAAAATGTCAACATATGCAGGAGTAA
ATTCCACTCTGCCTAATTTTTT
>894
GGGGGCATCTAAGGACATAATGTAAAAGAATTAAAATTTAAAAACAAAAACAAAATGT
CAACATATGCAGGAGTAAATTCCACTCTGCCTAATTTTTTTTTTTTTTTGAAATGAGATC
TTGTTCTGTCACCCAGGCTGGA
>895
GGCATCTAAGGACATAATGTAAAAGAATTAAAATTTAAAAACAAAAACAAAATGTCAA
CATATGCAGGAGTAAATTCCACTCTGCCTAATTTTTTTTTTTTTTTGAAATGAGATCTTGT
TCTGTCACCCAGGCTGGAGGG
>896
AAAAACAAAATGTCAACATATGCAGGAGTAAATTCCACTCTGCCTAATTTTTTTTTTTTT
TTGAAATGAGATCTTGTTCTGTCACCCAGGCTGGAGGGCAGTGGTGCAATCTCAGCTC
ACTGCAGCCACCACCTCCCAGG
>897
ATGCAGGAGTAAATTCCACTCTGCCTAATTTTTTTTTTTTTTTGAAATGAGATCTTGTTC
TGTCACCCAGGCTGGAGGGCAGTGGTGCAATCTCAGCTCACTGCAGCCACCACCTCCC
AGGTTCAAGCGATTCTCCCGCC
>898
ATTCCACTCTGCCTAATTTTTTTTTTTTTTTGAAATGAGATCTTGTTCTGTCACCCAGGC
TGGAGGGCAGTGGTGCAATCTCAGCTCACTGCAGCCACCACCTCCCAGGTTCAAGCGA
TTCTCCCGCCTCAGCCCCCTAA
>899
TTTTTTTGAAATGAGATCTTGTTCTGTCACCCAGGCTGGAGGGCAGTGGTGCAATCTCA
GCTCACTGCAGCCACCACCTCCCAGGTTCAAGCGATTCTCCCGCCTCAGCCCCCTAAG
TAGCTGGTATTACAGGCACGTGC
>900
GATCTTGTTCTGTCACCCAGGCTGGAGGGCAGTGGTGCAATCTCAGCTCACTGCAGCC
ACCACCTCCCAGGTTCAAGCGATTCTCCCGCCTCAGCCCCCTAAGTAGCTGGTATTACA
GGCACGTGCCACCACGCCTGGCT
>901
TTCTGTCACCCAGGCTGGAGGGCAGTGGTGCAATCTCAGCTCACTGCAGCCACCACCT
CCCAGGTTCAAGCGATTCTCCCGCCTCAGCCCCCTAAGTAGCTGGTATTACAGGCACGT
GCCACCACGCCTGGCTAATTTTT
>902
GGTGCAATCTCAGCTCACTGCAGCCACCACCTCCCAGGTTCAAGCGATTCTCCCGCCT
CAGCCCCCTAAGTAGCTGGTATTACAGGCACGTGCCACCACGCCTGGCTAATTTTTGTA
TTTTTAGTAGAGATAGGGTTTCA
>903
CACTGCAGCCACCACCTCCCAGGTTCAAGCGATTCTCCCGCCTCAGCCCCCTAAGTAG
CTGGTATTACAGGCACGTGCCACCACGCCTGGCTAATTTTTGTATTTTTAGTAGAGATAG
GGTTTCACCATGTTGGCCAGGC
>904
GCCTCAGCCCCCTAAGTAGCTGGTATTACAGGCACGTGCCACCACGCCTGGCTAATTTT

TGTATTTTTAGTAGAGATAGGGTTTCACCATGTTGGCCAGGCTGGTCTCAAACTCCTGA
CCTCAAGTGATCCGCCTGCCTC
>905
CCCTAAGTAGCTGGTATTACAGGCACGTGCCACCACGCCTGGCTAATTTTTGTATTTTTA
GTAGAGATAGGGTTTCACCATGTTGGCCAGGCTGGTCTCAAACTCCTGACCTCAAGTG
ATCCGCCTGCCTCGGCCTCCTA
>906
AGGCACGTGCCACCACGCCTGGCTAATTTTTGTATTTTTAGTAGAGATAGGGTTTCACC
ATGTTGGCCAGGCTGGTCTCAAACTCCTGACCTCAAGTGATCCGCCTGCCTCGGCCTC
CTAAAGTGCTGGGAGTACAGGCA
>907
TTTTTGTATTTTTAGTAGAGATAGGGTTTCACCATGTTGGCCAGGCTGGTCTCAAACTCC
TGACCTCAAGTGATCCGCCTGCCTCGGCCTCCTAAAGTGCTGGGAGTACAGGCATGAG
CCACTGCGCCCAGCCTACTCTG
>908
TTCACCATGTTGGCCAGGCTGGTCTCAAACTCCTGACCTCAAGTGATCCGCCTGCCTC
GGCCTCCTAAAGTGCTGGGAGTACAGGCATGAGCCACTGCGCCCAGCCTACTCTGCCC
AATTGTTGACATCACCTACAGCTC
>909
TTGGCCAGGCTGGTCTCAAACTCCTGACCTCAAGTGATCCGCCTGCCTCGGCCTCCTA
AAGTGCTGGGAGTACAGGCATGAGCCACTGCGCCCAGCCTACTCTGCCCAATTGTTGA
CATCACCTACAGCTCTATAGTAGA
>910
GTCTCAAACTCCTGACCTCAAGTGATCCGCCTGCCTCGGCCTCCTAAAGTGCTGGGAG
TACAGGCATGAGCCACTGCGCCCAGCCTACTCTGCCCAATTGTTGACATCACCTACAGC
TCTATAGTAGATGTCTGGAATGA
>911
AAGTGCTGGGAGTACAGGCATGAGCCACTGCGCCCAGCCTACTCTGCCCAATTGTTGA
CATCACCTACAGCTCTATAGTAGATGTCTGGAATGATGATCTGAAGGACTGTAAGACTG
TAAGATTTTTTGACTCCAGAAAT
>912
GGAGTACAGGCATGAGCCACTGCGCCCAGCCTACTCTGCCCAATTGTTGACATCACCT
ACAGCTCTATAGTAGATGTCTGGAATGATGATCTGAAGGACTGTAAGACTGTAAGATTT
TTTGACTCCAGAAATGGACACAA
>913
CAGCCTACTCTGCCCAATTGTTGACATCACCTACAGCTCTATAGTAGATGTCTGGAATGA
TGATCTGAAGGACTGTAAGACTGTAAGATTTTTTGACTCCAGAAATGGACACAATAGG
CTTGCGGTGTCTTCCCCTAACA
>914
TTGTTGACATCACCTACAGCTCTATAGTAGATGTCTGGAATGATGATCTGAAGGACTGT
AAGACTGTAAGATTTTTTGACTCCAGAAATGGACACAATAGGCTTGCGGTGTCTTCCCC
TAACAGTCCCTGGCAGGGTAAA
>915
ACAGCTCTATAGTAGATGTCTGGAATGATGATCTGAAGGACTGTAAGACTGTAAGATTT
TTTGACTCCAGAAATGGACACAATAGGCTTGCGGTGTCTTCCCCTAACAGTCCCTGGC
AGGGTAAAGGTCTGATGCCCTGG

>916
ATGTCTGGAATGATGATCTGAAGGACTGTAAGACTGTAAGATTTTTTGACTCCAGAAAT
GGACACAATAGGCTTGCGGTGTCTTCCCCTAACAGTCCCTGGCAGGGTAAAGGTCTGA
TGCCCTGGTACGTTCCCCAGTGG
>917
ACTGTAAGACTGTAAGATTTTTTGACTCCAGAAATGGACACAATAGGCTTGCGGTGTCT
TCCCCTAACAGTCCCTGGCAGGGTAAAGGTCTGATGCCCTGGTACGTTCCCCAGTGGC
AATCTGGAGTTCTCAACTGCAGA
>918
TGTAAGATTTTTTGACTCCAGAAATGGACACAATAGGCTTGCGGTGTCTTCCCCTAACA
GTCCCTGGCAGGGTAAAGGTCTGATGCCCTGGTACGTTCCCCAGTGGCAATCTGGAGT
TCTCAACTGCAGAGAATACTTTT
>919
TTTTTTGACTCCAGAAATGGACACAATAGGCTTGCGGTGTCTTCCCCTAACAGTCCCTG
GCAGGGTAAAGGTCTGATGCCCTGGTACGTTCCCCAGTGGCAATCTGGAGTTCTCAAC
TGCAGAGAATACTTTTCCTCTAC
>920
TAGGCTTGCGGTGTCTTCCCCTAACAGTCCCTGGCAGGGTAAAGGTCTGATGCCCTGGT
ACGTTCCCCAGTGGCAATCTGGAGTTCTCAACTGCAGAGAATACTTTTCCTCTACTCAG
GGGCGTCACTACCTGCATCTCT
>921
GTCCCTGGCAGGGTAAAGGTCTGATGCCCTGGTACGTTCCCCAGTGGCAATCTGGAGT
TCTCAACTGCAGAGAATACTTTTCCTCTACTCAGGGGCGTCACTACCTGCATCTCTCCC
TGAACATGCTTCATTTTCCACTC
>922
CCCTGGCAGGGTAAAGGTCTGATGCCCTGGTACGTTCCCCAGTGGCAATCTGGAGTTC
TCAACTGCAGAGAATACTTTTCCTCTACTCAGGGGCGTCACTACCTGCATCTCTCCCTG
AACATGCTTCATTTTCCACTCAT
>923
GATGCCCTGGTACGTTCCCCAGTGGCAATCTGGAGTTCTCAACTGCAGAGAATACTTTT
CCTCTACTCAGGGGCGTCACTACCTGCATCTCTCCCTGAACATGCTTCATTTTCCACTCA
TCACAAGTTTTTGGGGAAATG
>924
TTTCCTCTACTCAGGGGCGTCACTACCTGCATCTCTCCCTGAACATGCTTCATTTTCCAC
TCATCACAAGTTTTTGGGGAAATGCAAAGTGGCATCTTCTCCCCAAAAGGTAGGGATC
TTTGATTTTTTTAATCACGATA
>925
TCACTACCTGCATCTCTCCCTGAACATGCTTCATTTTCCACTCATCACAAGTTTTTGGGG
AAATGCAAAGTGGCATCTTCTCCCCAAAAGGTAGGGATCTTTGATTTTTTTAATCACGA
TAGAAAAGTTTACTTTGAAAT
>926
TGAACATGCTTCATTTTCCACTCATCACAAGTTTTTGGGGAAATGCAAAGTGGCATCTT
CTCCCCAAAAGGTAGGGATCTTTGATTTTTTTAATCACGATAGAAAAGTTTACTTTGAA
ATAAAAAAATTAAATATATGTT
>927
CATGCTTCATTTTCCACTCATCACAAGTTTTTGGGGAAATGCAAAGTGGCATCTTCTCC

CCAAAAGGTAGGGATCTTTGATTTTTTTAATCACGATAGAAAAGTTTACTTTGAAATAA
AAAAATTAAATATATGTTCCAA
>928
TTTTGGGGAAATGCAAAGTGGCATCTTCTCCCCAAAAGGTAGGGATCTTTGATTTTTTT
AATCACGATAGAAAAGTTTACTTTGAAATAAAAAAATTAAATATATGTTCCAAATTAAG
AGTTGGAACTTAAAGAGTTGCA
>929
TTGATTTTTTTAATCACGATAGAAAAGTTTACTTTGAAATAAAAAAATTAAATATATGTT
CCAAATTAAGAGTTGGAACTTAAAGAGTTGCAACATTCCATTCATACTCAGATACTCAA
AACACCACTGTATTCTTCTTC
>930
GATTTTTTTAATCACGATAGAAAAGTTTACTTTGAAATAAAAAAATTAAATATATGTTCC
AAATTAAGAGTTGGAACTTAAAGAGTTGCAACATTCCATTCATACTCAGATACTCAAAA
CACCACTGTATTCTTCTTCCT
>931
AAAAAATTAAATATATGTTCCAAATTAAGAGTTGGAACTTAAAGAGTTGCAACATTCCA
TTCATACTCAGATACTCAAAACACCACTGTATTCTTCTTCCTATCTCTTTAGTTGAAGTT
TTTTTTTGTAATTCAAAGCCC
>932
AAGAGTTGCAACATTCCATTCATACTCAGATACTCAAAACACCACTGTATTCTTCTTCCT
ATCTCTTTAGTTGAAGTTTTTTTTTGTAATTCAAAGCCCCCTCCCCAATTTCTGCCTGAG
ATTCTGAGGGAGGAGGAAAA
>933
TGCAACATTCCATTCATACTCAGATACTCAAAACACCACTGTATTCTTCTTCCTATCTCT
TTAGTTGAAGTTTTTTTTTGTAATTCAAAGCCCCCTCCCCAATTTCTGCCTGAGATTCTG
AGGGAGGAGGAAAAGTGTAA
>934
CAGATACTCAAAACACCACTGTATTCTTCTTCCTATCTCTTTAGTTGAAGTTTTTTTTTGT
AATTCAAAGCCCCCTCCCCAATTTCTGCCTGAGATTCTGAGGGAGGAGGAAAAGTGTA
AGATCCTGGGGGATCTAAGTC
>935
TCTTCTTCCTATCTCTTTAGTTGAAGTTTTTTTTTGTAATTCAAAGCCCCCTCCCCAATTT
CTGCCTGAGATTCTGAGGGAGGAGGAAAAGTGTAAGATCCTGGGGGATCTAAGTCTCT
TTACCTGGTGACCAAGCTGAG
>936
TTTAGTTGAAGTTTTTTTTTGTAATTCAAAGCCCCCTCCCCAATTTCTGCCTGAGATTCT
GAGGGAGGAGGAAAAGTGTAAGATCCTGGGGGATCTAAGTCTCTTTACCTGGTGACCA
AGCTGAGAACCATTTGGTCCTC
>937
TTTTTTTTGTAATTCAAAGCCCCCTCCCCAATTTCTGCCTGAGATTCTGAGGGAGGAGG
AAAAGTGTAAGATCCTGGGGGATCTAAGTCTCTTTACCTGGTGACCAAGCTGAGAACC
ATTTGGTCCTCAGCTTGGCTGGC
>938
AGCCCCCTCCCCAATTTCTGCCTGAGATTCTGAGGGAGGAGGAAAAGTGTAAGATCCT
GGGGGATCTAAGTCTCTTTACCTGGTGACCAAGCTGAGAACCATTTGGTCCTCAGCTT
GGCTGGCTGCTGCACAGATCTTGG

>939
CTGAGATTCTGAGGGAGGAGGAAAAGTGTAAGATCCTGGGGGATCTAAGTCTCTTTAC
CTGGTGACCAAGCTGAGAACCATTTGGTCCTCAGCTTGGCTGGCTGCTGCACAGATCT
TGGCCTCAGCAGAGTAAGAGAAGA
>940
AAAGTGTAAGATCCTGGGGGATCTAAGTCTCTTTACCTGGTGACCAAGCTGAGAACCA
TTTGGTCCTCAGCTTGGCTGGCTGCTGCACAGATCTTGGCCTCAGCAGAGTAAGAGAA
GAAGGAAAATTAAAAGCCCTTGCT
>941
ATCTAAGTCTCTTTACCTGGTGACCAAGCTGAGAACCATTTGGTCCTCAGCTTGGCTGG
CTGCTGCACAGATCTTGGCCTCAGCAGAGTAAGAGAAGAAGGAAAATTAAAAGCCCT
TGCTTCCTTCATCTATTTCACTAG
>942
TACCTGGTGACCAAGCTGAGAACCATTTGGTCCTCAGCTTGGCTGGCTGCTGCACAGA
TCTTGGCCTCAGCAGAGTAAGAGAAGAAGGAAAATTAAAAGCCCTTGCTTCCTTCATC
TATTTCACTAGAGGCTACATAATC
>943
GTCCTCAGCTTGGCTGGCTGCTGCACAGATCTTGGCCTCAGCAGAGTAAGAGAAGAA
GGAAAATTAAAAGCCCTTGCTTCCTTCATCTATTTCACTAGAGGCTACATAATCTAGAA
GTTAAGAGCATGAGCTTTGCAGTC
>944
ATCTTGGCCTCAGCAGAGTAAGAGAAGAAGGAAAATTAAAAGCCCTTGCTTCCTTCAT
CTATTTCACTAGAGGCTACATAATCTAGAAGTTAAGAGCATGAGCTTTGCAGTCAGAAT
GGTCTGATTTAAAGTTTCAGCTC
>945
AGTAAGAGAAGAAGGAAAATTAAAAGCCCTTGCTTCCTTCATCTATTTCACTAGAGGCT
ACATAATCTAGAAGTTAAGAGCATGAGCTTTGCAGTCAGAATGGTCTGATTTAAAGTTT
CAGCTCTGATGTTTAATAGATC
>946
AAATTAAAAGCCCTTGCTTCCTTCATCTATTTCACTAGAGGCTACATAATCTAGAAGTTA
AGAGCATGAGCTTTGCAGTCAGAATGGTCTGATTTAAAGTTTCAGCTCTGATGTTTAAT
AGATCTACAGTCTGCAGTAAG
>947
AGCCCTTGCTTCCTTCATCTATTTCACTAGAGGCTACATAATCTAGAAGTTAAGAGCATG
AGCTTTGCAGTCAGAATGGTCTGATTTAAAGTTTCAGCTCTGATGTTTAATAGATCTACA
GTCTGCAGTAAGGCACTTAA
>948
TTTCACTAGAGGCTACATAATCTAGAAGTTAAGAGCATGAGCTTTGCAGTCAGAATGGT
CTGATTTAAAGTTTCAGCTCTGATGTTTAATAGATCTACAGTCTGCAGTAAGGCACTTAA
TCTCTCTGAACCTCAGTTTTC
>949
TAGAAGTTAAGAGCATGAGCTTTGCAGTCAGAATGGTCTGATTTAAAGTTTCAGCTCTG
ATGTTTAATAGATCTACAGTCTGCAGTAAGGCACTTAATCTCTCTGAACCTCAGTTTTCA
TGTCCACAGAAATTGGGAAAA
>950
AAGAGCATGAGCTTTGCAGTCAGAATGGTCTGATTTAAAGTTTCAGCTCTGATGTTTAA

TAGATCTACAGTCTGCAGTAAGGCACTTAATCTCTCTGAACCTCAGTTTTCATGTCCAC
AGAAATTGGGAAAATAATAGCA
>951
TGAGCTTTGCAGTCAGAATGGTCTGATTTAAAGTTTCAGCTCTGATGTTTAATAGATCTA
CAGTCTGCAGTAAGGCACTTAATCTCTCTGAACCTCAGTTTTCATGTCCACAGAAATTG
GGAAAATAATAGCACCTGCAT
>952
TAAAGTTTCAGCTCTGATGTTTAATAGATCTACAGTCTGCAGTAAGGCACTTAATCTCTC
TGAACCTCAGTTTTCATGTCCACAGAAATTGGGAAAATAATAGCACCTGCATTAATAGG
ATTGTTATGTGGATTAAATAA
>953
AGTCTGCAGTAAGGCACTTAATCTCTCTGAACCTCAGTTTTCATGTCCACAGAAATTGG
GAAAATAATAGCACCTGCATTAATAGGATTGTTATGTGGATTAAATAAAACAACATAGGT
AATGTGTTGAGTTTCGTAGTC
>954
ACTTAATCTCTCTGAACCTCAGTTTTCATGTCCACAGAAATTGGGAAAATAATAGCACC
TGCATTAATAGGATTGTTATGTGGATTAAATAAAACAACATAGGTAATGTGTTGAGTTTC
GTAGTCAGTGCTCAGAATGAG
>955
CACAGAAATTGGGAAAATAATAGCACCTGCATTAATAGGATTGTTATGTGGATTAAATAA
AACAACATAGGTAATGTGTTGAGTTTCGTAGTCAGTGCTCAGAATGAGTTATTATTTTCG
CTGTCTCCTTCCTGTATGAT
>956
AAAATAATAGCACCTGCATTAATAGGATTGTTATGTGGATTAAATAAAACAACATAGGTA
ATGTGTTGAGTTTCGTAGTCAGTGCTCAGAATGAGTTATTATTTTCGCTGTCTCCTTCCT
GTATGATCTCTTAGAACTGC
>957
AGCACCTGCATTAATAGGATTGTTATGTGGATTAAATAAAACAACATAGGTAATGTGTTG
AGTTTCGTAGTCAGTGCTCAGAATGAGTTATTATTTTCGCTGTCTCCTTCCTGTATGATC
TCTTAGAACTGCTATACCCT
>958
GATTGTTATGTGGATTAAATAAAACAACATAGGTAATGTGTTGAGTTTCGTAGTCAGTG
CTCAGAATGAGTTATTATTTTCGCTGTCTCCTTCCTGTATGATCTCTTAGAACTGCTATAC
CCTCCTGTTCCACTAACTGA
>959
GTTGAGTTTCGTAGTCAGTGCTCAGAATGAGTTATTATTTTCGCTGTCTCCTTCCTGTAT
GATCTCTTAGAACTGCTATACCCTCCTGTTCCACTAACTGAGCTCTGAAAGGCTGCCAG
TGGTGGGAGGTACATGGGTAA
>960
TATTATTTTCGCTGTCTCCTTCCTGTATGATCTCTTAGAACTGCTATACCCTCCTGTTCCA
CTAACTGAGCTCTGAAAGGCTGCCAGTGGTGGGAGGTACATGGGTAAAGAGGGAGAT
GAGGGAGTAAGATTTAGCAATG
>961
CCTTCCTGTATGATCTCTTAGAACTGCTATACCCTCCTGTTCCACTAACTGAGCTCTGAA
AGGCTGCCAGTGGTGGGAGGTACATGGGTAAAGAGGGAGATGAGGGAGTAAGATTTA
GCAATGGAATTCCATGCAAAGGC

>962
ATGATCTCTTAGAACTGCTATACCCTCCTGTTCCACTAACTGAGCTCTGAAAGGCTGCC
AGTGGTGGGAGGTACATGGGTAAAGAGGGAGATGAGGGAGTAAGATTTAGCAATGGA
ATTCCATGCAAAGGCCAGCTCATC
>963
CTGCTATACCCTCCTGTTCCACTAACTGAGCTCTGAAAGGCTGCCAGTGGTGGGAGGT
ACATGGGTAAAGAGGGAGATGAGGGAGTAAGATTTAGCAATGGAATTCCATGCAAAGG
CCAGCTCATCTATTCCTCTGTCTC
>964
CTGAGCTCTGAAAGGCTGCCAGTGGTGGGAGGTACATGGGTAAAGAGGGAGATGAGG
GAGTAAGATTTAGCAATGGAATTCCATGCAAAGGCCAGCTCATCTATTCCTCTGTCTCT
AGCATGATCTCCCCCTAAGATTGA
>965
AGTGGTGGGAGGTACATGGGTAAAGAGGGAGATGAGGGAGTAAGATTTAGCAATGGA
ATTCCATGCAAAGGCCAGCTCATCTATTCCTCTGTCTCTAGCATGATCTCCCCCTAAGAT
TGAGATCTCCTGTATTTTTAGAA
>966
TGGGAGGTACATGGGTAAAGAGGGAGATGAGGGAGTAAGATTTAGCAATGGAATTCCA
TGCAAAGGCCAGCTCATCTATTCCTCTGTCTCTAGCATGATCTCCCCCTAAGATTGAGAT
CTCCTGTATTTTTAGAACTGAT
>967
GAGATGAGGGAGTAAGATTTAGCAATGGAATTCCATGCAAAGGCCAGCTCATCTATTCC
TCTGTCTCTAGCATGATCTCCCCCTAAGATTGAGATCTCCTGTATTTTTAGAACTGATAC
CCAGGTTTCCCTTAAAAGACG
>968
AATGGAATTCCATGCAAAGGCCAGCTCATCTATTCCTCTGTCTCTAGCATGATCTCCCCC
TAAGATTGAGATCTCCTGTATTTTTAGAACTGATACCCAGGTTTCCCTTAAAAGACGCTT
CAAGAAAAAATGTAAAAGAA
>969
AGGCCAGCTCATCTATTCCTCTGTCTCTAGCATGATCTCCCCCTAAGATTGAGATCTCCT
GTATTTTTAGAACTGATACCCAGGTTTCCCTTAAAAGACGCTTCAAGAAAAAATGTAAA
AGAAAAATCACTCTAAATACA
>970
TATTCCTCTGTCTCTAGCATGATCTCCCCCTAAGATTGAGATCTCCTGTATTTTTAGAACT
GATACCCAGGTTTCCCTTAAAAGACGCTTCAAGAAAAAATGTAAAAGAAAAATCACTC
TAAATACACGAACATGAGAGA
>971
AGATCTCCTGTATTTTTAGAACTGATACCCAGGTTTCCCTTAAAAGACGCTTCAAGAAA
AAATGTAAAAGAAAAATCACTCTAAATACACGAACATGAGAGAGACAGAAAGAGAGC
GGGCATGTGCGCGCGAGAGAGATT
>972
CAGGTTTCCCTTAAAAGACGCTTCAAGAAAAAATGTAAAAGAAAAATCACTCTAAATA
CACGAACATGAGAGAGACAGAAAGAGAGCGGGCATGTGCGCGCGAGAGAGATTGAG
TATTCTCTTGTAATTTTCCATCCTGC
>973
AAGACGCTTCAAGAAAAAATGTAAAAGAAAAATCACTCTAAATACACGAACATGAGA

GAGACAGAAAGAGAGCGGGCATGTGCGCGCGAGAGAGATTGAGTATTCTCTTGTAATT
TTCCATCCTGCTTGTTTTTAGGTGC
>974
GTAAAAGAAAAATCACTCTAAATACACGAACATGAGAGAGACAGAAAGAGAGCGGGC
ATGTGCGCGCGAGAGAGATTGAGTATTCTCTTGTAATTTTCCATCCTGCTTGTTTTTAGG
TGCTGCCATATGAAATCTCTGAA
>975
AAAAGAAAAATCACTCTAAATACACGAACATGAGAGAGACAGAAAGAGAGCGGGCAT
GTGCGCGCGAGAGAGATTGAGTATTCTCTTGTAATTTTCCATCCTGCTTGTTTTTAGGTG
CTGCCATATGAAATCTCTGAACT
>976
AGAGAGCGGGCATGTGCGCGCGAGAGAGATTGAGTATTCTCTTGTAATTTTCCATCCTG
CTTGTTTTTAGGTGCTGCCATATGAAATCTCTGAACTGAGGAGTCTCATCAGCCTTCCC
AGGACCCAAGGACATAGCTCTG
>977
TTGAGTATTCTCTTGTAATTTTCCATCCTGCTTGTTTTTAGGTGCTGCCATATGAAATCTC
TGAACTGAGGAGTCTCATCAGCCTTCCCAGGACCCAAGGACATAGCTCTGGCTATAAA
TGGTGCCAGTTTTGGTGGGGG
>978
AGTATTCTCTTGTAATTTTCCATCCTGCTTGTTTTTAGGTGCTGCCATATGAAATCTCTGA
ACTGAGGAGTCTCATCAGCCTTCCCAGGACCCAAGGACATAGCTCTGGCTATAAATGG
TGCCAGTTTTGGTGGGGGAGC
>979
TTTAGGTGCTGCCATATGAAATCTCTGAACTGAGGAGTCTCATCAGCCTTCCCAGGACC
CAAGGACATAGCTCTGGCTATAAATGGTGCCAGTTTTGGTGGGGGAGCTGCTGCTGCTT
TATCTGGGCCCAATAAACCTTA
>980
CTCTGAACTGAGGAGTCTCATCAGCCTTCCCAGGACCCAAGGACATAGCTCTGGCTAT
AAATGGTGCCAGTTTTGGTGGGGGAGCTGCTGCTGCTTTATCTGGGCCCAATAAACCTT
ATCAAACTGCTGTGCAGCCAGCC
>981
GTCTCATCAGCCTTCCCAGGACCCAAGGACATAGCTCTGGCTATAAATGGTGCCAGTTT
TGGTGGGGGAGCTGCTGCTGCTTTATCTGGGCCCAATAAACCTTATCAAACTGCTGTGC
AGCCAGCCTTCAGCGCTAAGTG
>982
TGCCAGTTTTGGTGGGGGAGCTGCTGCTGCTTTATCTGGGCCCAATAAACCTTATCAAA
CTGCTGTGCAGCCAGCCTTCAGCGCTAAGTGGAAATGCTTGGAGCCCAATGCTGGCAT
TCAGCCCACAGCCTCGCCTGCCT
>983
AGTTTTGGTGGGGGAGCTGCTGCTGCTTTATCTGGGCCCAATAAACCTTATCAAACTGC
TGTGCAGCCAGCCTTCAGCGCTAAGTGGAAATGCTTGGAGCCCAATGCTGGCATTCAG
CCCACAGCCTCGCCTGCCTTCTT
>984
ATCTGGGCCCAATAAACCTTATCAAACTGCTGTGCAGCCAGCCTTCAGCGCTAAGTGG
AAATGCTTGGAGCCCAATGCTGGCATTCAGCCCACAGCCTCGCCTGCCTTCTTCCTTTT
TGTTACCTTCTCCACCTCTTAAA

>985
TTATCAAACTGCTGTGCAGCCAGCCTTCAGCGCTAAGTGGAAATGCTTGGAGCCCAAT
GCTGGCATTCAGCCCACAGCCTCGCCTGCCTTCTTCCTTTTTGTTACCTTCTCCACCTCT
TAAATTCAGGCATTAATCTCTC
>986
ACTGCTGTGCAGCCAGCCTTCAGCGCTAAGTGGAAATGCTTGGAGCCCAATGCTGGCA
TTCAGCCCACAGCCTCGCCTGCCTTCTTCCTTTTTGTTACCTTCTCCACCTCTTAAATTC
AGGCATTAATCTCTCTTTAACA
>987
TGCTGGCATTCAGCCCACAGCCTCGCCTGCCTTCTTCCTTTTTGTTACCTTCTCCACCTC
TTAAATTCAGGCATTAATCTCTCTTTAACACTTACTAACAAGGGCTGGGCTTTTAATGGG
CAGCGTTTATGAAGCAAGGG
>988
TCTTCCTTTTTGTTACCTTCTCCACCTCTTAAATTCAGGCATTAATCTCTCTTTAACACTT
ACTAACAAGGGCTGGGCTTTTAATGGGCAGCGTTTATGAAGCAAGGGAGATTTTCACA
TAGAGTGAATATGGCACGGGG
>989
CCACCTCTTAAATTCAGGCATTAATCTCTCTTTAACACTTACTAACAAGGGCTGGGCTTT
TAATGGGCAGCGTTTATGAAGCAAGGGAGATTTTCACATAGAGTGAATATGGCACGGG
GGTGGGGAGGCCAGGGCCATAG
>990
ATTCAGGCATTAATCTCTCTTTAACACTTACTAACAAGGGCTGGGCTTTTAATGGGCAG
CGTTTATGAAGCAAGGGAGATTTTCACATAGAGTGAATATGGCACGGGGGTGGGGAGG
CCAGGGCCATAGAGGATTGTGCT
>991
TTAACACTTACTAACAAGGGCTGGGCTTTTAATGGGCAGCGTTTATGAAGCAAGGGAG
ATTTTCACATAGAGTGAATATGGCACGGGGGTGGGGAGGCCAGGGCCATAGAGGATTG
TGCTTTCTCTCCCATTGTTTCCAG
>992
TTTTAATGGGCAGCGTTTATGAAGCAAGGGAGATTTTCACATAGAGTGAATATGGCACG
GGGGTGGGGAGGCCAGGGCCATAGAGGATTGTGCTTTCTCTCCCATTGTTTCCAGAGA
TTCTCTCTTTCTCTGATTTGAGG
>993
ATGGGCAGCGTTTATGAAGCAAGGGAGATTTTCACATAGAGTGAATATGGCACGGGGG
TGGGGAGGCCAGGGCCATAGAGGATTGTGCTTTCTCTCCCATTGTTTCCAGAGATTCTC
TCTTTCTCTGATTTGAGGATTAA
>994
AGTGAATATGGCACGGGGGTGGGGAGGCCAGGGCCATAGAGGATTGTGCTTTCTCTCC
CATTGTTTCCAGAGATTCTCTCTTTCTCTGATTTGAGGATTAATAGGAGATTAGGATCAG
GGGTTAATTGTGTGCTTCTCCT
>995
GGCCAGGGCCATAGAGGATTGTGCTTTCTCTCCCATTGTTTCCAGAGATTCTCTCTTTCT
CTGATTTGAGGATTAATAGGAGATTAGGATCAGGGGTTAATTGTGTGCTTCTCCTGCTAC
TGCACTGAGGAGAGGCTGGT
>996
CATAGAGGATTGTGCTTTCTCTCCCATTGTTTCCAGAGATTCTCTCTTTCTCTGATTTGA

GGATTAATAGGAGATTAGGATCAGGGGTTAATTGTGTGCTTCTCCTGCTACTGCACTGA
GGAGAGGCTGGTAGGTGGATG
>997
TCTGATTTGAGGATTAATAGGAGATTAGGATCAGGGGTTAATTGTGTGCTTCTCCTGCTA
CTGCACTGAGGAGAGGCTGGTAGGTGGATGTGGACAGCAAAGCGGAAACCTACAGCA
GGCACTATCTAGGCAGAAGCTCA
>998
GATTTGAGGATTAATAGGAGATTAGGATCAGGGGTTAATTGTGTGCTTCTCCTGCTACTG
CACTGAGGAGAGGCTGGTAGGTGGATGTGGACAGCAAAGCGGAAACCTACAGCAGGC
ACTATCTAGGCAGAAGCTCAACA
>999
GGATCAGGGGTTAATTGTGTGCTTCTCCTGCTACTGCACTGAGGAGAGGCTGGTAGGT
GGATGTGGACAGCAAAGCGGAAACCTACAGCAGGCACTATCTAGGCAGAAGCTCAAC
AAGTGTAGTGATTCTTTCTTCTGTT
>1000
GTGCTTCTCCTGCTACTGCACTGAGGAGAGGCTGGTAGGTGGATGTGGACAGCAAAGC
GGAAACCTACAGCAGGCACTATCTAGGCAGAAGCTCAACAAGTGTAGTGATTCTTTCT
TCTGTTTCCCTGGTGAGGCACCAG
>1001
CTTCTCCTGCTACTGCACTGAGGAGAGGCTGGTAGGTGGATGTGGACAGCAAAGCGG
AAACCTACAGCAGGCACTATCTAGGCAGAAGCTCAACAAGTGTAGTGATTCTTTCTTCT
GTTTCCCTGGTGAGGCACCAGGAG
>1002
ACTGCACTGAGGAGAGGCTGGTAGGTGGATGTGGACAGCAAAGCGGAAACCTACAGC
AGGCACTATCTAGGCAGAAGCTCAACAAGTGTAGTGATTCTTTCTTCTGTTTCCCTGGT
GAGGCACCAGGAGGGTCTTTTCTC
>1003
GCTGGTAGGTGGATGTGGACAGCAAAGCGGAAACCTACAGCAGGCACTATCTAGGCA
GAAGCTCAACAAGTGTAGTGATTCTTTCTTCTGTTTCCCTGGTGAGGCACCAGGAGGG
TCTTTTCTCCTCTCCTTACATCCCT
>1004
CCTACAGCAGGCACTATCTAGGCAGAAGCTCAACAAGTGTAGTGATTCTTTCTTCTGTT
TCCCTGGTGAGGCACCAGGAGGGTCTTTTCTCCTCTCCTTACATCCCTCCACTCTTGCT
CTCCTTGCAGCCCAGTTCTTTC
>1005
CAGGCACTATCTAGGCAGAAGCTCAACAAGTGTAGTGATTCTTTCTTCTGTTTCCCTGG
TGAGGCACCAGGAGGGTCTTTTCTCCTCTCCTTACATCCCTCCACTCTTGCTCTCCTTG
CAGCCCAGTTCTTTCCTAACTT
>1006
TTCCCTGGTGAGGCACCAGGAGGGTCTTTTCTCCTCTCCTTACATCCCTCCACTCTTGC
TCTCCTTGCAGCCCAGTTCTTTCCTAACTTTCTTTAAATCCCTTTCCTCTAACAGGGTGT
ATAGACCTTAGTTAGAAAAAC
>1007
TGGTGAGGCACCAGGAGGGTCTTTTCTCCTCTCCTTACATCCCTCCACTCTTGCTCTCC
TTGCAGCCCAGTTCTTTCCTAACTTTCTTTAAATCCCTTTCCTCTAACAGGGTGTATAGA
CCTTAGTTAGAAAAACAGGTA

>1008
TCCCTCCACTCTTGCTCTCCTTGCAGCCCAGTTCTTTCCTAACTTTCTTTAAATCCCTTT
CCTCTAACAGGGTGTATAGACCTTAGTTAGAAAAACAGGTAGTCTCTAAATGGGATTGC
TCTTTATTGTTAATGAAATGA
>1009
CTCCTTGCAGCCCAGTTCTTTCCTAACTTTCTTTAAATCCCTTTCCTCTAACAGGGTGTA
TAGACCTTAGTTAGAAAAACAGGTAGTCTCTAAATGGGATTGCTCTTTATTGTTAATGA
AATGAATACCCAGGGACTGGG
>1010
TTGCAGCCCAGTTCTTTCCTAACTTTCTTTAAATCCCTTTCCTCTAACAGGGTGTATAGA
CCTTAGTTAGAAAAACAGGTAGTCTCTAAATGGGATTGCTCTTTATTGTTAATGAAATG
AATACCCAGGGACTGGGCTTC
>1011
CAGTTCTTTCCTAACTTTCTTTAAATCCCTTTCCTCTAACAGGGTGTATAGACCTTAGTT
AGAAAAACAGGTAGTCTCTAAATGGGATTGCTCTTTATTGTTAATGAAATGAATACCCA
GGGACTGGGCTTCCCCTCCGC
>1012
GACCTTAGTTAGAAAAACAGGTAGTCTCTAAATGGGATTGCTCTTTATTGTTAATGAAA
TGAATACCCAGGGACTGGGCTTCCCCTCCGCTTGCCCTGGGTTTGATGTGGTTGTATCC
CGTGCTATCAGAGGAGCCCTTC
>1013
TGAATACCCAGGGACTGGGCTTCCCCTCCGCTTGCCCTGGGTTTGATGTGGTTGTATCC
CGTGCTATCAGAGGAGCCCTTCCTTCACTCAAGTGTGTTCCCCTGCCCAGCTCTCTCCG
CAGACTCCTGCTGGGCTGAGCT
>1014
AATACCCAGGGACTGGGCTTCCCCTCCGCTTGCCCTGGGTTTGATGTGGTTGTATCCCG
TGCTATCAGAGGAGCCCTTCCTTCACTCAAGTGTGTTCCCCTGCCCAGCTCTCTCCGCA
GACTCCTGCTGGGCTGAGCTTT
>1015
CCCCTCCGCTTGCCCTGGGTTTGATGTGGTTGTATCCCGTGCTATCAGAGGAGCCCTTC
CTTCACTCAAGTGTGTTCCCCTGCCCAGCTCTCTCCGCAGACTCCTGCTGGGCTGAGC
TTTCCCTGCTCTTAAGAGTCAGG
>1016
GGTTTGATGTGGTTGTATCCCGTGCTATCAGAGGAGCCCTTCCTTCACTCAAGTGTGTT
CCCCTGCCCAGCTCTCTCCGCAGACTCCTGCTGGGCTGAGCTTTCCCTGCTCTTAAGAG
TCAGGAGTGGCTCTTGCTGGGA
>1017
AGAGGAGCCCTTCCTTCACTCAAGTGTGTTCCCCTGCCCAGCTCTCTCCGCAGACTCC
TGCTGGGCTGAGCTTTCCCTGCTCTTAAGAGTCAGGAGTGGCTCTTGCTGGGATGGAA
TGACCCGTCTTTGGGGCTGCCTCA
>1018
ACTCAAGTGTGTTCCCCTGCCCAGCTCTCTCCGCAGACTCCTGCTGGGCTGAGCTTTC
CCTGCTCTTAAGAGTCAGGAGTGGCTCTTGCTGGGATGGAATGACCCGTCTTTGGGGC
TGCCTCATGAGCGGCTCTTGTGAA
>1019
CCCTGCTCTTAAGAGTCAGGAGTGGCTCTTGCTGGGATGGAATGACCCGTCTTTGGGG

CTGCCTCATGAGCGGCTCTTGTGAACCCGGATCAGTTCCGATGTGTAAACTCTACCGCC
TGGCCTTCAGCGAACAGATACAG
>1020
GGCTCTTGCTGGGATGGAATGACCCGTCTTTGGGGCTGCCTCATGAGCGGCTCTTGTG
AACCCGGATCAGTTCCGATGTGTAAACTCTACCGCCTGGCCTTCAGCGAACAGATACA
GATTTCTGCCACCTTCCATGACCC
>1021
GCTGCCTCATGAGCGGCTCTTGTGAACCCGGATCAGTTCCGATGTGTAAACTCTACCGC
CTGGCCTTCAGCGAACAGATACAGATTTCTGCCACCTTCCATGACCCTACAGTTCATGG
GACTGGGTCTGGGGCAGTGCCA
>1022
CATGAGCGGCTCTTGTGAACCCGGATCAGTTCCGATGTGTAAACTCTACCGCCTGGCCT
TCAGCGAACAGATACAGATTTCTGCCACCTTCCATGACCCTACAGTTCATGGGACTGGG
TCTGGGGCAGTGCCAGAGGCAC
>1023
ATGTGTAAACTCTACCGCCTGGCCTTCAGCGAACAGATACAGATTTCTGCCACCTTCCA
TGACCCTACAGTTCATGGGACTGGGTCTGGGGCAGTGCCAGAGGCACGCATGGAGGT
GTGATTCTAGGTGAGTCCTGCGGA
>1024
TTCAGCGAACAGATACAGATTTCTGCCACCTTCCATGACCCTACAGTTCATGGGACTGG
GTCTGGGGCAGTGCCAGAGGCACGCATGGAGGTGTGATTCTAGGTGAGTCCTGCGGA
AAACCTCTGGCCCACCCGTGAGTC
>1025
GAACAGATACAGATTTCTGCCACCTTCCATGACCCTACAGTTCATGGGACTGGGTCTGG
GGCAGTGCCAGAGGCACGCATGGAGGTGTGATTCTAGGTGAGTCCTGCGGAAAACCT
CTGGCCCACCCGTGAGTCACGGAC
>1026
CCATGACCCTACAGTTCATGGGACTGGGTCTGGGGCAGTGCCAGAGGCACGCATGGAG
GTGTGATTCTAGGTGAGTCCTGCGGAAAACCTCTGGCCCACCCGTGAGTCACGGACAG
AACATGCAGACTCGGGCCTTGGTG
>1027
ACCCTACAGTTCATGGGACTGGGTCTGGGGCAGTGCCAGAGGCACGCATGGAGGTGT
GATTCTAGGTGAGTCCTGCGGAAAACCTCTGGCCCACCCGTGAGTCACGGACAGAACA
TGCAGACTCGGGCCTTGGTGACATA
>1028
AGTGCCAGAGGCACGCATGGAGGTGTGATTCTAGGTGAGTCCTGCGGAAAACCTCTG
GCCCACCCGTGAGTCACGGACAGAACATGCAGACTCGGGCCTTGGTGACATAAGCTCC
GCATTGCTAAAACCGCGTGACCTCG
>1029
GCACGCATGGAGGTGTGATTCTAGGTGAGTCCTGCGGAAAACCTCTGGCCCACCCGTG
AGTCACGGACAGAACATGCAGACTCGGGCCTTGGTGACATAAGCTCCGCATTGCTAAA
ACCGCGTGACCTCGAGGGCTGACT
>1030
CTGCGGAAAACCTCTGGCCCACCCGTGAGTCACGGACAGAACATGCAGACTCGGGCC
TTGGTGACATAAGCTCCGCATTGCTAAAACCGCGTGACCTCGAGGGCTGACTGGCCTG
AGAACCCTGGATGGCGCTCTCGGCC

>1031
ACGGACAGAACATGCAGACTCGGGCCTTGGTGACATAAGCTCCGCATTGCTAAAACCG
CGTGACCTCGAGGGCTGACTGGCCTGAGAACCCTGGATGGCGCTCTCGGCCACCCCC
ACCTCCCACCCCAACCTCCTGGGCT
>1032
GACTCGGGCCTTGGTGACATAAGCTCCGCATTGCTAAAACCGCGTGACCTCGAGGGCT
GACTGGCCTGAGAACCCTGGATGGCGCTCTCGGCCACCCCCACCTCCCACCCCAACCT
CCTGGGCTTCGGTCAGAATCCACA
>1033
GGCCTGAGAACCCTGGATGGCGCTCTCGGCCACCCCCACCTCCCACCCCAACCTCCTG
GGCTTCGGTCAGAATCCACAGCCCGTGCCCGAAGAGCGCTTCCCGCCTCTGGCACCCT
ACCTTCGCTCAGCTCCAGGGAAAA
>1034
ACCCTGGATGGCGCTCTCGGCCACCCCCACCTCCCACCCCAACCTCCTGGGCTTCGGT
CAGAATCCACAGCCCGTGCCCGAAGAGCGCTTCCCGCCTCTGGCACCCTACCTTCGCT
CAGCTCCAGGGAAAAGGGGAGAGG
>1035
CTCTCGGCCACCCCCACCTCCCACCCCAACCTCCTGGGCTTCGGTCAGAATCCACAGC
CCGTGCCCGAAGAGCGCTTCCCGCCTCTGGCACCCTACCTTCGCTCAGCTCCAGGGAA
AAGGGGAGAGGGCAGCTTTCTGCA
>1036
GGCTTCGGTCAGAATCCACAGCCCGTGCCCGAAGAGCGCTTCCCGCCTCTGGCACCCT
ACCTTCGCTCAGCTCCAGGGAAAAGGGGAGAGGGCAGCTTTCTGCAGTCAGAGGAAG
AGTACATTTTCTTTGGCTGCTCTAC
>1037
GGTCAGAATCCACAGCCCGTGCCCGAAGAGCGCTTCCCGCCTCTGGCACCCTACCTTC
GCTCAGCTCCAGGGAAAAGGGGAGAGGGCAGCTTTCTGCAGTCAGAGGAAGAGTACA
TTTTCTTTGGCTGCTCTACCCTCTG
>1038
ATCCACAGCCCGTGCCCGAAGAGCGCTTCCCGCCTCTGGCACCCTACCTTCGCTCAGC
TCCAGGGAAAAGGGGAGAGGGCAGCTTTCTGCAGTCAGAGGAAGAGTACATTTTCTT
TGGCTGCTCTACCCTCTGAAGTAGG
>1039
GCGCTTCCCGCCTCTGGCACCCTACCTTCGCTCAGCTCCAGGGAAAAGGGGAGAGGG
CAGCTTTCTGCAGTCAGAGGAAGAGTACATTTTCTTTGGCTGCTCTACCCTCTGAAGTA
GGGCGGCCAGCTGAAGGAGGACAC
>1040
AAAGGGGAGAGGGCAGCTTTCTGCAGTCAGAGGAAGAGTACATTTTCTTTGGCTGCTC
TACCCTCTGAAGTAGGGCGGCCAGCTGAAGGAGGACACACTTTTGAGGGGCCCAGAG
GTTGTCCAAGCTTCCCCCTGCCCCC
>1041
GGGGAGAGGGCAGCTTTCTGCAGTCAGAGGAAGAGTACATTTTCTTTGGCTGCTCTAC
CCTCTGAAGTAGGGCGGCCAGCTGAAGGAGGACACACTTTTGAGGGGCCCAGAGGTT
GTCCAAGCTTCCCCCTGCCCCCTGA
>1042
GAGTACATTTTCTTTGGCTGCTCTACCCTCTGAAGTAGGGCGGCCAGCTGAAGGAGGA

CACACTTTTGAGGGGCCCAGAGGTTGTCCAAGCTTCCCCCTGCCCCCTGAAGACTGTG
CACTGAGCTGGGCGCAGTTCTCGG
>1043
TTTCTTTGGCTGCTCTACCCTCTGAAGTAGGGCGGCCAGCTGAAGGAGGACACACTTT
TGAGGGGCCCAGAGGTTGTCCAAGCTTCCCCCTGCCCCCTGAAGACTGTGCACTGAG
CTGGGCGCAGTTCTCGGGAACTGTT
>1044
TCTTTGGCTGCTCTACCCTCTGAAGTAGGGCGGCCAGCTGAAGGAGGACACACTTTTG
AGGGGCCCAGAGGTTGTCCAAGCTTCCCCCTGCCCCCTGAAGACTGTGCACTGAGCT
GGGCGCAGTTCTCGGGAACTGTTTC
>1045
CTCTACCCTCTGAAGTAGGGCGGCCAGCTGAAGGAGGACACACTTTTGAGGGGCCCA
GAGGTTGTCCAAGCTTCCCCCTGCCCCCTGAAGACTGTGCACTGAGCTGGGCGCAGTT
CTCGGGAACTGTTTCCACCCAGATT
>1046
CGGCCAGCTGAAGGAGGACACACTTTTGAGGGGCCCAGAGGTTGTCCAAGCTTCCCC
CTGCCCCCTGAAGACTGTGCACTGAGCTGGGCGCAGTTCTCGGGAACTGTTTCCACCC
AGATTGCTGGGGGGCGGGGGGGGGT
>1047
TTTTGAGGGGCCCAGAGGTTGTCCAAGCTTCCCCCTGCCCCCTGAAGACTGTGCACTG
AGCTGGGCGCAGTTCTCGGGAACTGTTTCCACCCAGATTGCTGGGGGGCGGGGGGGG
GTGGGATGAGGGCAGAGCCGAGAGG
>1048
AAGCTTCCCCCTGCCCCCTGAAGACTGTGCACTGAGCTGGGCGCAGTTCTCGGGAACT
GTTTCCACCCAGATTGCTGGGGGGCGGGGGGGGGTGGGATGAGGGCAGAGCCGAGAG
GCTGTCCAAGGTTTGGGAGAGAGAA
>1049
CTGAGCTGGGCGCAGTTCTCGGGAACTGTTTCCACCCAGATTGCTGGGGGGCGGGGG
GGGGTGGGATGAGGGCAGAGCCGAGAGGCTGTCCAAGGTTTGGGAGAGAGAAAAGT
TTCTCCCAGGACTCGACCTTGGCCTCC
>1050
CTGGGCGCAGTTCTCGGGAACTGTTTCCACCCAGATTGCTGGGGGGCGGGGGGGGGT
GGGATGAGGGCAGAGCCGAGAGGCTGTCCAAGGTTTGGGAGAGAGAAAAGTTTCTCC
CAGGACTCGACCTTGGCCTCCAGCAA
>1051
GGGCGCAGTTCTCGGGAACTGTTTCCACCCAGATTGCTGGGGGGCGGGGGGGGGTGG
GATGAGGGCAGAGCCGAGAGGCTGTCCAAGGTTTGGGAGAGAGAAAAGTTTCTCCCA
GGACTCGACCTTGGCCTCCAGCAATC
>1052
CAGTTCTCGGGAACTGTTTCCACCCAGATTGCTGGGGGGCGGGGGGGGGTGGGATGA
GGGCAGAGCCGAGAGGCTGTCCAAGGTTTGGGAGAGAGAAAAGTTTCTCCCAGGACT
CGACCTTGGCCTCCAGCAATCGCGAC
>1053
ACTGTTTCCACCCAGATTGCTGGGGGGCGGGGGGGGGTGGGATGAGGGCAGAGCCGA
GAGGCTGTCCAAGGTTTGGGAGAGAGAAAAGTTTCTCCCAGGACTCGACCTTGGCCT
CCAGCAATCGCGACAGCTAAAAACGG

>1054
GCTGGGGGGCGGGGGGGGGTGGGATGAGGGCAGAGCCGAGAGGCTGTCCAAGGTTT
GGGAGAGAGAAAAGTTTCTCCCAGGACTCGACCTTGGCCTCCAGCAATCGCGACAGC
TAAAAACGGGTGTCTCGCTTCGACAAT
>1055
CAGAGCCGAGAGGCTGTCCAAGGTTTGGGAGAGAGAAAAGTTTCTCCCAGGACTCGA
CCTTGGCCTCCAGCAATCGCGACAGCTAAAAACGGGTGTCTCGCTTCGACAATAGATC
CCCGCGGACCTTCTGGCACCTGGTT
>1056
GCTGTCCAAGGTTTGGGAGAGAGAAAAGTTTCTCCCAGGACTCGACCTTGGCCTCCA
GCAATCGCGACAGCTAAAAACGGGTGTCTCGCTTCGACAATAGATCCCCGCGGACCTT
CTGGCACCTGGTTCACTAGCGCCCG
>1057
TCTCCCAGGACTCGACCTTGGCCTCCAGCAATCGCGACAGCTAAAAACGGGTGTCTCG
CTTCGACAATAGATCCCCGCGGACCTTCTGGCACCTGGTTCACTAGCGCCCGCGAACT
CTGCCTCGGGAGACTTATTGAAAT
>1058
CGACAGCTAAAAACGGGTGTCTCGCTTCGACAATAGATCCCCGCGGACCTTCTGGCAC
CTGGTTCACTAGCGCCCGCGAACTCTGCCTCGGGAGACTTATTGAAATCCGGATGCTC
AAGCCGGGAGGCGCGCAGTAACCA
>1059
CTTCGACAATAGATCCCCGCGGACCTTCTGGCACCTGGTTCACTAGCGCCCGCGAACT
CTGCCTCGGGAGACTTATTGAAATCCGGATGCTCAAGCCGGGAGGCGCGCAGTAACCA
GGAGGATGAGAGGGCCGGGTTTGG
>1060
TTCTGGCACCTGGTTCACTAGCGCCCGCGAACTCTGCCTCGGGAGACTTATTGAAATCC
GGATGCTCAAGCCGGGAGGCGCGCAGTAACCAGGAGGATGAGAGGGCCGGGTTTGGG
CTAGGAAAGCGGCCTTTTAAAACA
>1061
ACCTGGTTCACTAGCGCCCGCGAACTCTGCCTCGGGAGACTTATTGAAATCCGGATGC
TCAAGCCGGGAGGCGCGCAGTAACCAGGAGGATGAGAGGGCCGGGTTTGGGCTAGGA
AAGCGGCCTTTTAAAACAGATGTCA
>1062
GCCCGCGAACTCTGCCTCGGGAGACTTATTGAAATCCGGATGCTCAAGCCGGGAGGCG
CGCAGTAACCAGGAGGATGAGAGGGCCGGGTTTGGGCTAGGAAAGCGGCCTTTTAAA
ACAGATGTCAGGGGGACTGCAGCCC
>1063
TTGAAATCCGGATGCTCAAGCCGGGAGGCGCGCAGTAACCAGGAGGATGAGAGGGCC
GGGTTTGGGCTAGGAAAGCGGCCTTTTAAAACAGATGTCAGGGGGACTGCAGCCCCG
AGCCATGAGAAAAAAGTTAAAGGCGA
>1064
ATCCGGATGCTCAAGCCGGGAGGCGCGCAGTAACCAGGAGGATGAGAGGGCCGGGTT
TGGGCTAGGAAAGCGGCCTTTTAAAACAGATGTCAGGGGGACTGCAGCCCCGAGCCA
TGAGAAAAAAGTTAAAGGCGAGATGA
>1065
CGGGAGGCGCGCAGTAACCAGGAGGATGAGAGGGCCGGGTTTGGGCTAGGAAAGCG

GCCTTTTAAAACAGATGTCAGGGGGACTGCAGCCCCGAGCCATGAGAAAAAAGTTAA
AGGCGAGATGACACGCACTGAATTGGG
>1066
CAGGAGGATGAGAGGGCCGGGTTTGGGCTAGGAAAGCGGCCTTTTAAAACAGATGTC
AGGGGGACTGCAGCCCCGAGCCATGAGAAAAAAGTTAAAGGCGAGATGACACGCACT
GAATTGGGGCAAACATTGGAAGAGGA
>1067
TTTGGGCTAGGAAAGCGGCCTTTTAAAACAGATGTCAGGGGGACTGCAGCCCCGAGC
CATGAGAAAAAAGTTAAAGGCGAGATGACACGCACTGAATTGGGGCAAACATTGGAA
GAGGAGACAAAACTGCGTGCTTGAGC
>1068
CTAGGAAAGCGGCCTTTTAAAACAGATGTCAGGGGGACTGCAGCCCCGAGCCATGAG
AAAAAAGTTAAAGGCGAGATGACACGCACTGAATTGGGGCAAACATTGGAAGAGGAG
ACAAAACTGCGTGCTTGAGCACCGGG
>1069
AAAGCGGCCTTTTAAAACAGATGTCAGGGGGACTGCAGCCCCGAGCCATGAGAAAAA
AGTTAAAGGCGAGATGACACGCACTGAATTGGGGCAAACATTGGAAGAGGAGACAAA
ACTGCGTGCTTGAGCACCGGGGTGCG
>1070
GCAGCCCCGAGCCATGAGAAAAAAGTTAAAGGCGAGATGACACGCACTGAATTGGGG
CAAACATTGGAAGAGGAGACAAAACTGCGTGCTTGAGCACCGGGGTGCGGGGAGGG
GGGGACAAAACCCGTATCCAGTGCAAA
>1071
GTTAAAGGCGAGATGACACGCACTGAATTGGGGCAAACATTGGAAGAGGAGACAAAA
CTGCGTGCTTGAGCACCGGGGTGCGGGGAGGGGGGGACAAAACCCGTATCCAGTGCA
AATTAAAATCTTGGGAGTAGGTGGGG
>1072
ACGCACTGAATTGGGGCAAACATTGGAAGAGGAGACAAAACTGCGTGCTTGAGCACC
GGGGTGCGGGGAGGGGGGGACAAAACCCGTATCCAGTGCAAATTAAAATCTTGGGAG
TAGGTGGGGGCTGCTGCGCGCCCTTC
>1073
TGGAAGAGGAGACAAAACTGCGTGCTTGAGCACCGGGGTGCGGGGAGGGGGGGACA
AAACCCGTATCCAGTGCAAATTAAAATCTTGGGAGTAGGTGGGGGCTGCTGCGCGCCC
TTCACCCTCAGTTCCCCTATTAAGGA
>1074
GTGCTTGAGCACCGGGGTGCGGGGAGGGGGGGACAAAACCCGTATCCAGTGCAAATT
AAAATCTTGGGAGTAGGTGGGGGCTGCTGCGCGCCCTTCACCCTCAGTTCCCCTATTAA
GGATTCTGAGTCCCCATGCACTCC
>1075
CTTGAGCACCGGGGTGCGGGGAGGGGGGGACAAAACCCGTATCCAGTGCAAATTAAA
ATCTTGGGAGTAGGTGGGGGCTGCTGCGCGCCCTTCACCCTCAGTTCCCCTATTAAGGA
TTCTGAGTCCCCATGCACTCCTGT
>1076
AAAACCCGTATCCAGTGCAAATTAAAATCTTGGGAGTAGGTGGGGGCTGCTGCGCGCC
CTTCACCCTCAGTTCCCCTATTAAGGATTCTGAGTCCCCATGCACTCCTGTCCTCTGGCT
CCTTCCTTCCTCTCCGCTCGGC

>1077
AGTGCAAATTAAAATCTTGGGAGTAGGTGGGGGCTGCTGCGCGCCCTTCACCCTCAGT
TCCCCTATTAAGGATTCTGAGTCCCCATGCACTCCTGTCCTCTGGCTCCTTCCTTCCTCT
CCGCTCGGCCGGTGAGAGGCGG
>1078
TAAAATCTTGGGAGTAGGTGGGGGCTGCTGCGCGCCCTTCACCCTCAGTTCCCCTATTA
AGGATTCTGAGTCCCCATGCACTCCTGTCCTCTGGCTCCTTCCTTCCTCTCCGCTCGGC
CGGTGAGAGGCGGCCGCCGGCG
>1079
ATTAAGGATTCTGAGTCCCCATGCACTCCTGTCCTCTGGCTCCTTCCTTCCTCTCCGCTC
GGCCGGTGAGAGGCGGCCGCCGGCGCCCCAGCAGCAGCTAGATGTCAGGCGAAGCCC
GGAGCGCGGAGCGCGGGGAAGGG
>1080
TAAGGATTCTGAGTCCCCATGCACTCCTGTCCTCTGGCTCCTTCCTTCCTCTCCGCTCG
GCCGGTGAGAGGCGGCCGCCGGCGCCCCAGCAGCAGCTAGATGTCAGGCGAAGCCCG
GAGCGCGGAGCGCGGGGAAGGGAG
>1081
TGCACTCCTGTCCTCTGGCTCCTTCCTTCCTCTCCGCTCGGCCGGTGAGAGGCGGCCG
CCGGCGCCCCAGCAGCAGCTAGATGTCAGGCGAAGCCCGGAGCGCGGAGCGCGGGG
AAGGGAGGGGAAGGGAGGGGAGGGGA
>1082
GCTCCTTCCTTCCTCTCCGCTCGGCCGGTGAGAGGCGGCCGCCGGCGCCCCAGCAGCA
GCTAGATGTCAGGCGAAGCCCGGAGCGCGGAGCGCGGGGAAGGGAGGGGAAGGGAG
GGGAGGGGAAAGGAGGGGTGGGGCGG
>1083
TCGGCCGGTGAGAGGCGGCCGCCGGCGCCCCAGCAGCAGCTAGATGTCAGGCGAAGC
CCGGAGCGCGGAGCGCGGGGAAGGGAGGGGAAGGGAGGGGAGGGGAAAGGAGGGG
TGGGGCGGGGAGCGCGCGCGGGCTCGGC
>1084
AGAGGCGGCCGCCGGCGCCCCAGCAGCAGCTAGATGTCAGGCGAAGCCCGGAGCGCG
GAGCGCGGGGAAGGGAGGGGAAGGGAGGGGAGGGGAAAGGAGGGGTGGGGCGGGG
AGCGCGCGCGGGCTCGGCCAGTGCGGGG
>1085
GCGGCCGCCGGCGCCCCAGCAGCAGCTAGATGTCAGGCGAAGCCCGGAGCGCGGAGC
GCGGGGAAGGGAGGGGAAGGGAGGGGAGGGGAAAGGAGGGGTGGGGCGGGGAGCG
CGCGCGGGCTCGGCCAGTGCGGGGTGGT
>1086
CAGCTAGATGTCAGGCGAAGCCCGGAGCGCGGAGCGCGGGGAAGGGAGGGGAAGGG
AGGGGAGGGGAAAGGAGGGGTGGGGCGGGGAGCGCGCGCGGGCTCGGCCAGTGCG
GGGTGGTGGGCGGGCTGAGCCCGGGGACG
>1087
GGGGAAGGGAGGGGAAGGGAGGGGAGGGGAAAGGAGGGGTGGGGCGGGGAGCGCG
CGCGGGCTCGGCCAGTGCGGGGTGGTGGGCGGGCTGAGCCCGGGGACGGCGCGGGA
GGGGGGAGGGAAGGGGGGCAGCTGTGGGC
>1088
GAGGGGAAAGGAGGGGTGGGGCGGGGAGCGCGCGCGGGCTCGGCCAGTGCGGGGTG

GTGGGCGGGCTGAGCCCGGGGACGGCGCGGGAGGGGGGAGGGAAGGGGGGCAGCT
GTGGGCAGGGAGCCGGGCTCGGCCGCCAG
>1089
GGCGGGGAGCGCGCGCGGGCTCGGCCAGTGCGGGGTGGTGGGCGGGCTGAGCCCGG
GGACGGCGCGGGAGGGGGGAGGGAAGGGGGGCAGCTGTGGGCAGGGAGCCGGGCT
CGGCCGCCAGCACTAAAGATGGAGAGGCG
>1090
AGCGCGCGCGGGCTCGGCCAGTGCGGGGTGGTGGGCGGGCTGAGCCCGGGGACGGC
GCGGGAGGGGGGAGGGAAGGGGGGCAGCTGTGGGCAGGGAGCCGGGCTCGGCCGCC
AGCACTAAAGATGGAGAGGCGCCGGGGC
>1091
GCGCGGGCTCGGCCAGTGCGGGGTGGTGGGCGGGCTGAGCCCGGGGACGGCGCGGG
AGGGGGGAGGGAAGGGGGGCAGCTGTGGGCAGGGAGCCGGGCTCGGCCGCCAGCAC
TAAAGATGGAGAGGCGCCGGGGCCTTGC
>1092
TCGGCCAGTGCGGGGTGGTGGGCGGGCTGAGCCCGGGGACGGCGCGGGAGGGGGGA
GGGAAGGGGGGCAGCTGTGGGCAGGGAGCCGGGCTCGGCCGCCAGCACTAAAGATG
GAGAGGCGCCGGGGCCTTGCAGGGGAGG
>1093
CCCGGGGACGGCGCGGGAGGGGGGAGGGAAGGGGGGCAGCTGTGGGCAGGGAGCC
GGGCTCGGCCGCCAGCACTAAAGATGGAGAGGCGCCGGGGCCTTGCAGGGGAGGGG
GCTCGGCGTTGACGTGGGACGCGGCGGAG
>1094
GGGAGGGGGGAGGGAAGGGGGGCAGCTGTGGGCAGGGAGCCGGGCTCGGCCGCCAG
CACTAAAGATGGAGAGGCGCCGGGGCCTTGCAGGGGAGGGGGCTCGGCGTTGACGTG
GGACGCGGCGGAGGCGCAGCAGCCGGT
>1095
GAGGGGGGAGGGAAGGGGGGCAGCTGTGGGCAGGGAGCCGGGCTCGGCCGCCAGCA
CTAAAGATGGAGAGGCGCCGGGGCCTTGCAGGGGAGGGGGCTCGGCGTTGACGTGGG
ACGCGGCGGAGGCGCAGCAGCCGGTGG
>1096
GGGCAGCTGTGGGCAGGGAGCCGGGCTCGGCCGCCAGCACTAAAGATGGAGAGGCG
CCGGGGCCTTGCAGGGGAGGGGGCTCGGCGTTGACGTGGGACGCGGCGGAGGCGCA
GCAGCCGGTGGTGATTTGCTAACCTCGC
>1097
CACTAAAGATGGAGAGGCGCCGGGGCCTTGCAGGGGAGGGGGCTCGGCGTTGACGTG
GGACGCGGCGGAGGCGCAGCAGCCGGTGGTGATTTGCTAACCTCGCAGCAGAGAGGA
GTTGAGGGCGATGAGAGCGGGTACTG
>1098
AGATGGAGAGGCGCCGGGGCCTTGCAGGGGAGGGGGCTCGGCGTTGACGTGGGACG
CGGCGGAGGCGCAGCAGCCGGTGGTGATTTGCTAACCTCGCAGCAGAGAGGAGTTGA
GGGCGATGAGAGCGGGTACTGCGAACT
>1099
TGGAGAGGCGCCGGGGCCTTGCAGGGGAGGGGGCTCGGCGTTGACGTGGGACGCGG
CGGAGGCGCAGCAGCCGGTGGTGATTTGCTAACCTCGCAGCAGAGAGGAGTTGAGGG
CGATGAGAGCGGGTACTGCGAACTGCC

>1100
GAGGGGGCTCGGCGTTGACGTGGGACGCGGCGGAGGCGCAGCAGCCGGTGGTGATTT
GCTAACCTCGCAGCAGAGAGGAGTTGAGGGCGATGAGAGCGGGTACTGCGAACTGCC
GGGCGATGCTGTCGCTGCCGCCGTGA
>1101
TGACGTGGGACGCGGCGGAGGCGCAGCAGCCGGTGGTGATTTGCTAACCTCGCAGCA
GAGAGGAGTTGAGGGCGATGAGAGCGGGTACTGCGAACTGCCGGGCGATGCTGTCGC
TGCCGCCGTGATACGGAGAGCAACAG
>1102
GAGTTGAGGGCGATGAGAGCGGGTACTGCGAACTGCCGGGCGATGCTGTCGCTGCCG
CCGTGATACGGAGAGCAACAGTTCCCCAGCAACACCCCTCCCCGACACAGGCACACA
CCCCCCGACAGGCACGCACACCCACC
>1103
GTTGAGGGCGATGAGAGCGGGTACTGCGAACTGCCGGGCGATGCTGTCGCTGCCGCC
GTGATACGGAGAGCAACAGTTCCCCAGCAACACCCCTCCCCGACACAGGCACACACC
CCCCGACAGGCACGCACACCCACCCC
>1104
GAGCGGGTACTGCGAACTGCCGGGCGATGCTGTCGCTGCCGCCGTGATACGGAGAGC
AACAGTTCCCCAGCAACACCCCTCCCCGACACAGGCACACACCCCCCGACAGGCACG
CACACCCACCCCACAGTGCCCGGCTC
>1105
GGGCGATGCTGTCGCTGCCGCCGTGATACGGAGAGCAACAGTTCCCCAGCAACACCCC
TCCCCGACACAGGCACACACCCCCCGACAGGCACGCACACCCACCCCACAGTGCCCG
GCTCGGCTGCGGTGAGTGAGGGGCC
>1106
CGCCGTGATACGGAGAGCAACAGTTCCCCAGCAACACCCCTCCCCGACACAGGCACA
CACCCCCCGACAGGCACGCACACCCACCCCACAGTGCCCGGCTCGGCTGCGGTGAGT
GAGGGGCCGGGAAGAGGCGCACCGCC
>1107
AGTTCCCCAGCAACACCCCTCCCCGACACAGGCACACACCCCCCGACAGGCACGCAC
ACCCACCCCACAGTGCCCGGCTCGGCTGCGGTGAGTGAGGGGCCGGGAAGAGGCGC
ACCGCCCAGCGAGCGCCGCGCGCCGGG
>1108
CCGACACAGGCACACACCCCCCGACAGGCACGCACACCCACCCCACAGTGCCCGGCT
CGGCTGCGGTGAGTGAGGGGCCGGGAAGAGGCGCACCGCCCAGCGAGCGCCGCGCG
CCGGGGCTTCCCGGGGCTGGAGAGGTC
>1109
ACCCCCCGACAGGCACGCACACCCACCCCACAGTGCCCGGCTCGGCTGCGGTGAGTG
AGGGGCCGGGAAGAGGCGCACCGCCCAGCGAGCGCCGCGCGCCGGGGCTTCCCGGG
GCTGGAGAGGTCTGGGGGGCGCGCGCT
>1110
CACCCACCCCACAGTGCCCGGCTCGGCTGCGGTGAGTGAGGGGCCGGGAAGAGGCGC
ACCGCCCAGCGAGCGCCGCGCGCCGGGGCTTCCCGGGGCTGGAGAGGTCTGGGGGGC
GCGCGCTCGCTTCGGCCACTCGGCCG
>1111
GTGAGTGAGGGGCCGGGAAGAGGCGCACCGCCCAGCGAGCGCCGCGCGCCGGGGCT

TCCCGGGGCTGGAGAGGTCTGGGGGGCGCGCGCTCGCTTCGGCCACTCGGCCGCTGG
GCTTGTGCCTTTTTTATTTGGCGTGTT
>1112
CTCGCTTCGGCCACTCGGCCGCTGGGCTTGTGCCTTTTTTATTTGGCGTGTTCCCTTCCT
GCCGCTGCAGCCGTCGCCGCCACCGGTTGGGGGTCGGCTAGAAGGGGGAGCCCCGGC
TGTCAGCCTGGGCGCAGCTGCCT
>1113
GCCTTTTTTATTTGGCGTGTTCCCTTCCTGCCGCTGCAGCCGTCGCCGCCACCGGTTGG
GGGTCGGCTAGAAGGGGGAGCCCCGGCTGTCAGCCTGGGCGCAGCTGCCTCCCCAGC
CCTTCCTCTTCAGGGCACGGTCGG
>1114
TTTTTTATTTGGCGTGTTCCCTTCCTGCCGCTGCAGCCGTCGCCGCCACCGGTTGGGGG
TCGGCTAGAAGGGGGAGCCCCGGCTGTCAGCCTGGGCGCAGCTGCCTCCCCAGCCCT
TCCTCTTCAGGGCACGGTCGGGGT
>1115
CTGCAGCCGTCGCCGCCACCGGTTGGGGGTCGGCTAGAAGGGGGAGCCCCGGCTGTC
AGCCTGGGCGCAGCTGCCTCCCCAGCCCTTCCTCTTCAGGGCACGGTCGGGGTGAGA
GGTGGGGGGCGTAGCGGTGTGCGGGG
>1116
CCGCCACCGGTTGGGGGTCGGCTAGAAGGGGGAGCCCCGGCTGTCAGCCTGGGCGCA
GCTGCCTCCCCAGCCCTTCCTCTTCAGGGCACGGTCGGGGTGAGAGGTGGGGGGCGT
AGCGGTGTGCGGGGGGCTGAGGGCGTG
>1117
GGGTCGGCTAGAAGGGGGAGCCCCGGCTGTCAGCCTGGGCGCAGCTGCCTCCCCAGC
CCTTCCTCTTCAGGGCACGGTCGGGGTGAGAGGTGGGGGGCGTAGCGGTGTGCGGGG
GCTGAGGGCGTGAGCAGAGGGGTCGG
>1118
GCCTGGGCGCAGCTGCCTCCCCAGCCCTTCCTCTTCAGGGCACGGTCGGGGTGAGAG
GTGGGGGGCGTAGCGGTGTGCGGGGGGCTGAGGGCGTGAGCAGAGGGGTCGGGGATC
CGGAGGCTTTGTACCGCCAGGGGCTGG
>1119
CCCCAGCCCTTCCTCTTCAGGGCACGGTCGGGGTGAGAGGTGGGGGGCGTAGCGGTG
TGCGGGGGGCTGAGGGCGTGAGCAGAGGGGTCGGGGATCCGGAGGCTTTGTACCGCCA
GGGGCTGGCGGAGCAACAGAGCCCGT
>1120
CTTCCTCTTCAGGGCACGGTCGGGGTGAGAGGTGGGGGGCGTAGCGGTGTGCGGGGG
CTGAGGGCGTGAGCAGAGGGGTCGGGGATCCGGAGGCTTTGTACCGCCAGGGGCTGG
CGGAGCAACAGAGCCCGTGGGTGCTC
>1121
GGCGTAGCGGTGTGCGGGGGGCTGAGGGCGTGAGCAGAGGGGTCGGGGATCCGGAGG
CTTTGTACCGCCAGGGGCTGGCGGAGCAACAGAGCCCGTGGGTGCTCTTATGTATGCG
GACCGGTGCGCGGGCGCAAGATAAGG
>1122
TGCGGGGGGCTGAGGGCGTGAGCAGAGGGGTCGGGGATCCGGAGGCTTTGTACCGCCA
GGGGCTGGCGGAGCAACAGAGCCCGTGGGTGCTCTTATGTATGCGGACCGGTGCGCG
GGCGCAAGATAAGGTTGTGGTTTATT

>1123
CTGAGGGCGTGAGCAGAGGGGTCGGGGATCCGGAGGCTTTGTACCGCCAGGGGCTGG
CGGAGCAACAGAGCCCGTGGGTGCTCTTATGTATGCGGACCGGTGCGCGGGCGCAAG
ATAAGGTTGTGGTTTATTTATTTGTG
>1124
CGCCAGGGGCTGGCGGAGCAACAGAGCCCGTGGGTGCTCTTATGTATGCGGACCGGT
GCGCGGGCGCAAGATAAGGTTGTGGTTTATTTATTTGTGTGTTTATTCGCCGGCCGGCT
GGGAAGCTAGAATCGGAGGAGCTG
>1125
GGGGCTGGCGGAGCAACAGAGCCCGTGGGTGCTCTTATGTATGCGGACCGGTGCGCG
GGCGCAAGATAAGGTTGTGGTTTATTTATTTGTGTGTTTATTCGCCGGCCGGCTGGGAA
GCTAGAATCGGAGGAGCTGACGAG
>1126
CCGGTGCGCGGGCGCAAGATAAGGTTGTGGTTTATTTATTTGTGTGTTTATTCGCCGGC
CGGCTGGGAAGCTAGAATCGGAGGAGCTGACGAGTAGATCTGGGGGCGGAGGGGAGC
AGGACTGGGACTGCTTACGTTTTG
>1127
TGCGCGGGCGCAAGATAAGGTTGTGGTTTATTTATTTGTGTGTTTATTCGCCGGCCGGC
TGGGAAGCTAGAATCGGAGGAGCTGACGAGTAGATCTGGGGGCGGAGGGGAGCAGG
ACTGGGACTGCTTACGTTTTGTTTC
>1128
CGCAAGATAAGGTTGTGGTTTATTTATTTGTGTGTTTATTCGCCGGCCGGCTGGGAAGCT
AGAATCGGAGGAGCTGACGAGTAGATCTGGGGGCGGAGGGGAGCAGGACTGGGACT
GCTTACGTTTTGTTTCTCTTTGAG
>1129
GTTGTGGTTTATTTATTTGTGTGTTTATTCGCCGGCCGGCTGGGAAGCTAGAATCGGAG
GAGCTGACGAGTAGATCTGGGGGCGGAGGGGAGCAGGACTGGGACTGCTTACGTTTT
GTTTCTCTTTGAGAAAACGTGGTG
>1130
GAGCTGACGAGTAGATCTGGGGGCGGAGGGGAGCAGGACTGGGACTGCTTACGTTTT
GTTTCTCTTTGAGAAAACGTGGTGGGCTTTTTCTTGATTGGACTTGATCCCCACCCCCC
TTTTGCAGGGGAGGGAGGGAAGCT
>1131
TTTGTTTCTCTTTGAGAAAACGTGGTGGGCTTTTTCTTGATTGGACTTGATCCCCACCC
CCCTTTTGCAGGGGAGGGAGGGAAGCTCCAGAGGGTCTGCAGCGCTGCGGGCCCTCC
TCGGCTCTCGGCGGGACCGGCGGT
>1132
TTCTCTTTGAGAAAACGTGGTGGGCTTTTTCTTGATTGGACTTGATCCCCACCCCCCTT
TTGCAGGGGAGGGAGGGAAGCTCCAGAGGGTCTGCAGCGCTGCGGGCCCTCCTCGGC
TCTCGGCGGGACCGGCGGTGACAC
>1133
TTTTCTTGATTGGACTTGATCCCCACCCCCCTTTTGCAGGGGAGGGAGGGAAGCTCCA
GAGGGTCTGCAGCGCTGCGGGCCCTCCTCGGCTCTCGGCGGGACCGGCGGTGACACC
GGAGCTCGCCGTGCGCTCCCGGCCG
>1134
GAAGCTCCAGAGGGTCTGCAGCGCTGCGGGCCCTCCTCGGCTCTCGGCGGGACCGGC

GGTGACACCGGAGCTCGCCGTGCGCTCCCGGCCGCTCTCGGTGGGTGCCGGTCTCTGC
ACCTGATGCGTTCGGGATGCCTTTC
>1135
TCGGTGGGTGCCGGTCTCTGCACCTGATGCGTTCGGGATGCCTTTCCCACCCTGGCGC
GCCCGCCGCTAGCTCGCACAGCGCCTCGCACACTCCCGCACGCGCTTGAAATGCGCAC
GGTCCCGCCGGCCCGCGGAACCAC
>1136
GCCGGTCTCTGCACCTGATGCGTTCGGGATGCCTTTCCCACCCTGGCGCGCCCGCCGCT
AGCTCGCACAGCGCCTCGCACACTCCCGCACGCGCTTGAAATGCGCACGGTCCCGCC
GGCCCGCGGAACCACCCGGACGCA
>1137
CGCACACTCCCGCACGCGCTTGAAATGCGCACGGTCCCGCCGGCCCGCGGAACCACC
CGGACGCACGGAGCGCTCCGCACCGACTCGCTCGCCGCCTCCCCGAGACGCTCGCAC
CGTGCTTGGGCCGGGCGCGCTGGCCG
>1138
TCCCGCCGGCCCGCGGAACCACCCGGACGCACGGAGCGCTCCGCACCGACTCGCTCG
CCGCCTCCCCGAGACGCTCGCACCGTGCTTGGGCCGGGCGCGCTGGCCGCTGGCGCC
GCTGGCCAGAGGCCTGGGACCCAGCC
>1139
CTCGCCGCCTCCCCGAGACGCTCGCACCGTGCTTGGGCCGGGCGCGCTGGCCGCTGG
CGCCGCTGGCCAGAGGCCTGGGACCCAGCCGGTCGCTCCCAGGGGGTCACGGCCCTG
GGTCGGAGAGGGAGGGCGGGCAGACC
>1140
CGCCGCCTCCCCGAGACGCTCGCACCGTGCTTGGGCCGGGCGCGCTGGCCGCTGGCG
CCGCTGGCCAGAGGCCTGGGACCCAGCCGGTCGCTCCCAGGGGGTCACGGCCCTGGG
TCGGAGAGGGAGGGCGGGCAGACCCC
>1141
AGACGCTCGCACCGTGCTTGGGCCGGGCGCGCTGGCCGCTGGCGCCGCTGGCCAGAG
GCCTGGGACCCAGCCGGTCGCTCCCAGGGGGTCACGGCCCTGGGTCGGAGAGGGAGG
GCGGGCAGACCCCTTCTCGCCTTTCC
>1142
TGCTTGGGCCGGGCGCGCTGGCCGCTGGCGCCGCTGGCCAGAGGCCTGGGACCCAGC
CGGTCGCTCCCAGGGGGTCACGGCCCTGGGTCGGAGAGGGAGGGCGGGCAGACCCCT
TCTCGCCTTTCCTCCCACAACTCGCT
>1143
GCCGCTGGCCAGAGGCCTGGGACCCAGCCGGTCGCTCCCAGGGGGTCACGGCCCTGG
GTCGGAGAGGGAGGGCGGGCAGACCCCTTCTCGCCTTTCCTCCCACAACTCGCTGCG
GGGCTTTTGTGCTTCCCCTTCGCCGC
>1144
CAGAGGCCTGGGACCCAGCCGGTCGCTCCCAGGGGGTCACGGCCCTGGGTCGGAGAG
GGAGGGCGGGCAGACCCCTTCTCGCCTTTCCTCCCACAACTCGCTGCGGGGCTTTTGT
GCTTCCCCTTCGCCGCGGGGCGGGT
>1145
GCTGCGGGGCTTTTGTGCTTCCCCTTCGCCGCGGGGCGGGTCCGCCTCCCCTGCCGCT
CTCGCCGCGGAGTCCAGCCCGCCCGGACTGTCGCCGTTCCTCCCCGTCTCTTTCGCTTT
CCCTCGTCCCTAGCTCAGCTCTC

>1146
GGGGCTTTTGTGCTTCCCCTTCGCCGCGGGGCGGGTCCGCCTCCCCTGCCGCTCTCGC
CGCGGAGTCCAGCCCGCCCGGACTGTCGCCGTTCCTCCCCGTCTCTTTCGCTTTCCCTC
GTCCCTAGCTCAGCTCTCCTTCT
>1147
CGCCGCGGAGTCCAGCCCGCCCGGACTGTCGCCGTTCCTCCCCGTCTCTTTCGCTTTCC
CTCGTCCCTAGCTCAGCTCTCCTTCTTTCAGGAGTCTAGCTCCTCGGGAAAAGTTGCTT
CCCCAAGTTTGCTGAAGTCGTC
>1148
CGGAGTCCAGCCCGCCCGGACTGTCGCCGTTCCTCCCCGTCTCTTTCGCTTTCCCTCGT
CCCTAGCTCAGCTCTCCTTCTTTCAGGAGTCTAGCTCCTCGGGAAAAGTTGCTTCCCCA
AGTTTGCTGAAGTCGTCTCCAA
>1149
CCGGACTGTCGCCGTTCCTCCCCGTCTCTTTCGCTTTCCCTCGTCCCTAGCTCAGCTCT
CCTTCTTTCAGGAGTCTAGCTCCTCGGGAAAAGTTGCTTCCCCAAGTTTGCTGAAGTC
GTCTCCAAGTCTCGGTGGGGGTC
>1150
GACTGTCGCCGTTCCTCCCCGTCTCTTTCGCTTTCCCTCGTCCCTAGCTCAGCTCTCCTT
CTTTCAGGAGTCTAGCTCCTCGGGAAAAGTTGCTTCCCCAAGTTTGCTGAAGTCGTCT
CCAAGTCTCGGTGGGGGTCGCT
>1151
CGTCTCTTTCGCTTTCCCTCGTCCCTAGCTCAGCTCTCCTTCTTTCAGGAGTCTAGCTCC
TCGGGAAAAGTTGCTTCCCCAAGTTTGCTGAAGTCGTCTCCAAGTCTCGGTGGGGGTC
GCTGGGAACTGGGGGGGTGTGA
>1152
TCTCCTTCTTTCAGGAGTCTAGCTCCTCGGGAAAAGTTGCTTCCCCAAGTTTGCTGAAG
TCGTCTCCAAGTCTCGGTGGGGGTCGCTGGGAACTGGGGGGGTGTGAGAGCGCGGTC
GATCCCCGGAGCTCGGGCGGGTTA
>1153
TGCTTCCCCAAGTTTGCTGAAGTCGTCTCCAAGTCTCGGTGGGGGTCGCTGGGAACTG
GGGGGGTGTGAGAGCGCGGTCGATCCCCGGAGCTCGGGCGGGTTATCGCCGGCTCTTC
CCCGCGCCGCCTGGACTGGACGCT
>1154
AGTCGTCTCCAAGTCTCGGTGGGGGTCGCTGGGAACTGGGGGGGTGTGAGAGCGCGG
TCGATCCCCGGAGCTCGGGCGGGTTATCGCCGGCTCTTCCCCGCGCCGCCTGGACTGG
ACGCTGCAGCCAGCGCCAGCCGGAT
>1155
CGGTCGATCCCCGGAGCTCGGGCGGGTTATCGCCGGCTCTTCCCCGCGCCGCCTGGAC
TGGACGCTGCAGCCAGCGCCAGCCGGATCGCGGGCGCCGAGCGGCGGGTGGGGGAG
ACGGCTGCCCCGTGGGCGCCCAGATT
>1156
CGATCCCCGGAGCTCGGGCGGGTTATCGCCGGCTCTTCCCCGCGCCGCCTGGACTGGA
CGCTGCAGCCAGCGCCAGCCGGATCGCGGGCGCCGAGCGGCGGGTGGGGGAGACGG
CTGCCCCGTGGGCGCCCAGATTTCCC
>1157
CCGGAGCTCGGGCGGGTTATCGCCGGCTCTTCCCCGCGCCGCCTGGACTGGACGCTGC

AGCCAGCGCCAGCCGGATCGCGGGCGCCGAGCGGCGGGTGGGGGAGACGGCTGCCC
CGTGGGCGCCCAGATTTCCCTCAGGA
>1158
TCGCCGGCTCTTCCCCGCGCCGCCTGGACTGGACGCTGCAGCCAGCGCCAGCCGGATC
GCGGGCGCCGAGCGGCGGGTGGGGGAGACGGCTGCCCCGTGGGCGCCCAGATTTCCC
TCAGGACCCAAAAAGCTTGTTTCAG
>1159
CGCGCCGCCTGGACTGGACGCTGCAGCCAGCGCCAGCCGGATCGCGGGCGCCGAGCG
GCGGGTGGGGGAGACGGCTGCCCCGTGGGCGCCCAGATTTCCCTCAGGACCCAAAAA
GCTTGTTTCAGGGCGCCAAGCTTCCA
>1160
CAGCCAGCGCCAGCCGGATCGCGGGCGCCGAGCGGCGGGTGGGGGAGACGGCTGCC
CCGTGGGCGCCCAGATTTCCCTCAGGACCCAAAAAGCTTGTTTCAGGGCGCCAAGCTT
CCATTCTTTTGGCTGTTCCCTCCCTA
>1161
AGCCGGATCGCGGGCGCCGAGCGGCGGGTGGGGGAGACGGCTGCCCCGTGGGCGCC
CAGATTTCCCTCAGGACCCAAAAAGCTTGTTTCAGGGCGCCAAGCTTCCATTCTTTTG
GCTGTTCCCTCCCTAGTTCTCAGAAA
>1162
GGGCGCCGAGCGGCGGGTGGGGGAGACGGCTGCCCCGTGGGCGCCCAGATTTCCCTC
AGGACCCAAAAAGCTTGTTTCAGGGCGCCAAGCTTCCATTCTTTTGGCTGTTCCCTCC
CTAGTTCTCAGAAACCCCGATGTCT
>1163
TTTCCCTCAGGACCCAAAAAGCTTGTTTCAGGGCGCCAAGCTTCCATTCTTTTGGCTGT
TCCCTCCCTAGTTCTCAGAAACCCCGATGTCTGCGGGAAAGCACGCGGTGGCTCGGCT
CGGCTGAATGGTCATCCCAACCT
>1164
CAGGACCCAAAAAGCTTGTTTCAGGGCGCCAAGCTTCCATTCTTTTGGCTGTTCCCTC
CCTAGTTCTCAGAAACCCCGATGTCTGCGGGAAAGCACGCGGTGGCTCGGCTCGGCTG
AATGGTCATCCCAACCTTGACGGC
>1165
GGGCGCCAAGCTTCCATTCTTTTGGCTGTTCCCTCCCTAGTTCTCAGAAACCCCGATGT
CTGCGGGAAAGCACGCGGTGGCTCGGCTCGGCTGAATGGTCATCCCAACCTTGACGG
CCTCCGTCCCCACCCCGGGGCGGG
>1166
GCGGTGGCTCGGCTCGGCTGAATGGTCATCCCAACCTTGACGGCCTCCGTCCCCACCC
CGGGGCGGGCAGCAAAAATGTCTTGACCAGCCGTCCTGTGAATCCTTTCTGCGTGAGC
TTGGTGGCTCCTGAACTGATAGAG
>1167
TGAATGGTCATCCCAACCTTGACGGCCTCCGTCCCCACCCCGGGGCGGGCAGCAAAAA
TGTCTTGACCAGCCGTCCTGTGAATCCTTTCTGCGTGAGCTTGGTGGCTCCTGAACTGA
TAGAGCTCCTTCTGGGGGTGGAG
>1168
TCCGTCCCCACCCCGGGGCGGGCAGCAAAAATGTCTTGACCAGCCGTCCTGTGAATCC
TTTCTGCGTGAGCTTGGTGGCTCCTGAACTGATAGAGCTCCTTCTGGGGGTGGAGGCA
AGCAAGCAGTGTTCGAAGAGTGAG

>1169
AGCAAAAATGTCTTGACCAGCCGTCCTGTGAATCCTTTCTGCGTGAGCTTGGTGGCTC
CTGAACTGATAGAGCTCCTTCTGGGGGTGGAGGCAAGCAAGCAGTGTTCGAAGAGTG
AGGGAGAGACCTTGCTGTTCCTTGC
>1170
CAAAAATGTCTTGACCAGCCGTCCTGTGAATCCTTTCTGCGTGAGCTTGGTGGCTCCTG
AACTGATAGAGCTCCTTCTGGGGGTGGAGGCAAGCAAGCAGTGTTCGAAGAGTGAGG
GAGAGACCTTGCTGTTCCTTGCTC
>1171
CCGTCCTGTGAATCCTTTCTGCGTGAGCTTGGTGGCTCCTGAACTGATAGAGCTCCTTC
TGGGGGTGGAGGCAAGCAAGCAGTGTTCGAAGAGTGAGGGAGAGACCTTGCTGTTCC
TTGCTCTCAGTGCATTCACTGCAA
>1172
CGTGAGCTTGGTGGCTCCTGAACTGATAGAGCTCCTTCTGGGGGTGGAGGCAAGCAA
GCAGTGTTCGAAGAGTGAGGGAGAGACCTTGCTGTTCCTTGCTCTCAGTGCATTCACT
GCAATATTGGTCGCTGTAAGACAGA
>1173
TTCTGGGGGTGGAGGCAAGCAAGCAGTGTTCGAAGAGTGAGGGAGAGACCTTGCTGT
TCCTTGCTCTCAGTGCATTCACTGCAATATTGGTCGCTGTAAGACAGACACGGGGAGG
GTCCTGGGCCTGCGCGGTCTTTCTA
>1174
CGAAGAGTGAGGGAGAGACCTTGCTGTTCCTTGCTCTCAGTGCATTCACTGCAATATT
GGTCGCTGTAAGACAGACACGGGGAGGGTCCTGGGCCTGCGCGGTCTTTCTAACACTC
CTTTAAGCGAGGTTTGGAGTCTAG
>1175
GAGACCTTGCTGTTCCTTGCTCTCAGTGCATTCACTGCAATATTGGTCGCTGTAAGACA
GACACGGGGAGGGTCCTGGGCCTGCGCGGTCTTTCTAACACTCCTTTAAGCGAGGTTT
GGAGTCTAGGAGCCCTCTTATTG
>1176
ATATTGGTCGCTGTAAGACAGACACGGGGAGGGTCCTGGGCCTGCGCGGTCTTTCTAA
CACTCCTTTAAGCGAGGTTTGGAGTCTAGGAGCCCTCTTATTGATCCCGGACAGGCTCT
GGGAGAGGCCTTTGTTTCCCAGC
>1177
GTAAGACAGACACGGGGAGGGTCCTGGGCCTGCGCGGTCTTTCTAACACTCCTTTAAG
CGAGGTTTGGAGTCTAGGAGCCCTCTTATTGATCCCGGACAGGCTCTGGGAGAGGCCT
TTGTTTCCCAGCCCTGTCTTCCCT
>1178
ACAGACACGGGGAGGGTCCTGGGCCTGCGCGGTCTTTCTAACACTCCTTTAAGCGAGG
TTTGGAGTCTAGGAGCCCTCTTATTGATCCCGGACAGGCTCTGGGAGAGGCCTTTGTTT
CCCAGCCCTGTCTTCCCTTCGTA
>1179
GACACGGGGAGGGTCCTGGGCCTGCGCGGTCTTTCTAACACTCCTTTAAGCGAGGTTT
GGAGTCTAGGAGCCCTCTTATTGATCCCGGACAGGCTCTGGGAGAGGCCTTTGTTTCC
CAGCCCTGTCTTCCCTTCGTACTG
>1180
TGCGCGGTCTTTCTAACACTCCTTTAAGCGAGGTTTGGAGTCTAGGAGCCCTCTTATTG

ATCCCGGACAGGCTCTGGGAGAGGCCTTTGTTTCCCAGCCCTGTCTTCCCTTCGTACTG
GTTGTAACTCACACCACAGGTG
>1181
TTCTAACACTCCTTTAAGCGAGGTTTGGAGTCTAGGAGCCCTCTTATTGATCCCGGACA
GGCTCTGGGAGAGGCCTTTGTTTCCCAGCCCTGTCTTCCCTTCGTACTGGTTGTAACTC
ACACCACAGGTGCTGGTGCCAA
>1182
CAGGCTCTGGGAGAGGCCTTTGTTTCCCAGCCCTGTCTTCCCTTCGTACTGGTTGTAAC
TCACACCACAGGTGCTGGTGCCAATATCACTTCCAGCGCCTCTGAGCCATGTCCAGCAT
CCCTTGCTGCCCCTGGTCACAG
>1183
CCCAGCCCTGTCTTCCCTTCGTACTGGTTGTAACTCACACCACAGGTGCTGGTGCCAAT
ATCACTTCCAGCGCCTCTGAGCCATGTCCAGCATCCCTTGCTGCCCCTGGTCACAGGAC
TTGCAGACTCCTTGGAGCCTGT
>1184
TCTTCCCTTCGTACTGGTTGTAACTCACACCACAGGTGCTGGTGCCAATATCACTTCCA
GCGCCTCTGAGCCATGTCCAGCATCCCTTGCTGCCCCTGGTCACAGGACTTGCAGACT
CCTTGGAGCCTGTGGGACACACG
>1185
GTACTGGTTGTAACTCACACCACAGGTGCTGGTGCCAATATCACTTCCAGCGCCTCTGA
GCCATGTCCAGCATCCCTTGCTGCCCCTGGTCACAGGACTTGCAGACTCCTTGGAGCC
TGTGGGACACACGTGGAGTTCAC
>1186
GTGCTGGTGCCAATATCACTTCCAGCGCCTCTGAGCCATGTCCAGCATCCCTTGCTGCC
CCTGGTCACAGGACTTGCAGACTCCTTGGAGCCTGTGGGACACACGTGGAGTTCACA
CACCCAGCCCAGCATGAGCACTCC
>1187
TCCAGCGCCTCTGAGCCATGTCCAGCATCCCTTGCTGCCCCTGGTCACAGGACTTGCA
GACTCCTTGGAGCCTGTGGGACACACGTGGAGTTCACACACCCAGCCCAGCATGAGC
ACTCCTGGCTGTAGGGGACCATATT
>1188
TCCAGCATCCCTTGCTGCCCCTGGTCACAGGACTTGCAGACTCCTTGGAGCCTGTGGG
ACACACGTGGAGTTCACACACCCAGCCCAGCATGAGCACTCCTGGCTGTAGGGGACC
ATATTTAACTATGGTCTCTCTCCAT
>1189
GCTGCCCCTGGTCACAGGACTTGCAGACTCCTTGGAGCCTGTGGGACACACGTGGAG
TTCACACACCCAGCCCAGCATGAGCACTCCTGGCTGTAGGGGACCATATTTAACTATGG
TCTCTCTCCATACCTTAATGACCC
>1190
ACTCCTTGGAGCCTGTGGGACACACGTGGAGTTCACACACCCAGCCCAGCATGAGCA
CTCCTGGCTGTAGGGGACCATATTTAACTATGGTCTCTCTCCATACCTTAATGACCCATC
CTTCTTCGTTTTTTAGATCCTCC
>1191
GCTGTAGGGGACCATATTTAACTATGGTCTCTCTCCATACCTTAATGACCCATCCTTCTT
CGTTTTTTAGATCCTCCTGTCCACCGGGCAGTTGTGGGCAGGAGGGTAGGTACGACAC
CTTACGTAAGTGTGTGTGTGTA

>1192
CTATGGTCTCTCTCCATACCTTAATGACCCATCCTTCTTCGTTTTTTAGATCCTCCTGTCC
ACCGGGCAGTTGTGGGCAGGAGGGTAGGTACGACACCTTACGTAAGTGTGTGTGTGTA
AACACACACACACTTTTTTT
>1193
TCTCCATACCTTAATGACCCATCCTTCTTCGTTTTTTAGATCCTCCTGTCCACCGGGCAG
TTGTGGGCAGGAGGGTAGGTACGACACCTTACGTAAGTGTGTGTGTGTAAACACACAC
ACACTTTTTTTTTTTTTTTCTG
>1194
TTCGTTTTTTAGATCCTCCTGTCCACCGGGCAGTTGTGGGCAGGAGGGTAGGTACGAC
ACCTTACGTAAGTGTGTGTGTGTAAACACACACACACTTTTTTTTTTTTTTTCTGAGAC
GGAGTTTTGCTCCTGTTGCCCAG
>1195
GGGTAGGTACGACACCTTACGTAAGTGTGTGTGTGTAAACACACACACACTTTTTTTTT
TTTTTTCTGAGACGGAGTTTTGCTCCTGTTGCCCAGGCTGGAGTGCAGTGGCGCAATC
TCGGCTCACGGCAACCTCCGCCT
>1196
GTACGACACCTTACGTAAGTGTGTGTGTGTAAACACACACACACTTTTTTTTTTTTTTT
CTGAGACGGAGTTTTGCTCCTGTTGCCCAGGCTGGAGTGCAGTGGCGCAATCTCGGCT
CACGGCAACCTCCGCCTCCCGGT
>1197
TGTGTGTGTGTAAACACACACACACTTTTTTTTTTTTTTTCTGAGACGGAGTTTTGCTC
CTGTTGCCCAGGCTGGAGTGCAGTGGCGCAATCTCGGCTCACGGCAACCTCCGCCTCC
CGGTTTCAAGTGATTCTCCTGCC
>1198
GTGTAAACACACACACACTTTTTTTTTTTTTTTCTGAGACGGAGTTTTGCTCCTGTTGC
CCAGGCTGGAGTGCAGTGGCGCAATCTCGGCTCACGGCAACCTCCGCCTCCCGGTTTC
AAGTGATTCTCCTGCCTCAGCCT
>1199
TCCTGTTGCCCAGGCTGGAGTGCAGTGGCGCAATCTCGGCTCACGGCAACCTCCGCCT
CCCGGTTTCAAGTGATTCTCCTGCCTCAGCCTCCCGAGTAGCTGGGATTACAGGCGGG
TGCCACCGCGCCTGGCTAATTTTG
>1200
GAGTGCAGTGGCGCAATCTCGGCTCACGGCAACCTCCGCCTCCCGGTTTCAAGTGATT
CTCCTGCCTCAGCCTCCCGAGTAGCTGGGATTACAGGCGGGTGCCACCGCGCCTGGCT
AATTTTGTATTTTTTGTAGAGACG
>1201
GCAATCTCGGCTCACGGCAACCTCCGCCTCCCGGTTTCAAGTGATTCTCCTGCCTCAGC
CTCCCGAGTAGCTGGGATTACAGGCGGGTGCCACCGCGCCTGGCTAATTTTGTATTTTT
TGTAGAGACGGGGCTTCTCCAT
>1202
CTCAGCCTCCCGAGTAGCTGGGATTACAGGCGGGTGCCACCGCGCCTGGCTAATTTTGT
ATTTTTTGTAGAGACGGGGCTTCTCCATGTTGGTCAGGCTGGTCTCGAACTCCCGACCT
CAGGTGATCCGCCTGCCTGGGC
>1203
CAGCCTCCCGAGTAGCTGGGATTACAGGCGGGTGCCACCGCGCCTGGCTAATTTTGTAT

TTTTTGTAGAGACGGGGCTTCTCCATGTTGGTCAGGCTGGTCTCGAACTCCCGACCTCA
GGTGATCCGCCTGCCTGGGCCT
>1204
AATTTTGTATTTTTTGTAGAGACGGGGCTTCTCCATGTTGGTCAGGCTGGTCTCGAACT
CCCGACCTCAGGTGATCCGCCTGCCTGGGCCTCCCAAAGTGCTGGGATTAGAGGCGTG
AGCCACCGCGCCCTACCCCACAC
>1205
TTCTCCATGTTGGTCAGGCTGGTCTCGAACTCCCGACCTCAGGTGATCCGCCTGCCTGG
GCCTCCCAAAGTGCTGGGATTAGAGGCGTGAGCCACCGCGCCCTACCCCACACACTCT
TTCTGTCTGTCTCTGGCTGGTGT
>1206
GTCAGGCTGGTCTCGAACTCCCGACCTCAGGTGATCCGCCTGCCTGGGCCTCCCAAAG
TGCTGGGATTAGAGGCGTGAGCCACCGCGCCCTACCCCACACACTCTTTCTGTCTGTCT
CTGGCTGGTGTACATACACACAG
>1207
CAGGCTGGTCTCGAACTCCCGACCTCAGGTGATCCGCCTGCCTGGGCCTCCCAAAGTG
CTGGGATTAGAGGCGTGAGCCACCGCGCCCTACCCCACACACTCTTTCTGTCTGTCTCT
GGCTGGTGTACATACACACAGTT
>1208
GTCTCGAACTCCCGACCTCAGGTGATCCGCCTGCCTGGGCCTCCCAAAGTGCTGGGAT
TAGAGGCGTGAGCCACCGCGCCCTACCCCACACACTCTTTCTGTCTGTCTCTGGCTGG
TGTACATACACACAGTTCAGATTT
>1209
AGGCGTGAGCCACCGCGCCCTACCCCACACACTCTTTCTGTCTGTCTCTGGCTGGTGT
ACATACACACAGTTCAGATTTTATATTATACATACAATAACTTTGAGGCTTCCGTGGGTA
GAAATGACCCAAGTTAGTGTGT
>1210
TGAGCCACCGCGCCCTACCCCACACACTCTTTCTGTCTGTCTCTGGCTGGTGTACATAC
ACACAGTTCAGATTTTATATTATACATACAATAACTTTGAGGCTTCCGTGGGTAGAAATG
ACCCAAGTTAGTGTGTGGGTT
>1211
TTCTGTCTGTCTCTGGCTGGTGTACATACACACAGTTCAGATTTTATATTATACATACAAT
AACTTTGAGGCTTCCGTGGGTAGAAATGACCCAAGTTAGTGTGTGGGTTGTGTGTGTT
CCTGTTAGTACCTAGGGATGG
>1212
GGCTGGTGTACATACACACAGTTCAGATTTTATATTATACATACAATAACTTTGAGGCTT
CCGTGGGTAGAAATGACCCAAGTTAGTGTGTGGGTTGTGTGTGTTCCTGTTAGTACCTA
GGGATGGGGACAGGCCTTGGG
>1213
TACACACAGTTCAGATTTTATATTATACATACAATAACTTTGAGGCTTCCGTGGGTAGAA
ATGACCCAAGTTAGTGTGTGGGTTGTGTGTGTTCCTGTTAGTACCTAGGGATGGGGACA
GGCCTTGGGTCTGCCAGTGCT
>1214
CACAGTTCAGATTTTATATTATACATACAATAACTTTGAGGCTTCCGTGGGTAGAAATGA
CCCAAGTTAGTGTGTGGGTTGTGTGTGTTCCTGTTAGTACCTAGGGATGGGGACAGGC
CTTGGGTCTGCCAGTGCTTTGC

>1215
CAATAACTTTGAGGCTTCCGTGGGTAGAAATGACCCAAGTTAGTGTGTGGGTTGTGTG
TGTTCCTGTTAGTACCTAGGGATGGGGACAGGCCTTGGGTCTGCCAGTGCTTTGCAGC
CCTGAGAACAGTAGAAGGCCAGAG
>1216
GTGGGTAGAAATGACCCAAGTTAGTGTGTGGGTTGTGTGTGTTCCTGTTAGTACCTAGG
GATGGGGACAGGCCTTGGGTCTGCCAGTGCTTTGCAGCCCTGAGAACAGTAGAAGGC
CAGAGGCCAAGGCTGATGGTGAGA
>1217
CAAGTTAGTGTGTGGGTTGTGTGTGTTCCTGTTAGTACCTAGGGATGGGGACAGGCCTT
GGGTCTGCCAGTGCTTTGCAGCCCTGAGAACAGTAGAAGGCCAGAGGCCAAGGCTGA
TGGTGAGAGGATAGCCTTTGCGGA
>1218
TGTGGGTTGTGTGTGTTCCTGTTAGTACCTAGGGATGGGGACAGGCCTTGGGTCTGCC
AGTGCTTTGCAGCCCTGAGAACAGTAGAAGGCCAGAGGCCAAGGCTGATGGTGAGAG
GATAGCCTTTGCGGAGGAGAGCCCA
>1219
GGGATGGGGACAGGCCTTGGGTCTGCCAGTGCTTTGCAGCCCTGAGAACAGTAGAAG
GCCAGAGGCCAAGGCTGATGGTGAGAGGATAGCCTTTGCGGAGGAGAGCCCAGTGTC
CATAGATTTCTGTGGCCATGGCTACA
>1220
GGCCTTGGGTCTGCCAGTGCTTTGCAGCCCTGAGAACAGTAGAAGGCCAGAGGCCAA
GGCTGATGGTGAGAGGATAGCCTTTGCGGAGGAGAGCCCAGTGTCCATAGATTTCTGT
GGCCATGGCTACAGGCTCCTGGCCT
>1221
GGTCTGCCAGTGCTTTGCAGCCCTGAGAACAGTAGAAGGCCAGAGGCCAAGGCTGAT
GGTGAGAGGATAGCCTTTGCGGAGGAGAGCCCAGTGTCCATAGATTTCTGTGGCCATG
GCTACAGGCTCCTGGCCTCCAATGA
>1222
CCTGAGAACAGTAGAAGGCCAGAGGCCAAGGCTGATGGTGAGAGGATAGCCTTTGCG
GAGGAGAGCCCAGTGTCCATAGATTTCTGTGGCCATGGCTACAGGCTCCTGGCCTCCA
ATGAGTTTGACTCTGCTTCTGGGGC
>1223
CTGATGGTGAGAGGATAGCCTTTGCGGAGGAGAGCCCAGTGTCCATAGATTTCTGTGG
CCATGGCTACAGGCTCCTGGCCTCCAATGAGTTTGACTCTGCTTCTGGGGCTCCTGTAG
TGAGGCAGAGTTTGGAAAAACTG
>1224
CTTTGCGGAGGAGAGCCCAGTGTCCATAGATTTCTGTGGCCATGGCTACAGGCTCCTG
GCCTCCAATGAGTTTGACTCTGCTTCTGGGGCTCCTGTAGTGAGGCAGAGTTTGGAAA
AACTGTTTAAAGATAAGGCTTGAC
>1225
TCTGTGGCCATGGCTACAGGCTCCTGGCCTCCAATGAGTTTGACTCTGCTTCTGGGGCT
CCTGTAGTGAGGCAGAGTTTGGAAAAACTGTTTAAAGATAAGGCTTGACATCCAGGGG
GCTGGCGTGAAGCTGACAAGGAA
>1226
TGGCCATGGCTACAGGCTCCTGGCCTCCAATGAGTTTGACTCTGCTTCTGGGGCTCCTG

TAGTGAGGCAGAGTTTGGAAAAACTGTTTAAAGATAAGGCTTGACATCCAGGGGGCTG
GCGTGAAGCTGACAAGGAAGGGG
>1227
GCCTCCAATGAGTTTGACTCTGCTTCTGGGGCTCCTGTAGTGAGGCAGAGTTTGGAAA
AACTGTTTAAAGATAAGGCTTGACATCCAGGGGGCTGGCGTGAAGCTGACAAGGAAG
GGGCTAAAGTCTTGCTAATTTTAAG
>1228
TCTGCTTCTGGGGCTCCTGTAGTGAGGCAGAGTTTGGAAAAACTGTTTAAAGATAAGG
CTTGACATCCAGGGGGCTGGCGTGAAGCTGACAAGGAAGGGGCTAAAGTCTTGCTAA
TTTTAAGTAAGTGCACTTCATCATA
>1229
CCTGTAGTGAGGCAGAGTTTGGAAAAACTGTTTAAAGATAAGGCTTGACATCCAGGGG
GCTGGCGTGAAGCTGACAAGGAAGGGGCTAAAGTCTTGCTAATTTTAAGTAAGTGCAC
TTCATCATAATGAATGTTGGCCTT
>1230
GAGGCAGAGTTTGGAAAAACTGTTTAAAGATAAGGCTTGACATCCAGGGGGCTGGCG
TGAAGCTGACAAGGAAGGGGCTAAAGTCTTGCTAATTTTAAGTAAGTGCACTTCATCAT
AATGAATGTTGGCCTTGCCTAGGG
>1231
CAGAGTTTGGAAAAACTGTTTAAAGATAAGGCTTGACATCCAGGGGGCTGGCGTGAA
GCTGACAAGGAAGGGGCTAAAGTCTTGCTAATTTTAAGTAAGTGCACTTCATCATAATG
AATGTTGGCCTTGCCTAGGGAGCC
>1232
ATAAGGCTTGACATCCAGGGGGCTGGCGTGAAGCTGACAAGGAAGGGGCTAAAGTCT
TGCTAATTTTAAGTAAGTGCACTTCATCATAATGAATGTTGGCCTTGCCTAGGGAGCCCC
TACTCTCAGACACAGAGAAAGCT
>1233
AGGAAGGGGCTAAAGTCTTGCTAATTTTAAGTAAGTGCACTTCATCATAATGAATGTTG
GCCTTGCCTAGGGAGCCCCTACTCTCAGACACAGAGAAAGCTGAGGACTTATAGGACT
TCACTACAATTTTCATTTCACTT
>1234
AGGGGCTAAAGTCTTGCTAATTTTAAGTAAGTGCACTTCATCATAATGAATGTTGGCCTT
GCCTAGGGAGCCCCTACTCTCAGACACAGAGAAAGCTGAGGACTTATAGGACTTCACT
ACAATTTTCATTTCACTTCATC
>1235
TTTAAGTAAGTGCACTTCATCATAATGAATGTTGGCCTTGCCTAGGGAGCCCCTACTCTC
AGACACAGAGAAAGCTGAGGACTTATAGGACTTCACTACAATTTTCATTTCACTTCATC
TAACAGATGTCATAGAGTAGT
>1236
ATCATAATGAATGTTGGCCTTGCCTAGGGAGCCCCTACTCTCAGACACAGAGAAAGCT
GAGGACTTATAGGACTTCACTACAATTTTCATTTCACTTCATCTAACAGATGTCATAGAG
TAGTGAATTAGGCATCTGCTGT
>1237
CTCTCAGACACAGAGAAAGCTGAGGACTTATAGGACTTCACTACAATTTTCATTTCACT
TCATCTAACAGATGTCATAGAGTAGTGAATTAGGCATCTGCTGTGGCAGAACCTTCTTT
TTACTCTCAGCATCCCTGGCTT

>1238
ACAGAGAAAGCTGAGGACTTATAGGACTTCACTACAATTTTCATTTCACTTCATCTAAC
AGATGTCATAGAGTAGTGAATTAGGCATCTGCTGTGGCAGAACCTTCTTTTTACTCTCA
GCATCCCTGGCTTGAAAGGCAG
>1239
AGGACTTATAGGACTTCACTACAATTTTCATTTCACTTCATCTAACAGATGTCATAGAGT
AGTGAATTAGGCATCTGCTGTGGCAGAACCTTCTTTTTACTCTCAGCATCCCTGGCTTG
AAAGGCAGATTTGTAGATTTC
>1240
CACTACAATTTTCATTTCACTTCATCTAACAGATGTCATAGAGTAGTGAATTAGGCATCT
GCTGTGGCAGAACCTTCTTTTTACTCTCAGCATCCCTGGCTTGAAAGGCAGATTTGTAG
ATTTCTAGGAAGCTACTTCAA
>1241
TTCATCTAACAGATGTCATAGAGTAGTGAATTAGGCATCTGCTGTGGCAGAACCTTCTT
TTTACTCTCAGCATCCCTGGCTTGAAAGGCAGATTTGTAGATTTCTAGGAAGCTACTTC
AATTAAAATCTCTAAGATCCTC
>1242
AGTAGTGAATTAGGCATCTGCTGTGGCAGAACCTTCTTTTTACTCTCAGCATCCCTGGC
TTGAAAGGCAGATTTGTAGATTTCTAGGAAGCTACTTCAATTAAAATCTCTAAGATCCT
CTTGCTAAGGGCTTGGCAACAG
>1243
ATTAGGCATCTGCTGTGGCAGAACCTTCTTTTTACTCTCAGCATCCCTGGCTTGAAAGG
CAGATTTGTAGATTTCTAGGAAGCTACTTCAATTAAAATCTCTAAGATCCTCTTGCTAAG
GGCTTGGCAACAGGGAGGAGG
>1244
TCTGCTGTGGCAGAACCTTCTTTTTACTCTCAGCATCCCTGGCTTGAAAGGCAGATTTG
TAGATTTCTAGGAAGCTACTTCAATTAAAATCTCTAAGATCCTCTTGCTAAGGGCTTGGC
AACAGGGAGGAGGGGGCATGT
>1245
TCTCAGCATCCCTGGCTTGAAAGGCAGATTTGTAGATTTCTAGGAAGCTACTTCAATTA
AAATCTCTAAGATCCTCTTGCTAAGGGCTTGGCAACAGGGAGGAGGGGGCATGTTGTA
GTGGTTGAATTCTTACTTTTAGG
>1246
ATTTGTAGATTTCTAGGAAGCTACTTCAATTAAAATCTCTAAGATCCTCTTGCTAAGGGC
TTGGCAACAGGGAGGAGGGGGCATGTTGTAGTGGTTGAATTCTTACTTTTAGGGACAC
TGATGAATTTGTCTTTTGTTCT
>1247
GTAGATTTCTAGGAAGCTACTTCAATTAAAATCTCTAAGATCCTCTTGCTAAGGGCTTG
GCAACAGGGAGGAGGGGGCATGTTGTAGTGGTTGAATTCTTACTTTTAGGGACACTGA
TGAATTTGTCTTTTGTTCTCTCA
>1248
TACTTCAATTAAAATCTCTAAGATCCTCTTGCTAAGGGCTTGGCAACAGGGAGGAGGG
GGCATGTTGTAGTGGTTGAATTCTTACTTTTAGGGACACTGATGAATTTGTCTTTTGTTC
TCTCATCTCTTTTGCTTCTTCC
>1249
GATCCTCTTGCTAAGGGCTTGGCAACAGGGAGGAGGGGGCATGTTGTAGTGGTTGAAT

TCTTACTTTTAGGGACACTGATGAATTTGTCTTTTGTTCTCTCATCTCTTTTGCTTCTTCC
CCTCTTCTTTCTCCTAGCCTC
>1250
TTAGGGACACTGATGAATTTGTCTTTTGTTCTCTCATCTCTTTTGCTTCTTCCCCTCTTCT
TTCTCCTAGCCTCCTCTATTGGCCCAGGAAGCCCACCCAGCCCCGCCACGCAGAGCCC
AGAAGGAAAGAAAGCCTCATG
>1251
TTGTCTTTTGTTCTCTCATCTCTTTTGCTTCTTCCCCTCTTCTTTCTCCTAGCCTCCTCTAT
TGGCCCAGGAAGCCCACCCAGCCCCGCCACGCAGAGCCCAGAAGGAAAGAAAGCCT
CATGCCTGAGCCGAGGGGAGCA
>1252
CTTTTGTTCTCTCATCTCTTTTGCTTCTTCCCCTCTTCTTTCTCCTAGCCTCCTCTATTGG
CCCAGGAAGCCCACCCAGCCCCGCCACGCAGAGCCCAGAAGGAAAGAAAGCCTCAT
GCCTGAGCCGAGGGGAGCACCAT
>1253
GCTTCTTCCCCTCTTCTTTCTCCTAGCCTCCTCTATTGGCCCAGGAAGCCCACCCAGCC
CCGCCACGCAGAGCCCAGAAGGAAAGAAAGCCTCATGCCTGAGCCGAGGGGAGCAC
CATGGATCTGACAAAAATGGGCATG
>1254
CTATTGGCCCAGGAAGCCCACCCAGCCCCGCCACGCAGAGCCCAGAAGGAAAGAAAG
CCTCATGCCTGAGCCGAGGGGAGCACCATGGATCTGACAAAAATGGGCATGATCCAGC
TGCAGAACCCTAGCCACCCCACGGG
>1255
GGCCCAGGAAGCCCACCCAGCCCCGCCACGCAGAGCCCAGAAGGAAAGAAAGCCTC
ATGCCTGAGCCGAGGGGAGCACCATGGATCTGACAAAAATGGGCATGATCCAGCTGCA
GAACCCTAGCCACCCCACGGGGCTAC
>1256
GAAGGAAAGAAAGCCTCATGCCTGAGCCGAGGGGAGCACCATGGATCTGACAAAAAT
GGGCATGATCCAGCTGCAGAACCCTAGCCACCCCACGGGGCTACTGTGCAAGGCCAA
CCAGATGCGGCTGGCCGGGACTTTGT
>1257
AAGAAAGCCTCATGCCTGAGCCGAGGGGAGCACCATGGATCTGACAAAAATGGGCAT
GATCCAGCTGCAGAACCCTAGCCACCCCACGGGGCTACTGTGCAAGGCCAACCAGAT
GCGGCTGGCCGGGACTTTGTGCGATG
>1258
CTCATGCCTGAGCCGAGGGGAGCACCATGGATCTGACAAAAATGGGCATGATCCAGCT
GCAGAACCCTAGCCACCCCACGGGGCTACTGTGCAAGGCCAACCAGATGCGGCTGGC
CGGGACTTTGTGCGATGTGGTCATC
>1259
CATGGATCTGACAAAAATGGGCATGATCCAGCTGCAGAACCCTAGCCACCCCACGGGG
CTACTGTGCAAGGCCAACCAGATGCGGCTGGCCGGGACTTTGTGCGATGTGGTCATCA
TGGTGGACAGCCAGGAGTTCCACG
>1260
GGCTACTGTGCAAGGCCAACCAGATGCGGCTGGCCGGGACTTTGTGCGATGTGGTCAT
CATGGTGGACAGCCAGGAGTTCCACGCCCACCGGACGGTGCTGGCCTGCACCAGCAA
GATGTTTGAGATCCTCTTCCACCGC

>1261
ACTGTGCAAGGCCAACCAGATGCGGCTGGCCGGGACTTTGTGCGATGTGGTCATCATG
GTGGACAGCCAGGAGTTCCACGCCCACCGGACGGTGCTGGCCTGCACCAGCAAGATG
TTTGAGATCCTCTTCCACCGCAATA
>1262
TGTGCGATGTGGTCATCATGGTGGACAGCCAGGAGTTCCACGCCCACCGGACGGTGCT
GGCCTGCACCAGCAAGATGTTTGAGATCCTCTTCCACCGCAATAGTCAACACTATACTT
TGGACTTCCTCTCGCCAAAGACC
>1263
CGATGTGGTCATCATGGTGGACAGCCAGGAGTTCCACGCCCACCGGACGGTGCTGGCC
TGCACCAGCAAGATGTTTGAGATCCTCTTCCACCGCAATAGTCAACACTATACTTTGGA
CTTCCTCTCGCCAAAGACCTTCC
>1264
AGTTCCACGCCCACCGGACGGTGCTGGCCTGCACCAGCAAGATGTTTGAGATCCTCTT
CCACCGCAATAGTCAACACTATACTTTGGACTTCCTCTCGCCAAAGACCTTCCAGCAGA
TTCTGGAGTATGCATATACAGCC
>1265
CAGCAAGATGTTTGAGATCCTCTTCCACCGCAATAGTCAACACTATACTTTGGACTTCC
TCTCGCCAAAGACCTTCCAGCAGATTCTGGAGTATGCATATACAGCCACGCTGCAAGCC
AAGGCGGAGGACCTGGATGACC
>1266
GTTTGAGATCCTCTTCCACCGCAATAGTCAACACTATACTTTGGACTTCCTCTCGCCAA
AGACCTTCCAGCAGATTCTGGAGTATGCATATACAGCCACGCTGCAAGCCAAGGCGGA
GGACCTGGATGACCTGCTGTATG
>1267
AGTCAACACTATACTTTGGACTTCCTCTCGCCAAAGACCTTCCAGCAGATTCTGGAGTA
TGCATATACAGCCACGCTGCAAGCCAAGGCGGAGGACCTGGATGACCTGCTGTATGCG
GCCGAGATCCTGGAGATCGAGTA
>1268
ACTTTGGACTTCCTCTCGCCAAAGACCTTCCAGCAGATTCTGGAGTATGCATATACAGC
CACGCTGCAAGCCAAGGCGGAGGACCTGGATGACCTGCTGTATGCGGCCGAGATCCTG
GAGATCGAGTACCTGGAGGAACA
>1269
GGACTTCCTCTCGCCAAAGACCTTCCAGCAGATTCTGGAGTATGCATATACAGCCACGC
TGCAAGCCAAGGCGGAGGACCTGGATGACCTGCTGTATGCGGCCGAGATCCTGGAGAT
CGAGTACCTGGAGGAACAGTGCC
>1270
GGAGTATGCATATACAGCCACGCTGCAAGCCAAGGCGGAGGACCTGGATGACCTGCTG
TATGCGGCCGAGATCCTGGAGATCGAGTACCTGGAGGAACAGTGCCTGAAGATGCTGG
AGACCATCCAGGCCTCAGACGACA
>1271
AGCCACGCTGCAAGCCAAGGCGGAGGACCTGGATGACCTGCTGTATGCGGCCGAGAT
CCTGGAGATCGAGTACCTGGAGGAACAGTGCCTGAAGATGCTGGAGACCATCCAGGC
CTCAGACGACAATGACACGGAGGCCA
>1272
GCAAGCCAAGGCGGAGGACCTGGATGACCTGCTGTATGCGGCCGAGATCCTGGAGATC

GAGTACCTGGAGGAACAGTGCCTGAAGATGCTGGAGACCATCCAGGCCTCAGACGAC
AATGACACGGAGGCCACCATGGCCG
>1273
CAAGGCGGAGGACCTGGATGACCTGCTGTATGCGGCCGAGATCCTGGAGATCGAGTAC
CTGGAGGAACAGTGCCTGAAGATGCTGGAGACCATCCAGGCCTCAGACGACAATGAC
ACGGAGGCCACCATGGCCGATGGCG
>1274
TGACCTGCTGTATGCGGCCGAGATCCTGGAGATCGAGTACCTGGAGGAACAGTGCCTG
AAGATGCTGGAGACCATCCAGGCCTCAGACGACAATGACACGGAGGCCACCATGGCC
GATGGCGGGGCCGAGGAAGAAGAGG
>1275
CGAGTACCTGGAGGAACAGTGCCTGAAGATGCTGGAGACCATCCAGGCCTCAGACGA
CAATGACACGGAGGCCACCATGGCCGATGGCGGGGCCGAGGAAGAAGAGGACCGCA
AGGCTCGGTACCTCAAGAACATCTTCA
>1276
GATGCTGGAGACCATCCAGGCCTCAGACGACAATGACACGGAGGCCACCATGGCCGA
TGGCGGGGCCGAGGAAGAAGAGGACCGCAAGGCTCGGTACCTCAAGAACATCTTCAT
CTCGAAGCATTCCAGCGAGGAGAGTG
>1277
GACCATCCAGGCCTCAGACGACAATGACACGGAGGCCACCATGGCCGATGGCGGGGC
CGAGGAAGAAGAGGACCGCAAGGCTCGGTACCTCAAGAACATCTTCATCTCGAAGCA
TTCCAGCGAGGAGAGTGGGTATGCCA
>1278
TGACACGGAGGCCACCATGGCCGATGGCGGGGCCGAGGAAGAAGAGGACCGCAAGG
CTCGGTACCTCAAGAACATCTTCATCTCGAAGCATTCCAGCGAGGAGAGTGGGTATGC
CAGTGTGGCTGGACAGAGCCTCCCTG
>1279
GGCCGATGGCGGGGCCGAGGAAGAAGAGGACCGCAAGGCTCGGTACCTCAAGAACAT
CTTCATCTCGAAGCATTCCAGCGAGGAGAGTGGGTATGCCAGTGTGGCTGGACAGAGC
CTCCCTGGGCCCATGGTGGACCAGA
>1280
GGCCGAGGAAGAAGAGGACCGCAAGGCTCGGTACCTCAAGAACATCTTCATCTCGAA
GCATTCCAGCGAGGAGAGTGGGTATGCCAGTGTGGCTGGACAGAGCCTCCCTGGGCC
CATGGTGGACCAGAGCCCTTCAGTCT
>1281
GTACCTCAAGAACATCTTCATCTCGAAGCATTCCAGCGAGGAGAGTGGGTATGCCAGT
GTGGCTGGACAGAGCCTCCCTGGGCCCATGGTGGACCAGAGCCCTTCAGTCTCCACTT
CATTTGGTCTTTCAGCCATGAGTC
>1282
ACCTCAAGAACATCTTCATCTCGAAGCATTCCAGCGAGGAGAGTGGGTATGCCAGTGT
GGCTGGACAGAGCCTCCCTGGGCCCATGGTGGACCAGAGCCCTTCAGTCTCCACTTCA
TTTGGTCTTTCAGCCATGAGTCCC
>1283
GCATTCCAGCGAGGAGAGTGGGTATGCCAGTGTGGCTGGACAGAGCCTCCCTGGGCC
CATGGTGGACCAGAGCCCTTCAGTCTCCACTTCATTTGGTCTTTCAGCCATGAGTCCCA
CCAAGGCTGCAGTGGACAGTTTGA

>1284
GCTGGACAGAGCCTCCCTGGGCCCATGGTGGACCAGAGCCCTTCAGTCTCCACTTCAT
TTGGTCTTTCAGCCATGAGTCCCACCAAGGCTGCAGTGGACAGTTTGATGACCATAGG
ACAGTCTCTCCTGCAGGGAACTCT
>1285
AGAGCCTCCCTGGGCCCATGGTGGACCAGAGCCCTTCAGTCTCCACTTCATTTGGTCTT
TCAGCCATGAGTCCCACCAAGGCTGCAGTGGACAGTTTGATGACCATAGGACAGTCTC
TCCTGCAGGGAACTCTTCAGCCA
>1286
TGGGCCCATGGTGGACCAGAGCCCTTCAGTCTCCACTTCATTTGGTCTTTCAGCCATGA
GTCCCACCAAGGCTGCAGTGGACAGTTTGATGACCATAGGACAGTCTCTCCTGCAGGG
AACTCTTCAGCCACCTGCAGGGC
>1287
GTCTCCACTTCATTTGGTCTTTCAGCCATGAGTCCCACCAAGGCTGCAGTGGACAGTTT
GATGACCATAGGACAGTCTCTCCTGCAGGGAACTCTTCAGCCACCTGCAGGGCCCGAG
GAGCCAACTCTGGCTGGGGGTGG
>1288
TCTTTCAGCCATGAGTCCCACCAAGGCTGCAGTGGACAGTTTGATGACCATAGGACAG
TCTCTCCTGCAGGGAACTCTTCAGCCACCTGCAGGGCCCGAGGAGCCAACTCTGGCTG
GGGGTGGGCGGCACCCTGGGGTGG
>1289
GAGTCCCACCAAGGCTGCAGTGGACAGTTTGATGACCATAGGACAGTCTCTCCTGCAG
GGAACTCTTCAGCCACCTGCAGGGCCCGAGGAGCCAACTCTGGCTGGGGGTGGGCGG
CACCCTGGGGTGGCTGAGGTGAAGA
>1290
AGTGGACAGTTTGATGACCATAGGACAGTCTCTCCTGCAGGGAACTCTTCAGCCACCT
GCAGGGCCCGAGGAGCCAACTCTGGCTGGGGGTGGGCGGCACCCTGGGGTGGCTGA
GGTGAAGACGGAGATGATGCAGGTGG
>1291
TCTTCAGCCACCTGCAGGGCCCGAGGAGCCAACTCTGGCTGGGGGTGGGCGGCACCC
TGGGGTGGCTGAGGTGAAGACGGAGATGATGCAGGTGGATGAGGTGCCCAGCCAGGA
CAGCCCTGGGGCAGCCGAGTCCAGCA
>1292
GCCACCTGCAGGGCCCGAGGAGCCAACTCTGGCTGGGGGTGGGCGGCACCCTGGGGT
GGCTGAGGTGAAGACGGAGATGATGCAGGTGGATGAGGTGCCCAGCCAGGACAGCCC
TGGGGCAGCCGAGTCCAGCATCTCAG
>1293
CTGGGGGTGGGCGGCACCCTGGGGTGGCTGAGGTGAAGACGGAGATGATGCAGGTGG
ATGAGGTGCCCAGCCAGGACAGCCCTGGGGCAGCCGAGTCCAGCATCTCAGGAGGGA
TGGGGGACAAGGTTGAGGAAAGAGGC
>1294
GGGTGGGCGGCACCCTGGGGTGGCTGAGGTGAAGACGGAGATGATGCAGGTGGATGA
GGTGCCCAGCCAGGACAGCCCTGGGGCAGCCGAGTCCAGCATCTCAGGAGGGATGGG
GGACAAGGTTGAGGAAAGAGGCAAAG
>1295
GGCGGCACCCTGGGGTGGCTGAGGTGAAGACGGAGATGATGCAGGTGGATGAGGTGC

CCAGCCAGGACAGCCCTGGGGCAGCCGAGTCCAGCATCTCAGGAGGGATGGGGGACA
AGGTTGAGGAAAGAGGCAAAGAGGGG
>1296
GGCTGAGGTGAAGACGGAGATGATGCAGGTGGATGAGGTGCCCAGCCAGGACAGCCC
TGGGGCAGCCGAGTCCAGCATCTCAGGAGGGATGGGGGACAAGGTTGAGGAAAGAG
GCAAAGAGGGGCCTGGGACCCCGACTC
>1297
GGATGAGGTGCCCAGCCAGGACAGCCCTGGGGCAGCCGAGTCCAGCATCTCAGGAGG
GATGGGGGACAAGGTTGAGGAAAGAGGCAAAGAGGGGCCTGGGACCCCGACTCGAA
GCAGCGTCATCACCAGTGCTAGGGAGC
>1298
GGTGCCCAGCCAGGACAGCCCTGGGGCAGCCGAGTCCAGCATCTCAGGAGGGATGGG
GGACAAGGTTGAGGAAAGAGGCAAAGAGGGGCCTGGGACCCCGACTCGAAGCAGCG
TCATCACCAGTGCTAGGGAGCTACACT
>1299
CCAGCCAGGACAGCCCTGGGGCAGCCGAGTCCAGCATCTCAGGAGGGATGGGGGACA
AGGTTGAGGAAAGAGGCAAAGAGGGGCCTGGGACCCCGACTCGAAGCAGCGTCATC
ACCAGTGCTAGGGAGCTACACTATGGG
>1300
GACAGCCCTGGGGCAGCCGAGTCCAGCATCTCAGGAGGGATGGGGGACAAGGTTGAG
GAAAGAGGCAAAGAGGGGCCTGGGACCCCGACTCGAAGCAGCGTCATCACCAGTGCT
AGGGAGCTACACTATGGGCGAGAGGA
>1301
GCATCTCAGGAGGGATGGGGGACAAGGTTGAGGAAAGAGGCAAAGAGGGGCCTGGG
ACCCCGACTCGAAGCAGCGTCATCACCAGTGCTAGGGAGCTACACTATGGGCGAGAG
GAGAGTGCCGAGCAGGTGCCACCCCCA
>1302
ACAAGGTTGAGGAAAGAGGCAAAGAGGGGCCTGGGACCCCGACTCGAAGCAGCGTC
ATCACCAGTGCTAGGGAGCTACACTATGGGCGAGAGGAGAGTGCCGAGCAGGTGCCA
CCCCCAGCTGAGGCTGGCCAGGCCCCC
>1303
GGTTGAGGAAAGAGGCAAAGAGGGGCCTGGGACCCCGACTCGAAGCAGCGTCATCA
CCAGTGCTAGGGAGCTACACTATGGGCGAGAGGAGAGTGCCGAGCAGGTGCCACCCC
CAGCTGAGGCTGGCCAGGCCCCCACTG
>1304
AAAGAGGGGCCTGGGACCCCGACTCGAAGCAGCGTCATCACCAGTGCTAGGGAGCTA
CACTATGGGCGAGAGGAGAGTGCCGAGCAGGTGCCACCCCCAGCTGAGGCTGGCCAG
GCCCCCACTGGCCGACCTGAGCACCC
>1305
GACCCCGACTCGAAGCAGCGTCATCACCAGTGCTAGGGAGCTACACTATGGGCGAGA
GGAGAGTGCCGAGCAGGTGCCACCCCCAGCTGAGGCTGGCCAGGCCCCCACTGGCCG
ACCTGAGCACCCAGCACCCCCGCCTG
>1306
TGCTAGGGAGCTACACTATGGGCGAGAGGAGAGTGCCGAGCAGGTGCCACCCCCAGC
TGAGGCTGGCCAGGCCCCCACTGGCCGACCTGAGCACCCAGCACCCCCGCCTGAGAA
GCATCTGGGCATCTACTCCGTGTTGC

>1307
GCGAGAGGAGAGTGCCGAGCAGGTGCCACCCCCAGCTGAGGCTGGCCAGGCCCCCA
CTGGCCGACCTGAGCACCCAGCACCCCCGCCTGAGAAGCATCTGGGCATCTACTCCGT
GTTGCCCAACCACAAGGCTGACGCTG
>1308
GGTGCCACCCCCAGCTGAGGCTGGCCAGGCCCCCACTGGCCGACCTGAGCACCCAGC
ACCCCCGCCTGAGAAGCATCTGGGCATCTACTCCGTGTTGCCCAACCACAAGGCTGAC
GCTGTATTGAGCATGCCGTCTTCCG
>1309
CCGACCTGAGCACCCAGCACCCCCGCCTGAGAAGCATCTGGGCATCTACTCCGTGTTG
CCCAACCACAAGGCTGACGCTGTATTGAGCATGCCGTCTTCCGTGACCTCTGGCCTCC
ACGTGCAGCCTGCCCTGGCTGTCT
>1310
CACCCCCGCCTGAGAAGCATCTGGGCATCTACTCCGTGTTGCCCAACCACAAGGCTGA
CGCTGTATTGAGCATGCCGTCTTCCGTGACCTCTGGCCTCCACGTGCAGCCTGCCCTGG
CTGTCTCCATGGACTTCAGCACC
>1311
GTTGCCCAACCACAAGGCTGACGCTGTATTGAGCATGCCGTCTTCCGTGACCTCTGGC
CTCCACGTGCAGCCTGCCCTGGCTGTCTCCATGGACTTCAGCACCTATGGGGGGCTGC
TGCCCCAGGGCTTCATCCAGAGGG
>1312
TGAGCATGCCGTCTTCCGTGACCTCTGGCCTCCACGTGCAGCCTGCCCTGGCTGTCTCC
ATGGACTTCAGCACCTATGGGGGGCTGCTGCCCCAGGGCTTCATCCAGAGGGAGCTGT
TCAGCAAGCTGGGGGAGCTGGCT
>1313
TGGCCTCCACGTGCAGCCTGCCCTGGCTGTCTCCATGGACTTCAGCACCTATGGGGGG
CTGCTGCCCCAGGGCTTCATCCAGAGGGAGCTGTTCAGCAAGCTGGGGGAGCTGGCT
GTGGGCATGAAGTCAGAGAGCCGGA
>1314
GCAGCCTGCCCTGGCTGTCTCCATGGACTTCAGCACCTATGGGGGGCTGCTGCCCCAG
GGCTTCATCCAGAGGGAGCTGTTCAGCAAGCTGGGGGAGCTGGCTGTGGGCATGAAG
TCAGAGAGCCGGACCATCGGAGAGC
>1315
CTCCATGGACTTCAGCACCTATGGGGGGCTGCTGCCCCAGGGCTTCATCCAGAGGGAG
CTGTTCAGCAAGCTGGGGGAGCTGGCTGTGGGCATGAAGTCAGAGAGCCGGACCATC
GGAGAGCAGTGCAGCGTGTGTGGGG
>1316
GCTGCTGCCCCAGGGCTTCATCCAGAGGGAGCTGTTCAGCAAGCTGGGGGAGCTGGC
TGTGGGCATGAAGTCAGAGAGCCGGACCATCGGAGAGCAGTGCAGCGTGTGTGGGGT
CGAGCTTCCTGATAACGAGGCTGTGG
>1317
CTGCCCCAGGGCTTCATCCAGAGGGAGCTGTTCAGCAAGCTGGGGGAGCTGGCTGTG
GGCATGAAGTCAGAGAGCCGGACCATCGGAGAGCAGTGCAGCGTGTGTGGGGTCGAG
CTTCCTGATAACGAGGCTGTGGAGCA
>1318
CAGAGGGAGCTGTTCAGCAAGCTGGGGGAGCTGGCTGTGGGCATGAAGTCAGAGAGC

CGGACCATCGGAGAGCAGTGCAGCGTGTGTGGGGTCGAGCTTCCTGATAACGAGGCT
GTGGAGCAGCACAGGTAGGCCCCGCT
>1319
GGAGCTGGCTGTGGGCATGAAGTCAGAGAGCCGGACCATCGGAGAGCAGTGCAGCGT
GTGTGGGGTCGAGCTTCCTGATAACGAGGCTGTGGAGCAGCACAGGTAGGCCCCGCT
CCAGCCCCGCACCTGATGTAGGACTT
>1320
CATGAAGTCAGAGAGCCGGACCATCGGAGAGCAGTGCAGCGTGTGTGGGGTCGAGCT
TCCTGATAACGAGGCTGTGGAGCAGCACAGGTAGGCCCCGCTCCAGCCCCGCACCTGA
TGTAGGACTTGAGGCCCTCACACCC
>1321
AGAGAGCCGGACCATCGGAGAGCAGTGCAGCGTGTGTGGGGTCGAGCTTCCTGATAA
CGAGGCTGTGGAGCAGCACAGGTAGGCCCCGCTCCAGCCCCGCACCTGATGTAGGAC
TTGAGGCCCTCACACCCCTCCTTCAC
>1322
GTGCAGCGTGTGTGGGGTCGAGCTTCCTGATAACGAGGCTGTGGAGCAGCACAGGTA
GGCCCCGCTCCAGCCCCGCACCTGATGTAGGACTTGAGGCCCTCACACCCCTCCTTCA
CACCCTGGCTGCTCCCAAGTTAGGG
>1323
TCGAGCTTCCTGATAACGAGGCTGTGGAGCAGCACAGGTAGGCCCCGCTCCAGCCCCG
CACCTGATGTAGGACTTGAGGCCCTCACACCCCTCCTTCACACCCTGGCTGCTCCCAA
GTTAGGGGCCTGGCTCCCCTGTCT
>1324
CCTGATAACGAGGCTGTGGAGCAGCACAGGTAGGCCCCGCTCCAGCCCCGCACCTGAT
GTAGGACTTGAGGCCCTCACACCCCTCCTTCACACCCTGGCTGCTCCCAAGTTAGGGG
CCTGGCTCCCCTGTCTTGGCAATT
>1325
CTCCAGCCCCGCACCTGATGTAGGACTTGAGGCCCTCACACCCCTCCTTCACACCCTG
GCTGCTCCCAAGTTAGGGGCCTGGCTCCCCTGTCTTGGCAATTTGTGAAAAAAACCAGA
ACACTTCTTCTAAAGTTCTGGCGG
>1326
CCTTCACACCCTGGCTGCTCCCAAGTTAGGGGCCTGGCTCCCCTGTCTTGGCAATTTGT
GAAAAAACCAGAACACTTCTTCTAAAGTTCTGGCGGGGAGGGGAGCAGGTTTTTTAA
AGTGTAGTGAGGGGGCCTAGGATC
>1327
GCTCCCAAGTTAGGGGCCTGGCTCCCCTGTCTTGGCAATTTGTGAAAAAACCAGAACA
CTTCTTCTAAAGTTCTGGCGGGGAGGGGAGCAGGTTTTTTAAAGTGTAGTGAGGGGGC
CTAGGATCTTTCCAAAAAGCACCA
>1328
GGGCCTGGCTCCCCTGTCTTGGCAATTTGTGAAAAAACCAGAACACTTCTTCTAAAGT
TCTGGCGGGGAGGGGAGCAGGTTTTTTAAAGTGTAGTGAGGGGGCCTAGGATCTTTCC
AAAAAGCACCAGGGGACACTCATG
>1329
TGGCTCCCCTGTCTTGGCAATTTGTGAAAAAACCAGAACACTTCTTCTAAAGTTCTGG
CGGGGAGGGGAGCAGGTTTTTTAAAGTGTAGTGAGGGGGCCTAGGATCTTTCCAAAA
AGCACCAGGGGACACTCATGGGCGC

>1330
TGGCAATTTGTGAAAAAACCAGAACACTTCTTCTAAAGTTCTGGCGGGGAGGGGAGC
AGGTTTTTTAAAGTGTAGTGAGGGGGCCTAGGATCTTTCCAAAAAGCACCAGGGGACA
CTCATGGGCGCAGCTTCTTAATAGG
>1331
TCTGGCGGGGAGGGGAGCAGGTTTTTTAAAGTGTAGTGAGGGGGCCTAGGATCTTTCC
AAAAAGCACCAGGGGACACTCATGGGCGCAGCTTCTTAATAGGCCCTTCCCTTTGTTT
TTTGCTTTTGGGCCCCTGTTTGTT
>1332
GGTTTTTTAAAGTGTAGTGAGGGGGCCTAGGATCTTTCCAAAAAGCACCAGGGGACAC
TCATGGGCGCAGCTTCTTAATAGGCCCTTCCCTTTGTTTTTTGCTTTTGGGCCCCTGTTT
GTTTTTTCTGCTGTTTGGTCTG
>1333
GTGTAGTGAGGGGGCCTAGGATCTTTCCAAAAAGCACCAGGGGACACTCATGGGCGC
AGCTTCTTAATAGGCCCTTCCCTTTGTTTTTTGCTTTTGGGCCCCTGTTTGTTTTTTCTGC
TGTTTGGTCTGTTCTCCTTTGC
>1334
CCCTTTGTTTTTTGCTTTTGGGCCCCTGTTTGTTTTTTCTGCTGTTTGGTCTGTTCTCCTT
TGCTTGGAGTGACTGATAAAATGGAGAGAAGGAGAAAAGGAGTGGGATGAGGGTCTC
TTGGGCCCTGCATCTGACCATG
>1335
TTGGGCCCCTGTTTGTTTTTTCTGCTGTTTGGTCTGTTCTCCTTTGCTTGGAGTGACTGA
TAAAATGGAGAGAAGGAGAAAAGGAGTGGGATGAGGGTCTCTTGGGCCCTGCATCTG
ACCATGTTACTAGGTTTCAGGTC
>1336
GGGCCCCTGTTTGTTTTTTCTGCTGTTTGGTCTGTTCTCCTTTGCTTGGAGTGACTGATA
AAATGGAGAGAAGGAGAAAAGGAGTGGGATGAGGGTCTCTTGGGCCCTGCATCTGAC
CATGTTACTAGGTTTCAGGTCCT
>1337
TGTTTTTTCTGCTGTTTGGTCTGTTCTCCTTTGCTTGGAGTGACTGATAAAATGGAGAG
AAGGAGAAAAGGAGTGGGATGAGGGTCTCTTGGGCCCTGCATCTGACCATGTTACTAG
GTTTCAGGTCCTCGGGTTCCCAT
>1338
TAAAATGGAGAGAAGGAGAAAAGGAGTGGGATGAGGGTCTCTTGGGCCCTGCATCTG
ACCATGTTACTAGGTTTCAGGTCCTCGGGTTCCCATGCAGTGGTCCCTGTGAAGACAG
CCTGTCCCTTTCTGCAGCTTGGGAG
>1339
GAGAGAAGGAGAAAAGGAGTGGGATGAGGGTCTCTTGGGCCCTGCATCTGACCATGT
TACTAGGTTTCAGGTCCTCGGGTTCCCATGCAGTGGTCCCTGTGAAGACAGCCTGTCC
CTTTCTGCAGCTTGGGAGAATGGGA
>1340
GGGATGAGGGTCTCTTGGGCCCTGCATCTGACCATGTTACTAGGTTTCAGGTCCTCGGG
TTCCCATGCAGTGGTCCCTGTGAAGACAGCCTGTCCCTTTCTGCAGCTTGGGAGAATG
GGAGGGTCTGGGGGCTGCTTAAG
>1341
GTTACTAGGTTTCAGGTCCTCGGGTTCCCATGCAGTGGTCCCTGTGAAGACAGCCTGT

CCCTTTCTGCAGCTTGGGAGAATGGGAGGGTCTGGGGGCTGCTTAAGCCACAAGAGC
AGCTGCAGGTTCTGAACCTTATTCC
>1342
GTTTCAGGTCCTCGGGTTCCCATGCAGTGGTCCCTGTGAAGACAGCCTGTCCCTTTCTG
CAGCTTGGGAGAATGGGAGGGTCTGGGGGCTGCTTAAGCCACAAGAGCAGCTGCAGG
TTCTGAACCTTATTCCTGGTGAAA
>1343
TGGTCCCTGTGAAGACAGCCTGTCCCTTTCTGCAGCTTGGGAGAATGGGAGGGTCTGG
GGGCTGCTTAAGCCACAAGAGCAGCTGCAGGTTCTGAACCTTATTCCTGGTGAAAGTT
AATCTCCTTGCTCTGTCTCCCTTC
>1344
CCTGTCCCTTTCTGCAGCTTGGGAGAATGGGAGGGTCTGGGGGCTGCTTAAGCCACAA
GAGCAGCTGCAGGTTCTGAACCTTATTCCTGGTGAAAGTTAATCTCCTTGCTCTGTCTC
CCTTCTCATTCTCTGATCCTGCA
>1345
ATGGGAGGGTCTGGGGGCTGCTTAAGCCACAAGAGCAGCTGCAGGTTCTGAACCTTAT
TCCTGGTGAAAGTTAATCTCCTTGCTCTGTCTCCCTTCTCATTCTCTGATCCTGCACATG
CATTTAGGGGGAATTGATTTAA
>1346
TATTCCTGGTGAAAGTTAATCTCCTTGCTCTGTCTCCCTTCTCATTCTCTGATCCTGCAC
ATGCATTTAGGGGGAATTGATTTAAATGCAAGGGGAAAGGATGGGTATAAGAGTCTTG
GTCTTGCAGCCTGTGAAGTCTC
>1347
CCTTGCTCTGTCTCCCTTCTCATTCTCTGATCCTGCACATGCATTTAGGGGGAATTGATT
TAAATGCAAGGGGAAAGGATGGGTATAAGAGTCTTGGTCTTGCAGCCTGTGAAGTCTC
ATTCTTATCCCATCTCTGCTCA
>1348
TCATTCTCTGATCCTGCACATGCATTTAGGGGGAATTGATTTAAATGCAAGGGGAAAGG
ATGGGTATAAGAGTCTTGGTCTTGCAGCCTGTGAAGTCTCATTCTTATCCCATCTCTGCT
CACACTGAACTAAATGCCGCT
>1349
ATTCTCTGATCCTGCACATGCATTTAGGGGGAATTGATTTAAATGCAAGGGGAAAGGAT
GGGTATAAGAGTCTTGGTCTTGCAGCCTGTGAAGTCTCATTCTTATCCCATCTCTGCTCA
CACTGAACTAAATGCCGCTTC
>1350
CACATGCATTTAGGGGGAATTGATTTAAATGCAAGGGGAAAGGATGGGTATAAGAGTC
TTGGTCTTGCAGCCTGTGAAGTCTCATTCTTATCCCATCTCTGCTCACACTGAACTAAAT
GCCGCTTCAGGCTAGCATTGGC
>1351
TTGGTCTTGCAGCCTGTGAAGTCTCATTCTTATCCCATCTCTGCTCACACTGAACTAAAT
GCCGCTTCAGGCTAGCATTGGCTTCCCTCCGGCTTCCGTTTCCTTCTCTATGAAATAACC
ATTCATTGAGCTCTTATGAC
>1352
CTTGCAGCCTGTGAAGTCTCATTCTTATCCCATCTCTGCTCACACTGAACTAAATGCCG
CTTCAGGCTAGCATTGGCTTCCCTCCGGCTTCCGTTTCCTTCTCTATGAAATAACCATTC
ATTGAGCTCTTATGACATGTT

>1353
GCCGCTTCAGGCTAGCATTGGCTTCCCTCCGGCTTCCGTTTCCTTCTCTATGAAATAACC
ATTCATTGAGCTCTTATGACATGTTTGGTTCAGTACTAAGCACTTTCCATGAGTTGCATT
TTCATTACAACAGCCCTTTG
>1354
TCCCTCCGGCTTCCGTTTCCTTCTCTATGAAATAACCATTCATTGAGCTCTTATGACATG
TTTGGTTCAGTACTAAGCACTTTCCATGAGTTGCATTTTCATTACAACAGCCCTTTGAGA
TAGGTAATGTCCTTCTTTTT
>1355
TTCTCTATGAAATAACCATTCATTGAGCTCTTATGACATGTTTGGTTCAGTACTAAGCAC
TTTCCATGAGTTGCATTTTCATTACAACAGCCCTTTGAGATAGGTAATGTCCTTCTTTTT
TTTTTGGATGTGAAGTTTCG
>1356
CATTGAGCTCTTATGACATGTTTGGTTCAGTACTAAGCACTTTCCATGAGTTGCATTTTC
ATTACAACAGCCCTTTGAGATAGGTAATGTCCTTCTTTTTTTTTTGGATGTGAAGTTTCG
CTCTCCTTGCCCAGGCTGGA
>1357
CTTATGACATGTTTGGTTCAGTACTAAGCACTTTCCATGAGTTGCATTTTCATTACAACA
GCCCTTTGAGATAGGTAATGTCCTTCTTTTTTTTTTGGATGTGAAGTTTCGCTCTCCTTG
CCCAGGCTGGAGTGCAATGG
>1358
ATGAGTTGCATTTTCATTACAACAGCCCTTTGAGATAGGTAATGTCCTTCTTTTTTTTTT
GGATGTGAAGTTTCGCTCTCCTTGCCCAGGCTGGAGTGCAATGGTGTGATCTCTGCTCA
CCGCAACCTCTGCATCCCGGG
>1359
CAACAGCCCTTTGAGATAGGTAATGTCCTTCTTTTTTTTTTGGATGTGAAGTTTCGCTCT
CCTTGCCCAGGCTGGAGTGCAATGGTGTGATCTCTGCTCACCGCAACCTCTGCATCCC
GGGTTAAGGTGATTCTCCTGCC
>1360
CTCTCCTTGCCCAGGCTGGAGTGCAATGGTGTGATCTCTGCTCACCGCAACCTCTGCAT
CCCGGGTTAAGGTGATTCTCCTGCCTCAGCCTCCCGAGTAGCTCGGATTACAGGCACG
CACCACCATACCTGGCTAATTTT
>1361
GAGTGCAATGGTGTGATCTCTGCTCACCGCAACCTCTGCATCCCGGGTTAAGGTGATTC
TCCTGCCTCAGCCTCCCGAGTAGCTCGGATTACAGGCACGCACCACCATACCTGGCTAA
TTTTGAATTTTTAGTAGAGACG
>1362
CACCGCAACCTCTGCATCCCGGGTTAAGGTGATTCTCCTGCCTCAGCCTCCCGAGTAGC
TCGGATTACAGGCACGCACCACCATACCTGGCTAATTTTGAATTTTTAGTAGAGACGGG
CTTTCACCGTGTTGGTCAGGAT
>1363
TGCCTCAGCCTCCCGAGTAGCTCGGATTACAGGCACGCACCACCATACCTGGCTAATTT
TGAATTTTTAGTAGAGACGGGCTTTCACCGTGTTGGTCAGGATGGTCTCGAACTCCCG
ACCTCAGGTGATCGGCCTGCTTC
>1364
CAGGCACGCACCACCATACCTGGCTAATTTTGAATTTTTAGTAGAGACGGGCTTTCACC

GTGTTGGTCAGGATGGTCTCGAACTCCCGACCTCAGGTGATCGGCCTGCTTCGGCCTC
CCAAAGTGCTGGATTACAGGTAT
>1365
AATTTTGAATTTTTAGTAGAGACGGGCTTTCACCGTGTTGGTCAGGATGGTCTCGAACT
CCCGACCTCAGGTGATCGGCCTGCTTCGGCCTCCCAAAGTGCTGGATTACAGGTATGA
GCCACCGTGTCCAGCTGGTAATG
>1366
TTCACCGTGTTGGTCAGGATGGTCTCGAACTCCCGACCTCAGGTGATCGGCCTGCTTC
GGCCTCCCAAAGTGCTGGATTACAGGTATGAGCCACCGTGTCCAGCTGGTAATGTCCTT
CTTTTACATGGAACAATACTAAG
>1367
TCAGGATGGTCTCGAACTCCCGACCTCAGGTGATCGGCCTGCTTCGGCCTCCCAAAGT
GCTGGATTACAGGTATGAGCCACCGTGTCCAGCTGGTAATGTCCTTCTTTTACATGGAA
CAATACTAAGGCTCAGATGTCAA
>1368
GGTCTCGAACTCCCGACCTCAGGTGATCGGCCTGCTTCGGCCTCCCAAAGTGCTGGAT
TACAGGTATGAGCCACCGTGTCCAGCTGGTAATGTCCTTCTTTTACATGGAACAATACT
AAGGCTCAGATGTCAAGTCTCTT
>1369
TGCTGGATTACAGGTATGAGCCACCGTGTCCAGCTGGTAATGTCCTTCTTTTACATGGA
ACAATACTAAGGCTCAGATGTCAAGTCTCTTTTCTAGGGTCACATAGATAGTAAGAGGC
AGAGCTGGATTCAAATTTAGGT
>1370
ATTACAGGTATGAGCCACCGTGTCCAGCTGGTAATGTCCTTCTTTTACATGGAACAATA
CTAAGGCTCAGATGTCAAGTCTCTTTTCTAGGGTCACATAGATAGTAAGAGGCAGAGCT
GGATTCAAATTTAGGTCCTTAC
>1371
TGTCCAGCTGGTAATGTCCTTCTTTTACATGGAACAATACTAAGGCTCAGATGTCAAGT
CTCTTTTCTAGGGTCACATAGATAGTAAGAGGCAGAGCTGGATTCAAATTTAGGTCCTT
ACCCCCTGGTACTTTGCTGCCT
>1372
TCTTTTACATGGAACAATACTAAGGCTCAGATGTCAAGTCTCTTTTCTAGGGTCACATA
GATAGTAAGAGGCAGAGCTGGATTCAAATTTAGGTCCTTACCCCCTGGTACTTTGCTGC
CTCTTGGAGTCACTTAGACTTG
>1373
TTACATGGAACAATACTAAGGCTCAGATGTCAAGTCTCTTTTCTAGGGTCACATAGATA
GTAAGAGGCAGAGCTGGATTCAAATTTAGGTCCTTACCCCCTGGTACTTTGCTGCCTCT
TGGAGTCACTTAGACTTGAAGA
>1374
ACATGGAACAATACTAAGGCTCAGATGTCAAGTCTCTTTTCTAGGGTCACATAGATAGT
AAGAGGCAGAGCTGGATTCAAATTTAGGTCCTTACCCCCTGGTACTTTGCTGCCTCTTG
GAGTCACTTAGACTTGAAGAGG
>1375
AGGCTCAGATGTCAAGTCTCTTTTCTAGGGTCACATAGATAGTAAGAGGCAGAGCTGG
ATTCAAATTTAGGTCCTTACCCCCTGGTACTTTGCTGCCTCTTGGAGTCACTTAGACTTG
AAGAGGATCATGTCTATGGAAC

>1376
TAGATAGTAAGAGGCAGAGCTGGATTCAAATTTAGGTCCTTACCCCCTGGTACTTTGCT
GCCTCTTGGAGTCACTTAGACTTGAAGAGGATCATGTCTATGGAACAGAGCGTTTTGA
AGATTGCTTATAGGAAGTGGAGT
>1377
AAGAGGCAGAGCTGGATTCAAATTTAGGTCCTTACCCCCTGGTACTTTGCTGCCTCTTG
GAGTCACTTAGACTTGAAGAGGATCATGTCTATGGAACAGAGCGTTTTGAAGATTGCTT
ATAGGAAGTGGAGTAAATAACA
>1378
GAGCTGGATTCAAATTTAGGTCCTTACCCCCTGGTACTTTGCTGCCTCTTGGAGTCACT
TAGACTTGAAGAGGATCATGTCTATGGAACAGAGCGTTTTGAAGATTGCTTATAGGAAG
TGGAGTAAATAACAGGGGGGAT
>1379
CCTTACCCCCTGGTACTTTGCTGCCTCTTGGAGTCACTTAGACTTGAAGAGGATCATGT
CTATGGAACAGAGCGTTTTGAAGATTGCTTATAGGAAGTGGAGTAAATAACAGGGGGG
ATAAGTAGCGAGTTTGTTTTTTT
>1380
CCTCTTGGAGTCACTTAGACTTGAAGAGGATCATGTCTATGGAACAGAGCGTTTTGAA
GATTGCTTATAGGAAGTGGAGTAAATAACAGGGGGGATAAGTAGCGAGTTTGTTTTTTT
TTTCCTTGCTTCTGTCATAAACC
>1381
TTGGAGTCACTTAGACTTGAAGAGGATCATGTCTATGGAACAGAGCGTTTTGAAGATT
GCTTATAGGAAGTGGAGTAAATAACAGGGGGGATAAGTAGCGAGTTTGTTTTTTTTTTC
CTTGCTTCTGTCATAAACCACGT
>1382
GTCACTTAGACTTGAAGAGGATCATGTCTATGGAACAGAGCGTTTTGAAGATTGCTTAT
AGGAAGTGGAGTAAATAACAGGGGGGATAAGTAGCGAGTTTGTTTTTTTTTTCCTTGCT
TCTGTCATAAACCACGTGCCCA
>1383
CTTGAAGAGGATCATGTCTATGGAACAGAGCGTTTTGAAGATTGCTTATAGGAAGTGG
AGTAAATAACAGGGGGGATAAGTAGCGAGTTTGTTTTTTTTTTCCTTGCTTCTGTCATAA
ACCACGTGCCCATCATTTTATC
>1384
ATCATGTCTATGGAACAGAGCGTTTTGAAGATTGCTTATAGGAAGTGGAGTAAATAACA
GGGGGGATAAGTAGCGAGTTTGTTTTTTTTTTCCTTGCTTCTGTCATAAACCACGTGCC
CATCATTTTATCCTCTAGTTTC
>1385
TAAGTAGCGAGTTTGTTTTTTTTTTCCTTGCTTCTGTCATAAACCACGTGCCCATCATTT
TATCCTCTAGTTTCTGGGTCAGATGAATGGAGTCAAGAAATCCAGCTTTCTGTTGTCAT
TGACGACAGCATTTGGCTGCA
>1386
TTGTTTTTTTTTTCCTTGCTTCTGTCATAAACCACGTGCCCATCATTTTATCCTCTAGTTT
CTGGGTCAGATGAATGGAGTCAAGAAATCCAGCTTTCTGTTGTCATTGACGACAGCAT
TTGGCTGCATACCCTGTGGTC
>1387
GTCATAAACCACGTGCCCATCATTTTATCCTCTAGTTTCTGGGTCAGATGAATGGAGTCA

AGAAATCCAGCTTTCTGTTGTCATTGACGACAGCATTTGGCTGCATACCCTGTGGTCTG
CTGTTTTGCTGATCTTGGGTG
>1388
TTTATCCTCTAGTTTCTGGGTCAGATGAATGGAGTCAAGAAATCCAGCTTTCTGTTGTC
ATTGACGACAGCATTTGGCTGCATACCCTGTGGTCTGCTGTTTTGCTGATCTTGGGTGG
ATTCTCCACTTCCTCCCCCTTC
>1389
TGTTTTGCTGATCTTGGGTGGATTCTCCACTTCCTCCCCCTTCACCCCCATTCCCCTGTT
GCTCGTTCAGGTACGGGGGATGTGAGTGAGAGGGGTTGGGGTGGGGATATCTGGAAG
TTGATCTCATCACCCCTAGCCGG
>1390
GGTGGATTCTCCACTTCCTCCCCCTTCACCCCCATTCCCCTGTTGCTCGTTCAGGTACG
GGGGATGTGAGTGAGAGGGGTTGGGGTGGGGATATCTGGAAGTTGATCTCATCACCCC
TAGCCGGAATCTGCTCTGACCCT
>1391
CCATTCCCCTGTTGCTCGTTCAGGTACGGGGGATGTGAGTGAGAGGGGTTGGGGTGGG
GATATCTGGAAGTTGATCTCATCACCCCTAGCCGGAATCTGCTCTGACCCTGCCTCCTC
GACAGCTCAGAGGCTTAACTTGT
>1392
TCAGGTACGGGGGATGTGAGTGAGAGGGGTTGGGGTGGGGATATCTGGAAGTTGATCT
CATCACCCCTAGCCGGAATCTGCTCTGACCCTGCCTCCTCGACAGCTCAGAGGCTTAA
CTTGTGGAAAGTGTGGCAGAGCTA
>1393
GGTGGGGATATCTGGAAGTTGATCTCATCACCCCTAGCCGGAATCTGCTCTGACCCTGC
CTCCTCGACAGCTCAGAGGCTTAACTTGTGGAAAGTGTGGCAGAGCTAAACGTAGCCG
ACTTTTTCCAGTGGGTGCGAAAA
>1394
TCATCACCCCTAGCCGGAATCTGCTCTGACCCTGCCTCCTCGACAGCTCAGAGGCTTAA
CTTGTGGAAAGTGTGGCAGAGCTAAACGTAGCCGACTTTTTCCAGTGGGTGCGAAAAA
TAACCGCCACTAAGGTGTGTCTT
>1395
CCTAGCCGGAATCTGCTCTGACCCTGCCTCCTCGACAGCTCAGAGGCTTAACTTGTGG
AAAGTGTGGCAGAGCTAAACGTAGCCGACTTTTTCCAGTGGGTGCGAAAAATAACCGC
CACTAAGGTGTGTCTTCTTGATGC
>1396
CTGCTCTGACCCTGCCTCCTCGACAGCTCAGAGGCTTAACTTGTGGAAAGTGTGGCAG
AGCTAAACGTAGCCGACTTTTTCCAGTGGGTGCGAAAAATAACCGCCACTAAGGTGTG
TCTTCTTGATGCTTGTACCGCTCA
>1397
TGTGGAAAGTGTGGCAGAGCTAAACGTAGCCGACTTTTTCCAGTGGGTGCGAAAAATA
ACCGCCACTAAGGTGTGTCTTCTTGATGCTTGTACCGCTCAGAAATAGACTGAGGTTAT
GTTTGTTTTTGCTCAAACTTTCC
>1398
CGACTTTTTCCAGTGGGTGCGAAAAATAACCGCCACTAAGGTGTGTCTTCTTGATGCTT
GTACCGCTCAGAAATAGACTGAGGTTATGTTTGTTTTTGCTCAAACTTTCCTAAAGAGG
AAACCCGCTCCCCAAAGTTGAC

>1399
TCTTCTTGATGCTTGTACCGCTCAGAAATAGACTGAGGTTATGTTTGTTTTTGCTCAAAC
TTTCCTAAAGAGGAAACCCGCTCCCCAAAGTTGACCAGATAGATGTATCATCTTTCCGG
ATGTGAATTATTTCGGCTGTG
>1400
TTCTTGATGCTTGTACCGCTCAGAAATAGACTGAGGTTATGTTTGTTTTTGCTCAAACTT
TCCTAAAGAGGAAACCCGCTCCCCAAAGTTGACCAGATAGATGTATCATCTTTCCGGAT
GTGAATTATTTCGGCTGTGTG
>1401
CTGAGGTTATGTTTGTTTTTGCTCAAACTTTCCTAAAGAGGAAACCCGCTCCCCAAAGT
TGACCAGATAGATGTATCATCTTTCCGGATGTGAATTATTTCGGCTGTGTGCTATGAGAA
CTGGGTATGGTGGGAAGTAAT
>1402
GCTCCCCAAAGTTGACCAGATAGATGTATCATCTTTCCGGATGTGAATTATTTCGGCTGT
GTGCTATGAGAACTGGGTATGGTGGGAAGTAATTGTTTTTCTGGCTCTCATCAGGTCCT
GGTTCCTGGTTGCTTTTCTTT
>1403
ATAGATGTATCATCTTTCCGGATGTGAATTATTTCGGCTGTGTGCTATGAGAACTGGGTA
TGGTGGGAAGTAATTGTTTTTCTGGCTCTCATCAGGTCCTGGTTCCTGGTTGCTTTTCTT
TACCCGAGAGGGCCACAAAA
>1404
GAATTATTTCGGCTGTGTGCTATGAGAACTGGGTATGGTGGGAAGTAATTGTTTTTCTG
GCTCTCATCAGGTCCTGGTTCCTGGTTGCTTTTCTTTACCCGAGAGGGCCACAAAAGAT
ACCACTCTCCTGAGACTTCCAA
>1405
TATGGTGGGAAGTAATTGTTTTTCTGGCTCTCATCAGGTCCTGGTTCCTGGTTGCTTTTC
TTTACCCGAGAGGGCCACAAAAGATACCACTCTCCTGAGACTTCCAAGTTCCCCTTCA
GCAAGACCCTCATTCCTCTGGG
>1406
TGGTGGGAAGTAATTGTTTTTCTGGCTCTCATCAGGTCCTGGTTCCTGGTTGCTTTTCTT
TACCCGAGAGGGCCACAAAAGATACCACTCTCCTGAGACTTCCAAGTTCCCCTTCAGC
AAGACCCTCATTCCTCTGGGGA
>1407
AATTGTTTTTCTGGCTCTCATCAGGTCCTGGTTCCTGGTTGCTTTTCTTTACCCGAGAGG
GCCACAAAAGATACCACTCTCCTGAGACTTCCAAGTTCCCCTTCAGCAAGACCCTCAT
TCCTCTGGGGAGAGAGGGAGAG
>1408
CTCATCAGGTCCTGGTTCCTGGTTGCTTTTCTTTACCCGAGAGGGCCACAAAAGATACC
ACTCTCCTGAGACTTCCAAGTTCCCCTTCAGCAAGACCCTCATTCCTCTGGGGAGAGA
GGGAGAGTTGGCCATGTTATATT
>1409
GCCACAAAAGATACCACTCTCCTGAGACTTCCAAGTTCCCCTTCAGCAAGACCCTCAT
TCCTCTGGGGAGAGAGGGAGAGTTGGCCATGTTATATTCGAAAGAGCCTGGGTATTGG
AATTGGCCATATTTAGTGGTGAGA
>1410
CACAAAAGATACCACTCTCCTGAGACTTCCAAGTTCCCCTTCAGCAAGACCCTCATTC

CTCTGGGGAGAGAGGGAGAGTTGGCCATGTTATATTCGAAAGAGCCTGGGTATTGGAA
TTGGCCATATTTAGTGGTGAGAGC
>1411
AAGTTCCCCTTCAGCAAGACCCTCATTCCTCTGGGGAGAGAGGGAGAGTTGGCCATGT
TATATTCGAAAGAGCCTGGGTATTGGAATTGGCCATATTTAGTGGTGAGAGCCTGTGAT
ACTTATTTAACCTTTCTGGGCCT
>1412
CTGGGGAGAGAGGGAGAGTTGGCCATGTTATATTCGAAAGAGCCTGGGTATTGGAATT
GGCCATATTTAGTGGTGAGAGCCTGTGATACTTATTTAACCTTTCTGGGCCTTGTTTCAC
TCACTACATTTTTTTTTTTTTTT
>1413
GAGAGGGAGAGTTGGCCATGTTATATTCGAAAGAGCCTGGGTATTGGAATTGGCCATAT
TTAGTGGTGAGAGCCTGTGATACTTATTTAACCTTTCTGGGCCTTGTTTCACTCACTACA
TTTTTTTTTTTTTTTTTTTTT
>1414
CTGTGATACTTATTTAACCTTTCTGGGCCTTGTTTCACTCACTACATTTTTTTTTTTTTT
TTTTTTGAGACGGAGTCTCGCTCTGTCACCGGGCTGGAGTGCAATGGCGTGATCTCAG
CTCACTGCAGTCTCCACCTCC
>1415
TACTTATTTAACCTTTCTGGGCCTTGTTTCACTCACTACATTTTTTTTTTTTTTTTTTTTG
AGACGGAGTCTCGCTCTGTCACCGGGCTGGAGTGCAATGGCGTGATCTCAGCTCACTG
CAGTCTCCACCTCCCGGGTT
>1416
TGTTTCACTCACTACATTTTTTTTTTTTTTTTTTTTGAGACGGAGTCTCGCTCTGTCAC
CGGGCTGGAGTGCAATGGCGTGATCTCAGCTCACTGCAGTCTCCACCTCCCGGGTTCA
AGCGATTCTCCTGCCTCAGCCT
>1417
TTTTTTTTTTTTTTTTTTGAGACGGAGTCTCGCTCTGTCACCGGGCTGGAGTGCAATGG
CGTGATCTCAGCTCACTGCAGTCTCCACCTCCCGGGTTCAAGCGATTCTCCTGCCTCAG
CCTCCTGAGTAGCTGGGACTAC
>1418
TTTTTTTTGAGACGGAGTCTCGCTCTGTCACCGGGCTGGAGTGCAATGGCGTGATCTC
AGCTCACTGCAGTCTCCACCTCCCGGGTTCAAGCGATTCTCCTGCCTCAGCCTCCTGA
GTAGCTGGGACTACAGGCACCTGT
>1419
GAGTGCAATGGCGTGATCTCAGCTCACTGCAGTCTCCACCTCCCGGGTTCAAGCGATT
CTCCTGCCTCAGCCTCCTGAGTAGCTGGGACTACAGGCACCTGTCACCCCGCCCAGCT
AATTTTTTGTATTTTTAGTAGAGA
>1420
GGCGTGATCTCAGCTCACTGCAGTCTCCACCTCCCGGGTTCAAGCGATTCTCCTGCCTC
AGCCTCCTGAGTAGCTGGGACTACAGGCACCTGTCACCCCGCCCAGCTAATTTTTTGTA
TTTTTAGTAGAGACAGGGTTTC
>1421
GGGTTCAAGCGATTCTCCTGCCTCAGCCTCCTGAGTAGCTGGGACTACAGGCACCTGT
CACCCCGCCCAGCTAATTTTTTGTATTTTTAGTAGAGACAGGGTTTCACCATGTTGGCC
AGAATGGTCTCGATCTCTTGACC

>1422
CCTCAGCCTCCTGAGTAGCTGGGACTACAGGCACCTGTCACCCCGCCCAGCTAATTTTT
TGTATTTTTAGTAGAGACAGGGTTTCACCATGTTGGCCAGAATGGTCTCGATCTCTTGA
CCTTGCGATCCATCCACCTCGG
>1423
CAGCCTCCTGAGTAGCTGGGACTACAGGCACCTGTCACCCCGCCCAGCTAATTTTTTGT
ATTTTTAGTAGAGACAGGGTTTCACCATGTTGGCCAGAATGGTCTCGATCTCTTGACCT
TGCGATCCATCCACCTCGGCCT
>1424
GGCACCTGTCACCCCGCCCAGCTAATTTTTTGTATTTTTAGTAGAGACAGGGTTTCACC
ATGTTGGCCAGAATGGTCTCGATCTCTTGACCTTGCGATCCATCCACCTCGGCCTCCCA
AAGTGCTGGGATTACAGCTGTG
>1425
GTTTCACCATGTTGGCCAGAATGGTCTCGATCTCTTGACCTTGCGATCCATCCACCTCG
GCCTCCCAAAGTGCTGGGATTACAGCTGTGAGCCACCCCGCCCGGCCTTCACTCACTT
CTTAAAATGGAGACGGTCAACAG
>1426
GCCAGAATGGTCTCGATCTCTTGACCTTGCGATCCATCCACCTCGGCCTCCCAAAGTGC
TGGGATTACAGCTGTGAGCCACCCCGCCCGGCCTTCACTCACTTCTTAAAATGGAGAC
GGTCAACAGGGTTGGTGCTGAAT
>1427
GCCTCCCAAAGTGCTGGGATTACAGCTGTGAGCCACCCCGCCCGGCCTTCACTCACTT
CTTAAAATGGAGACGGTCAACAGGGTTGGTGCTGAATTAATGAAGTAAGATCCAGAAA
TCACTAGAGCTGTGATGGGCACAT
>1428
TGCTGGGATTACAGCTGTGAGCCACCCCGCCCGGCCTTCACTCACTTCTTAAAATGGA
GACGGTCAACAGGGTTGGTGCTGAATTAATGAAGTAAGATCCAGAAATCACTAGAGCT
GTGATGGGCACATAGTCACCGCAG
>1429
CACCCCGCCCGGCCTTCACTCACTTCTTAAAATGGAGACGGTCAACAGGGTTGGTGCT
GAATTAATGAAGTAAGATCCAGAAATCACTAGAGCTGTGATGGGCACATAGTCACCGC
AGCGGTGGAGAACTGACCATCTGT
>1430
CCGCCCGGCCTTCACTCACTTCTTAAAATGGAGACGGTCAACAGGGTTGGTGCTGAAT
TAATGAAGTAAGATCCAGAAATCACTAGAGCTGTGATGGGCACATAGTCACCGCAGCG
GTGGAGAACTGACCATCTGTCCCT
>1431
CACTTCTTAAAATGGAGACGGTCAACAGGGTTGGTGCTGAATTAATGAAGTAAGATCC
AGAAATCACTAGAGCTGTGATGGGCACATAGTCACCGCAGCGGTGGAGAACTGACCAT
CTGTCCCTTGTGTTGTTTAGGCCT
>1432
GGTTGGTGCTGAATTAATGAAGTAAGATCCAGAAATCACTAGAGCTGTGATGGGCACAT
AGTCACCGCAGCGGTGGAGAACTGACCATCTGTCCCTTGTGTTGTTTAGGCCTCATCTC
TGCATGGTCCTCATTGGCTCTG
>1433
CTGAATTAATGAAGTAAGATCCAGAAATCACTAGAGCTGTGATGGGCACATAGTCACC

GCAGCGGTGGAGAACTGACCATCTGTCCCTTGTGTTGTTTAGGCCTCATCTCTGCATGG
TCCTCATTGGCTCTGAGTTCTAG
>1434
CTAGAGCTGTGATGGGCACATAGTCACCGCAGCGGTGGAGAACTGACCATCTGTCCCT
TGTGTTGTTTAGGCCTCATCTCTGCATGGTCCTCATTGGCTCTGAGTTCTAGTGTGTAGT
GTCCACAGTGCCACCCTCCATG
>1435
GTGGAGAACTGACCATCTGTCCCTTGTGTTGTTTAGGCCTCATCTCTGCATGGTCCTCA
TTGGCTCTGAGTTCTAGTGTGTAGTGTCCACAGTGCCACCCTCCATGAGGGTTGTGTTG
ACTGTGAGGTGGTGCAGAGAAT
>1436
AACTGACCATCTGTCCCTTGTGTTGTTTAGGCCTCATCTCTGCATGGTCCTCATTGGCTC
TGAGTTCTAGTGTGTAGTGTCCACAGTGCCACCCTCCATGAGGGTTGTGTTGACTGTG
AGGTGGTGCAGAGAATGTCCCT
>1437
CTTGTGTTGTTTAGGCCTCATCTCTGCATGGTCCTCATTGGCTCTGAGTTCTAGTGTGTA
GTGTCCACAGTGCCACCCTCCATGAGGGTTGTGTTGACTGTGAGGTGGTGCAGAGAAT
GTCCCTGTCCTTGTGAGCATAT
>1438
TCATTGGCTCTGAGTTCTAGTGTGTAGTGTCCACAGTGCCACCCTCCATGAGGGTTGTG
TTGACTGTGAGGTGGTGCAGAGAATGTCCCTGTCCTTGTGAGCATATGACAGCAAGAC
ACTGGCCCTGGAATCCTCCGTGT
>1439
CTGAGTTCTAGTGTGTAGTGTCCACAGTGCCACCCTCCATGAGGGTTGTGTTGACTGTG
AGGTGGTGCAGAGAATGTCCCTGTCCTTGTGAGCATATGACAGCAAGACACTGGCCCT
GGAATCCTCCGTGTCACATGTGG
>1440
CACAGTGCCACCCTCCATGAGGGTTGTGTTGACTGTGAGGTGGTGCAGAGAATGTCCC
TGTCCTTGTGAGCATATGACAGCAAGACACTGGCCCTGGAATCCTCCGTGTCACATGTG
GGCTGCTGACAGGAAGAATGTGC
>1441
GTCCTTGTGAGCATATGACAGCAAGACACTGGCCCTGGAATCCTCCGTGTCACATGTG
GGCTGCTGACAGGAAGAATGTGCTTTTGTTGTTGTTGTTGTTGTTGAGGCAGAGTCTTA
CTCTGTCGCCCAGGCTGCAGTGC
>1442
TTGTGAGCATATGACAGCAAGACACTGGCCCTGGAATCCTCCGTGTCACATGTGGGCT
GCTGACAGGAAGAATGTGCTTTTGTTGTTGTTGTTGTTGTTGAGGCAGAGTCTTACTCT
GTCGCCCAGGCTGCAGTGCAGTG
>1443
GGAATCCTCCGTGTCACATGTGGGCTGCTGACAGGAAGAATGTGCTTTTGTTGTTGTTG
TTGTTGTTGAGGCAGAGTCTTACTCTGTCGCCCAGGCTGCAGTGCAGTGGCATGATCTT
GGCTCACTGCAACCTCTACTTC
>1444
TCCTCCGTGTCACATGTGGGCTGCTGACAGGAAGAATGTGCTTTTGTTGTTGTTGTTGT
TGTTGAGGCAGAGTCTTACTCTGTCGCCCAGGCTGCAGTGCAGTGGCATGATCTTGGC
TCACTGCAACCTCTACTTCCCGG

>1445
CTGCTGACAGGAAGAATGTGCTTTTGTTGTTGTTGTTGTTGTTGAGGCAGAGTCTTACT
CTGTCGCCCAGGCTGCAGTGCAGTGGCATGATCTTGGCTCACTGCAACCTCTACTTCCC
GGGTTCAAGTGATTCTCCTGCC
>1446
CTCTGTCGCCCAGGCTGCAGTGCAGTGGCATGATCTTGGCTCACTGCAACCTCTACTTC
CCGGGTTCAAGTGATTCTCCTGCCTCAGCCTCCTGAGCAGCTGGGACTACAGATGTGT
ACCACCATGCCCGACTAATTTTT
>1447
CAGTGCAGTGGCATGATCTTGGCTCACTGCAACCTCTACTTCCCGGGTTCAAGTGATTC
TCCTGCCTCAGCCTCCTGAGCAGCTGGGACTACAGATGTGTACCACCATGCCCGACTAA
TTTTTGTATTTTTAGTAGAGAT
>1448
ATGATCTTGGCTCACTGCAACCTCTACTTCCCGGGTTCAAGTGATTCTCCTGCCTCAGC
CTCCTGAGCAGCTGGGACTACAGATGTGTACCACCATGCCCGACTAATTTTTGTATTTTT
AGTAGAGATGGGGTTTCACCA
>1449
CACTGCAACCTCTACTTCCCGGGTTCAAGTGATTCTCCTGCCTCAGCCTCCTGAGCAGC
TGGGACTACAGATGTGTACCACCATGCCCGACTAATTTTTGTATTTTTAGTAGAGATGGG
GTTTCACCATGTTGGTCAGGC
>1450
GCCTCAGCCTCCTGAGCAGCTGGGACTACAGATGTGTACCACCATGCCCGACTAATTTT
TGTATTTTTAGTAGAGATGGGGTTTCACCATGTTGGTCAGGCTGGCCTGGAACTCCTGG
CCTTAAGTGATCCACCTGTCTC
>1451
AGATGTGTACCACCATGCCCGACTAATTTTTGTATTTTTAGTAGAGATGGGGTTTCACCA
TGTTGGTCAGGCTGGCCTGGAACTCCTGGCCTTAAGTGATCCACCTGTCTCGGCCTCCC
ATAGTGCTGGGATTATAGGTG
>1452
TTTTTGTATTTTTAGTAGAGATGGGGTTTCACCATGTTGGTCAGGCTGGCCTGGAACTC
CTGGCCTTAAGTGATCCACCTGTCTCGGCCTCCCATAGTGCTGGGATTATAGGTGTGTG
CCACCCTGCTGGGCCGAGAATG
>1453
TTCACCATGTTGGTCAGGCTGGCCTGGAACTCCTGGCCTTAAGTGATCCACCTGTCTCG
GCCTCCCATAGTGCTGGGATTATAGGTGTGTGCCACCCTGCTGGGCCGAGAATGTGCTT
TTGACTGAGGGTGGAAAGTCAG
>1454
CAGGCTGGCCTGGAACTCCTGGCCTTAAGTGATCCACCTGTCTCGGCCTCCCATAGTGC
TGGGATTATAGGTGTGTGCCACCCTGCTGGGCCGAGAATGTGCTTTTGACTGAGGGTG
GAAAGTCAGCAGCTGCAGCCCAA
>1455
TAAGTGATCCACCTGTCTCGGCCTCCCATAGTGCTGGGATTATAGGTGTGTGCCACCCT
GCTGGGCCGAGAATGTGCTTTTGACTGAGGGTGGAAAGTCAGCAGCTGCAGCCCAAG
AAGTTAGAGCCAGGGGCAGTGCCT
>1456
CGGCCTCCCATAGTGCTGGGATTATAGGTGTGTGCCACCCTGCTGGGCCGAGAATGTGC

TTTTGACTGAGGGTGGAAAGTCAGCAGCTGCAGCCCAAGAAGTTAGAGCCAGGGGCA
GTGCCTTTGATGGGAAGCTTGGGG
>1457
ATAGTGCTGGGATTATAGGTGTGTGCCACCCTGCTGGGCCGAGAATGTGCTTTTGACTG
AGGGTGGAAAGTCAGCAGCTGCAGCCCAAGAAGTTAGAGCCAGGGGCAGTGCCTTTG
ATGGGAAGCTTGGGGGTCTAAAGA
>1458
TGTGTGCCACCCTGCTGGGCCGAGAATGTGCTTTTGACTGAGGGTGGAAAGTCAGCA
GCTGCAGCCCAAGAAGTTAGAGCCAGGGGCAGTGCCTTTGATGGGAAGCTTGGGGGT
CTAAAGAAATCAGAAAGGGGAGAAAA
>1459
GACTGAGGGTGGAAAGTCAGCAGCTGCAGCCCAAGAAGTTAGAGCCAGGGGCAGTG
CCTTTGATGGGAAGCTTGGGGGTCTAAAGAAATCAGAAAGGGGAGAAAATAATCCTGC
GTGTGCTCAGCCCTCACTCAGATCAA
>1460
GTCAGCAGCTGCAGCCCAAGAAGTTAGAGCCAGGGGCAGTGCCTTTGATGGGAAGCT
TGGGGGTCTAAAGAAATCAGAAAGGGGAGAAAATAATCCTGCGTGTGCTCAGCCCTC
ACTCAGATCAACAGCCCCTCGGGTCC
>1461
TGCCTTTGATGGGAAGCTTGGGGGTCTAAAGAAATCAGAAAGGGGAGAAAATAATCCT
GCGTGTGCTCAGCCCTCACTCAGATCAACAGCCCCTCGGGTCCCAGCTAGATGTACTTT
GTAGAAGGCTTTTTTTCTCCCCC
>1462
GGAAGCTTGGGGGTCTAAAGAAATCAGAAAGGGGAGAAAATAATCCTGCGTGTGCTC
AGCCCTCACTCAGATCAACAGCCCCTCGGGTCCCAGCTAGATGTACTTTGTAGAAGGC
TTTTTTTCTCCCCCTCACCCTCAAA
>1463
TCAGAAAGGGGAGAAAATAATCCTGCGTGTGCTCAGCCCTCACTCAGATCAACAGCCC
CTCGGGTCCCAGCTAGATGTACTTTGTAGAAGGCTTTTTTTCTCCCCCTCACCCTCAAA
ACTCCAGGATTAATGTGCTATTC
>1464
TAATCCTGCGTGTGCTCAGCCCTCACTCAGATCAACAGCCCCTCGGGTCCCAGCTAGAT
GTACTTTGTAGAAGGCTTTTTTTCTCCCCCTCACCCTCAAAACTCCAGGATTAATGTGCT
ATTCCCTTTTTTCTACTGTTC
>1465
TCCTGCGTGTGCTCAGCCCTCACTCAGATCAACAGCCCCTCGGGTCCCAGCTAGATGT
ACTTTGTAGAAGGCTTTTTTTCTCCCCCTCACCCTCAAAACTCCAGGATTAATGTGCTAT
TCCCTTTTTTCTACTGTTCTCT
>1466
GCCCCTCGGGTCCCAGCTAGATGTACTTTGTAGAAGGCTTTTTTTCTCCCCCTCACCCT
CAAAACTCCAGGATTAATGTGCTATTCCCTTTTTTCTACTGTTCTCTGTCTCCCCCCAAC
CCATCAGCAGTCTGAGAAATG
>1467
CCTCAAAACTCCAGGATTAATGTGCTATTCCCTTTTTTCTACTGTTCTCTGTCTCCCCCC
AACCCATCAGCAGTCTGAGAAATGCTATGGTGGAAAAAAAAAAACAACAGCCCTATAA
CCAGTACAAACAAAAAGCCAAG

>1468
CAAAACTCCAGGATTAATGTGCTATTCCCTTTTTTCTACTGTTCTCTGTCTCCCCCCAAC
CCATCAGCAGTCTGAGAAATGCTATGGTGGAAAAAAAAAAACAACAGCCCTATAACCA
GTACAAACAAAAAGCCAAGACA
>1469
ACTGTTCTCTGTCTCCCCCCAACCCATCAGCAGTCTGAGAAATGCTATGGTGGAAAAA
AAAAAACAACAGCCCTATAACCAGTACAAACAAAAAGCCAAGACAAAAAAAAAAAA
AAAAAAAAATCAGAAGGGGAGATTTTG
>1470
CTCCCCCCAACCCATCAGCAGTCTGAGAAATGCTATGGTGGAAAAAAAAAAACAACA
GCCCTATAACCAGTACAAACAAAAAGCCAAGACAAAAAAAAAAAAAAAAAAAAATCA
GAAGGGGAGATTTTGAGGCCAGGAAGC
>1471
CAGTCTGAGAAATGCTATGGTGGAAAAAAAAAAACAACAGCCCTATAACCAGTACAA
ACAAAAAGCCAAGACAAAAAAAAAAAAAAAAAAAATCAGAAGGGGAGATTTTGAGG
CCAGGAAGCCATGGCATTCACTGGAAA
>1472
AAAAAAAACAACAGCCCTATAACCAGTACAAACAAAAAGCCAAGACAAAAAAAAAAA
AAAAAAAAAATCAGAAGGGGAGATTTTGAGGCCAGGAAGCCATGGCATTCACTGGAA
ATATATCAGTGATAAAATAACAGGCTA
>1473
AAAAACAACAGCCCTATAACCAGTACAAACAAAAAGCCAAGACAAAAAAAAAAAAA
AAAAAAATCAGAAGGGGAGATTTTGAGGCCAGGAAGCCATGGCATTCACTGGAAATAT
ATCAGTGATAAAATAACAGGCTACAG
>1474
CTATAACCAGTACAAACAAAAAGCCAAGACAAAAAAAAAAAAAAAAAAAATCAGAA
GGGGAGATTTTGAGGCCAGGAAGCCATGGCATTCACTGGAAATATATCAGTGATAAAAT
AACAGGCTACAGGGTTTATTTTATT
>1475
AAAAAAAAAAAAAATCAGAAGGGGAGATTTTGAGGCCAGGAAGCCATGGCATTCACT
GGAAATATATCAGTGATAAAATAACAGGCTACAGGGTTTATTTTATTTTATTTTTAAGTCA
ATAGGCCTGAGTGTTGGTAAAG
>1476
TCAGAAGGGGAGATTTTGAGGCCAGGAAGCCATGGCATTCACTGGAAATATATCAGTG
ATAAAATAACAGGCTACAGGGTTTATTTTATTTTATTTTTAAGTCAATAGGCCTGAGTGT
TGGTAAAGACTAGATACCCAGC
>1477
GGGAGATTTTGAGGCCAGGAAGCCATGGCATTCACTGGAAATATATCAGTGATAAAATA
ACAGGCTACAGGGTTTATTTTATTTTATTTTTAAGTCAATAGGCCTGAGTGTTGGTAAAG
ACTAGATACCCAGCAATCCTT
>1478
ATGGCATTCACTGGAAATATATCAGTGATAAAATAACAGGCTACAGGGTTTATTTTATTT
TATTTTTAAGTCAATAGGCCTGAGTGTTGGTAAAGACTAGATACCCAGCAATCCTTCTT
GCATTTCTTCTTGGGGTCGGG
>1479
AAATATATCAGTGATAAAATAACAGGCTACAGGGTTTATTTTATTTTATTTTTAAGTCAAT

AGGCCTGAGTGTTGGTAAAGACTAGATACCCAGCAATCCTTCTTGCATTTCTTCTTGGG
GTCGGGGGGCAGAAGGGAAT
>1480
TGATAAAATAACAGGCTACAGGGTTTATTTTATTTTATTTTTAAGTCAATAGGCCTGAGT
GTTGGTAAAGACTAGATACCCAGCAATCCTTCTTGCATTTCTTCTTGGGGTCGGGGGGC
AGAAGGGAATGGAGAGGAAAT
>1481
AAATAACAGGCTACAGGGTTTATTTTATTTTATTTTTAAGTCAATAGGCCTGAGTGTTGG
TAAAGACTAGATACCCAGCAATCCTTCTTGCATTTCTTCTTGGGGTCGGGGGGCAGAAG
GGAATGGAGAGGAAATGAAGG
>1482
GGCCTGAGTGTTGGTAAAGACTAGATACCCAGCAATCCTTCTTGCATTTCTTCTTGGGG
TCGGGGGGCAGAAGGGAATGGAGAGGAAATGAAGGCGGATTGCTGGTAAGTGAGGC
AGGAAGGCAGGGAGTTTATTATGGA
>1483
CTGAGTGTTGGTAAAGACTAGATACCCAGCAATCCTTCTTGCATTTCTTCTTGGGGTCG
GGGGGCAGAAGGGAATGGAGAGGAAATGAAGGCGGATTGCTGGTAAGTGAGGCAGG
AAGGCAGGGAGTTTATTATGGAATG
>1484
GGTAAAGACTAGATACCCAGCAATCCTTCTTGCATTTCTTCTTGGGGTCGGGGGGCAGA
AGGGAATGGAGAGGAAATGAAGGCGGATTGCTGGTAAGTGAGGCAGGAAGGCAGGG
AGTTTATTATGGAATGTCATCGGTA
>1485
GATACCCAGCAATCCTTCTTGCATTTCTTCTTGGGGTCGGGGGGCAGAAGGGAATGGA
GAGGAAATGAAGGCGGATTGCTGGTAAGTGAGGCAGGAAGGCAGGGAGTTTATTATG
GAATGTCATCGGTAAGTTTCCCCAT
>1486
TCTTGGGGTCGGGGGGCAGAAGGGAATGGAGAGGAAATGAAGGCGGATTGCTGGTAA
GTGAGGCAGGAAGGCAGGGAGTTTATTATGGAATGTCATCGGTAAGTTTCCCCATGATT
TGATCCCAAGCTGGTGTGGAGTGA
>1487
TCGGGGGGCAGAAGGGAATGGAGAGGAAATGAAGGCGGATTGCTGGTAAGTGAGGC
AGGAAGGCAGGGAGTTTATTATGGAATGTCATCGGTAAGTTTCCCCATGATTTGATCCC
AAGCTGGTGTGGAGTGAAGGGTAAG
>1488
GAAATGAAGGCGGATTGCTGGTAAGTGAGGCAGGAAGGCAGGGAGTTTATTATGGAAT
GTCATCGGTAAGTTTCCCCATGATTTGATCCCAAGCTGGTGTGGAGTGAAGGGTAAGCT
GTTGGCTCAACAGATAGTGAGAA
>1489
AGTGAGGCAGGAAGGCAGGGAGTTTATTATGGAATGTCATCGGTAAGTTTCCCCATGAT
TTGATCCCAAGCTGGTGTGGAGTGAAGGGTAAGCTGTTGGCTCAACAGATAGTGAGAA
TCAAACTTAGTGGAGTTGTAGAT
>1490
AAGGCAGGGAGTTTATTATGGAATGTCATCGGTAAGTTTCCCCATGATTTGATCCCAAG
CTGGTGTGGAGTGAAGGGTAAGCTGTTGGCTCAACAGATAGTGAGAATCAAACTTAGT
GGAGTTGTAGATCCTGTTTCCTG

>1491
AGGGAGTTTATTATGGAATGTCATCGGTAAGTTTCCCCATGATTTGATCCCAAGCTGGTG
TGGAGTGAAGGGTAAGCTGTTGGCTCAACAGATAGTGAGAATCAAACTTAGTGGAGTT
GTAGATCCTGTTTCCTGCAGCG
>1492
TTTATTATGGAATGTCATCGGTAAGTTTCCCCATGATTTGATCCCAAGCTGGTGTGGAGT
GAAGGGTAAGCTGTTGGCTCAACAGATAGTGAGAATCAAACTTAGTGGAGTTGTAGAT
CCTGTTTCCTGCAGCGGACAGC
>1493
CATCGGTAAGTTTCCCCATGATTTGATCCCAAGCTGGTGTGGAGTGAAGGGTAAGCTGT
TGGCTCAACAGATAGTGAGAATCAAACTTAGTGGAGTTGTAGATCCTGTTTCCTGCAGC
GGACAGCAAGATGTACAAGCCG
>1494
ATTTGATCCCAAGCTGGTGTGGAGTGAAGGGTAAGCTGTTGGCTCAACAGATAGTGAG
AATCAAACTTAGTGGAGTTGTAGATCCTGTTTCCTGCAGCGGACAGCAAGATGTACAA
GCCGTCCACTGCAGGAATGACGTG
>1495
AGGGTAAGCTGTTGGCTCAACAGATAGTGAGAATCAAACTTAGTGGAGTTGTAGATCC
TGTTTCCTGCAGCGGACAGCAAGATGTACAAGCCGTCCACTGCAGGAATGACGTGCTG
TAAAAGGGATGCAGTGTCAAGTGT
>1496
GCTGTTGGCTCAACAGATAGTGAGAATCAAACTTAGTGGAGTTGTAGATCCTGTTTCCT
GCAGCGGACAGCAAGATGTACAAGCCGTCCACTGCAGGAATGACGTGCTGTAAAAGG
GATGCAGTGTCAAGTGTAGTCATG
>1497
TCAAACTTAGTGGAGTTGTAGATCCTGTTTCCTGCAGCGGACAGCAAGATGTACAAGC
CGTCCACTGCAGGAATGACGTGCTGTAAAAGGGATGCAGTGTCAAGTGTAGTCATGGA
TGGGTGGGAAGACCAGACAAAGGC
>1498
AGATCCTGTTTCCTGCAGCGGACAGCAAGATGTACAAGCCGTCCACTGCAGGAATGAC
GTGCTGTAAAAGGGATGCAGTGTCAAGTGTAGTCATGGATGGGTGGGAAGACCAGAC
AAAGGCATCCAATGGGACTGACTTA
>1499
TTTCCTGCAGCGGACAGCAAGATGTACAAGCCGTCCACTGCAGGAATGACGTGCTGTA
AAAGGGATGCAGTGTCAAGTGTAGTCATGGATGGGTGGGAAGACCAGACAAAGGCAT
CCAATGGGACTGACTTACCTAAACA
>1500
CAGCGGACAGCAAGATGTACAAGCCGTCCACTGCAGGAATGACGTGCTGTAAAAGGG
ATGCAGTGTCAAGTGTAGTCATGGATGGGTGGGAAGACCAGACAAAGGCATCCAATGG
GACTGACTTACCTAAACAAACCAGA
>1501
CCGTCCACTGCAGGAATGACGTGCTGTAAAAGGGATGCAGTGTCAAGTGTAGTCATGG
ATGGGTGGGAAGACCAGACAAAGGCATCCAATGGGACTGACTTACCTAAACAAACCA
GAAGGAAGTCAGAACAGTGGGAAGA
>1502
GTGTAGTCATGGATGGGTGGGAAGACCAGACAAAGGCATCCAATGGGACTGACTTACC

TAAACAAACCAGAAGGAAGTCAGAACAGTGGGAAGATCTGATAGATAAGCAGTATTCT
GCAGGCAGATGAAGGAGCAAGCTT
>1503
TAGTCATGGATGGGTGGGAAGACCAGACAAAGGCATCCAATGGGACTGACTTACCTAA
ACAAACCAGAAGGAAGTCAGAACAGTGGGAAGATCTGATAGATAAGCAGTATTCTGC
AGGCAGATGAAGGAGCAAGCTTTGG
>1504
AAGGCATCCAATGGGACTGACTTACCTAAACAAACCAGAAGGAAGTCAGAACAGTGG
GAAGATCTGATAGATAAGCAGTATTCTGCAGGCAGATGAAGGAGCAAGCTTTGGGCCC
CCAAATCTTCACCAGTGCATCCACA
>1505
ATCCAATGGGACTGACTTACCTAAACAAACCAGAAGGAAGTCAGAACAGTGGGAAGA
TCTGATAGATAAGCAGTATTCTGCAGGCAGATGAAGGAGCAAGCTTTGGGCCCCCAAA
TCTTCACCAGTGCATCCACATTACA
>1506
CAATGGGACTGACTTACCTAAACAAACCAGAAGGAAGTCAGAACAGTGGGAAGATCT
GATAGATAAGCAGTATTCTGCAGGCAGATGAAGGAGCAAGCTTTGGGCCCCCAAATCT
TCACCAGTGCATCCACATTACAGAG
>1507
GACTTACCTAAACAAACCAGAAGGAAGTCAGAACAGTGGGAAGATCTGATAGATAAG
CAGTATTCTGCAGGCAGATGAAGGAGCAAGCTTTGGGCCCCCAAATCTTCACCAGTGC
ATCCACATTACAGAGGATAATGCCT
>1508
GATCTGATAGATAAGCAGTATTCTGCAGGCAGATGAAGGAGCAAGCTTTGGGCCCCCA
AATCTTCACCAGTGCATCCACATTACAGAGGATAATGCCTGAGGGAGGGGAAGATCAT
CTCAGGATTCTGGAACACTGGGCC
>1509
AGTATTCTGCAGGCAGATGAAGGAGCAAGCTTTGGGCCCCCAAATCTTCACCAGTGCA
TCCACATTACAGAGGATAATGCCTGAGGGAGGGGAAGATCATCTCAGGATTCTGGAAC
ACTGGGCCCTCCCTTTCTTATTCC
>1510
GATGAAGGAGCAAGCTTTGGGCCCCCAAATCTTCACCAGTGCATCCACATTACAGAGG
ATAATGCCTGAGGGAGGGGAAGATCATCTCAGGATTCTGGAACACTGGGCCCTCCCTT
TCTTATTCCAAAAGGCTGGAGCAG
>1511
AATGCCTGAGGGAGGGGAAGATCATCTCAGGATTCTGGAACACTGGGCCCTCCCTTTC
TTATTCCAAAAGGCTGGAGCAGTTCTGCATGTAGGCTTCTCTGGAAATGGTCTAGGAAC
CAGCTCATCCAGGCCAGGAGCAC
>1512
GAGGGAGGGGAAGATCATCTCAGGATTCTGGAACACTGGGCCCTCCCTTTCTTATTCC
AAAAGGCTGGAGCAGTTCTGCATGTAGGCTTCTCTGGAAATGGTCTAGGAACCAGCTC
ATCCAGGCCAGGAGCACCTGATCA
>1513
TGGGCCCTCCCTTTCTTATTCCAAAAGGCTGGAGCAGTTCTGCATGTAGGCTTCTCTGG
AAATGGTCTAGGAACCAGCTCATCCAGGCCAGGAGCACCTGATCATTCTCAGAGTTAC
GTACATATGCGATAAGATTGCAG

>1514
TCCCTTTCTTATTCCAAAAGGCTGGAGCAGTTCTGCATGTAGGCTTCTCTGGAAATGGT
CTAGGAACCAGCTCATCCAGGCCAGGAGCACCTGATCATTCTCAGAGTTACGTACATAT
GCGATAAGATTGCAGGGAACAG
>1515
AAGGCTGGAGCAGTTCTGCATGTAGGCTTCTCTGGAAATGGTCTAGGAACCAGCTCAT
CCAGGCCAGGAGCACCTGATCATTCTCAGAGTTACGTACATATGCGATAAGATTGCAGG
GAACAGTATAGTTCTATAGACTT
>1516
GGTCTAGGAACCAGCTCATCCAGGCCAGGAGCACCTGATCATTCTCAGAGTTACGTAC
ATATGCGATAAGATTGCAGGGAACAGTATAGTTCTATAGACTTAAGTGTGAACATAGTC
CTCATTTATAGTCCCTAGTCTCC
>1517
GAACCAGCTCATCCAGGCCAGGAGCACCTGATCATTCTCAGAGTTACGTACATATGCGA
TAAGATTGCAGGGAACAGTATAGTTCTATAGACTTAAGTGTGAACATAGTCCTCATTTAT
AGTCCCTAGTCTCCCATGGCT
>1518
GGAGCACCTGATCATTCTCAGAGTTACGTACATATGCGATAAGATTGCAGGGAACAGTA
TAGTTCTATAGACTTAAGTGTGAACATAGTCCTCATTTATAGTCCCTAGTCTCCCATGGC
TCACGCAGCTTGGTCTTTATG
>1519
CAGAGTTACGTACATATGCGATAAGATTGCAGGGAACAGTATAGTTCTATAGACTTAAG
TGTGAACATAGTCCTCATTTATAGTCCCTAGTCTCCCATGGCTCACGCAGCTTGGTCTTT
ATGTGTTTCTGAATTTAGTGC
>1520
ATATGCGATAAGATTGCAGGGAACAGTATAGTTCTATAGACTTAAGTGTGAACATAGTC
CTCATTTATAGTCCCTAGTCTCCCATGGCTCACGCAGCTTGGTCTTTATGTGTTTCTGAA
TTTAGTGCTGCAGAGGCACAA
>1521
ATAAGATTGCAGGGAACAGTATAGTTCTATAGACTTAAGTGTGAACATAGTCCTCATTTA
TAGTCCCTAGTCTCCCATGGCTCACGCAGCTTGGTCTTTATGTGTTTCTGAATTTAGTGC
TGCAGAGGCACAATGGGGTC
>1522
TAGTCCTCATTTATAGTCCCTAGTCTCCCATGGCTCACGCAGCTTGGTCTTTATGTGTTT
CTGAATTTAGTGCTGCAGAGGCACAATGGGGTCTGGTTTCTGGATCTTATACAGACAAG
CACTTAGGTCAGCTGCAGTGG
>1523
ATTTATAGTCCCTAGTCTCCCATGGCTCACGCAGCTTGGTCTTTATGTGTTTCTGAATTTA
GTGCTGCAGAGGCACAATGGGGTCTGGTTTCTGGATCTTATACAGACAAGCACTTAGG
TCAGCTGCAGTGGATGTCCTG
>1524
TGGTCTTTATGTGTTTCTGAATTTAGTGCTGCAGAGGCACAATGGGGTCTGGTTTCTGG
ATCTTATACAGACAAGCACTTAGGTCAGCTGCAGTGGATGTCCTGCACAGACACCGTC
GCAACTCTTGCCTGGTTCCTTTT
>1525
GTTTCTGAATTTAGTGCTGCAGAGGCACAATGGGGTCTGGTTTCTGGATCTTATACAGA

CAAGCACTTAGGTCAGCTGCAGTGGATGTCCTGCACAGACACCGTCGCAACTCTTGCC
TGGTTCCTTTTTGTTTTTGGAGA
>1526
TGAATTTAGTGCTGCAGAGGCACAATGGGGTCTGGTTTCTGGATCTTATACAGACAAGC
ACTTAGGTCAGCTGCAGTGGATGTCCTGCACAGACACCGTCGCAACTCTTGCCTGGTT
CCTTTTTGTTTTTGGAGATGGAG
>1527
CAATGGGGTCTGGTTTCTGGATCTTATACAGACAAGCACTTAGGTCAGCTGCAGTGGAT
GTCCTGCACAGACACCGTCGCAACTCTTGCCTGGTTCCTTTTTGTTTTTGGAGATGGAG
TCTCGCTCTGTCTCCCAGGCTG
>1528
GGTCAGCTGCAGTGGATGTCCTGCACAGACACCGTCGCAACTCTTGCCTGGTTCCTTT
TTGTTTTTGGAGATGGAGTCTCGCTCTGTCTCCCAGGCTGGAGTGCAGTGGCGCGATC
TCAGCTCACTGCAAGCTCTGCCTC
>1529
CTGCAGTGGATGTCCTGCACAGACACCGTCGCAACTCTTGCCTGGTTCCTTTTTGTTTT
TGGAGATGGAGTCTCGCTCTGTCTCCCAGGCTGGAGTGCAGTGGCGCGATCTCAGCTC
ACTGCAAGCTCTGCCTCCCAGGT
>1530
ACAGACACCGTCGCAACTCTTGCCTGGTTCCTTTTTGTTTTTGGAGATGGAGTCTCGCT
CTGTCTCCCAGGCTGGAGTGCAGTGGCGCGATCTCAGCTCACTGCAAGCTCTGCCTCC
CAGGTTCACGCTATTCTCCTGCC
>1531
GTTCCTTTTTGTTTTTGGAGATGGAGTCTCGCTCTGTCTCCCAGGCTGGAGTGCAGTGG
CGCGATCTCAGCTCACTGCAAGCTCTGCCTCCCAGGTTCACGCTATTCTCCTGCCTCAG
CCTCCCAAGTAGTTGGGACTAC
>1532
TTTTTGGAGATGGAGTCTCGCTCTGTCTCCCAGGCTGGAGTGCAGTGGCGCGATCTCA
GCTCACTGCAAGCTCTGCCTCCCAGGTTCACGCTATTCTCCTGCCTCAGCCTCCCAAGT
AGTTGGGACTACAGGCACCCGCC
>1533
AGTCTCGCTCTGTCTCCCAGGCTGGAGTGCAGTGGCGCGATCTCAGCTCACTGCAAGC
TCTGCCTCCCAGGTTCACGCTATTCTCCTGCCTCAGCCTCCCAAGTAGTTGGGACTACA
GGCACCCGCCACCACGCCCGGCT
>1534
GAGTGCAGTGGCGCGATCTCAGCTCACTGCAAGCTCTGCCTCCCAGGTTCACGCTATT
CTCCTGCCTCAGCCTCCCAAGTAGTTGGGACTACAGGCACCCGCCACCACGCCCGGCT
AATTTTTTGTATTTTTAGTAGAGA
>1535
GGCGCGATCTCAGCTCACTGCAAGCTCTGCCTCCCAGGTTCACGCTATTCTCCTGCCTC
AGCCTCCCAAGTAGTTGGGACTACAGGCACCCGCCACCACGCCCGGCTAATTTTTTGTA
TTTTTAGTAGAGACAGGGTTTC
>1536
GCGATCTCAGCTCACTGCAAGCTCTGCCTCCCAGGTTCACGCTATTCTCCTGCCTCAGC
CTCCCAAGTAGTTGGGACTACAGGCACCCGCCACCACGCCCGGCTAATTTTTTGTATTT
TTAGTAGAGACAGGGTTTCACC

>1537
CACTGCAAGCTCTGCCTCCCAGGTTCACGCTATTCTCCTGCCTCAGCCTCCCAAGTAGT
TGGGACTACAGGCACCCGCCACCACGCCCGGCTAATTTTTTGTATTTTTAGTAGAGACA
GGGTTTCACCGTGTTAGCCAGT
>1538
CCTCAGCCTCCCAAGTAGTTGGGACTACAGGCACCCGCCACCACGCCCGGCTAATTTT
TTGTATTTTTAGTAGAGACAGGGTTTCACCGTGTTAGCCAGTATGGTCTCGATCTCCTGA
ACTCGTGATCCACCTGCCTCGG
>1539
CAGCCTCCCAAGTAGTTGGGACTACAGGCACCCGCCACCACGCCCGGCTAATTTTTTG
TATTTTTAGTAGAGACAGGGTTTCACCGTGTTAGCCAGTATGGTCTCGATCTCCTGAAC
TCGTGATCCACCTGCCTCGGCCT
>1540
GCCTCCCAAGTAGTTGGGACTACAGGCACCCGCCACCACGCCCGGCTAATTTTTTGTAT
TTTTAGTAGAGACAGGGTTTCACCGTGTTAGCCAGTATGGTCTCGATCTCCTGAACTCG
TGATCCACCTGCCTCGGCCTCC
>1541
CCCAAGTAGTTGGGACTACAGGCACCCGCCACCACGCCCGGCTAATTTTTTGTATTTTT
AGTAGAGACAGGGTTTCACCGTGTTAGCCAGTATGGTCTCGATCTCCTGAACTCGTGAT
CCACCTGCCTCGGCCTCCCAAA
>1542
TACAGGCACCCGCCACCACGCCCGGCTAATTTTTTGTATTTTTAGTAGAGACAGGGTTT
CACCGTGTTAGCCAGTATGGTCTCGATCTCCTGAACTCGTGATCCACCTGCCTCGGCCT
CCCAAAGTGCTGGGATTACAGG
>1543
GTTTCACCGTGTTAGCCAGTATGGTCTCGATCTCCTGAACTCGTGATCCACCTGCCTCG
GCCTCCCAAAGTGCTGGGATTACAGGTGTGAGCCACCACGCCCGGCCTATTGCCTGGT
TCTTGAAGGGATTCTCAGGAACC
>1544
GCCAGTATGGTCTCGATCTCCTGAACTCGTGATCCACCTGCCTCGGCCTCCCAAAGTGC
TGGGATTACAGGTGTGAGCCACCACGCCCGGCCTATTGCCTGGTTCTTGAAGGGATTCT
CAGGAACCACAGGGAATGTCTG
>1545
CTCGGCCTCCCAAAGTGCTGGGATTACAGGTGTGAGCCACCACGCCCGGCCTATTGCC
TGGTTCTTGAAGGGATTCTCAGGAACCACAGGGAATGTCTGAGCCAGCTTTCTGGATC
CGTGTGTACAGCCCTTCCCAGAGG
>1546
CAAAGTGCTGGGATTACAGGTGTGAGCCACCACGCCCGGCCTATTGCCTGGTTCTTGA
AGGGATTCTCAGGAACCACAGGGAATGTCTGAGCCAGCTTTCTGGATCCGTGTGTACA
GCCCTTCCCAGAGGCTTTGTGGAG
>1547
GTGAGCCACCACGCCCGGCCTATTGCCTGGTTCTTGAAGGGATTCTCAGGAACCACAG
GGAATGTCTGAGCCAGCTTTCTGGATCCGTGTGTACAGCCCTTCCCAGAGGCTTTGTG
GAGCCAGCTGTACGGTGCATGAGC
>1548
CTGGTTCTTGAAGGGATTCTCAGGAACCACAGGGAATGTCTGAGCCAGCTTTCTGGAT

CCGTGTGTACAGCCCTTCCCAGAGGCTTTGTGGAGCCAGCTGTACGGTGCATGAGCTT
TGTGGCTAAGATATCAGAGTTCTG
>1549
AAGGGATTCTCAGGAACCACAGGGAATGTCTGAGCCAGCTTTCTGGATCCGTGTGTAC
AGCCCTTCCCAGAGGCTTTGTGGAGCCAGCTGTACGGTGCATGAGCTTTGTGGCTAAG
ATATCAGAGTTCTGCCTGGCCCTT
>1550
GAACCACAGGGAATGTCTGAGCCAGCTTTCTGGATCCGTGTGTACAGCCCTTCCCAGA
GGCTTTGTGGAGCCAGCTGTACGGTGCATGAGCTTTGTGGCTAAGATATCAGAGTTCTG
CCTGGCCCTTGGCCTTGGTCTCT
>1551
ATCCGTGTGTACAGCCCTTCCCAGAGGCTTTGTGGAGCCAGCTGTACGGTGCATGAGC
TTTGTGGCTAAGATATCAGAGTTCTGCCTGGCCCTTGGCCTTGGTCTCTCTCAGATGGT
CAGCTGCTCTGTTTTTAGTGGTT
>1552
TGTACAGCCCTTCCCAGAGGCTTTGTGGAGCCAGCTGTACGGTGCATGAGCTTTGTGG
CTAAGATATCAGAGTTCTGCCTGGCCCTTGGCCTTGGTCTCTCTCAGATGGTCAGCTGC
TCTGTTTTTAGTGGTTGATCATG
>1553
TGTACGGTGCATGAGCTTTGTGGCTAAGATATCAGAGTTCTGCCTGGCCCTTGGCCTTG
GTCTCTCTCAGATGGTCAGCTGCTCTGTTTTTAGTGGTTGATCATGTAGAAGGCCAACA
GGTGTTGACCTTGGTTTAGGCT
>1554
CTAAGATATCAGAGTTCTGCCTGGCCCTTGGCCTTGGTCTCTCTCAGATGGTCAGCTGC
TCTGTTTTTAGTGGTTGATCATGTAGAAGGCCAACAGGTGTTGACCTTGGTTTAGGCTA
CTTTTGGGATTTTTGAGGTTTC
>1555
TCTGCCTGGCCCTTGGCCTTGGTCTCTCTCAGATGGTCAGCTGCTCTGTTTTTAGTGGT
TGATCATGTAGAAGGCCAACAGGTGTTGACCTTGGTTTAGGCTACTTTTGGGATTTTTG
AGGTTTCCTGAGACTCTTGGTT
>1556
CAGATGGTCAGCTGCTCTGTTTTTAGTGGTTGATCATGTAGAAGGCCAACAGGTGTTGA
CCTTGGTTTAGGCTACTTTTGGGATTTTTGAGGTTTCCTGAGACTCTTGGTTTTCTTTCA
TCATCCCGTCCAGAACCTCTG
>1557
TTTAGTGGTTGATCATGTAGAAGGCCAACAGGTGTTGACCTTGGTTTAGGCTACTTTTG
GGATTTTTGAGGTTTCCTGAGACTCTTGGTTTTCTTTCATCATCCCGTCCAGAACCTCTG
ATGTCAAACTAAATTCTGTTT
>1558
GATCATGTAGAAGGCCAACAGGTGTTGACCTTGGTTTAGGCTACTTTTGGGATTTTTGA
GGTTTCCTGAGACTCTTGGTTTTCTTTCATCATCCCGTCCAGAACCTCTGATGTCAAACT
AAATTCTGTTTGGAAAAGGGT
>1559
TGGTTTAGGCTACTTTTGGGATTTTTGAGGTTTCCTGAGACTCTTGGTTTTCTTTCATCA
TCCCGTCCAGAACCTCTGATGTCAAACTAAATTCTGTTTGGAAAAGGGTATGCTGTAGA
ATAATGATAGCCACAGCAATG

>1560
GAGACTCTTGGTTTTCTTTCATCATCCCGTCCAGAACCTCTGATGTCAAACTAAATTCT
GTTTGGAAAAGGGTATGCTGTAGAATAATGATAGCCACAGCAATGATATTAATAATACCT
TTTATCGGTTGCAGCATTTTC
>1561
TTTCTTTCATCATCCCGTCCAGAACCTCTGATGTCAAACTAAATTCTGTTTGGAAAAGG
GTATGCTGTAGAATAATGATAGCCACAGCAATGATATTAATAATACCTTTTATCGGTTGCA
GCATTTTCTTCTTTGCAAAG
>1562
TCCAGAACCTCTGATGTCAAACTAAATTCTGTTTGGAAAAGGGTATGCTGTAGAATAAT
GATAGCCACAGCAATGATATTAATAATACCTTTTATCGGTTGCAGCATTTTCTTCTTTGCA
AAGCACCTCTAATGACTTTA
>1563
GGAAAAGGGTATGCTGTAGAATAATGATAGCCACAGCAATGATATTAATAATACCTTTTA
TCGGTTGCAGCATTTTCTTCTTTGCAAAGCACCTCTAATGACTTTATCTCTCTTGCCCTC
ACAGCCTTCCTTGAGTGGGT
>1564
AATAATGATAGCCACAGCAATGATATTAATAATACCTTTTATCGGTTGCAGCATTTTCTTC
TTTGCAAAGCACCTCTAATGACTTTATCTCTCTTGCCCTCACAGCCTTCCTTGAGTGGGT
ACAGGTCCATAATCCCATA
>1565
CTTTTATCGGTTGCAGCATTTTCTTCTTTGCAAAGCACCTCTAATGACTTTATCTCTCTTG
CCCTCACAGCCTTCCTTGAGTGGGTACAGGTCCATAATCCCATATCTGTGATAATGAAG
TCTGAAAAGCTCTGAAAACT
>1566
TGCAGCATTTTCTTCTTTGCAAAGCACCTCTAATGACTTTATCTCTCTTGCCCTCACAGC
CTTCCTTGAGTGGGTACAGGTCCATAATCCCATATCTGTGATAATGAAGTCTGAAAAGC
TCTGAAAACTAAAAAGTTTGC
>1567
TATCTCTCTTGCCCTCACAGCCTTCCTTGAGTGGGTACAGGTCCATAATCCCATATCTGT
GATAATGAAGTCTGAAAAGCTCTGAAAACTAAAAAGTTTGCAGTAAACTCATTTAGTG
GCAAAACCTGATCTGAACTGAT
>1568
TGCCCTCACAGCCTTCCTTGAGTGGGTACAGGTCCATAATCCCATATCTGTGATAATGA
AGTCTGAAAAGCTCTGAAAACTAAAAAGTTTGCAGTAAACTCATTTAGTGGCAAAACC
TGATCTGAACTGATGTGAGGCTA
>1569
GGTACAGGTCCATAATCCCATATCTGTGATAATGAAGTCTGAAAAGCTCTGAAAACTAA
AAAGTTTGCAGTAAACTCATTTAGTGGCAAAACCTGATCTGAACTGATGTGAGGCTATA
TTTATCCTGCTTAGTTTGAATA
>1570
AATCCCATATCTGTGATAATGAAGTCTGAAAAGCTCTGAAAACTAAAAAGTTTGCAGTA
AACTCATTTAGTGGCAAAACCTGATCTGAACTGATGTGAGGCTATATTTATCCTGCTTAG
TTTGAATATTCATATGTTTTG
>1571
AAAGCTCTGAAAACTAAAAAGTTTGCAGTAAACTCATTTAGTGGCAAAACCTGATCTG

AACTGATGTGAGGCTATATTTATCCTGCTTAGTTTGAATATTCATATGTTTTGCTGCAAAA
ATATTAATGTGTTTGATTACA
>1572
GTTTGCAGTAAACTCATTTAGTGGCAAAACCTGATCTGAACTGATGTGAGGCTATATTTA
TCCTGCTTAGTTTGAATATTCATATGTTTTGCTGCAAAAATATTAATGTGTTTGATTACAG
AGTGCTGCCCTAGACTCTG
>1573
CTATATTTATCCTGCTTAGTTTGAATATTCATATGTTTTGCTGCAAAAATATTAATGTGTTT
GATTACAGAGTGCTGCCCTAGACTCTGCTGGGGCTGTTAAACACGATAGGTGTCAGAG
TACCTTTCTTTTTTTTTTTT
>1574
ATATTTATCCTGCTTAGTTTGAATATTCATATGTTTTGCTGCAAAAATATTAATGTGTTTGA
TTACAGAGTGCTGCCCTAGACTCTGCTGGGGCTGTTAAACACGATAGGTGTCAGAGTA
CCTTTCTTTTTTTTTTTTTT
>1575
TGCAAAAATATTAATGTGTTTGATTACAGAGTGCTGCCCTAGACTCTGCTGGGGCTGTT
AAACACGATAGGTGTCAGAGTACCTTTCTTTTTTTTTTTTTTTTTTTTTTTTGAGACGGA
GTCTCGCTCTGTCGCCCAGGT
>1576
ATATTAATGTGTTTGATTACAGAGTGCTGCCCTAGACTCTGCTGGGGCTGTTAAACACG
ATAGGTGTCAGAGTACCTTTCTTTTTTTTTTTTTTTTTTTTTTTTGAGACGGAGTCTCGC
TCTGTCGCCCAGGTCGGACTG
>1577
TCTGCTGGGGCTGTTAAACACGATAGGTGTCAGAGTACCTTTCTTTTTTTTTTTTTTTTT
TTTTTTTGAGACGGAGTCTCGCTCTGTCGCCCAGGTCGGACTGCGGACTGCAGTGGCG
CAATCTCGGCTCACTGCAAGCT
>1578
GGGGCTGTTAAACACGATAGGTGTCAGAGTACCTTTCTTTTTTTTTTTTTTTTTTTTTTTT
GAGACGGAGTCTCGCTCTGTCGCCCAGGTCGGACTGCGGACTGCAGTGGCGCAATCT
CGGCTCACTGCAAGCTCCGCTT
>1579
AGGTGTCAGAGTACCTTTCTTTTTTTTTTTTTTTTTTTTTTTTGAGACGGAGTCTCGCTC
TGTCGCCCAGGTCGGACTGCGGACTGCAGTGGCGCAATCTCGGCTCACTGCAAGCTCC
GCTTCCCGGGTTCATGCCATTC
>1580
AGACGGAGTCTCGCTCTGTCGCCCAGGTCGGACTGCGGACTGCAGTGGCGCAATCTC
GGCTCACTGCAAGCTCCGCTTCCCGGGTTCATGCCATTCTCCTGCCTCAGCCTCCCGAG
TAGCTGGGACTACAGGCGCCCGCC
>1581
GACTGCAGTGGCGCAATCTCGGCTCACTGCAAGCTCCGCTTCCCGGGTTCATGCCATT
CTCCTGCCTCAGCCTCCCGAGTAGCTGGGACTACAGGCGCCCGCCACCGCGCCCGGCT
AATTTTTTGTATTTTTAGTAGAGA
>1582
GGCGCAATCTCGGCTCACTGCAAGCTCCGCTTCCCGGGTTCATGCCATTCTCCTGCCTC
AGCCTCCCGAGTAGCTGGGACTACAGGCGCCCGCCACCGCGCCCGGCTAATTTTTTGT
ATTTTTAGTAGAGACGGGGTTTC

>1583
GCAATCTCGGCTCACTGCAAGCTCCGCTTCCCGGGTTCATGCCATTCTCCTGCCTCAGC
CTCCCGAGTAGCTGGGACTACAGGCGCCCGCCACCGCGCCCGGCTAATTTTTTGTATTT
TTAGTAGAGACGGGGTTTCACC
>1584
CACTGCAAGCTCCGCTTCCCGGGTTCATGCCATTCTCCTGCCTCAGCCTCCCGAGTAGC
TGGGACTACAGGCGCCCGCCACCGCGCCCGGCTAATTTTTTGTATTTTTAGTAGAGACG
GGGTTTCACCTTGTTAGCCAGG
>1585
CAGCCTCCCGAGTAGCTGGGACTACAGGCGCCCGCCACCGCGCCCGGCTAATTTTTTG
TATTTTTAGTAGAGACGGGGTTTCACCTTGTTAGCCAGGATGGTCTCGATCTCCTGACC
TCATGATCCACCCGCCTCGGCCT
>1586
GCCTCCCGAGTAGCTGGGACTACAGGCGCCCGCCACCGCGCCCGGCTAATTTTTTGTAT
TTTTAGTAGAGACGGGGTTTCACCTTGTTAGCCAGGATGGTCTCGATCTCCTGACCTCA
TGATCCACCCGCCTCGGCCTCC
>1587
TACAGGCGCCCGCCACCGCGCCCGGCTAATTTTTTGTATTTTTAGTAGAGACGGGGTTT
CACCTTGTTAGCCAGGATGGTCTCGATCTCCTGACCTCATGATCCACCCGCCTCGGCCT
CCCAAAGTGCTGGGATTACAGG
>1588
GTTTCACCTTGTTAGCCAGGATGGTCTCGATCTCCTGACCTCATGATCCACCCGCCTCG
GCCTCCCAAAGTGCTGGGATTACAGGCGTGAGCACCGCGCCCGGCCCAGAGTACCTTT
CTAAAGTCTGAACAGTTTTGGTC
>1589
GCCAGGATGGTCTCGATCTCCTGACCTCATGATCCACCCGCCTCGGCCTCCCAAAGTGC
TGGGATTACAGGCGTGAGCACCGCGCCCGGCCCAGAGTACCTTTCTAAAGTCTGAACA
GTTTTGGTCGTTGAAGCACCTGA
>1590
CCTCATGATCCACCCGCCTCGGCCTCCCAAAGTGCTGGGATTACAGGCGTGAGCACCG
CGCCCGGCCCAGAGTACCTTTCTAAAGTCTGAACAGTTTTGGTCGTTGAAGCACCTGA
AGGTTTTGGATAAGGGATTATGAA
>1591
GCCTCGGCCTCCCAAAGTGCTGGGATTACAGGCGTGAGCACCGCGCCCGGCCCAGAG
TACCTTTCTAAAGTCTGAACAGTTTTGGTCGTTGAAGCACCTGAAGGTTTTGGATAAGG
GATTATGAACGATTATCATTCTCT
>1592
TCCCAAAGTGCTGGGATTACAGGCGTGAGCACCGCGCCCGGCCCAGAGTACCTTTCTA
AAGTCTGAACAGTTTTGGTCGTTGAAGCACCTGAAGGTTTTGGATAAGGGATTATGAA
CGATTATCATTCTCTATTGACAAA
>1593
ATTACAGGCGTGAGCACCGCGCCCGGCCCAGAGTACCTTTCTAAAGTCTGAACAGTTT
TGGTCGTTGAAGCACCTGAAGGTTTTGGATAAGGGATTATGAACGATTATCATTCTCTAT
TGACAAATGAGGAAAATGAGAT
>1594
GGTCGTTGAAGCACCTGAAGGTTTTGGATAAGGGATTATGAACGATTATCATTCTCTATT

GACAAATGAGGAAAATGAGATTTGGAGCAAGTAAGTGACTTCCCATGGTCACACAGCT
ATAAATGGGTAGAACCAGGCAC
>1595
AGCACCTGAAGGTTTTGGATAAGGGATTATGAACGATTATCATTCTCTATTGACAAATG
AGGAAAATGAGATTTGGAGCAAGTAAGTGACTTCCCATGGTCACACAGCTATAAATGG
GTAGAACCAGGCACTCAGCTTGC
>1596
GGATAAGGGATTATGAACGATTATCATTCTCTATTGACAAATGAGGAAAATGAGATTTG
GAGCAAGTAAGTGACTTCCCATGGTCACACAGCTATAAATGGGTAGAACCAGGCACTC
AGCTTGCCTTCTAATTCACAGTC
>1597
TATCATTCTCTATTGACAAATGAGGAAAATGAGATTTGGAGCAAGTAAGTGACTTCCCA
TGGTCACACAGCTATAAATGGGTAGAACCAGGCACTCAGCTTGCCTTCTAATTCACAGT
CCTGGGCGCTCTGCACCAGGCC
>1598
GACAAATGAGGAAAATGAGATTTGGAGCAAGTAAGTGACTTCCCATGGTCACACAGCT
ATAAATGGGTAGAACCAGGCACTCAGCTTGCCTTCTAATTCACAGTCCTGGGCGCTCTG
CACCAGGCCATATAAGAACATGA
>1599
AGTGACTTCCCATGGTCACACAGCTATAAATGGGTAGAACCAGGCACTCAGCTTGCCT
TCTAATTCACAGTCCTGGGCGCTCTGCACCAGGCCATATAAGAACATGAGCATTGCTCT
TCTGGGTCAAATCCATCTGACCT
>1600
CAGCTATAAATGGGTAGAACCAGGCACTCAGCTTGCCTTCTAATTCACAGTCCTGGGCG
CTCTGCACCAGGCCATATAAGAACATGAGCATTGCTCTTCTGGGTCAAATCCATCTGAC
CTAGTCTTTGGTTGCCATCACC
>1601
TGGGTAGAACCAGGCACTCAGCTTGCCTTCTAATTCACAGTCCTGGGCGCTCTGCACC
AGGCCATATAAGAACATGAGCATTGCTCTTCTGGGTCAAATCCATCTGACCTAGTCTTT
GGTTGCCATCACCATGCCTAAGA
>1602
CTGGGCGCTCTGCACCAGGCCATATAAGAACATGAGCATTGCTCTTCTGGGTCAAATCC
ATCTGACCTAGTCTTTGGTTGCCATCACCATGCCTAAGATACCACCATTGGAGGCTACA
ACCATCTTCCTTGACGTCAATC
>1603
GAACATGAGCATTGCTCTTCTGGGTCAAATCCATCTGACCTAGTCTTTGGTTGCCATCA
CCATGCCTAAGATACCACCATTGGAGGCTACAACCATCTTCCTTGACGTCAATCCTAAA
GGTCAGGTGTAGCTTCTCTTAG
>1604
TCTTCTGGGTCAAATCCATCTGACCTAGTCTTTGGTTGCCATCACCATGCCTAAGATACC
ACCATTGGAGGCTACAACCATCTTCCTTGACGTCAATCCTAAAGGTCAGGTGTAGCTTC
TCTTAGTAGCTATTTATGGCT
>1605
GTTGCCATCACCATGCCTAAGATACCACCATTGGAGGCTACAACCATCTTCCTTGACGT
CAATCCTAAAGGTCAGGTGTAGCTTCTCTTAGTAGCTATTTATGGCTGCCTTGTCATTCA
CAGATTTGCCAAAATCCTTTT

>1606
CATCACCATGCCTAAGATACCACCATTGGAGGCTACAACCATCTTCCTTGACGTCAATC
CTAAAGGTCAGGTGTAGCTTCTCTTAGTAGCTATTTATGGCTGCCTTGTCATTCACAGAT
TTGCCAAAATCCTTTTTGAAT
>1607
CCACCATTGGAGGCTACAACCATCTTCCTTGACGTCAATCCTAAAGGTCAGGTGTAGCT
TCTCTTAGTAGCTATTTATGGCTGCCTTGTCATTCACAGATTTGCCAAAATCCTTTTTGA
ATCCATTTCCATTTCCAGGAA
>1608
TTGACGTCAATCCTAAAGGTCAGGTGTAGCTTCTCTTAGTAGCTATTTATGGCTGCCTTG
TCATTCACAGATTTGCCAAAATCCTTTTTGAATCCATTTCCATTTCCAGGAAGTATCCTT
TTTAGGGGTGATGAATGACA
>1609
TAGCTATTTATGGCTGCCTTGTCATTCACAGATTTGCCAAAATCCTTTTTGAATCCATTTC
CATTTCCAGGAAGTATCCTTTTTAGGGGTGATGAATGACAAGTTTATAATCTCCTGGGCT
CCTGGAAATCTGGTCTTTT
>1610
TATTTATGGCTGCCTTGTCATTCACAGATTTGCCAAAATCCTTTTTGAATCCATTTCCATT
TCCAGGAAGTATCCTTTTTAGGGGTGATGAATGACAAGTTTATAATCTCCTGGGCTCCT
GGAAATCTGGTCTTTTGGAG
>1611
CCTTGTCATTCACAGATTTGCCAAAATCCTTTTTGAATCCATTTCCATTTCCAGGAAGTA
TCCTTTTTAGGGGTGATGAATGACAAGTTTATAATCTCCTGGGCTCCTGGAAATCTGGT
CTTTTGGAGAGAGGTTGTTGG
>1612
TATCCTTTTTAGGGGTGATGAATGACAAGTTTATAATCTCCTGGGCTCCTGGAAATCTGG
TCTTTTGGAGAGAGGTTGTTGGTTGTGAAGATAGGAAGATGGCAGAAGGCTTTGTAGT
GGTGAGGCCAGGTCTAGACCTA
>1613
TCCTTTTTAGGGGTGATGAATGACAAGTTTATAATCTCCTGGGCTCCTGGAAATCTGGT
CTTTTGGAGAGAGGTTGTTGGTTGTGAAGATAGGAAGATGGCAGAAGGCTTTGTAGTG
GTGAGGCCAGGTCTAGACCTAGC
>1614
GTTTATAATCTCCTGGGCTCCTGGAAATCTGGTCTTTTGGAGAGAGGTTGTTGGTTGTG
AAGATAGGAAGATGGCAGAAGGCTTTGTAGTGGTGAGGCCAGGTCTAGACCTAGCTCT
GCGTTTTTCAGCTCTGTCACCAT
>1615
CCTGGAAATCTGGTCTTTTGGAGAGAGGTTGTTGGTTGTGAAGATAGGAAGATGGCAG
AAGGCTTTGTAGTGGTGAGGCCAGGTCTAGACCTAGCTCTGCGTTTTTCAGCTCTGTCA
CCATTGACATCGTTTTTTTCATC
>1616
ATCTGGTCTTTTGGAGAGAGGTTGTTGGTTGTGAAGATAGGAAGATGGCAGAAGGCTT
TGTAGTGGTGAGGCCAGGTCTAGACCTAGCTCTGCGTTTTTCAGCTCTGTCACCATTGA
CATCGTTTTTTTCATCGTAAGCC
>1617
TGGAGAGAGGTTGTTGGTTGTGAAGATAGGAAGATGGCAGAAGGCTTTGTAGTGGTG

AGGCCAGGTCTAGACCTAGCTCTGCGTTTTTCAGCTCTGTCACCATTGACATCGTTTTT
TTCATCGTAAGCCTTAACTTCTTC
>1618
AGGCCAGGTCTAGACCTAGCTCTGCGTTTTTCAGCTCTGTCACCATTGACATCGTTTTT
TTCATCGTAAGCCTTAACTTCTTCATTTTTAAAACTAGGCACTTCAGTGAGCTCTTTCTG
GATTGTTGCGCTCTTTAAAAA
>1619
TTTTCAGCTCTGTCACCATTGACATCGTTTTTTTCATCGTAAGCCTTAACTTCTTCATTTT
TAAAACTAGGCACTTCAGTGAGCTCTTTCTGGATTGTTGCGCTCTTTAAAAAATTCTTTT
ATTCTTTGTGTACCCACTT
>1620
CACCATTGACATCGTTTTTTTCATCGTAAGCCTTAACTTCTTCATTTTTAAAACTAGGCA
CTTCAGTGAGCTCTTTCTGGATTGTTGCGCTCTTTAAAAAATTCTTTTATTCTTTGTGTA
CCCACTTATATCTTCATGAA
>1621
TCTTTCTGGATTGTTGCGCTCTTTAAAAAATTCTTTTATTCTTTGTGTACCCACTTATATC
TTCATGAAGTCCTTTTGGTTAATTGAATTTTGCTGTTTTGGAATCTGGATTGGCAGTTCA
GCCAGTTAAGTAGGGGGTC
>1622
TACCCACTTATATCTTCATGAAGTCCTTTTGGTTAATTGAATTTTGCTGTTTTGGAATCTG
GATTGGCAGTTCAGCCAGTTAAGTAGGGGGTCAGTTTGATTTGATTCTGTGTGTTCTGA
CTTTTTTTTTTTTTTTAAAT
>1623
ACTTATATCTTCATGAAGTCCTTTTGGTTAATTGAATTTTGCTGTTTTGGAATCTGGATTG
GCAGTTCAGCCAGTTAAGTAGGGGGTCAGTTTGATTTGATTCTGTGTGTTCTGACTTTT
TTTTTTTTTTTAAATACTTT
>1624
ATATCTTCATGAAGTCCTTTTGGTTAATTGAATTTTGCTGTTTTGGAATCTGGATTGGCA
GTTCAGCCAGTTAAGTAGGGGGTCAGTTTGATTTGATTCTGTGTGTTCTGACTTTTTTTT
TTTTTTTAAATACTTTAACC
>1625
CTTTTGGTTAATTGAATTTTGCTGTTTTGGAATCTGGATTGGCAGTTCAGCCAGTTAAGT
AGGGGGTCAGTTTGATTTGATTCTGTGTGTTCTGACTTTTTTTTTTTTTTTAAATACTTTA
ACCTGCTTAGGGTTACTGA
>1626
GGGGGTCAGTTTGATTTGATTCTGTGTGTTCTGACTTTTTTTTTTTTTTTAAATACTTTAA
CCTGCTTAGGGTTACTGAGCCATCTGGGATCTAGACTGAACTCTTGGAACTTTTGAAGA
TGATTTCCTTTATTTTATTT
>1627
TTGATTTGATTCTGTGTGTTCTGACTTTTTTTTTTTTTTTAAATACTTTAACCTGCTTAGG
GTTACTGAGCCATCTGGGATCTAGACTGAACTCTTGGAACTTTTGAAGATGATTTCCTT
TATTTTATTTTCAATGGAGG
>1628
TAAATACTTTAACCTGCTTAGGGTTACTGAGCCATCTGGGATCTAGACTGAACTCTTGG
AACTTTTGAAGATGATTTCCTTTATTTTATTTTCAATGGAGGAAAGTTGCCCACTCTTTC
ATCTCAGTGAGAAGTCCCATT

>1629
GCCATCTGGGATCTAGACTGAACTCTTGGAACTTTTGAAGATGATTTCCTTTATTTTATT
TTCAATGGAGGAAAGTTGCCCACTCTTTCATCTCAGTGAGAAGTCCCATTTTAATTTGC
CATCACTATTGGATGGAGCCG
>1630
TGGAACTTTTGAAGATGATTTCCTTTATTTTATTTTCAATGGAGGAAAGTTGCCCACTCT
TTCATCTCAGTGAGAAGTCCCATTTTAATTTGCCATCACTATTGGATGGAGCCGCTAATT
GCTGGGAGGGAGAAAGCTAT
>1631
ACTTTTGAAGATGATTTCCTTTATTTTATTTTCAATGGAGGAAAGTTGCCCACTCTTTCA
TCTCAGTGAGAAGTCCCATTTTAATTTGCCATCACTATTGGATGGAGCCGCTAATTGCTG
GGAGGGAGAAAGCTATAGTT
>1632
GAGGAAAGTTGCCCACTCTTTCATCTCAGTGAGAAGTCCCATTTTAATTTGCCATCACT
ATTGGATGGAGCCGCTAATTGCTGGGAGGGAGAAAGCTATAGTTTAGCAACATGTTTTC
CTCCTGCTCGTTCTAGGAAAAC
>1633
CTTTCATCTCAGTGAGAAGTCCCATTTTAATTTGCCATCACTATTGGATGGAGCCGCTAA
TTGCTGGGAGGGAGAAAGCTATAGTTTAGCAACATGTTTTCCTCCTGCTCGTTCTAGGA
AAACTATTATGAATCTTTAAA
>1634
CATCTCAGTGAGAAGTCCCATTTTAATTTGCCATCACTATTGGATGGAGCCGCTAATTGC
TGGGAGGGAGAAAGCTATAGTTTAGCAACATGTTTTCCTCCTGCTCGTTCTAGGAAAA
CTATTATGAATCTTTAAAATAG
>1635
TCCCATTTTAATTTGCCATCACTATTGGATGGAGCCGCTAATTGCTGGGAGGGAGAAAG
CTATAGTTTAGCAACATGTTTTCCTCCTGCTCGTTCTAGGAAAACTATTATGAATCTTTA
AAATAGTGATTATCCTAAACT
>1636
ATGGAGCCGCTAATTGCTGGGAGGGAGAAAGCTATAGTTTAGCAACATGTTTTCCTCCT
GCTCGTTCTAGGAAAACTATTATGAATCTTTAAAATAGTGATTATCCTAAACTGTTAAAA
TCAAATTCTATTCACAGCTCT
>1637
AAAGCTATAGTTTAGCAACATGTTTTCCTCCTGCTCGTTCTAGGAAAACTATTATGAATC
TTTAAAATAGTGATTATCCTAAACTGTTAAAATCAAATTCTATTCACAGCTCTTTGATTTC
TTGCTAATTCAGGAGAGAA
>1638
CTAGGAAAACTATTATGAATCTTTAAAATAGTGATTATCCTAAACTGTTAAAATCAAATT
CTATTCACAGCTCTTTGATTTCTTGCTAATTCAGGAGAGAAACTTTTCCAATCACTTTAA
TTTTTTTTATTAATTTAAAG
>1639
AAAATAGTGATTATCCTAAACTGTTAAAATCAAATTCTATTCACAGCTCTTTGATTTCTT
GCTAATTCAGGAGAGAAACTTTTCCAATCACTTTAATTTTTTTTATTAATTTAAAGCAAG
CCCTGCTGAAAGCCCAAGTA
>1640
AGTGATTATCCTAAACTGTTAAAATCAAATTCTATTCACAGCTCTTTGATTTCTTGCTAAT

TCAGGAGAGAAACTTTTCCAATCACTTTAATTTTTTTTATTAATTTAAAGCAAGCCCTGC
TGAAAGCCCAAGTATATAT
>1641
CTCTTTGATTTCTTGCTAATTCAGGAGAGAAACTTTTCCAATCACTTTAATTTTTTTTATT
AATTTAAAGCAAGCCCTGCTGAAAGCCCAAGTATATATGACTTAGAGTCTTACTAAATG
AATACAGGTGTGCCTCAGTT
>1642
TTGATTTCTTGCTAATTCAGGAGAGAAACTTTTCCAATCACTTTAATTTTTTTTATTAATT
TAAAGCAAGCCCTGCTGAAAGCCCAAGTATATATGACTTAGAGTCTTACTAAATGAATA
CAGGTGTGCCTCAGTTTGTC
>1643
AGGAGAGAAACTTTTCCAATCACTTTAATTTTTTTTATTAATTTAAAGCAAGCCCTGCTG
AAAGCCCAAGTATATATGACTTAGAGTCTTACTAAATGAATACAGGTGTGCCTCAGTTT
GTCCATTGGAGGGTTTCTGAA
>1644
TATTAATTTAAAGCAAGCCCTGCTGAAAGCCCAAGTATATATGACTTAGAGTCTTACTAA
ATGAATACAGGTGTGCCTCAGTTTGTCCATTGGAGGGTTTCTGAATTTTCCATAACTAA
AGATTTTCTATTCTGTTTTGG
>1645
AGCAAGCCCTGCTGAAAGCCCAAGTATATATGACTTAGAGTCTTACTAAATGAATACAG
GTGTGCCTCAGTTTGTCCATTGGAGGGTTTCTGAATTTTCCATAACTAAAGATTTTCTAT
TCTGTTTTGGCTGGGTGTGTG
>1646
AAAGCCCAAGTATATATGACTTAGAGTCTTACTAAATGAATACAGGTGTGCCTCAGTTT
GTCCATTGGAGGGTTTCTGAATTTTCCATAACTAAAGATTTTCTATTCTGTTTTGGCTGG
GTGTGTGGGGTGAAAGCTCTT
>1647
TCTTACTAAATGAATACAGGTGTGCCTCAGTTTGTCCATTGGAGGGTTTCTGAATTTTCC
ATAACTAAAGATTTTCTATTCTGTTTTGGCTGGGTGTGTGGGGTGAAAGCTCTTACTCTC
TGGCAGTTATTTAAGTAGTA
>1648
TGGAGGGTTTCTGAATTTTCCATAACTAAAGATTTTCTATTCTGTTTTGGCTGGGTGTGT
GGGGTGAAAGCTCTTACTCTCTGGCAGTTATTTAAGTAGTAAGAAATTTGCTACAACTC
TTCCCCACCCCCACGTTTTAC
>1649
TTCCATAACTAAAGATTTTCTATTCTGTTTTGGCTGGGTGTGTGGGGTGAAAGCTCTTAC
TCTCTGGCAGTTATTTAAGTAGTAAGAAATTTGCTACAACTCTTCCCCACCCCCACGTTT
TACAGATAGGAAATGAGCAC
>1650
TAAAGATTTTCTATTCTGTTTTGGCTGGGTGTGTGGGGTGAAAGCTCTTACTCTCTGGC
AGTTATTTAAGTAGTAAGAAATTTGCTACAACTCTTCCCCACCCCCACGTTTTACAGATA
GGAAATGAGCACTTTGACTGT
>1651
AGATTTTCTATTCTGTTTTGGCTGGGTGTGTGGGGTGAAAGCTCTTACTCTCTGGCAGT
TATTTAAGTAGTAAGAAATTTGCTACAACTCTTCCCCACCCCCACGTTTTACAGATAGG
AAATGAGCACTTTGACTGTCCA

>1652
CTTACTCTCTGGCAGTTATTTAAGTAGTAAGAAATTTGCTACAACTCTTCCCCACCCCCA
CGTTTTACAGATAGGAAATGAGCACTTTGACTGTCCAGGTCAGACTTCTGATTTTGCTA
CTTACTAACTGTGACAGTGGT
>1653
CAGTTATTTAAGTAGTAAGAAATTTGCTACAACTCTTCCCCACCCCCACGTTTTACAGAT
AGGAAATGAGCACTTTGACTGTCCAGGTCAGACTTCTGATTTTGCTACTTACTAACTGT
GACAGTGGTTGTGTTACTTAA
>1654
AAGAAATTTGCTACAACTCTTCCCCACCCCCACGTTTTACAGATAGGAAATGAGCACTT
TGACTGTCCAGGTCAGACTTCTGATTTTGCTACTTACTAACTGTGACAGTGGTTGTGTT
ACTTAACTTCTGTGTGCCTGTT
>1655
ACAGATAGGAAATGAGCACTTTGACTGTCCAGGTCAGACTTCTGATTTTGCTACTTACT
AACTGTGACAGTGGTTGTGTTACTTAACTTCTGTGTGCCTGTTTGCTCATTTGTGAAAC
AGAAATAATAGTAGTACCTACT
>1656
CTACTTACTAACTGTGACAGTGGTTGTGTTACTTAACTTCTGTGTGCCTGTTTGCTCATT
TGTGAAACAGAAATAATAGTAGTACCTACTTCATTGGGTTGTTATAAGGATTCATCAGA
AAATATTTATCCTGTGTGAGG
>1657
AACTGTGACAGTGGTTGTGTTACTTAACTTCTGTGTGCCTGTTTGCTCATTTGTGAAAC
AGAAATAATAGTAGTACCTACTTCATTGGGTTGTTATAAGGATTCATCAGAAAATATTTA
TCCTGTGTGAGGAACGGTGGC
>1658
TGTGACAGTGGTTGTGTTACTTAACTTCTGTGTGCCTGTTTGCTCATTTGTGAAACAGA
AATAATAGTAGTACCTACTTCATTGGGTTGTTATAAGGATTCATCAGAAAATATTTATCCT
GTGTGAGGAACGGTGGCACA
>1659
TCTGTGTGCCTGTTTGCTCATTTGTGAAACAGAAATAATAGTAGTACCTACTTCATTGGG
TTGTTATAAGGATTCATCAGAAAATATTTATCCTGTGTGAGGAACGGTGGCACATAGTA
AGTGCTCCATAAGTGTTTGCT
>1660
TGTTTGCTCATTTGTGAAACAGAAATAATAGTAGTACCTACTTCATTGGGTTGTTATAAG
GATTCATCAGAAAATATTTATCCTGTGTGAGGAACGGTGGCACATAGTAAGTGCTCCAT
AAGTGTTTGCTAGGGTATTTT
>1661
CTACTTCATTGGGTTGTTATAAGGATTCATCAGAAAATATTTATCCTGTGTGAGGAACGG
TGGCACATAGTAAGTGCTCCATAAGTGTTTGCTAGGGTATTTTTCTTGTCATTGTGTGAA
GTAGGTCATGCTAGCCATGC
>1662
TTCATTGGGTTGTTATAAGGATTCATCAGAAAATATTTATCCTGTGTGAGGAACGGTGGC
ACATAGTAAGTGCTCCATAAGTGTTTGCTAGGGTATTTTTCTTGTCATTGTGTGAAGTAG
GTCATGCTAGCCATGCAGGG
>1663
TTCATCAGAAAATATTTATCCTGTGTGAGGAACGGTGGCACATAGTAAGTGCTCCATAA

GTGTTTGCTAGGGTATTTTTCTTGTCATTGTGTGAAGTAGGTCATGCTAGCCATGCAGG
GTGAAAAATGTGTTCATGTTGG
>1664
GAGGAACGGTGGCACATAGTAAGTGCTCCATAAGTGTTTGCTAGGGTATTTTTCTTGTC
ATTGTGTGAAGTAGGTCATGCTAGCCATGCAGGGTGAAAAATGTGTTCATGTTGGTCCA
GATCCTGCTTTCTCCCTGAGAC
>1665
GAACGGTGGCACATAGTAAGTGCTCCATAAGTGTTTGCTAGGGTATTTTTCTTGTCATTG
TGTGAAGTAGGTCATGCTAGCCATGCAGGGTGAAAAATGTGTTCATGTTGGTCCAGATC
CTGCTTTCTCCCTGAGACCGT
>1666
ACATAGTAAGTGCTCCATAAGTGTTTGCTAGGGTATTTTTCTTGTCATTGTGTGAAGTAG
GTCATGCTAGCCATGCAGGGTGAAAAATGTGTTCATGTTGGTCCAGATCCTGCTTTCTC
CCTGAGACCGTTCTTCCGCAT
>1667
TTTTCTTGTCATTGTGTGAAGTAGGTCATGCTAGCCATGCAGGGTGAAAAATGTGTTCA
TGTTGGTCCAGATCCTGCTTTCTCCCTGAGACCGTTCTTCCGCATAGGCGTCAATCCGT
ACAGTGCTGTAAAACTGCTGCC
>1668
AGTAGGTCATGCTAGCCATGCAGGGTGAAAAATGTGTTCATGTTGGTCCAGATCCTGCT
TTCTCCCTGAGACCGTTCTTCCGCATAGGCGTCAATCCGTACAGTGCTGTAAAACTGCT
GCCTCGGGCACCTCATGTTTGG
>1669
ATGCAGGGTGAAAAATGTGTTCATGTTGGTCCAGATCCTGCTTTCTCCCTGAGACCGTT
CTTCCGCATAGGCGTCAATCCGTACAGTGCTGTAAAACTGCTGCCTCGGGCACCTCATG
TTTGGGGCTGAGACATTGCTTC
>1670
GTGTTCATGTTGGTCCAGATCCTGCTTTCTCCCTGAGACCGTTCTTCCGCATAGGCGTC
AATCCGTACAGTGCTGTAAAACTGCTGCCTCGGGCACCTCATGTTTGGGGCTGAGACA
TTGCTTCTGCGTCTTCCCCAGAA
>1671
TTCCGCATAGGCGTCAATCCGTACAGTGCTGTAAAACTGCTGCCTCGGGCACCTCATGT
TTGGGGCTGAGACATTGCTTCTGCGTCTTCCCCAGAAGCTGATCTCTTGTACTGTGGTG
GCCAGGGTACTCTTTGTCCCAT
>1672
AGTGCTGTAAAACTGCTGCCTCGGGCACCTCATGTTTGGGGCTGAGACATTGCTTCTG
CGTCTTCCCCAGAAGCTGATCTCTTGTACTGTGGTGGCCAGGGTACTCTTTGTCCCATT
GACCTGAGGCCTGGTGAGTCATT
>1673
TCATGTTTGGGGCTGAGACATTGCTTCTGCGTCTTCCCCAGAAGCTGATCTCTTGTACT
GTGGTGGCCAGGGTACTCTTTGTCCCATTGACCTGAGGCCTGGTGAGTCATTCCAGTTC
TCGTTTAAATTTAGCCGGGCAG
>1674
CTGCGTCTTCCCCAGAAGCTGATCTCTTGTACTGTGGTGGCCAGGGTACTCTTTGTCCC
ATTGACCTGAGGCCTGGTGAGTCATTCCAGTTCTCGTTTAAATTTAGCCGGGCAGCGCT
CCACTGCCATCGGGTCCAAATG

>1675
TGATCTCTTGTACTGTGGTGGCCAGGGTACTCTTTGTCCCATTGACCTGAGGCCTGGTG
AGTCATTCCAGTTCTCGTTTAAATTTAGCCGGGCAGCGCTCCACTGCCATCGGGTCCAA
ATGCCGGGGAGGCTCACTGATG
>1676
GTGGCCAGGGTACTCTTTGTCCCATTGACCTGAGGCCTGGTGAGTCATTCCAGTTCTCG
TTTAAATTTAGCCGGGCAGCGCTCCACTGCCATCGGGTCCAAATGCCGGGGAGGCTCA
CTGATGCGGTGTCCTGTTGCCTG
>1677
AGTCATTCCAGTTCTCGTTTAAATTTAGCCGGGCAGCGCTCCACTGCCATCGGGTCCAA
ATGCCGGGGAGGCTCACTGATGCGGTGTCCTGTTGCCTGTGCACGTGGCCAGGCGTGC
ACATCACTGTGCTTATGAGAGAG
>1678
CACTGCCATCGGGTCCAAATGCCGGGGAGGCTCACTGATGCGGTGTCCTGTTGCCTGT
GCACGTGGCCAGGCGTGCACATCACTGTGCTTATGAGAGAGATGAAGAGCACAGATAC
CACCTTACTGATGGGGGTCCTGGG
>1679
GGAGGCTCACTGATGCGGTGTCCTGTTGCCTGTGCACGTGGCCAGGCGTGCACATCAC
TGTGCTTATGAGAGAGATGAAGAGCACAGATACCACCTTACTGATGGGGGTCCTGGGG
CTGGGCTTGTGGGTGTTGGGGGTT
>1680
AGGCTCACTGATGCGGTGTCCTGTTGCCTGTGCACGTGGCCAGGCGTGCACATCACTG
TGCTTATGAGAGAGATGAAGAGCACAGATACCACCTTACTGATGGGGGTCCTGGGGCT
GGGCTTGTGGGTGTTGGGGGTTCC
>1681
GTGTCCTGTTGCCTGTGCACGTGGCCAGGCGTGCACATCACTGTGCTTATGAGAGAGA
TGAAGAGCACAGATACCACCTTACTGATGGGGGTCCTGGGGCTGGGCTTGTGGGTGTT
GGGGGTTCCAGAGCTCCTTCCCTT
>1682
ATGAAGAGCACAGATACCACCTTACTGATGGGGGTCCTGGGGCTGGGCTTGTGGGTGT
TGGGGGTTCCAGAGCTCCTTCCCTTTCCTGCCTGTCAGTGCAGGATGCTTCTAAAATGT
GGACTCAGCAGACATTGTTTTGA
>1683
GAAGAGCACAGATACCACCTTACTGATGGGGGTCCTGGGGCTGGGCTTGTGGGTGTTG
GGGGTTCCAGAGCTCCTTCCCTTTCCTGCCTGTCAGTGCAGGATGCTTCTAAAATGTGG
ACTCAGCAGACATTGTTTTGAGT
>1684
GGGTCCTGGGGCTGGGCTTGTGGGTGTTGGGGGTTCCAGAGCTCCTTCCCTTTCCTGC
CTGTCAGTGCAGGATGCTTCTAAAATGTGGACTCAGCAGACATTGTTTTGAGTGCTTGC
TACATGTCAGATTCTGTGCTAAG
>1685
TGTTGGGGGTTCCAGAGCTCCTTCCCTTTCCTGCCTGTCAGTGCAGGATGCTTCTAAAA
TGTGGACTCAGCAGACATTGTTTTGAGTGCTTGCTACATGTCAGATTCTGTGCTAAGTG
CACTCACAGCCAGCATCTCTGT
>1686
TGGGGGTTCCAGAGCTCCTTCCCTTTCCTGCCTGTCAGTGCAGGATGCTTCTAAAATGT

GGACTCAGCAGACATTGTTTTGAGTGCTTGCTACATGTCAGATTCTGTGCTAAGTGCAC
TCACAGCCAGCATCTCTGTAAG
>1687
CCTGTCAGTGCAGGATGCTTCTAAAATGTGGACTCAGCAGACATTGTTTTGAGTGCTTG
CTACATGTCAGATTCTGTGCTAAGTGCACTCACAGCCAGCATCTCTGTAAGACCTCTAG
CTGTCCTGTAAGGTGGGTATCA
>1688
GATGCTTCTAAAATGTGGACTCAGCAGACATTGTTTTGAGTGCTTGCTACATGTCAGAT
TCTGTGCTAAGTGCACTCACAGCCAGCATCTCTGTAAGACCTCTAGCTGTCCTGTAAGG
TGGGTATCAGTTTCACATTCTC
>1689
AATGTGGACTCAGCAGACATTGTTTTGAGTGCTTGCTACATGTCAGATTCTGTGCTAAG
TGCACTCACAGCCAGCATCTCTGTAAGACCTCTAGCTGTCCTGTAAGGTGGGTATCAGT
TTCACATTCTCAGATGGGCGGA
>1690
ACATTGTTTTGAGTGCTTGCTACATGTCAGATTCTGTGCTAAGTGCACTCACAGCCAGC
ATCTCTGTAAGACCTCTAGCTGTCCTGTAAGGTGGGTATCAGTTTCACATTCTCAGATG
GGCGGAGAGAAGCCCAAGAGGT
>1691
TTCTGTGCTAAGTGCACTCACAGCCAGCATCTCTGTAAGACCTCTAGCTGTCCTGTAAG
GTGGGTATCAGTTTCACATTCTCAGATGGGCGGAGAGAAGCCCAAGAGGTTAGTGGAC
TTTTCTCTAGTAAAGAGTTGTAC
>1692
CACTCACAGCCAGCATCTCTGTAAGACCTCTAGCTGTCCTGTAAGGTGGGTATCAGTTT
CACATTCTCAGATGGGCGGAGAGAAGCCCAAGAGGTTAGTGGACTTTTCTCTAGTAAA
GAGTTGTACAGGCCGGGCGCAGT
>1693
CAGCATCTCTGTAAGACCTCTAGCTGTCCTGTAAGGTGGGTATCAGTTTCACATTCTCA
GATGGGCGGAGAGAAGCCCAAGAGGTTAGTGGACTTTTCTCTAGTAAAGAGTTGTACA
GGCCGGGCGCAGTGGCTCATGCC
>1694
AGGTGGGTATCAGTTTCACATTCTCAGATGGGCGGAGAGAAGCCCAAGAGGTTAGTGG
ACTTTTCTCTAGTAAAGAGTTGTACAGGCCGGGCGCAGTGGCTCATGCCTGTAATCCCA
GCACTTTGGGAGGCCGAGGCGGG
>1695
GGTATCAGTTTCACATTCTCAGATGGGCGGAGAGAAGCCCAAGAGGTTAGTGGACTTT
TCTCTAGTAAAGAGTTGTACAGGCCGGGCGCAGTGGCTCATGCCTGTAATCCCAGCAC
TTTGGGAGGCCGAGGCGGGCGGAT
>1696
TCACATTCTCAGATGGGCGGAGAGAAGCCCAAGAGGTTAGTGGACTTTTCTCTAGTAA
AGAGTTGTACAGGCCGGGCGCAGTGGCTCATGCCTGTAATCCCAGCACTTTGGGAGGC
CGAGGCGGGCGGATCACGAGGTCA
>1697
AGGTTAGTGGACTTTTCTCTAGTAAAGAGTTGTACAGGCCGGGCGCAGTGGCTCATGC
CTGTAATCCCAGCACTTTGGGAGGCCGAGGCGGGCGGATCACGAGGTCAGGAGATCG
AGACCATCCTGGCTAACACAGTGAA

>1698
CTTTTCTCTAGTAAAGAGTTGTACAGGCCGGGCGCAGTGGCTCATGCCTGTAATCCCAG
CACTTTGGGAGGCCGAGGCGGGCGGATCACGAGGTCAGGAGATCGAGACCATCCTGG
CTAACACAGTGAAACCCCGTCTCT
>1699
TCTAGTAAAGAGTTGTACAGGCCGGGCGCAGTGGCTCATGCCTGTAATCCCAGCACTT
TGGGAGGCCGAGGCGGGCGGATCACGAGGTCAGGAGATCGAGACCATCCTGGCTAAC
ACAGTGAAACCCCGTCTCTACTAAA
>1700
ACAGGCCGGGCGCAGTGGCTCATGCCTGTAATCCCAGCACTTTGGGAGGCCGAGGCG
GGCGGATCACGAGGTCAGGAGATCGAGACCATCCTGGCTAACACAGTGAAACCCCGT
CTCTACTAAAAATACAAAAAATTAGC
>1701
GTGGCTCATGCCTGTAATCCCAGCACTTTGGGAGGCCGAGGCGGGCGGATCACGAGGT
CAGGAGATCGAGACCATCCTGGCTAACACAGTGAAACCCCGTCTCTACTAAAAATACA
AAAAATTAGCCGGGCGTGGTGGGG
>1702
GGTCAGGAGATCGAGACCATCCTGGCTAACACAGTGAAACCCCGTCTCTACTAAAAAT
ACAAAAAATTAGCCGGGCGTGGTGGGGGGCGCCTGTAGTCCCAGCTACTCGGGAGGC
TGAGGCAGGAGAATGGCGTGAACCC
>1703
TAACACAGTGAAACCCCGTCTCTACTAAAAATACAAAAAATTAGCCGGGCGTGGTGGG
GGGCGCCTGTAGTCCCAGCTACTCGGGAGGCTGAGGCAGGAGAATGGCGTGAACCCG
GGAGGCAGAGCTTGCAGTGAGCCGA
>1704
AGTGAAACCCCGTCTCTACTAAAAATACAAAAAATTAGCCGGGCGTGGTGGGGGGCGC
CTGTAGTCCCAGCTACTCGGGAGGCTGAGGCAGGAGAATGGCGTGAACCCGGGAGGC
AGAGCTTGCAGTGAGCCGAGATCAT
>1705
GTCTCTACTAAAAATACAAAAAATTAGCCGGGCGTGGTGGGGGGCGCCTGTAGTCCCA
GCTACTCGGGAGGCTGAGGCAGGAGAATGGCGTGAACCCGGGAGGCAGAGCTTGCAG
TGAGCCGAGATCATGCCACTCACTA
>1706
ACTAAAAATACAAAAAATTAGCCGGGCGTGGTGGGGGGCGCCTGTAGTCCCAGCTACT
CGGGAGGCTGAGGCAGGAGAATGGCGTGAACCCGGGAGGCAGAGCTTGCAGTGAGC
CGAGATCATGCCACTCACTACACTCC
>1707
CGTGGTGGGGGGCGCCTGTAGTCCCAGCTACTCGGGAGGCTGAGGCAGGAGAATGGC
GTGAACCCGGGAGGCAGAGCTTGCAGTGAGCCGAGATCATGCCACTCACTACACTCC
AGCCTGGGCAAAGGAGTGAGACTGTC
>1708
GCCTGTAGTCCCAGCTACTCGGGAGGCTGAGGCAGGAGAATGGCGTGAACCCGGGAG
GCAGAGCTTGCAGTGAGCCGAGATCATGCCACTCACTACACTCCAGCCTGGGCAAAG
GAGTGAGACTGTCTCAAAAAAAAAAA
>1709
GTAGTCCCAGCTACTCGGGAGGCTGAGGCAGGAGAATGGCGTGAACCCGGGAGGCAG

AGCTTGCAGTGAGCCGAGATCATGCCACTCACTACACTCCAGCCTGGGCAAAGGAGTG
AGACTGTCTCAAAAAAAAAAAAAAA
>1710
CCCAGCTACTCGGGAGGCTGAGGCAGGAGAATGGCGTGAACCCGGGAGGCAGAGCTT
GCAGTGAGCCGAGATCATGCCACTCACTACACTCCAGCCTGGGCAAAGGAGTGAGAC
TGTCTCAAAAAAAAAAAAAAAAAAAA
>1711
AGGAGAATGGCGTGAACCCGGGAGGCAGAGCTTGCAGTGAGCCGAGATCATGCCACT
CACTACACTCCAGCCTGGGCAAAGGAGTGAGACTGTCTCAAAAAAAAAAAAAAAAA
AAAAGAGTTGTACCAGGATTTATGTTC
>1712
CGGGAGGCAGAGCTTGCAGTGAGCCGAGATCATGCCACTCACTACACTCCAGCCTGG
GCAAAGGAGTGAGACTGTCTCAAAAAAAAAAAAAAAAAAAAAGAGTTGTACCAGGA
TTTATGTTCAGGTCTTCTGGCTCCATG
>1713
CATGCCACTCACTACACTCCAGCCTGGGCAAAGGAGTGAGACTGTCTCAAAAAAAAA
AAAAAAAAAAAAGAGTTGTACCAGGATTTATGTTCAGGTCTTCTGGCTCCATGCTCTCT
GCAGCTGGGTGACCAGGGGGCTCA
>1714
TGCCACTCACTACACTCCAGCCTGGGCAAAGGAGTGAGACTGTCTCAAAAAAAAAAA
AAAAAAAAAAGAGTTGTACCAGGATTTATGTTCAGGTCTTCTGGCTCCATGCTCTCTGC
AGCTGGGTGACCAGGGGGCTCATG
>1715
AAAAAAAAAAAAAAAAAAAGAGTTGTACCAGGATTTATGTTCAGGTCTTCTGGCTCCA
TGCTCTCTGCAGCTGGGTGACCAGGGGGCTCATGGCTTCACCAGCTCTGGCCTCCTCA
GTTCCTCTGGTGGCTTCTTGCGAG
>1716
AAAAAAAGAGTTGTACCAGGATTTATGTTCAGGTCTTCTGGCTCCATGCTCTCTGCAGC
TGGGTGACCAGGGGGCTCATGGCTTCACCAGCTCTGGCCTCCTCAGTTCCTCTGGTGG
CTTCTTGCGAGGAGGAAGGTTAC
>1717
ATTTATGTTCAGGTCTTCTGGCTCCATGCTCTCTGCAGCTGGGTGACCAGGGGGCTCAT
GGCTTCACCAGCTCTGGCCTCCTCAGTTCCTCTGGTGGCTTCTTGCGAGGAGGAAGGT
TACTGCAGAGGGTTCAGCTCTTT
>1718
CTGGGTGACCAGGGGGCTCATGGCTTCACCAGCTCTGGCCTCCTCAGTTCCTCTGGTG
GCTTCTTGCGAGGAGGAAGGTTACTGCAGAGGGTTCAGCTCTTTAGGGGCCCAGGCC
GCATGGATCCAAGGACTAGTACGTC
>1719
GGTGACCAGGGGGCTCATGGCTTCACCAGCTCTGGCCTCCTCAGTTCCTCTGGTGGCT
TCTTGCGAGGAGGAAGGTTACTGCAGAGGGTTCAGCTCTTTAGGGGCCCAGGCCGCAT
GGATCCAAGGACTAGTACGTCATC
>1720
CACCAGCTCTGGCCTCCTCAGTTCCTCTGGTGGCTTCTTGCGAGGAGGAAGGTTACTG
CAGAGGGTTCAGCTCTTTAGGGGCCCAGGCCGCATGGATCCAAGGACTAGTACGTCAT
CTCTTACCAACACAGAGAGATCAG

>1721
CTCAGTTCCTCTGGTGGCTTCTTGCGAGGAGGAAGGTTACTGCAGAGGGTTCAGCTCT
TTAGGGGCCCAGGCCGCATGGATCCAAGGACTAGTACGTCATCTCTTACCAACACAGA
GAGATCAGCTTCTAGTGATAGAGG
>1722
GCTTCTTGCGAGGAGGAAGGTTACTGCAGAGGGTTCAGCTCTTTAGGGGCCCAGGCC
GCATGGATCCAAGGACTAGTACGTCATCTCTTACCAACACAGAGAGATCAGCTTCTAGT
GATAGAGGCCTTTTATGCTGACTT
>1723
TGCGAGGAGGAAGGTTACTGCAGAGGGTTCAGCTCTTTAGGGGCCCAGGCCGCATGG
ATCCAAGGACTAGTACGTCATCTCTTACCAACACAGAGAGATCAGCTTCTAGTGATAGA
GGCCTTTTATGCTGACTTGAAAAA
>1724
GGTTCAGCTCTTTAGGGGCCCAGGCCGCATGGATCCAAGGACTAGTACGTCATCTCTTA
CCAACACAGAGAGATCAGCTTCTAGTGATAGAGGCCTTTTATGCTGACTTGAAAAACA
GAAAAAGAGAGAAAAAAGAAACC
>1725
GGGGCCCAGGCCGCATGGATCCAAGGACTAGTACGTCATCTCTTACCAACACAGAGAG
ATCAGCTTCTAGTGATAGAGGCCTTTTATGCTGACTTGAAAAACAGAAAAAGAGAGAA
AAAAGAAACCCATTTACAAAAATA
>1726
CAGGCCGCATGGATCCAAGGACTAGTACGTCATCTCTTACCAACACAGAGAGATCAGC
TTCTAGTGATAGAGGCCTTTTATGCTGACTTGAAAAACAGAAAAAGAGAGAAAAAAG
AAACCCATTTACAAAAATAAAAATC
>1727
GTCATCTCTTACCAACACAGAGAGATCAGCTTCTAGTGATAGAGGCCTTTTATGCTGAC
TTGAAAAACAGAAAAAGAGAGAAAAAAGAAACCCATTTACAAAAATAAAAATCCCAT
GCTGTTAATTTCATGCCAGCATAA
>1728
CAGAGAGATCAGCTTCTAGTGATAGAGGCCTTTTATGCTGACTTGAAAAACAGAAAAA
GAGAGAAAAAAGAAACCCATTTACAAAAATAAAAATCCCATGCTGTTAATTTCATGCC
AGCATAATCAGAAATTCCCCTTTC
>1729
AACAGAAAAAGAGAGAAAAAAGAAACCCATTTACAAAAATAAAAATCCCATGCTGTT
AATTTCATGCCAGCATAATCAGAAATTCCCCTTTCTACTGTTTCCCCTTAAAAGCCCCGG
TGTCCTTATTATGCTGGTGCAGT
>1730
AGAGAGAAAAAAGAAACCCATTTACAAAAATAAAAATCCCATGCTGTTAATTTCATGC
CAGCATAATCAGAAATTCCCCTTTCTACTGTTTCCCCTTAAAAGCCCCGGTGTCCTTATT
ATGCTGGTGCAGTCTGGAAAAA
>1731
AAAATCCCATGCTGTTAATTTCATGCCAGCATAATCAGAAATTCCCCTTTCTACTGTTTC
CCCTTAAAAGCCCCGGTGTCCTTATTATGCTGGTGCAGTCTGGAAAAACTATGGTTGGG
TTTTGCCTACGTGAGGCTTAT
>1732
GCATAATCAGAAATTCCCCTTTCTACTGTTTCCCCTTAAAAGCCCCGGTGTCCTTATTAT

GCTGGTGCAGTCTGGAAAAACTATGGTTGGGTTTTGCCTACGTGAGGCTTATCTCTGCA
GCTGCAAGGAGGGGATCCGGT
>1733
TAAAAGCCCCGGTGTCCTTATTATGCTGGTGCAGTCTGGAAAAACTATGGTTGGGTTTT
GCCTACGTGAGGCTTATCTCTGCAGCTGCAAGGAGGGGATCCGGTACTTTATTGTCAGC
ATGGAAACTTCCGCTAAATACT
>1734
TCCTTATTATGCTGGTGCAGTCTGGAAAAACTATGGTTGGGTTTTGCCTACGTGAGGCT
TATCTCTGCAGCTGCAAGGAGGGGATCCGGTACTTTATTGTCAGCATGGAAACTTCCGC
TAAATACTTAGAAAAACCTTAA
>1735
TGGTTGGGTTTTGCCTACGTGAGGCTTATCTCTGCAGCTGCAAGGAGGGGATCCGGTA
CTTTATTGTCAGCATGGAAACTTCCGCTAAATACTTAGAAAAACCTTAATTCTAAATTCC
TAGCTCACTTTCAACTGTGTCC
>1736
TATCTCTGCAGCTGCAAGGAGGGGATCCGGTACTTTATTGTCAGCATGGAAACTTCCGC
TAAATACTTAGAAAAACCTTAATTCTAAATTCCTAGCTCACTTTCAACTGTGTCCTCGGG
ACCTGATTTTCTTCGGTGTGA
>1737
TCCGGTACTTTATTGTCAGCATGGAAACTTCCGCTAAATACTTAGAAAAACCTTAATTCT
AAATTCCTAGCTCACTTTCAACTGTGTCCTCGGGACCTGATTTTCTTCGGTGTGAACAG
ATTGTGTTCAGCATTGCAGTA
>1738
ACCTTAATTCTAAATTCCTAGCTCACTTTCAACTGTGTCCTCGGGACCTGATTTTCTTCG
GTGTGAACAGATTGTGTTCAGCATTGCAGTAGAAAGGGTCCAAGTTAGGTAAGAGAAA
GGACTTCCTGAGACACTGGGGT
>1739
AAATTCCTAGCTCACTTTCAACTGTGTCCTCGGGACCTGATTTTCTTCGGTGTGAACAG
ATTGTGTTCAGCATTGCAGTAGAAAGGGTCCAAGTTAGGTAAGAGAAAGGACTTCCTG
AGACACTGGGGTGTTACTGAGAA
>1740
AGCTCACTTTCAACTGTGTCCTCGGGACCTGATTTTCTTCGGTGTGAACAGATTGTGTT
CAGCATTGCAGTAGAAAGGGTCCAAGTTAGGTAAGAGAAAGGACTTCCTGAGACACT
GGGGTGTTACTGAGAAAGTTGTTA
>1741
GTGTCCTCGGGACCTGATTTTCTTCGGTGTGAACAGATTGTGTTCAGCATTGCAGTAGA
AAGGGTCCAAGTTAGGTAAGAGAAAGGACTTCCTGAGACACTGGGGTGTTACTGAGA
AAGTTGTTAGAAGACTGTCACAGA
>1742
GTGTGAACAGATTGTGTTCAGCATTGCAGTAGAAAGGGTCCAAGTTAGGTAAGAGAAA
GGACTTCCTGAGACACTGGGGTGTTACTGAGAAAGTTGTTAGAAGACTGTCACAGACA
TTTTGATGAAGGGGGGACATTCTG
>1743
AGCATTGCAGTAGAAAGGGTCCAAGTTAGGTAAGAGAAAGGACTTCCTGAGACACTG
GGGTGTTACTGAGAAAGTTGTTAGAAGACTGTCACAGACATTTTGATGAAGGGGGGAC
ATTCTGGGCCATTTGAAGTGGTTTT

>1744
TTGCAGTAGAAAGGGTCCAAGTTAGGTAAGAGAAAGGACTTCCTGAGACACTGGGGT
GTTACTGAGAAAGTTGTTAGAAGACTGTCACAGACATTTTGATGAAGGGGGGACATTC
TGGGCCATTTGAAGTGGTTTTTAGA
>1745
GTTAGGTAAGAGAAAGGACTTCCTGAGACACTGGGGTGTTACTGAGAAAGTTGTTAG
AAGACTGTCACAGACATTTTGATGAAGGGGGGACATTCTGGGCCATTTGAAGTGGTTT
TTAGAGTTCTTTCCTCTTTAGGGCC
>1746
TACTGAGAAAGTTGTTAGAAGACTGTCACAGACATTTTGATGAAGGGGGGACATTCTG
GGCCATTTGAAGTGGTTTTTAGAGTTCTTTCCTCTTTAGGGCCATTCGGTTCCCCCAGA
AATAAGAAGAAAAGACATATACA
>1747
GTTGTTAGAAGACTGTCACAGACATTTTGATGAAGGGGGGACATTCTGGGCCATTTGA
AGTGGTTTTTAGAGTTCTTTCCTCTTTAGGGCCATTCGGTTCCCCCAGAAATAAGAAGA
AAAGACATATACACGCACATAAA
>1748
GGGGACATTCTGGGCCATTTGAAGTGGTTTTTAGAGTTCTTTCCTCTTTAGGGCCATTC
GGTTCCCCCAGAAATAAGAAGAAAAGACATATACACGCACATAAACCACCACCACCAG
GCTTGCTGAGAACAAGTCAAATG
>1749
TTCTGGGCCATTTGAAGTGGTTTTTAGAGTTCTTTCCTCTTTAGGGCCATTCGGTTCCCC
CAGAAATAAGAAGAAAAGACATATACACGCACATAAACCACCACCACCAGGCTTGCTG
AGAACAAGTCAAATGTGCCACC
>1750
CATTTGAAGTGGTTTTTAGAGTTCTTTCCTCTTTAGGGCCATTCGGTTCCCCCAGAAATA
AGAAGAAAAGACATATACACGCACATAAACCACCACCACCAGGCTTGCTGAGAACAA
GTCAAATGTGCCACCAAACCATC
>1751
TTAGGGCCATTCGGTTCCCCCAGAAATAAGAAGAAAAGACATATACACGCACATAAAC
CACCACCACCAGGCTTGCTGAGAACAAGTCAAATGTGCCACCAAACCATCGTCTTAGT
GGGAGATGGGGTGGCACTGCAGGG
>1752
TCGGTTCCCCCAGAAATAAGAAGAAAAGACATATACACGCACATAAACCACCACCACC
AGGCTTGCTGAGAACAAGTCAAATGTGCCACCAAACCATCGTCTTAGTGGGAGATGGG
GTGGCACTGCAGGGGCAGAAAGAC
>1753
CCCCCAGAAATAAGAAGAAAAGACATATACACGCACATAAACCACCACCACCAGGCTT
GCTGAGAACAAGTCAAATGTGCCACCAAACCATCGTCTTAGTGGGAGATGGGGTGGC
ACTGCAGGGGCAGAAAGACTGACGT
>1754
CACGCACATAAACCACCACCACCAGGCTTGCTGAGAACAAGTCAAATGTGCCACCAA
ACCATCGTCTTAGTGGGAGATGGGGTGGCACTGCAGGGGCAGAAAGACTGACGTCTG
ATGGACTTGGGTTTGAATACTAGTGC
>1755
AGGCTTGCTGAGAACAAGTCAAATGTGCCACCAAACCATCGTCTTAGTGGGAGATGGG

GTGGCACTGCAGGGGCAGAAAGACTGACGTCTGATGGACTTGGGTTTGAATACTAGTG
CTCCATGGTGAATTTGGGCAAATG
>1756
GCTGAGAACAAGTCAAATGTGCCACCAAACCATCGTCTTAGTGGGAGATGGGGTGGC
ACTGCAGGGGCAGAAAGACTGACGTCTGATGGACTTGGGTTTGAATACTAGTGCTCCA
TGGTGAATTTGGGCAAATGACTTCA
>1757
AGAACAAGTCAAATGTGCCACCAAACCATCGTCTTAGTGGGAGATGGGGTGGCACTGC
AGGGGCAGAAAGACTGACGTCTGATGGACTTGGGTTTGAATACTAGTGCTCCATGGTG
AATTTGGGCAAATGACTTCACCTC
>1758
TAGTGGGAGATGGGGTGGCACTGCAGGGGCAGAAAGACTGACGTCTGATGGACTTGG
GTTTGAATACTAGTGCTCCATGGTGAATTTGGGCAAATGACTTCACCTCTGTGAAGCAG
CAGTATCCTCTTCTGTGAAGTGAG
>1759
TCTGATGGACTTGGGTTTGAATACTAGTGCTCCATGGTGAATTTGGGCAAATGACTTCA
CCTCTGTGAAGCAGCAGTATCCTCTTCTGTGAAGTGAGGTTAGCCCCTACCTCTCAATG
TGGTTTTTAAAGTATGTTAATG
>1760
GATGGACTTGGGTTTGAATACTAGTGCTCCATGGTGAATTTGGGCAAATGACTTCACCT
CTGTGAAGCAGCAGTATCCTCTTCTGTGAAGTGAGGTTAGCCCCTACCTCTCAATGTGG
TTTTTAAAGTATGTTAATGAGA
>1761
CATGGTGAATTTGGGCAAATGACTTCACCTCTGTGAAGCAGCAGTATCCTCTTCTGTGA
AGTGAGGTTAGCCCCTACCTCTCAATGTGGTTTTTAAAGTATGTTAATGAGATCACTTGT
AACCTAAAGCACACAGAGTGT
>1762
CTCTGTGAAGCAGCAGTATCCTCTTCTGTGAAGTGAGGTTAGCCCCTACCTCTCAATGT
GGTTTTTAAAGTATGTTAATGAGATCACTTGTAACCTAAAGCACACAGAGTGTGAGGCC
ACCTCCCTACTTCCTGCTTCTC
>1763
GCAGTATCCTCTTCTGTGAAGTGAGGTTAGCCCCTACCTCTCAATGTGGTTTTTAAAGTA
TGTTAATGAGATCACTTGTAACCTAAAGCACACAGAGTGTGAGGCCACCTCCCTACTTC
CTGCTTCTCTCATGACCCCAG
>1764
GTTAGCCCCTACCTCTCAATGTGGTTTTTAAAGTATGTTAATGAGATCACTTGTAACCTA
AAGCACACAGAGTGTGAGGCCACCTCCCTACTTCCTGCTTCTCTCATGACCCCAGCCT
GACAAGGGAGCCGCTGAGCCCC
>1765
TAGCCCCTACCTCTCAATGTGGTTTTTAAAGTATGTTAATGAGATCACTTGTAACCTAAA
GCACACAGAGTGTGAGGCCACCTCCCTACTTCCTGCTTCTCTCATGACCCCAGCCTGA
CAAGGGAGCCGCTGAGCCCCCT
>1766
CTACCTCTCAATGTGGTTTTTAAAGTATGTTAATGAGATCACTTGTAACCTAAAGCACAC
AGAGTGTGAGGCCACCTCCCTACTTCCTGCTTCTCTCATGACCCCAGCCTGACAAGGG
AGCCGCTGAGCCCCCTGCCACC

>1767
ATCACTTGTAACCTAAAGCACACAGAGTGTGAGGCCACCTCCCTACTTCCTGCTTCTCT
CATGACCCCAGCCTGACAAGGGAGCCGCTGAGCCCCCTGCCACCCTCACCCGGCGTT
GTGCTCTAAGTGGATTAGCTCAGC
>1768
TAAAGCACACAGAGTGTGAGGCCACCTCCCTACTTCCTGCTTCTCTCATGACCCCAGC
CTGACAAGGGAGCCGCTGAGCCCCCTGCCACCCTCACCCGGCGTTGTGCTCTAAGTGG
ATTAGCTCAGCGTTGTGATCTTAA
>1769
ACAGAGTGTGAGGCCACCTCCCTACTTCCTGCTTCTCTCATGACCCCAGCCTGACAAG
GGAGCCGCTGAGCCCCCTGCCACCCTCACCCGGCGTTGTGCTCTAAGTGGATTAGCTC
AGCGTTGTGATCTTAATGCCATTA
>1770
TCTCATGACCCCAGCCTGACAAGGGAGCCGCTGAGCCCCCTGCCACCCTCACCCGGCG
TTGTGCTCTAAGTGGATTAGCTCAGCGTTGTGATCTTAATGCCATTAGGCCTGTTGGCA
CGGCTCTCCTTGCCATAGTGAGA
>1771
GCCTGACAAGGGAGCCGCTGAGCCCCCTGCCACCCTCACCCGGCGTTGTGCTCTAAGT
GGATTAGCTCAGCGTTGTGATCTTAATGCCATTAGGCCTGTTGGCACGGCTCTCCTTGC
CATAGTGAGAAGCAGATGTGGCC
>1772
CCCCTGCCACCCTCACCCGGCGTTGTGCTCTAAGTGGATTAGCTCAGCGTTGTGATCTT
AATGCCATTAGGCCTGTTGGCACGGCTCTCCTTGCCATAGTGAGAAGCAGATGTGGCC
GACCATAATGGCCCTTAGCGTGA
>1773
CTAAGTGGATTAGCTCAGCGTTGTGATCTTAATGCCATTAGGCCTGTTGGCACGGCTCT
CCTTGCCATAGTGAGAAGCAGATGTGGCCGACCATAATGGCCCTTAGCGTGATCCATCG
GGCCCACAGTGCTGGGGCAACC
>1774
ATTAGGCCTGTTGGCACGGCTCTCCTTGCCATAGTGAGAAGCAGATGTGGCCGACCATA
ATGGCCCTTAGCGTGATCCATCGGGCCCACAGTGCTGGGGCAACCATTATTAGTGTTAA
TTTGGGGGAGCTTTTTATGGTA
>1775
CTCCTTGCCATAGTGAGAAGCAGATGTGGCCGACCATAATGGCCCTTAGCGTGATCCAT
CGGGCCCACAGTGCTGGGGCAACCATTATTAGTGTTAATTTGGGGGAGCTTTTTATGGT
AGCTGGGGGAAATGTGGAGGGA
>1776
AGATGTGGCCGACCATAATGGCCCTTAGCGTGATCCATCGGGCCCACAGTGCTGGGGC
AACCATTATTAGTGTTAATTTGGGGGAGCTTTTTATGGTAGCTGGGGGAAATGTGGAGG
GATTGTTGGAAATTTATTTAAGC
>1777
AATGGCCCTTAGCGTGATCCATCGGGCCCACAGTGCTGGGGCAACCATTATTAGTGTTA
ATTTGGGGGAGCTTTTTATGGTAGCTGGGGGAAATGTGGAGGGATTGTTGGAAATTTAT
TTAAGCTGTTCAGCAGGTTAGG
>1778
GTGATCCATCGGGCCCACAGTGCTGGGGCAACCATTATTAGTGTTAATTTGGGGGAGCT

TTTTATGGTAGCTGGGGGAAATGTGGAGGGATTGTTGGAAATTTATTTAAGCTGTTCAG
CAGGTTAGGTGCTGCTGGGGTC
>1779
GTGTTAATTTGGGGGAGCTTTTTATGGTAGCTGGGGGAAATGTGGAGGGATTGTTGGA
AATTTATTTAAGCTGTTCAGCAGGTTAGGTGCTGCTGGGGTCTACTATGGCAAAACCCT
CAGCTCCCCCAACCCATCTCTTT
>1780
TTGGGGGAGCTTTTTATGGTAGCTGGGGGAAATGTGGAGGGATTGTTGGAAATTTATTT
AAGCTGTTCAGCAGGTTAGGTGCTGCTGGGGTCTACTATGGCAAAACCCTCAGCTCCC
CCAACCCATCTCTTTAAGGAACG
>1781
TTGTTGGAAATTTATTTAAGCTGTTCAGCAGGTTAGGTGCTGCTGGGGTCTACTATGGC
AAAACCCTCAGCTCCCCCAACCCATCTCTTTAAGGAACGTCCCTCCCCAACACCGAAG
CCCAGGCCTGATTACTTCCAGAG
>1782
TTCAGCAGGTTAGGTGCTGCTGGGGTCTACTATGGCAAAACCCTCAGCTCCCCCAACC
CATCTCTTTAAGGAACGTCCCTCCCCAACACCGAAGCCCAGGCCTGATTACTTCCAGA
GTTGGAGAGACAACTTAGCAGGCT
>1783
GTCTACTATGGCAAAACCCTCAGCTCCCCCAACCCATCTCTTTAAGGAACGTCCCTCCC
CAACACCGAAGCCCAGGCCTGATTACTTCCAGAGTTGGAGAGACAACTTAGCAGGCT
CTTGTTTCTTGCCGATGTACCTTA
>1784
AGCTCCCCCAACCCATCTCTTTAAGGAACGTCCCTCCCCAACACCGAAGCCCAGGCCT
GATTACTTCCAGAGTTGGAGAGACAACTTAGCAGGCTCTTGTTTCTTGCCGATGTACCT
TATTAGTAGCACCTGGCATCATA
>1785
CTCCCCCAACCCATCTCTTTAAGGAACGTCCCTCCCCAACACCGAAGCCCAGGCCTGA
TTACTTCCAGAGTTGGAGAGACAACTTAGCAGGCTCTTGTTTCTTGCCGATGTACCTTA
TTAGTAGCACCTGGCATCATAAC
>1786
TTTAAGGAACGTCCCTCCCCAACACCGAAGCCCAGGCCTGATTACTTCCAGAGTTGGA
GAGACAACTTAGCAGGCTCTTGTTTCTTGCCGATGTACCTTATTAGTAGCACCTGGCAT
CATAACCCACTAATGGGAACCAG
>1787
GGCCTGATTACTTCCAGAGTTGGAGAGACAACTTAGCAGGCTCTTGTTTCTTGCCGATG
TACCTTATTAGTAGCACCTGGCATCATAACCCACTAATGGGAACCAGTGCTCTCCTCCT
GCGTGGCCAGTGACAAGTAGCC
>1788
CTGATTACTTCCAGAGTTGGAGAGACAACTTAGCAGGCTCTTGTTTCTTGCCGATGTAC
CTTATTAGTAGCACCTGGCATCATAACCCACTAATGGGAACCAGTGCTCTCCTCCTGCG
TGGCCAGTGACAAGTAGCCCTG
>1789
CAGGCTCTTGTTTCTTGCCGATGTACCTTATTAGTAGCACCTGGCATCATAACCCACTAA
TGGGAACCAGTGCTCTCCTCCTGCGTGGCCAGTGACAAGTAGCCCTGTCAGCCGGAGC
TCGCCGTGCGAGGAAGTTCCTA

>1790
GTACCTTATTAGTAGCACCTGGCATCATAACCCACTAATGGGAACCAGTGCTCTCCTCC
TGCGTGGCCAGTGACAAGTAGCCCTGTCAGCCGGAGCTCGCCGTGCGAGGAAGTTCC
TATTAGCTTTATGGTCAGGAGAAC
>1791
ATTAGTAGCACCTGGCATCATAACCCACTAATGGGAACCAGTGCTCTCCTCCTGCGTGG
CCAGTGACAAGTAGCCCTGTCAGCCGGAGCTCGCCGTGCGAGGAAGTTCCTATTAGCT
TTATGGTCAGGAGAACTTGTCCT
>1792
TGGCATCATAACCCACTAATGGGAACCAGTGCTCTCCTCCTGCGTGGCCAGTGACAAG
TAGCCCTGTCAGCCGGAGCTCGCCGTGCGAGGAAGTTCCTATTAGCTTTATGGTCAGG
AGAACTTGTCCTGTTCAGGAAATG
>1793
TGGGAACCAGTGCTCTCCTCCTGCGTGGCCAGTGACAAGTAGCCCTGTCAGCCGGAGC
TCGCCGTGCGAGGAAGTTCCTATTAGCTTTATGGTCAGGAGAACTTGTCCTGTTCAGGA
AATGAATGTTGCCGCTAAAGGTC
>1794
GGCCAGTGACAAGTAGCCCTGTCAGCCGGAGCTCGCCGTGCGAGGAAGTTCCTATTAG
CTTTATGGTCAGGAGAACTTGTCCTGTTCAGGAAATGAATGTTGCCGCTAAAGGTCCG
CGGCCCTGACCCTGGCGGCATAAC
>1795
CAGTGACAAGTAGCCCTGTCAGCCGGAGCTCGCCGTGCGAGGAAGTTCCTATTAGCTT
TATGGTCAGGAGAACTTGTCCTGTTCAGGAAATGAATGTTGCCGCTAAAGGTCCGCGG
CCCTGACCCTGGCGGCATAACCTG
>1796
TGTCAGCCGGAGCTCGCCGTGCGAGGAAGTTCCTATTAGCTTTATGGTCAGGAGAACT
TGTCCTGTTCAGGAAATGAATGTTGCCGCTAAAGGTCCGCGGCCCTGACCCTGGCGGC
ATAACCTGTGCGGGCCGGGGAGAC
>1797
GAGGAAGTTCCTATTAGCTTTATGGTCAGGAGAACTTGTCCTGTTCAGGAAATGAATGT
TGCCGCTAAAGGTCCGCGGCCCTGACCCTGGCGGCATAACCTGTGCGGGCCGGGGAG
ACCCTGTGCAGGATGCTTGGGATT
>1798
AGGAAATGAATGTTGCCGCTAAAGGTCCGCGGCCCTGACCCTGGCGGCATAACCTGTG
CGGGCCGGGGAGACCCTGTGCAGGATGCTTGGGATTTCAGCCCCTCTCTGGTTCTTGT
CATATGTAAAGAGGGAATCATTGT
>1799
GTTGCCGCTAAAGGTCCGCGGCCCTGACCCTGGCGGCATAACCTGTGCGGGCCGGGG
AGACCCTGTGCAGGATGCTTGGGATTTCAGCCCCTCTCTGGTTCTTGTCATATGTAAAG
AGGGAATCATTGTGGTATGTTGCA
>1800
GCGGCCCTGACCCTGGCGGCATAACCTGTGCGGGCCGGGGAGACCCTGTGCAGGATG
CTTGGGATTTCAGCCCCTCTCTGGTTCTTGTCATATGTAAAGAGGGAATCATTGTGGTAT
GTTGCAGCCTTGAACTTTTGATT
>1801
GCGGGCCGGGGAGACCCTGTGCAGGATGCTTGGGATTTCAGCCCCTCTCTGGTTCTTG

TCATATGTAAAGAGGGAATCATTGTGGTATGTTGCAGCCTTGAACTTTTGATTTATGCAG
TGAGGGCGACTTAACCGTTTCC
>1802
ATGCTTGGGATTTCAGCCCCTCTCTGGTTCTTGTCATATGTAAAGAGGGAATCATTGTG
GTATGTTGCAGCCTTGAACTTTTGATTTATGCAGTGAGGGCGACTTAACCGTTTCCATG
CCTGGACGGCTGTGCAGTGACA
>1803
CTGGTTCTTGTCATATGTAAAGAGGGAATCATTGTGGTATGTTGCAGCCTTGAACTTTTG
ATTTATGCAGTGAGGGCGACTTAACCGTTTCCATGCCTGGACGGCTGTGCAGTGACAG
AGCCGTGTTGTGAGCGTATGTG
>1804
TTCTTGTCATATGTAAAGAGGGAATCATTGTGGTATGTTGCAGCCTTGAACTTTTGATTT
ATGCAGTGAGGGCGACTTAACCGTTTCCATGCCTGGACGGCTGTGCAGTGACAGAGCC
GTGTTGTGAGCGTATGTGCCTA
>1805
TGCAGCCTTGAACTTTTGATTTATGCAGTGAGGGCGACTTAACCGTTTCCATGCCTGGA
CGGCTGTGCAGTGACAGAGCCGTGTTGTGAGCGTATGTGCCTAGGTACATGTGTGTGT
GGGGTGGTCAAGGGGAAGGAGAG
>1806
CTTGAACTTTTGATTTATGCAGTGAGGGCGACTTAACCGTTTCCATGCCTGGACGGCTG
TGCAGTGACAGAGCCGTGTTGTGAGCGTATGTGCCTAGGTACATGTGTGTGTGGGGTG
GTCAAGGGGAAGGAGAGACCCCC
>1807
TTATGCAGTGAGGGCGACTTAACCGTTTCCATGCCTGGACGGCTGTGCAGTGACAGAG
CCGTGTTGTGAGCGTATGTGCCTAGGTACATGTGTGTGTGGGGTGGTCAAGGGGAAGG
AGAGACCCCCACGTGCTAGCAGGG
>1808
GCGACTTAACCGTTTCCATGCCTGGACGGCTGTGCAGTGACAGAGCCGTGTTGTGAGC
GTATGTGCCTAGGTACATGTGTGTGTGGGGTGGTCAAGGGGAAGGAGAGACCCCCACG
TGCTAGCAGGGGCTGTTGCTGGGA
>1809
CGGCTGTGCAGTGACAGAGCCGTGTTGTGAGCGTATGTGCCTAGGTACATGTGTGTGT
GGGGTGGTCAAGGGGAAGGAGAGACCCCCACGTGCTAGCAGGGGCTGTTGCTGGGA
GCGGGTGGATTTCATGGGAGGATTCC
>1810
TGCAGTGACAGAGCCGTGTTGTGAGCGTATGTGCCTAGGTACATGTGTGTGTGGGGTG
GTCAAGGGGAAGGAGAGACCCCCACGTGCTAGCAGGGGCTGTTGCTGGGAGCGGGTG
GATTTCATGGGAGGATTCCTTTTCT
>1811
AGTGACAGAGCCGTGTTGTGAGCGTATGTGCCTAGGTACATGTGTGTGTGGGGTGGTC
AAGGGGAAGGAGAGACCCCCACGTGCTAGCAGGGGCTGTTGCTGGGAGCGGGTGGAT
TTCATGGGAGGATTCCTTTTCTTTT
>1812
GTGAGCGTATGTGCCTAGGTACATGTGTGTGTGGGGTGGTCAAGGGGAAGGAGAGAC
CCCCACGTGCTAGCAGGGGCTGTTGCTGGGAGCGGGTGGATTTCATGGGAGGATTCCT
TTTCTTTTTTCTTTTTCTTGCATTC

>1813
GTCAAGGGGAAGGAGAGACCCCCACGTGCTAGCAGGGGCTGTTGCTGGGAGCGGGTG
GATTTCATGGGAGGATTCCTTTTCTTTTTTCTTTTTCTTGCATTCTTGCAGCATCTAAATT
TCCTGCGATTGTTGGCTGCAAA
>1814
CTGTTGCTGGGAGCGGGTGGATTTCATGGGAGGATTCCTTTTCTTTTTTCTTTTTCTTGC
ATTCTTGCAGCATCTAAATTTCCTGCGATTGTTGGCTGCAAAGTGGTAAGAAGCTGGAG
AGCATTGTGTCTGGAATTGAA
>1815
ATTCCTTTTCTTTTTTCTTTTTCTTGCATTCTTGCAGCATCTAAATTTCCTGCGATTGTTG
GCTGCAAAGTGGTAAGAAGCTGGAGAGCATTGTGTCTGGAATTGAACCTGGGTTCGAA
TTAATCTTCTGTCTCTTAGAG
>1816
TTCTTTTTTCTTTTTCTTGCATTCTTGCAGCATCTAAATTTCCTGCGATTGTTGGCTGCAA
AGTGGTAAGAAGCTGGAGAGCATTGTGTCTGGAATTGAACCTGGGTTCGAATTAATCT
TCTGTCTCTTAGAGGCTGTGT
>1817
AGTGGTAAGAAGCTGGAGAGCATTGTGTCTGGAATTGAACCTGGGTTCGAATTAATCT
TCTGTCTCTTAGAGGCTGTGTGACCGTGGCAAGATTCCTCACCACTCACTTCCTCATCT
GTGAGATGAAGTTAATAACTTCT
>1818
TGGTAAGAAGCTGGAGAGCATTGTGTCTGGAATTGAACCTGGGTTCGAATTAATCTTCT
GTCTCTTAGAGGCTGTGTGACCGTGGCAAGATTCCTCACCACTCACTTCCTCATCTGTG
AGATGAAGTTAATAACTTCTGA
>1819
TAATCTTCTGTCTCTTAGAGGCTGTGTGACCGTGGCAAGATTCCTCACCACTCACTTCC
TCATCTGTGAGATGAAGTTAATAACTTCTGATTCATACTGGTGGTAGAGCAGTGTTGTTA
AGTAGGTGATGTATAGAGAAT
>1820
TCTGTCTCTTAGAGGCTGTGTGACCGTGGCAAGATTCCTCACCACTCACTTCCTCATCT
GTGAGATGAAGTTAATAACTTCTGATTCATACTGGTGGTAGAGCAGTGTTGTTAAGTAG
GTGATGTATAGAGAATACATGG
>1821
AAGATTCCTCACCACTCACTTCCTCATCTGTGAGATGAAGTTAATAACTTCTGATTCATA
CTGGTGGTAGAGCAGTGTTGTTAAGTAGGTGATGTATAGAGAATACATGGCCCAGCGTC
CGGCCAGTGGTGTGGCCTCAG
>1822
TCACTTCCTCATCTGTGAGATGAAGTTAATAACTTCTGATTCATACTGGTGGTAGAGCA
GTGTTGTTAAGTAGGTGATGTATAGAGAATACATGGCCCAGCGTCCGGCCAGTGGTGTG
GCCTCAGGTTGTATACTTGAAA
>1823
CTTCCTCATCTGTGAGATGAAGTTAATAACTTCTGATTCATACTGGTGGTAGAGCAGTG
TTGTTAAGTAGGTGATGTATAGAGAATACATGGCCCAGCGTCCGGCCAGTGGTGTGGCC
TCAGGTTGTATACTTGAAAGGA
>1824
GTGAGATGAAGTTAATAACTTCTGATTCATACTGGTGGTAGAGCAGTGTTGTTAAGTAG

GTGATGTATAGAGAATACATGGCCCAGCGTCCGGCCAGTGGTGTGGCCTCAGGTTGTAT
ACTTGAAAGGATGTGTATACTT
>1825
CATACTGGTGGTAGAGCAGTGTTGTTAAGTAGGTGATGTATAGAGAATACATGGCCCAG
CGTCCGGCCAGTGGTGTGGCCTCAGGTTGTATACTTGAAAGGATGTGTATACTTCAGTA
TTTTTTCCATTGTAAAATTAAG
>1826
AGCAGTGTTGTTAAGTAGGTGATGTATAGAGAATACATGGCCCAGCGTCCGGCCAGTG
GTGTGGCCTCAGGTTGTATACTTGAAAGGATGTGTATACTTCAGTATTTTTTCCATTGTA
AAATTAAGGGTTTTTTTTCTTT
>1827
AGGTGATGTATAGAGAATACATGGCCCAGCGTCCGGCCAGTGGTGTGGCCTCAGGTTG
TATACTTGAAAGGATGTGTATACTTCAGTATTTTTTCCATTGTAAAATTAAGGGTTTTTTT
TCTTTTTTTCTTGAGACAGAG
>1828
ATACATGGCCCAGCGTCCGGCCAGTGGTGTGGCCTCAGGTTGTATACTTGAAAGGATGT
GTATACTTCAGTATTTTTTCCATTGTAAAATTAAGGGTTTTTTTTCTTTTTTTCTTGAGAC
AGAGTCCTACTTTGTTGCCC
>1829
TGGTGTGGCCTCAGGTTGTATACTTGAAAGGATGTGTATACTTCAGTATTTTTTCCATTG
TAAAATTAAGGGTTTTTTTTCTTTTTTTCTTGAGACAGAGTCCTACTTTGTTGCCCAGGC
TTGAGTGCAGTGGCGCGATC
>1830
TGAAAGGATGTGTATACTTCAGTATTTTTTCCATTGTAAAATTAAGGGTTTTTTTTCTTTT
TTTCTTGAGACAGAGTCCTACTTTGTTGCCCAGGCTTGAGTGCAGTGGCGCGATCTCA
GCTCACTGCAACTTCCGCCTC
>1831
AGGATGTGTATACTTCAGTATTTTTTCCATTGTAAAATTAAGGGTTTTTTTTCTTTTTTTC
TTGAGACAGAGTCCTACTTTGTTGCCCAGGCTTGAGTGCAGTGGCGCGATCTCAGCTC
ACTGCAACTTCCGCCTCCTGG
>1832
CATTGTAAAATTAAGGGTTTTTTTTCTTTTTTTCTTGAGACAGAGTCCTACTTTGTTGCC
CAGGCTTGAGTGCAGTGGCGCGATCTCAGCTCACTGCAACTTCCGCCTCCTGGGTCCA
AGTGATTCTCGTGCCTCAGCCT
>1833
TTTTTTCTTTTTTTCTTGAGACAGAGTCCTACTTTGTTGCCCAGGCTTGAGTGCAGTGG
CGCGATCTCAGCTCACTGCAACTTCCGCCTCCTGGGTCCAAGTGATTCTCGTGCCTCAG
CCTCCCAAGTACCTGGGACCAC
>1834
CTTTGTTGCCCAGGCTTGAGTGCAGTGGCGCGATCTCAGCTCACTGCAACTTCCGCCT
CCTGGGTCCAAGTGATTCTCGTGCCTCAGCCTCCCAAGTACCTGGGACCACAGGGGTG
CACCACCACACCTGGCTAATTTTT
>1835
GAGTGCAGTGGCGCGATCTCAGCTCACTGCAACTTCCGCCTCCTGGGTCCAAGTGATT
CTCGTGCCTCAGCCTCCCAAGTACCTGGGACCACAGGGGTGCACCACCACACCTGGCT
AATTTTTGTATATATATATATATA

>1836
GGCGCGATCTCAGCTCACTGCAACTTCCGCCTCCTGGGTCCAAGTGATTCTCGTGCCTC
AGCCTCCCAAGTACCTGGGACCACAGGGGTGCACCACCACACCTGGCTAATTTTTGTA
TATATATATATATATATATATTTAG
>1837
CACTGCAACTTCCGCCTCCTGGGTCCAAGTGATTCTCGTGCCTCAGCCTCCCAAGTACC
TGGGACCACAGGGGTGCACCACCACACCTGGCTAATTTTTGTATATATATATATATATATA
TTTAGTAGAGATGGGGTTTC
>1838
TACCTGGGACCACAGGGGTGCACCACCACACCTGGCTAATTTTTGTATATATATATATATA
TATATTTAGTAGAGATGGGGTTTCGCCATGTTGGCCAGGCTGGTCTTGAACTCCCGACC
TCAGGTGATCTGCCTGTCTC
>1839
CTGGGACCACAGGGGTGCACCACCACACCTGGCTAATTTTTGTATATATATATATATATAT
ATTTAGTAGAGATGGGGTTTCGCCATGTTGGCCAGGCTGGTCTTGAACTCCCGACCTCA
GGTGATCTGCCTGTCTCAGC
>1840
ACCTGGCTAATTTTTGTATATATATATATATATATATTTAGTAGAGATGGGGTTTCGCCATG
TTGGCCAGGCTGGTCTTGAACTCCCGACCTCAGGTGATCTGCCTGTCTCAGCCTCCTAA
AGTGCTGGGATTATAGGTG
>1841
ATATATATATATTTAGTAGAGATGGGGTTTCGCCATGTTGGCCAGGCTGGTCTTGAACTC
CCGACCTCAGGTGATCTGCCTGTCTCAGCCTCCTAAAGTGCTGGGATTATAGGTGTGAG
CCACTGCGCCTGGCCTAAGTT
>1842
GAGATGGGGTTTCGCCATGTTGGCCAGGCTGGTCTTGAACTCCCGACCTCAGGTGATC
TGCCTGTCTCAGCCTCCTAAAGTGCTGGGATTATAGGTGTGAGCCACTGCGCCTGGCCT
AAGTTTTTAAAAATTAAAAAGAA
>1843
CAGGCTGGTCTTGAACTCCCGACCTCAGGTGATCTGCCTGTCTCAGCCTCCTAAAGTG
CTGGGATTATAGGTGTGAGCCACTGCGCCTGGCCTAAGTTTTTAAAAATTAAAAAGAAT
GGCATAGTGCTTTTTTGCTCTTT
>1844
GTCTTGAACTCCCGACCTCAGGTGATCTGCCTGTCTCAGCCTCCTAAAGTGCTGGGATT
ATAGGTGTGAGCCACTGCGCCTGGCCTAAGTTTTTAAAAATTAAAAAGAATGGCATAGT
GCTTTTTTGCTCTTTAACAGAT
>1845
AGGTGATCTGCCTGTCTCAGCCTCCTAAAGTGCTGGGATTATAGGTGTGAGCCACTGCG
CCTGGCCTAAGTTTTTAAAAATTAAAAAGAATGGCATAGTGCTTTTTTGCTCTTTAACA
GATCAAGACCAAAAGTTTTCCC
>1846
AGCCTCCTAAAGTGCTGGGATTATAGGTGTGAGCCACTGCGCCTGGCCTAAGTTTTTAA
AAATTAAAAAGAATGGCATAGTGCTTTTTTGCTCTTTAACAGATCAAGACCAAAAGTTT
TCCCCCCATCATAGCCCAAAGT
>1847
AGCCACTGCGCCTGGCCTAAGTTTTTAAAAATTAAAAAGAATGGCATAGTGCTTTTTTG

CTCTTTAACAGATCAAGACCAAAAGTTTTCCCCCCATCATAGCCCAAAGTCCTAAAGA
AAGGCTACTTAAGTTGTTCGTTT
>1848
TGCGCCTGGCCTAAGTTTTTAAAAATTAAAAAGAATGGCATAGTGCTTTTTTGCTCTTTA
ACAGATCAAGACCAAAAGTTTTCCCCCCATCATAGCCCAAAGTCCTAAAGAAAGGCTA
CTTAAGTTGTTCGTTTTCTTTT
>1849
TTAAAAAGAATGGCATAGTGCTTTTTTGCTCTTTAACAGATCAAGACCAAAAGTTTTCC
CCCCATCATAGCCCAAAGTCCTAAAGAAAGGCTACTTAAGTTGTTCGTTTTCTTTTGTT
TCTCCCATGAGTAATGTTTCTA
>1850
GCTTTTTTGCTCTTTAACAGATCAAGACCAAAAGTTTTCCCCCCATCATAGCCCAAAGT
CCTAAAGAAAGGCTACTTAAGTTGTTCGTTTTCTTTTGTTTCTCCCATGAGTAATGTTTC
TAAGTGTGATTTTTGCAGAGA
>1851
TCTTTAACAGATCAAGACCAAAAGTTTTCCCCCCATCATAGCCCAAAGTCCTAAAGAA
AGGCTACTTAAGTTGTTCGTTTTCTTTTGTTTCTCCCATGAGTAATGTTTCTAAGTGTGA
TTTTTGCAGAGAACAGCAGTGT
>1852
CCCCATCATAGCCCAAAGTCCTAAAGAAAGGCTACTTAAGTTGTTCGTTTTCTTTTGTT
TCTCCCATGAGTAATGTTTCTAAGTGTGATTTTTGCAGAGAACAGCAGTGTCTGACCTC
ATTCTTGAGGGGAACTTTTCTG
>1853
CAAAGTCCTAAAGAAAGGCTACTTAAGTTGTTCGTTTTCTTTTGTTTCTCCCATGAGTA
ATGTTTCTAAGTGTGATTTTTGCAGAGAACAGCAGTGTCTGACCTCATTCTTGAGGGGA
ACTTTTCTGCCTGCCTCTTCTT
>1854
AAGGCTACTTAAGTTGTTCGTTTTCTTTTGTTTCTCCCATGAGTAATGTTTCTAAGTGTG
ATTTTTGCAGAGAACAGCAGTGTCTGACCTCATTCTTGAGGGGAACTTTTCTGCCTGCC
TCTTCTTGAGAAAAAAATGTTT
>1855
GGCTACTTAAGTTGTTCGTTTTCTTTTGTTTCTCCCATGAGTAATGTTTCTAAGTGTGAT
TTTTGCAGAGAACAGCAGTGTCTGACCTCATTCTTGAGGGGAACTTTTCTGCCTGCCT
CTTCTTGAGAAAAAAATGTTTGT
>1856
TTTCTCCCATGAGTAATGTTTCTAAGTGTGATTTTTGCAGAGAACAGCAGTGTCTGACC
TCATTCTTGAGGGGAACTTTTCTGCCTGCCTCTTCTTGAGAAAAAAATGTTTGTGAGTTG
TCTGGCAAATGGATGCGTTACT
>1857
GATTTTTGCAGAGAACAGCAGTGTCTGACCTCATTCTTGAGGGGAACTTTTCTGCCTG
CCTCTTCTTGAGAAAAAAATGTTTGTGAGTTGTCTGGCAAATGGATGCGTTACTTTTGCT
GCTGTTGCTGCCGCTGCCGTTTT
>1858
AGCAGTGTCTGACCTCATTCTTGAGGGGAACTTTTCTGCCTGCCTCTTCTTGAGAAAA
AATGTTTGTGAGTTGTCTGGCAAATGGATGCGTTACTTTTGCTGCTGTTGCTGCCGCTG
CCGTTTTAAAGGCCCCTGGTGAC

>1859
AATGTTTGTGAGTTGTCTGGCAAATGGATGCGTTACTTTTGCTGCTGTTGCTGCCGCTG
CCGTTTTAAAGGCCCCTGGTGACAAAGGAGTGAACTGCACTTTCGGGGAAAGCATGC
CGGAATCTGGCCCAGAAAAGAGGG
>1860
CTGGCAAATGGATGCGTTACTTTTGCTGCTGTTGCTGCCGCTGCCGTTTTAAAGGCCCC
TGGTGACAAAGGAGTGAACTGCACTTTCGGGGAAAGCATGCCGGAATCTGGCCCAGA
AAAGAGGGGCCGCTGGGGGAAGGG
>1861
CTGCTGTTGCTGCCGCTGCCGTTTTAAAGGCCCCTGGTGACAAAGGAGTGAACTGCAC
TTTCGGGGAAAGCATGCCGGAATCTGGCCCAGAAAAGAGGGGCCGCTGGGGGAAGG
GTTTGGAGATGACGCCTCCTCAGGAA
>1862
GTTTTAAAGGCCCCTGGTGACAAAGGAGTGAACTGCACTTTCGGGGAAAGCATGCCG
GAATCTGGCCCAGAAAAGAGGGGCCGCTGGGGGAAGGGTTTGGAGATGACGCCTCCT
CAGGAACTTTAAAAGAAATCTGTCCA
>1863
AAAGGCCCCTGGTGACAAAGGAGTGAACTGCACTTTCGGGGAAAGCATGCCGGAATC
TGGCCCAGAAAAGAGGGGCCGCTGGGGGAAGGGTTTGGAGATGACGCCTCCTCAGGA
ACTTTAAAAGAAATCTGTCCATCTGT
>1864
CTGCACTTTCGGGGAAAGCATGCCGGAATCTGGCCCAGAAAAGAGGGGCCGCTGGGG
GAAGGGTTTGGAGATGACGCCTCCTCAGGAACTTTAAAAGAAATCTGTCCATCTGTCT
CTGGCATTTATGGCATCGTTGACAG
>1865
AAGCATGCCGGAATCTGGCCCAGAAAAGAGGGGCCGCTGGGGGAAGGGTTTGGAGAT
GACGCCTCCTCAGGAACTTTAAAAGAAATCTGTCCATCTGTCTCTGGCATTTATGGCAT
CGTTGACAGCAGTTCAAGGAAAAA
>1866
ATCTGGCCCAGAAAAGAGGGGCCGCTGGGGGAAGGGTTTGGAGATGACGCCTCCTCA
GGAACTTTAAAAGAAATCTGTCCATCTGTCTCTGGCATTTATGGCATCGTTGACAGCAG
TTCAAGGAAAAATAAAAATAAGTT
>1867
ATGACGCCTCCTCAGGAACTTTAAAAGAAATCTGTCCATCTGTCTCTGGCATTTATGGC
ATCGTTGACAGCAGTTCAAGGAAAAATAAAAATAAGTTCTTATCTGATTGCGGGGCTGA
TTGCAAAGCCGAAGGACTAGAG
>1868
ACGCCTCCTCAGGAACTTTAAAAGAAATCTGTCCATCTGTCTCTGGCATTTATGGCATC
GTTGACAGCAGTTCAAGGAAAAATAAAAATAAGTTCTTATCTGATTGCGGGGCTGATTG
CAAAGCCGAAGGACTAGAGTGG
>1869
CCTCAGGAACTTTAAAAGAAATCTGTCCATCTGTCTCTGGCATTTATGGCATCGTTGAC
AGCAGTTCAAGGAAAAATAAAAATAAGTTCTTATCTGATTGCGGGGCTGATTGCAAAG
CCGAAGGACTAGAGTGGCCAGCG
>1870
AGAAATCTGTCCATCTGTCTCTGGCATTTATGGCATCGTTGACAGCAGTTCAAGGAAAA

ATAAAAATAAGTTCTTATCTGATTGCGGGGCTGATTGCAAAGCCGAAGGACTAGAGTG
GCCAGCGAGTCGGGCGGCTGGAT
>1871
GTTGACAGCAGTTCAAGGAAAAATAAAAATAAGTTCTTATCTGATTGCGGGGCTGATTG
CAAAGCCGAAGGACTAGAGTGGCCAGCGAGTCGGGCGGCTGGATCTGGAGTTTTGGA
GAAAGGGCCACTGGCTCATTTCTG
>1872
AGCAGTTCAAGGAAAAATAAAAATAAGTTCTTATCTGATTGCGGGGCTGATTGCAAAG
CCGAAGGACTAGAGTGGCCAGCGAGTCGGGCGGCTGGATCTGGAGTTTTGGAGAAAG
GGCCACTGGCTCATTTCTGGGCACG
>1873
AGGAAAAATAAAAATAAGTTCTTATCTGATTGCGGGGCTGATTGCAAAGCCGAAGGAC
TAGAGTGGCCAGCGAGTCGGGCGGCTGGATCTGGAGTTTTGGAGAAAGGGCCACTGG
CTCATTTCTGGGCACGCACGCTCAC
>1874
TCTGATTGCGGGGCTGATTGCAAAGCCGAAGGACTAGAGTGGCCAGCGAGTCGGGCG
GCTGGATCTGGAGTTTTGGAGAAAGGGCCACTGGCTCATTTCTGGGCACGCACGCTCA
CGGAGGCAAGTTCTCACCTCCTCGT
>1875
TCGGGCGGCTGGATCTGGAGTTTTGGAGAAAGGGCCACTGGCTCATTTCTGGGCACGC
ACGCTCACGGAGGCAAGTTCTCACCTCCTCGTGGGCTTCTCTTCACTTTACGTGGAAA
AACTGTGTACGTCTCAAATTAAAA
>1876
CGGCTGGATCTGGAGTTTTGGAGAAAGGGCCACTGGCTCATTTCTGGGCACGCACGCT
CACGGAGGCAAGTTCTCACCTCCTCGTGGGCTTCTCTTCACTTTACGTGGAAAAACTG
TGTACGTCTCAAATTAAAAGCAGC
>1877
CAAGTTCTCACCTCCTCGTGGGCTTCTCTTCACTTTACGTGGAAAAACTGTGTACGTCT
CAAATTAAAAGCAGCTCTTGCCCCGCTTAGAGCCGGTGAAAGCTGCTTCGAAGGCGTT
TGTAGAGTAGGGATTGGAAATGT
>1878
GTTCTCACCTCCTCGTGGGCTTCTCTTCACTTTACGTGGAAAAACTGTGTACGTCTCAA
ATTAAAAGCAGCTCTTGCCCCGCTTAGAGCCGGTGAAAGCTGCTTCGAAGGCGTTTGT
AGAGTAGGGATTGGAAATGTTCA
>1879
GGGCTTCTCTTCACTTTACGTGGAAAAACTGTGTACGTCTCAAATTAAAAGCAGCTCTT
GCCCCGCTTAGAGCCGGTGAAAGCTGCTTCGAAGGCGTTTGTAGAGTAGGGATTGGAA
ATGTTCAATGCAAAAGTTAAATC
>1880
GCTTCTCTTCACTTTACGTGGAAAAACTGTGTACGTCTCAAATTAAAAGCAGCTCTTGC
CCCGCTTAGAGCCGGTGAAAGCTGCTTCGAAGGCGTTTGTAGAGTAGGGATTGGAAAT
GTTCAATGCAAAAGTTAAATCGT
>1881
ACTTTACGTGGAAAAACTGTGTACGTCTCAAATTAAAAGCAGCTCTTGCCCCGCTTAG
AGCCGGTGAAAGCTGCTTCGAAGGCGTTTGTAGAGTAGGGATTGGAAATGTTCAATGC
AAAAGTTAAATCGTGACAGTTGTC

>1882
TACGTCTCAAATTAAAAGCAGCTCTTGCCCCGCTTAGAGCCGGTGAAAGCTGCTTCGA
AGGCGTTTGTAGAGTAGGGATTGGAAATGTTCAATGCAAAAGTTAAATCGTGACAGTT
GTCCCCTAAATGATCTGCTAAATT
>1883
CGCTTAGAGCCGGTGAAAGCTGCTTCGAAGGCGTTTGTAGAGTAGGGATTGGAAATGT
TCAATGCAAAAGTTAAATCGTGACAGTTGTCCCCTAAATGATCTGCTAAATTAAGGATC
CGCTAACATTTGTGAGTCTACCT
>1884
GAAAGCTGCTTCGAAGGCGTTTGTAGAGTAGGGATTGGAAATGTTCAATGCAAAAGTT
AAATCGTGACAGTTGTCCCCTAAATGATCTGCTAAATTAAGGATCCGCTAACATTTGTG
AGTCTACCTCCCTGCATTCCCCC
>1885
AGGGATTGGAAATGTTCAATGCAAAAGTTAAATCGTGACAGTTGTCCCCTAAATGATCT
GCTAAATTAAGGATCCGCTAACATTTGTGAGTCTACCTCCCTGCATTCCCCCCACCCCC
ACCCCCGCCCCCTTGGCAGAAG
>1886
GCAAAAGTTAAATCGTGACAGTTGTCCCCTAAATGATCTGCTAAATTAAGGATCCGCTA
ACATTTGTGAGTCTACCTCCCTGCATTCCCCCCACCCCCACCCCCGCCCCCTTGGCAGA
AGGGATAAAGATGGTACCTAAA
>1887
AAGGATCCGCTAACATTTGTGAGTCTACCTCCCTGCATTCCCCCCACCCCCACCCCCGC
CCCCTTGGCAGAAGGGATAAAGATGGTACCTAAAAACAATGTGAAGCGGCTGCTAAAT
CGAGTGACTCTAAAAACATTTTT
>1888
GATCCGCTAACATTTGTGAGTCTACCTCCCTGCATTCCCCCCACCCCCACCCCCGCCCC
CTTGGCAGAAGGGATAAAGATGGTACCTAAAAACAATGTGAAGCGGCTGCTAAATCGA
GTGACTCTAAAAACATTTTTCTT
>1889
AACATTTGTGAGTCTACCTCCCTGCATTCCCCCCACCCCCACCCCCGCCCCCTTGGCAG
AAGGGATAAAGATGGTACCTAAAAACAATGTGAAGCGGCTGCTAAATCGAGTGACTCT
AAAAACATTTTTCTTTCCTTGAA
>1890
CATTCCCCCCACCCCCACCCCCGCCCCCTTGGCAGAAGGGATAAAGATGGTACCTAAA
AACAATGTGAAGCGGCTGCTAAATCGAGTGACTCTAAAAACATTTTTCTTTCCTTGAAG
TTCAGTGTGGAAAACCAGACTGG
>1891
ACCCCCGCCCCCTTGGCAGAAGGGATAAAGATGGTACCTAAAAACAATGTGAAGCGGC
TGCTAAATCGAGTGACTCTAAAAACATTTTTCTTTCCTTGAAGTTCAGTGTGGAAAACC
AGACTGGACTTGTCAGGGATGCA
>1892
TGGTACCTAAAAACAATGTGAAGCGGCTGCTAAATCGAGTGACTCTAAAAACATTTTT
CTTTCCTTGAAGTTCAGTGTGGAAAACCAGACTGGACTTGTCAGGGATGCAGACCTAT
ATAGGGCCCATGTGCCTGGCCCCT
>1893
CCTAAAAACAATGTGAAGCGGCTGCTAAATCGAGTGACTCTAAAAACATTTTTCTTTCC

TTGAAGTTCAGTGTGGAAAACCAGACTGGACTTGTCAGGGATGCAGACCTATATAGGG
CCCATGTGCCTGGCCCCTTTCCT
>1894
AATCGAGTGACTCTAAAAACATTTTTCTTTCCTTGAAGTTCAGTGTGGAAAACCAGAC
TGGACTTGTCAGGGATGCAGACCTATATAGGGCCCATGTGCCTGGCCCCTTTCCTCACT
TTCTAGCACAATGGTTTTCATTG
>1895
ACATTTTTCTTTCCTTGAAGTTCAGTGTGGAAAACCAGACTGGACTTGTCAGGGATGC
AGACCTATATAGGGCCCATGTGCCTGGCCCCTTTCCTCACTTTCTAGCACAATGGTTTTC
ATTGCCACCTCTCCCGCGCCTT
>1896
CAGACTGGACTTGTCAGGGATGCAGACCTATATAGGGCCCATGTGCCTGGCCCCTTTCC
TCACTTTCTAGCACAATGGTTTTCATTGCCACCTCTCCCGCGCCTTTCCCCCCTTCATAA
TTATCCACTATTTTACTTTCT
>1897
CATTGCCACCTCTCCCGCGCCTTTCCCCCCTTCATAATTATCCACTATTTTACTTTCTGTG
TCCTGACAGCTACAAGAGTAGTTTTTGTTTTTTTGAGCTGAGTAGCTGCTAAATATTCCC
CAGCAGCCAGATTCAACGG
>1898
ACCTCTCCCGCGCCTTTCCCCCCTTCATAATTATCCACTATTTTACTTTCTGTGTCCTGAC
AGCTACAAGAGTAGTTTTTGTTTTTTTGAGCTGAGTAGCTGCTAAATATTCCCCAGCAG
CCAGATTCAACGGCTGGGGG
>1899
CCTTCATAATTATCCACTATTTTACTTTCTGTGTCCTGACAGCTACAAGAGTAGTTTTTGT
TTTTTTGAGCTGAGTAGCTGCTAAATATTCCCCAGCAGCCAGATTCAACGGCTGGGGGG
GAGAATAGGCCAAACCTTGG
>1900
TTCTGTGTCCTGACAGCTACAAGAGTAGTTTTTGTTTTTTTGAGCTGAGTAGCTGCTAA
ATATTCCCCAGCAGCCAGATTCAACGGCTGGGGGGGAGAATAGGCCAAACCTTGGCAT
CCCCTGTTGCAAAAGTGAAAAAT
>1901
TGTGTCCTGACAGCTACAAGAGTAGTTTTTGTTTTTTTGAGCTGAGTAGCTGCTAAATA
TTCCCCAGCAGCCAGATTCAACGGCTGGGGGGGAGAATAGGCCAAACCTTGGCATCCC
CTGTTGCAAAAGTGAAAAATATT
>1902
TCCTGACAGCTACAAGAGTAGTTTTTGTTTTTTTGAGCTGAGTAGCTGCTAAATATTCCC
CAGCAGCCAGATTCAACGGCTGGGGGGGAGAATAGGCCAAACCTTGGCATCCCCTGTT
GCAAAAGTGAAAAATATTTCTG
>1903
GTTTTTGTTTTTTTGAGCTGAGTAGCTGCTAAATATTCCCCAGCAGCCAGATTCAACGG
CTGGGGGGGAGAATAGGCCAAACCTTGGCATCCCCTGTTGCAAAAGTGAAAAATATTT
CTGTTTGCAGTTAATTTTAGTGG
>1904
TTCCCCAGCAGCCAGATTCAACGGCTGGGGGGGAGAATAGGCCAAACCTTGGCATCCC
CTGTTGCAAAAGTGAAAAATATTTCTGTTTGCAGTTAATTTTAGTGGCAAGCAGATCAT
GTTAACTGGATGTAATTGCTTCC

>1905
GGCTGGGGGGGAGAATAGGCCAAACCTTGGCATCCCCTGTTGCAAAAGTGAAAAATAT
TTCTGTTTGCAGTTAATTTTAGTGGCAAGCAGATCATGTTAACTGGATGTAATTGCTTCC
AGATATGGCCGAGGGTTTTGTG
>1906
GAGAATAGGCCAAACCTTGGCATCCCCTGTTGCAAAAGTGAAAAATATTTCTGTTTGC
AGTTAATTTTAGTGGCAAGCAGATCATGTTAACTGGATGTAATTGCTTCCAGATATGGCC
GAGGGTTTTGTGTGTTGAGATG
>1907
AAAAATATTTCTGTTTGCAGTTAATTTTAGTGGCAAGCAGATCATGTTAACTGGATGTAA
TTGCTTCCAGATATGGCCGAGGGTTTTGTGTGTTGAGATGAAGGGTCTGGTAAGTGCTT
ACTTCCTGTGTAAATTAGCCT
>1908
TGTTTGCAGTTAATTTTAGTGGCAAGCAGATCATGTTAACTGGATGTAATTGCTTCCAGA
TATGGCCGAGGGTTTTGTGTGTTGAGATGAAGGGTCTGGTAAGTGCTTACTTCCTGTGT
AAATTAGCCTATTCCATTCAG
>1909
TAGTGGCAAGCAGATCATGTTAACTGGATGTAATTGCTTCCAGATATGGCCGAGGGTTT
TGTGTGTTGAGATGAAGGGTCTGGTAAGTGCTTACTTCCTGTGTAAATTAGCCTATTCCA
TTCAGTCTTTCATTCTGGCCA
>1910
CAGATATGGCCGAGGGTTTTGTGTGTTGAGATGAAGGGTCTGGTAAGTGCTTACTTCCT
GTGTAAATTAGCCTATTCCATTCAGTCTTTCATTCTGGCCAGCCCTGGATTCTGTCATTC
TGCTCCCATTTTCCCCCTCCC
>1911
GGTTTTGTGTGTTGAGATGAAGGGTCTGGTAAGTGCTTACTTCCTGTGTAAATTAGCCT
ATTCCATTCAGTCTTTCATTCTGGCCAGCCCTGGATTCTGTCATTCTGCTCCCATTTTCC
CCCTCCCCAGCCTGCAGCCTG
>1912
ATTCCATTCAGTCTTTCATTCTGGCCAGCCCTGGATTCTGTCATTCTGCTCCCATTTTCC
CCCTCCCCAGCCTGCAGCCTGGTGTGAGAAGCCGCTTGCTATGTTCCTCTAAGTGTAAT
TCTTTTGTCACTTGGAGCCAC
>1913
TCAGTCTTTCATTCTGGCCAGCCCTGGATTCTGTCATTCTGCTCCCATTTTCCCCCTCCC
CAGCCTGCAGCCTGGTGTGAGAAGCCGCTTGCTATGTTCCTCTAAGTGTAATTCTTTTG
TCACTTGGAGCCACTCTCCTC
>1914
TTCTGCTCCCATTTTCCCCCTCCCCAGCCTGCAGCCTGGTGTGAGAAGCCGCTTGCTAT
GTTCCTCTAAGTGTAATTCTTTTGTCACTTGGAGCCACTCTCCTCTTGGTTCTTGATGTG
AGTTTGCCCTTAAATAATGAG
>1915
CCAGCCTGCAGCCTGGTGTGAGAAGCCGCTTGCTATGTTCCTCTAAGTGTAATTCTTTT
GTCACTTGGAGCCACTCTCCTCTTGGTTCTTGATGTGAGTTTGCCCTTAAATAATGAGA
AGGGTTTGAAAAGATGCCACCT
>1916
CTTGCTATGTTCCTCTAAGTGTAATTCTTTTGTCACTTGGAGCCACTCTCCTCTTGGTTC

TTGATGTGAGTTTGCCCTTAAATAATGAGAAGGGTTTGAAAAGATGCCACCTGGACCC
CAGGCCACCTCCCGCCTCTTTC
>1917
TGTAATTCTTTTGTCACTTGGAGCCACTCTCCTCTTGGTTCTTGATGTGAGTTTGCCCTT
AAATAATGAGAAGGGTTTGAAAAGATGCCACCTGGACCCCAGGCCACCTCCCGCCTCT
TTCCCAGCAAAGGCAGATCTGA
>1918
CACTTGGAGCCACTCTCCTCTTGGTTCTTGATGTGAGTTTGCCCTTAAATAATGAGAAG
GGTTTGAAAAGATGCCACCTGGACCCCAGGCCACCTCCCGCCTCTTTCCCAGCAAAGG
CAGATCTGAAGAGCATCTCCTGG
>1919
CCCTTAAATAATGAGAAGGGTTTGAAAAGATGCCACCTGGACCCCAGGCCACCTCCCG
CCTCTTTCCCAGCAAAGGCAGATCTGAAGAGCATCTCCTGGTCATGGGGGTTGCATCTT
GGAACCCAGAACTCCTTGTTTTC
>1920
AAATAATGAGAAGGGTTTGAAAAGATGCCACCTGGACCCCAGGCCACCTCCCGCCTCT
TTCCCAGCAAAGGCAGATCTGAAGAGCATCTCCTGGTCATGGGGGTTGCATCTTGGAA
CCCAGAACTCCTTGTTTTCTATGC
>1921
CGCCTCTTTCCCAGCAAAGGCAGATCTGAAGAGCATCTCCTGGTCATGGGGGTTGCAT
CTTGGAACCCAGAACTCCTTGTTTTCTATGCATTTTTTTTTCCTACTGCCCTCTGACCCC
CGACTCTCACCTGGGAGAACAC
>1922
CCCAGAACTCCTTGTTTTCTATGCATTTTTTTTTCCTACTGCCCTCTGACCCCCGACTCT
CACCTGGGAGAACACCTTCTCTTTCTCTCAATTAGTCCGACTCCTCCTCAGCCTGAACT
AAGCACGTTCCAGTGGGTCCG
>1923
TTTTTTTTTCCTACTGCCCTCTGACCCCCGACTCTCACCTGGGAGAACACCTTCTCTTTC
TCTCAATTAGTCCGACTCCTCCTCAGCCTGAACTAAGCACGTTCCAGTGGGTCCGAAAT
CTCAGAAAAAATGAAACGGCT
>1924
CCCTCTGACCCCCGACTCTCACCTGGGAGAACACCTTCTCTTTCTCTCAATTAGTCCGA
CTCCTCCTCAGCCTGAACTAAGCACGTTCCAGTGGGTCCGAAATCTCAGAAAAAATGA
AACGGCTGTTGCGCAGAGGAGAA
>1925
CCGACTCTCACCTGGGAGAACACCTTCTCTTTCTCTCAATTAGTCCGACTCCTCCTCAG
CCTGAACTAAGCACGTTCCAGTGGGTCCGAAATCTCAGAAAAAATGAAACGGCTGTTG
CGCAGAGGAGAAGGTTGTGGCCA
>1926
CCTGGGAGAACACCTTCTCTTTCTCTCAATTAGTCCGACTCCTCCTCAGCCTGAACTAA
GCACGTTCCAGTGGGTCCGAAATCTCAGAAAAAATGAAACGGCTGTTGCGCAGAGGA
GAAGGTTGTGGCCACAGAAAGGAG
>1927
TCCTCAGCCTGAACTAAGCACGTTCCAGTGGGTCCGAAATCTCAGAAAAAATGAAACG
GCTGTTGCGCAGAGGAGAAGGTTGTGGCCACAGAAAGGAGTTGCCTCATCAGACGTG
AAGCCGCCACCTTGCCTTGCAAAGC

>1928
CTCAGCCTGAACTAAGCACGTTCCAGTGGGTCCGAAATCTCAGAAAAAATGAAACGG
CTGTTGCGCAGAGGAGAAGGTTGTGGCCACAGAAAGGAGTTGCCTCATCAGACGTGA
AGCCGCCACCTTGCCTTGCAAAGCAG
>1929
GTTCCAGTGGGTCCGAAATCTCAGAAAAAATGAAACGGCTGTTGCGCAGAGGAGAAG
GTTGTGGCCACAGAAAGGAGTTGCCTCATCAGACGTGAAGCCGCCACCTTGCCTTGCA
AAGCAGGCTGCACCTCCTGACACAC
>1930
CTCAGAAAAAATGAAACGGCTGTTGCGCAGAGGAGAAGGTTGTGGCCACAGAAAGG
AGTTGCCTCATCAGACGTGAAGCCGCCACCTTGCCTTGCAAAGCAGGCTGCACCTCCT
GACACACCTGCCCCTCCCTGCAAGAA
>1931
GAGGAGAAGGTTGTGGCCACAGAAAGGAGTTGCCTCATCAGACGTGAAGCCGCCACC
TTGCCTTGCAAAGCAGGCTGCACCTCCTGACACACCTGCCCCTCCCTGCAAGAAAGG
GAGGGGAAATCCCACCCAAGAGAACC
>1932
GAGAAGGTTGTGGCCACAGAAAGGAGTTGCCTCATCAGACGTGAAGCCGCCACCTTG
CCTTGCAAAGCAGGCTGCACCTCCTGACACACCTGCCCCTCCCTGCAAGAAAGGGAG
GGGAAATCCCACCCAAGAGAACCTTC
>1933
AGACGTGAAGCCGCCACCTTGCCTTGCAAAGCAGGCTGCACCTCCTGACACACCTGC
CCCTCCCTGCAAGAAAGGGAGGGGAAATCCCACCCAAGAGAACCTTCTCCAGGTTCC
TGTGGGTTTCCAGGGTTGCAACTGAA
>1934
AGCCGCCACCTTGCCTTGCAAAGCAGGCTGCACCTCCTGACACACCTGCCCCTCCCTG
CAAGAAAGGGAGGGGAAATCCCACCCAAGAGAACCTTCTCCAGGTTCCTGTGGGTTT
CCAGGGTTGCAACTGAAGAAAAGTG
>1935
GCAAAGCAGGCTGCACCTCCTGACACACCTGCCCCTCCCTGCAAGAAAGGGAGGGGA
AATCCCACCCAAGAGAACCTTCTCCAGGTTCCTGTGGGTTTCCAGGGTTGCAACTGAA
GAAAAGTGTTAAGATTCATGTTCTC
>1936
ACCTCCTGACACACCTGCCCCTCCCTGCAAGAAAGGGAGGGGAAATCCCACCCAAGA
GAACCTTCTCCAGGTTCCTGTGGGTTTCCAGGGTTGCAACTGAAGAAAAGTGTTAAGA
TTCATGTTCTCCTAAATTCTGCTTT
>1937
AAGGGAGGGGAAATCCCACCCAAGAGAACCTTCTCCAGGTTCCTGTGGGTTTCCAGG
GTTGCAACTGAAGAAAAGTGTTAAGATTCATGTTCTCCTAAATTCTGCTTTTGTCCTGT
GGCCAGTCCCACATTCTTAGTGCT
>1938
AGGGGAAATCCCACCCAAGAGAACCTTCTCCAGGTTCCTGTGGGTTTCCAGGGTTGCA
ACTGAAGAAAAGTGTTAAGATTCATGTTCTCCTAAATTCTGCTTTTGTCCTGTGGCCAG
TCCCACATTCTTAGTGCTATTTG
>1939
CCAGGGTTGCAACTGAAGAAAAGTGTTAAGATTCATGTTCTCCTAAATTCTGCTTTTGT

CCTGTGGCCAGTCCCACATTCTTAGTGCTATTTGCATAAGAGGTTGCTGTTTATCTTCCT
ATATCAGCCCCACTGATAACC
>1940
GAAGAAAAGTGTTAAGATTCATGTTCTCCTAAATTCTGCTTTTGTCCTGTGGCCAGTCC
CACATTCTTAGTGCTATTTGCATAAGAGGTTGCTGTTTATCTTCCTATATCAGCCCCACTG
ATAACCCCTGTGGTCTGTTT
>1941
GATTCATGTTCTCCTAAATTCTGCTTTTGTCCTGTGGCCAGTCCCACATTCTTAGTGCTA
TTTGCATAAGAGGTTGCTGTTTATCTTCCTATATCAGCCCCACTGATAACCCCTGTGGTC
TGTTTAACAACCTATGAGGA
>1942
TGTCCTGTGGCCAGTCCCACATTCTTAGTGCTATTTGCATAAGAGGTTGCTGTTTATCTT
CCTATATCAGCCCCACTGATAACCCCTGTGGTCTGTTTAACAACCTATGAGGAGTAGATT
ACTTGATAAATGCCAGTTTT
>1943
AGAGGTTGCTGTTTATCTTCCTATATCAGCCCCACTGATAACCCCTGTGGTCTGTTTAAC
AACCTATGAGGAGTAGATTACTTGATAAATGCCAGTTTTTCCAATATGCAAAGATAAAC
TGTTGGGGTCAGGGTGAGGGG
>1944
GGTTGCTGTTTATCTTCCTATATCAGCCCCACTGATAACCCCTGTGGTCTGTTTAACAAC
CTATGAGGAGTAGATTACTTGATAAATGCCAGTTTTTCCAATATGCAAAGATAAACTGTT
GGGGTCAGGGTGAGGGGTGG
>1945
TCAGCCCCACTGATAACCCCTGTGGTCTGTTTAACAACCTATGAGGAGTAGATTACTTG
ATAAATGCCAGTTTTTCCAATATGCAAAGATAAACTGTTGGGGTCAGGGTGAGGGGTG
GTGTGTTATGAAGGAGGGGACAG
>1946
CCTGTGGTCTGTTTAACAACCTATGAGGAGTAGATTACTTGATAAATGCCAGTTTTTCCA
ATATGCAAAGATAAACTGTTGGGGTCAGGGTGAGGGGTGGTGTGTTATGAAGGAGGGG
ACAGGTGGGGAGCCAGGACCCT
>1947
ACCTATGAGGAGTAGATTACTTGATAAATGCCAGTTTTTCCAATATGCAAAGATAAACT
GTTGGGGTCAGGGTGAGGGGTGGTGTGTTATGAAGGAGGGGACAGGTGGGGAGCCAG
GACCCTCTTTTAGTGGGCTGGAGA
>1948
TAAATGCCAGTTTTTCCAATATGCAAAGATAAACTGTTGGGGTCAGGGTGAGGGGTGG
TGTGTTATGAAGGAGGGGACAGGTGGGGAGCCAGGACCCTCTTTTAGTGGGCTGGAG
AAGTGTGAAAGAGCCTGATGAAAGA
>1949
ATGCCAGTTTTTCCAATATGCAAAGATAAACTGTTGGGGTCAGGGTGAGGGGTGGTGT
GTTATGAAGGAGGGGACAGGTGGGGAGCCAGGACCCTCTTTTAGTGGGCTGGAGAAG
TGTGAAAGAGCCTGATGAAAGAAAA
>1950
TTTTTCCAATATGCAAAGATAAACTGTTGGGGTCAGGGTGAGGGGTGGTGTGTTATGAA
GGAGGGGACAGGTGGGGAGCCAGGACCCTCTTTTAGTGGGCTGGAGAAGTGTGAAAG
AGCCTGATGAAAGAAAACAGGTAG

>1951
ATATGCAAAGATAAACTGTTGGGGTCAGGGTGAGGGGTGGTGTGTTATGAAGGAGGGG
ACAGGTGGGGAGCCAGGACCCTCTTTTAGTGGGCTGGAGAAGTGTGAAAGAGCCTGA
TGAAAGAAAACAGGTAGCTTGTCAC
>1952
GTTGGGGTCAGGGTGAGGGGTGGTGTGTTATGAAGGAGGGGACAGGTGGGGAGCCAG
GACCCTCTTTTAGTGGGCTGGAGAAGTGTGAAAGAGCCTGATGAAAGAAAACAGGTA
GCTTGTCACATACAACAGCTGCATTT
>1953
GGAGGGGACAGGTGGGGAGCCAGGACCCTCTTTTAGTGGGCTGGAGAAGTGTGAAAG
AGCCTGATGAAAGAAAACAGGTAGCTTGTCACATACAACAGCTGCATTTATCATACGGT
TGATCAGCTTCCTGCCCTCACCTA
>1954
ACAGGTGGGGAGCCAGGACCCTCTTTTAGTGGGCTGGAGAAGTGTGAAAGAGCCTGA
TGAAAGAAAACAGGTAGCTTGTCACATACAACAGCTGCATTTATCATACGGTTGATCAG
CTTCCTGCCCTCACCTACTTTTCT
>1955
TCTTTTAGTGGGCTGGAGAAGTGTGAAAGAGCCTGATGAAAGAAAACAGGTAGCTTG
TCACATACAACAGCTGCATTTATCATACGGTTGATCAGCTTCCTGCCCTCACCTACTTTT
CTCCTCCTTCATGTGGACATGCA
>1956
AAAGAGCCTGATGAAAGAAAACAGGTAGCTTGTCACATACAACAGCTGCATTTATCAT
ACGGTTGATCAGCTTCCTGCCCTCACCTACTTTTCTCCTCCTTCATGTGGACATGCAGA
GTCTCTGAGAACATTTCATCAAG
>1957
TGCATTTATCATACGGTTGATCAGCTTCCTGCCCTCACCTACTTTTCTCCTCCTTCATGTG
GACATGCAGAGTCTCTGAGAACATTTCATCAAGTGGGTTGCCTATGCCCACATTAATAC
AAGATGGGAAAGGGTGGAGG
>1958
ATACGGTTGATCAGCTTCCTGCCCTCACCTACTTTTCTCCTCCTTCATGTGGACATGCAG
AGTCTCTGAGAACATTTCATCAAGTGGGTTGCCTATGCCCACATTAATACAAGATGGGA
AAGGGTGGAGGTTATCTTTGC
>1959
CTTCCTGCCCTCACCTACTTTTCTCCTCCTTCATGTGGACATGCAGAGTCTCTGAGAAC
ATTTCATCAAGTGGGTTGCCTATGCCCACATTAATACAAGATGGGAAAGGGTGGAGGTT
ATCTTTGCTTTTTAAGTTCCCA
>1960
TTCATGTGGACATGCAGAGTCTCTGAGAACATTTCATCAAGTGGGTTGCCTATGCCCAC
ATTAATACAAGATGGGAAAGGGTGGAGGTTATCTTTGCTTTTTAAGTTCCCATCTTTTTC
TCCCTTGGTCCTCTCCCACCC
>1961
GAGTCTCTGAGAACATTTCATCAAGTGGGTTGCCTATGCCCACATTAATACAAGATGGG
AAAGGGTGGAGGTTATCTTTGCTTTTTAAGTTCCCATCTTTTTCTCCCTTGGTCCTCTCC
CACCCCTACAAGTTGTTGGCT
>1962
ATCAAGTGGGTTGCCTATGCCCACATTAATACAAGATGGGAAAGGGTGGAGGTTATCTT

TGCTTTTTAAGTTCCCATCTTTTTCTCCCTTGGTCCTCTCCCACCCCTACAAGTTGTTGG
CTGGTAAGCAATTGATGCTCA
>1963
AGGGTGGAGGTTATCTTTGCTTTTTAAGTTCCCATCTTTTTCTCCCTTGGTCCTCTCCCA
CCCCTACAAGTTGTTGGCTGGTAAGCAATTGATGCTCAGAAAAATGATGTTCATCTGAA
ATCTTAAATGAGTGGACTTTT
>1964
TTTGCTTTTTAAGTTCCCATCTTTTTCTCCCTTGGTCCTCTCCCACCCCTACAAGTTGTT
GGCTGGTAAGCAATTGATGCTCAGAAAAATGATGTTCATCTGAAATCTTAAATGAGTGG
ACTTTTTCTACCTTCCCTGAC
>1965
TCCCATCTTTTTCTCCCTTGGTCCTCTCCCACCCCTACAAGTTGTTGGCTGGTAAGCAAT
TGATGCTCAGAAAAATGATGTTCATCTGAAATCTTAAATGAGTGGACTTTTTCTACCTTC
CCTGACTTTGCAGGTGCTTC
>1966
TGCTCAGAAAAATGATGTTCATCTGAAATCTTAAATGAGTGGACTTTTTCTACCTTCCCT
GACTTTGCAGGTGCTTCTTGAAGAGCCAAGGAGAAAAGAAAACTTCTGACCTCATCA
CCTAGTCAATAAGCTGTCTGAGA
>1967
GATGTTCATCTGAAATCTTAAATGAGTGGACTTTTTCTACCTTCCCTGACTTTGCAGGTG
CTTCTTGAAGAGCCAAGGAGAAAAGAAAACTTCTGACCTCATCACCTAGTCAATAAGC
TGTCTGAGAAACAAACAAAAGA
>1968
CCTTCCCTGACTTTGCAGGTGCTTCTTGAAGAGCCAAGGAGAAAAGAAAACTTCTGA
CCTCATCACCTAGTCAATAAGCTGTCTGAGAAACAAACAAAAGAGTTTAGACTGGCTG
CTAAAGCTAGGAACCCAGCTGATAG
>1969
GACTTTGCAGGTGCTTCTTGAAGAGCCAAGGAGAAAAGAAAACTTCTGACCTCATCA
CCTAGTCAATAAGCTGTCTGAGAAACAAACAAAAGAGTTTAGACTGGCTGCTAAAGCT
AGGAACCCAGCTGATAGCTTTGTTT
>1970
GCCAAGGAGAAAAGAAAACTTCTGACCTCATCACCTAGTCAATAAGCTGTCTGAGAA
ACAAACAAAAGAGTTTAGACTGGCTGCTAAAGCTAGGAACCCAGCTGATAGCTTTGTT
TTGATGTCCTTGTAATGTTTGCGGA
>1971
TTCTGACCTCATCACCTAGTCAATAAGCTGTCTGAGAAACAAACAAAAGAGTTTAGAC
TGGCTGCTAAAGCTAGGAACCCAGCTGATAGCTTTGTTTTGATGTCCTTGTAATGTTTG
CGGATCGCAGAGGGTGCTATTTA
>1972
GACCTCATCACCTAGTCAATAAGCTGTCTGAGAAACAAACAAAAGAGTTTAGACTGGC
TGCTAAAGCTAGGAACCCAGCTGATAGCTTTGTTTTGATGTCCTTGTAATGTTTGCGGA
TCGCAGAGGGTGCTATTTAGAGC
>1973
TCAATAAGCTGTCTGAGAAACAAACAAAAGAGTTTAGACTGGCTGCTAAAGCTAGGA
ACCCAGCTGATAGCTTTGTTTTGATGTCCTTGTAATGTTTGCGGATCGCAGAGGGTGCT
ATTTAGAGCAGGGGGCATCTACTG

>1974
CTGGCTGCTAAAGCTAGGAACCCAGCTGATAGCTTTGTTTTGATGTCCTTGTAATGTTT
GCGGATCGCAGAGGGTGCTATTTAGAGCAGGGGGCATCTACTGGGACAGACTCACTGG
GAAGTTGAAACAGCTAGCTCAAA
>1975
GGCTGCTAAAGCTAGGAACCCAGCTGATAGCTTTGTTTTGATGTCCTTGTAATGTTTGC
GGATCGCAGAGGGTGCTATTTAGAGCAGGGGGCATCTACTGGGACAGACTCACTGGGA
AGTTGAAACAGCTAGCTCAAAGT
>1976
TAGGAACCCAGCTGATAGCTTTGTTTTGATGTCCTTGTAATGTTTGCGGATCGCAGAGG
GTGCTATTTAGAGCAGGGGGCATCTACTGGGACAGACTCACTGGGAAGTTGAAACAGC
TAGCTCAAAGTCCAAGAGATGCT
>1977
TAATGTTTGCGGATCGCAGAGGGTGCTATTTAGAGCAGGGGGCATCTACTGGGACAGA
CTCACTGGGAAGTTGAAACAGCTAGCTCAAAGTCCAAGAGATGCTAAAGATAGTGAAA
TACAGCCCAGGAATTGAGGGGGAG
>1978
CGGATCGCAGAGGGTGCTATTTAGAGCAGGGGGCATCTACTGGGACAGACTCACTGGG
AAGTTGAAACAGCTAGCTCAAAGTCCAAGAGATGCTAAAGATAGTGAAATACAGCCCA
GGAATTGAGGGGGAGGGGGTTCCT
>1979
TCGCAGAGGGTGCTATTTAGAGCAGGGGGCATCTACTGGGACAGACTCACTGGGAAGT
TGAAACAGCTAGCTCAAAGTCCAAGAGATGCTAAAGATAGTGAAATACAGCCCAGGAA
TTGAGGGGGAGGGGGTTCCTCACT
>1980
TTTAGAGCAGGGGGCATCTACTGGGACAGACTCACTGGGAAGTTGAAACAGCTAGCTC
AAAGTCCAAGAGATGCTAAAGATAGTGAAATACAGCCCAGGAATTGAGGGGGAGGGG
GTTCCTCACTCAAGAAGGAGACTTT
>1981
GGGAAGTTGAAACAGCTAGCTCAAAGTCCAAGAGATGCTAAAGATAGTGAAATACAG
CCCAGGAATTGAGGGGGAGGGGGTTCCTCACTCAAGAAGGAGACTTTCTAGGAGGGG
GCGGGCGTGGTATTTTCTAGGGACAG
>1982
TTGAAACAGCTAGCTCAAAGTCCAAGAGATGCTAAAGATAGTGAAATACAGCCCAGGA
ATTGAGGGGGAGGGGGTTCCTCACTCAAGAAGGAGACTTTCTAGGAGGGGGCGGGCG
TGGTATTTTCTAGGGACAGTATGGA
>1983
AAGTCCAAGAGATGCTAAAGATAGTGAAATACAGCCCAGGAATTGAGGGGGAGGGGG
TTCCTCACTCAAGAAGGAGACTTTCTAGGAGGGGGCGGGCGTGGTATTTTCTAGGGAC
AGTATGGAATGAAGGGAAGAAGGTT
>1984
ATTGAGGGGGAGGGGGTTCCTCACTCAAGAAGGAGACTTTCTAGGAGGGGGCGGGCG
TGGTATTTTCTAGGGACAGTATGGAATGAAGGGAAGAAGGTTGGCTTTAGAAGGAGGC
ACCCTGTGGGAGGCTGAGACAGGTG
>1985
GGGGAGGGGGTTCCTCACTCAAGAAGGAGACTTTCTAGGAGGGGGCGGGCGTGGTAT

TTTCTAGGGACAGTATGGAATGAAGGGAAGAAGGTTGGCTTTAGAAGGAGGCACCCT
GTGGGAGGCTGAGACAGGTGGGTCAC
>1986
CCTCACTCAAGAAGGAGACTTTCTAGGAGGGGGCGGGCGTGGTATTTTCTAGGGACAG
TATGGAATGAAGGGAAGAAGGTTGGCTTTAGAAGGAGGCACCCTGTGGGAGGCTGAG
ACAGGTGGGTCACCTGAGGTCAGGA
>1987
TGGTATTTTCTAGGGACAGTATGGAATGAAGGGAAGAAGGTTGGCTTTAGAAGGAGGC
ACCCTGTGGGAGGCTGAGACAGGTGGGTCACCTGAGGTCAGGAGTTTGAGACCAGCC
TGGCCAACATGGTGAAACCCAGTCT
>1988
TTTCTAGGGACAGTATGGAATGAAGGGAAGAAGGTTGGCTTTAGAAGGAGGCACCCT
GTGGGAGGCTGAGACAGGTGGGTCACCTGAGGTCAGGAGTTTGAGACCAGCCTGGCC
AACATGGTGAAACCCAGTCTCTACTA
>1989
AATGAAGGGAAGAAGGTTGGCTTTAGAAGGAGGCACCCTGTGGGAGGCTGAGACAG
GTGGGTCACCTGAGGTCAGGAGTTTGAGACCAGCCTGGCCAACATGGTGAAACCCAG
TCTCTACTAAAAATACAAAAATTAGCT
>1990
GAGGCTGAGACAGGTGGGTCACCTGAGGTCAGGAGTTTGAGACCAGCCTGGCCAACA
TGGTGAAACCCAGTCTCTACTAAAAATACAAAAATTAGCTGGGTGGCAGGTGCCTGTA
ATCCCAGCTAGTCGGGGGAGGCTGA
>1991
AGGTCAGGAGTTTGAGACCAGCCTGGCCAACATGGTGAAACCCAGTCTCTACTAAAAA
TACAAAAATTAGCTGGGTGGCAGGTGCCTGTAATCCCAGCTAGTCGGGGGAGGCTGAG
GCAGGAAAATCGCTTGGGATTACA
>1992
CAACATGGTGAAACCCAGTCTCTACTAAAAATACAAAAATTAGCTGGGTGGCAGGTGC
CTGTAATCCCAGCTAGTCGGGGGAGGCTGAGGCAGGAAAATCGCTTGGGATTACAGGT
GCCTGTAATCCCAGCTAGTCGGGA
>1993
ATGGTGAAACCCAGTCTCTACTAAAAATACAAAAATTAGCTGGGTGGCAGGTGCCTGT
AATCCCAGCTAGTCGGGGGAGGCTGAGGCAGGAAAATCGCTTGGGATTACAGGTGCCT
GTAATCCCAGCTAGTCGGGAGAGG
>1994
CCCAGTCTCTACTAAAAATACAAAAATTAGCTGGGTGGCAGGTGCCTGTAATCCCAGCT
AGTCGGGGGAGGCTGAGGCAGGAAAATCGCTTGGGATTACAGGTGCCTGTAATCCCA
GCTAGTCGGGAGAGGCTGAGGCAG
>1995
TGGGTGGCAGGTGCCTGTAATCCCAGCTAGTCGGGGGAGGCTGAGGCAGGAAAATCG
CTTGGGATTACAGGTGCCTGTAATCCCAGCTAGTCGGGAGAGGCTGAGGCAGGAAAAT
TGCTTGAAGGAGGCGGAGGTTGCAG
>1996
GTAATCCCAGCTAGTCGGGGGAGGCTGAGGCAGGAAAATCGCTTGGGATTACAGGTGC
CTGTAATCCCAGCTAGTCGGGAGAGGCTGAGGCAGGAAAATTGCTTGAAGGAGGCGG
AGGTTGCAGTGAGCCCAGATCCTGC

>1997
TCCCAGCTAGTCGGGGGAGGCTGAGGCAGGAAAATCGCTTGGGATTACAGGTGCCTGT
AATCCCAGCTAGTCGGGAGAGGCTGAGGCAGGAAAATTGCTTGAAGGAGGCGGAGGT
TGCAGTGAGCCCAGATCCTGCCGTT
>1998
TAGTCGGGGGAGGCTGAGGCAGGAAAATCGCTTGGGATTACAGGTGCCTGTAATCCCA
GCTAGTCGGGAGAGGCTGAGGCAGGAAAATTGCTTGAAGGAGGCGGAGGTTGCAGTG
AGCCCAGATCCTGCCGTTGCACTCC
>1999
TCGCTTGGGATTACAGGTGCCTGTAATCCCAGCTAGTCGGGAGAGGCTGAGGCAGGAA
AATTGCTTGAAGGAGGCGGAGGTTGCAGTGAGCCCAGATCCTGCCGTTGCACTCCAGC
CCAAGACTTTGTCTCATTAAAAAA
>2000
CTGTAATCCCAGCTAGTCGGGAGAGGCTGAGGCAGGAAAATTGCTTGAAGGAGGCGG
AGGTTGCAGTGAGCCCAGATCCTGCCGTTGCACTCCAGCCCAAGACTTTGTCTCATTA
AAAAAAAAAAAAGAAGAAGAAG
>2001
ATCCCAGCTAGTCGGGAGAGGCTGAGGCAGGAAAATTGCTTGAAGGAGGCGGAGGTT
GCAGTGAGCCCAGATCCTGCCGTTGCACTCCAGCCCAAGACTTTGTCTCATTAAAAAA
AAAAAAAGAAGAAGAAGAAGAAGGA
>2002
GCTGAGGCAGGAAAATTGCTTGAAGGAGGCGGAGGTTGCAGTGAGCCCAGATCCTGC
CGTTGCACTCCAGCCCAAGACTTTGTCTCATTAAAAAAAAAAAAAAGAAGAAGAAGAA
GAAGGAGGCACCCTGGATGAAGACTC
>2003
GGTTGCAGTGAGCCCAGATCCTGCCGTTGCACTCCAGCCCAAGACTTTGTCTCATTAA
AAAAAAAAAAAGAAGAAGAAGAAGAAGGAGGCACCCTGGATGAAGACTCAGGCTCC
AGAATTTACTATCTGTATGTCTTGGG
>2004
TGCAGTGAGCCCAGATCCTGCCGTTGCACTCCAGCCCAAGACTTTGTCTCATTAAAAA
AAAAAAAAGAAGAAGAAGAAGAAGGAGGCACCCTGGATGAAGACTCAGGCTCCAGA
ATTTACTATCTGTATGTCTTGGGCAA
>2005
AGTGAGCCCAGATCCTGCCGTTGCACTCCAGCCCAAGACTTTGTCTCATTAAAAAAAA
AAAAAGAAGAAGAAGAAGAAGGAGGCACCCTGGATGAAGACTCAGGCTCCAGAATT
TACTATCTGTATGTCTTGGGCAAGTG
>2006
AGATCCTGCCGTTGCACTCCAGCCCAAGACTTTGTCTCATTAAAAAAAAAAAAAAGAAG
AAGAAGAAGAAGGAGGCACCCTGGATGAAGACTCAGGCTCCAGAATTTACTATCTGTA
TGTCTTGGGCAAGTGGCTTAGCTT
>2007
CCAGCCCAAGACTTTGTCTCATTAAAAAAAAAAAAAAGAAGAAGAAGAAGAAGGAGG
CACCCTGGATGAAGACTCAGGCTCCAGAATTTACTATCTGTATGTCTTGGGCAAGTGGC
TTAGCTTCTCTGAGCCCCTGTTTAC
>2008
AGAAGAAGAAGGAGGCACCCTGGATGAAGACTCAGGCTCCAGAATTTACTATCTGTAT

GTCTTGGGCAAGTGGCTTAGCTTCTCTGAGCCCCTGTTTACTTTTCTTTTGAAAATGAC
CACCTTGTCCATGGTAATGAGGA
>2009
AAGGAGGCACCCTGGATGAAGACTCAGGCTCCAGAATTTACTATCTGTATGTCTTGGGC
AAGTGGCTTAGCTTCTCTGAGCCCCTGTTTACTTTTCTTTTGAAAATGACCACCTTGTC
CATGGTAATGAGGATAAAAAAC
>2010
AGTGGCTTAGCTTCTCTGAGCCCCTGTTTACTTTTCTTTTGAAAATGACCACCTTGTCCA
TGGTAATGAGGATAAAAAACGTGTAAAGTGCATAGCAGAGTGCAGGTGTACTGGGTAG
ACATGTATGATTGCGCTGTAGC
>2011
AGCCCCTGTTTACTTTTCTTTTGAAAATGACCACCTTGTCCATGGTAATGAGGATAAAA
AACGTGTAAAGTGCATAGCAGAGTGCAGGTGTACTGGGTAGACATGTATGATTGCGCT
GTAGCCCTGTTATGGCCCGGGGA
>2012
CCACCTTGTCCATGGTAATGAGGATAAAAAACGTGTAAAGTGCATAGCAGAGTGCAGG
TGTACTGGGTAGACATGTATGATTGCGCTGTAGCCCTGTTATGGCCCGGGGAGAAACTA
TGAAAGGAACCAGTGAAATCTTT
>2013
GGATAAAAAACGTGTAAAGTGCATAGCAGAGTGCAGGTGTACTGGGTAGACATGTATG
ATTGCGCTGTAGCCCTGTTATGGCCCGGGGAGAAACTATGAAAGGAACCAGTGAAATC
TTTTGTTTTGGGCACTGCTAAATT
>2014
GCATAGCAGAGTGCAGGTGTACTGGGTAGACATGTATGATTGCGCTGTAGCCCTGTTAT
GGCCCGGGGAGAAACTATGAAAGGAACCAGTGAAATCTTTTGTTTTGGGCACTGCTAA
ATTGATGATAGTACTAACCCTGT
>2015
GCAGGTGTACTGGGTAGACATGTATGATTGCGCTGTAGCCCTGTTATGGCCCGGGGAGA
AACTATGAAAGGAACCAGTGAAATCTTTTGTTTTGGGCACTGCTAAATTGATGATAGTA
CTAACCCTGTGCTAGCAGCCAG
>2016
TACTGGGTAGACATGTATGATTGCGCTGTAGCCCTGTTATGGCCCGGGGAGAAACTATG
AAAGGAACCAGTGAAATCTTTTGTTTTGGGCACTGCTAAATTGATGATAGTACTAACCC
TGTGCTAGCAGCCAGCCGGTGA
>2017
TGGCCCGGGGAGAAACTATGAAAGGAACCAGTGAAATCTTTTGTTTTGGGCACTGCTA
AATTGATGATAGTACTAACCCTGTGCTAGCAGCCAGCCGGTGAAATAATAGGTGAATAA
TGTTAGTACATTGTGAATATAAA
>2018
ATGAAAGGAACCAGTGAAATCTTTTGTTTTGGGCACTGCTAAATTGATGATAGTACTAA
CCCTGTGCTAGCAGCCAGCCGGTGAAATAATAGGTGAATAATGTTAGTACATTGTGAAT
ATAAAAGTTTCCATTTGCAAAG
>2019
AAAGGAACCAGTGAAATCTTTTGTTTTGGGCACTGCTAAATTGATGATAGTACTAACCC
TGTGCTAGCAGCCAGCCGGTGAAATAATAGGTGAATAATGTTAGTACATTGTGAATATA
AAAGTTTCCATTTGCAAAGTGC

>2020
TTTGTTTTGGGCACTGCTAAATTGATGATAGTACTAACCCTGTGCTAGCAGCCAGCCGG
TGAAATAATAGGTGAATAATGTTAGTACATTGTGAATATAAAAGTTTCCATTTGCAAAGT
GCTACTGCTGTTCTAAAGAAG
>2021
CTAACCCTGTGCTAGCAGCCAGCCGGTGAAATAATAGGTGAATAATGTTAGTACATTGT
GAATATAAAAGTTTCCATTTGCAAAGTGCTACTGCTGTTCTAAAGAAGCCACATTTCTA
ATAAATGATTGCTTAATCCTGT
>2022
CAGCCAGCCGGTGAAATAATAGGTGAATAATGTTAGTACATTGTGAATATAAAAGTTTC
CATTTGCAAAGTGCTACTGCTGTTCTAAAGAAGCCACATTTCTAATAAATGATTGCTTAA
TCCTGTGATGCTGCCACAGCC
>2023
TAGGTGAATAATGTTAGTACATTGTGAATATAAAAGTTTCCATTTGCAAAGTGCTACTGC
TGTTCTAAAGAAGCCACATTTCTAATAAATGATTGCTTAATCCTGTGATGCTGCCACAGC
CCCACAGAGTTTGGGAATGT
>2024
AGTGCTACTGCTGTTCTAAAGAAGCCACATTTCTAATAAATGATTGCTTAATCCTGTGAT
GCTGCCACAGCCCCACAGAGTTTGGGAATGTTTGGGAATTCTTTCTGAAGTGTGTGCC
GCATTCTTTTGCTTTGCCTTTT
>2025
TGCTGTTCTAAAGAAGCCACATTTCTAATAAATGATTGCTTAATCCTGTGATGCTGCCAC
AGCCCCACAGAGTTTGGGAATGTTTGGGAATTCTTTCTGAAGTGTGTGCCGCATTCTTT
TGCTTTGCCTTTTGGGAGGGT
>2026
TGATTGCTTAATCCTGTGATGCTGCCACAGCCCCACAGAGTTTGGGAATGTTTGGGAAT
TCTTTCTGAAGTGTGTGCCGCATTCTTTTGCTTTGCCTTTTGGGAGGGTAGATGTGACTA
TTGGAAATGGTCAAAATAATG
>2027
CAGAGTTTGGGAATGTTTGGGAATTCTTTCTGAAGTGTGTGCCGCATTCTTTTGCTTTG
CCTTTTGGGAGGGTAGATGTGACTATTGGAAATGGTCAAAATAATGCAGTAGCAAATCT
GTTGACTATAGTGAGTGATAAG
>2028
GTGCCGCATTCTTTTGCTTTGCCTTTTGGGAGGGTAGATGTGACTATTGGAAATGGTCA
AAATAATGCAGTAGCAAATCTGTTGACTATAGTGAGTGATAAGCACTGTCGGCTGCTGT
GTCTGACTCGGAAGGCAGATTC
>2029
CCGCATTCTTTTGCTTTGCCTTTTGGGAGGGTAGATGTGACTATTGGAAATGGTCAAAA
TAATGCAGTAGCAAATCTGTTGACTATAGTGAGTGATAAGCACTGTCGGCTGCTGTGTC
TGACTCGGAAGGCAGATTCTGG
>2030
TTGGGAGGGTAGATGTGACTATTGGAAATGGTCAAAATAATGCAGTAGCAAATCTGTTG
ACTATAGTGAGTGATAAGCACTGTCGGCTGCTGTGTCTGACTCGGAAGGCAGATTCTG
GAAGGGGTGTGTGGGGAAGGCTT
>2031
TAATGCAGTAGCAAATCTGTTGACTATAGTGAGTGATAAGCACTGTCGGCTGCTGTGTC

TGACTCGGAAGGCAGATTCTGGAAGGGGTGTGTGGGGAAGGCTTGTTGGCTCAGCAC
TGGGGTGAGTGTTGGCCTCACTTC
>2032
GCAGTAGCAAATCTGTTGACTATAGTGAGTGATAAGCACTGTCGGCTGCTGTGTCTGAC
TCGGAAGGCAGATTCTGGAAGGGGTGTGTGGGGAAGGCTTGTTGGCTCAGCACTGGG
GTGAGTGTTGGCCTCACTTCTTAG
>2033
TGAGTGATAAGCACTGTCGGCTGCTGTGTCTGACTCGGAAGGCAGATTCTGGAAGGGG
TGTGTGGGGAAGGCTTGTTGGCTCAGCACTGGGGTGAGTGTTGGCCTCACTTCTTAGG
TGGGAAGTGGACAAGGCCAAGGGG
>2034
TGTCGGCTGCTGTGTCTGACTCGGAAGGCAGATTCTGGAAGGGGTGTGTGGGGAAGG
CTTGTTGGCTCAGCACTGGGGTGAGTGTTGGCCTCACTTCTTAGGTGGGAAGTGGACA
AGGCCAAGGGGCTTTTGATTTCCAG
>2035
TGTCTGACTCGGAAGGCAGATTCTGGAAGGGGTGTGTGGGGAAGGCTTGTTGGCTCA
GCACTGGGGTGAGTGTTGGCCTCACTTCTTAGGTGGGAAGTGGACAAGGCCAAGGGG
CTTTTGATTTCCAGCTACTATGGCTG
>2036
ATTCTGGAAGGGGTGTGTGGGGAAGGCTTGTTGGCTCAGCACTGGGGTGAGTGTTGG
CCTCACTTCTTAGGTGGGAAGTGGACAAGGCCAAGGGGCTTTTGATTTCCAGCTACTAT
GGCTGGACTCCCAGTGAGGAAGGA
>2037
AAGGCTTGTTGGCTCAGCACTGGGGTGAGTGTTGGCCTCACTTCTTAGGTGGGAAGTG
GACAAGGCCAAGGGGCTTTTGATTTCCAGCTACTATGGCTGGACTCCCAGTGAGGAAG
GAGTCGCTGCCATGGATTGCCTGG
>2038
CACTGGGGTGAGTGTTGGCCTCACTTCTTAGGTGGGAAGTGGACAAGGCCAAGGGGC
TTTTGATTTCCAGCTACTATGGCTGGACTCCCAGTGAGGAAGGAGTCGCTGCCATGGAT
TGCCTGGGACTGGGAAGACTCCTT
>2039
CACTTCTTAGGTGGGAAGTGGACAAGGCCAAGGGGCTTTTGATTTCCAGCTACTATGG
CTGGACTCCCAGTGAGGAAGGAGTCGCTGCCATGGATTGCCTGGGACTGGGAAGACT
CCTTTTAACTGCTGTAATGTTTCTG
>2040
TTTCCAGCTACTATGGCTGGACTCCCAGTGAGGAAGGAGTCGCTGCCATGGATTGCCT
GGGACTGGGAAGACTCCTTTTAACTGCTGTAATGTTTCTGCATTTGTGGTTTGGCCTCC
TGTGAGTTTGGGCTCCGGGGCGG
>2041
TGCCTGGGACTGGGAAGACTCCTTTTAACTGCTGTAATGTTTCTGCATTTGTGGTTTGG
CCTCCTGTGAGTTTGGGCTCCGGGGCGGCACTTCTGTTGTTAAAAGAACAGAAAAGA
AAACAAAACAAAACAAAAAACCAG
>2042
AATGTTTCTGCATTTGTGGTTTGGCCTCCTGTGAGTTTGGGCTCCGGGGCGGCACTTCT
GTTGTTAAAAGAACAGAAAAGAAAACAAAACAAAACAAAAAACCAGGACTGTCACT
GTTAGATGTTGGTTGAGGCTAGAAA

>2043
TTTGGGCTCCGGGGCGGCACTTCTGTTGTTAAAAGAACAGAAAAGAAAACAAAACAA
AACAAAAAACCAGGACTGTCACTGTTAGATGTTGGTTGAGGCTAGAAAGAATGGATTG
AGTTTTGACCCCCTACACCCCAGGG
>2044
GGCACTTCTGTTGTTAAAAGAACAGAAAAGAAAACAAAACAAAACAAAAAACCAGG
ACTGTCACTGTTAGATGTTGGTTGAGGCTAGAAAGAATGGATTGAGTTTTGACCCCCTA
CACCCCAGGGAGTAGGGAGGCCTGG
>2045
ACAGAAAAGAAAACAAAACAAAACAAAAAACCAGGACTGTCACTGTTAGATGTTGGT
TGAGGCTAGAAAGAATGGATTGAGTTTTGACCCCCTACACCCCAGGGAGTAGGGAGGC
CTGGGAGGCACAGTGGCCTGAGTTT
>2046
AACAAAACAAAACAAAAAACCAGGACTGTCACTGTTAGATGTTGGTTGAGGCTAGAA
AGAATGGATTGAGTTTTGACCCCCTACACCCCAGGGAGTAGGGAGGCCTGGGAGGCA
CAGTGGCCTGAGTTTGAATTCTGACT
>2047
AGGACTGTCACTGTTAGATGTTGGTTGAGGCTAGAAAGAATGGATTGAGTTTTGACCC
CCTACACCCCAGGGAGTAGGGAGGCCTGGGAGGCACAGTGGCCTGAGTTTGAATTCT
GACTCTGCCTCTTTGGAAGGCTCTG
>2048
TGTTAGATGTTGGTTGAGGCTAGAAAGAATGGATTGAGTTTTGACCCCCTACACCCCAG
GGAGTAGGGAGGCCTGGGAGGCACAGTGGCCTGAGTTTGAATTCTGACTCTGCCTCTT
TGGAAGGCTCTGGAAGGTTAAAG
>2049
AAGAATGGATTGAGTTTTGACCCCCTACACCCCAGGGAGTAGGGAGGCCTGGGAGGC
ACAGTGGCCTGAGTTTGAATTCTGACTCTGCCTCTTTGGAAGGCTCTGGAAGGTTAAA
GGAGCTTGTGTGTTCAAATCTTAGC
>2050
CCCCAGGGAGTAGGGAGGCCTGGGAGGCACAGTGGCCTGAGTTTGAATTCTGACTCT
GCCTCTTTGGAAGGCTCTGGAAGGTTAAAGGAGCTTGTGTGTTCAAATCTTAGCCTGG
TACCTGGCATATACTCAGCATGTAT
>2051
TAGGGAGGCCTGGGAGGCACAGTGGCCTGAGTTTGAATTCTGACTCTGCCTCTTTGGA
AGGCTCTGGAAGGTTAAAGGAGCTTGTGTGTTCAAATCTTAGCCTGGTACCTGGCATAT
ACTCAGCATGTATTTTTTCCCCT
>2052
GTTTGAATTCTGACTCTGCCTCTTTGGAAGGCTCTGGAAGGTTAAAGGAGCTTGTGTG
TTCAAATCTTAGCCTGGTACCTGGCATATACTCAGCATGTATTTTTTCCCCTTTCCTGAC
AATGTTCCTTGCCCCGAACACT
>2053
TTGGAAGGCTCTGGAAGGTTAAAGGAGCTTGTGTGTTCAAATCTTAGCCTGGTACCTG
GCATATACTCAGCATGTATTTTTTCCCCTTTCCTGACAATGTTCCTTGCCCCGAACACTT
GAATAGTGGAGAGAGGAATGTT
>2054
CCTGGCATATACTCAGCATGTATTTTTTCCCCTTTCCTGACAATGTTCCTTGCCCCGAAC

ACTTGAATAGTGGAGAGAGGAATGTTTGCTTTTCCTCTCCATTGAACCAACACTCGCCC
ATCCACTGAGCCCAAGATTCA
>2055
CATATACTCAGCATGTATTTTTTCCCCTTTCCTGACAATGTTCCTTGCCCCGAACACTTG
AATAGTGGAGAGAGGAATGTTTGCTTTTCCTCTCCATTGAACCAACACTCGCCCATCCA
CTGAGCCCAAGATTCACCTTA
>2056
CACTTGAATAGTGGAGAGAGGAATGTTTGCTTTTCCTCTCCATTGAACCAACACTCGCC
CATCCACTGAGCCCAAGATTCACCTTAGGATTGTCTGTTGGACTCGGCTCTTTTGTGAC
CTAACAGTGCAGGGGCCTTGTG
>2057
AATAGTGGAGAGAGGAATGTTTGCTTTTCCTCTCCATTGAACCAACACTCGCCCATCCA
CTGAGCCCAAGATTCACCTTAGGATTGTCTGTTGGACTCGGCTCTTTTGTGACCTAACA
GTGCAGGGGCCTTGTGCTGAGC
>2058
CGCCCATCCACTGAGCCCAAGATTCACCTTAGGATTGTCTGTTGGACTCGGCTCTTTTG
TGACCTAACAGTGCAGGGGCCTTGTGCTGAGCTGCACATGCTCGCTCTCGCTGGTGCT
CCCTCTCTCTCTCTCTCTCTGTC
>2059
ATCCACTGAGCCCAAGATTCACCTTAGGATTGTCTGTTGGACTCGGCTCTTTTGTGACC
TAACAGTGCAGGGGCCTTGTGCTGAGCTGCACATGCTCGCTCTCGCTGGTGCTCCCTC
TCTCTCTCTCTCTCTGTCTGCAT
>2060
GCTGAGCTGCACATGCTCGCTCTCGCTGGTGCTCCCTCTCTCTCTCTCTCTCTGTCTGC
ATTATTTATAGTAAACCATATTAGAACAGAGTGTTAAAGAGGAAGGATGAGTCATTCCC
ATTGAAACCATTGATGACATAA
>2061
GCTCTCGCTGGTGCTCCCTCTCTCTCTCTCTCTCTGTCTGCATTATTTATAGTAAACCATA
TTAGAACAGAGTGTTAAAGAGGAAGGATGAGTCATTCCCATTGAAACCATTGATGACA
TAAATAAAAGCAAAATGCTTA
>2062
TCTCGCTGGTGCTCCCTCTCTCTCTCTCTCTCTGTCTGCATTATTTATAGTAAACCATATT
AGAACAGAGTGTTAAAGAGGAAGGATGAGTCATTCCCATTGAAACCATTGATGACATA
AATAAAAGCAAAATGCTTACT
>2063
GTGCTCCCTCTCTCTCTCTCTCTCTGTCTGCATTATTTATAGTAAACCATATTAGAACAGA
GTGTTAAAGAGGAAGGATGAGTCATTCCCATTGAAACCATTGATGACATAAATAAAAG
CAAAATGCTTACTATGTGTTT
>2064
TATTAGAACAGAGTGTTAAAGAGGAAGGATGAGTCATTCCCATTGAAACCATTGATGA
CATAAATAAAAGCAAAATGCTTACTATGTGTTTTGTGCAGTATTTAAGGCCAGGTTGTG
CTAATTTGAATGCTCCCAAACTG
>2065
ATGAGTCATTCCCATTGAAACCATTGATGACATAAATAAAAGCAAAATGCTTACTATGT
GTTTTGTGCAGTATTTAAGGCCAGGTTGTGCTAATTTGAATGCTCCCAAACTGGATCTG
TGAAAGCATACATATGTGGGAA

>2066
TTCCCATTGAAACCATTGATGACATAAATAAAAGCAAAATGCTTACTATGTGTTTTGTGC
AGTATTTAAGGCCAGGTTGTGCTAATTTGAATGCTCCCAAACTGGATCTGTGAAAGCAT
ACATATGTGGGAAAAAATTCA
>2067
TATGTGTTTTGTGCAGTATTTAAGGCCAGGTTGTGCTAATTTGAATGCTCCCAAACTGGA
TCTGTGAAAGCATACATATGTGGGAAAAAATTCAGGTTTAACTAGAGGGAAGTTATTTA
TTGAAAATCCATTTCAGTATC
>2068
CCAGGTTGTGCTAATTTGAATGCTCCCAAACTGGATCTGTGAAAGCATACATATGTGGG
AAAAAATTCAGGTTTAACTAGAGGGAAGTTATTTATTGAAAATCCATTTCAGTATCTGCT
TTCTCCCTTCTATTTACTTCA
>2069
CTAATTTGAATGCTCCCAAACTGGATCTGTGAAAGCATACATATGTGGGAAAAAATTCA
GGTTTAACTAGAGGGAAGTTATTTATTGAAAATCCATTTCAGTATCTGCTTTCTCCCTTC
TATTTACTTCAGATCCATATT
>2070
AATTTGAATGCTCCCAAACTGGATCTGTGAAAGCATACATATGTGGGAAAAAATTCAGG
TTTAACTAGAGGGAAGTTATTTATTGAAAATCCATTTCAGTATCTGCTTTCTCCCTTCTAT
TTACTTCAGATCCATATTCA
>2071
GAATGCTCCCAAACTGGATCTGTGAAAGCATACATATGTGGGAAAAAATTCAGGTTTAA
CTAGAGGGAAGTTATTTATTGAAAATCCATTTCAGTATCTGCTTTCTCCCTTCTATTTACT
TCAGATCCATATTCATATCA
>2072
AAAGCATACATATGTGGGAAAAAATTCAGGTTTAACTAGAGGGAAGTTATTTATTGAAA
ATCCATTTCAGTATCTGCTTTCTCCCTTCTATTTACTTCAGATCCATATTCATATCATGCTT
GTTGTTCTGGCTAACGAGA
>2073
TTTAACTAGAGGGAAGTTATTTATTGAAAATCCATTTCAGTATCTGCTTTCTCCCTTCTAT
TTACTTCAGATCCATATTCATATCATGCTTGTTGTTCTGGCTAACGAGAGGGACTGGCTT
ATTCACTAGCTAAGGTTTG
>2074
GTATCTGCTTTCTCCCTTCTATTTACTTCAGATCCATATTCATATCATGCTTGTTGTTCTGG
CTAACGAGAGGGACTGGCTTATTCACTAGCTAAGGTTTGTTTATTTTCGGAAGGTTGAG
GAATAGCACAGAGAGGTTG
>2075
TTTACTTCAGATCCATATTCATATCATGCTTGTTGTTCTGGCTAACGAGAGGGACTGGCT
TATTCACTAGCTAAGGTTTGTTTATTTTCGGAAGGTTGAGGAATAGCACAGAGAGGTTG
TCTAAGTGTCCCTGTCTCCAT
>2076
TTCAGATCCATATTCATATCATGCTTGTTGTTCTGGCTAACGAGAGGGACTGGCTTATTC
ACTAGCTAAGGTTTGTTTATTTTCGGAAGGTTGAGGAATAGCACAGAGAGGTTGTCTAA
GTGTCCCTGTCTCCATTCCTC
>2077
CATGCTTGTTGTTCTGGCTAACGAGAGGGACTGGCTTATTCACTAGCTAAGGTTTGTTT

ATTTTCGGAAGGTTGAGGAATAGCACAGAGAGGTTGTCTAAGTGTCCCTGTCTCCATTC
CTCCTCTTGCCACCTGTTGAGG
>2078
TCTGGCTAACGAGAGGGACTGGCTTATTCACTAGCTAAGGTTTGTTTATTTTCGGAAGG
TTGAGGAATAGCACAGAGAGGTTGTCTAAGTGTCCCTGTCTCCATTCCTCCTCTTGCCA
CCTGTTGAGGAAGGCACTCTCC
>2079
GGTTGAGGAATAGCACAGAGAGGTTGTCTAAGTGTCCCTGTCTCCATTCCTCCTCTTGC
CACCTGTTGAGGAAGGCACTCTCCACCTGCTTTGCAGGCGTGGGCTCTCAATGTGTCC
AAGAATCACAGTCAGAGCTGAAG
>2080
GAGGAATAGCACAGAGAGGTTGTCTAAGTGTCCCTGTCTCCATTCCTCCTCTTGCCACC
TGTTGAGGAAGGCACTCTCCACCTGCTTTGCAGGCGTGGGCTCTCAATGTGTCCAAGA
ATCACAGTCAGAGCTGAAGGGCC
>2081
TCTAAGTGTCCCTGTCTCCATTCCTCCTCTTGCCACCTGTTGAGGAAGGCACTCTCCAC
CTGCTTTGCAGGCGTGGGCTCTCAATGTGTCCAAGAATCACAGTCAGAGCTGAAGGGC
CCTTGGAATCATTTATTGCAGCA
>2082
TCCTCTTGCCACCTGTTGAGGAAGGCACTCTCCACCTGCTTTGCAGGCGTGGGCTCTC
AATGTGTCCAAGAATCACAGTCAGAGCTGAAGGGCCCTTGGAATCATTTATTGCAGCAT
GCTCATTTGGGAGGAGTAAGTGG
>2083
CCACCTGTTGAGGAAGGCACTCTCCACCTGCTTTGCAGGCGTGGGCTCTCAATGTGTC
CAAGAATCACAGTCAGAGCTGAAGGGCCCTTGGAATCATTTATTGCAGCATGCTCATTT
GGGAGGAGTAAGTGGAGGTCCAG
>2084
AGGCGTGGGCTCTCAATGTGTCCAAGAATCACAGTCAGAGCTGAAGGGCCCTTGGAAT
CATTTATTGCAGCATGCTCATTTGGGAGGAGTAAGTGGAGGTCCAGAGAGGTTCTGTG
ATTTTCCCTAGGTCACACAGCAAG
>2085
TGTGTCCAAGAATCACAGTCAGAGCTGAAGGGCCCTTGGAATCATTTATTGCAGCATG
CTCATTTGGGAGGAGTAAGTGGAGGTCCAGAGAGGTTCTGTGATTTTCCCTAGGTCAC
ACAGCAAGCTAGAGGCAAAACCAG
>2086
TCACAGTCAGAGCTGAAGGGCCCTTGGAATCATTTATTGCAGCATGCTCATTTGGGAGG
AGTAAGTGGAGGTCCAGAGAGGTTCTGTGATTTTCCCTAGGTCACACAGCAAGCTAGA
GGCAAAACCAGGACCCTCACTCC
>2087
TCATTTATTGCAGCATGCTCATTTGGGAGGAGTAAGTGGAGGTCCAGAGAGGTTCTGT
GATTTTCCCTAGGTCACACAGCAAGCTAGAGGCAAAACCAGGACCCTCACTCCCAGCC
CACTTTAATTCTTCCTTCACAAGC
>2088
GCAGCATGCTCATTTGGGAGGAGTAAGTGGAGGTCCAGAGAGGTTCTGTGATTTTCCC
TAGGTCACACAGCAAGCTAGAGGCAAAACCAGGACCCTCACTCCCAGCCCACTTTAAT
TCTTCCTTCACAAGCCTGCCTGTG

>2089
CTCATTTGGGAGGAGTAAGTGGAGGTCCAGAGAGGTTCTGTGATTTTCCCTAGGTCAC
ACAGCAAGCTAGAGGCAAAACCAGGACCCTCACTCCCAGCCCACTTTAATTCTTCCTT
CACAAGCCTGCCTGTGACTGCCCA
>2090
CAGAGAGGTTCTGTGATTTTCCCTAGGTCACACAGCAAGCTAGAGGCAAAACCAGGA
CCCTCACTCCCAGCCCACTTTAATTCTTCCTTCACAAGCCTGCCTGTGACTGCCCAGGT
CTGTTCTCTGCTTCTACCAAGGGG
>2091
GGTCACACAGCAAGCTAGAGGCAAAACCAGGACCCTCACTCCCAGCCCACTTTAATTC
TTCCTTCACAAGCCTGCCTGTGACTGCCCAGGTCTGTTCTCTGCTTCTACCAAGGGGTA
GGCAAGACTAGAATCTGACTTGA
>2092
GGCAAAACCAGGACCCTCACTCCCAGCCCACTTTAATTCTTCCTTCACAAGCCTGCCT
GTGACTGCCCAGGTCTGTTCTCTGCTTCTACCAAGGGGTAGGCAAGACTAGAATCTGA
CTTGAGAGCAGTTTCAGATCTTTG
>2093
CAGCCCACTTTAATTCTTCCTTCACAAGCCTGCCTGTGACTGCCCAGGTCTGTTCTCTG
CTTCTACCAAGGGGTAGGCAAGACTAGAATCTGACTTGAGAGCAGTTTCAGATCTTTG
TCTGTGAGGGATGCTGGGGGGTT
>2094
ACTTTAATTCTTCCTTCACAAGCCTGCCTGTGACTGCCCAGGTCTGTTCTCTGCTTCTAC
CAAGGGGTAGGCAAGACTAGAATCTGACTTGAGAGCAGTTTCAGATCTTTGTCTGTGA
GGGATGCTGGGGGGTTTCTGGC
>2095
AATTCTTCCTTCACAAGCCTGCCTGTGACTGCCCAGGTCTGTTCTCTGCTTCTACCAAG
GGGTAGGCAAGACTAGAATCTGACTTGAGAGCAGTTTCAGATCTTTGTCTGTGAGGGA
TGCTGGGGGGTTTCTGGCATGGT
>2096
TTCCTTCACAAGCCTGCCTGTGACTGCCCAGGTCTGTTCTCTGCTTCTACCAAGGGGTA
GGCAAGACTAGAATCTGACTTGAGAGCAGTTTCAGATCTTTGTCTGTGAGGGATGCTG
GGGGGTTTCTGGCATGGTGACTT
>2097
CTGCCTGTGACTGCCCAGGTCTGTTCTCTGCTTCTACCAAGGGGTAGGCAAGACTAGA
ATCTGACTTGAGAGCAGTTTCAGATCTTTGTCTGTGAGGGATGCTGGGGGGTTTCTGGC
ATGGTGACTTGCCTCCTGGGACA
>2098
TCTGCTTCTACCAAGGGGTAGGCAAGACTAGAATCTGACTTGAGAGCAGTTTCAGATC
TTTGTCTGTGAGGGATGCTGGGGGGTTTCTGGCATGGTGACTTGCCTCCTGGGACACT
CATTAACCTCTCTGCTGCTGAGCC
>2099
GCTGGGGGGTTTCTGGCATGGTGACTTGCCTCCTGGGACACTCATTAACCTCTCTGCTG
CTGAGCCTGAGCAGACTAATGTACATCAACTCCTTAGGATTTACAGACAATGCCCTATG
TTGGGGGGTTTTGAGACCCAAAG
>2100
GGGGGGTTTCTGGCATGGTGACTTGCCTCCTGGGACACTCATTAACCTCTCTGCTGCTG

AGCCTGAGCAGACTAATGTACATCAACTCCTTAGGATTTACAGACAATGCCCTATGTTG
GGGGTTTTGAGACCCAAAGATT
>2101
TGCCTCCTGGGACACTCATTAACCTCTCTGCTGCTGAGCCTGAGCAGACTAATGTACAT
CAACTCCTTAGGATTTACAGACAATGCCCTATGTTGGGGGTTTTGAGACCCAAAGATTG
CTAAGACATGGGACTTTTCCTT
>2102
AGCCTGAGCAGACTAATGTACATCAACTCCTTAGGATTTACAGACAATGCCCTATGTTG
GGGGTTTTGAGACCCAAAGATTGCTAAGACATGGGACTTTTCCTTGAAGAGCTTATACT
TTGAGTTAAAATATGTATGTTG
>2103
CAGACTAATGTACATCAACTCCTTAGGATTTACAGACAATGCCCTATGTTGGGGGTTTT
GAGACCCAAAGATTGCTAAGACATGGGACTTTTCCTTGAAGAGCTTATACTTTGAGTTA
AAATATGTATGTTGATATCTAT
>2104
GTACATCAACTCCTTAGGATTTACAGACAATGCCCTATGTTGGGGGTTTTGAGACCCAA
AGATTGCTAAGACATGGGACTTTTCCTTGAAGAGCTTATACTTTGAGTTAAAATATGTAT
GTTGATATCTATGACAGAGAA
>2105
ATGTTGGGGGTTTTGAGACCCAAAGATTGCTAAGACATGGGACTTTTCCTTGAAGAGC
TTATACTTTGAGTTAAAATATGTATGTTGATATCTATGACAGAGAATAGGAAATGGAAAG
ACTATGATATATATATTCAGAG
>2106
TAAGACATGGGACTTTTCCTTGAAGAGCTTATACTTTGAGTTAAAATATGTATGTTGATA
TCTATGACAGAGAATAGGAAATGGAAAGACTATGATATATATATTCAGAGTTGAAATGA
CTTCTGTCTGGAGTGATTCAT
>2107
AGACATGGGACTTTTCCTTGAAGAGCTTATACTTTGAGTTAAAATATGTATGTTGATATC
TATGACAGAGAATAGGAAATGGAAAGACTATGATATATATATTCAGAGTTGAAATGACTT
CTGTCTGGAGTGATTCATTT
>2108
AGTTAAAATATGTATGTTGATATCTATGACAGAGAATAGGAAATGGAAAGACTATGATAT
ATATATTCAGAGTTGAAATGACTTCTGTCTGGAGTGATTCATTTTAAATTTTAAAACCTT
GTTGAGCACTTACTGTCTGC
>2109
TTAAAATATGTATGTTGATATCTATGACAGAGAATAGGAAATGGAAAGACTATGATATATA
TATTCAGAGTTGAAATGACTTCTGTCTGGAGTGATTCATTTTAAATTTTAAAACCTTGTT
GAGCACTTACTGTCTGCTG
>2110
TATGACAGAGAATAGGAAATGGAAAGACTATGATATATATATTCAGAGTTGAAATGACTT
CTGTCTGGAGTGATTCATTTTAAATTTTAAAACCTTGTTGAGCACTTACTGTCTGCTGGG
CACCTGGGTAGGCCCTGGGA
>2111
ATATATATATTCAGAGTTGAAATGACTTCTGTCTGGAGTGATTCATTTTAAATTTTAAAAC
CTTGTTGAGCACTTACTGTCTGCTGGGCACCTGGGTAGGCCCTGGGAATACTGTGATGA
GCAAGATAGACAAGACGTCT

>2112
TGTCTGGAGTGATTCATTTTAAATTTTAAAACCTTGTTGAGCACTTACTGTCTGCTGGGC
ACCTGGGTAGGCCCTGGGAATACTGTGATGAGCAAGATAGACAAGACGTCTACCCTCC
TGGGGCTTTCCTAATGGGGAAG
>2113
ATTTTAAAACCTTGTTGAGCACTTACTGTCTGCTGGGCACCTGGGTAGGCCCTGGGAAT
ACTGTGATGAGCAAGATAGACAAGACGTCTACCCTCCTGGGGCTTTCCTAATGGGGAA
GAAAGATCCTTACTTAGCTAGCA
>2114
ACCTTGTTGAGCACTTACTGTCTGCTGGGCACCTGGGTAGGCCCTGGGAATACTGTGAT
GAGCAAGATAGACAAGACGTCTACCCTCCTGGGGCTTTCCTAATGGGGAAGAAAGATC
CTTACTTAGCTAGCAGTCCTGTC
>2115
GTTGAGCACTTACTGTCTGCTGGGCACCTGGGTAGGCCCTGGGAATACTGTGATGAGC
AAGATAGACAAGACGTCTACCCTCCTGGGGCTTTCCTAATGGGGAAGAAAGATCCTTA
CTTAGCTAGCAGTCCTGTCCGTAA
>2116
GCCCTGGGAATACTGTGATGAGCAAGATAGACAAGACGTCTACCCTCCTGGGGCTTTC
CTAATGGGGAAGAAAGATCCTTACTTAGCTAGCAGTCCTGTCCGTAACTGTGTAAGGGT
GATTTTGATACATGTTGGGAGCC
>2117
AGCAAGATAGACAAGACGTCTACCCTCCTGGGGCTTTCCTAATGGGGAAGAAAGATCC
TTACTTAGCTAGCAGTCCTGTCCGTAACTGTGTAAGGGTGATTTTGATACATGTTGGGA
GCCTGGATGAAGCAGCACATGAG
>2118
AAGATAGACAAGACGTCTACCCTCCTGGGGCTTTCCTAATGGGGAAGAAAGATCCTTA
CTTAGCTAGCAGTCCTGTCCGTAACTGTGTAAGGGTGATTTTGATACATGTTGGGAGCC
TGGATGAAGCAGCACATGAGTAG
>2119
CTCCTGGGGCTTTCCTAATGGGGAAGAAAGATCCTTACTTAGCTAGCAGTCCTGTCCGT
AACTGTGTAAGGGTGATTTTGATACATGTTGGGAGCCTGGATGAAGCAGCACATGAGT
AGGATATTAAAGGATAAGTAGGT
>2120
AGAAAGATCCTTACTTAGCTAGCAGTCCTGTCCGTAACTGTGTAAGGGTGATTTTGATA
CATGTTGGGAGCCTGGATGAAGCAGCACATGAGTAGGATATTAAAGGATAAGTAGGTTT
TGAAGACGGGGACAGAAAGAAG
>2121
TACTTAGCTAGCAGTCCTGTCCGTAACTGTGTAAGGGTGATTTTGATACATGTTGGGAG
CCTGGATGAAGCAGCACATGAGTAGGATATTAAAGGATAAGTAGGTTTTGAAGACGGG
GACAGAAAGAAGGAAAATTGTGG
>2122
TTAGCTAGCAGTCCTGTCCGTAACTGTGTAAGGGTGATTTTGATACATGTTGGGAGCCT
GGATGAAGCAGCACATGAGTAGGATATTAAAGGATAAGTAGGTTTTGAAGACGGGGAC
AGAAAGAAGGAAAATTGTGGTCA
>2123
CAGTCCTGTCCGTAACTGTGTAAGGGTGATTTTGATACATGTTGGGAGCCTGGATGAAG

CAGCACATGAGTAGGATATTAAAGGATAAGTAGGTTTTGAAGACGGGGACAGAAAGAA
GGAAAATTGTGGTCAGGGCCAGG
>2124
AACTGTGTAAGGGTGATTTTGATACATGTTGGGAGCCTGGATGAAGCAGCACATGAGT
AGGATATTAAAGGATAAGTAGGTTTTGAAGACGGGGACAGAAAGAAGGAAAATTGTG
GTCAGGGCCAGGGGAGAAAACAAGG
>2125
TTGGGAGCCTGGATGAAGCAGCACATGAGTAGGATATTAAAGGATAAGTAGGTTTTGA
AGACGGGGACAGAAAGAAGGAAAATTGTGGTCAGGGCCAGGGGAGAAAACAAGGA
GTAACTTTCTTTGGCAGGAATGAGGGT
>2126
CCTGGATGAAGCAGCACATGAGTAGGATATTAAAGGATAAGTAGGTTTTGAAGACGGG
GACAGAAAGAAGGAAAATTGTGGTCAGGGCCAGGGGAGAAAACAAGGAGTAACTTT
CTTTGGCAGGAATGAGGGTGATTCAT
>2127
ACATGAGTAGGATATTAAAGGATAAGTAGGTTTTGAAGACGGGGACAGAAAGAAGGA
AAATTGTGGTCAGGGCCAGGGGAGAAAACAAGGAGTAACTTTCTTTGGCAGGAATGA
GGGTGATTCATGCTGGAATGGTAGGC
>2128
GTAGGATATTAAAGGATAAGTAGGTTTTGAAGACGGGGACAGAAAGAAGGAAAATTGT
GGTCAGGGCCAGGGGAGAAAACAAGGAGTAACTTTCTTTGGCAGGAATGAGGGTGAT
TCATGCTGGAATGGTAGGCTGGGGT
>2129
ACGGGGACAGAAAGAAGGAAAATTGTGGTCAGGGCCAGGGGAGAAAACAAGGAGTA
ACTTTCTTTGGCAGGAATGAGGGTGATTCATGCTGGAATGGTAGGCTGGGGTCAGACT
GTGGTGCTATAAATGCGGGGAGAGAG
>2130
CAGAAAGAAGGAAAATTGTGGTCAGGGCCAGGGGAGAAAACAAGGAGTAACTTTCTT
TGGCAGGAATGAGGGTGATTCATGCTGGAATGGTAGGCTGGGGTCAGACTGTGGTGCT
ATAAATGCGGGGAGAGAGATTAGAG
>2131
AGGGCCAGGGGAGAAAACAAGGAGTAACTTTCTTTGGCAGGAATGAGGGTGATTCAT
GCTGGAATGGTAGGCTGGGGTCAGACTGTGGTGCTATAAATGCGGGGAGAGAGATTAG
AGAAGGCAGCATGAAGAATGGAGAG
>2132
AGAAAACAAGGAGTAACTTTCTTTGGCAGGAATGAGGGTGATTCATGCTGGAATGGTA
GGCTGGGGTCAGACTGTGGTGCTATAAATGCGGGGAGAGAGATTAGAGAAGGCAGCA
TGAAGAATGGAGAGAAAATGAGTTT
>2133
GCAGGAATGAGGGTGATTCATGCTGGAATGGTAGGCTGGGGTCAGACTGTGGTGCTAT
AAATGCGGGGAGAGAGATTAGAGAAGGCAGCATGAAGAATGGAGAGAAAATGAGTTT
GAAGTTGAGCAGACCCGGGTTCAGA
>2134
AGGAATGAGGGTGATTCATGCTGGAATGGTAGGCTGGGGTCAGACTGTGGTGCTATAA
ATGCGGGGAGAGAGATTAGAGAAGGCAGCATGAAGAATGGAGAGAAAATGAGTTTGA
AGTTGAGCAGACCCGGGTTCAGATT

>2135
GAATGAGGGTGATTCATGCTGGAATGGTAGGCTGGGGTCAGACTGTGGTGCTATAAAT
GCGGGGAGAGAGATTAGAGAAGGCAGCATGAAGAATGGAGAGAAAATGAGTTTGAA
GTTGAGCAGACCCGGGTTCAGATTTT
>2136
AGGGTGATTCATGCTGGAATGGTAGGCTGGGGTCAGACTGTGGTGCTATAAATGCGGG
GAGAGAGATTAGAGAAGGCAGCATGAAGAATGGAGAGAAAATGAGTTTGAAGTTGAG
CAGACCCGGGTTCAGATTTTCATGC
>2137
GAATGGTAGGCTGGGGTCAGACTGTGGTGCTATAAATGCGGGGAGAGAGATTAGAGAA
GGCAGCATGAAGAATGGAGAGAAAATGAGTTTGAAGTTGAGCAGACCCGGGTTCAGA
TTTTCATGCTGCCACTTGCAAACTG
>2138
TGGTGCTATAAATGCGGGGAGAGAGATTAGAGAAGGCAGCATGAAGAATGGAGAGAA
AATGAGTTTGAAGTTGAGCAGACCCGGGTTCAGATTTTCATGCTGCCACTTGCAAACT
GTGTGATGTTGGCCATCAAGGACTG
>2139
GAGAGATTAGAGAAGGCAGCATGAAGAATGGAGAGAAAATGAGTTTGAAGTTGAGCA
GACCCGGGTTCAGATTTTCATGCTGCCACTTGCAAACTGTGTGATGTTGGCCATCAAGG
ACTGTGAACTGGCTGGGTGCAGTG
>2140
TGAAGTTGAGCAGACCCGGGTTCAGATTTTCATGCTGCCACTTGCAAACTGTGTGATG
TTGGCCATCAAGGACTGTGAACTGGCTGGGTGCAGTGGCTCACACCTGTCATCCCAGT
GCTTTGGGAGGCCAAGGCAGGAGG
>2141
AGATTTTCATGCTGCCACTTGCAAACTGTGTGATGTTGGCCATCAAGGACTGTGAACTG
GCTGGGTGCAGTGGCTCACACCTGTCATCCCAGTGCTTTGGGAGGCCAAGGCAGGAG
GATTGCGTGAGGCCAGAAGTTTGA
>2142
AAACTGTGTGATGTTGGCCATCAAGGACTGTGAACTGGCTGGGTGCAGTGGCTCACAC
CTGTCATCCCAGTGCTTTGGGAGGCCAAGGCAGGAGGATTGCGTGAGGCCAGAAGTT
TGAGACTGGCTTGGGCAATATAGCG
>2143
CAAGGACTGTGAACTGGCTGGGTGCAGTGGCTCACACCTGTCATCCCAGTGCTTTGGG
AGGCCAAGGCAGGAGGATTGCGTGAGGCCAGAAGTTTGAGACTGGCTTGGGCAATAT
AGCGAGACGATCTTTCCAAAAAAAA
>2144
GGACTGTGAACTGGCTGGGTGCAGTGGCTCACACCTGTCATCCCAGTGCTTTGGGAGG
CCAAGGCAGGAGGATTGCGTGAGGCCAGAAGTTTGAGACTGGCTTGGGCAATATAGC
GAGACGATCTTTCCAAAAAAAAAAA
>2145
TGGCTGGGTGCAGTGGCTCACACCTGTCATCCCAGTGCTTTGGGAGGCCAAGGCAGG
AGGATTGCGTGAGGCCAGAAGTTTGAGACTGGCTTGGGCAATATAGCGAGACGATCTT
TCCAAAAAAAAAAAAAAAAAAATTA
>2146
TGCAGTGGCTCACACCTGTCATCCCAGTGCTTTGGGAGGCCAAGGCAGGAGGATTGCG

TGAGGCCAGAAGTTTGAGACTGGCTTGGGCAATATAGCGAGACGATCTTTCCAAAAAA
AAAAAAAAAAAAAATTAAAAATGCA
>2147
AGTGCTTTGGGAGGCCAAGGCAGGAGGATTGCGTGAGGCCAGAAGTTTGAGACTGGC
TTGGGCAATATAGCGAGACGATCTTTCCAAAAAAAAAAAAAAAAAAAATTAAAAATGCA
GCTGTGCATGCCCATAATCCCAGCT
>2148
TTGAGACTGGCTTGGGCAATATAGCGAGACGATCTTTCCAAAAAAAAAAAAAAAAAAA
ATTAAAAATGCAGCTGTGCATGCCCATAATCCCAGCTTCTCGGGTGGCTGAAGAGGGA
GGATGGCTTGAACTTATAAGTTCAA
>2149
AGCGAGACGATCTTTCCAAAAAAAAAAAAAAAAAAAATTAAAAATGCAGCTGTGCATG
CCCATAATCCCAGCTTCTCGGGTGGCTGAAGAGGGAGGATGGCTTGAACTTATAAGTTC
AAGGCTGTGGTGAGCTATGATCAC
>2150
AAAAAAAAAAAAAAAAAAATTAAAAATGCAGCTGTGCATGCCCATAATCCCAGCTTCTC
GGGTGGCTGAAGAGGGAGGATGGCTTGAACTTATAAGTTCAAGGCTGTGGTGAGCTAT
GATCACGCCACTGCACTCCAGCCT
>2151
AAAAAAAAAAAAAAAAATTAAAAATGCAGCTGTGCATGCCCATAATCCCAGCTTCTCGG
GTGGCTGAAGAGGGAGGATGGCTTGAACTTATAAGTTCAAGGCTGTGGTGAGCTATGA
TCACGCCACTGCACTCCAGCCTGG
>2152
TGCAGCTGTGCATGCCCATAATCCCAGCTTCTCGGGTGGCTGAAGAGGGAGGATGGCT
TGAACTTATAAGTTCAAGGCTGTGGTGAGCTATGATCACGCCACTGCACTCCAGCCTGG
GCAACAAAGTGAGACCCTATCTC
>2153
AAGAGGGAGGATGGCTTGAACTTATAAGTTCAAGGCTGTGGTGAGCTATGATCACGCC
ACTGCACTCCAGCCTGGGCAACAAAGTGAGACCCTATCTCTATTTCAAAACAACAACA
ACAACAACAGCAACACACACCATG
>2154
CTTGAACTTATAAGTTCAAGGCTGTGGTGAGCTATGATCACGCCACTGCACTCCAGCCT
GGGCAACAAAGTGAGACCCTATCTCTATTTCAAAACAACAACAACAACAACAGCAAC
ACACACCATGAACTGTAGCTTCTT
>2155
CCACTGCACTCCAGCCTGGGCAACAAAGTGAGACCCTATCTCTATTTCAAAACAACAA
CAACAACAACAGCAACACACACCATGAACTGTAGCTTCTTCATTCCTACAGTGCAGTT
ATTGTGTGGGATAGCCTGAGGATT
>2156
ACAAAGTGAGACCCTATCTCTATTTCAAAACAACAACAACAACAACAGCAACACACA
CCATGAACTGTAGCTTCTTCATTCCTACAGTGCAGTTATTGTGTGGGATAGCCTGAGGA
TTTGGGGTCGTGTACTTAACACAC
>2157
TTTCAAAACAACAACAACAACAACAGCAACACACACCATGAACTGTAGCTTCTTCATT
CCTACAGTGCAGTTATTGTGTGGGATAGCCTGAGGATTTGGGGTCGTGTACTTAACACA
CTCTGCACAGTGCCTGTAACAGT

>2158
ACAACAACAGCAACACACACCATGAACTGTAGCTTCTTCATTCCTACAGTGCAGTTATT
GTGTGGGATAGCCTGAGGATTTGGGGTCGTGTACTTAACACACTCTGCACAGTGCCTGT
AACAGTGTAGTTGCTCAATAAA
>2159
ACAGCAACACACACCATGAACTGTAGCTTCTTCATTCCTACAGTGCAGTTATTGTGTGG
GATAGCCTGAGGATTTGGGGTCGTGTACTTAACACACTCTGCACAGTGCCTGTAACAG
TGTAGTTGCTCAATAAATATAAC
>2160
TCCTACAGTGCAGTTATTGTGTGGGATAGCCTGAGGATTTGGGGTCGTGTACTTAACAC
ACTCTGCACAGTGCCTGTAACAGTGTAGTTGCTCAATAAATATAACTGCTGTTTGTTTTA
ACTGCTGTTTCTTTTTGGACT
>2161
GTTATTGTGTGGGATAGCCTGAGGATTTGGGGTCGTGTACTTAACACACTCTGCACAGT
GCCTGTAACAGTGTAGTTGCTCAATAAATATAACTGCTGTTTGTTTTAACTGCTGTTTCT
TTTTGGACTTTATCCCGGGAG
>2162
TGTGTGGGATAGCCTGAGGATTTGGGGTCGTGTACTTAACACACTCTGCACAGTGCCT
GTAACAGTGTAGTTGCTCAATAAATATAACTGCTGTTTGTTTTAACTGCTGTTTCTTTTT
GGACTTTATCCCGGGAGAGTTG
>2163
TGTAGTTGCTCAATAAATATAACTGCTGTTTGTTTTAACTGCTGTTTCTTTTTGGACTTTA
TCCCGGGAGAGTTGGAGCCTTGGAAGGCATTTGAAAAGGAGAGTGACGTGACTGGAG
TGCTCCATACTGATGTGACATC
>2164
TAGTTGCTCAATAAATATAACTGCTGTTTGTTTTAACTGCTGTTTCTTTTTGGACTTTATC
CCGGGAGAGTTGGAGCCTTGGAAGGCATTTGAAAAGGAGAGTGACGTGACTGGAGTG
CTCCATACTGATGTGACATCCA
>2165
TTGTTTTAACTGCTGTTTCTTTTTGGACTTTATCCCGGGAGAGTTGGAGCCTTGGAAGG
CATTTGAAAAGGAGAGTGACGTGACTGGAGTGCTCCATACTGATGTGACATCCACTAG
GCATGTGTCTGACTGCCCCCTGC
>2166
TTTTTGGACTTTATCCCGGGAGAGTTGGAGCCTTGGAAGGCATTTGAAAAGGAGAGTG
ACGTGACTGGAGTGCTCCATACTGATGTGACATCCACTAGGCATGTGTCTGACTGCCCC
CTGCTCCTTCCCTGCCTTCACGA
>2167
AGCCTTGGAAGGCATTTGAAAAGGAGAGTGACGTGACTGGAGTGCTCCATACTGATGT
GACATCCACTAGGCATGTGTCTGACTGCCCCCTGCTCCTTCCCTGCCTTCACGAGCCTG
TCTGAAAAGGAGTTTAGTGCGTG
>2168
GCTCCATACTGATGTGACATCCACTAGGCATGTGTCTGACTGCCCCCTGCTCCTTCCCT
GCCTTCACGAGCCTGTCTGAAAAGGAGTTTAGTGCGTGATCATCAGCTCCTAAGGAGA
CCTCTCCCCTGTATCTTGTACCT
>2169
GTGACATCCACTAGGCATGTGTCTGACTGCCCCCTGCTCCTTCCCTGCCTTCACGAGCC

TGTCTGAAAAGGAGTTTAGTGCGTGATCATCAGCTCCTAAGGAGACCTCTCCCCTGTAT
CTTGTACCTGGCACTCTACCAA
>2170
CTGACTGCCCCCTGCTCCTTCCCTGCCTTCACGAGCCTGTCTGAAAAGGAGTTTAGTG
CGTGATCATCAGCTCCTAAGGAGACCTCTCCCCTGTATCTTGTACCTGGCACTCTACCA
AGATTTGATGAAAGGACATGAAA
>2171
CCCCTGCTCCTTCCCTGCCTTCACGAGCCTGTCTGAAAAGGAGTTTAGTGCGTGATCAT
CAGCTCCTAAGGAGACCTCTCCCCTGTATCTTGTACCTGGCACTCTACCAAGATTTGAT
GAAAGGACATGAAATGGACCCT
>2172
CTGCTCCTTCCCTGCCTTCACGAGCCTGTCTGAAAAGGAGTTTAGTGCGTGATCATCAG
CTCCTAAGGAGACCTCTCCCCTGTATCTTGTACCTGGCACTCTACCAAGATTTGATGAA
AGGACATGAAATGGACCCTTGC
>2173
AGTTTAGTGCGTGATCATCAGCTCCTAAGGAGACCTCTCCCCTGTATCTTGTACCTGGC
ACTCTACCAAGATTTGATGAAAGGACATGAAATGGACCCTTGCCACCTCTCGGTGGGC
GGGGAAGCTGGGAGAGGGGCCTG
>2174
GATCATCAGCTCCTAAGGAGACCTCTCCCCTGTATCTTGTACCTGGCACTCTACCAAGA
TTTGATGAAAGGACATGAAATGGACCCTTGCCACCTCTCGGTGGGCGGGGAAGCTGG
GAGAGGGGCCTGGGGAGAGGGGGT
>2175
CTGGCACTCTACCAAGATTTGATGAAAGGACATGAAATGGACCCTTGCCACCTCTCGG
TGGGCGGGGAAGCTGGGAGAGGGGCCTGGGGAGAGGGGGTCTTCAGCTGTCAGGAT
CAAAGGAAGTGACACTCTGAAGCTGG
>2176
TCTACCAAGATTTGATGAAAGGACATGAAATGGACCCTTGCCACCTCTCGGTGGGCGG
GGAAGCTGGGAGAGGGGCCTGGGGAGAGGGGGTCTTCAGCTGTCAGGATCAAAGGA
AGTGACACTCTGAAGCTGGTCTGCCG
>2177
AAATGGACCCTTGCCACCTCTCGGTGGGCGGGGAAGCTGGGAGAGGGGCCTGGGGAG
AGGGGGTCTTCAGCTGTCAGGATCAAAGGAAGTGACACTCTGAAGCTGGTCTGCCGG
ACTGTTACCCGTCTCTCTAACTCTCT
>2178
CCCTTGCCACCTCTCGGTGGGCGGGGAAGCTGGGAGAGGGGCCTGGGGAGAGGGGG
TCTTCAGCTGTCAGGATCAAAGGAAGTGACACTCTGAAGCTGGTCTGCCGGACTGTTA
CCCGTCTCTCTAACTCTCTTTTAATA
>2179
CTCGGTGGGCGGGGAAGCTGGGAGAGGGGCCTGGGGAGAGGGGGTCTTCAGCTGTC
AGGATCAAAGGAAGTGACACTCTGAAGCTGGTCTGCCGGACTGTTACCCGTCTCTCTA
ACTCTCTTTTAATAGAAACCCAGTCT
>2180
ATCAAAGGAAGTGACACTCTGAAGCTGGTCTGCCGGACTGTTACCCGTCTCTCTAACT
CTCTTTTAATAGAAACCCAGTCTCCCCTCTTGCCTCCCTGATAGTGAACAGCGACGCTA
TCTCCCGCGGGCCAGTCTCGGCT

>2181
AAGTGACACTCTGAAGCTGGTCTGCCGGACTGTTACCCGTCTCTCTAACTCTCTTTTAA
TAGAAACCCAGTCTCCCCTCTTGCCTCCCTGATAGTGAACAGCGACGCTATCTCCCGCG
GGCCAGTCTCGGCTGTGGTCGT
>2182
CCGGACTGTTACCCGTCTCTCTAACTCTCTTTTAATAGAAACCCAGTCTCCCCTCTTGCC
TCCCTGATAGTGAACAGCGACGCTATCTCCCGCGGGCCAGTCTCGGCTGTGGTCGTAG
CACTACGCAATGTCTGCAAACC
>2183
GTTACCCGTCTCTCTAACTCTCTTTTAATAGAAACCCAGTCTCCCCTCTTGCCTCCCTGA
TAGTGAACAGCGACGCTATCTCCCGCGGGCCAGTCTCGGCTGTGGTCGTAGCACTACG
CAATGTCTGCAAACCGACAGTG
>2184
TTTAATAGAAACCCAGTCTCCCCTCTTGCCTCCCTGATAGTGAACAGCGACGCTATCTC
CCGCGGGCCAGTCTCGGCTGTGGTCGTAGCACTACGCAATGTCTGCAAACCGACAGTG
GCATGGGGAGATAAGGGCCCCAA
>2185
TCCCCTCTTGCCTCCCTGATAGTGAACAGCGACGCTATCTCCCGCGGGCCAGTCTCGGC
TGTGGTCGTAGCACTACGCAATGTCTGCAAACCGACAGTGGCATGGGGAGATAAGGGC
CCCAACGTTGTGTAACGAGGGGC
>2186
AGCGACGCTATCTCCCGCGGGCCAGTCTCGGCTGTGGTCGTAGCACTACGCAATGTCT
GCAAACCGACAGTGGCATGGGGAGATAAGGGCCCCAACGTTGTGTAACGAGGGGCAT
GGGCTGTTTGCCCGAGTGGCTCCTG
>2187
TCCCGCGGGCCAGTCTCGGCTGTGGTCGTAGCACTACGCAATGTCTGCAAACCGACAG
TGGCATGGGGAGATAAGGGCCCCAACGTTGTGTAACGAGGGGCATGGGCTGTTTGCCC
GAGTGGCTCCTGGCCCCTGGGTAA
>2188
GTAGCACTACGCAATGTCTGCAAACCGACAGTGGCATGGGGAGATAAGGGCCCCAAC
GTTGTGTAACGAGGGGCATGGGCTGTTTGCCCGAGTGGCTCCTGGCCCCTGGGTAACT
GCTAACCTCAACGGAGGAGCGGGCC
>2189
AACCGACAGTGGCATGGGGAGATAAGGGCCCCAACGTTGTGTAACGAGGGGCATGGG
CTGTTTGCCCGAGTGGCTCCTGGCCCCTGGGTAACTGCTAACCTCAACGGAGGAGCGG
GCCGGCCTTGGAAACGCCGTTCCAG
>2190
TGTAACGAGGGGCATGGGCTGTTTGCCCGAGTGGCTCCTGGCCCCTGGGTAACTGCTA
ACCTCAACGGAGGAGCGGGCCGGCCTTGGAAACGCCGTTCCAGGGCCCCTCGCTGGT
TCCACGGATGCCTTTTTGTGGTTGG
>2191
TGGCTCCTGGCCCCTGGGTAACTGCTAACCTCAACGGAGGAGCGGGCCGGCCTTGGA
AACGCCGTTCCAGGGCCCCTCGCTGGTTCCACGGATGCCTTTTTGTGGTTGGATAAAG
GAAGCTCATGTAGGCCGCCCACAGC
>2192
GCCGGCCTTGGAAACGCCGTTCCAGGGCCCCTCGCTGGTTCCACGGATGCCTTTTTGT

GGTTGGATAAAGGAAGCTCATGTAGGCCGCCCACAGCGCAGCATGGCCTTGGGCTACG
GGTGTTTGGTTGTTTTGTTTTTCT
>2193
GCCTTGGAAACGCCGTTCCAGGGCCCCTCGCTGGTTCCACGGATGCCTTTTTGTGGTT
GGATAAAGGAAGCTCATGTAGGCCGCCCACAGCGCAGCATGGCCTTGGGCTACGGGTG
TTTGGTTGTTTTGTTTTTCTCTTT
>2194
GGGCCCCTCGCTGGTTCCACGGATGCCTTTTTGTGGTTGGATAAAGGAAGCTCATGTAG
GCCGCCCACAGCGCAGCATGGCCTTGGGCTACGGGTGTTTGGTTGTTTTGTTTTTCTCT
TTTCTTTGTCTTTTTTAAAAAA
>2195
CAGCATGGCCTTGGGCTACGGGTGTTTGGTTGTTTTGTTTTTCTCTTTTCTTTGTCTTTT
TTAAAAAAAGAAGTTGAAAGGGGGATAGTCTTGCTTGGCACCTACCACCTTTAACCCA
AAACCAAATAAAACAAACAGTT
>2196
CATGGCCTTGGGCTACGGGTGTTTGGTTGTTTTGTTTTTCTCTTTTCTTTGTCTTTTTTAA
AAAAAGAAGTTGAAAGGGGGATAGTCTTGCTTGGCACCTACCACCTTTAACCCAAAAC
CAAATAAAACAAACAGTTCCT
>2197
CGGGTGTTTGGTTGTTTTGTTTTTCTCTTTTCTTTGTCTTTTTTAAAAAAAGAAGTTGAA
AGGGGGATAGTCTTGCTTGGCACCTACCACCTTTAACCCAAAACCAAATAAAACAAAC
AGTTCCTAAATATAAATTAAAC
>2198
AGAAGTTGAAAGGGGGATAGTCTTGCTTGGCACCTACCACCTTTAACCCAAAACCAAA
TAAAACAAACAGTTCCTAAATATAAATTAAACTTCAACCTGCAGTATAGACATCCTCCTT
CCCTGCCCGAAACCCACTGGCC
>2199
CCTACCACCTTTAACCCAAAACCAAATAAAACAAACAGTTCCTAAATATAAATTAAACT
TCAACCTGCAGTATAGACATCCTCCTTCCCTGCCCGAAACCCACTGGCCACTGGAGGCT
AGCTTTAAGAAATTGGTTTTGG
>2200
CACCTTTAACCCAAAACCAAATAAAACAAACAGTTCCTAAATATAAATTAAACTTCAAC
CTGCAGTATAGACATCCTCCTTCCCTGCCCGAAACCCACTGGCCACTGGAGGCTAGCTT
TAAGAAATTGGTTTTGGAGACT
>2201
ATATAAATTAAACTTCAACCTGCAGTATAGACATCCTCCTTCCCTGCCCGAAACCCACT
GGCCACTGGAGGCTAGCTTTAAGAAATTGGTTTTGGAGACTCTGGCTTGAAAAGGAGC
CATGCCCACTTGTCTCACGTCCC
>2202
AATTAAACTTCAACCTGCAGTATAGACATCCTCCTTCCCTGCCCGAAACCCACTGGCCA
CTGGAGGCTAGCTTTAAGAAATTGGTTTTGGAGACTCTGGCTTGAAAAGGAGCCATGC
CCACTTGTCTCACGTCCCAGTCG
>2203
TAGACATCCTCCTTCCCTGCCCGAAACCCACTGGCCACTGGAGGCTAGCTTTAAGAAA
TTGGTTTTGGAGACTCTGGCTTGAAAAGGAGCCATGCCCACTTGTCTCACGTCCCAGT
CGCTGGCTCCAGGGGGCCGTGGTA

>2204
TAGCTTTAAGAAATTGGTTTTGGAGACTCTGGCTTGAAAAGGAGCCATGCCCACTTGT
CTCACGTCCCAGTCGCTGGCTCCAGGGGGCCGTGGTAGAACATATATTGTACTTCAGCT
GCCTTTCCCCTCCAAGCTTCCTC
>2205
TTGGTTTTGGAGACTCTGGCTTGAAAAGGAGCCATGCCCACTTGTCTCACGTCCCAGT
CGCTGGCTCCAGGGGGCCGTGGTAGAACATATATTGTACTTCAGCTGCCTTTCCCCTCC
AAGCTTCCTCAGCCACCCACAGC
>2206
CATGCCCACTTGTCTCACGTCCCAGTCGCTGGCTCCAGGGGGCCGTGGTAGAACATATA
TTGTACTTCAGCTGCCTTTCCCCTCCAAGCTTCCTCAGCCACCCACAGCCCTTTCAGAT
GAGCTGAGCCCTAGCACTGTGC
>2207
GTCCCAGTCGCTGGCTCCAGGGGGCCGTGGTAGAACATATATTGTACTTCAGCTGCCTT
TCCCCTCCAAGCTTCCTCAGCCACCCACAGCCCTTTCAGATGAGCTGAGCCCTAGCAC
TGTGCCCTCTCCCCTCTGCCCTC
>2208
GGGGGCCGTGGTAGAACATATATTGTACTTCAGCTGCCTTTCCCCTCCAAGCTTCCTCA
GCCACCCACAGCCCTTTCAGATGAGCTGAGCCCTAGCACTGTGCCCTCTCCCCTCTGC
CCTCTTAGGGTGATAGAATCCAC
>2209
AACATATATTGTACTTCAGCTGCCTTTCCCCTCCAAGCTTCCTCAGCCACCCACAGCCC
TTTCAGATGAGCTGAGCCCTAGCACTGTGCCCTCTCCCCTCTGCCCTCTTAGGGTGATA
GAATCCACTACCCTGACATCTG
>2210
ATATTGTACTTCAGCTGCCTTTCCCCTCCAAGCTTCCTCAGCCACCCACAGCCCTTTCA
GATGAGCTGAGCCCTAGCACTGTGCCCTCTCCCCTCTGCCCTCTTAGGGTGATAGAATC
CACTACCCTGACATCTGGGGTT
>2211
TACTTCAGCTGCCTTTCCCCTCCAAGCTTCCTCAGCCACCCACAGCCCTTTCAGATGAG
CTGAGCCCTAGCACTGTGCCCTCTCCCCTCTGCCCTCTTAGGGTGATAGAATCCACTAC
CCTGACATCTGGGGTTGCCCTG
>2212
CTCAGCCACCCACAGCCCTTTCAGATGAGCTGAGCCCTAGCACTGTGCCCTCTCCCCT
CTGCCCTCTTAGGGTGATAGAATCCACTACCCTGACATCTGGGGTTGCCCTGTTCTGCT
GTGGTGGGAGATGGGCTGGAAGG
>2213
TCTGCCCTCTTAGGGTGATAGAATCCACTACCCTGACATCTGGGGTTGCCCTGTTCTGC
TGTGGTGGGAGATGGGCTGGAAGGGACTTCGGAGGCTCTCCCGTGTTCTGTACCACCC
AGCTTCCCCTTGGATCCCTCCAC
>2214
GGGTGATAGAATCCACTACCCTGACATCTGGGGTTGCCCTGTTCTGCTGTGGTGGGAGA
TGGGCTGGAAGGGACTTCGGAGGCTCTCCCGTGTTCTGTACCACCCAGCTTCCCCTTG
GATCCCTCCACCCCCGCAGCCAG
>2215
TCCACTACCCTGACATCTGGGGTTGCCCTGTTCTGCTGTGGTGGGAGATGGGCTGGAA

GGGACTTCGGAGGCTCTCCCGTGTTCTGTACCACCCAGCTTCCCCTTGGATCCCTCCAC
CCCCGCAGCCAGGAGAGGGAAGT
>2216
CCTGTTCTGCTGTGGTGGGAGATGGGCTGGAAGGGACTTCGGAGGCTCTCCCGTGTTC
TGTACCACCCAGCTTCCCCTTGGATCCCTCCACCCCCGCAGCCAGGAGAGGGAAGTAA
CTGTTCTTTGAGCAGGACATTTCA
>2217
GAAGGGACTTCGGAGGCTCTCCCGTGTTCTGTACCACCCAGCTTCCCCTTGGATCCCTC
CACCCCCGCAGCCAGGAGAGGGAAGTAACTGTTCTTTGAGCAGGACATTTCATTTTCA
AAGTATTCTTGATGCCGCCCCCC
>2218
GGACTTCGGAGGCTCTCCCGTGTTCTGTACCACCCAGCTTCCCCTTGGATCCCTCCACC
CCCGCAGCCAGGAGAGGGAAGTAACTGTTCTTTGAGCAGGACATTTCATTTTCAAAGT
ATTCTTGATGCCGCCCCCCCGCC
>2219
CTTCGGAGGCTCTCCCGTGTTCTGTACCACCCAGCTTCCCCTTGGATCCCTCCACCCCC
GCAGCCAGGAGAGGGAAGTAACTGTTCTTTGAGCAGGACATTTCATTTTCAAAGTATT
CTTGATGCCGCCCCCCCGCCCCA
>2220
TCGGAGGCTCTCCCGTGTTCTGTACCACCCAGCTTCCCCTTGGATCCCTCCACCCCCGC
AGCCAGGAGAGGGAAGTAACTGTTCTTTGAGCAGGACATTTCATTTTCAAAGTATTCTT
GATGCCGCCCCCCCGCCCCACC
>2221
TGTACCACCCAGCTTCCCCTTGGATCCCTCCACCCCCGCAGCCAGGAGAGGGAAGTAA
CTGTTCTTTGAGCAGGACATTTCATTTTCAAAGTATTCTTGATGCCGCCCCCCCGCCCC
ACCGCCAAGAATTCTGCTCTGTG
>2222
GGATCCCTCCACCCCCGCAGCCAGGAGAGGGAAGTAACTGTTCTTTGAGCAGGACATT
TCATTTTCAAAGTATTCTTGATGCCGCCCCCCCGCCCCACCGCCAAGAATTCTGCTCTG
TGGGTGATACTTATAAGGTAACT
>2223
AACTGTTCTTTGAGCAGGACATTTCATTTTCAAAGTATTCTTGATGCCGCCCCCCCGCC
CCACCGCCAAGAATTCTGCTCTGTGGGTGATACTTATAAGGTAACTATATGGCTTCCTTA
TTTAGGCAGCAGTTTCTCTTC
>2224
TCAAAGTATTCTTGATGCCGCCCCCCCGCCCCACCGCCAAGAATTCTGCTCTGTGGGTG
ATACTTATAAGGTAACTATATGGCTTCCTTATTTAGGCAGCAGTTTCTCTTCTATGATTTT
AGTTCCTCTCATGATTCTTA
>2225
CCGCCCCACCGCCAAGAATTCTGCTCTGTGGGTGATACTTATAAGGTAACTATATGGCTT
CCTTATTTAGGCAGCAGTTTCTCTTCTATGATTTTAGTTCCTCTCATGATTCTTATAACCC
TCTCTCAGATACTTCCAGG
>2226
CCCACCGCCAAGAATTCTGCTCTGTGGGTGATACTTATAAGGTAACTATATGGCTTCCTT
ATTTAGGCAGCAGTTTCTCTTCTATGATTTTAGTTCCTCTCATGATTCTTATAACCCTCTC
TCAGATACTTCCAGGATGA

>2227
GTGGGTGATACTTATAAGGTAACTATATGGCTTCCTTATTTAGGCAGCAGTTTCTCTTCTA
TGATTTTAGTTCCTCTCATGATTCTTATAACCCTCTCTCAGATACTTCCAGGATGATAAAT
CTTCTATCACTGTTTCAT
>2228
TCCTTATTTAGGCAGCAGTTTCTCTTCTATGATTTTAGTTCCTCTCATGATTCTTATAACC
CTCTCTCAGATACTTCCAGGATGATAAATCTTCTATCACTGTTTCATCTGATCACATCAG
GGTGATTATATTTCGTGAC
>2229
GGCAGCAGTTTCTCTTCTATGATTTTAGTTCCTCTCATGATTCTTATAACCCTCTCTCAGA
TACTTCCAGGATGATAAATCTTCTATCACTGTTTCATCTGATCACATCAGGGTGATTATAT
TTCGTGACATAACCAGGT
>2230
TCTTATAACCCTCTCTCAGATACTTCCAGGATGATAAATCTTCTATCACTGTTTCATCTGA
TCACATCAGGGTGATTATATTTCGTGACATAACCAGGTGGCTTTAATCCGTTGGTGGAA
GAACCAGGCTCTCACCTCCA
>2231
AGGATGATAAATCTTCTATCACTGTTTCATCTGATCACATCAGGGTGATTATATTTCGTGA
CATAACCAGGTGGCTTTAATCCGTTGGTGGAAGAACCAGGCTCTCACCTCCATTGTTTA
AGATGTTATGTTTCTACTAA
>2232
TTTCATCTGATCACATCAGGGTGATTATATTTCGTGACATAACCAGGTGGCTTTAATCCG
TTGGTGGAAGAACCAGGCTCTCACCTCCATTGTTTAAGATGTTATGTTTCTACTAATCTG
TCCACTGAGTGTCTCCATCC
>2233
CTGATCACATCAGGGTGATTATATTTCGTGACATAACCAGGTGGCTTTAATCCGTTGGTG
GAAGAACCAGGCTCTCACCTCCATTGTTTAAGATGTTATGTTTCTACTAATCTGTCCACT
GAGTGTCTCCATCCTATTGT
>2234
ATTTCGTGACATAACCAGGTGGCTTTAATCCGTTGGTGGAAGAACCAGGCTCTCACCTC
CATTGTTTAAGATGTTATGTTTCTACTAATCTGTCCACTGAGTGTCTCCATCCTATTGTTT
CCATTGCATGCTCTCAGGTT
>2235
GTTGGTGGAAGAACCAGGCTCTCACCTCCATTGTTTAAGATGTTATGTTTCTACTAATCT
GTCCACTGAGTGTCTCCATCCTATTGTTTCCATTGCATGCTCTCAGGTTATTTCCTTTGTT
CTTGGCTGTCTGATTGCTC
>2236
AAGATGTTATGTTTCTACTAATCTGTCCACTGAGTGTCTCCATCCTATTGTTTCCATTGCA
TGCTCTCAGGTTATTTCCTTTGTTCTTGGCTGTCTGATTGCTCCCAGAGGCTGCAGTTAT
TTTATTTTATTTATTTCAT
>2237
TCCATCCTATTGTTTCCATTGCATGCTCTCAGGTTATTTCCTTTGTTCTTGGCTGTCTGAT
TGCTCCCAGAGGCTGCAGTTATTTTATTTTATTTATTTCATTTTTTGGTGGGAGTAGGGG
ATTGATCTCTCTCTTGCCT
>2238
CATCCTATTGTTTCCATTGCATGCTCTCAGGTTATTTCCTTTGTTCTTGGCTGTCTGATTG

CTCCCAGAGGCTGCAGTTATTTATTTATTTATTTCATTTTTTGGTGGGAGTAGGGGATT
GATCTCTCTCTTGCCTTT
>2239
TTCTTGGCTGTCTGATTGCTCCCAGAGGCTGCAGTTATTTATTTATTTATTTCATTTTT
TGGTGGGAGTAGGGGATTGATCTCTCTCTTGCCTTTAGCTTGAATGGGTGGTCATTTCC
AGGCTTACACATTGCTCCAT
>2240
TGCAGTTATTTATTTATTTATTTCATTTTTTGGTGGGAGTAGGGGATTGATCTCTCTCT
TGCCTTTAGCTTGAATGGGTGGTCATTTCCAGGCTTACACATTGCTCCATGCCCACTGTA
GGGGTCTGGCTTAGAGATG
>2241
TTCATTTTTTGGTGGGAGTAGGGGATTGATCTCTCTCTTGCCTTTAGCTTGAATGGGTGG
TCATTTCCAGGCTTACACATTGCTCCATGCCCACTGTAGGGGTCTGGCTTAGAGATGGT
CGGGGGAGAGAAGGTGGTGAT
>2242
TCTCTCTCTTGCCTTTAGCTTGAATGGGTGGTCATTTCCAGGCTTACACATTGCTCCATG
CCCACTGTAGGGGTCTGGCTTAGAGATGGTCGGGGGAGAGAAGGTGGTGATTTAAAA
GATTTGGAGGCGGTGGCTTACAT
>2243
TTTAGCTTGAATGGGTGGTCATTTCCAGGCTTACACATTGCTCCATGCCCACTGTAGGG
GTCTGGCTTAGAGATGGTCGGGGGAGAGAAGGTGGTGATTTAAAAGATTTGGAGGCG
GTGGCTTACATTGATTTTAGCTTA
>2244
GTCATTTCCAGGCTTACACATTGCTCCATGCCCACTGTAGGGGTCTGGCTTAGAGATGG
TCGGGGGAGAGAAGGTGGTGATTTAAAAGATTTGGAGGCGGTGGCTTACATTGATTTT
AGCTTATCTAACCCTTTAGCTAG
>2245
CATTGCTCCATGCCCACTGTAGGGGTCTGGCTTAGAGATGGTCGGGGGAGAGAAGGTG
GTGATTTAAAAGATTTGGAGGCGGTGGCTTACATTGATTTTAGCTTATCTAACCCTTTAG
CTAGAGGCTGCTCTTCCCTCAG
>2246
CATGCCCACTGTAGGGGTCTGGCTTAGAGATGGTCGGGGGAGAGAAGGTGGTGATTTA
AAAGATTTGGAGGCGGTGGCTTACATTGATTTTAGCTTATCTAACCCTTTAGCTAGAGG
CTGCTCTTCCCTCAGAGTGTCCA
>2247
TTAGAGATGGTCGGGGGAGAGAAGGTGGTGATTTAAAAGATTTGGAGGCGGTGGCTTA
CATTGATTTTAGCTTATCTAACCCTTTAGCTAGAGGCTGCTCTTCCCTCAGAGTGTCCAA
CTAGCTGTAACTTAAAGCAACA
>2248
AGAGAAGGTGGTGATTTAAAAGATTTGGAGGCGGTGGCTTACATTGATTTTAGCTTATC
TAACCCTTTAGCTAGAGGCTGCTCTTCCCTCAGAGTGTCCAACTAGCTGTAACTTAAAG
CAACAGAGAAATGTATATCATC
>2249
ATTTGGAGGCGGTGGCTTACATTGATTTTAGCTTATCTAACCCTTTAGCTAGAGGCTGCT
CTTCCCTCAGAGTGTCCAACTAGCTGTAACTTAAAGCAACAGAGAAATGTATATCATCC
CTTTTCTTGCATTAATTAGGG

>2250
TTGGAGGCGGTGGCTTACATTGATTTTAGCTTATCTAACCCTTTAGCTAGAGGCTGCTCT
TCCCTCAGAGTGTCCAACTAGCTGTAACTTAAAGCAACAGAGAAATGTATATCATCCCT
TTTCTTGCATTAATTAGGGCT
>2251
TTTAGCTTATCTAACCCTTTAGCTAGAGGCTGCTCTTCCCTCAGAGTGTCCAACTAGCT
GTAACTTAAAGCAACAGAGAAATGTATATCATCCCTTTTCTTGCATTAATTAGGGCTGAG
AAATTCATTATTTAGAGGGAT
>2252
AGAGTGTCCAACTAGCTGTAACTTAAAGCAACAGAGAAATGTATATCATCCCTTTTCTT
GCATTAATTAGGGCTGAGAAATTCATTATTTAGAGGGATAAGAGCTCCGGAGGAGGAG
GGTTTTTTGAACTAATGTAGAGA
>2253
CCAACTAGCTGTAACTTAAAGCAACAGAGAAATGTATATCATCCCTTTTCTTGCATTAAT
TAGGGCTGAGAAATTCATTATTTAGAGGGATAAGAGCTCCGGAGGAGGAGGGTTTTTT
GAACTAATGTAGAGATCTGTGG
>2254
CAGAGAAATGTATATCATCCCTTTTCTTGCATTAATTAGGGCTGAGAAATTCATTATTTAG
AGGGATAAGAGCTCCGGAGGAGGAGGGTTTTTTGAACTAATGTAGAGATCTGTGGCCA
GCTGTGTGATTCTCAGGTAGC
>2255
TATATCATCCCTTTTCTTGCATTAATTAGGGCTGAGAAATTCATTATTTAGAGGGATAAGA
GCTCCGGAGGAGGAGGGTTTTTTGAACTAATGTAGAGATCTGTGGCCAGCTGTGTGAT
TCTCAGGTAGCAGGTGAGGGG
>2256
ATCATCCCTTTTCTTGCATTAATTAGGGCTGAGAAATTCATTATTTAGAGGGATAAGAGC
TCCGGAGGAGGAGGGTTTTTTGAACTAATGTAGAGATCTGTGGCCAGCTGTGTGATTC
TCAGGTAGCAGGTGAGGGGGTG
>2257
TAGGGCTGAGAAATTCATTATTTAGAGGGATAAGAGCTCCGGAGGAGGAGGGTTTTTT
GAACTAATGTAGAGATCTGTGGCCAGCTGTGTGATTCTCAGGTAGCAGGTGAGGGGGT
GGGGGTTGATGAGAGGGGTGAGAA
>2258
TCATTATTTAGAGGGATAAGAGCTCCGGAGGAGGAGGGTTTTTTGAACTAATGTAGAGA
TCTGTGGCCAGCTGTGTGATTCTCAGGTAGCAGGTGAGGGGGTGGGGGTTGATGAGAG
GGGTGAGAAGGAGCAGGTGCCAG
>2259
GAGCTCCGGAGGAGGAGGGTTTTTTGAACTAATGTAGAGATCTGTGGCCAGCTGTGTG
ATTCTCAGGTAGCAGGTGAGGGGGTGGGGGTTGATGAGAGGGGTGAGAAGGAGCAGG
TGCCAGGGTCAGGCAGGGGAGGGAG
>2260
CTCCGGAGGAGGAGGGTTTTTTGAACTAATGTAGAGATCTGTGGCCAGCTGTGTGATT
CTCAGGTAGCAGGTGAGGGGGTGGGGGTTGATGAGAGGGGTGAGAAGGAGCAGGTG
CCAGGGTCAGGCAGGGGAGGGAGCCC
>2261
GAGGAGGAGGGTTTTTTGAACTAATGTAGAGATCTGTGGCCAGCTGTGTGATTCTCAG

GTAGCAGGTGAGGGGGTGGGGGTTGATGAGAGGGGTGAGAAGGAGCAGGTGCCAGG
GTCAGGCAGGGGAGGGAGCCCCCACT
>2262
AACTAATGTAGAGATCTGTGGCCAGCTGTGTGATTCTCAGGTAGCAGGTGAGGGGGTG
GGGGTTGATGAGAGGGGTGAGAAGGAGCAGGTGCCAGGGTCAGGCAGGGGAGGGAG
CCCCCACTCCGTGCATCATCCCAGAG
>2263
TGGCCAGCTGTGTGATTCTCAGGTAGCAGGTGAGGGGGTGGGGGTTGATGAGAGGGG
TGAGAAGGAGCAGGTGCCAGGGTCAGGCAGGGGAGGGAGCCCCCACTCCGTGCATCA
TCCCAGAGCCCTGTTCCTCCAAGCCC
>2264
CTCAGGTAGCAGGTGAGGGGGTGGGGGTTGATGAGAGGGGTGAGAAGGAGCAGGTG
CCAGGGTCAGGCAGGGGAGGGAGCCCCCACTCCGTGCATCATCCCAGAGCCCTGTTC
CTCCAAGCCCAGATTAGACACCCTTCT
>2265
GTAGCAGGTGAGGGGGTGGGGGTTGATGAGAGGGGTGAGAAGGAGCAGGTGCCAGG
GTCAGGCAGGGGAGGGAGCCCCCACTCCGTGCATCATCCCAGAGCCCTGTTCCTCCAA
GCCCAGATTAGACACCCTTCTCCACG
>2266
CAGGTGAGGGGGTGGGGGTTGATGAGAGGGGTGAGAAGGAGCAGGTGCCAGGGTCA
GGCAGGGGAGGGAGCCCCCACTCCGTGCATCATCCCAGAGCCCTGTTCCTCCAAGCCC
AGATTAGACACCCTTCTCCACGTGGG
>2267
AGAGGGGTGAGAAGGAGCAGGTGCCAGGGTCAGGCAGGGGAGGGAGCCCCCACTCC
GTGCATCATCCCAGAGCCCTGTTCCTCCAAGCCCAGATTAGACACCCTTCTCCACGTGG
GGCCTCAAGTTAGAAATCCTGGGAG
>2268
GGTCAGGCAGGGGAGGGAGCCCCCACTCCGTGCATCATCCCAGAGCCCTGTTCCTCCA
AGCCCAGATTAGACACCCTTCTCCACGTGGGGCCTCAAGTTAGAAATCCTGGGAGGAG
GCCTTATTGCTCAGGATCATCTGG
>2269
CCGTGCATCATCCCAGAGCCCTGTTCCTCCAAGCCCAGATTAGACACCCTTCTCCACGT
GGGGCCTCAAGTTAGAAATCCTGGGAGGAGGCCTTATTGCTCAGGATCATCTGGGAAT
GTTGCTTTCTGGGTTTAAAATAC
>2270
GCATCATCCCAGAGCCCTGTTCCTCCAAGCCCAGATTAGACACCCTTCTCCACGTGGG
GCCTCAAGTTAGAAATCCTGGGAGGAGGCCTTATTGCTCAGGATCATCTGGGAATGTTG
CTTTCTGGGTTTAAAATACAGTC
>2271
CCCAGATTAGACACCCTTCTCCACGTGGGGCCTCAAGTTAGAAATCCTGGGAGGAGGC
CTTATTGCTCAGGATCATCTGGGAATGTTGCTTTCTGGGTTTAAAATACAGTCAATGTTT
AGATGAGCTGCTGTTCAGGCAC
>2272
AGAAATCCTGGGAGGAGGCCTTATTGCTCAGGATCATCTGGGAATGTTGCTTTCTGGGT
TTAAAATACAGTCAATGTTTAGATGAGCTGCTGTTCAGGCACATGGTCTGGTTAGCAAA
GGGTTAAATCCTGCATCCCGTT

>2273
GAGGAGGCCTTATTGCTCAGGATCATCTGGGAATGTTGCTTTCTGGGTTTAAAATACAG
TCAATGTTTAGATGAGCTGCTGTTCAGGCACATGGTCTGGTTAGCAAAGGGTTAAATCC
TGCATCCCGTTTTTAGTCATCA
>2274
TCAGGATCATCTGGGAATGTTGCTTTCTGGGTTTAAAATACAGTCAATGTTTAGATGAG
CTGCTGTTCAGGCACATGGTCTGGTTAGCAAAGGGTTAAATCCTGCATCCCGTTTTTAG
TCATCAAGCTAGCTCAGATCTG
>2275
TGTTGCTTTCTGGGTTTAAAATACAGTCAATGTTTAGATGAGCTGCTGTTCAGGCACAT
GGTCTGGTTAGCAAAGGGTTAAATCCTGCATCCCGTTTTTAGTCATCAAGCTAGCTCAG
ATCTGCCCACCTTGGAGCCCTG
>2276
GTTTAGATGAGCTGCTGTTCAGGCACATGGTCTGGTTAGCAAAGGGTTAAATCCTGCAT
CCCGTTTTTAGTCATCAAGCTAGCTCAGATCTGCCCACCTTGGAGCCCTGTGCTTTCCT
GCCTTAGTGGGCAGAGTCTAGG
>2277
GAGCTGCTGTTCAGGCACATGGTCTGGTTAGCAAAGGGTTAAATCCTGCATCCCGTTTT
TAGTCATCAAGCTAGCTCAGATCTGCCCACCTTGGAGCCCTGTGCTTTCCTGCCTTAGT
GGGCAGAGTCTAGGGGACCTGT
>2278
TGCTGTTCAGGCACATGGTCTGGTTAGCAAAGGGTTAAATCCTGCATCCCGTTTTTAGT
CATCAAGCTAGCTCAGATCTGCCCACCTTGGAGCCCTGTGCTTTCCTGCCTTAGTGGGC
AGAGTCTAGGGGACCTGTGGCA
>2279
GTTAGCAAAGGGTTAAATCCTGCATCCCGTTTTTAGTCATCAAGCTAGCTCAGATCTGC
CCACCTTGGAGCCCTGTGCTTTCCTGCCTTAGTGGGCAGAGTCTAGGGGACCTGTGGC
ATGTTCAATGATTGAGGACTCTT
>2280
GCATCCCGTTTTTAGTCATCAAGCTAGCTCAGATCTGCCCACCTTGGAGCCCTGTGCTT
TCCTGCCTTAGTGGGCAGAGTCTAGGGGACCTGTGGCATGTTCAATGATTGAGGACTC
TTGTCCAGATTCCAAAAACCTAC
>2281
TTTTAGTCATCAAGCTAGCTCAGATCTGCCCACCTTGGAGCCCTGTGCTTTCCTGCCTT
AGTGGGCAGAGTCTAGGGGACCTGTGGCATGTTCAATGATTGAGGACTCTTGTCCAGA
TTCCAAAAACCTACTGTGATGAT
>2282
GTCATCAAGCTAGCTCAGATCTGCCCACCTTGGAGCCCTGTGCTTTCCTGCCTTAGTGG
GCAGAGTCTAGGGGACCTGTGGCATGTTCAATGATTGAGGACTCTTGTCCAGATTCCA
AAAACCTACTGTGATGATGCTGT
>2283
CTTGGAGCCCTGTGCTTTCCTGCCTTAGTGGGCAGAGTCTAGGGGACCTGTGGCATGT
TCAATGATTGAGGACTCTTGTCCAGATTCCAAAAACCTACTGTGATGATGCTGTTCTGA
AGCTGTGGTAGGCGGTGATTGAG
>2284
GCTTTCCTGCCTTAGTGGGCAGAGTCTAGGGGACCTGTGGCATGTTCAATGATTGAGG

ACTCTTGTCCAGATTCCAAAAACCTACTGTGATGATGCTGTTCTGAAGCTGTGGTAGGC
GGTGATTGAGTGGAGTGTGTAGT
>2285
GTGGCATGTTCAATGATTGAGGACTCTTGTCCAGATTCCAAAAACCTACTGTGATGATG
CTGTTCTGAAGCTGTGGTAGGCGGTGATTGAGTGGAGTGTGTAGTGACCCTCAGGAAT
TCTTCAGAAGTTAGGATTTTGAC
>2286
TCAATGATTGAGGACTCTTGTCCAGATTCCAAAAACCTACTGTGATGATGCTGTTCTGA
AGCTGTGGTAGGCGGTGATTGAGTGGAGTGTGTAGTGACCCTCAGGAATTCTTCAGAA
GTTAGGATTTTGACGATACCTCT
>2287
GATTCCAAAAACCTACTGTGATGATGCTGTTCTGAAGCTGTGGTAGGCGGTGATTGAGT
GGAGTGTGTAGTGACCCTCAGGAATTCTTCAGAAGTTAGGATTTTGACGATACCTCTGA
TAAGTTGGGTTTTTAATTTGCT
>2288
ACCTACTGTGATGATGCTGTTCTGAAGCTGTGGTAGGCGGTGATTGAGTGGAGTGTGTA
GTGACCCTCAGGAATTCTTCAGAAGTTAGGATTTTGACGATACCTCTGATAAGTTGGGT
TTTTAATTTGCTGGTTTATCTT
>2289
TGATGCTGTTCTGAAGCTGTGGTAGGCGGTGATTGAGTGGAGTGTGTAGTGACCCTCA
GGAATTCTTCAGAAGTTAGGATTTTGACGATACCTCTGATAAGTTGGGTTTTTAATTTGC
TGGTTTATCTTAGAGTGGAGGC
>2290
GTTCTGAAGCTGTGGTAGGCGGTGATTGAGTGGAGTGTGTAGTGACCCTCAGGAATTC
TTCAGAAGTTAGGATTTTGACGATACCTCTGATAAGTTGGGTTTTTAATTTGCTGGTTTA
TCTTAGAGTGGAGGCTGTGGCC
>2291
ATTGAGTGGAGTGTGTAGTGACCCTCAGGAATTCTTCAGAAGTTAGGATTTTGACGATA
CCTCTGATAAGTTGGGTTTTTAATTTGCTGGTTTATCTTAGAGTGGAGGCTGTGGCCCA
CAGCAAGTAGTACATTTTCCAT
>2292
ATTCTTCAGAAGTTAGGATTTTGACGATACCTCTGATAAGTTGGGTTTTTAATTTGCTGG
TTTATCTTAGAGTGGAGGCTGTGGCCCACAGCAAGTAGTACATTTTCCATCTCAGCCCA
GTGCATGGACAAACGTACCTC
>2293
TCTTCAGAAGTTAGGATTTTGACGATACCTCTGATAAGTTGGGTTTTTAATTTGCTGGTT
TATCTTAGAGTGGAGGCTGTGGCCCACAGCAAGTAGTACATTTTCCATCTCAGCCCAGT
GCATGGACAAACGTACCTCAA
>2294
TGACGATACCTCTGATAAGTTGGGTTTTTAATTTGCTGGTTTATCTTAGAGTGGAGGCTG
TGGCCCACAGCAAGTAGTACATTTTCCATCTCAGCCCAGTGCATGGACAAACGTACCTC
AAACCAAAGGTTCAATGAAGC
>2295
TTTTTAATTTGCTGGTTTATCTTAGAGTGGAGGCTGTGGCCCACAGCAAGTAGTACATTT
TCCATCTCAGCCCAGTGCATGGACAAACGTACCTCAAACCAAAGGTTCAATGAAGCTC
TATTTATCTTTACTATATGCTC

>2296
AATTTGCTGGTTTATCTTAGAGTGGAGGCTGTGGCCCACAGCAAGTAGTACATTTTCCA
TCTCAGCCCAGTGCATGGACAAACGTACCTCAAACCAAAGGTTCAATGAAGCTCTATT
TATCTTTACTATATGCTCTGGTA
>2297
TGTGGCCCACAGCAAGTAGTACATTTTCCATCTCAGCCCAGTGCATGGACAAACGTAC
CTCAAACCAAAGGTTCAATGAAGCTCTATTTATCTTTACTATATGCTCTGGTATCTTCTCT
TCCATTCTATCTTATTTCATT
>2298
AATGAAGCTCTATTTATCTTTACTATATGCTCTGGTATCTTCTCTTCCATTCTATCTTATTT
CATTTAAGAACGTTAATGAAGACCCACTAATGGATTGCCACCCATGGTTCGAGGAGGTC
CCCCAAACAGTGAGGCCTT
>2299
TTATCTTTACTATATGCTCTGGTATCTTCTCTTCCATTCTATCTTATTTCATTTAAGAACGT
TAATGAAGACCCACTAATGGATTGCCACCCATGGTTCGAGGAGGTCCCCCAAACAGTG
AGGCCTTAGCCATAGGTATT
>2300
TCCATTCTATCTTATTTCATTTAAGAACGTTAATGAAGACCCACTAATGGATTGCCACCC
ATGGTTCGAGGAGGTCCCCCAAACAGTGAGGCCTTAGCCATAGGTATTCAATTCAAAA
AGCATTTACTGAGCATCACTGT
>2301
TCATTTAAGAACGTTAATGAAGACCCACTAATGGATTGCCACCCATGGTTCGAGGAGGT
CCCCCAAACAGTGAGGCCTTAGCCATAGGTATTCAATTCAAAAAGCATTTACTGAGCAT
CACTGTGTACTAGGTGGTTGCT
>2302
CGTTAATGAAGACCCACTAATGGATTGCCACCCATGGTTCGAGGAGGTCCCCCAAACA
GTGAGGCCTTAGCCATAGGTATTCAATTCAAAAAGCATTTACTGAGCATCACTGTGTAC
TAGGTGGTTGCTAAATGCTATGG
>2303
TGAAGACCCACTAATGGATTGCCACCCATGGTTCGAGGAGGTCCCCCAAACAGTGAGG
CCTTAGCCATAGGTATTCAATTCAAAAAGCATTTACTGAGCATCACTGTGTACTAGGTG
GTTGCTAAATGCTATGGGGGTAA
>2304
ATTGCCACCCATGGTTCGAGGAGGTCCCCCAAACAGTGAGGCCTTAGCCATAGGTATTC
AATTCAAAAAGCATTTACTGAGCATCACTGTGTACTAGGTGGTTGCTAAATGCTATGGG
GGTAATGCAGAGAAATTAATAA
>2305
CCCAAACAGTGAGGCCTTAGCCATAGGTATTCAATTCAAAAAGCATTTACTGAGCATCA
CTGTGTACTAGGTGGTTGCTAAATGCTATGGGGGTAATGCAGAGAAATTAATAAATCCC
TGTAAGAGTTCACTGTAAAACA
>2306
CAATTCAAAAAGCATTTACTGAGCATCACTGTGTACTAGGTGGTTGCTAAATGCTATGG
GGGTAATGCAGAGAAATTAATAAATCCCTGTAAGAGTTCACTGTAAAACAGAAATGAC
AGCCATATGTGTATGACTTTGTG
>2307
ATTCAAAAAGCATTTACTGAGCATCACTGTGTACTAGGTGGTTGCTAAATGCTATGGGG

GTAATGCAGAGAAATTAATAAATCCCTGTAAGAGTTCACTGTAAAACAGAAATGACAG
CCATATGTGTATGACTTTGTGTG
>2308
TGGTTGCTAAATGCTATGGGGGTAATGCAGAGAAATTAATAAATCCCTGTAAGAGTTCA
CTGTAAAACAGAAATGACAGCCATATGTGTATGACTTTGTGTGTGTGTGTATATATATATA
TCAGACTGTATGATTAAATA
>2309
AATGCTATGGGGGTAATGCAGAGAAATTAATAAATCCCTGTAAGAGTTCACTGTAAAAC
AGAAATGACAGCCATATGTGTATGACTTTGTGTGTGTGTGTATATATATATATCAGACTGT
ATGATTAAATATTATATAAG
>2310
GTTCACTGTAAAACAGAAATGACAGCCATATGTGTATGACTTTGTGTGTGTGTGTATATA
TATATATCAGACTGTATGATTAAATATTATATAAGTGAGGTATGGGGGAGCAGAGGAGAG
GAATTGAATTTTGAATTTTG
>2311
CCATATGTGTATGACTTTGTGTGTGTGTGTATATATATATATCAGACTGTATGATTAAATAT
TATATAAGTGAGGTATGGGGGAGCAGAGGAGAGGAATTGAATTTTGAATTTTGATTGAA
GATTCAGGAGGGCTTCATG
>2312
ATGTGTATGACTTTGTGTGTGTGTGTATATATATATATCAGACTGTATGATTAAATATTATA
TAAGTGAGGTATGGGGGAGCAGAGGAGAGGAATTGAATTTTGAATTTTGATTGAAGAT
TCAGGAGGGCTTCATGGAGG
>2313
GATTAAATATTATATAAGTGAGGTATGGGGGAGCAGAGGAGAGGAATTGAATTTTGAAT
TTTGATTGAAGATTCAGGAGGGCTTCATGGAGGAGGTAGCATTTGGCCAGGTTTTAGA
GAATGGCTAGGCTTTGGACAAGT
>2314
ATATTATATAAGTGAGGTATGGGGGAGCAGAGGAGAGGAATTGAATTTTGAATTTTGAT
TGAAGATTCAGGAGGGCTTCATGGAGGAGGTAGCATTTGGCCAGGTTTTAGAGAATGG
CTAGGCTTTGGACAAGTGGAGAC
>2315
ATGGGGGAGCAGAGGAGAGGAATTGAATTTTGAATTTTGATTGAAGATTCAGGAGGGC
TTCATGGAGGAGGTAGCATTTGGCCAGGTTTTAGAGAATGGCTAGGCTTTGGACAAGT
GGAGACAGGAACTGCCATTTGGAG
>2316
TTGAATTTTGAATTTTGATTGAAGATTCAGGAGGGCTTCATGGAGGAGGTAGCATTTGG
CCAGGTTTTAGAGAATGGCTAGGCTTTGGACAAGTGGAGACAGGAACTGCCATTTGGA
GGTAGGAGATGAGCTAATGTGTG
>2317
GAATTTTGAATTTTGATTGAAGATTCAGGAGGGCTTCATGGAGGAGGTAGCATTTGGCC
AGGTTTTAGAGAATGGCTAGGCTTTGGACAAGTGGAGACAGGAACTGCCATTTGGAG
GTAGGAGATGAGCTAATGTGTGGC
>2318
ATTTTGATTGAAGATTCAGGAGGGCTTCATGGAGGAGGTAGCATTTGGCCAGGTTTTAG
AGAATGGCTAGGCTTTGGACAAGTGGAGACAGGAACTGCCATTTGGAGGTAGGAGAT
GAGCTAATGTGTGGCAGTGTATGG

>2319
GATTCAGGAGGGCTTCATGGAGGAGGTAGCATTTGGCCAGGTTTTAGAGAATGGCTAG
GCTTTGGACAAGTGGAGACAGGAACTGCCATTTGGAGGTAGGAGATGAGCTAATGTGT
GGCAGTGTATGGGTGGGGGCAGGT
>2320
GTAGCATTTGGCCAGGTTTTAGAGAATGGCTAGGCTTTGGACAAGTGGAGACAGGAAC
TGCCATTTGGAGGTAGGAGATGAGCTAATGTGTGGCAGTGTATGGGTGGGGGCAGGTG
CCTGTATACAAGGTAGTGTGCTGG
>2321
CAGGTTTTAGAGAATGGCTAGGCTTTGGACAAGTGGAGACAGGAACTGCCATTTGGAG
GTAGGAGATGAGCTAATGTGTGGCAGTGTATGGGTGGGGGCAGGTGCCTGTATACAAG
GTAGTGTGCTGGAATGGGGCGATG
>2322
TGGCTAGGCTTTGGACAAGTGGAGACAGGAACTGCCATTTGGAGGTAGGAGATGAGCT
AATGTGTGGCAGTGTATGGGTGGGGGCAGGTGCCTGTATACAAGGTAGTGTGCTGGAA
TGGGGCGATGGGCATGGGGTAGGA
>2323
AGTGGAGACAGGAACTGCCATTTGGAGGTAGGAGATGAGCTAATGTGTGGCAGTGTAT
GGGTGGGGGCAGGTGCCTGTATACAAGGTAGTGTGCTGGAATGGGGCGATGGGCATGG
GGTAGGATGGGAGATCAAGCAGGG
>2324
ATTTGGAGGTAGGAGATGAGCTAATGTGTGGCAGTGTATGGGTGGGGGCAGGTGCCTG
TATACAAGGTAGTGTGCTGGAATGGGGCGATGGGCATGGGGTAGGATGGGAGATCAAG
CAGGGAAAGTGGATTGGGGCCAGG
>2325
AGTGTATGGGTGGGGGCAGGTGCCTGTATACAAGGTAGTGTGCTGGAATGGGGCGATG
GGCATGGGGTAGGATGGGAGATCAAGCAGGGAAAGTGGATTGGGGCCAGGTCCTAGA
ATGATTTGAATACCAAGCTAGCTTT
>2326
GGTGGGGGCAGGTGCCTGTATACAAGGTAGTGTGCTGGAATGGGGCGATGGGCATGGG
GTAGGATGGGAGATCAAGCAGGGAAAGTGGATTGGGGCCAGGTCCTAGAATGATTTGA
ATACCAAGCTAGCTTTCTGGACAT
>2327
GGAATGGGGCGATGGGCATGGGGTAGGATGGGAGATCAAGCAGGGAAAGTGGATTGG
GGCCAGGTCCTAGAATGATTTGAATACCAAGCTAGCTTTCTGGACATTTGTAAGGCGCA
CTTGATAGGGGTTGGATCATTGCG
>2328
TGGGGTAGGATGGGAGATCAAGCAGGGAAAGTGGATTGGGGCCAGGTCCTAGAATGA
TTTGAATACCAAGCTAGCTTTCTGGACATTTGTAAGGCGCACTTGATAGGGGTTGGATC
ATTGCGATATTATTGGAAGACAAA
>2329
AGGGAAAGTGGATTGGGGCCAGGTCCTAGAATGATTTGAATACCAAGCTAGCTTTCTG
GACATTTGTAAGGCGCACTTGATAGGGGTTGGATCATTGCGATATTATTGGAAGACAAA
ACTGGGGAGGGTGTGAGACCATG
>2330
TGGGGCCAGGTCCTAGAATGATTTGAATACCAAGCTAGCTTTCTGGACATTTGTAAGGC

GCACTTGATAGGGGTTGGATCATTGCGATATTATTGGAAGACAAAACTGGGGAGGGTG
TGAGACCATGTGGCTGCTCAGAA
>2331
CCAAGCTAGCTTTCTGGACATTTGTAAGGCGCACTTGATAGGGGTTGGATCATTGCGAT
ATTATTGGAAGACAAAACTGGGGAGGGTGTGAGACCATGTGGCTGCTCAGAATGCAG
AGCCCCTCTGATGTCCTCAGCCTT
>2332
TGGACATTTGTAAGGCGCACTTGATAGGGGTTGGATCATTGCGATATTATTGGAAGACA
AAACTGGGGAGGGTGTGAGACCATGTGGCTGCTCAGAATGCAGAGCCCCTCTGATGTC
CTCAGCCTTCTACGTATTTAGTG
>2333
AGGGGTTGGATCATTGCGATATTATTGGAAGACAAAACTGGGGAGGGTGTGAGACCAT
GTGGCTGCTCAGAATGCAGAGCCCCTCTGATGTCCTCAGCCTTCTACGTATTTAGTGAT
GGGGGTGGTGGTGGTAAAGAGCC
>2334
GGATCATTGCGATATTATTGGAAGACAAAACTGGGGAGGGTGTGAGACCATGTGGCTG
CTCAGAATGCAGAGCCCCTCTGATGTCCTCAGCCTTCTACGTATTTAGTGATGGGGGTG
GTGGTGGTAAAGAGCCTTGCAGA
>2335
GAAGACAAAACTGGGGAGGGTGTGAGACCATGTGGCTGCTCAGAATGCAGAGCCCCT
CTGATGTCCTCAGCCTTCTACGTATTTAGTGATGGGGGTGGTGGTGGTAAAGAGCCTTG
CAGACAGCCTGGCTTGGGTGCTTG
>2336
TCAGAATGCAGAGCCCCTCTGATGTCCTCAGCCTTCTACGTATTTAGTGATGGGGGTGG
TGGTGGTAAAGAGCCTTGCAGACAGCCTGGCTTGGGTGCTTGCTTGAAGTTCTTACGC
CCACTAACTCCTGCCCTGCTCTG
>2337
AGAATGCAGAGCCCCTCTGATGTCCTCAGCCTTCTACGTATTTAGTGATGGGGGTGGTG
GTGGTAAAGAGCCTTGCAGACAGCCTGGCTTGGGTGCTTGCTTGAAGTTCTTACGCCC
ACTAACTCCTGCCCTGCTCTGTG
>2338
CGTATTTAGTGATGGGGGTGGTGGTGGTAAAGAGCCTTGCAGACAGCCTGGCTTGGGT
GCTTGCTTGAAGTTCTTACGCCCACTAACTCCTGCCCTGCTCTGTGGAGCCTGCTGAAT
GGGGCATTAGGCTAGTCACACAC
>2339
TGCAGACAGCCTGGCTTGGGTGCTTGCTTGAAGTTCTTACGCCCACTAACTCCTGCCCT
GCTCTGTGGAGCCTGCTGAATGGGGCATTAGGCTAGTCACACACCTGTCCTTTTTCACA
TTCATGCTGTACCCTTTGCAAA
>2340
TGCTTGCTTGAAGTTCTTACGCCCACTAACTCCTGCCCTGCTCTGTGGAGCCTGCTGAA
TGGGGCATTAGGCTAGTCACACACCTGTCCTTTTTCACATTCATGCTGTACCCTTTGCA
AACATTACGCAGCAGTGCAAAC
>2341
GGGGCATTAGGCTAGTCACACACCTGTCCTTTTTCACATTCATGCTGTACCCTTTGCAA
ACATTACGCAGCAGTGCAAACTCAGCCCTGGGCCTCAGAGGAGCCAGGCGTGGCCAG
GAGAGTGGGATTTGGAACGGAAGT

>2342
GCATTAGGCTAGTCACACACCTGTCCTTTTTCACATTCATGCTGTACCCTTTGCAAACAT
TACGCAGCAGTGCAAACTCAGCCCTGGGCCTCAGAGGAGCCAGGCGTGGCCAGGAGA
GTGGGATTTGGAACGGAAGTGCT
>2343
GTCACACACCTGTCCTTTTTCACATTCATGCTGTACCCTTTGCAAACATTACGCAGCAG
TGCAAACTCAGCCCTGGGCCTCAGAGGAGCCAGGCGTGGCCAGGAGAGTGGGATTTG
GAACGGAAGTGCTTTTGTTTTTGG
>2344
CCTTTTTCACATTCATGCTGTACCCTTTGCAAACATTACGCAGCAGTGCAAACTCAGCC
CTGGGCCTCAGAGGAGCCAGGCGTGGCCAGGAGAGTGGGATTTGGAACGGAAGTGCT
TTTGTTTTTGGCATTCACTACTCC
>2345
TTCACATTCATGCTGTACCCTTTGCAAACATTACGCAGCAGTGCAAACTCAGCCCTGGG
CCTCAGAGGAGCCAGGCGTGGCCAGGAGAGTGGGATTTGGAACGGAAGTGCTTTTGT
TTTTGGCATTCACTACTCCCCCAC
>2346
GCAGTGCAAACTCAGCCCTGGGCCTCAGAGGAGCCAGGCGTGGCCAGGAGAGTGGG
ATTTGGAACGGAAGTGCTTTTGTTTTTGGCATTCACTACTCCCCCACCCCCACTGGCAC
TTGGATTGTCCTTCTTTTTCTCCTT
>2347
CTACTCCCCCACCCCCACTGGCACTTGGATTGTCCTTCTTTTTCTCCTTGGTGTCTCTCA
TCCTGTCCAGCCTTCAAGACCTCCTCAAGTCAGGCTTCTCTGGGAAGCGCTGTCTGTC
TGTCTTGGTGCTTGCTGGCTTC
>2348
CCACCCCCACTGGCACTTGGATTGTCCTTCTTTTTCTCCTTGGTGTCTCTCATCCTGTCC
AGCCTTCAAGACCTCCTCAAGTCAGGCTTCTCTGGGAAGCGCTGTCTGTCTGTCTTGG
TGCTTGCTGGCTTCCTCTTCTG
>2349
CTTTTTCTCCTTGGTGTCTCTCATCCTGTCCAGCCTTCAAGACCTCCTCAAGTCAGGCT
TCTCTGGGAAGCGCTGTCTGTCTGTCTTGGTGCTTGCTGGCTTCCTCTTCTGTCTCCTG
TGCCATCTCCAGTTTGCACCAT
>2350
CTCTGGGAAGCGCTGTCTGTCTGTCTTGGTGCTTGCTGGCTTCCTCTTCTGTCTCCTGT
GCCATCTCCAGTTTGCACCATTTGAGTATAAGCTCTGCTGCCTCATTTGAGTTTAAGTTT
TATCCCCCCAGCCACAACCTA
>2351
TCTGTCTGTCTTGGTGCTTGCTGGCTTCCTCTTCTGTCTCCTGTGCCATCTCCAGTTTGC
ACCATTTGAGTATAAGCTCTGCTGCCTCATTTGAGTTTAAGTTTTATCCCCCCAGCCACA
ACCTAAGTTCCATGAGGATA
>2352
TTCCTCTTCTGTCTCCTGTGCCATCTCCAGTTTGCACCATTTGAGTATAAGCTCTGCTGC
CTCATTTGAGTTTAAGTTTTATCCCCCCAGCCACAACCTAAGTTCCATGAGGATAGTGAT
TGTGGTTTATCTTCTCTCCC
>2353
CCATCTCCAGTTTGCACCATTTGAGTATAAGCTCTGCTGCCTCATTTGAGTTTAAGTTTT

ATCCCCCCAGCCACAACCTAAGTTCCATGAGGATAGTGATTGTGGTTTATCTTCTCTCCC
TCAGCTGTCTGCTGACACCA
>2354
TGCACCATTTGAGTATAAGCTCTGCTGCCTCATTTGAGTTTAAGTTTTATCCCCCCAGCC
ACAACCTAAGTTCCATGAGGATAGTGATTGTGGTTTATCTTCTCTCCCTCAGCTGTCTGC
TGACACCATGCTGAATACAT
>2355
TGAGTATAAGCTCTGCTGCCTCATTTGAGTTTAAGTTTTATCCCCCCAGCCACAACCTAA
GTTCCATGAGGATAGTGATTGTGGTTTATCTTCTCTCCCTCAGCTGTCTGCTGACACCAT
GCTGAATACATAGTAGGTAC
>2356
AGTTTTATCCCCCCAGCCACAACCTAAGTTCCATGAGGATAGTGATTGTGGTTTATCTTC
TCTCCCTCAGCTGTCTGCTGACACCATGCTGAATACATAGTAGGTACTCAGTAAATAATA
GCTAATTGAGCATTTACTAT
>2357
CCATGAGGATAGTGATTGTGGTTTATCTTCTCTCCCTCAGCTGTCTGCTGACACCATGCT
GAATACATAGTAGGTACTCAGTAAATAATAGCTAATTGAGCATTTACTATGGACAAAGCA
CTTTACTTGCATTAACACAT
>2358
TGAGGATAGTGATTGTGGTTTATCTTCTCTCCCTCAGCTGTCTGCTGACACCATGCTGAA
TACATAGTAGGTACTCAGTAAATAATAGCTAATTGAGCATTTACTATGGACAAAGCACTT
TACTTGCATTAACACATCCC
>2359
TCTCCCTCAGCTGTCTGCTGACACCATGCTGAATACATAGTAGGTACTCAGTAAATAATA
GCTAATTGAGCATTTACTATGGACAAAGCACTTTACTTGCATTAACACATCCCATCCTCA
CAACCACCTGTGAGGTGAAT
>2360
TGACACCATGCTGAATACATAGTAGGTACTCAGTAAATAATAGCTAATTGAGCATTTACT
ATGGACAAAGCACTTTACTTGCATTAACACATCCCATCCTCACAACCACCTGTGAGGTG
AATCCTATTAAAAGGAATACT
>2361
ATTGAGCATTTACTATGGACAAAGCACTTTACTTGCATTAACACATCCCATCCTCACAAC
CACCTGTGAGGTGAATCCTATTAAAAGGAATACTTGGGTGCTTTCAGAGTTATTCTCTC
CAGCTTTGGACTTCTTGTTTC
>2362
GACAAAGCACTTTACTTGCATTAACACATCCCATCCTCACAACCACCTGTGAGGTGAAT
CCTATTAAAAGGAATACTTGGGTGCTTTCAGAGTTATTCTCTCCAGCTTTGGACTTCTTG
TTTCATTTGCCCTGAGAGCTC
>2363
TTAACACATCCCATCCTCACAACCACCTGTGAGGTGAATCCTATTAAAAGGAATACTTG
GGTGCTTTCAGAGTTATTCTCTCCAGCTTTGGACTTCTTGTTTCATTTGCCCTGAGAGCT
CCTCAACTCTGTTGATGCTGT
>2364
AAAAGGAATACTTGGGTGCTTTCAGAGTTATTCTCTCCAGCTTTGGACTTCTTGTTTCA
TTTGCCCTGAGAGCTCCTCAACTCTGTTGATGCTGTTCTTTCAAAGGTTGCTTGTGACC
TCTTAGTTCTCAAGAGCACTGT

>2365
AAGGAATACTTGGGTGCTTTCAGAGTTATTCTCTCCAGCTTTGGACTTCTTGTTTCATTT
GCCCTGAGAGCTCCTCAACTCTGTTGATGCTGTTCTTTCAAAGGTTGCTTGTGACCTCT
TAGTTCTCAAGAGCACTGTAT
>2366
TCCAGCTTTGGACTTCTTGTTTCATTTGCCCTGAGAGCTCCTCAACTCTGTTGATGCTG
TTCTTTCAAAGGTTGCTTGTGACCTCTTAGTTCTCAAGAGCACTGTATTAGGTTGGTGC
AAAAGTAATTGCTATTTTAGAC
>2367
TTTCATTTGCCCTGAGAGCTCCTCAACTCTGTTGATGCTGTTCTTTCAAAGGTTGCTTG
TGACCTCTTAGTTCTCAAGAGCACTGTATTAGGTTGGTGCAAAAGTAATTGCTATTTTA
GACATTACTTTCAATGGCAAAA
>2368
GCCCTGAGAGCTCCTCAACTCTGTTGATGCTGTTCTTTCAAAGGTTGCTTGTGACCTCT
TAGTTCTCAAGAGCACTGTATTAGGTTGGTGCAAAAGTAATTGCTATTTTAGACATTACT
TTCAATGGCAAAAACTGCAAT
>2369
ATGCTGTTCTTTCAAAGGTTGCTTGTGACCTCTTAGTTCTCAAGAGCACTGTATTAGGT
TGGTGCAAAAGTAATTGCTATTTTAGACATTACTTTCAATGGCAAAAACTGCAATTACTT
TTGCGCCAACTAATACTTTCC
>2370
AGGTTGCTTGTGACCTCTTAGTTCTCAAGAGCACTGTATTAGGTTGGTGCAAAAGTAAT
TGCTATTTTAGACATTACTTTCAATGGCAAAAACTGCAATTACTTTTGCGCCAACTAATA
CTTTCCCAGACTCCAGTATTG
>2371
TTGCTATTTTAGACATTACTTTCAATGGCAAAAACTGCAATTACTTTTGCGCCAACTAAT
ACTTTCCCAGACTCCAGTATTGGCTCCATTGACCCCTCCTGTTCCTGCCAACTTCTCCC
CCTCCTCCTCTGGTATTTCCT
>2372
TTTAGACATTACTTTCAATGGCAAAAACTGCAATTACTTTTGCGCCAACTAATACTTTCC
CAGACTCCAGTATTGGCTCCATTGACCCCTCCTGTTCCTGCCAACTTCTCCCCCTCCTC
CTCTGGTATTTCCTGGAACTC
>2373
CATTGACCCCTCCTGTTCCTGCCAACTTCTCCCCCTCCTCCTCTGGTATTTCCTGGAACT
CCCTCCCTAGGGTCAGTGCCATGTGGTGCCTGGAACTCCCTGGGATCTGTGTTTGAATC
CTGACATTGCCACTTTCTAAG
>2374
CCCCTCCTGTTCCTGCCAACTTCTCCCCCTCCTCCTCTGGTATTTCCTGGAACTCCCTCC
CTAGGGTCAGTGCCATGTGGTGCCTGGAACTCCCTGGGATCTGTGTTTGAATCCTGACA
TTGCCACTTTCTAAGTGGCAA
>2375
GGTCAGTGCCATGTGGTGCCTGGAACTCCCTGGGATCTGTGTTTGAATCCTGACATTGC
CACTTTCTAAGTGGCAAACACTTTGGCCACCTCCCCCTCTTCCTGAGTCTGTTTCATCA
TCTGCAAAATGCTGGTAGTTTC
>2376
CTGTGTTTGAATCCTGACATTGCCACTTTCTAAGTGGCAAACACTTTGGCCACCTCCCC

CTCTTCCTGAGTCTGTTTCATCATCTGCAAAATGCTGGTAGTTTCTCTTCTCACAGGAA
AGATGGTGGTTGTTCATCGATG
>2377
TAAGTGGCAAACACTTTGGCCACCTCCCCCTCTTCCTGAGTCTGTTTCATCATCTGCAA
AATGCTGGTAGTTTCTCTTCTCACAGGAAAGATGGTGGTTGTTCATCGATGTCTGTTGA
AACCGTGACCCTCTCTGCTGAA
>2378
TTGGCCACCTCCCCCTCTTCCTGAGTCTGTTTCATCATCTGCAAAATGCTGGTAGTTTCT
CTTCTCACAGGAAAGATGGTGGTTGTTCATCGATGTCTGTTGAAACCGTGACCCTCTCT
GCTGAATGTAGAAGAAGATGA
>2379
CACCTCCCCCTCTTCCTGAGTCTGTTTCATCATCTGCAAAATGCTGGTAGTTTCTCTTCT
CACAGGAAAGATGGTGGTTGTTCATCGATGTCTGTTGAAACCGTGACCCTCTCTGCTG
AATGTAGAAGAAGATGACCTTA
>2380
CTTCTCACAGGAAAGATGGTGGTTGTTCATCGATGTCTGTTGAAACCGTGACCCTCTCT
GCTGAATGTAGAAGAAGATGACCTTACCAGTTCTCCTGTCTCTCCAGTGTCTTTCTTGA
CAATCTTGTGGTTTCCACAAGC
>2381
CTCACAGGAAAGATGGTGGTTGTTCATCGATGTCTGTTGAAACCGTGACCCTCTCTGCT
GAATGTAGAAGAAGATGACCTTACCAGTTCTCCTGTCTCTCCAGTGTCTTTCTTGACAA
TCTTGTGGTTTCCACAAGCAAC
>2382
TGGTTGTTCATCGATGTCTGTTGAAACCGTGACCCTCTCTGCTGAATGTAGAAGAAGAT
GACCTTACCAGTTCTCCTGTCTCTCCAGTGTCTTTCTTGACAATCTTGTGGTTTCCACA
AGCAACCCTCTGTGGGTGATTT
>2383
CTGTTGAAACCGTGACCCTCTCTGCTGAATGTAGAAGAAGATGACCTTACCAGTTCTC
CTGTCTCTCCAGTGTCTTTCTTGACAATCTTGTGGTTTCCACAAGCAACCCTCTGTGGG
TGATTTCCAGATCTCTACCTCTA
>2384
GAAGAAGATGACCTTACCAGTTCTCCTGTCTCTCCAGTGTCTTTCTTGACAATCTTGTG
GTTTCCACAAGCAACCCTCTGTGGGTGATTTCCAGATCTCTACCTCTAGTTGTTTTTCGT
TTTTTGTCTTTTCTTGTGTTT
>2385
CCTGTCTCTCCAGTGTCTTTCTTGACAATCTTGTGGTTTCCACAAGCAACCCTCTGTGG
GTGATTTCCAGATCTCTACCTCTAGTTGTTTTTCGTTTTTTGTCTTTTCTTGTGTTTCACA
CATTCTTTTCCAGTAGCCTC
>2386
TTTCCAGATCTCTACCTCTAGTTGTTTTTCGTTTTTTGTCTTTTCTTGTGTTTCACACATT
CTTTTCCAGTAGCCTCACGGGCACACCAGTCCTGCAAGGATCTAGAATGTGATGGTTTG
AGCTAGTTCAACACCTTCCT
>2387
CCAGATCTCTACCTCTAGTTGTTTTTCGTTTTTTGTCTTTTCTTGTGTTTCACACATTCTT
TTCCAGTAGCCTCACGGGCACACCAGTCCTGCAAGGATCTAGAATGTGATGGTTTGAG
CTAGTTCAACACCTTCCTCTC

>2388
GTTGTTTTTCGTTTTTTGTCTTTTCTTGTGTTTCACACATTCTTTTCCAGTAGCCTCACG
GGCACACCAGTCCTGCAAGGATCTAGAATGTGATGGTTTGAGCTAGTTCAACACCTTC
CTCTCGAATCGTCTCCTGTGTC
>2389
CGTTTTTTGTCTTTTCTTGTGTTTCACACATTCTTTTCCAGTAGCCTCACGGGCACACCA
GTCCTGCAAGGATCTAGAATGTGATGGTTTGAGCTAGTTCAACACCTTCCTCTCGAATC
GTCTCCTGTGTCCTCATGTTT
>2390
TGTCTTTTCTTGTGTTTCACACATTCTTTTCCAGTAGCCTCACGGGCACACCAGTCCTG
CAAGGATCTAGAATGTGATGGTTTGAGCTAGTTCAACACCTTCCTCTCGAATCGTCTCC
TGTGTCCTCATGTTTTGTGGTC
>2391
TCACACATTCTTTTCCAGTAGCCTCACGGGCACACCAGTCCTGCAAGGATCTAGAATGT
GATGGTTTGAGCTAGTTCAACACCTTCCTCTCGAATCGTCTCCTGTGTCCTCATGTTTTG
TGGTCTCTGCCCTCTTCCCAG
>2392
CTAGTTCAACACCTTCCTCTCGAATCGTCTCCTGTGTCCTCATGTTTTGTGGTCTCTGCC
CTCTTCCCAGGCACCCAACTGCAGACTTTGGCATCATCTGTGGCTCCTGTCTTCCCTTT
GCCTTCCATATCCACCATTTG
>2393
TCCTCTCGAATCGTCTCCTGTGTCCTCATGTTTTGTGGTCTCTGCCCTCTTCCCAGGCAC
CCAACTGCAGACTTTGGCATCATCTGTGGCTCCTGTCTTCCCTTTGCCTTCCATATCCAC
CATTTGCCTCTCCAGCCTAC
>2394
TGCAGACTTTGGCATCATCTGTGGCTCCTGTCTTCCCTTTGCCTTCCATATCCACCATTT
GCCTCTCCAGCCTACTTAACCAGATGGCTGTCCTGATTTCCTCACCAGCCTTCCTGCAG
TGCACCCCTGCAAGGCTCATC
>2395
ATCATCTGTGGCTCCTGTCTTCCCTTTGCCTTCCATATCCACCATTTGCCTCTCCAGCCT
ACTTAACCAGATGGCTGTCCTGATTTCCTCACCAGCCTTCCTGCAGTGCACCCCTGCAA
GGCTCATCACCCCACTTCCCT
>2396
TTGCCTTCCATATCCACCATTTGCCTCTCCAGCCTACTTAACCAGATGGCTGTCCTGATT
TCCTCACCAGCCTTCCTGCAGTGCACCCCTGCAAGGCTCATCACCCCACTTCCCTGAG
CAGAGTAGGGCTCTGATGGGGT
>2397
TCTCCAGCCTACTTAACCAGATGGCTGTCCTGATTTCCTCACCAGCCTTCCTGCAGTGC
ACCCCTGCAAGGCTCATCACCCCACTTCCCTGAGCAGAGTAGGGCTCTGATGGGGTTG
CTCTCCTGCTCTGAACCCTGCAG
>2398
GCTGTCCTGATTTCCTCACCAGCCTTCCTGCAGTGCACCCCTGCAAGGCTCATCACCCC
ACTTCCCTGAGCAGAGTAGGGCTCTGATGGGGTTGCTCTCCTGCTCTGAACCCTGCAG
TGGCTCACTATGGCCTATTCTAG
>2399
GATTTCCTCACCAGCCTTCCTGCAGTGCACCCCTGCAAGGCTCATCACCCCACTTCCCT

GAGCAGAGTAGGGCTCTGATGGGGTTGCTCTCCTGCTCTGAACCCTGCAGTGGCTCAC
TATGGCCTATTCTAGAGAAGAAA
>2400
GCTCATCACCCCACTTCCCTGAGCAGAGTAGGGCTCTGATGGGGTTGCTCTCCTGCTCT
GAACCCTGCAGTGGCTCACTATGGCCTATTCTAGAGAAGAAACGAGAGAATTCTTAGC
GTGGCATTCAAAGCCATCCGGGA
>2401
CAGAGTAGGGCTCTGATGGGGTTGCTCTCCTGCTCTGAACCCTGCAGTGGCTCACTAT
GGCCTATTCTAGAGAAGAAACGAGAGAATTCTTAGCGTGGCATTCAAAGCCATCCGGG
ATCCTAGATTCCAGCTACATTTTC
>2402
GCTCTCCTGCTCTGAACCCTGCAGTGGCTCACTATGGCCTATTCTAGAGAAGAAACGA
GAGAATTCTTAGCGTGGCATTCAAAGCCATCCGGGATCCTAGATTCCAGCTACATTTTC
ACTCTTCATCCCTTTCTCTGCAT
>2403
AACCCTGCAGTGGCTCACTATGGCCTATTCTAGAGAAGAAACGAGAGAATTCTTAGCG
TGGCATTCAAAGCCATCCGGGATCCTAGATTCCAGCTACATTTTCACTCTTCATCCCTTT
CTCTGCATATTTCTTAGAATGT
>2404
ACTATGGCCTATTCTAGAGAAGAAACGAGAGAATTCTTAGCGTGGCATTCAAAGCCATC
CGGGATCCTAGATTCCAGCTACATTTTCACTCTTCATCCCTTTCTCTGCATATTTCTTAGA
ATGTACACCCCTGAAGGCAG
>2405
CAAAGCCATCCGGGATCCTAGATTCCAGCTACATTTTCACTCTTCATCCCTTTCTCTGCA
TATTTCTTAGAATGTACACCCCTGAAGGCAGGGACCATCTTTTTCACATCTGTGTTACA
ACCTTTTAGTCCACTCTCTAG
>2406
CTAGATTCCAGCTACATTTTCACTCTTCATCCCTTTCTCTGCATATTTCTTAGAATGTACA
CCCCTGAAGGCAGGGACCATCTTTTTCACATCTGTGTTACAACCTTTTAGTCCACTCTC
TAGCATATGGCAACTCAGCA
>2407
CATATTTCTTAGAATGTACACCCCTGAAGGCAGGGACCATCTTTTTCACATCTGTGTTAC
AACCTTTTAGTCCACTCTCTAGCATATGGCAACTCAGCAGATGCTGCTGTTGAGTAAAC
TCTTATGCTTCCCTTCCCATG
>2408
AATGTACACCCCTGAAGGCAGGGACCATCTTTTTCACATCTGTGTTACAACCTTTTAGT
CCACTCTCTAGCATATGGCAACTCAGCAGATGCTGCTGTTGAGTAAACTCTTATGCTTC
CCTTCCCATGCCATATCCTTCT
>2409
TTCACATCTGTGTTACAACCTTTTAGTCCACTCTCTAGCATATGGCAACTCAGCAGATGC
TGCTGTTGAGTAAACTCTTATGCTTCCCTTCCCATGCCATATCCTTCTCTGGTCATCGCA
ACAGGGCCAAATCTCTCATG
>2410
AGATGCTGCTGTTGAGTAAACTCTTATGCTTCCCTTCCCATGCCATATCCTTCTCTGGTC
ATCGCAACAGGGCCAAATCTCTCATGCTTCAGTGTAAATGCCCTCTCCTTGCAGCATCC
TTTCCTGGGGCATTCTTCCTC

>2411
CTTATGCTTCCCTTCCCATGCCATATCCTTCTCTGGTCATCGCAACAGGGCCAAATCTCT
CATGCTTCAGTGTAAATGCCCTCTCCTTGCAGCATCCTTTCCTGGGGCATTCTTCCTCCT
TGCATCCAGTTGGAAATAAT
>2412
ATATCCTTCTCTGGTCATCGCAACAGGGCCAAATCTCTCATGCTTCAGTGTAAATGCCCT
CTCCTTGCAGCATCCTTTCCTGGGGCATTCTTCCTCCTTGCATCCAGTTGGAAATAATAT
GTCCCTCCATTGGAGCTCCC
>2413
TCATGCTTCAGTGTAAATGCCCTCTCCTTGCAGCATCCTTTCCTGGGGCATTCTTCCTCC
TTGCATCCAGTTGGAAATAATATGTCCCTCCATTGGAGCTCCCACACCAGAAGAGGTAT
AGATAAAGCCAGATACTTCTT
>2414
TGCAGCATCCTTTCCTGGGGCATTCTTCCTCCTTGCATCCAGTTGGAAATAATATGTCCC
TCCATTGGAGCTCCCACACCAGAAGAGGTATAGATAAAGCCAGATACTTCTTTTTTTTT
TTTTTTTTTTGATACAGAGTC
>2415
GGGCATTCTTCCTCCTTGCATCCAGTTGGAAATAATATGTCCCTCCATTGGAGCTCCCAC
ACCAGAAGAGGTATAGATAAAGCCAGATACTTCTTTTTTTTTTTTTTTTTTTGATACAGA
GTCTCACTCTGTTGCCCAGG
>2416
TCTTCCTCCTTGCATCCAGTTGGAAATAATATGTCCCTCCATTGGAGCTCCCACACCAG
AAGAGGTATAGATAAAGCCAGATACTTCTTTTTTTTTTTTTTTTTTTGATACAGAGTCTC
ACTCTGTTGCCCAGGCTGGAG
>2417
TCCTTGCATCCAGTTGGAAATAATATGTCCCTCCATTGGAGCTCCCACACCAGAAGAGG
TATAGATAAAGCCAGATACTTCTTTTTTTTTTTTTTTTTTTGATACAGAGTCTCACTCTGT
TGCCCAGGCTGGAGTGTAGT
>2418
GGAGCTCCCACACCAGAAGAGGTATAGATAAAGCCAGATACTTCTTTTTTTTTTTTTTTT
TTTGATACAGAGTCTCACTCTGTTGCCCAGGCTGGAGTGTAGTGGCATGAACTTGGCTC
ACTGCAGCCTCTGCCTCTTGG
>2419
GGTATAGATAAAGCCAGATACTTCTTTTTTTTTTTTTTTTTTTGATACAGAGTCTCACTCT
GTTGCCCAGGCTGGAGTGTAGTGGCATGAACTTGGCTCACTGCAGCCTCTGCCTCTTG
GGTTCAAGTGATTCTTGCGTC
>2420
ATAAAGCCAGATACTTCTTTTTTTTTTTTTTTTTTTGATACAGAGTCTCACTCTGTTGCCC
AGGCTGGAGTGTAGTGGCATGAACTTGGCTCACTGCAGCCTCTGCCTCTTGGGTTCAA
GTGATTCTTGCGTCTCAGCCT
>2421
TTTTTTTGATACAGAGTCTCACTCTGTTGCCCAGGCTGGAGTGTAGTGGCATGAACTTG
GCTCACTGCAGCCTCTGCCTCTTGGGTTCAAGTGATTCTTGCGTCTCAGCCTCCCGAGT
AGCTGAGATTACAGACATGCGC
>2422
CTCTGTTGCCCAGGCTGGAGTGTAGTGGCATGAACTTGGCTCACTGCAGCCTCTGCCT

CTTGGGTTCAAGTGATTCTTGCGTCTCAGCCTCCCGAGTAGCTGAGATTACAGACATGC
GCTACCATGCCTGGCTAATTTTT
>2423
GAGTGTAGTGGCATGAACTTGGCTCACTGCAGCCTCTGCCTCTTGGGTTCAAGTGATTC
TTGCGTCTCAGCCTCCCGAGTAGCTGAGATTACAGACATGCGCTACCATGCCTGGCTAA
TTTTTGTATTTTTAGTAGAGAT
>2424
GGCATGAACTTGGCTCACTGCAGCCTCTGCCTCTTGGGTTCAAGTGATTCTTGCGTCTC
AGCCTCCCGAGTAGCTGAGATTACAGACATGCGCTACCATGCCTGGCTAATTTTTGTATT
TTTAGTAGAGATGCGGTTTCA
>2425
GTCTCAGCCTCCCGAGTAGCTGAGATTACAGACATGCGCTACCATGCCTGGCTAATTTT
TGTATTTTTAGTAGAGATGCGGTTTCACCATGCTGGCCAGGCTGGTCCCCAACTCCTCG
CCTCAAGTGATCTTCCTGCCTT
>2426
TCAGCCTCCCGAGTAGCTGAGATTACAGACATGCGCTACCATGCCTGGCTAATTTTTGT
ATTTTTAGTAGAGATGCGGTTTCACCATGCTGGCCAGGCTGGTCCCCAACTCCTCGCCT
CAAGTGATCTTCCTGCCTTAGC
>2427
TTTTTGTATTTTTAGTAGAGATGCGGTTTCACCATGCTGGCCAGGCTGGTCCCCAACTC
CTCGCCTCAAGTGATCTTCCTGCCTTAGCCTCCCAGAGTGCTAGGATTACAGGTGTGAG
TCACTGTGCCCGGCCTGGATCT
>2428
GAGATGCGGTTTCACCATGCTGGCCAGGCTGGTCCCCAACTCCTCGCCTCAAGTGATC
TTCCTGCCTTAGCCTCCCAGAGTGCTAGGATTACAGGTGTGAGTCACTGTGCCCGGCCT
GGATCTTTTATTGTTTGTATTTC
>2429
CAGGCTGGTCCCCAACTCCTCGCCTCAAGTGATCTTCCTGCCTTAGCCTCCCAGAGTGC
TAGGATTACAGGTGTGAGTCACTGTGCCCGGCCTGGATCTTTTATTGTTTGTATTTCTGT
TCATTTCTTCCCTAAGAAACC
>2430
GTCCCCAACTCCTCGCCTCAAGTGATCTTCCTGCCTTAGCCTCCCAGAGTGCTAGGATT
ACAGGTGTGAGTCACTGTGCCCGGCCTGGATCTTTTATTGTTTGTATTTCTGTTCATTTC
TTCCCTAAGAAACCCCACGTT
>2431
TACAGGTGTGAGTCACTGTGCCCGGCCTGGATCTTTTATTGTTTGTATTTCTGTTCATTT
CTTCCCTAAGAAACCCCACGTTTTTGTGGGCAGGACCTGGAATCCTATTCAGTTATACA
GCCCCGCTTCGTCCACTGCTC
>2432
GGCCTGGATCTTTTATTGTTTGTATTTCTGTTCATTTCTTCCCTAAGAAACCCCACGTTTT
TGTGGGCAGGACCTGGAATCCTATTCAGTTATACAGCCCCGCTTCGTCCACTGCTCCCC
TTCAGAGTCTAGTGCCCTGG
>2433
TTGTATTTCTGTTCATTTCTTCCCTAAGAAACCCCACGTTTTTGTGGGCAGGACCTGGA
ATCCTATTCAGTTATACAGCCCCGCTTCGTCCACTGCTCCCCTTCAGAGTCTAGTGCCCT
GGACATCATAGGTATTCAGTA

>2434
CGTTTTTGTGGGCAGGACCTGGAATCCTATTCAGTTATACAGCCCCGCTTCGTCCACTG
CTCCCCTTCAGAGTCTAGTGCCCTGGACATCATAGGTATTCAGTACCTCTTGCTGGCTTA
GGAACAGGAGGATGCCTGATG
>2435
TTTTTGTGGGCAGGACCTGGAATCCTATTCAGTTATACAGCCCCGCTTCGTCCACTGCT
CCCCTTCAGAGTCTAGTGCCCTGGACATCATAGGTATTCAGTACCTCTTGCTGGCTTAG
GAACAGGAGGATGCCTGATGGC
>2436
AGTTATACAGCCCCGCTTCGTCCACTGCTCCCCTTCAGAGTCTAGTGCCCTGGACATCA
TAGGTATTCAGTACCTCTTGCTGGCTTAGGAACAGGAGGATGCCTGATGGCCAGGGTG
TTCCTCTGAAGCAGTGGTGGCTG
>2437
CGTCCACTGCTCCCCTTCAGAGTCTAGTGCCCTGGACATCATAGGTATTCAGTACCTCT
TGCTGGCTTAGGAACAGGAGGATGCCTGATGGCCAGGGTGTTCCTCTGAAGCAGTGGT
GGCTGAGCTTTTTGAATATCTGC
>2438
CTGCTCCCCTTCAGAGTCTAGTGCCCTGGACATCATAGGTATTCAGTACCTCTTGCTGG
CTTAGGAACAGGAGGATGCCTGATGGCCAGGGTGTTCCTCTGAAGCAGTGGTGGCTGA
GCTTTTTGAATATCTGCATTGTG
>2439
AGTGCCCTGGACATCATAGGTATTCAGTACCTCTTGCTGGCTTAGGAACAGGAGGATGC
CTGATGGCCAGGGTGTTCCTCTGAAGCAGTGGTGGCTGAGCTTTTTGAATATCTGCATT
GTGCTAAGGGTTAACTACCCAG
>2440
CCTCTTGCTGGCTTAGGAACAGGAGGATGCCTGATGGCCAGGGTGTTCCTCTGAAGCA
GTGGTGGCTGAGCTTTTTGAATATCTGCATTGTGCTAAGGGTTAACTACCCAGGCAGTA
CTGGTGGGGAAGGGCCCCAGAAG
>2441
TGCCTGATGGCCAGGGTGTTCCTCTGAAGCAGTGGTGGCTGAGCTTTTTGAATATCTGC
ATTGTGCTAAGGGTTAACTACCCAGGCAGTACTGGTGGGGAAGGGCCCCAGAAGCAG
GCCCAGACTCAGGTATTGTTAAAG
>2442
GGTGTTCCTCTGAAGCAGTGGTGGCTGAGCTTTTTGAATATCTGCATTGTGCTAAGGGT
TAACTACCCAGGCAGTACTGGTGGGGAAGGGCCCCAGAAGCAGGCCCAGACTCAGGT
ATTGTTAAAGCAGAGTCATCTTTG
>2443
TGGTGGCTGAGCTTTTTGAATATCTGCATTGTGCTAAGGGTTAACTACCCAGGCAGTAC
TGGTGGGGAAGGGCCCCAGAAGCAGGCCCAGACTCAGGTATTGTTAAAGCAGAGTCA
TCTTTGCTCTGAAGTTTTTTCTTT
>2444
GAGCTTTTTGAATATCTGCATTGTGCTAAGGGTTAACTACCCAGGCAGTACTGGTGGGG
AAGGGCCCCAGAAGCAGGCCCAGACTCAGGTATTGTTAAAGCAGAGTCATCTTTGCTC
TGAAGTTTTTTCTTTTATCTTTT
>2445
TATCTGCATTGTGCTAAGGGTTAACTACCCAGGCAGTACTGGTGGGGAAGGGCCCCAG

AAGCAGGCCCAGACTCAGGTATTGTTAAAGCAGAGTCATCTTTGCTCTGAAGTTTTTTC
TTTTATCTTTTCTTTTCTTTTTT
>2446
GGTGGGGAAGGGCCCCAGAAGCAGGCCCAGACTCAGGTATTGTTAAAGCAGAGTCAT
CTTTGCTCTGAAGTTTTTTCTTTTATCTTTTCTTTTCTTTTTTAATTGGGGGAAACCTTGT
CCTAATTGGCATCTTTATTGTG
>2447
TTCTTTTATCTTTTCTTTTCTTTTTTAATTGGGGGAAACCTTGTCCTAATTGGCATCTTTA
TTGTGGCAGGAATAGGCCAGGCAGGGCTGGGGGAGTGTGAAGTCTGTGAAGGGAGGT
GGCCTGGGGGCCCCCCCACACA
>2448
TATCTTTTCTTTTCTTTTTTAATTGGGGGAAACCTTGTCCTAATTGGCATCTTTATTGTGG
CAGGAATAGGCCAGGCAGGGCTGGGGGAGTGTGAAGTCTGTGAAGGGAGGTGGCCTG
GGGGCCCCCCCACACAATGCCT
>2449
GGAAACCTTGTCCTAATTGGCATCTTTATTGTGGCAGGAATAGGCCAGGCAGGGCTGG
GGGAGTGTGAAGTCTGTGAAGGGAGGTGGCCTGGGGGCCCCCCCACACAATGCCTGA
TTGTGCAGGCATAGGTATGAACACA
>2450
GTCCTAATTGGCATCTTTATTGTGGCAGGAATAGGCCAGGCAGGGCTGGGGGAGTGTG
AAGTCTGTGAAGGGAGGTGGCCTGGGGGCCCCCCCACACAATGCCTGATTGTGCAGG
CATAGGTATGAACACATTGCAGCCT
>2451
TAATTGGCATCTTTATTGTGGCAGGAATAGGCCAGGCAGGGCTGGGGGAGTGTGAAGT
CTGTGAAGGGAGGTGGCCTGGGGGCCCCCCCACACAATGCCTGATTGTGCAGGCATAG
GTATGAACACATTGCAGCCTGTGT
>2452
GCTGGGGGAGTGTGAAGTCTGTGAAGGGAGGTGGCCTGGGGGCCCCCCCACACAATG
CCTGATTGTGCAGGCATAGGTATGAACACATTGCAGCCTGTGTGCACCCACAGGGTGA
CCACGAGCAAGGCCTGGGGACGAGG
>2453
AAGGGAGGTGGCCTGGGGGCCCCCCCACACAATGCCTGATTGTGCAGGCATAGGTATG
AACACATTGCAGCCTGTGTGCACCCACAGGGTGACCACGAGCAAGGCCTGGGGACGA
GGACTACGAGGCCTGCAGCTGGGTC
>2454
GGCCCCCCCACACAATGCCTGATTGTGCAGGCATAGGTATGAACACATTGCAGCCTGT
GTGCACCCACAGGGTGACCACGAGCAAGGCCTGGGGACGAGGACTACGAGGCCTGC
AGCTGGGTCCTCTTTTCTCCCTCCTC
>2455
GCCTGATTGTGCAGGCATAGGTATGAACACATTGCAGCCTGTGTGCACCCACAGGGTG
ACCACGAGCAAGGCCTGGGGACGAGGACTACGAGGCCTGCAGCTGGGTCCTCTTTTC
TCCCTCCTCACCCCCACCCAGTACC
>2456
ATGAACACATTGCAGCCTGTGTGCACCCACAGGGTGACCACGAGCAAGGCCTGGGGA
CGAGGACTACGAGGCCTGCAGCTGGGTCCTCTTTTCTCCCTCCTCACCCCCACCCAGT
ACCCACGTGATTGGGCCACATCCCA

>2457
ATTGCAGCCTGTGTGCACCCACAGGGTGACCACGAGCAAGGCCTGGGGACGAGGACT
ACGAGGCCTGCAGCTGGGTCCTCTTTTCTCCCTCCTCACCCCCACCCAGTACCCACGT
GATTGGGCCACATCCCAGTAACAAG
>2458
CAAGGCCTGGGGACGAGGACTACGAGGCCTGCAGCTGGGTCCTCTTTTCTCCCTCCTC
ACCCCCACCCAGTACCCACGTGATTGGGCCACATCCCAGTAACAAGCAGTCCTTGTGG
AAGAGCCTTCATGAAGACCTTTGC
>2459
CCTGCAGCTGGGTCCTCTTTTCTCCCTCCTCACCCCCACCCAGTACCCACGTGATTGGG
CCACATCCCAGTAACAAGCAGTCCTTGTGGAAGAGCCTTCATGAAGACCTTTGCTGGG
AGTGGCGGGCAGGGATCTGCGGG
>2460
CTGGGTCCTCTTTTCTCCCTCCTCACCCCCACCCAGTACCCACGTGATTGGGCCACATC
CCAGTAACAAGCAGTCCTTGTGGAAGAGCCTTCATGAAGACCTTTGCTGGGAGTGGCG
GGCAGGGATCTGCGGGGAGCGTT
>2461
CCACCCAGTACCCACGTGATTGGGCCACATCCCAGTAACAAGCAGTCCTTGTGGAAGA
GCCTTCATGAAGACCTTTGCTGGGAGTGGCGGGCAGGGATCTGCGGGGAGCGTTGCT
GGTTCAAGCTGGGGCTTTCCTATGG
>2462
CGTGATTGGGCCACATCCCAGTAACAAGCAGTCCTTGTGGAAGAGCCTTCATGAAGAC
CTTTGCTGGGAGTGGCGGGCAGGGATCTGCGGGGAGCGTTGCTGGTTCAAGCTGGGG
CTTTCCTATGGATGATGTCCTGTGA
>2463
CCACATCCCAGTAACAAGCAGTCCTTGTGGAAGAGCCTTCATGAAGACCTTTGCTGGG
AGTGGCGGGCAGGGATCTGCGGGGAGCGTTGCTGGTTCAAGCTGGGGCTTTCCTATGG
ATGATGTCCTGTGAAGGGTAGATC
>2464
CAAGCAGTCCTTGTGGAAGAGCCTTCATGAAGACCTTTGCTGGGAGTGGCGGGCAGG
GATCTGCGGGGAGCGTTGCTGGTTCAAGCTGGGGCTTTCCTATGGATGATGTCCTGTGA
AGGGTAGATCCTGGAGGGACAGAC
>2465
GCGGGCAGGGATCTGCGGGGAGCGTTGCTGGTTCAAGCTGGGGCTTTCCTATGGATGA
TGTCCTGTGAAGGGTAGATCCTGGAGGGACAGACAGGTGTCTGATCTTTGGCAGGCAG
GGATGTCTCATTTGTACATTATAG
>2466
CAGGGATCTGCGGGGAGCGTTGCTGGTTCAAGCTGGGGCTTTCCTATGGATGATGTCCT
GTGAAGGGTAGATCCTGGAGGGACAGACAGGTGTCTGATCTTTGGCAGGCAGGGATG
TCTCATTTGTACATTATAGGTATT
>2467
GCTTTCCTATGGATGATGTCCTGTGAAGGGTAGATCCTGGAGGGACAGACAGGTGTCT
GATCTTTGGCAGGCAGGGATGTCTCATTTGTACATTATAGGTATTCAGTATCTCTTGCTG
GCTTAGGCTTCATAATGTCTCA
>2468
TCCTATGGATGATGTCCTGTGAAGGGTAGATCCTGGAGGGACAGACAGGTGTCTGATC

TTTGGCAGGCAGGGATGTCTCATTTGTACATTATAGGTATTCAGTATCTCTTGCTGGCTT
AGGCTTCATAATGTCTCATTTG
>2469
GGTAGATCCTGGAGGGACAGACAGGTGTCTGATCTTTGGCAGGCAGGGATGTCTCATT
TGTACATTATAGGTATTCAGTATCTCTTGCTGGCTTAGGCTTCATAATGTCTCATTTGTAC
ATCATAGCCTTCAGTACCTCT
>2470
GTCTGATCTTTGGCAGGCAGGGATGTCTCATTTGTACATTATAGGTATTCAGTATCTCTT
GCTGGCTTAGGCTTCATAATGTCTCATTTGTACATCATAGCCTTCAGTACCTCTTGCTGA
TTTGGTTAACCCAGTTGTTA
>2471
TTTGTACATTATAGGTATTCAGTATCTCTTGCTGGCTTAGGCTTCATAATGTCTCATTTGT
ACATCATAGCCTTCAGTACCTCTTGCTGATTTGGTTAACCCAGTTGTTATACCACGATAT
TGATTTTTCATGCCATTTT
>2472
ATTATAGGTATTCAGTATCTCTTGCTGGCTTAGGCTTCATAATGTCTCATTTGTACATCAT
AGCCTTCAGTACCTCTTGCTGATTTGGTTAACCCAGTTGTTATACCACGATATTGATTTT
TCATGCCATTTTCTTTAGG
>2473
GTACCTCTTGCTGATTTGGTTAACCCAGTTGTTATACCACGATATTGATTTTTCATGCCAT
TTTCTTTAGGAAGATTGGAAGATCATTGAGGATAGGGACTGTGACCTTTTCTTTTTTTCT
ACCCCAGTACCCAACCTGG
>2474
CTCTTGCTGATTTGGTTAACCCAGTTGTTATACCACGATATTGATTTTTCATGCCATTTTC
TTTAGGAAGATTGGAAGATCATTGAGGATAGGGACTGTGACCTTTTCTTTTTTTCTACC
CCAGTACCCAACCTGGCAGG
>2475
AACCCAGTTGTTATACCACGATATTGATTTTTCATGCCATTTTCTTTAGGAAGATTGGAA
GATCATTGAGGATAGGGACTGTGACCTTTTCTTTTTTTCTACCCCAGTACCCAACCTGG
CAGGTAGCACAGCGCAATGAT
>2476
AGTTGTTATACCACGATATTGATTTTTCATGCCATTTTCTTTAGGAAGATTGGAAGATCAT
TGAGGATAGGGACTGTGACCTTTTCTTTTTTTCTACCCCAGTACCCAACCTGGCAGGTA
GCACAGCGCAATGATCCACG
>2477
CATTTTCTTTAGGAAGATTGGAAGATCATTGAGGATAGGGACTGTGACCTTTTCTTTTTT
TCTACCCCAGTACCCAACCTGGCAGGTAGCACAGCGCAATGATCCACGATGTTTAATAG
GTTGAATTGTAGAGGGGGCCT
>2478
GATTGGAAGATCATTGAGGATAGGGACTGTGACCTTTTCTTTTTTTCTACCCCAGTACC
CAACCTGGCAGGTAGCACAGCGCAATGATCCACGATGTTTAATAGGTTGAATTGTAGA
GGGGGCCTCTGATTCCAAAGATG
>2479
ATCATTGAGGATAGGGACTGTGACCTTTTCTTTTTTTCTACCCCAGTACCCAACCTGGC
AGGTAGCACAGCGCAATGATCCACGATGTTTAATAGGTTGAATTGTAGAGGGGGCCTCT
GATTCCAAAGATGGGGGCAGAA

>2480
TTTTCTTTTTTTCTACCCCAGTACCCAACCTGGCAGGTAGCACAGCGCAATGATCCACG
ATGTTTAATAGGTTGAATTGTAGAGGGGGCCTCTGATTCCAAAGATGGGGGCAGAATAT
CAGTCCTCTCCAGACTGCCTGG
>2481
CTACCCCAGTACCCAACCTGGCAGGTAGCACAGCGCAATGATCCACGATGTTTAATAGG
TTGAATTGTAGAGGGGGCCTCTGATTCCAAAGATGGGGGCAGAATATCAGTCCTCTCC
AGACTGCCTGGGAATCCTCCTCT
>2482
CAGGTAGCACAGCGCAATGATCCACGATGTTTAATAGGTTGAATTGTAGAGGGGGCCT
CTGATTCCAAAGATGGGGGCAGAATATCAGTCCTCTCCAGACTGCCTGGGAATCCTCCT
CTCTCCAGCCTGGGCCTGGCCAG
>2483
AGCGCAATGATCCACGATGTTTAATAGGTTGAATTGTAGAGGGGGCCTCTGATTCCAAA
GATGGGGGCAGAATATCAGTCCTCTCCAGACTGCCTGGGAATCCTCCTCTCTCCAGCCT
GGGCCTGGCCAGTGGGAGACAG
>2484
GATCCACGATGTTTAATAGGTTGAATTGTAGAGGGGGCCTCTGATTCCAAAGATGGGGG
CAGAATATCAGTCCTCTCCAGACTGCCTGGGAATCCTCCTCTCTCCAGCCTGGGCCTGG
CCAGTGGGAGACAGAGGCTGCT
>2485
CCTCTGATTCCAAAGATGGGGGCAGAATATCAGTCCTCTCCAGACTGCCTGGGAATCCT
CCTCTCTCCAGCCTGGGCCTGGCCAGTGGGAGACAGAGGCTGCTGAAGTAGCTGGCA
AGGAGGCAAAGAAGATGATGACCT
>2486
ATGGGGGCAGAATATCAGTCCTCTCCAGACTGCCTGGGAATCCTCCTCTCTCCAGCCTG
GGCCTGGCCAGTGGGAGACAGAGGCTGCTGAAGTAGCTGGCAAGGAGGCAAAGAAG
ATGATGACCTAGCTACGGTCCTCAT
>2487
GCAGAATATCAGTCCTCTCCAGACTGCCTGGGAATCCTCCTCTCTCCAGCCTGGGCCTG
GCCAGTGGGAGACAGAGGCTGCTGAAGTAGCTGGCAAGGAGGCAAAGAAGATGATG
ACCTAGCTACGGTCCTCATGTGCCC
>2488
CTCCAGACTGCCTGGGAATCCTCCTCTCTCCAGCCTGGGCCTGGCCAGTGGGAGACAG
AGGCTGCTGAAGTAGCTGGCAAGGAGGCAAAGAAGATGATGACCTAGCTACGGTCCT
CATGTGCCCACTGGGGAATCATCTT
>2489
TGGGCCTGGCCAGTGGGAGACAGAGGCTGCTGAAGTAGCTGGCAAGGAGGCAAAGA
AGATGATGACCTAGCTACGGTCCTCATGTGCCCACTGGGGAATCATCTTTTTGCATCTG
AGTTAGTCAATGTGCTGTGCATTCT
>2490
AGGCAAAGAAGATGATGACCTAGCTACGGTCCTCATGTGCCCACTGGGGAATCATCTT
TTTGCATCTGAGTTAGTCAATGTGCTGTGCATTCTCTGCAGAATTTGTGAACCTGGTATA
GGTATGAGGTGAGAGAGGCCAG
>2491
TCCTCATGTGCCCACTGGGGAATCATCTTTTTGCATCTGAGTTAGTCAATGTGCTGTGCA

TTCTCTGCAGAATTTGTGAACCTGGTATAGGTATGAGGTGAGAGAGGCCAGGAGGTCT
GGCTGCTCCCCCTCTAGACTTC
>2492
AATCATCTTTTTGCATCTGAGTTAGTCAATGTGCTGTGCATTCTCTGCAGAATTTGTGAA
CCTGGTATAGGTATGAGGTGAGAGAGGCCAGGAGGTCTGGCTGCTCCCCCTCTAGACT
TCGCAGGTAAAACCCAAGGTGT
>2493
CTGAGTTAGTCAATGTGCTGTGCATTCTCTGCAGAATTTGTGAACCTGGTATAGGTATG
AGGTGAGAGAGGCCAGGAGGTCTGGCTGCTCCCCCTCTAGACTTCGCAGGTAAAACC
CAAGGTGTGTGCCACGTAAAGGTT
>2494
TTAGTCAATGTGCTGTGCATTCTCTGCAGAATTTGTGAACCTGGTATAGGTATGAGGTG
AGAGAGGCCAGGAGGTCTGGCTGCTCCCCCTCTAGACTTCGCAGGTAAAACCCAAGG
TGTGTGCCACGTAAAGGTTCAGAG
>2495
GTCAATGTGCTGTGCATTCTCTGCAGAATTTGTGAACCTGGTATAGGTATGAGGTGAGA
GAGGCCAGGAGGTCTGGCTGCTCCCCCTCTAGACTTCGCAGGTAAAACCCAAGGTGT
GTGCCACGTAAAGGTTCAGAGGTT
>2496
TGCAGAATTTGTGAACCTGGTATAGGTATGAGGTGAGAGAGGCCAGGAGGTCTGGCTG
CTCCCCCTCTAGACTTCGCAGGTAAAACCCAAGGTGTGTGCCACGTAAAGGTTCAGAG
GTTTTATAGGCATCCAGATAGCAC
>2497
ATAGGTATGAGGTGAGAGAGGCCAGGAGGTCTGGCTGCTCCCCCTCTAGACTTCGCAG
GTAAAACCCAAGGTGTGTGCCACGTAAAGGTTCAGAGGTTTTATAGGCATCCAGATAG
CACATGTGGGTCTCTGCCCCTGCT
>2498
GAGGCCAGGAGGTCTGGCTGCTCCCCCTCTAGACTTCGCAGGTAAAACCCAAGGTGT
GTGCCACGTAAAGGTTCAGAGGTTTTATAGGCATCCAGATAGCACATGTGGGTCTCTGC
CCCTGCTTGCAGCCTCCCTGGGGC
>2499
AGGAGGTCTGGCTGCTCCCCCTCTAGACTTCGCAGGTAAAACCCAAGGTGTGTGCCAC
GTAAAGGTTCAGAGGTTTTATAGGCATCCAGATAGCACATGTGGGTCTCTGCCCCTGCT
TGCAGCCTCCCTGGGGCAGCCAG
>2500
CTGCTCCCCCTCTAGACTTCGCAGGTAAAACCCAAGGTGTGTGCCACGTAAAGGTTCA
GAGGTTTTATAGGCATCCAGATAGCACATGTGGGTCTCTGCCCCTGCTTGCAGCCTCCC
TGGGGCAGCCAGCCAGGCTCAGG
>2501
TGCCACGTAAAGGTTCAGAGGTTTTATAGGCATCCAGATAGCACATGTGGGTCTCTGCC
CCTGCTTGCAGCCTCCCTGGGGCAGCCAGCCAGGCTCAGGGCAGGAAGCTGCTGATT
CTGGGTCACTTGGCTGCTCTTGCT
>2502
TCAGAGGTTTTATAGGCATCCAGATAGCACATGTGGGTCTCTGCCCCTGCTTGCAGCCT
CCCTGGGGCAGCCAGCCAGGCTCAGGGCAGGAAGCTGCTGATTCTGGGTCACTTGGC
TGCTCTTGCTTGCTGCTGCTGTGA

>2503
TTTATAGGCATCCAGATAGCACATGTGGGTCTCTGCCCCTGCTTGCAGCCTCCCTGGGG
CAGCCAGCCAGGCTCAGGGCAGGAAGCTGCTGATTCTGGGTCACTTGGCTGCTCTTGC
TTGCTGCTGCTGTGATTGTTCCT
>2504
AGGGCAGGAAGCTGCTGATTCTGGGTCACTTGGCTGCTCTTGCTTGCTGCTGCTGTGA
TTGTTCCTACAGTTAAGATCTTAGCTCCATAAGGGACATACGGAGGTCATCTAGGTCAT
CTCATGGCTCTCAGGCAAGAAAA
>2505
AGGAAGCTGCTGATTCTGGGTCACTTGGCTGCTCTTGCTTGCTGCTGCTGTGATTGTTC
CTACAGTTAAGATCTTAGCTCCATAAGGGACATACGGAGGTCATCTAGGTCATCTCATG
GCTCTCAGGCAAGAAAAGTAGC
>2506
TGGGTCACTTGGCTGCTCTTGCTTGCTGCTGCTGTGATTGTTCCTACAGTTAAGATCTTA
GCTCCATAAGGGACATACGGAGGTCATCTAGGTCATCTCATGGCTCTCAGGCAAGAAA
AGTAGCATTATTGCTCTCATGC
>2507
CTGCTCTTGCTTGCTGCTGCTGTGATTGTTCCTACAGTTAAGATCTTAGCTCCATAAGGG
ACATACGGAGGTCATCTAGGTCATCTCATGGCTCTCAGGCAAGAAAAGTAGCATTATTG
CTCTCATGCCCACTTTATGGA
>2508
CTTGCTGCTGCTGTGATTGTTCCTACAGTTAAGATCTTAGCTCCATAAGGGACATACGG
AGGTCATCTAGGTCATCTCATGGCTCTCAGGCAAGAAAAGTAGCATTATTGCTCTCATG
CCCACTTTATGGATAAGTGACC
>2509
TTCCTACAGTTAAGATCTTAGCTCCATAAGGGACATACGGAGGTCATCTAGGTCATCTCA
TGGCTCTCAGGCAAGAAAAGTAGCATTATTGCTCTCATGCCCACTTTATGGATAAGTGA
CCGAGGCCTGCAGAGGCAGAG
>2510
TCTAGGTCATCTCATGGCTCTCAGGCAAGAAAAGTAGCATTATTGCTCTCATGCCCACT
TTATGGATAAGTGACCGAGGCCTGCAGAGGCAGAGTGGCTATTCCAGCCATTAAATGT
GGCTCCCTGCTATTCTGTTCCCT
>2511
ATCTCATGGCTCTCAGGCAAGAAAAGTAGCATTATTGCTCTCATGCCCACTTTATGGATA
AGTGACCGAGGCCTGCAGAGGCAGAGTGGCTATTCCAGCCATTAAATGTGGCTCCCTG
CTATTCTGTTCCCTGCCAGGAT
>2512
GCATTATTGCTCTCATGCCCACTTTATGGATAAGTGACCGAGGCCTGCAGAGGCAGAGT
GGCTATTCCAGCCATTAAATGTGGCTCCCTGCTATTCTGTTCCCTGCCAGGATATTCTGT
GGATGGTTAGGGGAGGGGTCT
>2513
GAGGCCTGCAGAGGCAGAGTGGCTATTCCAGCCATTAAATGTGGCTCCCTGCTATTCTG
TTCCCTGCCAGGATATTCTGTGGATGGTTAGGGGAGGGGTCTTACCTCAGCTAACGAGG
TGCATTTTCTGTGGGGTGAGGC
>2514
GGCTATTCCAGCCATTAAATGTGGCTCCCTGCTATTCTGTTCCCTGCCAGGATATTCTGT

GGATGGTTAGGGGAGGGGTCTTACCTCAGCTAACGAGGTGCATTTTCTGTGGGGTGAG
GCCATTCTTGTCACTTAGGTGT
>2515
TTCCAGCCATTAAATGTGGCTCCCTGCTATTCTGTTCCCTGCCAGGATATTCTGTGGATG
GTTAGGGGAGGGGTCTTACCTCAGCTAACGAGGTGCATTTTCTGTGGGGTGAGGCCAT
TCTTGTCACTTAGGTGTCTGAG
>2516
TGTGGCTCCCTGCTATTCTGTTCCCTGCCAGGATATTCTGTGGATGGTTAGGGGAGGGG
TCTTACCTCAGCTAACGAGGTGCATTTTCTGTGGGGTGAGGCCATTCTTGTCACTTAGG
TGTCTGAGGCCTGATGGTGTTG
>2517
AGGATATTCTGTGGATGGTTAGGGGAGGGGTCTTACCTCAGCTAACGAGGTGCATTTTC
TGTGGGGTGAGGCCATTCTTGTCACTTAGGTGTCTGAGGCCTGATGGTGTTGGGGAAT
GTCCTGGTTCTATTCCCAGGGCT
>2518
TTAGGGGAGGGGTCTTACCTCAGCTAACGAGGTGCATTTTCTGTGGGGTGAGGCCATT
CTTGTCACTTAGGTGTCTGAGGCCTGATGGTGTTGGGGAATGTCCTGGTTCTATTCCCA
GGGCTGTAACGTGGGGGAAGGAT
>2519
GGGTCTTACCTCAGCTAACGAGGTGCATTTTCTGTGGGGTGAGGCCATTCTTGTCACTT
AGGTGTCTGAGGCCTGATGGTGTTGGGGAATGTCCTGGTTCTATTCCCAGGGCTGTAAC
GTGGGGGAAGGATGGAGGAAGA
>2520
TGAGGCCATTCTTGTCACTTAGGTGTCTGAGGCCTGATGGTGTTGGGGAATGTCCTGGT
TCTATTCCCAGGGCTGTAACGTGGGGGAAGGATGGAGGAAGACAGAATGGCCTCTGG
AACTTTTGGATCTTTCTGAGTTGA
>2521
TAGGTGTCTGAGGCCTGATGGTGTTGGGGAATGTCCTGGTTCTATTCCCAGGGCTGTAA
CGTGGGGGAAGGATGGAGGAAGACAGAATGGCCTCTGGAACTTTTGGATCTTTCTGA
GTTGAGGCCTTGTCTGGCCAACTC
>2522
CTGAGGCCTGATGGTGTTGGGGAATGTCCTGGTTCTATTCCCAGGGCTGTAACGTGGG
GGAAGGATGGAGGAAGACAGAATGGCCTCTGGAACTTTTGGATCTTTCTGAGTTGAGG
CCTTGTCTGGCCAACTCCGGGAGG
>2523
CCAGGGCTGTAACGTGGGGGAAGGATGGAGGAAGACAGAATGGCCTCTGGAACTTTT
GGATCTTTCTGAGTTGAGGCCTTGTCTGGCCAACTCCGGGAGGCTGCTGGCATGGCAA
GCAGGGTAGGGATGCCTGGCCTAGC
>2524
GCTGTAACGTGGGGGAAGGATGGAGGAAGACAGAATGGCCTCTGGAACTTTTGGATC
TTTCTGAGTTGAGGCCTTGTCTGGCCAACTCCGGGAGGCTGCTGGCATGGCAAGCAGG
GTAGGGATGCCTGGCCTAGCCAGGG
>2525
GGAAGACAGAATGGCCTCTGGAACTTTTGGATCTTTCTGAGTTGAGGCCTTGTCTGGC
CAACTCCGGGAGGCTGCTGGCATGGCAAGCAGGGTAGGGATGCCTGGCCTAGCCAGG
GGAGCTCCCTCTCTCCCCTCTGCTG

>2526
CTGGAACTTTTGGATCTTTCTGAGTTGAGGCCTTGTCTGGCCAACTCCGGGAGGCTGC
TGGCATGGCAAGCAGGGTAGGGATGCCTGGCCTAGCCAGGGGAGCTCCCTCTCTCCCC
TCTGCTGCCCCTCTTTCCACCCCC
>2527
GTTGAGGCCTTGTCTGGCCAACTCCGGGAGGCTGCTGGCATGGCAAGCAGGGTAGGG
ATGCCTGGCCTAGCCAGGGGAGCTCCCTCTCTCCCCTCTGCTGCCCCTCTTTCCACCCC
CATCATAAACACGTGTGGACTTTC
>2528
AGGCCTTGTCTGGCCAACTCCGGGAGGCTGCTGGCATGGCAAGCAGGGTAGGGATGC
CTGGCCTAGCCAGGGGAGCTCCCTCTCTCCCCTCTGCTGCCCCTCTTTCCACCCCCATC
ATAAACACGTGTGGACTTTCTGGT
>2529
TCCCTCTCTCCCCTCTGCTGCCCCTCTTTCCACCCCCATCATAAACACGTGTGGACTTTC
TGGTGGTGAGAAGATGGTTTTGGAGGGCTGGGCTGAGGTCTAGGCAGGCAAGAGCTT
CCAAATGTGTGGCTTTGATTCTC
>2530
CTCTCTCCCCTCTGCTGCCCCTCTTTCCACCCCCATCATAAACACGTGTGGACTTTCTG
GTGGTGAGAAGATGGTTTTGGAGGGCTGGGCTGAGGTCTAGGCAGGCAAGAGCTTCC
AAATGTGTGGCTTTGATTCTCAGC
>2531
CCCCATCATAAACACGTGTGGACTTTCTGGTGGTGAGAAGATGGTTTTGGAGGGCTGG
GCTGAGGTCTAGGCAGGCAAGAGCTTCCAAATGTGTGGCTTTGATTCTCAGCCTTTTCA
TCCCTCAATCCTGGGAGACTCAT
>2532
ATCATAAACACGTGTGGACTTTCTGGTGGTGAGAAGATGGTTTTGGAGGGCTGGGCTG
AGGTCTAGGCAGGCAAGAGCTTCCAAATGTGTGGCTTTGATTCTCAGCCTTTTCATCCC
TCAATCCTGGGAGACTCATTTTA
>2533
ACACGTGTGGACTTTCTGGTGGTGAGAAGATGGTTTTGGAGGGCTGGGCTGAGGTCTA
GGCAGGCAAGAGCTTCCAAATGTGTGGCTTTGATTCTCAGCCTTTTCATCCCTCAATCC
TGGGAGACTCATTTTAAAACTTA
>2534
GATGGTTTTGGAGGGCTGGGCTGAGGTCTAGGCAGGCAAGAGCTTCCAAATGTGTGGC
TTTGATTCTCAGCCTTTTCATCCCTCAATCCTGGGAGACTCATTTTAAAACTTAAAAAA
GGACTTCATTCTATTGGATGCTA
>2535
GTCTAGGCAGGCAAGAGCTTCCAAATGTGTGGCTTTGATTCTCAGCCTTTTCATCCCTC
AATCCTGGGAGACTCATTTTAAAACTTAAAAAAGGACTTCATTCTATTGGATGCTATGT
GGGCAGGCCCTGGGCTGTTGTA
>2536
AATGTGTGGCTTTGATTCTCAGCCTTTTCATCCCTCAATCCTGGGAGACTCATTTTAAAA
CTTAAAAAAGGACTTCATTCTATTGGATGCTATGTGGGCAGGCCCTGGGCTGTTGTAGA
GAGGCCTGAAACCCACTTCAG
>2537
CCCTCAATCCTGGGAGACTCATTTTAAAACTTAAAAAAGGACTTCATTCTATTGGATGC

TATGTGGGCAGGCCCTGGGCTGTTGTAGAGAGGCCTGAAACCCACTTCAGTTTCCTGG
ACAGGCTGGGAGTGTGTACCCAT
>2538
CTCATTTTAAAACTTAAAAAAGGACTTCATTCTATTGGATGCTATGTGGGCAGGCCCTG
GGCTGTTGTAGAGAGGCCTGAAACCCACTTCAGTTTCCTGGACAGGCTGGGAGTGTGT
ACCCATGGGTTCCCTGCTGCCCT
>2539
GACTTCATTCTATTGGATGCTATGTGGGCAGGCCCTGGGCTGTTGTAGAGAGGCCTGAA
ACCCACTTCAGTTTCCTGGACAGGCTGGGAGTGTGTACCCATGGGTTCCCTGCTGCCCT
ATTCCTGGAAGACACTAATATC
>2540
TATGTGGGCAGGCCCTGGGCTGTTGTAGAGAGGCCTGAAACCCACTTCAGTTTCCTGG
ACAGGCTGGGAGTGTGTACCCATGGGTTCCCTGCTGCCCTATTCCTGGAAGACACTAAT
ATCCCTCCCAAACTCTTGGTTGG
>2541
ACCCACTTCAGTTTCCTGGACAGGCTGGGAGTGTGTACCCATGGGTTCCCTGCTGCCCT
ATTCCTGGAAGACACTAATATCCCTCCCAAACTCTTGGTTGGTTTCTGCTTTTTTTCTGT
AGCATTTTGTCTTTGTGGGAA
>2542
GCTGCCCTATTCCTGGAAGACACTAATATCCCTCCCAAACTCTTGGTTGGTTTCTGCTTT
TTTTCTGTAGCATTTTGTCTTTGTGGGAAATTTTAAGAAACATATGTATGGCTTTTTGGT
TTCCACTTATTTATTTTCCC
>2543
TCTTTGTGGGAAATTTTAAGAAACATATGTATGGCTTTTTGGTTTCCACTTATTTATTTTC
CCCTCTGAGTTTCAGATTTCATCCTTCTCCACCCCTTCCCCCTGCCCTAAGGTGGGGCT
AGTGCAAATCGCAGTGTGTG
>2544
TGGGAAATTTTAAGAAACATATGTATGGCTTTTTGGTTTCCACTTATTTATTTTCCCCTCT
GAGTTTCAGATTTCATCCTTCTCCACCCCTTCCCCCTGCCCTAAGGTGGGGCTAGTGCA
AATCGCAGTGTGTGTGTAGT
>2545
TTTCCACTTATTTATTTTCCCCTCTGAGTTTCAGATTTCATCCTTCTCCACCCCTTCCCCC
TGCCCTAAGGTGGGGCTAGTGCAAATCGCAGTGTGTGTGTAGTGGGATGGTTAAAGAG
CCGTACATCCTCACACCTGGT
>2546
TTTATTTTCCCCTCTGAGTTTCAGATTTCATCCTTCTCCACCCCTTCCCCCTGCCCTAAG
GTGGGGCTAGTGCAAATCGCAGTGTGTGTGTAGTGGGATGGTTAAAGAGCCGTACATC
CTCACACCTGGTGACGCAGACT
>2547
GATTTCATCCTTCTCCACCCCTTCCCCCTGCCCTAAGGTGGGGCTAGTGCAAATCGCAG
TGTGTGTGTAGTGGGATGGTTAAAGAGCCGTACATCCTCACACCTGGTGACGCAGACT
AAGGCAATTAACCTTCTTTAGTA
>2548
CCACCCCTTCCCCCTGCCCTAAGGTGGGGCTAGTGCAAATCGCAGTGTGTGTGTAGTG
GGATGGTTAAAGAGCCGTACATCCTCACACCTGGTGACGCAGACTAAGGCAATTAACC
TTCTTTAGTAAAGACCCTTTCAGC

>2549
TAGTGCAAATCGCAGTGTGTGTGTAGTGGGATGGTTAAAGAGCCGTACATCCTCACAC
CTGGTGACGCAGACTAAGGCAATTAACCTTCTTTAGTAAAGACCCTTTCAGCTGGGTG
CGGTGGGAGGCACTTCGGGAGGCC
>2550
AGTGGGATGGTTAAAGAGCCGTACATCCTCACACCTGGTGACGCAGACTAAGGCAATT
AACCTTCTTTAGTAAAGACCCTTTCAGCTGGGTGCGGTGGGAGGCACTTCGGGAGGCC
GAGGTGGAAGGATTGCTTGAGCCC
>2551
GATGGTTAAAGAGCCGTACATCCTCACACCTGGTGACGCAGACTAAGGCAATTAACCT
TCTTTAGTAAAGACCCTTTCAGCTGGGTGCGGTGGGAGGCACTTCGGGAGGCCGAGGT
GGAAGGATTGCTTGAGCCCTGGAG
>2552
CACCTGGTGACGCAGACTAAGGCAATTAACCTTCTTTAGTAAAGACCCTTTCAGCTGG
GTGCGGTGGGAGGCACTTCGGGAGGCCGAGGTGGAAGGATTGCTTGAGCCCTGGAGT
TGGAGACCAGCTTGGGCAGCATGGT
>2553
AAGGCAATTAACCTTCTTTAGTAAAGACCCTTTCAGCTGGGTGCGGTGGGAGGCACTT
CGGGAGGCCGAGGTGGAAGGATTGCTTGAGCCCTGGAGTTGGAGACCAGCTTGGGCA
GCATGGTGAAACCACCTCTCTAAAA
>2554
TTAACCTTCTTTAGTAAAGACCCTTTCAGCTGGGTGCGGTGGGAGGCACTTCGGGAGG
CCGAGGTGGAAGGATTGCTTGAGCCCTGGAGTTGGAGACCAGCTTGGGCAGCATGGT
GAAACCACCTCTCTAAAAAAATATA
>2555
TAGTAAAGACCCTTTCAGCTGGGTGCGGTGGGAGGCACTTCGGGAGGCCGAGGTGGA
AGGATTGCTTGAGCCCTGGAGTTGGAGACCAGCTTGGGCAGCATGGTGAAACCACCT
CTCTAAAAAAATATAAAAATTAACGG
>2556
TCAGCTGGGTGCGGTGGGAGGCACTTCGGGAGGCCGAGGTGGAAGGATTGCTTGAGC
CCTGGAGTTGGAGACCAGCTTGGGCAGCATGGTGAAACCACCTCTCTAAAAAAATATA
AAAATTAACGGGACGTTGTGGCATG
>2557
GGCAGCATGGTGAAACCACCTCTCTAAAAAAATATAAAAATTAACGGGACGTTGTGGC
ATGCACCTATAGTCCCAGCTACTTGGGAGGCTGGGGCGCGAGAAGTGCTTGAACCTGA
GAGATGGAGACTGCGGTGAGGCGA
>2558
ATGGTGAAACCACCTCTCTAAAAAAATATAAAAATTAACGGGACGTTGTGGCATGCAC
CTATAGTCCCAGCTACTTGGGAGGCTGGGGCGCGAGAAGTGCTTGAACCTGAGAGATG
GAGACTGCGGTGAGGCGAGATTGC
>2559
ACCTCTCTAAAAAAATATAAAAATTAACGGGACGTTGTGGCATGCACCTATAGTCCCAG
CTACTTGGGAGGCTGGGGCGCGAGAAGTGCTTGAACCTGAGAGATGGAGACTGCGGT
GAGGCGAGATTGCACCACTGCACT
>2560
AATATAAAAATTAACGGGACGTTGTGGCATGCACCTATAGTCCCAGCTACTTGGGAGGC

TGGGGCGCGAGAAGTGCTTGAACCTGAGAGATGGAGACTGCGGTGAGGCGAGATTGC
ACCACTGCACTTCAGCTAGGTGAC
>2561
GACGTTGTGGCATGCACCTATAGTCCCAGCTACTTGGGAGGCTGGGGCGCGAGAAGTG
CTTGAACCTGAGAGATGGAGACTGCGGTGAGGCGAGATTGCACCACTGCACTTCAGCT
AGGTGACAGAGTGGGACCCTGTCT
>2562
CCCAGCTACTTGGGAGGCTGGGGCGCGAGAAGTGCTTGAACCTGAGAGATGGAGACT
GCGGTGAGGCGAGATTGCACCACTGCACTTCAGCTAGGTGACAGAGTGGGACCCTGT
CTCAAAACAAAACAAAACAACAACAA
>2563
GGGCGCGAGAAGTGCTTGAACCTGAGAGATGGAGACTGCGGTGAGGCGAGATTGCAC
CACTGCACTTCAGCTAGGTGACAGAGTGGGACCCTGTCTCAAAACAAAACAAAACAA
CAACAAAAAAAGCATGTTTTCCTGTC
>2564
GTGCTTGAACCTGAGAGATGGAGACTGCGGTGAGGCGAGATTGCACCACTGCACTTC
AGCTAGGTGACAGAGTGGGACCCTGTCTCAAAACAAAACAAAACAACAACAAAAAA
AGCATGTTTTCCTGTCTTTCCCTTCCT
>2565
CCACTGCACTTCAGCTAGGTGACAGAGTGGGACCCTGTCTCAAAACAAAACAAAACA
ACAACAAAAAAAGCATGTTTTCCTGTCTTTCCCTTCCTCTCCTCCACCCCCTGGCATGA
ACTCTCAACTCTTAGCCTGTGGAT
>2566
CAAAAAAAGCATGTTTTCCTGTCTTTCCCTTCCTCTCCTCCACCCCCTGGCATGAACTC
TCAACTCTTAGCCTGTGGATGTCCGTTTCTATAAATGCTGGTGCTCGGTGGTCACCGTA
GACGTCCGAGCAGCCCCTGGGG
>2567
GCATGAACTCTCAACTCTTAGCCTGTGGATGTCCGTTTCTATAAATGCTGGTGCTCGGT
GGTCACCGTAGACGTCCGAGCAGCCCCTGGGGCTTTGGCTTGTGTTTTCTCTGTACTTG
TGAGGGTGGTTGCCACTTCTGG
>2568
CTCAACTCTTAGCCTGTGGATGTCCGTTTCTATAAATGCTGGTGCTCGGTGGTCACCGT
AGACGTCCGAGCAGCCCCTGGGGCTTTGGCTTGTGTTTTCTCTGTACTTGTGAGGGTG
GTTGCCACTTCTGGCCTTCTTTG
>2569
GCTCGGTGGTCACCGTAGACGTCCGAGCAGCCCCTGGGGCTTTGGCTTGTGTTTTCTC
TGTACTTGTGAGGGTGGTTGCCACTTCTGGCCTTCTTTGTGCTTTCTTTTTCTTTCCTTT
CCCCAATCTTTTGTTCCTCCCT
>2570
CAATCTTTTGTTCCTCCCTCCCTCCCTCCCTCCCTTCCTCCCTTCCTTCCTTCTGCTTTCC
TGTTTAAAGTATATGGCTGCACCTAGCATGACACAGTTCCTATTTATTCCTCAGACTTGC
TGGGACAGTCTCCTCTGTA
>2571
CTCCCTCCCTCCCTCCCTTCCTCCCTTCCTTCCTTCTGCTTTCCTGTTTAAAGTATATGGC
TGCACCTAGCATGACACAGTTCCTATTTATTCCTCAGACTTGCTGGGACAGTCTCCTCT
GTATGCGGCGACTGAGGGTG

>2572
CCCTCCCTTCCTCCCTTCCTTCCTTCTGCTTTCCTGTTTAAAGTATATGGCTGCACCTAG
CATGACACAGTTCCTATTTATTCCTCAGACTTGCTGGGACAGTCTCCTCTGTATGCGGC
GACTGAGGGTGGGTTCCAGTG
>2573
CCTGTTTAAAGTATATGGCTGCACCTAGCATGACACAGTTCCTATTTATTCCTCAGACTT
GCTGGGACAGTCTCCTCTGTATGCGGCGACTGAGGGTGGGTTCCAGTGCCCCAGCCCA
CTCCCCTTCCCCTTGTTTTTGT
>2574
CTAGCATGACACAGTTCCTATTTATTCCTCAGACTTGCTGGGACAGTCTCCTCTGTATGC
GGCGACTGAGGGTGGGTTCCAGTGCCCCAGCCCACTCCCCTTCCCCTTGTTTTTGTCC
ACTTGGCTGGACAAAGAGAACA
>2575
AGTTCCTATTTATTCCTCAGACTTGCTGGGACAGTCTCCTCTGTATGCGGCGACTGAGG
GTGGGTTCCAGTGCCCCAGCCCACTCCCCTTCCCCTTGTTTTTGTCCACTTGGCTGGAC
AAAGAGAACAAAACCAAATGGC
>2576
ACTGAGGGTGGGTTCCAGTGCCCCAGCCCACTCCCCTTCCCCTTGTTTTTGTCCACTTG
GCTGGACAAAGAGAACAAAACCAAATGGCCTCCCCTCCCCCTCTCCTAAACCGGTCA
AGCAGTGGTATTCTGGGATGATTT
>2577
TGAGGGTGGGTTCCAGTGCCCCAGCCCACTCCCCTTCCCCTTGTTTTTGTCCACTTGGC
TGGACAAAGAGAACAAAACCAAATGGCCTCCCCTCCCCCTCTCCTAAACCGGTCAAG
CAGTGGTATTCTGGGATGATTTTC
>2578
TTGTCCACTTGGCTGGACAAAGAGAACAAAACCAAATGGCCTCCCCTCCCCCTCTCCT
AAACCGGTCAAGCAGTGGTATTCTGGGATGATTTTCCCTGCAACCATTTAAACAAGTGC
TTTGATGCTTACGAGTCATTGGA
>2579
TCCACTTGGCTGGACAAAGAGAACAAAACCAAATGGCCTCCCCTCCCCCTCTCCTAAA
CCGGTCAAGCAGTGGTATTCTGGGATGATTTTCCCTGCAACCATTTAAACAAGTGCTTT
GATGCTTACGAGTCATTGGAATG
>2580
CCTCCCCCTCTCCTAAACCGGTCAAGCAGTGGTATTCTGGGATGATTTTCCCTGCAACC
ATTTAAACAAGTGCTTTGATGCTTACGAGTCATTGGAATGACCAGGCCTGTGCGCCCCT
TCTCACTGACACCCTTAGACCA
>2581
CGGTCAAGCAGTGGTATTCTGGGATGATTTTCCCTGCAACCATTTAAACAAGTGCTTTG
ATGCTTACGAGTCATTGGAATGACCAGGCCTGTGCGCCCCTTCTCACTGACACCCTTAG
ACCAGGCTGGGCCTCACCCTTT
>2582
TTCTGGGATGATTTTCCCTGCAACCATTTAAACAAGTGCTTTGATGCTTACGAGTCATTG
GAATGACCAGGCCTGTGCGCCCCTTCTCACTGACACCCTTAGACCAGGCTGGGCCTCA
CCCTTTTGTCTTGCTGCCCCTC
>2583
CAAGTGCTTTGATGCTTACGAGTCATTGGAATGACCAGGCCTGTGCGCCCCTTCTCACT

GACACCCTTAGACCAGGCTGGGCCTCACCCTTTTGTCTTGCTGCCCCTCGGTGCCCAG
TGGGCGTGGGCAGTAGGACTGAG
>2584
GCTTTGATGCTTACGAGTCATTGGAATGACCAGGCCTGTGCGCCCCTTCTCACTGACAC
CCTTAGACCAGGCTGGGCCTCACCCTTTTGTCTTGCTGCCCCTCGGTGCCCAGTGGGC
GTGGGCAGTAGGACTGAGCAGAG
>2585
CCCCTTCTCACTGACACCCTTAGACCAGGCTGGGCCTCACCCTTTTGTCTTGCTGCCCC
TCGGTGCCCAGTGGGCGTGGGCAGTAGGACTGAGCAGAGGGGCCGAGGCTGTGCTGG
CCGGCAGCTCTGTTACAGTAGAGT
>2586
ACACCCTTAGACCAGGCTGGGCCTCACCCTTTTGTCTTGCTGCCCCTCGGTGCCCAGT
GGGCGTGGGCAGTAGGACTGAGCAGAGGGGCCGAGGCTGTGCTGGCCGGCAGCTCTG
TTACAGTAGAGTGGAAGGAGGTCCC
>2587
CCCTTAGACCAGGCTGGGCCTCACCCTTTTGTCTTGCTGCCCCTCGGTGCCCAGTGGG
CGTGGGCAGTAGGACTGAGCAGAGGGGCCGAGGCTGTGCTGGCCGGCAGCTCTGTTA
CAGTAGAGTGGAAGGAGGTCCCGGC
>2588
AGGCTGGGCCTCACCCTTTTGTCTTGCTGCCCCTCGGTGCCCAGTGGGCGTGGGCAGT
AGGACTGAGCAGAGGGGCCGAGGCTGTGCTGGCCGGCAGCTCTGTTACAGTAGAGTG
GAAGGAGGTCCCGGCCAGAGGGGCA
>2589
TGCCCCTCGGTGCCCAGTGGGCGTGGGCAGTAGGACTGAGCAGAGGGGCCGAGGCTG
TGCTGGCCGGCAGCTCTGTTACAGTAGAGTGGAAGGAGGTCCCGGCCAGAGGGGCAG
GTCAAACAGGCCTCTTTTATTTACAG
>2590
CAGTGGGCGTGGGCAGTAGGACTGAGCAGAGGGGCCGAGGCTGTGCTGGCCGGCAG
CTCTGTTACAGTAGAGTGGAAGGAGGTCCCGGCCAGAGGGGCAGGTCAAACAGGCCT
CTTTTATTTACAGTGCTCTCTTTCACG
>2591
GTGGGCGTGGGCAGTAGGACTGAGCAGAGGGGCCGAGGCTGTGCTGGCCGGCAGCTC
TGTTACAGTAGAGTGGAAGGAGGTCCCGGCCAGAGGGGCAGGTCAAACAGGCCTCTT
TTATTTACAGTGCTCTCTTTCACGGC
>2592
GTGGGCAGTAGGACTGAGCAGAGGGGCCGAGGCTGTGCTGGCCGGCAGCTCTGTTAC
AGTAGAGTGGAAGGAGGTCCCGGCCAGAGGGGCAGGTCAAACAGGCCTCTTTTATTT
ACAGTGCTCTCTTTCACGGCCTCACT
>2593
TGAGCAGAGGGGCCGAGGCTGTGCTGGCCGGCAGCTCTGTTACAGTAGAGTGGAAGG
AGGTCCCGGCCAGAGGGGCAGGTCAAACAGGCCTCTTTTATTTACAGTGCTCTCTTTC
ACGGCCTCACTGAAACAGGAGACCC
>2594
GCTGTGCTGGCCGGCAGCTCTGTTACAGTAGAGTGGAAGGAGGTCCCGGCCAGAGGG
GCAGGTCAAACAGGCCTCTTTTATTTACAGTGCTCTCTTTCACGGCCTCACTGAAACAG
GAGACCCGAGAGGCTCCGGTGAAA

>2595
CGGCCAGAGGGGCAGGTCAAACAGGCCTCTTTTATTTACAGTGCTCTCTTTCACGGCC
TCACTGAAACAGGAGACCCGAGAGGCTCCGGTGAAAGGGCACTGTGTTTGTGCATTC
AGGCCTTCCAGGGCTGGCTACAAAG
>2596
CCAGAGGGGCAGGTCAAACAGGCCTCTTTTATTTACAGTGCTCTCTTTCACGGCCTCA
CTGAAACAGGAGACCCGAGAGGCTCCGGTGAAAGGGCACTGTGTTTGTGCATTCAGG
CCTTCCAGGGCTGGCTACAAAGGTG
>2597
CTTTCACGGCCTCACTGAAACAGGAGACCCGAGAGGCTCCGGTGAAAGGGCACTGTG
TTTGTGCATTCAGGCCTTCCAGGGCTGGCTACAAAGGTGGGGGCACCCGGAGCACTGA
AGGGGCTTTTTGTCATTGGCAGTTT
>2598
CCTCACTGAAACAGGAGACCCGAGAGGCTCCGGTGAAAGGGCACTGTGTTTGTGCAT
TCAGGCCTTCCAGGGCTGGCTACAAAGGTGGGGGCACCCGGAGCACTGAAGGGGCTT
TTTGTCATTGGCAGTTTGGCTTAGGG
>2599
CGGTGAAAGGGCACTGTGTTTGTGCATTCAGGCCTTCCAGGGCTGGCTACAAAGGTGG
GGGCACCCGGAGCACTGAAGGGGCTTTTTGTCATTGGCAGTTTGGCTTAGGGGAGAA
GCGAGGGAGAGCCAGTGTGGGAGCC
>2600
GGGCACTGTGTTTGTGCATTCAGGCCTTCCAGGGCTGGCTACAAAGGTGGGGGCACCC
GGAGCACTGAAGGGGCTTTTTGTCATTGGCAGTTTGGCTTAGGGGAGAAGCGAGGGA
GAGCCAGTGTGGGAGCCGCCTCTAG
>2601
CAGGCCTTCCAGGGCTGGCTACAAAGGTGGGGGCACCCGGAGCACTGAAGGGGCTTT
TTGTCATTGGCAGTTTGGCTTAGGGGAGAAGCGAGGGAGAGCCAGTGTGGGAGCCGC
CTCTAGGATTGTGAGCAAGTGCCTGT
>2602
AGGGCTGGCTACAAAGGTGGGGGCACCCGGAGCACTGAAGGGGCTTTTTGTCATTGG
CAGTTTGGCTTAGGGGAGAAGCGAGGGAGAGCCAGTGTGGGAGCCGCCTCTAGGATT
GTGAGCAAGTGCCTGTTTAGACAGCT
>2603
AGCACTGAAGGGGCTTTTTGTCATTGGCAGTTTGGCTTAGGGGAGAAGCGAGGGAGA
GCCAGTGTGGGAGCCGCCTCTAGGATTGTGAGCAAGTGCCTGTTTAGACAGCTCCACG
CTGCTCCTTAAAACCTATTTTGTGG
>2604
GGGCTTTTTGTCATTGGCAGTTTGGCTTAGGGGAGAAGCGAGGGAGAGCCAGTGTGG
GAGCCGCCTCTAGGATTGTGAGCAAGTGCCTGTTTAGACAGCTCCACGCTGCTCCTTA
AAACCTATTTTGTGGTCTGGGGTTG
>2605
GTCATTGGCAGTTTGGCTTAGGGGAGAAGCGAGGGAGAGCCAGTGTGGGAGCCGCCT
CTAGGATTGTGAGCAAGTGCCTGTTTAGACAGCTCCACGCTGCTCCTTAAAACCTATTT
TGTGGTCTGGGGTTGGGGCGGGGG
>2606
GCTTAGGGGAGAAGCGAGGGAGAGCCAGTGTGGGAGCCGCCTCTAGGATTGTGAGCA

AGTGCCTGTTTAGACAGCTCCACGCTGCTCCTTAAAACCTATTTTGTGGTCTGGGGTTG
GGGCGGGGGCACACTGCCTCGATC
>2607
CCTTAAAACCTATTTTGTGGTCTGGGGTTGGGGCGGGGGCACACTGCCTCGATCCTCTT
GTTGTGTGGAGCAAAGTGATTCCTCTCTTCTTGTGTTCTCCAGATGGTTCCGTTGTCCT
CACATCCATTGTGGTTGAGGAT
>2608
AAACCTATTTTGTGGTCTGGGGTTGGGGCGGGGGCACACTGCCTCGATCCTCTTGTTGT
GTGGAGCAAAGTGATTCCTCTCTTCTTGTGTTCTCCAGATGGTTCCGTTGTCCTCACAT
CCATTGTGGTTGAGGATACAGT
>2609
GCGGGGGCACACTGCCTCGATCCTCTTGTTGTGTGGAGCAAAGTGATTCCTCTCTTCTT
GTGTTCTCCAGATGGTTCCGTTGTCCTCACATCCATTGTGGTTGAGGATACAGTGCAGA
GAAGACCCCGCTGTCCTCTGCT
>2610
GAGCAAAGTGATTCCTCTCTTCTTGTGTTCTCCAGATGGTTCCGTTGTCCTCACATCCAT
TGTGGTTGAGGATACAGTGCAGAGAAGACCCCGCTGTCCTCTGCTCCCCCTCCTTTTCT
CTCCAAAGAGAAGTTTCAGGG
>2611
GTGATTCCTCTCTTCTTGTGTTCTCCAGATGGTTCCGTTGTCCTCACATCCATTGTGGTT
GAGGATACAGTGCAGAGAAGACCCCGCTGTCCTCTGCTCCCCCTCCTTTTCTCTCCAA
AGAGAAGTTTCAGGGACAGCAA
>2612
TCTTCTTGTGTTCTCCAGATGGTTCCGTTGTCCTCACATCCATTGTGGTTGAGGATACAG
TGCAGAGAAGACCCCGCTGTCCTCTGCTCCCCCTCCTTTTCTCTCCAAAGAGAAGTTT
CAGGGACAGCAATAGACTTCTG
>2613
CATTGTGGTTGAGGATACAGTGCAGAGAAGACCCCGCTGTCCTCTGCTCCCCCTCCTTT
TCTCTCCAAAGAGAAGTTTCAGGGACAGCAATAGACTTCTGTGGGGCCTTTGTTCCTT
GTAGCTTTCATTTTTCAATTCTT
>2614
TTGTGGTTGAGGATACAGTGCAGAGAAGACCCCGCTGTCCTCTGCTCCCCCTCCTTTTC
TCTCCAAAGAGAAGTTTCAGGGACAGCAATAGACTTCTGTGGGGCCTTTGTTCCTTGT
AGCTTTCATTTTTCAATTCTTCT
>2615
AGGATACAGTGCAGAGAAGACCCCGCTGTCCTCTGCTCCCCCTCCTTTTCTCTCCAAA
GAGAAGTTTCAGGGACAGCAATAGACTTCTGTGGGGCCTTTGTTCCTTGTAGCTTTCAT
TTTTCAATTCTTCTTCTCCCTGT
>2616
CCTCCTTTTCTCTCCAAAGAGAAGTTTCAGGGACAGCAATAGACTTCTGTGGGGCCTT
TGTTCCTTGTAGCTTTCATTTTTCAATTCTTCTTCTCCCTGTATTATCTTGCTAGGCCTTA
GTTTGTGCATCTCTAAAATGG
>2617
CTTCTGTGGGGCCTTTGTTCCTTGTAGCTTTCATTTTTCAATTCTTCTTCTCCCTGTATTA
TCTTGCTAGGCCTTAGTTTGTGCATCTCTAAAATGGGCTGGGAGGTGCTAGCCCATACC
TAATATATAGGGCCGTCGTA

>2618
GGGGCCTTTGTTCCTTGTAGCTTTCATTTTTCAATTCTTCTTCTCCCTGTATTATCTTGCT
AGGCCTTAGTTTGTGCATCTCTAAAATGGGCTGGGAGGTGCTAGCCCATACCTAATATAT
AGGGCCGTCGTAAGGCTTA
>2619
TTTCAATTCTTCTTCTCCCTGTATTATCTTGCTAGGCCTTAGTTTGTGCATCTCTAAAATG
GGCTGGGAGGTGCTAGCCCATACCTAATATATAGGGCCGTCGTAAGGCTTAGGTCCCAT
AACTCATGGGGTTGGCTTGC
>2620
TCTTCTTCTCCCTGTATTATCTTGCTAGGCCTTAGTTTGTGCATCTCTAAAATGGGCTGG
GAGGTGCTAGCCCATACCTAATATATAGGGCCGTCGTAAGGCTTAGGTCCCATAACTCAT
GGGGTTGGCTTGCACAGGGC
>2621
ATCTTGCTAGGCCTTAGTTTGTGCATCTCTAAAATGGGCTGGGAGGTGCTAGCCCATAC
CTAATATATAGGGCCGTCGTAAGGCTTAGGTCCCATAACTCATGGGGTTGGCTTGCACA
GGGCTCTCTCCTTTCAGACTGG
>2622
TAGCCCATACCTAATATATAGGGCCGTCGTAAGGCTTAGGTCCCATAACTCATGGGGTTG
GCTTGCACAGGGCTCTCTCCTTTCAGACTGGTCAGGCCATGGTCCTTTTATGAGTCTGA
AGAGCCAAAATGAAGACCTCA
>2623
TATAGGGCCGTCGTAAGGCTTAGGTCCCATAACTCATGGGGTTGGCTTGCACAGGGCTC
TCTCCTTTCAGACTGGTCAGGCCATGGTCCTTTTATGAGTCTGAAGAGCCAAAATGAA
GACCTCACTTTCTAGTATCCCCA
>2624
ATAACTCATGGGGTTGGCTTGCACAGGGCTCTCTCCTTTCAGACTGGTCAGGCCATGGT
CCTTTTATGAGTCTGAAGAGCCAAAATGAAGACCTCACTTTCTAGTATCCCCAATCTTC
CTCCCACCTTTCCCTGGCTCTT
>2625
ATGGGGTTGGCTTGCACAGGGCTCTCTCCTTTCAGACTGGTCAGGCCATGGTCCTTTTA
TGAGTCTGAAGAGCCAAAATGAAGACCTCACTTTCTAGTATCCCCAATCTTCCTCCCAC
CTTTCCCTGGCTCTTGAGGGTT
>2626
GCACAGGGCTCTCTCCTTTCAGACTGGTCAGGCCATGGTCCTTTTATGAGTCTGAAGA
GCCAAAATGAAGACCTCACTTTCTAGTATCCCCAATCTTCCTCCCACCTTTCCCTGGCT
CTTGAGGGTTCTTTGGGCATGTT
>2627
GAAGAGCCAAAATGAAGACCTCACTTTCTAGTATCCCCAATCTTCCTCCCACCTTTCCC
TGGCTCTTGAGGGTTCTTTGGGCATGTTCCCCTCTGGTCTCCATTATTATATAGGGAAGA
GAAATTTTCCAGAAGAATATT
>2628
TCCTCCCACCTTTCCCTGGCTCTTGAGGGTTCTTTGGGCATGTTCCCCTCTGGTCTCCAT
TATTATATAGGGAAGAGAAATTTTCCAGAAGAATATTTGAAGGATTAAAGTTTGAGGTT
GTAGAATATGTGAAAACACAC
>2629
CCACCTTTCCCTGGCTCTTGAGGGTTCTTTGGGCATGTTCCCCTCTGGTCTCCATTATTA

TATAGGGAAGAGAAATTTTCCAGAAGAATATTTGAAGGATTAAAGTTTGAGGTTGTAGA
ATATGTGAAAACACACACACA
>2630
GCTCTTGAGGGTTCTTTGGGCATGTTCCCCTCTGGTCTCCATTATTATATAGGGAAGAGA
AATTTTCCAGAAGAATATTTGAAGGATTAAAGTTTGAGGTTGTAGAATATGTGAAAACA
CACACACACCACACCACACCC
>2631
TGTTCCCCTCTGGTCTCCATTATTATATAGGGAAGAGAAATTTTCCAGAAGAATATTTGA
AGGATTAAAGTTTGAGGTTGTAGAATATGTGAAAACACACACACACCACACCACACCC
CAAAATCACCCTTTTAAAGAAG
>2632
TCTCCATTATTATATAGGGAAGAGAAATTTTCCAGAAGAATATTTGAAGGATTAAAGTTT
GAGGTTGTAGAATATGTGAAAACACACACACACCACACCACACCCCAAAATCACCCTT
TTAAAGAAGATTAAAAAACCAC
>2633
AAGTTTGAGGTTGTAGAATATGTGAAAACACACACACACCACACCACACCCCAAAATC
ACCCTTTTAAAGAAGATTAAAAAACCACCAGTGCCTGTCCTACATTGGTAAACTCTGG
CAAGCCTCATAATGTTGACTTTAC
>2634
TTTGAGGTTGTAGAATATGTGAAAACACACACACACCACACCACACCCCAAAATCACC
CTTTTAAAGAAGATTAAAAAACCACCAGTGCCTGTCCTACATTGGTAAACTCTGGCAA
GCCTCATAATGTTGACTTTACTGA
>2635
ATGTGAAAACACACACACACCACACCACACCCCAAAATCACCCTTTTAAAGAAGATTA
AAAAACCACCAGTGCCTGTCCTACATTGGTAAACTCTGGCAAGCCTCATAATGTTGACT
TTACTGACCTGCCTGACTTCAGT
>2636
CCAAAATCACCCTTTTAAAGAAGATTAAAAAACCACCAGTGCCTGTCCTACATTGGTA
AACTCTGGCAAGCCTCATAATGTTGACTTTACTGACCTGCCTGACTTCAGTGGCCGAGT
CTACATGGGGTGTTAAGGCCCCA
>2637
GTGCCTGTCCTACATTGGTAAACTCTGGCAAGCCTCATAATGTTGACTTTACTGACCTG
CCTGACTTCAGTGGCCGAGTCTACATGGGGTGTTAAGGCCCCACGTGTTTTTGCCTGTG
CCCTTTAATTGTATCTTCAGGA
>2638
CCTACATTGGTAAACTCTGGCAAGCCTCATAATGTTGACTTTACTGACCTGCCTGACTT
CAGTGGCCGAGTCTACATGGGGTGTTAAGGCCCCACGTGTTTTTGCCTGTGCCCTTTAA
TTGTATCTTCAGGACCATATTT
>2639
AGTGGCCGAGTCTACATGGGGTGTTAAGGCCCCACGTGTTTTTGCCTGTGCCCTTTAAT
TGTATCTTCAGGACCATATTTTCAGAACCAGAAATCACAGCCACAGTTTGGCAGTGGC
ACAGAATGGTTCAATTTAGGCCT
>2640
CATGGGGTGTTAAGGCCCCACGTGTTTTTGCCTGTGCCCTTTAATTGTATCTTCAGGAC
CATATTTTCAGAACCAGAAATCACAGCCACAGTTTGGCAGTGGCACAGAATGGTTCAA
TTTAGGCCTATCCCAGAGAGGCT

>2641
GTGTTTTTGCCTGTGCCCTTTAATTGTATCTTCAGGACCATATTTTCAGAACCAGAAATC
ACAGCCACAGTTTGGCAGTGGCACAGAATGGTTCAATTTAGGCCTATCCCAGAGAGGC
TTGGGTTGTCTGTGAAGTTCTG
>2642
GCCTGTGCCCTTTAATTGTATCTTCAGGACCATATTTTCAGAACCAGAAATCACAGCCA
CAGTTTGGCAGTGGCACAGAATGGTTCAATTTAGGCCTATCCCAGAGAGGCTTGGGTT
GTCTGTGAAGTTCTGGGTGGTCT
>2643
CCTTTAATTGTATCTTCAGGACCATATTTTCAGAACCAGAAATCACAGCCACAGTTTGG
CAGTGGCACAGAATGGTTCAATTTAGGCCTATCCCAGAGAGGCTTGGGTTGTCTGTGA
AGTTCTGGGTGGTCTGTATTGTG
>2644
TTTTCAGAACCAGAAATCACAGCCACAGTTTGGCAGTGGCACAGAATGGTTCAATTTA
GGCCTATCCCAGAGAGGCTTGGGTTGTCTGTGAAGTTCTGGGTGGTCTGTATTGTGGA
GAGGTGAACACCGTATTGCTGGGG
>2645
CACAGTTTGGCAGTGGCACAGAATGGTTCAATTTAGGCCTATCCCAGAGAGGCTTGGG
TTGTCTGTGAAGTTCTGGGTGGTCTGTATTGTGGAGAGGTGAACACCGTATTGCTGGG
GGTGTGGAGGGGGGATGTTGCTGT
>2646
GGTTCAATTTAGGCCTATCCCAGAGAGGCTTGGGTTGTCTGTGAAGTTCTGGGTGGTCT
GTATTGTGGAGAGGTGAACACCGTATTGCTGGGGGTGTGGAGGGGGGATGTTGCTGTT
TCCCTGCTACAGAAAGGCCAGGC
>2647
GGGTTGTCTGTGAAGTTCTGGGTGGTCTGTATTGTGGAGAGGTGAACACCGTATTGCT
GGGGGTGTGGAGGGGGGATGTTGCTGTTTCCCTGCTACAGAAAGGCCAGGCTTCCTTT
TCAGCCTTTTGTTCCCCGATTCCC
>2648
GTATTGTGGAGAGGTGAACACCGTATTGCTGGGGGTGTGGAGGGGGGATGTTGCTGTT
TCCCTGCTACAGAAAGGCCAGGCTTCCTTTTCAGCCTTTTGTTCCCCGATTCCCCTCTC
TTCCTCCACTACACTCAGCCCTG
>2649
AGAGGTGAACACCGTATTGCTGGGGGTGTGGAGGGGGGATGTTGCTGTTTCCCTGCTA
CAGAAAGGCCAGGCTTCCTTTTCAGCCTTTTGTTCCCCGATTCCCCTCTCTTCCTCCAC
TACACTCAGCCCTGCTGCTGCTT
>2650
TACAGAAAGGCCAGGCTTCCTTTTCAGCCTTTTGTTCCCCGATTCCCCTCTCTTCCTCC
ACTACACTCAGCCCTGCTGCTGCTTCCAAGGGACAACTCCCTCTCTTGTCCTTGCCCAT
CTTTGAGAGAGGCGGTGAATAA
>2651
CTTTTCAGCCTTTTGTTCCCCGATTCCCCTCTCTTCCTCCACTACACTCAGCCCTGCTGC
TGCTTCCAAGGGACAACTCCCTCTCTTGTCCTTGCCCATCTTTGAGAGAGGCGGTGAAT
AAACACCAAGGCAAGAAGAAG
>2652
CTCCACTACACTCAGCCCTGCTGCTGCTTCCAAGGGACAACTCCCTCTCTTGTCCTTGC

CCATCTTTGAGAGAGGCGGTGAATAAACACCAAGGCAAGAAGAAGCAACGGGACCCC
TAGAAGGAGATGAGTGTGTGGCCC
>2653
CCACTACACTCAGCCCTGCTGCTGCTTCCAAGGGACAACTCCCTCTCTTGTCCTTGCCC
ATCTTTGAGAGAGGCGGTGAATAAACACCAAGGCAAGAAGAAGCAACGGGACCCCTA
GAAGGAGATGAGTGTGTGGCCCAA
>2654
TCCAAGGGACAACTCCCTCTCTTGTCCTTGCCCATCTTTGAGAGAGGCGGTGAATAAA
CACCAAGGCAAGAAGAAGCAACGGGACCCCTAGAAGGAGATGAGTGTGTGGCCCAA
GGATGGTGGAGCATTGTCCTGAAAGG
>2655
AAGGGACAACTCCCTCTCTTGTCCTTGCCCATCTTTGAGAGAGGCGGTGAATAAACAC
CAAGGCAAGAAGAAGCAACGGGACCCCTAGAAGGAGATGAGTGTGTGGCCCAAGGA
TGGTGGAGCATTGTCCTGAAAGGTAG
>2656
GGACAACTCCCTCTCTTGTCCTTGCCCATCTTTGAGAGAGGCGGTGAATAAACACCAA
GGCAAGAAGAAGCAACGGGACCCCTAGAAGGAGATGAGTGTGTGGCCCAAGGATGGT
GGAGCATTGTCCTGAAAGGTAGAAC
>2657
TTGTCCTTGCCCATCTTTGAGAGAGGCGGTGAATAAACACCAAGGCAAGAAGAAGCA
ACGGGACCCCTAGAAGGAGATGAGTGTGTGGCCCAAGGATGGTGGAGCATTGTCCTG
AAAGGTAGAACCAAGACTATAGCCTC
>2658
GGCGGTGAATAAACACCAAGGCAAGAAGAAGCAACGGGACCCCTAGAAGGAGATGA
GTGTGTGGCCCAAGGATGGTGGAGCATTGTCCTGAAAGGTAGAACCAAGACTATAGCC
TCCGAGGCTCTGTTTACCATATTCTT
>2659
GAAGAAGCAACGGGACCCCTAGAAGGAGATGAGTGTGTGGCCCAAGGATGGTGGAGC
ATTGTCCTGAAAGGTAGAACCAAGACTATAGCCTCCGAGGCTCTGTTTACCATATTCTT
GGCTCTTGTGCCACTTCTGTGTCC
>2660
AAGCAACGGGACCCCTAGAAGGAGATGAGTGTGTGGCCCAAGGATGGTGGAGCATTG
TCCTGAAAGGTAGAACCAAGACTATAGCCTCCGAGGCTCTGTTTACCATATTCTTGGCT
CTTGTGCCACTTCTGTGTCCAGCA
>2661
CTAGAAGGAGATGAGTGTGTGGCCCAAGGATGGTGGAGCATTGTCCTGAAAGGTAGA
ACCAAGACTATAGCCTCCGAGGCTCTGTTTACCATATTCTTGGCTCTTGTGCCACTTCTG
TGTCCAGCAGCTGGAGCTTGTAG
>2662
AGAACCAAGACTATAGCCTCCGAGGCTCTGTTTACCATATTCTTGGCTCTTGTGCCACT
TCTGTGTCCAGCAGCTGGAGCTTGTAGAAGAAAGATGGGAAAGACATAGTACAGGAA
ATGTTCCCATTCAGCCAGCACTGA
>2663
ACCAAGACTATAGCCTCCGAGGCTCTGTTTACCATATTCTTGGCTCTTGTGCCACTTCTG
TGTCCAGCAGCTGGAGCTTGTAGAAGAAAGATGGGAAAGACATAGTACAGGAAATGT
TCCCATTCAGCCAGCACTGATTT

>2664
TACCATATTCTTGGCTCTTGTGCCACTTCTGTGTCCAGCAGCTGGAGCTTGTAGAAGAA
AGATGGGAAAGACATAGTACAGGAAATGTTCCCATTCAGCCAGCACTGATTTATCTGCT
GCCTGGTTTCTCAGGAGGCCTA
>2665
ATTCTTGGCTCTTGTGCCACTTCTGTGTCCAGCAGCTGGAGCTTGTAGAAGAAAGATG
GGAAAGACATAGTACAGGAAATGTTCCCATTCAGCCAGCACTGATTTATCTGCTGCCTG
GTTTCTCAGGAGGCCTAATCTGT
>2666
TGGCTCTTGTGCCACTTCTGTGTCCAGCAGCTGGAGCTTGTAGAAGAAAGATGGGAAA
GACATAGTACAGGAAATGTTCCCATTCAGCCAGCACTGATTTATCTGCTGCCTGGTTTC
TCAGGAGGCCTAATCTGTAAGAA
>2667
CTGTGTCCAGCAGCTGGAGCTTGTAGAAGAAAGATGGGAAAGACATAGTACAGGAAA
TGTTCCCATTCAGCCAGCACTGATTTATCTGCTGCCTGGTTTCTCAGGAGGCCTAATCT
GTAAGAACAGTCGAGAACTTTATT
>2668
TGGGAAAGACATAGTACAGGAAATGTTCCCATTCAGCCAGCACTGATTTATCTGCTGCC
TGGTTTCTCAGGAGGCCTAATCTGTAAGAACAGTCGAGAACTTTATTTCTGCCCTTTAA
CCTATGTCCATTTTCTAGATAG
>2669
AGGAAATGTTCCCATTCAGCCAGCACTGATTTATCTGCTGCCTGGTTTCTCAGGAGGCC
TAATCTGTAAGAACAGTCGAGAACTTTATTTCTGCCCTTTAACCTATGTCCATTTTCTAG
ATAGTTTCTAAAAAAGGCATC
>2670
ATGTTCCCATTCAGCCAGCACTGATTTATCTGCTGCCTGGTTTCTCAGGAGGCCTAATCT
GTAAGAACAGTCGAGAACTTTATTTCTGCCCTTTAACCTATGTCCATTTTCTAGATAGTT
TCTAAAAAAGGCATCACACC
>2671
TCAGGAGGCCTAATCTGTAAGAACAGTCGAGAACTTTATTTCTGCCCTTTAACCTATGT
CCATTTTCTAGATAGTTTCTAAAAAAGGCATCACACCAGCTCTTTGAGACAACATGTCA
TATGCAACTGTTGGTTTTGGGT
>2672
GAGGCCTAATCTGTAAGAACAGTCGAGAACTTTATTTCTGCCCTTTAACCTATGTCCATT
TTCTAGATAGTTTCTAAAAAAGGCATCACACCAGCTCTTTGAGACAACATGTCATATGC
AACTGTTGGTTTTGGGTGGAA
>2673
GTAAGAACAGTCGAGAACTTTATTTCTGCCCTTTAACCTATGTCCATTTTCTAGATAGTT
TCTAAAAAAGGCATCACACCAGCTCTTTGAGACAACATGTCATATGCAACTGTTGGTTT
TGGGTGGAATGAAACTGAATT
>2674
GAGAACTTTATTTCTGCCCTTTAACCTATGTCCATTTTCTAGATAGTTTCTAAAAAAGGC
ATCACACCAGCTCTTTGAGACAACATGTCATATGCAACTGTTGGTTTTGGGTGGAATGA
AACTGAATTGTCAACGGACAT
>2675
GGTTTTGGGTGGAATGAAACTGAATTGTCAACGGACATTTTTTCTTTCTGTATTCATGTG

TTTGGAATAGTGACAACGTCATTGGTTCAGACTTTGAGCCCCCTTCTGGTTAGGCTCTT
CAGTATAAGCCCTTAAAAATA
>2676
TGAATTGTCAACGGACATTTTTTCTTTCTGTATTCATGTGTTTGGAATAGTGACAACGTC
ATTGGTTCAGACTTTGAGCCCCCTTCTGGTTAGGCTCTTCAGTATAAGCCCTTAAAAATA
TTTTTGACTTTTCAGGGCCA
>2677
CTTTCTGTATTCATGTGTTTGGAATAGTGACAACGTCATTGGTTCAGACTTTGAGCCCC
CTTCTGGTTAGGCTCTTCAGTATAAGCCCTTAAAAATATTTTTGACTTTTCAGGGCCAGG
CGCGGTGGCTCACGCCTGTAA
>2678
TTCATGTGTTTGGAATAGTGACAACGTCATTGGTTCAGACTTTGAGCCCCCTTCTGGTT
AGGCTCTTCAGTATAAGCCCTTAAAAATATTTTTGACTTTTCAGGGCCAGGCGCGGTGG
CTCACGCCTGTAATCCCAGCAC
>2679
TGTTTGGAATAGTGACAACGTCATTGGTTCAGACTTTGAGCCCCCTTCTGGTTAGGCTC
TTCAGTATAAGCCCTTAAAAATATTTTTGACTTTTCAGGGCCAGGCGCGGTGGCTCACG
CCTGTAATCCCAGCACTTTGGG
>2680
TTCAGACTTTGAGCCCCCTTCTGGTTAGGCTCTTCAGTATAAGCCCTTAAAAATATTTTT
GACTTTTCAGGGCCAGGCGCGGTGGCTCACGCCTGTAATCCCAGCACTTTGGGAGGCC
GAGGCAGGTGGATCACGAGGTC
>2681
CAGTATAAGCCCTTAAAAATATTTTTGACTTTTCAGGGCCAGGCGCGGTGGCTCACGCC
TGTAATCCCAGCACTTTGGGAGGCCGAGGCAGGTGGATCACGAGGTCAGGAGATCGA
GACCATCCTGGCTAACACGGTGAA
>2682
CTTAAAAATATTTTTGACTTTTCAGGGCCAGGCGCGGTGGCTCACGCCTGTAATCCCAG
CACTTTGGGAGGCCGAGGCAGGTGGATCACGAGGTCAGGAGATCGAGACCATCCTGG
CTAACACGGTGAAAGCCCGTCTCT
>2683
CAGGGCCAGGCGCGGTGGCTCACGCCTGTAATCCCAGCACTTTGGGAGGCCGAGGCA
GGTGGATCACGAGGTCAGGAGATCGAGACCATCCTGGCTAACACGGTGAAAGCCCGT
CTCTACTAAAAATACAAAAAATTAGC
>2684
CTTTGGGAGGCCGAGGCAGGTGGATCACGAGGTCAGGAGATCGAGACCATCCTGGCT
AACACGGTGAAAGCCCGTCTCTACTAAAAATACAAAAAATTAGCCAGGTGTGGTGGCG
GGCGCCTGTAGTCCCAGCTACTCGA
>2685
GGTCAGGAGATCGAGACCATCCTGGCTAACACGGTGAAAGCCCGTCTCTACTAAAAAT
ACAAAAAATTAGCCAGGTGTGGTGGCGGGCGCCTGTAGTCCCAGCTACTCGAGAGGCT
GAGGCTGGAGAATGGCGTGAACCC
>2686
TAACACGGTGAAAGCCCGTCTCTACTAAAAATACAAAAAATTAGCCAGGTGTGGTGGC
GGGCGCCTGTAGTCCCAGCTACTCGAGAGGCTGAGGCTGGAGAATGGCGTGAACCCG
GAGGCAGAGTTTGCAGTGAGCTGAG

>2687
GGTGAAAGCCCGTCTCTACTAAAAATACAAAAAATTAGCCAGGTGTGGTGGCGGGCGC
CTGTAGTCCCAGCTACTCGAGAGGCTGAGGCTGGAGAATGGCGTGAACCCGGAGGCA
GAGTTTGCAGTGAGCTGAGGTTGTG
>2688
ATACAAAAAATTAGCCAGGTGTGGTGGCGGGCGCCTGTAGTCCCAGCTACTCGAGAGG
CTGAGGCTGGAGAATGGCGTGAACCCGGAGGCAGAGTTTGCAGTGAGCTGAGGTTGT
GCCACTGCACTCCAGCCTGGGAGAC
>2689
GTGTGGTGGCGGGCGCCTGTAGTCCCAGCTACTCGAGAGGCTGAGGCTGGAGAATGG
CGTGAACCCGGAGGCAGAGTTTGCAGTGAGCTGAGGTTGTGCCACTGCACTCCAGCC
TGGGAGACAGAGCAAGACTCCGTCTC
>2690
GGTGGCGGGCGCCTGTAGTCCCAGCTACTCGAGAGGCTGAGGCTGGAGAATGGCGTG
AACCCGGAGGCAGAGTTTGCAGTGAGCTGAGGTTGTGCCACTGCACTCCAGCCTGGG
AGACAGAGCAAGACTCCGTCTCAAAA
>2691
CGCCTGTAGTCCCAGCTACTCGAGAGGCTGAGGCTGGAGAATGGCGTGAACCCGGAG
GCAGAGTTTGCAGTGAGCTGAGGTTGTGCCACTGCACTCCAGCCTGGGAGACAGAGC
AAGACTCCGTCTCAAAAAAATAAAAA
>2692
GAGGCTGGAGAATGGCGTGAACCCGGAGGCAGAGTTTGCAGTGAGCTGAGGTTGTGC
CACTGCACTCCAGCCTGGGAGACAGAGCAAGACTCCGTCTCAAAAAAATAAAAAATA
AAAAACATTAAAAAATTGACTTTTTG
>2693
TGGCGTGAACCCGGAGGCAGAGTTTGCAGTGAGCTGAGGTTGTGCCACTGCACTCCA
GCCTGGGAGACAGAGCAAGACTCCGTCTCAAAAAAATAAAAAATAAAAAACATTAAA
AAATTGACTTTTTGAGTAAAACAAAA
>2694
GCGTGAACCCGGAGGCAGAGTTTGCAGTGAGCTGAGGTTGTGCCACTGCACTCCAGC
CTGGGAGACAGAGCAAGACTCCGTCTCAAAAAAATAAAAAATAAAAAACATTAAAAA
ATTGACTTTTTGAGTAAAACAAAAAC
>2695
GAACCCGGAGGCAGAGTTTGCAGTGAGCTGAGGTTGTGCCACTGCACTCCAGCCTGG
GAGACAGAGCAAGACTCCGTCTCAAAAAAATAAAAAATAAAAAACATTAAAAAATTG
ACTTTTTGAGTAAAACAAAAACCAAA
>2696
TGGGAGACAGAGCAAGACTCCGTCTCAAAAAAATAAAAAATAAAAAACATTAAAAAA
TTGACTTTTTGAGTAAAACAAAAACCAAACGAGTCCGTGTAAAGTCTAAGATGTTGGT
TCATCTCAACATTGGGATATTGGAA
>2697
CGTCTCAAAAAAATAAAAAATAAAAAACATTAAAAAATTGACTTTTTGAGTAAAACAA
AAACCAAACGAGTCCGTGTAAAGTCTAAGATGTTGGTTCATCTCAACATTGGGATATTG
GAATAGGCCACTTTGGAAGGTTT
>2698
AATAAAAAATAAAAAACATTAAAAAATTGACTTTTTGAGTAAAACAAAAACCAAACGA

GTCCGTGTAAAGTCTAAGATGTTGGTTCATCTCAACATTGGGATATTGGAATAGGCCAC
TTTGGAAGGTTTCTCTAATTGAT
>2699
AACAAAAACCAAACGAGTCCGTGTAAAGTCTAAGATGTTGGTTCATCTCAACATTGGG
ATATTGGAATAGGCCACTTTGGAAGGTTTCTCTAATTGATGAAGGGCATGGGCCACCCC
ATGGATTGCAATGCTGTGGCTGT
>2700
CGAGTCCGTGTAAAGTCTAAGATGTTGGTTCATCTCAACATTGGGATATTGGAATAGGC
CACTTTGGAAGGTTTCTCTAATTGATGAAGGGCATGGGCCACCCCATGGATTGCAATGC
TGTGGCTGTTTGGGTGGCATCC
>2701
AGATGTTGGTTCATCTCAACATTGGGATATTGGAATAGGCCACTTTGGAAGGTTTCTCT
AATTGATGAAGGGCATGGGCCACCCCATGGATTGCAATGCTGTGGCTGTTTGGGTGGC
ATCCAGCTTCTAGGGGATGGTGG
>2702
TTCTCTAATTGATGAAGGGCATGGGCCACCCCATGGATTGCAATGCTGTGGCTGTTTGG
GTGGCATCCAGCTTCTAGGGGATGGTGGCAAACTTGGGTGTGCAGGCAGTAGGAAGG
AGGAGGTAGCTTCCAGAGAGAGCC
>2703
ATTGATGAAGGGCATGGGCCACCCCATGGATTGCAATGCTGTGGCTGTTTGGGTGGCAT
CCAGCTTCTAGGGGATGGTGGCAAACTTGGGTGTGCAGGCAGTAGGAAGGAGGAGGT
AGCTTCCAGAGAGAGCCATTGCTG
>2704
GGATTGCAATGCTGTGGCTGTTTGGGTGGCATCCAGCTTCTAGGGGATGGTGGCAAAC
TTGGGTGTGCAGGCAGTAGGAAGGAGGAGGTAGCTTCCAGAGAGAGCCATTGCTGGG
GCTCAAGAAGTTTCTTTCTGGGCTC
>2705
TGCAATGCTGTGGCTGTTTGGGTGGCATCCAGCTTCTAGGGGATGGTGGCAAACTTGG
GTGTGCAGGCAGTAGGAAGGAGGAGGTAGCTTCCAGAGAGAGCCATTGCTGGGGCTC
AAGAAGTTTCTTTCTGGGCTCAAGC
>2706
TTGGGTGGCATCCAGCTTCTAGGGGATGGTGGCAAACTTGGGTGTGCAGGCAGTAGGA
AGGAGGAGGTAGCTTCCAGAGAGAGCCATTGCTGGGGCTCAAGAAGTTTCTTTCTGG
GCTCAAGCATGGTTGCTAGTGGTCC
>2707
GCAAACTTGGGTGTGCAGGCAGTAGGAAGGAGGAGGTAGCTTCCAGAGAGAGCCATT
GCTGGGGCTCAAGAAGTTTCTTTCTGGGCTCAAGCATGGTTGCTAGTGGTCCACACAG
TGCTTTCGTGTGAATTAGAAAAAGC
>2708
AACTTGGGTGTGCAGGCAGTAGGAAGGAGGAGGTAGCTTCCAGAGAGAGCCATTGCT
GGGGCTCAAGAAGTTTCTTTCTGGGCTCAAGCATGGTTGCTAGTGGTCCACACAGTGC
TTTCGTGTGAATTAGAAAAAGCACC
>2709
GTAGGAAGGAGGAGGTAGCTTCCAGAGAGAGCCATTGCTGGGGCTCAAGAAGTTTCT
TTCTGGGCTCAAGCATGGTTGCTAGTGGTCCACACAGTGCTTTCGTGTGAATTAGAAA
AAGCACCCTTGTTGTGGATGGATGT

>2710
GAGAGAGCCATTGCTGGGGCTCAAGAAGTTTCTTTCTGGGCTCAAGCATGGTTGCTAG
TGGTCCACACAGTGCTTTCGTGTGAATTAGAAAAAGCACCCTTGTTGTGGATGGATGT
GGCTCTTAACTAGGGTCTATCCCA
>2711
GCTCAAGAAGTTTCTTTCTGGGCTCAAGCATGGTTGCTAGTGGTCCACACAGTGCTTTC
GTGTGAATTAGAAAAAGCACCCTTGTTGTGGATGGATGTGGCTCTTAACTAGGGTCTAT
CCCACAGTGAGCTGGTTGGGGC
>2712
GAAGTTTCTTTCTGGGCTCAAGCATGGTTGCTAGTGGTCCACACAGTGCTTTCGTGTGA
ATTAGAAAAAGCACCCTTGTTGTGGATGGATGTGGCTCTTAACTAGGGTCTATCCCACA
GTGAGCTGGTTGGGGCTGGATC
>2713
GGTCCACACAGTGCTTTCGTGTGAATTAGAAAAAGCACCCTTGTTGTGGATGGATGTG
GCTCTTAACTAGGGTCTATCCCACAGTGAGCTGGTTGGGGCTGGATCTCAAACCTCTTT
GCCCCTCAATCAGAGGCAGGAGG
>2714
TTTCGTGTGAATTAGAAAAAGCACCCTTGTTGTGGATGGATGTGGCTCTTAACTAGGGT
CTATCCCACAGTGAGCTGGTTGGGGCTGGATCTCAAACCTCTTTGCCCCTCAATCAGAG
GCAGGAGGTCCCTGGCCCAAGG
>2715
GTGTGAATTAGAAAAAGCACCCTTGTTGTGGATGGATGTGGCTCTTAACTAGGGTCTAT
CCCACAGTGAGCTGGTTGGGGCTGGATCTCAAACCTCTTTGCCCCTCAATCAGAGGCA
GGAGGTCCCTGGCCCAAGGGGCT
>2716
TCTTAACTAGGGTCTATCCCACAGTGAGCTGGTTGGGGCTGGATCTCAAACCTCTTTGC
CCCTCAATCAGAGGCAGGAGGTCCCTGGCCCAAGGGGCTTCCATGGTTGGAGTGGTC
GTCAGCCGAAAGCTGTTCTGGTGA
>2717
GTGAGCTGGTTGGGGCTGGATCTCAAACCTCTTTGCCCCTCAATCAGAGGCAGGAGGT
CCCTGGCCCAAGGGGCTTCCATGGTTGGAGTGGTCGTCAGCCGAAAGCTGTTCTGGTG
AGATGTTTTGAGCTATCAGTTCCA
>2718
GATCTCAAACCTCTTTGCCCCTCAATCAGAGGCAGGAGGTCCCTGGCCCAAGGGGCTT
CCATGGTTGGAGTGGTCGTCAGCCGAAAGCTGTTCTGGTGAGATGTTTTGAGCTATCA
GTTCCAAGTTTCCAAAGGCCAAGG
>2719
CCCCTCAATCAGAGGCAGGAGGTCCCTGGCCCAAGGGGCTTCCATGGTTGGAGTGGTC
GTCAGCCGAAAGCTGTTCTGGTGAGATGTTTTGAGCTATCAGTTCCAAGTTTCCAAAG
GCCAAGGCCTCTGGGATCAGTTTC
>2720
GGCAGGAGGTCCCTGGCCCAAGGGGCTTCCATGGTTGGAGTGGTCGTCAGCCGAAAG
CTGTTCTGGTGAGATGTTTTGAGCTATCAGTTCCAAGTTTCCAAAGGCCAAGGCCTCTG
GGATCAGTTTCACTGATCCTTCAA
>2721
CCCTGGCCCAAGGGGCTTCCATGGTTGGAGTGGTCGTCAGCCGAAAGCTGTTCTGGTG

AGATGTTTTGAGCTATCAGTTCCAAGTTTCCAAAGGCCAAGGCCTCTGGGATCAGTTTC
ACTGATCCTTCAACCAGCTTATG
>2722
CCAAGGGGCTTCCATGGTTGGAGTGGTCGTCAGCCGAAAGCTGTTCTGGTGAGATGTT
TTGAGCTATCAGTTCCAAGTTTCCAAAGGCCAAGGCCTCTGGGATCAGTTTCACTGATC
CTTCAACCAGCTTATGTGCCCTG
>2723
GTTGGAGTGGTCGTCAGCCGAAAGCTGTTCTGGTGAGATGTTTTGAGCTATCAGTTCC
AAGTTTCCAAAGGCCAAGGCCTCTGGGATCAGTTTCACTGATCCTTCAACCAGCTTATG
TGCCCTGTTAGCCCAGATACCCC
>2724
TTTGAGCTATCAGTTCCAAGTTTCCAAAGGCCAAGGCCTCTGGGATCAGTTTCACTGAT
CCTTCAACCAGCTTATGTGCCCTGTTAGCCCAGATACCCCTCCAGAGTGCTTCGCACTG
GGCTGCAGGTCACTGTGGCCCC
>2725
AAGTTTCCAAAGGCCAAGGCCTCTGGGATCAGTTTCACTGATCCTTCAACCAGCTTATG
TGCCCTGTTAGCCCAGATACCCCTCCAGAGTGCTTCGCACTGGGCTGCAGGTCACTGT
GGCCCCACACCATAGAAGCTGGG
>2726
GGCCTCTGGGATCAGTTTCACTGATCCTTCAACCAGCTTATGTGCCCTGTTAGCCCAGA
TACCCCTCCAGAGTGCTTCGCACTGGGCTGCAGGTCACTGTGGCCCCACACCATAGAA
GCTGGGGGCCTGTCTGCATGTTC
>2727
GATCCTTCAACCAGCTTATGTGCCCTGTTAGCCCAGATACCCCTCCAGAGTGCTTCGCA
CTGGGCTGCAGGTCACTGTGGCCCCACACCATAGAAGCTGGGGGCCTGTCTGCATGTT
CTGGCATCCATAAAGCTGCCTGC
>2728
CCTGTTAGCCCAGATACCCCTCCAGAGTGCTTCGCACTGGGCTGCAGGTCACTGTGGC
CCCACACCATAGAAGCTGGGGGCCTGTCTGCATGTTCTGGCATCCATAAAGCTGCCTGC
AAACGTGAGAGGGGGTTTATGGC
>2729
GGCTGCAGGTCACTGTGGCCCCACACCATAGAAGCTGGGGGCCTGTCTGCATGTTCTG
GCATCCATAAAGCTGCCTGCAAACGTGAGAGGGGGTTTATGGCACCCTTTTAGCATGA
CTGTCTCTTGTGACCAAGGCCAAG
>2730
GCCCCACACCATAGAAGCTGGGGGCCTGTCTGCATGTTCTGGCATCCATAAAGCTGCCT
GCAAACGTGAGAGGGGGTTTATGGCACCCTTTTAGCATGACTGTCTCTTGTGACCAAG
GCCAAGAAAAATAGCACAAAGAT
>2731
CCTGTCTGCATGTTCTGGCATCCATAAAGCTGCCTGCAAACGTGAGAGGGGGTTTATGG
CACCCTTTTAGCATGACTGTCTCTTGTGACCAAGGCCAAGAAAAATAGCACAAAGATG
TAGGAGTGGTGAGGAATGAAGAT
>2732
ATAAAGCTGCCTGCAAACGTGAGAGGGGGTTTATGGCACCCTTTTAGCATGACTGTCTC
TTGTGACCAAGGCCAAGAAAAATAGCACAAAGATGTAGGAGTGGTGAGGAATGAAGA
TGCACGTGCACGCGCCCTGCAGTC

>2733
CTGCCTGCAAACGTGAGAGGGGGTTTATGGCACCCTTTTAGCATGACTGTCTCTTGTGA
CCAAGGCCAAGAAAAATAGCACAAAGATGTAGGAGTGGTGAGGAATGAAGATGCACG
TGCACGCGCCCTGCAGTCTCAGTC
>2734
GGTTTATGGCACCCTTTTAGCATGACTGTCTCTTGTGACCAAGGCCAAGAAAAATAGCA
CAAAGATGTAGGAGTGGTGAGGAATGAAGATGCACGTGCACGCGCCCTGCAGTCTCA
GTCAATGCGCTCTTTAGGGTTTAG
>2735
AGCATGACTGTCTCTTGTGACCAAGGCCAAGAAAAATAGCACAAAGATGTAGGAGTG
GTGAGGAATGAAGATGCACGTGCACGCGCCCTGCAGTCTCAGTCAATGCGCTCTTTAG
GGTTTAGTTTGGAAAACCACAGTGT
>2736
AGGCCAAGAAAAATAGCACAAAGATGTAGGAGTGGTGAGGAATGAAGATGCACGTGC
ACGCGCCCTGCAGTCTCAGTCAATGCGCTCTTTAGGGTTTAGTTTGGAAAACCACAGT
GTCGTGTCTTATGTAACCAATTTCT
>2737
AGAAAAATAGCACAAAGATGTAGGAGTGGTGAGGAATGAAGATGCACGTGCACGCGC
CCTGCAGTCTCAGTCAATGCGCTCTTTAGGGTTTAGTTTGGAAAACCACAGTGTCGTGT
CTTATGTAACCAATTTCTTTTCTT
>2738
AAGATGCACGTGCACGCGCCCTGCAGTCTCAGTCAATGCGCTCTTTAGGGTTTAGTTTG
GAAAACCACAGTGTCGTGTCTTATGTAACCAATTTCTTTTCTTTTCTTTTCTTTTCTTTTT
TTTTTTTTTTTTTTGTGATG
>2739
CGTGTCTTATGTAACCAATTTCTTTTCTTTTCTTTTCTTTTCTTTTTTTTTTTTTTTTTTTG
TGATGGAGTCTCGTGTCTTGCTCTGTCACCCAGGCTGGAGTGCAGTGGCTCGATCTCG
GCTCACTGCAACCTCCGCCT
>2740
TCTTTTCTTTTCTTTTCTTTTTTTTTTTTTTTTTTTGTGATGGAGTCTCGTGTCTTGCTCTG
TCACCCAGGCTGGAGTGCAGTGGCTCGATCTCGGCTCACTGCAACCTCCGCCTCCCAG
GTTCAAGCGACTATCCTGCC
>2741
TGTCTTGCTCTGTCACCCAGGCTGGAGTGCAGTGGCTCGATCTCGGCTCACTGCAACC
TCCGCCTCCCAGGTTCAAGCGACTATCCTGCCTCAGCCTCCTGAGTAGCTGGGACTAC
AGGCGCCCGCCACCACGCCCAGCT
>2742
CTCTGTCACCCAGGCTGGAGTGCAGTGGCTCGATCTCGGCTCACTGCAACCTCCGCCT
CCCAGGTTCAAGCGACTATCCTGCCTCAGCCTCCTGAGTAGCTGGGACTACAGGCGCC
CGCCACCACGCCCAGCTAATTTTT
>2743
GAGTGCAGTGGCTCGATCTCGGCTCACTGCAACCTCCGCCTCCCAGGTTCAAGCGACT
ATCCTGCCTCAGCCTCCTGAGTAGCTGGGACTACAGGCGCCCGCCACCACGCCCAGCT
AATTTTTGTGTATTTAGTAGAGA
>2744
GGCTCGATCTCGGCTCACTGCAACCTCCGCCTCCCAGGTTCAAGCGACTATCCTGCCTC

AGCCTCCTGAGTAGCTGGGACTACAGGCGCCCGCCACCACGCCCAGCTAATTTTTTGT
GTATTTAGTAGAGACGGGGTTTC
>2745
TCGATCTCGGCTCACTGCAACCTCCGCCTCCCAGGTTCAAGCGACTATCCTGCCTCAGC
CTCCTGAGTAGCTGGGACTACAGGCGCCCGCCACCACGCCCAGCTAATTTTTTGTGTAT
TTAGTAGAGACGGGGTTTCACC
>2746
CACTGCAACCTCCGCCTCCCAGGTTCAAGCGACTATCCTGCCTCAGCCTCCTGAGTAG
CTGGGACTACAGGCGCCCGCCACCACGCCCAGCTAATTTTTTGTGTATTTAGTAGAGAC
GGGGTTTCACCGTGTTAGCCAGG
>2747
CCTCAGCCTCCTGAGTAGCTGGGACTACAGGCGCCCGCCACCACGCCCAGCTAATTTT
TTGTGTATTTAGTAGAGACGGGGTTTCACCGTGTTAGCCAGGATGGTCACAATCTCCTG
ACCTCGTGGTCCGCCCGCCTTGG
>2748
CAGCCTCCTGAGTAGCTGGGACTACAGGCGCCCGCCACCACGCCCAGCTAATTTTTTG
TGTATTTAGTAGAGACGGGGTTTCACCGTGTTAGCCAGGATGGTCACAATCTCCTGACC
TCGTGGTCCGCCCGCCTTGGCCT
>2749
TACAGGCGCCCGCCACCACGCCCAGCTAATTTTTTGTGTATTTAGTAGAGACGGGGTTT
CACCGTGTTAGCCAGGATGGTCACAATCTCCTGACCTCGTGGTCCGCCCGCCTTGGCCT
CCCACAGTGCTGGGATTACAGG
>2750
GTTTCACCGTGTTAGCCAGGATGGTCACAATCTCCTGACCTCGTGGTCCGCCCGCCTTG
GCCTCCCACAGTGCTGGGATTACAGGCGTGAGCCACCACGCCCGGCCACCAATTTCTT
ATACATAGGTTATGGAGCAGGGA
>2751
GCCAGGATGGTCACAATCTCCTGACCTCGTGGTCCGCCCGCCTTGGCCTCCCACAGTG
CTGGGATTACAGGCGTGAGCCACCACGCCCGGCCACCAATTTCTTATACATAGGTTATG
GAGCAGGGAGAAAGAGACATGCA
>2752
CTTGGCCTCCCACAGTGCTGGGATTACAGGCGTGAGCCACCACGCCCGGCCACCAATT
TCTTATACATAGGTTATGGAGCAGGGAGAAAGAGACATGCAAACAAGGCAGAATTGGG
ACCTTTGCAAGAAGACCTTCTTCA
>2753
CCACAGTGCTGGGATTACAGGCGTGAGCCACCACGCCCGGCCACCAATTTCTTATACAT
AGGTTATGGAGCAGGGAGAAAGAGACATGCAAACAAGGCAGAATTGGGACCTTTGCA
AGAAGACCTTCTTCACTCGCCCAC
>2754
GCCACCACGCCCGGCCACCAATTTCTTATACATAGGTTATGGAGCAGGGAGAAAGAGA
CATGCAAACAAGGCAGAATTGGGACCTTTGCAAGAAGACCTTCTTCACTCGCCCACTT
TCTCATACTTTTAGTCACCAAGGC
>2755
TTCTTATACATAGGTTATGGAGCAGGGAGAAAGAGACATGCAAACAAGGCAGAATTGG
GACCTTTGCAAGAAGACCTTCTTCACTCGCCCACTTTCTCATACTTTTAGTCACCAAGG
CAGGGTCTTTTTAAACAACTTCG

>2756
TTATACATAGGTTATGGAGCAGGGAGAAAGAGACATGCAAACAAGGCAGAATTGGGAC
CTTTGCAAGAAGACCTTCTTCACTCGCCCACTTTCTCATACTTTTAGTCACCAAGGCAG
GGTCTTTTTAAACAACTTCGAGG
>2757
TGCAAACAAGGCAGAATTGGGACCTTTGCAAGAAGACCTTCTTCACTCGCCCACTTTC
TCATACTTTTAGTCACCAAGGCAGGGTCTTTTTAAACAACTTCGAGGCTCAGGCCTAGG
GGGTTAGGAGAGTAAACCCTAAA
>2758
AGGCAGAATTGGGACCTTTGCAAGAAGACCTTCTTCACTCGCCCACTTTCTCATACTTT
TAGTCACCAAGGCAGGGTCTTTTTAAACAACTTCGAGGCTCAGGCCTAGGGGGTTAGG
AGAGTAAACCCTAAATCCCTTTC
>2759
AGAATTGGGACCTTTGCAAGAAGACCTTCTTCACTCGCCCACTTTCTCATACTTTTAGT
CACCAAGGCAGGGTCTTTTTAAACAACTTCGAGGCTCAGGCCTAGGGGGTTAGGAGA
GTAAACCCTAAATCCCTTTCACCC
>2760
GACCTTCTTCACTCGCCCACTTTCTCATACTTTTAGTCACCAAGGCAGGGTCTTTTTAA
ACAACTTCGAGGCTCAGGCCTAGGGGGTTAGGAGAGTAAACCCTAAATCCCTTTCACC
CTAGCATGCTTGGGTGCCGTGTT
>2761
TCGCCCACTTTCTCATACTTTTAGTCACCAAGGCAGGGTCTTTTTAAACAACTTCGAGG
CTCAGGCCTAGGGGGTTAGGAGAGTAAACCCTAAATCCCTTTCACCCTAGCATGCTTGG
GTGCCGTGTTGACAAGGTCTCT
>2762
CTTTTTAAACAACTTCGAGGCTCAGGCCTAGGGGGTTAGGAGAGTAAACCCTAAATCC
CTTTCACCCTAGCATGCTTGGGTGCCGTGTTGACAAGGTCTCTGAAGTGCCCATGGGA
AGCAGACCAGGCTCTGAGAATAAT
>2763
GCCTAGGGGGTTAGGAGAGTAAACCCTAAATCCCTTTCACCCTAGCATGCTTGGGTGCC
GTGTTGACAAGGTCTCTGAAGTGCCCATGGGAAGCAGACCAGGCTCTGAGAATAATTT
CCATGGAATTAATTATTGTTCTT
>2764
CCCTAAATCCCTTTCACCCTAGCATGCTTGGGTGCCGTGTTGACAAGGTCTCTGAAGTG
CCCATGGGAAGCAGACCAGGCTCTGAGAATAATTTCCATGGAATTAATTATTGTTCTTA
CTCCAATAGATTTGAAATCTAT
>2765
TAAATCCCTTTCACCCTAGCATGCTTGGGTGCCGTGTTGACAAGGTCTCTGAAGTGCCC
ATGGGAAGCAGACCAGGCTCTGAGAATAATTTCCATGGAATTAATTATTGTTCTTACTCC
AATAGATTTGAAATCTATTTA
>2766
CCCTAGCATGCTTGGGTGCCGTGTTGACAAGGTCTCTGAAGTGCCCATGGGAAGCAGA
CCAGGCTCTGAGAATAATTTCCATGGAATTAATTATTGTTCTTACTCCAATAGATTTGAA
ATCTATTTAACTTTTCAGGGTC
>2767
TGCCCATGGGAAGCAGACCAGGCTCTGAGAATAATTTCCATGGAATTAATTATTGTTCTT

ACTCCAATAGATTTGAAATCTATTTAACTTTTCAGGGTCCCCTAGCTTTTTAAATGTTTC
AAGTGTATTCTCTCTGATAG
>2768
TGAGAATAATTTCCATGGAATTAATTATTGTTCTTACTCCAATAGATTTGAAATCTATTTA
ACTTTTCAGGGTCCCCTAGCTTTTTAAATGTTTCAAGTGTATTCTCTCTGATAGGTTGCC
TTTTCTCTCCCTGTAGGCT
>2769
TTCCATGGAATTAATTATTGTTCTTACTCCAATAGATTTGAAATCTATTTAACTTTTCAGG
GTCCCCTAGCTTTTTAAATGTTTCAAGTGTATTCTCTCTGATAGGTTGCCTTTTCTCTCCC
TGTAGGCTATAATTAGTG
>2770
ATTTGAAATCTATTTAACTTTTCAGGGTCCCCTAGCTTTTTAAATGTTTCAAGTGTATTCT
CTCTGATAGGTTGCCTTTTCTCTCCCTGTAGGCTATAATTAGTGTCCTTTACACAAAATT
GGCTTAGAATTGGATTTAG
>2771
CAGGGTCCCCTAGCTTTTTAAATGTTTCAAGTGTATTCTCTCTGATAGGTTGCCTTTTCT
CTCCCTGTAGGCTATAATTAGTGTCCTTTACACAAAATTGGCTTAGAATTGGATTTAGAG
AAGATAAGAATCAAATTACC
>2772
TCTCTCTGATAGGTTGCCTTTTCTCTCCCTGTAGGCTATAATTAGTGTCCTTTACACAAA
ATTGGCTTAGAATTGGATTTAGAGAAGATAAGAATCAAATTACCAGTATTTATCTTGTAA
ATTCCAAAAGATCTACTTAA
>2773
GTTGCCTTTTCTCTCCCTGTAGGCTATAATTAGTGTCCTTTACACAAAATTGGCTTAGAA
TTGGATTTAGAGAAGATAAGAATCAAATTACCAGTATTTATCTTGTAAATTCCAAAAGAT
CTACTTAAGACACTGTATTA
>2774
TATAATTAGTGTCCTTTACACAAAATTGGCTTAGAATTGGATTTAGAGAAGATAAGAATC
AAATTACCAGTATTTATCTTGTAAATTCCAAAAGATCTACTTAAGACACTGTATTAAAAA
AGGAGTATAAAAGAAAAGTT
>2775
ATTGGCTTAGAATTGGATTTAGAGAAGATAAGAATCAAATTACCAGTATTTATCTTGTAA
ATTCCAAAAGATCTACTTAAGACACTGTATTAAAAAAGGAGTATAAAAGAAAAGTTTAT
TCTATGAAAAAGTAAATTCCT
>2776
ATTGGATTTAGAGAAGATAAGAATCAAATTACCAGTATTTATCTTGTAAATTCCAAAAGA
TCTACTTAAGACACTGTATTAAAAAAGGAGTATAAAAGAAAAGTTTATTCTATGAAAAA
GTAAATTCCTCATGGTCTGAG
>2777
TAAGAATCAAATTACCAGTATTTATCTTGTAAATTCCAAAAGATCTACTTAAGACACTGT
ATTAAAAAAGGAGTATAAAAGAAAAGTTTATTCTATGAAAAAGTAAATTCCTCATGGTC
TGAGCAACCAGGGGTATTTTA
>2778
TTCCAAAAGATCTACTTAAGACACTGTATTAAAAAAGGAGTATAAAAGAAAAGTTTATT
CTATGAAAAAGTAAATTCCTCATGGTCTGAGCAACCAGGGGTATTTTAATATCAAATCTC
CAGACTTCCCTTGGAGGCACC

>2779
ACACTGTATTAAAAAAGGAGTATAAAAGAAAAGTTTATTCTATGAAAAAGTAAATTCCT
CATGGTCTGAGCAACCAGGGGTATTTTAATATCAAATCTCCAGACTTCCCTTGGAGGCA
CCTAAAATTGCCTTTTCCCTTG
>2780
GTTTATTCTATGAAAAAGTAAATTCCTCATGGTCTGAGCAACCAGGGGTATTTTAATATC
AAATCTCCAGACTTCCCTTGGAGGCACCTAAAATTGCCTTTTCCCTTGTCGCTCGTTTTT
ATCTCCAGTTGAGCTTCTGA
>2781
AAAAGTAAATTCCTCATGGTCTGAGCAACCAGGGGTATTTTAATATCAAATCTCCAGAC
TTCCCTTGGAGGCACCTAAAATTGCCTTTTCCCTTGTCGCTCGTTTTTATCTCCAGTTGA
GCTTCTGAAATATGTTGCCAG
>2782
TCAAATCTCCAGACTTCCCTTGGAGGCACCTAAAATTGCCTTTTCCCTTGTCGCTCGTT
TTTATCTCCAGTTGAGCTTCTGAAATATGTTGCCAGGCTTTGGATATTGGAGAGCTGGT
GTGGTTTCAGCTTTCATTAAAA
>2783
TCTCCAGACTTCCCTTGGAGGCACCTAAAATTGCCTTTTCCCTTGTCGCTCGTTTTTATC
TCCAGTTGAGCTTCTGAAATATGTTGCCAGGCTTTGGATATTGGAGAGCTGGTGTGGTT
TCAGCTTTCATTAAAATTCGG
>2784
GCACCTAAAATTGCCTTTTCCCTTGTCGCTCGTTTTTATCTCCAGTTGAGCTTCTGAAAT
ATGTTGCCAGGCTTTGGATATTGGAGAGCTGGTGTGGTTTCAGCTTTCATTAAAATTCG
GAGGTTTTGACTTATCTATTA
>2785
TTTCCCTTGTCGCTCGTTTTTATCTCCAGTTGAGCTTCTGAAATATGTTGCCAGGCTTTG
GATATTGGAGAGCTGGTGTGGTTTCAGCTTTCATTAAAATTCGGAGGTTTTGACTTATCT
ATTATTTCTGATTTGTGGTA
>2786
TTTTATCTCCAGTTGAGCTTCTGAAATATGTTGCCAGGCTTTGGATATTGGAGAGCTGGT
GTGGTTTCAGCTTTCATTAAAATTCGGAGGTTTTGACTTATCTATTATTTCTGATTTGTGG
TAAGACATAAGCCTCTAAA
>2787
TCTGAAATATGTTGCCAGGCTTTGGATATTGGAGAGCTGGTGTGGTTTCAGCTTTCATTA
AAATTCGGAGGTTTTGACTTATCTATTATTTCTGATTTGTGGTAAGACATAAGCCTCTAA
AATATGCTATCTTAAAGAGA
>2788
CTGGTGTGGTTTCAGCTTTCATTAAAATTCGGAGGTTTTGACTTATCTATTATTTCTGATT
TGTGGTAAGACATAAGCCTCTAAAATATGCTATCTTAAAGAGATACACACAAATTTTATC
TTAGCATGTTAAGTTTTTA
>2789
GGTTTCAGCTTTCATTAAAATTCGGAGGTTTTGACTTATCTATTATTTCTGATTTGTGGTA
AGACATAAGCCTCTAAAATATGCTATCTTAAAGAGATACACACAAATTTTATCTTAGCAT
GTTAAGTTTTTAAGCCGTT
>2790
GAGGTTTTGACTTATCTATTATTTCTGATTTGTGGTAAGACATAAGCCTCTAAAATATGCT

ATCTTAAAGAGATACACACAAATTTTATCTTAGCATGTTAAGTTTTTAAGCCGTTGAGAG
ATGGCAGAATAGACCTCTG
>2791
CTGATTTGTGGTAAGACATAAGCCTCTAAAATATGCTATCTTAAAGAGATACACACAAAT
TTTATCTTAGCATGTTAAGTTTTTAAGCCGTTGAGAGATGGCAGAATAGACCTCTGTGG
TCTCCTGTGTGCTTCTTCGTC
>2792
GGTAAGACATAAGCCTCTAAAATATGCTATCTTAAAGAGATACACACAAATTTTATCTTA
GCATGTTAAGTTTTTAAGCCGTTGAGAGATGGCAGAATAGACCTCTGTGGTCTCCTGTG
TGCTTCTTCGTCCTAAAATGT
>2793
ATAAGCCTCTAAAATATGCTATCTTAAAGAGATACACACAAATTTTATCTTAGCATGTTA
AGTTTTTAAGCCGTTGAGAGATGGCAGAATAGACCTCTGTGGTCTCCTGTGTGCTTCTT
CGTCCTAAAATGTCAGCATGC
>2794
CTAAAATATGCTATCTTAAAGAGATACACACAAATTTTATCTTAGCATGTTAAGTTTTTA
AGCCGTTGAGAGATGGCAGAATAGACCTCTGTGGTCTCCTGTGTGCTTCTTCGTCCTAA
AATGTCAGCATGCTGGTTTTC
>2795
CTTAAAGAGATACACACAAATTTTATCTTAGCATGTTAAGTTTTTAAGCCGTTGAGAGAT
GGCAGAATAGACCTCTGTGGTCTCCTGTGTGCTTCTTCGTCCTAAAATGTCAGCATGCT
GGTTTTCATGTGCTGCCTCGT
>2796
TTAAGCCGTTGAGAGATGGCAGAATAGACCTCTGTGGTCTCCTGTGTGCTTCTTCGTCC
TAAAATGTCAGCATGCTGGTTTTCATGTGCTGCCTCGTACAGTAAGTCCCCAGCAGATT
TAAATAAGGTTGTGTCAAAACA
>2797
CTGTGGTCTCCTGTGTGCTTCTTCGTCCTAAAATGTCAGCATGCTGGTTTTCATGTGCTG
CCTCGTACAGTAAGTCCCCAGCAGATTTAAATAAGGTTGTGTCAAAACATATGCCTATG
TTTTTTTTAACATCGTTGTCT
>2798
TCCTAAAATGTCAGCATGCTGGTTTTCATGTGCTGCCTCGTACAGTAAGTCCCCAGCAG
ATTTAAATAAGGTTGTGTCAAAACATATGCCTATGTTTTTTTTAACATCGTTGTCTCATAA
GTGTGCAGTAATGGCATATT
>2799
CCCAGCAGATTTAAATAAGGTTGTGTCAAAACATATGCCTATGTTTTTTTTAACATCGTT
GTCTCATAAGTGTGCAGTAATGGCATATTGAGACTGTTCATAGACTGGTCAGCTGCATT
TCCTGCAGCCGAGGAACCCTT
>2800
GATTTAAATAAGGTTGTGTCAAAACATATGCCTATGTTTTTTTTAACATCGTTGTCTCATA
AGTGTGCAGTAATGGCATATTGAGACTGTTCATAGACTGGTCAGCTGCATTTCCTGCAG
CCGAGGAACCCTTTATTAAG
